# Broadly Applicable Copper(I)-Catalyzed Alkyne Semihydrogenation and Hydrogenation of α,β-Unsaturated Amides Enabled by Bifunctional Iminopyridine Ligands

**DOI:** 10.1021/jacs.5c01339

**Published:** 2025-04-16

**Authors:** Mahadeb Gorai, Jonas H. Franzen, Philipp Rotering, Tobias Rüffer, Fabian Dielmann, Johannes F. Teichert

**Affiliations:** † Institut für Chemie, Technische Universität Chemnitz, Straße der Nationen 62, 09111 Chemnitz, Germany; ‡ Department of General, Inorganic and Theoretical Chemistry, Universität Innsbruck, Innrain 80/82, 6020 Innsbruck, Austria

## Abstract

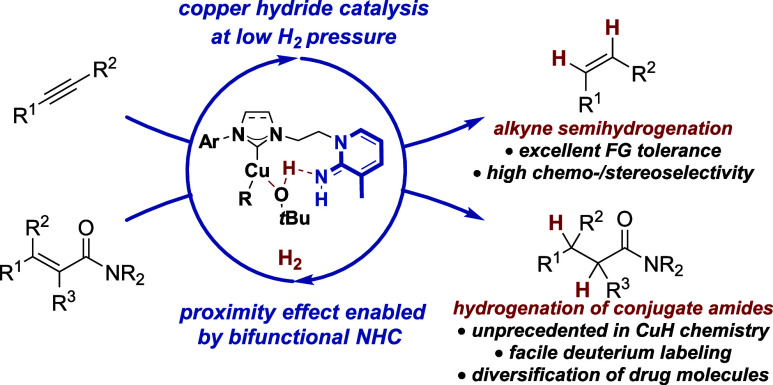

A highly active bifunctional catalyst consisting of a copper(I)/N-heterocyclic carbene complex and a basic 2-iminopyridine subunit allows for copper hydride chemistry under low H_2_ pressure, achieving efficient catalysis reaching 1 bar (balloon pressure). The bifunctional catalyst tolerates a remarkable variety of functional groups in catalytic alkyne semihydrogenations. Furthermore, this catalyst design gives rise to a high reactivity that allows for the catalytic hydrogenation of α,β-unsaturated amides (a substrate class hitherto unreactive in copper hydride catalysis) at a low H_2_ pressure for the first time. In this manner, late-stage modification and isotope labeling of α,β-unsaturated amides, common subunits in biologically active compounds, can be realized through catalytic hydrogenation using a first-row transition metal catalyst based on abundant copper. Preliminary mechanistic experiments indicate that the bifunctional catalyst operates via an iminopyridine-mediated proximity effect. We hypothesize that the coordination of an alcohol as a proton source on the copper(I) complex facilitates the overall reactions through a rapid proto-decupration step.

## Introduction

**Scheme 1:**
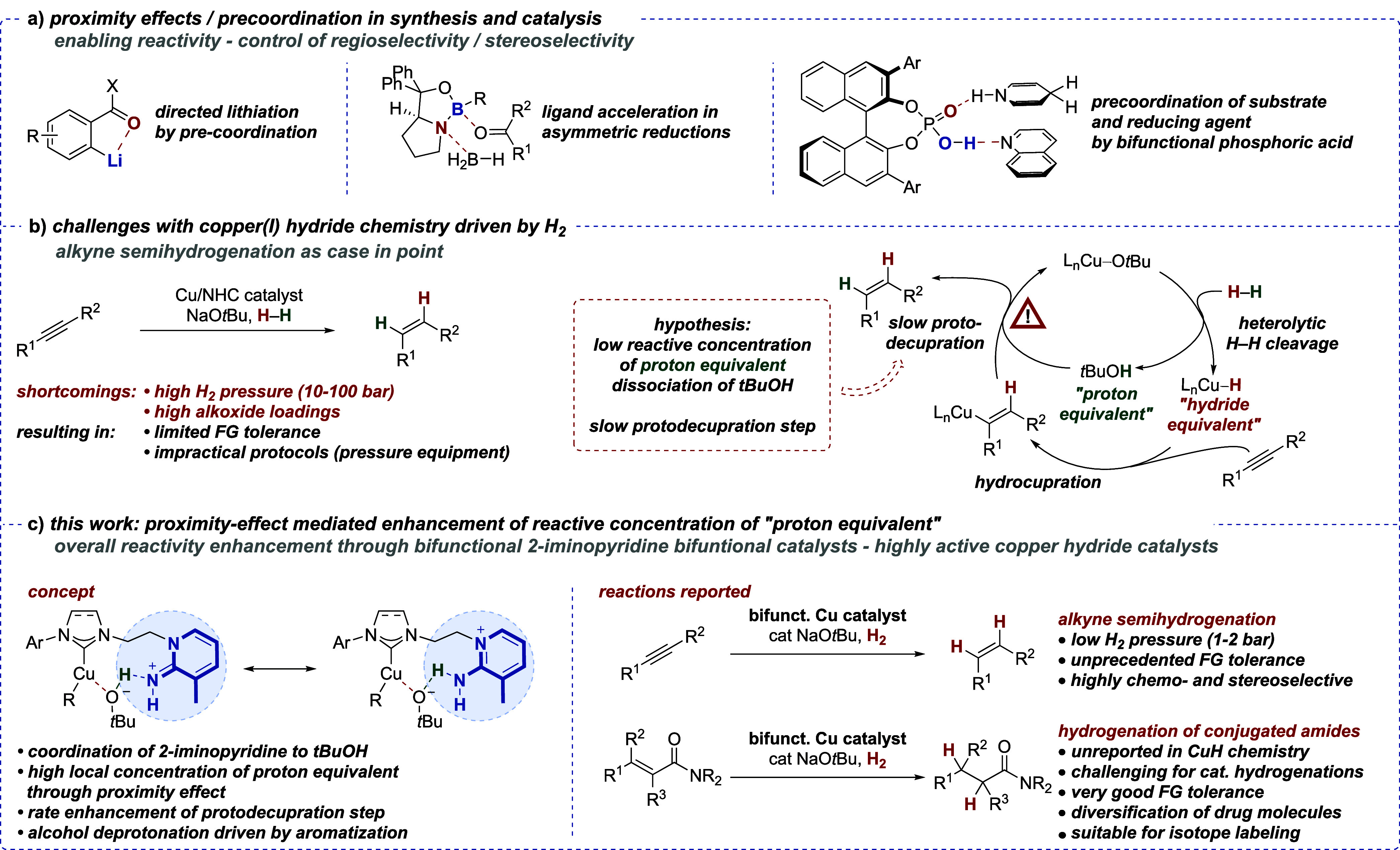
Examples of Proximity Effects in Organometallic Chemistry and Catalysis—Application to H_2_-Driven Copper(I) Hydride-Based Transformations (a) Reported examples of proximity effects. (b) Challenges in copper(I)-catalyzed alkyne semihydrogenations and catalytic cycle highlighting the fate of the proton and hydride equivalent. (c) Design of bifunctional copper(I)/NHC complexes for H_2_-driven copper(I) hydride catalysis.

Positioning the reactants of a chemical reaction in close proximity through noncovalent interactions can significantly enhance reaction rates and improve selectivity.^1^ For instance, this “proximity effect”^2,3^ enables the selective metalation of benzene derivatives by placing alkyllithium reagents close to the reactive site by a directing group (Scheme 1a).^4^ In a similar vein, bifunctional catalysts with multiple binding sites promote a reaction of two substrates by orienting them in a close spatial relationship,^5−7^ as exemplified by the rate enhancement through cooperativity^5,8,9^ observed with the CBS catalyst in asymmetric reductions^10,11^ or chiral phosphoric acid derivatives in transfer hydrogenations (Scheme 1a).^12^ In other words, multifunctional catalysts can compensate for a low reactant concentration. This process resembles enzymatic reactivity, raising the effective concentration of a substrate within a catalytic pocket even under high overall dilution.

Bifunctional catalysts have also been shown to be effective in controlling reactivity and selectivity^13−15^ in the realm of H_2_-driven copper hydride chemistry.^16,17^ While copper(I)-catalyzed alkyne semihydrogenations (Scheme 1b^16,18^) and other H_2_-driven reactions^19,20^ are synthetically appealing, they have some shortcomings: (i) These processes generally require elevated H_2_ pressure (ranging from 10 to 100 bar), lowering overall practicability due to the need for high-pressure equipment.^21^ (ii) Often, stoichiometric amounts of alkoxide additives are required, limiting the overall tolerance of functional groups.^13,14,20^ The necessity for these stoichiometric alkoxide additives is in contrast to the catalytic cycle (Scheme 1b),^16^ which demonstrates that catalytic amounts of alkoxide should suffice. These limitations with H_2_-driven copper hydride chemistry, albeit highly atom-economic and therefore desirable, are in stark contrast to hydrosilane-based methods, which are highly functional group tolerant.^17,22^

We hypothesized that a key challenge of the H_2_-based methods could be a sluggish final proto-decupration step utilizing alcohol as a proton source.^16^ The alcohol can be seen as a “proton equivalent” that is formed alongside the “hydride equivalent” (i.e., the respective copper(I) hydride complex) during the heterolytic H–H bond cleavage along a Cu–O bond.^23^ Since the alcohol can dissociate from the catalyst but is required for the proto-decupration step, we surmised that a low local concentration of the “proton equivalent” could be the reason for the slow closing of the catalytic cycle (Scheme 1b).

We therefore envisaged the construction of a bifunctional catalyst to raise the effective concentration of the “proton equivalent” through a proximity effect: A 2-iminopyridine moiety would act as both a proton shuttle driven by aromatization and a coordinating unit to the alcohol (Scheme 1c). Such a catalyst would position the alcohol close to the copper(I) atom and effect a rate enhancement of the overall H_2_-driven transformation by facilitating the key proto-decupration step (Scheme 1c). As an additional benefit, the bifunctional catalyst design could allow for a generally lower amount of alkoxide additive.

Inspired by the development of highly electron-rich phosphine ligands bearing basic imine functions (driven by aromatization) adjacent to phosphorus atoms,^24,25^ we herein report the development of bifunctional copper(I) catalysts based on N-heterocyclic carbene ligands (NHCs)^26^ with a basic 2-iminopyridine moiety (Scheme 1c). The resulting catalysts outperform all previously known copper(I) complexes for H_2_-driven catalytic reactions, enabling catalysis to be conducted for the first time in standard laboratory glassware under a low H_2_ pressure. Moreover, the new bifunctional catalysts exhibit exceptional functional group tolerance in chemo- and stereoselective alkyne semihydrogenations.^27^ Significantly extending the scope of copper(I) hydride chemistry, the bifunctional catalysts facilitate the hydrogenation of α,β-unsaturated amides (conjugated amides). This substrate class is challenging and unreported in copper(I) hydride catalysis in particular. In addition, it represents a rarely realized catalytic hydrogenation in general and an important example of catalytic hydrogenation with well-accessible 3d metal catalysts.^28^ The resulting catalytic protocol based on copper(I) allows for late-stage modification and isotope labeling of biologically active conjugated amides under a low H_2_/D_2_ pressure.

## Results and Discussion

**Scheme 2:**
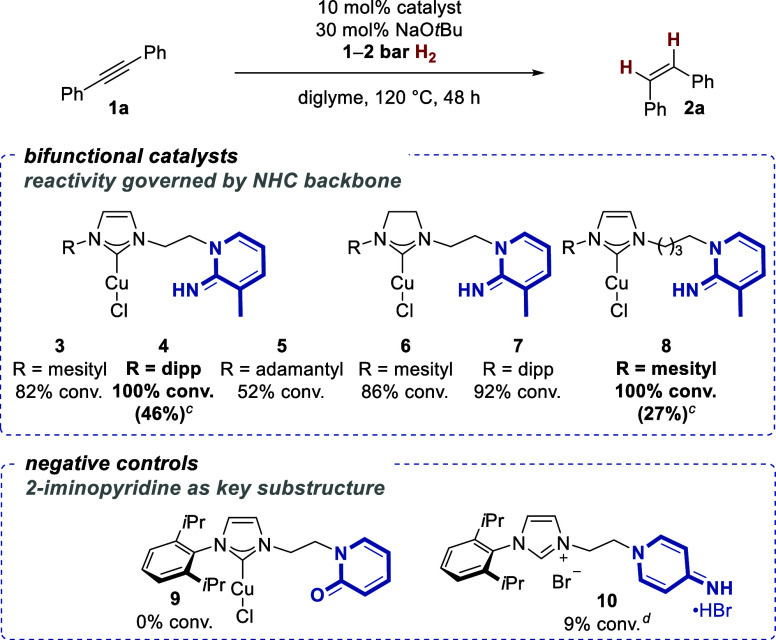
Identification of Bifunctional Catalysts for Alkyne Semihydrogenation at a Low H_2_ Pressure and Catalytic Base Loading^,^ All reactions were performed according to the general procedure 1 (**GP1**, see the Supporting Information for details) with 0.2 mmol of tolane (**1a**). Conversion and *Z*/*E* ratio were measured by GC/GC-MS and/or ^1^H NMR analysis; in all cases, *Z*/*E* selectivity is >99:1. Catalysis was performed with a H_2_ balloon (1 bar). Catalysis was performed by *in situ* generation of the active catalyst with 10 mol % CuCl, 10 mol % **10**, and 50 mol % NaO*t*Bu (abbreviations: mesityl = 2,4,6-*tri*-Me-C_6_H_2_, dipp = 2,6-*di*-*i*Pr–C_6_H_3_).

A variety of new imidazolium- and imidazolinium-based copper(I) complexes **3**–**8** were synthesized in a modular, high-yielding three-step protocol from commercially available building blocks (Scheme 2).^29^ The actual copper(I)/NHC complexation could be achieved by deprotonation of the respective azolium salts in the presence of copper sources or by transmetalation from the corresponding silver(I)/NHC complexes.^30,31^

We decided to employ copper(I)-catalyzed alkyne semihydrogenations as model reaction^16^ to validate our working hypothesis of efficient copper hydride catalysis through proximity effect. In this manner, a direct comparison to literature-known methods and their limitations in terms of functional group tolerance required H_2_ pressure, and stereoselectivity could be drawn. With 10 mol % of newly synthesized bifunctional copper(I)/NHC complexes **3**–**8** in diglyme at 120 °C,^29^ the formation of (*Z*)-stilbene (**2a**) from tolane (**1a**) could be affected at 1–2 bar H_2_ pressure in standard laboratory glassware (Scheme 2) (to do so, we initially pressurize the glass vessel with a slight overpressure of ∼1.5 bar H_2_, seal the vessel and then heat the mixture to the appropriate temperature). Importantly, this circumvents the need for high-pressure vessels and realizes a practical overall protocol.^32^ In all cases, complete (*Z*)-selectivity and no detectable over-reduction to the corresponding alkane were observed. Imidazolium-derived complexes **4** and **8** turned out to be the most active, with the former giving an acceptable turnover of 1a even at balloon pressure.

As a negative control and to underscore the necessity of a bifunctional catalyst^5−7,33^ that bears both active components (the copper(I)/NHC and the 2-iminopyridine subunit, respectively) within the same molecule, several standard copper(I)/NHC complexes in combination with the appropriate aminopyridine gave insignificant catalytic turnovers in the same transformation.^29,34^ In the same vein, we investigated the geometric requirements for the bifunctional catalysts: as an important negative geometric control, the 4-iminopyridine-derived regioisomer **10** led to only a 9% conversion of **1a**. Furthermore, the 2-pyridone unit in **9**, which can in principle undergo aromatization but has significantly lower basicity^35^ compared to the 2-iminopyridines,^24^ did not lead to an active catalyst. Combined, these results show that the precise geometric positioning of the basic imine unit gives rise to exceptionally high activity in copper hydride catalysis. It should be noted that the described copper(I) complexes **3**–**8** can be stored in a glovebox without decomposition. However, a more practical *in situ* protocol has been developed by forming the copper(I)/NHC complexes from the corresponding imidazoli(ni)um salts and CuCl, thereby eliminating the need for air-free and oxygen-free storage.^29^

**Scheme 3:**
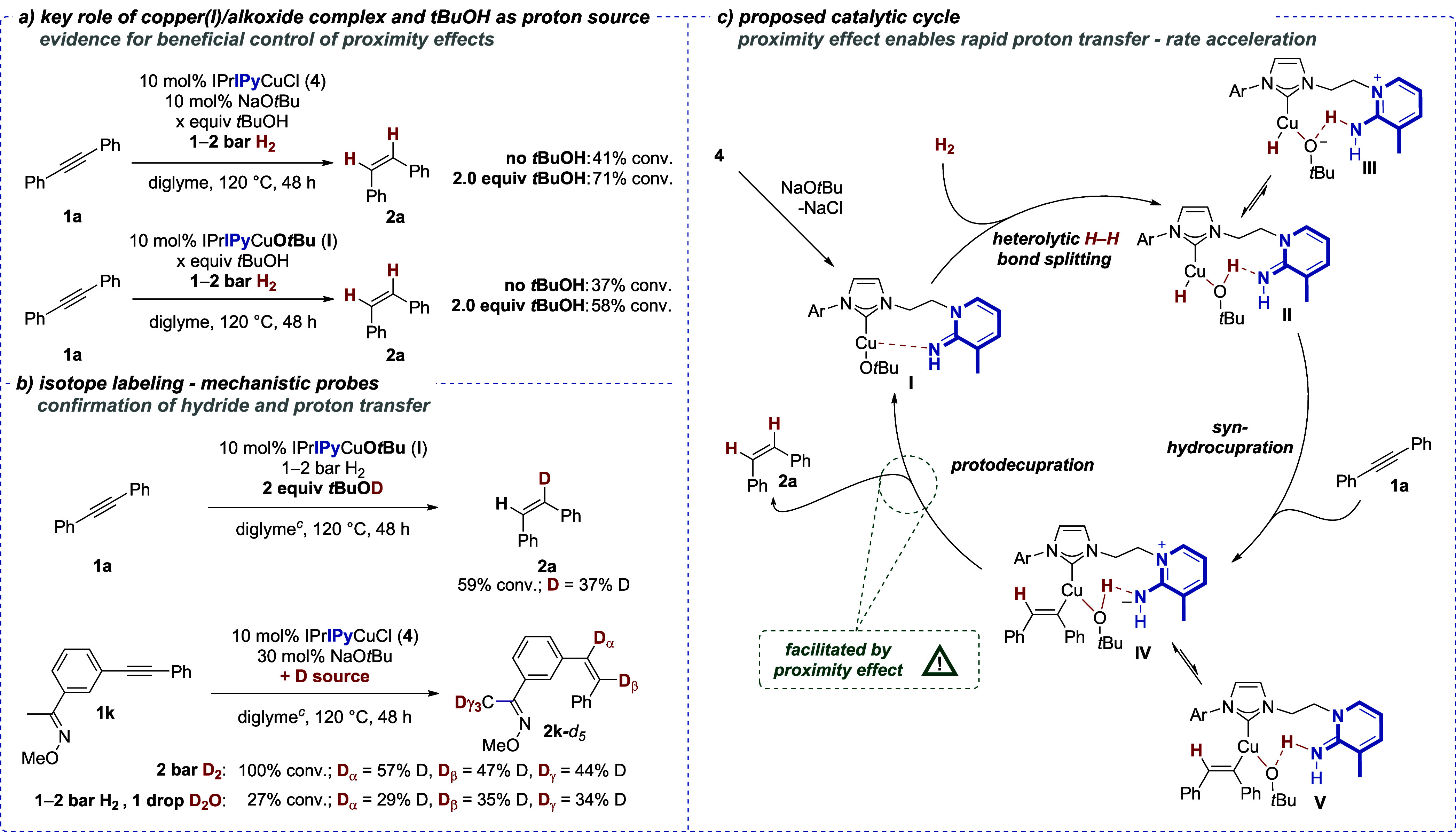
Control Experiments, Mechanistic Probes, and Proposed Catalytic Cycle for the Copper(I)-Catalyzed Alkyne Semihydrogenation with Bifunctional Catalysts^,^ All reactions were performed according to the general procedure 1 (**GP1**, see the Supporting Information for details) with 0.2 mmol of tolane (**1a**). Conversion and *Z*/*E* ratio were measured by GC/GC-MS and/or ^1^H NMR analysis; in all cases, *Z*/*E* selectivity is >99:1. Contains 83 ppm H_2_O.

With these initial encouraging results in terms of reactivity and selectivity, we turned our attention to probing the key mechanistic hypothesis of rate acceleration by proton transfer through a proximity effect. When the semihydrogenation of **1a** was performed with only 10 mol % of NaO*t*Bu as an additive, 41% conversion was observed *in the absence* of *t*BuOH, in contrast to a significantly higher conversion (71%) of **1a***in the presence* of 2.0 equiv *t*BuOH as an additive (Scheme 3a). This result supports the role of *t*BuOH as a proton source in the proto-decupration step, originating in the catalytic setting from the heterolytic splitting of the H–H bond along the Cu–O bond (*cf*. Scheme 1b).^16,23^ In the absence of both NaO*t*Bu and HO*t*Bu (not shown), no conversion was observed, confirming the essential role of the polar Cu–O bond for dihydrogen activation.^29^ Importantly, simple, non-bifunctional copper(I)/NHC complexes such as IPrCuCl and SIMesCuCl lose their activity in the catalytic alkyne semihydrogenation upon the addition of *t*BuOH as a protic additive.^29^ This is in stark contrast to the conversion boosting that is observed with the bifunctional iminopyridine-bearing complexes such as **4**.^29^ This supports the involvement of the iminopyridine subunit in a proton transfer step in the overall catalytic cycle. To further investigate the role of *tert*-butoxide in the catalytic protocol, *tert*-butoxide complex **I**(38) was employed in the semihydrogenation of **1a**, leading to 37% conversion without *t*BuOH additive and 58% conversion in the presence of *t*BuOH (Scheme 3a). These results show that a base-free protocol is feasible, although complex **I** is much less tolerant of air and moisture. Importantly, these results also underscore that the overall catalysis likely runs through copper(I)/alkoxide complex **I**.

These mechanistic experiments are corroborated by deuterium labeling experiments (Scheme 3b): First, the use of *t*BuOD led to significant deuterium incorporation in *Z*-stilbene (**2a**-*d*_1_) of 37%, indicating that a proto-decupration step involving *t*BuOD as proton/deuteron source is operative. Second, under optimized conditions employing D_2_, stilbene **2k**-*d*_5_ was isolated from the alkyne semihydrogenation of **1k** with 57 and 47% deuterium incorporation at the alkene positions. Two key conclusions can be drawn from this result: (i) the initial hydrocupration step proceeds without regioselectivity and (ii) the two-step mechanism of hydrocupration/proto-decupration (see Scheme 1c) is indeed operative. While the copper(I) hydride stems directly from H_2_/D_2_, the ensuing proto/deutero-decupration step is facilitated by the resulting *t*BuOH/*t*BuOD (originating from the heterolytic H–H/D–D bond cleavage). Notably, a proton could also stem from residual water (83 ppm of H_2_O was detected in the diglyme used) or from the deprotonation of acidic positions such as the γ-position of the oxime in **1k**. To probe the role of residual water as a proton source for proto-decupration, a drop of D_2_O was added during the semihydrogenation of **1k** with H_2_: although conversion significantly dropped, the deuteration incorporation of 29 and 35% at the olefinic positions of **2k**-*d*_5_ is strongly supportive of a proto-decupration pathway. For more labeling studies, see the Supporting Information.

These experiments, in conjunction with the screening of structurally related catalysts illustrated in Scheme 2, show that the basic 2-iminopyridine moiety is involved in the catalysis, significantly enhancing the overall rate of the H_2_-driven copper(I) hydride chemistry. Also drawing upon preliminary density functional theory (DFT) analysis, we propose a catalytic cycle for the copper(I)-catalyzed alkyne semihydrogenation in which the 2-iminopyridine moiety facilitates proton transfer via a proximity effect (Scheme 3c): after the initial formation of catalytically active copper(I) alkoxide complex **I** from **4**, iminopyridine acts as a hemilabile ligand.^36^ The optimized geometries of **4** and **I**, obtained by DFT calculations, reveal a preorganized catalyst structure characterized by a weak N···Cu interaction (see the Supporting Information).^37^ Heterolytic H–H bond cleavage along the Cu–O bond^16,23^ leads to copper(I) hydride complex **II**, which could be facilitated by the basic iminopyridine nitrogen atom coordinating to the *in situ* generated *tert*-butanol and thus effectively acting as a proton shuttle. When considering the two possible tautomers **II** and **III** as shown in Scheme 3, DFT calculations reveal that during geometry optimization, both tautomers converge into **II** as minimum structure. From this, we conclude that **II**, in which the proton is bound to the alcohol oxygen atom, is more stable. Subsequent *syn*-hydrocupration^38^ would lead to vinylcopper(I) complex **IV** that again can appear as two tautomers **IV** and **V**, depending on the protonation state of iminopyridine. As in the earlier case, DFT analysis reveals that tautomer **IV** is the minimum structure, with the proton bound to the alcohol function. Hence, in both structures **II** and **IV**, the *tert-*butanol molecule coordinates to the Cu atom while simultaneously forming a hydrogen-bonding interaction with the imino nitrogen atom. We hypothesize that the coordination of the alcohol/alkoxide in close proximity to the copper atom is the main factor for the rate acceleration of the overall process, as in the subsequent proto-decupration step (**IV** → **I**), the proton is delivered to the carbon atom in an intramolecular fashion. In this step, *tert*-butanol plays the key role as a proton source, as established in mechanistic experiments, and also reforms copper(I) alkoxide complex **I**. Therefore, the overall reaction could be sped up by this proximity-driven proto-decupration, although further mechanistic investigations to fully elucidate the exact role of the iminopyridine group are required.

Next, we probed the functional group tolerance for the catalytic alkyne semihydrogenation as a benchmark for homogeneous copper(I)-catalyzed hydrogenations. All alkyne semihydrogenations (**1** → **2**; Table 1) took place without the formation of any detectable side products (such as over-reduced alkanes) and with consistently high (*Z*)-stereoselectivity. This shows that the present catalyst indeed displays the hallmarks of typical copper(I) hydride catalysis.^17^

**Table 1:**
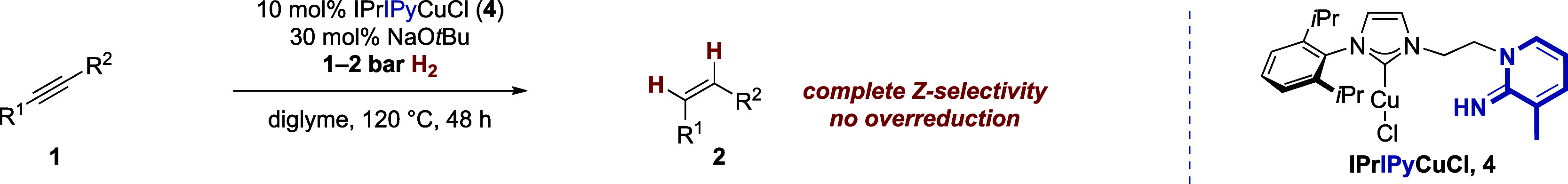

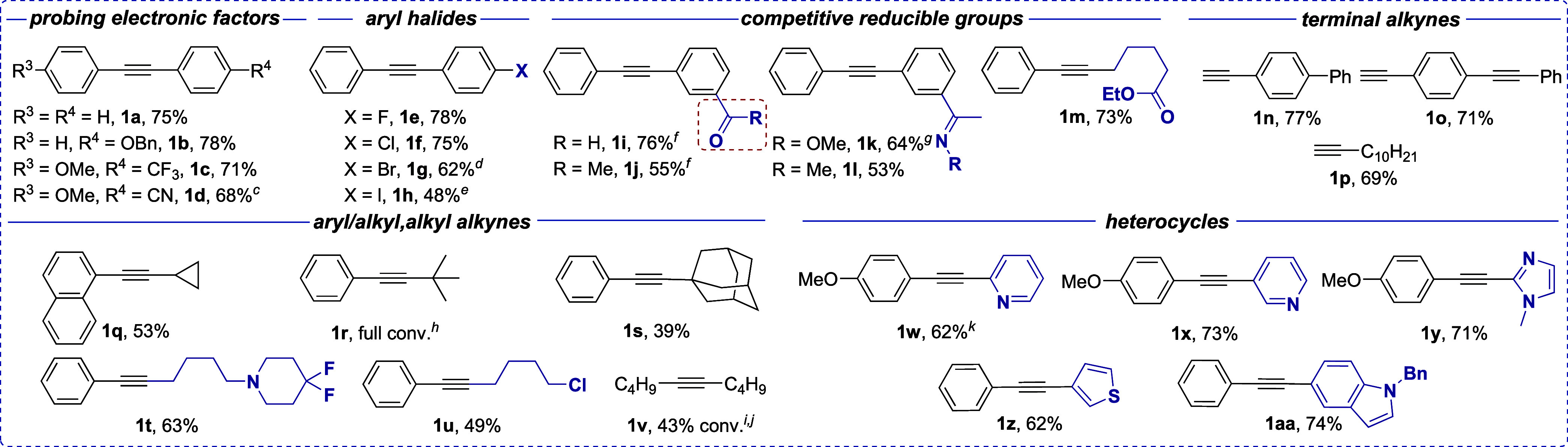
Copper(I)-Catalyzed H_2_-Driven Alkyne Semihydrogenation, Scope^a^^,^^b^

a All reactions were performed according to the general procedure 1 (**GP1**, see the Supporting Information for details) with 0.2 mmol of the substrate; isolated yields are given.

b Conversion and *Z*/*E* ratio were measured by GC/GC-MS and/or ^1^H NMR analysis; in all cases, *Z*/*E* selectivity = 99:1.

c 7% Formation of the corresponding alkane was observed.

d 13% Proto-debromination of **1g** was observed.

e 27% Proto-deiodination of **1h** was observed.

f Complete reduction of the respective carbonyl groups was observed.

g Gram scale reaction on a 4.5 mmol scale was also performed.

h Product **2r** is volatile.

i 84% Conversion was achieved at 100 bar H_2_.

j Product **2v** is volatile.

k 10% Formation of the corresponding alkane was observed.

The semihydrogenations took place regardless of the electronic predisposition of the alkynes **1**, as showcased by the successful generation of (*Z*)-stilbenes **2a**–**2d**. Noteworthy, nitrile-containing substrates such as **1d** had been shown to be problematic in earlier studies,^18a,18c^ possibly due to coordination to the copper(I) catalyst. With bifunctional catalyst **4**, earlier observed proto-dehalogenations^18a^ of halogen-containing compounds were largely suppressed, as witnessed by the formation of bromides and iodides **2g**,**h** (isolated yield of the halogen-containing products 62 and 48%, respectively). We found that the expected 1,2-reductions of ketones and aldehydes took place concomitantly to the alkyne semihydrogenations of **1i** and **1j**, respectively. Remarkably, for the less reactive imines and oximes **1l** and **1k**, the corresponding (*Z*)-alkenes could be isolated with the C=N double bond intact. No reduction of the ester group^13^ in **1m** was observed. For the first time with copper(I)/NHC complexes, terminal alkynes **1n**–**1p** could successfully be converted to the corresponding alkenes. This transformation had generally not been possible with copper(I)/NHC-based hydrogenation catalysts due to the use of larger amounts of alkoxide additives, leading to deprotonation of the terminal alkynes.^18d,39^ Of particular note is the reaction of diyne **1o**, which was doubly semihydrogenated to the corresponding (*Z*)-diene **2o** (not shown). Among the tested aryl,alkyl alkynes, particularly the successful conversion of cyclopropane **1q** is of note, indicating the absence of a radical pathway. Furthermore, the potential electrophilic alkyl chloride **1u** remained intact during the alkyne semihydrogenation. The alkyl,alkyl alkyne **1v** also showed significant conversion (43% at 1–2 bar). The present bifunctional catalyst **4** is resistant to other potentially coordinating groups: in this vein, a variety of heterocycles such as pyridines **1w** and **1x**, imidazole **1y**, thiophene **1z**, and indole **1aa** underwent successful semihydrogenation. Collectively, these results demonstrate that the new protocol surpasses earlier catalysts in terms of functional group tolerance and reduced H_2_ pressure required.

The catalytic alkyne semihydrogenation served as a model reaction to benchmark the new bifunctional catalyst **4** to earlier reported complexes in the realm of H_2_-driven copper hydride catalysis. Going further, we sought to challenge the catalytic protocol with a class of poorly reactive compounds: we therefore chose to investigate the reduction of α,β-unsaturated amides **11** (conjugated amides, Table 2) next. These compounds are characterized by an intrinsically low reactivity in conjugate additions (with carbon or heteroatom nucleophiles alike) catalyzed by copper,^40,41^ or other transition metals,^42^ giving rise to several synthetic work-around strategies.^43^ In a similar vein, catalytic homogeneous hydrogenations of conjugated amides have rarely been realized in general and require the use of precious transition metals in combination with a high H_2_ pressure.^44,45^ Underlining this low general reactivity, conjugated amide substructures often appear in biologically active compounds.^46,47^ Of particular relevance to the present study, however, is the fact that conjugated amides were found to be unreactive toward copper(I) hydride complexes in the past.^48,49^ If realized, the selective reduction of conjugated amides would thus offer (i) an attractive manner to structurally diversify and potentially enhance pharmacological profiles of biologically relevant compounds at a late stage in synthesis and (ii) an attractive manner to introduce isotope labels for drug discovery, study of reaction mechanisms, or biosynthetic pathways if carried out with D_2_ (or T_2_) gas.^50^

**Table 2:**
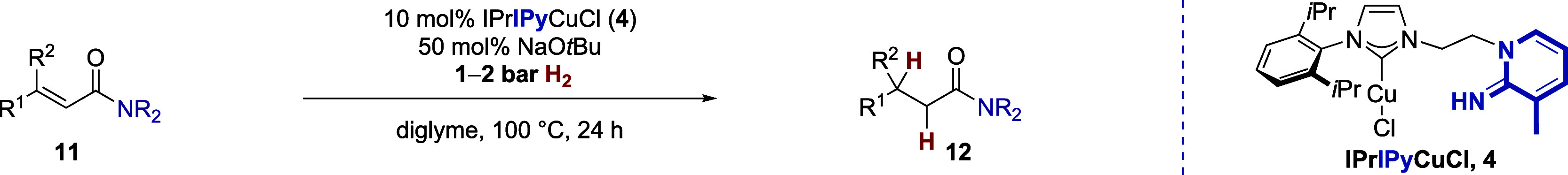

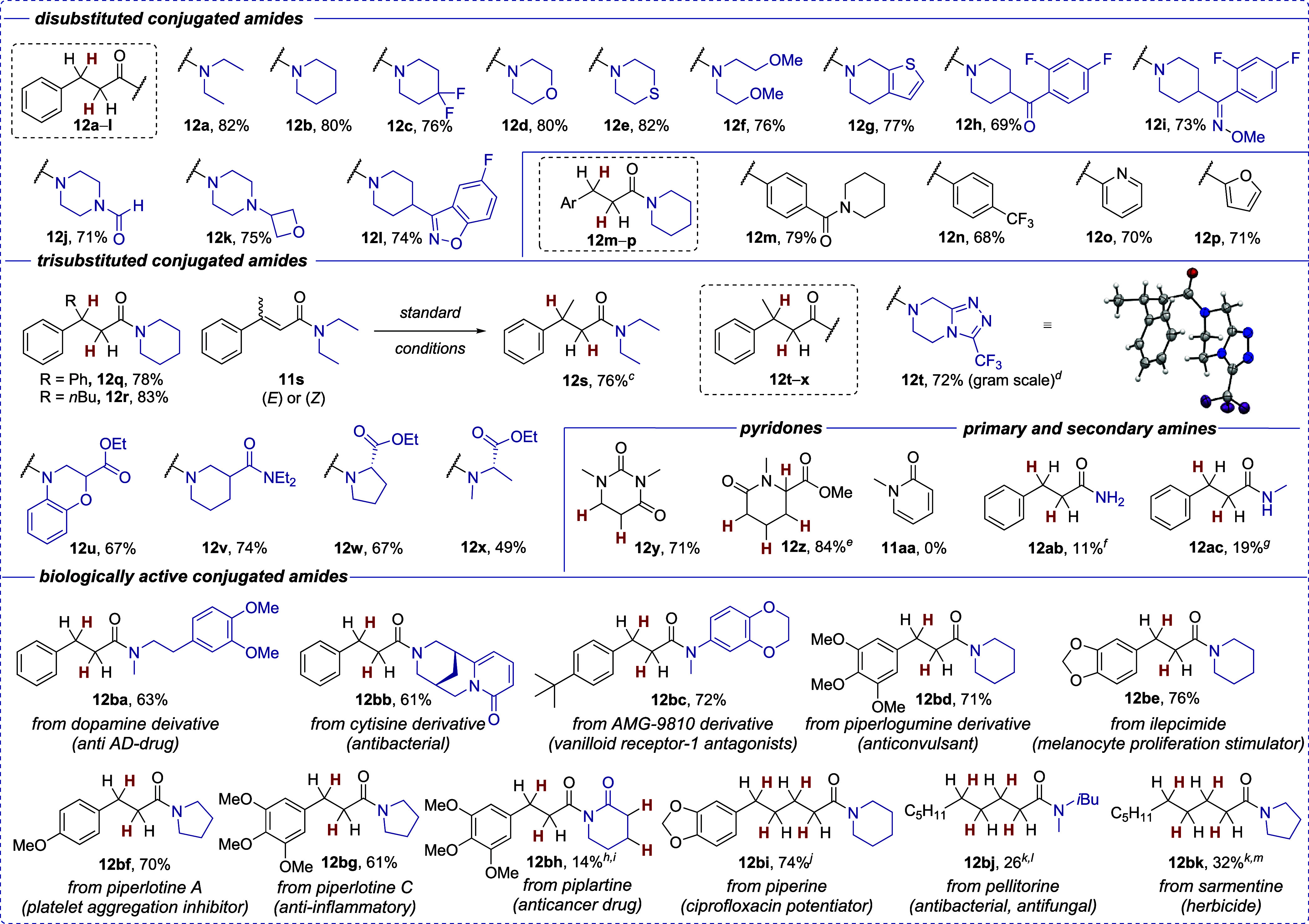
Copper(I)-Catalyzed H_2_-Mediated 1,4-Reduction of Conjugated Amides, Scope^a^^,^^b^

a All reactions were performed according to the general procedure 2 (**GP2**, see the Supporting Information for details) with 0.2 mmol of the substrate; isolated yields are given.

b Conversion was measured by GC/GC-MS and/or ^1^H NMR analysis.

c For the (*Z*)-isomer, 71% of amide **12s** was isolated.

d Gram scale reaction on a 3.4 mmol scale was also performed (75% isolated yield); CCDC: 2415987.

e Doubly 1,4-reduced product of lactam **11z** was obtained.

f 19% Conversion of **11ab** was observed.

g 29% Conversion of **11ac** was observed.

h Double 1,4-reduced product of enamide **11ah** was obtained.

i 23% Conversion to amide **12ah** was observed along with 46% of transesterification product (not isolated).

j Complete 1,6 and 1,4-reduction of **11ai** occurred.

k Hydrogenation was performed at a 0.4 mmol scale.

l Additional 18% only 1,4-reduced product was isolated (see the Supporting Information).

m Additional 17% only 1,4-reduced product was isolated (see the Supporting Information).

We found that the highly reactive copper(I)/NHC catalyst **4** undergoes clean and high-yielding 1,4-reductions of a large variety of conjugated amides **11** at a low H_2_ pressure (Table 2). In order to achieve full conversion to the respective reduced alkyl amides **12** after 24 h, a slightly elevated amount of 50 mol % NaO*t*Bu was used. Noteworthy, also for the 1,4-reductions of conjugated amides, we have observed a similar trend that lowering of the NaO*t*Bu loading is possible when *t*BuOH is used as an additive.^29^ Also, a complete deactivation of non-bifunctional catalysts in the presence of *t*BuOH as an additive for the alkyne semihydrogenation was observed for the reduction of conjugated amides.^29^ Combined, these results indicate that in both cases, similar reaction mechanisms are operative.

Using this protocol, a variety of cinnamides (**11a–n**) were successfully reduced to the corresponding saturated amides (**12a–n**) with good isolated yields. A representative ketone and an oxime (**12h** and **i**, respectively) remained intact during the conjugate reduction, underscoring a remarkably high chemoselectivity of the present protocol. Similarly, the formamide in **12j** was also tolerated. Mirroring the high functional group tolerance identified in the alkyne semihydrogenations, a large variety of heterocyclic conjugated amides **11o–p** (including the sterically more demanding trisubstituted conjugated amides **11q–x**) could successfully be converted to the reduced amides **12o–x**. Noteworthy, both *E*- and *Z*-configured diastereomers of **11s** gave similar results in terms of yields of the corresponding hydrogenation products. No racemization was found during the reduction of the amino acid derivatives **12w** and **12x**, a feat that could not been achieved with related bifunctional catalysts that operate under more basic conditions.^13^ The catalytic reduction of conjugated amides also includes lactams **11y,z** as substrates. This is particularly noteworthy, as lactones had been unreactive in a related earlier study of catalytic ester hydrogenation with bifunctional catalysts.^13^ Primary and secondary conjugated amides (**12ab** and **12ac**, respectively) were also tolerated in the 1,4-reduction under standard conditions.

With regard to the reaction of conjugated amide subunits within biologically active compounds, the simple and selective reduction protocol with catalyst **4** showed remarkable efficacy in modifying several drug molecules **11ba–bk** with good yields. Notably, biologically active derivatives such as the dopamine analogue **11ba**,^51^ cytisine derivative **11bb**,^52^ and AMG-9810 derivative (**11ac**)^53^ were successfully converted. Furthermore, plant-derived piperamides, including piperlogumine (**11bd**),^54^ ilepcimide (**11be**),^55^ piperlotine A (**11bf**),^56^ and piperlotine C (**11bg**),^57^ in addition to conjugated amides piplartine (**11bh**),^58^ all underwent clean hydrogenation to afford the corresponding saturated amides with generally high yields. A limitation was found for dienamide **11bh**, which gave only a poor isolated yield (14%) due to competitive transesterification with NaO*t*Bu. Turning our attention to α,β,γ,δ-diunsaturated enamides, piperine (**11bi**)^59^ underwent complete 1,6- and 1,4-reductions to amide **12bi**,^59^ while pellitorine (**11bj**)^46^ and sarmentine **11bk**(46,60) gave both monoreduced 1,4- and doubly reduced 1,4- and 1,6-reduced products. This indicates that the 1,4-reduction is faster than the respective 1,6-reduction. The reduction of the conjugated amides could be carried out on gram scale, as sitagliptin analogue **12t** could be isolated with 75%. Furthermore, a crystal structure analysis of **12t** fully confirmed the expected connectivity.^29^

**Scheme 4:**
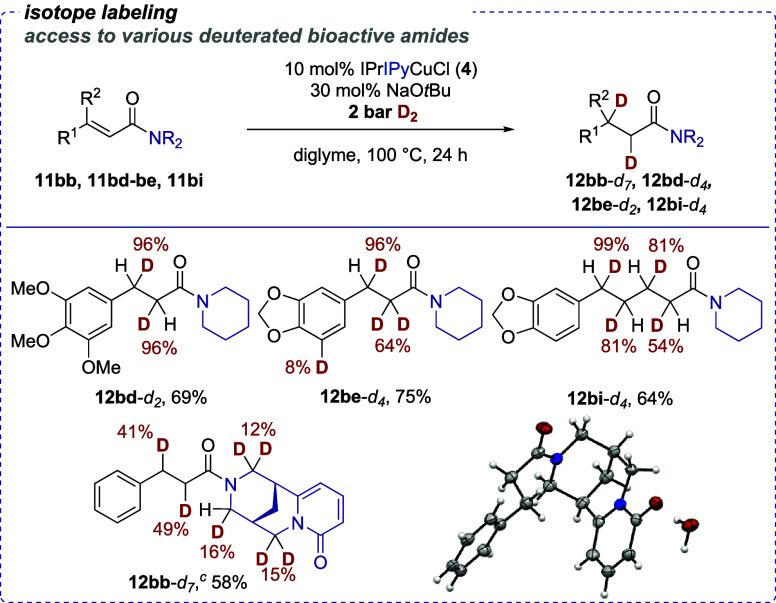
Copper(I)-Catalyzed D_2_-Mediated 1,4-Reduction of Conjugated Amides, Isotope Labeling^,^ All reactions were performed according to the general procedure 3 (**GP3**, see the Supporting Information for details) with 0.2 mmol of the substrate; isolated yields are given. Conversion was measured by GC/GC-MS and/or NMR analysis. CCDC: 2415988; see the Supporting Information for details.

We were able to demonstrate that the conjugate reduction protocol with bifunctional catalysts such as **4** could be rendered a simple method to incorporate isotope labels into relevant molecules. In this vein, a simple switch to D_2_ gas (2 bar) under otherwise unchanged standard conditions gave the deuterated products of enamides **11bb** and **11bd**–**be** as well as dienamide **11bi** (Scheme 4). The corresponding alkyl amides were deuterated with high fidelity at the β-positions (96% D for **12bd**-*d*_2_ and 96% D for **12be**-*d*_4_, respectively) and the δ-position (99% D for **12bi**-*d*_4_). These are the positions that the deuteride would be transferred to in a conjugate addition mechanism. The respective α-positions (for **12bd**-*d*_2_) and/or γ-positions (for **12bd**-*d*_2_ and **12bi**-*d*_4_, respectively) would then be deuterated in a potential proto-decupration step with *t*BuOD. Lower deuterium incorporation at the potentially enolizeable positions (81 and 54% D, respectively, for **12bi**-*d*_4_) eludes to the fact that there are trace proton sources present. These could stem from residual water or from other potentially acidic positions in the substrates. Indeed, for **12be**-*d*_4_ and **12bb**-*d*_7_, we could identify small deuterium incorporation at the aromatic ring in **12be**-*d*_4_ and at the α-positions of the nitrogen atoms in the cytisine backbone of **12bb**-*d*_7_. This indicates that other processes effectively leading to a H/D exchange could take place depending on the substrate structure and, therefore, explain the lower D incorporation rates at the enolizeable positions in **12be**-*d*_4_, **12bi**-*d*_4_, and **12bb**-*d*_7_. Notwithstanding, the present method can be used for selective deuterium labeling at a late stage in synthesis using D_2_ as a readily available deuterium source at a low pressure.

## Conclusions

In this study, we have demonstrated that incorporating a basic 2-iminopyridine moiety into standard copper(I)/NHC complexes results in catalysts with the highest reported activity for H_2_-driven copper(I)-catalyzed reductive transformations. We hypothesize that by placing the basic imine function in a close spatial relationship to the copper atom, a proximity effect enables a rapid proto-decupration step, facilitating reactions with hitherto unreported reactivity: for the first time, a low H_2_ pressure (reaching balloon pressure) is sufficient to drive copper(I) hydride-based reactions with H_2_ as a terminal reducing agent. Furthermore, the bifunctional catalyst design permits the use of catalytic amounts of alkoxide additive, providing excellent overall functional group tolerance.

We employed a stereoselective alkyne semihydrogenation as a model reaction to elucidate the mode of action of the bifunctional catalyst and to assess its functional group tolerance. The catalyst can be conveniently prepared *in situ* without the need for a glovebox, leading to an easy-to-apply protocol. Furthermore, we demonstrate that for the first time, a catalytic hydride transfer to the challenging substrate class of α,β-unsaturated amides is possible. Several biologically active compounds were reduced under mild reaction conditions. Switching to D_2_ gas as the deuteride source allows for facile isotope labeling of the conjugated amides, which occur frequently in biologically active molecules.

We believe that this work bears implication for multifunctional ligand design of NHC complexes, for method development based on copper(I)hydride complexes using only H_2_ as a terminal reducing agent, and finally the development of synthetic methods and isotope labeling strategies.

## Supporting Information Available

Detailed starting material synthesis; general procedures; characterization data; NMR; IR spectra for all compounds; and computational methods (PDF)
Spectral data for catalyst precursors and catalyst; ^13^C NMR and ^1^H and ^1^H COSY (PDF)
