# Correction to “Broadly Applicable Copper(I)-Catalyzed Alkyne Semihydrogenation and Hydrogenation of α,β-Unsaturated Amides Enabled by Bifunctional Iminopyridine Ligands”

**DOI:** 10.1021/jacs.5c07662

**Published:** 2025-06-04

**Authors:** Mahadeb Gorai, Jonas H. Franzen, Philipp Rotering, Tobias Rüffer, Fabian Dielmann, Johannes F. Teichert

There were two mistakes in the chemical drawings in Table 2 in the published article. Compound **12o** was mistakenly depicted as the 2-substituted pyridine derivative; however, the compound prepared was the 3-pyridine derivative. Furthermore, in compound **12z** the methyl ester was mistakenly shown in the 6-position of the lactam, whereas it must be correctly depicted as the regioisomeric compound with the ester in the 3-position.

The complete corrected Table 2 is presented below. The correction has no influence on the data or the conclusions of the work. All data in the original Supporting Information documents are correct.

**Table 2:**
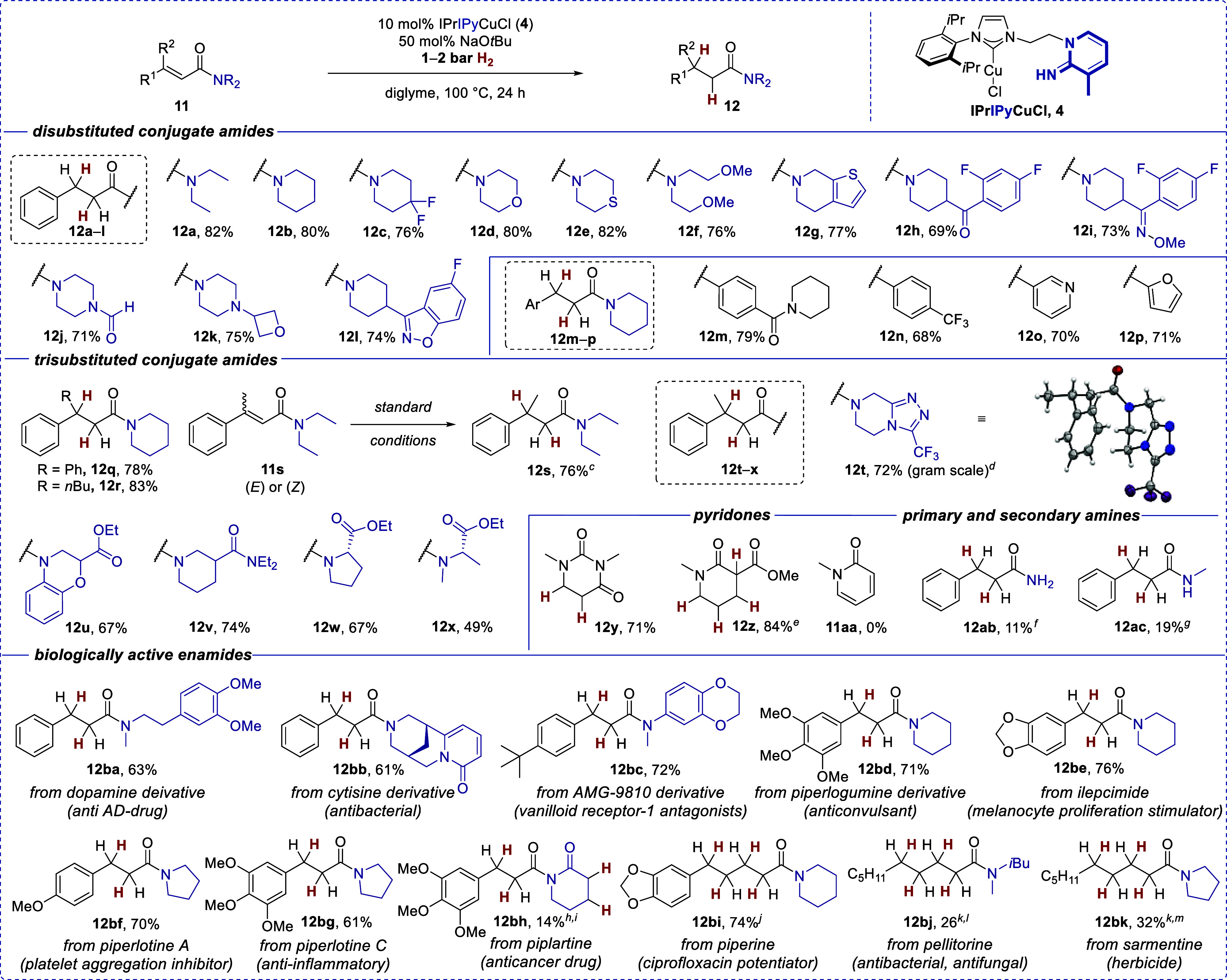
Copper­(I)-Catalyzed H_2_-Mediated 1,4-Reduction of Conjugated Amides, Scope^a^,^b^

a All reactions were performed according to the general procedure 2 (**GP2**, see the Supporting Information for details) with 0.2 mmol of the substrate; isolated yields are given.

b Conversion was measured by GC/GC-MS and/or ^1^H NMR analysis.

c For the (*Z*)-isomer, 71% of amide **12s** was isolated.

d Gram scale reaction on a 3.4 mmol scale was also performed (75% isolated yield); CCDC: 2415987.

e Doubly 1,4-reduced product of lactam **11z** was obtained.

f 19% Conversion of **11ab** was observed.

g 29% Conversion of **11ac** was observed.

h Double 1,4-reduced product of enamide **11ah** was obtained.

i 23% Conversion to amide **12ah** was observed along with 46% of transesterification product (not isolated).

j Complete 1,6 and 1,4-reduction of **11ai** occurred.

k Hydrogenation was performed in 0.4 mmol scale.

l Additional 18% only 1,4-reduced product was isolated (see Supporting Information).

m Additional 17% only 1,4-reduced product was isolated (see Supporting Information).

