# Supplementary material for: Broadly Applicable Copper(I)-Catalyzed Alkyne Semihydrogenation and Hydrogenation of α,β-Unsaturated Amides Enabled by Bifunctional Iminopyridine Ligands
Source: J Am Chem Soc. 2025 Apr 16;147(17):14481–90. doi: 10.1021/jacs.5c01339 (PMC12046561; doi:10.1021/jacs.5c01339)
Supplement: Supplementary file 1 — ja5c01339_si_001.pdf [file ja5c01339_si_001.pdf]

Supporting Information

Broadly applicable copper(I)-catalyzed alkyne semihydrogenation and hydrogenation of α,β-unsaturated amides enabled by bifunctional iminopyridine ligands

Mahadeb Gorai[a], Jonas H. Franzen[b], Philipp Rotering[b], Tobias Rüffer[a], Fabian Dielmann[b] and Johannes F. Teichert*

[a]Technische Universität Chemnitz, Institut für Chemie, Straße der Nationen 62, 09111 Chemnitz, Germany.
[b]IDepartment of General, Inorganic and Theoretical Chemistry, Universität Innsbruck, Innrain 80/82, 6020 Innsbruck, Austria.

johannes.teichert@chemie.tu-chemnitz.de

Contents

1.1 General Information 4

2 Additional data 7

2.1 Additional optimization data 7

3 General procedures 12

3.1 General Procedure 1 – H_2-Mediated alkyne semihydrogenation (GP1) 12

3.2 General Procedure 2 – 1,4-reduction of α,β-unsaturated amides with IPrIPyCuCl (GP2) 13

3.3 General Procedure 3 – 1,4-reduction of α,β-unsaturated amides with IPrIPyCuCl using D_2 as reductant (GP3) 13

3.4 Overview: Synthesis of bifunctional Cu-IPy/NHC complex 3-7 14

3.5 General Procedure 7 – Synthesis of Alkynes (GP7) 16

3.6 General Procedure 8 – Synthesis of conjugate amides (GP8) 16

3.7 General Procedure 9 – Synthesis of conjugate amides from cinnamyl chloride (GP9) 17

4 Control experiments 17

4.1 Deuteration experiments and role of water: 17

4.2 Probing hydrogenation with in situ IR spectroscopy 28

4.3 Alkyne semihydrogenation with copper(I)-*tert*-butoxide complex I 31

5 Alkyne semihydrogenation (in situ protocol) directly from ligand precursors 32

6 Gram scale reaction 33

6.1 1-(3-((*Z*)-Styryl)phenyl)ethan-1-one *O*-methyl oxime (2k) 33

6.2 3-phenyl-1-(3-(trifluoromethyl)-5,6-dihydro-[1,2,4]triazolo[4,3-a]pyrazin-7(8*H*)-yl)butan-1-one (12t) 34

7 Experimental Details 35

7.1 Synthesis of IMesIPyCuCl (3) 35

7.2 Synthesis of IPrIPyCuCl (4) 36

7.3 Synthesis of IAdIPyCuCl (5) 38

7.4 Synthesis of SIMesIPyCuCl (6) 40

7.5 Synthesis of SIPrIPyCuCl (7) 42

7.6 Synthesis of 4-carbon linker complex IMesIPy*CuCl (8) 44
7.7 Synthesis of IPrIPOCuCl (9) 46
7.8 Synthesis of ligand precursor IPr[4]IPyHBr (10) 48
7.9 Synthesis of IMesEtCuCl (15) 50
7.10 Alternate synthetic approach for complex IPrIPyCuCl (4) 61
7.11 Alkyne semihydrogention products 63
7.12 Products of conjugate reduction of α,β-unsaturated amides 78
7.13 Synthesis of starting materials for the catalytic alkyne semihydrogenation 110
7.14 Synthesis of starting materials for catalytic hydrogenation of enamide 123
8 X-ray Diffraction Study of S4d 145
9 Crystallographic Data 153
9.1 3-Phenyl-1-(3-(trifluoromethyl)-5,6-dihydro-[1,2,4]triazolo[4,3-*a*]pyrazin-7(8*H*)-yl)butan-1-one (12t) 153
9.2 (1*R*,5*S*)-3-(3-Phenylpropanoyl-2,3-d_2)-1,2,3,4,5,6-hexahydro-8*H*-1,5-methanopyrido[1,2-*a*][1,5]diazocin-8-one (12ab-d_7) 159
10 Computational Details 165
11 References: 175

1.1 General Information

The numbering of the molecules in the main manuscript and Supporting Information do not necessarily follow IUPAC recommendations and were done at our own discretion to guide the reader.

All reactions were carried out in flame dried glassware under a nitrogen atmosphere using standard Schlenk techniques. Glassware and stir bars contaminated with transition metals were treated with *aqua regia* (conc. HCl/conc. HNO_3 3:1) prior to cleaning. For cleaning, glassware and stir bars were kept in an *iso*-PrOH/KOH bath overnight, rinsed with H_2O, kept in a citric acid/H_2O bath overnight and finally rinsed with deionized H_2O and dried at 120 °C. Solutions and reagents were added with nitrogen-flushed disposable syringes/needles. Solvents were added using glass syringes and stainless-steel needles (stored at 120 °C).

Analytical thin layer chromatography (TLC) was performed on silica gel 60 G/UV_{254} polyester sheets (*Macherey-Nagel*). The indication of the analytes performed by different methods:

- Irradiation of the TLC plate with UV light (λ = 254 and 366 nm) with UV absorption by the analytes.
- Dipping the TLC plate in a solution of $12MoO_3{\cdot}H_3PO_4{\cdot}nH_2O$ (25 g), $Ce(SO_4)_2$ (10 g) and conc. H_2SO_4 (60 mL) in H_2O (940 mL) followed by heating with a heat gun.[1]
- Dipping the TLC plate in a solution of $KMnO_4$ (4.0 g), K_2CO_3 (20 g), and KOH (0.30 g) in H_2O (200 mL) followed by heating with a heat gun.

Flash column chromatography was performed on silica gel Davisil LC60A (40-63 µm, pore size 60 Å, *Grace*) using the indicated solvents. The required amounts of silica gel, column diameter, and fraction size were based on elaborated parameters known in the literature[2]and were adjusted if necessary.

NMR spectra were recorded on Avance III 600 (*Bruker*) at the Institute for Chemistry of *Technische Universität Chemnitz.* Chemical shifts (δ) are reported in parts per million (ppm) and are referenced to the residual solvent resonance as the internal standard according to the standard literature.[3,4] Data are reported as follows: chemical shift, multiplicity (br s = broad singlet, s = singlet, d = doublet, t = triplet, q = quartet, m = multiplet, m_{c} = centrosymmetric multiplet), coupling constants (Hz), integration and – if possible – atom assignment. The assignment refers to the atom number shown in the corresponding molecule figure and was achieved *via* analysis of 2D NMR spectra (COSY, HMQC, HSQC, HMBC, NOESY). Diastereotopic atoms are differentiated by a & b in necessity. If a distinct assignment was not

possible, atoms were marked with "*" and are interchangeable. Designation "*Ar*" refers to atoms of an aromatic system where a distinct assignment was not possible.

Melting points (m.p.) were determined using a Melting Point System MP70 (*Mettler Toledo*).

Infrared (IR) spectra were recorded on a Cary 630 FT-IR spectrometer equipped with an ATR unit (*Agilent Technologies).*

High resolution mass spectra (HRMS) were obtained from the Analytical Facility at the Institute for Chemistry at *Technische Universität Chemnitz* (ESI/APCI: micrOTOF QII (*Bruker Daltonik*)).

Analytical gas chromatography (GC) of reaction mixtures was performed using a gas chromatograph *Agilent* 8890 GC System. The instrument was equipped with an *Agilent* J&W HP-5 GC column (length: 30 m, inner diameter: 0.32 mm, film thickness of the stationary phase: 0.25 µm). The following temperature program was used for the analysis: carrier gas N_2, detector temperature 320 °C, flow rate 6.0 mL/min, temperature program: 40 °C start temperature, 20 °C/min heating rate to 250° C and remain for 10 min at 250 °C. The data was recorded with the program OpenLab CDS (*Agilent softwares*).

Analytical gas chromatography coupled with mass spectrometry (GC-MS) of reaction mixtures was performed using a 5975C system (*Agilent Technologies*), which was equipped with an EI ionization source. The following temperature program was used for analysis: carrier gas He; flow rate 0.8 mL/min; temperature program: 40 °C starting temperature, 20 °C/min heating rate to final temperature 250 °C for 10 min. Data were recorded using the MSD ChemStation E.02.02.1432 program (*Agilent softwares*).

1.1.1 Solvents

Tetrahydrofuran (THF), 1,4-dioxane and diglyme were dried over sodium/benzophenone and distilled under N_2-atmosphere prior to use. The water content (in ppm) of solvents were measured with *Karl-Fischer-Titration*.

The solvents used for extraction, thin-layer chromatography, and flash column chromatography were cyclohexane, ethyl acetate (EtOAc), CH_2Cl_2, methanol (MeOH), Et_2O, acetone, and *n*-pentane were obtained in technical grade and distilled under reduced pressure before use.

1.1.2 High-pressure reactions (H_2)

All high-pressure reactions were performed in vials (50 × 14 mm, *Schuett-Biotec*, hereafter referred to as "hydrogenation vials") that were stored in a drying oven at 120 °C before use

and used directly unless otherwise indicated. The vessels were fitted with magnetic stir bars and septa (*Saint-Gobain Performance Plastics*). The respective autoclave (BR-100 or BR-300 including the associated heating blocks, *Berghof*) was purged with N_2 (3 × 5 bar) before the hydrogenation vessel was placed in the autoclave under nitrogen counterflow and the septum was pierced with a needle (0.90 × 59 mm, *Braun*). The autoclave was then flushed successively with N_2 (3 × 5 bar) and H_2/D_2 (3 × 2 bar) before the desired H_2 pressure was set. Now the autoclave was stirred in the already preheated heating block at 750 rpm. At the end of the reaction time, the autoclave was cooled to room temperature and H_2 overpressure was released. After purging with N_2 (3 × 5 bar) the hydrogenation vessels were removed. All hydrogenation reactions were performed in a separate safety room using a *Swagelok* system.

1.1.3 Reagents

All reagents were purchased from common suppliers (*Sigma-Aldrich*, *Alfa Aesar*, *TCI*, *Acros*, *Strem*, *Merck*, *ABCR*, *Fluka*, *Fisher Scientific*, *Carbolution*, *BLD Pharm*) and used without further purification unless otherwise stated. H_2 gas (99.999%) was purchased from *Air Liquide*. D_2 gas (99.8%) was purchased from *Sigma Aldrich*.

1-Mesityl-1*H*-imidazole (**S3a**),[5] 1-(2,6-diisopropylphenyl)-1*H*-imidazole (**S4a**),[6] 1-mesityl-4,5-dihydro-1*H*-imidazole (**S6a**),[7] 1-(2,6-diisopropylphenyl)-4,5-dihydro-1*H*-imidazole (**S7a**),[7] 1-adamentyll-1*H*-imidazole (**S5a**),[8] (*E*)-3-phenylbut-2-enoic acid (**S11s**),[9] 3,3-diphenylprop-2-enoic acid (**S11q**),[10] (*E*)-3-phenylhept-2-enoic acid (**S11r**),[10] (*E*)-3-(furan-2-yl)prop-2-enoic acid (**S11p**),[10] *N*,*N*-diethylcinnamamide (**11a**),[11] (1*R*,5*S*)-3-cinnamoyl-1,2,3,4,5,6-hexahydro-8*H*-1,5-methanopyrido[1,2-*a*][1,5]diazocin-8-one (**11bb**),[12] (*E*)-1-(3-(3,4,5-trimethoxyphenyl)acryloyl)-5,6-dihydropyridin-2(1*H*)-one (**11bh**),[13] (*E*)-1-(piperidin-1-yl)-3-(3,4,5-trimethoxyphenyl)prop-2-en-1-one (**11bd**)[14] and (2*E*,4*E*)-deca-2,4-dienoic acid (**S11bj**),[15] 1-Chloro-4-(phenylethynyl)benzene (**1f**)[16] were prepared according to literature procedure.

2 Additional data

2.1 Additional optimization data

2.1.1 Influence of catalyst structure

Table S1: Influence of catalyst on copper catalyzed H_2-mediated stereoselective alkyne semihydrogenation.

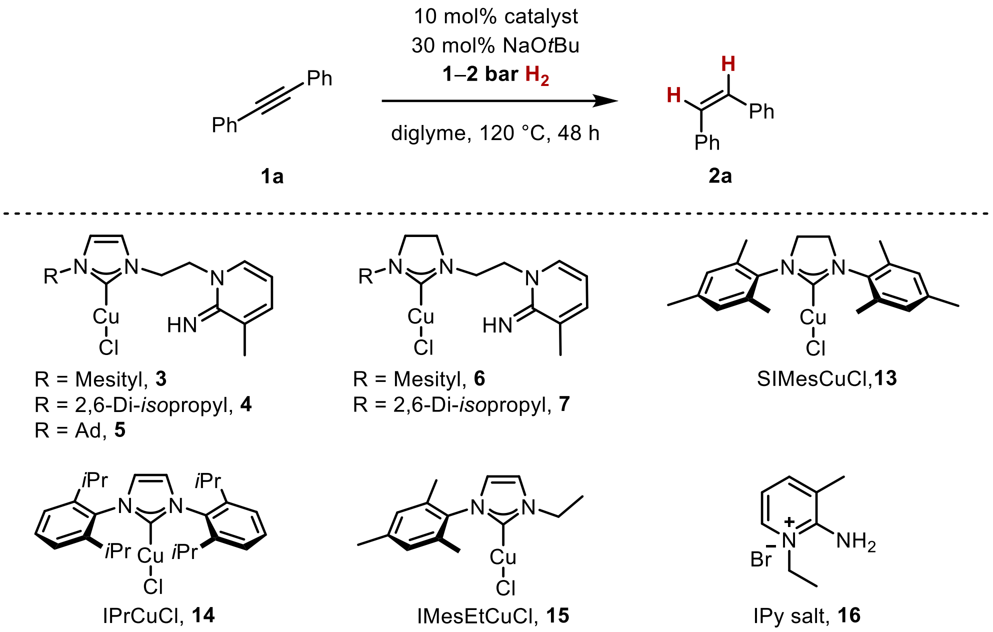

entry	catalyst	conversion of 1a
1	[IMesIPyCuCl] (**3**)	82%
**2**	**[IPrIPyCuCl] (4)**	**100%**
3	[SIMesCuCl] (**13**)	17%
4	[IPrCuCl] (**14**)	13%
5	[IMesEtCuCl] (**15**)	19%
6	[SIMesCuCl] (**13**) + IPy salt (**16**)	20%[a]
7	[IPrCuCl] (**14**) + IPy salt (**16**)	15%[a]
8	[IMesEtCuCl] (**15**) + IPy salt (**16**)	13%[a]
9	[IAdPyCuCl] (**5**)	52%
10	[SIMesIPyCuCl] (**6**)	86%
11	[SIPrIPyCuCl] (**7**)	92%

All reactions were performed according to **GP1** with 0.20 mmol tolane (**1a**) in 2.0 mL of diglyme. The conversion and *Z*/*E* ratio were measured by GC/GC-MS and/or ^{1}H NMR analysis. In all cases *Z*/*E* selectivity is > 99:1. [a]Catalysis was performed with additional 10 mol% IPy salt (**16**) and 10 mol% KHMDS.

2.1.2 Influence of pyridine substituent on bifunctional ligand

Table S2: Influence of iminopyridine backbone on copper catalyzed H_2-mediated stereoselective alkyne semihydrogenation.

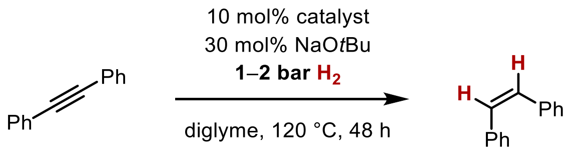

1a **2a**

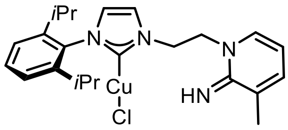

IPrIPyCuCl, **4**

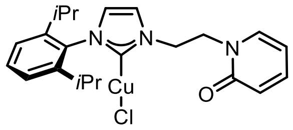

IPrIPOCuCl, **9**

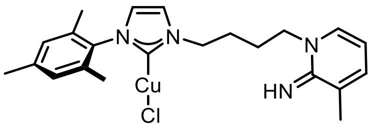

IMesIPy*CuCl, **8**

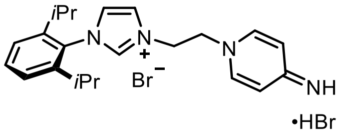

IPr[4]IPyHBr, **10**

entry	catalyst	conversion of 1a
1	[IPrIPyCuCl] (**4**)	100%
2	[IPrIPOCuCl] (**9**)	0%
3	[IMesIPy*CuCl] (**8**)	100%
4	[IPy[4]IPyHBr] (**10**)	9%[a]

All reactions were performed according to **GP1** with 0.20 mmol tolane (**1a**) in in 2.0 mL of diglyme. The conversion and *Z*/*E* ratio were measured by GC/GC-MS and/or ^{1}H NMR analysis. In all cases *Z*/*E* selectivity is > 99:1. [a]Catalysis was performed by generating Cu/NHC catalyst *in situ* with 10 mol% CuCl, 10 mol% **10** & 50 mol% NaO*t*Bu.

2.1.3 Influence of base

Table S3: Influence of base on copper catalyzed H_2-mediated stereoselective alkyne semihydrogenation.

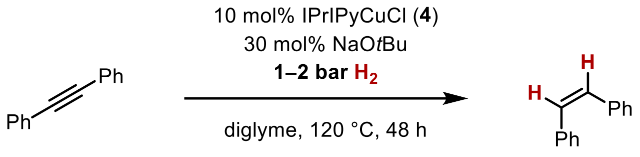

1a **2a**

entry	deviation from standard conditons	conversion of 1a
1	NaO*t*Bu (20 mol%) + LiHMDS (10 mol%)	100%
2	NaO*t*Bu (20 mol%) + KHMDS (10 mol%)	100%
**3**	**NaO*t*Bu (30 mol%)**	**100%**
4	KHMDS (30 mol%)	0%
5	LDA (30 mol%)	0%
6	KO*t*Bu (30 mol%)	0%
7	NaO*t*Bu (10 mol%) + KO*t*Bu (20 mol%)	58%

entry	deviation from standard conditons	conversion of 1a
8	NaBARF (10 mol%) + KO*t*Bu (30 mol%)	0%
9	NaO*t*Bu (30 mol%) + 15-crown-5 (30 mol%)	100%

All reactions were performed according to **GP1** with 0.20 mmol tolane (**1a**) in 2.0 mL of diglyme. The conversion and *Z*/*E* ratio were measured by GC/GC-MS and/or ^{1}H NMR analysis. In all cases *Z*/*E* selectivity is > 99:1.

In order to investigate the influence of base in stereoselective alkyne semihydrogenation, the aforementioned experiments were conducted. The experiment results suggest that the *tert*-butoxide based bases are crucial for the catalytic activity (Table S3, entries 1–5). Further investigation on the importance of counter ion revealed that the catalytic activity is only present when NaO*t*Bu is used as a base (Table S3, entries 6–8). These results signifiy that at least the first catalytic cycle of the reaction mechanism involves both Na^+ and *tert*-butoxide. In an attempt to eliminate Na^+ involvement, 15-crown-5 was introduced as an additive (Table S3, entry 9), which strongly binds Na^+. A complete turnover was observed, indicating the catalytic mechanism is more complex and reliant on NaO*t*Bu role as a base, rather than solely the Na^+ ion itself.

2.1.4 Influence of solvent, pressure, temperature and time

Table S4: Influence of solvent, pressure, temperature and time on copper catalyzed H_2-mediated alkyne semihydrogenation.

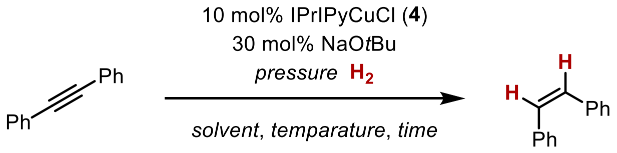

1a 2a

entry	solvent	pressure	temperature	time	conversion of 1a
1	THF	5 bar	80 °C	24 h	100%[a]
2	THF	2 bar	80 °C	24 h	48%[a]
3	1,4-dioxane	2 bar	100 °C	24 h	80%[a]
4	1,4-dioxane	2 bar	100 °C	48 h	82%[a]
5	diglyme	2 bar	120 °C	24 h	100%[a]
6	diglyme	1 bar (balloon)	120 °C	48 h	46%[b]
**7**	**diglyme**	**1.5 bar**	**120 °C**	**48 h**	**100%**
8	1,2-dichlorobenzene	1.5 bar	120 °C	48 h	0%
9	DMF	1.5 bar	120 °C	48 h	78%

All reactions were performed according to **GP1** with 0.20 mmol tolane (**1a**) in 2.0 mL of diglyme. The conversion and *Z*/*E* ratio were measured by GC/GC-MS and/or ^{1}H NMR analysis. In all cases *Z*/*E* selectivity is > 99:1. [a]Catalysis was performed in autoclave. [b]Catalysis was performed with H_2-balloon.

2.1.5 Influence of *tert*-butanol (*t*BuOH) as additive in alkyne semihydrogenation

Table S5: Influence of *tert*-butanol on copper catalyzed H_2-mediated stereoselective alkyne semihydrogenation.

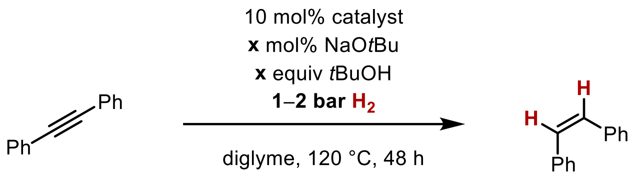

1a **2a**

entry	catalyst	NaO*t*Bu (mol%)	*t*BuOH (equiv)	conversion of 1a
1	IPrIPyCuCl (**4**)	30 mol%	0 equiv	100%
2	IPrIPyCuCl (**4**)	10 mol%	0 equiv	41%
3	IPrIPyCuCl (**4**)	10 mol%	2 equiv	71%
4	IPrIPyCuCl (**4**)	0 mol%	2 equiv	0%
5	IPrIPyCuCl (**4**)	0 mol%	2 equiv[a]	0%
6	SIMesCuCl (**13**)	30 mol%	2 equiv	<5%
7	IPrCuCl (**14**)	30 mol%	2 equiv	0%

All reactions were performed according to **GP1** with 0.20 mmol tolane (**1a**) in 2.0 mL of diglyme. The conversion and *Z*/*E* ratio were measured by GC/GC-MS and/or ^{1}H NMR analysis. In all cases *Z*/*E* selectivity is > 99:1. [a]The alkyne semihydrogenation was performed with additional 10 mol% $AgNO_3$.

In order to probe our hypothesis regarding the proximity effect facilitated by the 2-iminopyridine subunit, a series of experiments were conducted. When 2 equivalent of *tert*-butanol (*t*BuOH) was added alongside 10 mol% sodium *tert*-butoxide (NaO*t*Bu), the conversion of tolane (**1a**) significantly increased from 41% to 71% (Table S5, entries 2–3). These findings highlight the critical role of the 2-iminopyridine subunit, which likely coordinates with *t*BuOH and positions it close to the copper(I) center, enhancing catalytic activity. Notably, using *t*BuOH alone as the *tert*-butoxide source resulted in no observable conversion under standard conditions (Table S5, entries 4–5). This underscores the necessity of NaO*t*Bu for the catalytic efficacy of the Cu/NHC complex **4** in alkyne semihydrogenation, further supported by investigations into the influence of the base (Table S3). Moreover, when alkyne semihydrogenation of **1a** was performed using standard copper(I)/NHC complexes (**13** and **14**), the presence of *t*BuOH led to no catalytic activity (Table S5, entries 6–7). These findings further support our hypothesis that the 2-iminopyridine subunit in copper(I)/NHC complex **4** coordinates with *t*BuOH, positioning it close to the copper(I) center and thereby enhancing catalytic activity.

2.1.6 Influence of concentration

Table S6: Influence of concentration on copper catalyzed H_2-mediated alkyne semihydrogenation.

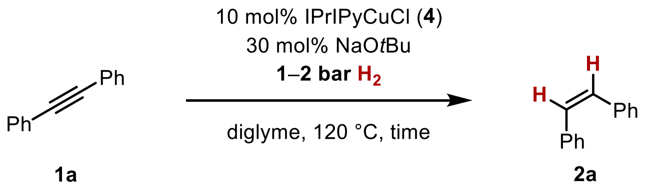

entry	concentration	time	conversion of 1a
1	0.05M	48 h	100%
**2**	**0.10M**	**48 h**	**100%**
3	0.20M	48 h	100%
4	0.40M	48 h	100%
5	0.10M	24 h	67%
6	0.40M	24 h	74%
7	0.01M	24 h	37%

All reactions were performed according to **GP1** with 0.20 mmol tolane (**1a**). The conversion and *Z*/*E* ratio were measured by GC/GC-MS and/or ^{1}H NMR analysis. In all cases *Z*/*E* selectivity is > 99:1.

2.1.7 Optimization for H_2-mediated 1,4-reduction of conjugated amides

Table S7: Influence of catalyst, pressure and base loading on the copper(I)-catalyzed H_2-mediated 1,4-reduction of α,β-unsaturated amides

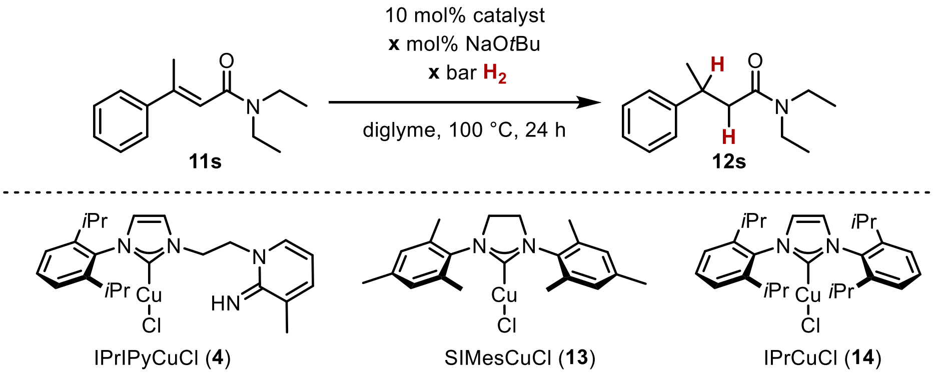

entry	catalyst	NaO*t*Bu loading	H_2 pressure	conversion of 11s
1	[IPrIPyCuCl] (**4**)	30 mol%	1.5 bar	80%
2	[IPrIPyCuCl] (**4**)	30 mol%	2 bar	100%[a]
3	[SIMesCuCl] (**13**)	30 mol%	2 bar	21%[a]
4	[IPrCuCl] (**14**)	30 mol%	2 bar	16%[a]
**5**	**[IPrIPyCuCl] (4)**	**50 mol%**	**1.5 bar**	**100%**
6	[SIMesCuCl] (**13**)	50 mol%	1.5 bar	26%
7	[IPrCuCl] (**14**)	50 mol%	1.5 bar	20%

All reactions were performed with 0.20 mmol enamide **11s** (0.1M). The conversion was measured by GC/GC-MS and/or ^{1}H NMR analysis. [a]Catalysis was performed in autoclave.

2.1.8 Influence of *tert*-butanol (*t*BuOH) as additive in 1,4-reduction of conjugated amides

Table S8: Influence of *tert*-butanol on copper catalyzed H_2-mediated 1,4-reduction of conjugated amides.

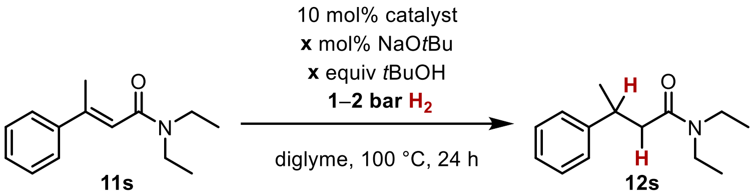

entry	catalyst	NaO*t*Bu (mol%)	*t*BuOH (equiv)	conversion of 11s
1	IPrIPyCuCl (**4**)	10 mol%	0 equiv	37%
2	IPrIPyCuCl (**4**)	10 mol%	2 equiv	53%
3	IPrIPyCuCl (**4**)	30 mol%	0 equiv	85%
4	IPrIPyCuCl (**4**)	30 mol%	2 equiv	100%
5	SIMesCuCl (**13**)	50 mol%	2 equiv	8%
6	IPrCuCl (**14**)	50 mol%	2 equiv	0%

All reactions were performed according to **GP2** with 0.20 mmol tolane (**11s**) in 2.0 mL of diglyme. The conversion was measured by GC/GC-MS and/or ^{1}H NMR analysis.

The rate enhancing effect of the 2-iminopyridine-based catalyst in the presence of *t*BuOH was also evident in the 1,4-reduction of conjugated amides. When 2 equivalents of *tert*-butanol (*t*BuOH) were added alongside 10 mol% NaO*t*Bu, the conversion of conjugated amide **11s** increased significantly from 37% to 53% (Table S8, entries 1–2). A similar reactivity trend was observed when 30 mol% *t*BuOH was used, with the conversion of **11s** rising from 85% to 100% in the presence of two equivalents of *t*BuOH (Table XX, entries 3–4). In contrast, when standard copper(I)/NHC complexes (**13** and **14**) were employed, the reactivity dropped significantly in the presence of *t*BuOH as an additive (Table S8, entries 5–6). Similar results were observed in the case of alkyne semihydrogenation (Table S5, entries 6–7), further supporting the critical role of the 2-iminopyridine subunit in enhancing catalytic activity.

3 General procedures

3.1 General Procedure 1 – H_2-Mediated alkyne semihydrogenation (GP1)

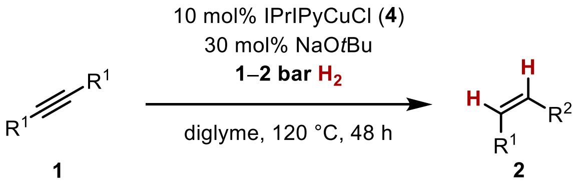

In an Ar-filled glovebox, [IPrIPyCuCl] (**4**, 9.30 mg, 20.0 µmol, 10.0 mol%) and NaO*t*Bu (5.95 mg, 60.0 µmol, 30.0 mol%) are placed in a 5 mL glass vial with a stirring bar. The vial is sealed with septa inside the glovebox and transferred outside. The solids are dissolved in diglyme (0.5 mL) and the mixture is stirred for 10 min at 40 °C. The alkyne (**1**, 0.200 mmol, 1.00 equiv), dissolved

in diglyme (1.5 mL) in another 5 mL vial is transferred to the reaction mixture vial. The reaction mixture is then transferred to a 25 mL pressure tube under N_2 counterflow. The N_2 atmosphere is removed under vaccum (2 × 10^{-2} bar) and then backfilled with H_2 (1.5 bar). The reaction mixture is stirred for 48 h at 120 °C under H_2 atmosphere (1.5 bar). The reaction mixture is allowed to cool to room temperature and H_2 is replaced with N_2. The crude reaction mixture is filtered over a plug of silica (1 × 5 cm, eluent: CH_2Cl_2, 5 mL) and all volatiles are removed under reduced pressure. The crude product is purified by flash column chromatography on silica gel.

3.2 General Procedure 2 – 1,4-reduction of α,β-unsaturated amides with IPrIPyCuCl (GP2)

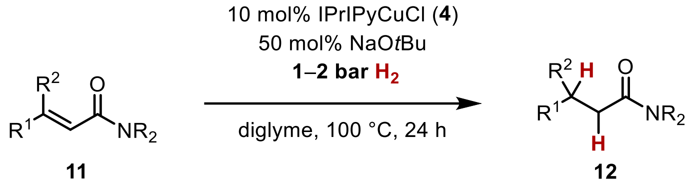

In an Ar-filled glovebox, [IPrIPyCuCl] (**4**, 9.30 mg, 20.0 μmol, 10.0 mol%) and NaO*t*Bu (9.62 mg, 100 μmol, 50.0 mol%) are placed in a 5 mL glass vial with a stirring bar. The vial is sealed with septa inside the glovebox and transferred outside. The solids are dissolved in diglyme (0.5 mL) and the mixture is stirred for 10 min at 40 °C. The appropriate enamide **11** (0.200 mmol, 1.00 equiv), dissolved in diglyme (1.5 mL) in another 5 mL vial is then transferred to the reaction mixture vial. The reaction mixture is transferred to a 25 mL pressure tube under N_2 counterflow. The N_2 atmosphere is removed under vacuum (2 × 10^{-2} bar) and then backfilled with H_2 (1.5 bar). The reaction mixture is stirred for 24 h at 100 °C under H_2 atmosphere (1.5 bar). The reaction mixture is allowed cool to room temperature and H_2 is replaced with N_2. The crude reaction mixture is filtered over a plug of silica (1 × 5 cm, eluent: CH_2Cl_2, 5 mL) and all volatiles are removed under reduced pressure. The crude product is purified by flash column chromatography on silica gel.

3.3 General Procedure 3 – 1,4-reduction of α,β-unsaturated amides with IPrIPyCuCl using D_2 as reductant (GP3)

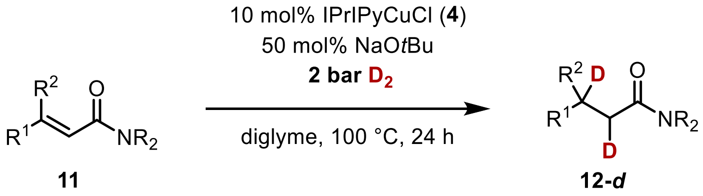

In an Ar-filled glovebox, [IPrIPyCuCl] (**4**, 9.30 mg, 20.0 μmol, 10.0 mol%) and NaO*t*Bu (9.62 mg, 100 μmol, 50.0 mol%) are placed in a 5 mL glass vial with a stirring bar. The vial is sealed with septa inside the glovebox and transferred outside. The solids are dissolved in diglyme (0.5 mL)

and the mixture is stirred for 10 min at 40 °C. The enamide **11** (0.200 mmol, 1.00 equiv), dissolved in diglyme (1.5 mL) in another 5 mL vial is then transferred to the reaction mixture vial. The reaction mixture is placed in an autoclave N_2 counterflow. The N_2 atmosphere is replaced by D_2 (2 bar). The reaction mixture is stirred for 24 h at 100 °C under D_2 atmosphere (2 bar). The reaction mixture is cooled to room temperature and D_2 is replaced with N_2. The crude reaction mixture is filtered over a plug of silica (1 × 5 cm, eluent: CH_2Cl_2, 5 mL) and all volatiles are removed under reduced pressure. The crude product is purified by flash column chromatography on silica gel.

Note: In case of deuteration study, all ^{1}H NMR experiments were measured with 40 seconds relaxation time.

3.4 Overview: Synthesis of bifunctional Cu-IPy/NHC complex 3-7

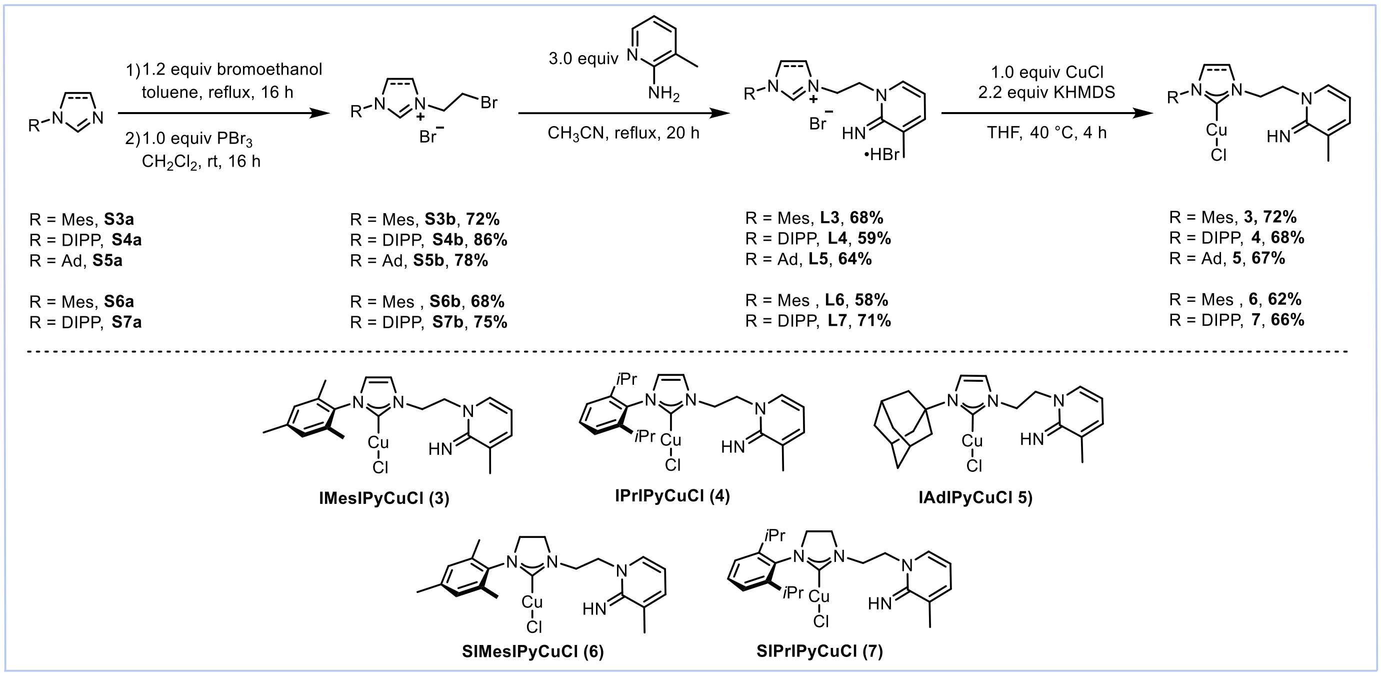

3.4.1 General procedure 4 – Synthesis of imidazolium/dihydro-imidazolium bromide salts S3b–S7b (GP4)

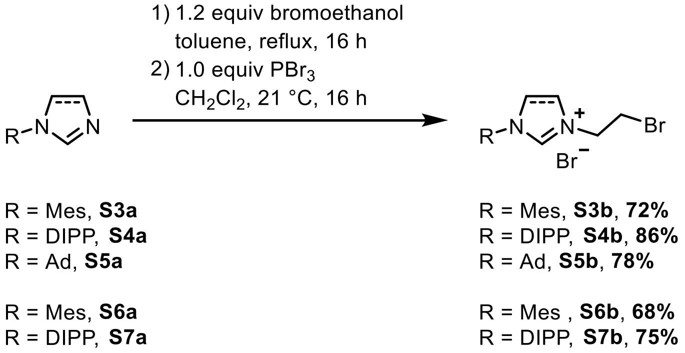

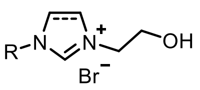

A Schlenk flask equipped with magnetic stirring bar is charged with imidazole derivatives **S3a–S7a** (1.00 equiv) and toluene (2 mL/mmol). Bromoethanol (1.20 equiv) is added and the reaction mixture is refluxed at 120 °C for 16 h. After that, the reaction mixture is allowed to cool to room temperature and all the volatiles are removed under reduced pressure. The resulting crude is then washed with Et_2O (5 × 5 mL/mmol) and dried under reduced pressure to afford the corresponding alcohol, which is used for the next step without further purification.

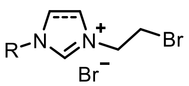

S3b-S7b

A Schlenk flask equipped with magnetic stirring bar is charged with crude alcohol (1.00 equiv) and dichloromethane (5 mL/mmol). Phosphorus tribromide (1.00 equiv) is added dropwise over 10 min at 0 °C. The reaction mixture is warmed to room temperature and stirred for 16 h at room temperature. The reaction is quenched by slow addition of water (2 mL/mmol of alcohol) over 10 min at 0 °C, the layers are separated, and the aqueous layer is extracted with dichloromethane (2 × 10 mL/mmol). The combined organic layer is dried over Na_2SO_4. After filtration and removal of all volatiles under reduced pressure resulted the **S3b–S7b** as brown solid.

3.4.2 General procedure 5 – Synthesis of 2-iminopyridinium salts L3–L7 (GP5)

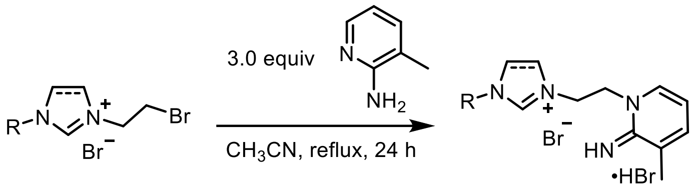

S3b-S7b

R = Mes, **L3**, **68%**
R = DIPP, **L4**, **59%**
R = Ad, **L5**, **64%**

R = Mes , **L6**, **58%**
R = DIPP, **L7**, **71%**

A Schlenk flask equipped with magnetic stirring bar is charged with **S3b–S7b** (1.00 equiv) and CH_3CN (2 mL/mmol). 3-methyl-2-aminopyridine (3.00 equiv) is added and the reaction mixture is refluxed at 90 °C for 24 h. The reaction mixture is allowed to cool to room temperature and acetone (5 mL/mmol of **S3b–S7b**) is added. The resulting precipitate is filtered with glass frit (P4), washed with acetone (3 × 5 mL/mmol of **S3b–S7b**) and dried under reduced pressure to afford the **L3–L7** as colorless solid.

3.4.3 General procedure 6 – synthesis of copper complexes 3-7 (GP6)

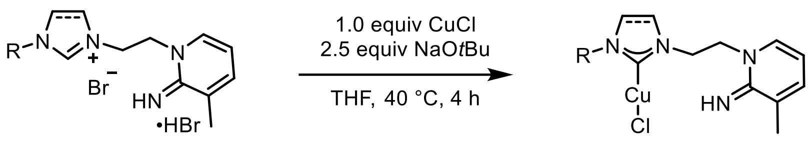

L3-L7

R = Mes, **3, 72%**
R = DIPP, **4**, **68%**
R = Ad, **5**, **67%**

R = Mes , **6**, **62%**
R = DIPP, **7**, **66%**

A Schlenk flask equipped with magnetic stirring bar is charged with **L3–L7** (1.00 equiv). CuCl (1.00 equiv) and NaO*t*Bu (2.50 equiv). THF (20 mL/mmol) is added. The reaction mixture is stirred for 4 h at 40 °C. The resulting yellow mixture is filtered under N_2 atmosphere with Schlenk frit (P4). The filtrate is concentrated under vacuum using oil pump (10^{-2} bar), resulting **3–7** as yellow solid, which is stored in glove box.

3.5 General Procedure 7 – Synthesis of Alkynes (GP7)

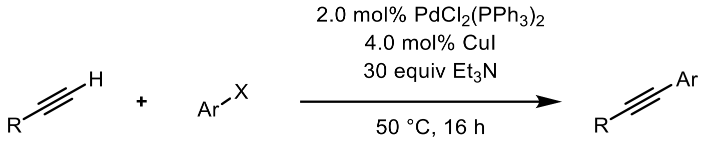

S1 1.2 equiv | **S2** 1.0 equiv | **1**

Based on literature procedure,[16] a Schlenk tube is equipped with a magnetic stir bar is charged with aryl halide (**S2**, 1.00 equiv), $Pd(PPh_3)_2Cl_2$ (2.00 mol%), CuI (4.00 mol%) and triethyl amine (30.0 equiv). Terminal alkyne (**S1**, 1.20 equiv) is added to the reaction mixture at room temperature. The reaction mixture is stirred at 50 °C for 16 h. The reaction mixture is allowed to cool to room temperature, filtered over celite (4 × 3 cm) and washed with EtOAc (5 mL/mmol of aryl halide). All volatiles are removed under reduced pressure. The crude product is purified by flash column chromatography on silica gel.

3.6 General Procedure 8 – Synthesis of conjugate amides (GP8)

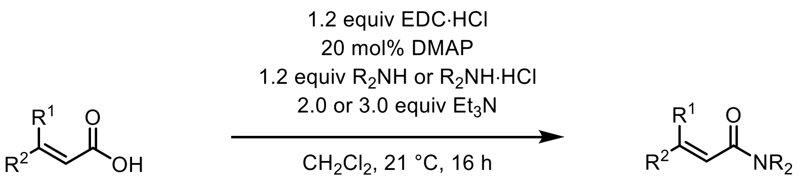

R^2 = aryl, alkyl
R^1 = H, alkyl

S11m-S11x
S11be, S11bj, S11bk

R^2 = aryl, alkyl
R^1 = H, alkyl

11m-11x
11be, 11bj, 11bk

A Schlenk flask equipped with magnetic stirring bar is charged with acid **S11** (1.00 equiv) and CH_2Cl_2 (5 mL/mmol of acid). EDC·HCl (1.20 equiv), DMAP (20.0 mol%), Et_3N (2.00 equiv for

R_2NH or 3.00 equiv for $R_2NH·HCl$) and the corresponding amine (1.20 equiv) are added at room temperature. The reaction mixture is stirred at room temperature for 16 h. The reaction is quenched by addition of sat. aq. NH_4Cl. The organic phase is washed with sat. aq. NH_4Cl (2 × 5 mL/mmol of acid **S11**). The organic layer is dried over $MgSO_4$, all the volatiles are removed under reduced pressure. The crude product is purified on silica gel by flash column chromatography.

3.7 General Procedure 9 – Synthesis of conjugate amides from cinnamyl chloride (GP9)

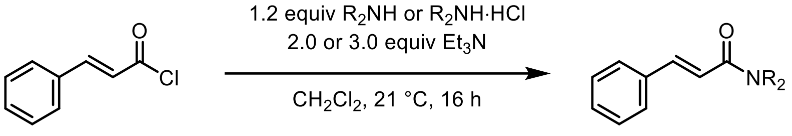

S17 **11b-11h, 11j-11k**

A Schlenk flask equipped with magnetic stirring bar is charged with cinnamyl chloride (**S17**, 1.00 equiv) and CH_2Cl_2 (5 mL/mmol of cinnamyl chloride). Et_3N (2.00 equiv for R_2NH or 3.00 equiv for $R_2NH·HCl$) and the corresponding amine (1.20 equiv) are added at room temperature. The reaction mixture is stirred at room temperature for 16 h. The reaction is quenched by addition of sat. aq. NH_4Cl. The organic phase is washed with sat. aq. NH_4Cl (2 × 5 mL/mmol of **S17**). The organic layer is dried over $MgSO_4$, all the volatiles are removed under reduced pressure. The crude product is purified on silica gel by flash column chromatography.

4 Control experiments

4.1 Deuteration experiments and role of water:

All the experiments below were conducted according to **GP1** with 0.20 mmol of respective alkyne. All the ^1H NMRs for the reactions below were measured with 40 second relaxation time.

4.1.1 Deuteration experiments with symmetrical alkyne, tolane (1a)

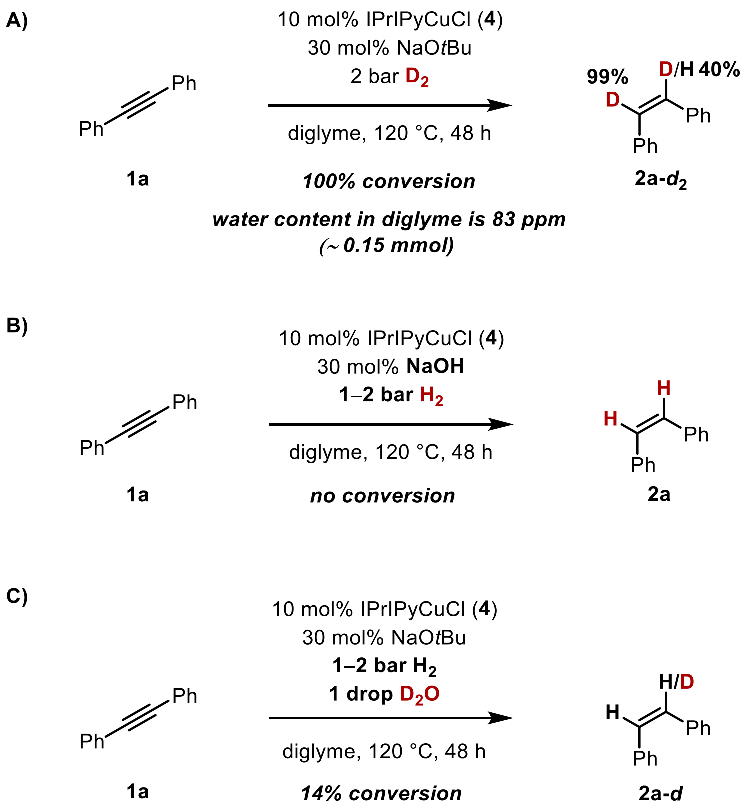

Scheme S1: Alkyne semihydrogenation of tolane (**1a**) with D_2 (**A**), NaOH (**B**) and (H_2 + D_2O) (**C**).

The reduced deuterium incorporation (Scheme S1, **A**) indicates that water from the solvent acts as an additional proton source during the proto-decupration step. When the catalysis was performed using NaOH instead of NaO*t*Bu, no conversion was observed (Scheme S1, **B**), ruling out the possibility of a [Cu–OH] as the active catalyst[17,18] and suggesting the role of water as an extra proton source instead. To elucidate this, an experiment was conducted with the addition of one drop of D_2O (Scheme S1, **C**). Although the conversion decreased due to the presence of excess H_2O/D_2O, GC/GCMS and ^{2}H-NMR analyses showed deuterium incorporation in the alkene product. This strongly indicates the involvement of water as a proton source in the reaction mechanism.

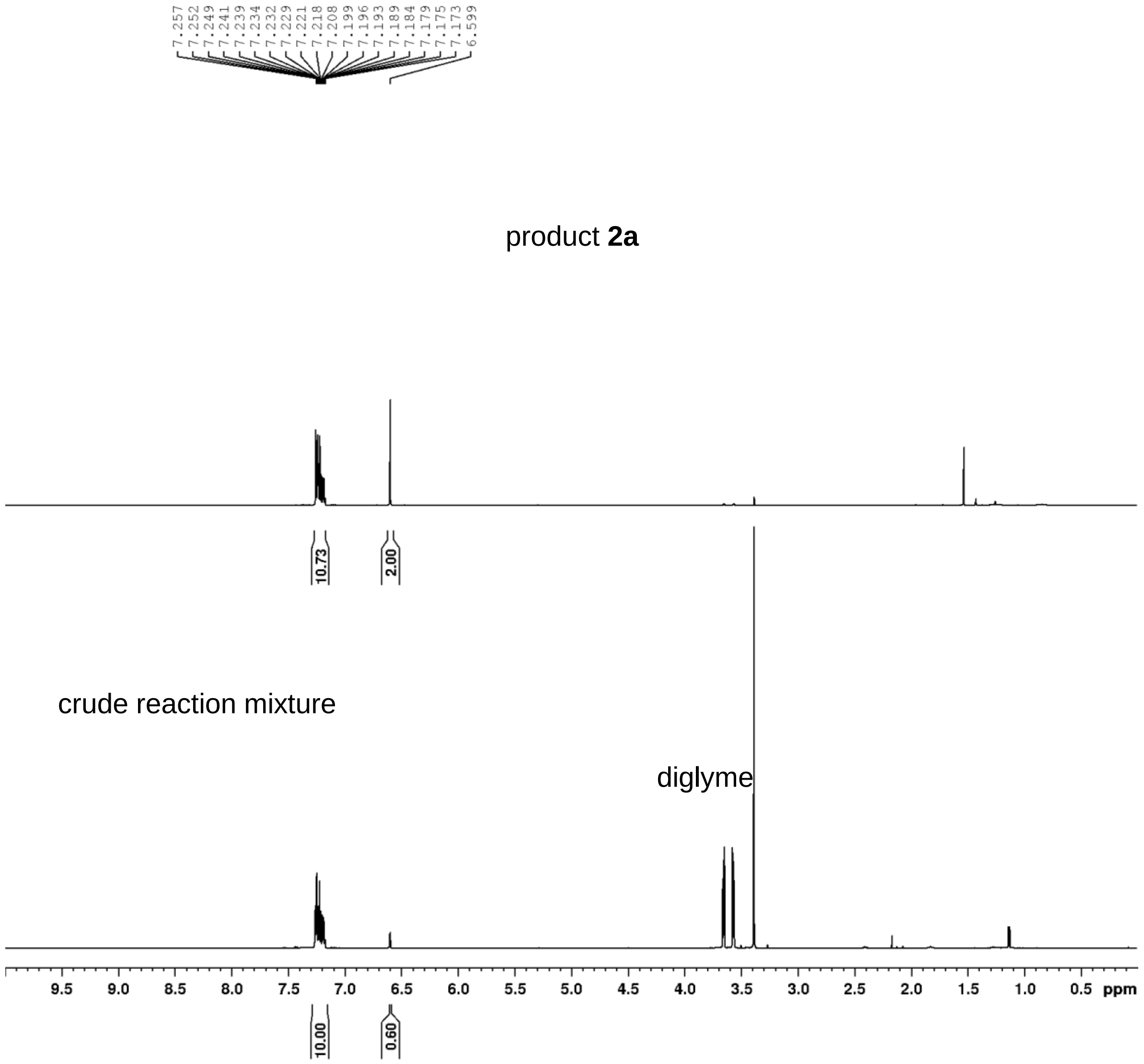

Figure S1: ^{1}H NMR ($CDCl_3$, 600 MHz) comparison between product **2a** (top) and crude mixture for Scheme S1 (**A**) (bottom).

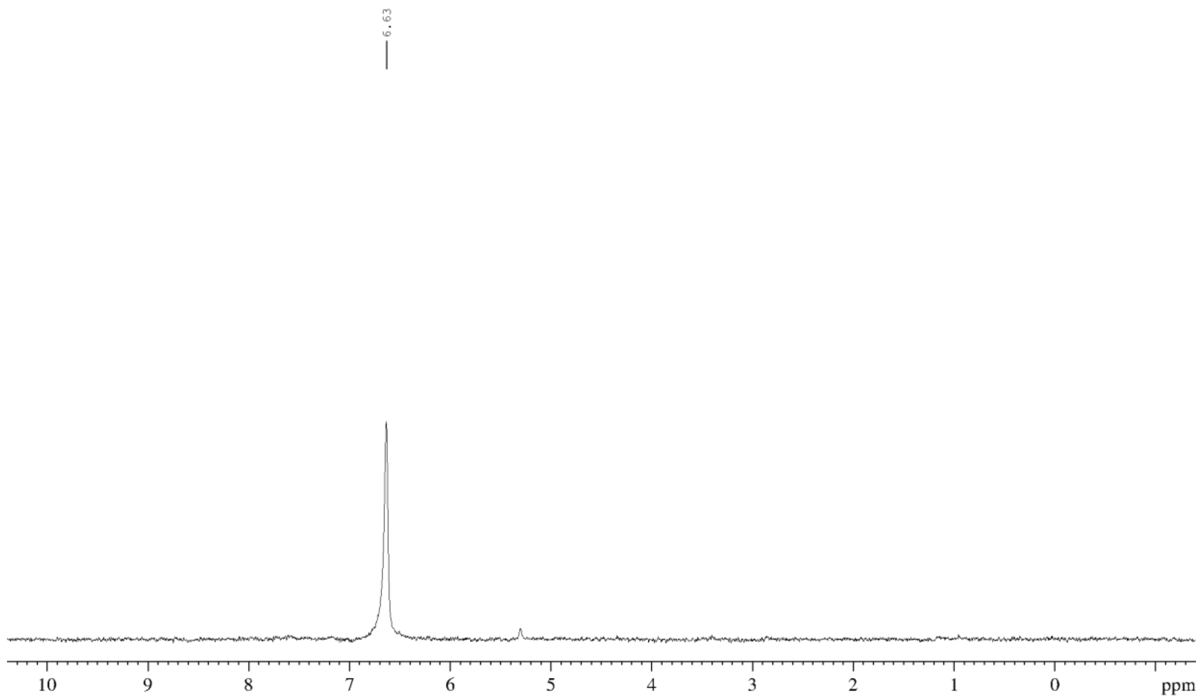

Figure S2: ^{2}H NMR ($CHCl_3$, 92 MHz) of crude mixture for Scheme S1 (**A**) (CD_2Cl_2 is used for calibration).

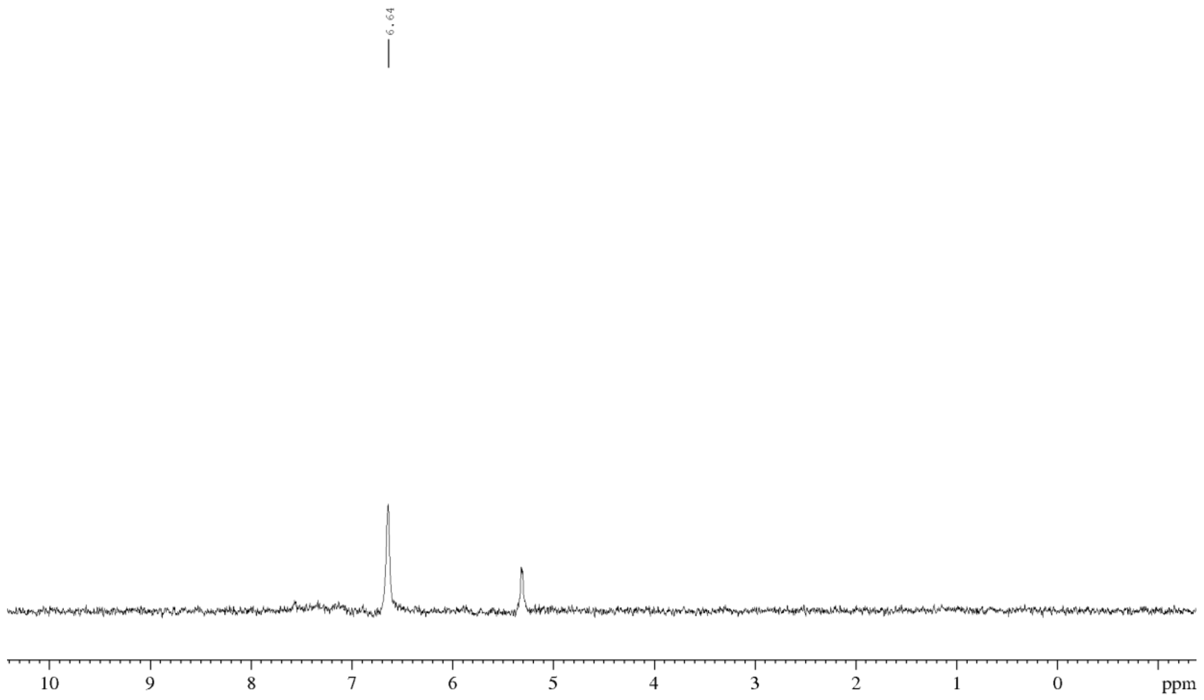

Figure S3: ^{2}H NMR ($CHCl_3$, 92 MHz) of crude mixture for Scheme S1 (**C**). (CD_2Cl_2 is used for calibration).

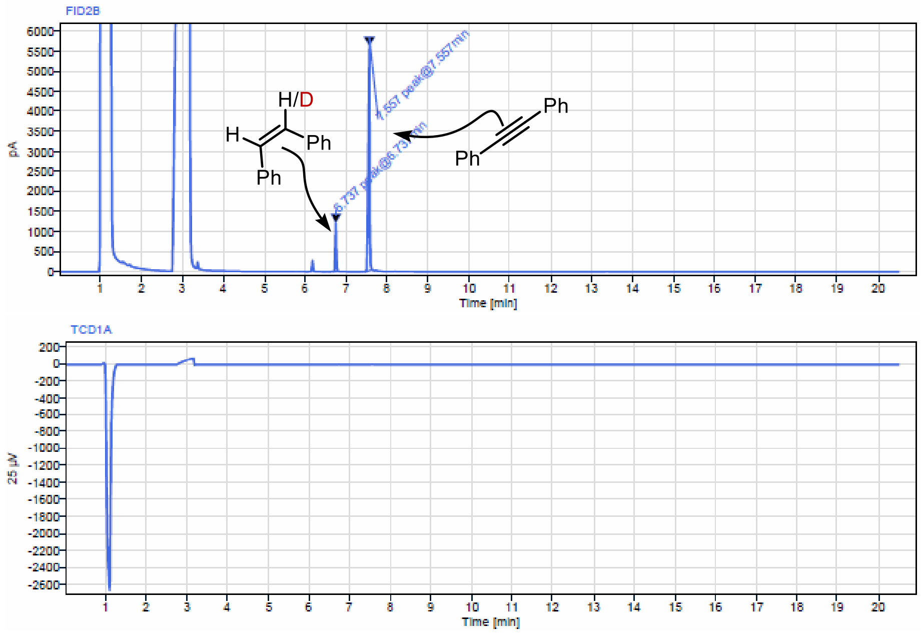

Signal: FID2B

Name	RT [min]	RF	Area	Peak Area Percent	Concentration []	Group
peak@6.737min	6.74		2165.350	14.09		
peak@7.557min	7.56		13199.030	85.91		

Figure S4: GC chromatogram of crude mixture for Scheme 1 (**C**).

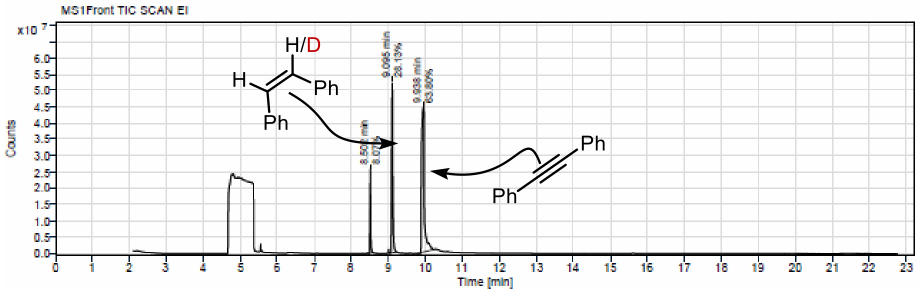

Signal: MS1Front TIC SCAN EI

RT	m/z	Spectral purity (%)	Peak area	Area %
8.502			29820000	8.07

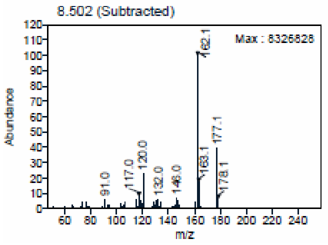

RT	m/z	Spectral purity (%)	Peak area	Area %
9.095			104000000	28.13

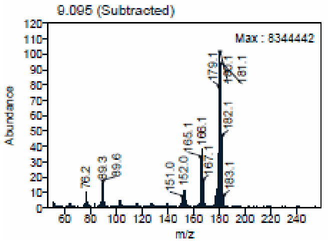

RT	m/z	Spectral purity (%)	Peak area	Area %
9.938			235800000	63.80

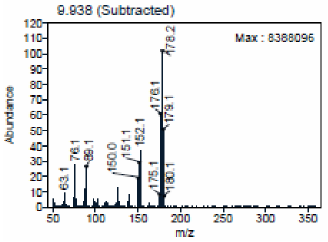

Figure S5: GC-MS chromatogram of crude mixture for Scheme 1 (**C**).

4.1.2 Deuteration experiments with unsymmetrical alkyne, 1k

A)

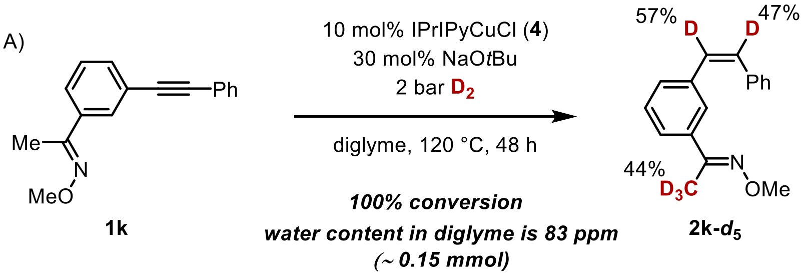

B)

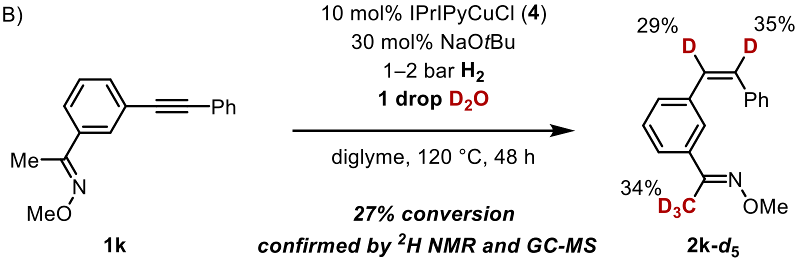

Scheme S2: Alkyne semihydrogenation of **1k** with D_2 (**A**) and (H_2 + D_2O) (**B**).

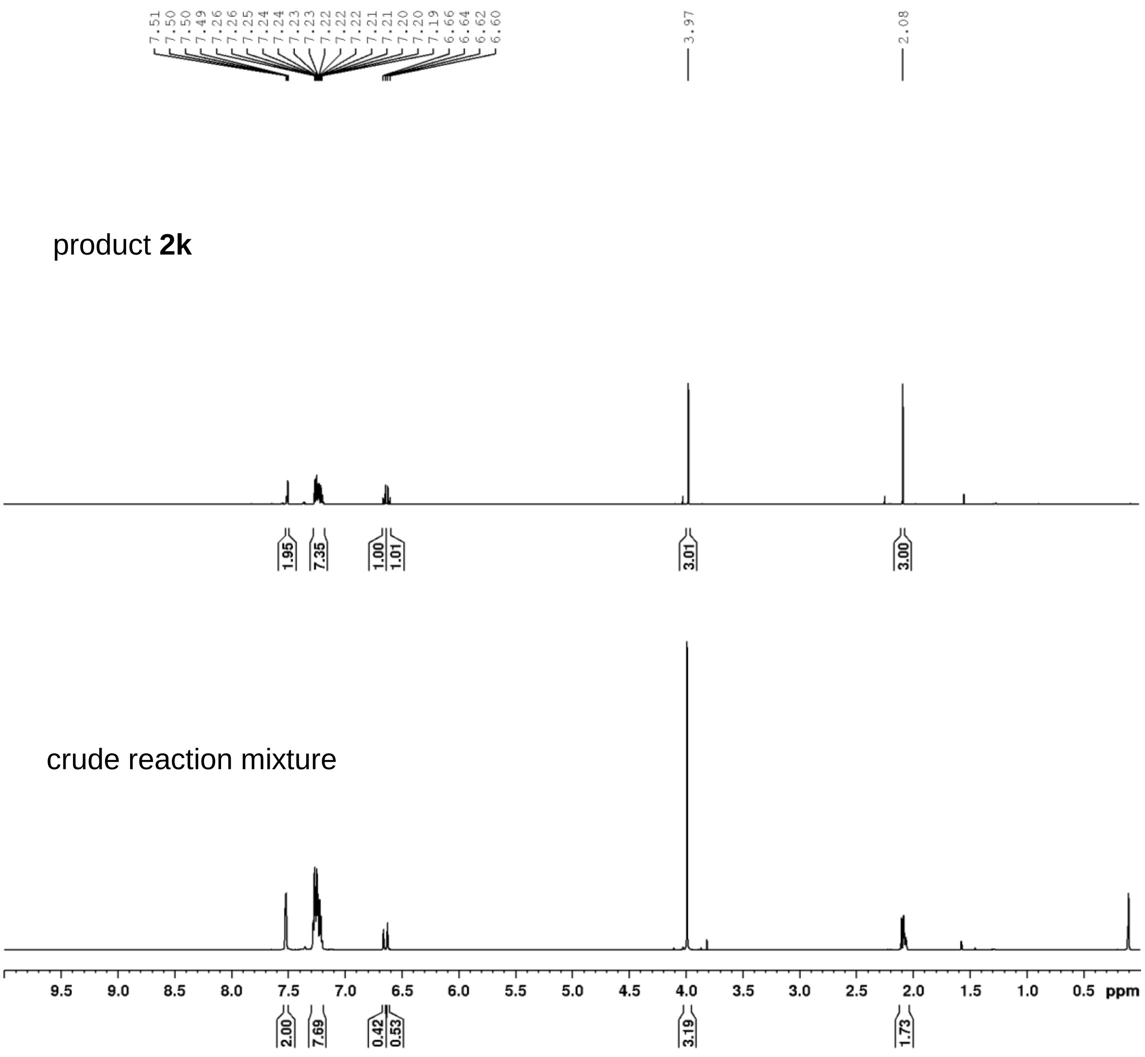

Figure S6: ^{1}H NMR ($CDCl_3$, 600 MHz) comparison between product **2k** (top) and crude mixture for Scheme S2 (**A**) (bottom).

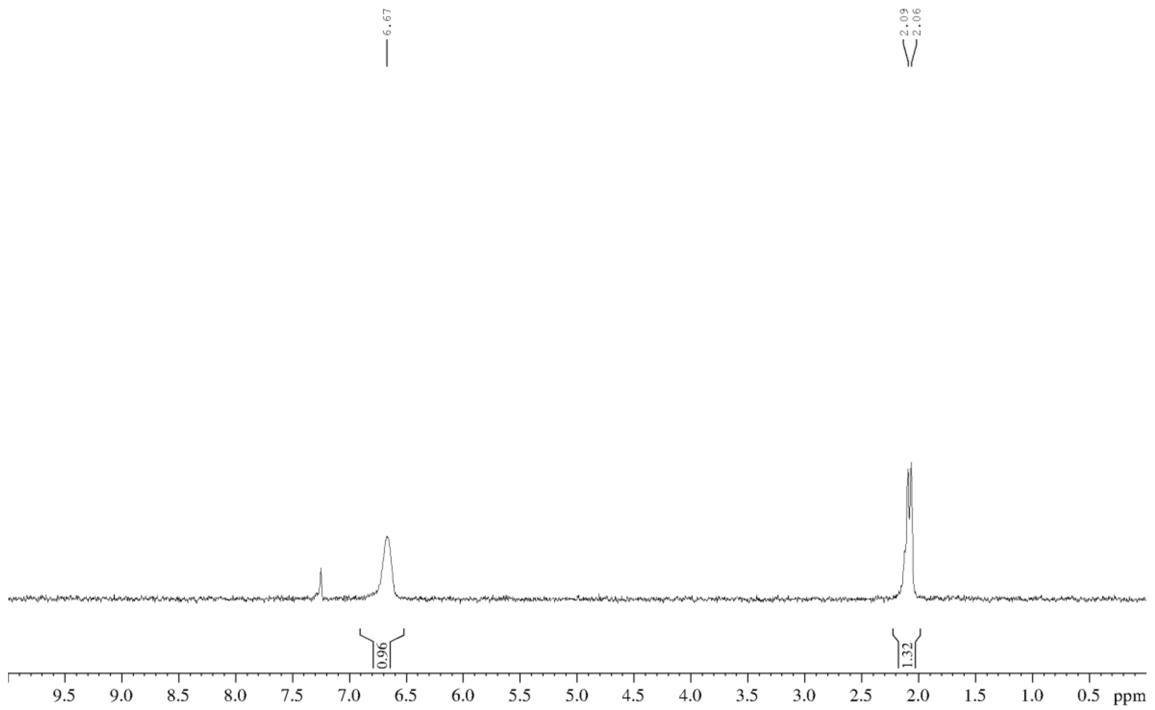

Figure S7: ^{2}H NMR ($CHCl_3$, 92 MHz) of crude mixture for Scheme S2 (**A**).

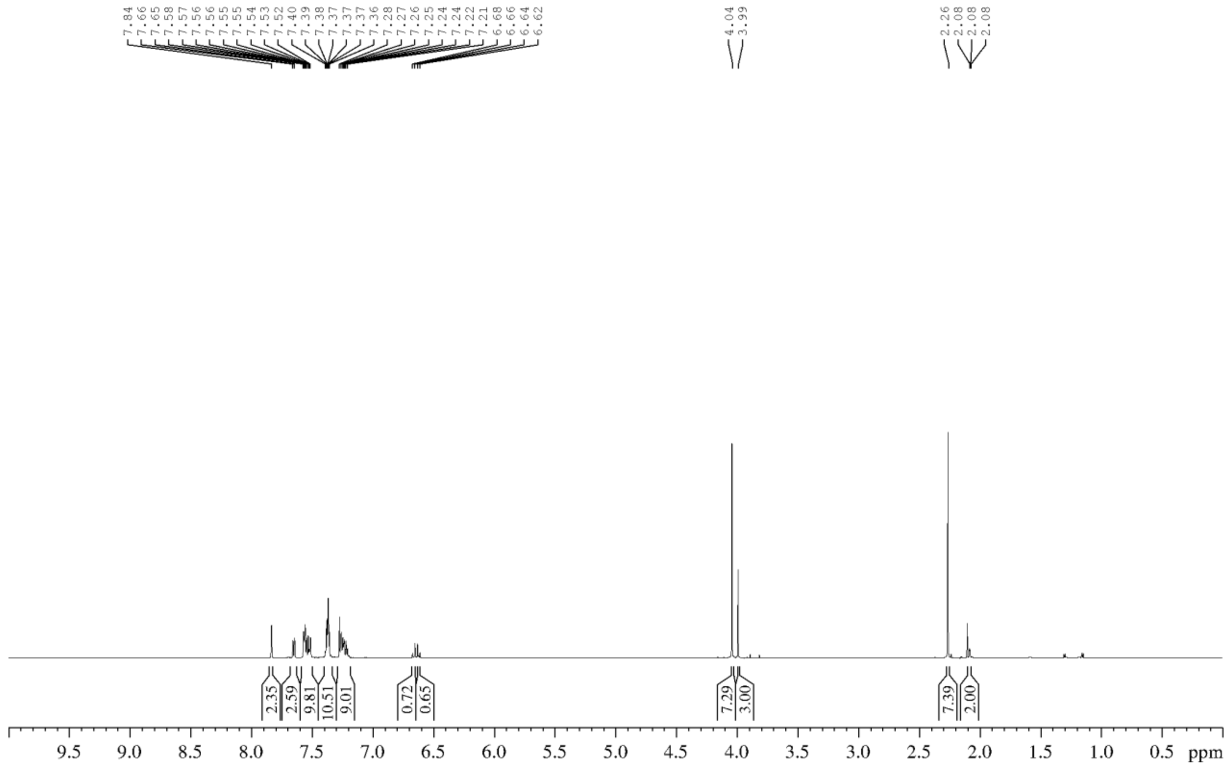

Figure S8: ^{1}H NMR ($CHCl_3$, 600 MHz) of crude mixture for Scheme S2 (**B**).

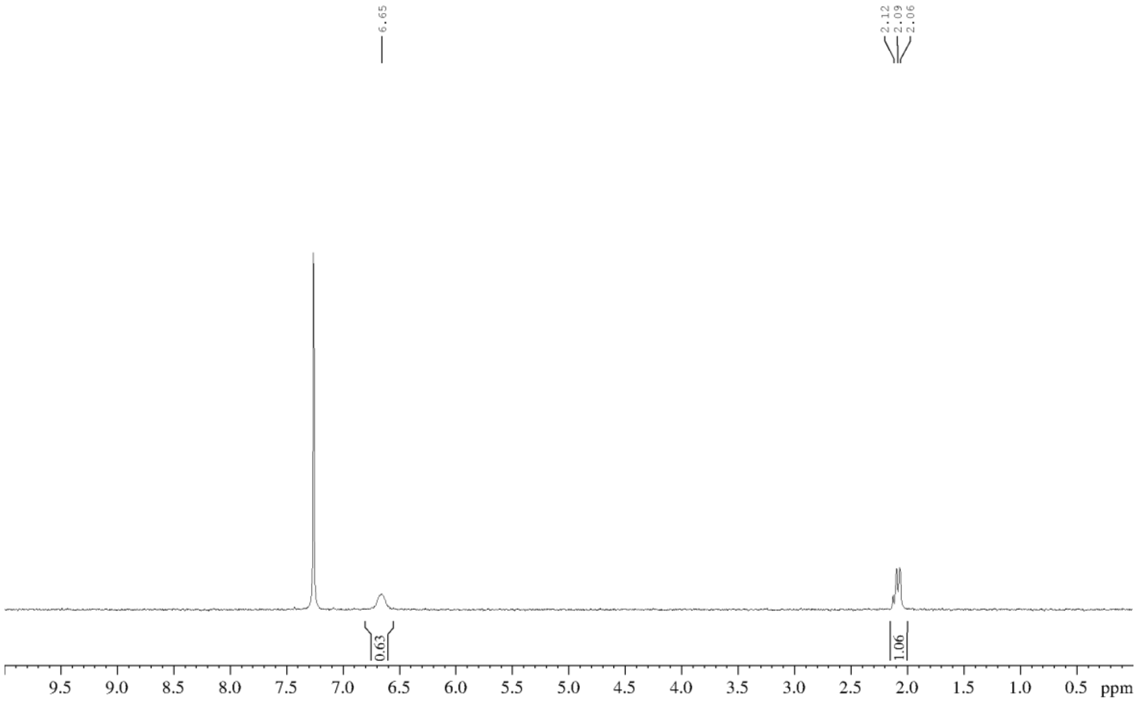

Figure S9: ^{2}H NMR ($CHCl_3$, 92 MHz) of crude mixture for Scheme S2 (**B**).

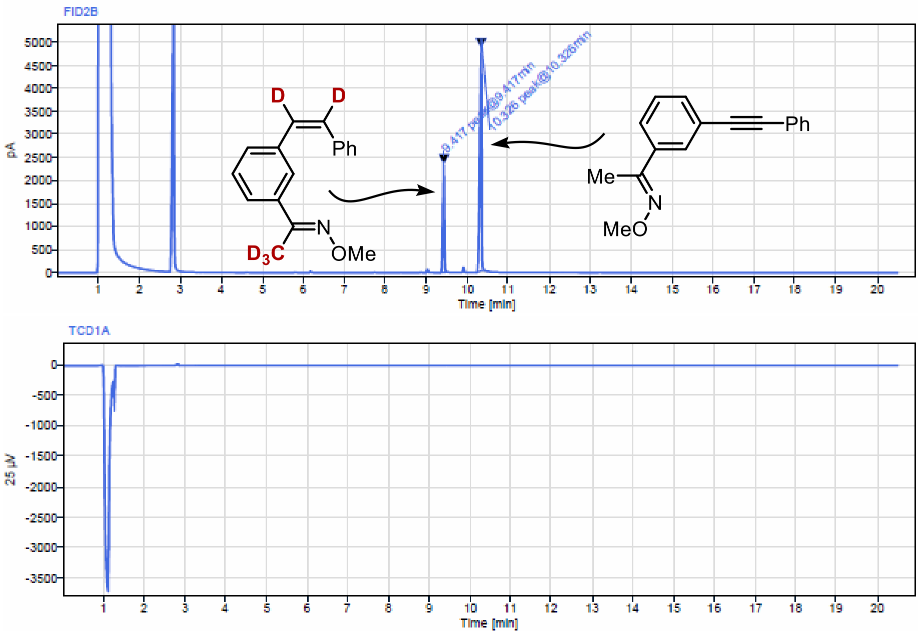

Signal:	FID2B					
Name	RT [min]	RF	Area	Peak Area Percent	Concentration []	Group
peak@9.417min	9.42		5349.230	27.67		
peak@10.326min	10.33		13984.980	72.33		

Figure S10: GC chromatogram of crude mixture for Scheme 2 (**B**).

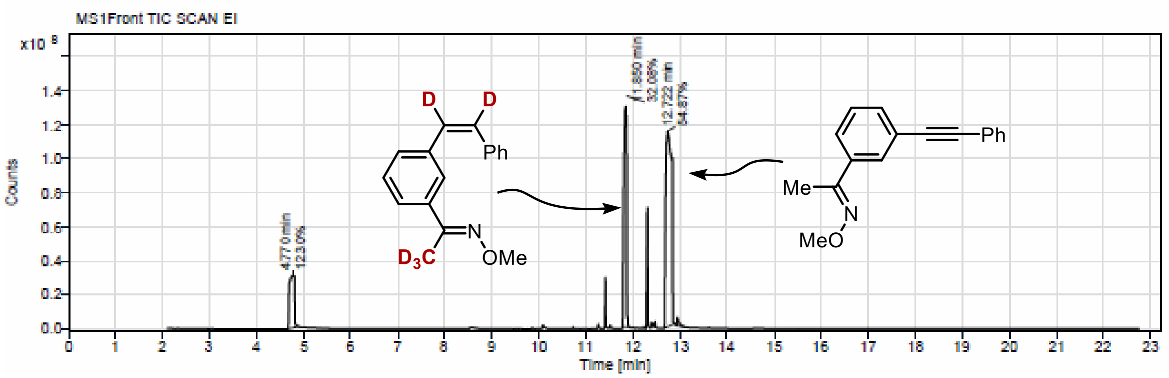

Signal: MS1Front TIC SCAN EI

RT	m/z	Spectral purity (%)	Peak area	Area %
4.770			227100000	12.30

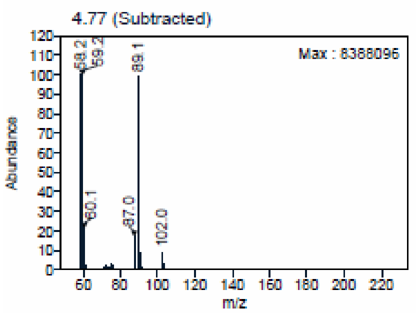

RT	m/z	Spectral purity (%)	Peak area	Area %
11.850			592100000	32.08

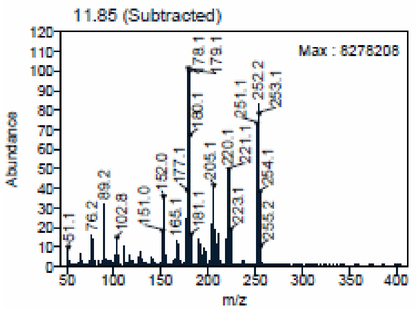

RT	m/z	Spectral purity (%)	Peak area	Area %
12.722			1013000000	54.87

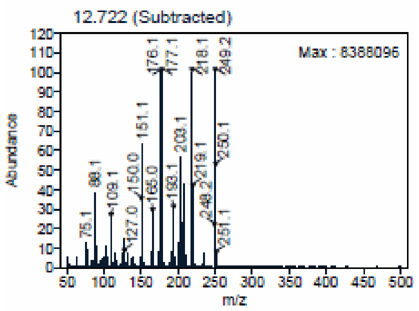

Figure S11: GC-MS chromatogram of crude mixture for Scheme 2 (**B**).

4.2 Alkyne semihydrogenation with *t*BuOD as additive

The alkyne semihydrogenation was conducted according to **GP1** with 0.20 mmol of tolane (**1a**). The ^{1}H NMR was measured with 40 second relaxation time.

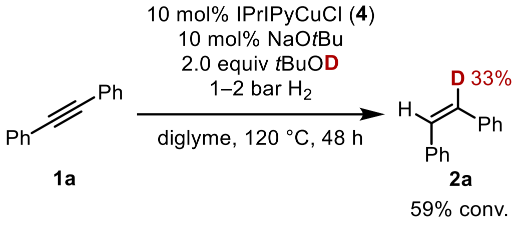

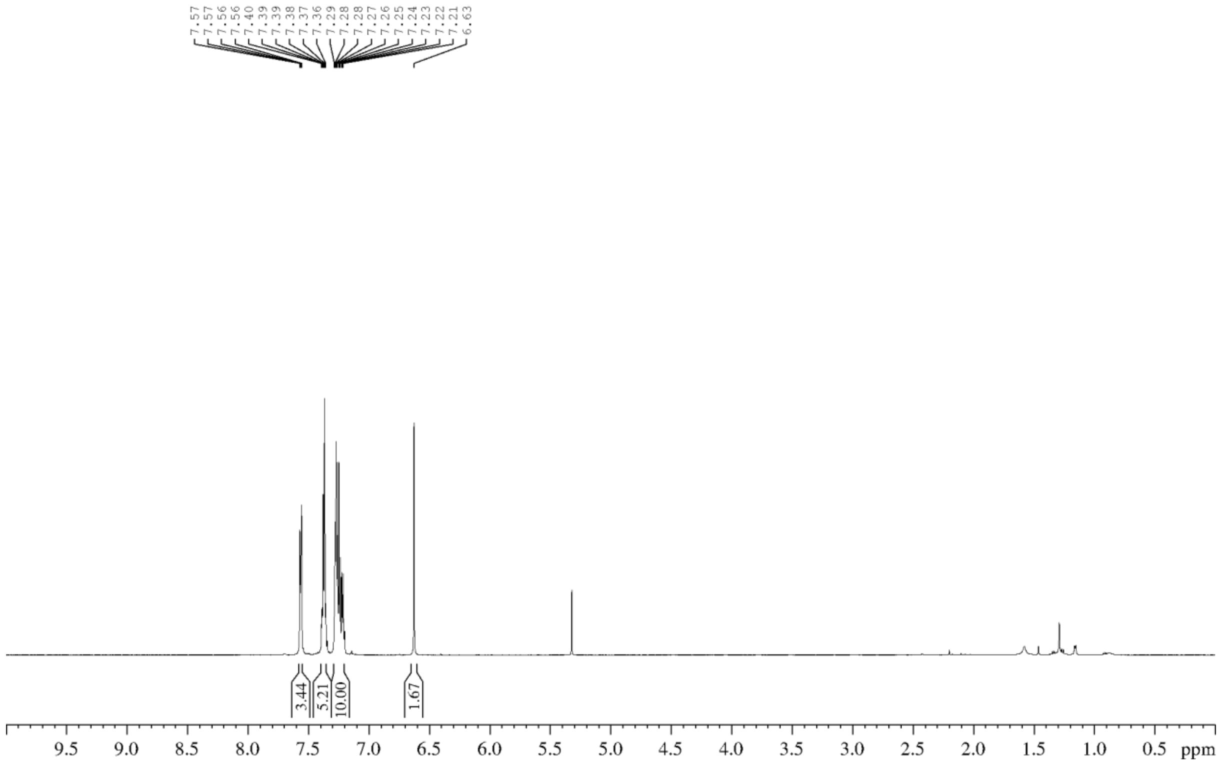

Figure S12: ^{1}H NMR ($CDCl_3$, 600 MHz) after alkyne semihydrogenation of tolane (**1a**) with 2.0 equiv *t*BuOD additive.

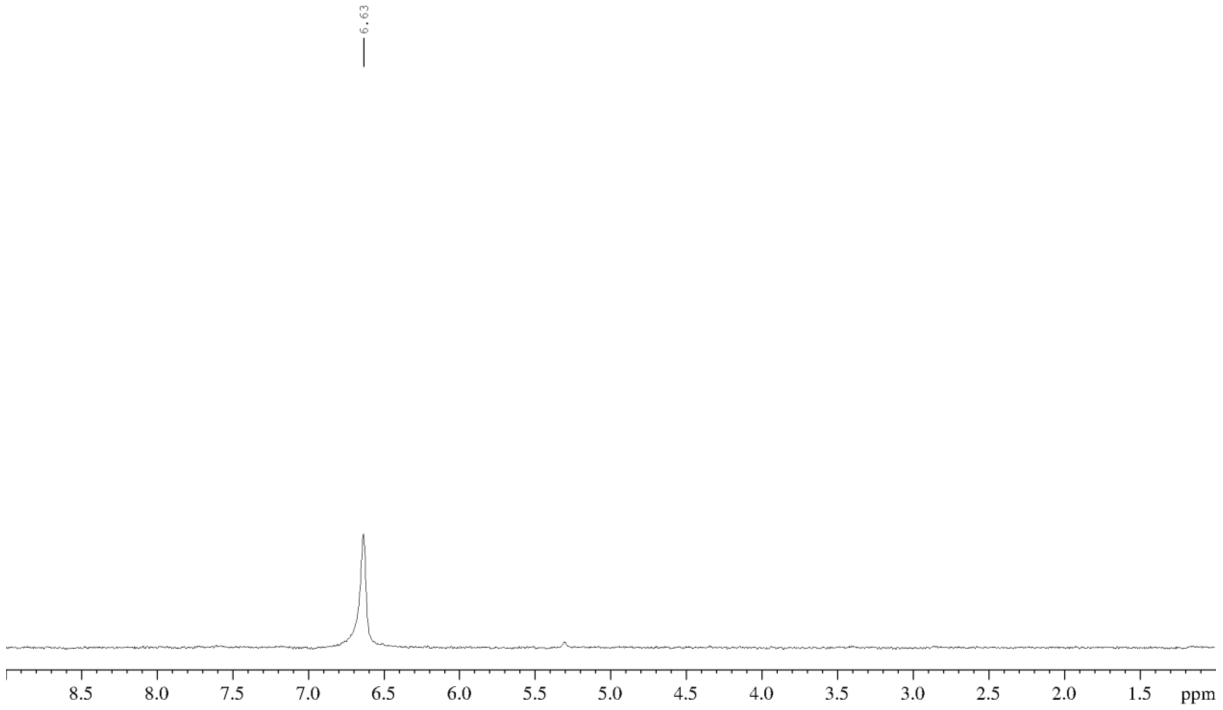

Figure S13: ^2H NMR ($CHCl_3$, 92 MHz) after alkyne semihydrogenation of tolane (**1a**) with 2.0 equiv *t*BuOD additive. (CD_2Cl_2 is used for calibration).

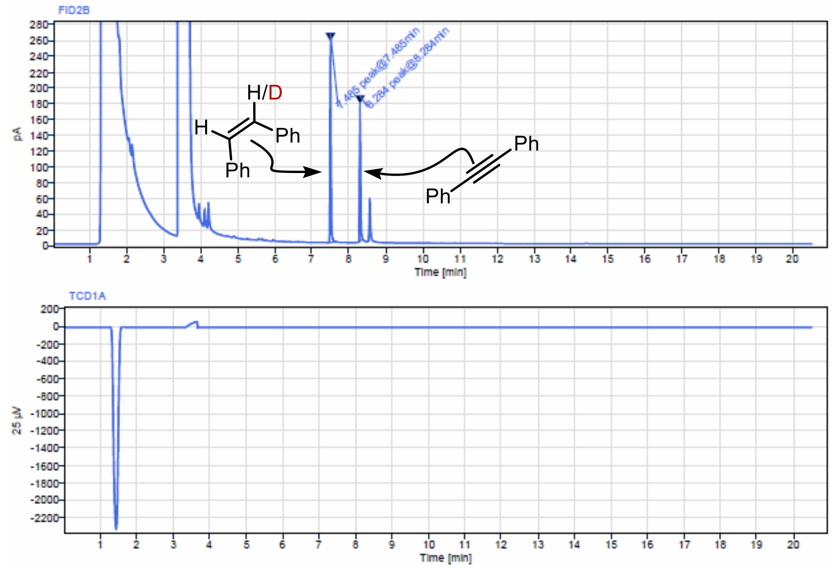

Signal: FID2B

Name	RT [min]	RF	Area	Peak Area Percent	Concentration []	Group
peak@7.485min	7.48		388.377	58.82		
peak@8.284min	8.28		271.953	41.18		

Figure S14: GC chromatogram after alkyne semihydrogenation of tolane (**1a**) with 2.0 equiv *t*BuOD additive.

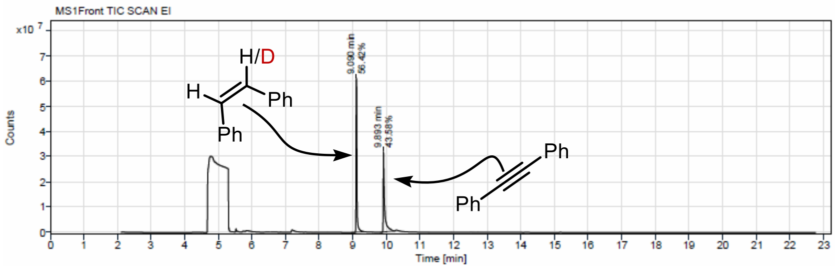

Signal: MS1Front TIC SCAN EI

RT	m/z	Spectral purity (%)	Peak area	Area %
9.090			116600000	56.42

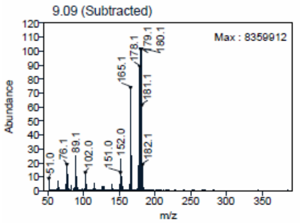

RT	m/z	Spectral purity (%)	Peak area	Area %
9.893			90070000	43.58

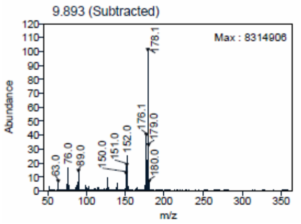

Figure S15: GC-MS chromatogram after alkyne semihydrogenation of tolane (**1a**) with 2.0 equiv *t*BuOD additive.

4.3 Probing hydrogenation with in situ IR spectroscopy

In situ FT-IR spectroscopic measurements were conducted using a Mettler-Toledo ReactIR 15 spectrometer. Additionally, the DS AgX Fiber Conduit technology with a probe size of 6.3 mm and

a DiComp ATR Sensor were employed. Materials of the probe head which came in contact with the reaction mixtures are the Hastelloy C22 alloy of the jacket, the gold seal and the diamond window at the probe tip. Spectra were recorded with 128 scans per minute.

Spectra were analyzed with the Mettler-Toledo iC IR 7.0 (7.0.297.0) program.

4.3.1 Procedure for the alkyne semihydrogenation with *in situ* IR spectroscopy

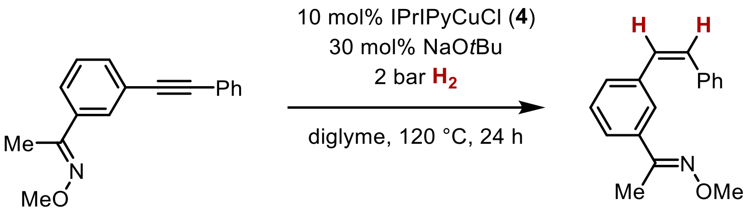

1k **2k**

A 10 mL two-necked flask was charged with [IPrIPyCuCl] (**4,** 37.2 mg, 80.0 μmol, 10.0 mol%) and NaO*t*Bu (23.8 mg, 0.240 mmol, 30.0 mol%) in an Ar glovebox, sealed with septa and transferred outside. The solids were dissolved in diglyme (1 mL). The flask was then connected to the preheated (120 °C) reactIR probe under N_2-counterflow (balloon) and three consecutive baseline spectra were measured (every 1 min a 128 scans). The alkyne, 1-(3-(Phenylethynyl)phenyl)ethan-1-one *O*-methyl oxime (**1k**, 200 mg, 0.800 mmol. 1.00 equiv), dissolved in diglyme (2 mL) in another 5 mL vial was transferred to the reaction flask. The septa of the reaction flask was pinched with a needle for gas exchange. After closing, the autoclave was purged with N_2 (3 x 5 bar) and H_2 (5 x 2 bar). The reaction mixture was stirred for 24 h at 120 °C (heating block temperature) under H_2-atmosphere (2 bar). The crude reaction mixture was filtered over a plug silica (1 × 5 cm, eluent: CH_2Cl_2, 10 mL) and all volatiles were removed under reduced pressure. Conversion was determined by GC/GC-MS and NMR-analysis. IR spectra were analyzed and processed with Mettler-Toledo iC IR 7.0 (7.0.297.0) and QtiPlot 1.0.0 (5.12.8).

4.3.2 Results of the alkyne semihydrogenation with *in situ* IR spectroscopy

To follow the conversion of starting material **1k**, the area of the fingerprint region IR band at $\tilde{\nu}$ = 758 cm^{-1} was monitored (Figure S16). The area was determined from $\tilde{\nu}$ = 775–755 cm^{-1} to two baseline points at $\tilde{\nu}$ = 755 $cm^{-1}$and $\tilde{\nu}$ = 775 cm^{-1}.

To plot the trend of the area of the IR band at $\tilde{\nu}$ = 758 cm^{-1} against the elapsed time, the area between $\tilde{\nu}$ = 755–775 cm^{-1} in the baseline spectrum was set to zero and the data points were normalized to the area of the IR band at $\tilde{\nu}$ = 758 cm^{-1} at the start of the reaction (t ~ 0 min) (Figure S16).

Under standard condition the area of the alkyne (**1k**) IR band in the fingerprint region $\tilde{\nu}$ = 758 cm^{-1} decreased quickly and slowed down after ~4.5 h (Figure S12). Afterwards the reaction proceeds until full conversion was reached after 24 h.

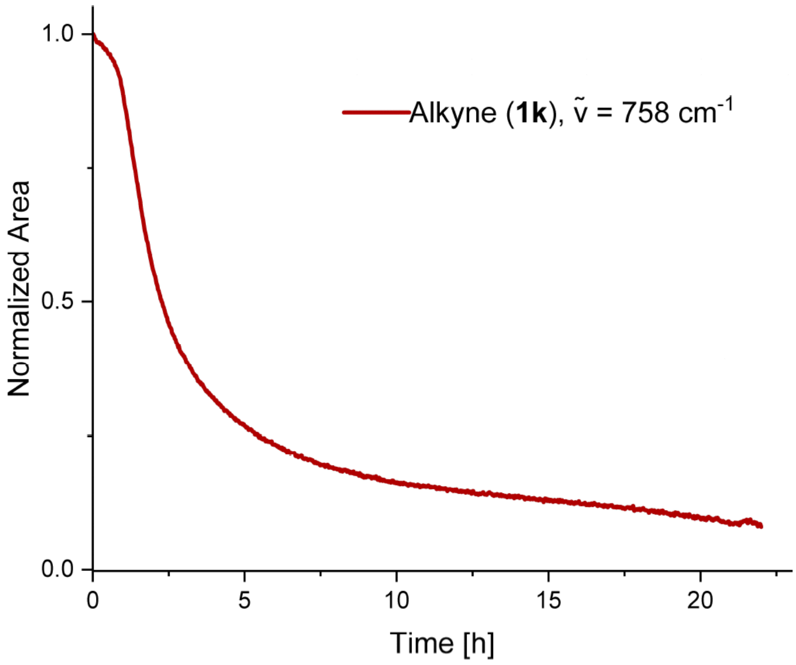

Figure S16: Plot of the normalized area of the alkyne (**1k**) fingerprint region band at ṽ = 758 cm^{-1} against the elapsed time.

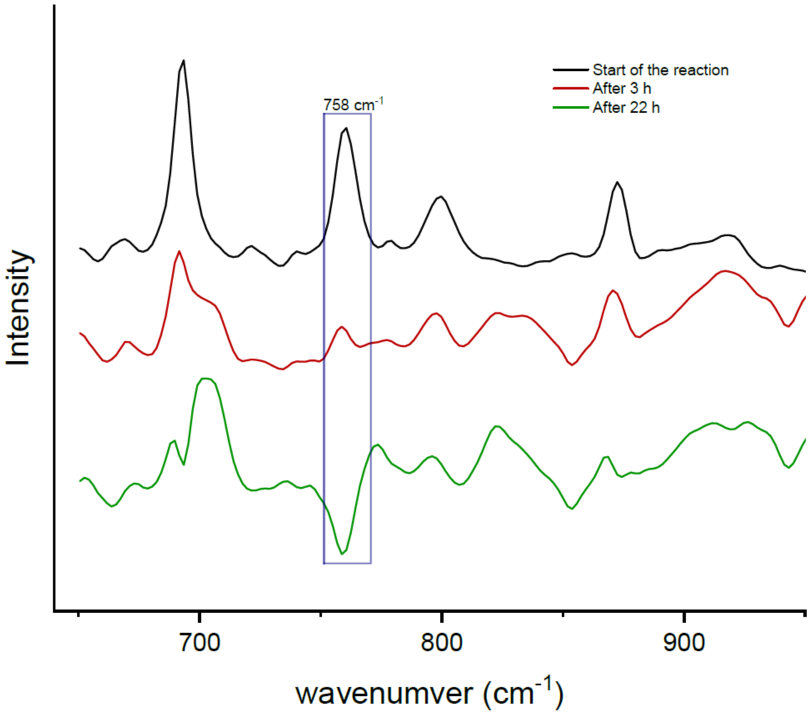

Figure S17: IR-spectra cut-out of the bands from wavenumber (ṽ) 650 to 950 cm^{-1} of the reaction after addition of alkyne, **1k** (black), after 3 h (red) and after 22 h (green).

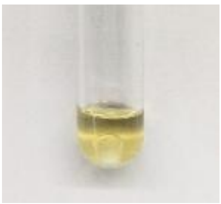
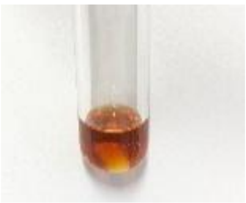

Figure S18: The reaction mixture before (left, t = 0 min) and after (right, t = 24 h) catalysis.

Several of the catalytic protocols aimed at H_2 activation with copper(I)/NHC complexes have been reported to be heterogeneous mixtures when catalytically active.[19] Importantly, heterogeneous copper particles could themselves be catalytically active and could potentially lead to lower chemo- and stereoselectivity. In our case, the reaction mixture remained fully homogeneous and in addition, no significant induction period was observed in the catalytic alkyne semihydrogenation when followed by *in situ* IR spectroscopy.

4.4 Alkyne semihydrogenation with copper(I)-*tert*-butoxide complex I

In an Ar-filled glovebox, [IPrIPyCuCl] (**4**, 9.30 mg, 20.0 µmol, 1.00 equiv) and NaO*t*Bu (3.85 mg, 40.0 µmol, 2.00 equiv) were placed in a 5 mL glass vial with a stiring bar. THF (2 mL) was added and the reaction mixture was stirred at room temperature (21 °C) for 1 h. The ^{1}H NMR was recorded after 20 min and after 1 h (Figure S15). ^{1}H NMR analysis of the crude mixture showed complete conversion of IPrIPyCuCl (**4**) to IPrIPyCuO*t*Bu (**I**) after 1 h.

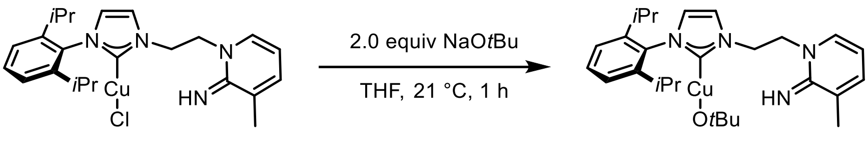

IPrIPyCuCl, **4** IPrIPyCuO*t*Bu, **I**

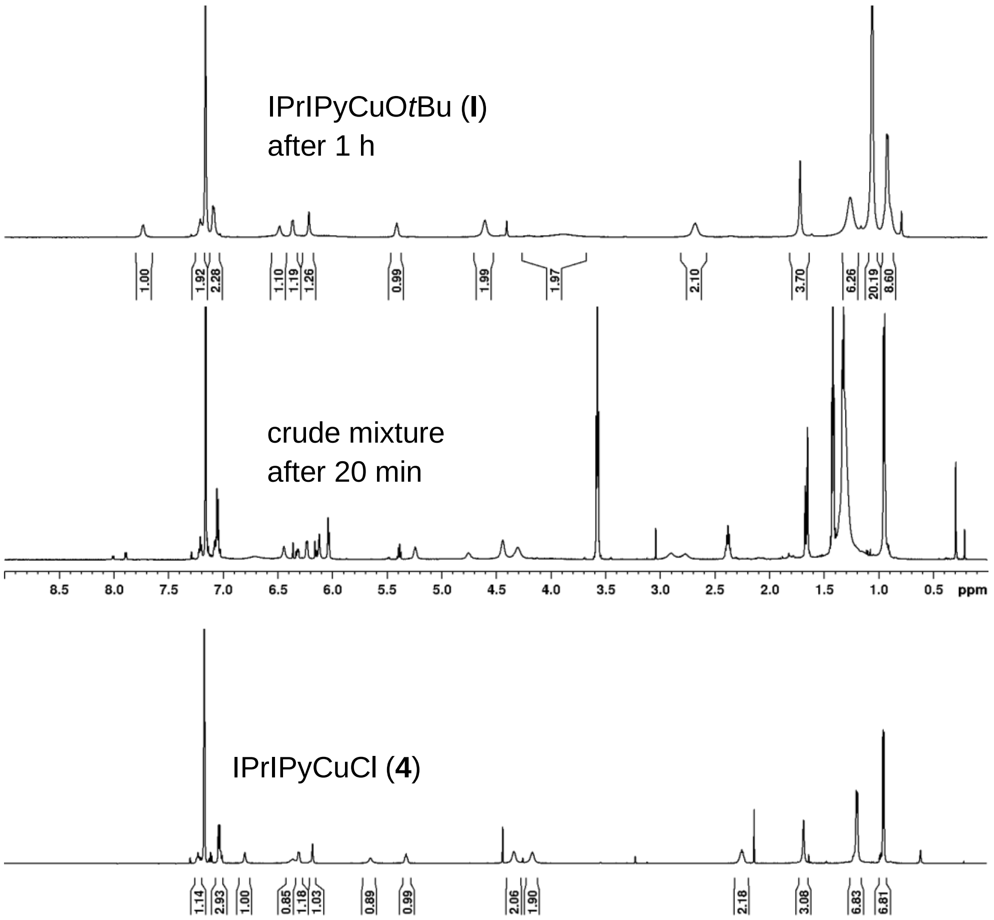

Figure S19: ^1H NMR (C_6D_6, 600 MHz) of IPrIPyCuCl (**4**) (bottom), crude mixture after 20 min (middle) and crude mixture after 1 h (top).

After 1 h, the crude reaction mixture was filtered with PTFE (0.45 µM) to remove excess of NaO*t*Bu. The filtrate was concentrated under oil pump vacuum (2×10^{-2}). The crude IPrIPyCuO*t*Bu (**I**) was then subjected for alkyne semihydrogenation of tolane (**1a**) according to **GP1**. After 48 h, 37% conversion of tolane (**1a**) to (*Z*)-stilbene (**2a**) was observed by GC/GC-MS analysis. Upon performing alkyne semihydrogenation of tolane (**1a**) with 2 equiv *t*BuOH as additive, the conversion increased to 58%, further supporting the proximity effect enabled by 2-iminopyridine subunit.

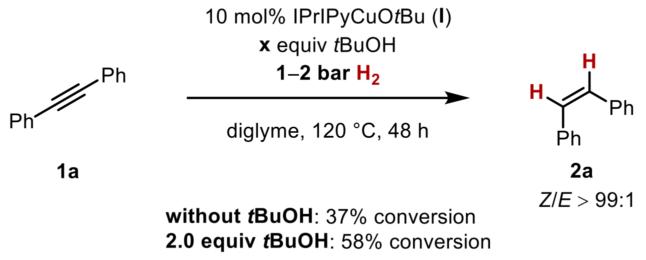

5 Alkyne semihydrogenation (in situ protocol) directly from ligand precursors

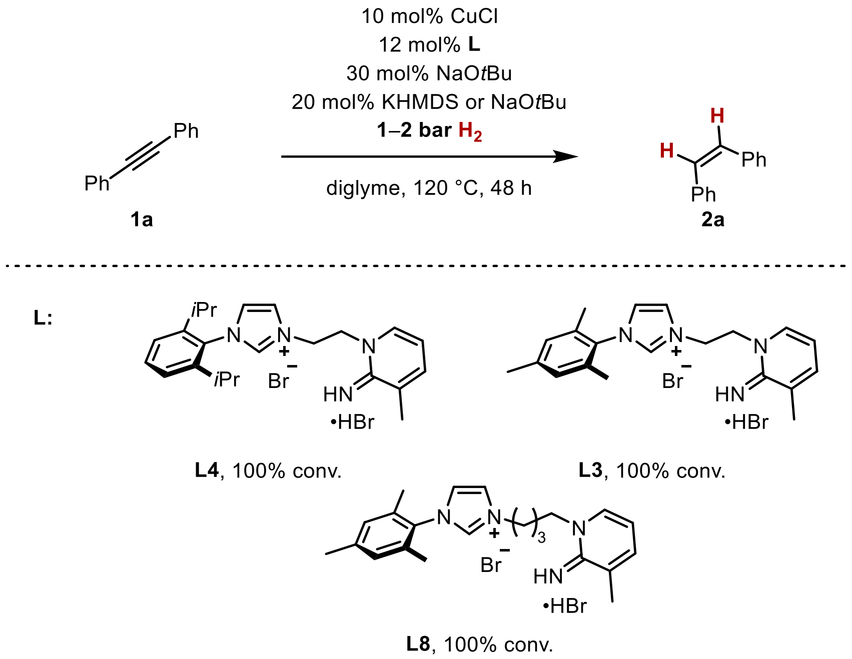

In a 5 mL reaction vial, copper(I)chloride (3.00 mg, 30.0 µmol, 10.0 mol%), ligand precursor (**L**, 12.6 mg for **L4**/ 11.8 mg for **L3**/ 12.2 mg for **L8**, 36.0 µmol, 12 mol%), NaO*t*Bu (8.70 mg, 90.0 µmol, 30.0 mol%) and KHMDS (12.0 mg, 60.0 µmol, 20.0 mol%) / NaO*t*Bu (5.80 mg, 60.0 µmol, 20.0 mol%) were placed with a stirring bar. The vial was then evacuated under vacuum and back-flushed with N_2 gas. This process was repeated for three times. Diglyme (0.5 mL) was added and the mixture was stirred for 10 min at 40 °C. The alkyne, **1a** (53.0 mg, 0.300 mmol, 1.00 equiv) dissolved in diglyme (2.5 mL) in another 5 mL vial was added to the reaction vial. The

reaction mixture was transferred to a pressure tube under N_2 counterflow. The N_2 atmosphere was removed under vaccum (2 × 10^{-2} bar) and then backfilled with H_2 (1.5 bar). The reaction mixture was stirred for 48 h at 120 °C under H_2 atmosphere (1.5 bar). The reaction mixture was allowed to cool down to room temperature and H_2 gas was replaced with N_2 (3 times purging). The crude reaction mixture was filtered over a plug of silica (1 × 5 cm, eluent: CH_2Cl_2, 5 mL). The conversion was monitored by GC/GC-MS and NMR analysis. The analytical data (GC/GC-MS and ^{1}H NMR analysis of the crude mixture) showed complete conversion of the alkyne, **1a** to the alkene **2a** with exclusive *Z*-selectivity in all cases.

Note: For the catalysis above as the copper(I)/NHC complexes were generated *in situ,* 20 mol% additional KHMDS or NaO*t*Bu was added.

6 Gram scale reaction

6.1 1-(3-((*Z*)-Styryl)phenyl)ethan-1-one *O*-methyl oxime (2k)

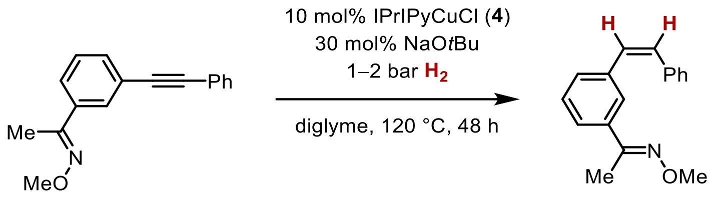

1k **2k**

In an Ar-filled glovebox, [IPrIPyCuCl] (**4**, 210 mg, 0 450 mmol, 10.0 mol%) and NaO*t*Bu (130 mg, 1.35 mmol, 30.0 mol%) were placed in a 50 mL round bottom flask with a stirring bar. The flask was capped inside the glovebox with a septum and then transferred outside. The solids were dissolved in diglyme (10 mL) and the mixture was stirred for 10 min at 40 °C. The alkyne, **1k** (1.13 g, 4.50 mmol, 1.00 equiv), dissolved in diglyme (15 mL) in another 50 mL Schlenk flask was transferred to the reaction mixture flask. The reaction mixture was then transferred to a 100 mL pressure tube under N_2 counterflow. The N_2 atmosphere was removed under vaccum (2 × 10^{-2} bar) and then backfilled with H_2 (1.5 bar). The reaction mixture was stirred for 48 h at 120 °C under H_2 atmosphere (1.5 bar). The reaction mixture was cooled down to room temperature and H_2 gas was replaced with N_2. The crude reaction mixture was quenched with sat. aq. NH_4Cl-solution (10 mL) and the aqueous phase was extracted with CH_2Cl_2 (3 x 15 mL). The combined organic layers were dried over $MgSO_4$ and filtered. All volatiles removed under reduced pressure and the crude product was purified by flash column chromatography on silica gel (cyclohexane/EtOAc = 40:1) to yield **2k** as orange oil (816 mg, 3.24 mmol, 72%).

6.2 3-phenyl-1-(3-(trifluoromethyl)-5,6-dihydro-[1,2,4]triazolo[4,3-a]pyrazin-7(8*H*)-yl)butan-1-one (12t)

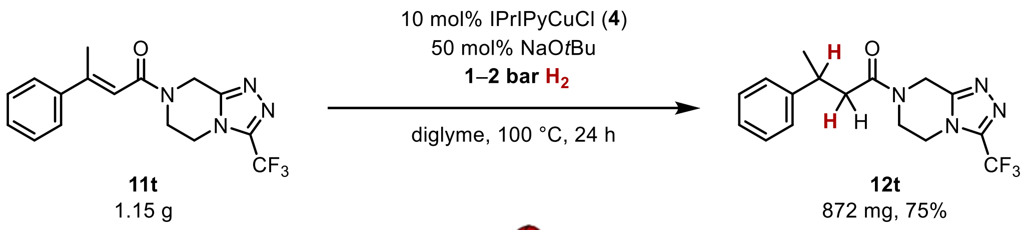

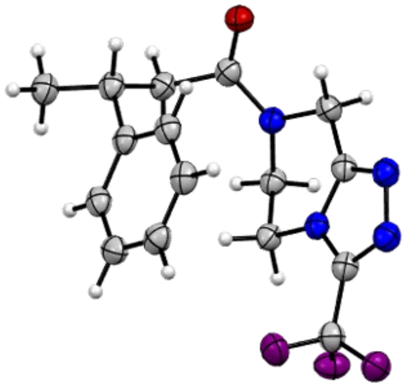

[*X-ray*], **12t**

In an Ar-filled glovebox, [IPrIPyCuCl] (**4**, 160 mg, 0 342 mmol, 10.0 mol%) and NaO*t*Bu (164 mg, 1.71 mmol, 30.0 mol%) were placed in a 50 mL round bottom flask with a stirring bar. The flask was sealed inside the glovebox with a septum and then transferred outside. The solids were dissolved in diglyme (10 mL) and the mixture was stirred for 10 min at 40 °C. The enamide **11t** (1.15 g, 3.42 mmol, 1.00 equiv), dissolved in diglyme (15 mL) in another 50 mL Schlenk flask was transferred to the reaction mixture flask. The reaction mixture was then transferred to a 100 mL pressure tube under N_2 counterflow. The N_2 atmosphere was removed under vacuum (2×10^{-2} bar) and then backfilled with H_2 (1.5 bar). The reaction mixture was stirred for 24 h at 100 °C under H_2 atmosphere (1.5 bar). The reaction mixture was cooled down to room temperature and H_2 gas was replaced with N_2. The crude reaction mixture was quenched with sat. aq. NH_4Cl-solution (10 mL) and the aqueous phase was extracted with CH_2Cl_2 (3 x 15 mL). The combined organic layers were dried over $MgSO_4$ and filtered. All volatiles removed under reduced pressure and the crude product was purified by flash column chromatography on silica gel (EtOAc/MeOH = 95:5) to yield **12t** as colorless solid (872 mg, 2.56 mmol, 75%).

The synthesized amide **12t** was crystallized from CH_2Cl_2.

7 Experimental Details

7.1 Synthesis of IMesIPyCuCl (3)

7.1.1 3-(2-Bromoethyl)-1-mesityl-1*H*-imidazol-3-ium bromide (S3b)

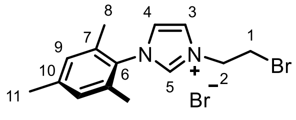

S3b
$C_{14}H_{18}Br_2N_2$
Mw = 374.12 gmol^{-1}

Prepared according to **GP4** from 1-mesityl-1*H*-imidazole (**S3a**, 1.86 g, 10.0 mmol, 1.00 equiv) and bromoethanol (0.850 mL, 12.0 mmol, 1.20 equiv) in toluene (20 mL). For the bromination step, PBr_3 (0.950 mL, 10.0 mmol, 1.00 equiv) was used in CH_2Cl_2 (50 mL). The filtrate was concentrated under reduced pressure to afford **S3b** as brown solid (2.70 g, 7.20 mmol, 72%).

^{1}H NMR (600 MHz, $CDCl_3$) δ = 10.21 (s, 1H, H-5), 8.08 (t, $^3J_{4,3/5}$ = 1.7 Hz, 1H, H-4), 7.16 (t, $^3J_{3,4/5}$ = 1.6 Hz, 1H, H-3), 7.02 (s, 2H, H-9), 5.26 (t, $^3J_{2,1}$ = 5.4 Hz, 2H, H-2), 4.06 (t, $^3J_{1,2}$ = 5.4 Hz, 2H, H-1), 2.35 (s, 3H, H-11), 2.09 (s, 6H, H-8) ppm.

^{13}C NMR (151 MHz, $CDCl_3$) δ = 141.8 (C-6), 138.3 (C-5), 134.4 (C-7), 130.7 (C-10), 130.1 (C-9), 124.1 (C-3), 122.7 (C-4), 51.9 (C-2), 32.0 (C-1), 21.3 (C-11), 17.8 (C-8) ppm.

HRMS (APCI) for $C_{14}H_{18}{}^{79}BrN_2^+$ [$(M–Br^-)^+$] calculated: 293.0648, found: 293.0647.

The data is in accordance with literature.[20]

7.1.2 3-(2-(2-Imino-3-methylpyridin-1(2*H*)-yl)ethyl)-1-mesityl-1*H*-imidazol-3-ium bromide hydrobromide (L3)

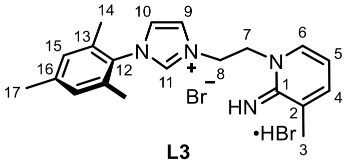

L3
$C_{20}H_{26}Br_2N_4$
Mw = 482.26 gmol^{-1}

Prepared according to **GP5** from 3-(2-bromoethyl)-1-mesityl-1*H*-imidazol-3-ium bromide (**S3b**, 2.61 g, 7.00 mmol. 1.00 equiv) and 3-methyl-2-aminopyridine (2.28 g, 21.0 mmol, 3.00 equiv) in CH_3CN (16 mL). Purification by washing the resulting solid with acetone (3 × 35 mL) yielded **L3** as colorless solid (2.29 g, 4.76 mmol, 68%).

Mp = 296 °C (acetone).

^{1}H NMR (600 MHz, DMSO-*d*$_6$) δ = 9.38 (app t, ^3J = 1.6 Hz, 1H, H-11), 8.42 (br s, 2H, N–*H*$_2$), 8.12 (app t, ^3J = 1.6 Hz, 1H, H-9), 7.97 (app t, ^3J = 1.7 Hz, 1H, H-10), 7.78 (d, $^3J_{4,5}$ = 7.2 Hz, 1H, H-4), 7.67 (d, $^3J_{6,5}$ = 6.5 Hz, 1H, H-6), 7.13 (s, 2H, H-15), 6.77 (t, $^3J_{5,4/6}$ = 6.9 Hz, 1H, H-5), 4.89 (t, $^3J_{8,7}$ = 5.5 Hz, 2H, H-8), 4.75 (t, $^3J_{7,8}$ = 5.5 Hz, 2H, H-7), 2.31 (s, 3H, H-17), 2.25 (s, 3H, H-3), 1.95 (s, 6H, H-14) ppm.

^{13}C NMR (151 MHz, DMSO-*d*$_6$) δ = 153.2 (C-1), 141.4 (C-4), 140.4 (C-16), 138.2 (C-11), 137.3 (C-6), 134.2 (C-13), 130.9 (C-12), 129.3 (C-15), 124.4 (C-10), 124.2 (C-9), 123.6 (C-2), 112.6 (C-5), 52.7 (C-8), 46.4 (C-7), 20.6 (C-17), 17.6 (C-3), 17.0 (C-14) ppm.

HRMS (APCI) for $C_{20}H_{25}N_4^+$ [(M–2Br–H$^+$)$^+$] calculated: 321.2074, found: 321.2074.

IR (ATR): ṽ = 3202 (w), 3015 (br), 2657 (w), 1654.9 (m), 1572 (m), 1207 (m), 1159 (w), 1047 (w), 849 (w), 771 (w) cm^{-1}.

7.1.3 (1-(2-(2-Imino-3-methylpyridin-1(2*H*)-yl)ethyl)-3-mesityl-1,3-dihydro-2*H*-imidazol-2-ylidene)copper(I) chloride (3)

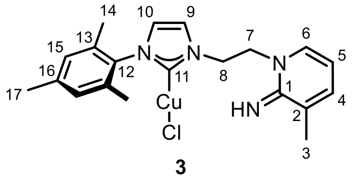

3

$C_{20}H_{24}ClCuN_4$

Mw = 420.44 gmol^{-1}

Prepared according to **GP6** from 3-(2-(2-imino-3-methylpyridin-1(2*H*)-yl)ethyl)-1-mesityl-1*H*-imidazol-3-ium bromide hydrobromide (**L3**, 150 mg, 0.300 mmol, 1.00 equiv), copper(I) chloride (30.0 mg, 0.300 mmol, 1.00 equiv) and NaO*t*Bu (75.0 mg, 0.780 mmol, 2.50 equiv) in THF (6 mL). The filtrate was concentrated under oil pump vacuum (2 × 10^{-2} mbar) yielding **3** as yellow solid (94.2 mg, 0.220 mmol, 72%).

^{1}H NMR (600 MHz, CD_2Cl_2) δ = 6.93 (d, $^3J_{4,5}$ = 6.9 Hz, 1H, H-4), 6.89 (s, 2H, H-15), 6.76 (d, $^3J_{9,10}$ = 1.3 Hz, 1H, H-9), 6.72 (d, $^3J_{6,5}$ = 6.5 Hz, 1H, H-6), 6.64 (d, $^3J_{10,9}$ = 1.6 Hz, 1H, H-10), 5.67 (s, 1H, N–*H*), 5.50 (t, $^3J_{5,6/4}$ = 6.7 Hz, 1H, H-5), 4.10 (t, $^3J_{8,7}$ = 6.1 Hz, 2H, H-8), 4.05 (t, $^3J_{7,8}$ = 6.1 Hz, 2H, H-7), 2.24 (s, 3H, H-17), 2.00 (s, 6H, H-14), 1.98 (s, 3H, H-3) ppm.

^{13}C NMR (151 MHz, CD_2Cl_2) δ = 186.0 (C-11), 160.5 (C-1), 139.2 (C-16), 137.4 (C-4), 137.1 (C-2), 135.9 (C-13), 132.4 (C-6), 129.2 (C-15), 125.8 (C-12), 121.7 (C-9), 120.1 (C-10), 101.7 (C-5), 51.0 (C-8), 47.0 (C-7), 21.0 (C-17), 18.4 (C-14), 18.2 (C-3) ppm.

HRMS (APCI) for $C_{20}H_{24}CuN_4^+$ [(M–Cl)$^+$] calculated: 383.1291, found: 383.1287.

7.2 Synthesis of IPrIPyCuCl (4)

7.2.1 3-(2-Bromoethyl)-1-(2,6-diisopropylphenyl)-1*H*-imidazol-3-ium bromide (S4b)

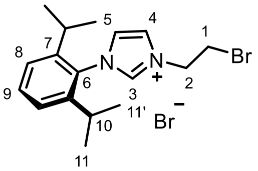

S4b

$C_{17}H_{24}Br_2N_2$

Mw = 416.20 gmol^{-1}

Prepared according to **GP4** from 1-(2,6-diisopropylphenyl)-1*H*-imidazole (**S4a**, 2.28 g, 10.0 mmol, 1.00 equiv), bromoethanol (0.850 mL, 12.0 mmol, 1.20 equiv) in toluene (20 mL). For the bromination step, PBr_3 (0.950 mL, 10.0 mmol, 1.00 equiv) was used in CH_2Cl_2 (50 mL). The filtrate was concentrated under reduced pressure to afford **S4b** as brown solid (3.57 g, 8.60 mmol, 86%).

Mp = 118 °C (CH_2Cl_2).

^{1}H NMR (600 MHz, $CDCl_3$) δ = 10.05 (s, 1H, H-3), 8.34 (s, 1H, H-4), 7.55 (t, $^3J_{9,8}$ = 7.9 Hz, 1H, H-9), 7.32 (d, $^3J_{8,9}$ = 7.9 Hz, 2H, H-8), 7.19 (s, 1H, H-5), 5.32 (t, $^3J_{2,1}$ = 5.3 Hz, 2H, H-2), 4.09 (t, $^3J_{1,2}$ = 5.3 Hz, 2H, H-1), 2.34 (sept, $^3J_{10,11/11'}$ = 6.8 Hz, 2H, H-10), 1.24 (d, $^3J_{11,10}$ = 6.8 Hz, 6H, H-11), 1.17 (d, $^3J_{11',10}$ = 6.8 Hz, 6H, H-11') ppm.

^{13}**C NMR** (151 MHz, $CDCl_3$) δ = 145.6 (C-7), 138.3 (C-3), 132.3 (C-9), 130.1 (C-6), 124.9 (C-8), 124.3 (C-4), 123.9 (C-5), 51.9 (C-2), 32.4 (C-1), 28.9 (C-10), 24.6 (C-11), 24.3 (C-11') ppm.

HRMS (APCI) for $C_{17}H_{24}{}^{79}BrN_2^+$ [$(M–Br^-)^+$] calculated: 335.1117, found: 335.1117.

IR (ATR): ṽ = 3459 (br) (w), 3384 (br), 3131 (w), 3067 (w), 2967 (m), 2929 (w), 2870 (w), 2452 (w), 2370 (w), 1550 (s), 1449 (m), 1312 (w), 1274 (w), 1192 (s), 1107 (w), 1066 (w), 998 (w), 950 (w), 857 (w), 812 (s), 764 (s), 670 (w) cm^{-1}.

7.2.2 1-(2,6-Diisopropylphenyl)-3-(2-(2-imino-3-methylpyridin-1(2*H*)-yl)ethyl)-1*H*-imidazol-3-ium bromide hydrobromide (L4)

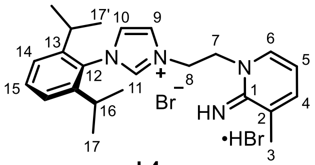

L4

$C_{23}H_{32}Br_2N_4$

Mw = 524.34 $gmol^{-1}$

Prepared according to **GP5** from 3-(2-bromoethyl)-1-(2,6-diisopropylphenyl)-1*H*-imidazol-3-ium bromide (**S4b**, 3.30 g, 8.00 mmol. 1.00 equiv) and 3-methyl-2-aminopyridine (2.60 g, 24.0 mmol, 3.00 equiv) in CH_3CN (16 mL). Purification by washing the resulting solid with acetone (3 × 40 mL) yielded **L4** as colorless solid (2.46 g, 4.7 mmol, 59%).

Mp = >300 °C (acetone).

^{1}**H NMR** (600 MHz, DMSO-d_6) δ = 9.44 (s, 1H, H-11), 8.44 (br s, 2H, N–H_2), 8.25 (s, 1H, H-9)*, 8.15 (s, 1H, H-10)*, 7.81 (d, $^3J_{4,5}$ = 7.2 Hz, 1H, H-4), 7.68 (d, $^3J_{6,5}$ = 6.6 Hz, 1H, H-6), 7.60 (t, $^3J_{15,14}$ = 7.8 Hz, 1H, H-15), 7.43 (d, $^3J_{14,15}$ = 7.8 Hz, 2H, H-14), 6.79 (t, $^3J_{5,4/6}$ = 6.9 Hz, 1H, H-5), 4.92 (t, $^3J_{8,7}$ = 5.3 Hz, 2H, H-8), 4.78 (t, $^3J_{7,8}$ = 5.3 Hz, 2H, H-7), 2.24 (s, 3H, H-3), 2.07 (hept, $^3J_{16,17/17'}$ = 6.8 Hz, 2H, H-16), 1.10 (d, $^3J_{17,16}$ = 6.8 Hz, 6H, H-17), 1.07 (d, $^3J_{17',16}$ = 6.8 Hz, 6H, H-17') ppm.

^{13}**C NMR** (151 MHz, DMSO-d_6) δ = 153.1 (C-1), 145.0 (C-13), 141.4 (C-4), 138.3 (C-11), 137.3 (C-6), 131.6 (C-15), 130.1 (C-12), 125.6 (C-10)*, 124.5 (C-14), 124.3 (C-2), 123.7 (C-9)*, 112.5 (C-5), 52.8 (C-8), 46.4 (C-7), 27.9 (C-16), 24.1 (C-17), 23.6 (C-17'), 17.5 (C-3) ppm.

HRMS (APCI) for $C_{23}H_{31}N_4^+$ [$(M–2Br^-–H)^+$] calculated: 363.2543, found: 363.2540.

IR (ATR): ṽ = 3287 (w), 3108 (br), 2967 (w), 2866 (w), 2378 (w), 2344 (w) 1651 (m), 1528 (m), 1461 (m), 1192 (w) cm^{-1}.

7.2.3 (1-(2,6-Diisopropylphenyl)-3-(2-(2-imino-3-methylpyridin-1(2*H*)-yl)ethyl)-1,3-dihydro-2*H*-imidazol-2-ylidene)copper(I) chloride (4)

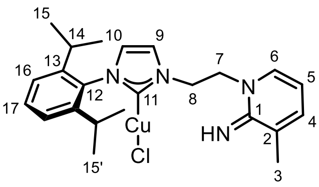

4
$C_{23}H_{30}ClCuN_4$
Mw = 461.52 gmol^{-1}

Prepared according to **GP6** 1-(2,6-diisopropylphenyl)-3-(2-(2-imino-3-methylpyridin-1(2*H*)-yl)ethyl)-1H-imidazol-3-ium bromide hydrobromide (**L4**, 162 mg, 0.310 mmol, 1.00 equiv), copper(I) chloride (30.0 mg, 0.310 mmol, 1.00 equiv) and NaO*t*Bu (75.0 mg, 0.780 mmol, 2.50 equiv) in THF (6 mL The filtrate was concentrated under oil pump vacuum (2 × 10^{-2} mbar) yielding **4** as yellow solid (98.1 mg, 0.210 mmol, 68%).

^{1}H NMR (600 MHz, CD_2Cl_2) δ = 7.48 (t, $^3J_{17,16}$ = 7.8 Hz, 1H, H-17), 7.28 (d, $^3J_{16,17}$ = 7.8 Hz, 2H, H-16), 7.18 (s, 1H, H-10)*, 6.89 (s, 1H, H-9)*, 6.81 (d, $^3J_{6,5}$ = 6.4 Hz, 1H, H-6), 6.69 (br s, 1H, H-4), 5.91 (s, 1H, N–*H*), 5.65 (t, $^3J_{5,4/6}$ = 6.6 Hz, 1H, H-5), 4.79 (br s, 2H, H-8), 4.53 (br s, 2H, H-7),
2.40–2.27 (m, 2H, H-14), 2.06 (s, 3H, H-3), 1.20 (d, $^3J_{15,14}$ = 6.9 Hz, 6H, H-15), 1.09 (d, $^3J_{15',14}$ = 6.9 Hz, 6H, H-15') ppm.

^{13}C NMR (151 MHz, CD_2Cl_2) δ = 179.5 (C-11), 160.3 (C-1), 146.2 (C-13), 136.1 (C-4), 135.2 (C-12), 132.9 (C-6), 130.7 (C-17), 127.4 (C-2), 124.5 (C-16), 124.0 (C-10)*, 121.7 (C-9)*, 103.3 (C-5), 53.3 (C-7), 47.6 (C-8), 28.6 (C-14), 24.6 (C-15), 24.5 (C-15'), 18.5 (C-3) ppm.

HRMS (APCI) for $C_{23}H_{31}CuN_4{}^{35}Cl^+$ [$(M+H)^+$] calculated: 461.1528, found: 461.1531.

7.3 Synthesis of IAdIPyCuCl (5)

7.3.1 1-(Adamantan-1-yl)-3-(2-bromoethyl)-1*H*-imidazol-3-ium bromide (S5b)

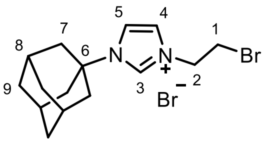

S5b
$C_{15}H_{22}Br_2N_2$
Mw = 390.16 gmol^{-1}

Prepared according to **GP4** from 1-adamantyl-1*H*-imidazole (**S5a**, 1.00 g, 4.94 mmol, 1.00 equiv), bromoethanol (0.420 mL, 5.93 mmol, 1.20 equiv) in toulene (10 mL). For the bromination, PBr_3 (0.470 mL, 4.94 mmol, 1.00 equiv) was used in CH_2Cl_2 (25 mL). The filtrate is concentrated under reduced pressure to afford **S5b** as colorless solid (1.45 g, 3.74 mmol, 75%).

Mp = 95 °C (CH_2Cl_2).

^{1}H NMR (600 MHz, $CDCl_3$) δ = 10.76 (s, 1H, H-3), 7.56 (s, 1H, H-4), 7.34 (s, 1H, H-5), 5.05 (t, $^3J_{2,1}$ = 5.3 Hz, 2H, H-2), 3.97 (t, $^3J_{1,2}$ = 5.3 Hz, 2H, H-1), 2.36–2.30 (m, 3H, H-8), 2.22 (d, $^3J_{7,8}$ = 2.8 Hz, 6H, H-7), 1.82–1.76 (m, 6H, H-9) ppm.

^{13}C NMR (151 MHz, $CDCl_3$): δ = 136.2 (C-3), 123.2 (C-5), 117.4 (C-4), 61.2 (C-2), 51.6 (C-1), 43.0 (C-6), 35.4 (C-9), 31.6 (C-6), 29.5 (C-7) ppm.

HRMS (APCI) for $C_{15}H_{22}{}^{79}BrN_2^+$ [(M–Br$^-$)$^+$] calculated: 309.0961, found: 309.0960.

IR (ATR): ṽ = 3395 (w), 3022 (w), 2914 (m), 2855 (m), 2683 (w), 2430 (w), 2370 (w), 2087 (br), 1994 (w), 1550 (m), 1453 (m), 1364 (w), 1308 (w), 1267 (w), 1162 (m), 1103 (w), 984 (w), 730 (s) cm^{-1}.

7.3.2 1-(Adamantan-1-yl)-3-(2-(2-imino-3-methylpyridin-1(2*H*)-yl)ethyl)-1*H*-imidazol-3-ium bromide (L5)

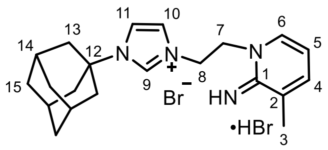

L5
$C_{21}H_{30}Br_2N_4$
Mw = 497.14 gmol^{-1}

Prepared according to **GP5** from 1-(adamantan-1-yl)-3-(2-bromoethyl)-1H-imidazol-3-ium bromide (**S5b**, 0.600 g, 1.53 mmol, 1.00 equiv), 3-methyl-2-aminopyridine (0.470 g, 4.61 mmol, 3.00 equiv) in CH_3CN (3 mL). Purification by washing the resulting solid with acetone (3 × 10 mL) yielded **L5** as colorless solid (0.520 g, 1.05 mmol, 68%).

Mp = >300 °C (acetone).

^{1}H NMR (600 MHz, DMSO-d_6) δ = 9.49 (app t, $^3J_{9,10/11}$ = 1.8 Hz, 1H, H-9), 8.41 (s, 2H, N–H_2), 8.10 (s, 1H, H-10,), 7.84 (s, 1H, H-11), 7.78 (d, $^3J_{4,5}$ = 7.4 Hz, 1H, H-4), 7.65 (d, $^3J_{6,5}$ = 6.6 Hz, 1H, H-6), 6.76 (t, $^3J_{5,4/6}$ = 6.9 Hz, 1H, H-5), 4.87 (d, $^3J_{8,7}$ = 5.7 Hz, 2H, H-8), 4.64 (d, $^3J_{7,8}$ = 5.7 Hz, 2H, H-7), 2.25 (s, 3H, H-3), 2.22–2.18 (m, 3H, H-14), 2.06 (d, $^3J_{13,14}$ = 2.9 Hz, 6H, H-13), 1.75–1.67 (m, 6H, H-15) ppm.

^{13}C NMR (151 MHz, DMSO-d_6) δ = 153.2 (C-1), 141.3 (C-4), 137.2 (C-2), 135.2 (C-9), 124.2 (C-2), 123.0 (C-10), 119.4 (C-11), 112.4 (C-6), 59.2 (C-12), 52.6 (C-8), 46.0 (C-7), 41.5 (C-13), 34.8 (C-15), 28.8 (C-14), 17.7 (C-3) ppm

HRMS (APCI) for $C_{21}H_{29}N_4^+$ [(M–2Br$^-$–H)$^+$] calculated: 337.2387, found: 337.2388.

IR (ATR): ṽ = 3063 (br), 2989 (w), 2907 (m), 2847 (w), 1651 (s), 1591 (w), 1531 (m), 1479 (w), 1449 (w), 1159 (m), 1099 (w), 745 (w) cm^{-1}.

7.3.3 (Adamantan-1-yl)-3-(2-(2-imino-3-methylpyridin-1(2*H*)-yl)ethyl)-1,3-dihydro-2*H*-imidazol-2-ylidene)copper(I) chloride (5)

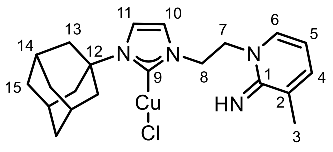

5

$C_{21}H_{28}ClCuN_4$
Mw = 435.49 gmol^{-1}

Prepared according to **GP6** 1-(adamantan-1-yl)-3-(2-(2-imino-3-methylpyridin-1(2*H*)-yl)ethyl)-1*H*-imidazol-3-ium bromide (**L5**, 154 mg, 0.310 mmol, 1.00 equiv), copper(I) chloride (30.0 mg, 0.310 mmol, 1.00 equiv) and NaO*t*Bu (75.0 mg, 0.780 mmol, 2.50 equiv) in THF (6 mL). The filtrate was concentrated under oil pump vacuum (2 × 10^{-2} mbar) yielding **5** as yellow solid (89.2 mg, 0.200 mmol, 66%).

^{1}H NMR (600 MHz, CD_2Cl_2) δ = 7.08 (d, $^3J_{10,11}$ = 1.9 Hz, 1H, H-10), 6.99 (d, $^3J_{11,10}$ = 2.0 Hz, 1H, H-11), 6.83 (d, $^3J_{4,5}$ = 6.5 Hz, 1H, H-4), 6.60 (d, $^3J_{6,5}$ = 6.4 Hz, 1H, H-6), 5.85 (s, 1H, N–*H*), 5.67 (t, $^3J_{5,4/6}$ = 6.8 Hz, 1H, H-5), 4.62 (t, $^3J_{8,7}$ = 6.2 Hz, 2H, H-8), 4.48 (t, $^3J_{7,8}$ = 6.2 Hz, 2H, H-7), 2.32 (s, 6H, H-13), 2.24 (m, 3H, H-14), 2.09 (s, 3H, H-3), 1.77 (s, 6H, H-15) ppm.

^{1}H NMR shows traces of toluene at 7.24, 7.17, 7.13 and 2.33 ppm.

^{13}C NMR (151 MHz, CD_2Cl_2) δ = 175.5 (C-9), 160.0 (C-1), 135.9 (C-5), 133.2 (C-4), 127.4 (C-2), 119.9 (C-10), 117.9 (C-11), 103.8 (C-6), 58.4 (C-8), 53.4 (C-12), 48.8 (C-7), 45.2 (C-13), 36.1 (C-15), 30.3 (C-13), 18.6 (C-3) ppm.

^{13}C NMR shows traces of toluene at 138.3, 129,3, 128.5, 125.6 and 21.5 ppm.

HRMS (APCI) for. $C_{21}H_{29}CuN_4{}^{35}Cl^+$ [(M+H$^+$)$^+$] calculated: 435.1371, found: 435.1378.

7.4 Synthesis of SIMesIPyCuCl (6)

7.4.1 3-(2-Bromoethyl)-1-mesityl-4,5-dihydro-1*H*-imidazol-3-ium bromide (S6b)

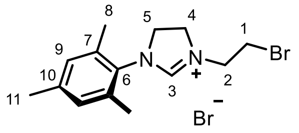

S6b

$C_{14}H_{20}Br_2N_2$
Mw = 376.14 gmol^{-1}

Prepared according to **GP4** from 1-mesityl-4,5-dihydro-1*H*-imidazole (**S6a**, 1.00 g, 5.31 mmol, 1.00 equiv), bromoethanol (0.450 mL, 6.37 mmol, 1.20 equiv) in toluene (9 mL). For the bromination, PBr_3 (0.500 mL, 5.31 mmol, 1.00 equiv) was used in CH_2Cl_2 (20 mL). The filtrate was concentrated under reduced pressure to afford **S6b** as brown solid (1.35 g, 3.61 mmol, 68%).

Mp = 121 °C (CH_2Cl_2).

^{1}H NMR (600 MHz, $CDCl_3$) δ = 9.24 (s, 1H, H-3), 6.93 (s, 2H, H-9), 4.47 (br s, 4H, H-2, H-5), 4.22 (br s, 2H, H-4), 3.82 (br s, 2H, H-1), 2.35 (s, 6H, H-8), 2.28 (s, 3H, H-11) ppm.

^{13}C NMR (151 MHz, $CDCl_3$) δ = 160.0 (C-3), 140.6 (C-10), 135.5 (C-7), 130.4 (C-6), 130.1 (C-9), 51.4 (C-5), 50.2 (C-2), 49.7 (C-4), 30.1 (C-1), 21.2 (C-11), 18.5 (C-8) ppm.

HRMS (APCI) for $C_{14}H_{20}{}^{79}BrN_2^+$ [(M–Br$^-$)$^+$] calculated: 295.0804, found: 295.0798.

IR (ATR): ṽ = 3391 (br), 2952 (br), 2415 (w), 2378 (w), 2344 (w), 1636 (s), 1483 (m), 1442 (m), 1297 (w), 1256 (m), 1200 (m), 842 (w) cm^{-1}.

7.4.2 3-(2-(2-Imino-3-methylpyridin-1(2*H*)-yl)ethyl)-1-mesityl-4,5-dihydro-1*H*-imidazol-3-ium bromide hydrobromide (L6)

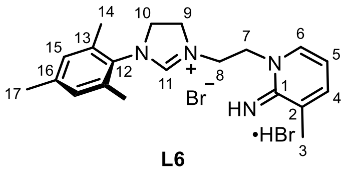

L6
$C_{20}H_{28}Br_2N_4$
Mw = 484.28 gmol^{-1}

Prepared according to **GP5** from 3-(2-bromoethyl)-1-mesityl-4,5-dihydro-1*H*-imidazol-3-ium bromide (**S6b**, 1.10 g, 2.92 mmol, 1.00 equiv), 3-methyl-2-aminopyridine (0.950 g, 8.77 mmol, 3.00 equiv) in CH_3CN (6 mL). Purification by washing the resulting solid with acetone (3 × 15 mL) yielded **L6** as colorless solid (0.820 g, 1.69 mmol, 58%).

Mp = 190 °C (acetone).

^{1}H NMR (600 MHz, DMSO-*d*$_6$) δ = 8.64 (s, 1H, H-11), 8.31 (br s, 2H, N–*H*$_2$), 8.13 (d, $^3J_{6,5}$ = 6.6 Hz, 1H, H-6), 7.80 (d, $^3J_{4,5}$ = 7.2 Hz, 1H, H-4), 7.00 (s, 2H, H-15), 6.87 (t, $^3J_{5,4/6}$ = 6.9 Hz, 1H, H-5), 4.61 (t, $^3J_{9,10}$ = 5.4 Hz, 2H, H-9), 4.33 (t, $^3J_{8,7}$ = 9.6 Hz, 2H, H-8), 4.19 (t, $^3J_{7,8}$ = 9.6 Hz, 2H, H-7), 3.96 (t, $^3J_{10,9}$ = 5.2 Hz, 2H, H-10), 2.24 (s, 3H, H-17), 2.23 (s, 3H, H-3), 2.13 (s, 6H, H-14) ppm.

^{13}C NMR (151 MHz, DMSO-*d*$_6$) δ = 160.0 (C-11), 153.1 (C-1), 141.3 (C-4), 139.5 (C-16), 138.0 (C-6), 135.3 (C-13), 130.8 (C-12), 129.3 (C-15), 124.3 (C-2), 112.6 (C-5), 50.7 (C-8), 50.5 (C-9), 48.7 (C-7), 45.2 (C-10), 20.5 (C-17), 17.5 (C-3), 17.2 (C-14) ppm.

HRMS (ESI) for $C_{20}H_{27}N_4^+$ [(M–2Br$^-$–H)$^+$] calculated: 323.2230, found: 323.2233.

IR (ATR): ṽ = 3265 (br), 3078 (br), 2911 (w), 1632 (s), 1591 (w), 1528 (w), 1483 (w), 1446 (w), 1356 (w), 1256 (w), 1215 (m), 1162 (w), 857 (w), 782 (w), 749 (w) cm^{-1}.

7.4.3 (1-(2-(2-Imino-3-methylpyridin-1(2*H*)-yl)ethyl)-3-mesitylimidazolidin-2-ylidene)copper(I) chloride (6)

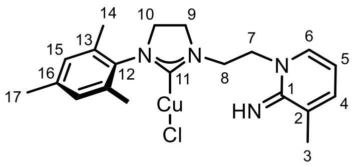

6
$C_{20}H_{26}ClCuN_4$
Mw = 421.45 gmol^{-1}

Prepared according to **GP6** 3-(2-(2-imino-3-methylpyridin-1(2*H*)-yl)ethyl)-1-mesityl-4,5-dihydro-1*H*-imidazol-3-ium bromide hydrobromide (**L6**, 152 mg, 0.310 mmol, 1.00 equiv), copper(I) chloride (30.0 mg, 0.310 mmol, 1.00 equiv) and NaO*t*Bu (75.0 mg, 0.780 mmol, 2.50 equiv) in THF (6 mL). The filtrate was concentrated under oil pump vacuum (2 × 10^{-2} mbar) yielding **6** as yellow solid (81.2 mg, 0.190 mmol, 62%).

^{1}H NMR (600 MHz, CD_2Cl_2) δ = 7.23 (d, $^3J_{6,5}$ = 6.7 Hz, 1H, H-6), 7.16 (d, $^3J_{4,5}$ = 6.8 Hz, 1H, H-4), 6.93 (s, 2H, H-15), 6.25 (t, $^3J_{5,4/6}$ = 6.8 Hz, 1H, H-5), 5.48 (br s, 1H, N–*H*), 4.92 (br s, 2H, H-8),

4.24 (br s, 2H, H-7), 4.00 (t, $^3J_{10,9}$ = 10.4 Hz, 2H, H-10), 3.82 (t, $^3J_{9,10}$ = 10.4 Hz, 2H, H-9), 2.28 (s, 3H, H-17), 2.15 (s, 9H, H-3, H-14) ppm.

^{13}C NMR (151 MHz, CD_2Cl_2) δ = 202.4 (C-11), 160.7 (C-1), 139.1 (C-16), 136.9 (C-4), 136.8 (C-6), 136.1 (C-13), 135.4 (C-12), 130.0 (C-2), 129.9 (C-15), 108.6 (C-5), 51.7 (C-8), 51.4 (C-9), 50.2 (C-10), 47.4 (C-7), 21.1 (C-17), 18.3 (C-14, C-3) ppm.

C-1 peak is assigned from ^{1}H, ^{13}C HMBC NMR.

HRMS (APCI) for $C_{20}H_{26}CuN_4^+$ [(M–Cl$^-$)$^+$] calculated: 385.1448, found: 385.1451.

7.5 Synthesis of SIPrIPyCuCl (7)

7.5.1 3-(2-Bromoethyl)-1-(2,6-diisopropylphenyl)-4,5-dihydro-1*H*-imidazol-3-ium bromide (S7b)

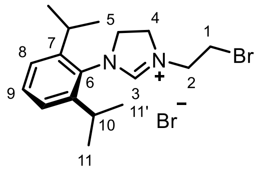

S7b

$C_{17}H_{26}Br_2N_2$

Mw = 418.22 gmol^{-1}

Prepared according to **GP4** from 1-(2,6-diisopropylphenyl)-4,5-dihydro-1*H*-imidazole (**S7a**, 1.50 g, 6.51 mmol, 1.00 equiv), bromoethanol (0.560 mL, 7.84 mmol, 1.20 equiv) in toluene (12 mL). For the bromination, PBr_3 (0.620 mL, 6.50 mmol, 1.00 equiv) was used in CH_2Cl_2 (30 mL). The filtrate was concentrated under reduced pressure to afford **S7b** as brown solid (1.92 g, 4.60 mmol, 71%).

Mp = 102 °C (CH_2Cl_2).

^{1}H NMR (600 MHz, $CDCl_3$) δ = 9.08 (s, 1H, H-3), 7.44 (t, $^3J_{9,8}$ = 7.8 Hz, 1H, H-9), 7.25 (d, $^3J_{8,9}$ = 7.8 Hz, 2H, H-8), 4.55 (t, $^3J_{4,5}$ = 5.5 Hz, 2H, H-4), 4.49 (t, $^3J_{2,1}$ = 10.2 Hz, 2H, H-2), 4.23 (t, $^3J_{1,2}$ = 10.2 Hz, 2H, H-1), 3.87 (t, $^3J_{5,4}$ = 5.5 Hz, 2H, H-5), 3.02 (sept, $^3J_{10,11/11'}$ = 6.6 Hz, 2H, H-10), 1.29 (d, $^3J_{11/11',10}$ = 6.6 Hz, 12H, H-11, H-11') ppm.

^{13}C NMR (151 MHz, $CDCl_3$**)** δ = 159.4 (C-3), 146.8 (C-7), 131.5 (C-9), 129.7 (C-6), 125.2 (C-8), 53.6 (C-1), 50.1 (C-4), 49.3 (C-2), 30.0 (C-5), 28.9 (C-10), 25.3 (C-11), 24.4 (C-11') ppm.

HRMS (APCI) for $C_{17}H_{26}{}^{79}BrN_2^+$ [(M–Br$^-$)$^+$] calculated: 337.1274, found: 337.1270.

IR (ATR): ṽ = 3459 (br), 3384 (br), 3131 (w), 3067 (w), 2967 (w), 2929 (w), 2870 (w), 2452 (w), 2370 (w), 1550 (m), 1449 (m), 1312 (w),.1274 (w), 1192 (s),.1107 (w), 998 (w), 950 (w), 857 (w), 812 (w), 764 (s), 670 (w) cm^{-1}.

7.5.2 1-(2,6-Diisopropylphenyl)-3-(2-(2-imino-3-methylpyridin-1(2*H*)-yl)ethyl)-4,5-dihydro-1*H*-imidazol-3-ium bromide hydrobromide (L7)

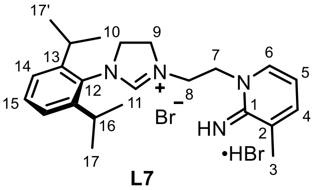

L7

$C_{23}H_{34}Br_2N_4$

Mw = 434.36 gmol^{-1}

Prepared according to **GP5** from 3-(2-bromoethyl)-1-(2,6-diisopropylphenyl)-4,5-dihydro-1*H*-imidazol-3-ium bromide (**S7b**, 0.800 g, 1.91 mmol, 1.00 equiv), 3-methyl-2-aminopyridine (0.620 g, 5.74 mmol, 3.00 equiv) in CH_3CN (4 mL). Purification by washing the resulting solid with acetone (3 × 10 mL) yielded **L7** as colorless solid (0.710 g, 1.35 mmol, 71%).

Mp = >300 °C (acetone).

^{1}H NMR (600 MHz, DMSO-d_6) δ = 8.79 (s, 1H, H-11), 8.36 (br s, 2H, N–H_2), 8.21 (d, $^3J_{6,5}$ = 6.6 Hz, 1H, H-6), 7.84 (d, $^3J_{4,5}$ = 7.2 Hz, 1H, H-4), 7.47 (t, $^3J_{15,14}$ = 7.8 Hz, 1H, H-15), 7.32 (d, $^3J_{14,15}$ = 7.8 Hz, 2H, H-14), 6.91 (t, $^3J_{5,6/4}$ = 6.9 Hz, 1H, H-5), 4.64 (t, $^3J_{9,10}$ = 4.8 Hz, 2H, H-9), 4.43 (t, $^3J_{8,7}$ = 10.2 Hz, 2H, H-8), 4.16 (t, $^3J_{7,8}$ = 10.4 Hz, 2H, H-7), 4.01 (t, $^3J_{10,9}$ = 4.8 Hz, 2H, H-10), 2.76 (hept, $^3J_{16,17}$ = 6.8 Hz, 2H, H-16), 2.23 (s, 3H, H-3), 1.22 (d, $^3J_{17,16}$ = 6.7 Hz, 6H, H-17), 1.04 (d, $^3J_{17',16}$ = 6.7 Hz, 6H, H-17') ppm.

^{13}C NMR (151 MHz, DMSO-d_6) δ = 159.5 (C-11), 153.0 (C-1), 146.3 (C-13), 141.3 (C-4), 138.0 (C-6), 130.8 (C-15), 129.8 (C-12), 124.8 (C-14), 124.1 (C-2), 112.5 (C-5), 53.5 (C-7), 50.5 (C-9), 48.8 (C-8), 45.5 (C-10), 27.7 (C-16), 24.7 (C-17), 24.0 (C-17'), 17.5 (C-3) ppm.

HRMS (APCI) for $C_{23}H_{32}N_4^+$ [(M–2Br$^-$–H)$^+$] calculated: 363.2543, found: 363.2540.

IR (ATR): ṽ = 3291 (w), 3123 (br), 2959 (w), 2929 (w), 2866 (w), 1628 (m), 1591 (w), 1528 (w), 1461 (w), 1394 (w), 1349 (w), 1315 (w), 1256 (m), 1054 (w), 808 (w), 764 (w) cm^{-1}.

7.5.3 (1-(2,6-Diisopropylphenyl)-3-(2-(2-imino-3-methylpyridin-1(2*H*)-yl)ethyl)imidazolidin-2-yl)copper(I) chloride (7)

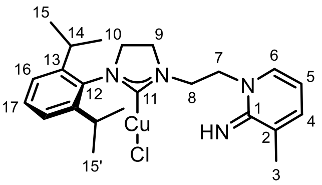

7

$C_{23}H_{32}ClCuN_4$

Mw = 464.54 gmol^{-1}

Prepared according to **GP6** 1-(2,6-diisopropylphenyl)-3-(2-(2-imino-3-methylpyridin-1(2*H*)-yl)ethyl)-4,5-dihydro-1*H*-imidazol-3-ium bromide hydrobromide (**L7**, 132 mg, 0.250 mmol, 1.00 equiv), copper(I) chloride (24.6 mg, 0.250 mmol, 1.00 equiv) and NaO*t*Bu (60.0 mg, 0.630 mmol, 2.50 equiv) in THF (5 mL). The filtrate was concentrated under oil pump vacuum (2 × 10^{-2} mbar) yielding **7** as yellow solid (76.0 mg, 0.170 mmol, 66%).

^{1}H NMR (600 MHz, CD_2Cl_2) δ = 7.38 (t, $^3J_{17,16}$ = 7.8 Hz, 1H, H-17), 7.21 (d, $^3J_{16,17}$ = 7.8 Hz, 2H, H-16), 7.09 (d, $^3J_{6,5}$ = 6.9 Hz, 1H, H-6), 6.95 (d, $^3J_{4,5}$ = 6.6 Hz, 1H, H-4), 5.97 (br s, 1H, H-5), 5.70 (s, 1H, N–*H*), 4.58 (br s, 2H, H-8), 4.20 (br s, 2H, H-7), 3.95 (t, $^3J_{9,10}$ = 9.6 Hz, 2H, H-9)*, 3.77 (t,

$^3J_{10,9}$ = 9.6 Hz, 2H, H-10), 2.82 (p, $^3J_{14,15}$ = 6.9 Hz, 2H, H-14), 2.08 (s, 3H, H-3), 1.21 (d, $^3J_{15,14}$ = 6.9 Hz, 12H, H-15, H-15') ppm.

^{13}C NMR (151 MHz, CD_2Cl_2) δ = 202.3 (C-11), 160.6 (C-1), 147.3 (C-13), 136.2 (C-4), 135.0 (C-12), 134.1 (C-6), 129.9 (C-17), 127.5 (C-2), 124.8 (C-16), 105.3 (C-5), 54.4 (C-8), 50.2 (C-9), 50.1 (C-10), 47.6 (C-7), 28.6 (C-14), 25.4 (C-15), 24.4 (C-15'), 18.7 (C-3) ppm.

C-1 is assigned from ^{1}H, ^{13}C HMBC NMR.

HRMS (APCI) for. $C_{23}H_{32}CuN_4^+$ [(M–Cl$^-$)$^+$] calculated: 427.1917, found: 427.1915.

7.6 Synthesis of 4-carbon linker complex IMesIPy*CuCl (8)

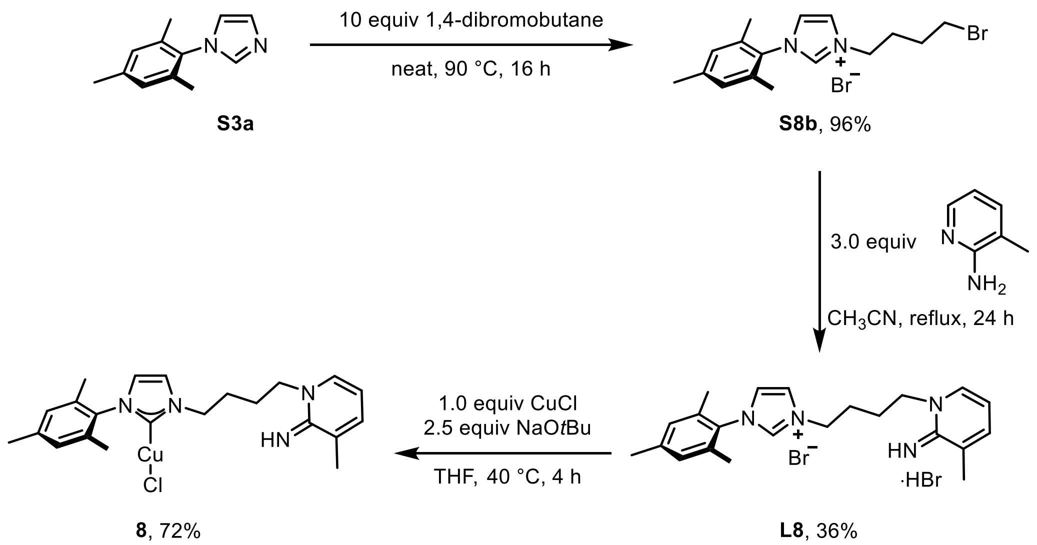

7.6.1 3-(4-bromobutyl)-1-mesityl-1*H*-imidazol-3-ium bromide (S8b)

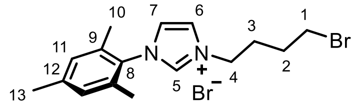

S8b
$C_{16}H_{22}Br_2N_2$
Mw = 402.17 gmol^{-1}

According to a literature procedure,[21] a 50 mL round bottom flask equipped with a magnetic stir bar was charged with 1-mesityl-1*H*-imidazole (**S3a**, 1.20 g, 6.40 mmol, 1.00 equiv) and 1,4-dibromobutane (14.0 g, 64.0 mmol, 10.0 equiv). The mixture was stirred at 90 °C for 16 h. After allowing the reaction mixture to cool to room temperature, the solid was filtered off with glass frit (P4). The filtrate was then dried under reduced pressure to afford **S8b** as brown solid (2.50 g, 6.30 mmol, 96% yield).

^{1}H NMR (600 MHz, $CDCl_3$) δ = 10.51 (s, 1H, H-5), 7.75 (s, 1H, H-6), 7.16 (s, 1H, H-7), 7.01 (s, 2H, H-11), 4.86 (t, $^3J_{4,3}$ = 7.3 Hz, 2H, H-4), 3.50 (t, $^3J_{1,2}$ = 6.2 Hz, 2H, H-1), 2.34 (s, 3H, H-13), 2.21 (p, $^3J_{3,2/4}$ = 7.4 Hz, 2H, H-3), 2.08 (s, 6H, H-10), 2.04–2.00 (m, 2H, H-2) ppm.

The ^{1}H NMR spectrum shows traces Et_2O (resonances at 3.50 and 1.96 ppm).

^{13}C NMR (151 MHz, $CDCl_3$) δ = 141.6 (C-12), 138.5 (C-5), 134.3 (C-9), 130.7 (C-8), 130.1 (C-11), 123.3 (C-6), 122.6 (C-7), 49.5 (C-4), 33.1 (C-1), 29.3 (C-3), 29.1 (C-2), 21.2 (C-13), 17.8 (C-10) ppm.

HRMS (APCI) for $C_{16}H_{22}{}^{79}BrN_2^+$ [(M–Br$^-$)$^+$] calculated: 321.0961, found: 321.0963.

The data is in accordance with literature.[21]

7.6.2 3-(4-(2-Imino-3-methylpyridin-1(2*H*)-yl)butyl)-1-mesityl-1*H*-imidazol-3-ium bromide (L8)

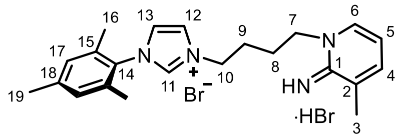

L8
$C_{22}H_{30}Br_2N_4$
Mw = 510.32 gmol^{-1}

Prepared according to **GP5** from 3-(4-bromobutyl)-1-mesityl-1*H*-imidazol-3-ium bromide (**S8b**, 2.50 g, 6.30 mmol, 1.00 equiv), 3-methyl-2-aminopyridine (2.00 g, 19.0 mmol, 3.00 equiv) in CH_3CN (12 mL). Purification by washing the resulting solid with acetone (3 × 30 mL) yielded **L8** as colorless solid (1.20 g, 2.30 mmol, 36%).

Mp = 180 °C (acetone, decomposition).

^{1}H NMR (600 MHz, DMSO-d_6) δ = 9.51 (s, 1H, H-11), 8.14 (br s, 2H, N–H_2), 8.12 (app t, J = 1.8 Hz, 1H, H-13), 8.05 (d, $^3J_{4.5}$ = 6.7 Hz, 1H, H-4), 7.96 (app t, J = 1.8 Hz, 1H, H-12), 7.79 (d, $^3J_{6,5}$ = 6.3 Hz, 1H, H-6), 7.15 (s, 2H, H-17), 6.90 (t, $^3J_{5,4/6}$ = 6.9 Hz, 1H, H-5), 4.38 (t, $^3J_{10,9}$ = 6.7 Hz, 2H, H-10), 4.32 (t, $^3J_{7,8}$ = 7.6 Hz, 2H, H-7), 2.33 (s, 3H, H-19), 2.23 (s, 3H, H-3), 2.01 (s, 6H, H-16), 2.00–1.94 (m, 2H, H-9), 1.6–1.63 (m, 2H, H-8) ppm.

^{13}C NMR (151 MHz, DMSO-d_6) δ = 152.6 (C-1), 141.2 (C-6), 140.3 (C-18), 138.0 (C-4), 137.3 (C-11), 134.3 (C-15), 131.1 (C-14), 129.2 (C-17), 124.0 (C-12), 123.5 (C-2), 123.3 (C-13), 112.7 (C-5), 52.8 (C-10), 48.8 (C-7), 25.7 (C-9), 24.2 (C-8), 20.6 (C-19), 17.4 (C-3), 16.9 (C-16) ppm.

HRMS (APCI) for $C_{22}H_{29}N_4^+$ [(M–2Br$^-$–H$^+$)$^+$] calculated: 349.2387, found: 349.2389.

IR (ATR): ṽ = 3485 (w), 3313 (w), 3123 (w), 3026 (br), 2940 (w), 2862 (w), 2750 (w), 1647 (s), 1513 (m), 1200 (w), 1162 (w), 1110 (w), 1066 (m), 857 (m), 797 (w) cm^{-1}.

7.6.3 (1-(4-(2-Imino-3-methylpyridin-1(2H)-yl)butyl)-3-mesityl-1,3-dihydro-2*H*-imidazol-2-ylidene)copper(I) chloride (8)

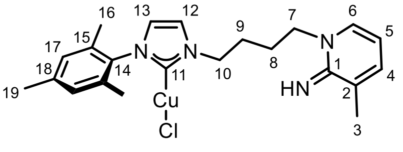

8
$C_{22}H_{28}ClCuN_4$
Mw = 447.49 gmol^{-1}

Prepared according to **GP6** 3-(4-(2-imino-3-methylpyridin-1(2*H*)-yl)butyl)-1-mesityl-1*H*-imidazol-3-ium bromide (**L8**, 127 mg, 0.250 mmol, 1.00 equiv), copper(I) chloride (24.6 mg, 0.250 mmol, 1.00 equiv) and NaO*t*Bu (60.0 mg, 0.630 mmol, 2.50 equiv) in THF (5 mL). The filtrate was concentrated under oil pump vacuum (2 × 10^{-2} mbar) yielding **8** as yellow solid (82.0 mg, 0.180 mmol, 72%).

^{1}H NMR (600 MHz, CD_2Cl_2) δ = 7.23 (s, 1H, H-12), 7.07 (br s, 1H, H-4), 7.00 (s, 2H, H-17), 6.95 (d, $^{3}J_{6,5}$ = 6.6 Hz, 1H, H-6), 6.88 (s, 1H, H-13), 5.97 (br s, 1H, H-6), 5.32 (br s, 1H, N–*H*), 4.38 (s, 4H, H-10, H-7), 2.35 (s, 3H, H-19), 2.05–2.01 (m, 11H, H-3, H-9, H-16), 1.95–1.94 (m, 2H, H-8) ppm.

^{13}C NMR (151 MHz, CD_2Cl_2) δ = 201.1 (C-11), 159.6 (C-1), 146.6 (C-18), 139.5 (C-14), 136.3 (C-4), 136.2 (C-2), 135.6 (C-15), 134.2 (C-6), 129.5 (C-17), 122.3 (C-13), 121.0 (C-12), 105.6 (C-5), 51.2 (C-10), 49.9 (C-7), 28.5 (C-9), 24.9 (C-8), 21.2 (C-19), 18.6 (C-16), 17.9 (C-3) ppm.

C-1, C-11 and C-18 are assigned from ^{1}H, ^{13}C HMBC NMR.

HRMS (APCI) for. $C_{22}H_{28}CuN_4^+$ [$(M–Cl^-)^+$] calculated: 411.1604, found: 411.1605.

7.7 Synthesis of IPrIPOCuCl (9)

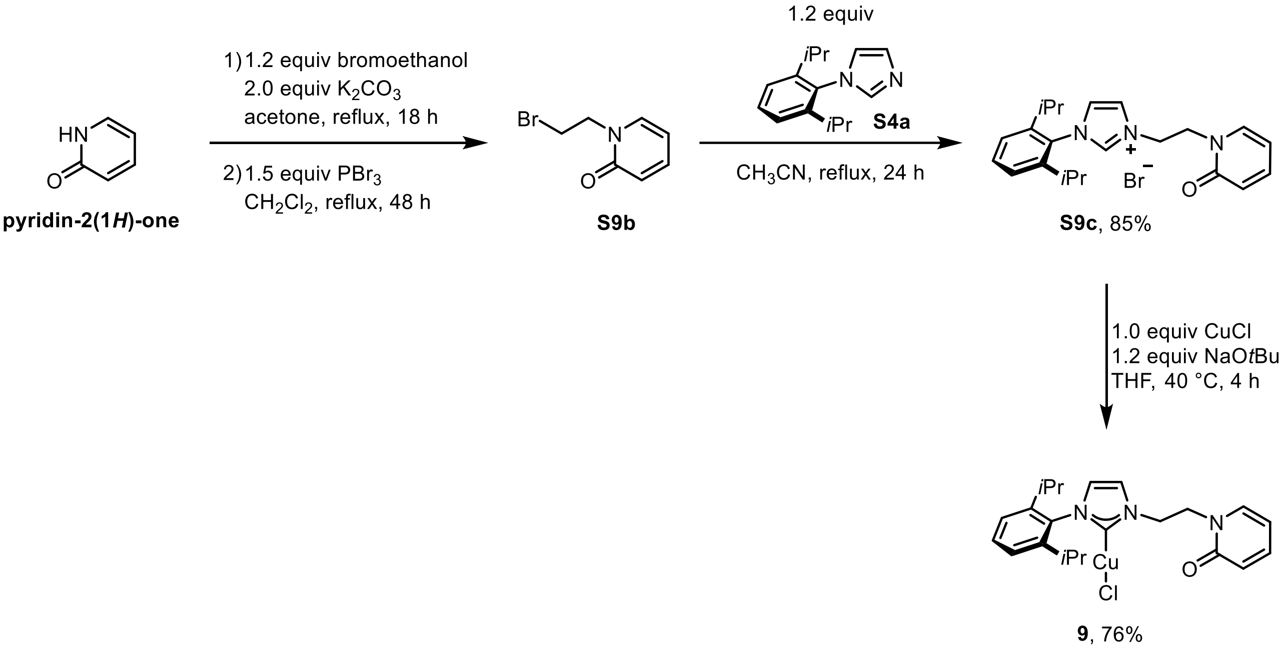

7.7.1 1-(2-Bromoethyl)pyridin-2(1*H*)-one (S9b)

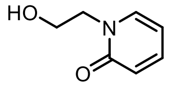

A 100 mL round bottom flask fitted with reflux condenser was charged with pyridin-2(1*H*)-one (1.90 g, 20.0 mmol, 1.00 equiv), bromoethanol (1.70 mL, 24.0 mmol, 1.20 equiv), K_2CO_3 (5.50 g, 40.0 mmol, 2.00 equiv) and acetone (40 mL). The mixture was refluxed for 18 h. After allowing the reaction mixture to cool to room temperature, all solid was filtered off over a glass frit (P4). The filtrate was concentrated under reduced pressure to afford the corresponding alcohol, which was used directly for the next step.

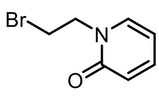

S9b
C_7H_8BrNO
Mw = 202.05 gmol^{-1}

A 100 mL round bottom flask fitted with reflux condenser was charged with the crude alcohol (2.10 g, 20.0 mmol, 1.00 equiv) and CH_2Cl_2 (100 mL). PBr_3 (2.90 mL, 30.0 mmol, 1.50 equiv) was added dropwise over 10 min at 0 °C. The mixture was warmed to room temperature and then refluxed for 48 h After

allowing the reaction mixture to cool to room temperature, water (20 mL) was added slowly over 10 min. The layers were separated and the organic phase was washed with brine (3 × 10 mL). The organic layer was dried over $MgSO_4$. All volatiles were removed under reduced pressure to afford **S9b** as yellow solid.

Note: The precise yield could not be calculated as the prepared compound **S9b** contained some unidentified impurities. The crude product mixture **S9b** was used for the next step directly without further purification.

7.7.2 1-(2,6-Diisopropylphenyl)-3-(2-(2-oxopyridin-1(2*H*)-yl)ethyl)-1*H*-imidazol-3-ium bromide (S9c)

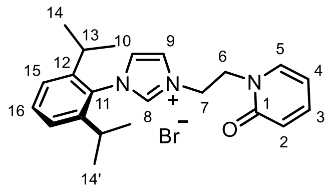

S9c
$C_{22}H_{28}BrN_3O$
Mw = 430.39 gmol^{-1}

In deference to a literature procedure,[5] a 100 mL round bottom flask equipped with a magnetic stir bar was charged with 1-(2,6-diisopropylphenyl)-1*H*-imidazole (**S4a**, 2.70 g, 12.0 mmol, 1.20 equiv), 1-(2-bromoethyl)pyridin-2(1*H*)-one (**S9b**, 2.00 g, 10.0 mmol, 1.00 equiv) and CH_3CN (20 mL). The mixture was refluxed for 24 h and then allowed to cool to room temperature. The resulting solid was filtered off with glass frit (P4) and washed with acetone (20 mL). The solid was dried under reduced pressure to afford **S9c** as colorless solid (3.60 g, 8.50 mmol, 85% yield).

Mp = 280 °C (acetone).

^{1}H NMR (600 MHz, $CDCl_3$) δ = 10.34 (app t, *J* = 1.6 Hz, 1H, H-8), 8.02 (dd, $^3J_{5,4}$ = 6.8 Hz, $^4J_{5,3}$ = 2.0 Hz, 1H, H-4), 8.00 (app t, *J* = 1.7 Hz, 1H, H-9), 7.54 (t, $^3J_{16,15}$ = 7.9 Hz, 1H, H-16), 7.37 (ddd, $^3J_{3,2}$ = 9.0 Hz, $^3J_{3,4}$ = 6.6 Hz, $^4J_{3,5}$ = 2.1 Hz, 1H, H-3), 7.31 (d, $^3J_{15,16}$ = 7.9 Hz, 2H, H-15), 7.09 (app t, *J* = 1.8 Hz, 1H, H-10), 6.50 (d, $^3J_{2,3}$ = 9.0 Hz 1H, H-2), 6.22 (td, $^3J_{4,5/3}$ = 6.7 Hz, $^4J_{4,2}$ = 1.4 Hz, 1H, H-4), 5.20 (t, $^3J_{7,6}$ = 6.7 Hz, 2H, H-7), 4.86 (t, $^3J_{6,7}$ = 6.7 Hz, 2H, H-6), 2.16 (sept, $^3J_{13,14}$ = 6.8 Hz, 2H, H-13), 1.23 (d, $^3J_{14,13}$ = 6.8 Hz, 6H, H-14), 1.12 (d, $^3J_{14',13}$ = 6.8 Hz, 6H, H-14') ppm.

The ^{1}H NMR shows traces of acetone at 2.16 ppm.

^{13}C NMR (151 MHz, $CDCl_3$) δ = 163.0 (C-1), 145.4 (C-12), 140.8 (C-3), 139.4 (C-4), 138.6 (C-8), 132.3 (C-16), 130.0 (C-11), 124.9 (C-15), 124.1 (C-9), 124.0 (C-10), 120.4 (C-2), 106.9 (C-4), 48.9 (C-7), 47.9 (C-6), 31.1 (C-13), 24.5 (C-14), 24.4 (C-14') ppm.

The ^{13}C NMR shows traces of acetone at 207.6 and 28.9 ppm.

HRMS (APCI) for $C_{22}H_{38}N_3O^+$ [(M–Br$^-$)$^+$] calculated: 350.2227, found: 350.2230.

IR (ATR): ṽ = 3019 (br), 2967 (m), 2870 (w), 1658 (s), 1584 (m), 1539 (m), 1461 (m), 1386 (w), 1256 (w), 1189 (w), 1148 (w), 1058 (w), 957 (w), 846 (w), 805 (m), 730 (m), 697 (w) cm^{-1}.

7.7.3 (1-(2,6-Diisopropylphenyl)-3-(2-(2-oxopyridin-1(2*H*)-yl)ethyl)-1,3-dihydro-2*H*-imidazol-2-ylidene)copper(I) chloride (9)

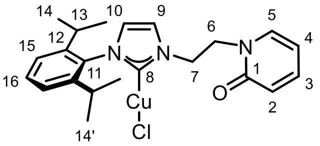

9
$C_{22}H_{27}ClCuN_3O$
MW = 448.47 gmol^{-1}

Prepared according to **GP6** from 1-(2,6-diisopropylphenyl)-3-(2-(2-oxopyridin-1(2*H*)-yl)ethyl)-1*H*-imidazol-3-ium bromide (**S9c**, 133 mg, 0.310 mmol, 1.00 equiv), copper(I) chloride (30.0 mg, 0.310 mmol, 1.00 equiv) and NaO*t*Bu (35.7 mg, 0.372 mmol, 1.20 equiv) in THF (6 mL). The filtrate was concentrated under oil pump vacuum (2×10^{-2} mbar) yielding **9** as brown solid (120 mg, 0.23 mmol, 76%).

^{1}H NMR (600 MHz, CD_2Cl_2) δ = 7.49 (t, $^3J_{16,15}$ = 7.9 Hz, 1H, H-16), 7.34 (ddd, $^3J_{3,2}$ = 9.0 Hz, $^3J_{3,4}$ = 6.6 Hz, $^4J_{3,5}$ = 2.1 Hz, 1H, H-3), 7.29 (d, $^3J_{15,16}$ = 7.9 Hz, 2H, H-15), 7.12 (br s, 1H, H-5), 7.02 (s, 1H, H-10), 6.87 (s, 1H, H-9), 6.49 (d, $^3J_{2,3}$ = 9.0 Hz, 1H, H-2), 6.08 (td, $^3J_{4,5/3}$ = 6.7 Hz, $^4J_{4,2}$ = 1.4 Hz, 1H, H-4), 4.63 (br s, 2H, H-7), 4.44 (t, $^3J_{6,7}$ = 6.0 Hz, 2H, H-6), 2.32 (sept, $^3J_{13,14}$ = 6.8 Hz, 2H, H-13), 1.23 ($^3J_{14,13}$ = 6.8 Hz, 6H, H-14), 1.10 ($^3J_{14',13}$ = 6.8 Hz, 6H, H-14') ppm.

^{13}C NMR (151 MHz, CD_2Cl_2) δ = 179.0 (C-8), 162.7 (C-1), 146.1 (C-12), 140.4 (C-3), 135.0 (C-11), 130.7 (C-16), 125.6 (C-5), 124.5 (C-15), 123.9 (C-9), 121.9 (C-10), 121.3 (C-2), 106.3 (C-4), 51.4 (C-7), 49.2 (C-6), 28.6 (C-13), 24.7 (C-14), 24.4 (C-14') ppm.

C-8 is assigned from ^{1}H,^{13}C HMBC NMR.

HRMS (APCI) for. $C_{22}H_{27}CuN_3O^+$ [(M–Cl$^-$)$^+$] calculated: 412.1445, found: 412.1440.

7.8 Synthesis of ligand precursor IPr^{4}IPyHBr (10)

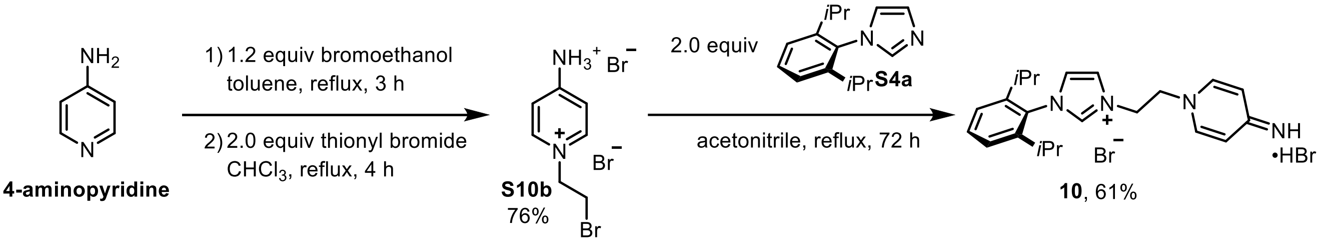

7.8.1 4-Amino-1-(2-bromoethyl)pyridin-1-ium bromide (S10b)

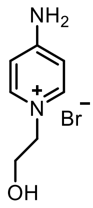

A 100 mL round bottom flask equipped with a magnetic stir bar was charged with 4-aminopyridine (2.00 g, 21.0 mmol, 1.00 equiv), bromoethanol (3.20 mL, 25.0 mmol, 1.20 equiv) and toluene (60 mL). The mixture was refluxed for 3 h and then allowed to cool to room temperature. The resulting solid was filtered off with glass frit (P4) and washed with Et_2O (20 mL). The solid was dried under reduced pressure to afford the corresponding alcohol, which was used for the next step without further purification.

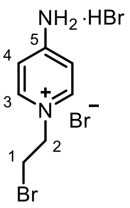

A 100 mL round bottom flask equipped with a magnetic stir bar was charged with the crude alcohol (4.60 g, 21.0 mmol, 1.00 equiv) and chloroform (40 mL) and thionyl bromide (3.30 mL, 42.0 mmol, 2.00 equiv) was added dropwise at 0 °C over 10 min. The reaction mixture was allowed slowly to warm to room temperature. The mixture was refluxed for 4 h and then allowed to cool to room temperature. The resulting solid was filtered off with glass frit (P4) and washed with acetone (20 mL). The solid was dried under reduced pressure to afford **S10b** as brown solid (4.60 g, 16.0 mmol, 75% yield).

Mp = 187 °C (acetone)

^{1}H NMR (600 MHz, DMSO-*d*$_6$) δ = 8.23 (s, 2H, N–*H*$_2$), 8.21 (d, $^3J_{3,4}$ = 7.4 Hz, 2H, H-3), 6.86 (d, $^3J_{4,3}$ = 7.4 Hz, 2H, H-4), 4.55 (t, $^3J_{2,1}$ = 5.9 Hz, 2H, H-2), 3.92 (t, $^3J_{1,2}$ = 5.9 Hz, 2H, H-1), 3.45 (s, 1H, N–*H*) ppm.

^{13}C NMR (151 MHz, DMSO-*d*$_6$) δ = 158.9 (C-5), 143.1 (C-3), 109.1 (C-4), 57.4 (C-2), 32.3 (C-1) ppm.

HRMS (APCI) for $C_7H_{10}{}^{79}BrN_2^+$ [(M–Br$^-$)$^+$] calculated: 201.0022, found: 201.0022.

IR (ATR): ṽ = 3287 (br), 3134 (br), 3052 (w), 2993 (m), 2944 (m), 2851 (w), 2750 (w), 2687 (w), 1666 (s), 1546 (m), 1196 (m), 1170 (m), 816 (m) cm^{-1}.

7.8.2 1-(2,6-Diisopropylphenyl)-3-(2-(4-iminopyridin-1(4*H*)-yl)ethyl)-1*H*-imidazol-3-ium bromide hydrobromide (10)

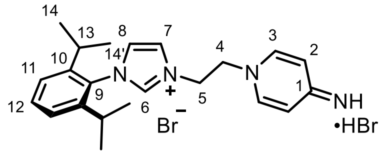

In deference of a literature procedure,[5] a 100 mL round bottom flask equipped with a magnetic stir bar was charged with 4-amino-1-(2-bromoethyl)pyridin-1-ium bromide (**S10b**, 2.00 g, 7.10 mmol, 1.00 equiv), 1-(2,6-diisopropylphenyl)-1*H*-imidazole (**S4a**, 3.15 g, 14.0 mmol, 2.00 equiv) and CH_3CN (20 mL). The mixture was refluxed for 72 h and then allowed to cool to room temperature. The resulting solid was filtered off with glass frit (P4) and washed with acetone (20 mL). The solid was dried under reduced pressure to afford **10** as yellow solid (2.20 g, 4.33 mmol, 61% yield).

Mp = >300 °C (acetone, decomposition).

^{1}H NMR (600 MHz, DMSO-*d*$_6$) δ = 9.55 (s, 1H, H-6), 8.35 (s, 2H, N–*H*$_2$), 8.30 (s, 1H, H-7), 8.19–8.16 (m, 3H, H-8, H-3), 7.61 (t, $^3J_{12,11}$ = 7.8 Hz, 1H, H-12), 7.43 (d, $^3J_{11,12}$ = 7.8 Hz, 2H, H-11), 6.84 (d, $^3J_{2,3}$ = 7.4 Hz, 2H, H-2), 4.88–4.84 (m, 4H, H-4, H-5), 2.03 (p, $^3J_{13,14/14'}$ = 6.9 Hz, 2H, H-13), 1.10 (d, $^3J_{14,13}$ = 6.9 Hz, 6H, H-14), 1.08 (d, $^3J_{14',13}$ = 6.9 Hz, 6H, H-14’) ppm.

The ^{1}H NMR shows impurity of acetone at 2.08 ppm.

^{13}C NMR (151 MHz, DMSO-d_6) δ = 159.0 (C-1), 145.0 (C-9), 142.7 (C-3), 138.1 (C-6), 131.6 (C-12), 130.1 (C-10), 125.8 (C-8), 124.5 (C-11), 123.3 (C-7), 109.7 (C-2), 55.3 (C-5), 49.4 (C-4), 28.1 (C-13), 23.9 (C-14), 23.7 (C-14') ppm.

The ^{13}C NMR shows impurity of acetone at 206.6 and 30.7 ppm.

HRMS (ESI) for $C_{22}H_{29}N_4^+$ [(M–2Br$^-$–H$^+$)$^+$] calculated: 349.2387, found: 349.2385.

IR (ATR): ṽ = 3291 (br), 3153 (br), 3019 (w), 2967 (w), 1654 (m), 1539 (w), 1461 (w), 1356 (w), 1185 (w), 1118 (w), 846 (w), 767 (w), 719 (w) cm^{-1}.

7.9 Synthesis of IMesEtCuCl (15)

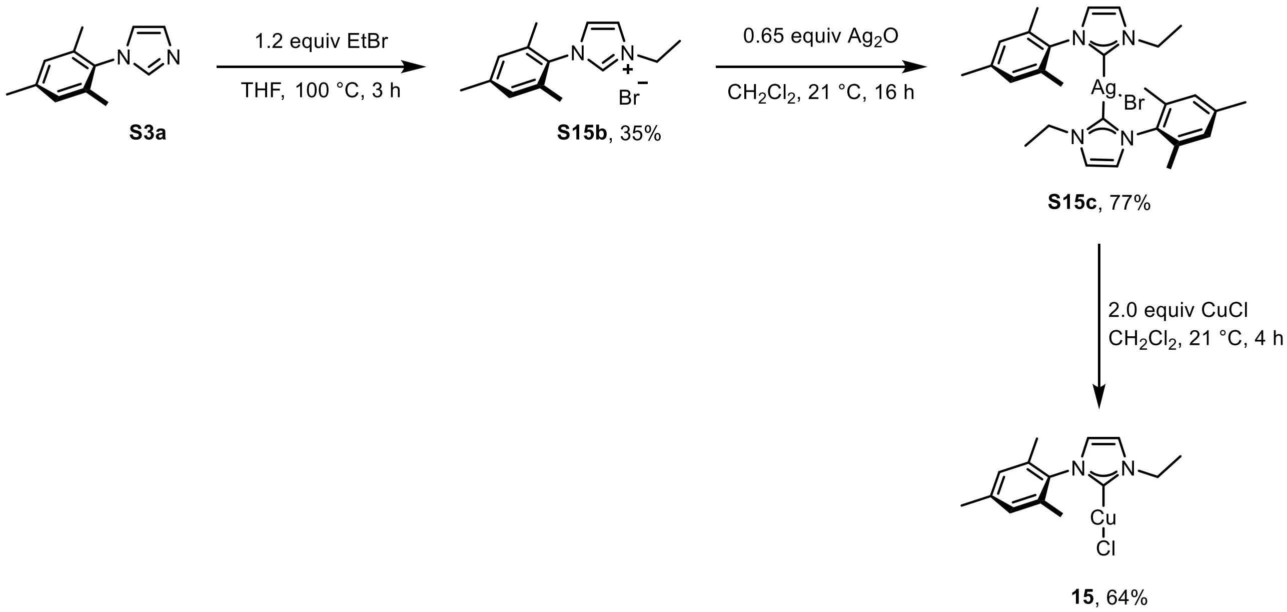

7.9.1 3-Ethyl-1-mesityl-1*H*-imidazol-3-ium bromide (S15b)

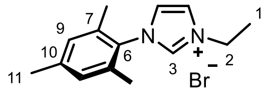

S15b
$C_{14}H_{19}BrN_2$
Mw = 295.22 gmol^{-1}

Following literature procedure,[22] In an ACE pressure tube, 1-mesityl-1*H*-imidazole (**S3a**, 1.00 g, 9.18 mmol, 1.00 equiv) was dissolved in THF (20 mL). Ethyl bromide (1.00 mL, 18.4 mmol, 1.20 equiv) was added. The pressure vessel was capped tightly and the mixture was stirred at 100 °C for 3 h. The reaction mixture was allowed to cool to room temperature. The resulting precipitate was filtered over a glass frit (P4), washed with THF (3 × 5 mL) and dried under reduced pressure to afford **S15b** as brown solid (0.960 g, 3.25 mmol, 35%).

^{1}H NMR (600 MHz, DMSO-d_6) δ = 9.41 (s, 1H, H-3), 8.10 (app t, J = 1.8 Hz, 1H, H-4), 7.92 (app t, J = 1.8 Hz, 1H, H-5), 7.15 (s, 2H, H-9), 4.31 (q, $^3J_{2,1}$ = 7.3 Hz, 2H, H-2), 2.33 (s, 3H, H-11), 2.02 (s, 6H, H-8), 1.51 (t, $^3J_{1,2}$ = 7.2 Hz, 3H, H-1) ppm.

^{13}**C NMR** (151 MHz, DMSO-d_6) δ = 140.2 (C-10), 136.9 (C-3), 134.3 (C-7), 131.2 (C-6), 129.2 (C-9), 123.8 (C-5), 122.8 (C-4), 44.7 (C-2), 20.6 (C-11), 16.9 (C-8), 14.9 (C-1) ppm.

HRMS (APCI) for $C_{14}H_{19}N_2^+$ [(M–Br$^-$)$^+$] calculated: 215.1543, found: 215.1549.

The data is in accordance with literature.[22]

7.9.2 Bis(1-ethyl-3-mesityl-1,3-dihydro-2*H*-imidazol-2-ylidene)silver(I) bromide (S15c)

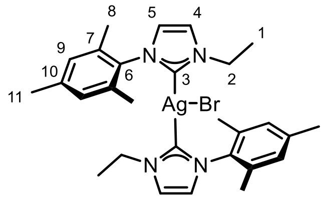

S15c
$C_{28}H_{36}AgBrN_4$
Mw = 618.41 gmol^{-1}

In deference to a literature procedure,[5] a 100 mL-Schlenk flask equipped with magnetic stir bar was charged with 3-ethyl-1-mesityl-1*H*-imidazol-3-ium bromide (**S15b**, 0.200 g, 0.678 mmol, 1.00 equiv), Ag_2O (0.102 g, 0.440 mmol, 0.650 equiv) and CH_2Cl_2 (5 mL). The reaction mixture was stirred at room temperature for 16 h. The reaction mixture was filtered over a Schlenk frit (P4) under N_2-atmosphere. The filtrate was concentrated under vacuum using oil pump vacuum (2 x 10^{-2} mbar). The product **S15c** was obtained as brown solid (0.209 g, 0.520 mmol, 77%).

^{1}**H NMR** (600 MHz, $CDCl_3$) δ = 7.26 (s, 2H, H-4), 6.94 (s, 4H, H-9), 6.92 (d, $^3J_{5,4}$ = 1.8 Hz, 2H, H-5), 4.23 (br s, 4H, H-2), 2.33 (s, 6H, H-11), 1.93 (s, 12H, H-8), 1.49 (t, $^3J_{1,2}$ = 7.4 Hz, 6H, H-1) ppm.

^{13}**C NMR** (151 MHz, $CDCl_3$) δ = 167.5 (C-3), 139.6 (C-10), 135.6 (C-7), 134.8 (C-6), 129.5 (C-9), 122.8 (C-4), 120.6 (C-5), 47.1 (C-2), 21.2 (C-11), 17.8 (C-8), 17.2 (C-1) ppm.

C-3 is assigned from ^{1}H, ^{13}C HMBC NMR.

HRMS (APCI) for $C_{28}H_{36}AgN_4^+$ [(M–Br$^-$)$^+$] calculated: 535.1985, found: 535.1988.

7.9.3 1-Ethyl-3-mesityl-1,3-dihydro-2*H*-imidazol-2-ylidene copper(I) chloride (15)

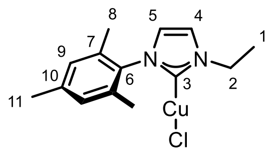

15
$C_{14}H_{18}ClCuN_2$
Mw = 314.32 gmol^{-1}

In deference to a literature procedure,[5] in an Ar glovebox a 5 mL vial, CuCl (40.0 mg, 0.400 mmol, 2.00 equiv) and bis(1-ethyl-3-mesityl-1,3-dihydro-2*H*-imidazol-2-ylidene)silver(I) bromide (**S15c**, 123 mg, 0.200 mmol, 1.00 equiv) were dissolved in CH_2Cl_2 (3 mL). The vial was closed with a septum and wrapped in aluminium foil. The reaction mixture was stirred at room temperature for 4 h. The reaction mixture was filtered over a PTFE syringe filter (0.45 µm). The filtrate was concentrated under vacuum using oil pump vacuum (2 x 10^{-2} mbar). The product **15** was obtained as colorless solid (40.2 mg, 0.130 mmol, 64%).

^{1}**H NMR** (600 MHz, $CDCl_3$) δ = 7.10 (s, 1H, H-4), 6.94 (s, 2H, H-9), 6.85 (s, 1H, H-5), 4.30 (q, $^3J_{2,1}$ = 7.4 Hz, 2H, H-2), 2.32 (s, 3H, H-11), 1.99 (s, 6H, H-8), 1.55 (t, $^3J_{1,2}$ = 7.5 Hz, 3H, H-1) ppm.

^{13}C NMR (151 MHz, $CDCl_3$) δ = 177.5 (C-3), 139.5 (C-10), 135.4 (C-7), 134.8 (C-6), 129.5 (C-9), 122.4 (C-5), 120.0 (C-4), 46.7 (C-2), 21.2 (C-11), 17.9 (C-8), 17.3 (C-1) ppm.

HRMS (APCI) for $C_{14}H_{19}N_2^+$ [(M–CuCl$^-$)$^+$]calculated: 215.1543, found: 215.1548.

7.10 Attempted synthesis of *N*-methyl derived 2-iminopyridine-based copper(I)/NHC complex 16

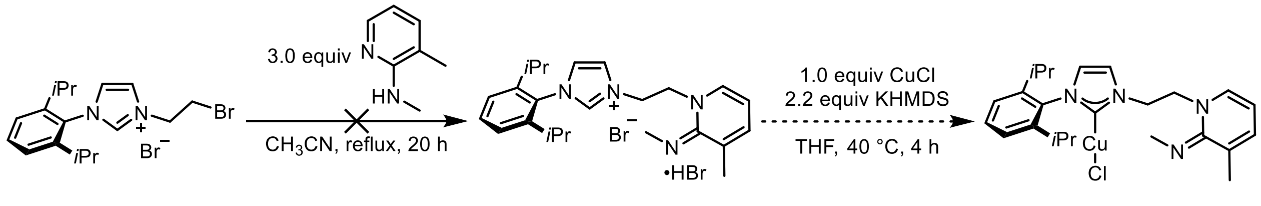

S4b **16a** **16**

7.10.1 1-(2,6-Di*iso*propylphenyl)-3-(2-(3-methyl-2-(methylimino)pyridin-1(2*H*)-yl)ethyl)-1*H*-imidazol-3-ium bromide hydrobromide (15a)

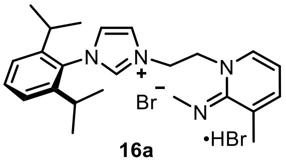

16a

Prepared according to **GP5** from 3-(2-bromoethyl)-1-(2,6-diisopropylphenyl)-1*H*-imidazol-3-ium bromide (**S4b**, 0.560 g, 1.35 mmol. 1.00 equiv) and *N*,3-dimethyl-2-aminopyridine (0.49 g, 4.03 mmol, 3.00 equiv) in CH_3CN (6 mL). After refluxing the reaction mixure for 24 h, no desired product **16a** formation was observed. After the workup according to **GP5**, the elimination product **16aa** along with the protonated salt of *N*,3-dimethyl-2-aminopyridine **16ab** was isolated.

Note: Upon performing the reaction at room temperature to until 40 °C, no conversion of **S4b** was observed in ^{1}H NMR analysis.

7.10.1.1 *N*,3-Dimethylpyridin-2-amine hydrobromide (16ab)

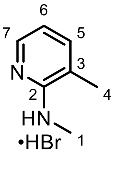

16ab
$C_7H_{11}BrN_2$
Mw = 203.08 gmol^{-1}

Prepared according to **GP5** from 3-(2-bromoethyl)-1-(2,6-diisopropylphenyl)-1*H*-imidazol-3-ium bromide (**S4b**, 0.560 g, 1.35 mmol. 1.00 equiv) and *N*,3-dimethyl-2-aminopyridine (0.49 g, 4.03 mmol, 3.00 equiv) in CH_3CN (6 mL). After 24 h, the reaction mixture was cooled to room temperature. The solid was filtered off and washed with acetone (10 mL). The soild was then dried under reduced pressure to afford **16ab** (167 mg, 0.832 mmol, 61% yield) as colorless solid.

Mp = 137 °C (acetone).

^{1}H NMR (600 MHz, DMSO-d_6) δ = 12.93 (brs, 1H, N–*H*), 8.10 (brs, 1H, N–*H*), 7.81 (d, $^3J_{7,6}$ = 6.8 Hz, 1H, H-7), 7.73 (d, $^3J_{5,6}$ = 6.8 Hz, 1H, H-5), 6.81 (t, $^3J_{6,5/7}$ = 6.8 Hz, 1H, H-6), 2.99 (d, $^3J_{1,N–H}$ = 4.7 Hz, 3H, H-1), 2.18 (s, 3H, H-4) ppm.

^{13}C NMR (151 MHz, DMSO-d_6) δ =152.7 (C-2), 140.5 (C-7), 134.0 (C-5), 122.3 (C-3), 111.7 (C-6), 29.0 (C-1), 16.6 (C-4) ppm.

7.10.1.2 1-(2,6-Di*iso*propylphenyl)-3-vinyl-1*H*-imidazol-3-ium bromide (16aa)

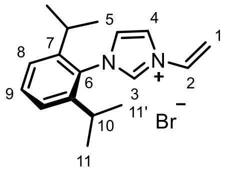

16aa
$C_{17}H_{23}BrN_2$
Mw = 335.29 gmol^{-1}

Prepared according to **GP5** from 3-(2-bromoethyl)-1-(2,6-diisopropylphenyl)-1*H*-imidazol-3-ium bromide (**S4b**, 0.560 g, 1.35 mmol. 1.00 equiv) and *N*,3-dimethyl-2-aminopyridine (0.49 g, 4.03 mmol, 3.00 equiv) in CH_3CN (6 mL). After 24 h, the reaction mixture was cooled to room temperature. The solid was filtered off and washed with acetone (10 mL). The filtrate was then concentrated under reduced pressure. The crude product was purified on silica gel by flash column chromatography (CH_2Cl_2/MeOH = 90:10) to afford **16aa** (145 mg, 0.432 mmol, 32% yield) as brown solid.

Mp = 81 °C (CH_2Cl_2).

^{1}H NMR (600 MHz, $CDCl_3$) δ = 10.85 (s, 1H, H-3), 8.58 (s, 1H, H-4), 8.18 (dd, $^3J_{2,1a}$ = 15.6 Hz, $^3J_{2,1b}$ = 8.7 Hz, 1H, H-2), 7.52 (t, $^3J_{9,8}$ = 7.9 Hz, 1H, H-9), 7.29–7.28 (m, 3H, H-5, H-8), 6.19 (dd, $^3J_{1a,2}$ = 15.6 Hz, $^2J_{1a,1b}$ = 3.0 Hz, 1H, H-1a), 5.40 (dd, $^3J_{1b,2}$ = 8.7 Hz, $^2J_{1b,1a}$ = 3.0 Hz, 1H, H-1b), 2.29 (sept, $^3J_{10,11/11'}$ = 6.8 Hz, 2H, H-10), 1.21 (d, $^3J_{11,10}$ = 6.8 Hz, 6H, H-11), 1.13 (d, $^3J_{11',10}$ = 6.8 Hz, 6H, H-11') ppm.

^{1}H NMR shows impurities of acetone at 2.10 ppm.

^{13}C NMR (151 MHz, $CDCl_3$) δ =145.3 (C-7), 137.1 (C-3), 130.0 (C-6), 128.8 (C-2), 125.0 (C-5), 124.9 (C-8), 123.0 (C-9), 119.9 (C-4), 110.1 (C-1), 28.8 (C-10), 24.5 (C-11), 24.3 (C-11') ppm.

^{13}C NMR shows impurities of acetone at 207.1 and 31.0 ppm.

7.10.2 Attempted direct methylation of 2-iminopyridine-based copper(I)/NHC complex 4 for the synthesis complex 16

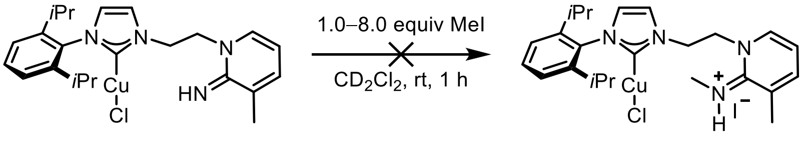

4 **16**

In an Ar-filled glovebox, [IPrIPyCuCl] (**4**, 9.30 mg, 20.0 µmol, 10.0 mol%) is placed in a 5 mL glass vial with a stirring bar. The vial is sealed with septa inside the glovebox and transferred outside. CD_2Cl_2 (1 mL) was added. Methyl iodide (16.0 mg, 160 µmol, 8.00 equiv) was added and the mixture is stirred for 1 h at room tempearture. The progress of the reaction was monitored by ^{1}H NMR analysis. After 1 h, no methylation at the iminopyridine nitrogen was observed in ^{1}H NMR.

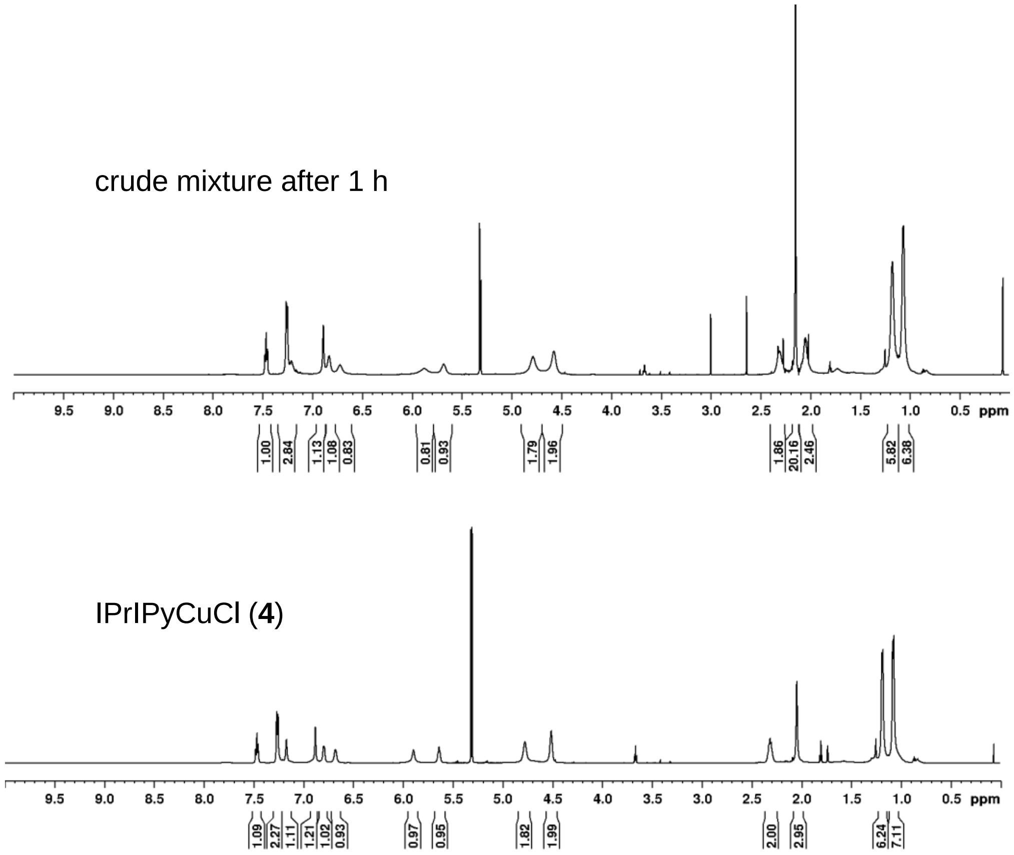

Figure S20: ^{1}H NMR (CD_2Cl_2, 600 MHz) of IPrIPyCuCl (**4**) (bottom) and crude mixture after 1 h (top).

7.11 Attempted synthesis of 4-crabon linker based copper(I)/NHC complex 17

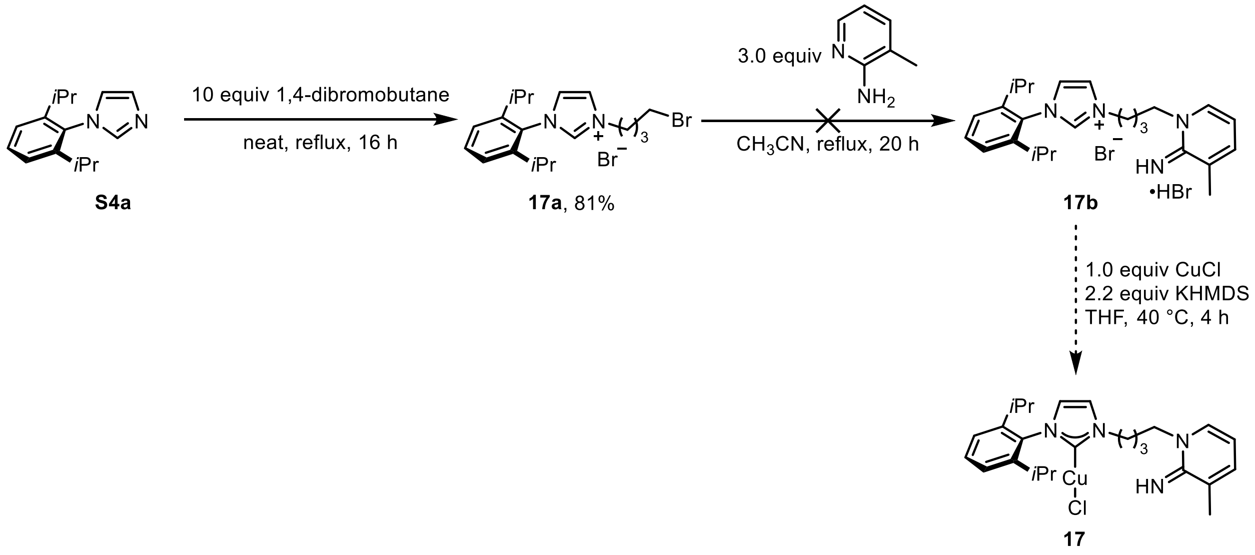

7.11.1 3-(4-Bromobutyl)-1-(2,6-di*iso*propylphenyl)-1*H*-imidazol-3-ium bromide (17a)

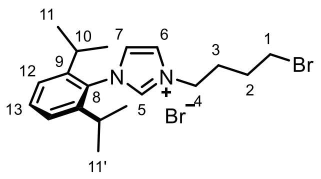

17a
$C_{19}H_{28}Br_2N_2$
Mw = 444.25 gmol^{-1}

According to a literature procedure,[21] a 50 mL round bottom flask equipped with a magnetic stir bar was charged with 1-(2,6-di*iso*propylphenyl)-1*H*-imidazole (**S4a**, 1.00 g, 4.38 mmol, 1.00 equiv) and 1,4-dibromobutane (9.45 g, 43.8 mmol, 10.0 equiv). The mixture was stirred at 90 °C for 16 h. After allowing the reaction mixture to cool to room temperature, the solid was filtered off with glass frit (P4). The filtrate was then dried under reduced pressure to afford **17a** as brown solid (1.57 g, 3.53 mmol, 81% yield).

^{1}H NMR (600 MHz, $CDCl_3$) δ = 10.58 (app t, *J* = 1.7 Hz, 1H, H-5), 7.85 (app t, *J* = 1.7 Hz, 1H, H-6), 7.54 (t, $^3J_{13,12}$ = 7.8 Hz, 1H, H-13), 7.31 (d, $^3J_{12,13}$ = 7.9 Hz, 2H, H-12), 7.18 (app t, *J* = 1.7 Hz, 1H, H-7), 4.95 (t, $^3J_{4,3}$ = 7.1 Hz, 2H, H-4), 3.51 (t, $^3J_{1,2}$ = 6.3 Hz, 2H, H-1), 2.28 (p, $^3J_{10,11/11'}$ = 6.8 Hz, 2H, H-10), 2.21 (p, $^3J_{3,2/4}$ = 7.2 Hz, 2H, H-3), 2.07–2.02 (m, 2H, H-2), 1.25 (d, $^3J_{11,10}$ = 6.8 Hz, 6H, H-11), 1.16 (d, $^3J_{11',10}$ = 6.8 Hz, 6H, H-11') ppm.

^{13}C NMR (151 MHz, $CDCl_3$) δ = 145.5 (C-13), 138.8 (C-9), 132.2 (C-5), 130.2 (C-8), 124.9 (C-12), 124.3 (C-7)*, 122.7 (C-6)*, 49.5 (C-4), 33.1 (C-1), 29.4 (C-3), 29.1 (C-2), 29.0 (C-10), 24.6 (C-11), 24.3 (C-11') ppm.

HRMS (APCI) for $C_{19}H_{29}{}^{79}BrN_2^+$ $[(M–Br^-)^+]$ calculated: 363.1430, found: 363.1437.

7.11.2 1-(2,6-Di*iso*propylphenyl)-3-(4-(2-imino-3-methylpyridin-1(2*H*)-yl)butyl)-1*H*-imidazol-3-ium bromide (17b)

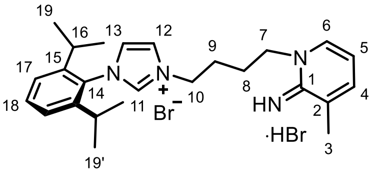

17b
$C_{25}H_{35}BrN_4$
Mw = 471.49 gmol^{-1}

Prepared according to **GP5** from 3-(4-Bromobutyl)-1-(2,6-di*iso*propylphenyl)-1*H*-imidazol-3-ium bromide (**17a**, 1.50 g, 3.37 mmol, 1.00 equiv), 3-methyl-2-aminopyridine (1.06 g, 10.1 mmol, 3.00 equiv) in CH_3CN (8 mL). After 24 h of reflux, the formation of the desired product **17b** was confirmed by crude ^{1}H NMR analysis. However, the purification of **17b** using the usual precipitation method with acetone as the solvent was unsuccessful. Several alternative solvent mixtures, including CH_2Cl_2/Et_2O, CH_2Cl_2/EtOAc and MeOH/Et_2O were tested in an attempt to isolate pure **17b** from the crude reaction mixture. Unfortunately, in all cases, unidentified impurities were obtained along with the desired product.

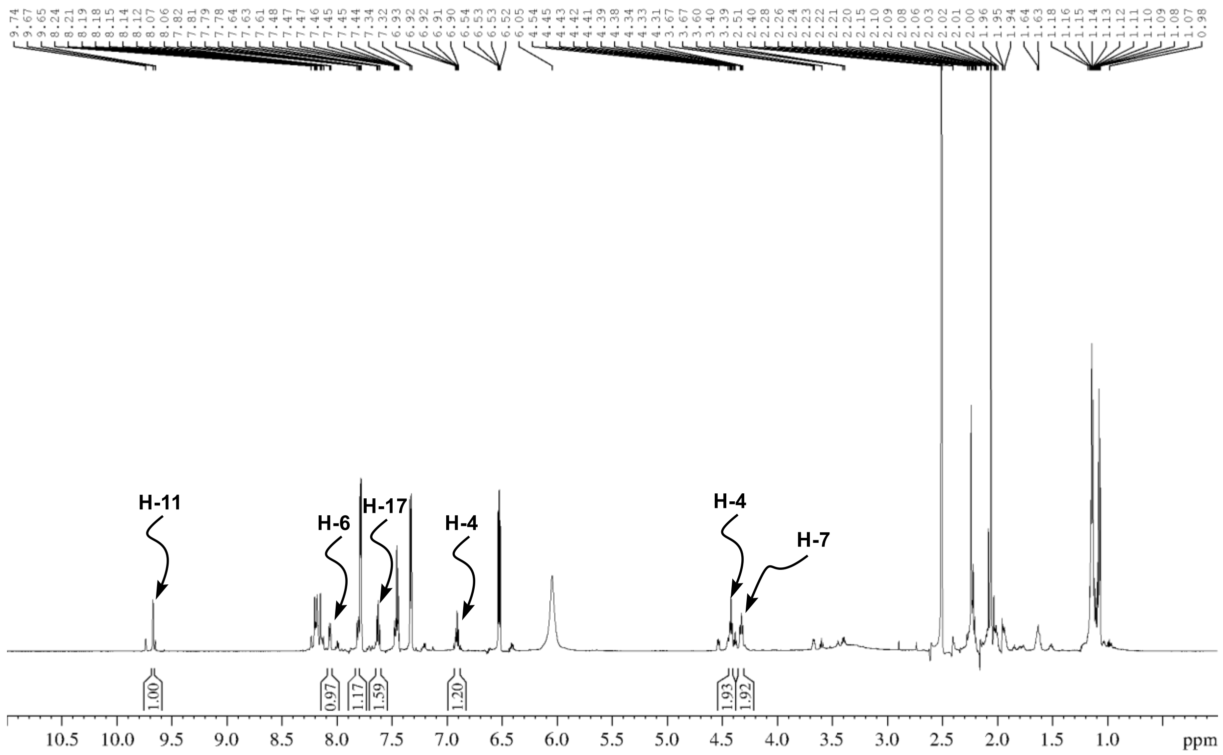

Figure S21: ^{1}H NMR (DMSO-d_6, 600 MHz) of crude reaction mixture for **17b** after washing with acetone (20 mL).

7.12 Attempted synthesis of 8-crabon linker based copper(I)/NHC complex 18

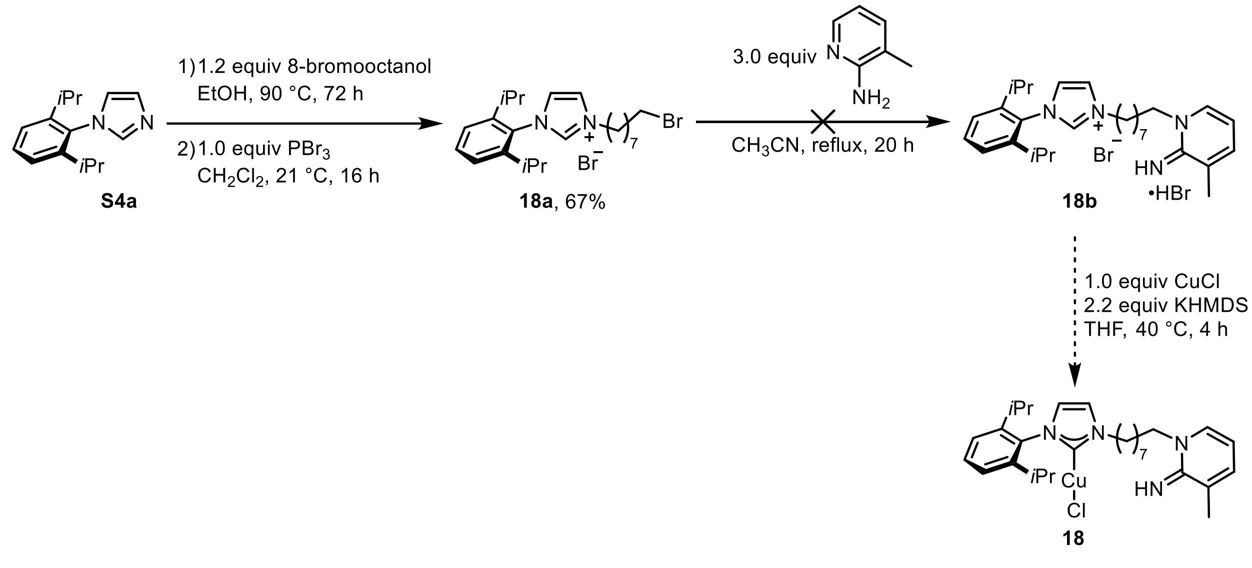

7.12.1 3-(8-Bromooctyl)-1-(2,6-di*iso*propylphenyl)-1*H*-imidazol-3-ium bromide (18a)

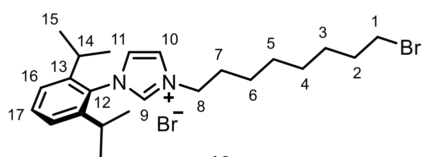

18a

$C_{23}H_{36}Br_2N_2$

Mw = 500.36 gmol^{-1}

A 50 mL round bottom flask equipped with a magnetic stir bar was charged with 1-(2,6-di*iso*propylphenyl)-1*H*-imidazole (**S4a**, 1.00 g, 4.38 mmol, 1.00 equiv), 8-bromooctanol (1.09 g, 5.25 mmol, 1.20 equiv) and ethanol (15 mL). The mixture was stirred at 90 °C for 72 h. After allowing the reaction mixture to cool to room temperature, all the volatiles are removed under reduced pressure. The resulting

crude is then washed with Et_2O (5 × 5 mL/mmol) and dried under reduced pressure to afford the corresponding alcohol, which is used for the next step without further purification.

A Schlenk flask equipped with magnetic stirring bar is charged with crude alcohol (1.92 g, 4.38 mmol, 1.00 equiv) and dichloromethane (20 mL). Phosphorus tribromide (0.416 mL, 4.38 mmol, 1.00 equiv) was added dropwise over 10 min at 0 °C. The reaction mixture was warmed to room temperature and stirred for 16 h at room temperature. The reaction was quenched by slow addition of water (10 mL) over 10 min at 0 °C, the layers are separated, and the aqueous layer was extracted with dichloromethane (2 × 10 mL). The combined organic layer was dried over Na_2SO_4. After filtration and removal of all volatiles under reduced pressure resulted the **18a** as brown oil (1.47 g, 2.94 mmol, 67% yield).

^{1}H NMR (600 MHz, $CDCl_3$) δ = 10.57 (s, 1H, H-9), 7.77 (s, 1H, H-10), 7.53 (t, $^3J_{17,16}$ = 7.8 Hz, 1H, H-17), 7.30 (t, $^3J_{16,17}$ = 7.8 Hz, 2H, H-16), 7.17 (s, 1H, H-11), 4.84 (t, $^3J_{7,8}$ = 7.1 Hz, 2H, H-8), 3.40 (t, $^3J_{1,2}$ = 6.3 Hz, 2H, H-1), 2.28 (p, $^3J_{14,15/15'}$ = 6.8 Hz, 2H, H-14), 2.04–1.98 (m, 2H, H-7), 1.85–1.81 (m, 2H, H-2), 1.43–1.36 (m, 6H, H-3, H-5, H-6), 1.33–1.30 (m, 2H, H-4), 1.24 (d, $^3J_{15,14}$ = 6.8 Hz, 6H, H-15), 1.15 (d, $^3J_{15',14}$ = 6.8 Hz, 6H, H-15') ppm.

^{13}C NMR (151 MHz, $CDCl_3$) δ = 145.5 (C-17), 138.9 (C-13), 132.1 (C-9), 130.2 (C-12), 124.7 (C-16), 124.2 (C-10)*, 122.6 (C-11)*, 50.6 (C-8), 34.1 (C-1), 32.8 (C-7), 30.7 (C-2), 29.0 (C-14), 28.9 (C-6), 28.7 (C-5), 28.0 (C-3), 26.0 (C-4), 24.6 (C-15), 24.3 (C-15') ppm.

HRMS (APCI) for $C_{23}H_{36}{}^{79}BrN_2^+$ [$(M–Br^-)^+$] calculated: 419.2062, found: 419.2056.

7.12.2 1-(2,6-Di*iso*propylphenyl)-3-(4-(2-imino-3-methylpyridin-1(2*H*)-yl)octyl)-1*H*-imidazol-3-ium bromide (18b)

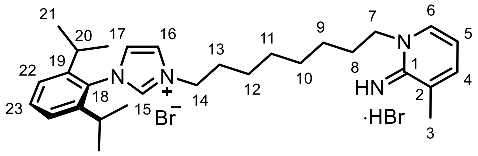

18b
$C_{29}H_{44}Br_2N_4$
Mw = 527.59 gmol^{-1}

Prepared according to **GP5** from 3-(4-Bromooctyl)-1-(2,6-di*iso*propylphenyl)-1*H*-imidazol-3-ium bromide (**18a**, 1.40 g, 2.79 mmol, 1.00 equiv), 3-methyl-2-aminopyridine (0.880 g, 8.37 mmol, 3.00 equiv) in CH_3CN (8 mL). After 24 h of reflux, the formation of the desired product **18b** was confirmed by crude ^{1}H NMR analysis. However, the purification of **18b** using the usual precipitation method with acetone as the solvent was unsuccessful. Several alternative solvent mixtures, including CH_2Cl_2/Et_2O, CH_2Cl_2/EtOAc and MeOH/Et_2O were tested in an attempt to isolate pure **18b** from the crude reaction mixture. Unfortunately, in all cases, unidentified impurities were obtained along with the desired product.

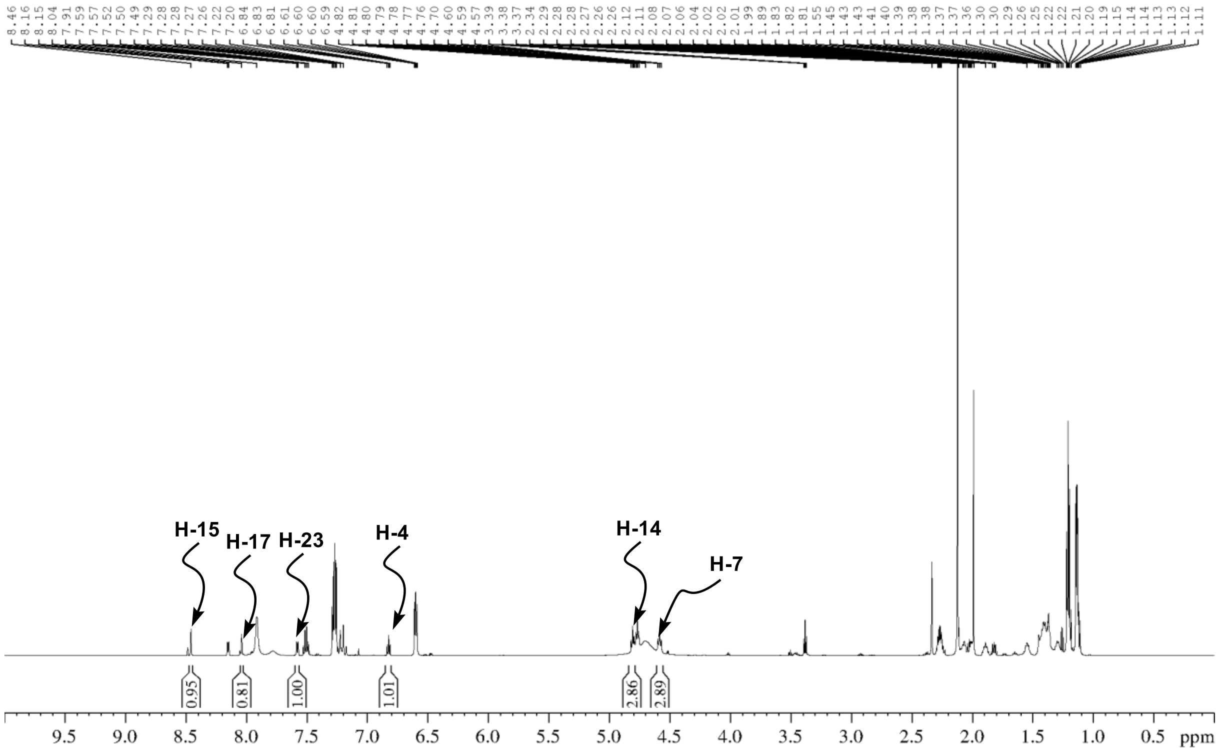

Figure S22: ^{1}H NMR ($CDCl_3$, 600 MHz) of crude reaction mixture for **18b** after washing with acetone (20 mL).

7.13 Synthesis of complex IPrIPyCuOtBu (I)

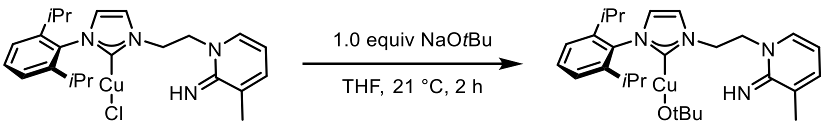

IPrIPyCuCl, **4** IPrIPyCuO*t*Bu, **I**

7.13.1 *tert*-Butoxy(1-(2,6-di*iso*propylphenyl)-3-(2-(2-imino-3-methylpyridin-1(2*H*)-yl)ethyl)-2,3-dihydro-1*H*-imidazol-2-yl)copper (I) (I)

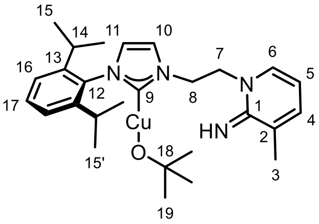

XX

$C_{27}H_{39}CuN_4O$

Mw = 499.18 gmol^{-1}

In an Ar-filled glovebox, [IPrIPyCuCl] (**4**, 37.2 mg, 80.0 µmol, 1.00 equiv) and NaO*t*Bu (7.70 mg, 80.0 µmol, 1.00 equiv) were placed in a 5 mL glass vial with a stirring bar. THF (2 mL) was added and the reaction mixture was stirred at room temperature (21 °C) for 2 h. The progress of the reaction was monitored by ^{1}H NMR. After completion, the crude reaction mixture was filtered filtered over a PTFE syringe filter (0.45 µm). The filtrate was concentrated under oil pump vacuum (2 × 10^{-2} mbar). The resulting yellow solid was dissolved in dry benzene (2 mL) and filtered over a PTFE syringe filter (0.45 µm). The filtrate was then concentrated under oil pump vacuum (2 × 10^{-2} mbar). The resulting yellow solid was washed with *n*-pentane (3 × 1 mL). The solid was dried under oil pump vacuum (2 × 10^{-2} mbar) to afford *tert*-butoxide complex **I** as yellow solid (25.6 mg, 0.051 mmol, 64% yield).

Note: For the preparation and isolation of *tert*-butoxide complex **I**, the used solvents (THF, benzene and *n*-pentane) were freshly dried and degassed before use.

^{1}H NMR (600 MHz, C_6D_6) δ = 7.89 (d, $^{3}J_{6,5}$ = 6.9 Hz, 1H, H-6), 7.14 (t, $^{3}J_{17,16}$ = 7.8 Hz, 1H, H-17), 7.07 (d, J = 8.0 Hz, 2H), 6.34 (s, 1H), 6.31 (d, J = 6.5 Hz, 1H), 6.15 (d, J = 1.7 Hz, 1H), 6.03 (s, 1H), 5.38 (t, J = 6.7 Hz, 1H), 4.75 (s, 2H), 4.01 (s, 2H), 2.77 (s, 2H), 1.67 (s, 3H), 1.32 (s, 12H), 1.11 (s, 4H), 0.94 (d, J = 6.9 Hz, 7H) ppm.

^{13}C NMR (151 MHz, C_6D_6) δ = 190.2 (C-9), 160.7 (C-1), 138.7 (C-13), 136.9 (C-4), 132.1 (C-6), 129.9 (C-17), 129.3 (C-2), 124.7 (C-12), 124.0 (C-16), 121.4 (C-10)*, 120.6 (C-11)*, 100.8 (C-5), 51.0 (C-8), 46.4 (C-7), 30.2 (C-19), 28.6 (C-14), 28.3 (C-15), 23.4 (C-15'), 18.2 (C-3) ppm.

C-9 was assigned from ^{1}H,^{13}C HMBC NMR. C-18 peak could not be found.

HRMS (APCI) for $C_{23}H_{30}CuN^{+}$ [$(M–C_4H_9O^{-})^{+}$] calculated: 425.1761, found: 425.1753.

The most abundant peak was obtained for $C_{46}H_{60}CuN_8^{+}$ calculated: 787.4237, found: 787.4228.

7.13.1.1 Alkyne semihydrogenation with isolated *tert*-butoxide complex I

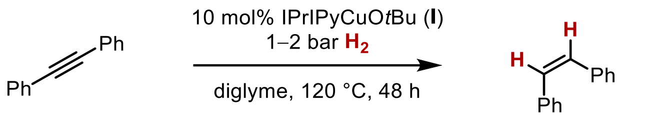

1a **2a** 33% conversion

Accorging to **GP1**, in an Ar-filled glovebox, [IPrIPyCuO*t*Bu] (**I**, 9.98 mg, 20.0 µmol, 10.0 mol%) was placed in a 5 mL glass vial with a stirring bar. The vial was sealed with septa inside the glovebox and transferred outside. The alkyne (**1**, 0.200 mmol, 1.00 equiv), dissolved in diglyme (2.0 mL) was added. The reaction mixture was then transferred to a 25 mL pressure tube under N_2 counterflow. The N_2 atmosphere is removed under vaccum (2 × 10^{-2} bar) and then backfilled with H_2 (1.5 bar). The reaction mixture is stirred for 48 h at 120 °C under H_2 atmosphere (1.5 bar). The reaction mixture is allowed to cool to room temperature and H_2 is replaced with N_2. The crude reaction mixture is filtered over a plug of silica (1 × 5 cm, eluent: CH_2Cl_2, 5 mL) and all volatiles are removed under reduced pressure. The crude product is purified by flash column chromatography on silica gel.

After 48 h, 33% conversion of tolane (**1a**) to (*Z*)-silbene (**2a**) was observed in GC/GC-MS analysis.

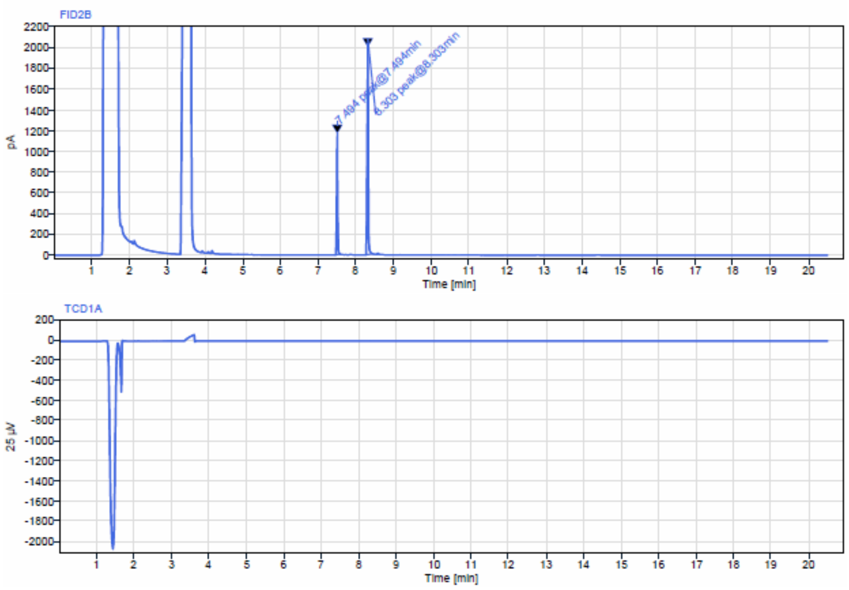

Signal: FID2B

Name	RT [min]	RF	Area	Peak Area Percent	Concentration []	Group
peak@7.494min	7.49		1796.756	32.95		
peak@8.303min	8.30		3656.605	67.05		

Figure S23: GC chromatogram of crude mixture after alkyne semihydrogenation of tolane (**1a**) with IPrIPyCuO*t*Bu (**I**)

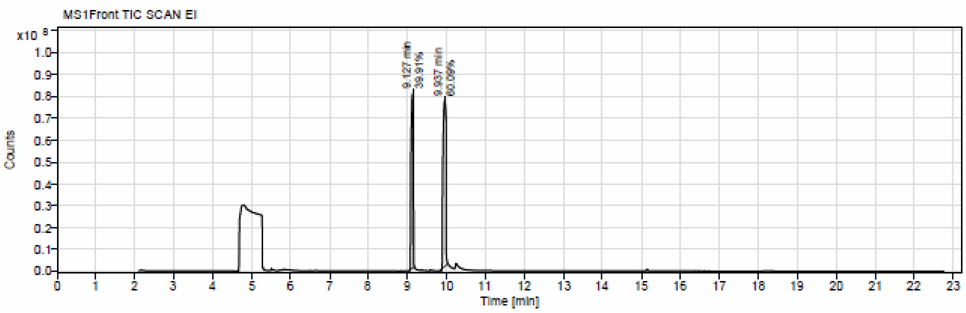

Signal: MS1Front TIC SCAN EI

RT	m/z	Spectral purity (%)	Peak area	Area %
9.127			269400000	39.91

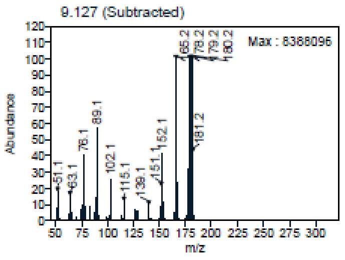

RT	m/z	Spectral purity (%)	Peak area	Area %
9.937			405600000	60.09

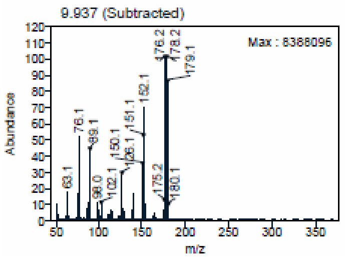

Figure S24: GC-MS chromatogram of crude mixture after alkyne semihydrogenation of tolane (**1a**) with IPrIPyCuO*t*Bu (**I**)

7.14 Alternate synthetic approach for complex IPrIPyCuCl (4)

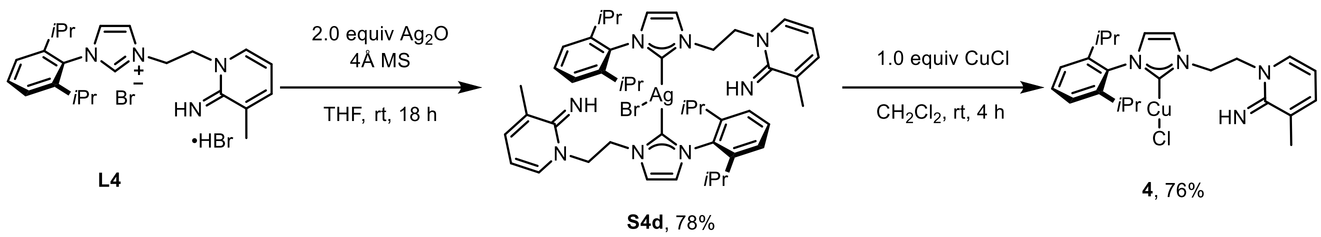

7.14.1 Bis(1-(2,6-diisopropylphenyl)-3-(2-(2-imino-3-methylpyridin-1(2*H*)-yl)ethyl)-1,3-dihydro-2*H*-imidazol-2-ylidene)silver(I) bromide (S4d)

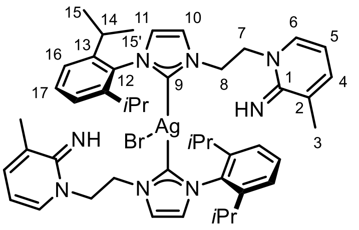

S4d
$C_{46}H_{60}AgBrN_8$
Mw = 912.83 gmol^{-1}

In deference to a literature procedure,[5] a 50 mL Schlenk flask equipped with a magnetic stir bar was charged with 1-(2,6-diisopropylphenyl)-3-(2-(2-imino-3-methylpyridin-1(2*H*)-yl)ethyl)-1*H*-imidazol-3-ium bromide hydrobromide (**L4**, 524 mg, 1.00 mmol, 300 mg/mmol of **L4**) and THF (20 mL). The flask was covered with aluminium foil. The mixture was stirred at room temperature for 18 h and then filtered through a Schlenk frit (P4) under N_2 atmosphere and washed with THF (10 mL). The filtrate was concentrated under oil pump vacuum (2×10^{-2} mbar) yielding the product **S4d** as colorless solid (356 mg, 0.390 mmol, 78% yield).

^{1}H NMR (600 MHz, $CDCl_3$) δ = 7.45 (t, $^3J_{17,16}$ = 7.8 Hz, 2H, H-17), 7.23 (d, $^3J_{16,17}$ = 7.8 Hz, 4H, H-16), 7.11 (d, $^3J_{10,11}$ = 1.7 Hz, 2H, H-10), 6.91 (d, $^3J_{11,10}$ = 1.7 Hz, 2H, H-11), 6.73 (d, $^3J_{4,5}$ = 6.5 Hz, 2H, H-4), 6.52 (d, $^3J_{6,5}$ = 6.9 Hz, 2H, H-6), 6.01 (s, 2H, N–*H*), 5.53 (t, $^3J_{5,4/6}$ = 6.7 Hz, 2H, H-5), 4.79 (t, $^3J_{8,7}$ = 5.8 Hz, 4H, H-8), 4.37 (t, $^3J_{7,8}$ = 5.8 Hz, 4H, H-7), 2.29 (sept, $^3J_{14,15/15'}$ = 6.9 Hz, 4H, H-14), 2.08 (s, 6H, H-3), 1.20 (d, $^3J_{15,14}$ =6.9 Hz, 12H, H-15), 1.08 (d, $^3J_{15,14}$ =6.9 Hz, 12H, H-15') ppm.

^{1}H NMR (600 MHz, CD_2Cl_2) δ = 7.47 (t, $^3J_{17,16}$ = 7.9 Hz, 2H, H-17), 7.27 (d, $^3J_{16,17}$ = 7.9 Hz, 4H, H-16), 7.22 (s, 2H, H-10), 6.98 (s, 2H, H-11), 6.72 (d, $^3J_{4,5}$ = 6.5 Hz, 2H, H-4), 6.52 (s, 2H, H-6), 6.01 (s, 2H, N–*H*), 5.53 (t, $^3J_{5,4/6}$ = 6.7 Hz, 2H, H-5), 4.79 (br s, 4H, H-8), 4.31 (br s, 4H, H-7), 2.32 – 2.24 (m, 4H, H-14), 2.07 (s, 6H, H-3), 1.20 (d, $^3J_{15,14}$ =6.9 Hz, 12H, H-15), 1.08 (d, $^3J_{15,14}$ = 6.9 Hz, 12H, H-15') ppm.

The ^{1}H NMR shows traces of toluene at 7.24, 7.15 and 2.34 ppm.

^{13}C NMR (151 MHz, CD_2Cl_2) δ = 185.2 (C-9), 160.3 (C-1), 146.2 (C-13), 135.8 (C-4), 135.2 (C-12), 131.9 (C-6), 130.7 (C-17), 127.4 (C-2), 124.6 (C-16), 124.5 (C-10), 121.7 (C-11), 102.0 (C-5), 53.2 (C-8), 47.6 (C-7), 28.5 (C-14), 24.6 (C-15), 24.5 (C-15'), 18.6 (C-3) ppm.

C-9 is assigned from ^{1}H, ^{13}C HMBC NMR.

The ^{13}C NMR shows traces of toluene at 138.2, 129.5, 128.7, 125.8 and 21.3 ppm.

HRMS (APCI) for $C_{46}H_{60}AgN_8^+$ [(M–Br$^-$)$^+$] calculated: 831.3986, found: 831.3984.

SC-XRD single-crystals suitable for x-ray diffraction analysis were obtained by gas phase diffusion of Et_2O into a solution of **S4d** (mixture of CH_2Cl_2/*n*-hexane) at –40 °C. A selected representation of the molecular structure in the solid state is depicted in Figure S25. Section 9 "X-ray Diffraction Study of **S4d**" is presented for further details.

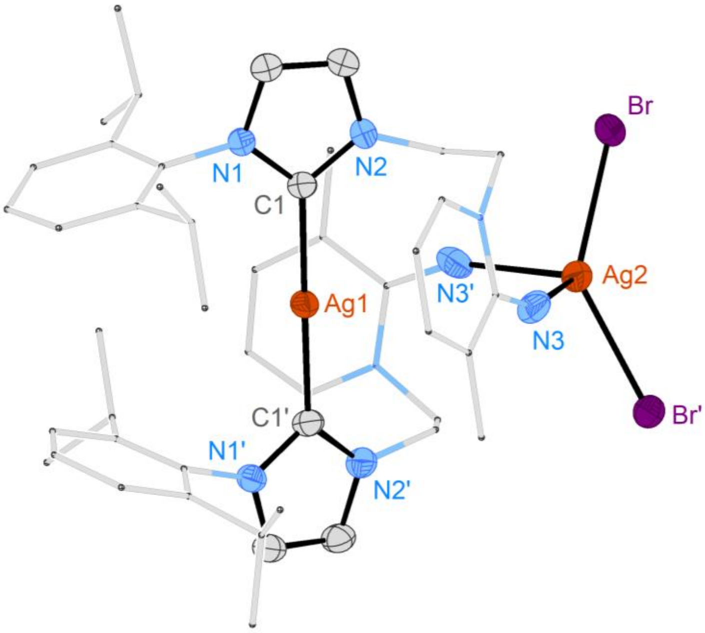

Figure S25: Molecular structure of **S4d** in the solid state with ellipsoids set at 30% probability. For clarity, hydrogen atoms and solvent molecules are omitted, and parts of the ligand are depicted in the "wires-and-sticks" model. Selected bond lengths [Å] and angles [°]: C1–Ag1 2.088(7), N3–Ag2 2.458(8), Ag2–Br 2.631(1); C1–Ag1–C1' 179.3(4), N3–Ag2–N3' 104.4(3), Br–Ag2–Br' 137.1(6), N1–C1–N2 102.4(6).

7.14.2 (1-(2,6-Diisopropylphenyl)-3-(2-(2-imino-3-methylpyridin-1(2*H*)-yl)ethyl)-1,3-dihydro-2*H*-imidazol-2-ylidene)copper(I) chloride (4)

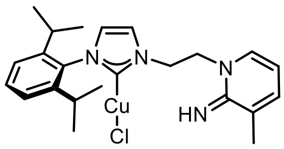

4
$C_{23}H_{30}ClCuN_4$
Mw = 461.52 gmol^{-1}

In deference of literature procedure,[5] in an Ar glovebox a 5 mL vial, CuCl (17.0 mg, 0.180 mmol, 1.00 equiv) and bis(1-(2,6-diisopropylphenyl)-3-(2-(2-imino-3-methylpyridin-1(2*H*)-yl)ethyl)-1,3-dihydro-2*H*-imidazol-2-ylidene)silver(I) bromide (**S4d**, 100 mg, 0.180 mmol, 1.00 equiv), were dissolved in CH_2Cl_2 (2.0 mL). The vial was closed with a septum and covered with aluminium foil. The reaction mixture was stirred at room temperature for 4 h and after that time filtered over a PTFE syringe filter (0.45 µm). The filtrate

was concentrated under oil pump vacuum (2 × 10^{-2} mbar). The product **4** was obtained as yellow solid (63.0 mg, 0.140 mmol, 76% yield).

The analytical data is provided in page no. S35.

7.15 Alkyne semihydrogention products

7.15.1 (*Z*)-1,2-Diphenylethene (2a)

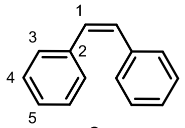

2a
$C_{14}H_{12}$
Mw = 180.25 gmol^{-1}

Prepared according to **GP1** from 1,2-diphenylethyne (**1a**, 35.6 mg, 0.200 mmol, 1.00 equiv), [IPrIPyCuCl] (**4**, 9.30 mg, 20.0 µmol, 10.0 mol%), NaO*t*Bu (5.95 mg, 60.0 µmol, 30.0 mol%) in diglyme (2.0 mL). The reaction mixture was stirred for 48 h at 120 °C under H_2 atmosphere (1.5 bar). Purification by flash column chromatography on silica gel yielded **2a** as colorless oil (27.0 mg, 0.150 mmol, 75%).

R_f = 0.40 (SiO_2, cyclohexane/EtOAc = 100:0).

^{1}H NMR (600 MHz, $CDCl_3$): δ = 7.26–7.17 (m, 10H, H-3, H-4; H-5), 6.60 (s, 2H, H-1) ppm.

^{13}C NMR (151 MHz, $CDCl_3$): δ = 137.4 (C-2), 130.4 (C-1), 129.0 (C-3)*, 128.3 (C-4)*, 127.2 (C-5) ppm.

HRMS (APCI) for $C_{14}H_{12}^+$ [$(M)^+$] calculated: 180.0934, found: 180.0936.

The data is in accordance with literature.[16]

7.15.2 (*Z*)-1-(Benzyloxy)-4-styrylbenzene (2b)

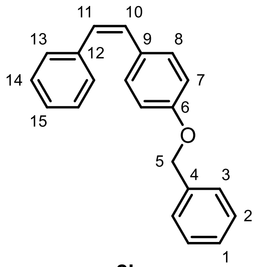

2b
$C_{21}H_{18}O$
Mw = 286.37 gmol^{-1}

Prepared according to **GP1** from 1-(benzyloxy)-4-(phenylethynyl)benzene (**1b**, 56.9 mg, 0.200 mmol, 1.00 equiv), [IPrIPyCuCl] (**4**, 9.30 mg, 20.0 µmol, 10.0 mol%), NaO*t*Bu (5.95 mg, 60.0 µmol, 30.0 mol%) in diglyme (2.0 mL). The reaction mixture was stirred for 48 h at 120 °C under H_2 atmosphere (1.5 bar). Purification by flash column chromatography on silica gel yielded **2b** as colorless oil (44.7 mg, 0.156 mmol, 78%).

R_f = 0.20 (SiO_2, cyclohexane/EtOAc = 40:1).

^{1}H NMR (600 MHz, $CDCl_3$): δ = 7.43–7.42 (m, 2H, H-13), 7.38 (t, $^3J_{14,13/15}$ = 7.5 Hz, 2H, H-14), 7.34–7.32 (m, 1H, H-15), 7.28–7.27 (m, 2H, H-3), 7.26–7.23 (m, 2H, H-2), 7.23–7.17 (m, 3H, H-1, H-8), 6.83 (d, $^3J_{7,8}$ = 8.8 Hz, 2H, H-7), 6.53 (d, $^3J_{11,10}$ = 12.2 Hz, 1H, H-11), 6.51 (d, $^3J_{10,11}$ = 12.2 Hz, 1H, H-10), 5.04 (s, 2H, H-5) ppm.

^{13}**C NMR** (151 MHz, $CDCl_3$): δ = 158.1 (C-6), 137.8 (C-12), 137.1 (C-9), 130.3 (C-8), 130.1 (C-4), 129.9 (C-11), 129.0 (C-10), 128.9 (C-2), 128.8 (C-3), 128.4 (C-14), 128.2 (C-15), 127.7 (C-13), 127.1 (C-1), 114.7 (C-7), 70.1 (C-5) ppm.

HRMS (APCI) for $C_{21}H_{19}O^+$ [$(M+H)^+$] calculated 287.1430, found 287.1429.

IR (ATR): ṽ = 3060 (w), 3026 (w), 2926 (w), 2862 (w), 1602 (m), 1509 (s), 1449 (w), 1382 (w), 1293 (w), 1244 (s), 1177 (m), 1110 (w), 1013 (br), 969 (w), 916 (w), 872 (w), 771 (w), 738 (w), 697 (m) cm^{-1}.

7.15.3 (*Z*)-1-Methoxy-4-(4-(trifluoromethyl)styryl)benzene (2c)

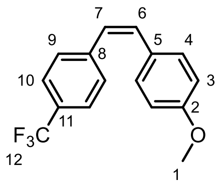

2c
$C_{16}H_{13}F_3O$
Mw = 278.27 gmol^{-1}

Prepared according to **GP1** from 1-methoxy-4-((4-(trifluoromethyl)phenyl)ethynyl)benzene (**1c**, 55.3 mg, 0.200 mmol, 1.00 equiv), [IPrIPyCuCl] (**4**, 9.30 mg, 20.0 µmol, 10.0 mol%), NaO*t*Bu (5.95 mg, 60.0 µmol, 30.0 mol%) in diglyme (2.0 mL). The reaction mixture was stirred for 48 h at 120 °C under H_2 atmosphere (1.5 bar). Purification by flash column chromatography on silica gel yielded **2c** as colorless oil (39.6 mg, 0.140 mmol, 71%).

$\mathbf{R}_f$ = 0.20 (SiO_2, cyclohexane/EtOAc = 30:1).

^{1}**H NMR** (600 MHz, $CDCl_3$): δ = 7.48 (d, $^3J_{10,9}$ = 8.2 Hz, 2H, H-10), 7.36 (d, $^3J_{9,10}$ = 8.4 Hz, 2H, H-9), 7.15 (d, $^3J_{4,3}$ = 8.7 Hz, 2H, H-4), 6.77 (d, $^3J_{3,4}$ = 8.7 Hz, 2H, H-3), 6.64 (d, $^3J_{6,7}$ = 12.1 Hz, 1H, H-6), 6.50 (d, $^3J_{7,6}$ = 12.1 Hz, 1H, H-7), 3.80 (s, 3H, H-1) ppm.

^{13}**C NMR** (151 MHz, $CDCl_3$): δ = 159.2 (C-2), 141.5 (C-8), 132.0 (C-6), 130.3 (C-4), 129.2 (C-9), 129.0 (C-5), 128.6 (q, $^2J_{11,F}$ = 129 Hz, C-11), 127.4 (C-7), 125.3 (q, $^3J_{10,F}$ = 3.7 Hz, C-10), 123.4 (q, $^1J_{12,F}$ = 272.6 Hz, C-12), 113.9 (C-3), 55.4 (C-1) ppm.

^{19}**F NMR** (473 MHz, $CDCl_3$): δ = –62.5 (s) ppm.

HRMS (APCI) for $C_{16}H_{13}F_3O^+$ [$(M)^+$] calculated: 278.0913, found: 278.0912.

IR (ATR): ṽ = 3056 (w), 3011 (w), 2944 (m), 2870 (w), 2363 (w), 1606 (m), 1509 (m), 1464 (w), 1412 (w), 1323 (s), 1252 (m), 1166 (m), 1121 (m), 1066 (w), 1036 (w), 969 (w), 883 (w), 831 (w), 738 (s), 704 (w) cm^{-1}.

7.15.4 (*Z*)-4-(4-Methoxystyryl)benzonitrile (2d)

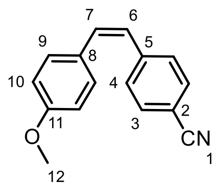

2d
$C_{16}H_{13}NO$
Mw = 235.29 gmol^{-1}

Prepared according to **GP1** from 4-((4-methoxyphenyl)ethynyl)benzonitrile (**1d**, 46.7 mg, 0.200 mmol, 1.00 equiv), [IPrIPyCuCl] (**4**, 9.30 mg, 20.0 µmol, 10.0 mol%), NaO*t*Bu (5.95 mg, 60.0 µmol, 30.0 mol%) in diglyme (2.0 mL). The reaction mixture was stirred for 48 h at 120 °C under H_2 atmosphere (1.5 bar). Purification by flash column chromatography on silica gel yielded **2d** as yellow solid (32.0 mg, 0.136 mmol, 68%).

$\mathbf{R}_f$ = 0.20 (SiO_2, cyclohexane/EtOAc = 20:1).

^{1}**H NMR** (600 MHz, $CDCl_3$): δ = 7.62 (d, $^3J_{3,4}$ = 8.4 Hz, 2H, H-3), 7.55 (d, $^3J_{4,3}$ = 8.4 Hz, 2H, H-4), 7.48 (d, $^3J_{9,10}$ = 8.7 Hz, 2H, H-9), 7.17 (d, $^3J_{7,6}$ = 12.2 Hz, 1H, H-7), 6.95 (d, $^3J_{6,7}$ = 12.2 Hz, 1H, H-6), 6.92 (d, $^3J_{10,9}$ = 8.8 Hz, 2H, H-10), 3.85 (s, 3H, H-12) ppm.

^{13}**C NMR** (151 MHz, $CDCl_3$): δ = 160.2 (C-11), 142.4 (C-5), 132.6 (C-3), 132.1 (C-7), 129.2 (C-8), 128.4 (C-9), 126.7 (C-4), 124.7 (C-6), 119.3 (C-1), 114.5 (C-10), 110.2 (C-2), 55.5 (C-12) ppm.

HRMS (APCI) for $C_{16}H_{14}NO^+$ [$(M+H)^+$] calculated 236.1070, found 230.1064.

The data is in accordance with literature.[23]

7.15.5 (*Z*)-1-Fluoro-4-styrylbenzene (2e)

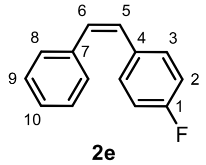

2e
$C_{14}H_{11}F$
Mw = 198.24 gmol^{-1}

Prepared according to **GP1** from 1-fluoro-4-(phenylethynyl)benzene (**1e**, 39.2 mg, 0.200 mmol, 1.00 equiv), [IPrIPyCuCl] (**4**, 9.30 mg, 20.0 µmol, 10.0 mol%), NaO*t*Bu (5.95 mg, 60.0 µmol, 30.0 mol%) in diglyme (2.0 mL). The reaction mixture was stirred for 48 h at 120 °C under H_2 atmosphere (1.5 bar). Purification by flash column chromatography on silica gel yielded **2e** as colorless oil (30.9 mg, 0.156 mmol, 78%).

$\mathbf{R}_f$ = 0.30 (SiO_2, cyclohexane/EtOAc = 100:0).

^{1}**H NMR** (600 MHz, $CDCl_3$): δ = 7.26–7.19 (m, 7H, H-3, H-8, H-9, H-10), 6.91 (t, $^3J_{2,3/F}$ = 8.7 Hz, 2H, H-2), 6.60 (d, $^3J_{5,6}$ = 12.2 Hz, 1H, H-5), 6.56 (d, $^3J_{6,5}$ = 12.2 Hz, 1H, H-6) ppm.

^{13}**C NMR** (151 MHz, $CDCl_3$): δ = 161.9 (d, $^1J_{1,F}$ = 246.8 Hz, C-1), 137.2 (C-7), 133.3 (d, $^5J_{4,F}$ = 3.7 Hz, C-4), 130.6 (d, $^4J_{3,F}$ = 7.8 Hz, C-3), 130.4 (C-5), 129.2 (C-6), 129.0 (C-8), 128.4 (C-9), 127.3 (C-10), 115.3 (d, $^3J_{2,F}$ = 21.4 Hz, C-2) ppm.

^{19}**F NMR** (473 MHz, $CDCl_3$): δ = –114.7 (s) ppm.

HRMS (APCI) for $C_{14}H_{11}F^+$ [$(M)^+$] calculated: 198.0839, found: 198.0834.

IR (ATR): ṽ = 3056 (w), 3019 (w), 2929 (w), 1602 (s), 1509 (s), 1446 (w), 1267 (w), 1222 (s), 1159 (m), 1095 (w), 1013 (w), 920 (w), 872 (m), 834 (m), 771 (w), 738 (m), 697 (m) cm^{-1}.

7.15.6 (*Z*)-1-Chloro-4-styrylbenzene (2f)

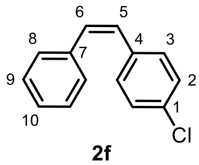

2f
$C_{14}H_{11}Cl$
Mw = 214.69 gmol^{-1}

Prepared according to **GP1** from 1-chloro-4-(phenylethynyl)benzene (**1f**, 42.5 mg, 0.200 mmol, 1.00 equiv), [IPrIPyCuCl] (**4**, 9.30 mg, 20.0 µmol, 10.0 mol%), NaO*t*Bu (5.95 mg, 60.0 µmol, 30.0 mol%) in diglyme (2.0 mL). The reaction mixture was stirred for 48 h at 120 °C under H_2 atmosphere (1.5 bar). Purification by flash column chromatography on silica gel yielded **2f** as colorless oil (32.0 mg, 0.150 mmol, 75%).

$\mathbf{R}_f$ = 0.30 (SiO_2, cyclohexane/EtOAc = 100:0).

^{1}H NMR (600 MHz, $CDCl_3$): δ = 7.26–7.20 (m, 5H, H-2, H-8, H-10), 7.20–7.16 (m, 4H, H-3, H-9), 6.63 (d, $^3J_{5,6}$ = 12.2 Hz, 1H, H-5), 6.53 (d, $^3J_{6,5}$ = 12.2 Hz, 1H, H-6) ppm.

^{13}C NMR (151 MHz, $CDCl_3$): δ = 137.0 (C-1), 135.8 (C-4), 132.9 (C-7), 131.1 (C-5), 130.4 (C-2), 129.1 (C-6), 128.9 (C-3), 128.5 (C-8), 128.4 (C-9), 127.5 (C-10) ppm.

HRMS (APCI) for $C_{14}H_{11}{}^{35}Cl^+$ [$(M)^+$] calculated: 214.0544, found: 214.0540.

IR (ATR): ṽ = 3056 (w), 3015 (w), 2926 (w), 2855 (w), 2650 (w), 2374 (w), 1490 (s), 1397 (w), 1088 (s), 1010 (s), 965 (w), 920 (w), 872 (w), 823 (m), 756 (w), 697 (s) cm^{-1}.

7.15.7 (*Z*)-1-Bromo-4-styrylbenzene (2g)

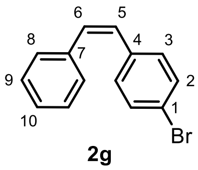

2g
$C_{14}H_{11}Br$
Mw = 259.14 gmol^{-1}

Prepared according to **GP1** from 1-bromo-4-(phenylethynyl)benzene (**1g**, 51.4 mg, 0.200 mmol, 1.00 equiv), [IPrIPyCuCl] (**4**, 9.30 mg, 20.0 µmol, 10.0 mol%), NaO*t*Bu (5.95 mg, 60.0 µmol, 30.0 mol%) in diglyme (2.0 mL). The reaction mixture was stirred for 48 h at 120 °C under H_2 atmosphere (1.5 bar). Purification by flash column chromatography on silica gel yielded **2g** as colorless oil (32.0 mg, 0.120 mmol, 62%).

$\mathbf{R}_f$ = 0.30 (SiO_2, cyclohexane/EtOAc = 100:0).

^{1}H NMR (600 MHz, $CDCl_3$): δ = 7.34 (d, $^3J_{2,3}$ = 8.4 Hz, 2H, H-2), 7.26–7.20 (m, 5H, H-3, H-8, H-10), 7.12–7.10 (m, 2H, H-9), 6.63 (d, $^3J_{5,6}$ = 12.2 Hz, 1H, H-5), 6.51 (d, $^3J_{5,6}$ = 12.2 Hz, 1H, H-5) ppm.

^{13}C NMR (151 MHz, $CDCl_3$): δ = 137.0 (C-7), 136.2 (C-4), 131.5 (C-2), 131.2 (C-5), 130.7 (C-8), 129.1 (C-6), 128.9 (C-3), 128.5 (C-9), 127.5 (C-10), 121.1 (C-1) ppm.

HRMS (APCI) for $C_{14}H_{11}{}^{79}Br^+$ [$(M)^+$] calculated: 258.0039, found: 258.0038.

IR (ATR): ṽ = 3056 (w), 3015 (w), 2959 (w), 2926 (w), 2855 (w), 2650 (w), 2370 (w), 1587 (w), 1483 (s), 1394 (w), 1263 (w), 1177 (w), 1107 (w), 1069 (s), 1010 (s), 961 (w), 820 (m), 697 (s) cm^{-1}.

The data is in accordance with literature.[24]

7.15.8 (*Z*)-1-Iodo-4-styrylbenzene (2h)

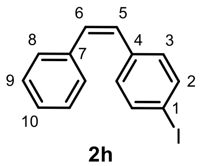

2h
$C_{14}H_{11}I$
Mw = 304.12 gmol^{-1}

Prepared according to **GP1** from 1-iodo-4-(phenylethynyl)benzene (**1h**, 60.8 mg, 0.200 mmol, 1.00 equiv), [IPrIPyCuCl] (**4**, 9.30 mg, 20.0 µmol, 10.0 mol%), NaO*t*Bu (5.95 mg, 60.0 µmol, 30.0 mol%) in diglyme (2.0 mL). The reaction mixture was stirred for 48 h at 120 °C under H_2 atmosphere (1.5 bar). Purification by flash column chromatography on silica gel yielded **2h** as colorless oil (29.6 mg, 0.096 mmol, 48%).

R*f* = 0.40 (SiO_2, cyclohexane/EtOAc = 100:0).

^{1}H NMR (600 MHz, $CDCl_3$): δ = 7.57 (d, $^3J_{2,3}$ = 8.4 Hz, 2H, H-2), 7.29–7.23 (m, 5H, H-8, H-9, H-10), 7.00 (d, $^3J_{3,2}$ = 8.4 Hz, 2H, H-3), 6.66 (d, $^3J_{5,6}$ = 12.2 Hz, 1H, H-5), 6.52 (d, $^3J_{6,5}$ = 12.2 Hz, 1H, H-6) ppm.

^{13}C NMR (151 MHz, $CDCl_3$): δ = 137.4 (C-2), 137.0 (C-7), 136.8 (C-4), 131.2 (C-6), 130.9 (C-3), 129.1 (C-5), 128.9 (C-8), 128.5 (C-9), 127.5 (C-10), 92.6 (C-1) ppm.

HRMS (APCI) for $C_{14}H_{11}I^+$ [$(M)^+$] calculated: 305.9900, found: 305.9899.

IR (ATR): ṽ = 3056 (w), 2926 (m), 2855 (w), 1580 (w), 1449 (w), 1267 (m), 1062 (w), 969 (m), 812 (s), 745 (m), 693 (m) cm^{-1}.

7.15.9 (*Z*)-(3-Styrylphenyl)methanol (2i)

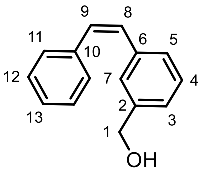

2i
$C_{15}H_{14}O$
Mw = 210.28 gmol^{-1}

Prepared according to **GP1** from 3-(phenylethynyl)benzaldehyde (**1i**, 41.3 mg, 0.200 mmol, 1.00 equiv), [IPrIPyCuCl] (**4**, 9.30 mg, 20.0 µmol, 10.0 mol%), NaO*t*Bu (5.95 mg, 60.0 µmol, 30.0 mol%) in diglyme (2.0 mL). The reaction mixture was stirred for 48 h at 120 °C under H_2 atmosphere (1.5 bar). Purification by flash column chromatography on silica gel yielded **2i** as colorless oil (31.5 mg, 0.152 mmol, 76%).

R*f* = 0.20 (SiO_2, cyclohexane/EtOAc = 10:1).

^{1}H NMR (600 MHz, $CDCl_3$): δ = 7.26–7.17 (m, 9H, H-3, H-4, H-5, H-7, H-11, H-12, H-13), 6.63 (d, $^3J_{9,8}$ = 12.3 Hz, 1H, H-9), 6.60 (d, $^3J_{8,9}$ = 12.3 Hz, 1H, H-8), 4.60 (d, $^3J_{1,O\text{–}H}$ = 5.0 Hz, 2H, H-1), 1.53 (br s, 1H, O–*H*) ppm.

^{13}C NMR (151 MHz, $CDCl_3$): δ = 141.0 (C-2), 137.7(C-10), 137.3 (C-6), 130.7 (C-9), 130.1 (C-8), 129.0 (C-11)*, 128.6 (C-4)**, 128.4 (C-12)*, 128.3 (C-7)**, 127.7 (C-13), 127.3 (C-7), 126.0 (C-3), 65.4 (C-1) ppm.

HRMS (APCI) for $C_{15}H_{14}O^+$ [M^+] calculated 210.1039, found 210.1039.

IR (ATR): ṽ = 3574 (w), 3347 (br), 3056 (w), 3015 (w), 2929 (w), 2877 (w), 2344 (w), 2087 (w), 1945 (w), 1886 (w), 1707 (w), 1602 (m), 1494 (w), 1446 (w), 1267 (s), 1222 (w), 1136 (w), 1017 (br), 793 (w), 734 (s), 702 (w) 697 (s) cm^{-1}.

7.15.10 (*Z*)-1-(3-Styrylphenyl)ethan-1-ol (2j)

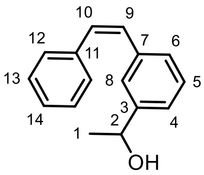

2j
$C_{16}H_{16}O$
Mw = 224.30 $gmol^{-1}$

Prepared according to **GP1** from 1-(3-(phenylethynyl)phenyl)ethan-1-one (**1j**, 44.0 mg, 0.200 mmol, 1.00 equiv), [IPrIPyCuCl] (**4**, 9.30 mg, 20.0 µmol, 10.0 mol%), NaO*t*Bu (5.95 mg, 60.0 µmol, 30.0 mol%) in diglyme (2.0 mL). The reaction mixture was stirred for 48 h at 120 °C under H_2 atmosphere (1.5 bar). Purification by flash column chromatography on silica gel yielded **2j** as colorless oil (24.6 mg, 0.110 mmol, 55%).

$\mathbf{R_f}$ = 0.20 (SiO_2, cyclohexane/EtOAc = 20:1).

^{1}H NMR (600 MHz, $CDCl_3$): δ = 7.28–7.15 (m, 9H, H-4, H-5. H-6, H-8, H-12, H-13, H-14), 6.62 (d, $^3J_{10,9}$ = 12.3 Hz, 1H, H-10), 6.60 (d, $^3J_{9,10}$ = 12.3 Hz, 1H, H-9), 4.78 (dd, $^3J_{2,1}$ = 6.5 Hz, $^3J_{2,O\text{-}H}$ = 3.2 Hz, 1H, H-2), 1.65 (d, $^3J_{O\text{–}H,2}$ = 3.2 Hz, 1H, O–*H*), 1.40 (d, $^3J_{1,2}$ = 6.5 Hz, 3H, H-1) ppm.

^{13}C NMR (151 MHz, $CDCl_3$): δ = 145.8 (C-3), 137.5 (C-7), 137.4 (C-11), 130.7 (C-10), 130.2 (C-9), 129.0 (C-12)*, 128.5 (C-14)**, 128.3 (C-13)*, 128.1 (C-5)**, 127.3 (C-6)**, 126.2 (C-8), 124.3 (C-4), 70.4 (C-2), 25.2 (C-1) ppm.

HRMS (APCI) for $C_{16}H_{16}O^+$ [M^+] calculated 224.1196, found 224.1194.

IR (ATR): ṽ = 3347 (br), 3056 (w), 3022 (w), 2974 (m), 2929 (w), 2870 (w), 1602 (m), 1490 (w), 1446 (w), 1401 (w), 1367 (w), 1259 (w), 1073 (m), 1013 (w), 961 (w), 916 (w), 872 (w), 767 (m), 693 (s) cm^{-1}.

7.15.11 1-(3-((*Z*)-Styryl)phenyl)ethan-1-one *O*-methyl oxime (2k)

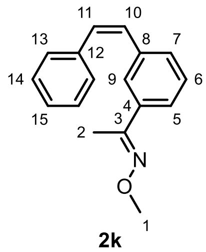

2k
$C_{17}H_{17}NO$
Mw = 251.33 $gmol^{-1}$

Prepared according to **GP1** from 1-(3-(phenylethynyl)phenyl)ethan-1-one *O*-methyl oxime (**1k**, 49.9 mg, 0.200 mmol, 1.00 equiv), [IPrIPyCuCl] (**4**, 9.30 mg, 20.0 µmol, 10.0 mol%), NaO*t*Bu (5.95 mg, 60.0 µmol, 30.0 mol%) in diglyme (2.0 mL). The reaction mixture was stirred for 48 h at 120 °C under H_2 atmosphere (1.5 bar). Purification by flash column chromatography on silica gel yielded **2k** as orange oil (32.2 mg, 0.128 mmol, 64%).

$\mathbf{R_f}$ = 0.20 (SiO_2, cyclohexane/EtOAc = 20:1).

^{1}H NMR (600 MHz, $CDCl_3$): δ = 7.51–7.50 (m, 2H, H-5, H-9), 7.26–7.19 (m, 7H, H-6, H-7, H-13, H-14, H-15), 6.65 (d, $^3J_{11,10}$ = 12.2 Hz, 1H, H-11), 6.61 (d, $^3J_{10,11}$ = 12.2 Hz, 1H, H-10), 3.97 (s, 3H, H-1), 2.08 (s, 3H, H-2) ppm.

^{13}C NMR (151 MHz, $CDCl_3$): δ = 154.6 (C-3), 137.4 (C-4), 137.3 (C-12), 136.7 (C-8), 130.9 (C-11), 130.0 (C-10), 129.7 (C-6)**, 129.0 (C-13)*, 128.4 (C-14)*, 128.4 (C-7)**, 127.3 (C-15)**, 126.9 (C-9), 124.8 (C-5), 62.0 (C-1), 12.6 (C-2) ppm.

HRMS (APCI) for $C_{17}H_{18}NO^+$ [$(M+H)^+$] calculated 252.1383, found 252.1379.

IR (ATR): ṽ = 3060 (w), 2959 (w), 2817 (w), 1599 (m), 1490 (m), 1371 (m), 1047 (s), 872 (s), 752 (s), 685 (s) cm^{-1}.

7.15.12 *N*-Methyl-1-(3-((*Z*)-styryl)phenyl)ethan-1-imine (2l)

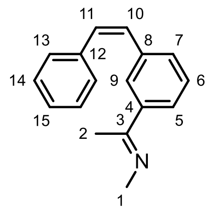

2l

$C_{17}H_{17}N$

Mw = 235.33 $gmol^{-1}$

Prepared according to **GP1** from *N*-methyl-1-(3-(phenylethynyl)phenyl)ethan-1-imine (**1l**, 46.7 mg, 0.200 mmol, 1.00 equiv), [IPrIPyCuCl] (**4**, 9.30 mg, 20.0 µmol, 10.0 mol%), NaO*t*Bu (5.95 mg, 60.0 µmol, 30.0 mol%) in diglyme (2.0 mL). The reaction mixture was stirred for 48 h at 120 °C under H_2 atmosphere (1.5 bar). Purification by flash column chromatography on silica gel yielded **2l** as orange oil (24.9 mg, 0.106 mmol, 53%).

R_f = 0.20 (SiO_2, cyclohexane/EtOAc = 20:1)

^{1}H NMR (600 MHz, $CDCl_3$): δ = 7.61–7.60 (m, 2H, H-5, H-9), 7.26–7.18 (m, 7H, H-6, H-7, H-13, H-14, H-15), 6.63 (s, 2H, H-10, H-11)), 3.32 (s, 3H, H-1), 2.09 (s, 3H, H-2) ppm.

^{13}C NMR (151 MHz, $CDCl_3$): δ = 167.1 (C-3), 141.4 (C-4), 137.4 (C-12), 137.3 (C-8), 130.7 (C-11), 130.2 (C-10), 130.0 (C-15)**, 129.0 (C-13)*, 128.4 (C-14)*, 128.3 (C-7)**, 127.4 (C-6)**, 127.3 (C-5), 125.3 (C-9), 39.6 (C-1), 15.1 (C-2) ppm.

C-4 is assigned from ^{1}H, ^{13}C HMBC NMR.

HRMS (APCI) for $C_{17}H_{18}N^+$ [$(M+H^+)^+$] calculated 236.1434, found 236.1435.

IR (ATR): ṽ = 3056 (w), 3015 (w), 2963 (w), 2926 (w), 2855 (w), 1684 (s), 1595 (w), 1494 (w), 1427 (m), 1356 (m), 1263 (s), 1196 (w), 1077 (w), 1028 (w), 954 (w), 928 (w), 797 (w), 771 (w), 738 (w) 693 (s) cm^{-1}.

7.15.13 Ethyl (*Z*)-6-phenylhex-5-enoate (2m)

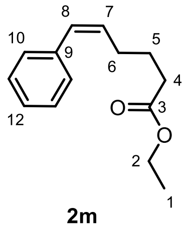

2m
$C_{14}H_{18}O_2$
Mw = 218.30 gmol^{-1}

Prepared according to **GP1** from 1ethyl 6-phenylhex-5-ynoate (**1m**, 44.0 mg, 0.200 mmol, 1.00 equiv), [IPrIPyCuCl] (**4**, 9.30 mg, 20.0 µmol, 10.0 mol%), NaO*t*Bu (5.95 mg, 60.0 µmol, 30.0 mol%) in diglyme (2.0 mL). The reaction mixture was stirred for 48 h at 120 °C under H_2 atmosphere (1.5 bar). Purification by flash column chromatography on silica gel yielded **2m** as colorless oil (32.3 mg, 0.148 mmol, 73%).

R*f* = 0.30 (SiO_2, cyclohexane/EtOAc = 20:1).

^{1}H NMR (600 MHz, $CDCl_3$): δ = 7.33 (t, $^3J_{11,10/12}$ = 7.1 Hz, 2H, H-11), 7.26 (d, $^3J_{10,11}$ = 7.1 Hz, 2H, H-10), 7.22 (t, $^3J_{12,11}$ = 7.2 Hz, 1H, H-12), 6.46 (d, $^3J_{8,7}$ = 11.6 Hz, 1H, H-8), 5.63 (dt, $^3J_{7,8}$ = 11.6 Hz, $^3J_{7,6}$ = 7.3 Hz, 1H, H-7), 4.10 (q, $^3J_{2,1}$ = 7.1 Hz, 2H, H-2), 2.39–2.35 (m, 2H, H-6), 2.32 (t, $^3J_{4,5}$ = 7.5 Hz, 2H, H-4), 1.79 (p, $^3J_{5,4/6}$ = 7.5 Hz, 2H, H-5), 1.22 (t, $^3J_{1,2}$ = 7.1 Hz, 3H, H-1) ppm.

^{13}C NMR (151 MHz, $CDCl_3$): δ = 173.7 (C-3), 137.6 (C-9), 131.8 (C-7), 129.9 (C-8), 128.9 (C-11), 128.3 (C-10), 126.7 (C-12), 60.4 (C-2), 34.0 (C-4), 28.1 (C-6), 25.3 (C-5), 14.4 (C-1) ppm.

HRMS (APCI) for $C_{14}H_{19}O_2^+$ [(M+H)$^+$] calculated 219.1380, found 219.1382.

IR (ATR): ṽ = 3056 (w), 2985 (w), 2937 (w), 2873 (w), 1729 (s), 1494 (w), 1446 (w), 1375 (w), 1267 (m), 1181 (w), 1073 (w), 1028 (w), 961 (w), 916 (w), 734 (s) cm^{-1}.

7.15.14 4-Vinyl-1,1'-biphenyl (2n)

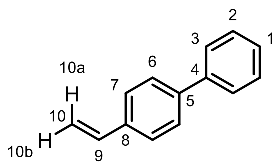

2n
$C_{14}H_{12}$
Mw = 180.25 gmol^{-1}

Prepared according to **GP1** from 4-ethynyl-1,1'-biphenyl (**1n**, 35.6 mg, 0.200 mmol, 1.00 equiv), [IPrIPyCuCl] (**4**, 9.30 mg, 20.0 µmol, 10.0 mol%), NaO*t*Bu (5.95 mg, 60.0 µmol, 30.0 mol%) in diglyme (2.0 mL). The reaction mixture was stirred for 48 h at 120 °C under H_2 atmosphere (1.5 bar). Purification by flash column chromatography on silica gel yielded **2n** as white solid (27.6 mg, 0.15 mmol, 77%).

R*f* = 0.35 (SiO_2, cyclohexane).

^{1}H NMR (600 MHz, $CDCl_3$): δ = 7.61 (dd, $^3J_{3,2}$ = 8.2 Hz, $^4J_{3,1}$ = 1.3 Hz, 2H, H-3), 7.58 (d, $^3J_{6,7}$ = 8.3 Hz, 2H, H-6), 7.49 (d, $^3J_{7,6}$ = 8.3 Hz, 2H, H-7), 7.45 (dd, $^3J_{2,3}$ = 8.3 Hz, $^3J_{2,1}$ = 7.8 Hz, 2H, H-2), 7.35 (tt, $^3J_{1,2}$ = 7.8 Hz, $^4J_{1,3}$ = 1.3 Hz, 1H, H-1), 6.77 (dd, $^3J_{9,10a}$ = 17.6 Hz, $^3J_{9,10b}$ = 10.9 Hz, 1H, H-9), 5.80 (dd, $^3J_{10a,9}$ = 17.6 Hz, $^1J_{10a,10b}$ = 0.9 Hz, 1H, H-10a), 5.28 (dd, $^3J_{10b,9}$ = 10.8 Hz, $^1J_{10b,10a}$ = 0.9 Hz, 1H, H-10b) ppm.

^{13}C NMR (151 MHz, $CDCl_3$): δ = 140.9 (C-8), 140.7 (C-4), 136.7 (C-5), 136.6 (C-9), 128.9 (C-2), 127.5 (C-1), 127.4 (C-6), 127.1 (C-3), 126.8 (C-7), 114.0 (C-10) ppm.

HRMS (APCI) for $C_{14}H_{12}^+$ [M^+] calculated 180.0934, found 180.0928.

The data is in accordance with literature.[25]

7.15.15 (*Z*)-1-Styryl-4-vinylbenzene (2o)

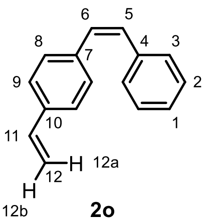

2o
$C_{16}H_{14}$
Mw = 206.29 gmol^{-1}

Prepared according to **GP1** from 1-ethynyl-4-(phenylethynyl)benzene(**1o**, 42.0 mg, 0.20 mmol, 1.00 equiv), [IPrIPyCuCl] (**4**, 9.30 mg, 20.0 µmol, 10.0 mol%), NaO*t*Bu (5.95 mg, 60.0 µmol, 30.0 mol%) in diglyme (2.0 mL). The reaction mixture was stirred for 48 h at 120 °C under H_2 atmosphere (1.5 bar). Purification by flash column chromatography on silica gel yielded **2o** as colorless oil (29.2 mg, 0.142 mmol, 71%).

R*f* = 0.35 (SiO_2, cyclohexane).

^{1}H NMR (600 MHz, $CDCl_3$): δ = 7.29–7.18 (m, 9H, H-1, H-2, H-3, H-8, H-9), 6.67 (dd, $^3J_{11,12a}$ = 17.6 Hz, $^3J_{11,12b}$ = 10.9 Hz, 1H, H-11), 6.60 (d, $^3J_{5,6}$ = 12.3 Hz, 1H, H-5), 6.57 (d, $^3J_{6,5}$ = 12.3 Hz, 1H, H-6), 5.72 (dd, $^3J_{12a,11}$ = 17.6 Hz, $^1J_{12a,12b}$ = 0.9 Hz, 1H, H-12a), 5.22 (dd, $^3J_{12b,11}$ = 10.8 Hz, $^1J_{12b,12a}$ = 0.9 Hz, 1H, H-12b) ppm.

^{13}C NMR (151 MHz, $CDCl_3$): δ = 137.4 (C-10), 136.9 (C-4), 136.6 (C-11), 136.5 (C-10), 130.5 (C-6), 130.0 (C-5), 129.2 (C-9)*, 129.0 (C-8)*, 128.4 (C-3)*, 127.3 (C-1), 126.2 (C-2)*, 113.8 (C-12) ppm.

HRMS (APCI) for $C_{16}H_{14}^+$ [M^+] calculated 206.1090, found 206.1085.

IR (ATR): ṽ = 3082 (w), 3052 (w), 3015 (w), 2967 (w), 2873 (w), 1509 (w), 1446 (w), 1412 (w), 1267 (m), 1181 (w), 1118 (w), 1073 (w), 991 (w), 909 (w), 834 (m), 771 (w), 738 (s), 697 (s) cm^{-1}.

7.15.16 Dodec-1-ene (2p)

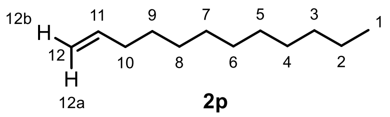

2p
$C_{12}H_{24}$
Mw = 168.32 gmol^{-1}

Prepared according to **GP1** from dodec-1-yne (**1p**, 34.0 mg, 0.200 mmol, 1.00 equiv), [IPrIPyCuCl] (**4**, 9.30 mg, 20.0 µmol, 10.0 mol%), NaO*t*Bu (5.95 mg, 60.0 µmol, 30.0 mol%) in diglyme (2.0 mL). The reaction mixture was stirred for 48 h at 120 °C under H_2 atmosphere (1.5 bar). Purification by flash column chromatography on silica gel yielded **2p** (25.2 mg, 0.154 mmol, 69%) as colorless oil.

R*f* = 0.30 (SiO_2, *n*-pentane).

^{1}H NMR (600 MHz, $CDCl_3$): δ = 5.82 (tdd, $^3J_{11,12a}$ = 16.9 Hz, $^3J_{11,12b}$ = 10.2 Hz, $^3J_{11,10}$ = 6.7 Hz, 1H, H-11), 4.99 (qd, $^3J_{12a,11}$ = 17.1 Hz, $^{2/4}J_{12a,12b/10}$ = 1.8 Hz, 1H, H-12a), 4.93 (qd, $^3J_{12b,11}$ = 10.1 Hz, $^{2/4}J_{12b,12a/10}$ = 1.9 Hz, 1H, H-12b), 2.04 (dtd, $^3J_{10,11}$ = 6.7 Hz, $^3J_{10,9}$ = 5.4 Hz, $^4J_{10,12}$ = 1.5 Hz, 2H,

H-10), 1.37 (q, $^3J_{9,10/8}$ = 7.1 Hz, 2H, H-9), 1.31–1.23 (m, 14H, H-2, H-3, H-4, H-5, H-6, H-7, H-8), 0.88 (t, $^3J_{1,2}$ = 7.0 Hz, 3H, H-1) ppm.

^{13}C NMR (151 MHz, $CDCl_3$): δ = 139.4 (C-12), 114.2 (C-11), 34.0 (C-10), 32.1 (C-3), 29.8 (C-7, C-8)*, 29.7 (C-6)*, 29.5 (C-5)*, 29.3 (C-4)*, 29.1 (C-9), 22.8 (C-2), 14.3 (C-1) ppm.

GC-MS (EI) for $C_{12}H_{24}^+$ [M^+] calculated 168.2, found 168.1.

The data is in accordance with literature.[26]

7.15.17 (*Z*)-1-(2-Cyclopropylvinyl)naphthalene (2q)

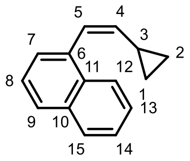

2q
$C_{15}H_{14}$
Mw = 194.28 gmol^{-1}

Prepared according to **GP1** from 1-(2-cyclopropylethynyl)naphthalene (**1q**, 38.4 mg, 0.200 mmol, 1.00 equiv), [IPrIPyCuCl] (**4**, 9.30 mg, 20.0 µmol, 10.0 mol%), NaO*t*Bu (5.95 mg, 60.0 µmol, 30.0 mol%) in diglyme (2.0 mL). The reaction mixture was stirred for 48 h at 120 °C under H_2 atmosphere (1.5 bar). Purification by flash column chromatography on silica gel yielded **2q** as yellow oil (20.5 mg, 0.11 mmol, 53%).

R*f* = 0.25 (SiO_2, cyclohexane).

^{1}H NMR (600 MHz, $CDCl_3$): δ = 8.09–8.08 (m, 1H, H-9), 7.86 (dd, $^3J_{15,14}$ = 7.7 Hz, $^4J_{15,13}$ = 1.8 Hz, 1H, H-15), 7.76 (d, $^3J_{7,8}$ = 8.2 Hz, 1H, H-7), 7.58 (dd, $^3J_{12,13}$ = 7.0 Hz, $^4J_{12,14}$ = 1.1 Hz, 1H, H-12), 7.52–7.45 (m, 3H, H-8, H-14, H-13), 6.80 (d, $^3J_{5,4}$ = 11.3 Hz, 1H, H-5), 5.31–5.27 (m, 1H, H-4), 1.69–1.63 (m, 1H, H-3), 0.76–0.73 (m, 2H, H-2), 0.51–0.50 (m, 2H, H-1) ppm.

^{13}C NMR (151 MHz, $CDCl_3$): δ = 138.6 (C-4), 135.2 (C-6), 133.8 (C-10), 132.1 (C-11), 128.5 (C-15), 127.2 (C-7), 126.7 (C-12), 125.9 (C-9), 125.8 (C-8)*, 125.4 (C-14)*, 125.3 (C-13)*, 124.9 (C-5), 11.5 (C-3), 7.8 (C-1, C-2) ppm.

HRMS (APCI) for $C_{15}H_{14}^+$ [M^+] calculated 194.1090, found 194.1085.

IR (ATR): ṽ = 3045 (w), 3004 (w), 2926 (m), 2855 (w), 2646 (w), 1640 (w), 1591 (w), 1509 (w), 1427 (w), 1386 (m), 1259 (w), 1166 (w), 1099 (w), 1013 (w), 935 (m), 887 (w), 779 (s), 749 (w), 689 (w) cm^{-1}.

7.15.18 (Z)-(3,3-dimethylbut-1-en-1-yl)benzene (2r)

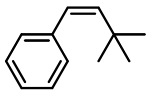

2r
$C_{12}H_{16}$
Mw = 160.26 gmol^{-1}

Prepared according to **GP1** from (3,3-dimethylbut-1-yn-1-yl)benzene (**1r**, 31.6 mg, 0.200 mmol, 1.00 equiv), [IPrIPyCuCl] (**4**, 9.30 mg, 20.0 µmol, 10.0 mol%), NaO*t*Bu (5.95 mg, 60.0 µmol, 30.0 mol%) in diglyme (2.0 mL). The reaction mixture was stirred for 48 h at 120 °C under H_2 atmosphere (1.5 bar).

Due to the low boiling point, product **2r** could not be fully purified. The crude GC/GCMS spectra are provided, which are in agreement of full conversion of alkyne **1r** to alkene **2r** with exclusive *Z*-selectivity.

7.15.19 1-((*Z*)-Styryl)adamantane (2s)

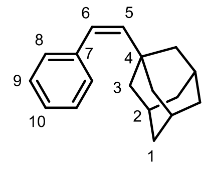

2s
$C_{18}H_{22}$
Mw = 238.37 gmol^{-1}

Prepared according to **GP1** from 1-(phenylethynyl)adamantane (**1s**, 47.3 mg, 0.200 mmol, 1.00 equiv), [IPrIPyCuCl] (**4**, 9.30 mg, 20.0 µmol, 10.0 mol%), NaO*t*Bu (5.95 mg, 60.0 µmol, 30.0 mol%) in diglyme (2.0 mL). The reaction mixture was stirred for 48 h at 120 °C under H_2 atmosphere (1.5 bar). Purification by flash column chromatography on silica gel yielded **2s** as colorless oil (18.6 mg, 0.08 mmol, 39%).

R*f* = 0.30 (SiO_2, cyclohexane).

^{1}H NMR (600 MHz, $CDCl_3$): δ = 7.28–7.25 (m, 2H, H-9), 7.21–7.18 (m, 3H, H-8, H-10), 6.40 (d, $^3J_{6,5}$ = 12.7 Hz, 1H, H-6), 5.36 (d, $^3J_{6,5}$ = 12.7 Hz, 1H, H-5), 1.86 (br s, 3H, H-2), 1.63–1.55 (m, 12H, H-1, H-3) ppm.

^{13}C NMR (151 MHz, $CDCl_3$): δ = 143.1 (C-5), 139.9 (C-7), 129.1 (C-8), 127.6 (C-9), 127.2 (C-10), 126.2 (C-6), 43.3 (C-3), 36.9 (C-1), 36.8 (C-4), 28.7 (C-2) ppm.

HRMS (APCI) for $C_{18}H_{22}^+$ [M$^+$] calculated 238.1716, found 238.1717.

IR (ATR): ṽ = 3056 (w), 3022 (w), 2933 (w), 2903 (s), 2847 (m), 1490 (w), 1449 (w), 1408 (w), 1345 (w), 1315 (w), 1263 (m), 1181 (w), 1103 (w), 1069 (w), 1028 (w), 987 (w), 916 (w), 834 (w), 738 (s), 700 (s) cm^{-1}.

7.15.20 (*Z*)-4,4-Difluoro-1-(6-phenylhex-5-en-1-yl)piperidine (2t)

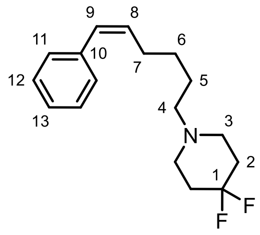

2t
$C_{17}H_{23}F_2N$
Mw = 279.37 gmol^{-1}

Prepared according to **GP1** from 4,4-difluoro-1-(6-phenylhex-5-yn-1-yl)piperidine (**1t**, 55.5 mg, 0.200 mmol, 1.00 equiv), [IPrIPyCuCl] (**4**, 9.30 mg, 20.0 µmol, 10.0 mol%), NaO*t*Bu (5.95 mg, 60.0 µmol, 60.0 mol%) in diglyme (2.0 mL). The reaction mixture was stirred for 48 h at 120 °C under H_2 atmosphere (1.5 bar). Purification by flash column chromatography on silica gel yielded **2t** as yellow oil (35.2 mg, 0.126 mmol, 63%).

R*f* = 0.20 (SiO_2, cyclohexane/EtOAc = 10:1).

^{1}H NMR (600 MHz, $CDCl_3$): δ = 7.34 (d, $^3J_{11,12}$ = 8.2 Hz, 2H, H-11), 7.29 (t, $^3J_{12,11/13}$ = 7.7 Hz, 2H, H-12), 7.21–7.18 (m, 1H, H-13), 6.39 (d, $^3J_{9,8}$ =12.0 Hz, 1H, H-9), 6.21 (dt, $^3J_{8,9}$ = 12.0 Hz, $^3J_{8,7}$ =6.9 Hz, 1H, H-8), 2.54 (br s, 4H, H-3), 2.40 (t, $^3J_{4,5}$ = 6.6 Hz, 2H, H-4), 2.24–2.22 (m, 2H, H-7), 2.03–1.96 (m, 4H, H-2), 1.56–1.48 (m, 4H, H-5, H-6) ppm.

^{13}C NMR (151 MHz, $CDCl_3$): δ = 137.9 (C-10), 130.7 (C-8), 130.2 (C-9), 128.6 (C-11), 127.0 (C-13), 126.1 (C-12), 122.3 (t, $^1J_{1,F}$ = 241.4 Hz, C-1), 57.8 (C-4), 50.2 (t, $^3J_{3,F}$ = 18.0 Hz, C-3), 34.2 (t, $^2J_{2,F}$ = 22.8 Hz, C-2), 33.0 (C-7), 27.4 (C-5), 27.0 (C-6) ppm.

^{19}F NMR (473 MHz, $CDCl_3$): δ = –98.2 (s) ppm.

HRMS (APCI) for C17H24F_2N^+ [$(M+H)^+$] calculated 280.1871, found 280.1867.

IR (ATR): ṽ = 3060 (w), 3026 (w), 2937 (w), 2862 (w), 2817 (w), 1364 (m), 1312 (w), 1267 (w), 1155 (w), 1095 (s), 1077 (w), 954 (m), 738 (s), 693 (m) cm^{-1}.

7.15.21 (*Z*)-(6-Chlorohex-1-en-1-yl)benzene (2u)

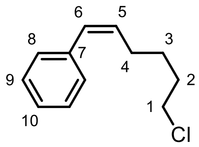

2u
$C_{12}H_{15}Cl$
Mw = 194.70 $gmol^{-1}$

Prepared according to **GP1** from (6-chlorohex-1-yn-1-yl)benzene (**1u**, 38.5 mg, 0.200 mmol, 1.00 equiv), [IPrIPyCuCl] (**4**, 9.30 mg, 2.00 µmol, 10.0 mol%), NaO*t*Bu (5.95 mg, 2.00 µmol, 30.0 mol%) in diglyme (2.0 mL). The reaction mixture was stirred for 48 h at 120 °C under H_2 atmosphere (1.5 bar). Purification by flash column chromatography on silica gel yielded **2u** as colorless oil (19.0 mg, 0.095 mmol, 49%).

R_f = 0.30 (SiO_2, cyclohexane/EtOAc = 50:1).

^{1}H NMR (600 MHz, $CDCl_3$): δ = 7.34 (t, $^3J_{9,8/10}$ = 7.1 Hz, 2H, H-9), 7.27 (d, $^3J_{8,9}$ = 6.7 Hz, 2H, H-8) 7.22 (tt, $^3J_{10,9}$ = 7.2 Hz, $^4J_{10,8}$ = 1.2 Hz, 1H, H-10), 6.45 (td, $^3J_{6,5}$ = 11.6 Hz, $^4J_{6,4}$ = 1.9 Hz, 1H, H-6), 5.64 (td, $^3J_{5,6}$ = 11.6 Hz, $^3J_{5,4}$ = 7.2 Hz, 1H, H-5), 3.52 (t, $^3J_{1,2}$ = 6.6 Hz, 2H, H-1), 2.37 (dq, $^3J_{4,5/3}$ = 7.4 Hz, $^4J_{4,6}$ = 1.8 Hz, 2H, H-4), 1.83–1.79 (m, 2H, H-2), 1.63–1.60 (m, 2H, H-3) ppm.

^{13}C NMR (151 MHz, $CDCl_3$): δ = 137.7 (C-7), 132.2 (C-5), 129.6 (C-6), 128.9 (C-9), 128.3 (C-8), 126.7 (C-10), 45.0 (C-1), 32.3 (C-4), 27.9 (C-2), 27.3 (C-3) ppm.

HRMS (APCI) for $C_{12}H_{15}{}^{35}Cl^+$ [M^+] calculated 194.0857, found 194.0861.

IR (ATR): ṽ = 3011 (w), 2937 (m), 2862 (w), 1725 (w), 1494 (w), 1446 (m), 1274 (m), 1073 (w), 916 (w), 767 (w), 700 (m) cm^{-1}.

7.15.22 (*Z*)-Dec-5-ene (2v)

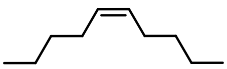

2v
$C_{10}H_{20}$
Mw = 140.27 $gmol^{-1}$

Prepared according to **GP1** from 5-dodecyne (**1v**, 27.6 mg, 0.200 mmol, 1.00 equiv), [IPrIPyCuCl] (**4**, 9.30 mg, 2.00 µmol, 10.0 mol%), NaO*t*Bu (5.95 mg, 2.00 µmol, 30.0 mol%) in diglyme (2.0 mL). The reaction mixture was stirred for 48 h at 120 °C under H_2 atmosphere (1.5 bar). After hydrogenation, 43% conversion of alkyne **1v** to alkene **2v** was observed. Upon performing the alkyne semihydrogenation at 100 bar, 85% conversion alkyne **1v** to alkene **2v** was observed.

Due to the low boiling point, product **2v** could not be purified. The crude GC and ^{1}H NMR spectra are provided, which are in agreement alkyne semihydrogenation of **1v** to alkene **2v** with exclusive *Z*-selectivity.

7.15.23 (*Z*)-2-(4-Methoxystyryl)pyridine (2w)

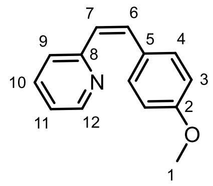

2w
$C_{14}H_{13}NO$
Mw = 211.26 gmol^{-1}

Prepared according to **GP1** from 2-((4-methoxyphenyl)ethynyl)pyridine (**1w**, 41.9 mg, 0.200 mmol, 1.00 equiv), [IPrIPyCuCl] (**4**, 9.30 mg, 20.0 µmol, 10.0 mol%), NaO*t*Bu (5.95 mg, 60.0 µmol, 30.0 mol%) in diglyme (2.0 mL). The reaction mixture was stirred for 48 h at 120 °C under H_2 atmosphere (1.5 bar). Purification by flash column chromatography on silica gel yielded **2w** & **2w'** (90:10 ratio) as colorless oil (32.0 mg, 0.124 mmol, 62%).

The yield of the product **2v** is calculated with exclusion of the over reduction product (**2v'**) amnout using ^{1}H NMR.

R*f* = 0.20 (SiO_2, cyclohexane/EtOAc = 20:1).

^{1}H NMR (600 MHz, $CDCl_3$): δ = 8.59 (d, $^{3}J_{12,11}$ = 5.4 Hz 1H, H-12), 7.65 (td, $^{3}J_{10,11/9}$ = 7.7 Hz, $^{4}J_{10,12}$ = 1.8 Hz, 1H, H-10), 7.60 (d, $^{3}J_{6,7}$ =12.1 Hz, 1H, H-6), 7.53 (d, $^{3}J_{4,3}$ = 8.5 Hz, 2H, H-4), 7.36 (d, $^{3}J_{9,10}$ = 7.9 Hz, 1H, H-9), 7.16–7.09 (m, 1H, H-11), 7.05 (d, $^{3}J_{6,7}$ =12.1 Hz, 1H, H-7), 6.91 (d, $^{3}J_{3,4}$ = 8.6 Hz, 2H, H-3), 3.84 (s, 3H, H-1) ppm.

^{13}C NMR (151 MHz, $CDCl_3$): δ = 160.0 (C-2), 156.1 (C-8), 149.6 (C-12), 136.7 (C-10), 132.6 (C-6), 130.5 (C-5), 128.6 (C-4), 125.8 (C-7), 121.8 (C-9), 121.6 (C-11), 114.3 (C-3), 55.5 (C-1) ppm.

HRMS (APCI) for $C_{14}H_{14}NO^{+}$ [$(M+H)^{+}$] calculated 212.1070, found 212.1064.

IR (ATR): ṽ = 3056 (w), 3008 (w), 2959 (w), 2933 (w), 2840 (w), 2307 (w), 2221 (w), 1606 (w), 1513 (m), 1464 (w), 1431 (w), 1248 (s), 1177 (w), 1092 (w), 1032 (m), 831 (w), 734 (s) cm^{-1}.

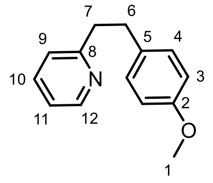

2w'
$C_{14}H_{15}NO$
Mw = 213.26 gmol^{-1}

The ^{1}H NMR and ^{13}C NMR shows impurities of the corresponding over-reduction product **2w'** (10%)

7.15.24 (*Z*)-3-(4-Methoxystyryl)pyridine (2x)

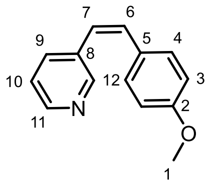

2x
$C_{14}H_{13}NO$
Mw = 211.26 gmol^{-1}

Prepared according to **GP1** from 3-((4-methoxyphenyl)ethynyl)pyridine (**1x**, 41.9 mg, 0.200 mmol, 1.00 equiv), [IPrIPyCuCl] (**4**, 9.30 mg, 20.0 µmol, 10.0 mol%), NaO*t*Bu (5.95 mg, 60.0 µmol, 60.0 mol%) in diglyme (2.0 mL). The reaction mixture was stirred for 48 h at 120 °C under H_2 atmosphere (1.5 bar). Purification by flash column chromatography on silica gel yielded **2x** as colorless oil (30.8 mg, 0.146 mmol, 73%).

R*f* = 0.20 (SiO_2, cyclohexane/EtOAc = 20:1).

^{1}H NMR (600 MHz, $CDCl_3$): δ = 8.50 (d, $^4J_{12,11}$ = 2.1 Hz 1H, H-12), 8.41 (dd, $^3J_{11,10}$ = 4.8 Hz, $^4J_{11,9}$ = 1.4 Hz, 1H, H-11), 7.54 (dt, $^3J_{10,11/9}$ = 7.9 Hz, $^4J_{10,12}$ = 12.1 Hz, 1H, H-10), 7.15–7.12 (m, 3H, H-4, H-9), 6.77 (d, $^3J_{3,4}$ = 8.7 Hz, 2H, H-3), 6.67 (d, $^3J_{7,6}$ = 12.1 Hz, 1H, H-7), 6.45 (d, $^3J_{6,7}$ = 12.1 Hz, 1H, H-6), 3.79 (s, 3H, H-1) ppm.

^{13}C NMR (151 MHz, $CDCl_3$): δ = 159.2 (C-12), 150.3 (C-11), 148.1 (C-9), 135.9 (C-10), 133.5 (C-8), 132.3 (C-7), 130.2 (C-4), 129.0 (C-5), 125.1 (C-6), 123.2 (C-9), 114.0 (C-3), 55.4 (C-1) ppm.

HRMS (APCI) for $C_{14}H_{14}NO^+$ [(M+H)$^+$] calculated 212.1070, found 212.1064.

IR (ATR): ṽ = 3056 (w), 3008 (w), 2959 (w), 2933 (w), 2840 (w), 2307 (w), 2221 (w) 1606 (m), 1513 (s), 1464 (w), 1431 (w), 1248 (s), 1177 (m), 1092 (w), 1032 (s), 831 (m), 734 (w) cm^{-1}.

7.15.25 (*Z*)-2-(4-Methoxystyryl)-1-methyl-1*H*-imidazole (2y)

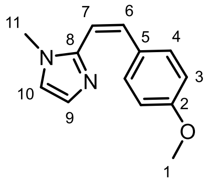

2y
$C_{13}H_{14}N_2O$
Mw = 214.27 gmol^{-1}

Prepared according to **GP1** from 2-((4-methoxyphenyl)ethynyl)-1-methyl-1*H*-imidazole (**1y**, 42.4 mg, 0.200 mmol, 1.00 equiv), [IPrIPyCuCl] (**4**, 9.30 mg, 20.0 µmol, 10.0 mol%), NaO*t*Bu (5.95 mg, 60.0 µmol, 30.0 mol%) in diglyme (2.0 mL). The reaction mixture was stirred for 48 h at 120 °C under H_2 atmosphere (1.5 bar). Purification by flash column chromatography on silica gel yielded **2y** as orange oil (30.5 mg, 0.142 mmol, 71%).

R*f* = 0.30 (SiO_2, CH_2Cl_2/MeOH = 95:5)

^{1}H NMR (600 MHz, $CDCl_3$): δ = 7.26 (d, $^3J_{4,3}$ = 8.7 Hz, 2H, H-4), 7.10 (s, 1H, H-9), 6.82 (s, 1H, H-10), 6.78 (d, $^3J_{3,4}$ = 8.7 Hz, 2H, H-3), 6.73 (d, $^3J_{7,6}$ = 12.2 Hz, 1H, H-7), 6.28 (d, $^3J_{6,7}$ = 12.1 Hz, 1H, H-6), 3.81 (s, 3H, H-1), 3.34 (s, 3H, H-11) ppm.

^{13}C NMR (151 MHz, $CDCl_3$): δ = 159.6 (C-2), 145.0 (C-8), 135.3 (C-6), 130.5 (C-4), 129.1 (C-5), 128.7 (C-9), 120.7 (C-10), 115.1 (C-7), 113.9 (C-3), 55.4 (C-1), 33.2 (C-11) ppm.

HRMS (APCI) for $C_{13}H_{15}N_2O^+$ [(M+H)$^+$] calculated 215.1179, found 215.1179.

IR (ATR): ṽ = 3108 (w), 3052 (w), 3004 (w), 2937 (m), 2840 (w), 2560 (w), 1740 (m), 1684 (w), 1602 (m), 1513 (m), 1461 (w), 1423 (w), 1304 (w), 1252 (s), 1177 (w), 1136 (w), 1028 (m), 969 (w), 935 (w), 730 (s) cm^{-1}.

7.15.26 (*Z*)-3-Styrylthiophene (2z)

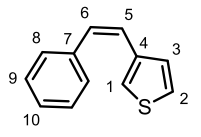

2z
$C_{12}H_{10}S$
Mw = 186.27 $gmol^{-1}$

Prepared according to **GP1** from 3-(phenylethynyl)thiophene (**1z**, 36.8 mg, 0.200 mmol, 1.00 equiv), [IPrIPyCuCl] (**4**, 9.30 mg, 20.0 µmol, 10.0 mol%), NaO*t*Bu (5.95 mg, 60.0 µmol, 30.0 mol%) in diglyme (2.0 mL). The reaction mixture was stirred for 48 h at 120 °C under H_2 atmosphere (1.5 bar). Purification by flash column chromatography on silica gel yielded **2z** as colorless oil (23.0 mg, 0.124 mmol, 62%).

R*f* = 0.20 (SiO_2, cyclohexane/EtOAc = 40:1).

^{1}H NMR (600 MHz, $CDCl_3$): δ = 7.27–7.20 (m, 4H, H-8. H-9), 7.20–7.19 (m, 1H, H-10), 7.09 (dd, $^3J_{3,2}$ = 5.0 Hz, $^4J_{3,1}$ = 3.0 Hz, 1H, H-3), 7.07 (dd, $^4J_{1,3}$ = 3.0 Hz, $^4J_{1,2}$ = 1.2 Hz, 1H, H-1), 6.83 (dd, $^3J_{2,3}$ = 5.0 Hz, $^4J_{2,1}$ = 1.3 Hz, 1H, H-2), 6.53 (d, $^3J_{6,5}$ = 12.1 Hz, 1H, H-6), 6.51 (d, $^3J_{5,6}$ = 12.1 Hz, 1H, H-5) ppm.

^{13}C NMR (151 MHz, $CDCl_3$): δ = 138.4 (C-7), 137.9 (C-4), 129.6 (C-6), 128.9 (C-8), 128.4 (C-9), 128.1 (C-2), 127.3 (C-10), 125.0 (C-3), 124.5 (C-5), 124.2 (C-1) ppm.

HRMS (APCI) for $C_{12}H_{10}S^+$ [M^+] calculated 186.0498, found 186.0493.

IR (ATR): ṽ = 3104 (w), 3056 (w), 3022 (w), 2926 (w), 2855 (w), 1599 (w), 1490 (m), 1420 (w), 1353 (w), 1263 (m), 1073 (m), 961 (w), 916 (w), 868 (w), 779 (s), 693 (m) cm^{-1}.

7.15.27 (*Z*)-1-Benzyl-5-styryl-1*H*-indole (2aa)

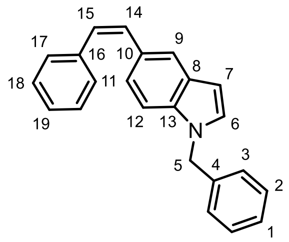

2aa
$C_{23}H_{19}N$
Mw = 309.41 $gmol^{-1}$

Prepared according to **GP1** from 1-benzyl-5-(phenylethynyl)-1*H*-indole (**1aa**, 61.5 mg, 0.200 mmol, 1.00 equiv), [IPrIPyCuCl] (**4**, 9.30 mg, 20.0 µmol, 10.0 mol%), NaO*t*Bu (5.95 mg, 60.0 µmol, 30.0 mol%) in diglyme (2.0 mL). The reaction mixture was stirred for 48 h at 120 °C under H_2 atmosphere (1.5 bar). Purification by flash column chromatography on silica gel yielded **2aa** as colorless solid (45.8 mg, 0.15 mmol, 74%).

R*f* = 0.20 (SiO_2, cyclohexane/EtOAc = 10:1).

Mp = 68 °C (cyclohexane).

^{1}H NMR (600 MHz, $CDCl_3$): δ = 7.57 (s, 1H, H-9), 7.32–7.26 (m, 5H, H-12, H-17, H-18), 7.23–7.17 (m, 3H, H-1, H-6, H-19), 7.12–7.10 (m, 4H, H-2, H-3), 7.09–7.08 (m, 1H, H-11) 6.72 (d,

$^{3}J_{14,15}$ = 12.1 Hz, 1H, H-14), 6.53 (d, $^{3}J_{15,14}$ = 12.1 Hz, 1H, H-15), 6.46 (d, $^{3}J_{7,6}$ = 3.1 Hz, 1H, H-7), 5.28 (s, 2H, H-5) ppm.

^{13}C NMR (151 MHz, $CDCl_3$): δ = 138.1 (C-16), 137.5 (C-10), 135.7 (C-4), 131.5 (C-15), 129.1 (C-3)*, 128.9 (C-17)***, 128.8 (C-19)**, 128.7 (C-8)** 128.6 (C-12), 128.3 (C-18)***, 128.2 (C-14), 127.8 (C-6)**, 126.9 (C-2)*, 126.8 (C-1), 123.3 (C-11), 121.7 (C-13), 109.5 (C-9), 102.2 (C-7), 50.3 (C-5) ppm.

HRMS (APCI) for $C_{23}H_{20}N^+$ [$(M+H)^+$] calculated 310.1590, found 310.1584.

IR (ATR): ṽ = 3060 (w), 3026 (m), 2922 (w), 2858 (w), 2657 (w), 1599 (m), 1483 (m), 1446 (s), 1394 (w), 1356 (w), 1263 (m), 1181 (m), 1121 (w), 1073 (m), 1028 (m), 961 (w), 916 (w), 890 (w) cm^{-1}.

7.16 Products of conjugate reduction of α,β-unsaturated amides

7.16.1 *N*,*N*-Diethyl-3-phenylpropanamide (12a)

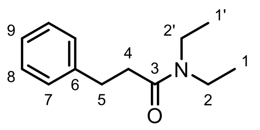

12a
$C_{13}H_{19}NO$
Mw = 205.30 $gmol^{-1}$

Prepared according to **GP2** from *N*,*N*-diethylcinnamamide (**11a**, 40.6 mg, 0.200 mmol, 1.00 equiv), [IPrIPyCuCl] (**4**, 9.30 mg, 20.0 µmol, 10.0 mol%), NaO*t*Bu (9.62 mg, 100 µmol, 50.0 mol%) in diglyme (2.0 mL). The reaction mixture was stirred for 24 h at 100 °C under H_2 atmosphere (1.5 bar). Purification by flash column chromatography on silica gel (cyclohexane/EtOAc = 4:1) yielded **12a** as colorless oil (33.6 mg, 0.164 mmol, 82%).

R_f = 0.20 (SiO_2, cyclohexane/EtOAc = 4:1).

^{1}H NMR (600 MHz, $CDCl_3$): δ = 7.29 (t, $^{3}J_{8,7/9}$ = 7.6 Hz, 2H, H-8), 7.23–7.18 (m, 3H, H-7, H-9), 3.38 (q, $^{3}J_{2,1}$ = 7.1 Hz, 2H, H-2), 3.22 (q, $^{3}J_{2',1'}$ = 7.1 Hz, 2H, H-2'), 2.98 (t, $^{3}J_{5,4}$ = 7.8 Hz, 2H, H-5), 2.59 (t, $^{3}J_{4,5}$ = 7.8 Hz, 2H, H-4), 1.12–1.09 (m, 6H, H-1, H-1') ppm.

^{13}C NMR (151 MHz, $CDCl_3$): δ = 171.4 (C-3), 141.7 (C-6), 128.6 (C-7)*, 128.6 (C-8)*, 126.2 (C-9), 42.0 (C-2), 40.3 (C-2'), 35.2 (C-5), 31.8 (C-4), 14.4 (C-1), 13.2 (C-1') ppm.

HRMS (ESI) for $C_{13}H_{20}NO^+$ [$(M+H^+)^+$] calculated: 206.1539, found: 206.1541.

IR (ATR): ṽ = 3027 (w), 2974 (w), 2933 (w), 1636 (s), 1453 (m), 1431 (m), 1379 (w), 1267 (w), 1137 (m), 1073 (w), 749 (w), 701 (m) cm^{-1}.

The data is in accordance with literature.[27]

7.16.2 3-Phenyl-1-(piperidin-1-yl)propan-1-one (12b)

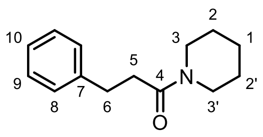

12b
$C_{14}H_{19}NO$
Mw = 217.32 gmol^{-1}

Prepared according to **GP2** from (*E*)-3-phenyl-1-(piperidin-1-yl)prop-2-en-1-one (**11b**, 43.1 mg, 0.200 mmol, 1.00 equiv), [IPrIPyCuCl] (**4**, 9.30 mg, 20.0 μmol, 10.0 mol%), NaO*t*Bu (9.62 mg, 100 μmol, 50.0 mol%) in diglyme (2.0 mL). The reaction mixture was stirred for 24 h at 100 °C under H_2 atmosphere (1.5 bar). Purification by flash column chromatography on silica gel (cyclohexane/EtOAc = 4:1) yielded **12b** as colorless oil (34.8 mg, 0.160 mmol, 80%).

R*f* = 0.15 (SiO_2, cyclohexane/EtOAc = 4:1).

^{1}H NMR (600 MHz, $CDCl_3$): δ = 7.28 (t, $^3J_{9,8/10}$ = 7.6 Hz, 2H, H-9), 7.22–7.18 (m, 3H, H-8, H-10), 3.56 (t, $^3J_{3,2}$ = 6.0 Hz, 2H, H-3), 3.33 (t, $^3J_{3',2'}$ = 6.0 Hz, 2H, H-3’), 2.96 (t, $^3J_{6,5}$ = 7.8 Hz, 2H, H-6), 2.61 (t, $^3J_{5,6}$ = 7.8 Hz, 2H, H-5), 1.63–1.59 (m, 2H, H-2), 1.54–1.50 (m, 2H, H-2’), 1.47–1.43 (m, 2H, H-1) ppm.

^{13}C NMR (151 MHz, $CDCl_3$): δ = 170.5 (C-4), 141.6 (C-7), 128.6 (C-8)*, 128.6 (C-9)*, 126.2 (C-10), 46.7 (C-3), 42.8 (C-3’), 35.3 (C-6), 31.7 (C-5), 26.5 (C-2), 25.7 (C-2’), 24.7 (C-1) ppm.

HRMS (ESI) for $C_{14}H_{20}NO^+$ [(M+H$^+$)$^+$] calculated: 218.1539, found: 218.1539.

IR (ATR): ṽ = 2937 (w), 2855 (w), 1636 (s), 1438 (m), 1252 (w), 1215 (w), 1137 (m), 1010 (w), 853 (w), 731 (m), 700 (m) cm^{-1}.

The data is in accordance with literature.[28]

7.16.3 1-(4,4-Difluoropiperidin-1-yl)-3-phenylpropan-1-one (12c)

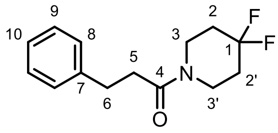

12c
$C_{14}H_{17}F_2NO$
Mw = 253.30 gmol^{-1}

Prepared according to **GP2** from (*E*)-1-(4,4-difluoropiperidin-1-yl)-3-phenylprop-2-en-1-one (**11c**, 50.2 mg, 0.200 mmol, 1.00 equiv), [IPrIPyCuCl] (**4**, 9.30 mg, 20.0 μmol, 10.0 mol%), NaO*t*Bu (9.62 mg, 100 μmol, 50.0 mol%) in diglyme (2.0 mL). The reaction mixture was stirred for 24 h at 100 °C under H_2 atmosphere (1.5 bar). Purification by flash column chromatography on silica gel (cyclohexane/EtOAc = 4:1) yielded **12c** as colorless oil (38.5 mg, 0.152 mmol, 76%).

R*f* = 0.15 (SiO_2, cyclohexane/EtOAc = 4:1).

^{1}H NMR (600 MHz, $CDCl_3$): δ = 7.29 (dd, $^3J_{9,10}$ = 8.1 Hz, $^3J_{9,8}$ = 7.0 Hz, 2H, H-9), 7.22–7.20 (m, 3H, H-8, H-10), 3.73 (t, $^3J_{3,2}$ = 6.0 Hz, 2H, H-3), 3.47 (t, $^3J_{3',2'}$ = 6.0 Hz, 2H, H-3’), 2.98 (t, $^3J_{6,5}$ = 7.2 Hz, 2H, H-6), 2.65 (t, $^3J_{5,6}$ = 7.2 Hz, 2H, H-5), 1.91 (tt, $^3J_{2,F}$ = 12.8 Hz, $^3J_{2,3}$ = 6.0 Hz, 2H, H-2), 1.76 (tt, $^3J_{2',F}$ = 12.8 Hz, $^3J_{2',3'}$ = 6.0 Hz, 2H, H-2’) ppm.

^{13}C NMR (151 MHz, $CDCl_3$): δ = 170.8 (C-4), 141.1 (C-7), 128.7 (C-8), 128.6 (C-9), 126.5 (C-10), 121.6 (t, $^{1}J_{1,F}$ = 242.2 Hz, C-1), 42.5 (t, $^{4}J_{3,F}$ = 5.5 Hz, C-3), 38.7 (t, $^{4}J_{3',F}$ = 5.5 Hz, C-3'), 35.0 (C-6), 34.5 (t, $^{3}J_{2,F}$ = 23.6 Hz, C-2), 33.9 (t, $^{3}J_{2',F}$ = 23.6 Hz, C-2'), 31.7 (C-5) ppm.

^{19}F NMR (473 MHz, $CDCl_3$): δ = –98.0 (s) ppm.

HRMS (ESI) for $C_{14}H_{18}F_2NO^+$ [$(M+H^+)^+$] calculated: 254.1351, found 254.1353.

IR (ATR): ṽ = 3055 (w), 3030 (w), 2981 (w), 2944 (w), 1643 (s), 1442 (m), 1360 (w), 1236 (w), 1218 (w), 1110 (m), 980 (w), 950 (w), 734 (s) cm^{-1}.

7.16.4 1-Morpholino-3-phenylpropan-1-one (12d)

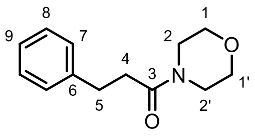

12d
$C_{13}H_{17}NO_2$
Mw = 219.28 $gmol^{-1}$

Prepared according to **GP2** from (*E*)-1-morpholino-3-phenylprop-2-en-1-one (**11d**, 43.4 mg, 0.200 mmol, 1.00 equiv), [IPrIPyCuCl] (**4**, 9.30 mg, 20.0 µmol, 10.0 mol%), NaO*t*Bu (9.62 mg, 100 µmol, 50.0 mol%) in diglyme (2.0 mL). The reaction mixture was stirred for 24 h at 100 °C under H_2 atmosphere (1.5 bar). Purification by flash column chromatography on silica gel (cyclohexane/EtOAc = 3:1) yielded **12d** as colorless oil (33.2 mg, 0.160 mmol, 80%).

R_f = 0.20 (SiO_2, cyclohexane/EtOAc = 3:1).

^{1}H NMR (600 MHz, $CDCl_3$): δ = 7.30–7.28 (m, 2H, H-8), 7.22–7.20 (m, 3H, H-7, H-9), 3.64–3.61 (m, 4H, H-1, H-1'), 3.51 (t, $^{3}J_{2,1}$ = 4.8 Hz, 2H, H-2), 3.35 (t, $^{3}J_{2',1'}$ = 4.8 Hz, 2H, H-2'), 2.98 (t, $^{3}J_{5,4}$ = 7.2 Hz, 2H, H-5), 2.61 (t, $^{3}J_{4,5}$ = 7.2 Hz, 2H, H-4) ppm.

^{13}C NMR (151 MHz, $CDCl_3$): δ = 171.0 (C-3), 141.2 (C-6), 128.7 (C-7)*, 128.6 (C-8)*, 126.4 (C-9), 67.0 (C-1), 66.6 (C-1'), 46.1 (C-2), 42.1 (C-2'), 35.0 (C-5), 31.6 (C-4) ppm.

HRMS (ESI) for $C_{13}H_{18}NO_2^+$ [$(M+H^+)^+$] calculated: 220.1332, found: 220.1332.

IR (ATR): ṽ = 2963 (w), 2922 (w), 2859 (w), 1647 (s), 1431 (m), 1274 (w), 1230 (w), 1118 (m), 1028 (w), 700 (w) cm^{-1}.

The data is in accordance with literature.[28]

7.16.5 3-Phenyl-1-thiomorpholinopropan-1-one (12e)

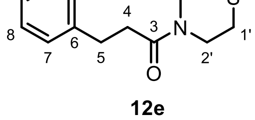

12e
$C_{13}H_{17}NOS$
Mw = 235.35 $gmol^{-1}$

Prepared according to **GP2** from (*E*)-3-phenyl-1-thiomorpholinoprop-2-en-1-one (**11e**, 50.2 mg, 0.200 mmol, 1.00 equiv), [IPrIPyCuCl] (**4**, 9.30 mg, 20.0 µmol, 10.0 mol%), NaO*t*Bu (9.62 mg, 100 µmol, 50.0 mol%) in diglyme (2.0 mL). The reaction mixture was stirred for 24 h at 100 °C under H_2 atmosphere (1.5 bar). Purification by flash column chromatography on silica gel (cyclohexane/EtOAc = 3:1) yielded **12e** as colorless oil (43.6 mg, 0.154 mmol, 82%).

R$_f$ = 0.20 (SiO_2, cyclohexane/EtOAc = 3:1).

^{1}H NMR (600 MHz, $CDCl_3$): δ = 7.29 (dd, $^3J_{8,9}$ = 8.2 Hz, $^3J_{8,7}$ = 7.0 Hz, 2H, H-8), 7.21–7.19 (m, 3H, H-7, H-9), 3.87 (t, $^3J_{2,1}$ = 5.4 Hz, 2H, H-2), 3.65 (t, $^3J_{2',1'}$ = 4.8 Hz, 2H, H-2'), 2.97 (t, $^3J_{5,4}$ = 7.2 Hz, 2H, H-5), 2.61–2.57 (m, 4H, H-4, H-1'), 2.45 (t, $^3J_{1,2}$ = 5.4 Hz, 2H, H-1) ppm.

^{13}C NMR (151 MHz, $CDCl_3$): δ = 170.7 (C-3), 141.2 (C-6), 128.6 (C-7)*, 128.5 (C-8)*, 126.4 (C-9), 48.3 (C-2), 44.4 (C-2'), 35.2 (C-5), 31.6 (C-4), 27.8 (C-1), 27.4 (C-1') ppm.

HRMS (ESI) for $C_{13}H_{18}NOS^+$ [(M+H$^+$)$^+$] calculated: 236.1104, found: 236.1111.

IR (ATR): ṽ = 3064 (w), 3027 (w), 2911 (w), 1640 (s), 1420 (m), 1289 (w), 1185 (m), 954 (w), 749 (w), 701 (m) cm^{-1}.

7.16.6 *N*,*N*-Bis(2-methoxyethyl)-3-phenylpropanamide (12f)

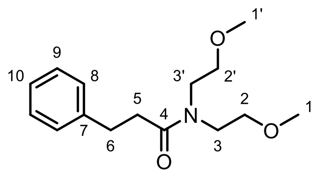

12f
$C_{15}H_{23}NO_3$
Mw = 265.35 gmol^{-1}

Prepared according to **GP2** from *N*,*N*-bis(2-methoxyethyl)cinnamamide (**11f**, 52.7 mg, 0.200 mmol, 1.00 equiv), [IPrIPyCuCl] (**4**, 9.30 mg, 20.0 µmol, 10.0 mol%), NaO*t*Bu (9.62 mg, 100 µmol, 50.0 mol%) in diglyme (2.0 mL). The reaction mixture was stirred for 24 h at 100 °C under H_2 atmosphere (1.5 bar). Purification by flash column chromatography on silica gel (cyclohexane/EtOAc = 3:1) yielded **12f** as colorless oil (40.3 mg, 0.152 mmol, 76%).

R$_f$ = 0.20 (SiO_2, cyclohexane/EtOAc = 3:1).

^{1}H NMR (600 MHz, $CDCl_3$): δ = 7.28 (t, $^3J_{9,8/10}$ = 7.6 Hz, 2H, H-9), 7.22–7.18 (m, 3H, H-8, H-10), 3.56 (t, $^3J_{2,3}$ = 5.4 Hz, 2H, H-2), 3.51 (t, $^3J_{2',3'}$ = 5.4 Hz, 2H, H-2'), 3.41 (t, $^3J_{3,2}$ = 5.7 Hz, 2H, H-3), 3.31 (t, $^3J_{3',2'}$ = 5.7 Hz, 2H, H-3'), 3.29 (s, 3H, H-1), 3.28 (s, 3H, H-1'), 2.97 (t, $^3J_{6,5}$ = 7.8 Hz, 2H, H-6), 2.69 (t, $^3J_{5,6}$ = 7.8 Hz, 2H, H-5) ppm.

^{13}C NMR (151 MHz, $CDCl_3$): δ = 172.9 (C-4), 141.7 (C-7), 128.6 (C-8)*, 128.5 (C-9)*, 126.1 (C-10), 71.3 (C-2), 70.8 (C-2'), 59.2 (C-1), 58.9 (C-1'), 48.9 (C-3), 46.5 (C-3'), 35.1 (C-6), 31.6 (C-5) ppm.

HRMS (ESI) for $C_{15}H_{24}NO_3^+$ [(M+H$^+$)$^+$] calculated: 266.1751, found: 266.1755.

IR (ATR): ṽ = 3030 (w), 2985 (w), 2930 (w), 2885 (w), 1640 (s), 1453 (m), 1420 (w), 1267 (w), 1192 (w), 1118 (m), 734 (m) cm^{-1}.

7.16.7 1-(4,7-Dihydrothieno[2,3-*c*]pyridin-6(5*H*)-yl)-3-phenylpropan-1-one (12g)

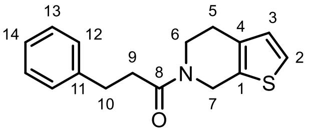

12g
$C_{16}H_{17}NOS$
Mw = 271.36 gmol^{-1}

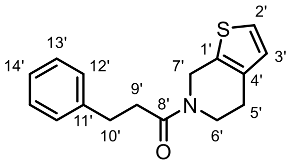

12g_rotamer
$C_{16}H_{17}NOS$
Mw = 271.36 gmol^{-1}

Prepared according to **GP2** from (*E*)-1-(4,7-dihydrothieno[2,3-c]pyridin-6(5*H*)-yl)-3-phenylprop-2-en-1-one (**11g**, 53.8 mg, 0.200 mmol, 1.00 equiv), [IPrIPyCuCl] (**4**, 9.30 mg, 20.0 µmol, 10.0 mol%), NaO*t*Bu (9.62 mg, 100 µmol, 50.0 mol%) in diglyme (2.0 mL). The reaction mixture was stirred for 24 h at 100 °C under H_2 atmosphere (1.5 bar). Purification by flash column chromatography on silica gel (cyclohexane/EtOAc = 1:1) yielded **12g** and **12g_rotamer** (50:50 ratio) as colorless oil (42.0 mg, 0.154 mmol, 77%).

$\mathbf{R_f}$ = 0.30 (cyclohexane/EtOAc = 1:1).

^{1}H NMR (600 MHz, $CDCl_3$): δ = 7.31–7.17 (m, 5H, H-12, H-13, H-14), 7.13 (d, $^3J_{3,2}$ = 5.2 Hz, 1H, H-3), 6.81 (d, $^3J_{2,3}$ = 5.1 Hz, 1H, H-2), 4.69 (s, 2H, H-7), 3.93 (t, $^3J_{6,5}$ = 5.7 Hz, 2H, H-6), 3.04–2.97 (m, 2H, H-10), 2.85 (t, $^3J_{5,6}$ = 5.9 Hz, 2H, H-5), 2.76–2.67 (m, 2H, H-9) ppm.

^{13}C NMR (151 MHz, $CDCl_3$): δ = 171.4 (C-8), 141.4 (C-11), 134.6 (C-4), 132.7 (C-1), 128.7 (C-12)*, 128.6 (C-13)*, 126.4 (C-14), 125.4 (C-2), 123.7 (C-3), 42.8 (C-7), 39.9 (C-6), 36.0 (C-9), 31.6 (C-10), 24.9 (C-5) ppm.

The ^{1}H and ^{13}C NMR spectrum for the two rotamers are well separated, which is why both ^{1}H and ^{13}C NMR are assigned separately.

^{1}H NMR of **12g_rotamer** (600 MHz, $CDCl_3$): δ = 7.31–7.17 (m, 5H, H-12', H-13', H-14'), 7.13 (d, $^3J_{3',2'}$ = 5.2 Hz, 1H, H-3'), 6.71 (d, $^3J_{2',3'}$ = 5.1 Hz, 1H, H-2'), 4.47 (s, 2H, H-7'), 3.67 (t, $^3J_{6',5'}$ = 5.7 Hz, 2H, H-6'), 3.04–2.97 (m, 2H, H-10'), 2.81 (t, $^3J_{5',6'}$ = 5.8 Hz, 2H, H-5'), 2.76–2.67 (m, 2H, H-9') ppm.

^{13}C NMR of **12g_rotamer** (151 MHz, $CDCl_3$): δ = 171.3 (C-8'), 141.4 (C-11'), 132.3 (C-4'), 131.2 (C-1'), 128.6 (C-12')*, 128.5 (C-13')*, 126.3 (C-14'), 124.6 (C-2'), 123.5 (C-3'), 45.7 (C-7'), 43.5 (C-6'), 35.6 (C-9'), 31.5 (C-10'), 25.8 (C-5') ppm.

Rotamer ratio is determined from the integration value of H-2 (at 6.81 ppm) and H-2' (at 6.71 ppm).

HRMS (ESI) for $C_{16}H_{18}NOS^+$ [(M+H$^+$)$^+$] calculated: 272.1104, found: 272.1097.

IR (ATR): ṽ = 3064 (w), 3026 (w), 2930 (w), 2859 (w), 1636 (s), 1494 (w), 1438 (s), 1349 (w), 1271 (m), 1207 (w), 1121 (w), 998 (w), 954 (w), 834 (m), 752 (w), 700 (m) cm^{-1}.

7.16.8 1-(4-(2,4-Difluorobenzoyl)piperidin-1-yl)-3-phenylpropan-1-one (12h)

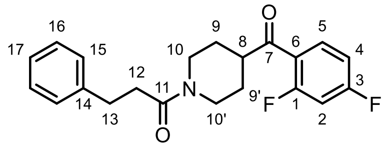

12h
$C_{21}H_{21}F_2NO_2$
Mw = 357.40 gmol^{-1}

Prepared according to **GP2** from (*E*)-1-(4-(2,4-difluorobenzoyl)piperidin-1-yl)-3-phenylprop-2-en-1-one (**11h**, 71.9 mg, 0.200 mmol, 1.00 equiv), [IPrIPyCuCl] (**4**, 9.30 mg, 20.0 µmol, 10.0 mol%), NaO*t*Bu (9.62 mg, 100 µmol, 50.0 mol%) in diglyme (2.0 mL). The reaction mixture was stirred for 24 h at 100 °C under H_2 atmosphere (1.5 bar). Purification by flash column chromatography on silica gel (cyclohexane/EtOAc = 3:1) yielded **12h** as colorless oil (51.7 mg, 0.138 mmol, 69%).

R*f* = 0.20 (cyclohexane/EtOAc = 3:1).

^{1}H NMR (600 MHz, $CDCl_3$): δ = 7.83 (q, $^{3/4}J_{5,4/3F,1F}$ = 7.8 Hz, 1H, H-5), 7.27 (t, $^3J_{16,15/17}$ = 7.2 Hz, 2H, H-16), 7.21–7.17 (m, 3H, H-15, H-17), 6.96 (t, $^3J_{4,5/3F}$ = 8.4 Hz, 1H, H-4), 6.86 (t, $^3J_{2,1F/3F}$ = 10.2 Hz, 1H, H-2), 4.56 (d, $^3J_{10a,9a}$ = 13.4 Hz, 1H, H-10a), 3.83 (d, $^3J_{10'a,9'a}$ = 13.4 Hz, 1H, H-10'a), 3.28 (t, $^3J_{8,9a/9'a}$ = 11.6 Hz, 1H, H-8), 3.07 (t, $^3J_{10'b,9'}$ = 13.6 Hz, 1H, H-10'b), 2.96 (t, $^3J_{13,12}$ = 7.9 Hz, 2H, H-13), 2.78 (t, $^3J_{10b,9}$ = 12.2 Hz, 1H, H-10b), 2.63 (t, $^3J_{12,13}$ = 7.9 Hz, 2H, H-12), 1.93–1.83 (m, 2H, H-9) 1.56–1.53 (m, 2H, H-9') ppm.

^{13}C NMR (151 MHz, $CDCl_3$): δ = 198.7 (d, $^3J_{7,1F}$ = 4.8 Hz, C-7), 170.6 (C-11), 165.8 (dd, $^1J_{1,1F}$ = 257.2 Hz, $^3J_{1,3F}$ = 12.5 Hz, C-1), 162.0 (dd, $^1J_{3,3F}$ = 255.7 Hz, $^3J_{3,1F}$ = 12.5 Hz, C-3), 141.3 (C-14), 133.1 (dd, $^3J_{5,1F}$ = 10.4 Hz, $^3J_{5,3F}$ = 4.4 Hz, C-5), 128.6 (C-15)*, 128.5 (C-16)*, 126.2 (C-17), 121.5 (dd, $^2J_{6,1F}$ = 13.6 Hz, $^4J_{6,3F}$ = 3.7 Hz, C-6), 112.6 (dd, $^2J_{4,3F}$ = 21.5 Hz, $^4J_{4,1F}$ = 3.4 Hz, C-4), 104.8 (dd, $^2J_{2,3F}$ = 28.2 Hz, $^2J_{2,1F}$ = 25.5 Hz, C-2), 47.8 (d, $^4J_{8,1F}$ = 6.8 Hz, C-8), 45.0 (C-10'), 41.3 (C-10), 35.2 (C-12), 31.6 (C-13), 28.1 (C-9), 27.9 (C-9') ppm.

^{19}F NMR (473 MHz, $CDCl_3$): δ = –101.7 (d, $^4J_{1F,3F}$ = 12.0 Hz, F-1), –106.5 (d, $^4J_{3F,31F}$ = 12.0 Hz, F-3) ppm.

HRMS (ESI) for $C_{21}H_{22}F_2NO_2^+$ [(M+H$^+$)$^+$] calculated: 358.1613, found: 358.1612.

IR (ATR): ṽ = 3063 (w), 3026 (w), 2930 (w), 2863 (w), 1781 (s), 1684 (m), 1640 (s), 1606 (s), 1494 (w), 1423 (w), 1375 (w), 1308 (w), 1267 (w), 1237 (w), 1200 (w), 1144 (w), 976 (m), 853 (w), 753 (w), 700 (w) cm^{-1}.

7.16.9 1-(4-((2,4-Difluorophenyl)(methoxyimino)methyl)piperidin-1-yl)-3-phenylpropan-1-one (12i)

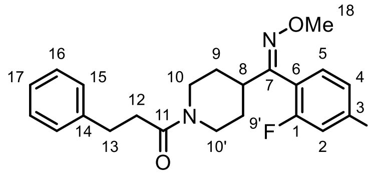

12i
$C_{22}H_{24}F_2N_2O_2$
Mw = 386.44 gmol^{-1}

Prepared according to **GP2** from (*E*)-1-(4-(2,4-difluorophenyl)(methoxyimino)methyl)piperidin-1-yl)-3-phenylprop-2-en-1-one (**11i**, 76.8 mg, 0.200 mmol, 1.00 equiv), [IPrIPyCuCl] (9.30 mg, 20.0 µmol, 10.0 mol%), NaO*t*Bu (9.62 mg, 100 µmol, 50.0 mol%) in diglyme (2.0 mL). The reaction mixture was stirred for 24 h at 100 °C under H_2 atmosphere (1.5 bar). Purification by flash column chromatography on silica gel (cyclohexane/EtOAc = 3:1) yielded **12i** as colorless oil (56.4 mg, 0.146 mmol, 73%).

R*f* = 0.20 (cyclohexane/EtOAc = 3:1).

^{1}H NMR (600 MHz, $CDCl_3$): δ = 7.25 (t, $^3J_{16,15/17}$ = 7.2 Hz, 2H, H-16), 7.19–7.15 (m, 3H, H-15, H-17), 7.03 (q, $^{3/4}J_{5,4/3F,1F}$ = 7.8 Hz, 1H, H-5), 6.89 (t, $^3J_{4,5/3F}$ = 8.4 Hz, 1H, H-4), 6.83 (t, $^3J_{2,1F/3F}$ = 10.2 Hz, 1H, H-2), 4.58 (d, $^2J_{10a,10b}$ = 12.9 Hz, 1H, H-10a), 3.80–3.79 (m, 4H, H-10'a, H-18), 2.97–2.92 (m, 3H, H-10'b, H-13), 2.63–2.57 (m, 4H, H-10b, H-8, H-12), 1.79–1.77 (m, 2H, H-9), 1.48–1.35 (m, 2H, H-9') ppm.

^{13}C NMR (151 MHz, $CDCl_3$): δ = 170.5 (C-11), 163.1 (dd, $^1J_{1,1F}$ = 250.9 Hz, $^3J_{1,3F}$ = 11.9 Hz, C-1), 159.0 (dd, $^1J_{3,3F}$ = 250.6 Hz, $^3J_{3,1F}$ = 12.4 Hz, C-3), 154.3 (d, $^3J_{7,1F}$ = 2.1 Hz, C-7), 141.4 (C-14), 129.8 (dd, $^3J_{5,1F}$ = 9.4 Hz, $^3J_{5,3F}$ = 6.3 Hz, C-5), 128.5 (C-15)*, 128.4 (C-16)* 126.2 (C-17), 117.7 (dd, $^2J_{6,1F}$ = 18.2 Hz, $^4J_{6,3F}$ = 4.4 Hz, C-6), 111.5 (dd, $^2J_{4,3F}$ = 21.5 Hz, $^4J_{4,1F}$ = 3.4 Hz, C-4), 104.4 (t, $^2J_{2,1F/3F}$ = 25.6 Hz, C-2), 62.0 (C-18), 45.4 (C-10'), 41.9 (C-8), 41.5 (C-10), 35.2 (C-12), 31.6 (C-13), 29.6 (C-9'), 29.1 (C-9') ppm.

^{19}F NMR (473 MHz, $CDCl_3$): δ = –108.8 (d, $^4J_{1F,3F}$ = 8.7 Hz, F-1), –108.9 (d, $^4J_{3F,31F}$ = 8.7 Hz, F-3) ppm.

HRMS (ESI) for $C_{22}H_{25}F_2N_2O_2^+$ [(M+H$^+$)$^+$] calculated: 387.1879, found: 387.1872.

IR (ATR): ṽ = 3027 (w), 2937 (w), 2858 (w), 2822 (w), 1633 (s), 1502 (m), 1446 (s), 1267 (w), 1207 (w), 1140 (w), 1084 (w), 1036 (w), 8995 (w), 965 (w), 917 (w), 850 (w), 731 (w) cm$^{–1}$.

7.16.10 4-(3-Phenylpropanoyl)piperazine-1-carbaldehyde (12j)

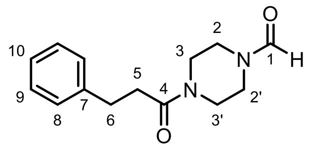

12j
$C_{14}H_{18}N_2O_2$
Mw = 246.31 gmol^{-1}

Prepared according to **GP2** from 4-cinnamoylpiperazine-1-carbaldehyde (**11j**, 48.8 mg, 0.200 mmol, 1.00 equiv), [IPrIPyCuCl] (**4**, 9.30 mg, 20.0 µmol, 10.0 mol%), NaO*t*Bu (9.62 mg, 100 µmol, 50.0 mol%) in diglyme (2.0 mL). The reaction mixture was stirred for 24 h at 100 °C under H_2 atmosphere (1.5 bar). Purification by flash

column chromatography on silica gel (cyclohexane/EtOAc = 2:3) yielded **12j** and **12j_rotamer** (60:40 ratio) as colorless oil (31.0 mg, 0.126 mmol, 63%).

R*f* = 0.20 (cyclohexane/EtOAc = 2:3).

^{1}H NMR (600 MHz, $CDCl_3$): δ = 8.05–8.04 (m, 1H, H-1), 7.29 (t, $^3J_{9,8/10}$ = 7.5 Hz, 2H, H-9), 7.22–7.20 (m, 3H, H-8, H-10), 3.66–3.60 (m, 2H, H-3)*, 3.49 (t, $^3J_{3'a,2'}$ = 5.3 Hz, 1H, H-3'a)*, 3.38–3.31 (m, 4H, H-2, H-2'), 3.15 (t, $^3J_{3'b,2'}$ = 5.1 Hz, 1H, H-3'b)*, 2.99 (t, $^3J_{6,5}$ = 7.8 Hz, 2H, H-6), 2.66 (t, $^3J_{5,6}$ = 7.8 Hz, 2H, H-5) ppm.

The ^{1}H NMR spectrum is not well separated for the two rotamers, which is why only ^{13}C NMR is assigned separately.

^{13}C NMR (151 MHz, $CDCl_3$): δ = 171.1 (C-4), 161.0 (C-1), 141.0 (C-7), 128.7 (C-8, C-9), 126.6 (C-10), 46.3 (C-3), 45.6 (C-3'), 42.3 (C-2), 40.0 (C-2'), 35.1 (C-5), 31.7 (C-6) ppm.

^{13}C NMR of **12j_rotamer** (151 MHz, $CDCl_3$): δ = 171.0 (C-4), 160.8 (C-1), 141.0 (C-7), 128.6 (C-8, C-9), 126.6 (C-10), 45.4 (C-3), 45.2 (C-3'), 41.2 (C-2), 40.1 (C-2'), 35.1 (C-5), 31.7 (C-6) ppm.

Rotamer ratio is determined from the integration value of H-1 at 8.06–8.05 ppm.

HRMS (ESI) for $C_{14}H_{19}N_2O_2^+$ [(M+H$^+$)$^+$] calculated: 247.1441, found: 247.1443.

IR (ATR): ṽ = 2922 (w), 2867 (w), 1645 (s), 1640 (s), 1431 (s), 1278 (w), 1248 (w), 1196 (w), 1002 (m), 752 (w), 701 (w) cm^{-1}.

7.16.11 1-(4-(Oxetan-3-yl)piperidin-1-yl)-3-phenylpropan-1-one (12k)

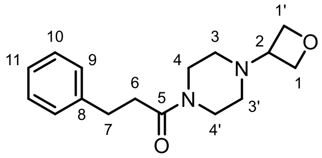

12k
$C_{16}H_{22}N_2O_2$
Mw = 274.36 gmol^{-1}

Prepared according to **GP2** from (*E*)-1-(4-(oxetan-3-yl)piperidin-1-yl)-3-phenylprop-2-en-1-one (**11k**, 54.3 mg, 0.200 mmol, 1.00 equiv), [IPrIPyCuCl] (**4**, 9.30 mg, 20.0 µmol, 10.0 mol%), NaO*t*Bu (9.62 mg, 100 µmol, 50.0 mol%) in diglyme (2.0 mL). The reaction mixture was stirred for 24 h at 100 °C under H_2 atmosphere (1.5 bar). Purification by flash column chromatography on silica gel (CH_2Cl_2/MeOH = 95:5) yielded **12k** as yellow oil (41.0 mg, 0.150 mmol, 75%).

R*f* = 0.25 (CH_2Cl_2/MeOH = 95:5).

^{1}H NMR (600 MHz, $CDCl_3$): δ = 7.29–7.26 (m, 2H, H-10), 7.21–7.18 (m, 3H, H-9, H-11), 4.64 (t, $^3J_{1,2/1'}$ = 6.6 Hz, 2H, H-1), 4.57 (t, $^3J_{1',2/1}$ = 6.6 Hz, 2H, H-1'), 3.66 (t, $^3J_{4,3}$ = 5.3 Hz, 2H, H-4), 3.47–3.40 (m, 3H, H-2, H-4') 2.96 (t, $^3J_{7,8}$ = 7.2 Hz, 2H, H-7), 2.61 (t, $^3J_{6,7}$ = 7.2 Hz, 2H, H-6), 2.25 (t, $^3J_{3,4}$ = 5.1 Hz, 2H, H-3), 2.16 (t, $^3J_{3',4'}$ = 5.1 Hz, 2H, H-3') ppm.

^{13}C NMR (151 MHz, $CDCl_3$): δ = 170.7 (C-5), 141.3 (C-8), 128.6 (C-9)*, 128.6 (C-10)*, 126.3 (C-11), 75.4 (C-1, C-1'), 59.1 (C-2), 49.6 (C-3), 49.4 (C-3'), 45.3 (C-4'), 41.3 (C-4), 35.0 (C-6), 31.6 (C-7) ppm.

HRMS (ESI) for $C_{16}H_{23}N_2O_2^+$ [$(M+H^+)^+$] calculated: 275.1754, found: 275.1753.

IR (ATR): ṽ = 2945 (w), 2874 (w), 2821 (w), 1640 (s), 1435 (s), 1230 (w), 1155 (w), 1118 (w), 1021 (w), 977 (w), 890 (w), 731 (m) cm^{-1}.

7.16.12 1-(4-(6-Fluorobenzo[*d*]isoxazol-3-yl)piperidin-1-yl)-3-phenylpropan-1-one (12l)

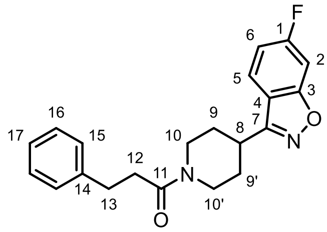

12l
$C_{21}H_{21}FN_2O_2$
Mw = 352.41 $gmol^{-1}$

Prepared according to **GP2** from (*E*)-1-(4-(6-fluorobenzo[*d*]isoxazol-3-yl)piperidin-1-yl)-3-phenylprop-2-en-1-one (**11l**, 70.1 mg, 0.200 mmol, 1.00 equiv), [IPrIPyCuCl] (**4**, 9.30 mg, 20.0 µmol, 10.0 mol%), NaO*t*Bu (9.62 mg, 100 µmol, 50.0 mol%) in diglyme (2.0 mL). The reaction mixture was stirred for 24 h at 100 °C under H_2 atmosphere (1.5 bar). Purification by flash column chromatography on silica gel (cyclohexane/EtOAc = 2:1) yielded **12l** as colorless oil (52.0 mg, 0.148 mmol, 74%).

R_f = 0.15 (cyclohexane/EtOAc = 2:1).

^{1}H NMR (600 MHz, $CDCl_3$): δ = 7.59 (dd, $^3J_{5,6}$ = 8.7 Hz, $^4J_{5,F}$ = 5.0 Hz, 1H, H-5), 7.30 (t, $^3J_{16,15/17}$ = 7.5 Hz, 2H, H-16), 7.26–7.23 (m, 3H, H-6, H-15), 7.20 (t, $^3J_{17,16}$ = 7.3 Hz, 1H, H-17), 7.07 (td, $^3J_{2,F}$ = 8.9 Hz, $^4J_{2,6}$ = 2.1 Hz, 1H, H-2), 4.68 (dt, $^2J_{10'a,10'b}$ = 13.6 Hz, $^3J_{10'a,9'}$ = 4.2 Hz, 1H, H-10'a), 3.95 (dt, $^2J_{10a,10b}$ = 13.9 Hz, $^3J_{10a,9}$ = 4.2 Hz, 1H, H-10a), 3.28 (tt, $^3J_{8,9}$ = 11.2 Hz, $^3J_{8,9'}$ = 3.9 Hz, 1H, H-8), 3.18 (dt, $^2J_{10'b,10'a}$ = 12.9 Hz, $^3J_{10'b,9'}$ = 2.8 Hz, 1H, H-10'b), 3.00 (t, $^3J_{13,12}$ = 7.9 Hz, 2H, H-13), 2.89 (dt, $^2J_{10b,10a}$ = 13.6 Hz, $^3J_{10b,9}$ = 3.0 Hz, 1H, H-10b), 2.73–2.64 (m, 2H, H-12), 2.12–2.03 (m, 2H, H-9), 1.89–1.81 (m, 2H, H-9') ppm.

^{13}C NMR (151 MHz, $CDCl_3$): δ = 170.8 (C-11), 165.2 (C-4), 164.1 (C-3), 163.8 (d, $^1J_{1,F}$ = 78.1 Hz, C-1), 160.3 (C-7), 141.4 (C-14), 128.7 (C-15)*, 128.6 (C-16)*, 126.4 (C-17), 122.3 (d, $^3J_{5,F}$ = 11.0 Hz, C-5), 112.7 (d, $^2J_{6,F}$ = 25.5 Hz, C-6), 97.7 (d, $^2J_{2,F}$ = 26.8 Hz, C-2), 45.5 (C-10), 41.6 (C-10'), 35.3 (C-12), 34.4 (C-8), 31.7 (C-13), 30.6 (C-9), 30.3 (C-9') ppm.

^{19}F NMR (473 MHz, $CDCl_3$): δ = –109.1 (s) ppm.

HRMS (ESI) for $C_{21}H_{22}FN_2O_2^+$ [$(M+H^+)^+$] calculated: 353.1660, found: 353.1657.

IR (ATR): ṽ = 3027 (w), 2907 (w), 2848 (w), 1640 (s), 1423 (m), 1259 (w), 1215 (w), 1017 (w), 831 (m), 752 (w), 700 (m) cm^{-1}.

7.16.13 1-(Piperidin-1-yl)-3-(4-(piperidine-1-carbonyl)phenyl)propan-1-one (12m)

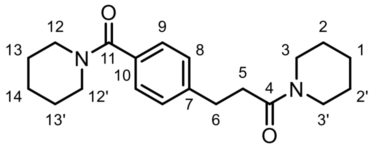

12m
$C_{20}H_{28}N_2O_2$
Mw = 328.46 gmol^{-1}

Prepared according to **GP2** from (*E*)-1-(piperidin-1-yl)-3-(4-(piperidine-1-carbonyl)phenyl)prop-2-en-1-one (**11m**, 66.1 mg, 0.200 mmol, 1.00 equiv), [IPrIPyCuCl] (**4**, 9.30 mg, 20.0 µmol, 10.0 mol%), NaO*t*Bu (9.62 mg, 100 µmol, 50.0 mol%) in diglyme (2.0 mL). The reaction mixture was stirred for 24 h at 100 °C under H_2 atmosphere (1.5 bar). Purification by flash column chromatography on silica gel (cyclohexane/EtOAc = 0:100) yielded **12m** as colorless oil (51.9 mg, 0.16 mmol, 79%).

R*f* = 0.25 (cyclohexane/EtOAc = 0:100).

^{1}H NMR (600 MHz, $CDCl_3$): δ = 7.28 (d, $^3J_{9,8}$ = 8.1 Hz, 2H, H-9), 7.21 (d, $^3J_{8,9}$ = 8.1 Hz, 2H, H-8), 3.66 (br s, 2H, H-12), 3.52 (t, $^3J_{12',13'}$ = 5.4 Hz, 2H, H-12'), 3.38–3.30 (m, 4H, H-3, H-3'), 2.95 (t, $^3J_{6,5}$ = 7.8 Hz, 2H, H-6), 2.58 (t, $^3J_{5,6}$ = 7.8 Hz, 2H, H-5), 1.64–1.58 (m, 6H, H-13, H-13', H-14), 1.51–1.43 (m, 6H, H-2, H-2', H-1) ppm.

^{13}C NMR (151 MHz, $CDCl_3$): δ = 170.3 (C-11), 170.1 (C-4), 143.1 (C-10), 134.3 (C-7), 128.5 (C-9), 127.1 (C-8), 48.8 (C-3), 46.6 (C-3'), 43.2 (C-2), 42.8 (C-2'), 34.9 (C-5), 31.3 (C-6), 26.6 (C-13), 26.4 (C-13')*, 25.6 (C-2, C-2')*, 24.7 (C-14)**, 24.5 (C-1)** ppm.

HRMS (ESI) for $C_{20}H_{29}N_2O_2^+$ [(M+H$^+$)$^+$] calculated: 329.2224, found: 329.2221.

IR (ATR): ṽ = 2937 (w), 2855 (w), 1625 (s), 1431 (s), 1357 (w), 1275 (w), 1107 (w), 1002 (w), 921 (w), 854 (w), 731 (m) cm^{-1}.

7.16.14 1-(Piperidin-1-yl)-3-(4-(trifluoromethyl)phenyl)propan-1-one (12n)

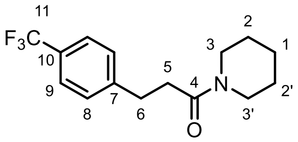

12n
$C_{15}H_{18}F_3NO$
Mw = 285.31 gmol^{-1}

Prepared according to **GP2** from (*E*)-1-(piperidin-1-yl)-3-(4-(trifluoromethyl)phenyl)prop-2-en-1-one (**11n**, 56.7 mg, 0.200 mmol, 1.00 equiv), [IPrIPyCuCl] (**4**, 9.30 mg, 20.0 µmol, 10.0 mol%), NaO*t*Bu (9.62 mg, 100 µmol, 50.0 mol%) in diglyme (2.0 mL). The reaction mixture was stirred for 24 h at 100 °C under H_2 atmosphere (1.5 bar). Purification by flash column chromatography on silica gel (cyclohexane/EtOAc = 3:1) yielded **12n** as colorless oil (39.0 mg, 0.136 mmol, 68%).

R*f* = 0.25 (SiO_2, cyclohexane/EtOAc = 3:1).

^{1}H NMR (600 MHz, $CDCl_3$): δ = 7.53 (d, $^3J_{9,8}$ = 8.0 Hz, 2H, H-9), 7.34 (d, $^3J_{8,9}$ = 8.0 Hz, 2H, H-8), 3.55 (t, $^3J_{3,2}$ = 5.4 Hz, 2H, H-3), 3.34 (t, $^3J_{3',2'}$ = 5.4 Hz, 2H, H-3'), 3.03 (t, $^3J_{6,5}$ = 7.8 Hz, 2H, H-6), 2.62 (t, $^3J_{5,6}$ = 7.8 Hz, 2H, H-5), 1.64–1.60 (m, 2H, H-2), 1.54–1.50 (m, 2H, H-2'), 1.49–1.45 (m, 2H, H-1) ppm.

^{13}C NMR (151 MHz, $CDCl_3$): δ = 169.9 (C-4), 145.9 (C-7), 129.0 (C-8), 128.6 (q, $^{2}J_{10,F}$ = 32.4 Hz, C-10), 125.5 (q, $^{3}J_{9,F}$ = 3.8 Hz, C-9), 124.4 (q, $^{3}J_{11,F}$ = 271.9 Hz, C-11), 46.7 (C-3), 42.9 (C-3'), 34.7 (C-6), 31.4 (C-5), 26.5 (C-2), 25.7 (C-2'), 24.6 (C-1) ppm.

^{19}F NMR (473 MHz, $CDCl_3$): δ = –62.4 (s) ppm.

HRMS (ESI) for $C_{15}H_{19}F_3NO^+$ [(M+H$^+$)$^+$] calculated: 286.1413, found: 286.1407.

IR (ATR): ṽ = 2937 (w), 2859 (w), 1640 (s), 1438 (m), 1323 (s), 1162 (w), 1107 (m), 1066 (m), 1017 (w), 831 (m) cm^{-1}.

7.16.15 1-(Piperidin-1-yl)-3-(pyridin-3-yl)propan-1-one (12o)

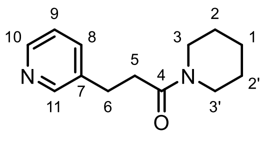

12o
$C_{13}H_{18}N_2O$
Mw = 218.30 gmol^{-1}

Prepared according to **GP2** from (*E*)-1-(piperidin-1-yl)-3-(pyridin-3-yl)prop-2-en-1-one (**11o**, 43.3 mg, 0.200 mmol, 1.00 equiv), [IPrIPyCuCl] (**4**, 9.30 mg, 20.0 µmol, 10.0 mol%), NaO*t*Bu (9.62 mg, 100 µmol, 50.0 mol%) in diglyme (2.0 mL). The reaction mixture was stirred for 24 h at 100 °C under H_2 atmosphere (1.5 bar). Purification by flash column chromatography on silica gel (CH_2Cl_2/MeOH = 95:5) yielded **12o** as yellow oil (30.7 mg, 0.140 mmol, 70%).

R*f* = 0.30 (SiO_2, CH_2Cl_2/MeOH = 95:5).

^{1}H NMR (600 MHz, $CDCl_3$): δ = 8.48 (d, $^{4}J_{11,8}$ = 2.3 Hz, 1H, H-11), 8.44 (dd, $^{3}J_{10,9}$ = 4.8 Hz, $^{4}J_{10,8}$ = 1.6 Hz, 1H, H-10), 7.56 (dd, $^{3}J_{8,9}$ = 7.8 Hz, $^{4}J_{8,11}$ = 2.0 Hz, 1H, H-8), 7.20 (dd, $^{3}J_{9,8}$ = 7.8 Hz, $^{3}J_{9,10}$ = 4.8 Hz, 1H, H-9), 3.55 (t, $^{3}J_{3,2}$ = 5.4 Hz, 2H, H-3), 3.33 (t, $^{3}J_{3',2'}$ = 5.4 Hz, 2H, H-3'), 2.97 (t, $^{3}J_{6,5}$ = 7.8 Hz, 2H, H-6), 2.61 (t, $^{3}J_{5,6}$ = 7.8 Hz, 2H, H-5), 1.63–1.59 (m, 2H, H-2), 1.53–1.50 (m, 2H, H-2'), 1.49–1.45 (m, 2H, H-1) ppm.

^{13}C NMR (151 MHz, $CDCl_3$): δ = 169.8 (C-4), 150.0 (C-11), 147.7 (C-10), 137.0 (C-7), 136.3 (C-8), 123.5 (C-9), 46.7 (C-3), 42.9 (C-3'), 34.6 (C-6), 28.7 (C-5), 26.5 (C-2), 25.7 (C-2'), 24.6 (C-1) ppm.

HRMS (ESI) for $C_{13}H_{19}N_2O^+$ [(M+H$^+$)$^+$] calculated: 219.1492, found: 219.1492.

IR (ATR): ṽ = 2937 (w), 2859 (w), 1644 (s), 1595 (s), 1423 (m), 1297 (w), 1244 (w), 1215 (w), 1136 (w), 1021 (w), 998 (m), 797 (m), 719 (m) cm^{-1}.

7.16.16 3-(Furan-2-yl)-1-(piperidin-1-yl)propan-1-one (12p)

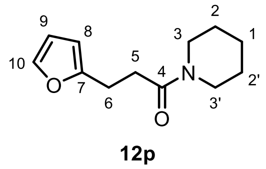

12p
$C_{12}H_{17}NO_2$
Mw = 207.27 gmol^{-1}

Prepared according to **GP2** from (*E*)-3-(furan-2-yl)-1-(piperidin-1-yl)prop-2-en-1-one (**11p**, 41.0 mg, 0.200 mmol, 1.00 equiv), [IPrIPyCuCl] (**4**, 9.30 mg, 20.0 µmol, 10.0 mol%), NaO*t*Bu (9.62 mg, 100 µmol, 50.0 mol%) in diglyme (2.0 mL). The reaction mixture was stirred for 24 h at 100 °C

under H_2 atmosphere (1.5 bar). Purification by flash column chromatography on silica gel (cyclohexane/EtOAc = 4:1) yielded **12p** as colorless oil (29.4 mg, 0.142 mmol, 71%).

R*f* = 0.25 (SiO_2, cyclohexane/EtOAc = 4:1).

^{1}H NMR (600 MHz, $CDCl_3$): δ = 7.30 (dd, $^3J_{10,9}$ = 1.9 Hz, $^4J_{10,8}$ = 0.9 Hz, 1H, H-10), 6.28 (dd, $^3J_{9,8}$ = 3.2 Hz, $^3J_{9,10}$ = 1.9 Hz, 1H, H-9), 6.02 (dd, $^3J_{8,9}$ = 3.2 Hz, $^4J_{8,10}$ = 0.9 Hz, 1H, H-8), 3.56 (t, $^3J_{3,2}$ = 6.0 Hz, 2H, H-3), 3.37 (t, $^3J_{3',2'}$ = 6.0 Hz, 2H, H-3'), 2.98 (t, $^3J_{6,5}$ = 7.2 Hz, 2H, H-6), 2.65 (t, $^3J_{5,6}$ = 7.2 Hz, 2H, H-5), 1.66–1.61 (m, 2H, H-2), 1.55–1.50 (m, 4H, H-2', H-1) ppm.

^{13}C NMR (151 MHz, $CDCl_3$): δ = 170.0 (C-4), 155.2 (C-7), 141.1 (C-10), 110.4 (C-9), 105.4 (C-8), 46.7 (C-3), 42.9 (C-3'), 31.8 (C-6), 26.6 (C-5), 25.7 (C-2), 24.7 (C-2'), 24.1(C-1) ppm.

HRMS (ESI) for $C_{12}H_{18}NO_2^+$ [(M+H$^+$)$^+$] calculated: 208.1332, found: 208.1338.

IR (ATR): ṽ = 2933 (w), 2855 (w), 1640 (s), 1438 (m), 1278 (w), 1218 (w), 1140 (w), 1010 (w), 853 (w), 801 (w), 730 (m) cm^{-1}.

7.16.17 3,3-Diphenyl-1-(piperidin-1-yl)propan-1-one (12q)

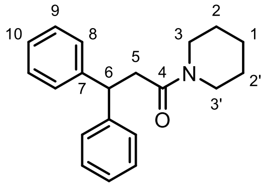

12q
$C_{20}H_{23}NO$
Mw = 293.41 gmol^{-1}

Prepared according to **GP2** from (*E*)-3-phenyl-1-(piperidin-1-yl)prop-2-en-1-one (**11q**, 58.2 mg, 0.200 mmol, 1.00 equiv), [IPrIPyCuCl] (**4**, 9.30 mg, 20.0 µmol, 10.0 mol%), NaO*t*Bu (9.62 mg, 100 µmol, 50.0 mol%) in diglyme (2.0 mL). The reaction mixture was stirred for 24 h at 100 °C under H_2 atmosphere (1.5 bar). Purification by flash column chromatography on silica gel (cyclohexane/EtOAc = 4:1) yielded **12q** as colorless oil (41.7 mg, 0.142 mmol, 71%).

R*f* = 0.25 (SiO_2, cyclohexane/EtOAc = 4:1).

^{1}H NMR (600 MHz, $CDCl_3$): δ = 7.28–7.23 (m, 8H, H-8, H-9), 7.19–7.16 (m, 2H, H-10), 4.68 (t, $^3J_{6,5}$ = 7.5 Hz, 1H, H-6), 3.48 (t, $^3J_{3,2}$ = 5.4 Hz, 2H, H-3), 3.30 (t, $^3J_{3',2'}$ = 5.4 Hz, 2H, H-3'), 3.05 (d, $^3J_{5,6}$ = 7.5 Hz, 2H, H-5), 1.56–1.52 (m, 2H, H-2), 1.44–1.40 (m, 2H, H-2'), 1.33–1.29 (m, 2H, H-1) ppm.

^{13}C NMR (151 MHz, $CDCl_3$): δ = 169.5 (C-4), 144.5 (C-7), 128.6 (C-8)*, 128.1 (C-9)*, 126.5 (C-10), 47.5 (C-6), 46.9 (C-3), 43.0 (C-3'), 39.0 (C-5), 26.5 (C-2), 25.6 (C-2'), 24.6 (C-1) ppm.

C-4 peak is assigned from ^{1}H,^{13}C HMBC NMR.

HRMS (ESI) for $C_{20}H_{24}NO^+$ [(M+H$^+$)$^+$] calculated: 294.1852, found: 294.1850.

IR (ATR): ṽ = 2952 (w), 2914 (w), 2855 (w), 1625 (s), 1494 (m), 1442 (w), 1256 (w), 1133 (w), 1018 (w), 749 (w), 730 (m) cm^{-1}.

7.16.18 3-Phenyl-1-(piperidin-1-yl)heptan-1-one (12r)

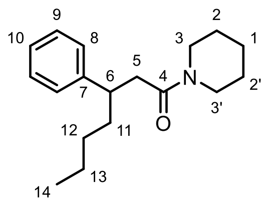

12r
$C_{18}H_{27}NO$
Mw = 273.42 gmol^{-1}

Prepared according to **GP2** from (*E*)-3-phenyl-1-(piperidin-1-yl)prop-2-en-1-one (**11r**, 54.3 mg, 0.200 mmol, 1.00 equiv), [IPrIPyCuCl] (**4**, 9.30 mg, 20.0 µmol, 10.0 mol%), NaO*t*Bu (9.62 mg, 100 µmol, 50.0 mol%) in diglyme (2.0 mL). The reaction mixture was stirred for 24 h at 100 °C under H_2 atmosphere (1.5 bar). Purification by flash column chromatography on silica gel (cyclohexane/EtOAc = 4:1) yielded **12r** as colorless oil (45.6 mg, 0.166 mmol, 83%).

R*f* = 0.20 (SiO_2, cyclohexane/EtOAc = 4:1).

^{1}H NMR (600 MHz, $CDCl_3$): δ = 7.28 (t, $^3J_{9,8/10}$ = 7.6 Hz, 2H, H-9), 7.21–7.17 (m, 3H, H-8, H-10), 3.56–3.54 (m, 1H, H-3a), 3.43–3.38 (m, 1H, H-3b), 3.26–3.23 (m, 2H, H-3’), 3.14–3.12 (m, 1H, H-6), 2.60 (dd, $^2J_{5a,5b}$ = 14.7 Hz, $^3J_{5a,6}$ = 7.2 Hz, 1H, H-5a), 2.54 (dd, $^2J_{5b,5a}$ = 14.7 Hz, $^3J_{5b,6}$ = 7.2 Hz, 1H, H-5b), 1.75–1.72 (m, 1H, H-11a), 1.63–1.60 (m, 1H, H-11b), 1.56–1.53 (m, 2H, H-2), 1.43–1.40 (m, 3H, H-2’, H-12a), 1.30–1.10 (m, 5H, H-1, H-12b, H-13), 0.82 (t, $^3J_{14,13}$ = 7.3 Hz, 3H, H-14) ppm.

^{13}C NMR (151 MHz, $CDCl_3$): δ = 170.2 (C-4), 145.2 (C-7), 128.5 (C-8)*, 127.8 (C-9)*, 126.4 (C-10), 47.0 (C-3), 42.8 (C-3’, C-6), 40.8 (C-5), 35.8 (C-11), 29.9 (C-12), 26.5 (C-13), 25.7 (C-2’), 24.7 (C-2), 22.8 (C-1), 14.1 (C-14) ppm.

HRMS (ESI) for $C_{18}H_{28}NO^+$ [(M+H$^+$)$^+$] calculated: 274.2165, found: 274.2166.

IR (ATR): ṽ = 2933 (w), 2859 (w), 1633 (s), 1442 (m), 1256 (w), 1222 (w), 1110 (w), 1025 (w), 734 (m) cm^{-1}.

7.16.19 *N*,*N*-Diethyl-3-phenylbutanamide (12s)

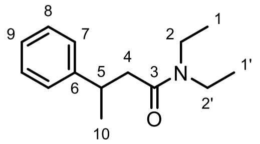

12s
$C_{14}H_{21}NO$
Mw = 219.33 gmol^{-1}

Prepared according to **GP2** from (*E*)-*N*,*N*-diethyl-3-phenylbut-2-enamide (**11s**, 43.4 mg, 0.200 mmol, 1.00 equiv), [IPrIPyCuCl] (**4**, 9.30 mg, 20.0 µmol, 10.0 mol%), NaO*t*Bu (9.62 mg, 100 µmol, 50.0 mol%) in diglyme (2.0 mL). The reaction mixture was stirred for 24 h at 100 °C under H_2 atmosphere (1.5 bar). Purification by flash column chromatography on silica gel (cyclohexane/EtOAc = 4:1) yielded **12s** as colorless oil (33.2 mg, 0.152 mmol, 76%).

R*f* = 0.20 (SiO_2, cyclohexane/EtOAc = 4:1).

^{1}H NMR (600 MHz, $CDCl_3$): δ = 7.29 (dd, $^3J_{8,7}$ = 8.1 Hz, $^3J_{8,9}$ = 7.0 Hz, 2H, H-8), 7.25 (dd, $^3J_{7,8}$ = 8.3 Hz, $^4J_{7,9}$ = 1.6 Hz, 2H, H-7), 7.20–7.17 (m, 1H, H-9), 3.45–3.34 (m, 2H, H-2), 3.31–3.25 (m, 1H, H-5), 3.22–3.13 (m, 2H, H-2’), 2.56 (dd, $^2J_{4a,4b}$ = 14.7 Hz, $^3J_{4a,5}$ = 6.4 Hz, 1H, H-4a), 2.49

(dd, $^2J_{4b,4a}$ = 14.7 Hz, $^3J_{4b,5}$ = 8.1 Hz, 1H, H-4b), 1.33 (d, $^3J_{10,5}$ = 6.9 Hz, 3H, H-10), 1.08–1.04 (m, 6H, H-1, H-1') ppm.

^{13}C NMR (151 MHz, $CDCl_3$): δ = 170.9 (C-3), 146.7 (C-6), 128.5 (C-8), 127.1 (C-7), 126.3 (C-9), 42.1 (C-2), 41.8 (C-2'), 40.3 (C-5), 36.8 (C-4), 21.5 (C-10), 14.5 (C-1), 13.2 (C-1') ppm.

HRMS (ESI) for $C_{14}H_{22}NO^+$ [$(M+H^+)^+$] calculated: 220.1696, found: 220.1704.

IR (ATR): ṽ = 2970 (w), 2933 (w), 1632 (s), 1453(m), 1427 (m), 1379 (w), 1274 (w), 1222 (w), 1140 (m), 1080 (w), 760 (w), 700 (s) cm^{-1}.

The data is in accordance with literature.[29]

7.16.20 *N*,*N*-Diethyl-3-phenylbutanamide (12s)

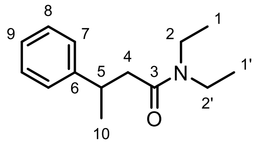

12s
$C_{14}H_{21}NO$
Mw = 219.33 gmol^{-1}

Prepared according to **GP2** from (*Z*)-*N*,*N*-diethyl-3-phenylbut-2-enamide (*Z*-**11s**, 43.4 mg, 0.200 mmol, 1.00 equiv), [IPrIPyCuCl] (**4**, 9.30 mg, 20.0 μmol, 10.0 mol%), NaO*t*Bu (9.62 mg, 100 μmol, 50.0 mol%) in diglyme (2.0 mL). The reaction mixture was stirred for 24 h at 100 °C under H_2 atmosphere (1.5 bar). After hydrogenation, complete conversion of (*Z*)-**11s** to saturated amide **12s** was obtained. Purification by flash column chromatography on silica gel (cyclohexane/EtOAc = 4:1) yielded **12s** as colorless oil (31.1 mg, 0.142 mmol, 76%).

R*f* = 0.20 (SiO_2, cyclohexane/EtOAc = 4:1).

The analysis data for **12s** is already provided in page no 91–92.

7.16.21 3-Phenyl-1-(3-(trifluoromethyl)-5,6-dihydro-[1,2,4]triazolo[4,3-*a*]pyrazin-7(8*H*)-yl)butan-1-one (12t)

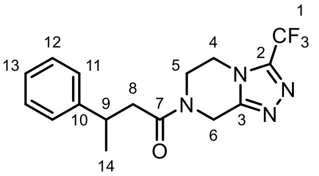

12t
$C_{16}H_{17}F_3N_4O$
Mw = 338.33 gmol^{-1}

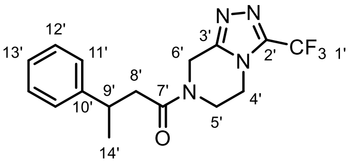

12t_rotamer
$C_{16}H_{17}F_3N_4O$
Mw = 338.33 gmol^{-1}

Prepared according to **GP2** from ethyl (*E*)-3-phenyl-1-(3-(trifluoromethyl)-5,6-dihydro-[1,2,4]triazolo[4,3-*a*]pyrazin-7(8*H*)-yl)but-2-en-1-one (**11t**, 70.3 mg, 0.200 mmol, 1.00 equiv), [IPrIPyCuCl] (**4**, 9.30 mg, 20.0 μmol, 10.0 mol%), NaO*t*Bu (9.62 mg, 100 μmol, 50.0 mol%) in diglyme (2.0 mL). The reaction mixture was stirred for 24 h at 100 °C under H_2 atmosphere (1.5 bar). Purification by flash column chromatography on silica gel (EtOAc/MeOH = 95:5) yielded **12t** and **12t_rotamer** (70:30 ratio) as colorless solid (48.7 mg, 0.144 mmol, 72%).

M.p. = 61 °C (CH_2Cl_2)

R*f* = 0.20 (EtOAc/MeOH = 95:5).

^{1}H NMR (600 MHz, $CDCl_3$): δ = 7.22–6.97 (m, 5H, H-11, H-12, H-13), 5.07–4.62 (m, 2H, H-4), 4.21–3.89 (m, 2H, H-5), 3.75–3.61 (m, 2H, H-6), 3.34–3.27 (m, 1H, H-9), 2.72–2.68 (m, 1H, H-8a), 260–2.57 (m, 1H, H-8b), 1.35 (d, $^{3}J_{14,9}$ = 7.0 Hz, 3H, H-14) ppm.

The ^{1}H NMR spectrum is not well separated for the two rotamers, which is why only ^{13}C and ^{19}F NMR are assigned separately.

^{13}C NMR (151 MHz, $CDCl_3$): δ = 171.0 (C-7), 149.3 (C-3), 144.5 (C-10), 143.4 (d, $^{2}J_{2,F}$ = 40.0 Hz, C-2), 128.4 (C-11), 126.7 (C-12), 126.5 (C-13), 118.2 (q, $^{1}J_{1,F}$ = 270.5 Hz, C-1), 43.1 (C-6), 42.7 (C-5), 41.4 (C-4), 37.9 (C-9), 37.5 (C-8), 21.4 (C-14) ppm.

^{19}F NMR (473 MHz, $CDCl_3$): δ = –63.1 (s) ppm.

^{13}C NMR of **12t_rotamer** (151 MHz, $CDCl_3$): δ = 170.8 (C-7'), 150.4 (C-3'), 145.4 (C-10'), 143.1 (d, $^{2}J_{2,F}$ = 40.0 Hz, C-2'), 128.7 (C-11'), 126.8 (C-12'), 126.5 (C-13'), 118.17 (q, $^{1}J_{1,F}$ = 270.5 Hz, C-1'), 43.4 (C-6'), 42.7 (C-5'), 41.8 (C-4'), 39.0 (C-9'), 37.1 (C-8'), 21.4 (C-14') ppm.

^{19}F NMR of 12t_rotamer (473 MHz, $CDCl_3$): δ = –63.0 (s) ppm.

Rotamer ratio is determined from the ^{19}F NMR integration value at –63.1 (**12t**) and –63.0 (**12t_rotamer**) ppm.

HRMS (ESI) for $C_{16}H_{18}F_3N_3O^+$ [(M+H$^+$)$^+$] calculated: 339.1427, found: 339.1426.

IR (ATR): ṽ = 2982 (w), 2937 (w), 1640 (s), 1495 (w), 1438 (w), 1267 (w), 1192 (w), 1155 (w), 1013 (w), 797 (w), 775 (w), 708 (w) cm^{-1}.

7.16.22 Ethyl 4-(3-phenylbutanoyl)-3,4-dihydro-2*H*-benzo[*b*][1,4]oxazine-2-carboxylate (12u)

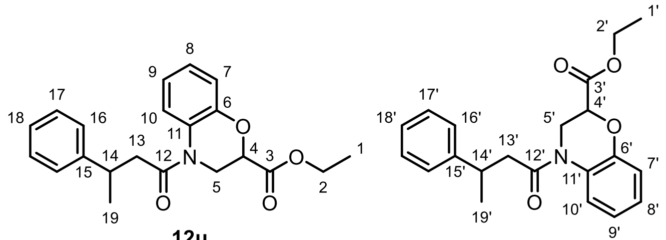

Prepared according to **GP2** from ethyl (*E*)-4-(3-phenylbut-2-enoyl)-3,4-dihydro-2H-benzo[*b*][1,4]oxazine-2-carboxylate (**11u**, 70.3 mg, 0.200 mmol, 1.00 equiv), [IPrIPyCuCl] (**4**, 9.30 mg, 20.0 μmol, 10.0 mol%), NaO*t*Bu (9.62 mg, 100 μmol, 50.0 mol%) in diglyme (2.0 mL). The reaction mixture was stirred for 24 h at 100 °C under H_2 atmosphere (1.5 bar). Purification by flash column chromatography on silica gel (cyclohexane/EtOAc = 5:1) yielded **12u** and **12u_rotamer** (50:50 ratio) as yellow oil (47.4 mg, 0.134 mmol, 67%).

R*f* = 0.15 (cyclohexane/EtOAc = 5:1).

^{1}H NMR (600 MHz, $CDCl_3$): δ = 7.29–7.23 (m, 3H, H-16, H-18), 7.19–7.18 (m, 2H, H-17), 7.12–7.08 (m, 2H, H-10, H-8), 7.04–7.01 (m, 1H, H-9), 6.93–6.88 (m, 1H, H-7), 4.72 (br s, 1H,

H-4), 4.21–4.16 (m, 2H, H-2), 4.06–3.96 (m, 2H, H-5), 3.44–3.37 (m, 1H, H-14), 3.06 (br s, 1H, H-13a), 2.87–2.84 (m, 1H, H-13b), 1.29–1.22 (m, 6H, H-1, H-19,) ppm.

^{13}C NMR (151 MHz, $CDCl_3$): δ = 168.7 (C-3), 168.6 (C-12), 146.0 (C-15), 128.6 (C-16)*, 127.1 (C-17)*, 126.8 (C-11), 126.5 (C-18), 126.4 (C-8), 124.4 (C-6), 120.8 (C-9), 120.6 (C-10), 117.6 (C-7), 74.0 (C-4), 62.0 (C-2), 42.1 (C-5), 37.3 (C-14), 22.2 (C-13), 14.2 (C-19)**, 14.2 (C-1)** ppm.

The ^{1}H and ^{13}C NMR spectrum is well separated for the two rotamers, which is why both ^{1}H and ^{13}C NMR are assigned separately.

^{1}H NMR of **12u_rotamar** (600 MHz, $CDCl_3$): δ = 7.29–7.23 (m, 3H, H-16', H-18'), 7.19–7.18 (m, 2H, H-17'), 7.12–7.08 (m, 2H, H-10', H-8'), 7.04–7.01 (m, 1H, H-9'), 6.93–6.88 (m, 1H, H-7'), 4.34 (br s, 1H, H-4'), 4.21–4.16 (m, 2H, H-2'), 3.96–3.89 (m, 2H, H-5'), 3.44–3.37 (m, 1H, H-14'), 2.92 (br s, 1H, H-13'a), 2.72–2.69 (m, 1H, H-13'b),1.29–1.22 (m, 6H, H-1', H-19') ppm.

^{13}C NMR 12u_rotamar (151 MHz, $CDCl_3$): δ = 168.7 (C-3'), 168.6 (C-12'), 146.1 (C-15'), 128.6 (C-16')*, 127.0 (C-17')*, 126.8 (C-11'), 126.5 (C-18'), 126.4 (C-8'), 124.4 (C-6'), 120.8 (C-9'), 120.6 (C-10'), 117.6 (C-7'), 74.0 (C-4'), 62.0 (C-2'), 41.0 (C-5'), 36.6 (C-14'), 21.8 (C-13'), 14.2 (C-19')**, 14.2 (C-1')** ppm.

Rotamer ratio is determined from the integration value of H-4 (at 4.72 ppm) and H-4' (at 4.34 ppm).

HRMS (ESI) for $C_{21}H_{24}NO_4^+$ [(M+H$^+$)$^+$] calculated: 354.1700, found: 354.1698.

IR (ATR): ṽ = 3030 (w), 2967 (w), 2937 (w), 2877 (w), 1751 (s), 1662 (s), 1494 (s), 1371 (w), 1304 (w), 1244 (w), 1185 (m), 1125 (w), 1088 (w), 1017 (w), 753 (m), 701 (w) cm^{-1}.

7.16.23 *N*,*N*-Diethyl-1-(3-phenylbutanoyl)piperidine-3-carboxamide (12v)

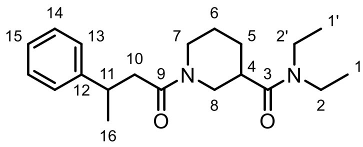

12v
$C_{20}H_{30}N_2O_2$
Mw = 330.47 gmol^{-1}

Prepared according to **GP2** from (*E*)-*N*,*N*-diethyl-1-(3-phenylbut-2-enoyl)piperidine-3-carboxamide (**11v**, 65.6 mg, 0.200 mmol, 1.00 equiv), [IPrIPyCuCl] (**4**, 9.30 mg, 20.0 µmol, 10.0 mol%), NaO*t*Bu (9.62 mg, 100 µmol, 50.0 mol%) in diglyme (2.0 mL). The reaction mixture was stirred for 24 h at 100 °C under H_2 atmosphere (1.5 bar). Purification by flash column chromatography on silica gel (cyclohexane/EtOAc = 0:100) yielded **12v** and **12v_rotamer** (60:40 mixture) as yellow oil (48.9 mg, 0.148 mmol, 74% yield).

R*f* = 0.20 (cyclohexane/EtOAc = 0:100).

^{1}H NMR (600 MHz, $CDCl_3$): δ = 7.27 (d, $^{3}J_{13,14}$ = 7.5 Hz, 2H, H-13), 7.22 (t, $^{3}J_{14,13/15}$ = 7.0 Hz, 2H, H-14), 7.17 (t, $^{3}J_{15,14}$ = 7.3 Hz, 1H, H-15), 4.63–4.60 (m, 1H, H-8a), 3.78–3.74 (m, 1H, H-8b),

3.45–3.16 (m, 5H, H-7, H-11, H-2), 2.82–2.10 (m, 5H, H-2', H-4, H-6), 1.84–1.38 (m, 4H, H-10, H-5), 1.33–1.30 (m, 3H, H-16), 1.19–1.13 (m, 3H, H-1)*, 1.10–1.05 (m, 3H, H-1')* ppm.

The ^{1}H NMR spectrum is not well separated for the two rotamers, which is why only ^{13}C NMR is assigned separately.

^{13}C NMR (151 MHz, $CDCl_3$): δ = 172.1 (C-9), 170.3 (C-3), 146.2 (C-12), 128.5 (C-13), 126.8 (C-14), 126.3 (C-15), 46.3 (C-8), 45.0 (C-7), 41.7 (C-2), 41.6 (C-2'), 40.1 (C-11), 39.2 (C-4), 36.7 (C-10), 28.6 (C-5), 24.5 (C-6), 21.6 (C-1), 14.9 (C-16), 13.1 (C-1') ppm.

^{13}C NMR of **12v_rotamer** (151 MHz, $CDCl_3$): δ = 172.3 (C-9), 170.0 (C-3), 146.6 (C-12), 128.4 (C-13), 126.9 (C-14), 126.2 (C-15), 48.5 (C-8), 42.0 (C-7), 41.9 (C-2), 41.5 (C-2'), 39.9 (C-11), 36.7 (C-10), 27.7 (C-4), 25.6 (C-5), 21.9 (C-6), 14.9 (C-16), 13.0 (C-1') ppm.

Rotamer ratio is determined from the integration value of H-8 at 4.63–4.60 ppm.

HRMS (ESI) for $C_{20}H_{31}N_2O_2^+$ [(M+H$^+$)$^+$] calculated: 331.2380, found: 331.2380.

IR (ATR): ṽ = 2967 (w), 2937 (w), 2870 (w), 1629 (s), 1428 (m), 1218 (w), 1144 (w), 1088 (w), 764 (w), 730 (w), 701 (w) cm^{-1}.

7.16.24 Ethyl (3-phenylbutanoyl)-*L*-prolinate (12w)

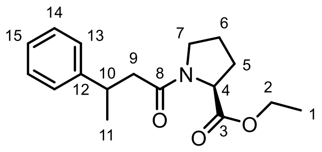

12w
$C_{17}H_{23}NO_3$
Mw = 289.37 gmol^{-1}

Prepared according to **GP2** from ethyl (*E*)-(3-phenylbut-2-enoyl)-*L*-prolinate (**11w**, 57.5 mg, 0.200 mmol, 1.00 equiv), [IPrIPyCuCl] (**4**, 9.30 mg, 20.0 µmol, 10.0 mol%), NaO*t*Bu (9.62 mg, 100 µmol, 50.0 mol%) in diglyme (2.0 mL). The reaction mixture was stirred for 24 h at 100 °C under H_2 atmosphere (1.5 bar). Purification by flash column chromatography on silica gel (cyclohexane/EtOAc = 3:1) yielded **12w** (50:50 d.r.) as colorless oil (38.8 mg, 0.134 mmol, 67%).

R*f* = 0.20 (cyclohexane/EtOAc = 3:1).

^{1}H NMR (600 MHz, $CDCl_3$): δ = 7.26–7.12 (m, 5H, H-13, H-14, H-15), 4.44–3.95 (m, 3H, H-2, H-4), 3.59–3.20 (m, 3H, H-7, H-10), 2.59–2.31 (m, 2H, H-9), 2.21–1.70 (m, 4H, H-5, H-6), 1.31–1.13 (m, 6H, H-1, H-11) ppm.

The ^{1}H NMR spectrum is not well separated for the two diasteromers and additionally each diasteroisomer is present as 77:23 rotamar ratio (originated from the staring material **11w**, see Section 7.18.22), resulting 3 to 4 signals per carbon atom in ^{13}C NMR spectrum.

^{13}C NMR (151 MHz, $CDCl_3$): δ = 172.2 (C-3), 172.2 (C-3), 172.1 (C-3), 172.0 (C-3), 170.6 (C-8), 170.5 (C-8), 170.4 (C-8), 146.5 (C-12), 146.4 (C-12), 146.3 (C-12), 145.9 (C-12), 128.3 (C-13), 128.3 (C-13), 128.2 (C-13), 126.7 (C-14), 126.7 (C-14), 126.6 (C-14), 126.2 (C-15), 126.1 (C-15), 126.0 (C-15), 61.4 (C-2), 60.8 (C-2), 60.7 (C-2), 59.4 (C-4), 59.2 (C-4), 58.7 (C-4), 58.6 (C-4),

47.0 (C-7), 46.7 (C-7), 46.2 (C-7), 46.0 (C-7), 43.4 (C-10), 43.0 (C-10), 42.6 (C-10), 36.5 (C-9), 36.3 (C-9), 35.8 (C-9), 31.3 (C-5), 31.0 (C-5), 29.1 (C-5), 24.7 (C-6), 24.6 (C-6), 22.4 (C-6), 22.3 (C-6), 21.3 (C-11), 21.2 (C-11), 21.2 (C-11), 21.2 (C-11), 14.1 (C-1), 14.0 (C-1), 14.0 (C-1), 14.0 (C-1) ppm.

Diastereomeric ratio (d.r.) is determined from the integration value of H-2 at 2.59–2.31 ppm.

HRMS (ESI) for $C_{17}H_{24}NO_3^+$ $[(M+H^+)^+]$ calculated: 290.1751, found: 290.1746.

IR (ATR): $\tilde{\nu}$ = 2967 (w), 2877 (w), 1740 (s), 1640 (s), 1416 (m), 1371 (w), 1185 (s), 1095 (w), 1028 (w), 760 (w), 700 (m) cm^{-1}.

7.16.25 Ethyl *N*-methyl-*N*-(3-phenylbutanoyl)-*L*-alaninate (12x)

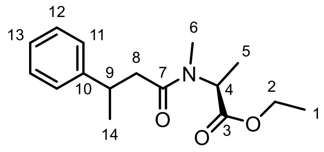

12x
$C_{16}H_{23}NO_3$
Mw = 277.37

Prepared according to **GP2** from ethyl ethyl (*E*)-*N*-methyl-*N*-(3-phenylbut-2-enoyl)-*L*-alaninate (**11x**, 55.2 mg, 0.200 mmol, 1.00 equiv), [IPrIPyCuCl] (**4**, 9.30 mg, 20.0 µmol, 10.0 mol%), NaO*t*Bu (9.62 mg, 100 µmol, 50.0 mol%) in diglyme (2.0 mL). The reaction mixture was stirred for 24 h at 100 °C under H_2 atmosphere (1.5 bar). Purification by flash column chromatography on silica gel (cyclohexane/EtOAc = 4:1) yielded **12x** (50:50 d.r.) as colorless oil (27.2 mg, 0.098 mmol, 49%).

$\mathbf{R}_f$ = 0.15 (cyclohexane/EtOAc = 4:1).

^{1}H NMR (600 MHz, $CDCl_3$): δ = 7.29–7.26 (m, 2H, H-11), 7.25–7.22 (m, 2H, H-12), 7.19–7.16 (m, 1H, H-13), 5.24–4.40 (m, 1H, H-4), 4.19–4.07 (m, 2H, H-2), 3.39–3.33 (m, 1H, H-9), 2.86–2.76 (m, 3H, H-6), 2.67–2.52 (m, 2H, H-8), 1.43–1.18 (m, 9H, H-1, H-5, H-14) ppm.

The ^{1}H NMR spectrum is not well separated for the two diasteromers and additionally each diasteroisomer is present as 70:30 rotamar ratio (originated from the staring material **11x**, see Section 7.18.23), resulting 3 to 4 signals per carbon atom in ^{13}C NMR spectrum.

^{13}C NMR (151 MHz, $CDCl_3$): δ = 172.1 (C-3), 172.0 (C-3), 171.9 (C-3), 171.6 (C-7), 171.1 (C-7), 171.0 (C-7), 146.6 (C-10), 146.5 (C-10), 146.4 (C-10), 146.3 (C-10), 128.6 (C-12), 128.5 (C-12), 128.4 (C-12), 126.9 (C-11), 126.8 (C-11), 126.7 (C-11), 126.4 (C-13), 126.3 (C-13), 126.2 (C-13), 61.5 (C-2), 61.1 (C-2), 61.0 (C-2), 55.2 (C-4), 55.1 (C-4), 52.3 (C-4), 52.0 (C-4), 42.0 (C-6), 41.9 (C-6), 41.8 (C-6), 41.6 (C-6), 36.8 (C-9), 36.7 (C-9), 36.3 (C-9), 36.2 (C-9), 31.8 (C-8), 31.6 (C-8), 28.7 (C-8), 28.6 (C-8), 21.8 (C-14), 21.7 (C-14), 21.6 (C-14), 21.5 (C-14), 15.6 (C-5), 15.3 (C-5), 14.5 (C-5), 14.4 (C-5), 14.2 (C-1), 14.2 (C-1), 14.1 (C-1) ppm.

Diastereomeric ratio (d.r.) is determined from the integration value of H-4 at 5.24–4.40 ppm.

HRMS (ESI) for $C_{16}H_{24}NO_3^+$ $[(M+H^+)^+]$ calculated: 278.1751, found: 278.1746.

IR (ATR): ṽ = 2981 (w), 2937 (w), 1736 (s), 1643 (s), 1453 (w), 1401 (w), 1304 (w), 1200 (w), 1129 (w), 1129 (w), 1084 (w), 1017 (w), 760 (w), 700 (m) cm^{-1}.

7.16.26 1,3-Dimethyldihydropyrimidine-2,4(1*H*,3*H*)-dione (12y)

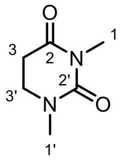

12y
$C_6H_{10}N_2O_2$
Mw = 142.16 $gmol^{-1}$

Prepared according to **GP2** from 1,3-dimethylpyrimidine-2,4(1*H*,3*H*)-dione (**11y**, 28.0 mg, 0.200 mmol, 1.00 equiv), [IPrIPyCuCl] (**4**, 9.30 mg, 20.0 µmol, 10.0 mol%), NaO*t*Bu (9.62 mg, 100 µmol, 50.0 mol%) in diglyme (2.0 mL). The reaction mixture was stirred for 24 h at 100 °C under H_2 atmosphere (1.5 bar). Purification by flash column chromatography on silica gel (cyclohexane/EtOAc = 1:1) yielded **12y** as yellow oil (48.0 mg, 0.168 mmol, 84%).

R*f* = 0.30 (cyclohexane/EtOAc = 1:1).

¹H NMR (600 MHz, $CDCl_3$): δ = 3.36 (t, $^3J_{3',3}$ = 6.9 Hz, 2H, H-3'), 3.18 (s, 3H, H-1), 3.05 (s, 3H, H-1'), 2.73 (t, $^3J_{3,3'}$ = 6.9 Hz, 2H, H-3) ppm.

¹³C NMR (151 MHz, $CDCl_3$): δ = 169.6 (C-2), 154.3 (C-2'), 43.1 (C-3'), 36.1 (C-1), 31.6 (C-1'), 27.8 (C-3) ppm.

HRMS (ESI) for $C_6H_{11}N_2O_2^+$ [(M+H⁺)⁺] calculated: 143.0815, found: 143.0811.

IR (ATR): ṽ = 2959 (w), 2922 (w), 2855 (w), 1714 (m), 1670 (s), 1494 (w), 1457 (w), 1375 (w), 1263 (w), 1129 (w), 1084 (w), 1021 (w), 798 (w), 734 (m) cm^{-1}.

The data is in accordance with literature.[30]

7.16.27 Methyl-1-methyl-2-oxopiperidine-3-carboxylate (12z)

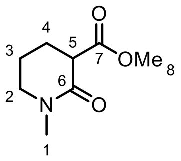

12z
$C_8H_{13}NO_3$
Mw = 171.20 $gmol^{-1}$

Prepared according to **GP2** from methyl 1-methyl-2-oxo-1,2-dihydropyridine-3-carboxylate (**11z**, 33.4 mg, 0.200 mmol, 1.00 equiv), [IPrIPyCuCl] (**4**, 9.30 mg, 20.0 µmol, 10.0 mol%), NaO*t*Bu (9.62 mg, 100 µmol, 50.0 mol%) in diglyme (2.0 mL). The reaction mixture was stirred for 24 h at 100 °C under H_2 atmosphere (1.5 bar). Purification by flash column chromatography on silica gel (cyclohexane/EtOAc = 0:100) yielded **12z** as yellow oil (48.0 mg, 0.168 mmol, 84%).

R*f* = 0.30 (cyclohexane/EtOAc = 0:100).

¹H NMR (600 MHz, $CDCl_3$): δ = 3.74 (s, 3H, H-8), 3.41 (t, $^3J_{5,4}$ = 7.0 Hz, 1H, H-5), 3.38–3.34 (m, 1H, H-2a), 3.29–3.25 (m, 1H, H-2b), 2.96 (s, 3H, H-1), 2.15–2.10 (m, 1H, H-4a), 2.07–2.01 (m, 1H, H-4b), 1.99–1.93 (m, 1H, H-3a), 1.82–1.76 (m, 1H, H-3b) ppm.

^{13}C NMR (151 MHz, $CDCl_3$): δ = 171.7 (C-7), 165.8 (C-6), 52.6 (C-8), 49.9 (C-5), 49.1 (C-1), 35.1 (C-2), 25.4 (C-4), 21.1 (C-3) ppm.

HRMS (ESI) for $C_8H_{14}NO_3^+$ [$(M+H^+)^+$] calculated: 172.0968, found: 172.0980.

IR (ATR): ṽ = 2955 (w), 2877 (w), 2247 (w), 1736 (s), 1640 (s), 1505 (w), 1438 (w), 1405 (w), 1356 (w), 1330 (w), 1263 (w), 1203 (w), 1162 (w), 909 (s), 723 (s) cm^{-1}.

The data is in accordance with literature.[31]

7.16.28 3-phenylpropanamide (12aa)

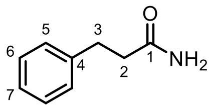

12aa
$C_9H_{11}NO$
Mw = 149.19 $gmol^{-1}$

Prepared according to **GP2** from cinnamamide (**11aa**, 29.4 mg, 0.200 mmol, 1.00 equiv), [IPrIPyCuCl] (**4**, 9.30 mg, 20.0 µmol, 10.0 mol%), NaO*t*Bu (9.62 mg, 100 µmol, 50.0 mol%) in diglyme (2.0 mL). The reaction mixture was stirred for 24 h at 100 °C under H_2 atmosphere (1.5 bar). After hydrogenation, 19% conversion of **11aa** to saturated amide **12aa** was obtained. Purification by flash column chromatography on silica gel (cyclohexane/EtOAc = 4:1) yielded **12aa** as colorless solid (3.43 mg, 0.023 mmol, 11%).

R_f = 0.20 (SiO_2, cyclohexane/EtOAc = 1:5).

^{1}H NMR (600 MHz, $CDCl_3$): δ = 7.31–7.28 (m, 2H, H-6), 7.23–7.20 (m, 3H, H-5, H-7), 5.41 (brs, 1H, N–*H*), 5.33 (brs, 1H, N–*H*), 2.98 (t, $^3J_{3,2}$ = 7.8 Hz, 2H, H-3), 2.54 (t, $^3J_{2,3}$ = 7.8 Hz, 2H, H-2) ppm.

^{13}C NMR (151 MHz, $CDCl_3$): δ = 174.5 (C-1), 140.8 (C-4), 128.7 (C-5)*, 128.5 (C-6)*, 126.5 (C-7), 37.7 (C-3), 31.5 (C-2) ppm.

GC-MS (EI) for $C_9H_{11}NO^+$ [M^+] calculated: 149.1, found: 149.1.

7.16.29 *N*-methyl-3-phenylpropanamide (12ab)

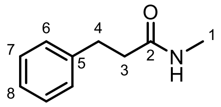

12ab
$C_{10}H_{13}NO$
Mw = 163.22 $gmol^{-1}$

Prepared according to **GP2** from *N*-methylcinnamamide (**11ab**, 32.2 mg, 0.200 mmol, 1.00 equiv), [IPrIPyCuCl] (**4**, 9.30 mg, 20.0 µmol, 10.0 mol%), NaO*t*Bu (9.62 mg, 100 µmol, 50.0 mol%) in diglyme (2.0 mL). The reaction mixture was stirred for 24 h at 100 °C under H_2 atmosphere (1.5 bar). After hydrogenation, 30% conversion of **11ab** to saturated amide **12ab** was obtained. Purification by flash column chromatography on silica gel (cyclohexane/EtOAc = 4:1) yielded **12ab** as colorless solid (6.10 mg, 0.037 mmol, 19%).

R_f = 0.20 (SiO_2, cyclohexane/EtOAc = 1:5).

^{1}H NMR (600 MHz, $CDCl_3$): δ = 7.30–7.27 (m, 2H, H-7), 7.21–7.19 (m, 3H, H-6, H-8), 5.36 (brs, 1H, N–*H*), 2.97 (t, $^{3}J_{3,4}$ = 7.8 Hz, 2H, H-3), 2.77 (d, $^{3}J_{1,N\text{-}H}$ = 4.8 Hz, 3H, H-1), 2.46 (t, $^{3}J_{4,3}$ = 7.8 Hz, 2H, H-4) ppm.

^{13}C NMR (151 MHz, $CDCl_3$): δ = 172.8 (C-2), 141.1 (C-5), 128.7 (C-6)*, 128.5 (C-7)*, 126.4 (C-8), 38.6 (C-1), 31.9 (C-4), 26.4 (C-3) ppm.

HRMS (ESI) for $C_{10}H_{14}NO^+$ [$(M+H^+)^+$] calculated: 164.1070, found: 164.1072.

7.16.30 *N*-(3,4-Dimethoxyphenethyl)-*N*-methyl-3-phenylpropanamide (12ba)

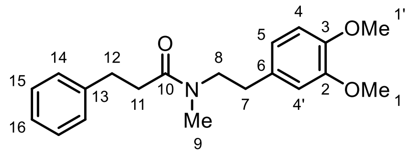

12ba
$C_{20}H_{25}NO_3$
Mw = 327.42 gmol^{-1}

Prepared according to **GP2** from *N*-(3,4-dimethoxyphenethyl)-*N*-methylcinnamamide (**11ba**, 65.1 mg, 0.200 mmol, 1.00 equiv), [IPrIPyCuCl] (**4**, 9.30 mg, 20.0 µmol, 10.0 mol%), NaO*t*Bu (9.65 mg, 100 µmol, 50.0 mol%) in diglyme (2.0 mL). The reaction mixture was stirred for 24 h at 100 °C under H_2 atmosphere (1.5 bar). Purification by flash column chromatography on silica gel (cyclohexane/EtOAc = 1:1) yielded **12ba** and **12ba_rotamer** (55:45 ratio) as colorless oil (41.0 mg, 0.125 mmol, 63%).

R_f = 0.20 (cyclohexane/EtOAc = 1:1).

^{1}H NMR (600 MHz, $CDCl_3$): δ = 7.30–7.10 (m, 5H, H-14, H-15, H-16), 6.80–6.73 (m, 2H, H-4, H-5), 6.57–6.59 (m, 1H, H-4'), 3.87–3.83 (m, 6H, H-1, H-1'), 3.58–3.43 (m, 2H, H-8), 2.98–2.82 (m, 5H, H-7, H-9), 2.79–2.71 (m, 2H, H-12), 2.61–2.33 (m, 2H, H-11) ppm.

The ^{1}H NMR spectrum is not well separated for the two rotamers, which is why only ^{13}C NMR is assigned separately.

^{13}C NMR (151 MHz, $CDCl_3$): δ = 172.3 (C-10), 149.2 (C-2), 148.0 (C-3), 141.7 (C-13), 131.9 (C-6), 128.6 (C-14), 128.5 (C-15), 126.2 (C-16), 120.9 (C-4'), 112.1 (C-4), 111.6 (C-5), 56.0 (C-1, C-1'), 51.7 (C-9), 36.1 (C-7), 35.0 (C-11), 33.7 (C-12), 31.6 (C-8) ppm.

^{13}C NMR of **12ba_rotamer** (151 MHz, $CDCl_3$): δ = 172.0 (C-10), 149.1 (C-2), 147.7 (C-3), 141.6 (C-13), 130.9 (C-6), 128.5 (C-14), 128.4 (C-15), 126.2 (C-16), 120.8 (C-4'), 112.0 (C-4), 111.4 (C-5), 56.0 (C-1, C-1'), 50.3 (C-9), 35.7 (C-7), 34.5 (C-11), 33.5 (C-12), 31.4 (C-8) ppm.

Rotamer ratio is determined from the integration value of H-8 at 3.58–3.43 ppm.

HRMS (ESI) for $C_{20}H_{26}NO_3^+$ [$(M+H^+)^+$] calculated: 328.1907, found: 328.1904.

IR (ATR): ṽ = 3060 (w), 2985 (w), 2937 (w), 2840 (w), 1736 (w), 1640 (s), 1513 (m), 1449 (w), 1397 (w), 1300 (w), 1263 (w), 1196 (w), 1140 (m), 1028 (w), 760 (w), 700 (w) cm^{-1}.

7.16.31 (1*R*,5*S*)-3-(3-Phenylpropanoyl)-1,2,3,4,5,6-hexahydro-8*H*-1,5-methanopyrido[1,2-*a*][1,5]diazocin-8-one (12bb)

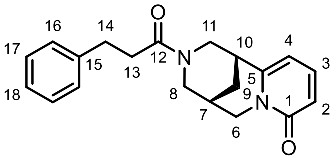

12bb
$C_{20}H_{22}N_2O_2$
Mw = 322.41 gmol^{-1}

Prepared according to **GP2** from (1*R*,5*S*)-3-cinnamoyl-1,2,3,4,5,6-hexahydro-8*H*-1,5-methanopyrido[1,2-*a*][1,5]diazocin-8-one (**11bb**, 64.1 mg, 0.200 mmol, 1.00 equiv), [IPrIPyCuCl] (**4**, 9.30 mg, 20.0 µmol, 10.0 mol%), NaO*t*Bu (9.62 mg, 100 µmol, 50.0 mol%) in diglyme (2.0 mL). The reaction mixture was stirred for 24 h at 100 °C under H_2 atmosphere (1.5 bar). Purification by flash column chromatography on silica gel (CH_2Cl_2/MeOH = 97:3) yielded **12bb** and **12bb_rotamer** (54:46 ratio) as colorless soild (39.2 mg, 0.121 mmol, 61%).

M.p. = 51 °C (CH_2Cl_2).

R*f* = 0.20 (CH_2Cl_2/MeOH = 97:3).

^{1}H NMR (600 MHz, $CDCl_3$): δ = 7.27–7.23 (m, 3H, H-3, H-17*), 7.18–7.14 (m, 2H, H-16)*, 7.05–7.03 (m, 1H, H-18), 6.44 (t, $^{3/4}J_{2,3/4}$ = 10.2 Hz, 1H, H-2), 6.07–6.01 (m, 1H, H-4), 4.84–4.68 (m, 1H, H-8_{eq})**, 4.12–4.08 (m, 1H, H-11_{eq})**, 3.93–3.83 (m, 2H, H-6), 3.30–3.21 (m, 1H, H-9a)***, 3.08–3.04 (m, 1H, H-7)***, 2.84–2.48 (m, 4H, H-13, H-14), 2.38–1.92 (m. 4H, H-9b, H-8_{ax}, H-11_{ax}) ppm.

The ^{1}H NMR spectrum is not well separated for the two rotamers, which is why only ^{13}C NMR is assigned separately.

^{13}C NMR (151 MHz, $CDCl_3$): δ = 171.5 (C-12), 163.3 (C-1), 148.7 (C-5), 141.2 (C-15), 138.5 (C-3), 128.6 (C-17)*, 128.5 (C-16)*, 128.3 (C-18), 118.1 (C-2), 104.9 (C-4), 52.9 (C-6), 49.0 (C-8)**, 47.9 (C-11)**, 35.1 (C-14)***, 34.7 (C-13)***, 31.1 (C-7)****, 27.5 (C-10)****, 26.3 (C-9) ppm.

^{13}C NMR of 12bb_rotamer(151 MHz, $CDCl_3$): δ = 171.6 (C-12), 163.5 (C-1), 148.5 (C-5), 141.1 (C-15), 139.2 (C-3), 128.6 (C-17)*, 128.3 (C-18), 126.2 (C-16)*, 117.5 (C-2), 105.9 (C-4), 51.7 (C-6), 49.0 (C-11)**, 48.8 (C-8)**, 35.0 (C-14)***, 34.5 (C-13)***, 31.2 (C-7)****, 27.7 (C-10)****, 26.2 (C-9) ppm.

Rotamer ratio is determined from the integration value of H-4 at 6.07–6.01 ppm.

HRMS (ESI) for $C_{20}H_{23}N_2O_2^+$ [(M+H$^+$)$^+$] calculated: 323.1754, found: 323.1747.

IR (ATR): ṽ = 3052 (w), 2937 (w), 2862 (w), 1647 (s), 1576 (w), 1543 (m), 1423 (w), 1263 (w), 1215 (w), 1181 (w), 1095 (w), 797 (w), 730 (m), 700 (m) cm^{-1}.

7.16.32 3-(4-(*tert*-Butyl)phenyl)-*N*-(2,3-dihydrobenzo[*b*][1,4]dioxin-6-yl)-*N*-methylpropanamide (12bc)

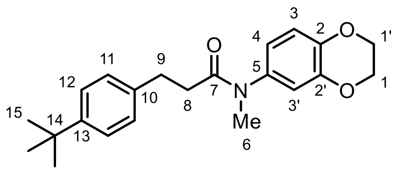

12bc
$C_{22}H_{27}NO_3$
Mw = 53.46

Prepared according to **GP2** from (*E*)-3-(4-(tert-butyl)phenyl)-*N*-(2,3-dihydrobenzo[*b*][1,4]dioxin-6-yl)-*N*-methylacrylamide (**11bc**, 70.3 mg, 0.200 mmol, 1.00 equiv), [IPrIPyCuCl] (**4**, 9.30 mg, 20.0 µmol, 10.0 mol%), NaO*t*Bu (9.65 mg, 100 µmol, 50.0 mol%) in diglyme (2.0 mL). The reaction mixture was stirred for 24 h at 100 °C under H_2 atmosphere (1.5 bar). Purification by flash column chromatography on silica gel (cyclohexane/EtOAc = 1:1) yielded **12bc** (51.0 mg, 0.144 mmol, 72%) as colorless solid.

Mp = 72 °C (cyclohexane).

R_f = 0.25 (cyclohexane/EtOAc = 1:1).

^{1}H NMR (600 MHz, $CDCl_3$): δ = 7.27–7.25 (m, 2H, H-12), 7.02 (d, $^3J_{11,12}$ = 7.9 Hz, 2H, H-11), 6.80 (d, $^3J_{3,4}$ = 8.5 Hz, 1H, H-3), 6.55 (d, $^4J_{3',4}$ = 2.5 Hz, 1H, H-3'), 6.48 (dd, $^3J_{4,3}$ = 8.5 Hz, $^4J_{4,3'}$ = 2.5 Hz, 1H, H-4), 4.26 (s, 4H, H-1, H-1'), 3.20 (s, 3H, H-6), 2.88 (t, $^3J_{9,8}$ = 8.0 Hz, 2H, H-9), 2.38 (t, $^3J_{8,9}$ = 8.0 Hz, 2H, H-8), 1.29 (s, 9H, H-15) ppm.

^{13}C NMR (151 MHz, $CDCl_3$): δ = 172.8 (C-7), 148.9 (C-13), 144.0 (C-2)*, 143.2 (C-2')*, 138.4 (C-10), 137.5 (C-5), 128.2 (C-12), 125.3 (C-11), 120.4 (C-4), 118.0 (C-3), 116.3 (C-3'), 64.4 (C-1, C-1'), 37.5 (C-6), 36.0 (C-8), 34.5 (C-9), 31.5 (C-15), 31.4 (C-14) ppm.

HRMS (ESI) for $C_{22}H_{28}NO_3^+$ [(M+H$^+$)$^+$] calculated: 354.2064, found: 354.2061.

IR (ATR): ṽ = 2959 (w), 2873 (w), 1736 (w), 1654 (s), 1587 (m), 1505 (s), 1461 (w), 1420 (w), 1379 (w), 1300 (w), 1244 (w), 1192 (w), 1110 (m), 1066 (m), 1017 (w), 928 (w), 894 (w), 816 (w), 749 (w) cm^{-1}.

7.16.33 1-(Piperidin-1-yl)-3-(3,4,5-trimethoxyphenyl)propan-1-one (12bd)

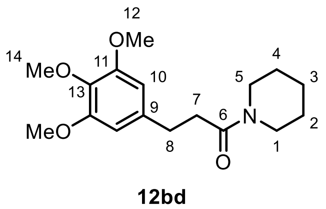

12bd
$C_{17}H_{25}NO_4$
Mw = 307.39 gmol^{-1}

Prepared according to **GP2** from (*E*)-1-(piperidin-1-yl)-3-(3,4,5-trimethoxyphenyl)prop-2-en-1-one (**11bd**, 61.1 mg, 0.200 mmol, 1.00 equiv), [IPrIPyCuCl] (**4**, 9.30 mg, 20.0 µmol, 10.0 mol%), NaO*t*Bu (9.62 mg, 100 µmol, 50.0 mol%) in diglyme (2.0 mL). The reaction mixture was stirred for 24 h at 100 °C under H_2 atmosphere (1.5 bar). Purification by flash column chromatography on silica gel (cyclohexane/EtOAc = 1:1) yielded **12bd** as colorless oil (43.6 mg, 0.142 mmol, 71%).

R_f = 0.30 (cyclohexane/EtOAc = 1:1).

^{1}H NMR (600 MHz, $CDCl_3$): δ = 6.43 (s, 2H, H-10), 3.84 (s, 6H, H-12), 3.81 (s, 3H, H-14), 3.56 (t, $^3J_{5,4}$ = 5.6 Hz, 2H, H-5), 3.34 (t, $^3J_{1,2}$ = 5.6 Hz, 2H, H-1), 2.90 (t, $^3J_{8,7}$ = 7.2 Hz, 2H, H-8), 2.60 (t, $^3J_{7,8}$ = 7.2 Hz, 2H, H-7), 1.63–1.59 (m, 2H, H-2), 1.53–1.49 (m, 2H, H-4), 1.48–1.44 (m, 2H, H-3) ppm.

^{13}C NMR (151 MHz, $CDCl_3$): δ = 170.5 (C-6), 153.3 (C-11), 137.4 (C-13), 136.4 (C-9), 105.5 (C-10), 61.0 (C-14), 56.2 (C-12), 46.8 (C-1), 42.9 (C-5), 35.4 (C-7), 32.2 (C-8), 26.6 (C-2), 25.7 (C-4), 24.6 (C-3) ppm.

HRMS (ESI) for $C_{17}H_{26}NO_4^+$ [(M+H$^+$)$^+$] calculated: 308.1856, found: 308.1850.

IR (ATR): ṽ = 2937 (w), 2855 (w), 1632 (s), 1587 (s), 1505 (w), 1453 (m), 1345 (w), 1237 (w), 1118 (s), 1006 (m), 853 (w), 775 (w) cm^{-1}.

7.16.34 3-(benzo[*d*][1,3]dioxol-5-yl)-1-(piperidin-1-yl)propan-1-one (12be)

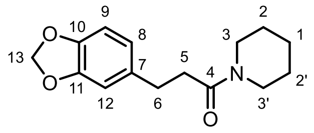

12be
$C_{15}H_{19}NO_3$
Mw = 261.32 gmol^{-1}

Prepared according to **GP2** from (*E*)-3-(benzo[*d*][1,3]dioxol-5-yl)-1-(piperidin-1-yl)prop-2-en-1-one (**11be**, 51.9 mg, 0.200 mmol, 1.00 equiv), [IPrIPyCuCl] (**4**, 9.30 mg, 20.0 µmol, 10.0 mol%), NaO*t*Bu (9.62 mg, 100 µmol, 50.0 mol%) in diglyme (2.0 mL). The reaction mixture was stirred for 24 h at 100 °C under H_2 atmosphere (1.5 bar). Purification by flash column chromatography on silica gel (cyclohexane/EtOAc = 1:1) yielded **12be** as colorless oil (39.6 mg, 0.151 mmol, 76%).

R$_f$ = 0.30 (cyclohexane/EtOAc = 1:1).

^{1}H NMR (600 MHz, $CDCl_3$): δ = 6.73–6.71 (m, 2H, H-9, H-12), 6.66 (dd, $^3J_{8,9}$ = 7.9 Hz, $^4J_{8,12}$ = 1.7 Hz, 1H, H-8), 5.91 (s, 2H, H-13), 3.55 (t, $^3J_{3,2}$ = 5.6 Hz, 2H, H-3), 3.34 (t, $^3J_{3',2'}$ = 5.6 Hz, 2H, H-3'), 2.88 (t, $^3J_{5,4}$ = 7.8 Hz, 2H, H-6), 2.56 (t, $^3J_{4,5}$ = 7.8 Hz, 2H, H-5), 1.61 (p, $^3J_{1,2/2'}$ = 5.9 Hz, 2H, H-1), 1.53–1.50 (m, 2H, H-2), 1.48–1.47 (m, 2H, H-2') ppm.

^{13}C NMR (151 MHz, $CDCl_3$): δ = 170.4 (C-4), 147.7 (C-10)*, 145.9 (C-11)*, 135.5 (C-7), 121.3 (C-7), 109.1 (C-8), 108.4 (C-12), 100.9 (C-13), 46.7 (C-3), 42.8 (C-3'), 35.5 (C-5), 31.4 (C-6), 26.6 (C-2'), 25.7 (C-2), 24.7 (C-1) ppm.

HRMS (ESI) for $C_{15}H_{20}NO_3^+$ [(M+H$^+$)$^+$] calculated: 262.1438, found: 262.1433.

IR (ATR): ṽ = 2937 (w), 2858 (w), 1632 (s), 1490 (w), 1438 (w), 1244 (m), 1189 (w), 1036 (w), 939 (w), 808 (w), 734 (m) cm^{-1}.

7.16.35 3-(4-Methoxyphenyl)-1-(pyrrolidin-1-yl)propan-1-one (12bf)

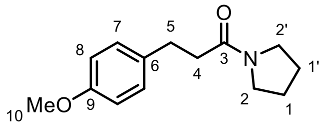

12bf
$C_{14}H_{19}NO_2$
Mw = 233.32 gmol^{-1}

Prepared according to **GP2** from ((E)-3-(4-methoxyphenyl)-1-(pyrrolidin-1-yl)prop-2-en-1-one (**11bf**, 46.3 mg, 0.200 mmol, 1.00 equiv), [IPrIPyCuCl] (**4**, 9.30 mg, 20.0 µmol, 10.0 mol%), NaO*t*Bu (9.65 mg, 100 µmol, 50.0 mol%) in diglyme (2.0 mL). The reaction mixture was stirred for 24 h at 100 °C under H_2 atmosphere (1.5 bar). Purification by flash column chromatography on silica gel (cyclohexane/EtOAc = 2:1) yielded **12bf** (32.5 mg, 0.139 mmol, 70%) as colorless oil.

R*f* = 0.20 (cyclohexane/EtOAc = 2:1).

^{1}H NMR (600 MHz, $CDCl_3$): δ = 7.14 (d, ${}^3J_{7,8}$ = 8.6 Hz, 2H, H-7), 6.82 (d, ${}^3J_{8,7}$ = 8.6 Hz, 2H, H-8), 3.78 (s, 3H, H-10), 3.46 (t, ${}^3J_{2,1}$ = 6.9 Hz, 2H, H-2)*, 3.29 (t, ${}^3J_{2',1'}$ = 6.9 Hz, 2H, H-2')*, 2.92 (t, ${}^3J_{5,4}$ = 7.2 Hz, 2H, H-5), 2.52 (t, ${}^3J_{4,5}$ = 7.2 Hz, 2H, H-4), 1.88 (p, ${}^3J_{1,2/1'}$ = 6.6 Hz, 2H, H-1), 1.82 (p, ${}^3J_{1',2'/1}$ = 6.6 Hz, 2H, H-1') ppm.

^{13}C NMR (151 MHz, $CDCl_3$): δ = 171.0 (C-3), 158.1 (C-9), 133.8 (C-6), 129.5 (C-8), 114.0 (C-7), 55.4 (C-10), 46.7 (C-2), 45.8 (C-2'), 37.2 (C-4), 30.5 (C-5), 26.2 (C-1), 24.5 (C-1') ppm.

HRMS (ESI) for $C_{16}H_{24}NO_4^+$ [(M+H$^+$)$^+$] calculated: 234.1489, found: 234.1491.

IR (ATR): ṽ = 2952 (w), 2873 (w), 2836 (w), 1636 (s), 1513 (m), 1431 (m), 1341 (w), 1300 (w), 1177 (w), 1032 (m), 823 (m) cm^{-1}.

7.16.36 1-(Pyrrolidin-1-yl)-3-(3,4,5-trimethoxyphenyl)propan-1-one (12bg)

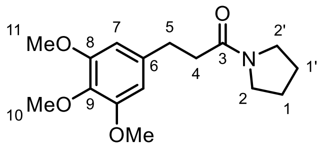

12bg
$C_{16}H_{23}NO_4$
Mw = 293.36 gmol^{-1}

Prepared according to **GP2** from (*E*)-1-(pyrrolidin-1-yl)-3-(3,4,5-trimethoxyphenyl)prop-2-en-1-one (**11bg**, 58.3 mg, 0.200 mmol, 1.00 equiv), [IPrIPyCuCl] (**4**, 9.30 mg, 20.0 µmol, 10.0 mol%), NaO*t*Bu (9.65 mg, 100 µmol, 50.0 mol%) in diglyme (2.0 mL). The reaction mixture was stirred for 24 h at 100 °C under H_2 atmosphere (1.5 bar). Purification by flash column chromatography on silica gel (cyclohexane/EtOAc = 2:1) yielded **12bg** (35.8 mg, 0.122 mmol, 61%) as colorless oil.

R*f* = 0.20 (cyclohexane/EtOAc = 2:1).

^{1}H NMR (600 MHz, $CDCl_3$): δ = 6.45 (s, 2H, H-7), 3.84 (s, 6H, H-11), 3.82 (s, 3H, H-10), 3.47 (t, ${}^3J_{2,1}$ = 6.9 Hz, 2H, H-2)*, 3.30 (t, ${}^3J_{2',1'}$ = 6.9 Hz, 2H, H-2')*, 2.93 (t, ${}^3J_{5,4}$ = 7.2 Hz, 2H, H-5), 2.55 (t, ${}^3J_{4,5}$ = 7.2 Hz, 2H, H-4), 1.89 (p, ${}^3J_{1,2/1'}$ = 6.6 Hz, 2H, H-1), 1.82 (, ${}^3J_{1',2'/1}$ = 6.6 Hz, 2H, H-1') ppm.

^{13}**C NMR** (151 MHz, $CDCl_3$): δ = 170.8 (C-3), 153.3 (C-8), 137.6 (C-9), 136.4 (C-6), 105.5 (C-7), 61.0 (C-11), 56.2 (C-10), 46.8 (C-2)*, 45.8 (C-2')*, 37.1 (C-4), 31.2 (C-5), 26.2 (C-1), 24.5 (C-1') ppm.

HRMS (ESI) for $C_{16}H_{24}NO_4^+$ [(M+H$^+$)$^+$] calculated: 294.1700, found: 294.1701.

IR (ATR): ṽ = 2974 (w), 2944 (w), 2877 (w), 1632 (s), 1587 (m), 1505 (w), 1453 (m), 1420 (s), 1338 (w), 1241 (w), 1192 (w), 1118 (s), 1013 (w), 827 (w), 775 (w) cm^{-1}.

7.16.37 1-(3-(3,4,5-Trimethoxyphenyl)propanoyl)piperidin-2-one (12bh)

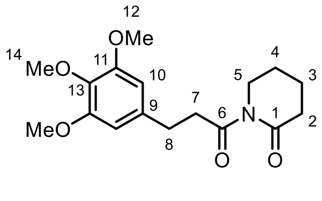

12bh
$C_{17}H_{23}NO_5$
Mw = 321.37 gmol^{-1}

Prepared according to **GP2** from (*E*)-1-(3-(3,4,5-trimethoxyphenyl)acryloyl)-5,6-dihydropyridin-2(1*H*)-one (**11bh**, 63.5 mg, 0.200 mmol, 1.00 equiv), [IPrIPyCuCl] (**4**, 9.30 mg, 20.0 µmol, 10.0 mol%), NaO*t*Bu (9.62 mg, 100 µmol, 50.0 mol%) in diglyme (2.0 mL). The reaction mixture was stirred for 24 h at 100 °C under H_2 atmosphere (1.5 bar). Purification by flash column chromatography on silica gel (cyclohexane/EtOAc = 1:3) yielded **12ah** as colorless oil (9.21 mg, 0.028 mmol, 14%).

R_f = 0.20 (cyclohexane/EtOAc = 1:3).

^{1}**H NMR** (600 MHz, $CDCl_3$): δ = 6.45 (s, 2H, H-10), 3.84 (s, 6H, H-12), 3.81 (s, 3H, H-14), 3.71 (t, $^3J_{5,4}$ = 5.9 Hz, 2H, H-5), 3.22 (t, $^3J_{7,8}$ = 7.7 Hz, 2H, H-7), 2.91 (t, $^3J_{8,7}$ = 7.7 Hz, 2H, H-8), 2.53 (t, $^3J_{2,3}$ = 6.6 Hz, 2H, H-2), 1.83–1.80 (m, 4H, H-3, H-4) ppm.

^{13}**C NMR** (151 MHz, $CDCl_3$): δ = 176.3 (C-6), 173.6 (C-1), 153.2 (C-11), 137.2 (C-9), 136.3 (C-13), 105.6 (C-10), 61.0 (C-14), 56.2 (C-12), 44.2 (C-5), 41.5 (C-7), 35.0 (C-2), 31.7 (C-8), 22.5 (C-3)*, 20.4 (C-4)* ppm.

HRMS (ESI) for $C_{17}H_{24}NO_5^+$ [(M+H$^+$)$^+$] calculated: 322.1649, found: 322.1642.

IR (ATR): ṽ = 2940 (w), 2840 (w), 1688 (s), 1587 (m), 1505 (w), 1457 (w), 1423 (w), 1289 (w), 1237 (w), 1121 (s), 1006 (w), 827 (w), 775 (w) cm^{-1}.

7.16.38 5-(Benzo[*d*][1,3]dioxol-5-yl)-1-(piperidin-1-yl)pentan-1-one (12bi)

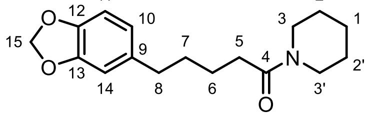

12bi
$C_{17}H_{23}NO_3$
Mw = 289.37 gmol^{-1}

Prepared according to **GP2** from (2*E*,4*E*)-5-(benzo[*d*][1,3]dioxol-5-yl)-1-(piperidin-1-yl)penta-2,4-dien-1-one (**11bi**, 57.1 mg, 0.200 mmol, 1.00 equiv), [IPrIPyCuCl] (**4**, 9.30 mg, 20.0 µmol, 10.0 mol%), NaO*t*Bu (9.62 mg, 100 µmol, 50.0 mol%) in diglyme (2.0 mL). The reaction mixture was stirred for 24 h at 100 °C under H_2 atmosphere (1.5 bar). Purification by flash column chromatography on silica gel (cyclohexane/EtOAc = 1:3) yielded **12bi** as yellow oil (43.0 mg, 0.148 mmol, 74%).

R*f* = 0.30 (cyclohexane/EtOAc = 1:3).

^{1}H NMR (600 MHz, $CDCl_3$): δ = 6.71 (d, $^3J_{10,11}$ = 7.9 Hz, 1H, H-10), 6.66 (s, 1H, H-14), 6.61 (d, $^3J_{11,10}$ = 7.9 Hz, 1H, H-11), 5.90 (s, 2H, H-15), 3.53 (t, $^3J_{3,2}$ = 5.6 Hz, 2H, H-3), 3.35 (t, $^3J_{3',2'}$ = 5.6 Hz, 2H, H-3'), 2.56 (t, $^3J_{5,6}$ = 7.0 Hz, 2H, H-5), 2.31 (t, $^3J_{8,7}$ = 7.0 Hz, 2H, H-8), 1.63–1.60 (m, 6H, H-1, H-6, H-7), 1.52–1.51 (m, 4H, H-2, H-2') ppm.

^{1}H NMR shows impurities of H_2O at 1.54 ppm.

^{13}C NMR (151 MHz, $CDCl_3$): δ = 171.3 (C-4), 147.6 (C-13)*, 145.6 (C-12)*, 136.3 (C-9), 121.2 (C-11), 109.0 (C-14), 108.2 (C-10), 100.8 (C-15), 46.7 (C-3), 42.7 (C-3'), 35.6 (C-8), 33.4 (C-5), 31.6 (C-7), 26.7 (C-2), 25.7 (C-2'), 25.1 (C-1), 24.7 (C-6) ppm.

HRMS (ESI) for $C_{17}H_{24}NO_3^+$ [(M+H$^+$)$^+$] calculated: 290.1751, found: 290.1749.

IR (ATR): ṽ = 2937 (w), 2859 (w), 1633 (s), 1491 (m), 1438 (s), 1245 (m), 1189 (w), 1036 (m), 939 (w), 809 (m), 734 (m) cm^{-1}.

7.16.39 *N*-Isobutyl-*N*-methyldecanamide (12bj-1) and (*E*)-1-(pyrrolidin-1-yl)dec-4-en-1-one (12bj-2)

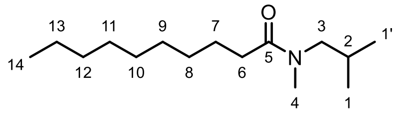

12bj-1
$C_{15}H_{31}NO$
Mw = 241.42 gmol^{-1}

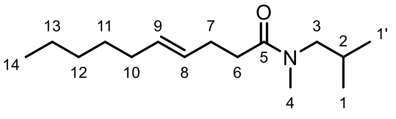

12bj-2
$C_{15}H_{29}NO$
Mw = 239.40 gmol^{-1}

Prepared according to **GP2** from (2*E*,4*E*)-*N*-isobutyl-*N*-methyldeca-2,4-dienamide (**11bj**, 88.4 mg, 0.400 mmol, 1.00 equiv), [IPrIPyCuCl] (**4**, 18.6 mg, 40.0 µmol, 10.0 mol%), NaO*t*Bu (19.3 mg, 200 µmol, 50.0 mol%) in diglyme (2.0 mL). The reaction mixture was stirred for 24 h at 100 °C under H_2 atmosphere (1.5 bar). Purification by flash column chromatography on silica gel (cyclohexane/EtOAc = 1:1) yielded **12bj-1** (50:50 rotamer ratio) (25.0 mg, 0.104 mmol, 26%) and **12bj-2** (50:50 rotamer ratio) (16.8 mg, 0.070 mmol, 18%) as colorless oil.

12bj-1:

R*f* = 0.20 (cyclohexane/EtOAc = 1:1).

^{1}H NMR (600 MHz, $CDCl_3$): δ = 3.20–3.07 (m, 2H, H-3), 2.97–2.90 (m, 3H, H-4), 2.31–2.28 (m, 2H, H-6), 1.97–1.90 (m, 1H, H-2), 1.63 (p, $^{3,}J_{7,8/6}$ = 7.5 Hz, 2H, H-7), 1.34–1.22 (m, 12H, H-8, H-9, H-10, H-11, H-12, H-13), 0.91 (d, $^3J_{1,2}$ = 6.7 Hz, 3H, H-1), 0.88–0.86 (m, 6H, H-1', H-14) ppm.

The ^{1}H NMR spectrum is not well separated for the two rotamers, which is why only ^{13}C NMR is assigned separately.

^{13}C NMR (151 MHz, $CDCl_3$): δ = 173.5 (C-5), 57.5 (C-3), 36.2 (C-4), 34.1 (C-6), 32.0 (C-8), 29.7 (C-9), 29.7 (C-10), 29.6 (C-11), 29.4 (C-12), 27.8 (C-2), 25.6 (C-7), 22.8 (C-13), 20.2 (C-1), 20.1 (C-1'), 14.3 (C-14) ppm.

^{13}C NMR of **12bj-1_rotamer** (151 MHz, $CDCl_3$): δ = 173.4 (C-5), 55.1 (C-3), 33.9 (C-4), 33.3 (C-6), 32.0 (C-8), 29.7 (C-9), 29.7 (C-10), 29.6 (C-11), 29.4 (C-12), 26.9 (C-2), 25.3 (C-7), 22.8 (C-13), 20.2 (C-1), 20.1 (C-1'), 14.3 (C-14) ppm.

Rotamer ratio is determined from the integration value of H-3 at 3.20–3.07 ppm.

HRMS (ESI) for $C_{15}H_{32}NO^+$ [(M+H$^+$)$^+$] calculated: 242.2478, found: 242.2481.

IR (ATR): ṽ = 2926 (w), 2855 (w), 1647 (s), 1464 (w), 1401 (w), 1341 (w), 1278 (w), 1148 (w), 1088 (w) cm^{-1}.

12bj-2:

R*f* = 0.20 (cyclohexane/EtOAc = 1:1).

^{1}H NMR (600 MHz, $CDCl_3$): δ = 5.49–5.38 (m, 2H, H-8, H-9), 3.20–3.06 (m, 2H, H-3), 2.96–2.90 (m, 3H, H-4), 2.37–2.30 (m, 4H, H-6, H-7), 1.97–1.90 (m, 3H, H-2, H-10), 1.34–1.23 (m, 6H, H-11, H-12, H-13), 0.91–0.86 (m, 9H, H-1, H-1', H-14) ppm.

The ^{1}H NMR spectrum is not well separated for the two rotamers, which is why only ^{13}C NMR is assigned separately.

^{13}C NMR (151 MHz, $CDCl_3$): δ = 172.8 (C-5), 131.6 (C-8), 129.0 (C-9), 57.5 (C-3), 36.2 (C-4), 33.9 (C-6), 32.6 (C-10), 31.5 (C-11), 29.3 (C-12), 28.6 (C-7), 27.8 (C-2), 22.7 (C-13), 20.2 (C-1), 20.1 (C-1'), 14.2 (C-14) ppm.

^{13}C NMR of **12bj-2_rotamer** (151 MHz, $CDCl_3$): δ = 172.7 (C-5), 131.5 (C-8), 129.0 (C-9), 55.2 (C-3), 34.1 (C-4), 33.4 (C-6), 32.6 (C-10), 31.5 (C-11), 29.3 (C-12), 28.4 (C-7), 26.9 (C-2), 22.7 (C-13), 20.2 (C-1), 20.1 (C-1'), 14.2 (C-14) ppm.

Rotamer ratio is determined from the integration value of H-3 at 3.20–3.06 ppm.

HRMS (ESI) for $C_{15}H_{30}NO^+$ [(M+H$^+$)$^+$] calculated: 240.2322, found: 240.2323.

IR (ATR): ṽ = 2959 (w), 2926 (w), 2855 (w), 1647 (s), 1464 (w), 1401 (w), 1278 (w), 1148 (w), 969 (w) cm^{-1}.

7.16.40 1-(Pyrrolidin-1-yl)decan-1-one (12bk-1) and (*E*)-1-(pyrrolidin-1-yl)dec-4-en-1-one (12bk-2)

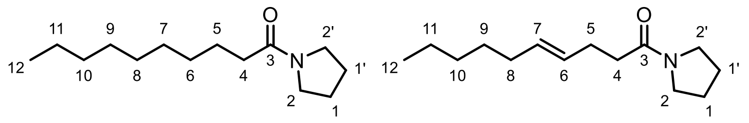

Prepared according to **GP2** from (2*E*,4*E*)-1-(Pyrrolidin-1-yl)deca-2,4-dien-1-one **(11bk**, 88.4 mg, 0.400 mmol, 1.00 equiv), [IPrIPyCuCl] (**4**, 18.6 mg, 40.0 µmol, 10.0 mol%), NaO*t*Bu (19.3 mg, 200 µmol, 50.0 mol%) in diglyme (2.0 mL). The reaction mixture was stirred for 24 h at 100 °C under H_2 atmosphere (1.5 bar). Purification by flash column chromatography on silica gel (cyclohexane/EtOAc = 1:1) yielded **12bk-1** (28.8 mg, 0.128 mmol, 32%) and **12bk-2** (15.2 mg, 0.068 mmol, 17%) as colorless oil.

12ak-1:

$\mathbf{R}_f$ = 0.20 (cyclohexane/EtOAc = 1:1).

^{1}H NMR (600 MHz, $CDCl_3$): δ = 3.45 (t, $^3J_{2,1}$ = 6.9 Hz, 2H, H-2)*, 3.40 (t, $^3J_{2',1'}$ = 6.9 Hz, 2H, H-2')*, 2.24 (t, $^3J_{4,5}$ = 7.8 Hz, 2H, H-4), 1.94 (p, $^3J_{1,1'/2}$ = 7.2 Hz, 2H, H-1)*, 1.83 (p, $^3J_{1',1/2'}$ = 7.2 Hz, 2H, H-1'), 1.63 (p, $^3J_{5,4/6}$ = 7.4 Hz, 2H, H-5), 1.35–1.21 (m, 12H, H-6, H-7, H-8, H-9, H-10, H-11), 0.87 (t, $^3J_{12,11}$ = 7.0 Hz, 3H, H-12) ppm.

^{13}C NMR (151 MHz, $CDCl_3$): δ = 172.1 (C-3), 46.8 (C-2)*, 45.7 (C-2')*, 35.0 (C-4), 32.0 (C-6)*, 29.7 (C-7)*, 29.6 (C-8)*, 29.6 (C-9)*, 29.4 (C-10), 26.3 (C-1), 25.1 (C-5), 24.5 (C-1'), 22.8 (C-11), 14.2 (C-12) ppm.

HRMS (ESI) for $C_{14}H_{28}NO^+$ [$(M+H^+)^+$] calculated: 226.2165, found: 226.2167.

IR (ATR): ṽ = 2959 (w), 2929 (w), 2870 (w), 1654 (s), 1602 (s), 1416 (s), 1338 (w), 1192 (w), 998 (m), 864 (w) cm^{-1}.

12ak-2:

$\mathbf{R}_f$ = 0.20 (cyclohexane/EtOAc = 1:1).

^{1}H NMR (600 MHz, $CDCl_3$): δ = 5.49–5.41 (m, 2H, H-6, H-7), 3.46 (t, $^3J_{2,1}$ = 6.9 Hz, 2H, H-2)*, 3.40 (t, $^3J_{2',1'}$ = 6.9 Hz, 2H, H-2')*, 2.35–2.29 (m, 4H, H-4, H-5), 1.98–1.91 (m, 4H, H-1*, H-8), 1.84 (p, $^3J_{1',1/2'}$ = 7.2 Hz, 2H, H-1')*, 1.35–1.22 (m, 6H, H-9, H-10, H-11), 0.87 (t, $^3J_{12,11}$ = 7.0 Hz, 3H, H-12) ppm.

^{13}C NMR (151 MHz, $CDCl_3$): δ = 171.3 (C-3), 131.6 (C-6), 128.9 (C-7), 46.7 (C-2)*, 45.7 (C-2')*, 35.1 (C-4), 32.7 (C-5), 31.5 (C-8), 29.3 (C-9), 28.2 (C-10), 26.3 (C-1), 24.6 (C-1'), 22.7 (C-11), 14.2 (C-12) ppm.

HRMS (ESI) for $C_{14}H_{26}NO^+$ [$(M+H^+)^+$] calculated: 224.2009, found: 224.2012.

IR (ATR): ṽ = 2955 (w), 2926 (w), 2873 (w), 1640 (s), 1427 (m), 1341 (w), 1253 (w), 1192 (w), 969 (w) cm^{-1}.

7.16.41 1-(Piperidin-1-yl)-3-(3,4,5-trimethoxyphenyl)propan-1-one-2,3-*d*₂ (12bd-*d*₂)

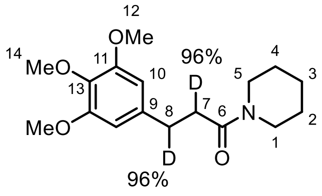

12bd-d_2
$C_{17}H_{23}D_2NO_4$
Mw = 309.40 $gmol^{-1}$

Prepared according to **GP2** from (*E*)-1-(piperidin-1-yl)-3-(3,4,5-trimethoxyphenyl)prop-2-en-1-one (**11bd**, 61.1 mg, 0.200 mmol, 1.00 equiv), [IPrIPyCuCl] (**4**, 9.30 mg, 20.0 µmol, 10.0 mol%), NaO*t*Bu (9.62 mg, 100 µmol, 50.0 mol%) in diglyme (2.0 mL). The reaction mixture was stirred for 24 h at 100 °C under D_2 atmosphere (2 bar). Purification by flash column chromatography on silica gel (cyclohexane/EtOAc = 1:1) yielded **12bd**-d_2 as colorless oil (42.7 mg, 0.138 mmol, 69%).

R*f* = 0.30 (cyclohexane/EtOAc = 1:1).

¹H NMR (600 MHz, $CDCl_3$): δ = 6.44 (s, 2H, H-10), 3.85 (s, 6H, H-12), 3.81 (s, 3H, H-14), 3.56 (t, $^3J_{5,4}$ = 5.6 Hz, 2H, H-5), 3.34 (t, $^3J_{1,2}$ = 5.6 Hz, 2H, H-1), 2.90–2.88 (m, 1.04H, H-8), 2.60–2.57 (m, 1.04H, H-7), 1.63–1.60 (m, 2H, H-2), 1.53–1.50 (m, 2H, H-4), 1.48–1.44 (m, 2H, H-3) ppm.

^{1}H NMR spectrum shows an impurity of H_2O at 1.54 ppm.

²H NMR (92.2 MHz, $CDCl_3$): δ = 2.89 (s, 0.96D, D-8), 2.59 (s, 0.96D, D-7) ppm.

¹³C NMR (151 MHz, $CDCl_3$): δ = 170.5 (C-6), 153.3 (C-11), 137.4 (C-13), 136.5 (C-9), 105.5 (C-10), 61.0 (C-14), 56.3 (C-12), 46.8 (C-1), 42.9 (C-5), 35.1 (m, C-7), 31.8 (m, C-8), 26.6 (C-2), 25.7 (C-4), 24.7 (C-1) ppm.

HRMS (ESI) for $C_{17}H_{24}D_2NO_4^+$ [$(M+H^+)^+$] calculated: 310.1982, found: 310.1982.

IR (ATR): ṽ = 2940 (w), 2858 (w), 1632 (s), 1587 (m), 1505 (w), 1442 (m), 1267 (m), 1125 (s), 1006 (w), 730 (m) cm^{-1}.

7.16.42 3-(Benzo[*d*][1,3]dioxol-5-yl)-1-(piperidin-1-yl)propan-1-one-2,3-*d*₄ (12be-*d*₄)

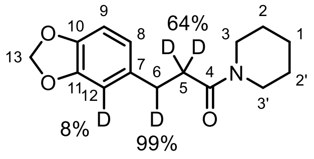

12be-d_4
$C_{15}H_{17}D_2NO_3$
Mw = 263.33 $gmol^{-1}$

Prepared according to **GP2** from (*E*)-3-(benzo[*d*][1,3]dioxol-5-yl)-1-(piperidin-1-yl)prop-2-en-1-one (**11be**, 51.9 mg, 0.200 mmol, 1.00 equiv), [IPrIPyCuCl] (**4**, 9.30 mg, 20.0 µmol, 10.0 mol%), NaO*t*Bu (9.62 mg, 100 µmol, 50.0 mol%) in diglyme (2.0 mL). The reaction mixture was stirred for 24 h at 100 °C under H_2 atmosphere (1.5 bar). Purification by flash column chromatography on silica gel (cyclohexane/EtOAc = 1:1) yielded **12be**-d_4 as colorless oil (39.5 mg, 0.150 mmol, 75%).

R*f* = 0.30 (cyclohexane/EtOAc = 1:1).

^{1}H NMR (600 MHz, $CDCl_3$): δ = 6.73–6.70 (m, 1.92H, H-9, H-12), 6.66 (dd, $^{3}J_{8,9}$ = 7.8 Hz, 1H, H-8), 5.91 (s, 2H, H-13), 3.55 (t, $^{3}J_{3,2}$ = 5.6 Hz, 2H, H-3), 3.33 (t, $^{3}J_{3',2'}$ = 5.6 Hz, 2H, H-3'), 2.87–2.84 (m, 1H, H-6), 2.56–2.52 (m, 0.73H, H-5), 1.61 (p, $^{3}J_{1,2/2'}$ = 5.9 Hz, 2H, H-1), 1.53–1.50 (m, 2H, H-2), 1.49–1.45 (m, 2H, H-2') ppm.

^{2}H NMR (92.2 MHz, $CDCl_3$): δ = 6.74 (s, 0.08D, D-12), 2.83 (s, 1.00D, D-6), 2.51 (s, 1.27D, D-5) ppm.

^{13}C NMR (151 MHz, $CDCl_3$): δ = 170.4 (C-4), 147.7 (C-10)*, 145.9 (C-11)*, 135.4 (C-7), 121.3 (C-9), 109.0 (C-8), 108.4 (C-12), 100.9 (C-13), 46.7 (C-3), 42.8 (C-3'), 35.8–34.7 (m, C-5), 31.4–30.6 (m, C-6), 26.5 (C-2'), 25.7 (C-2), 24.7 (C-1) ppm.

HRMS (ESI) for $C_{15}H_{18}D_2NO_3^+$ [(M+H$^+$)$^+$] calculated: 264.1563, found: 264.1557.

IR (ATR): ṽ = 3000 (w), 2937 (w), 2855 (w), 2780 (w), 1628 (s), 1490 (m), 1438 (s), 1353 (w), 1233 (s), 1125 (w), 1036 (m), 931 (w), 853 (w), 805 (w), 749 (m), 663 (w) cm^{-1}.

7.16.43 5-(Benzo[*d*][1,3]dioxol-5-yl)-1-(piperidin-1-yl)pentan-1-one-2,3,4,5-*d*$_4$ (12bi-*d*$_4$)

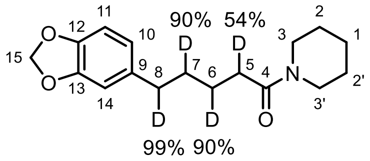

12bi-*d*$_4$
$C_{17}H_{19}D_4NO_3$
Mw = 293.40 gmol^{-1}

Prepared according to **GP2** from (2*E*,4*E*)-5-(benzo[*d*][1,3]dioxol-5-yl)-1-(piperidin-1-yl)penta-2,4-dien-1-one (**11bi**, 57.1 mg, 0.200 mmol, 1.00 equiv), [IPrIPyCuCl] (**4**, 9.30 mg, 20.0 µmol, 10.0 mol%), NaO*t*Bu (9.62 mg, 100 µmol, 50.0 mol%) in diglyme (2.0 mL). The reaction mixture was stirred for 24 h at 100 °C under D_2 atmosphere (2 bar). Purification by flash column chromatography on silica gel (cyclohexane/EtOAc = 1:3) yielded **12bi**-*d*$_4$ as yellow oil (37.5 mg, 0.128 mmol, 64%).

R$_f$ = 0.30 (cyclohexane/EtOAc = 1:3).

^{1}H NMR (600 MHz, $CDCl_3$): δ = 6.71 (d, $^{3}J_{10,11}$ = 7.9 Hz, 1H, H-10), 6.67 (s, 1H, H-14), 6.62 (d, $^{3}J_{11,10}$ = 7.9 Hz, 1H, H-11), 5.91 (s, 2H, H-15), 3.53 (t, $^{3}J_{3,2}$ = 5.6 Hz, 2H, H-3), 3.36 (t, $^{3}J_{3',2'}$ = 5.6 Hz, 2H, H-3'), 2.57–2.53 (m, 1.46H, H-5), 2.31–2.28 (m, 1H, H-8), 1.61–1.36 (m, 4.2H, H-1, H-6, H-7), 1.26–1.15 (m, 4H, H-2, H-2') ppm.

^{1}H NMR spectrum shows traces of H_2O at 1.54 ppm.

^{2}H NMR (92.2 MHz, $CDCl_3$): δ = 2.55 (s, 0.54D, D-5), 2.30 (s, 0.99D, D-8), 1.61 (s, 1.80D, D-6, D-7) ppm.

^{13}C NMR (151 MHz, $CDCl_3$): δ = 171.3 (C-4), 147.6 (C-13)*, 145.6 (C-12)*, 136.3 (C-9), 121.2 (C-11), 109.0 (C-14), 108.2 (C-10), 100.8 (C-15), 46.8 (C-3), 42.7 (C-3'), 35.8–35.1 (m, C-8), 33.3–32.8 (m, C-5), 31.5–31.4 (m, C-7), 26.7 (C-2), 25.7 (C-2'), 24.7 (C-1), 24.8–24.2 (m, C-6) ppm.

HRMS (ESI) for $C_{17}H_{20}D_4NO_3^+$ [(M+H$^+$)$^+$] calculated: 294.2002, found: 294.2001.

IR (ATR): ṽ = 2929 (w), 2855 (w), 1632 (s), 1487 (m), 1438 (s), 1244 (s), 1189 (w), 1036 (m), 931 (w), 853 (w), 805 (w), 734 (w) cm^{-1}.

7.16.44 (1*R*,5*S*)-3-(3-Phenylpropanoyl-2,3-*d*$_2$)-1,2,3,4,5,6-hexahydro-8*H*-1,5-methanopyrido[1,2-*a*][1,5]diazocin-8-one-*d*$_7$ (12bb-*d*$_7$)

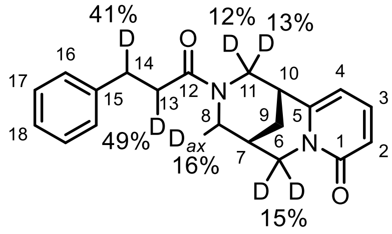

12bb-*d*$_7$
$C_{20}H_{20}D_2N_2O_2$
Mw = 324.42 gmol^{-1}

Prepared according to **GP2** from (1*R*,5*S*)-3-cinnamoyl-1,2,3,4,5,6-hexahydro-8*H*-1,5-methanopyrido[1,2-*a*][1,5]diazocin-8-one (**11bb**, 64.1 mg, 0.200 mmol, 1.00 equiv), [IPrIPyCuCl] (**4**, 9.30 mg, 20.0 µmol, 10.0 mol%), NaO*t*Bu (9.62 mg, 100 µmol, 50.0 mol%) in diglyme (2.0 mL). The reaction mixture was stirred for 24 h at 100 °C under D_2 atmosphere (2 bar). Purification by flash column chromatography on silica gel (CH_2Cl_2/MeOH = 97:3) yielded **12bb**-*d*$_7$ and **12bb**-*d*$_7$**_rotamer** (54:46 ratio) as colorless solid (39.2 mg, 0.121 mmol, 61%).

M.p. = 51 °C (CH_2Cl_2).

R*f* = 0.20 (CH_2Cl_2/MeOH = 97:3).

^{1}H NMR (600 MHz, $CDCl_3$): δ = 7.27–7.23 (m, 3H, H-3, H-17*), 7.18–7.16 (m, 2H, H-16)*, 7.05–7.04 (m, 1H, H-18), 6.44 (t, $^{3/4}J_{2,3/4}$ = 10.2 Hz, 1H, H-2), 6.08–6.01 (m, 1H, H-4), 4.84–4.69 (m, 1H, H-8$_{eq}$)**, 4.12–4.08 (m, 0.88H, H-11$_{eq}$)**, 3.94–3.84 (m, 1.69H, H-6), 3.31–3.21 (m, 1H, H-9a)**, 3.08–3.05 (m, 1H, H-7)***, 2.85–1.96 (m, 6.8H, H-13, H-14, H-9, H-8$_{ax}$, H-11$_{ax}$) ppm.

^{2}H NMR (92.2 MHz, $CDCl_3$): δ = 4.08 (s, 0.12D, D-11$_{eq}$), 3.86 (s, 0.29D, D-6), 2.79 (s, 0.49D, D-13), 2.56 (s, 0.41D, D-14), 2.34 (s, 0.16D, D-8$_{ax}$)*, 2.17 (s, 0.13D, D-11$_{ax}$) ppm.

The ^{1}H NMR spectrum is not well separated for the two rotamers, which is why only ^{13}C NMR is assigned separately.

^{13}C NMR (151 MHz, $CDCl_3$): δ = 171.5 (C-12), 163.3 (C-1), 148.7 (C-5), 141.2 (C-15), 138.5 (C-3), 128.6 (C-17)*, 128.5 (C-16)*, 128.3 (C-18), 118.2 (C-2), 104.9 (C-4), 52.9 (C-6), 49.1 (C-8)**, 48.9–48.6 (m, C-11)**, 35.2 (C-7)***, 34.5 (C-10)***, 30.7–30.5 (m, C-14), 27.8–27.5 (m, C-13), 26.4 (C-9) ppm.

^{13}C NMR of **12ab**-*d*$_7$**_rotamer** (151 MHz, $CDCl_3$): δ = 171.7 (C-12), 163.5 (C-1), 148.6 (C-5), 141.1 (C-15), 139.3 (C-3), 128.6 (C-17)*, 128.3 (C-18), 126.2 (C-16)*, 117.6 (C-2), 106.0 (C-4), 51.7 (C-6), 49.1 (C-8)**, 47.9–47.8 (m, C-11)**, 35.0 (C-7)*** 34.7 (C-10)***, 30.7–30.5 (m, C-14), 27.8–27.5 (m, C-13), 26.3 (C-9) ppm.

Rotamer ratio is determined from the integration value of H-4 at 6.08–6.01 ppm.

HRMS (ESI) for $C_{20}H_{21}D_2N_2O_2^+$ [(M+H$^+$)$^+$] calculated: 325.1880, found: 325.1879.

IR (ATR): ṽ = 3052 (w), 2937 (w), 2862 (w), 1647 (s), 1576 (m), 1543 (m), 1423 (m), 1263 (w), 1215 (s), 1181 (w), 1095 (w), 797 (w), 730 (m), 700 (m) cm^{-1}.

7.16.45 Unsuccessful substrate for the conjugate reduction of α,β-unsaturated amides

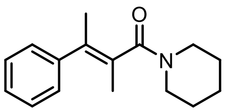

11ac, 0% conv.

7.17 Synthesis of starting materials for the catalytic alkyne semihydrogenation

7.17.1 1-(Benzyloxy)-4-(phenylethynyl)benzene (1b)

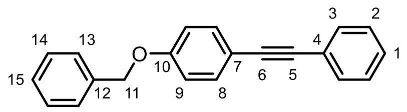

1b
$C_{21}H_{16}O$
Mw = 284.36 gmol^{-1}

Prepared according to **GP7** from 1-(benzyloxy)-4-iodobenzene (930 mg, 3.00 mmol, 1.00 equiv), ethynylbenzene (0.400 mL, 3.60 mmol, 1.20 equiv), $Pd(PPh_3)_2Cl_2$ (42.1 mg, 60.0 µmol, 2.00 mol%) and CuI (22.8 mg, 120 µmol, 4.00 mol%) in Et_3N (12.5 mL, 47.3 mmol, 30.0 equiv). Purification by flash column chromatography on silica gel yielded **1b** as colorless soild (613 mg, 2.16 mmol, 72% yield).

$\mathbf{R}_f$ = 0.30 (SiO_2, cyclohexane/EtOAc = 20:1).

^{1}H NMR (600 MHz, $CDCl_3$): δ = 7.51 (dd, $^3J_{13,14}$ = 8.0 Hz, $^4J_{13,15}$ = 1.6 Hz, 2H, H-13), 7.47 (d, $^3J_{8,9}$ = 8.7 Hz, 2H, H-8), 7.44 (d, $^3J_{3,2}$ = 6.9 Hz, 2H, H-3), 7.40 (t, $^3J_{2,3/1}$ = 7.5 Hz, 2H, H-2), 7.35–7.31 (m, 4H, H-1, H-14, H-15), 6.95 (d, $^3J_{9,8}$ = 8.7 Hz, 2H, H-9), 5.09 (s, 2H, H-11) ppm.

^{13}C NMR (151 MHz, $CDCl_3$): δ = 158.9 (C-10), 136.7 (C-12), 133.2 (C-8), 131.6 (C-13), 128.8 (C-14), 128.5 (C-2), 128.2 (C-15), 128.1 (C-1), 127.6 (C-3), 123.7 (C-4), 115.8 (C-7), 115.1 (C-9), 89.5 (C-6), 88.3 (C-5), 70.2 (C-11) ppm.

HRMS (APCI) for $C_{21}H_{17}O^+$ [(M+H$^+$)$^+$] calculated: 285.1274, found: 285.1272.

The data is in accordance with literature.[32]

7.17.2 1-Methoxy-4-((4-(trifluoromethyl)phenyl)ethynyl)benzene (1c)

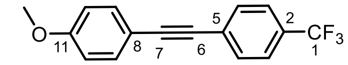

1c
$C_{16}H_{11}F_3O$
Mw = 276.26 gmol^{-1}

Prepared according to **GP7** from 1-iodo-4-(trifluoromethyl)benzene (816 mg, 3.00 mmol, 1.00 equiv), 1-ethynyl-4-methoxybenzene (476 mg, 3.60 mmol, 1.20 equiv), $Pd(PPh_3)_2Cl_2$ (42.1 mg, 60.0 µmol, 2.00 mol%) and CuI (22.8 mg, 120 µmol, 4.00 mol%) in Et_3N (12.5 mL, 90.0 mmol, 30.0 equiv). Purification by flash column chromatography on silica gel yielded **1c** as yellow solid (704 mg, 2.55 mmol, 85% yield).

R*f* = 0.30 (SiO_2, cyclohexane/EtOAc = 40:1).

^{1}H NMR (600 MHz, $CDCl_3$): δ = 7.61 (d, $^3J_{3,4}$ = 8.6 Hz, 2H, H-3), 7.59 (d, $^3J_{4,3}$ = 8.7 Hz, 2H, H-4), 7.49 (d, $^3J_{9,10}$ = 8.8 Hz, 2H, H-9), 6.90 (d, $^3J_{10,9}$ = 8.6 Hz, 2H, H-10), 3.84 (s, 3H, H-12) ppm.

^{13}C NMR (151 MHz, $CDCl_3$): δ = 160.2 (C-11), 133.4 (C-4), 131.7 (C-9), 129.7 (q, $^3J_{2,F}$ = 32.5 Hz, C-2), 127.6 (C-5), 125.4 (q, $^3J_{3,F}$ = 3.7 Hz, C-3), 123.2 (q, $^1J_{1,F}$ = 272.0 Hz, C-1), 114.8 (C-8), 114.2 (C-10), 92.1 (C-7), 87.0 (C-6), 55.5 (C-12) ppm.

^{19}F NMR (473 MHz, $CDCl_3$): δ = –62.7(s) ppm.

HRMS (APCI) for $C_{16}H_{11}F_3O^+$ [$(M)^+$] calculated: 276.0757, found: 276.0755.

The data is in accordance with literature.[33]

7.17.3 4-((4-methoxyphenyl)ethynyl)benzonitrile (1d)

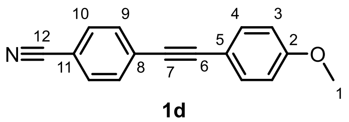

1d
$C_{16}H_{11}NO$
Mw = 233.27 gmol^{-1}

Prepared according to **GP7** from 1-iodo-4-methoxybenzene (702 mg, 3.00 mmol, 1.00 equiv), 4-ethynylbenzonitrile (458 mg, 3.60 mmol, 1.20 equiv), $Pd(PPh_3)_2Cl_2$ (42.1 mg, 60.0 µmol, 2.00 mol%) and CuI (22.8 mg, 120 µmol, 4.00 mol%) in Et_3N (12.5 mL, 90.0 mmol, 30.0 equiv). Purification by flash column chromatography on silica gel yielded **1d** as yellow solid (602 mg, 2.57 mmol, 86% yield).

R*f* = 0.30 (SiO_2, cyclohexane/EtOAc = 3:1).

^{1}H NMR (600 MHz, $CDCl_3$): δ = 7.62 (d, $^3J_{10,9}$ = 8.4 Hz, 2H, H-10), 7.58 (d, $^3J_{9,10}$ = 8.5 Hz, 2H, H-9), 7.48 (d, $^3J_{4,3}$ = 8.7 Hz, 2H, H-4), 6.90 (d, $^3J_{3,4}$ = 8.7 Hz, 2H, H-3), 3.84 (s, 3H, H-1) ppm.

^{13}C NMR (151 MHz, $CDCl_3$): δ = 160.4 (C-2), 133.5 (C-4), 132.2 (C-10), 132.0 (C-9), 128.8 (C-8), 118.8 (C-12), 114.4 (C-5), 114.3 (C-3), 111.2 (C-11), 94.2 (C-6), 86.9 (C-7), 55.5 (C-1) ppm.

HRMS (APCI) for $C_{16}H_{12}NO^+$ [$(M+H^+)^+$] calculated 234.0913, found 234.0910.

The data is in accordance with literature.[34]

7.17.4 1-Fluoro-4-(phenylethynyl)benzene (1e)

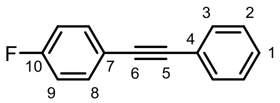

1e
$C_{14}H_9F$
Mw = 196.21 gmol^{-1}

Prepared according to **GP7** from 1-fluro-4-iodobenzene (666 mg, 3.00 mmol, 1.00 equiv), ethynylbenzene (0.400 mL, 3.60 mmol, 1.20 equiv), $Pd(PPh_3)_2Cl_2$ (42.1 mg, 60.0 µmol, 2.00 mol%) and CuI (22.8 mg, 120 µmol, 4.00 mol%) in Et_3N (12.5 mL, 47.3 mmol, 30.0 equiv). Purification by flash column chromatography on silica gel yielded **1e** as colorless solid (580 mg, 2.95 mmol, 82% yield).

R*f* = 0.25 (SiO_2, cyclohexane).

^{1}H NMR (600 MHz, $CDCl_3$): δ = 7.53–7.50 (m, 4H, H-2, H-8), 7.37–7.33 (m, 3H, H-3, H-1), 7.05 (t, $^{3}J_{2,1/3}$ = 8.7 Hz, 2H, H-9) ppm.

^{13}C NMR (151 MHz, $CDCl_3$): δ = 163.6 (d, $^{1}J_{10,F}$ = 249.6 Hz, C-10), 133.6 (d, $^{3}J_{8,F}$ = 8.3 Hz, C-8), 131.7 (C-3), 128.5 (C-2), 128.4 (C-1), 123.2 (C-4), 119.5 (d, $^{4}J_{7,F}$ = 3.4 Hz, C-7), 115.9 (d, $^{2}J_{9,F}$ = 21.9 Hz, C-9), 89.2 (C-6), 88.4 (C-5) ppm.

^{19}F NMR (473 MHz, $CDCl_3$): δ = –111.0(s) ppm

HRMS (APCI) for $C_{14}H_9F^+$ [$(M)^+$] calculated: 196.0683, found: 196.0680.

The data is in accordance with literature.[35]

7.17.5 1-Bromo-4-(phenylethynyl)benzene (1g)

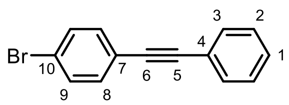

1g
$C_{14}H_9Br$
Mw = 257.13 gmol^{-1}

Prepared according to **GP7** from 1-bromo-4-iodobenzene (848 mg, 3.00 mmol, 1.00 equiv), ethynylbenzene (0.400 mL, 3.60 mmol, 1.20 equiv), $Pd(PPh_3)_2Cl_2$ (42.1 mg, 60.0 µmol, 2.00 mol%) and CuI (22.8 mg, 120 µmol, 4.00 mol%) in Et_3N (12.5 mL, 47.3 mmol, 30.0 equiv). Purification by flash column chromatography on silica gel yielded **1g** as colorless solid (493 mg, 1.92 mmol, 68% yield).

R*f* = 0.30 (SiO_2, cyclohexane).

^{1}H NMR (600 MHz, $CDCl_3$): δ = 7.53–7.52 (m, 2H, H-3), 7.48 (d, $^{3}J_{9,8}$ = 8.3 Hz, 2H, H-9), 7.39 (d, $^{3}J_{8,9}$ = 8.3 Hz, 2H, H-8), 7.36–7.35 (m, 3H, H-1, H-2) ppm.

^{13}C NMR (151 MHz, $CDCl_3$): δ = 133.2 (C-8), 131.8 (C-3), 131.7 (C-9), 128.7 (C-1), 128.5 (C-2), 123.1 (C-4), 122.6 (C-10), 122.4 (C-7), 90.6 (C-5), 88.4 (C-6) ppm.

HRMS (APCI) for $C_{14}H_9{}^{79}Br^+$ [$(M)^+$] calculated: 255.9882, found: 255.9885.

The data is in accordance with literature.[35]

7.17.6 1-Iodo-4-(phenylethynyl)benzene (1h)

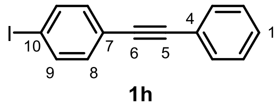

1h
$C_{14}H_9I$
Mw = 304.13 gmol^{-1}

Prepared according to **GP7** from 1,4-diiodobenzene (990 mg, 3.00 mmol, 1.00 equiv), ethynylbenzene (0.400 mL, 3.60 mmol, 1.20 equiv), $Pd(PPh_3)_2Cl_2$ (42.1 mg, 60.0 µmol, 2.00 mol%) and CuI (22.8 mg, 120 µmol, 4.00 mol%) in Et_3N (12.5 mL, 47.3 mmol, 30.0 equiv). Purification by flash column chromatography on silica gel yielded **1h** as yellow solid (340 mg, 1.12 mmol, 37% yield).

R*f* = 0.30 (SiO_2, cyclohexane).

^{1}H NMR (600 MHz, $CDCl_3$): δ = 7.71 (d, $^3J_{9,8}$ = 8.5 Hz, 2H, H-9), 7.54–7.53 (m, 2H, H-3), 7.37–7.36 (m, 3H, H-1, H-2), 7.28–7.26 (m, 2H, H-8) ppm.

^{13}C NMR (151 MHz, $CDCl_3$): δ = 137.7 (C-9), 133.2 (C-3), 131.7 (C-2), 128.7 (C-1), 128.5 (C-8), 123.1 (C-4), 123.0 (C-7), 94.2 (C-10), 90.9 (C-6), 88.6 (C-5) ppm.

HRMS (APCI) for $C_{14}H_{10}I$ [$(M+H^+)^+$] calculated 303.9743, found 303.9740.

The data is in accordance with literature.[36]

7.17.7 3-(Phenylethynyl)benzaldehyde (1i)

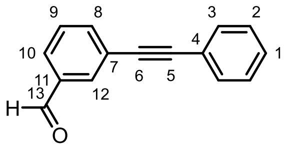

1i
$C_{15}H_{10}O$
Mw = 206.24 gmol^{-1}

Prepared according to **GP7** from 3-iodobenzaldehyde (696 mg, 3.00 mmol, 1.00 equiv), ethynylbenzene (0.400 mL, 3.60 mmol, 1.20 equiv), $Pd(PPh_3)_2Cl_2$ (42.1 mg, 60.0 µmol, 2.00 mol%) and CuI (22.8 mg, 120 µmol, 4.00 mol%) in Et_3N (12.5 mL, 47.3 mmol, 30.0 equiv). Purification by flash column chromatography on silica gel yielded **1i** as brown soild (576 mg, 2.79 mmol, 93% yield).

R$_f$ = 0.20 (SiO_2, cyclohexane/EtOAc = 20:1).

^{1}H NMR (600 MHz, $CDCl_3$): δ = 10.02 (s, 1H, H-13), 8.04 (d, $^4J_{12,10}$ = 1.8 Hz, 1H, H-12), 7.85 (dd, $^3J_{10,9}$ = 7.8 Hz, $^4J_{10,12}$ = 1.6 Hz, 1H, H-10), 7.78 (dd, $^3J_{8,9}$ = 7.7 Hz, $^4J_{8,10}$ = 1.6 Hz, 1H, H-8), 7.56–7.52 (m, 3H, H-3, H-1), 7.38–7.37 (m, 3H, H-2, H-9) ppm.

^{13}C NMR (151 MHz, $CDCl_3$): δ = 191.7 (C-13), 137.3 (C-8), 136.6 (C-11), 133.1 (C-12), 131.9 (C-3), 129.3 (C-1), 129.0 (C-9), 128.9 (C-10), 128.6 (C-2), 124.7 (C-7), 122.8 (C-4), 91.1 (C-5), 88.0 (C-6) ppm.

HRMS (APCI) for $C_{15}H_{11}O^+$ [$(M+H^+)^+$] calculated: 207.0804, found: 207.0802.

The data is in accordance with literature.[37]

7.17.8 1-(3-(Phenylethynyl)phenyl)ethan-1-one (1j)

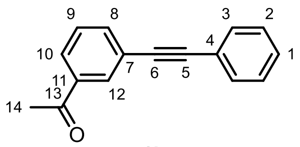

1j
$C_{16}H_{12}O$
Mw = 220.27 gmol^{-1}

Prepared according to **GP7** from 1-(3-bromophenyl)ethan-1-one (299 mg, 1.50 mmol, 1.00 equiv), ethynylbenzene (0.200 mL, 1.60 mmol, 1.20 equiv), $Pd(PPh_3)_2Cl_2$ (21.1 mg, 30.0 µmol, 2.00 mol%) and CuI (11.5 mg, 60.0 µmol, 4.00 mol%) in Et_3N (7.00 mL, 45.0 mmol, 30.0 equiv). Purification by flash column chromatography on silica gel yielded **1j** as brown solid (248 mg, 1.13 mmol, 75% yield).

R$_f$ = 0.20 (SiO_2, cyclohexane/EtOAc = 10:1).

^{1}H NMR (600 MHz, $CDCl_3$): δ = 8.11 (t, $^4J_{12,8/10}$ = 1.6 Hz, 1H, H-12), 7.92 (dt, $^3J_{10,9}$ = 7.9 Hz, $^4J_{10,12/8}$ = 1.4 Hz, 1H, H-10), 7.72 (dt, $^3J_{8,9}$ = 7.6 Hz, $^4J_{8,10/12}$ = 1.4 Hz, 1H, H-8), 7.56–7.54 (m, 2H, H-3), 7.46 (t, $^3J_{9,8/10}$ = 7.7 Hz, 1H, H-9), 7.38–7.36 (m, 3H, H-2, H-1), 2.63 (s, 3H, H-14) ppm.

^{13}C NMR (151 MHz, $CDCl_3$): δ = 197.6 (C-13), 137.4 (C-11), 136.0 (C-8), 131.8 (C-3), 131.7 (C-10), 128.9 (C-9), 128.8 (C-1), 128.6 (C-2), 128.0 (C-12), 124.1 (C-7), 122.9 (C-4), 90.5 (C-5), 88.4 (C-6), 26.8 (C-14) ppm.

HRMS (APCI) for $C_{16}H_{13}O^+$ [$(M+H^+)^+$] calculated 221.0961, found 221.0968.

The data is in accordance with literature.[37]

7.17.9 Synthesis of 1-(3-(phenylethynyl)phenyl)ethan-1-one *O*-methyl oxime (1k)

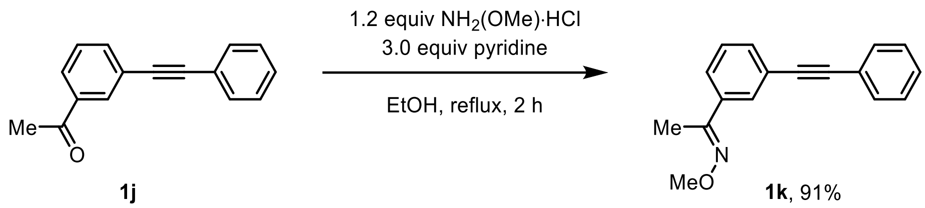

1j **1k**, 91%

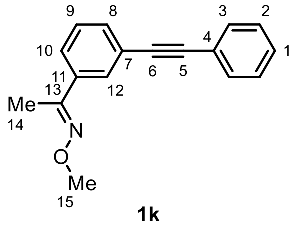

1k
$C_{17}H_{15}NO$
Mw = 249.31 gmol^{-1}

In deference to a literature procedure,[38] a Schlenk tube equipped with a magnetic stir bar was charged with 1-(3-(phenylethynyl)phenyl)ethan-1-one (**1j**, 0.660 g, 3.00 mmol, 1.00 equiv), pyridine (0.720 mL, 9.00 mmol, 3.00 equiv) and ethanol (15 mL). *O*-methylhydroxylamine hydrochloride (300 mg, 3.60 mmol, 1.20 equiv) was added and the reaction mixture was refluxed for 2 h (conversion monitored *via* TLC, cyclohexane/EtOAc = 20:1). The reaction mixture was allowed to cool to room temperature. All volatiles were removed under reduced pressure. The crude product was purified by flash column chromatography on silica gel to afford **1k** as orange oil (680 mg, 2.71 mmol, 91% yield).

R_f = 0.30 (cyclohexane/EtOAc = 20:1).

^{1}H NMR (600 MHz, $CDCl_3$): δ = 7.81 (t, $^4J_{12,8/10}$ = 1.8 Hz, 1H, H-12), 7.63 (dt, $^3J_{10,9}$ = 8.0 Hz, $^4J_{10,8/12}$ = 1.5 Hz, 1H, H-10), 7.55–7.53 (m, 2H, H-2), 7.52 (dt, $^3J_{8,9}$ = 7.7 Hz, $^4J_{8,10/12}$ = 1.8 Hz, 1H, H-8), 7.36–7.34 (m, 4H, H-1, H-3, H-9), 4.02 (s, 3H, H-15), 2.24 (s, 3H, H-14) ppm.

^{13}C NMR (151 MHz, $CDCl_3$): δ = 154.0 (C-13), 137.0 (C-11), 132.2 (C-8), 131.8 (C-3), 129.4 (C-12), 128.6 (C-9, C-10), 128.5 (C-2), 126.0 (C-7)*, 123.6 (C-4)*, 123.3 (C-1), 89.8 (C-6), 89.2 (C-5), 62.2 (C-15), 12.7 (C-14) ppm.

HRMS (APCI) for $C_{17}H_{16}NO^+$ [$(M+H)^+$] calculated 250.1226, found 250.1229.

IR (ATR): ṽ = 3060 (w), 2937 (w), 2899 (w), 2817 (w), 1599 (w), 1490 (m), 1442 (w), 1367 (w), 1330 (w), 1047 (s), 793 (m), 752 (s), 689 (s) cm^{-1}.

7.17.10 Synthesis of *N*-methyl-1-(3-(phenylethynyl)phenyl)ethan-1-imine (1l)

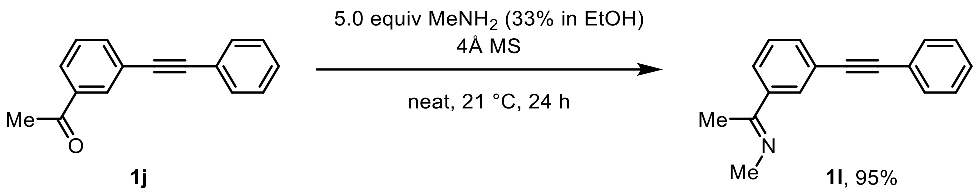

1j **1l**, 95%

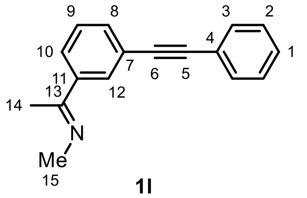

1l
$C_{17}H_{15}N$
Mw = 233.31 gmol^{-1}

In deference to a literature procedure,[39] a Schlenk tube equipped with a magnetic stir bar was charged with 1-(3-(phenylethynyl)phenyl)ethan-1-one (**1j**, 0.660 g, 3.00 mmol, 1.00 equiv), 500 mg 4 Å MS and $MeNH_2$ (7.50 mL, 33% in EtOH, 15.0 mmol, 5.00 equiv). The reaction mixture was stirred at room temperature for 24 h (conversion monitored *via* TLC, cyclohexane/EtOAc = 15:1). The mixture was then filtered through Celite (4 × 3 cm) and washed with CH_2Cl_2 (20 mL). All the volatiles were removed under reduced pressure. The crude product was purified by flash column chromatography on silica gel to afford **1l** as orange oil (664 mg, 2.85 mmol, 95% yield).

R*f* = 0.20 (cyclohexane/EtOAc = 15:1).

^{1}H NMR (600 MHz, $CDCl_3$): δ = 7.93 (t, $^4J_{12,8/10}$ = 1.8 Hz, 1H, H-12), 7.74 (dt, $^3J_{10,9}$ = 8.0 Hz, $^4J_{10,8/12}$ = 1.5 Hz, 1H, H-10), 7.55–7.53 (m, 3H, H-2, H-8), 7.37–7.33 (m, 4H, H-1, H-3, H-9), 3.37 (s, 3H, H-15), 2.25 (s, 3H, H-14) ppm.

^{13}C NMR (151 MHz, $CDCl_3$): δ = 166.3 (C-13), 141.4 (C-11), 132.5 (C-8), 131.7 (C-3), 129.9 (C-12), 128.5 (C-2), 128.4 (C-9, C-10), 126.4 (C-1), 123.4 (C-4), 123.3 (C-7), 89.6 (C-6)*, 89.3 (C-5)*, 39.7 (C-15), 15.1 (C-14) ppm.

HRMS (APCI) for $C_{17}H_{16}N^+$ [$(M+H)^+$] calculated 234.1277, found 234.1276.

IR (ATR): ṽ = 3060 (w), 2862 (w), 2370 (w), 2344 (w), 1688 (m), 1636 (m), 1599 (w), 1490 (m), 1438 (w), 1364 (w), 1319 (w), 1244 (w), 1118 (w), 1069 (w), 913 (w), 793 (m), 752 (s), 723 (w), 685(s) cm^{-1}.

7.17.11 Ethyl 6-phenylhex-5-ynoate (1m)

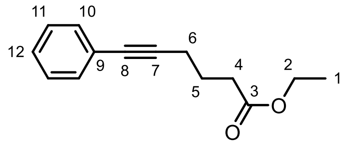

1m
$C_{14}H_{16}O_2$
Mw = 216.28 gmol^{-1}

Prepared according to **GP7** from iodobenzene (0.340 mL, 3.00 mmol, 1.00 equiv), ethyl hex-5-ynoate (504 mg, 3.60 mmol, 1.20 equiv), $Pd(PPh_3)_2Cl_2$ (42.1 mg, 60.0 µmol, 2.00 mol%) and CuI (22.8 mg, 120 µmol, 4.00 mol%) in Et_3N (12.5 mL, 47.3 mmol, 30.0 equiv). Purification by flash column chromatography on silica gel yielded **1m** as colorless oil (450 mg, 2.08 mmol, 69% yield)

R*f* = 0.20 (SiO_2, cyclohexane/EtOAc = 20:1).

^{1}H NMR (600 MHz, $CDCl_3$): δ = 7.40–7.38 (m, 2H, H-11), 7.29–7.26 (m, 3H, H-10, H-12), 4.14 (q, $^3J_{2,1}$ = 7.2 Hz, 2H, H-2), 2.49 (t, $^3J_{6/4,5}$ = 7.1 Hz, 4H, H-6, H-4), 1.93 (p, $^3J_{5,6/4}$ = 7.1 Hz, 2H, H-5), 1.26 (t, $^3J_{1,2}$ = 7.2 Hz, 3H, H-1) ppm.

^{13}C NMR (151 MHz, $CDCl_3$): δ = 173.3 (C-3), 131.7 (C-11), 128.3 (C-10), 127.8 (C-12), 123.9 (C-9), 89.0 (C-8), 81.5 (C-7), 60.5 (C-2), 33.3 (C-4), 24.1 (C-5), 19.0 (C-6), 14.4 (C-1) ppm.

HRMS (APCI) for $C_{14}H_{17}O_2^+$ [$(M+H^+)^+$] calculated: 217.1223, found: 217.1219.

The data is in accordance with literature.[40]

7.17.12 1-(Phenylethynyl)-4-(prop-1-yn-1-yl)benzene (1o)

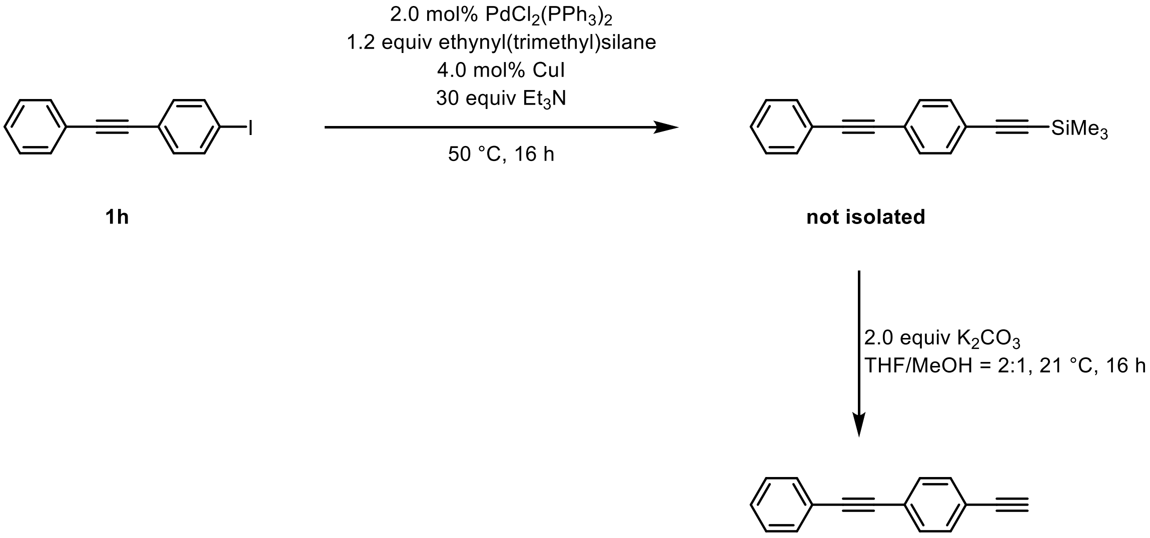

1h **not isolated**

1o, 86% overall yield

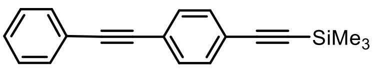

Prepared according to **GP7** from 1-iodo-4-(2-phenylethynyl)benzene (**1h**, 438 mg, 1.44 mmol, 1.00 equiv), ethynyl(trimethyl)silane (0.250 mL, 1.73 mmol, 1.20 equiv), $Pd(PPh_3)_2Cl_2$ (20.1 mg, 29.0 µmol, 2.00 mol%) and CuI (13.2 mg, 580 µmol, 4.00 mol%) in Et_3N (6.20 mL, 43.2 mmol, 30.0 equiv). Purification by flash column chromatography on silica gel (cyclohexane) quantitatively yielded trimethyl((4-(phenylethynyl)phenyl)ethynyl)silane as colorless solid, which was used directly for the desilylation step.

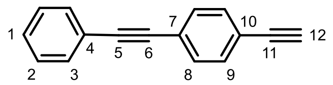

1o
$C_{16}H_{10}$
Mw = 202.26 gmol^{-1}

According to a literature procedure,[41] a Schlenk flask equipped with a magnetic stir bar was charged with trimethyl((4-(phenylethynyl)phenyl)ethynyl)silane (384 mg, 1.40 mmol, 1.00 equiv) and K_2CO_3 (386 mg, 2.80 mmol, 2.00 equiv). THF/MeOH (15 mL, 2:1) was added and the mixture was stirred for 16 h at room temperature. The soilds were filtered off with glass frit (P4) and all the volatiles were removed under reduced pressure. The

crude product was purified by flash column chromatography on silica gel to afford **1o** as colorless solid (250 mg, 1.24 mmol, 86% yield).

R*f* = 0.40 (cyclohexane).

^{1}H NMR (600 MHz, $CDCl_3$): δ = 7.54–7.52 (m, 2H, H-2), 7.49–7.48 (m, 4H, H-8, H-3), 7.36–7.35 (m, 3H, H-9, H-1), 3.17 (s, 1H, H-12) ppm.

^{13}C NMR (151 MHz, $CDCl_3$): δ = 132.3 (C-2), 131.9 (C-3), 131.7 (C-8), 128.8 (C-1), 128.6 (C-9), 124.0 (C-4)*, 123.2 (C-7)*, 122.1 (C-10), 91.6 (C-6), 89.1 (C-5), 83.5 (C-11), 79.1 (C-12) ppm.

HRMS (APCI) for $C_{18}H_{20}^+$ [M^+] calculated 202.0777, found 202.0771.

The data is in accordance with literature.[41]

7.17.13 1-(Cyclopropylethynyl)naphthalene (1q)

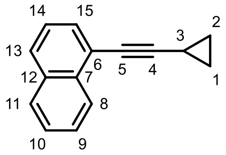

1q
$C_{15}H_{12}$
Mw = 192.26 gmol^{-1}

Prepared according to **GP7** from 1-iodonaphthalene (0.230 mL, 1.57 mmol, 1.00 equiv), ethynylcyclopropane (0.160 mL, 1.89 mmol, 1.20 equiv), $Pd(PPh_3)_2Cl_2$ (22.1 mg, 31.4 µmol, 2.00 mol%) and CuI (19.0 mg, 63.0 µmol, 4.00 mol%) in Et_3N (6.60 mL, 47.3 mmol, 30.0 equiv). Purification by flash column chromatography on silica gel yielded **1q** as colorless oil (250 mg, 1.30 mmol, 83% yield).

R*f* = 0.30 (SiO_2, cyclohexane).

^{1}H NMR (600 MHz, $CDCl_3$): δ = 8.30 (d, $^3J_{8,9}$ = 8.4 Hz, 1H, H-8), 7.82 (d, $^3J_{11,10}$ = 7.8 Hz, 1H, H-11), 7.76 (d, $^3J_{13,14}$ = 8.2 Hz, 1H, H-13), 7.60 (dd, $^3J_{15,14}$ = 7.1 Hz, $^4J_{15,13}$ = 1.2 Hz, 1H, H-15), 7.54 (ddd, $^3J_{9,8}$ = 8.3 Hz, $^3J_{9,10}$ = 6.8 Hz, $^4J_{9,11}$ = 1.4 Hz, 1H, H-9), 7.49 (ddd, $^3J_{10,11}$ = 8.1 Hz, $^3J_{10,9}$ = 6.8 Hz, $^4J_{10,8}$ = 1.3 Hz, 1H, H-10), 7.38 (dd, $^3J_{14,13}$ = 8.3 Hz, $^3J_{14,15}$ = 7.1 Hz, 1H, H-14), 1.61 (tt, $^3J_{3,2}$ = 8.2 Hz, $^4J_{3,1}$ = 5.1 Hz, 1H, H-3), 0.98–0.92 (m, 4H, H-1, H-2) ppm.

^{13}C NMR (151 MHz, $CDCl_3$): δ = 133.7 (C-7), 133.3 (C-12), 130.2 (C-15), 128.3 (C-11), 128.0 (C-13), 126.6 (C-8), 126.4 (C-9), 126.3 (C-10), 125.4 (C-14), 121.7 (C-6), 98.7 (C-5), 73.9 (C-4), 9.1 (C-1, C-2), 0.7 (C-3) ppm.

HRMS (APCI) for $C_{16}H_{13}^+$ [$(M+H^+)^+$] calculated 193.1012, found 193.1006.

The data is in accordance with literature.[35]

7.17.14 1-(Phenylethynyl)adamantine (1s)

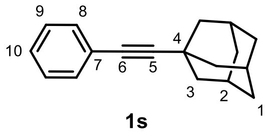

1s
$C_{18}H_{20}$
Mw = 236.36 gmol^{-1}

Prepared according to **GP7** from iodobenzene (0.340 mL, 3.00 mmol, 1.00 equiv), 1-ethynyladamantane (577 mg, 3.60 mmol, 1.20 equiv), $Pd(PPh_3)_2Cl_2$ (42.1 mg, 60.0 µmol, 2.00 mol%) and CuI (22.8 mg, 120 µmol, 4.00 mol%) in Et_3N (12.5 mL, 47.3 mmol, 30.0 equiv). Purification by flash column chromatography on silica gel yielded **1s** as colorless oil (400 mg, 1.69 mmol, 56% yield).

R*f* = 0.30 (cyclohexane).

^{1}H NMR (600 MHz, $CDCl_3$): δ = 7.41–7.39 (m, 2H, H-9), 7.28–7.25 (m, 3H, H-8, H-10), 2.01 (s, 3H, H-2), 1.98–1.97 (m, 6H, H-3)*, 1.74–1.73 (m, 6H, H-2)* ppm.

^{13}C NMR (151 MHz, $CDCl_3$): δ = 131.8 (C-9), 128.2 (C-8), 127.5 (C-10), 124.2 (C-7), 98.5 (C-6), 79.5 (C-5), 43.0 (C-3)*, 36.5 (C-1)*, 30.2 (C-4), 28.2 (C-2) ppm.

HRMS (APCI) for $C_{18}H_{20}^+$ [M$^+$] calculated 237.1638, found 237.1635.

The data is in accordance with literature.[42]

7.17.15 6-Phenylhex-5-yn-1-ol (1ab)

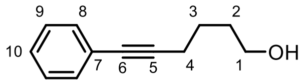

1ab
$C_{12}H_{14}O$
Mw = 174.24 gmol^{-1}

Prepared according to **GP7** from iodobenzene (0.560 mL, 5.00 mmol, 1.00 equiv), hex-5-yn-1-ol (0.660 mL, 6.00 mmol, 1.20 equiv), $Pd(PPh_3)_2Cl_2$ (70.3 mg, 0.100 mmol, 2.00 mol%) and CuI (38.3 mg, 0.200 mol, 4.00 mol%) in Et_3N (24.0 mL, 150 mmol, 30.0 equiv). Purification by flash column chromatography on silica gel yielded **1ab** as orange oil (600 mg, 3.34 mmol, 69% yield).

R*f* = 0.20 (SiO_2, cyclohexane/EtOAc = 4:1).

^{1}H NMR (600 MHz, $CDCl_3$): δ = 7.40–7.38 (m, 2H, H-8), 7.28–7.26 (m, 3H, H-9, H-10), 3.71 (q, $^3J_{1,2/O\text{–}H}$ = 5.7 Hz, 2H, H-1), 2.46 (t, $^3J_{4,3}$ = 6.8 Hz, 2H, H-4), 1.76–1.69 (m, 4H, H-2, H-3), 1.33 (br s, 1H, O–*H*) ppm.

^{13}C NMR (151 MHz, $CDCl_3$): δ = 131.7 (C-8), 128.3 (C-9), 127.7 (C-10), 124.0 (C-7), 90.0 (C-5), 81.1 (C-6), 62.6 (C-1), 32.1 (C-4), 25.2 (C-2), 19.3 (C-3) ppm.

HRMS (APCI) for $C_{12}H_{15}O^+$ [(M+H$^+$)$^+$] calculated 175.1117, found 175.1116.

The data is in accordance with literature.[43]

7.17.16 Synthesis of 4,4-difluoro-1-(6-phenylhex-5-yn-1-yl)piperidine (1t)

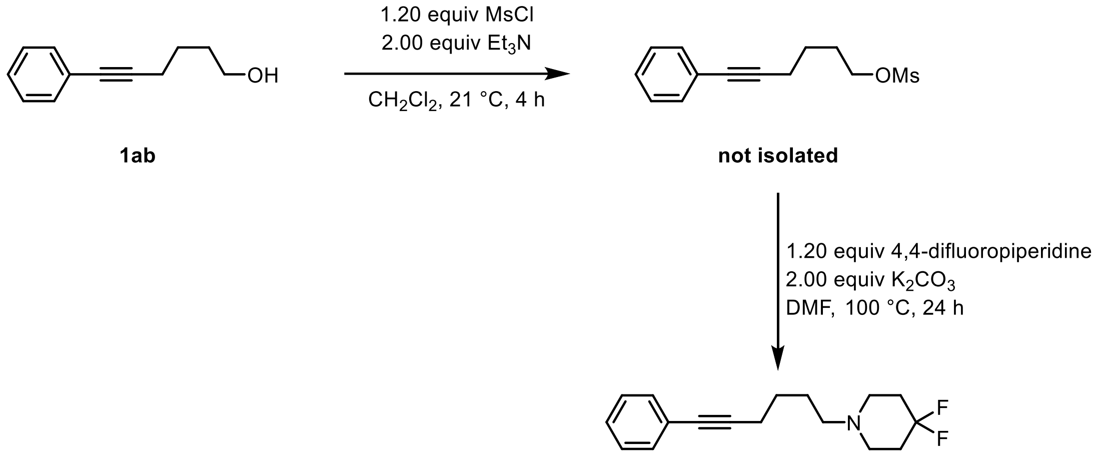

1t, 70% overall yield

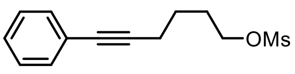

According to a literature procedure,[43] a Schlenk tube equipped with a magnetic stir bar was charged with 6-phenylhex-5-yn-1-ol (**1ab**, 348 mg, 2.00 mmol, 1.00 equiv) and CH_2Cl_2 (10 mL). Et_3N (0.600 mL, 4.00 mmol, 2.00 equiv) and methanesulfonyl chloride (0.190 mL, 2.40 mmol, 1.20 equiv) were added to the reaction mixture at 0 °C. The reaction mixture was allowed to warm to room temperature and stirred for additional 4 h at room temperature. The reaction mixture was quenched with 5% (w/v) citric acid solution (10 mL). The organic layer was separated and dried over $MgSO_4$. All volatiles were removed under reduced pressure to afford the corresponding mesylated product, which was directly used for the next step without further purification.

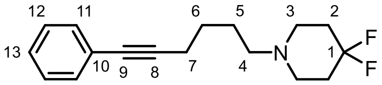

1t
$C_{17}H_{21}F_2N$
Mw = 277.36 gmol^{-1}

A Schlenk tube equipped with a magnetic stir bar was charged with crude mesylated product (500 mg, 2.00 mmol, 1.00 equiv), 4,4-difluoropiperidine (291 mg, 2.40 mmol, 1.20 equiv), K_2CO_3 (276 mg, 4.00 mmol, 2.00 equiv) and DMF (5 mL). The reaction mixture was stirred at 100 °C for 16 h. The reaction mixture was allowed to cool to room temperature. The crude mixture was diluted with EtOAc (20 mL) and water (10 mL) was added. The organic phase was separated and the aqueous phase was extracted with EtOAc (2 × 10 mL). The combined organic layer was then extracted with brine (3 × 10 mL) and dried over $MgSO_4$. All the volatiles were removed under reduced pressure and the crude product was purified by flash column chromatography on silica gel to afford **1t** as orange oil (377 mg, 1.40 mmol, 70% overall yield).

R_f = 0.20 (cyclohexane/EtOAc = 9:1).

^{1}H NMR (600 MHz, $CDCl_3$): δ = 7.39–7.37 (m, 2H, H-12), 7.28–7.26 (m, 3H, H-11, H-13), 2.55 (br s, 4H, H-3), 2.47–2.42 (m, 4H, H-4, H-7), 2.03–1.96 (m, 4H, H-2), 1.67–1.62 (m, 4H, H-5, H-6) ppm.

^{13}**C NMR** (151 MHz, $CDCl_3$): δ = 131.6 (C-10), 128.3 (C-12), 127.7 (C-11), 124.1 (C-13), 122.3 (t, $^{1}J_{C,F}$ = 241.3 Hz, C-1), 90.0 (C-9), 81.0 (C-8), 57.3 (C-4), 50.2 (t, $^{3}J_{C,F}$ = 5.3 Hz, C-3), 34.1 (t, $^{2}J_{C,F}$ = 22.9 Hz, C-2), 26.8 (C-5), 26.6 (C-6), 19.5 (C-7) ppm.

^{19}**F NMR** (473 MHz, $CDCl_3$): δ = –98.1(s) ppm.

HRMS (APCI) for $C_{17}H_{22}F_2N^+$ [$(M+H^+)^+$] calculated: 278.1715, found: 278.1714.

IR (ATR): ṽ = 2940 (m), 2866 (w), 2817 (w), 1364 (s), 1312 (w), 1095 (s), 954 (m), 756 (s), 693 (m) cm^{-1}.

7.17.17 Synthesis of (6-chlorohex-1-yn-1-yl)benzene (1u)

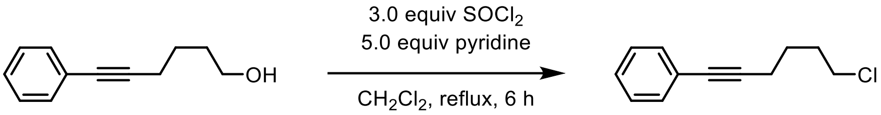

1ab **1u**, 81%

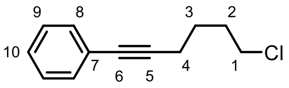

1u
$C_{12}H_{13}Cl$
Mw = 192.69 $gmol^{-1}$

According to a literature procedure,[44] a Schlenk tube equipped with a magnetic stir bar was charged with 6-phenylhex-5-yn-1-ol (**1ab**, 378 mg, 2.17 mmol, 1.00 equiv) and CH_2Cl_2 (10 mL). Thionyl chloride (0.470 mL, 6.51 mmol, 3.00 equiv) and pyridine (0.870 mL, 10.8 mmol, 5.00 equiv) were added to the reaction mixture at 0 °C. The reaction mixture was allowed to slowly warm to room temperature and then refluxed for 6 h. The reaction mixture was allowed to cool to room temperature and the reaction quenched by slow addition of sat. aq. NH_4Cl solution (10 mL) over 10 min at 0 °C. The organic layer was separated and dried over $MgSO_4$. All volatiles were removed under reduced pressure and the crude product was purified by flash column chromatography on silica gel (cyclohexane) to afford **1u** as colorless oil (340 mg, 1.76 mmol, 81% yield).

$\mathbf{R}_f$ = 0.20 (cyclohexane/EtOAc = 50:1).

^{1}**H NMR** (600 MHz, $CDCl_3$): δ = 7.40–7.38 (m, 2H, H-9), 7.29–7.26 (m, 3H, H-8, H-10), 3.61 (t, $^{3}J_{1,2}$ = 6.6 Hz, 2H, H-1), 2.47 (t, $^{3}J_{4,3}$ = 6.9 Hz, 2H, H-4), 2.00–1.95 (m, 2H, H-2), 1.77 (p, $^{3}J_{3,2/4}$ = 7.1 Hz, 2H, H-3) ppm.

^{13}**C NMR** (151 MHz, $CDCl_3$): δ = 131.7 (C-8), 128.4 (C-9), 127.8 (C-10), 123.9 (C-7), 89.4 (C-6), 81.4 (C-5), 44.7 (C-1), 31.8 (C-4), 26.0 (C-2), 18.9 (C-3) ppm.

HRMS (APCI) for $C_{12}H_{14}{}^{35}Cl^+$ [$(M+H)^+$] calculated 193.0779, found 193.0772.

The data is in accordance with literature.[44]

7.17.18 2-((4-methoxyphenyl)ethynyl)pyridine (1w)

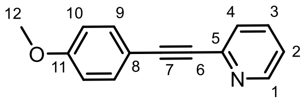

1w
$C_{14}H_{11}NO$
Mw = 209.25 $gmol^{-1}$

Prepared according to **GP7** from 2-bromopyridine (474 mg, 3.00 mmol, 1.00 equiv), 1-ethynyl-4-methoxybenzene (476 mg, 3.60 mmol, 1.20 equiv), $Pd(PPh_3)_2Cl_2$ (42.1 mg, 60.0 µmol, 2.00 mol%) and CuI (22.8 mg, 120 µmol, 4.00 mol%) in Et_3N (12.5 mL, 90.0 mmol, 30.0 equiv). Purification by flash column chromatography on silica gel yielded **1w** as yellow solid (400 mg, 1.91 mmol, 64% yield).

R*f* = 0.25 (SiO_2, cyclohexane/EtOAc = 3:1).

^{1}H NMR (600 MHz, $CDCl_3$): δ = 8.60 (d, $^3J_{1,2}$ = 4.9 Hz, 1H, H-1), 7.65 (td, $^3J_{3,2/4}$ = 7.7 Hz, $^4J_{3,1}$ = 1.8 Hz, 1H, H-3), 7.54 (d, $^3J_{9,10}$ = 8.7 Hz, 2H, H-9), 7.49 (d, $^3J_{4,3}$ = 7.8 Hz, 1H, H-4), 7.20 (ddd, $^3J_{2,3}$ = 7.6 Hz, $^3J_{2,1}$ = 4.9 Hz, $^4J_{2,4}$ = 1.1 Hz, 1H, H-2), 6.88 (d, $^3J_{10,9}$ = 8.7 Hz, 2H, H-10), 3.82 (s, 3H, H-12) ppm.

^{13}C NMR (151 MHz, $CDCl_3$): δ = 160.3 (C-11), 150.1 (C-1), 143.9 (C-5), 136.2 (C-3), 133.7 (C-9), 127.0 (C-4), 122.5 (C-2), 114.4 (C-8), 114.2 (C-10), 89.6 (C-7), 87.7 (C-6), 55.4 (C-12) ppm.

HRMS (APCI) for $C_{14}H_{12}NO^+$ [$(M+H^+)^+$] calculated 210.0913, found 210.0915.

The data is in accordance with literature.[45]

7.17.19 3-((4-Methoxyphenyl)ethynyl)pyridine (1x)

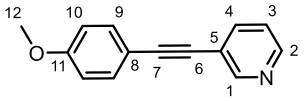

1x
$C_{14}H_{11}NO$
Mw = 209.25 $gmol^{-1}$

Prepared according to **GP7** from 3-bromopyridine (474 mg, 3.00 mmol, 1.00 equiv), 1-ethynyl-4-methoxybenzene (476 mg, 3.60 mmol, 1.20 equiv), $Pd(PPh_3)_2Cl_2$ (42.1 mg, 60.0 µmol, 2.00 mol%) and CuI (22.8 mg, 120 µmol, 4.00 mol%) in Et_3N (12.5 mL, 90.0 mmol, 30.0 equiv). Purification by flash column chromatography on silica gel yielded **1x** as yellow solid (363 mg, 1.74 mmol, 58% yield).

R*f* = 0.25 (SiO_2, cyclohexane/EtOAc = 3:1).

^{1}H NMR (600 MHz, $CDCl_3$): δ = 8.76 (s, 1H, H-1), 8.54 (d, $^3J_{2,3}$ = 4.9 Hz, 1H, H-2), 7.78 (d, $^3J_{4,3}$ = 7.9 Hz, 1H, H-4), 7.49 (d, $^3J_{9,10}$ = 8.8 Hz, 2H, H-9), 7.27 (dd, $^3J_{3,4}$ = 7.9 Hz, $^3J_{3,2}$ = 4.9 Hz, 1H, H-3), 6.90 (d, $^3J_{10,9}$ = 8.7 Hz, 2H, H-10), 3.84 (s, 3H, H-12) ppm.

^{13}C NMR (151 MHz, $CDCl_3$): δ = 160.2 (C-11), 152.3 (C-1), 148.4 (C-2), 138.4 (C-4), 133.3 (C-10), 123.2 (C-3), 114.7 (C-8, C-5) 114.2 (C-9), 92.9 (C-7), 84.9 (C-6), 55.5 (C-12) ppm.

HRMS (APCI) for $C_{14}H_{12}NO^+$ [$(M+H^+)^+$] calculated 210.0913, found 210.0915.

The data is in accordance with literature.[46]

7.17.20 Synthesis of 2-((4-methoxyphenyl)ethynyl)-1-methyl-1*H*-imidazole (1y)

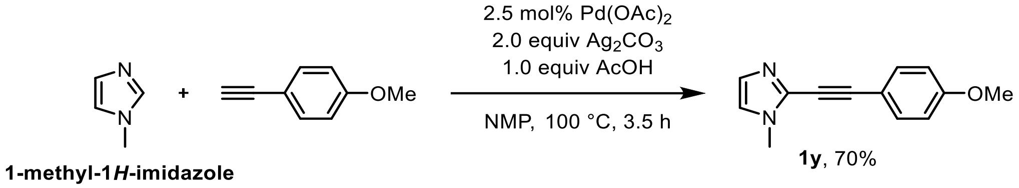

1-methyl-1*H*-imidazole **1y**, 70%

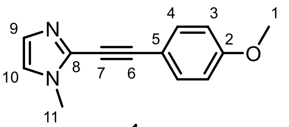

1x
$C_{13}H_{12}N_2O$
Mw = 212.25 gmol^{-1}

According to a literature procedure,[47] a Schlenk flask equipped with a magnetic stir bar was charged with 1-methyl-1*H*-imidazole (410 mg, 5.00 mmol, 1.00 equiv), $Pd(OAc)_2$ (23.0 mg, 0.130 mmol, 2.50 mol%), Ag_2CO_3 (2.75 g, 10.0 mmol, 2.00 equiv) and AcOH (0.300 mL, 5.00 mmol, 1.00 equiv). To this reaction mixture, 1-ethynyl-4-methoxybenzene (1.98 g, 15.0 mmol, 3.00 equiv), dissolved in NMP (20 mL) was added dropwise over 45 min at room temperature. The reaction mixture was stirred further at 100 °C for 3.5 h. The reaction mixture was allowed to cool to room temperature. The crude mixture was diluted with EtOAc (30 mL), filtered over Celite (4 × 3 cm) and eluted with EtOAc (20 mL). The filtrate was concentrated under reduced pressure. The crude product was purified by flash column chromatography on silica gel to afford **1y** as yellow soild (743 mg, 3.5 mmol, 70% yield).

R*f* = 0.40 (EtOAc/CH_2Cl_2 = 4:1).

^{1}H NMR (600 MHz, $CDCl_3$): δ = 7.50 (d, $^3J_{4,3}$ = 8.8 Hz, 2H, H-4), 7.07 (d, $^3J_{10,9}$ = 1.2 Hz, 1H, H-10), 6.92 (d, $^3J_{9,10}$ = 1.2 Hz, 1H, H-9), 6.88 (d, $^3J_{3,4}$ = 8.8 Hz, 2H, H-3), 3.83 (s, 3H, H-1), 3.77 (s, 3H, H-11) ppm.

^{13}C NMR (151 MHz, $CDCl_3$): δ = 160.3 (C-2), 133.4 (C-4), 133.0 (C-8), 129.7 (C-10), 121.3 (C-9), 114.2 (C-3), 114.1 (C-5), 92.9 (C-6), 77.5 (C-7), 55.5 (C-1), 33.6 (C-11) ppm.

HRMS (APCI) for $C_{13}H_{12}N_2O^+$ [(M)$^+$] calculated 213.1022, found 213.1015.

The data is in accordance with literature.[47]

7.17.21 3-(Phenylethynyl)thiophene (1z)

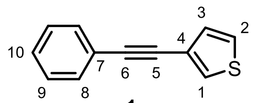

1z
$C_{12}H_8S$
Mw = 184.26 gmol^{-1}

Prepared according to **GP7** from 3-iodothiophene (0.310 mL, 3.00 mmol, 1.00 equiv), ethynylbenzene (0.400 mL, 3.60 mmol, 1.20 equiv), $Pd(PPh_3)_2Cl_2$ (42.1 mg, 60.0 µmol, 2.00 mol%) and CuI (22.8 mg, 120 µmol, 4.00 mol%) in Et_3N (12.5 mL, 47.3 mmol, 30.0 equiv). Purification by flash column chromatography on silica gel yielded **1z** as colorless solid (387 mg, 2.10 mmol, 70% yield).

R*f* = 0.30 (SiO_2, cyclohexane).

^{1}H NMR (600 MHz, $CDCl_3$): δ = 7.53–7.51 (m, 3H, H-1, H-8), 7.36–7.32 (m, 3H, H-9, H-10), 7.31 (dd, $^{3}J_{2,3}$ = 5.0 Hz, $^{4}J_{2,1}$ = 3.0 Hz, 1H, H-2)*, 7.20 (dd, $^{3}J_{3,2}$ = 5.0 Hz, $^{4}J_{3,1}$ = 1.1 Hz, 1H, H-3)* ppm.

^{13}C NMR (151 MHz, $CDCl_3$): δ = 131.7 (C-8), 130.0 (C-3)*, 128.7 (C-1), 128.5 (C-9), 128.4 (C-10), 125.5 (C-2)*, 123.4 (C-7), 122.5 (C-4), 89.0 (C-6), 84.6 (C-5) ppm.

HRMS (APCI) for $C_{12}H_8S^+$ [$(M)^+$] calculated: 184.0341, found: 184.0342.

The data is in accordance with literature.[48]

7.17.22 1-Benzyl-5-(phenylethynyl)-1*H*-indole (1aa)

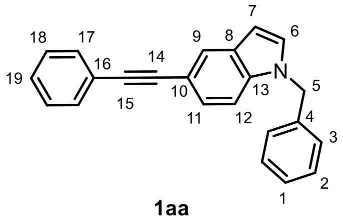

1aa
$C_{23}H_{17}N$
Mw = 307.40 gmol^{-1}

Prepared according to **GP7** from 1-benzyl-5-iodo-1*H*-indole[49] (1.00 g, 3.00 mmol, 1.00 equiv), ethynylbenzene (0.400 mL, 3.60 mmol, 1.20 equiv), $Pd(PPh_3)_2Cl_2$ (42.1 mg, 60.0 μmol, 2.00 mol%) and CuI (22.8 mg, 120 μmol, 4.00 mol%) in Et_3N (12.5 mL, 47.3 mmol, 30.0 equiv). Purification by flash column chromatography on silica gel to afford product **1aa** as colorless solid (645 mg, 2.10 mmol, 70% yield).

R*f* = 0.20 (cyclohexane/EtOAc = 20:1).

^{1}H NMR (600 MHz, $CDCl_3$): δ = 7.89 (d, $^{4}J_{9,11}$ = 1.5 Hz, 1H, H-9), 7.55 (d, $^{3}J_{17,18}$ = 7.4 Hz, 2H, H-17) 7.37–7.26 (m, 8H, H-1, H-2, H-3, H-11, H-12, H-19), 7.17 (d, $^{3}J_{6,7}$ = 3.2 Hz, 1H, H-6), 7.13–7.12 (m, 2H, H-18), 6.57 (d, $^{3}J_{7,6}$ = 3.6 Hz, 1H, H-7), 5.35 (s, 2H, H-5) ppm.

^{13}C NMR (151 MHz, $CDCl_3$): δ = 137.3 (C-4), 136.1 (C-16), 131.6 (C-17), 129.4 (C-6), 129.0 (C-2)**, 128.8 (C-10)*, 128.4 (C-3)**, 127.9 (C-12)*, 127.8 (C-1)*, 126.9 (C-18), 125.5 (C-9), 125.0 (C-19)*, 124.2 (C-8), 114.3 (C-13), 109.9 (C-11)*, 102.2 (C-7), 91.2 (C-15), 87.2 (C-14), 50.4 (C-5) ppm.

HRMS (APCI) for $C_{23}H_{18}N^+$ [$(M+H)^+$] calculated 308.1434, found 308.1435.

The data is in accordance with literature.[49]

7.18 Synthesis of starting materials for catalytic hydrogenation of α,β-unsaturated amides

7.18.1 (*E*)-3-Phenyl-1-(piperidin-1-yl)prop-2-en-1-one (11b)

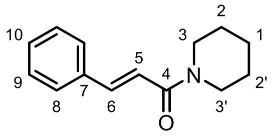

11b
$C_{14}H_{17}NO$
Mw = 215.30 gmol^{-1}

Prepared according to **GP9** from cinnamyl chloride (**S17**, 0.830 g, 5.00 mmol, 1.00 equiv), Et_3N (1.40 mL, 10.0 mmol, 2.00 equiv) and piperidine (0.600 mL, 6.00 mmol, 1.20 equiv) in CH_2Cl_2 (25 mL). The reaction mixture was stirred for 16 h at room temperature. Purification by

flash column chromatography on silica gel (cyclohexane/EtOAc = 3:1) yielded **11b** as colorless solid (780 mg, 3.62 mmol, 72%).

Mp = 146 °C (cyclohexane).

R*f* = 0.25 (cyclohexane/EtOAc = 3:1).

^{1}H NMR (600 MHz, $CDCl_3$): δ = 7.64 (d, $^3J_{6,5}$ = 15.4 Hz, 1H, H-6), 7.51 (d, $^3J_{8,9}$ = 6.8 Hz, 2H, H-8), 7.37–7.31 (m, 3H, H-9, H-10), 6.90 (d, $^3J_{5,6}$ = 15.4 Hz, 1H, H-5), 3.64–3.59 (m, 4H, H-3, H-3'), 1.69–1.64 (m, 2H, H-2)*, 1.63–1.59 (m, 4H, H-2'*, H-1) ppm.

^{13}C NMR (151 MHz, $CDCl_3$): δ = 165.4 (C-4), 142.2 (C-6), 135.6 (C-7), 129.5 (C-10), 128.8 (C-8), 127.8 (C-9), 117.9 (C-5), 47.1 (C-3), 43.4 (C-3'), 26.8 (C-2)*, 25.7 (C-2')*, 24.7 (C-1) ppm.

HRMS (ESI) for $C_{14}H_{18}NO^+$ [(M+H$^+$)$^+$] calculated: 216.1383, found: 216.1377.

IR (ATR): ṽ = 2937 (w), 2863 (w), 1587 (s), 1498 (w), 1439 (m), 1282 (w), 1249 (w), 1017 (w), 980 (w), 853 (w), 764 (m), 708 (w) cm^{-1}.

The data is in accordance with literature.[10]

7.18.2 (*E*)-1-(4,4-Difluoropiperidin-1-yl)-3-phenylprop-2-en-1-one (11c)

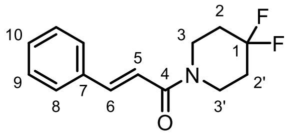

11c
$C_{14}H_{15}F_2NO$
Mw = 251.28 gmol^{-1}

Prepared according to **GP9** from cinnamyl chloride (**S17**, 0.830 g, 5.00 mmol, 1.00 equiv), Et_3N (1.40 mL, 10.0 mmol, 2.00 equiv) and 4,4-difluoropiperidine (0.730 g, 6.00 mmol, 1.20 equiv) in CH_2Cl_2 (25 mL). The reaction mixture was stirred for 16 h at room temperature. Purification by flash column chromatography on silica gel (cyclohexane/EtOAc = 2:1) yielded **11c** as colorless solid (820 mg, 3.26 mmol, 65%).

Mp = 123 °C (cyclohexane).

R*f* = 0.15 (cyclohexane/EtOAc = 2:1).

^{1}H NMR (600 MHz, $CDCl_3$): δ = 7.69 (d, $^3J_{6,5}$ = 15.4 Hz, 1H, H-6), 7.53–7.52 (m, 2H, H-8), 7.40–7.35 (m, 3H, H-9, H-10), 6.88 (d, $^3J_{5,6}$ = 15.4 Hz, 1H, H-5), 3.83 (br s, 2H, H-3), 3.77 (br s, 2H, H-3'), 2.07–2.00 (m, 4H, H-2, H-2') ppm.

^{13}C NMR (151 MHz, $CDCl_3$): δ = 165.7 (C-4), 143.8 (C-6), 135.1 (C-7), 130.0 (C-10), 129.0 (C-9)*, 127.9 (C-8)*, 121.6 (t, $^1J_{1,F}$ = 242.3 Hz, C-1), 116.6 (C-5), 42.8 (C-3), 39.3 (C-3'), 35.0 (C-2), 34.0 (C-2') ppm.

^{19}F NMR (472 MHz, $CDCl_3$): δ = –97.8 (s) ppm

HRMS (ESI) for $C_{14}H_{16}F_2NO^+$ [(M+H$^+$)$^+$] calculated: 252.1194, found 252.1194.

IR (ATR): ṽ = 3071 (w), 3034 (w), 2978 (w), 2944 (w), 2877 (w), 1595 (s), 1461 (m), 1356 (m), 1256 (w), 1218 (w), 1103 (s), 976 (w), 864 (w), 764 (m), 704 (w) cm^{-1}.

7.18.3 (*E*)-1-Morpholino-3-phenylprop-2-en-1-one (11d)

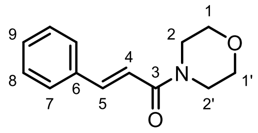

11d
$C_{13}H_{15}NO_2$
Mw = 217.27 $gmol^{-1}$

Prepared according to **GP9** from cinnamyl chloride (**S17**, 0.830 g, 5.00 mmol, 1.00 equiv), Et_3N (1.40 mL, 10.0 mmol, 2.00 equiv) and morpholine (0.530 g, 6.00 mmol, 1.20 equiv) in CH_2Cl_2 (25 mL). The reaction mixture was stirred for 16 h at room temperature. Purification by flash column chromatography on silica gel (cyclohexane/EtOAc = 2:1) yielded **11d** as colorless solid (980 mg, 4.51 mmol, 90%).

Mp = 83 °C (cyclohexane).

R*f* = 0.20 (cyclohexane/EtOAc = 2:1).

^{1}H NMR (600 MHz, $CDCl_3$): δ = 7.70 (d, $^3J_{5,4}$ = 15.4 Hz, 1H, H-5), 7.52 (dd, $^3J_{7,8}$ = 7.8 Hz, $^4J_{7,9}$ = 1.8 Hz, 2H, H-7), 7.39–7.34 (m, 3H, H-8, H-9), 6.84 (d, $^3J_{4,5}$ = 15.4 Hz, 1H, H-4), 3.73–3.66 (m, 8H, H-2, H-2', H-1, H-1') ppm.

^{13}C NMR (151 MHz, $CDCl_3$): δ = 165.7 (C-3), 143.4 (C-5), 135.3 (C-6), 129.9 (C-9), 129.0 (C-7), 127.9 (C-8), 116.7 (C-4), 67.0 (C-1, C-1'), 46.4 (C-2)*, 42.6 (C-2')* ppm.

HRMS (ESI) for $C_{13}H_{16}NO_2^+$ [(M+H$^+$)$^+$] calculated: 218.1176, found: 218.1175.

IR (ATR): ṽ = 2967 (w), 2926 (w), 2859 (w), 1595 (s), 1453 (m), 1267 (w), 1226 (w), 1114 (s), 1043 (w), 972 (m), 760 (s), 700 (m) cm^{-1}.

The data is in accordance with literature.[50]

7.18.4 (*E*)-3-Phenyl-1-thiomorpholinoprop-2-en-1-one (11e)

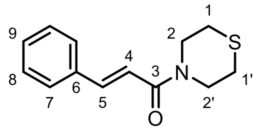

11e
$C_{13}H_{15}NOS$
Mw = 233.33 $gmol^{-1}$

Prepared according to **GP9** from cinnamyl chloride (**S17**, 0.830 g, 5.00 mmol, 1.00 equiv), Et_3N (1.40 mL, 10.0 mmol, 2.00 equiv) and thiomorpholine (0.600 mL, 6.00 mmol, 1.20 equiv) in CH_2Cl_2 (25 mL). The reaction mixture was stirred for 16 h at room temperature. Purification by flash column chromatography on silica gel (cyclohexane/EtOAc = 3:1) yielded **11e** as colourless solid (830 mg, 3.56 mmol, 71%).

Mp = 83 °C (cyclohexane).

R*f* = 0.20 (cyclohexane/EtOAc = 3:1).

^{1}H NMR (600 MHz, $CDCl_3$): δ = 7.66 (d, $^{3}J_{5,4}$ = 15.4 Hz, 1H, H-5), 7.51 (d, $^{3}J_{7,8}$ = 7.8 Hz, 2H, H-7), 7.38–7.33 (m, 3H, H-8, H-9), 6.83 (d, $^{3}J_{4,5}$ = 15.4 Hz, 1H, H-4), 3.97 (br s, 2H, H-2)*, 3.91 (br s, 2H, H-2')* 2.66 (br s, 4H, H-1, H-1') ppm.

^{13}C NMR (151 MHz, $CDCl_3$): δ = 165.7 (C-3), 143.2 (C-5), 135.2 (C-6), 129.8 (C-9), 128.9 (C-7), 127.8 (C-8), 117.1 (C-4), 48.8 (C-2)*, 45.1 (C-2')*, 28.3 (C-1)**, 27.5 (C-1')** ppm.

HRMS (ESI) for $C_{13}H_{16}NOS^+$ [(M+H$^+$)$^+$] calculated: 234.0947, found: 234.0949.

IR (ATR): ṽ = 2907 (w), 2866 (w), 1595 (s), 1424 (m), 1297 (w), 1252 (w), 1185 (s), 954 (w), 854 (w), 678 (w) cm^{-1}.

7.18.5 *N*,*N*-Bis(2-methoxyethyl)cinnamamide (11f)

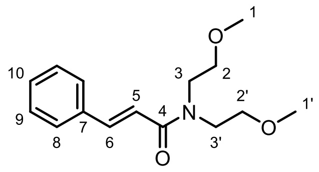

11f
$C_{15}H_{21}NO_3$
Mw = 263.34 gmol^{-1}

Prepared according to **GP9** from cinnamyl chloride (**S17**, 0.830 g, 5.00 mmol, 1.00 equiv), Et_3N (1.40 mL, 10.0 mmol, 2.00 equiv) and bis(2-methoxyethyl)amine (0.890 mL, 6.00 mmol, 1.20 equiv) in CH_2Cl_2 (25 mL). The reaction mixture was stirred for 16 h at room temperature. Purification by flash column chromatography on silica gel (cyclohexane/EtOAc = 2:1) yielded **11f** as colorless solid (1.02 g, 3.87 mmol, 77%).

Mp = 50 °C (cyclohexane).

R*f* = 0.20 (cyclohexane/EtOAc = 2:1).

^{1}H NMR (600 MHz, $CDCl_3$): δ = 7.67 (d, $^{3}J_{6,5}$ = 15.4 Hz, 1H, H-6), 7.52–7.50 (m, 2H, H-8), 7.37–7.33 (m, 3H, H-9, H-10), 6.97 (d, $^{3}J_{5,6}$ = 15.4 Hz, 1H, H-5), 3.71 (t, $^{3}J_{2,3}$ = 5.9 Hz, 2H, H-2), 3.67 (t, $^{3}J_{2',3'}$ = 5.4 Hz, 2H, H-2'), 3.59 (t, $^{3}J_{3',2'}$ = 5.4 Hz, 2H, H-3'), 3.55 (t, $^{3}J_{3,2}$ = 5.9 Hz, 2H, H-3), 3.35 (s, 3H, H-1), 3.34 (s, 3H, H-1') ppm.

^{13}C NMR (151 MHz, $CDCl_3$): δ = 167.0 (C-4), 142.5 (C-6), 135.6 (C-7), 129.6 (C-10), 128.9 (C-8), 127.9 (C-9), 118.1 (C-5), 71.4 (C-2), 71.3 (C-2'), 59.3 (C-1), 58.9 (C-1'), 49.2 (C-3), 47.5 (C-3') ppm.

HRMS (ESI) for $C_{15}H_{22}NO_3^+$ [(M+H$^+$)$^+$] calculated: 264.1594, found: 264.1595.

IR (ATR): ṽ = 2982 (w), 2929 (w), 2881 (w), 2829 (w), 1651 (s), 1606 (s), 1453 (m), 1416 (m), 1189 (w), 1110 (s), 1013 (w), 976 (w), 764 (w), 708 (w) cm^{-1}.

7.18.6 (*E*)-1-(4,7-dihydrothieno[2,3-c]pyridin-6(5*H*)-yl)-3-phenylprop-2-en-1-one (11g)

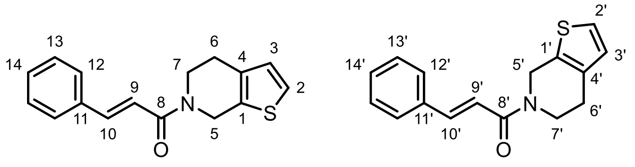

11g
$C_{16}H_{15}NOS$
Mw = 269.36 gmol^{-1}

11g_rotamer
$C_{16}H_{15}NOS$
Mw = 269.36 gmol^{-1}

Prepared according to **GP9** from cinnamyl chloride (**S17**, 0.830 g, 5.00 mmol, 1.00 equiv), Et_3N (2.10 mL, 15.0 mmol, 3.00 equiv) and 4,5,6,7-tetrahydrothieno[2,3-*c*]pyridine hydrochloride (1.00 g, 6.00 mmol, 1.20 equiv) in CH_2Cl_2 (25 mL). The reaction mixture was stirred for 16 h at room temperature. Purification by flash column chromatography on silica gel (cyclohexane/EtOAc = 1:1) yielded **11g** and **11g_rotamer** (50:50 ratio) as colorless solid (1.20 g, 4.55 mmol, 89%).

Mp = 104 °C (cyclohexane).

R*f* = 0.30 (cyclohexane/EtOAc = 1:1).

^{1}H NMR (600 MHz, $CDCl_3$): δ = 7.70 (d, $^3J_{10,9}$ = 15.5 Hz, 1H, H-10), 7.54 (d, $^3J_{12,13}$ = 7.8 Hz, 2H, H-12), 7.40–7.35 (m, 3H, H-13, H-14), 7.16 (d, $^3J_{2,3}$ = 5.1 Hz, 1H, H-2), 6.94 (d, $^3J_{9,10}$ = 15.1 Hz, 1H, H-9), 6.83 (d, $^3J_{3,2}$ = 5.1 Hz, 1H, H-3), 4.79 (br s, 2H, H-5), 4.03 (br s, 2H, H-7), , 2.97 (s, 2H, H-6) ppm.

^{13}C NMR (151 MHz, $CDCl_3$): δ = 166.2 (C-8), 143.1 (C-10), 135.4 (C-11), 132.6 (C-1), 132.3 (C-4), 129.8 (C-14), 129.0 (C-12), 127.9 (C-13), 125.4 (C-2), 123.7 (C-3), 117.8 (C-9), 46.1 (C-5), 44.1 (C-7), 26.2 (C-6) ppm.

The ^{1}H and ^{13}C NMR spectrum is well separated for the two rotamers, which is why both ^{1}H and ^{13}C NMR are assigned separately for both the rotamers.

^{1}H NMR of **11g_rotamer** (600 MHz, $CDCl_3$): δ = 7.70 (d, $^3J_{10',9'}$ = 15.5 Hz, 1H, H-10'), 7.54 (d, $^3J_{12',13'}$ = 7.8 Hz, 2H, H-12'), 7.40–7.35 (m, 3H, H-13', H-14'), 7.16 (d, $^3J_{2',3'}$ = 5.1 Hz, 1H, H-2'), 6.94 (d, $^3J_{9',10'}$ = 15.1 Hz, 1H, H-9'), 6.83 (d, $^3J_{3',2'}$ = 5.1 Hz, 1H, H-3'), 4.75 (br s, 2H, H-5'), 3.93 (br s, 2H, H-7'), 2.93 (s, 2H, H-6') ppm.

^{13}C NMR of **11g_rotamer** (151 MHz, $CDCl_3$): δ = 166.2 (C-8'), 143.1 (C-10'), 134.6 (C-11'), 132.6 (C-1'), 131.3 (C-4'), 129.8 (C-14'), 129.0 (C-12'), 127.9 (C-13'), 124.6 (C-2'), 123.7 (C-3'), 117.5 (C-9'), 43.4 (C-5'), 40.5 (C-7'), 24.9 (C-6') ppm.

Rotamer ratio is determined from the integration value of H-5 (at 4.79 ppm) and H-5' (at 4.75 ppm).

HRMS (ESI) for $C_{16}H_{16}NOS^+$ [$(M+H^+)^+$] calculated: 270.0947, found: 270.0942.

IR (ATR): ṽ = 3056 (w), 2940 (w), 2877 (w), 2829 (w), 1613 (s), 1431 (m), 1215 (w), 1140 (w), 1059 (w), 984 (w), 831 (w), 701 (w) cm^{-1}.

7.18.7 (*E*)-1-(4-(2,4-difluorobenzoyl)piperidin-1-yl)-3-phenylprop-2-en-1-one (11h)

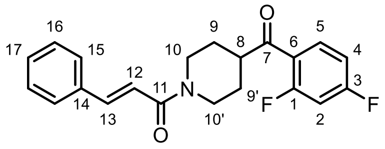

11h
$C_{21}H_{19}F_2NO_2$
Mw = 355.39 gmol^{-1}

Prepared according to **GP9** from cinnamyl chloride (**S17**, 0.830 g, 5.00 mmol, 1.00 equiv), Et_3N (2.10 mL, 15.0 mmol, 3.00 equiv) and (2,4-difluorophenyl)(piperidin-4-yl)methanone hydrochloride (1.57 g, 6.00 mmol, 1.20 equiv) in CH_2Cl_2 (25 mL). The reaction mixture was stirred for 16 h at room temperature. Purification by flash column chromatography on silica gel (cyclohexane/EtOAc = 2:1) yielded **11h** as colorless solid (1.38 g, 3.88 mmol, 78% yield).

Mp = 109 °C (cyclohexane).

R*f* = 0.15 (cyclohexane/EtOAc = 2:1).

^{1}H NMR (600 MHz, $CDCl_3$): δ = 7.86 (td, $^3J_{5,4}$ = 8.6 Hz, $^4J_{5,1F/3F}$ = 6.9 Hz, 1H, H-5), 7.65 (d, $^3J_{13,12}$ = 15.4 Hz, 1H, H-13), 7.52 (d, $^3J_{15,16}$ = 7.3 Hz, 2H, H-15), 7.38–7.32 (m, 3H, H-16, H-17), 6.98 (t, $^3J_{4,5/3F}$ = 8.4 Hz, 1H, H-4), 6.90–6.87 (m, 2H, H-2, H-12), 4.63 (br s, 1H, H-10a), 4.15 (br s, 1H, H-10'a), 3.39–3.36 (m, 1H, H-8), 3.28 (br s, 1H, H-10b), 2.94 (br s, 1H, H-10'b), 2.00–1.98 (m, 2H, H-9)*, 1.78–1.66 (m, 2H, H-9')* ppm.

^{13}C NMR (151 MHz, $CDCl_3$): δ = 198.7 (d, $^3J_{7,1F}$ = 4.8 Hz, C-7), 165.9 (dd, $^1J_{1,1F}$ = 257.2 Hz, $^3J_{1,3F}$ = 12.5 Hz, C-1) 165.6 (C-11), 162.1 (dd, $^1J_{3,3F}$ = 255.6 Hz, $^3J_{3,1F}$ = 12.5 Hz, C-3), 142.8 (C-13), 135.4 (C-14), 133.2 (dd, $^3J_{5,1F}$ = 10.5 Hz, $^3J_{5,3F}$ = 4.5 Hz, C-5), 129.7 (C-17), 128.9 (C-15), 127.8 (C-16), 121.6 (dd, $^2J_{6,1F}$ = 13.6 Hz, $^4J_{6,3F}$ = 3.8 Hz, C-6), 117.4 (C-12), 112.7 (dd, $^2J_{4,3F}$ = 21.3 Hz, $^4J_{4,1F}$ = 3.3 Hz, C-4), 104.9 (dd, $^2J_{2,3F}$ = 28.1 Hz, $^2J_{2,1F}$ = 25.4 Hz, C-2), 47.9 (d, $^4J_{8,1F}$ = 6.9 Hz, C-8), 45.5 (C-10), 42.0 (C-10'), 28.4 (C-9)*, 28.0 (C-9')* ppm.

^{19}F NMR (473 MHz, $CDCl_3$): δ = –101.7 (d, $^4J_{1F,3F}$ = 12.1 Hz, F-1), –106.5 (d, $^4J_{3F,31F}$ = 12.1 Hz, F-3) ppm.

HRMS (ESI) for $C_{21}H_{20}F_2NO_2^+$ [(M+H$^+$)$^+$] calculated: 356.1457, found: 356.1453.

IR (ATR): ṽ = 2933 (w), 2855 (w), 1651 (s), 1610 (s), 1450 (w), 1412 (s), 1323 (w), 1263 (w), 1189 (w), 1110 (w), 980 (w), 854 (w), 768 (w), 708 (w) cm^{-1}.

7.18.8 Synthesis of (*E*)-1-(4-(-(2,4-difluorophenyl)(methoxyimino)methyl)piperidin-1-yl)-3-phenylprop-2-en-1-one (11i)

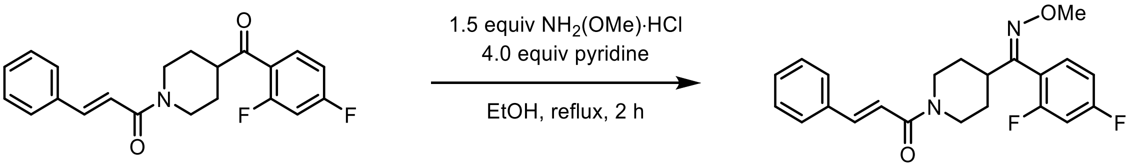

11h **11i**, 73%

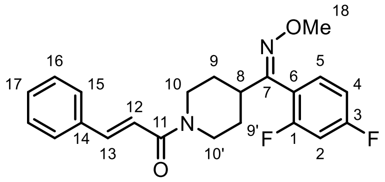

11i
$C_{22}H_{22}F_2N_2O_2$
Mw = 384.43 gmol^{-1}

In deference to a literature procedure,[38] a Schlenk tube equipped with a magnetic stir bar was charged with (*E*)-1-(4-(2,4-difluorobenzoyl)piperidin-1-yl)-3-phenylprop-2-en-1-one (**11h**, 0.750 g, 2.11 mmol, 1.00 equiv), pyridine (0.680 mL, 8.44 mmol, 4.00 equiv) and ethanol (15 mL). *O*-methylhydroxylamine hydrochloride (264 mg, 3.17 mmol, 1.50 equiv) was added and the reaction mixture was refluxed for 2 h (conversion monitored *via* TLC, cyclohexane/EtOAc = 2:1). The reaction mixture was allowed to cool to room temperature. All volatiles were removed under reduced pressure. The crude product was purified by flash column chromatography on silica gel (cyclohexane/EtOAc = 2:1) to afford **11i** as colorless oil (590 mg, 1.53 mmol, 73% yield).

R*f* = 0.20 (cyclohexane/EtOAc = 2:1).

^{1}H NMR (600 MHz, $CDCl_3$): δ = 7.63 (d, $^3J_{13,12}$ = 15.4 Hz, 1H, H-13), 7.50–7.49 (m, 2H, H-15), 7.37–7.32 (m, 3H, H-16, H-17), 7.18 (td, $^3J_{5,4}$ = 8.3 Hz, $^4J_{5,1F/3F}$ = 6.3 Hz, 1H, H-5), 6.89–6.81 (m, 3H, H-2, H-4, H-12), 4.80 (d, $^2J_{10a,10b}$ = 13.1 Hz, 1H, H-10a), 4.14 (d, $^2J_{10'a,10'b}$ = 13.6 Hz, 1H, H-10’a), 3.96 (s, 3H, H-18), 3.44 (tt, $^3J_{8,9a/9'a}$ = 12.3 Hz, $^3J_{8,9b/9'b}$ = 3.7 Hz, 1H, H-8), 3.16 (t, $^{2/3}J_{10b,10a/9b}$ = 13.1 Hz, 1H, H-10b), 2.68 (t, $^{2/3}J_{10'b,10'a/9'b}$ = 13.6 Hz, 1H, H-10’b), 1.88–1.85 (m, 2H, H-9)*, 1.57–1.50 (m, 2H, H-9’)* ppm.

^{13}C NMR (151 MHz, $CDCl_3$): δ = 165.4 (C-11), 163.4 (dd, $^1J_{1,1F}$ = 250.9 Hz, $^3J_{1,3F}$ = 11.5 Hz, C-1), 160.6 (dd, $^1J_{3,3F}$ = 250.0 Hz, $^3J_{3,1F}$ = 12.5 Hz, C-3), 157.8 (C-7), 142.8 (C-13), 135.4 (C-14), 132.0 (dd, $^3J_{5,1F}$ = 9.6 Hz, $^3J_{5,3F}$ = 4.9 Hz, C-5), 129.7 (C-17), 128.9 (C-15), 127.8 (C-16), 119.4 (dd, $^2J_{6,1F}$ = 17.0 Hz, $^4J_{6,3F}$ = 4.0 Hz, C-6), 117.3 (C-12), 111.6 (dd, $^2J_{4,3F}$ = 21.7 Hz, $^4J_{4,1F}$ = 3.7 Hz, C-4), 104.4 (t, $^2J_{2,1F/3F}$ = 25.8 Hz, C-2), 62.3 (C-18), 46.1 (C-10), 42.5 (C-10’), 37.5 (C-8), 28.6 (C-9)*, 27.6 (C-9’)* ppm.

^{19}F NMR (473 MHz, $CDCl_3$): δ = –108.5 (d, $^4J_{1F,3F}$ = 8.7 Hz, F-1), –108.9 (d, $^4J_{3F,31F}$ = 8.7 Hz, F-3) ppm.

HRMS (ESI) for $C_{22}H_{23}F_2N_2O_2^+$ [(M+H$^+$)$^+$] calculated: 385.1722, found: 385.1718.

IR (ATR): ṽ = 3056 (w), 2997 (w), 2922 (w), 1640 (s), 1494 (w), 1420 (m), 1275 (w), 1211 (w), 1133 (w), 1092 (w), 1013 (w), 760 (w), 734 (w), 697 (w) cm^{-1}.

7.18.9 4-Cinnamoylpiperazine-1-carbaldehyde (11j)

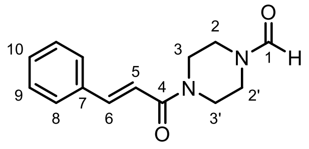

11j

$C_{14}H_{16}N_2O_2$

Mw = 244.29 gmol^{-1}

Prepared according to **GP9** from cinnamyl chloride (**S17**, 333 mg, 2.00 mmol, 1.00 equiv), Et_3N (0.560 mL, 4.00 mmol, 3.00 equiv) and piperazine-1-carbaldehyde (274 mg, 2.40 mmol, 1.20 equiv) in CH_2Cl_2 (10 mL). The reaction mixture was stirred for 16 h at room temperature. Purification by flash column chromatography on silica gel (cyclohexane/EtOAc = 1:1) yielded **11j** as colorless solid (360 mg, 1.47 mmol, 74% yield).

Mp = 115 °C (cyclohexane).

R*f* = 0.20 (cyclohexane/EtOAc = 1:1).

^{1}H NMR (600 MHz, $CDCl_3$): δ = 8.12 (s, 1H, H-1), 7.71 (d, $^3J_{6,5}$ = 15.4 Hz, 1H, H-6), 7.53 (dd, $^3J_{8,9}$ = 7.5 Hz, $^4J_{8,10}$ = 2.1 Hz, 2H, H-8), 7.40–7.36 (m, 3H, H-9, H-10), 6.86 (d, $^3J_{5,6}$ = 15.4 Hz, 1H, H-5), 3.76–3.62 (m, 4H, H-3, H-3'), 3.63–3.61 (m, 2H, H-2), 3.45–3.43 (m, 2H, H-2') ppm.

^{13}C NMR (151 MHz, $CDCl_3$): δ = 165.9* (C-4), 161.0 (C-1), 144.0 (C-6), 135.0 (C-7), 130.1 (C-10), 129.0 (C-8), 128.0 (C-9), 116.4 (C-5), 45.5 (C-3, C-2), 42.9 (C-3'), 40.5 (C-2') ppm.

C-3' is assigned from ^{1}H,^{13}C HSQC NMR.

HRMS (ESI) for $C_{14}H_{17}N_2O_2^+$ [(M+H$^+$)$^+$] calculated: 245.1285, found: 245.1281.

IR (ATR): ṽ = 3023 (w), 2967 (w), 2922 (w), 2855 (w), 1662 (s), 1599 (s), 1427 (s), 1211 (w), 1166 (w), 1002 (w), 965 (w), 760 (w), 704 (w) cm^{-1}.

7.18.10 (*E*)-1-(4-(Oxetan-3-yl)piperazin-1-yl)-3-phenylprop-2-en-1-one (11k)

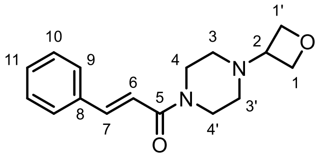

11k

$C_{16}H_{20}N_2O_2$

Mw = 272.35 gmol^{-1}

Prepared according to **GP9** from cinnamyl chloride (**S17**, 333 mg, 2.00 mmol, 1.00 equiv), Et_3N (0.560 mL, 4.00 mmol, 3.00 equiv) and 1-(oxetan-3-yl)piperazine (172 mg, 2.40 mmol, 1.20 equiv) in CH_2Cl_2 (10 mL). The reaction mixture was stirred for 16 h at room temperature. Purification by flash column chromatography on silica gel (cyclohexane/EtOAc = 1:1) yielded **11k** as colorless solid (357 mg, 1.31 mmol, 66% yield).

Mp = 120 °C (cyclohexane).

R*f* = 0.25 (cyclohexane/EtOAc = 1:1).

^{1}H NMR (600 MHz, $CDCl_3$): δ = 7.66 (d, $^3J_{7,6}$ = 15.4 Hz, 1H, H-7), 7.51 (d, $^3J_{9,10}$ = 6.8 Hz, 2H, H-9), 7.38–7.33 (m, 3H, H-10, H-11), 6.86 (d, $^3J_{6,7}$ = 15.4 Hz, 1H, H-6), 4.67 (t, $^3J_{1,2}$ = 6.5 Hz, 2H,

H-1), 4.62 (t, $^3J_{1',2}$ = 6.1 Hz, 2H, H-1'), 3.78 (br s, 2H, H-4), 3.69 (br s, 2H, H-4'), 3.50 (p, $^3J_{2,1/1'}$ = 6.4 Hz, 1H, H-2), 2.36–2.35 (m, 4H, H-3, H-3') ppm.

^{13}C NMR (151 MHz, $CDCl_3$): δ = 165.6 (C-5), 143.1 (C-7), 135.3 (C-8), 129.8 (C-11), 128.9 (C-9), 127.9 (C-10), 117 (C-6), 75.4 (C-1, C-1'), 59.1 (C-2), 50.0 (C-4), 49.4 (C-4'), 45.6 (C-3), 41.9 (C-3') ppm.

HRMS (ESI) for $C_{16}H_{21}N_2O_2^+$ [(M+H$^+$)$^+$] calculated: 273.1598, found: 273.1591.

IR (ATR): ṽ = 2941 (w), 2877 (w), 2777 (w), 1599 (s), 1422 (m), 1364 (w), 1203 (w), 1148 (w), 1118 (w), 1029 (w), 976 (w), 890 (w), 760 (w), 704 (w) cm^{-1}.

7.18.11 (*E*)-1-(4-(6-fluorobenzo[*d*]isoxazol-3-yl)piperidin-1-yl)-3-phenylprop-2-en-1-one (11l)

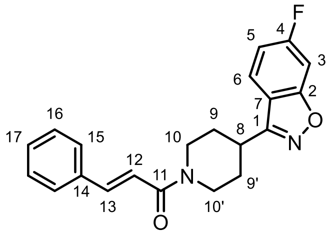

11l
$C_{21}H_{19}FN_2O_2$
Mw = 350.39 gmol^{-1}

Prepared according to **GP9** from cinnamyl chloride (**S17**, 0.830 g, 5.00 mmol, 1.00 equiv), Et_3N (2.10 mL, 15.0 mmol, 3.00 equiv) and 6-fluoro-3-(piperidin-4-yl)benzo[*d*]isoxazole hydrochloride (1.42 g, 6.00 mmol, 1.20 equiv) in CH_2Cl_2 (25 mL). The reaction mixture was stirred for 16 h at room temperature. Purification by flash column chromatography on silica gel (cyclohexane/EtOAc = 2:1) yielded **11l** as colorless solid (0.970 g, 2.77 mmol, 55%).

Mp = 146 °C (cyclohexane).

R*f* = 0.20 (cyclohexane/EtOAc = 2:1).

^{1}H NMR (600 MHz, $CDCl_3$): δ = 7.70 (d, $^3J_{13,12}$ = 15.5 Hz, 1H, H-13), 7.64 (dd, $^3J_{6,5}$ = 8.7 Hz, $^4J_{6,F}$ = 5.0 Hz, 1H, H-6), 7.54 (d, $^3J_{15,16}$ = 7.8 Hz, 2H, H-15), 7.40–7.34 (m, 3H, H-16, H-17), 7.27–7.25 (m, 1H, H-5), 7.08 (td, $^3J_{3,F}$ = 8.9 Hz, $^4J_{3,5}$ = 2.1 Hz, 1H, H-3), 6.94 (d, $^3J_{12,13}$ = 15.5 Hz, 1H, H-12), 4.76 (br s, 1H, H-10'a), 4.27 (br s, 1H, H-10a), 3.41–3.36 (m, 2H, H-10'b, H-8), 3.04 (br s, 1H, H-10b), 2.20–2.17 (m, 2H, H-9), 2.04–2.00 (m, 2H, H-9') ppm.

^{13}C NMR (151 MHz, $CDCl_3$): δ = 165.7 (C-11), 165.2 (C-2), 164.1 (d, $^1J_{4,F}$ = 54.6 Hz, C-4), 163.5 (C-7), 160.3 (C-1), 143.1 (C-13), 135.4 (C-14), 129.8 (C-17), 129.0 (C-15), 127.9 (C-16), 122.3 (d, $^3J_{6,F}$ = 11.0 Hz, C-6), 117.3 (C-12), 112.8 (d, $^2J_{3,F}$ = 25.5 Hz, C-3), 97.7 (d, $^2J_{5,F}$ = 26.8 Hz, C-5), 45.9 (C-10), 42.2 (C-10'), 34.5 (C-8), 30.9 (C-9), 30.4 (C-9') ppm.

^{19}F NMR (473 MHz, $CDCl_3$): δ = –109.1 (s) ppm.

HRMS (ESI) for $C_{21}H_{20}FN_2O_2^+$ [(M+H$^+$)$^+$] calculated: 351.1503, found: 351.1497.

IR (ATR): ṽ = 2944 (w), 2859 (w), 1610 (s), 1413 (m), 1350 (w), 1271 (w), 1211 (w), 1122 (w), 940 (w), 850 (w), 809 (w), 768 (w), 704 (w) cm^{-1}.

7.18.12 Synthesis of (*E*)-1-(piperidin-1-yl)-3-(4-(piperidine-1-carbonyl)phenyl)prop-2-en-1-one (11m)

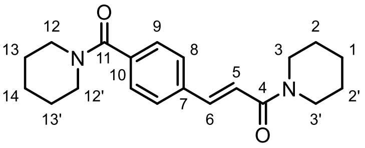

11m
$C_{20}H_{26}N_2O_2$
Mw = 326.44 gmol^{-1}

Prepared according to **GP8** from (*E*)-4-(2-carboxyvinyl)benzoic acid (**S11m**, 576 mg, 3.00 mmol, 1.00 equiv), EDC·HCl (1.26 g, 6.60 mmol, 2.20 equiv), DMAP (147 mg, 1.20 mmol, 40.0 mol%), Et_3N (1.67 mL, 12.0 mmol, 4.00 equiv) and morpholine (0.710 mL, 7.20 mmol, 2.40 equiv) in CH_2Cl_2 (15 mL). The reaction mixture was stirred for 16 h at room temperature. Purification by flash column chromatography on silica gel (cyclohexane/EtOAc = 0:100) yielded **11m** as colorless solid (0.430 g, 1.31 mmol, 65% yield)

Mp = 188 °C (EtOAc).

R*f* = 0.15 (cyclohexane/EtOAc = 0:100).

^{1}H NMR (600 MHz, $CDCl_3$): δ = 7.61 (d, $^3J_{6,5}$ = 15.5 Hz, 1H, H-6), 7.52 (d, $^3J_{8,9}$ = 7.9 Hz, 2H, H-8), 7.37 (d, $^3J_{9,8}$ = 7.9 Hz, 2H, H-9), 6.92 (d, $^3J_{5,6}$ = 15.5 Hz, 1H, H-5), 3.68–3.65 (m, 4H, H-3, H-3')*, 3.57 (br s, 2H, H-12)*, 3.32 (br s, 2H, H-12')*, 1.67–1.59 (m, 10H, H-2, H-2', H-13, H-13', H-1**), 1.50 (br s, 2H, H-14)** ppm.

^{13}C NMR (151 MHz, $CDCl_3$): δ = 169.7 (C-11), 165.1 (C-4), 141.2 (C-6), 137.3 (C-10), 136.6 (C-7), 127.7 (C-8), 127.4 (C-9), 118.9 (C-5), 48.8 (C-3)*, 47.1 (C-3')*, 43.4 (C-12)*, 43.2 (C-12')*, 26.8 (C-2)**, 26.6 (C-2')**, 25.7 (C-13, C-13')**, 24.7 (C-1)***, 24.6 (C-14)*** ppm.

HRMS (ESI) for $C_{20}H_{27}N_2O_2^+$ [(M+H$^+$)$^+$] calculated: 327.2067, found: 327.2062.

IR (ATR): ṽ = 2993 (w), 2847 (w), 1613 (s), 1431 (s), 1271 (m), 1110 (w), 849 (w), 768 (w) cm^{-1}.

7.18.13 (*E*)-1-(Piperidin-1-yl)-3-(4-(trifluoromethyl)phenyl)prop-2-en-1-one (11n)

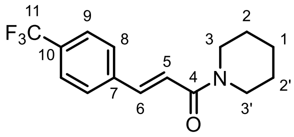

11n
$C_{15}H_{16}F_3NO$
Mw = 283.29 gmol^{-1}

Prepared according to **GP8** from (*E*)-3-(4-(trifluoromethyl)phenyl)acrylic acid (**S11n**, 468 mg, 2.16 mmol, 1.00 equiv), EDC·HCl (500 mg, 2.60 mmol, 1.20 equiv), DMAP (52.9 mg, 0.430 mmol, 20.0 mol%), Et_3N (0.600 mL, 4.32 mmol, 2.00 equiv) and piperidine (0.260 mL, 2.60 mmol, 1.20 equiv) in CH_2Cl_2 (12 mL). The reaction mixture was stirred for 16 h at room temperature. Purification by flash column chromatography on silica gel (cyclohexane/EtOAc = 2:1) yielded **11n** as colorless solid (0.390 g, 1.36 mmol, 63%).

Mp = 92 °C (cyclohexane).

R*f* = 0.25 (cyclohexane/EtOAc = 2:1).

^{1}H NMR (600 MHz, $CDCl_3$): δ = 7.64 (d, $^{3}J_{6,5}$ = 15.4 Hz, 1H, H-6), 7.61 (s, 4H, H-8, H-9), 6.98 (d, $^{3}J_{5,6}$ = 15.4 Hz, 1H, H-5), 3.67 (br s, 2H, H-3), 3.58 (br s, 2H, H-3’), 1.70–1.67 (m, 2H, H-2), 1.62 (br s, 4H, H-2’, H-1) ppm.

^{13}C NMR (151 MHz, $CDCl_3$): δ = 164.8 (C-4), 140.5 (C-6), 139.1 (C-7), 131.1 (q, $^{2}J_{10,F}$ = 32.5 Hz, C-10), 127.9 (C-8), 125.8 (q, $^{3}J_{9,F}$ = 4.8 Hz, C-9), 124.08 (q, $^{1}J_{11,F}$ = 272.2 Hz, C-11), 120.5 (C-5), 47.2 (C-3), 43.5 (C-3’), 26.9 (C-2), 25.7 (C-2’), 24.7 (C-1) ppm.

^{19}F NMR (473 MHz, $CDCl_3$): δ = –62.73 (s) ppm.

HRMS (ESI) for $C_{15}H_{17}F_3NO^+$ [$(M+H^+)^+$] calculated: 284.1257, found: 284.1251.

IR (ATR): ṽ = 2952 (w), 2926 (w), 2859 (w), 1606 (s), 1435 (m), 1327 (w), 1282 (w), 1167 (w), 1107 (m), 1066 (w), 1017 (w), 977 (m), 827 (m), 749 (w) cm^{-1}.

7.18.14 (*E*)-1-(Piperidin-1-yl)-3-(pyridin-3-yl)prop-2-en-1-one (11o)

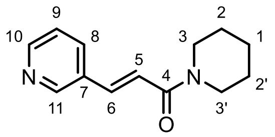

11o
$C_{13}H_{16}N_2O$
Mw = 216.28 $gmol^{-1}$

Prepared according to **GP8** from (*E*)-3-(pyridin-3-yl)acrylic acid (**S11o**, 615 mg, 4.12 mmol, 1.00 equiv), EDC·HCl (950 mg, 4.95 mmol, 1.20 equiv), DMAP (100 mg, 0.830 mmol, 20.0 mol%), Et_3N (1.20 mL, 8.40 mmol, 2.00 equiv) and piperidine (0.500 mL, 4.95 mmol, 1.20 equiv) in CH_2Cl_2 (25 mL). The reaction mixture was stirred for 16 h at room temperature. Purification by flash column chromatography on silica gel (CH_2Cl_2/MeOH = 95:5) yielded **11o** as yellow solid (0.780 g, 3.60 mmol, 87%).

Mp = 102 °C (CH_2Cl_2).

R*f* = 0.30 (CH_2Cl_2/MeOH = 95:5).

^{1}H NMR (600 MHz, $CDCl_3$): δ = 8.73 (s, 1H, H-11), 8.54 (d, $^{3}J_{10,9}$ = 4.9 Hz, 1H, H-10), 7.79 (d, $^{3}J_{8,9}$ = 7.8 Hz, 1H, H-8), 7.60 (d, $^{3}J_{6,5}$ = 15.5 Hz, 1H, H-6), 7.29 (dd, $^{3}J_{9,8}$ = 8.2 Hz, $^{3}J_{9,10}$ = 5.1 Hz, 1H, H-9), 6.96 (d, $^{3}J_{5,6}$ = 15.5 Hz, 1H, H-5), 3.66 (br s, 2H, H-3), 3.57 (br s, 2H, H-3’), 1.67 (br s, 2H, H-2), 1.61 (br s, 4H, H-2’, H-1) ppm.

^{13}C NMR (151 MHz, $CDCl_3$): δ = 164.7 (C-4), 150.3 (C-10), 149.3 (C-11), 138.6 (C-6), 134.3 (C-8), 131.3 (C-7), 123.7 (C-9), 120.0 (C-5), 47.2 (C-3), 43.5 (C-3’), 26.9 (C-2), 25.7 (C-2’), 24.7 (C-1) ppm.

HRMS (ESI) for $C_{13}H_{17}N_2O^+$ [$(M+H^+)^+$] calculated: 217.1335, found: 217.1332.

IR (ATR): ṽ = 2937 (w), 2859 (w), 1644 (s), 1595 (s), 1423 (m), 1297 (w), 1244 (w), 1215 (w), 1136 (w), 1021 (w), 998 (m), 797 (m), 719 (m) cm^{-1}.

7.18.15 (*E*)-3-(Furan-2-yl)-1-(piperidin-1-yl)prop-2-en-1-one (11p)

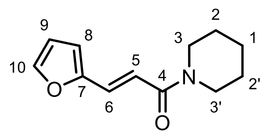

11p
$C_{12}H_{15}NO_2$
Mw = 205.26 gmol^{-1}

Prepared according to **GP8** from ethyl (*E*)-3-(furan-2-yl)prop-2-enoic acid (**S11p**, 690 mg, 5.00 mmol, 1.00 equiv), EDC·HCl (1.15 g, 6.00 mmol, 1.20 equiv), DMAP (122 mg, 1.00 mmol, 20.0 mol%), Et_3N (1.40 mL, 10.0 mmol, 2.00 equiv) and piperidine (0.600 mL, 6.00 mmol, 1.20 equiv) in CH_2Cl_2 (25 mL). The reaction mixture was stirred for 16 h at room temperature. Purification by flash column chromatography on silica gel (cyclohexane/EtOAc = 2:1) yielded **11p** as brown solid (0.800 g, 3.90 mmol, 78%).

Mp = 73 °C (cyclohexane).

R*f* = 0.25 (cyclohexane/EtOAc = 2:1).

^{1}H NMR (600 MHz, $CDCl_3$): δ = 7.44–7.41 (m, 2H, H-6, H-10), 6.82 (d, $^3J_{5,6}$ = 15.1 Hz, 1H, H-5), 6.51 (d, $^3J_{8,9}$ = 3.4 Hz, 1H, H-8), 6.44–6.43 (m, 1H, H-9), 3.64 (br s, 2H, H-3), 3.57 (br s, 2H, H-3’), 1.66–1.65 (m, 2H, H-2), 1.59 (br s, 4H, H-2’, H-1) ppm.

^{13}C NMR (151 MHz, $CDCl_3$): δ = 165.2 (C-4), 152.0 (C-7), 143.8 (C-6), 129.3 (C-10), 115.3 (C-5), 113.5 (C-8), 112.2 (C-9), 47.1 (C-3), 43.5 (C-3’), 26.9 (C-2), 25.8 (C-2’), 24.8 (C-1) ppm.

HRMS (ESI) for $C_{12}H_{16}NO_2^+$ [(M+H$^+$)$^+$] calculated: 206.1176, found: 206.1179.

IR (ATR): ṽ = 3097 (w), 3004 (w), 2937 (w), 2862 (w), 2735 (w), 1640 (s), 1584 (s), 1439 (m), 1278 (w), 1249 (w), 1140 (w), 1013 (m), 980 (w), 883 (w), 850 (m), 749 (m), 716 (m) cm^{-1}.

7.18.16 3,3-Diphenyl-1-(piperidin-1-yl)prop-2-en-1-one (11q)

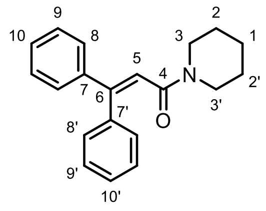

11q
$C_{20}H_{21}NO$
Mw = 291.40 gmol^{-1}

Prepared according to **GP8** from ethyl 3,3-diphenylprop-2-enoic acid (**S11q**, 1.12 g, 5.00 mmol, 1.00 equiv), EDC·HCl (1.15 g, 6.00 mmol, 1.20 equiv), DMAP (122 mg, 1.00 mmol, 20.0 mol%), Et_3N (1.40 mL, 10.0 mmol, 2.00 equiv) and piperidine (0.600 mL, 6.00 mmol, 1.20 equiv) in CH_2Cl_2 (25 mL). The reaction mixture was stirred for 16 h at room temperature. Purification by flash column chromatography on silica gel (cyclohexane/EtOAc = 2:1) yielded **11q** as colorless solid (0.991 g, 3.40 mmol, 68%).

Mp = 120 °C (cyclohexane).

R*f* = 0.30 (cyclohexane/EtOAc = 3:1).

^{1}H NMR (600 MHz, $CDCl_3$): δ = 7.34–7.31 (m, 6H, H-8, H-8’, H-10, H-10’), 7.30–7.27 (m, 4H, H-9, H-9’), 6.32 (s, 1H, H-5), 3.46 (t, $^3J_{3,2}$ = 5.5 Hz, 2H, H-3), 3.23 (t, $^3J_{3',2'}$ = 5.6 Hz, 2H, H-3’), 1.46–1.42 (m, 2H, H-2), 1.40–1.37 (m, 2H, H-2’), 1.05–1.01 (m, 2H, H-1) ppm.

^{13}C NMR (151 MHz, $CDCl_3$): δ = 167.0 (C-4), 146.5 (C-6), 141.4 (C-7)*, 139.1 (C-7')*, 129.7 (C-8)**, 128.5 (C-10)***, 128.4 (C-10')***, 128.4 (C-8')**, 128.3 (C-9)**, 128.3 (C-9')**, 121.7 (C-5), 47.4 (C-3), 42.1 (C-3'), 25.9 (C-2), 25.2 (C-2'), 24.5 (C-1) ppm.

HRMS (ESI) for $C_{20}H_{22}NO^+$ [(M+H$^+$)$^+$] calculated: 292.1696, found: 292.1691.

IR (ATR): ṽ = 2855 (w), 1591 (s), 1435 (m), 1263 (w), 1025 (w), 973 (w), 846 (w) cm^{-1}.

7.18.17 (*E*)-3-Phenyl-1-(piperidin-1-yl)hept-2-en-1-one (11r)

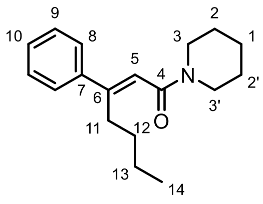

11r
$C_{18}H_{25}NO$
Mw = 271.40 gmol^{-1}

Prepared according to **GP8** from ethyl (*E*)-3-phenylhept-2-enoic acid (**S11r**, 1.02 g, 5.00 mmol, 1.00 equiv), EDC·HCl (1.15 g, 6.00 mmol, 1.20 equiv), DMAP (122 mg, 1.00 mmol, 20.0 mol%), Et_3N (1.40 mL, 10.0 mmol, 2.00 equiv) and piperidine (0.600 mL, 6.00 mmol, 1.20 equiv) in CH_2Cl_2 (25 mL). The reaction mixture was stirred for 16 h at room temperature. Purification by flash column chromatography on silica gel (cyclohexane/EtOAc = 3:1) yielded **11r** as colorless oil (0.990 g, 3.65 mmol, 73%).

R*f* = 0.30 (cyclohexane/EtOAc = 3:1).

^{1}H NMR (600 MHz, $CDCl_3$): δ = 7.32–7.26 (m, 5H, H-8, H-9, H-10), 5.87 (s, 1H, H-5), 3.39 (t, $^3J_{3,2}$ = 5.5 Hz, 2H, H-3), 3.09 (t, $^3J_{3',2'}$ = 5.6 Hz, 2H, H-3'), 2.47 (t, $^3J_{11,12}$ = 7.8 Hz, 2H, H-11), 1.40–1.32 (m, 8H, H-2, H-2', H-1, H-12), 0.90 (p, $^3J_{13,12/14}$ = 5.9 Hz, 2H, H-13), 0.87 (t, $^3J_{14,13}$ = 7.1 Hz, 3H, H-14) ppm.

^{13}C NMR (151 MHz, $CDCl_3$): δ = 167.7 (C-4), 146.5 (C-6), 139.8 (C-7), 128.3 (C-8), 127.9 (C-10), 127.7 (C-9), 120.3 (C-5), 47.4 (C-3), 42.0 (C-3'), 37.5 (C-11), 30.2 (C-2), 25.7 (C-2'), 25.1 (C-12), 24.4 (C-13), 22.5 (C-1), 14.0 (C-14) ppm.

HRMS (ESI) for $C_{18}H_{26}NO^+$ [(M+H$^+$)$^+$] calculated: 272.2009, found: 272.2004.

IR (ATR): ṽ = 2933 (w), 2855 (w), 1610 (s), 1439 (m), 1371 (w), 1259 (m), 1233 (m), 1029 (w), 969 (w), 850 (w), 768 (w), 701 (m) cm^{-1}.

7.18.18 (*E*)-*N*,*N*-Diethyl-3-phenylbut-2-enamide (11s)

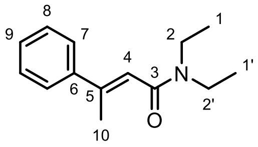

11s
$C_{14}H_{19}NO$
Mw = 217.32 gmol^{-1}

Prepared according to **GP8** from (*E*)-3-phenylbut-2-enoic acid (**S11s**, 810 mg, 5.00 mmol, 1.00 equiv), EDC·HCl (1.15 g, 6.00 mmol, 1.20 equiv), DMAP (122 mg, 1.00 mmol, 20.0 mol%), Et_3N (1.40 mL, 10.0 mmol, 2.00 equiv) and piperidine (0.600 mL, 6.00 mmol, 1.20 equiv) in CH_2Cl_2 (25 mL). The reaction mixture was stirred for 16 h at room temperature. Purification by flash column chromatography on silica gel (cyclohexane/EtOAc = 3:1) yielded **11s** as colorless solid (0.740 g, 3.48 mmol, 68%).

Mp = 56 °C (cyclohexane).

R$_f$ = 0.30 (cyclohexane/EtOAc = 3:1).

^{1}H NMR (600 MHz, $CDCl_3$): δ = 7.46–7.44 (m, 2H, H-7), 7.38–7.35 (m, 2H, H-8), 7.35–7.30 (m, 1H, H-9), 6.29 (d, $^4J_{4,10}$ = 1.3 Hz, 1H, H-4), 3.48 (q, $^3J_{2',1'}$ = 7.1 Hz, 2H, H-2'), 3.39 (q, $^3J_{2,1}$ = 7.1 Hz, 2H, H-2), 2.30 (d, $^4J_{10,4}$ = 1.2 Hz, 3H, H-10), 1.21–1.16 (m, 6H, H-1', H-1) ppm.

^{13}C NMR (151 MHz, $CDCl_3$): δ = 167.8 (C-3), 145.6 (C-5), 142.3 (C-6), 128.6 (C-7), 128.1 (C-9), 126.1 (C-8), 120.4 (C-4), 42.7 (C-2), 39.7 (C-2'), 18.0 (C-10), 14.5 (C-1'), 13.3 (C-1) ppm.

HRMS (ESI) for $C_{14}H_{20}NO^+$ [(M+H$^+$)$^+$] calculated: 218.1539, found: 218.1543.

IR (ATR): ṽ = 2967 (w), 2926 (w), 2859 (w), 1595 (s), 1453 (m), 1267 (w), 1226 (w), 1114 (s), 1043 (w), 972 (m), 760 (s), 700 (m) cm^{-1}.

The data is in accordance with literature.[51]

7.18.19 (*E*)-3-Phenyl-1-(3-(trifluoromethyl)-5,6-dihydro-[1,2,4]triazolo[4,3-*a*]pyrazin-7(8*H*)-yl)but-2-en-1-one (11t)

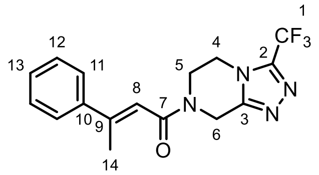

11t

$C_{16}H_{15}F_3N_4O$

Mw = 336.32 gmol^{-1}

Prepared according to **GP8** from (*E*)-3-phenylbut-2-enoic acid (**S11s**, 324 mg, 2.00 mmol, 1.00 equiv), EDC·HCl (460 mg, 2.40 mmol, 1.20 equiv), DMAP (49.0 mg, 0.400 mmol, 20.0 mol%), Et_3N (0.840 mL, 6.00 mmol, 3.00 equiv) and 3-(trifluoromethyl)-5,6,7,8-tetrahydro-[1,2,4]triazolo[4,3-*a*]pyrazine hydrochloride (0.590 g, 2.40 mmol, 1.20 equiv) in CH_2Cl_2 (10 mL). The reaction mixture was stirred for 16 h at room temperature. Purification by flash column chromatography on silica gel (EtOAc/MeOH = 95:5) yielded **11t** as colorless oil (0.560 g, 1.66 mmol, 83%).

R$_f$ = 0.20 (EtOAc/MeOH = 95:5).

^{1}H NMR (600 MHz, $CDCl_3$): δ = 7.46–7.44 (m, 2H, H-11), 7.41–7.36 (m, 3H, H-12, H-13), 6.29 (d, $^4J_{8,14}$ = 1.3 Hz, 1H, H-8), 5.07–5.06 (m, 2H, H-6), 4.21 (t, $^3J_{4,5}$ = 5.3 Hz, 2H, H-4), 4.17–4.06 (m, 2H, H-5), 2.36 (s, 3H, H-14) ppm.

^{13}C NMR (151 MHz, $CDCl_3$): δ = 167.2 (C-7), 151.5 (C-3), 149.9 (C-2), 141.4 (C-9), 129.2 (C-13), 128.8 (C-11), 126.2 (C-12), 121.1 (C-10), 118.4 (q, $^3J_{1,F}$ = 270.8 Hz, C-1), 117.0 (C-8), 43.5 (C-6), 43.3 (C-4), 38.1 (C-5), 18.5 (C-14) ppm.

^{19}F NMR (473 MHz, $CDCl_3$): δ = –63.0 (s) ppm.

HRMS (ESI) for $C_{16}H_{16}F_3N_3O^+$ [(M+H$^+$)$^+$] calculated: 337.1271, found: 337.1270.

IR (ATR): ṽ = 3026 (w), 2922 (w), 1640 (s), 1498 (w), 1420 (w), 1274 (w), 1211 (w), 1129 (s), 1013 (w), 939 (w), 756 (w), 693 (w) cm^{-1}.

7.18.20 Ethyl (*E*)-4-(3-phenylbut-2-enoyl)-3,4-dihydro-2*H*-benzo[*b*][1,4]oxazine-2-carboxylate (11u)

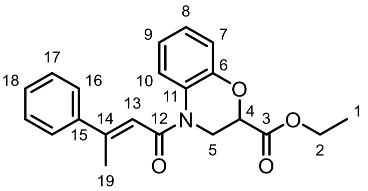

11u
$C_{21}H_{21}NO_4$
Mw = 351.53 gmol^{-1}

Prepared according to **GP8** from (*E*)-3-phenylbut-2-enoic acid (**S11s**, 324 mg, 2.00 mmol, 1.00 equiv), EDC·HCl (460 mg, 2.40 mmol, 1.20 equiv), DMAP (49.0 mg, 0.400 mmol, 20.0 mol%), Et_3N (0.560 mL, 4.00 mmol, 2.00 equiv) and ethyl 3,4-dihydro-2*H*-benzo[*b*][1,4]oxazine-2-carboxylate (0.420 g, 2.40 mmol, 1.20 equiv) in CH_2Cl_2 (10 mL). The reaction mixture was stirred for 16 h at room temperature. Purification by flash column chromatography on silica gel (cyclohexane/EtOAc = 5:1) yielded **11u** as yellow oil (0.400 g, 1.14 mmol, 57%).

$\mathbf{R_f}$ = 0.20 (cyclohexane/EtOAc = 5:1).

^{1}H NMR (600 MHz, $CDCl_3$): δ = 7.42 (d, $^3J_{16,17}$ = 7.2 Hz, 2H, H-16), 7.36–7.30 (m, 3H, H-17, H-18), 7.30–7.26 (m, 1H, H-10), 7.11 (t, $^3J_{8,9/7}$ = 7.4 Hz, 1H, H-8), 7.07 (d, $^3J_{7,8}$ = 8.2 Hz, 1H, H-7), 6.91 (t, $^3J_{9,8/10}$ = 7.7 Hz, 1H, H-9), 6.44 (s, 1H, H-13), 4.95 (t, $^3J_{4,5}$ = 4.0 Hz, 1H, H-4), 4.61 (d, $^2J_{5a,5b}$ = 12.8 Hz, 1H, H-5a), 4.21 (q, $^3J_{2,1}$ = 7.1 Hz, 2H, H-2), 3.87 (dd, $^2J_{5b,5a}$ = 13.4 Hz, $^3J_{5b,4}$ = 3.6 Hz, 1H, H-5b), 2.51 (s, 3H, H-19), 1.26 (t, $^3J_{1,2}$ = 7.1 Hz, 3H, H-1) ppm.

^{13}C NMR (151 MHz, $CDCl_3$): δ = 168.8 (C-3), 165.6 (C-12), 145.7 (C-14), 142.0 (C-15), 128.9 (C-18), 128.8 (C-11), 128.7 (C-16), 126.5 (C-8), 126.4 (C-6), 126.3 (C-17), 124.1 (C-10), 120.7 (C-9), 119.2 (C-13), 117.5 (C-7), 74.0 (C-4), 62.1 (C-2), 40.8 (C-5), 18.2 (C-19), 14.2 (C-1) ppm.

C-12 and C-6 were assigned from ^{1}H,^{13}C HMBC and C-5 was assigned from ^{1}H,^{13}C HSQC NMR.

HRMS (ESI) for $C_{21}H_{22}NO_4^+$ [(M+H$^+$)$^+$] calculated: 352.1543, found: 352.1540.

IR (ATR): $\tilde{\nu}$ = 2982 (w), 2930 (w), 2855 (w), 1751 (s), 1651 (s), 1495 (s), 1364 (w), 1244 (w), 1185 (w), 1125 (w), 1092 (w), 753 (w), 701 (w) cm^{-1}.

7.18.21 (*E*)-*N*,*N*-Diethyl-1-(3-phenylbut-2-enoyl)piperidine-3-carboxamide (11v)

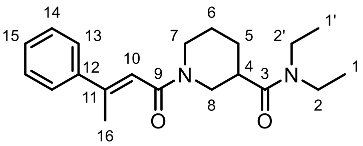

11v
$C_{20}H_{28}N_2O_2$
Mw = 328.46 gmol^{-1}

Prepared according to **GP8** from (*E*)-3-phenylbut-2-enoic acid (**S11s**, 324 mg, 2.00 mmol, 1.00 equiv), EDC·HCl (460 mg, 2.40 mmol, 1.20 equiv), DMAP (49.0 mg, 0.400 mmol, 20.0 mol%), Et_3N (0.560 mL, 4.00 mmol, 2.00 equiv) and ethyl 3,4-dihydro-2*H*-benzo[*b*][1,4]oxazine-2-carboxylate (0.422 g, 2.40 mmol, 1.20 equiv) in CH_2Cl_2 (10 mL). The reaction mixture was stirred for 16 h at room temperature. Purification by flash column chromatography on silica gel (cyclohexane/EtOAc = 0:100) yielded **11v** and **11v_rotamer** (60:40 ratio) as yellow oil (0.430 g, 1.31 mmol, 65%).

R_f = 0.20 (cyclohexane/EtOAc = 0:100).

^{1}H NMR (600 MHz, $CDCl_3$): δ = 7.46–7.42 (m, 2H, H-13), 7.38–7.31 (m, 3H, H-14, H-15), 6.28–6.27 (m, 1H, H-10), 4.73–4.70 (m, 1H, H-8a), 3.99–3.94 (m, 1H, H-8b), 3.51–3.19 (m, 5H, H-7, H-2, H-2'a*), 2.83–2.50 (m, 2H, H-2'b, H-4)*, 2.27–2.25 (m, 3H, H-16), 1.94–1.78 (m, 3H, H-5, H-6a), 1.57–1.42 (m, 1H, H-6b), 1.24–1.07 (m, 6H, H-1, H-1') ppm.

The ^{1}H NMR spectrum is not well separated for the two rotamers, which is why only ^{13}C NMR is assigned separately.

^{13}C NMR (151 MHz, $CDCl_3$): δ = 172.2 (C-3), 167.4 (C-9), 145.5 (C-11), 141.8 (C-12), 128.6 (C-13, C-14), 126.1 (C-15), 119.9 (C-10), 47.1 (C-8), 44.8 (C-2), 41.9 (C-7), 40.5 (C-2'), 40.3 (C-4), 28.7 (C-6), 25.9 (C-16), 18.1 (C-5), 15.1 (C-1), 13.2 (C-1') ppm.

^{13}C NMR for 11v_rotamer (151 MHz, $CDCl_3$): δ = 172.2 (C-3), 167.3 (C-9), 144.9 (C-11), 141.6 (C-12), 128.4 (C-13, C-14), 126.0 (C-15), 120.2 (C-10), 49.4 (C-8), 42.1 (C-2), 41.9 (C-7), 40.3 (C-2'), 39.4 (C-4), 28.0 (C-6), 24.8 (C-16), 18.0 (C-5), 15.1 (C-1), 13.2 (C-1') ppm.

Rotamer ratio is determined from the integration value of H-10 at 6.28–6.27 ppm.

HRMS (ESI) for $C_{20}H_{29}N_2O_2^+$ [$(M+H^+)^+$] calculated: 329.2224, found: 329.2224.

IR (ATR): ṽ = 2937 (w), 2862 (w), 1636 (s), 1423 (s), 1297 (w), 1244 (w), 1215 (w), 1140 (w), 991 (w), 798 (w), 768 (w), 693 (w) cm^{-1}.

7.18.22 Ethyl (*E*)-(3-phenylbut-2-enoyl)-L-prolinate (11w)

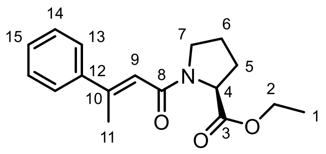

11w
$C_{17}H_{21}NO_3$
Mw = 287.36 gmol^{-1}

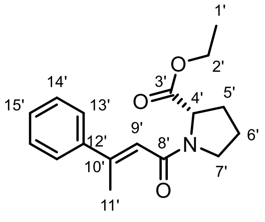

11w_rotamer
$C_{17}H_{21}NO_3$
Mw = 287.36 gmol^{-1}

Prepared according to **GP8** from (*E*)-3-phenylbut-2-enoic acid (**S11s**, 324 mg, 2.00 mmol, 1.00 equiv), EDC·HCl (460 mg, 2.40 mmol, 1.20 equiv), DMAP (49.0 mg, 0.400 mmol, 20.0 mol%), Et_3N (0.840 mL, 6.00 mmol, 3.00 equiv) and ethyl *L*-prolinate hydrochloride (0.431 g, 2.40 mmol, 1.20 equiv) in CH_2Cl_2 (10 mL). The reaction mixture was stirred for 16 h at room temperature. Purification by flash column chromatography on silica gel (cyclohexane/EtOAc = 3:2) yielded **11w** and **11w_rotamer** (77:23 ratio) as colorless soild (0.322 g, 1.12 mmol, 56%).

M.p. = 87 °C (cyclohexane).

R_f = 0.20 (cyclohexane/EtOAc = 3:2).

^{1}H NMR (600 MHz, $CDCl_3$): δ = 7.42–7.40 (m, 2H, H-13), 7.33–7.26 (m, 3H, H-14, H-15), 6.24 (s, 1H, H-9), 4.52 (dd, $^3J_{4,5a}$ = 8.5 Hz, $^3J_{4,5b}$ = 4.3 Hz, 1H, H-4), 4.17 (q, $^3J_{2,1}$ = 7.1 Hz, 2H, H-2),

3.56–3.37 (m, 2H, H-7), 2.42 (s, 3H, H-11), 2.31–1.86 (m, 4H, H-5, H-6), 1.25 (t, $^{3}J_{1,2}$ = 7.1 Hz, 3H, H-1) ppm.

^{13}C NMR (151 MHz, $CDCl_3$): δ = 172.4 (C-3), 166.3 (C-8), 149.8 (C-10), 142.6 (C-12), 128.4 (C-13, C-14), 126.1 (C-15), 118.9 (C-9), 61.0 (C-2), 58.7 (C-4), 47.4 (C-7), 29.3 (C-5), 24.9 (C-6), 18.0 (C-11), 14.2 (C-1) ppm.

The ^{1}H and ^{13}C NMR spectrum is well separated for the two rotamers, which is why both ^{1}H and ^{13}C NMR are assigned separately.

11w_rotamer:

^{1}H NMR (600 MHz, $CDCl_3$): δ = 7.36–7.35 (m, 2H, H-13'), 7.33–7.26 (m, 3H, H-14', H-15'), 6.09 (s, 1H, H-9'), 4.41 (dd, $^{3}J_{4',5a'}$ = 8.6 Hz, $^{3}J_{4',5b'}$ = 3.3 Hz, 1H, H-4'), 4.12–4.06 (m, 2H, H-2'), 3.56–3.37 (m, 2H, H-7'), 2.36 (s, 3H, H-11'), 2.31–1.86 (m, 4H, H-5', H-6'), 1.14 (t, $^{3}J_{1,2}$ = 7.1 Hz, 3H, H-1') ppm.

^{13}C NMR (151 MHz, $CDCl_3$): δ = 172.4 (C-3'), 166.8 (C-8'), 149.0 (C-10'), 142.2 (C-12'), 128.4 (C-13', C-14'), 126.0 (C-15'), 119.3 (C-9'), 61.4 (C-2'), 59.8 (C-4'), 46.2 (C-7'), 31.4 (C-5'), 22.8 (C-6'), 17.9 (C-11'), 14.0 (C-1') ppm.

Rotamer ratio is determined from the integration value of H-9 at 6.24 and 6.09 ppm.

HRMS (ESI) for $C_{17}H_{22}NO_3^+$ [(M+H$^+$)$^+$] calculated: 288.1594, found: 288.1589.

IR (ATR): ṽ = 2978 (w), 1773 (s), 1668 (m), 1453 (m), 1408 (m), 1330 (w), 1263 (w), 1151 (w), 1095 (w), 1032 (w), 861 (w), 801 (w), 767 (w), 704 (w) cm^{-1}.

7.18.23 Ethyl *(E)*-*N*-methyl-*N*-(3-phenylbut-2-enoyl)-*L*-alaninate (11x)

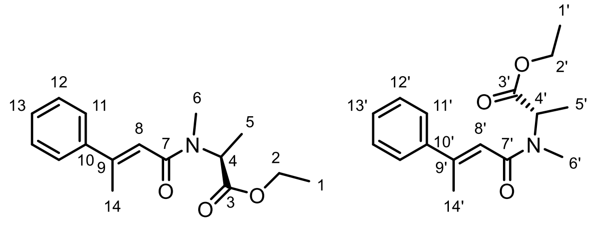

11x
$C_{16}H_{21}NO_3$
Mw = 275.35 gmol^{-1}

11x_rotamer
$C_{16}H_{21}NO_3$
Mw = 275.35 gmol^{-1}

Prepared according to **GP8** from (*E*)-3-phenylbut-2-enoic acid (**S11s**, 324 mg, 2.00 mmol, 1.00 equiv), EDC·HCl (460 mg, 2.40 mmol, 1.20 equiv), DMAP (49.0 mg, 0.400 mmol, 20.0 mol%), Et_3N (0.840 mL, 6.00 mmol, 3.00 equiv) and ethyl methyl-*L*-alaninate hydrochloride (0.402 g, 2.40 mmol, 1.20 equiv) in CH_2Cl_2 (10 mL). The reaction mixture was stirred for 16 h at room temperature. Purification by flash column chromatography on silica gel (cyclohexane/EtOAc = 3:2) yielded **11x** and **11x_rotamer** (70:30 ratio) as yellow oil (0.438 g, 1.59 mmol, 80%).

R*f* = 0.15 (cyclohexane/EtOAc = 4:1).

^{1}H NMR (600 MHz, $CDCl_3$): δ = 7.47–7.44 (m, 2H, H-11), 7.38–7.31 (m, 3H, H-12, H-13), 6.30 (q, $^{4}J_{8,14}$ = 1.2 Hz, 1H, H-8), 5.31 (q, $^{3}J_{4,5}$ = 7.3 Hz, 1H, H-4), 4.24–4.16 (m, 2H, H-2), 3.02 (s, 3H,

H-6),, 2.31 (d, $^4J_{14,8}$ = 1.2 Hz, 3H, H-14), 1.46 (d, $^3J_{5,4}$ = 7.3 Hz, 3H, H-5), 1.29 (t, $^3J_{1,2}$ = 7.1 Hz, 3H, H-1) ppm.

^{13}C NMR (151 MHz, $CDCl_3$): δ = 172.0 (C-3), 168.9 (C-7), 146.4 (C-9), 142.0 (C-10), 128.6 (C-11), 128.5 (C-12), 126.1 (C-13), 119.8 (C-8), 61.3 (C-2), 51.9 (C-4), 32.1 (C-6), 18.0 (C-14), 14.6 (C-5), 14.4 (C-1) ppm.

The ^{1}H and ^{13}C NMR spectrum is well separated for the two rotamers, which is why both ^{1}H and ^{13}C NMR are assigned separately.

11x_rotamer:

^{1}H NMR (600 MHz, $CDCl_3$): δ = 7.47–7.44 (m, 2H, H-11'), 7.38–7.31 (m, 3H, H-12', H-13'), 6.27 (q, $^4J_{8',14'}$ = 1.2 Hz, 1H, H-8'), 4.73 (q, $^3J_{4',5'}$ = 7.1 Hz, 1H, H-4'), 4.24–4.16 (m, 2H, H-2'), 2.93 (s, 3H, H-6'), 2.25 (d, $^4J_{14',8'}$ = 1.2 Hz, 3H, H-14'), 1.45 (d, $^3J_{5',4'}$ = 7.3 Hz, 3H, H-5'), 1.27 (t, $^3J_{1',2'}$ = 7.1 Hz, 3H, H-1') ppm.

^{13}C NMR (151 MHz, $CDCl_3$): δ = 171.3 (C-3'), 169.4 (C-7'), 145.5 (C-9'), 141.4 (C-10'), 128.6 (C-11'), 128.5 (C-12'), 126.1 (C-13'), 119.9 (C-8'), 61.7 (C-2'), 56.3 (C-4'), 28.4 (C-6'), 18.1 (C-14'), 15.6 (C-5'), 14.3 (C-1') ppm.

Rotamer ratio is determined from the integration value of H-8 at 6.30 and 6.27 ppm.

HRMS (ESI) for $C_{16}H_{22}NO_3^+$ [(M+H$^+$)$^+$] calculated: 276.1594, found: 276.1589.

IR (ATR): ṽ = 2985 (w), 2940 (w), 1736 (s), 1636 (s), 1446 (w), 1394 (w), 1304 (w), 1200 (m), 1080 (m), 1021 (w), 861 (w), 760 (w), 697 (w) cm^{-1}.

7.18.24 *N*-(3,4-Dimethoxyphenethyl)-*N*-methylcinnamamide (11ba)

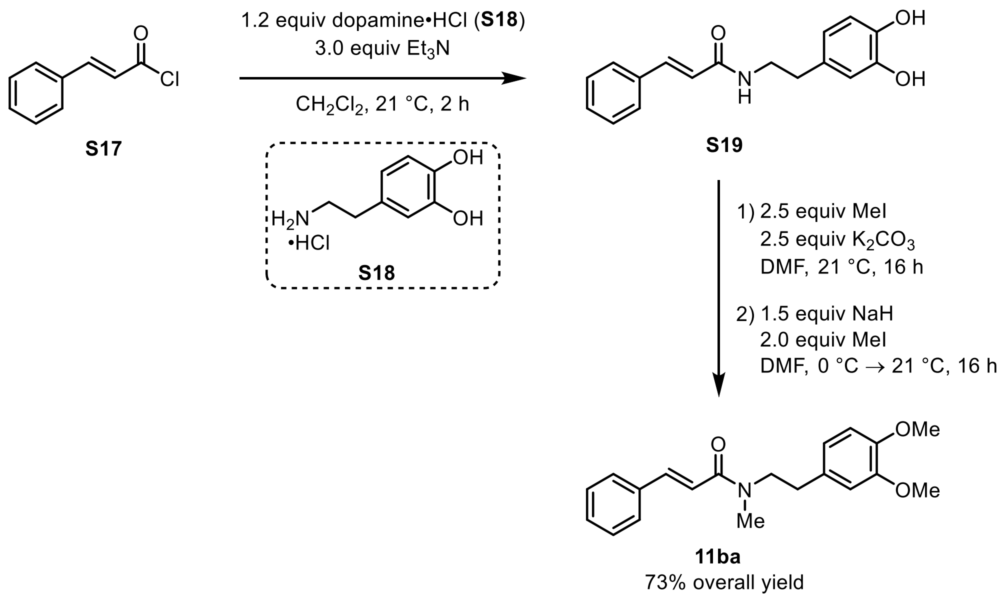

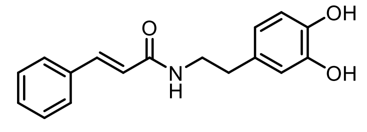

N-(3,4-dihydroxyphenethyl)cinnamamide
S19

Prepared according to **GP9** from cinnamyl chloride (**S17**, 0.830 g, 5.00 mmol, 1.00 equiv), Et_3N (2.10 mL, 15.0 mmol, 3.00 equiv) and 4-(2-aminoethyl)benzene-1,2-diol hydrochloride or dopamine·HCl (**S18**, 1.14 g, 6.00 mmol, 1.20 equiv) in CH_2Cl_2 (50 mL). The reaction mixture was stirred for 16 h at room temperature. Purification by flash column chromatography on silica gel (cyclohexane/EtOAc = 1:1) yielded **S19** as yellow solid (1.10 g, 3.88 mmol, 78% yield), which was used directly for the next step.

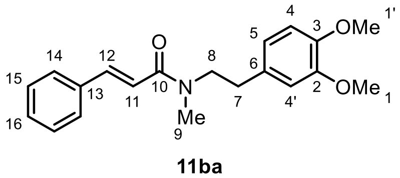

11ba
$C_{20}H_{23}NO_3$
Mw = 325.41 gmol^{-1}

In deference to a literature procedure,[52,53] a Schlenk tube equipped with a magnetic stir bar was charged with *N*-(3,4-dihydroxyphenethyl)cinnamamide (**S19**, 1.10 g, 3.88 mmol, 1.00 equiv), K_2CO_3 (1.10 g, 7.76 mmol, 2.00 equiv) and DMF (15 mL). Methyl iodide (0.600 mL, 9.70 mmol, 2.50 equiv) was added and the reaction mixture was stirred at room temperature for 16 h. After completion (monitored *via* TLC, EtOAc/cyclohexane = 1:1), the reaction mixture was filtered through glass frit (P4). The filtrate was then transferred into a 25 mL Schlenk flask and the reaction mixture was cooled to 0 °C. NaH (233 mg, 5.82 mmol, 1.50 equiv) was added portion wise over 10 min at 0 °C. The reaction mixture was stirred at the same temperature for additional 30 min. Methyl iodide (0.630 mL, 10.0 mmol, 2.00 equiv) was added and the reaction mixture was allowed to warm to room temperature and stirred for 16 h at room temperature. The reaction mixture was quenched by dropwise addition of water (10 mL) and diluted with EtOAc (50 mL). The organic layer was separated and the aqueous phase was extracted with EtOAc (3 × 20 mL). The combined organic layer was washed with brine (3 × 20 mL) and dried over $MgSO_4$. All volatiles were removed under reduced pressure. The crude product was purified by flash column chromatography on silica gel (cyclohexane/EtOAc = 1:1) to afford **11ba** and **11ba_rotamer** (50:50 rotamer ratio) as yellow solid (0.800 g, 2.72 mmol, 70% yield after 2 steps).

R*f* = 0.25 (cyclohexane/EtOAc = 1:1).

^{1}H NMR (600 MHz, $CDCl_3$): δ = 7.61–7.22 (m, 6H, H-12, H-14, H-15; H-16), 6.77–6.42 (m, 4H, H-4, H-4’, H-5, H-11), 3.77–3.66 (m, 6H, H-1, H-1’), 3.60–3.55 (m, 2H, H-8), 2.95 (s, 3H, H-9), 2.78–2.74 (m, 2H, H-7) ppm.

^{1}H NMR shows impurities of ethyl acetate at 4.12, 2.05 and 1.26 ppm.

The ^{1}H NMR spectrum is not well separated for the two rotamers, which is why only ^{13}C NMR is assigned separately.

^{13}C NMR (151 MHz, $CDCl_3$): δ = 167.0 (C-10), 149.3 (C-2), 148.1 (C-3), 142.7 (C-12), 141.7, 135.5 (C-13), 131.9 (C-6), 129.7 (C-16), 128.9 (C-15)*, 127.8 (C-14)*, 120.9 (C-11), 117.7 (C-4), 112.2 (C-4’), 111.7 (C-5), 56.0 (C-1, C-1’), 52.2 (C-8), 36.5 (C-9), 34.5 (C-7) ppm.

^{13}**C NMR** of **11ba_rotame**r (151 MHz, $CDCl_3$): δ = 166.4 (C-9), 149.1 (C-2), 147.7 (C-3), 141.8 (C-12), 135.5 (C-13), 130.9 (C-6), 129.5 (C-16), 128.9 (C-15)*, 127.8 (C-14), 120.9 (C-11), 117.7 (C-4), 112.2 (C-4'), 111.4 (C-5), 56.0 (C-1, C-1'), 50.9 (C-8), 35.1 (C-9), 33.5 (C-7) ppm.

^{13}C NMR shows impurities of ethyl acetate at 171.3, 21.2 and 14.4 ppm.

HRMS (ESI) for $C_{20}H_{24}NO_3^+$ [(M+H$^+$)$^+$] calculated: 326.1751, found: 326.1748.

IR (ATR): ṽ = 2926 (w), 2855 (w), 1651 (w), 1610 (w), 1502 (w), 1461 (w), 1375 (w), 1319 (w), 1244 (w), 1185 (w), 1114 (w), 1066 (w), 995 (w), 905 (w), 816 (w), 745 (w) cm^{-1}.

7.18.25 (*E*)-3-(4-(*tert*-Butyl)phenyl)-*N*-(2,3-dihydrobenzo[*b*][1,4]dioxin-6-yl)-*N*-methylacrylamide (11bc)

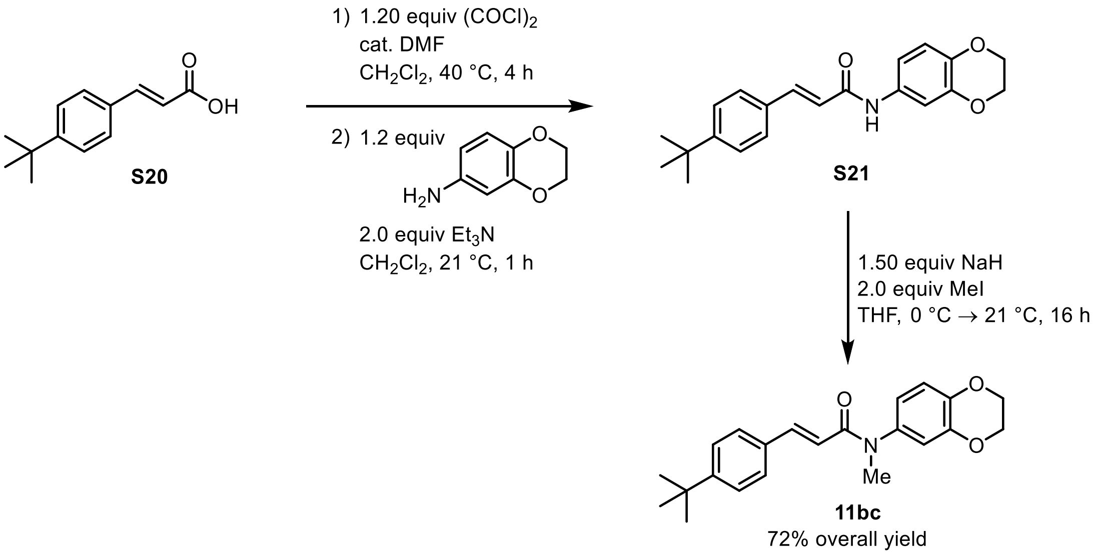

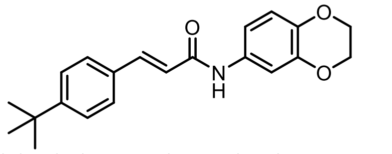

(*E*)-3-(4-(*tert*-butyl)phenyl)-*N*-(2,3-dihydrobenzo[*b*][1,4]dioxin-6-yl)acrylamide

S21

In deference to a literature procedure,[54] a Schlenk tube equipped with a magnetic stir bar was charged with (*E*)-3-(4-(*tert*-butyl)phenyl)acrylic acid (**S20**, 0.810 g, 5.00 mmol, 1.00 equiv), DMF (1 drop) and CH_2Cl_2 (25 mL). Oxalyl chloride (480 µL, 6.00 mmol, 1.20 equiv) was added at room temperature and the reaction mixture was stirred for 4 h at 40 °C. The reaction mixture was allowed to cool to room temperature. All volatiles were removed under reduced pressure. The crude acid chloride was dissolved in CH_2Cl_2 (25 mL). 2,3-Dihydrobenzo[*b*][1,4]dioxin-6-amine (1.15 g, 6.00 mmol, 1.20 equiv) and Et_3N (1.20 mL, 10.0 mmol, 2.00 equiv) were added at room temperature and the reaction mixture was stirred at room temperature for 1 h. All volatiles were removed under reduced pressure. The crude product was purified by flash column chromatography on silica gel (cyclohexane/EtOAc = 1:1) to afford **S21** as brown solid, which was directly used for the next step.

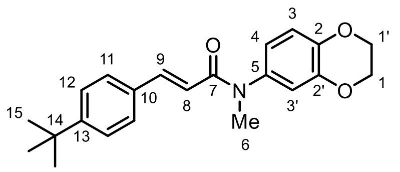

11bc
$C_{22}H_{25}NO_3$
Mw = 351.45 gmol^{-1}

In deference to a literature procedure,[55] a Schlenk tube equipped with a magnetic stir bar was charged with NaH (300 mg, 7.50 mmol, 1.50 equiv) and THF (20 mL). The reaction mixture was cooled to 0 °C. (*E*)-3-(4-(*tert*-butyl)phenyl)-*N*-(2,3-dihydrobenzo[*b*][1,4]dioxin-6-yl)acrylamide (**S21**, 1.69 g, 5.00 mmol, 1.00 equiv), dissolved in THF (10 mL) was added dropwise at 0 °C over 10 min. The reaction mixture was stirred at the same temperature for additional 30 min. Methyl iodide (0.630 mL, 10.0 mmol, 2.00 equiv) was added and the reaction mixture was allowed to warm to room temperature and stirred for 16 h. The reaction mixture was quenched by dropwise addition of water (10 mL) and diluted with EtOAc (50 mL). The organic layer was separated and the aqueous phase was extracted with EtOAc (2 × 20 mL). The combined organic layer was washed with brine (3 × 20 mL) and dried over $MgSO_4$. All volatiles were removed under reduced pressure. The crude product was purified by flash column chromatography on silica gel (cyclohexane/EtOAc = 2:1) to afford **11bc** as brown solid (1.26 g, 3.76 mmol, 72% yield after 2 steps).

R*f* = 0.30 (cyclohexane/EtOAc = 2:1).

^{1}H NMR (600 MHz, $CDCl_3$): δ = 7.64 (d, $^3J_{9,10}$ = 15.5 Hz, 1H, H-9), 7.33–7.28 (m, 4H, H-11, H-12), 6.88 (d, $^3J_{3,4}$ = 8.5 Hz, 1H, H-3), 6.75 (d, $^4J_{3',4}$ = 2.5 Hz, 1H, H-3'), 6.69 (dd, $^3J_{4,3}$ = 8.5 Hz, $^4J_{4,3'}$ = 2.5 Hz, 1H, H-4), 6.37 (d, $^3J_{10,9}$ = 15.5 Hz, 1H, H-8), 4.30 (s, 4H, H-1, H-1'), 3.34 (s, 3H, H-6), 1.29 (s, 9H, H-15) ppm.

^{13}C NMR (151 MHz, $CDCl_3$): δ = 166.6 (C-7), 153.0 (C-5), 144.0 (C-2)*, 143.1 (C-1')*, 141.5 (C-9), 137.2 (C-10), 132.8 (C-13), 127.8 (C-11)**, 125.7 (C-12)**, 120.8 (C-3'), 118.2 (C-8), 117.9 (C-3), 116.4 (C-4), 64.5 (C-1, C-1'), 37.8 (C-6), 34.9 (C-14), 31.3 (C-15) ppm.

HRMS (ESI) for $C_{22}H_{26}NO_3^+$ [(M+H$^+$)$^+$] calculated: 352.1907, found: 352.1905.

IR (ATR): ṽ = 2926 (w), 2855 (w), 1651 (w), 1610 (w), 1502 (w), 1461 (w), 1375 (w), 1319 (w), 1244 (w), 1185 (w), 1114 (w), 1066 (w), 995 (w), 905 (w), 816 (w), 745 (w) cm^{-1}.

7.18.26 (*E*)-3-(Benzo[*d*][1,3]dioxol-5-yl)-1-(piperidin-1-yl)prop-2-en-1-one (11be)

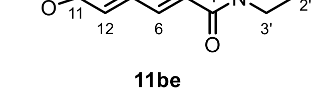

11be
$C_{15}H_{17}NO_3$
Mw = 259.30 gmol^{-1}

Prepared according to **GP8** from (*E*)-3-(benzo[*d*][1,3]dioxol-5-yl)acrylic acid (**S11be**, 384 mg, 2.00 mmol, 1.00 equiv), EDC·HCl (460 mg, 2.40 mmol, 1.20 equiv), DMAP (49.0 mg, 0.400 mmol, 20.0 mol%), Et_3N (0.560 mL, 4.00 mmol, 2.00 equiv) and piperidine (0.240 mL, 2.40 mmol, 1.20 equiv) in CH_2Cl_2 (10 mL). The reaction mixture was stirred for 16 h at room temperature. Purification by flash column chromatography on silica gel (cyclohexane/EtOAc = 1:1) yielded **11be** as yellow solid (387 mg, 1.49 mmol, 75%).

R_f = 0.20 (cyclohexane/EtOAc = 1:1).

^{1}H NMR (600 MHz, $CDCl_3$): δ = 7.56 (d, $^3J_{6,5}$ = 15.3 Hz, 1H, H-6), 7.03 (s, 1H, H-12), 6.98 (d, $^3J_{9,8}$ = 8.0 Hz, 1H, H-9), 6.79 (d, $^3J_{8,9}$ = 8.0 Hz, 1H, H-8), 6.73 (d, $^3J_{5,6}$ = 15.3 Hz, 1H, H-5), 5.98 (s, 2H, H-13), 3.65 (br s, 2H, H-3), 3.56 (br s, 2H, H-3'), 1.67–1.66 (m, 2H, H-1), 1.60–1.59 (m, 4H, H-2, H-2') ppm.

^{13}C NMR (151 MHz, $CDCl_3$): δ = 165.5 (C-4), 148.9 (C-10)*, 148.3 (C-11)*, 142.1 (C-6), 130.1 (C-7), 123.7 (C-9), 115.8 (C-5), 108.6 (C-8), 106.5 (C-12), 101.5 (C-13), 47.1 (C-3'), 43.5 (C-3), 26.9 (C-2), 25.7 (C-2'), 24.8 (C-1) ppm.

HRMS (ESI) for $C_{15}H_{18}NO_3^+$ [$(M+H^+)^+$] calculated: 260.1281, found: 260.1281.

The data is in accordance with literature.[56]

7.18.27 (2*E*,4*E*)-*N*-Isobutyl-*N*-methyldeca-2,4-dienamide (11bj)

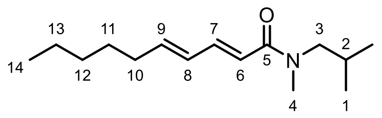

11bj
$C_{15}H_{27}NO$
Mw = 237.39 gmol^{-1}

Prepared according to **GP8** from (2*E*,4*E*)-deca-2,4-dienoic acid (**S11bj**, 336 mg, 2.00 mmol, 1.00 equiv), EDC·HCl (460 mg, 2.40 mmol, 1.20 equiv), DMAP (49.0 mg, 0.400 mmol, 20.0 mol%), Et_3N (0.840 mL, 6.00 mmol, 3.00 equiv) and *N*-methylisobutylamine (0.250 mL, 2.40 mmol, 1.20 equiv) in CH_2Cl_2 (10 mL). The reaction mixture was stirred for 16 h at room temperature. Purification by flash column chromatography on silica gel (cyclohexane/EtOAc = 1:1) yielded **11bj** and **11bj_rotamer** (60:40 ratio) as colorless oil (0.200 g, 0.840 mmol, 42%).

R_f = 0.20 (cyclohexane/EtOAc = 1:1).

^{1}H NMR (600 MHz, $CDCl_3$): δ = 7.28–7.24 (m, 1H, H-7), 6.25–6.16 (m, 2H, H-9, H-8), 6.08–6.03 (m, 1H, H-6), 3.27–3.14 (m, 2H, H-3), 3.04–2.99 (m, 3H, H-4), 2.15–2.12 (m, 2H, H-10), 1.99–1.91 (m, 1H, H-2), 1.41 (p, $^3J_{11,10/12}$ = 7.4 Hz, 2H, H-11), 1.32–1.24 (m, 4H, H-12, H-13), 0.91–0.87 (m, 9H, H-1, H-14) ppm.

The ^{1}H NMR spectrum is not well separated for the two rotamers, which is why only ^{13}C NMR is assigned separately.

^{13}C NMR (151 MHz, $CDCl_3$): δ = 167.4 (C-5), 143.2 (C-7), 142.9 (C-9), 128.9 (C-8), 118.8 (C-6), 57.6 (C-3) 36.2 (C-4), 33.1 (C-10), 28.6 (C-11), 28.5 (C-2), 28.4 (C-12), 22.6 (C-13), 20.2 (C-1), 14.1 (C-14) ppm.

^{13}C NMR of **11aj_rotamer** (151 MHz, $CDCl_3$): δ = 167.1 (C-5), 143.1 (C-7), 142.8 (C-9), 128.9 (C-8), 118.7 (C-6), 55.5 (C-3), 35.1 (C-4), 31.5 (C-10), 28.6 (C-11), 28.4 (C-12), 27.1 (C-2), 22.6 (C-13), 20.17 (C-1), 14.1 (C-14) ppm.

Rotamer ratio is determined from the integration value of H-3 at 3.27–3.14 ppm.

HRMS (ESI) for $C_{15}H_{28}NO^+$ $[(M+H^+)^+]$ calculated: 238.2165, found: 238.2166.

IR (ATR): $\tilde{\nu}$ = 2959 (w), 2929 (w), 2873 (w), 1654 (s), 1464 (m), 1397 (w), 1274 (w), 1244 (w), 1133 (w), 1084 (w), 998 (w) cm^{-1}.

7.18.28 (2*E*,4*E*)-1-(Pyrrolidin-1-yl)deca-2,4-dien-1-one (11bk)

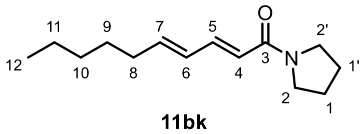

11bk
$C_{14}H_{23}NO$
Mw = 221.33 $gmol^{-1}$

Prepared according to **GP8** from (2*E*,4*E*)-deca-2,4-dienoic acid (**S11bj**, 336 mg, 2.00 mmol, 1.00 equiv), EDC·HCl (460 mg, 2.40 mmol, 1.20 equiv), DMAP (49.0 mg, 0.400 mmol, 20.0 mol%), Et_3N (0.840 mL, 6.00 mmol, 3.00 equiv) and pyrrolidine (0.200 mL, 2.40 mmol, 1.20 equiv) in CH_2Cl_2 (10 mL). The reaction mixture was stirred for 16 h at room temperature. Purification by flash column chromatography on silica gel (cyclohexane/EtOAc = 1:1) yielded **11bk** as colorless oil (0.221 g, 1.00 mmol, 50%).

$\mathbf{R_f}$ = 0.20 (cyclohexane/EtOAc = 1:1).

^{1}H NMR (600 MHz, $CDCl_3$): δ = 7.25 (dd, $^3J_{5,4}$ = 14.8 Hz, $^3J_{5,6}$ = 10.9 Hz, 1H, H-5), 6.18–6.13 (m, 1H, H-7), 6.08–6.07 (m, 1H, H-4), 6.06–6.03 (m, 1H, H-6), 3.50 (m, 4H, H-2, H-2’), 2.14–2.10 (m, 2H, H-8), 1.94 (p, $^3J_{1,1'/2}$ = 7.2 Hz, 2H, H-1), 1.84 (p, $^3J_{1',1/2'}$ = 7.2 Hz, 2H, H-1’), 1.40 (p, $^3J_{9,8/10}$ = 7.8 Hz, 2H, H-9), 1.32–1.22 (m, 4H, H-10, H-11), 0.86 (t, $^3J_{12,11}$ = 7.0 Hz, 3H, H-12) ppm.

^{13}C NMR (151 MHz, $CDCl_3$): δ = 165.3 (C-3), 143.2 (C-7), 142.2 (C-5), 128.8 (C-6), 119.9 (C-4), 46.5 (C-2)*, 45.9 (C-2’), 33.0 (C-8), 31.5 (C-9), 28.6 (C-10), 26.2 (C-1), 24.4 (C-1’), 22.6 (C-11), 14.1 (C-12) ppm.

HRMS (ESI) for $C_{14}H_{24}NO^+$ $[(M+H^+)^+]$ calculated: 222.1852, found: 222.1854.

IR (ATR): $\tilde{\nu}$ = 2926 (w), 2855 (w), 1640 (s), 1423 (m), 1341 (w), 1253 (w), 1226 (w), 1192 (w), 861 (w), 723 (w) cm^{-1}.

8 X-ray Diffraction Study of S4d

General information: Single-crystal X-ray diffraction (SC XRD) data was collected on a XtaLAB Synergy R Diffractometer with Dual-Wavelength System with Cu-K$_\alpha$ radiation (λ = 1.54184 Å) and a HyPix-Arc 150° detector. The crystal was submerged under oil, selected under a microscope, mounted on a MiTeGen MicroLoop and then immediately placed in a cold stream of N_2 on the diffractometer, where it was kept at a constant temperature of T = 123.00 K during data collection. The CrysAlisPro suite[57] was employed for data collection, data reduction and cell refinement. Using Ole4[58] as the graphical interface, the structure was solved with the ShelXT[59] solution program using dual methods and refined with ShelXL 2018/3[60] using full matrix least squares minimisation on F^2. All non-hydrogen atoms were refined anisotropically. Hydrogen atom

positions were calculated geometrically and refined using the riding model. The Figures depicting the molecular structures were generated using Diamond (v 4.6.8).

SC XRD studiy of S4d:

Colorless single crystals were obtained by gas phase diffusion of Et_2O into a solution of **S4d** (CH_2Cl_2/*n*-hexane) at –40 °C. The analysis revealed that **S4d** crystallizes in the monoclinic space group *C*2/*c*. Since *Z*' = 0.5, the asymmetric unit contains only half of the molecular formula (Figure S26) while the other half constits of symmetry-equivalent atoms (Figure S27). The Ag2 atom and the Br atom are partly occupied (Ag2: 0.4; Br: 0.9). Furthermore, the CH_2Cl_2 solvent molecule is disordered over two positions with occupancies of 0.4 and 0.1, respectively. the Br atom are partly occupied (Ag2: 0.4; Br: 0.9). Furthermore, the CH_2Cl_2 solvent molecule is disordered over two positions with occupancies of 0.4 and 0.1, respectively.

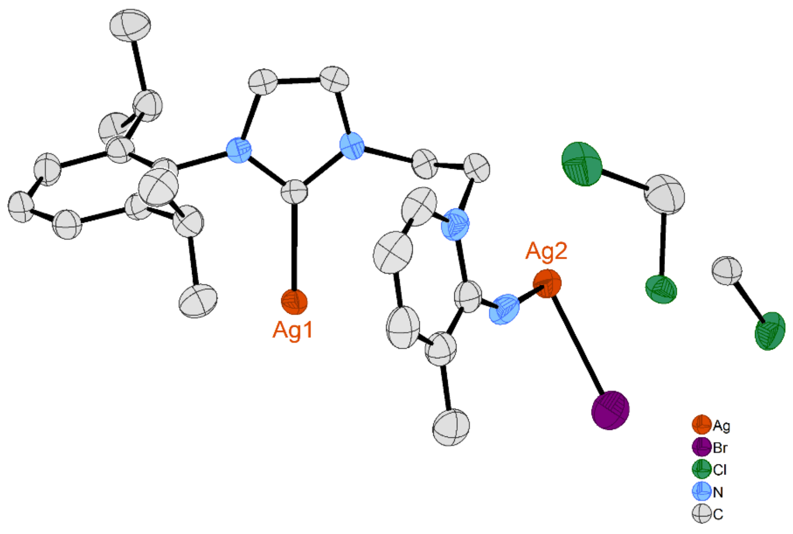

Figure S26: Depiction of the asymmetric unit of **S4d**. Hydrogen atoms are omitted for clarity. Ellipsoids are set at 30% probability.

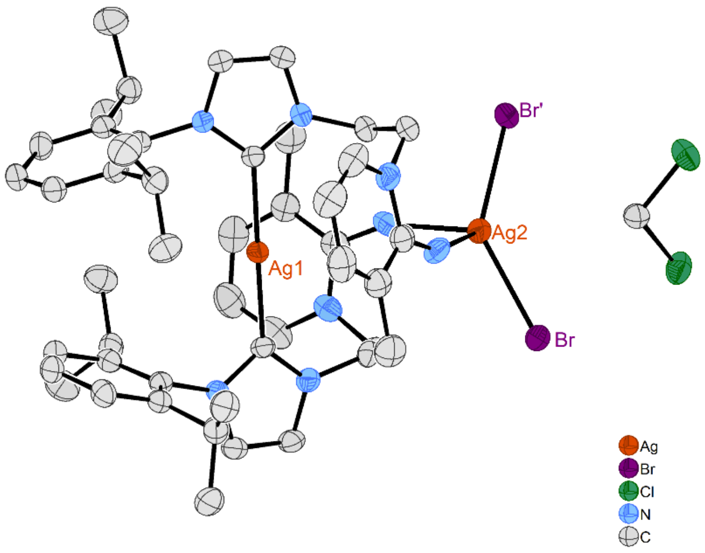

Figure S27: Full molecular view of **S4d** in the solid-state. Hydrogen atoms are omitted for clarity. Ellipsoids are set at 30% probability.

The partly occupied Ag2 and Br/Br' positions as well as the disorder of the CH_2Cl_2 solvent molecule are best explainded by the presence of two different complexes. The major component (80%) is shown in Figure S28. It features the linear coordination of the Ag1 atom by the NHC moieties of two ligands and the coordination of the Ag2 atom by two imine moieties and two Br atoms.

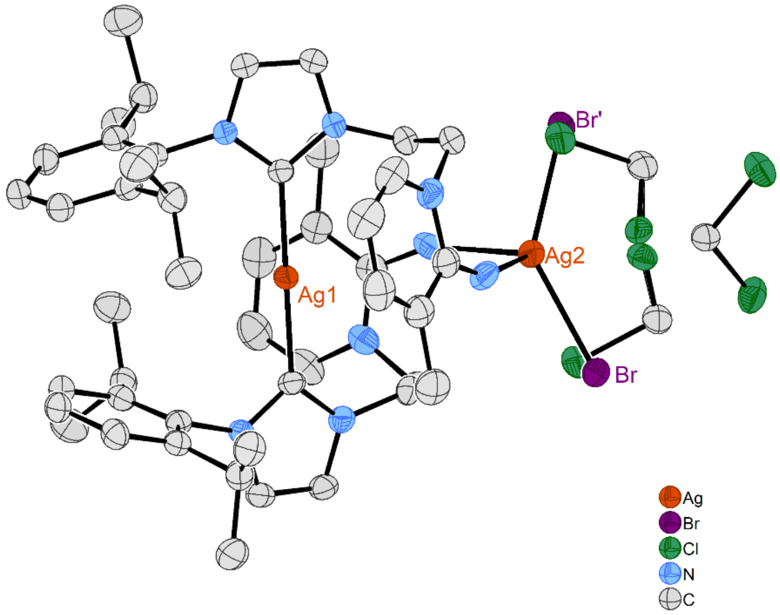

Figure S28: Molecular structure of the major component (occupancy 0.8) of **S4d**. Hydrogen atoms are omitted for clarity. Ellipsoids are set at 50% probability.

The minor component (20%) is depicted in Figure S29. Again, the Ag1 atom is linearly coordinated by the NHC moieties of two ligands. However, the Ag2 atom is missing. Now, the Br atom is a separate anion, no longer attached to an Ag center. Further, the Br anion and the CH_2Cl_2 molecule are disordered along a symmetry element (2-fold axis).

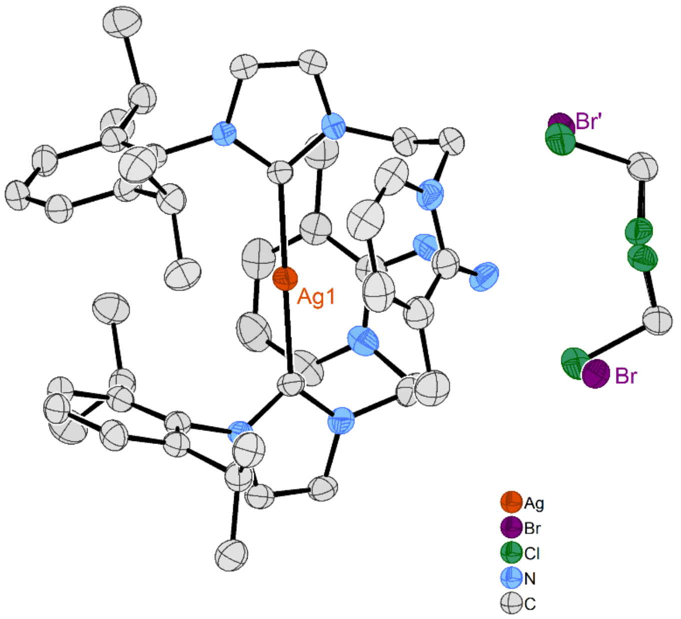

Figure S29: Molecular structure of the minor component (occupancy 0.2) of **S4d**. Hydrogen atoms are omitted for clarity. Ellipsoids are set at 30% probability.

Table S9: Crystallographic data of **S4d**.

CCDC identification code: 2389422			
Empirical formula	$C_{47}H_{62}Ag_{1.8}Br_{1.8}Cl_2N_8$	*Z'*	0.5
$\rho_{calc.}$/ g·cm^{-3}	1.553	Wavelength / Å	1.54184
μ / mm^{-1}	8.811	Radiation type	Cu K$_\alpha$
Formula Weight	1147.95	Q_{min} / °	3.797
Colour	clear colourless	Q_{max} / °	66.598
Shape	block-shaped	Measured Refl's.	18453
Size / mm^3	0.07×0.05×0.04	Indep't Refl's	4181
T / K	123.00(10)	Refl's I≥2 *s*(I)	3548
Crystal System	monoclinic	R_{int}	0.0715
Space Group	*C*2/*c*	Parameters	312
a / Å	15.4130(5)	Restraints	35
b / Å	17.7624(4)	Largest Peak	1.677
c / Å	17.9298(6)	Deepest Hole	-1.159
α / °	90	GooF	1.174
β / °	90.064(3)	wR_2 (all data)	0.2164
γ / °	90	wR_2	0.2114
Volume / Å^3	4908.7(3)	R_1 (all data)	0.0780
Z	4	R_1	0.0717

Bond lengths and bond angles are given in the tables below. Figure S30 depicts the asymmetric unit of **S4d** and all atoms are labelled for reference to the table. Thus, symmetry generated atoms

are not depicted for better clarity. Nevertheless, Bond lengths and angles including symmetry generated atoms are reported and marked accordingly.

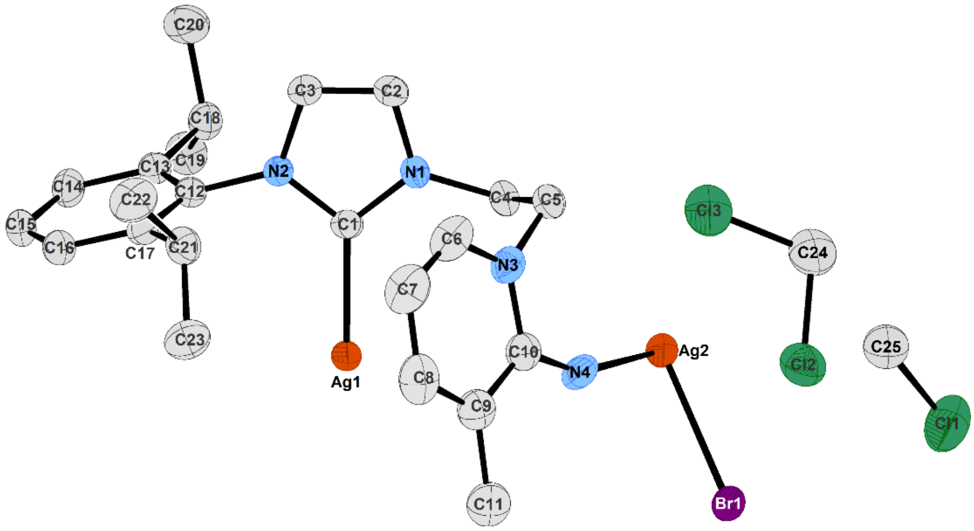

Figure S30: Asymmetric unit of **S4d**. Hydrogen atoms are omitted for clarity. Ellipsoids are set at 30% probability. The labels are used as reference for the reported bond lengths and angles in table S10, table S11 and table S12.

Table S10: Bond Lengths in Å for **S4d**.

Ag1	C1	2.088(7)	N3	C5	1.475(12)
Ag1	C1[1]	2.088(7)	N3	C6	1.362(11)
Ag2	Br1	2.6308(11)	C12	C13	1.407(11)
Ag2	Br1[1]	2.6308(11)	C12	C17	1.385(11)
Ag2	N4[1]	2.458(8)	C13	C14	1.403(10)
Ag2	N4	2.458(8)	C13	C18	1.526(11)
Ag2	Cl3	2.32(3)	C14	C15	1.372(12)
Ag2	Cl2	2.225(16)	C17	C16	1.387(11)
Br1	Cl2	2.521(17)	C17	C21	1.518(11)
Cl1	C25	1.743(9)	C16	C15	1.386(12)
N2	C1	1.377(9)	C10	C9	1.419(13)
N2	C12	1.434(9)	C18	C19	1.545(11)
N2	C3	1.397(9)	C18	C20	1.541(13)
C1	N1	1.374(9)	C4	C5	1.503(12)
N1	C4	1.463(9)	C3	C2	1.347(12)
N1	C2	1.383(10)	C21	C23	1.510(14)
N4	C10	1.316(11)	C21	C22	1.532(12)
N3	C10	1.399(11)	C9	C11	1.511(14)

C9	C8	1.371(14)	C25	C24	1.88(3)
C6	C7	1.298(15)	Cl3	C24	1.79(2)
C7	C8	1.434(16)	Cl2	C24	1.77(2)
C25	Cl2	1.34(2)			

Table S11: Bond Angles in ° for **S4d**.

C1	Ag1	C1^{1}	179.3(4)	C17	C12	N2	118.6(7)
Br1^{1}	Ag2	Br1	137.10(6)	C17	C12	C13	123.7(7)
N4	Ag2	Br1	88.93(16)	C12	C13	C18	123.1(6)
N4	Ag2	Br1^{1}	117.82(17)	C14	C13	C12	116.5(7)
N4^{1}	Ag2	Br1^{1}	88.93(16)	C14	C13	C18	120.4(7)
N4^{1}	Ag2	Br1	117.82(17)	C15	C14	C13	121.4(8)
N4	Ag2	N4^{1}	104.4(3)	C12	C17	C16	116.4(7)
Cl3	Ag2	Br1	137.3(6)	C12	C17	C21	121.5(7)
Cl3	Ag2	Br1^{1}	13.7(6)	C16	C17	C21	122.0(7)
Cl3	Ag2	N4	105.2(6)	C15	C16	C17	122.4(7)
Cl3	Ag2	N4^{1}	97.7(6)	N4	C10	N3	117.5(8)
Cl2	Ag2	Br1^{1}	75.2(5)	N4	C10	C9	126.3(8)
Cl2	Ag2	Br1	61.9(5)	N3	C10	C9	116.3(8)
Cl2	Ag2	N4^{1}	129.4(8)	C13	C18	C19	113.4(7)
Cl2	Ag2	N4	125.6(8)	C13	C18	C20	109.8(7)
Cl2	Ag2	Cl3	77.6(7)	C20	C18	C19	109.5(7)
Cl2	Br1	Ag2	51.1(4)	N1	C4	C5	112.1(7)
C1	N2	C12	125.5(6)	C14	C15	C16	119.4(7)
C1	N2	C3	111.3(6)	C2	C3	N2	107.4(7)
C3	N2	C12	123.1(6)	N3	C5	C4	110.4(6)
N2	C1	Ag1	128.2(5)	C17	C21	C22	111.0(7)
N1	C1	Ag1	128.5(5)	C23	C21	C17	112.3(7)
N1	C1	N2	102.4(6)	C23	C21	C22	108.7(8)
C1	N1	C4	124.0(6)	C3	C2	N1	106.0(6)
C1	N1	C2	112.9(6)	C10	C9	C11	118.2(8)
C2	N1	C4	123.1(6)	C8	C9	C10	119.9(10)
C10	N4	Ag2	155.1(6)	C8	C9	C11	121.8(9)
C10	N3	C5	119.0(7)	C7	C6	N3	124.4(10)
C6	N3	C10	121.2(8)	C6	C7	C8	117.0(10)
C6	N3	C5	119.7(8)	C9	C8	C7	121.2(10)
C13	C12	N2	117.5(6)	Cl1	C25	Cl1^{1}	108.1(8)

Cl1	C25	C24	78.4(14)	C25	Cl2	Br1	130.8(11)
Cl1	C25	C24	155(2)	C25	Cl2	C24	72.9(12)
Cl2	C25	Cl1	116.9(9)	C24	Cl2	Ag2	89.3(12)
Cl2	C25	Cl1[1]	134.4(11)	C24	Cl2	Br1	155.0(14)
Cl2	C25	C24	64.1(11)	Cl3	C24	C25	149(2)
C24	Cl3	Ag2	86.0(13)	Cl2	C24	C25	42.9(8)
Ag2	Cl2	Br1	67.0(4)	Cl2	C24	Cl3	106.2(18)
C25	Cl2	Ag2	162.2(12)				

Table S12: Torsion Angles in ° for **S4d**.

Ag1	C1	N1	C4	12.1(10)	N4	C10	C9	C8	-179.4(8)
Ag1	C1	N1	C2	-168.6(6)	N3	C10	C9	C11	177.8(7)
Ag2	N4	C10	N3	-27.8(17)	N3	C10	C9	C8	1.0(12)
Ag2	N4	C10	C9	152.5(11)	N3	C6	C7	C8	-0.3(16)
Ag2	Cl3	C24	C25	-15(9)	C12	N2	C1	Ag1	-14.4(10)
Ag2	Cl3	C24	Cl2	-7(3)	C12	N2	C1	N1	175.5(6)
Ag2	Cl2	C24	C25	-178(2)	C12	N2	C3	C2	-176.0(7)
Ag2	Cl2	C24	Cl3	8(4)	C12	C13	C14	C15	0.7(11)
Br1	Cl2	C24	C25	163(7)	C12	C13	C18	C19	139.4(8)
Br1	Cl2	C24	Cl3	-11(10)	C12	C13	C18	C20	-97.7(9)
Cl1	C25	Cl2	Ag2	-146(6)	C12	C17	C16	C15	-2.5(11)
Cl1	C25	Cl2	Ag2	44(8)	C12	C17	C21	C23	-116.1(8)
Cl1	C25	Cl2	Br1	-132.8(12)	C12	C17	C21	C22	122.1(8)
Cl1	C25	Cl2	Br1	37(3)	C13	C12	C17	C16	4.6(11)
Cl1	C25	Cl2	C24	38(3)	C13	C12	C17	C21	-174.0(7)
Cl1	C25	Cl2	C24	-152(3)	C13	C14	C15	C16	1.2(12)
Cl1	C25	C24	Cl3	-143(9)	C14	C13	C18	C19	-42.0(10)
Cl1	C25	C24	Cl3	109(8)	C14	C13	C18	C20	80.9(9)
Cl1	C25	C24	Cl2	-153(2)	C17	C12	C13	C14	-3.8(11)
Cl1	C25	C24	Cl2	99(3)	C17	C12	C13	C18	174.8(7)
N2	C1	N1	C4	-177.8(7)	C17	C16	C15	C14	-0.3(12)
N2	C1	N1	C2	1.5(8)	C16	C17	C21	C23	65.4(10)
N2	C12	C13	C14	-178.7(6)	C16	C17	C21	C22	-56.5(11)
N2	C12	C13	C18	-0.1(11)	C10	N3	C5	C4	76.4(8)
N2	C12	C17	C16	179.5(6)	C10	N3	C6	C7	-1.1(14)
N2	C12	C17	C21	0.9(11)	C10	C9	C8	C7	-2.4(14)
N2	C3	C2	N1	-0.1(9)	C18	C13	C14	C15	-177.9(8)
C1	N2	C12	C13	-95.2(9)	C4	N1	C2	C3	178.4(7)
C1	N2	C12	C17	89.6(9)	C3	N2	C1	Ag1	168.6(6)
C1	N2	C3	C2	1.1(9)	C3	N2	C1	N1	-1.5(8)
C1	N1	C4	C5	-113.6(8)	C3	N2	C12	C13	81.5(9)
C1	N1	C2	C3	-0.9(9)	C3	N2	C12	C17	-93.7(9)
N1	C4	C5	N3	61.6(9)	C5	N3	C10	N4	5.4(10)
N4	C10	C9	C11	-2.5(13)	C5	N3	C10	C9	-174.9(7)

C5	N3	C6	C7	174.6(9)	C6	N3	C5	C4	-99.4(9)
C21	C17	C16	C15	176.1(7)	C6	C7	C8	C9	2.1(15)
C2	N1	C4	C5	67.3(9)	C25	Cl2	C24	Cl3	-174(4)
C11	C9	C8	C7	-179.2(9)	Cl2	C25	C24	Cl3	11(8)
C6	N3	C10	N4	-178.9(7)	C24	C25	Cl2	Ag2	6(7)
C6	N3	C10	C9	0.8(11)	C24	C25	Cl2	Br1	-171(4)

9 Crystallographic Data

9.1 3-Phenyl-1-(3-(trifluoromethyl)-5,6-dihydro-[1,2,4]triazolo[4,3-*a*]pyrazin-7(8*H*)-yl)butan-1-one (12t)

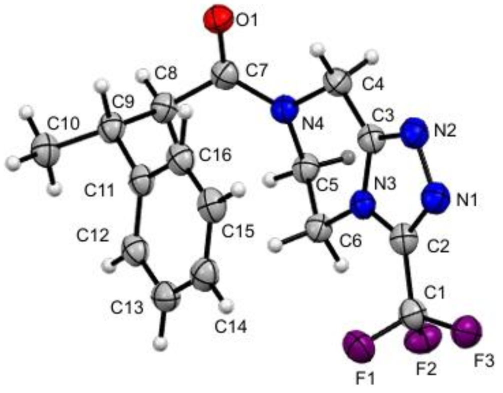

CCDC identification code: 2415987.

Table S13: Crystal data and structure refinement for **12t**.

Empirical formula	$C_{16}H_{17}F_3N_4O$	
Formula weight	338.33	
Temperature	100 K	
Wavelength	1.54178 A	
Crystal system, space group	Monoclinic, P 21	
Unit cell dimensions	a = 10.0740(3) A	alpha = 90 deg.
	b = 7.3884(2) A	beta = 106.609(1) deg.
	c = 10.5955(3) A	gamma = 90 deg.

Volume	755.73(4) A^3
Z, Calculated density	2, 1.487 Mg/m^3
Absorption coefficient	1.043 mm^-1
F(000)	352
Crystal size	0.170 x 0.130 x 0.100 mm
Theta range for data collection	4.354 to 74.459 deg.
Limiting indices	–12<=h<=12, –9<=k<=9, –13<=l<=13
Reflections collected / unique	33476 / 3081 [R(int) = 0.0460]
Completeness to theta = 67.679	99.8 %
Absorption correction	Semi-empirical from equivalents
Max. and min. transmission	0.90 and 0.69
Refinement method	Full-matrix least-squares on F^2
Data / restraints / parameters	3081 / 1 / 218
Goodness-of-fit on F^2	1.078
Final R indices [I>2sigma(I)]	R1 = 0.0318, wR2 = 0.0834
R indices (all data)	R1 = 0.0328, wR2 = 0.0845
Absolute structure parameter	0.06(4)
Extinction coefficient	n/a
Largest diff. peak and hole	0.212 and -0.156 e.A^-3

Table S14: Bond lengths [A] and angles [deg] for **12t**.

C(1)-F(3)	1.331(3)	C(1)-C(2)	1.494(3)
C(1)-F(1)	1.337(3)	C(2)-N(1)	1.311(3)
C(1)-F(2)	1.339(3)	C(2)-N(3)	1.355(3)

C(3)-N(2)	1.311(3)	C(10)-H(10C)	0.9800
C(3)-N(3)	1.355(3)	C(11)-C(12)	1.399(3)
C(3)-C(4)	1.499(3)	C(11)-C(16)	1.400(3)
C(4)-N(4)	1.455(3)	C(12)-C(13)	1.390(3)
C(4)-H(4A)	0.9900	C(12)-H(12)	0.9500
C(4)-H(4B)	0.9900	C(13)-C(14)	1.386(4)
C(5)-N(4)	1.457(3)	C(13)-H(13)	0.9500
C(5)-C(6)	1.521(3)	C(14)-C(15)	1.390(4)
C(5)-H(5A)	0.9900	C(14)-H(14)	0.9500
C(5)-H(5B)	0.9900	C(15)-C(16)	1.387(3)
C(6)-N(3)	1.474(3)	C(15)-H(15)	0.9500
C(6)-H(6A)	0.9900	C(16)-H(16)	0.9500
C(6)-H(6B)	0.9900	N(1)-N(2)	1.387(3)
C(7)-O(1)	1.228(3)		
C(7)-N(4)	1.364(3)	F(3)-C(1)-F(1)	107.48(19)
C(7)-C(8)	1.512(3)	F(3)-C(1)-F(2)	107.29(19)
C(8)-C(9)	1.550(3)	F(1)-C(1)-F(2)	106.68(19)
C(8)-H(8A)	0.9900	F(3)-C(1)-C(2)	110.56(18)
C(8)-H(8B)	0.9900	F(1)-C(1)-C(2)	112.33(19)
C(9)-C(10)	1.524(3)	F(2)-C(1)-C(2)	112.22(19)
C(9)-C(11)	1.527(3)	N(1)-C(2)-N(3)	111.4(2)
C(9)-H(9)	1.0000	N(1)-C(2)-C(1)	124.0(2)
C(10)-H(10A)	0.9800	N(3)-C(2)-C(1)	124.60(19)
C(10)-H(10B)	0.9800	N(2)-C(3)-N(3)	110.99(19)

N(2)-C(3)-C(4)	126.7(2)	C(7)-C(8)-H(8A)	109.7
N(3)-C(3)-C(4)	122.28(18)	C(9)-C(8)-H(8A)	109.7
N(4)-C(4)-C(3)	109.50(17)	C(7)-C(8)-H(8B)	109.7
N(4)-C(4)-H(4A)	109.8	C(9)-C(8)-H(8B)	109.7
C(3)-C(4)-H(4A)	109.8	H(8A)-C(8)-H(8B)	108.2
N(4)-C(4)-H(4B)	109.8	C(10)-C(9)-C(11)	113.28(19)
C(3)-C(4)-H(4B)	109.8	C(10)-C(9)-C(8)	110.08(18)
H(4A)-C(4)-H(4B)	108.2	C(11)-C(9)-C(8)	110.33(18)
N(4)-C(5)-C(6)	110.37(17)	C(10)-C(9)-H(9)	107.6
N(4)-C(5)-H(5A)	109.6	C(11)-C(9)-H(9)	107.6
C(6)-C(5)-H(5A)	109.6	C(8)-C(9)-H(9)	107.6
N(4)-C(5)-H(5B)	109.6	C(9)-C(10)-H(10A)	109.5
C(6)-C(5)-H(5B)	109.6	C(9)-C(10)-H(10B)	109.5
H(5A)-C(5)-H(5B)	108.1	H(10A)-C(10)-H(10B)	109.5
N(3)-C(6)-C(5)	107.70(17)	C(9)-C(10)-H(10C)	109.5
N(3)-C(6)-H(6A)	110.2	H(10A)-C(10)-H(10C)	109.5
C(5)-C(6)-H(6A)	110.2	H(10B)-C(10)-H(10C)	109.5
N(3)-C(6)-H(6B)	110.2	C(12)-C(11)-C(16)	118.1(2)
C(5)-C(6)-H(6B)	110.2	C(12)-C(11)-C(9)	122.6(2)
H(6A)-C(6)-H(6B)	108.5	C(16)-C(11)-C(9)	119.2(2)
O(1)-C(7)-N(4)	120.43(19)	C(13)-C(12)-C(11)	120.7(2)
O(1)-C(7)-C(8)	119.52(19)	C(13)-C(12)-H(12)	119.6
N(4)-C(7)-C(8)	119.82(19)	C(11)-C(12)-H(12)	119.6
C(7)-C(8)-C(9)	109.70(18)	C(14)-C(13)-C(12)	120.5(2)

C(14)-C(13)-H(13)	119.8	C(11)-C(16)-H(16)	119.5
C(12)-C(13)-H(13)	119.8	C(2)-N(1)-N(2)	106.35(18)
C(13)-C(14)-C(15)	119.4(2)	C(3)-N(2)-N(1)	107.00(18)
C(13)-C(14)-H(14)	120.3	C(3)-N(3)-C(2)	104.27(17)
C(15)-C(14)-H(14)	120.3	C(3)-N(3)-C(6)	124.62(17)
C(16)-C(15)-C(14)	120.3(2)	C(2)-N(3)-C(6)	131.09(18)
C(16)-C(15)-H(15)	119.8	C(7)-N(4)-C(4)	118.13(18)
C(14)-C(15)-H(15)	119.8	C(7)-N(4)-C(5)	127.82(18)
C(15)-C(16)-C(11)	120.9(2)	C(4)-N(4)-C(5)	113.79(17)
C(15)-C(16)-H(16)	119.5		

Symmetry transformations used to generate equivalent atoms.

Table S15: Torsion angles [deg] **12t**.

F(3)-C(1)-C(2)-N(1)	–12.6(3)	C(7)-C(8)-C(9)-C(10)	170.80(18)
F(1)-C(1)-C(2)-N(1)	107.4(3)	C(7)-C(8)-C(9)-C(11)	–63.4(2)
F(2)-C(1)-C(2)-N(1)	–132.4(2)	C(10)-C(9)-C(11)-C(12)	33.0(3)
F(3)-C(1)-C(2)-N(3)	166.6(2)	C(8)-C(9)-C(11)-C(12)	–90.9(2)
F(1)-C(1)-C(2)-N(3)	–73.3(3)	C(10)-C(9)-C(11)-C(16)	–150.4(2)
F(2)-C(1)-C(2)-N(3)	46.9(3)	C(8)-C(9)-C(11)-C(16)	85.7(2)
N(2)-C(3)-C(4)-N(4)	–169.8(2)	C(16)-C(11)-C(12)-C(13)	0.4(3)
N(3)-C(3)-C(4)-N(4)	13.4(3)	C(9)-C(11)-C(12)-C(13)	176.99(19)
N(4)-C(5)-C(6)-N(3)	–49.3(2)	C(11)-C(12)-C(13)-C(14)	–1.1(3)
O(1)-C(7)-C(8)-C(9)	–67.7(3)	C(12)-C(13)-C(14)-C(15)	0.9(3)
N(4)-C(7)-C(8)-C(9)	106.9(2)	C(13)-C(14)-C(15)-C(16)	0.0(3)

C(14)-C(15)-C(16)-C(11)	–0.7(3)	C(1)-C(2)-N(3)-C(3)	–178.9(2)
C(12)-C(11)-C(16)-C(15)	0.5(3)	N(1)-C(2)-N(3)-C(6)	178.6(2)
C(9)-C(11)-C(16)-C(15)	–176.24(19)	C(1)-C(2)-N(3)-C(6)	–0.7(3)
N(3)-C(2)-N(1)-N(2)	–0.2(2)	C(5)-C(6)-N(3)-C(3)	19.3(3)
C(1)-C(2)-N(1)-N(2)	179.2(2)	C(5)-C(6)-N(3)-C(2)	–158.7(2)
N(3)-C(3)-N(2)-N(1)	0.4(2)	O(1)-C(7)-N(4)-C(4)	7.0(3)
C(4)-C(3)-N(2)-N(1)	–176.7(2)	C(8)-C(7)-N(4)-C(4)	–167.45(19)
C(2)-N(1)-N(2)-C(3)	–0.1(2)	O(1)-C(7)-N(4)-C(5)	–166.7(2)
N(2)-C(3)-N(3)-C(2)	–0.4(2)	C(8)-C(7)-N(4)-C(5)	18.8(3)
C(4)-C(3)-N(3)-C(2)	176.8(2)	C(3)-C(4)-N(4)-C(7)	139.6(2)
N(2)-C(3)-N(3)-C(6)	–178.86(19)	C(3)-C(4)-N(4)-C(5)	–45.8(2)
C(4)-C(3)-N(3)-C(6)	–1.6(3)	C(6)-C(5)-N(4)-C(7)	–118.8(2)
N(1)-C(2)-N(3)-C(3)	0.4(2)	C(6)-C(5)-N(4)-C(4)	67.2(2)

Symmetry transformations used to generate equivalent atoms.

9.2 (1*R*,5*S*)-3-(3-Phenylpropanoyl-2,3-*d*$_2$)-1,2,3,4,5,6-hexahydro-8*H*-1,5-methanopyrido[1,2-*a*][1,5]diazocin-8-one (12bb-*d*$_7$)

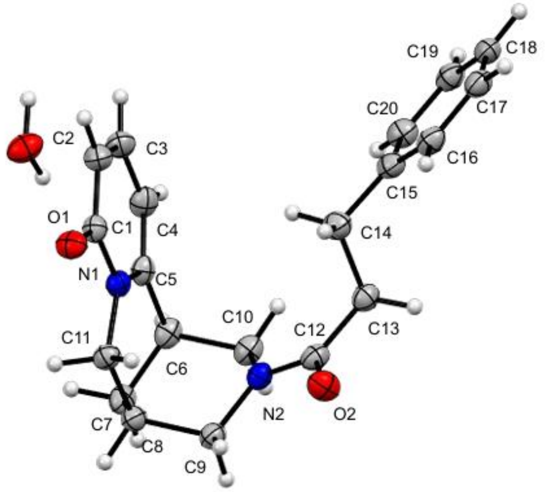

CCDC identification code: 2415988.

Table S16. Crystal data and structure refinement for **12bb**-*d*$_7$.

Empirical formula	$C_{20}H_{24}N_2O_3$	
Formula weight	340.41	
Temperature	100 K	
Wavelength	1.54178 A	
Crystal system, space group	Monoclinic, P 21	
Unit cell dimensions	a = 8.1358(2) A	alpha = 90 deg.
	b = 10.5343(3) A	beta = 107.258(1) deg.
	c = 10.6370(3) A	gamma = 90 deg.
Volume	870.60(4) A^3	
Z, Calculated density	2, 1.299 Mg/m^3	
Absorption coefficient	0.706 mm^-1	
F(000)	364	
Crystal size	0.200 x 0.110 x 0.060 mm	

Theta range for data collection	4.352 to 74.442 deg.
Limiting indices	–9<=h<=9, –13<=k<=13, –12<=l<=13
Reflections collected / unique	29110 / 3508 [R(int) = 0.0468]
Completeness to theta = 67.679	99.9 %
Absorption correction	Semi-empirical from equivalents
Max. and min. transmission	0.96 and 0.80
Refinement method	Full-matrix least-squares on F^2
Data / restraints / parameters	3508 / 4 / 232
Goodness-of-fit on F^2	1.066
Final R indices [I>2sigma(I)]	R1 = 0.0318, wR2 = 0.0818
R indices (all data)	R1 = 0.0330, wR2 = 0.0830
Absolute structure parameter	0.11(7)
Extinction coefficient	n/a
Largest diff. peak and hole	0.205 and -0.134 e.A^-3

Table S17: Bond lengths [A] and angles [deg] for **12bb**-d_7.

O(2)-C(12)	1.243(3)	C(8)-C(11)	1.531(3)
O(3)-H(1)	0.99(2)	C(8)-H(8)	1.0000
O(3)-H(2)	0.98(2)	C(9)-N(2)	1.467(3)
C(1)-O(1)	1.243(3)	C(9)-H(9A)	0.9900
C(1)-N(1)	1.401(3)	C(9)-H(9B)	0.9900
C(1)-C(2)	1.431(3)	C(10)-N(2)	1.465(3)
C(8)-C(9)	1.523(3)	C(10)-C(6)	1.546(3)
C(8)-C(7)	1.528(3)	C(10)-H(10A)	0.9900

C(10)-H(10B)	0.9900	C(2)-C(3)	1.354(3)
C(11)-N(1)	1.484(3)	C(2)-H(2A)	0.9500
C(11)-H(11A)	0.9900	C(3)-C(4)	1.412(3)
C(11)-H(11B)	0.9900	C(3)-H(3)	0.9500
C(12)-N(2)	1.351(3)	C(4)-C(5)	1.364(3)
C(12)-C(13)	1.514(3)	C(4)-H(4)	0.9500
C(13)-C(14)	1.536(3)	C(5)-C(6)	1.508(3)
C(13)-H(13A)	0.9900	C(6)-C(7)	1.520(3)
C(13)-H(13B)	0.9900	C(6)-H(6)	1.0000
C(14)-C(15)	1.512(3)	C(7)-H(7A)	0.9900
C(14)-H(14A)	0.9900	C(7)-H(7B)	0.9900
C(14)-H(14B)	0.9900		
C(15)-C(20)	1.396(3)	H(1)-O(3)-H(2)	100(2)
C(15)-C(16)	1.397(3)	O(1)-C(1)-N(1)	119.13(18)
C(16)-C(17)	1.384(3)	O(1)-C(1)-C(2)	124.8(2)
C(16)-H(16)	0.9500	N(1)-C(1)-C(2)	116.02(18)
C(17)-C(18)	1.384(3)	C(9)-C(8)-C(7)	109.87(18)
C(17)-H(17)	0.9500	C(9)-C(8)-C(11)	112.53(17)
C(18)-C(19)	1.382(3)	C(7)-C(8)-C(11)	109.96(17)
C(18)-H(18)	0.9500	C(9)-C(8)-H(8)	108.1
C(19)-C(20)	1.391(3)	C(7)-C(8)-H(8)	108.1
C(19)-H(19)	0.9500	C(11)-C(8)-H(8)	108.1
C(20)-H(20)	0.9500	N(2)-C(9)-C(8)	111.28(17)
N(1)-C(5)	1.378(3)	N(2)-C(9)-H(9A)	109.4

C(8)-C(9)-H(9A)	109.4	H(13A)-C(13)-H(13B)	108.3
N(2)-C(9)-H(9B)	109.4	C(15)-C(14)-C(13)	112.40(17)
C(8)-C(9)-H(9B)	109.4	C(15)-C(14)-H(14A)	109.1
H(9A)-C(9)-H(9B)	108.0	C(13)-C(14)-H(14A)	109.1
N(2)-C(10)-C(6)	110.85(16)	C(15)-C(14)-H(14B)	109.1
N(2)-C(10)-H(10A)	109.5	C(13)-C(14)-H(14B)	109.1
C(6)-C(10)-H(10A)	109.5	H(14A)-C(14)-H(14B)	107.9
N(2)-C(10)-H(10B)	109.5	C(20)-C(15)-C(16)	118.4(2)
C(6)-C(10)-H(10B)	109.5	C(20)-C(15)-C(14)	121.14(19)
H(10A)-C(10)-H(10B)	108.1	C(16)-C(15)-C(14)	120.4(2)
N(1)-C(11)-C(8)	115.25(16)	C(17)-C(16)-C(15)	120.7(2)
N(1)-C(11)-H(11A)	108.5	C(17)-C(16)-H(16)	119.7
C(8)-C(11)-H(11A)	108.5	C(15)-C(16)-H(16)	119.7
N(1)-C(11)-H(11B)	108.5	C(18)-C(17)-C(16)	120.3(2)
C(8)-C(11)-H(11B)	108.5	C(18)-C(17)-H(17)	119.8
H(11A)-C(11)-H(11B)	107.5	C(16)-C(17)-H(17)	119.8
O(2)-C(12)-N(2)	121.23(19)	C(19)-C(18)-C(17)	119.8(2)
O(2)-C(12)-C(13)	118.64(19)	C(19)-C(18)-H(18)	120.1
N(2)-C(12)-C(13)	120.03(18)	C(17)-C(18)-H(18)	120.1
C(12)-C(13)-C(14)	109.15(17)	C(18)-C(19)-C(20)	120.2(2)
C(12)-C(13)-H(13A)	109.9	C(18)-C(19)-H(19)	119.9
C(14)-C(13)-H(13A)	109.9	C(20)-C(19)-H(19)	119.9
C(12)-C(13)-H(13B)	109.9	C(19)-C(20)-C(15)	120.5(2)
C(14)-C(13)-H(13B)	109.9	C(19)-C(20)-H(20)	119.7

C(15)-C(20)-H(20)	119.7	C(4)-C(5)-N(1)	120.06(19)
C(5)-N(1)-C(1)	122.44(17)	C(4)-C(5)-C(6)	121.74(19)
C(5)-N(1)-C(11)	123.34(16)	N(1)-C(5)-C(6)	118.20(18)
C(1)-N(1)-C(11)	113.88(15)	C(5)-C(6)-C(7)	110.64(17)
C(12)-N(2)-C(10)	125.28(18)	C(5)-C(6)-C(10)	111.58(16)
C(12)-N(2)-C(9)	119.58(17)	C(7)-C(6)-C(10)	110.03(18)
C(10)-N(2)-C(9)	114.19(17)	C(5)-C(6)-H(6)	108.2
C(3)-C(2)-C(1)	121.5(2)	C(7)-C(6)-H(6)	108.2
C(3)-C(2)-H(2A)	119.3	C(10)-C(6)-H(6)	108.2
C(1)-C(2)-H(2A)	119.3	C(6)-C(7)-C(8)	106.86(17)
C(2)-C(3)-C(4)	120.3(2)	C(6)-C(7)-H(7A)	110.3
C(2)-C(3)-H(3)	119.9	C(8)-C(7)-H(7A)	110.3
C(4)-C(3)-H(3)	119.9	C(6)-C(7)-H(7B)	110.3
C(5)-C(4)-C(3)	119.7(2)	C(8)-C(7)-H(7B)	110.3
C(5)-C(4)-H(4)	120.1	H(7A)-C(7)-H(7B)	108.6
C(3)-C(4)-H(4)	120.1		

Symmetry transformations used to generate equivalent atoms.

Table S18: Torsion angles [deg] for **12bb**-*d*$_7$.

C(7)-C(8)-C(9)-N(2)	57.6(2)	N(2)-C(12)-C(13)-C(14)	90.4(2)
C(11)-C(8)-C(9)-N(2)	–65.3(2)	C(12)-C(13)-C(14)-C(15)	169.43(18)
C(9)-C(8)-C(11)-N(1)	86.9(2)	C(13)-C(14)-C(15)-C(20)	–102.9(2)
C(7)-C(8)-C(11)-N(1)	–35.9(2)	C(13)-C(14)-C(15)-C(16)	76.5(2)
O(2)-C(12)-C(13)-C(14)	–86.0(2)	C(20)-C(15)-C(16)-C(17)	–2.3(3)

C(14)-C(15)-C(16)-C(17)	178.20(19)	O(1)-C(1)-C(2)-C(3)	–176.2(2)
C(15)-C(16)-C(17)-C(18)	–0.3(3)	N(1)-C(1)-C(2)-C(3)	2.2(3)
C(16)-C(17)-C(18)-C(19)	2.0(3)	C(1)-C(2)-C(3)-C(4)	–2.4(3)
C(17)-C(18)-C(19)-C(20)	–1.1(3)	C(2)-C(3)-C(4)-C(5)	0.8(3)
C(18)-C(19)-C(20)-C(15)	–1.5(3)	C(3)-C(4)-C(5)-N(1)	0.9(3)
C(16)-C(15)-C(20)-C(19)	3.2(3)	C(3)-C(4)-C(5)-C(6)	–179.31(19)
C(14)-C(15)-C(20)-C(19)	–177.32(19)	C(1)-N(1)-C(5)-C(4)	–1.1(3)
O(1)-C(1)-N(1)-C(5)	178.09(19)	C(11)-N(1)-C(5)-C(4)	–171.79(19)
C(2)-C(1)-N(1)-C(5)	–0.4(3)	C(1)-N(1)-C(5)-C(6)	179.10(17)
O(1)-C(1)-N(1)-C(11)	4.6(3)	C(11)-N(1)-C(5)-C(6)	–8.0(3)
C(2)-C(1)-N(1)-C(11)	–173.92(18)	C(4)-C(5)-C(6)-C(7)	–144.0(2)
C(8)-C(11)-N(1)-C(5)	8.2(3)	N(1)-C(5)-C(6)-C(7)	35.8(2)
C(8)-C(11)-N(1)-C(1)	–178.33(16)	C(4)-C(5)-C(6)-C(10)	93.2(2)
O(2)-C(12)-N(2)-C(10)	–174.47(19)	N(1)-C(5)-C(6)-C(10)	–87.1(2)
C(13)-C(12)-N(2)-C(10)	9.2(3)	N(2)-C(10)-C(6)-C(5)	66.6(2)
O(2)-C(12)-N(2)-C(9)	–6.3(3)	N(2)-C(10)-C(6)-C(7)	–56.7(2)
C(13)-C(12)-N(2)-C(9)	177.35(18)	C(5)-C(6)-C(7)-C(8)	–62.8(2)
C(6)-C(10)-N(2)-C(12)	–138.9(2)	C(10)-C(6)-C(7)-C(8)	60.9(2)
C(6)-C(10)-N(2)-C(9)	52.4(2)	C(9)-C(8)-C(7)-C(6)	–61.5(2)
C(8)-C(9)-N(2)-C(12)	137.30(19)	C(11)-C(8)-C(7)-C(6)	62.9(2)
C(8)-C(9)-N(2)-C(10)	–53.3(2)		

Symmetry transformations used to generate equivalent atoms.

10 Computational Details

DFT calculations were carried out to gain a first insight into the influence of an adjacent imine function on the structures of typical intermediates of the catalytic cycle of alkyne semihydrogenation.

All geometry optimizations and frequency calculations were performed using the Gaussian16 program package.[61] The input geometries for these calculations were generated by first building the molecule using the Avogadro (v. 1.2.0)[62,63] program. At first, a universal force field (UFF) optimization implemented in the Avogadro suite was performed. Afterwards, a preliminary refinement of the structure was performed using the xtb (v 6.3.2) program.[64] The obtained cartesian coordinates were used to generate the input file for subsequent DFT calculations. Geometry optimizations and frequency calculations were performed using the B3LYP[65,66] functional. A split basis set with the def2SVP[67] basis set for Cu and 6-31G(d,p)[68–72] basis set for all other atoms was chosen. The absence of imaginary frequencies confirmed the stationary point as local minimum of each geometry. To account for dispersion effects, Grimme's dispersion correction with the Becke-Johnson damping (GD3BJ)[73] was applied in all calculations. The Avogadro (v. 1.2.0) software was employed to render and depict the molecules.

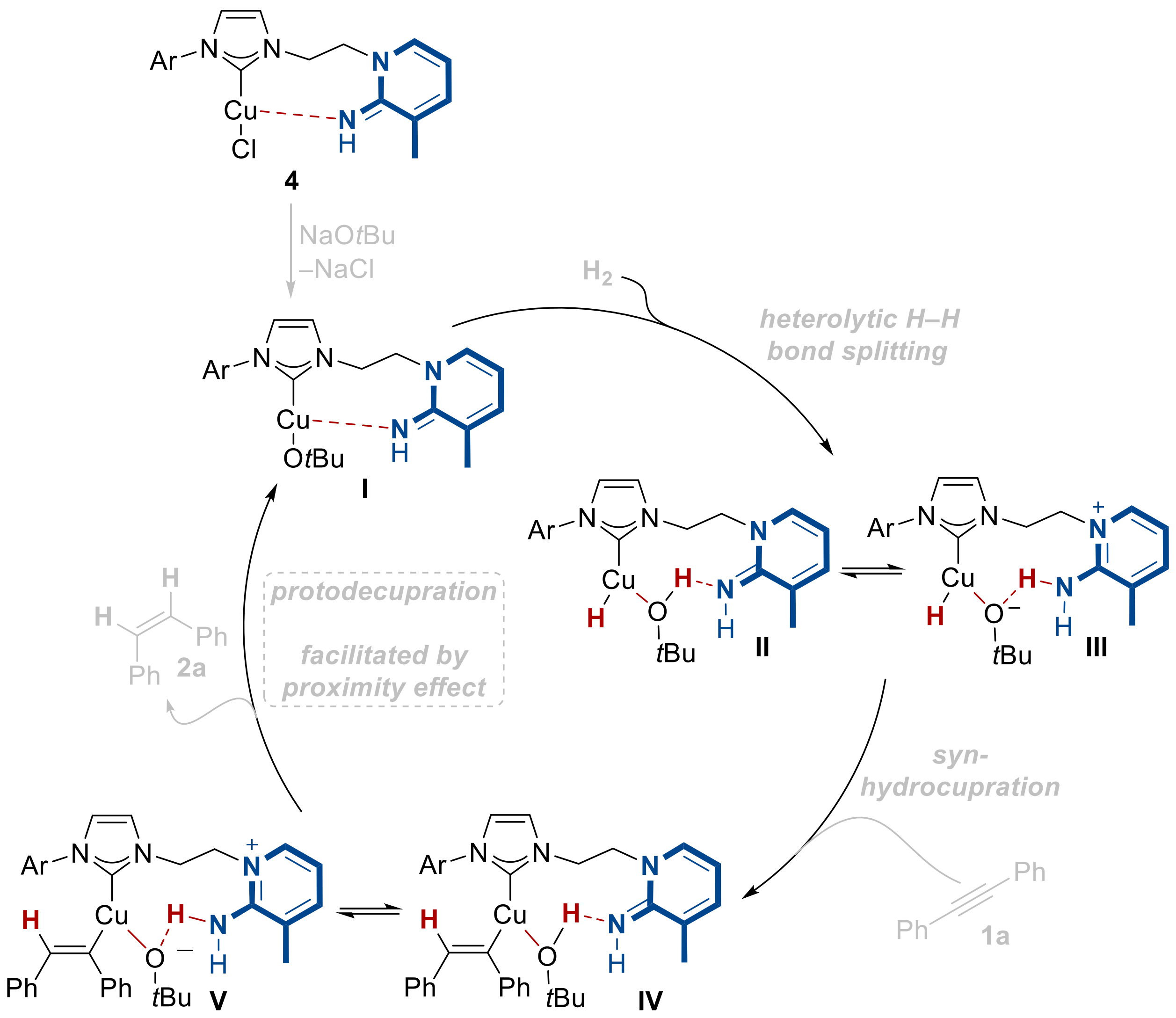

Scheme S2: Proposed catalytic cycle for the copper(I) catalyzed alkyne semihydrogenation with complex **4**. Complex **4** and intermediates **I–V,** investigated herein by DFT calculations, are highlighted.

Complex 4 and **intermediate I**:

The optimized geometries of the isolated precatalyst **4** and the intermediate **I** are depicted in Figure S31. The structures reveal a short contact between the imino N atom and the copper(I) center leading to a preorganization of the catalyst. The imino N—Cu distance are 2.440 Å (**4**) and 2.506 Å (**I**) which, in both cases, is lower than the sum of the Van-der-Waals radii: $\sum_{VdW}$(N,Cu) = 1.55 Å + 1.40 Å = 2.95 Å.[74] Notably, exchanging the chloride with a *tert*-butoxide ligand leads to the elongation of the N—Cu distance, indicating a weakening of the bond.

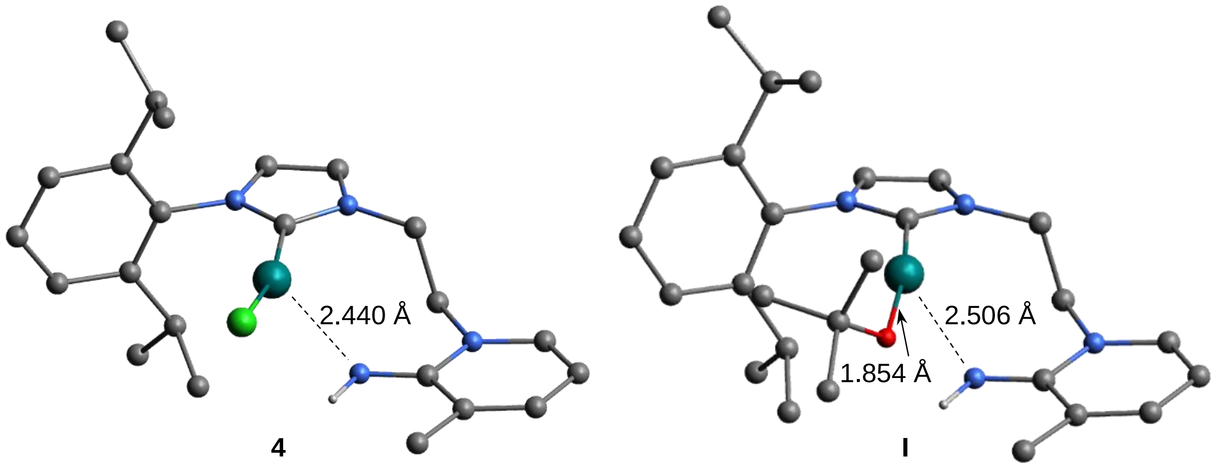

4 **I**

Figure S31: Optimized geometry of complex **4** (left) and the intermediate **I** (right). Hydrogen atoms except for the imino N–H atom are omitted for clarity.

Tautomer II:

We tried to assess the optimized geometry for the tautomers (**II** and **III**) seperately and provide the corresponding energy difference. Yet, during geometry optimizations, the tautomers converged into **II**. This suggests that **II** is the minimum of both starting geometries and therefore the energetically favoured structure. The optimized geometry is depicted in Figure S32, showing that the structure **II,** having the proton bound to the oxygen atom, is energetically more favoured. In this minimum structure, *tert*-butanol is coordinating to the copper(I) center via its oxygen atom and is additionally forming a hydrogen bond with the adjacent imine group. The O—Cu distance is 2.465 Å and the imino N—H–O*t*Bu distance is 1.805 Å (**I**) which, in both cases, is lower than the sum of the Van-der-Waals radii, respectively: $\sum_{VdW}$(O,Cu) = 1.52 Å + 1.40 Å = 2.92 Å[14] and $\sum_{VdW}$(H,N) = 1.10 Å + 1.55 Å = 2.65 Å.[74,75]

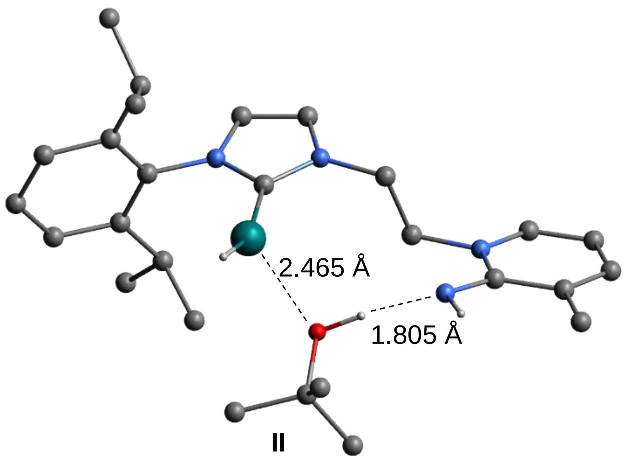

II

Figure S32: Optimized geometry of the tautomer **II**. Hydrogen atoms are omitted for clarity except for the H atom at the Cu center, of the *tert*-butanol and the imino N–H atom.

Tautomer III:

Like for the tautomers of **II**, we tried to assess the optimized geometry for tautomers (**IV** and **V**) seperately and provide the corresponding energy difference. Yet, during geometry optimizations, the tautomers converged into **IV.** This suggests that **IV** is the energetically favoured structure. The optimized geometry is depicted in Figure S33. Like for the tautomer**II**, the proton is preferentially bound to the oxygen atom and the *tert*-butanol is interacting with both the copper(I) center and the imine function. **IV** features O—Cu and imino N—HO*t*Bu distances of 2.244 Å and 1.757 Å, respectively.

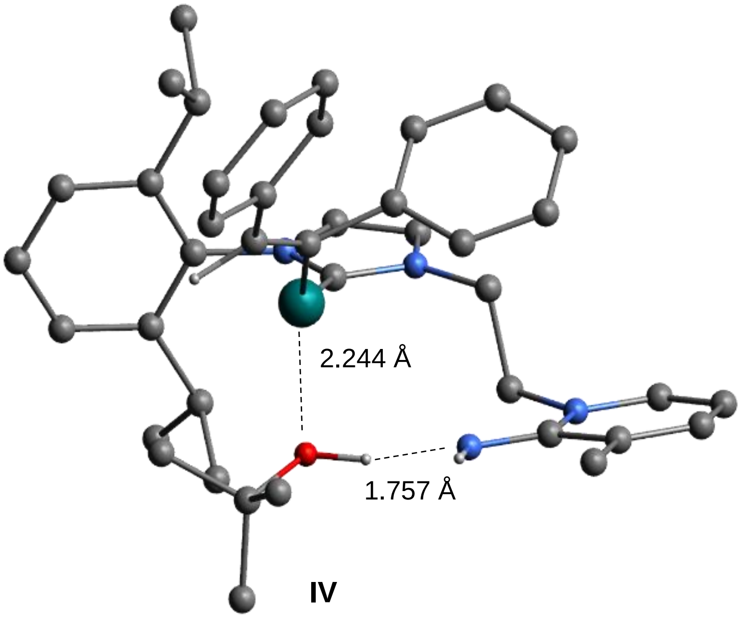

IV

Figure S33: Optimized geometry for **IV**. Hydrogen atoms are omitted for clarity except for the H atoms of the 1,2-diphenylethenyl group, the *tert*-butanol and the imino N–H atom.

Table S19: Cartesian coordinates of the optimized geometry of the precatalyst **4**.

atom	X	Y	Z	atom	X	Y	Z
C	1.76979	-2.06234	1.73431	H	0.16944	-2.95977	2.98576
C	0.53563	-2.23522	2.27586	H	-1.9634	-1.38829	2.95294
N	1.67565	-0.96255	0.8873	H	-1.91784	-0.01186	1.83858
C	0.42111	-0.43468	0.88191	H	-2.82054	-2.83387	1.2433
N	-0.26671	-1.23042	1.74939	H	-1.90353	-1.96795	-0.01798
N	-3.71009	-1.105	0.49559	H	-6.76809	1.07921	-0.29543
C	-1.70175	-1.07603	1.9383	H	-6.94929	-0.82688	1.34778
C	-2.51642	-1.85057	0.87638	H	-4.90092	-2.17175	1.82528
C	-3.54197	-0.08254	-0.46371	H	-2.36136	0.8224	-1.66385
C	-4.72487	0.73219	-0.73005	H	-3.74892	2.53065	-1.40497
C	-5.89866	0.46076	-0.08945	H	-4.31694	1.49189	-2.70589
C	-6.01298	-0.60873	0.85049	H	-5.48855	2.44536	-1.77866
C	-4.90703	-1.3503	1.11795	H	5.13559	1.92469	0.01107
N	-2.36832	0.05169	-1.0005	H	5.51902	0.9198	-2.21093
C	-4.5669	1.86388	-1.70393	H	4.10706	-0.94287	-2.99995
Cu	-0.28538	1.0016	-0.15647	H	2.54678	0.63944	2.42394
C	2.73777	-0.45973	0.05685	H	4.26204	1.84016	3.69728
C	3.51502	0.60884	0.52825	H	5.00581	0.45882	2.86884
C	4.52227	1.09127	-0.3149	H	5.23426	2.1003	2.24903
C	4.73543	0.52825	-1.56916	H	2.29806	3.09704	2.60375
C	3.94014	-0.52592	-2.01292	H	1.6272	2.51454	1.06393
C	2.9192	-1.04256	-1.20963	H	3.20151	3.31745	1.09445
C	3.24022	1.27555	1.86621	H	1.53382	-2.6232	-0.85693
C	4.51156	1.42368	2.71613	H	0.1819	-2.29795	-2.9277
C	2.54713	2.63239	1.64371	H	0.26354	-0.80944	-1.9727
C	1.99213	-2.13532	-1.7229	H	1.2629	-0.98088	-3.42353
C	0.85564	-1.51654	-2.55973	H	2.04535	-4.0388	-2.7606
C	2.73217	-3.22253	-2.51575	H	3.56904	-3.63752	-1.94523
Cl	-0.72406	2.90258	-1.153	H	3.12658	-2.83878	-3.46195
H	2.69303	-2.601	1.87127				

Table S20: Cartesian coordinates of the optimized geometry of intermediate **I**.

atom	X	Y	Z	atom	X	Y	Z
C	1.6358	-3.08689	-0.24325	H	-1.92182	-1.75293	1.61695
C	0.45255	-3.61702	0.16245	H	-3.20344	-3.57311	-0.32474
N	1.54184	-1.71528	-0.03259	H	-2.13107	-2.39692	-1.12753
C	0.33002	-1.36557	0.48379	H	-6.23244	1.22786	0.65962
N	-0.3249	-2.55332	0.60777	H	-6.77367	-1.16636	1.24796
N	-3.71345	-1.57474	-0.08179	H	-5.05946	-2.91524	0.76668
C	-1.7268	-2.62624	0.99303	H	-2.05733	0.87168	-1.28306
C	-2.69578	-2.61128	-0.21981	H	-2.98231	2.4308	-0.11186
C	-3.36367	-0.26853	-0.48953	H	-3.88569	2.30273	-1.62108
C	-4.34241	0.77238	-0.17806	H	-4.71767	2.88704	-0.16868
C	-5.51797	0.44169	0.43058	H	5.33966	0.74439	0.74493
C	-5.83216	-0.90762	0.78052	H	5.1702	1.85636	-1.45157
C	-4.91047	-1.87007	0.52031	H	3.28085	1.3577	-2.9605
N	-2.21394	-0.11111	-1.06967	H	3.08029	-2.02911	1.82256
C	-3.96712	2.18095	-0.53235	H	4.95777	-1.86529	3.35309
Cu	-0.32753	0.40526	0.49679	H	5.59977	-1.92592	1.70085
C	2.52843	-0.74081	-0.41846	H	5.55353	-0.39476	2.58952
C	3.57341	-0.45395	0.47679	H	2.74495	-0.60779	3.82947
C	4.52268	0.49123	0.07858	H	1.78055	5.6E-4	2.46385
C	4.42357	1.12412	-1.15902	H	3.27969	0.81942	2.91939
C	3.36184	0.8395	-2.0108	H	0.47793	-0.97814	-2.05855
C	2.37968	-0.09247	-1.65534	H	-0.41239	0.70089	-3.55721
C	3.59565	-1.06428	1.86971	H	0.15583	1.48387	-2.0715
C	5.01041	-1.32651	2.40187	H	1.11113	1.60025	-3.56648
C	2.79828	-0.16029	2.83101	H	0.81648	-1.35255	-4.48449
C	1.20642	-0.35985	-2.5864	H	2.13867	-2.08583	-3.5563
C	0.47651	0.9401	-2.96502	H	2.39732	-0.55795	-4.41005
C	1.66826	-1.13578	-3.83152	H	1.53623	3.05228	-0.79361
O	-0.80535	2.17083	0.19578	H	2.04318	3.93619	0.66121
C	-0.0523	3.25013	0.68195	H	1.83655	2.1723	0.70821
C	1.43207	3.10234	0.29412	H	0.24042	2.40934	2.65922
C	-0.17187	3.32338	2.216	H	-1.22676	3.39035	2.50129

C	-0.62001	4.5362	0.05397	H	0.36082	4.18399	2.63944
H	2.52391	-3.54497	-0.64671	H	-1.66802	4.66391	0.34488
H	0.1073	-4.6384	0.18731	H	-0.06471	5.43	0.36162
H	-1.87234	-3.52232	1.60328	H	-0.57974	4.45844	-1.03733

Table S21: Cartesian coordinates of the optimized geometry of intermediate **II**.

atom	X	Y	Z	atom	X	Y	Z
C	1.80898	-2.54641	-1.01674	H	-2.06389	-1.72485	-1.88474
C	0.51443	-2.91363	-0.84638	H	-1.98744	-0.15531	-1.06732
N	1.95087	-1.31297	-0.39058	H	-7.51213	-1.62413	-0.11025
C	0.78567	-0.88773	0.18453	H	-6.35815	-3.08712	-1.81174
N	-0.0876	-1.89681	-0.11022	H	-3.9057	-2.78987	-2.14573
N	-3.73488	-1.27816	-0.73311	H	-4.0348	0.93312	1.54545
C	-1.50359	-1.83684	0.2057	H	-2.00101	1.23767	0.39495
C	-2.28669	-1.1986	-0.95334	H	-6.25374	1.35723	1.20739
C	-4.30847	-0.43874	0.2357	H	-7.46995	0.10557	1.51505
C	-5.74441	-0.59374	0.44137	H	-5.97068	0.14503	2.45729
C	-6.44323	-1.52168	-0.27664	H	0.83267	1.63179	2.52222
C	-5.80504	-2.35441	-1.2388	H	5.82376	0.08274	1.63923
C	-4.46741	-2.20137	-1.4303	H	6.41135	1.51801	-0.28373
N	-3.52178	0.38479	0.86295	H	4.92822	1.63674	-2.25121
C	-6.39668	0.29845	1.4577	H	2.68406	-1.99094	1.76944
Cu	0.60495	0.68654	1.31124	H	4.41201	-3.29226	2.97082
C	3.17003	-0.55185	-0.35794	H	4.77443	-3.24871	1.23422
C	3.99302	-0.63965	0.77416	H	5.68307	-2.22723	2.35639
C	5.16747	0.12241	0.77586	H	3.20664	-1.32607	4.08349
C	5.49758	0.93138	-0.30572	H	2.70913	0.07432	3.10672
C	4.65975	0.9972	-1.41792	H	4.4247	-0.17664	3.51151
C	3.4763	0.25585	-1.46841	H	1.97483	-0.57967	-2.72678
C	3.64954	-1.51843	1.96577	H	0.77199	1.54836	-3.21022

C	4.69093	-2.63702	2.13896	H	0.90699	1.28121	-1.46394
C	3.4892	-0.68064	3.24457	H	1.97642	2.44035	-2.26183
C	2.51692	0.36829	-2.64523	H	2.50342	0.54148	-4.80517
C	1.47756	1.47206	-2.37503	H	4.00824	-0.14569	-4.16453
C	3.22696	0.59992	-3.98593	H	3.68827	1.59159	-4.03544
O	-1.22494	1.72652	0.02729	H	-1.05234	2.97963	2.32275
C	-1.468	3.13432	0.21566	H	-1.92418	4.47462	1.87284
C	-1.83151	3.40016	1.68145	H	-2.78895	2.93241	1.93795
C	-2.60053	3.56104	-0.72733	H	-3.51859	3.01144	-0.49276
C	-0.15951	3.83987	-0.13524	H	-2.32887	3.34417	-1.76532
H	2.63458	-3.03848	-1.50443	H	-2.80985	4.63276	-0.63922
H	-0.0184	-3.79816	-1.158	H	0.10706	3.65655	-1.1797
H	-1.86979	-2.85077	0.39506	H	0.63942	3.45726	0.50615
H	-1.62771	-1.23935	1.10823	H	-0.25105	4.92037	0.01451

Table S22: Cartesian coordinates of the optimized geometry of rotamer **IV**.

atom	X	Y	Z	atom	X	Y	Z
C	-3.17682	-1.5369	-2.31087	H	-4.10098	3.25286	-3.28401
C	-2.06844	-2.27978	-2.56484	H	-1.04084	3.51585	-2.58003
N	-2.83545	-0.66787	-1.27775	H	-1.10891	2.99113	-0.89532
C	-1.54134	-0.82921	-0.88136	H	-2.30175	4.15568	-1.52445
N	-1.08543	-1.83067	-1.6895	H	-4.28665	-2.18412	-0.0641
N	1.7736	-3.7599	-0.24882	H	-3.76546	-2.86451	2.26359
C	0.26737	-2.35088	-1.5674	H	-2.70441	-1.53773	1.74968
C	0.36739	-3.40739	-0.45777	H	-4.07368	-1.21331	2.82422
C	2.53658	-2.9856	0.63972	H	-6.10532	-3.11375	1.24539
C	3.97343	-3.24385	0.62766	H	-6.76727	-1.831	0.21407
C	4.49209	-4.20284	-0.19366	H	-6.50874	-1.54753	1.94223
C	3.66379	-4.97238	-1.05942	H	1.44952	0.81792	2.72451
C	2.33043	-4.71213	-1.06176	H	1.18184	0.91545	4.48039
N	1.92872	-2.11297	1.38586	H	1.79313	-0.5839	3.77605
C	4.8335	-2.42379	1.54493	H	-0.09622	-2.03995	4.5723
Cu	-0.41647	0.26479	0.26866	H	-1.77659	-1.51184	4.36936

C	-3.69642	0.311	-0.67448	H	-0.70735	-0.65961	5.50487
C	-3.56444	1.6536	-1.06585	H	-2.29302	0.61584	3.06643
C	-4.37565	2.5933	-0.4192	H	-0.95841	1.6053	2.44644
C	-5.26916	2.20588	0.57463	H	-1.28996	1.55332	4.19341
C	-5.37799	0.86586	0.94046	C	1.03905	1.56701	0.08091
C	-4.5933	-0.11256	0.32212	C	1.35232	2.70583	0.7394
C	-2.62	2.08549	-2.17756	C	2.47614	3.64182	0.54674
C	-3.42124	2.41027	-3.45114	C	1.88201	0.96705	-0.97132
C	-1.72033	3.25825	-1.76066	C	3.24671	3.72349	-0.63006
C	-4.66302	-1.56925	0.75886	C	2.79588	4.5286	1.59303
C	-3.73968	-1.80961	1.96725	C	3.84825	5.43463	1.48803
C	-6.09579	-2.03606	1.05378	C	4.29795	4.63066	-0.73662
O	-0.42795	-0.94917	2.15642	C	4.61123	5.48887	0.32029
C	-0.32115	-0.22714	3.40045	H	2.20369	4.49303	2.50459
C	1.11725	0.26168	3.6049	H	4.07188	6.10113	2.31699
C	-0.75267	-1.16424	4.53372	H	4.87393	4.67281	-1.65742
C	-1.27655	0.96161	3.27288	H	5.43197	6.19481	0.23127
H	-4.15605	-1.53725	-2.76105	H	0.7015	3.00604	1.56446
H	-1.89119	-3.06119	-3.28675	H	3.01232	3.07921	-1.46743
H	0.57915	-2.76623	-2.52939	C	1.44591	0.88933	-2.31317
H	0.92836	-1.51622	-1.33437	C	3.08452	0.30221	-0.64908
H	-0.18366	-4.31442	-0.72172	C	2.16856	0.18344	-3.27203
H	-0.04142	-3.01005	0.46894	C	3.34024	-0.49635	-2.92293
H	5.56355	-4.38299	-0.18477	C	3.79333	-0.4251	-1.60274
H	4.07282	-5.74086	-1.70219	H	4.70695	-0.93954	-1.31636
H	1.62814	-5.23941	-1.69688	H	3.44357	0.36491	0.37316
H	2.60261	-1.5841	1.92926	H	0.51909	1.38474	-2.58536
H	-4.30156	3.63931	-0.6974	H	1.8079	0.15125	-4.29739
H	-5.88481	2.95182	1.06866	H	3.89302	-1.06315	-3.66607
H	-6.07541	0.57904	1.71993	H	4.76712	-1.35626	1.30361
H	-1.96063	1.24487	-2.40451	H	5.88096	-2.72229	1.46677
H	-2.7464	2.67897	-4.27055	H	4.52427	-2.53823	2.5915
H	-4.02407	1.55375	-3.7709	H	0.40503	-1.45422	1.96053

11 References:

[1] D. Seebach, R. Imwinkelried, G. Stucky, *Helvetica Chimica Acta* **1987**, *70*, 448–464.
[2] W. C. Still, M. Kahn, A. Mitra, *J. Org. Chem.* **1978**, *43*, 2923–2925.
[3] G. R. Fulmer, A. J. M. Miller, N. H. Sherden, H. E. Gottlieb, A. Nudelman, B. M. Stoltz, J. E. Bercaw, K. I. Goldberg, *Organometallics* **2010**, *29*, 2176–2179.
[4] H. E. Gottlieb, V. Kotlyar, A. Nudelman, *J. Org. Chem.* **1997**, *62*, 7512–7515.
[5] B. M. Zimmermann, T. T. Ngoc, D.-I. Tzaras, T. Kaicharla, J. F. Teichert, *J. Am. Chem. Soc.* **2021**, *143*, 16865–16873.
[6] A. J. Huckaba, A. Senes, S. Aghazada, A. Babaei, S. C. J. Meskers, I. Zimmermann, P. Schouwink, N. Gasilova, R. A. J. Janssen, H. J. Bolink, M. K. Nazeeruddin, *ACS Omega* **2018**, *3*, 2673–2682.
[7] C. Marshall, M. F. Ward, J. M. S. Skakle, *Synthesis* **2006**, *2006*, 1040–1044.
[8] M. Brill, A. Collado, D. B. Cordes, A. M. Z. Slawin, M. Vogt, H. Grützmacher, S. P. Nolan, *Organometallics* **2015**, *34*, 263–274.
[9] Q. Yan, D. Kong, W. Zhao, G. Zi, G. Hou, *J. Org. Chem.* **2016**, *81*, 2070–2077.
[10] C. U. Brzezinski, A. R. LeBlanc, M. G. Clerici, W. M. Wuest, *Org. Lett.* **2024**, *26*, 5534–5538.
[11] A. Chardon, T. Mohy El Dine, R. Legay, M. De Paolis, J. Rouden, J. Blanchet, *Chem. Eur. J* **2017**, *23*, 2005–2009.
[12] O. A. Nurkenov, Zh. S. Nurmaganbetov, T. M. Seilkhanov, S. D. Fazylov, Zh. B. Satpayeva, K. M. Turdybekov, S. A. Talipov, R. B. Seydakhmetova, *Russ J Gen Chem* **2019**, *89*, 2044–2051.
[13] W. I. Lone, A. Rashid, B. A. Bhat, S. Rashid, *Chem. Commun.* **2024**, *60*, 6544–6547.
[14] M. Subramani, S. K. Rajendran, *Eur. J. Org. Chem.* **2019**, *2019*, 3677–3686.
[15] T. Varlet, M. Matišić, E. Van Elslande, L. Neuville, V. Gandon, G. Masson, *J. Am. Chem. Soc.* **2021**, *143*, 11611–11619.
[16] L. T. Brechmann, B. Kaewmee, J. F. Teichert, *ACS Catal.* **2023**, *13*, 12634–12642.
[17] N. O. Thiel, J. F. Teichert, *Org. Biomol. Chem.* **2016**, *14*, 10660–10666.
[18] G. C. Fortman, A. M. Z. Slawin, S. P. Nolan, *Organometallics* **2010**, *29*, 3966–3972.
[19] T. Wakamatsu, K. Nagao, H. Ohmiya, M. Sawamura, *Organometallics* **2016**, *35*, 1354–1357.
[20] W. J. Kerr, G. J. Knox, M. Reid, T. Tuttle, J. Bergare, R. A. Bragg, *ACS Catal.* **2020**, *10*, 11120–11126.
[21] Q. Teng, C. Singh, Y. Han, H. V. Huynh, *Org. Biomol. Chem.* **2020**, *18*, 2487–2491.
[22] S. Ahrens, A. Peritz, T. Strassner, *Angew. Chem. Int. Ed.* **2009**, *48*, 7908–7910.
[23] D.-J. Dong, H.-H. Li, S.-K. Tian, *J. Am. Chem. Soc.* **2010**, *132*, 5018–5020.
[24] E. Richmond, J. Moran, *J. Org. Chem.* **2015**, *80*, 6922–6929.
[25] M. Majchrzak, G. Wilkowski, M. Kubicki, *Eur. J. Org. Chem.* **2017**, *2017*, 4291–4299.
[26] S.-S. Li, L. Tao, F.-Z.-R. Wang, Y.-M. Liu, Y. Cao, *Adv. Synth. Catal.* **2016**, *358*, 1410–1416.
[27] P. Adler, C. J. Teskey, D. Kaiser, M. Holy, H. H. Sitte, N. Maulide, *Nat. Chem.* **2019**, *11*, 329–334.
[28] P. Wheeler, H. U. Vora, T. Rovis, *Chem. Sci.* **2013**, *4*, 1674.
[29] B. M. Zimmermann, S. C. K. Kobosil, J. F. Teichert, *Chem. Commun.* **2019**, *55*, 2293–2296.
[30] J. Bruffaerts, N. von Wolff, Y. Diskin-Posner, Y. Ben-David, D. Milstein, *J. Am. Chem. Soc.* **2019**, *141*, 16486–16493.
[31] A. J. M. Farley, P. Jakubec, A. M. Goldys, D. J. Dixon, *Tetrahedron* **2018**, *74*, 5206–5212.
[32] Y. Thummala, A. K. Morri, G. V. Karunakar, V. R. Doddi, *Eur. J. Org. Chem.* **2018**, *2018*, 6280–6285.
[33] H. Zhong, J. Wang, L. Li, R. Wang, *Dalton Trans.* **2013**, *43*, 2098–2103.
[34] R. Bernini, S. Cacchi, G. Fabrizi, G. Forte, F. Petrucci, A. Prastaro, S. Niembro, A. Shafir, A. Vallribera, *Org. Biomol. Chem.* **2009**, *7*, 2270–2273.

[35] M. Deponti, S. I. Kozhushkov, D. S. Yufit, L. Ackermann, *Org. Biomol. Chem.* **2012**, *11*, 142–148.
[36] J. Yang, M. Chen, J. Ma, W. Huang, H. Zhu, Y. Huang, W. Wang, *J. Mater. Chem. C* **2015**, *3*, 10074–10078.
[37] C.-H. Lin, Y.-J. Wang, C.-F. Lee, *Eur. J. Org. Chem.* **2010**, *2010*, 4368–4371.
[38] P. C. Too, Y.-F. Wang, S. Chiba, *Org. Lett.* **2010**, *12*, 5688–5691.
[39] M. Tait, M. Donnard, A. Minassi, J. Lefranc, B. Bechi, G. Carbone, P. O'Brien, J. Clayden, *Org. Lett.* **2013**, *15*, 34–37.
[40] M. Chen, X. Zheng, W. Li, J. He, A. Lei, *J. Am. Chem. Soc.* **2010**, *132*, 4101–4103.
[41] X. Lou, J. Lin, C. Y. Kwok, H. Lyu, *Angewandte Chemie International Edition* **2023**, *62*, e202312633.
[42] P. Thangsan, T. Rukkijakan, B. Thanaussavadate, K. Yiamsawat, J. Sirijaraensre, K. P. Gable, P. Chuawong, *Org. Biomol. Chem.* **2023**, *21*, 1501–1513.
[43] B. D. Stubbert, T. J. Marks, *J. Am. Chem. Soc.* **2007**, *129*, 4253–4271.
[44] V. K. Chenniappan, R. J. Rahaim, *Org. Lett.* **2016**, *18*, 5090–5093.
[45] S. Cacchi, G. Fabrizi, A. Fochetti, F. Ghirga, A. Goggiamani, A. Iazzetti, *Org. Biomol. Chem.* **2019**, *17*, 527–532.
[46] Y. Yang, X. Chew, C. W. Johannes, E. G. Robins, H. Jong, Y. H. Lim, *Eur. J. Org. Chem.* **2014**, *2014*, 7184–7192.
[47] F. Bellina, M. Biagetti, S. Guariento, M. Lessi, M. Fausti, P. Ronchi, E. Rosadoni, *RSC Adv.* **2021**, *11*, 25504–25509.
[48] B. E. Moulton, A. C. Whitwood, A. K. Duhme-Klair, J. M. Lynam, I. J. S. Fairlamb, *J. Org. Chem.* **2011**, *76*, 5320–5334.
[49] C.-L. Sun, Q. Liao, T. Li, J. Li, J.-Q. Jiang, Z.-Z. Xu, X.-D. Wang, R. Shen, D.-C. Bai, Q. Wang, S.-X. Zhang, H.-B. Fu, H.-L. Zhang, *Chem. Sci.* **2014**, *6*, 761–769.
[50] W. I. Nicholson, F. Barreteau, J. A. Leitch, R. Payne, I. Priestley, E. Godineau, C. Battilocchio, D. L. Browne, *Angew. Chem. Int. Ed* **2021**, *60*, 21868–21874.
[51] C. Clarasó, L. Vicens, A. Polo, M. Costas, *Org. Lett.* **2019**, *21*, 2430–2435.
[52] J. Barrios-Rivera, Y. Xu, M. Wills, *Org. Lett.* **2020**, *22*, 6283–6287.
[53] E. Manoni, A. Gualandi, L. Mengozzi, M. Bandini, P. G. Cozzi, *RSC Adv.* **2015**, *5*, 10546–10550.
[54] Y. Kadosh, S. Muthuraman, K. Yaniv, Y. Baruch, J. Gopas, A. Kushmaro, R. S. Kumar, *Molecules* **2021**, *26*, 2293.
[55] H.-Z. Wu, Z.-S. Teng, Y.-X. Ke, Y. Zou, P. Gao, Y. Li, C.-H. Zhou, Z.-L. Zang, *Org. Biomol. Chem.* **2023**, *21*, 8579–8583.
[56] W.-L. Yu, Z.-G. Ren, K.-X. Ma, H.-Q. Yang, J.-J. Yang, H. Zheng, W. Wu, P.-F. Xu, *Chem. Sci.* **2022**, *13*, 7947–7954.
[57] CrysAlisPro Software System, Rigaku Oxford Diffraction, (2023), **n.d.**
[58] O. V. Dolomanov, L. J. Bourhis, R. J. Gildea, J. a. K. Howard, H. Puschmann, *J. Appl. Cryst.* **2009**, *42*, 339–341.
[59] G. M. Sheldrick, *Acta Crystallogr A* **2015**, *71*, 3–8.
[60] G. M. Sheldrick, *Acta Crystallogr C* **2015**, *71*, 3–8.
[61] Gaussian 16, Revision C.01 M. J. Frisch, G. W. Trucks, H. B. Schlegel, G. E. Scuseria, M. A. Robb, J. R. Cheeseman, G. Scalmani, V. Barone, G. A. Petersson, H. Nakatsuji, X. Li, M. Caricato, A. V. Marenich, J. Bloino, B. G. Janesko, R. Gomperts, B. Mennucci, H. P. Hratchian, J. V. Ortiz, A. F. Izmaylov, J. L. Sonnenberg, D. Williams-Young, F. Ding, F. Lipparini, F. Egidi, J. Goings, B. Peng, A. Petrone, T. Henderson, D. Ranasinghe, V. G. Zakrzewski, J. Gao, N. Rega, G. Zheng, W. Liang, M. Hada, M. Ehara, K. Toyota, R. Fukuda, J. Hasegawa, M. Ishida, T. Nakajima, Y. Honda, O. Kitao, H. Nakai, T. Vreven, K. Throssell, J. A. Montgomery, Jr., J. E. Peralta, F. Ogliaro, M. J. Bearpark, J. J. Heyd, E. N. Brothers, K. N. Kudin, V. N. Staroverov, T. A. Keith, R. Kobayashi, J. Normand, K. Raghavachari, A. P. Rendell, J. C. Burant, S. S.

Iyengar, J. Tomasi, M. Cossi, J. M. Millam, M. Klene, C. Adamo, R. Cammi, J. W. Ochterski, R. L. Martin, K. Morokuma, O. Farkas, J. B. Foresman and D. J. Fox, Inc., Wallingford CT, **2016**.
[62] M. D. Hanwell, D. E. Curtis, D. C. Lonie, T. Vandermeersch, E. Zurek, G. R. Hutchison, *J Cheminform* **2012**, *4*, 17.
[63] Avogadro an open-source molecular builder and visualization tool. Version 1.2.0. http://avogadro.cc/.
[64] S. Grimme, C. Bannwarth, S. Ehlert, *grimme-lab/xtb: xtb version 6.3.2* **2020**.
[65] C. Lee, W. Yang, R. G. Parr, *Phys Rev B Condens Matter* **1988**, *37*, 785–789.
[66] A. D. Becke, *J. Chem. Phys.* **1993**, *98*, 5648–5652.
[67] F. Weigend, R. Ahlrichs, *Phys. Chem. Chem. Phys.* **2005**, *7*, 3297–3305.
[68] R. Ditchfield, W. J. Hehre, J. A. Pople, *J. Chem. Phys.* **1971**, *54*, 724–728.
[69] W. J. Hehre, R. Ditchfield, J. A. Pople, *J. Chem. Phys.* **1972**, *56*, 2257–2261.
[70] P. C. Hariharan, J. A. Pople, *Theoret. Chim. Acta* **1973**, *28*, 213–222.
[71] M. S. Gordon, J. S. Binkley, J. A. Pople, W. J. Pietro, W. J. Hehre, *J. Am. Chem. Soc.* **1982**, *104*, 2797–2803.
[72] M. M. Francl, W. J. Pietro, W. J. Hehre, J. S. Binkley, M. S. Gordon, D. J. DeFrees, J. A. Pople, *J. Chem. Phys.* **1982**, *77*, 3654–3665.
[73] S. Grimme, S. Ehrlich, L. Goerigk, *J. Comput. Chem.* **2011**, *32*, 1456–1465.
[74] A. Bondi, *J. Phys. Chem.* **1964**, *68*, 441–451.
[75] R. S. Rowland, R. Taylor, *J. Phys. Chem.* **1996**, *100*, 7384–7391.
