# Supplementary material for: Broadly Applicable Copper(I)-Catalyzed Alkyne Semihydrogenation and Hydrogenation of α,β-Unsaturated Amides Enabled by Bifunctional Iminopyridine Ligands
Source: J Am Chem Soc. 2025 Apr 16;147(17):14481–90. doi: 10.1021/jacs.5c01339 (PMC12046561; doi:10.1021/jacs.5c01339)
Supplement: Supplementary file 2 — ja5c01339_si_002.pdf [file ja5c01339_si_002.pdf]

Spectral data

Broadly applicable copper(I)-catalyzed alkyne semihydrogenation and hydrogenation of α,β-unsaturated amides enabled by bifunctional iminopyridine ligands

Mahadeb Gorai[a], Jonas H. Franzen[b], Philipp Rotering[b], Tobias Rüffer[a], Fabian Dielmann[b] and Johannes F. Teichert*

[a]Technische Universität Chemnitz, Institut für Chemie, Straße der Nationen 62, 09111 Chemnitz, Germany.
[b]Department of General, Inorganic and Theoretical Chemistry, Universität Innsbruck, Innrain 80/82, 6020 Innsbruck, Austria.

johannes.teichert@chemie.tu-chemnitz.de

1 Spectral data for catalyst precursors and catalysts

1.1 3-(2-Bromoethyl)-1-mesityl-1*H*-imidazol-3-ium bromide (S3b) (600 MHz, $CDCl_3$)

1.1.1 ^1H NMR

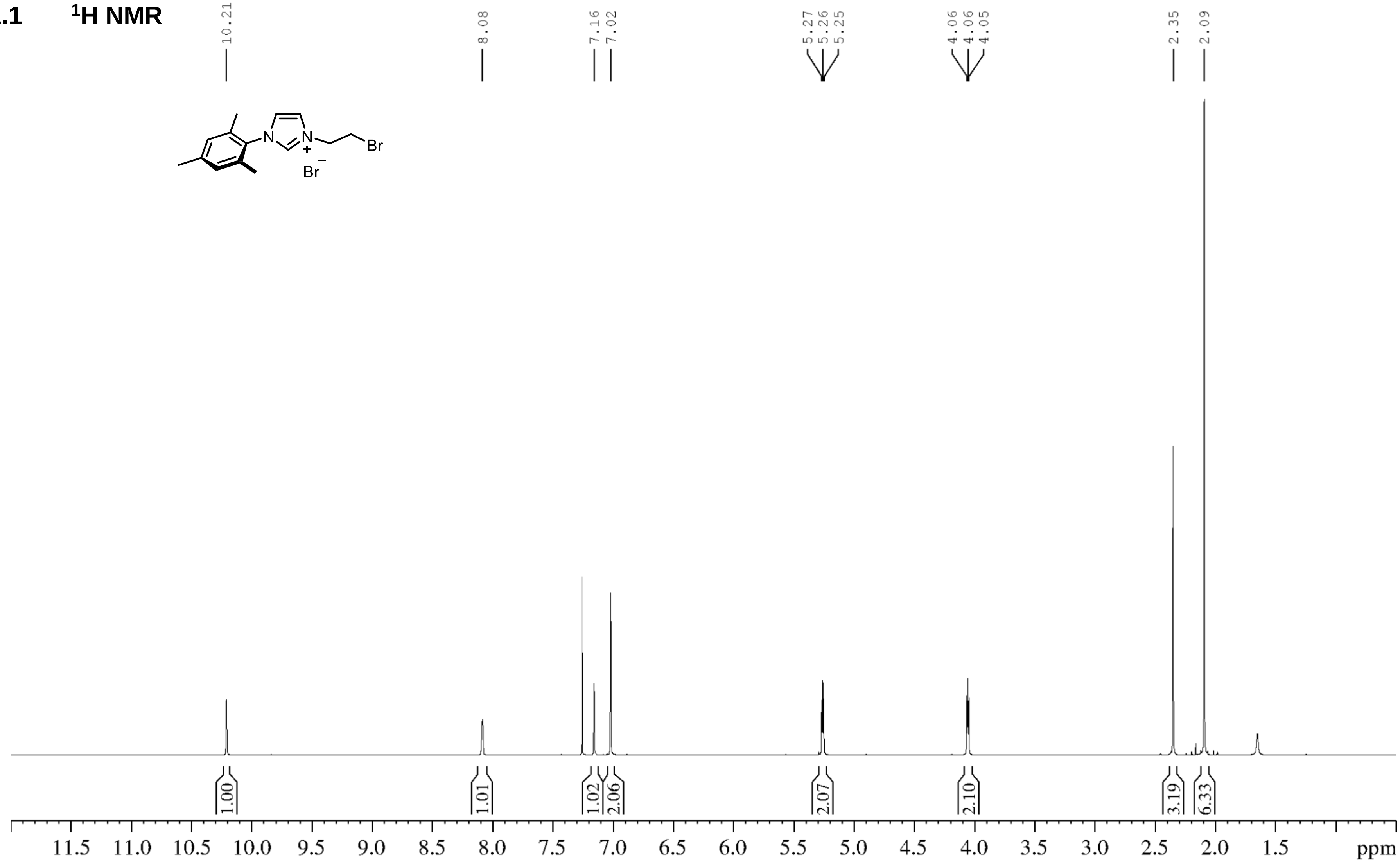

1.1.2 ^{13}C NMR

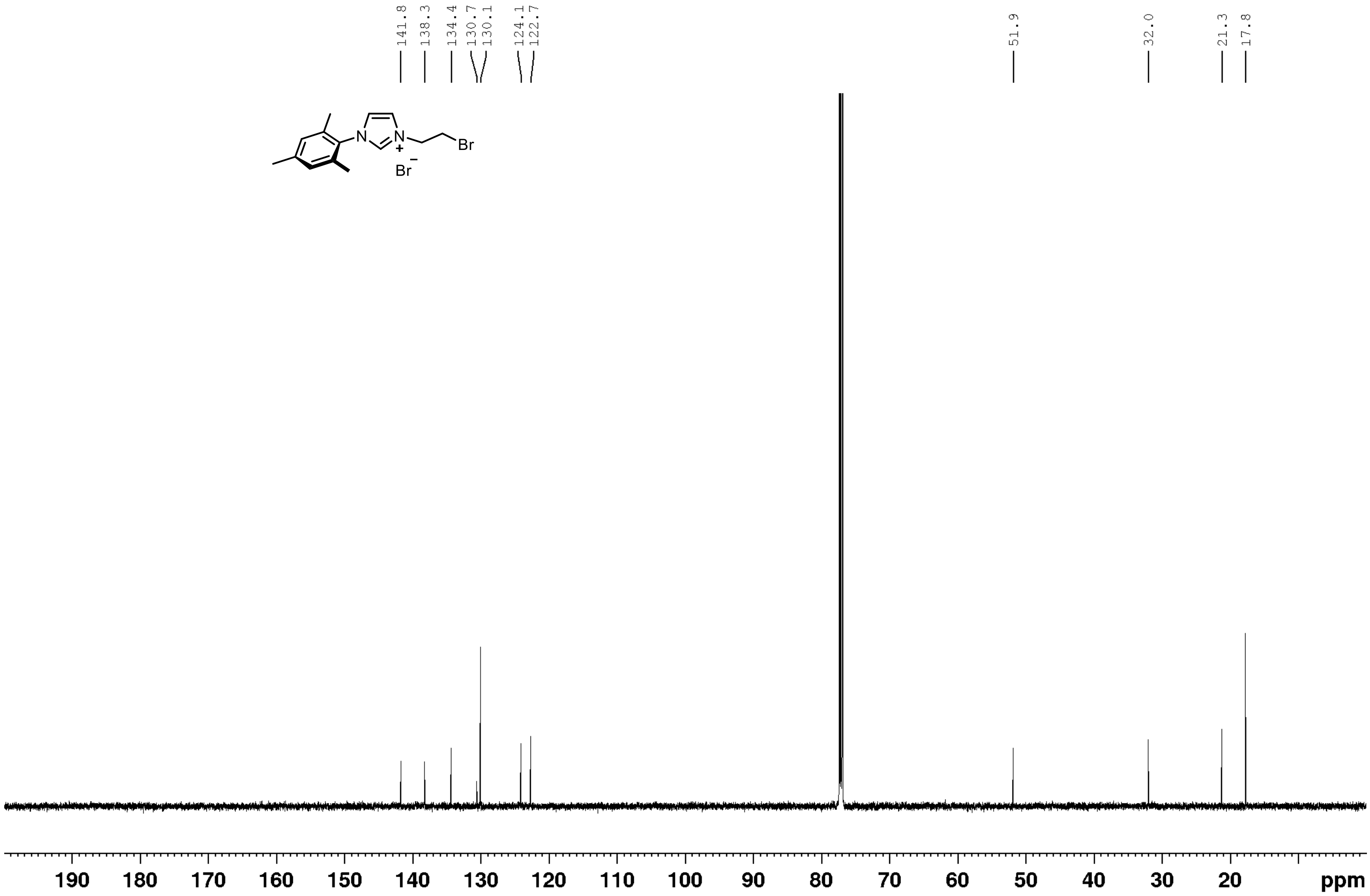
141.8
138.3
134.4
130.7
130.1
124.1
122.7
51.9
32.0
21.3
17.8
N
N
+
Br
Br
−
190
180
170
160
150
140
130
120
110
100
90
80
70
60
50
40
30
20
ppm

1.1.3 ^{1}H, ^{1}H COSY

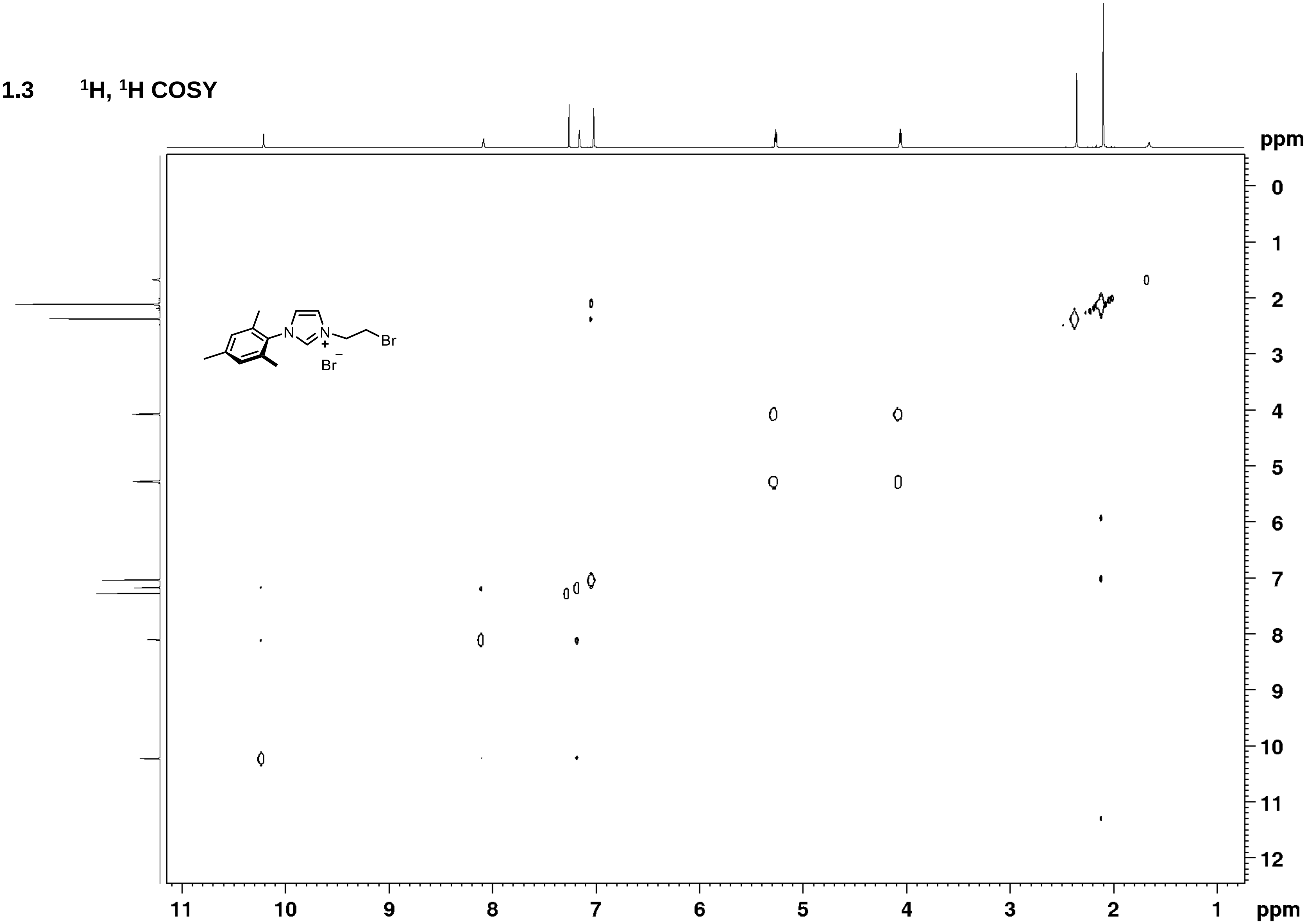

1.1.4 ^{1}H, ^{13}C HMBC

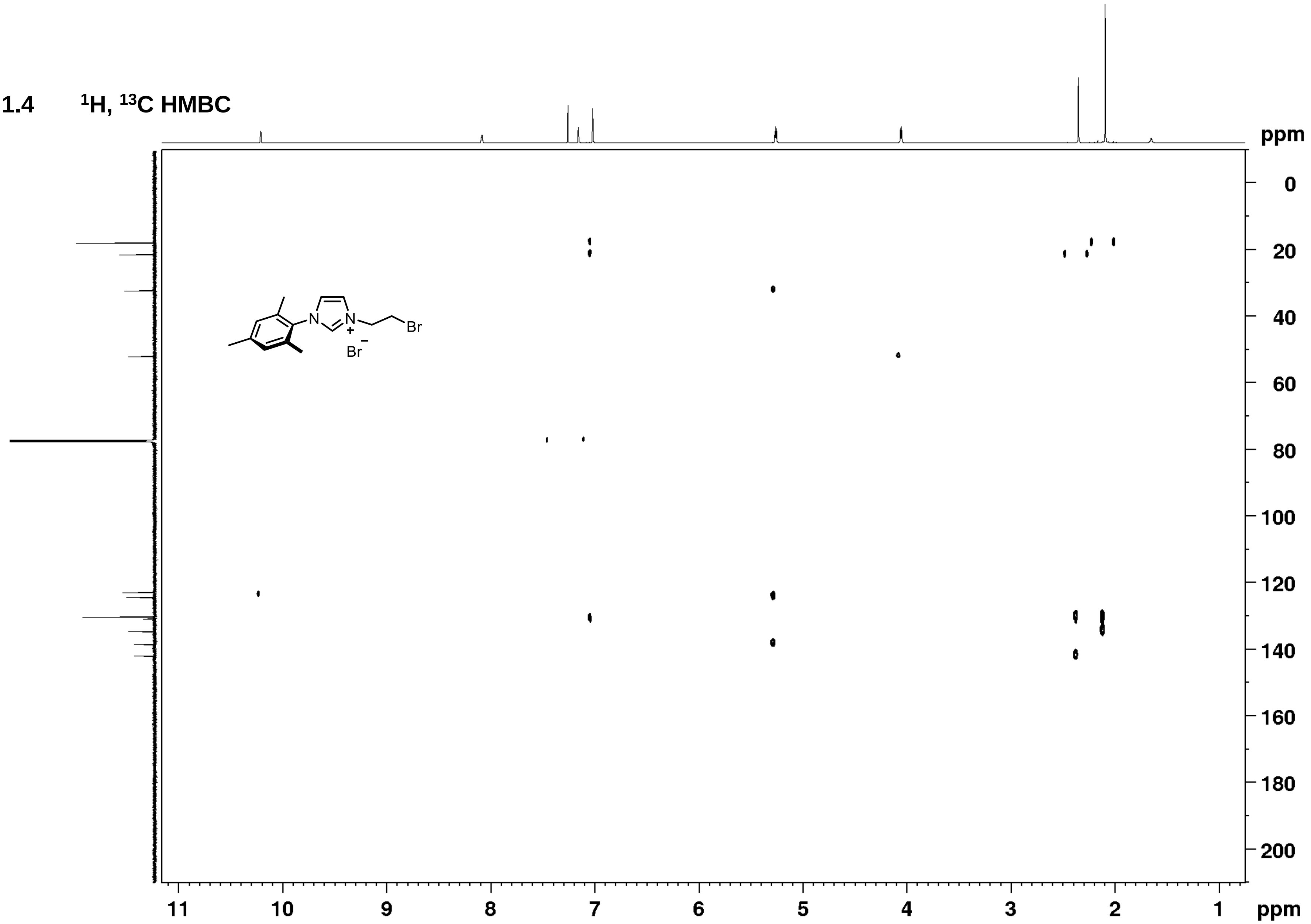

1.1.5 ^{1}H, ^{13}C HSQC

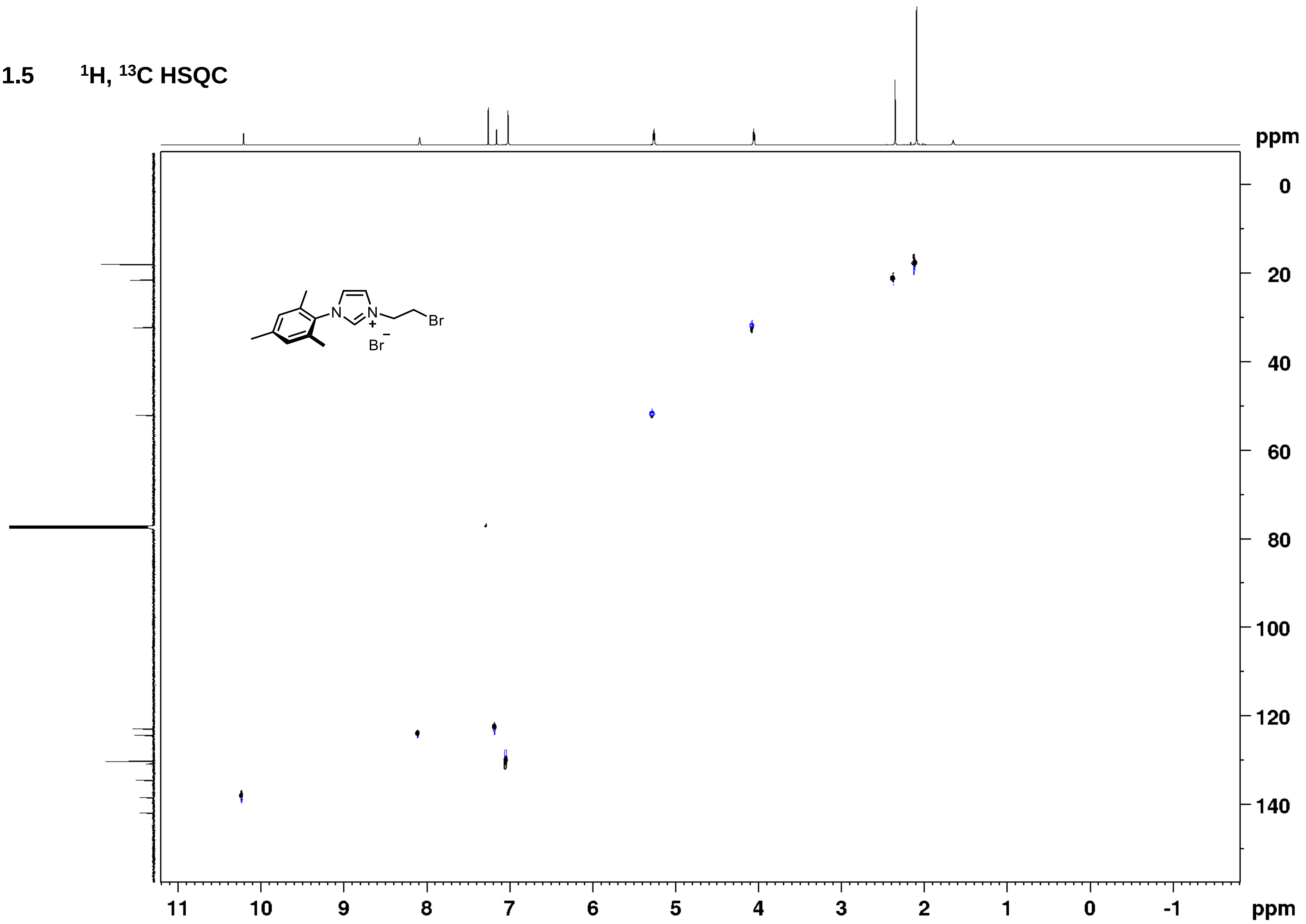

1.1.6 HRMS

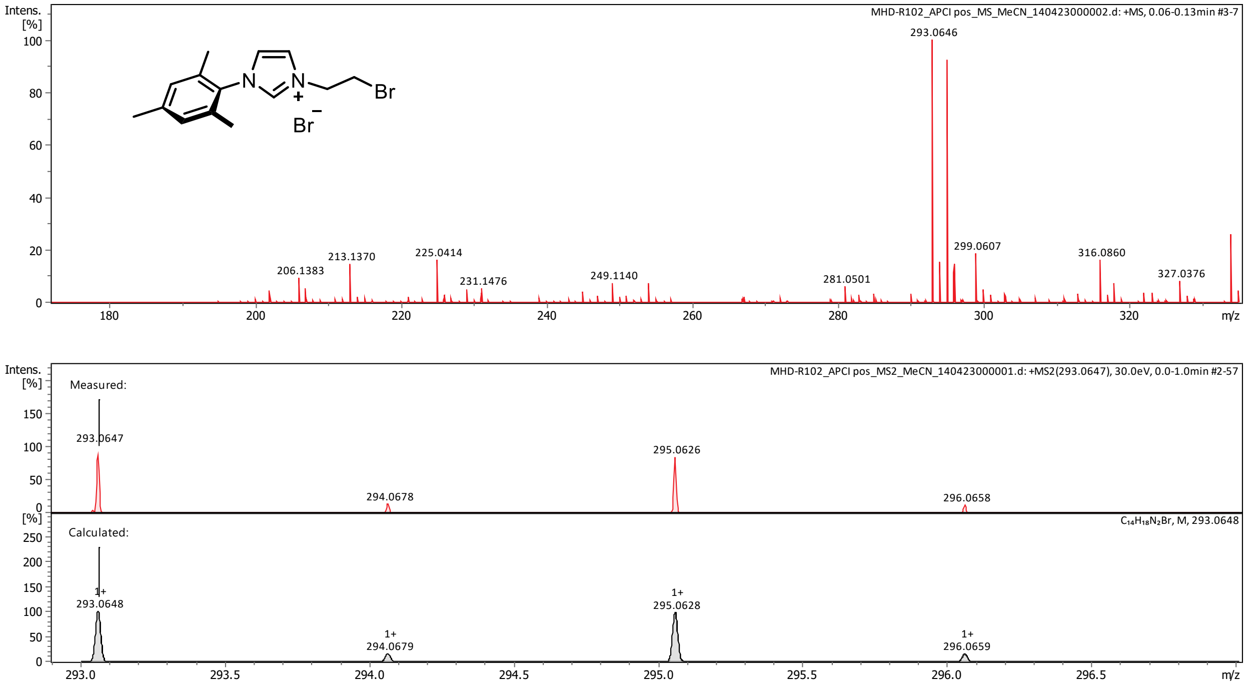

MHD-R102_APCI pos_MS_MeCN_140423000002.d: +MS, 0.06-0.13min #3-7
Intens. [%]
293.0646
299.0607
316.0860
327.0376
281.0501
249.1140
231.1476
225.0414
213.1370
206.1383
m/z
Br
Br
MHD-R102_APCI pos_MS2_MeCN_140423000001.d: +MS2(293.0647), 30.0eV, 0.0-1.0min #2-57
Measured:
293.0647
294.0678
295.0626
296.0658
Calculated:
C14H18N2Br, M, 293.0648
1+ 293.0648
1+ 294.0679
1+ 295.0628
1+ 296.0659

1.2 3-(2-(2-Imino-3-methylpyridin-1(2*H*)-yl)ethyl)-1-mesityl-1*H*-imidazol-3-ium bromide hydrobromide (L3) (600 MHz, DMSO-d_6)

1.2.1 ^{1}H NMR

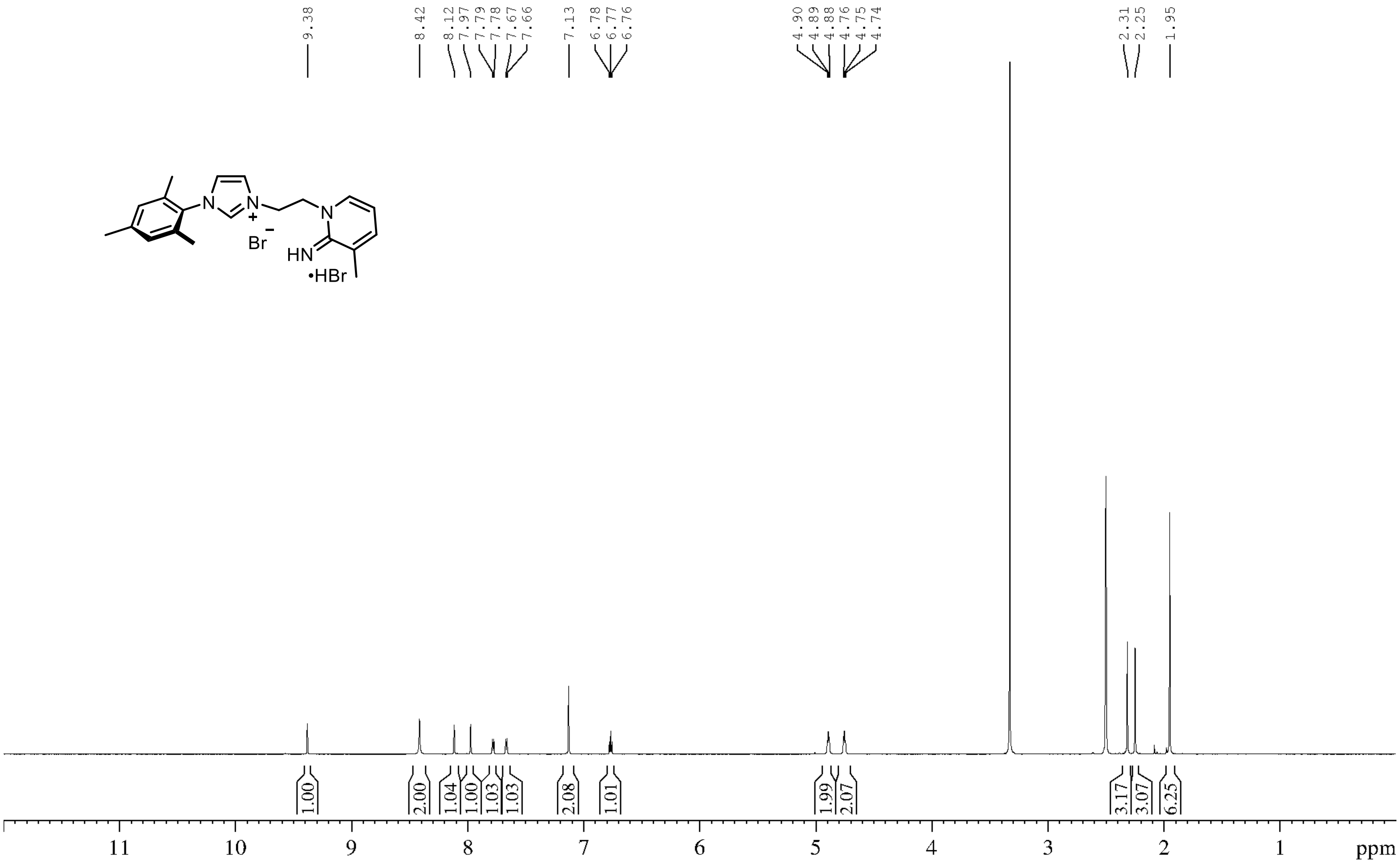

1.2.2 ^{13}C NMR

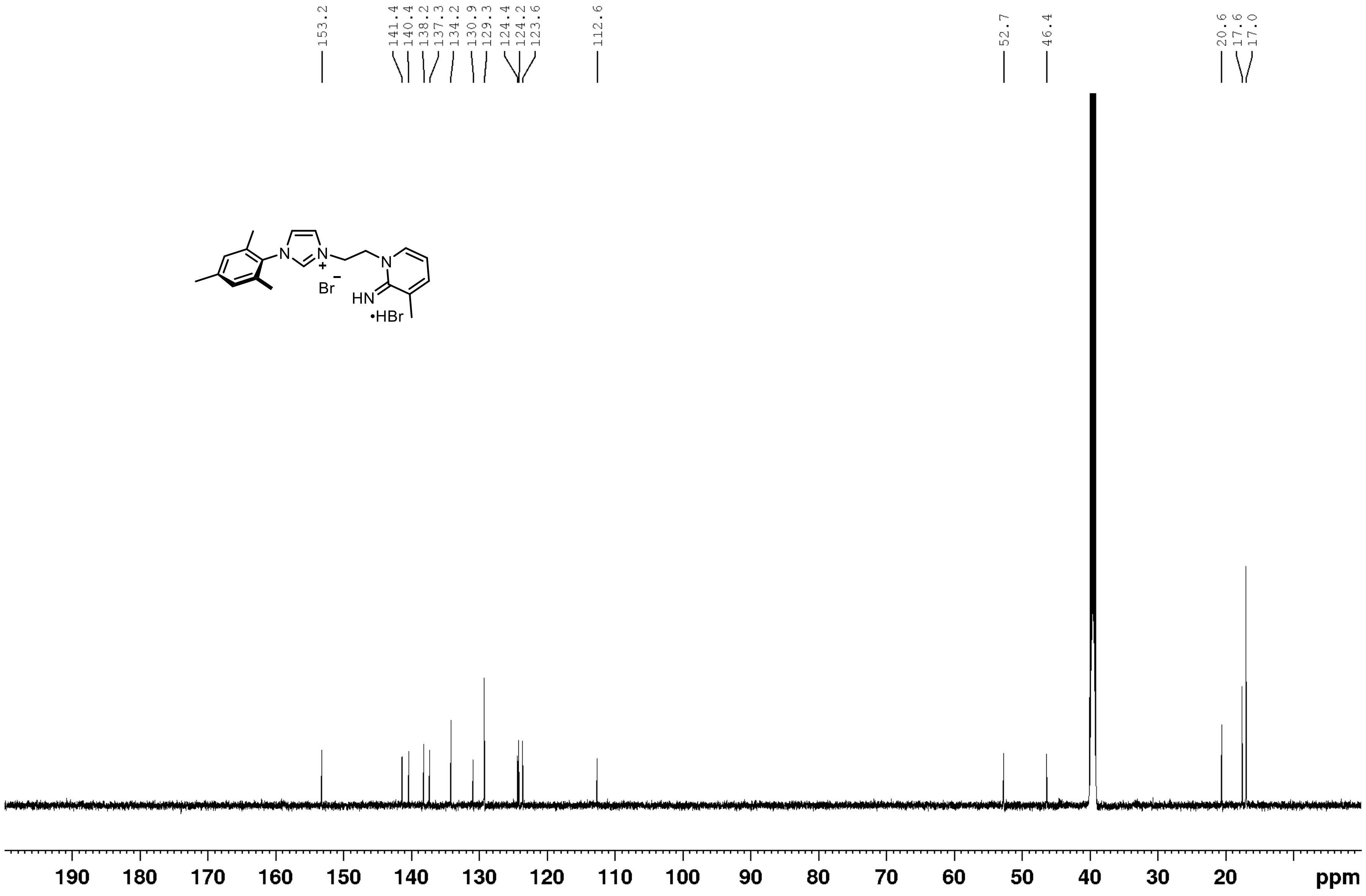
153.2
141.4
140.4
138.2
137.3
134.2
130.9
129.3
124.4
124.2
123.6
112.6
52.7
46.4
20.6
17.6
17.0
Br
HN
•HBr
190 180 170 160 150 140 130 120 110 100 90 80 70 60 50 40 30 20 ppm

1.2.3 ^{1}H, ^{1}H COSY

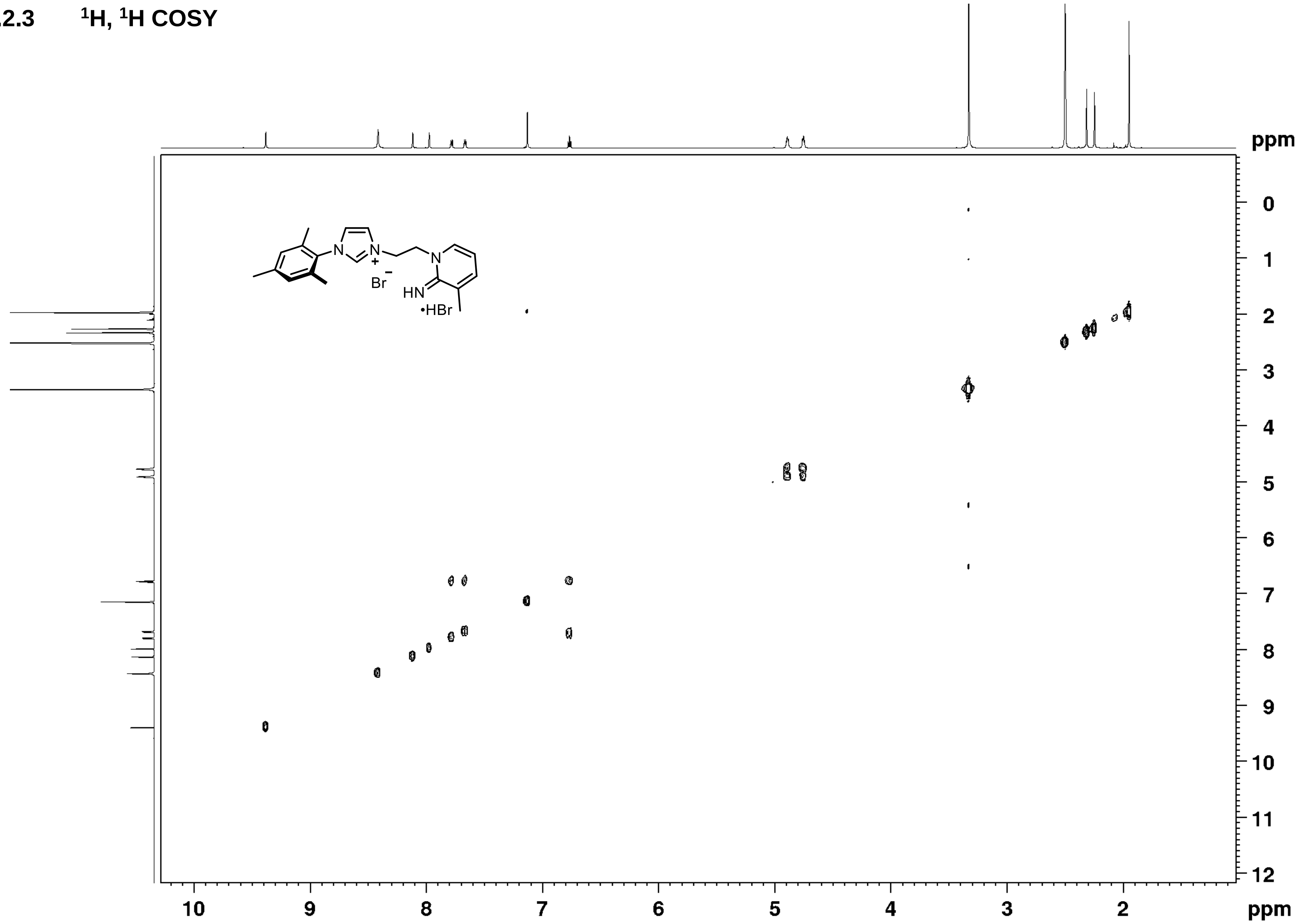

1.2.4 ^{1}H, ^{13}C HMBC

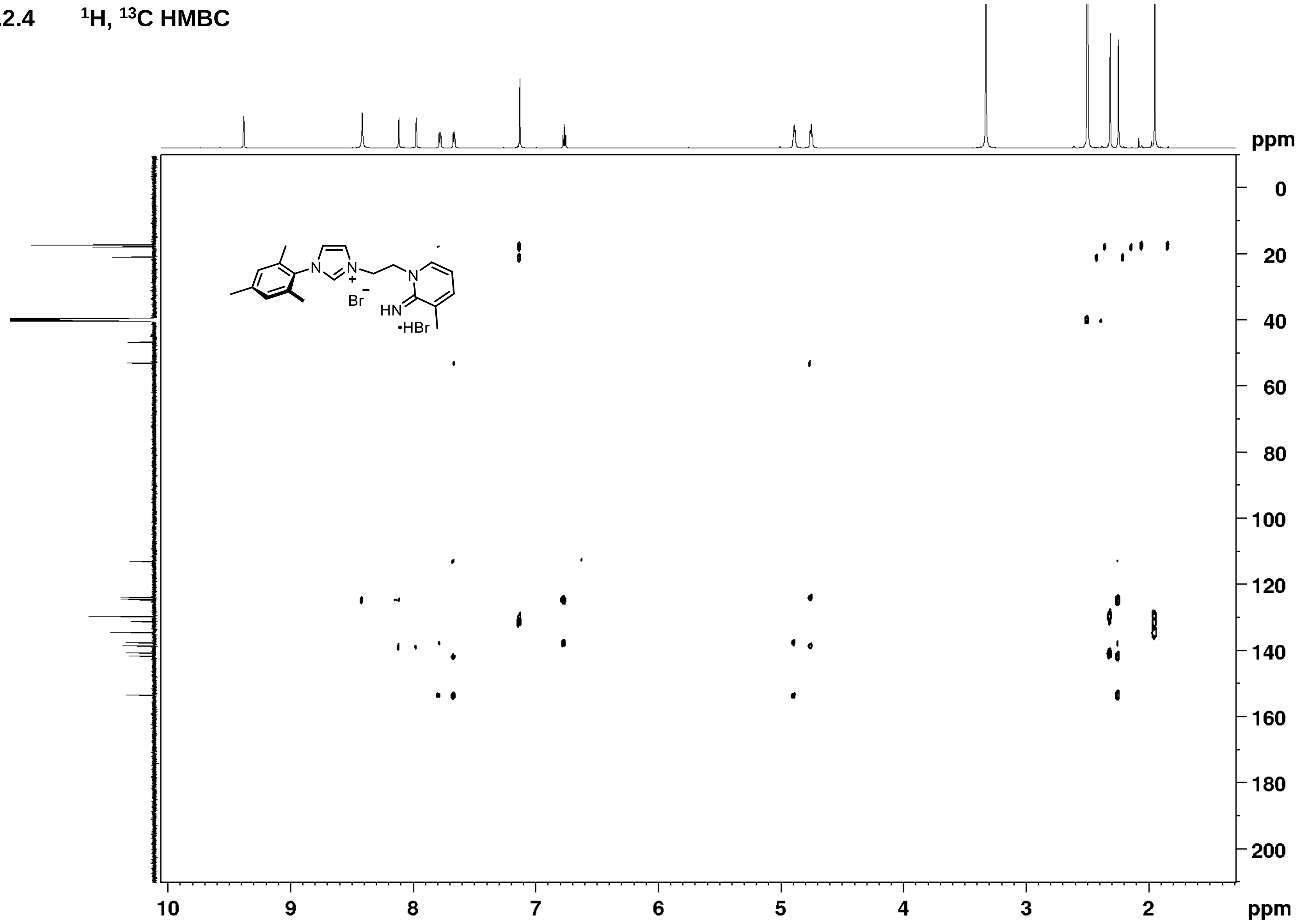

1.2.5 ^{1}H, ^{13}C HSQC

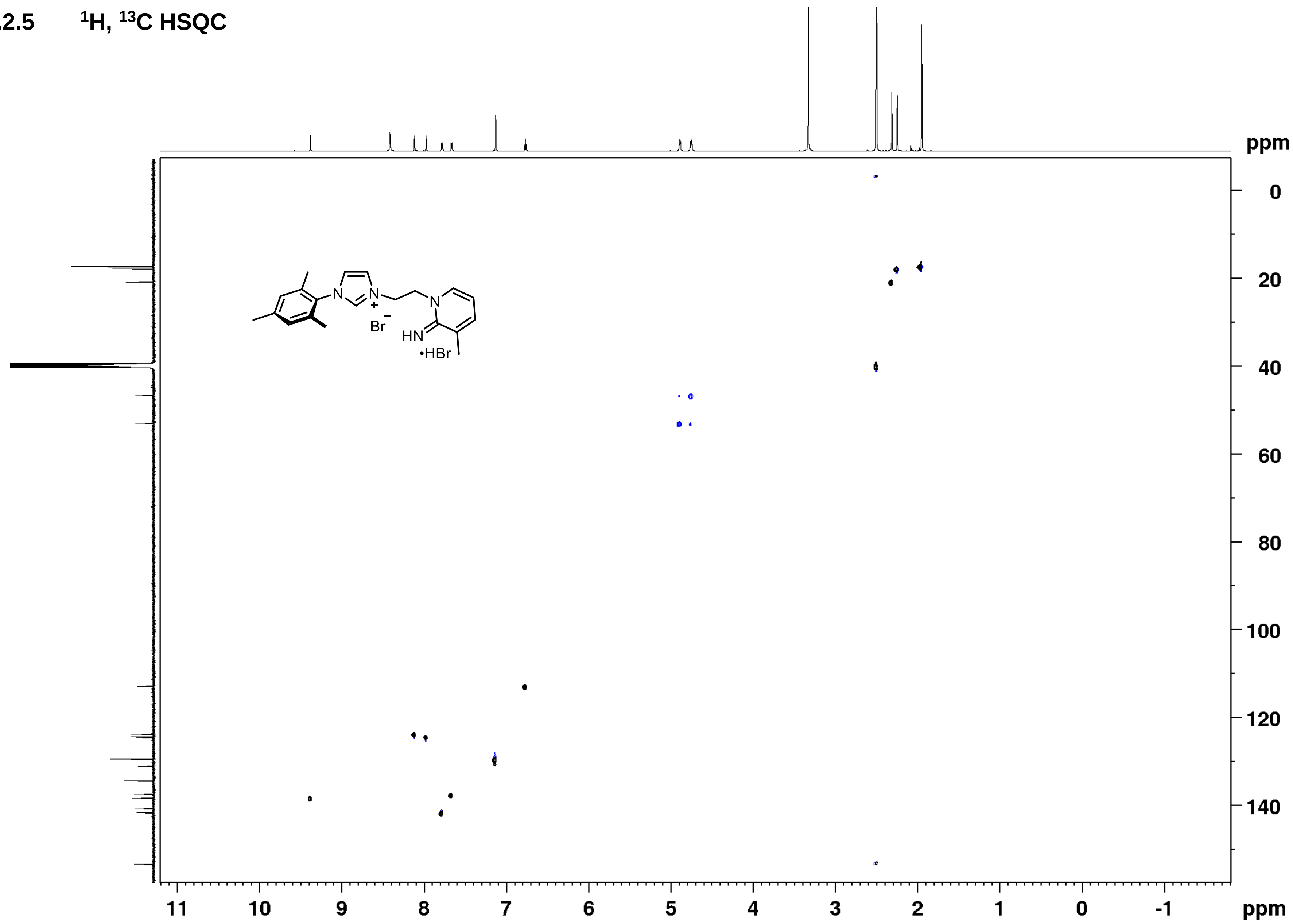

1.2.6 HRMS

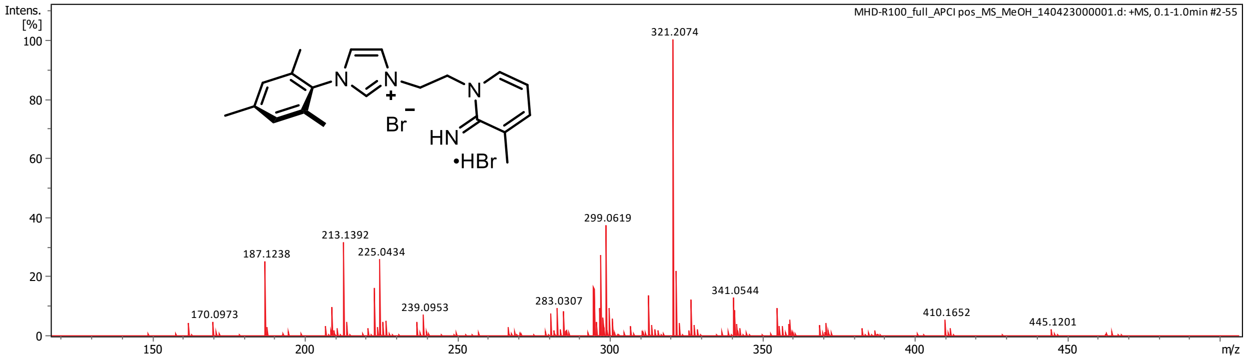
Intens.
[%]
MHD-R100_full_APCI pos_MS_MeOH_140423000001.d: +MS, 0.1-1.0min #2-55
321.2074
299.0619
213.1392
225.0434
187.1238
341.0544
239.0953
283.0307
170.0973
410.1652
445.1201
Br
HN
•HBr
m/z

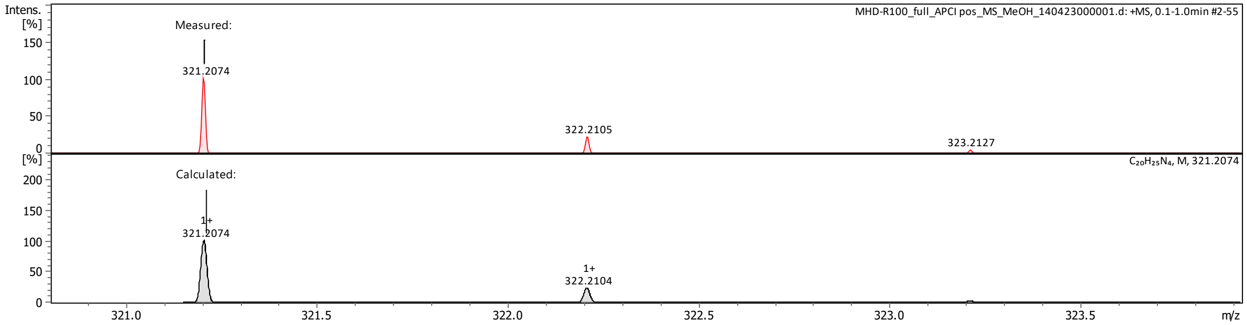
Intens.
[%]
MHD-R100_full_APCI pos_MS_MeOH_140423000001.d: +MS, 0.1-1.0min #2-55
Measured:
321.2074
322.2105
323.2127
$C_{20}H_{25}N_4$, M, 321.2074
Calculated:
1+
321.2074
1+
322.2104
m/z

1.2.7 IR

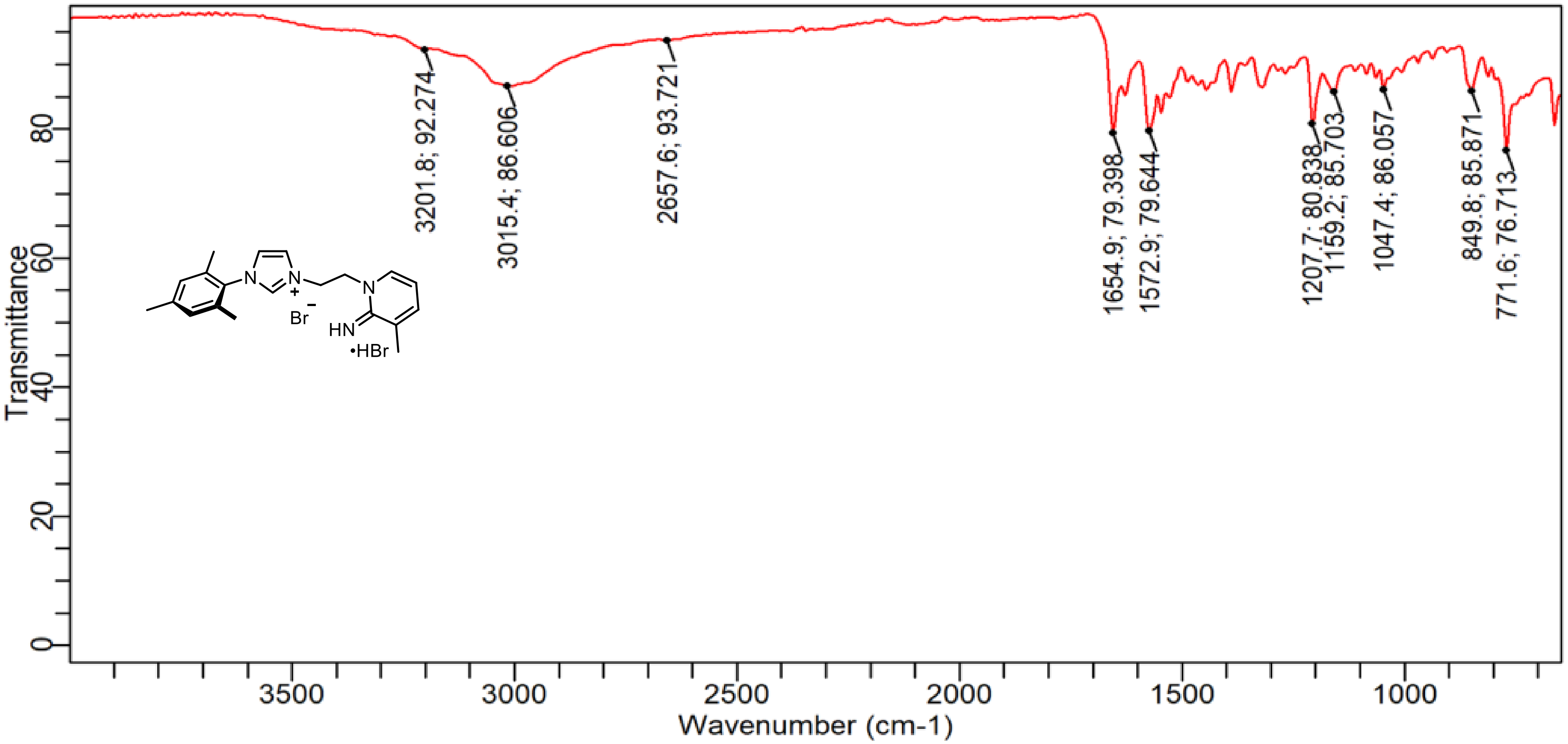

Transmittance
Wavenumber (cm-1)
0
20
40
60
80
3500
3000
2500
2000
1500
1000
3201.8; 92.274
3015.4; 86.606
2657.6; 93.721
1654.9; 79.398
1572.9; 79.644
1207.7; 80.838
1159.2; 85.703
1047.4; 86.057
849.8; 85.871
771.6; 76.713
N
N
+
Br
−
N
HN
•HBr

1.3 (1-(2-(2-Imino-3-methylpyridin-1(2*H*)-yl)ethyl)-3-mesityl-1,3-dihydro-2*H*-imidazol-2-ylidene)copper(I) chloride (3) (600 MHz, CD_2Cl_2)

1.3.1 ^{1}H NMR

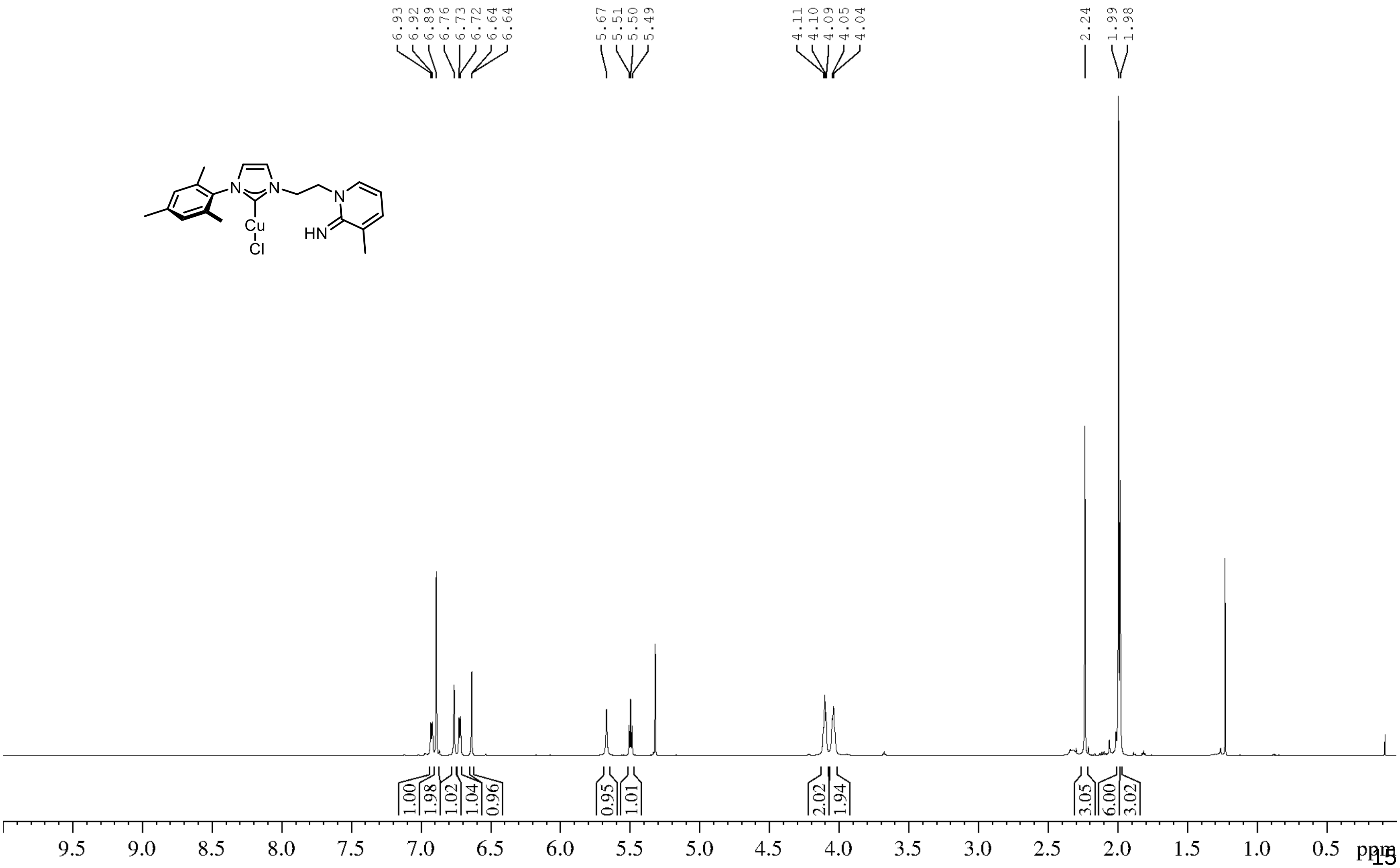

1.3.2 ^{13}C NMR

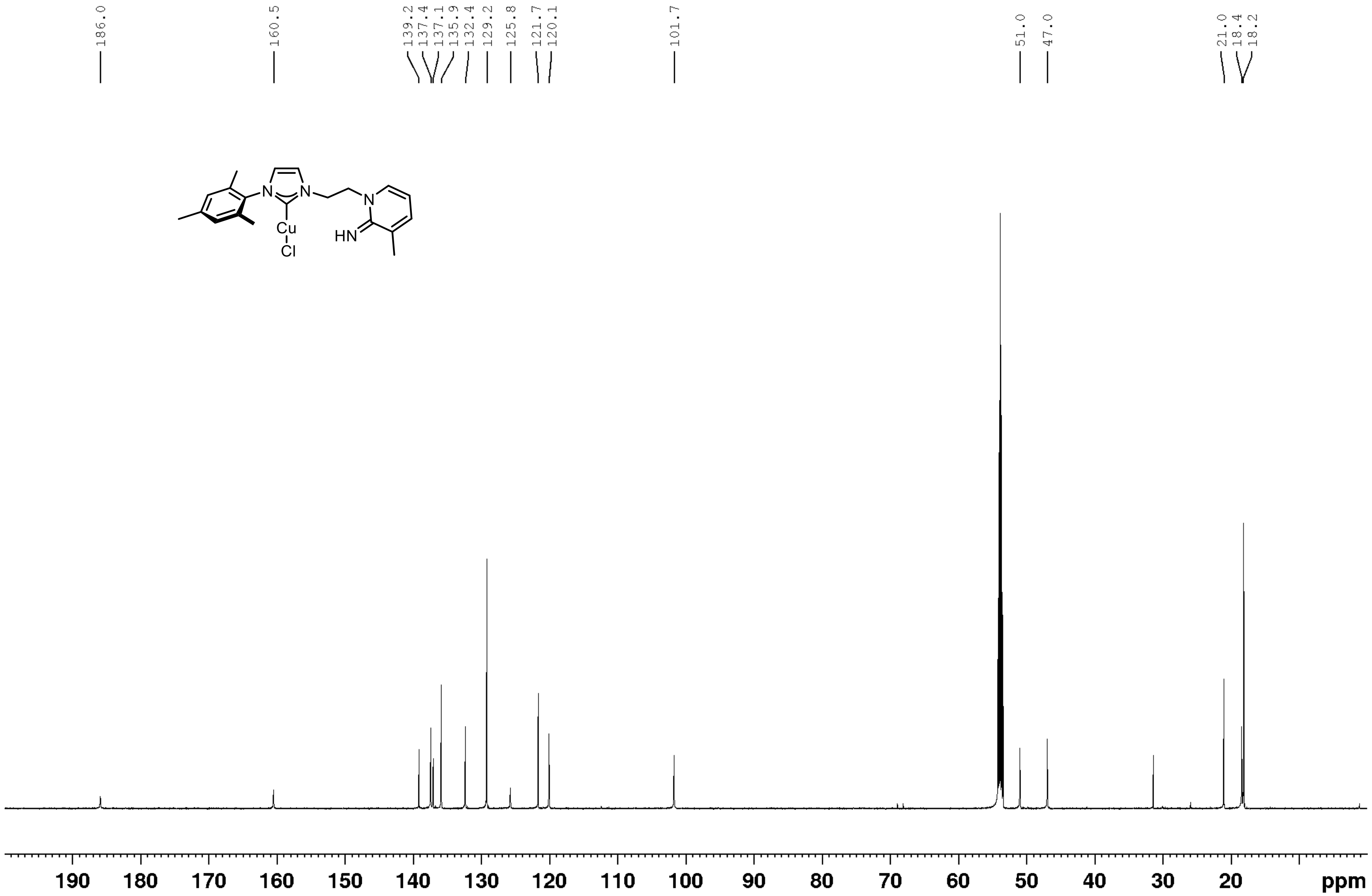

186.0
160.5
139.2
137.4
137.1
135.9
132.4
129.2
125.8
121.7
120.1
101.7
51.0
47.0
21.0
18.4
18.2
N
N
Cu
Cl
N
HN
190
180
170
160
150
140
130
120
110
100
90
80
70
60
50
40
30
20
ppm

1.3.3 ^{1}H, ^{1}H COSY

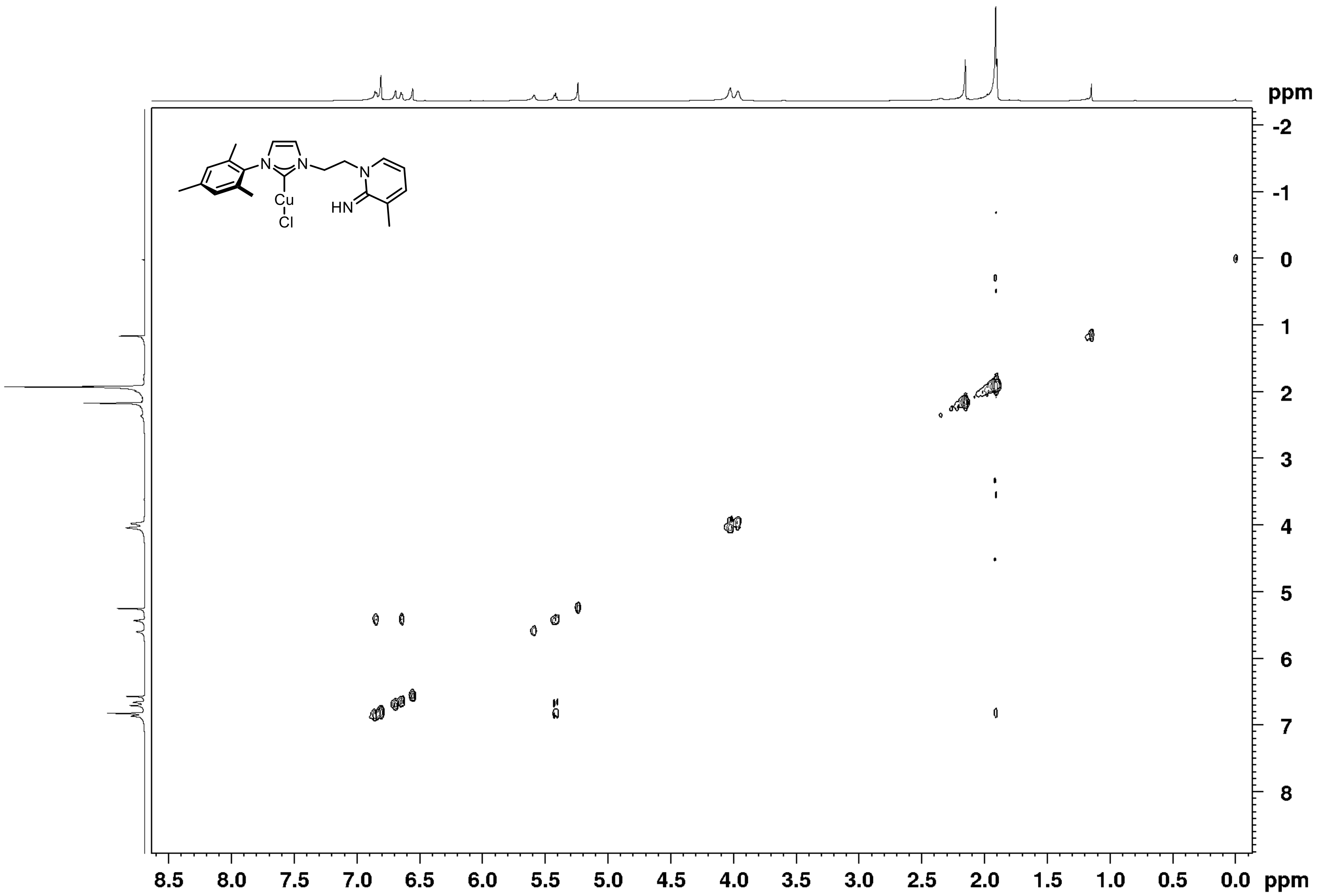

1.3.4 ^{1}H, ^{13}C HMBC

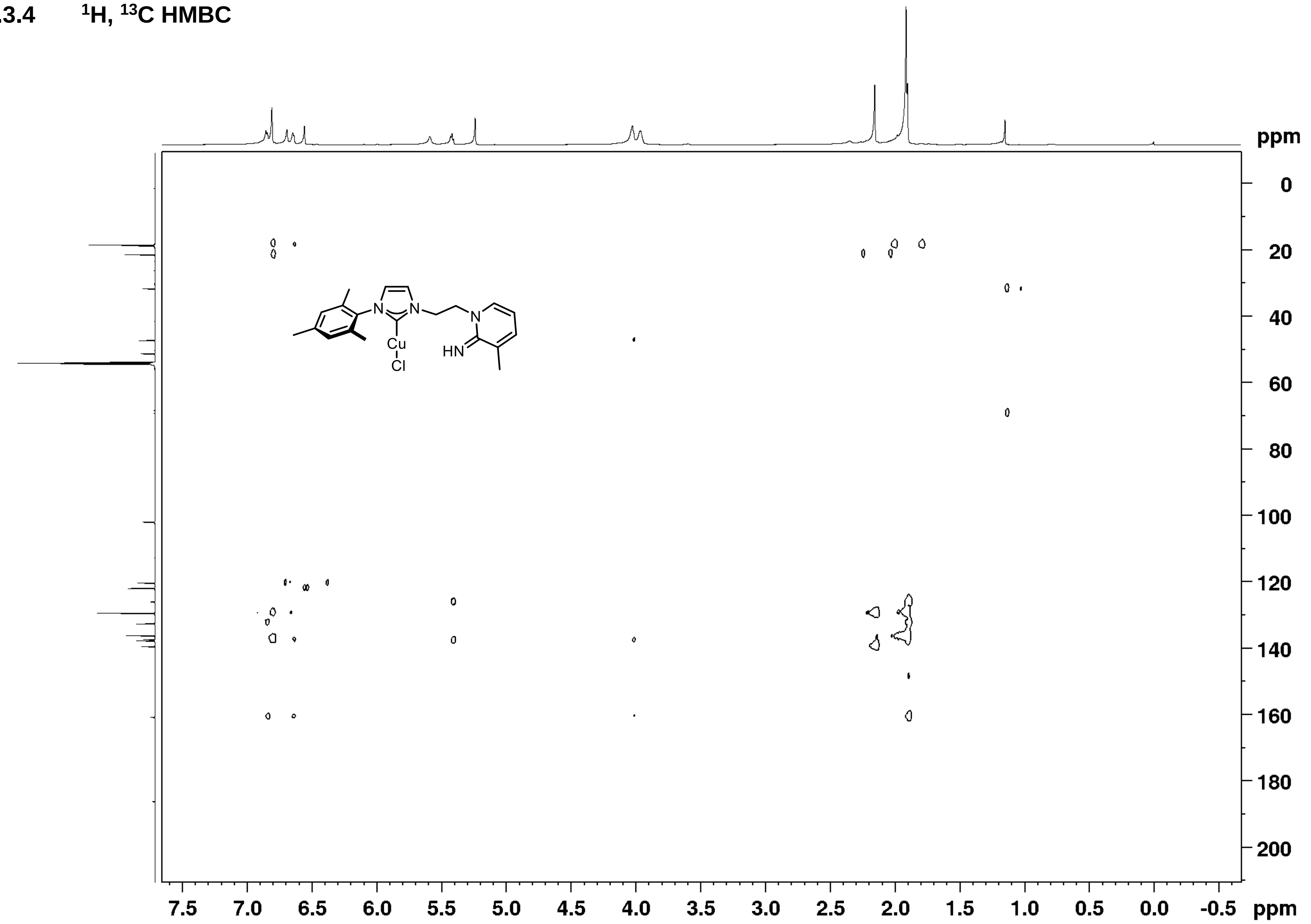

1.3.5 ^{1}H, ^{13}C HSQC

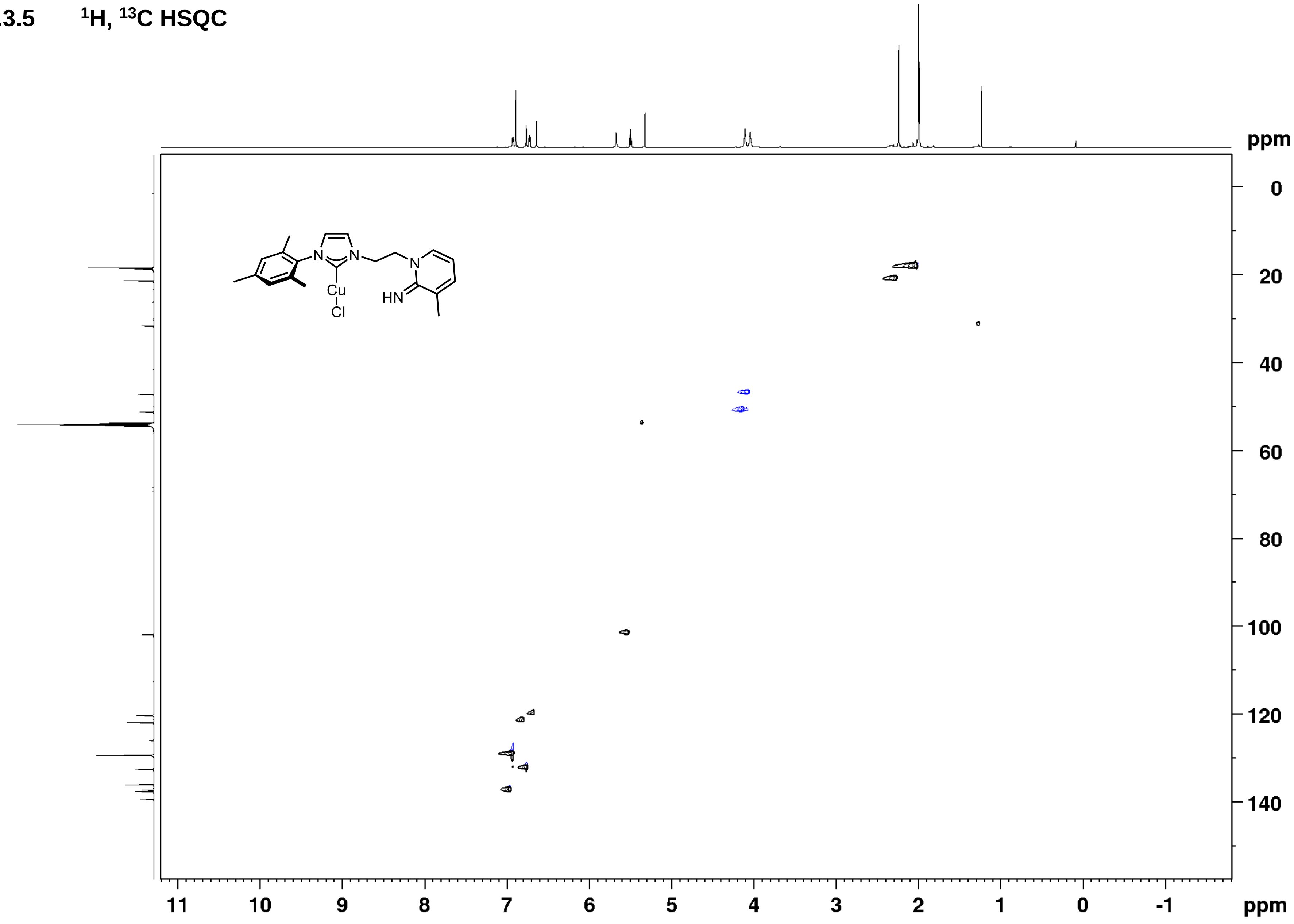

1.3.6 HRMS

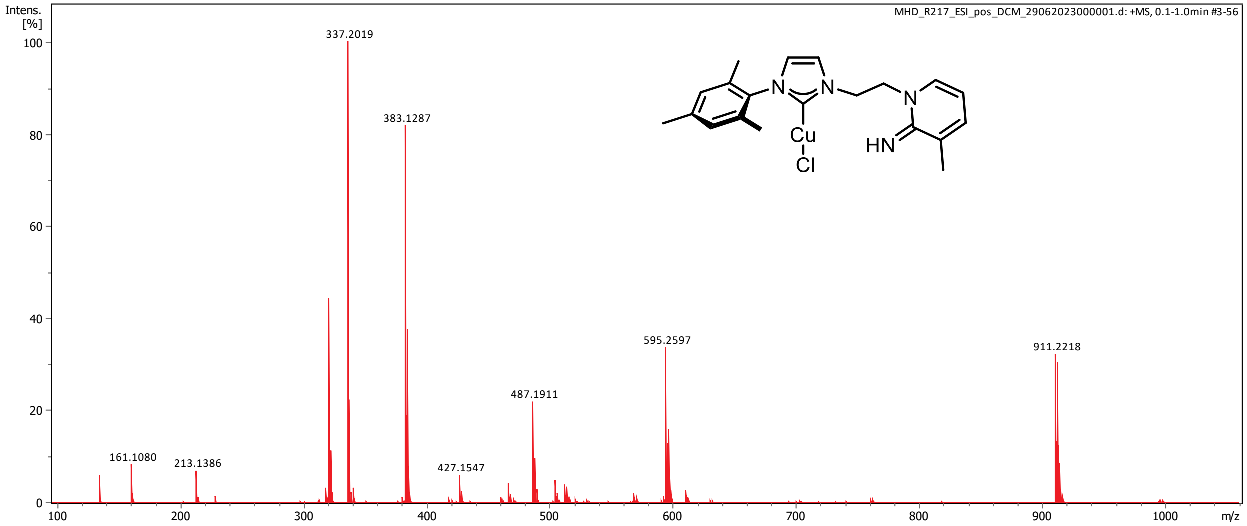

MHD_R217_ESI_pos_DCM_29062023000001.d: +MS, 0.1-1.0min #3-56
Intens. [%]
337.2019
383.1287
595.2597
911.2218
487.1911
161.1080
213.1386
427.1547
m/z
N
N
N
HN
Cu
Cl

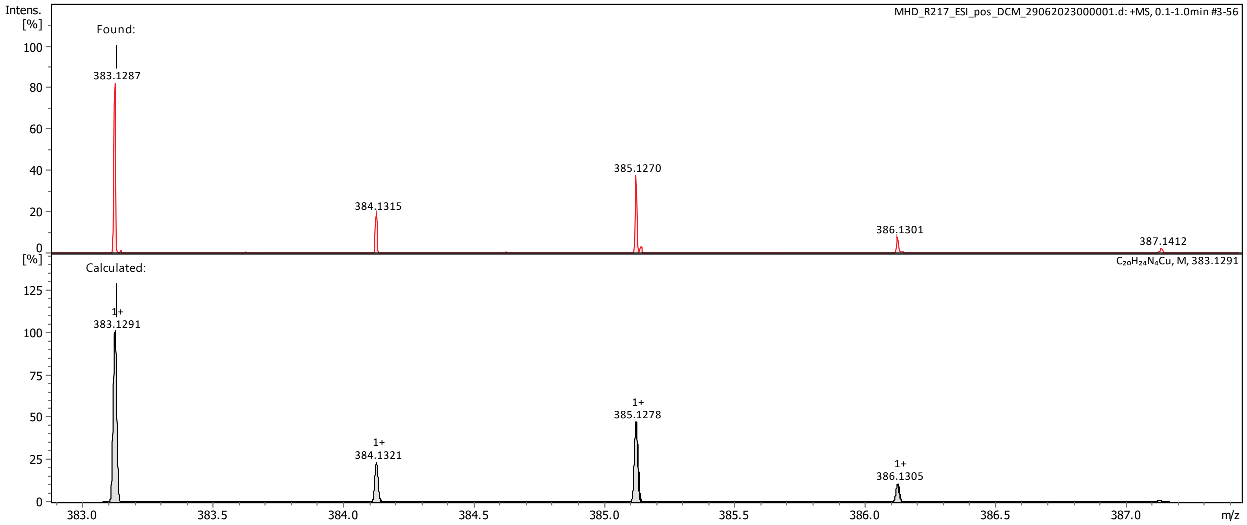

MHD_R217_ESI_pos_DCM_29062023000001.d: +MS, 0.1-1.0min #3-56
Intens. [%]
Found:
383.1287
384.1315
385.1270
386.1301
387.1412
Calculated:
$C_{20}H_{24}N_4Cu$, M, 383.1291
1+ 383.1291
1+ 384.1321
1+ 385.1278
1+ 386.1305
m/z

1.4 3-(2-Bromoethyl)-1-(2,6-diisopropylphenyl)-1*H*-imidazol-3-ium bromide (S4b) (600 MHz, $CDCl_3$)

1.4.1 ^{1}H NMR

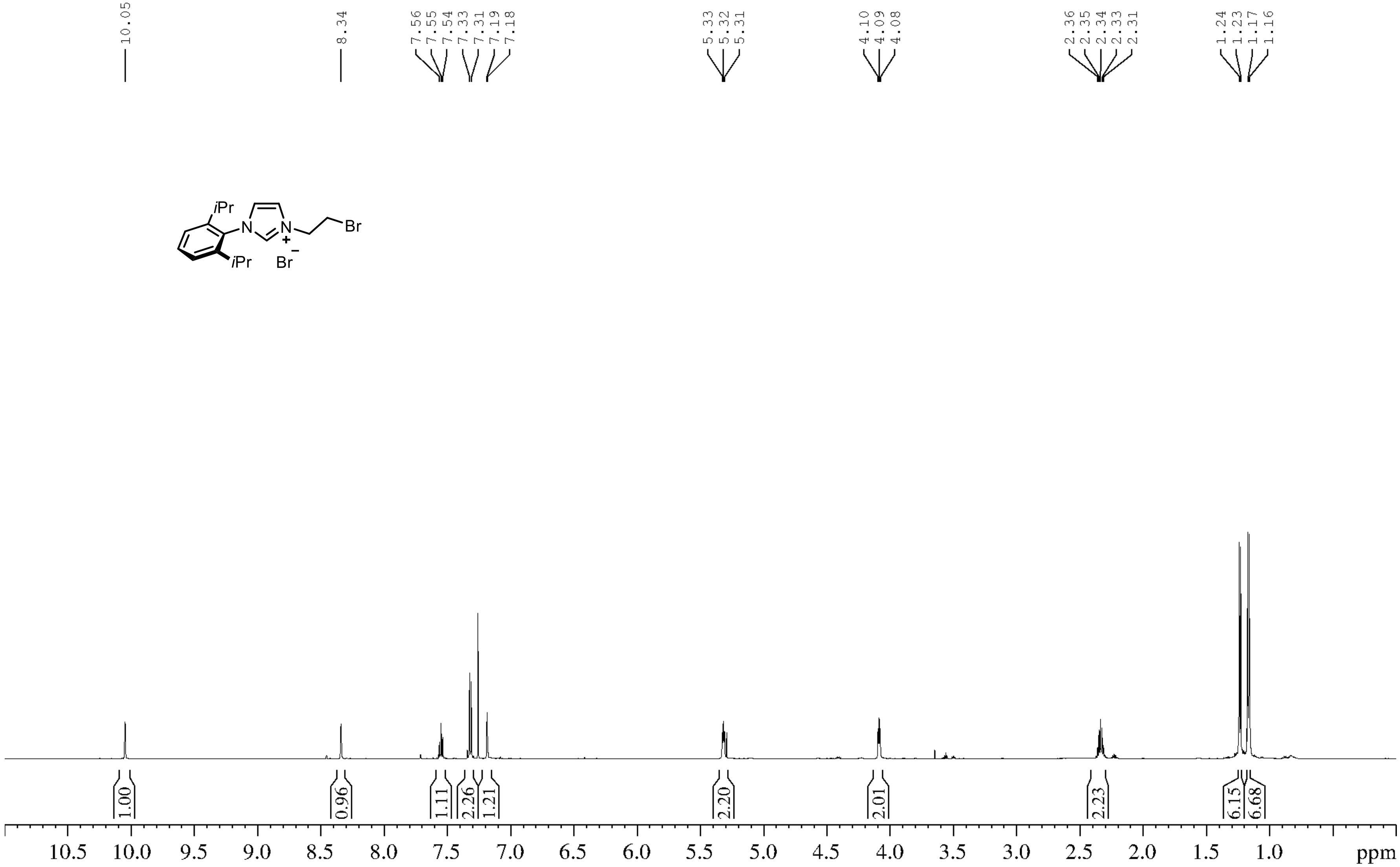

1.4.2 ^{13}C NMR

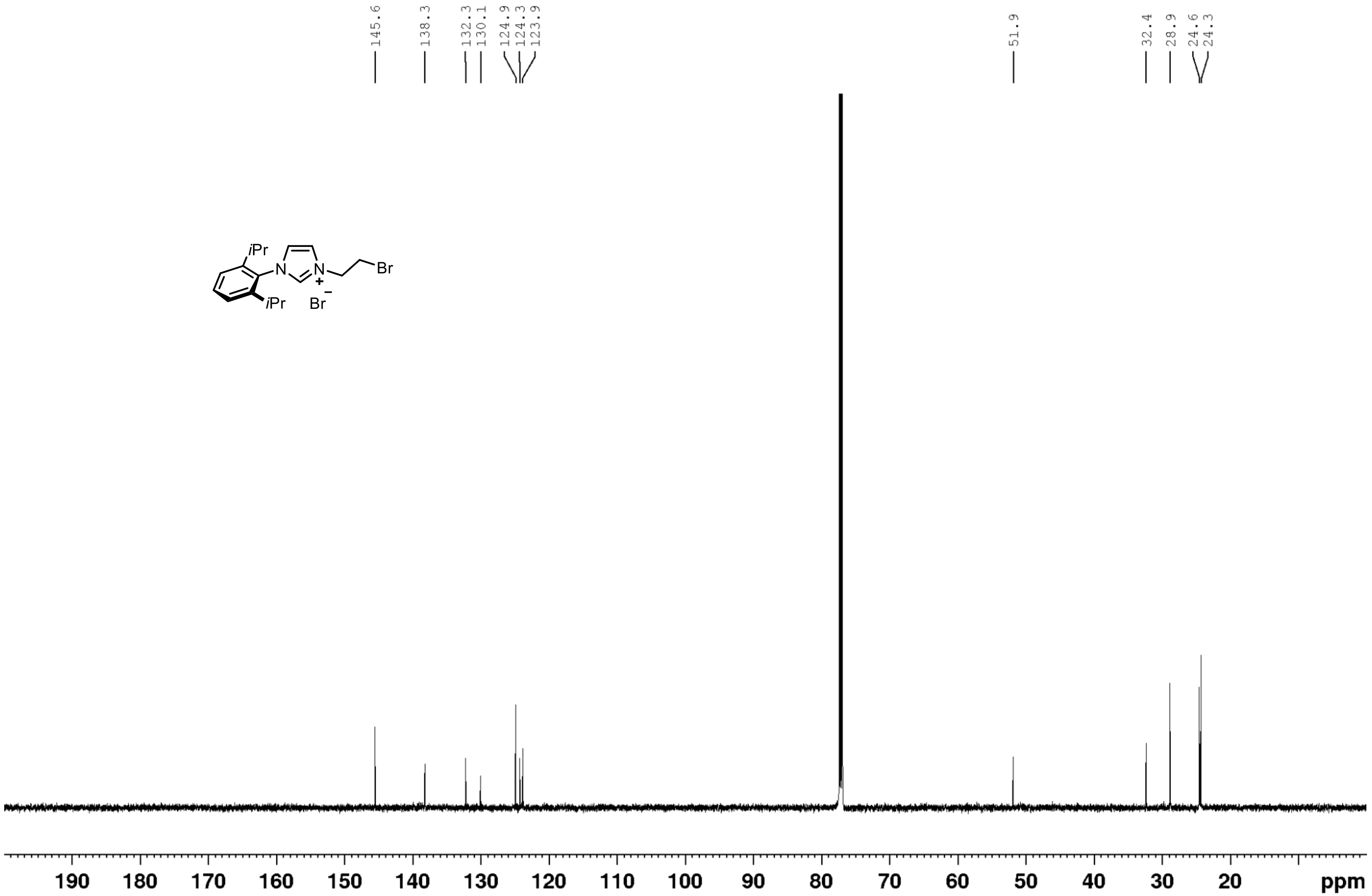

145.6
138.3
132.3
130.1
124.9
124.3
123.9
51.9
32.4
28.9
24.6
24.3
iPr
N
N
+
Br
iPr
Br−
190
180
170
160
150
140
130
120
110
100
90
80
70
60
50
40
30
20
ppm

1.4.3 $^{1}H,^{1}H$ COSY

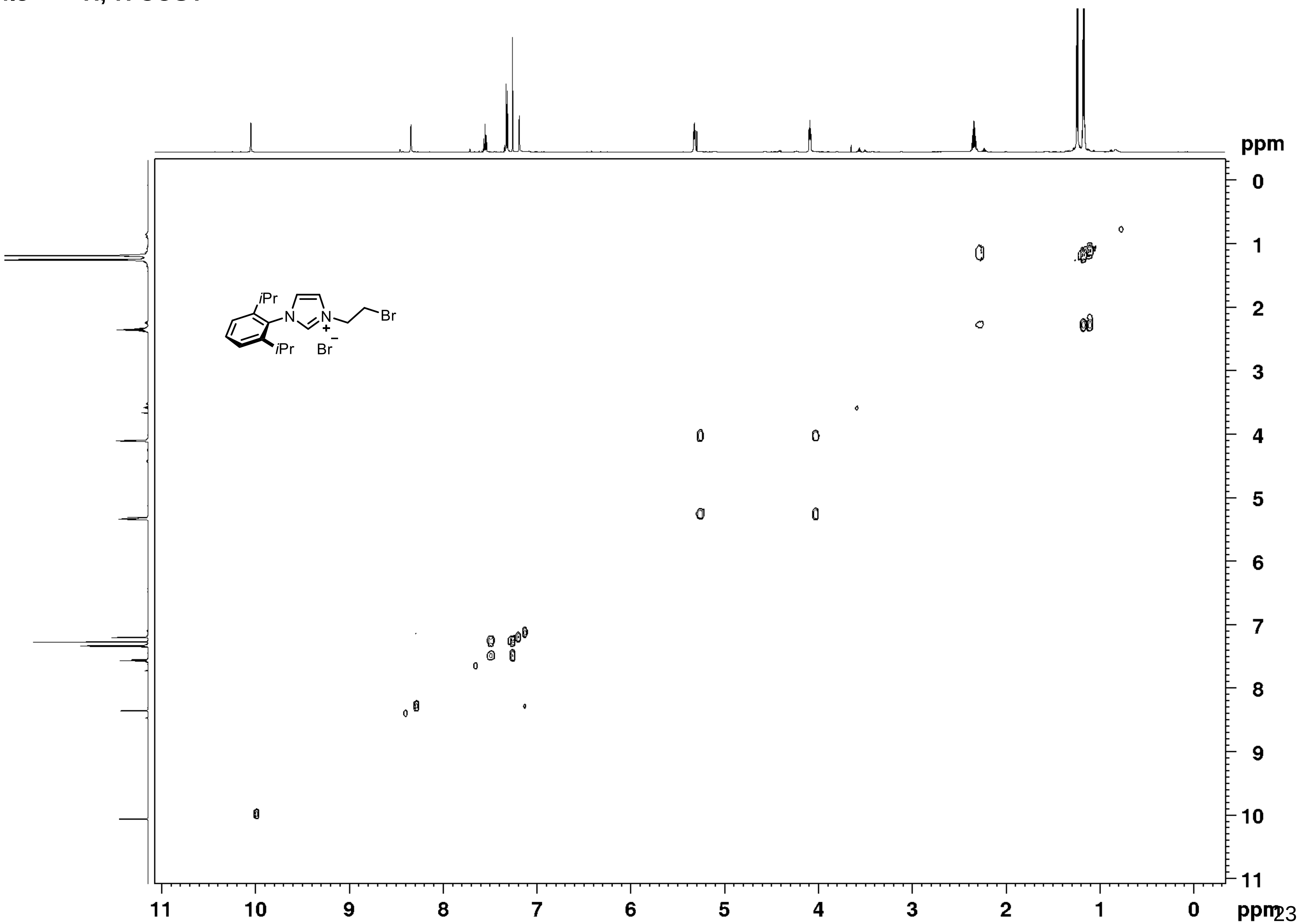

1.4.4 ^{1}H, ^{13}C HMBC

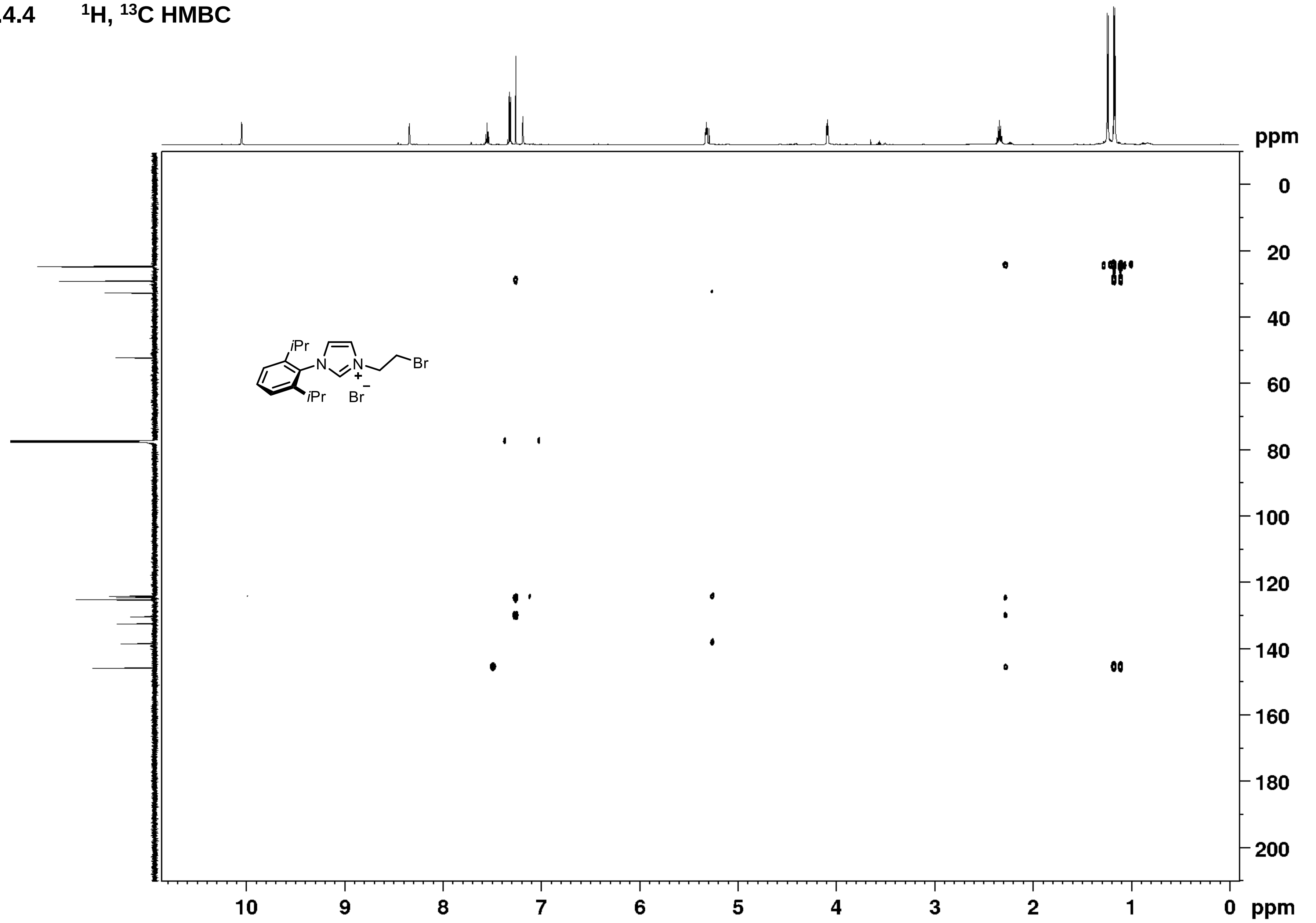

1.4.5 ^{1}H, ^{13}C HSQC

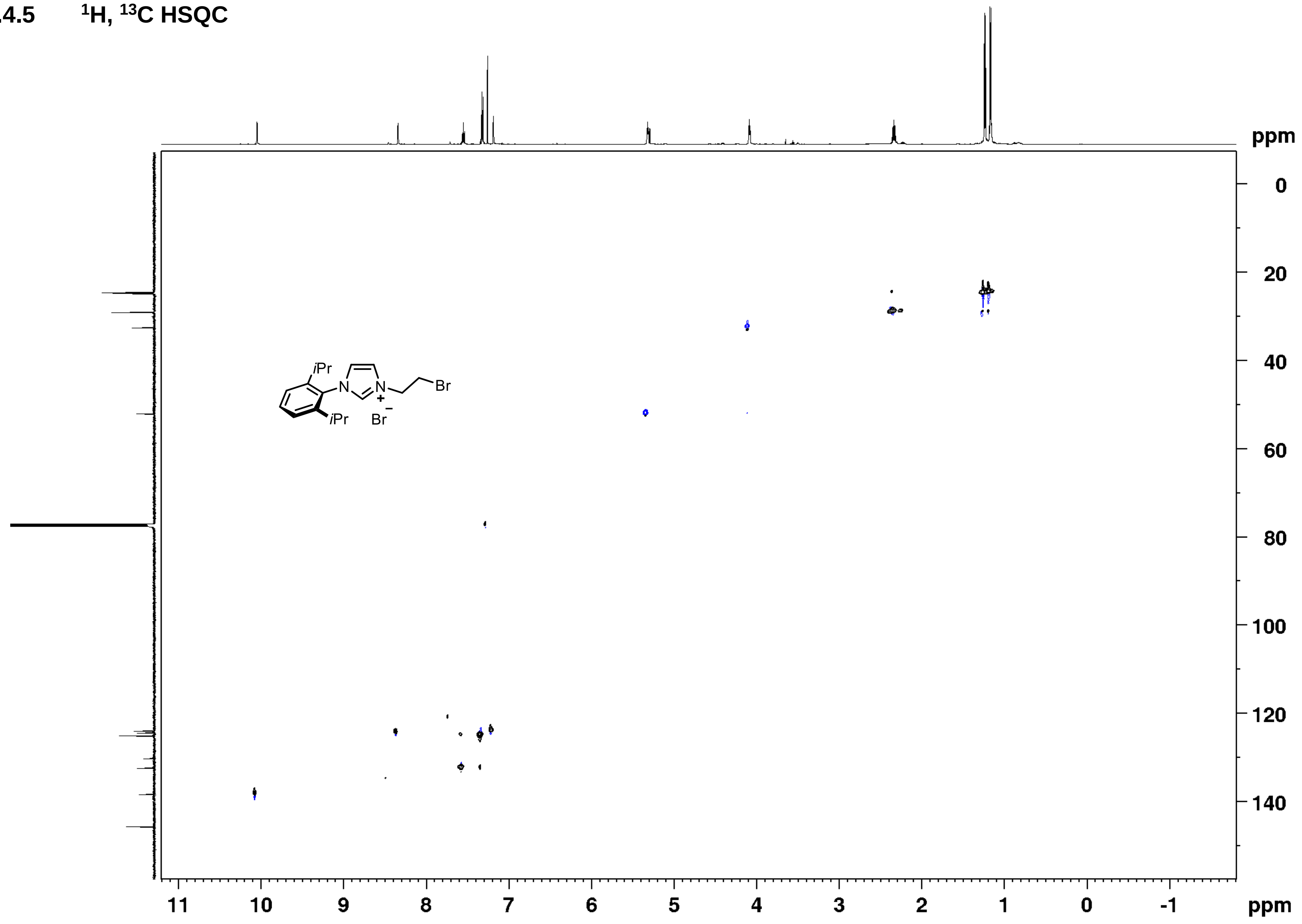

1.4.6 HRMS

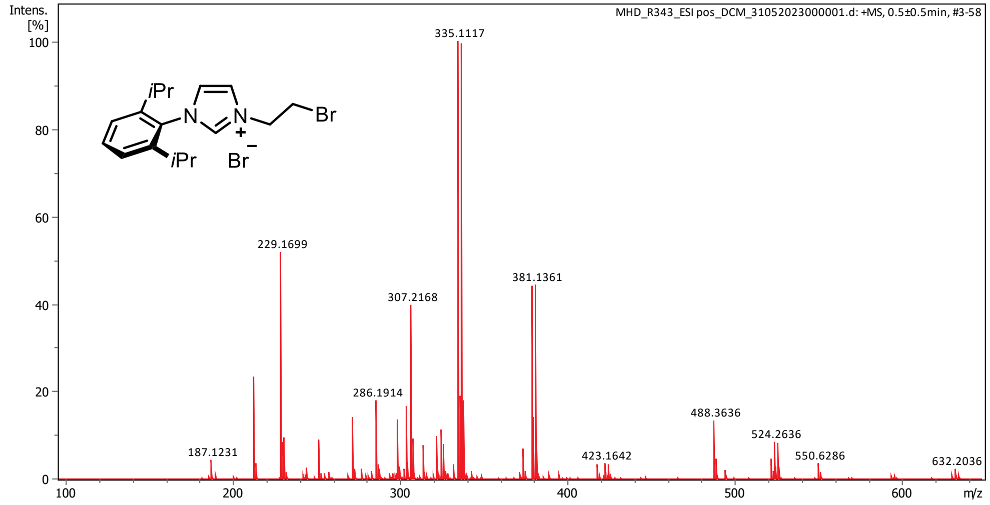

MHD_R343_ESI pos_DCM_31052023000001.d: +MS, 0.5±0.5min, #3-58
Intens. [%]
335.1117
229.1699
381.1361
307.2168
286.1914
187.1231
423.1642
488.3636
524.2636
550.6286
632.2036
m/z
iPr
N
N
+
Br
Br
−

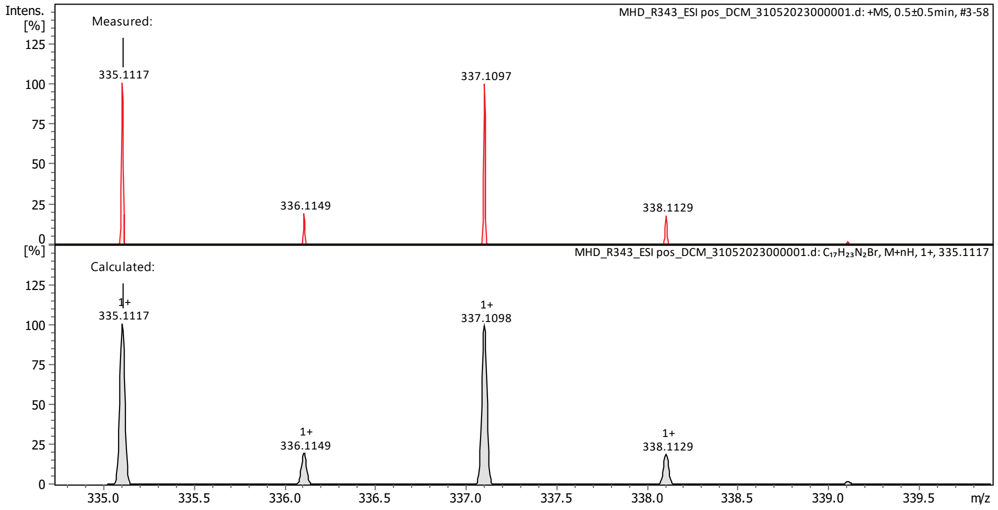

Measured:
MHD_R343_ESI pos_DCM_31052023000001.d: +MS, 0.5±0.5min, #3-58
335.1117
336.1149
337.1097
338.1129
Calculated:
MHD_R343_ESI pos_DCM_31052023000001.d: $C_{17}H_{23}N_2Br$, M+nH, 1+, 335.1117
1+ 335.1117
1+ 336.1149
1+ 337.1098
1+ 338.1129
Intens. [%]
m/z

1.4.7 IR

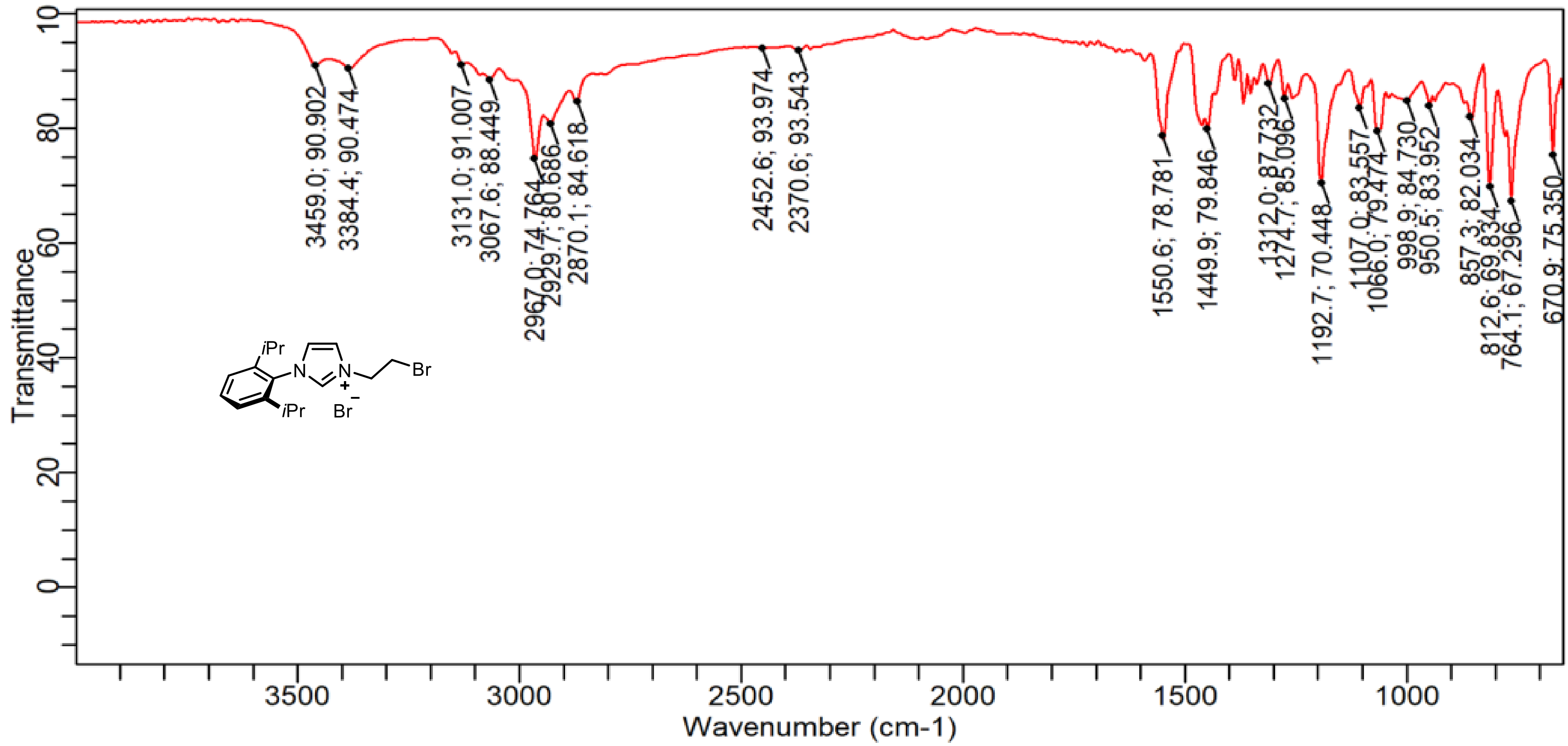

Transmittance
Wavenumber (cm-1)
3459.0; 90.902
3384.4; 90.474
3131.0; 91.007
3067.6; 88.449
2967.0; 74.764
2929.7; 80.686
2870.1; 84.618
2452.6; 93.974
2370.6; 93.543
1550.6; 78.781
1449.9; 79.846
1312.0; 87.732
1274.7; 85.096
1192.7; 70.448
1107.0; 83.557
1066.0; 79.474
998.9; 84.730
950.5; 83.952
857.3; 82.034
812.6; 69.834
764.1; 67.296
670.9; 75.350
iPr
N
N
Br
Br

1.5 1-(2,6-Diisopropylphenyl)-3-(2-(2-imino-3-methylpyridin-1(2*H*)-yl)ethyl)-1*H*-imidazol-3-ium bromide hydrobromide (L4) (600 MHz, DMSO-d_6)

1.5.1 ^{1}H NMR

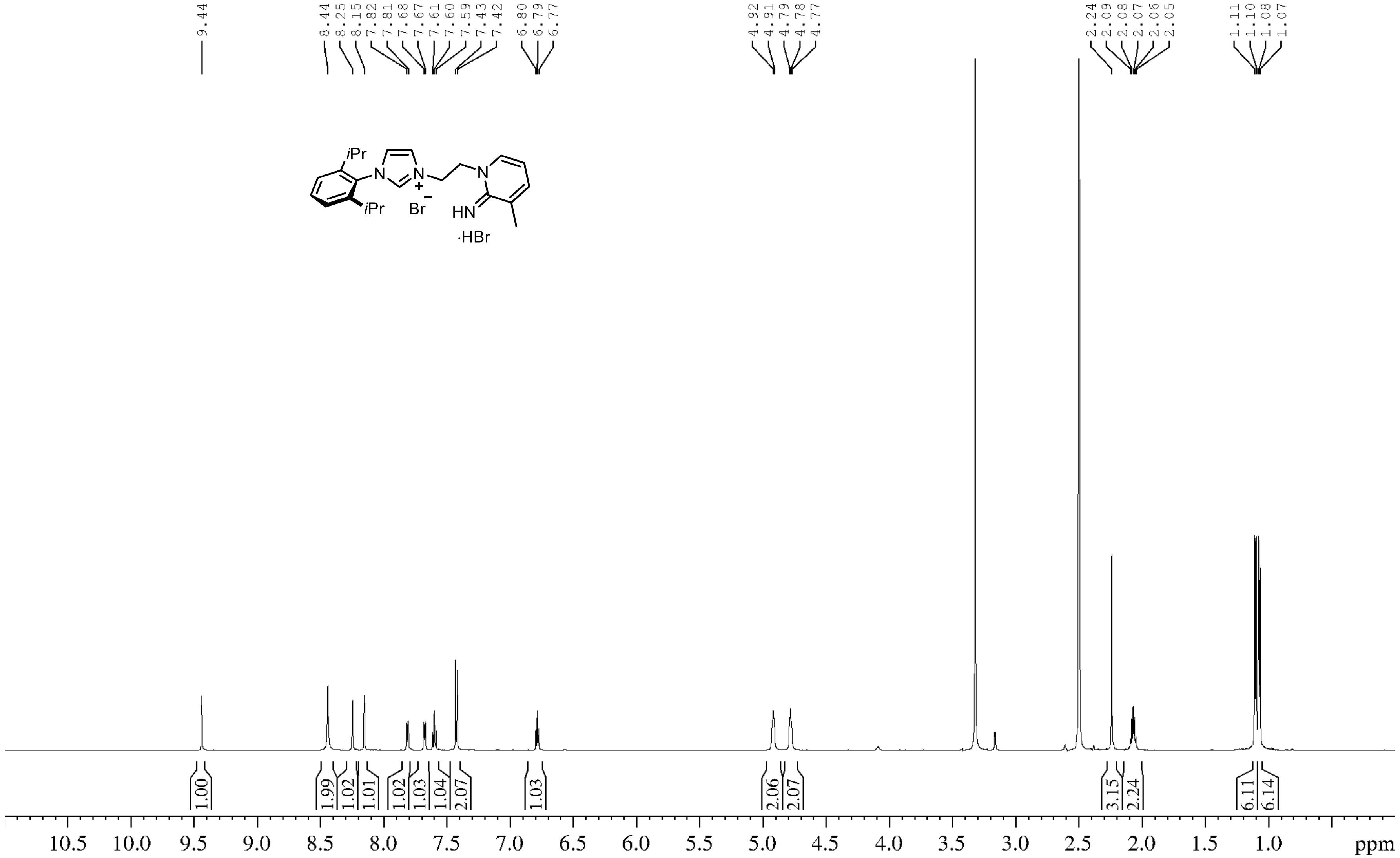

1.5.2 ^{13}C NMR

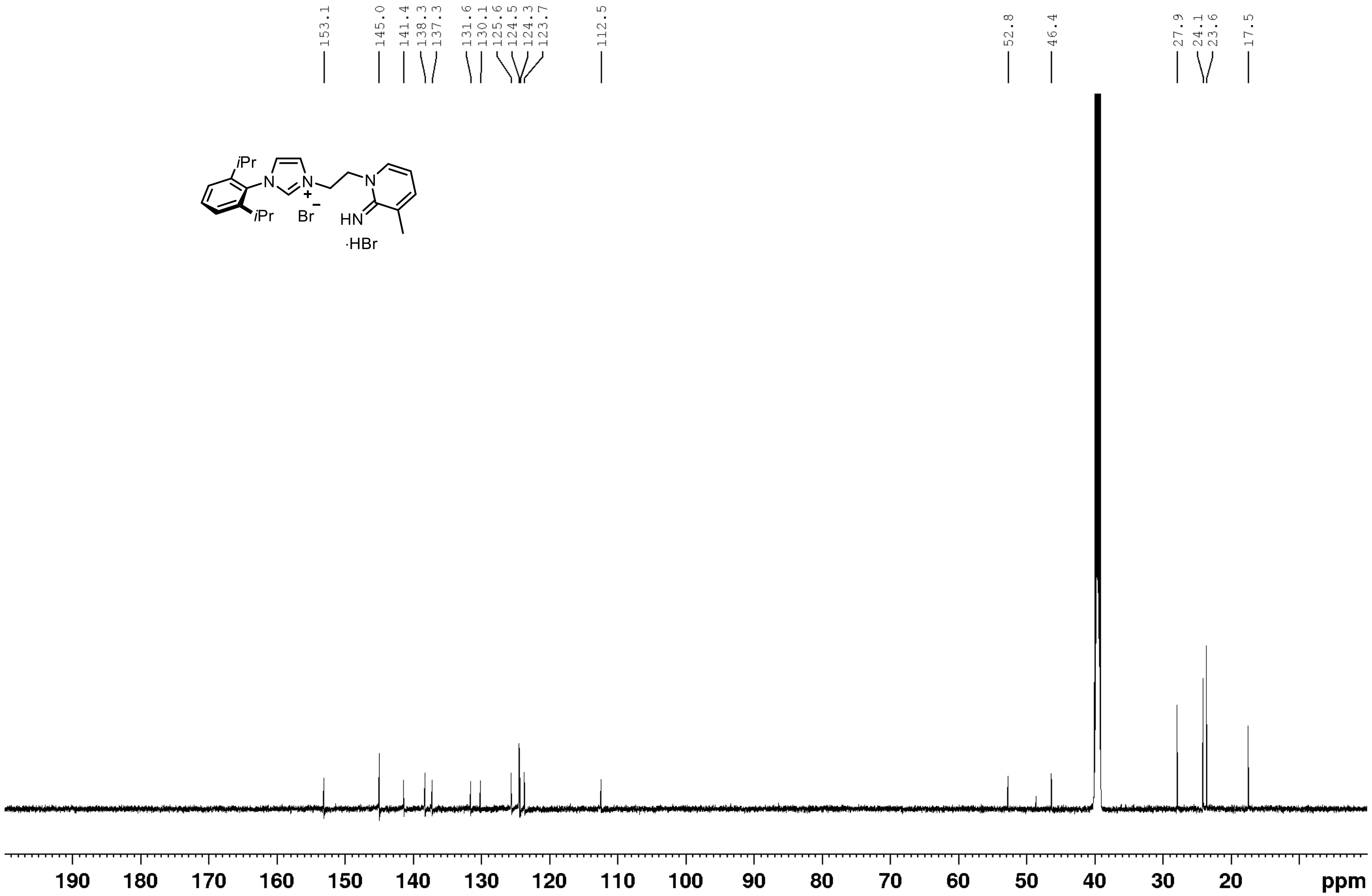

153.1
145.0
141.4
138.3
137.3
131.6
130.1
125.6
124.5
124.3
123.7
112.5
52.8
46.4
27.9
24.1
23.6
17.5
iPr
N
N
+
Br
−
HN
·HBr
190 180 170 160 150 140 130 120 110 100 90 80 70 60 50 40 30 20
ppm

1.5.3 ^{1}H, ^{1}H COSY

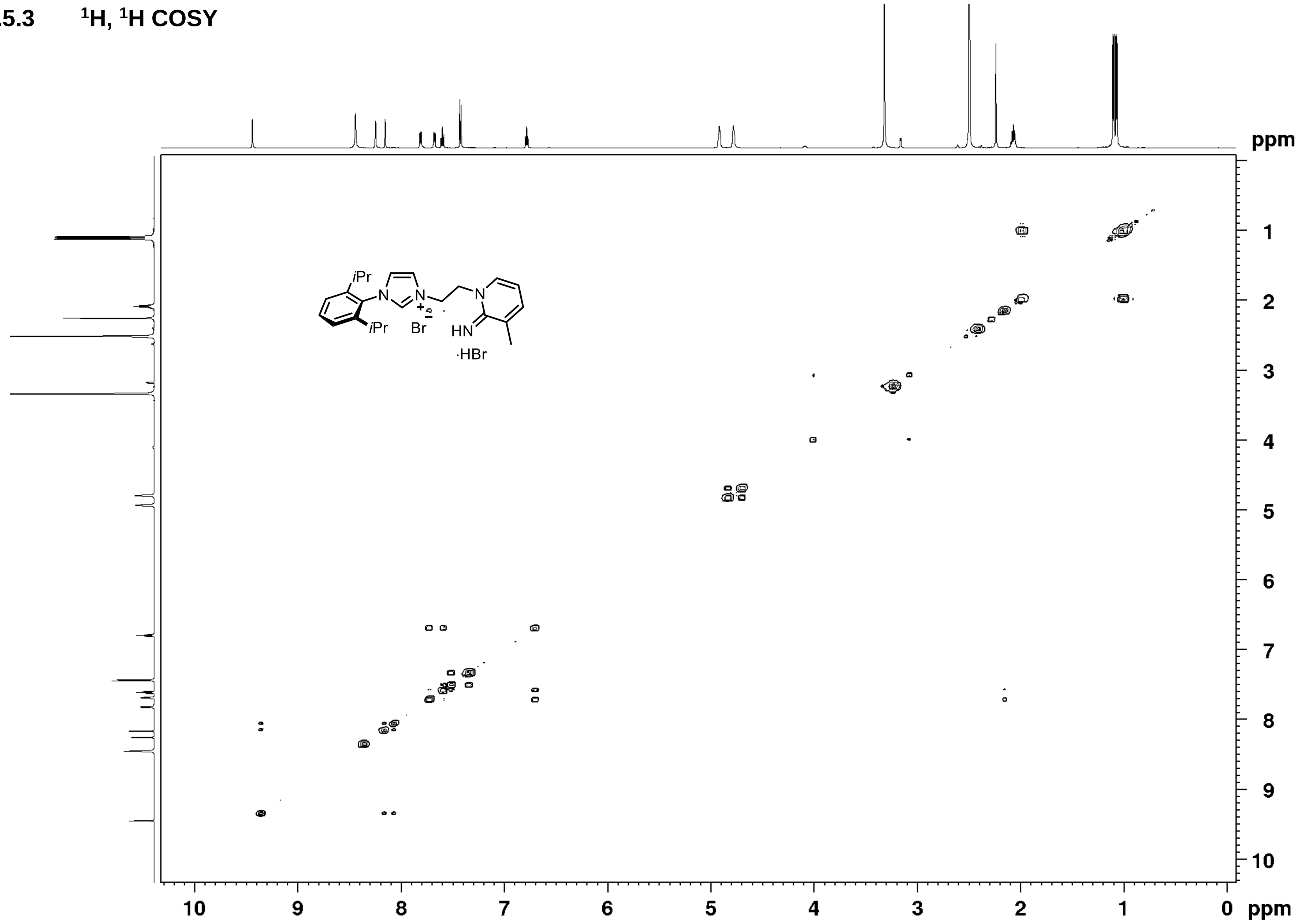

1.5.4 ^{1}H, ^{13}C HMBC

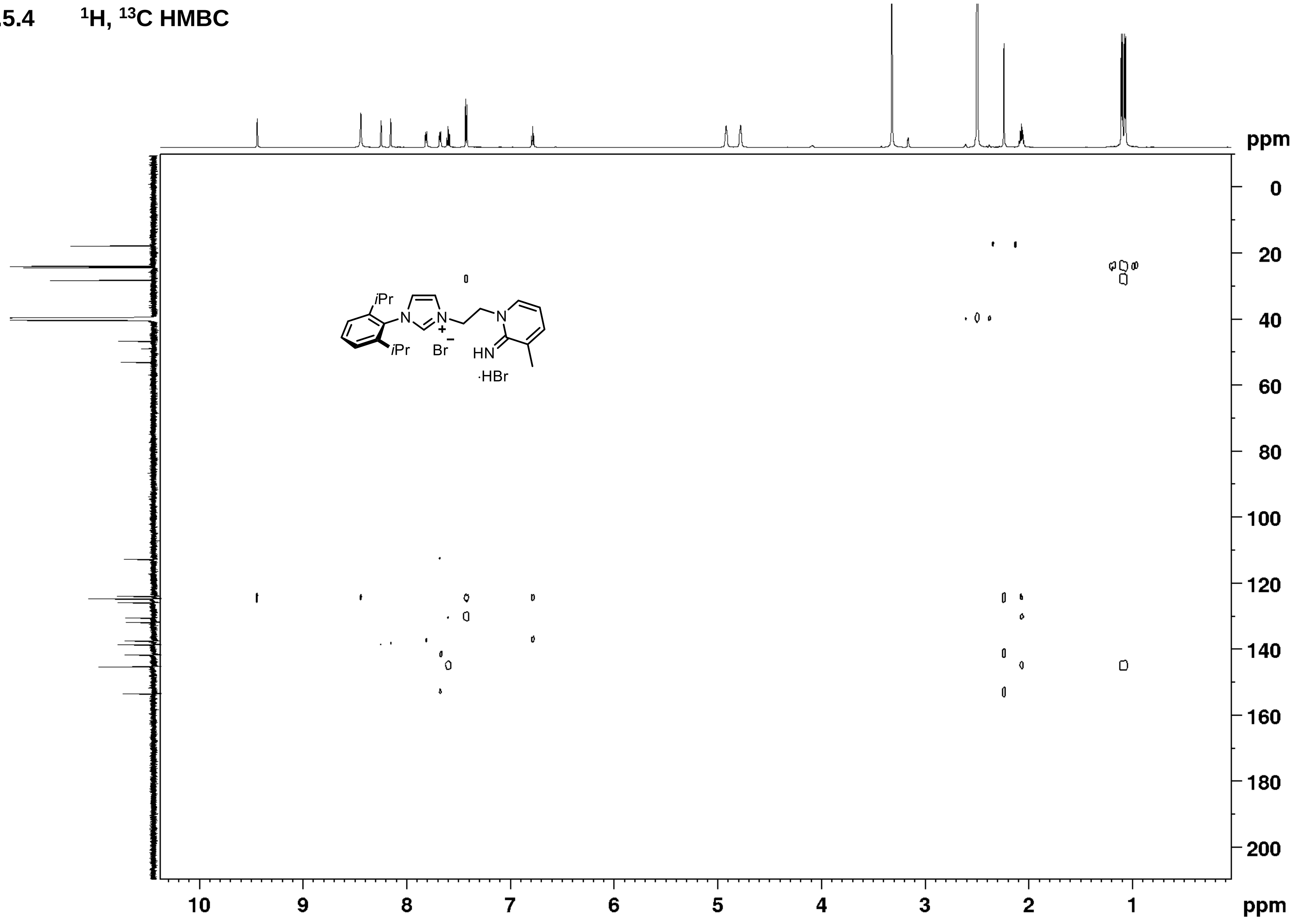

1.5.5 ^{1}H, ^{13}C HSQC

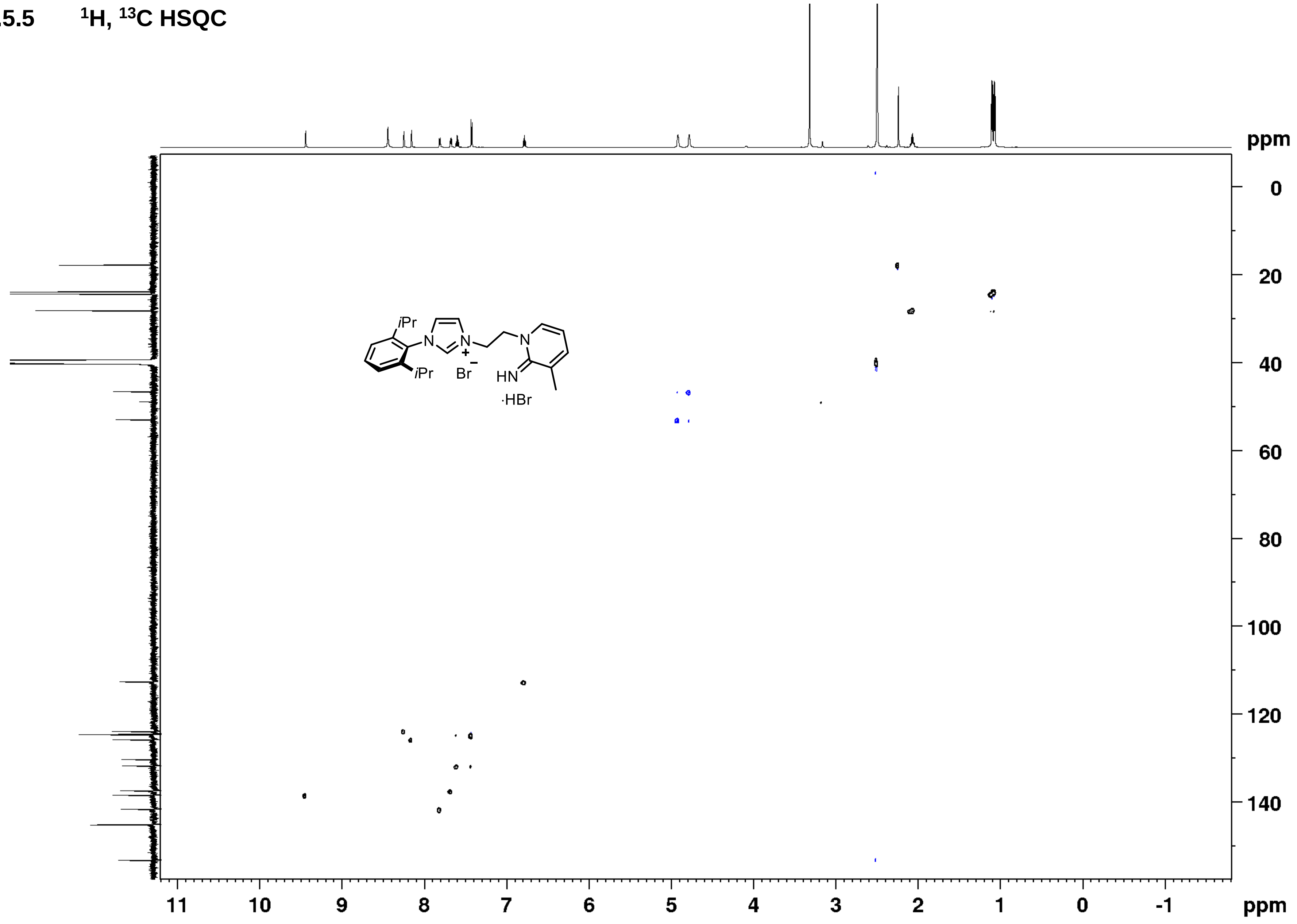

1.5.6 HRMS

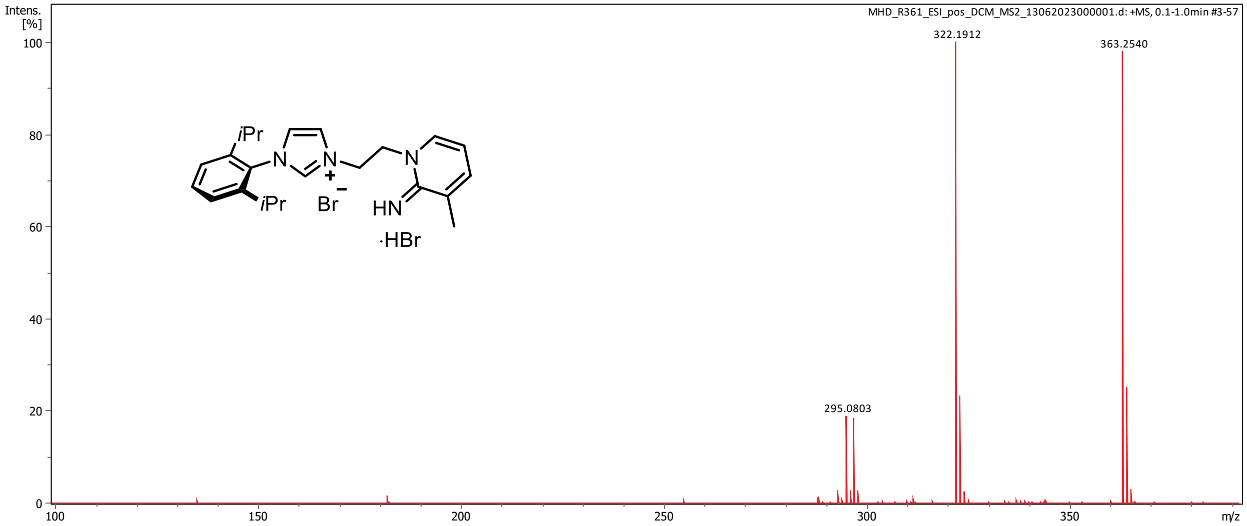
Intens. [%]
MHD_R361_ESI_pos_DCM_MS2_13062023000001.d: +MS, 0.1-1.0min #3-57
322.1912
363.2540
295.0803
iPr
iPr
Br
HN
·HBr
m/z

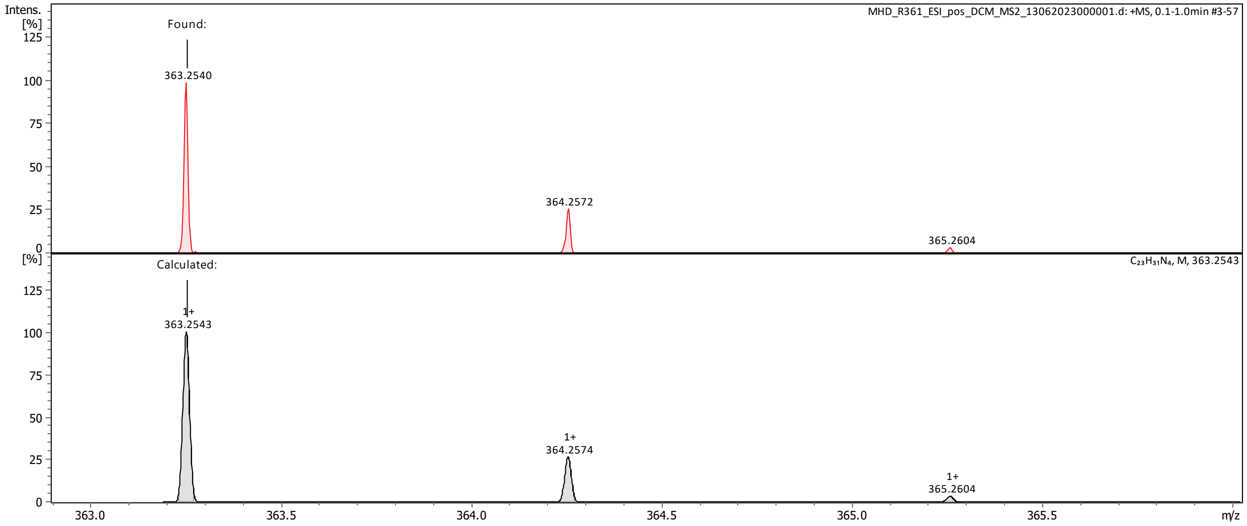
Intens. [%]
MHD_R361_ESI_pos_DCM_MS2_13062023000001.d: +MS, 0.1-1.0min #3-57
Found:
363.2540
364.2572
365.2604
Calculated:
C23H31N4, M, 363.2543
1+
363.2543
1+
364.2574
1+
365.2604
m/z

1.5.7 IR

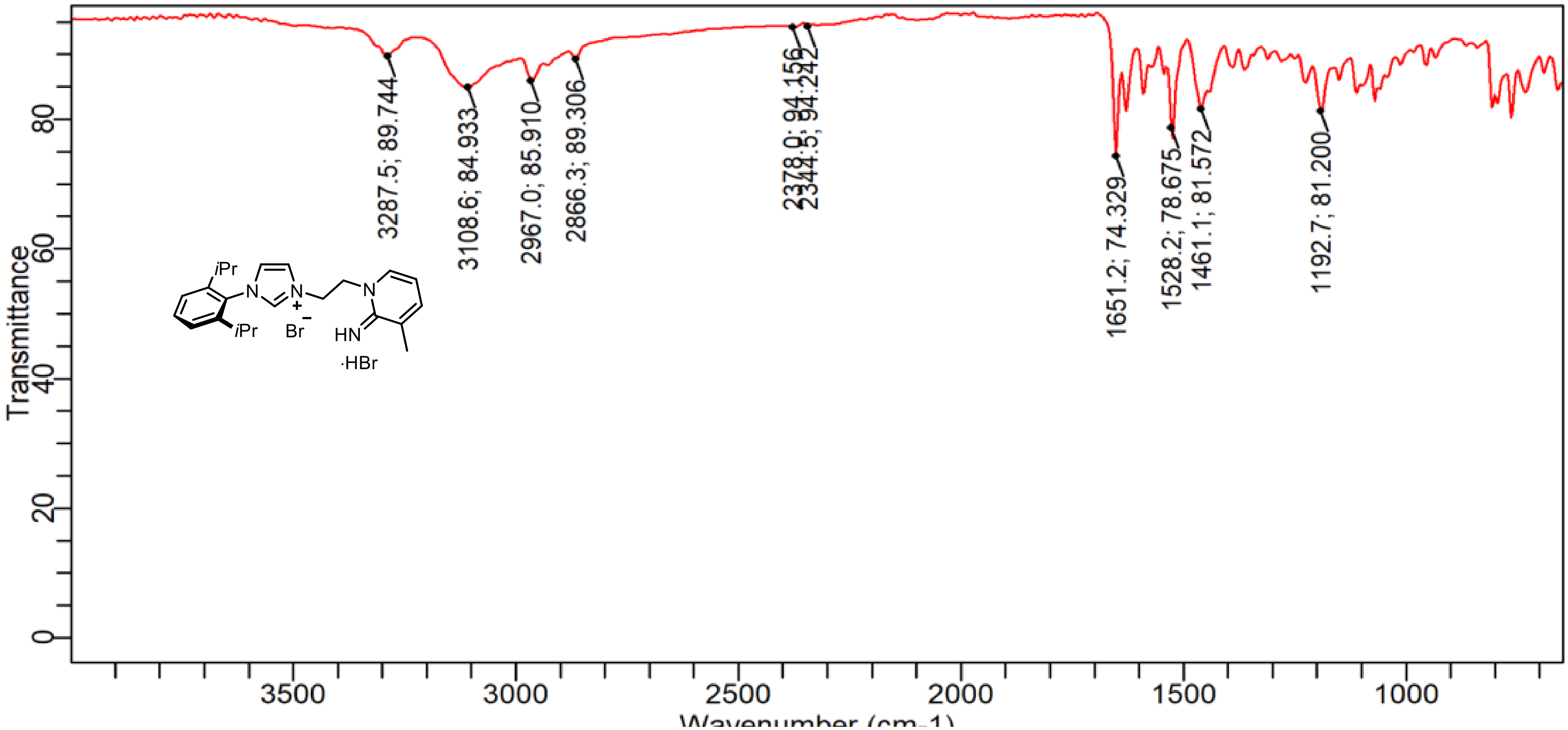

Transmittance
Wavenumber (cm-1)
3287.5; 89.744
3108.6; 84.933
2967.0; 85.910
2866.3; 89.306
2378.0; 94.156
2344.5; 94.242
1651.2; 74.329
1528.2; 78.675
1461.1; 81.572
1192.7; 81.200
iPr
iPr
Br
HN
·HBr

1.6 (1-(2,6-Diisopropylphenyl)-3-(2-(2-imino-3-methylpyridin-1(2*H*)-yl)ethyl)-1,3-dihydro-2*H*-imidazol-2-ylidene)copper(I) chloride (4) (600 MHz, CD_2Cl_2)

1.6.1 ^{1}H NMR

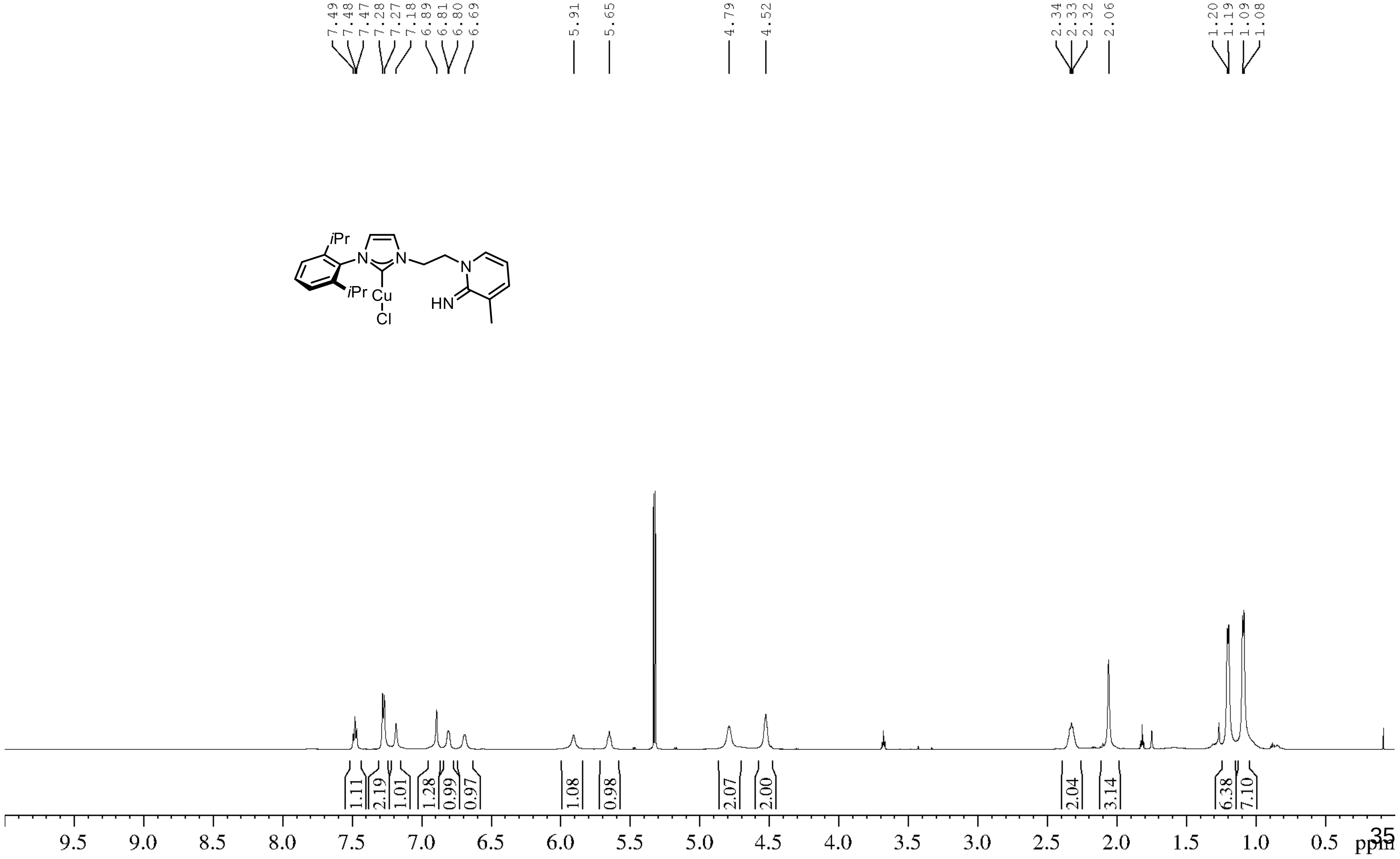

1.6.2 ^{13}C NMR

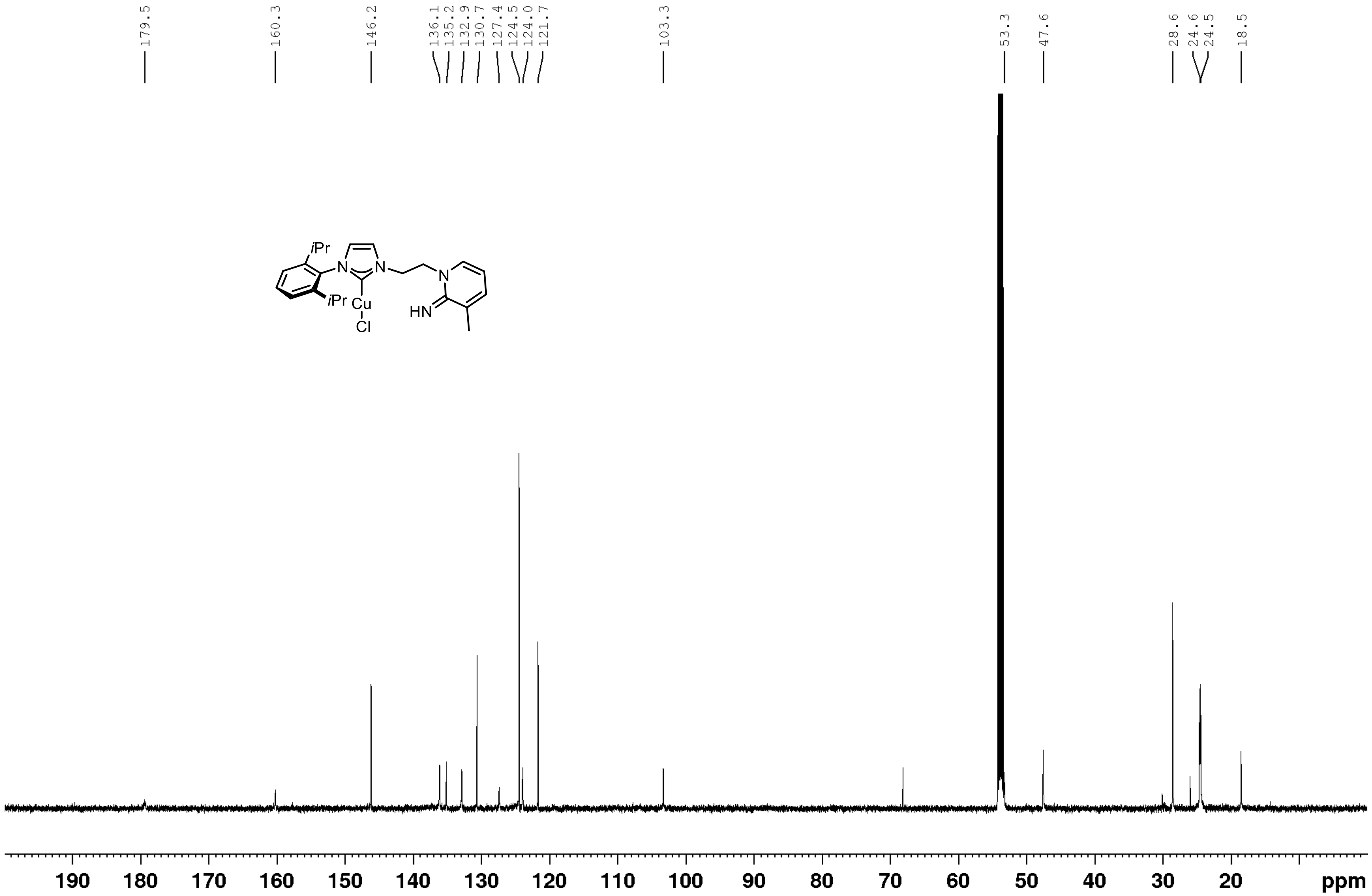
179.5
160.3
146.2
136.1
135.2
132.9
130.7
127.4
124.5
124.0
121.7
103.3
53.3
47.6
28.6
24.6
24.5
18.5
iPr
N
N
N
iPr
Cu
Cl
HN
190 180 170 160 150 140 130 120 110 100 90 80 70 60 50 40 30 20 ppm

1.6.3 ^{1}H, ^{1}H COSY

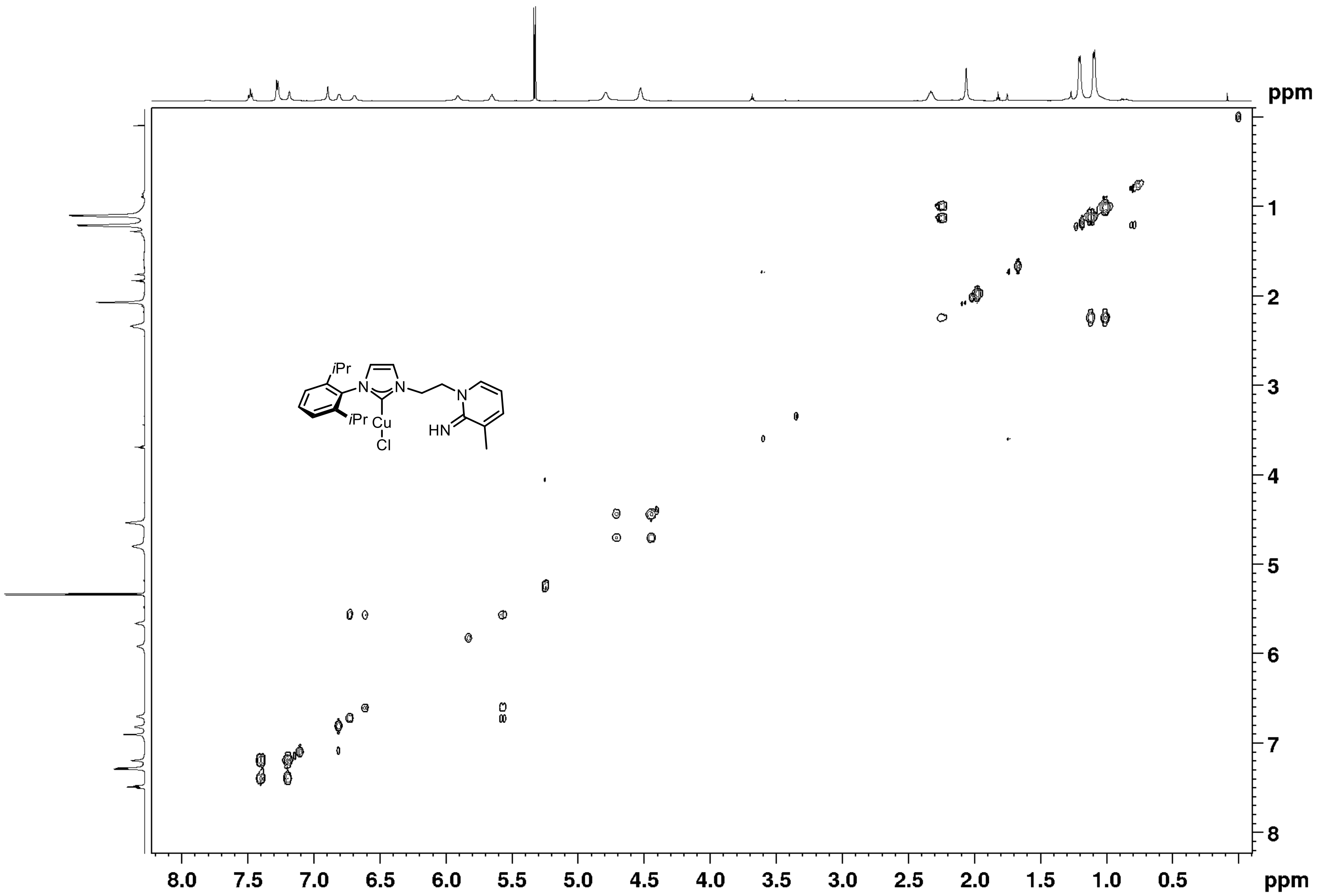

1.6.4 ^{1}H, ^{13}C HMBC

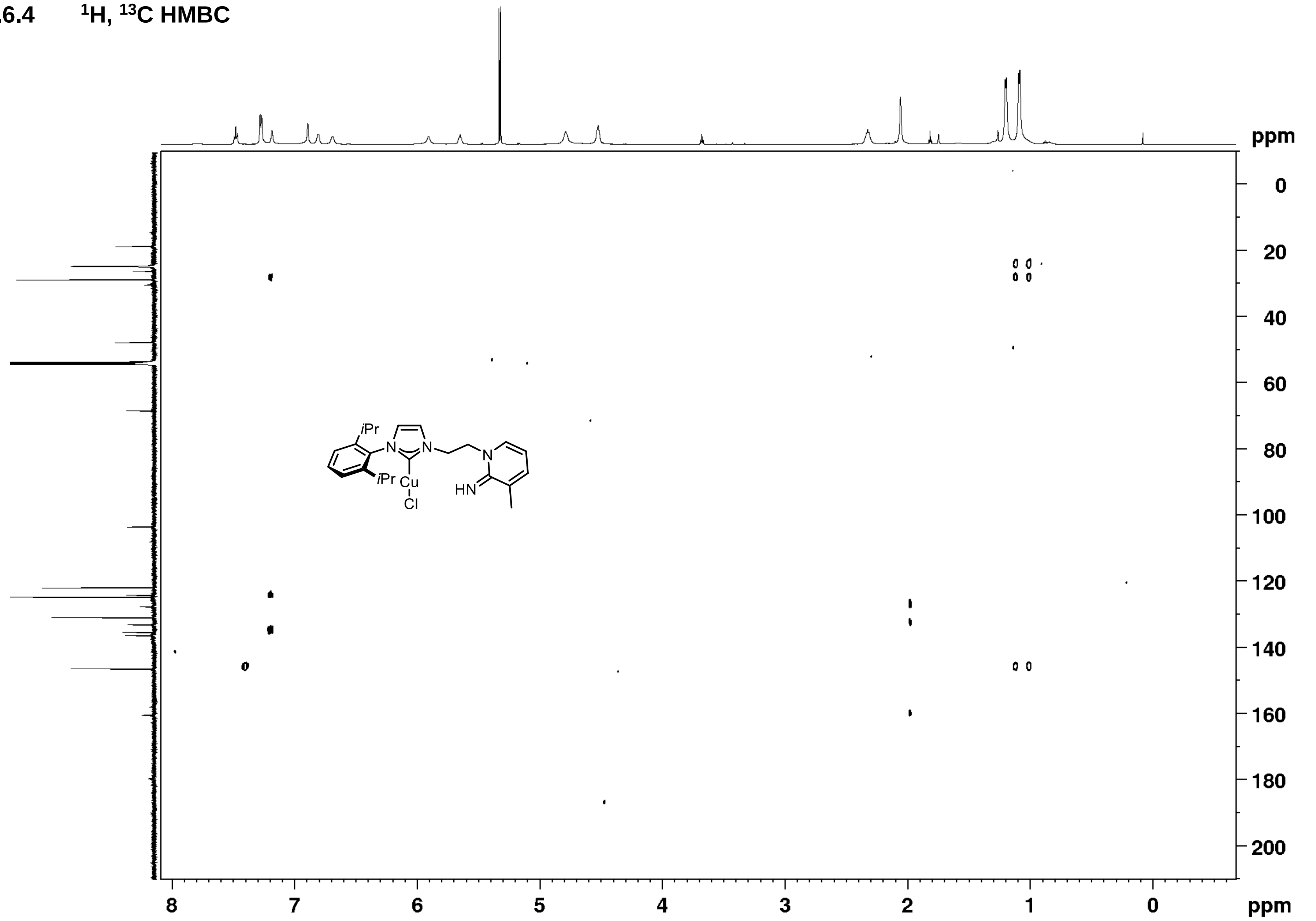

1.6.5 ^{1}H, ^{13}C HSQC

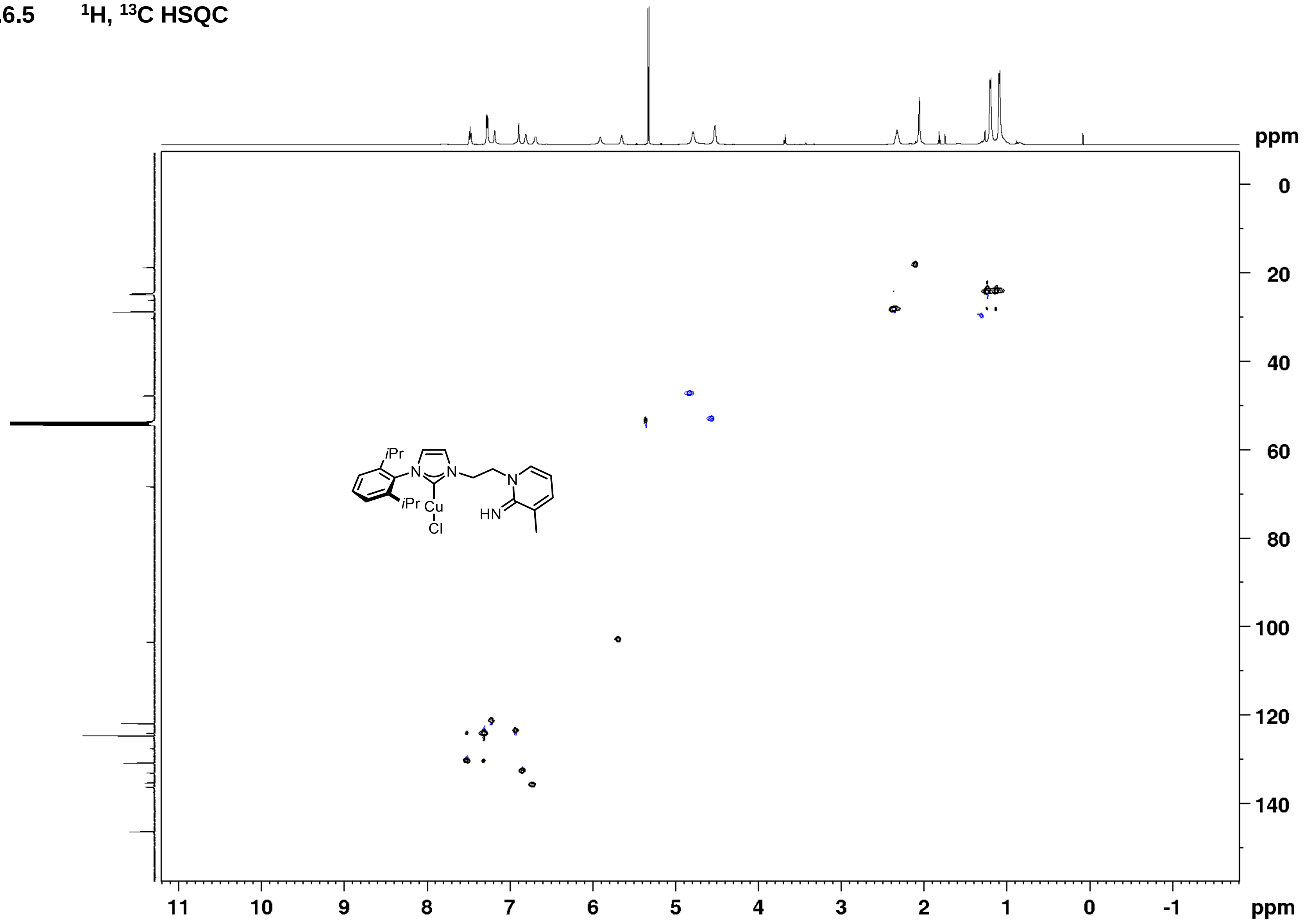

1.6.6 HRMS

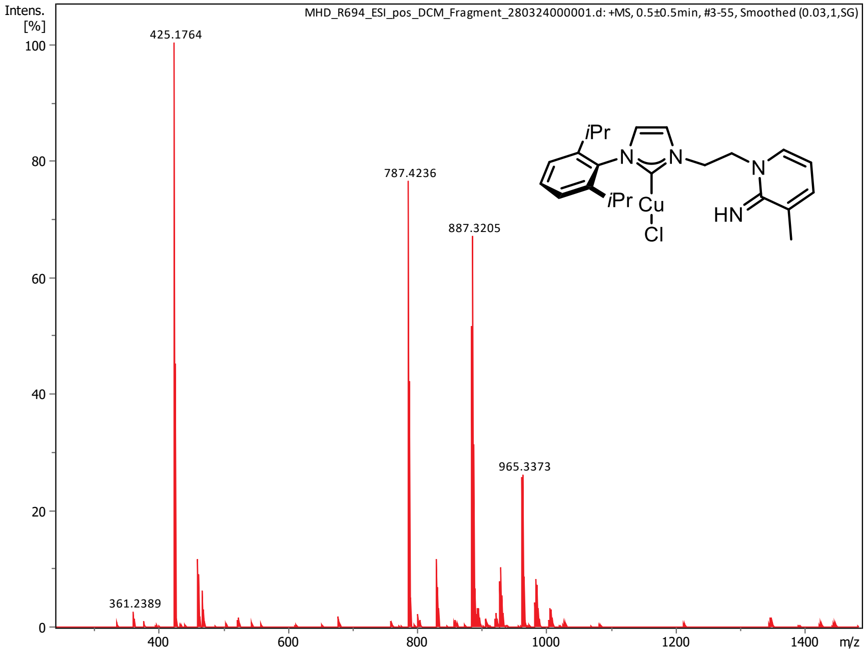
Intens. [%]
MHD_R694_ESI_pos_DCM_Fragment_280324000001.d: +MS, 0.5±0.5min, #3-55, Smoothed (0.03,1,SG)
425.1764
787.4236
887.3205
965.3373
361.2389
iPr
N
N
N
iPr
Cu
Cl
HN
m/z

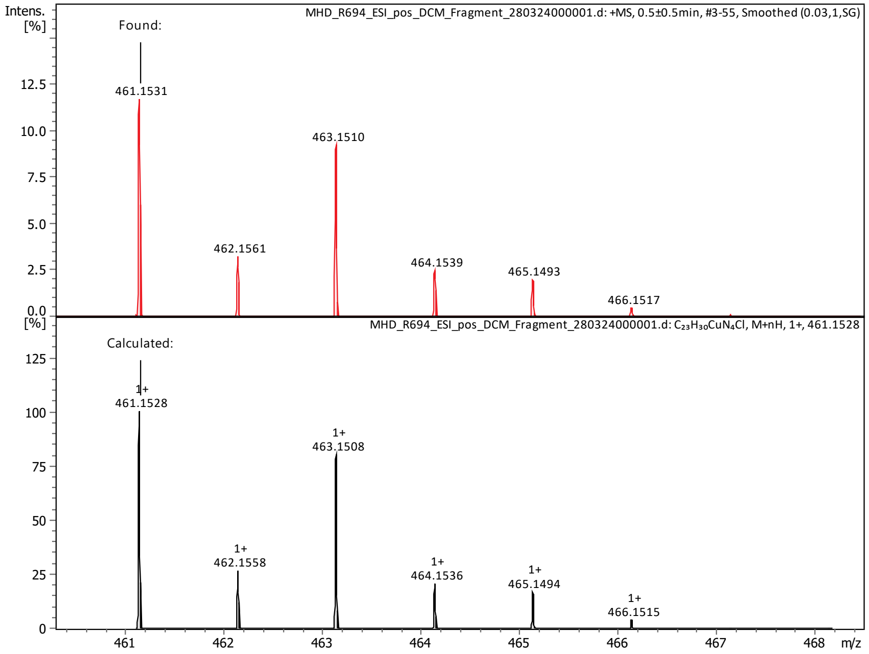
Intens. [%]
Found:
MHD_R694_ESI_pos_DCM_Fragment_280324000001.d: +MS, 0.5±0.5min, #3-55, Smoothed (0.03,1,SG)
461.1531
462.1561
463.1510
464.1539
465.1493
466.1517
Calculated:
MHD_R694_ESI_pos_DCM_Fragment_280324000001.d: C23H30CuN4Cl, M+nH, 1+, 461.1528
1+ 461.1528
1+ 462.1558
1+ 463.1508
1+ 464.1536
1+ 465.1494
1+ 466.1515
m/z

1.7 1-(adamantan-1-yl)-3-(2-bromoethyl)-1*H*-imidazol-3-ium bromide (S5b) (600 MHz, $CDCl_3$)

1.7.1 ^{1}H NMR

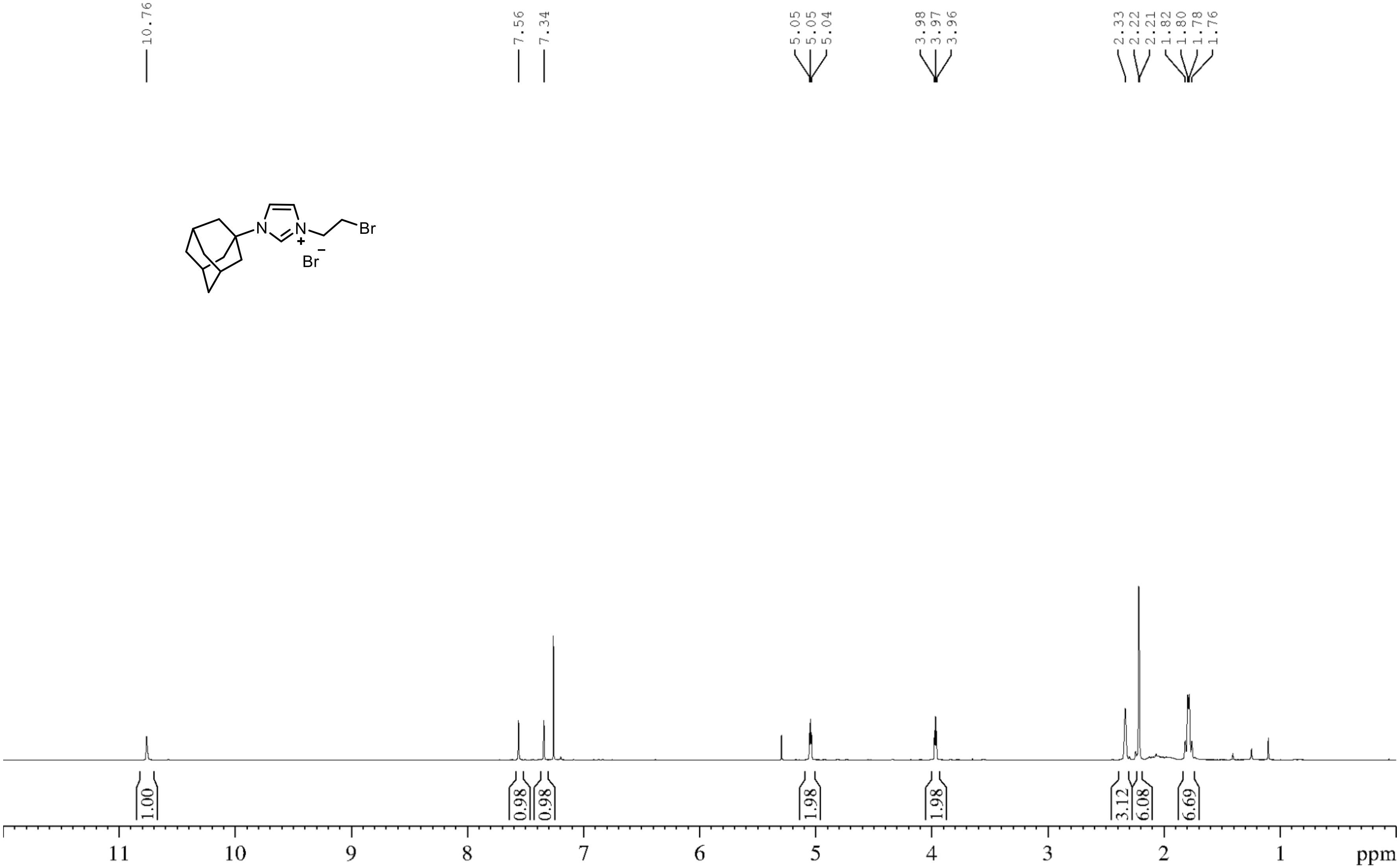

1.7.2 ^{13}C NMR

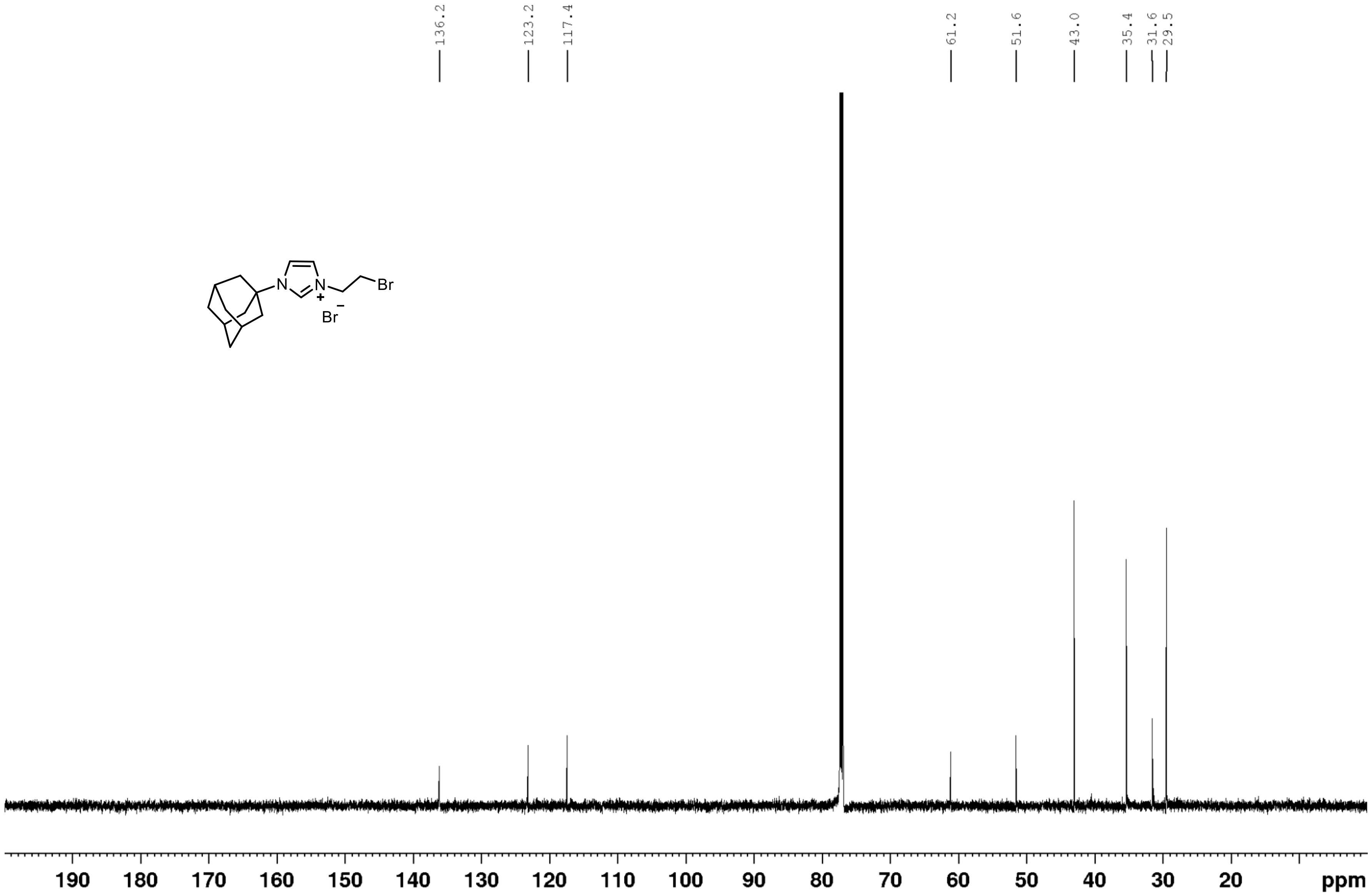

136.2
123.2
117.4
61.2
51.6
43.0
35.4
31.6
29.5
N
N
+
Br
Br
−
190
180
170
160
150
140
130
120
110
100
90
80
70
60
50
40
30
20
ppm

1.7.3 ^{1}H, ^{1}H COSY

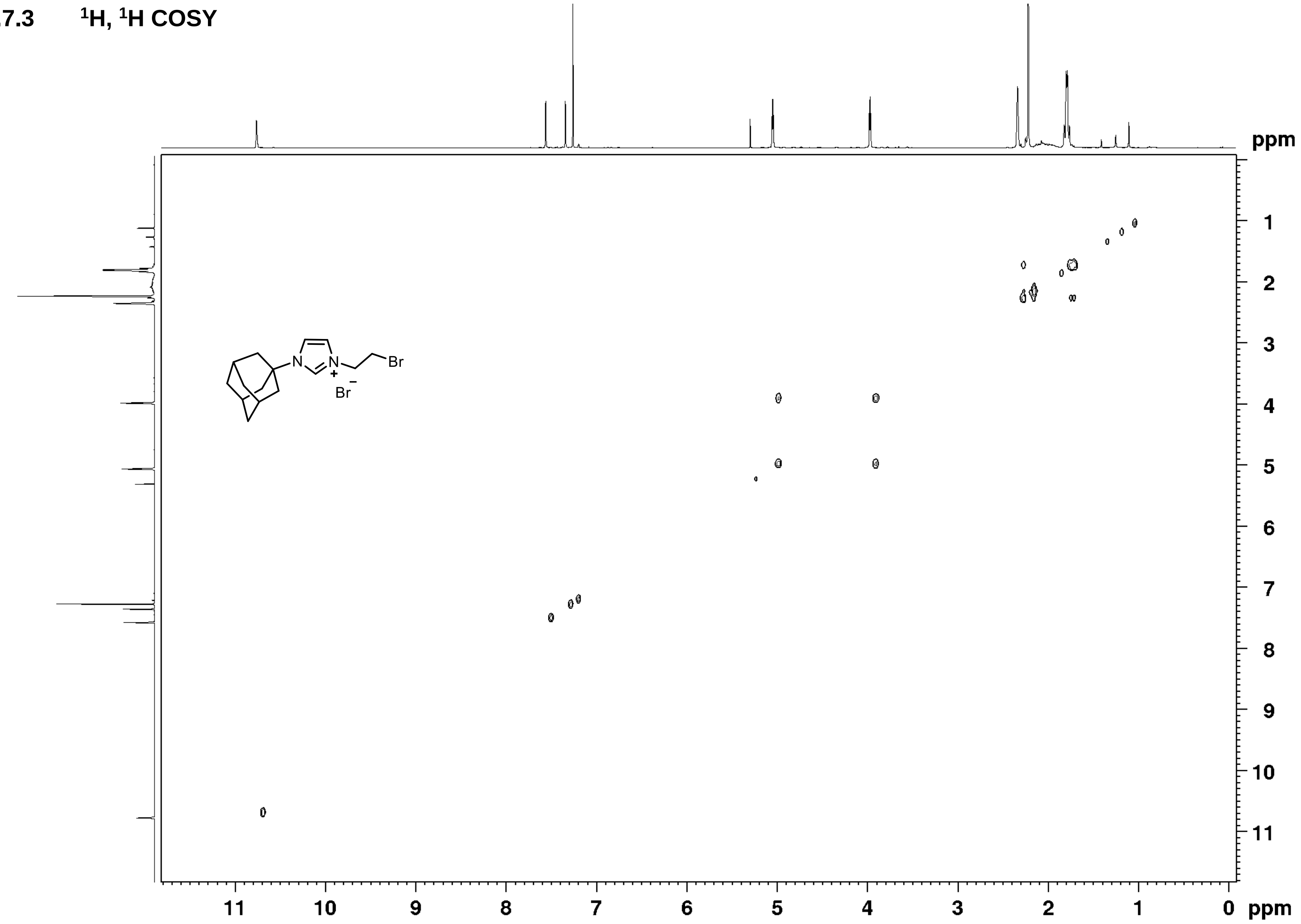

1.7.4 ^{1}H, ^{13}C HMBC

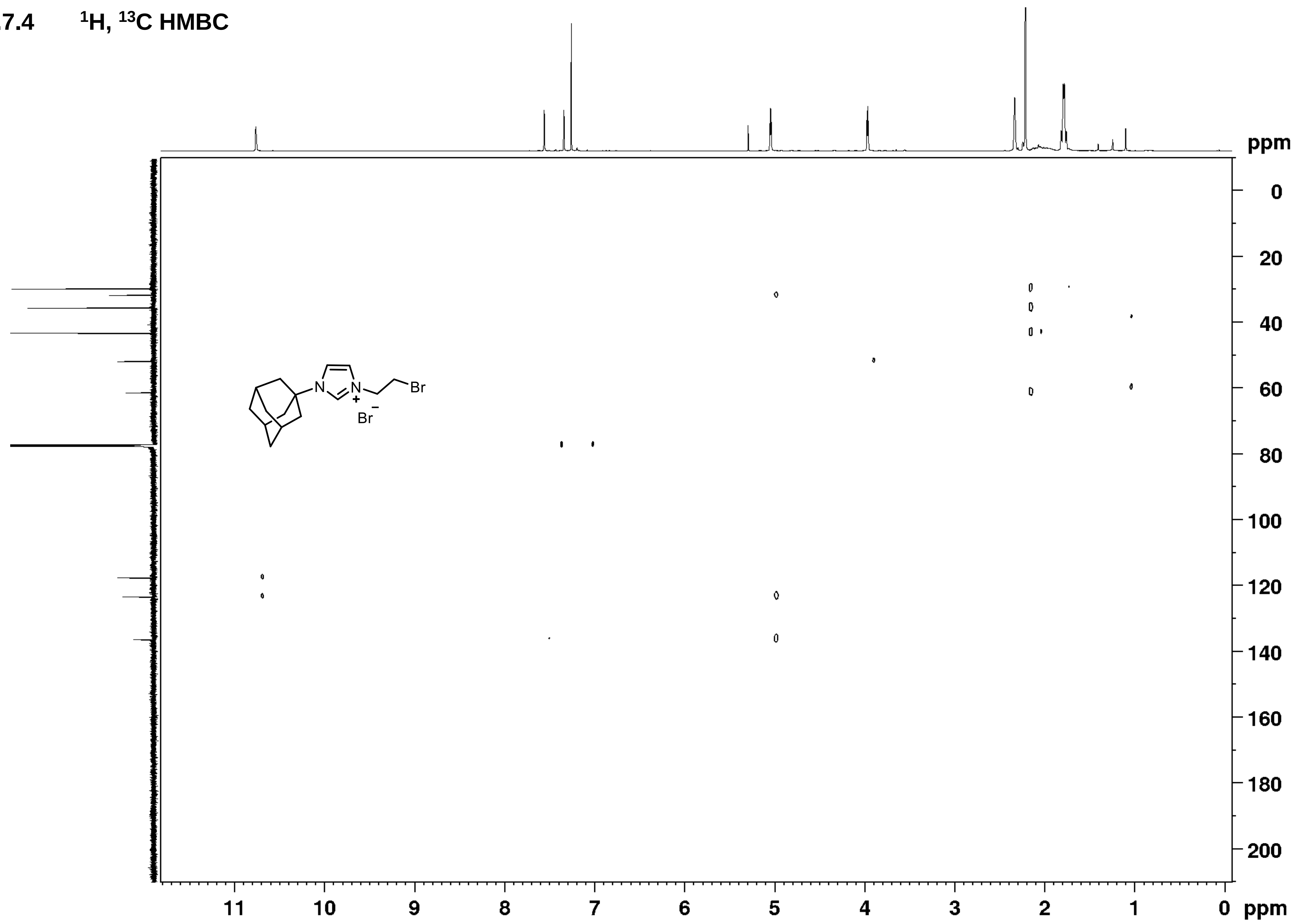

1.7.5 ^{1}H, ^{13}C HSQC

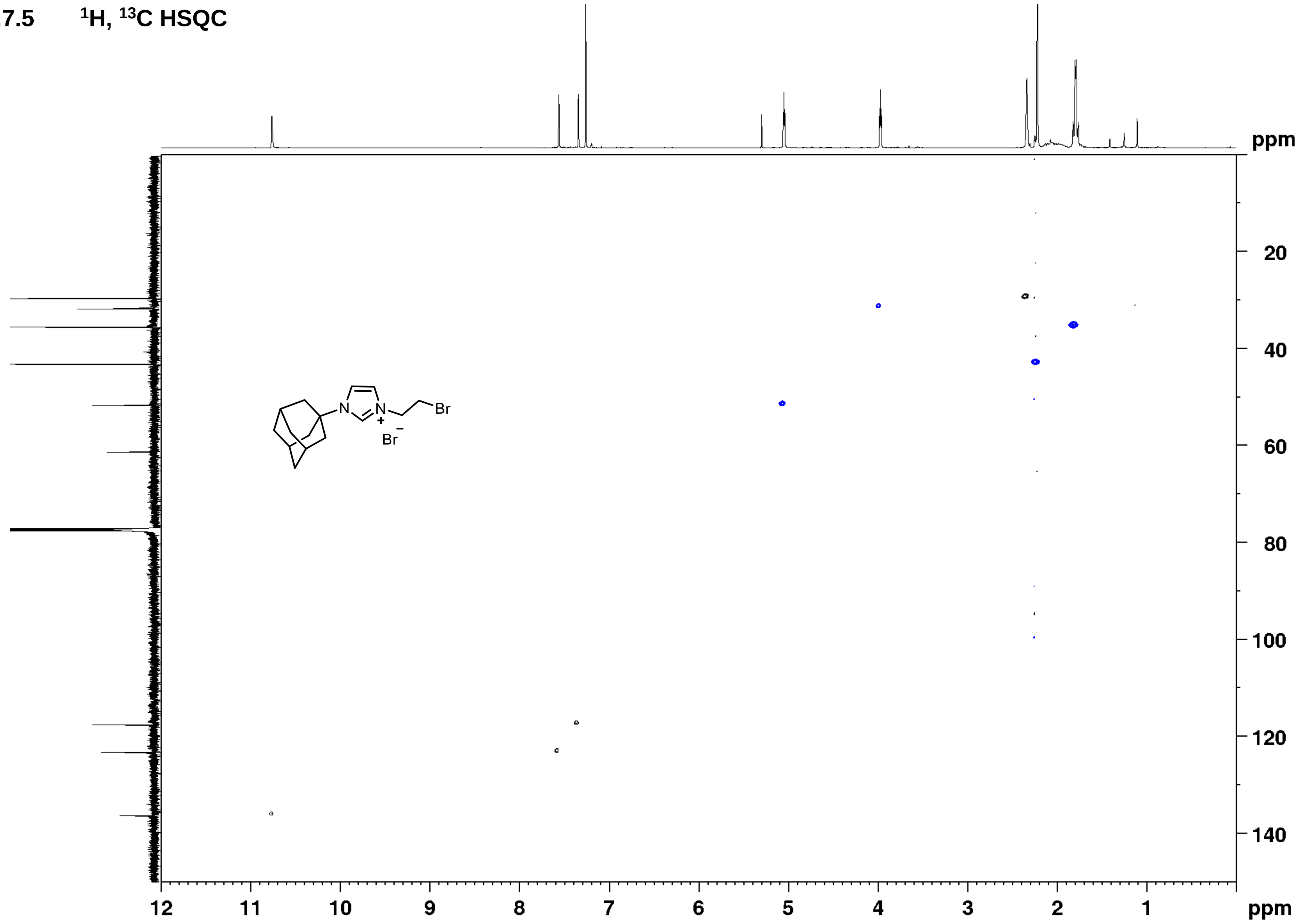

1.7.6 HRMS

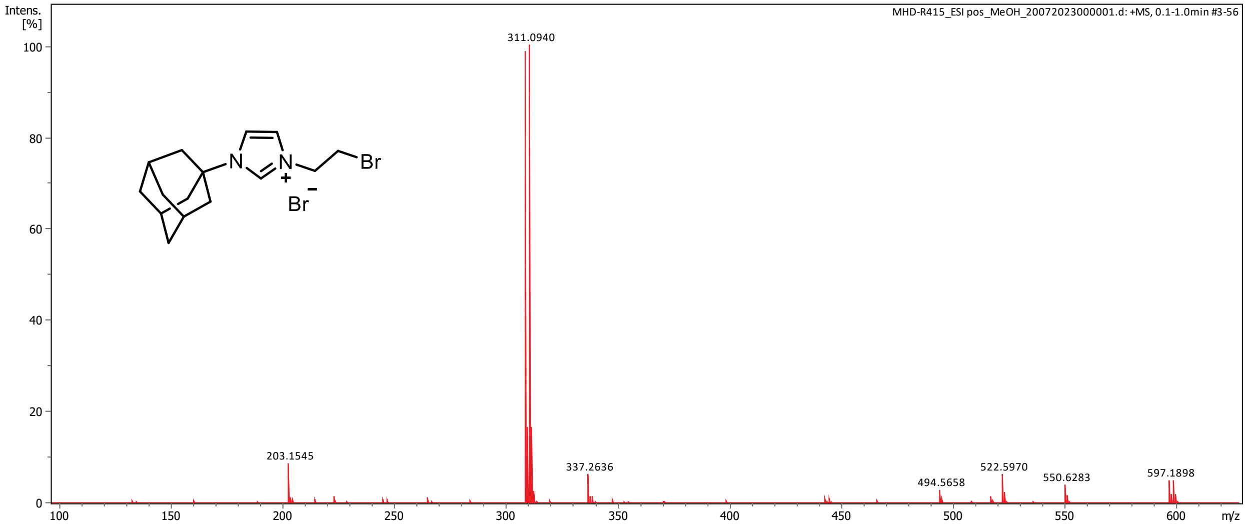

MHD-R415_ESI pos_MeOH_20072023000001.d: +MS, 0.1-1.0min #3-56
Intens. [%]
311.0940
203.1545
337.2636
494.5658
522.5970
550.6283
597.1898
m/z
N
N
Br
Br

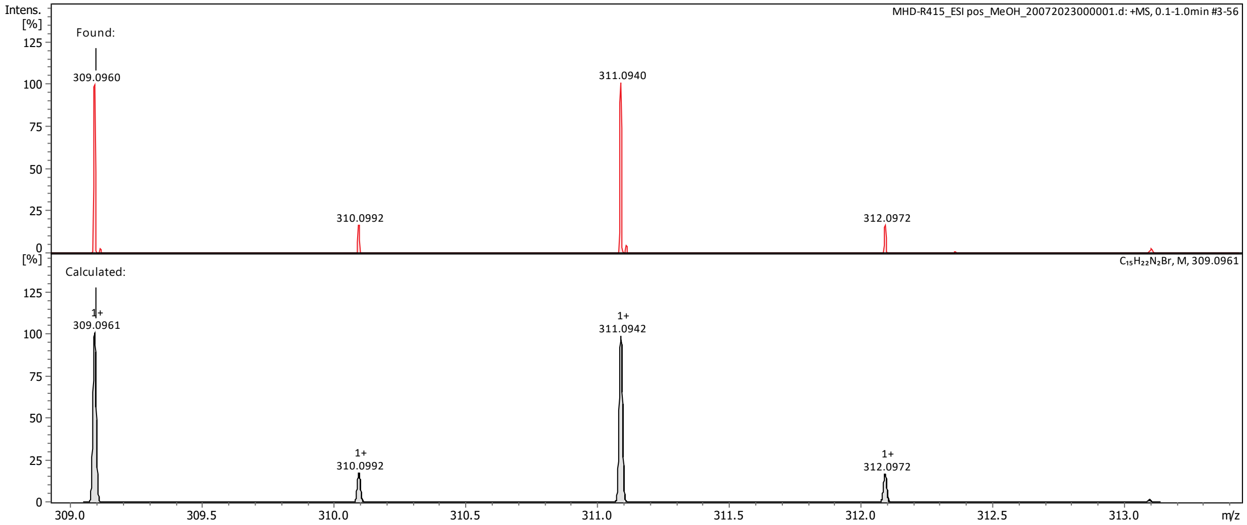

MHD-R415_ESI pos_MeOH_20072023000001.d: +MS, 0.1-1.0min #3-56
Found:
309.0960
310.0992
311.0940
312.0972
Calculated:
C15H22N2Br, M, 309.0961
309.0961
310.0992
311.0942
312.0972
m/z

1.7.7 IR

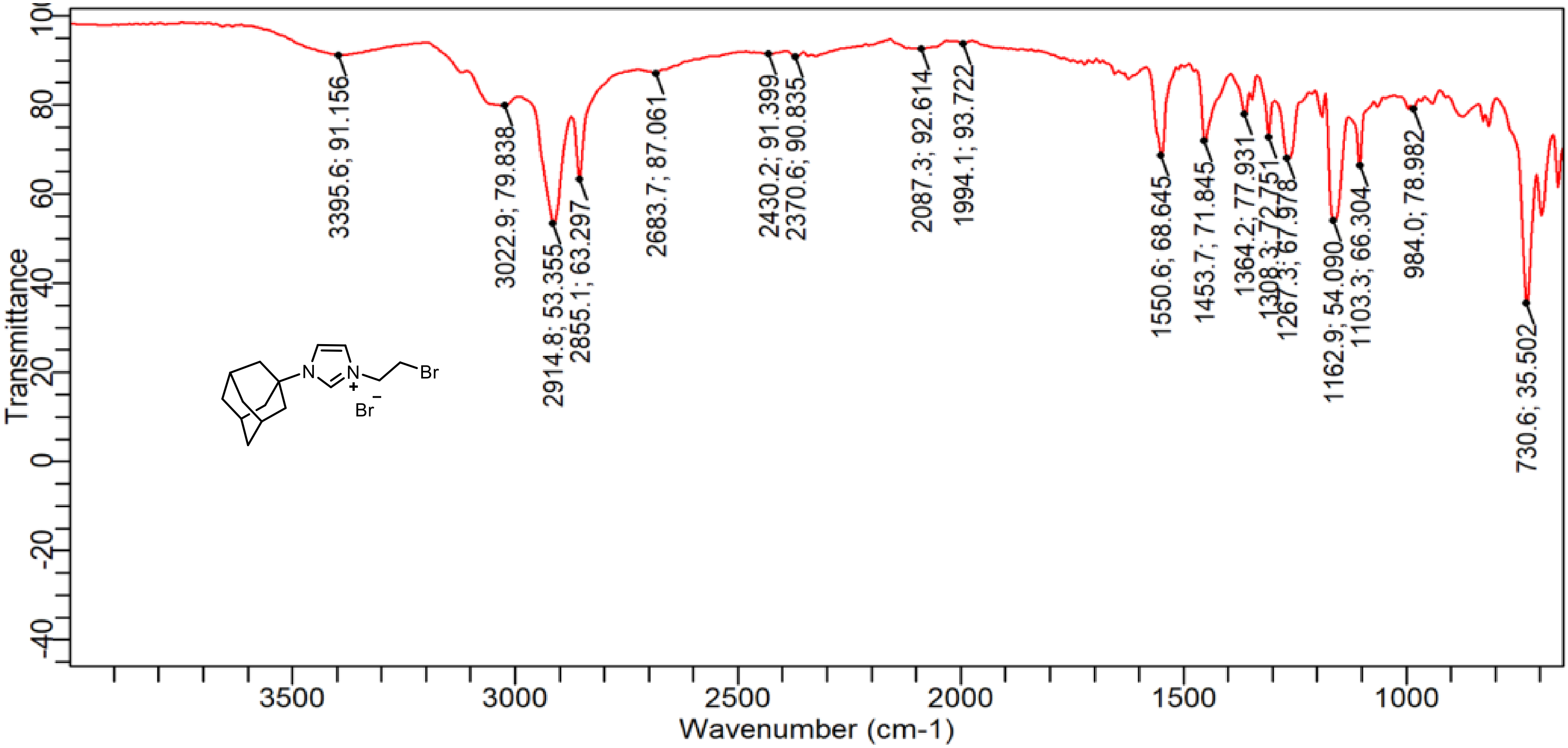

Transmittance
Wavenumber (cm-1)
3395.6; 91.156
3022.9; 79.838
2914.8; 53.355
2855.1; 63.297
2683.7; 87.061
2430.2; 91.399
2370.6; 90.835
2087.3; 92.614
1994.1; 93.722
1550.6; 68.645
1453.7; 71.845
1364.2; 77.931
1162.9; 54.090
1103.3; 66.304
984.0; 78.982
730.6; 35.502
N
N
Br
Br

1.8 1-(Adamantan-1-yl)-3-(2-(2-imino-3-methylpyridin-1(2*H*)-yl)ethyl)-1*H*-imidazol-3-ium bromide (L5) (600 MHz, DMSO-d_6)

1.8.1 ^{1}H NMR

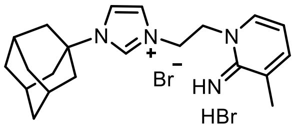

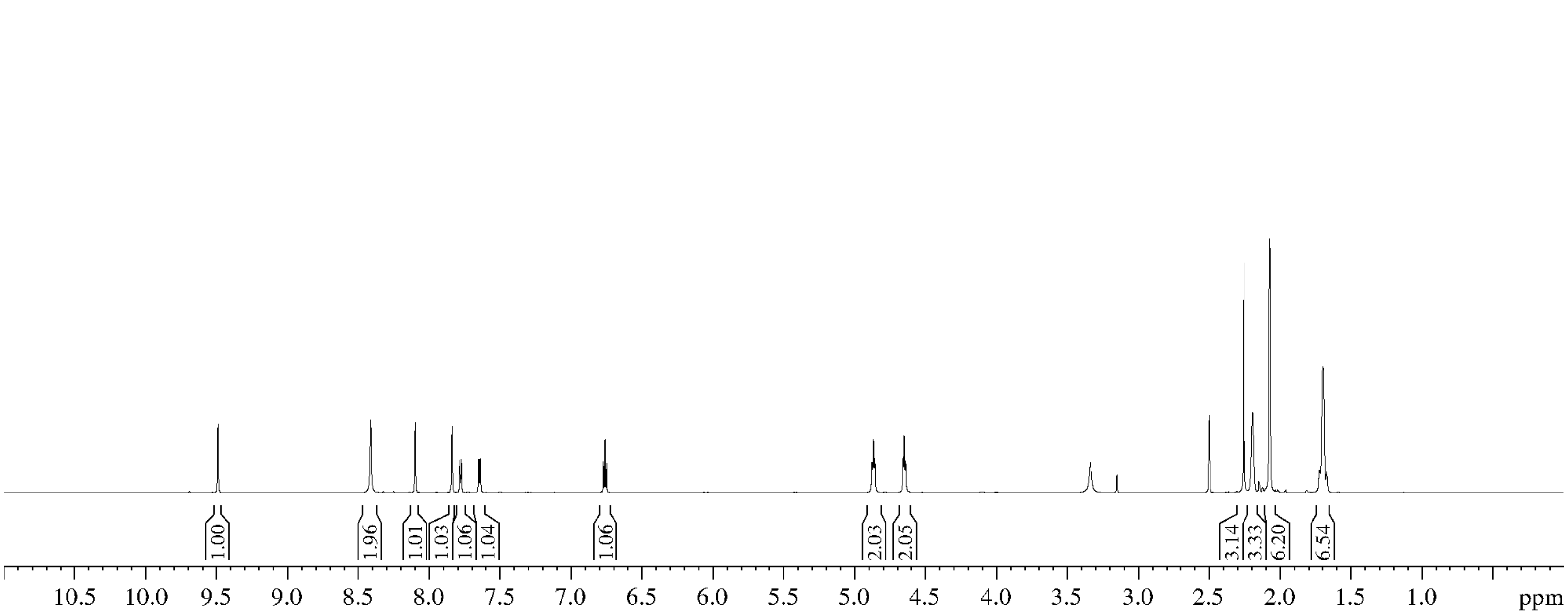

1.8.2 ^{13}C NMR

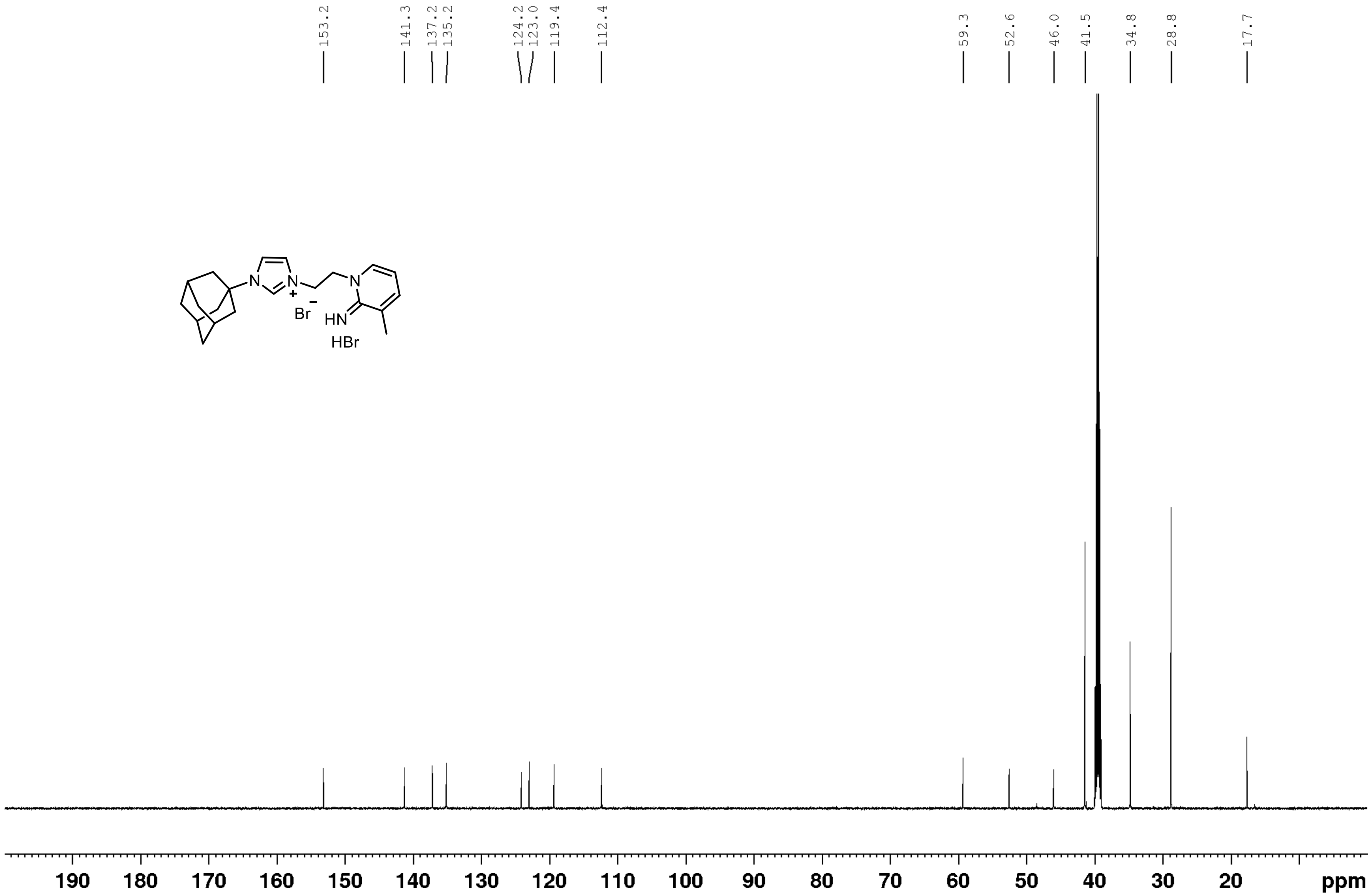
153.2
141.3
137.2
135.2
124.2
123.0
119.4
112.4
59.3
52.6
46.0
41.5
34.8
28.8
17.7
N
N
+
Br
−
N
HN
HBr
190
180
170
160
150
140
130
120
110
100
90
80
70
60
50
40
30
20
ppm

1.8.3 ^{1}H, ^{1}H COSY

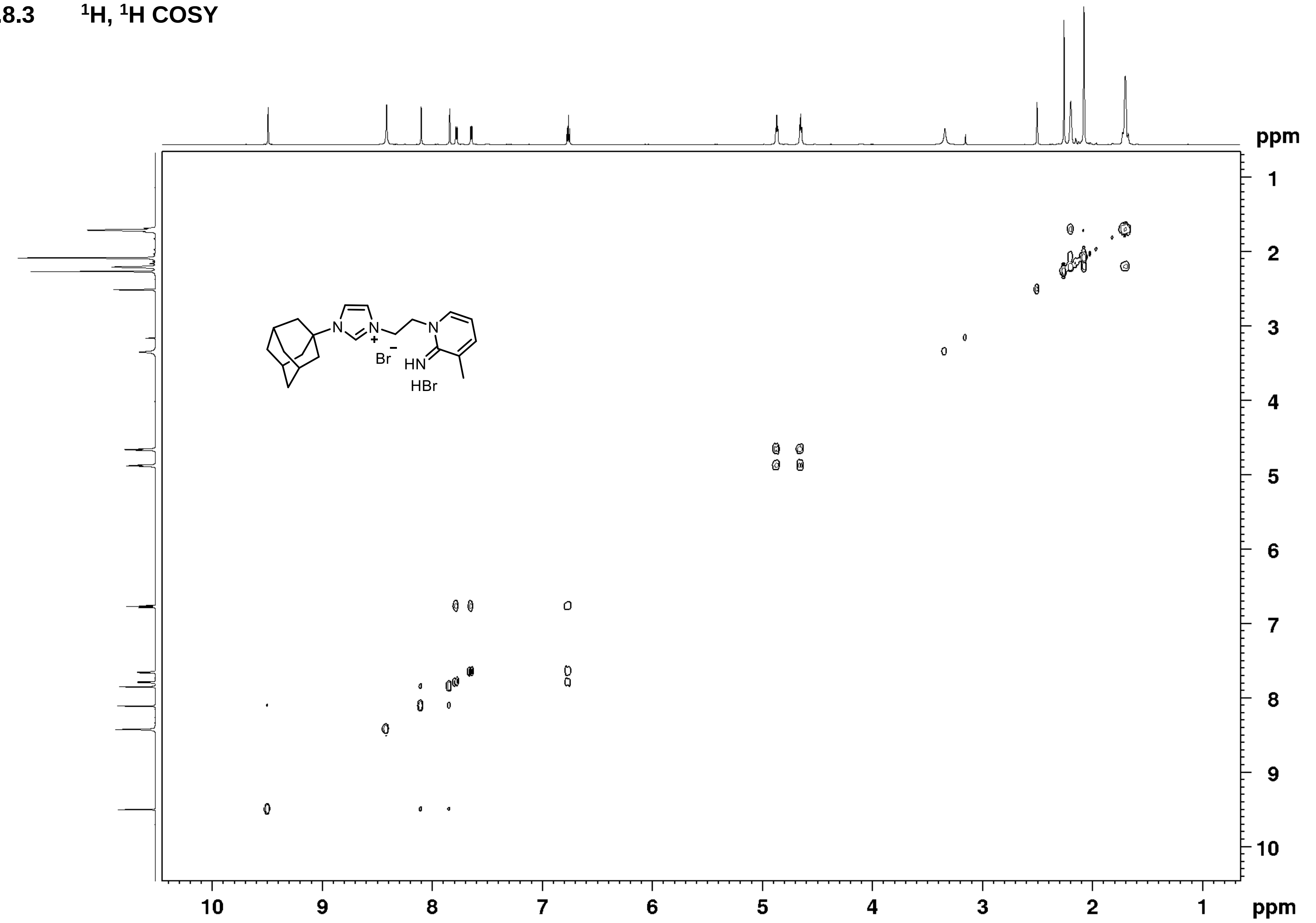

1.8.4 ¹H, ¹³C HMBC

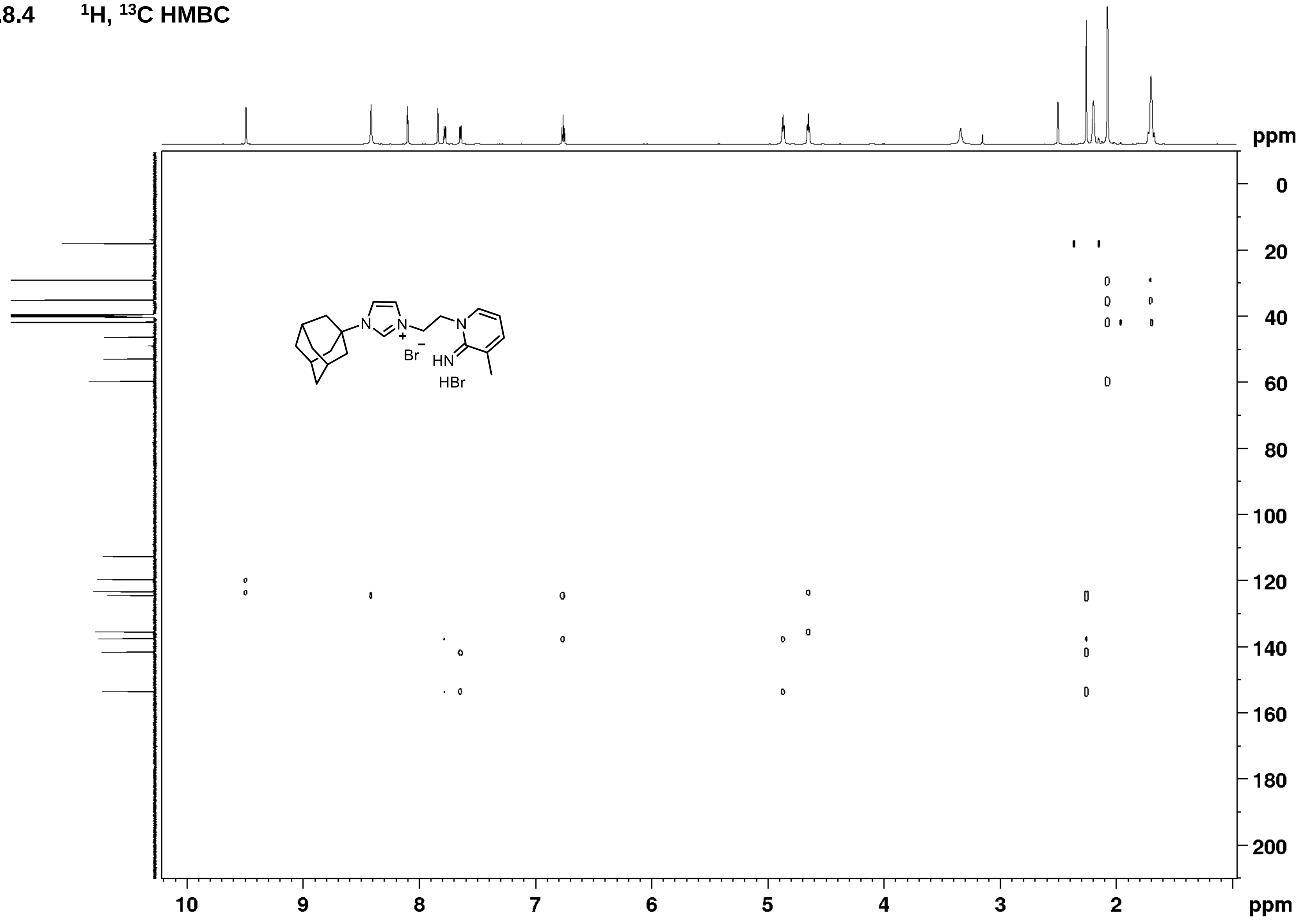

1.8.5 ^{1}H, ^{13}C HSQC

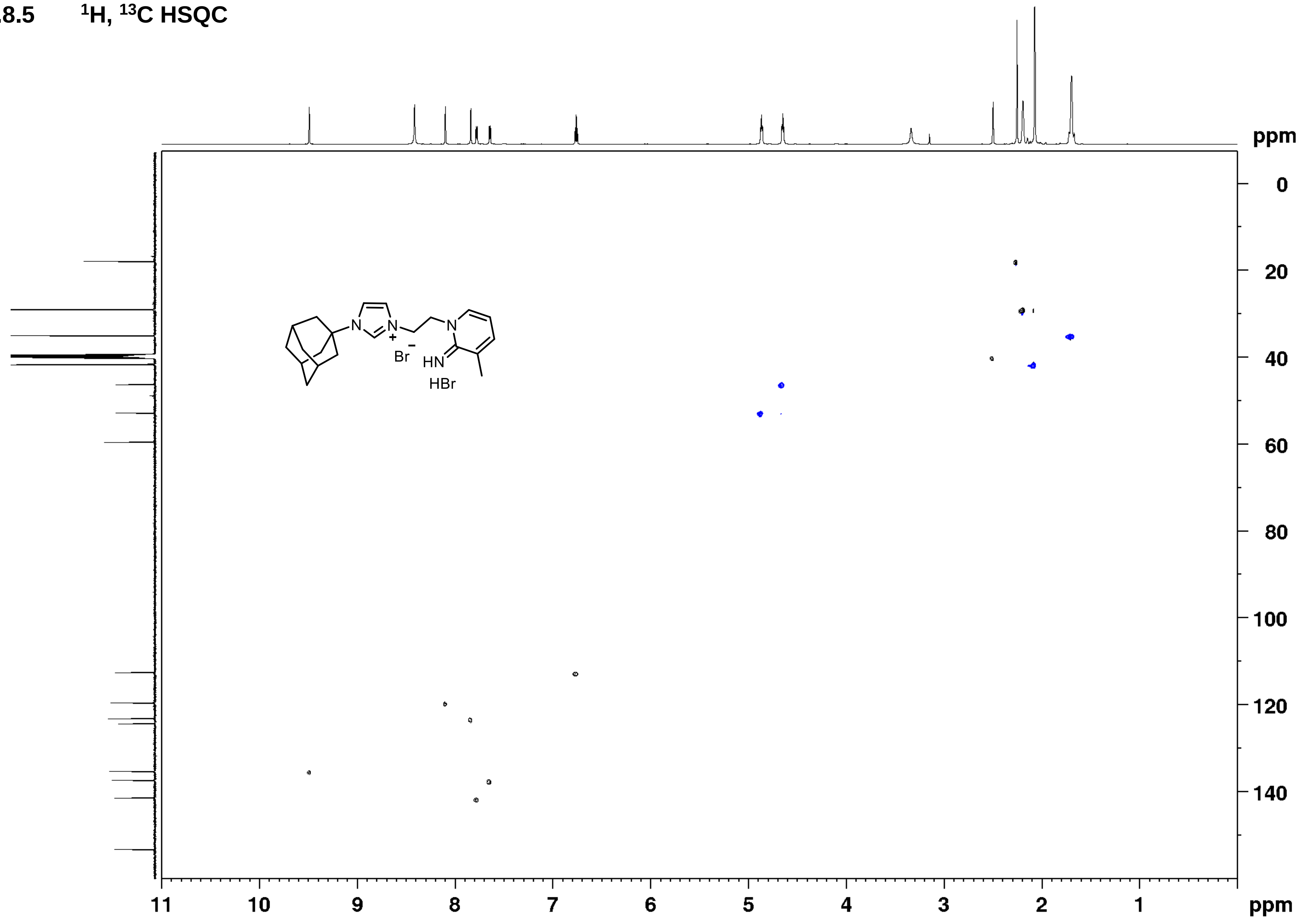

1.8.6 HRMS

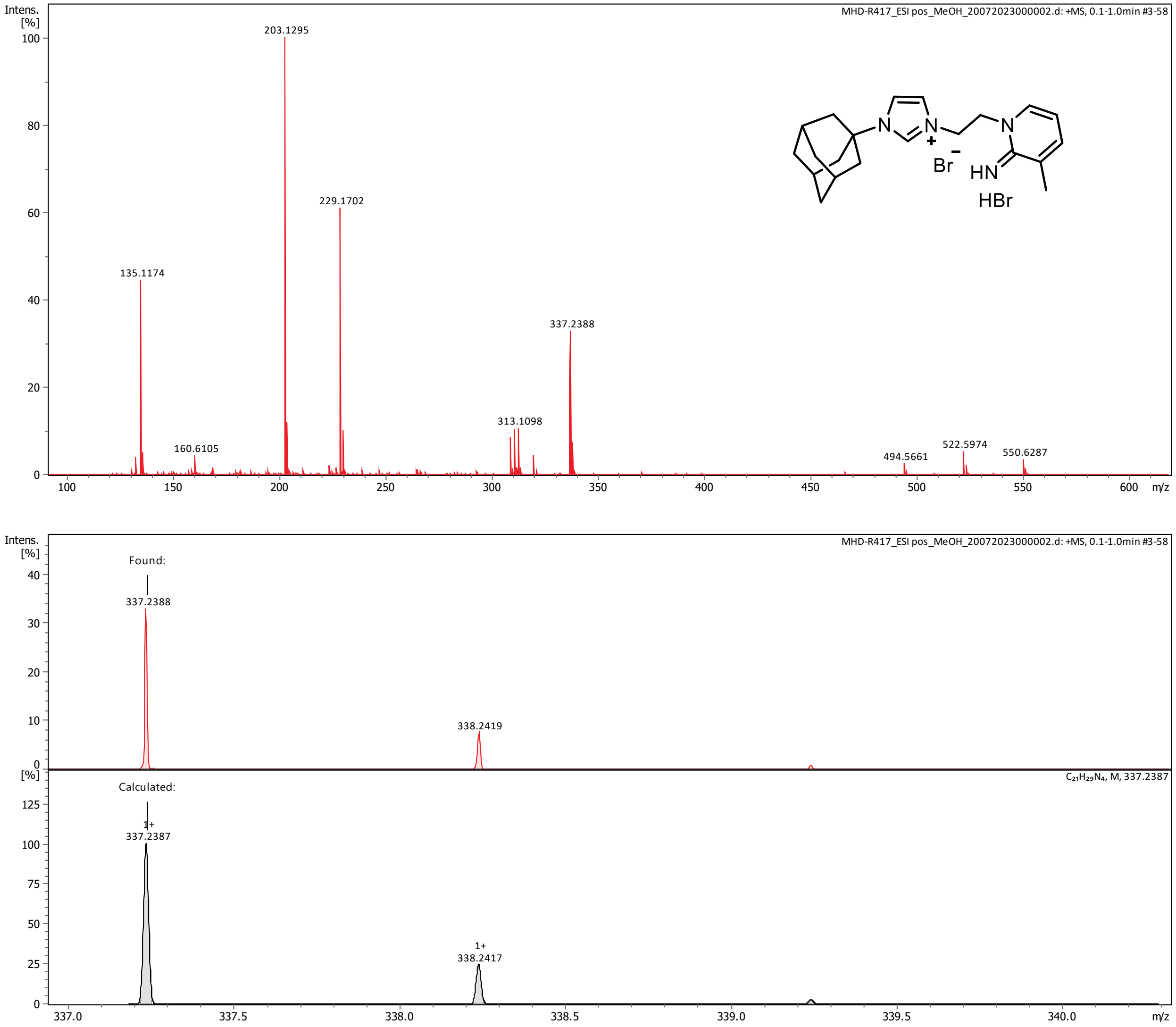

MHD-R417_ESI pos_MeOH_20072023000002.d: +MS, 0.1-1.0min #3-58
Intens. [%]
135.1174
160.6105
203.1295
229.1702
313.1098
337.2388
494.5661
522.5974
550.6287
m/z
Br
HN
HBr
Found:
337.2388
338.2419
Calculated:
1+
337.2387
1+
338.2417
C₂₁H₂₉N₄, M, 337.2387

1.8.7 IR

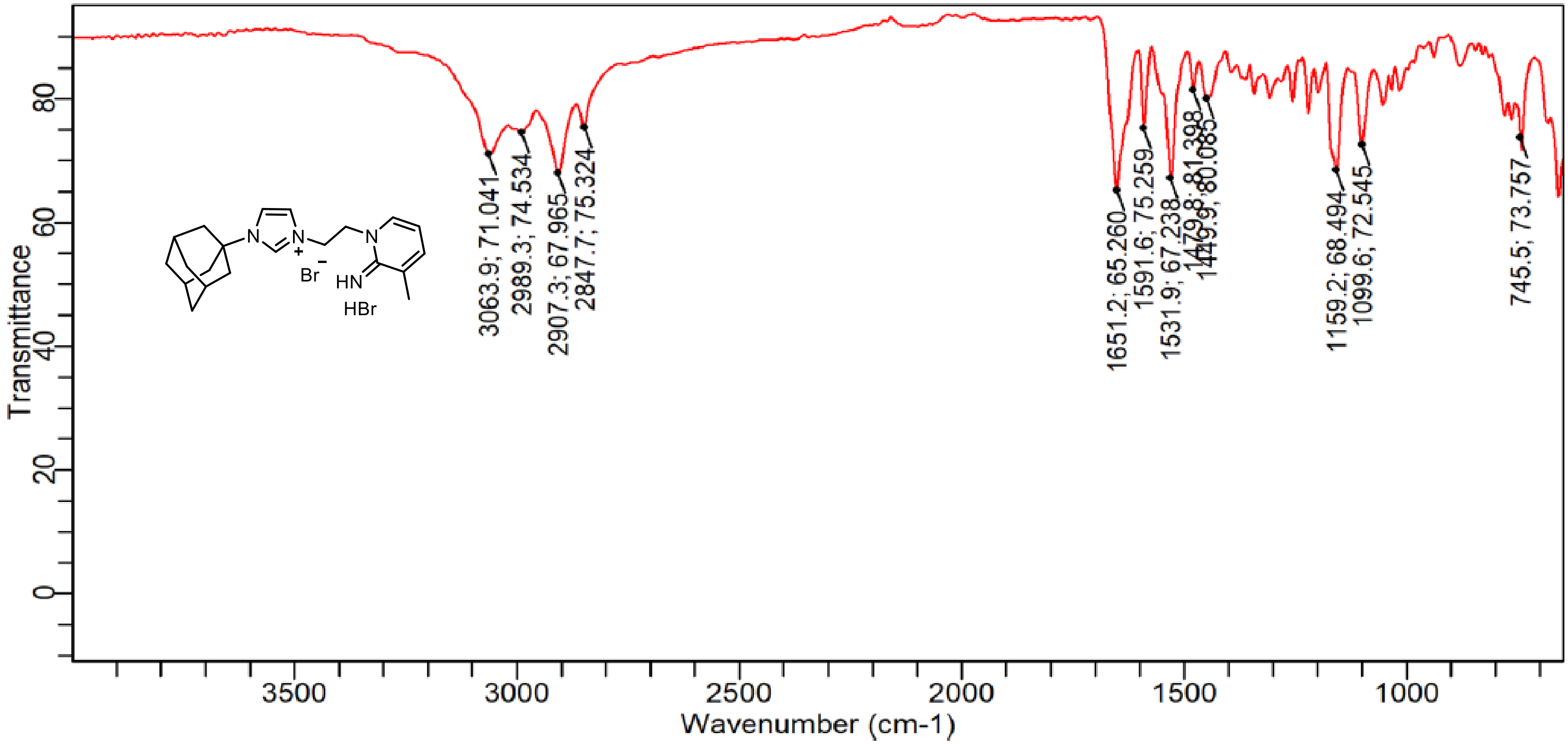

Transmittance
Wavenumber (cm-1)
3063.9; 71.041
2989.3; 74.534
2907.3; 67.965
2847.7; 75.324
1651.2; 65.260
1591.6; 75.259
1531.9; 67.238
1479.8; 81.398
1449.9; 80.085
1159.2; 68.494
1099.6; 72.545
745.5; 73.757
Br
HN
HBr

1.9 (Adamantan-1-yl)-3-(2-(2-imino-3-methylpyridin-1(2*H*)-yl)ethyl)-1,3-dihydro-2*H*-imidazol-2-ylidene)copper(I) chloride (5) (600 MHz, CD_2Cl_2)

1.9.1 ^{1}H NMR

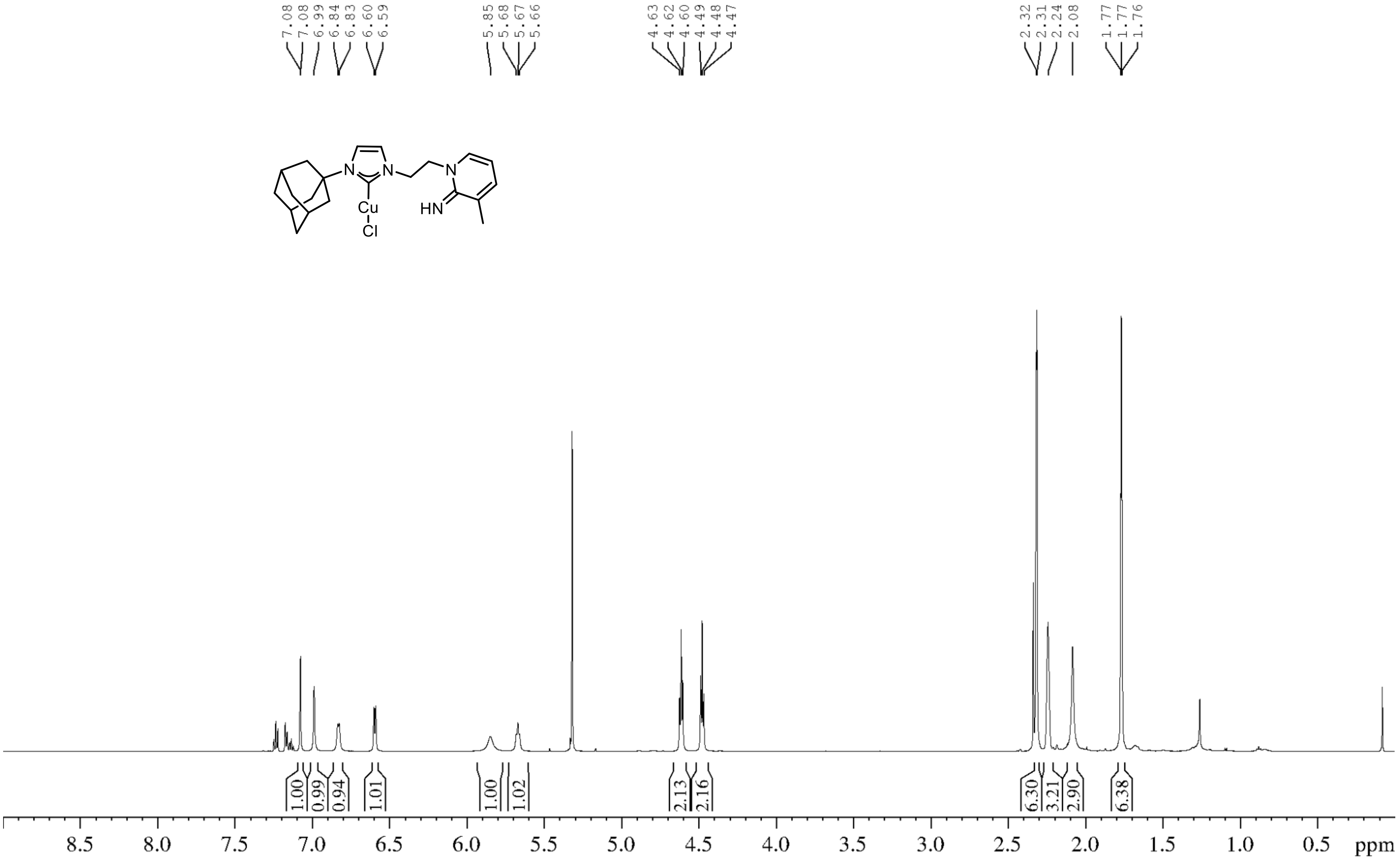

1.9.2 ^{13}C NMR

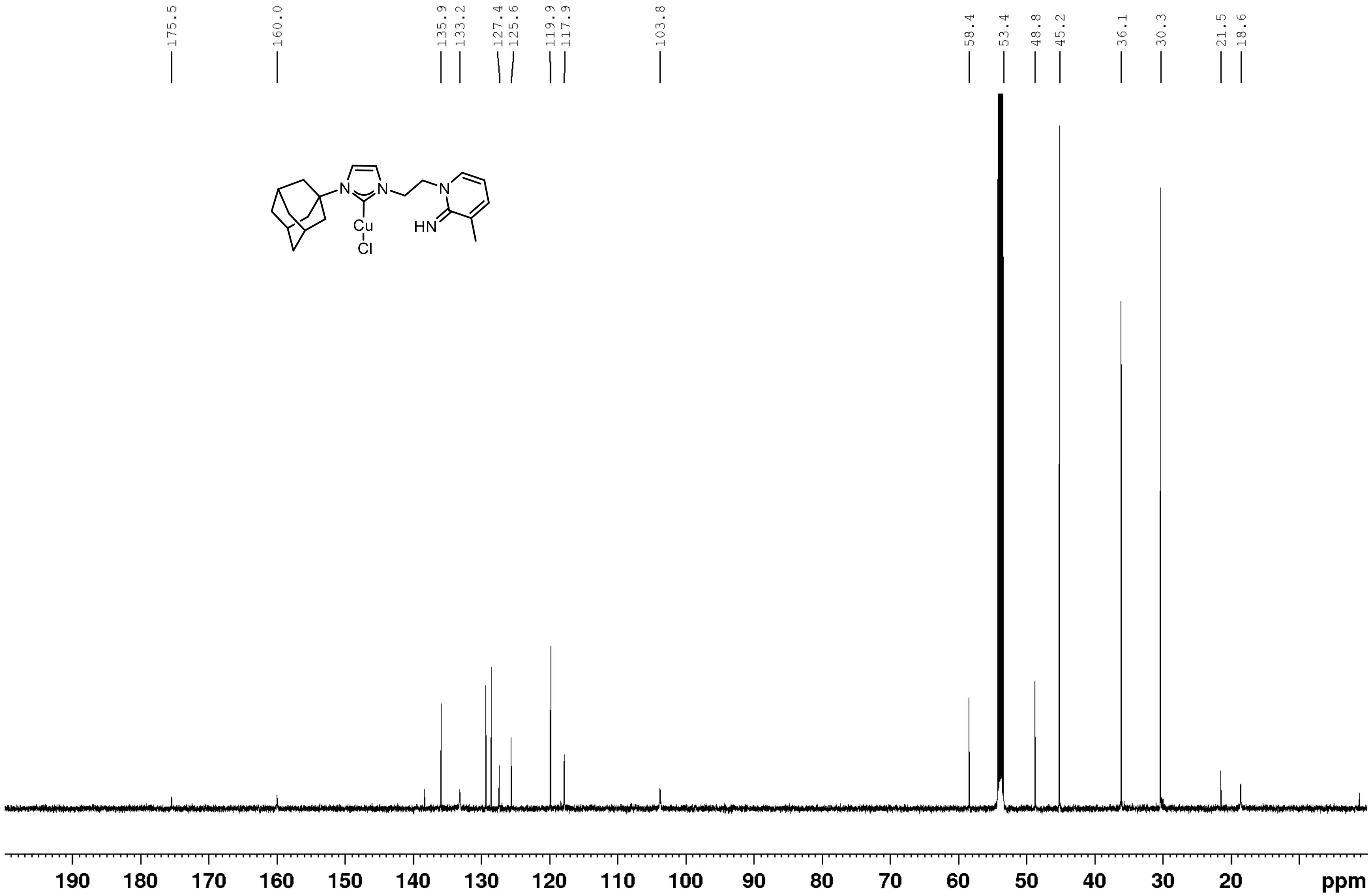

175.5
160.0
135.9
133.2
127.4
125.6
119.9
117.9
103.8
58.4
53.4
48.8
45.2
36.1
30.3
21.5
18.6
Cu
Cl
HN
N
190
180
170
160
150
140
130
120
110
100
90
80
70
60
50
40
30
20
ppm

1.9.3 ^{1}H, ^{1}H COSY

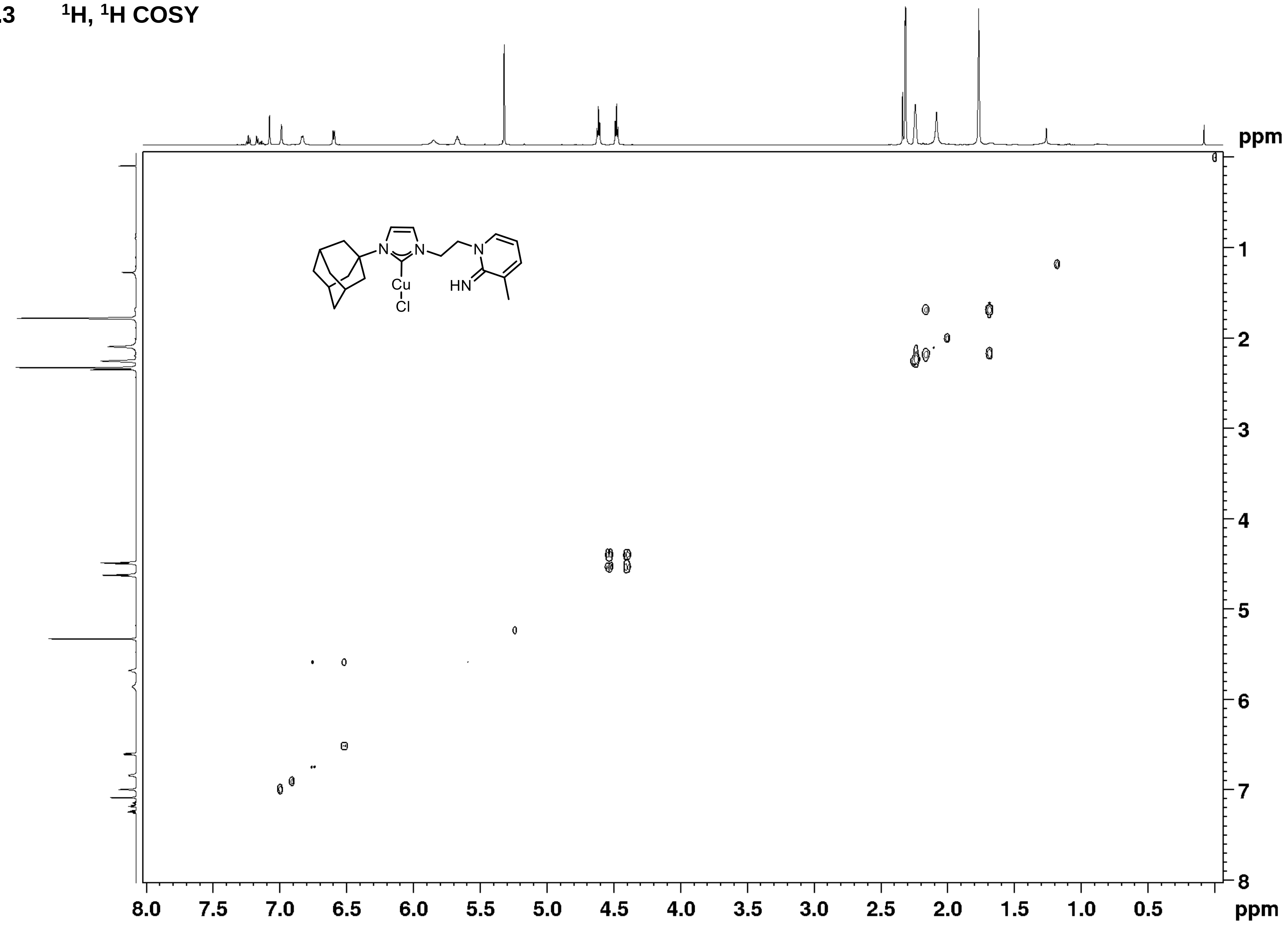

1.9.4 ¹H, ¹³C HMBC

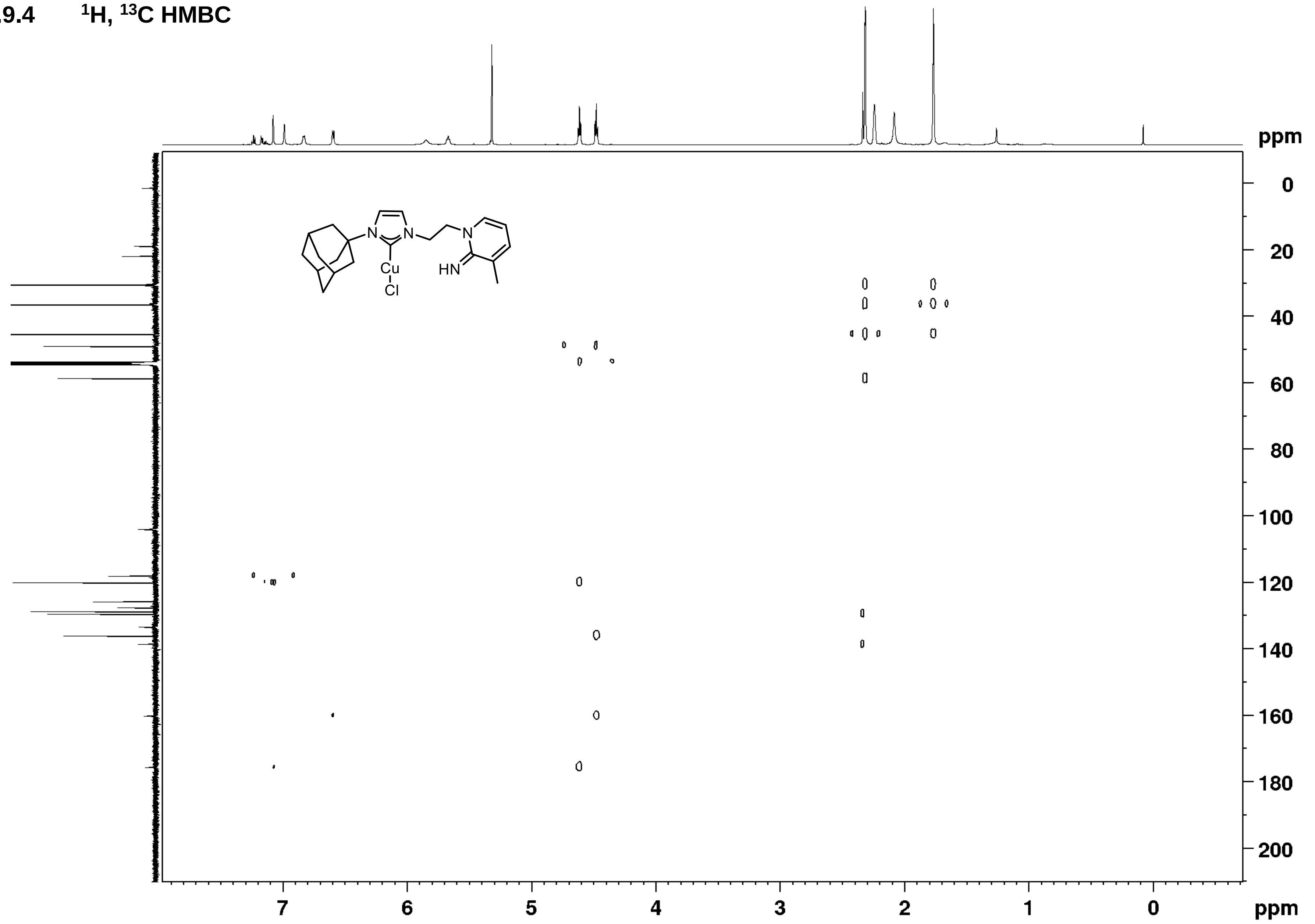

1.9.5 ^{1}H, ^{13}C HSQC

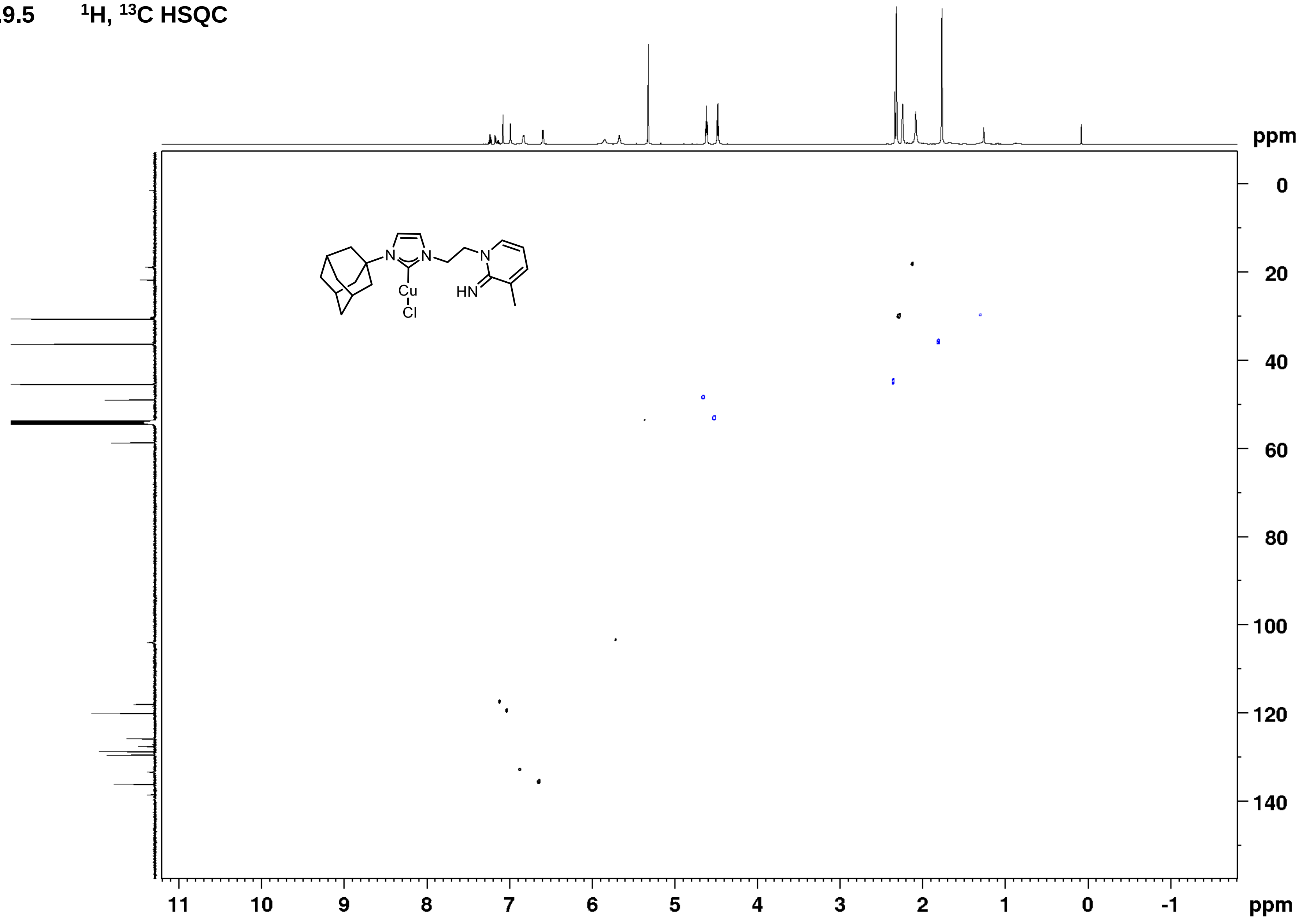

1.9.6 HRMS

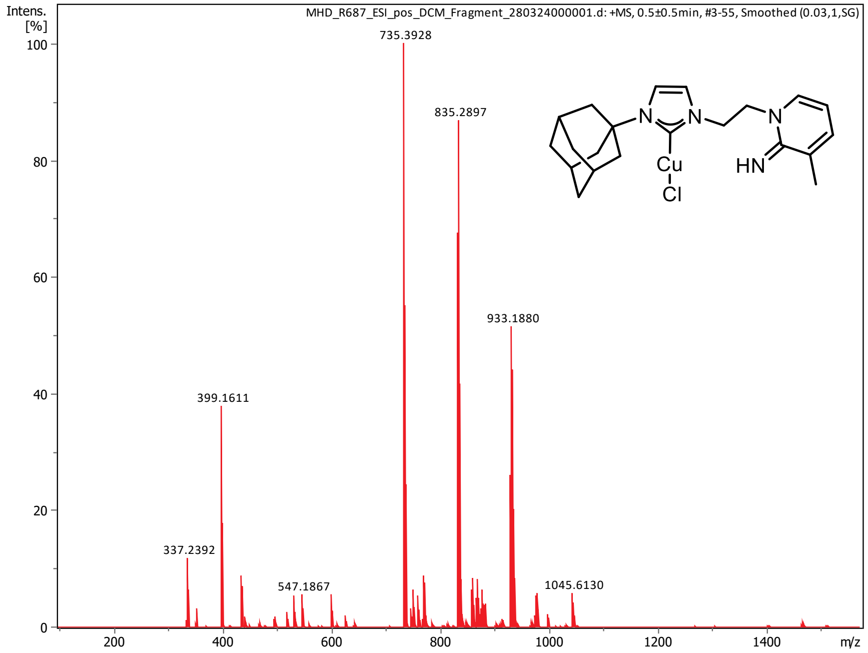

Intens. [%]
MHD_R687_ESI_pos_DCM_Fragment_280324000001.d: +MS, 0.5±0.5min, #3-55, Smoothed (0.03,1,SG)
735.3928
835.2897
933.1880
399.1611
337.2392
547.1867
1045.6130
N
N
N
Cu
Cl
HN
m/z

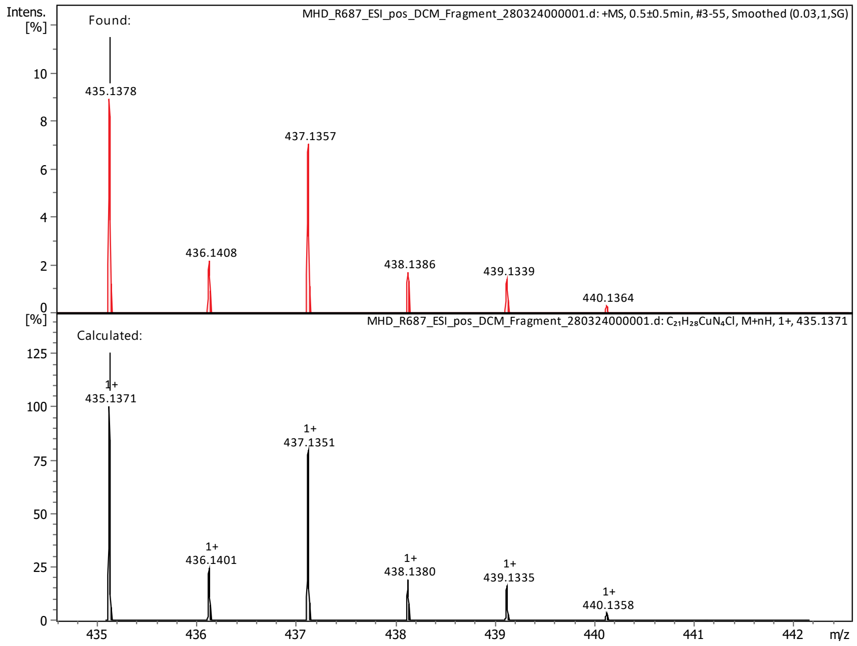

Intens. [%]
Found:
MHD_R687_ESI_pos_DCM_Fragment_280324000001.d: +MS, 0.5±0.5min, #3-55, Smoothed (0.03,1,SG)
435.1378
436.1408
437.1357
438.1386
439.1339
440.1364
Calculated:
MHD_R687_ESI_pos_DCM_Fragment_280324000001.d: C21H28CuN4Cl, M+nH, 1+, 435.1371
1+ 435.1371
1+ 436.1401
1+ 437.1351
1+ 438.1380
1+ 439.1335
1+ 440.1358
m/z

1.10 3-(2-Bromoethyl)-1-mesityl-4,5-dihydro-1*H*-imidazol-3-ium bromide (S6b) (600 MHz, $CDCl_3$)

1.10.1 ^{1}H NMR

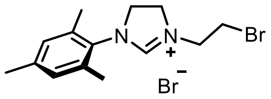

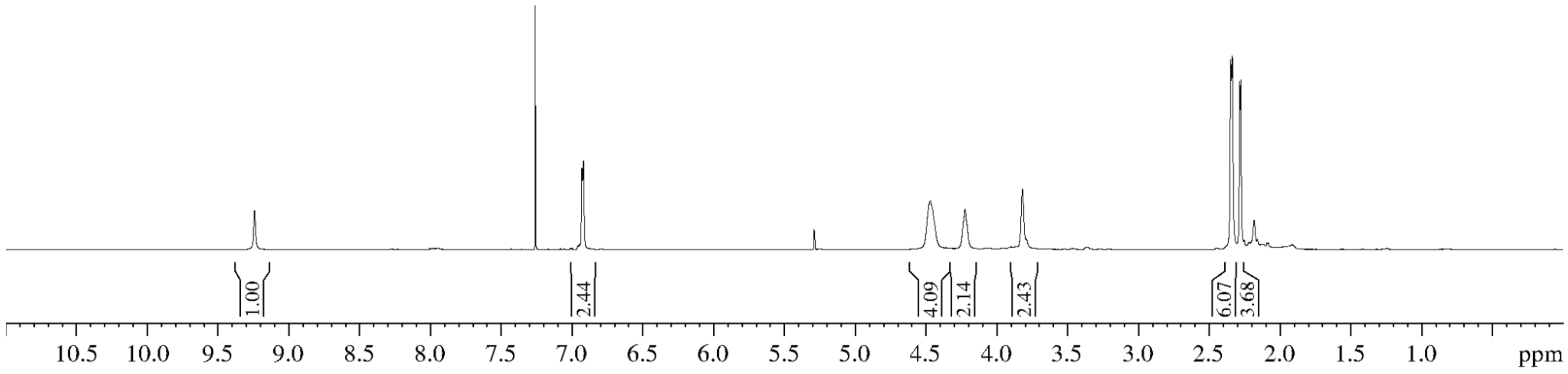

1.10.2 ^{13}C NMR

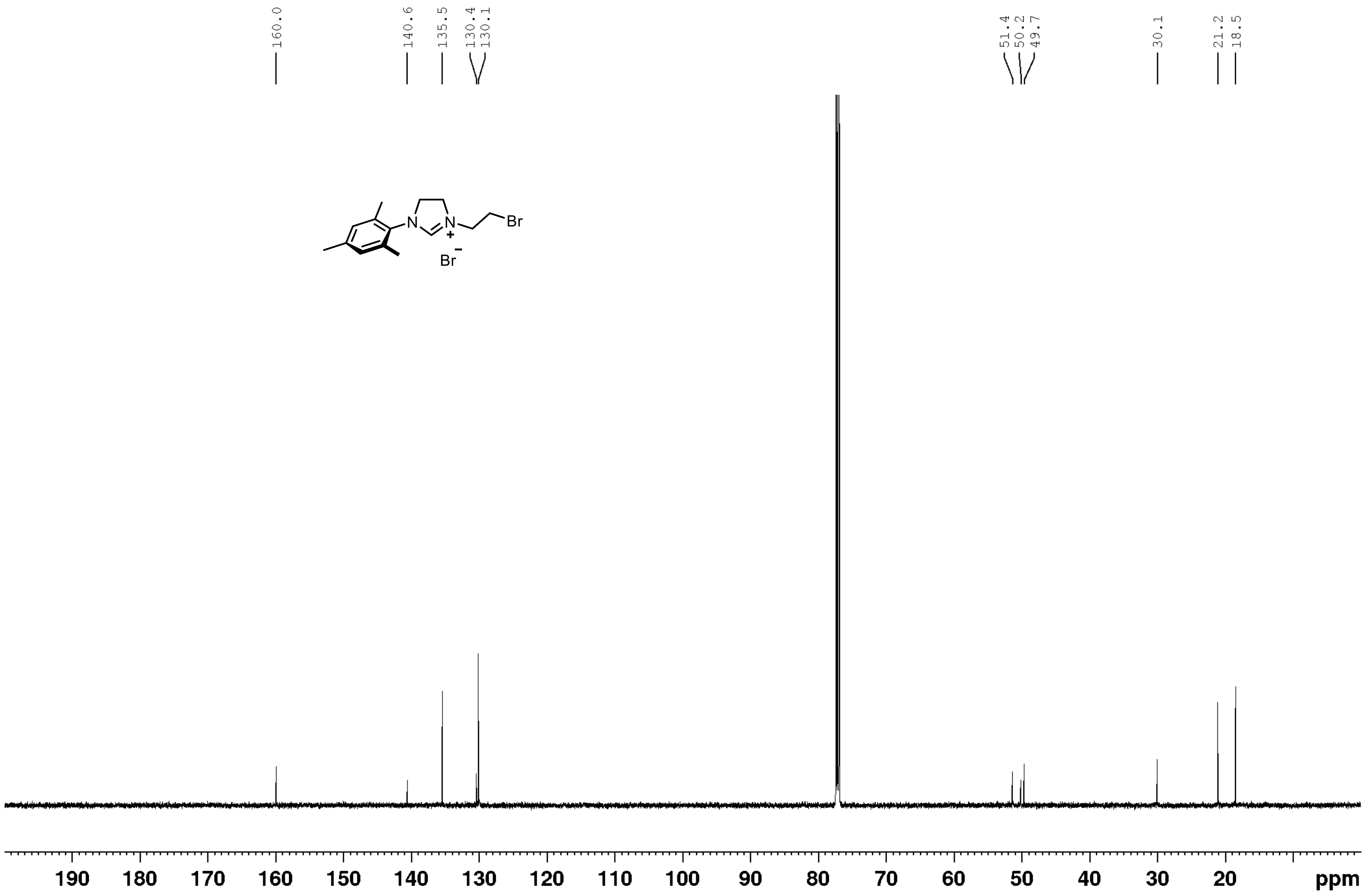

160.0
140.6
135.5
130.4
130.1
51.4
50.2
49.7
30.1
21.2
18.5
N
N
+
Br
Br
190
180
170
160
150
140
130
120
110
100
90
80
70
60
50
40
30
20
ppm

1.10.3 ¹H, ¹H COSY

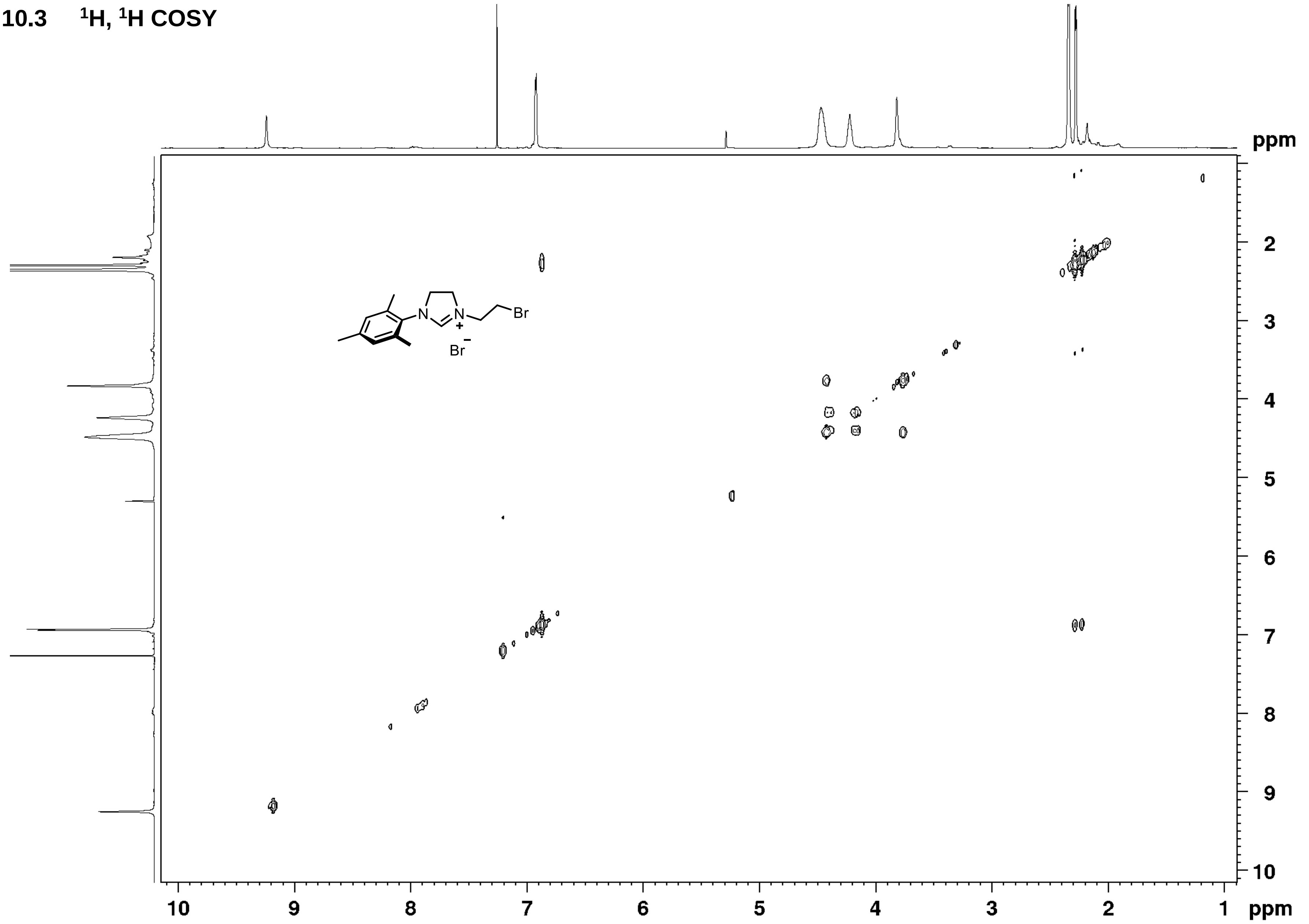

1.10.4 ^{1}H, ^{13}C HMBC

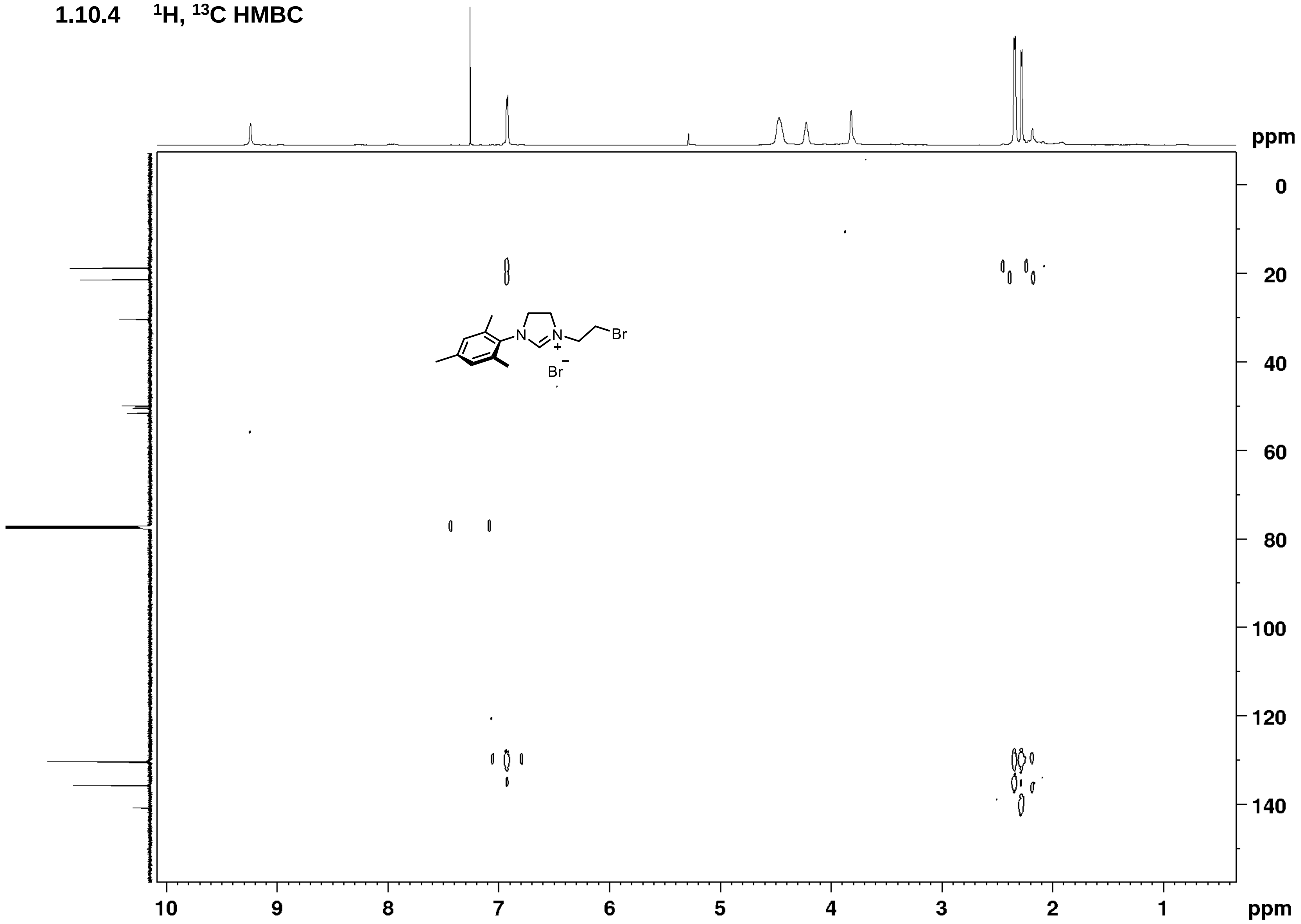

1.10.5 ^{1}H, ^{13}C HSQC

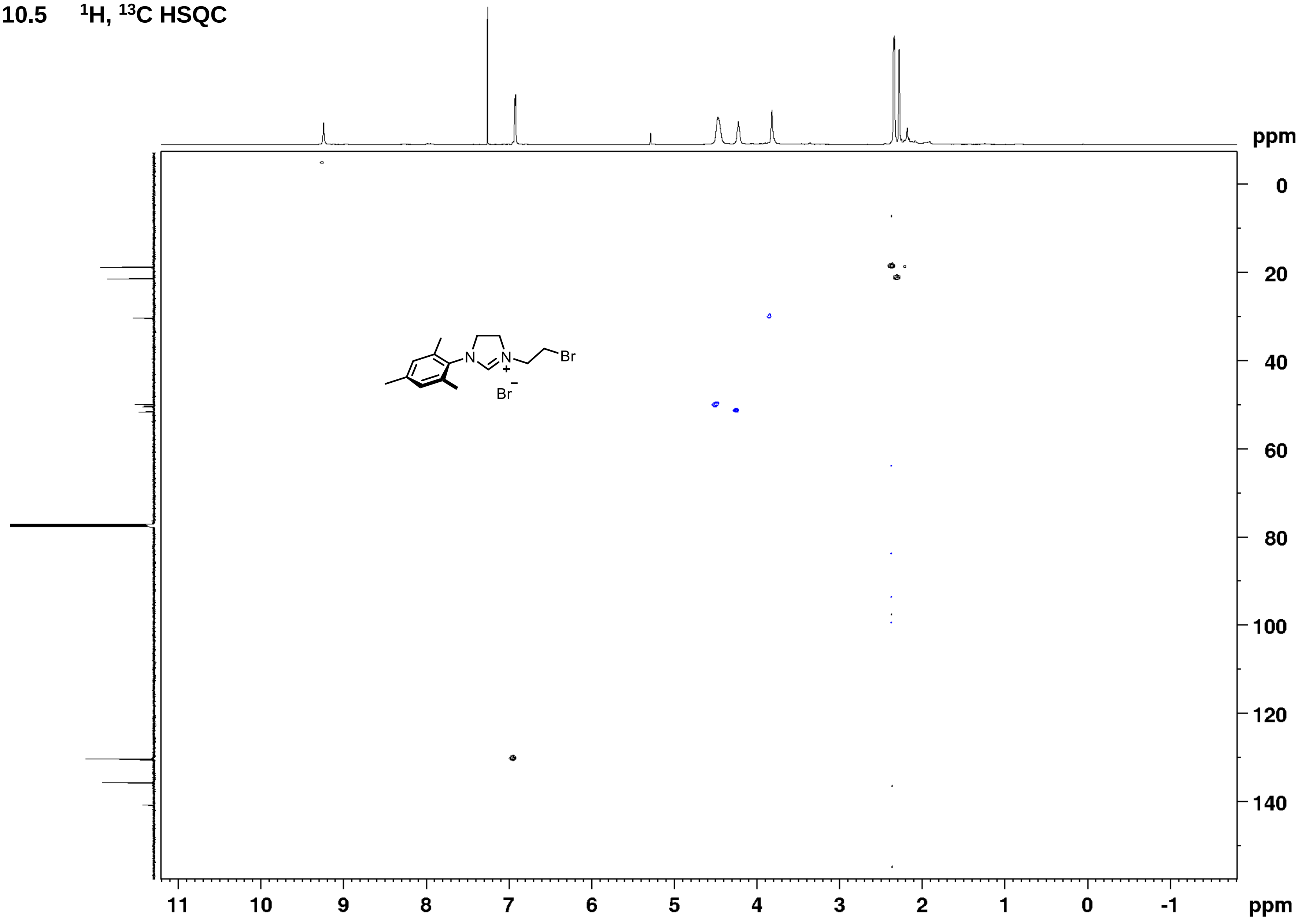

1.10.6 HRMS

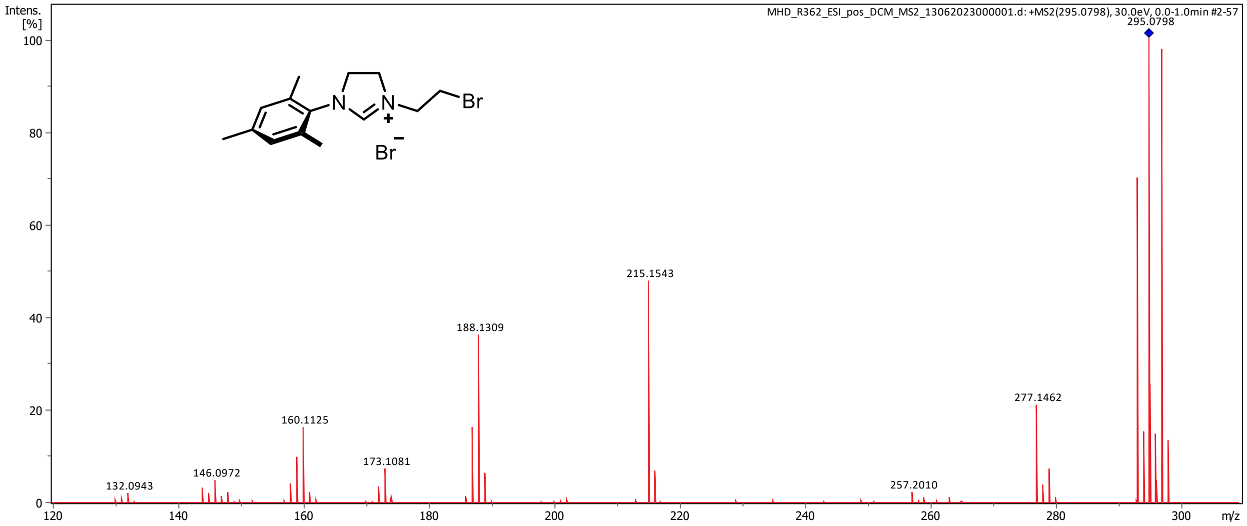

Intens. [%]
MHD_R362_ESI_pos_DCM_MS2_13062023000001.d: +MS2(295.0798), 30.0eV, 0.0-1.0min #2-57
295.0798
277.1462
257.2010
215.1543
188.1309
173.1081
160.1125
146.0972
132.0943
Br
N
N
+
Br
−
m/z

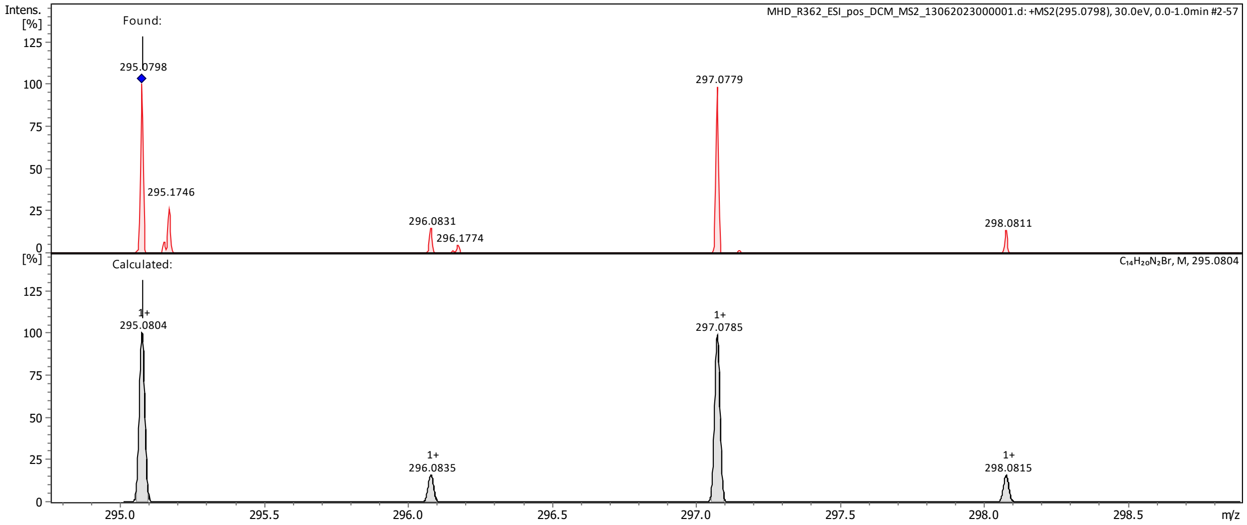

Intens. [%]
Found:
MHD_R362_ESI_pos_DCM_MS2_13062023000001.d: +MS2(295.0798), 30.0eV, 0.0-1.0min #2-57
295.0798
295.1746
296.0831
296.1774
297.0779
298.0811
Calculated:
C14H20N2Br, M, 295.0804
1+ 295.0804
1+ 296.0835
1+ 297.0785
1+ 298.0815
m/z

1.10.7 IR

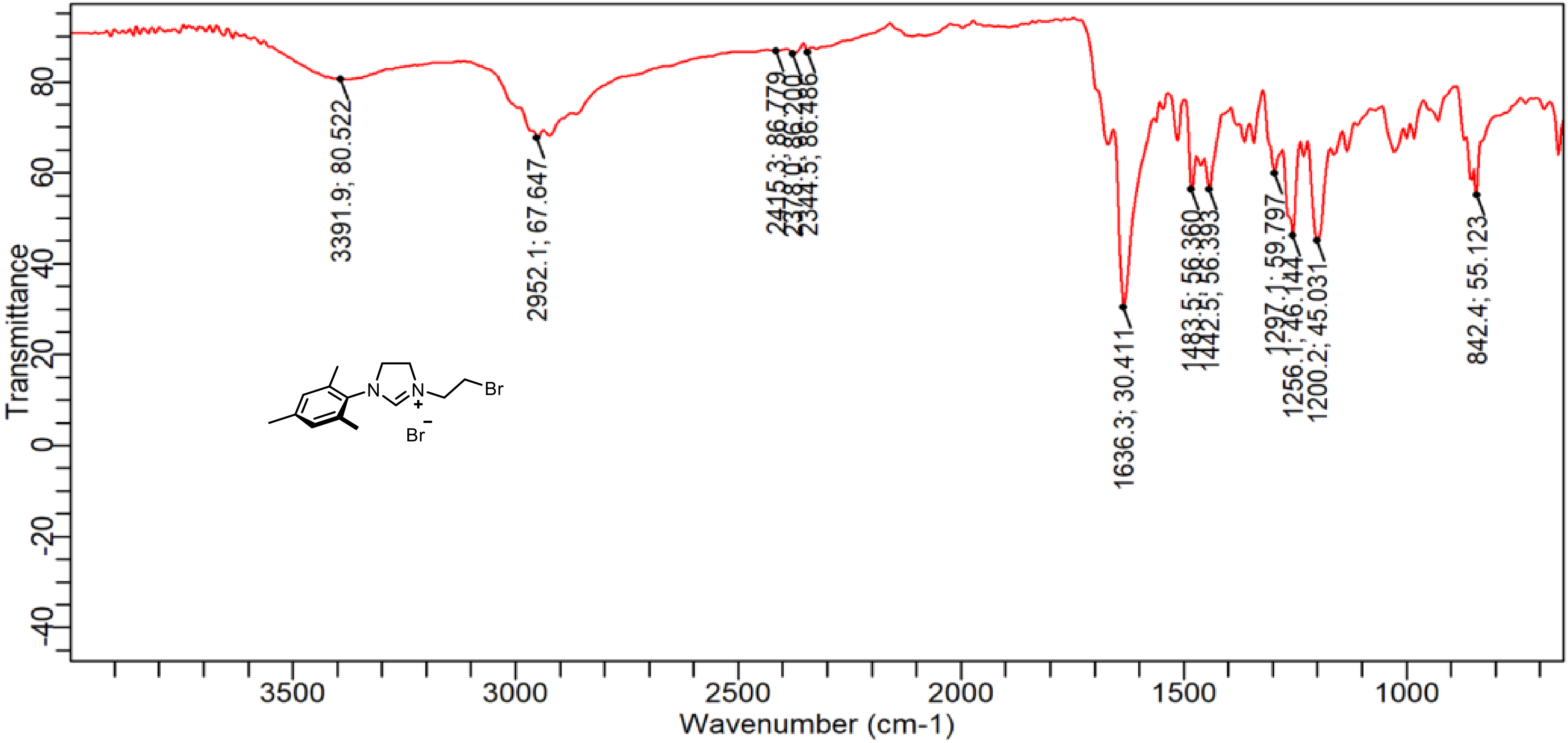
3391.9; 80.522
2952.1; 67.647
2415.3; 86.779
2378.0; 86.200
2344.5; 86.486
1636.3; 30.411
1483.5; 56.360
1442.5; 56.393
1297.1; 59.797
1256.1; 46.144
1200.2; 45.031
842.4; 55.123
Transmittance
Wavenumber (cm-1)
Br
Br

1.11 3-(2-(2-Imino-3-methylpyridin-1(2*H*)-yl)ethyl)-1-mesityl-4,5-dihydro-1*H*-imidazol-3-ium bromide hydrobromide (L6) (600 MHz, DMSO-d_6)

1.11.1 ^{1}H NMR

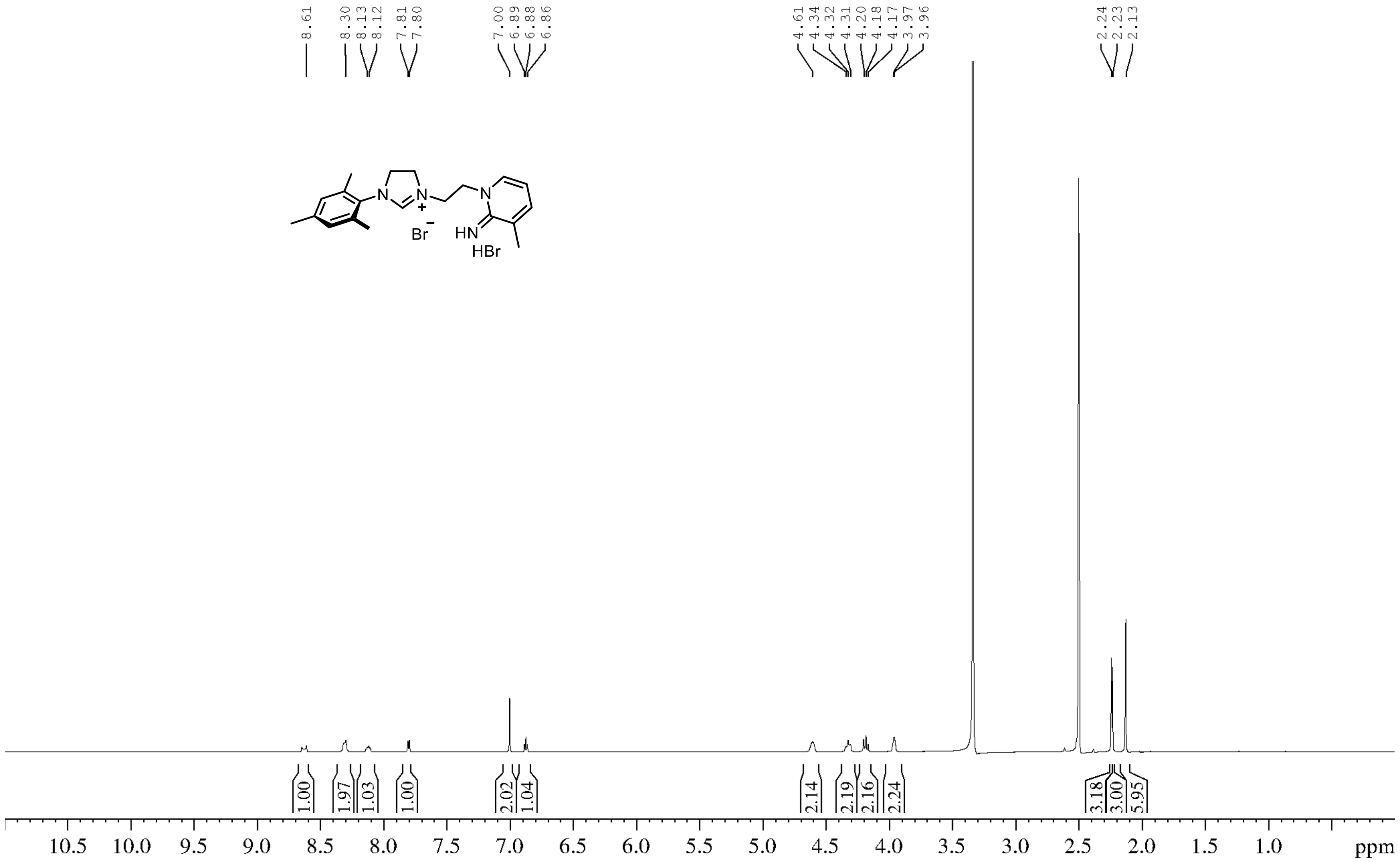

1.11.2 ^{13}C NMR

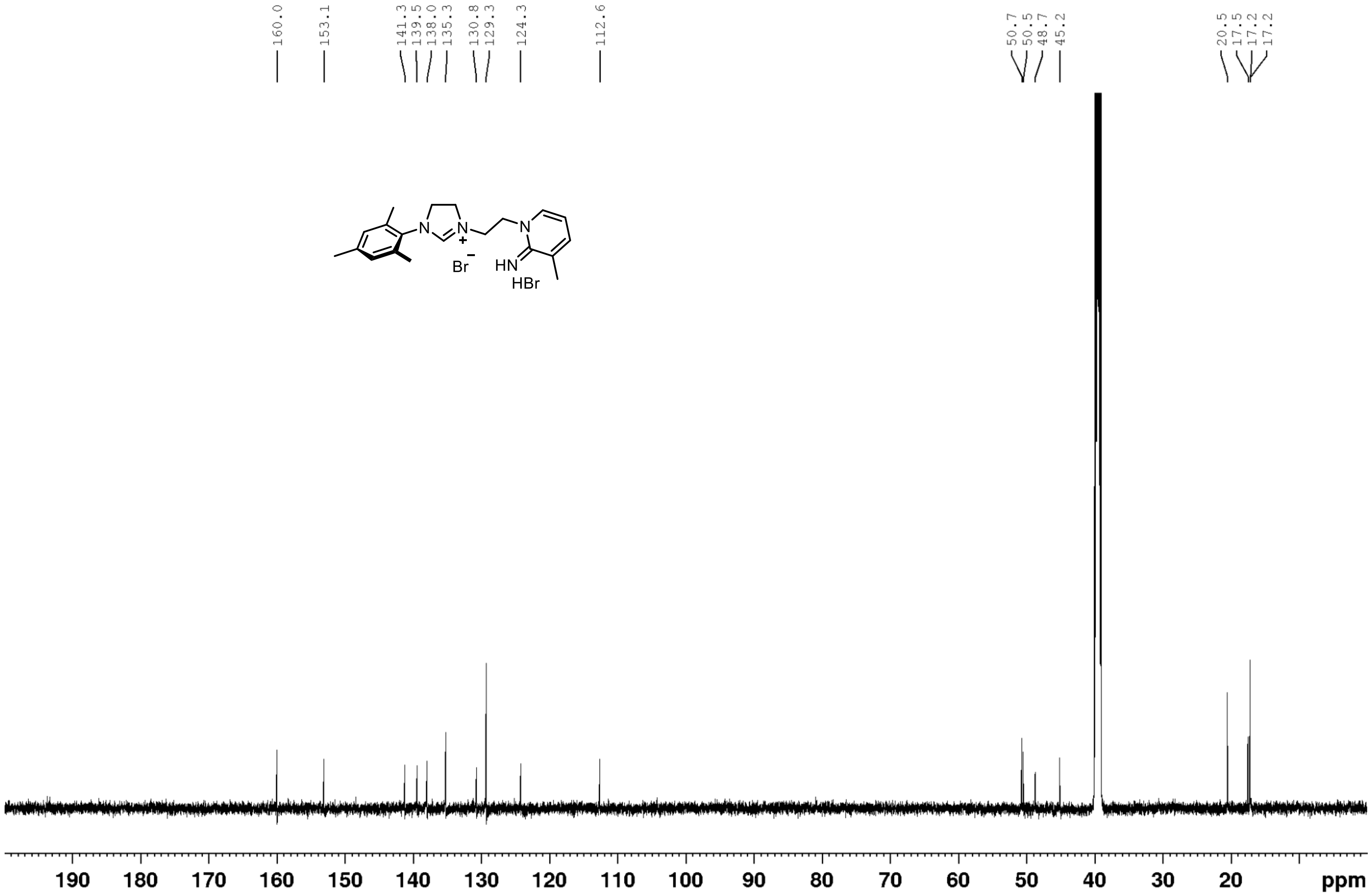

160.0
153.1
141.3
139.5
138.0
135.3
130.8
129.3
124.3
112.6
50.7
50.5
48.7
45.2
20.5
17.5
17.2
17.2
N
N
+
Br
N
HN
HBr
190 180 170 160 150 140 130 120 110 100 90 80 70 60 50 40 30 20 ppm

1.11.3 ^{1}H, ^{1}H COSY

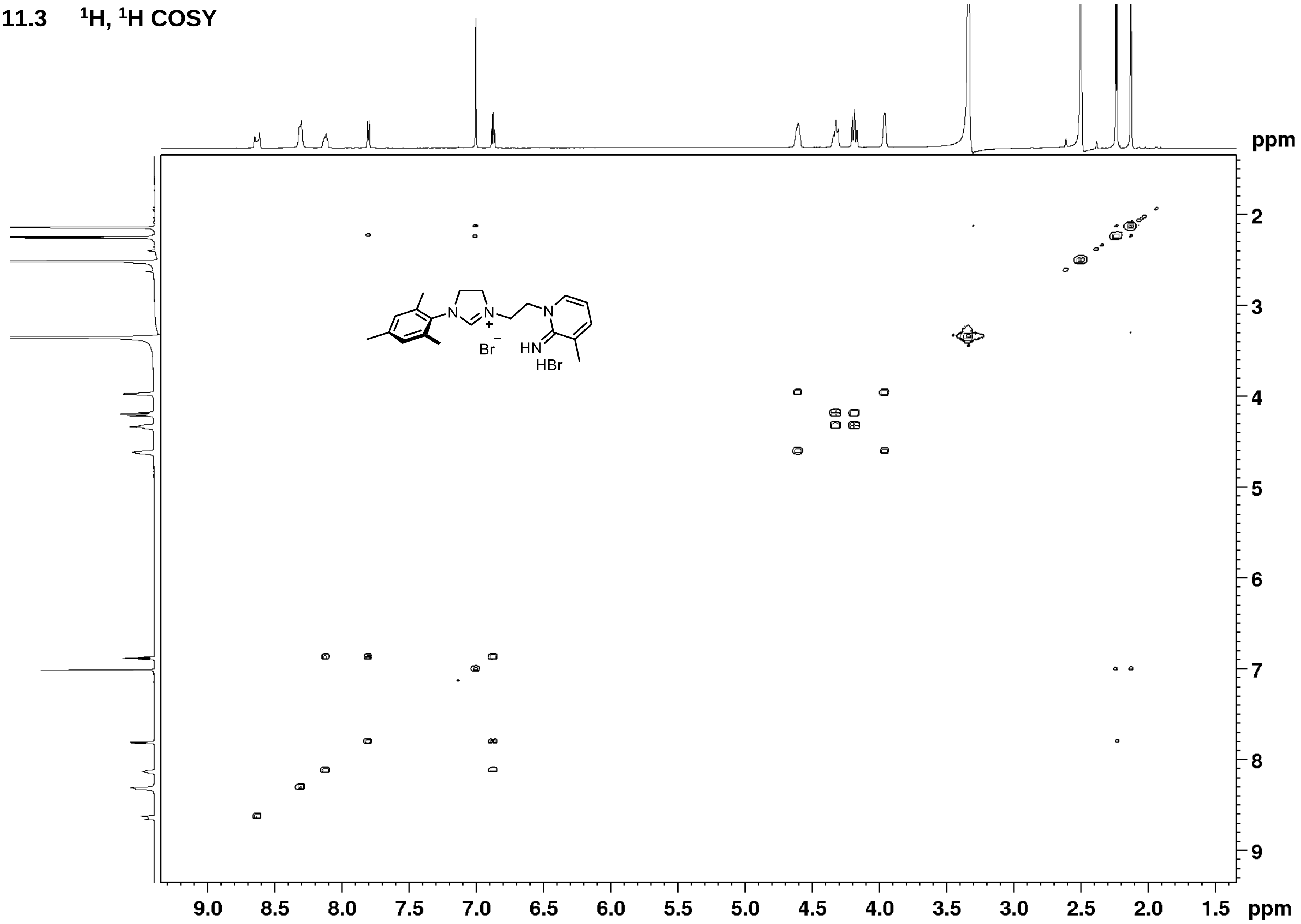

1.11.4 ^{1}H, ^{13}C HMBC

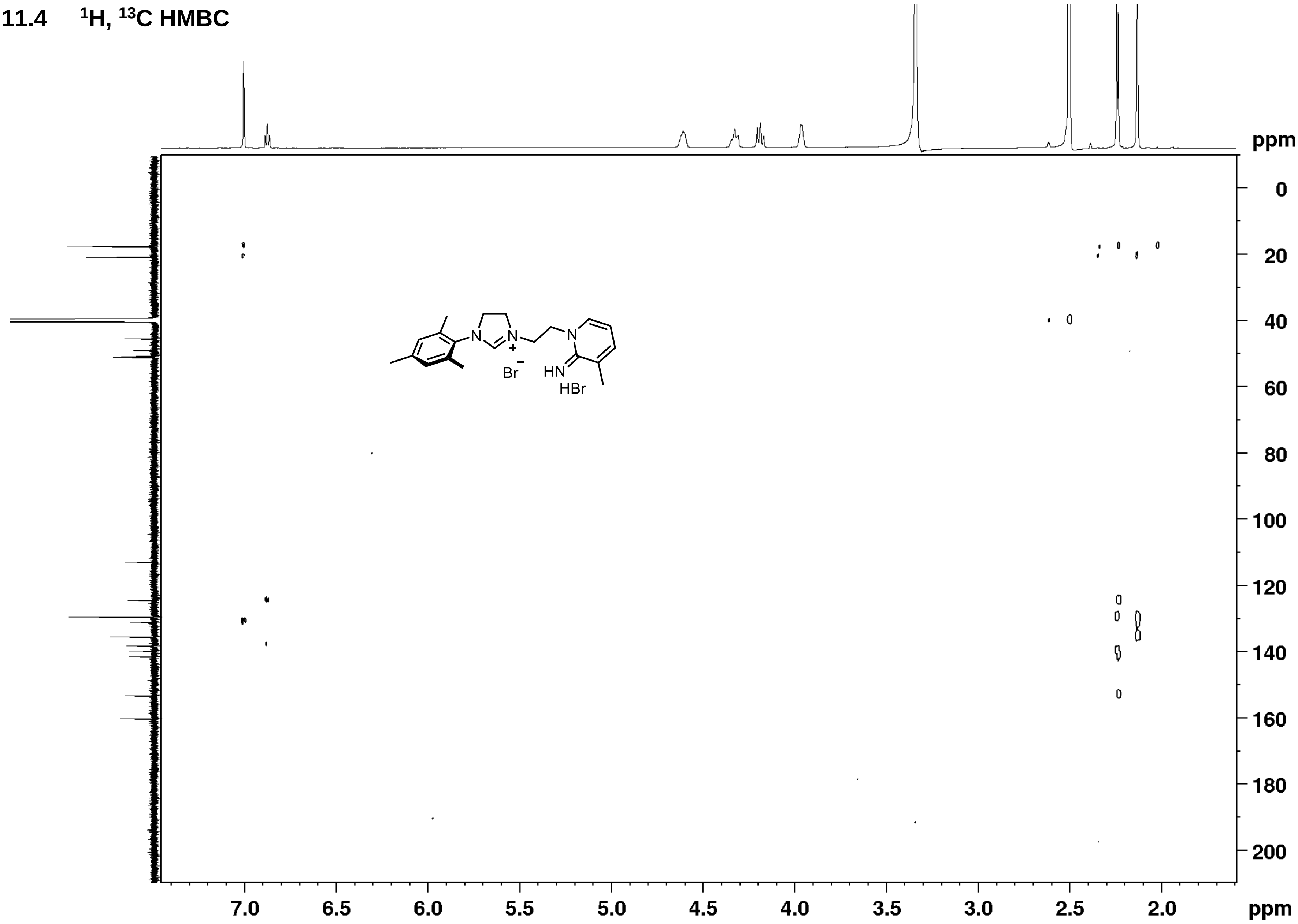

1.11.5 ^{1}H, ^{13}C HSQC

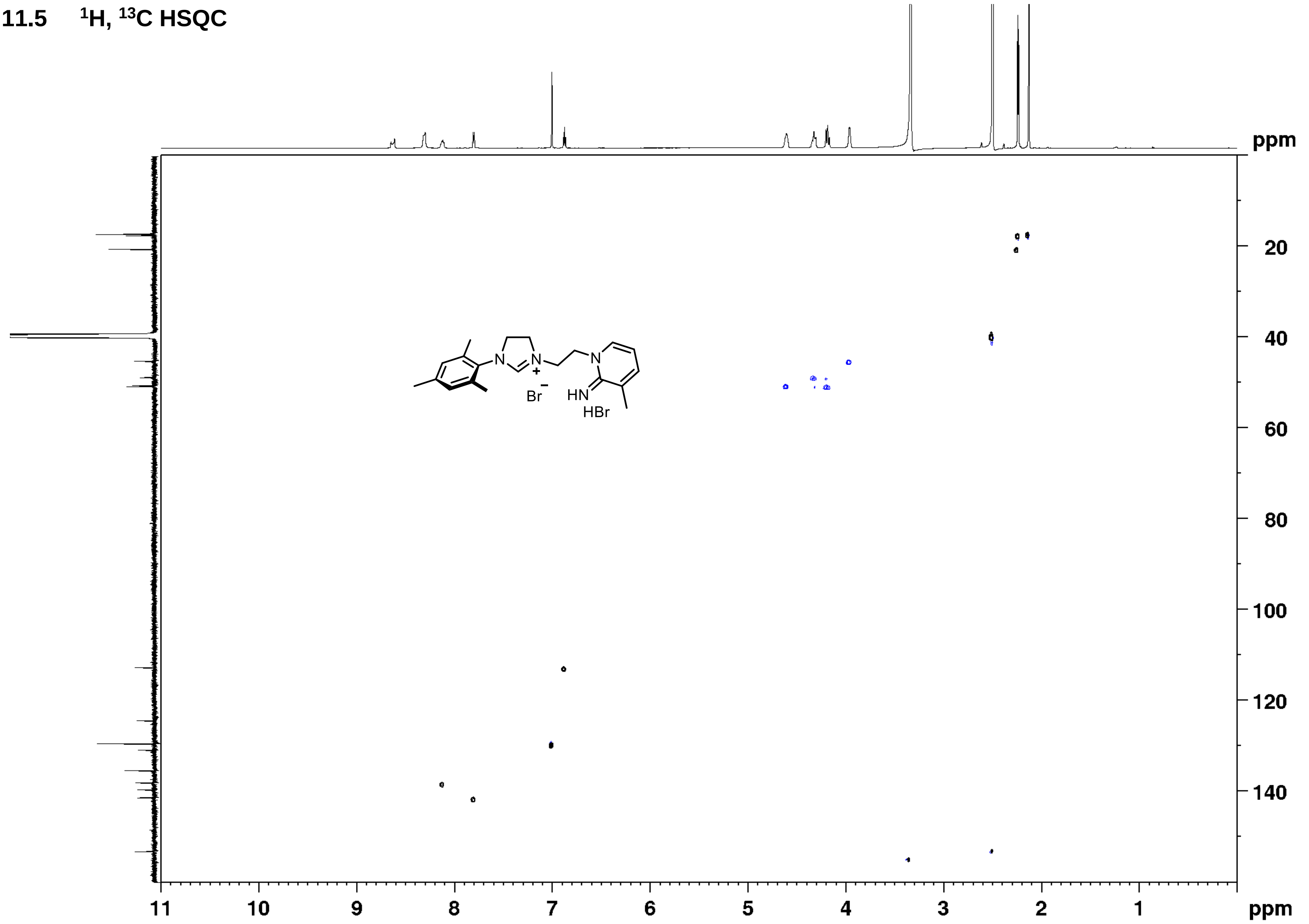

1.11.6 HRMS

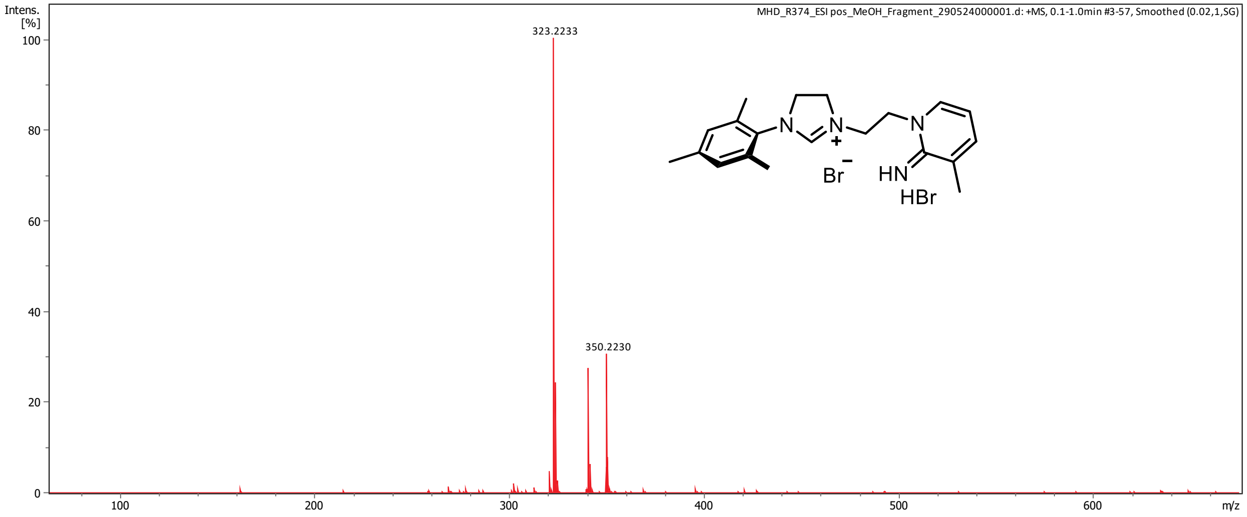

Intens.
[%]
MHD_R374_ESI pos_MeOH_Fragment_290524000001.d: +MS, 0.1-1.0min #3-57, Smoothed (0.02,1,SG)
323.2233
350.2230
N
N
+
Br
−
N
HN
HBr
100
80
60
40
20
0
100
200
300
400
500
600
m/z

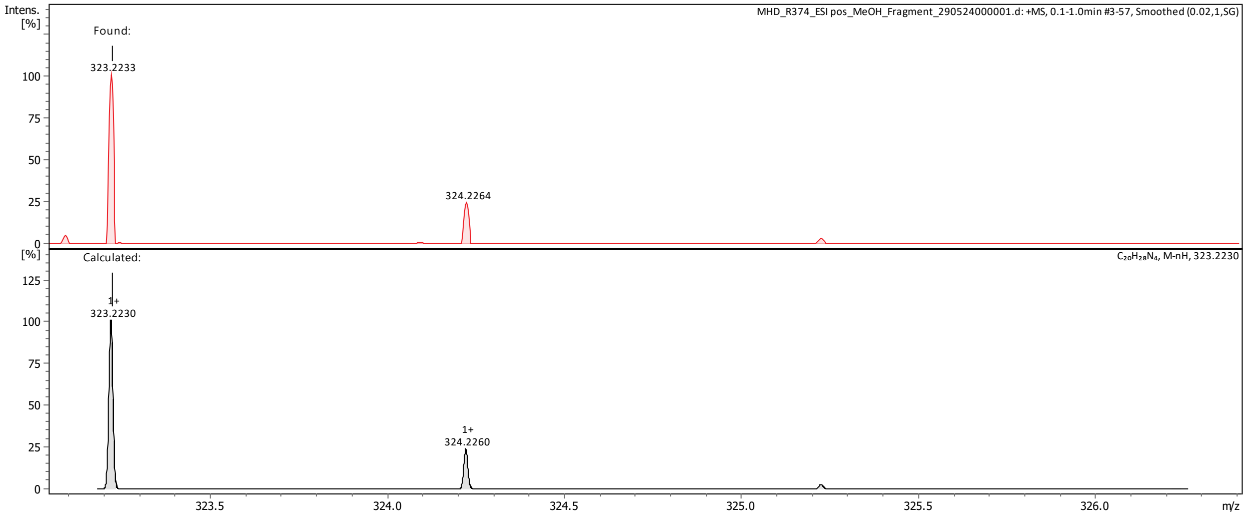

Intens.
[%]
MHD_R374_ESI pos_MeOH_Fragment_290524000001.d: +MS, 0.1-1.0min #3-57, Smoothed (0.02,1,SG)
Found:
323.2233
324.2264
Calculated:
$C_{20}H_{28}N_4$, M-nH, 323.2230
1+
323.2230
1+
324.2260
323.5
324.0
324.5
325.0
325.5
326.0
m/z

1.11.7 IR

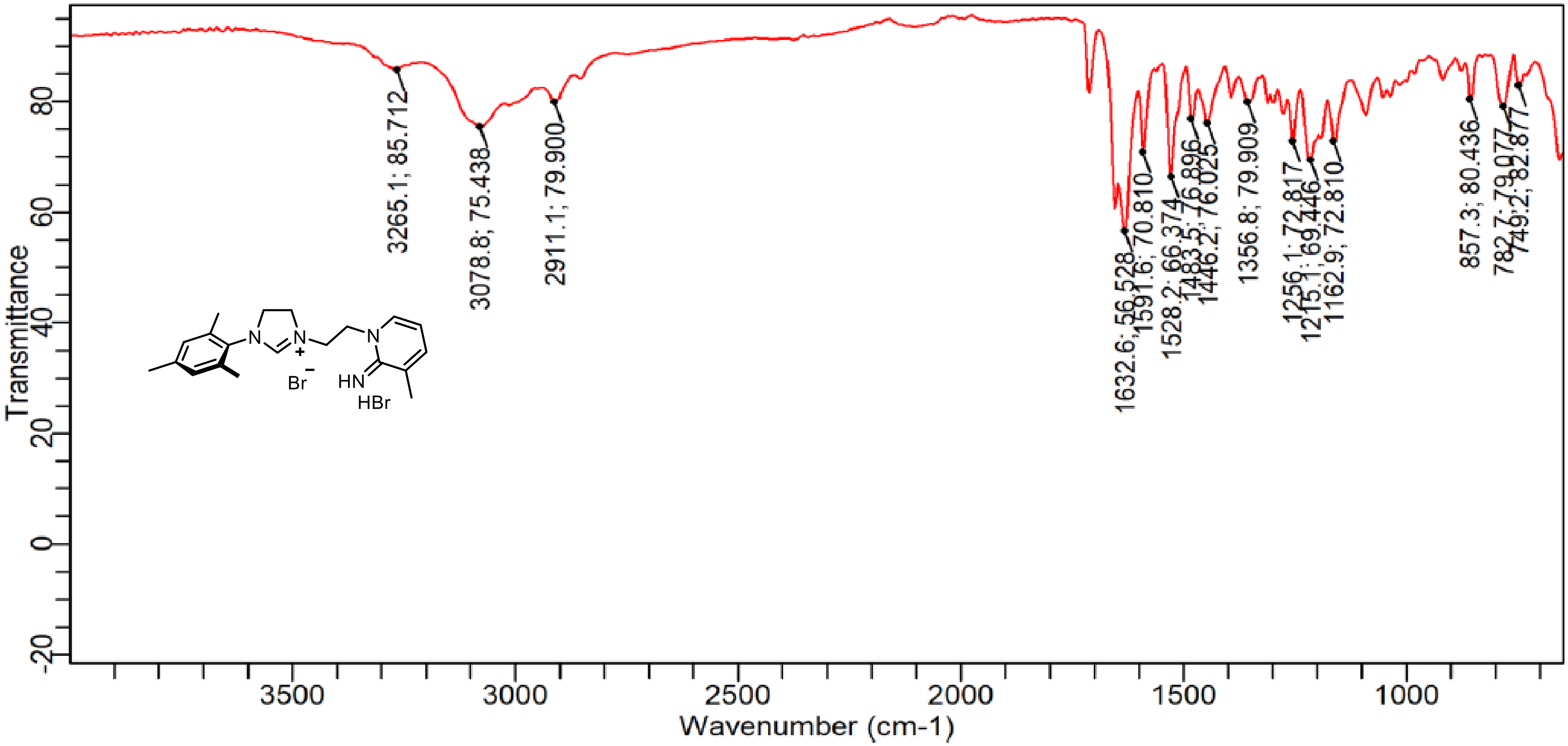

3265.1; 85.712
3078.8; 75.438
2911.1; 79.900
1632.6; 56.528
1591.6; 70.810
1528.2; 66.374
1483.5; 76.896
1446.2; 76.025
1356.8; 79.909
1256.1; 72.817
1215.1; 69.446
1162.9; 72.810
857.3; 80.436
782.7; 79.077
749.2; 82.877
Transmittance
Wavenumber (cm-1)
Br
HN
HBr

1.12 (1-(2-(2-Imino-3-methylpyridin-1(2*H*)-yl)ethyl)-3-mesitylimidazolidin-2-ylidene)copper(I) chloride (6) (600 MHz, CD_2Cl_2)

1.12.1 ^{1}H NMR

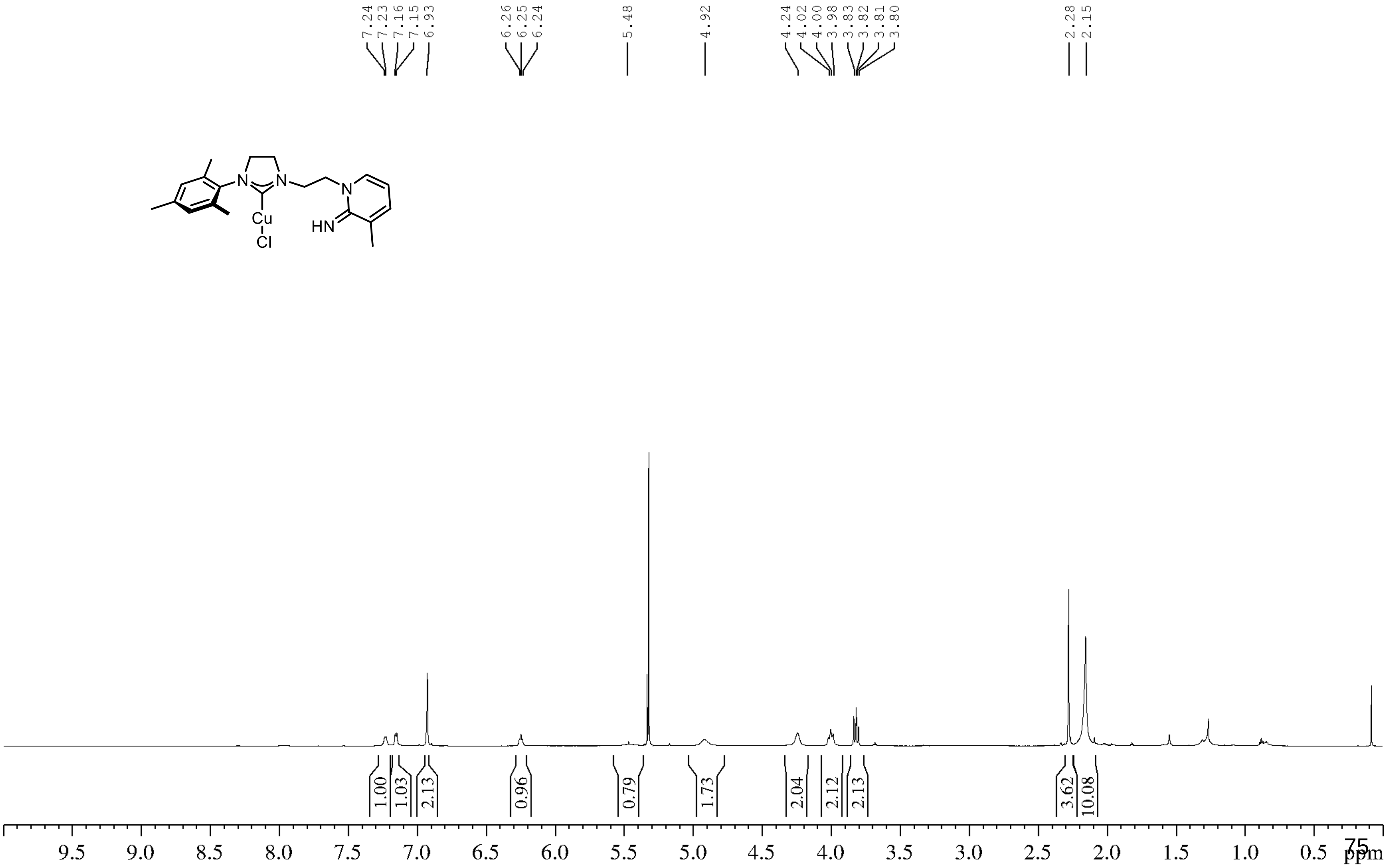

1.12.2 ^{13}C NMR

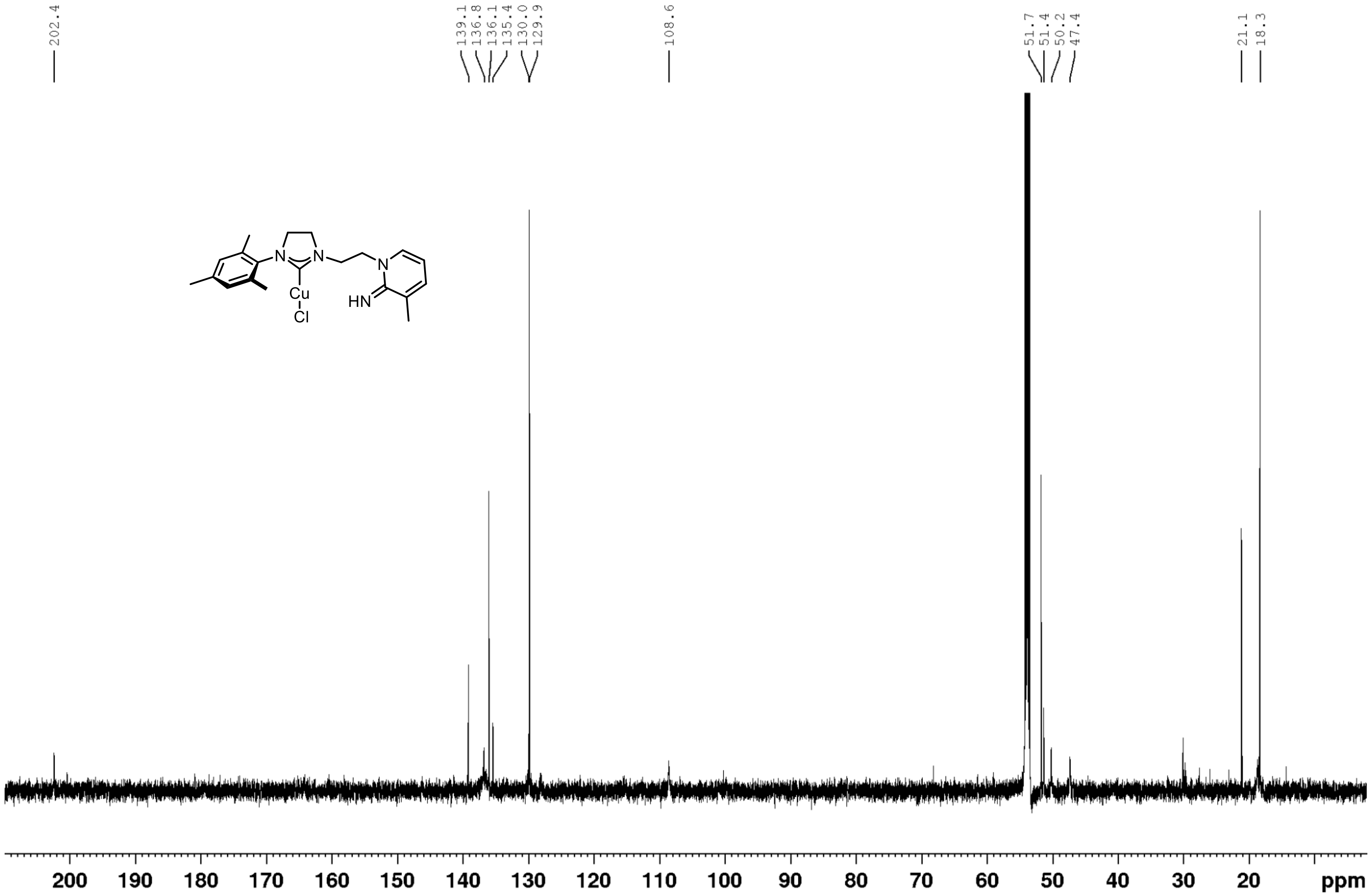

202.4
139.1
136.8
136.1
135.4
130.0
129.9
108.6
51.7
51.4
50.2
47.4
21.1
18.3
N
N
N
Cu
Cl
HN
200
190
180
170
160
150
140
130
120
110
100
90
80
70
60
50
40
30
20
ppm

1.12.3 ^{1}H, ^{1}H COSY

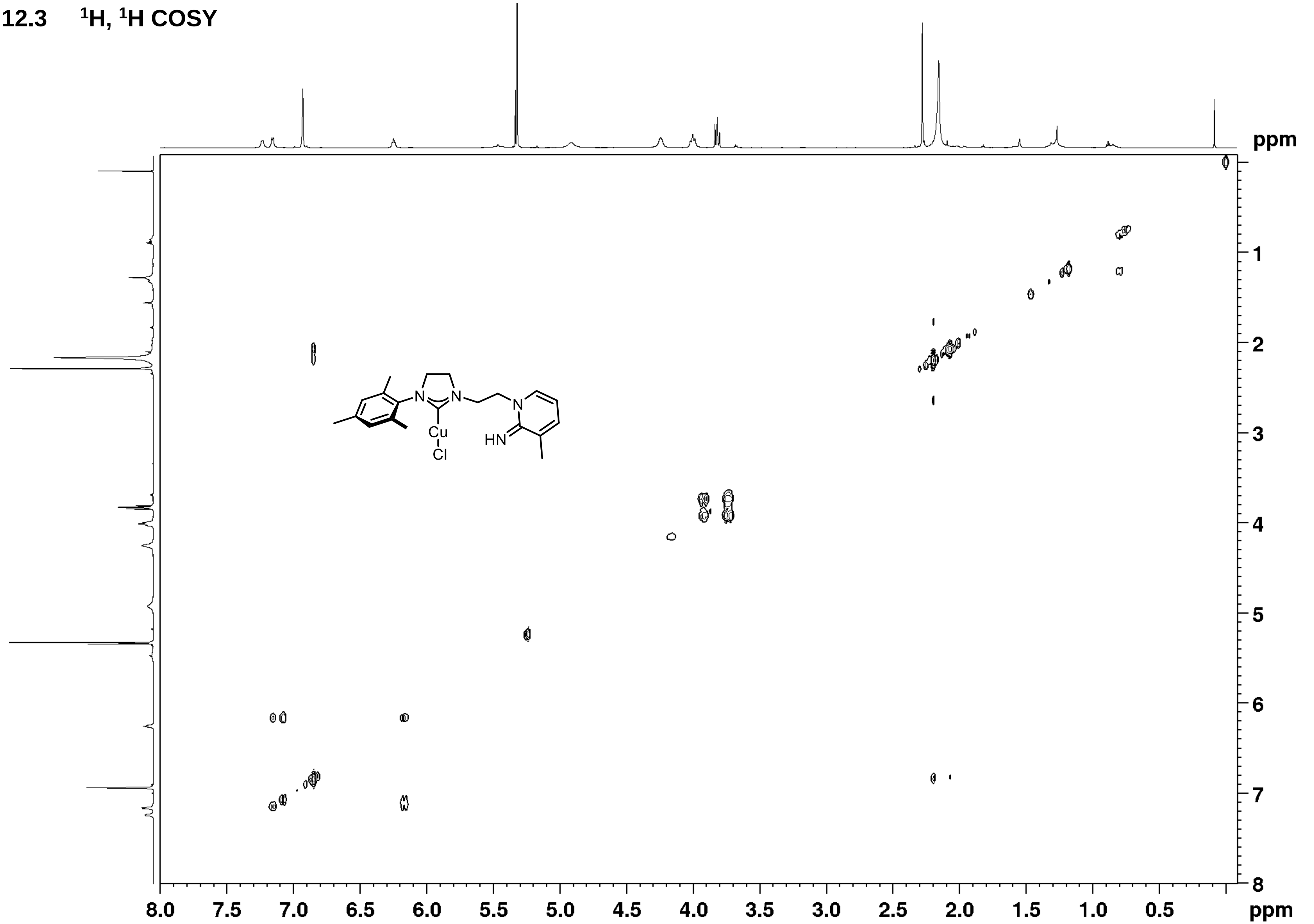

1.12.4 ^{1}H, ^{13}C HMBC

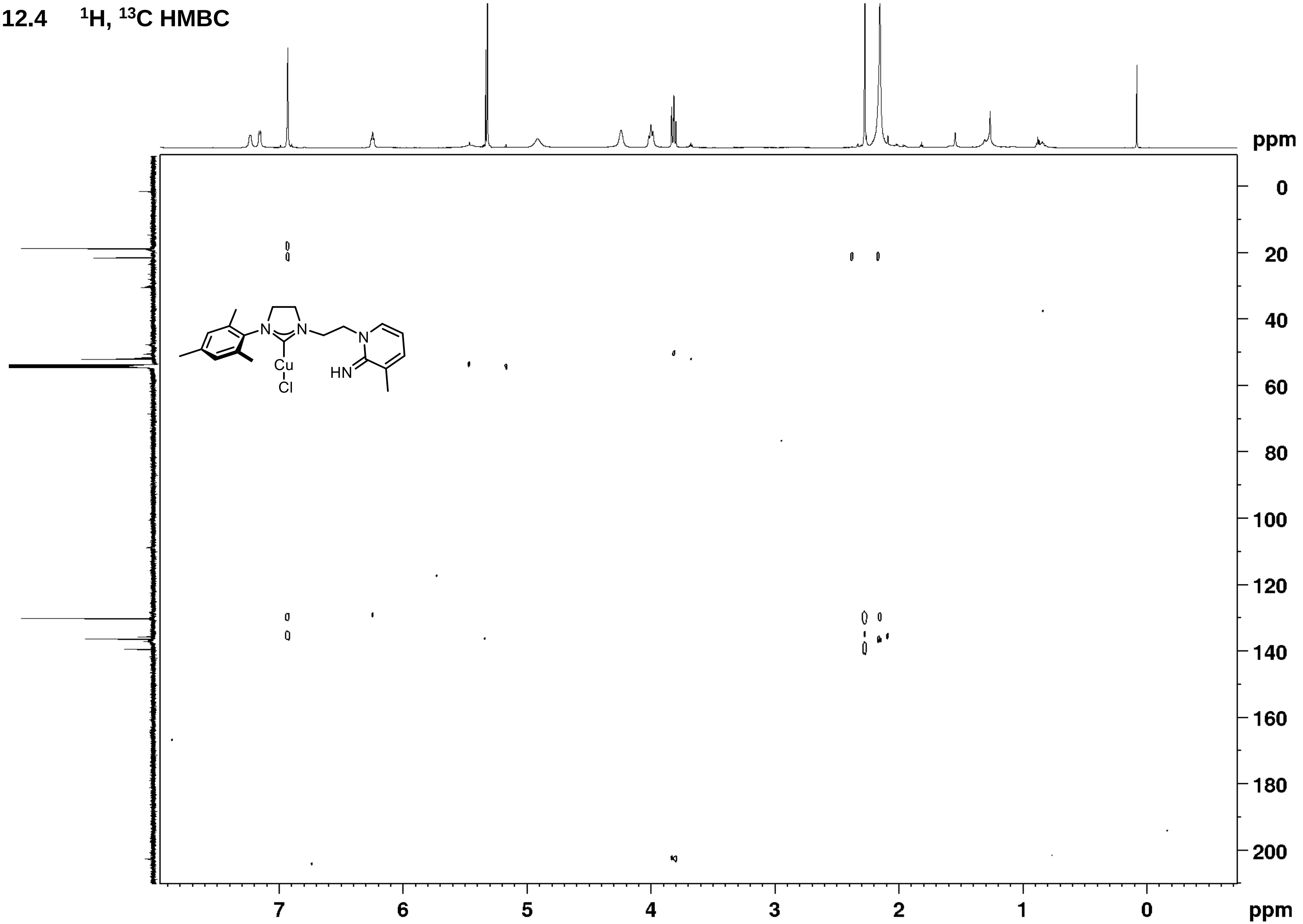

1.12.5 ^{1}H, ^{13}C HSQC

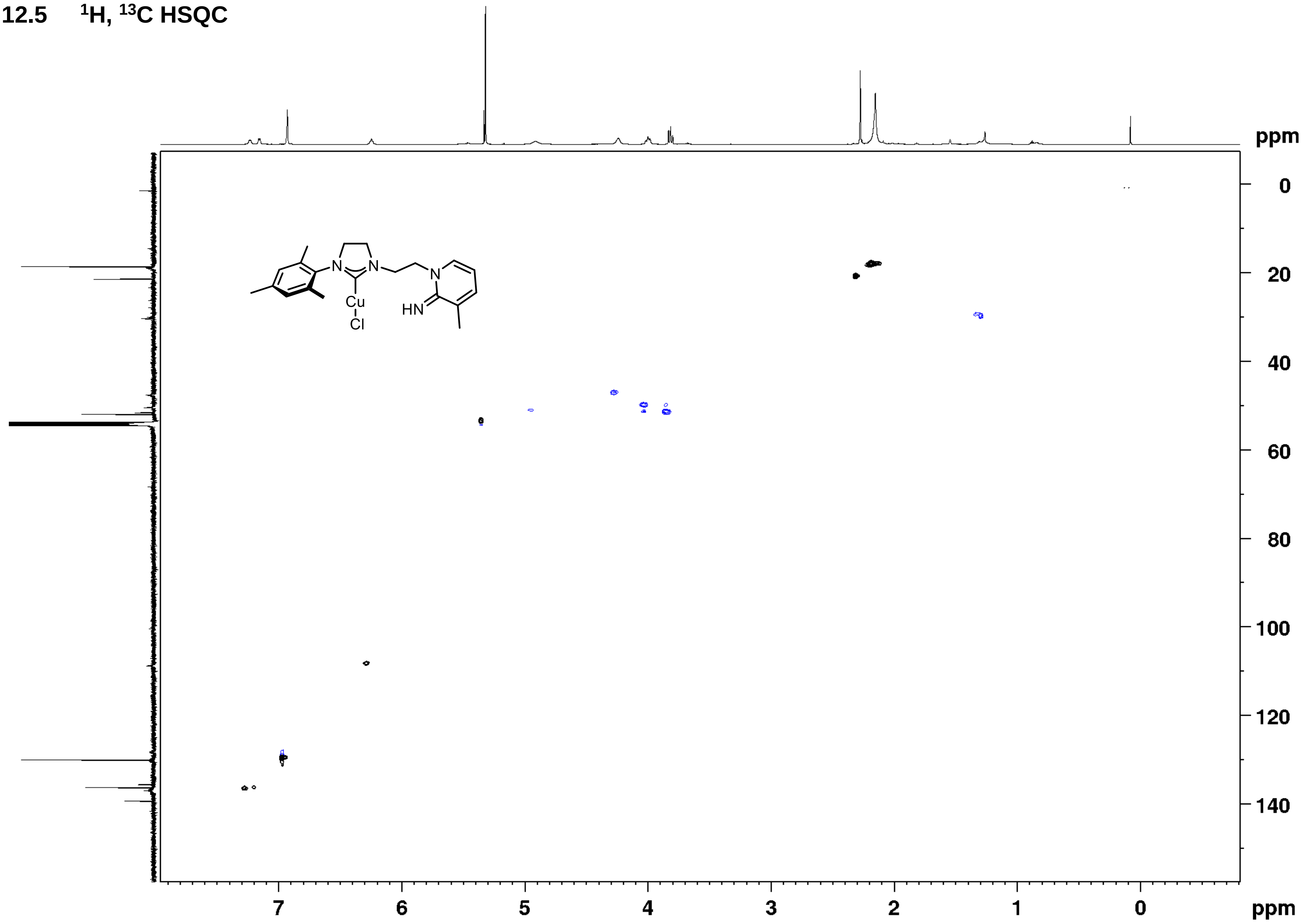

1.12.6 HRMS

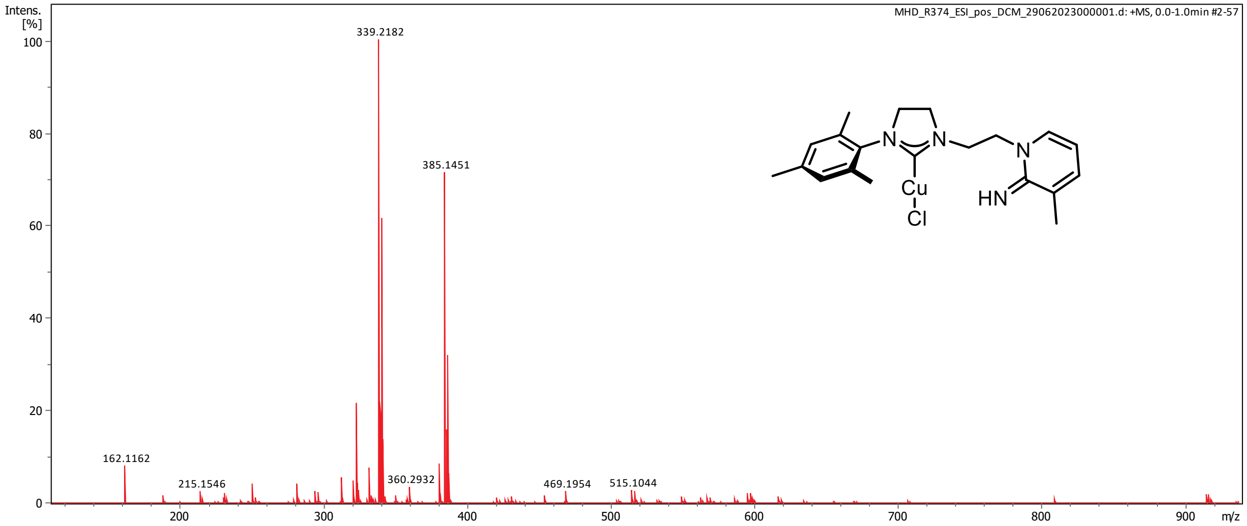

Intens. [%]
MHD_R374_ESI_pos_DCM_29062023000001.d: +MS, 0.0-1.0min #2-57
339.2182
385.1451
162.1162
215.1546
360.2932
469.1954
515.1044
m/z
Cu
Cl
HN

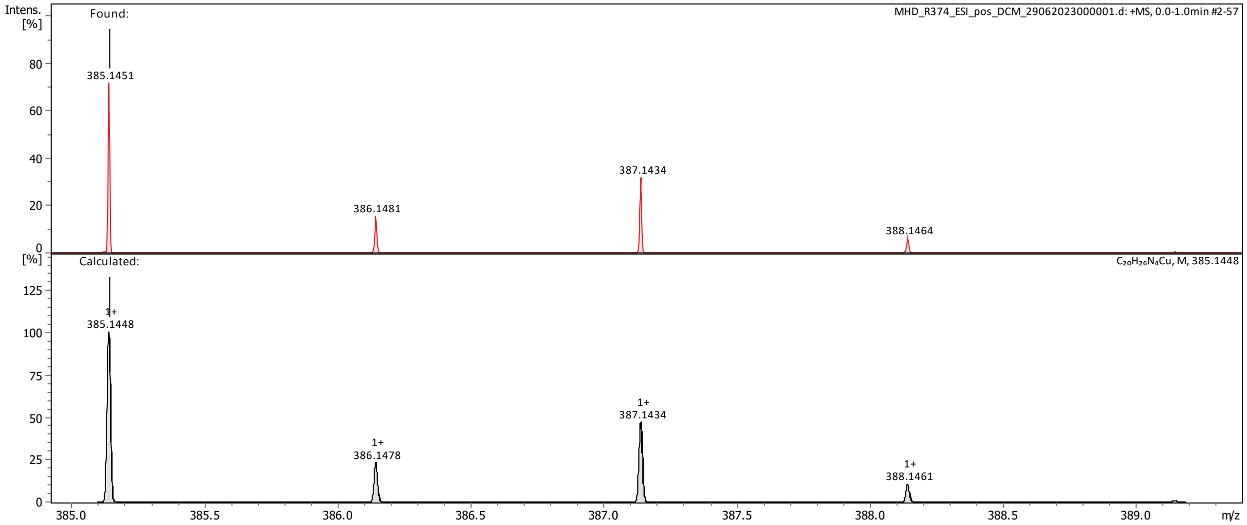

Intens. [%]
Found:
MHD_R374_ESI_pos_DCM_29062023000001.d: +MS, 0.0-1.0min #2-57
385.1451
386.1481
387.1434
388.1464
Calculated:
C20H26N4Cu, M, 385.1448
1+ 385.1448
1+ 386.1478
1+ 387.1434
1+ 388.1461
m/z

1.13 3-(2-Bromoethyl)-1-(2,6-diisopropylphenyl)-4,5-dihydro-1*H*-imidazol-3-ium bromide (S7b) (600 MHz, $CDCl_3$)

1.13.1 ^{1}H NMR

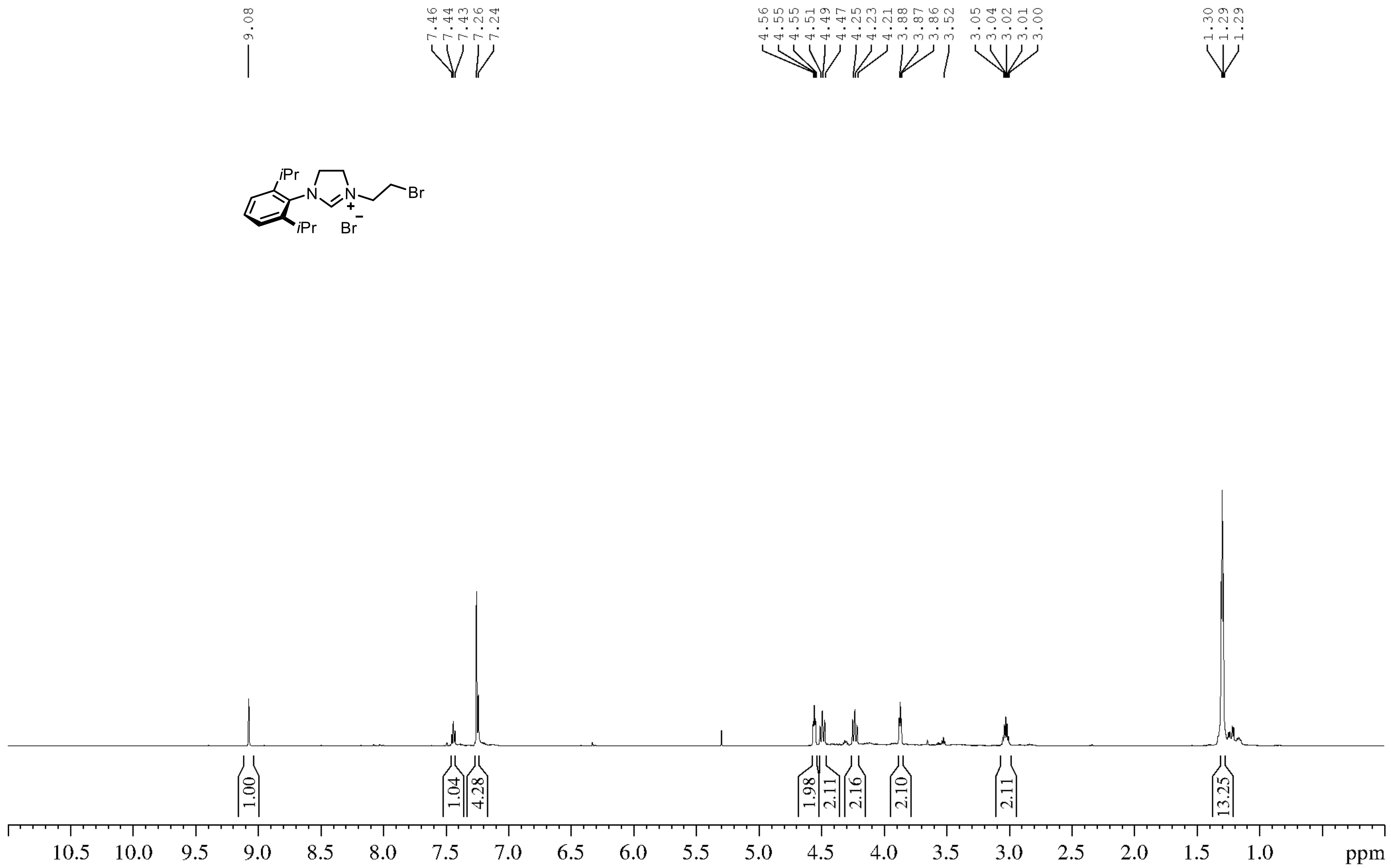

1.13.2 ^{13}C NMR

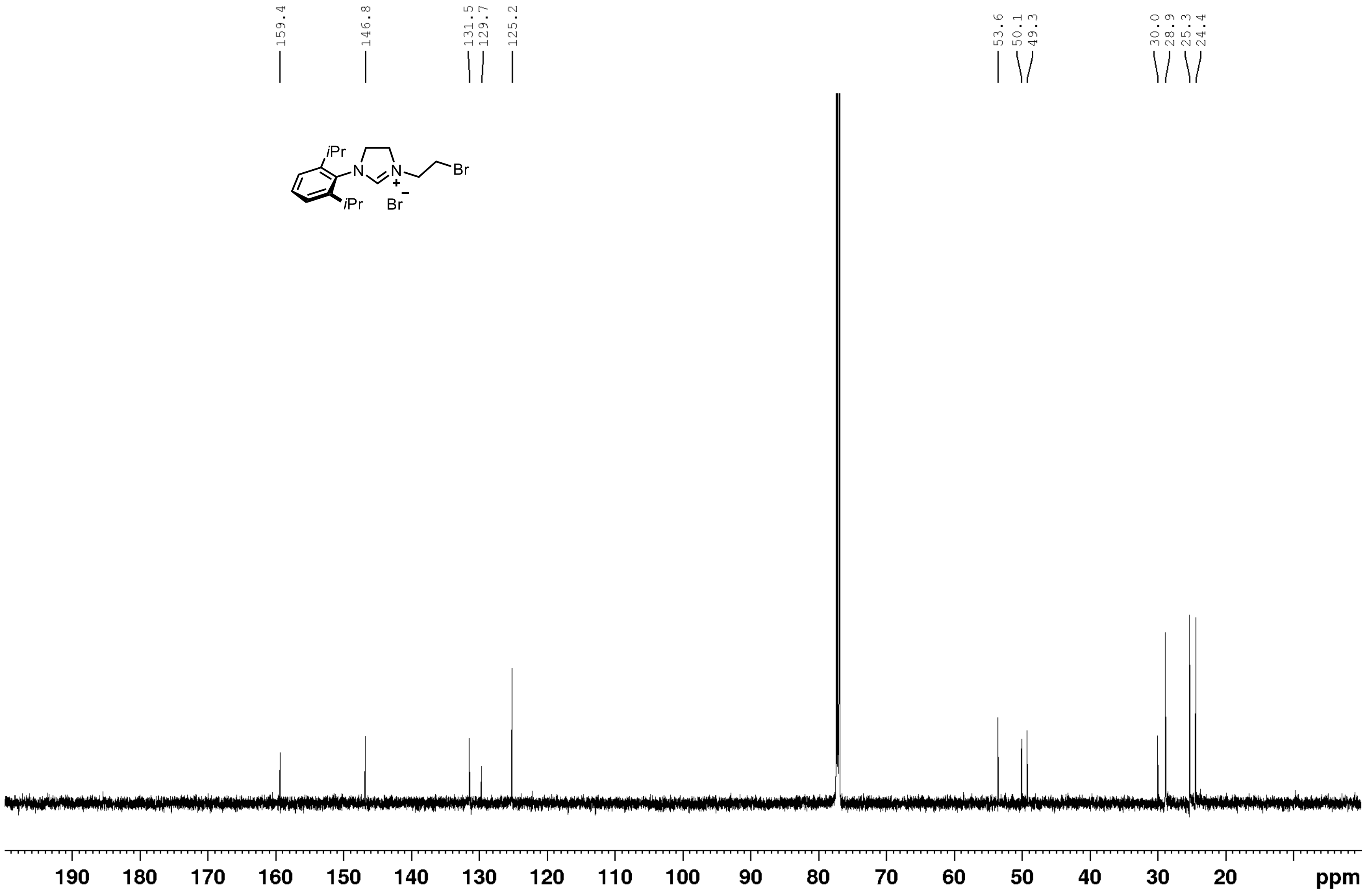
159.4
146.8
131.5
129.7
125.2
53.6
50.1
49.3
30.0
28.9
25.3
24.4
iPr
N
N
Br
Br
iPr
190
180
170
160
150
140
130
120
110
100
90
80
70
60
50
40
30
20
ppm

1.13.3 ^{1}H, ^{1}H COSY

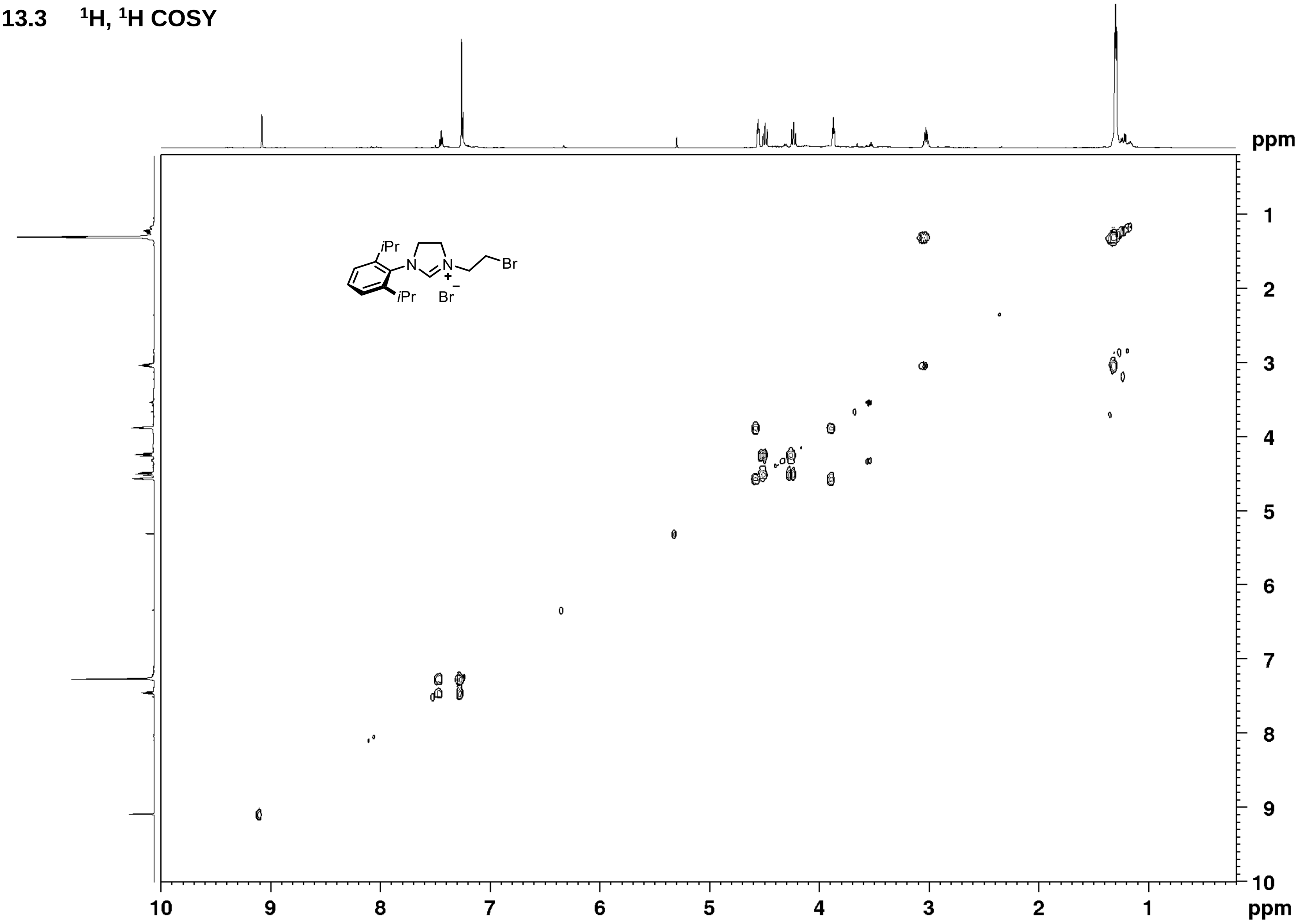

1.13.4 ^{1}H, ^{13}C HMBC

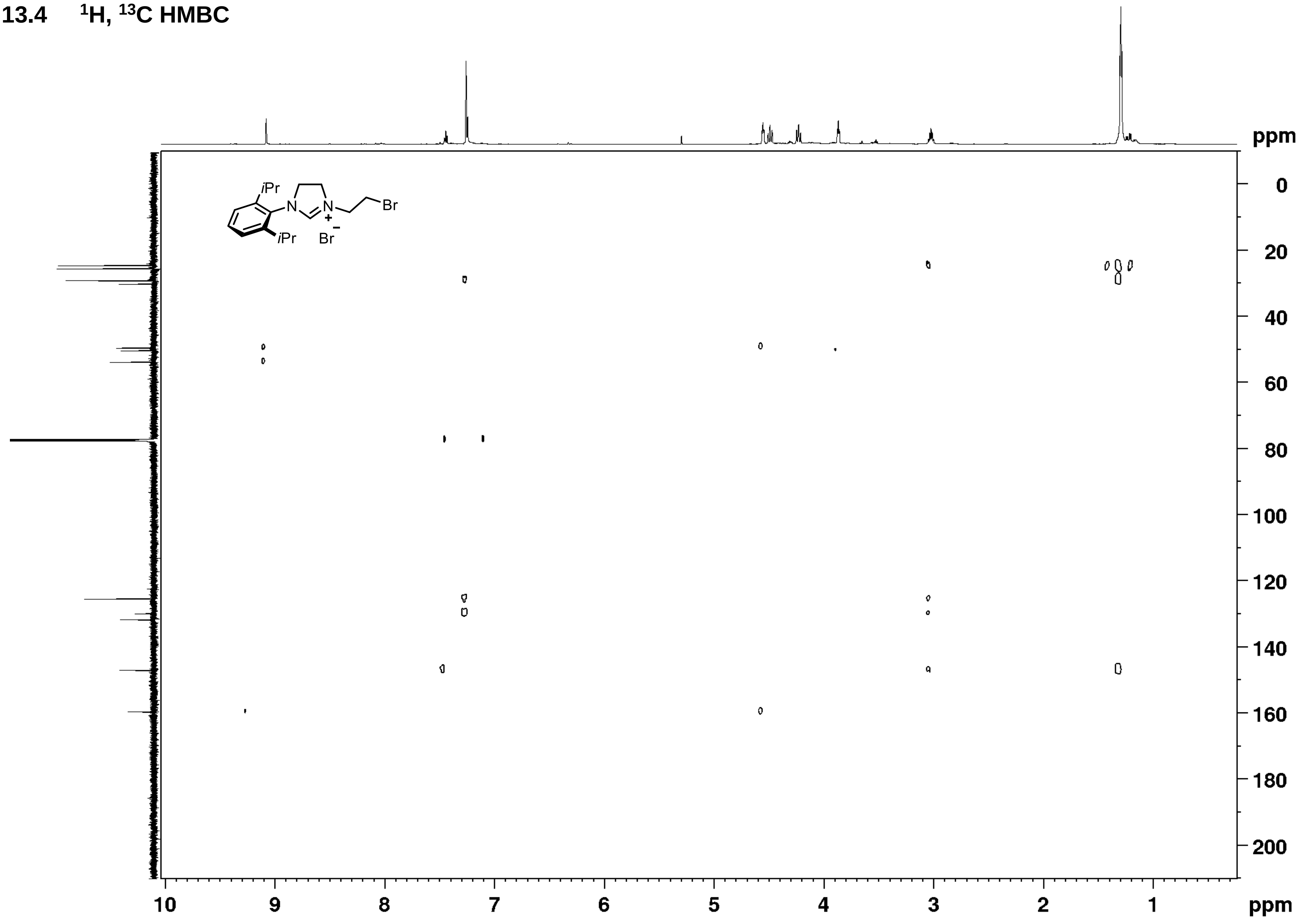

1.13.5 ^{1}H, ^{13}C HSQC

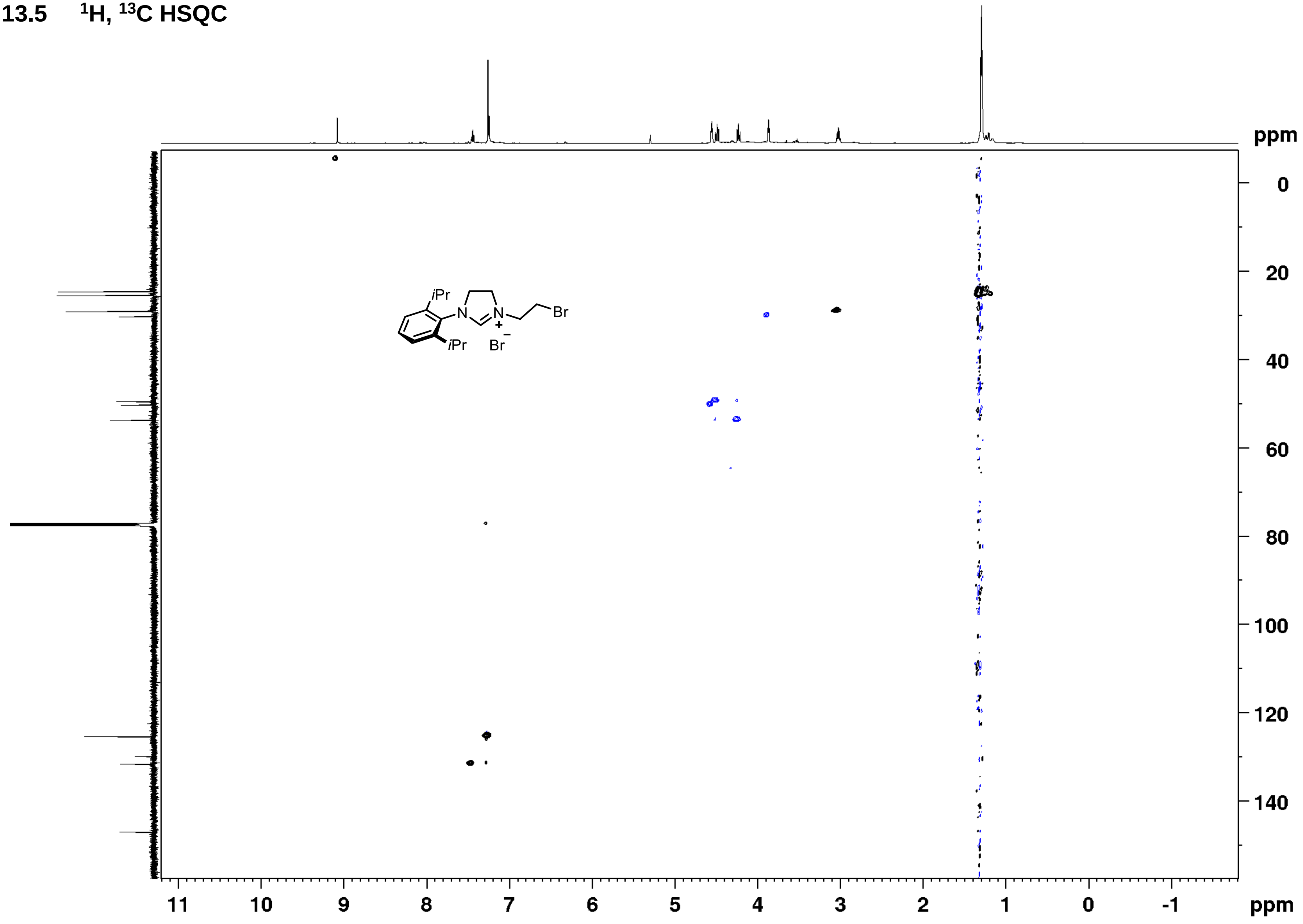

1.13.6 HRMS

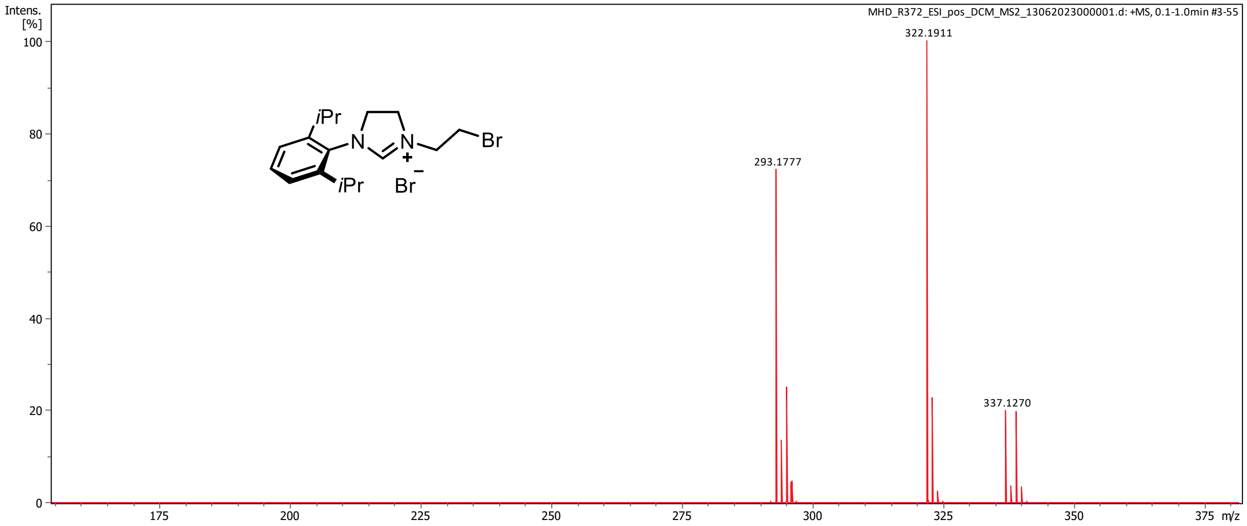

Intens. [%]
MHD_R372_ESI_pos_DCM_MS2_13062023000001.d: +MS, 0.1-1.0min #3-55
322.1911
293.1777
337.1270
iPr
N
N
Br
Br
iPr
m/z

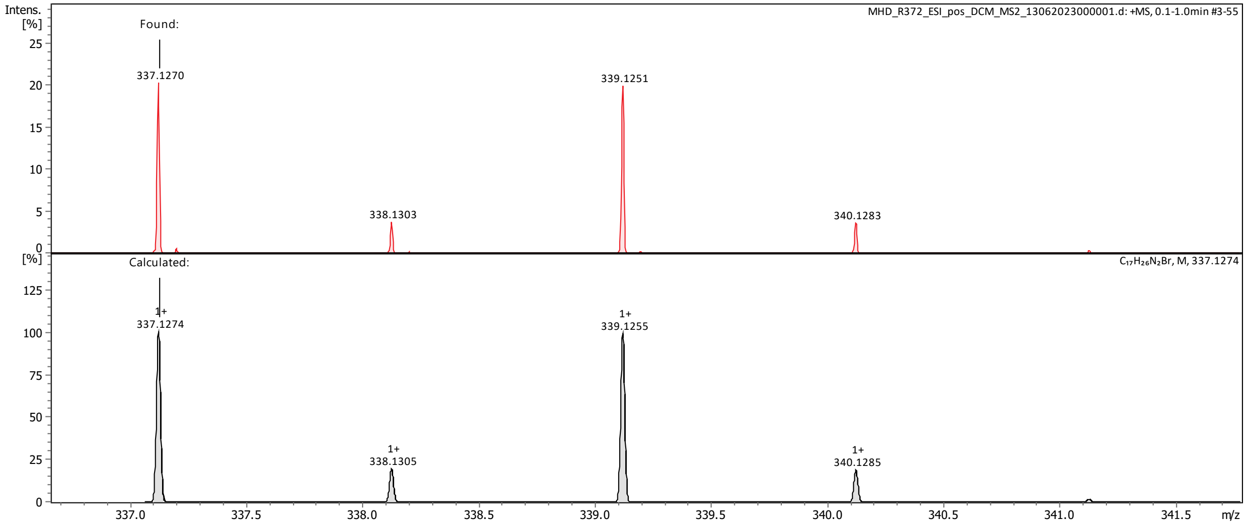

Intens. [%]
MHD_R372_ESI_pos_DCM_MS2_13062023000001.d: +MS, 0.1-1.0min #3-55
Found:
337.1270
338.1303
339.1251
340.1283
Calculated:
C17H26N2Br, M, 337.1274
1+ 337.1274
1+ 338.1305
1+ 339.1255
1+ 340.1285
m/z

1.13.7 IR

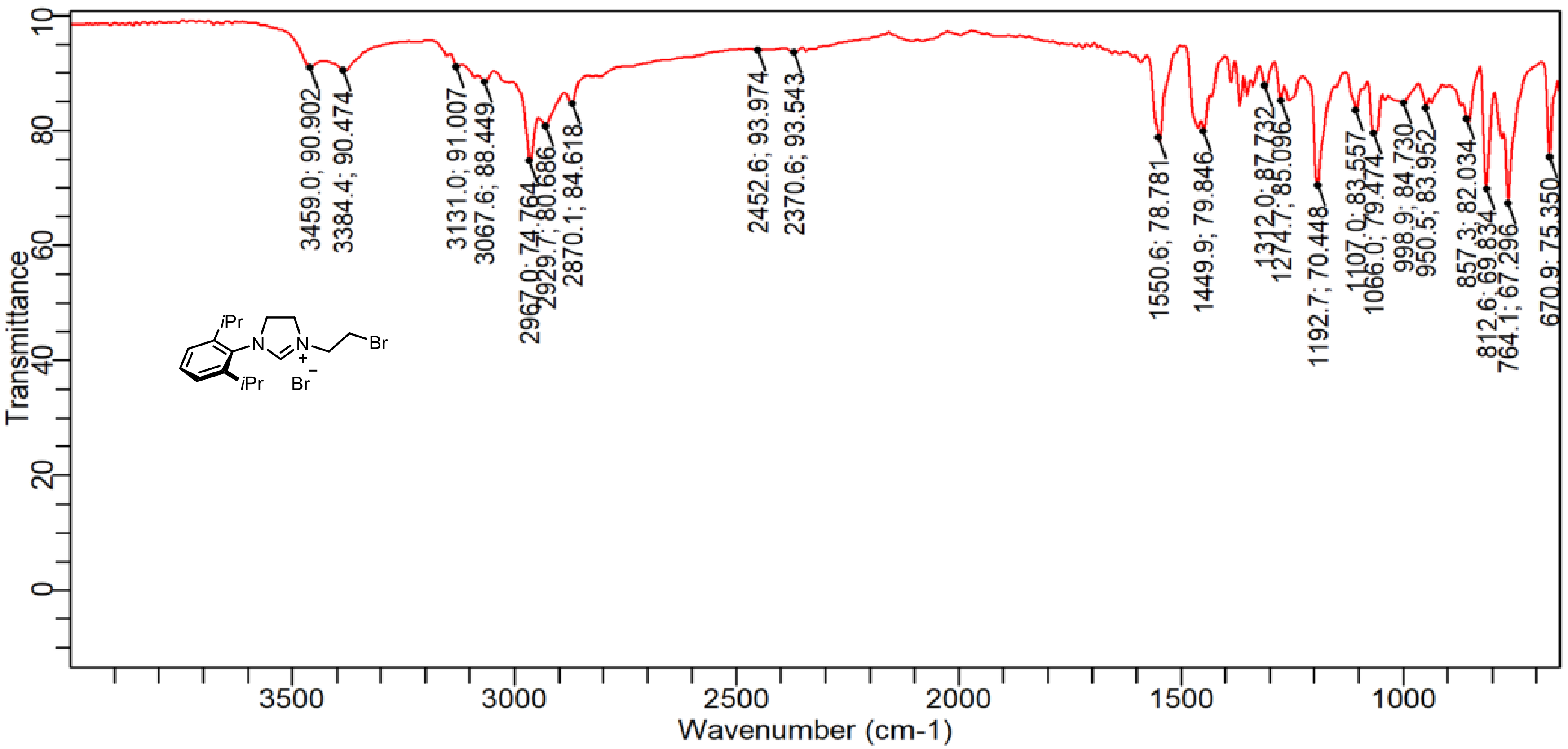
Transmittance
Wavenumber (cm-1)
3459.0; 90.902
3384.4; 90.474
3131.0; 91.007
3067.6; 88.449
2967.0; 74.764
2929.7; 80.686
2870.1; 84.618
2452.6; 93.974
2370.6; 93.543
1550.6; 78.781
1449.9; 79.846
1312.0; 87.732
1274.7; 85.096
1192.7; 70.448
1107.0; 83.557
1066.0; 79.474
998.9; 84.730
950.5; 83.952
857.3; 82.034
812.6; 69.834
764.1; 67.296
670.9; 75.350
iPr
N
N
Br
iPr
Br

1.14 1-(2,6-Diisopropylphenyl)-3-(2-(2-imino-3-methylpyridin-1(2*H*)-yl)ethyl)-4,5-dihydro-1*H*-imidazol-3-ium bromide hydrobromide (L7) (600 MHz, DMSO-d_6)

1.14.1 ^{1}H NMR

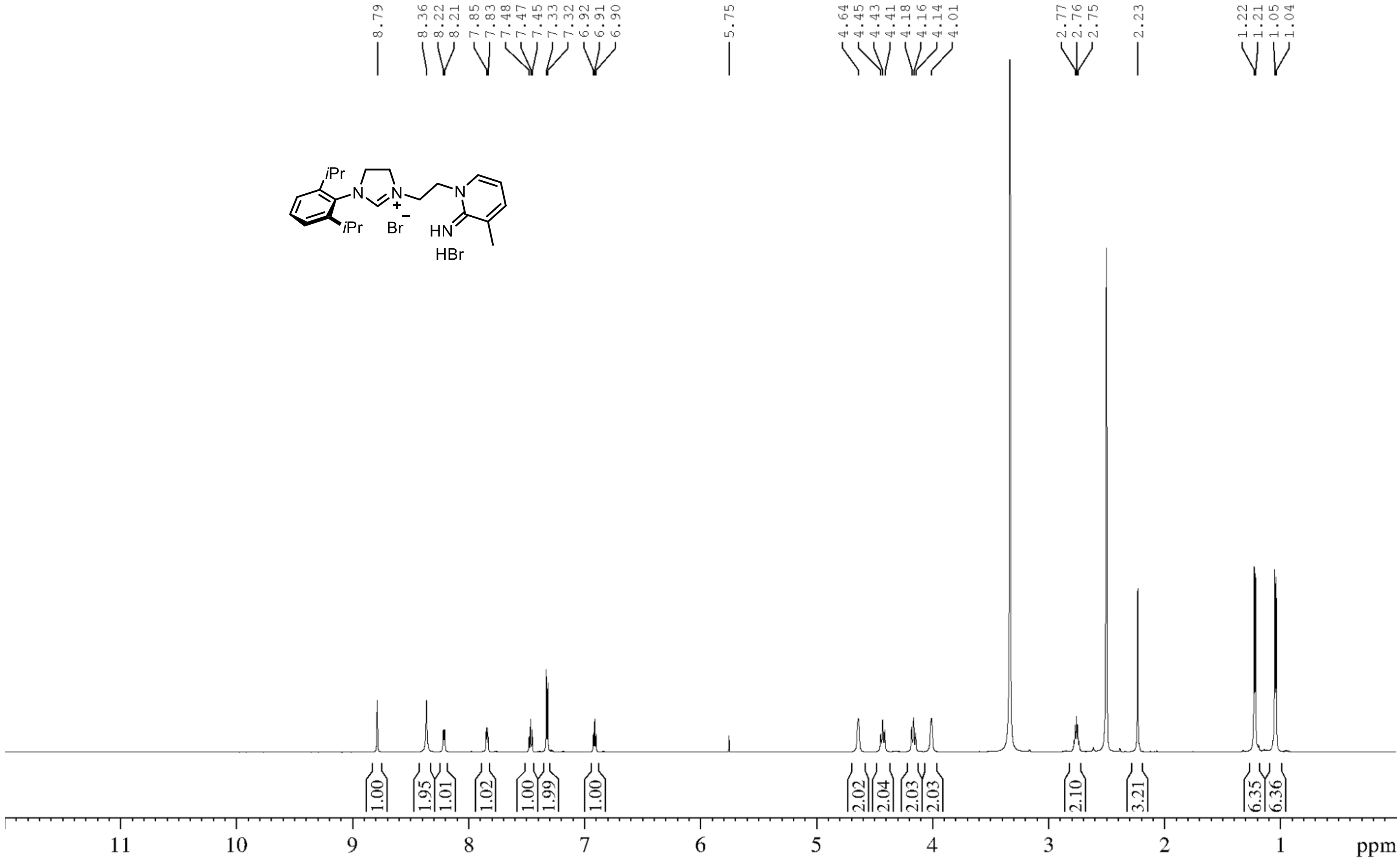

1.14.2 ^{13}C NMR

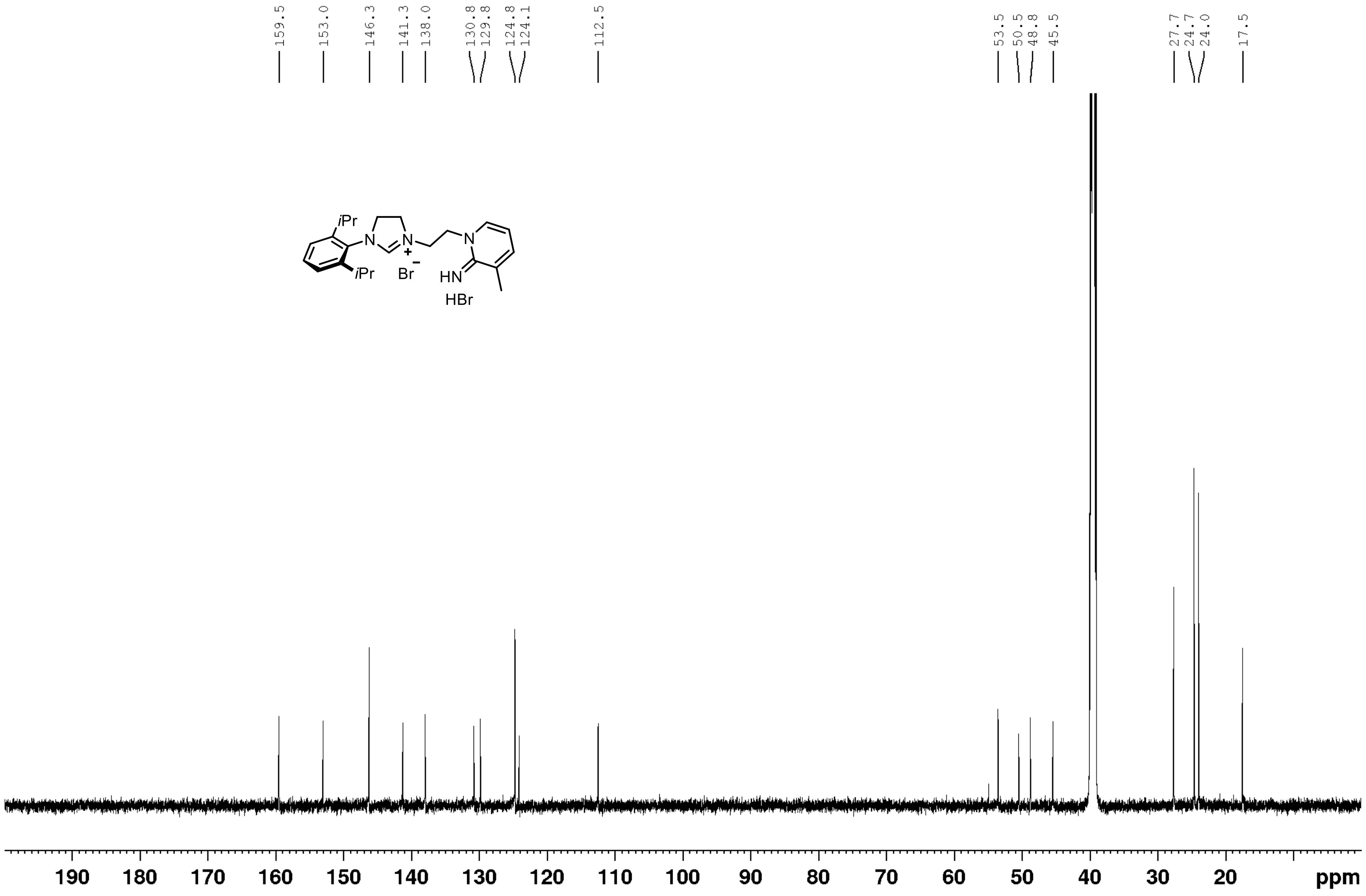
159.5
153.0
146.3
141.3
138.0
130.8
129.8
124.8
124.1
112.5
53.5
50.5
48.8
45.5
27.7
24.7
24.0
17.5
iPr
N
N
+
Br
-
N
HN
HBr
iPr
190
180
170
160
150
140
130
120
110
100
90
80
70
60
50
40
30
20
ppm

1.14.3 ^{1}H, ^{1}H COSY

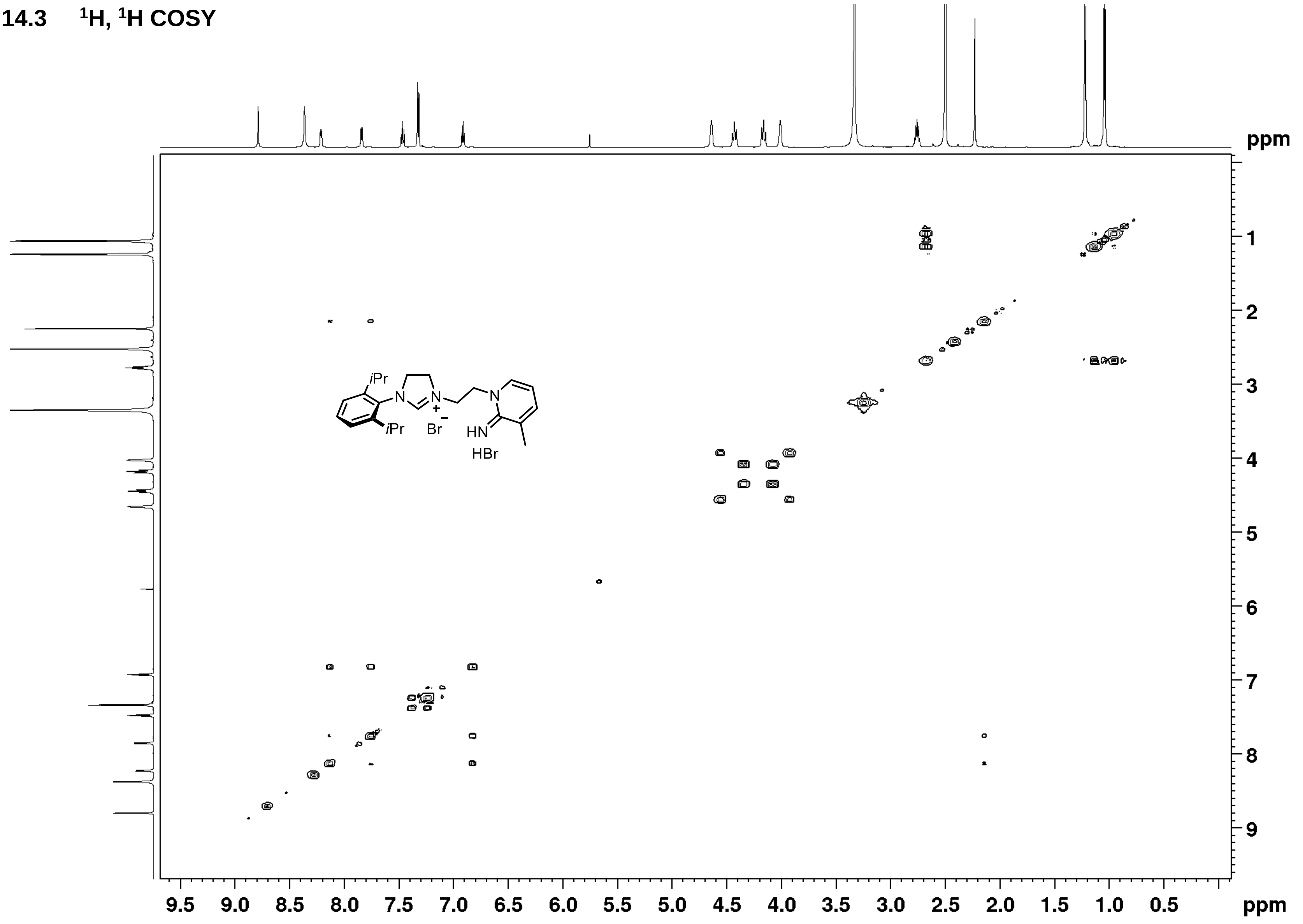

1.14.4 ^{1}H, ^{13}C HMBC

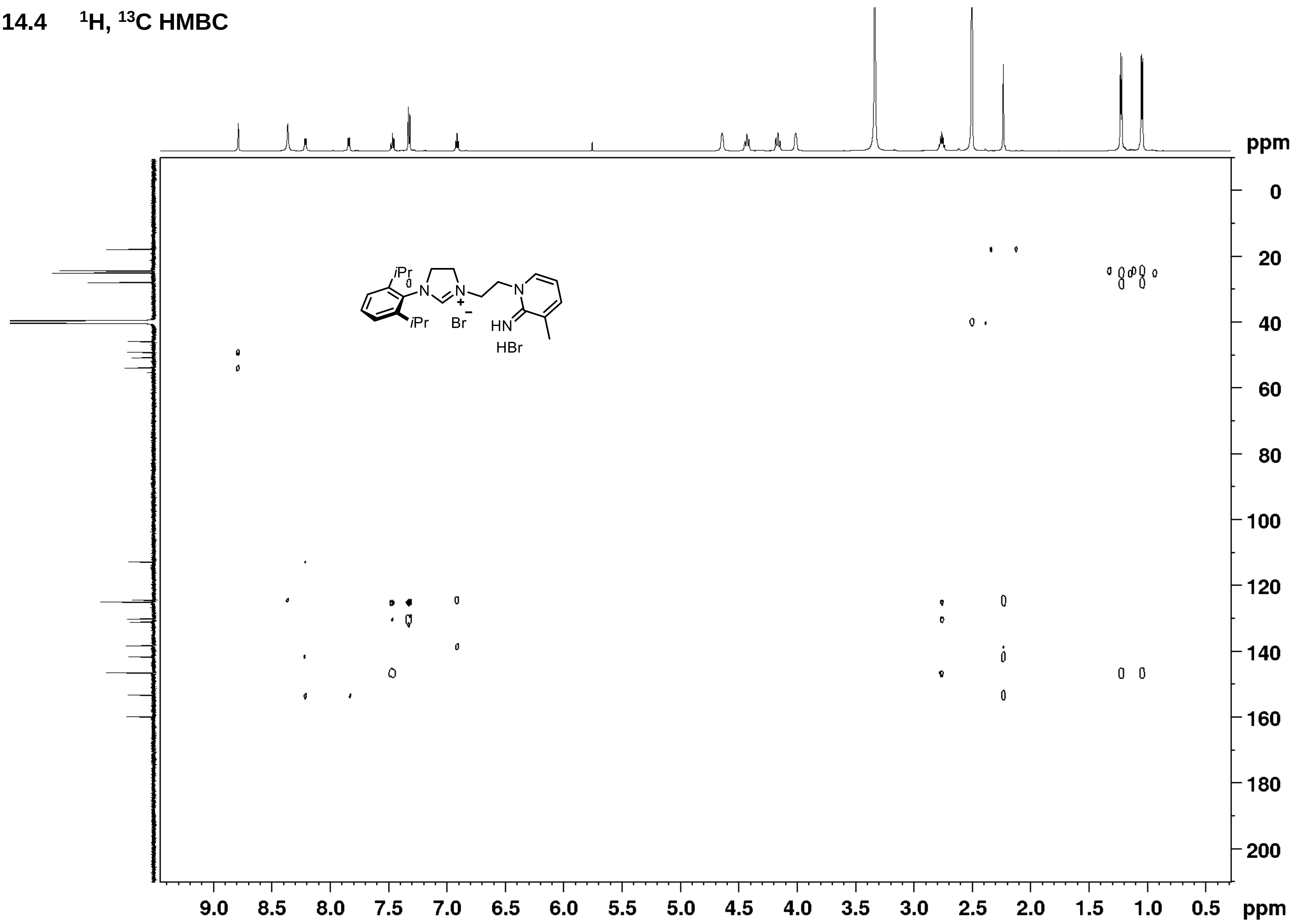

1.14.5 ^{1}H, ^{13}C HSQC

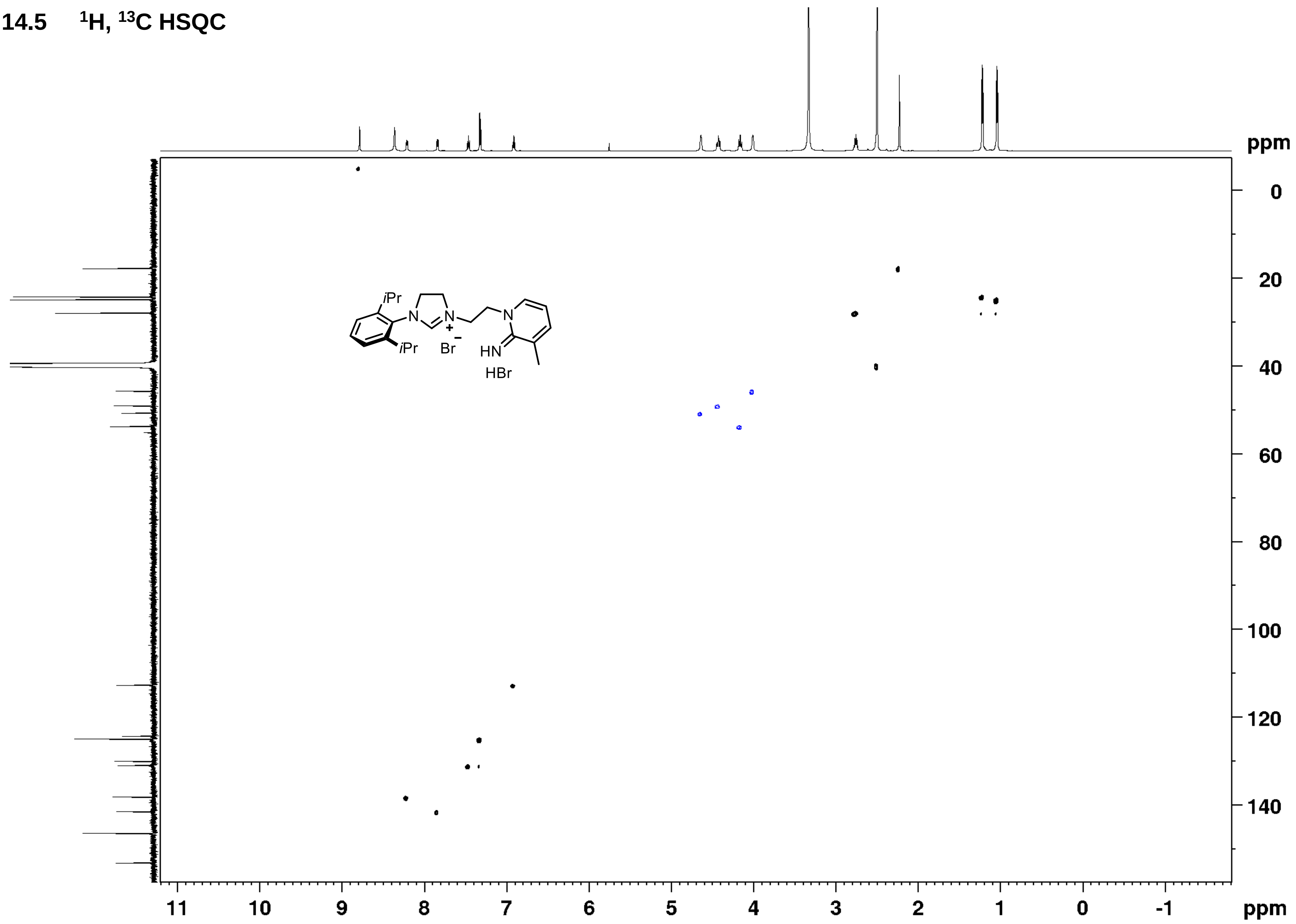

1.14.6 HRMS

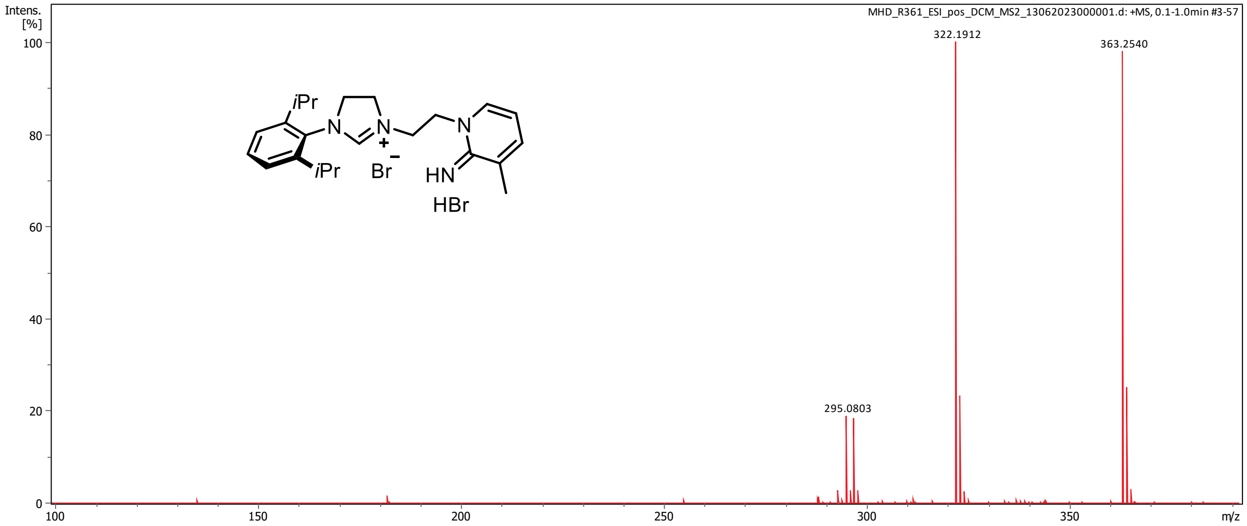

MHD_R361_ESI_pos_DCM_MS2_13062023000001.d: +MS, 0.1-1.0min #3-57
Intens. [%]
322.1912
363.2540
295.0803
iPr
iPr
Br
HN
HBr
m/z

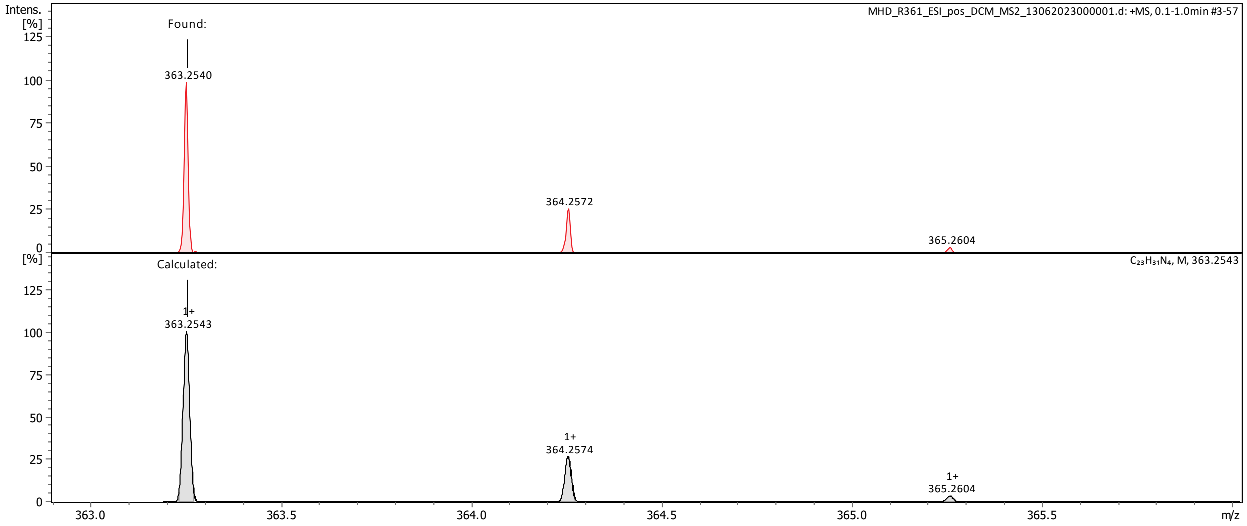

MHD_R361_ESI_pos_DCM_MS2_13062023000001.d: +MS, 0.1-1.0min #3-57
Intens. [%]
Found:
363.2540
364.2572
365.2604
Calculated:
$C_{23}H_{31}N_4$, M, 363.2543
1+ 363.2543
1+ 364.2574
1+ 365.2604
m/z

1.14.7 IR

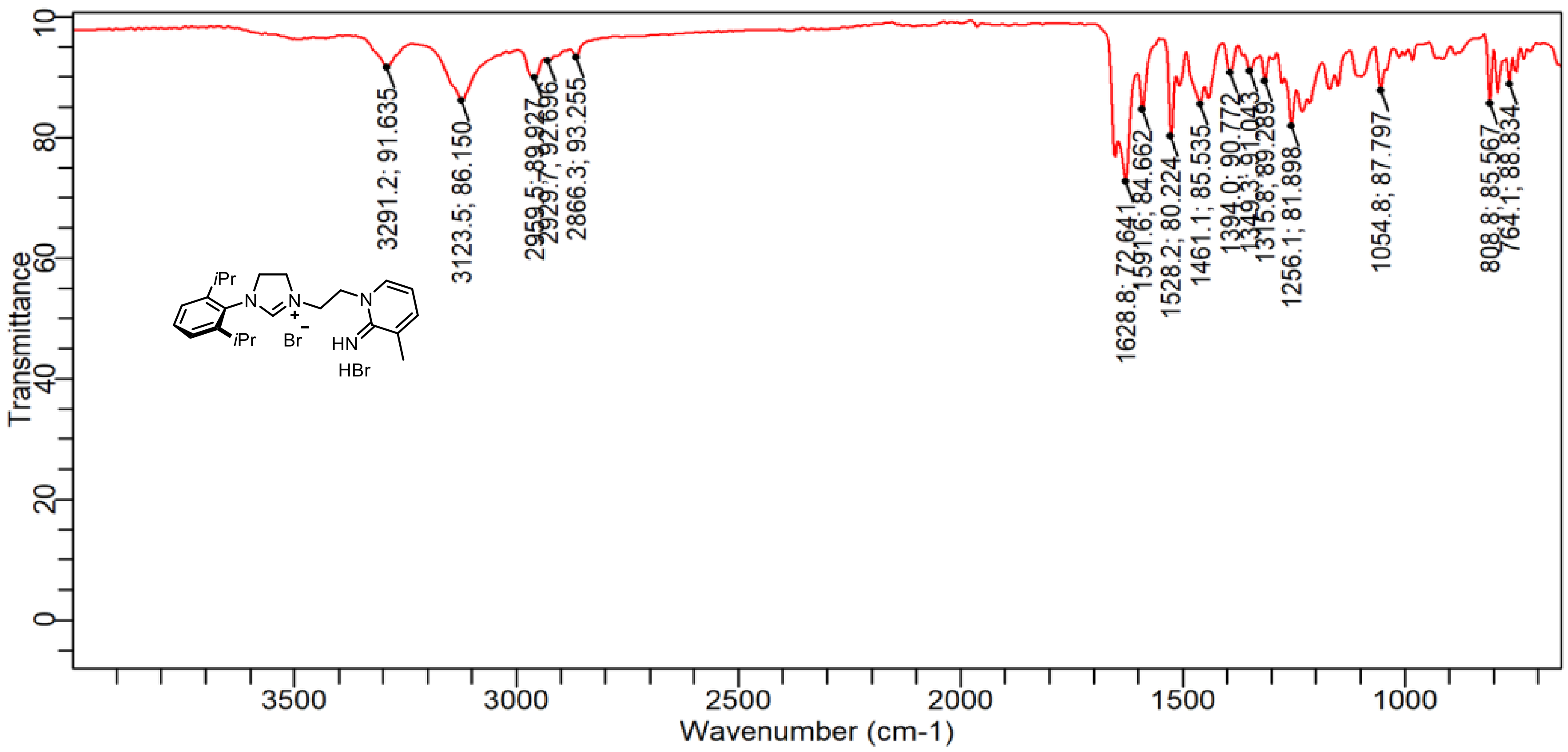

Transmittance
Wavenumber (cm-1)
3291.2; 91.635
3123.5; 86.150
2959.5; 89.927
2929.7; 92.696
2866.3; 93.255
1628.8; 72.641
1591.6; 84.662
1528.2; 80.224
1461.1; 85.535
1394.0; 90.772
1349.3; 91.043
1315.8; 89.289
1256.1; 81.898
1054.8; 87.797
808.8; 85.567
764.1; 88.834
iPr
N
N
Br
HN
HBr

1.15 (1-(2,6-Diisopropylphenyl)-3-(2-(2-imino-3-methylpyridin-1(2*H*)-yl)ethyl)imidazolidin-2-yl)copper(I) chloride (7) (600 MHz, CD_2Cl_2)

1.15.1 ^{1}H NMR

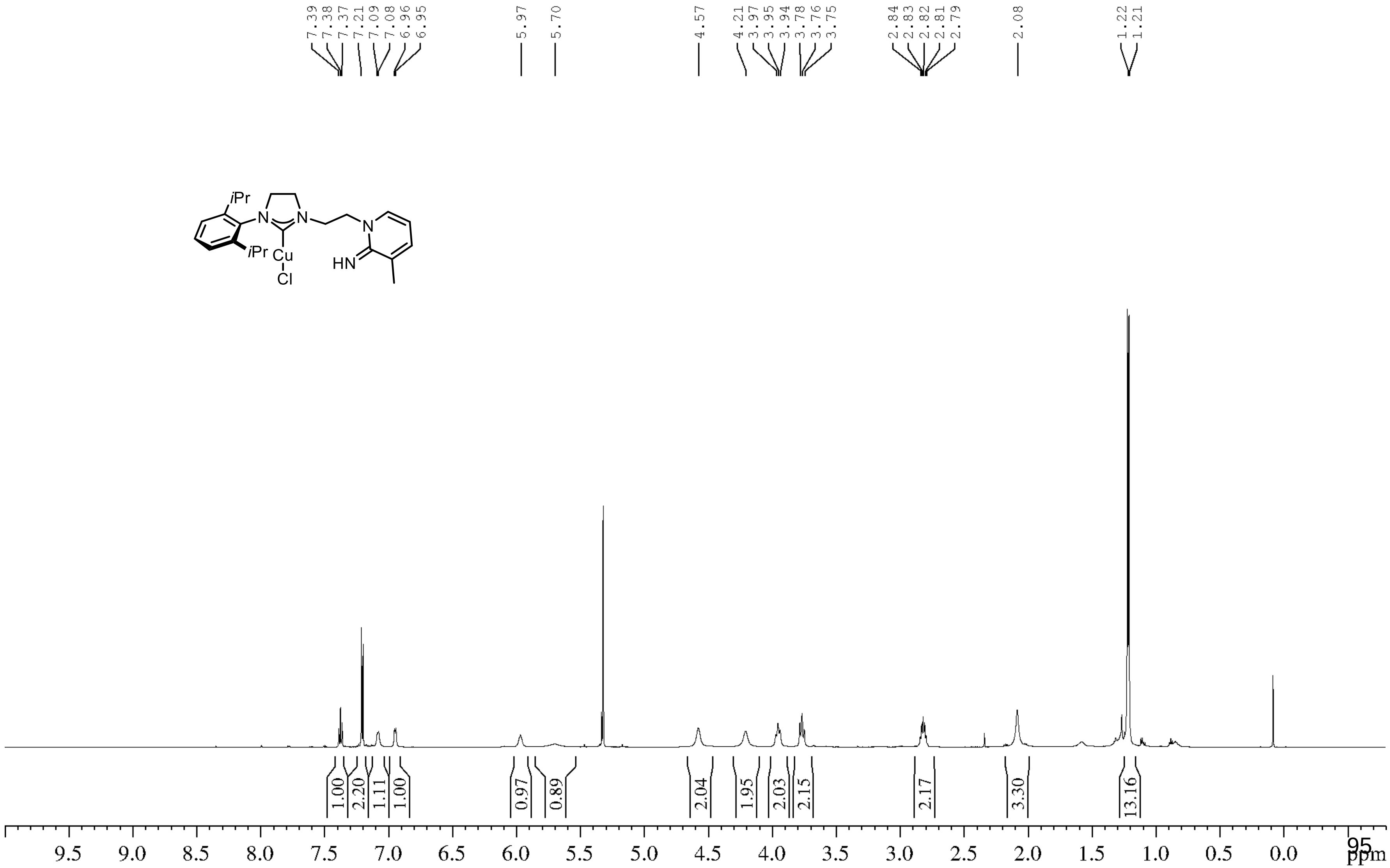

1.15.2 ^{13}C NMR

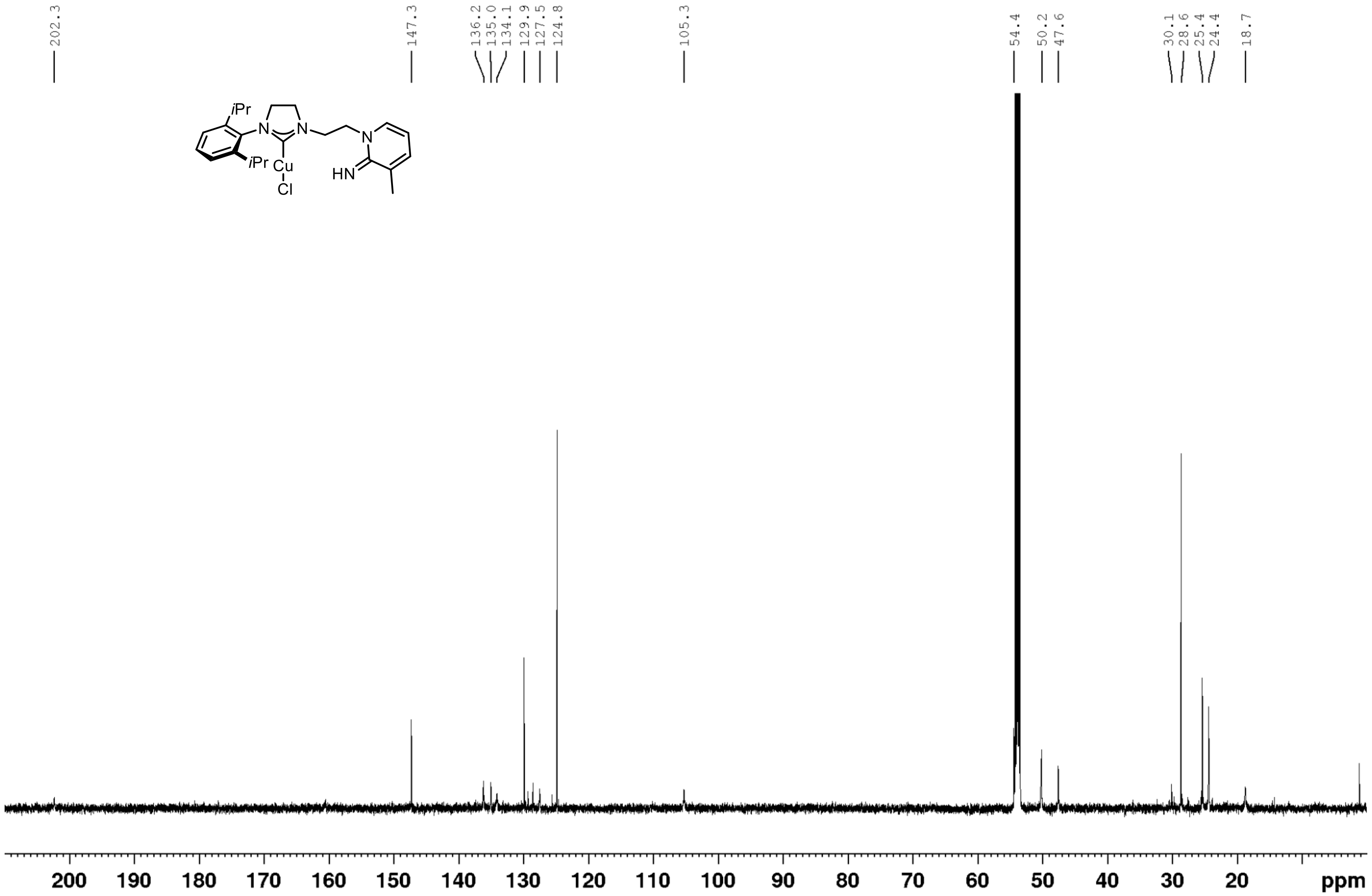
202.3
147.3
136.2
135.0
134.1
129.9
127.5
124.8
105.3
54.4
50.2
47.6
30.1
28.6
25.4
24.4
18.7
iPr
iPr
N
N
N
Cu
Cl
HN
200 190 180 170 160 150 140 130 120 110 100 90 80 70 60 50 40 30 20
ppm

1.15.3 ^{1}H, ^{1}H COSY

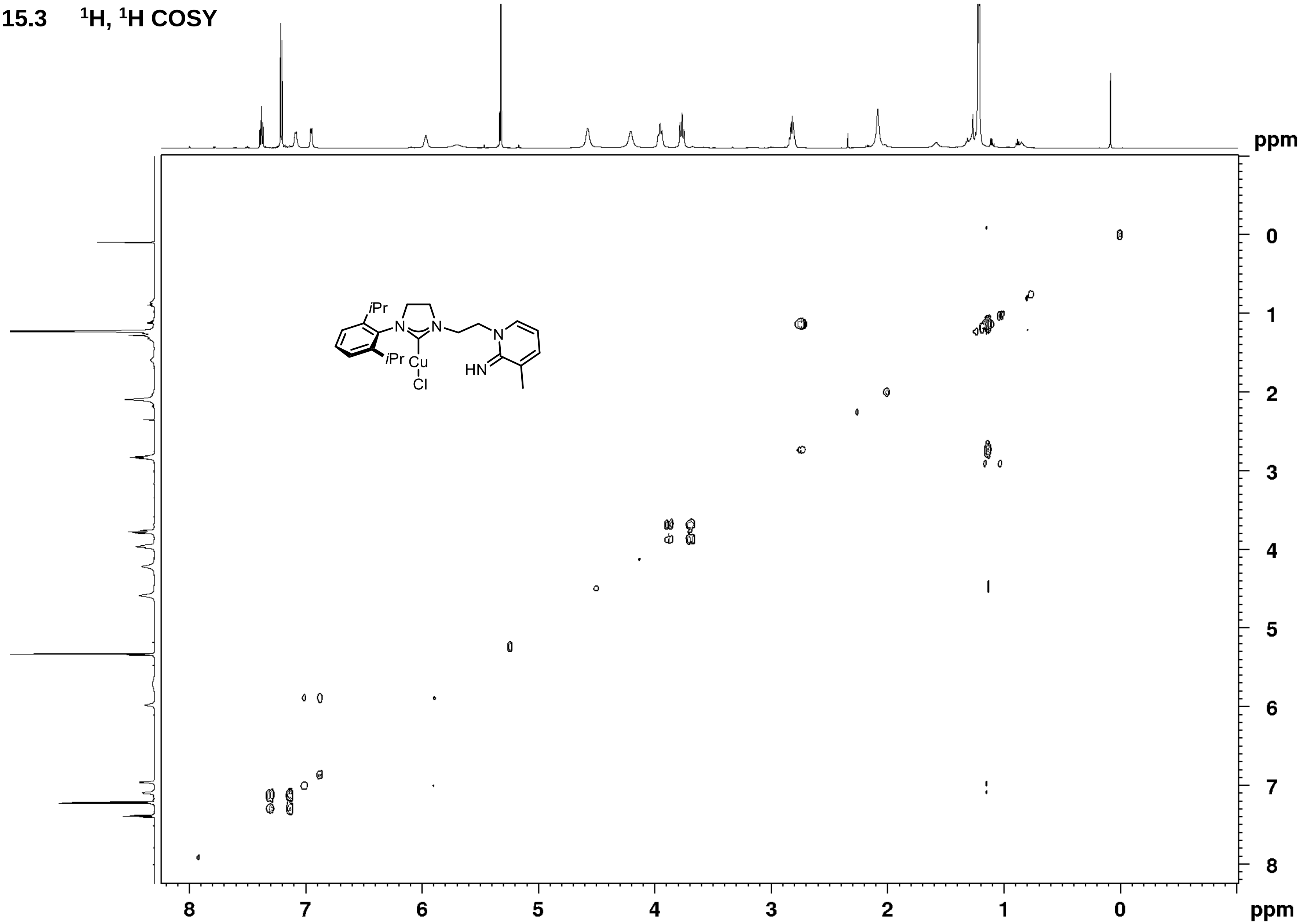

1.15.4 ^{1}H, ^{13}C HMBC

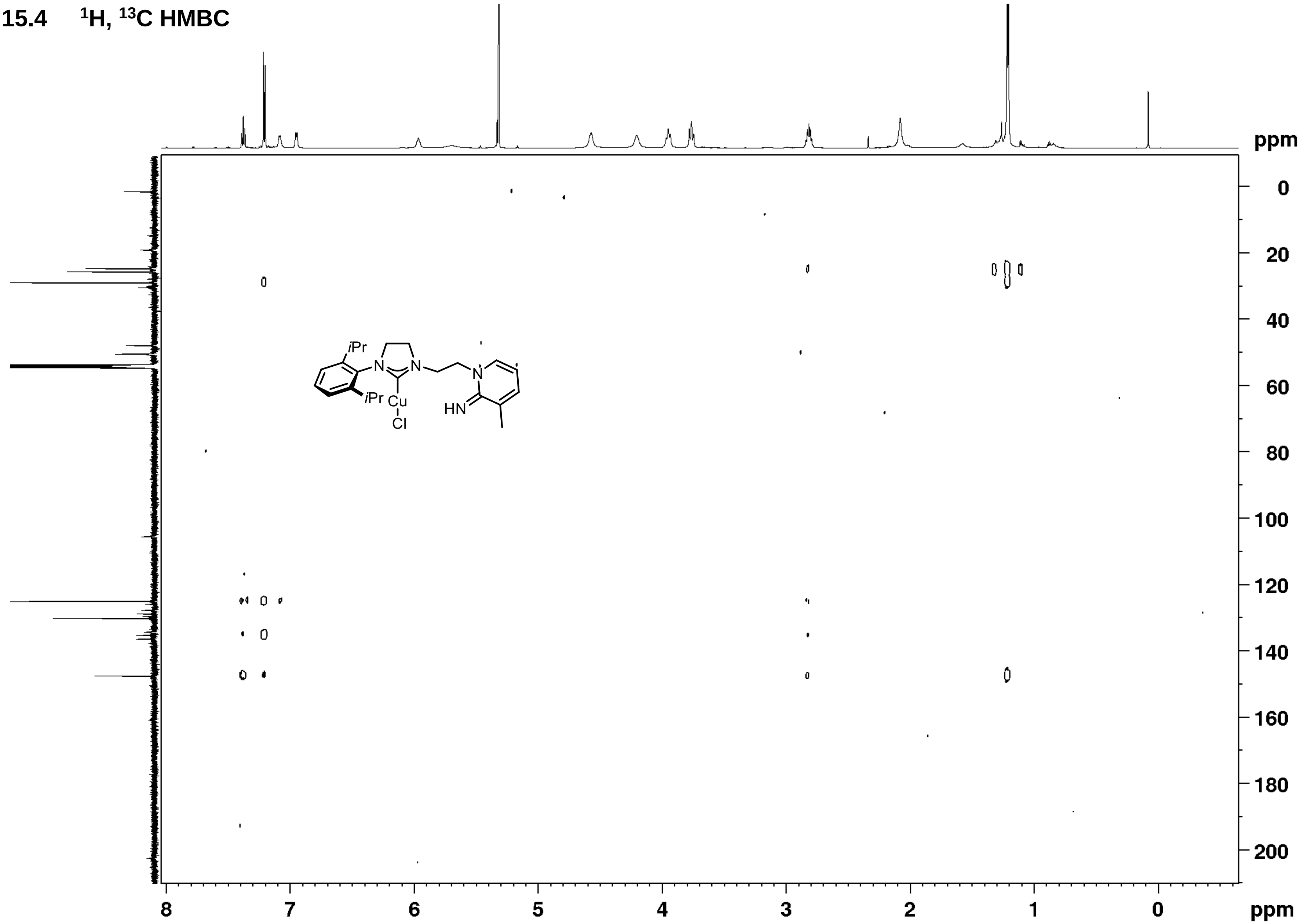

1.15.5 ^{1}H, ^{13}C HSQC

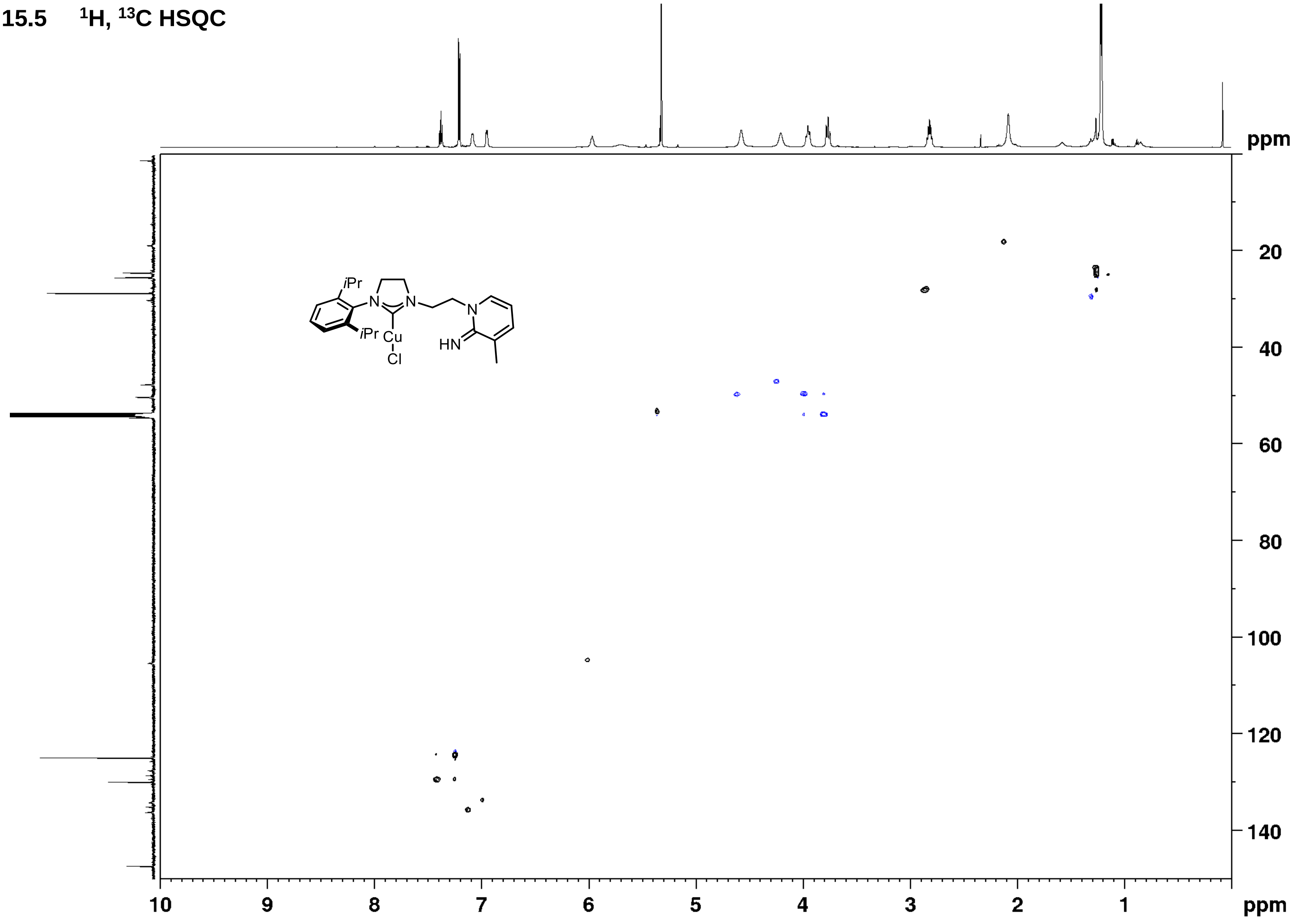

1.15.6 HRMS

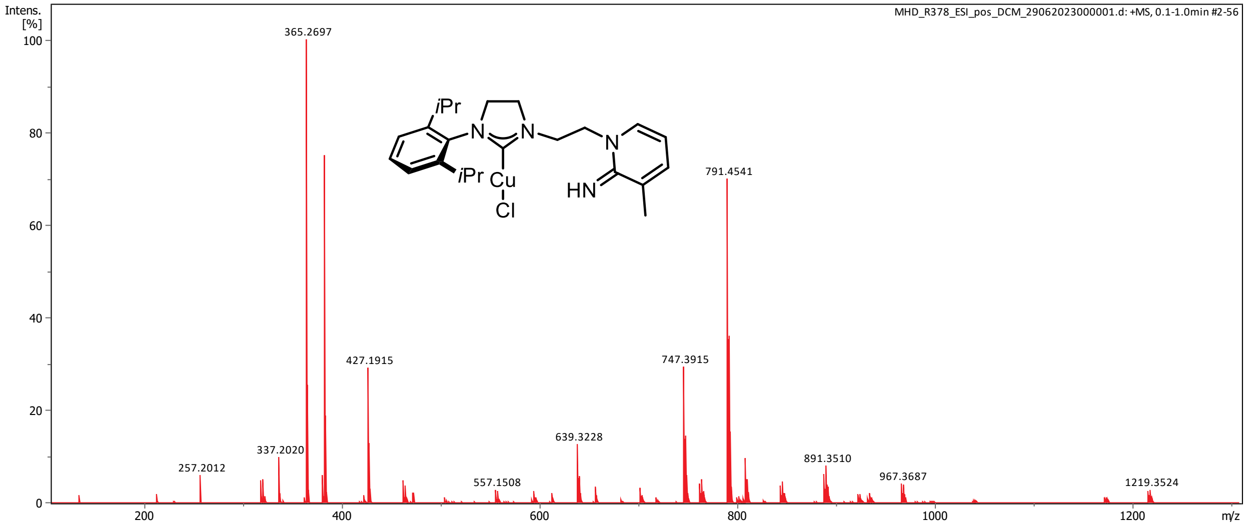
Intens.
[%]
MHD_R378_ESI_pos_DCM_29062023000001.d: +MS, 0.1-1.0min #2-56
365.2697
791.4541
427.1915
747.3915
639.3228
337.2020
257.2012
891.3510
967.3687
557.1508
1219.3524
iPr
N
N
N
iPr
Cu
Cl
HN
m/z

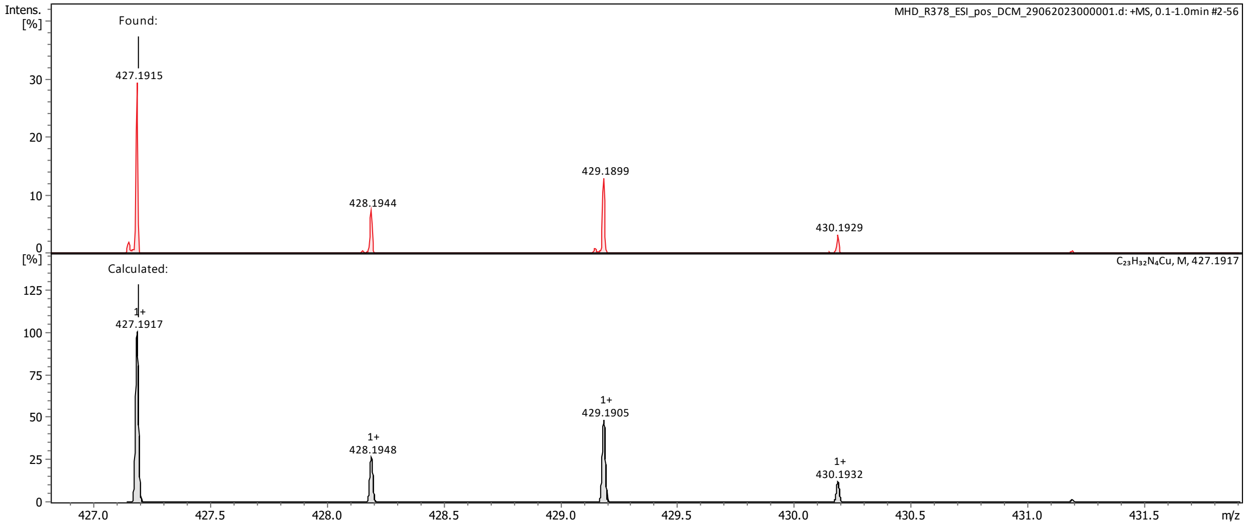
Intens.
[%]
MHD_R378_ESI_pos_DCM_29062023000001.d: +MS, 0.1-1.0min #2-56
Found:
427.1915
428.1944
429.1899
430.1929
Calculated:
$C_{23}H_{32}N_4Cu$, M, 427.1917
1+
427.1917
1+
428.1948
1+
429.1905
1+
430.1932
m/z

1.16 3-(4-Bromobutyl)-1-mesityl-1*H*-imidazol-3-ium bromide (S8b) (600 MHz, $CDCl_3$)

1.16.1 ^{1}H NMR

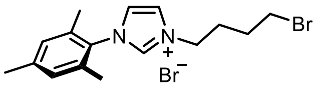

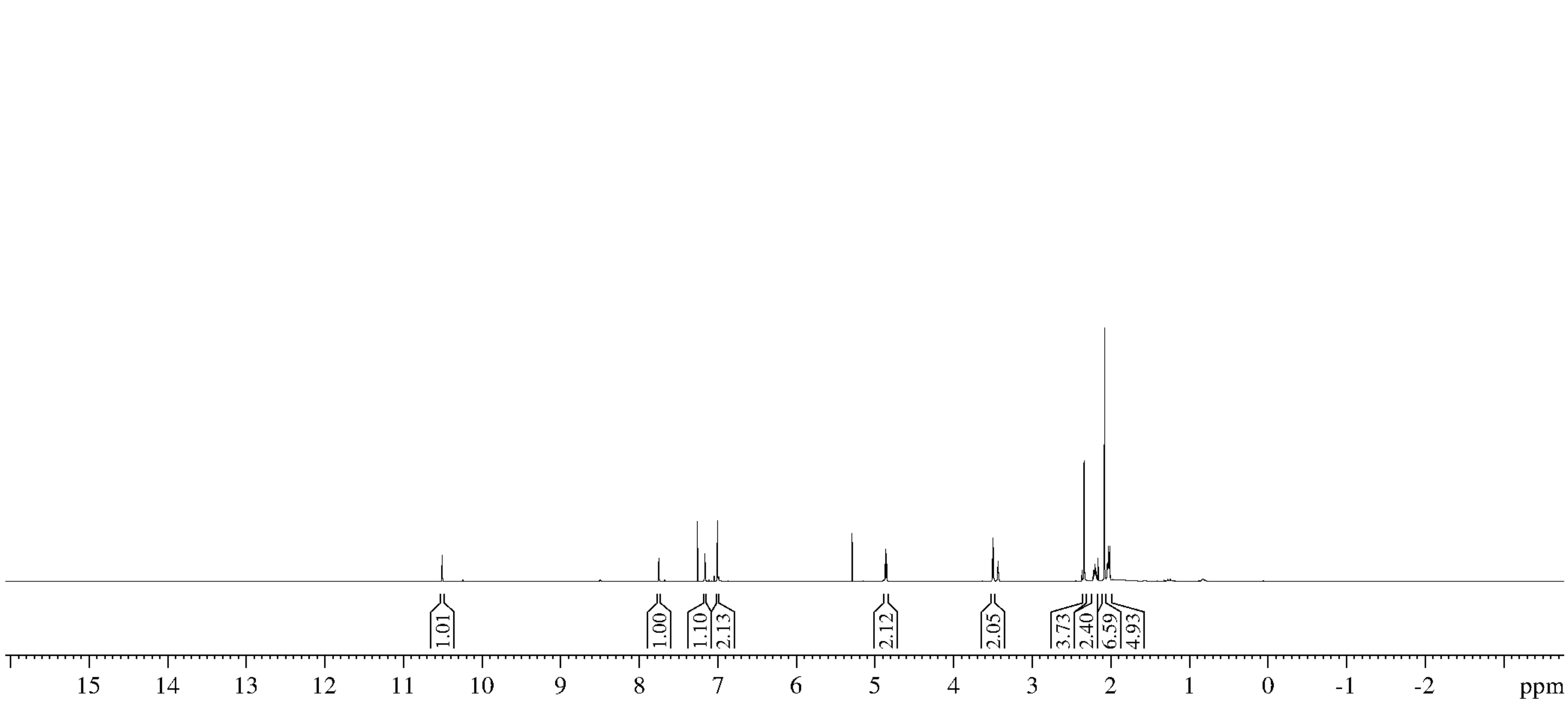

1.16.2 ^{13}C NMR

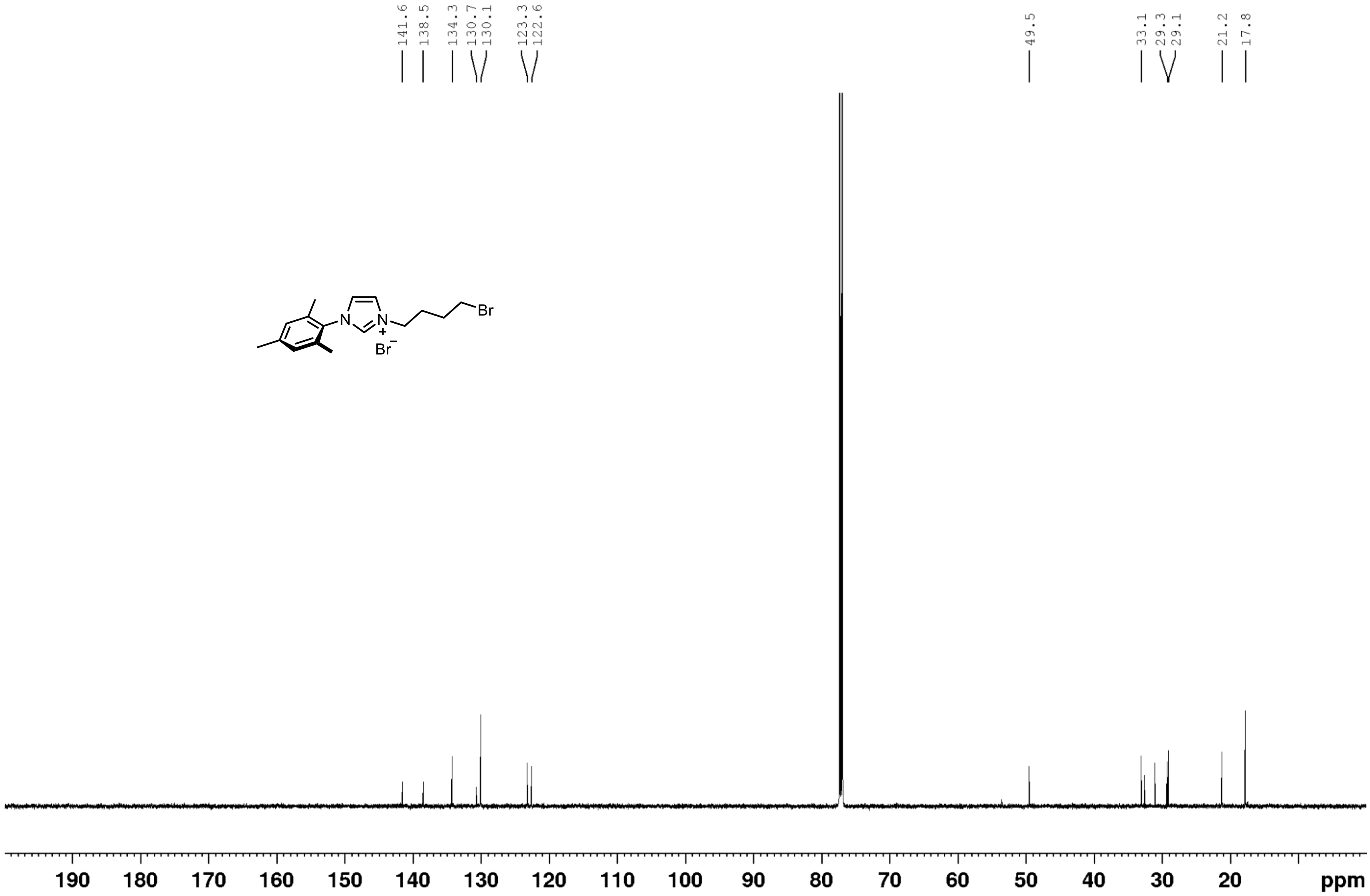

141.6
138.5
134.3
130.7
130.1
123.3
122.6
49.5
33.1
29.3
29.1
21.2
17.8
N
N
+
Br
Br
−
190
180
170
160
150
140
130
120
110
100
90
80
70
60
50
40
30
20
ppm

1.16.3 ^{1}H, ^{1}H COSY

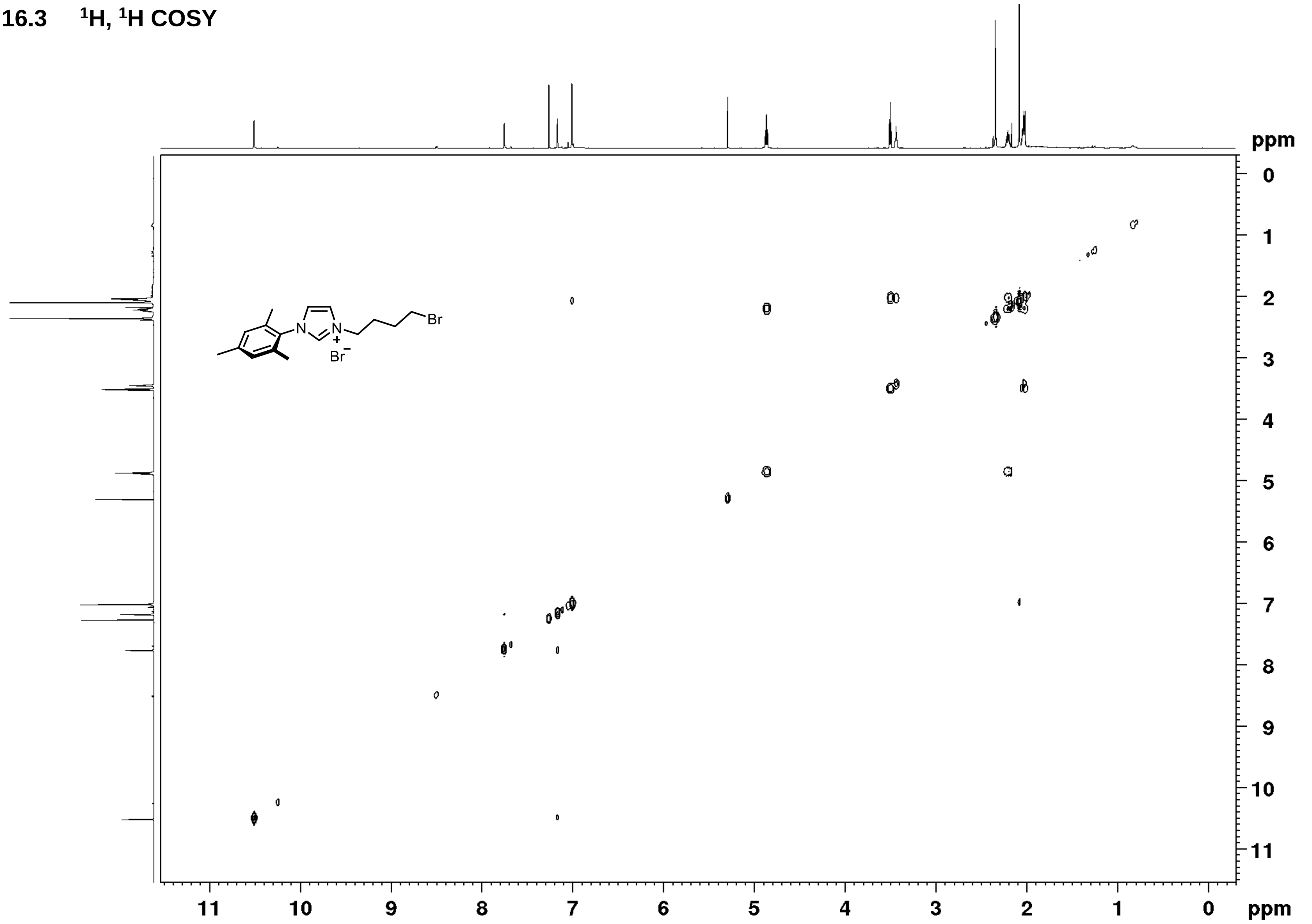

1.16.4 ^1H, ^{13}C HMBC

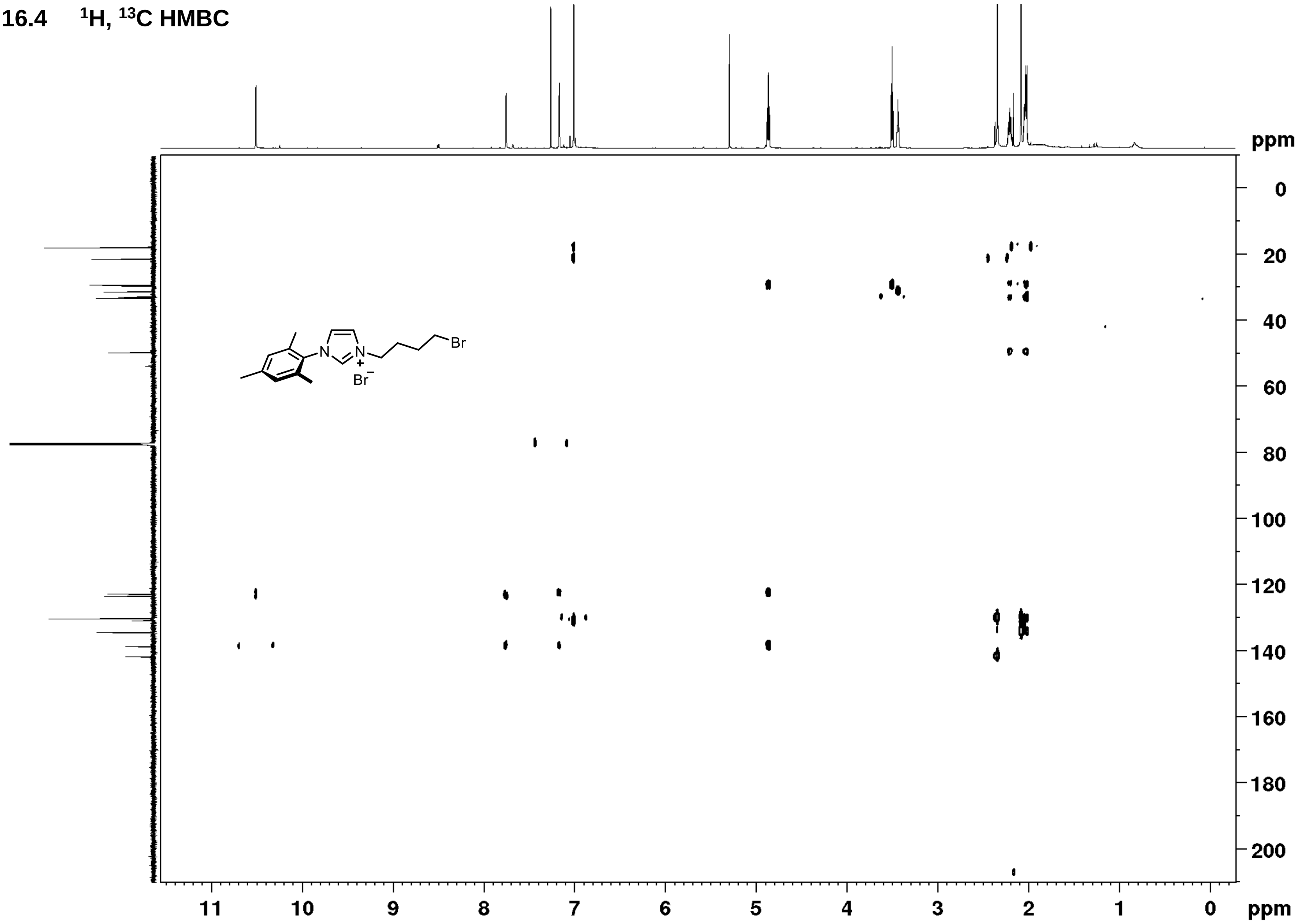

1.16.5 ^{1}H, ^{13}C HSQC

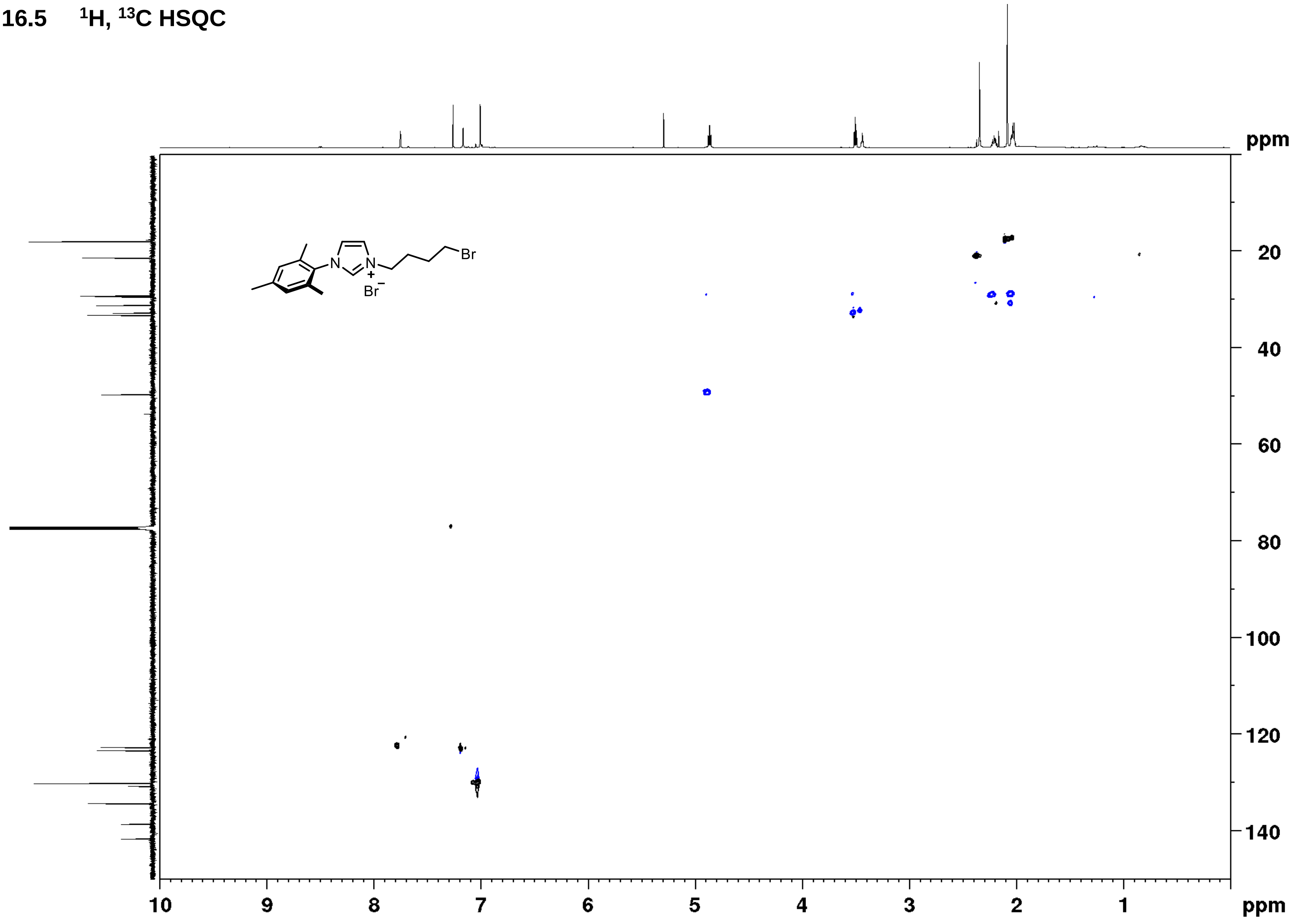

1.16.6 HRMS

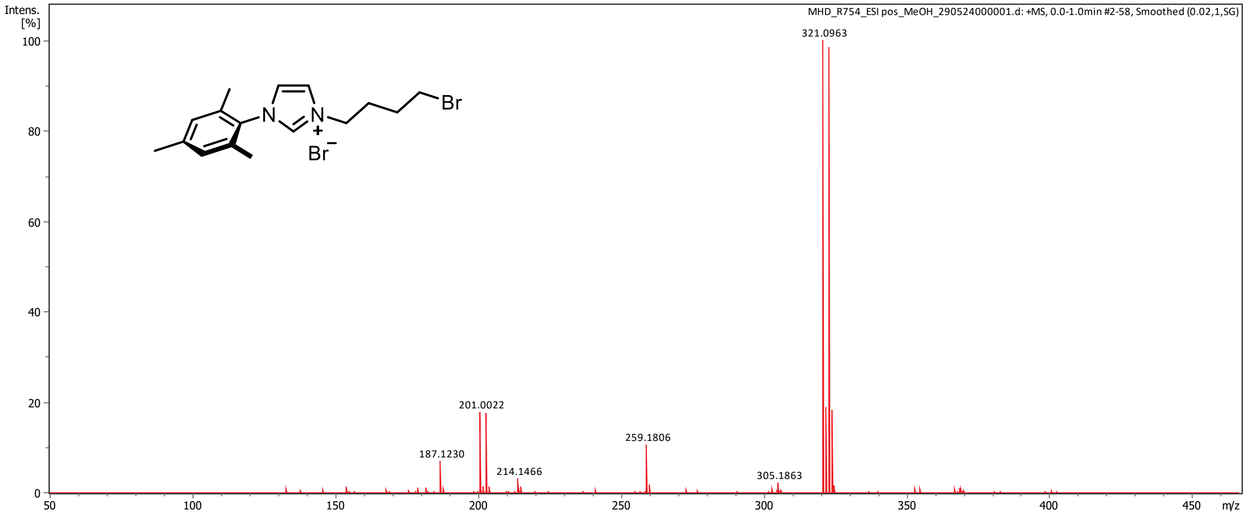

MHD_R754_ESI pos_MeOH_290524000001.d: +MS, 0.0-1.0min #2-58, Smoothed (0.02,1,SG)
Intens. [%]
321.0963
201.0022
187.1230
214.1466
259.1806
305.1863
Br
N
N
+
Br
−
m/z

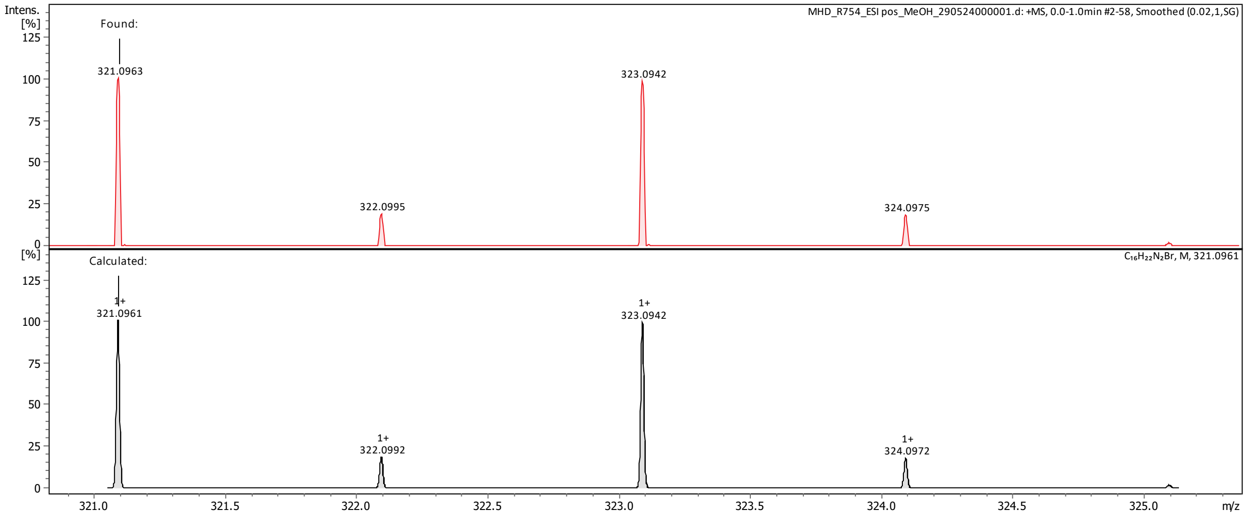

MHD_R754_ESI pos_MeOH_290524000001.d: +MS, 0.0-1.0min #2-58, Smoothed (0.02,1,SG)
Intens. [%]
Found:
321.0963
323.0942
322.0995
324.0975
Calculated:
C16H22N2Br, M, 321.0961
1+ 321.0961
1+ 323.0942
1+ 322.0992
1+ 324.0972
m/z

1.17 3-(4-(2-Imino-3-methylpyridin-1(2*H*)-yl)butyl)-1-mesityl-1*H*-imidazol-3-ium bromide (L8) (600 MHz, DMSO-*d*$_6$)

1.17.1 ^{1}H NMR

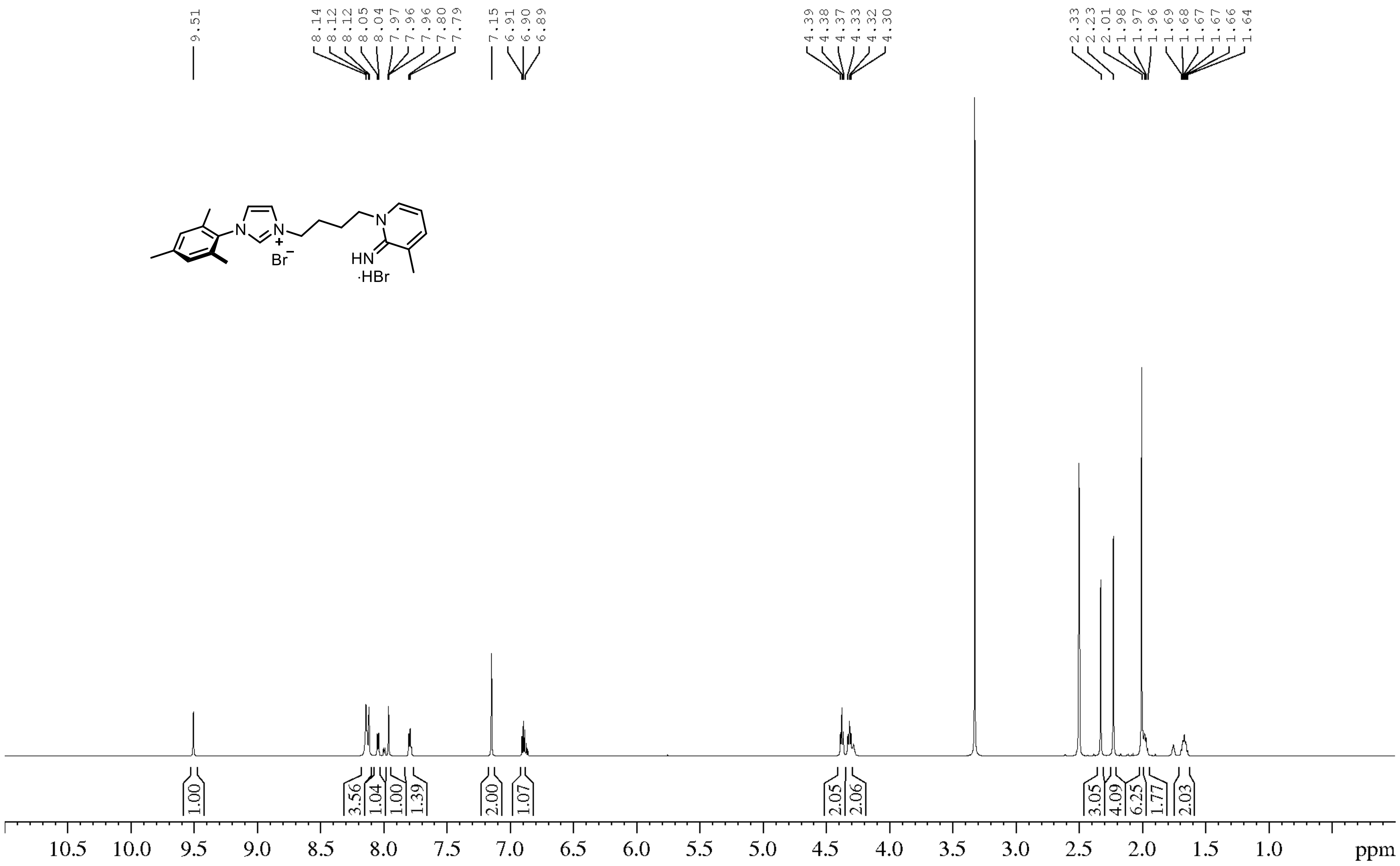

1.17.2 ^{13}C NMR

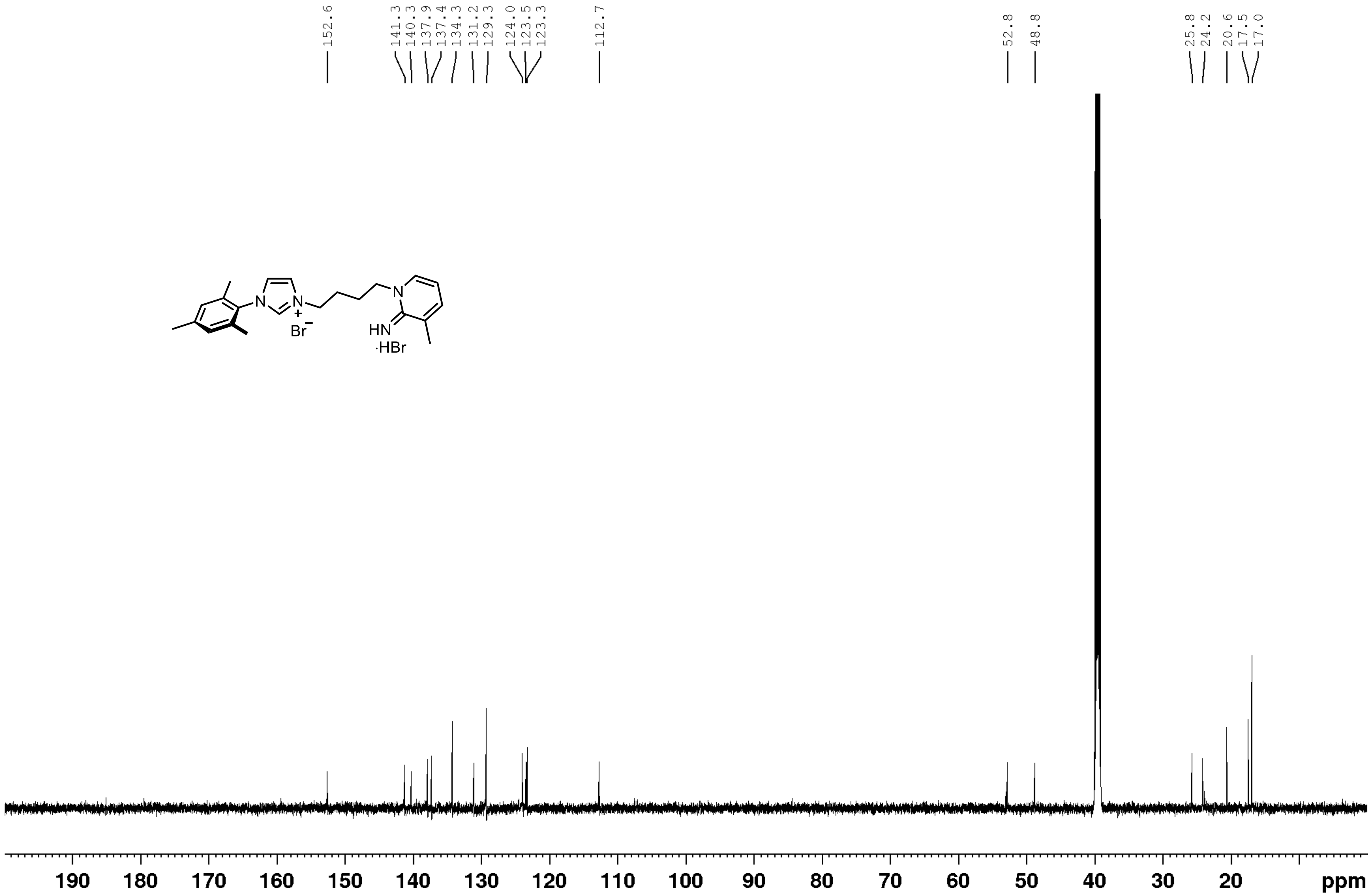
152.6
141.3
140.3
137.9
137.4
134.3
131.2
129.3
124.0
123.5
123.3
112.7
52.8
48.8
25.8
24.2
20.6
17.5
17.0
HN
·HBr
Br
190 180 170 160 150 140 130 120 110 100 90 80 70 60 50 40 30 20
ppm

1.17.3 ^{1}H, ^{1}H COSY

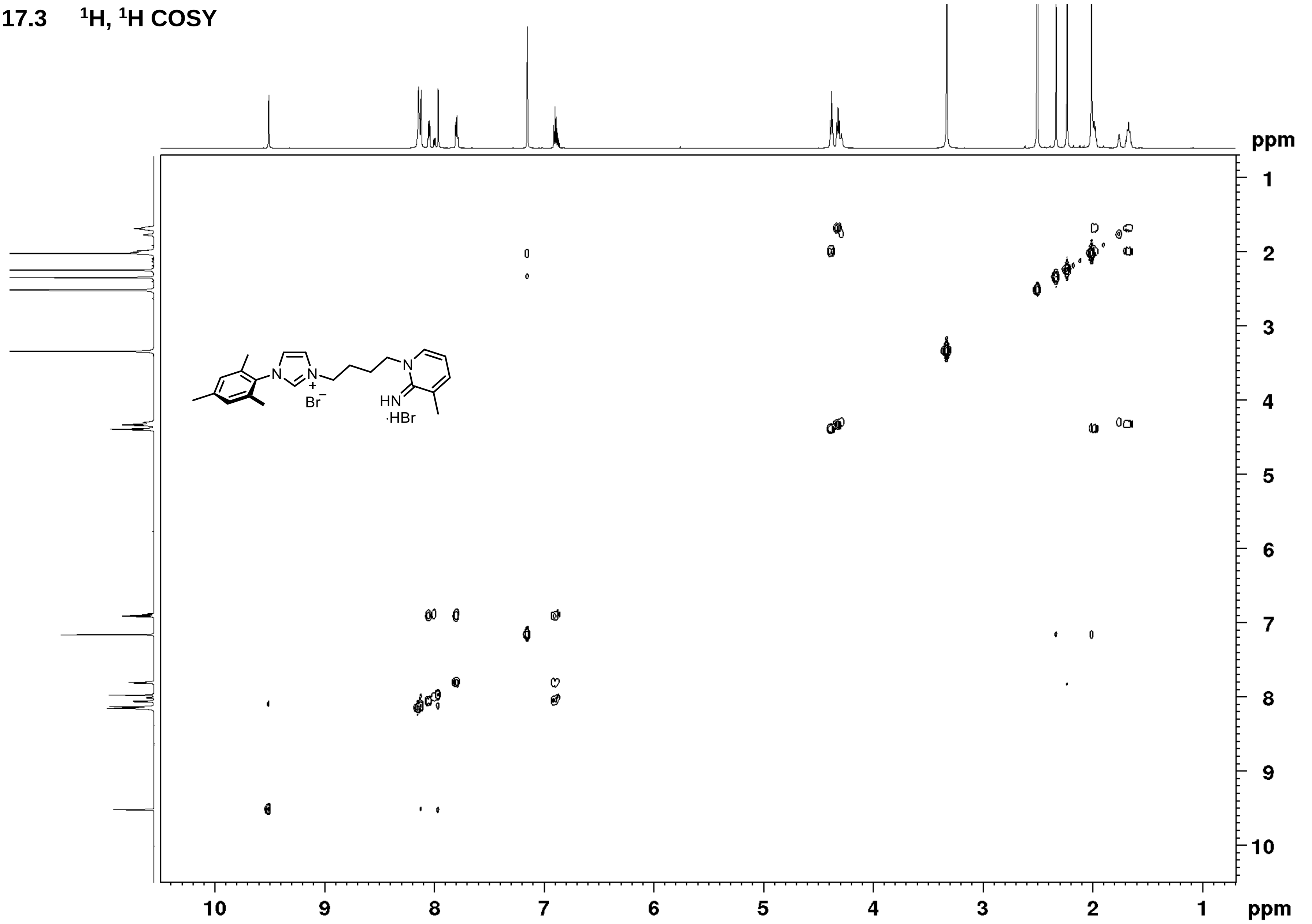

1.17.4 ^{1}H, ^{13}C HMBC

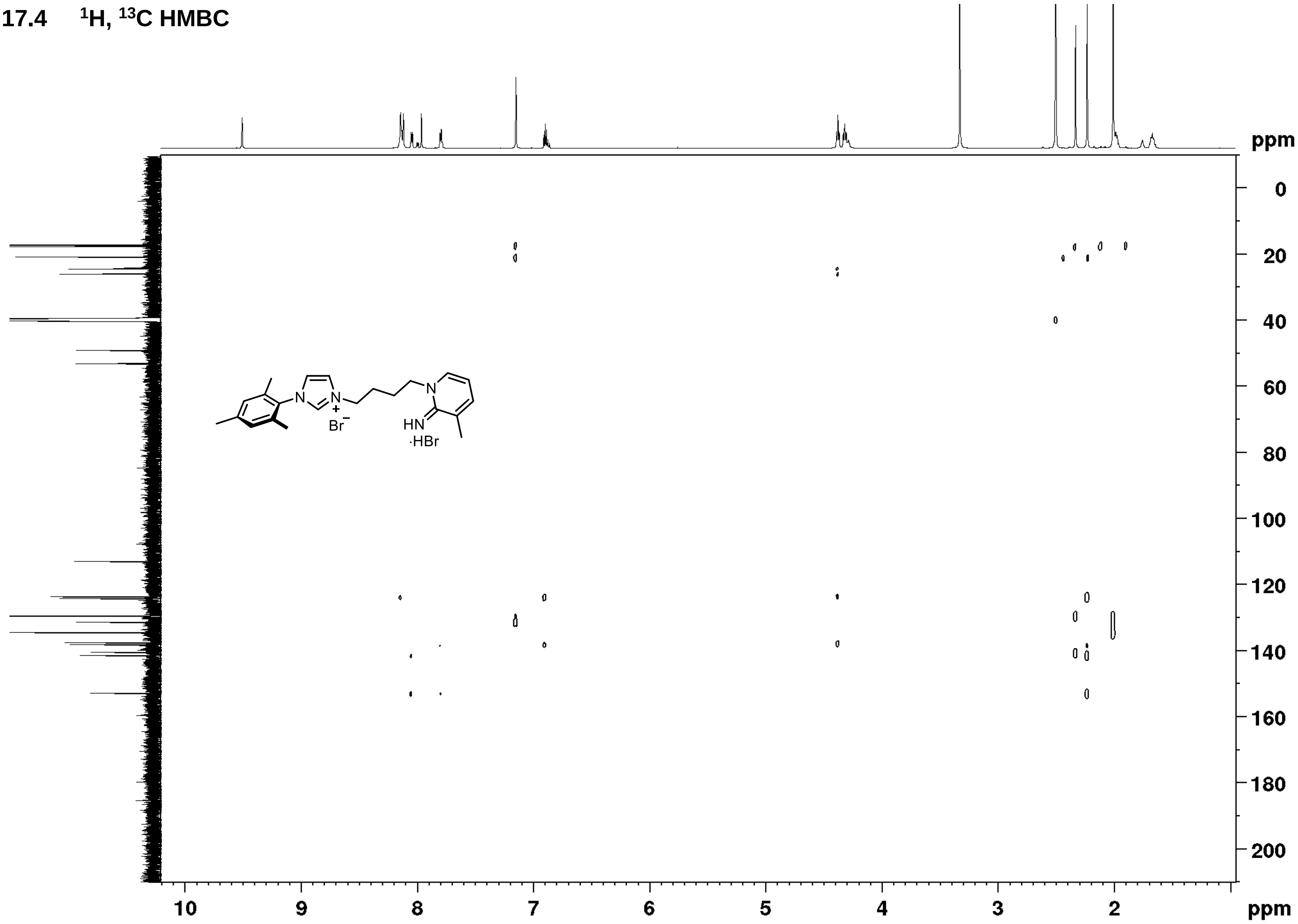

1.17.5 ¹H, ¹³C HSQC

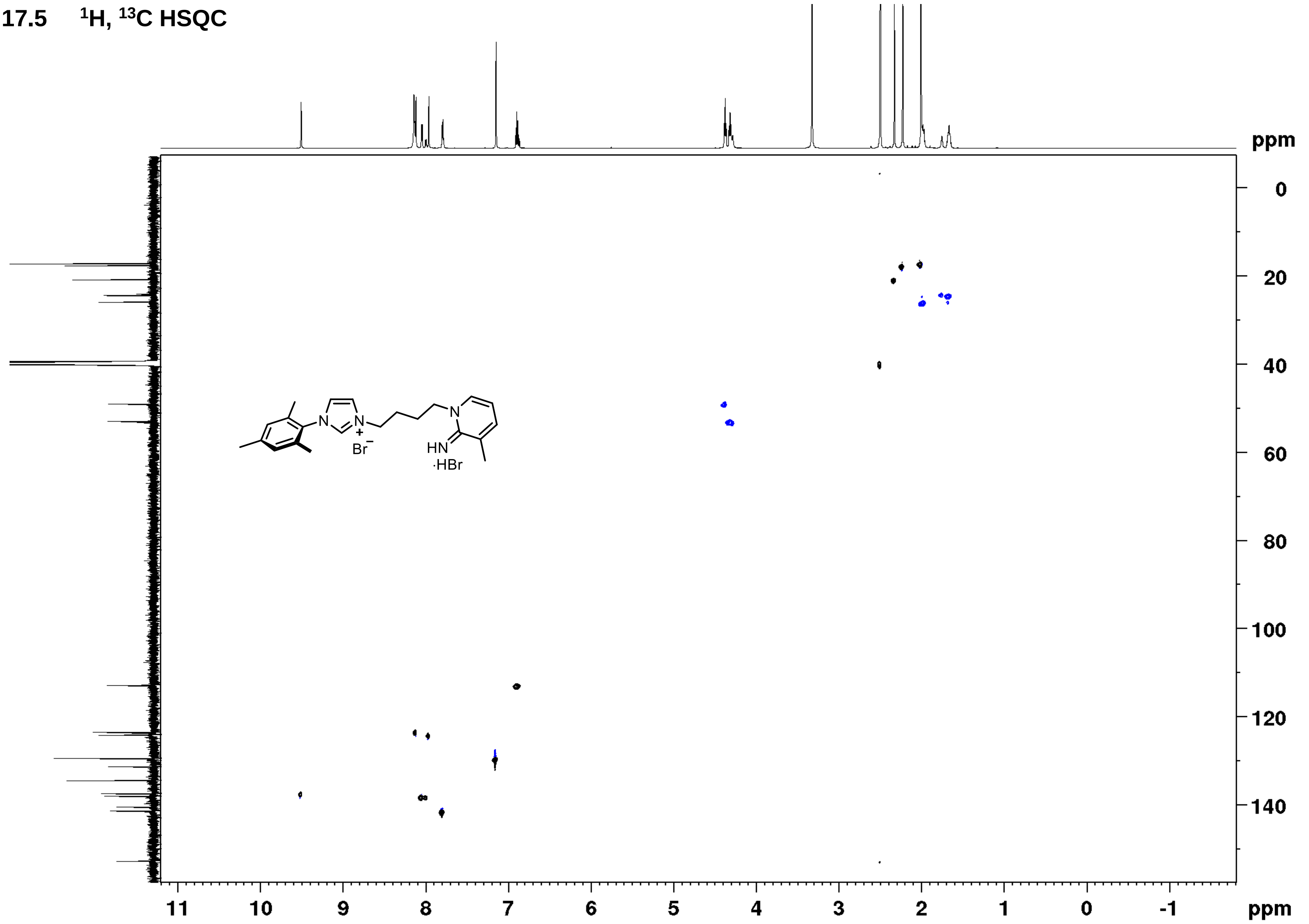

1.17.6 HRMS

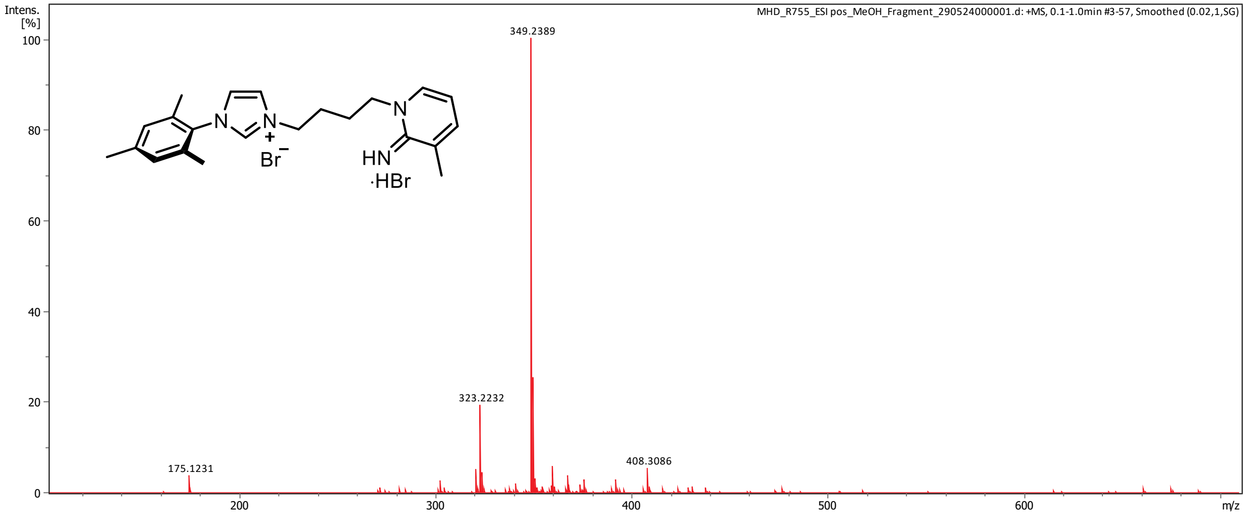

MHD_R755_ESI pos_MeOH_Fragment_290524000001.d: +MS, 0.1-1.0min #3-57, Smoothed (0.02,1,SG)
Intens. [%]
349.2389
323.2232
408.3086
175.1231
m/z
N
N
Br
HN
·HBr

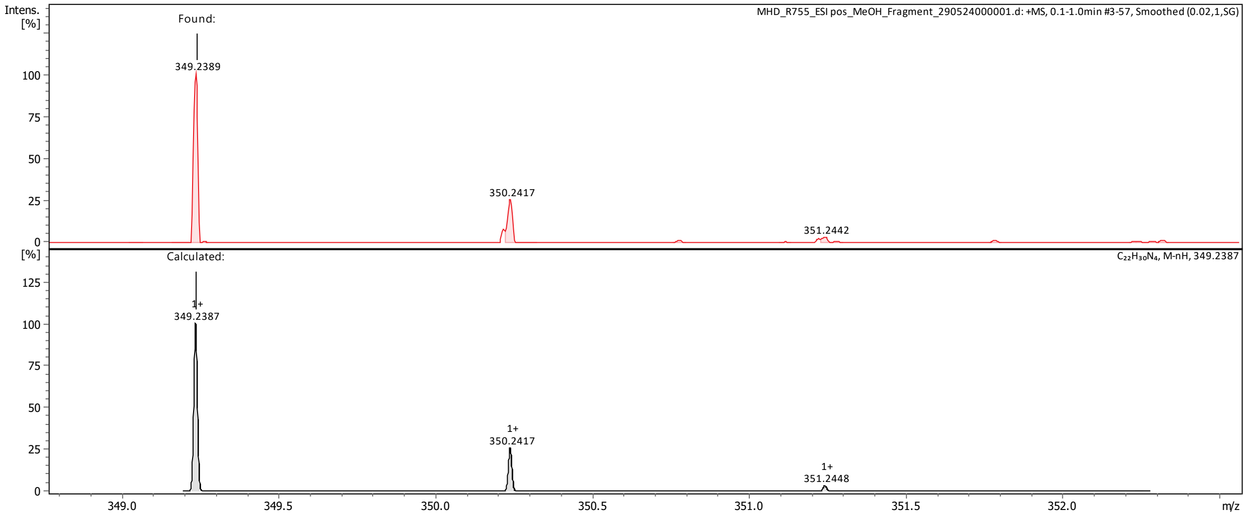

MHD_R755_ESI pos_MeOH_Fragment_290524000001.d: +MS, 0.1-1.0min #3-57, Smoothed (0.02,1,SG)
Found:
349.2389
350.2417
351.2442
Calculated:
C22H30N4, M-nH, 349.2387
1+ 349.2387
1+ 350.2417
1+ 351.2448
m/z

1.17.7 IR

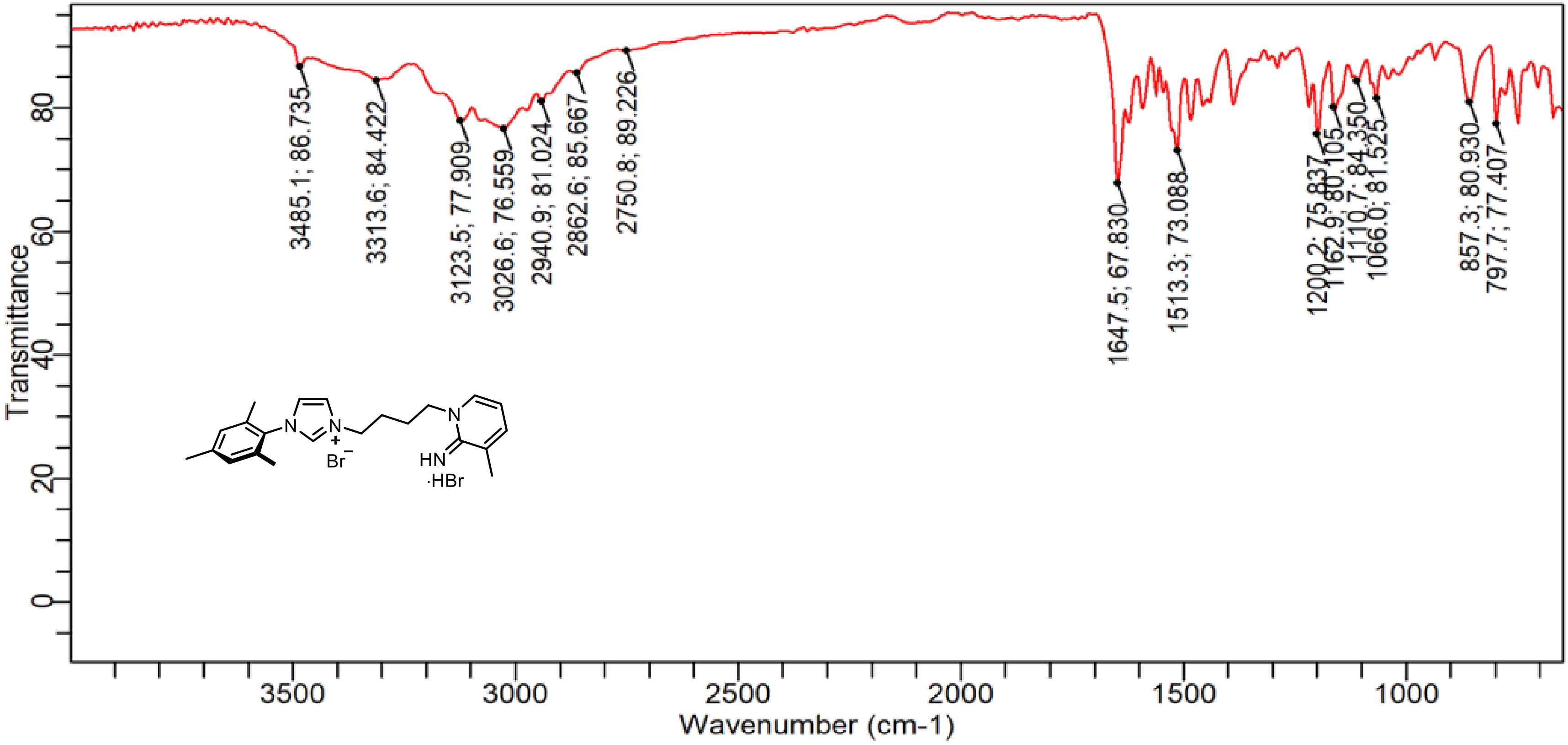

Transmittance
Wavenumber (cm-1)
3485.1; 86.735
3313.6; 84.422
3123.5; 77.909
3026.6; 76.559
2940.9; 81.024
2862.6; 85.667
2750.8; 89.226
1647.5; 67.830
1513.3; 73.088
1200.2; 75.837
1162.9; 80.105
1110.7; 84.350
1066.0; 81.525
857.3; 80.930
797.7; 77.407
Br
HN
·HBr

1.18 (1-(4-(2-Imino-3-methylpyridin-1(2H)-yl)butyl)-3-mesityl-1,3-dihydro-2*H*-imidazol-2-ylidene)copper(I) chloride (8) (600 MHz, CD_2Cl_2)

1.18.1 ^{1}H NMR

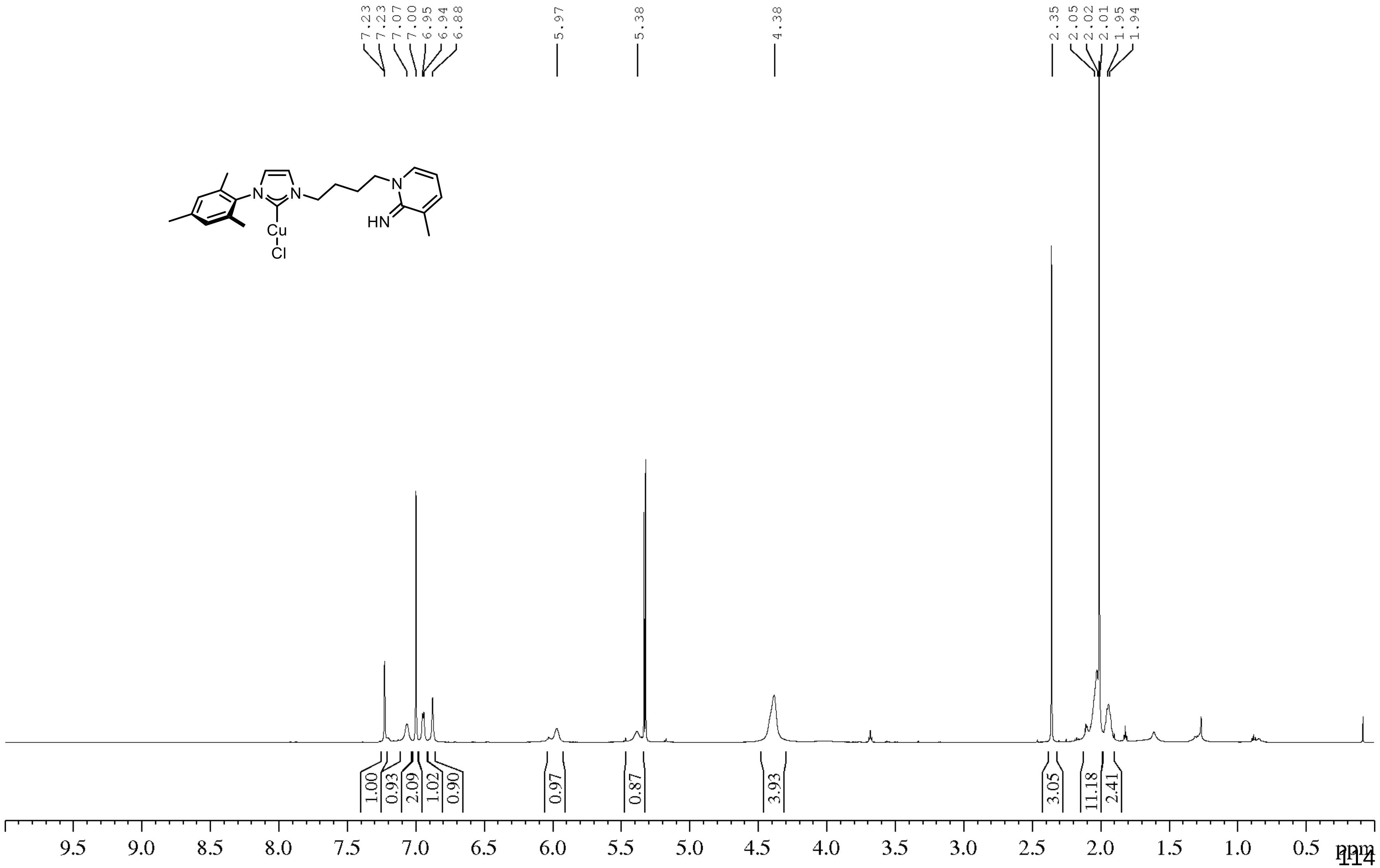

1.18.2 ^{13}C NMR

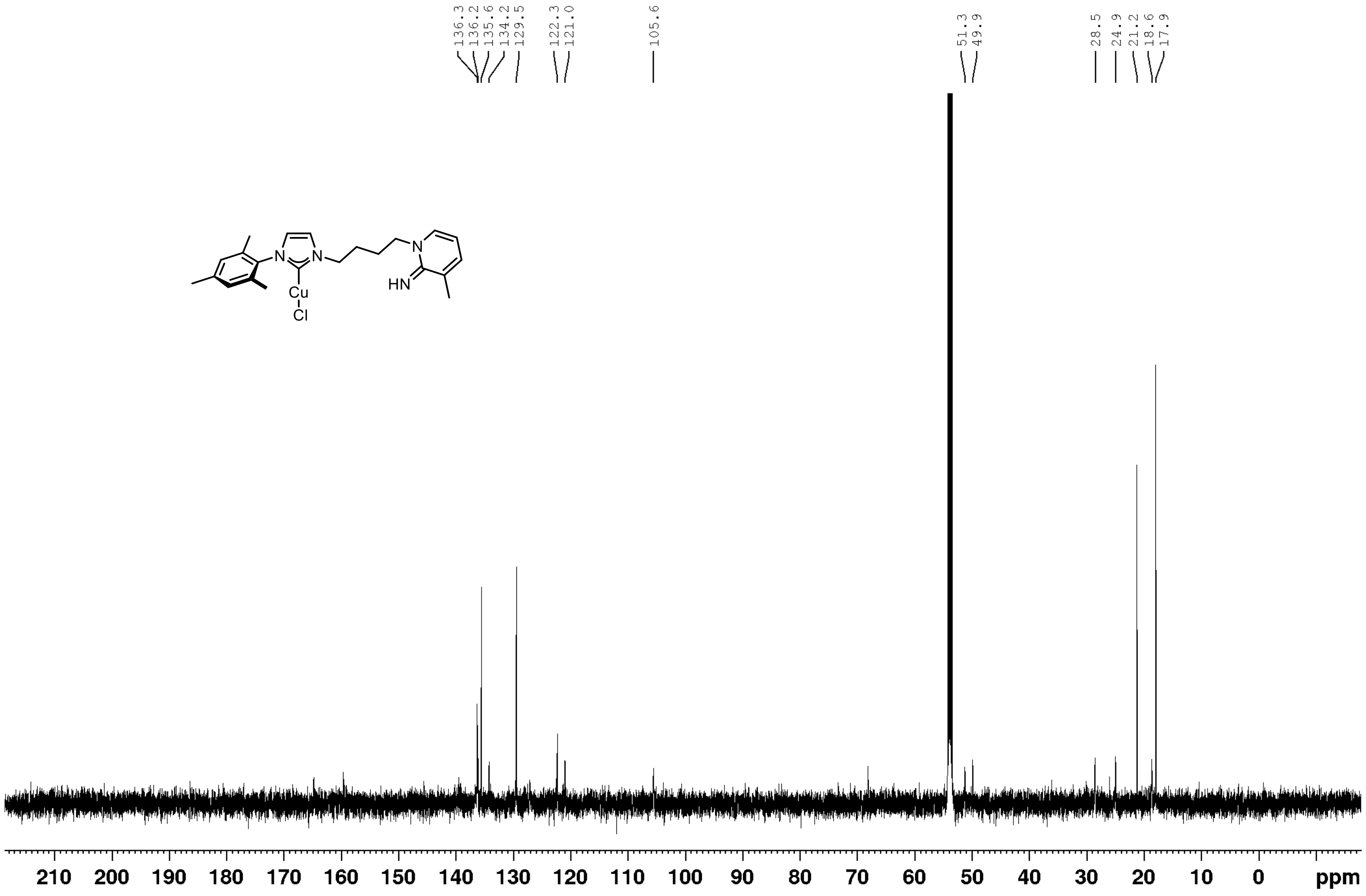
136.3
136.2
135.6
134.2
129.5
122.3
121.0
105.6
51.3
49.9
28.5
24.9
21.2
18.6
17.9
N
N
Cu
Cl
N
HN
210 200 190 180 170 160 150 140 130 120 110 100 90 80 70 60 50 40 30 20 10 0 ppm

1.18.3 ^{1}H, ^{1}H COSY

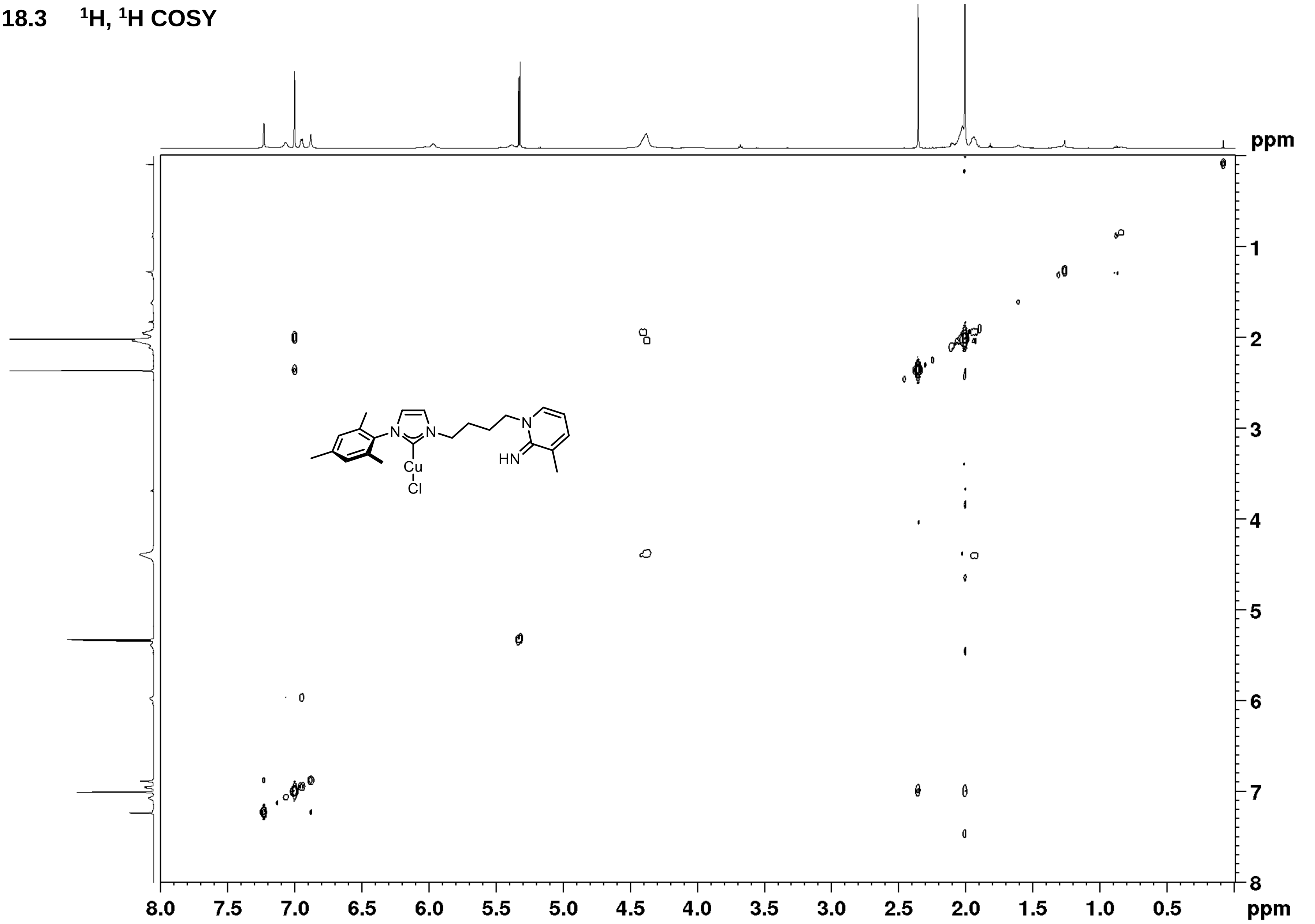

1.18.4 ^{1}H, ^{13}C HSQC

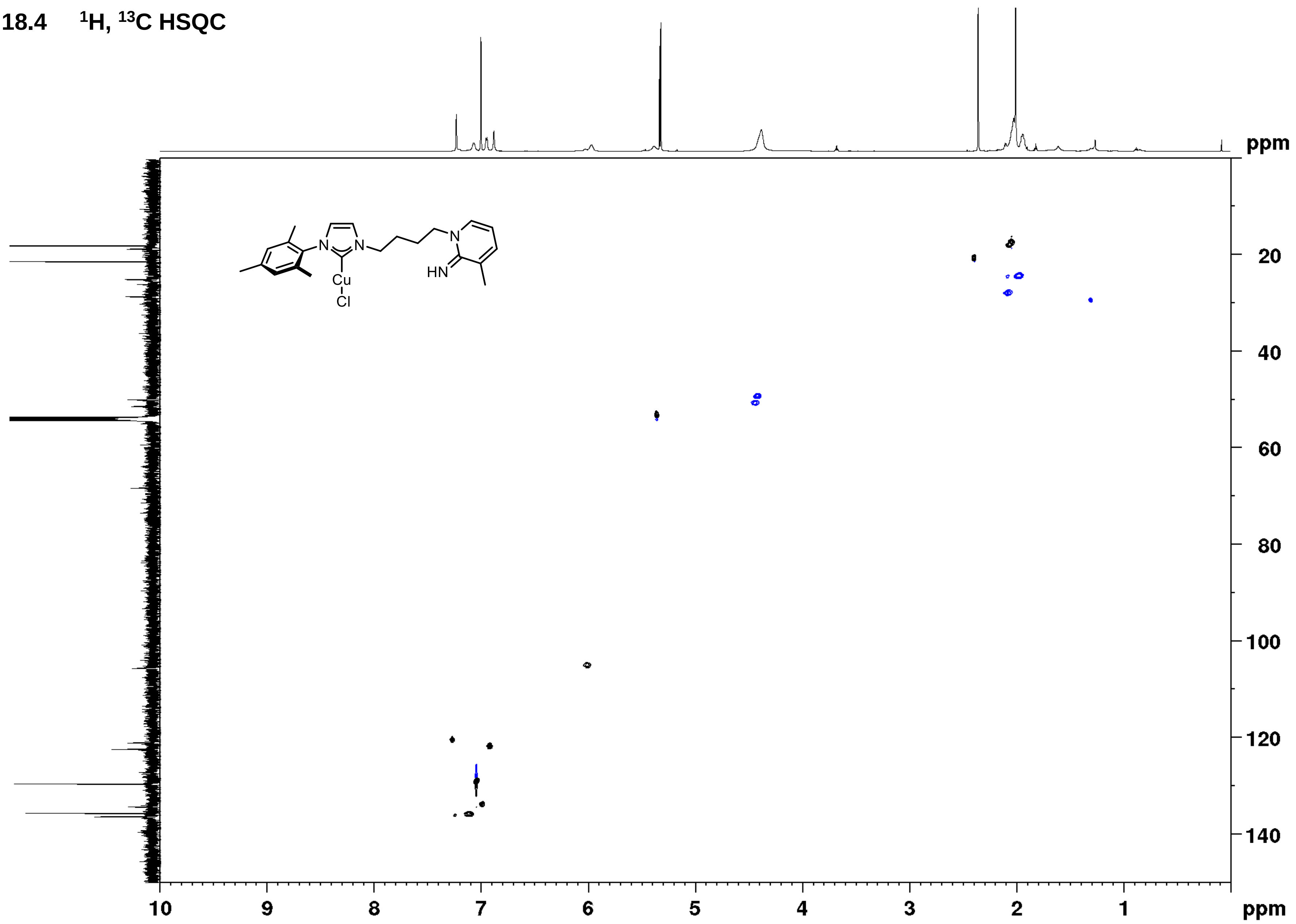

1.18.5 HRMS

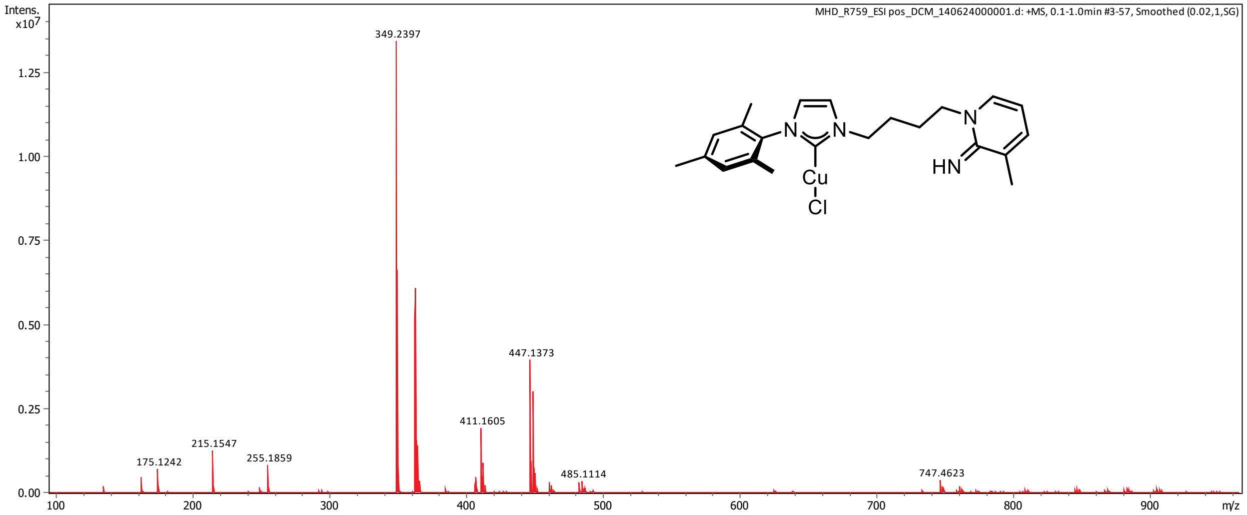
Intens.
x10^7
MHD_R759_ESI pos_DCM_140624000001.d: +MS, 0.1-1.0min #3-57, Smoothed (0.02,1,SG)
349.2397
447.1373
411.1605
215.1547
255.1859
175.1242
485.1114
747.4623
N
N
Cu
Cl
N
HN
m/z

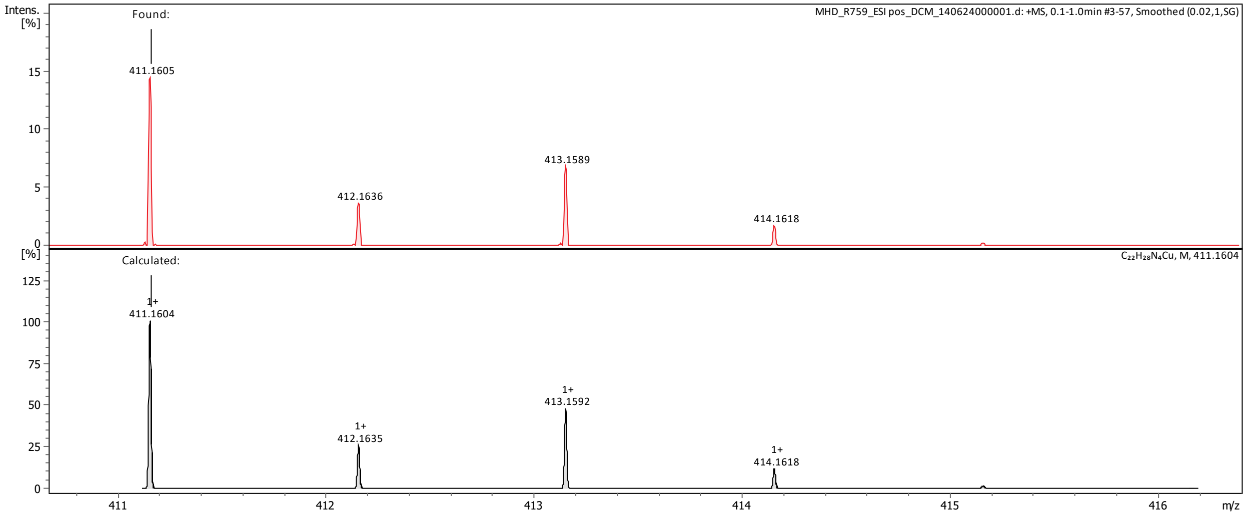
Intens.
[%]
Found:
411.1605
412.1636
413.1589
414.1618
MHD_R759_ESI pos_DCM_140624000001.d: +MS, 0.1-1.0min #3-57, Smoothed (0.02,1,SG)
Calculated:
C22H28N4Cu, M, 411.1604
1+
411.1604
1+
412.1635
1+
413.1592
1+
414.1618
m/z

1.19 1-(2,6-Diisopropylphenyl)-3-(2-(2-oxopyridin-1(2*H*)-yl)ethyl)-1*H*-imidazol-3-ium bromide (S9c) (600 MHz, $CDCl_3$)

1.19.1 ^{1}H NMR

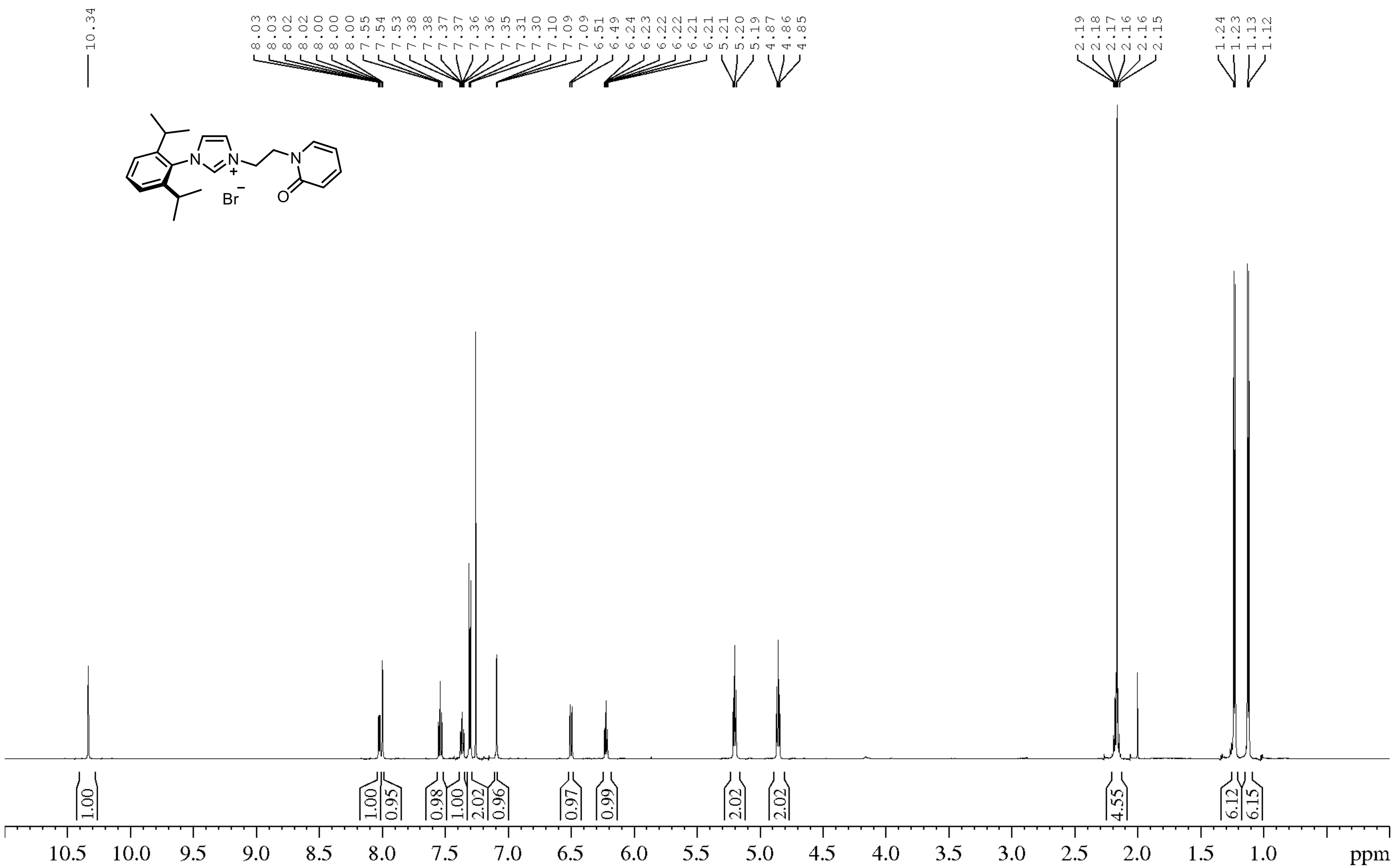

1.19.2 ^{13}C NMR

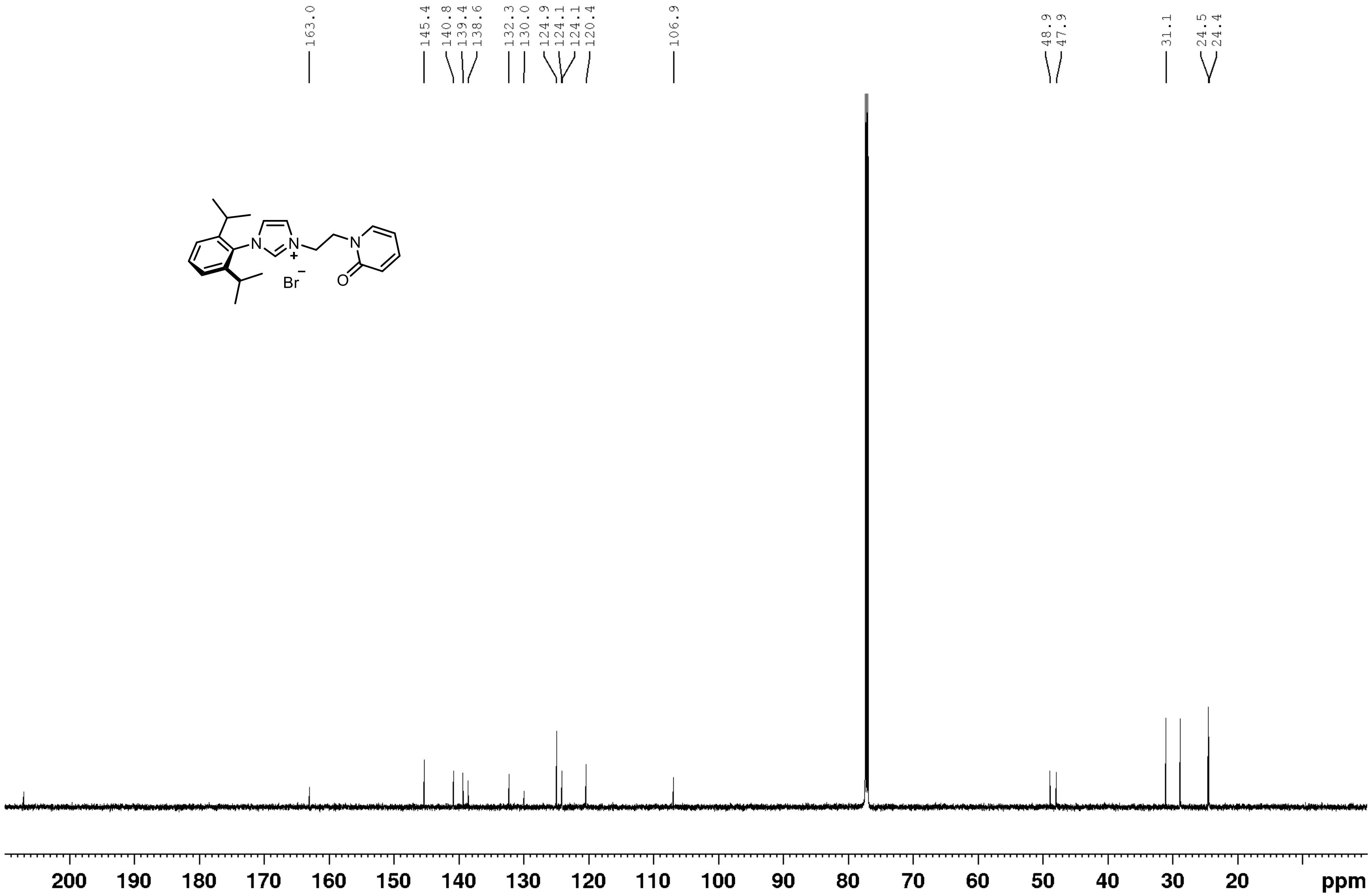

163.0
145.4
140.8
139.4
138.6
132.3
130.0
124.9
124.1
124.1
120.4
106.9
48.9
47.9
31.1
24.5
24.4
Br
O
N
200 190 180 170 160 150 140 130 120 110 100 90 80 70 60 50 40 30 20 ppm

1.19.3 ^{1}H, ^{1}H COSY

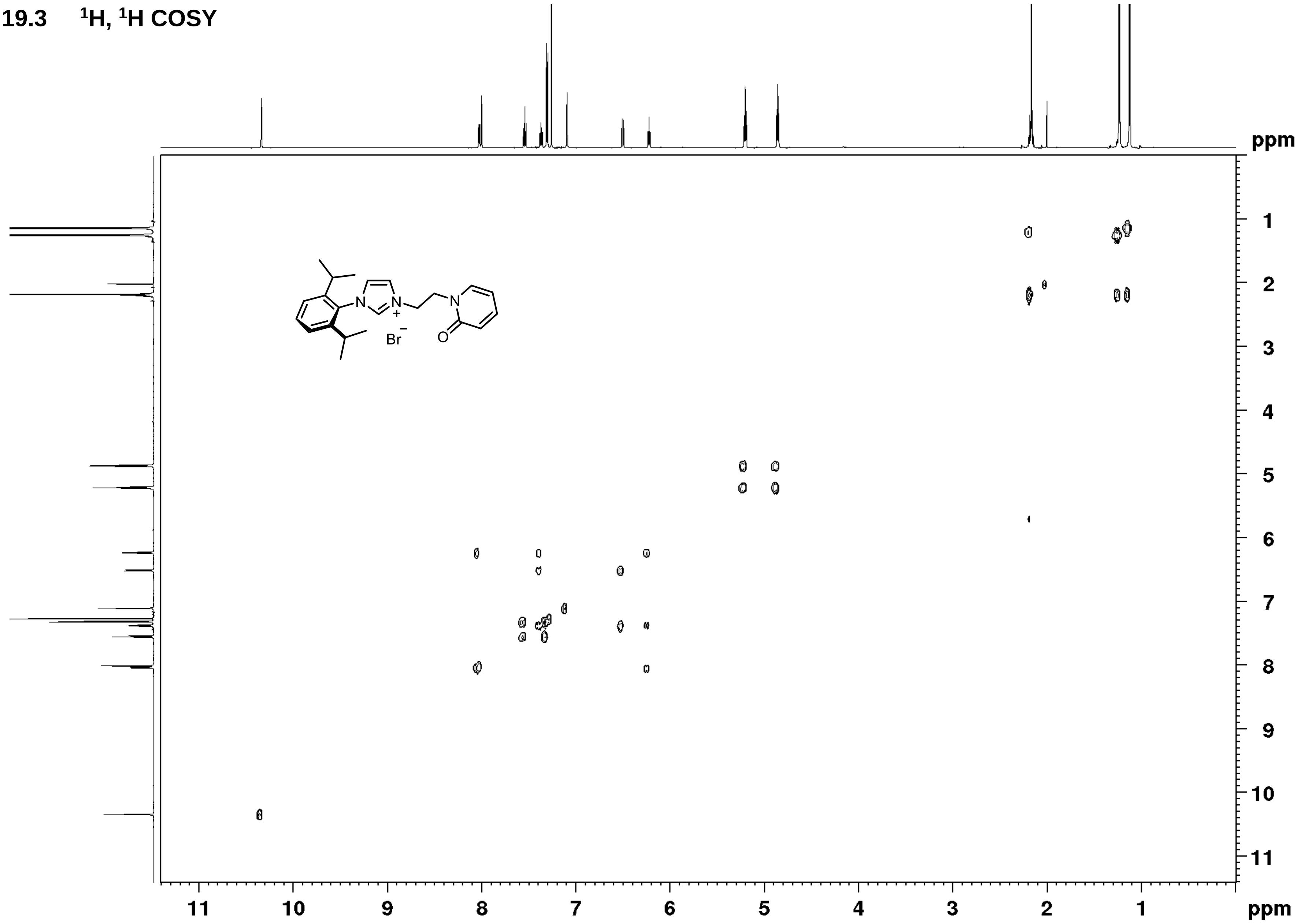

1.19.4 ^{1}H, ^{13}C HMBC

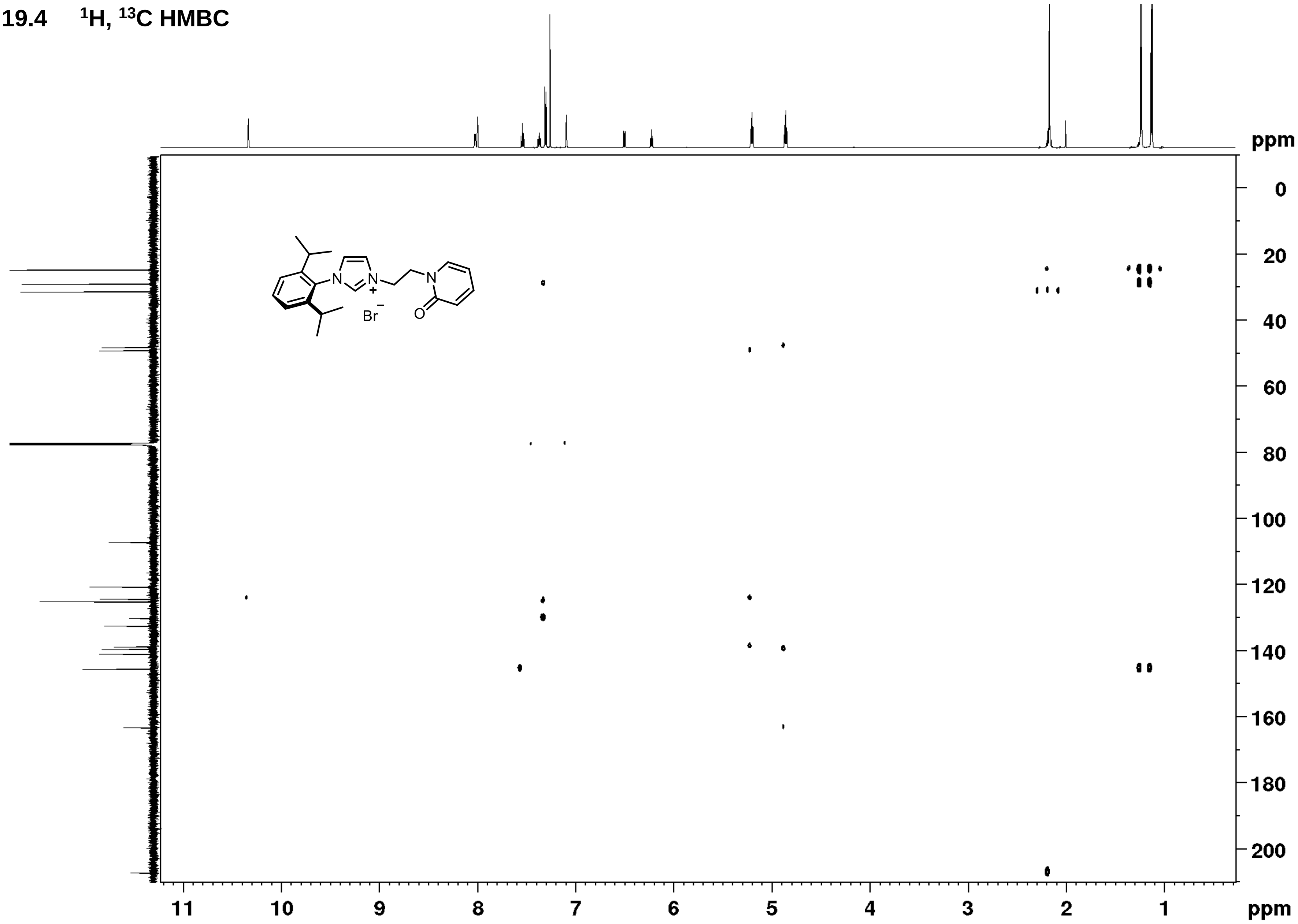

1.19.5 ^{1}H, ^{13}C HSQC

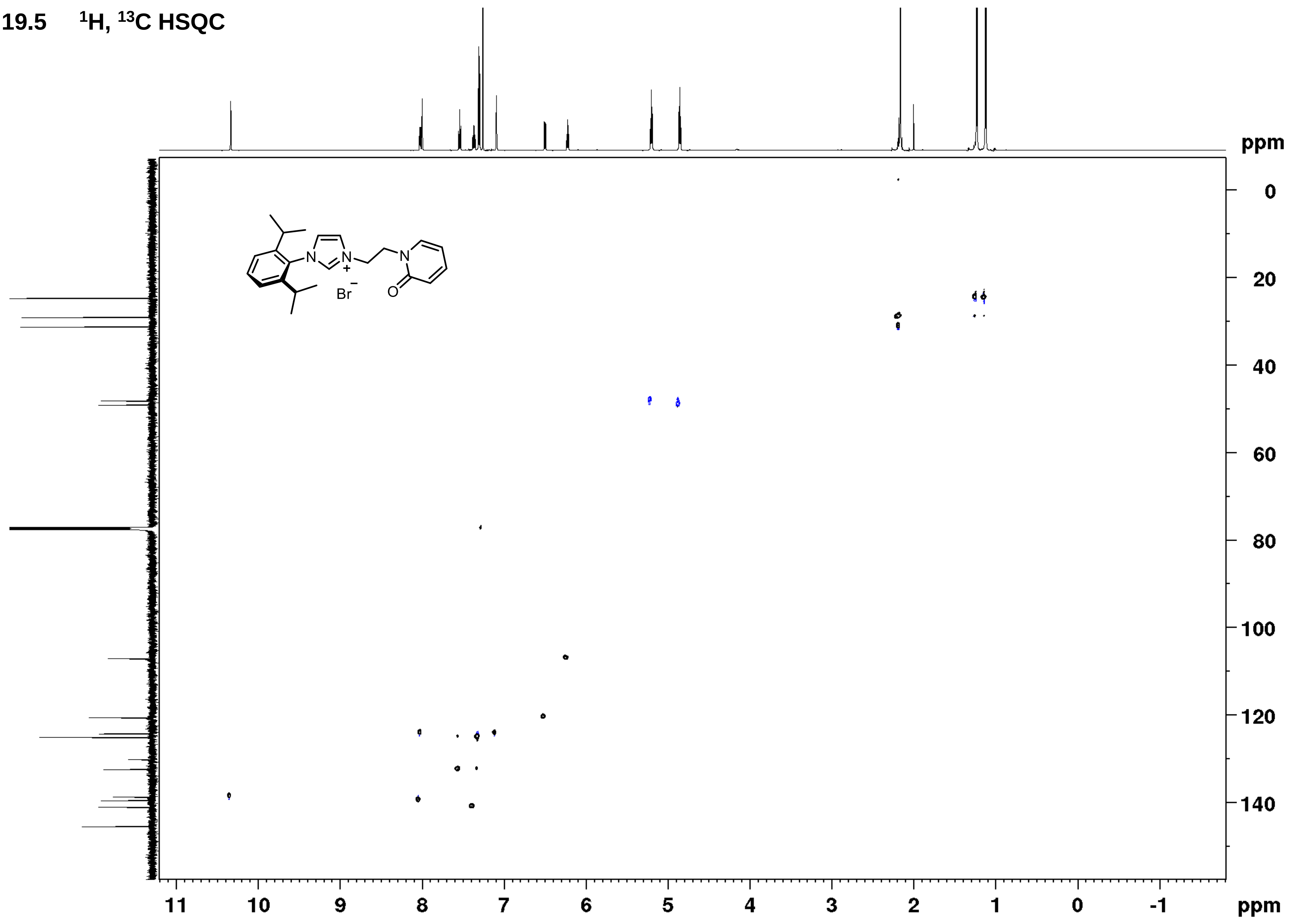

1.19.6 HRMS

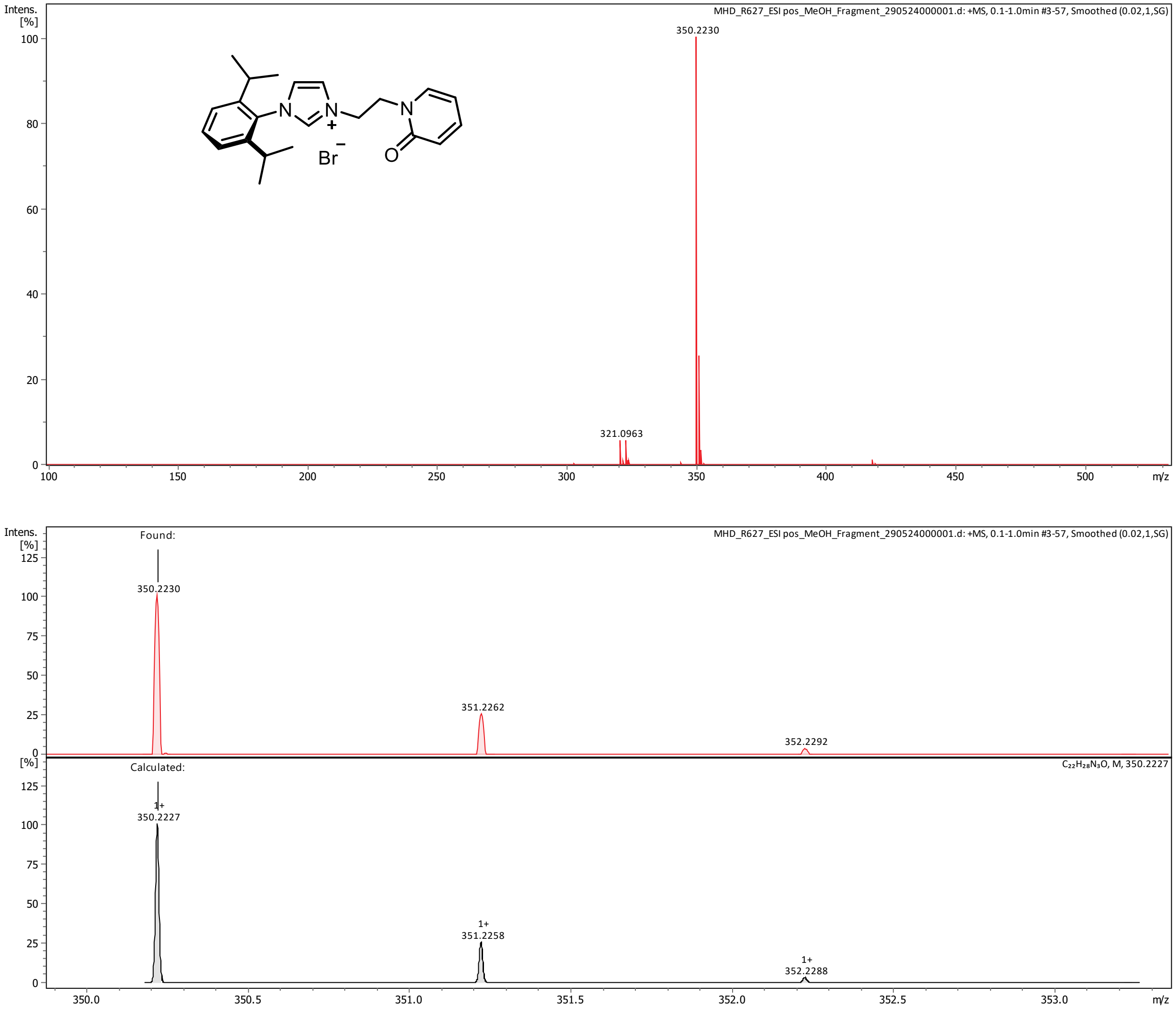
MHD_R627_ESI pos_MeOH_Fragment_290524000001.d: +MS, 0.1-1.0min #3-57, Smoothed (0.02,1,SG)
350.2230
321.0963
Intens. [%]
m/z
Br
O
N
Found:
350.2230
351.2262
352.2292
Calculated:
$C_{22}H_{28}N_3O$, M, 350.2227
1+ 350.2227
1+ 351.2258
1+ 352.2288

1.19.7 IR

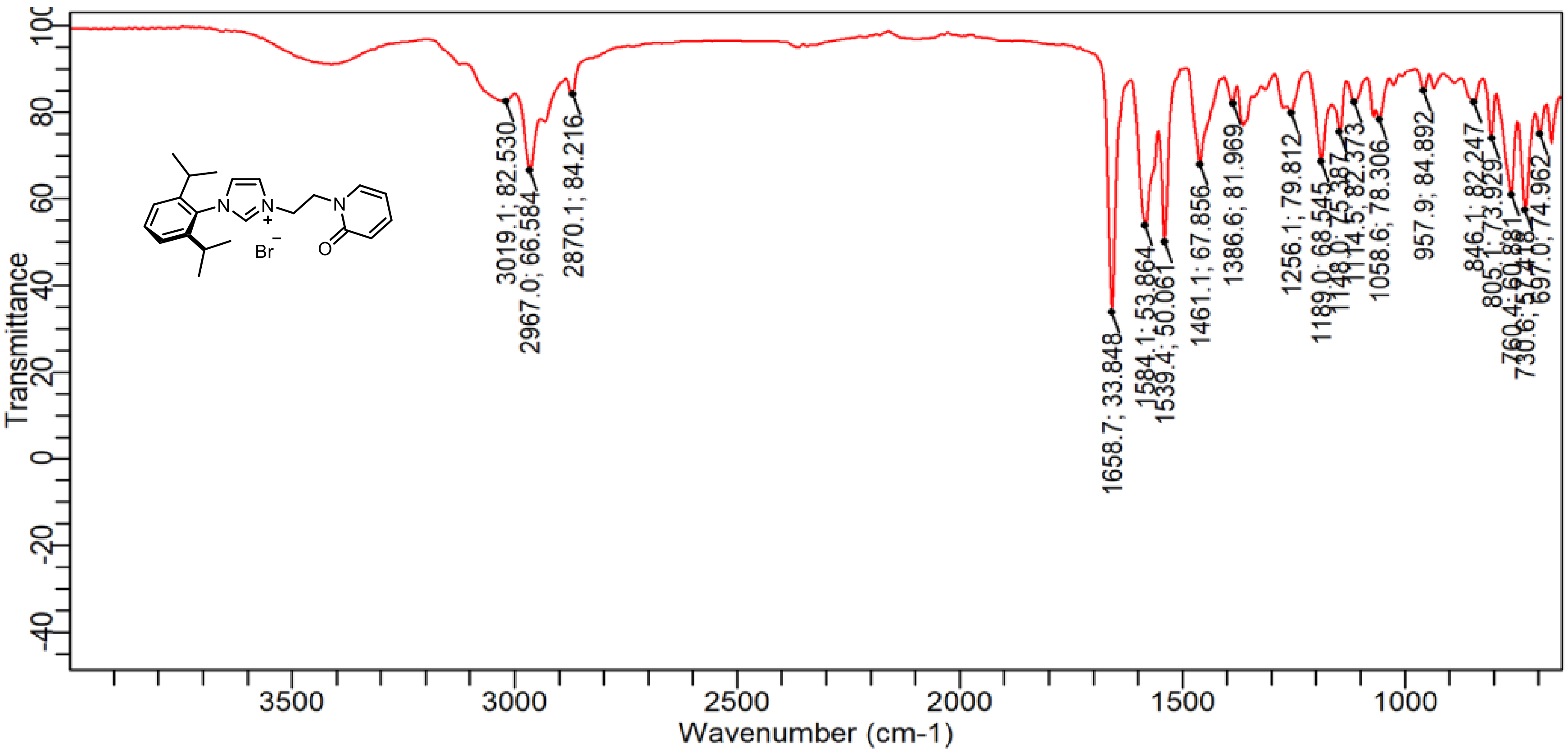

Transmittance
Wavenumber (cm-1)
3019.1; 82.530
2967.0; 66.584
2870.1; 84.216
1658.7; 33.848
1584.1; 53.864
1539.4; 50.061
1461.1; 67.856
1386.6; 81.969
1256.1; 79.812
1189.0; 68.545
1058.6; 78.306
957.9; 84.892
846.1; 82.247
697.0; 74.962
Br
O
N

1.20 (1-(2,6-Diisopropylphenyl)-3-(2-(2-oxopyridin-1(2*H*)-yl)ethyl)-1,3-dihydro-2*H*-imidazol-2-ylidene)copper(I) chloride (9) (600 MHz, CD_2Cl_2)

1.20.1 ^{1}H NMR

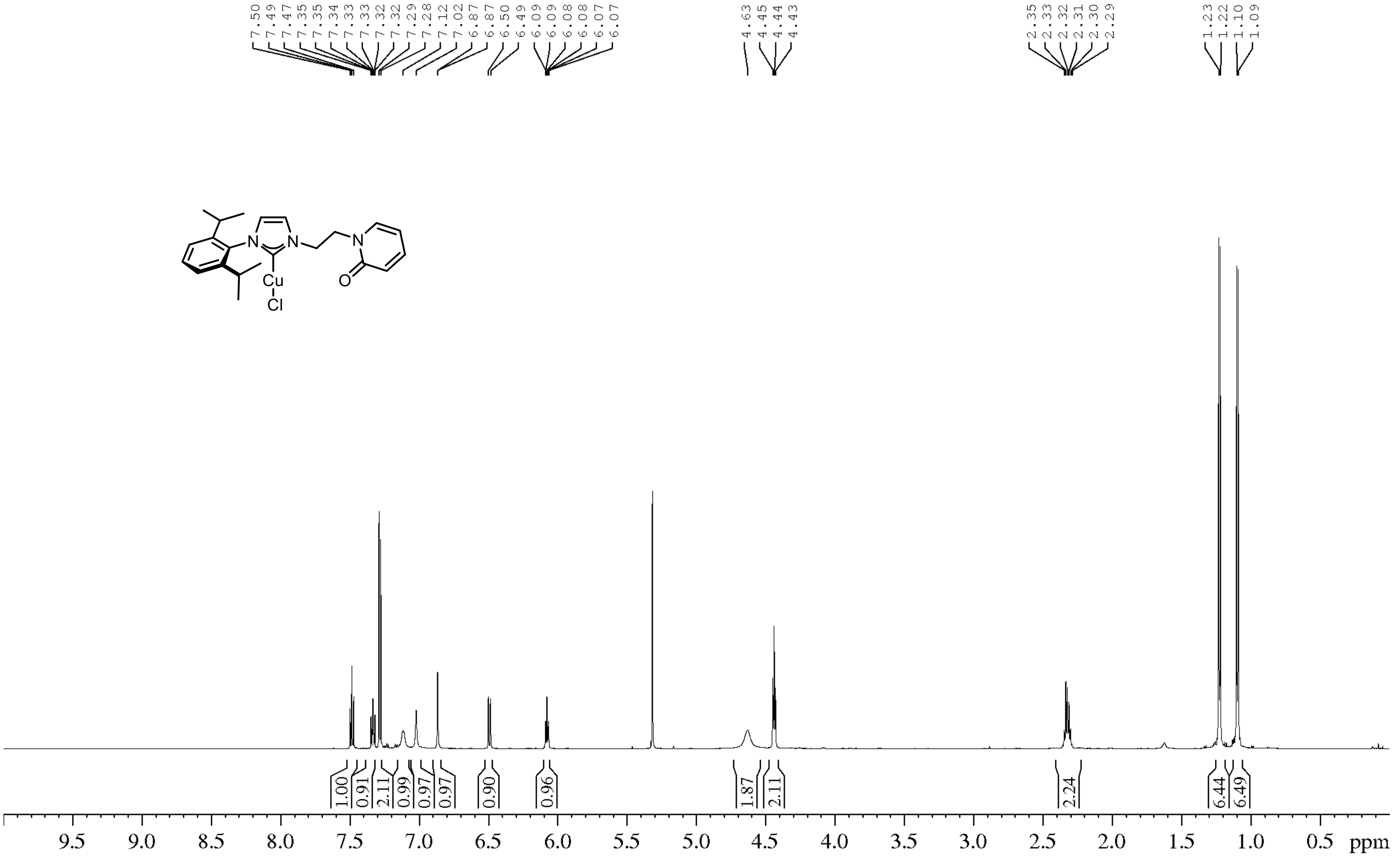

1.20.2 ^{13}C NMR

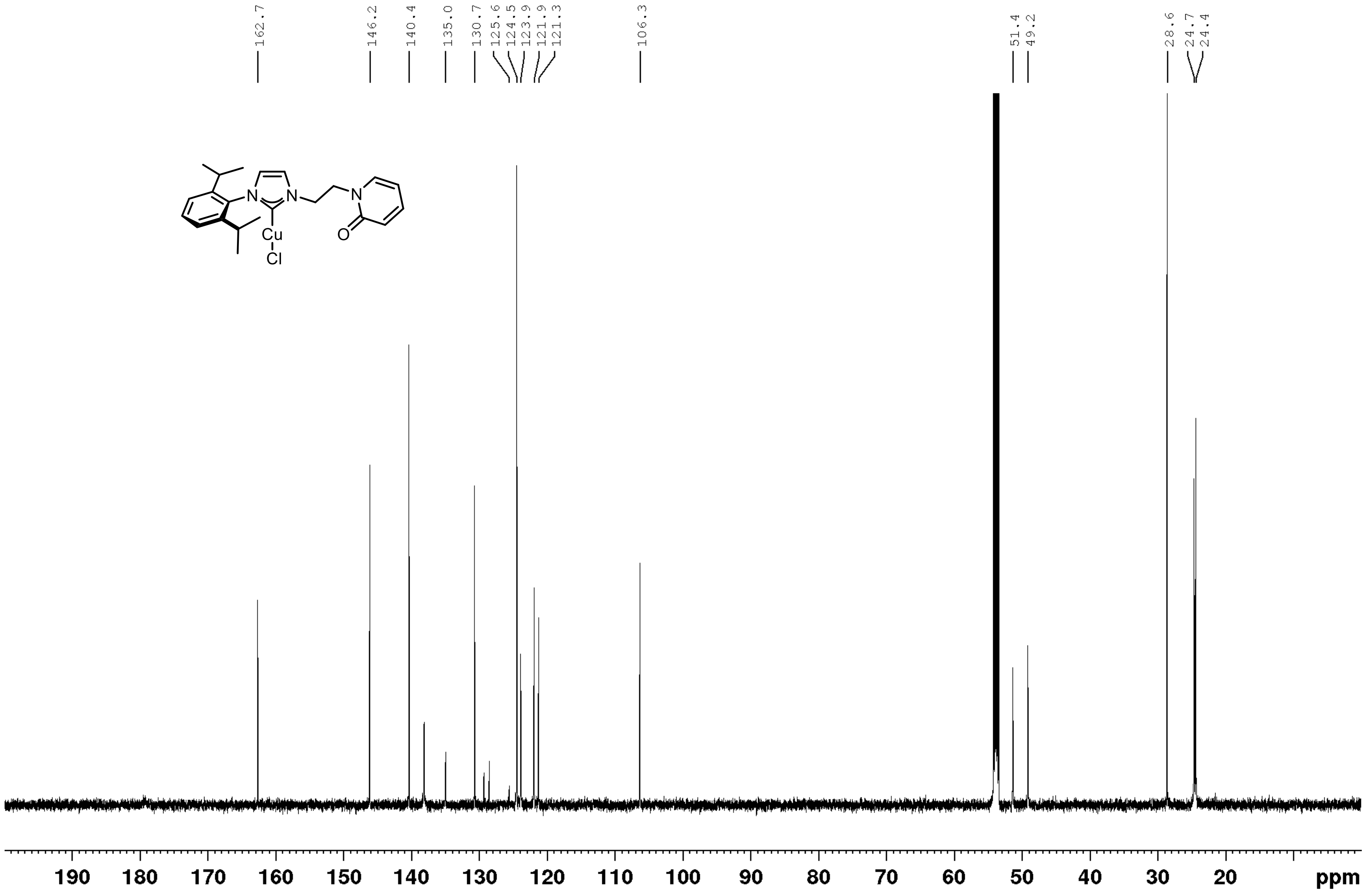

162.7
146.2
140.4
135.0
130.7
125.6
124.5
123.9
121.9
121.3
106.3
51.4
49.2
28.6
24.7
24.4
N
N
N
Cu
Cl
O
190
180
170
160
150
140
130
120
110
100
90
80
70
60
50
40
30
20
ppm

1.20.3 ^{1}H, ^{1}H COSY

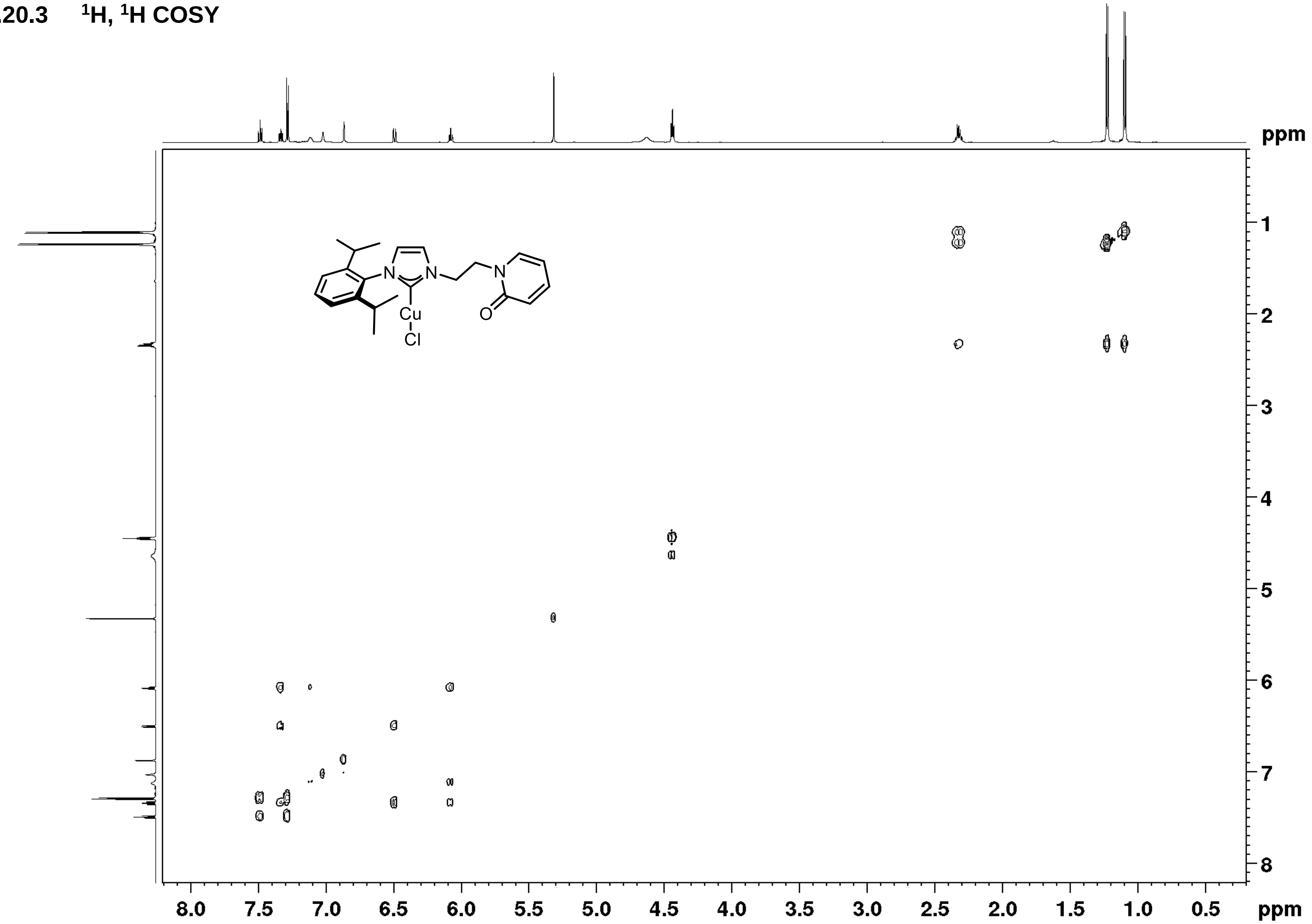

1.20.4 ^{1}H, ^{13}C HMBC

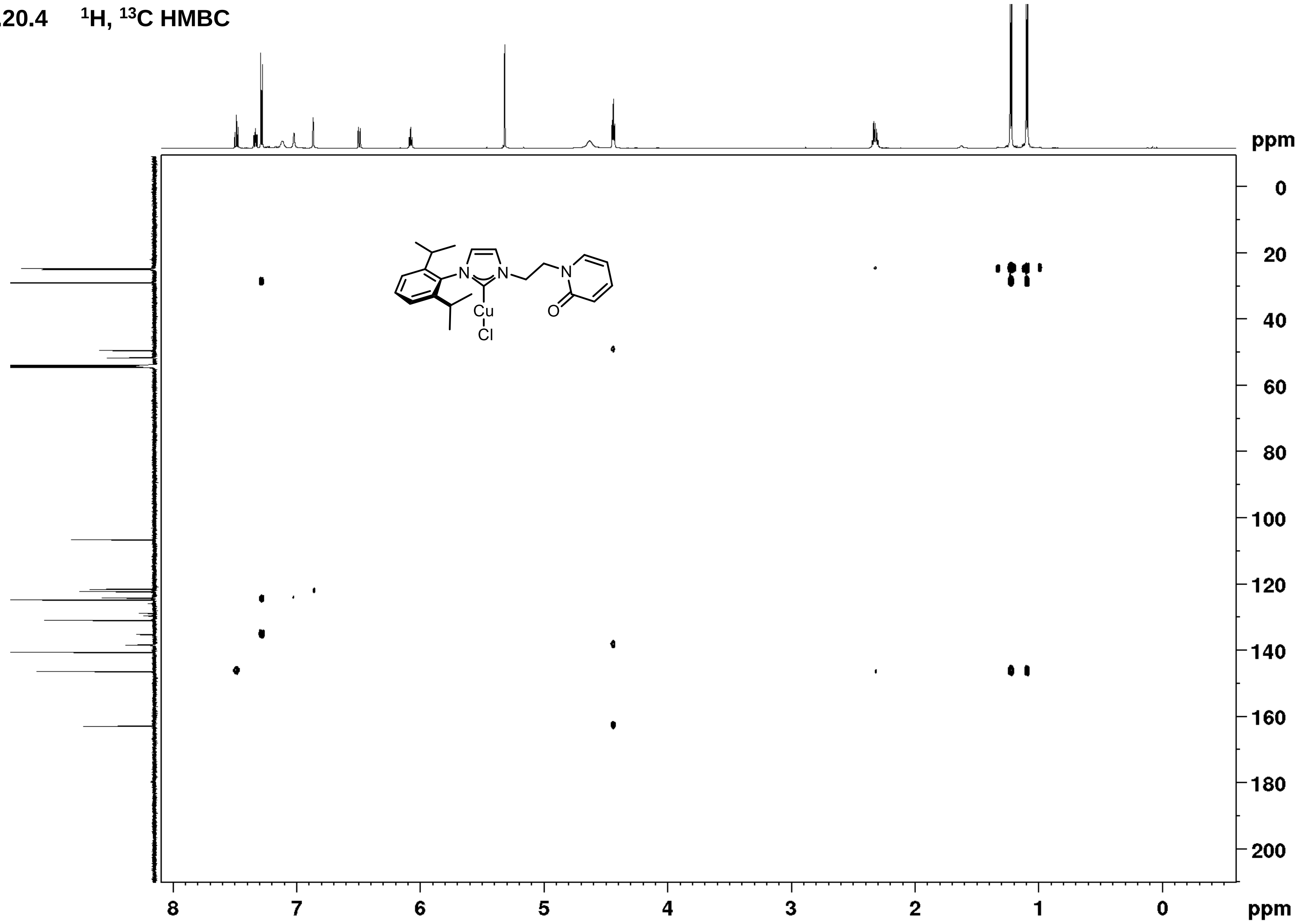

1.20.5 ^{1}H, ^{13}C HSQC

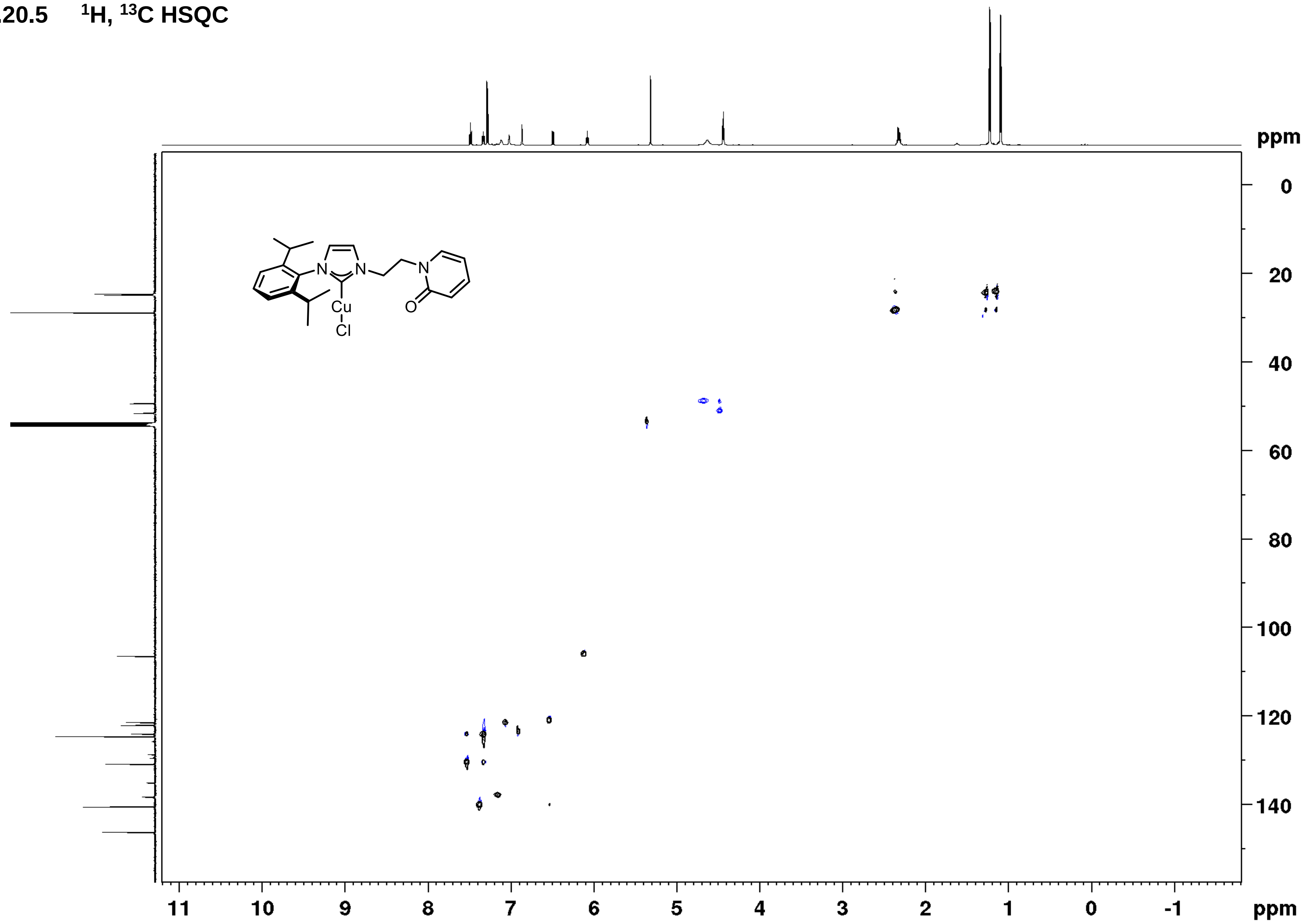

1.20.6 HRMS

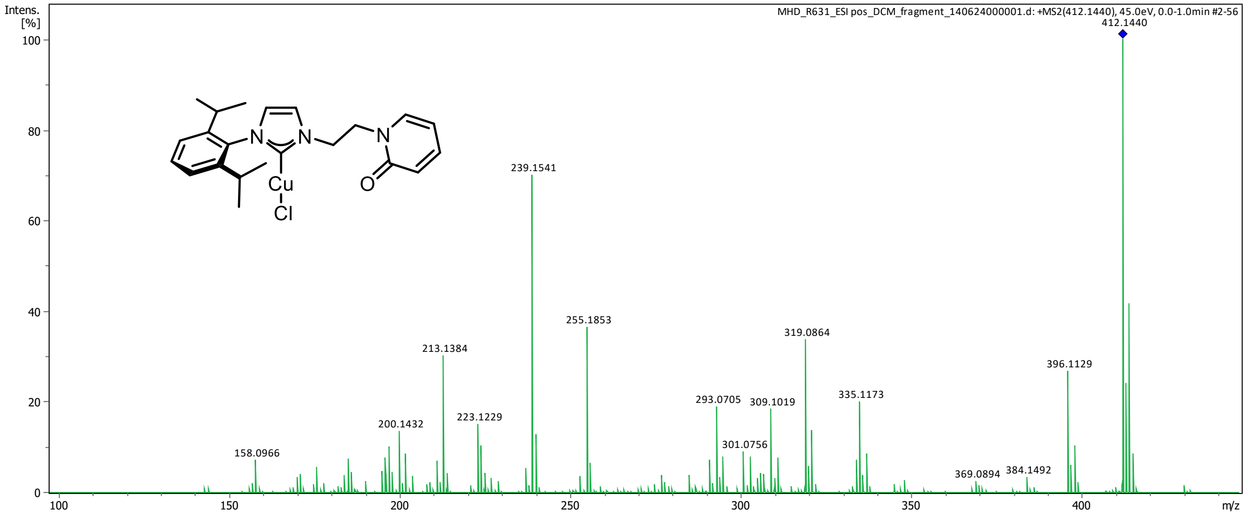

Intens. [%]
MHD_R631_ESI pos_DCM_fragment_140624000001.d: +MS2(412.1440), 45.0eV, 0.0-1.0min #2-56
412.1440
396.1129
369.0894
384.1492
335.1173
319.0864
309.1019
301.0756
293.0705
255.1853
239.1541
223.1229
213.1384
200.1432
158.0966
N
Cu
Cl
O
m/z

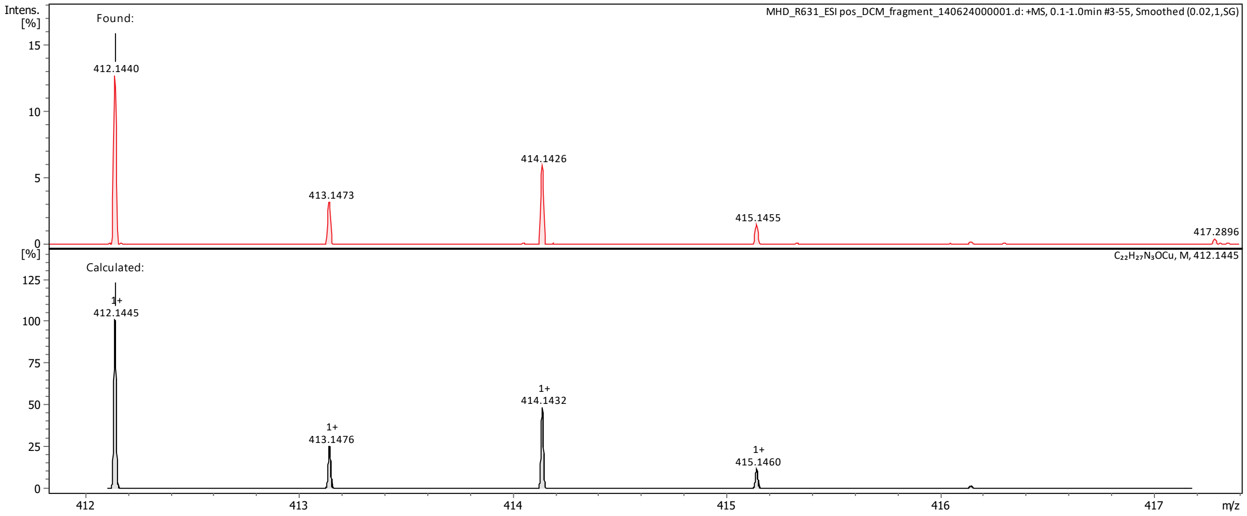

Intens. [%]
Found:
MHD_R631_ESI pos_DCM_fragment_140624000001.d: +MS, 0.1-1.0min #3-55, Smoothed (0.02,1,SG)
412.1440
413.1473
414.1426
415.1455
417.2896
Calculated:
C22H27N3OCu, M, 412.1445
1+ 412.1445
1+ 413.1476
1+ 414.1432
1+ 415.1460
m/z

1.21 4-Amino-1-(2-bromoethyl)pyridin-1-ium bromide (10b) (600 MHz, DMSO-d_6)

1.21.1 ^{1}H NMR

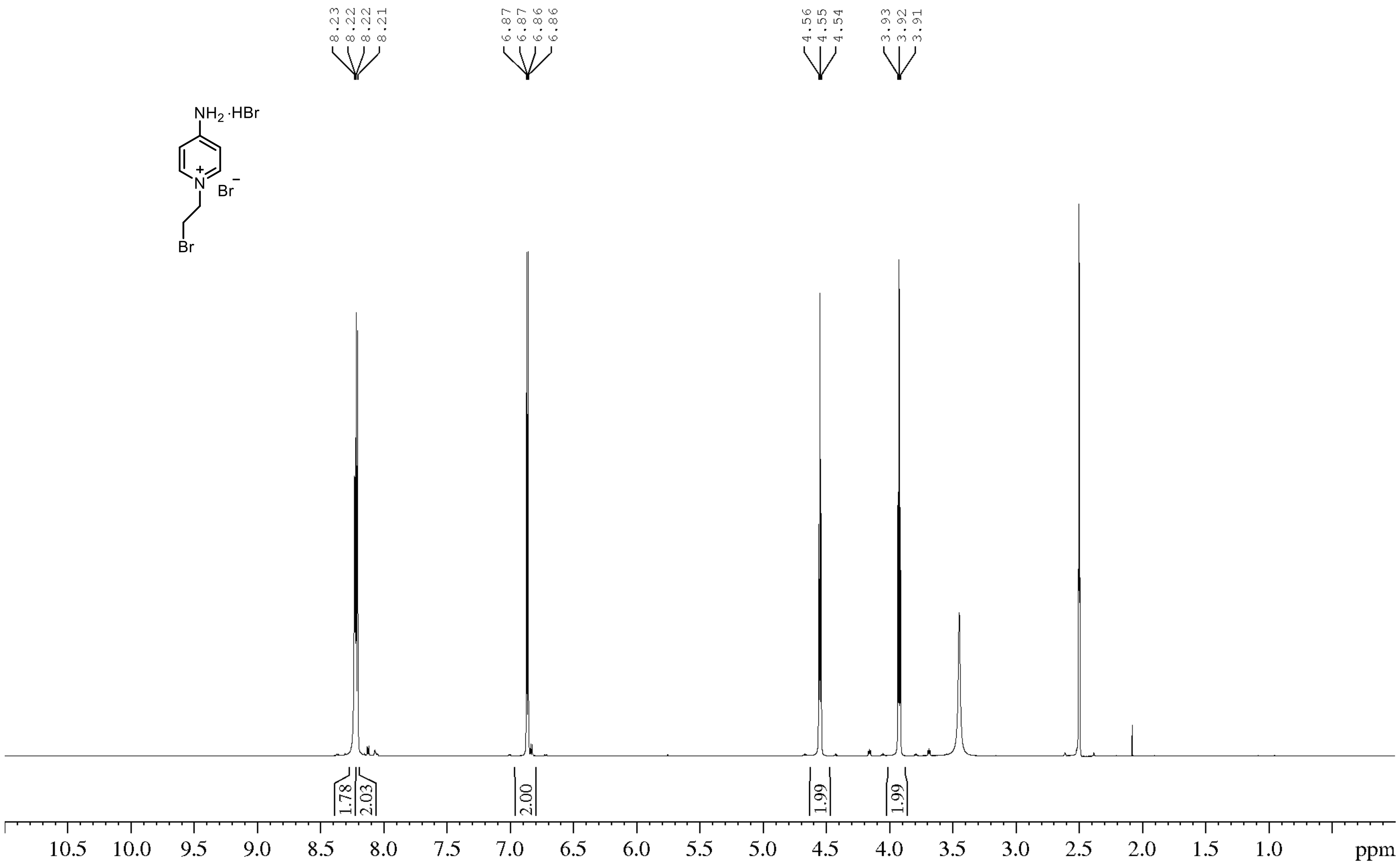

1.21.2 ^{13}C NMR

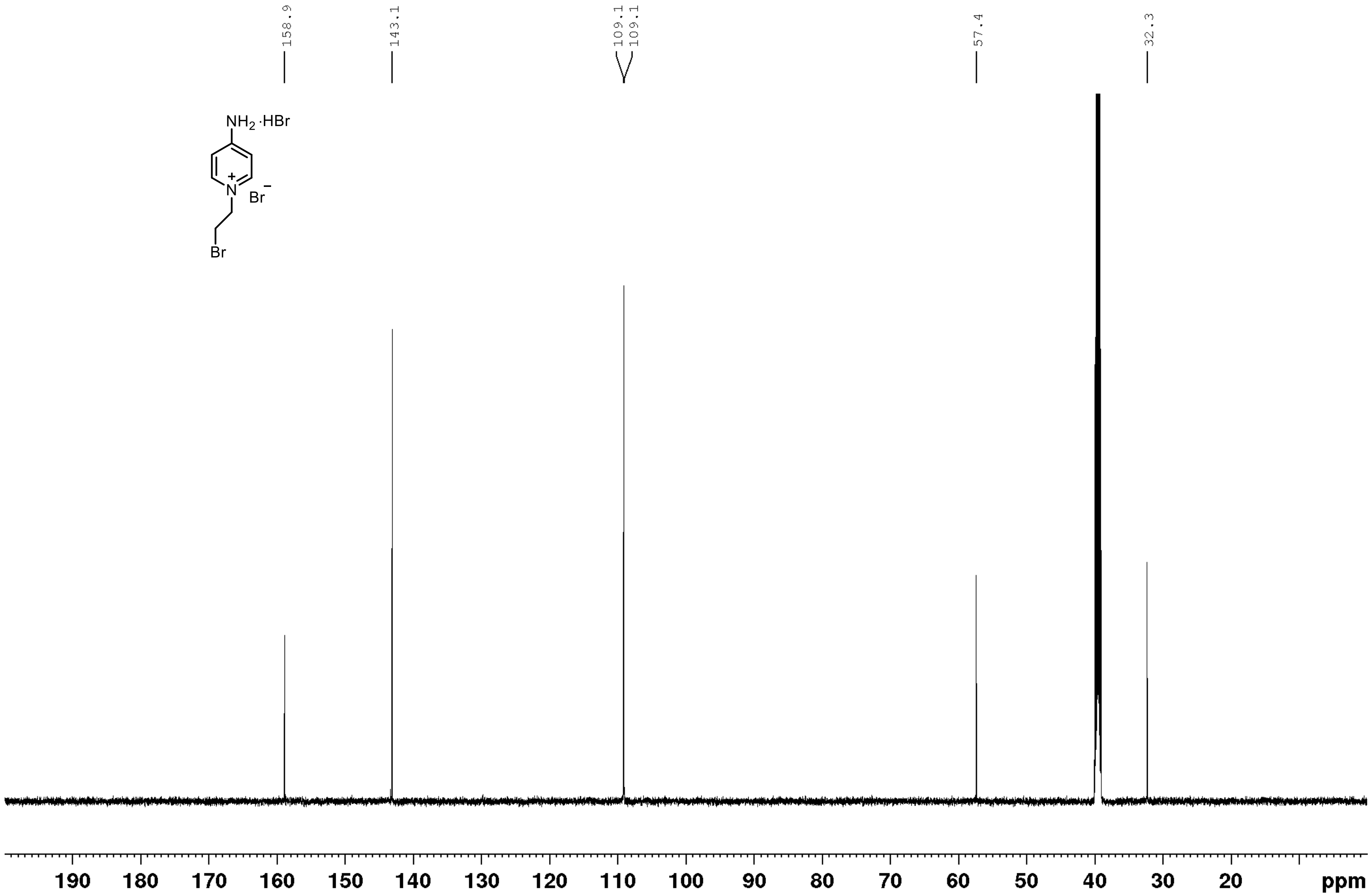
158.9
143.1
109.1
109.1
57.4
32.3
NH2·HBr
N
Br
Br
190
180
170
160
150
140
130
120
110
100
90
80
70
60
50
40
30
20
ppm

1.21.3 ^{1}H, ^{1}H COSY

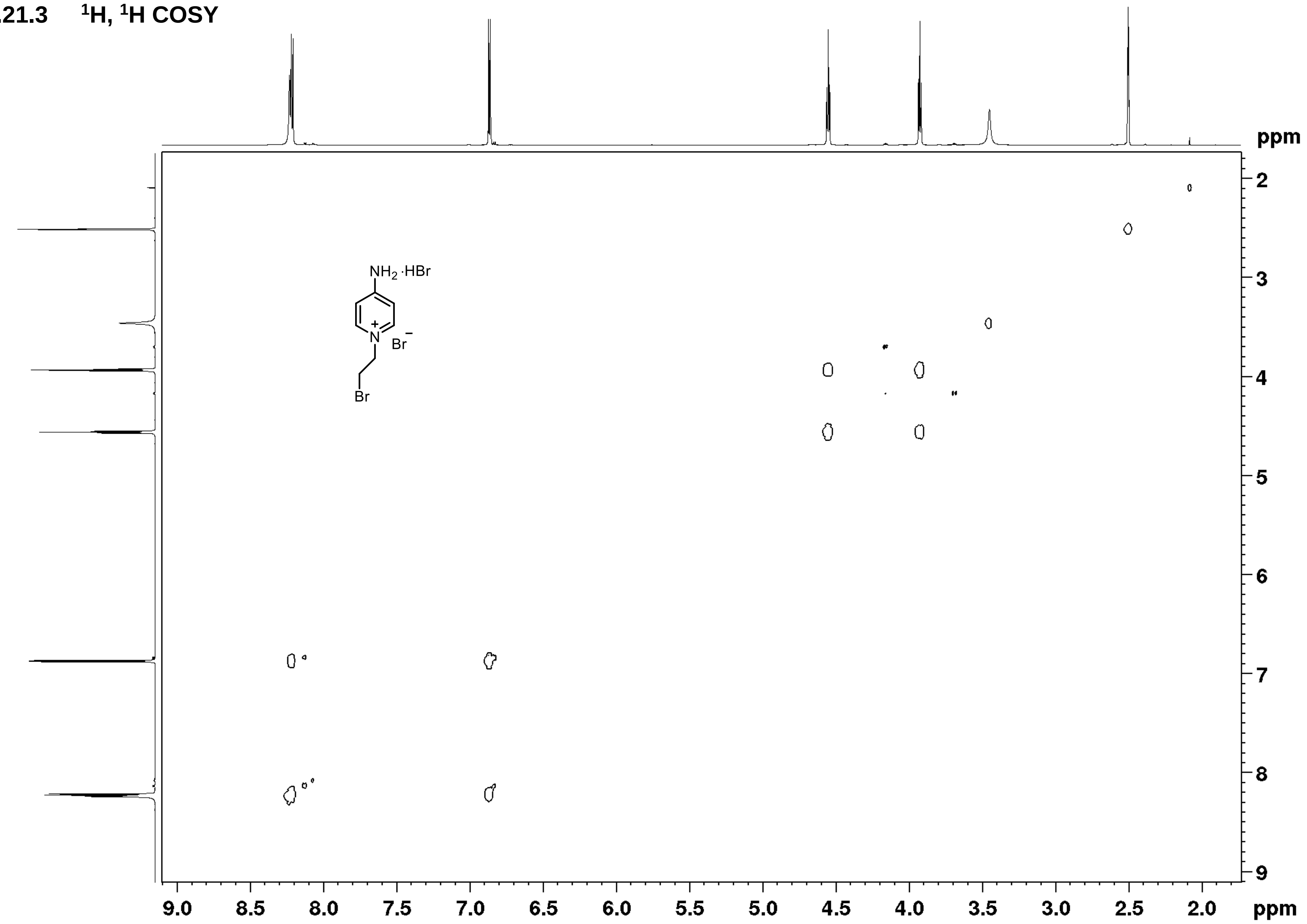

1.21.4 ^{1}H, ^{13}C HMBC

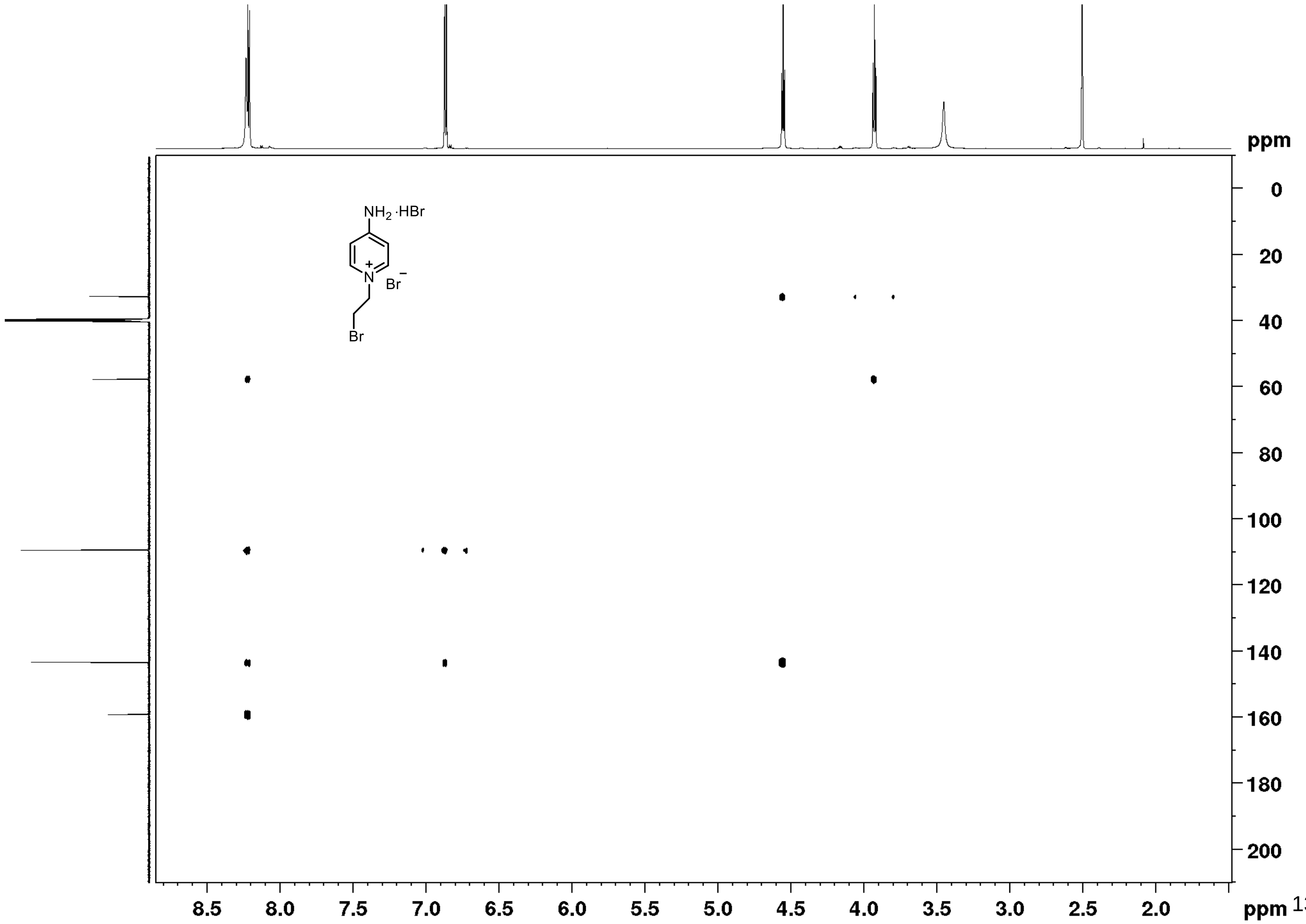

1.21.5 ^{1}H, ^{13}C HSQC

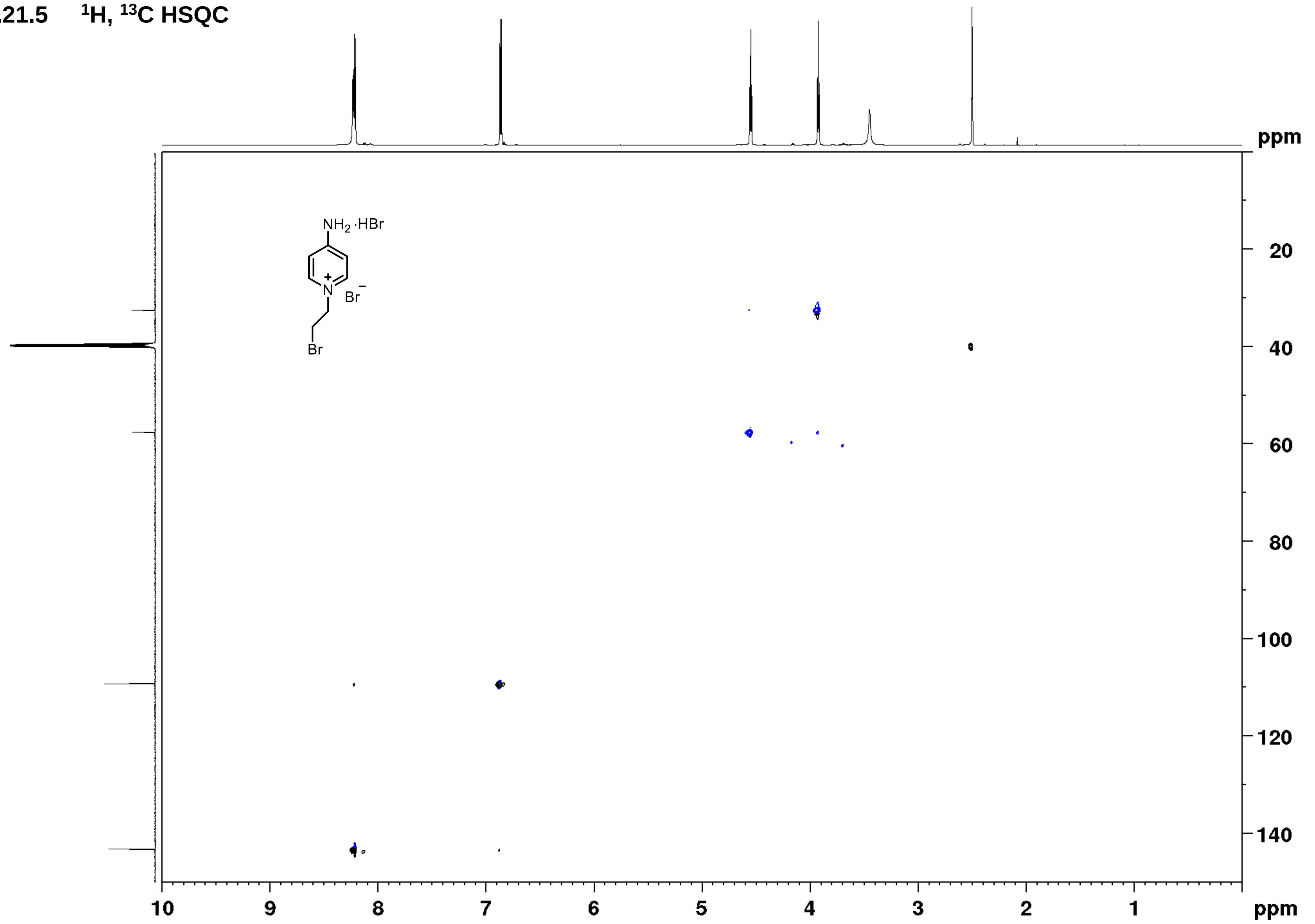

1.21.6 HRMS

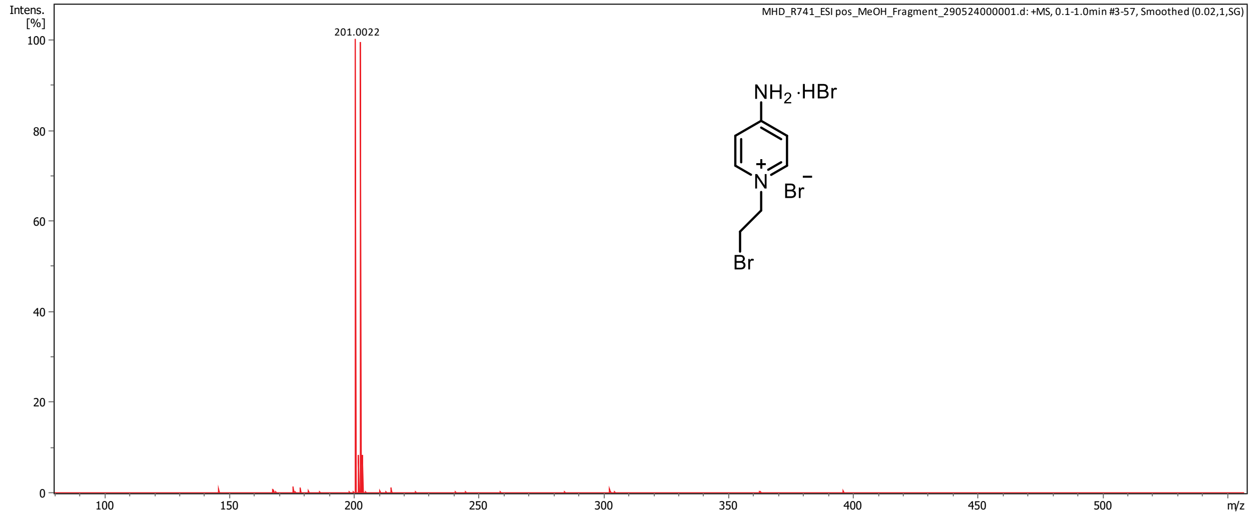
MHD_R741_ESI pos_MeOH_Fragment_290524000001.d: +MS, 0.1-1.0min #3-57, Smoothed (0.02,1,SG)
Intens. [%]
201.0022
NH2 ·HBr
N
+
Br
−
Br
m/z

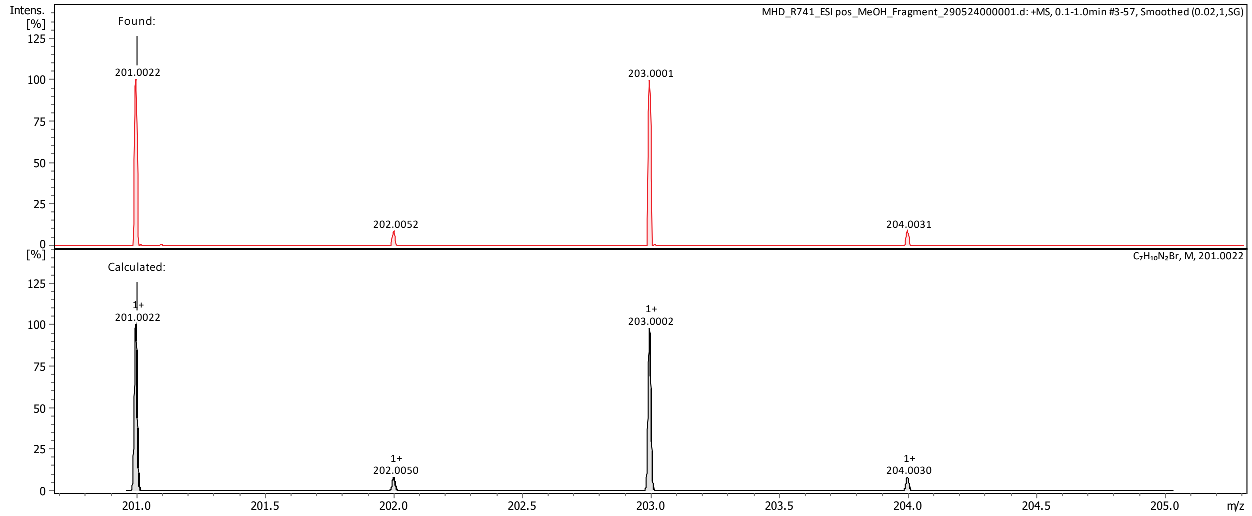
MHD_R741_ESI pos_MeOH_Fragment_290524000001.d: +MS, 0.1-1.0min #3-57, Smoothed (0.02,1,SG)
Intens. [%]
Found:
201.0022
202.0052
203.0001
204.0031
C7H10N2Br, M, 201.0022
Calculated:
1+ 201.0022
1+ 202.0050
1+ 203.0002
1+ 204.0030
m/z

1.21.7 IR

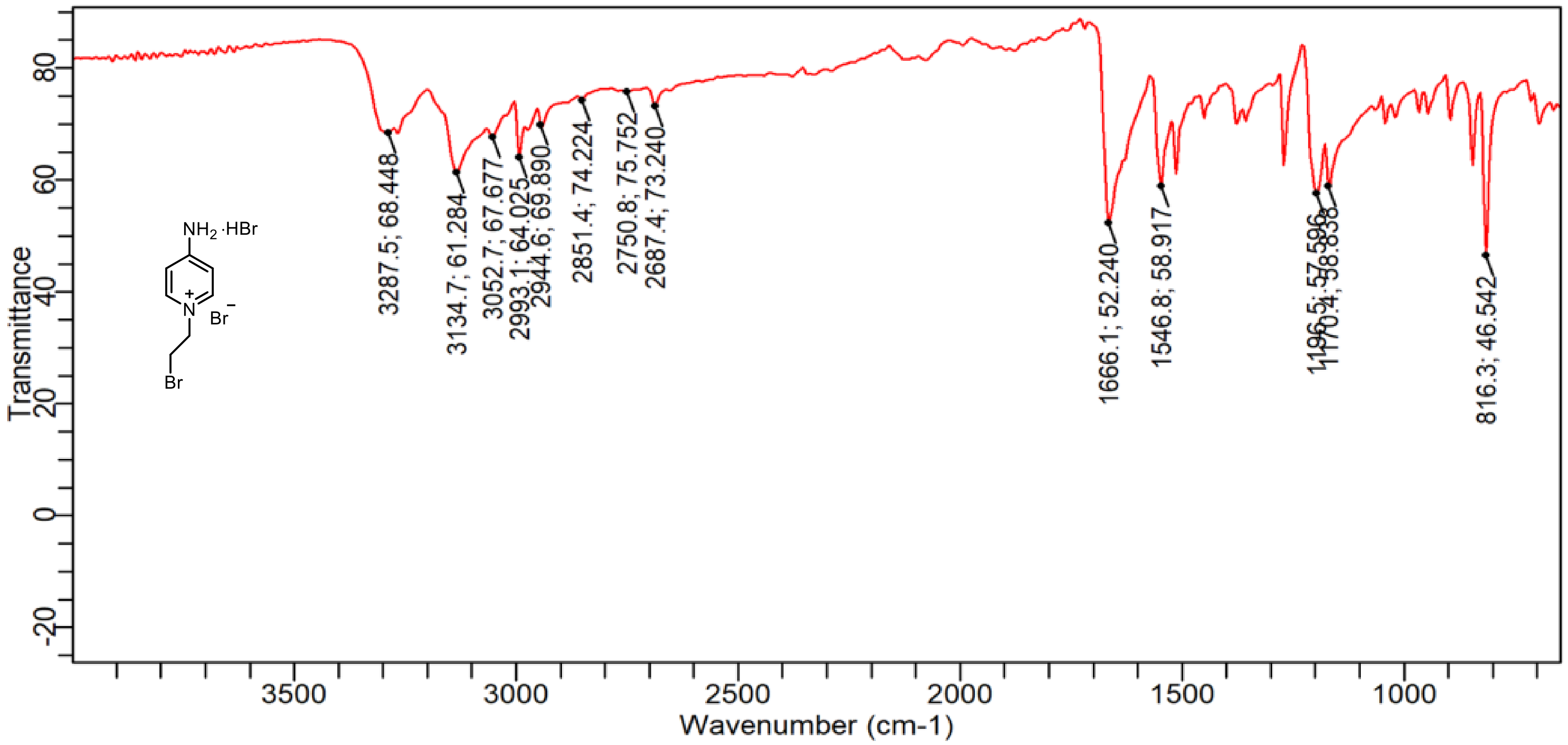

3287.5; 68.448
3134.7; 61.284
3052.7; 67.677
2993.1; 64.025
2944.6; 69.890
2851.4; 74.224
2750.8; 75.752
2687.4; 73.240
1666.1; 52.240
1546.8; 58.917
1196.5; 57.596
1170.4; 58.858
816.3; 46.542
Transmittance
Wavenumber (cm-1)

1.22 1-(2,6-Diisopropylphenyl)-3-(2-(4-iminopyridin-1(4*H*)-yl)ethyl)-1*H*-imidazol-3-ium bromide hydrobromide (10) (600 MHz, DMSO-d_6)

1.22.1 ^{1}H NMR

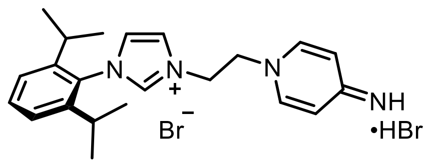

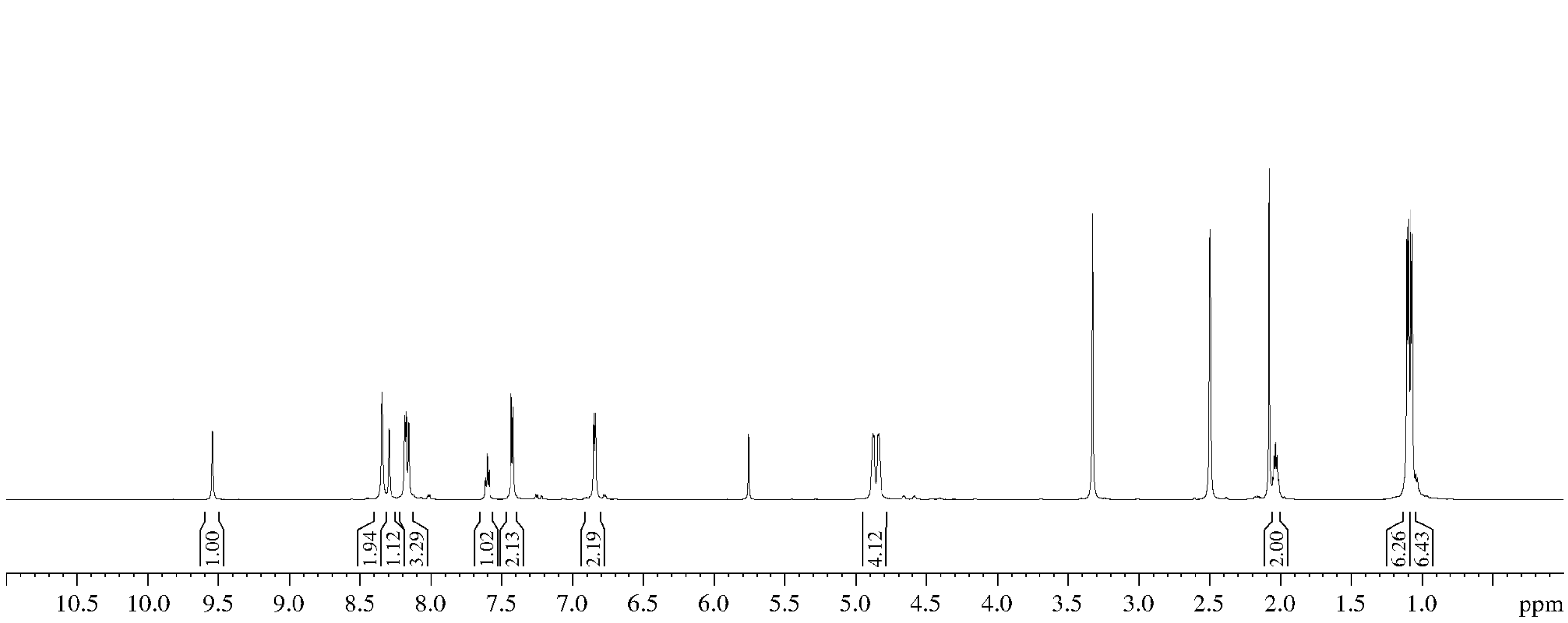

1.22.2 ^{13}C NMR

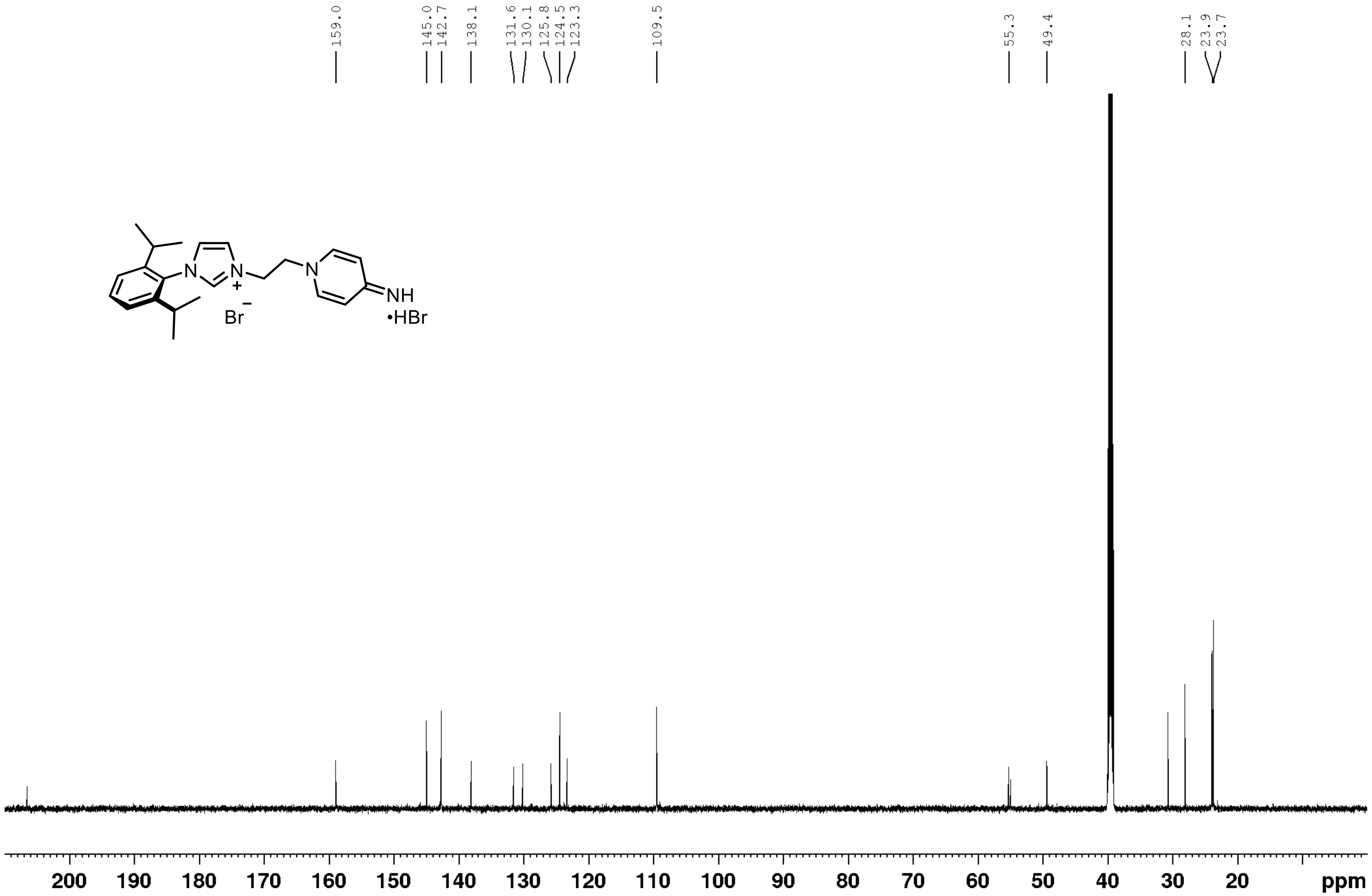
159.0
145.0
142.7
138.1
131.6
130.1
125.8
124.5
123.3
109.5
55.3
49.4
28.1
23.9
23.7
N
N
+
Br−
NH
•HBr
200 190 180 170 160 150 140 130 120 110 100 90 80 70 60 50 40 30 20 ppm

1.22.3 ^{1}H, ^{1}H COSY

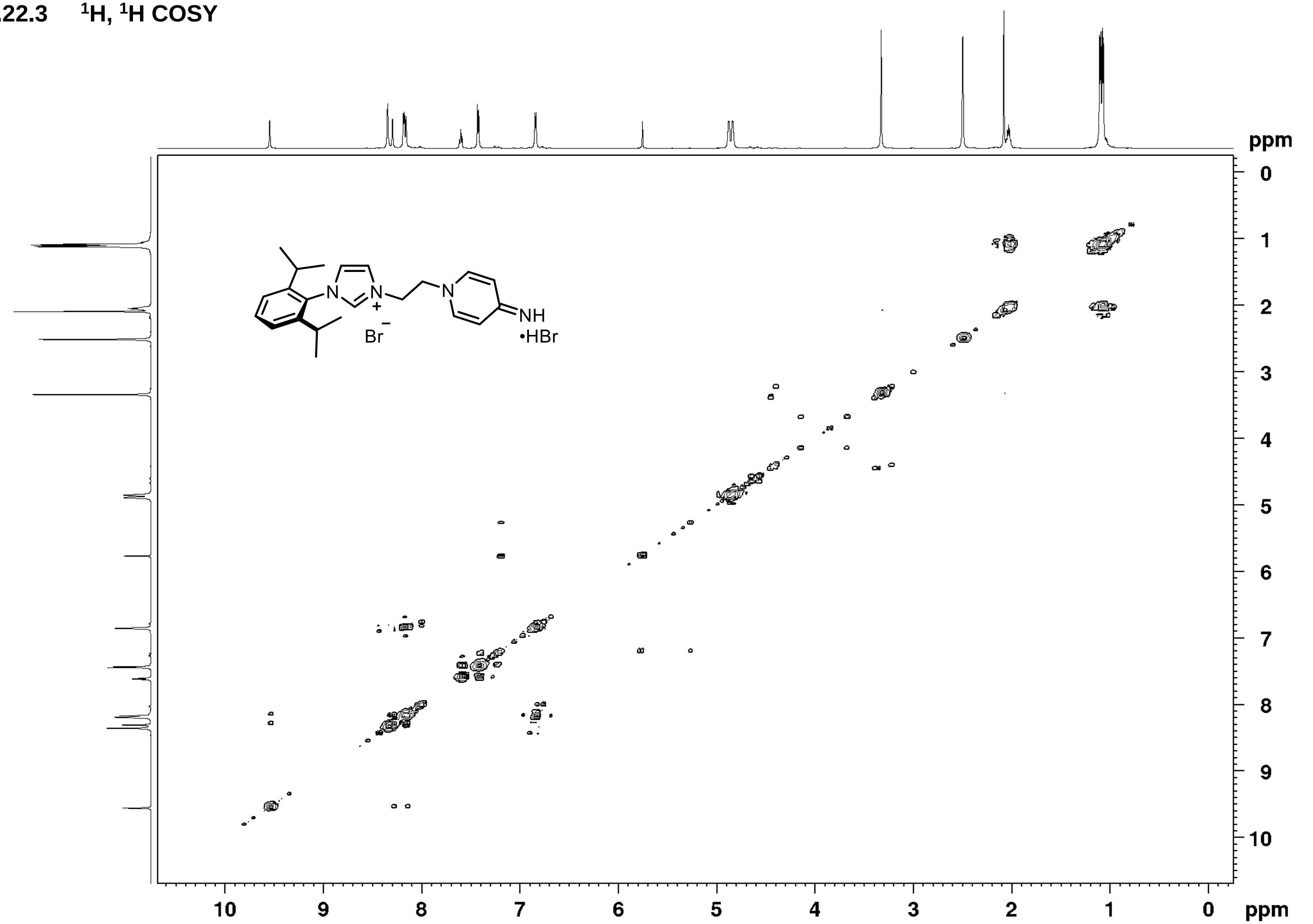

1.22.4 ^{1}H, ^{13}C HMBC

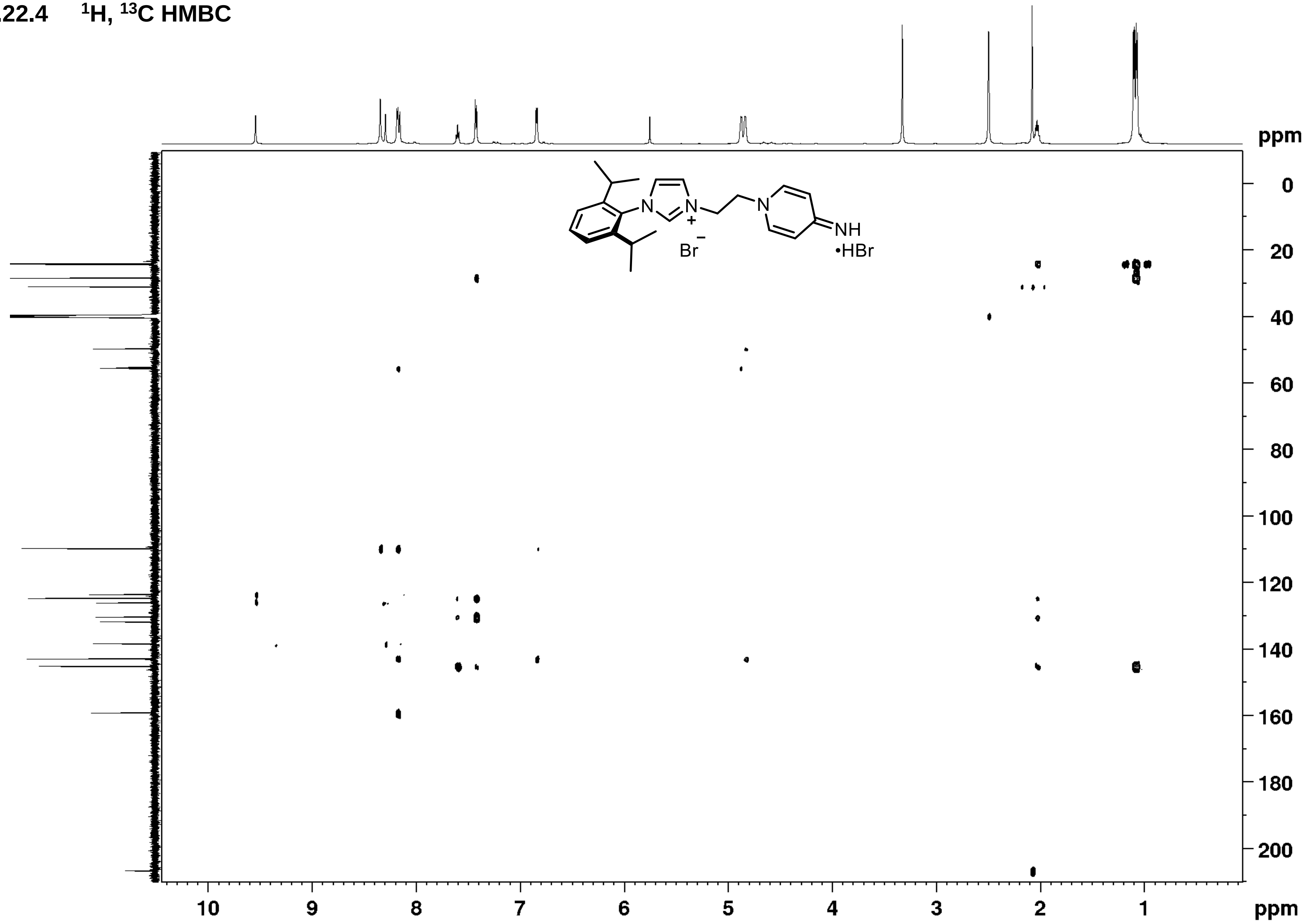

1.22.5 ^{1}H, ^{13}C HSQC

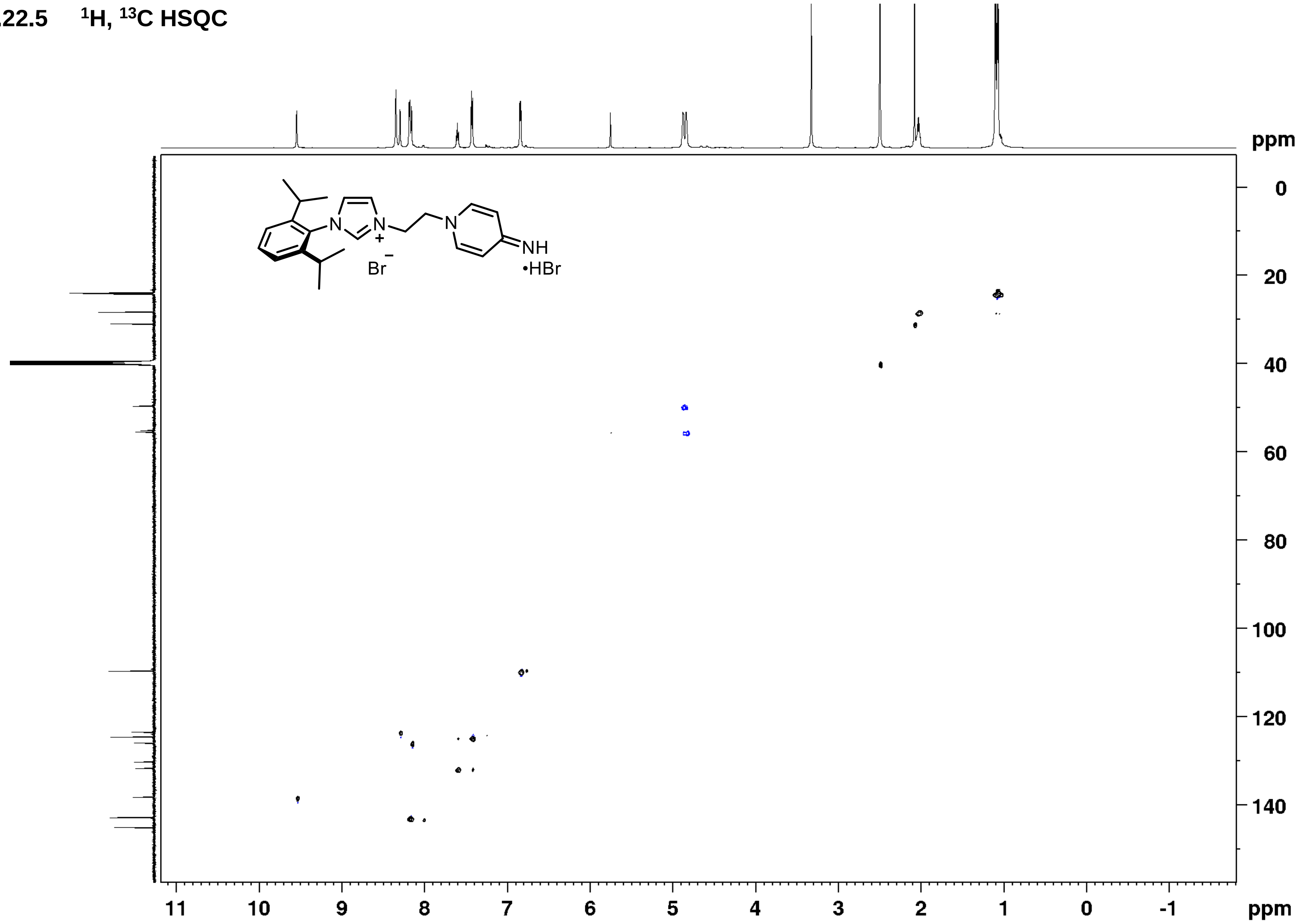

1.22.6 HRMS

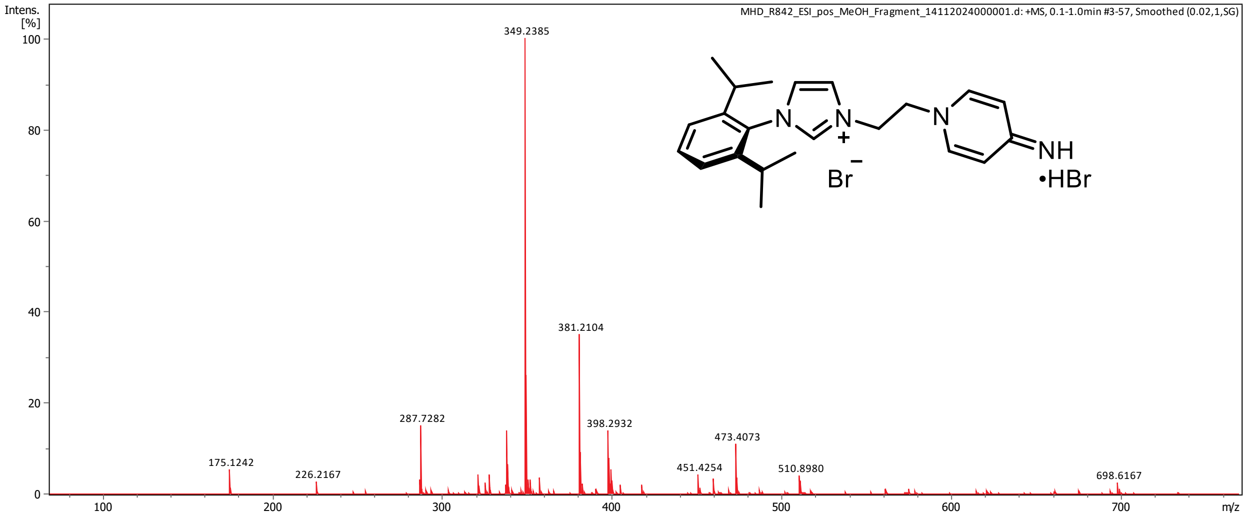
MHD_R842_ESI_pos_MeOH_Fragment_14112024000001.d: +MS, 0.1-1.0min #3-57, Smoothed (0.02,1,SG)
Intens. [%]
349.2385
381.2104
287.7282
398.2932
473.4073
175.1242
226.2167
451.4254
510.8980
698.6167
m/z
NH
•HBr
Br

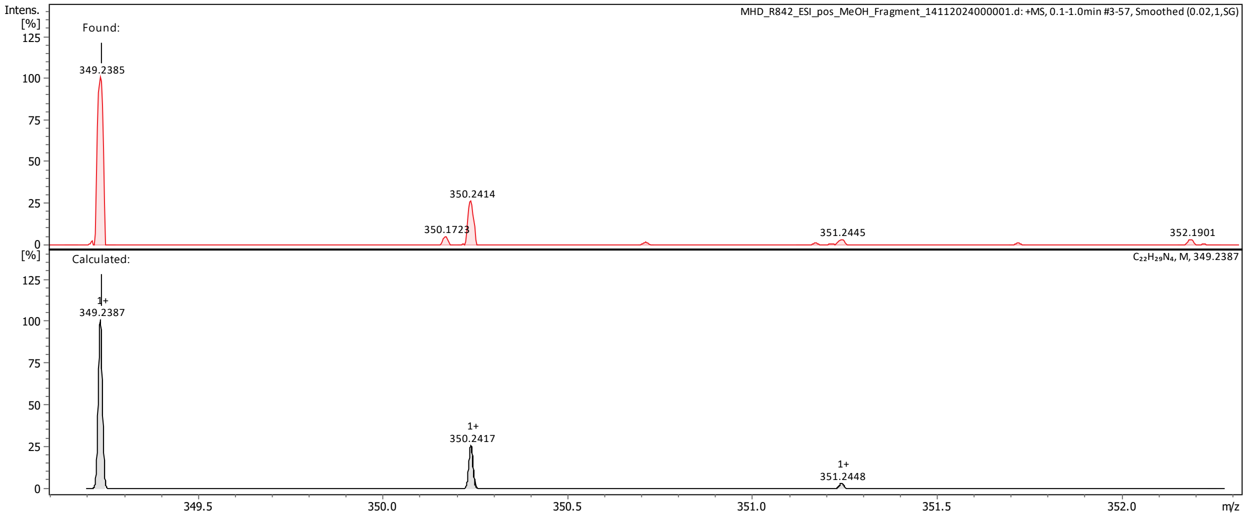
MHD_R842_ESI_pos_MeOH_Fragment_14112024000001.d: +MS, 0.1-1.0min #3-57, Smoothed (0.02,1,SG)
Found:
349.2385
350.2414
350.1723
351.2445
352.1901
Calculated:
C22H29N4, M, 349.2387
1+ 349.2387
1+ 350.2417
1+ 351.2448
m/z

1.22.7 IR

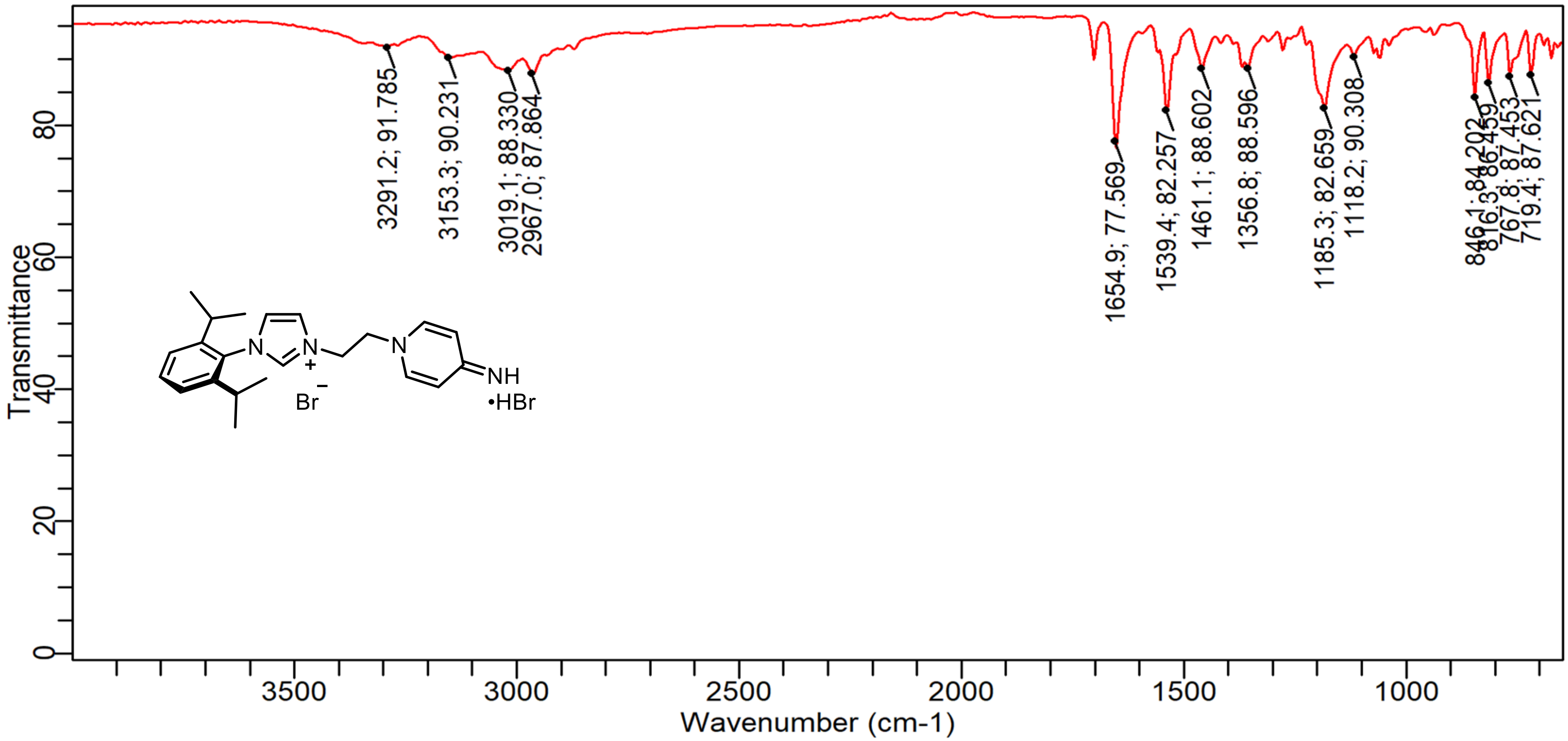
3291.2; 91.785
3153.3; 90.231
3019.1; 88.330
2967.0; 87.864
1654.9; 77.569
1539.4; 82.257
1461.1; 88.602
1356.8; 88.596
1185.3; 82.659
1118.2; 90.308
846.1; 84.202
816.3; 86.459
767.8; 87.453
719.4; 87.621
Transmittance
Wavenumber (cm-1)
Br
•HBr
NH

1.23 3-Ethyl-1-mesityl-1*H*-imidazol-3-ium bromide (S15b) (600 MHz, DMSO-d_6)

1.23.1 ^{1}H NMR

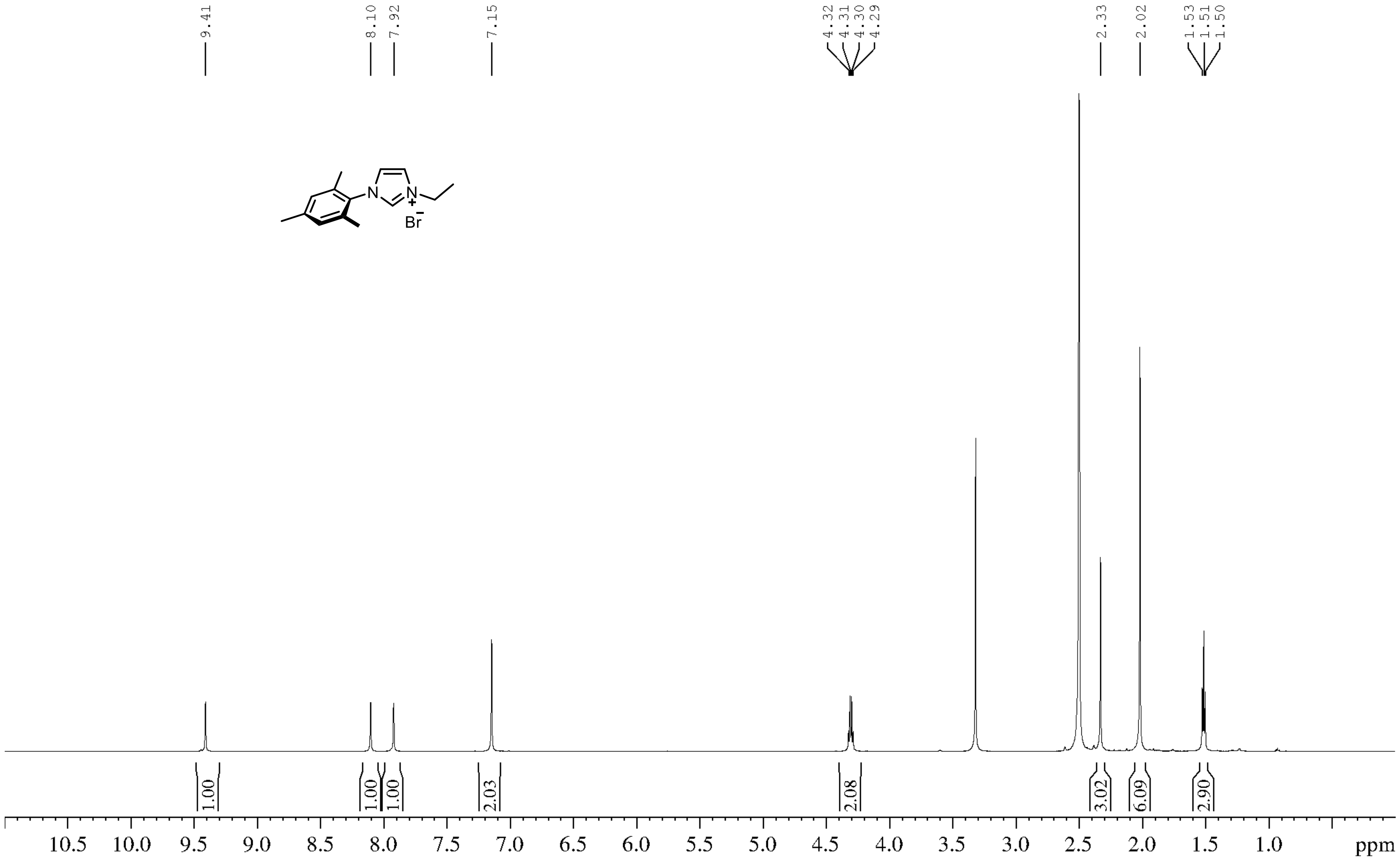

1.23.2 ^{13}C NMR

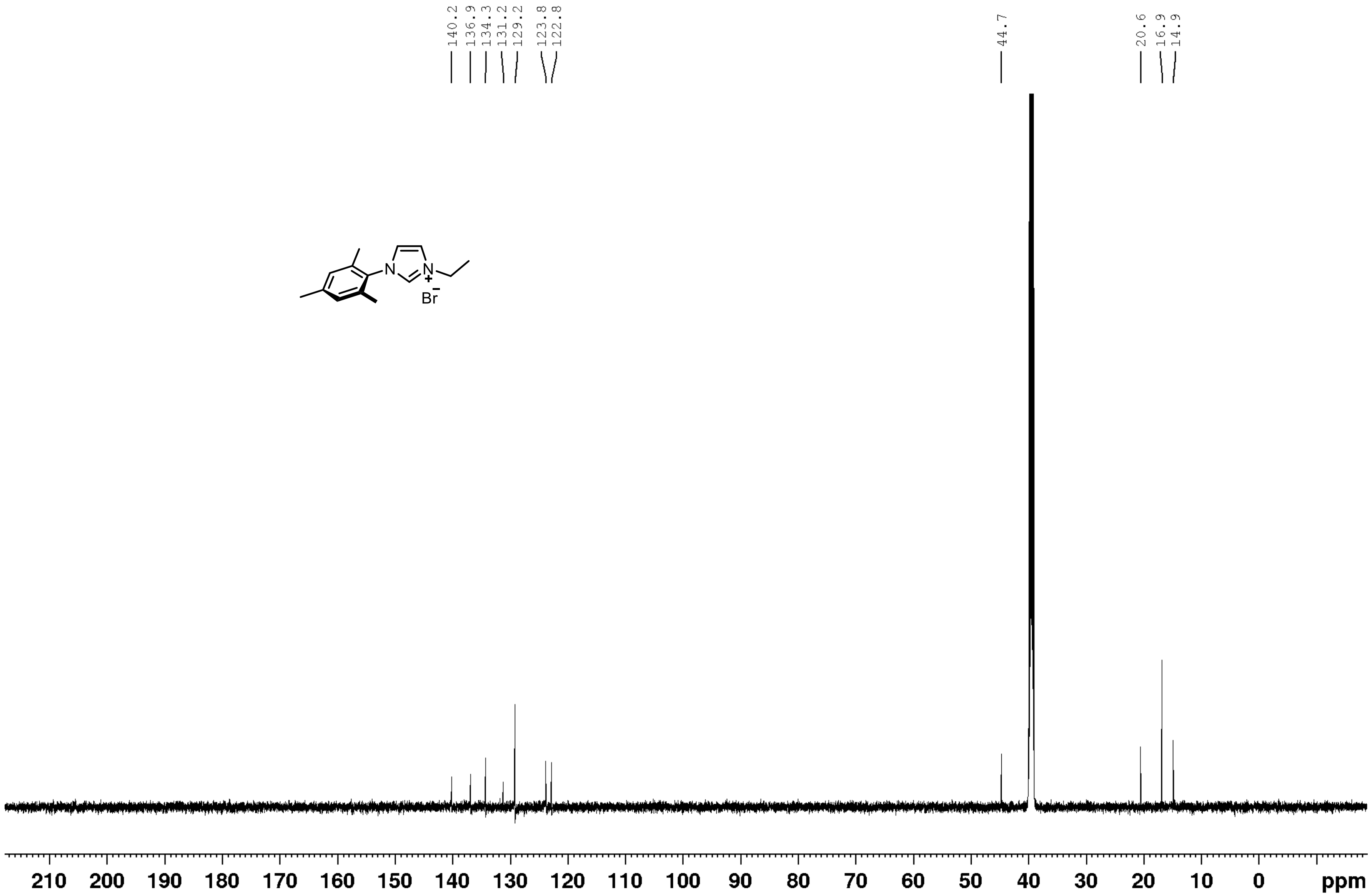

140.2
136.9
134.3
131.2
129.2
123.8
122.8
44.7
20.6
16.9
14.9
N
N
+
Br
210 200 190 180 170 160 150 140 130 120 110 100 90 80 70 60 50 40 30 20 10 0
ppm

1.23.3 ^{1}H, ^{1}H COSY

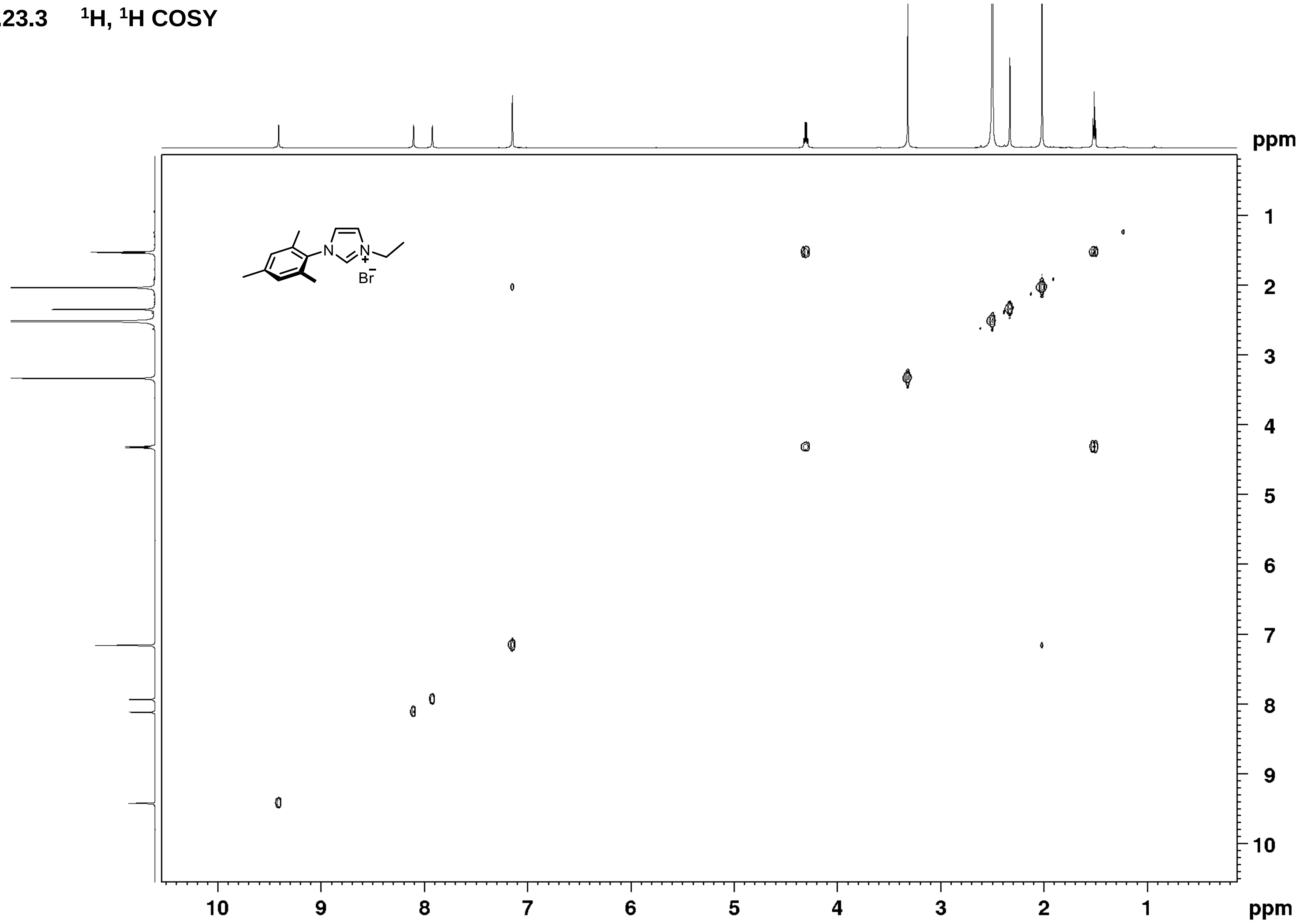

1.23.4 ^{1}H, ^{13}C HMBC

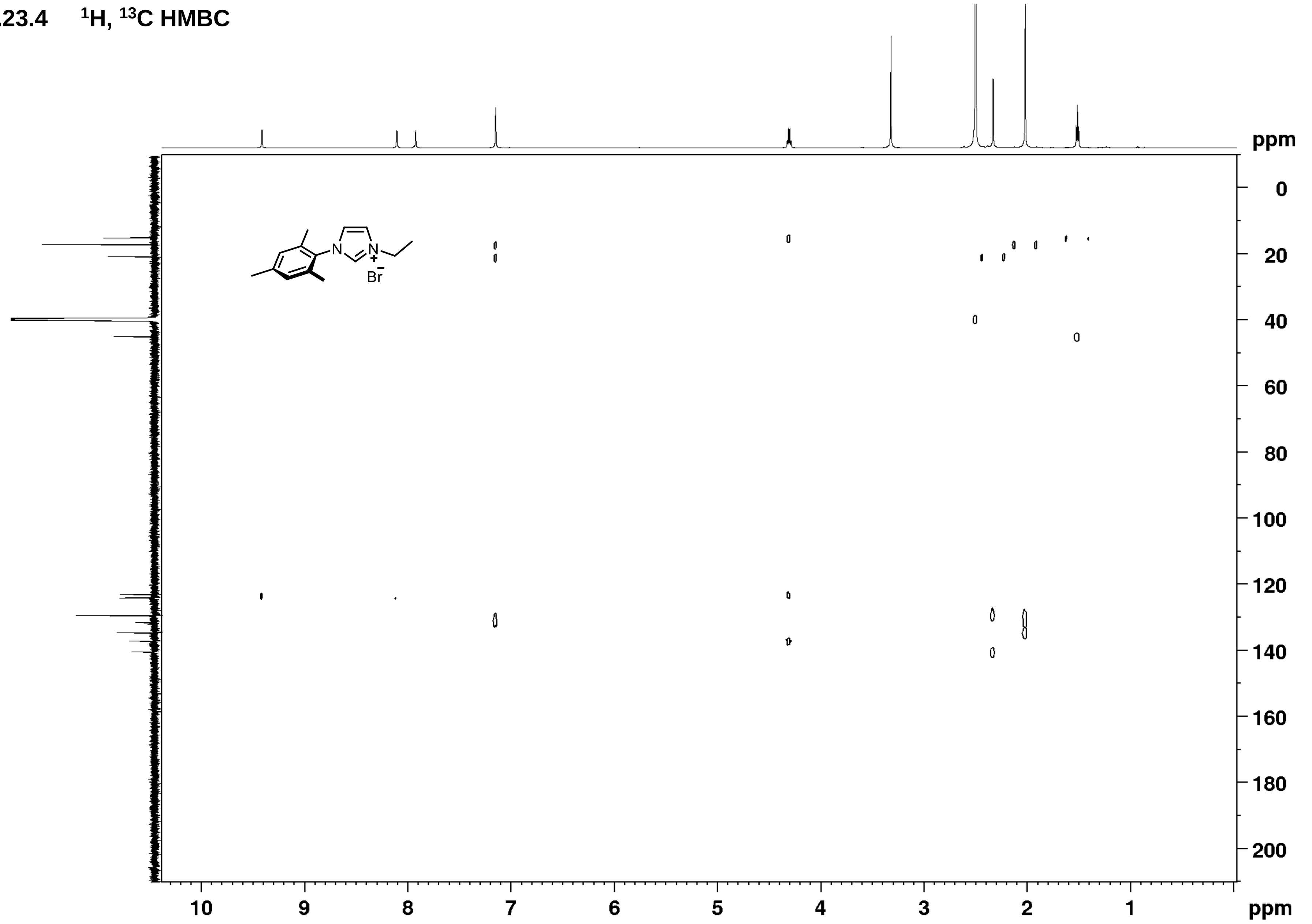

1.23.5 ¹H, ¹³C HSQC

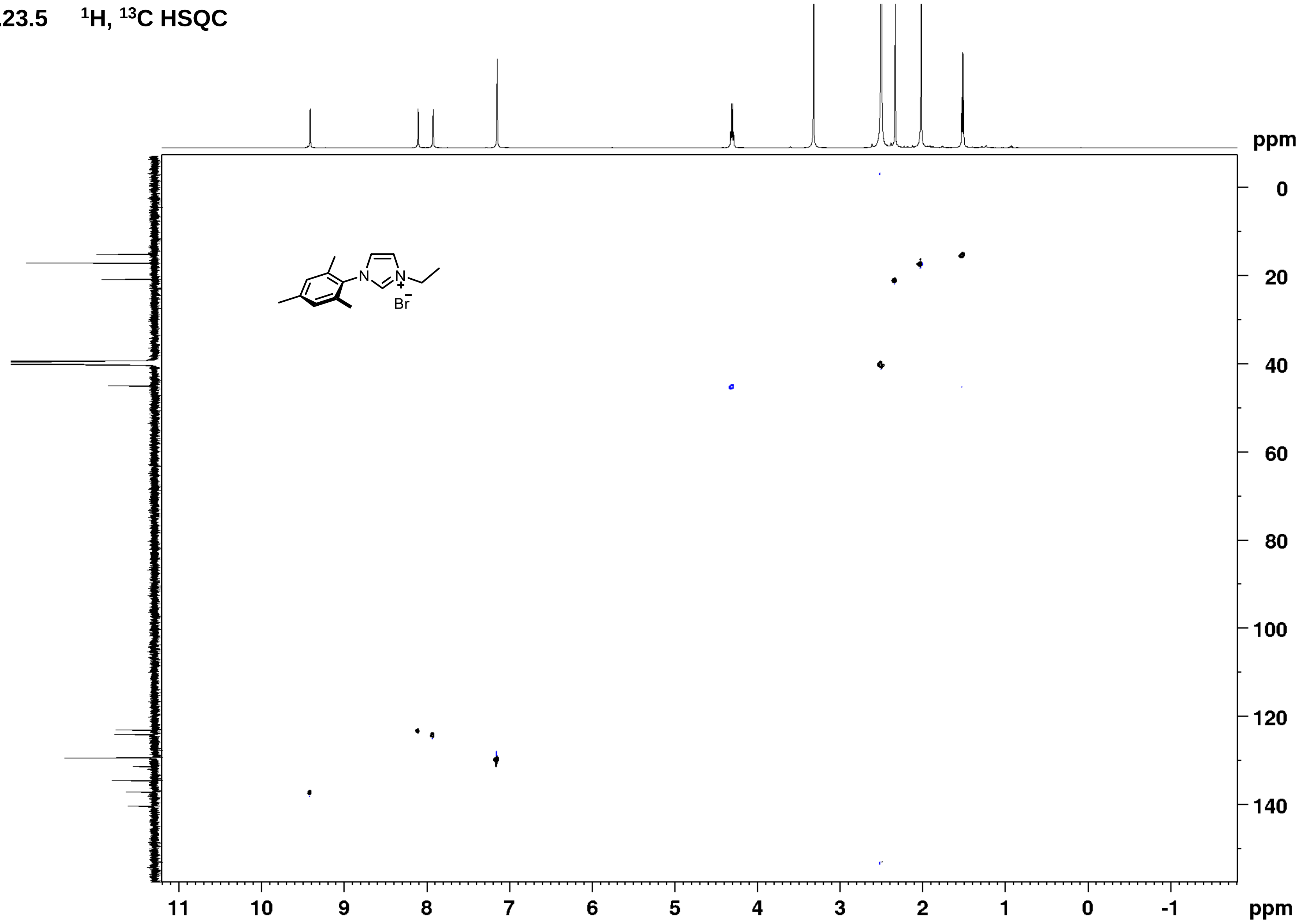

1.23.6 HRMS

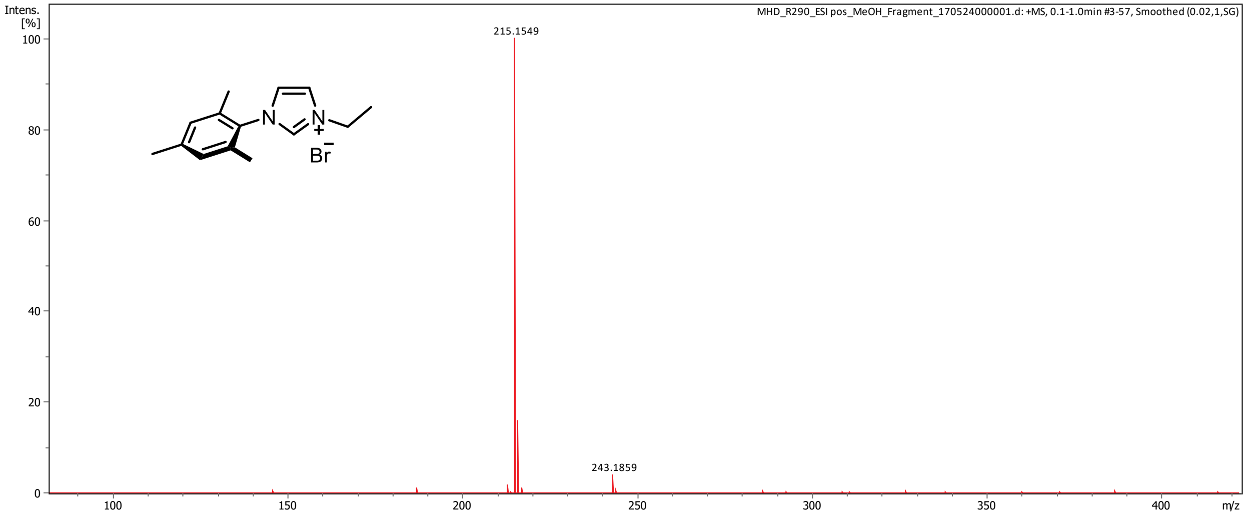

Intens. [%]
MHD_R290_ESI pos_MeOH_Fragment_170524000001.d: +MS, 0.1-1.0min #3-57, Smoothed (0.02,1,SG)
215.1549
243.1859
N
N
+
Br
m/z

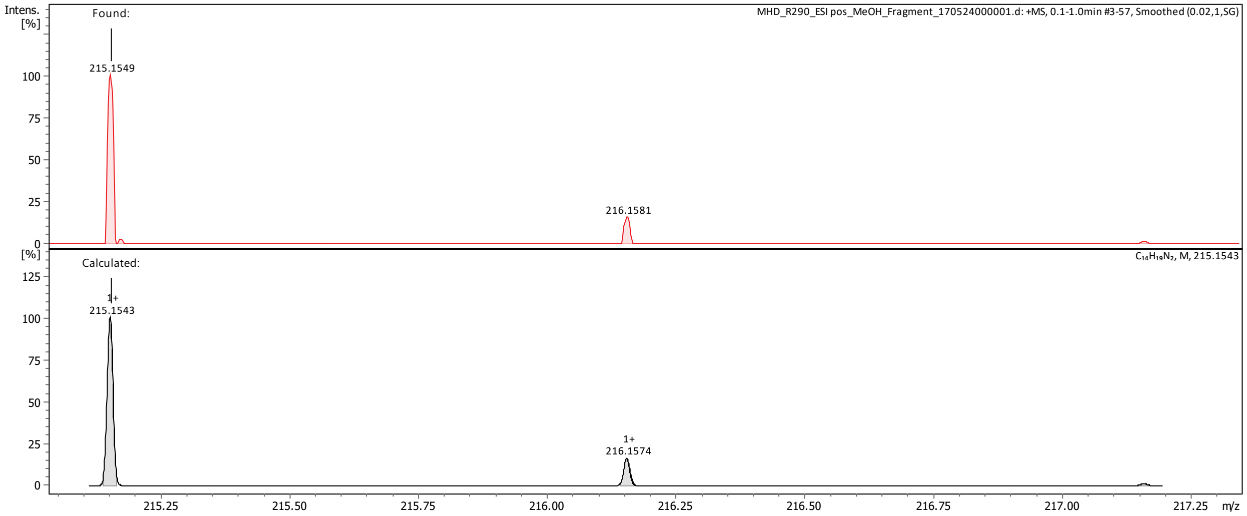

Intens. [%]
Found:
215.1549
216.1581
MHD_R290_ESI pos_MeOH_Fragment_170524000001.d: +MS, 0.1-1.0min #3-57, Smoothed (0.02,1,SG)
Calculated:
1+
215.1543
1+
216.1574
$C_{14}H_{19}N_2$, M, 215.1543
m/z

1.24 Bis(1-ethyl-3-mesityl-1,3-dihydro-2*H*-imidazol-2-ylidene)silver(I) bromide (S15c) (600 MHz, $CDCl_3$)

1.24.1 ^{1}H NMR

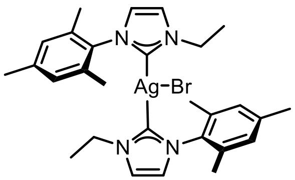

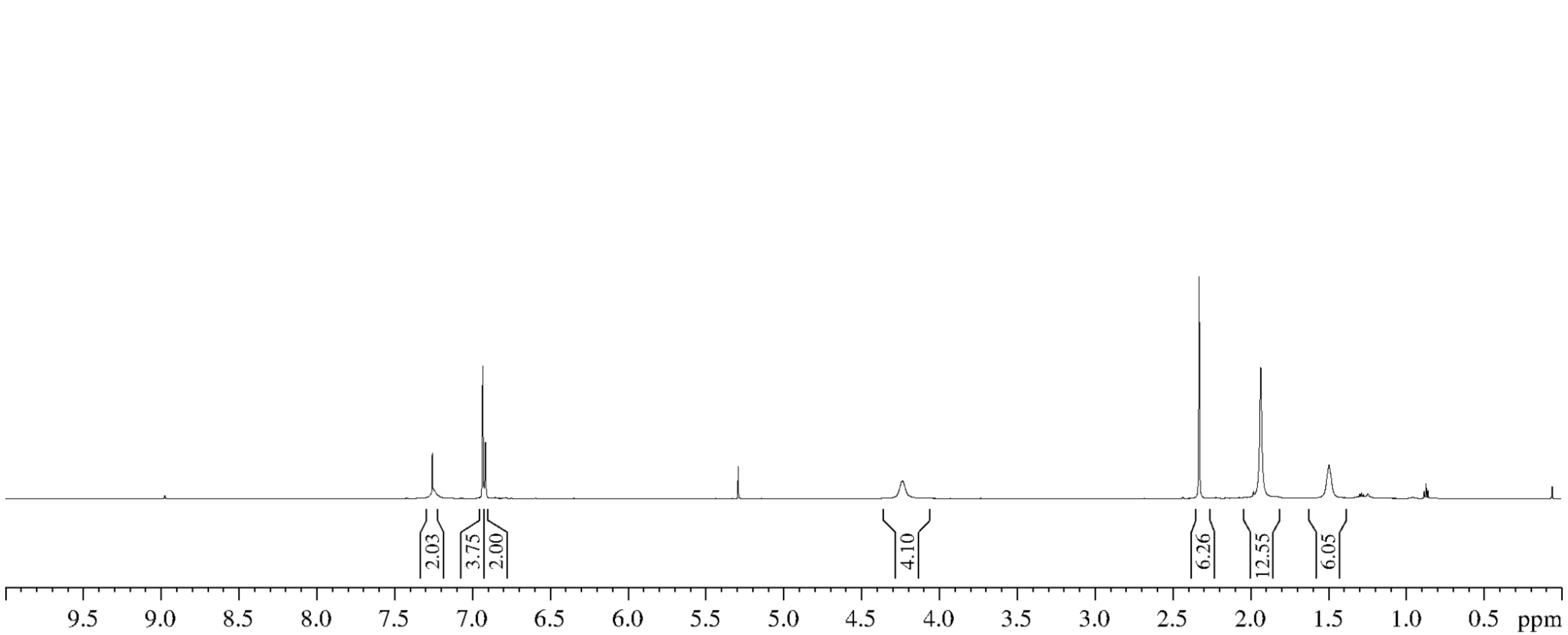

1.24.2 ^{13}C NMR

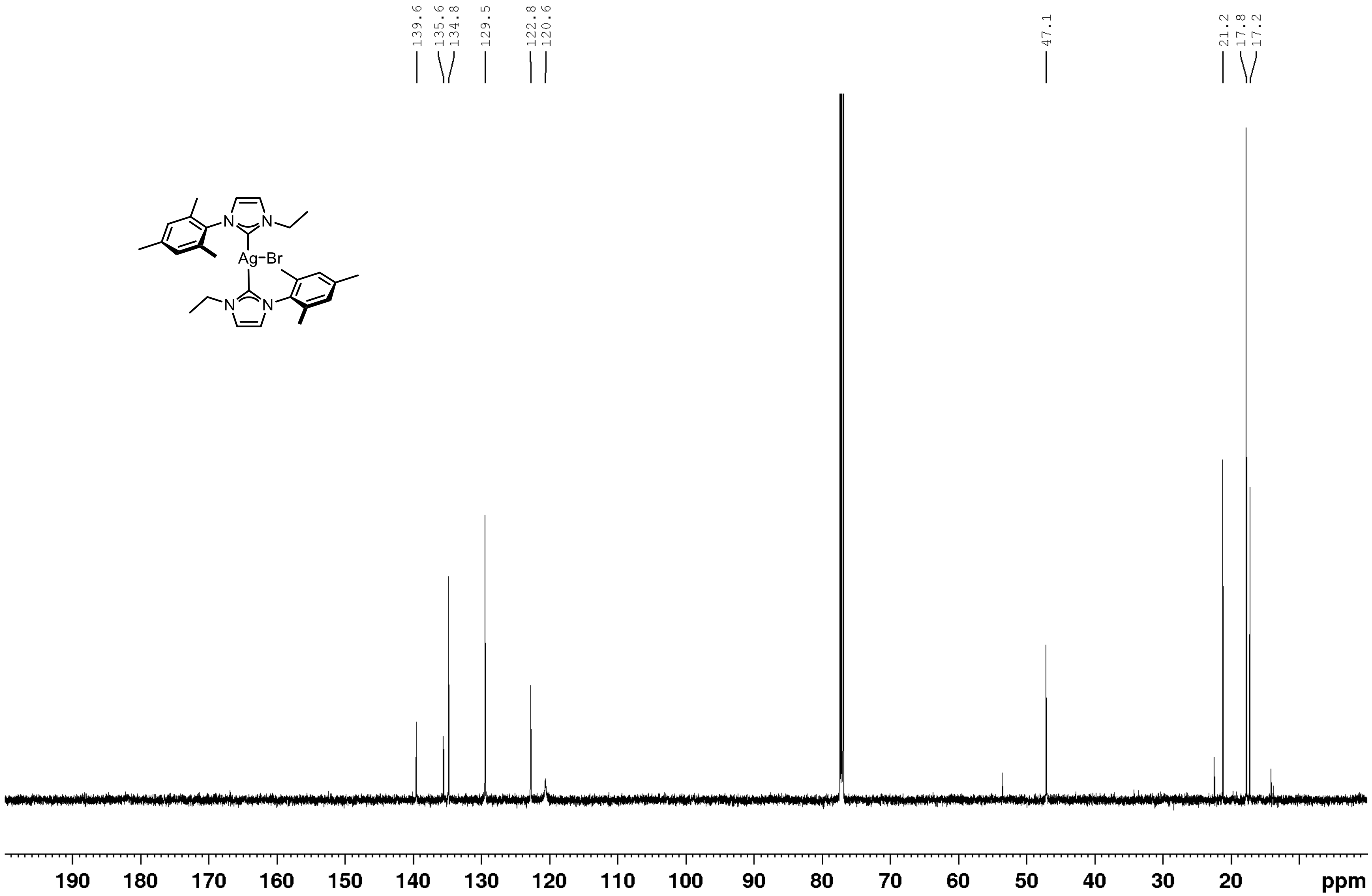
139.6
135.6
134.8
129.5
122.8
120.6
47.1
21.2
17.8
17.2
Ag
Br
N
190 180 170 160 150 140 130 120 110 100 90 80 70 60 50 40 30 20 ppm

1.24.3 ^{1}H, ^{1}H COSY

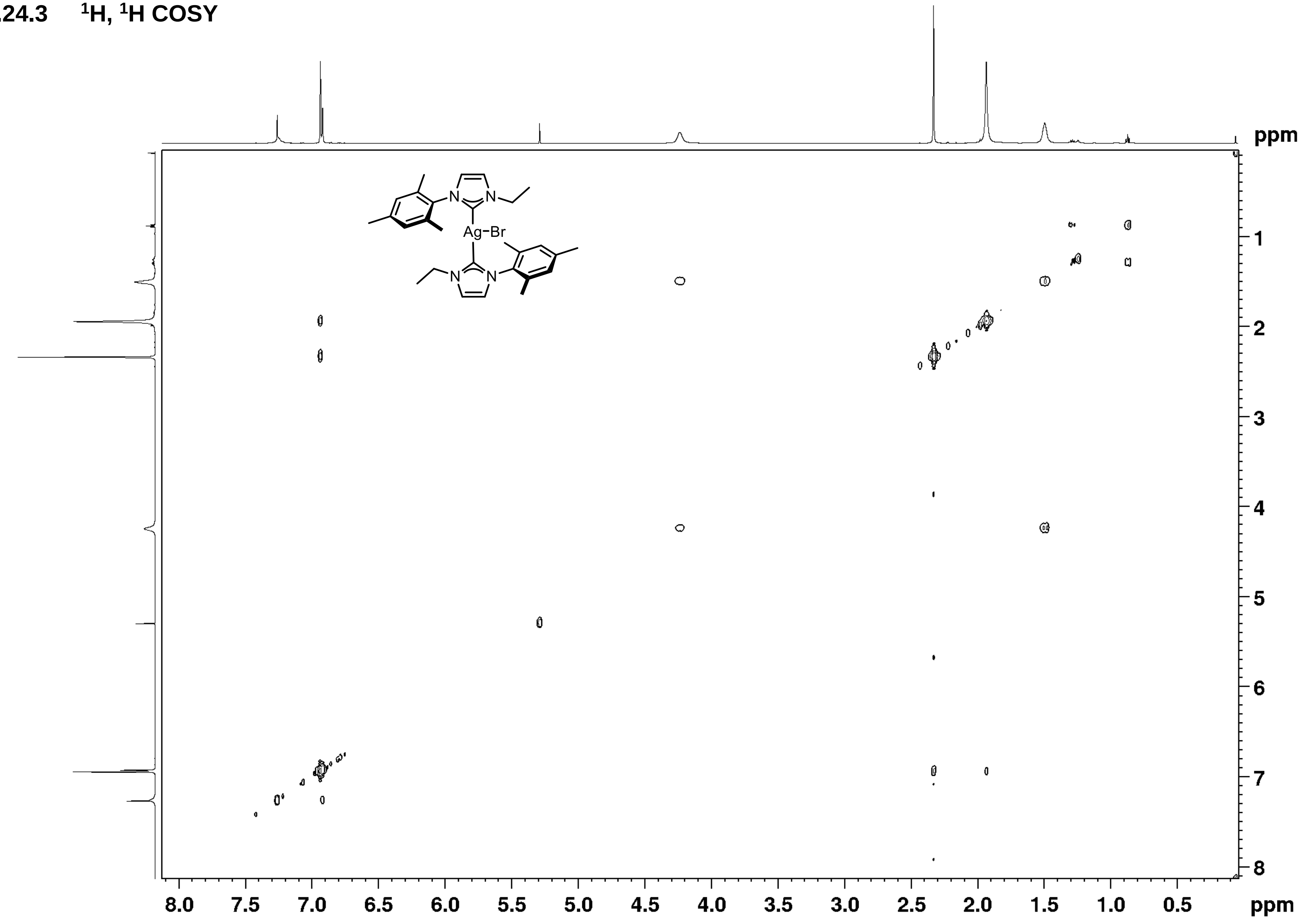

1.24.4 ^{1}H, ^{13}C HMBC

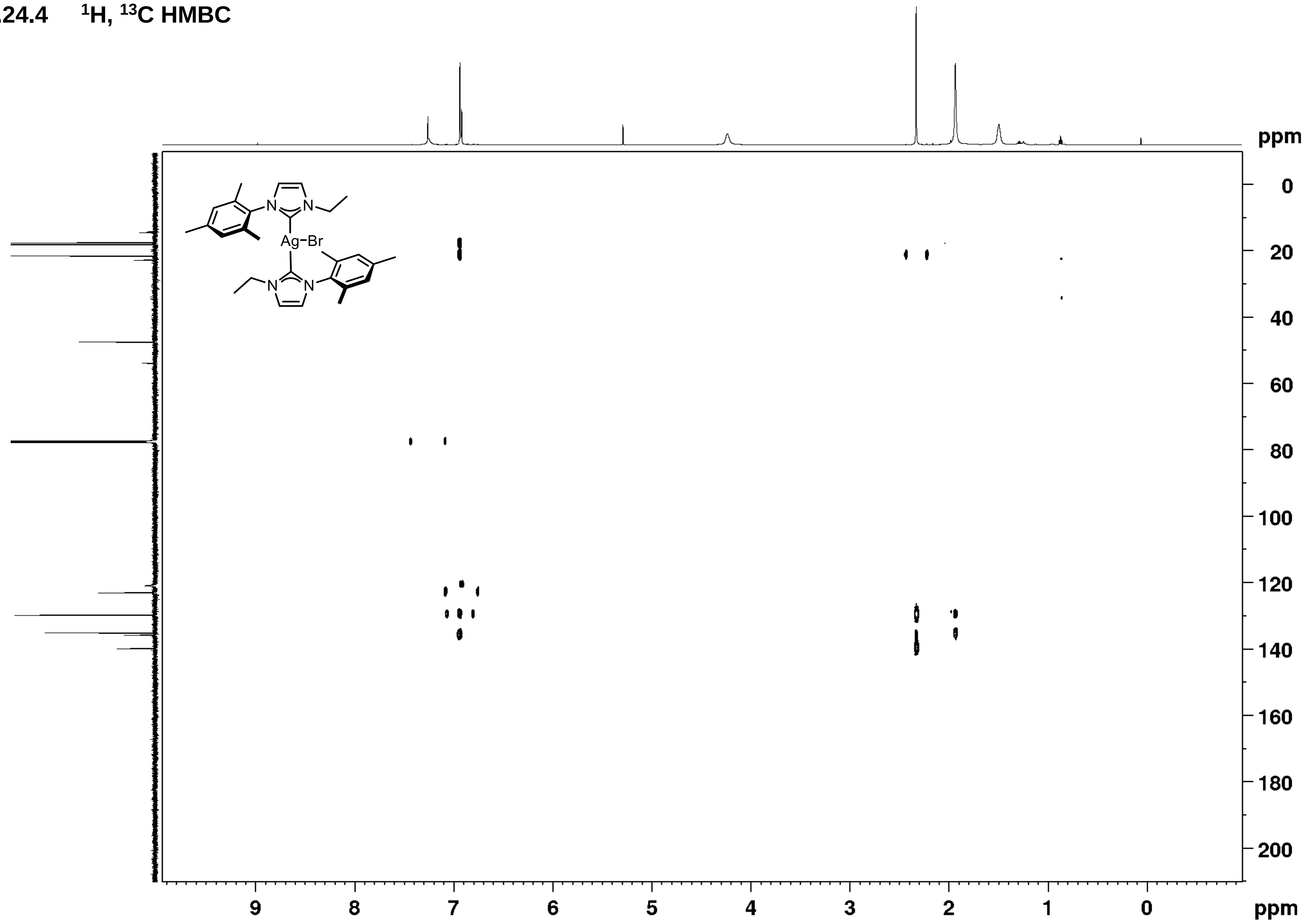

1.24.5 ^{1}H, ^{13}C HSQC

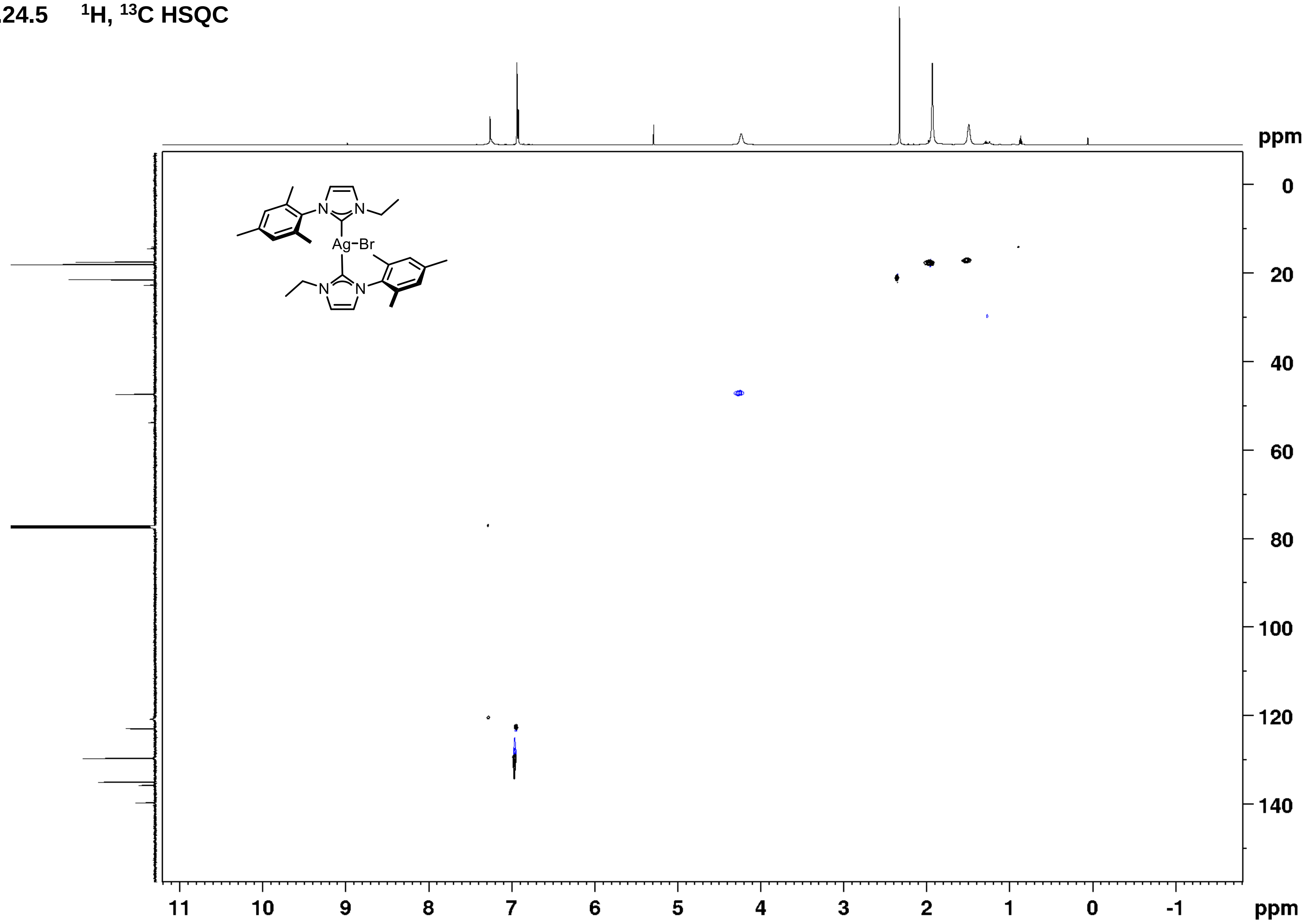

1.24.6 HRMS

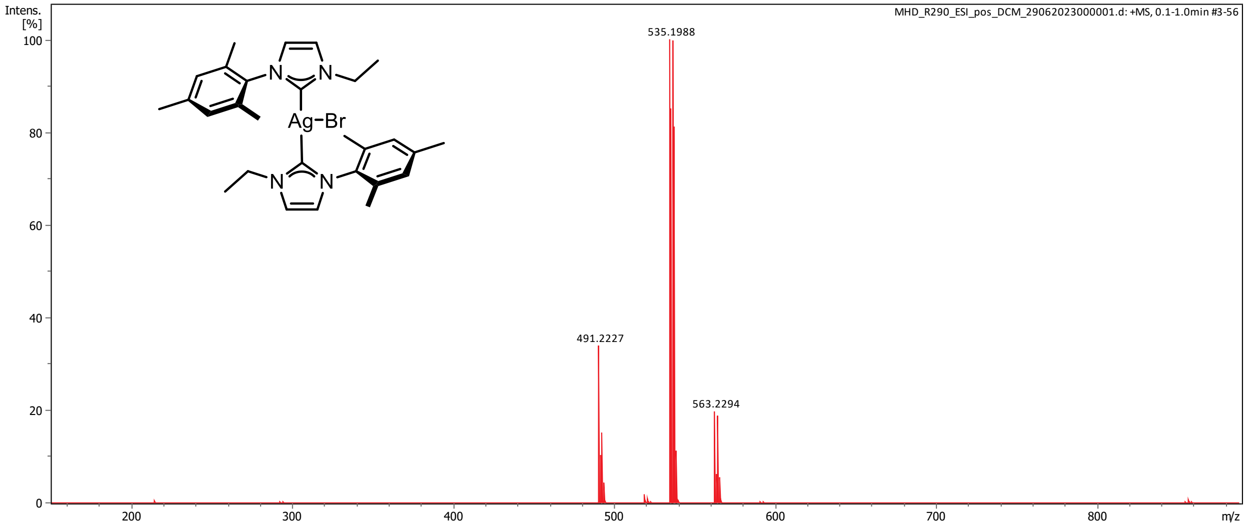
MHD_R290_ESI_pos_DCM_29062023000001.d: +MS, 0.1-1.0min #3-56
Intens. [%]
535.1988
491.2227
563.2294
Ag-Br
m/z

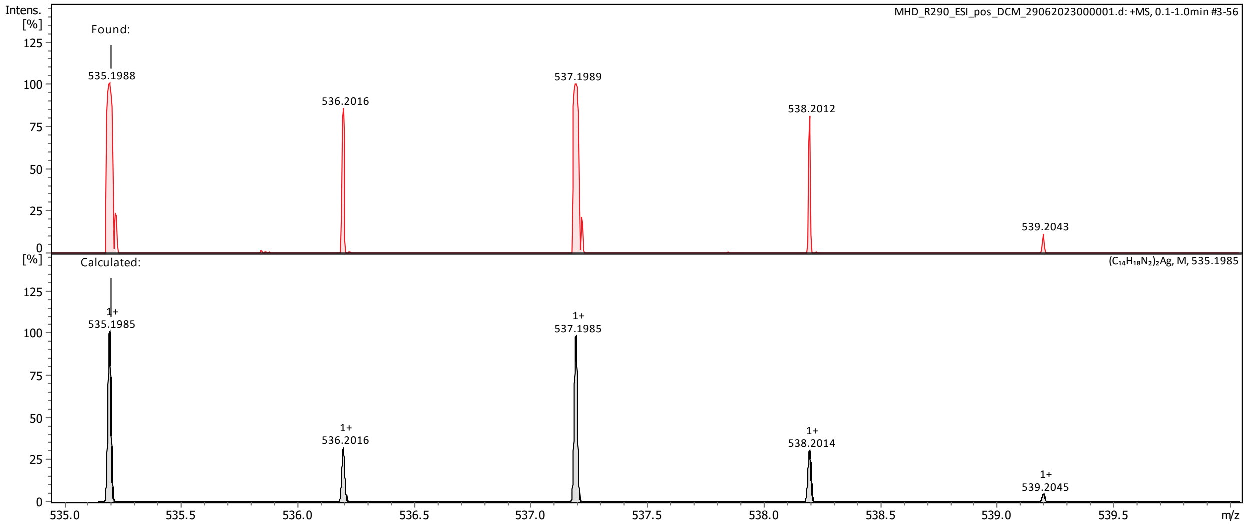
MHD_R290_ESI_pos_DCM_29062023000001.d: +MS, 0.1-1.0min #3-56
Found:
535.1988
536.2016
537.1989
538.2012
539.2043
Calculated:
$(C_{14}H_{18}N_2)_2Ag$, M, 535.1985
1+ 535.1985
1+ 536.2016
1+ 537.1985
1+ 538.2014
1+ 539.2045
m/z

1.25 1-Ethyl-3-mesityl-1,3-dihydro-2*H*-imidazol-2-ylidene copper(I) chloride (15) (600 MHz, $CDCl_3$)

1.25.1 ^{1}H NMR

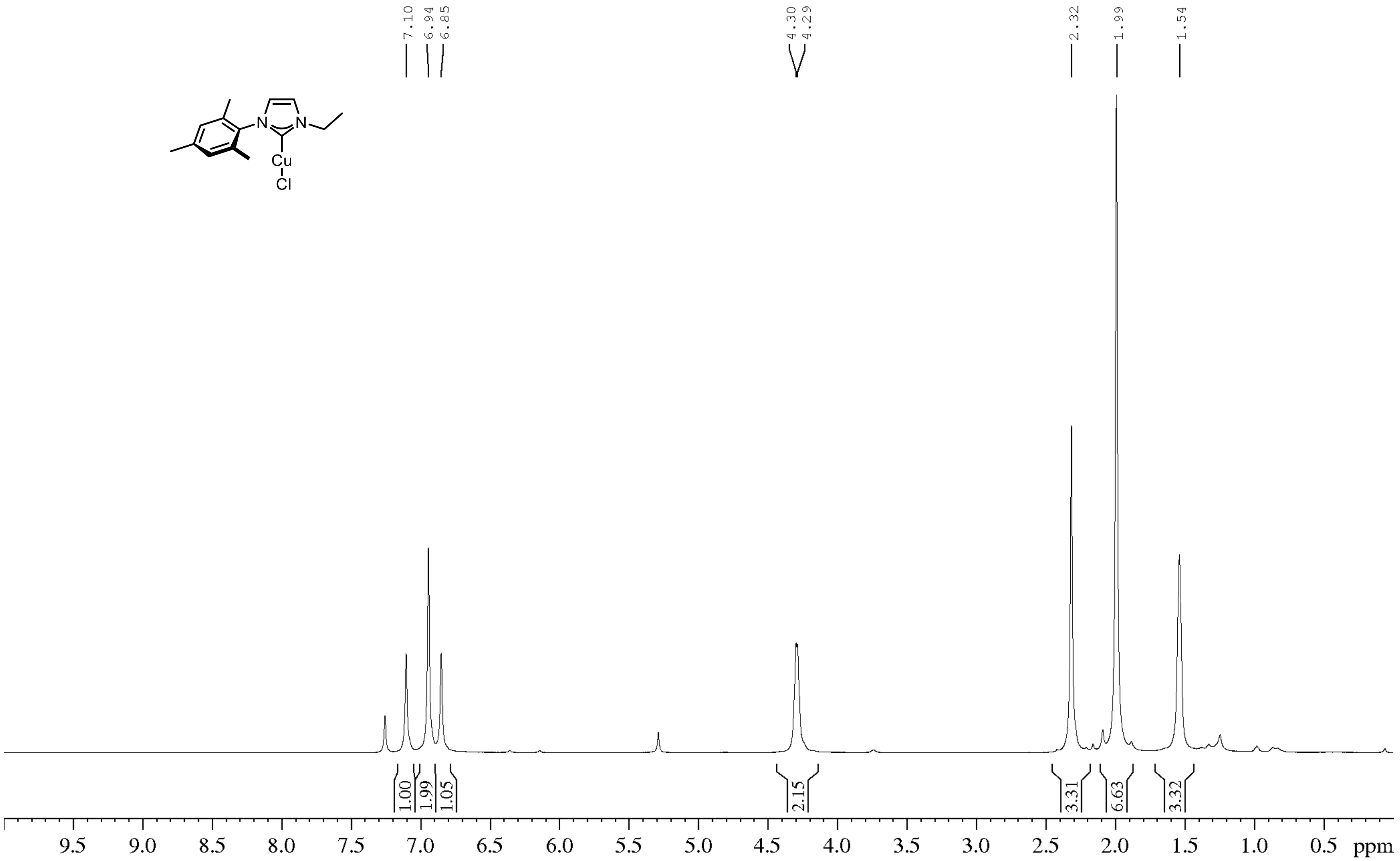

1.25.2 ^{13}C NMR

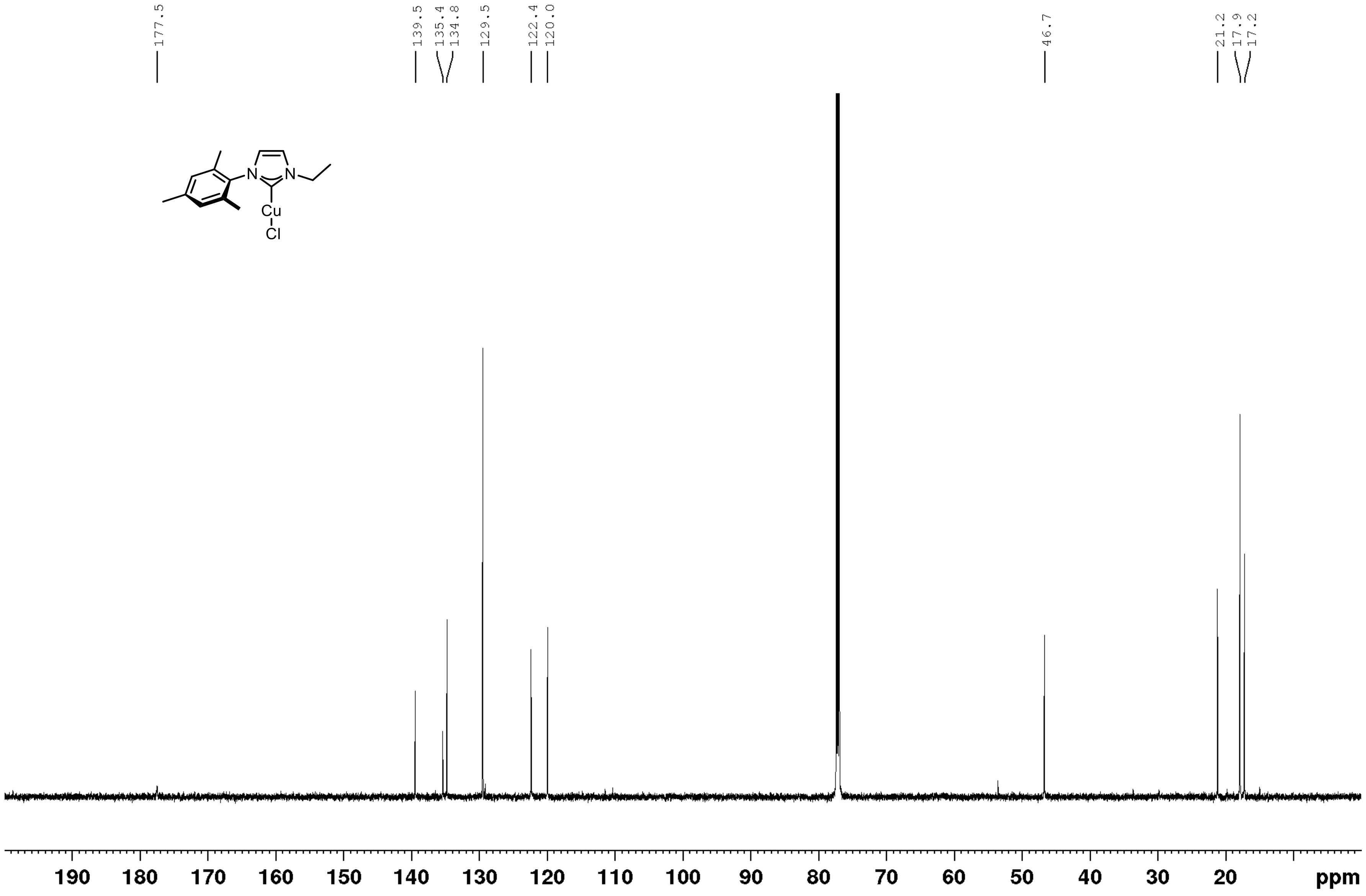
177.5
139.5
135.4
134.8
129.5
122.4
120.0
46.7
21.2
17.9
17.2
N
N
Cu
Cl
190
180
170
160
150
140
130
120
110
100
90
80
70
60
50
40
30
20
ppm

1.25.3 ^{1}H, ^{1}H COSY

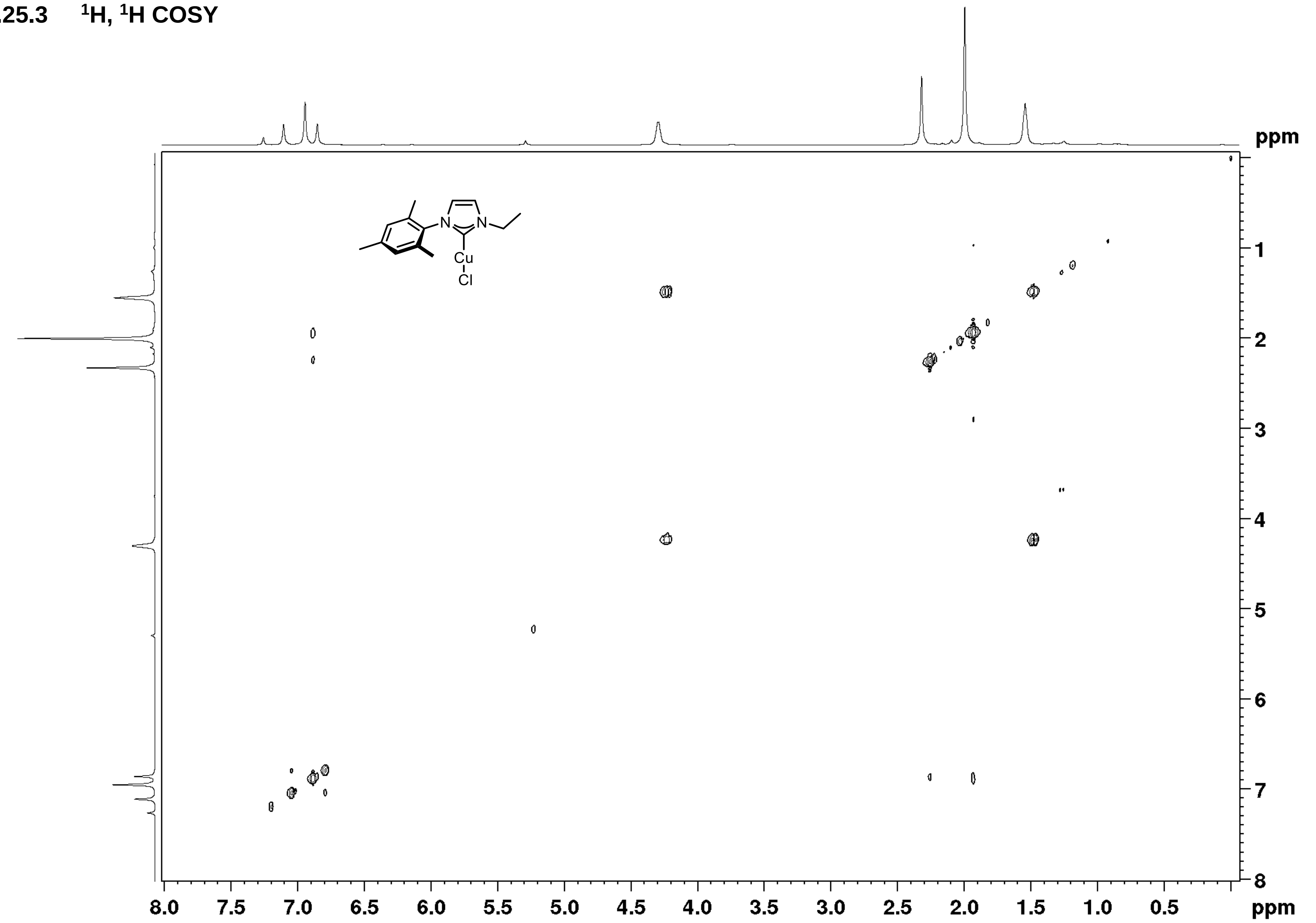

1.25.4 ^{1}H, ^{13}C HMBC

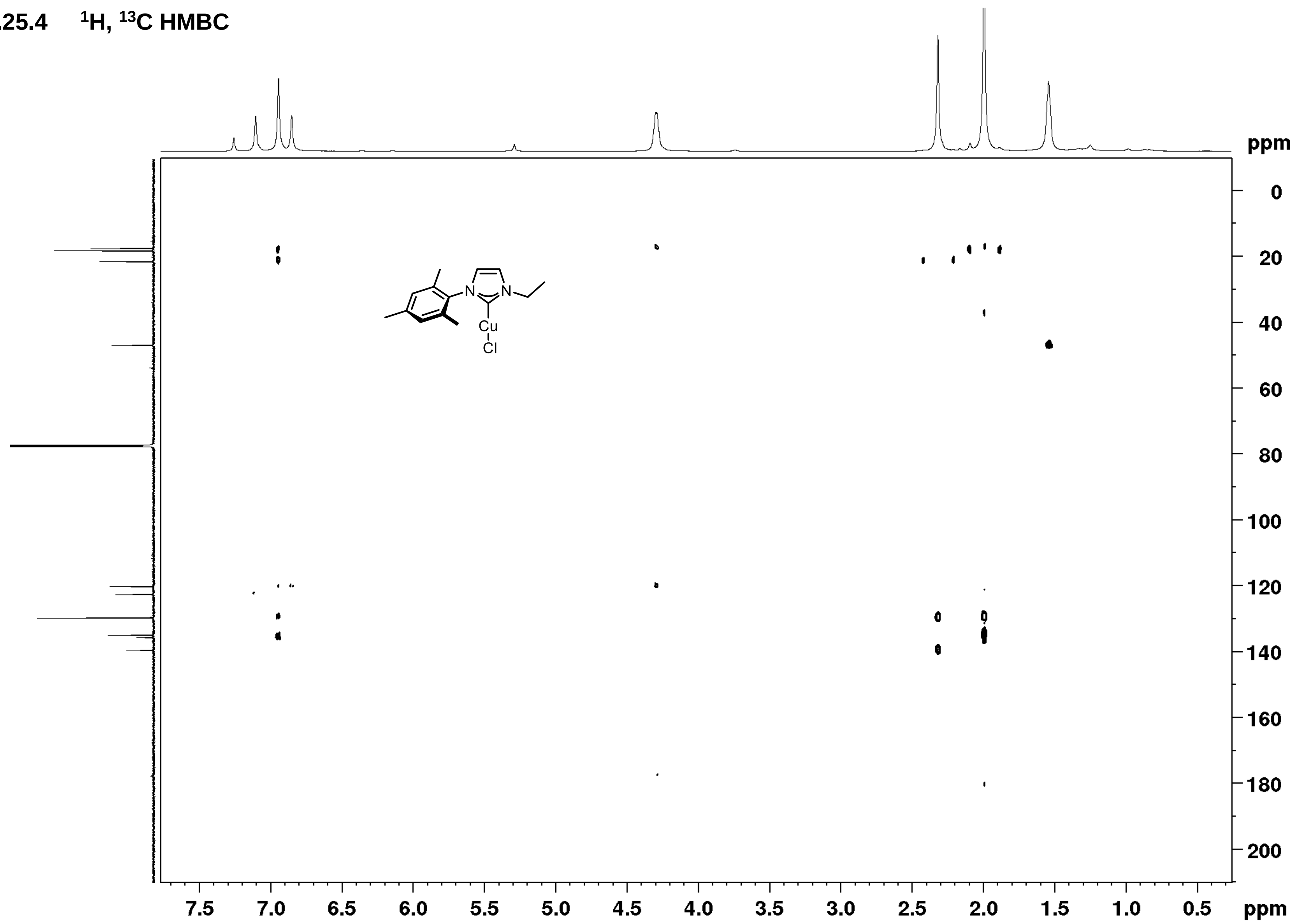

1.25.5 ^{1}H, ^{13}C HSQC

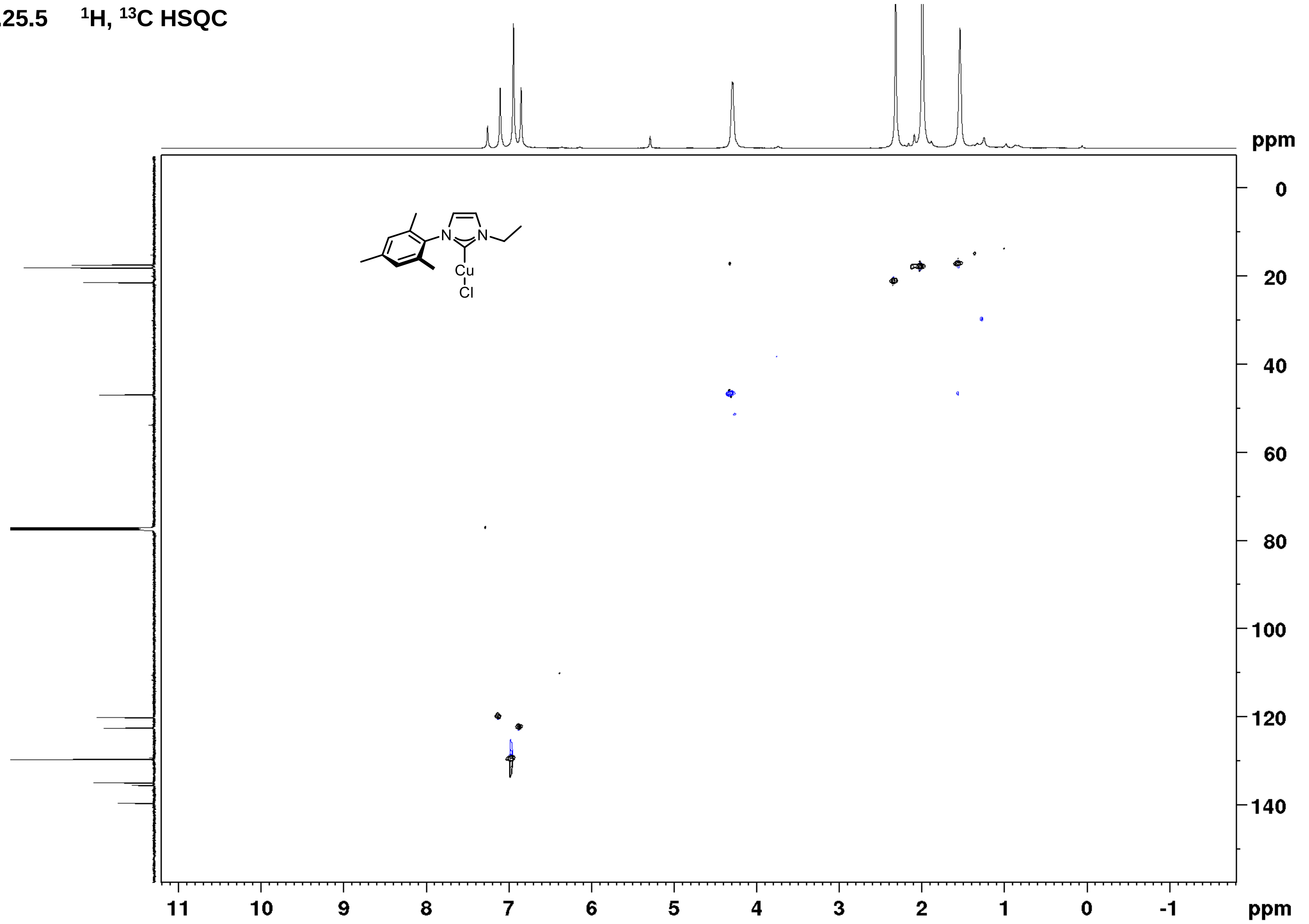

1.25.6 HRMS

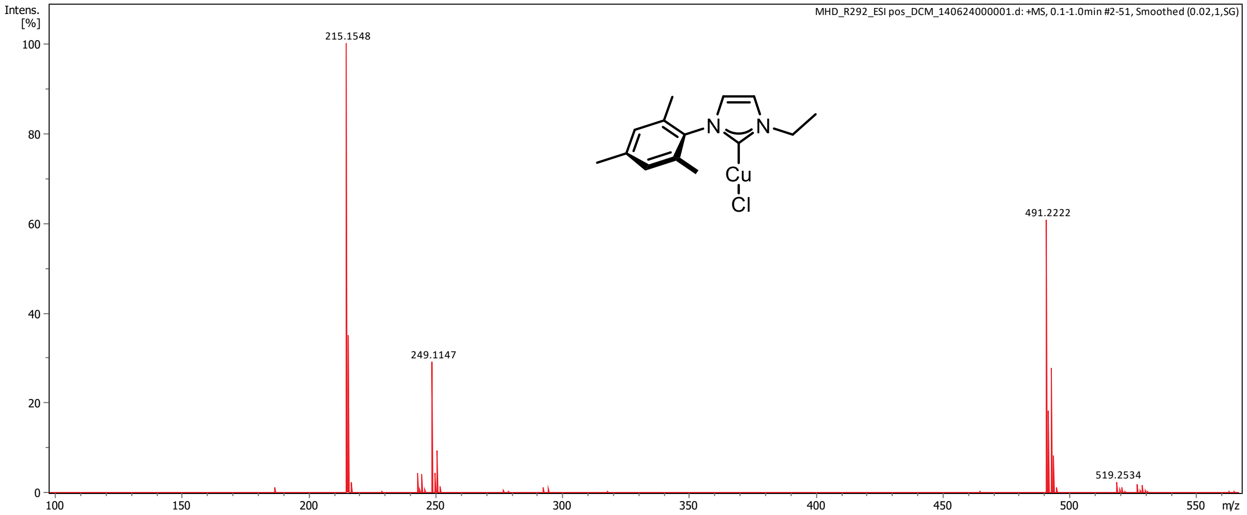
Intens. [%]
MHD_R292_ESI pos_DCM_140624000001.d: +MS, 0.1-1.0min #2-51, Smoothed (0.02,1,SG)
215.1548
249.1147
491.2222
519.2534
N
N
Cu
Cl
m/z

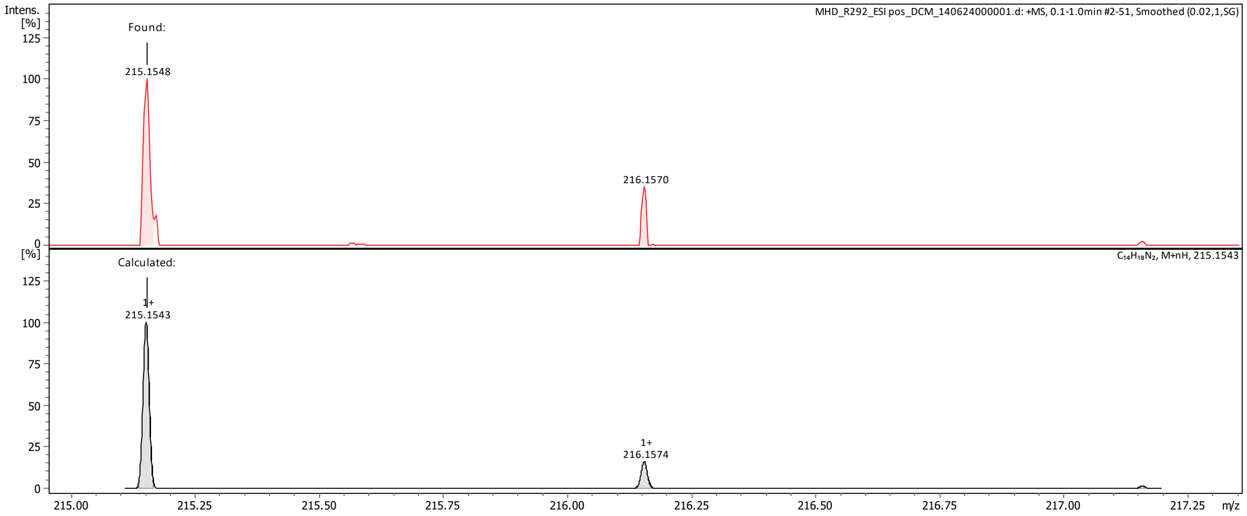
Intens. [%]
MHD_R292_ESI pos_DCM_140624000001.d: +MS, 0.1-1.0min #2-51, Smoothed (0.02,1,SG)
Found:
215.1548
216.1570
Calculated:
1+
215.1543
1+
216.1574
C14H18N2, M+nH, 215.1543
m/z

1.26 1-(2,6-Di*iso*propylphenyl)-3-vinyl-1*H*-imidazol-3-ium bromide 16aa ($CDCl_3$, 600 MHz)

1.26.1 ^1H NMR

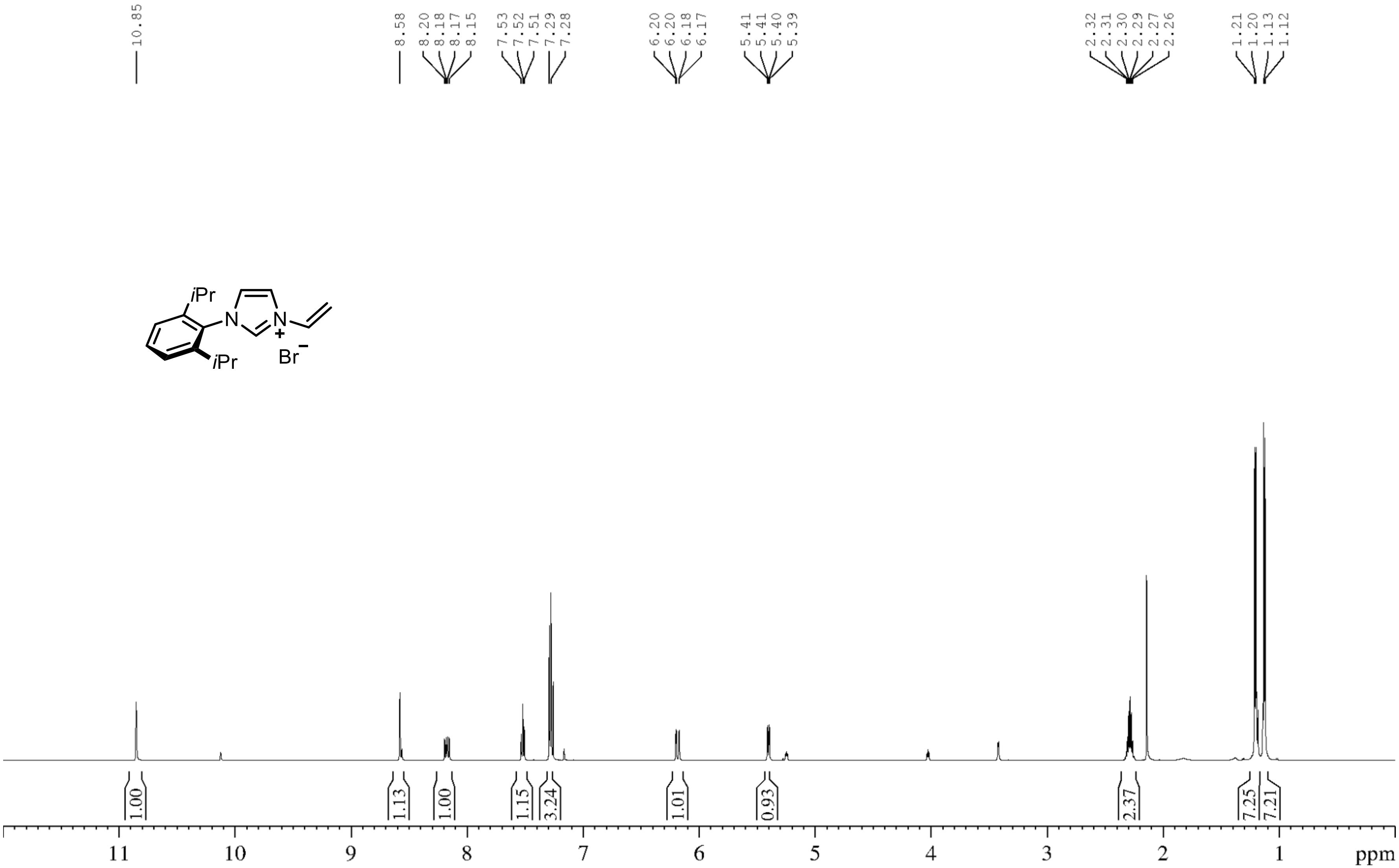

1.26.2 ^{13}C NMR

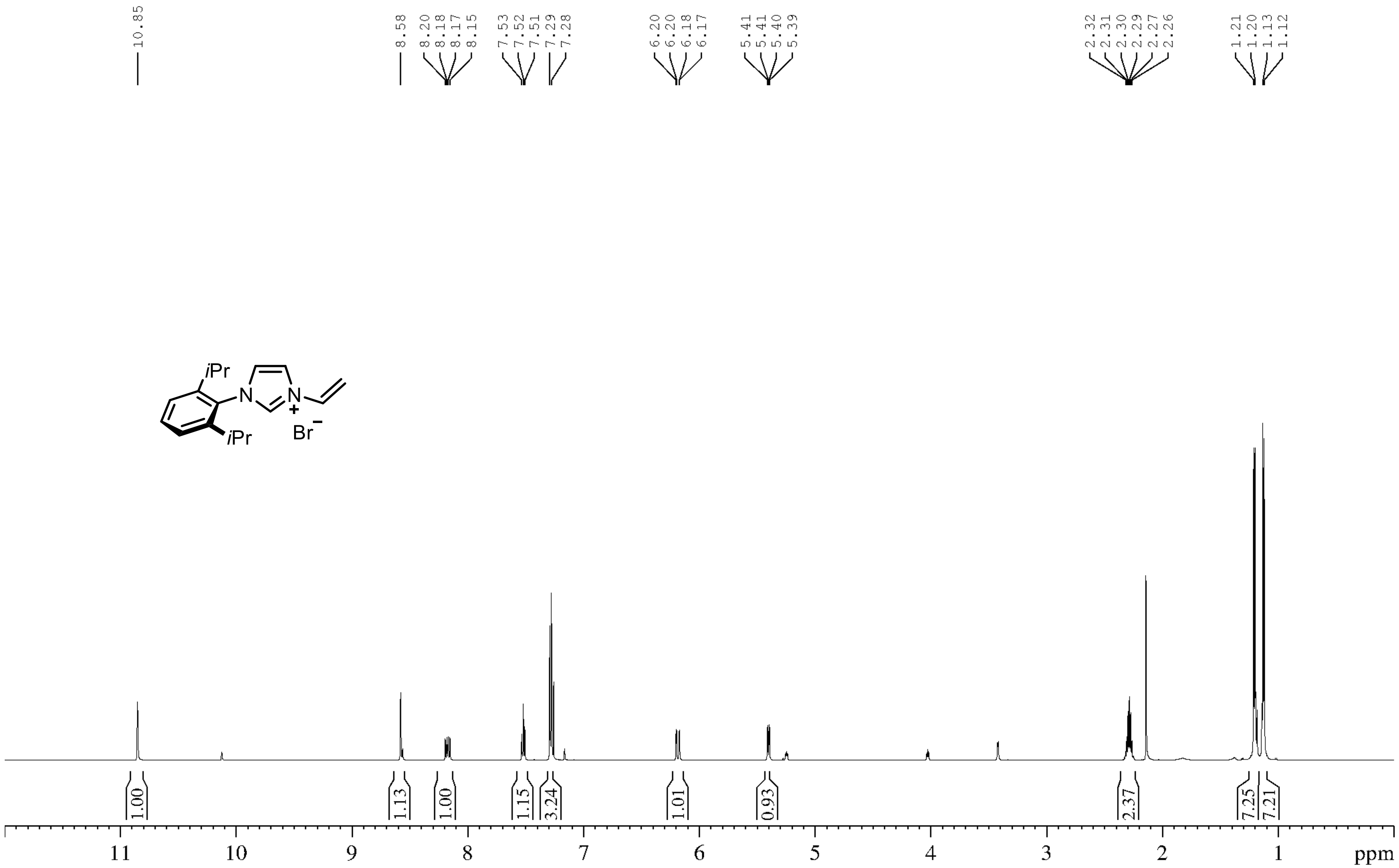

10.85
8.58
8.20
8.18
8.17
8.15
7.53
7.52
7.51
7.29
7.28
6.20
6.20
6.18
6.17
5.41
5.41
5.40
5.39
2.32
2.31
2.30
2.29
2.27
2.26
1.21
1.20
1.13
1.12
iPr
N
N
+
Br−
iPr
1.00
1.13
1.00
1.15
3.24
1.01
0.93
2.37
7.25
7.21
11
10
9
8
7
6
5
4
3
2
1
ppm

1.26.3 ^{1}H ^{1}H COSY

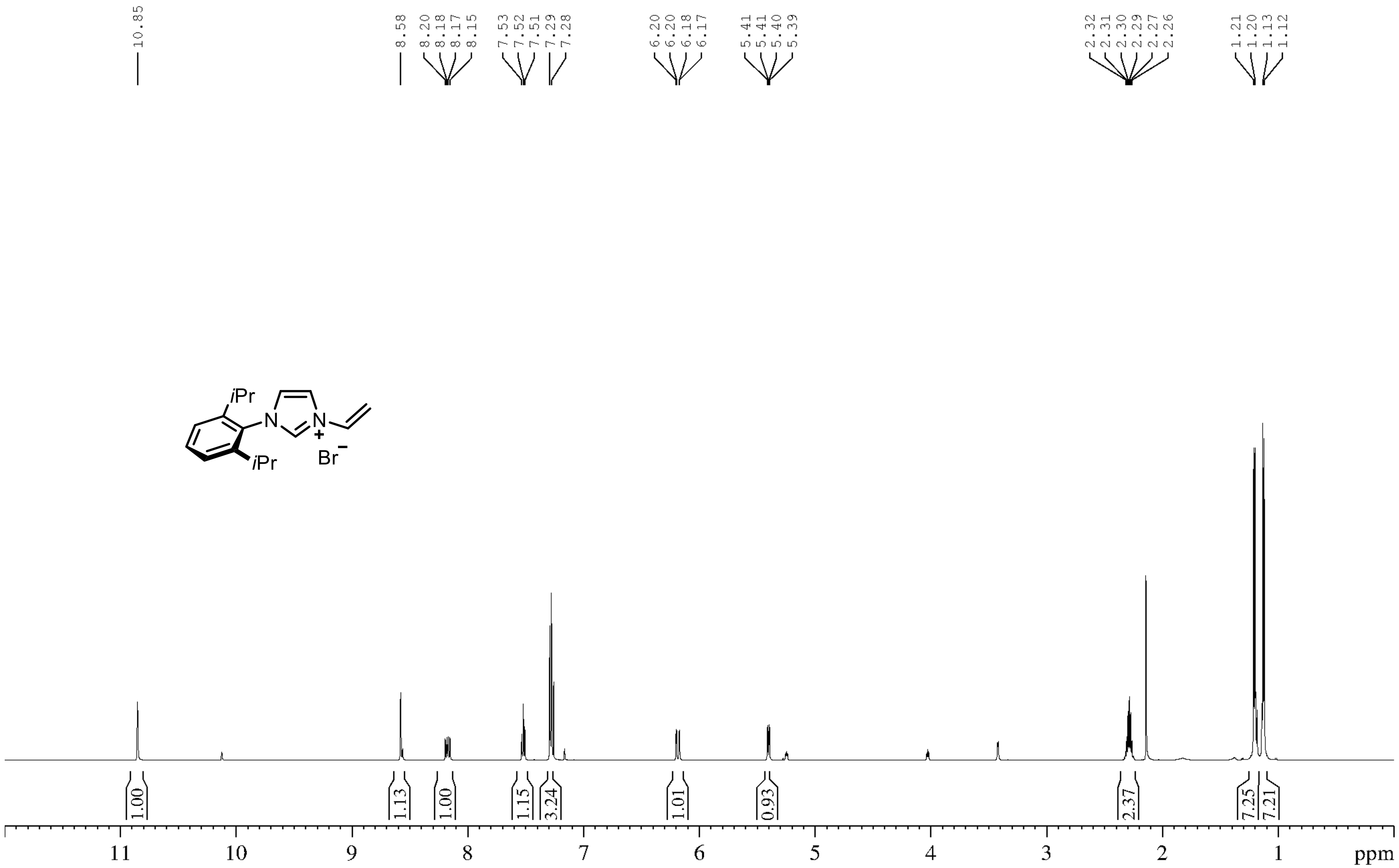

1.26.4 ^{1}H ^{13}C HMBC

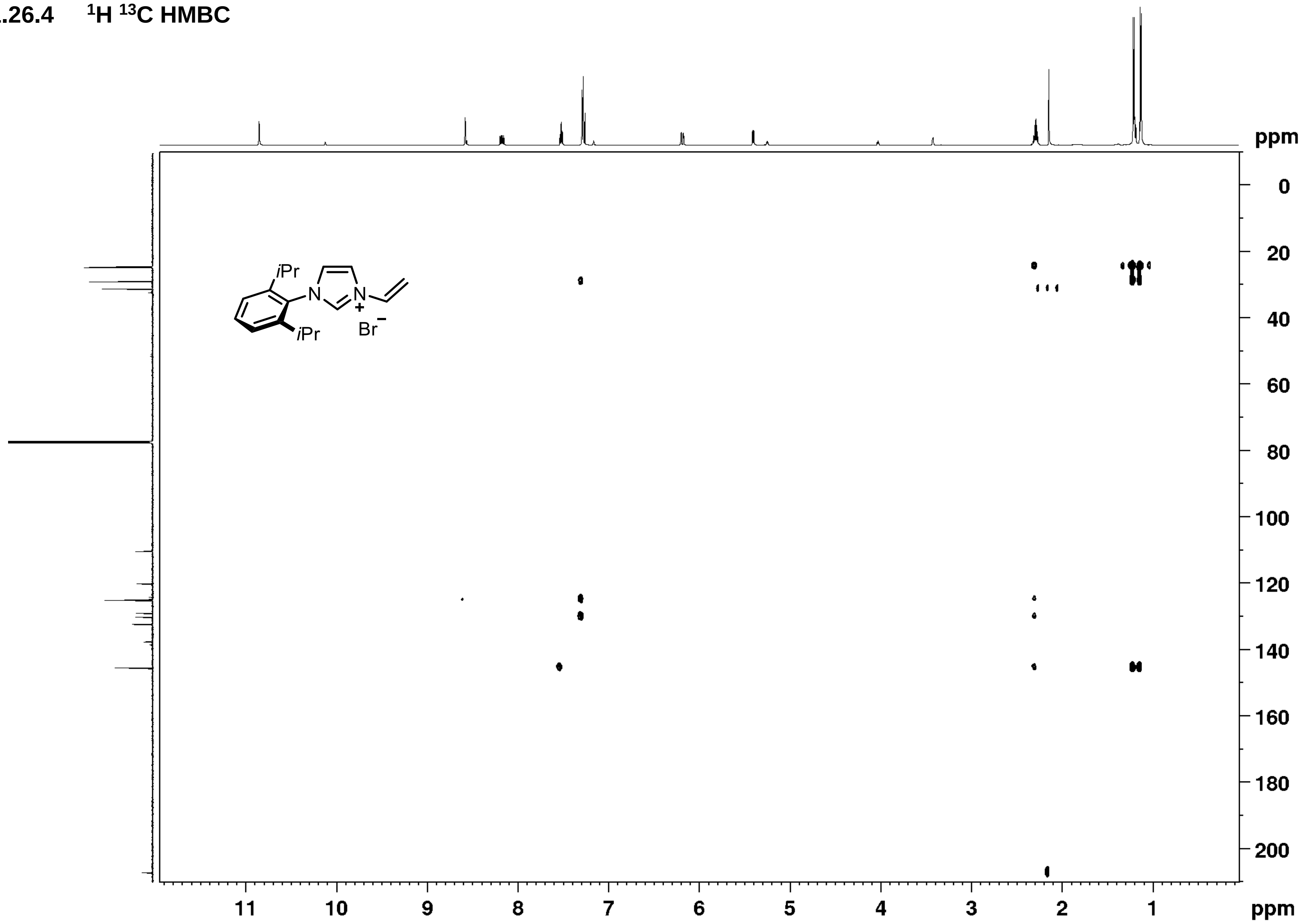

1.26.5 ^{1}H ^{13}C HSQC

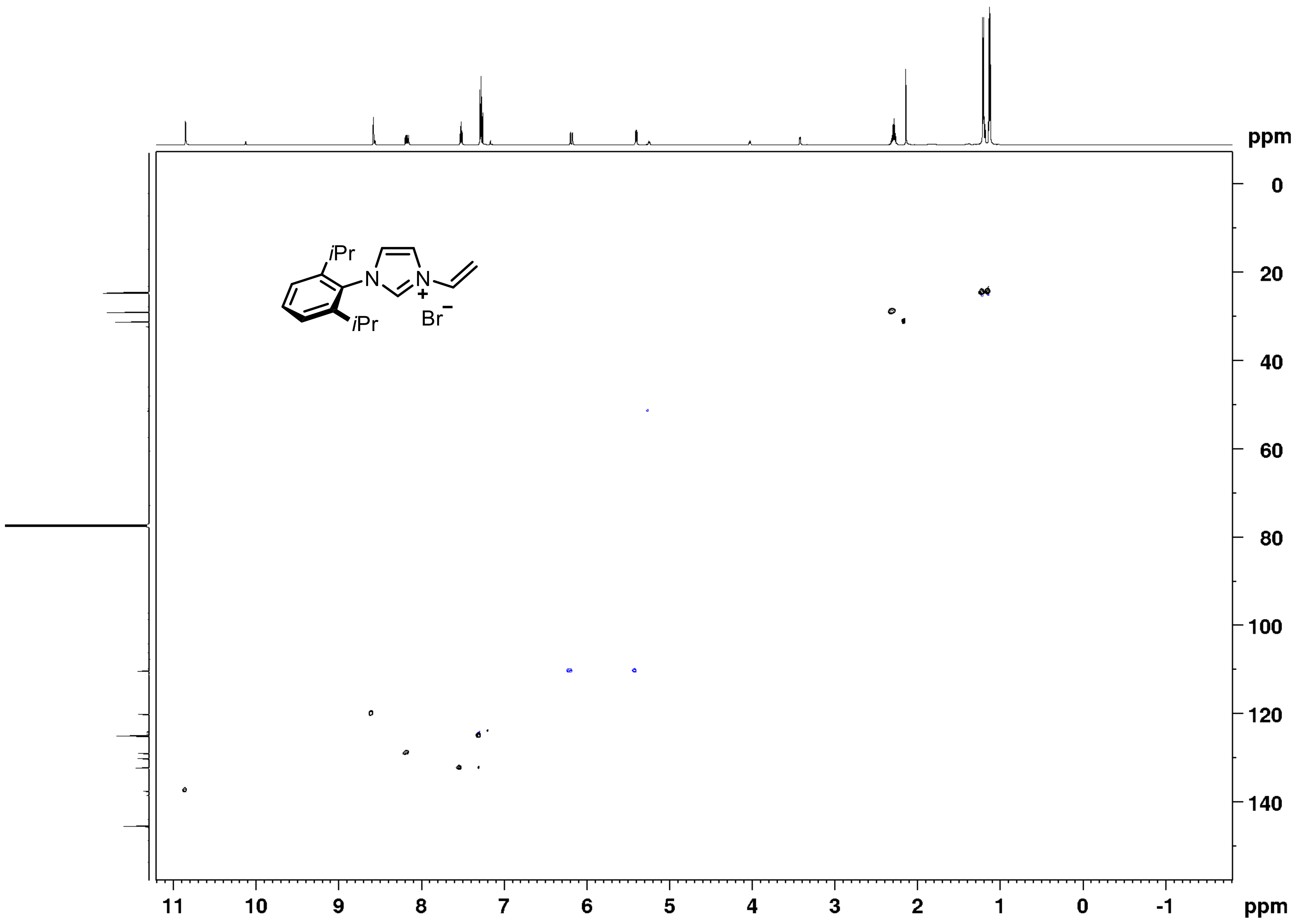

1.27 *N*,3-Dimethylpyridin-2-amine hydrobromide 16ab ($CDCl_3$, 600 MHz)

1.27.1 ^{1}H NMR

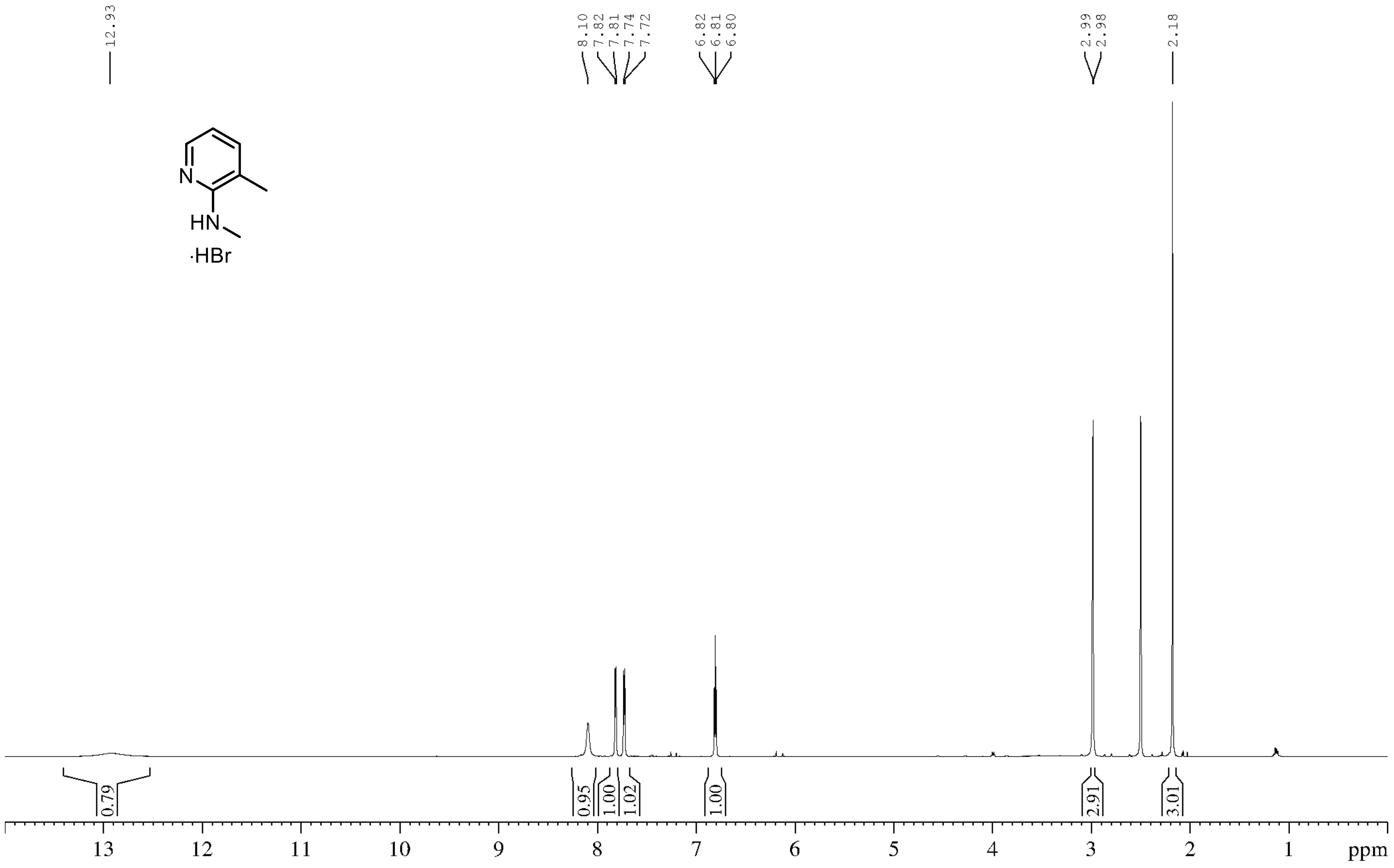

1.27.2 ^{13}C NMR

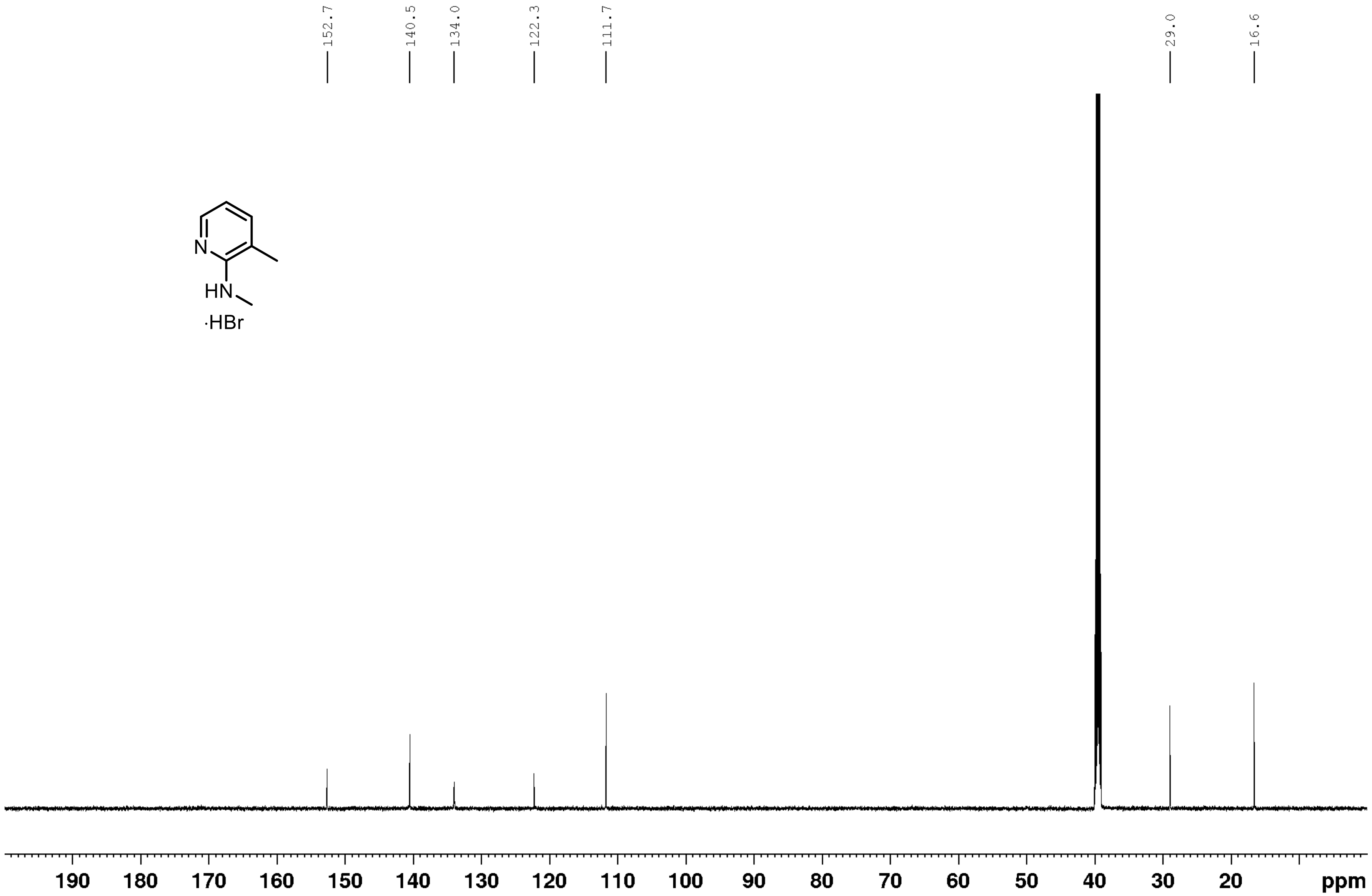
152.7
140.5
134.0
122.3
111.7
29.0
16.6
N
HN
·HBr
190
180
170
160
150
140
130
120
110
100
90
80
70
60
50
40
30
20
ppm

1.27.3 ^{1}H ^{1}H COSY

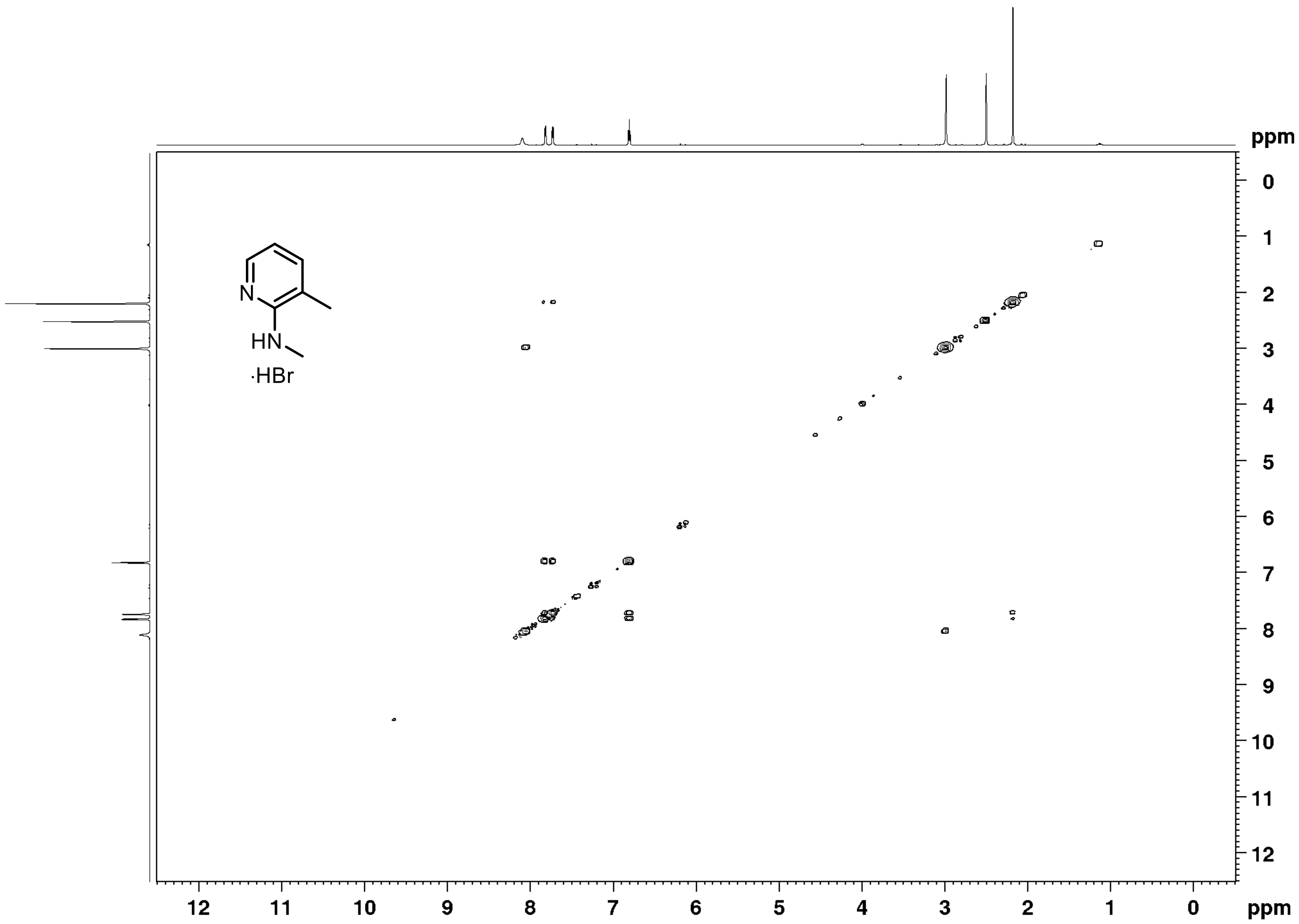

1.27.4 ^{1}H ^{13}C HMBC

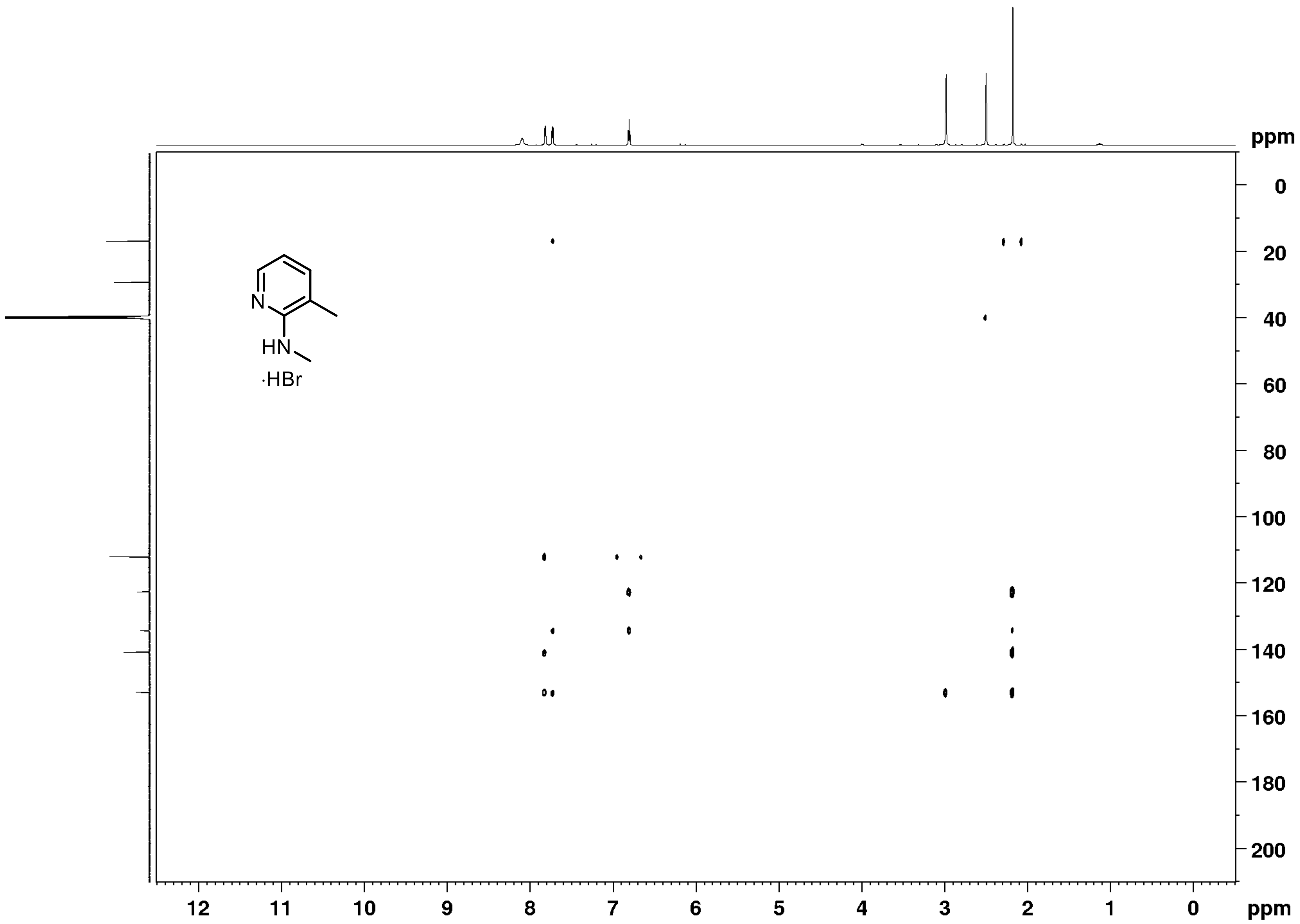

1.27.5 ^{1}H ^{13}C HSQC

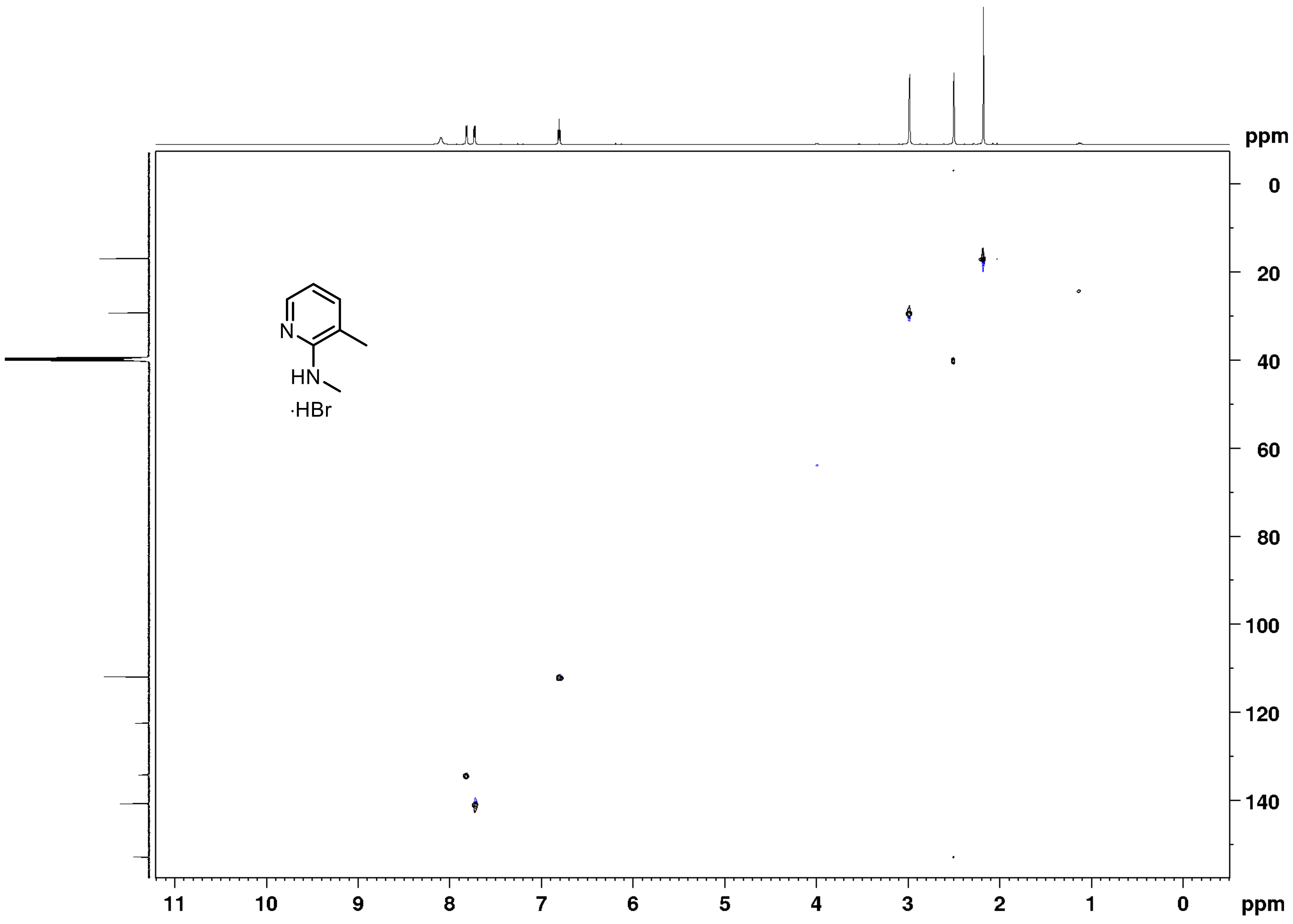

1.28 3-(4-Bromobutyl)-1-(2,6-di*iso*propylphenyl)-1*H*-imidazol-3-ium bromide (17a) ($CDCl_3$, 600 MHz)

1.28.1 ^{1}H NMR

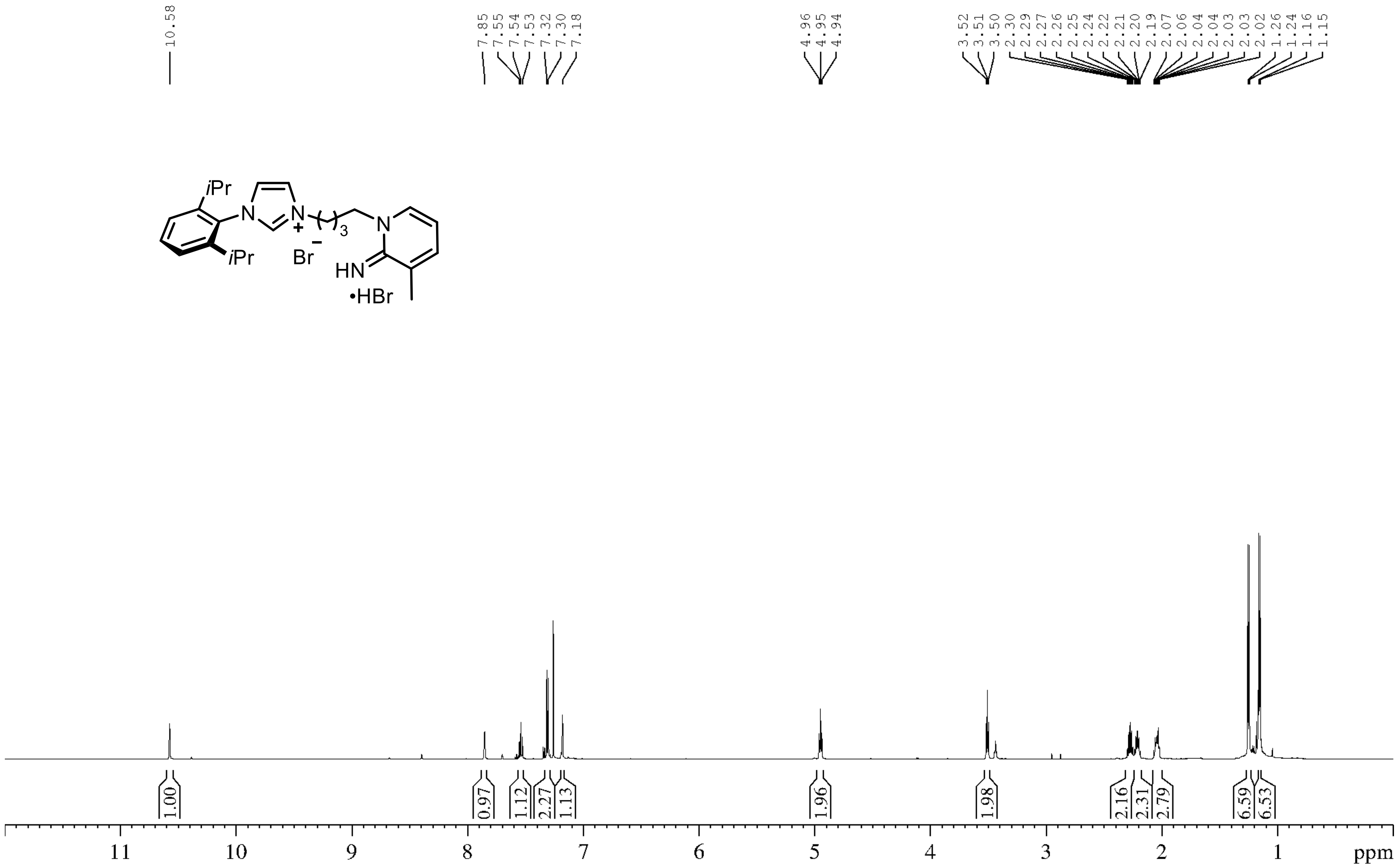

1.28.2 ^{13}C NMR

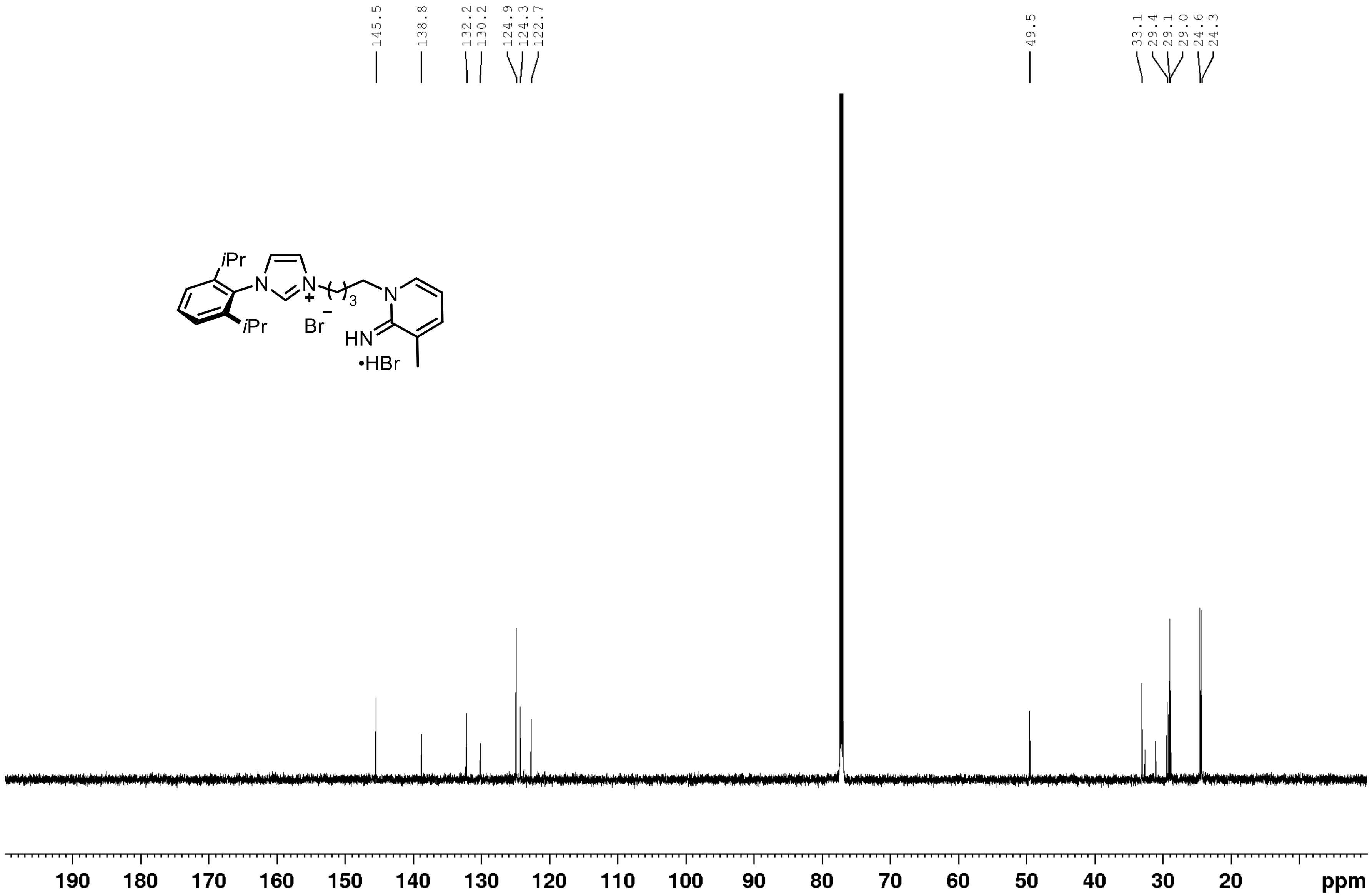
145.5
138.8
132.2
130.2
124.9
124.3
122.7
49.5
33.1
29.4
29.1
29.0
24.6
24.3
iPr
N
N
+
3
N
iPr
Br
HN
•HBr
190
180
170
160
150
140
130
120
110
100
90
80
70
60
50
40
30
20
ppm

1.28.3 ^{1}H ^{1}H COSY

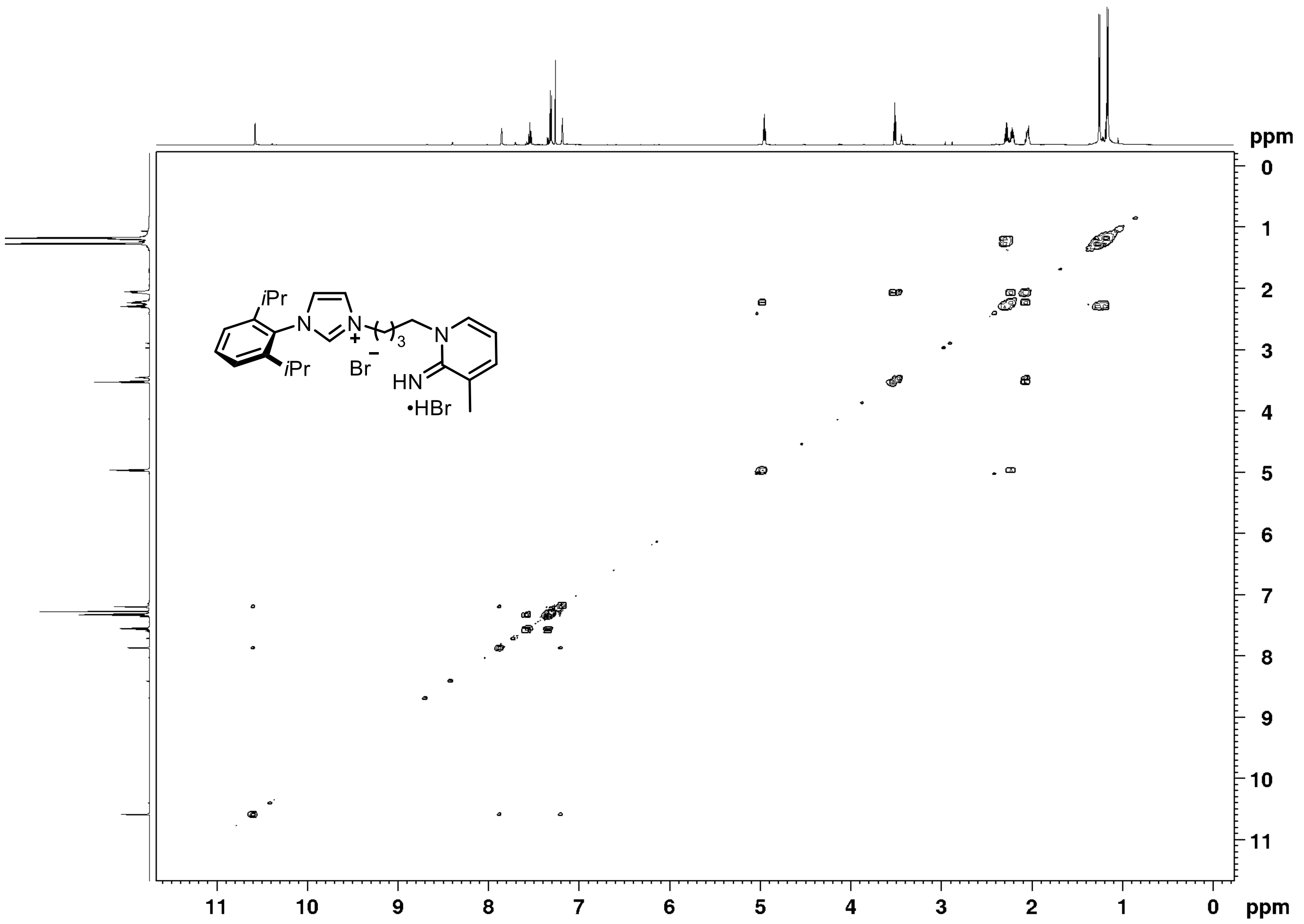

1.28.4 ^{1}H ^{13}C HMBC

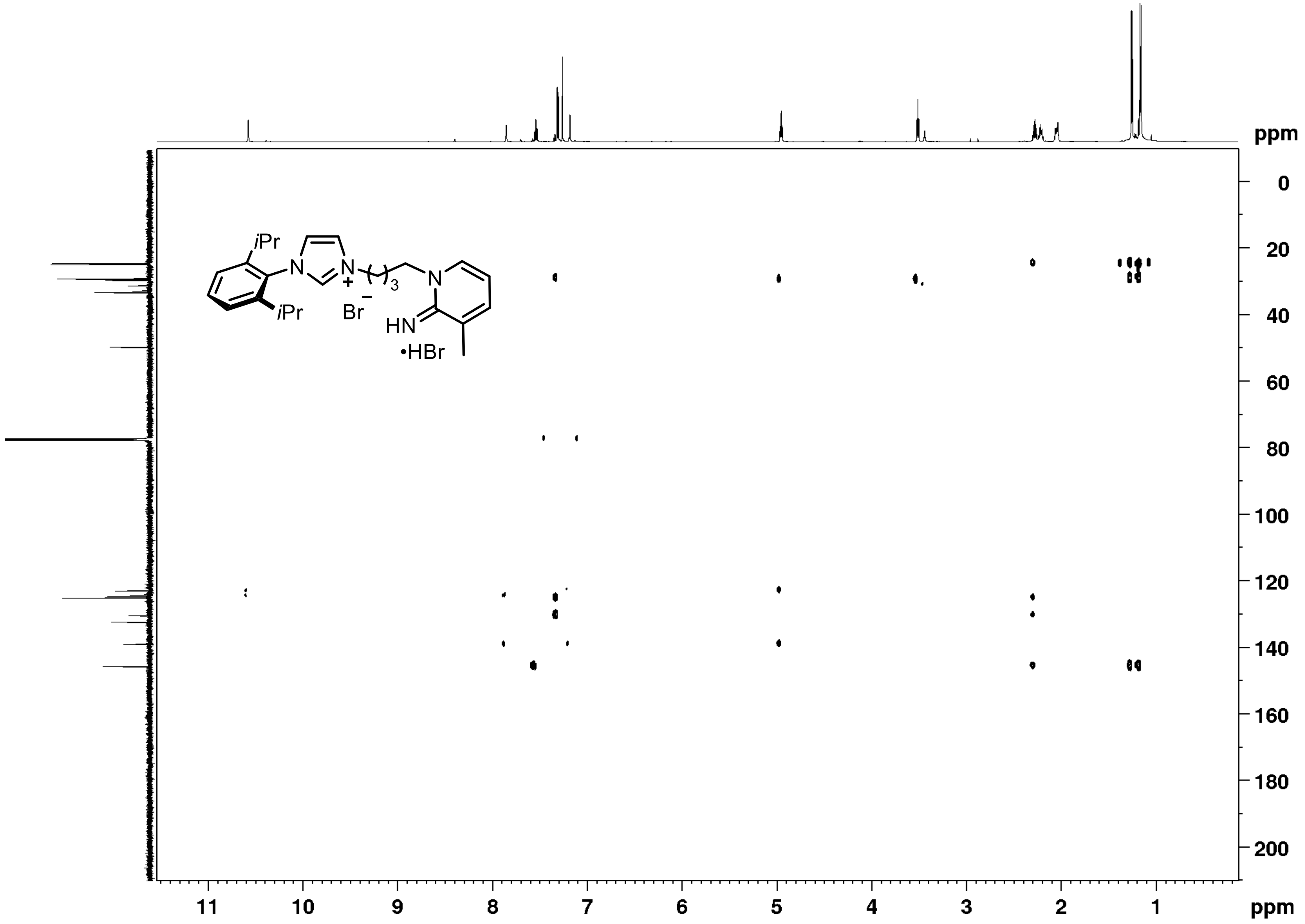

1.28.5 ^{1}H ^{13}C HSQC

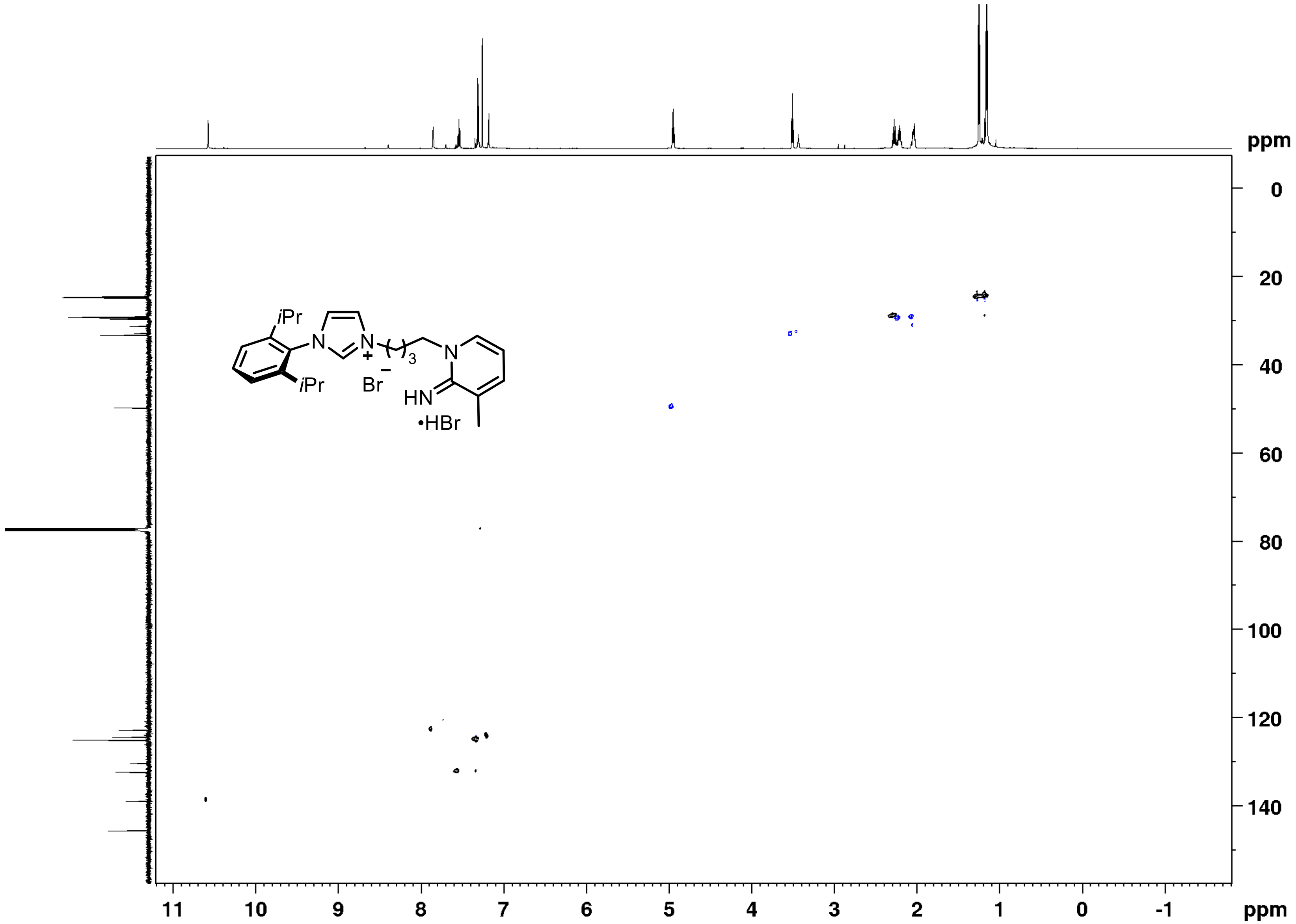

1.28.6 HRMS

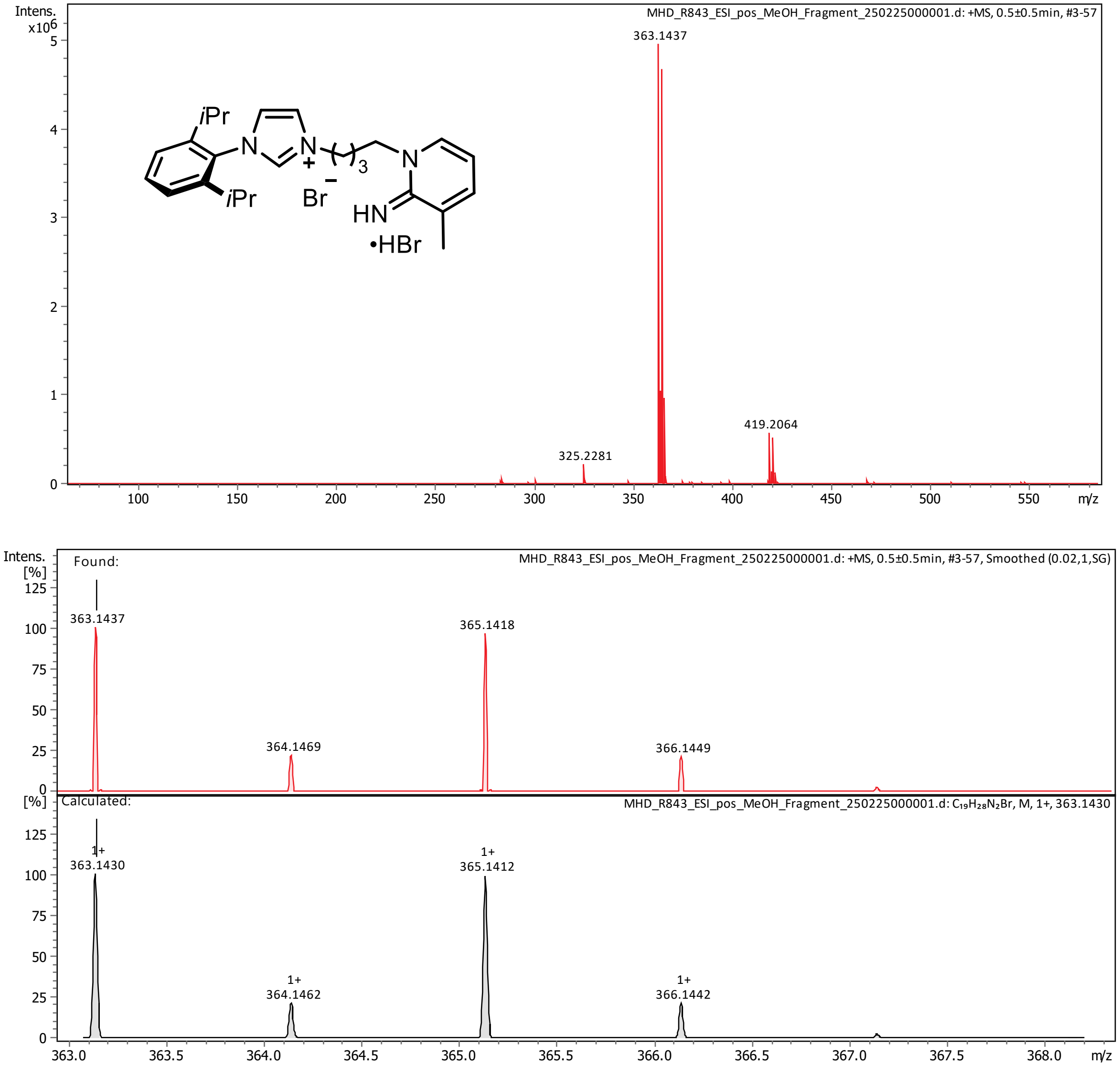

Intens.
x10^6
MHD_R843_ESI_pos_MeOH_Fragment_250225000001.d: +MS, 0.5±0.5min, #3-57
363.1437
419.2064
325.2281
iPr
N
N
+
Br
-
3
N
HN
•HBr
iPr
m/z
Intens.
[%]
Found:
MHD_R843_ESI_pos_MeOH_Fragment_250225000001.d: +MS, 0.5±0.5min, #3-57, Smoothed (0.02,1,SG)
363.1437
365.1418
364.1469
366.1449
Calculated:
MHD_R843_ESI_pos_MeOH_Fragment_250225000001.d: C19H28N2Br, M, 1+, 363.1430
1+
363.1430
1+
365.1412
1+
364.1462
1+
366.1442
m/z

1.29 3-(8-Bromooctyl)-1-(2,6-di*iso*propylphenyl)-1*H*-imidazol-3-ium bromide (18a) ($CDCl_3$, 600 MHz)

1.29.1 ^{1}H NMR

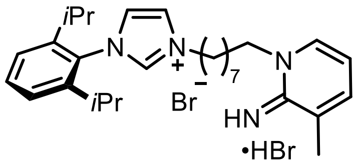

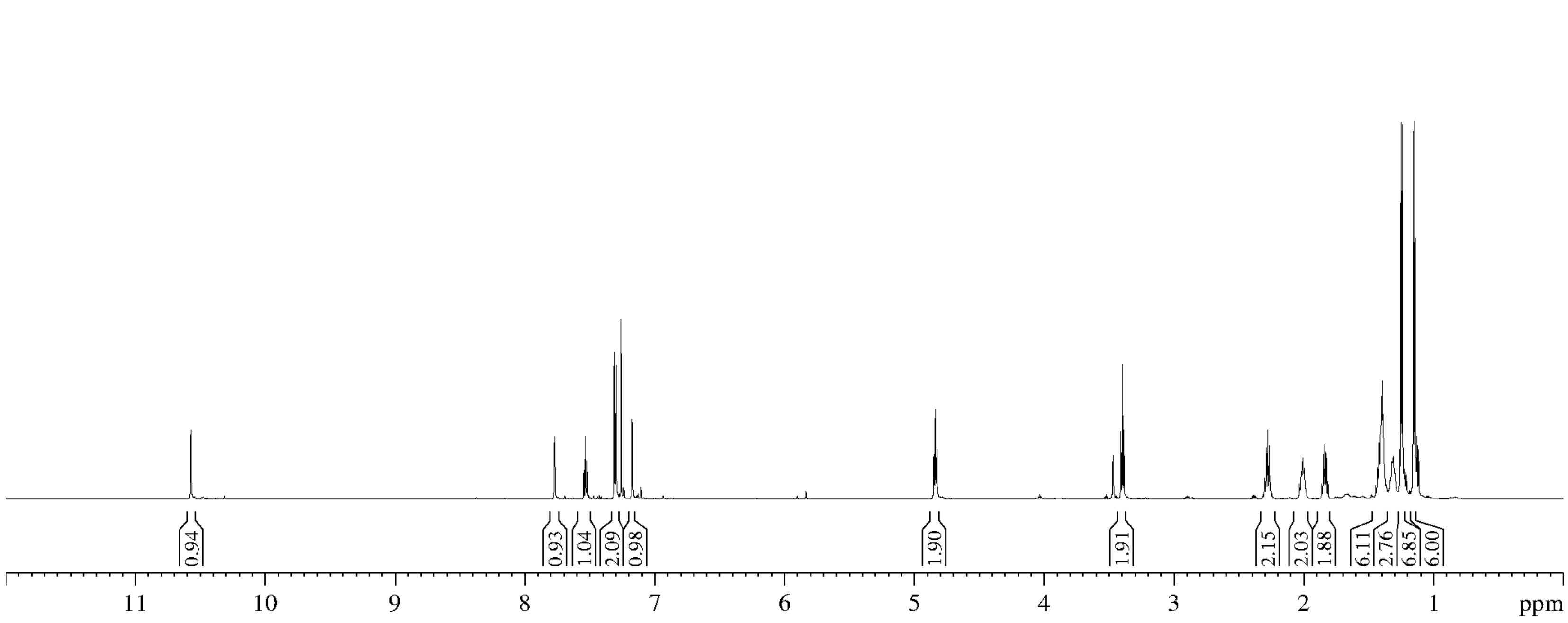

1.29.2 ^{13}C NMR

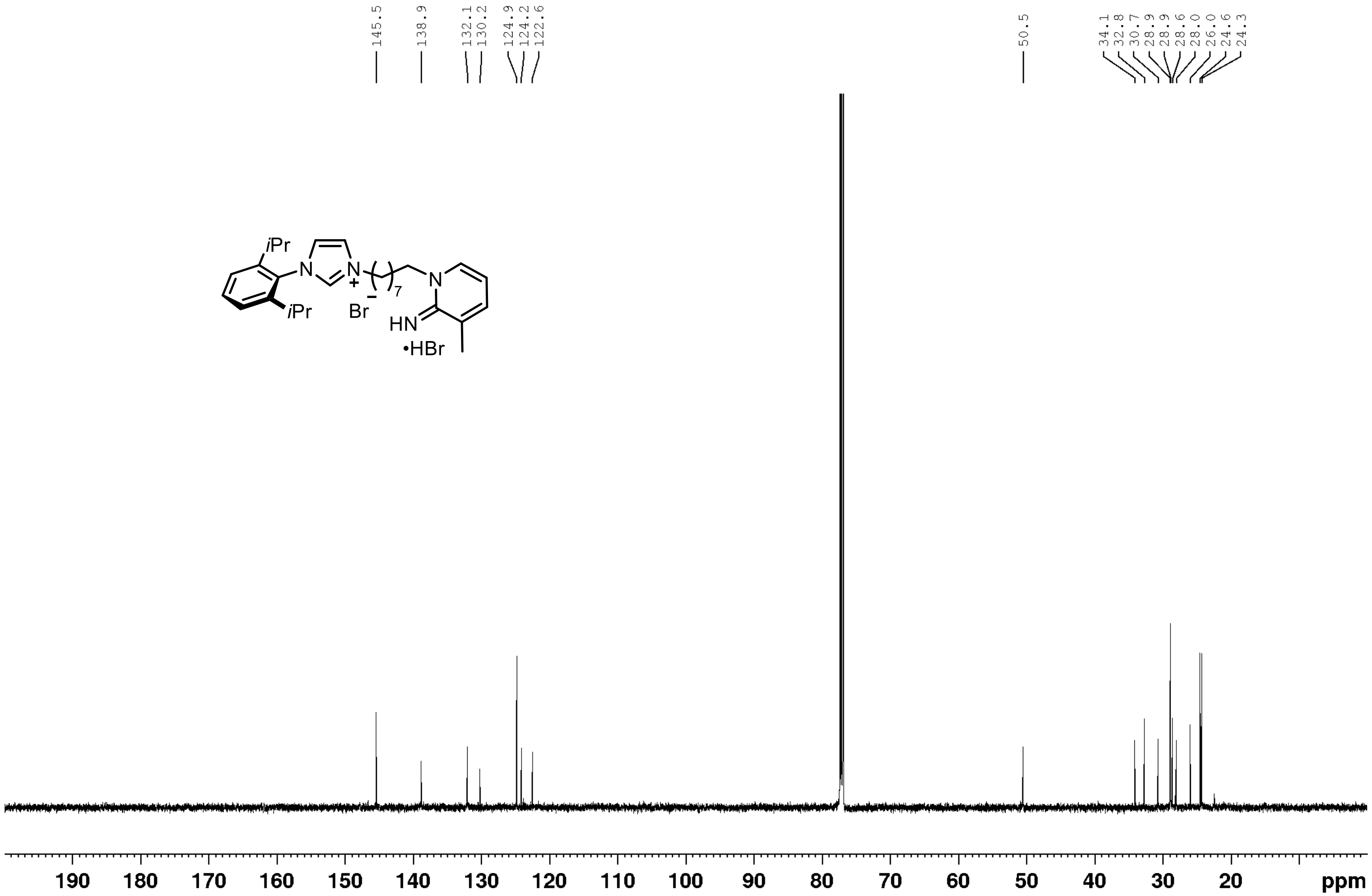

145.5
138.9
132.1
130.2
124.9
124.2
122.6
50.5
34.1
32.8
30.7
28.9
28.9
28.6
28.0
26.0
24.6
24.3
iPr
N
N
+
7
N
iPr
Br
HN
•HBr
190
180
170
160
150
140
130
120
110
100
90
80
70
60
50
40
30
20
ppm

1.29.3 ^{1}H ^{1}H COSY

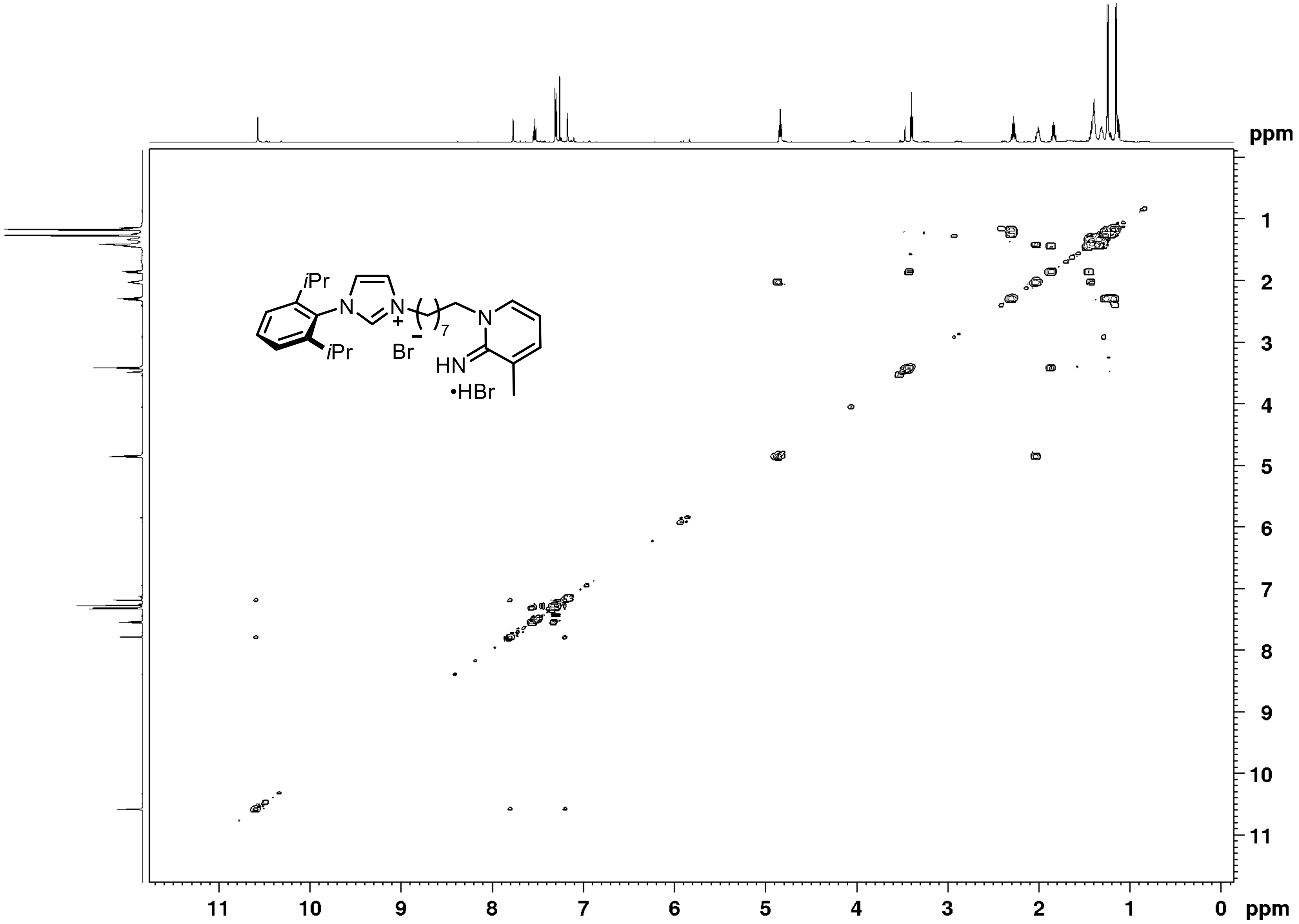

1.29.4 ^{1}H ^{13}C HMBC

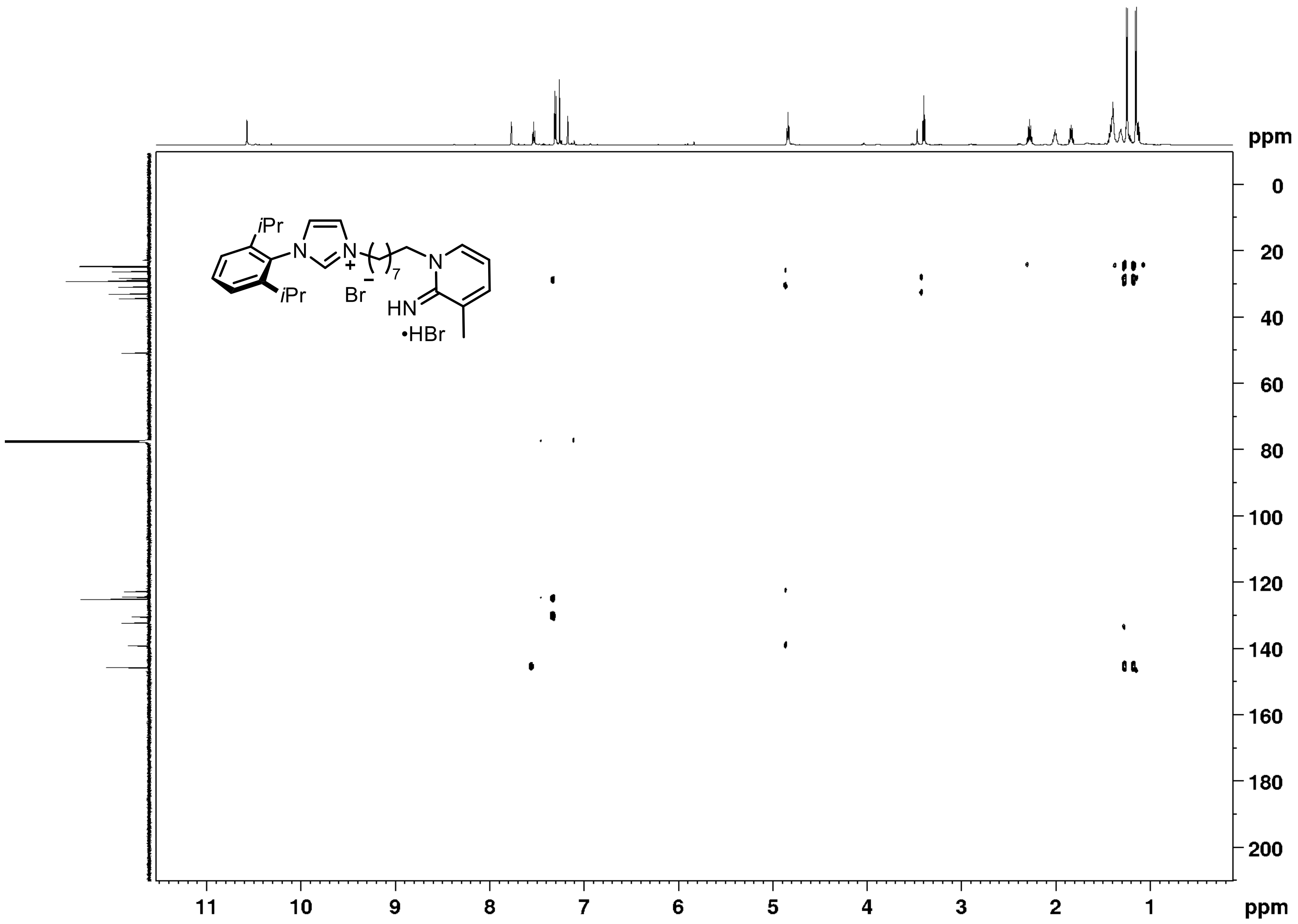

1.29.5 1H 13C HSQC

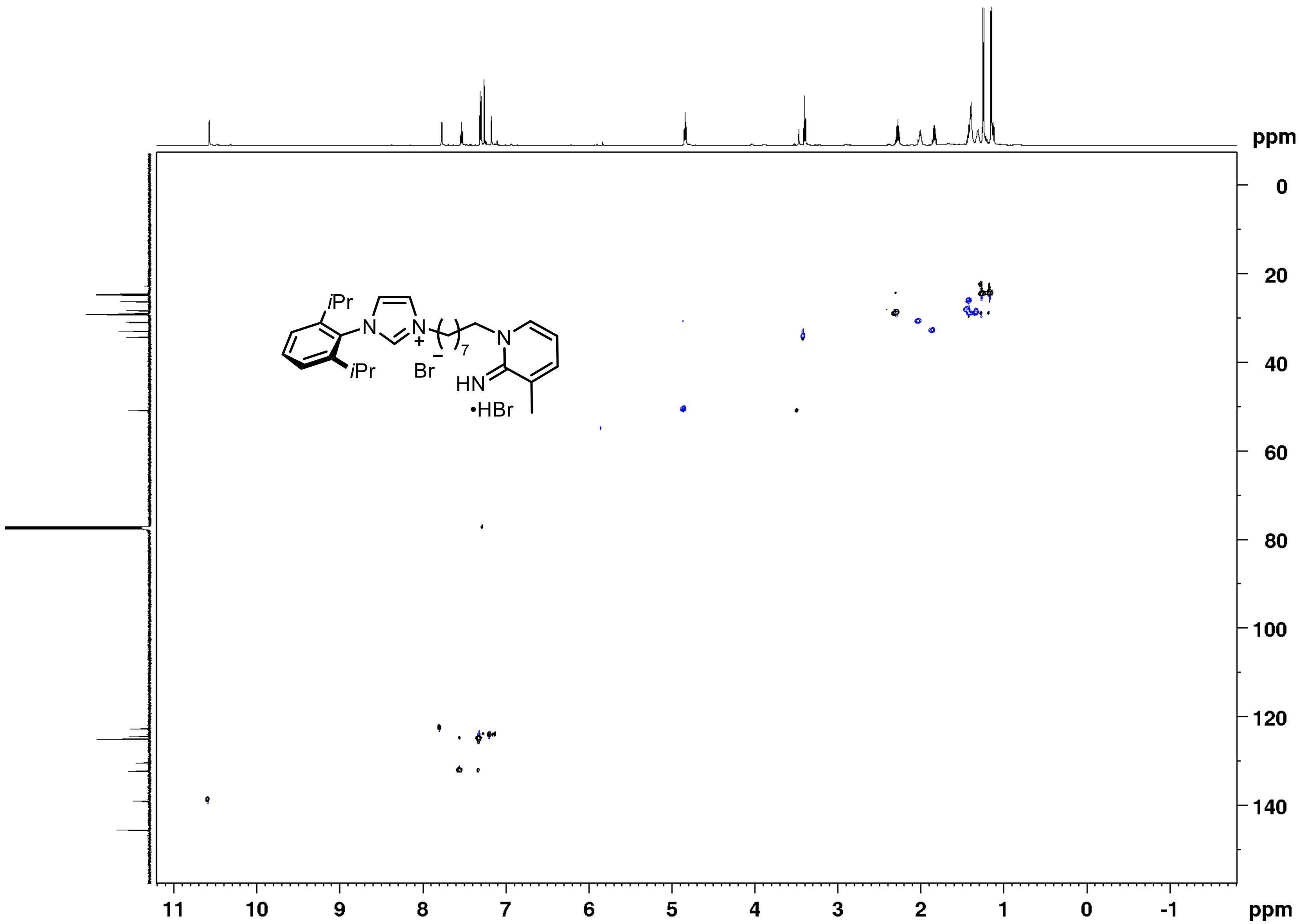

1.29.6 HRMS

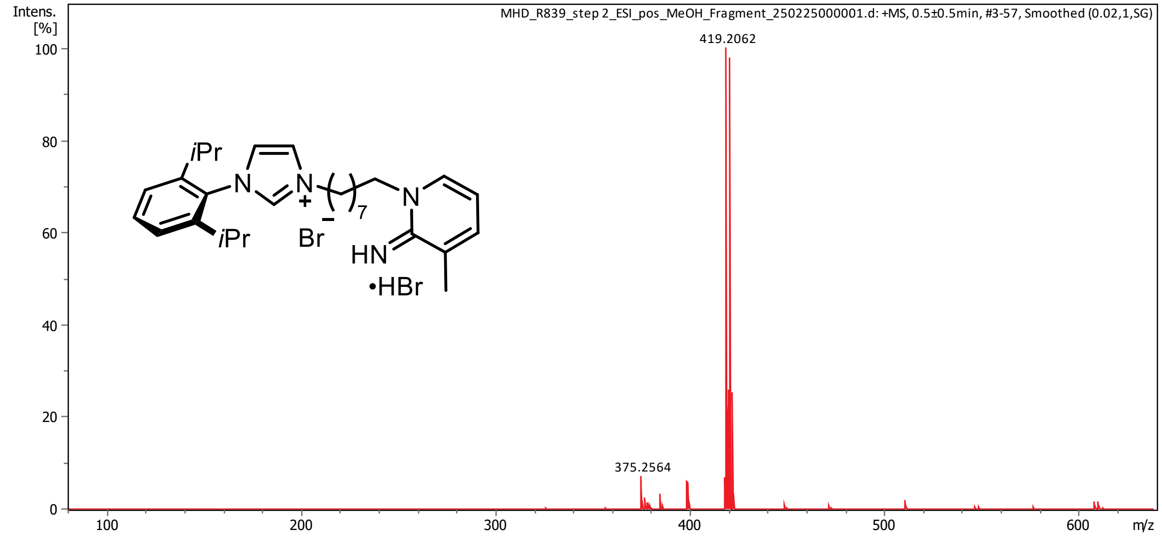

MHD_R839_step 2_ESI_pos_MeOH_Fragment_250225000001.d: +MS, 0.5±0.5min, #3-57, Smoothed (0.02,1,SG)
Intens. [%]
419.2062
375.2564
m/z
iPr
iPr
N
N
+
Br
–
7
N
HN
•HBr

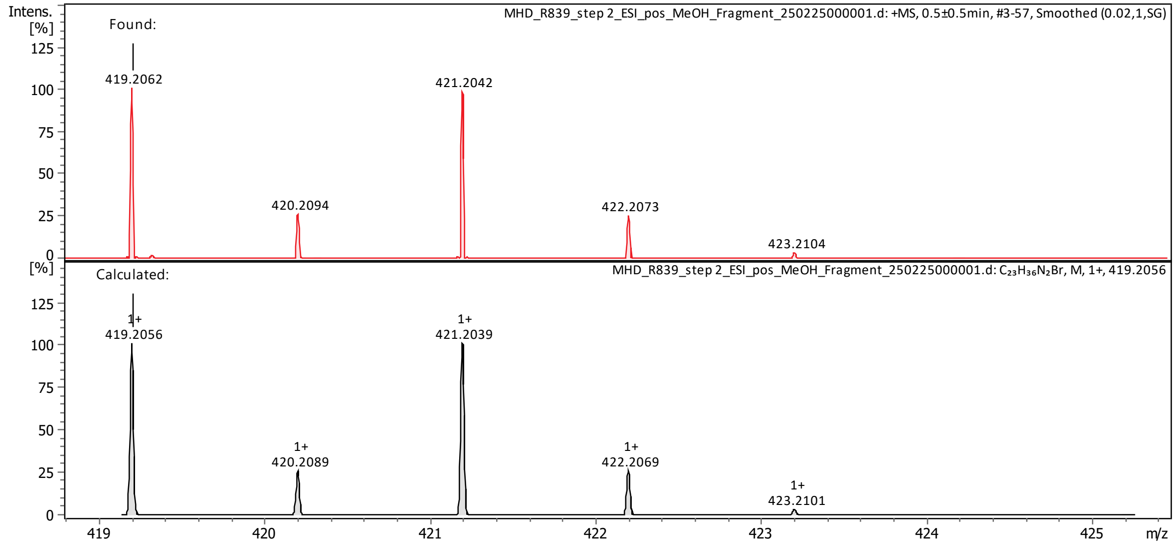

Found:
MHD_R839_step 2_ESI_pos_MeOH_Fragment_250225000001.d: +MS, 0.5±0.5min, #3-57, Smoothed (0.02,1,SG)
419.2062
420.2094
421.2042
422.2073
423.2104
Calculated:
MHD_R839_step 2_ESI_pos_MeOH_Fragment_250225000001.d: C23H36N2Br, M, 1+, 419.2056
1+ 419.2056
1+ 420.2089
1+ 421.2039
1+ 422.2069
1+ 423.2101
m/z

1.30 *tert*-Butoxy(1-(2,6-di*iso*propylphenyl)-3-(2-(2-imino-3-methylpyridin-1(2*H*)-yl)ethyl)-2,3-dihydro-1*H*-imidazol-2-yl)copper (I) (I) (C_6D_6, 600 MHz)

1.30.1 ^{1}H NMR

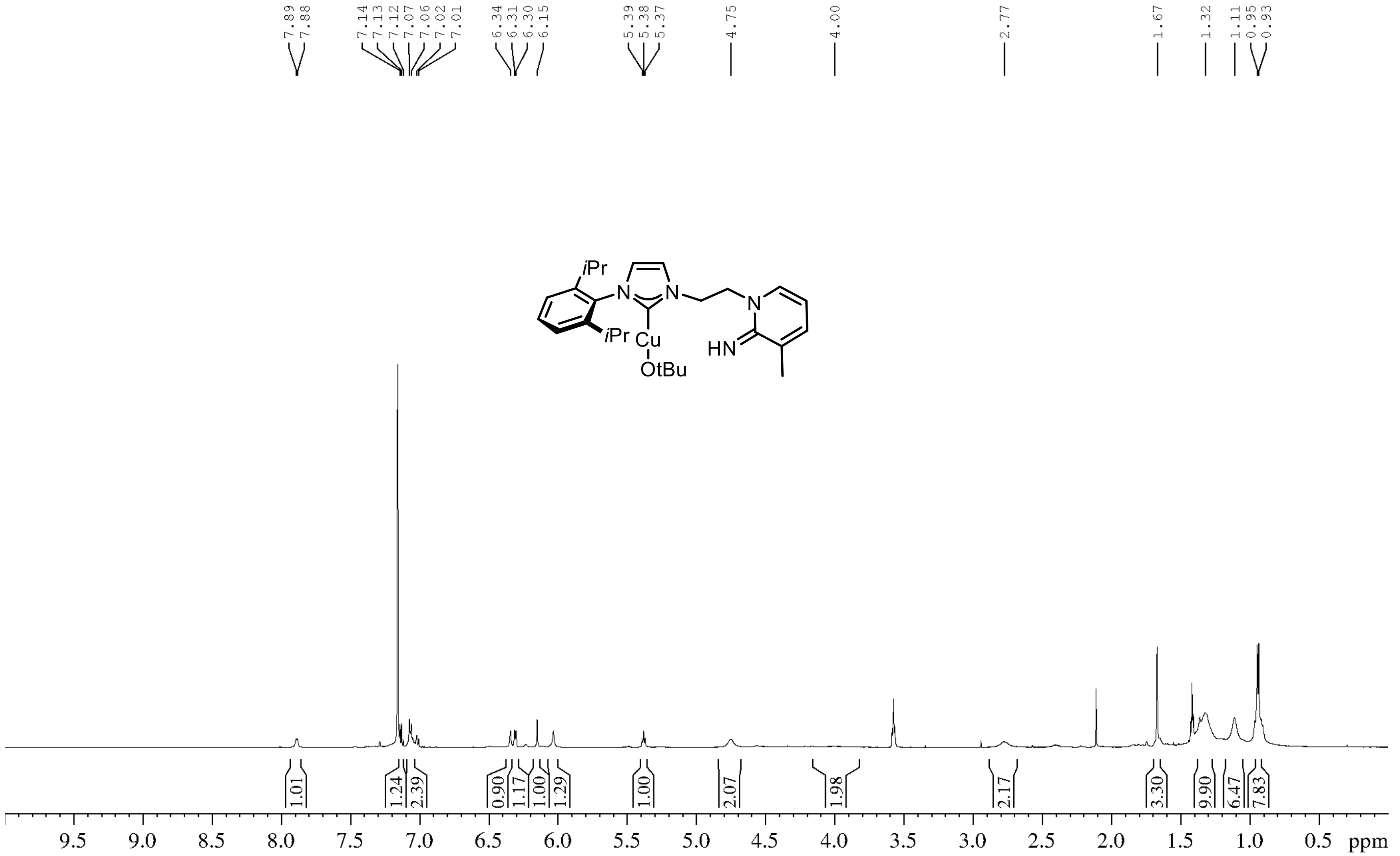

1.30.2 ^{13}C NMR

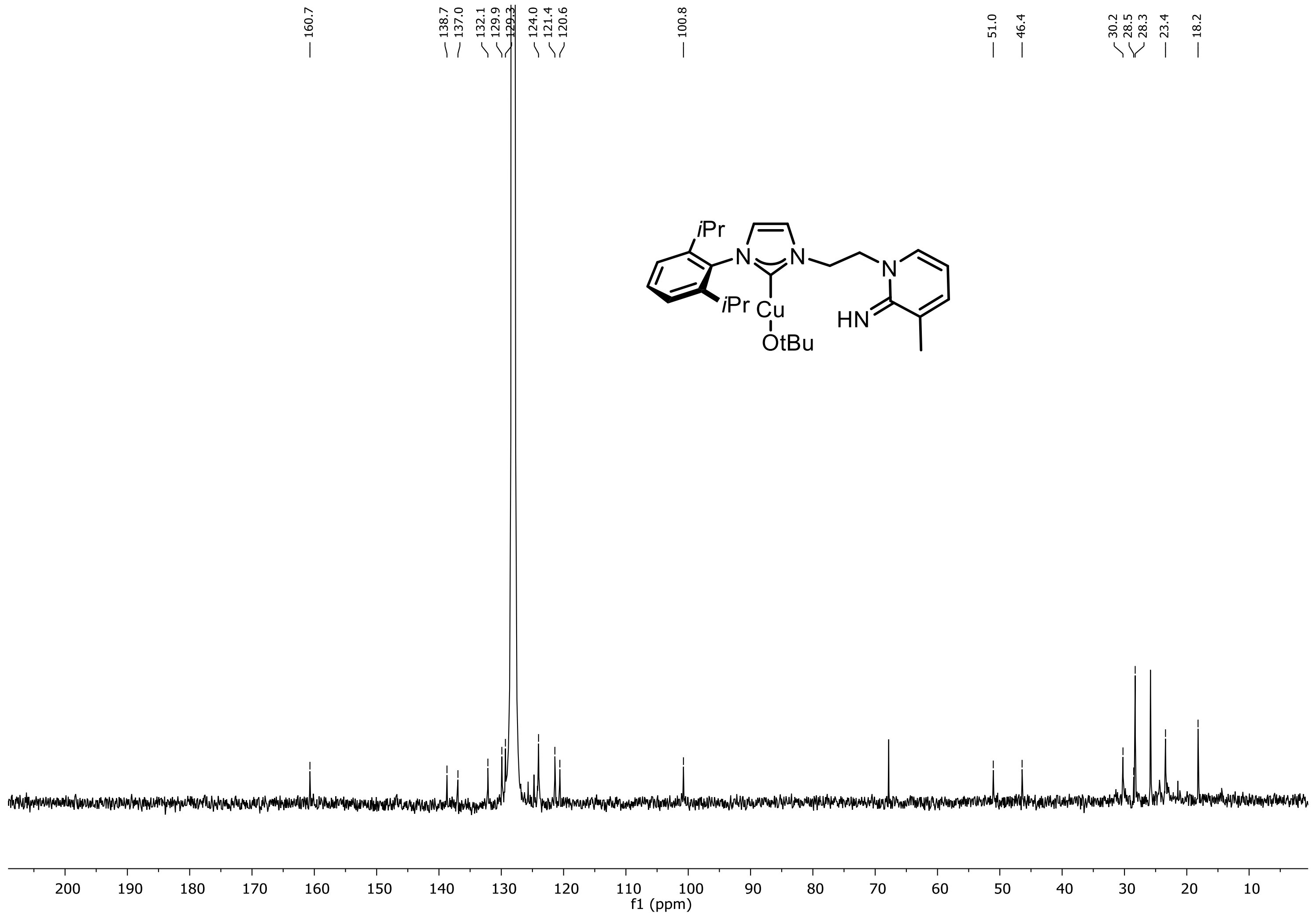

160.7
138.7
137.0
132.1
129.9
129.2
124.0
121.4
120.6
100.8
51.0
46.4
30.2
28.5
28.3
23.4
18.2
iPr
N
N
N
iPr
Cu
OtBu
HN
200
190
180
170
160
150
140
130
120
110
100
90
80
70
60
50
40
30
20
10
f1 (ppm)

1.30.3 ^{1}H, ^{13}C HSQC

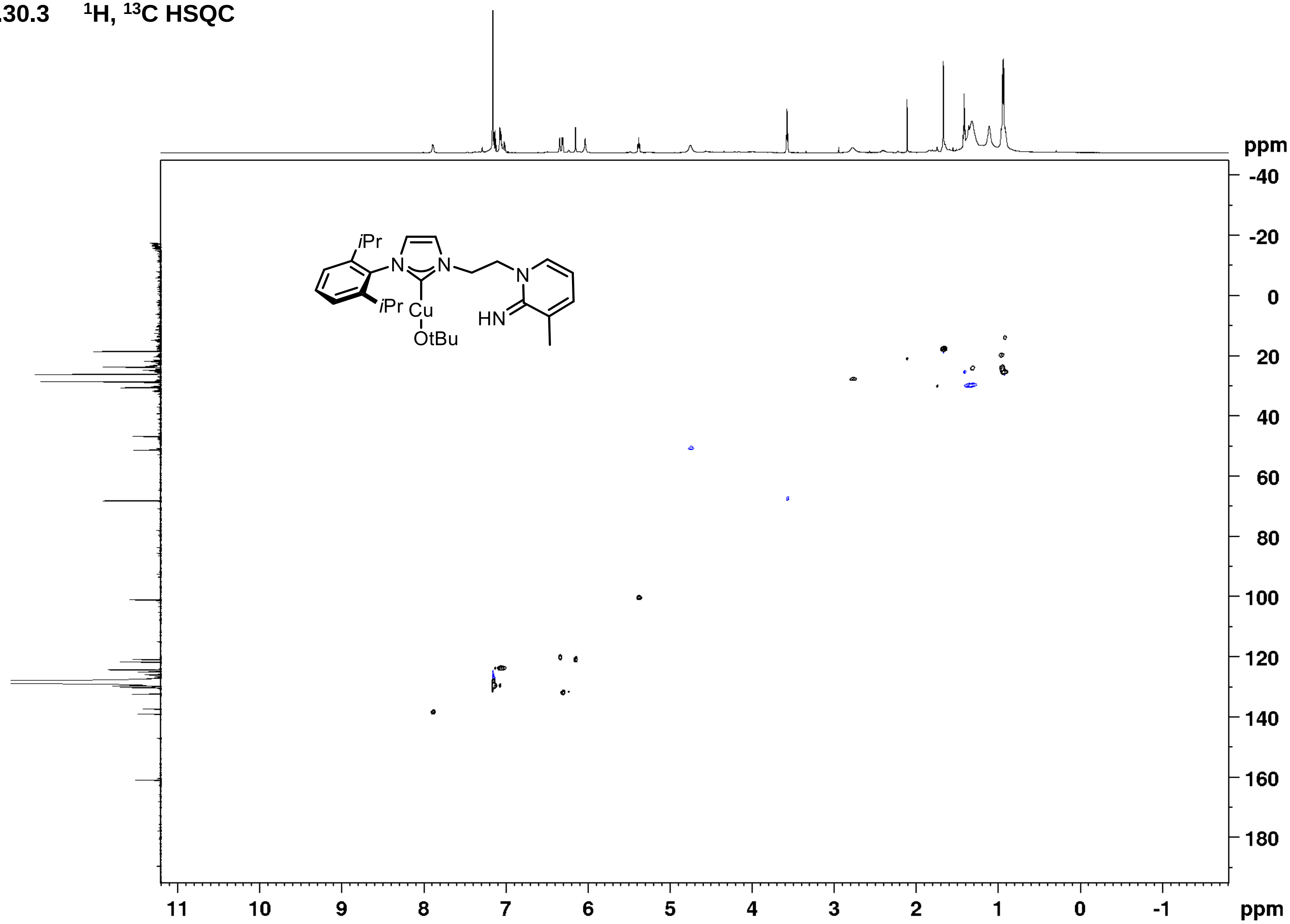

1.30.4 ^{1}H, ^{13}C HMBC

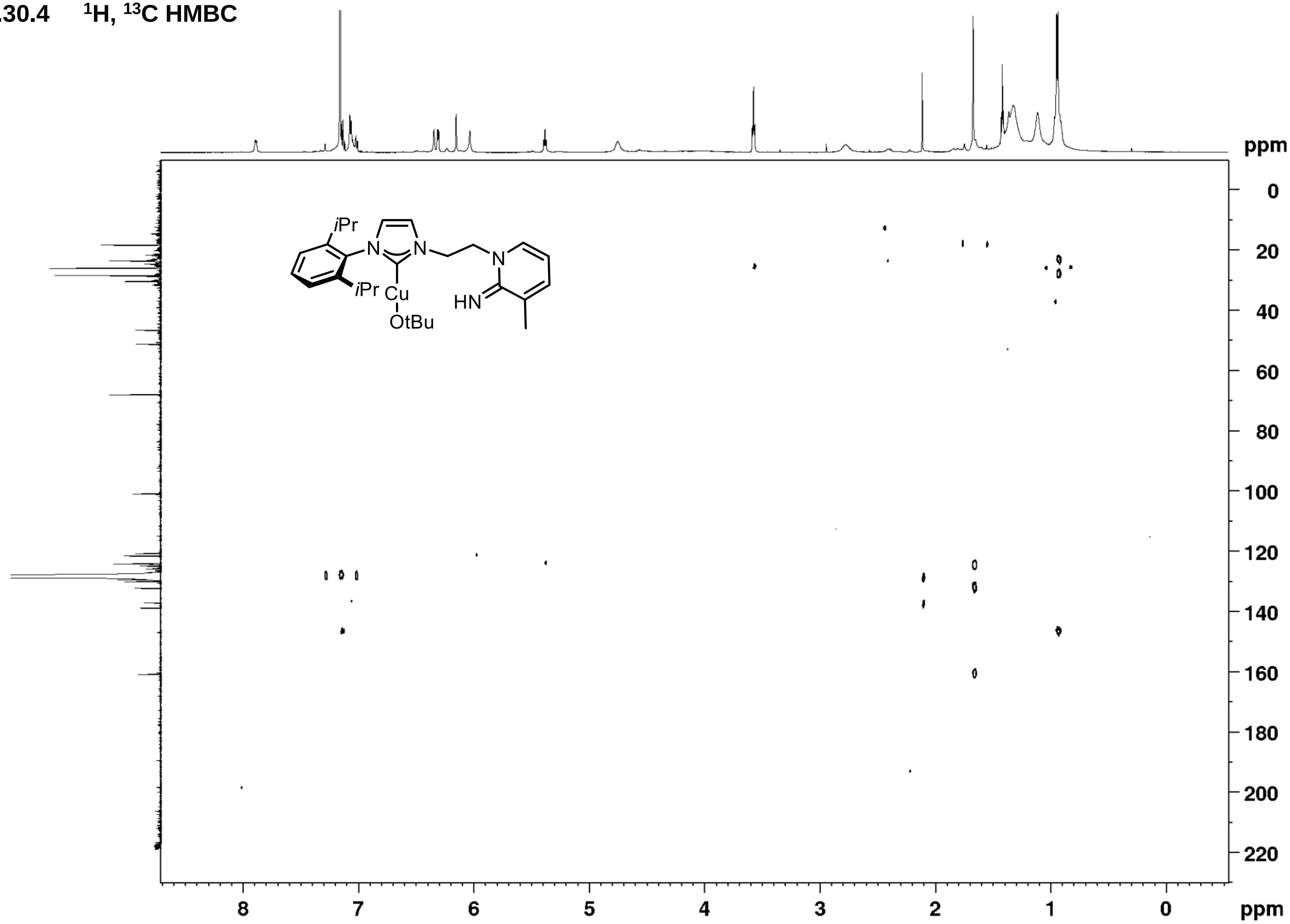

1.30.5 HRMS

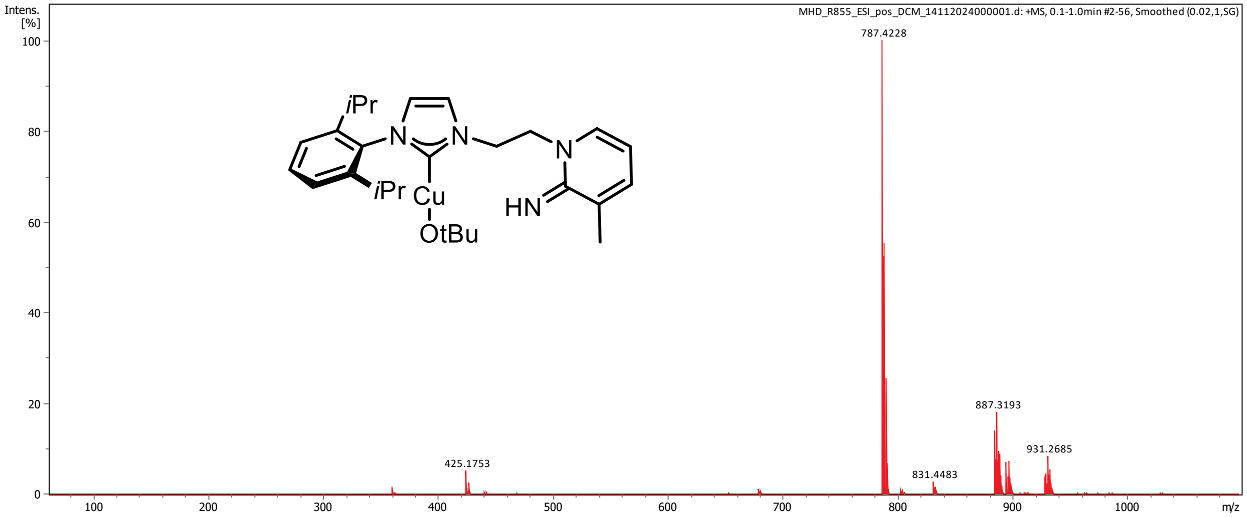

Intens. [%]
MHD_R855_ESI_pos_DCM_14112024000001.d: +MS, 0.1-1.0min #2-56, Smoothed (0.02,1,SG)
787.4228
887.3193
931.2685
831.4483
425.1753
iPr
iPr
N
N
N
Cu
OtBu
HN
m/z

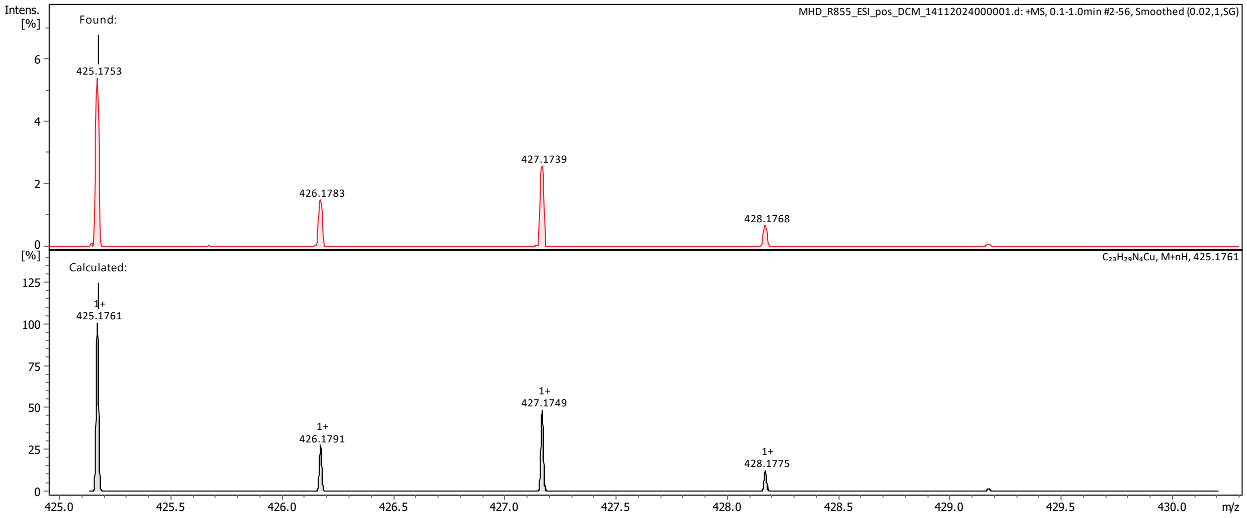

Intens. [%]
MHD_R855_ESI_pos_DCM_14112024000001.d: +MS, 0.1-1.0min #2-56, Smoothed (0.02,1,SG)
Found:
425.1753
426.1783
427.1739
428.1768
Calculated:
C23H29N4Cu, M+nH, 425.1761
1+
425.1761
1+
426.1791
1+
427.1749
1+
428.1775
m/z

1.31 1-(2,6-Diisopropylphenyl)-3-(2-(2-imino-3-methylpyridin-1(2*H*)-yl)ethyl)-1,3-dihydro-2*H*-imidazol-2-ylidene)silver(I) bromide (S4d) (600 MHz, CD_2Cl_2)

1.31.1 ^1H NMR in $CDCl_3$

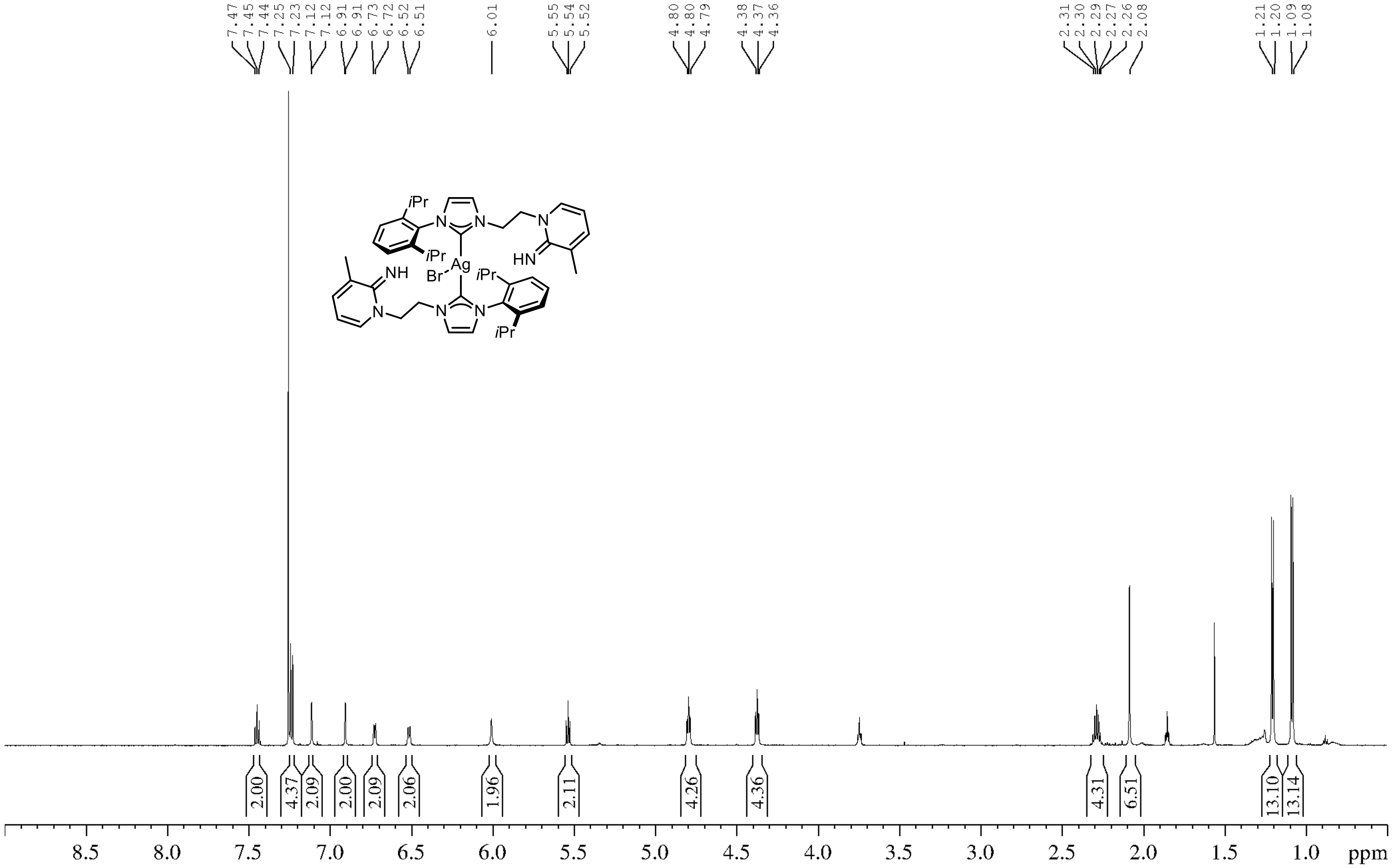

1.31.2 ^{1}H NMR in CD_2Cl_2

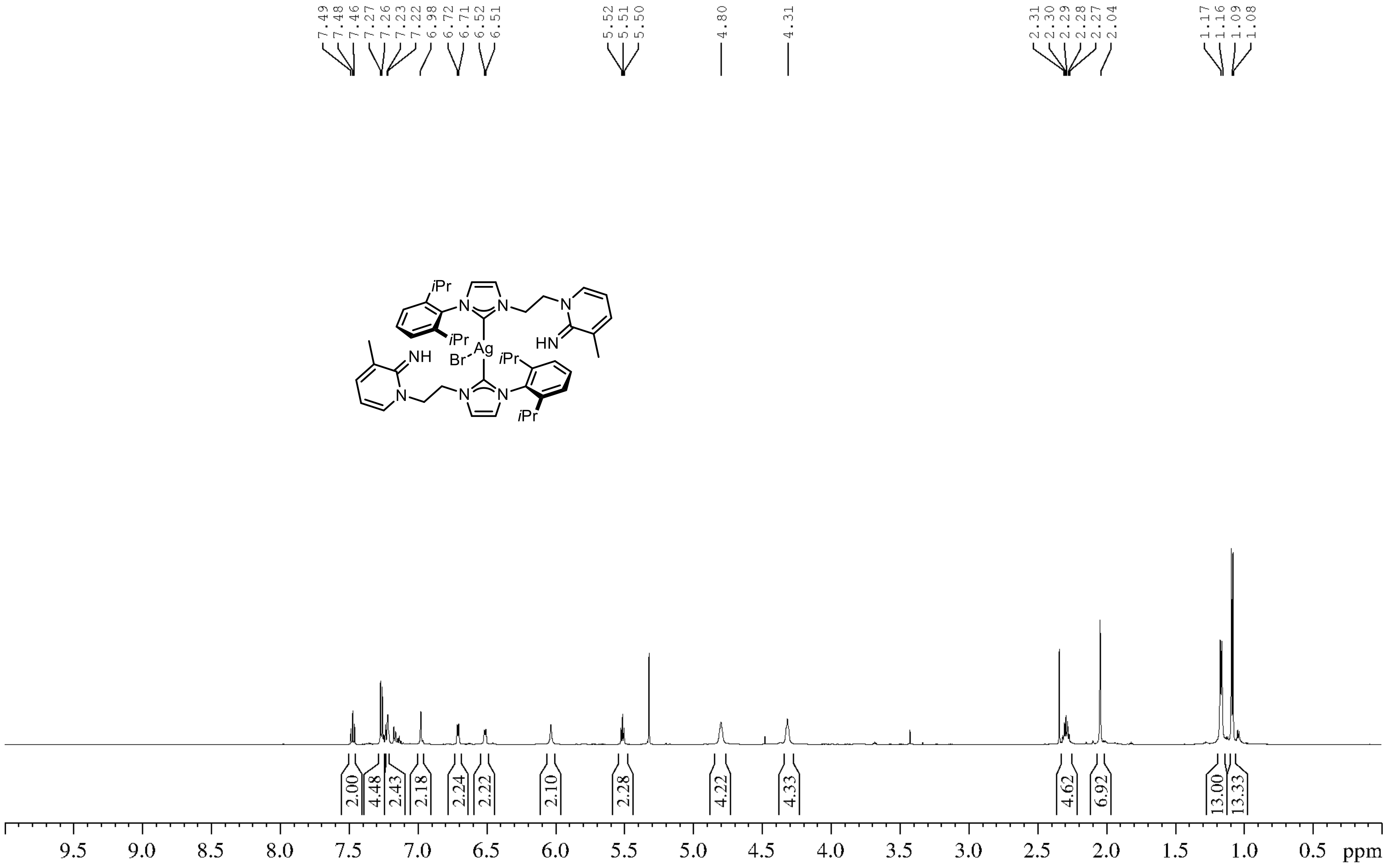

1.31.3 ^{13}C NMR

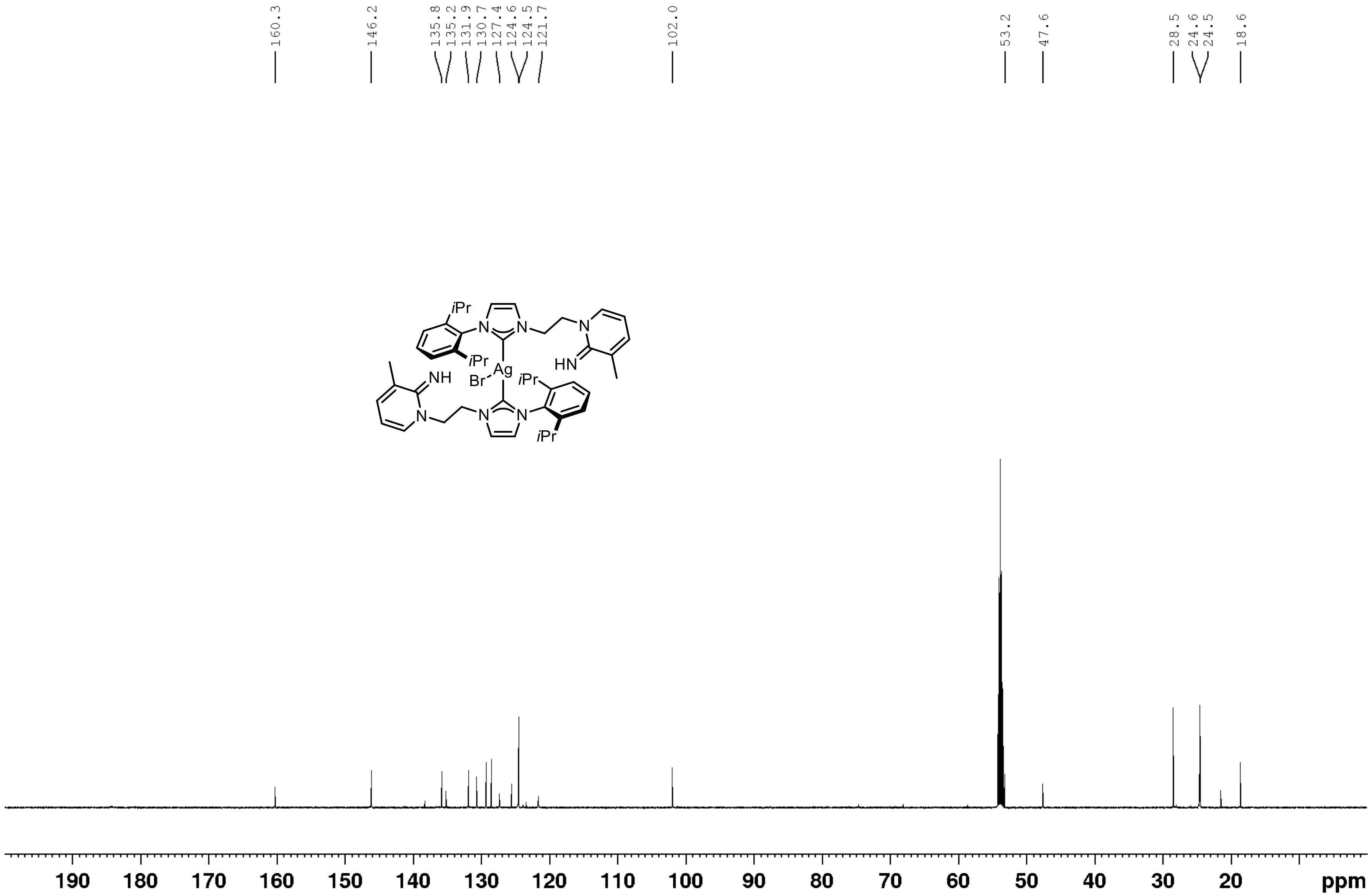
160.3
146.2
135.8
135.2
131.9
130.7
127.4
124.6
124.5
121.7
102.0
53.2
47.6
28.5
24.6
24.5
18.6
iPr
N
N
N
iPr
HN
NH
Br
Ag
iPr
N
N
N
iPr
190
180
170
160
150
140
130
120
110
100
90
80
70
60
50
40
30
20
ppm

1.31.4 ¹H, ¹H COSY

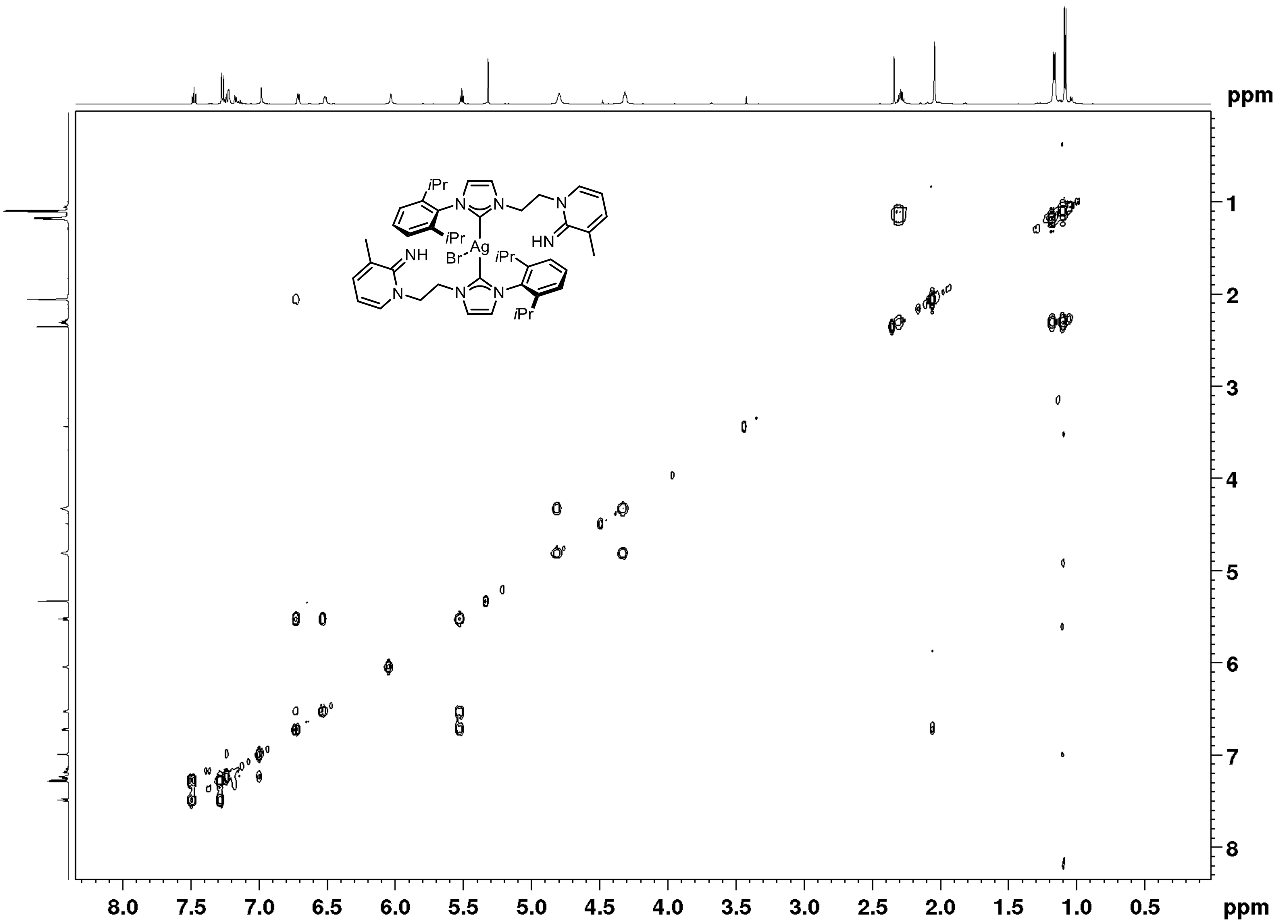

1.31.5 ^1H ^{13}C HMBC

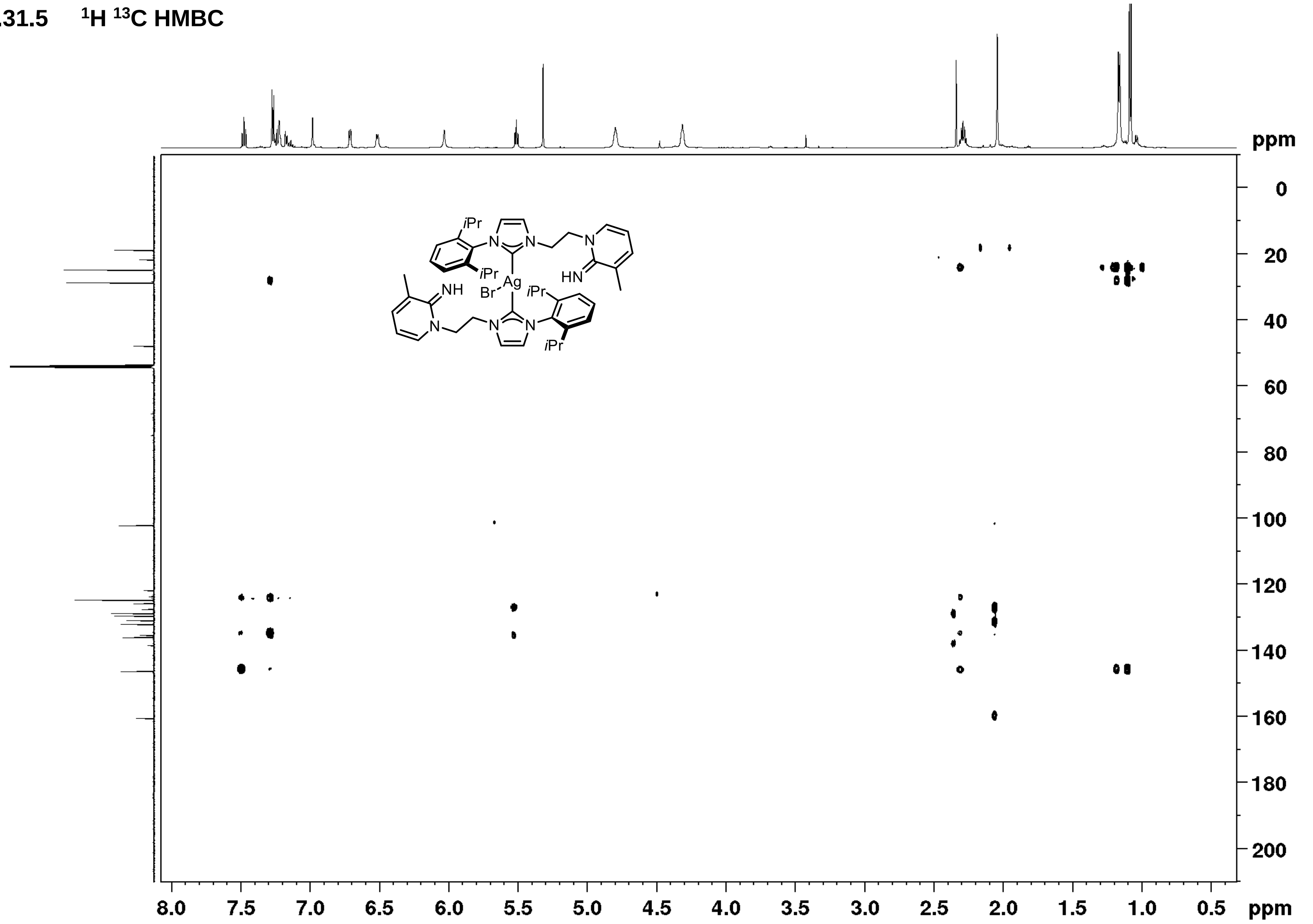

1.31.6 ^{1}H ^{13}C HMBC

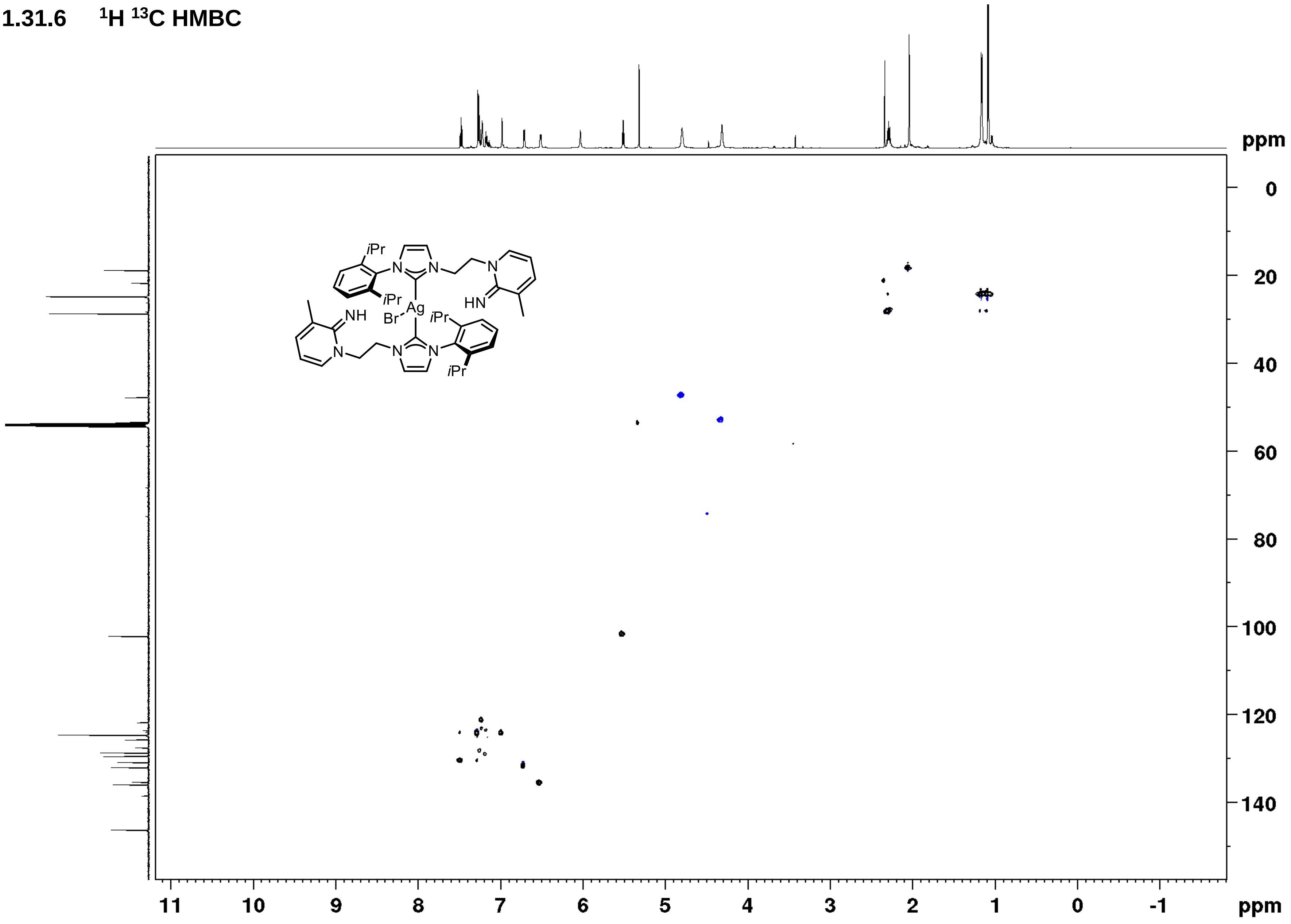

1.31.7 HRMS

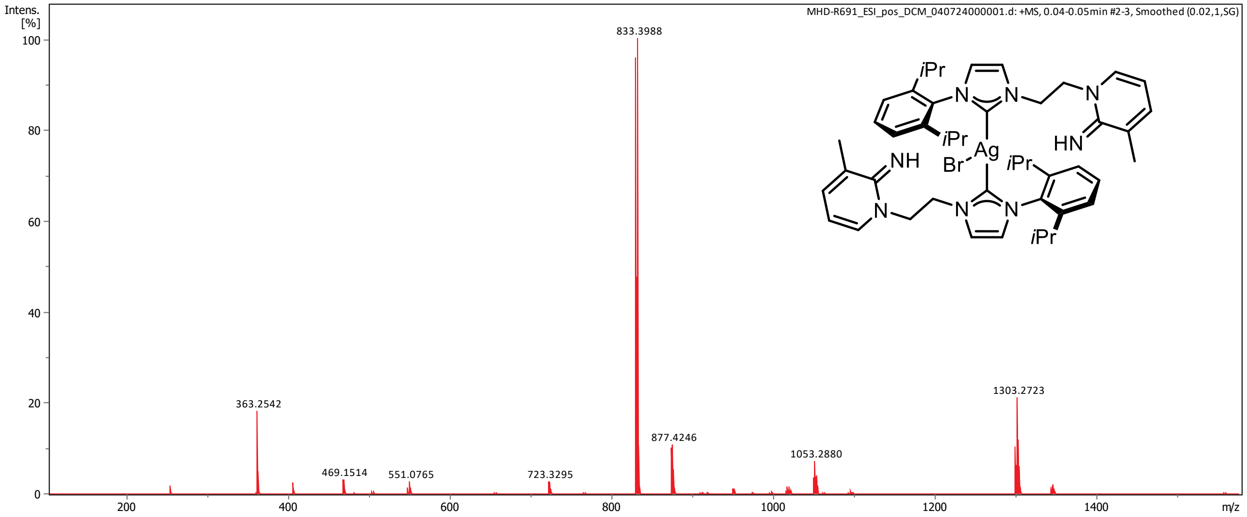

MHD-R691_ESI_pos_DCM_040724000001.d: +MS, 0.04-0.05min #2-3, Smoothed (0.02,1,SG)
Intens. [%]
833.3988
363.2542
469.1514
551.0765
723.3295
877.4246
1053.2880
1303.2723
m/z
iPr
NH
HN
Ag
Br

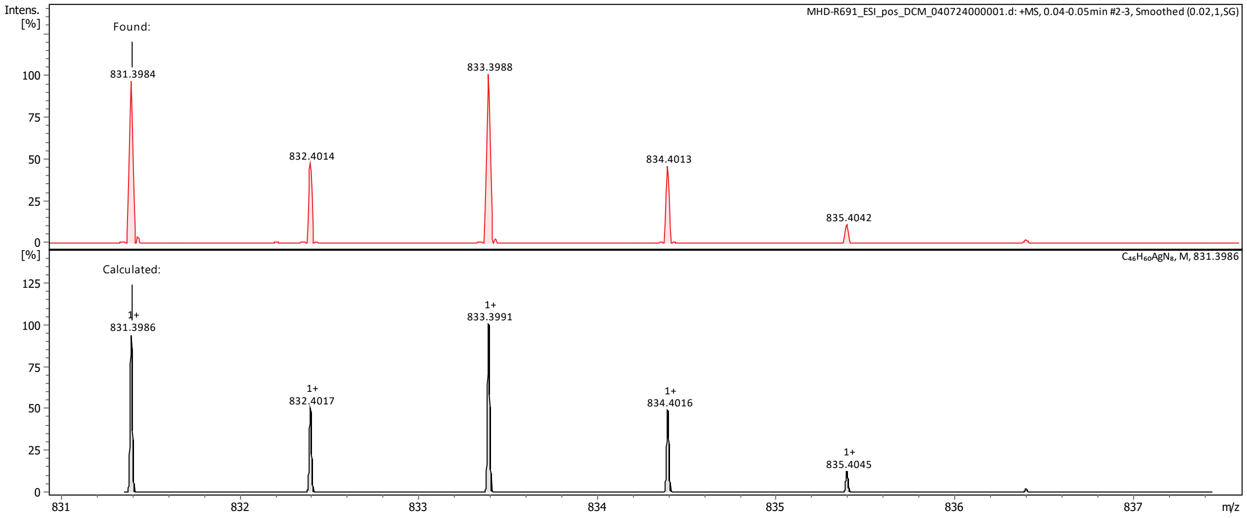

MHD-R691_ESI_pos_DCM_040724000001.d: +MS, 0.04-0.05min #2-3, Smoothed (0.02,1,SG)
Found:
831.3984
832.4014
833.3988
834.4013
835.4042
Calculated:
C46H60AgN8, M, 831.3986
1+ 831.3986
1+ 832.4017
1+ 833.3991
1+ 834.4016
1+ 835.4045
m/z

2 Alkyne semihydrogenation products

2.1 (*Z*)-1,2-Diphenylethene (2a) (600 MHz, $CDCl_3$)

2.1.1 ^{1}H NMR

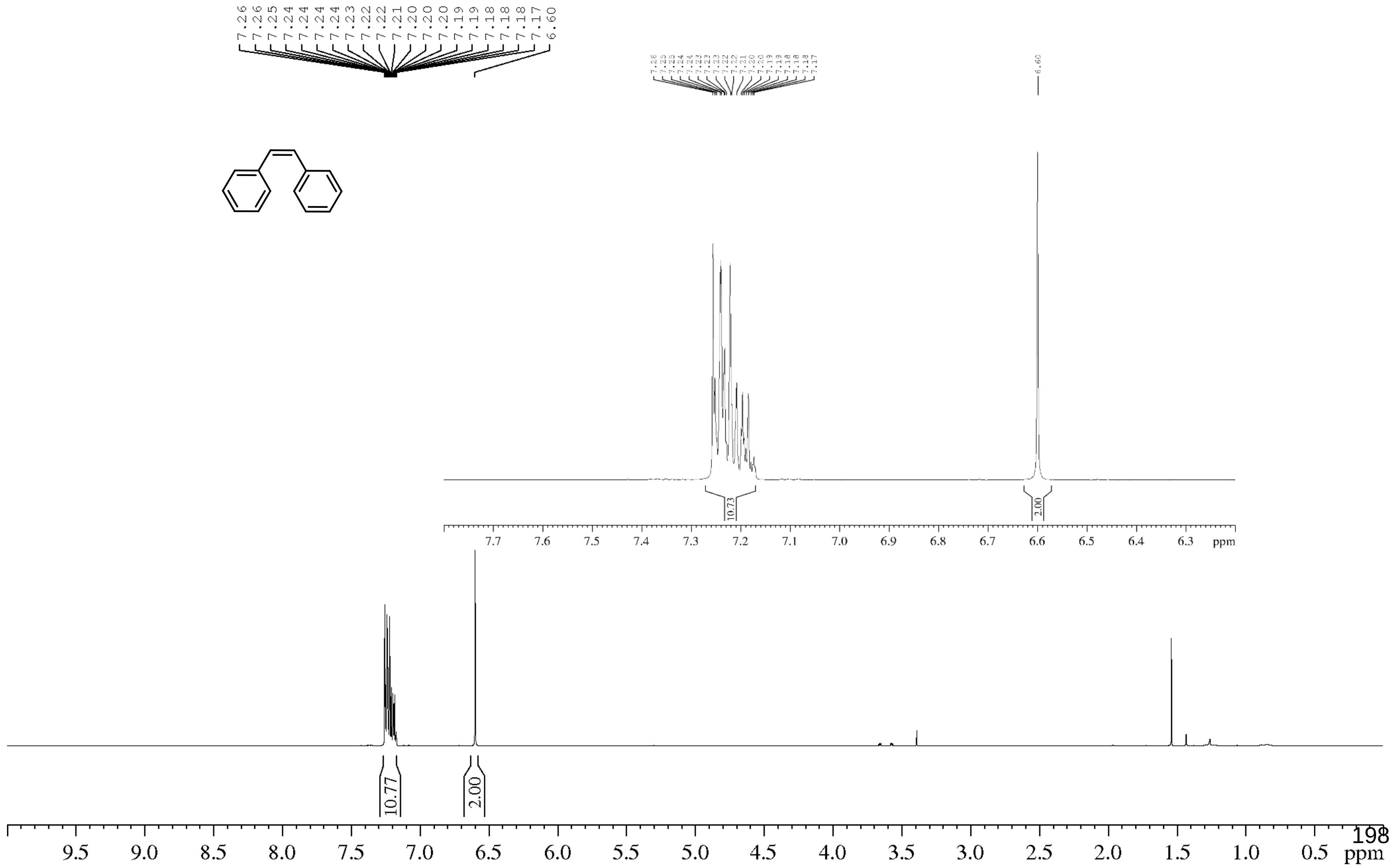

2.1.2 ^{13}C NMR

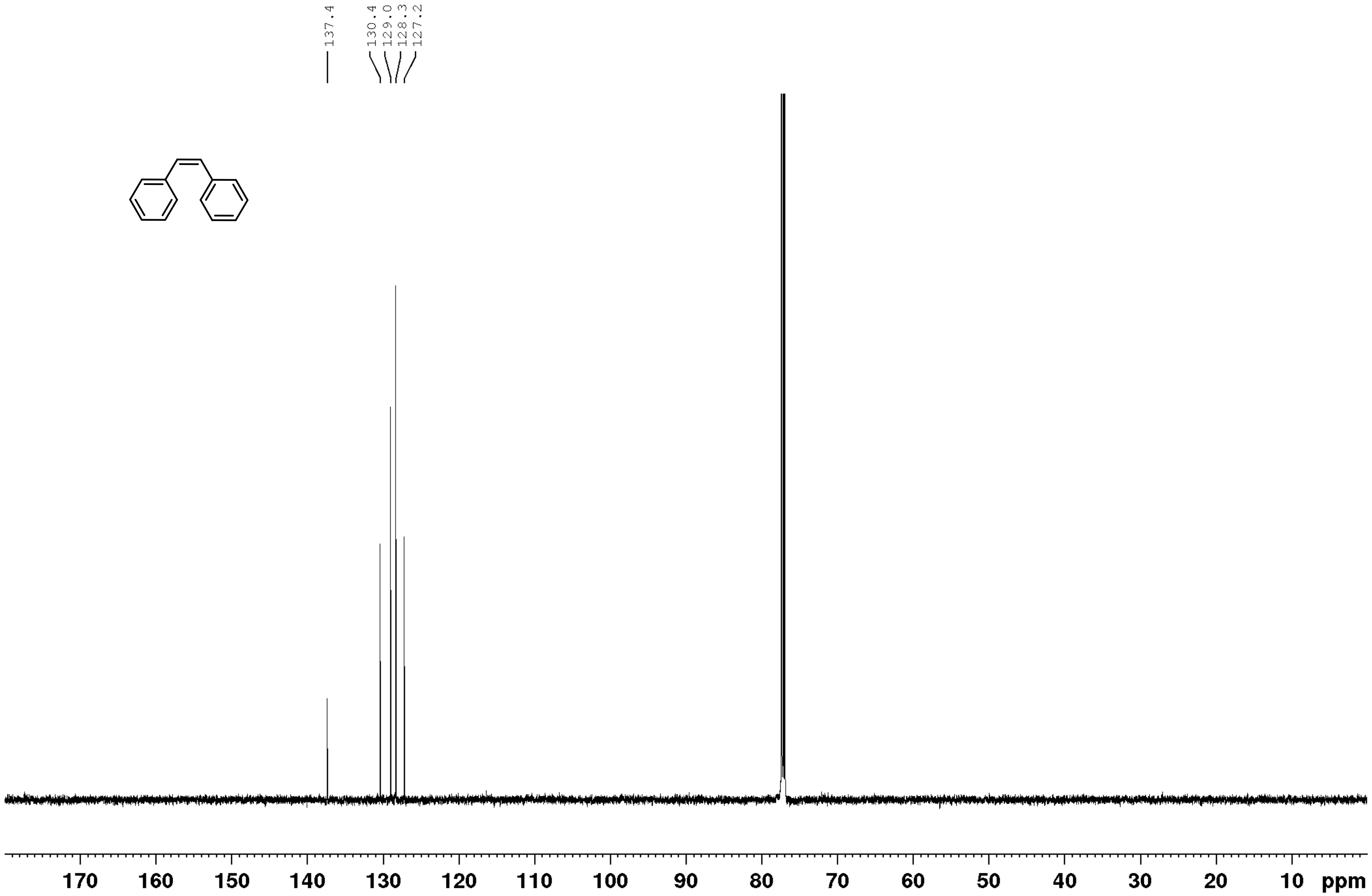
137.4
130.4
129.0
128.3
127.2
170
160
150
140
130
120
110
100
90
80
70
60
50
40
30
20
10
ppm

2.1.3 $^{1}H, ^{1}H$ COSY

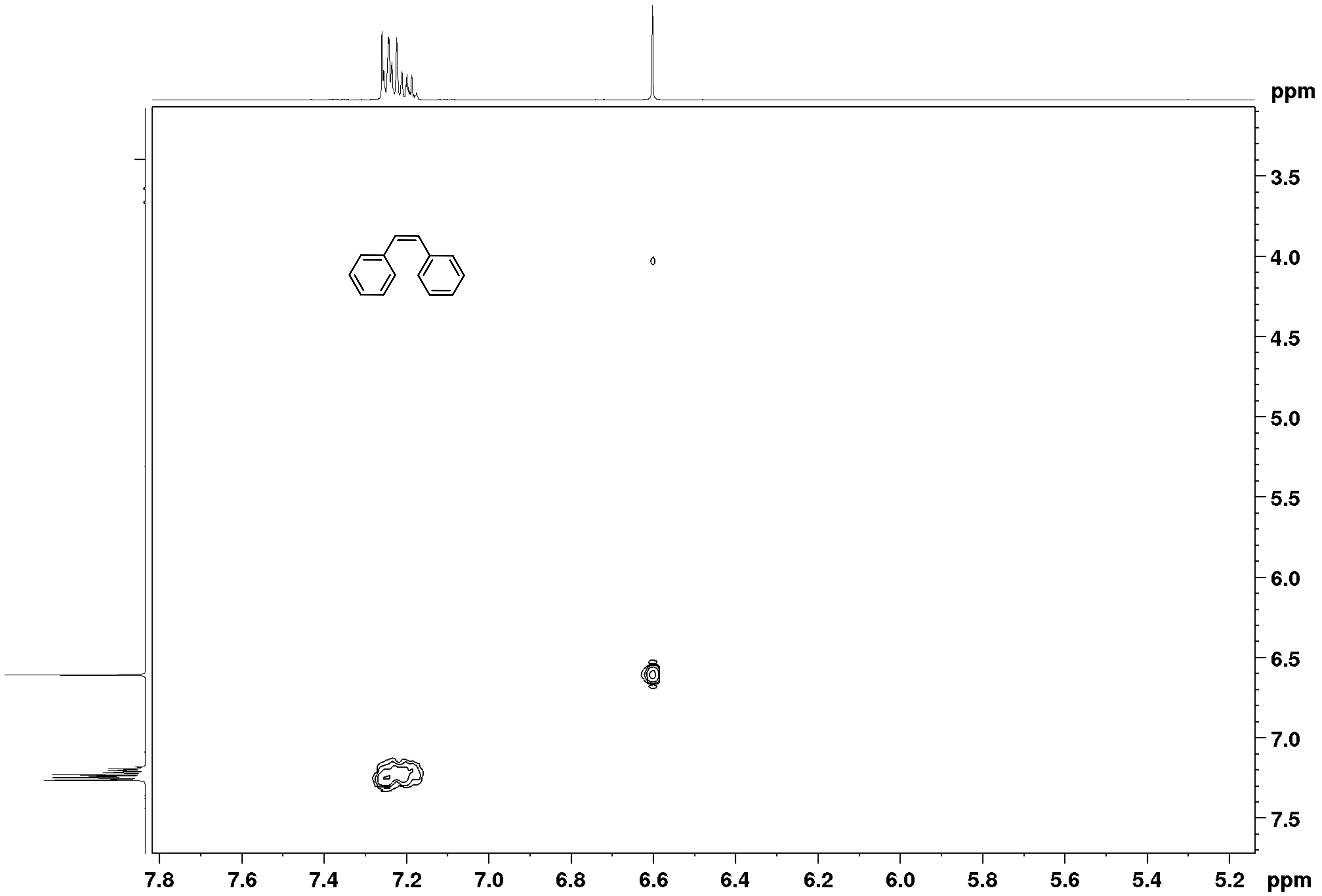

2.1.4 ^{1}H ^{13}C HMBC

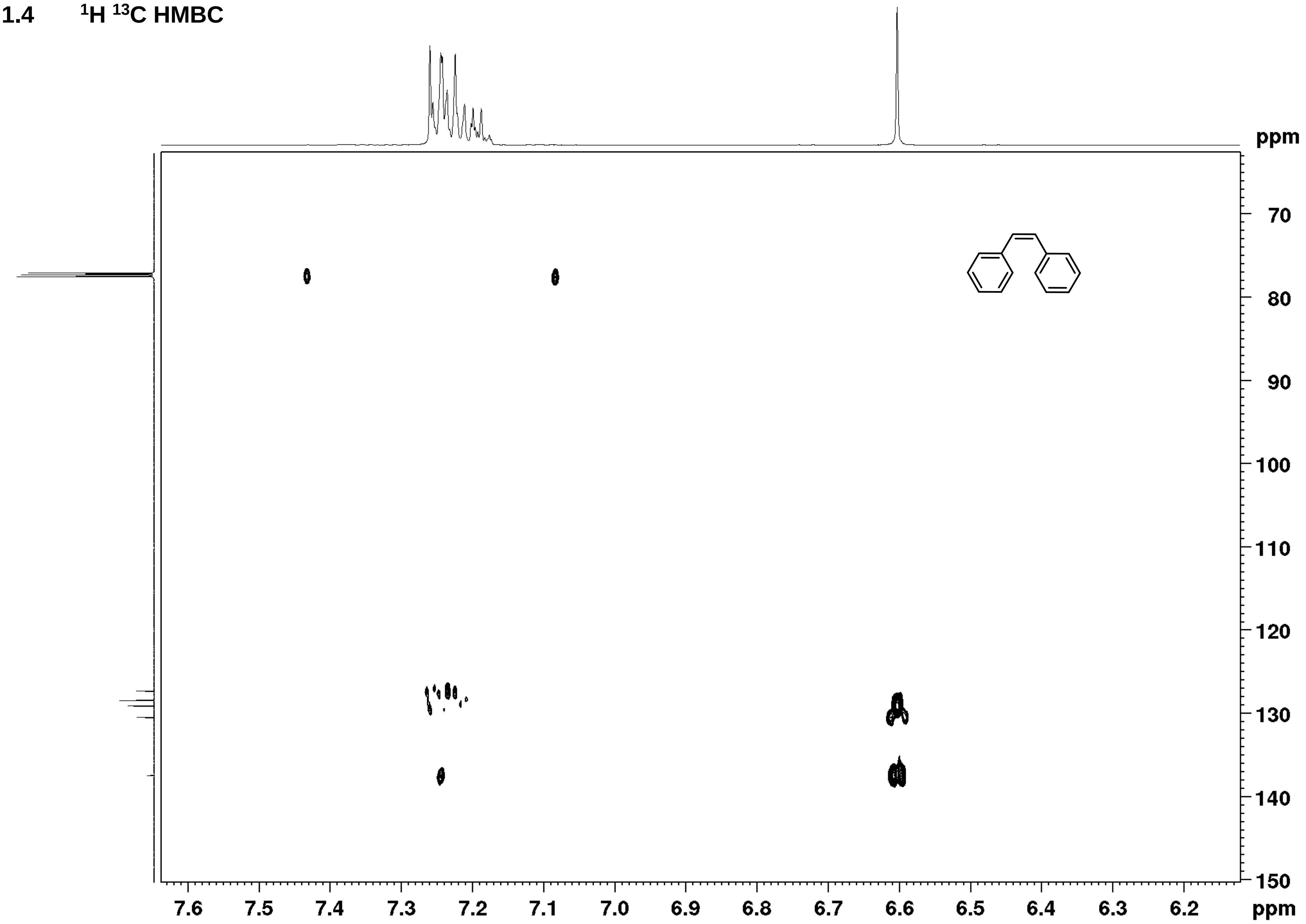

2.1.5 ^{1}H ^{13}C HSQC

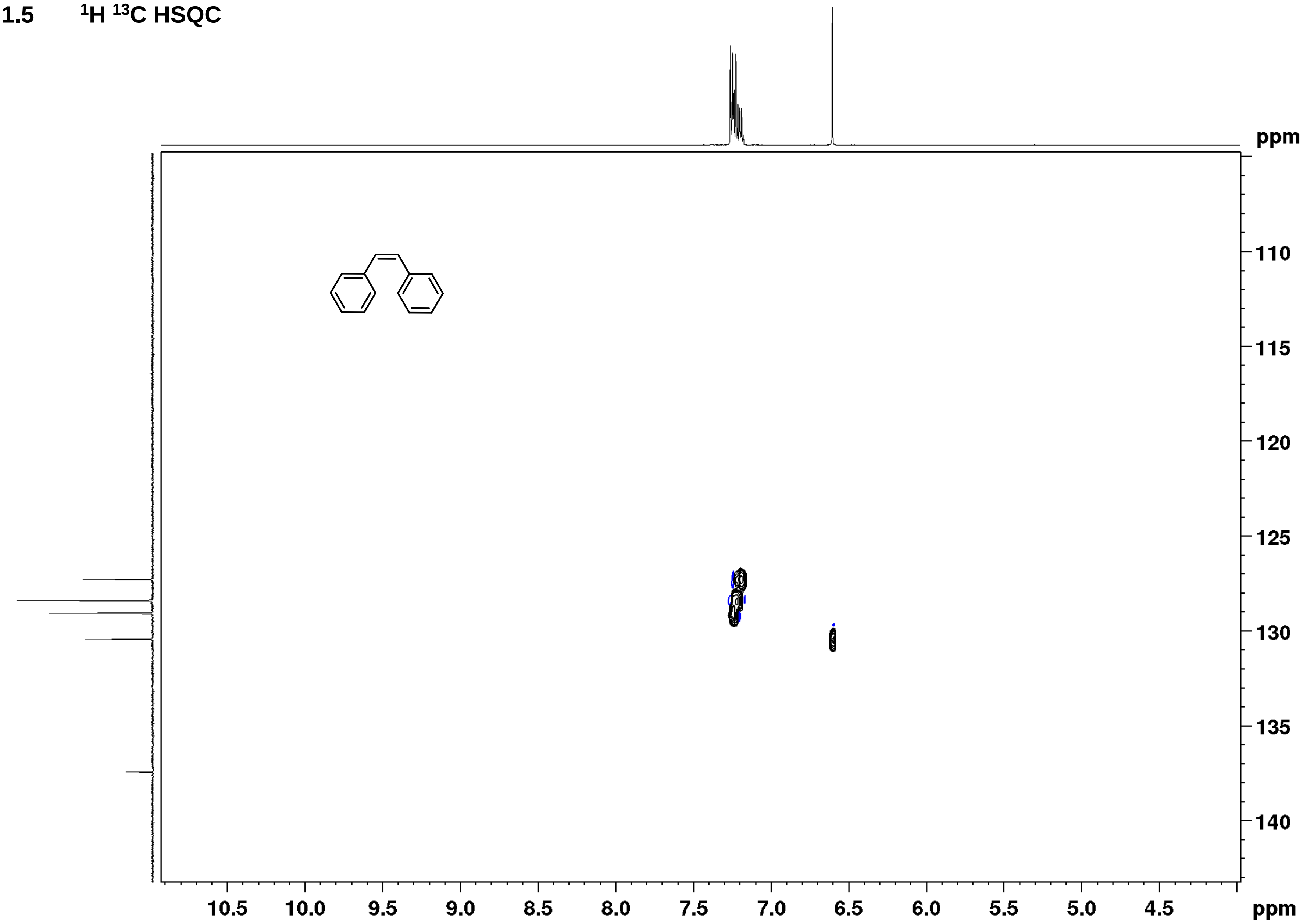

2.1.6 HRMS

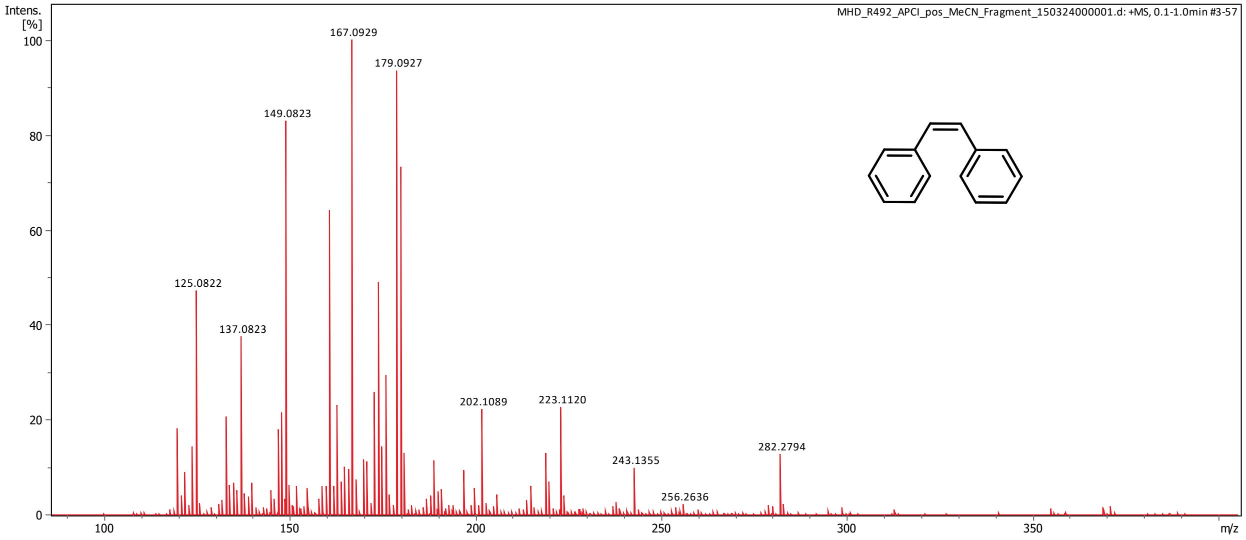
Intens. [%]
MHD_R492_APCI_pos_MeCN_Fragment_150324000001.d: +MS, 0.1-1.0min #3-57
167.0929
179.0927
149.0823
125.0822
137.0823
202.1089
223.1120
243.1355
256.2636
282.2794
m/z

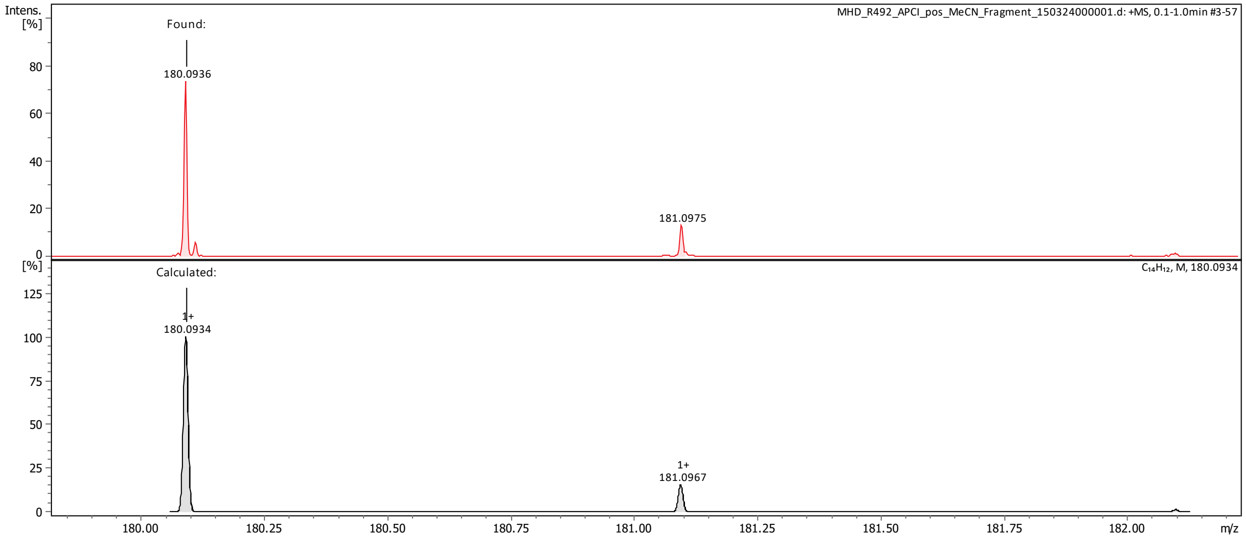
Intens. [%]
MHD_R492_APCI_pos_MeCN_Fragment_150324000001.d: +MS, 0.1-1.0min #3-57
Found:
180.0936
181.0975
Calculated:
C14H12, M, 180.0934
1+
180.0934
1+
181.0967
m/z

2.2 (*Z*)-1-(Benzyloxy)-4-styrylbenzene (2b) (600 MHz, $CDCl_3$)

2.2.1 ^{1}H NMR

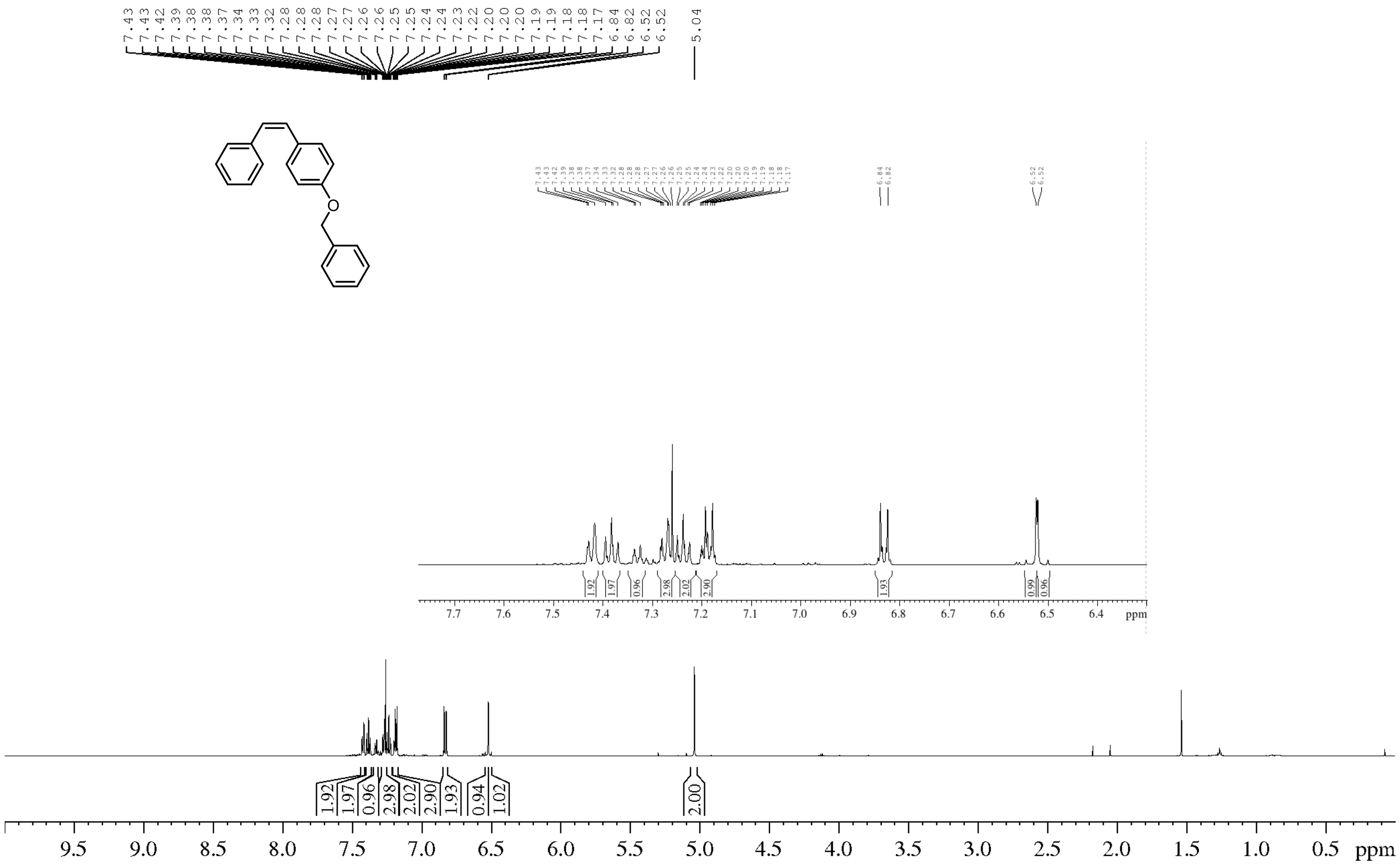

2.2.2 ^{13}C NMR

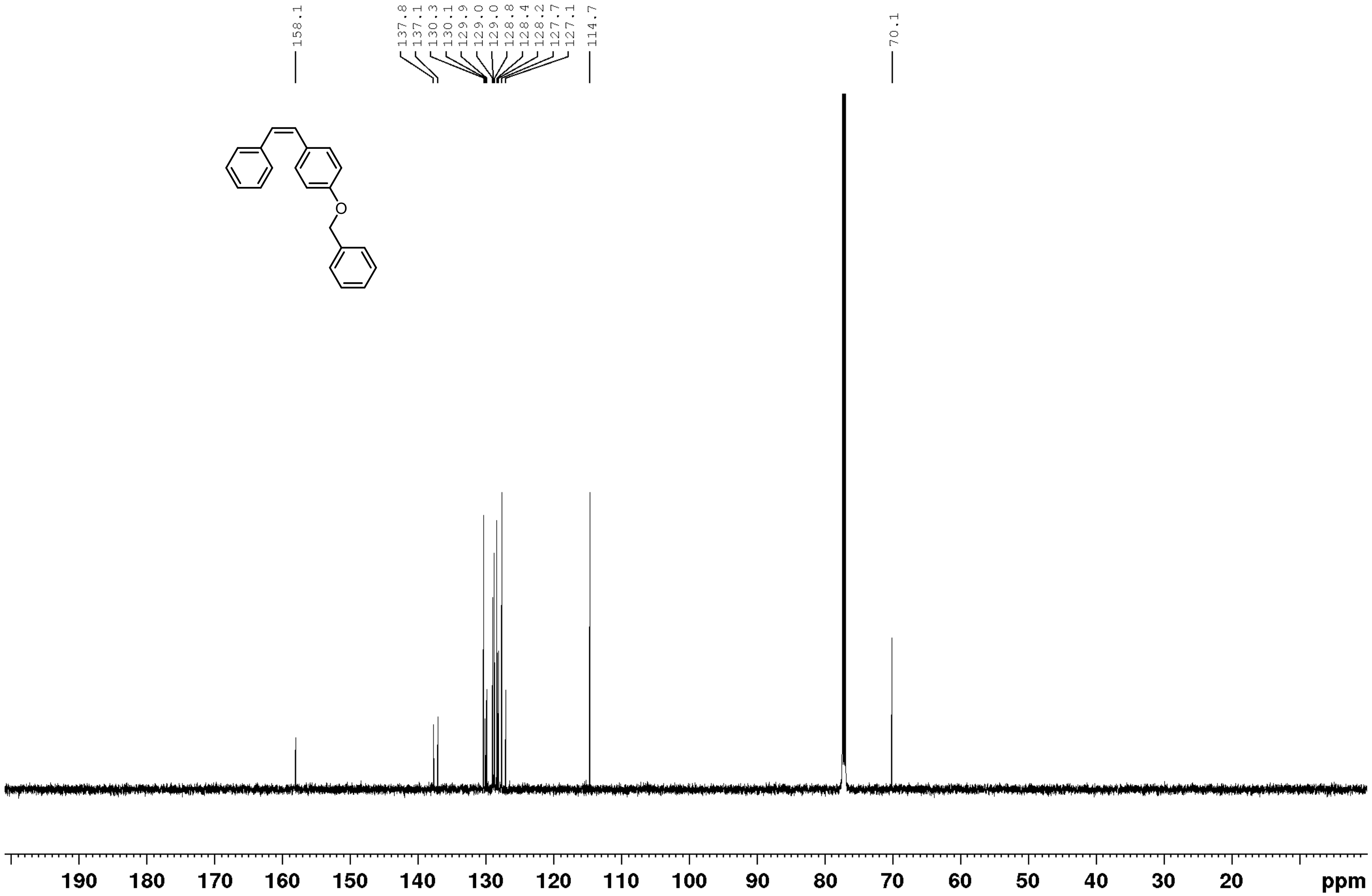
158.1
137.8
137.1
130.3
130.1
129.9
129.0
129.0
128.8
128.4
128.2
127.7
127.1
114.7
70.1
190
180
170
160
150
140
130
120
110
100
90
80
70
60
50
40
30
20
ppm

2.2.3 ^1H, ^1H COSY

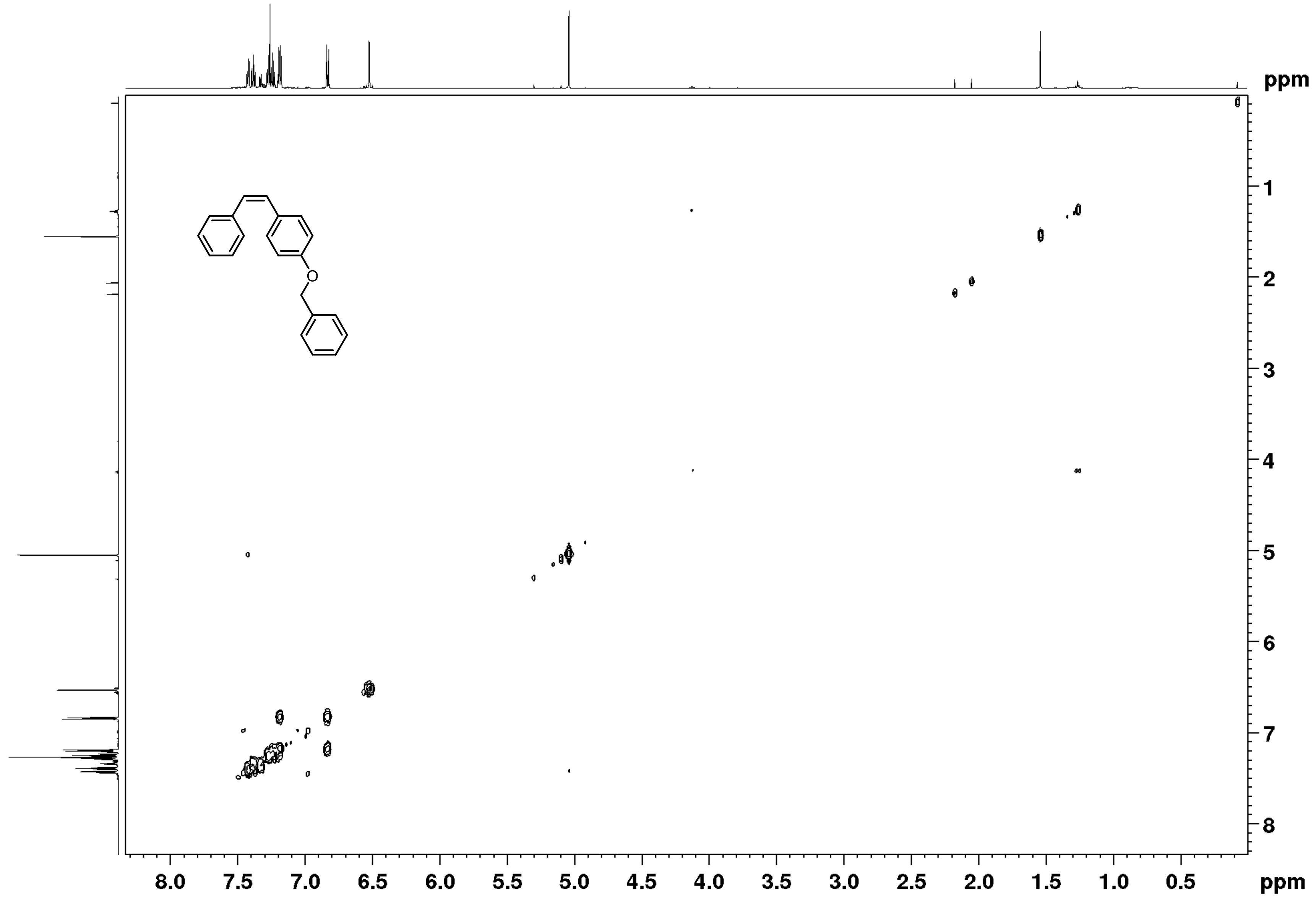

2.2.4 ^1H, ^{13}C HMBC

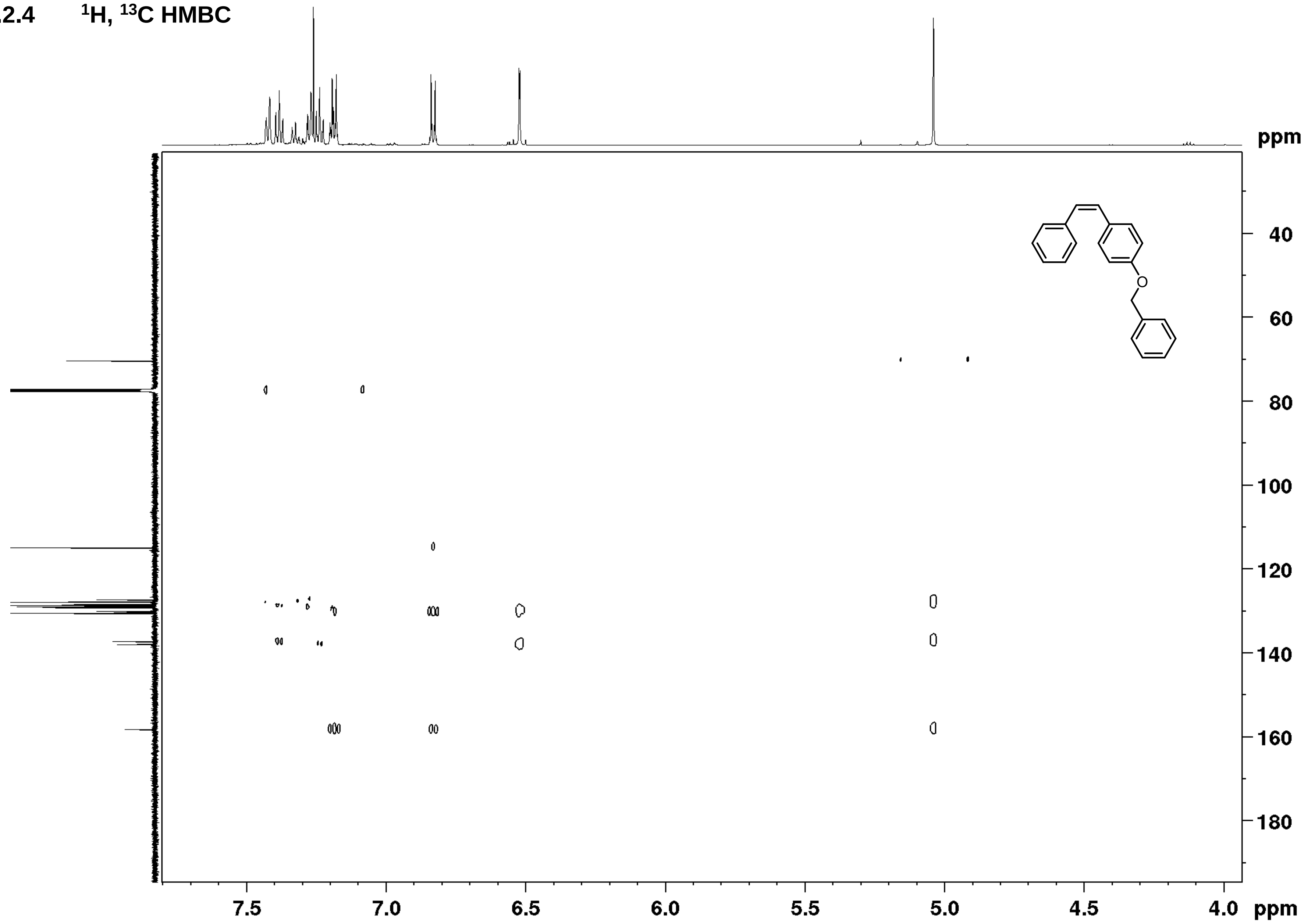

2.2.5 ^{1}H, ^{13}C HSQC

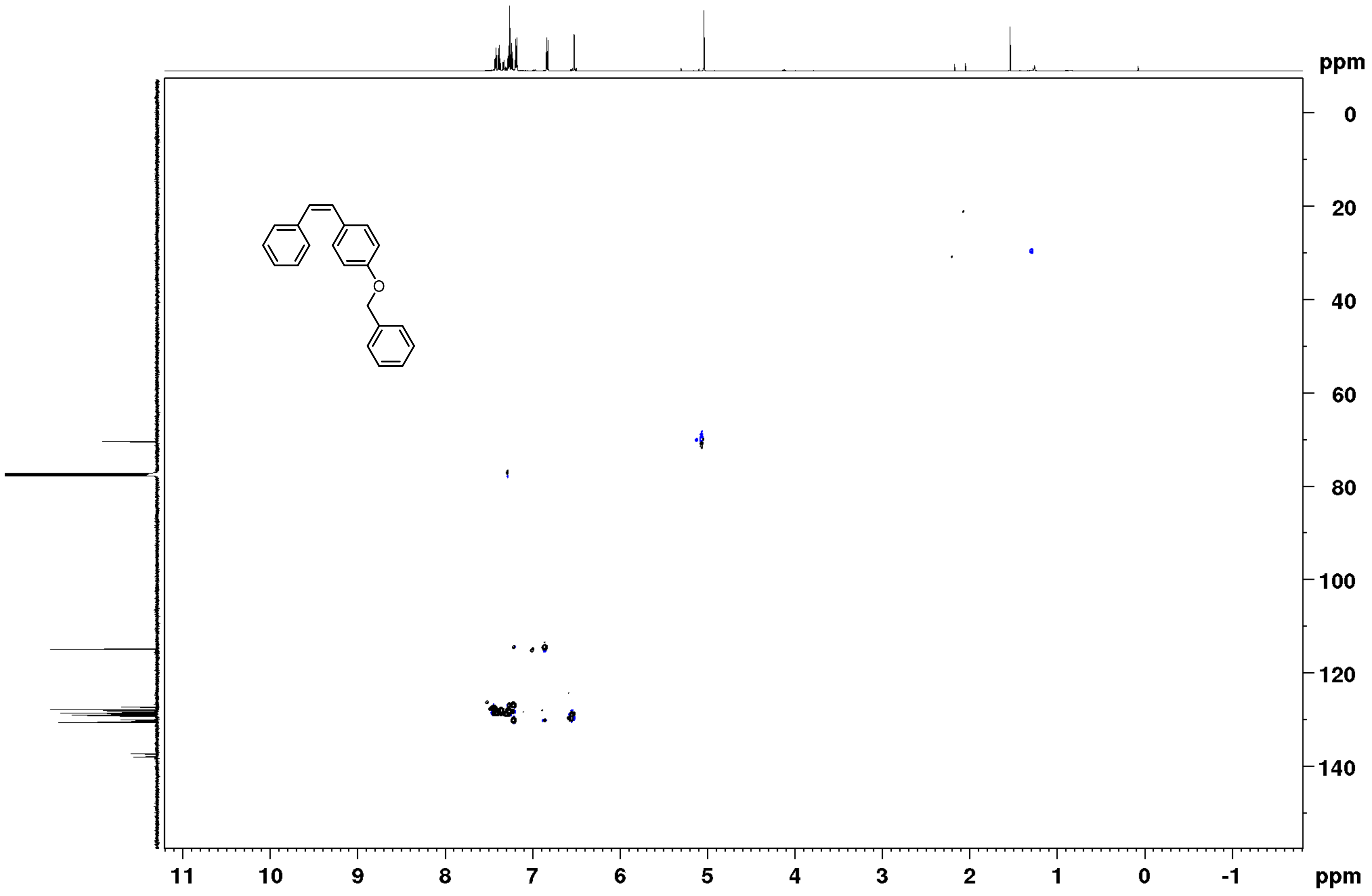

2.2.6 HRMS

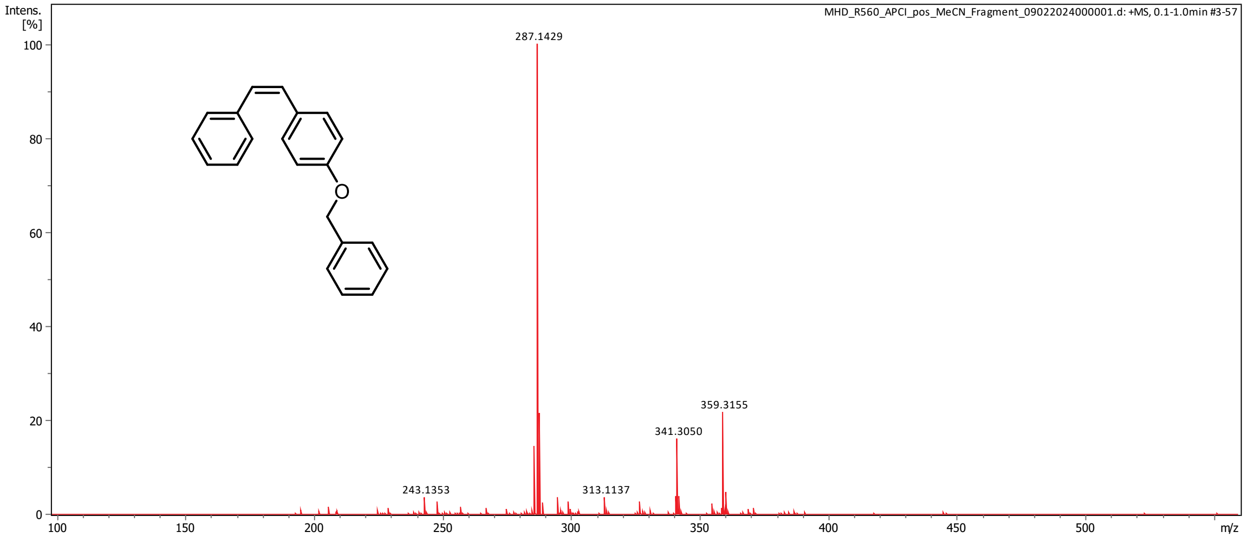

MHD_R560_APCI_pos_MeCN_Fragment_09022024000001.d: +MS, 0.1-1.0min #3-57
Intens. [%]
287.1429
359.3155
341.3050
243.1353
313.1137
m/z

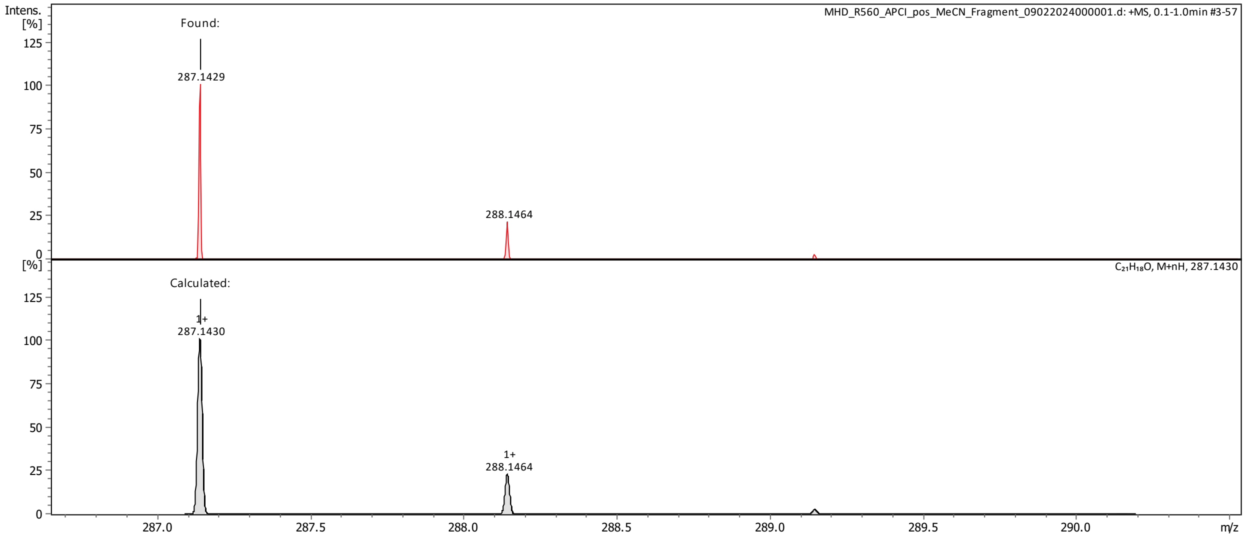

MHD_R560_APCI_pos_MeCN_Fragment_09022024000001.d: +MS, 0.1-1.0min #3-57
Intens. [%]
Found:
287.1429
288.1464
C21H18O, M+nH, 287.1430
Calculated:
1+ 287.1430
1+ 288.1464
m/z

2.2.7 IR

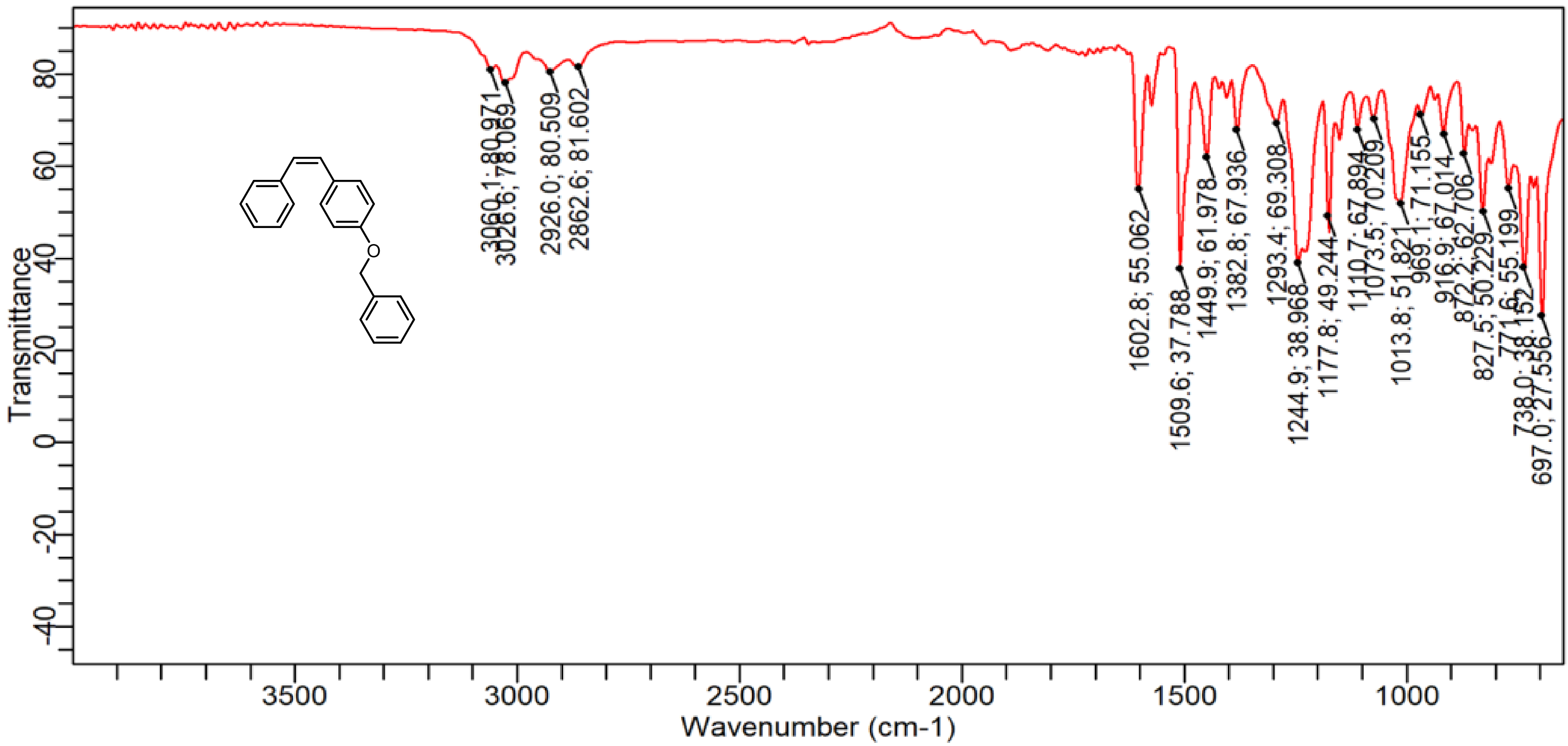

Transmittance
Wavenumber (cm-1)
3060.1; 80.971
3026.6; 78.069
2926.0; 80.509
2862.6; 81.602
1602.8; 55.062
1509.6; 37.788
1449.9; 61.978
1382.8; 67.936
1293.4; 69.308
1244.9; 38.968
1177.8; 49.244
1110.7; 67.894
1073.5; 70.209
1013.8; 51.821
969.1; 71.155
916.9; 67.014
872.2; 62.706
827.5; 50.229
771.6; 55.199
738.0; 38.152
697.0; 27.556

2.3 (*Z*)-1-Methoxy-4-(4-(trifluoromethyl)styryl)benzene (2c) (600 MHz, $CDCl_3$)

2.3.1 ^{1}H NMR

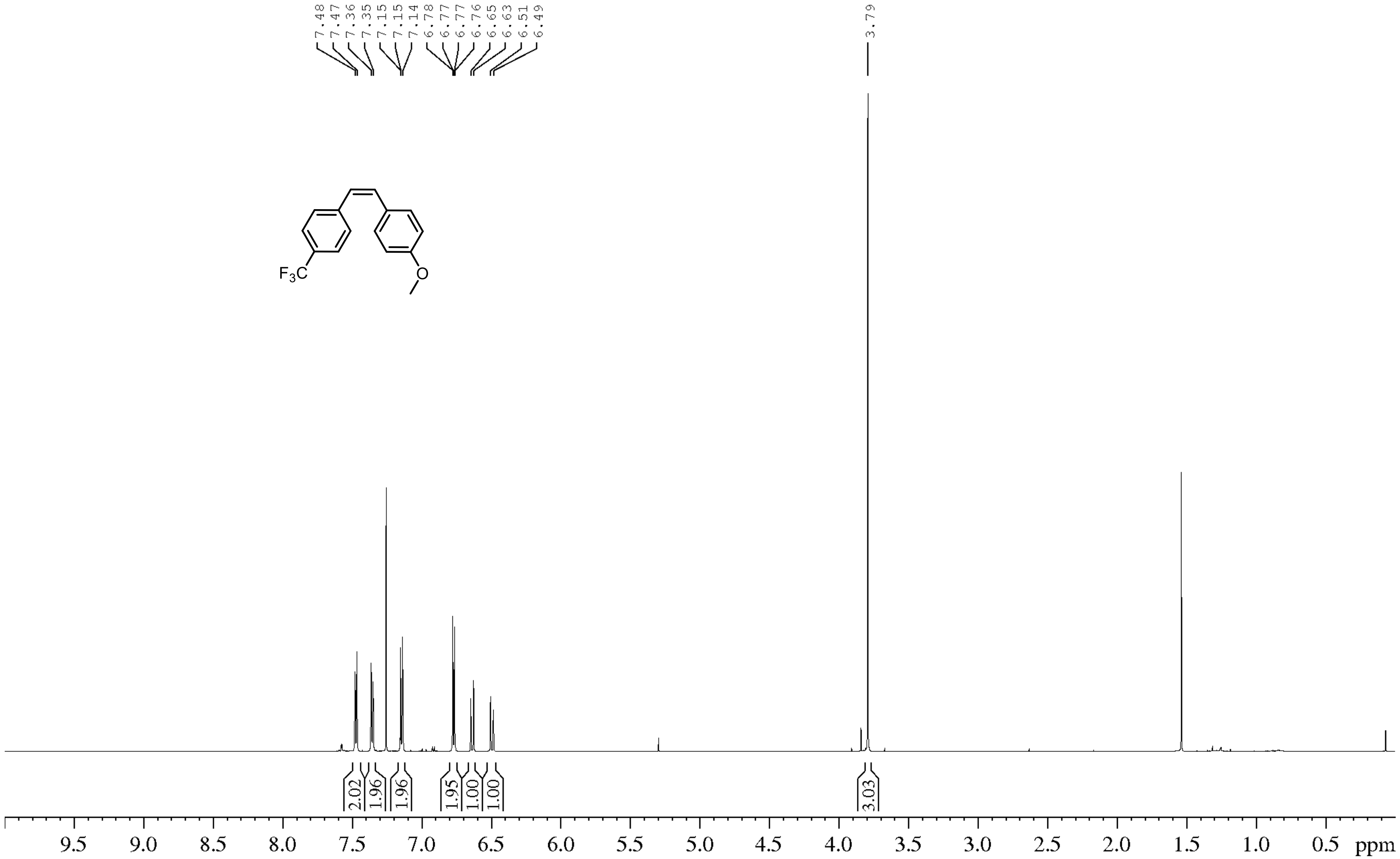

2.3.2 ^{13}C NMR

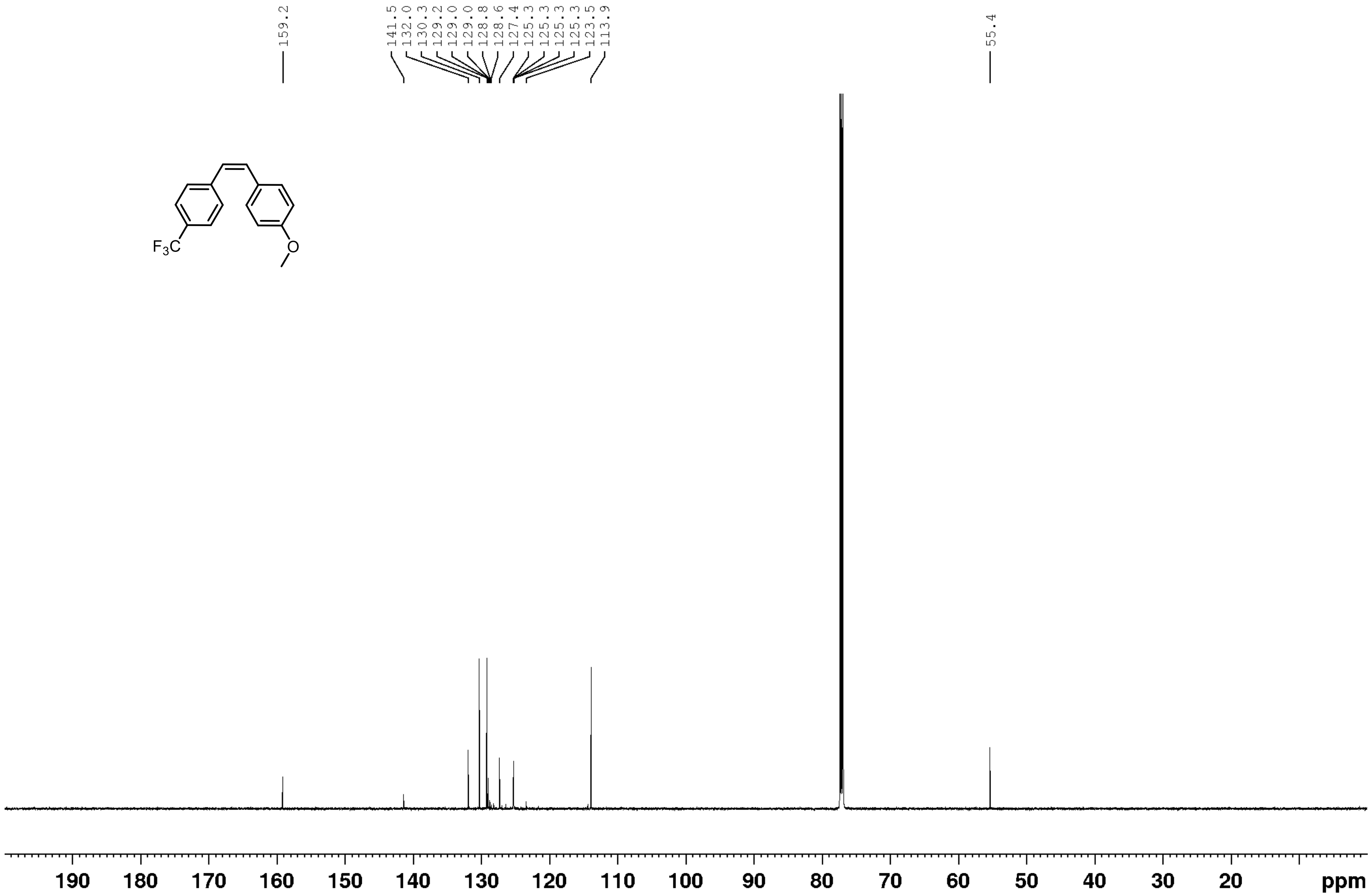
159.2
141.5
132.0
130.3
129.2
129.0
129.0
128.8
128.6
127.4
125.3
125.3
125.3
125.3
123.5
113.9
55.4
F3C
O
190
180
170
160
150
140
130
120
110
100
90
80
70
60
50
40
30
20
ppm

2.3.3 ^{1}H, ^{1}H COSY

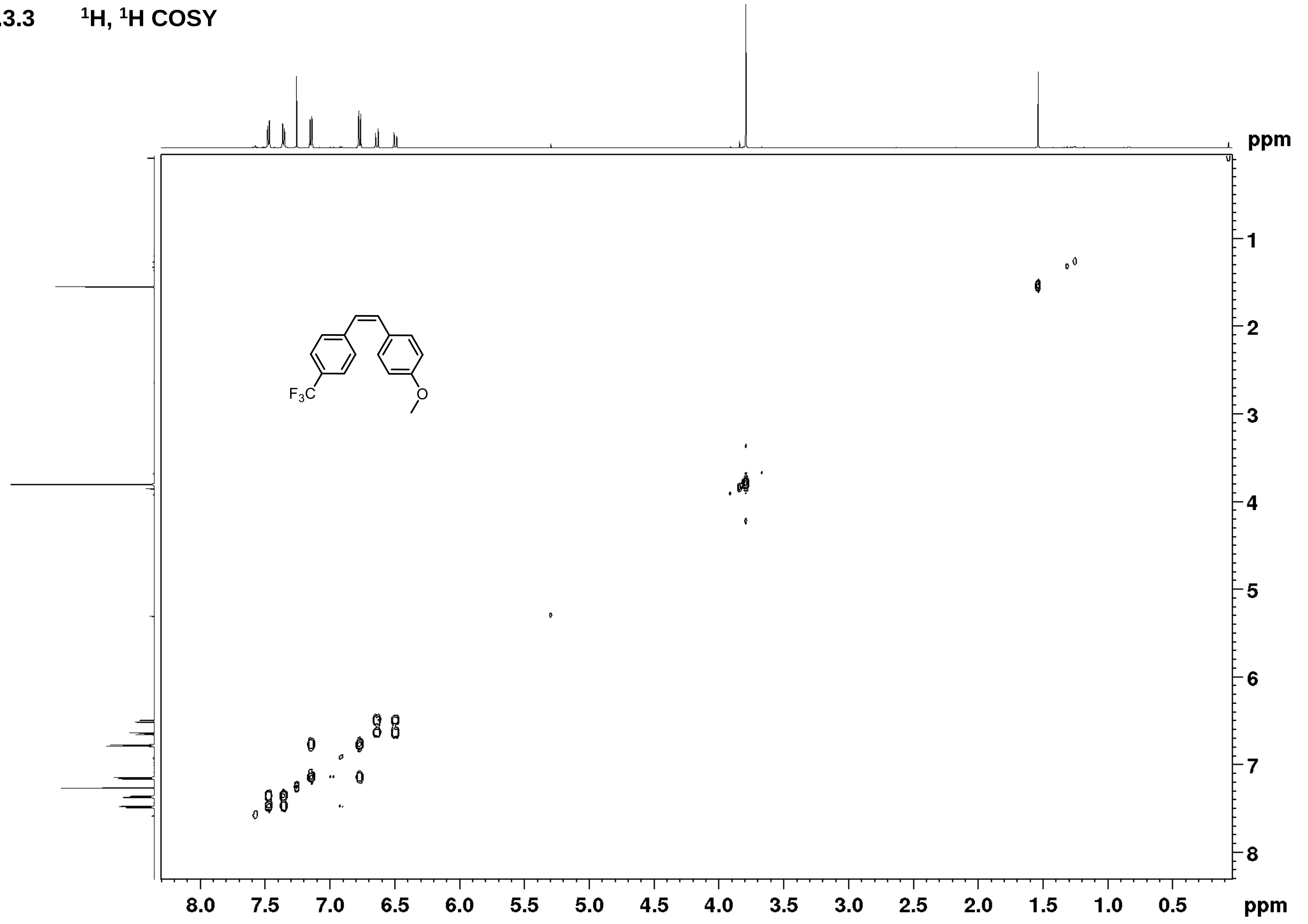

2.3.4 ^{1}H, ^{13}C HMBC

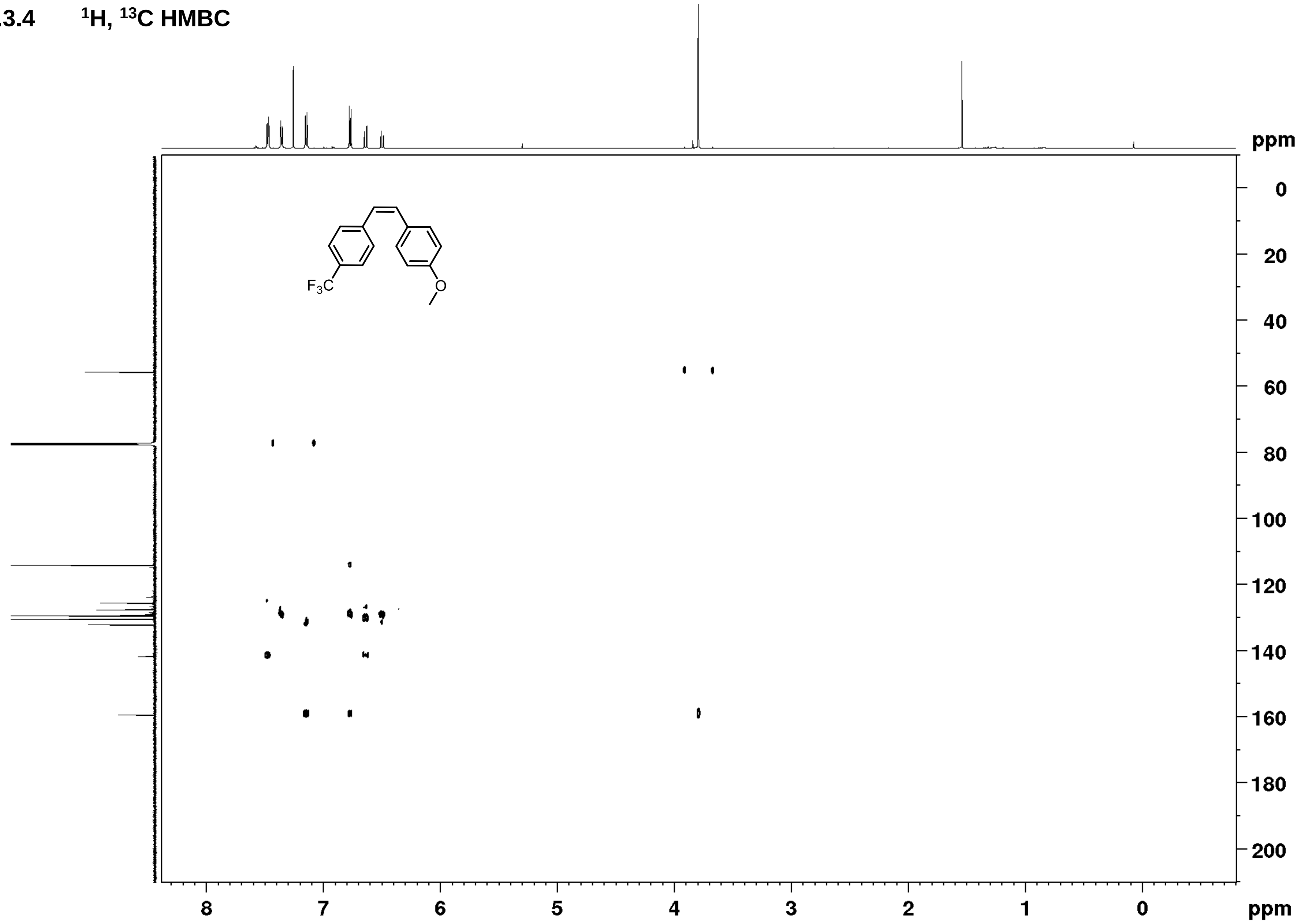

2.3.5 ^{1}H, ^{13}C HSQC

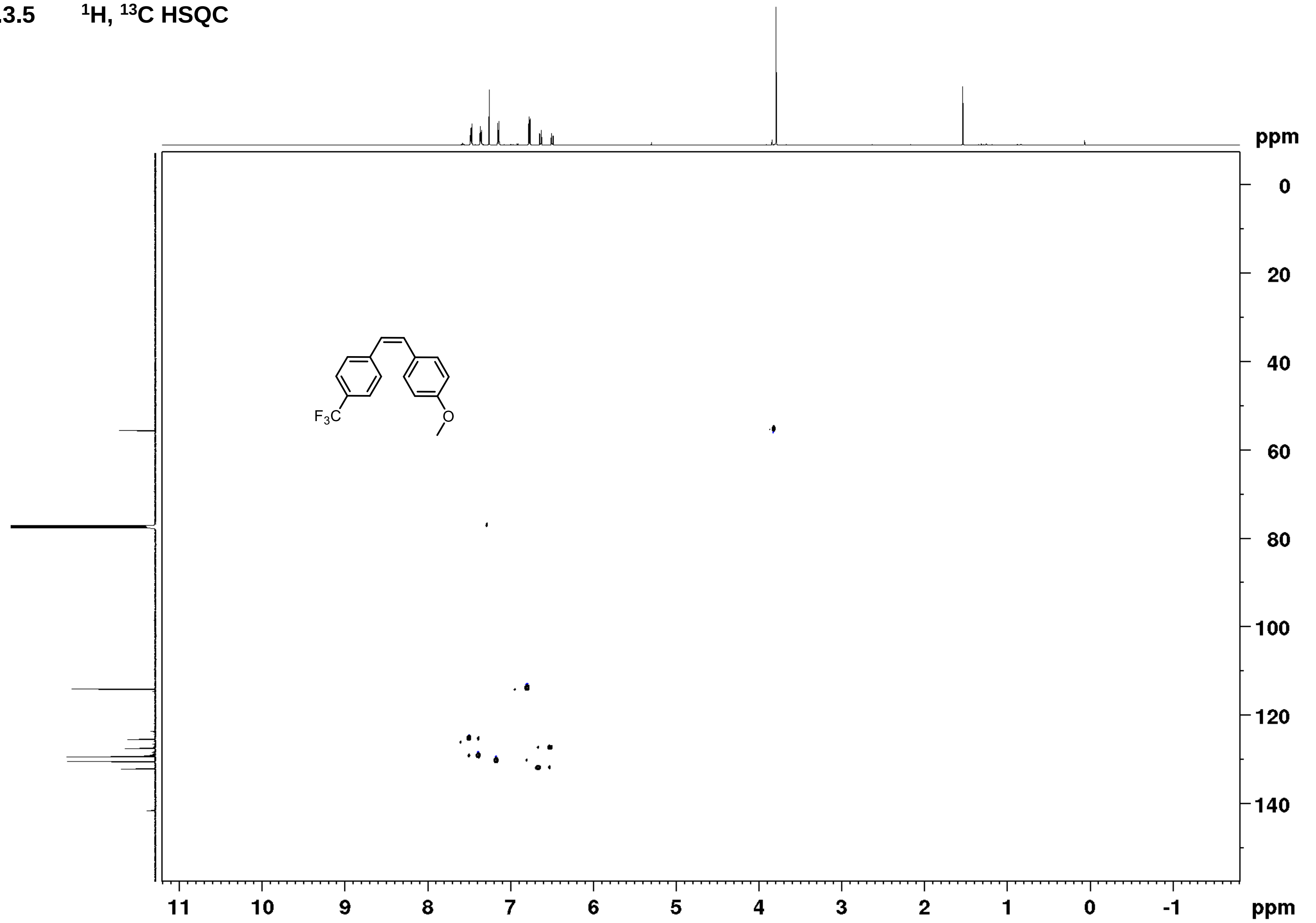

2.3.6 ^{19}F NMR

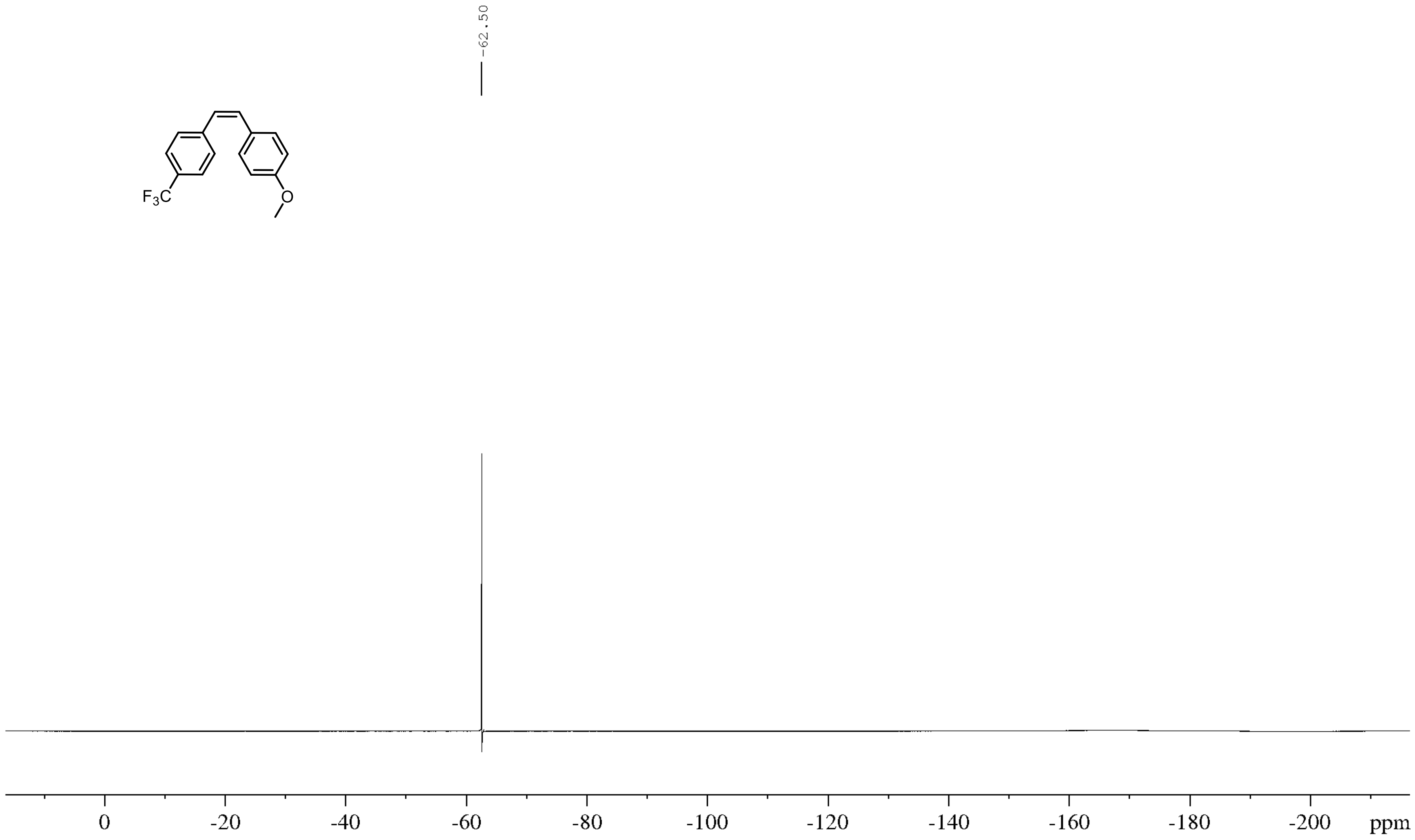

2.3.7 HRMS

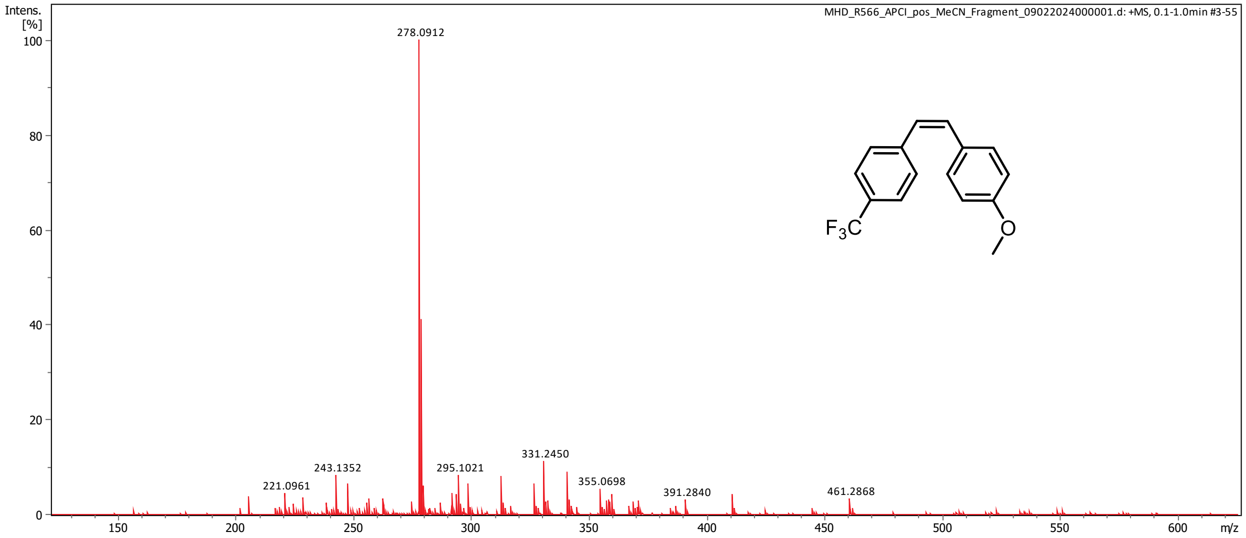

MHD_R566_APCI_pos_MeCN_Fragment_09022024000001.d: +MS, 0.1-1.0min #3-55
Intens. [%]
278.0912
243.1352
221.0961
295.1021
331.2450
355.0698
391.2840
461.2868
m/z
F3C
O

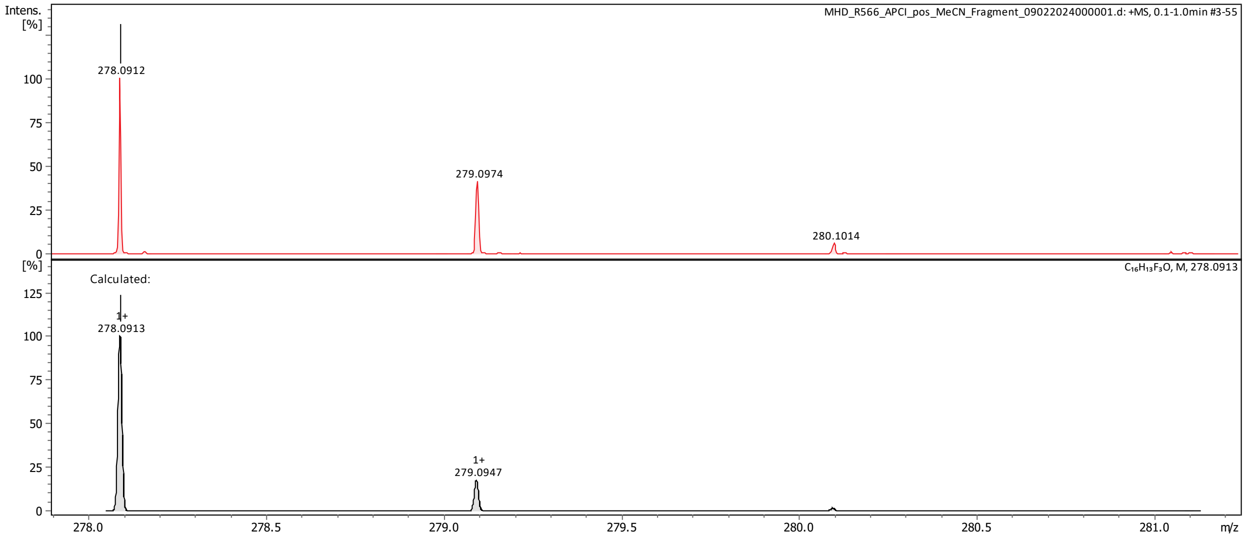

MHD_R566_APCI_pos_MeCN_Fragment_09022024000001.d: +MS, 0.1-1.0min #3-55
Intens. [%]
278.0912
279.0974
280.1014
Calculated:
C16H13F3O, M, 278.0913
1+ 278.0913
1+ 279.0947
m/z

2.3.8 IR

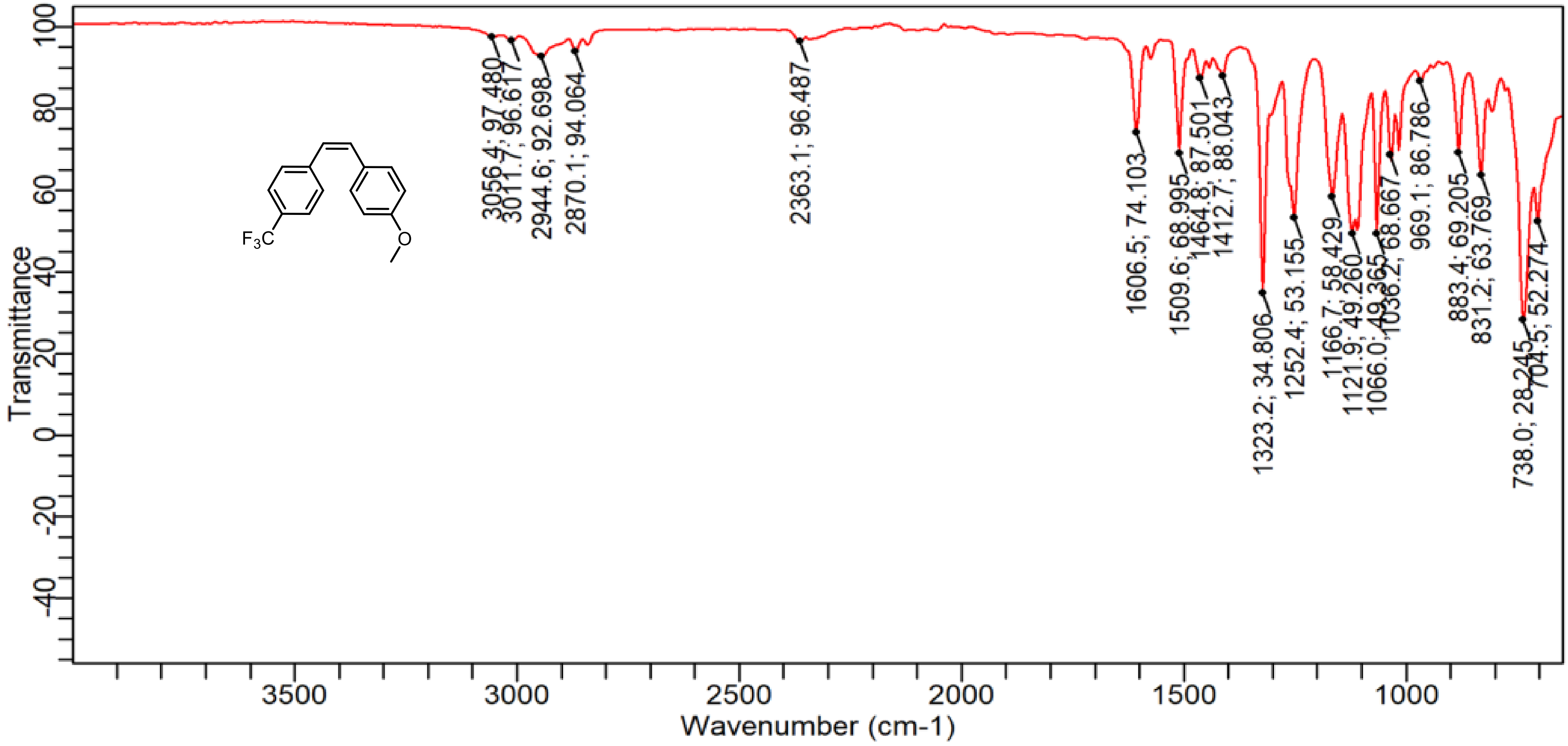
Transmittance
Wavenumber (cm-1)
F3C
O
3056.4; 97.480
3011.7; 96.617
2944.6; 92.698
2870.1; 94.064
2363.1; 96.487
1606.5; 74.103
1509.6; 68.995
1464.8; 87.501
1412.7; 88.043
1323.2; 34.806
1252.4; 53.155
1166.7; 58.429
1121.9; 49.260
1066.0; 49.365
1036.2; 68.667
969.1; 86.786
883.4; 69.205
831.2; 63.769
738.0; 28.245
704.5; 52.274
100
80
60
40
20
0
-20
-40
3500
3000
2500
2000
1500
1000

2.4 (*Z*)-4-(4-Methoxystyryl)benzonitrile (2d) (600 MHz, $CDCl_3$)

2.4.1 ^{1}H NMR

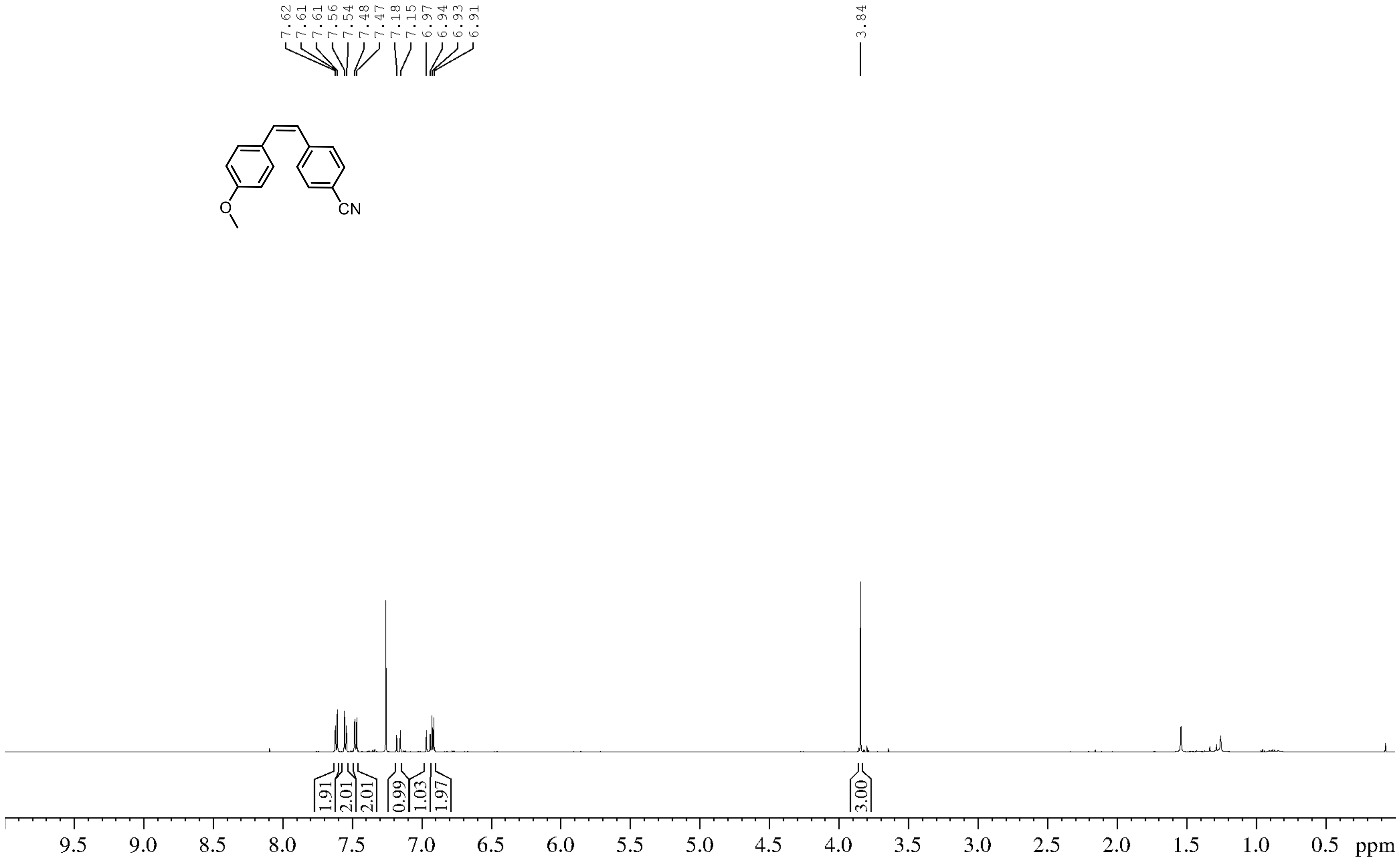

2.4.2 ^{13}C NMR

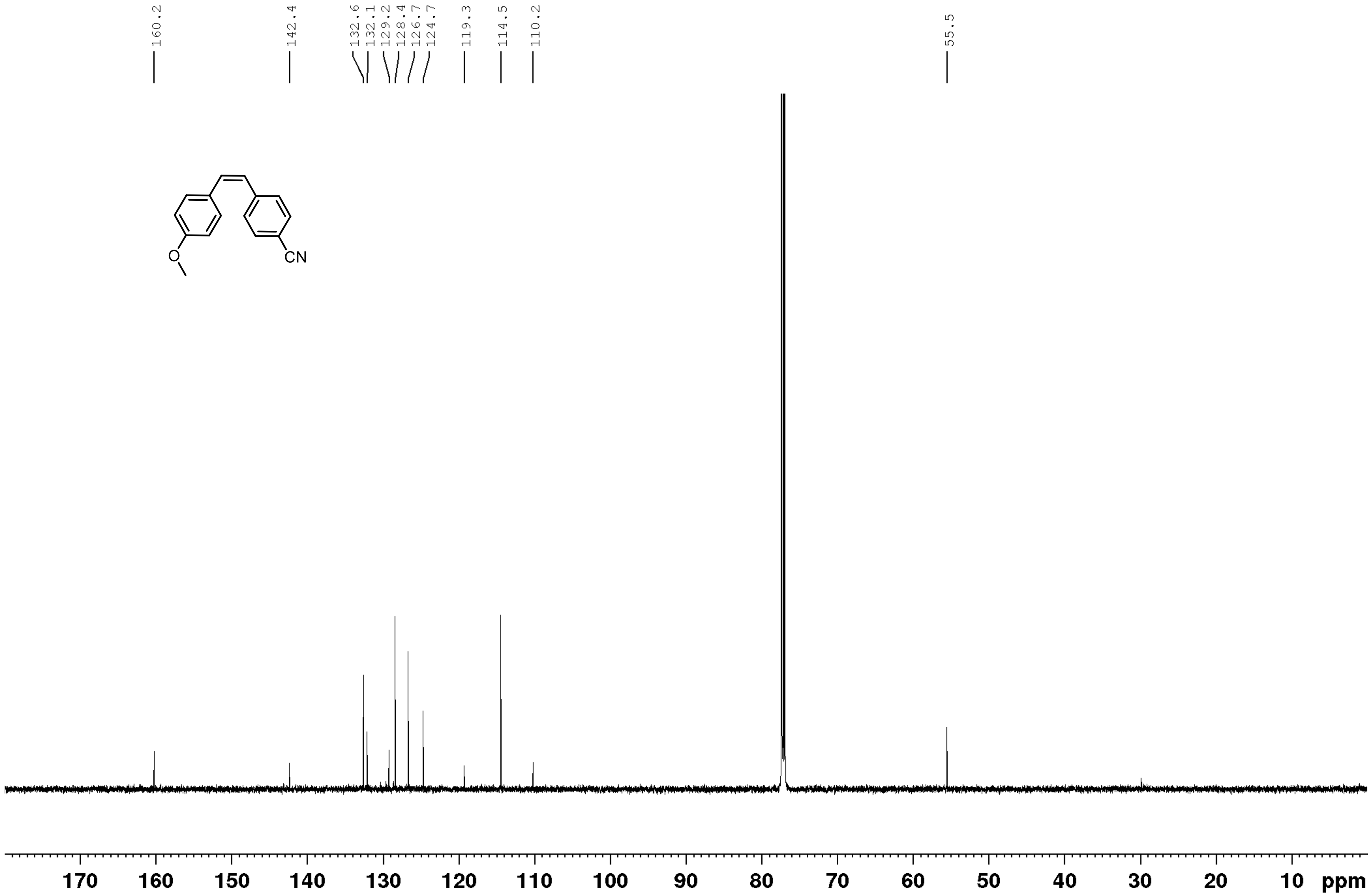

160.2
142.4
132.6
132.1
129.2
128.4
126.7
124.7
119.3
114.5
110.2
55.5
O
CN
170
160
150
140
130
120
110
100
90
80
70
60
50
40
30
20
10
ppm

2.4.3 ¹H, ¹H COSY

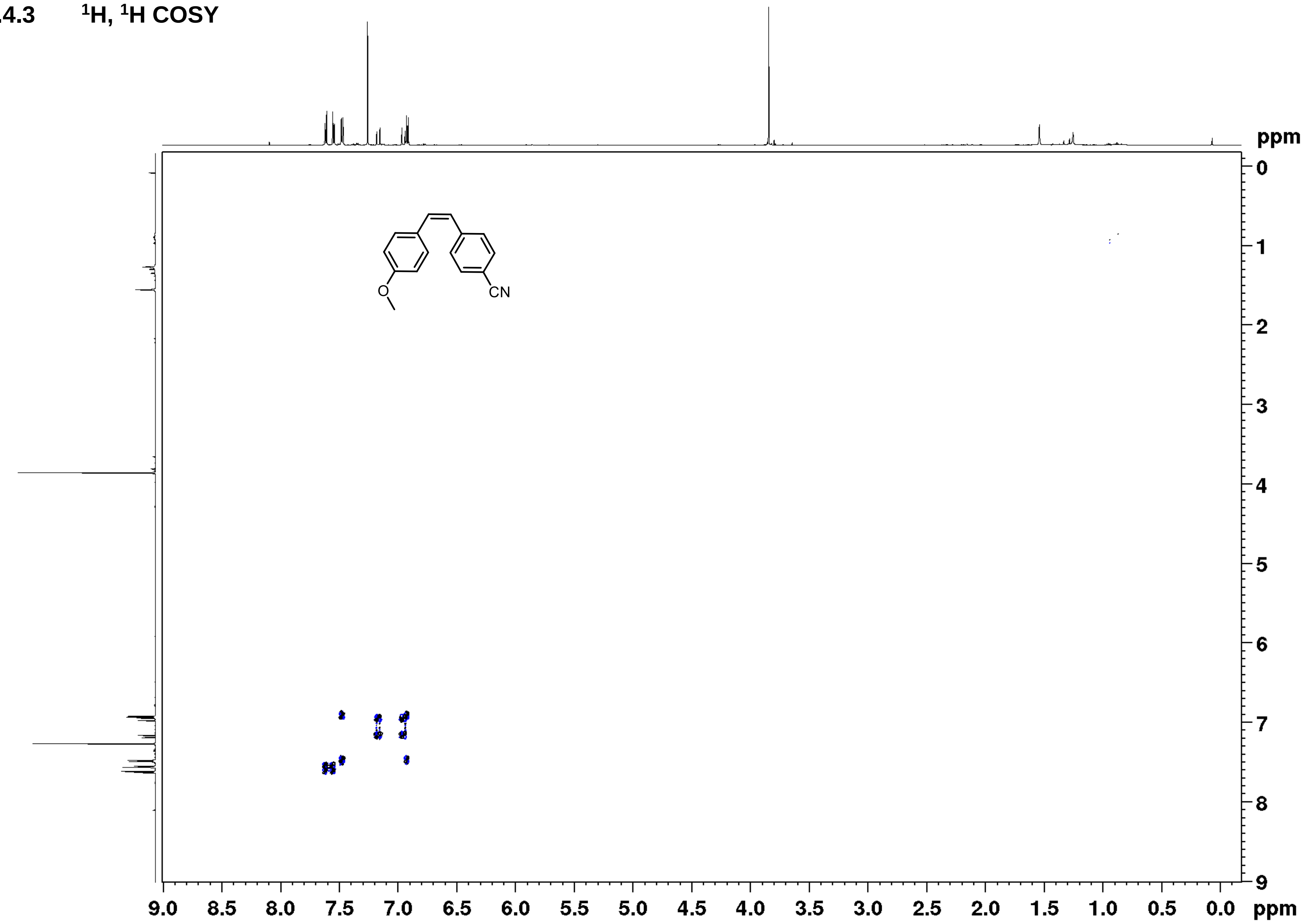

2.4.4 ^{1}H, ^{13}C HMBC

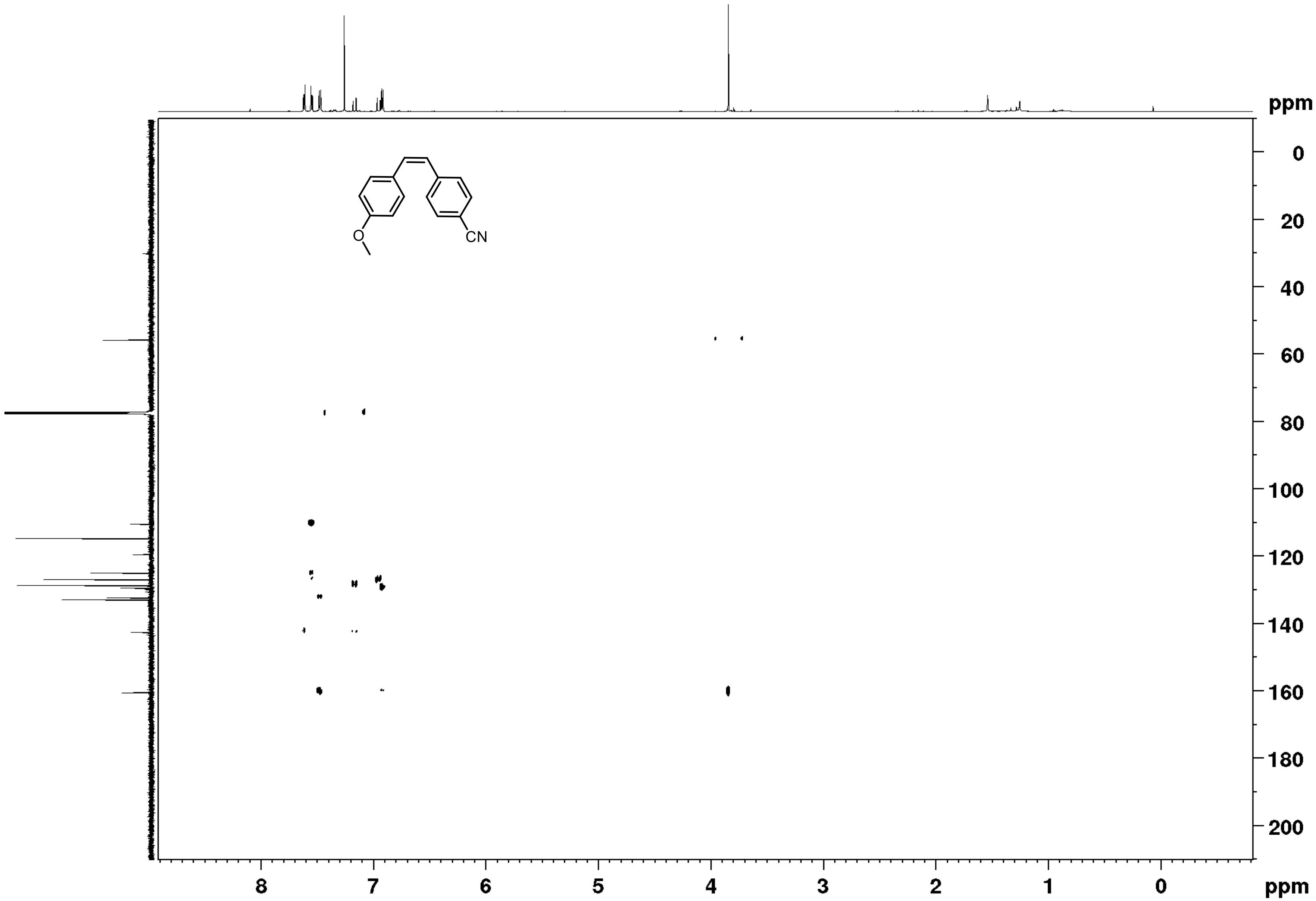

2.4.5 ^{1}H, ^{13}C HSQC

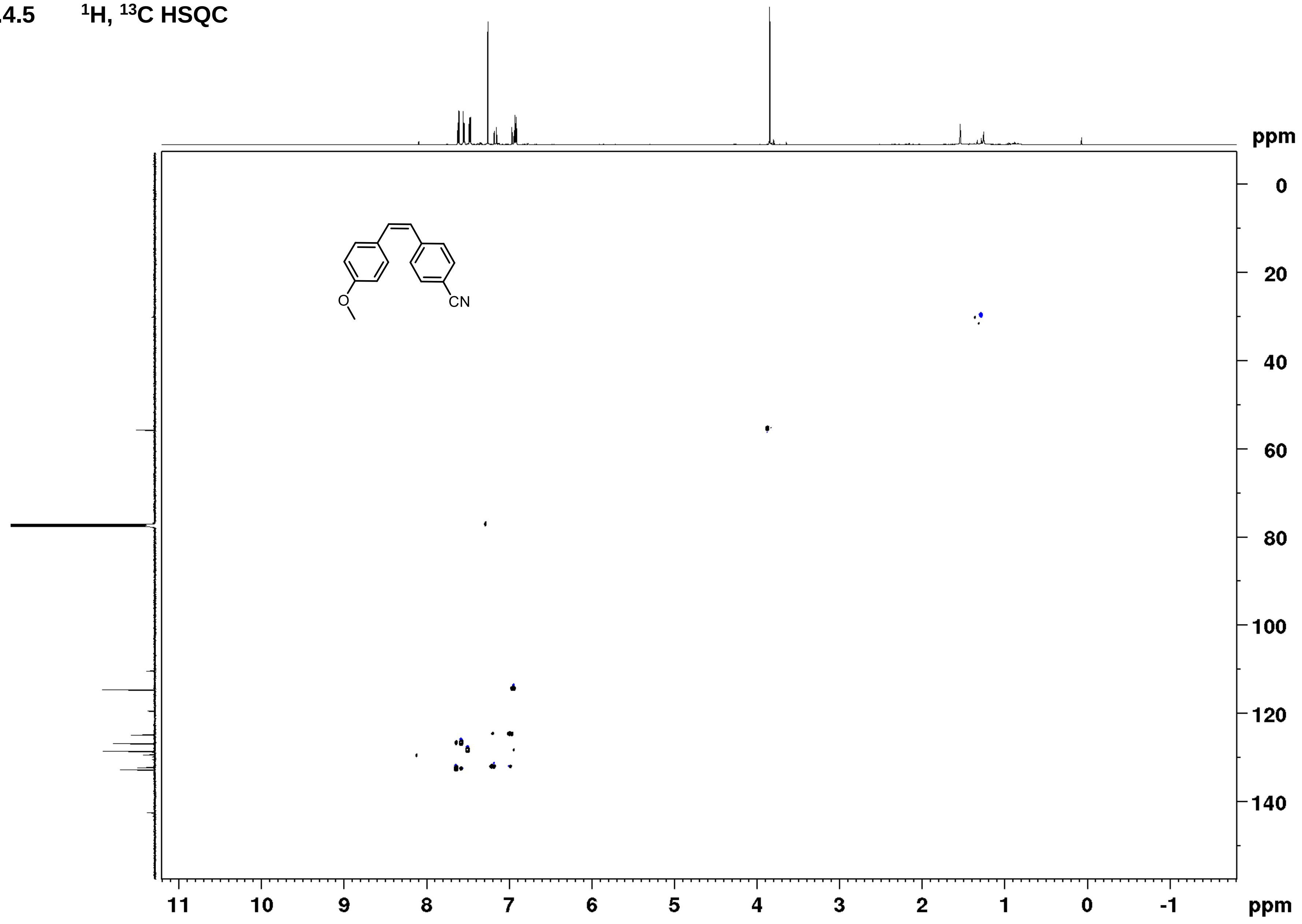

2.4.6 HRMS

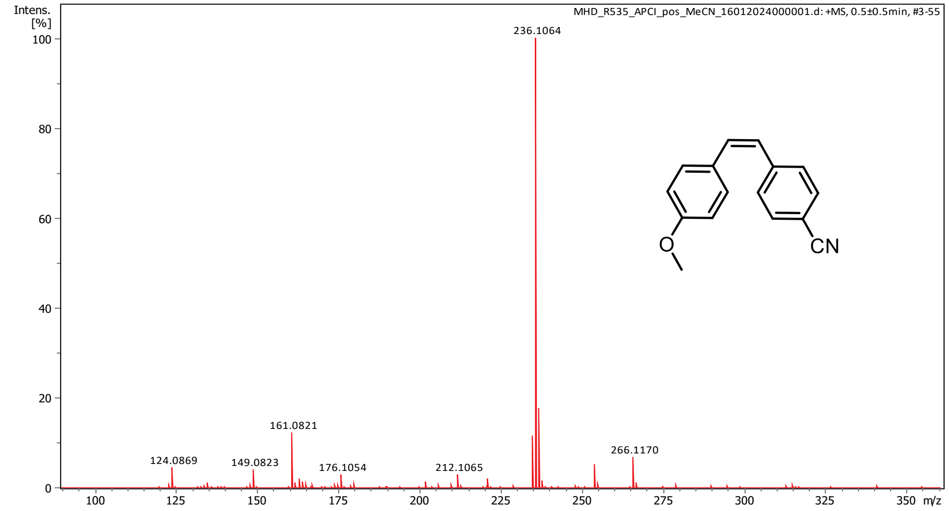

Intens. [%]
MHD_R535_APCI_pos_MeCN_16012024000001.d: +MS, 0.5±0.5min, #3-55
236.1064
161.0821
124.0869
149.0823
176.1054
212.1065
266.1170
O
CN
m/z

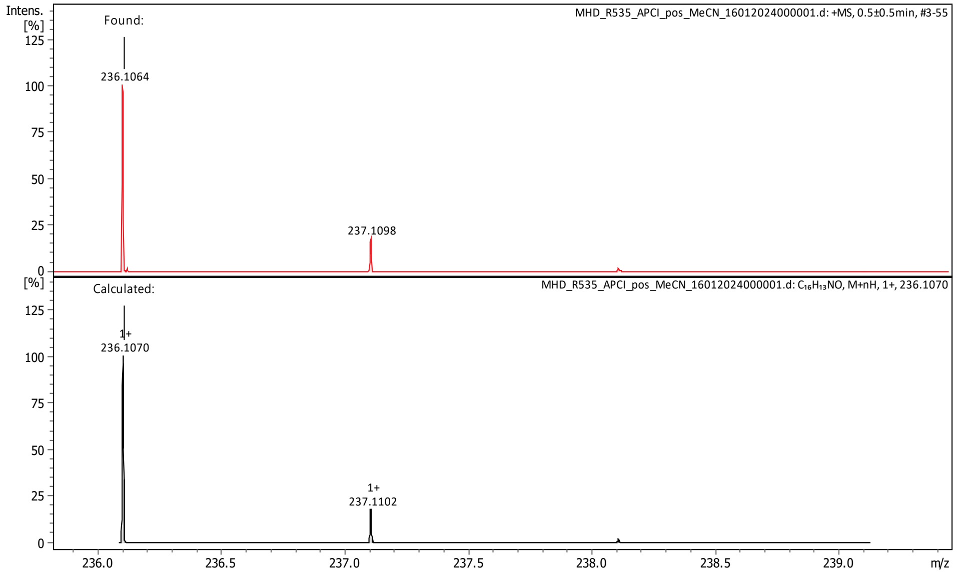

Intens. [%]
Found:
MHD_R535_APCI_pos_MeCN_16012024000001.d: +MS, 0.5±0.5min, #3-55
236.1064
237.1098
Calculated:
MHD_R535_APCI_pos_MeCN_16012024000001.d: C₁₆H₁₃NO, M+nH, 1+, 236.1070
1+
236.1070
1+
237.1102
m/z

2.5 (*Z*)-1-Fluoro-4-styrylbenzene (2e) (600 MHz, $CDCl_3$)

2.5.1 ^{1}H NMR

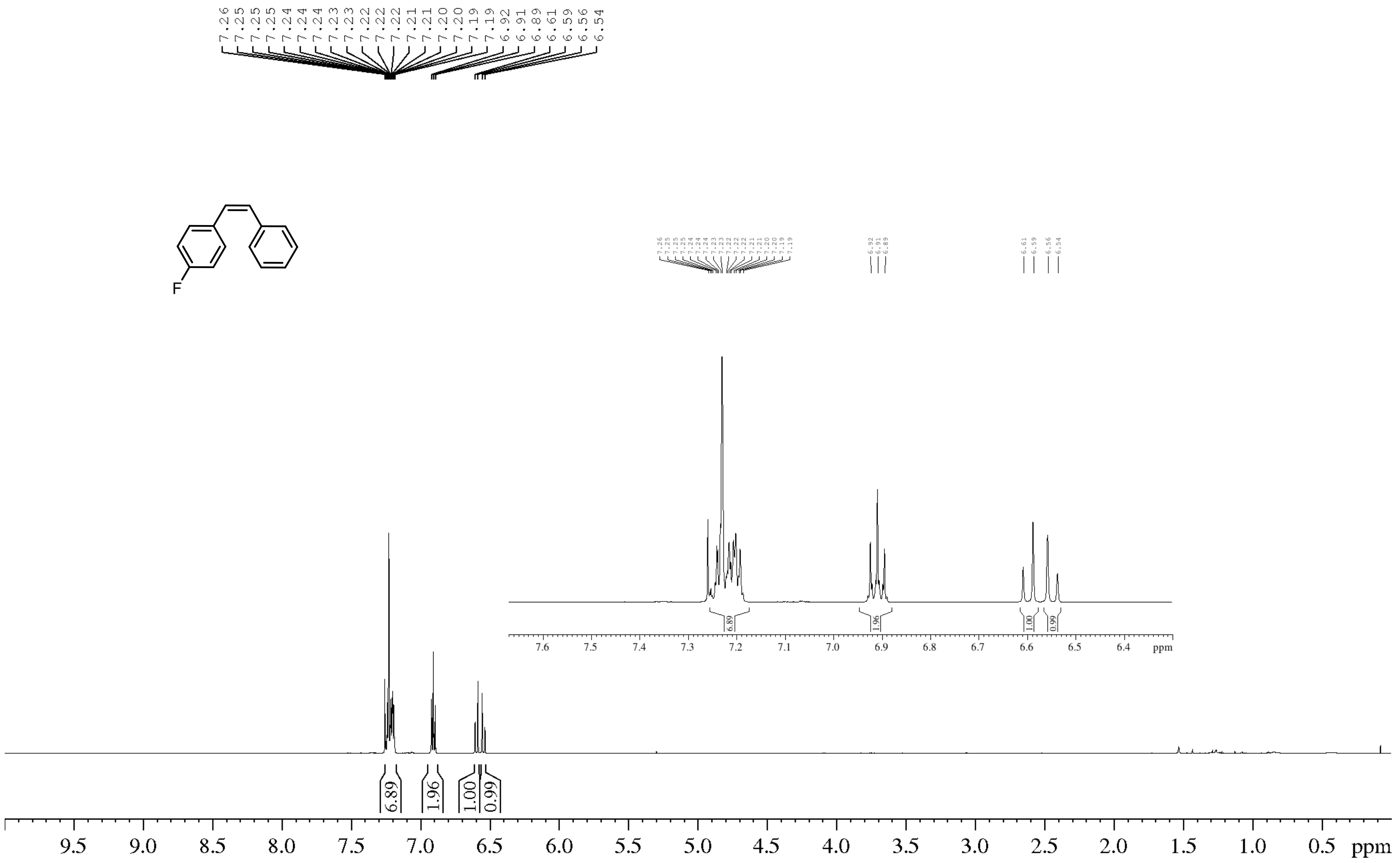

2.5.2 ^{13}C NMR

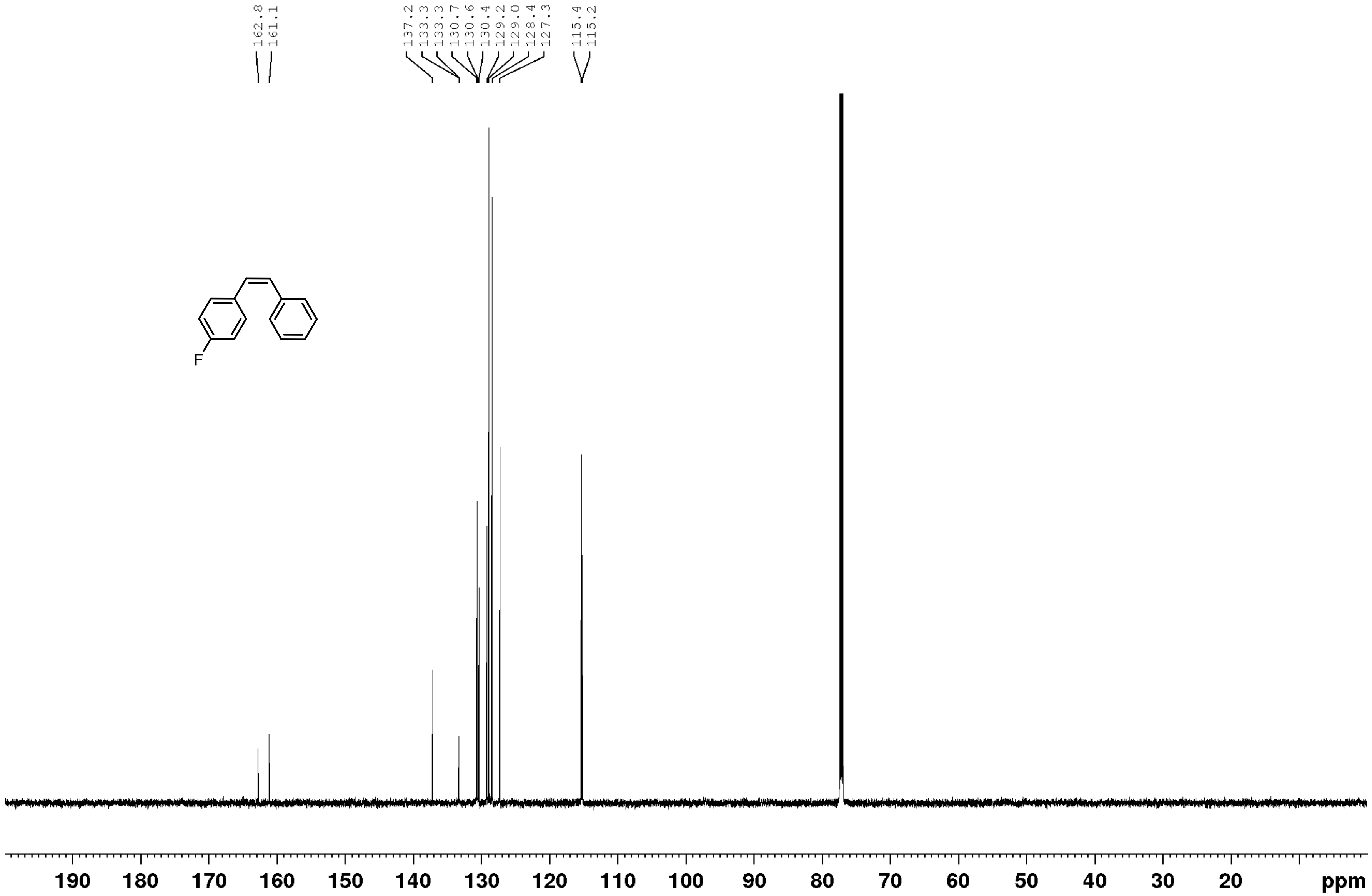

162.8
161.1
137.2
133.3
133.3
130.7
130.6
130.4
129.2
129.0
128.4
127.3
115.4
115.2
F
190
180
170
160
150
140
130
120
110
100
90
80
70
60
50
40
30
20
ppm

2.5.3 ^{1}H, ^{1}H COSY

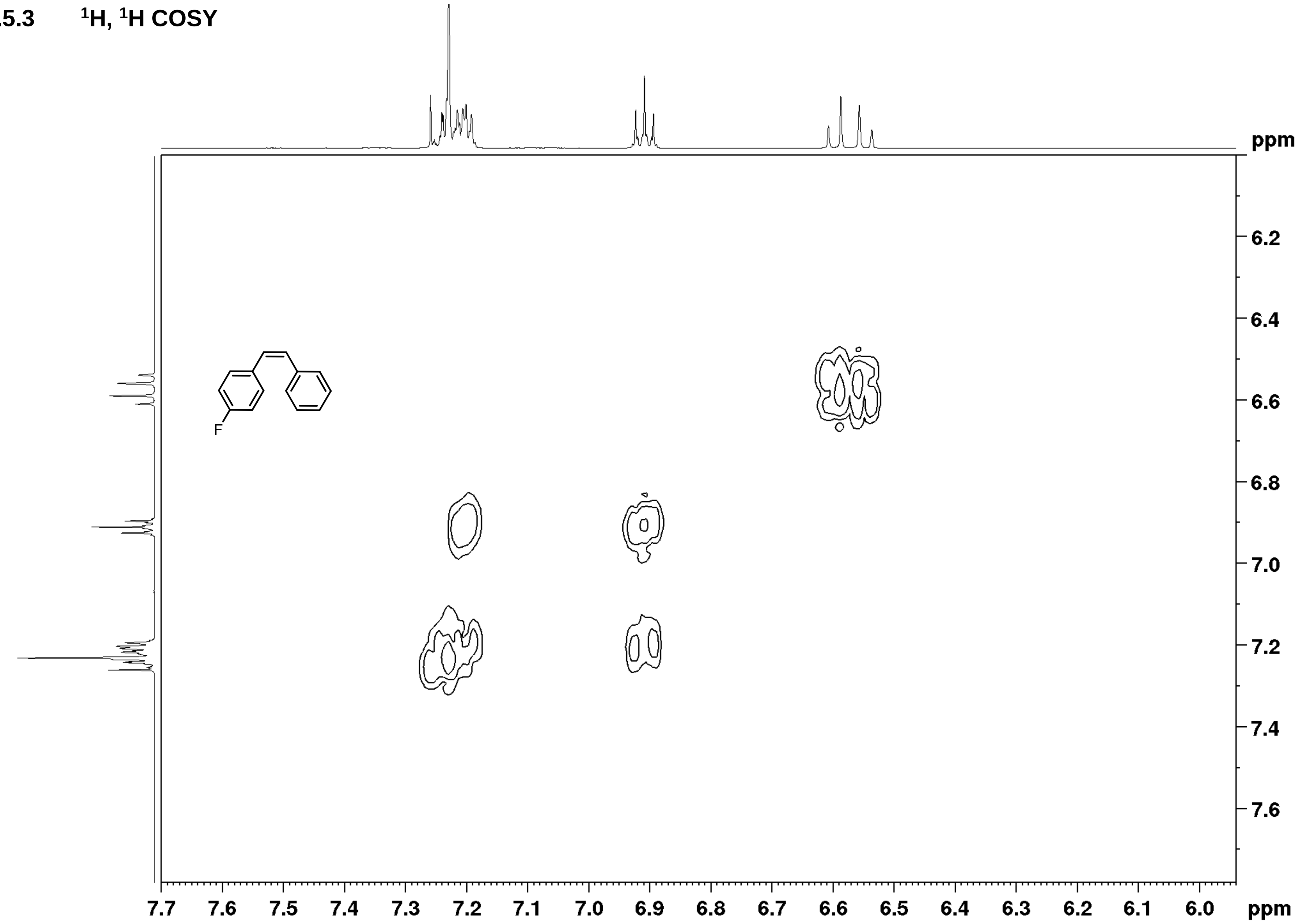

2.5.4 ^{1}H, ^{13}C HMBC

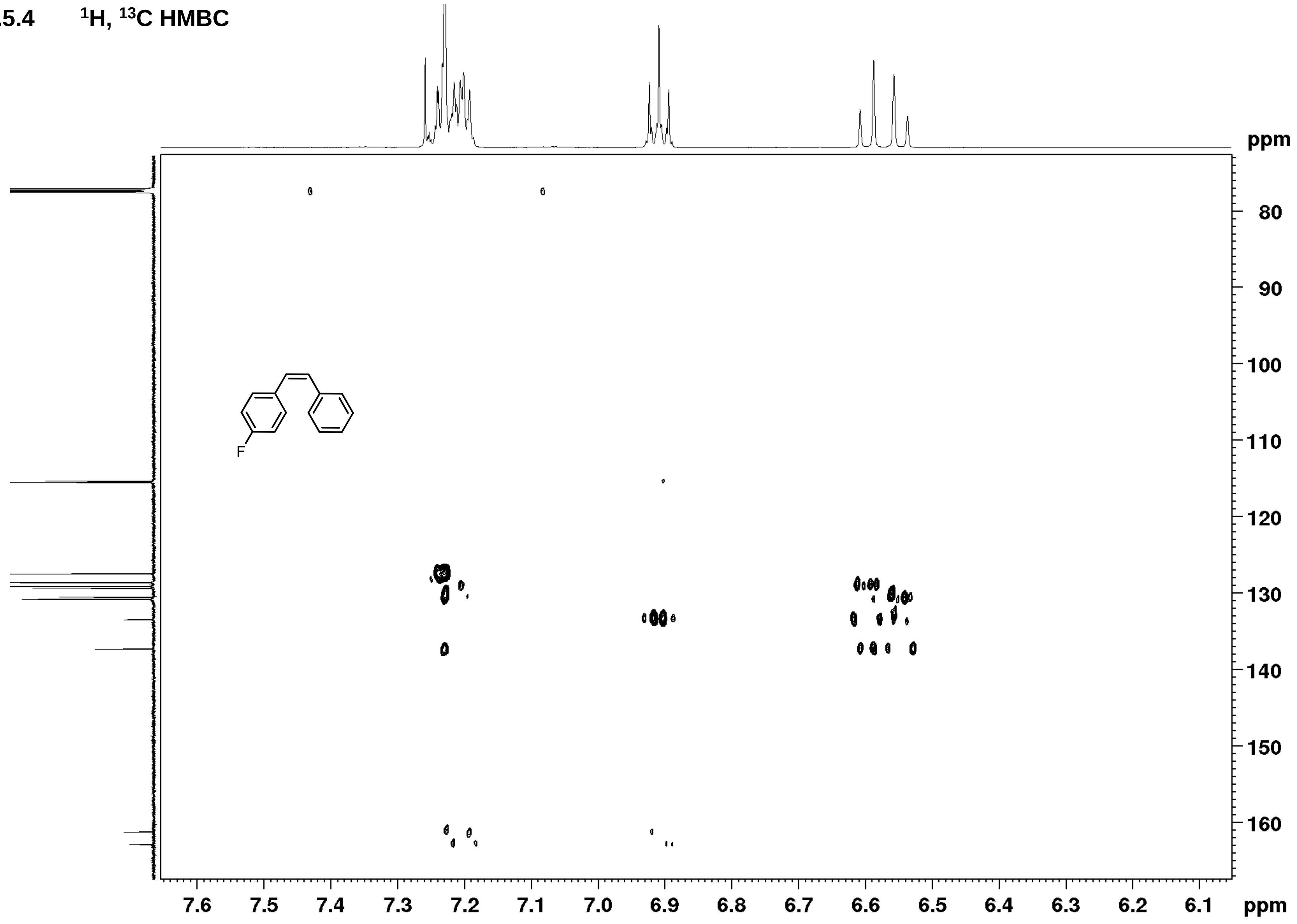

2.5.5 ^{1}H, ^{13}C HSQC

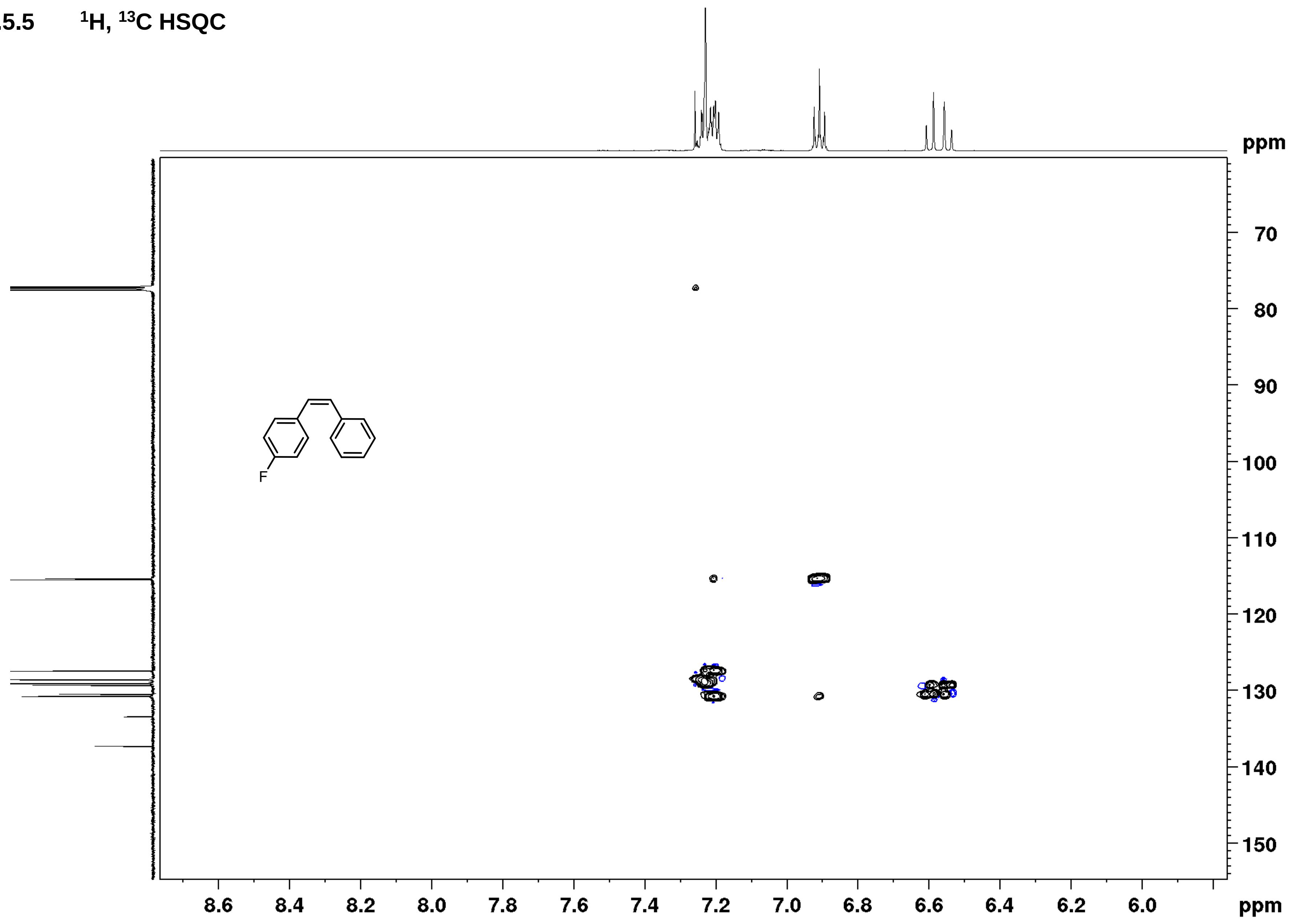

2.5.6 ^{19}F NMR

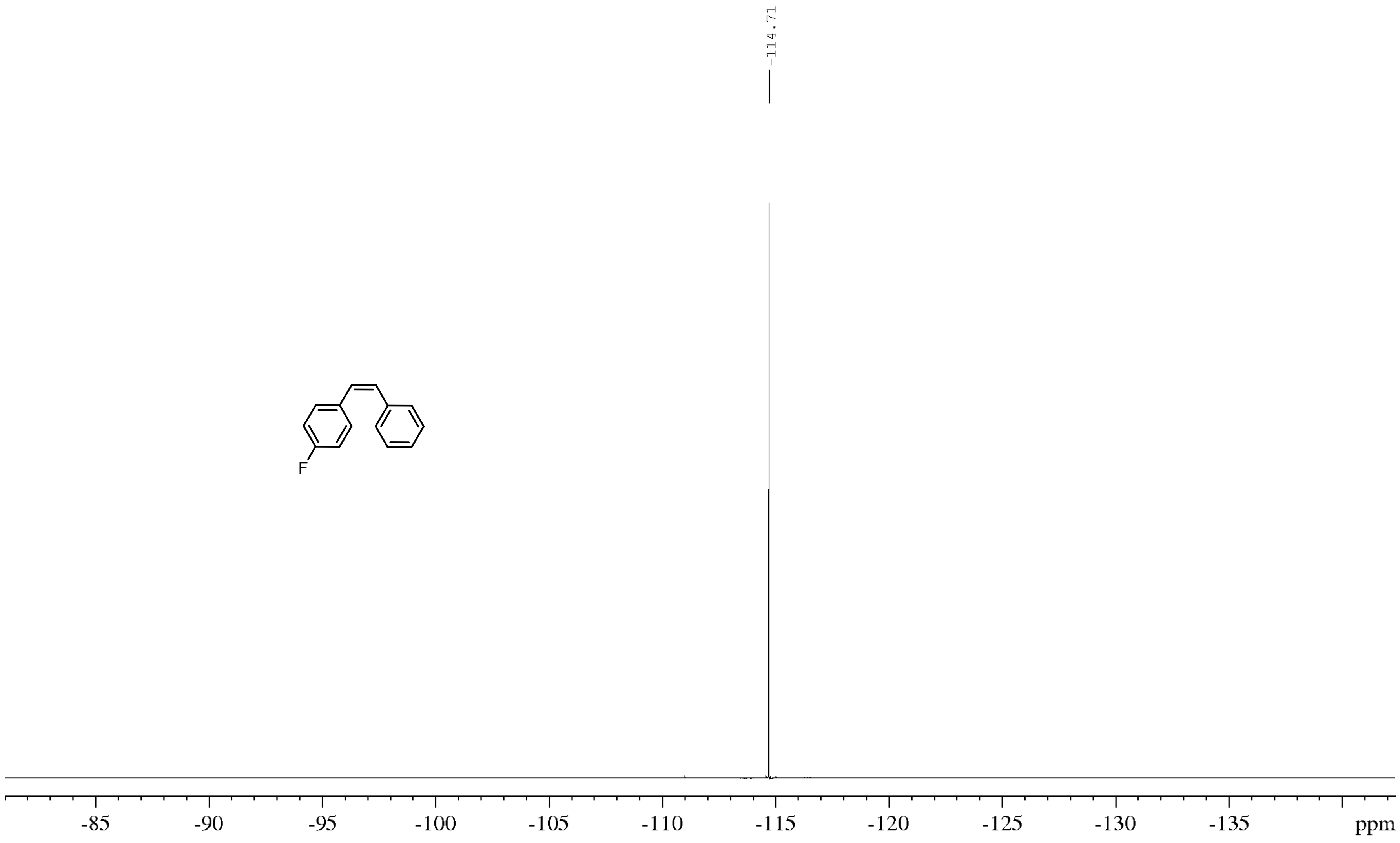

2.5.7 HRMS

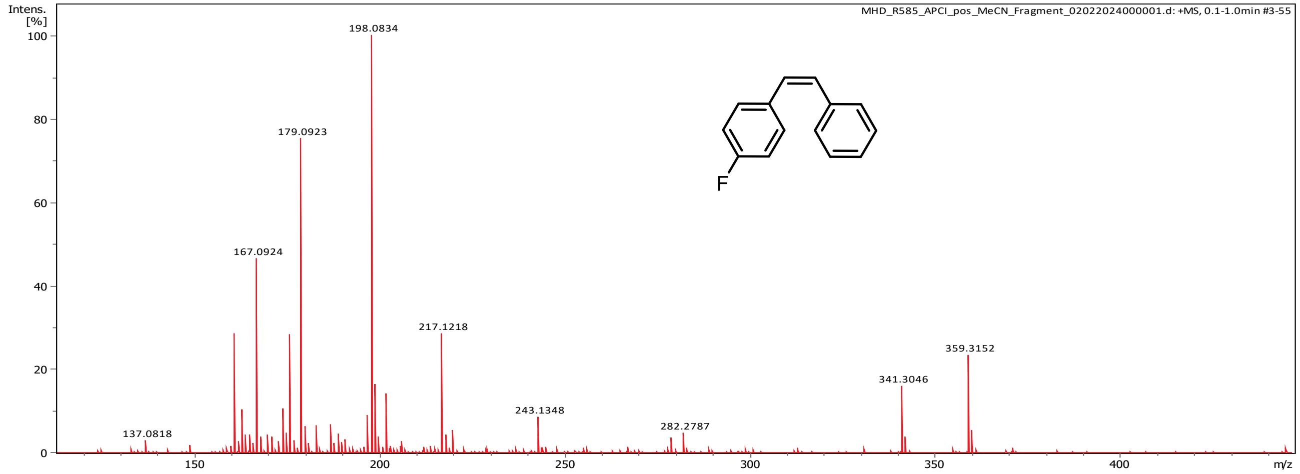

Intens. [%]
MHD_R585_APCI_pos_MeCN_Fragment_02022024000001.d: +MS, 0.1-1.0min #3-55
198.0834
179.0923
167.0924
217.1218
359.3152
341.3046
243.1348
282.2787
137.0818
F
m/z

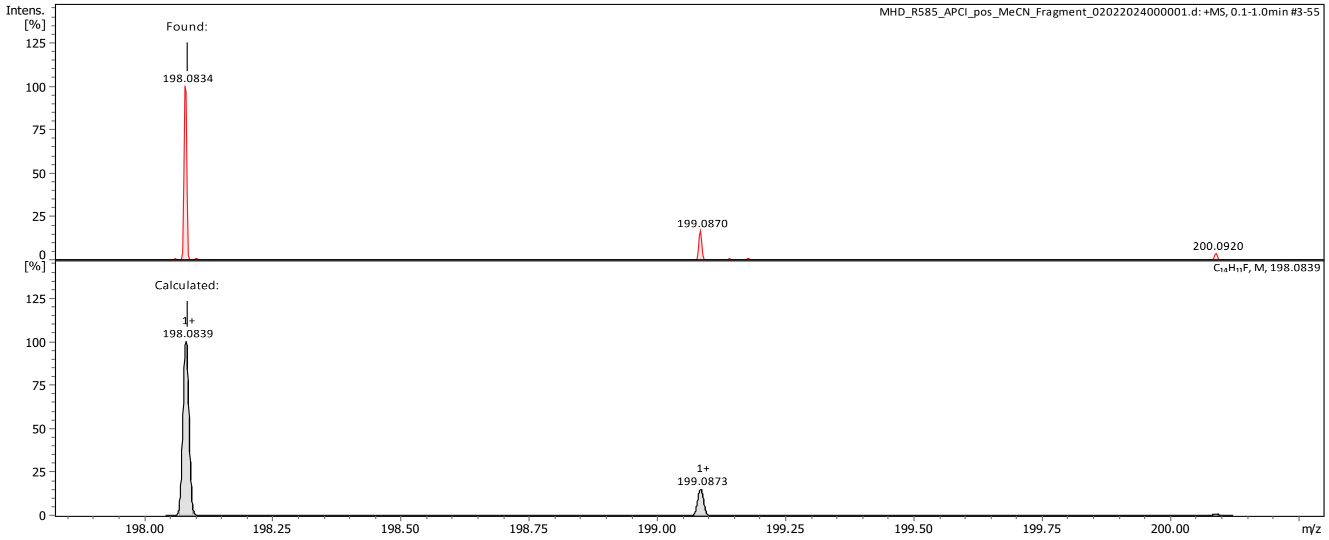

Intens. [%]
MHD_R585_APCI_pos_MeCN_Fragment_02022024000001.d: +MS, 0.1-1.0min #3-55
Found:
198.0834
199.0870
200.0920
C14H11F, M, 198.0839
Calculated:
1+
198.0839
1+
199.0873
m/z

2.5.8 IR

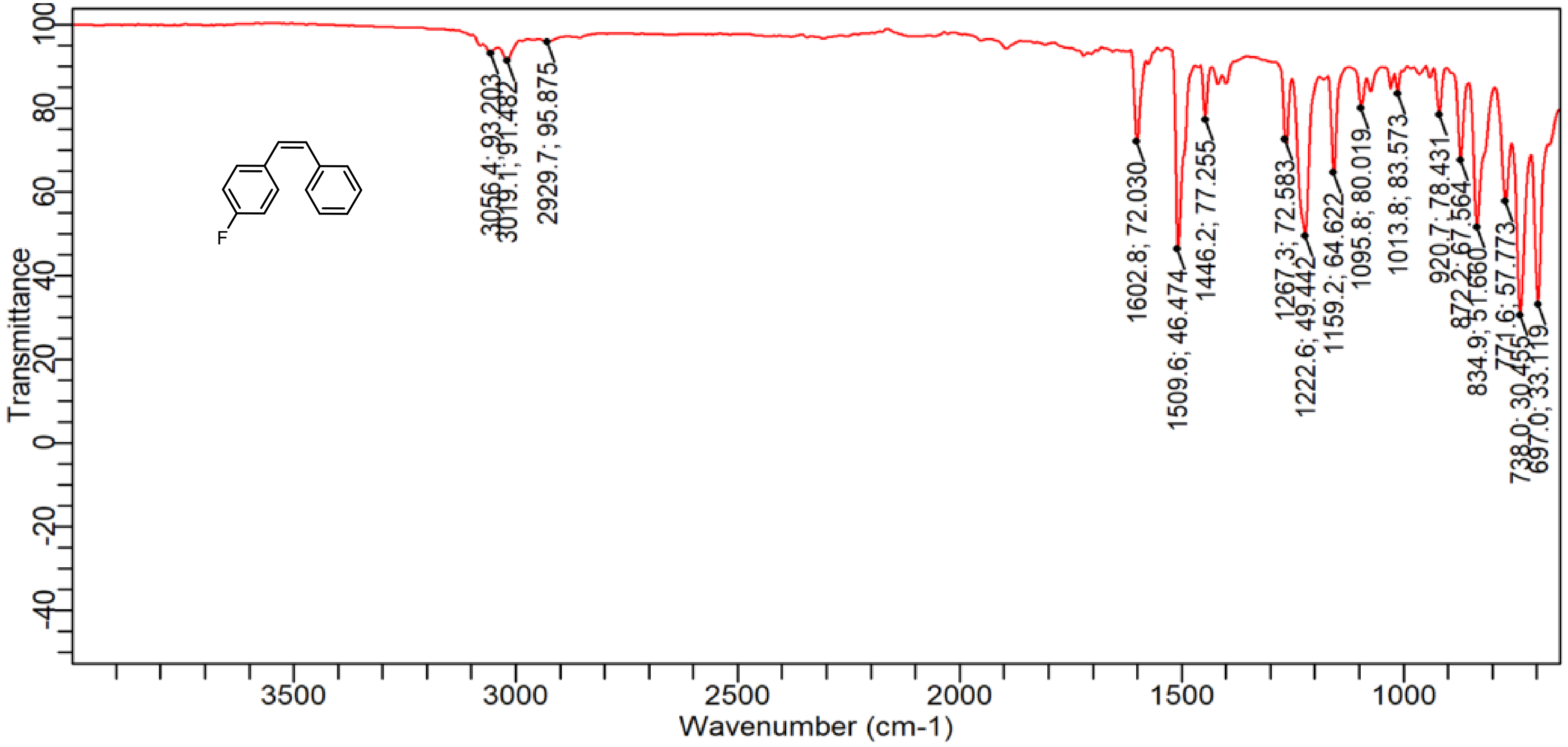

Transmittance
Wavenumber (cm-1)
F
3056.4; 93.203
3019.1; 91.482
2929.7; 95.875
1602.8; 72.030
1509.6; 46.474
1446.2; 77.255
1267.3; 72.583
1222.6; 49.442
1159.2; 64.622
1095.8; 80.019
1013.8; 83.573
920.7; 78.431
872.2; 67.564
834.9; 51.660
771.6; 57.773
738.0; 30.455
697.0; 33.119

2.6 (*Z*)-1-Chloro-4-styrylbenzene (2f) (600 MHz, $CDCl_3$)

2.6.1 ^{1}H NMR

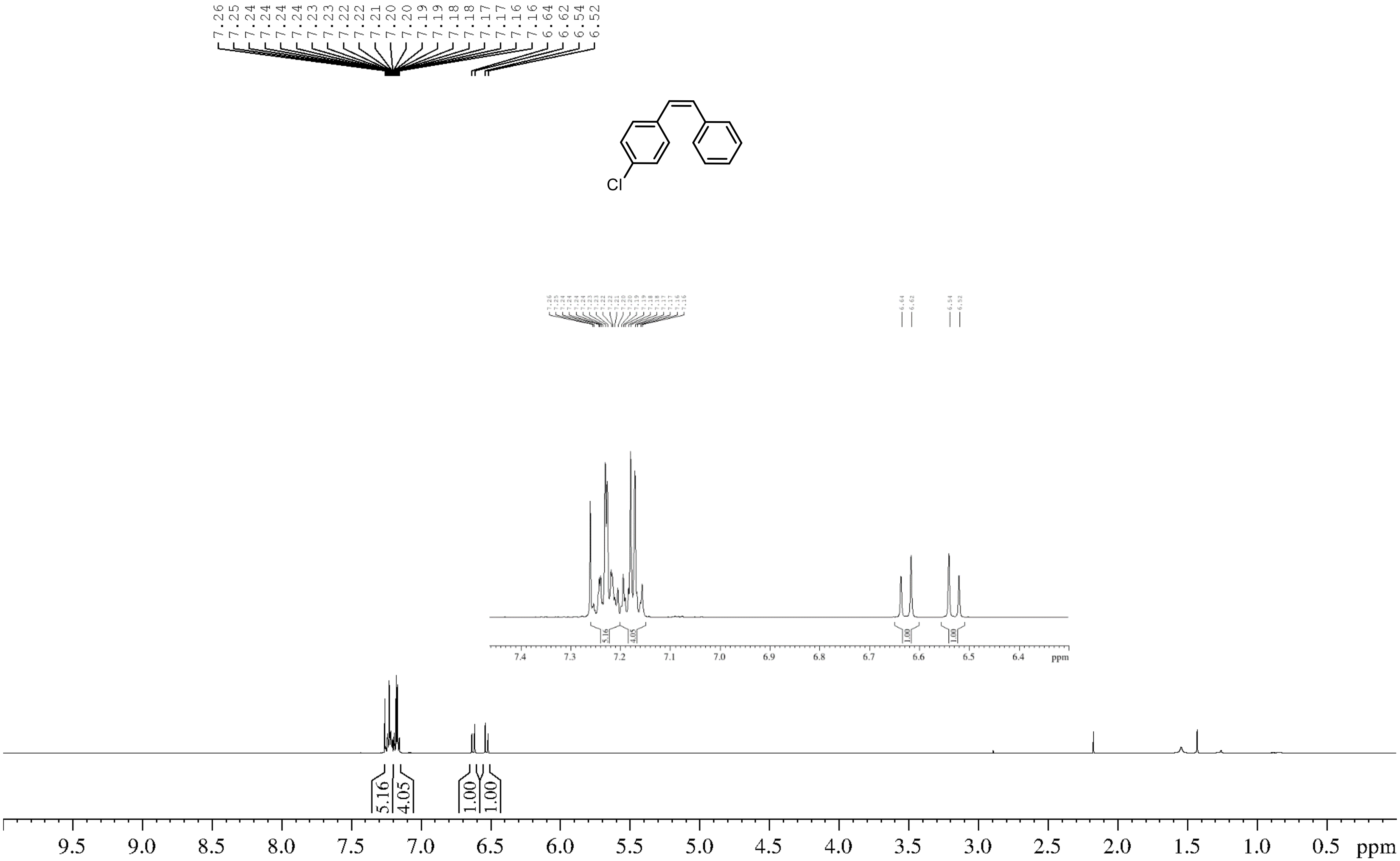

2.6.2 ^{13}C NMR

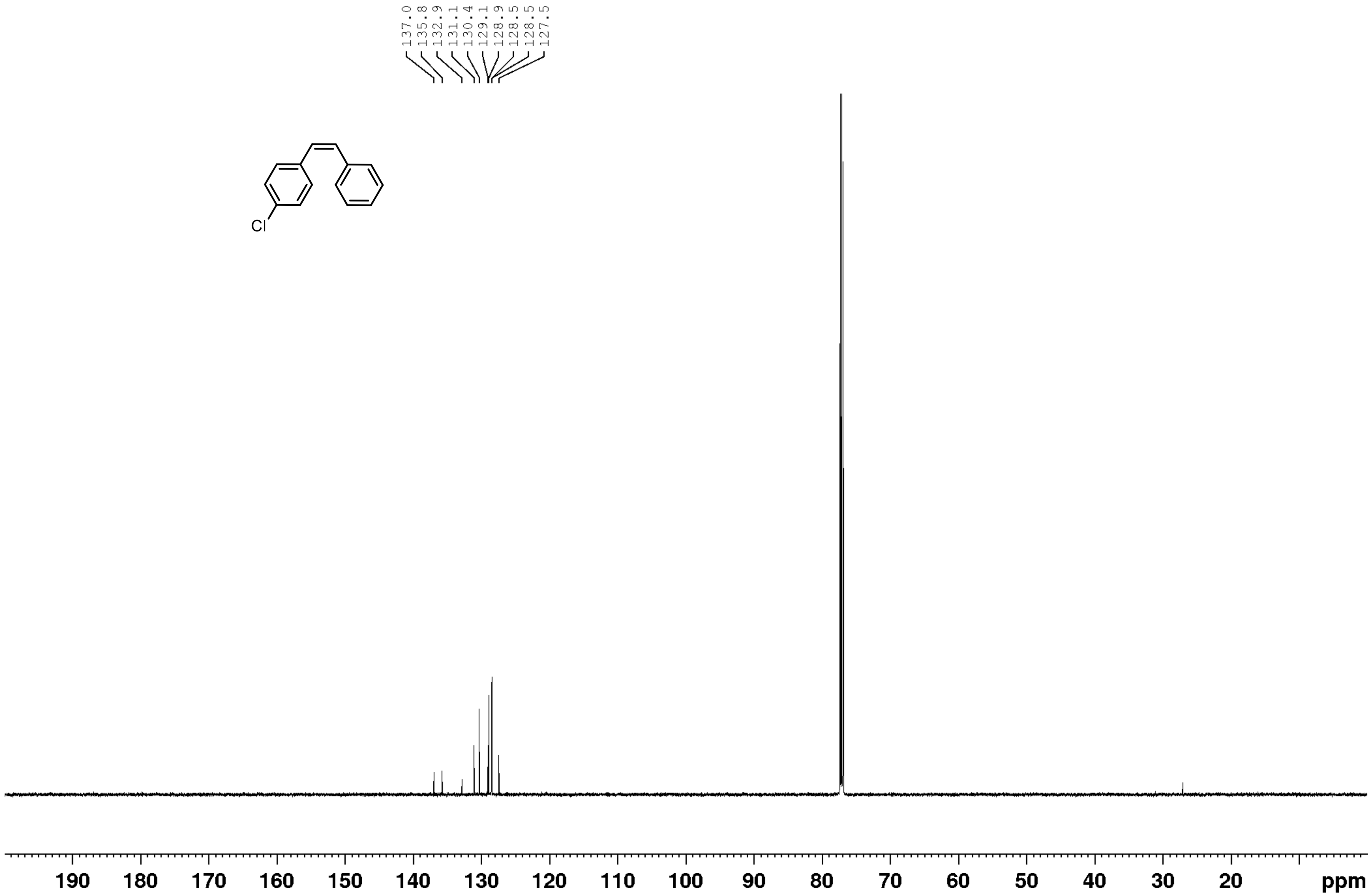

137.0
135.8
132.9
131.1
130.4
129.1
128.9
128.5
128.5
127.5
Cl
190
180
170
160
150
140
130
120
110
100
90
80
70
60
50
40
30
20
ppm

2.6.3 ^{1}H, ^{1}H COSY

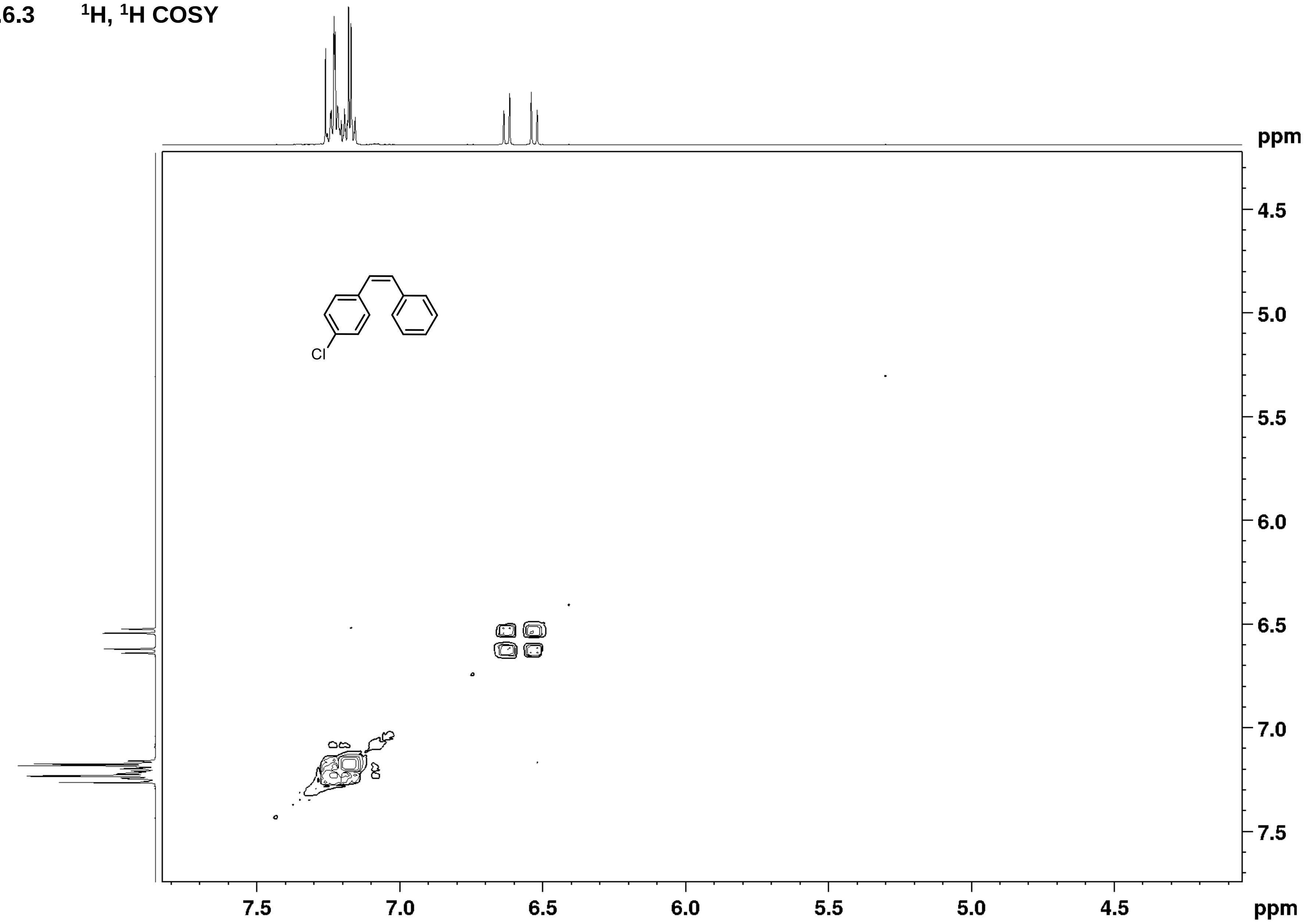

2.6.4 ¹H, ¹³C HMBC

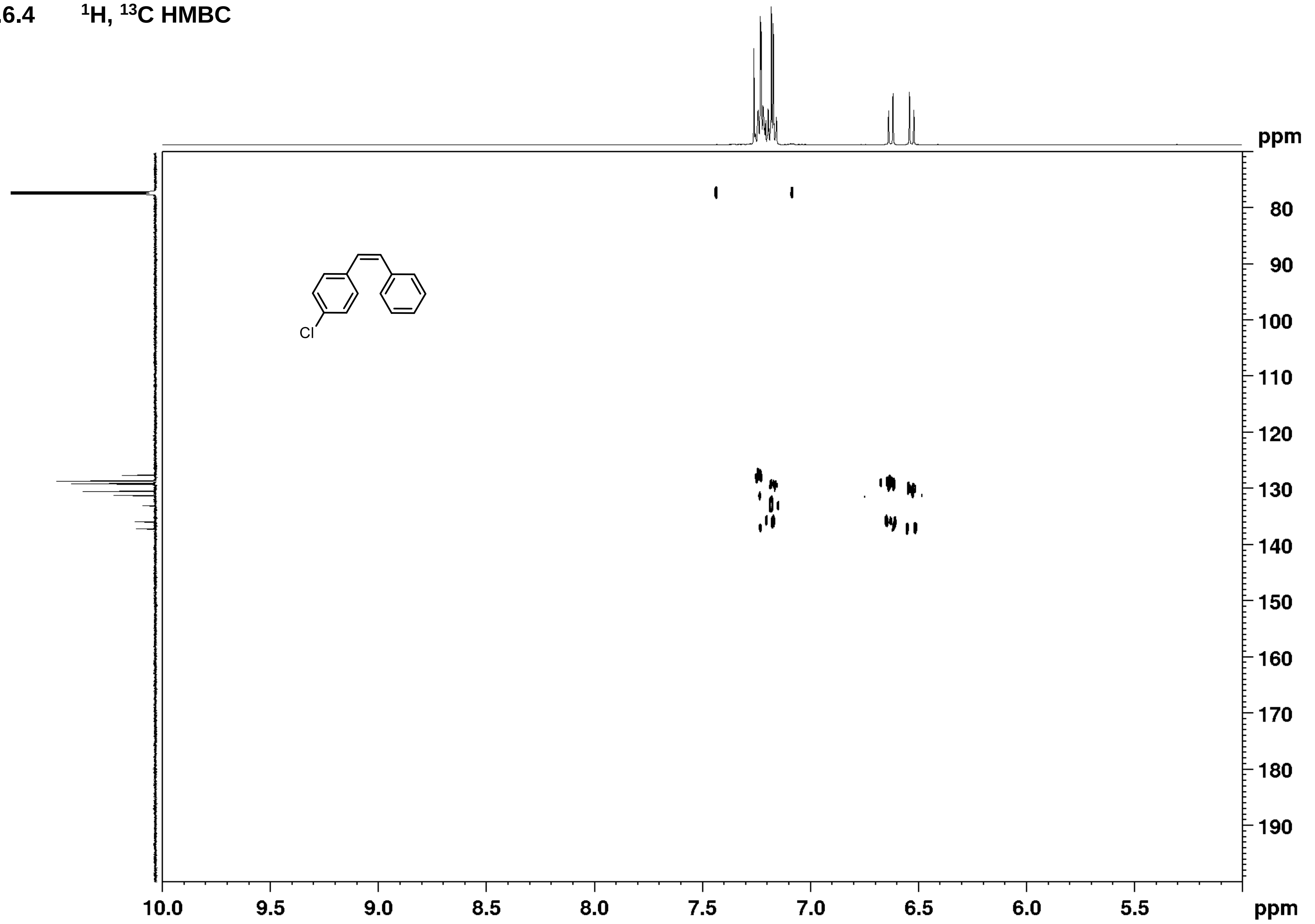

2.6.5 ^{1}H, ^{13}C HSQC

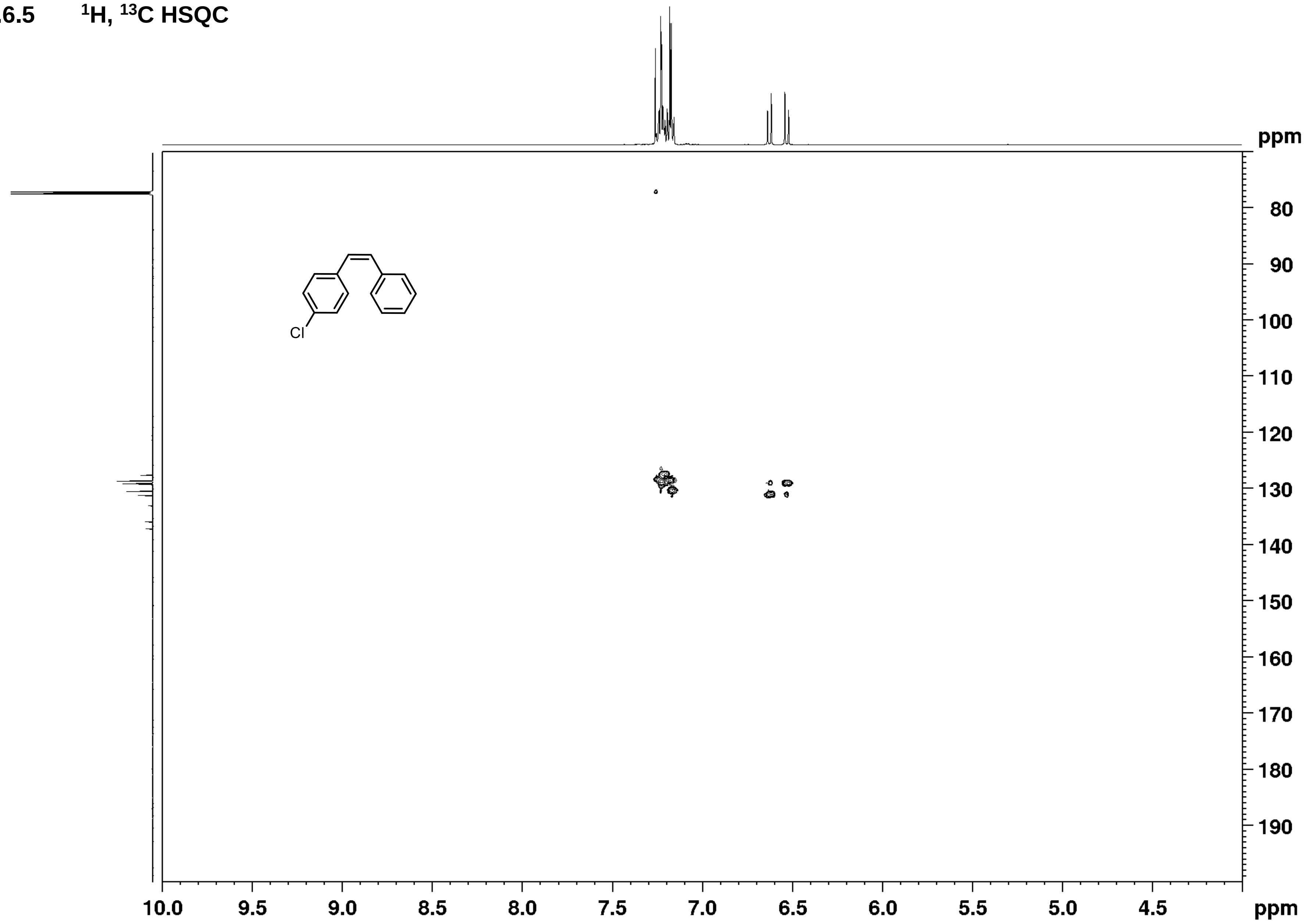

2.6.6 HRMS

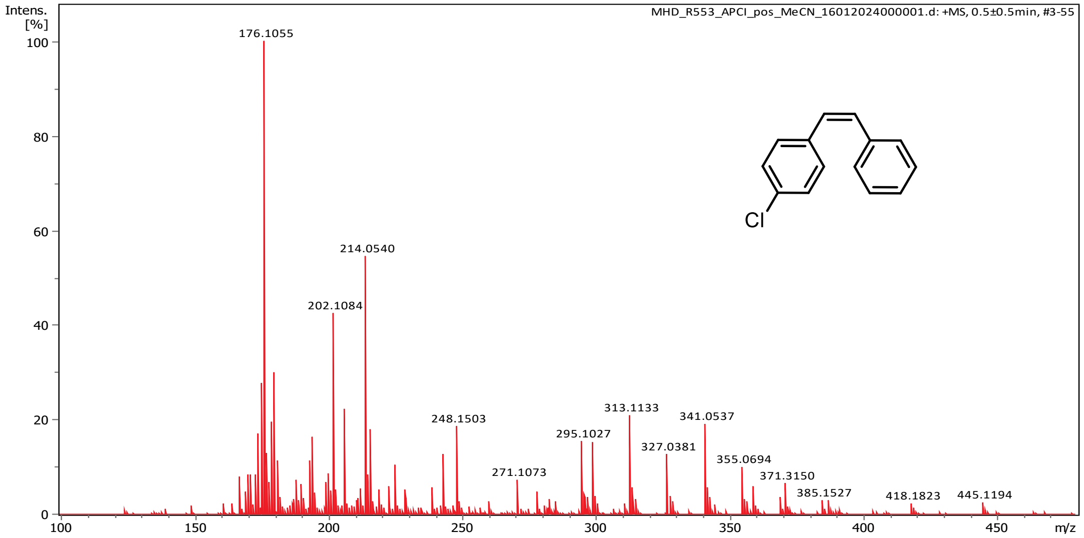
Intens. [%]
MHD_R553_APCI_pos_MeCN_16012024000001.d: +MS, 0.5±0.5min, #3-55
176.1055
214.0540
202.1084
248.1503
271.1073
295.1027
313.1133
327.0381
341.0537
355.0694
371.3150
385.1527
418.1823
445.1194
Cl
m/z

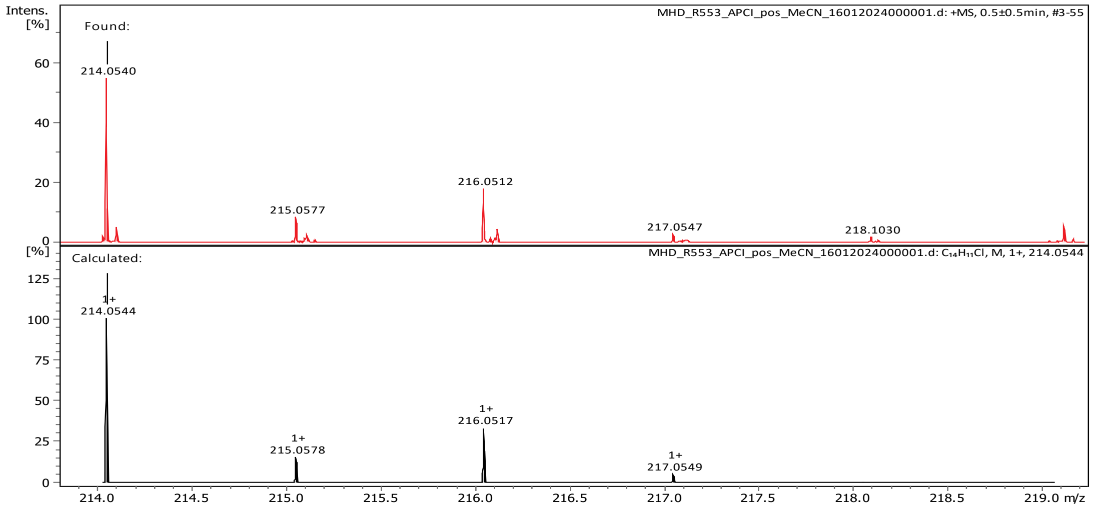
Intens. [%]
Found:
MHD_R553_APCI_pos_MeCN_16012024000001.d: +MS, 0.5±0.5min, #3-55
214.0540
215.0577
216.0512
217.0547
218.1030
Calculated:
MHD_R553_APCI_pos_MeCN_16012024000001.d: $C_{14}H_{11}Cl$, M, 1+, 214.0544
1+ 214.0544
1+ 215.0578
1+ 216.0517
1+ 217.0549
m/z

2.6.7 IR

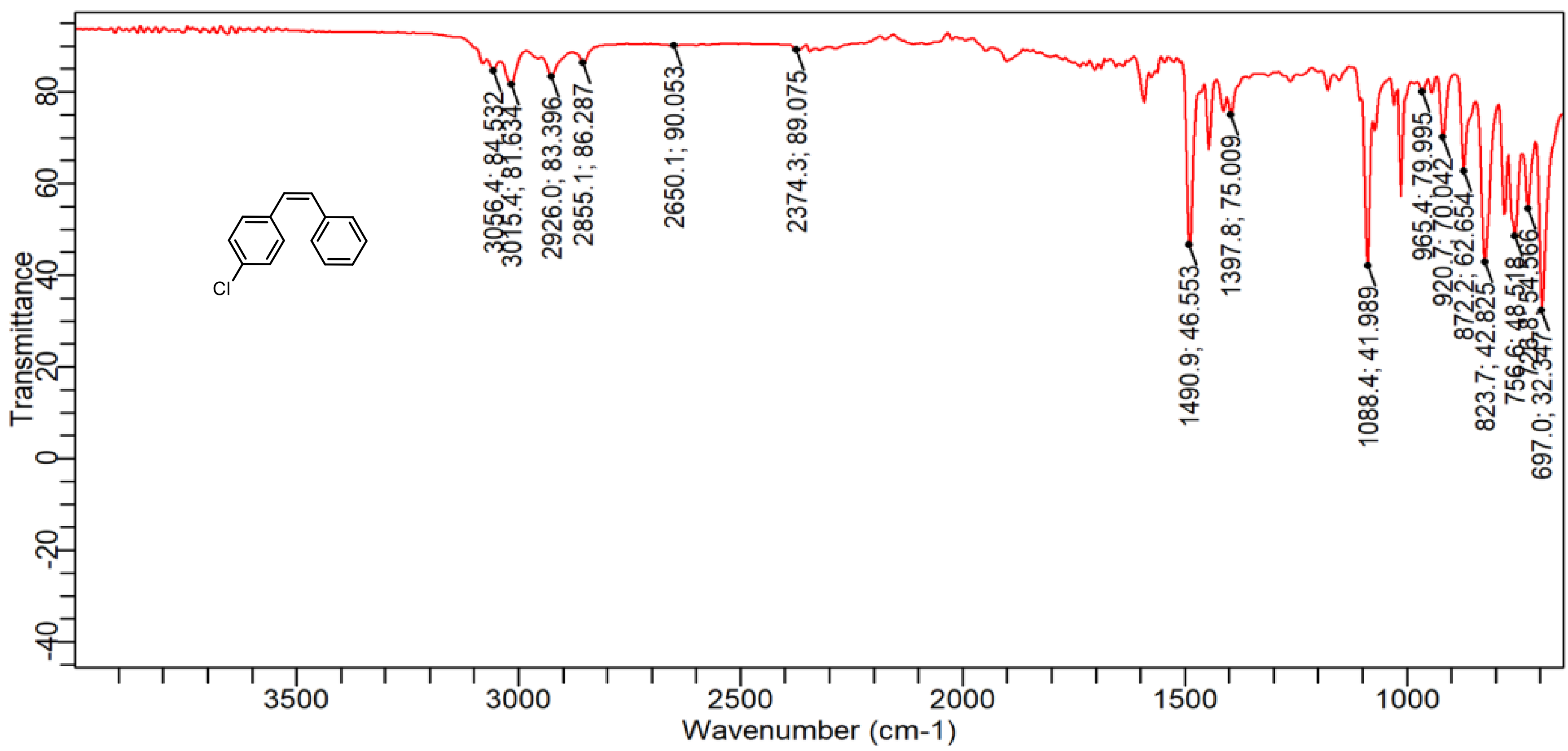

Transmittance
Wavenumber (cm-1)
Cl
3056.4; 84.532
3015.4; 81.634
2926.0; 83.396
2855.1; 86.287
2650.1; 90.053
2374.3; 89.075
1490.9; 46.553
1397.8; 75.009
1088.4; 41.989
965.4; 79.995
920.7; 70.042
872.2; 62.654
823.7; 42.825
756.6; 48.518
697.0; 32.347

2.7 (*Z*)-1-Bromo-4-styrylbenzene (2g) (600 MHz, $CDCl_3$)

2.7.1 ^{1}H NMR

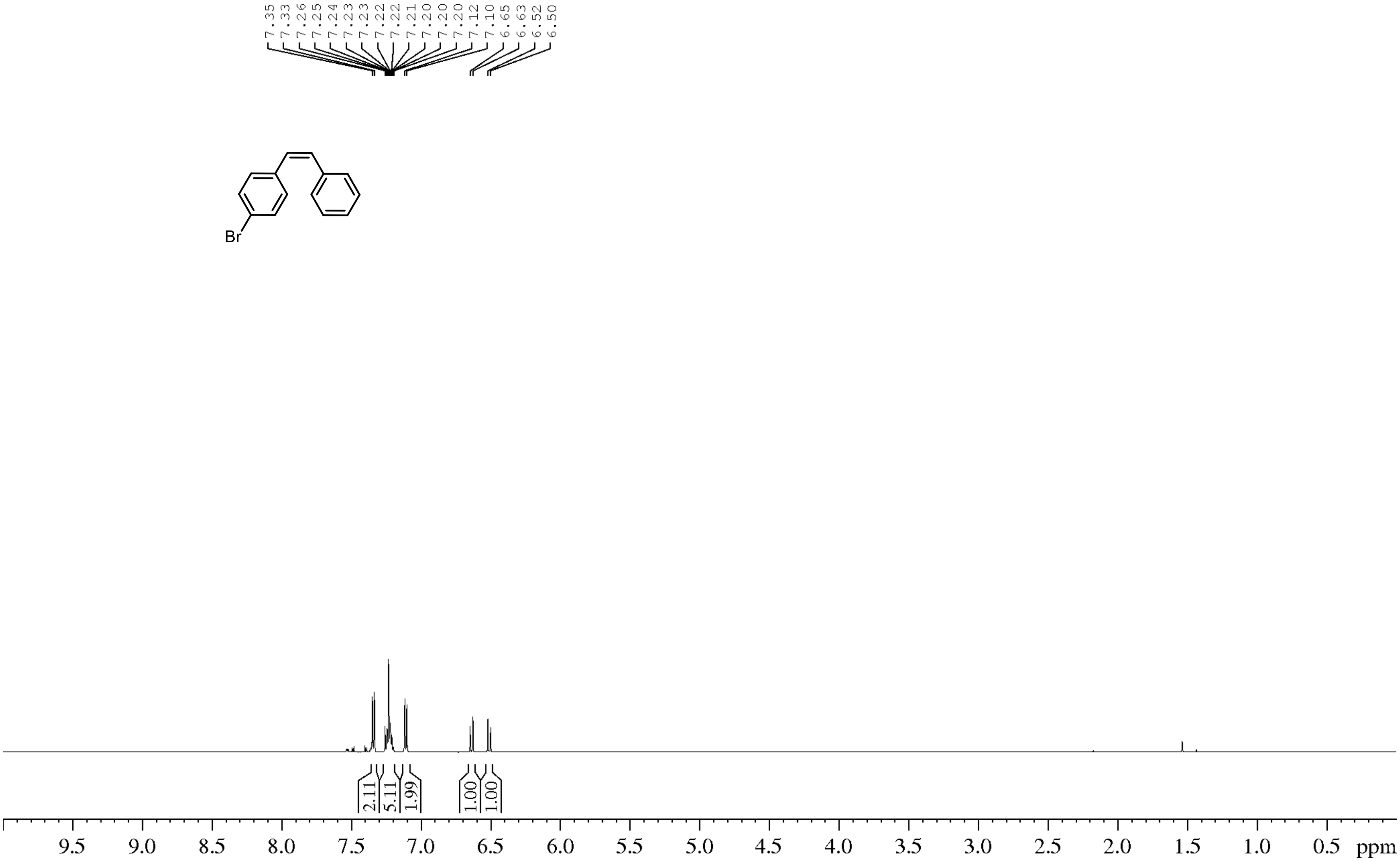

2.7.2 ^{13}C NMR

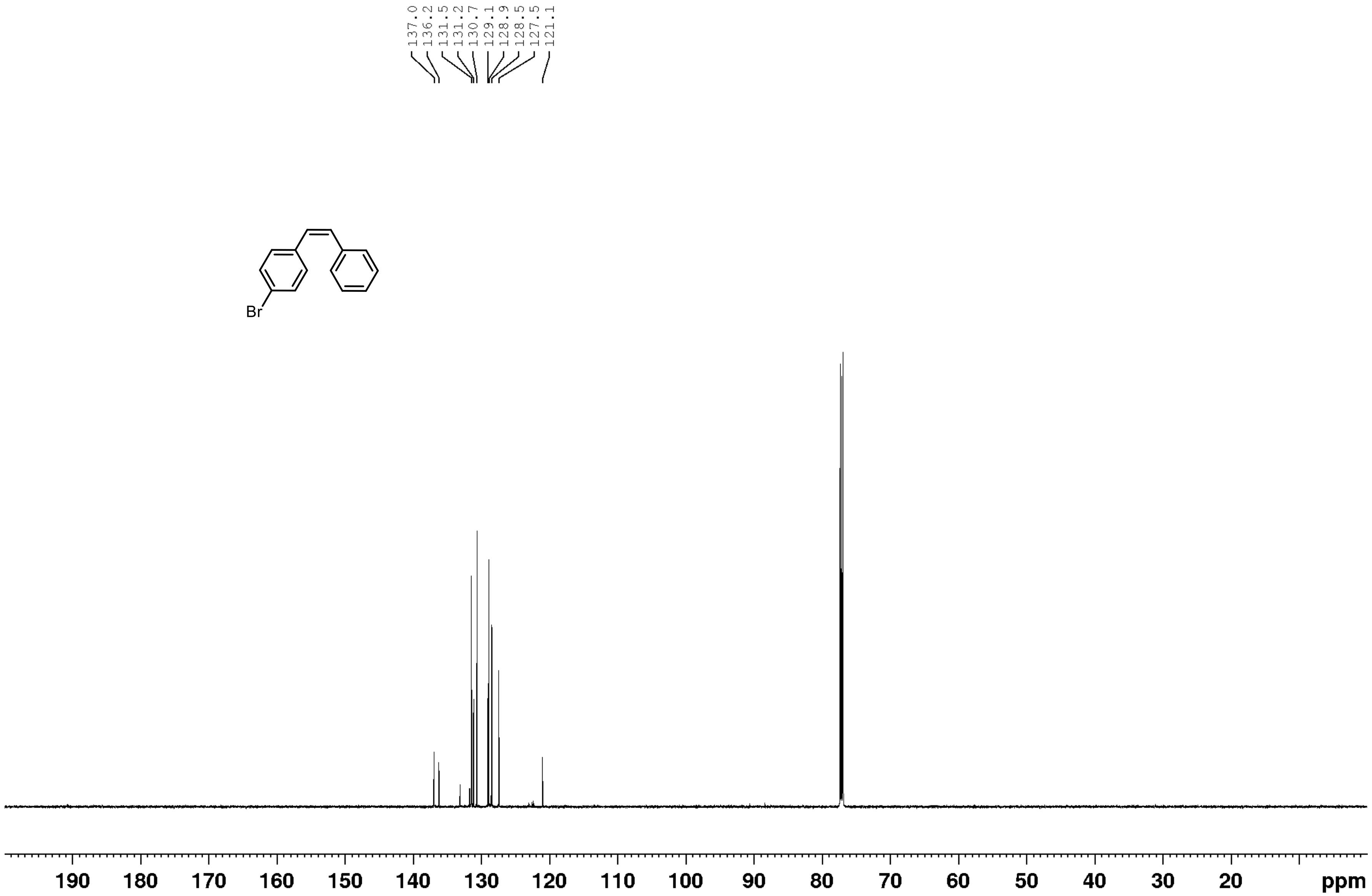

137.0
136.2
131.5
131.2
130.7
129.1
128.9
128.5
127.5
121.1
Br
190
180
170
160
150
140
130
120
110
100
90
80
70
60
50
40
30
20
ppm

2.7.3 ^{1}H, ^{1}H COSY

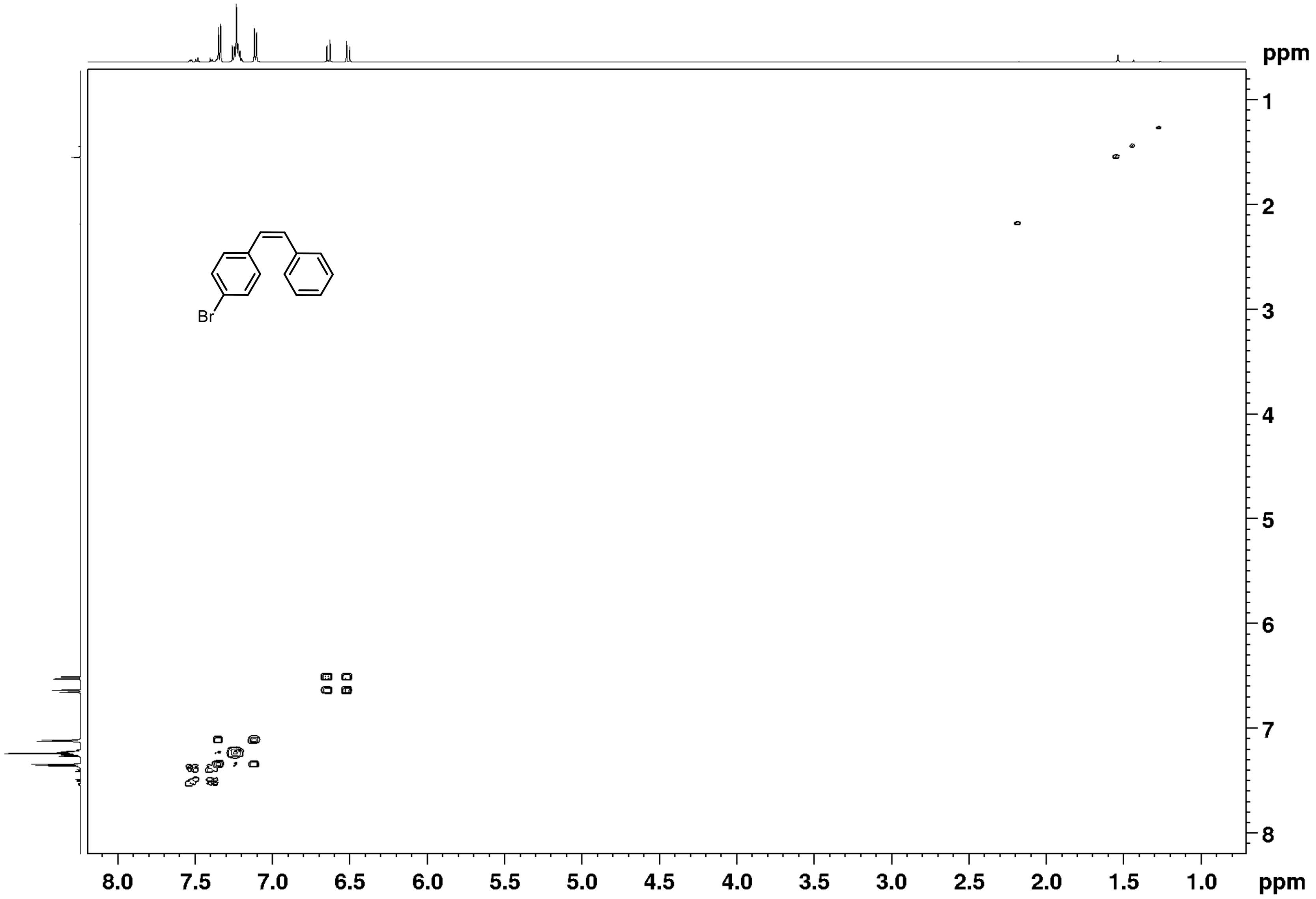

2.7.4 ^{1}H ^{13}C HMBC

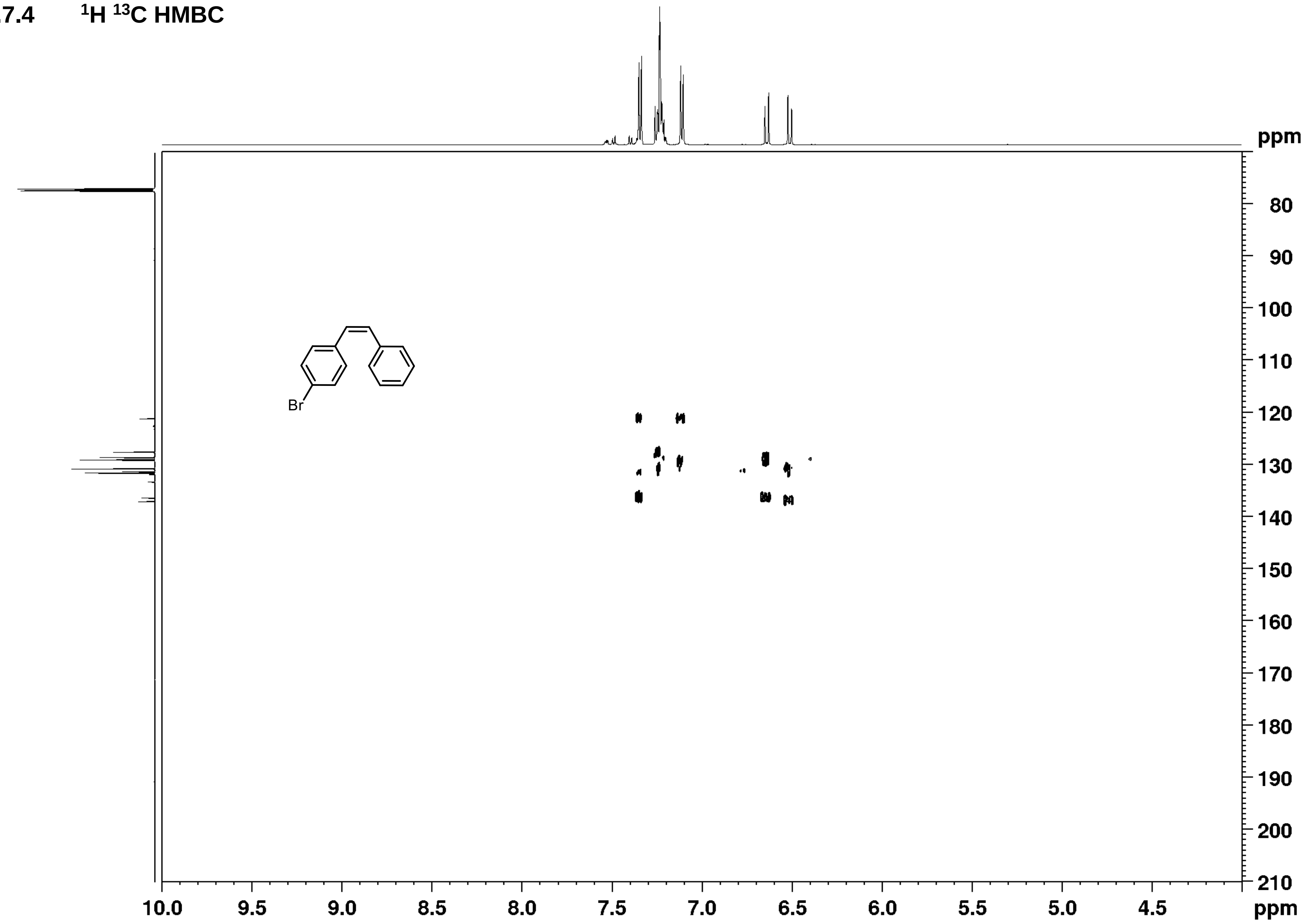

2.7.5 ¹H, ¹³C HSQC

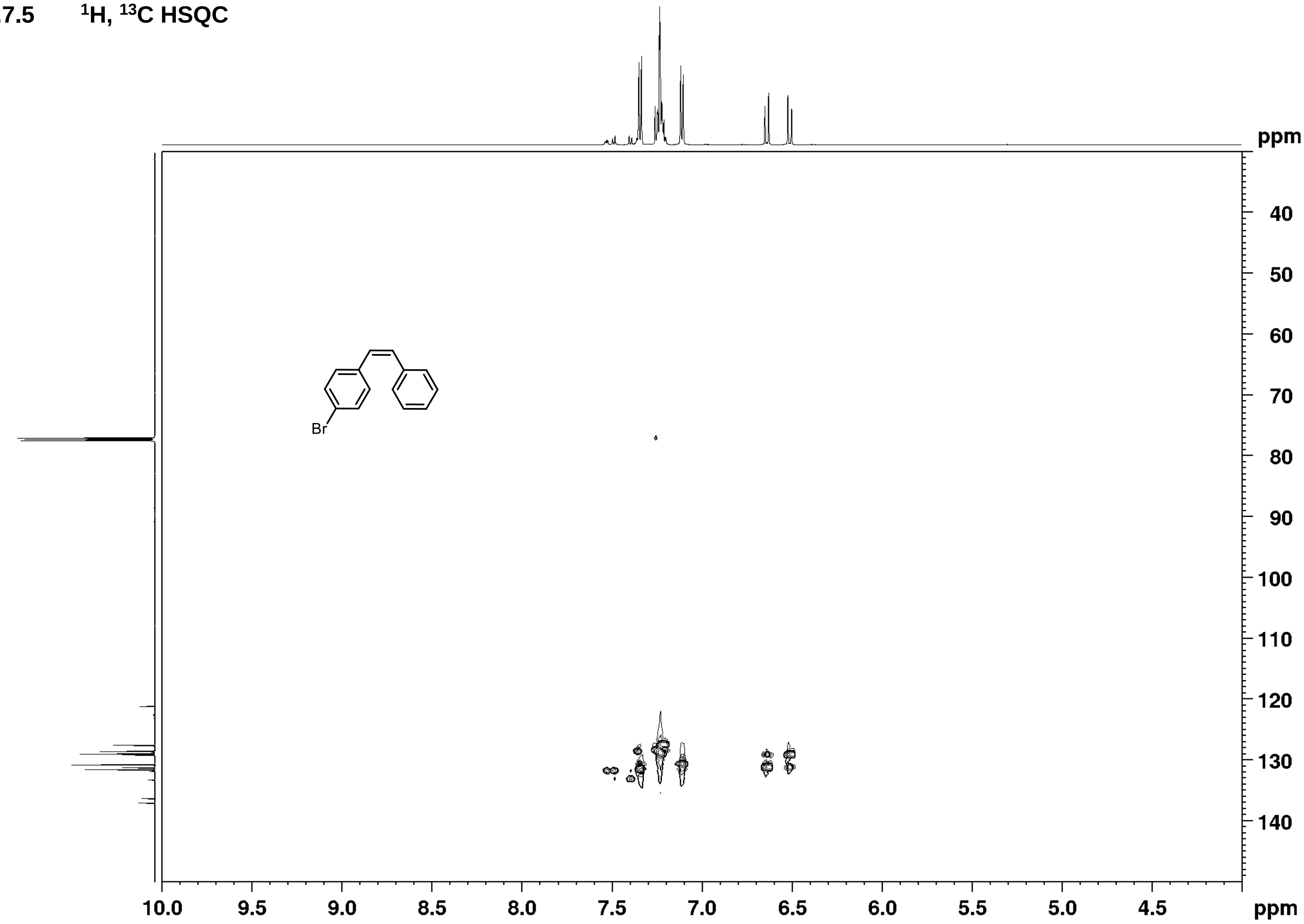

2.7.6 HRMS

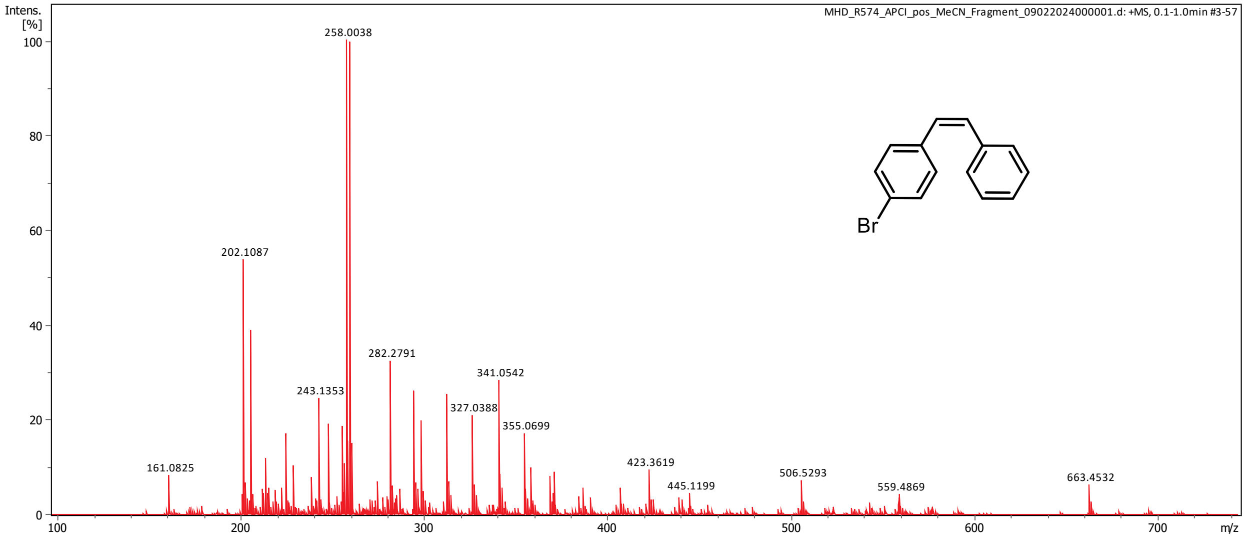

Intens. [%]
MHD_R574_APCI_pos_MeCN_Fragment_09022024000001.d: +MS, 0.1-1.0min #3-57
258.0038
202.1087
282.2791
341.0542
243.1353
327.0388
355.0699
161.0825
423.3619
445.1199
506.5293
559.4869
663.4532
Br
m/z

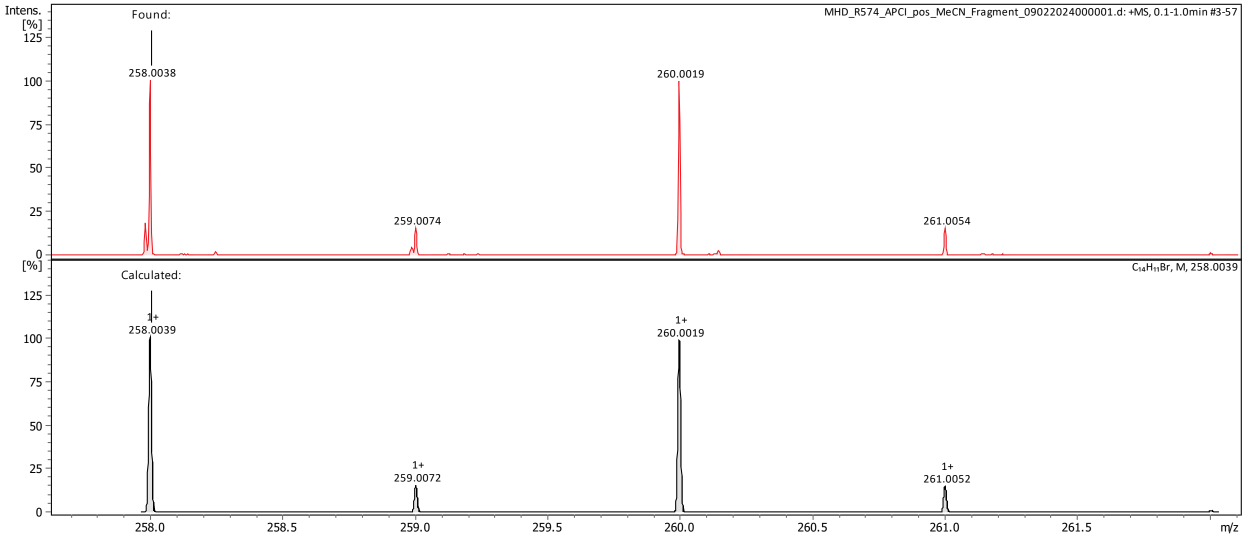

Intens. [%]
Found:
MHD_R574_APCI_pos_MeCN_Fragment_09022024000001.d: +MS, 0.1-1.0min #3-57
258.0038
260.0019
259.0074
261.0054
Calculated:
C14H11Br, M, 258.0039
1+ 258.0039
1+ 260.0019
1+ 259.0072
1+ 261.0052
m/z

2.7.7 IR

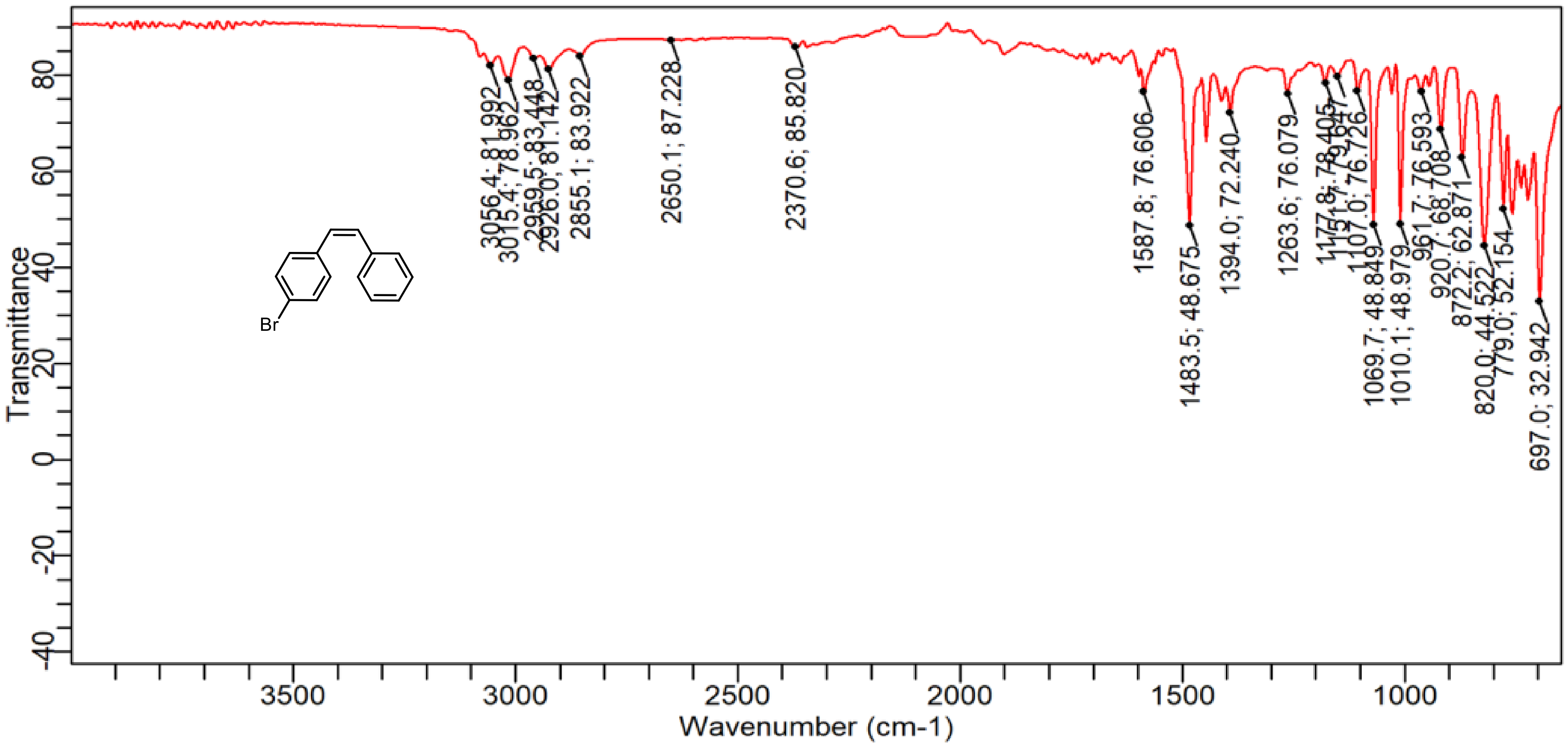

Transmittance
Wavenumber (cm-1)
Br
3056.4; 81.992
3015.4; 78.962
2959.5; 83.448
2926.0; 81.142
2855.1; 83.922
2650.1; 87.228
2370.6; 85.820
1587.8; 76.606
1483.5; 48.675
1394.0; 72.240
1263.6; 76.079
1069.7; 48.849
1010.1; 48.979
961.7; 76.593
872.2; 62.871
820.0; 44.522
779.0; 52.154
697.0; 32.942

2.8 (*Z*)-1-Iodo-4-styrylbenzene (2h) (600 MHz, $CDCl_3$)

2.8.1 ^{1}H NMR

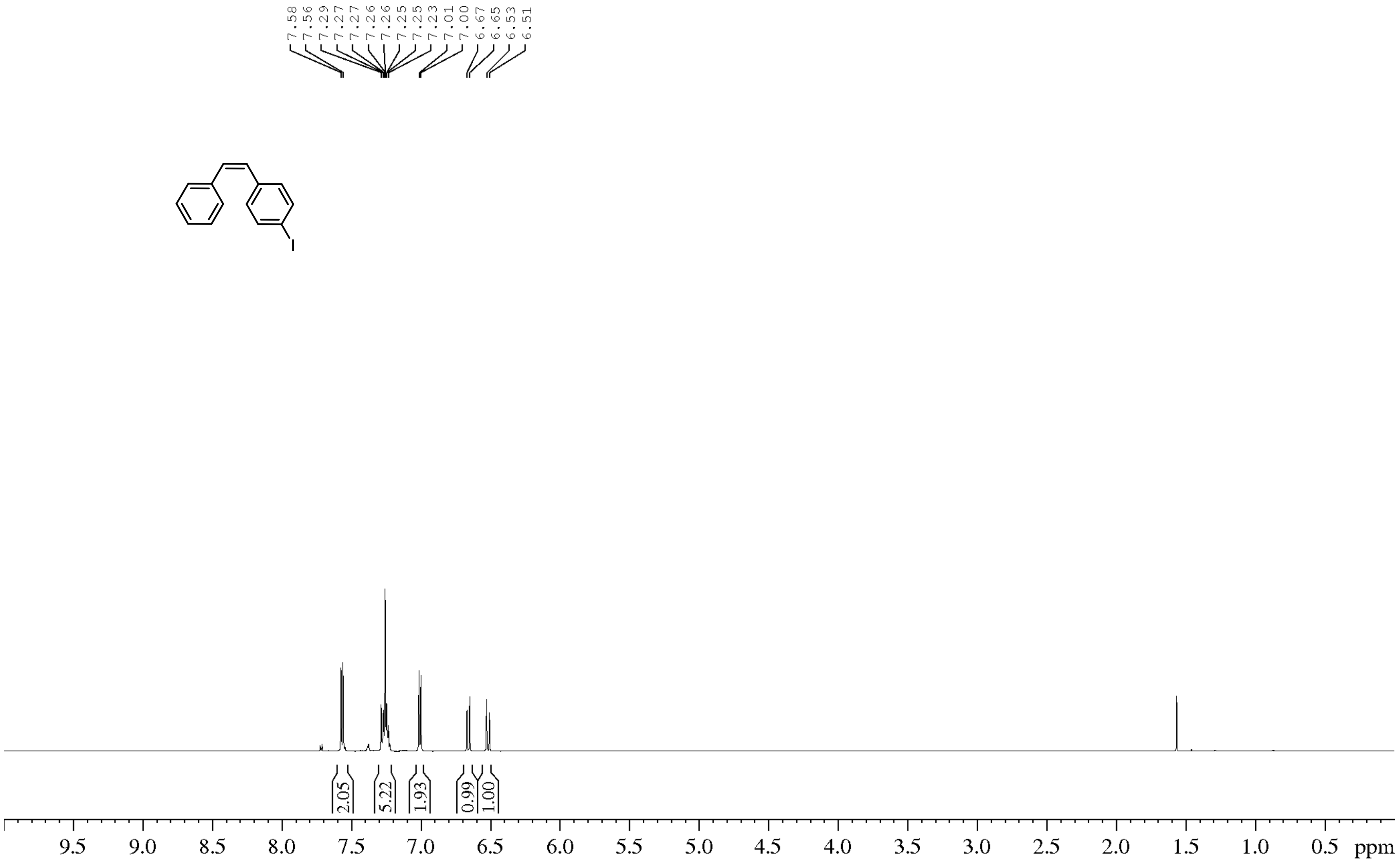

2.8.2 ^{13}C NMR

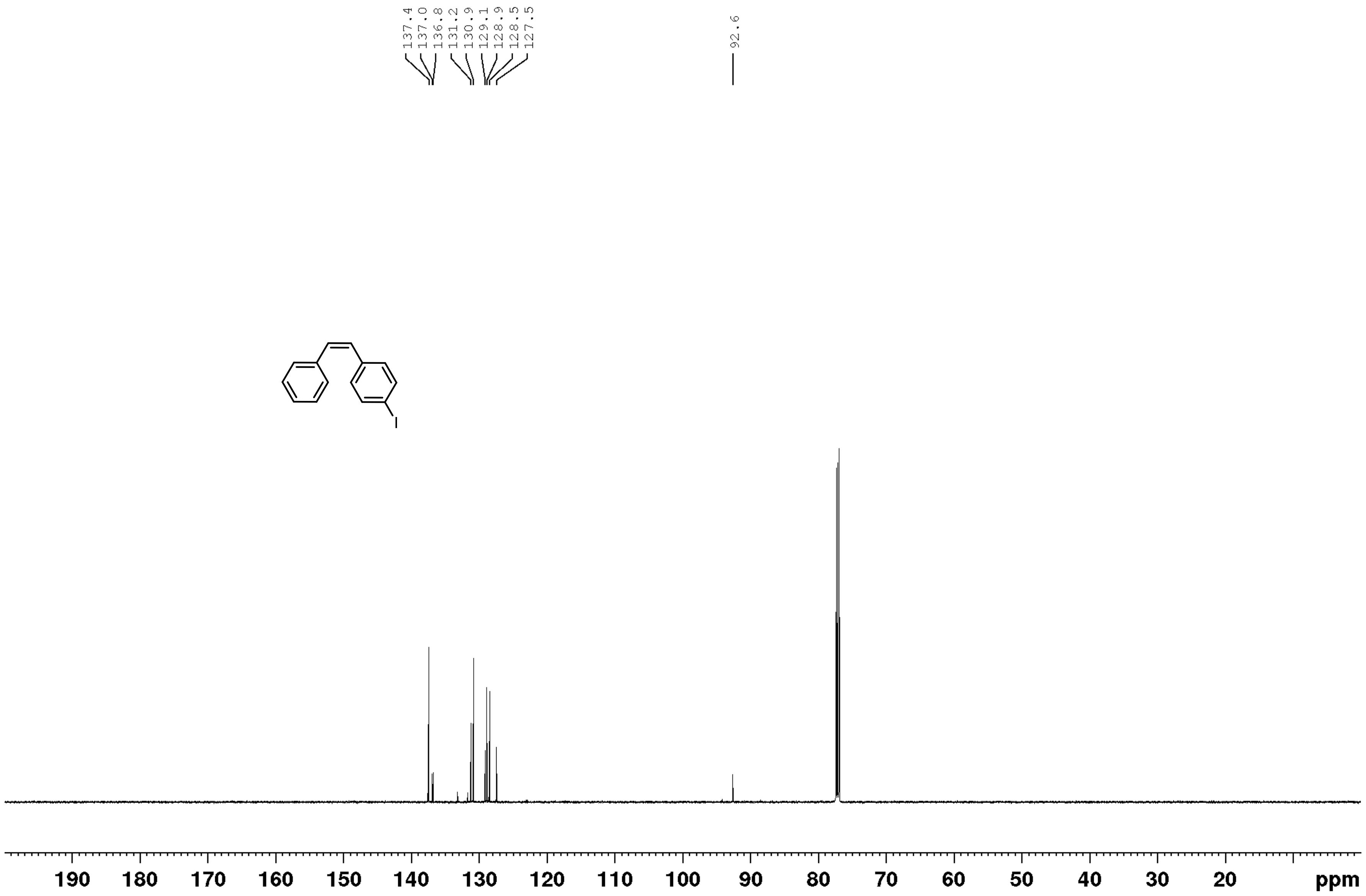
137.4
137.0
136.8
131.2
130.9
129.1
128.9
128.5
127.5
92.6
I
190
180
170
160
150
140
130
120
110
100
90
80
70
60
50
40
30
20
ppm

2.8.3 ¹H, ¹H COSY

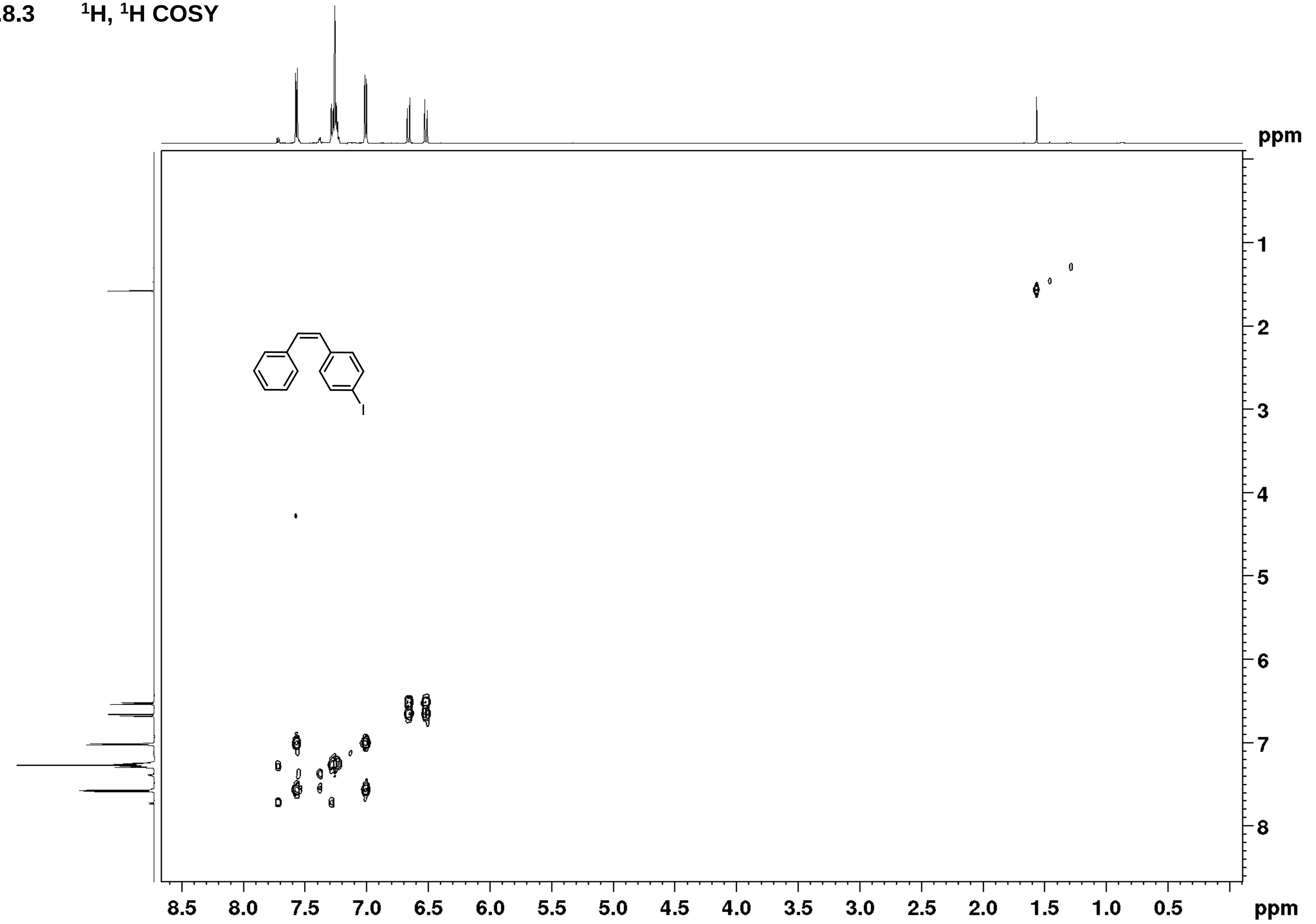

2.8.4 ^{1}H, ^{13}C HMBC

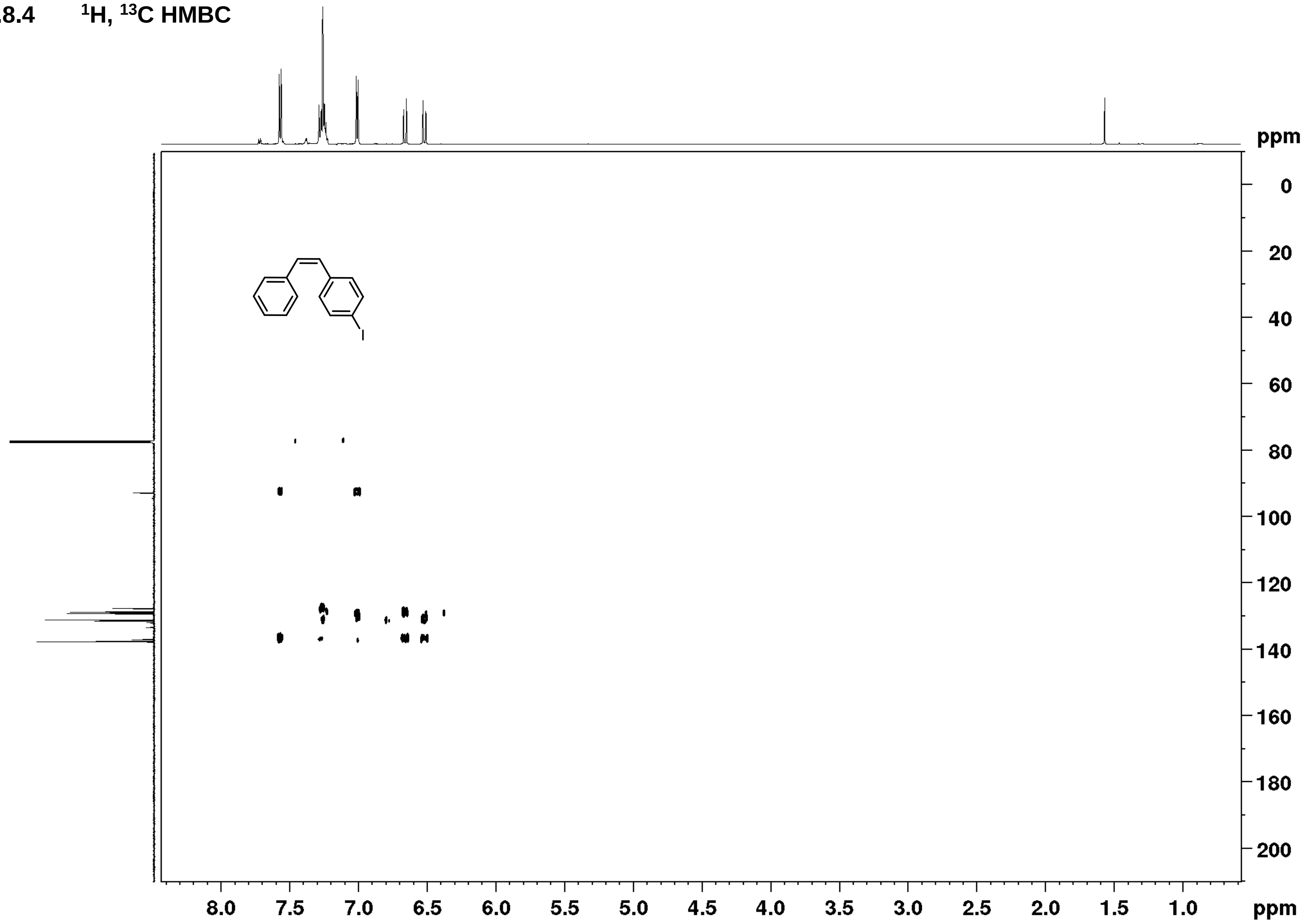

2.8.5 ^{1}H, ^{13}C HSQC

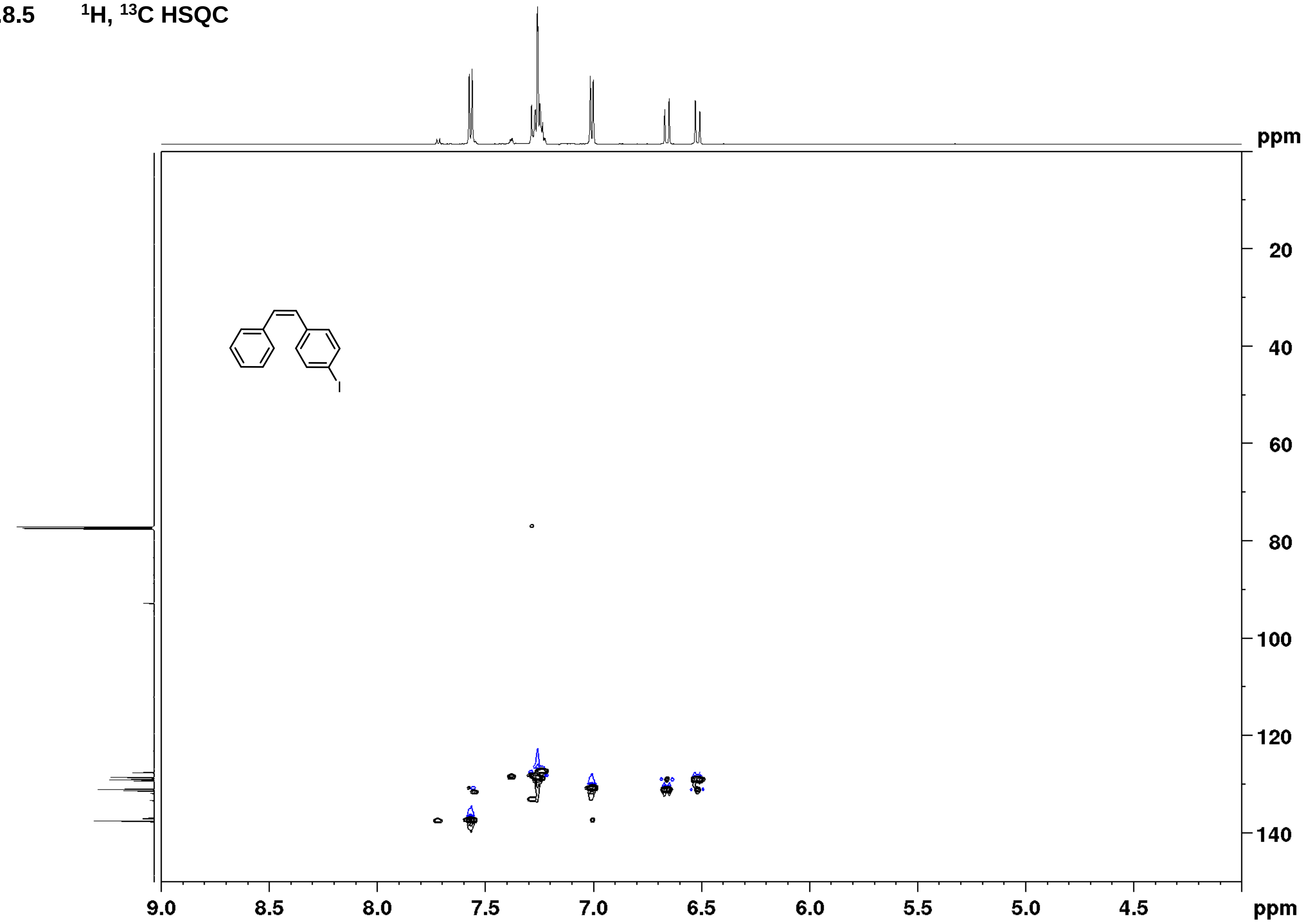

2.8.6 HRMS

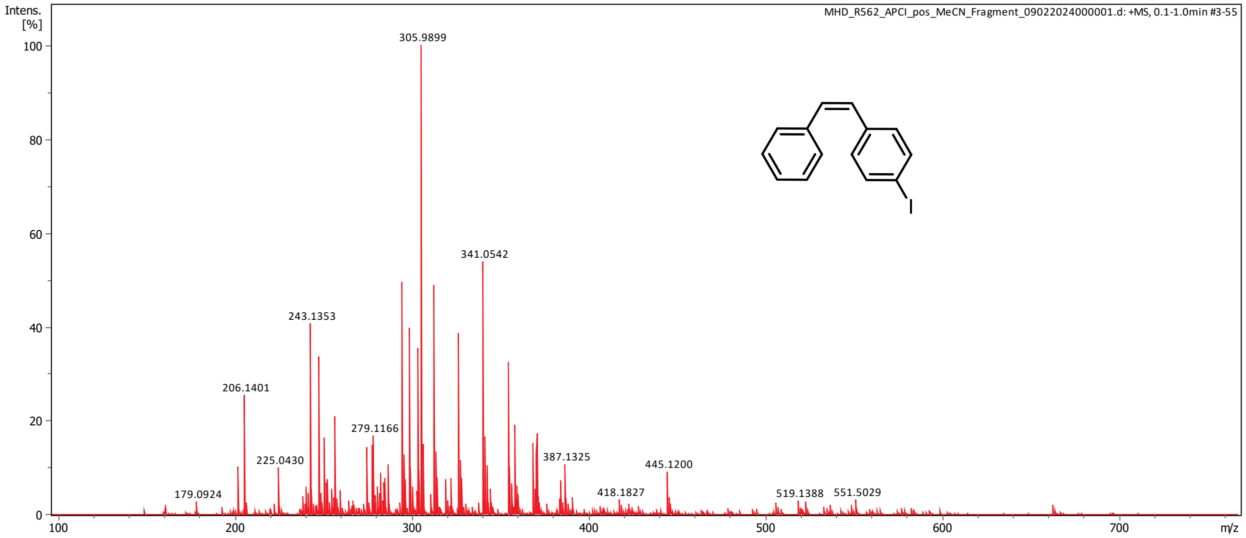
Intens. [%]
MHD_R562_APCI_pos_MeCN_Fragment_09022024000001.d: +MS, 0.1-1.0min #3-55
305.9899
341.0542
243.1353
206.1401
279.1166
225.0430
387.1325
445.1200
179.0924
418.1827
519.1388
551.5029
I
m/z

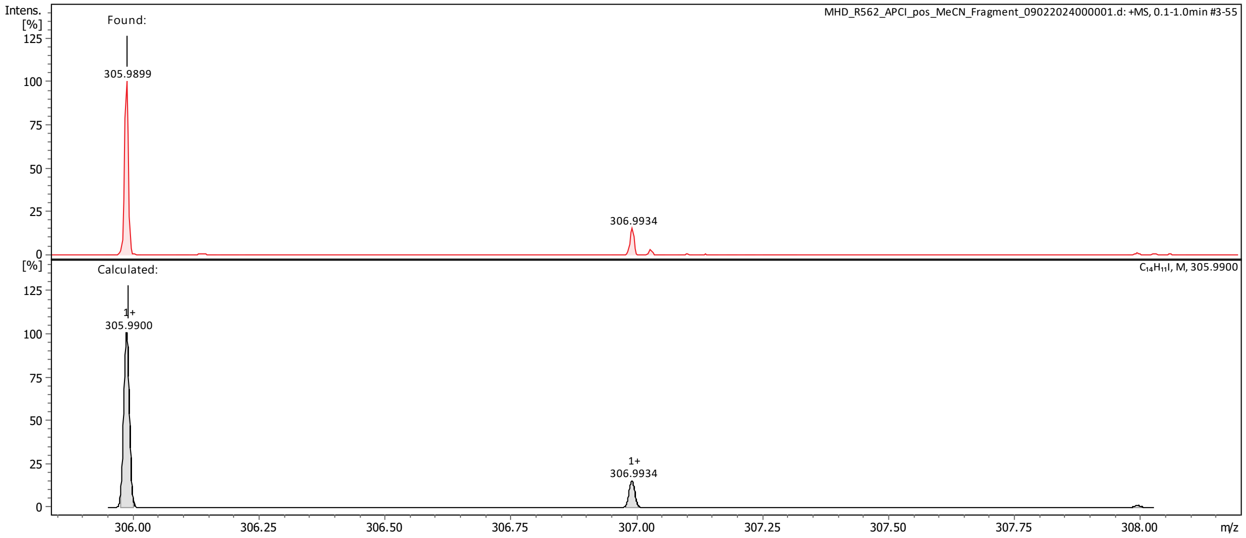
Intens. [%]
MHD_R562_APCI_pos_MeCN_Fragment_09022024000001.d: +MS, 0.1-1.0min #3-55
Found:
305.9899
306.9934
Calculated:
C₁₄H₁₁I, M, 305.9900
1+
305.9900
1+
306.9934
m/z

2.8.7 IR

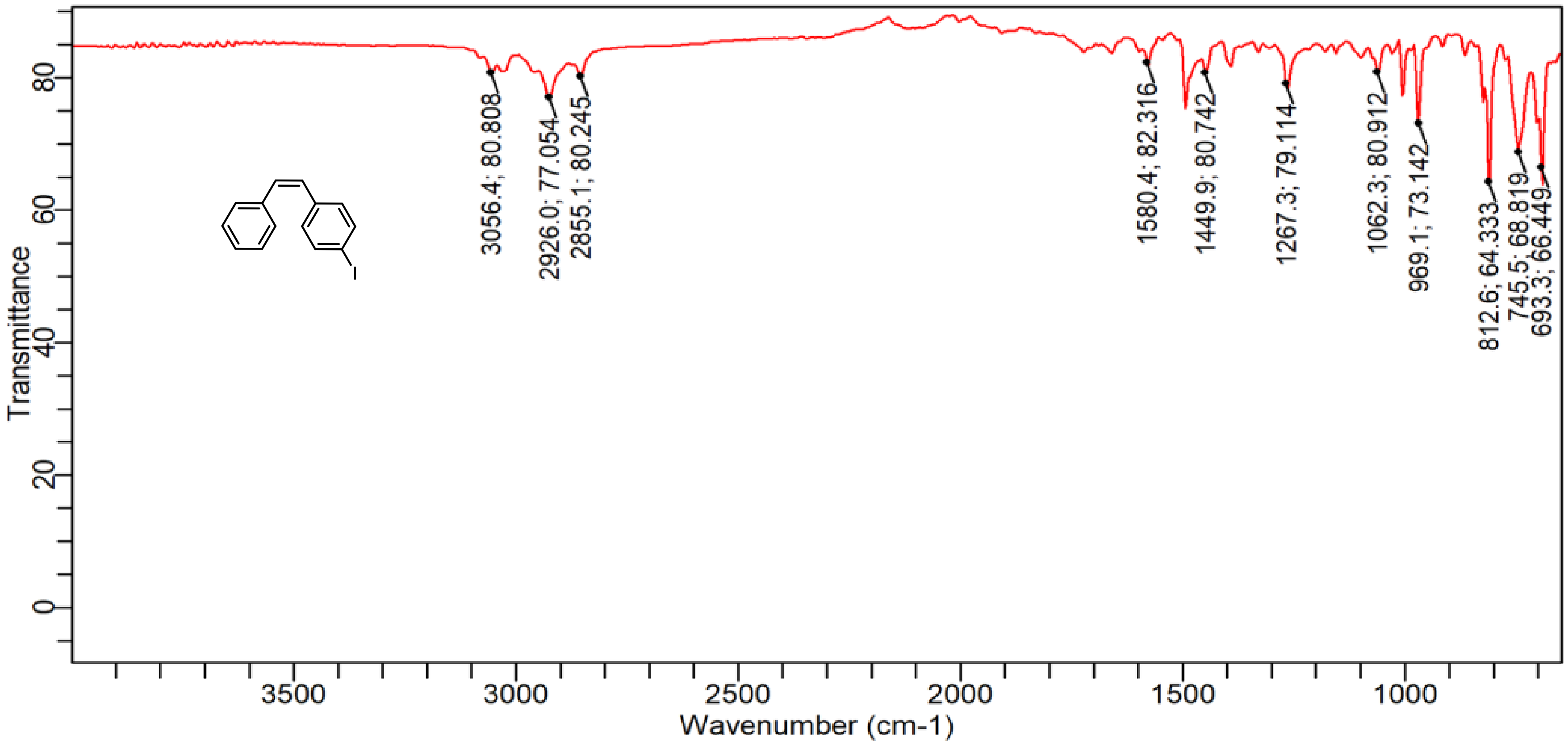

3056.4; 80.808
2926.0; 77.054
2855.1; 80.245
1580.4; 82.316
1449.9; 80.742
1267.3; 79.114
1062.3; 80.912
969.1; 73.142
812.6; 64.333
745.5; 68.819
693.3; 66.449
I
Transmittance
Wavenumber (cm-1)

2.9 (*Z*)-(3-Styrylphenyl)methanol (2i) (600 MHz, $CDCl_3$)

2.9.1 ^{1}H NMR

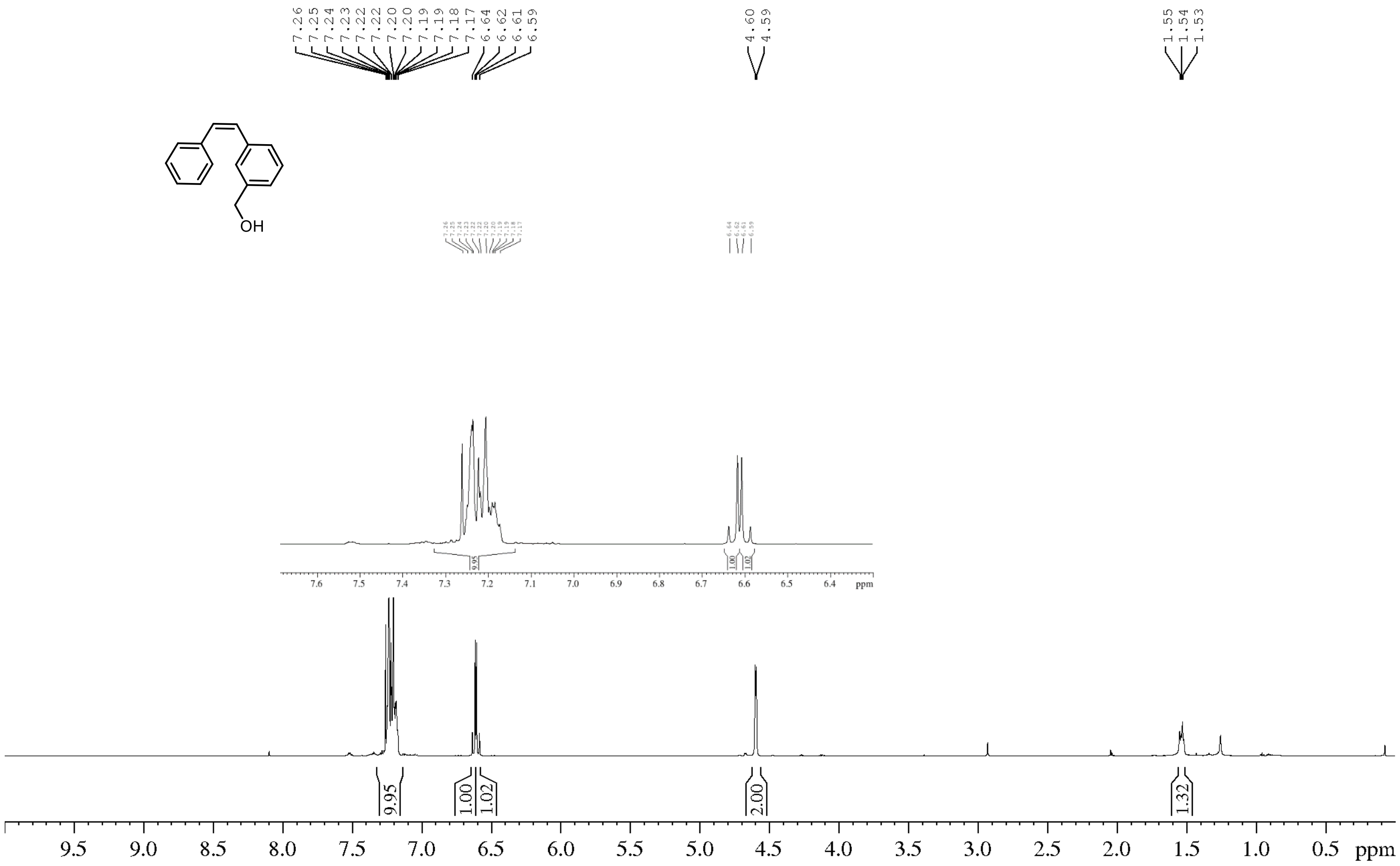

2.9.2 ^{13}C NMR

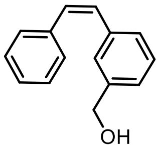
OH

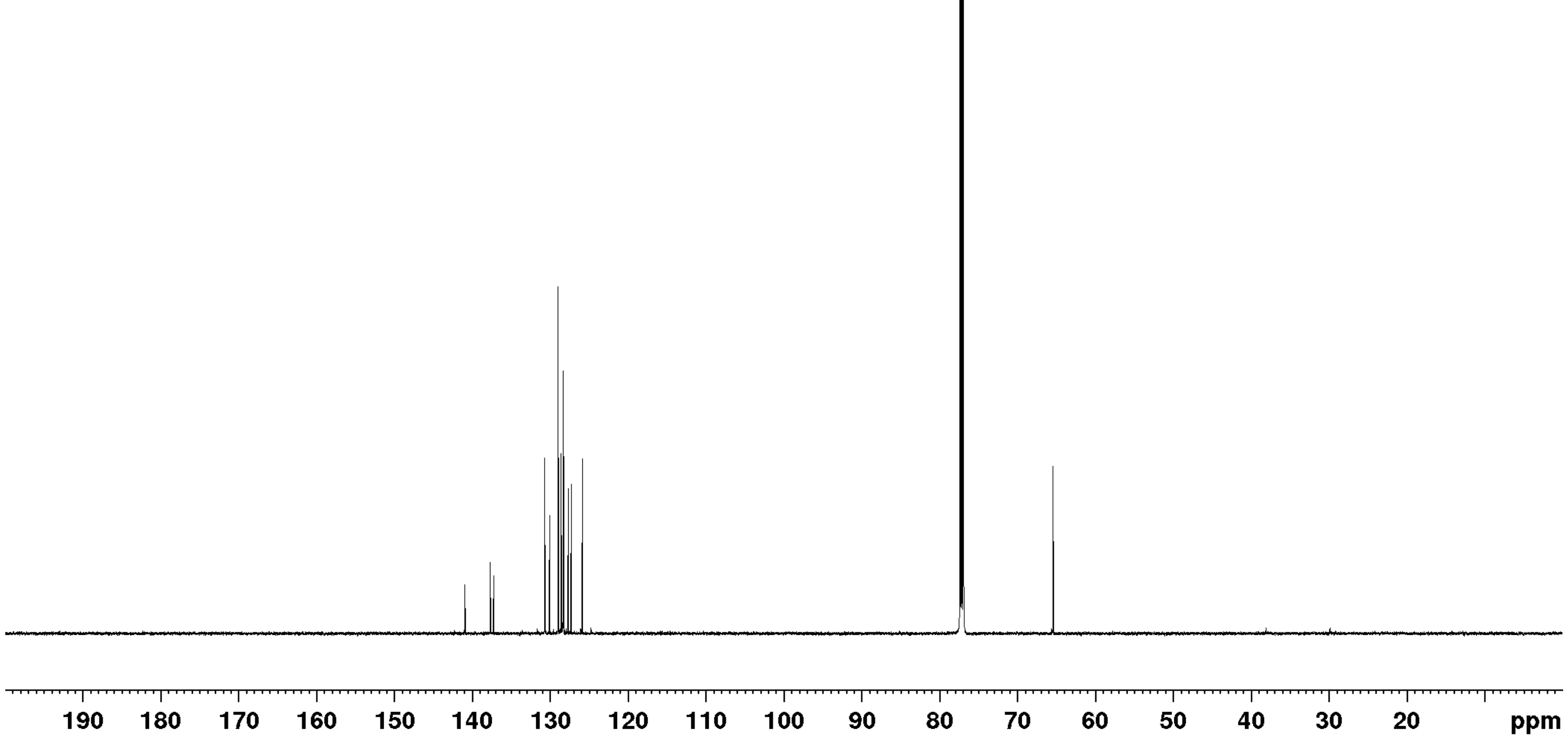
141.0
137.7
137.3
130.7
130.1
129.0
128.6
128.4
128.3
127.7
127.3
125.9
65.4
190
180
170
160
150
140
130
120
110
100
90
80
70
60
50
40
30
20
ppm

2.9.3 ^{1}H, ^{1}H COSY

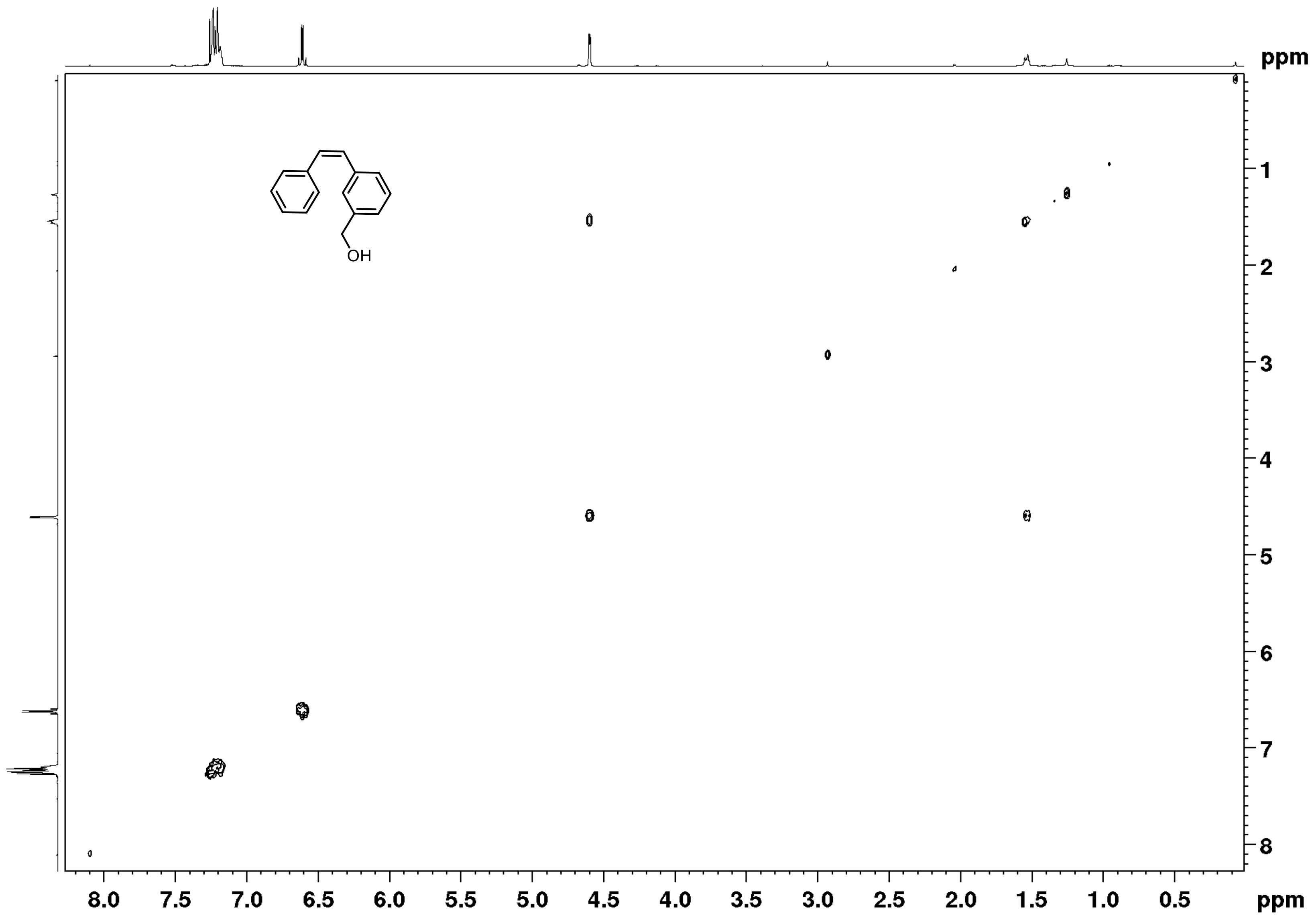

2.9.4 ^{1}H, ^{13}C HMBC

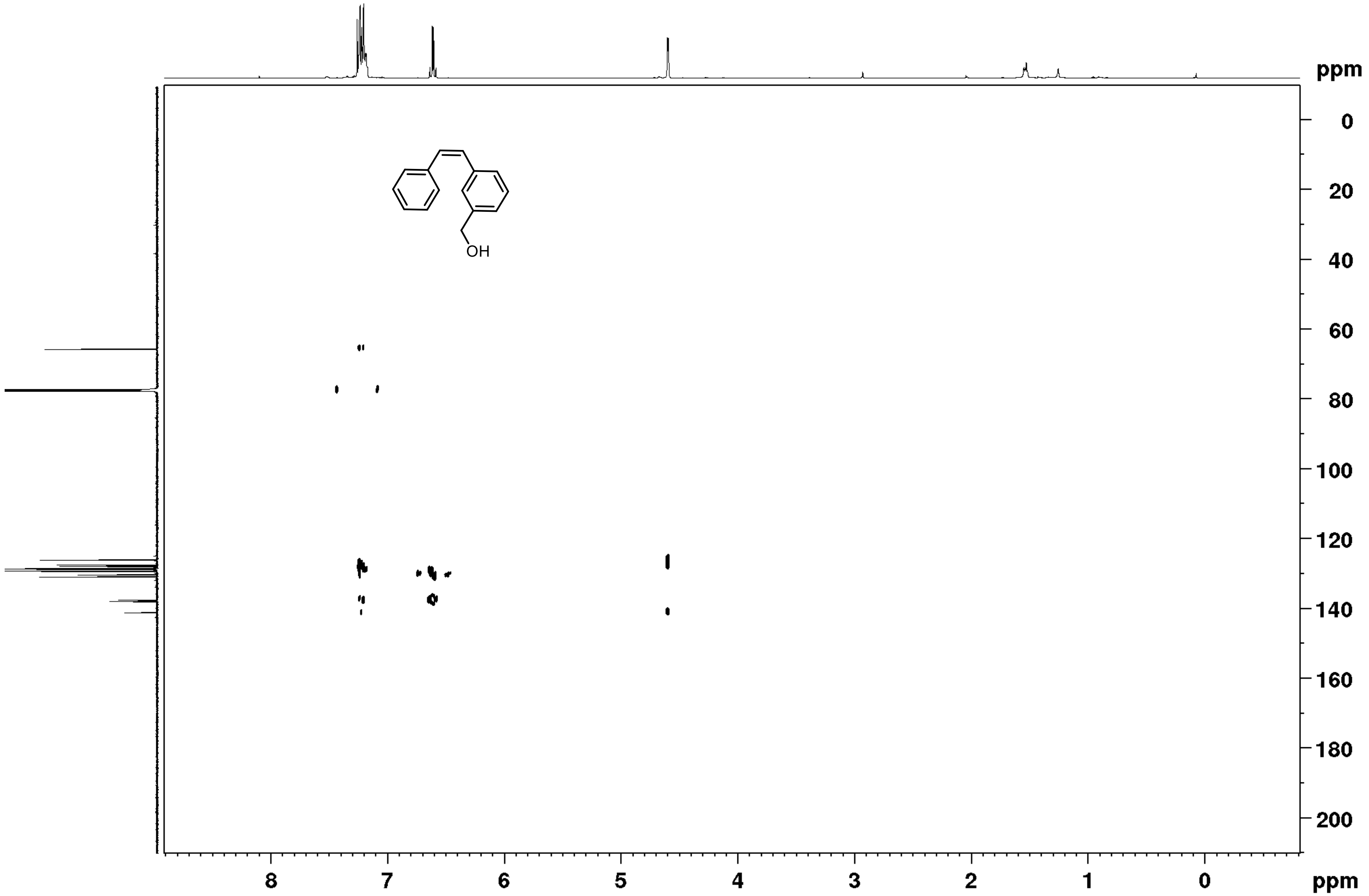

2.9.5 ^{1}H, ^{13}C HSQC

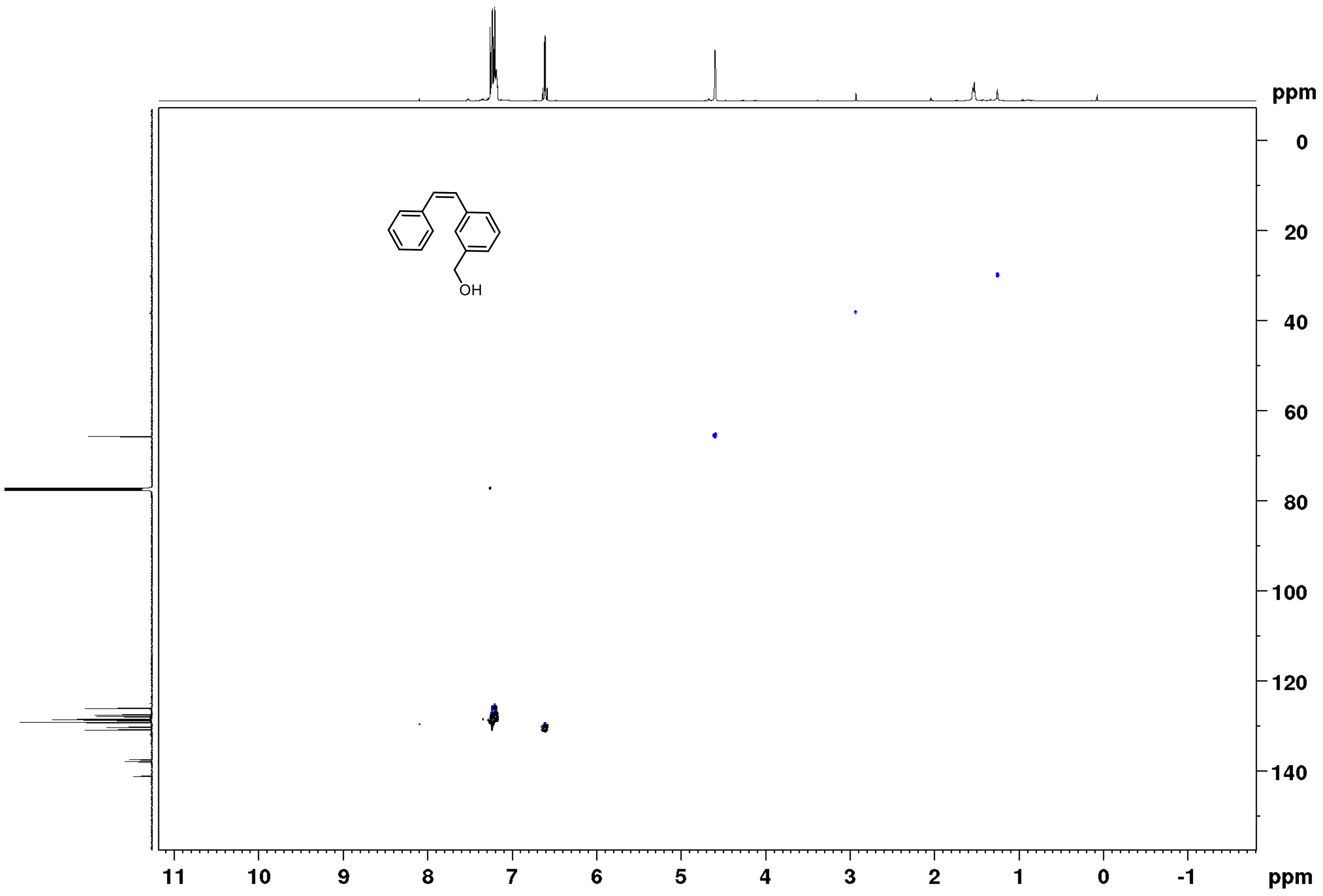

2.9.6 HRMS

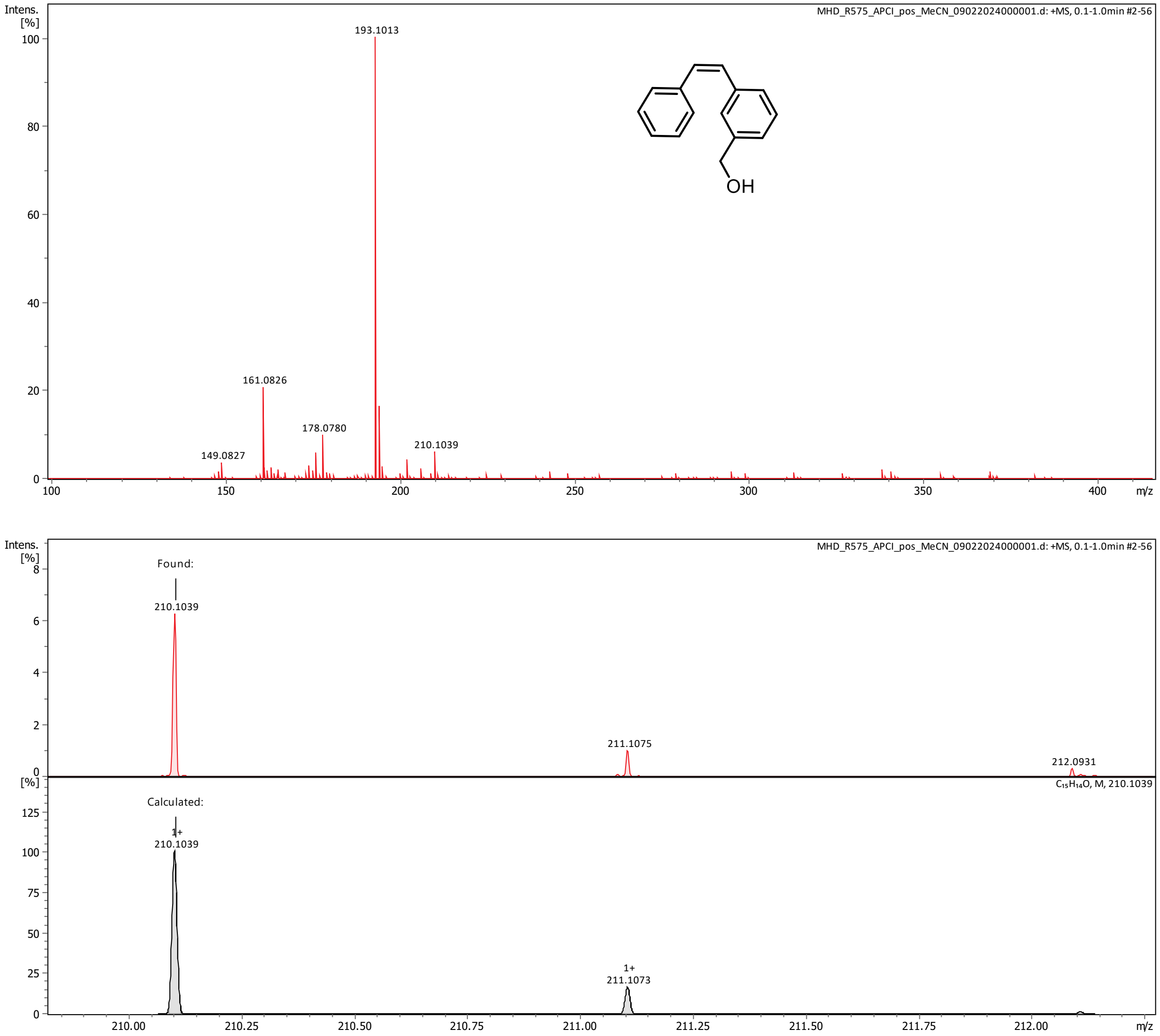

MHD_R575_APCI_pos_MeCN_09022024000001.d: +MS, 0.1-1.0min #2-56
Intens. [%]
193.1013
161.0826
178.0780
149.0827
210.1039
OH
m/z
Found:
210.1039
211.1075
212.0931
Calculated:
1+
210.1039
1+
211.1073
C15H14O, M, 210.1039

2.9.7 IR

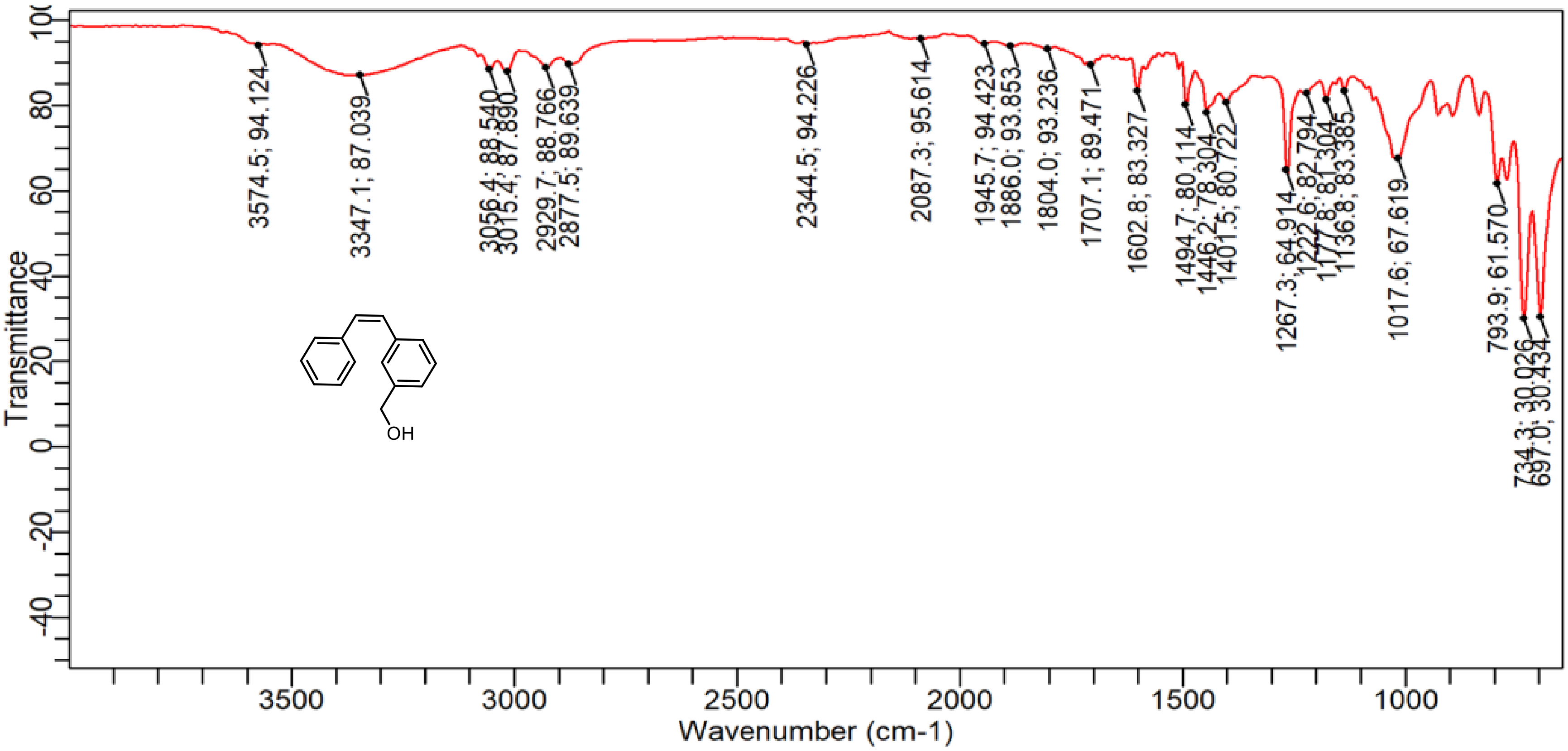

3574.5; 94.124
3347.1; 87.039
3056.4; 88.540
3015.4; 87.890
2929.7; 88.766
2877.5; 89.639
2344.5; 94.226
2087.3; 95.614
1945.7; 94.423
1886.0; 93.853
1804.0; 93.236
1707.1; 89.471
1602.8; 83.327
1494.7; 80.114
1446.2; 78.304
1401.5; 80.722
1267.3; 64.914
1222.6; 82.794
1177.8; 81.304
1136.8; 83.385
1017.6; 67.619
793.9; 61.570
734.3; 30.026
697.0; 30.434
OH
Transmittance
Wavenumber (cm-1)

2.10 (*Z*)-1-(3-Styrylphenyl)ethan-1-ol (2j) (600 MHz, $CDCl_3$)

2.10.1 ^{1}H NMR

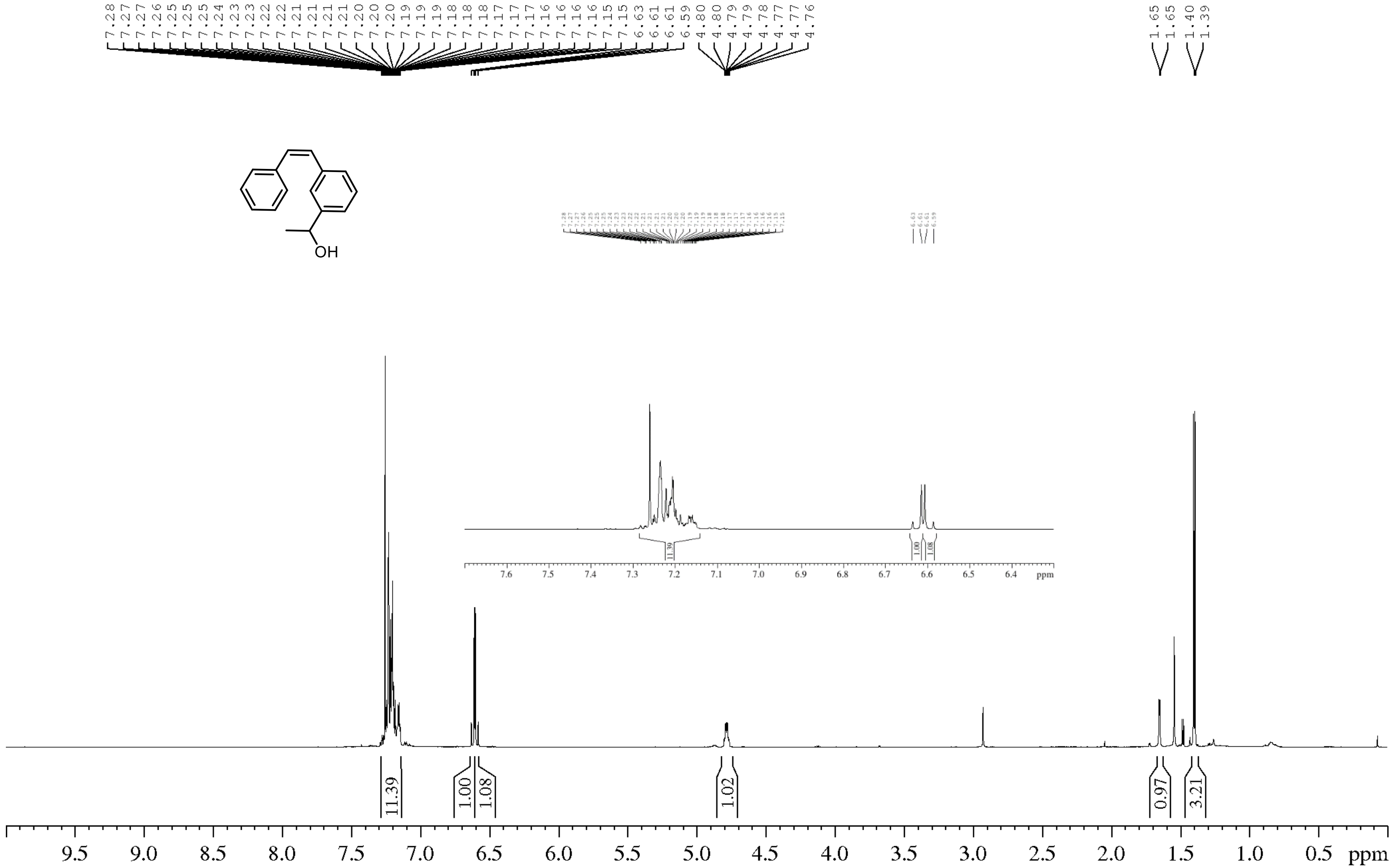

2.10.2 ^{13}C NMR

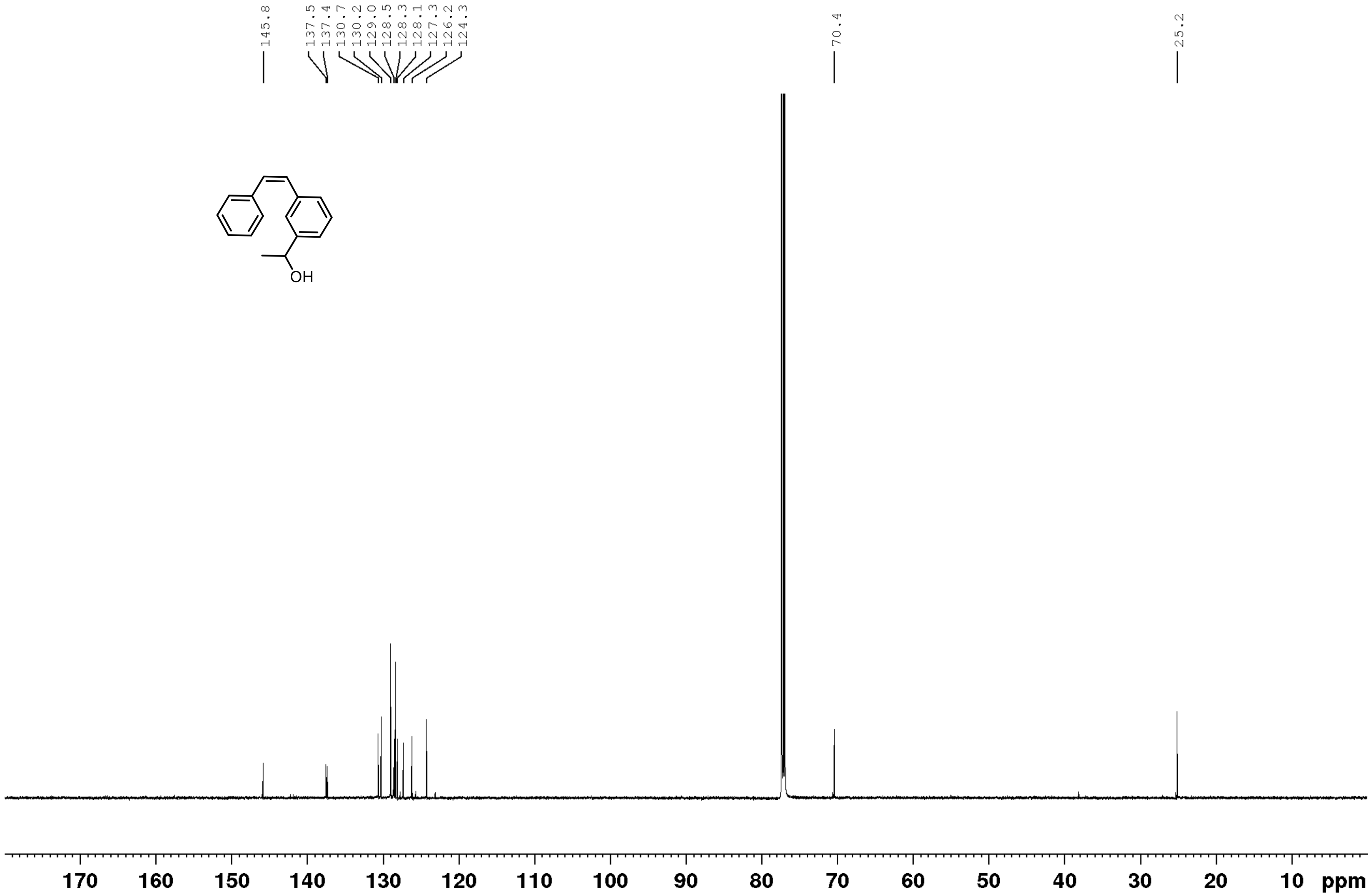
145.8
137.5
137.4
130.7
130.2
129.0
128.5
128.3
128.1
127.3
126.2
124.3
70.4
25.2
OH
170
160
150
140
130
120
110
100
90
80
70
60
50
40
30
20
10
ppm

2.10.3 ^{1}H, ^{1}H COSY

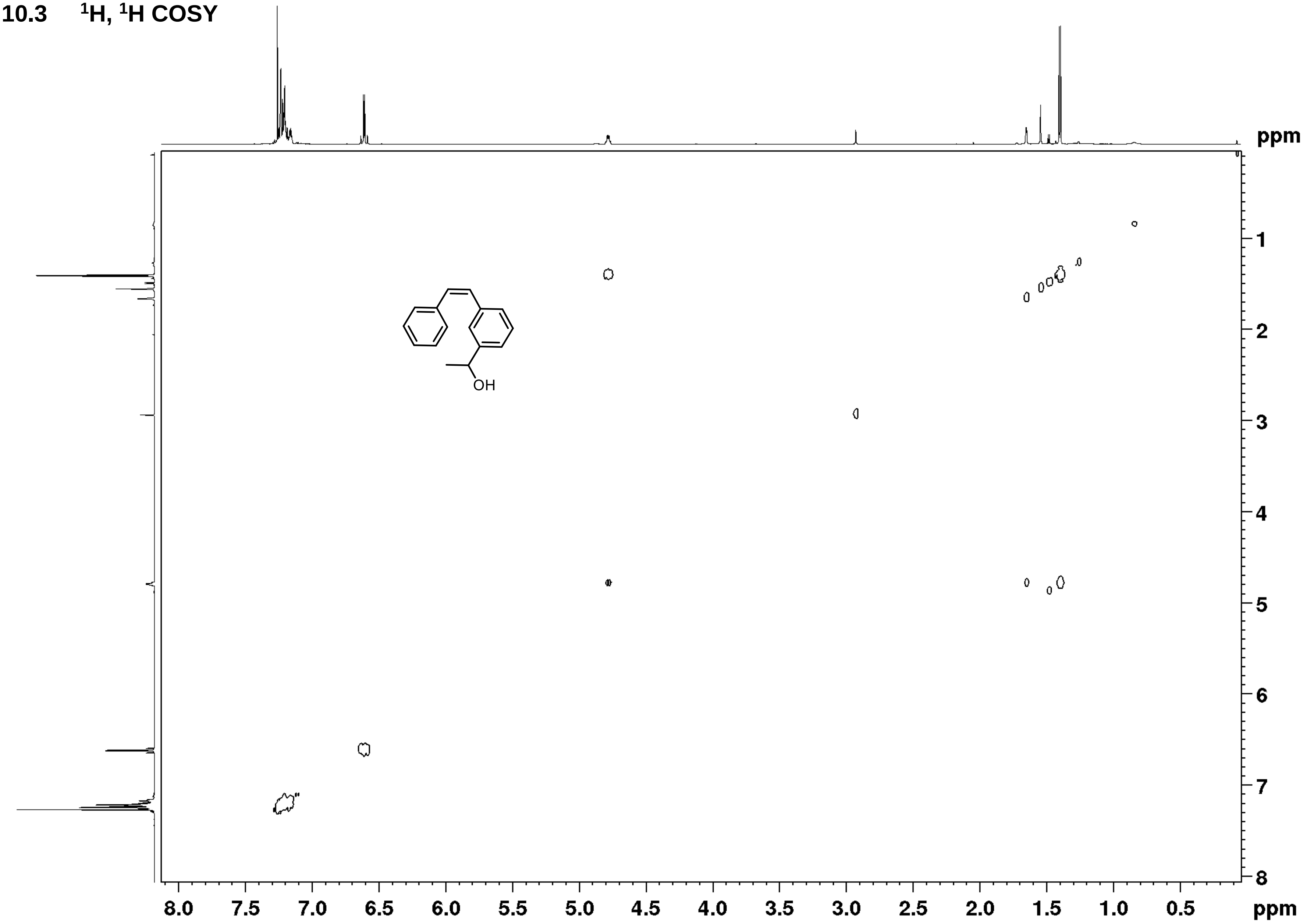

2.10.4 ^{1}H, ^{13}C HMBC

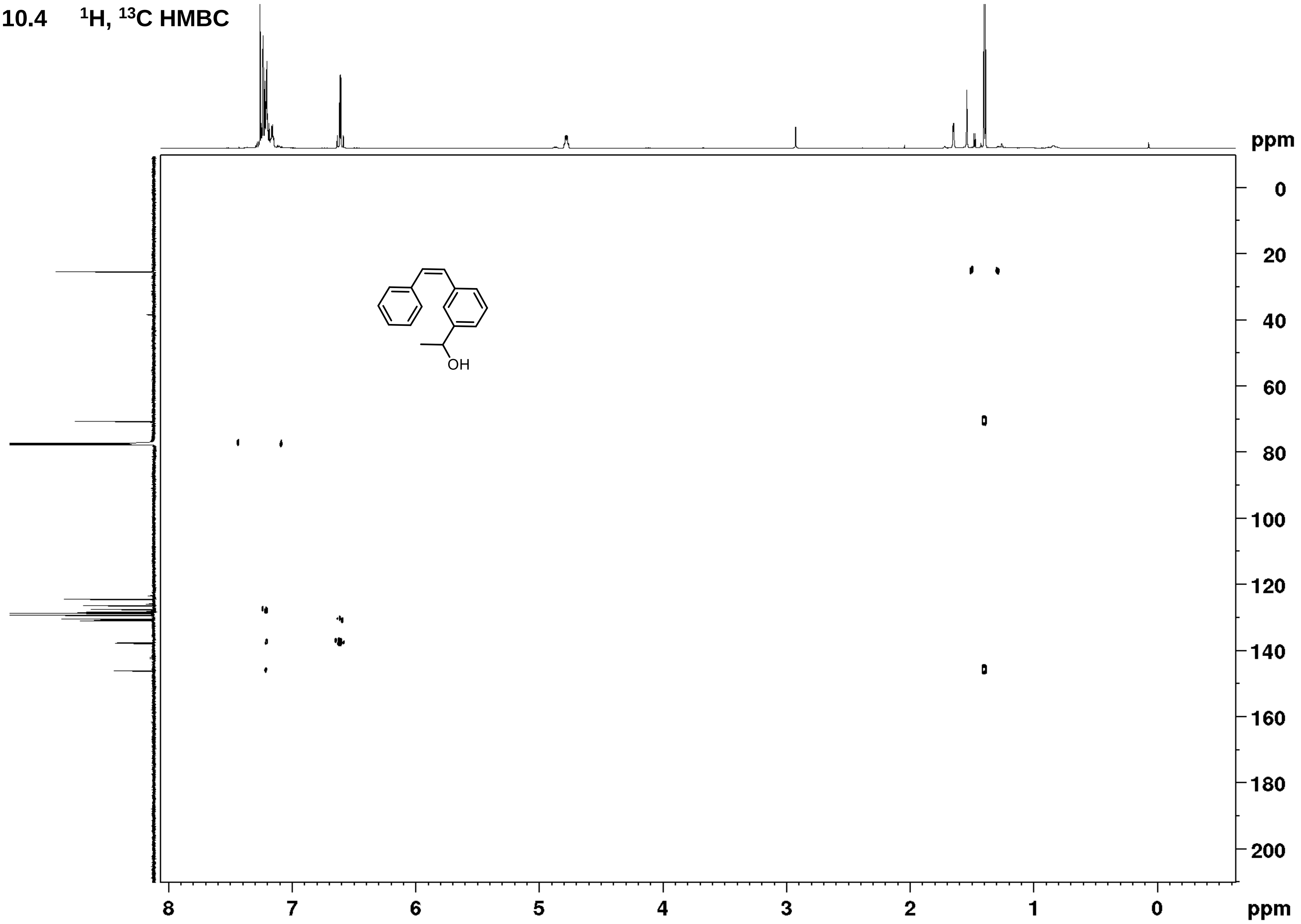

2.10.5 ^{1}H, ^{13}C HSQC

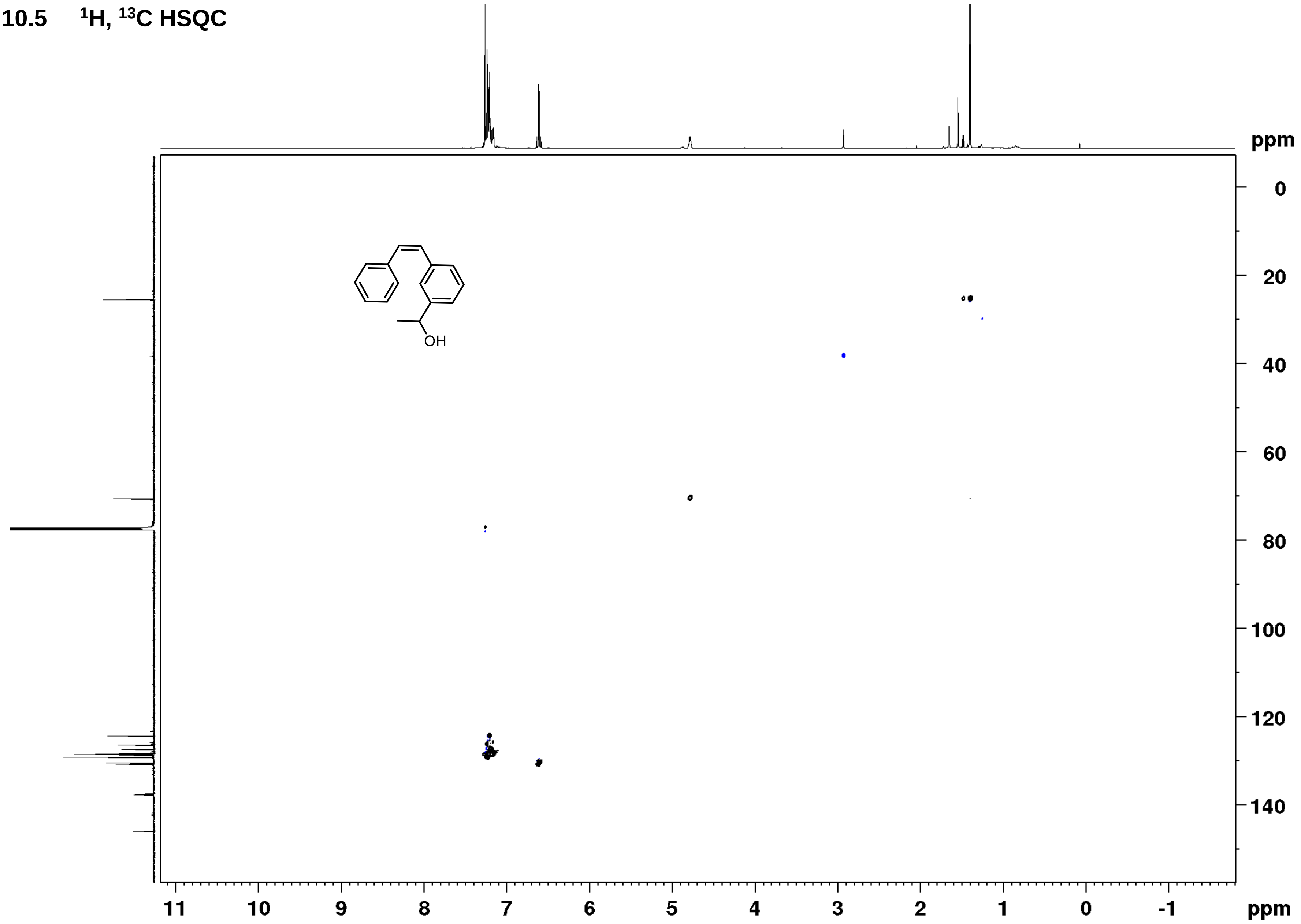

2.10.6 HRMS

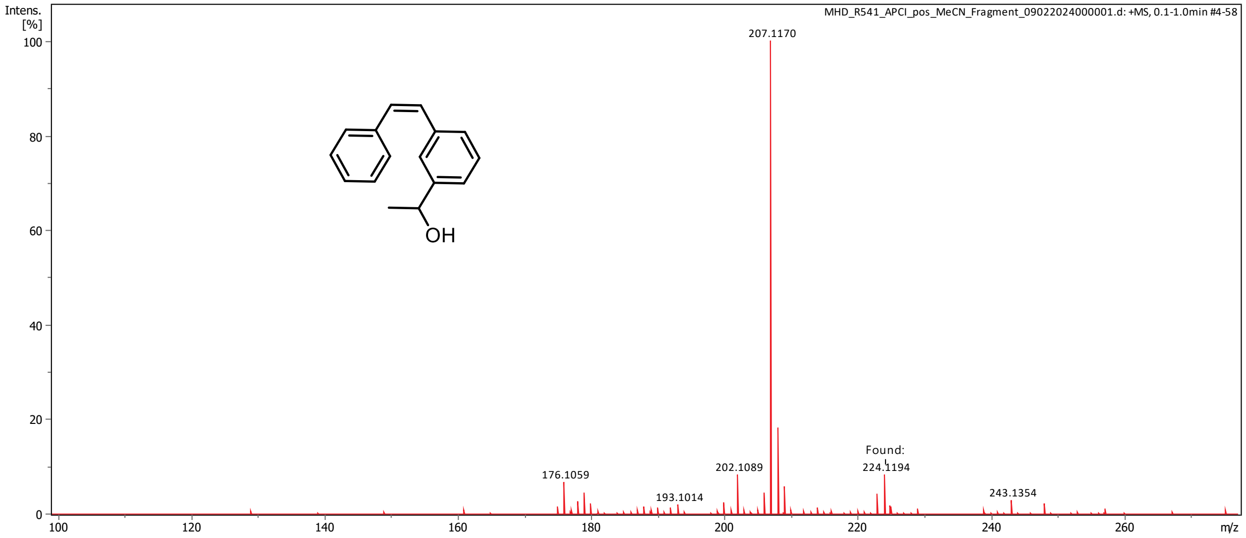

Intens. [%]
MHD_R541_APCI_pos_MeCN_Fragment_09022024000001.d: +MS, 0.1-1.0min #4-58
207.1170
176.1059
193.1014
202.1089
Found:
224.1194
243.1354
OH
m/z

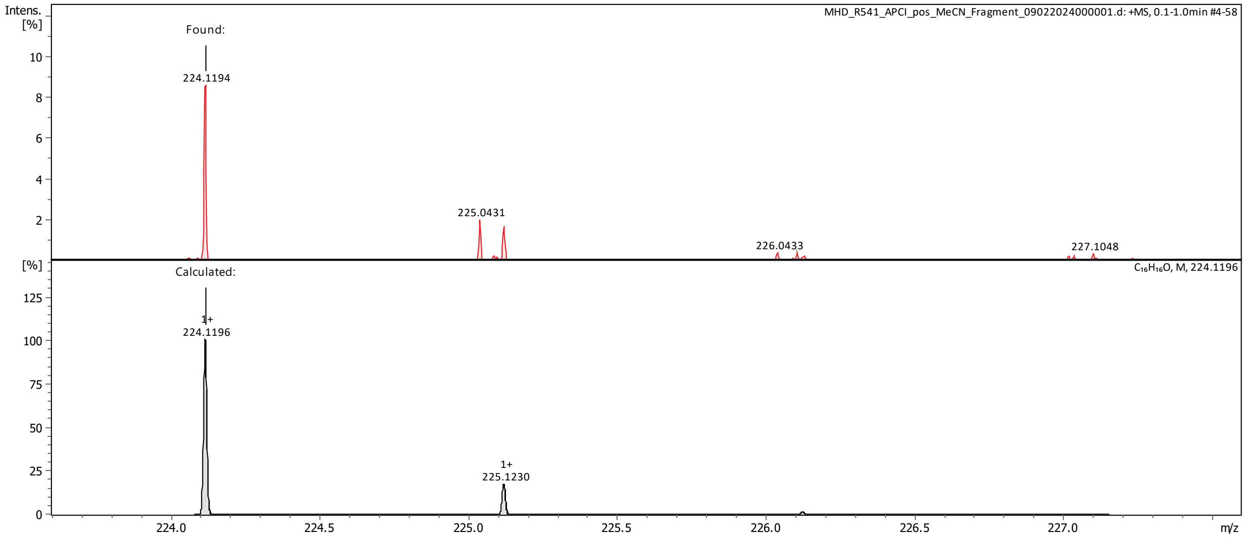

Intens. [%]
MHD_R541_APCI_pos_MeCN_Fragment_09022024000001.d: +MS, 0.1-1.0min #4-58
Found:
224.1194
225.0431
226.0433
227.1048
[%]
Calculated:
C16H16O, M, 224.1196
1+
224.1196
1+
225.1230
m/z

2.10.7 IR

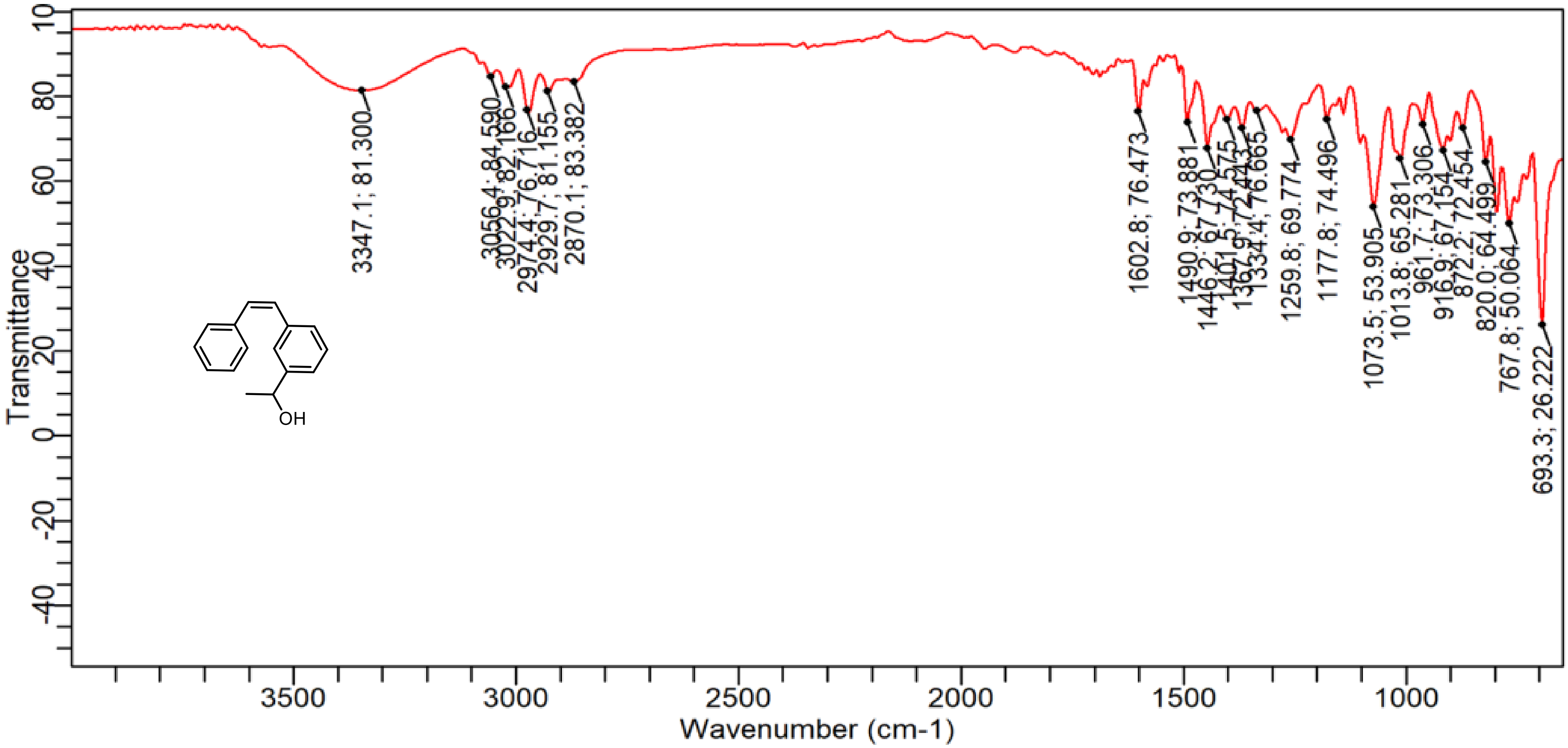
Transmittance
Wavenumber (cm-1)
OH
3347.1; 81.300
2870.1; 83.382
1602.8; 76.473
1259.8; 69.774
1177.8; 74.496
1073.5; 53.905
1013.8; 65.281
767.8; 50.064
693.3; 26.222

2.11 1-(3-((*Z*)-Styryl)phenyl)ethan-1-one *O*-methyl oxime (2k) (600 MHz, $CDCl_3$)

2.11.1 ^{1}H NMR

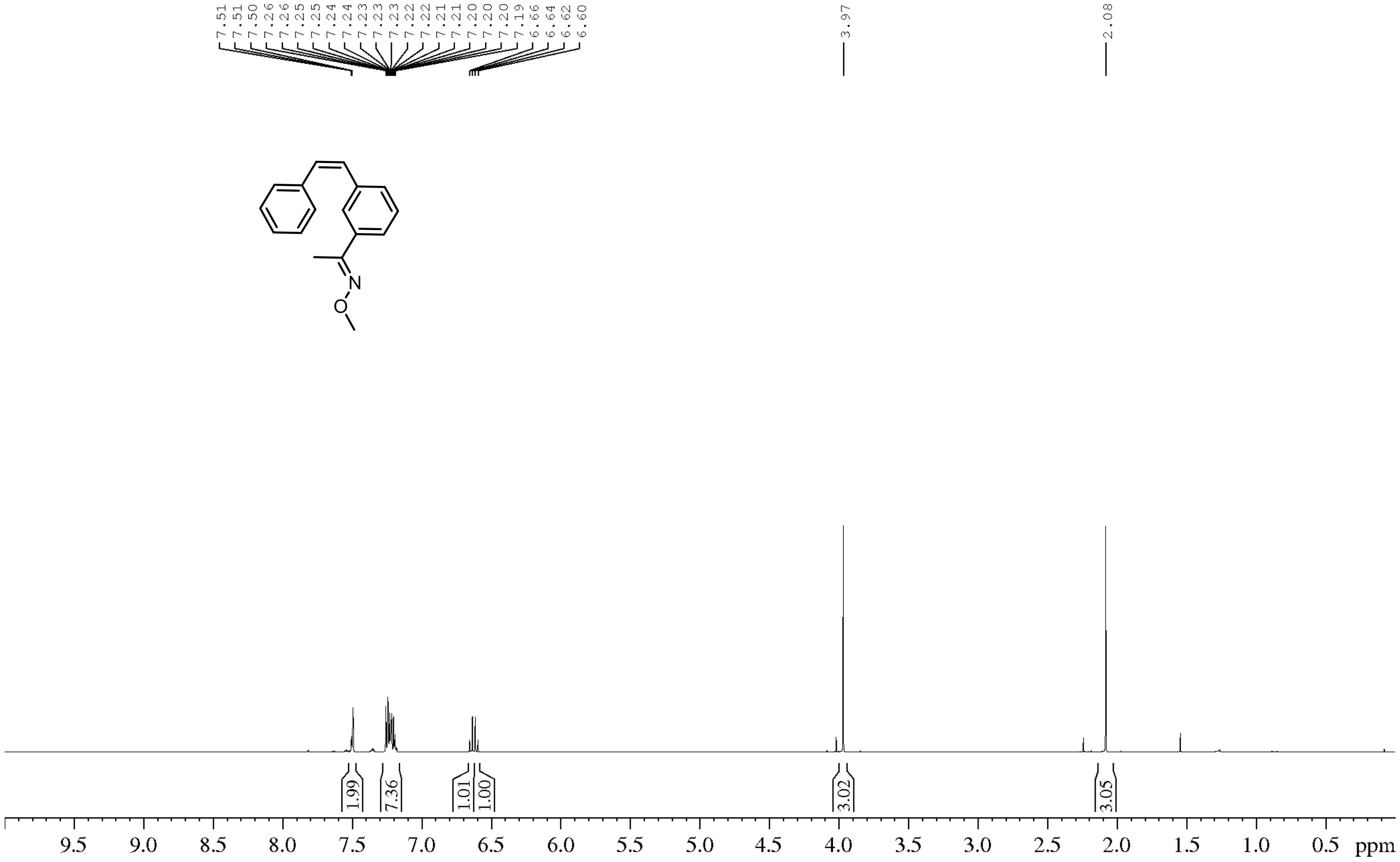

2.11.2 ^{13}C NMR

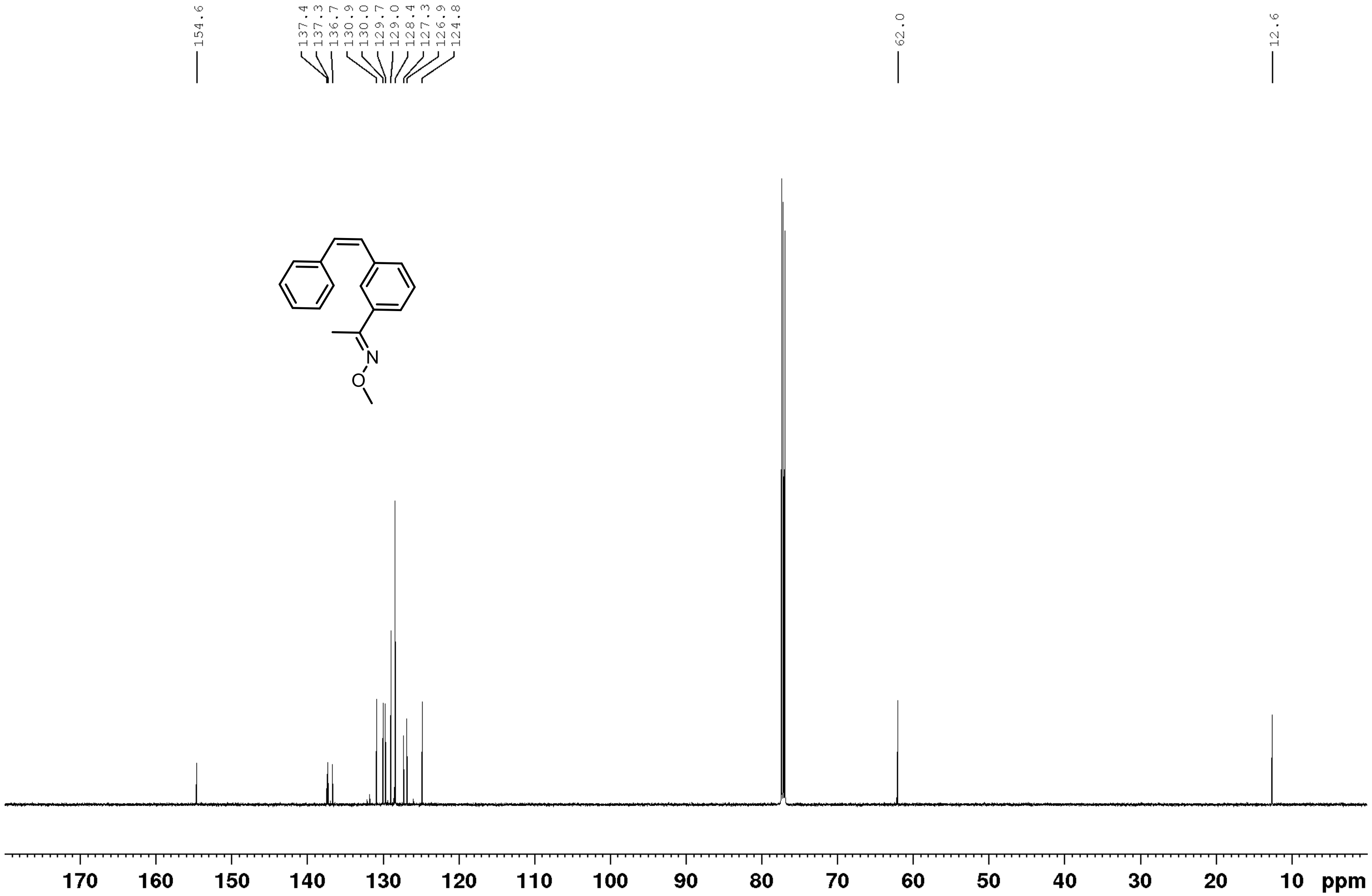

154.6
137.4
137.3
136.7
130.9
130.0
129.7
129.0
128.4
127.3
126.9
124.8
62.0
12.6
N
O
170
160
150
140
130
120
110
100
90
80
70
60
50
40
30
20
10
ppm

2.11.3 ¹H, ¹H COSY

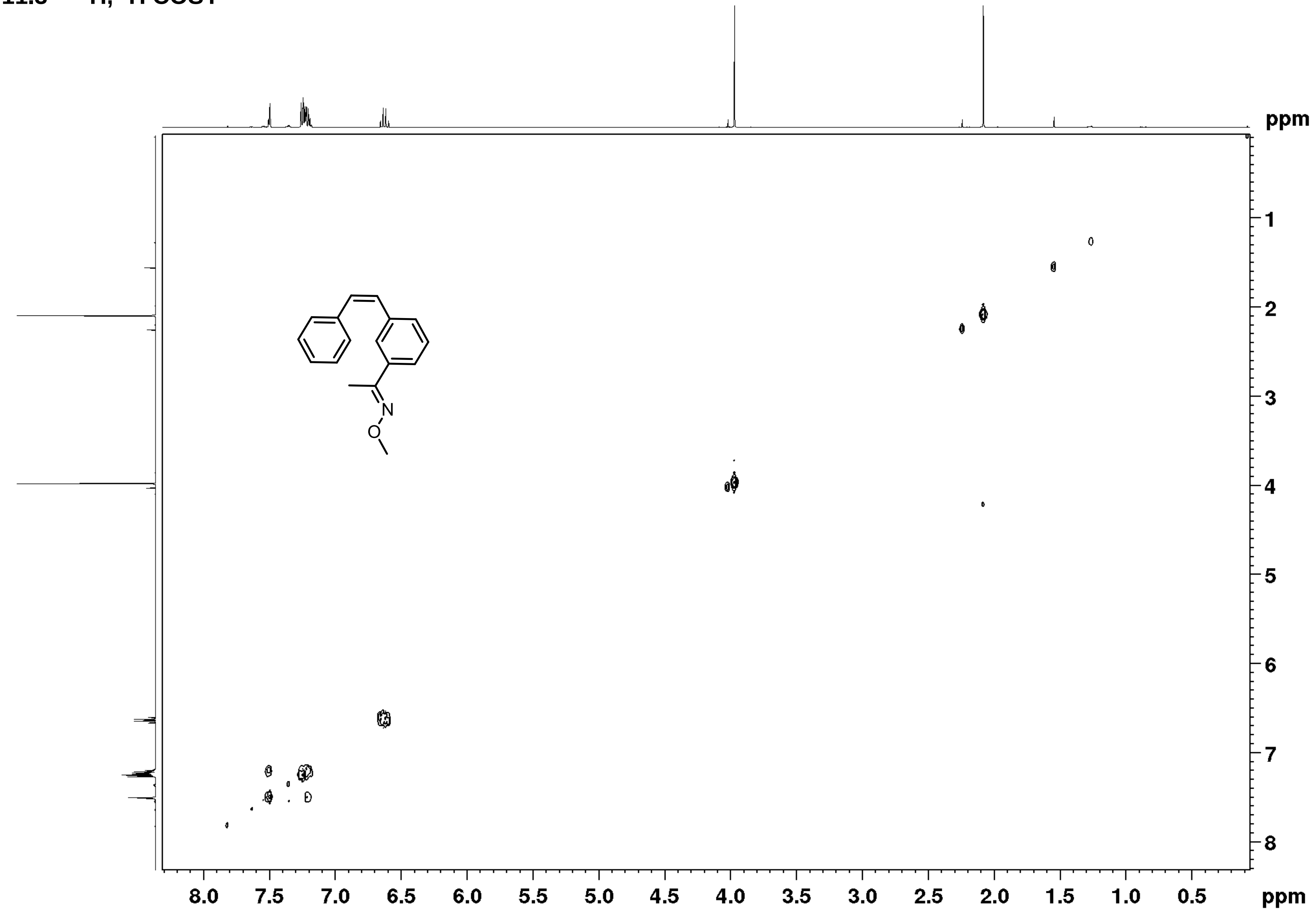

2.11.4 ^{1}H, ^{13}C HMBC

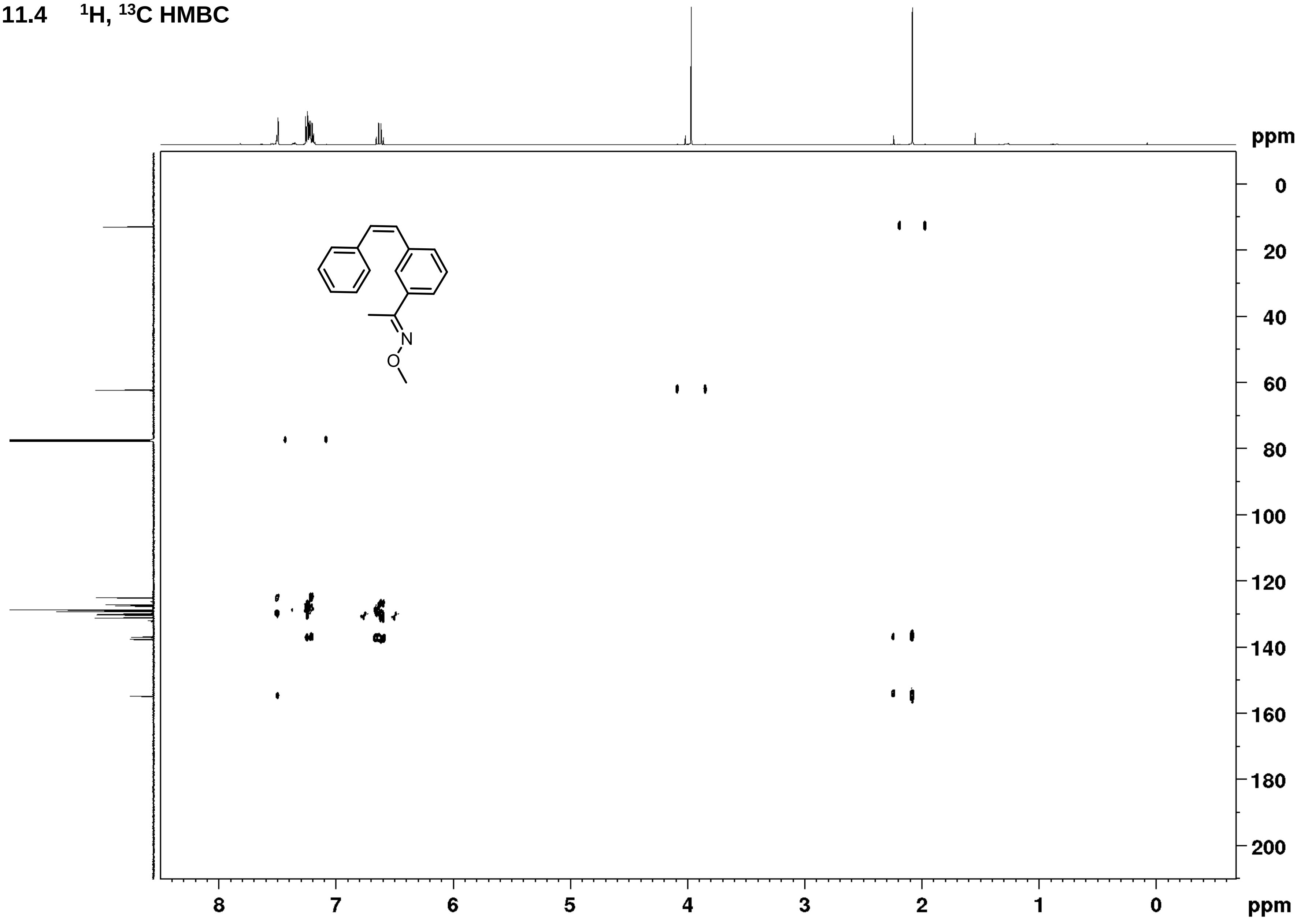

2.11.5 ^{1}H, ^{13}C HSQC

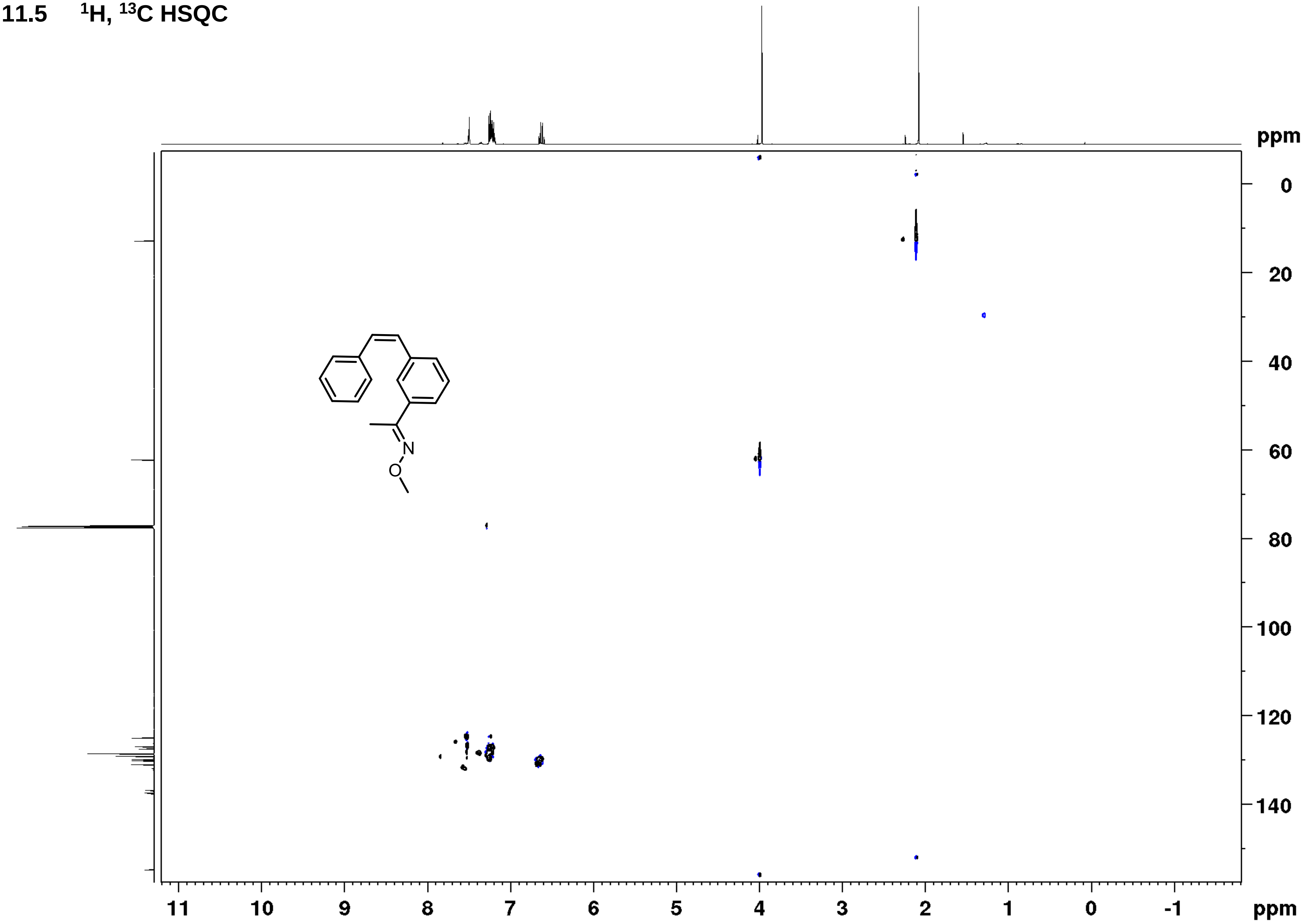

2.11.6 HRMS

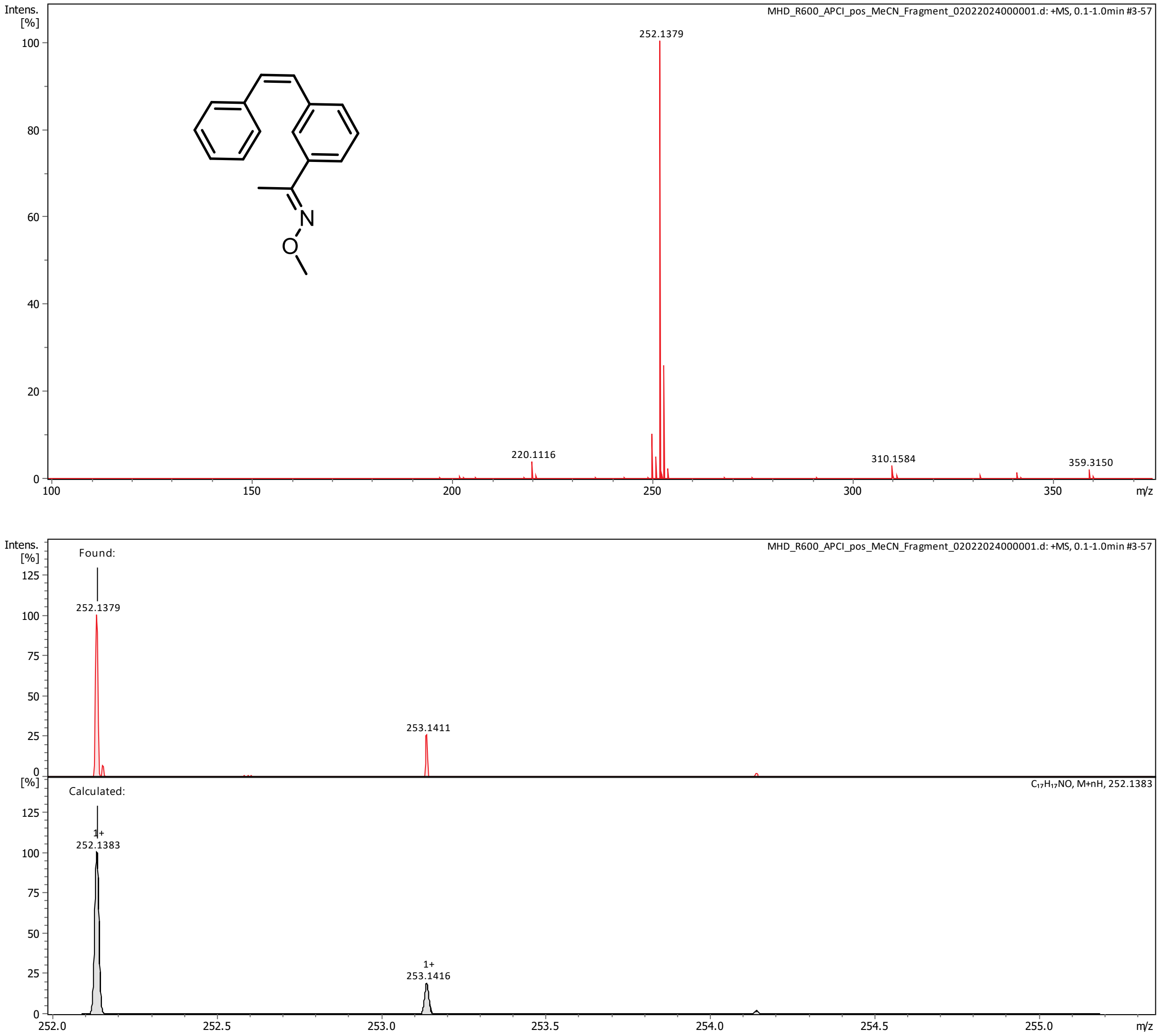

MHD_R600_APCI_pos_MeCN_Fragment_02022024000001.d: +MS, 0.1-1.0min #3-57
Intens. [%]
252.1379
220.1116
310.1584
359.3150
m/z
N
O
Found:
252.1379
253.1411
Calculated:
C₁₇H₁₇NO, M+nH, 252.1383
1+ 252.1383
1+ 253.1416

2.11.7 IR

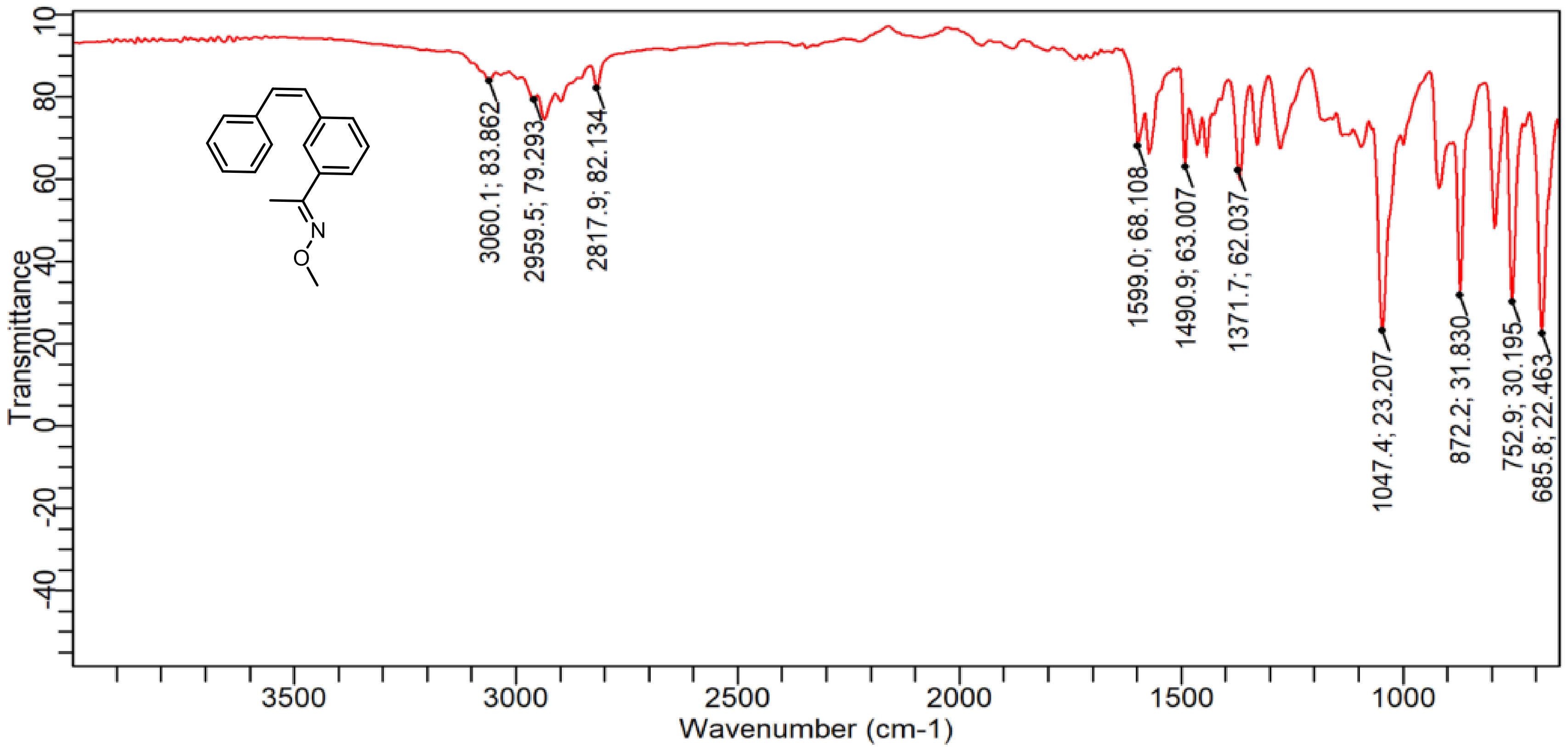
3060.1; 83.862
2959.5; 79.293
2817.9; 82.134
1599.0; 68.108
1490.9; 63.007
1371.7; 62.037
1047.4; 23.207
872.2; 31.830
752.9; 30.195
685.8; 22.463
Transmittance
Wavenumber (cm-1)
N
O

2.12 *N*-Methyl-1-(3-((*Z*)-styryl)phenyl)ethan-1-imine (2l) (600 MHz, $CDCl_3$)

2.12.1 ^{1}H NMR

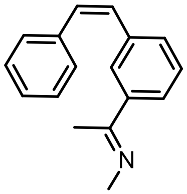

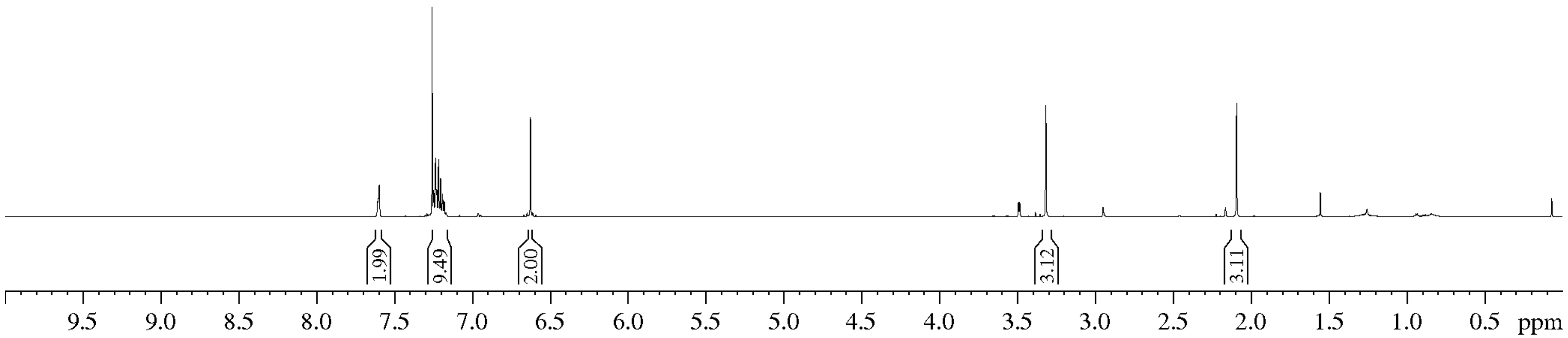

2.12.2 ^{13}C NMR

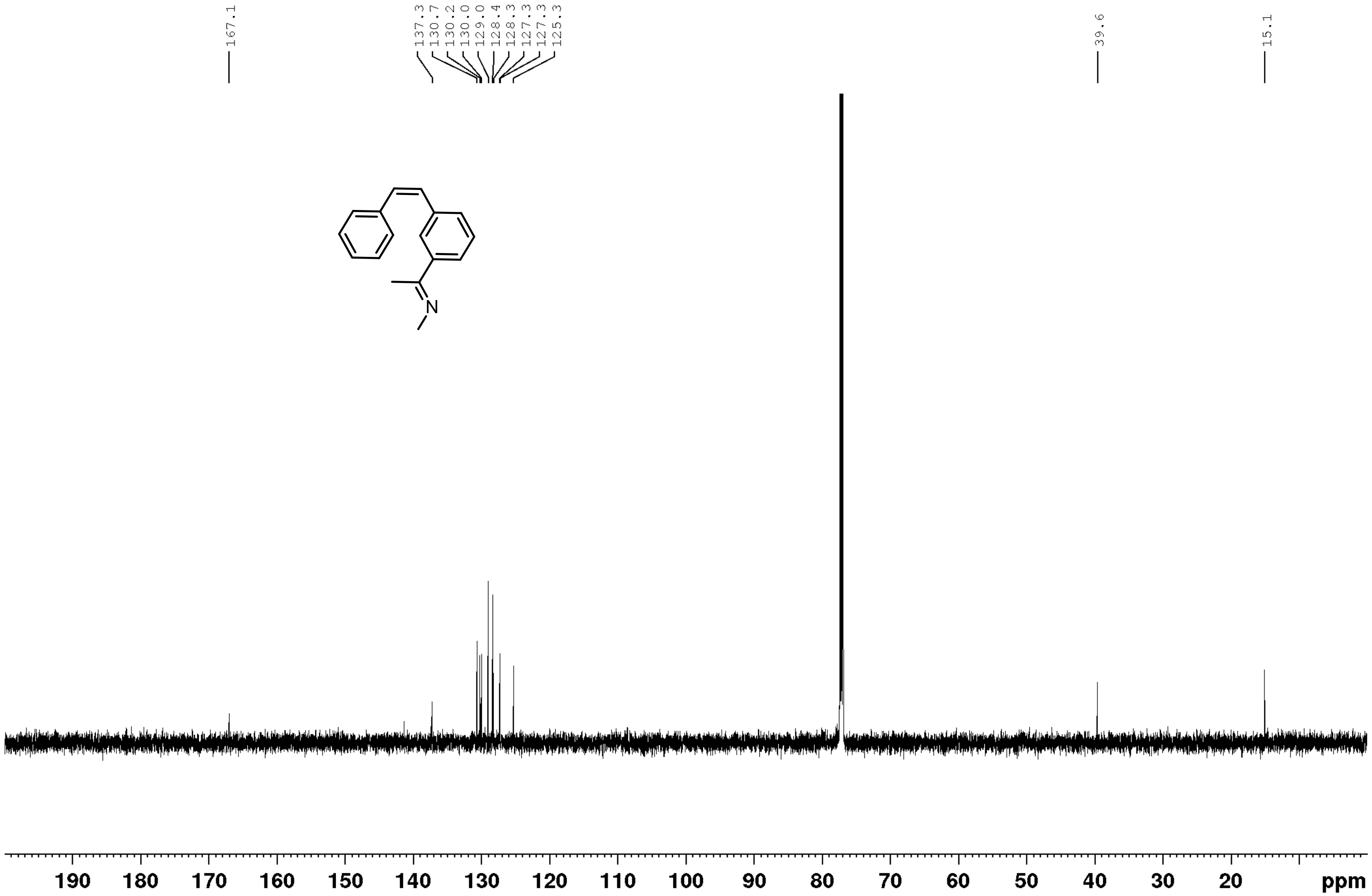

167.1
137.3
130.7
130.2
130.0
129.0
128.4
128.3
127.3
127.3
125.3
39.6
15.1
N
190
180
170
160
150
140
130
120
110
100
90
80
70
60
50
40
30
20
ppm

2.12.3 $^1H, ^1H$ COSY

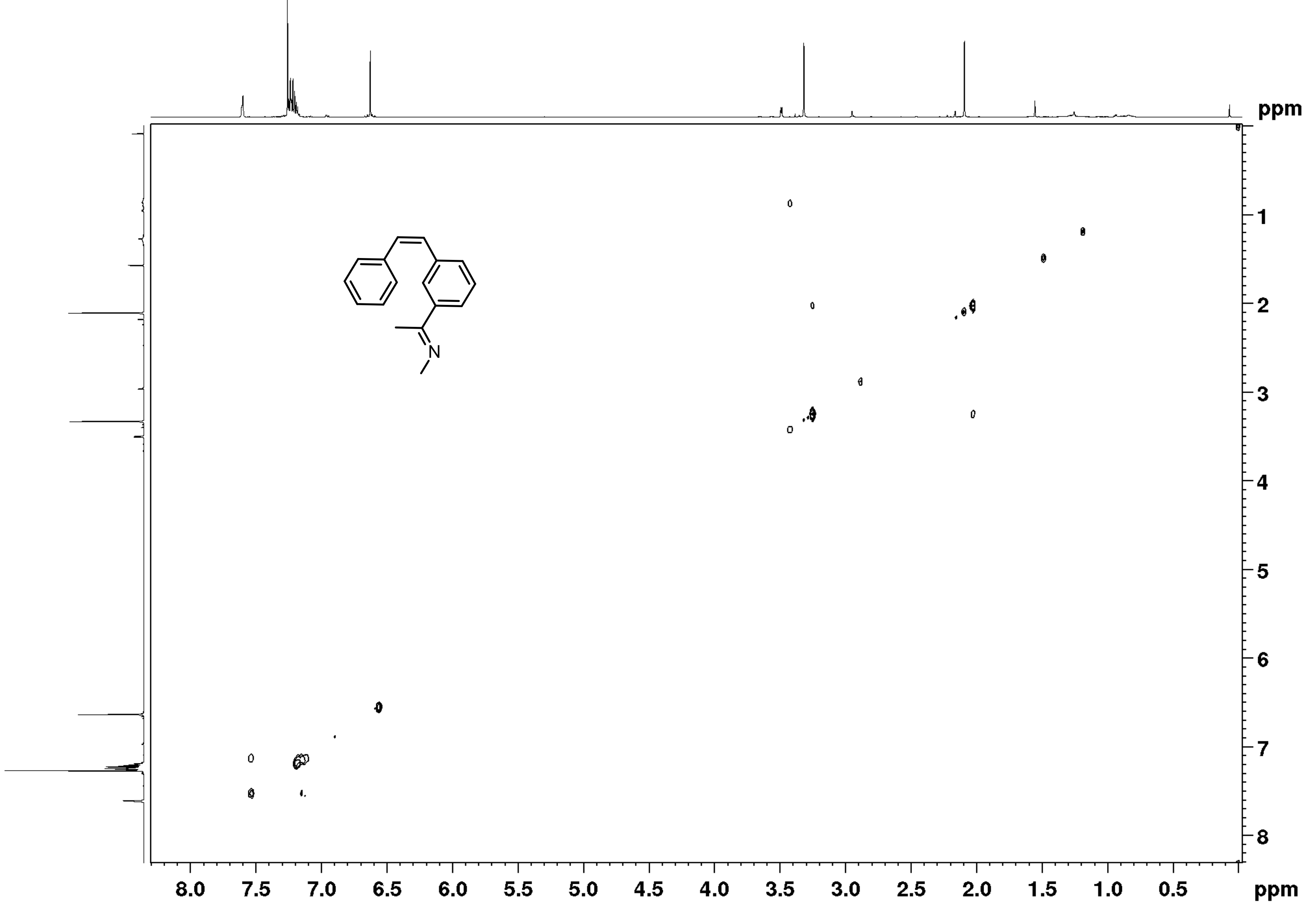

2.12.4 ^{1}H, ^{13}C HMBC

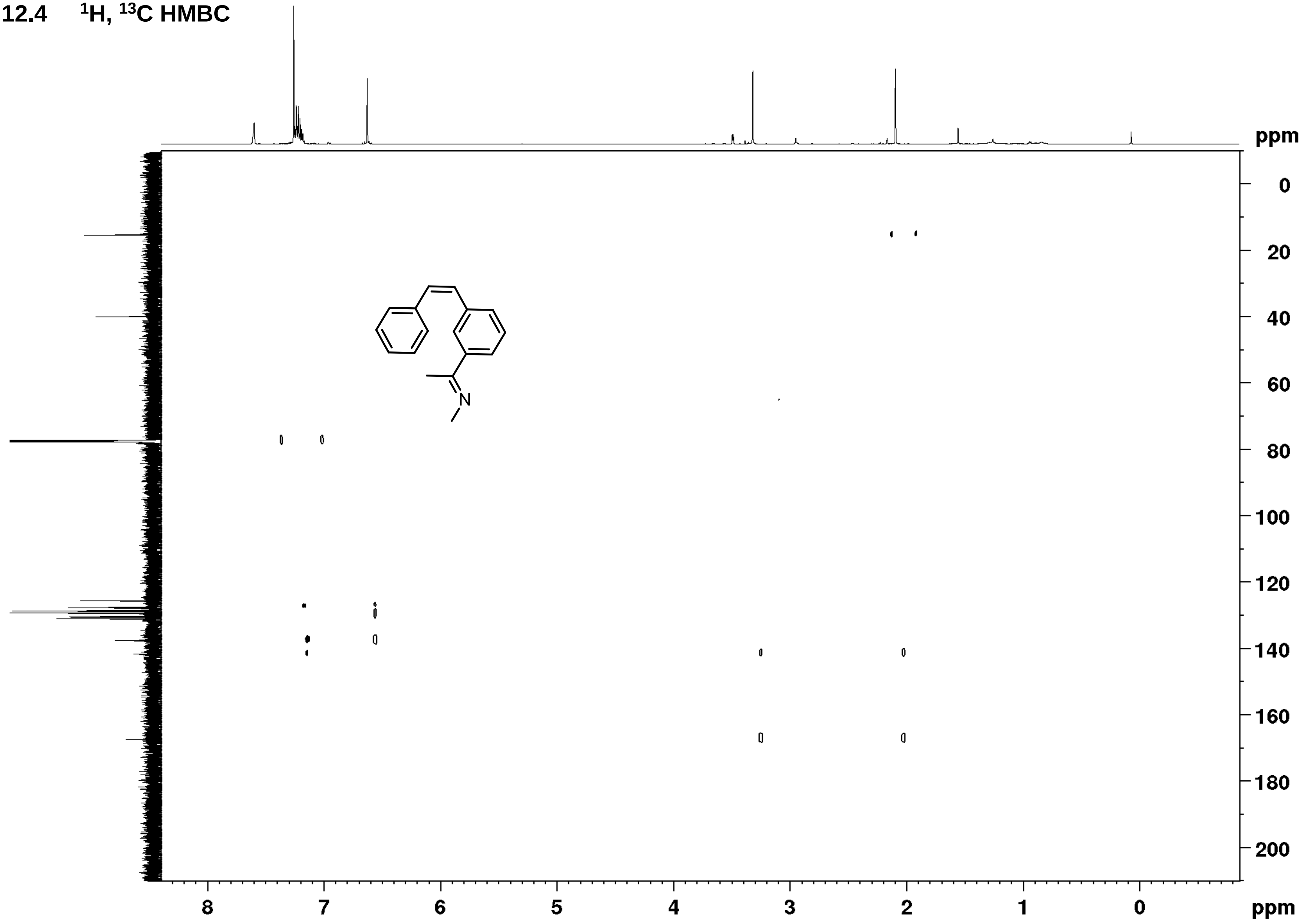

2.12.5 ^{1}H, ^{13}C HSQC

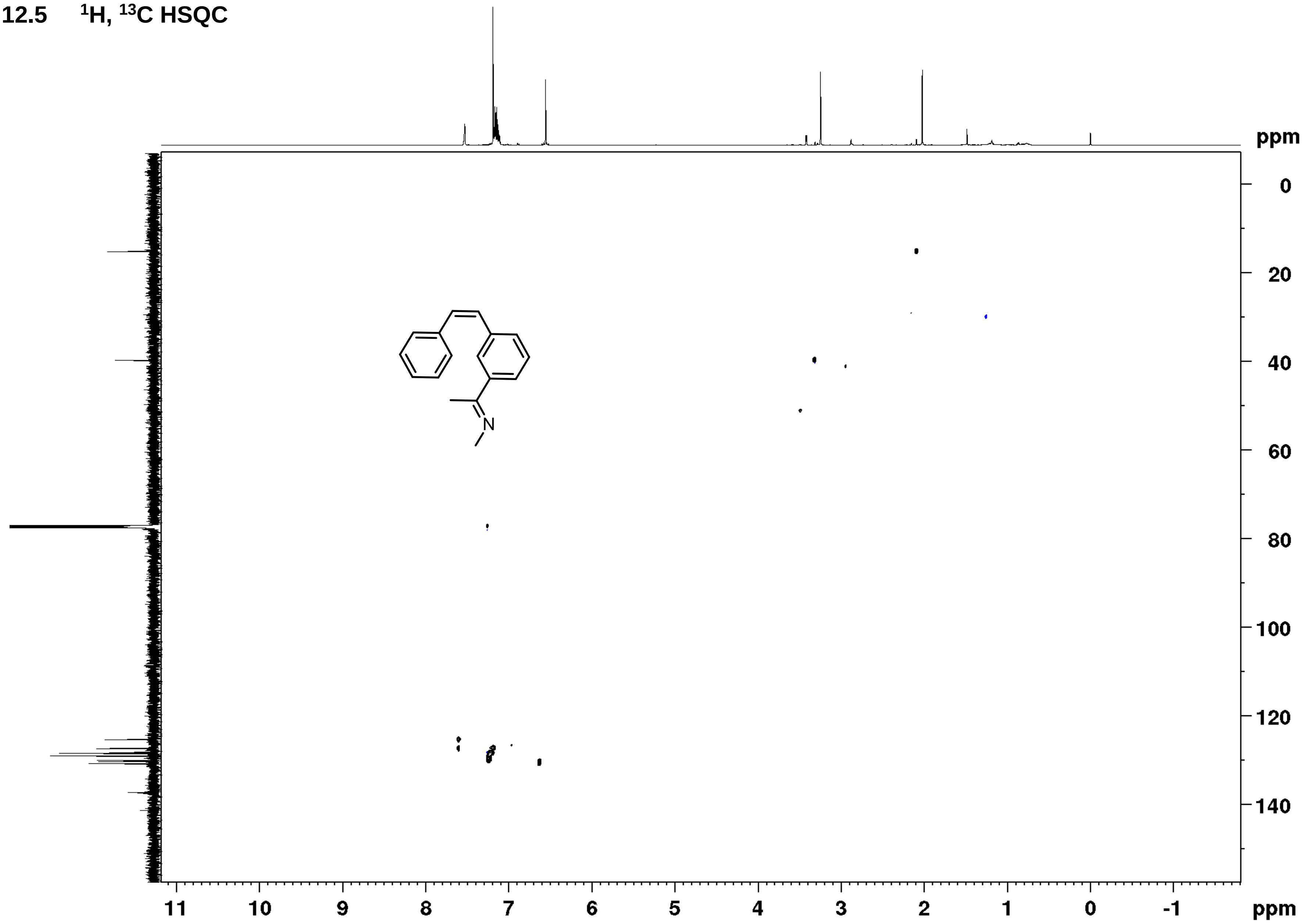

2.12.6 HRMS

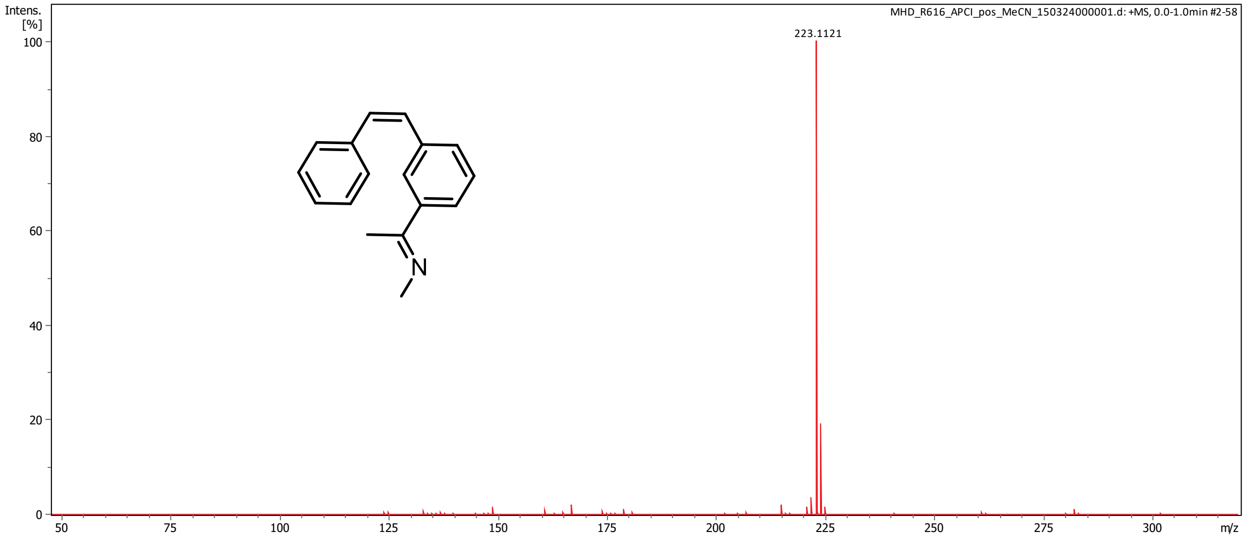

Intens. [%]
MHD_R616_APCI_pos_MeCN_150324000001.d: +MS, 0.0-1.0min #2-58
223.1121
N
m/z

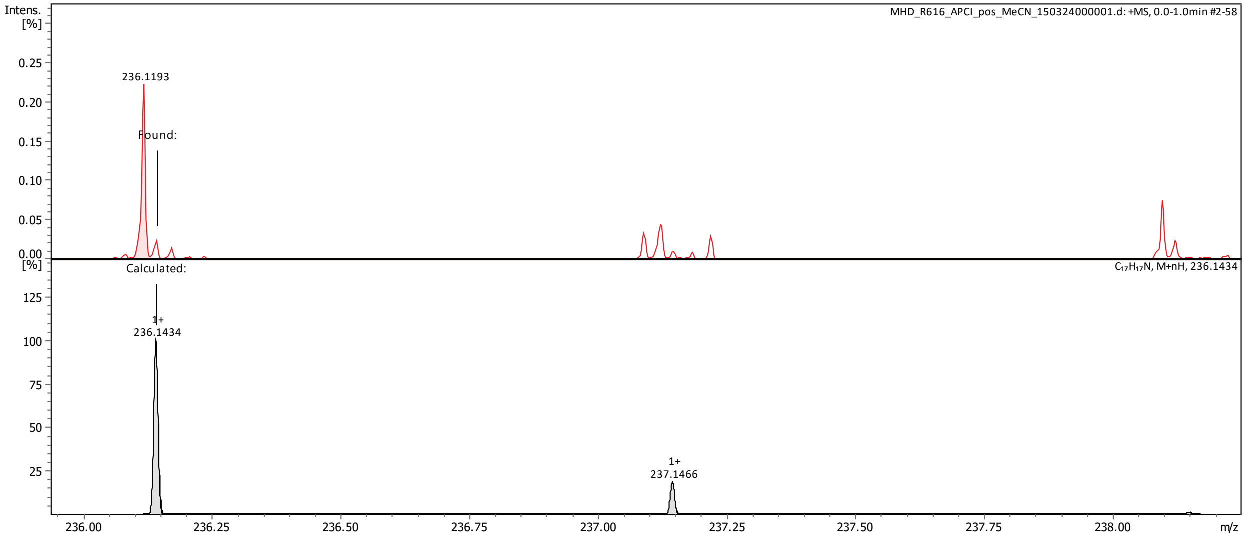

Intens. [%]
MHD_R616_APCI_pos_MeCN_150324000001.d: +MS, 0.0-1.0min #2-58
236.1193
Found:
Calculated:
C17H17N, M+nH, 236.1434
1+ 236.1434
1+ 237.1466
m/z

2.12.7 IR

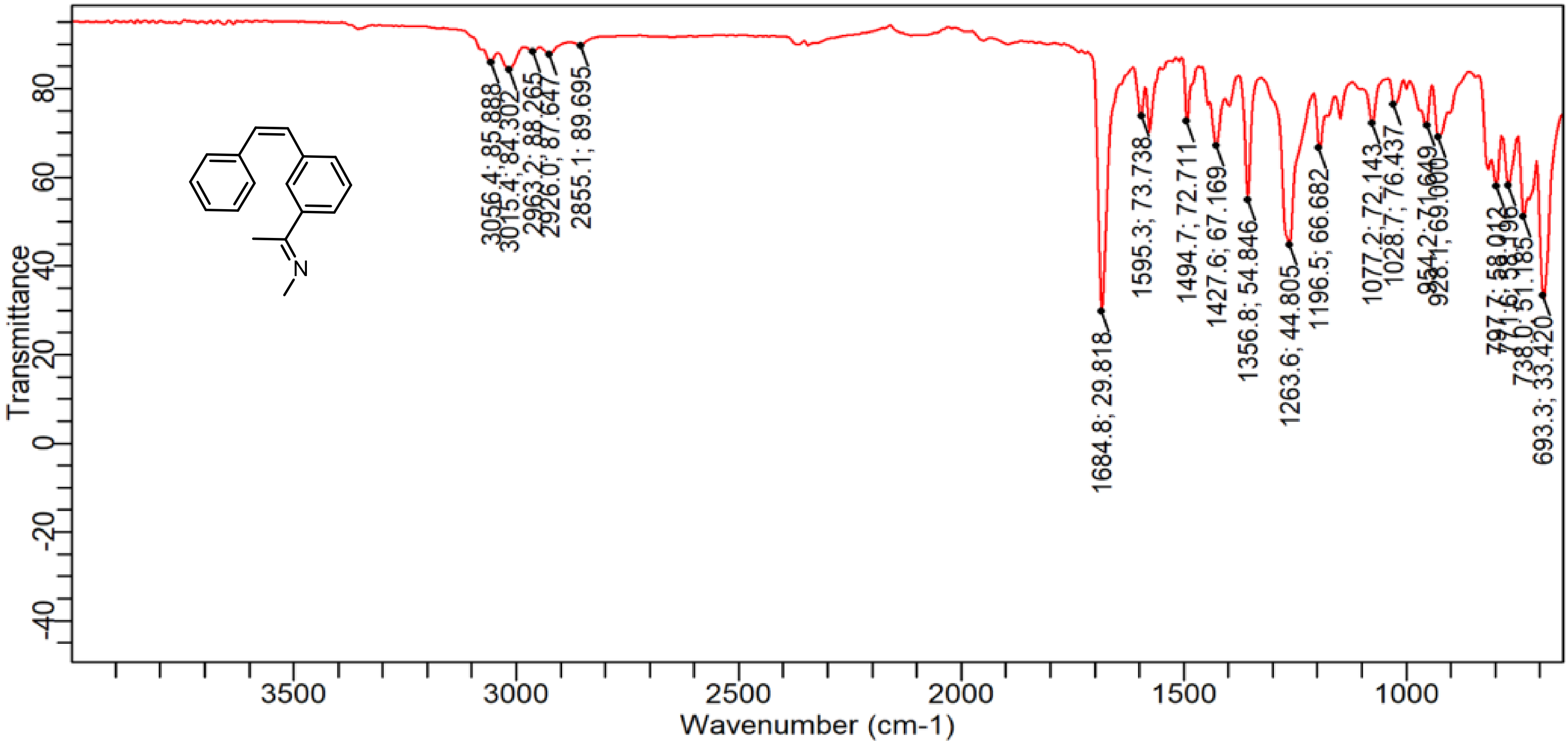

3056.4; 85.888
3015.4; 84.302
2963.2; 88.265
2926.0; 87.647
2855.1; 89.695
1684.8; 29.818
1595.3; 73.738
1494.7; 72.711
1427.6; 67.169
1356.8; 54.846
1263.6; 44.805
1196.5; 66.682
1077.2; 72.143
1028.7; 76.437
954.2; 71.649
928.1; 69.000
797.7; 58.012
771.6; 58.196
738.0; 51.185
693.3; 33.420
N
Transmittance
Wavenumber (cm-1)

2.13 Ethyl (*Z*)-6-phenylhex-5-enoate (2m) (600 MHz, $CDCl_3$)

2.13.1 ^{1}H NMR

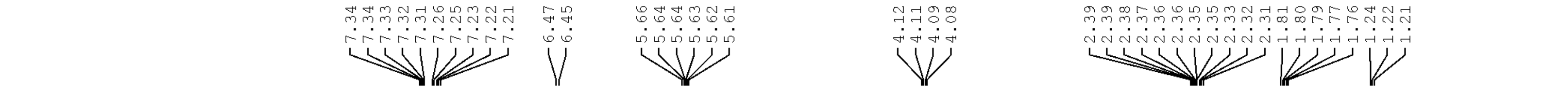

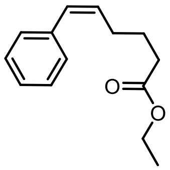

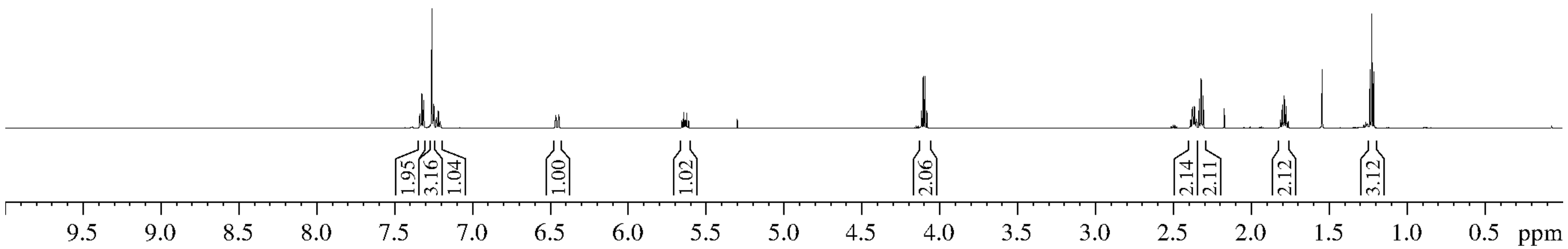

2.13.2 ^{13}C NMR

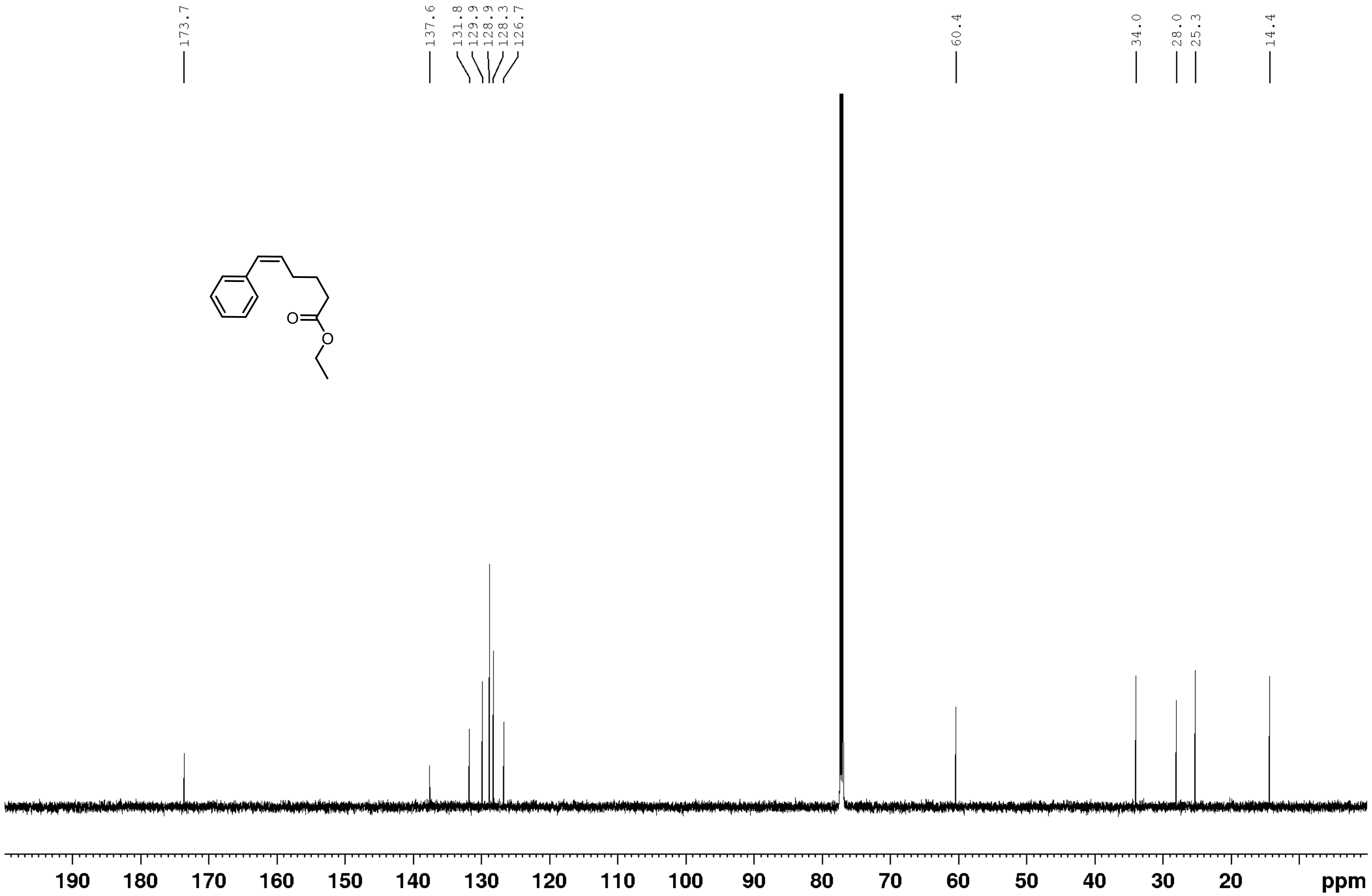
173.7
137.6
131.8
129.9
128.9
128.3
126.7
60.4
34.0
28.0
25.3
14.4
O
O
190 180 170 160 150 140 130 120 110 100 90 80 70 60 50 40 30 20
ppm

2.13.3 ^{1}H, ^{1}H COSY

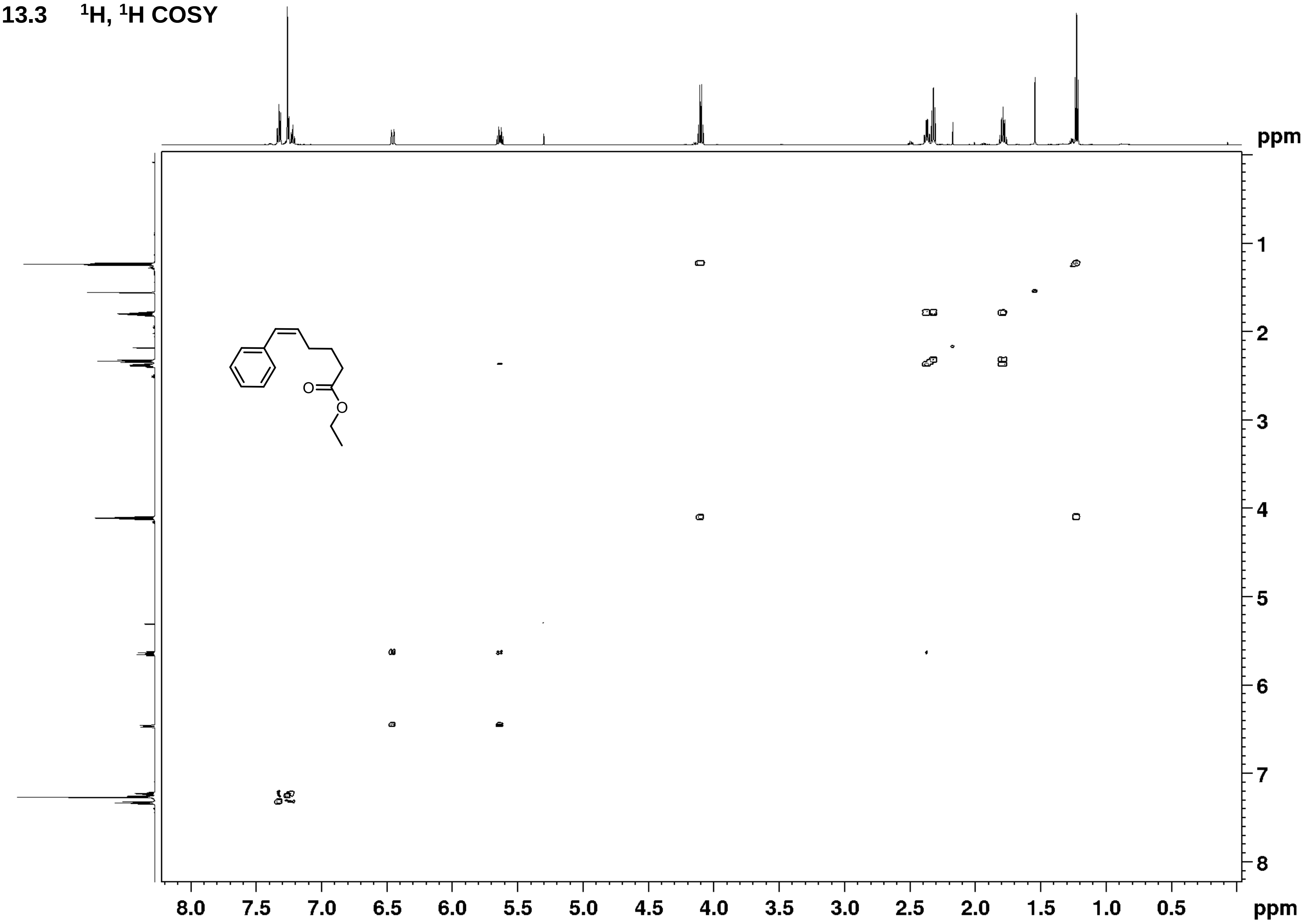

2.13.4 ^{1}H ^{13}C HMBC

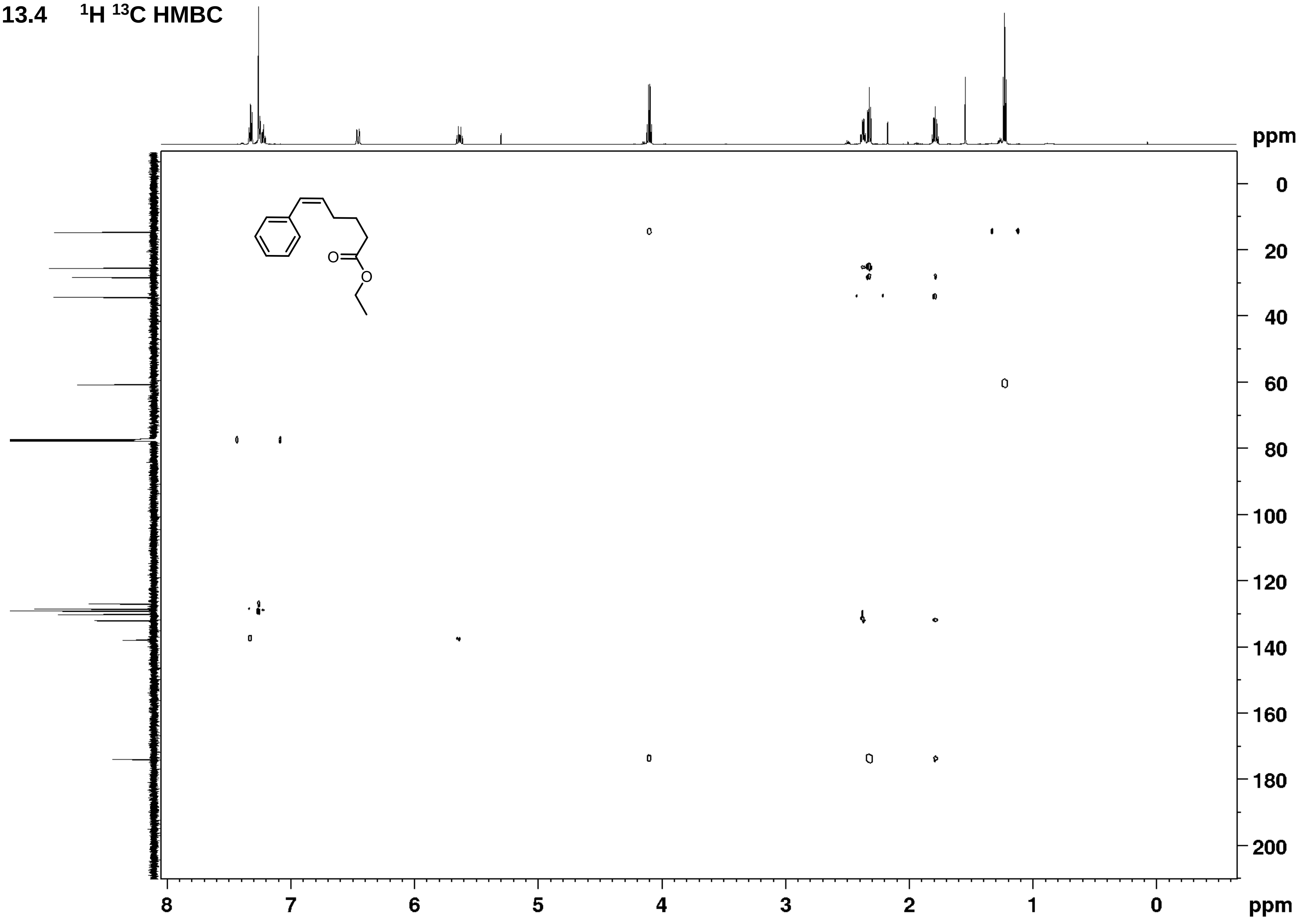

2.13.5 ^{1}H ^{13}C HSQC

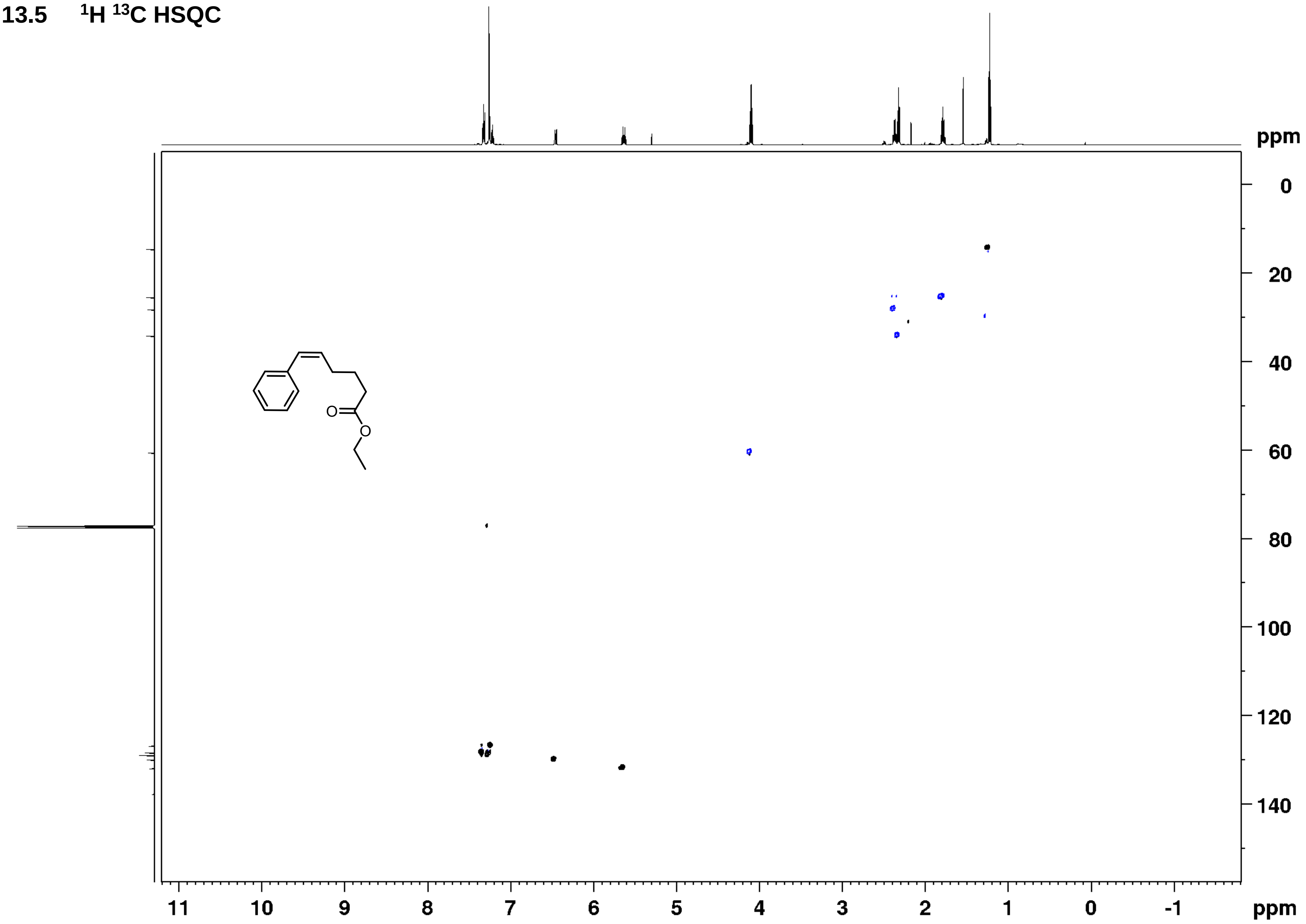

2.13.6 HRMS

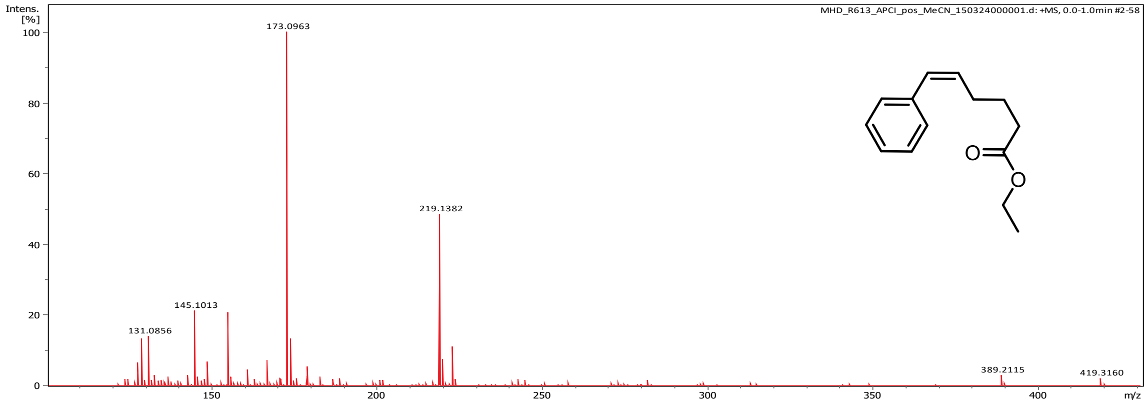

Intens. [%]
MHD_R613_APCI_pos_MeCN_150324000001.d: +MS, 0.0-1.0min #2-58
173.0963
219.1382
145.1013
131.0856
389.2115
419.3160
m/z

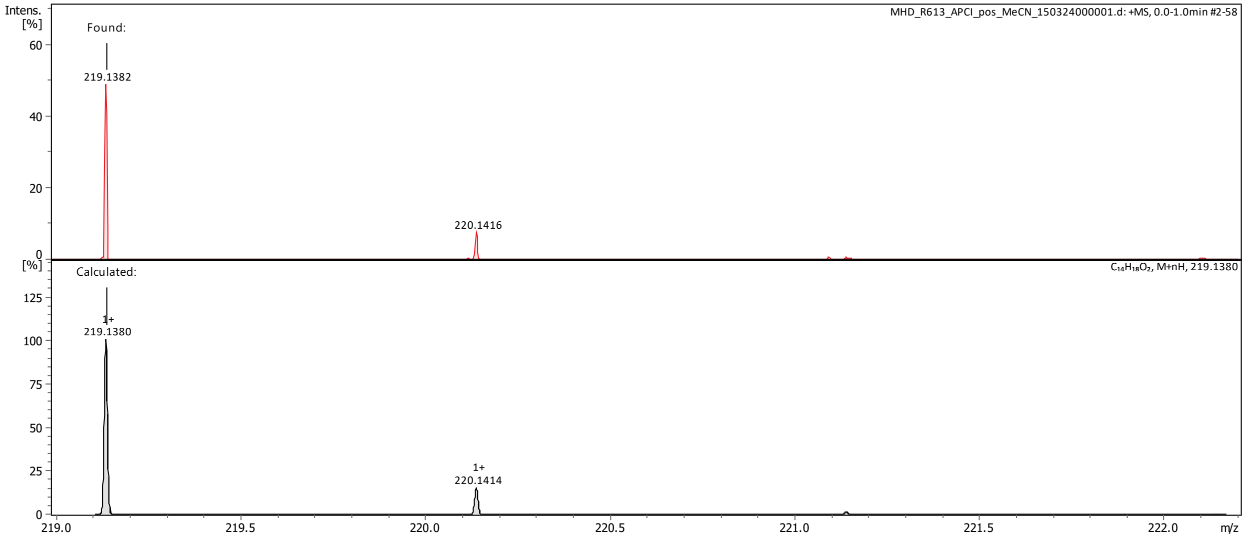

Intens. [%]
MHD_R613_APCI_pos_MeCN_150324000001.d: +MS, 0.0-1.0min #2-58
Found:
219.1382
220.1416
Calculated:
C14H18O2, M+nH, 219.1380
1+
219.1380
1+
220.1414
m/z

2.13.7 IR

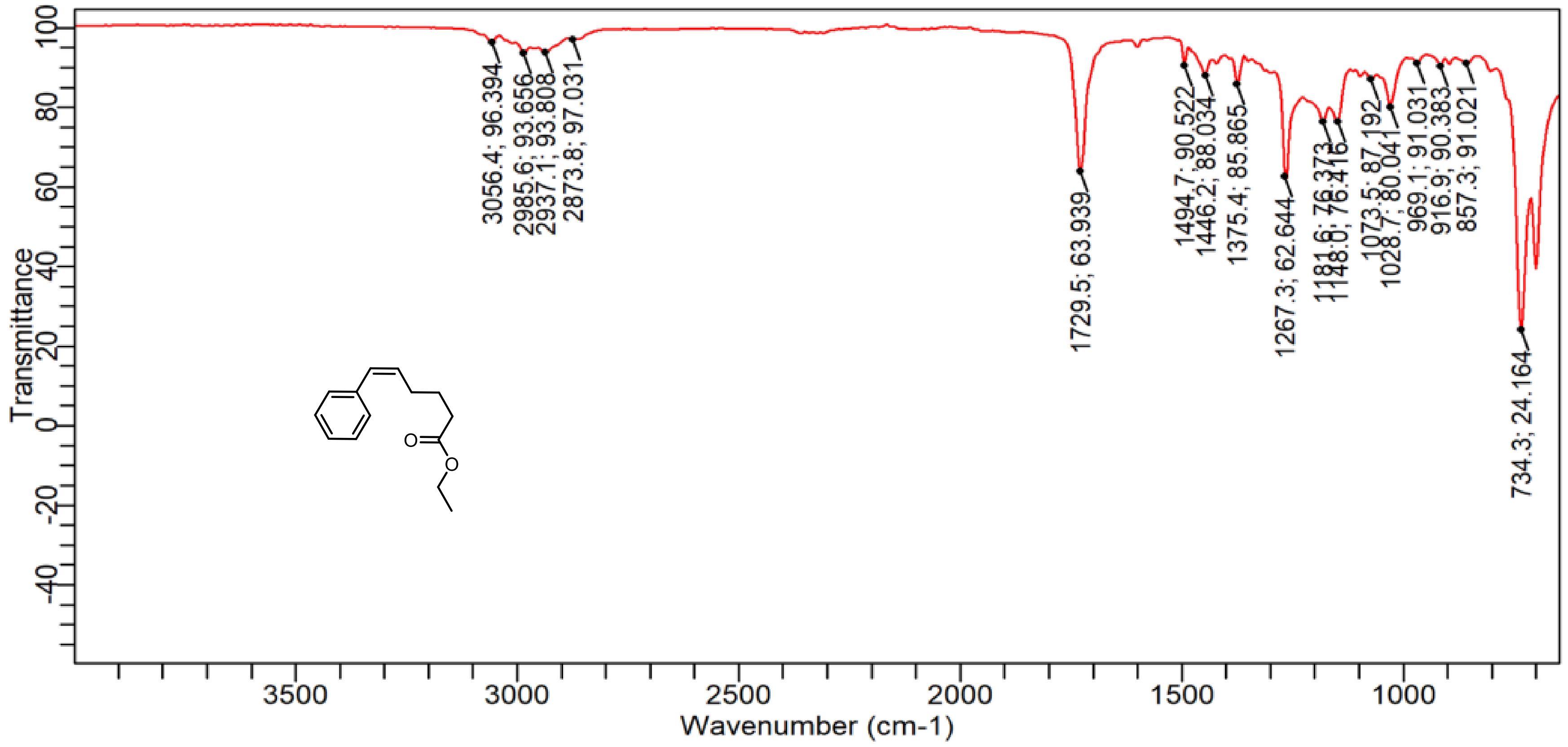

3056.4; 96.394
2985.6; 93.656
2937.1; 93.808
2873.8; 97.031
1729.5; 63.939
1494.7; 90.522
1446.2; 88.034
1375.4; 85.865
1267.3; 62.644
1181.6; 76.373
1148.0; 76.416
1073.5; 87.192
1028.7; 80.041
969.1; 91.031
916.9; 90.383
857.3; 91.021
734.3; 24.164
Transmittance
Wavenumber (cm-1)
O
O

2.14 4-Vinyl-1,1'-biphenyl (2n) (600 MHz, $CDCl_3$)

2.14.1 ^{1}H NMR

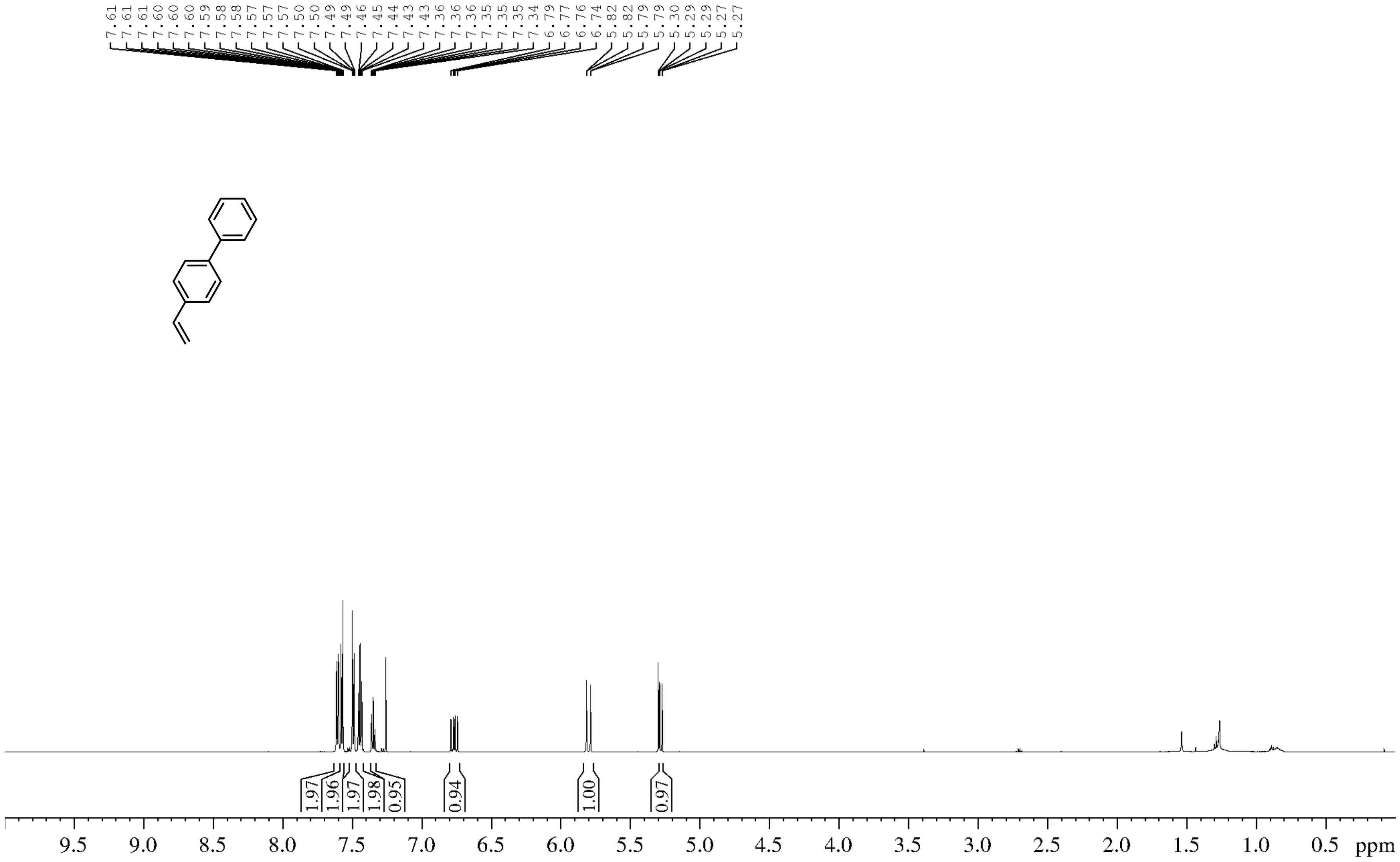

2.14.2 ^{13}C NMR

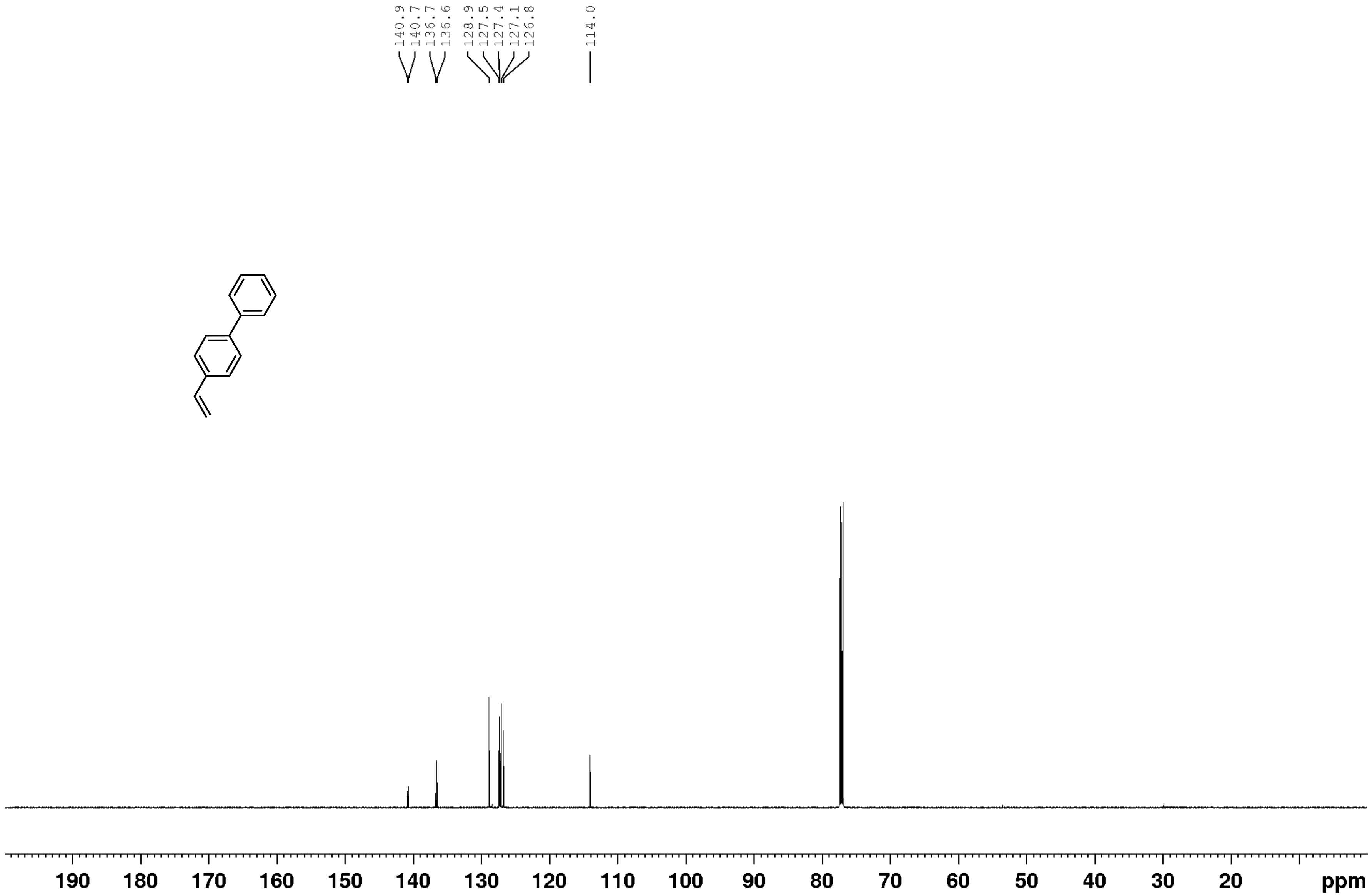
140.9
140.7
136.7
136.6
128.9
127.5
127.4
127.1
126.8
114.0
190 180 170 160 150 140 130 120 110 100 90 80 70 60 50 40 30 20 ppm

2.14.3 ¹H, ¹H COSY

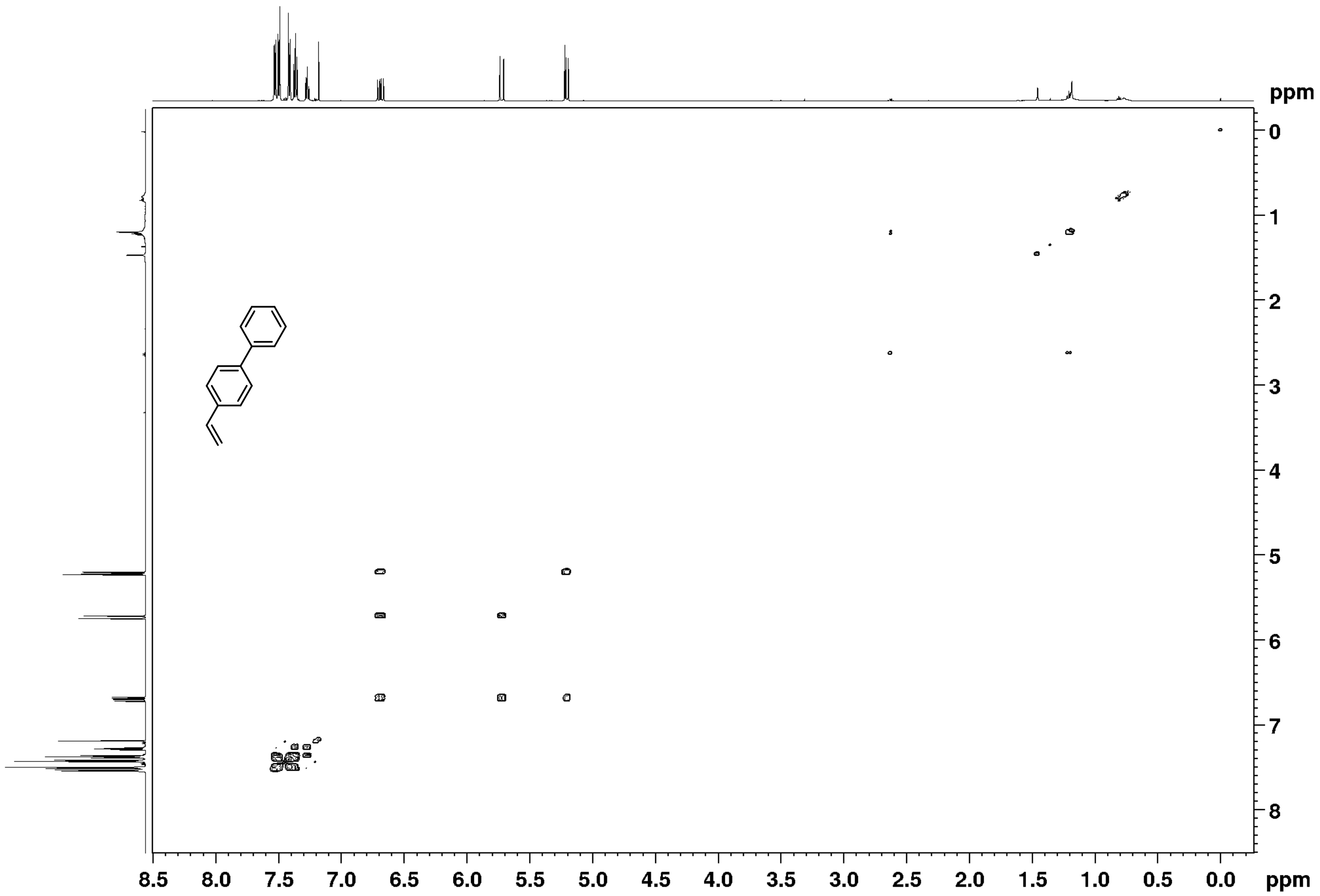

2.14.4 ^{1}H, ^{13}C HMBC

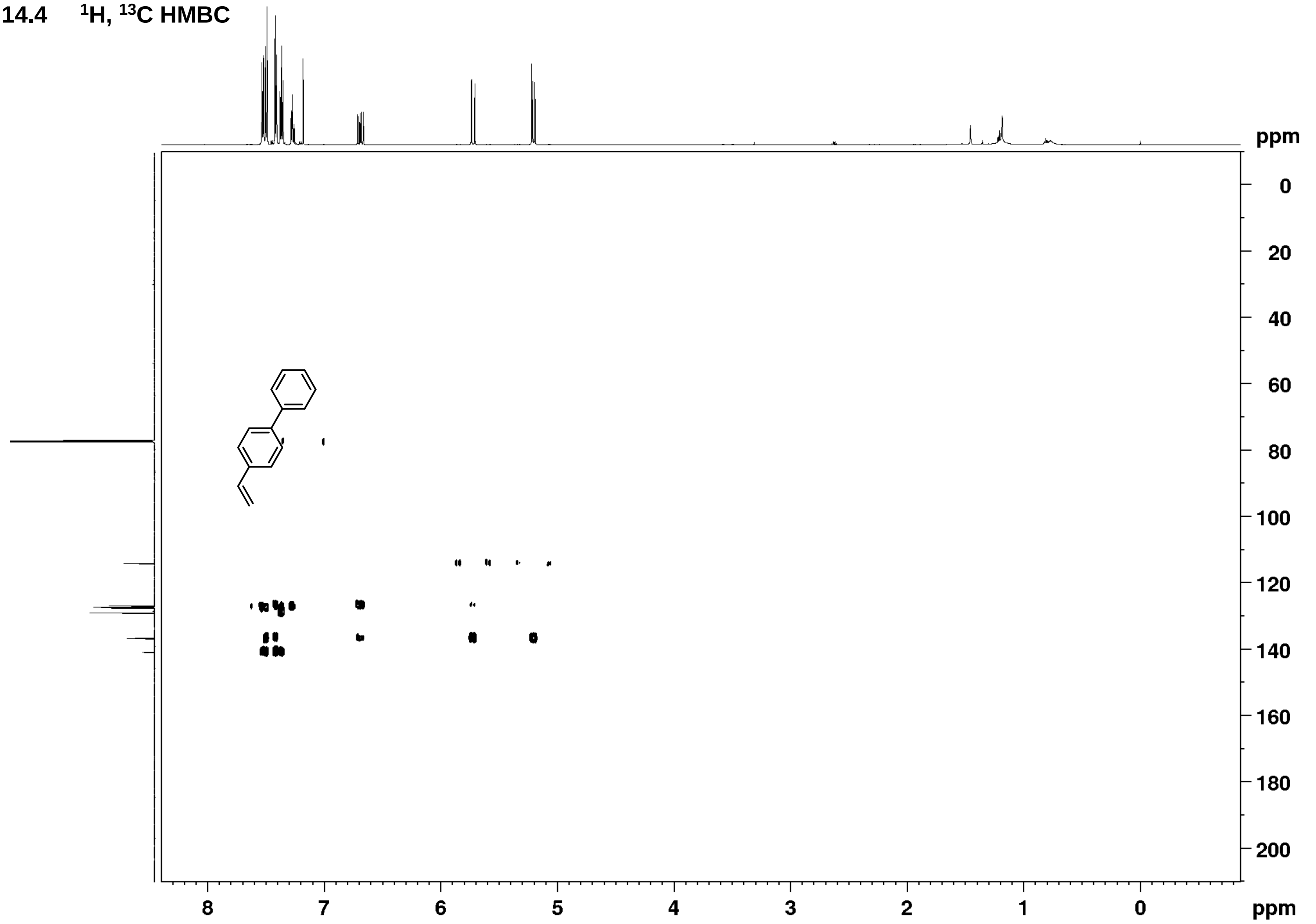

2.14.5 ^{1}H, ^{13}C HSQC

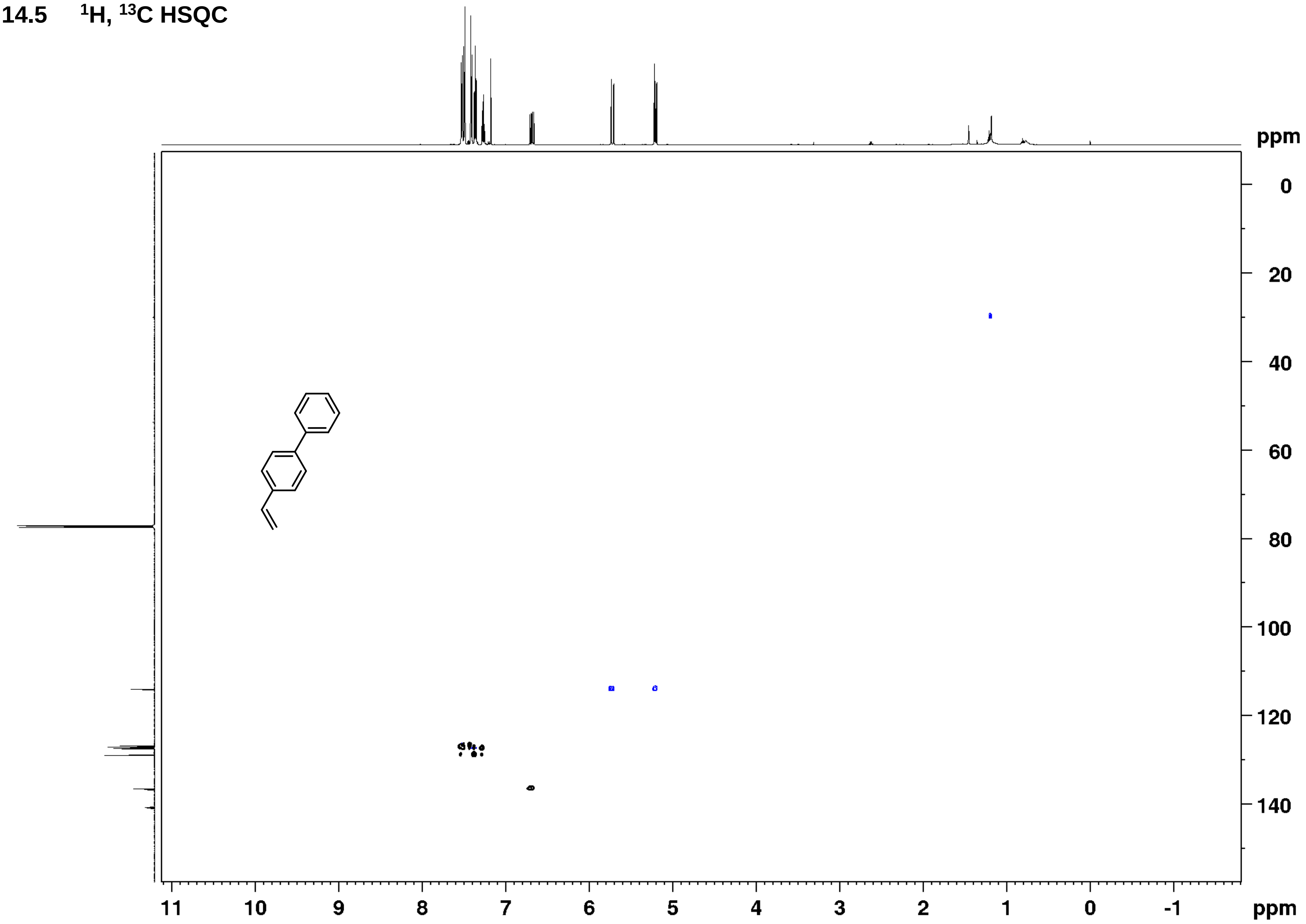

2.14.6 HRMS

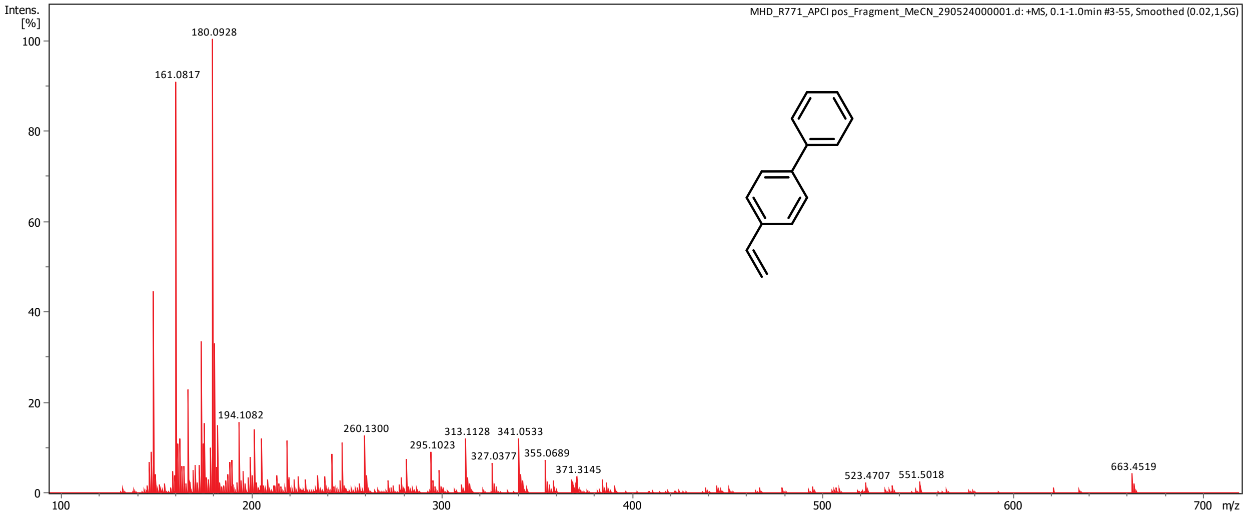

Intens. [%]
MHD_R771_APCI pos_Fragment_MeCN_290524000001.d: +MS, 0.1-1.0min #3-55, Smoothed (0.02,1,SG)
180.0928
161.0817
194.1082
260.1300
295.1023
313.1128
327.0377
341.0533
355.0689
371.3145
523.4707
551.5018
663.4519
m/z

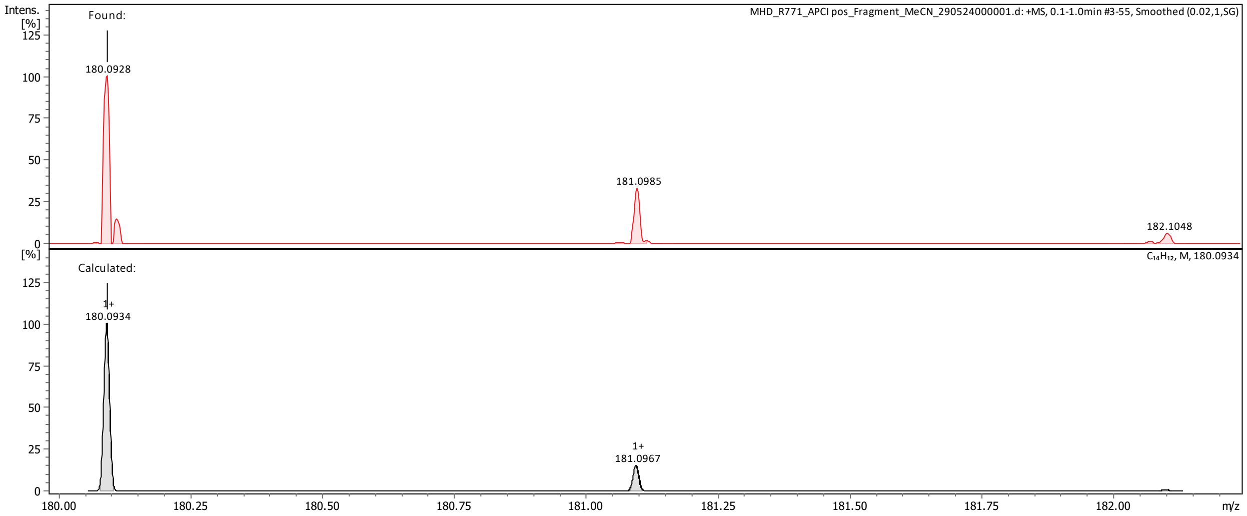

Intens. [%]
Found:
180.0928
181.0985
182.1048
MHD_R771_APCI pos_Fragment_MeCN_290524000001.d: +MS, 0.1-1.0min #3-55, Smoothed (0.02,1,SG)
Calculated:
1+ 180.0934
1+ 181.0967
C₁₄H₁₂, M, 180.0934
m/z

2.15 (*Z*)-1-Styryl-4-vinylbenzene (2o) (600 MHz, $CDCl_3$)

2.15.1 ^{1}H NMR

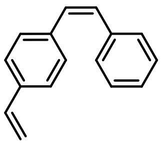

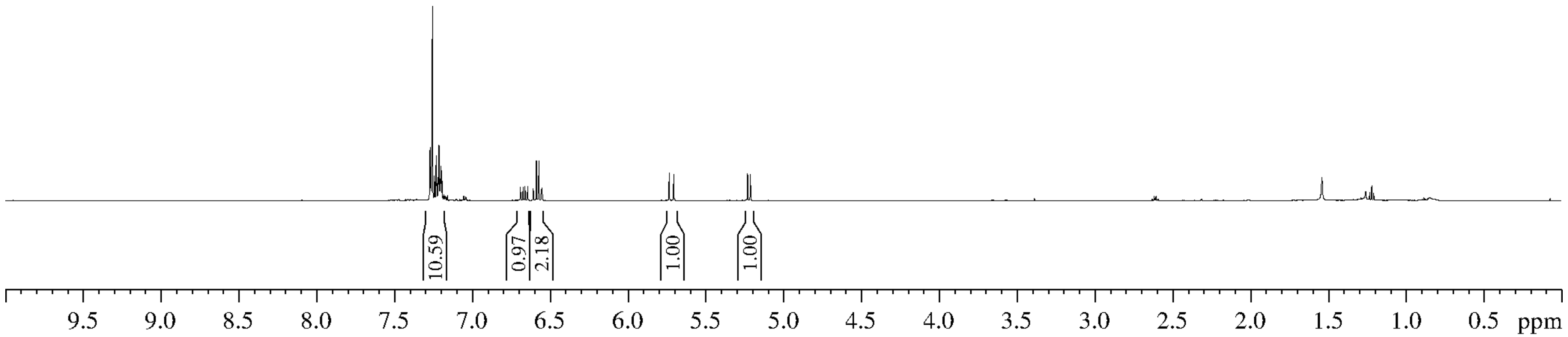

2.15.2 ^{13}C NMR

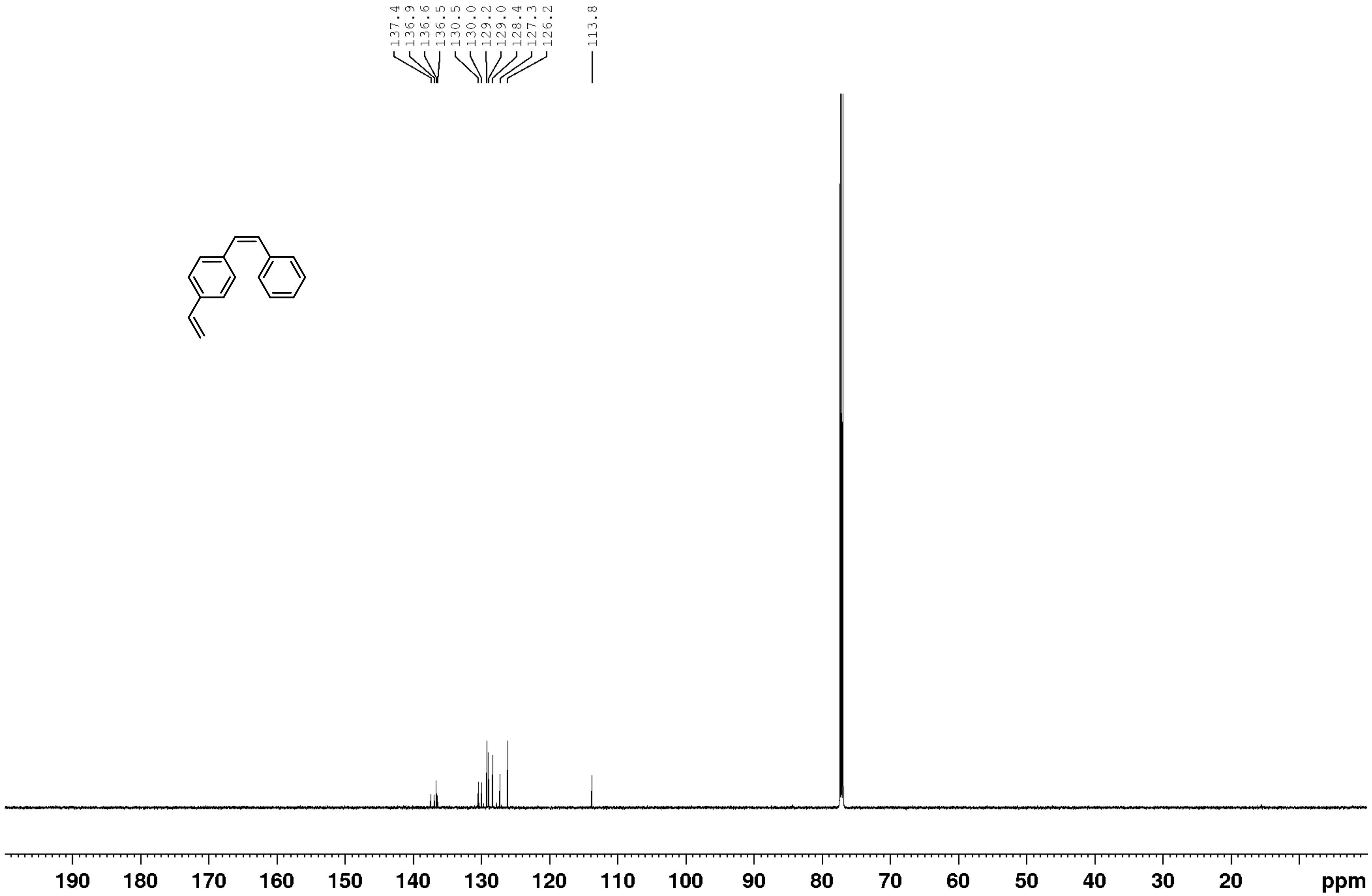

137.4
136.9
136.6
136.5
130.5
130.0
129.2
129.0
128.4
127.3
126.2
113.8
190
180
170
160
150
140
130
120
110
100
90
80
70
60
50
40
30
20
ppm

2.15.3 ^{1}H, ^{1}H COSY

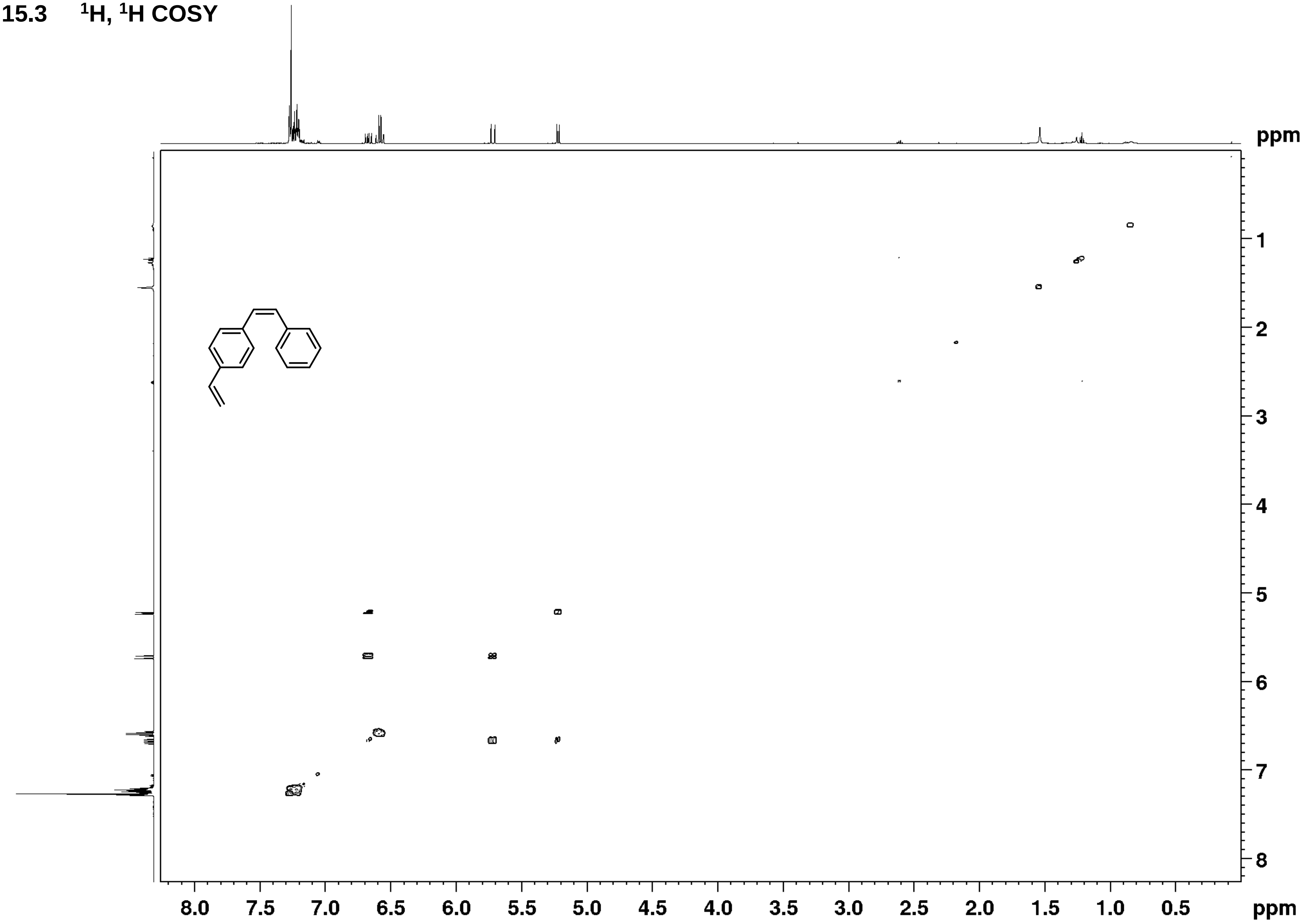

2.15.4 ^{1}H, ^{13}C HMBC

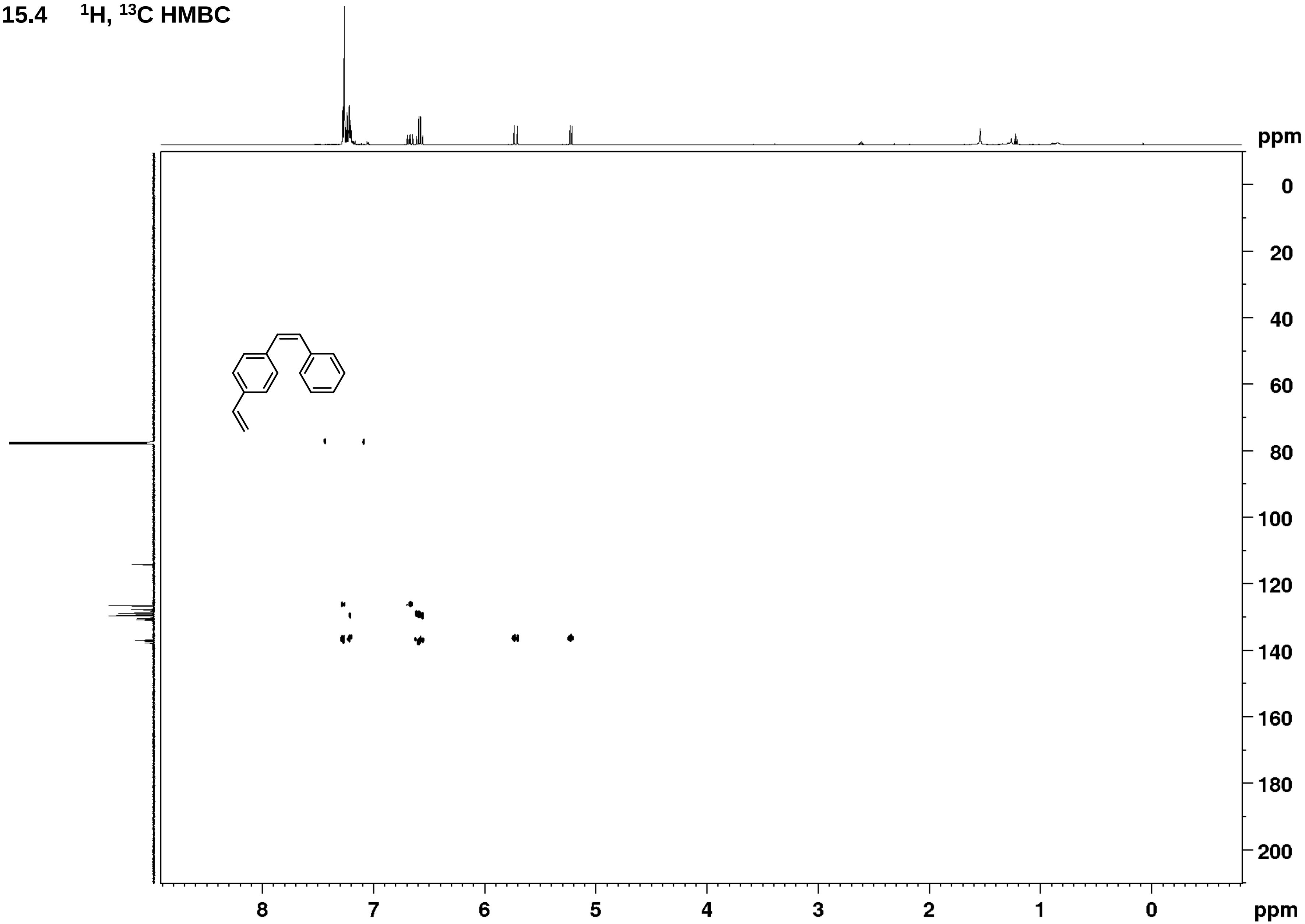

2.15.5 ^{1}H, ^{13}C HSQC

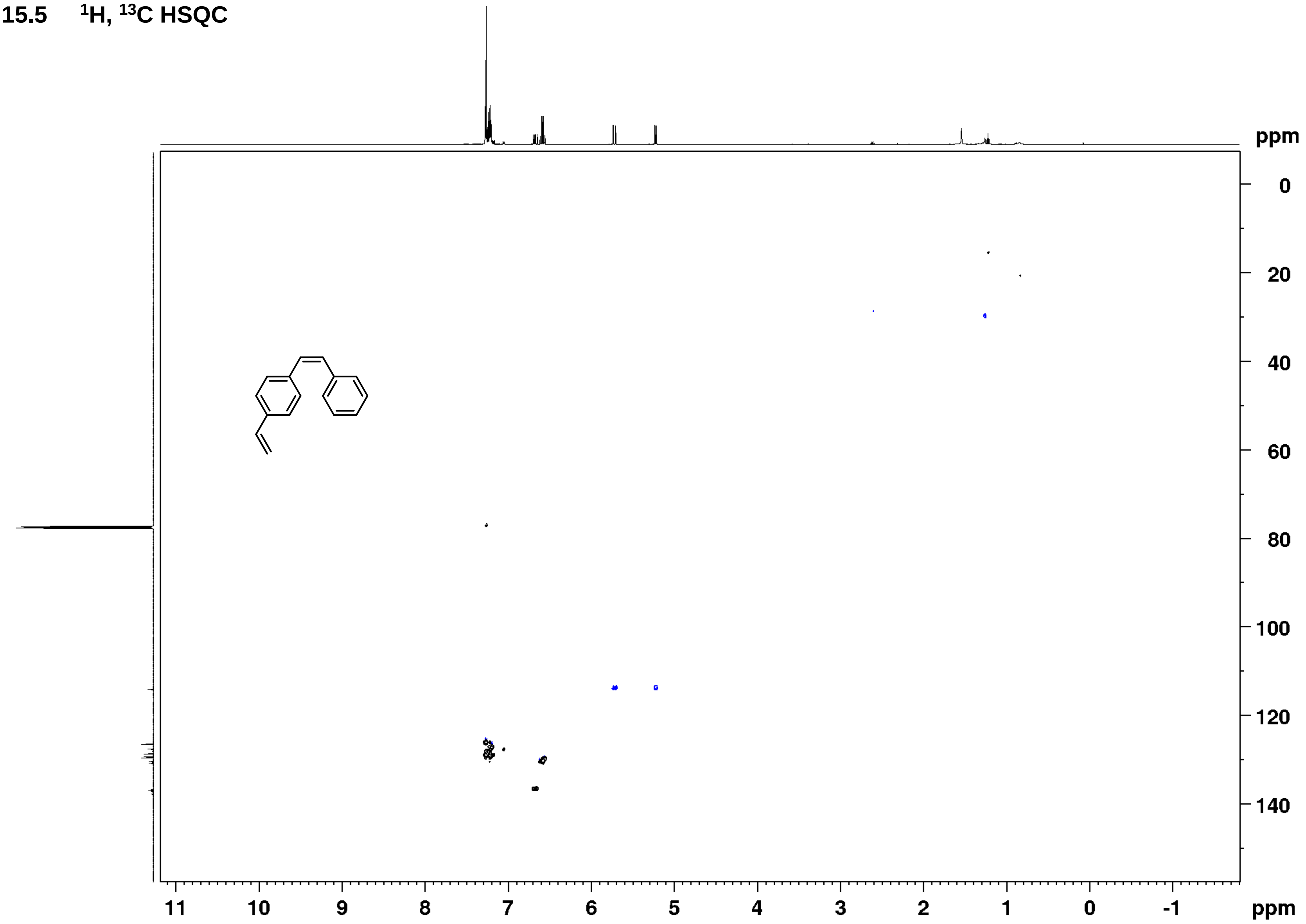

2.15.6 HRMS

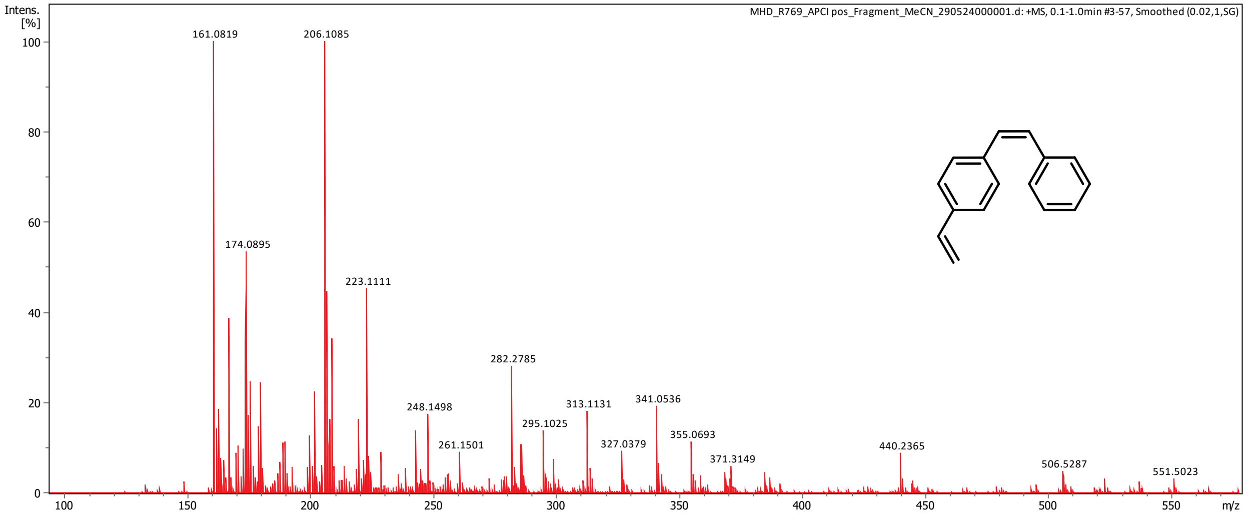

MHD_R769_APCI pos_Fragment_MeCN_290524000001.d: +MS, 0.1-1.0min #3-57, Smoothed (0.02,1,SG)
Intens. [%]
161.0819
206.1085
174.0895
223.1111
282.2785
248.1498
313.1131
341.0536
295.1025
261.1501
327.0379
355.0693
371.3149
440.2365
506.5287
551.5023
m/z

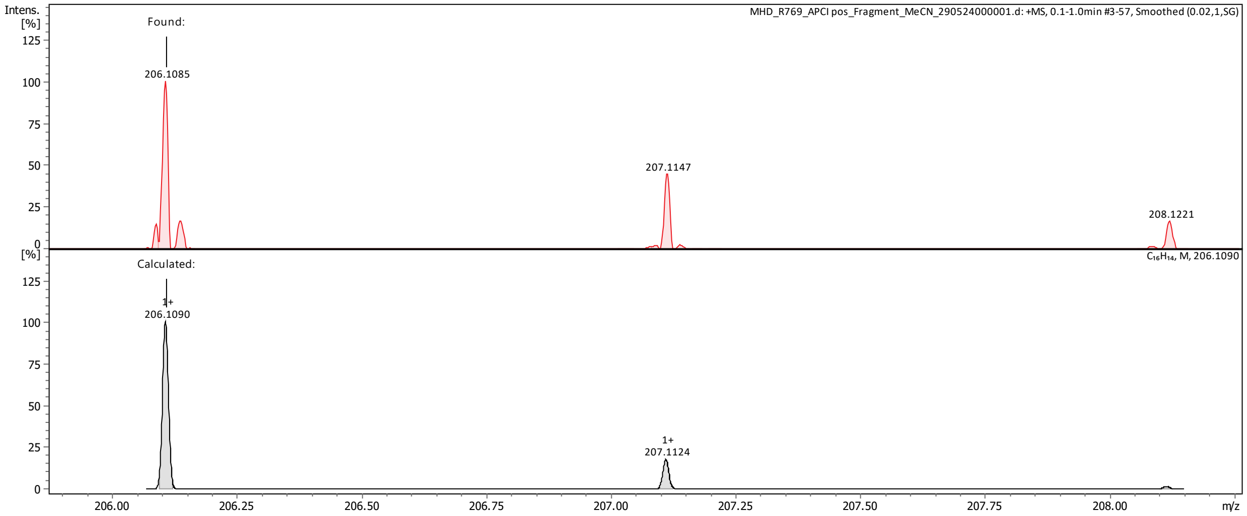

MHD_R769_APCI pos_Fragment_MeCN_290524000001.d: +MS, 0.1-1.0min #3-57, Smoothed (0.02,1,SG)
Found:
206.1085
207.1147
208.1221
Calculated:
C16H14, M, 206.1090
1+
206.1090
1+
207.1124
m/z

2.16 Dodec-1-ene (2p) (600 MHz, $CDCl_3$)

2.16.1 ^{1}H NMR

2.16.2

2.16.3 ^{13}C NMR

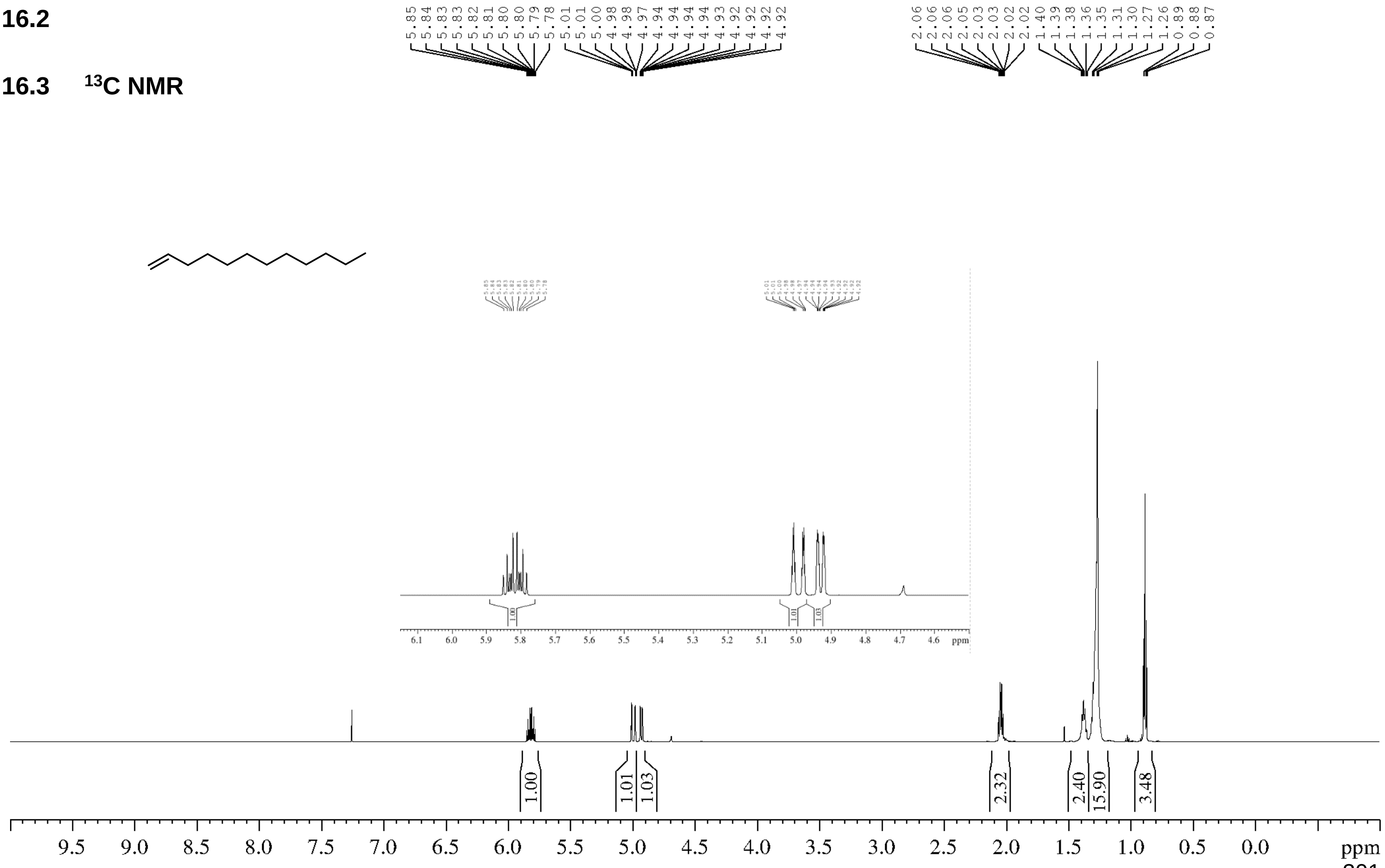

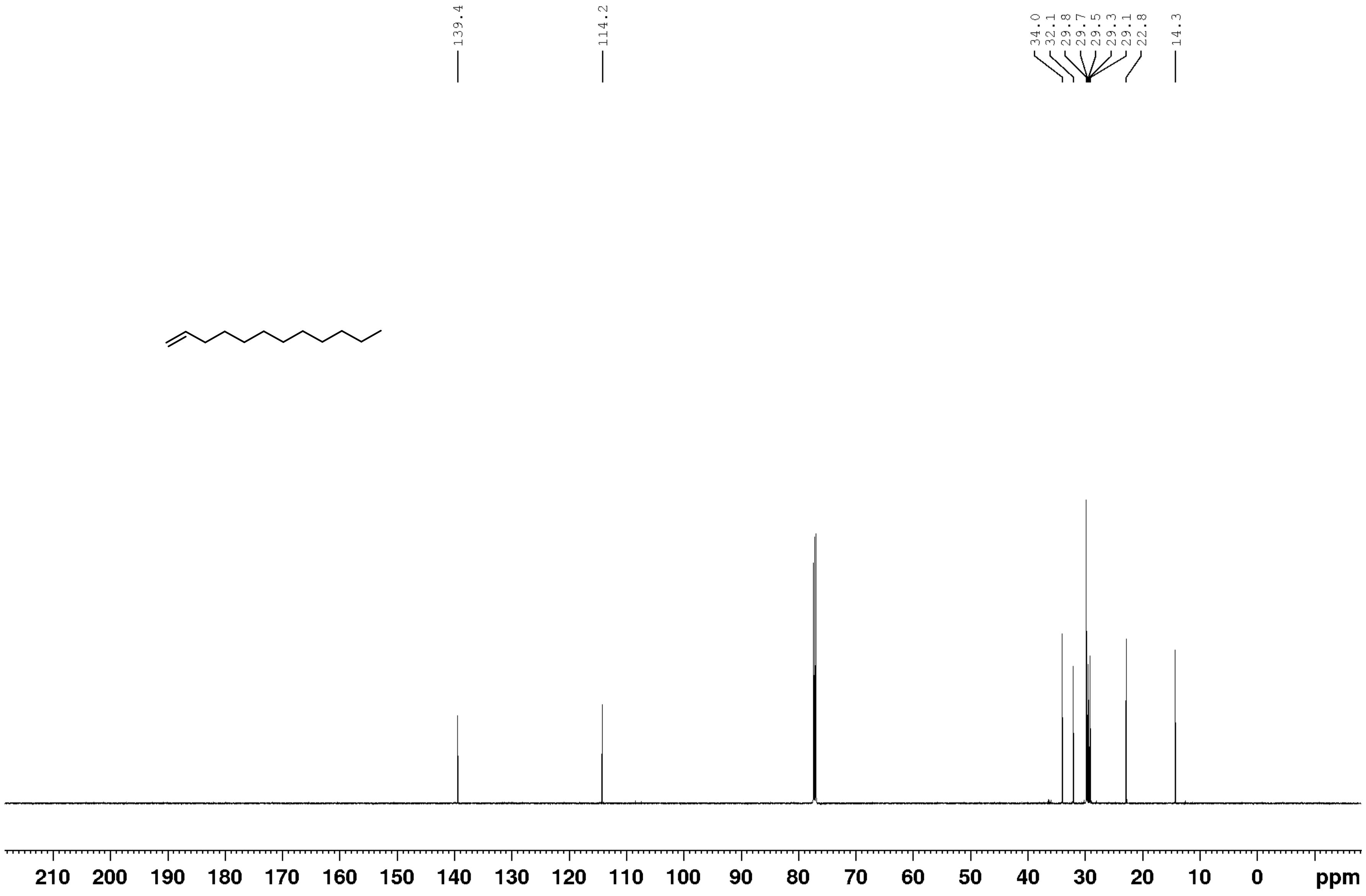
139.4
114.2
34.0
32.1
29.8
29.7
29.5
29.3
29.1
22.8
14.3
210 200 190 180 170 160 150 140 130 120 110 100 90 80 70 60 50 40 30 20 10 0 ppm

2.16.4 ^{1}H, ^{1}H COSY

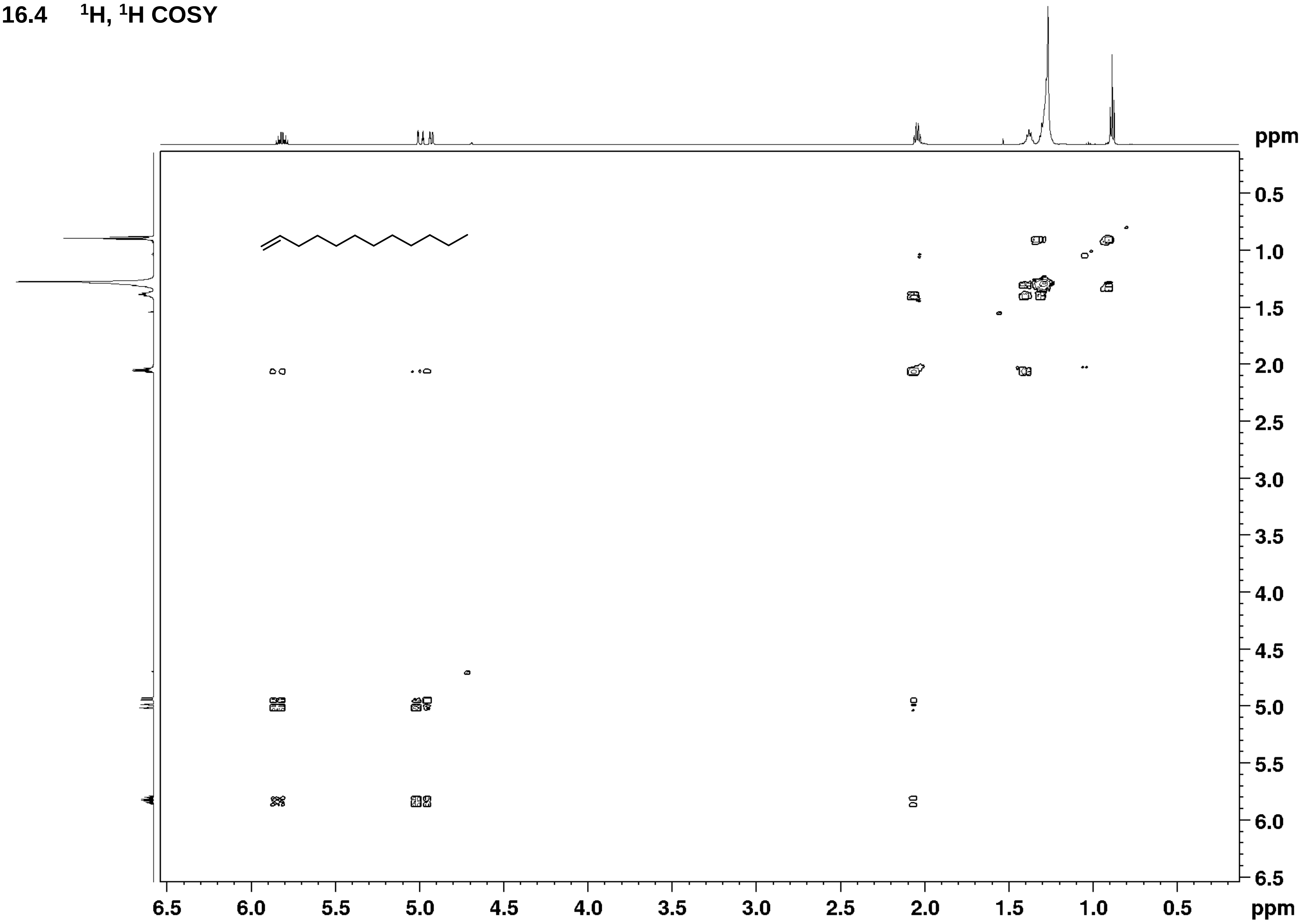

2.16.5 ^{1}H, ^{13}C HMBC

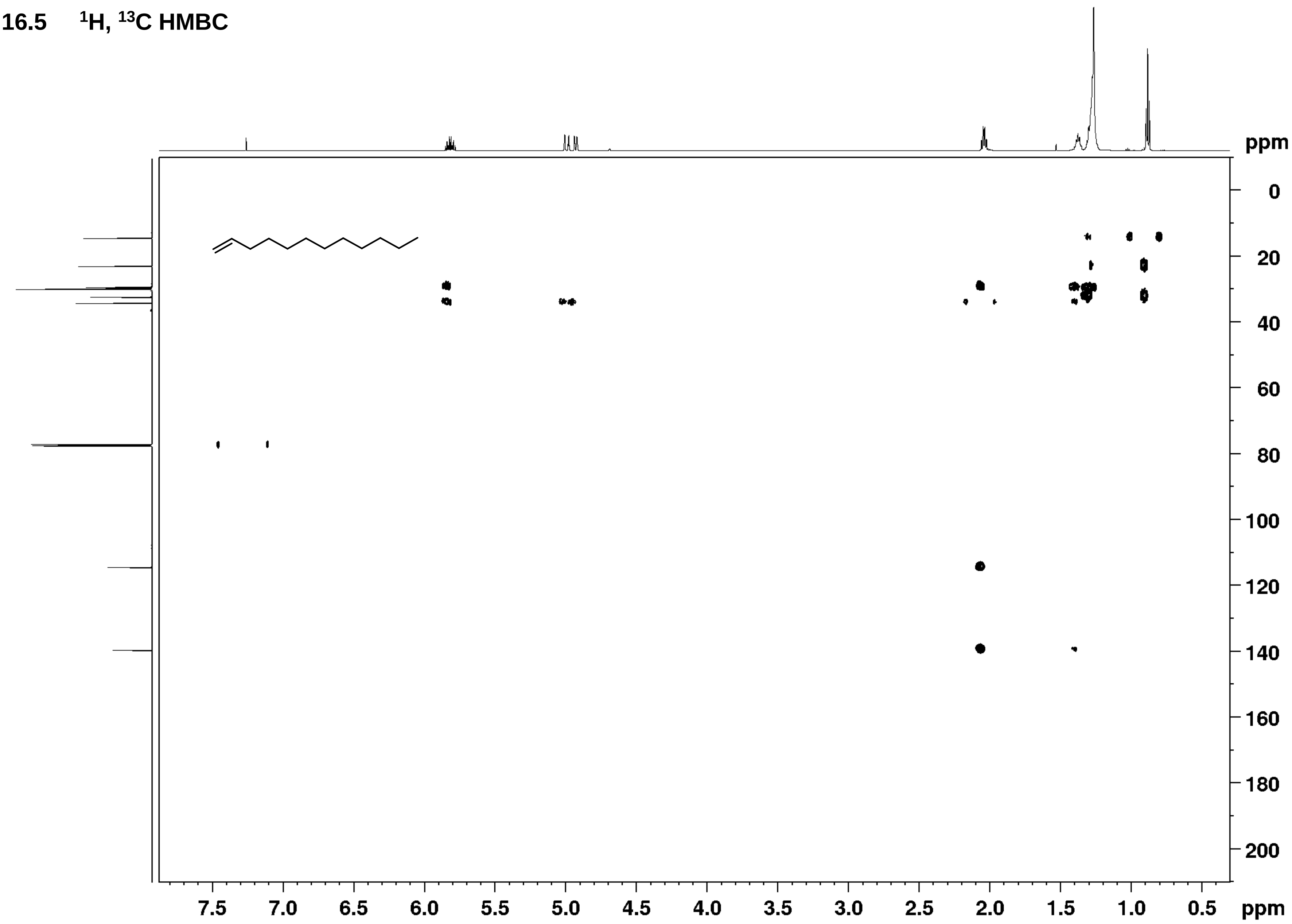

2.16.6 ^{1}H, ^{13}C HSQC

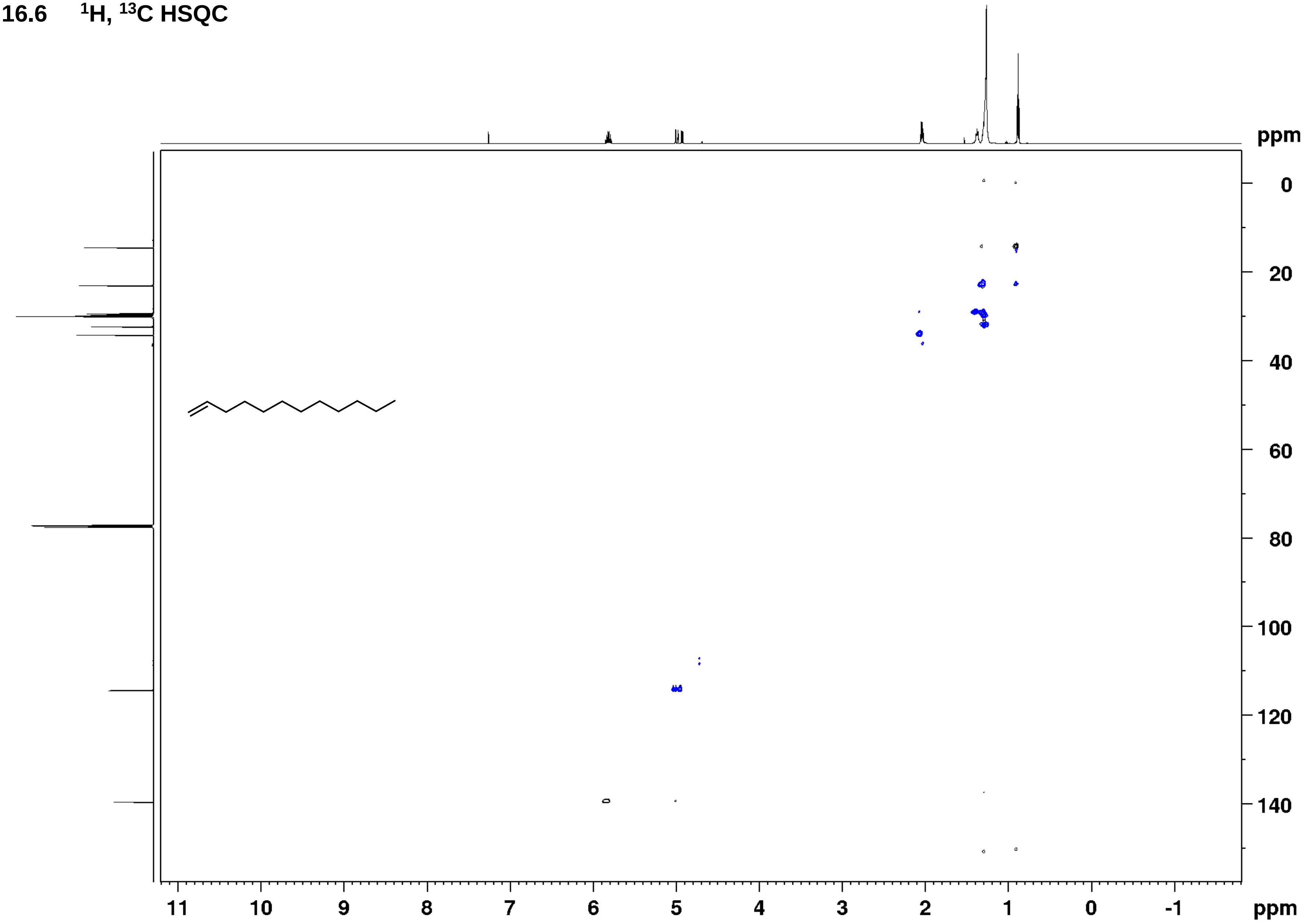

2.16.7 GC-MS

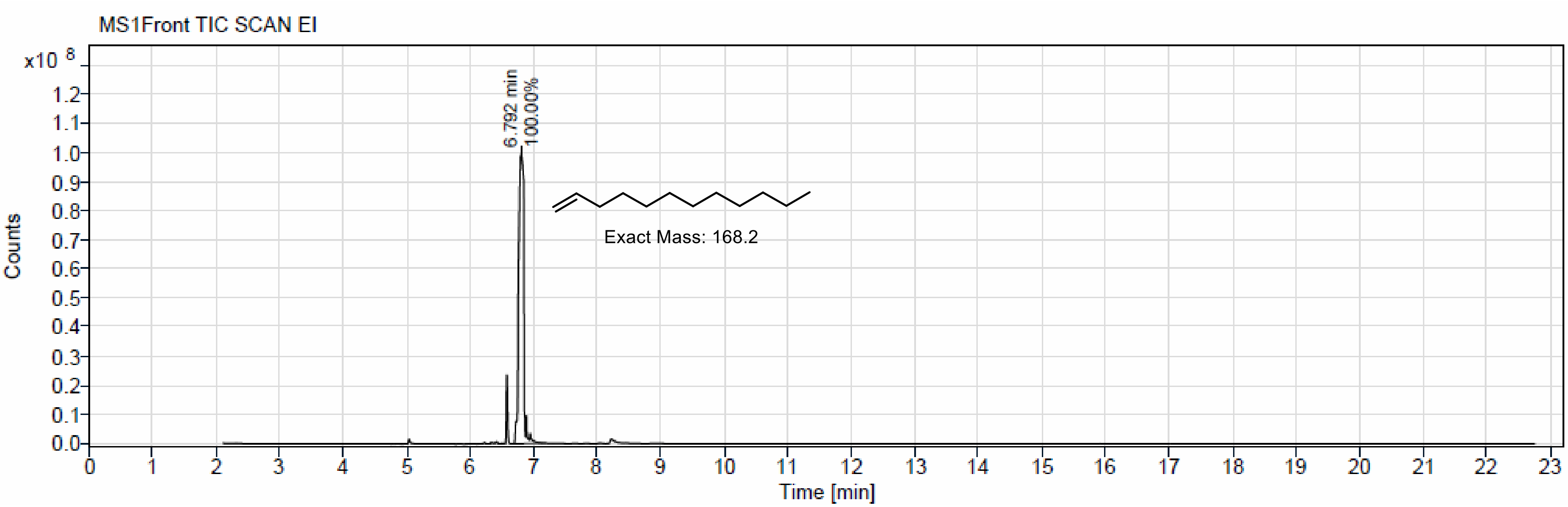

Signal: MS1Front TIC SCAN EI

RT	m/z	Spectral purity (%)	Peak area	Area %
6.792			581200000	100.00

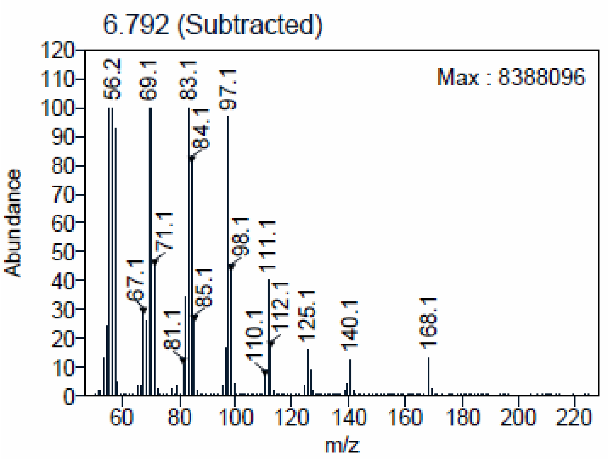

2.17 (*Z*)-1-(2-Cyclopropylvinyl)naphthalene (2q) (600 MHz, $CDCl_3$)

2.17.1 ^{1}H NMR

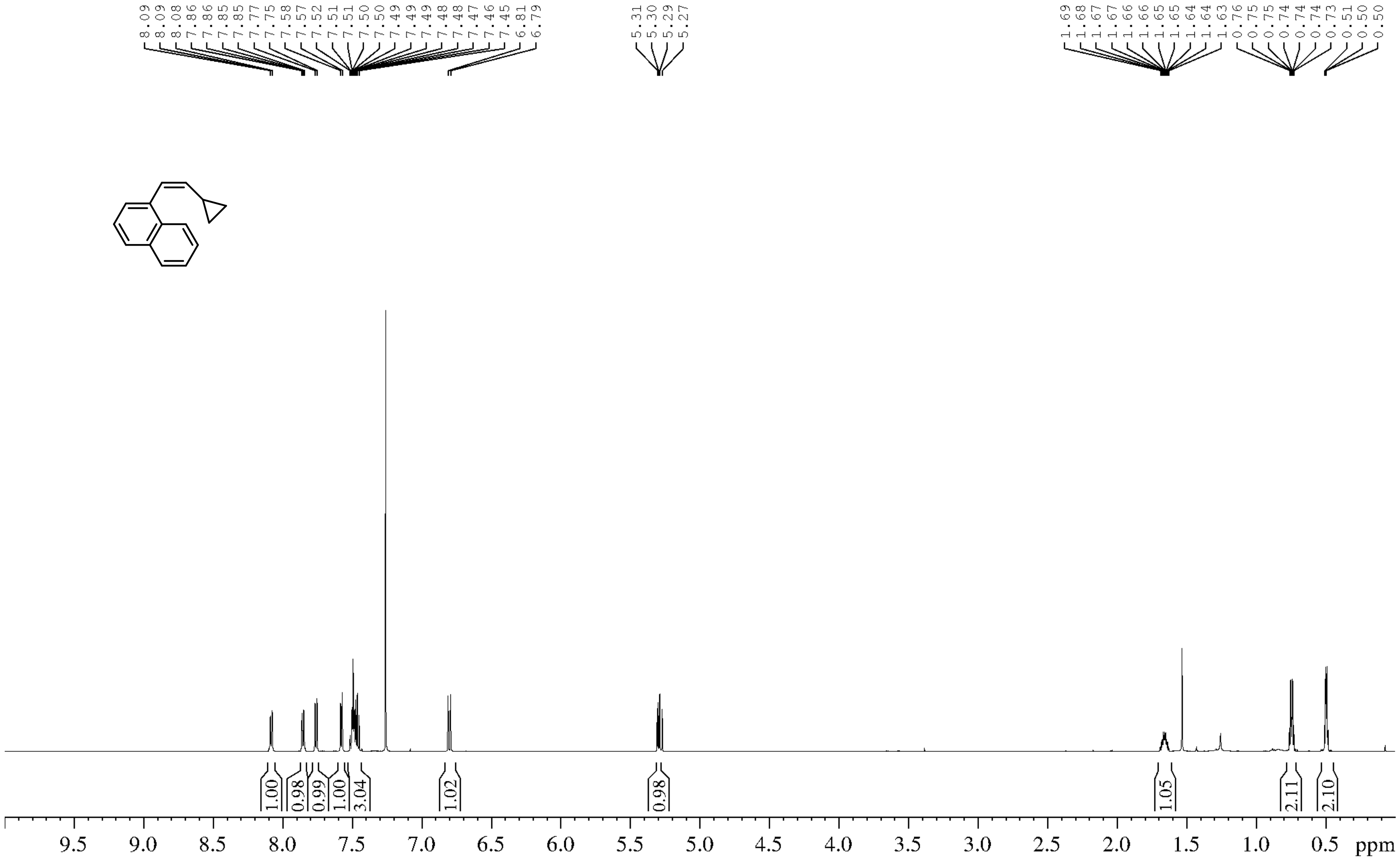

2.17.2 ^{13}C NMR

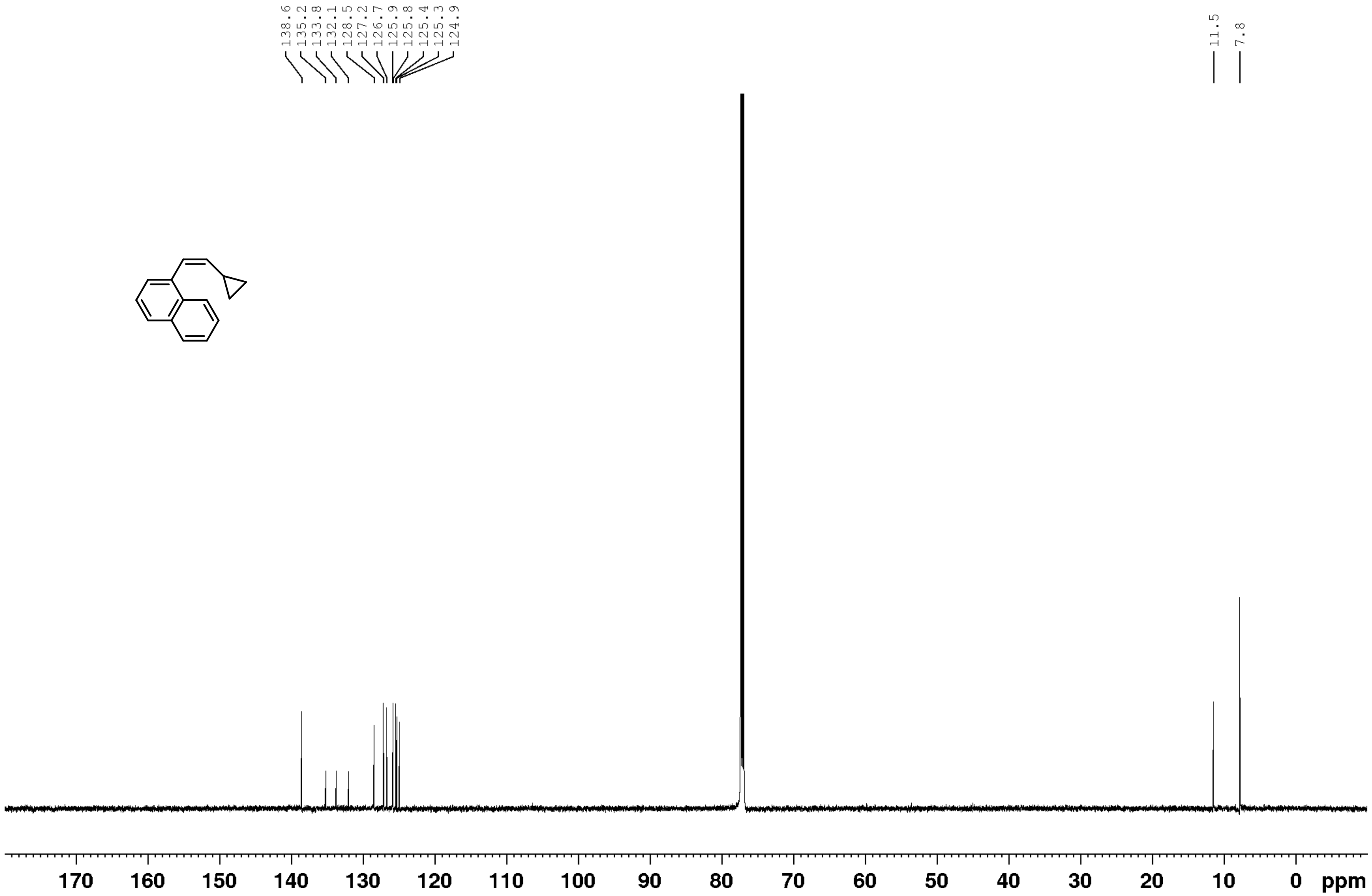
138.6
135.2
133.8
132.1
128.5
127.2
126.7
125.9
125.8
125.4
125.3
124.9
11.5
7.8
170 160 150 140 130 120 110 100 90 80 70 60 50 40 30 20 10 0 ppm

2.17.3 ^{1}H, ^{1}H COSY

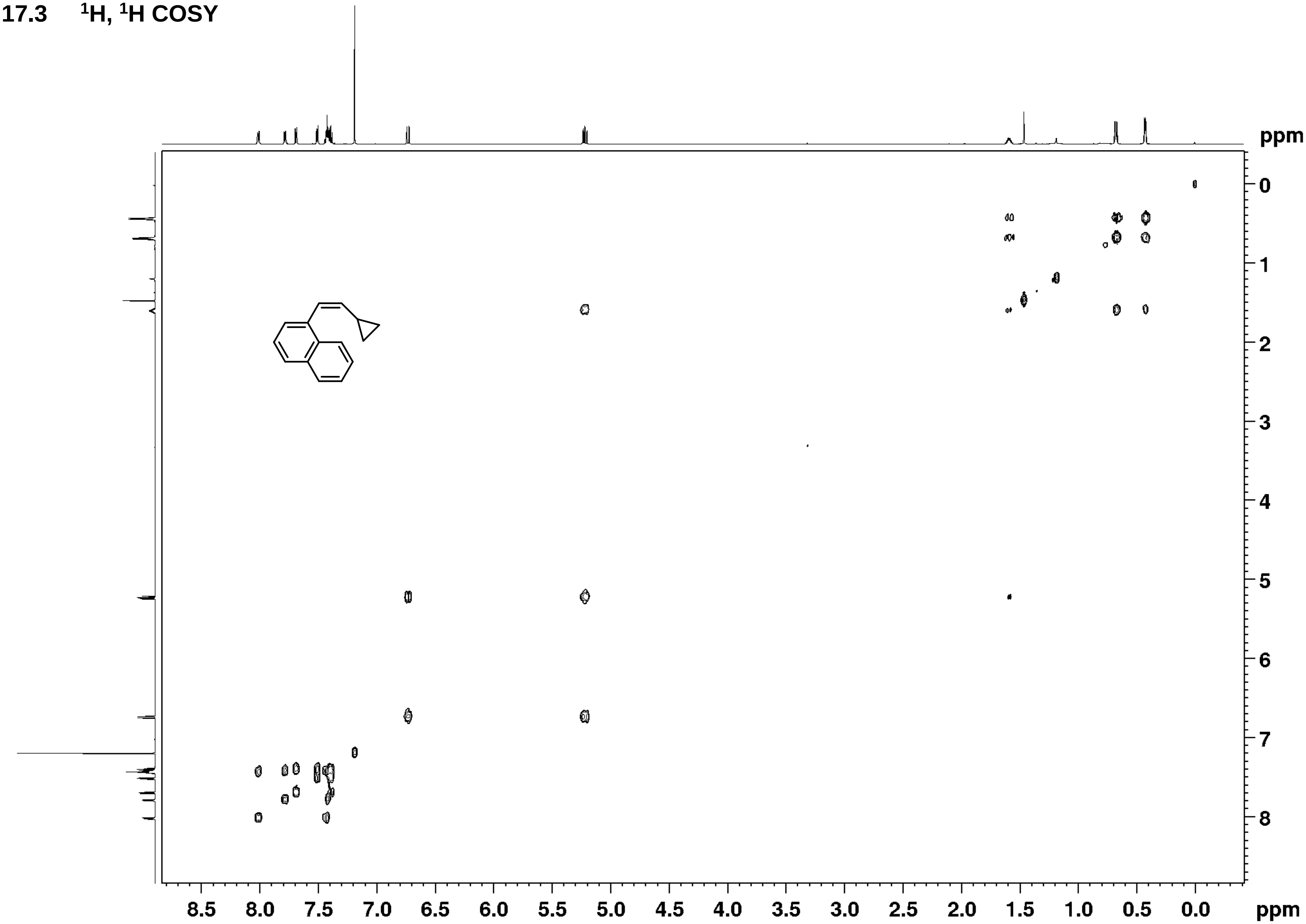

2.17.4 ^{1}H ^{13}C HMBC

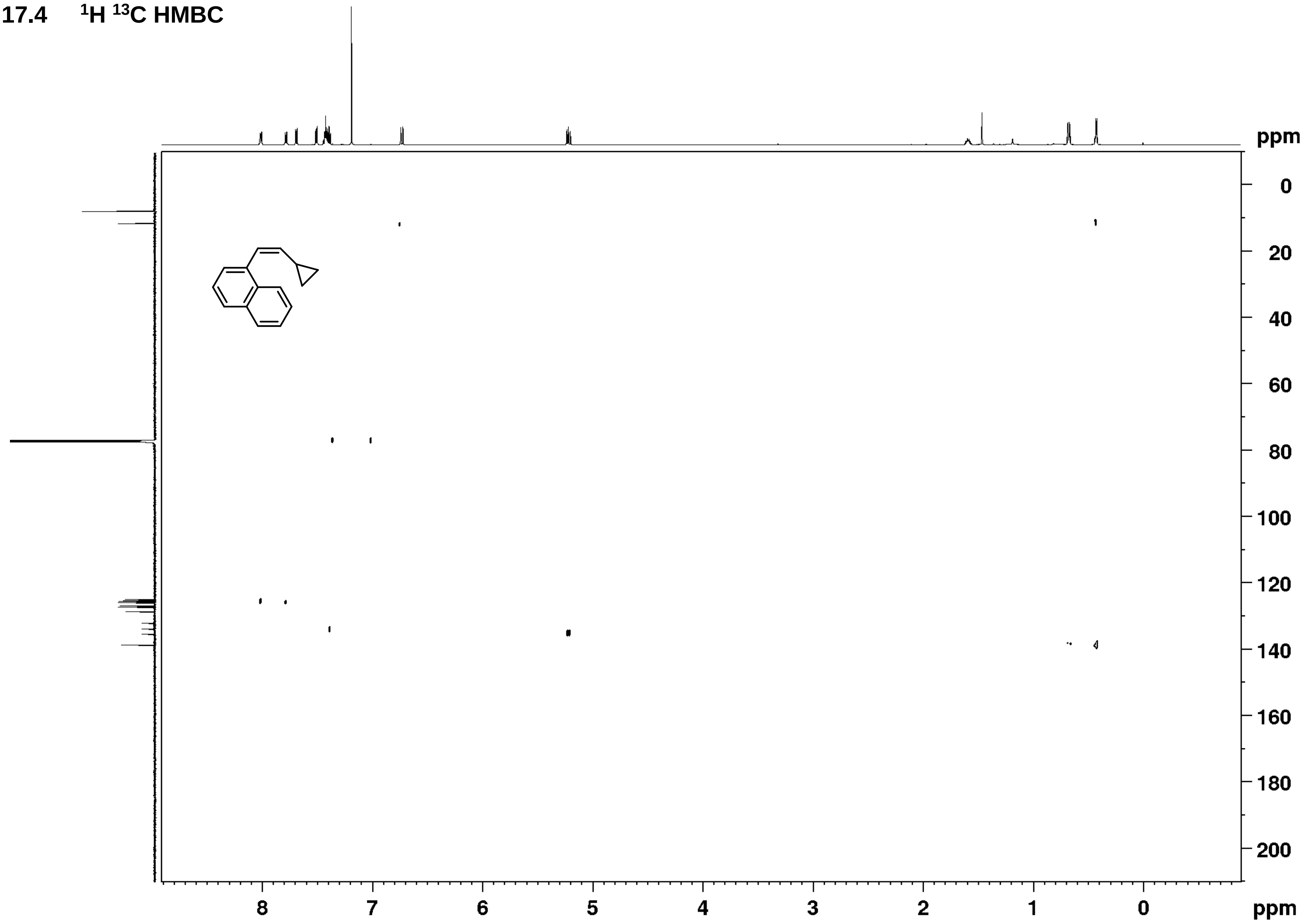

2.17.5 ^{1}H ^{13}C HSQC

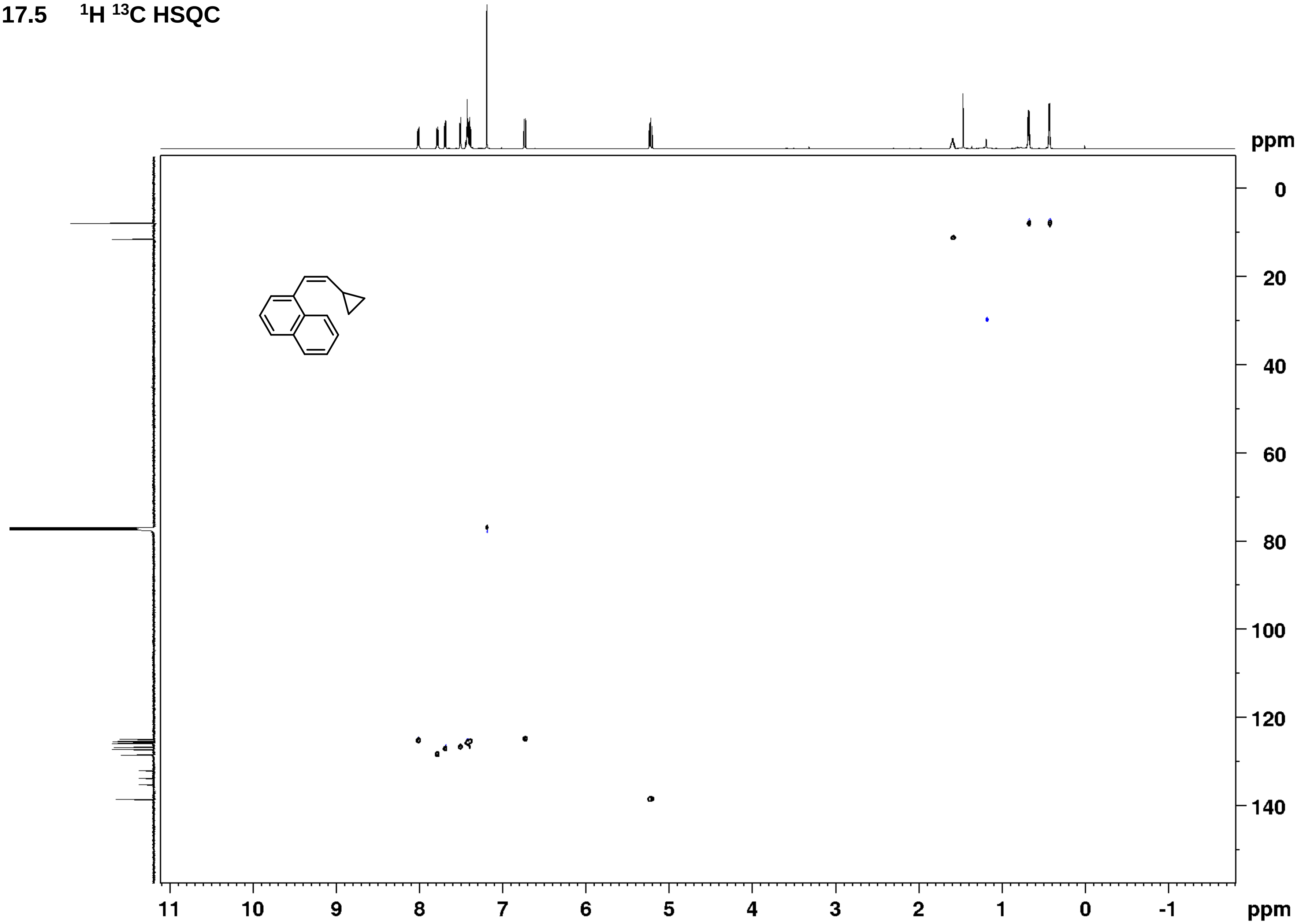

2.17.6 HRMS

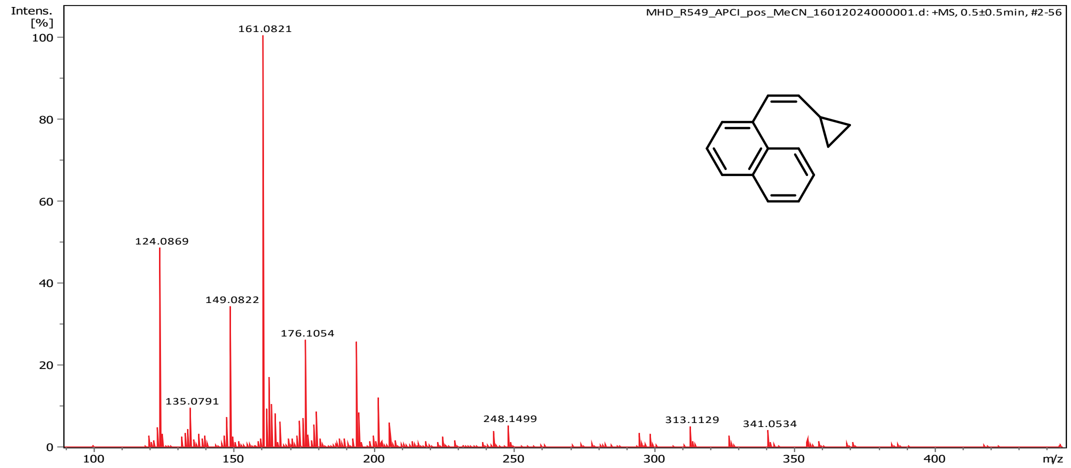
Intens.
[%]
MHD_R549_APCI_pos_MeCN_16012024000001.d: +MS, 0.5±0.5min, #2-56
161.0821
124.0869
149.0822
176.1054
135.0791
248.1499
313.1129
341.0534
m/z

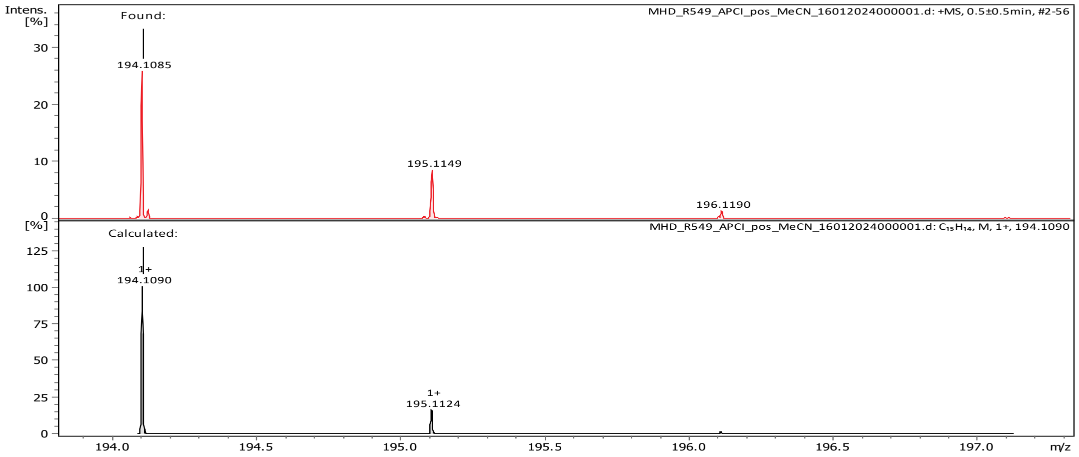
Intens.
[%]
Found:
MHD_R549_APCI_pos_MeCN_16012024000001.d: +MS, 0.5±0.5min, #2-56
194.1085
195.1149
196.1190
Calculated:
MHD_R549_APCI_pos_MeCN_16012024000001.d: C15H14, M, 1+, 194.1090
1+
194.1090
1+
195.1124
m/z

2.17.7 IR

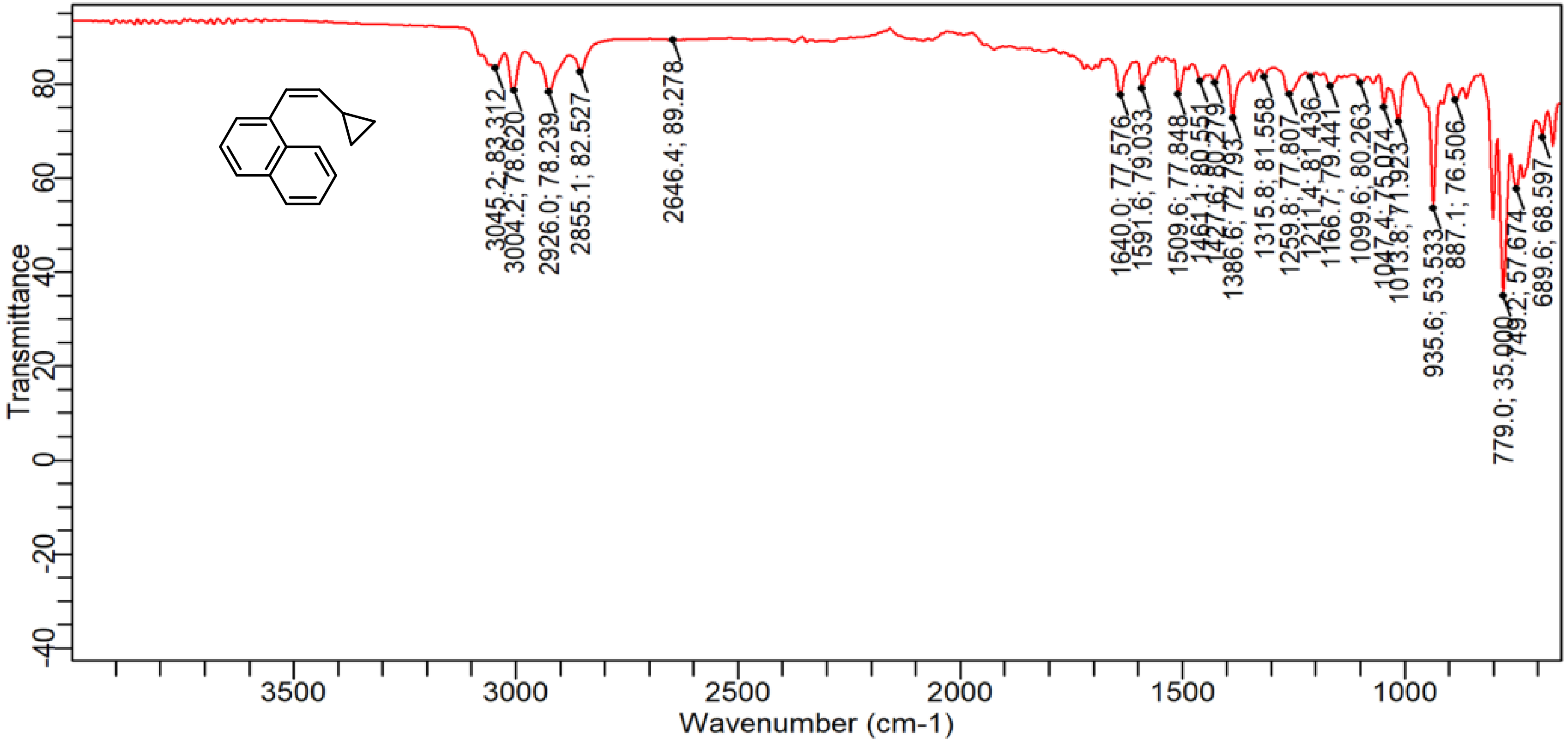

Transmittance
Wavenumber (cm-1)
3045.2; 83.312
3004.2; 78.620
2926.0; 78.239
2855.1; 82.527
2646.4; 89.278
1640.0; 77.576
1591.6; 79.033
1509.6; 77.848
1461.1; 80.551
1427.6; 80.279
1386.6; 72.793
1315.8; 81.558
1259.8; 77.807
1211.4; 81.436
1166.7; 79.441
1099.6; 80.263
1047.4; 75.074
1013.8; 71.923
935.6; 53.533
887.1; 76.506
779.0; 35.000
749.2; 57.674
689.6; 68.597

2.18 (Z)-(3,3-dimethylbut-1-en-1-yl)benzene (2r)

2.18.1 GC spectra of the crude mixture

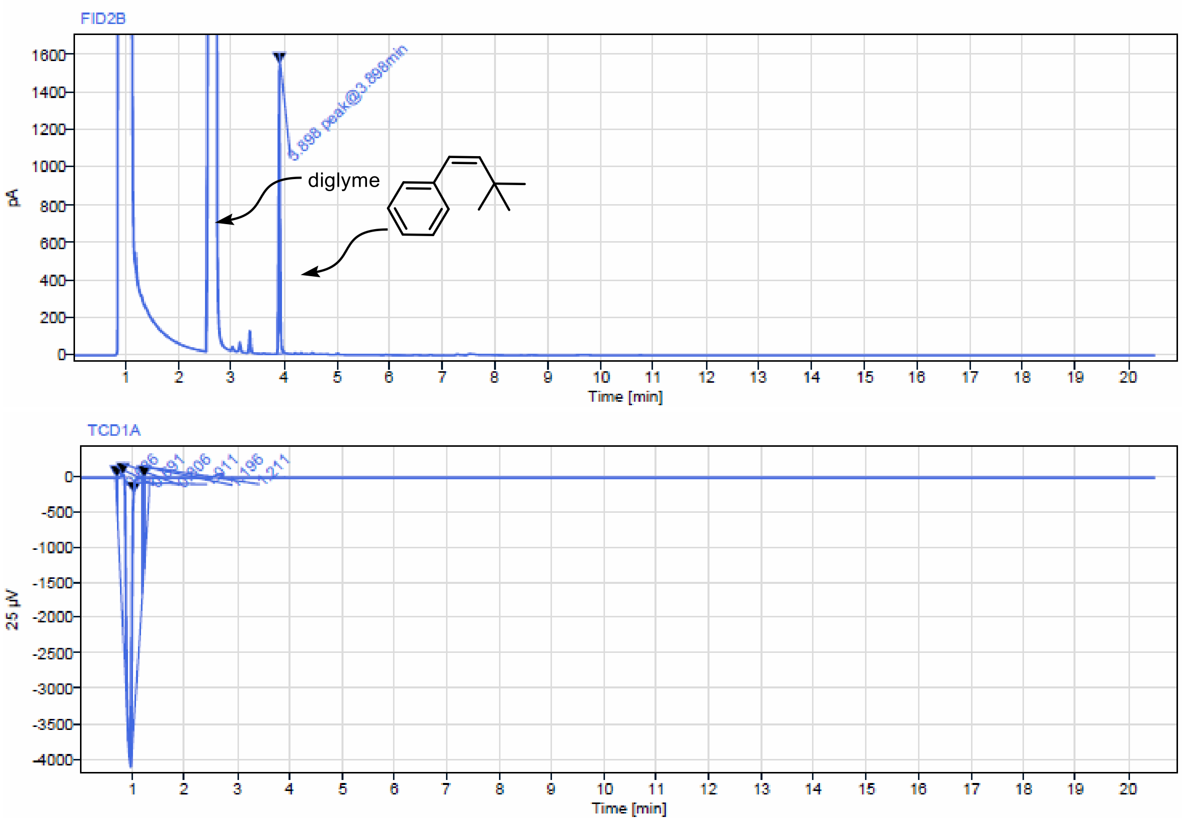

Signal: FID2B

Name	RT [min]	RF	Area	Peak Area Percent	Concentration []	Group
peak@3.898min	3.90		2922.451	100.00		

2.18.2 GCMS spectra of the crude mixture

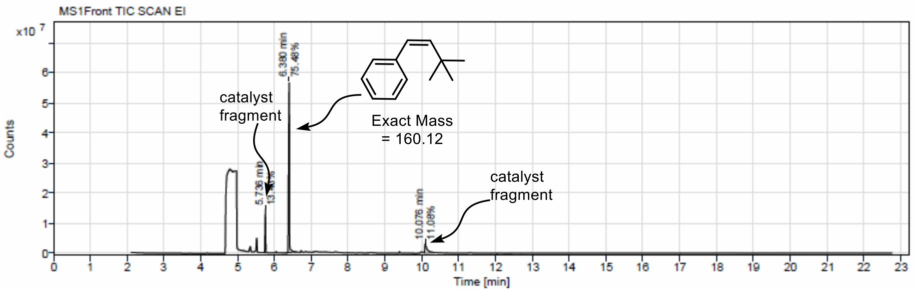

Signal: MS1Front TIC SCAN EI

RT	m/z	Spectral purity (%)	Peak area	Area %
5.736			16520000	13.43

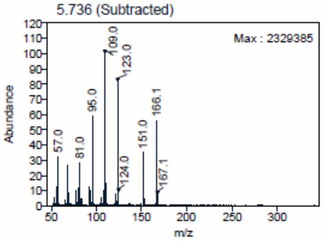

RT	m/z	Spectral purity (%)	Peak area	Area %
6.380			92850000	75.48

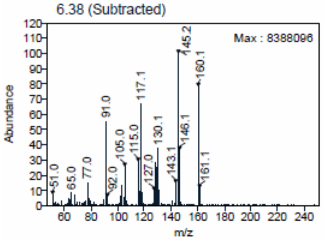

RT	m/z	Spectral purity (%)	Peak area	Area %
10.076			13630000	11.08

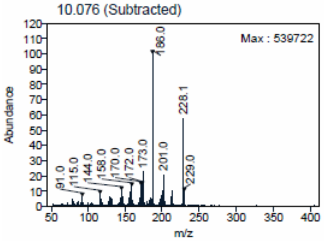

2.18.3 GC spectra of alkyne 1r

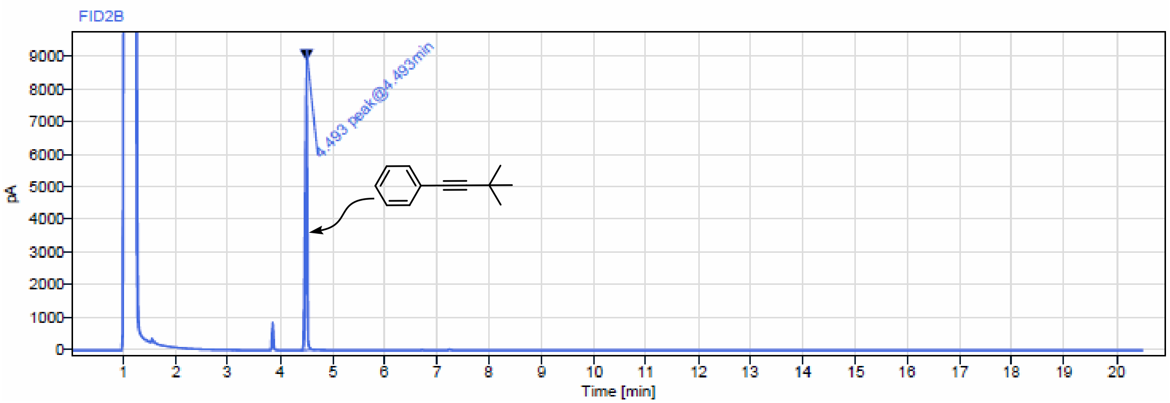

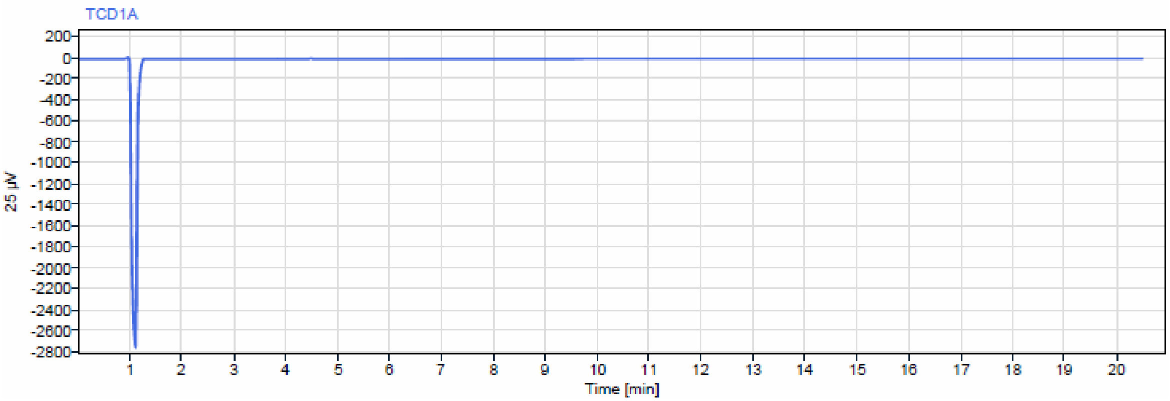

Signal: FID2B

Name	RT [min]	RF	Area	Peak Area Percent	Concentration []	Group
peak@4.493min	4.49		19432.778	100.00		

2.18.4 GCMS spectra of alkyne 1r

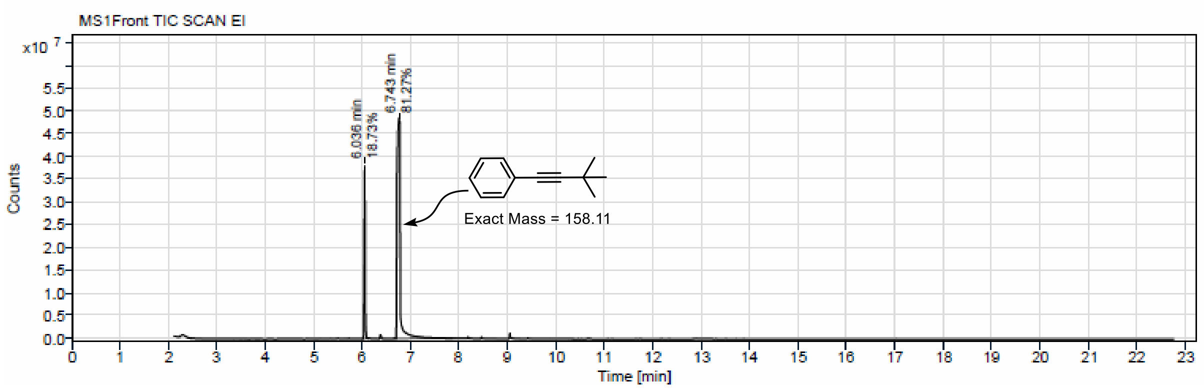

Signal: MS1Front TIC SCAN EI

RT	m/z	Spectral purity (%)	Peak area	Area %
6.036			52520000	18.73

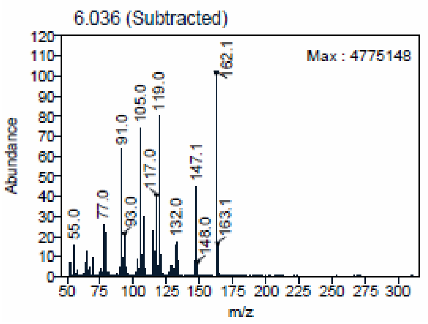

RT	m/z	Spectral purity (%)	Peak area	Area %
6.743			227900000	81.27

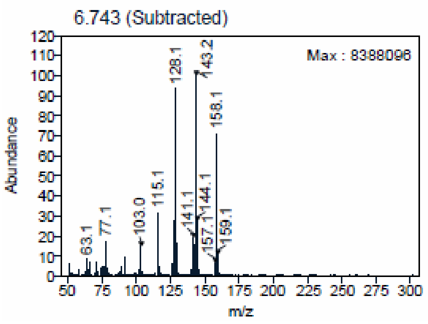

2.19 1-((*Z*)-Styryl)adamantane (2s) (600 MHz, $CDCl_3$)

2.19.1 ^{1}H NMR

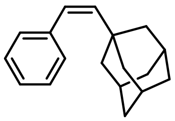

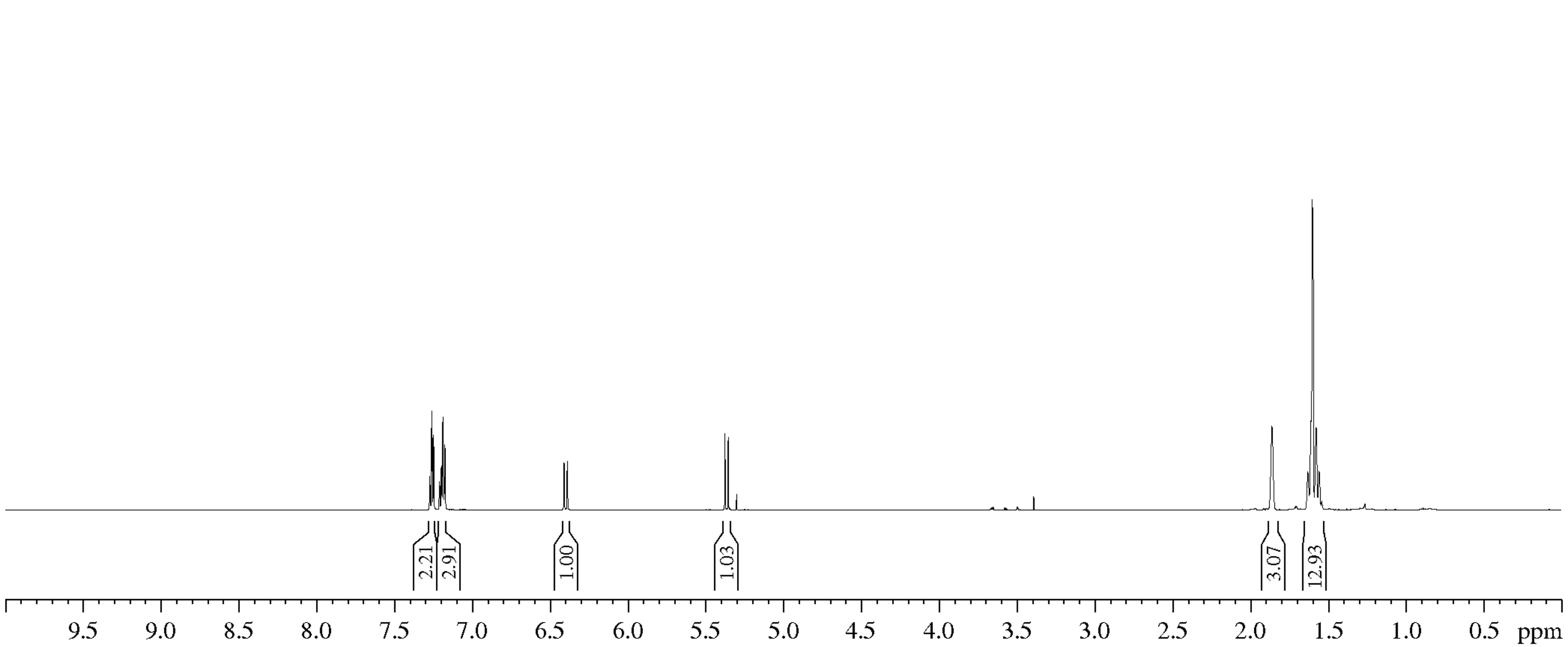

2.19.2 ^{13}C NMR

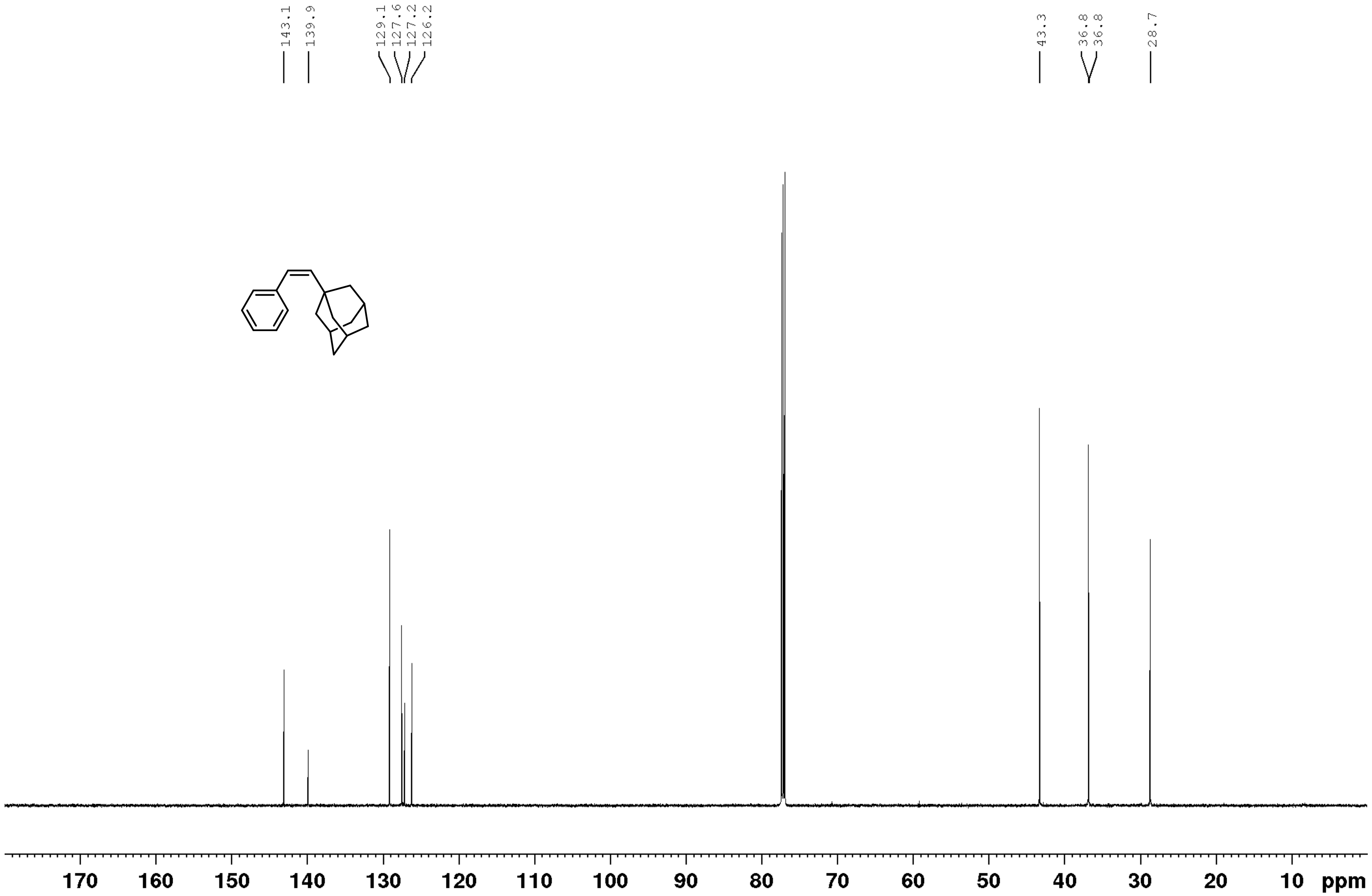
143.1
139.9
129.1
127.6
127.2
126.2
43.3
36.8
36.8
28.7
170
160
150
140
130
120
110
100
90
80
70
60
50
40
30
20
10
ppm

2.19.3 ^{1}H, ^{1}H COSY

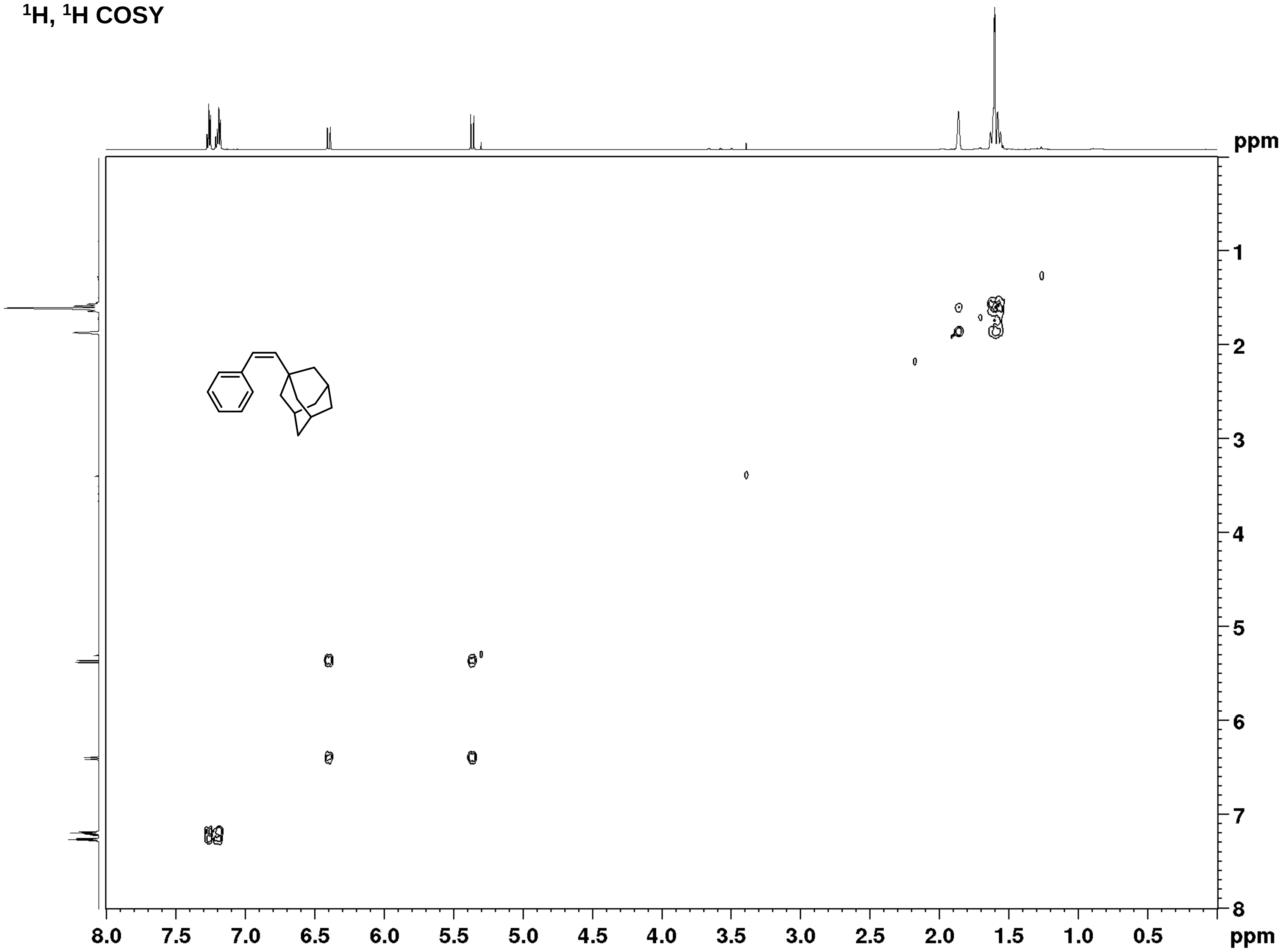

2.19.4 ^{1}H, ^{13}C HMBC

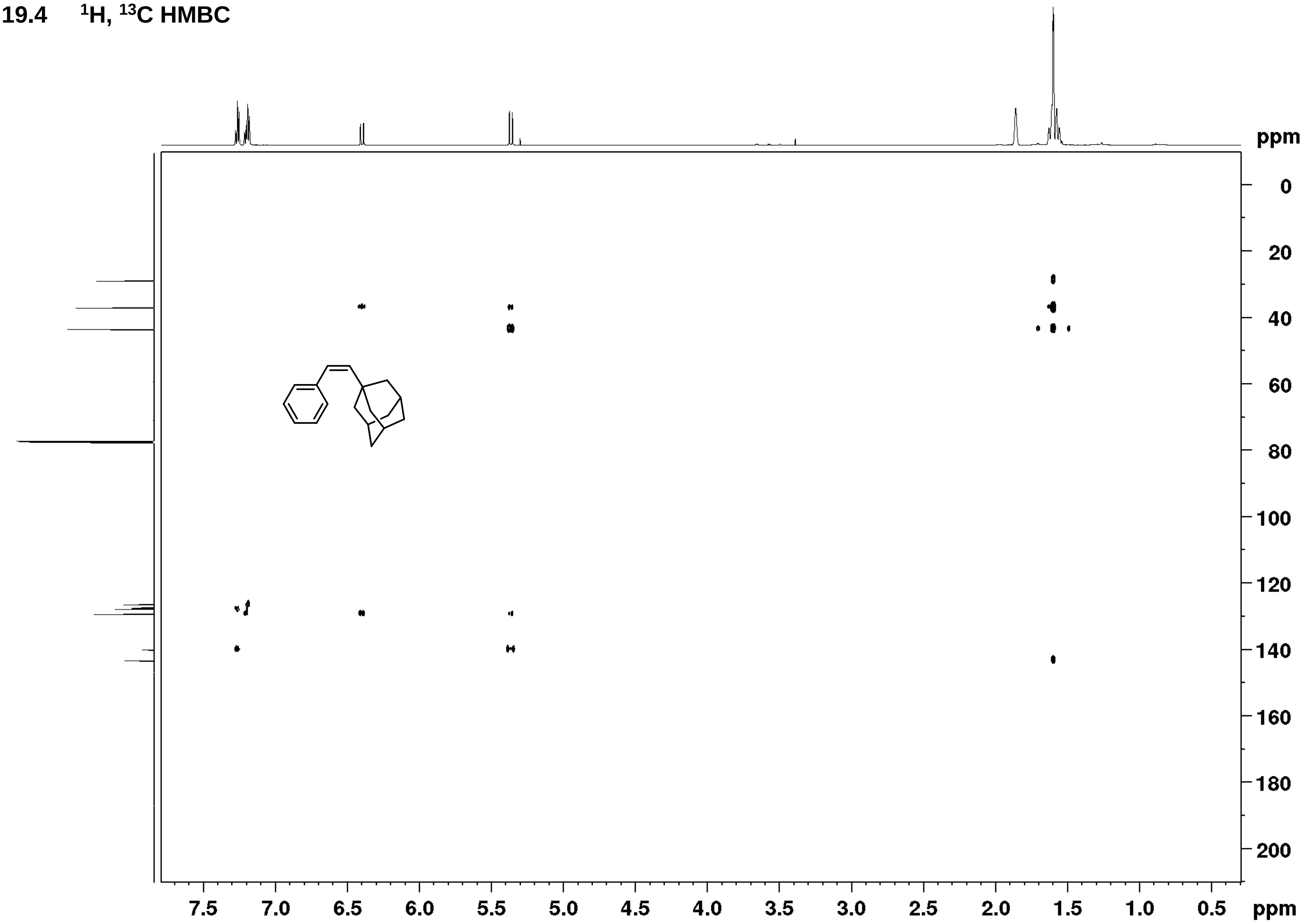

2.19.5 ^{1}H, ^{13}C HSQC

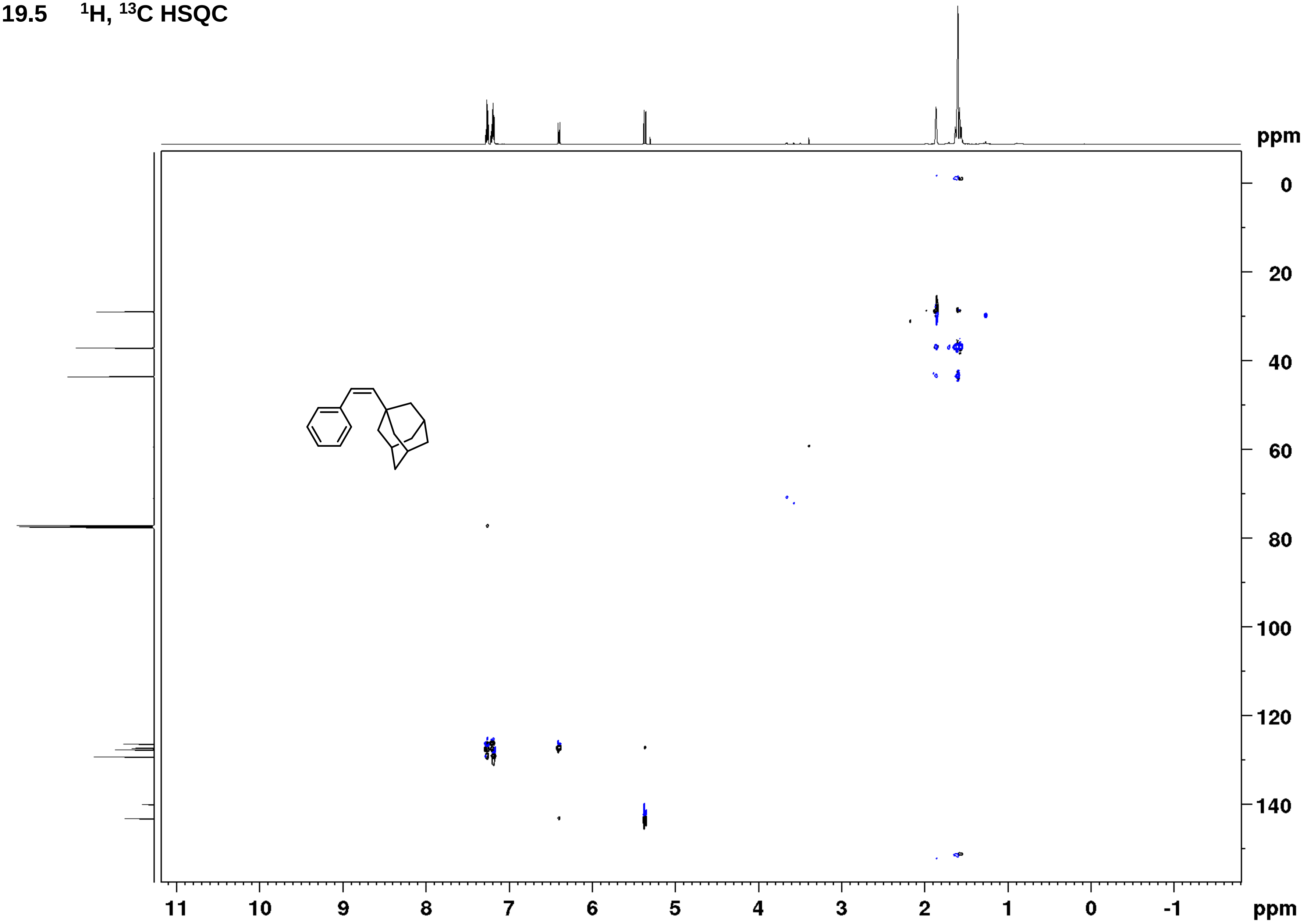

2.19.6 HRMS

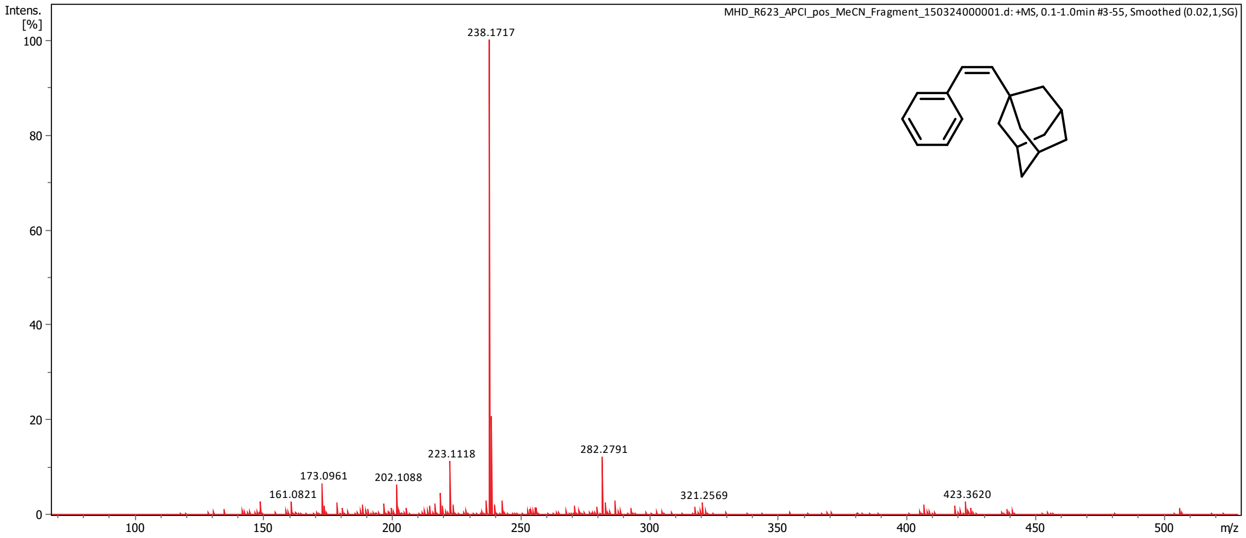

Intens. [%]
MHD_R623_APCI_pos_MeCN_Fragment_150324000001.d: +MS, 0.1-1.0min #3-55, Smoothed (0.02,1,SG)
238.1717
282.2791
223.1118
173.0961
202.1088
161.0821
321.2569
423.3620
m/z

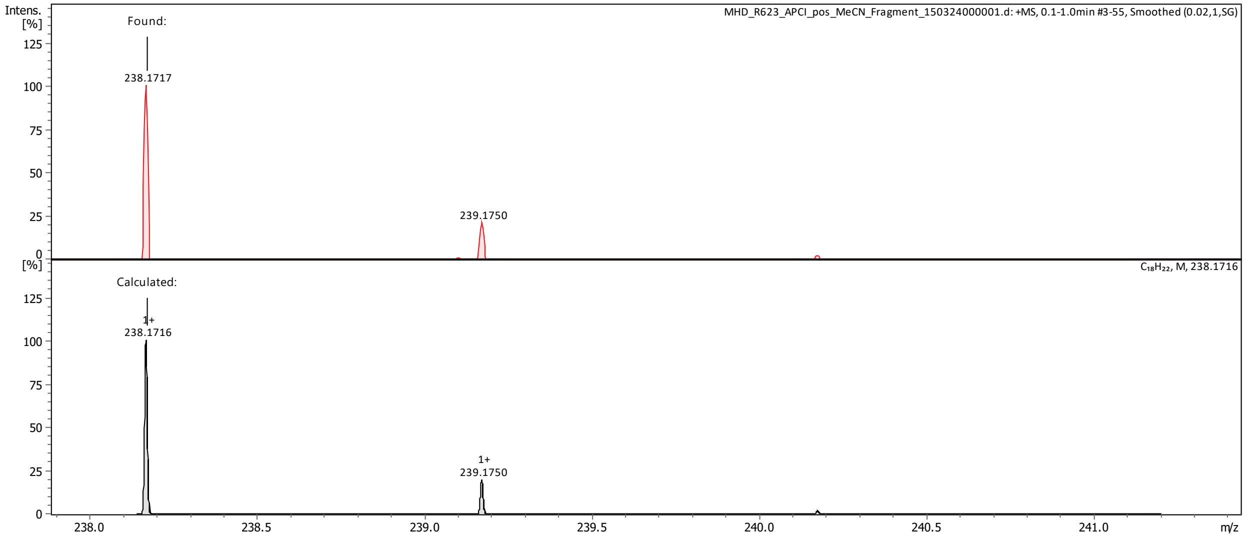

Intens. [%]
MHD_R623_APCI_pos_MeCN_Fragment_150324000001.d: +MS, 0.1-1.0min #3-55, Smoothed (0.02,1,SG)
Found:
238.1717
239.1750
Calculated:
$C_{18}H_{22}$, M, 238.1716
1+
238.1716
1+
239.1750
m/z

2.19.7 IR

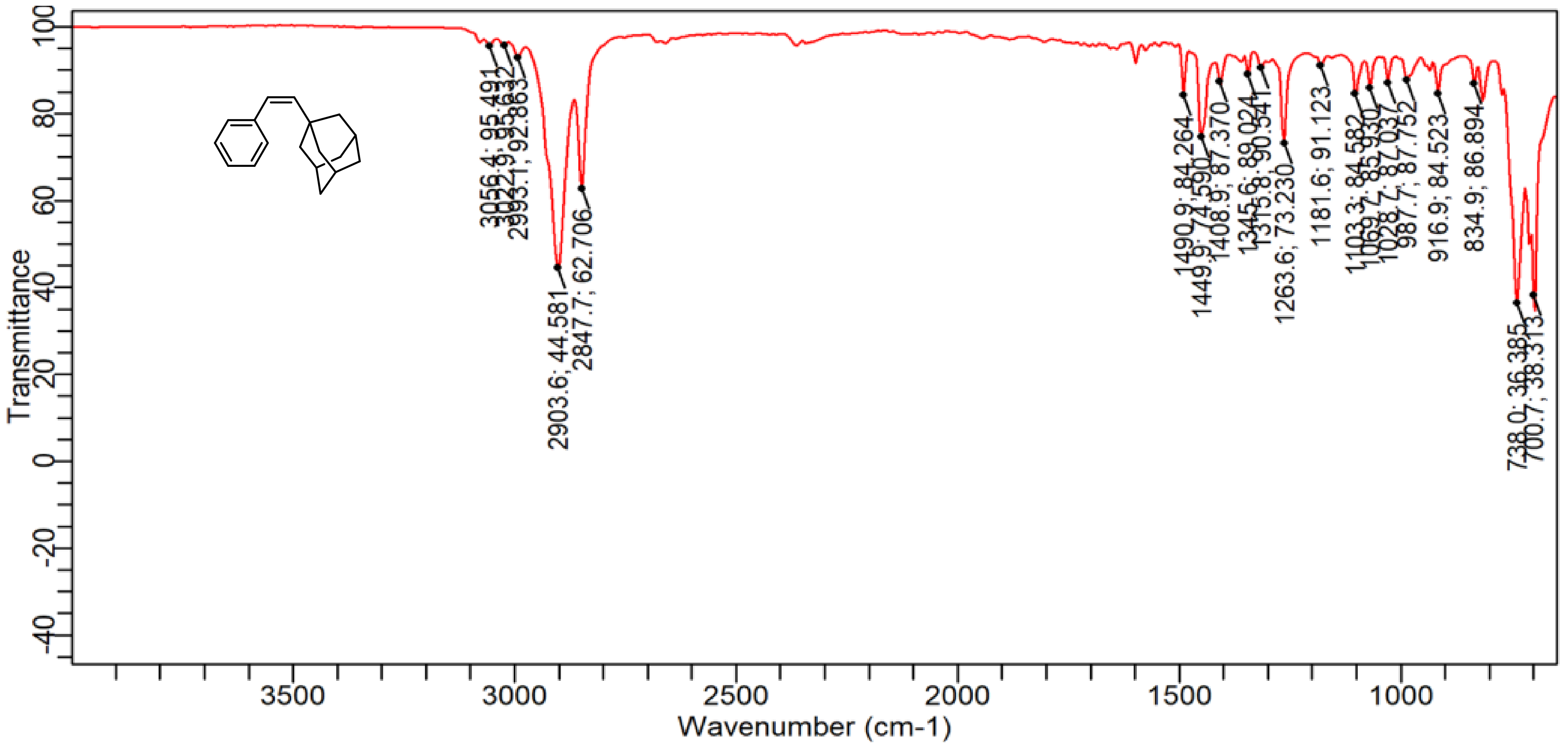

2.20 (*Z*)-4,4-Difluoro-1-(6-phenylhex-5-en-1-yl)piperidine (2t) (600 MHz, $CDCl_3$)

2.20.1 ^{1}H NMR

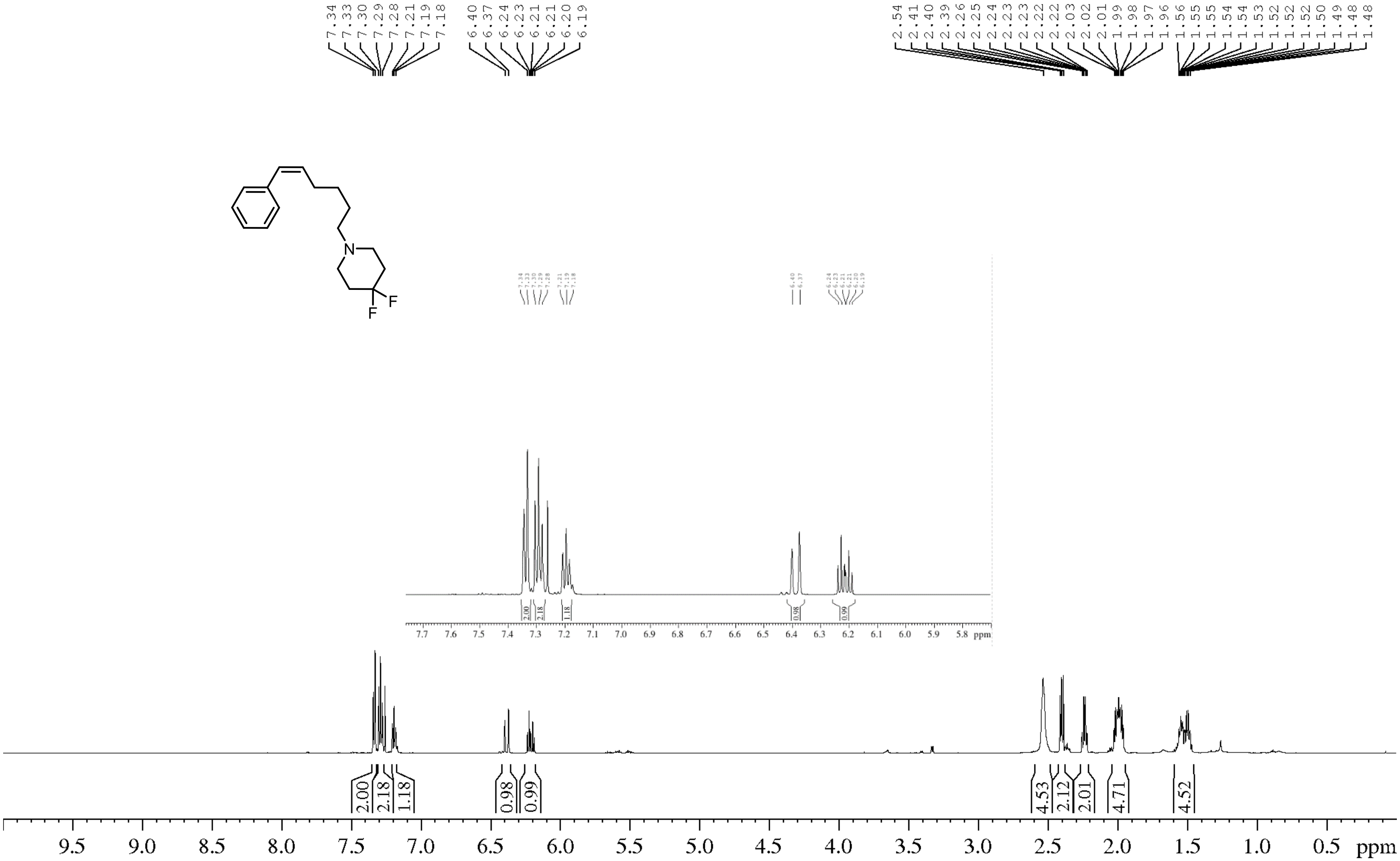

2.20.2 ^{13}C NMR

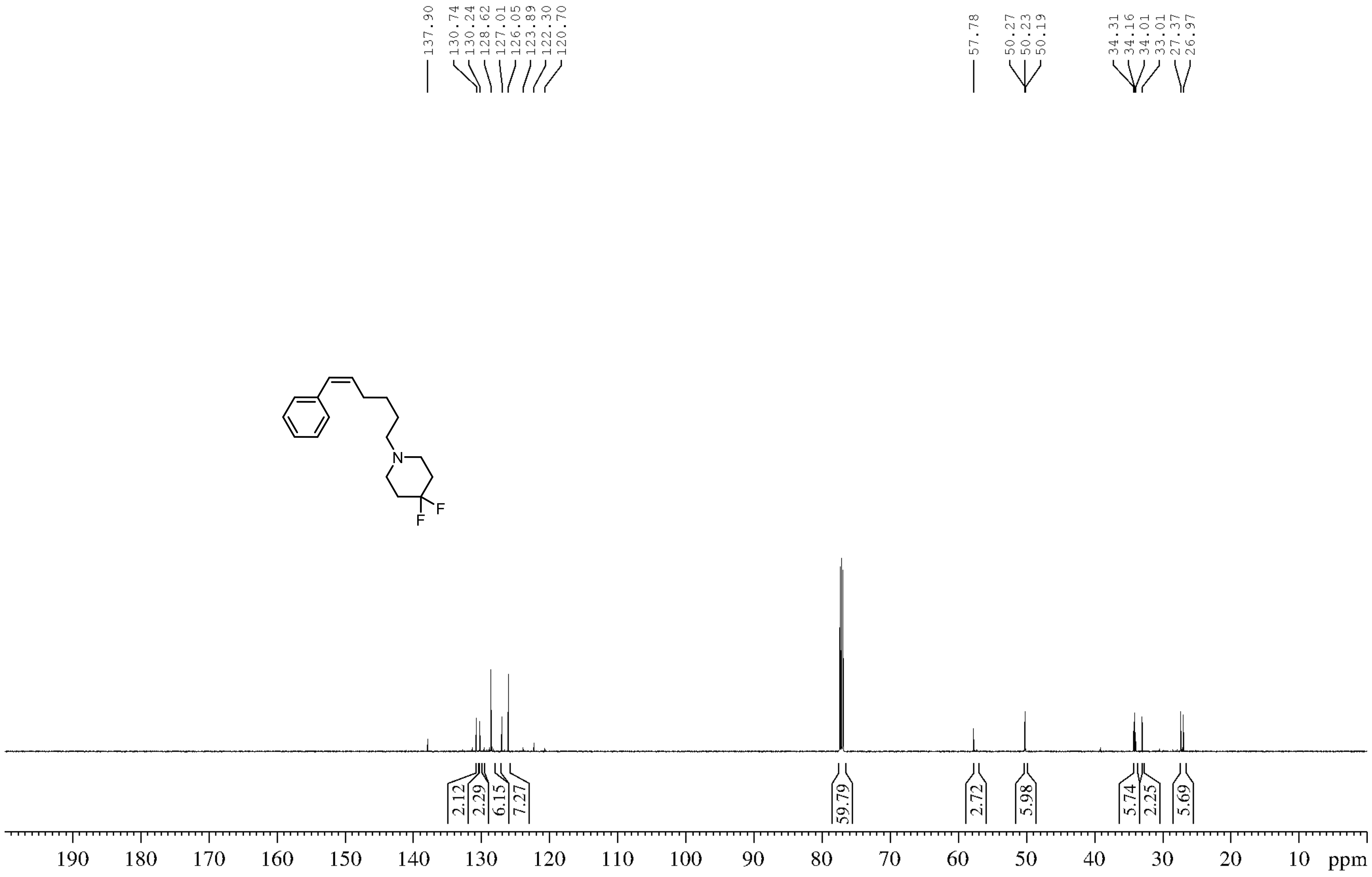

137.90
130.74
130.24
128.62
127.01
126.05
123.89
122.30
120.70
57.78
50.27
50.23
50.19
34.31
34.16
34.01
33.01
27.37
26.97
2.12
2.29
6.15
7.27
59.79
2.72
5.98
5.74
2.25
5.69
190 180 170 160 150 140 130 120 110 100 90 80 70 60 50 40 30 20 10 ppm

2.20.3 ¹H, ¹H COSY

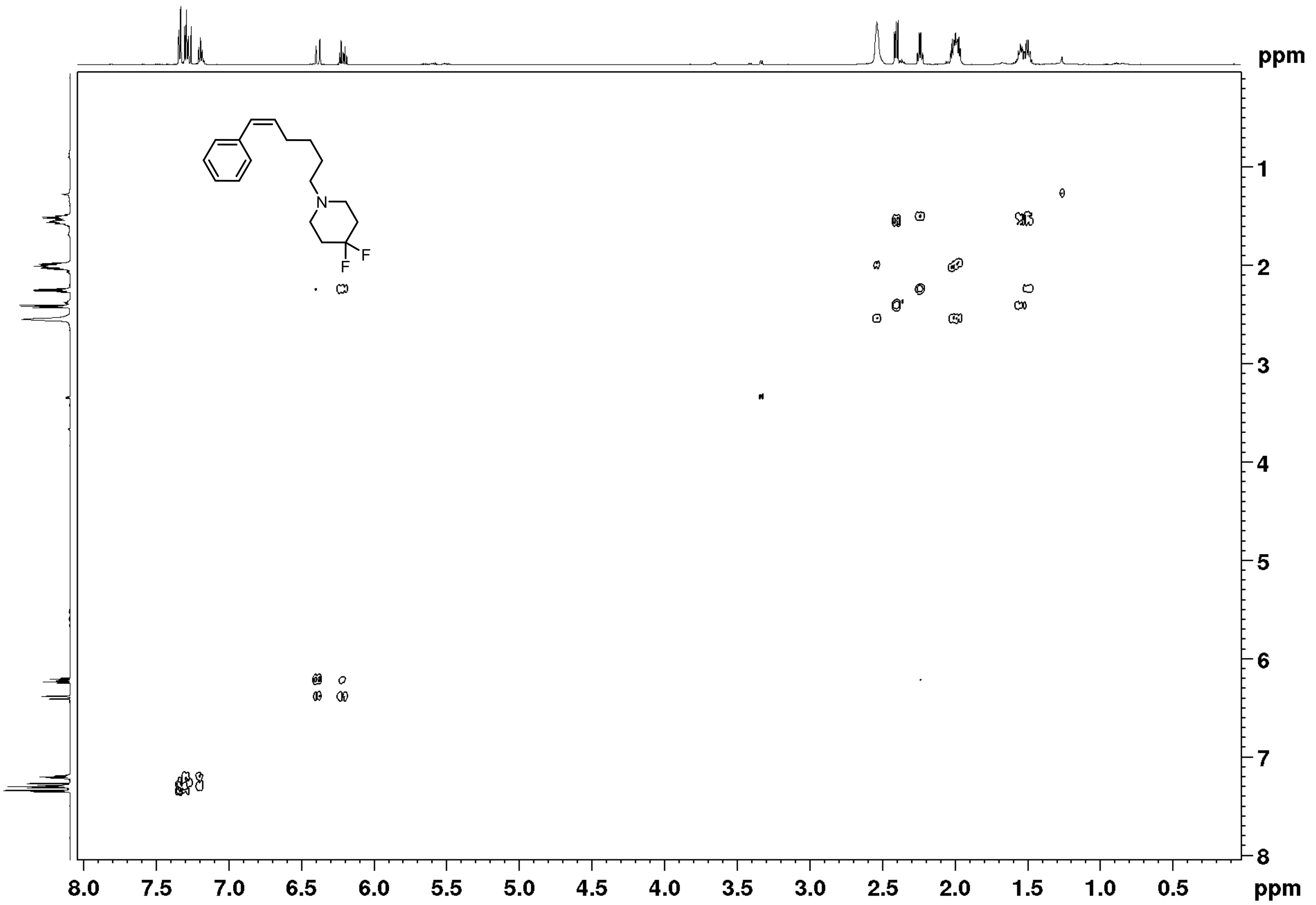

2.20.4 ^{1}H, ^{13}C HMBC

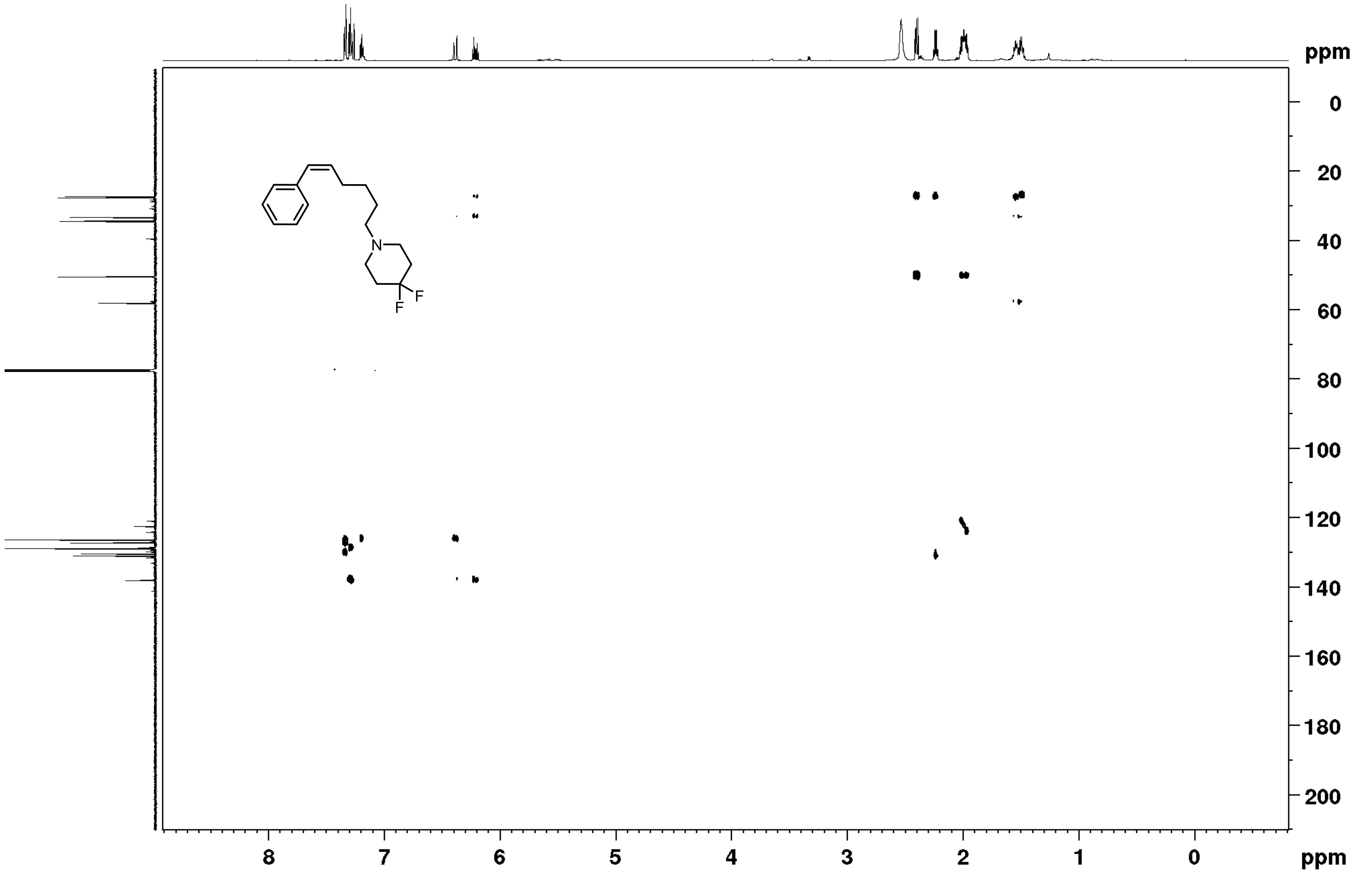

2.20.5 ^{1}H, ^{13}C HSQC

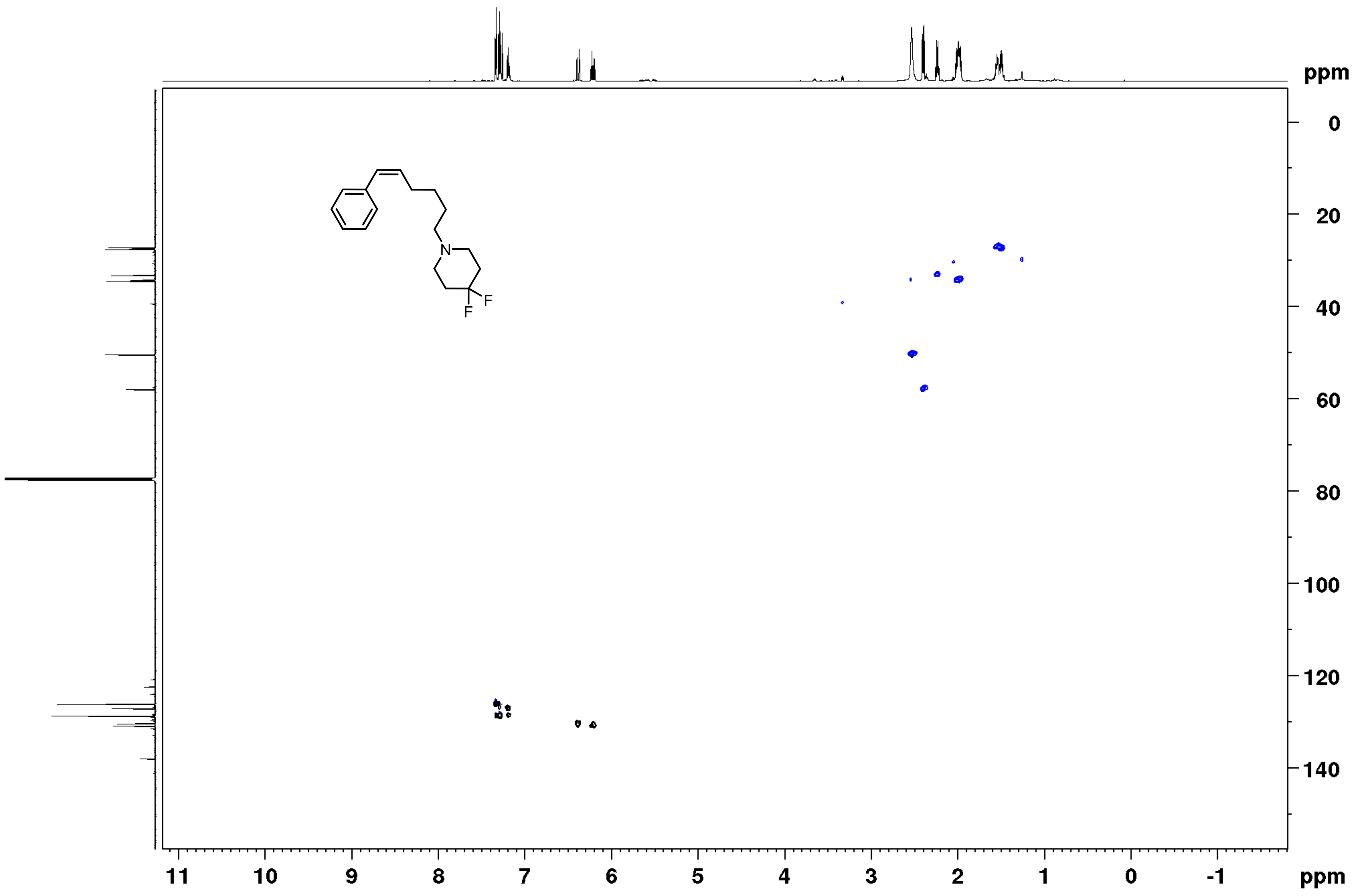

2.20.6 ^{19}F NMR

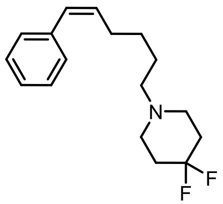

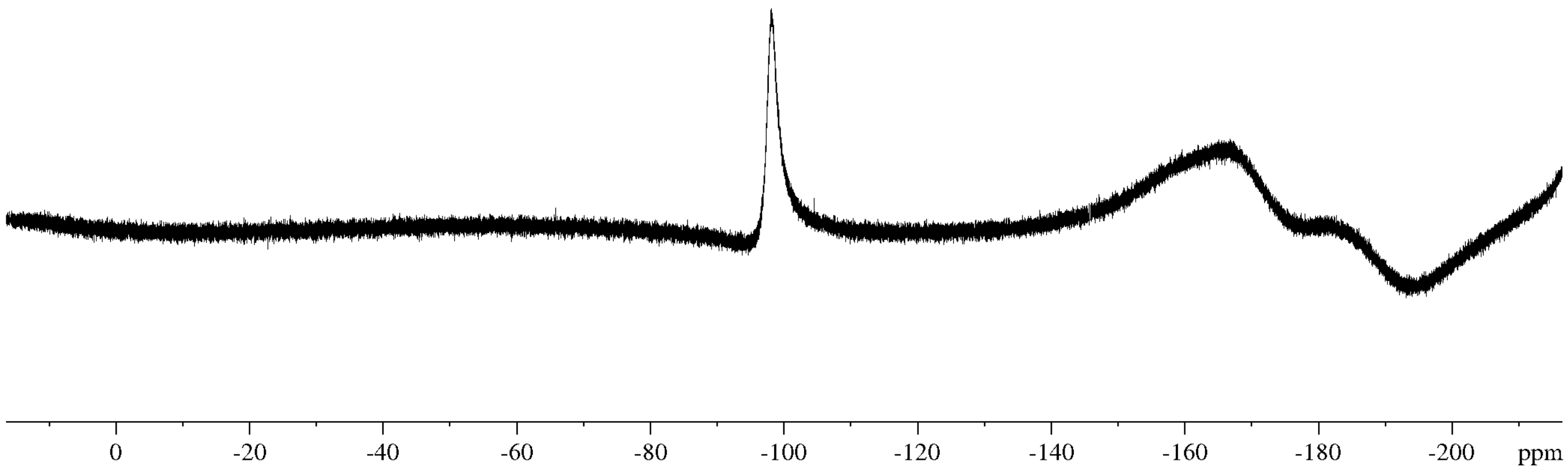

2.20.7 HRMS

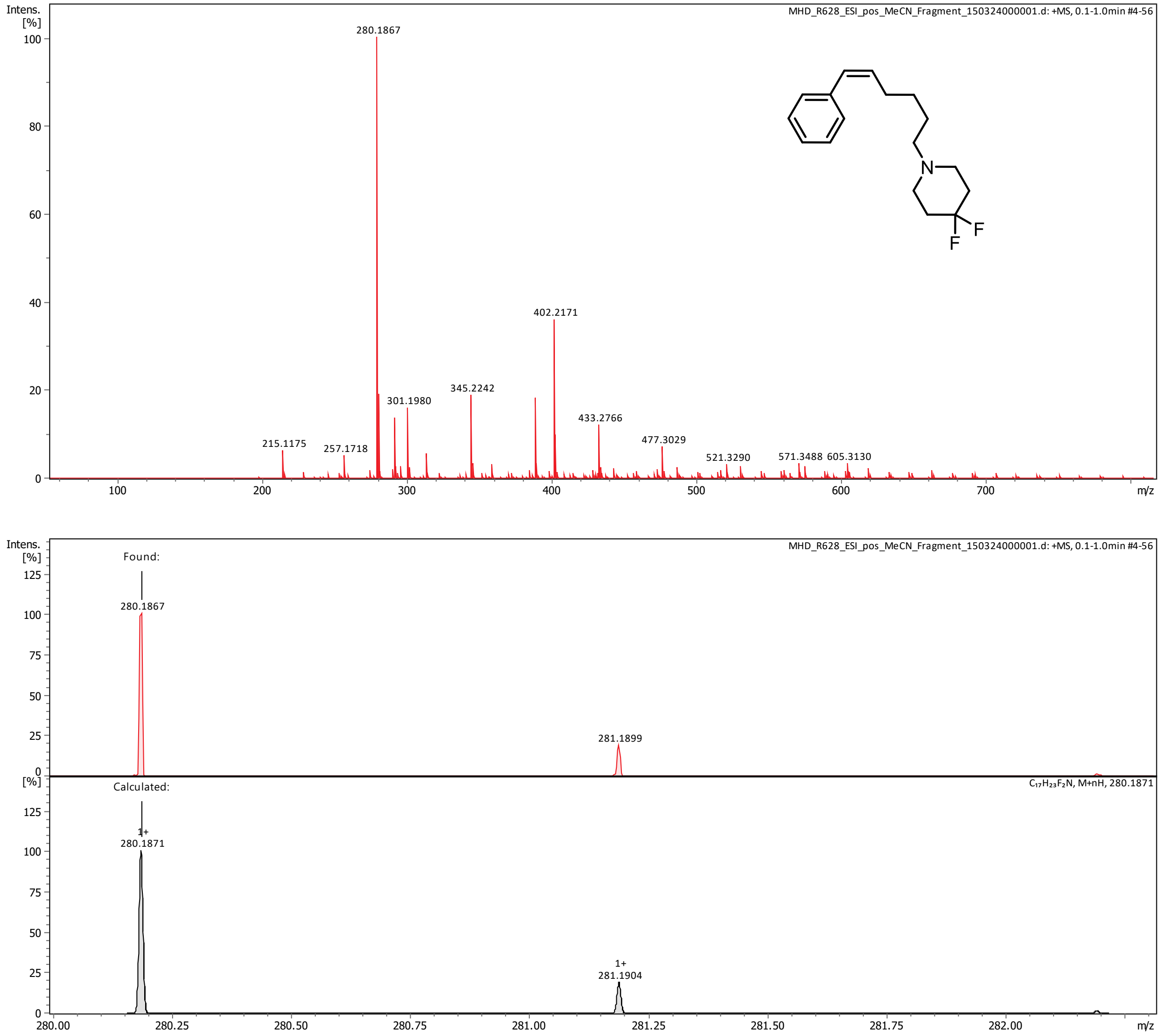

Intens. [%]
MHD_R628_ESI_pos_MeCN_Fragment_150324000001.d: +MS, 0.1-1.0min #4-56
280.1867
402.2171
345.2242
301.1980
433.2766
215.1175
257.1718
477.3029
521.3290
571.3488 605.3130
F
F
N
m/z
Found:
280.1867
281.1899
Calculated:
C17H23F2N, M+nH, 280.1871
1+
280.1871
1+
281.1904

2.20.8 IR

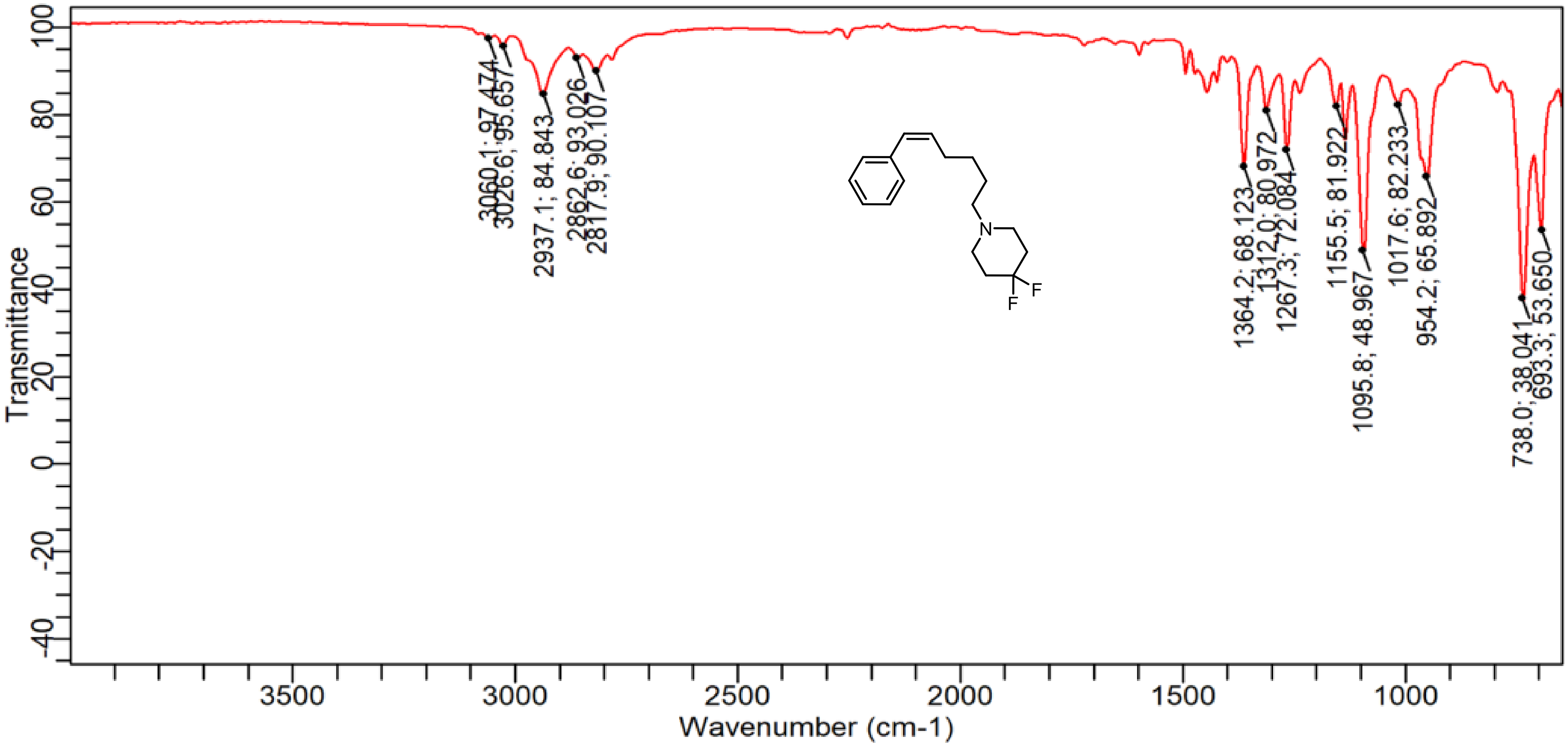

Transmittance
Wavenumber (cm-1)
3060.1; 97.474
3026.6; 95.657
2937.1; 84.843
2862.6; 93.026
2817.9; 90.107
1364.2; 68.123
1312.0; 80.972
1267.3; 72.084
1155.5; 81.922
1095.8; 48.967
1017.6; 82.233
954.2; 65.892
738.0; 38.041
693.3; 53.650
N
F
F

2.21 (*Z*)-(6-Chlorohex-1-en-1-yl)benzene (2u) (600 MHz, $CDCl_3$)

2.21.1 ^{1}H NMR

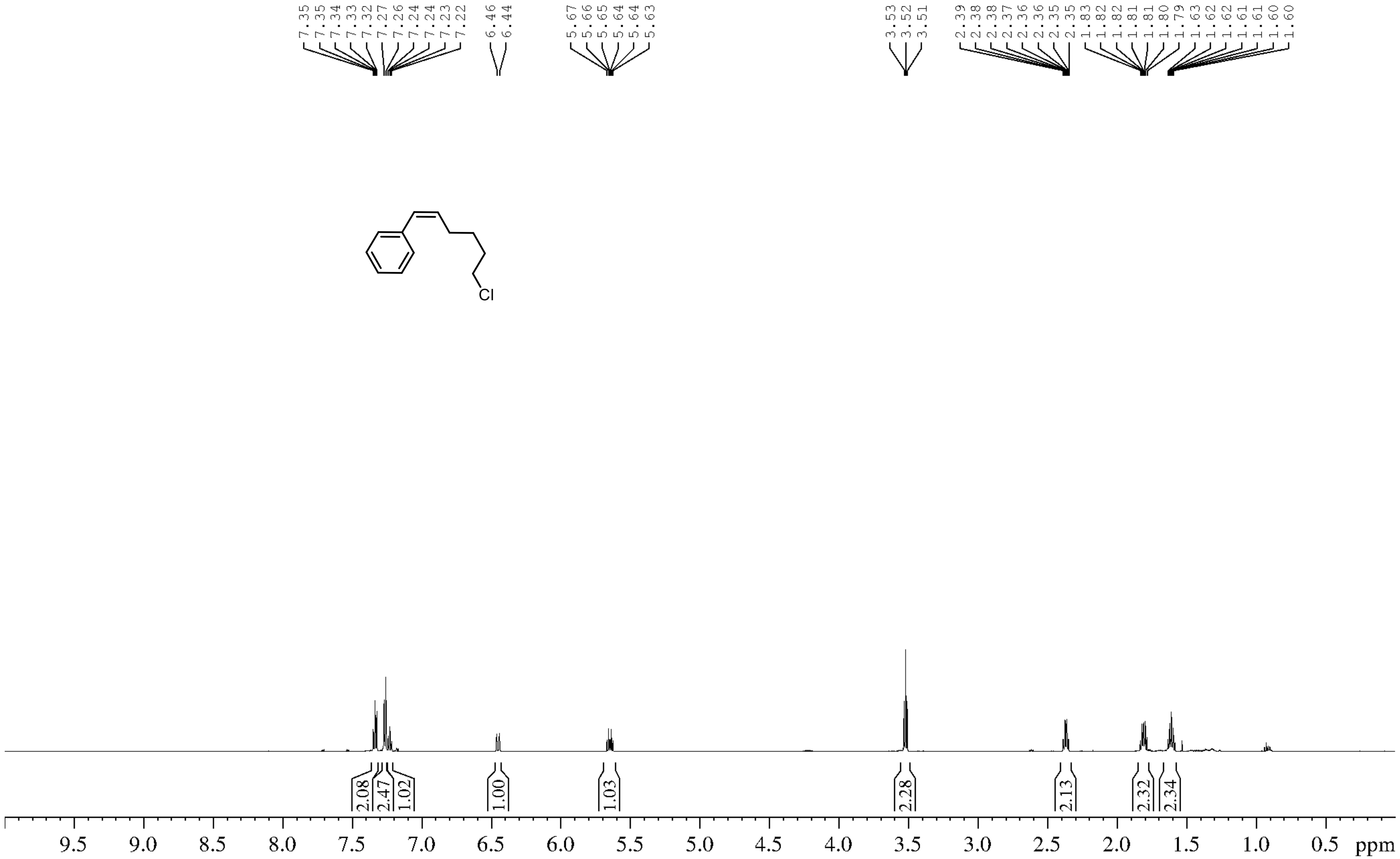

2.21.2 ^{13}C NMR

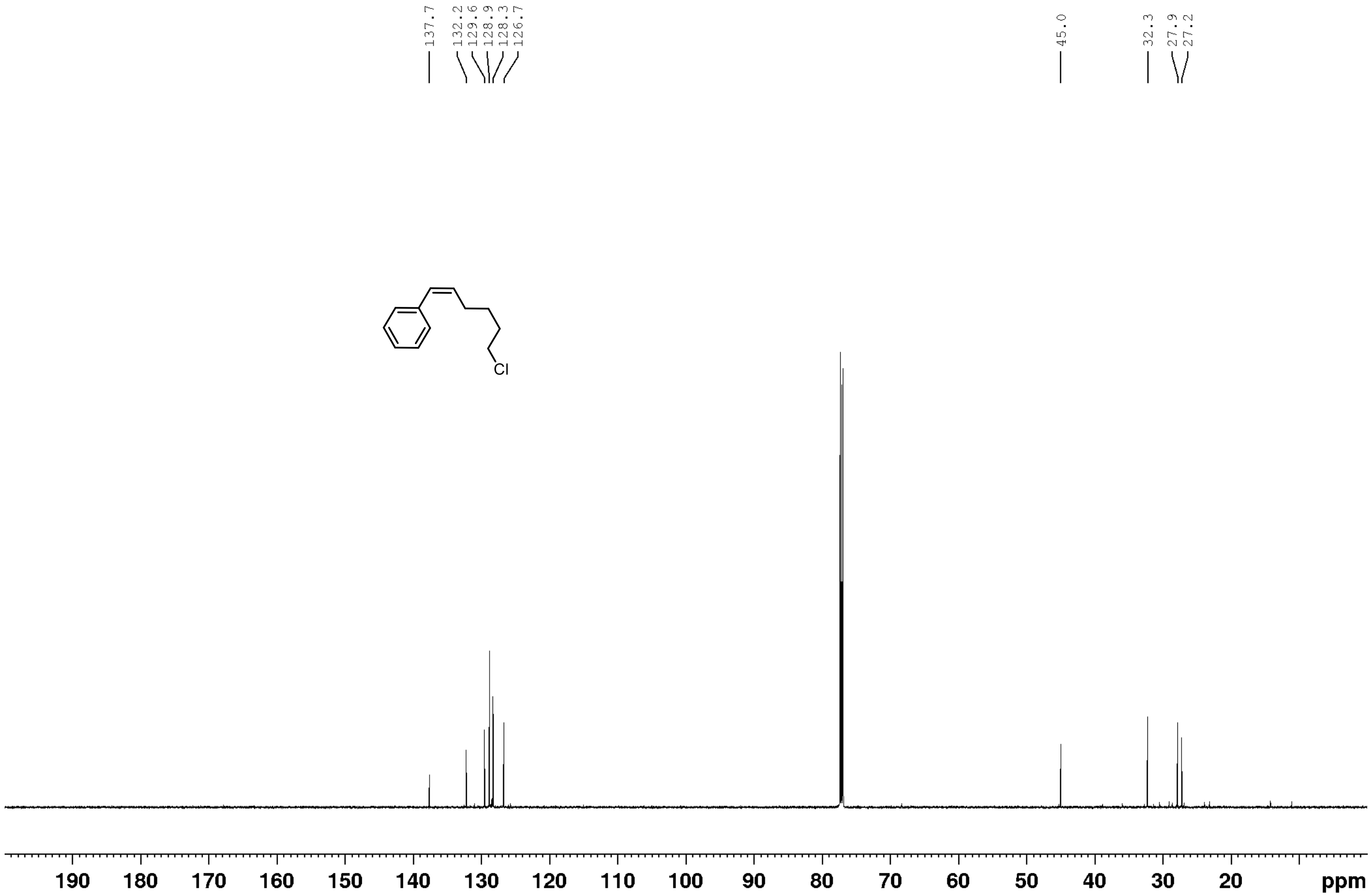
137.7
132.2
129.6
128.9
128.3
126.7
45.0
32.3
27.9
27.2
Cl
190 180 170 160 150 140 130 120 110 100 90 80 70 60 50 40 30 20
ppm

2.21.3 ^{1}H, ^{1}H COSY

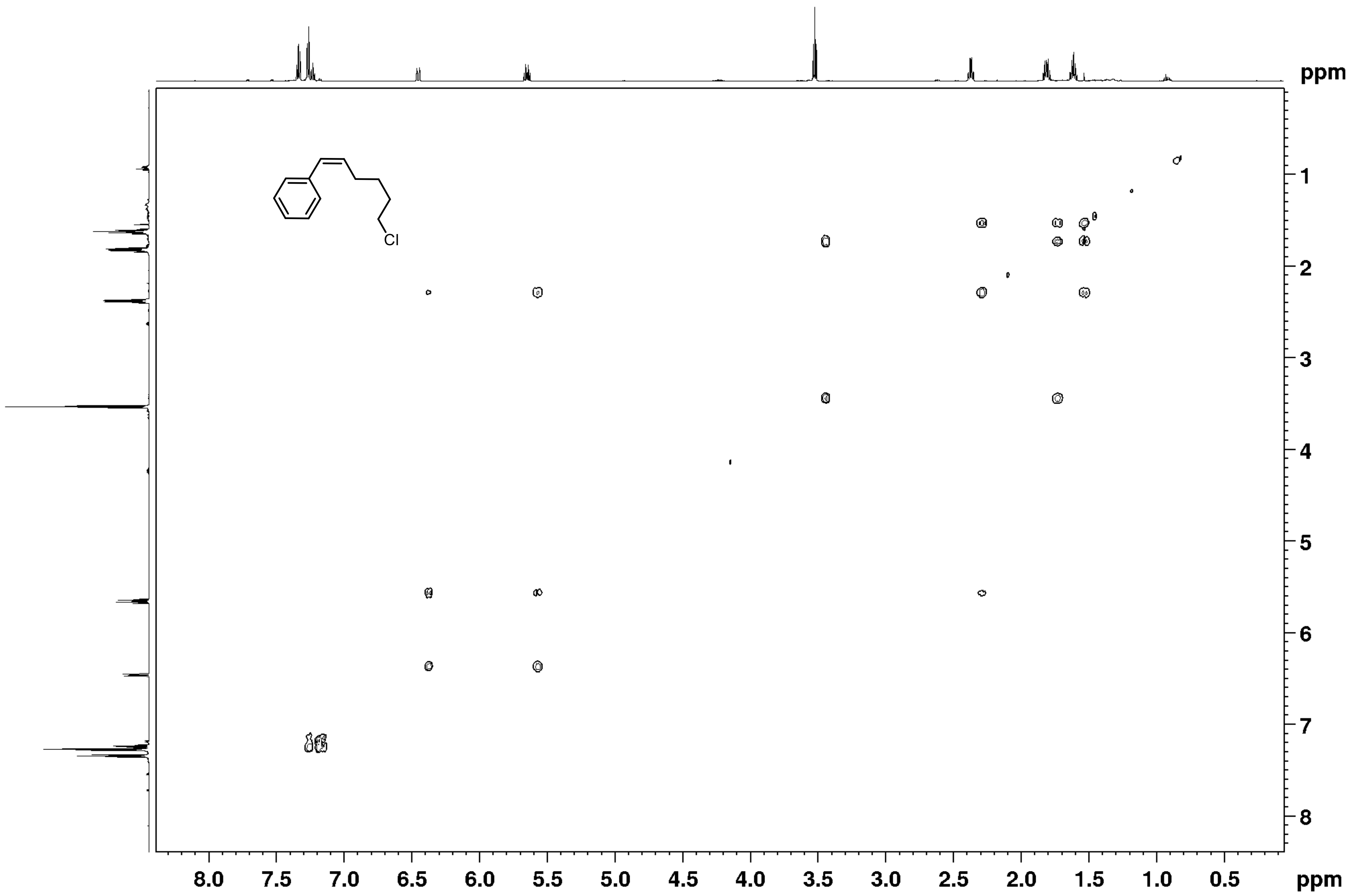

2.21.4 ^{1}H, ^{13}C HMBC

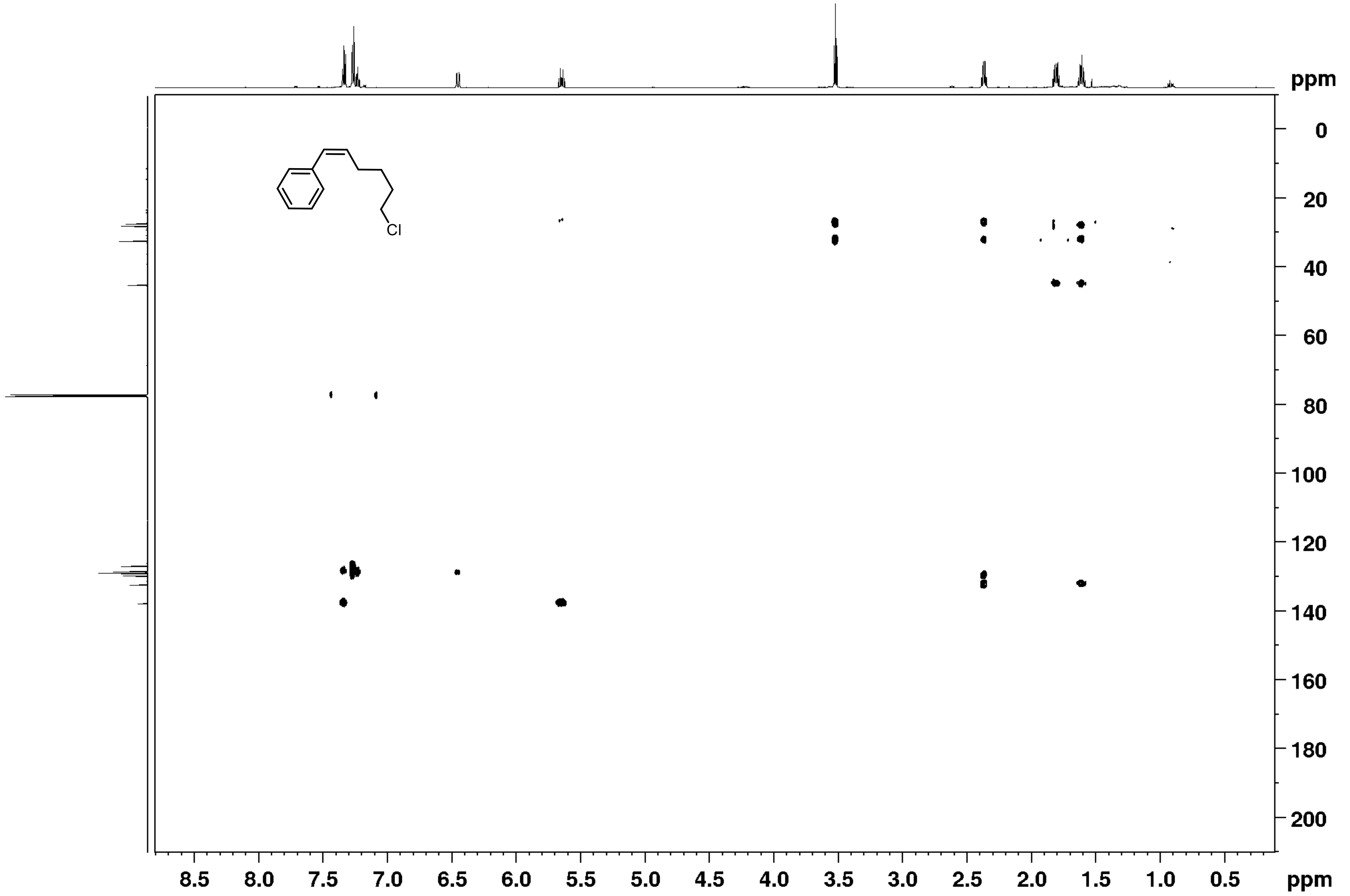

2.21.5 ^{1}H, ^{13}C HSQC

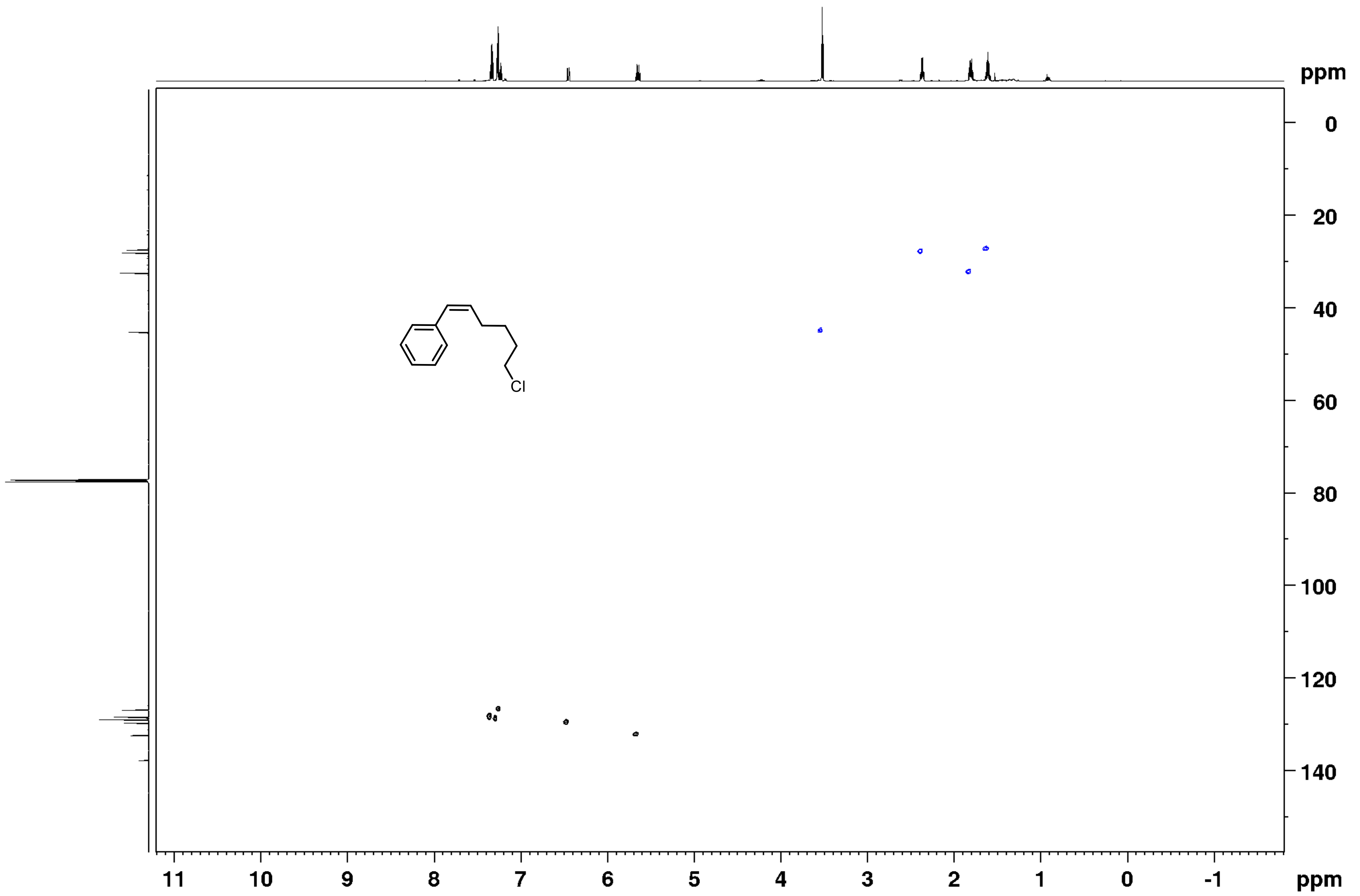

2.21.6 HRMS

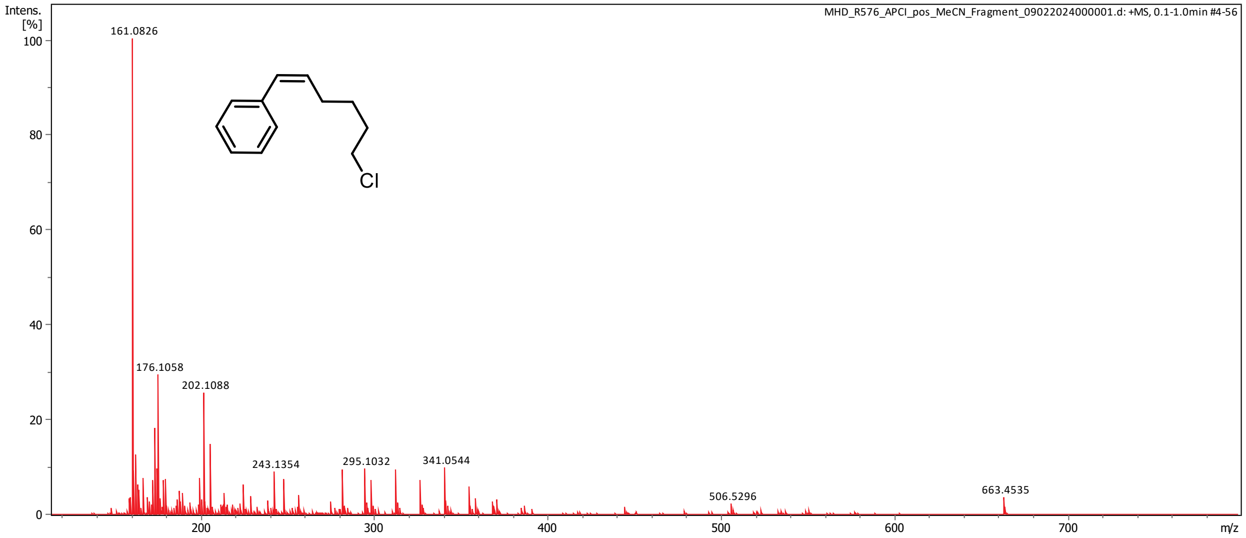

MHD_R576_APCI_pos_MeCN_Fragment_09022024000001.d: +MS, 0.1-1.0min #4-56
Intens. [%]
161.0826
176.1058
202.1088
243.1354
295.1032
341.0544
506.5296
663.4535
Cl
m/z

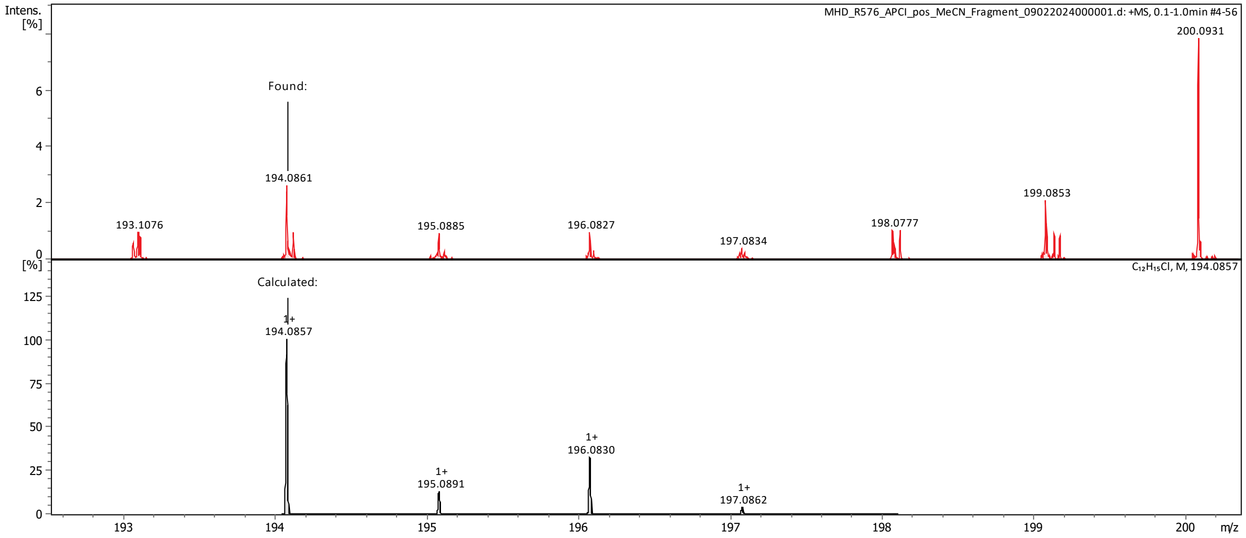

MHD_R576_APCI_pos_MeCN_Fragment_09022024000001.d: +MS, 0.1-1.0min #4-56
Intens. [%]
Found:
194.0861
193.1076
195.0885
196.0827
197.0834
198.0777
199.0853
200.0931
$C_{12}H_{15}Cl$, M, 194.0857
Calculated:
1+ 194.0857
1+ 195.0891
1+ 196.0830
1+ 197.0862
m/z

2.21.7 IR

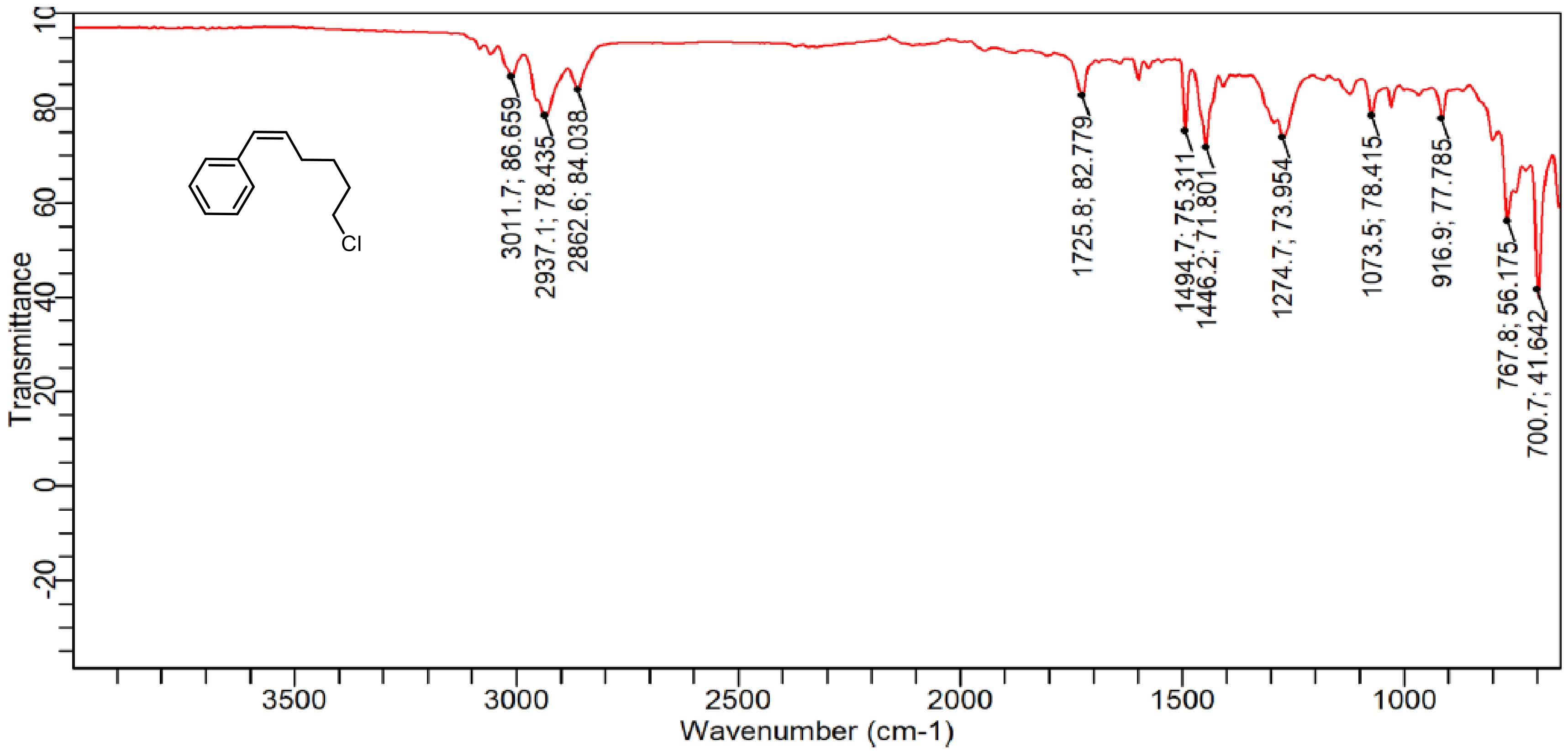

Cl
3011.7; 86.659
2937.1; 78.435
2862.6; 84.038
1725.8; 82.779
1494.7; 75.311
1446.2; 71.801
1274.7; 73.954
1073.5; 78.415
916.9; 77.785
767.8; 56.175
700.7; 41.642
Transmittance
Wavenumber (cm-1)

2.22 (*Z*)-Dec-5-ene (2v)

2.22.1 Crude GC chromatogram after hydrogenation at 1–2 bar

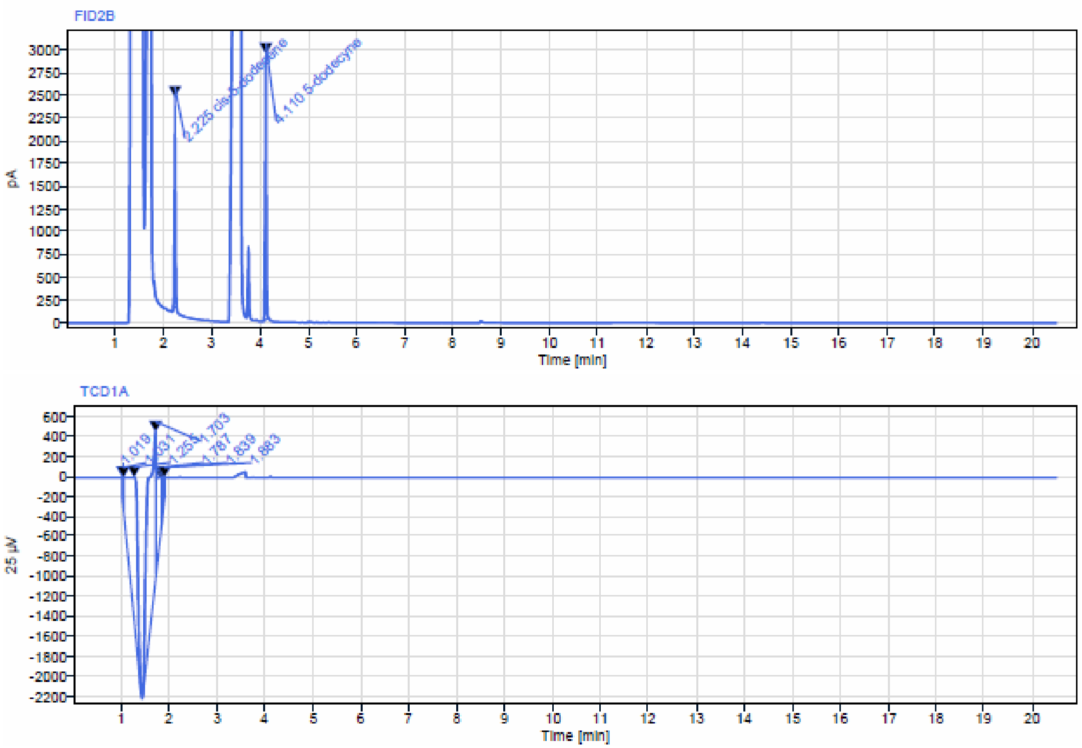

Signal: FID2B

Name	RT [min]	RF	Area	Peak Area Percent	Concentration []	Group
cis-5-dodecene	2.22		3225.568	43.09		
5-dodecyne	4.11		4259.523	56.91		

2.22.2 Crude GC chromatogram after hydrogenation at 100 bar

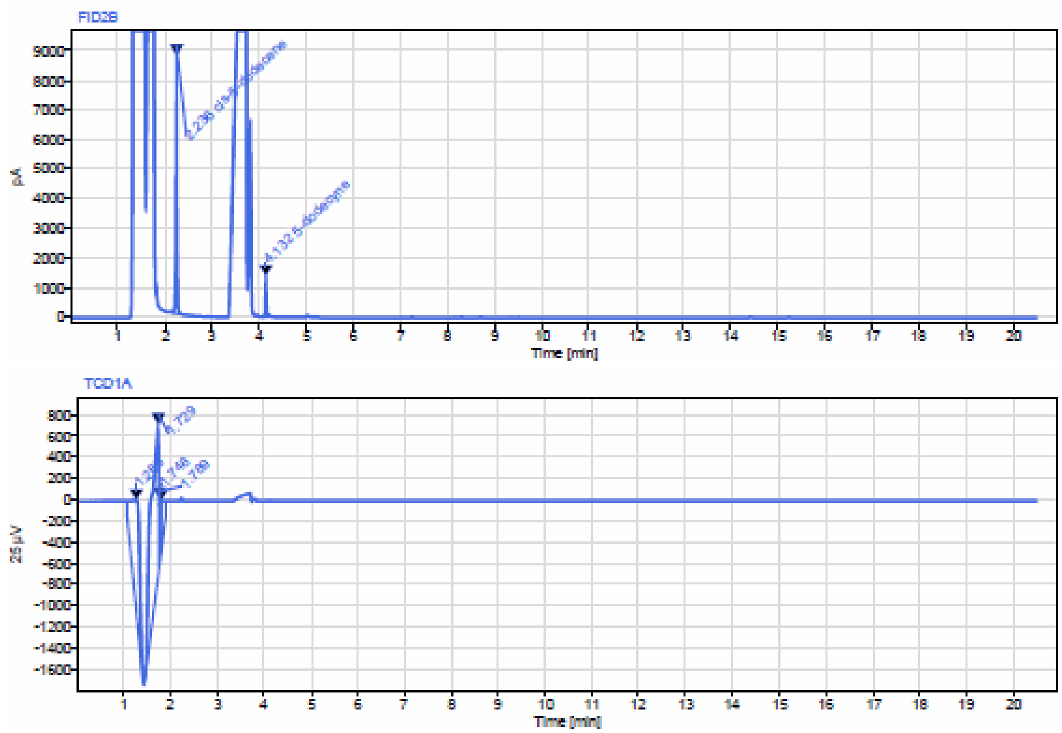

Signal:	FID2B					
Name	RT [min]	RF	Area	Peak Area Percent	Concentration []	Group
cis-5-dodecene	2.24		11629.636	85.33		
5-dodecyne	4.13		2000.030	14.67		

2.22.3 ^{1}H NMR

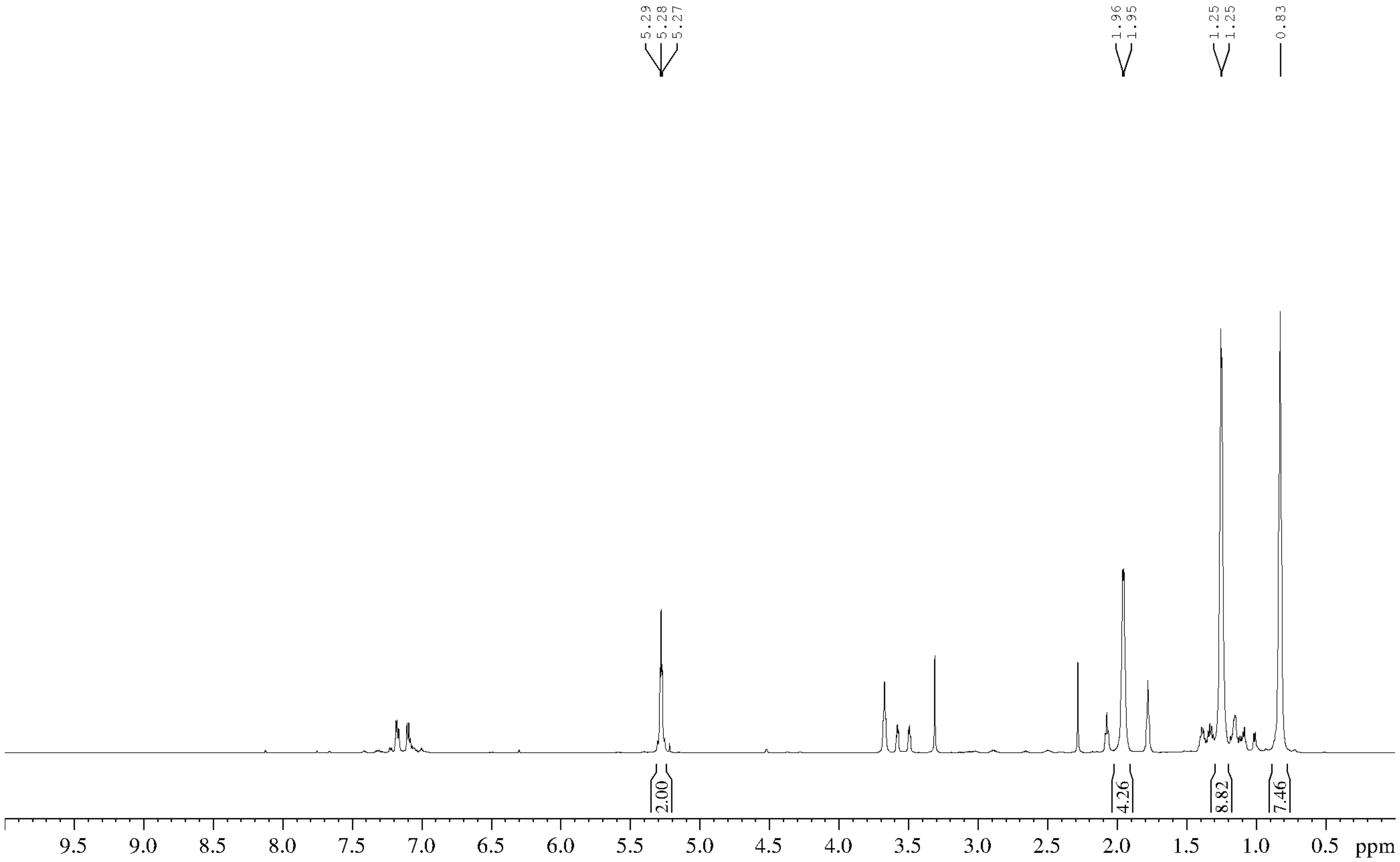
5.29
5.28
5.27
1.96
1.95
1.25
1.25
0.83
2.00
4.26
8.82
7.46
9.5
9.0
8.5
8.0
7.5
7.0
6.5
6.0
5.5
5.0
4.5
4.0
3.5
3.0
2.5
2.0
1.5
1.0
0.5
ppm

2.23 (*Z*)-2-(4-Methoxystyryl)pyridine (2w) (600 MHz, $CDCl_3$)

2.23.1 ^{1}H NMR

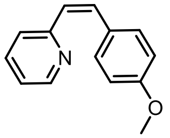

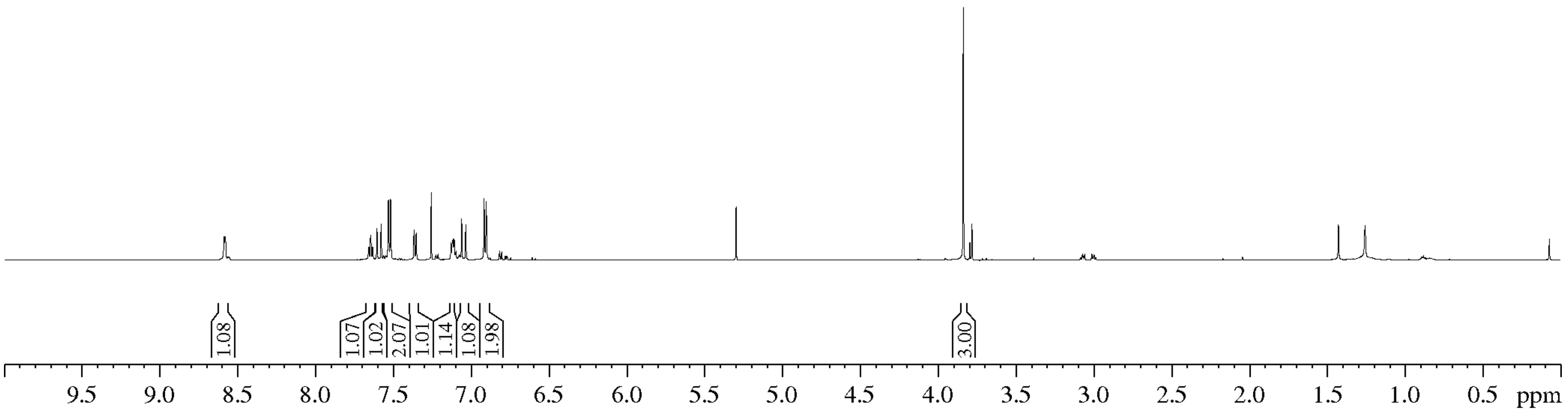

2.23.2 ^{13}C NMR

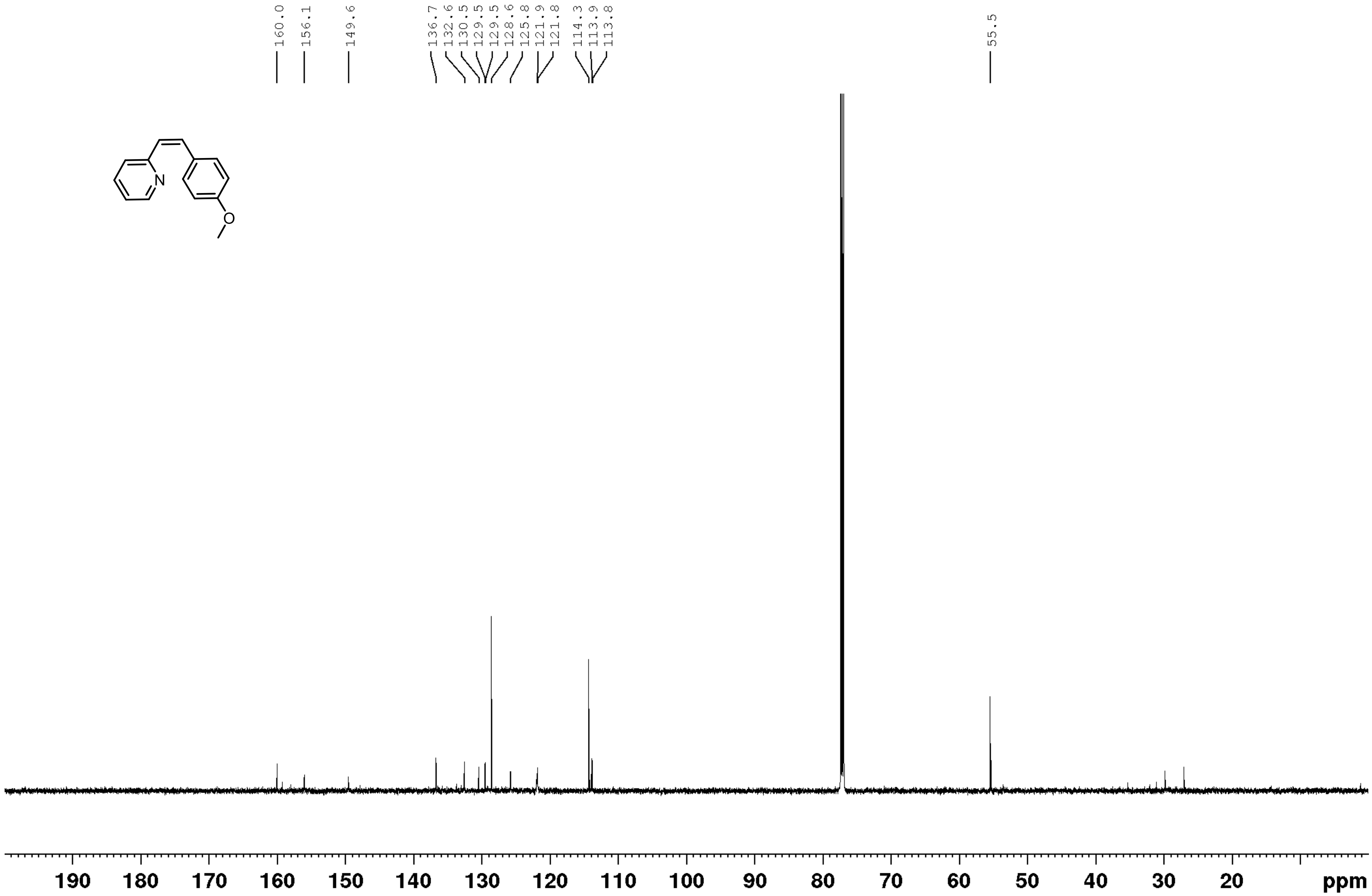
160.0
156.1
149.6
136.7
132.6
130.5
129.5
129.5
128.6
125.8
121.9
121.8
114.3
113.9
113.8
55.5
190 180 170 160 150 140 130 120 110 100 90 80 70 60 50 40 30 20 ppm

2.23.3 ^{1}H, ^{1}H COSY

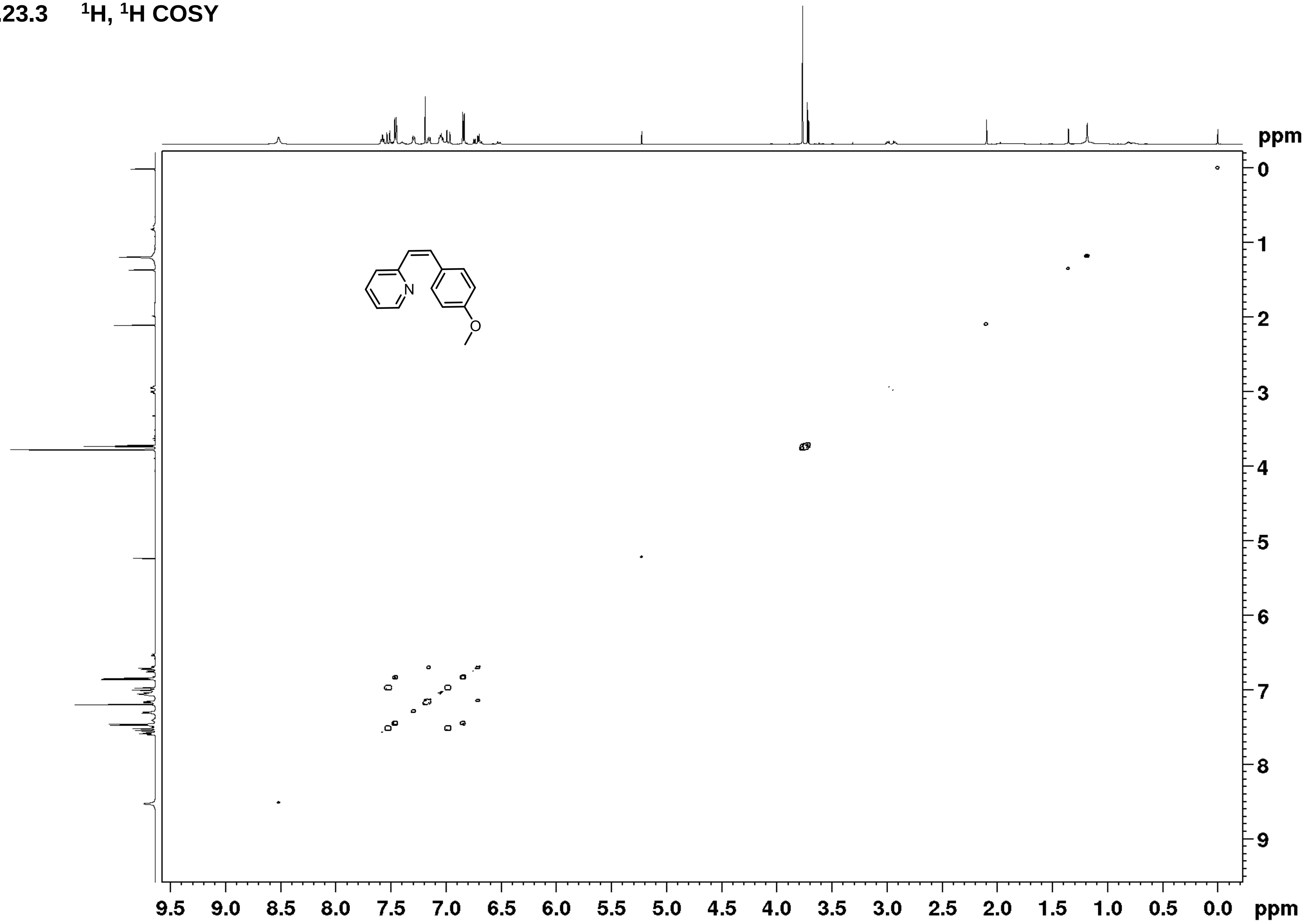

2.23.4 ^{1}H, ^{13}C HMBC

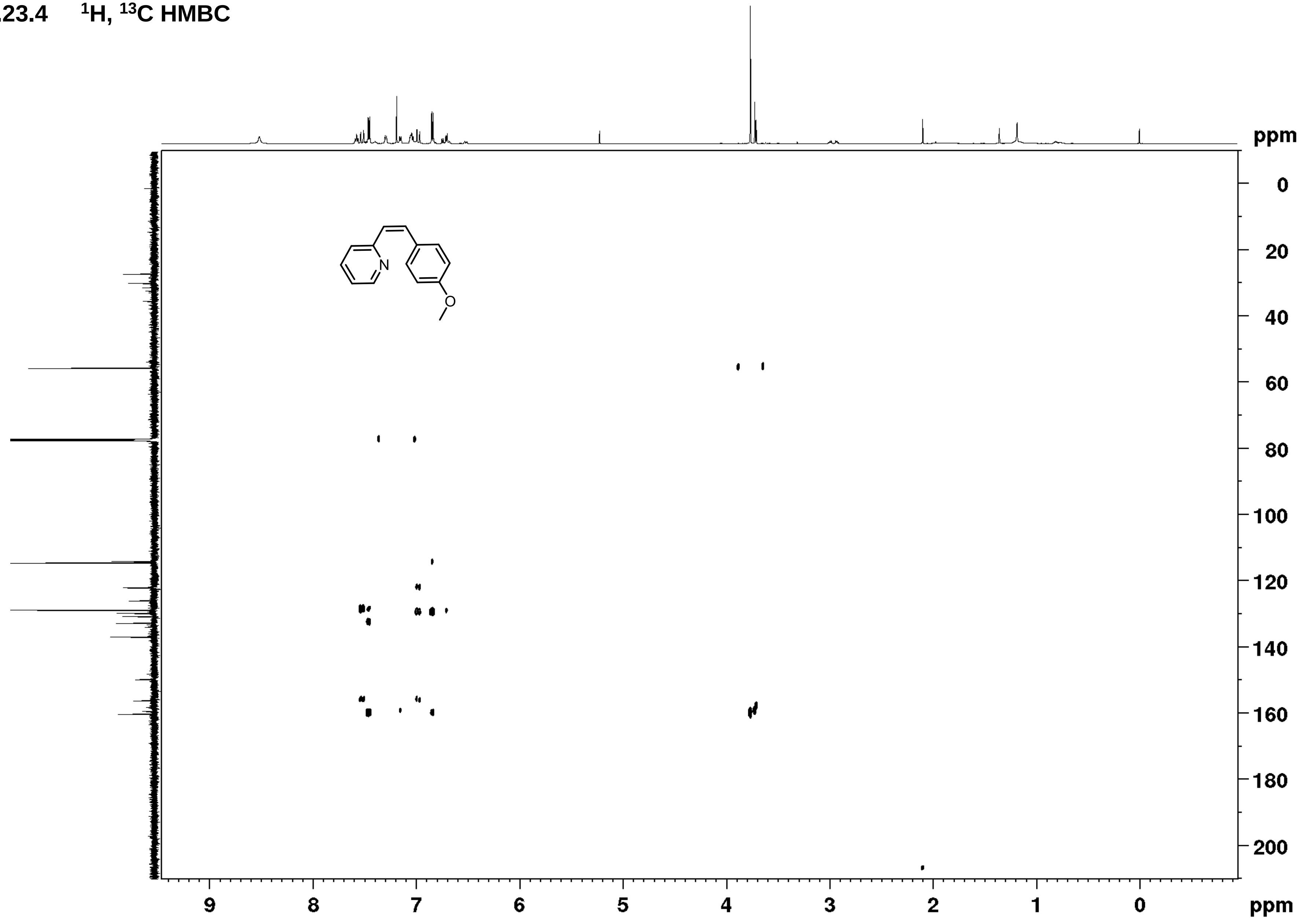

2.23.5 ^{1}H, ^{13}C HSQC

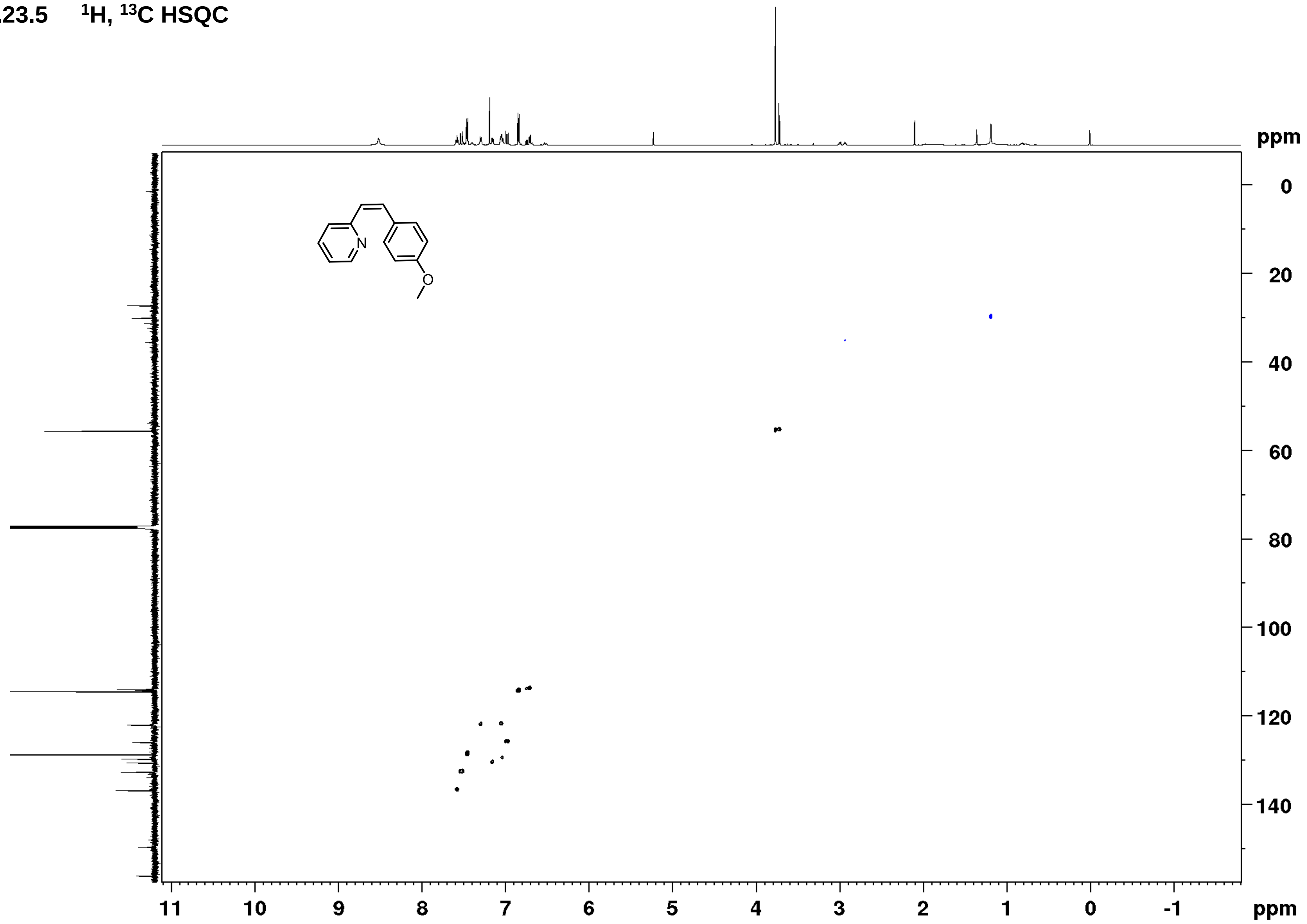

2.23.6 HRMS

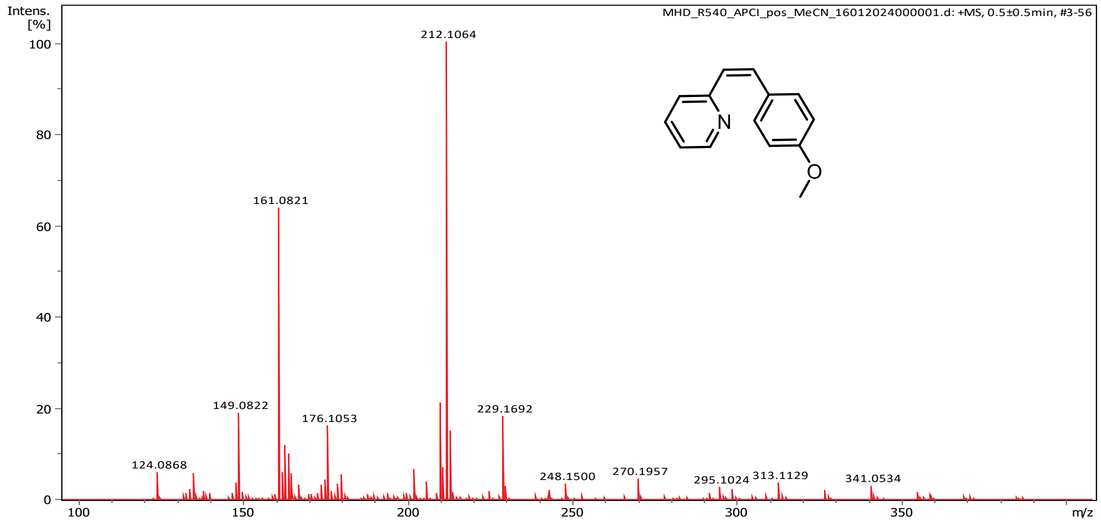

Intens. [%]
MHD_R540_APCI_pos_MeCN_16012024000001.d: +MS, 0.5±0.5min, #3-56
212.1064
161.0821
149.0822
176.1053
229.1692
124.0868
248.1500
270.1957
295.1024
313.1129
341.0534
N
O
m/z

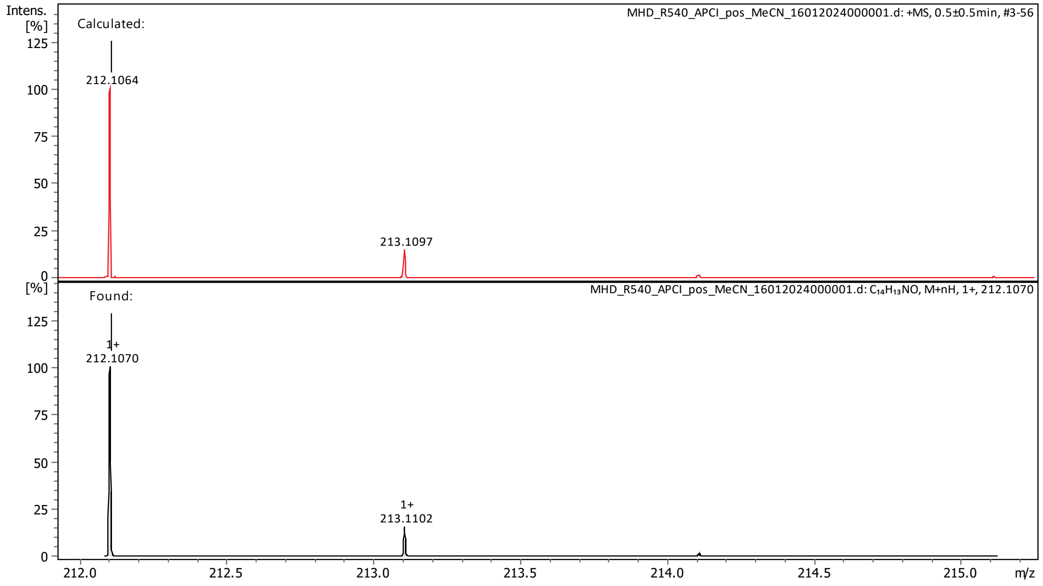

Intens. [%]
Calculated:
MHD_R540_APCI_pos_MeCN_16012024000001.d: +MS, 0.5±0.5min, #3-56
212.1064
213.1097
Found:
MHD_R540_APCI_pos_MeCN_16012024000001.d: C14H13NO, M+nH, 1+, 212.1070
1+
212.1070
1+
213.1102
m/z

2.23.7 IR

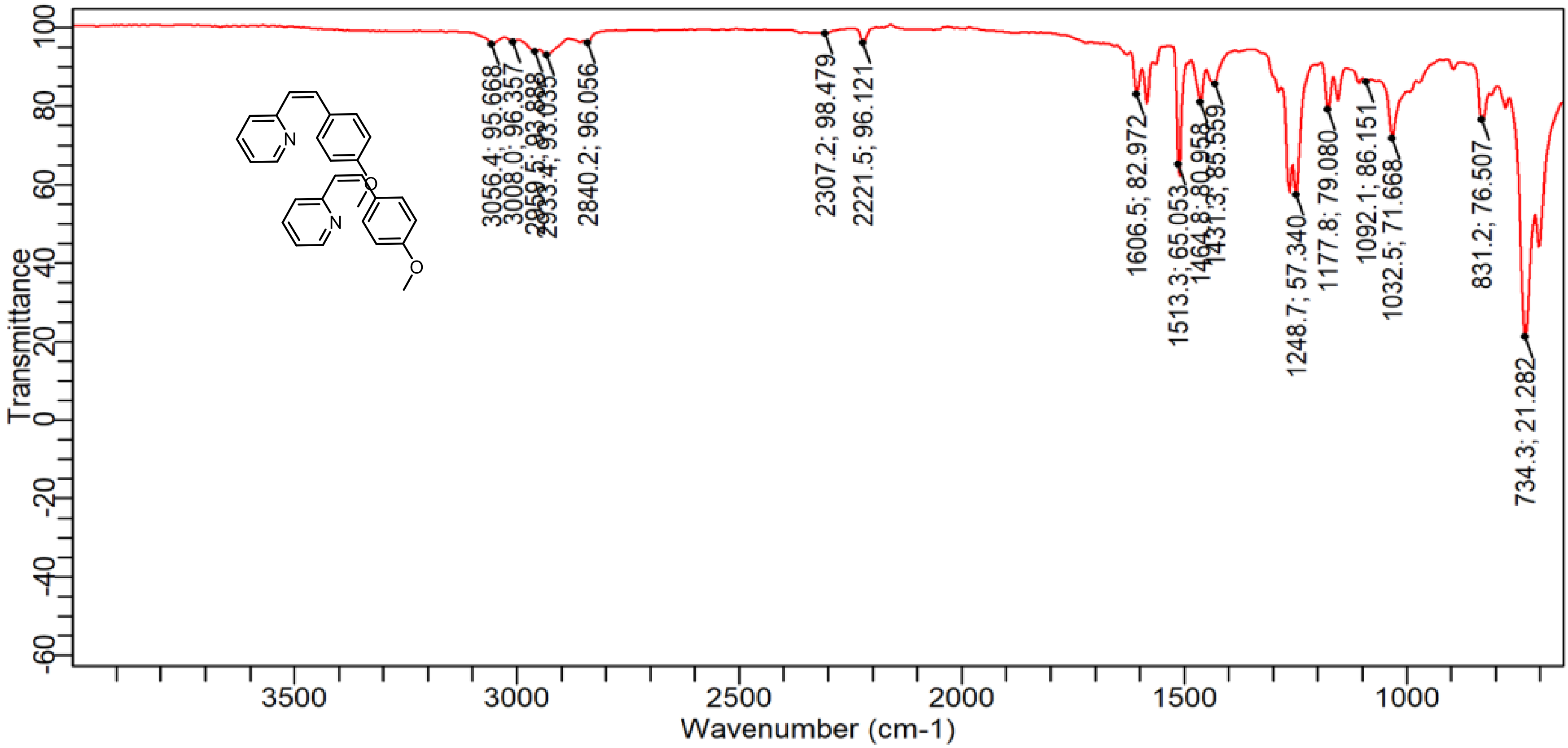

Transmittance
Wavenumber (cm-1)
3056.4; 95.668
3008.0; 96.357
2959.5; 93.888
2933.4; 93.035
2840.2; 96.056
2307.2; 98.479
2221.5; 96.121
1606.5; 82.972
1513.3; 65.053
1464.8; 80.958
1431.3; 85.559
1248.7; 57.340
1177.8; 79.080
1092.1; 86.151
1032.5; 71.668
831.2; 76.507
734.3; 21.282

2.24 (*Z*)-3-(4-Methoxystyryl)pyridine (2x) (600 MHz, $CDCl_3$)

2.24.1 ^{1}H NMR

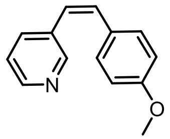

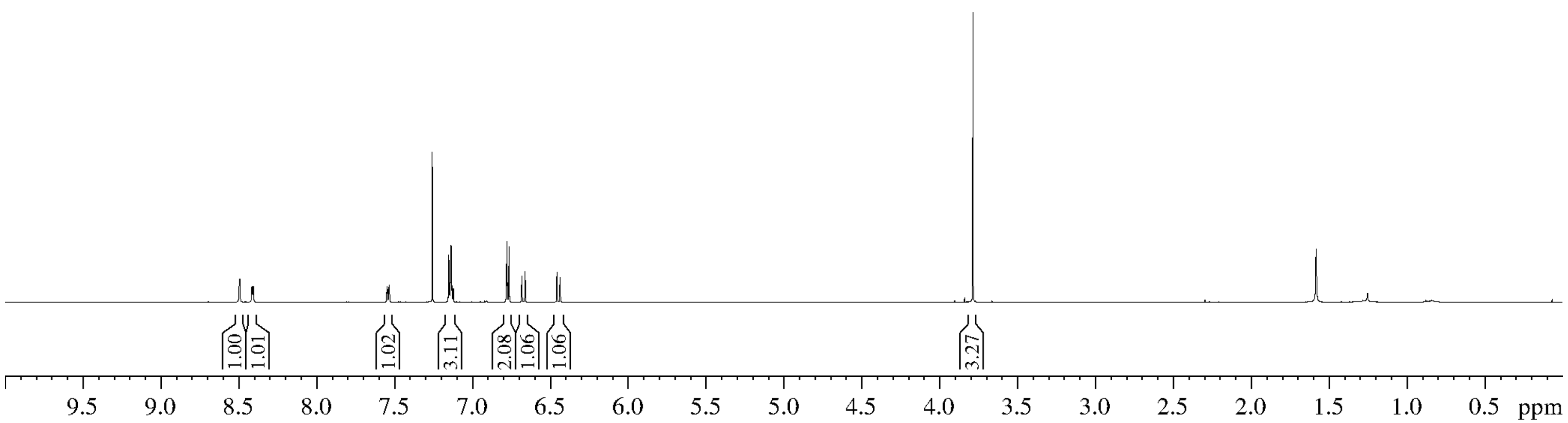

2.24.2 ^{13}C NMR

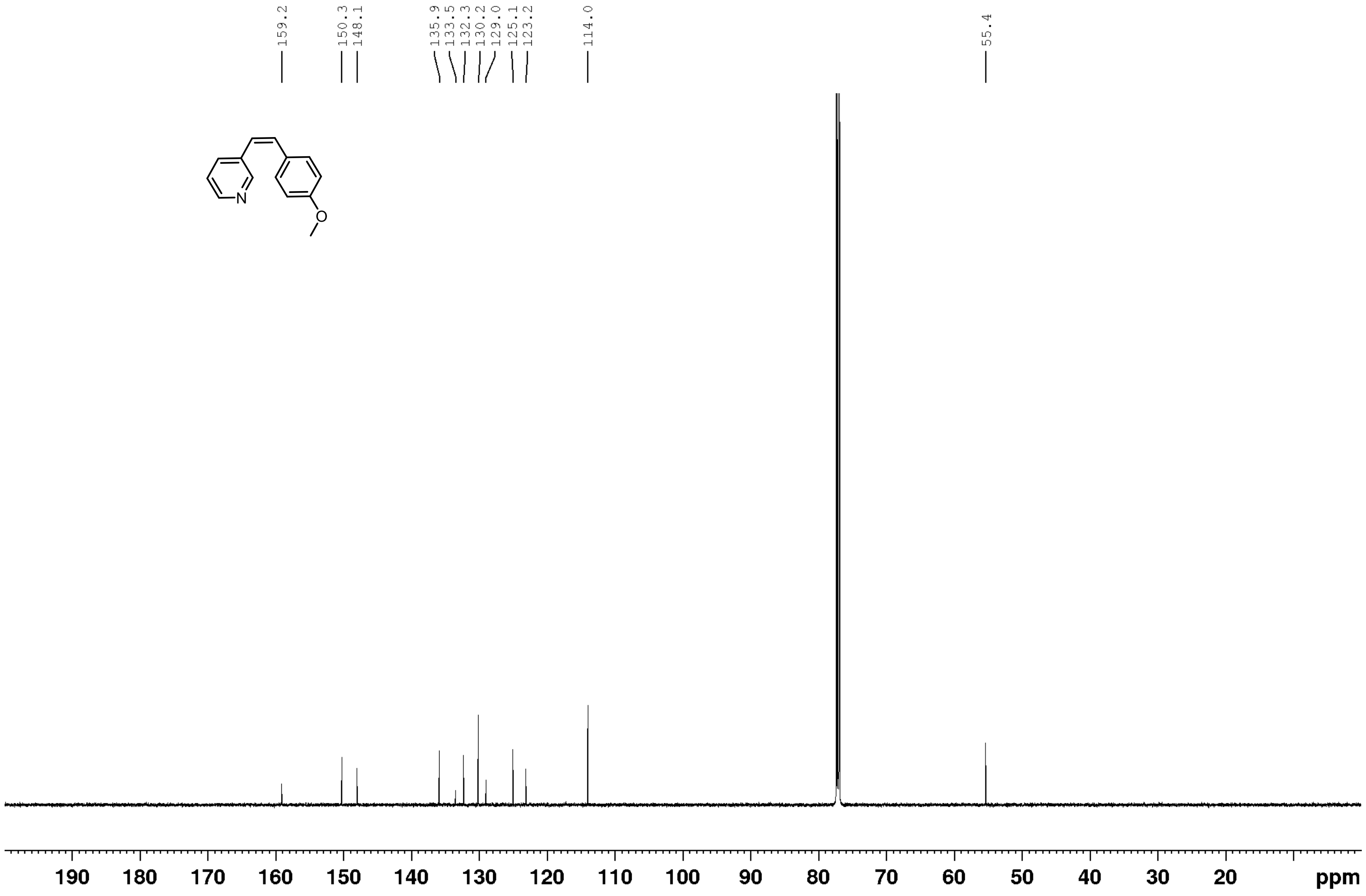

159.2
150.3
148.1
135.9
133.5
132.3
130.2
129.0
125.1
123.2
114.0
55.4
N
O
190
180
170
160
150
140
130
120
110
100
90
80
70
60
50
40
30
20
ppm

2.24.3 ^{1}H, ^{1}H COSY

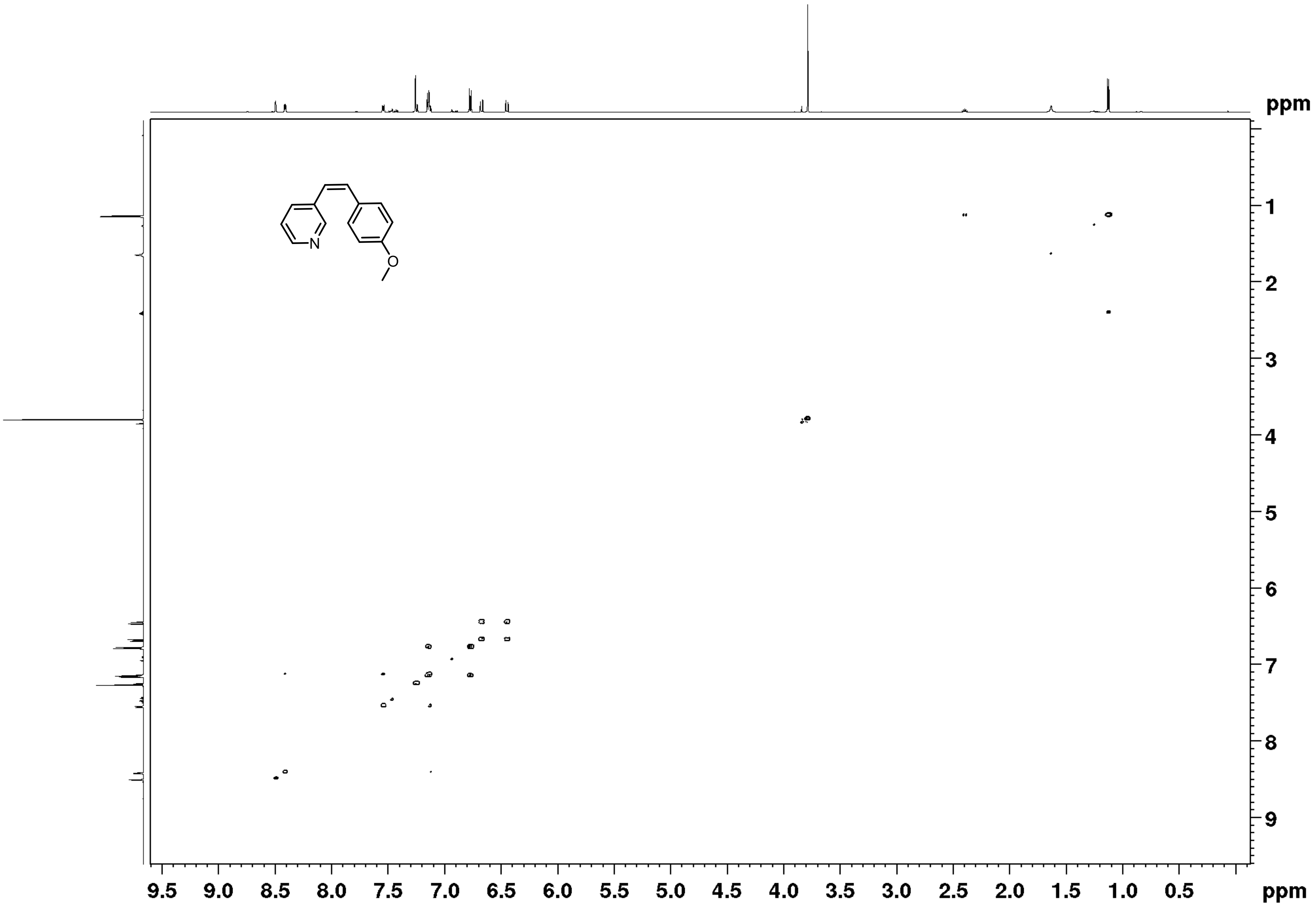

2.24.4 ^{1}H, ^{13}C HMBC

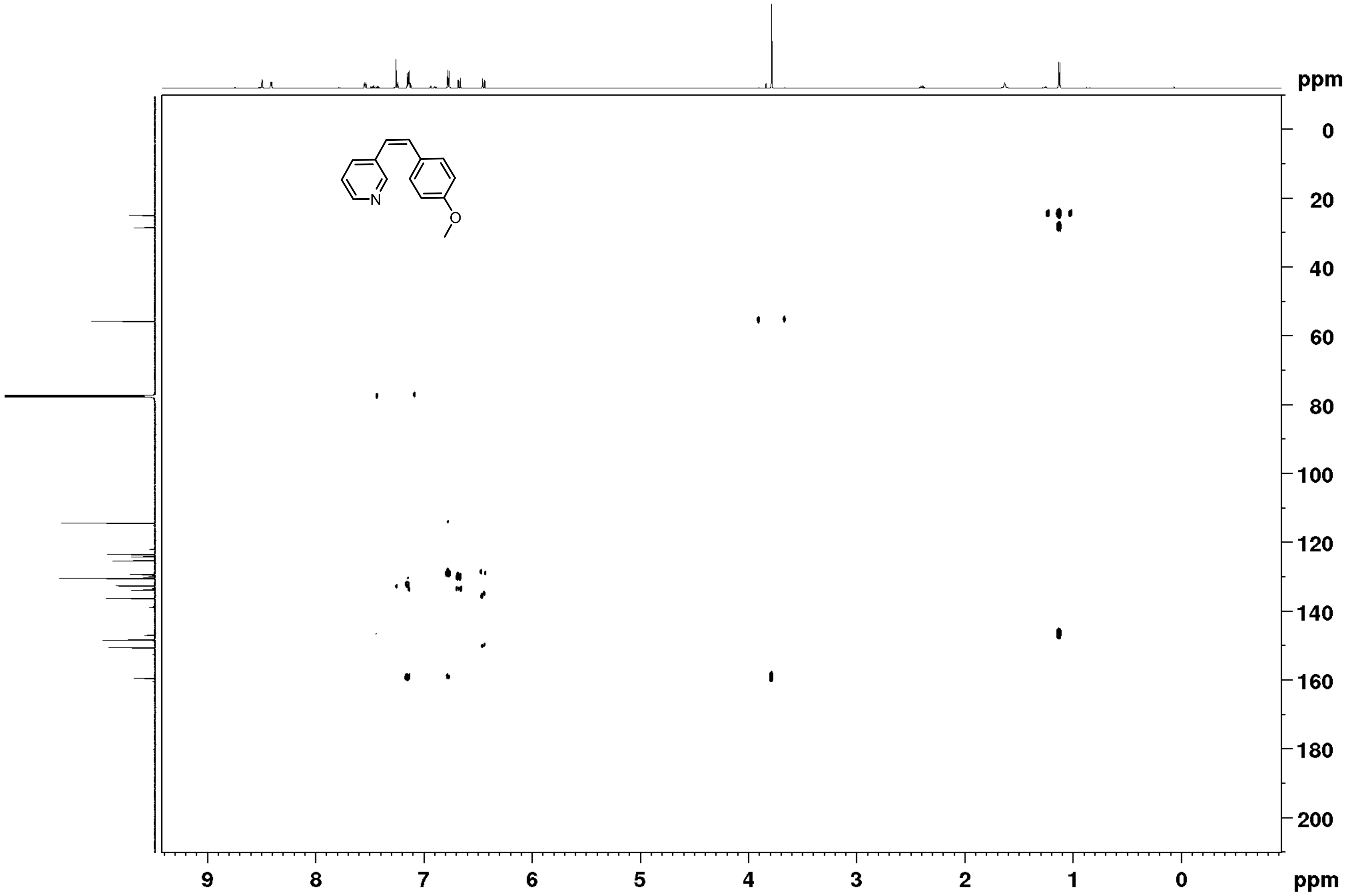

2.24.5 ^{1}H, ^{13}C HSQC

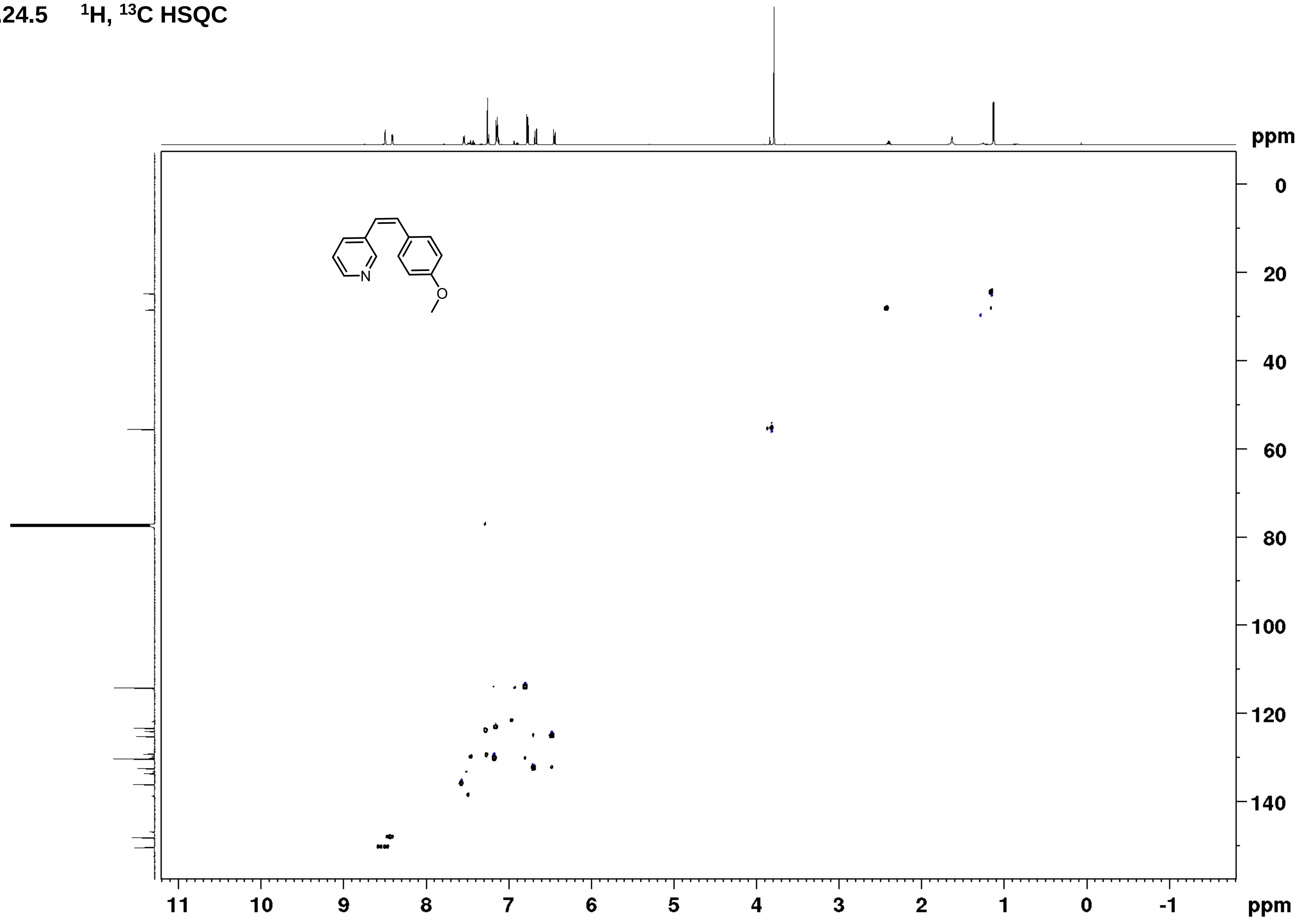

2.24.6 HRMS

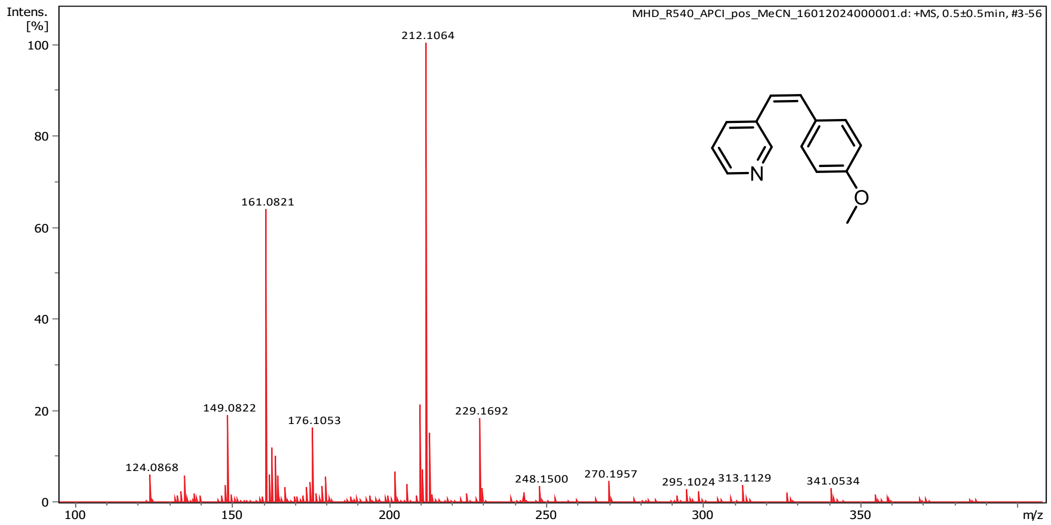

Intens. [%]
MHD_R540_APCI_pos_MeCN_16012024000001.d: +MS, 0.5±0.5min, #3-56
212.1064
161.0821
149.0822
176.1053
229.1692
124.0868
248.1500
270.1957
295.1024
313.1129
341.0534
N
O
m/z

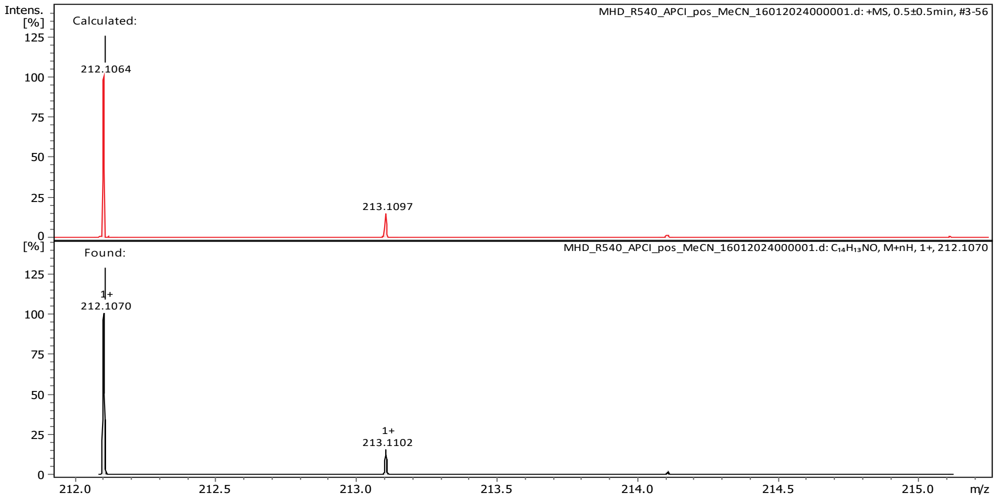

Intens. [%]
Calculated:
MHD_R540_APCI_pos_MeCN_16012024000001.d: +MS, 0.5±0.5min, #3-56
212.1064
213.1097
Found:
MHD_R540_APCI_pos_MeCN_16012024000001.d: C₁₄H₁₃NO, M+nH, 1+, 212.1070
1+
212.1070
1+
213.1102
m/z

2.24.7 IR

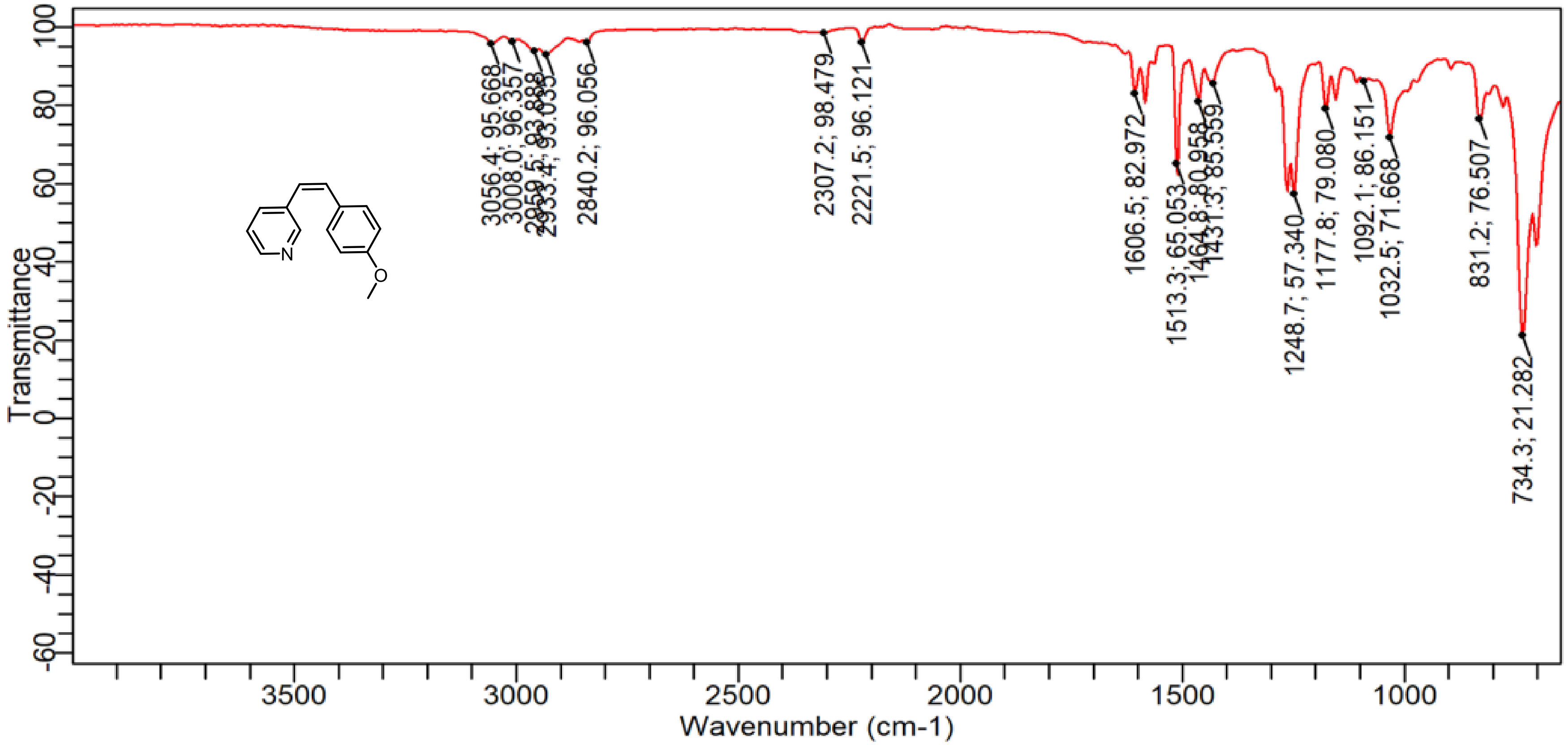

3056.4; 95.668
3008.0; 96.357
2959.5; 93.888
2933.4; 93.035
2840.2; 96.056
2307.2; 98.479
2221.5; 96.121
1606.5; 82.972
1513.3; 65.053
1464.8; 80.958
1431.3; 85.559
1248.7; 57.340
1177.8; 79.080
1092.1; 86.151
1032.5; 71.668
831.2; 76.507
734.3; 21.282
Transmittance
Wavenumber (cm-1)
N
O

2.25 (*Z*)-2-(4-Methoxystyryl)-1-methyl-1*H*-imidazole (2y) (600 MHz, $CDCl_3$)

2.25.1 ^{1}H NMR

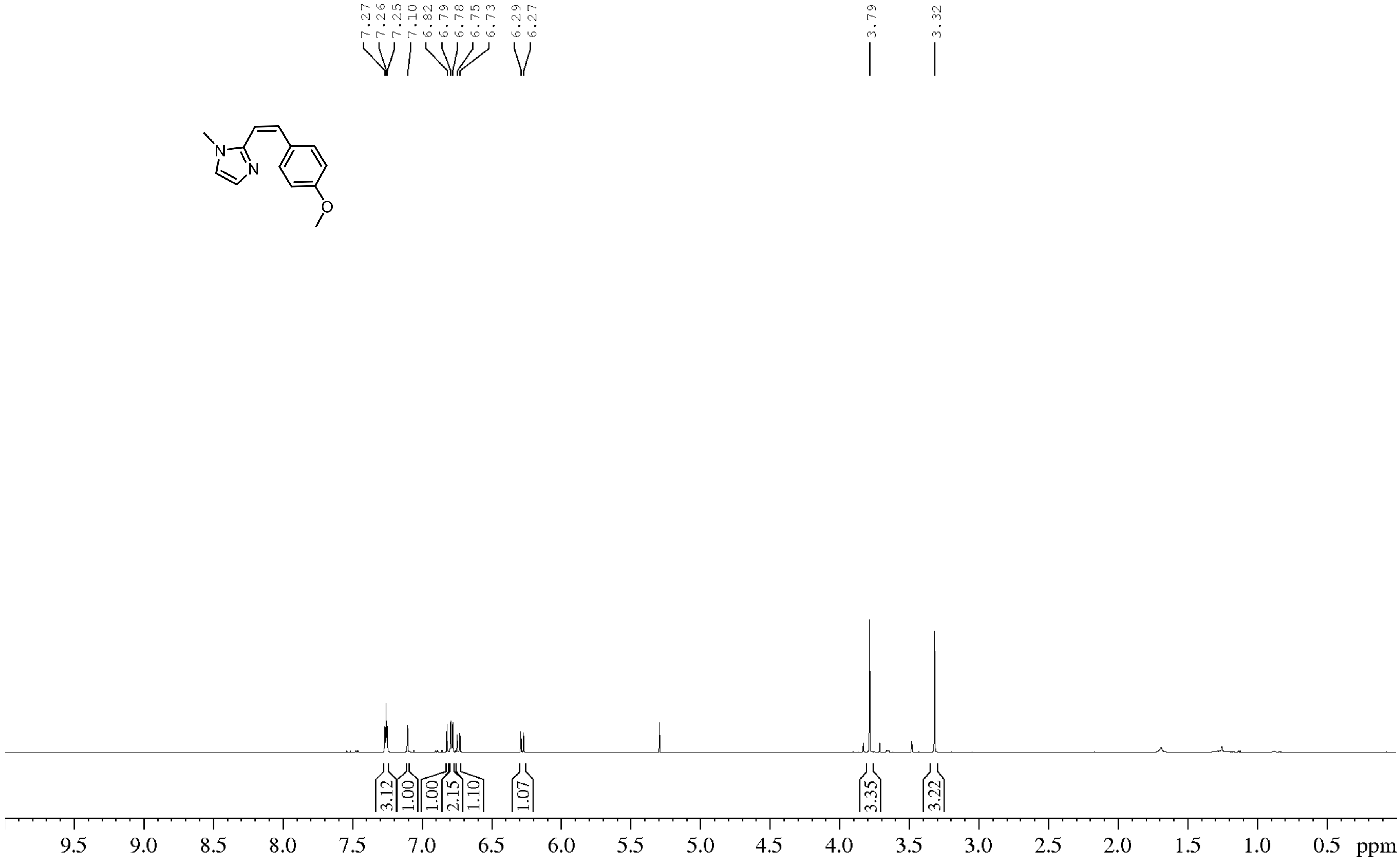

2.25.2 ^{13}C NMR

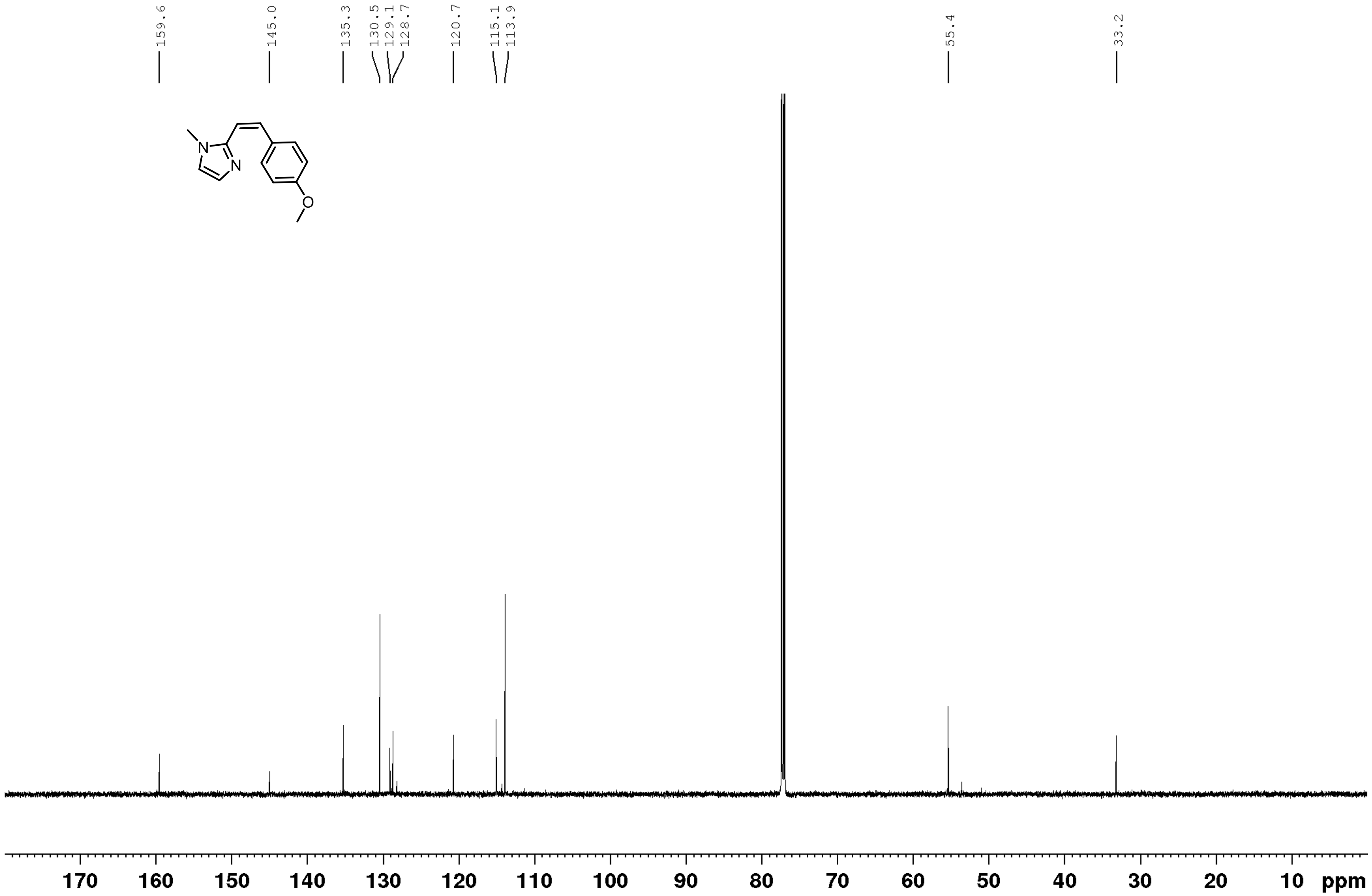
159.6
145.0
135.3
130.5
129.1
128.7
120.7
115.1
113.9
55.4
33.2
N
N
O
170
160
150
140
130
120
110
100
90
80
70
60
50
40
30
20
10
ppm

2.25.3 ^{1}H, ^{1}H COSY

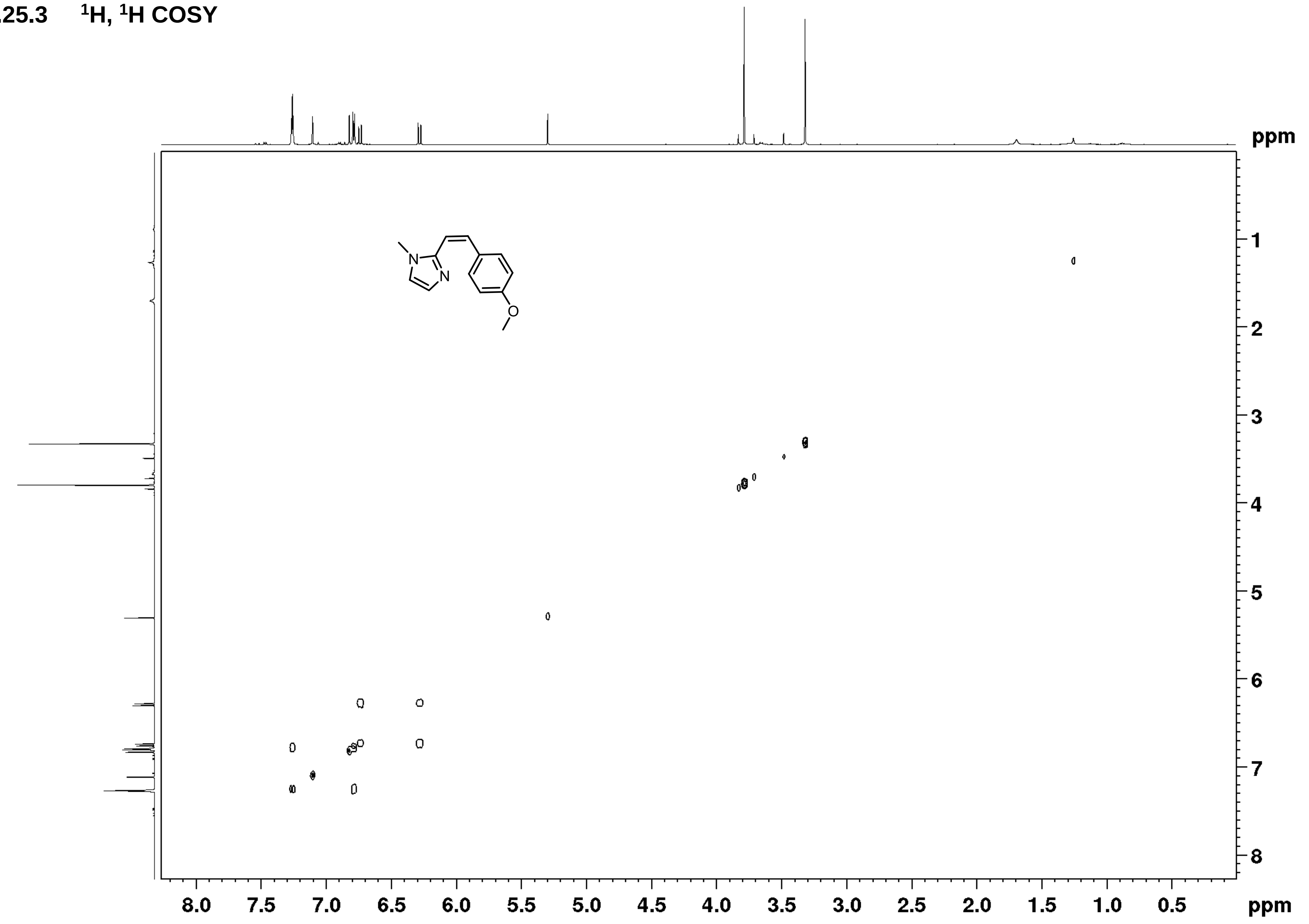

2.25.4 ^{1}H, ^{13}C HMBC

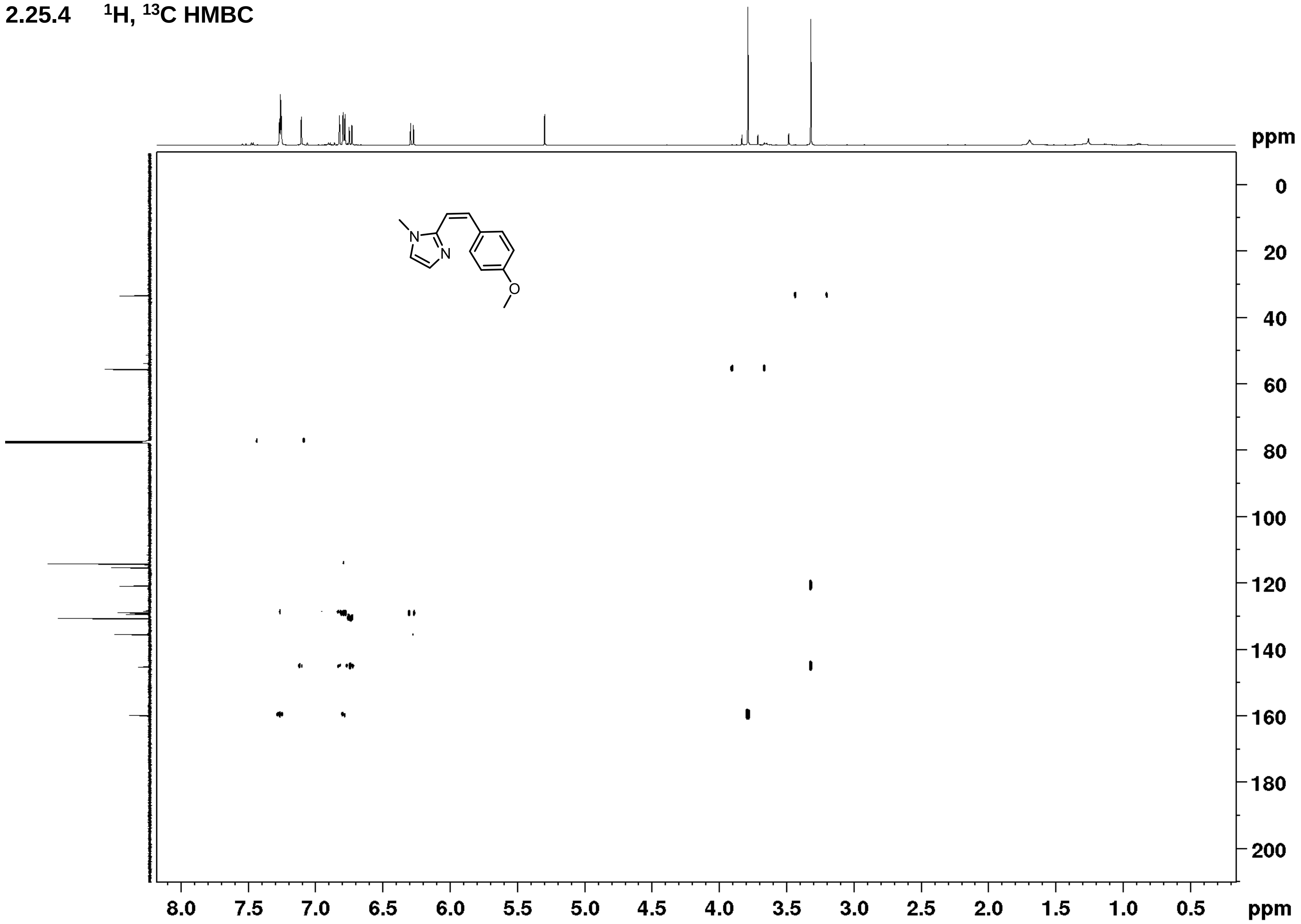

2.25.5 ^{1}H, ^{13}C HSQC

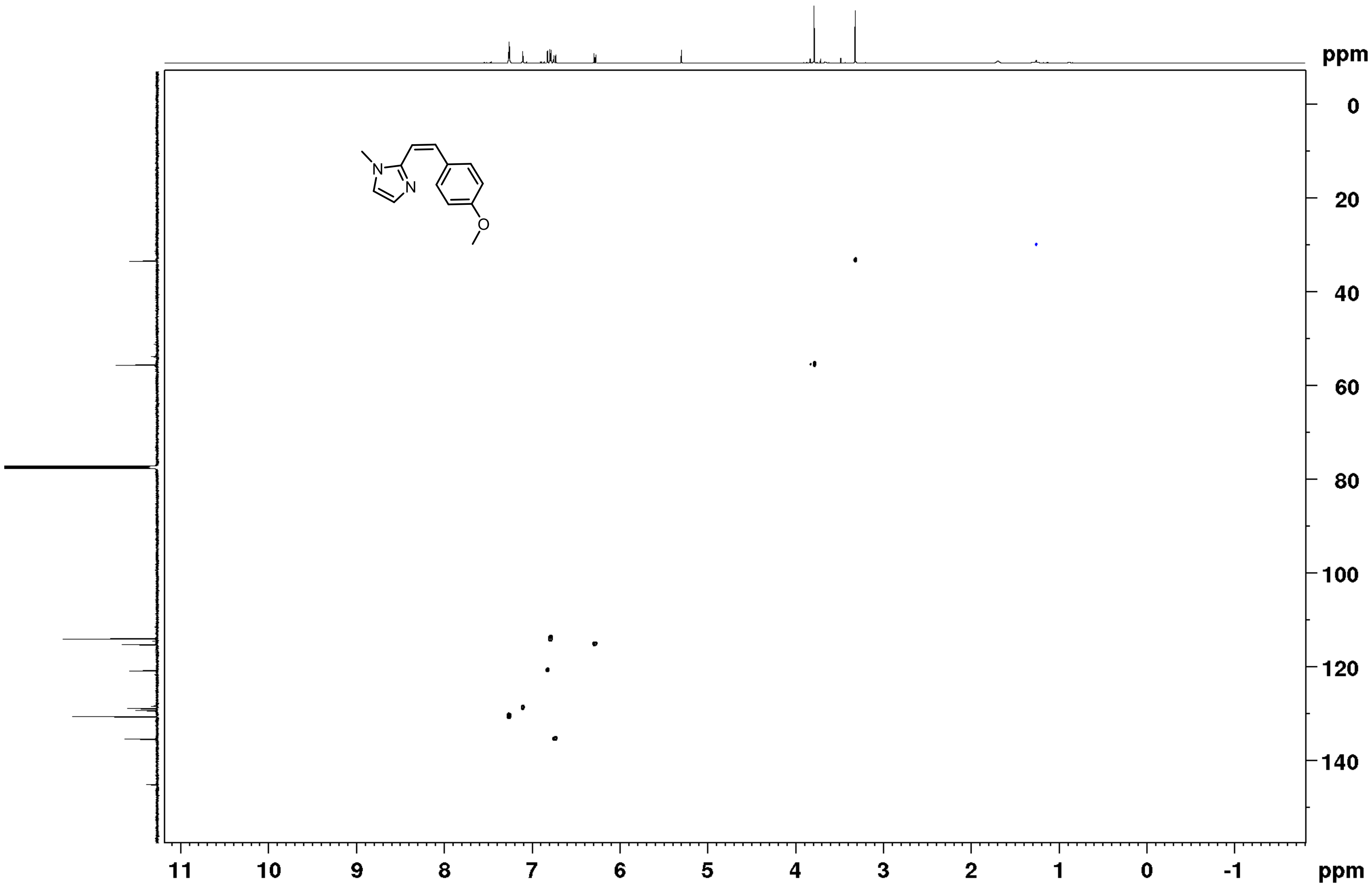

2.25.6 HRMS

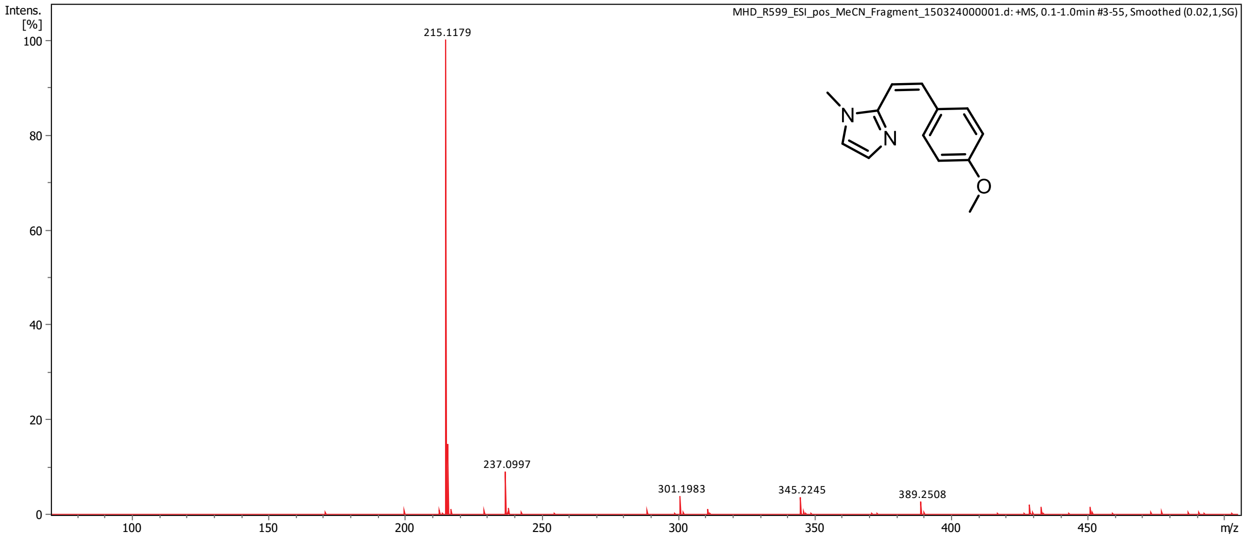
Intens. [%]
MHD_R599_ESI_pos_MeCN_Fragment_150324000001.d: +MS, 0.1-1.0min #3-55, Smoothed (0.02,1,SG)
215.1179
237.0997
301.1983
345.2245
389.2508
m/z

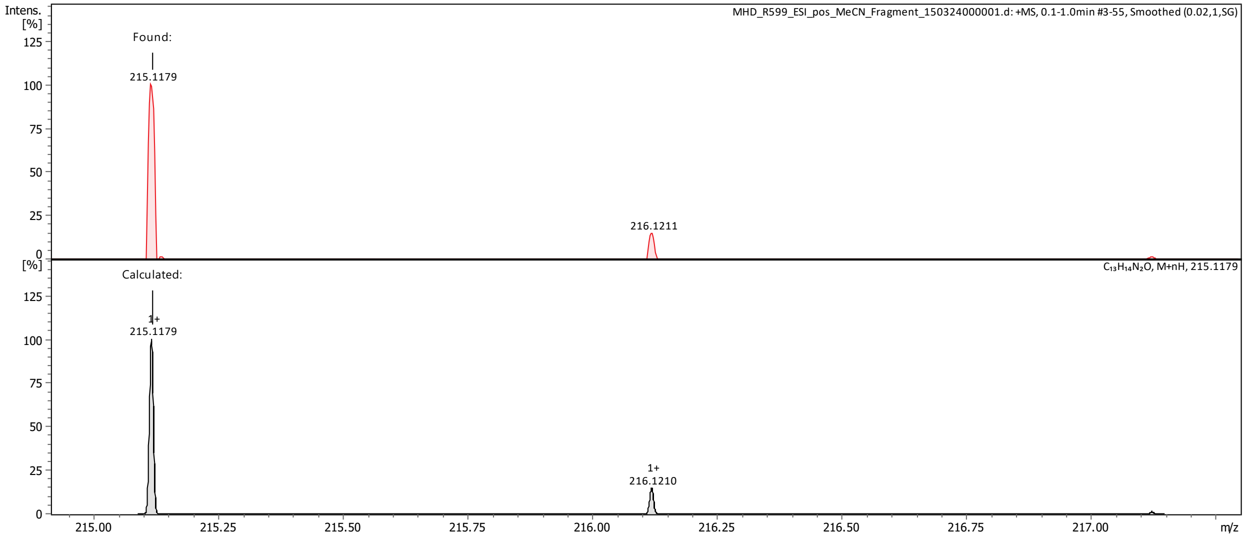
Intens. [%]
MHD_R599_ESI_pos_MeCN_Fragment_150324000001.d: +MS, 0.1-1.0min #3-55, Smoothed (0.02,1,SG)
Found:
215.1179
216.1211
Calculated:
$C_{13}H_{14}N_2O$, M+nH, 215.1179
1+ 215.1179
1+ 216.1210
m/z

2.25.7 IR

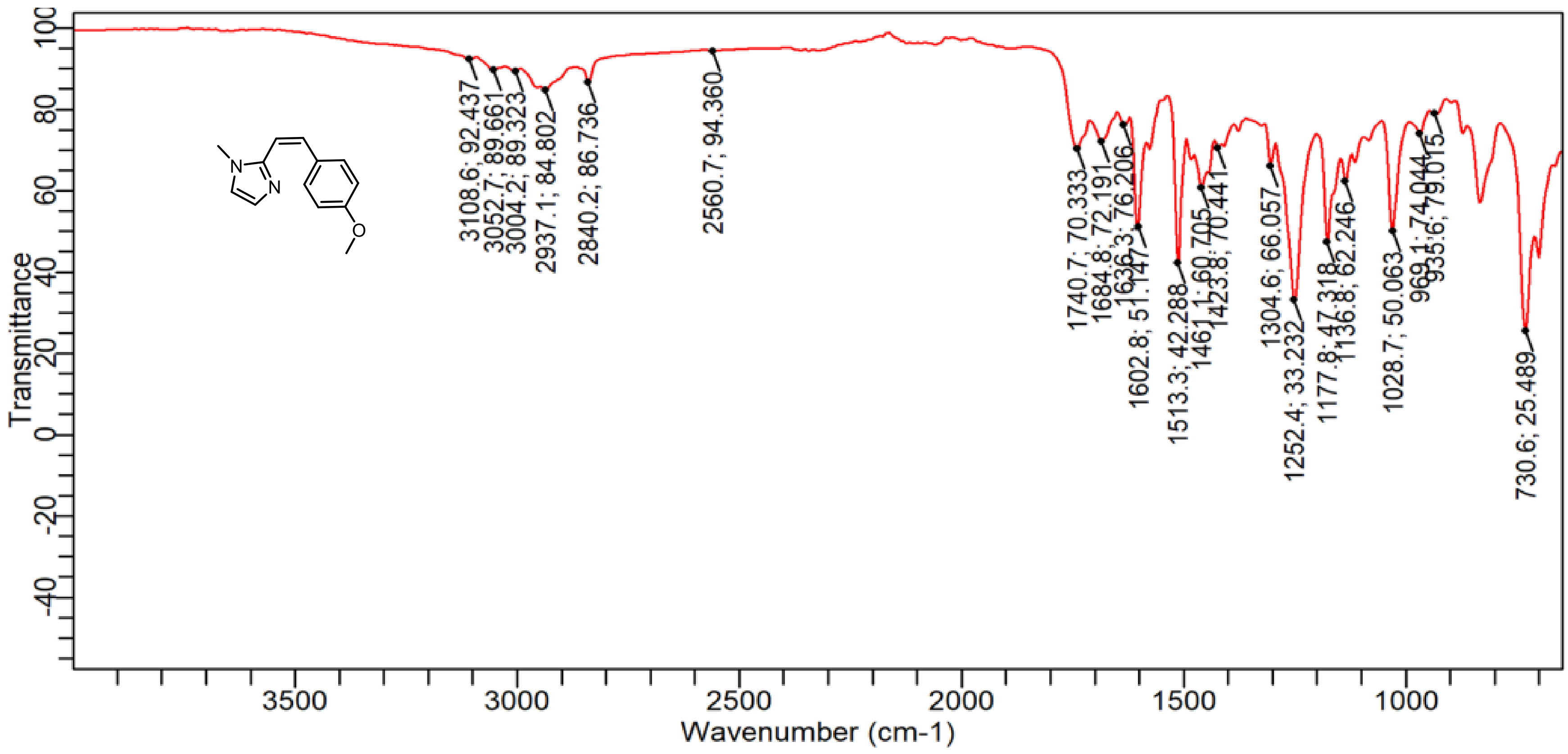
Transmittance
Wavenumber (cm-1)
3108.6; 92.437
3052.7; 89.661
3004.2; 89.323
2937.1; 84.802
2840.2; 86.736
2560.7; 94.360
1740.7; 70.333
1684.8; 72.191
1636.3; 76.206
1602.8; 51.147
1513.3; 42.288
1461.1; 60.705
1423.8; 70.441
1304.6; 66.057
1252.4; 33.232
1177.8; 47.318
1136.8; 62.246
1028.7; 50.063
969.1; 74.044
935.6; 79.015
730.6; 25.489
N
N
O

2.26 (*Z*)-3-Styrylthiophene (2z) (600 MHz, $CDCl_3$)

2.26.1 ^{1}H NMR

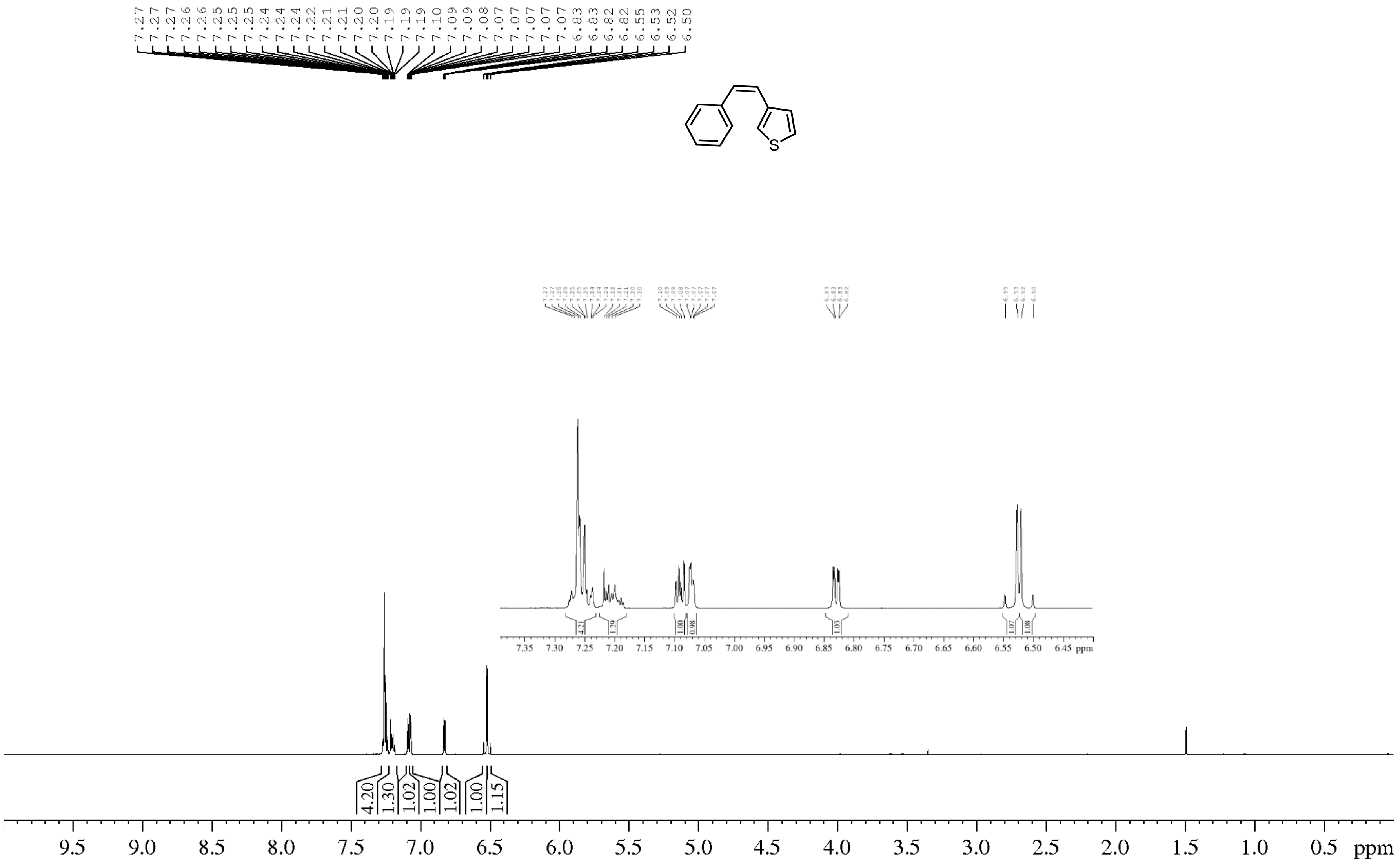

2.26.2 ^{13}C NMR

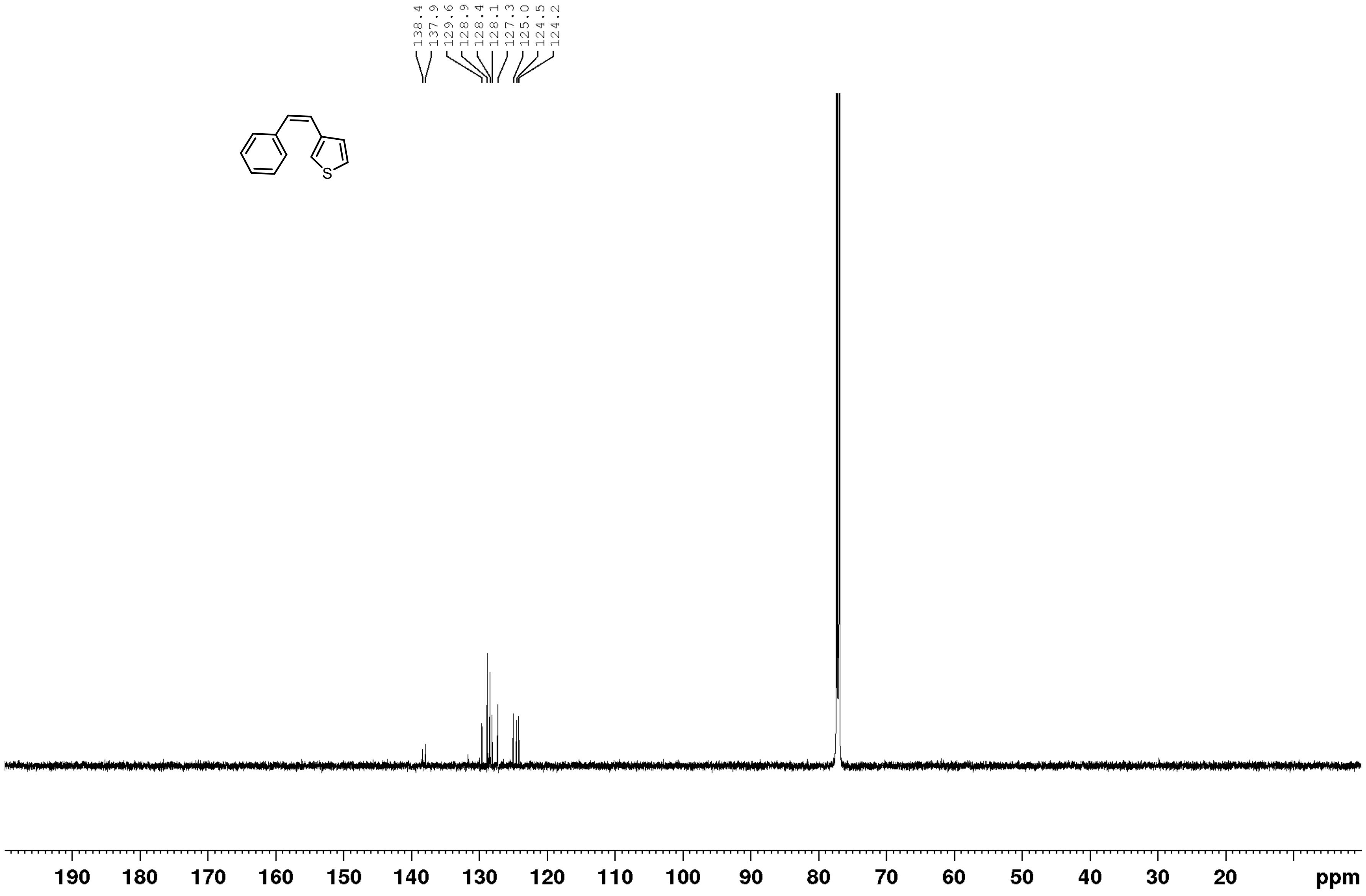

138.4
137.9
129.6
128.9
128.4
128.1
127.3
125.0
124.5
124.2
S
190 180 170 160 150 140 130 120 110 100 90 80 70 60 50 40 30 20 ppm

2.26.3 ^{1}H, ^{1}H COSY

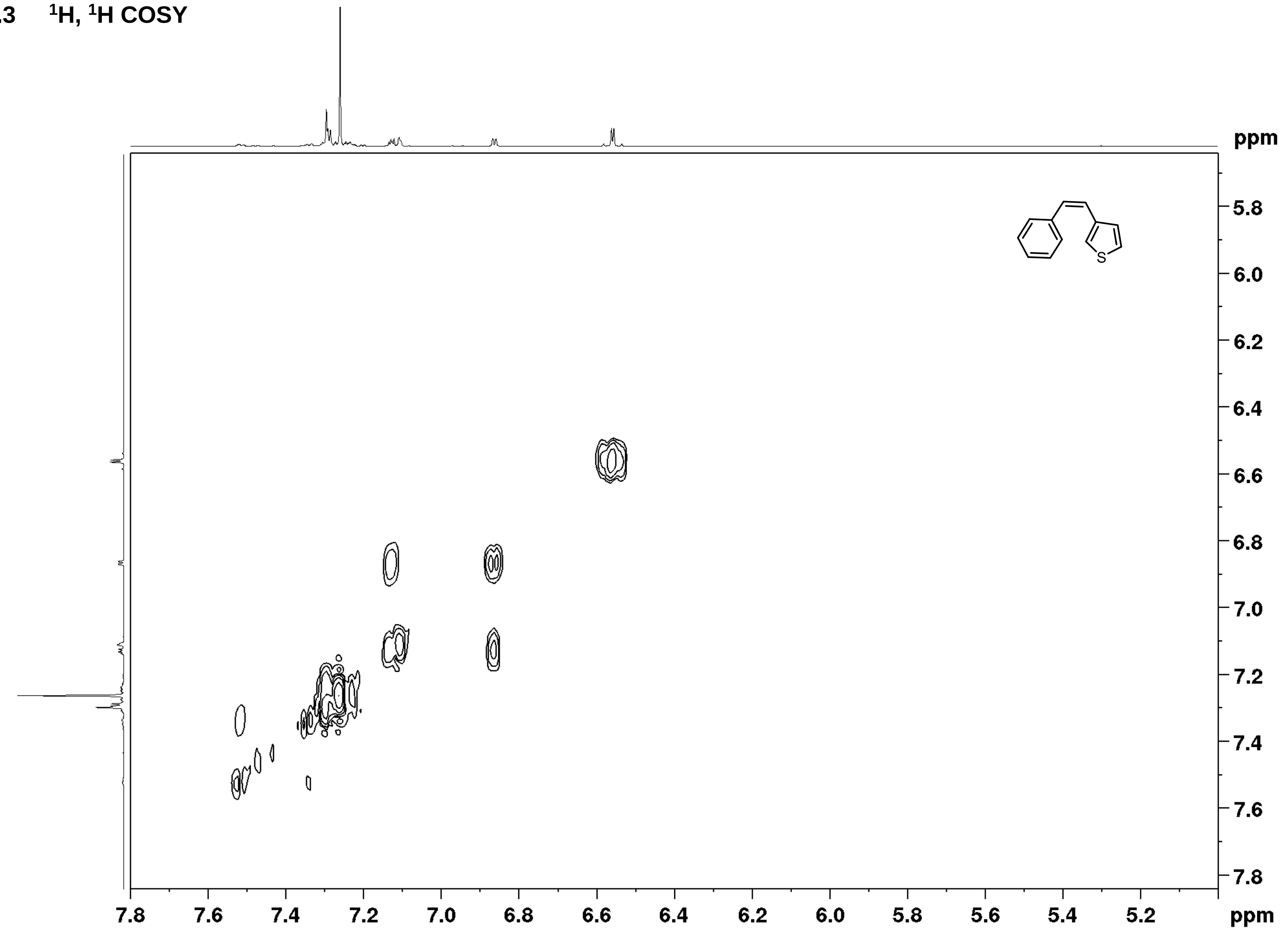

2.26.4 ^{1}H, ^{13}C HMBC

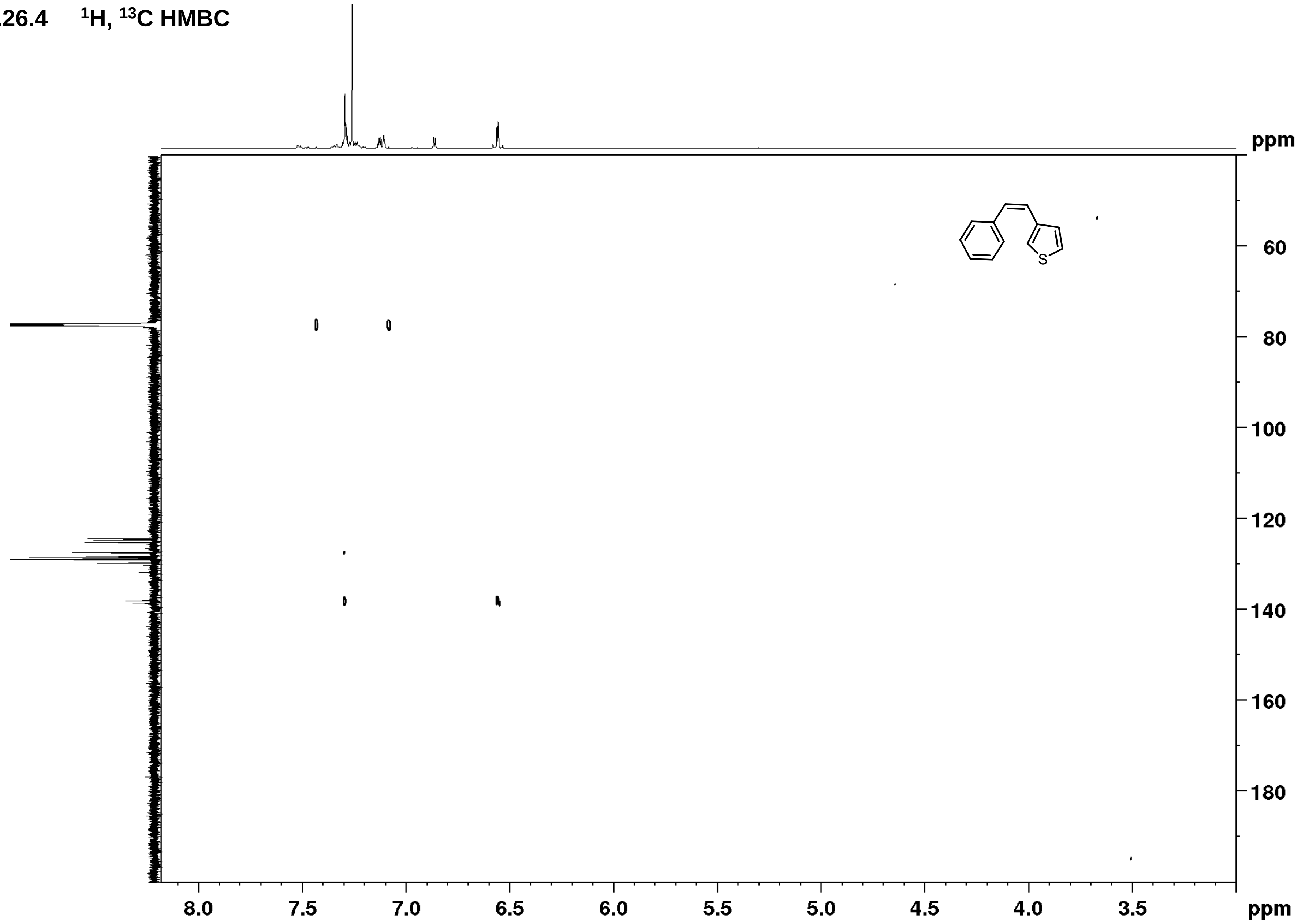

2.26.5 ^{1}H, ^{13}C HSQC

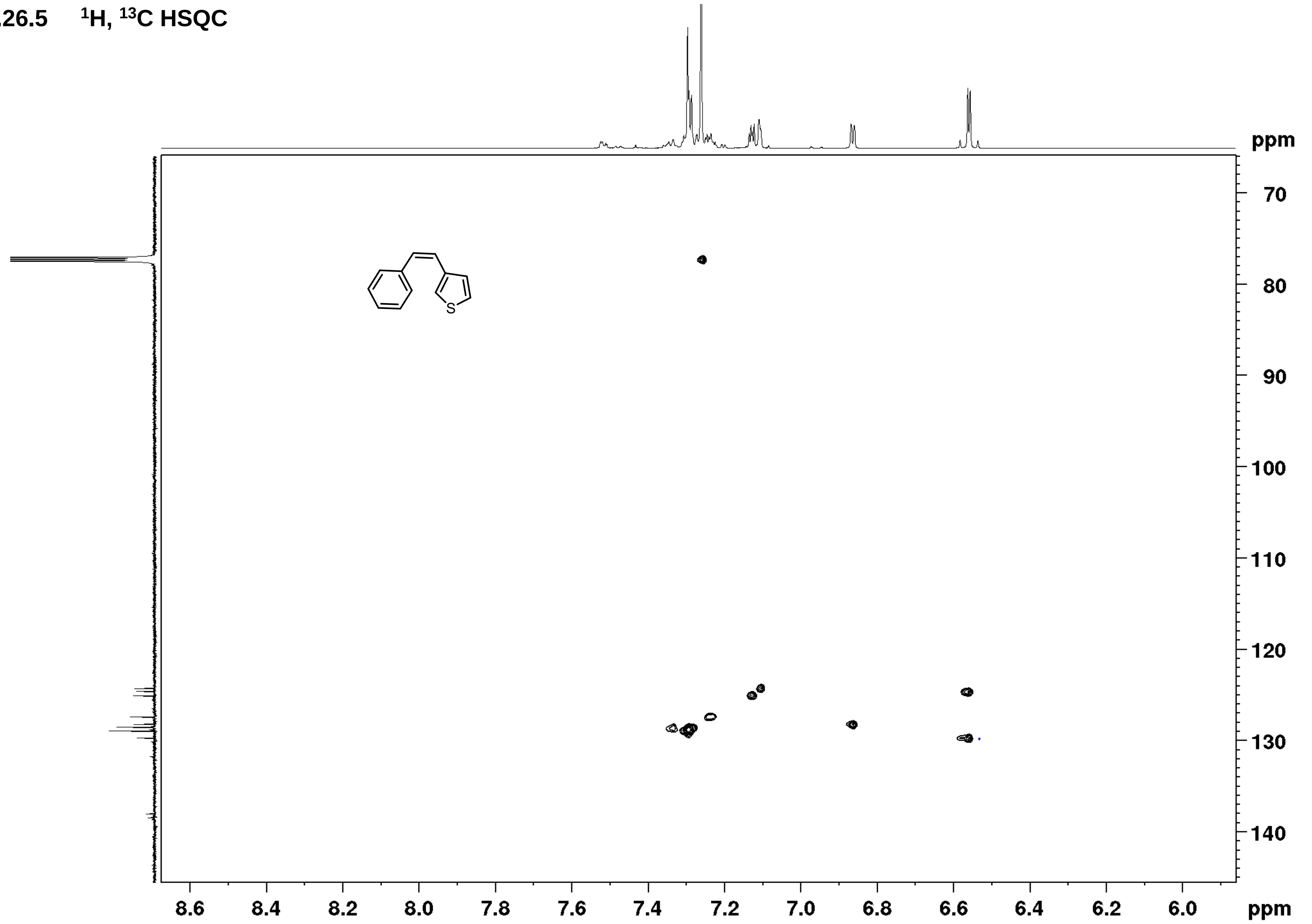

2.26.6 HRMS

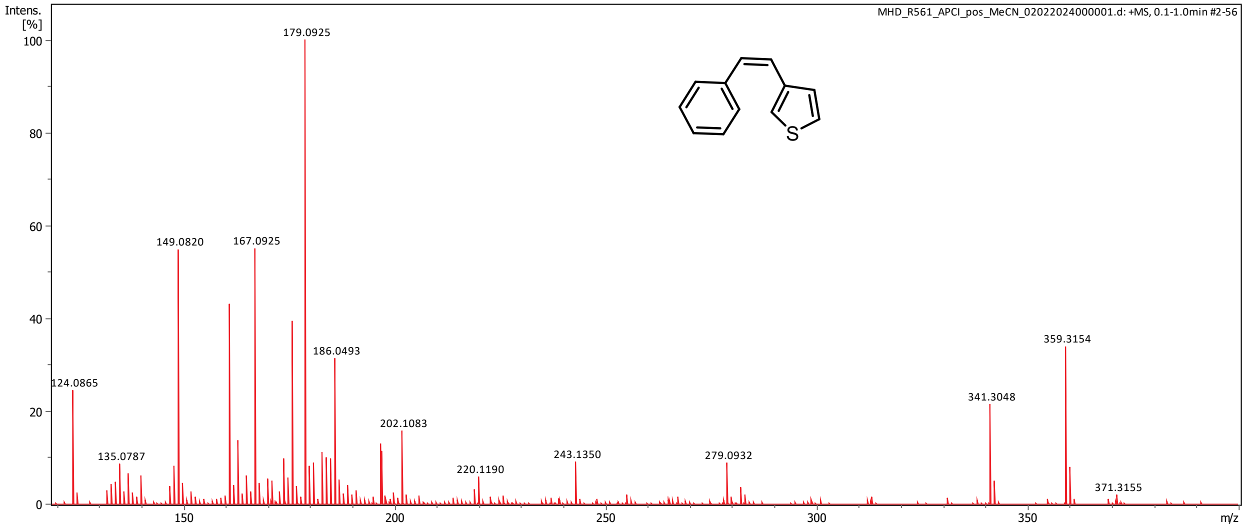
Intens. [%]
MHD_R561_APCI_pos_MeCN_02022024000001.d: +MS, 0.1-1.0min #2-56
179.0925
149.0820
167.0925
186.0493
124.0865
135.0787
202.1083
220.1190
243.1350
279.0932
341.3048
359.3154
371.3155
S
m/z

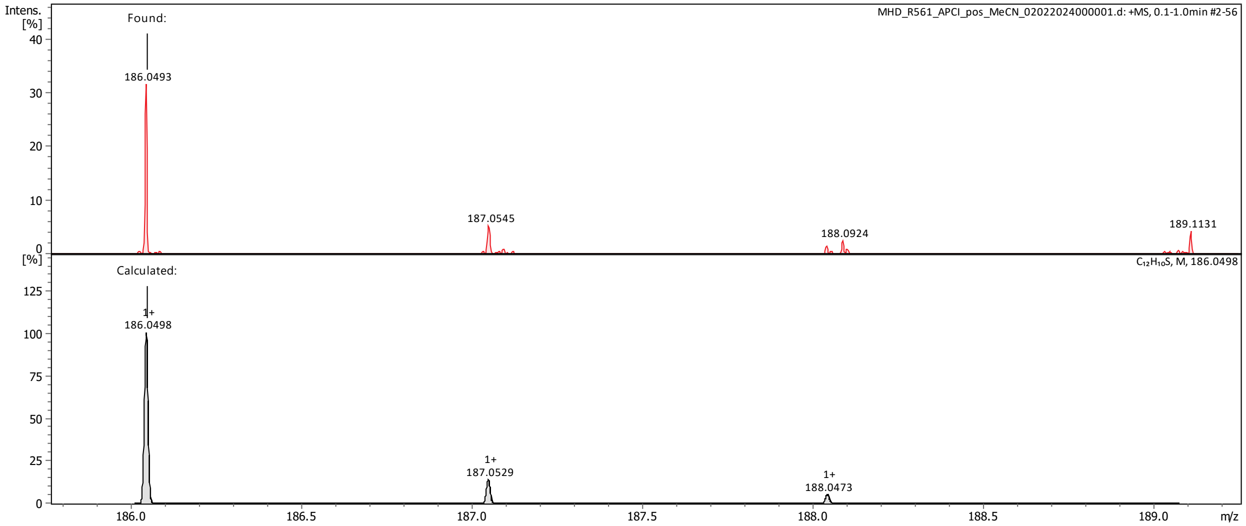
Intens. [%]
MHD_R561_APCI_pos_MeCN_02022024000001.d: +MS, 0.1-1.0min #2-56
Found:
186.0493
187.0545
188.0924
189.1131
Calculated:
C12H10S, M, 186.0498
1+ 186.0498
1+ 187.0529
1+ 188.0473
m/z

2.26.7 IR

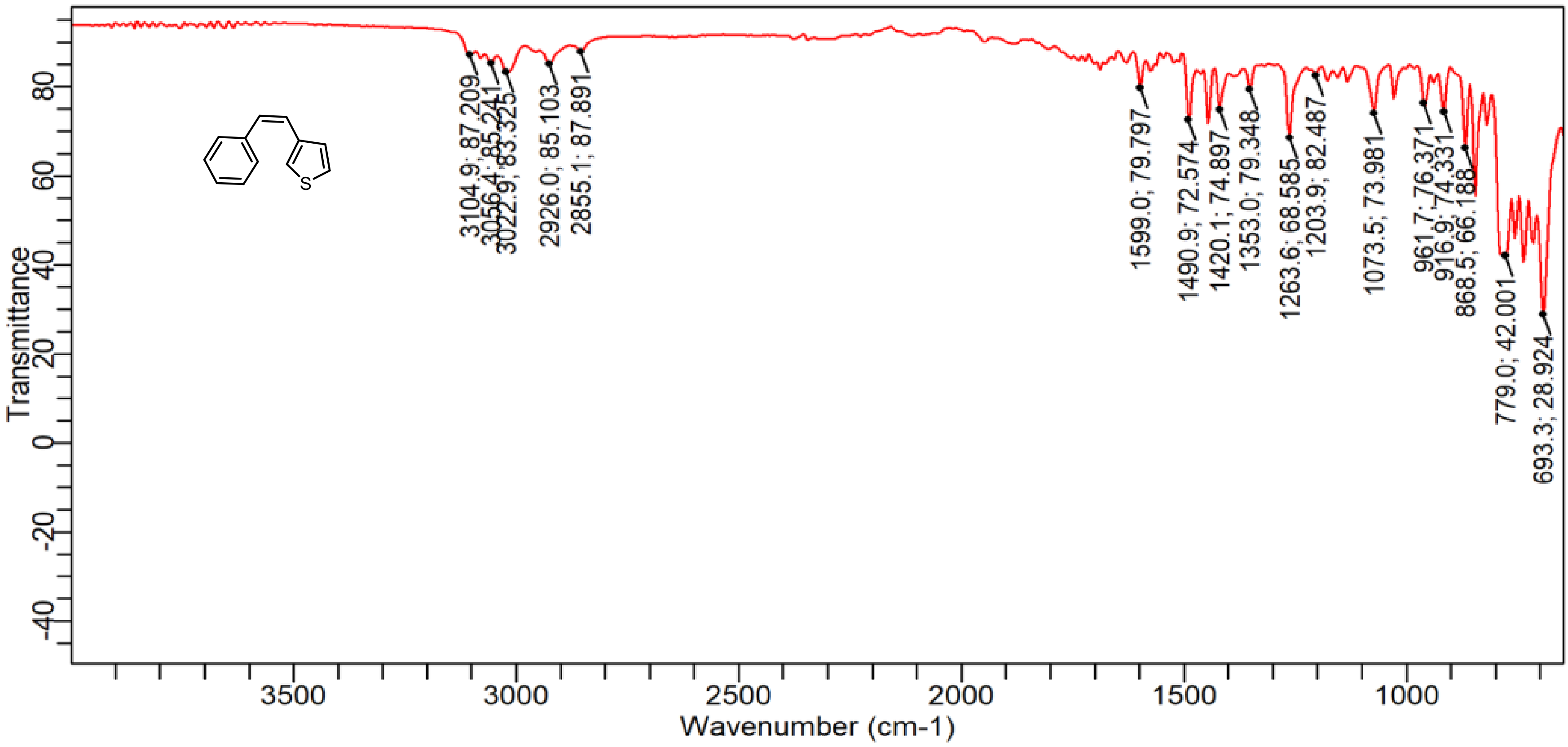

Transmittance
Wavenumber (cm-1)
3104.9; 87.209
3056.4; 85.241
3022.9; 83.325
2926.0; 85.103
2855.1; 87.891
1599.0; 79.797
1490.9; 72.574
1420.1; 74.897
1353.0; 79.348
1263.6; 68.585
1203.9; 82.487
1073.5; 73.981
961.7; 76.371
916.9; 74.331
868.5; 66.188
779.0; 42.001
693.3; 28.924

2.27 (*Z*)-1-Benzyl-5-styryl-1*H*-indole (2aa) (600 MHz, $CDCl_3$)

2.27.1 ^1H NMR

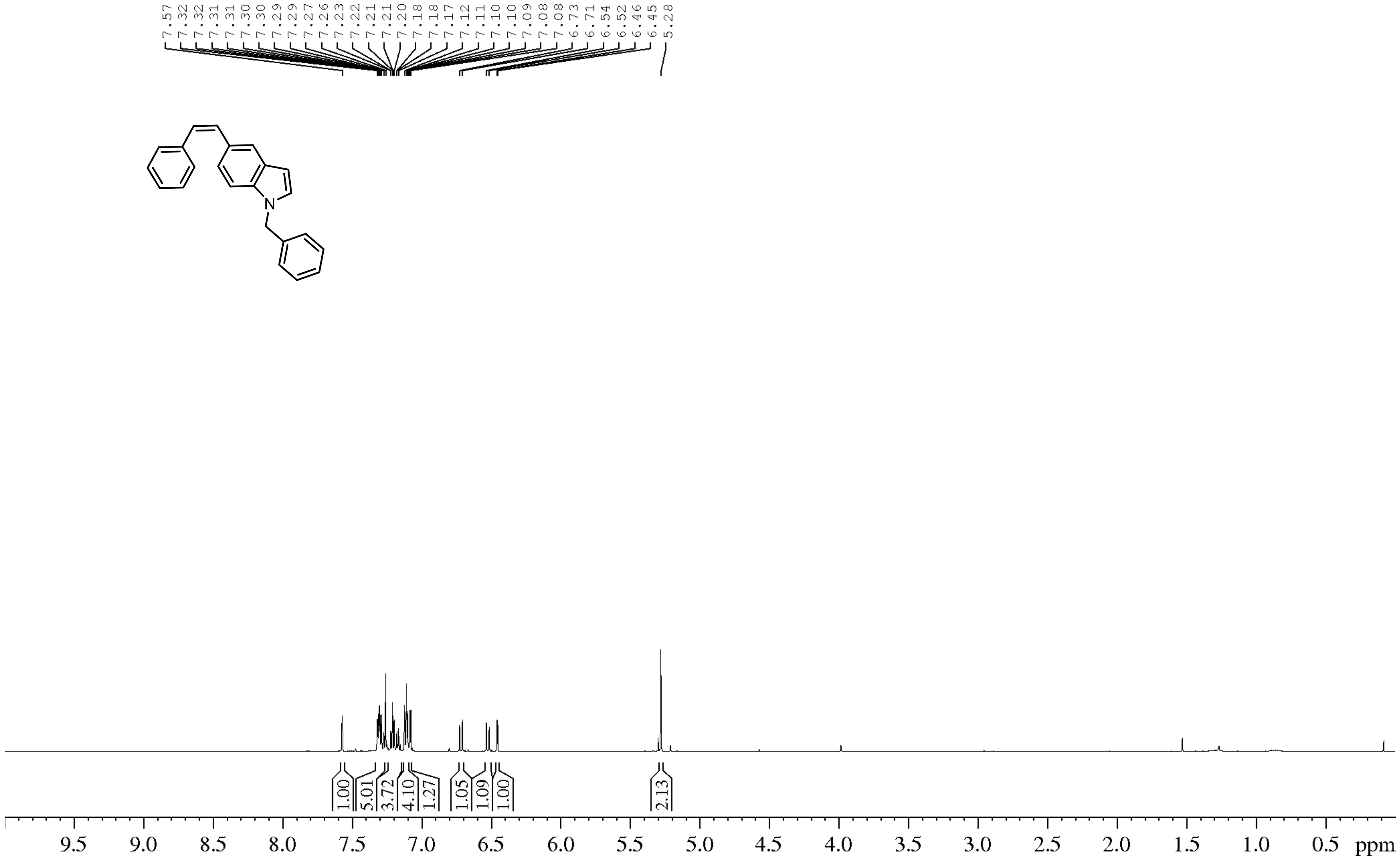

2.27.2 ^{13}C NMR

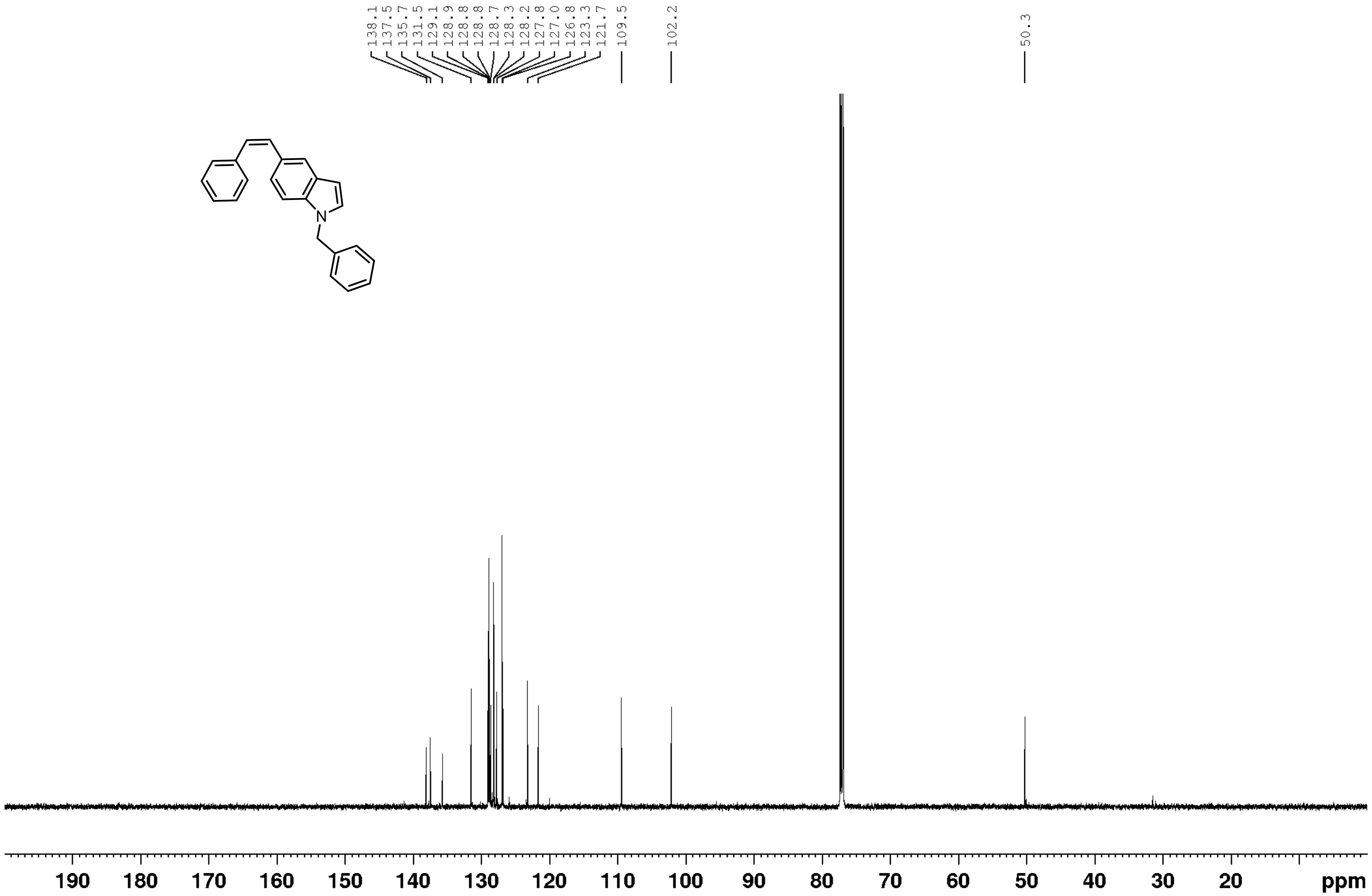
138.1
137.5
135.7
131.5
129.1
128.9
128.8
128.8
128.7
128.3
128.2
127.8
127.0
126.8
123.3
121.7
109.5
102.2
50.3
190
180
170
160
150
140
130
120
110
100
90
80
70
60
50
40
30
20
ppm

2.27.3 ¹H, ¹H COSY

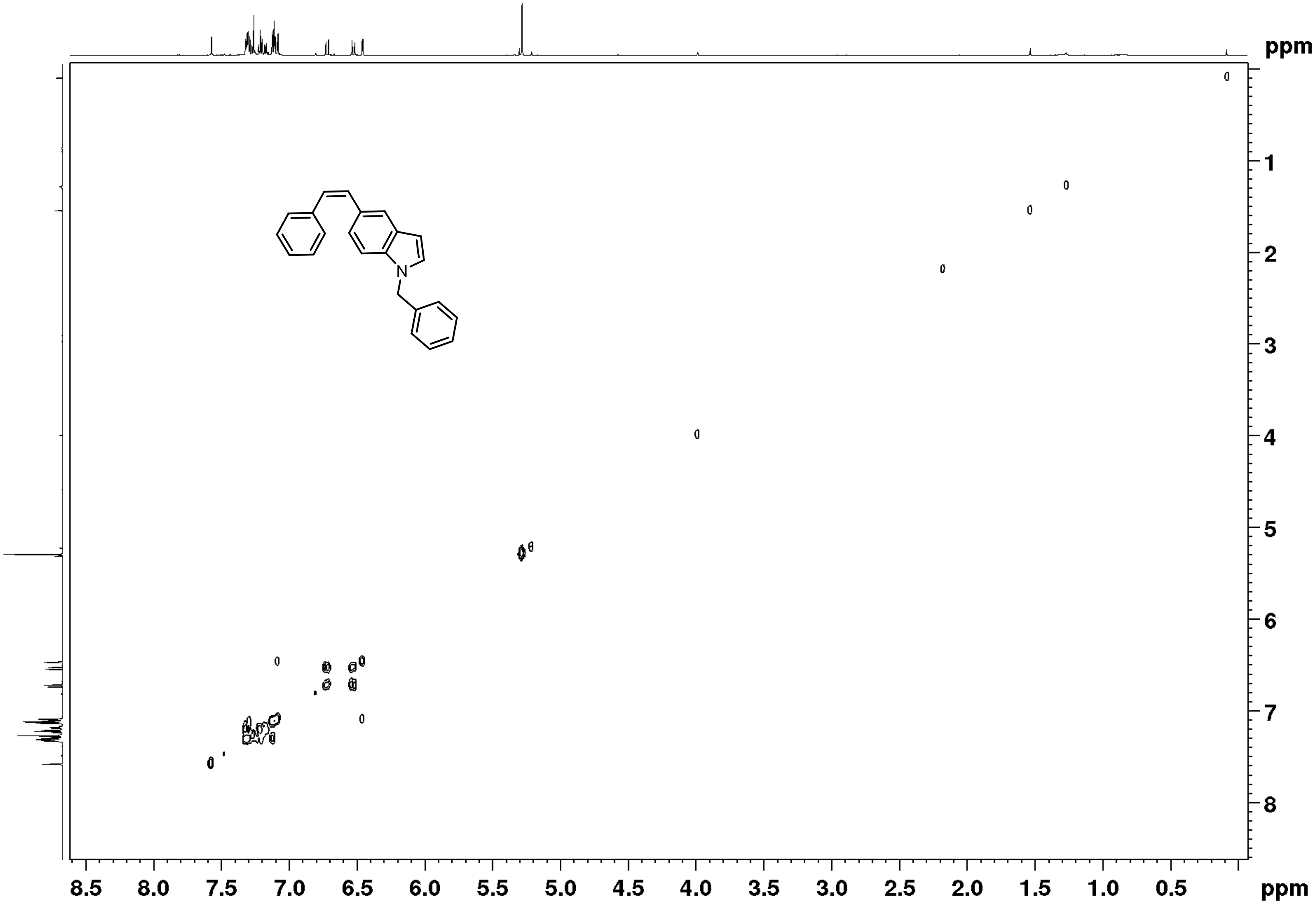

2.27.4 ^{1}H, ^{13}C HMBC

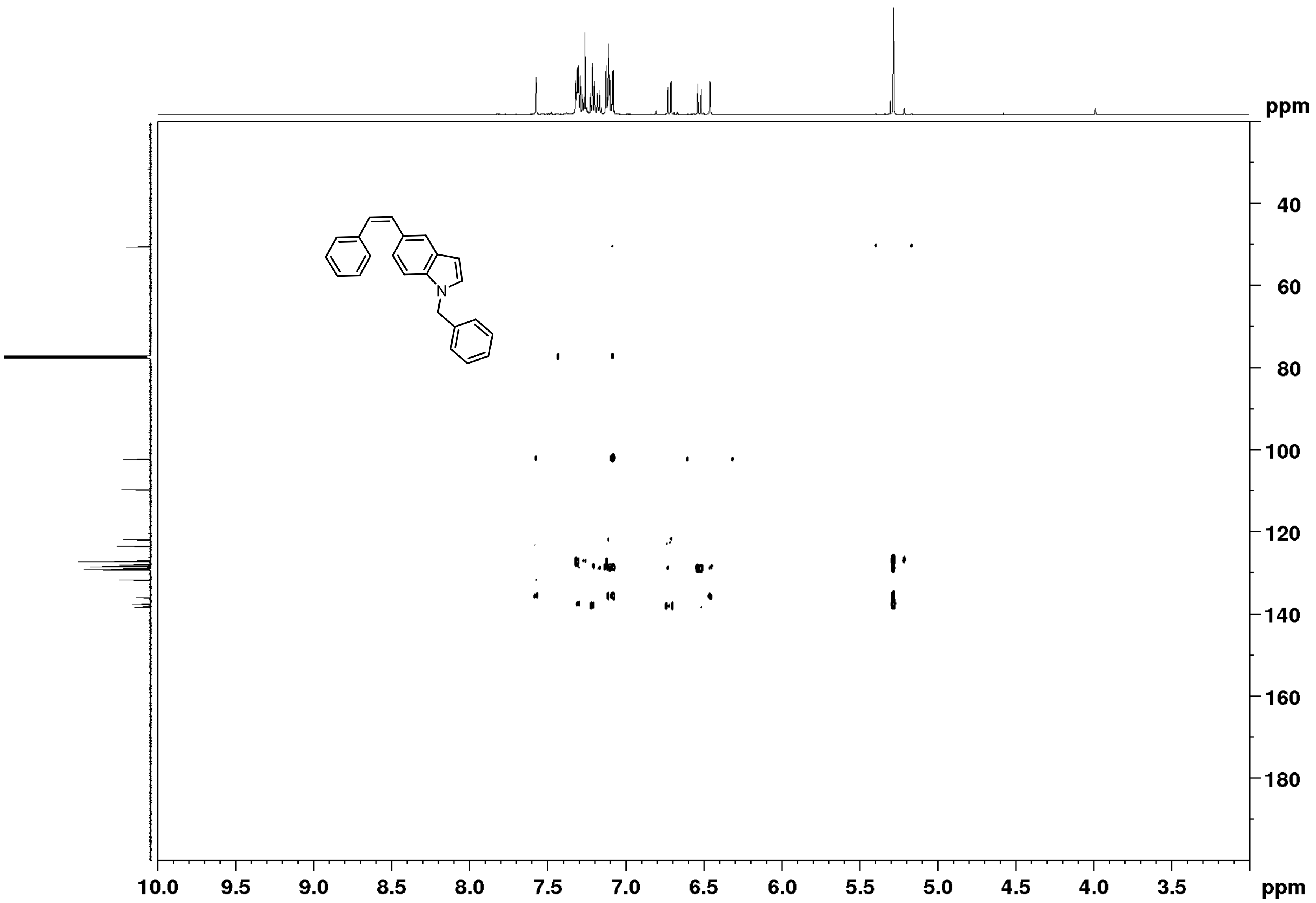

2.27.5 ^{1}H, ^{13}C HSQC

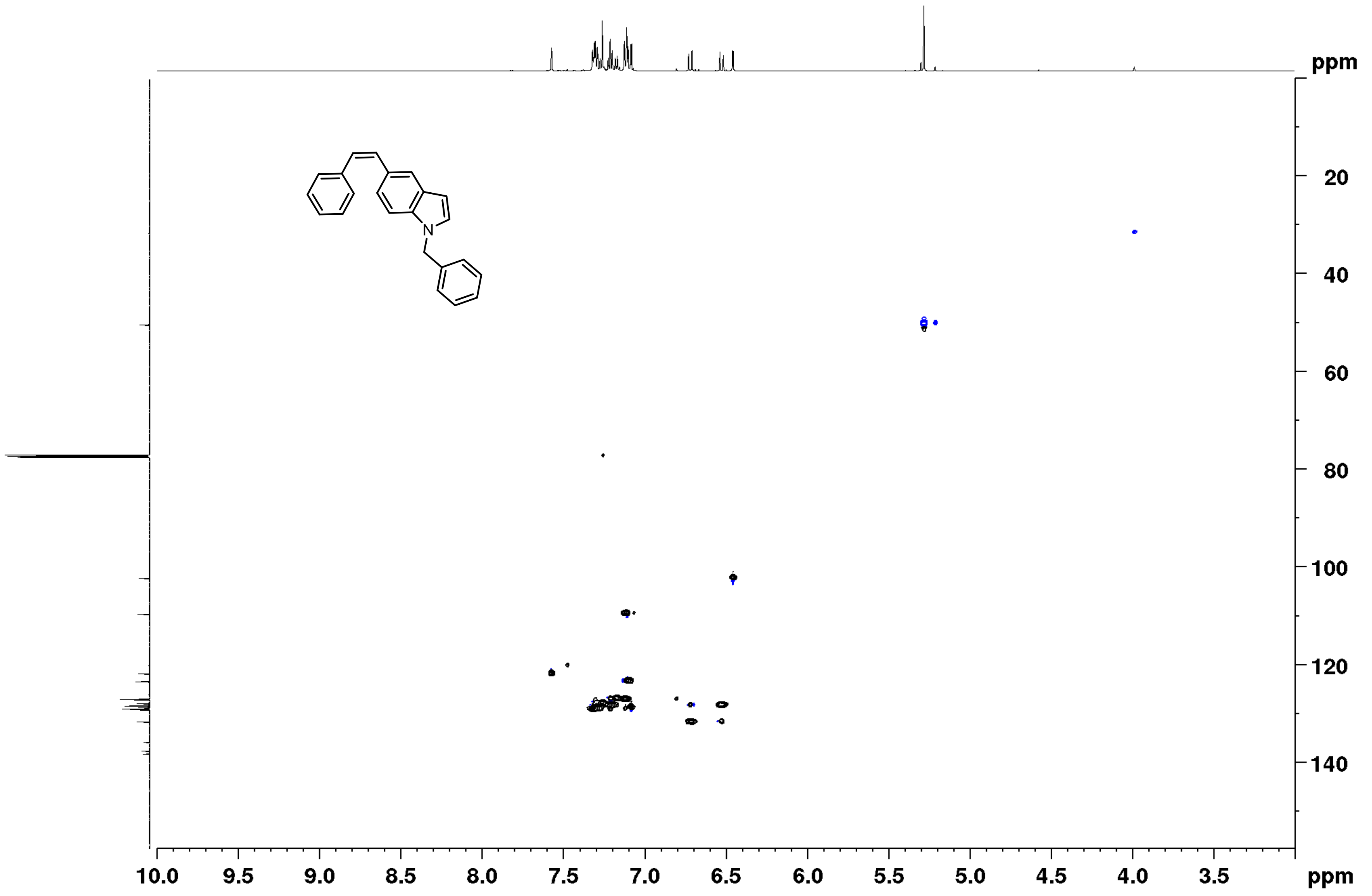

2.27.6 HRMS

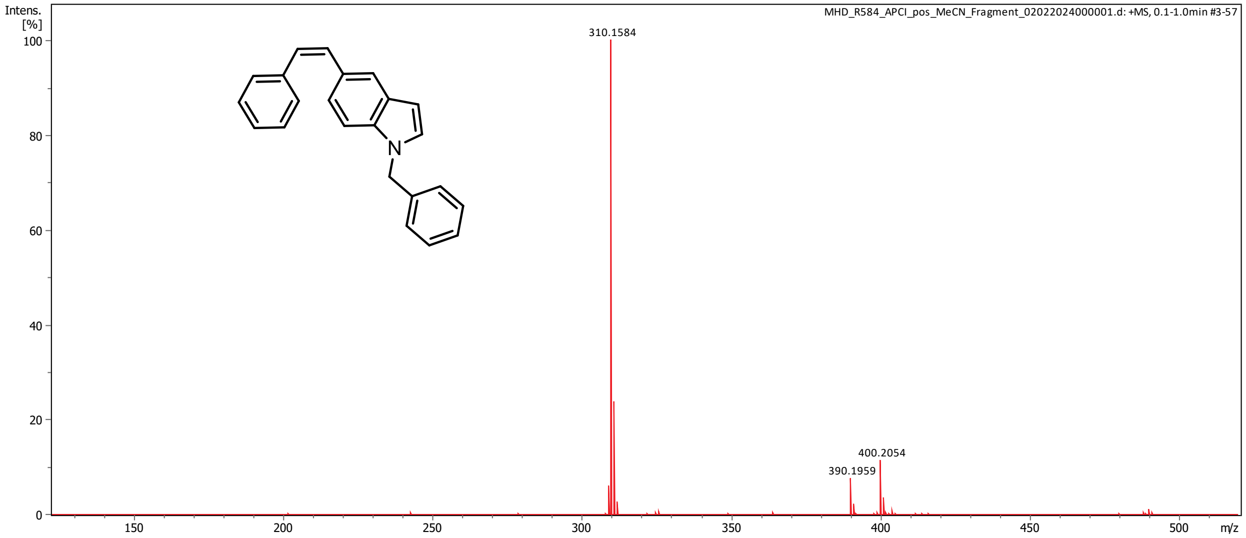

Intens. [%]
MHD_R584_APCI_pos_MeCN_Fragment_02022024000001.d: +MS, 0.1-1.0min #3-57
310.1584
400.2054
390.1959
N
m/z

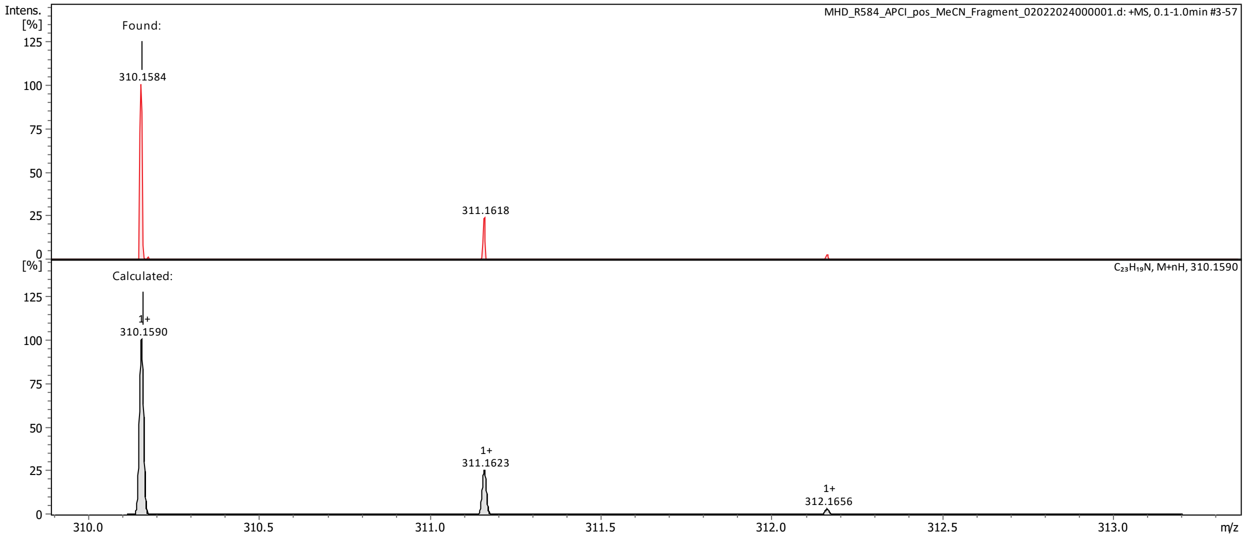

Intens. [%]
MHD_R584_APCI_pos_MeCN_Fragment_02022024000001.d: +MS, 0.1-1.0min #3-57
Found:
310.1584
311.1618
Calculated:
$C_{23}H_{19}N$, M+nH, 310.1590
1+ 310.1590
1+ 311.1623
1+ 312.1656
m/z

2.27.7 IR

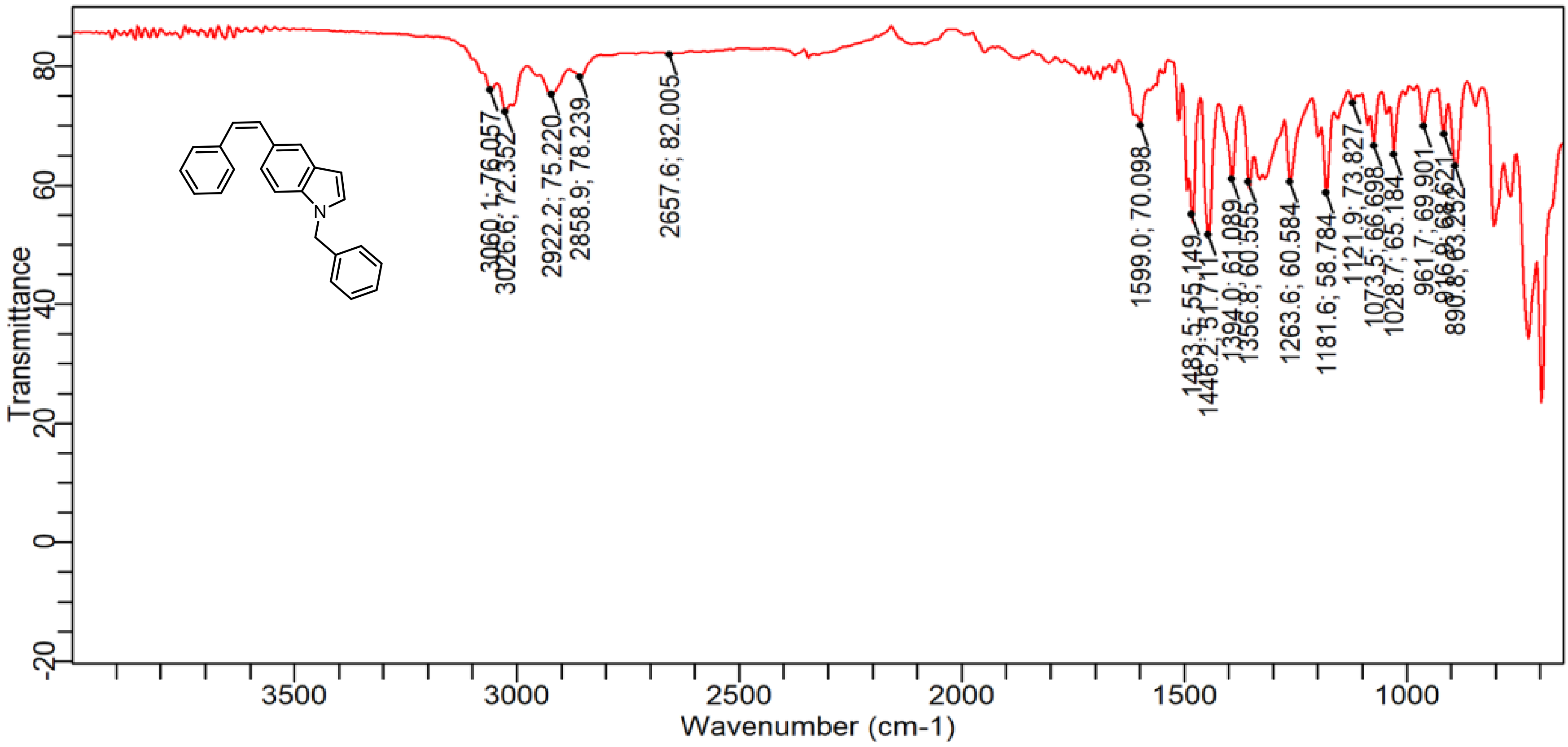

3060.1; 76.057
3026.6; 72.352
2922.2; 75.220
2858.9; 78.239
2657.6; 82.005
1599.0; 70.098
1483.5; 55.149
1446.2; 51.711
1394.0; 61.089
1356.8; 60.555
1263.6; 60.584
1181.6; 58.784
1121.9; 73.827
1073.5; 66.698
1028.7; 65.184
961.7; 69.901
916.9; 68.621
890.8; 63.252
Transmittance
Wavenumber (cm-1)
N

3 Spectra of products from conjugate reduction of unsaturated amides

3.1 *N*,*N*-Diethyl-3-phenylpropanamide (12a) (600 MHz, $CDCl_3$)

3.1.1 ^{1}H NMR

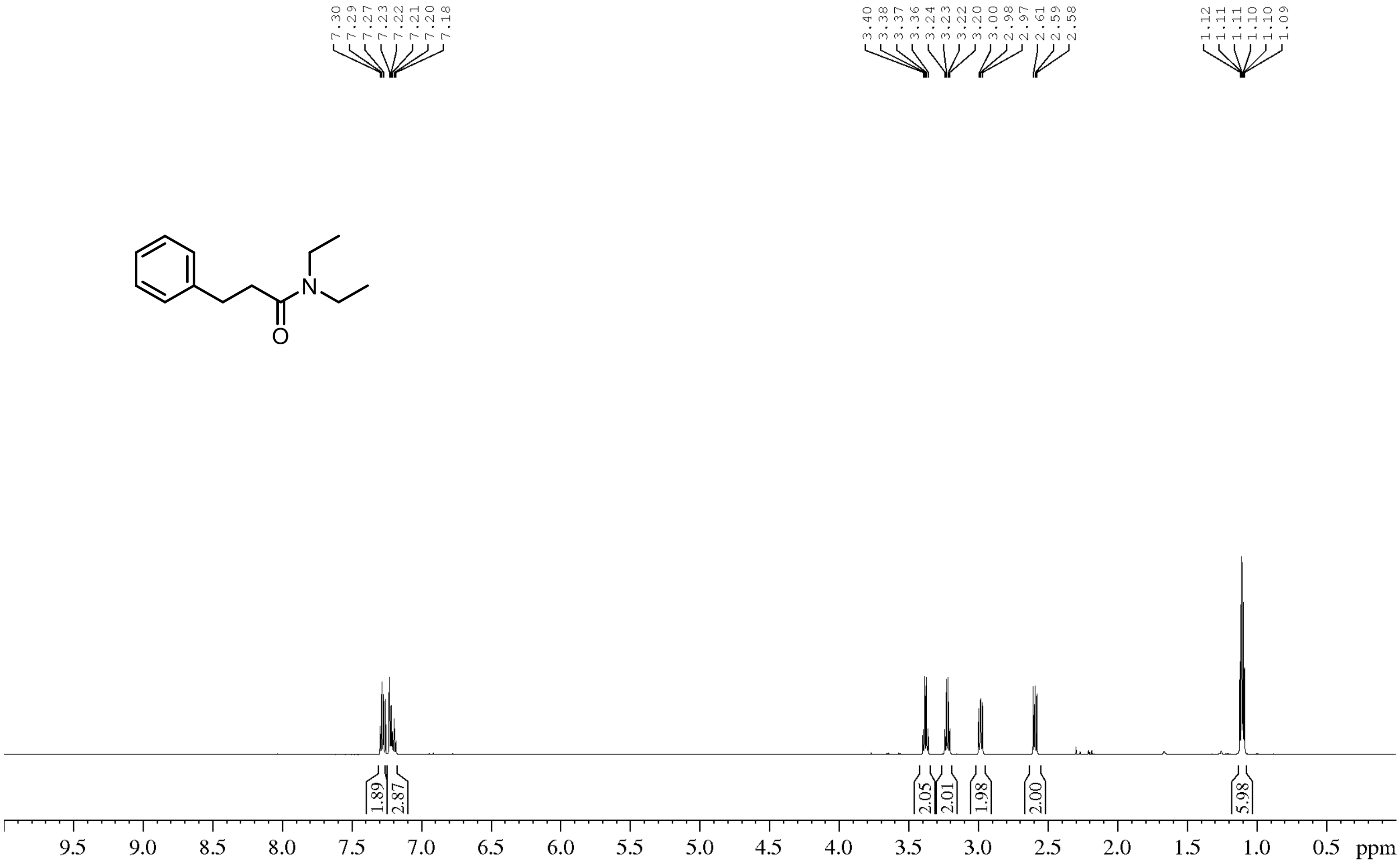

3.1.2 ^{13}C NMR

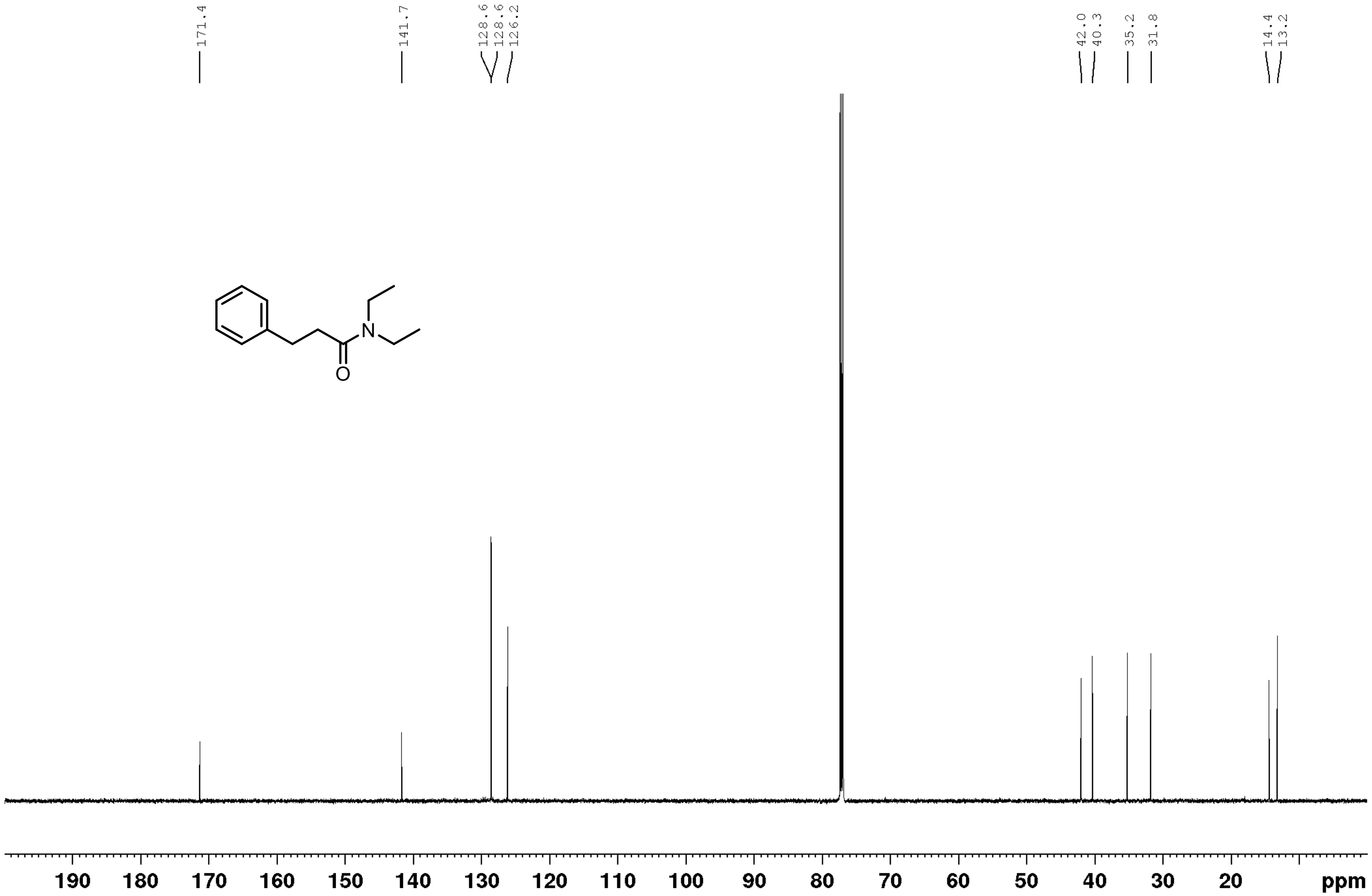
171.4
141.7
128.6
128.6
126.2
42.0
40.3
35.2
31.8
14.4
13.2
N
O
190 180 170 160 150 140 130 120 110 100 90 80 70 60 50 40 30 20
ppm

3.1.3 ^{1}H, ^{1}H COSY

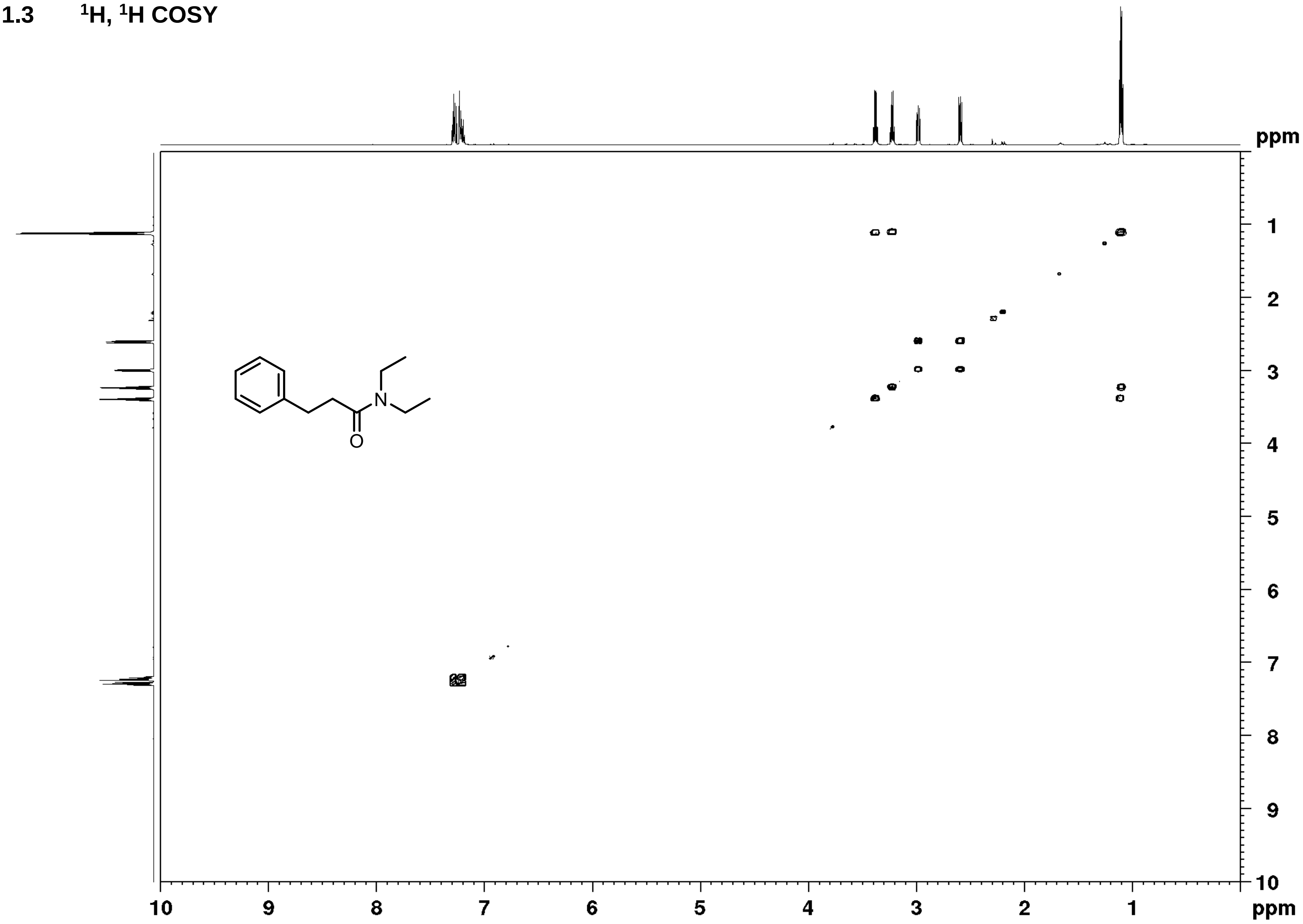

3.1.4 ^{1}H, ^{13}C HMBC

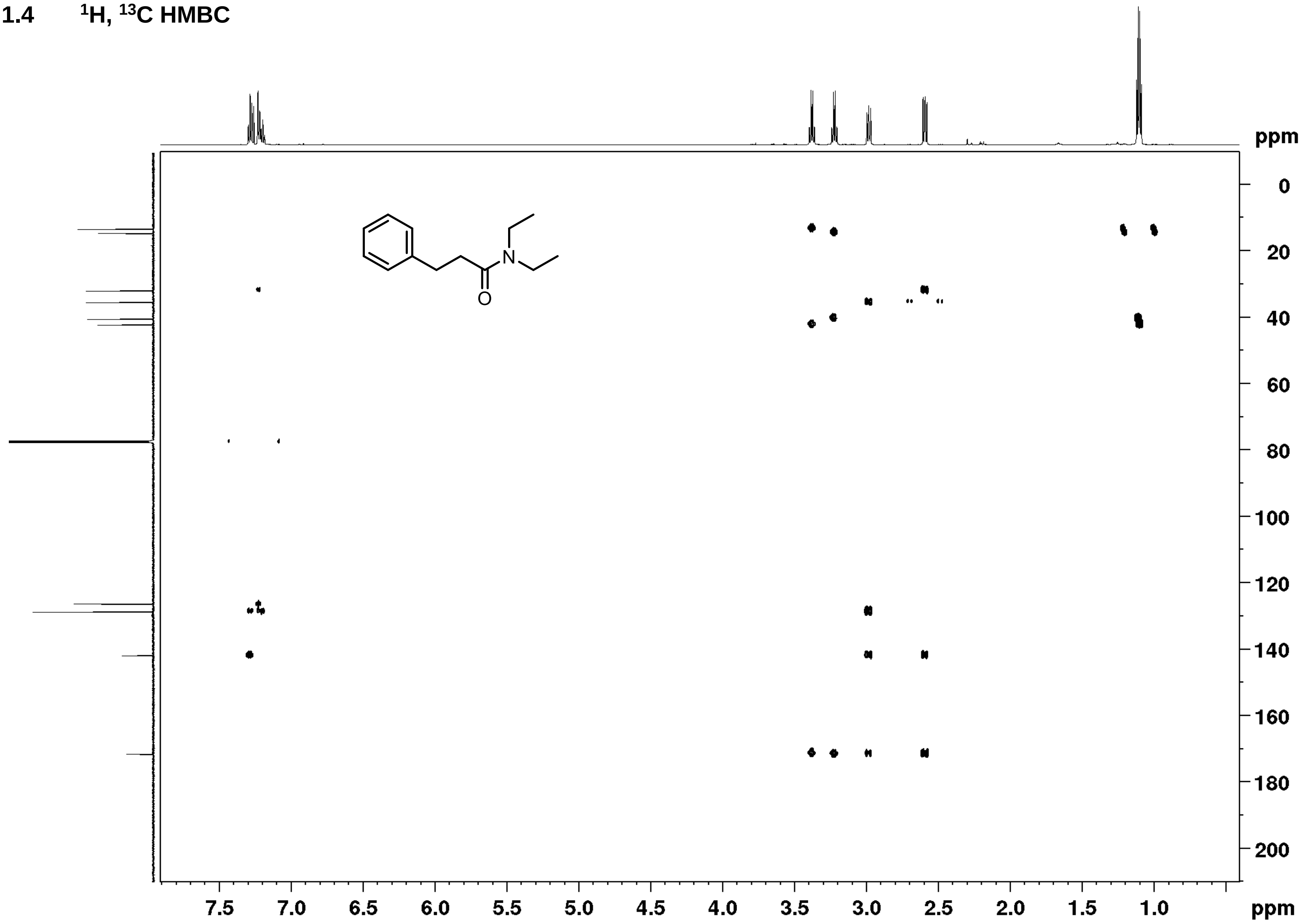

3.1.5 ^{1}H, ^{13}C HSQC

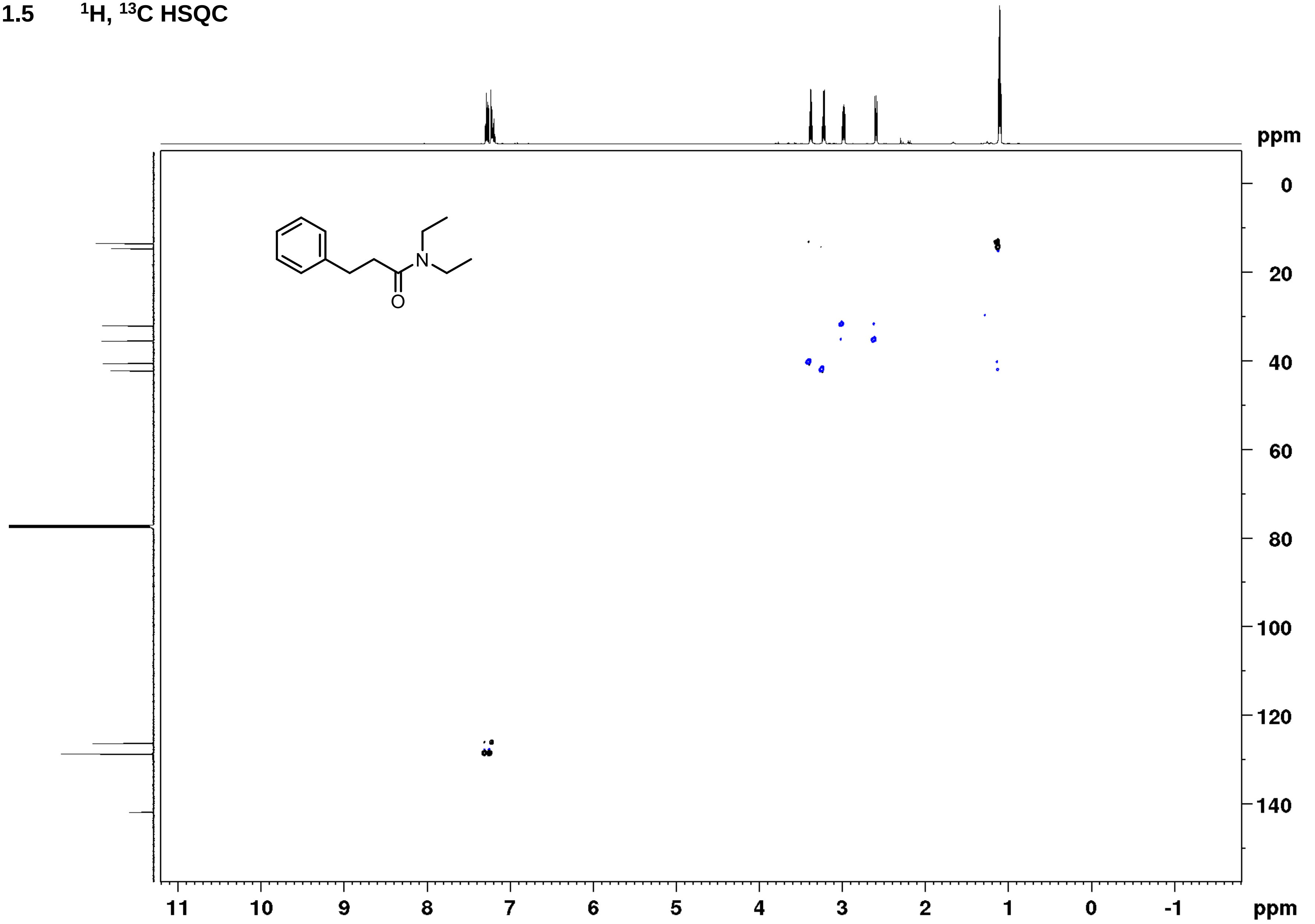

3.1.6 HRMS

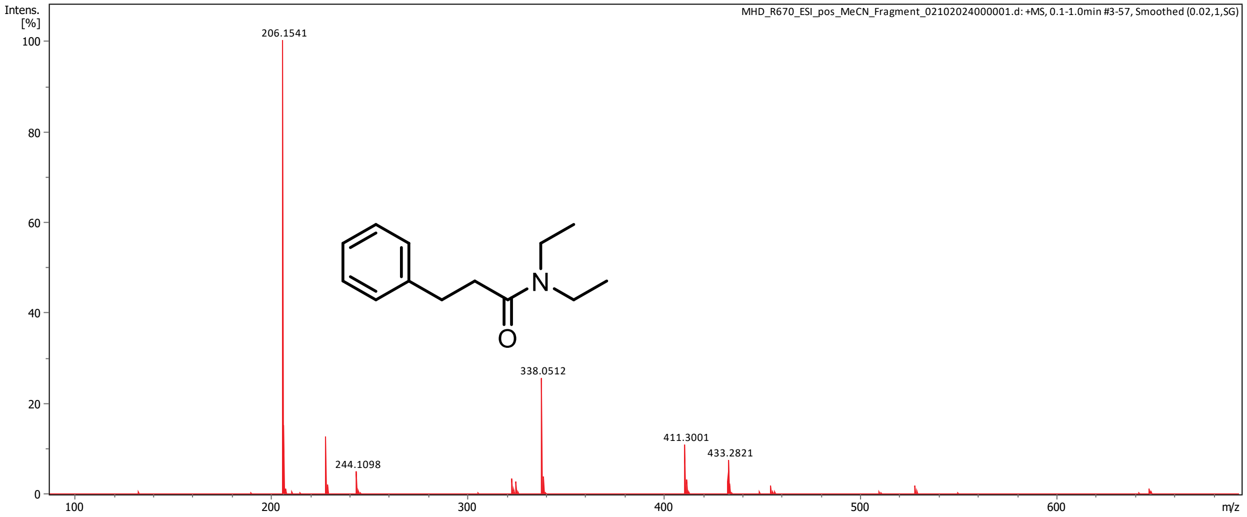
MHD_R670_ESI_pos_MeCN_Fragment_02102024000001.d: +MS, 0.1-1.0min #3-57, Smoothed (0.02,1,SG)
Intens. [%]
206.1541
244.1098
338.0512
411.3001
433.2821
N
O
m/z

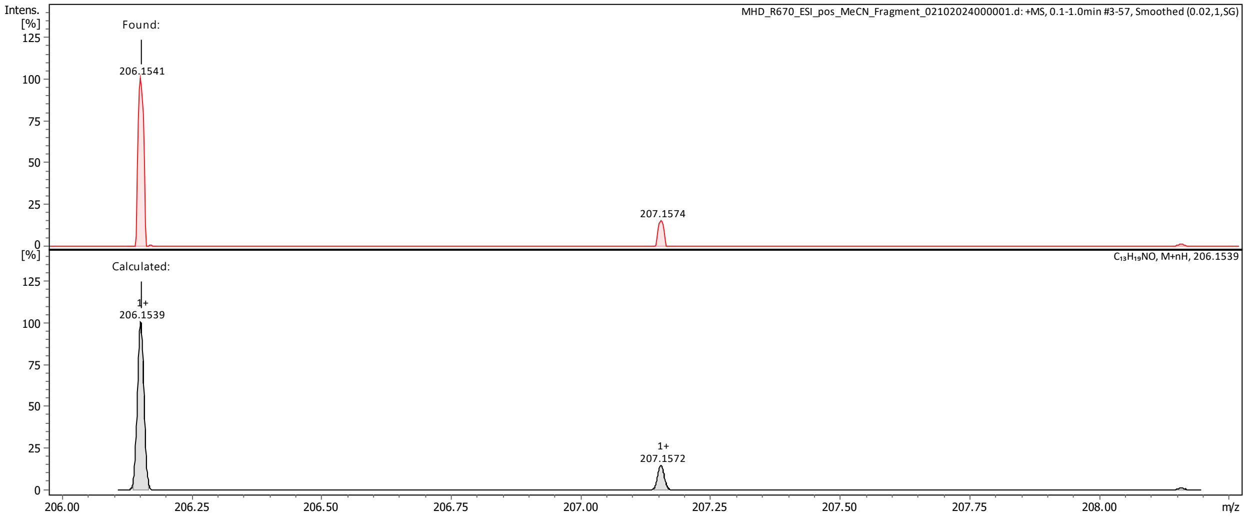
MHD_R670_ESI_pos_MeCN_Fragment_02102024000001.d: +MS, 0.1-1.0min #3-57, Smoothed (0.02,1,SG)
Intens. [%]
Found:
206.1541
207.1574
$C_{13}H_{19}NO$, M+nH, 206.1539
Calculated:
1+
206.1539
1+
207.1572
m/z

3.1.7 IR

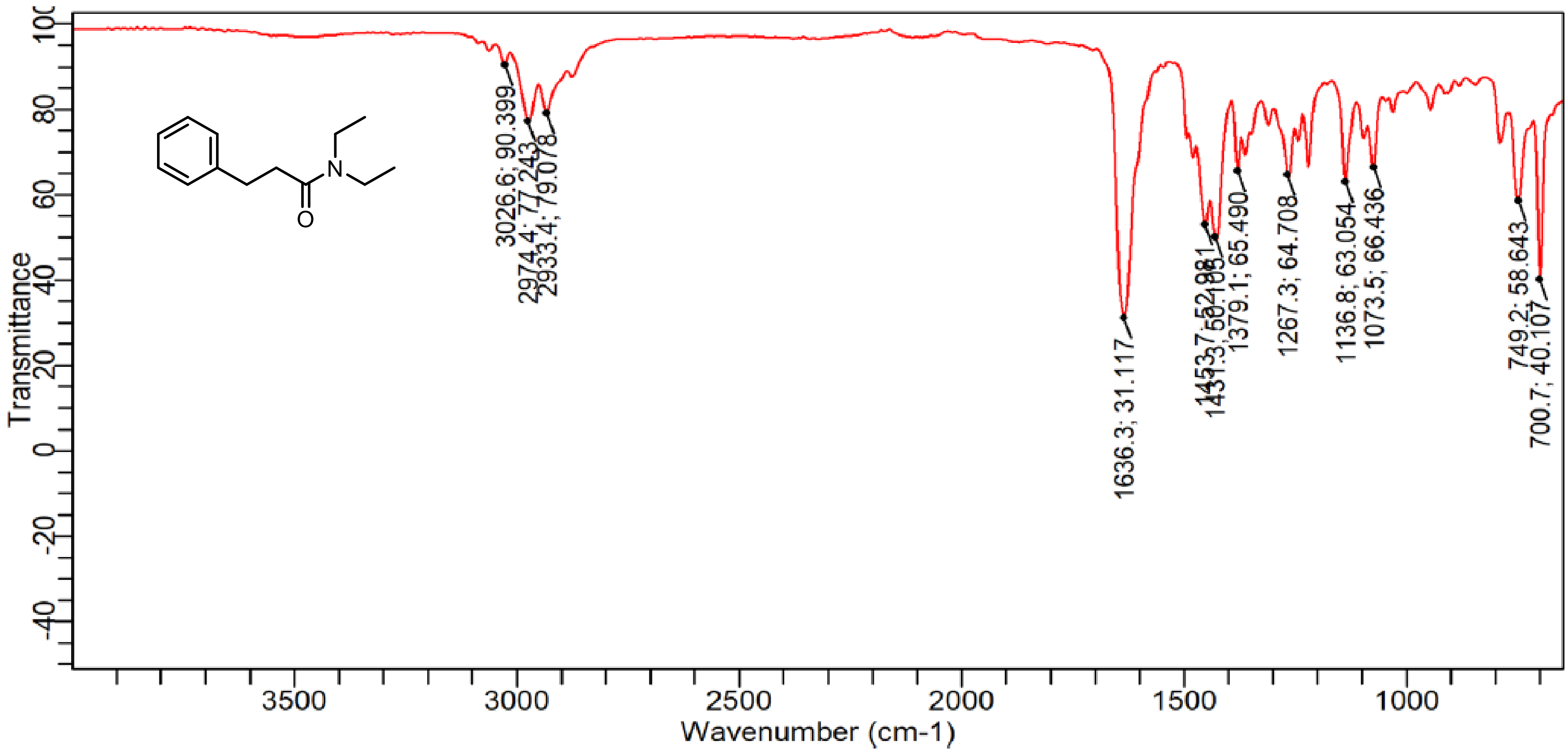

Transmittance
Wavenumber (cm-1)
3026.6; 90.399
2974.4; 77.243
2933.4; 79.078
1636.3; 31.117
1453.7; 52.981
1431.3; 50.165
1379.1; 65.490
1267.3; 64.708
1136.8; 63.054
1073.5; 66.436
749.2; 58.643
700.7; 40.107

3.2 3-Phenyl-1-(piperidin-1-yl)propan-1-one (12b) (600 MHz, $CDCl_3$)

3.2.1 ^{1}H NMR

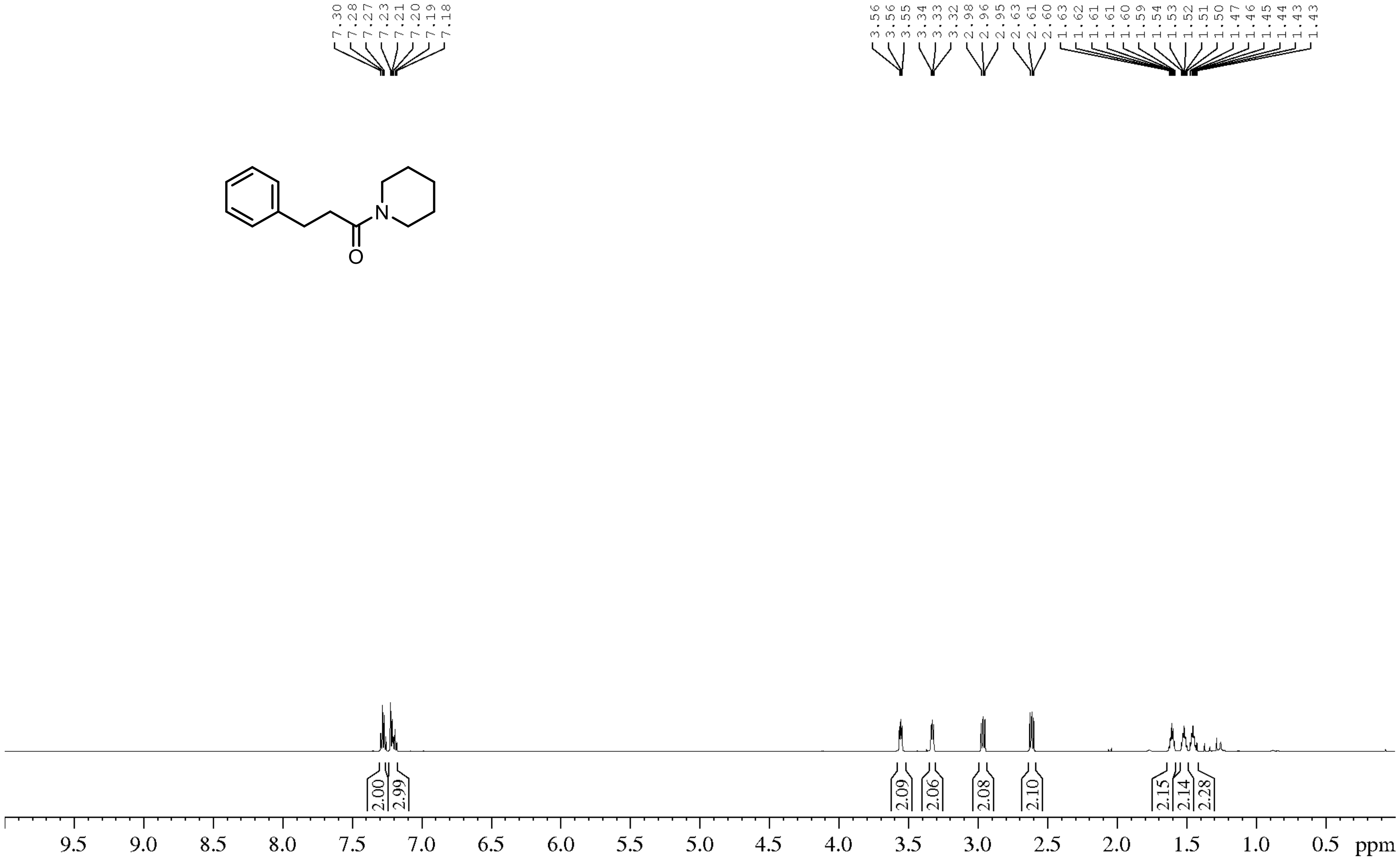

3.2.2 ^{13}C NMR

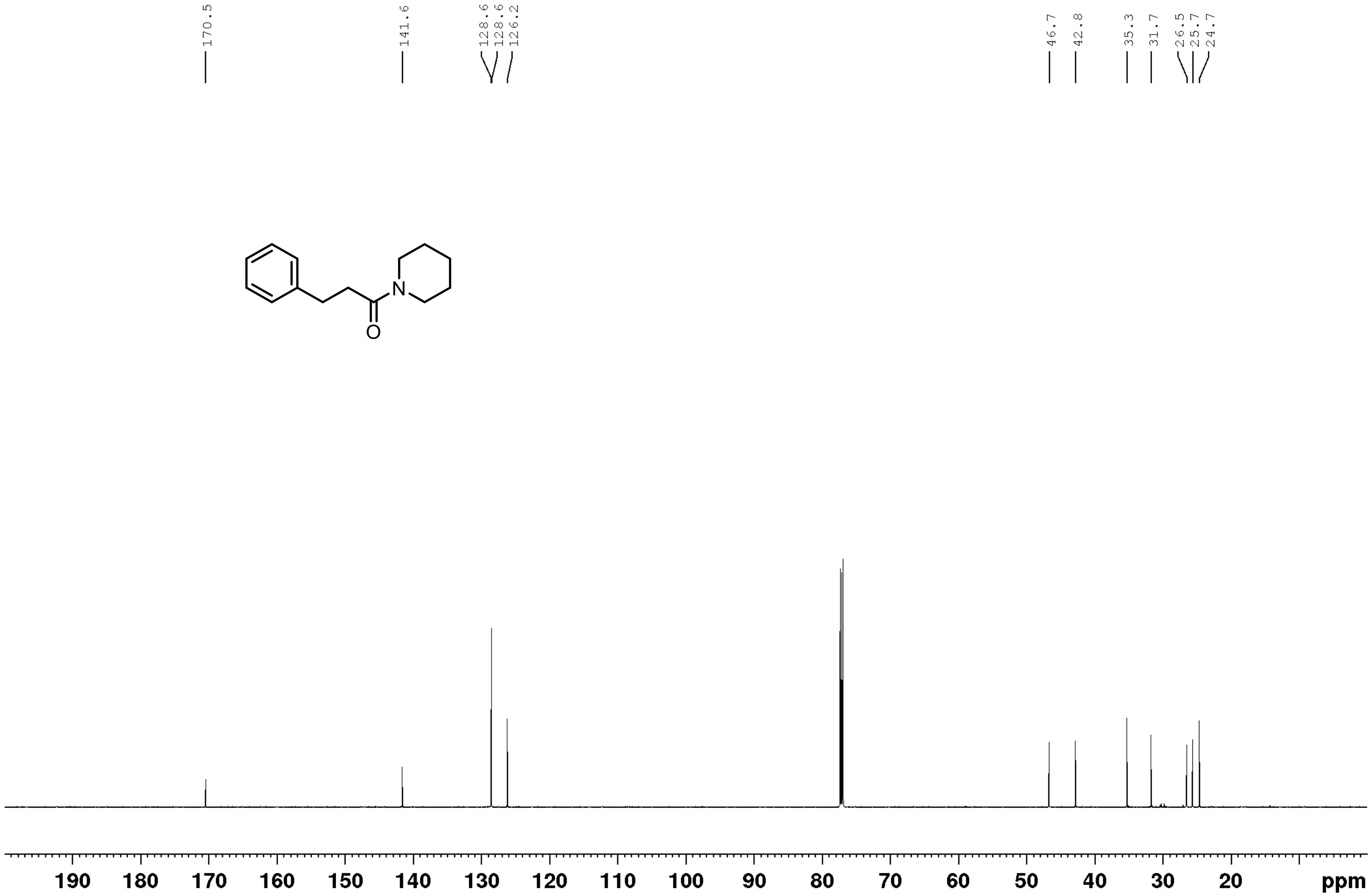
170.5
141.6
128.6
128.6
126.2
46.7
42.8
35.3
31.7
26.5
25.7
24.7
190
180
170
160
150
140
130
120
110
100
90
80
70
60
50
40
30
20
ppm

3.2.3 ^{1}H, ^{1}H COSY

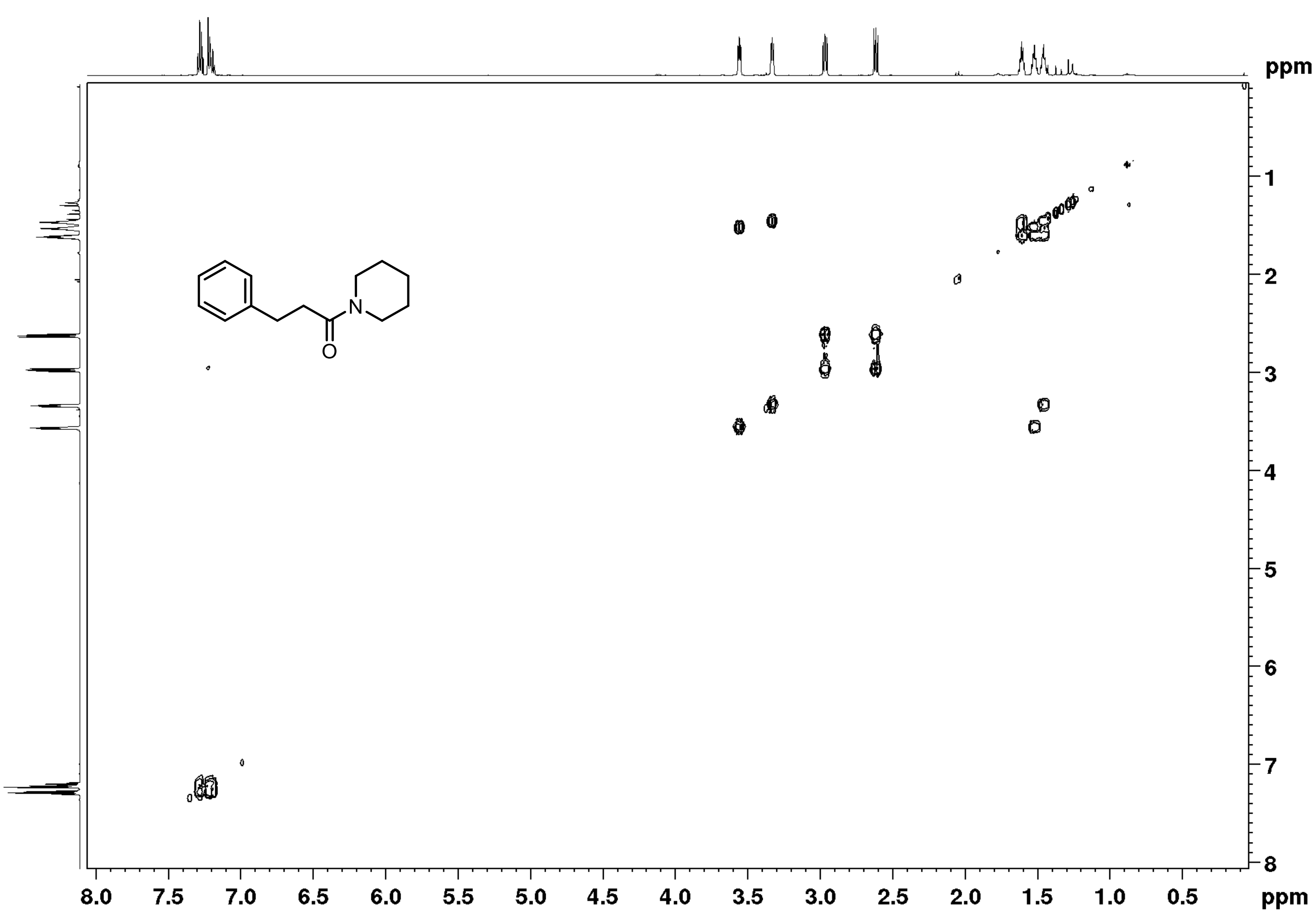

3.2.4 ^{1}H, ^{13}C HMBC

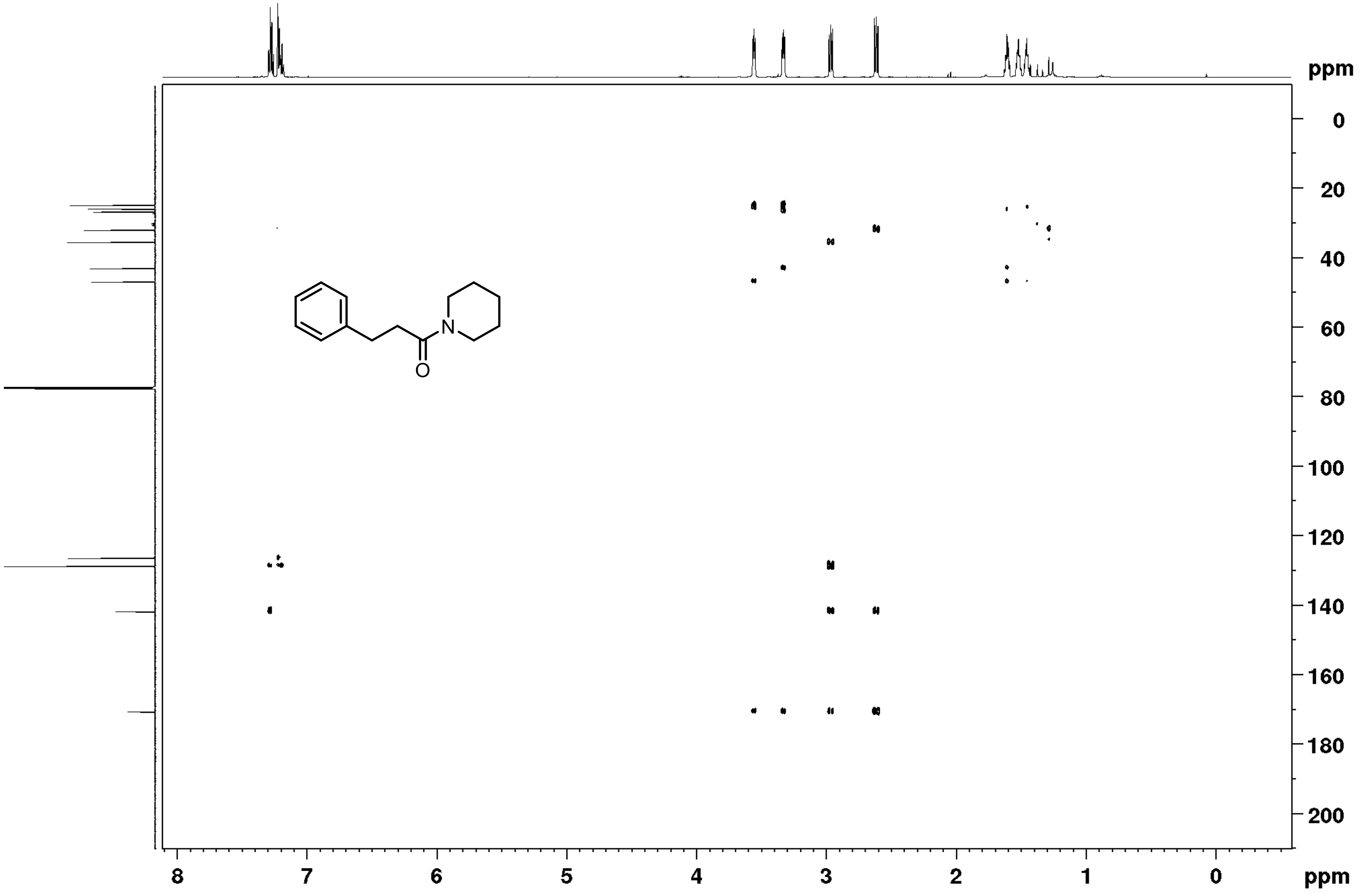

3.2.5 ^1H, ^{13}C HSQC

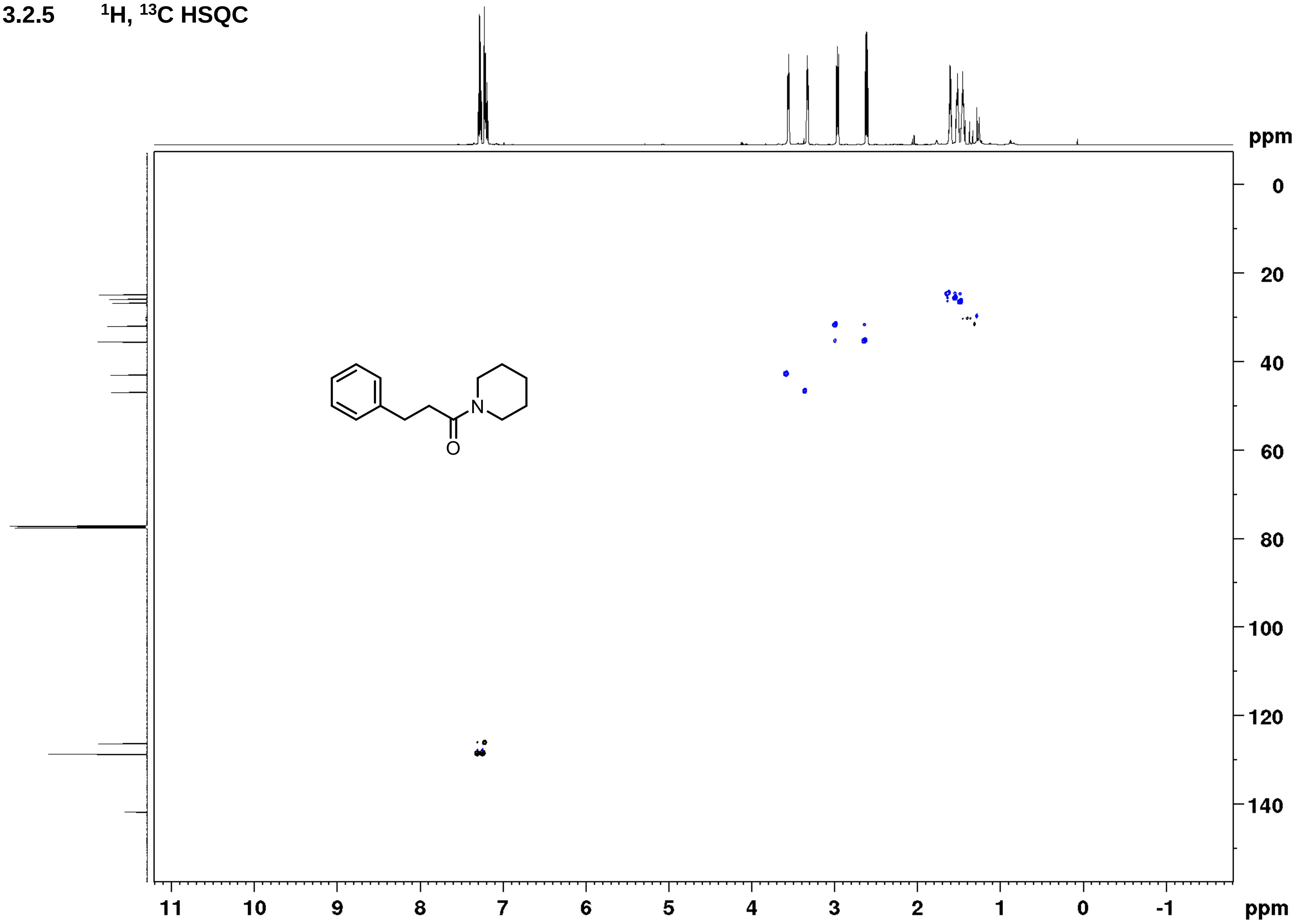

3.2.6 HRMS

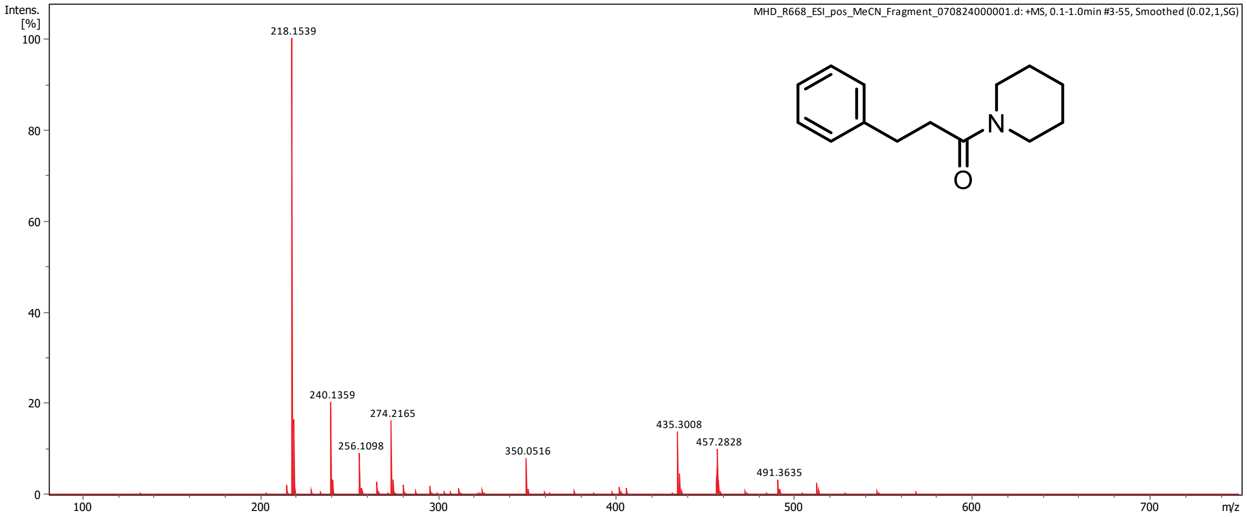

Intens. [%]
MHD_R668_ESI_pos_MeCN_Fragment_070824000001.d: +MS, 0.1-1.0min #3-55, Smoothed (0.02,1,SG)
218.1539
240.1359
274.2165
256.1098
350.0516
435.3008
457.2828
491.3635
N
O
m/z

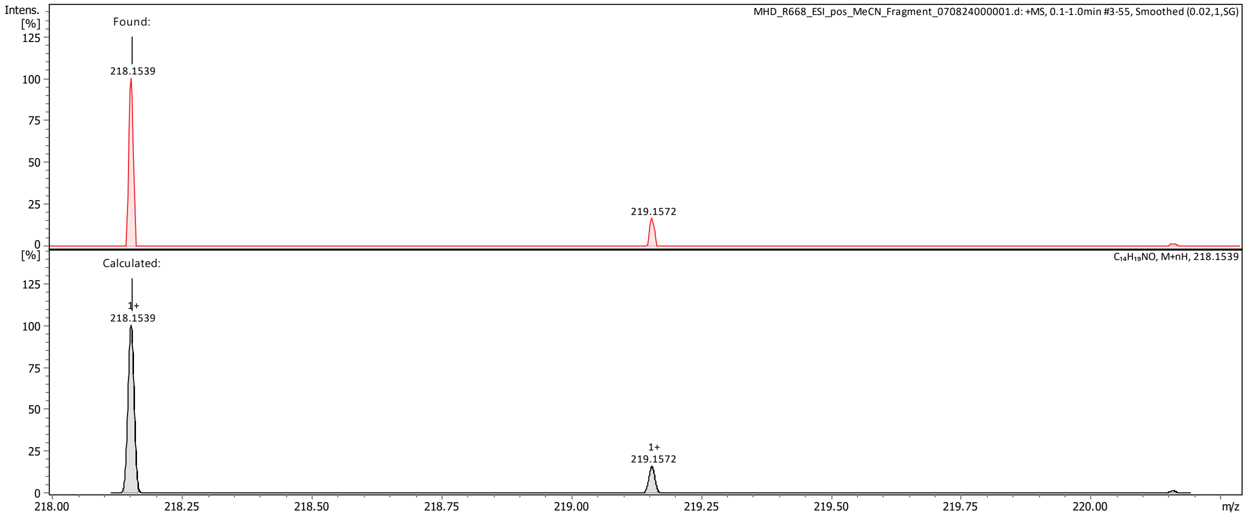

Intens. [%]
MHD_R668_ESI_pos_MeCN_Fragment_070824000001.d: +MS, 0.1-1.0min #3-55, Smoothed (0.02,1,SG)
Found:
218.1539
219.1572
Calculated:
C14H19NO, M+nH, 218.1539
1+
218.1539
1+
219.1572
m/z

3.2.7 IR

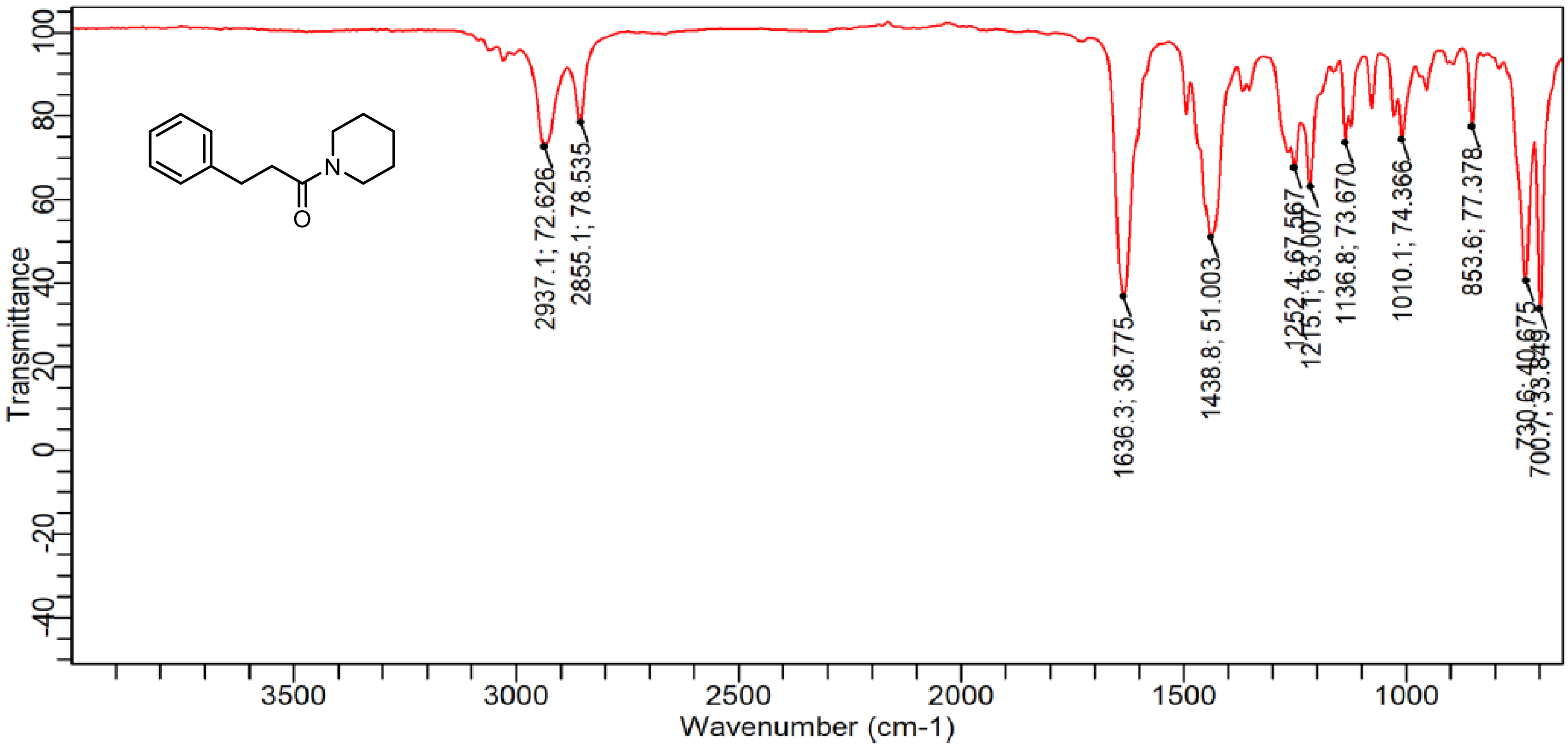

O
N
2937.1; 72.626
2855.1; 78.535
1636.3; 36.775
1438.8; 51.003
1252.4; 67.567
1215.1; 63.007
1136.8; 73.670
1010.1; 74.366
853.6; 77.378
730.6; 40.675
700.7; 33.849
Transmittance
Wavenumber (cm-1)

3.3 1-(4,4-Difluoropiperidin-1-yl)-3-phenylpropan-1-one (12c) (600 MHz, $CDCl_3$)

3.3.1 ^{1}H NMR

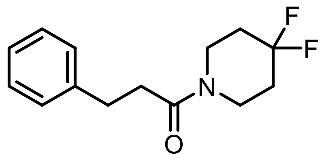

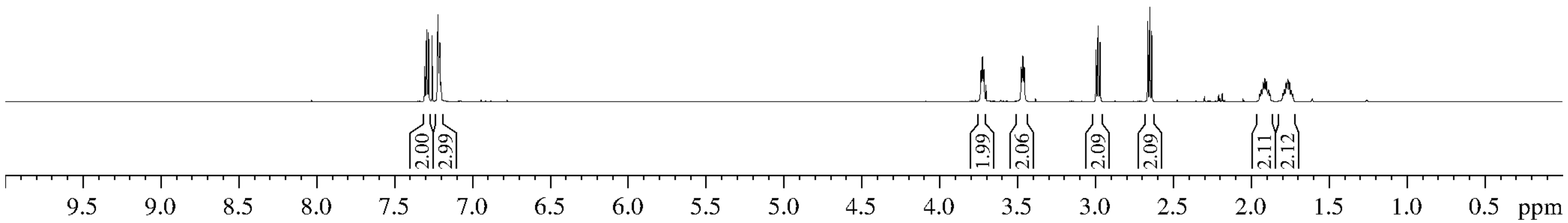

3.3.2 ^{13}C NMR

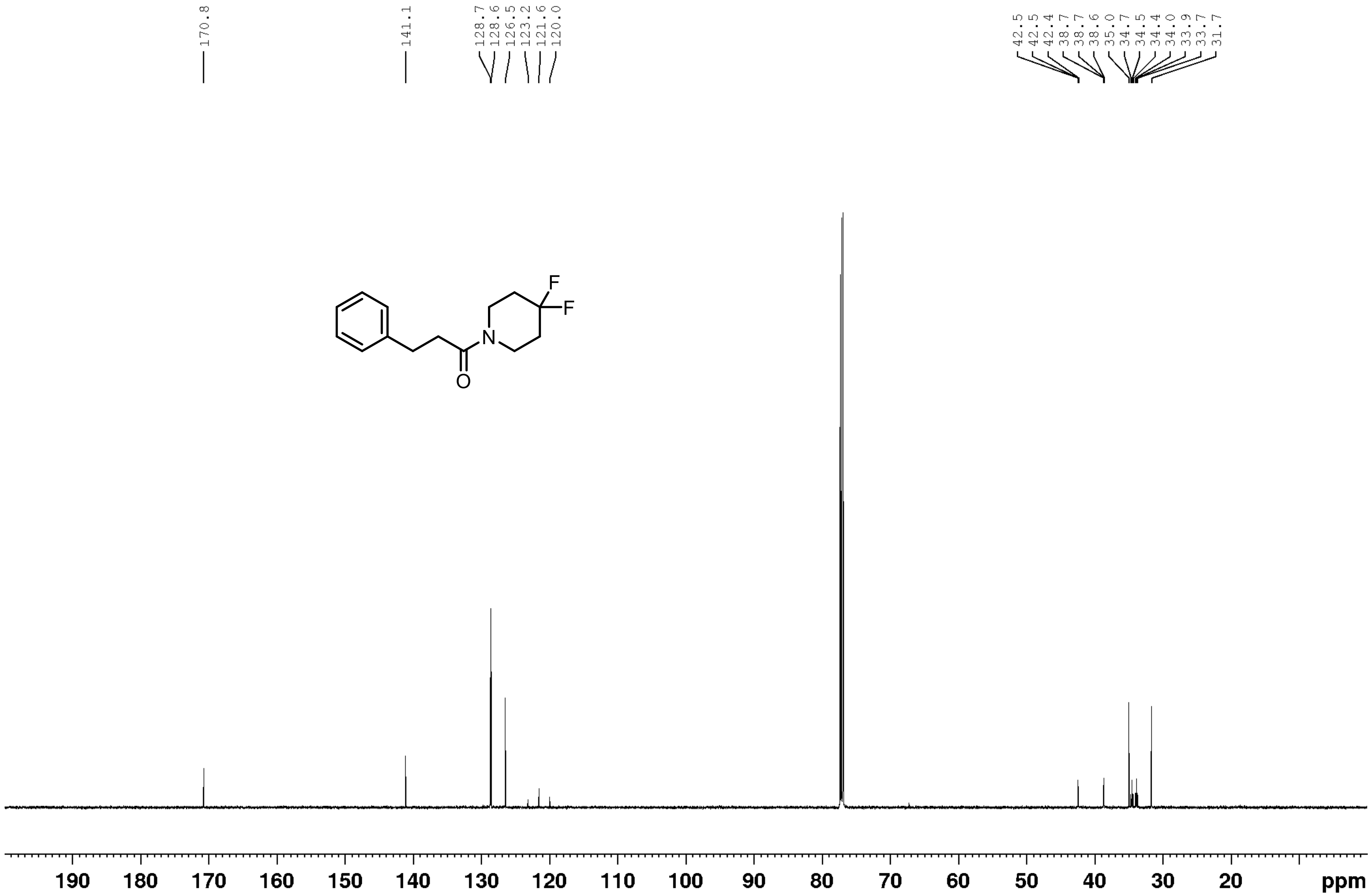

170.8
141.1
128.7
128.6
126.5
123.2
121.6
120.0
42.5
42.5
42.4
38.7
38.7
38.6
35.0
34.7
34.5
34.4
34.0
33.9
33.7
31.7
F
F
N
O
190
180
170
160
150
140
130
120
110
100
90
80
70
60
50
40
30
20
ppm

3.3.3 ^{19}F NMR

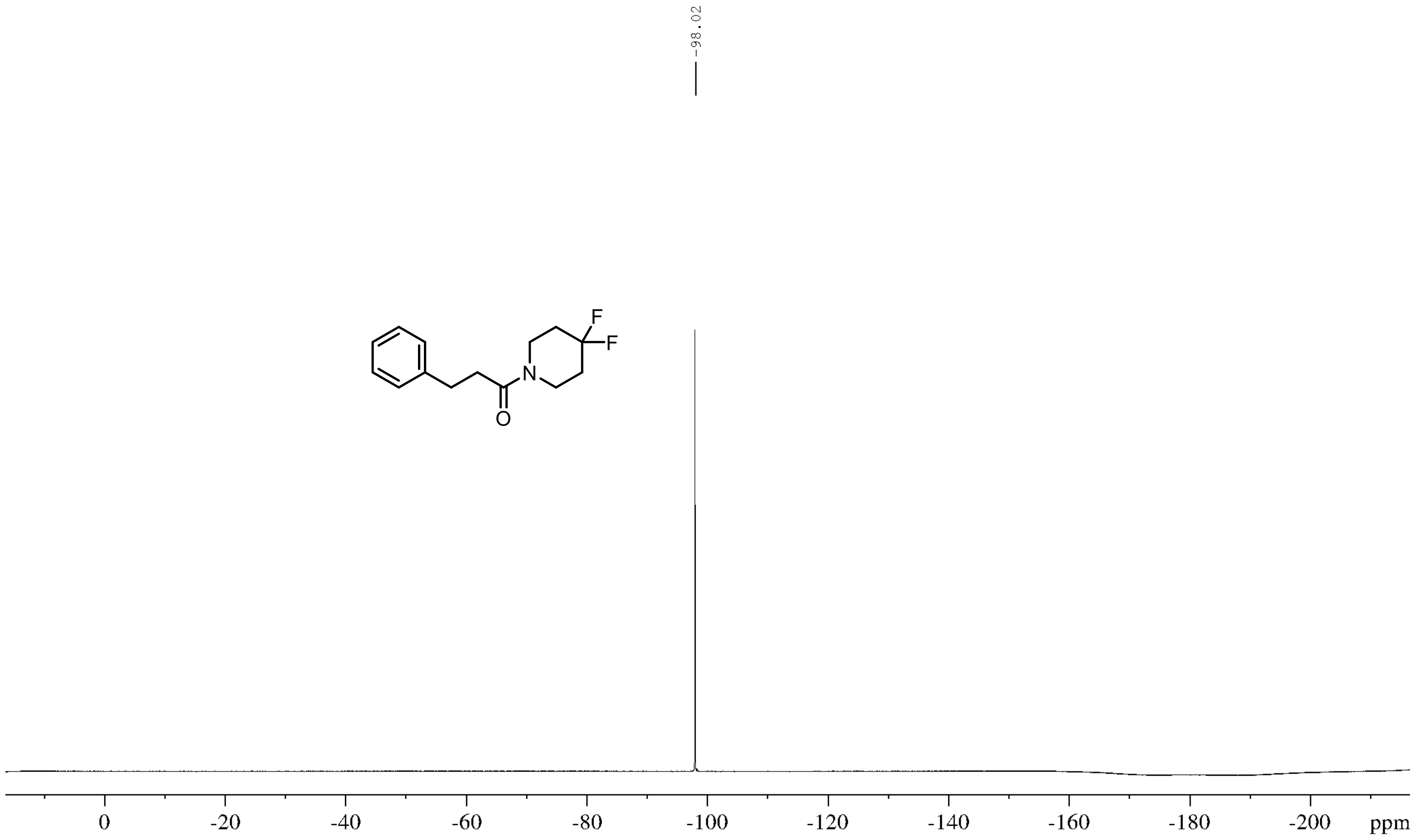

3.3.4 ^{1}H, ^{1}H COSY

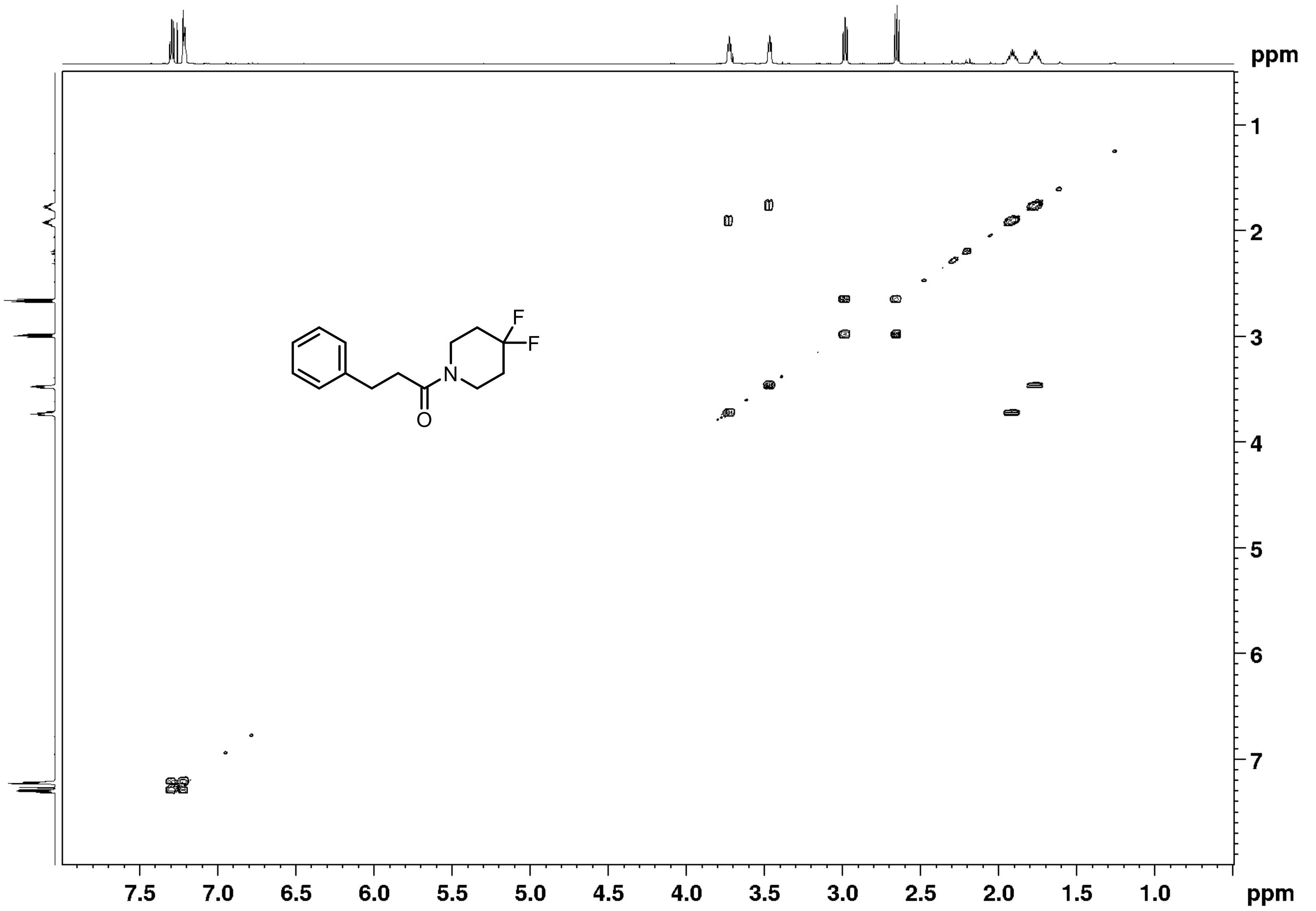

3.3.5 ^{1}H, ^{13}C HMBC

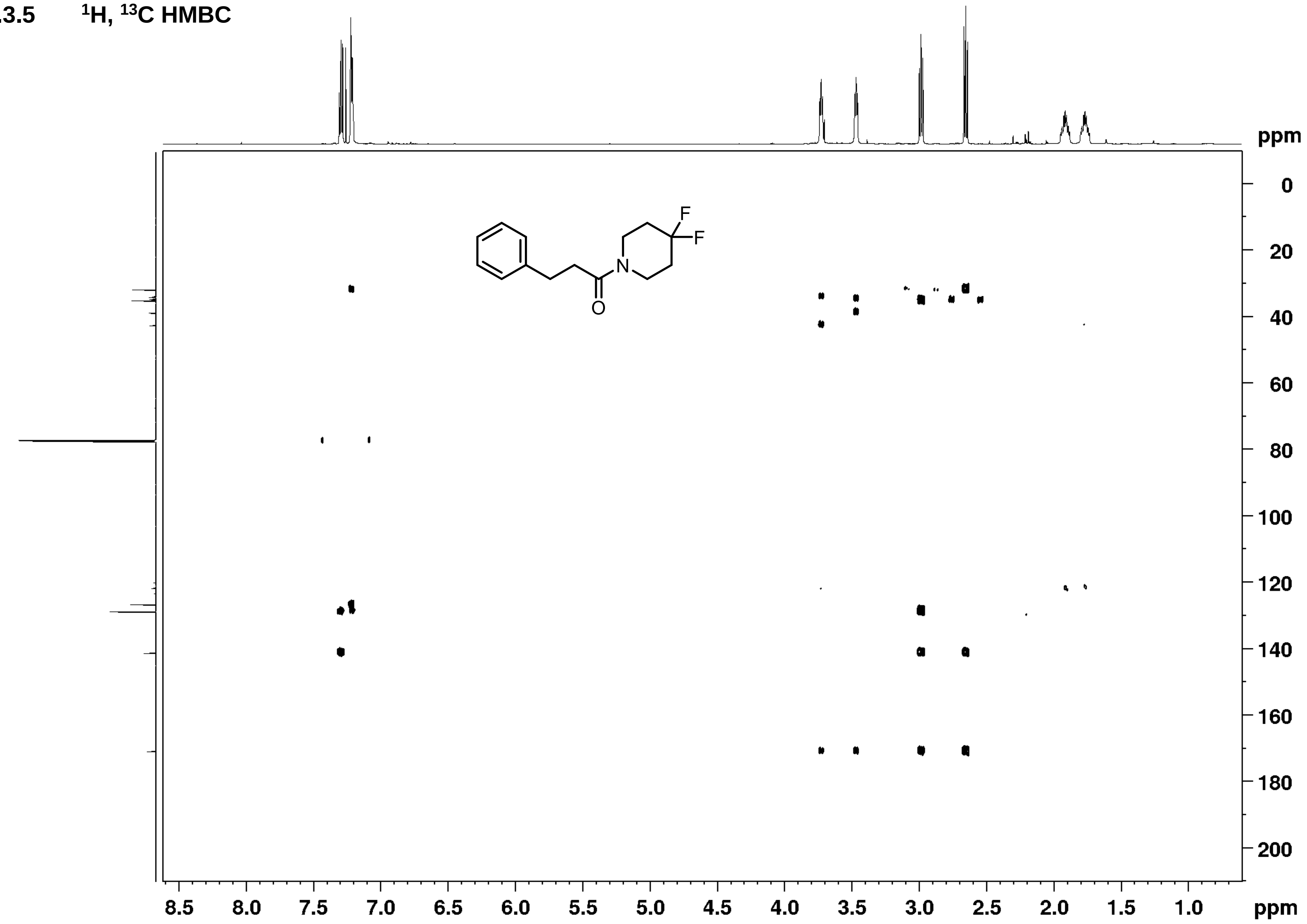

3.3.6 ^1H, ^{13}C HSQC

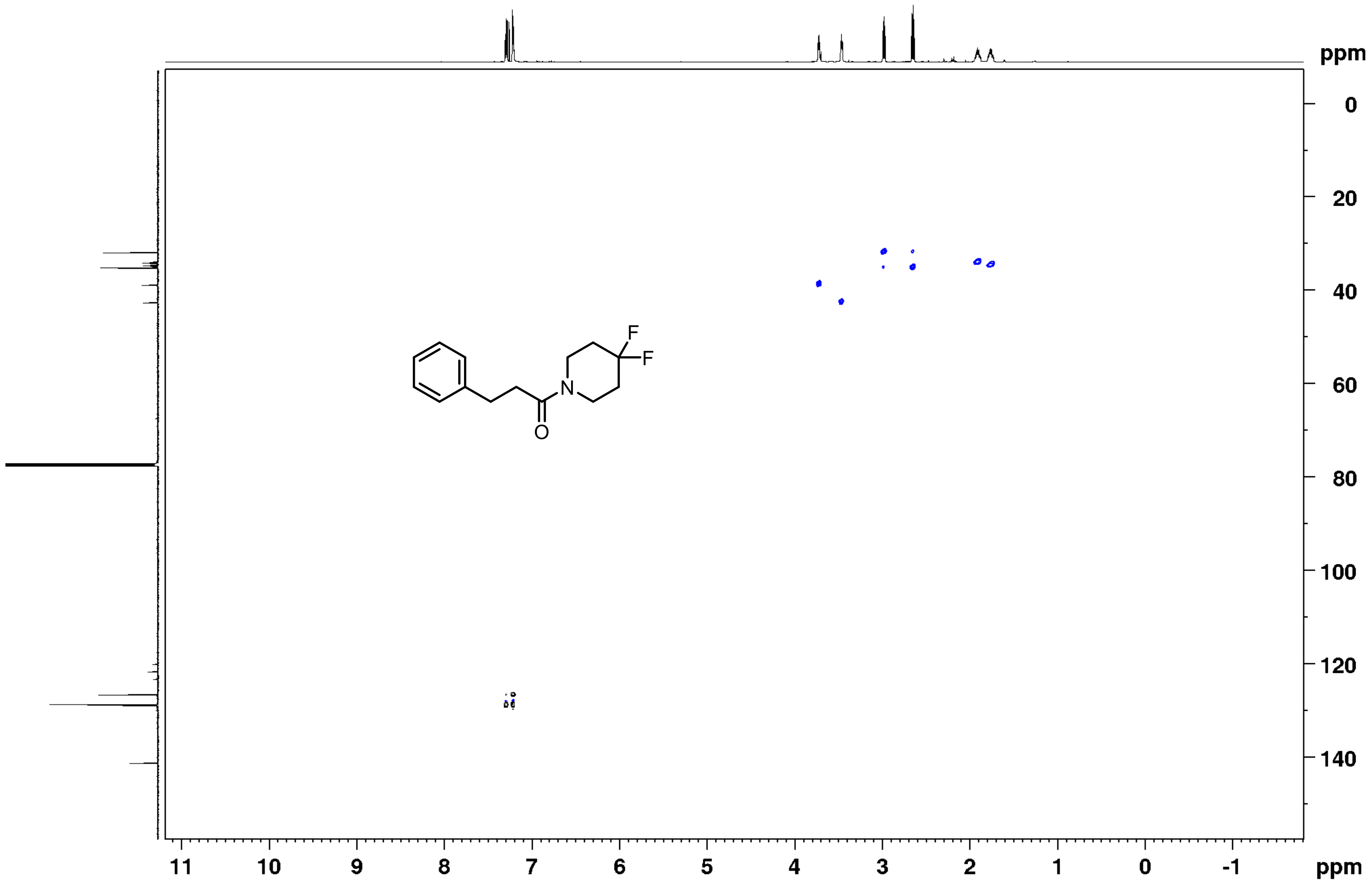

3.3.7 HRMS

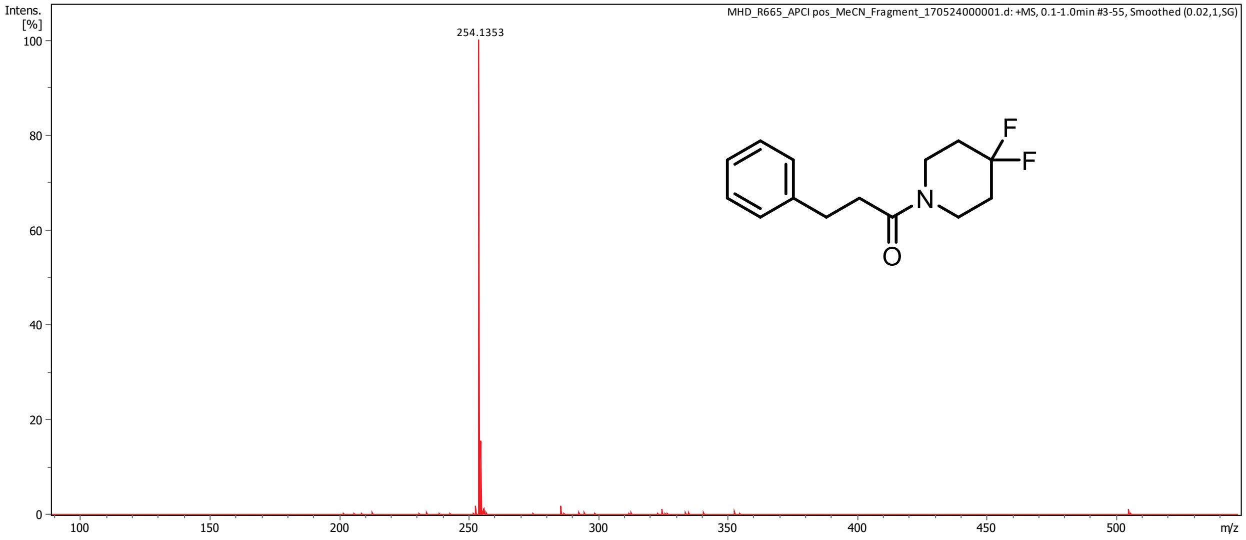
Intens.
[%]
MHD_R665_APCI pos_MeCN_Fragment_170524000001.d: +MS, 0.1-1.0min #3-55, Smoothed (0.02,1,SG)
254.1353
F
F
N
O
m/z

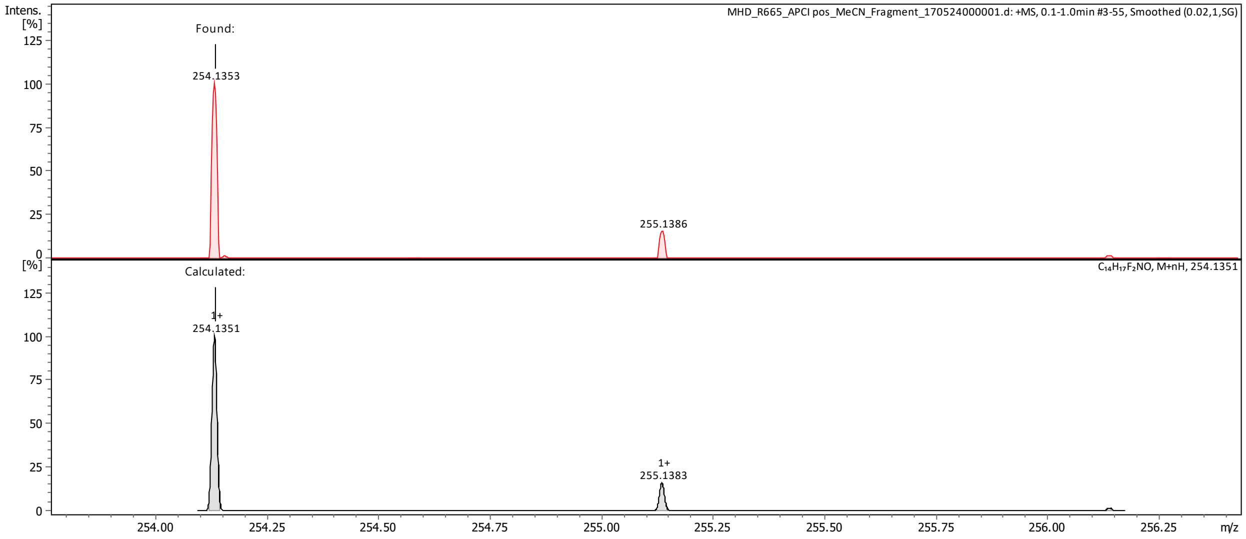
Intens.
[%]
MHD_R665_APCI pos_MeCN_Fragment_170524000001.d: +MS, 0.1-1.0min #3-55, Smoothed (0.02,1,SG)
Found:
254.1353
255.1386
Calculated:
C14H17F2NO, M+nH, 254.1351
1+
254.1351
1+
255.1383
m/z

3.3.8 IR

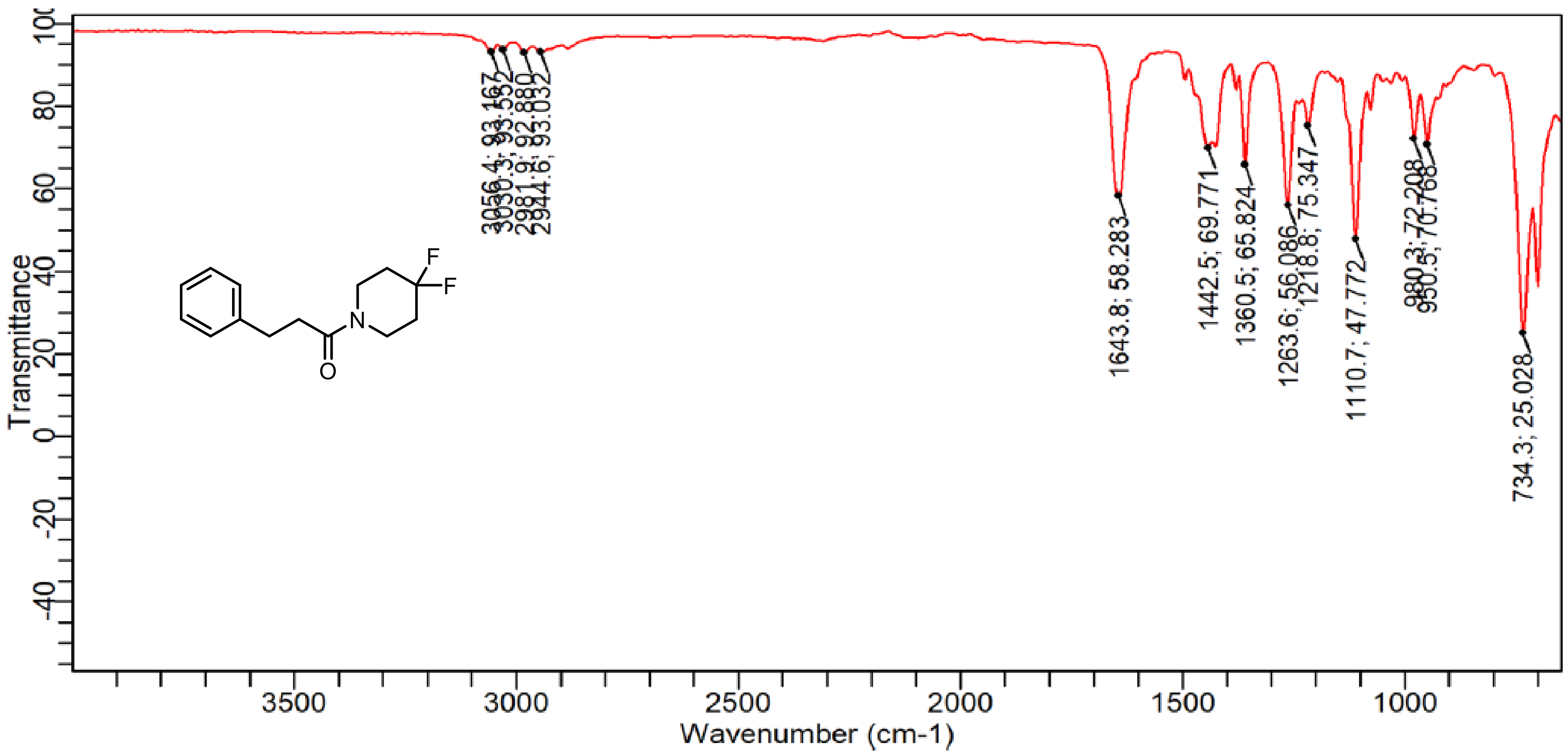

Transmittance
Wavenumber (cm-1)
3056.4; 93.167
3030.3; 93.552
2981.9; 92.880
2944.6; 93.032
1643.8; 58.283
1442.5; 69.771
1360.5; 65.824
1263.6; 56.086
1218.8; 75.347
1110.7; 47.772
980.3; 72.208
950.5; 70.768
734.3; 25.028

3.4 1-Morpholino-3-phenylpropan-1-one (12d) (600 MHz, $CDCl_3$)

3.4.1 ^1H NMR

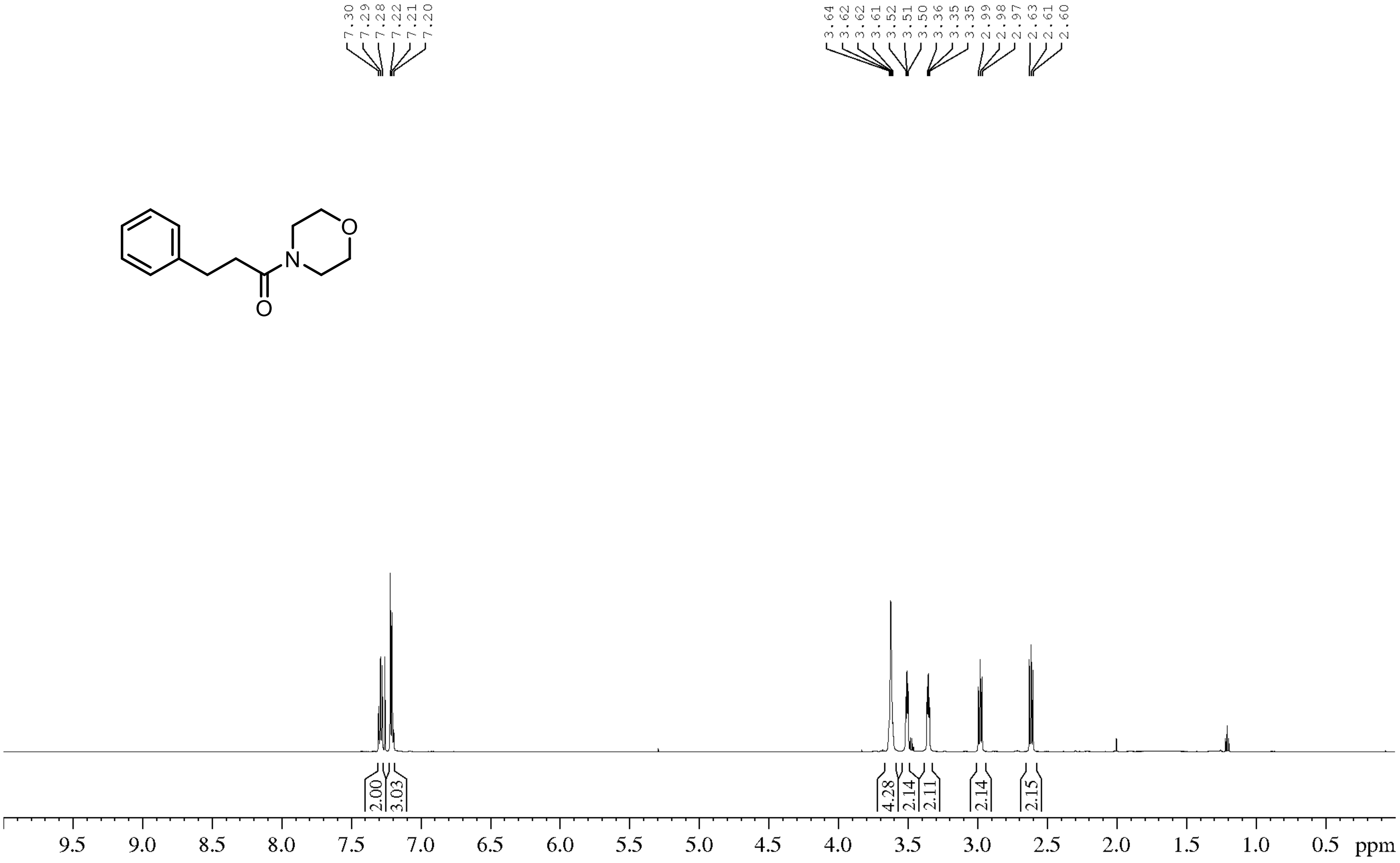

3.4.2 ^{13}C NMR

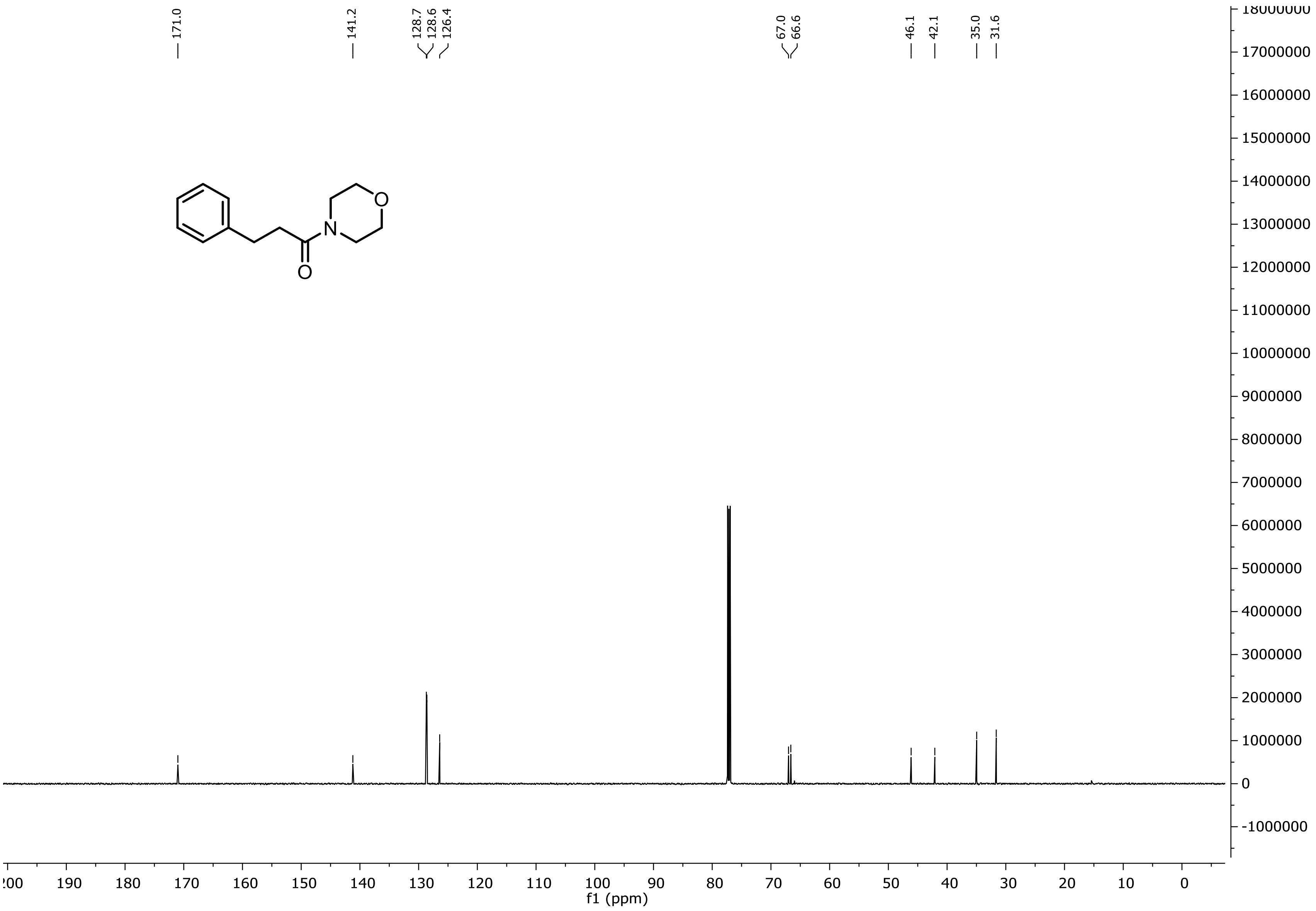

3.4.3 ^{1}H, ^{1}H COSY

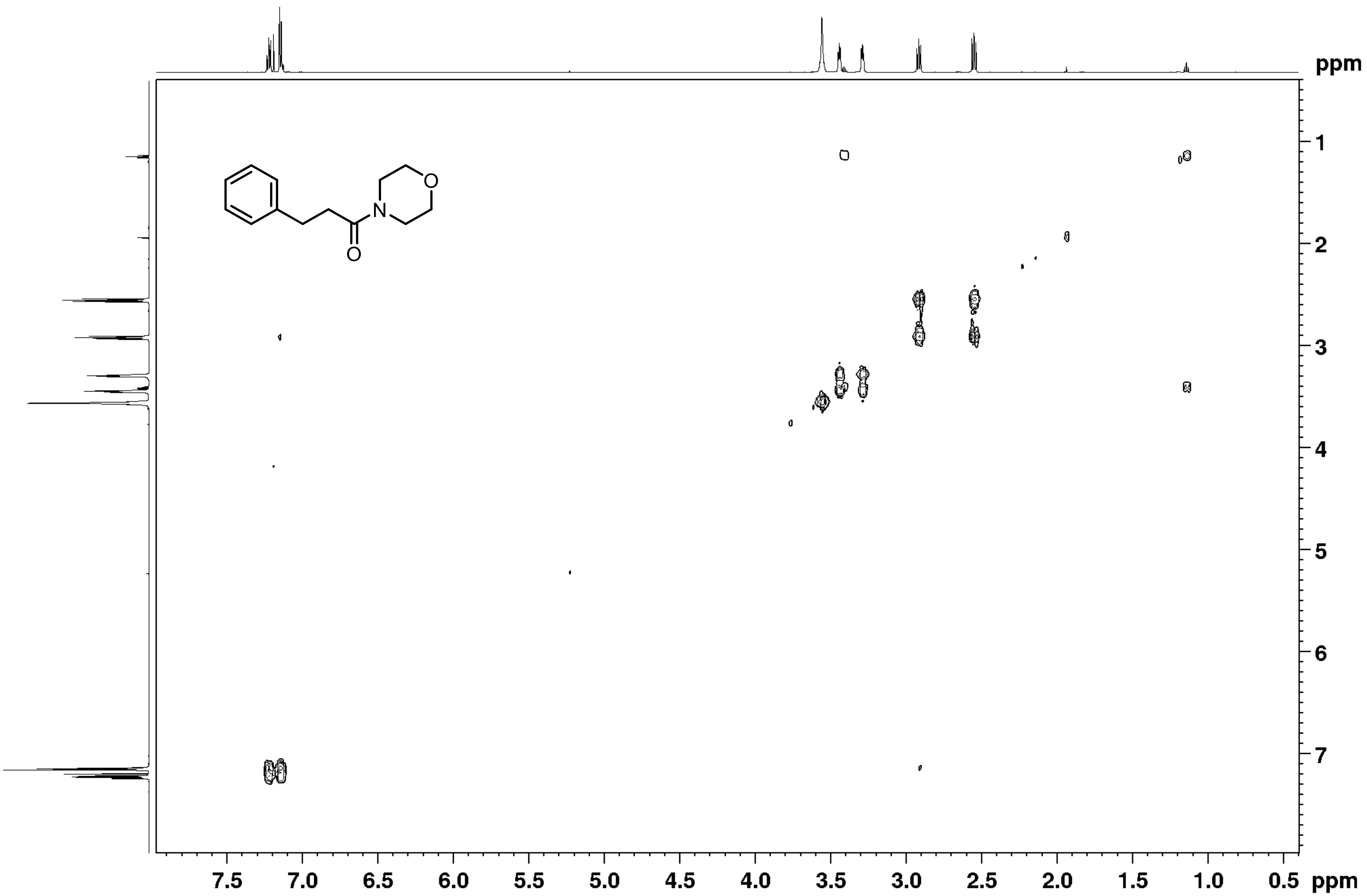

3.4.4 ^{1}H, ^{13}C HMBC

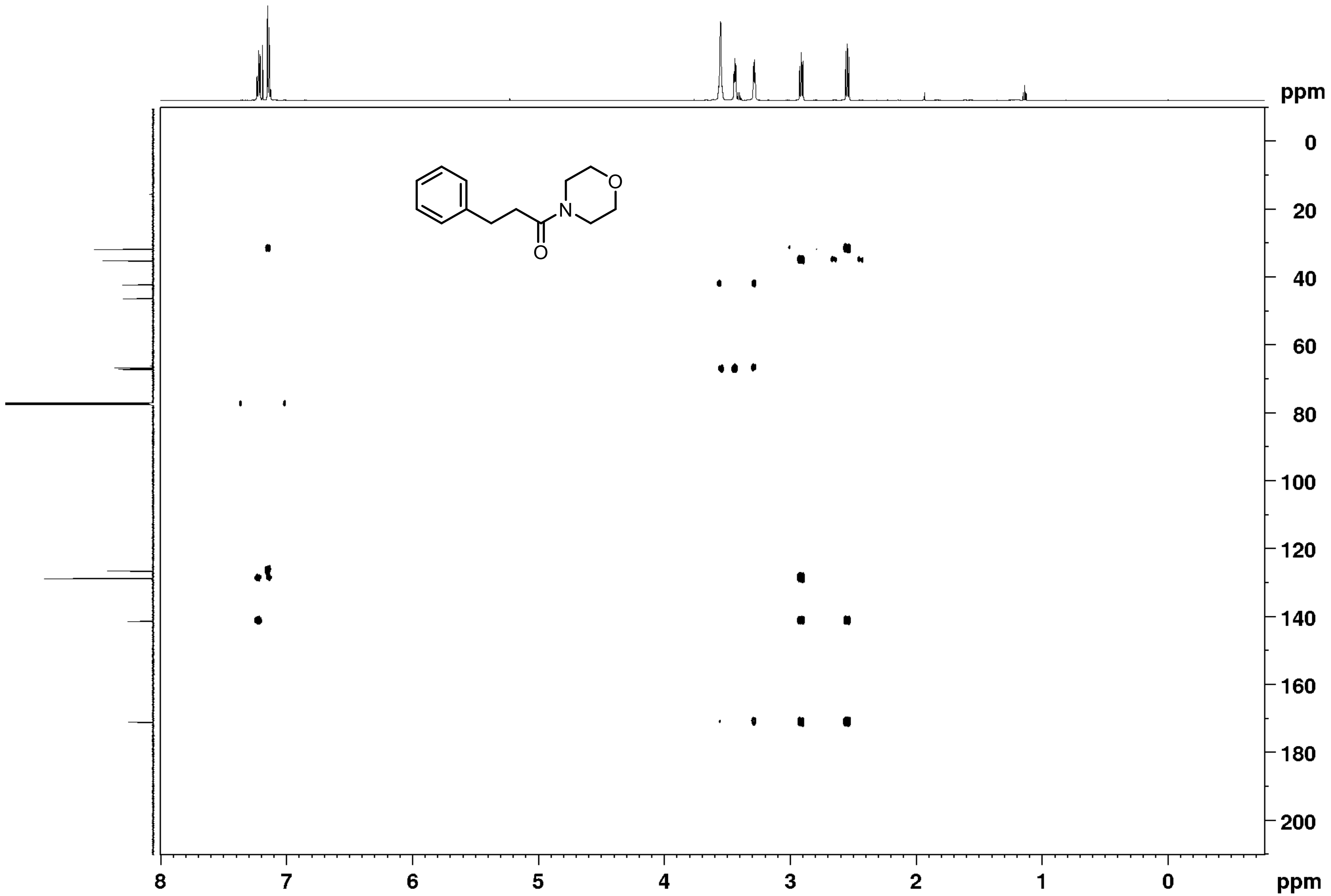

3.4.5 ^{1}H, ^{13}C HSQC

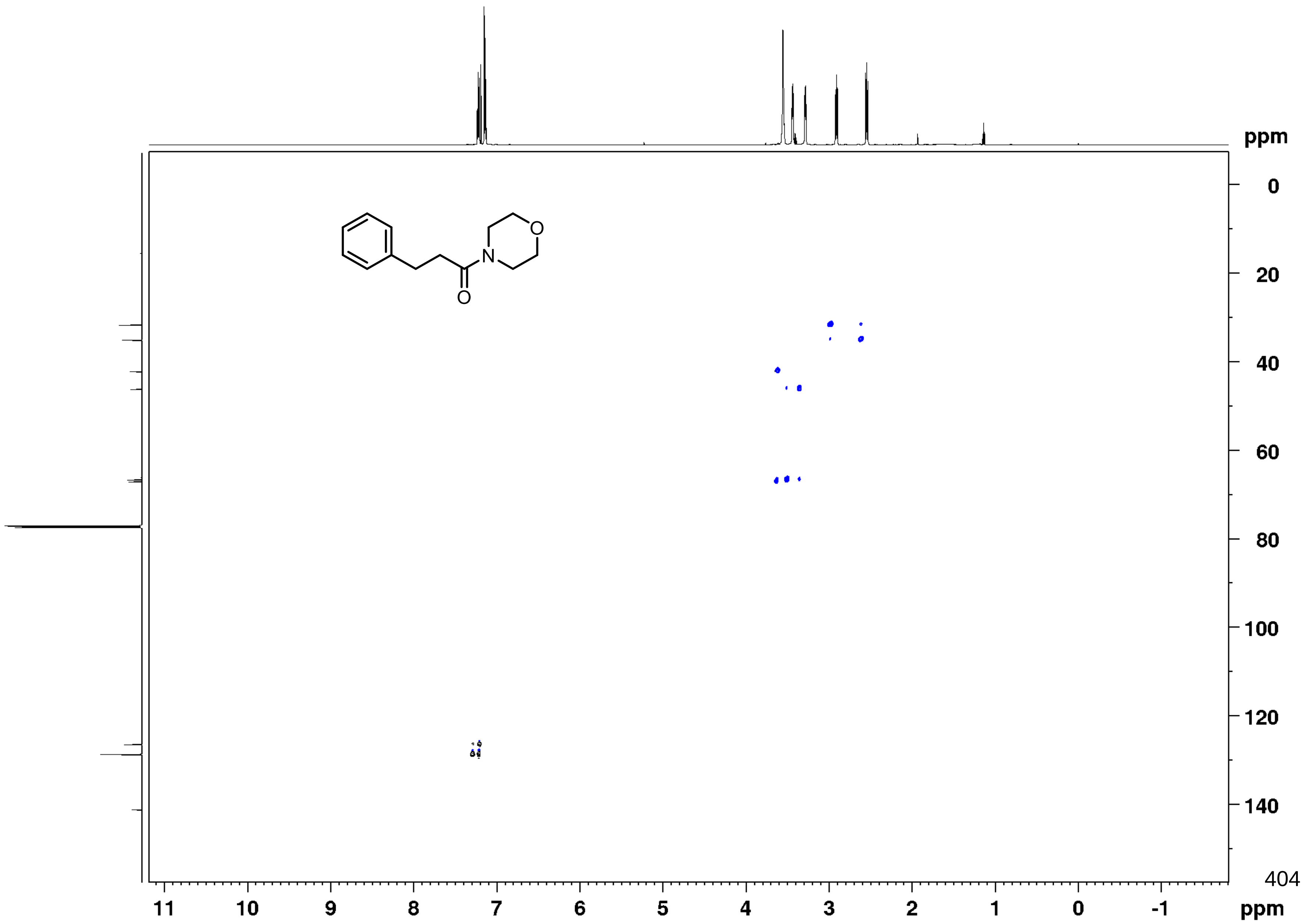

3.4.6 HRMS

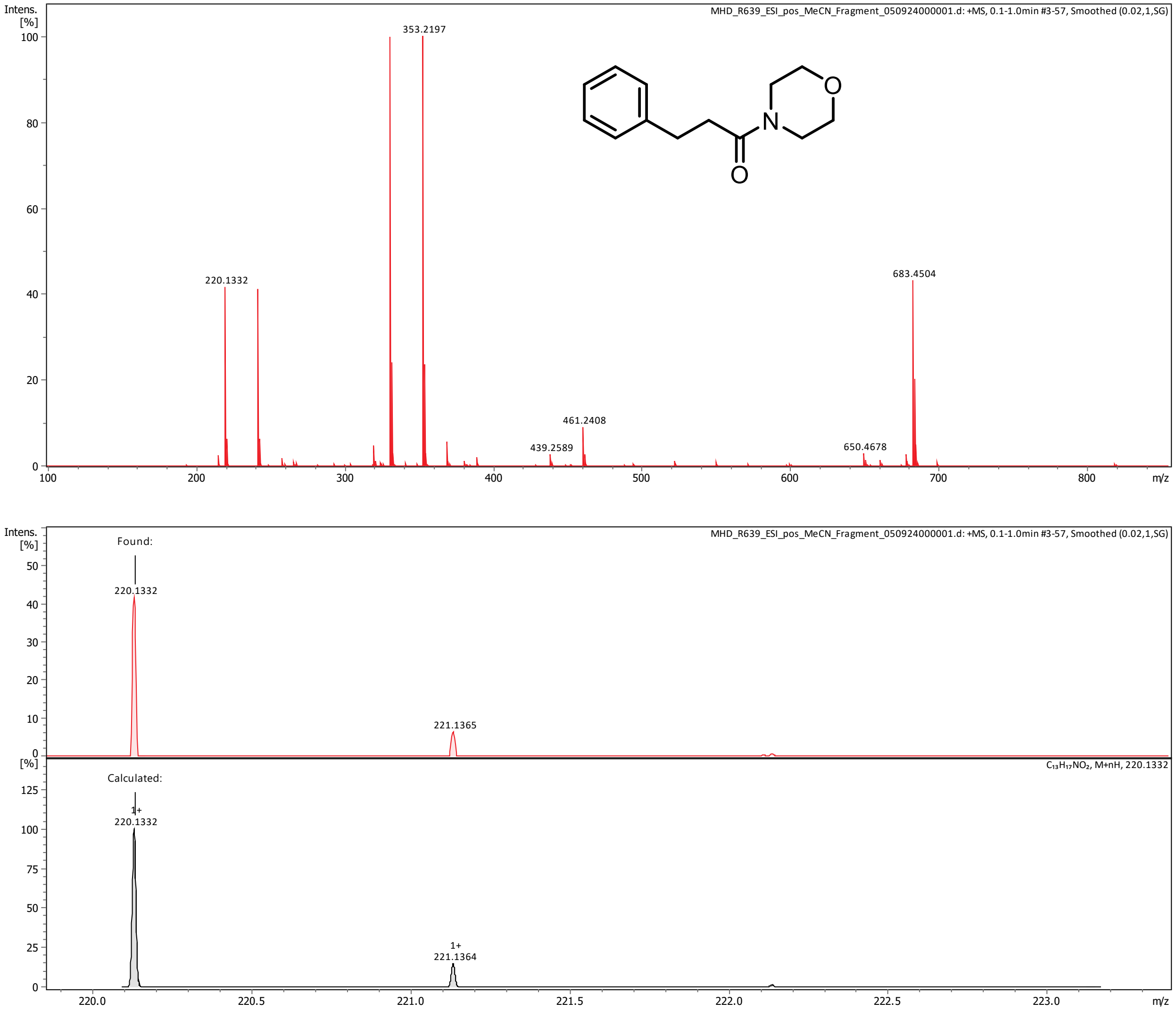

MHD_R639_ESI_pos_MeCN_Fragment_050924000001.d: +MS, 0.1-1.0min #3-57, Smoothed (0.02,1,SG)
Intens. [%]
353.2197
220.1332
683.4504
461.2408
439.2589
650.4678
m/z
Found:
220.1332
221.1365
Calculated:
1+
220.1332
1+
221.1364
$C_{13}H_{17}NO_2$, M+nH, 220.1332
N
O
O

3.4.7 IR

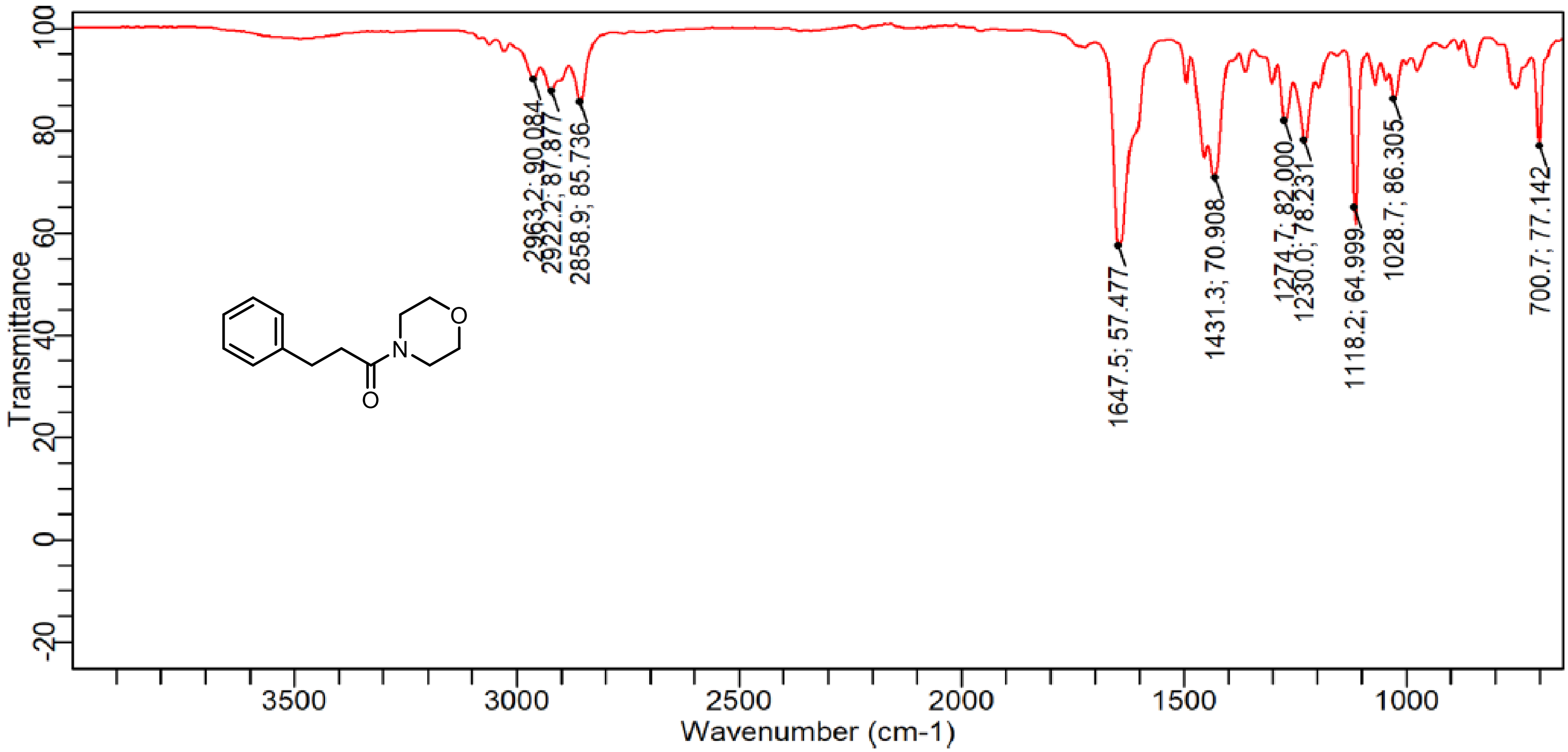

Transmittance
Wavenumber (cm-1)
2963.2; 90.084
2922.2; 87.877
2858.9; 85.736
1647.5; 57.477
1431.3; 70.908
1274.7; 82.000
1230.0; 78.231
1118.2; 64.999
1028.7; 86.305
700.7; 77.142
N
O
O

3.5 3-Phenyl-1-thiomorpholinopropan-1-one (12e) (600 MHz, $CDCl_3$)

3.5.1 ^{1}H NMR

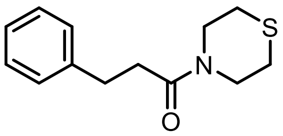

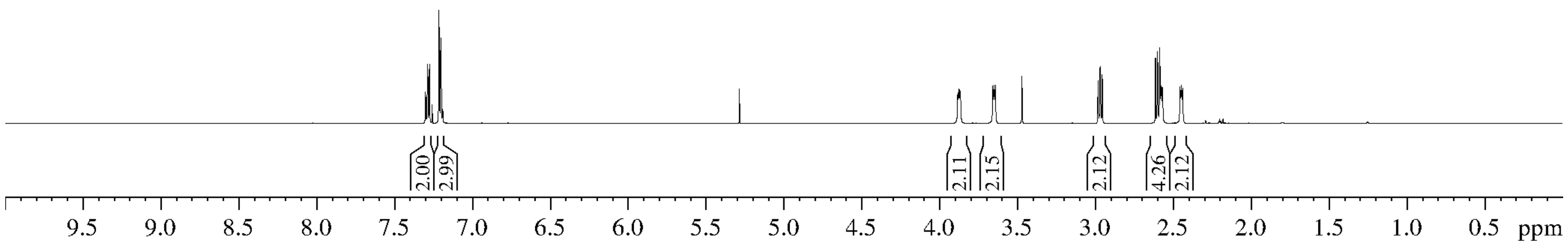

3.5.2 ^{13}C NMR

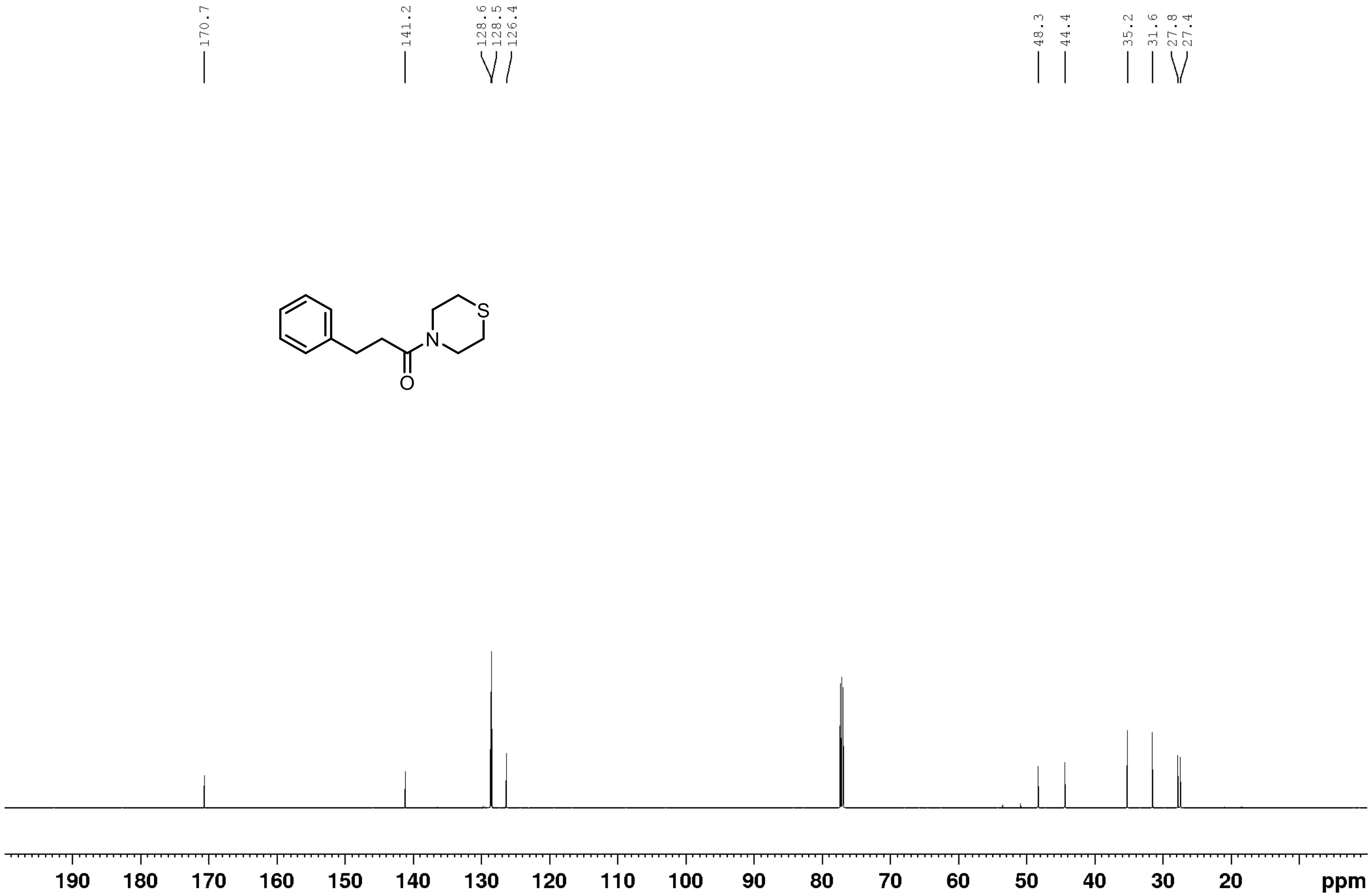
170.7
141.2
128.6
128.5
126.4
48.3
44.4
35.2
31.6
27.8
27.4
S
N
O
190 180 170 160 150 140 130 120 110 100 90 80 70 60 50 40 30 20
ppm

3.5.3 ¹H, ¹H COSY

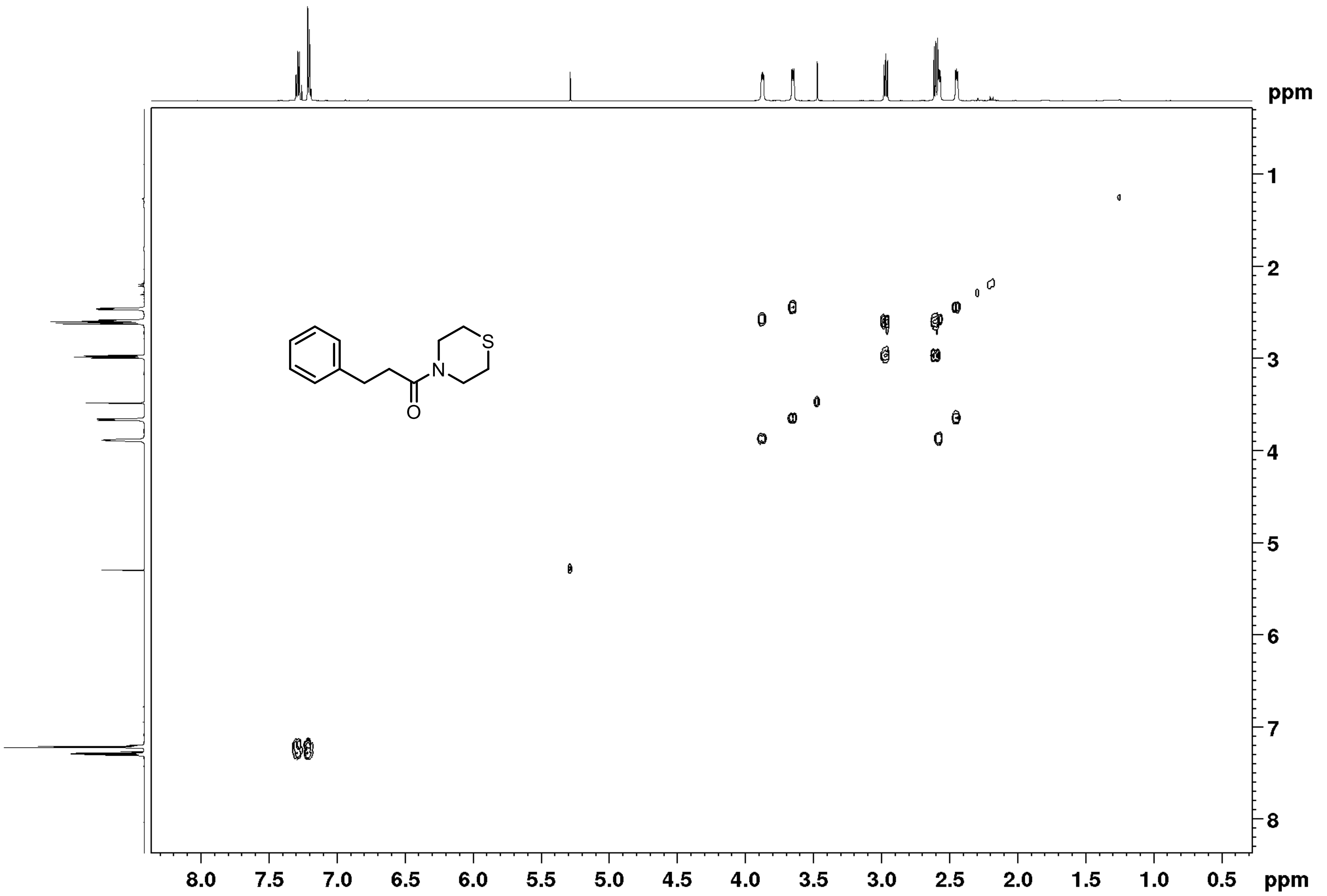

3.5.4 ^{1}H, ^{13}C HMBC

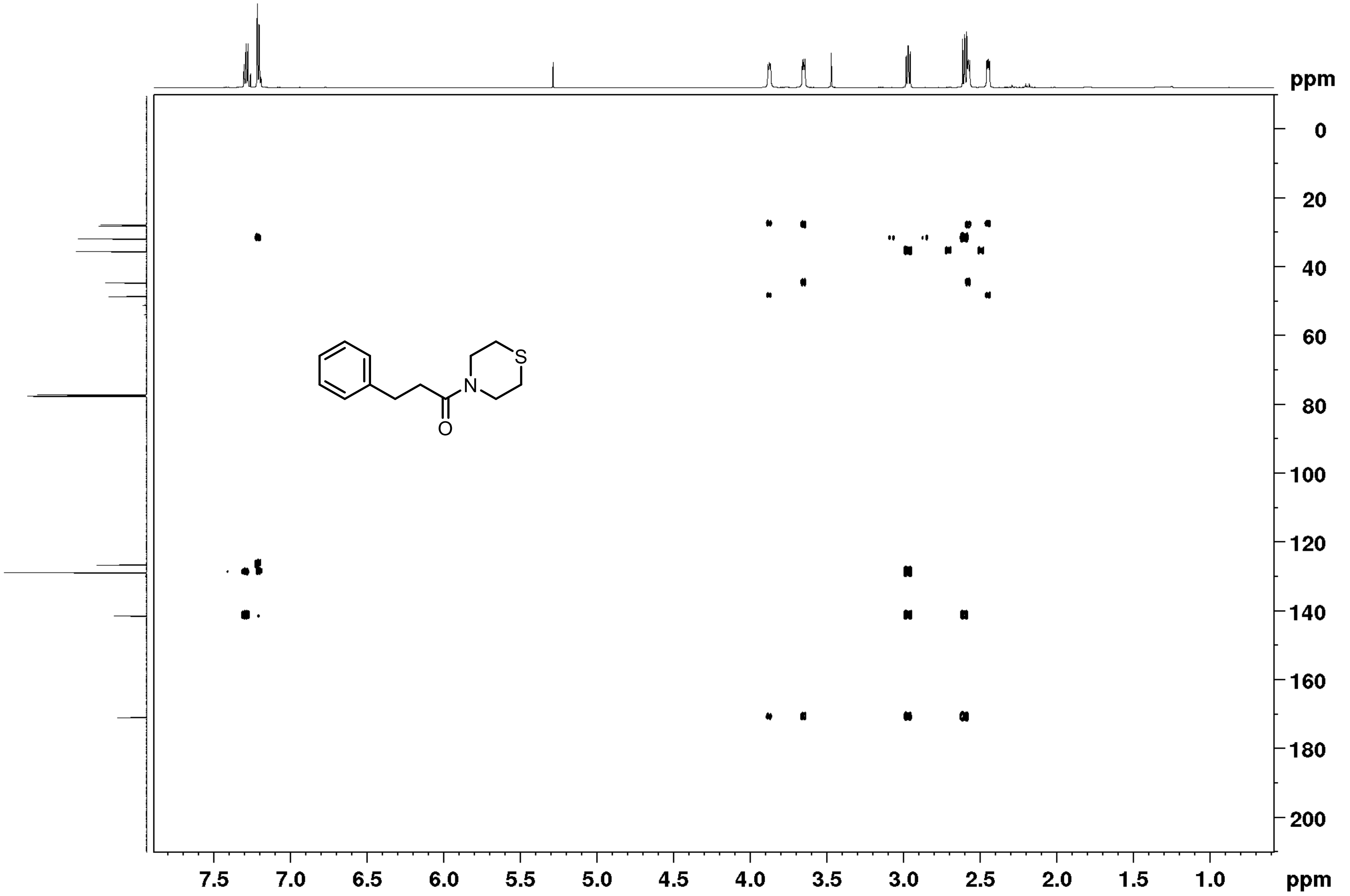

3.5.5 ^{1}H, ^{13}C HSQC

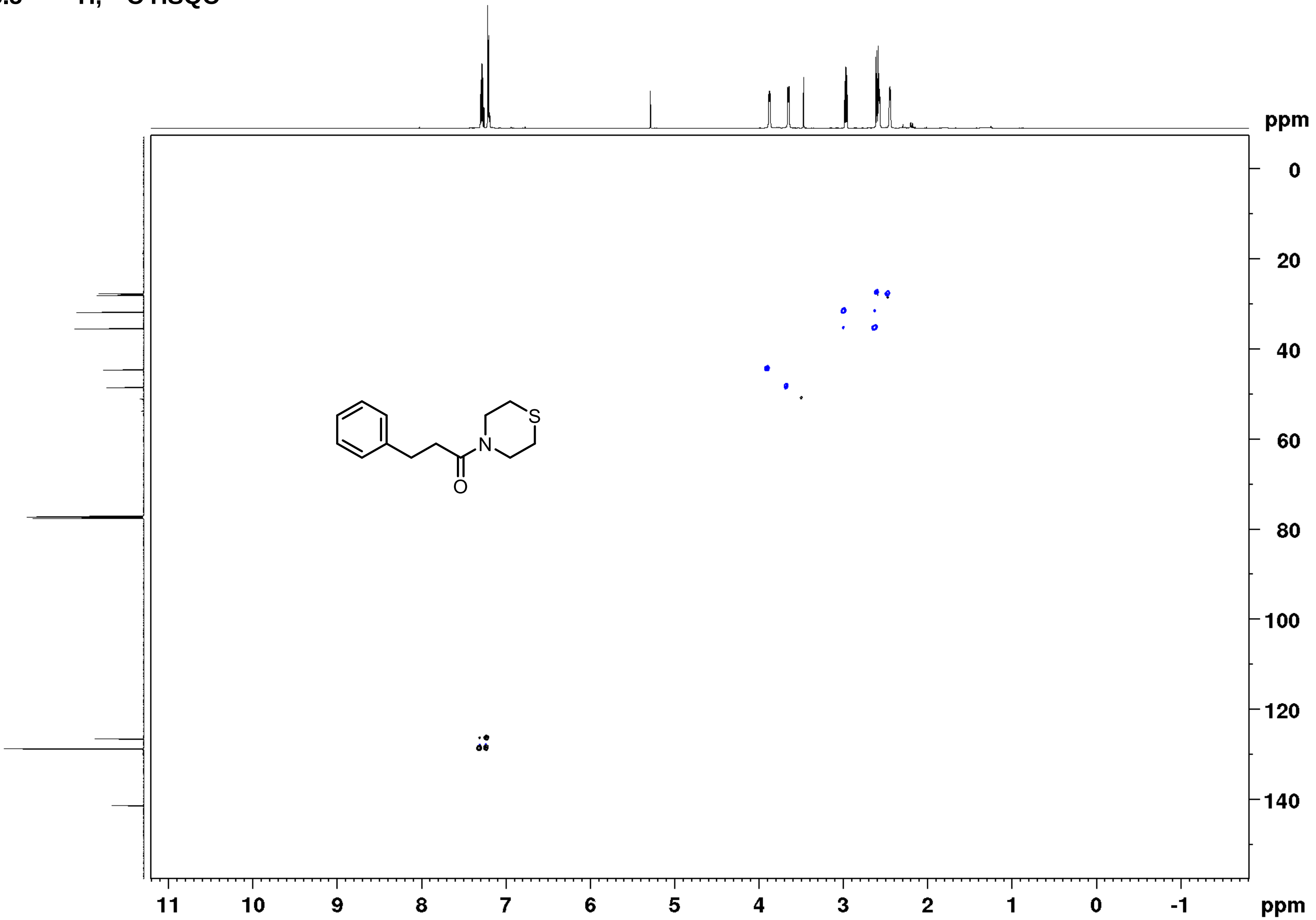

3.5.6 HRMS

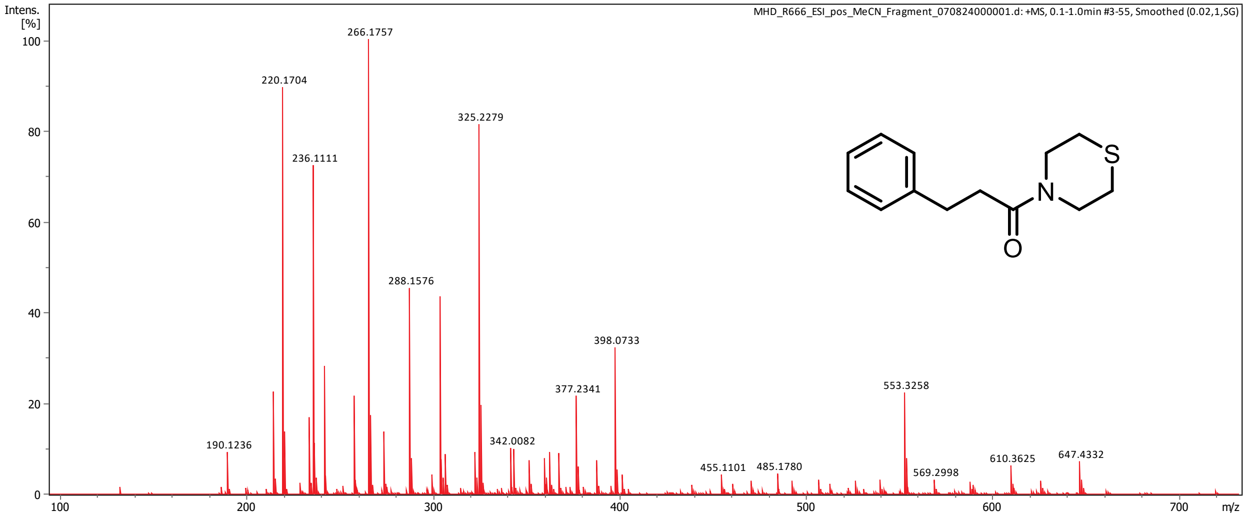

Intens. [%]
MHD_R666_ESI_pos_MeCN_Fragment_070824000001.d: +MS, 0.1-1.0min #3-55, Smoothed (0.02,1,SG)
266.1757
220.1704
325.2279
236.1111
288.1576
398.0733
377.2341
553.3258
342.0082
190.1236
455.1101
485.1780
569.2998
610.3625
647.4332
m/z

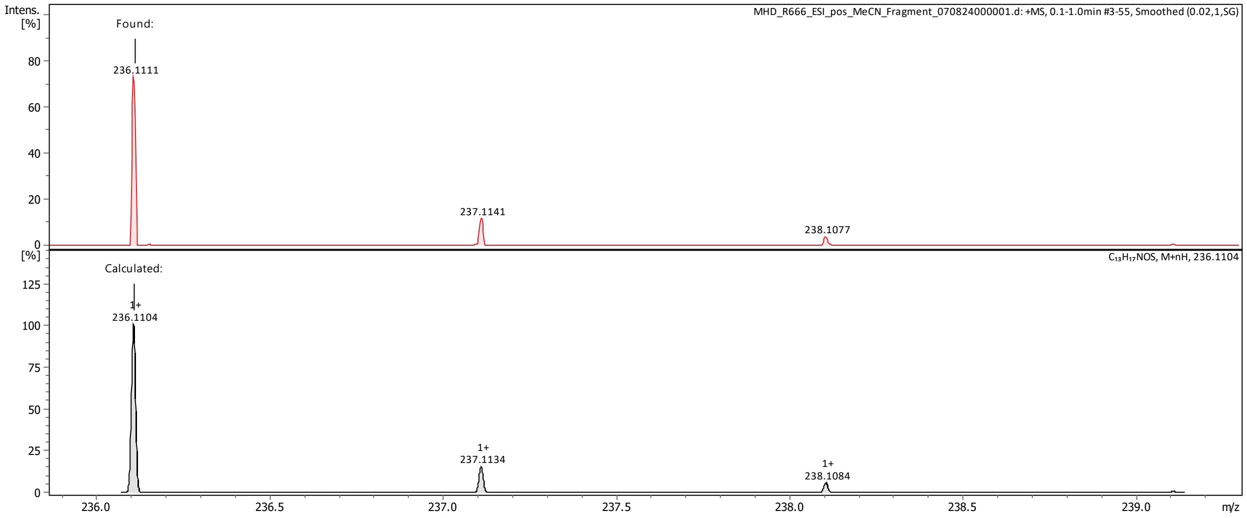

Intens. [%]
MHD_R666_ESI_pos_MeCN_Fragment_070824000001.d: +MS, 0.1-1.0min #3-55, Smoothed (0.02,1,SG)
Found:
236.1111
237.1141
238.1077
$C_{13}H_{17}NOS$, M+nH, 236.1104
Calculated:
1+
236.1104
1+
237.1134
1+
238.1084
m/z

3.5.7 IR

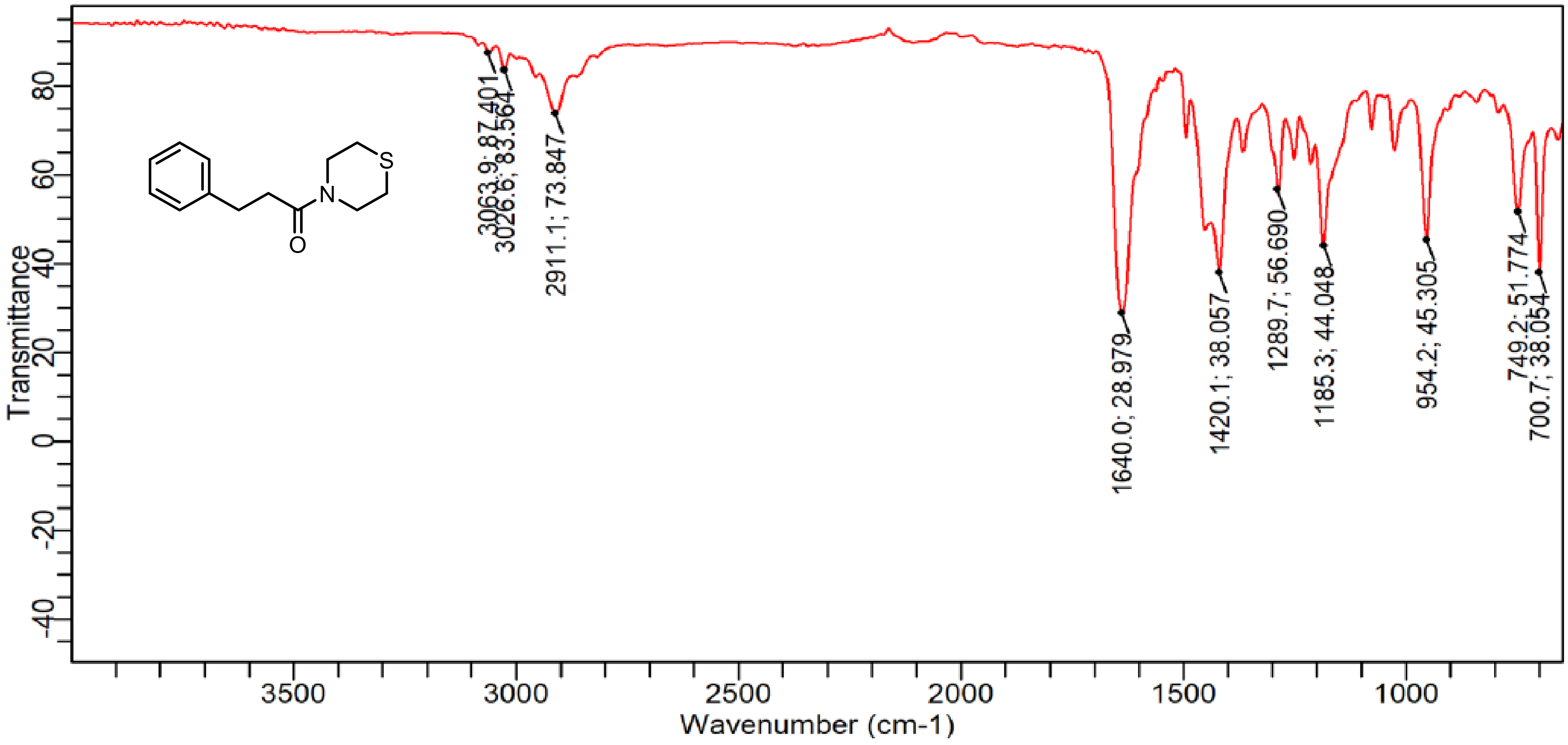

3063.9; 87.401
3026.6; 83.564
2911.1; 73.847
1640.0; 28.979
1420.1; 38.057
1289.7; 56.690
1185.3; 44.048
954.2; 45.305
749.2; 51.774
700.7; 38.054
Transmittance
Wavenumber (cm-1)
S
N
O

3.6 *N*,*N*-Bis(2-methoxyethyl)-3-phenylpropanamide (12f) (600 MHz, $CDCl_3$)

3.6.1 ^{1}H NMR

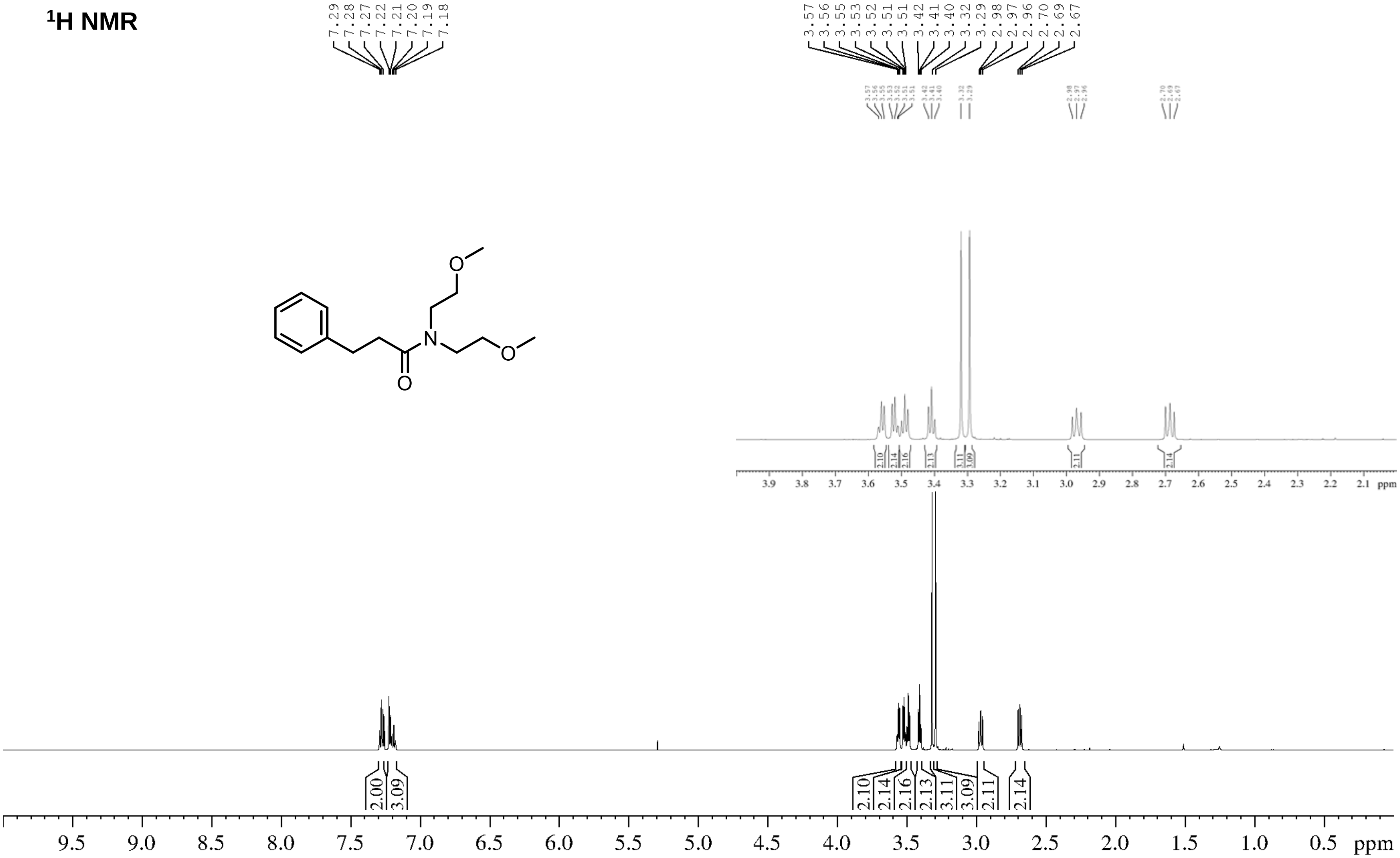

3.6.2 ^13^C NMR

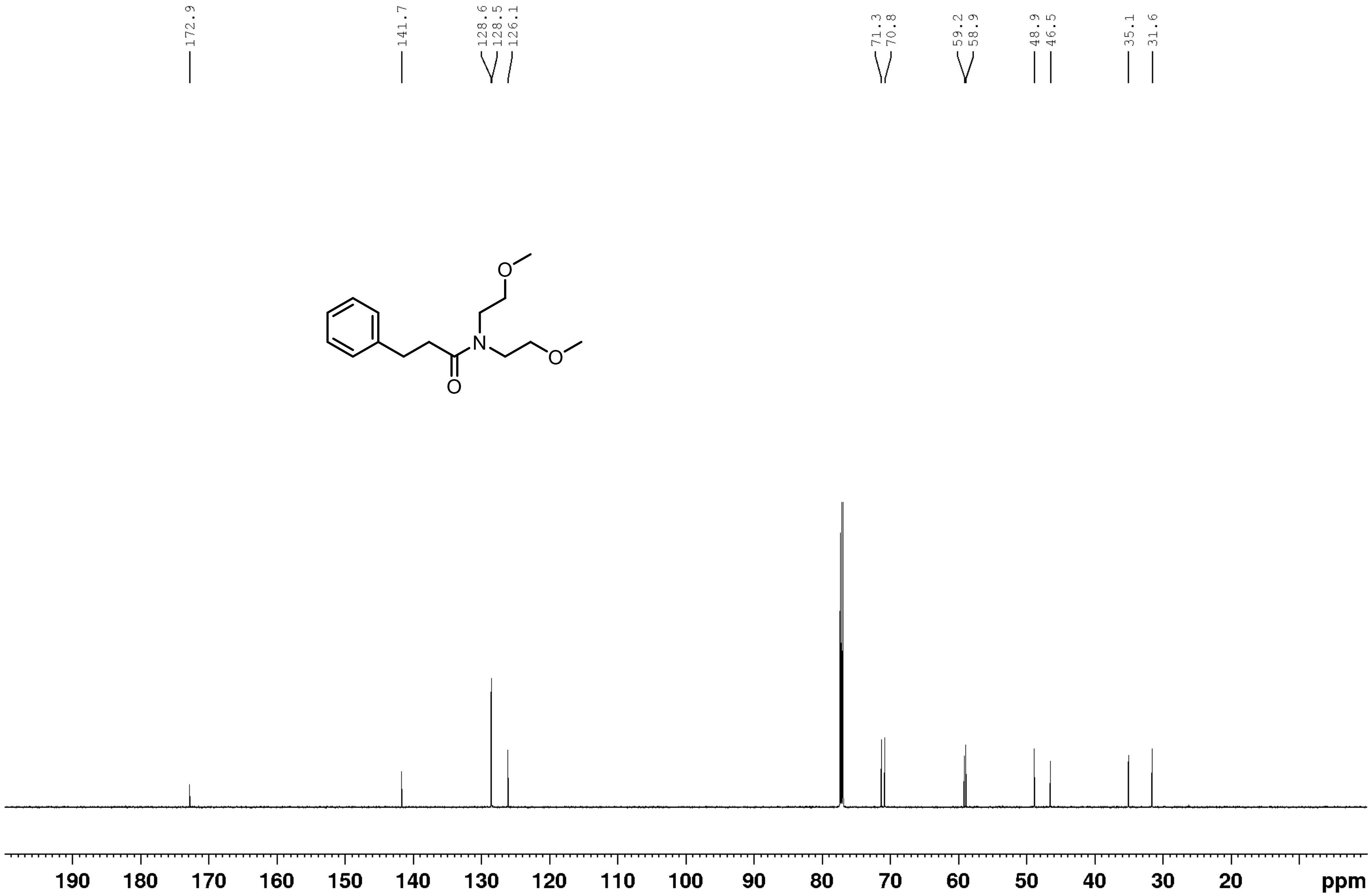
172.9
141.7
128.6
128.5
126.1
71.3
70.8
59.2
58.9
48.9
46.5
35.1
31.6
O
N
O
O
190
180
170
160
150
140
130
120
110
100
90
80
70
60
50
40
30
20
ppm

3.6.3 ^{1}H, ^{1}H COSY

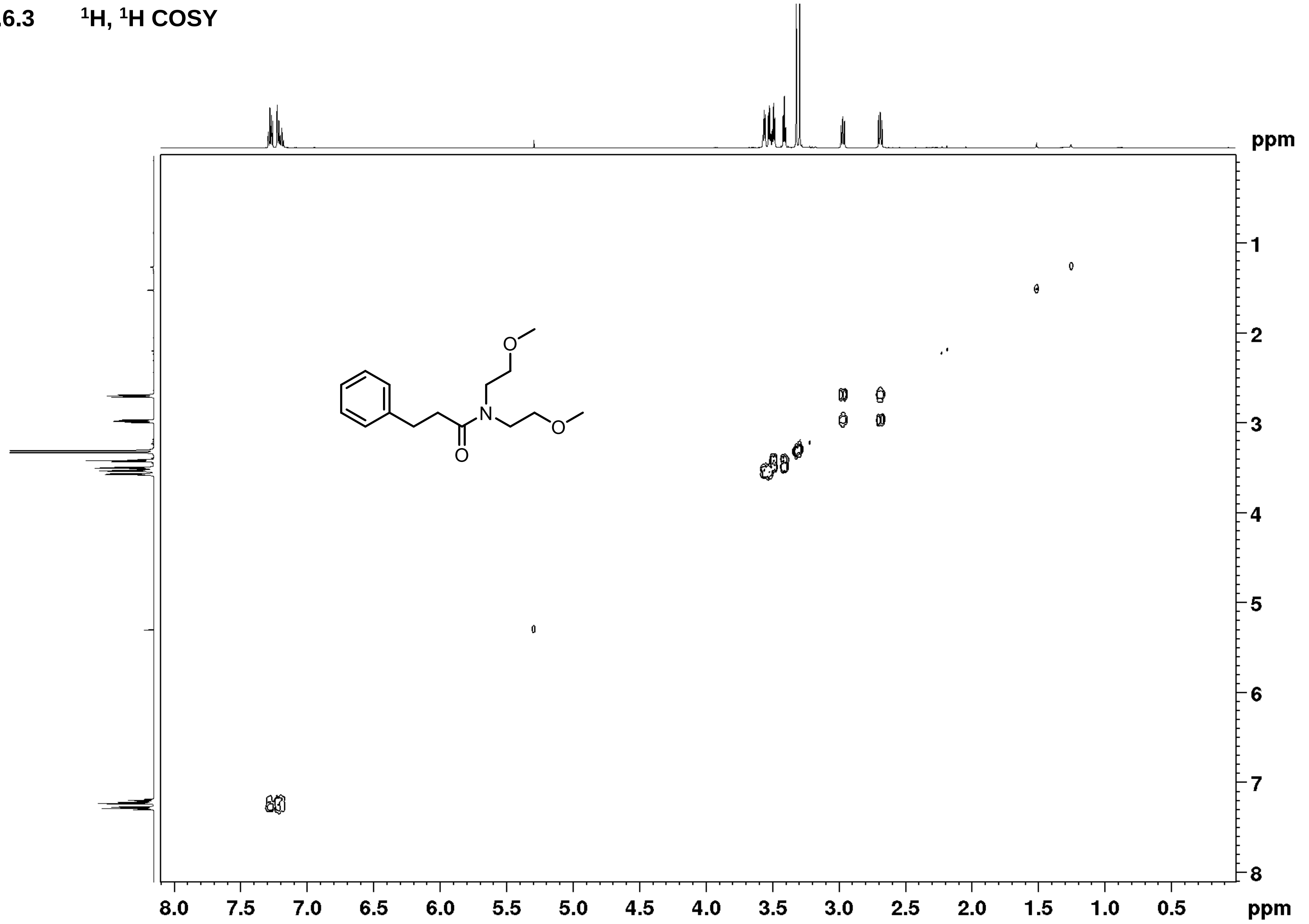

3.6.4 ^{1}H, ^{13}C HMBC

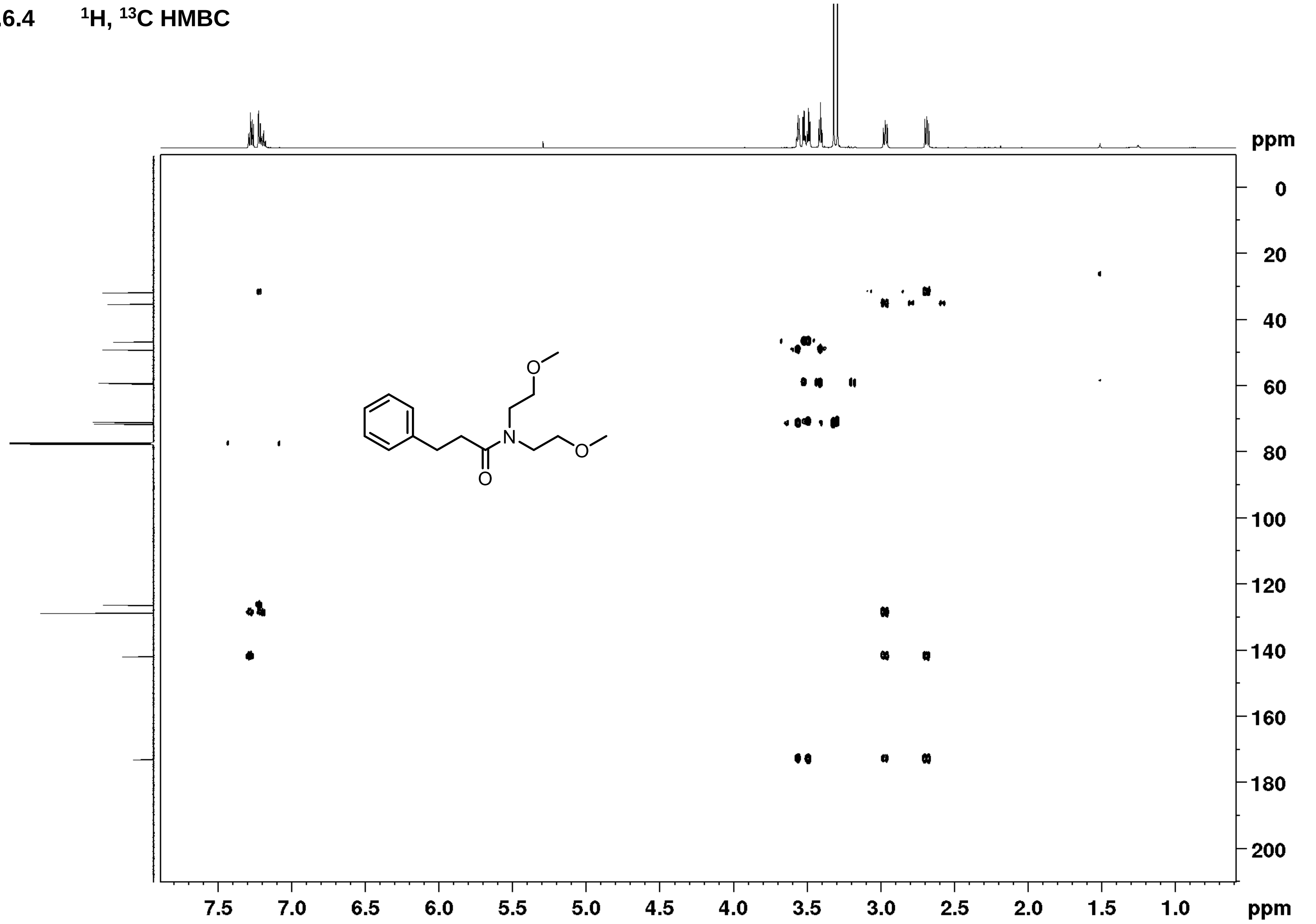

3.6.5 ^{1}H, ^{13}C HSQC

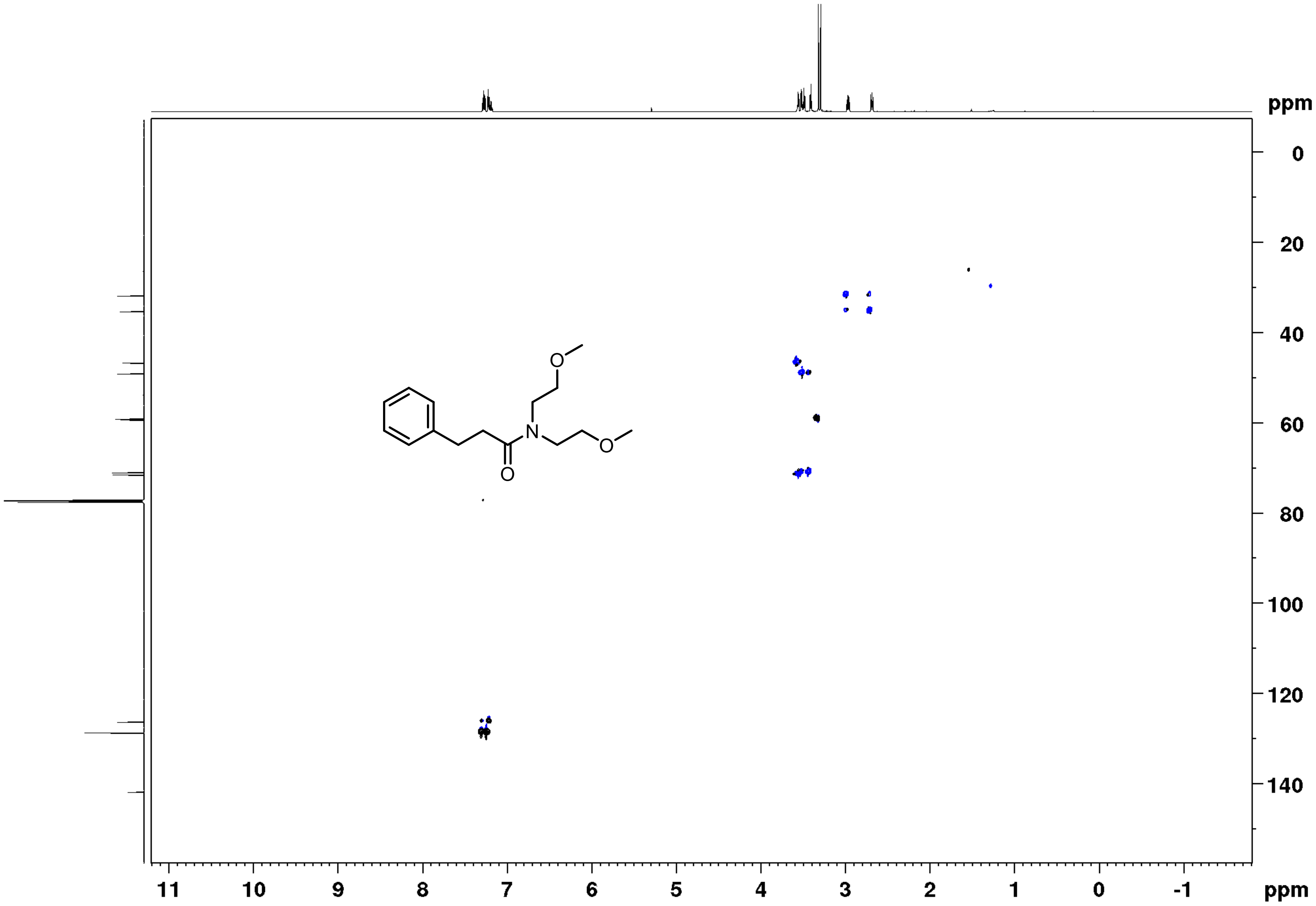

3.6.6 HRMS

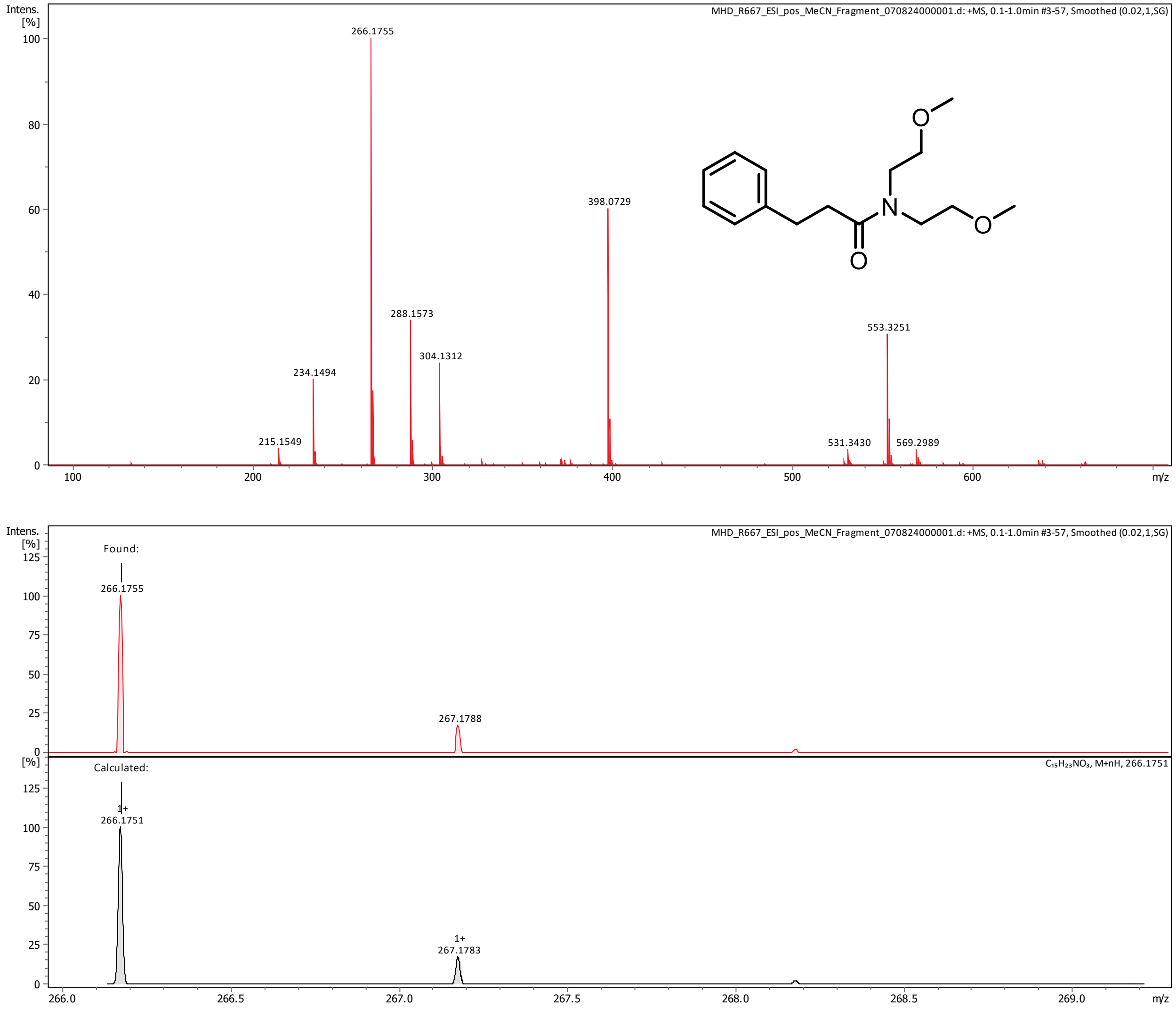

Intens. [%]
MHD_R667_ESI_pos_MeCN_Fragment_070824000001.d: +MS, 0.1-1.0min #3-57, Smoothed (0.02,1,SG)
266.1755
398.0729
288.1573
304.1312
234.1494
553.3251
215.1549
531.3430
569.2989
m/z
Found:
266.1755
267.1788
Calculated:
$C_{15}H_{23}NO_3$, M+nH, 266.1751
1+
266.1751
1+
267.1783

3.6.7 IR

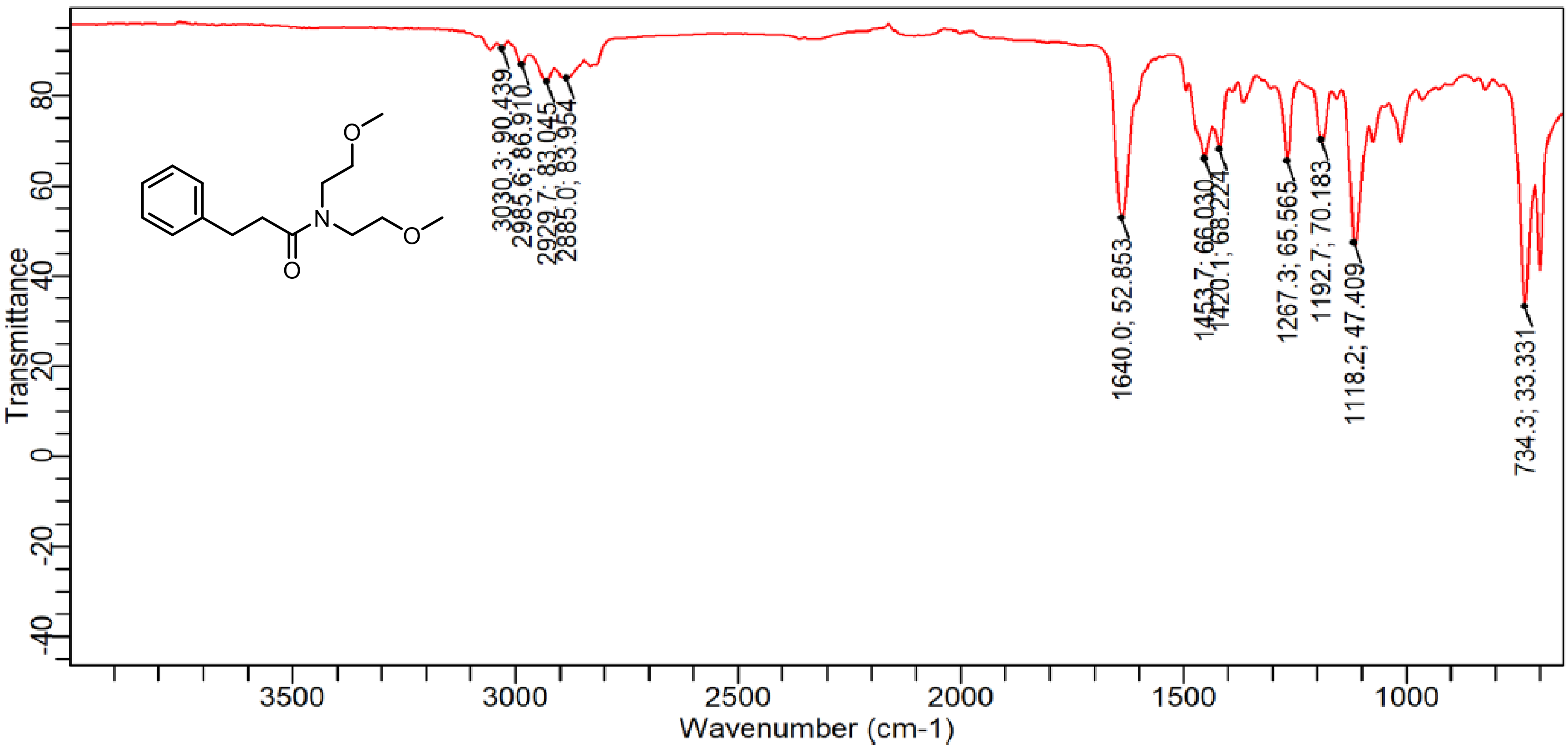

3030.3; 90.439
2985.6; 86.910
2929.7; 83.045
2885.0; 83.954
1640.0; 52.853
1453.7; 66.030
1420.1; 68.224
1267.3; 65.565
1192.7; 70.183
1118.2; 47.409
734.3; 33.331
Transmittance
Wavenumber (cm-1)

3.7 1-(4,7-Dihydrothieno[2,3-*c*]pyridin-6(5*H*)-yl)-3-phenylpropan-1-one (12g & 12g_rotamer) (600 MHz, $CDCl_3$)

3.7.1 ^{1}H NMR

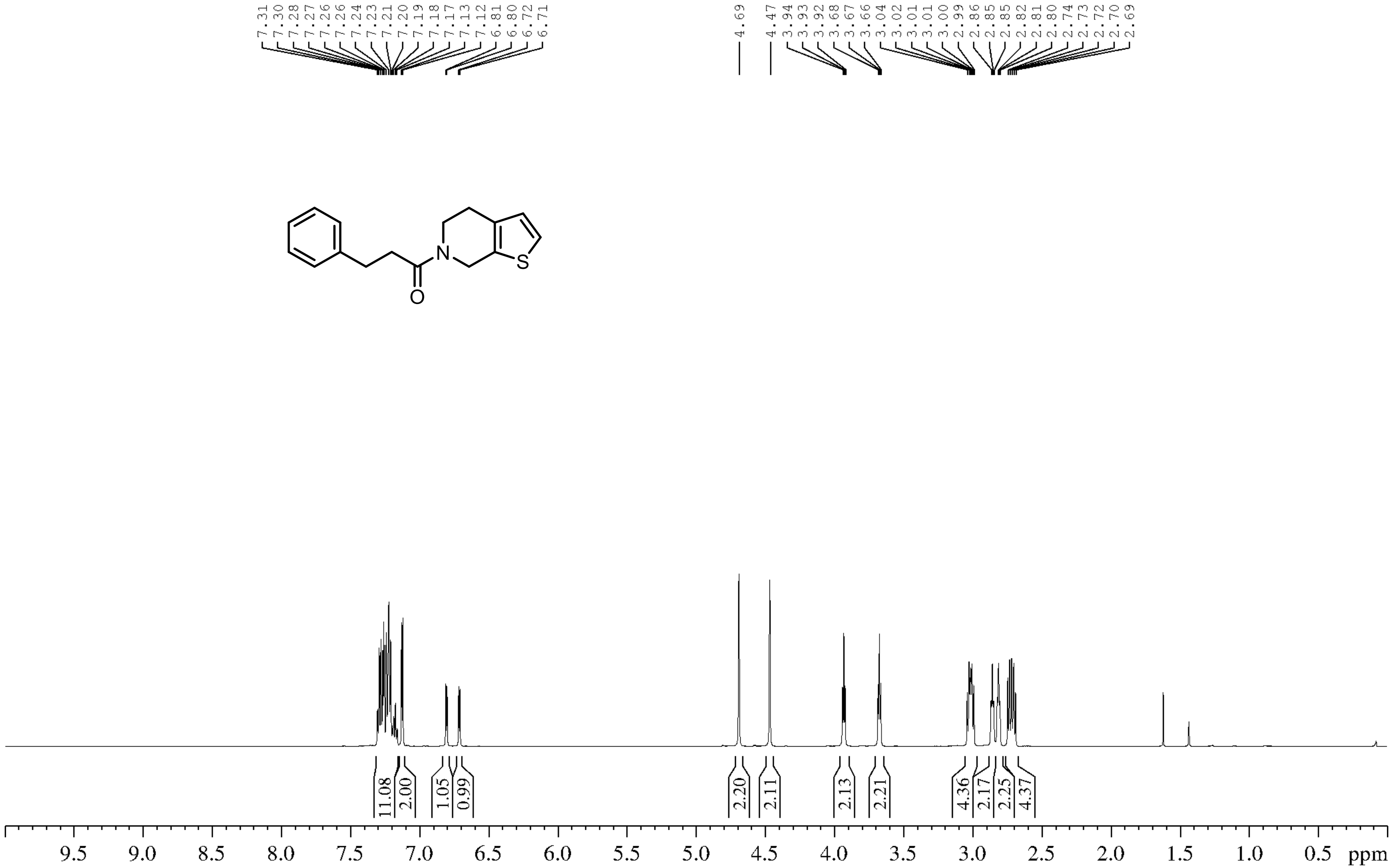

3.7.2 ^{13}C NMR

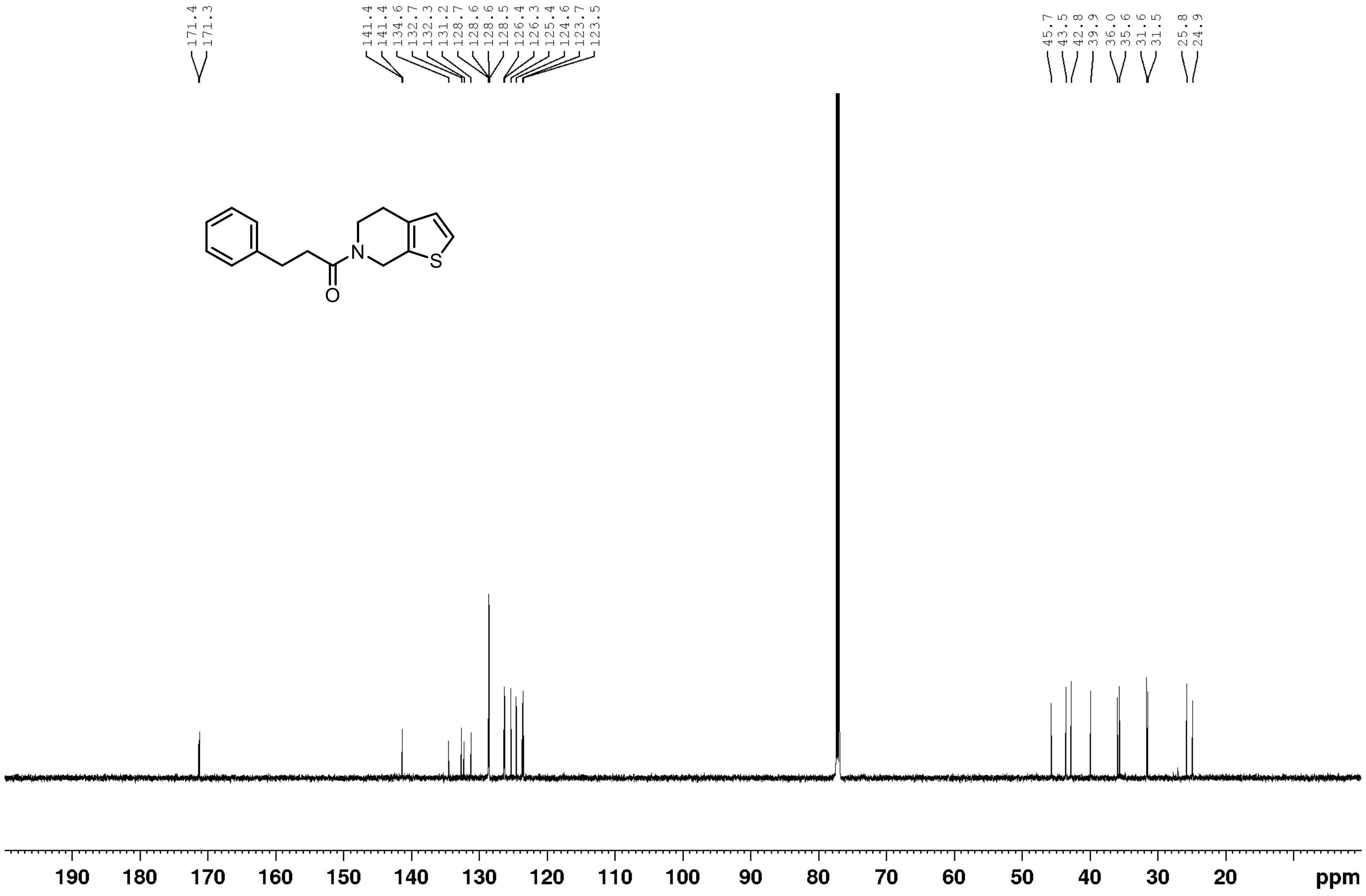

171.4
171.3
141.4
141.4
134.6
132.7
132.3
131.2
128.7
128.6
128.6
128.5
126.4
126.3
125.4
124.6
123.7
123.5
45.7
43.5
42.8
39.9
36.0
35.6
31.6
31.5
25.8
24.9
N
O
S
190 180 170 160 150 140 130 120 110 100 90 80 70 60 50 40 30 20 ppm

3.7.3 ^{1}H, ^{1}H COSY

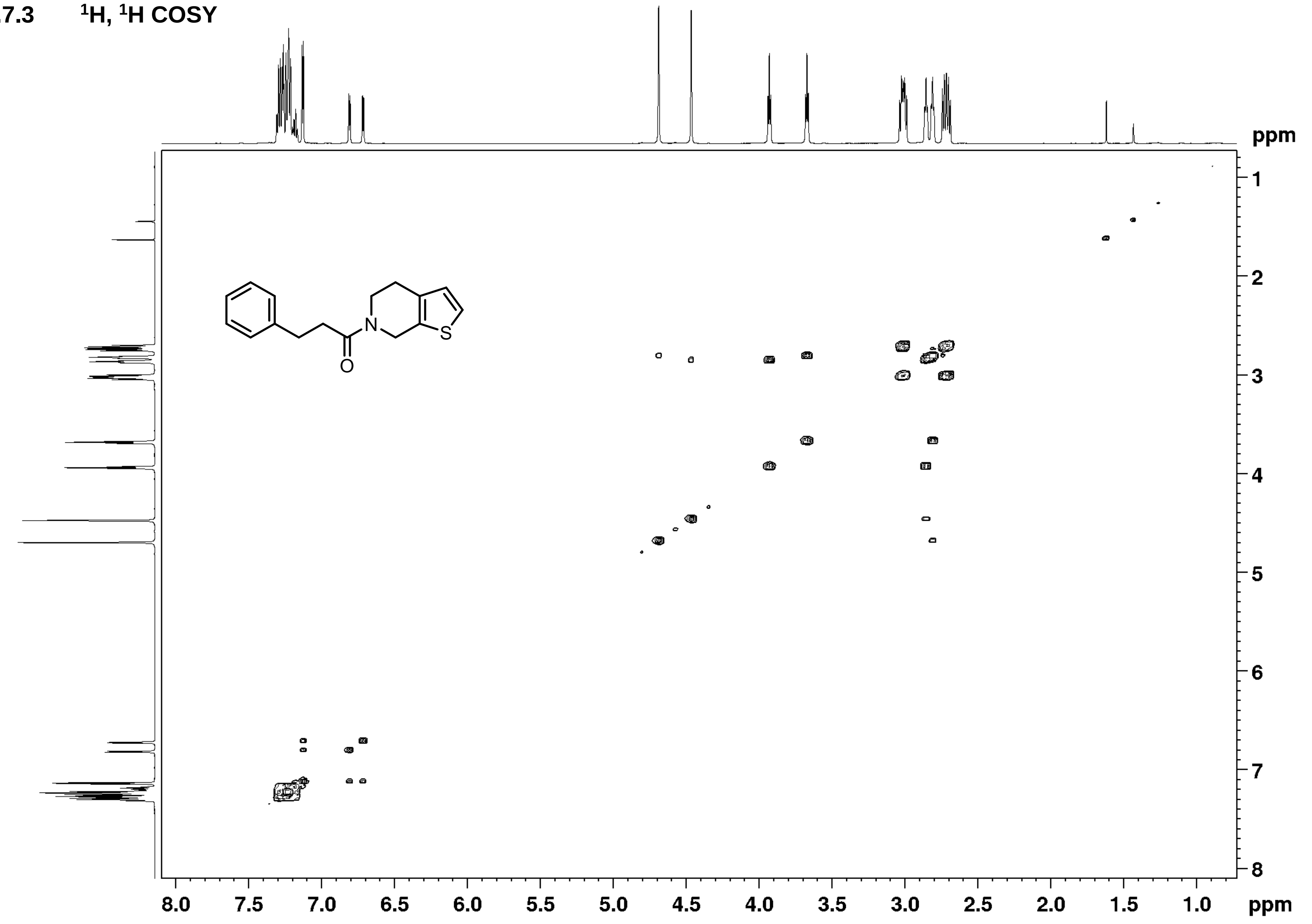

3.7.4 ^{1}H, ^{13}C HMBC

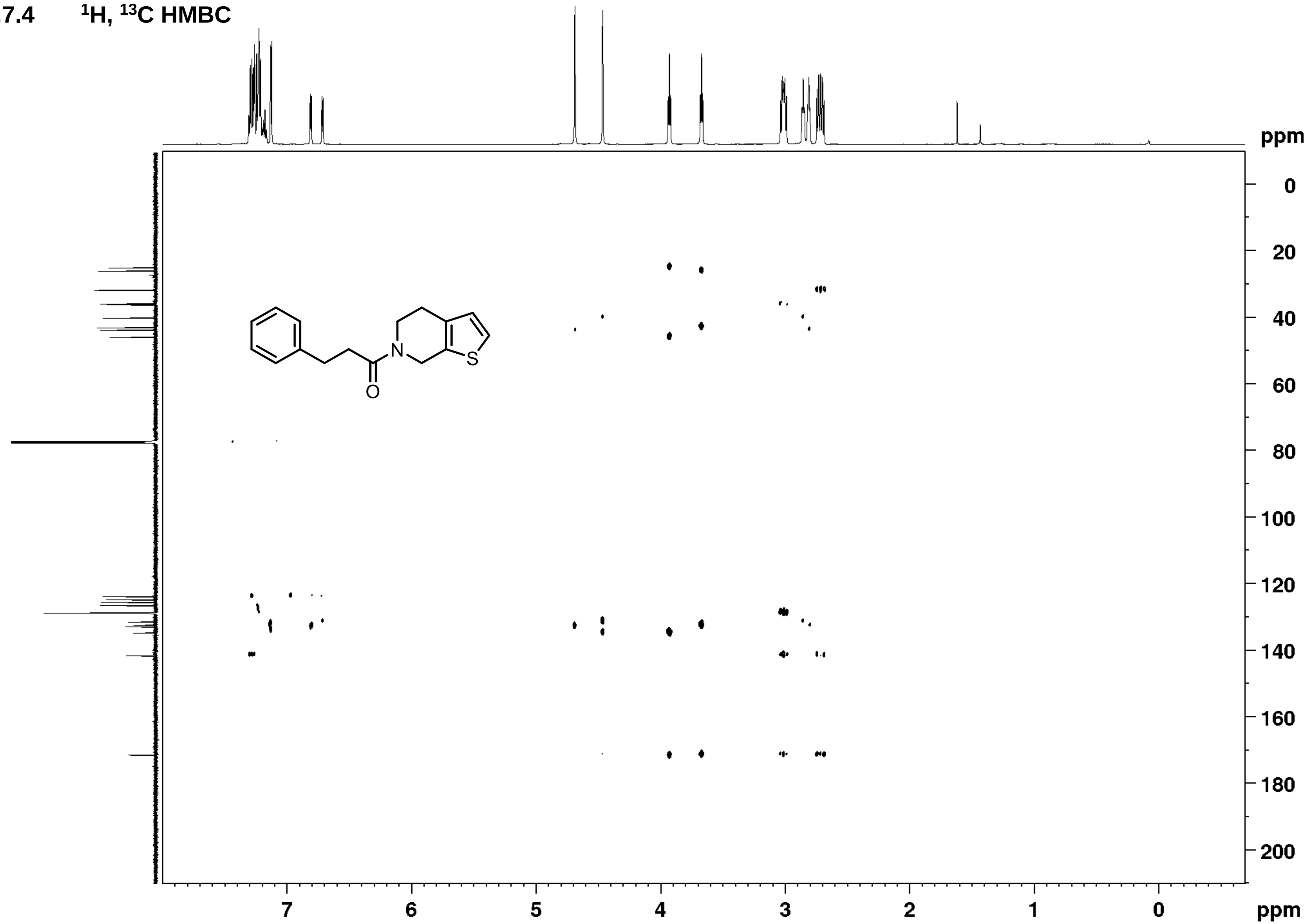

3.7.5 ¹H, ¹³C HSQC

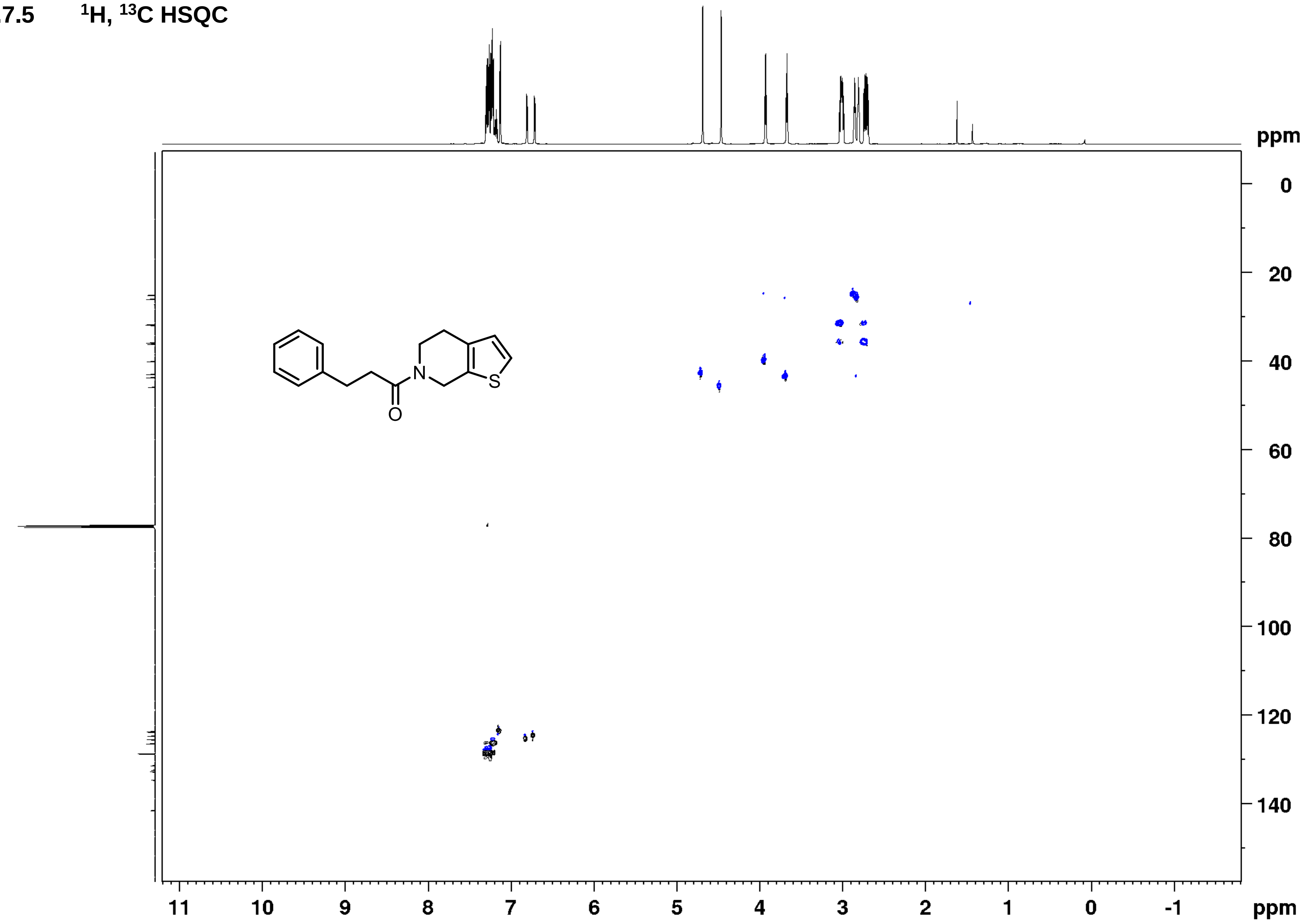

3.7.6 HRMS

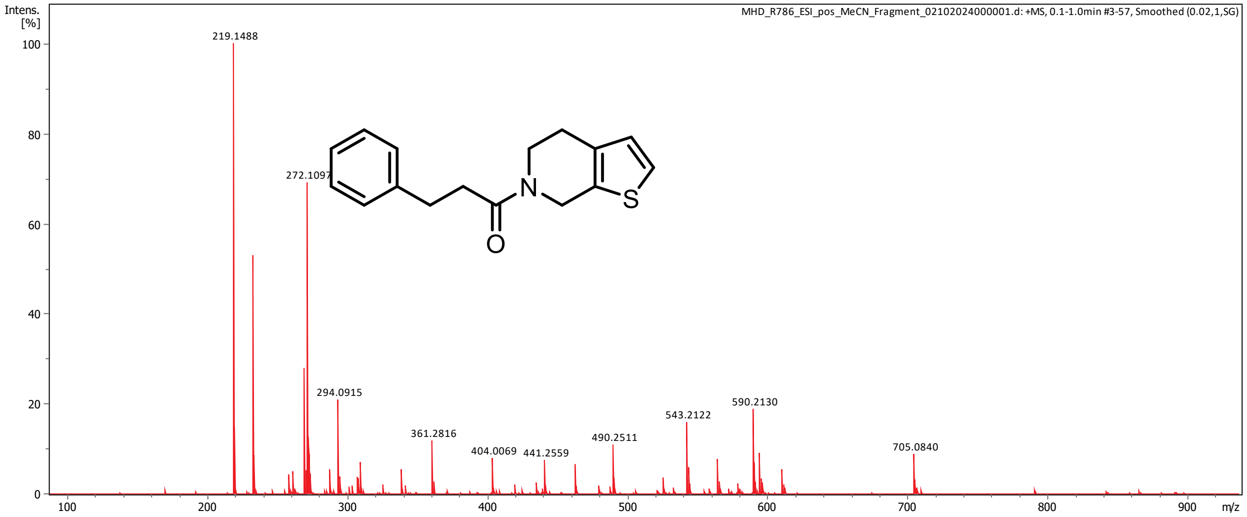

Intens. [%]
MHD_R786_ESI_pos_MeCN_Fragment_02102024000001.d: +MS, 0.1-1.0min #3-57, Smoothed (0.02,1,SG)
219.1488
272.1097
294.0915
361.2816
404.0069
441.2559
490.2511
543.2122
590.2130
705.0840
m/z
N
O
S

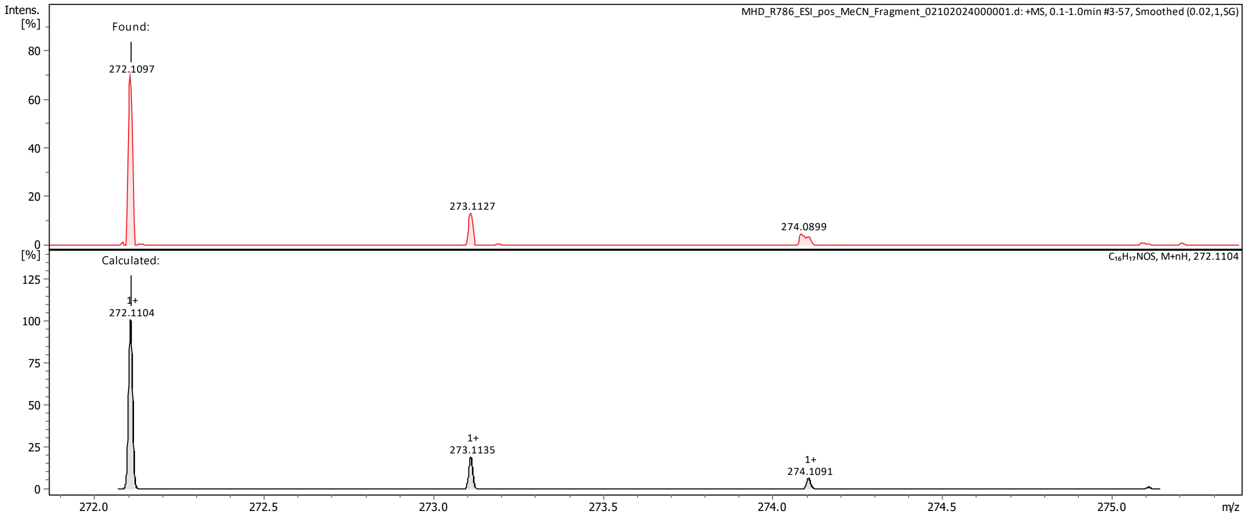

Intens. [%]
MHD_R786_ESI_pos_MeCN_Fragment_02102024000001.d: +MS, 0.1-1.0min #3-57, Smoothed (0.02,1,SG)
Found:
272.1097
273.1127
274.0899
Calculated:
C16H17NOS, M+nH, 272.1104
1+ 272.1104
1+ 273.1135
1+ 274.1091
m/z

3.7.7 IR

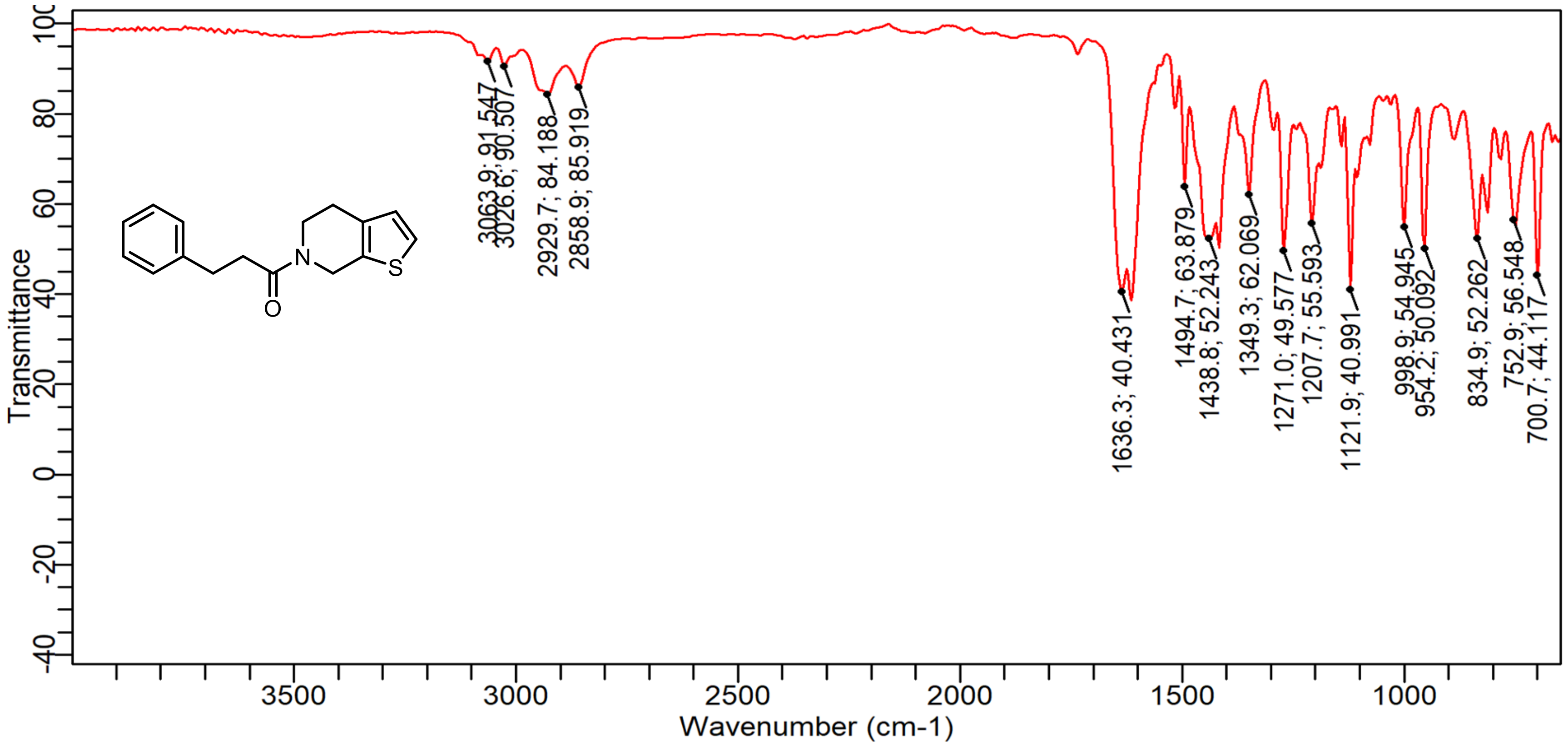
Transmittance
Wavenumber (cm-1)
3063.9; 91.547
3026.6; 90.507
2929.7; 84.188
2858.9; 85.919
1636.3; 40.431
1494.7; 63.879
1438.8; 52.243
1349.3; 62.069
1271.0; 49.577
1207.7; 55.593
1121.9; 40.991
998.9; 54.945
954.2; 50.092
834.9; 52.262
752.9; 56.548
700.7; 44.117

3.8 1-(4-(2,4-Difluorobenzoyl)piperidin-1-yl)-3-phenylpropan-1-one (12h) (600 MHz, $CDCl_3$)

3.8.1 ^1H NMR

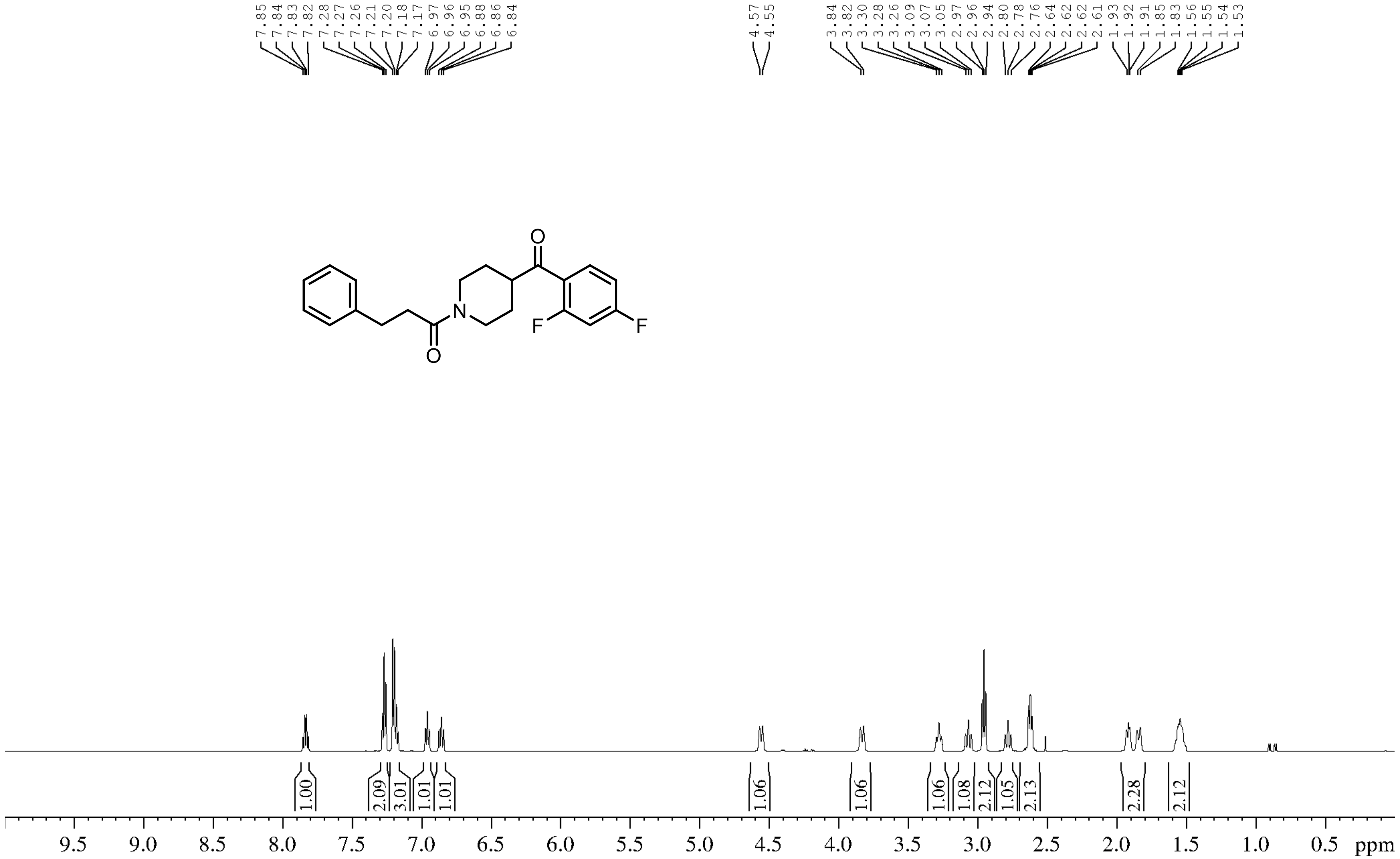

3.8.2 ^{13}C NMR

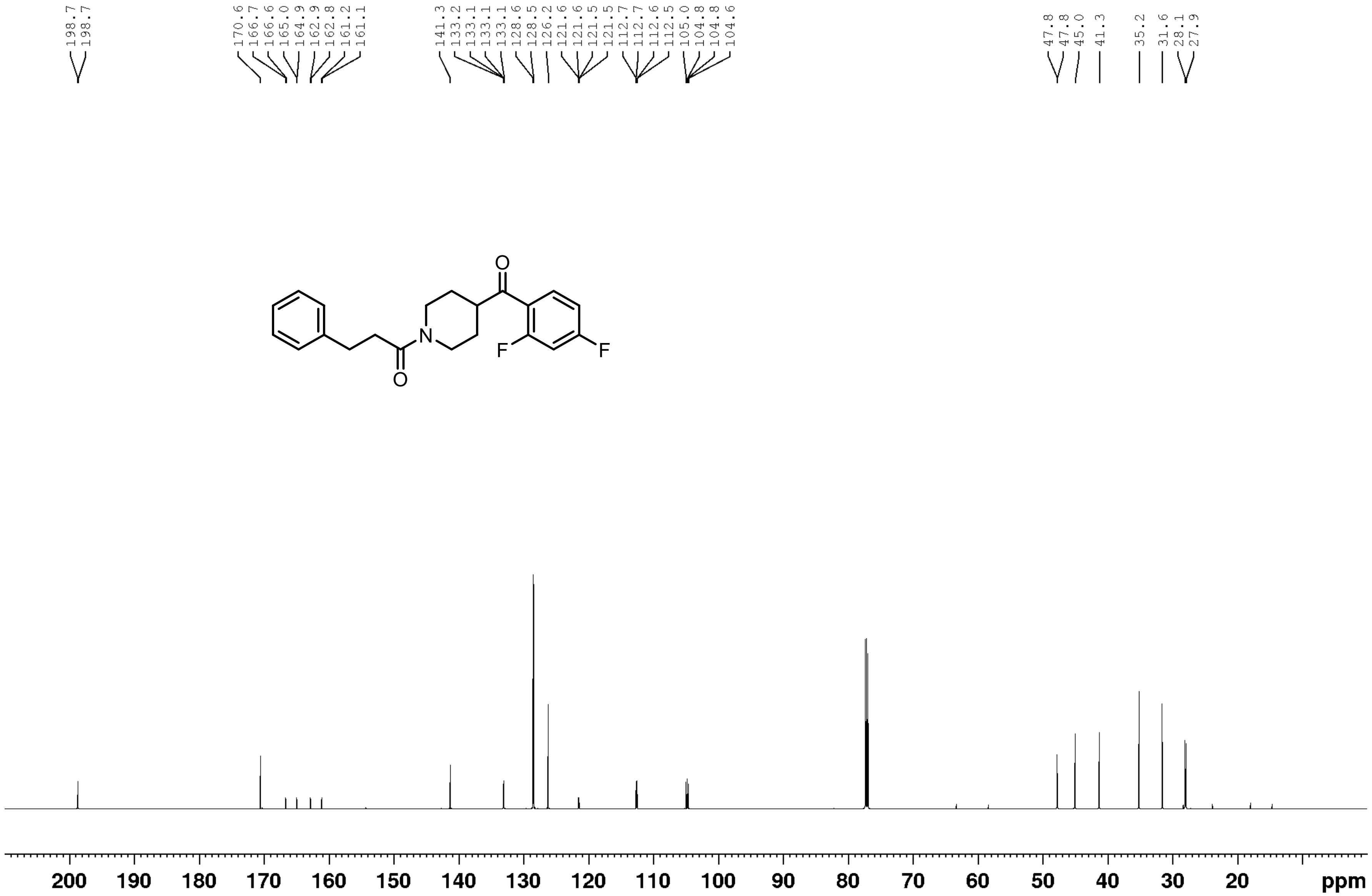
198.7
198.7
170.6
166.7
166.6
165.0
164.9
162.9
162.8
161.2
161.1
141.3
133.2
133.1
133.1
133.1
128.6
128.5
126.2
121.6
121.6
121.5
121.5
112.7
112.7
112.6
112.5
105.0
104.8
104.8
104.6
47.8
47.8
45.0
41.3
35.2
31.6
28.1
27.9
O
N
O
F
F
200 190 180 170 160 150 140 130 120 110 100 90 80 70 60 50 40 30 20
ppm

3.8.3 ¹⁹F NMR

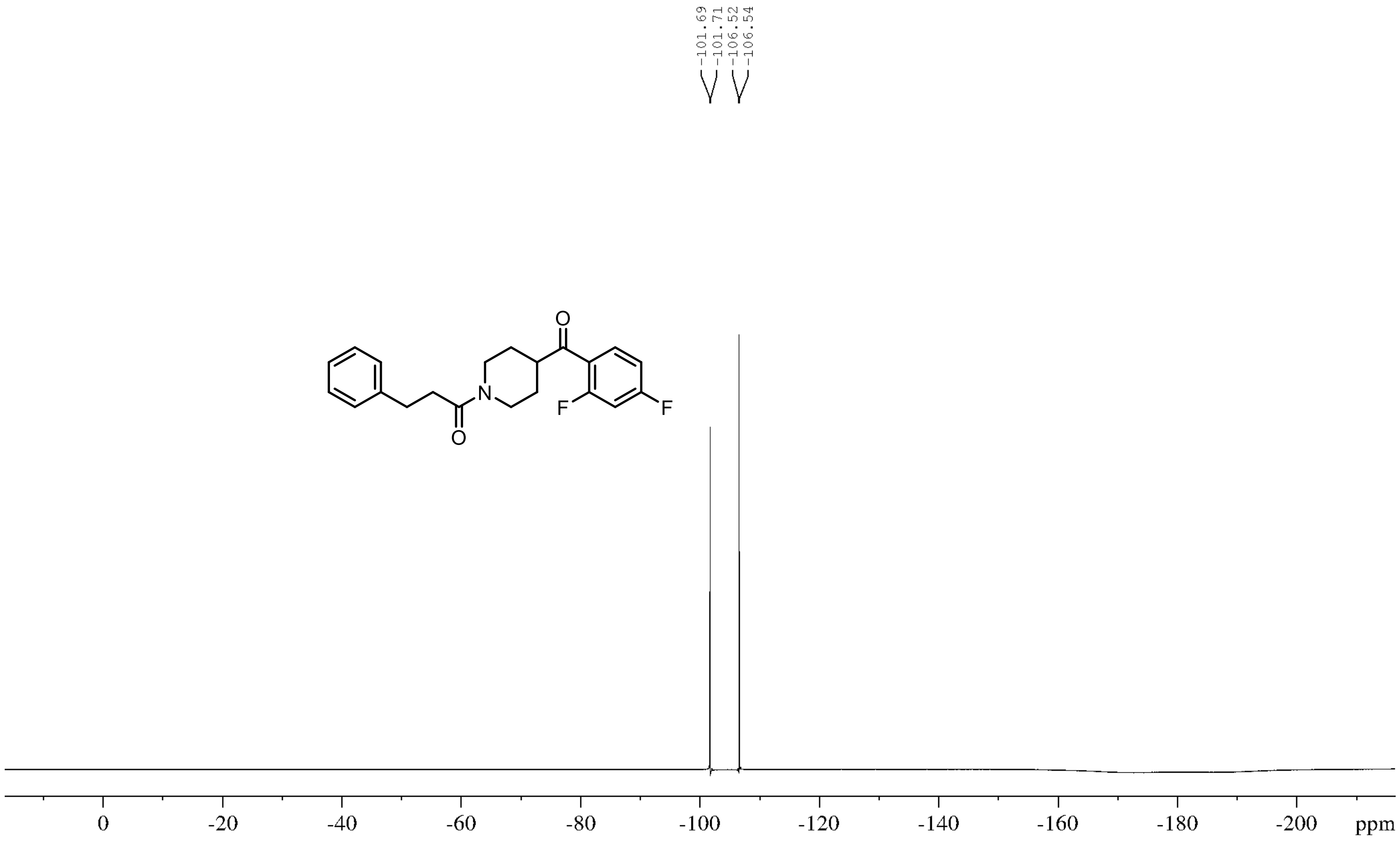

-101.69
-101.71
-106.52
-106.54
O
N
O
F
F
0
-20
-40
-60
-80
-100
-120
-140
-160
-180
-200
ppm

3.8.4 ^{1}H, ^{1}H COSY

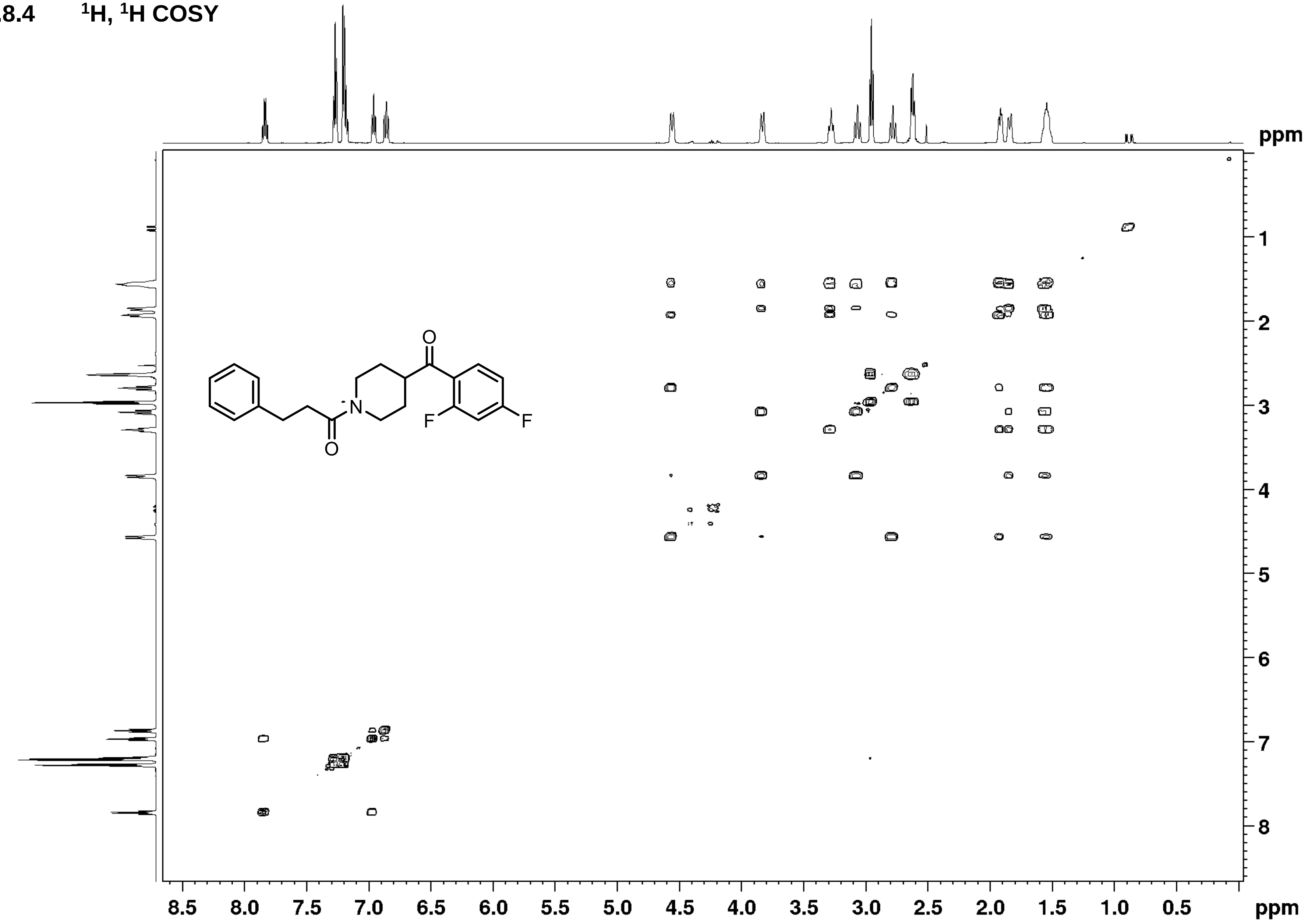

3.8.5 ^{1}H, ^{13}C HMBC

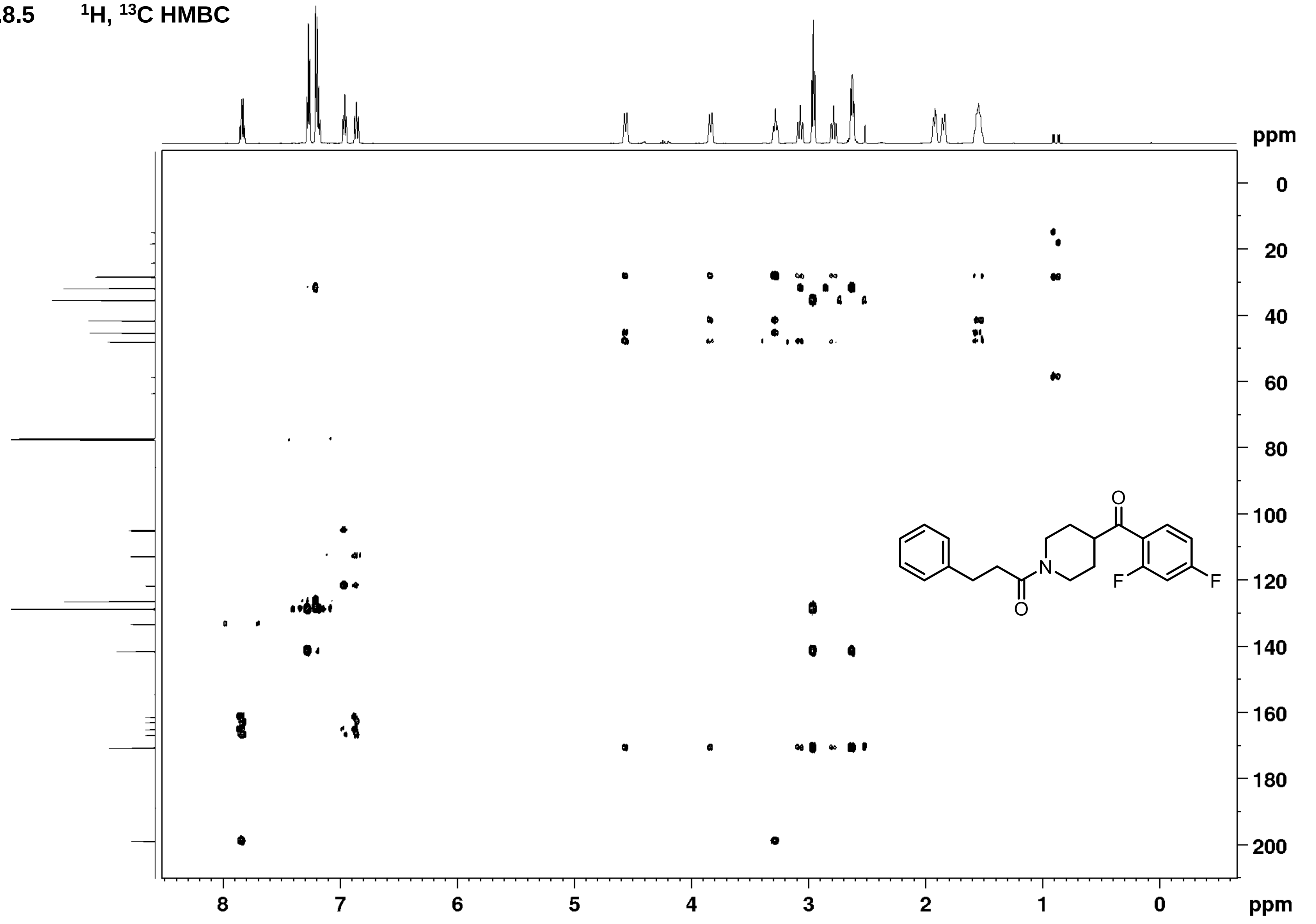

3.8.6 ^{1}H, ^{13}C HSQC

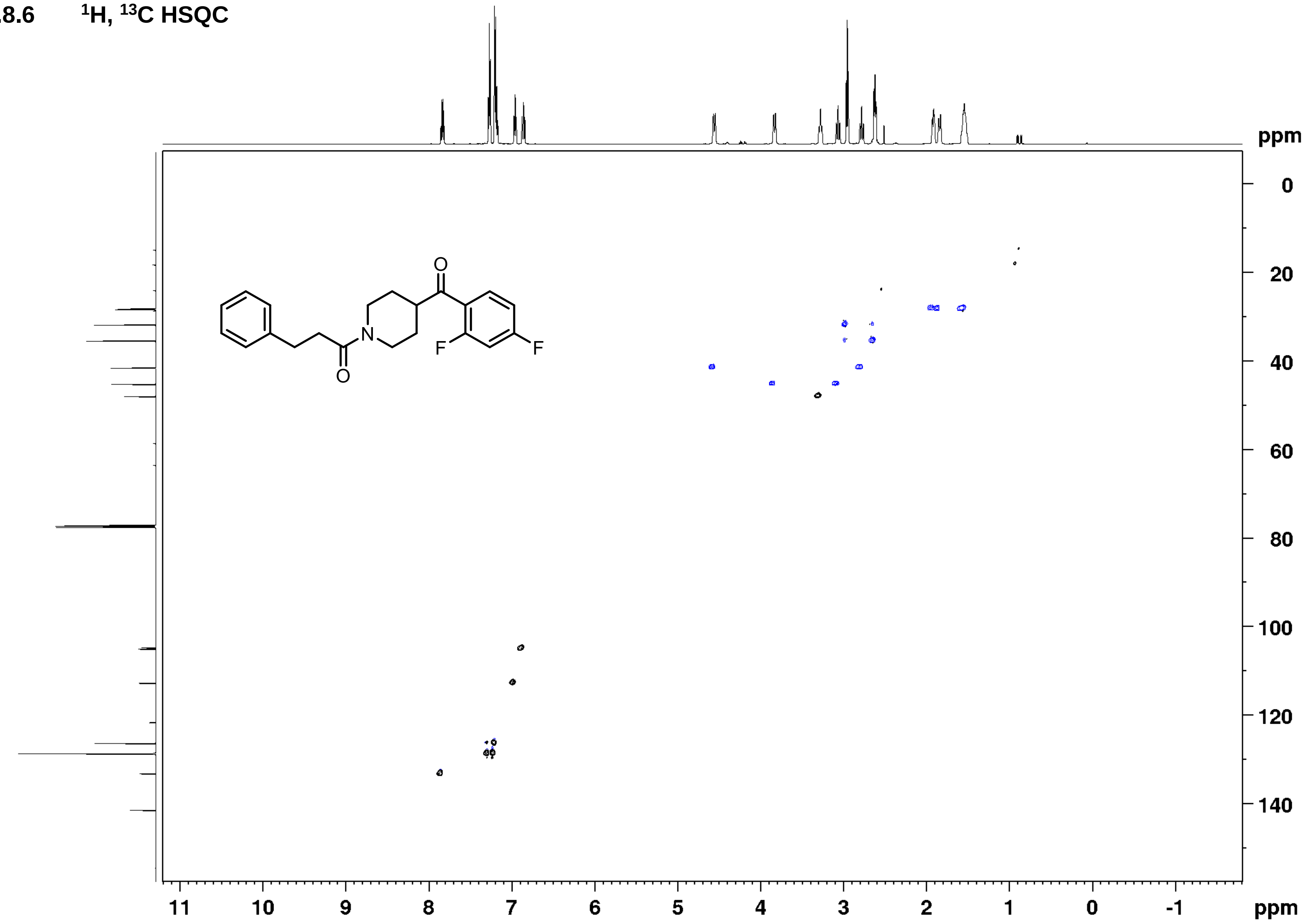

3.8.7 HRMS

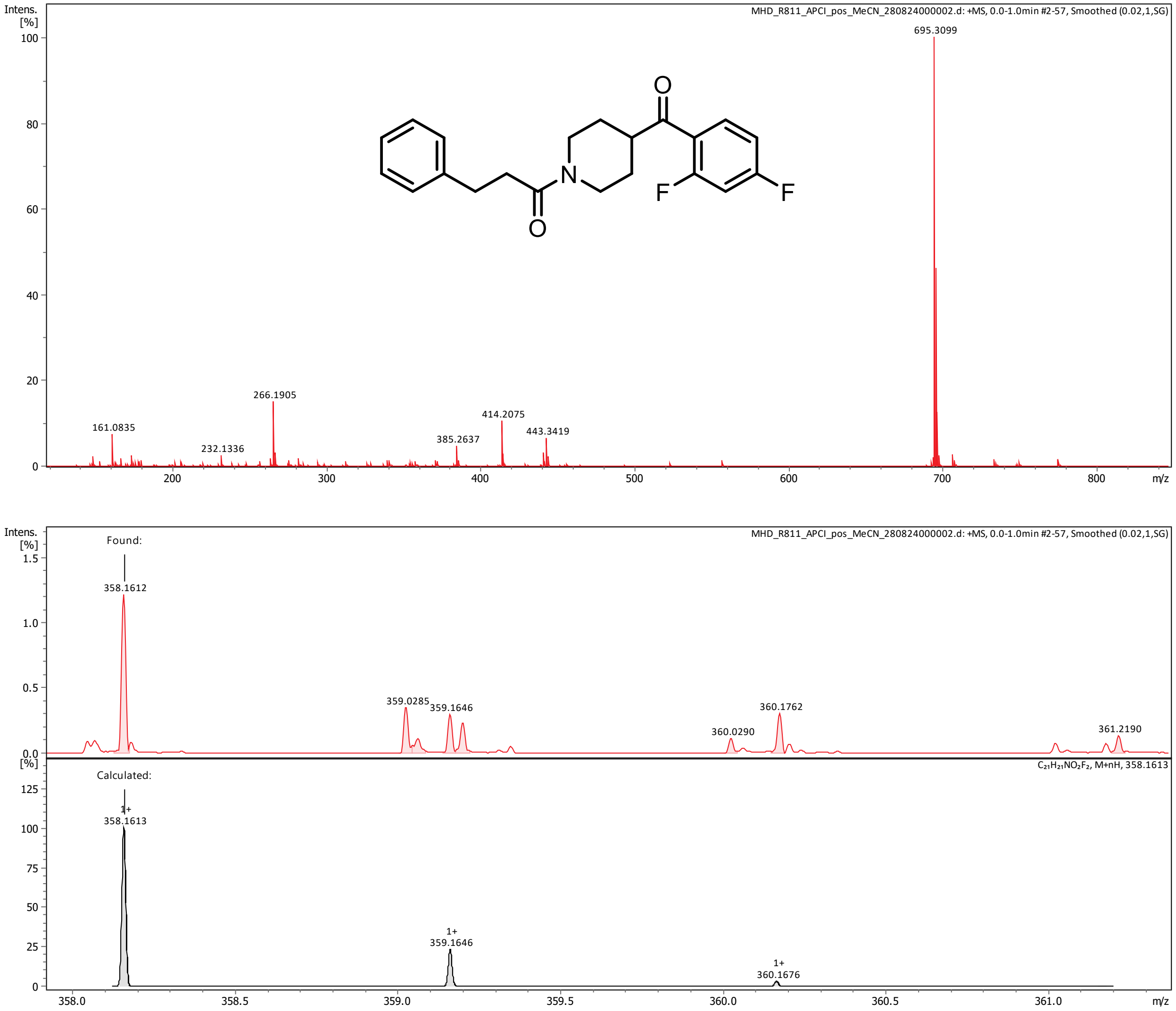

MHD_R811_APCI_pos_MeCN_280824000002.d: +MS, 0.0-1.0min #2-57, Smoothed (0.02,1,SG)
Intens. [%]
695.3099
266.1905
161.0835
232.1336
385.2637
414.2075
443.3419
m/z
Found:
358.1612
359.0285
359.1646
360.0290
360.1762
361.2190
Calculated:
1+ 358.1613
1+ 359.1646
1+ 360.1676

3.8.8 IR

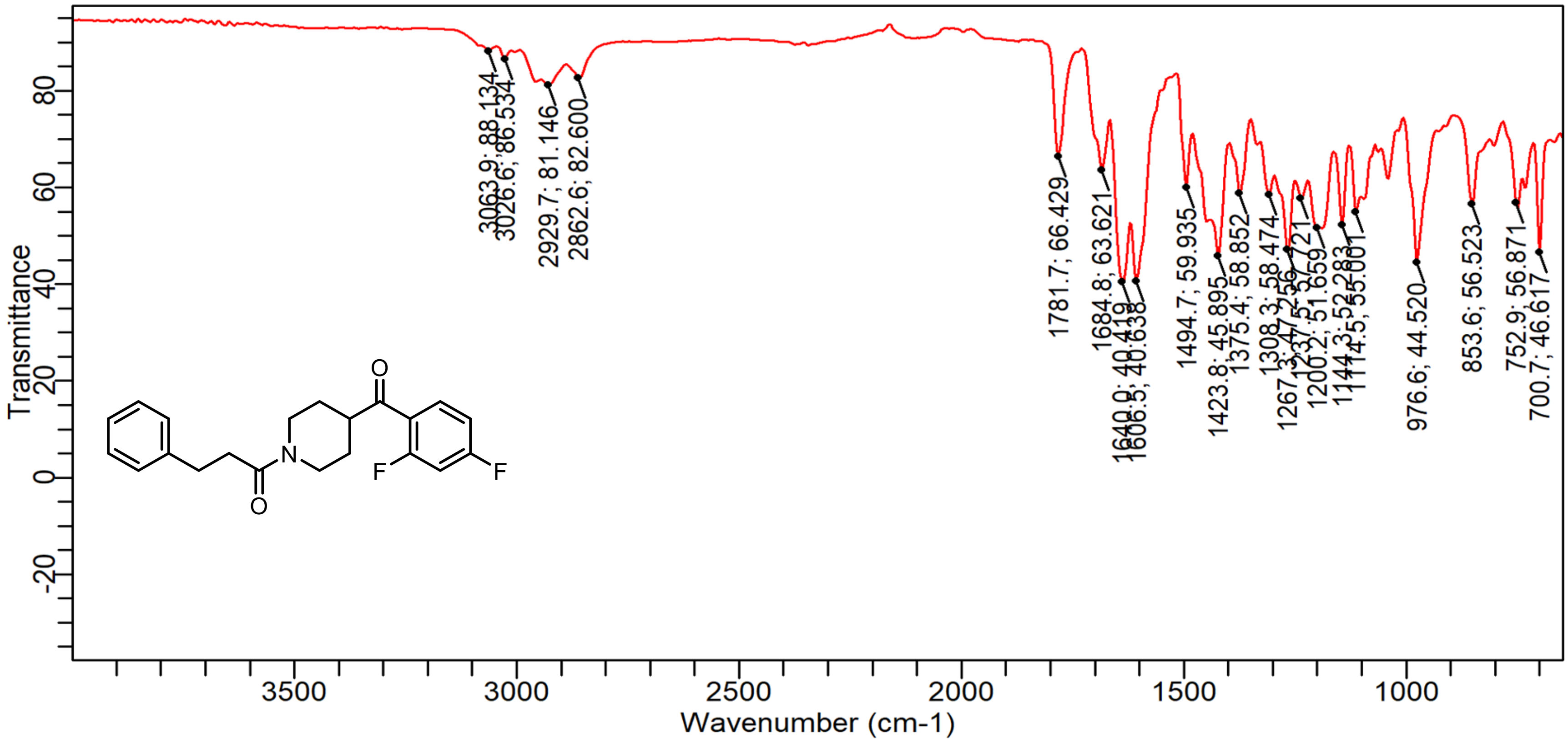

Transmittance
Wavenumber (cm-1)
3063.9; 88.134
3026.6; 86.534
2929.7; 81.146
2862.6; 82.600
1781.7; 66.429
1684.8; 63.621
1640.0; 40.419
1606.5; 40.638
1494.7; 59.935
1423.8; 45.895
1375.4; 58.852
1308.3; 58.474
1237.5; 57.721
1200.2; 51.659
1144.3; 52.283
1114.5; 55.001
976.6; 44.520
853.6; 56.523
752.9; 56.871
700.7; 46.617
F
F
N
O
O

3.9 1-(4-((2,4-Difluorophenyl)(methoxyimino)methyl)piperidin-1-yl)-3-phenylpropan-1-one (12i) (600 MHz, $CDCl_3$)

3.9.1 ^{1}H NMR

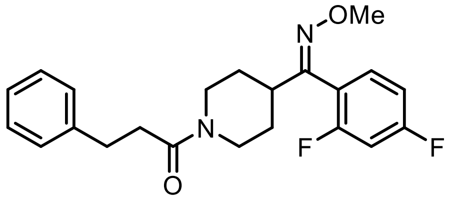

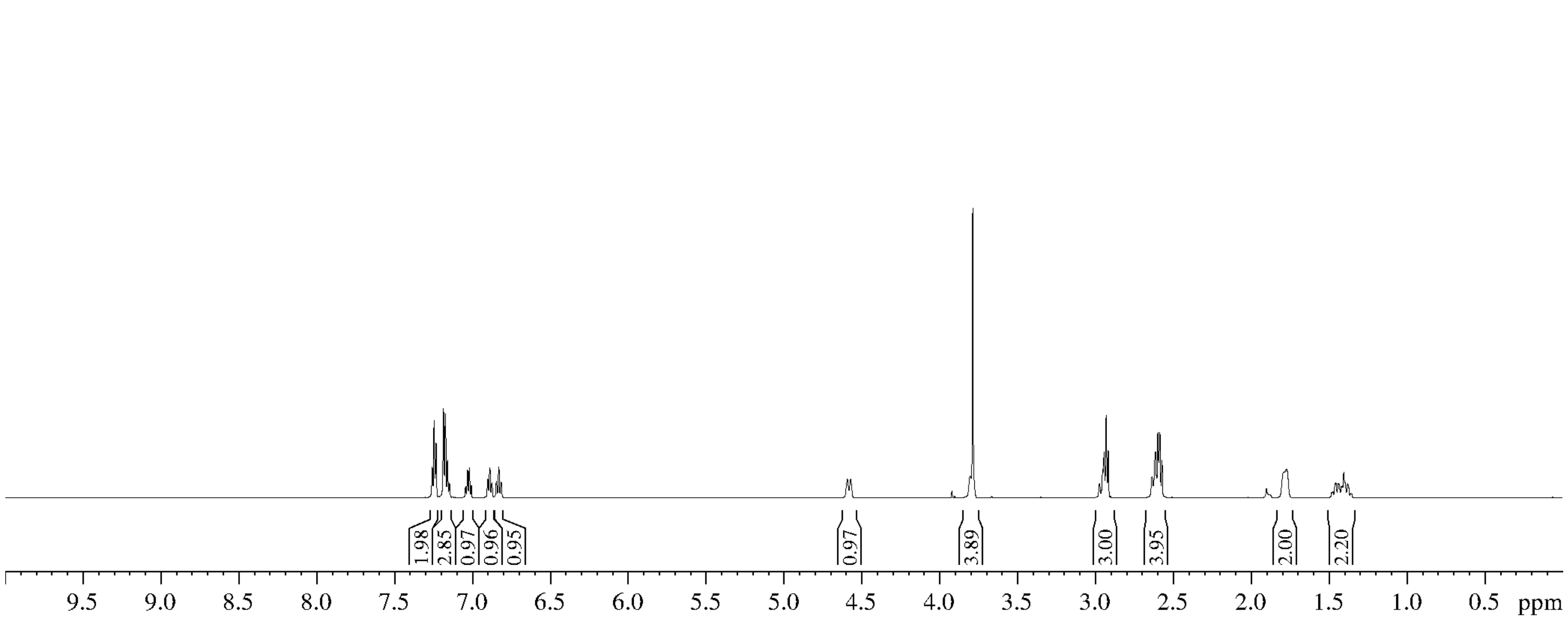

3.9.2 ^{13}C NMR

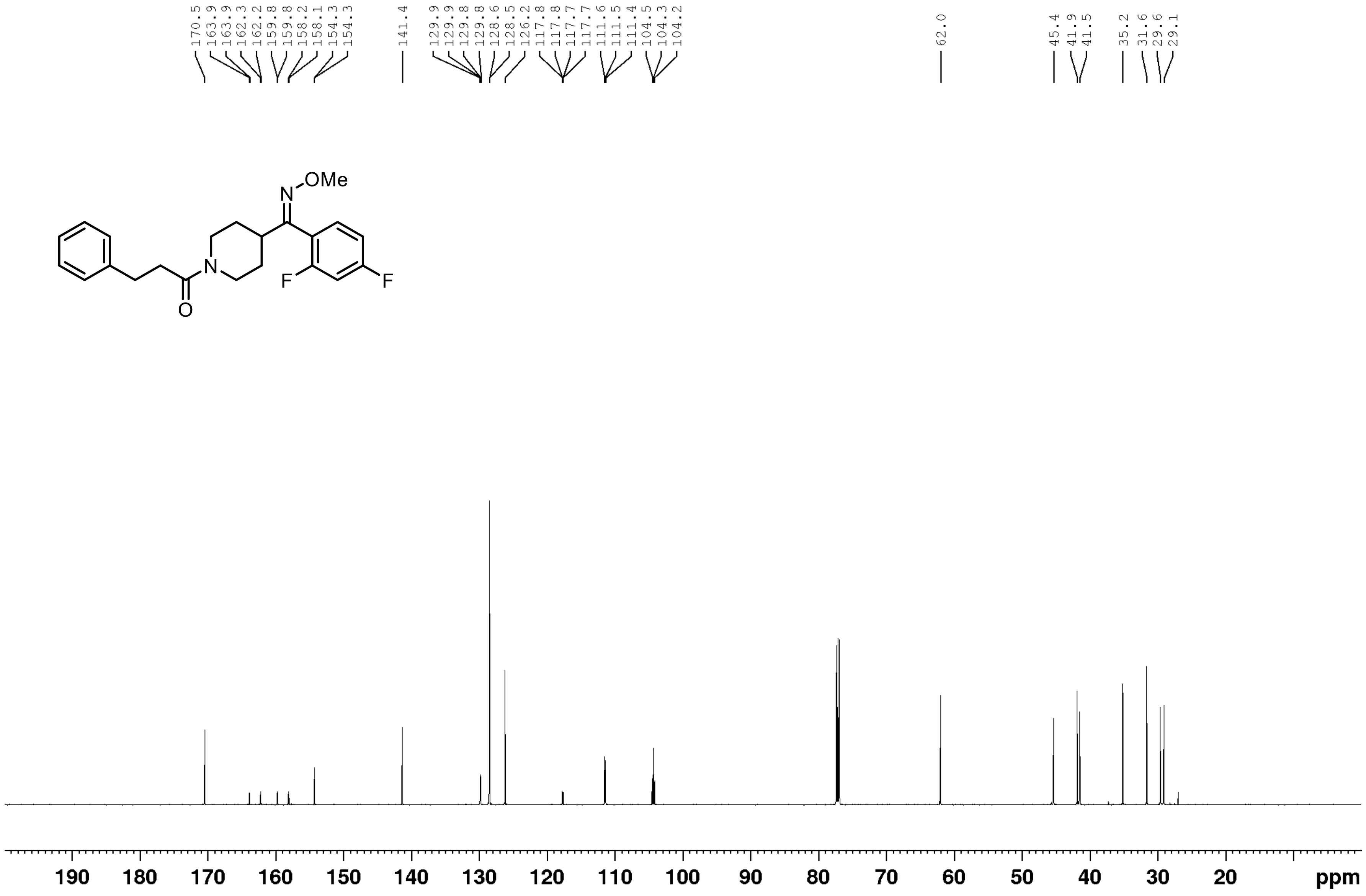

170.5
163.9
163.9
162.3
162.2
159.8
159.8
158.2
158.1
154.3
154.3
141.4
129.9
129.9
129.8
129.8
128.6
128.5
126.2
117.8
117.8
117.7
117.7
111.6
111.5
111.4
104.5
104.3
104.2
62.0
45.4
41.9
41.5
35.2
31.6
29.6
29.1
OMe
N
N
O
F
F
190
180
170
160
150
140
130
120
110
100
90
80
70
60
50
40
30
20
ppm

3.9.3 ^{19}F

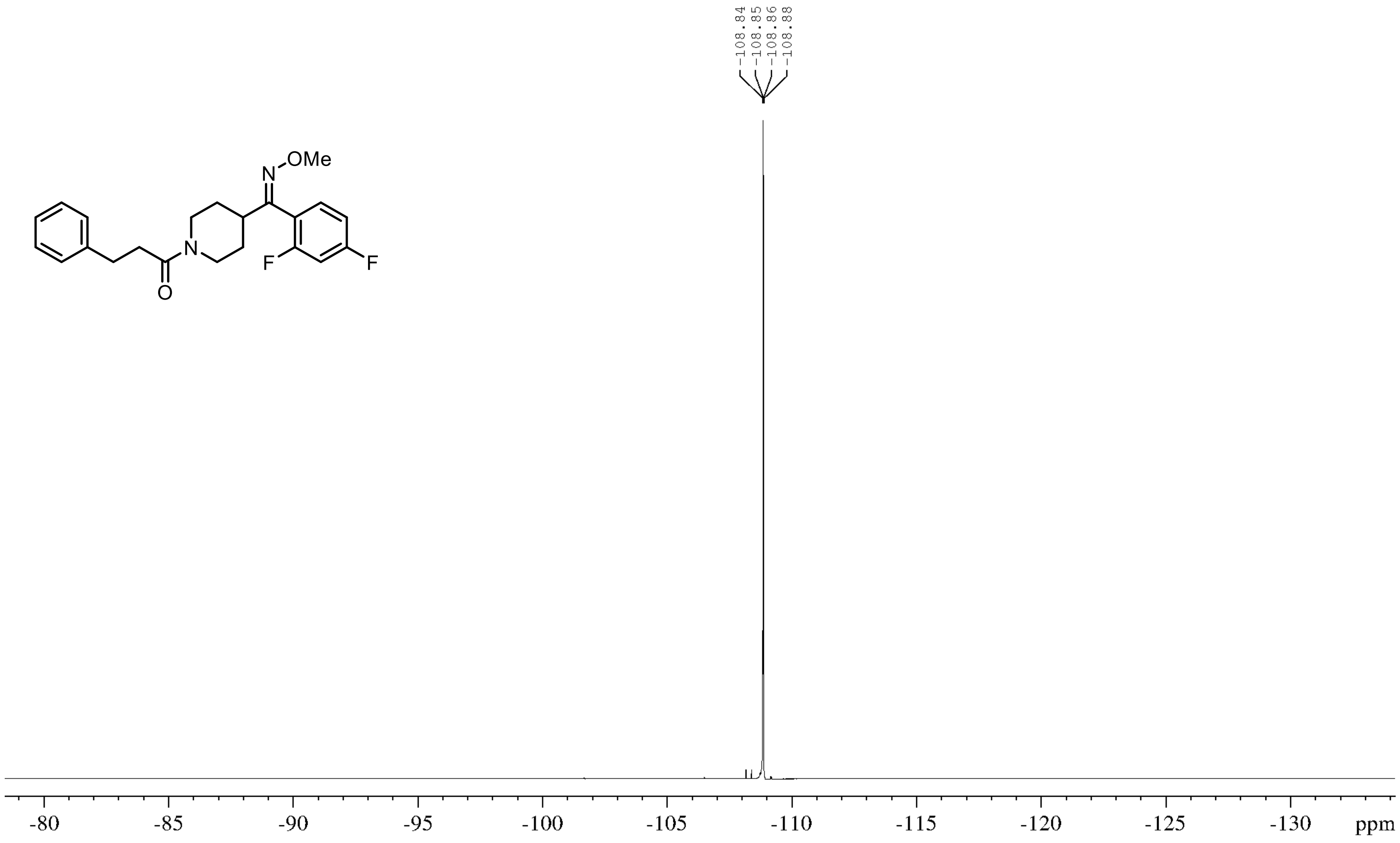

OMe
N
N
O
F
F
-108.84
-108.85
-108.86
-108.88
-80
-85
-90
-95
-100
-105
-110
-115
-120
-125
-130
ppm

3.9.4 ^{1}H, ^{1}H COSY

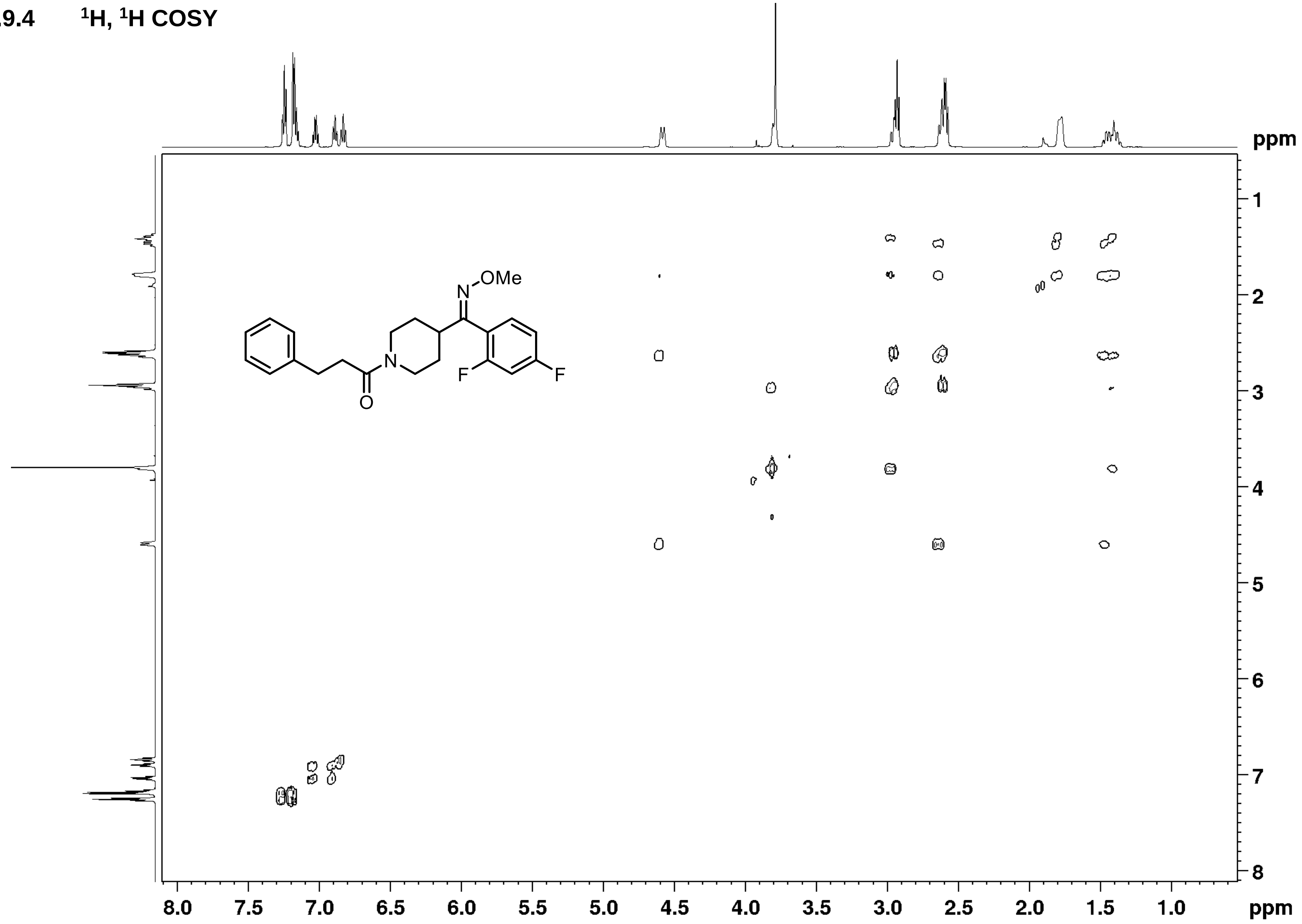

3.9.5 ^{1}H, ^{13}C HMBC

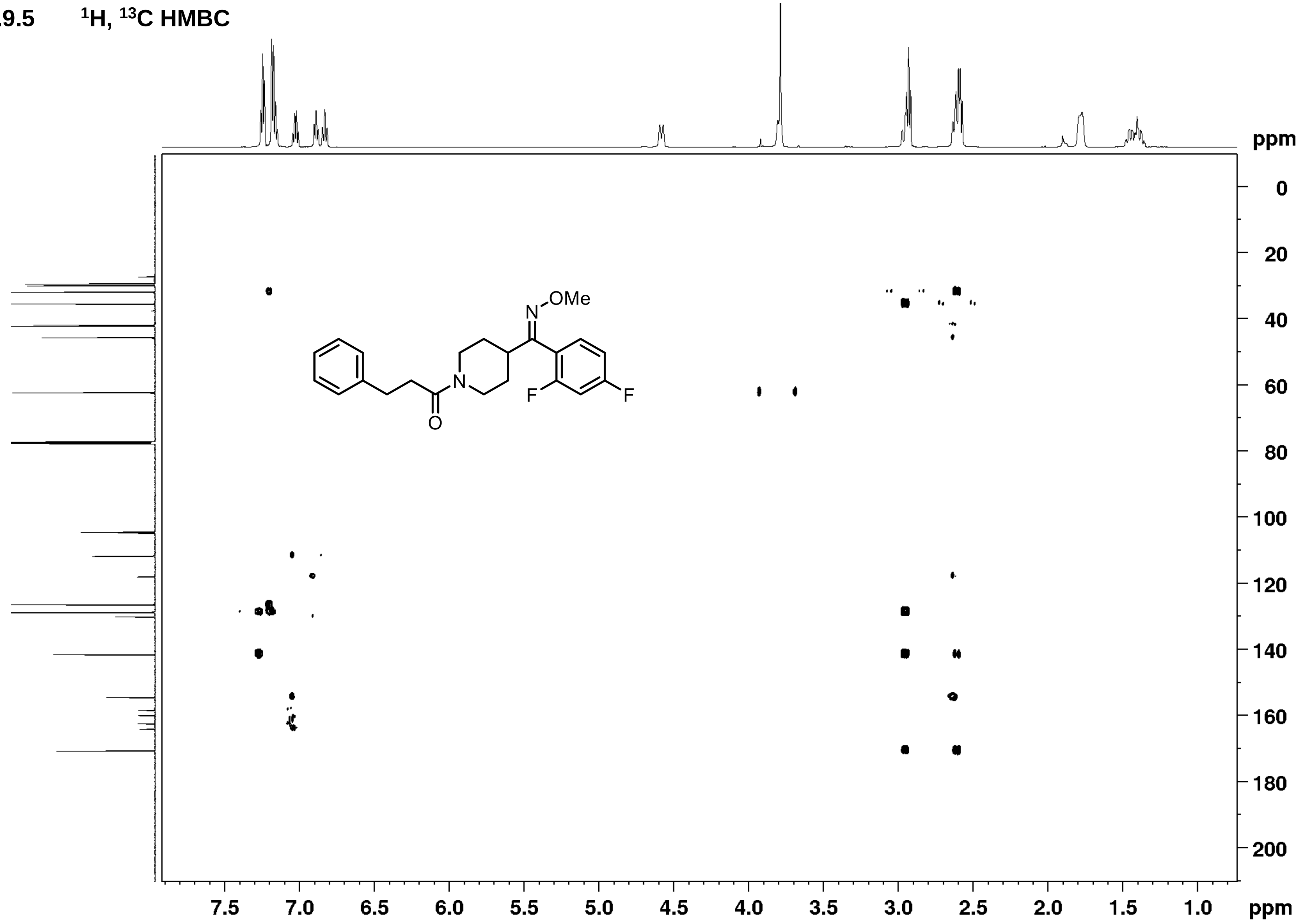

3.9.6 ^{1}H, ^{13}C HSQC

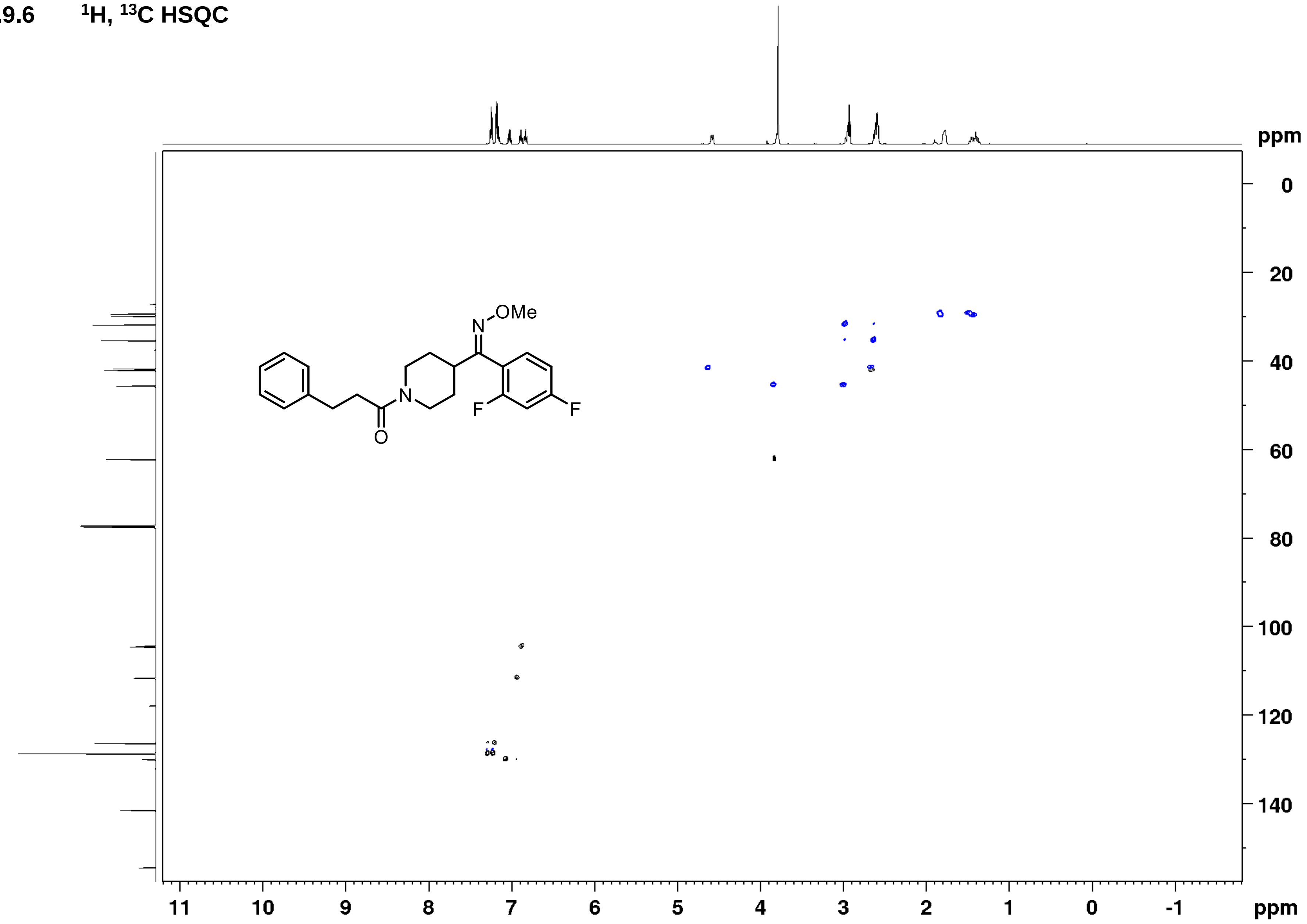

3.9.7 HRMS

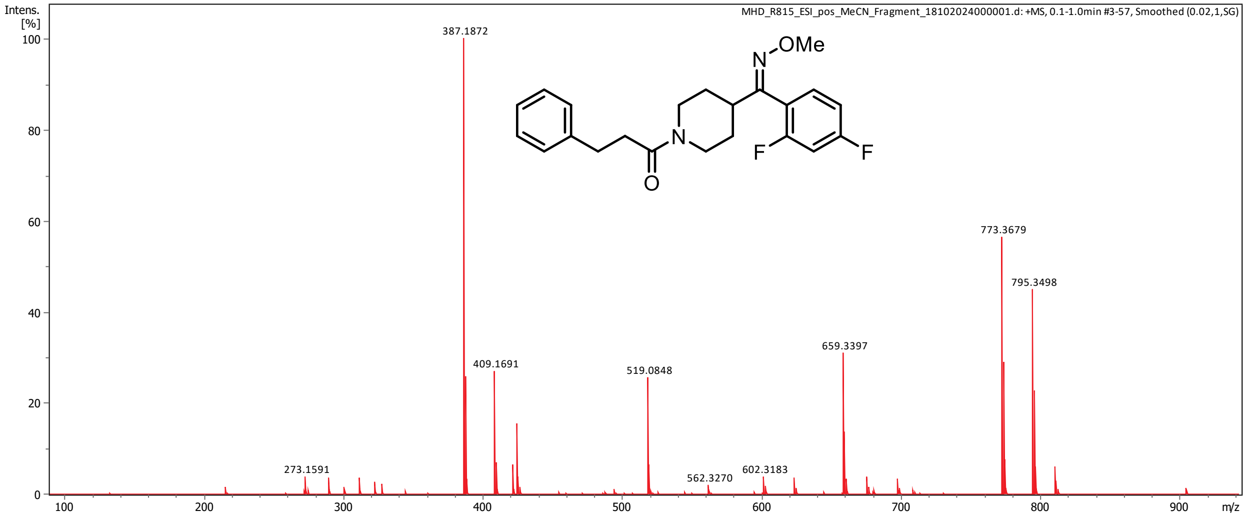

MHD_R815_ESI_pos_MeCN_Fragment_18102024000001.d: +MS, 0.1-1.0min #3-57, Smoothed (0.02,1,SG)
Intens. [%]
387.1872
409.1691
519.0848
659.3397
773.3679
795.3498
273.1591
562.3270
602.3183
m/z
N
OMe
O
F
F

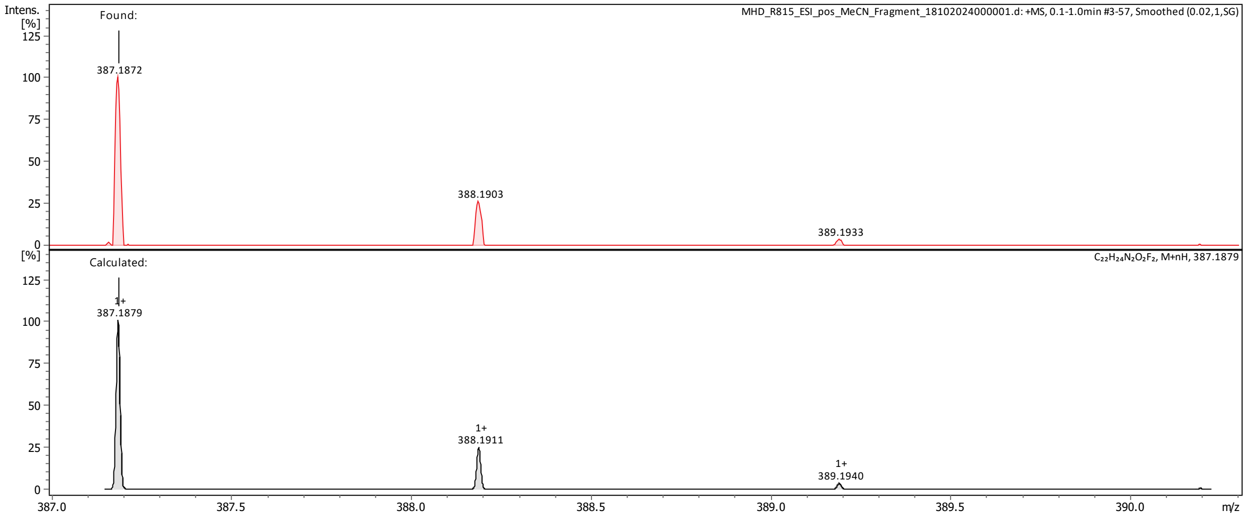

MHD_R815_ESI_pos_MeCN_Fragment_18102024000001.d: +MS, 0.1-1.0min #3-57, Smoothed (0.02,1,SG)
Found:
387.1872
388.1903
389.1933
Calculated:
$C_{22}H_{24}N_2O_2F_2$, M+nH, 387.1879
1+
387.1879
1+
388.1911
1+
389.1940
m/z

3.9.8 IR

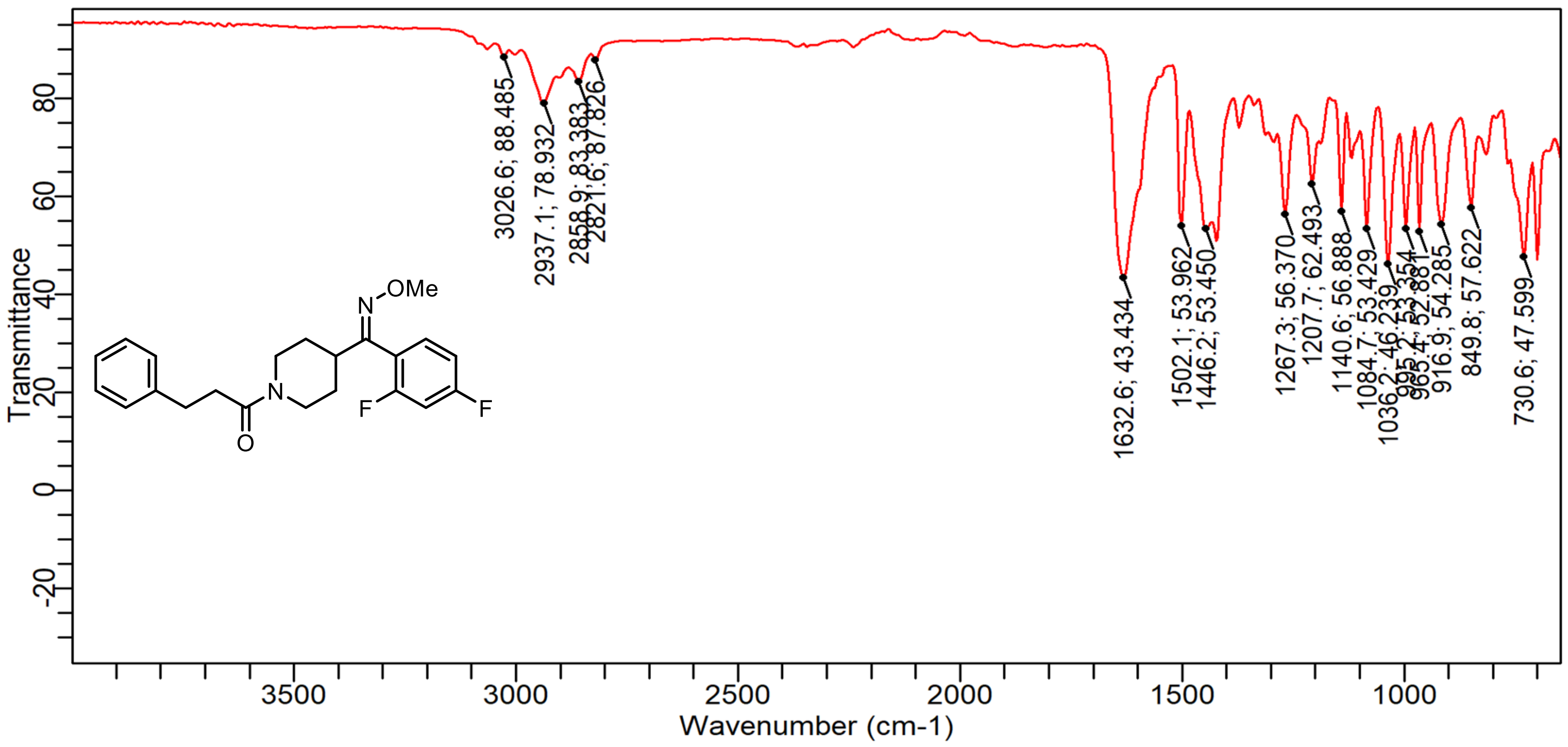

Transmittance
Wavenumber (cm-1)
3026.6; 88.485
2937.1; 78.932
2858.9; 83.383
2821.6; 87.826
1632.6; 43.434
1502.1; 53.962
1446.2; 53.450
1267.3; 56.370
1207.7; 62.493
1140.6; 56.888
1084.7; 53.429
1036.2; 46.239
916.9; 54.285
849.8; 57.622
730.6; 47.599
N
OMe
N
O
F
F

3.10 4-(3-Phenylpropanoyl)piperazine-1-carbaldehyde (12j & 12j rotamer) (600 MHz, $CDCl_3$)

3.10.1 ^{1}H NMR

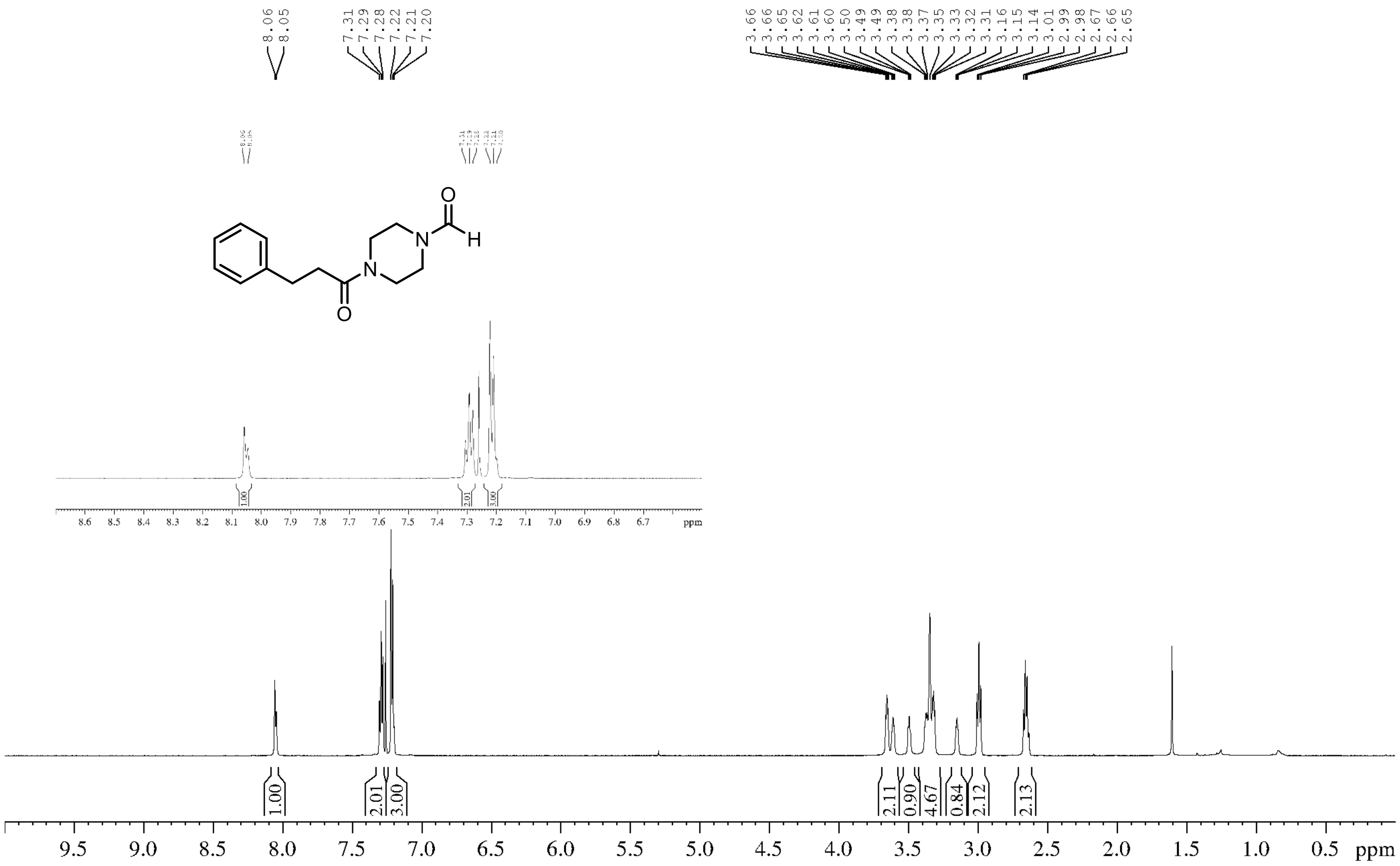

3.10.2 ^{13}C NMR

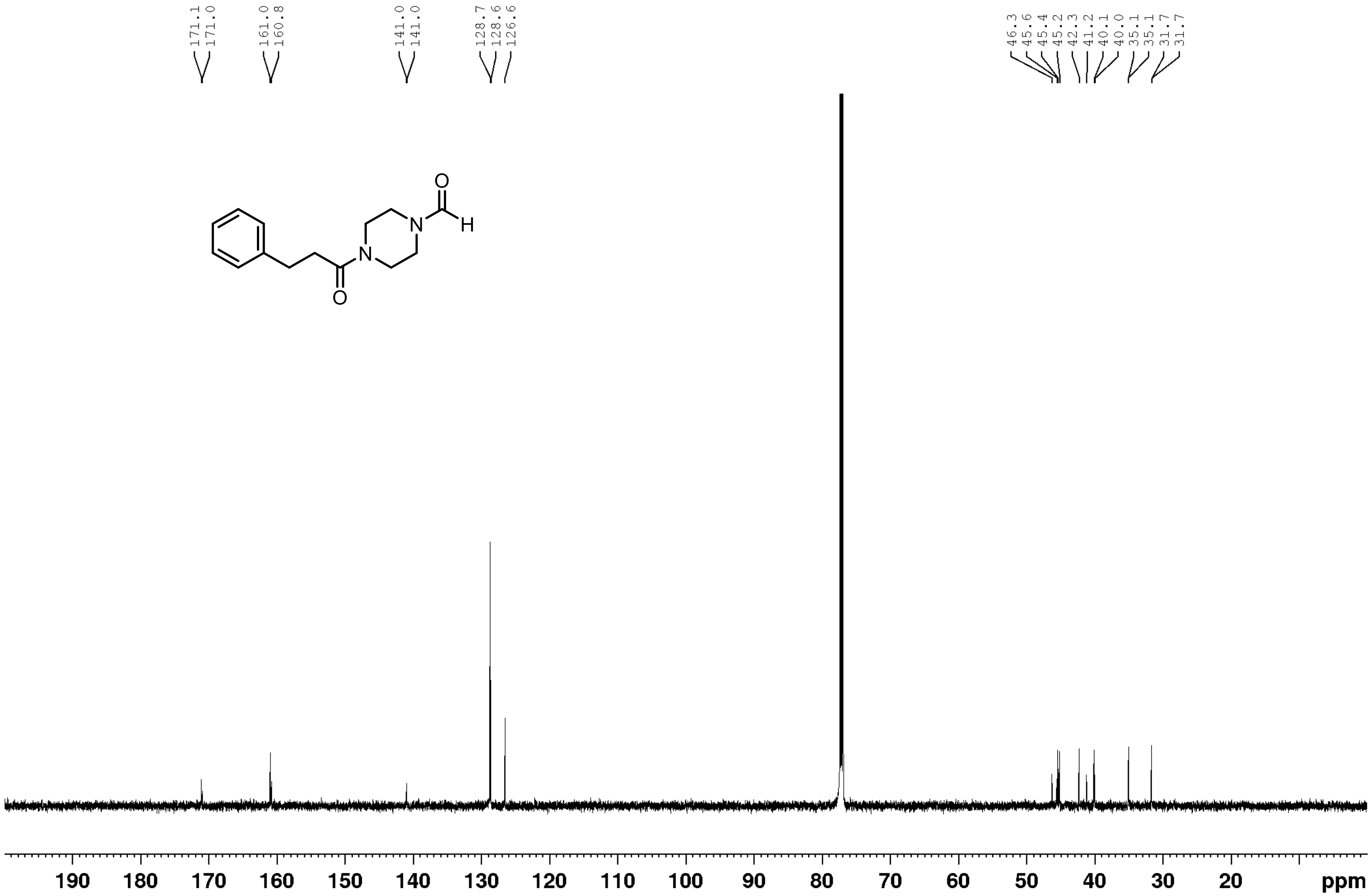
171.1
171.0
161.0
160.8
141.0
141.0
128.7
128.6
126.6
46.3
45.6
45.4
45.2
42.3
41.2
40.1
40.0
35.1
35.1
31.7
31.7
O
N
N
O
H
190
180
170
160
150
140
130
120
110
100
90
80
70
60
50
40
30
20
ppm

3.10.3 ¹H, ¹H COSY

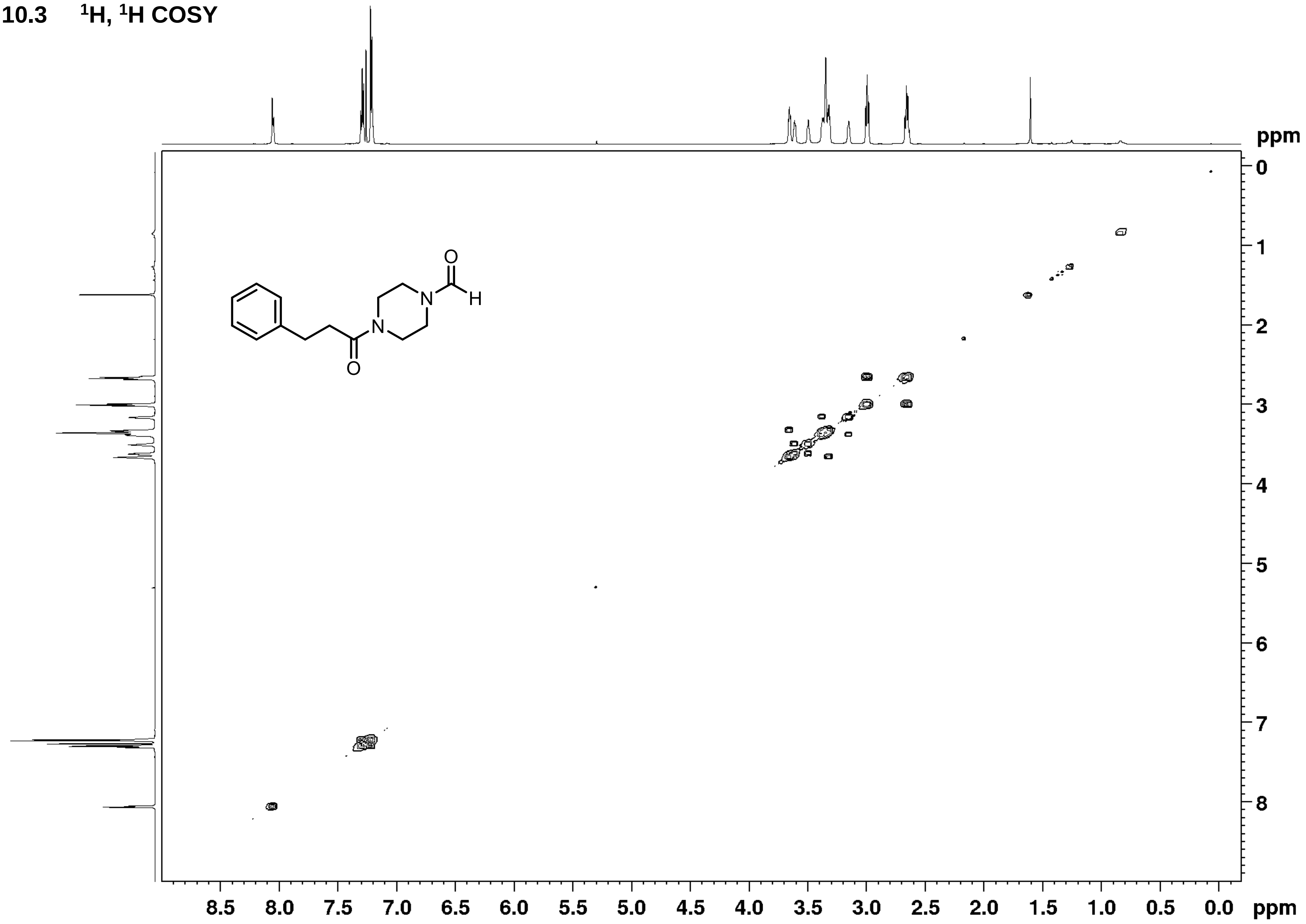

3.10.4 ^{1}H, ^{13}C HMBC

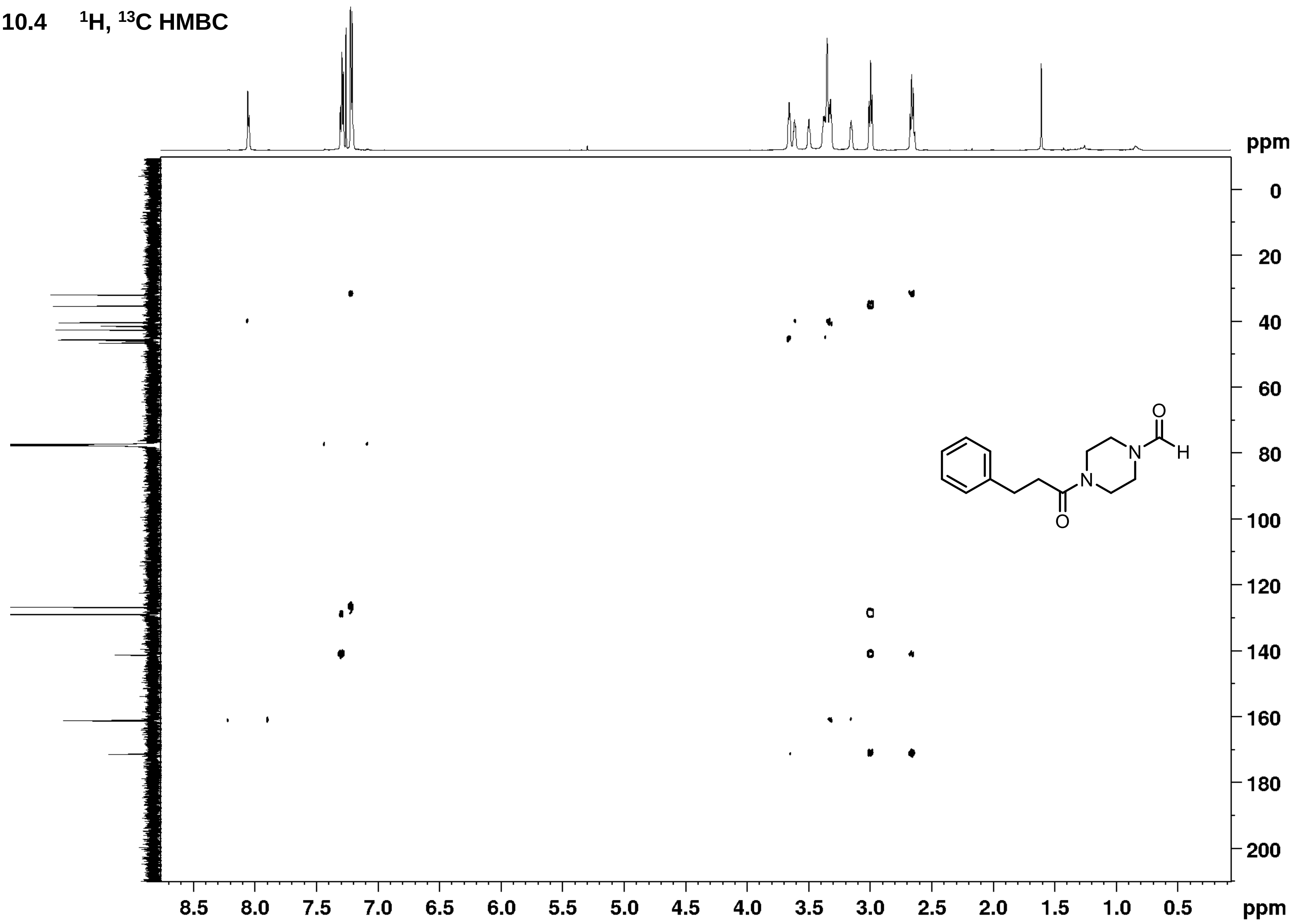

3.10.5 ^{1}H, ^{13}C HSQC

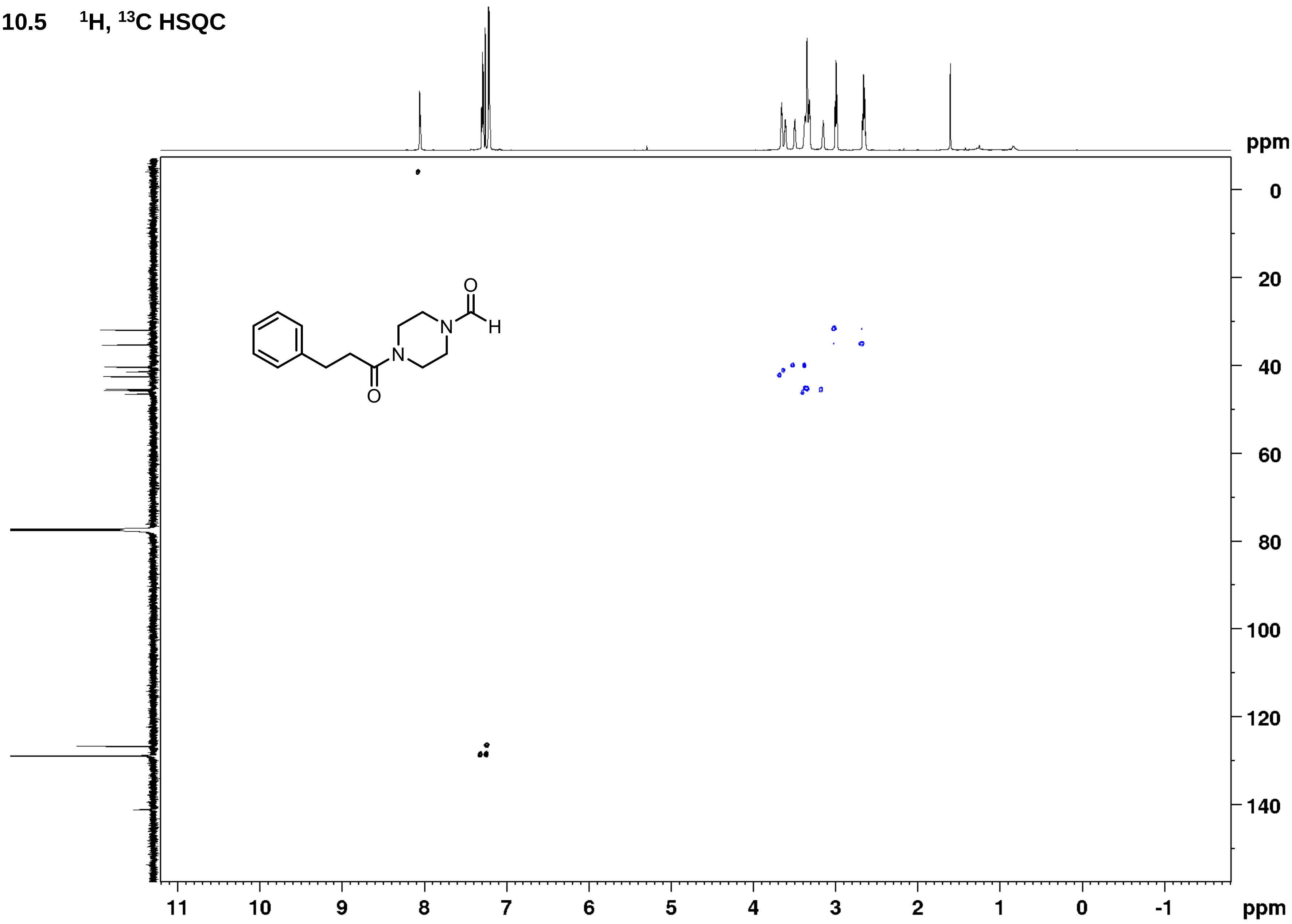

3.10.6 HRMS

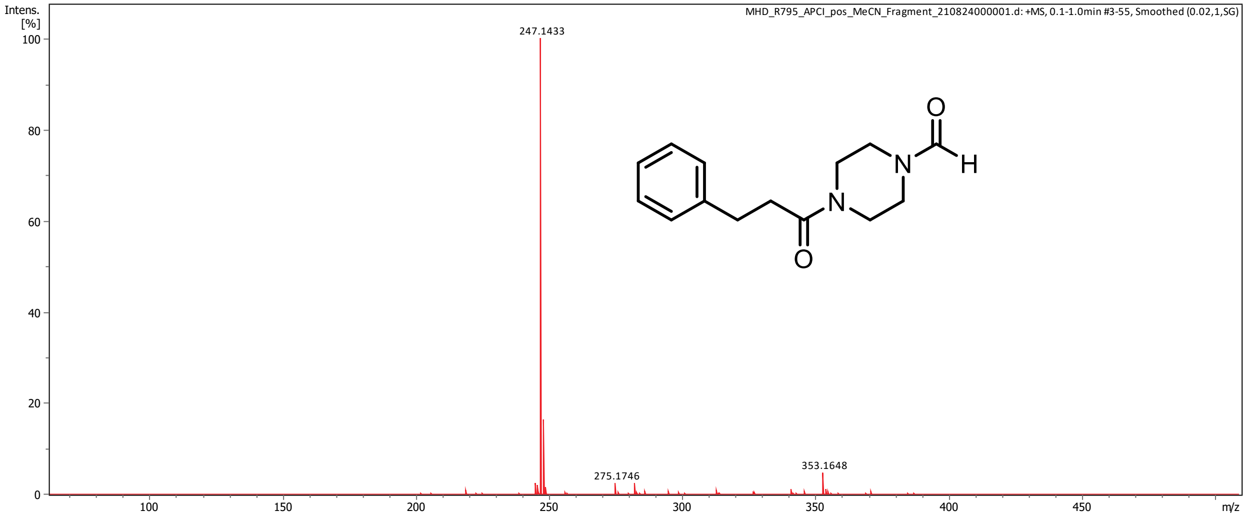
MHD_R795_APCI_pos_MeCN_Fragment_210824000001.d: +MS, 0.1-1.0min #3-55, Smoothed (0.02,1,SG)
Intens. [%]
247.1433
275.1746
353.1648
m/z
O
N
N
H
O

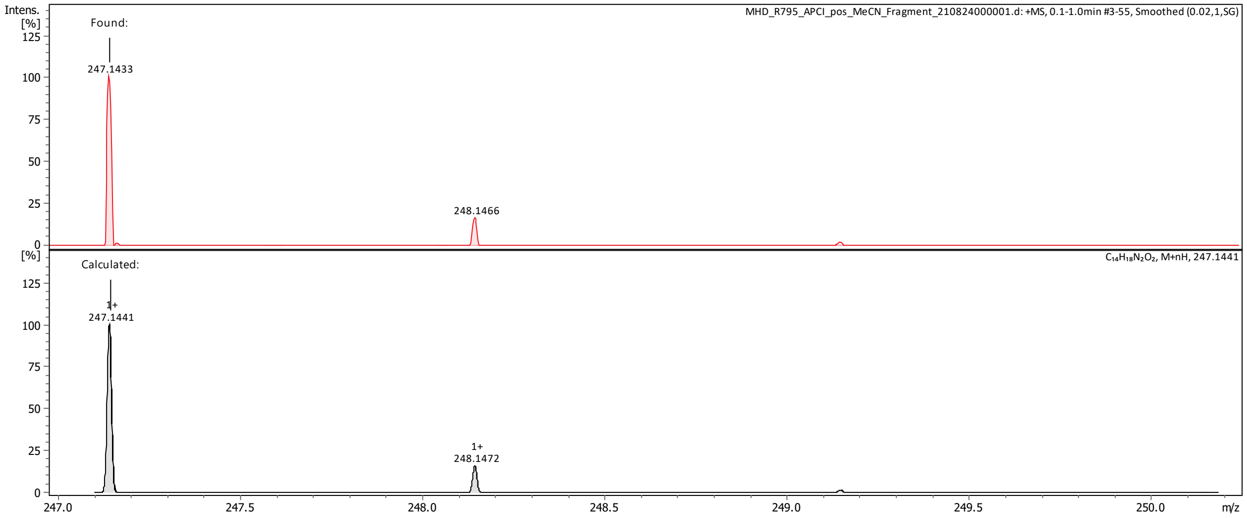
MHD_R795_APCI_pos_MeCN_Fragment_210824000001.d: +MS, 0.1-1.0min #3-55, Smoothed (0.02,1,SG)
Intens. [%]
Found:
247.1433
248.1466
Calculated:
C14H18N2O2, M+nH, 247.1441
1+ 247.1441
1+ 248.1472
m/z

3.10.7 IR

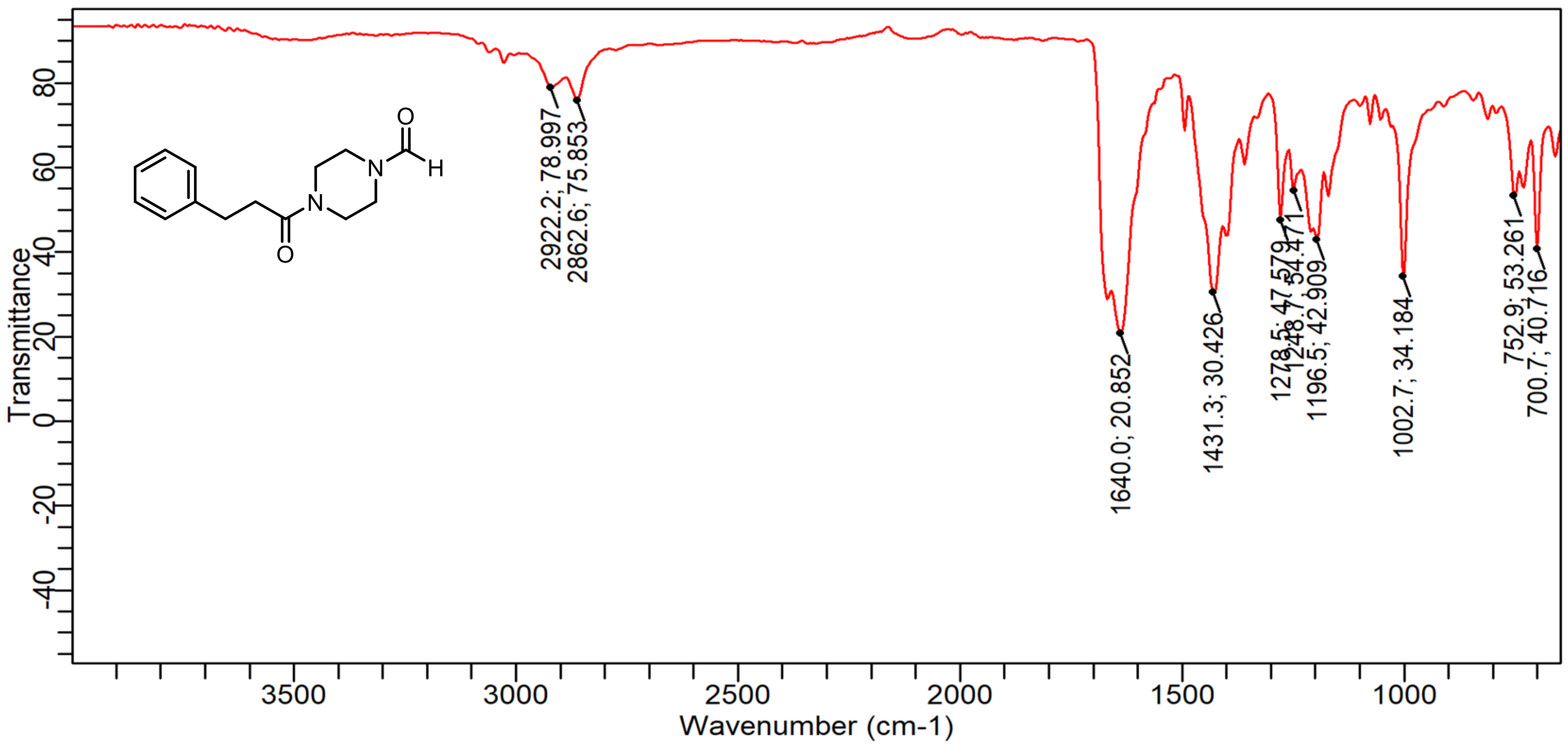

2922.2; 78.997
2862.6; 75.853
1640.0; 20.852
1431.3; 30.426
1278.5; 47.579
1248.7; 54.471
1196.5; 42.909
1002.7; 34.184
752.9; 53.261
700.7; 40.716
Transmittance
Wavenumber (cm-1)

3.11 1-(4-(Oxetan-3-yl)piperidin-1-yl)-3-phenylpropan-1-one (12k) (600 MHz, $CDCl_3$)

3.11.1 ^{1}H NMR

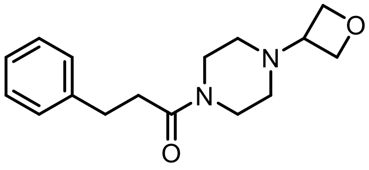

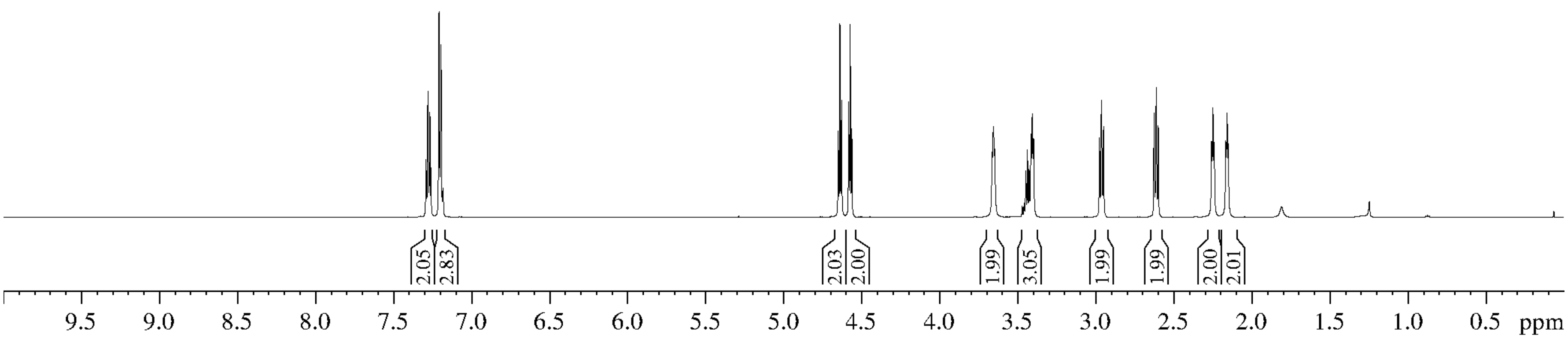

3.11.2 ^{13}C NMR

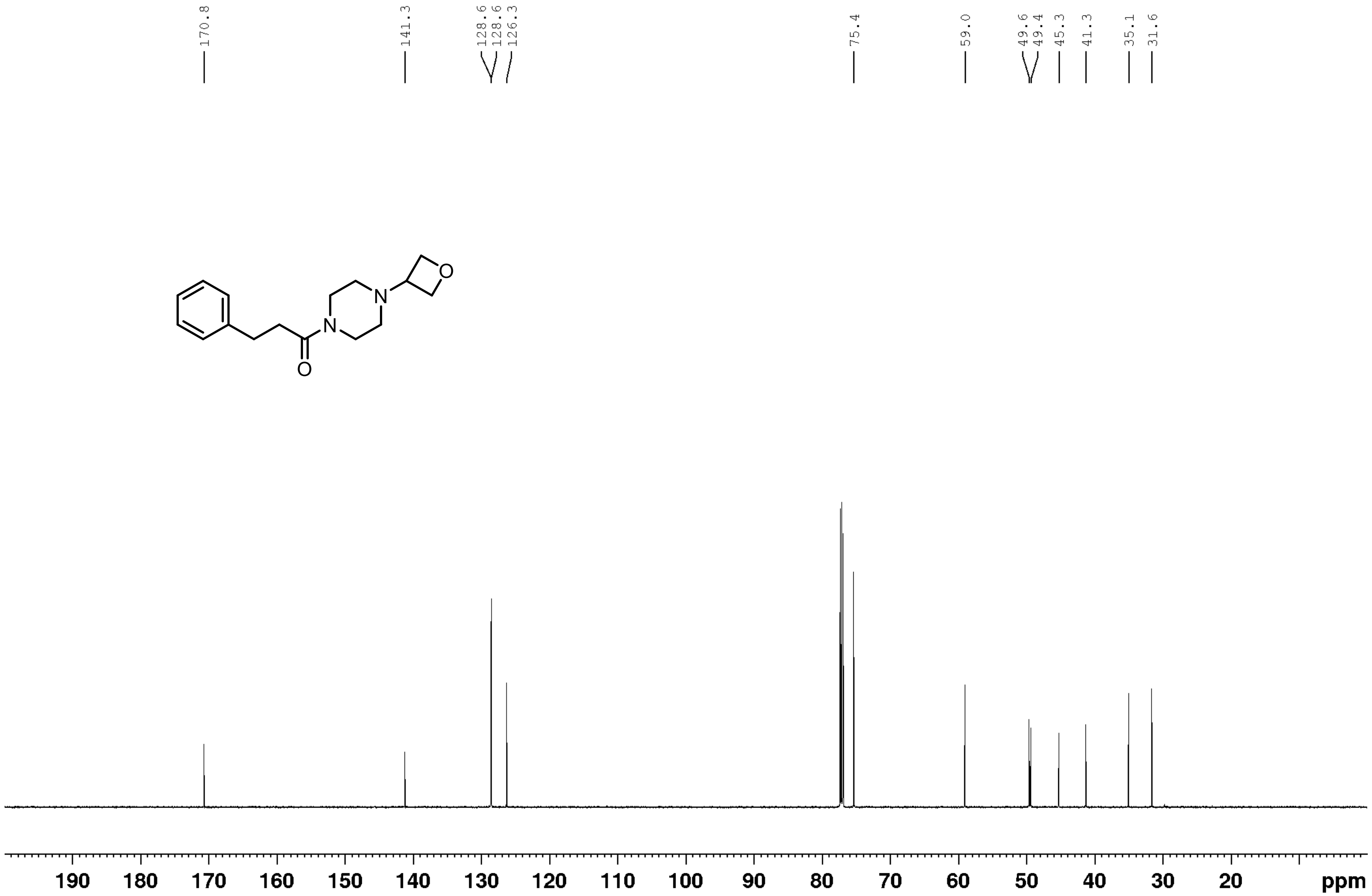

3.11.3 ^{1}H, ^{1}H COSY

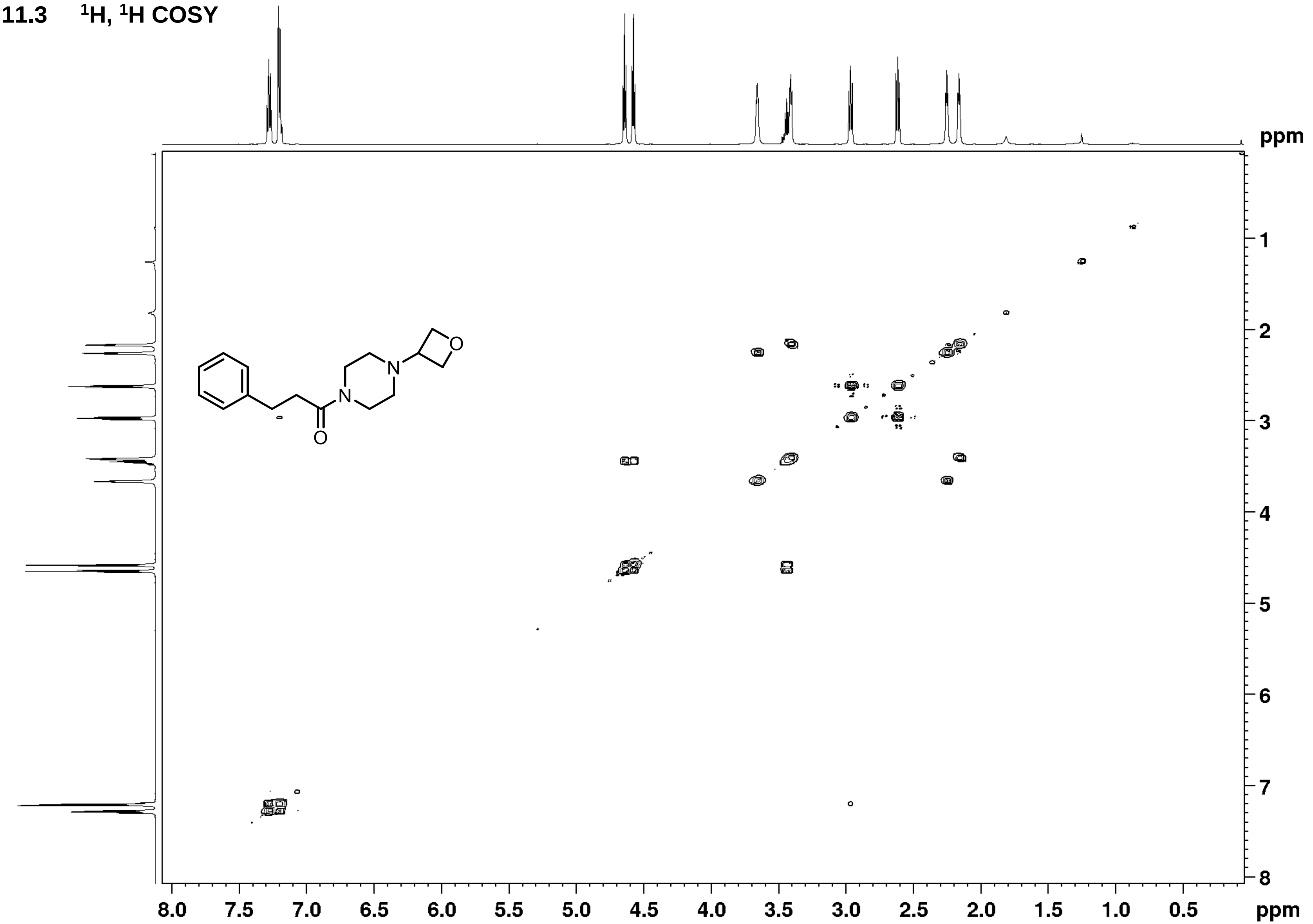

3.11.4 ¹H, ¹³C HMBC

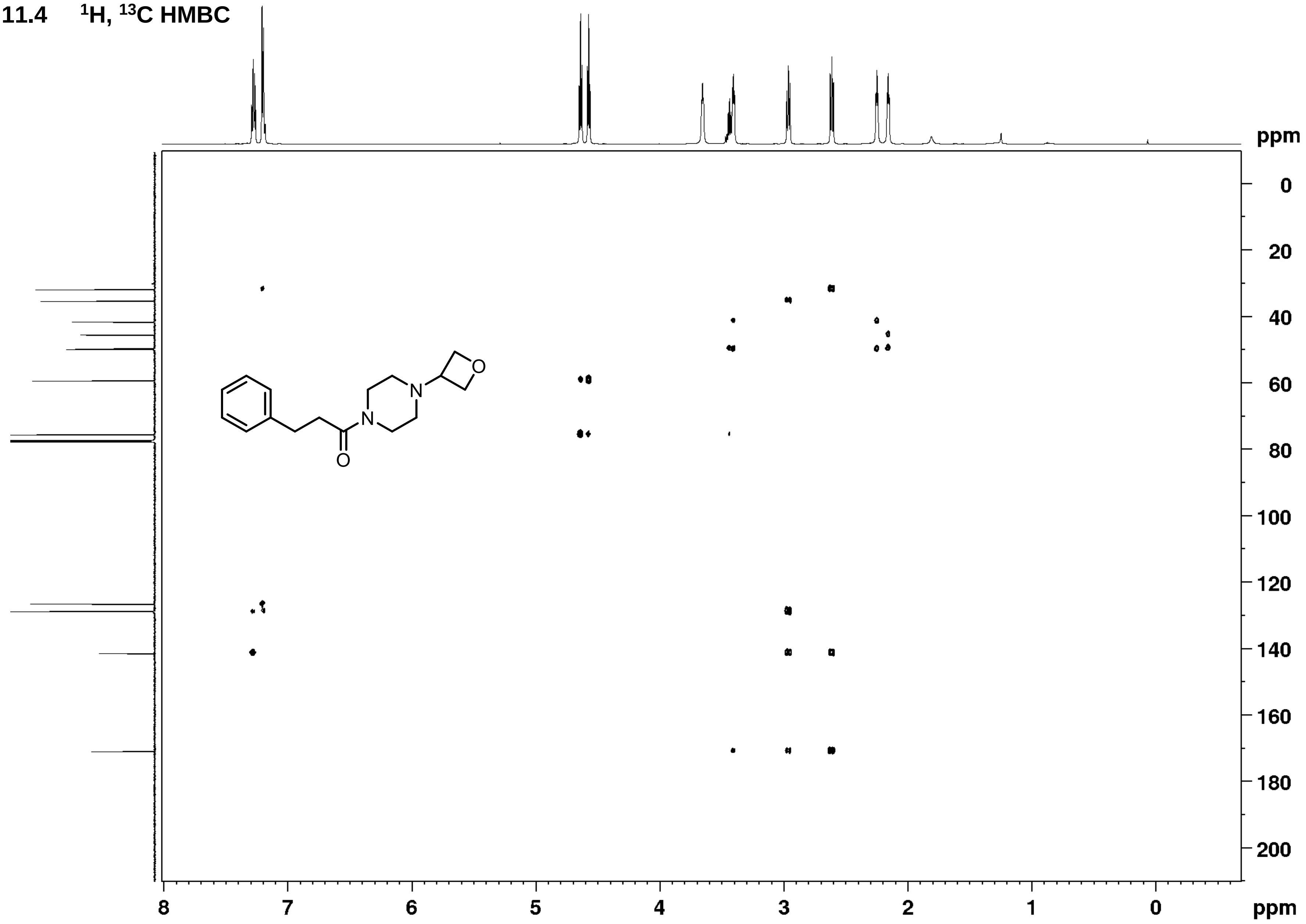

3.11.5 ^{1}H, ^{13}C HSQC

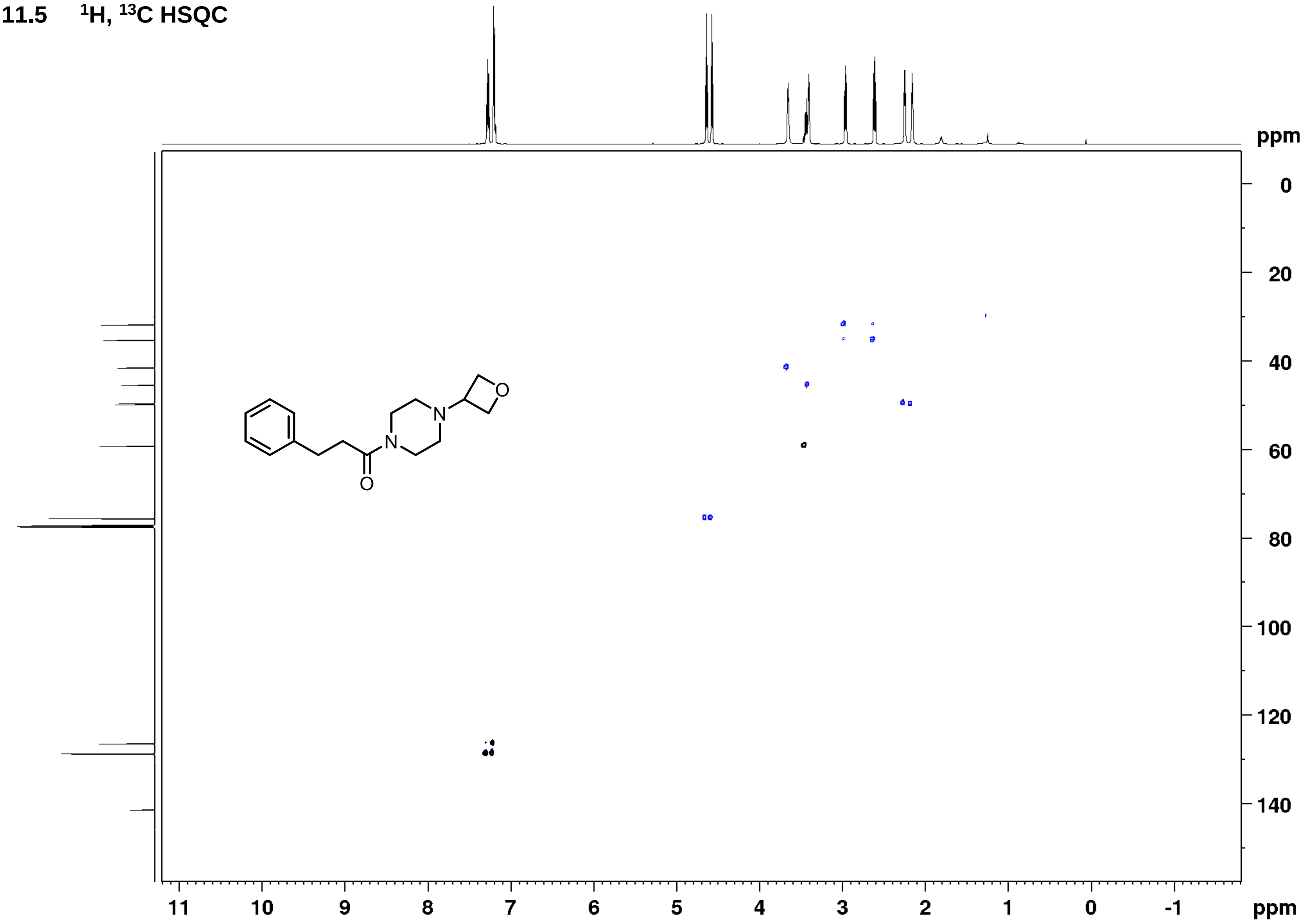

3.11.6 HRMS

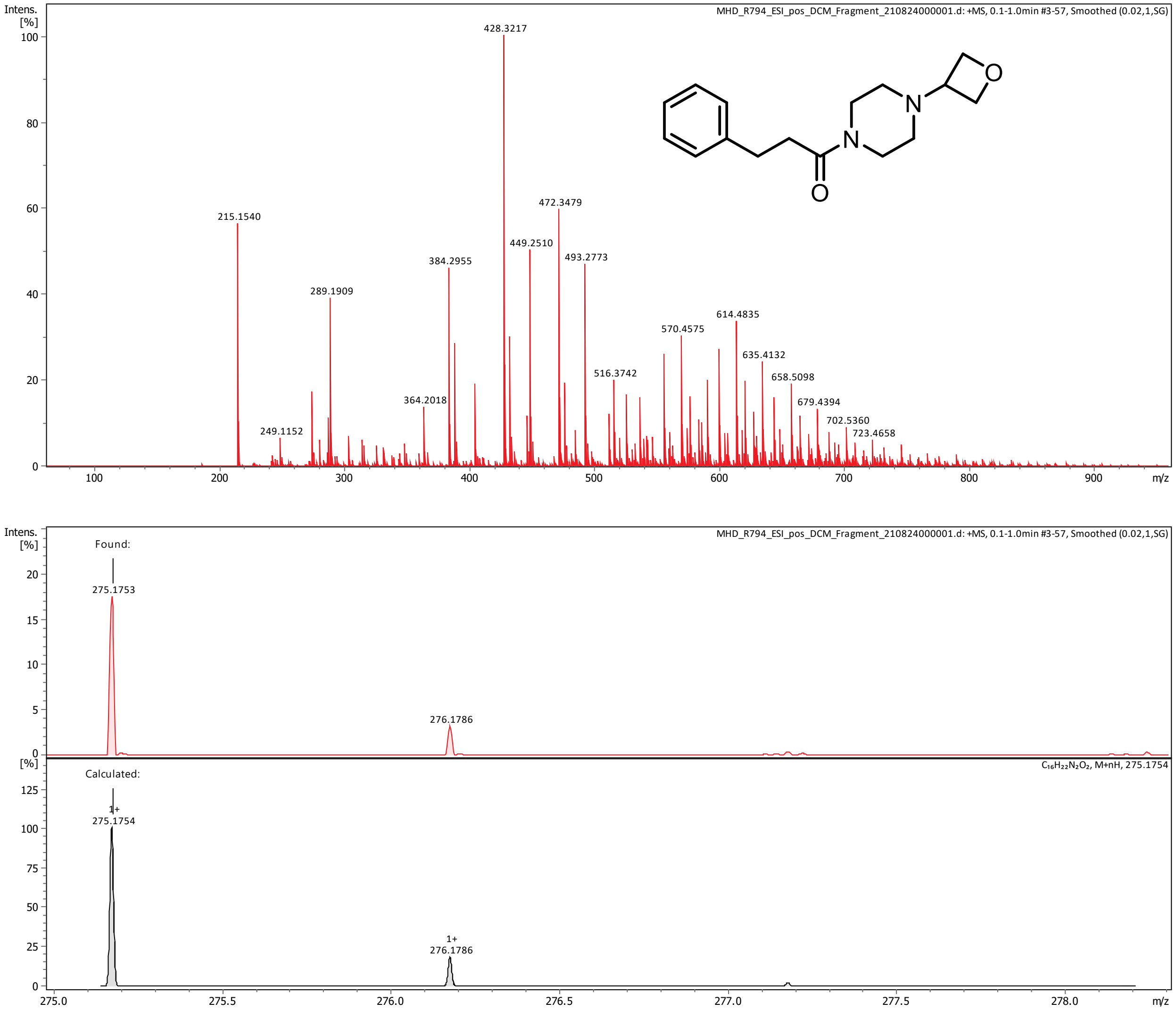
MHD_R794_ESI_pos_DCM_Fragment_210824000001.d: +MS, 0.1-1.0min #3-57, Smoothed (0.02,1,SG)
Intens. [%]
428.3217
472.3479
215.1540
449.2510
384.2955
493.2773
289.1909
614.4835
570.4575
635.4132
516.3742
658.5098
364.2018
679.4394
702.5360
723.4658
249.1152
m/z
O
N
N
O
MHD_R794_ESI_pos_DCM_Fragment_210824000001.d: +MS, 0.1-1.0min #3-57, Smoothed (0.02,1,SG)
Found:
275.1753
276.1786
Calculated:
$C_{16}H_{22}N_2O_2$, M+nH, 275.1754
1+
275.1754
1+
276.1786

3.11.7 IR

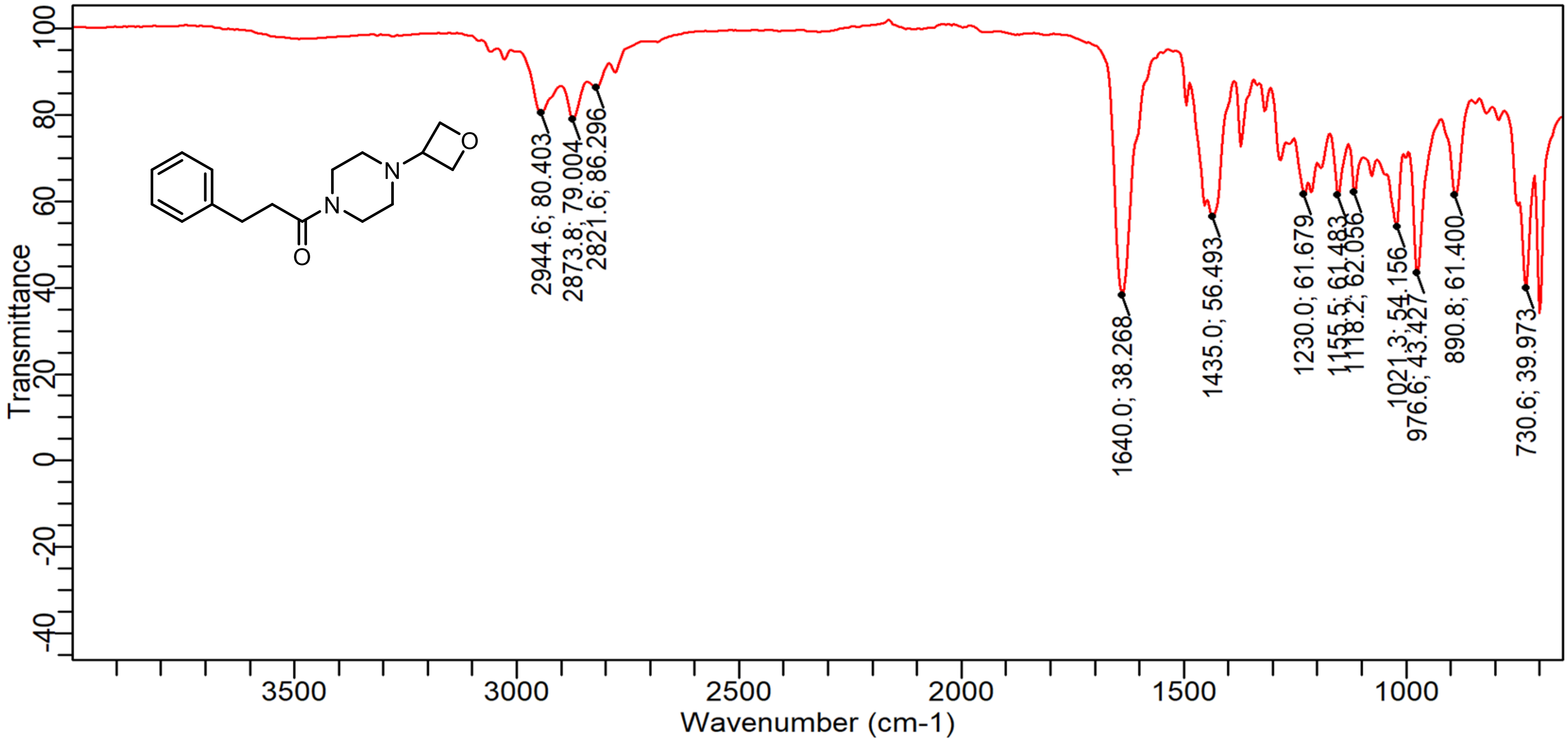

Transmittance
Wavenumber (cm-1)
2944.6; 80.403
2873.8; 79.004
2821.6; 86.296
1640.0; 38.268
1435.0; 56.493
1230.0; 61.679
1155.5; 61.483
1118.2; 62.056
1021.3; 54.156
976.6; 43.427
890.8; 61.400
730.6; 39.973
N
N
O
O

3.12 1-(4-(6-Fluorobenzo[*d*]isoxazol-3-yl)piperidin-1-yl)-3-phenylpropan-1-one (12l) (600 MHz, $CDCl_3$)

3.12.1 ^{1}H NMR

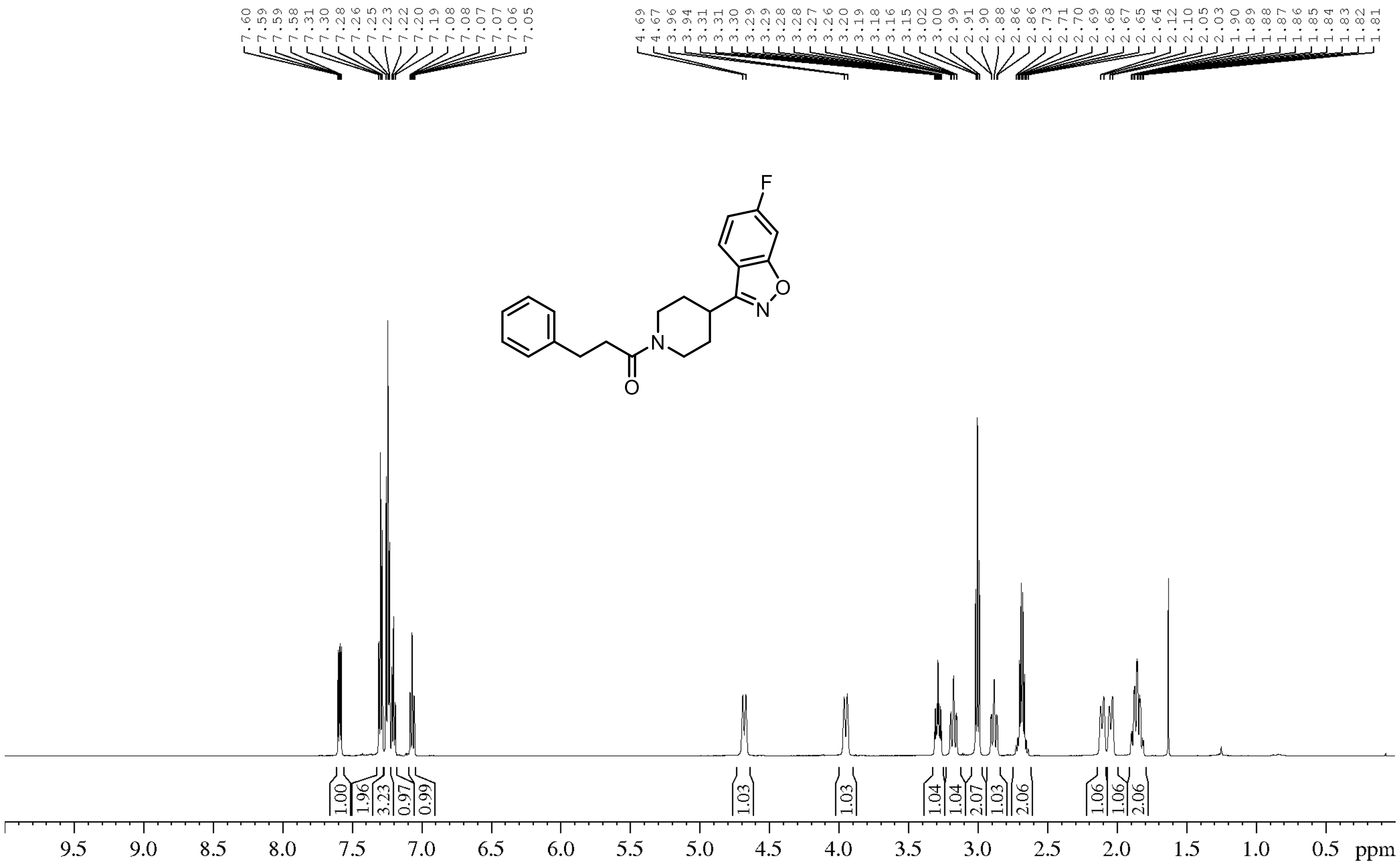

3.12.2 ^{13}C NMR

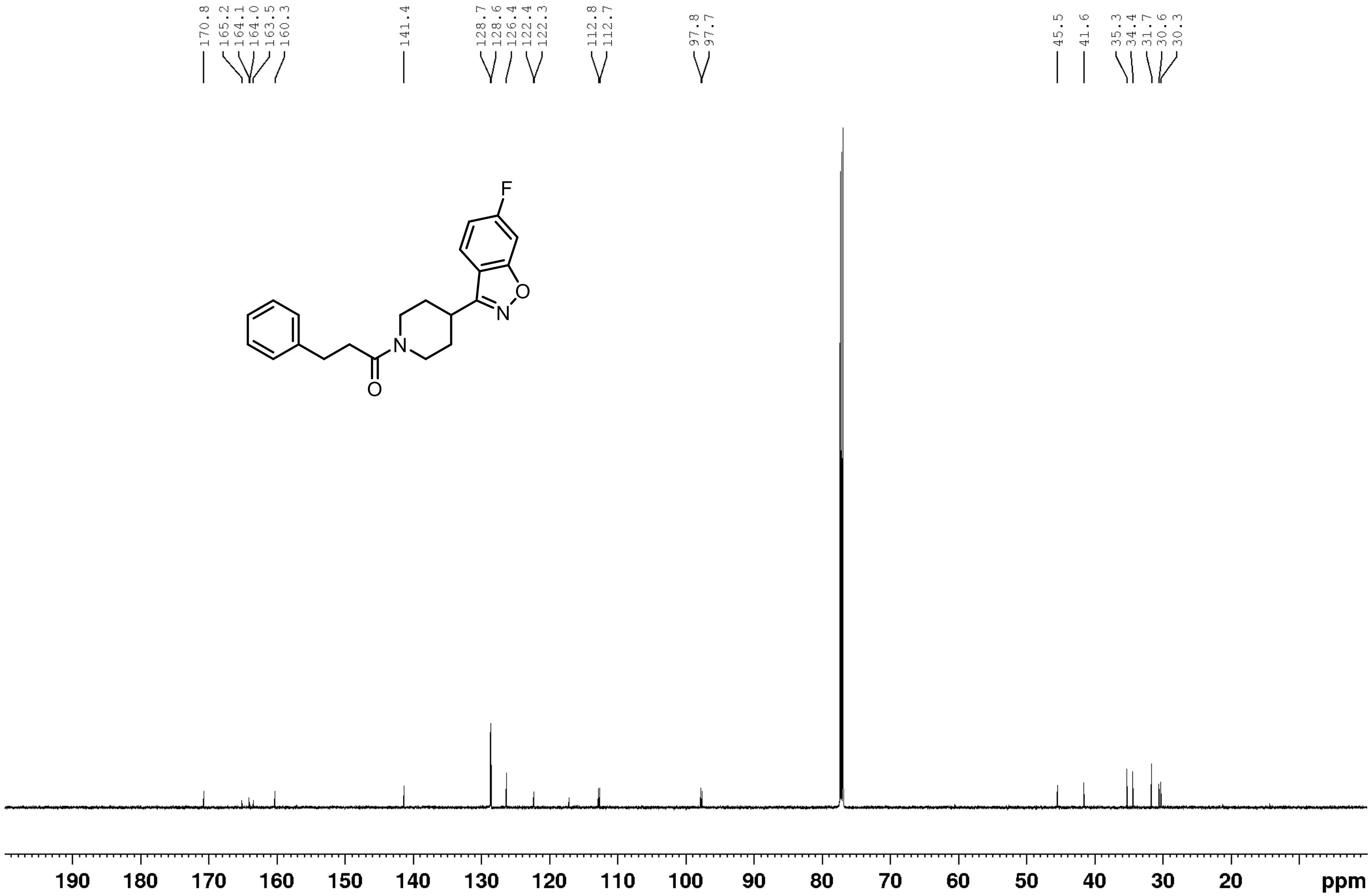
170.8
165.2
164.1
164.0
163.5
160.3
141.4
128.7
128.6
126.4
122.4
122.3
112.8
112.7
97.8
97.7
45.5
41.6
35.3
34.4
31.7
30.6
30.3
F
O
N
190 180 170 160 150 140 130 120 110 100 90 80 70 60 50 40 30 20 ppm

3.12.3 ^{19}F NMR

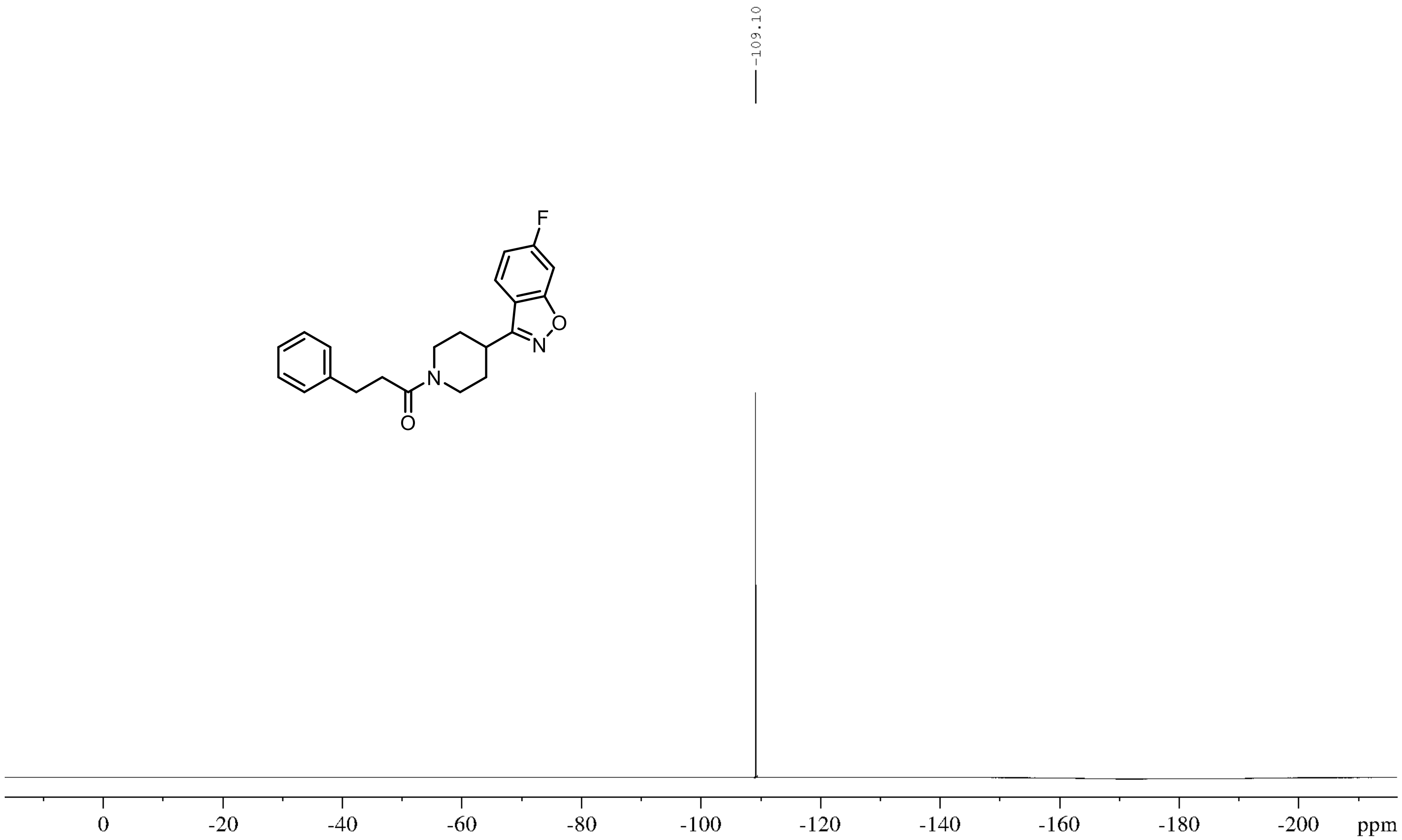

3.12.4 ^{1}H, ^{1}H COSY

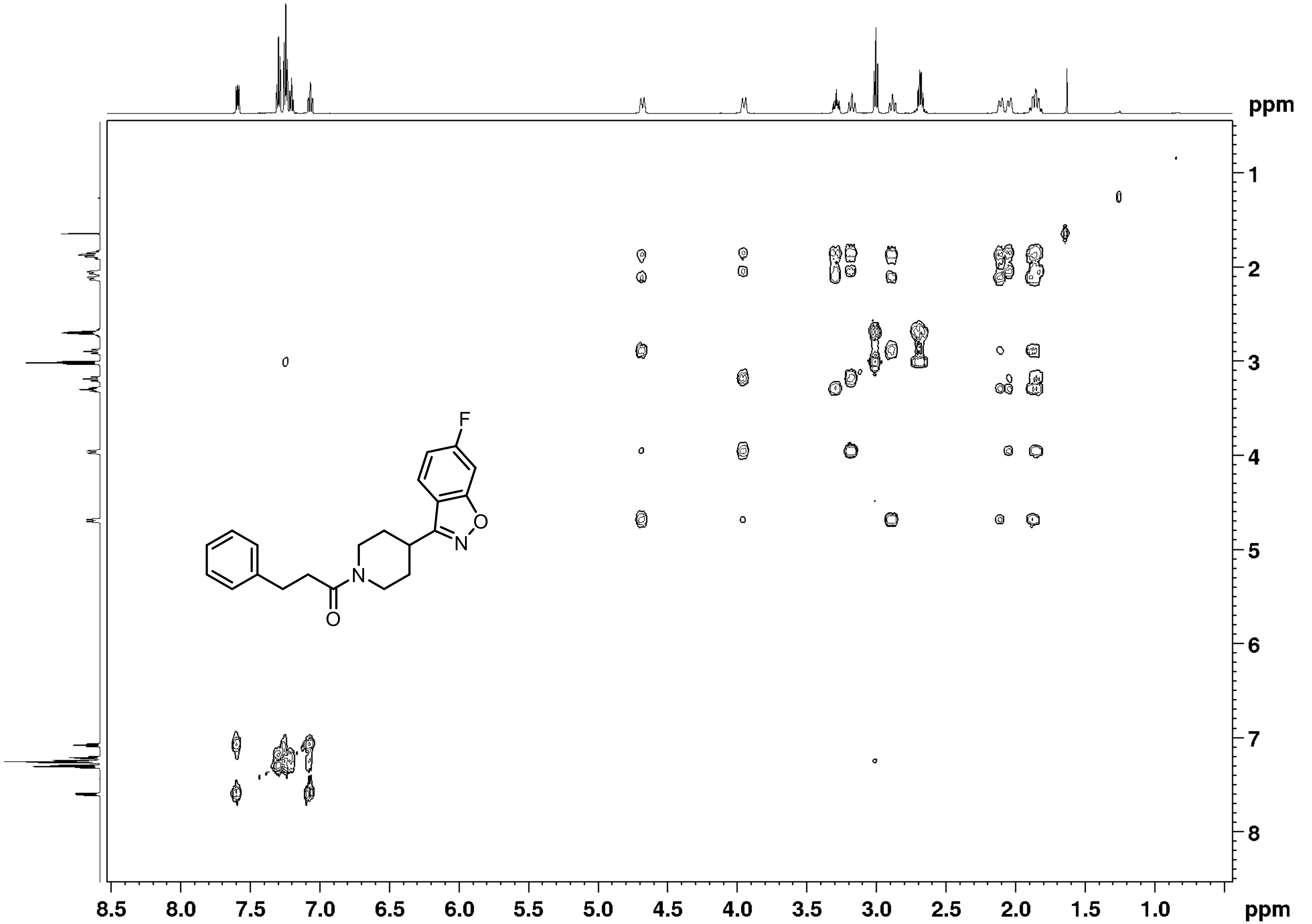

3.12.5 ^{1}H, ^{13}C HMBC

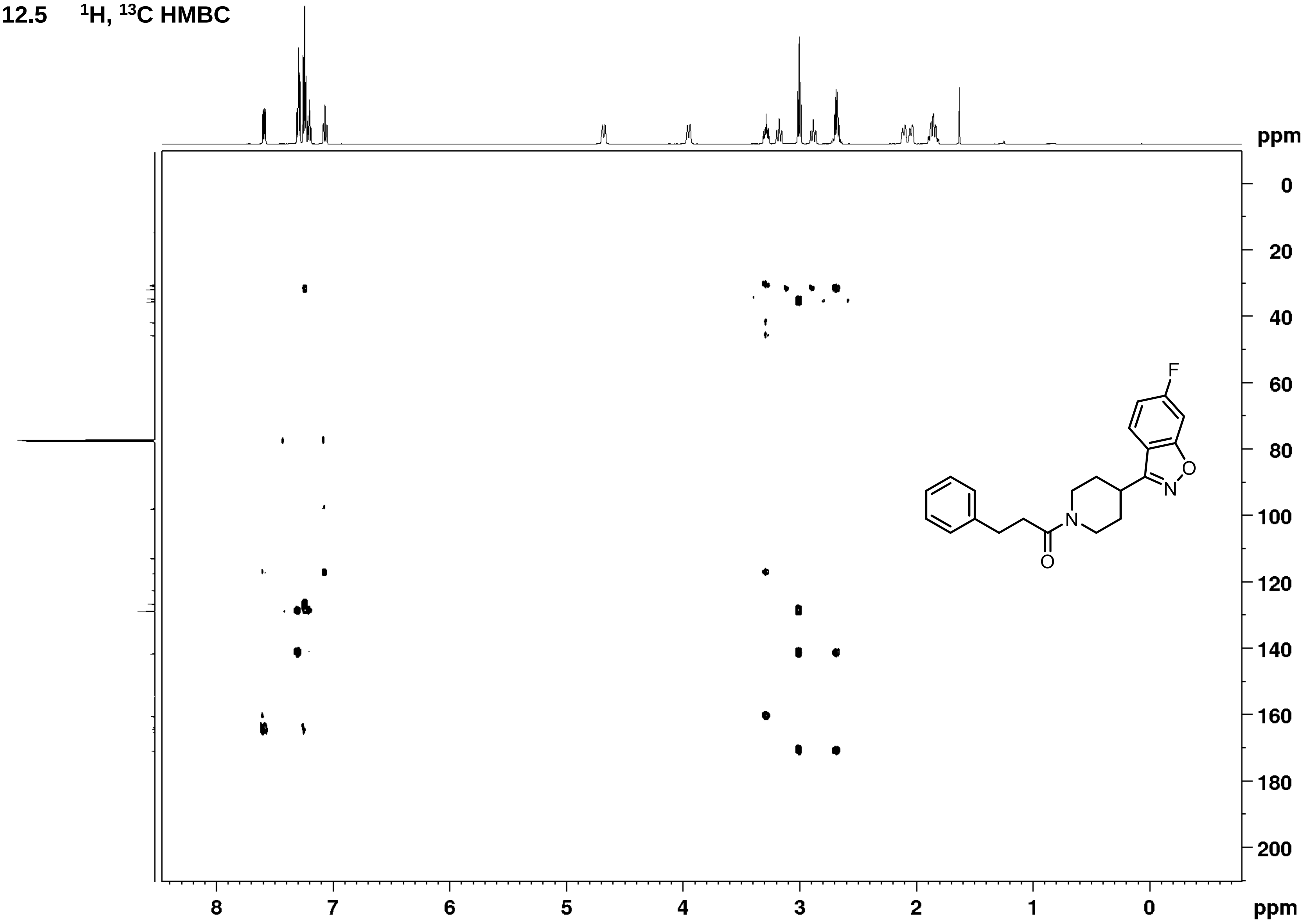

3.12.6 ^{1}H, ^{13}C HSQC

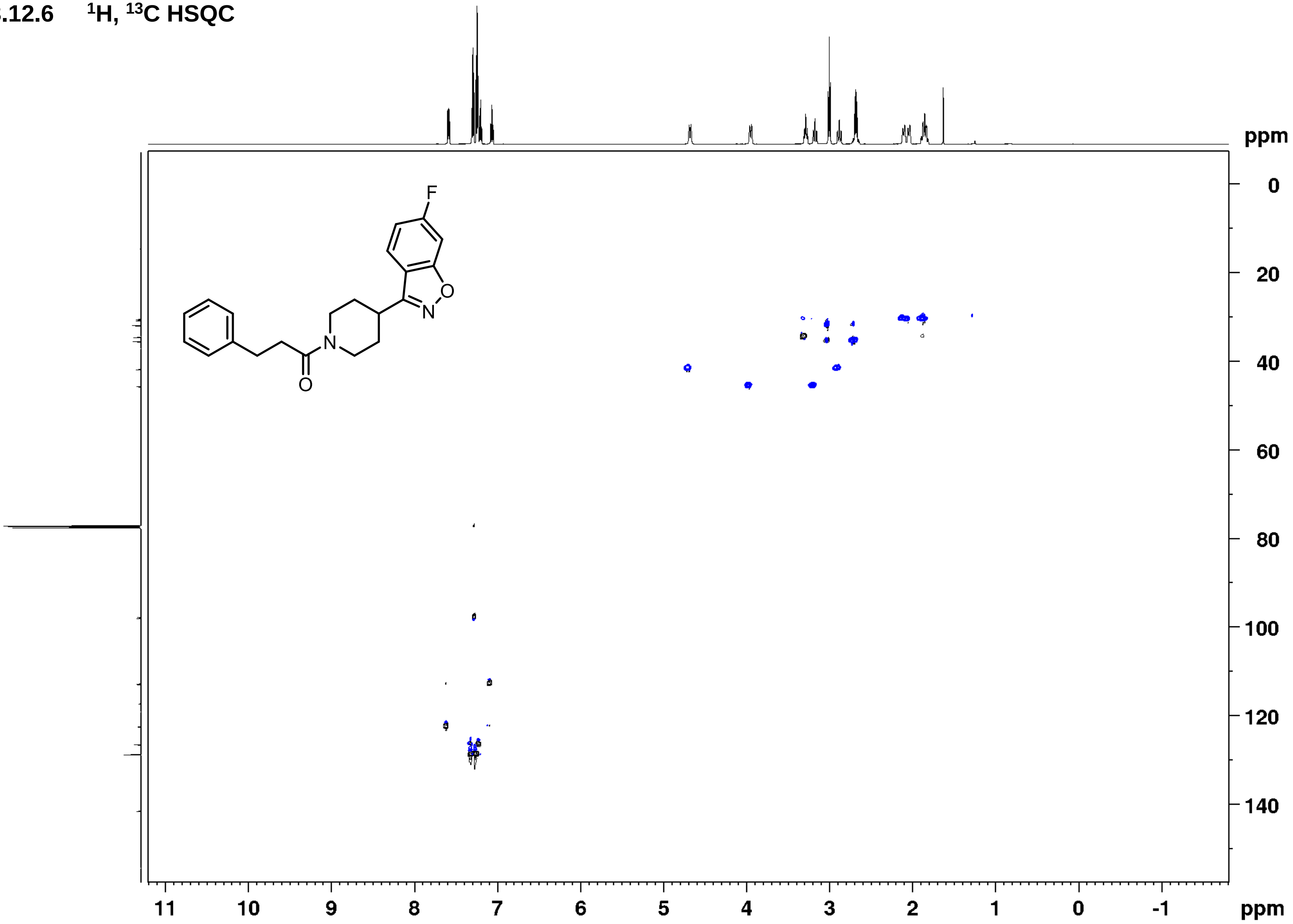

3.12.7 HRMS

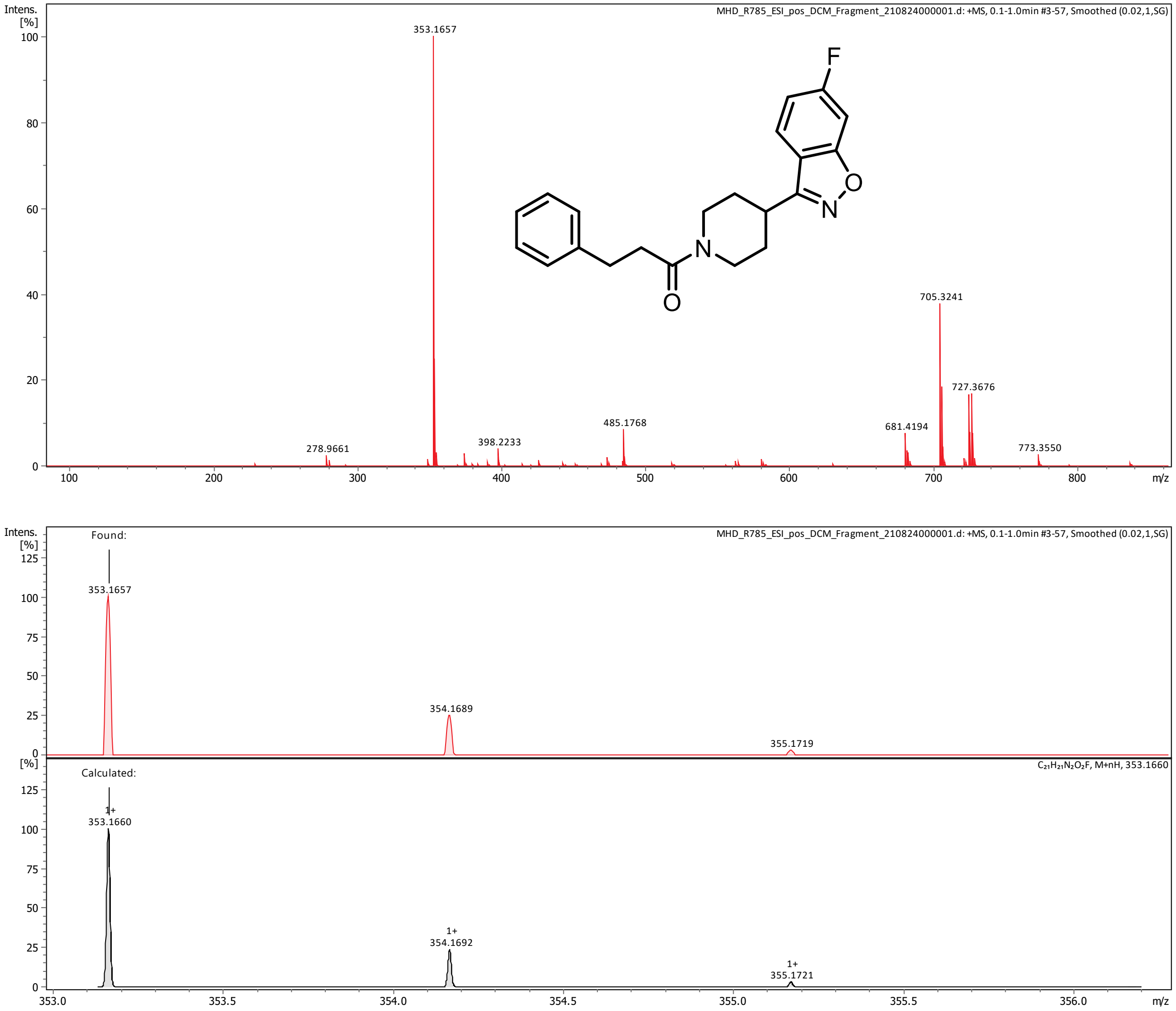
MHD_R785_ESI_pos_DCM_Fragment_210824000001.d: +MS, 0.1-1.0min #3-57, Smoothed (0.02,1,SG)
353.1657
705.3241
727.3676
485.1768
681.4194
398.2233
278.9661
773.3550
Found:
354.1689
355.1719
Calculated:
$C_{21}H_{21}N_2O_2F$, M+nH, 353.1660
353.1660
354.1692
355.1721

3.12.8 IR

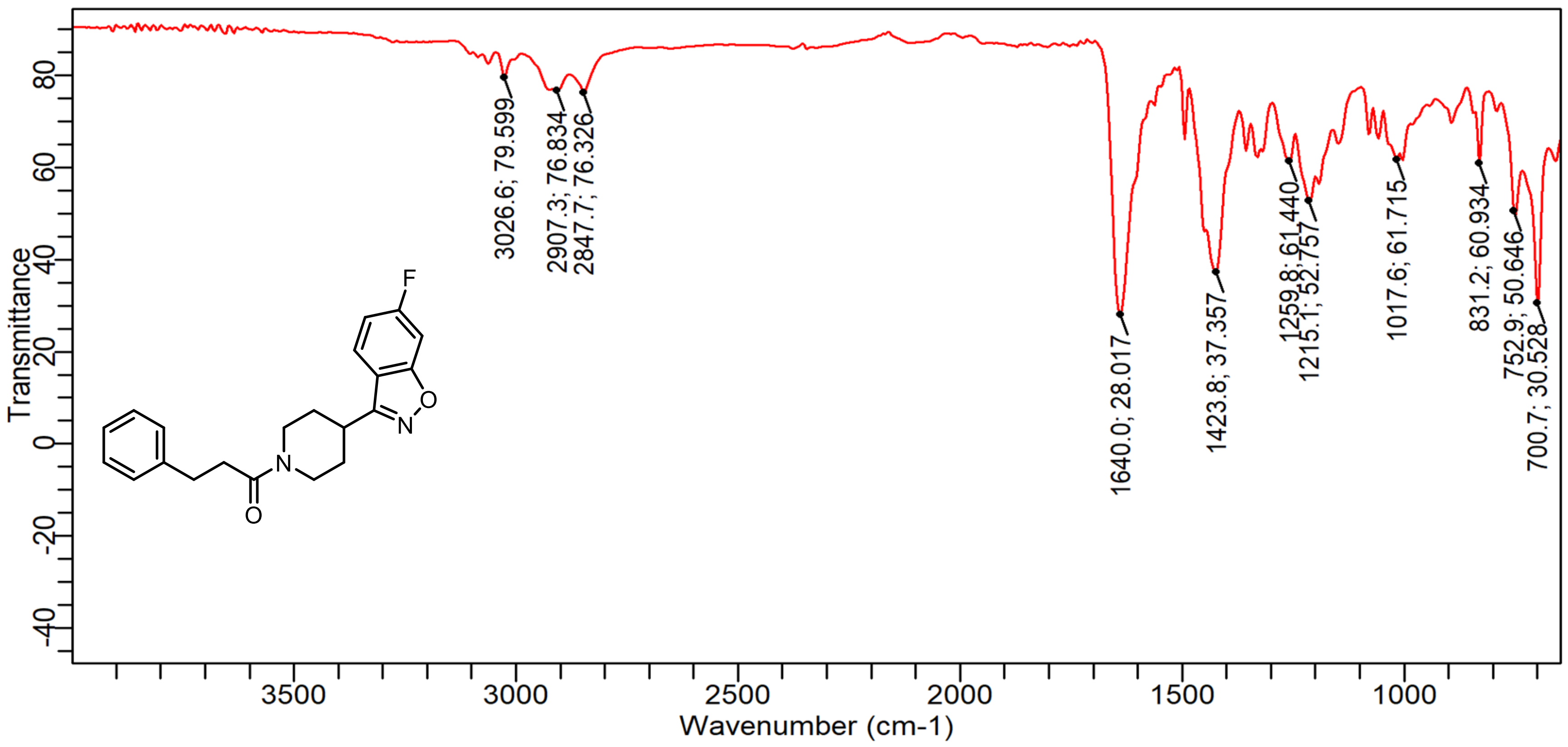
3026.6; 79.599
2907.3; 76.834
2847.7; 76.326
1640.0; 28.017
1423.8; 37.357
1259.8; 61.440
1215.1; 52.757
1017.6; 61.715
831.2; 60.934
752.9; 50.646
700.7; 30.528
Transmittance
Wavenumber (cm-1)
3500
3000
2500
2000
1500
1000
80
60
40
20
0
-20
-40
F
O
N
N
O

3.13 1-(Piperidin-1-yl)-3-(4-(piperidine-1-carbonyl)phenyl)propan-1-one (12m) (600 MHz, $CDCl_3$)

3.13.1 ^1H NMR

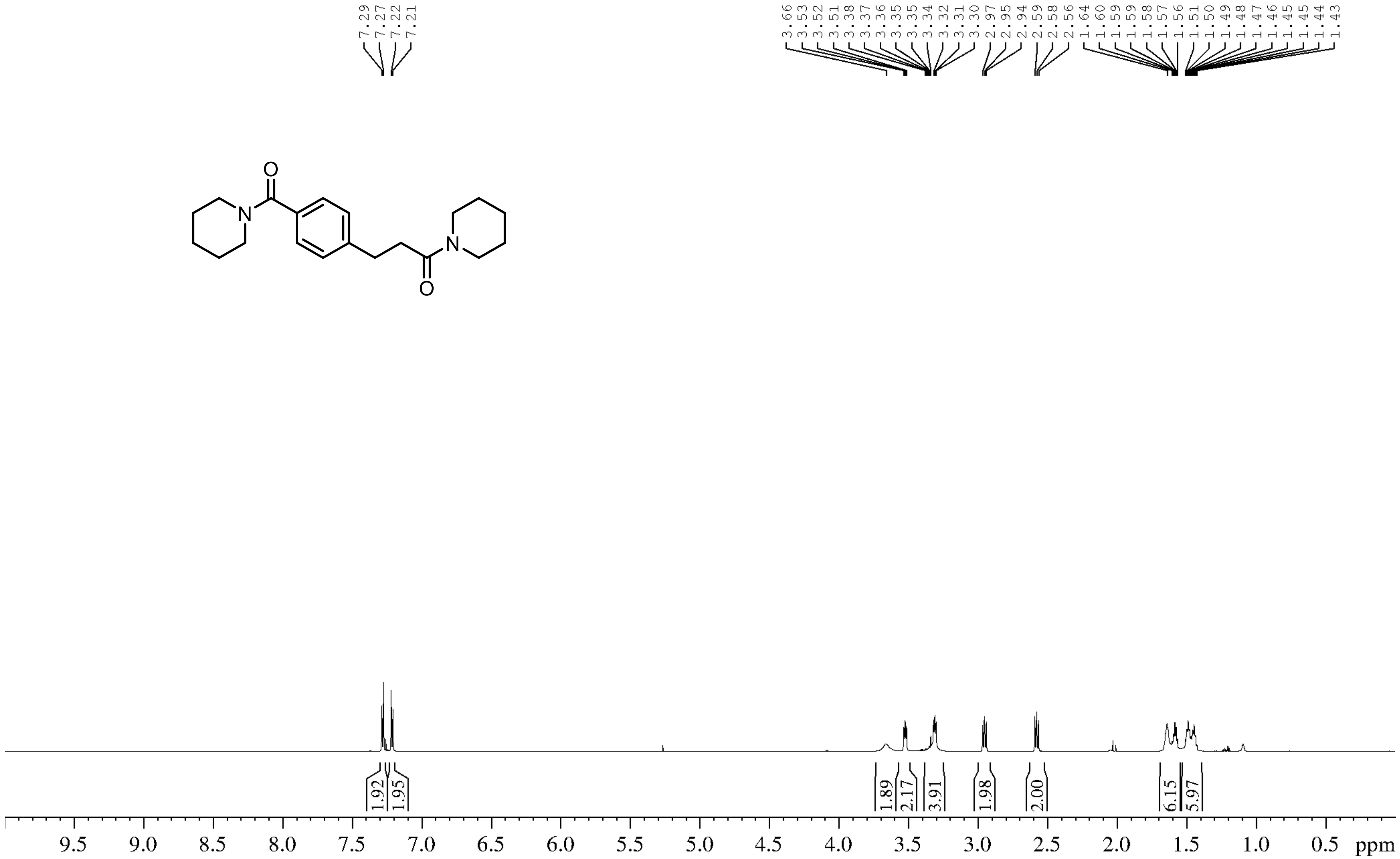

3.13.2 ^{13}C NMR

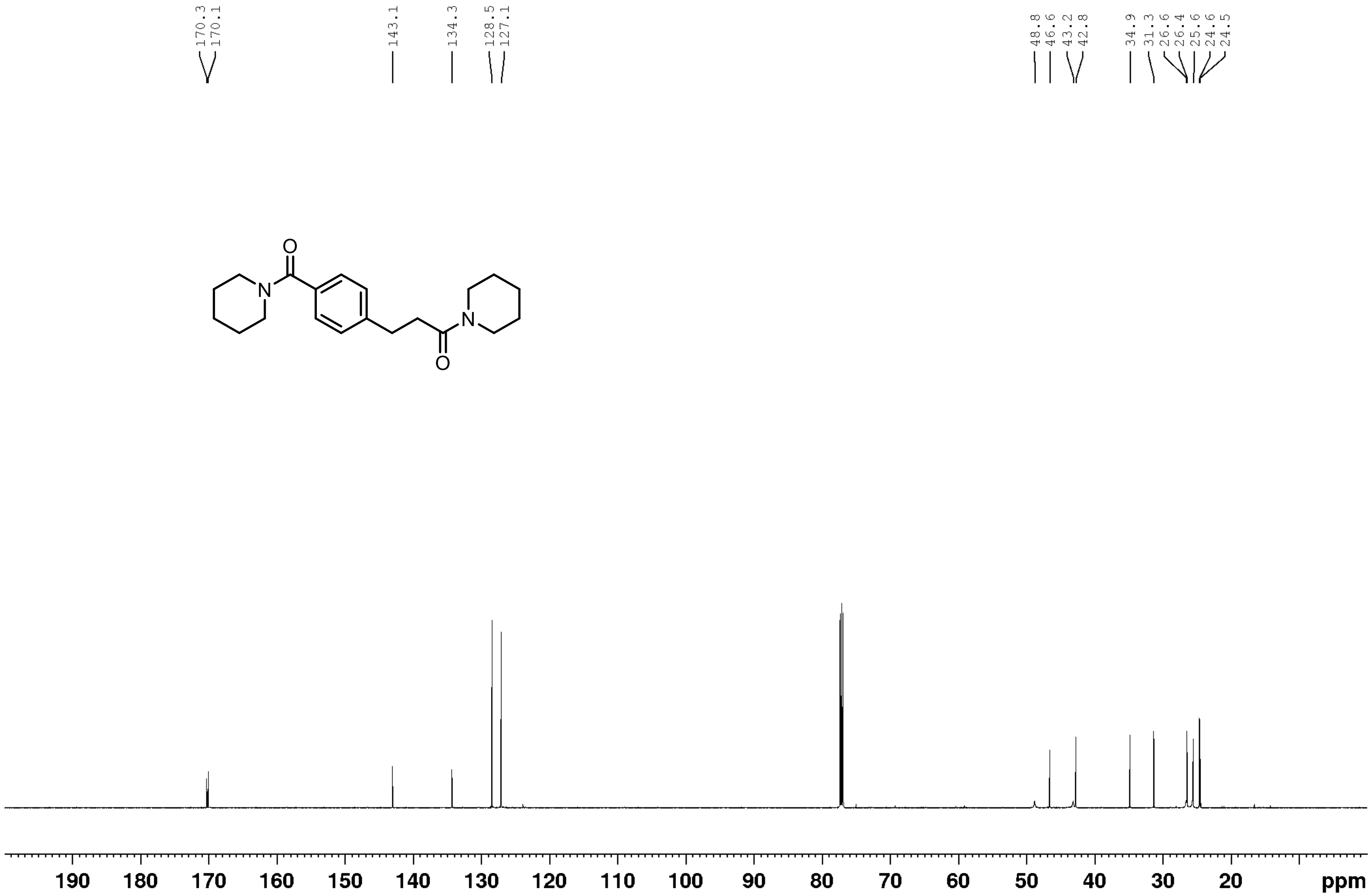
170.3
170.1
143.1
134.3
128.5
127.1
48.8
46.6
43.2
42.8
34.9
31.3
26.6
26.4
25.6
24.6
24.5
O
N
N
O
190
180
170
160
150
140
130
120
110
100
90
80
70
60
50
40
30
20
ppm

3.13.3 1H, 1H COSY

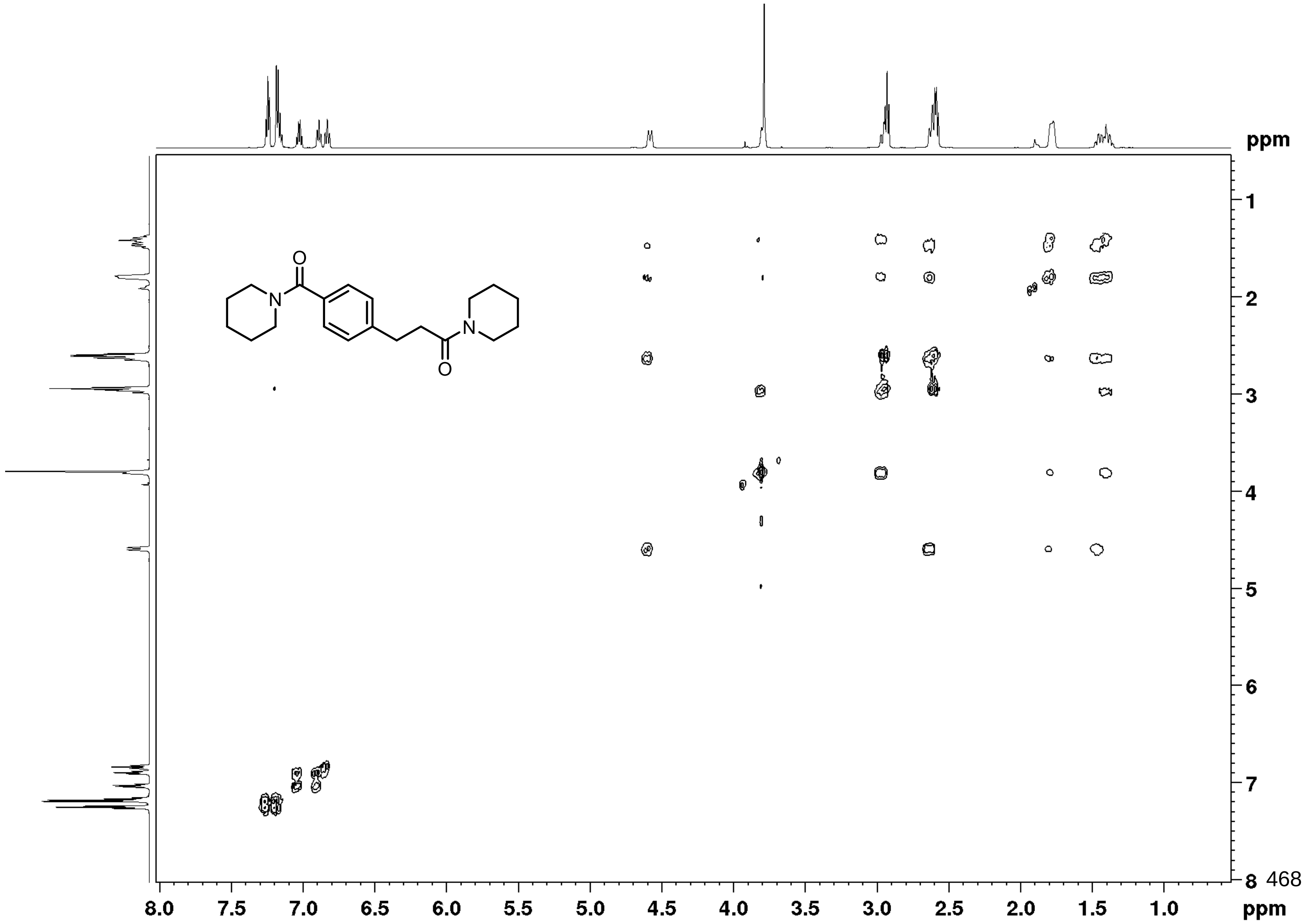

3.13.4 ¹H, ¹³C HMBC

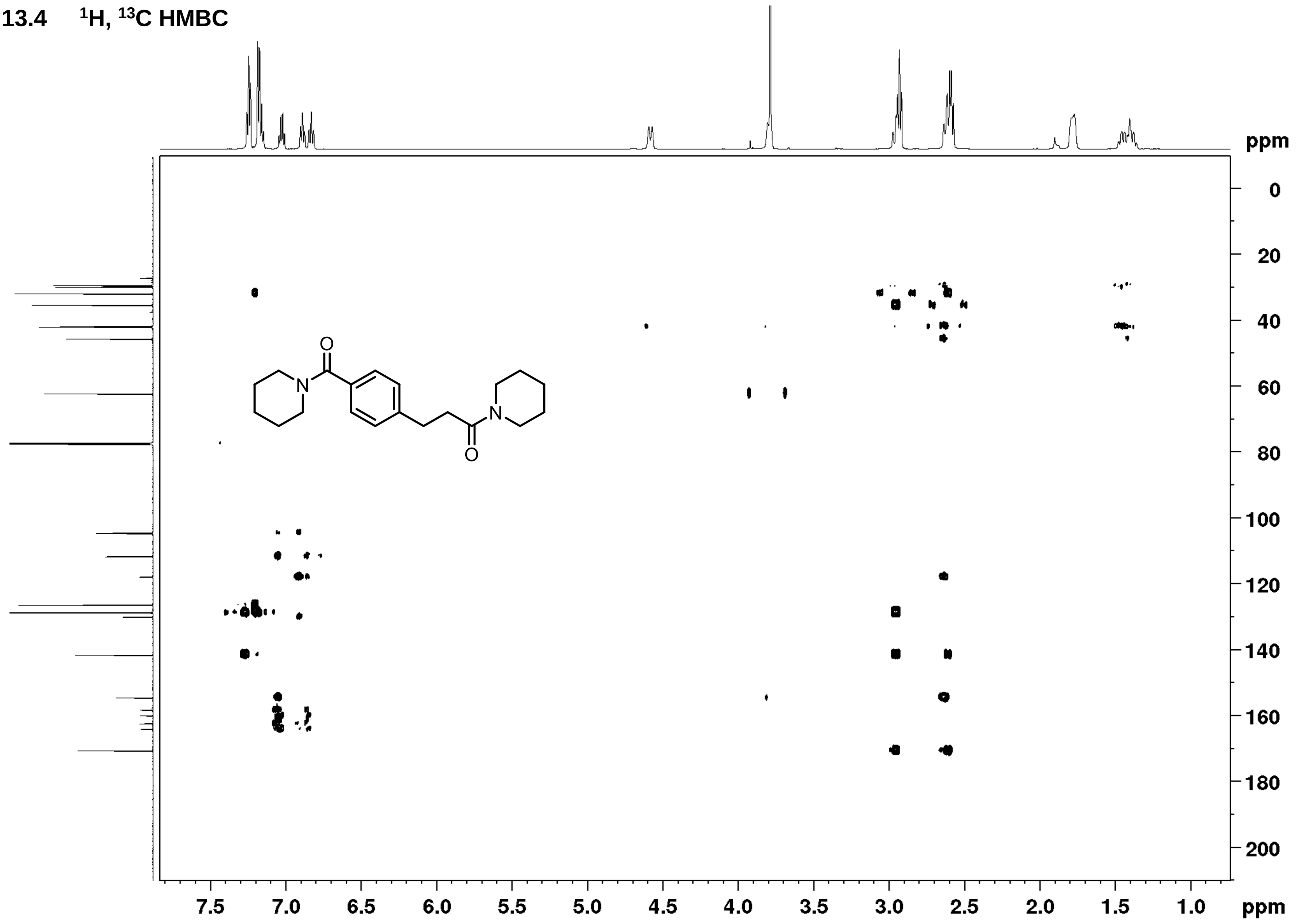

3.13.5 ^{1}H, ^{13}C HSQC

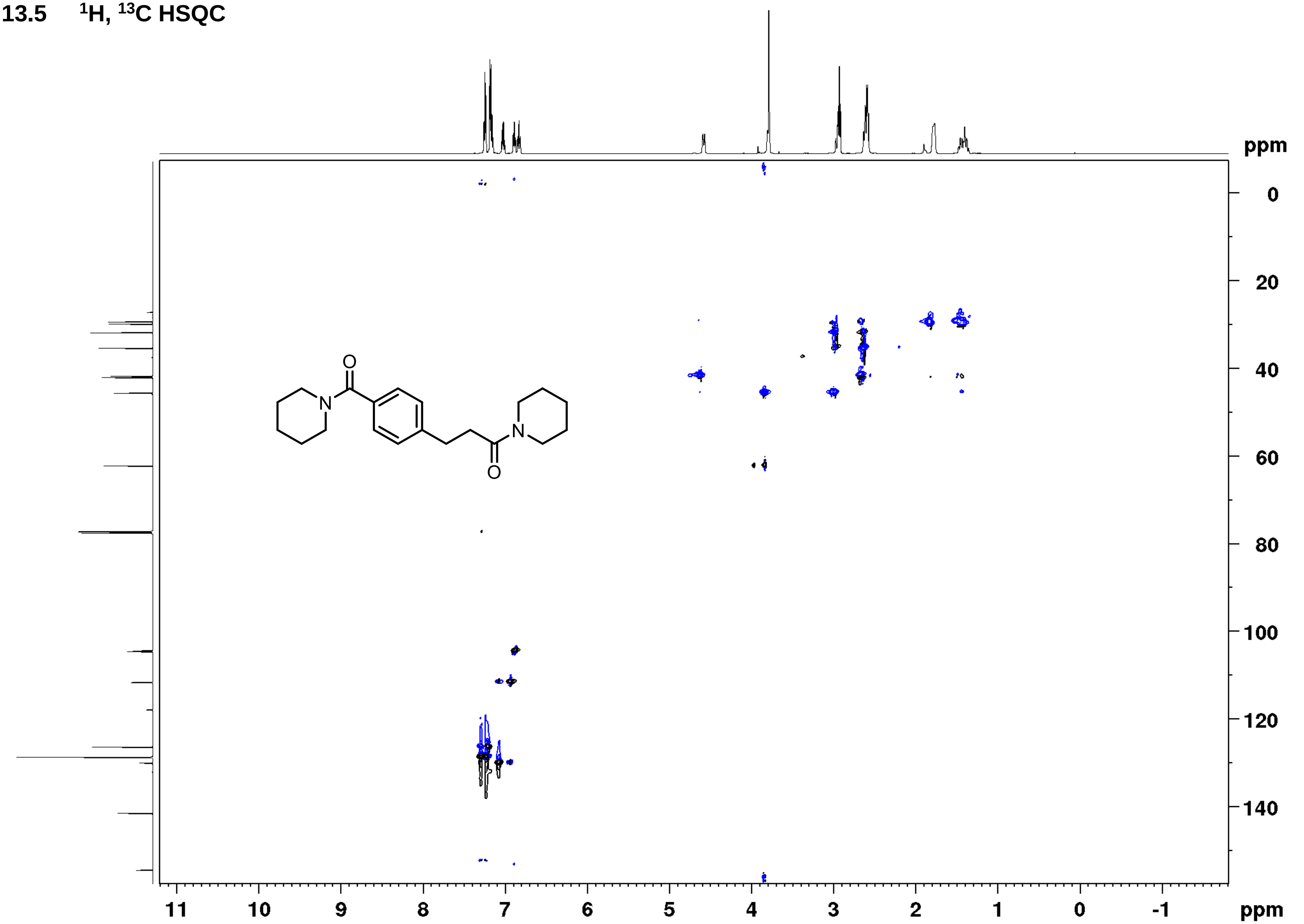

3.13.6 HRMS

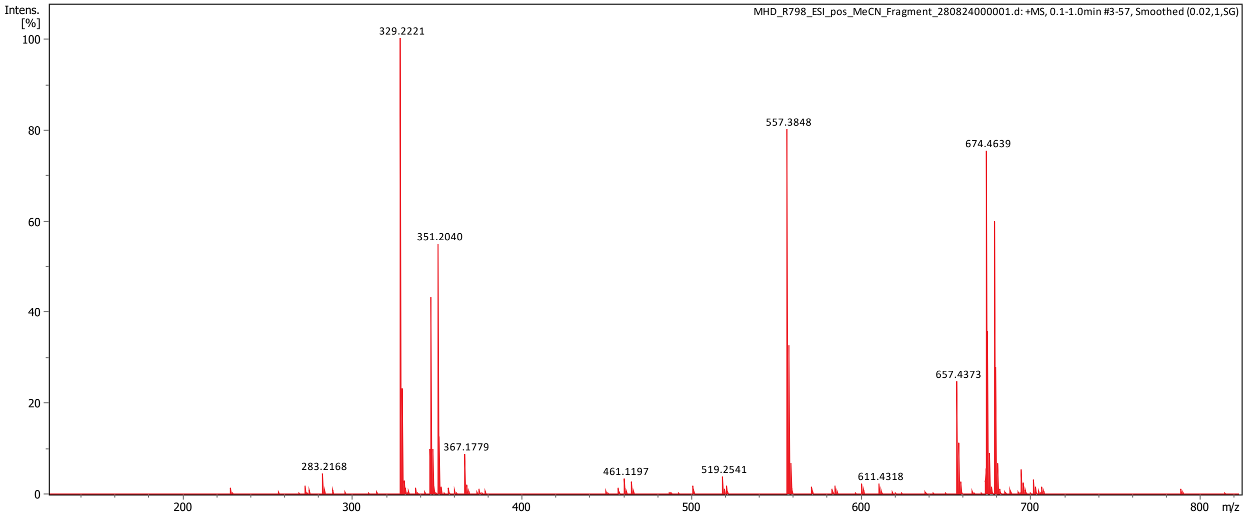
MHD_R798_ESI_pos_MeCN_Fragment_280824000001.d: +MS, 0.1-1.0min #3-57, Smoothed (0.02,1,SG)
Intens. [%]
329.2221
557.3848
674.4639
351.2040
657.4373
367.1779
283.2168
461.1197
519.2541
611.4318
m/z

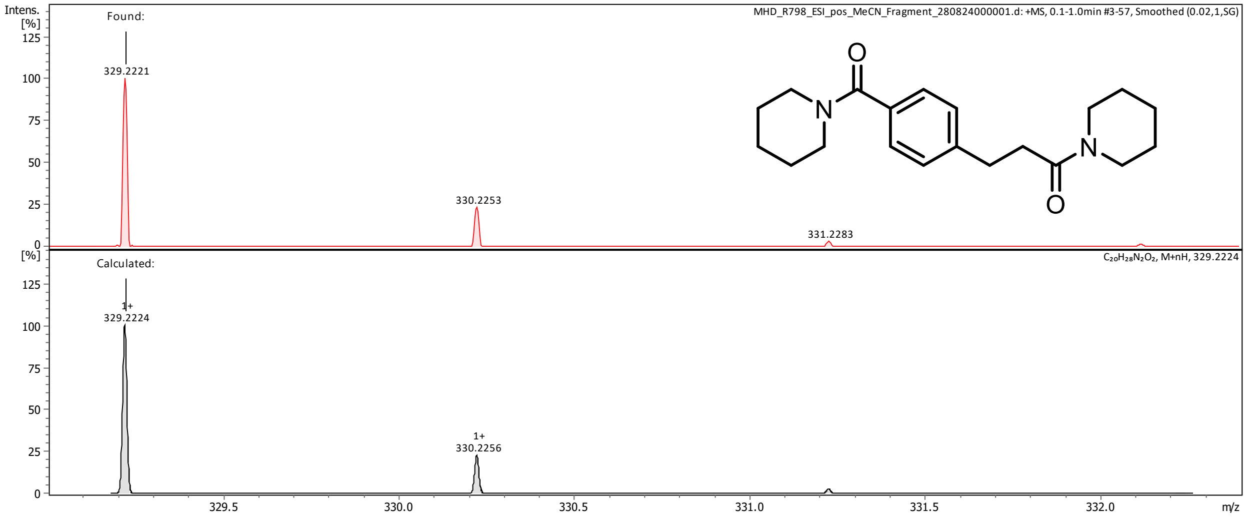
MHD_R798_ESI_pos_MeCN_Fragment_280824000001.d: +MS, 0.1-1.0min #3-57, Smoothed (0.02,1,SG)
Found:
329.2221
330.2253
331.2283
Calculated:
$C_{20}H_{28}N_2O_2$, M+nH, 329.2224
1+ 329.2224
1+ 330.2256
m/z

3.13.7 IR

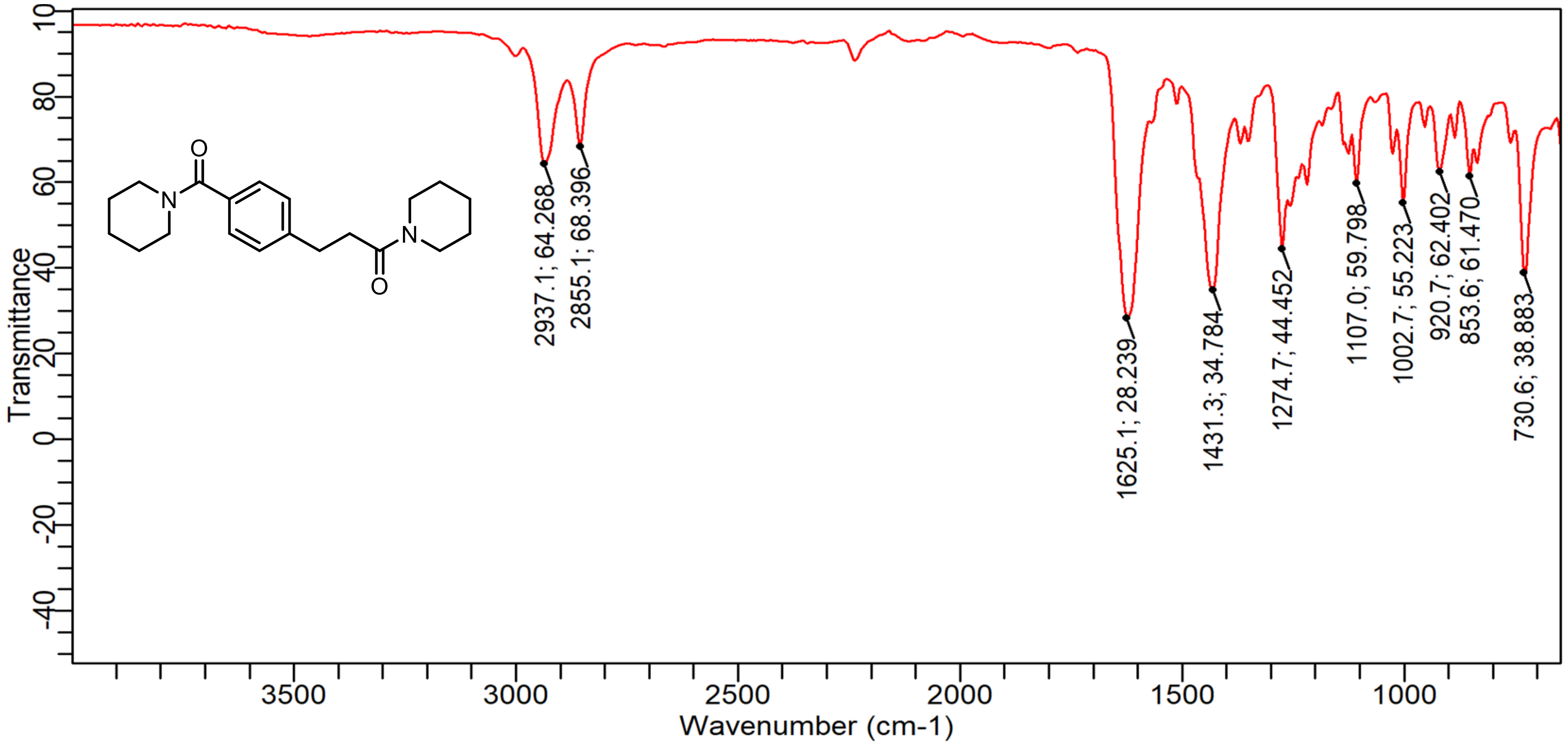

Transmittance
Wavenumber (cm-1)
2937.1; 64.268
2855.1; 68.396
1625.1; 28.239
1431.3; 34.784
1274.7; 44.452
1107.0; 59.798
1002.7; 55.223
920.7; 62.402
853.6; 61.470
730.6; 38.883

3.14 1-(Piperidin-1-yl)-3-(4-(trifluoromethyl)phenyl)propan-1-one (12n) (600 MHz, $CDCl_3$)

3.14.1 ^{1}H NMR

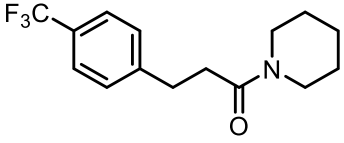

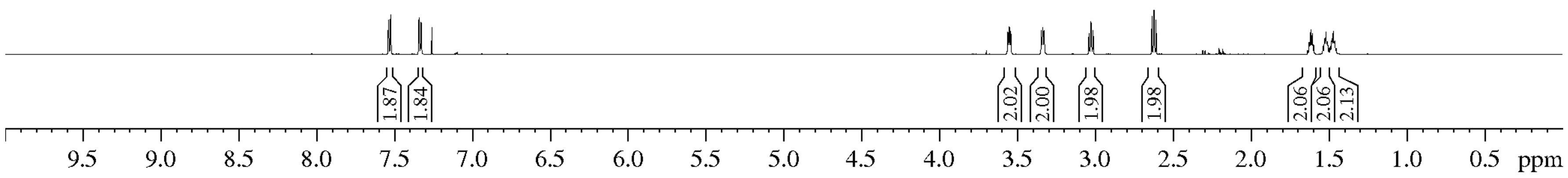

3.14.2 ^{13}C NMR

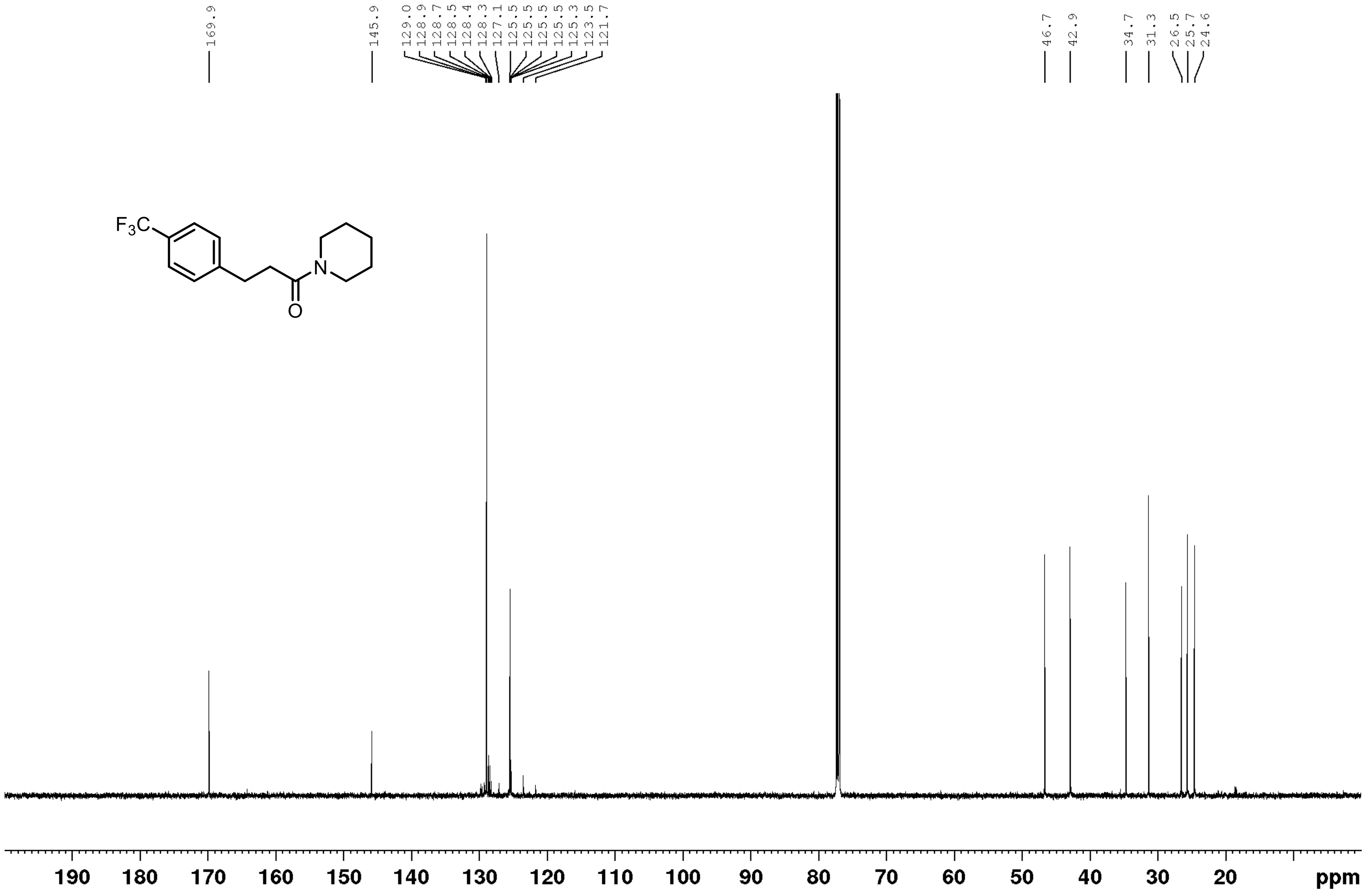

169.9
145.9
129.0
128.9
128.7
128.5
128.4
128.3
127.1
125.5
125.5
125.5
125.5
125.3
123.5
121.7
46.7
42.9
34.7
31.3
26.5
25.7
24.6
F3C
N
O
190
180
170
160
150
140
130
120
110
100
90
80
70
60
50
40
30
20
ppm

3.14.3 ^{19}F NMR

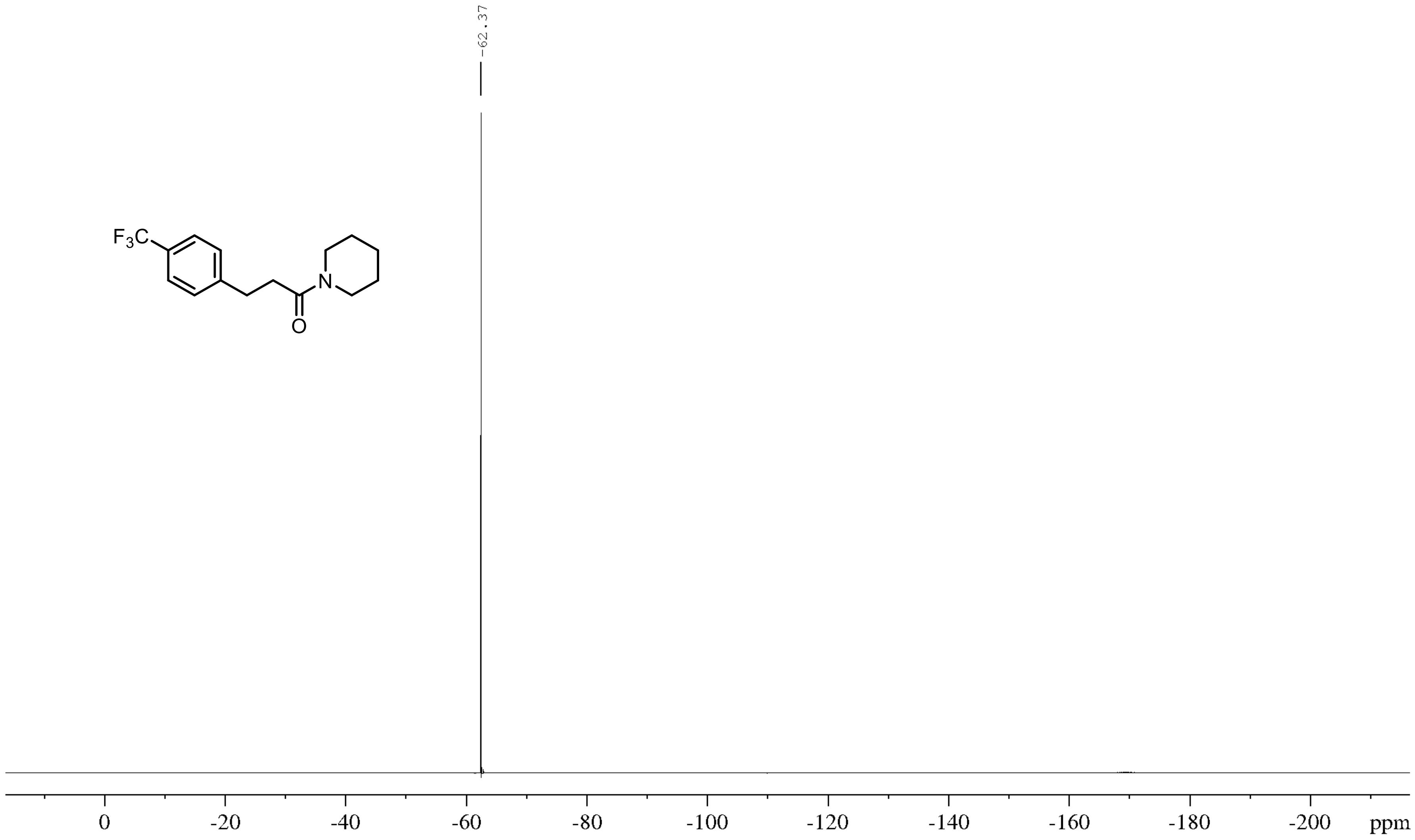

3.14.4 ¹H, ¹H COSY

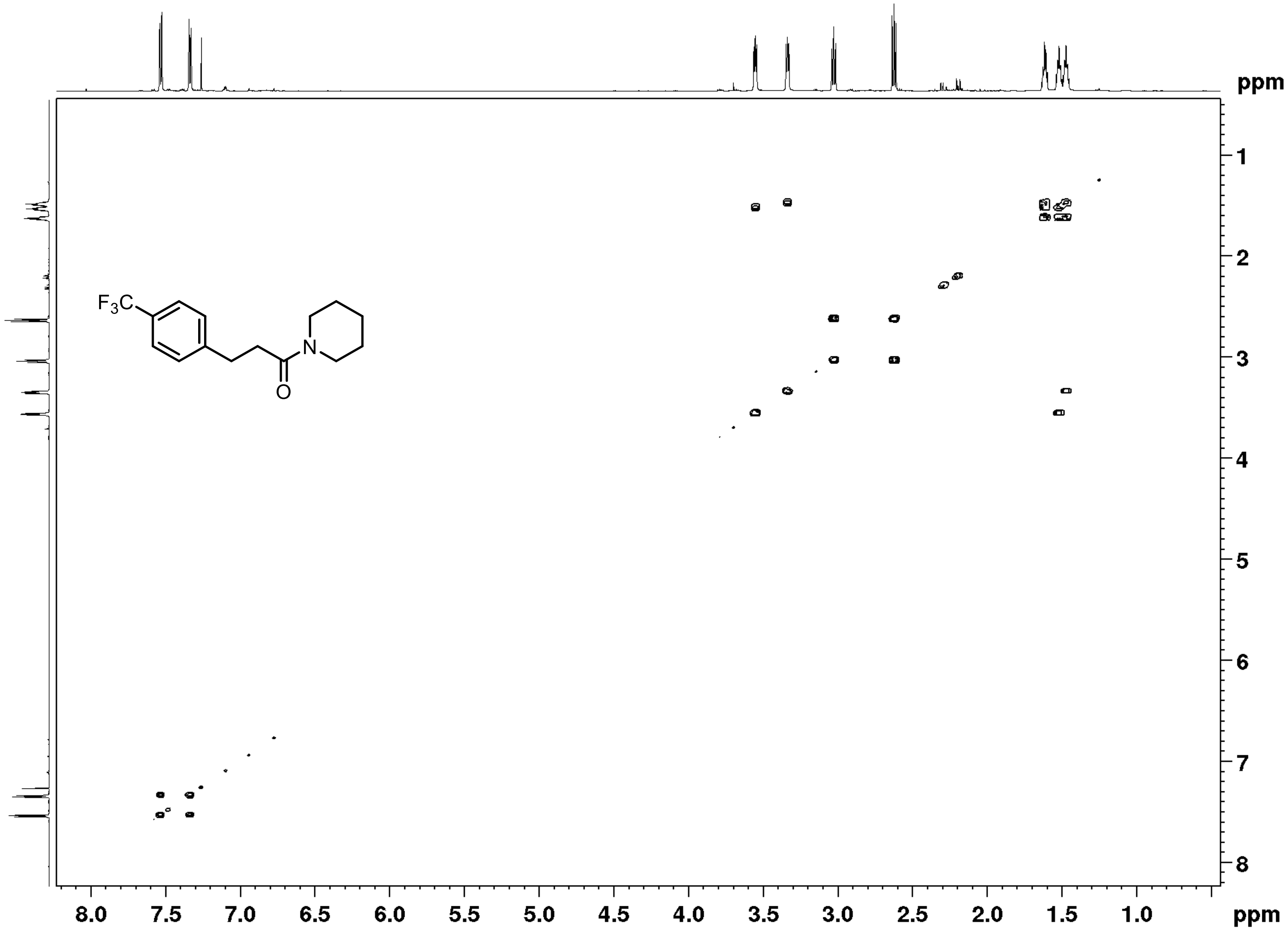

3.14.5 ^{1}H, ^{13}C HMBC

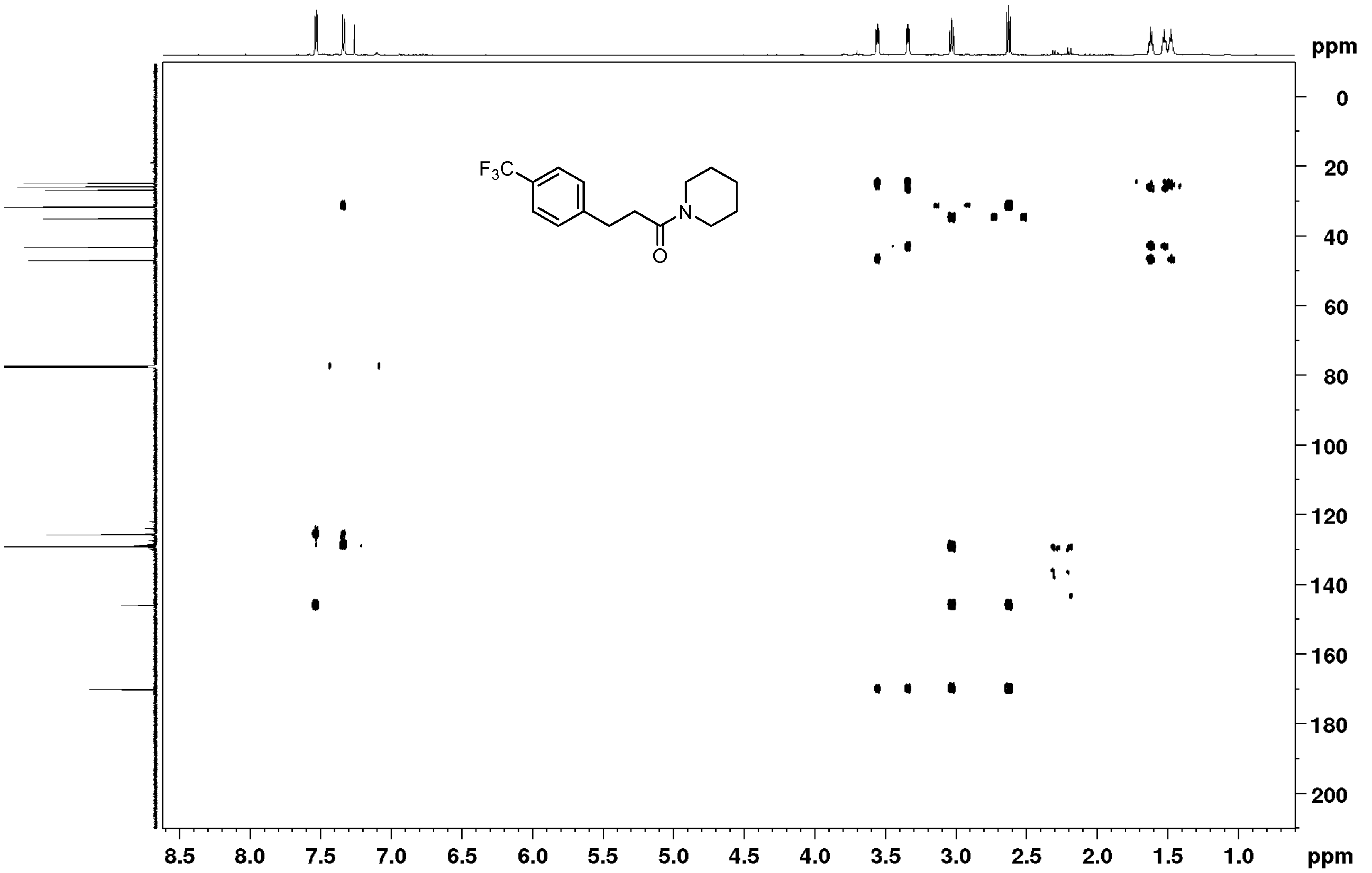

3.14.6 ^{1}H, ^{13}C HSQC

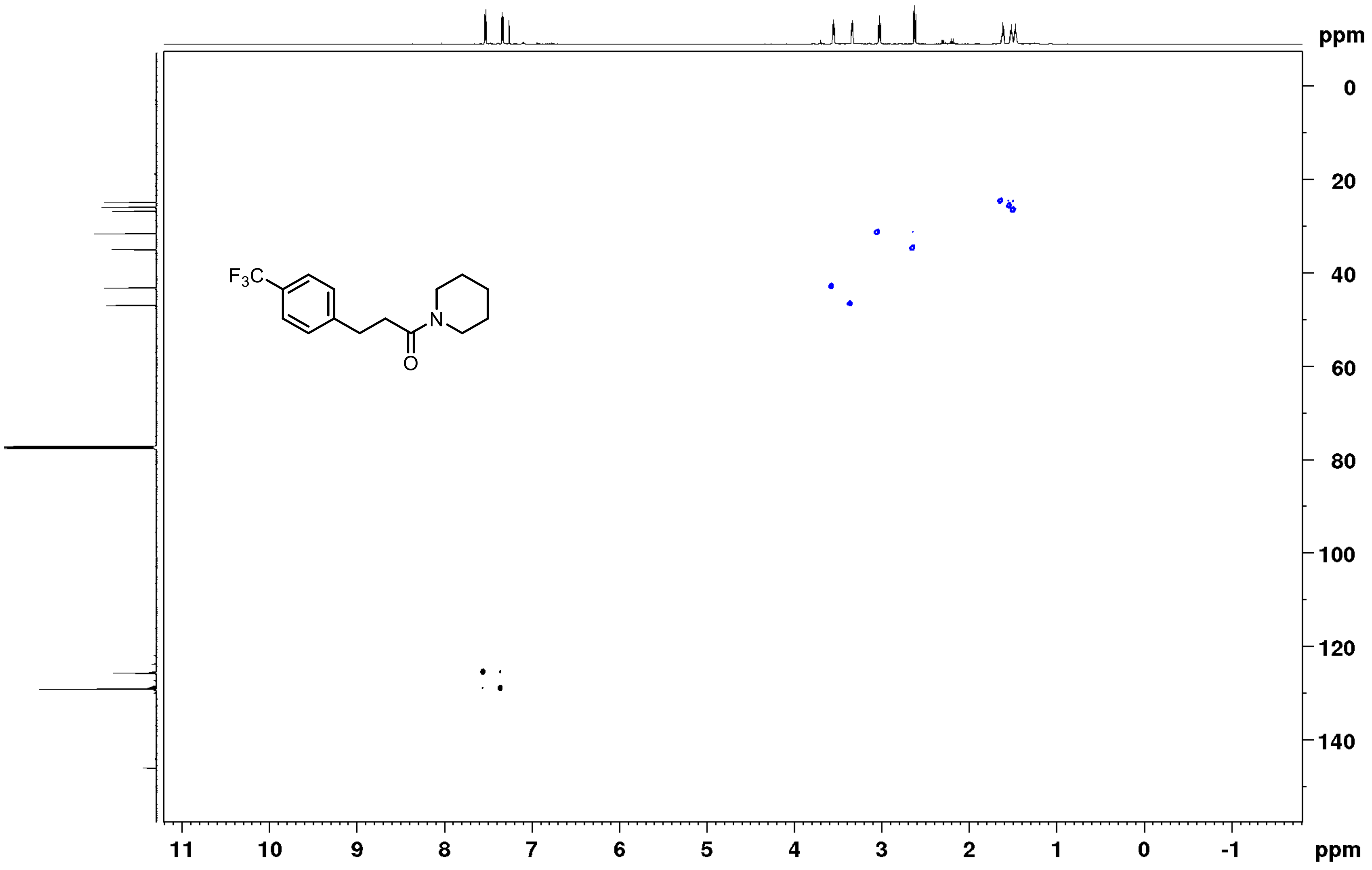

3.14.7 HRMS

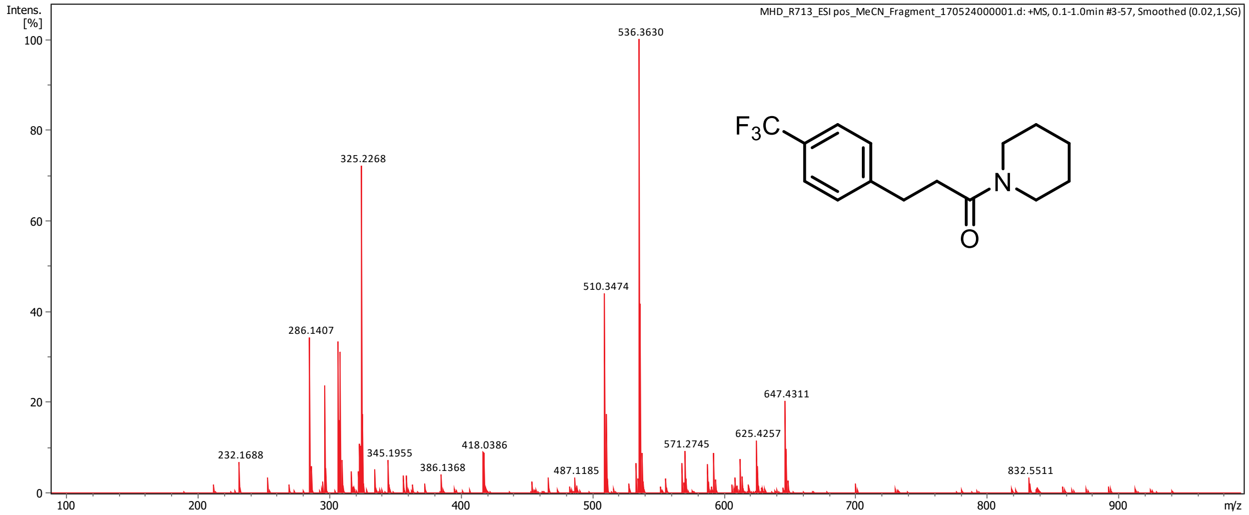

MHD_R713_ESI pos_MeCN_Fragment_170524000001.d: +MS, 0.1-1.0min #3-57, Smoothed (0.02,1,SG)
Intens. [%]
536.3630
325.2268
510.3474
286.1407
647.4311
625.4257
571.2745
418.0386
345.1955
386.1368
232.1688
487.1185
832.5511
m/z
F3C
N
O

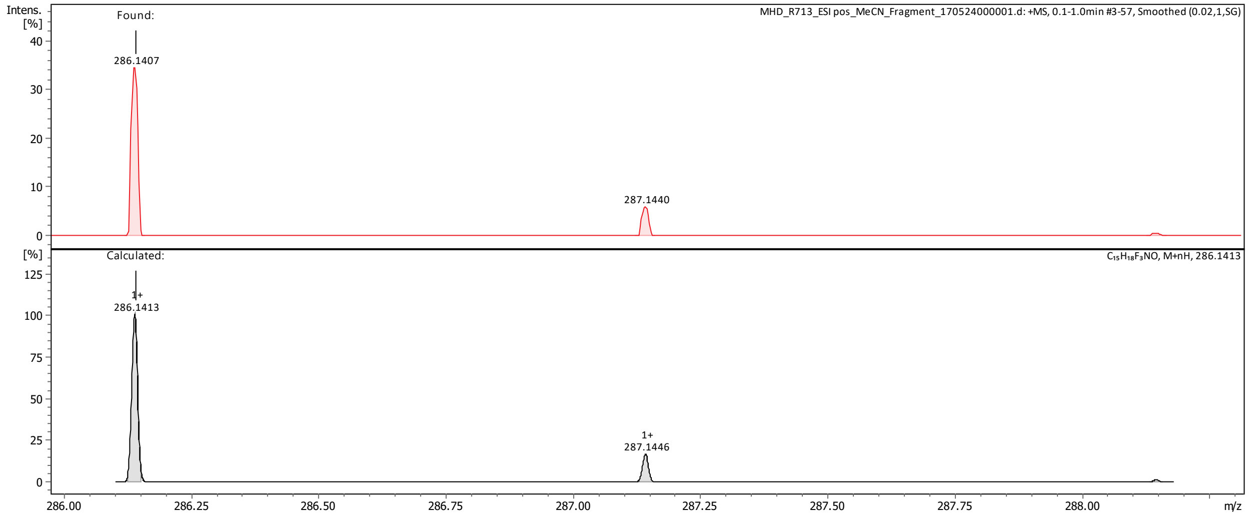

MHD_R713_ESI pos_MeCN_Fragment_170524000001.d: +MS, 0.1-1.0min #3-57, Smoothed (0.02,1,SG)
Intens. [%]
Found:
286.1407
287.1440
Calculated:
C15H18F3NO, M+nH, 286.1413
1+
286.1413
1+
287.1446
m/z

3.14.8 IR

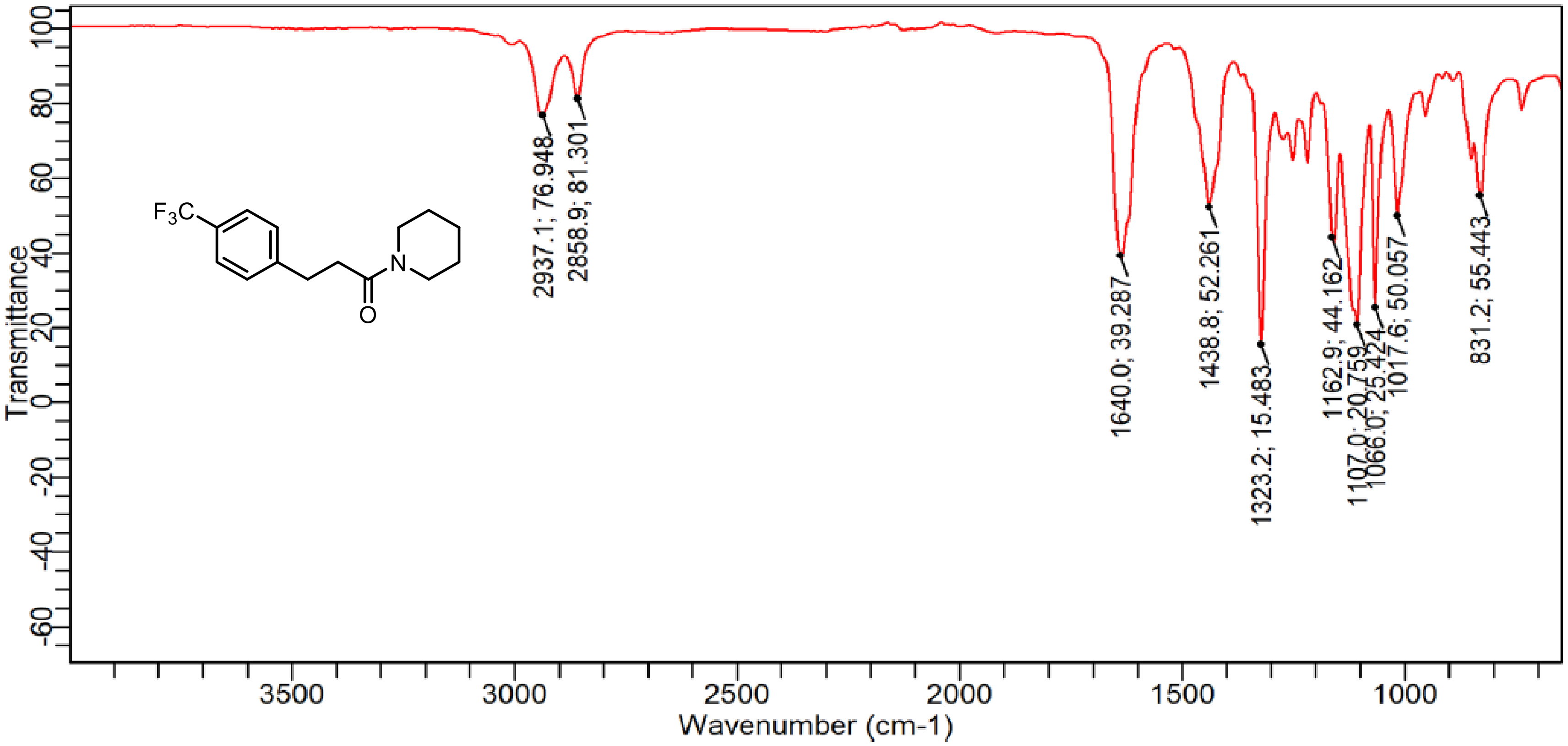

Transmittance
Wavenumber (cm-1)
100
80
60
40
20
0
-20
-40
-60
3500
3000
2500
2000
1500
1000
F3C
N
O
2937.1; 76.948
2858.9; 81.301
1640.0; 39.287
1438.8; 52.261
1323.2; 15.483
1162.9; 44.162
1107.0; 20.759
1066.0; 25.424
1017.6; 50.057
831.2; 55.443

3.15 1-(Piperidin-1-yl)-3-(pyridin-3-yl)propan-1-one (12o) (600 MHz, $CDCl_3$)

3.15.1 ^{1}H NMR

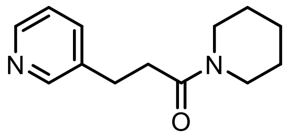

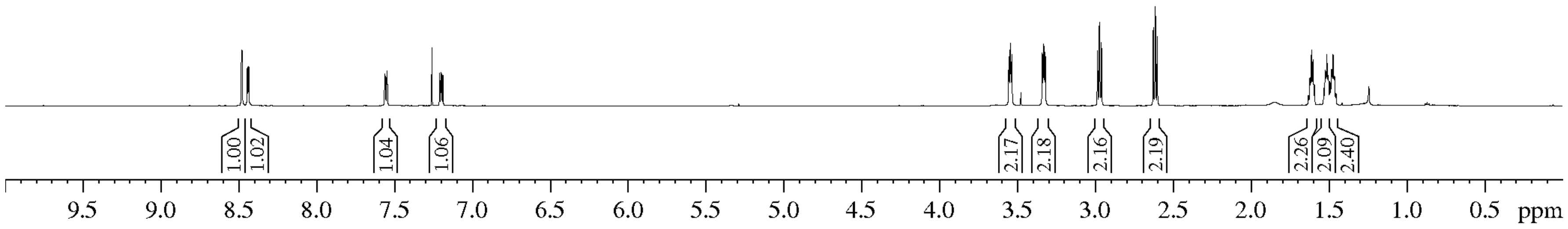

3.15.2 ^{13}C NMR

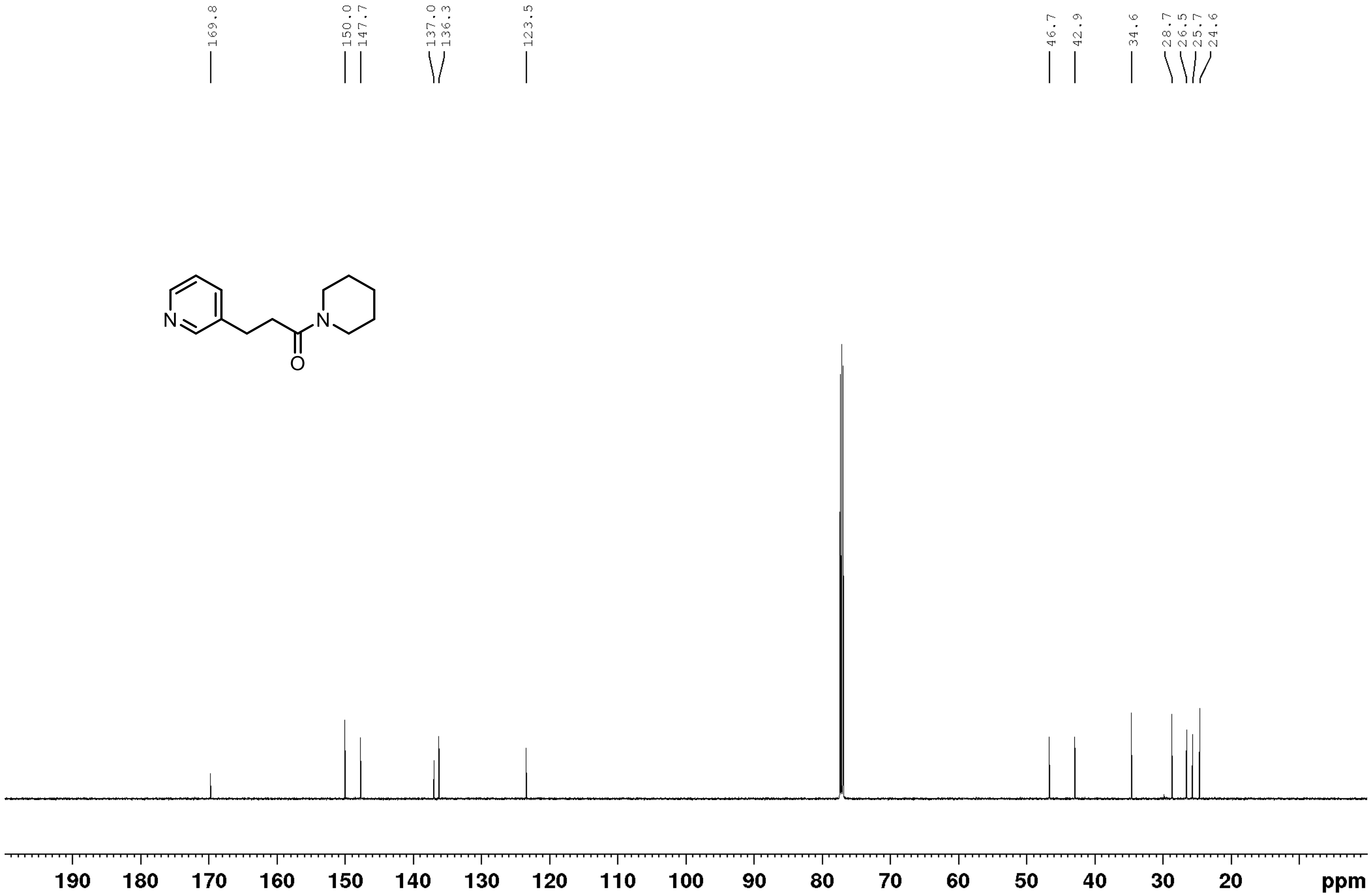

169.8
150.0
147.7
137.0
136.3
123.5
46.7
42.9
34.6
28.7
26.5
25.7
24.6
N
N
O
190
180
170
160
150
140
130
120
110
100
90
80
70
60
50
40
30
20
ppm

3.15.3 ^{1}H, ^{1}H COSY

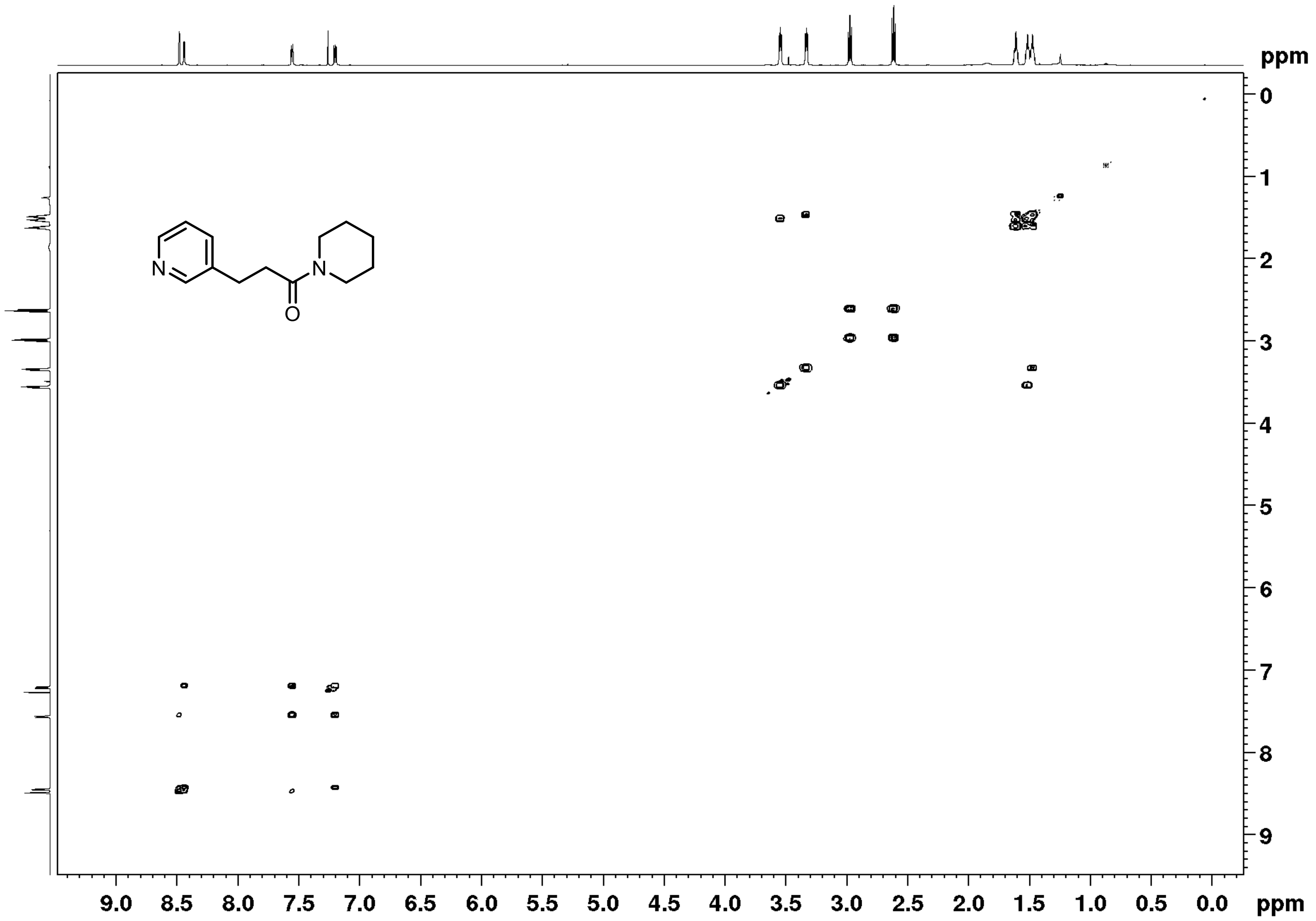

3.15.4 ^{1}H, ^{13}C HMBC

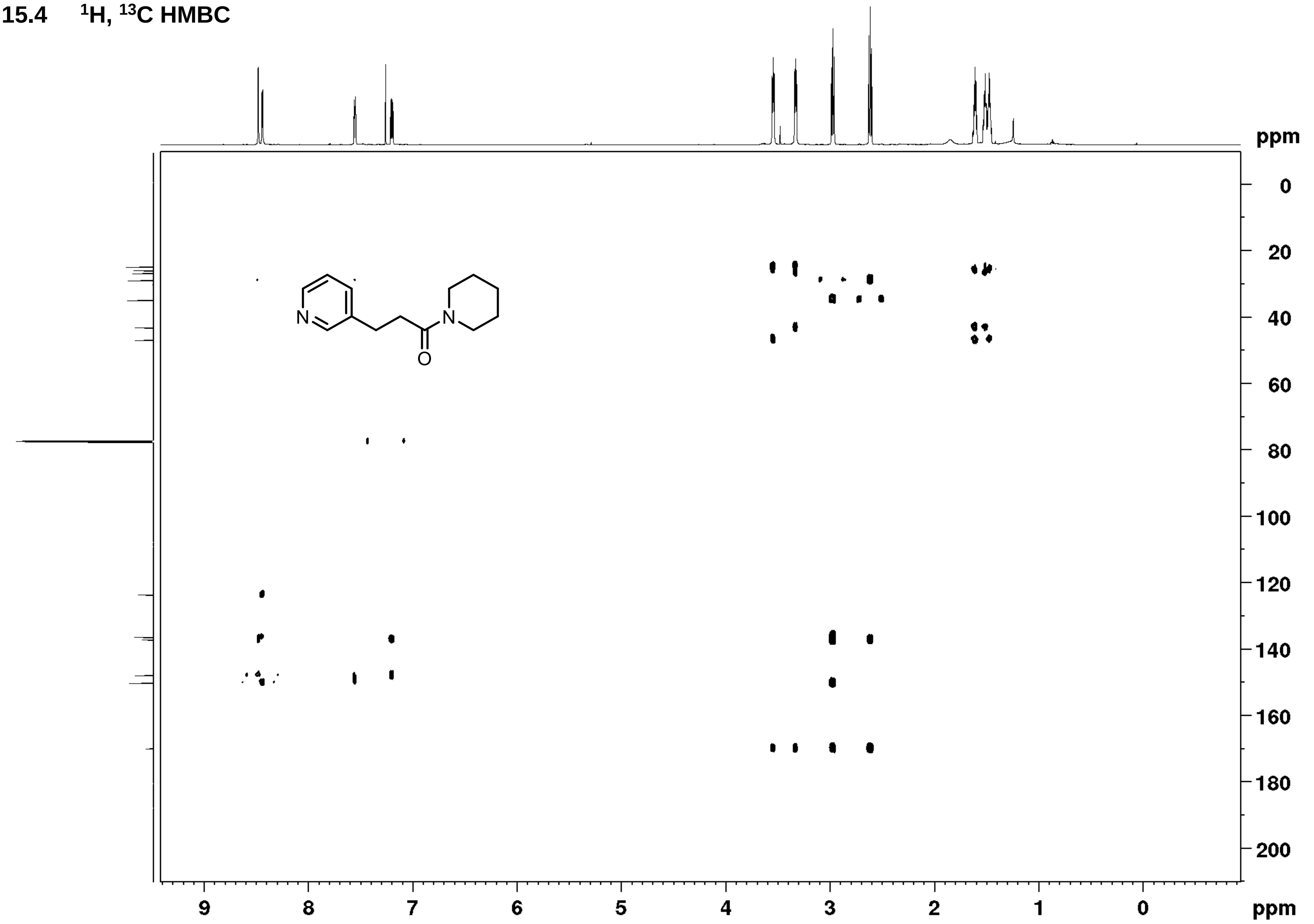

3.15.5 ^{1}H, ^{13}C HSQC

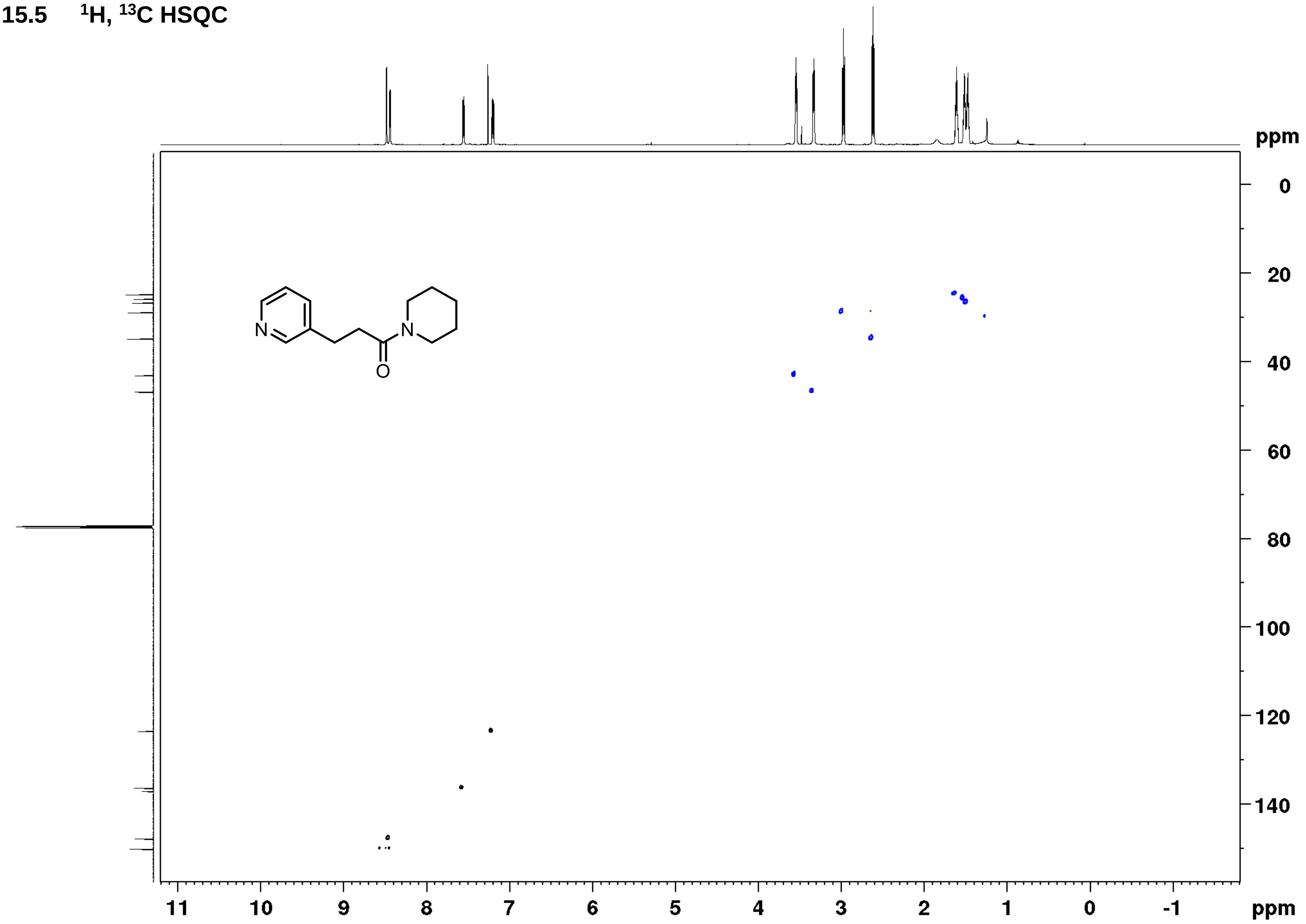

3.15.6 HRMS

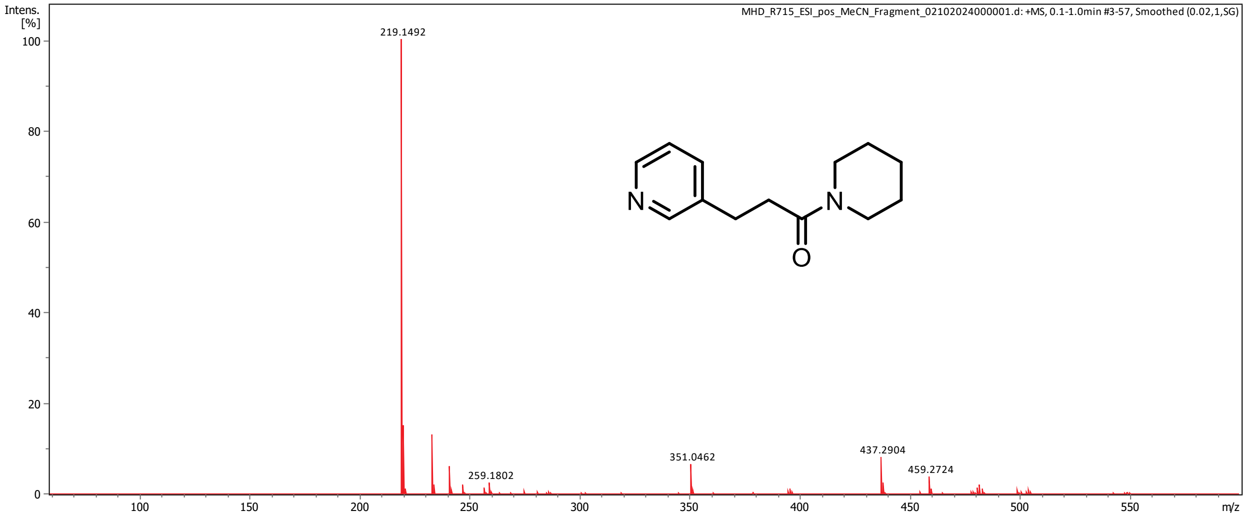
Intens. [%]
MHD_R715_ESI_pos_MeCN_Fragment_02102024000001.d: +MS, 0.1-1.0min #3-57, Smoothed (0.02,1,SG)
219.1492
259.1802
351.0462
437.2904
459.2724
m/z
N
N
O

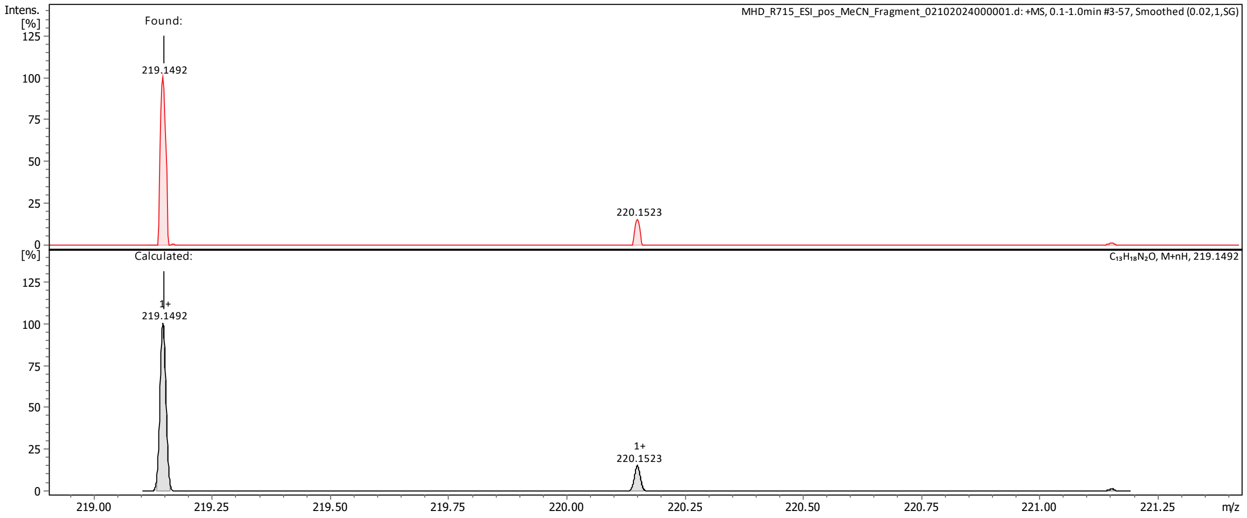
Intens. [%]
MHD_R715_ESI_pos_MeCN_Fragment_02102024000001.d: +MS, 0.1-1.0min #3-57, Smoothed (0.02,1,SG)
Found:
219.1492
220.1523
Calculated:
$C_{13}H_{18}N_2O$, M+nH, 219.1492
1+
219.1492
1+
220.1523
m/z

3.15.7 IR

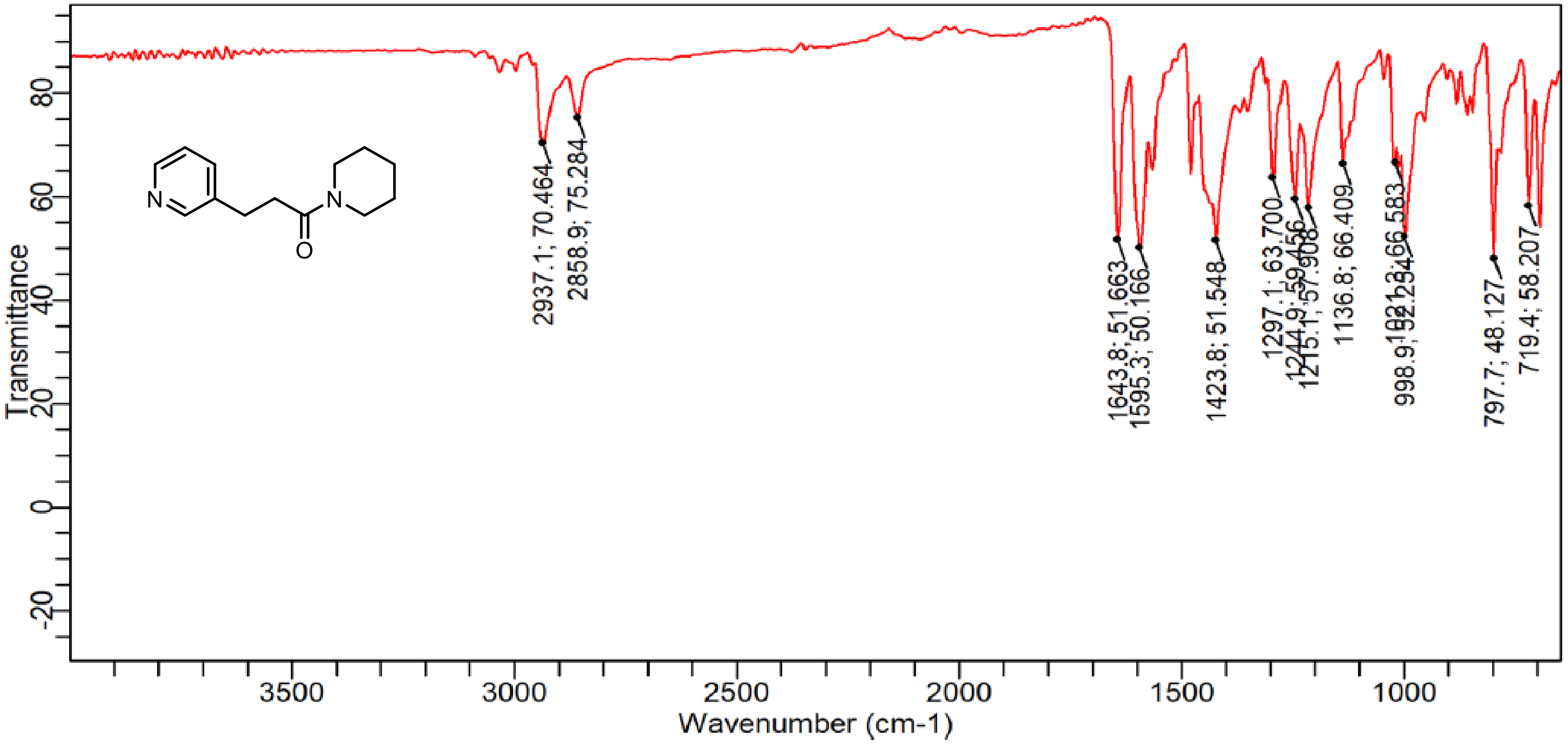

Transmittance
Wavenumber (cm-1)
2937.1; 70.464
2858.9; 75.284
1643.8; 51.663
1595.3; 50.166
1423.8; 51.548
1297.1; 63.700
1244.9; 59.456
1215.1; 57.908
1136.8; 66.409
1021.3; 66.583
998.9; 52.254
797.7; 48.127
719.4; 58.207

3.16 3-(Furan-2-yl)-1-(piperidin-1-yl)propan-1-one (12p) (600 MHz, $CDCl_3$)

3.16.1 ^{1}H NMR

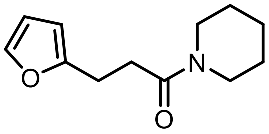

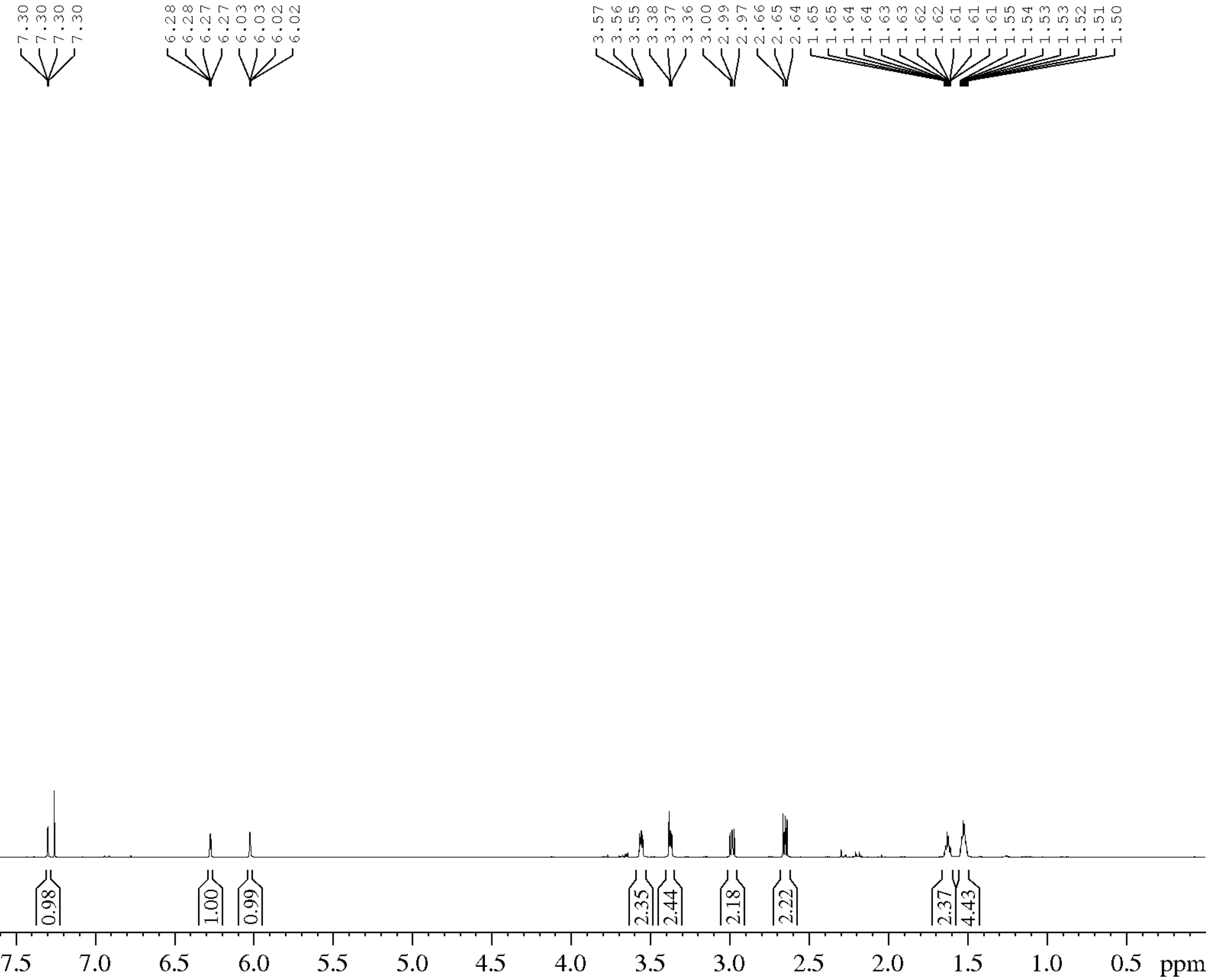

3.16.2 ^{13}C NMR

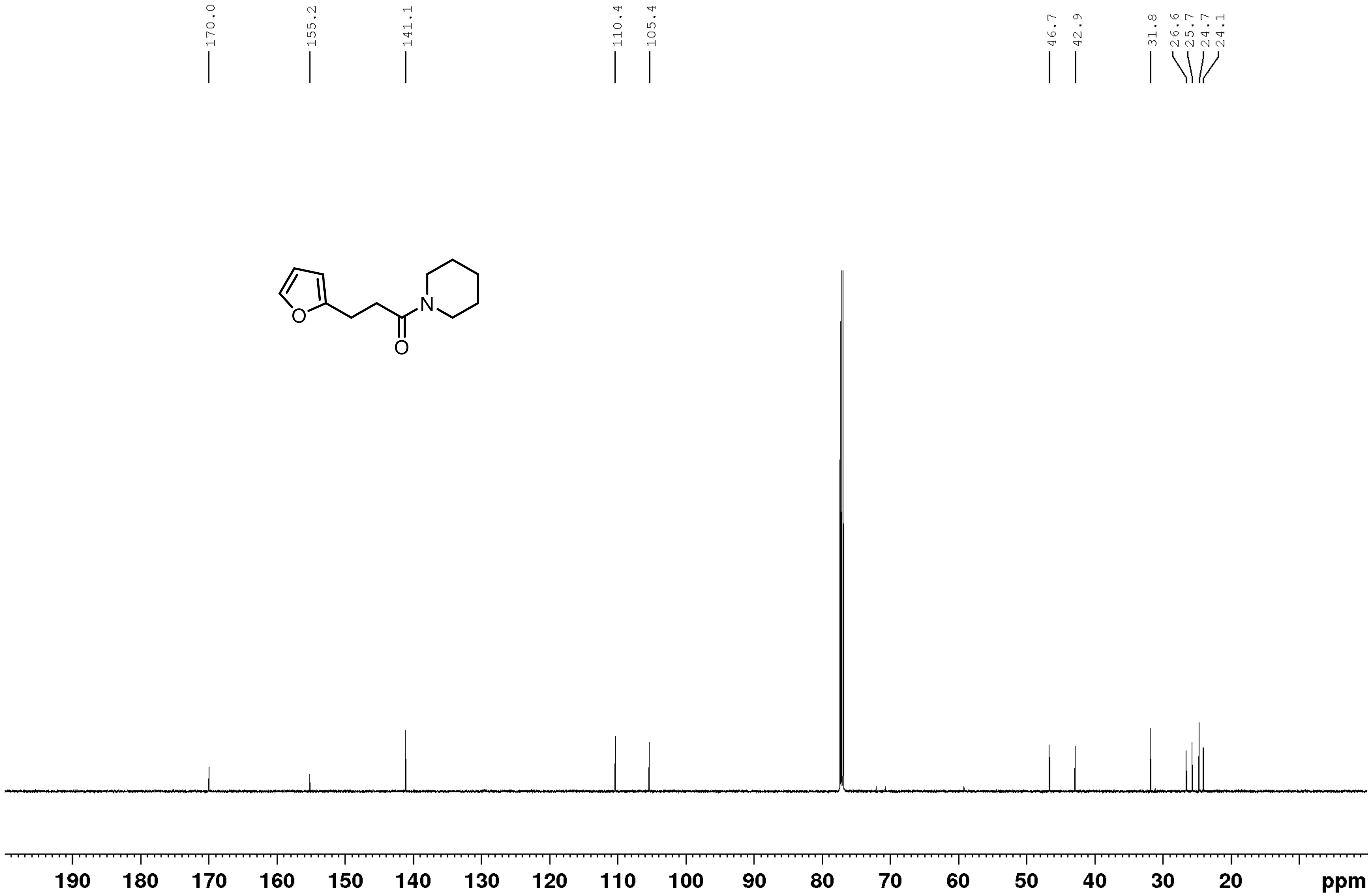
170.0
155.2
141.1
110.4
105.4
46.7
42.9
31.8
26.6
25.7
24.7
24.1
O
N
O
190 180 170 160 150 140 130 120 110 100 90 80 70 60 50 40 30 20
ppm

3.16.3 ¹H, ¹H COSY

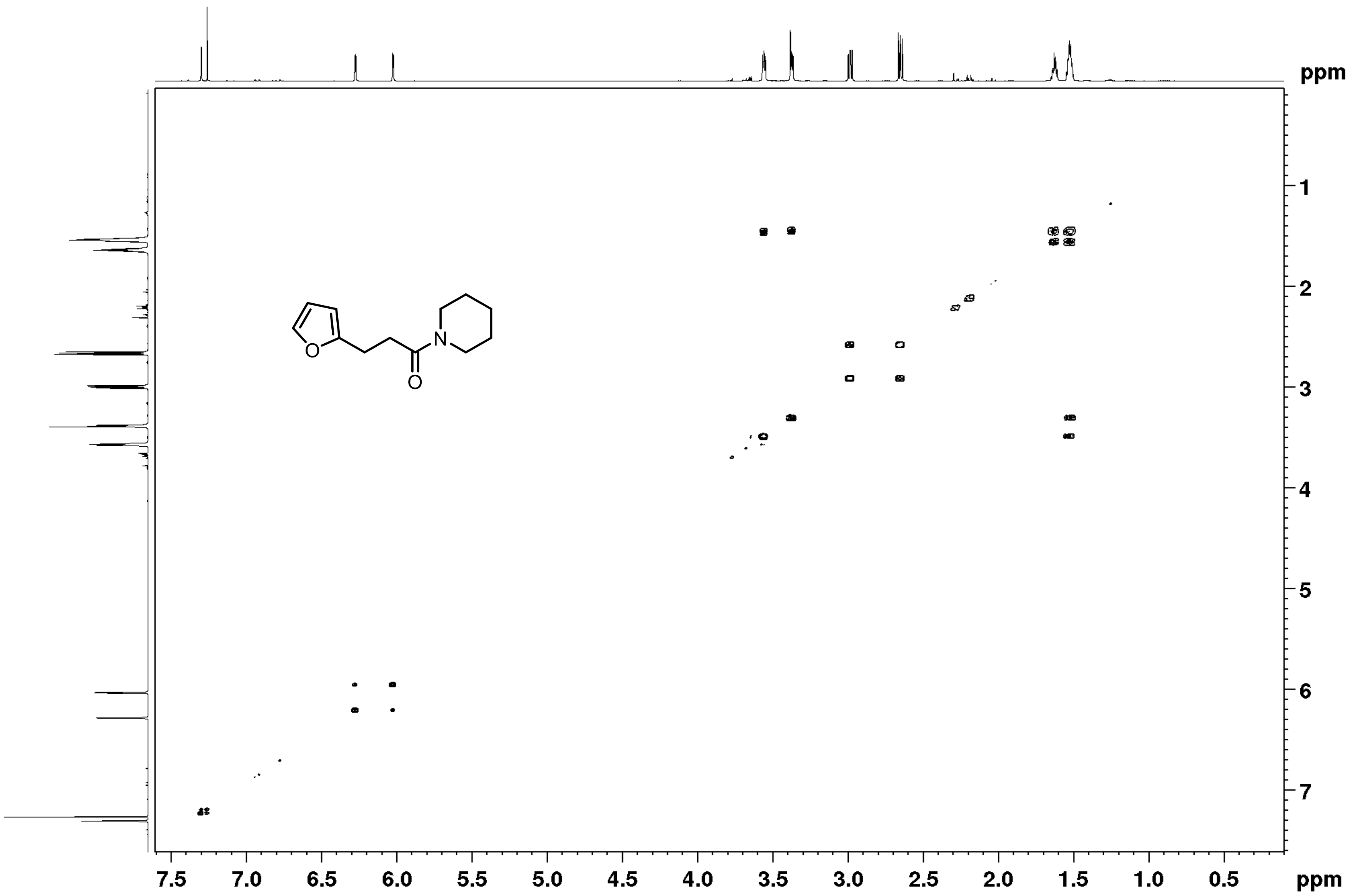

3.16.4 ^{1}H, ^{13}C HMBC

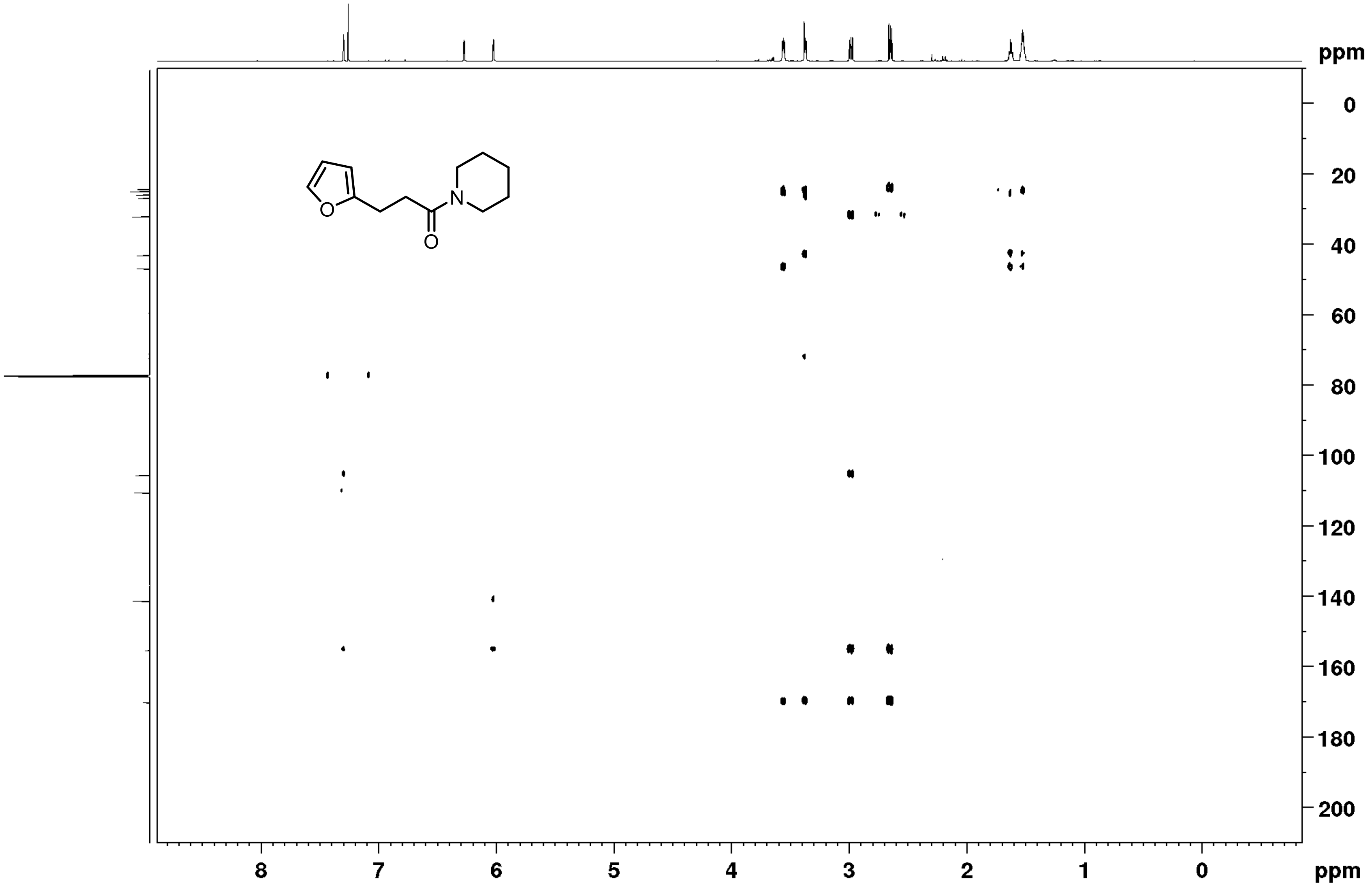

3.16.5 ^{1}H, ^{13}C HSQC

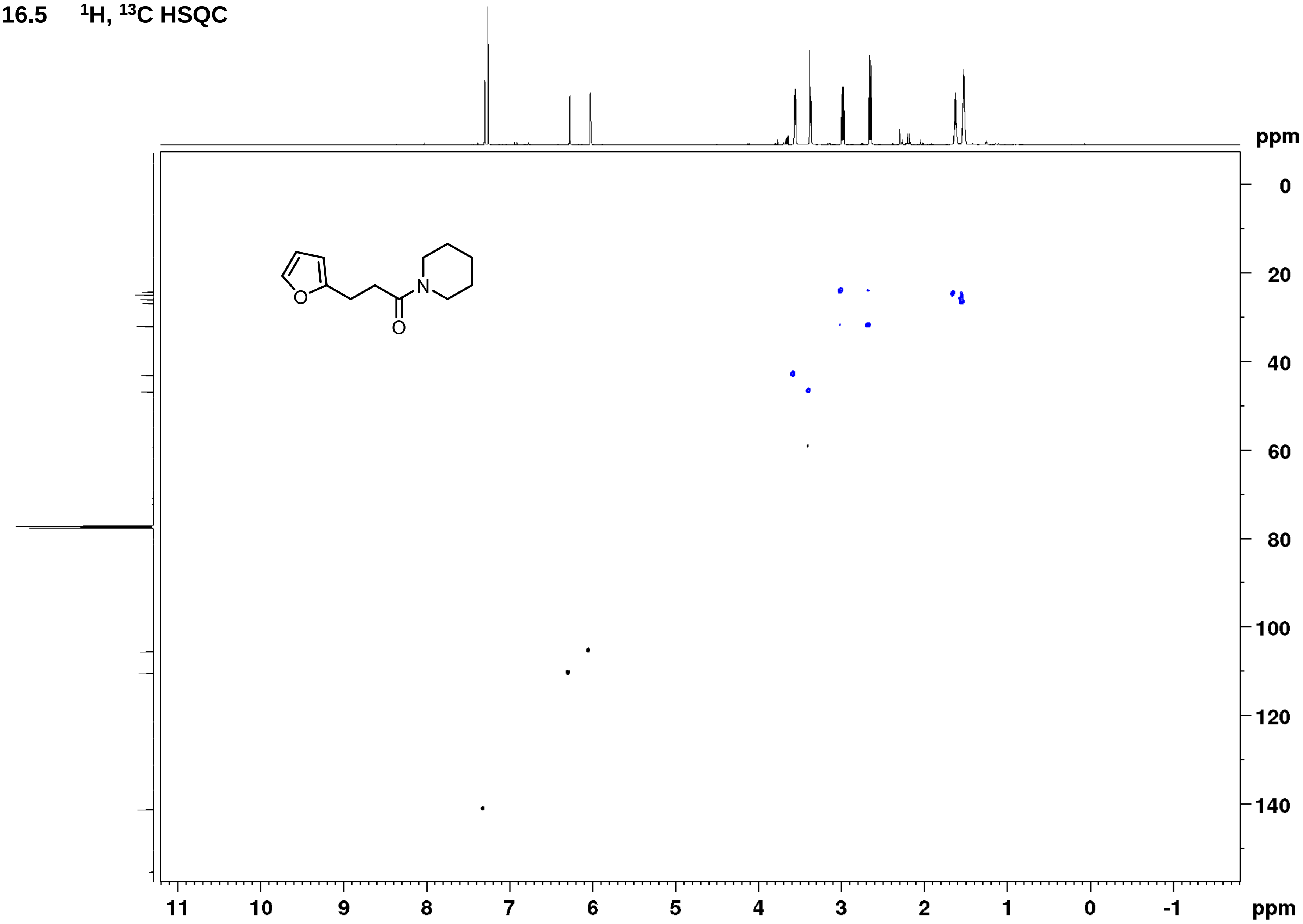

3.16.6 HRMS

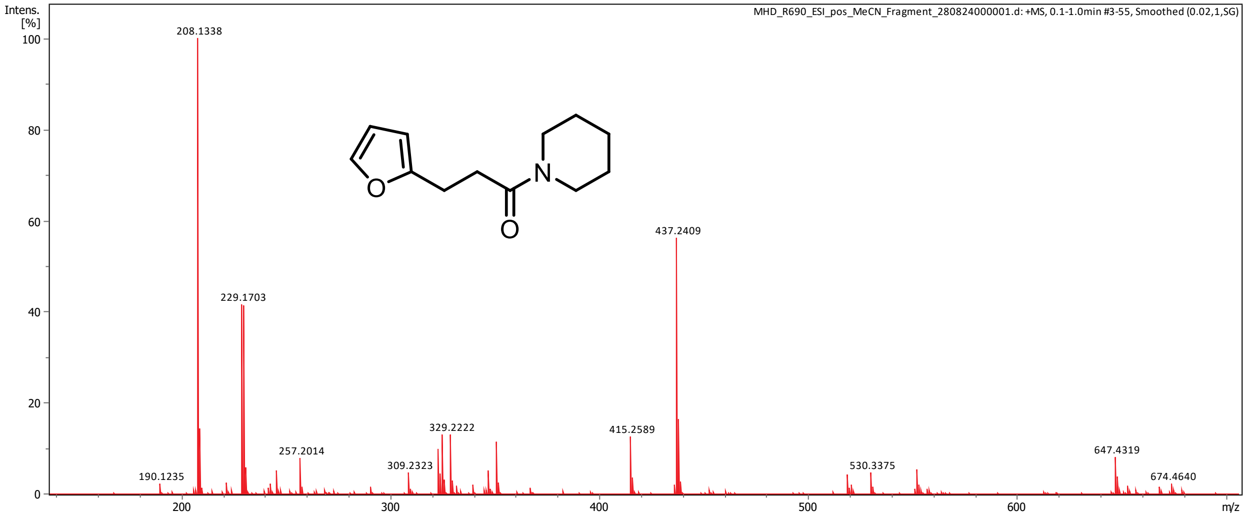

MHD_R690_ESI_pos_MeCN_Fragment_280824000001.d: +MS, 0.1-1.0min #3-55, Smoothed (0.02,1,SG)
Intens. [%]
208.1338
229.1703
437.2409
190.1235
257.2014
309.2323
329.2222
415.2589
530.3375
647.4319
674.4640
m/z

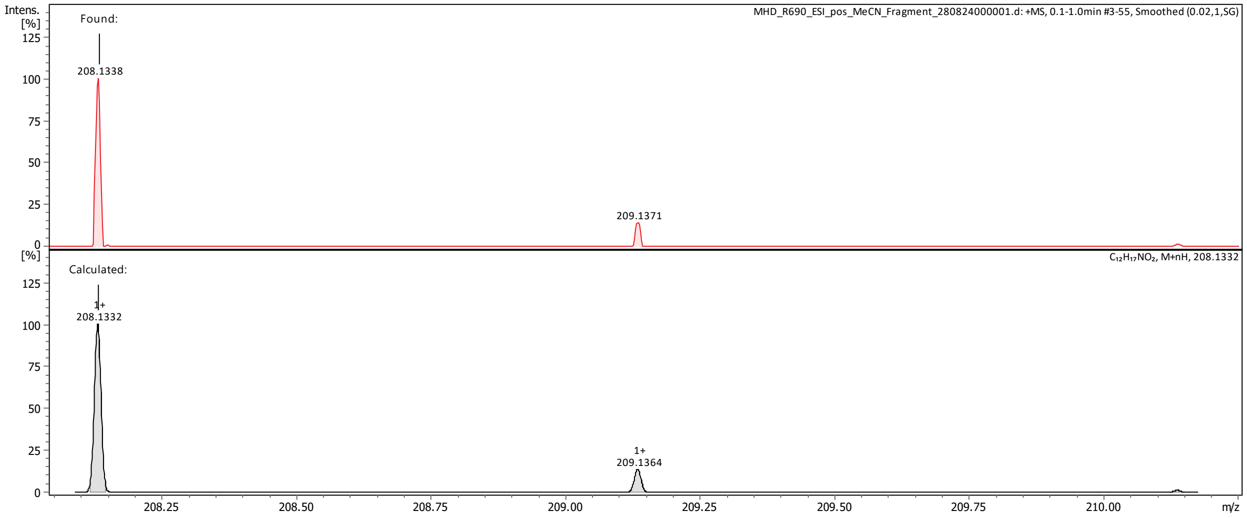

MHD_R690_ESI_pos_MeCN_Fragment_280824000001.d: +MS, 0.1-1.0min #3-55, Smoothed (0.02,1,SG)
Found:
208.1338
209.1371
Calculated:
$C_{12}H_{17}NO_2$, M+nH, 208.1332
1+
208.1332
1+
209.1364
m/z

3.16.7 IR

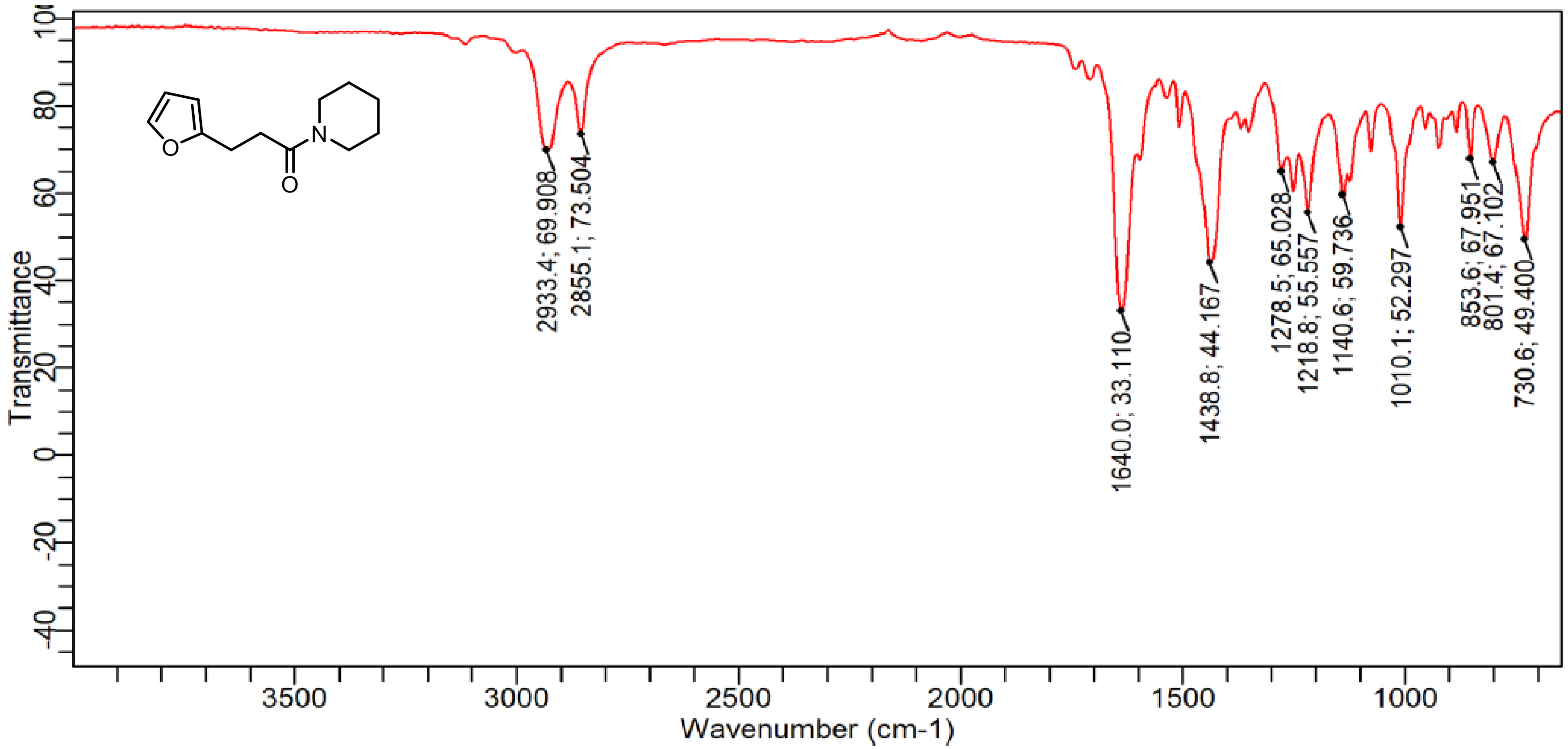

Transmittance
Wavenumber (cm-1)
2933.4; 69.908
2855.1; 73.504
1640.0; 33.110
1438.8; 44.167
1278.5; 65.028
1218.8; 55.557
1140.6; 59.736
1010.1; 52.297
853.6; 67.951
801.4; 67.102
730.6; 49.400
O
N

3.17 3,3-Diphenyl-1-(piperidin-1-yl)propan-1-one (12q) (600 MHz, CDCl$_3$)

3.17.1 ^{1}H NMR

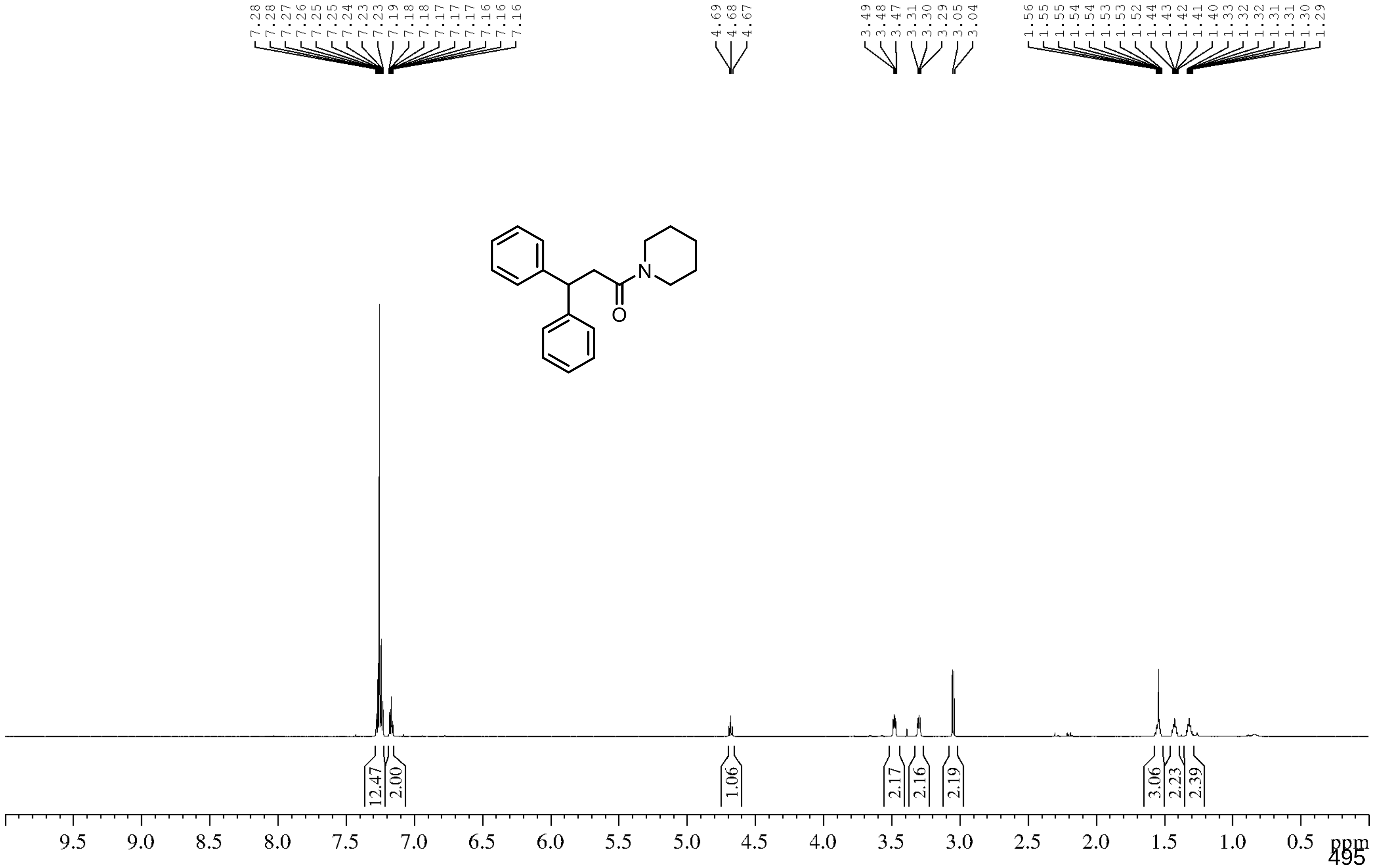

3.17.2 ^{13}C NMR

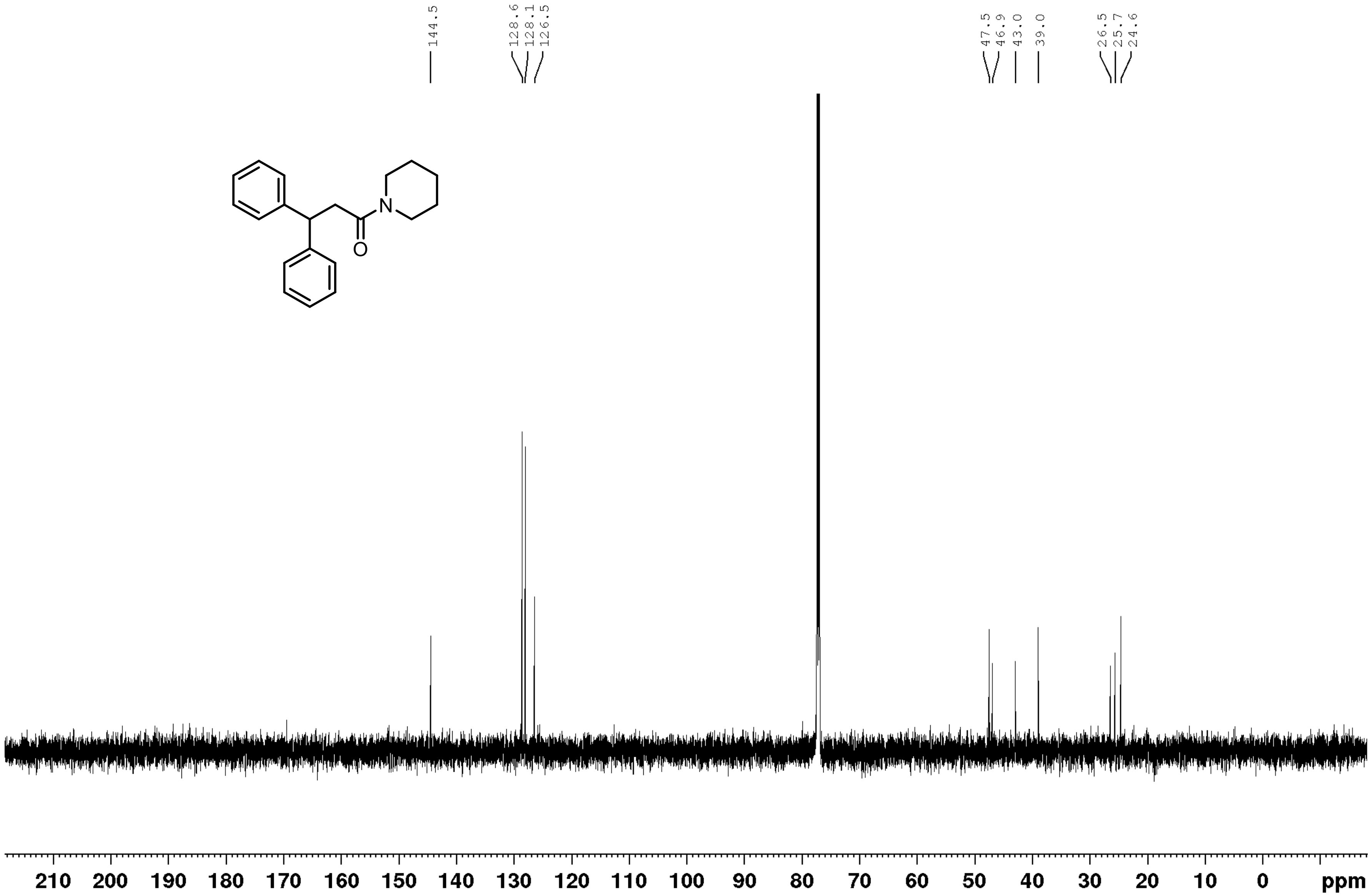

144.5
128.6
128.1
126.5
47.5
46.9
43.0
39.0
26.5
25.7
24.6
N
O
210 200 190 180 170 160 150 140 130 120 110 100 90 80 70 60 50 40 30 20 10 0 ppm

3.17.3 ^{1}H, ^{1}H COSY

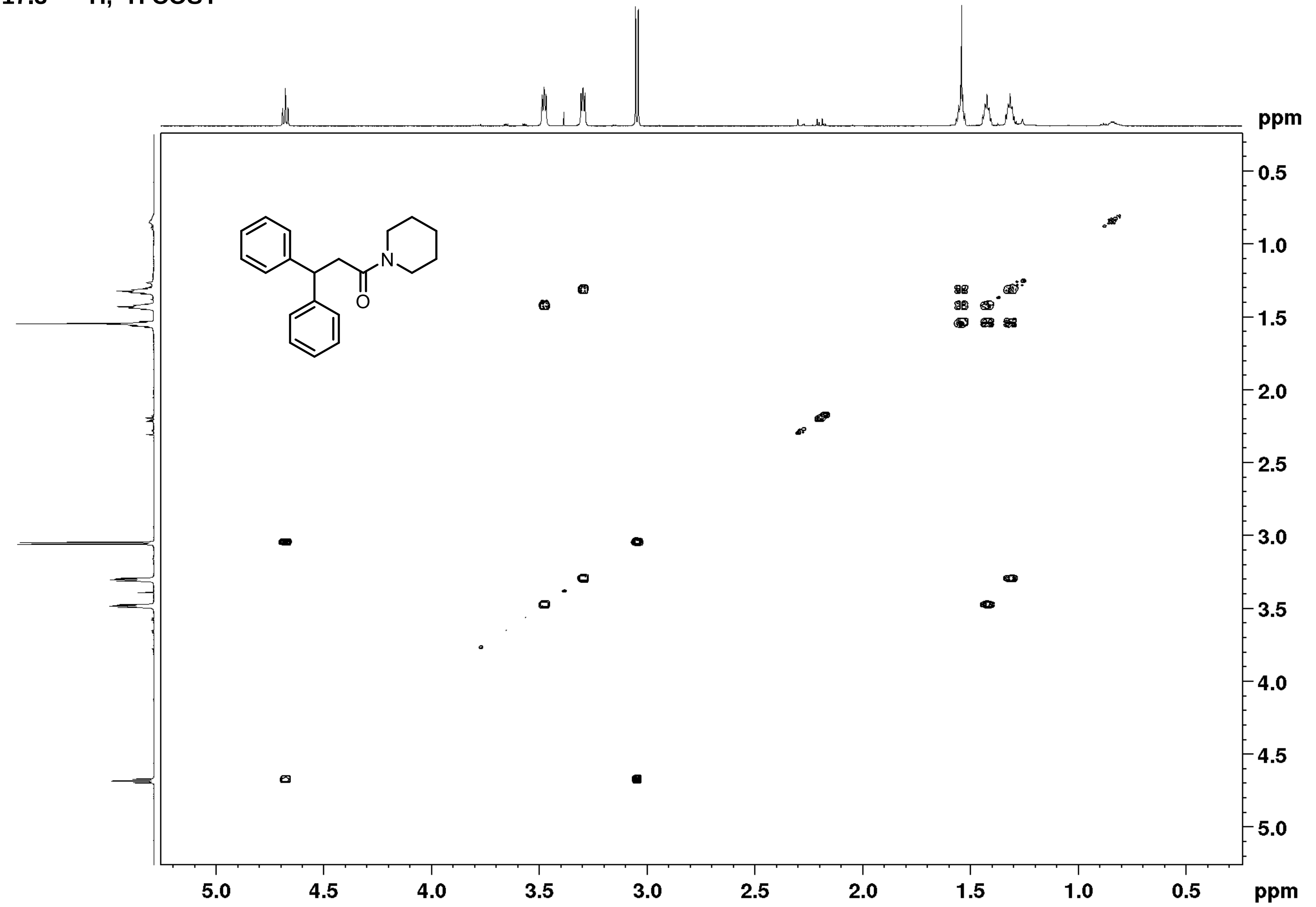

3.17.4 ^{1}H, ^{13}C HMBC

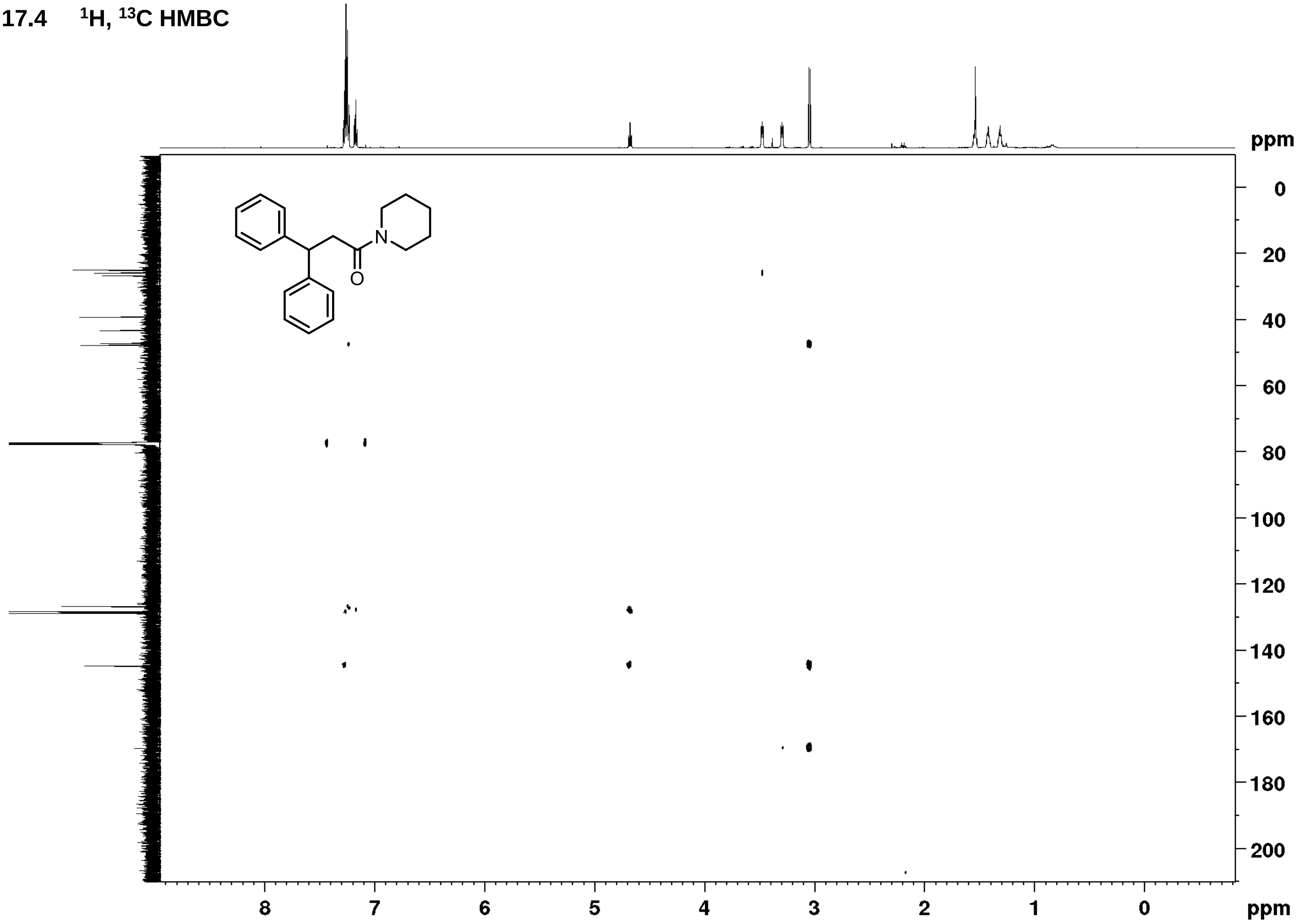

3.17.5 ^{1}H, ^{13}C HSQC

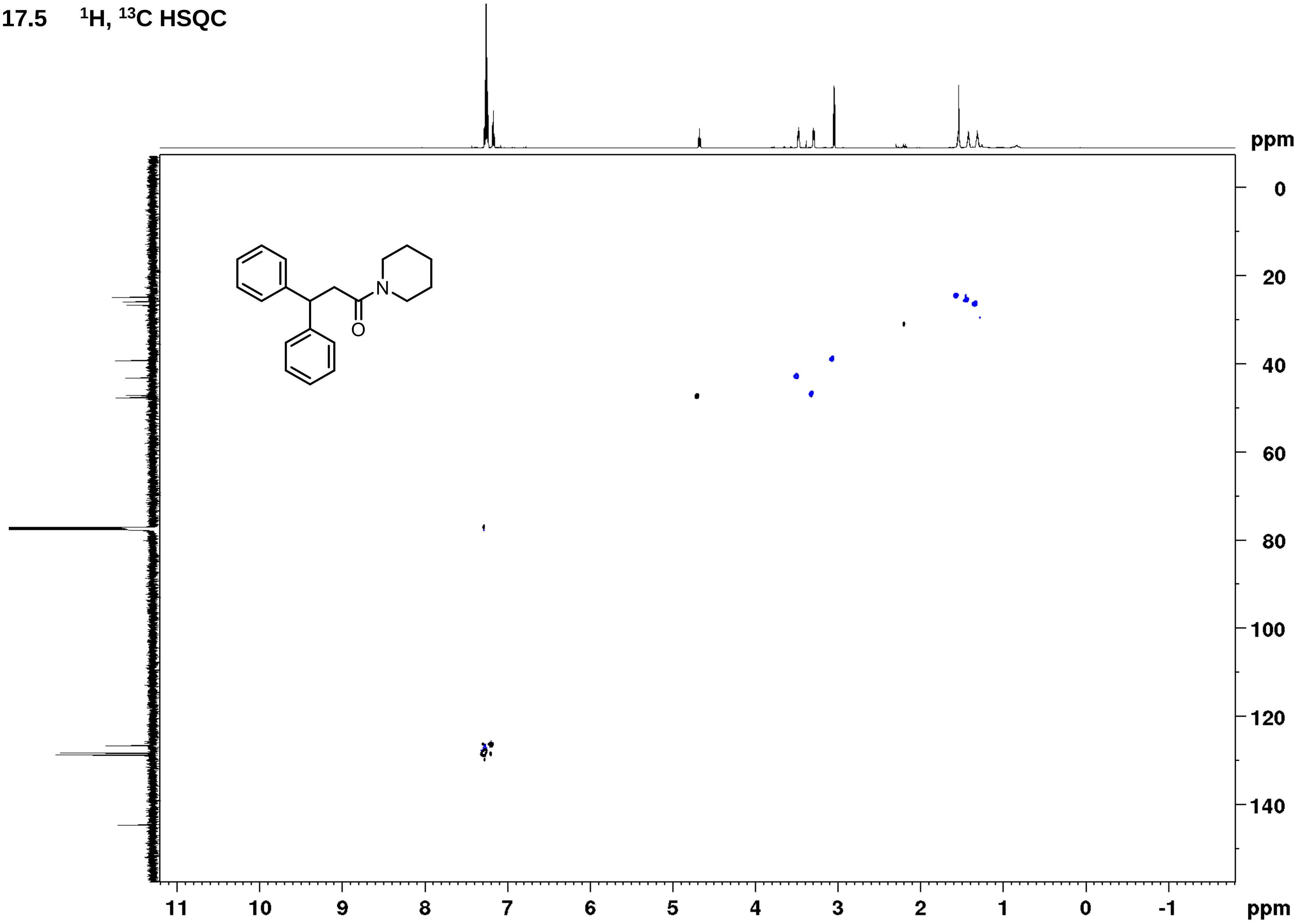

3.17.6 HRMS

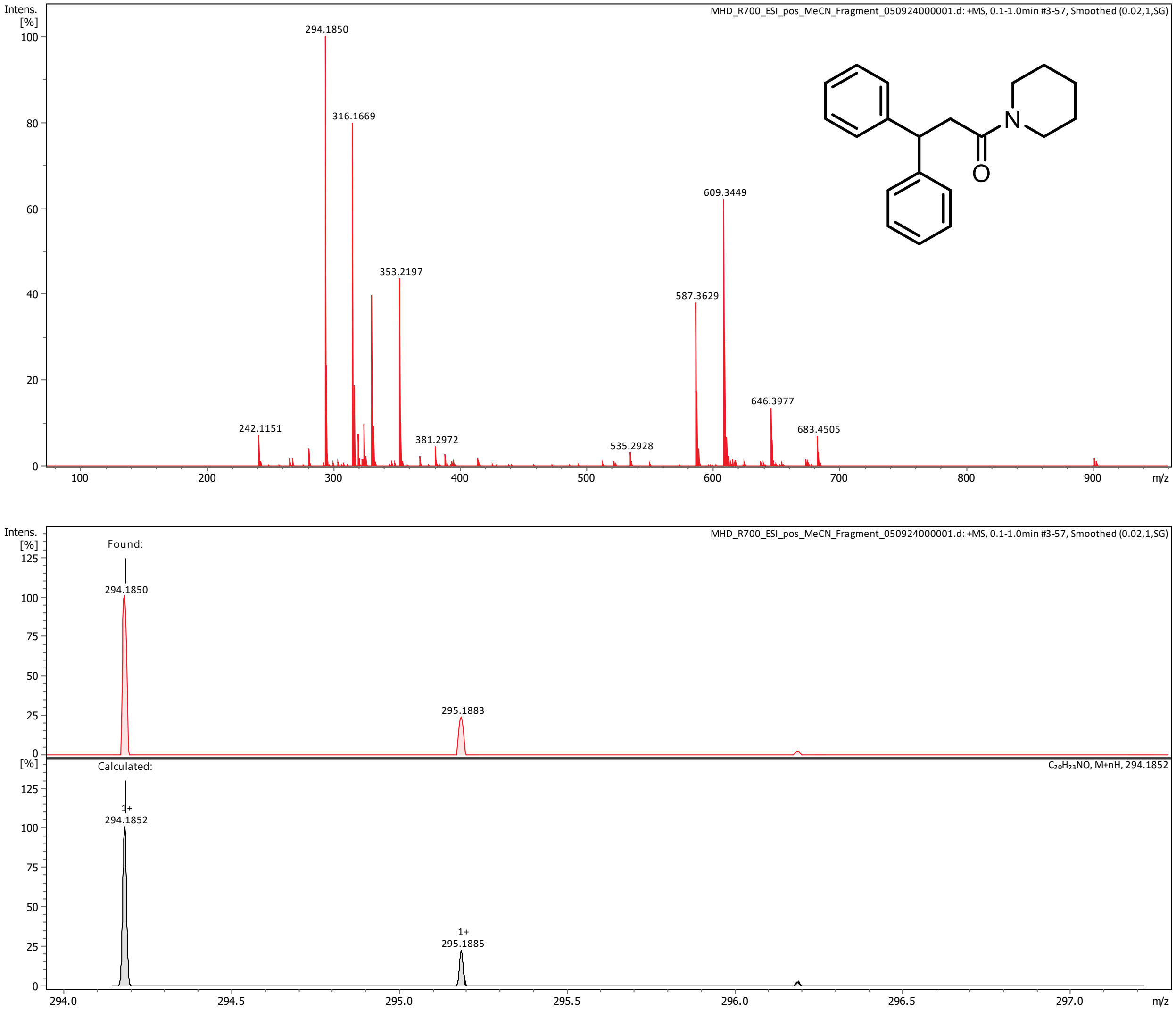

Intens. [%]
MHD_R700_ESI_pos_MeCN_Fragment_050924000001.d: +MS, 0.1-1.0min #3-57, Smoothed (0.02,1,SG)
294.1850
316.1669
353.2197
609.3449
587.3629
646.3977
683.4505
242.1151
381.2972
535.2928
m/z
N
O
Found:
294.1850
295.1883
Calculated:
$C_{20}H_{23}NO$, M+nH, 294.1852
1+
294.1852
1+
295.1885

3.17.7 IR

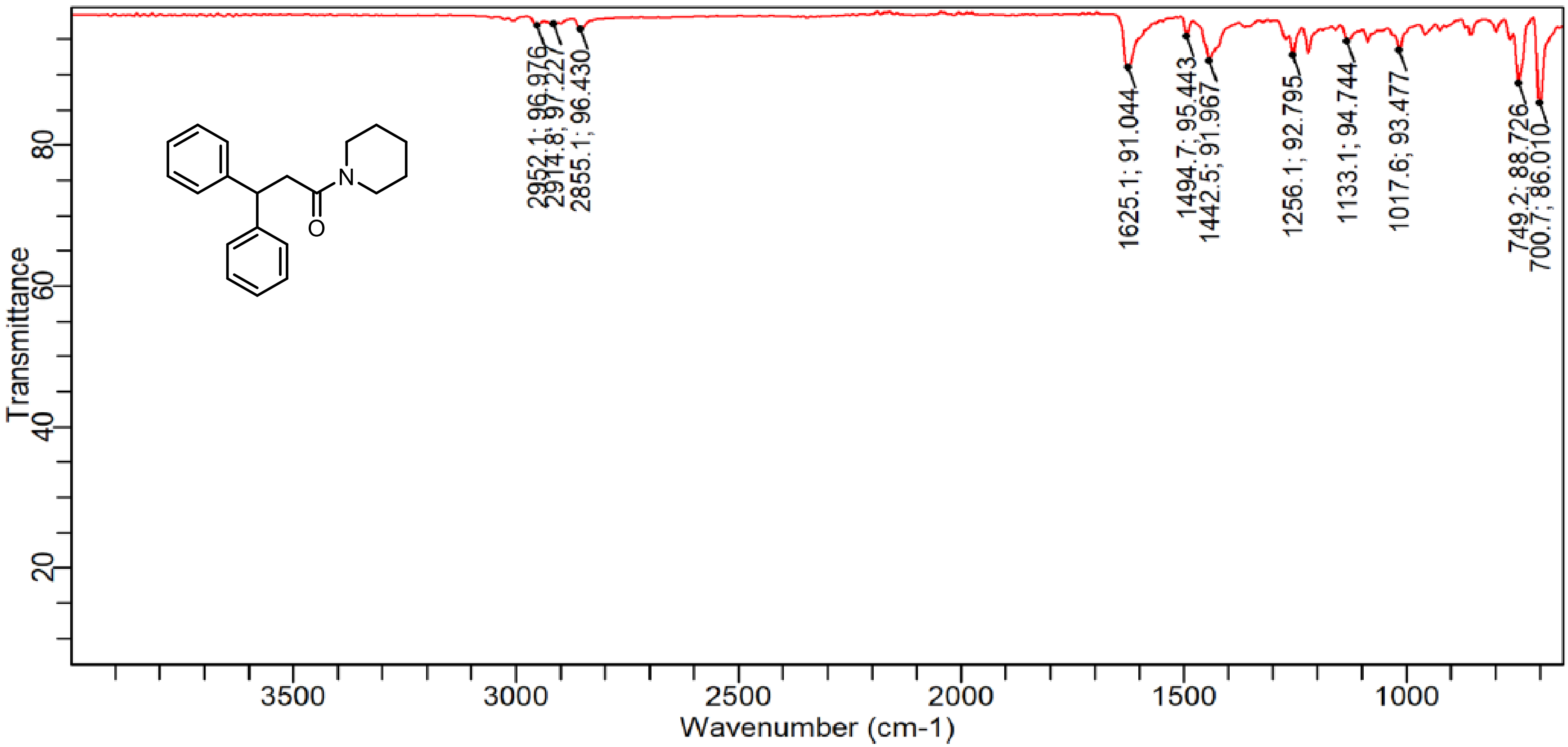

Transmittance
Wavenumber (cm-1)
20
40
60
80
3500
3000
2500
2000
1500
1000
2952.1; 96.976
2914.8; 97.227
2855.1; 96.430
1625.1; 91.044
1494.7; 95.443
1442.5; 91.967
1256.1; 92.795
1133.1; 94.744
1017.6; 93.477
749.2; 88.726
700.7; 86.010
N
O

3.18 3-Phenyl-1-(piperidin-1-yl)heptan-1-one (12r) (600 MHz, $CDCl_3$)

3.18.1 ^{1}H NMR

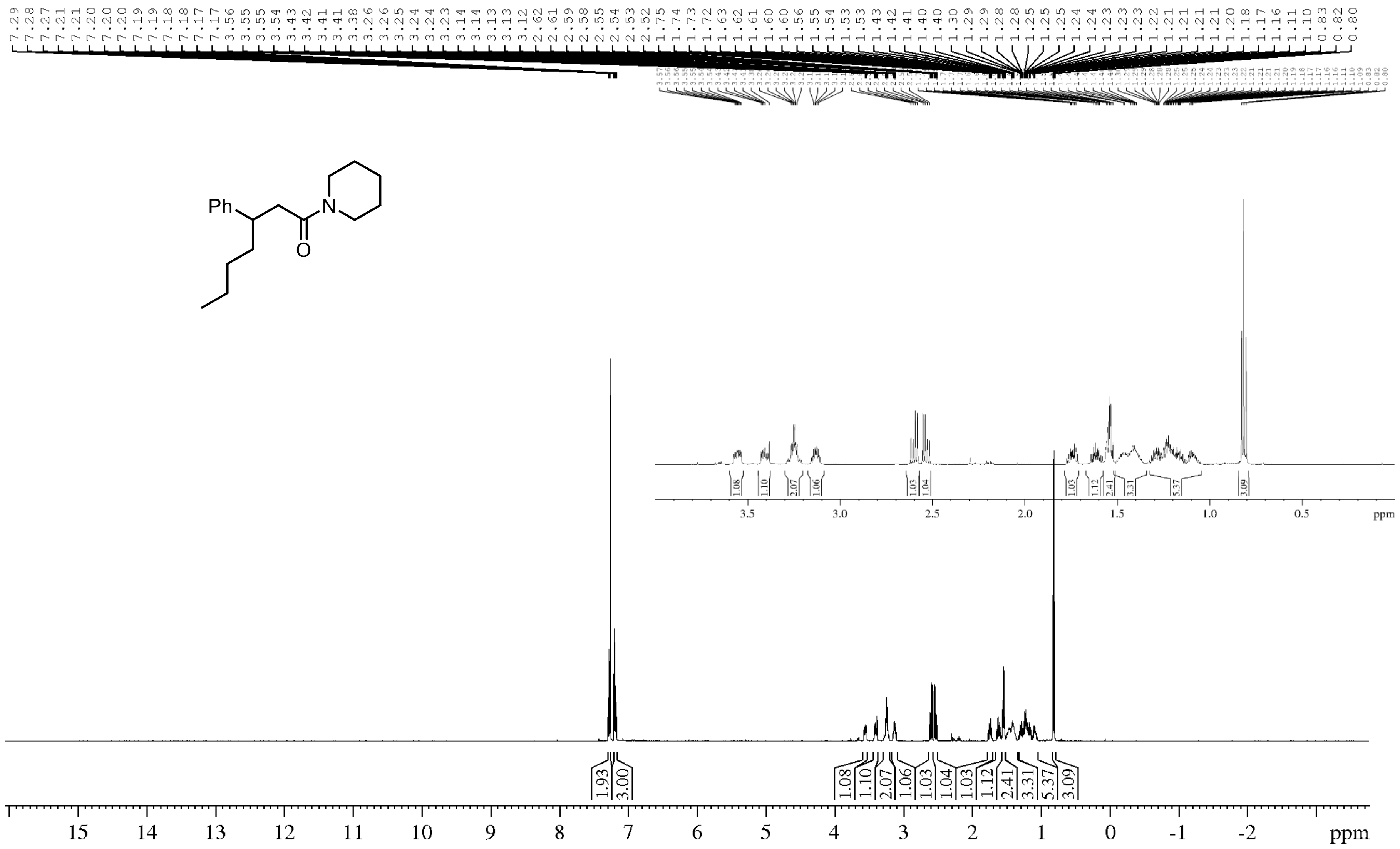

3.18.2 ^{13}C NMR

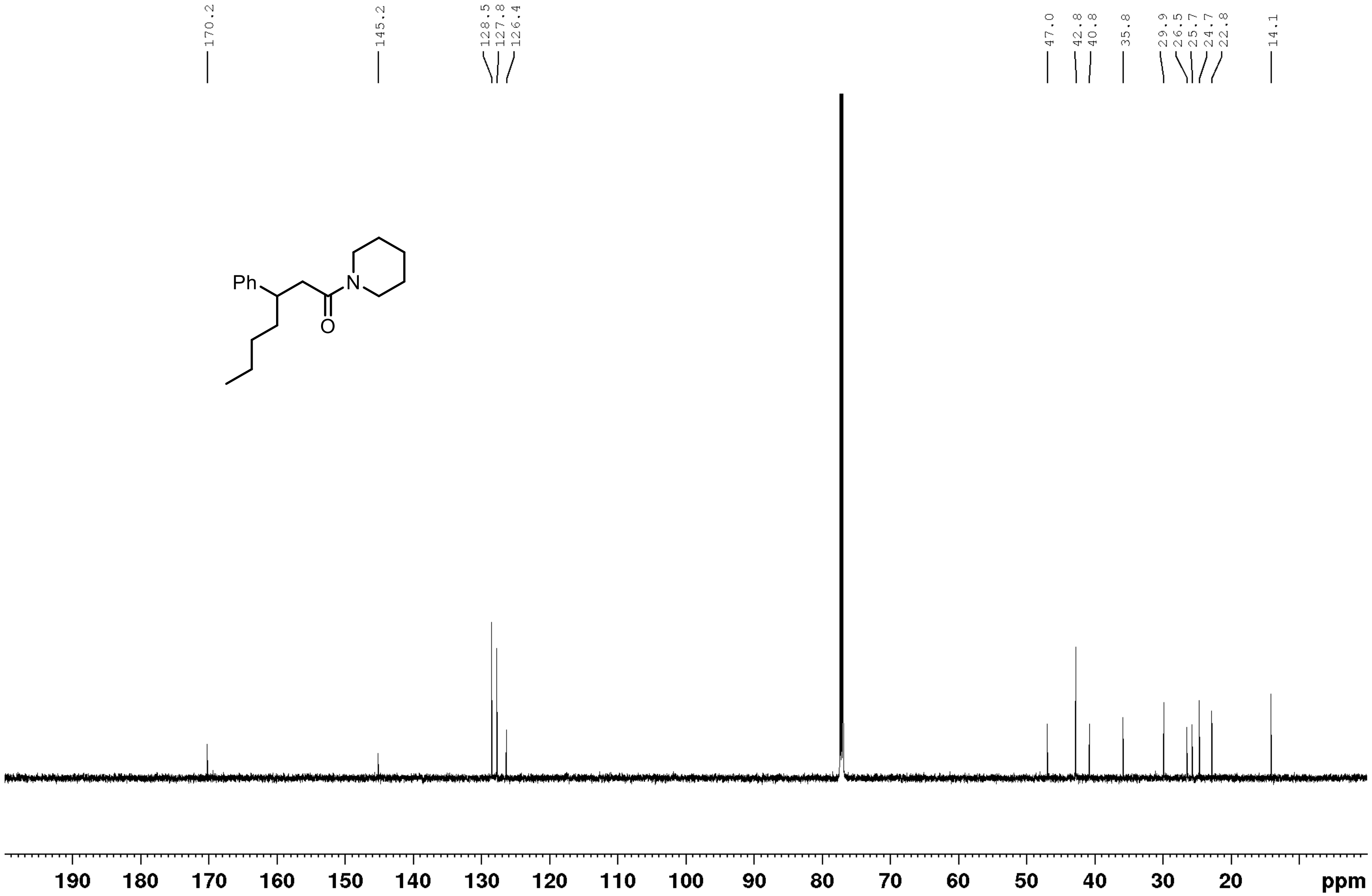

170.2
145.2
128.5
127.8
126.4
47.0
42.8
40.8
35.8
29.9
26.5
25.7
24.7
22.8
14.1
Ph
N
O
190
180
170
160
150
140
130
120
110
100
90
80
70
60
50
40
30
20
ppm

3.18.3 ^{1}H, ^{1}H COSY

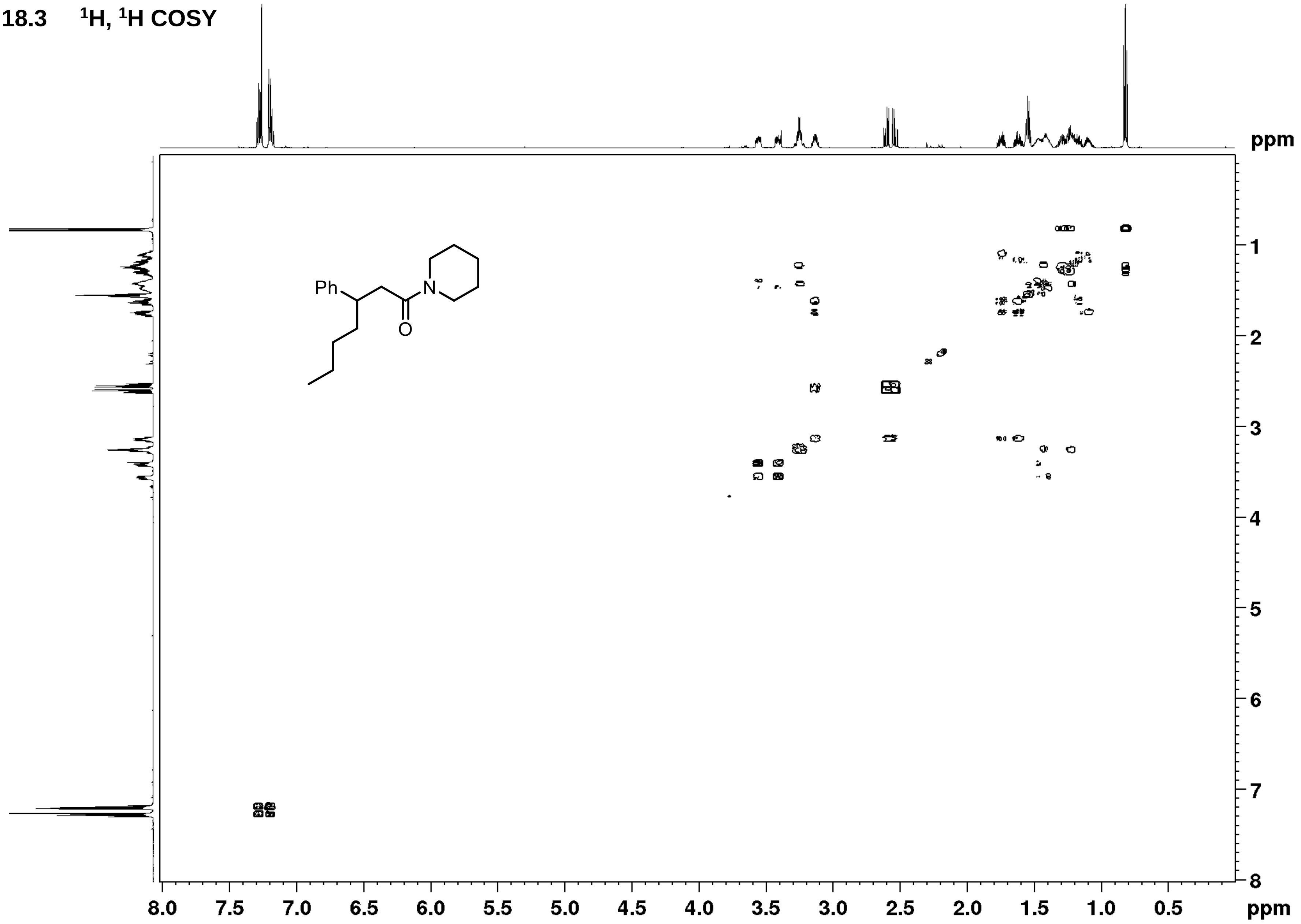

3.18.4 ^{1}H, ^{13}C HMBC

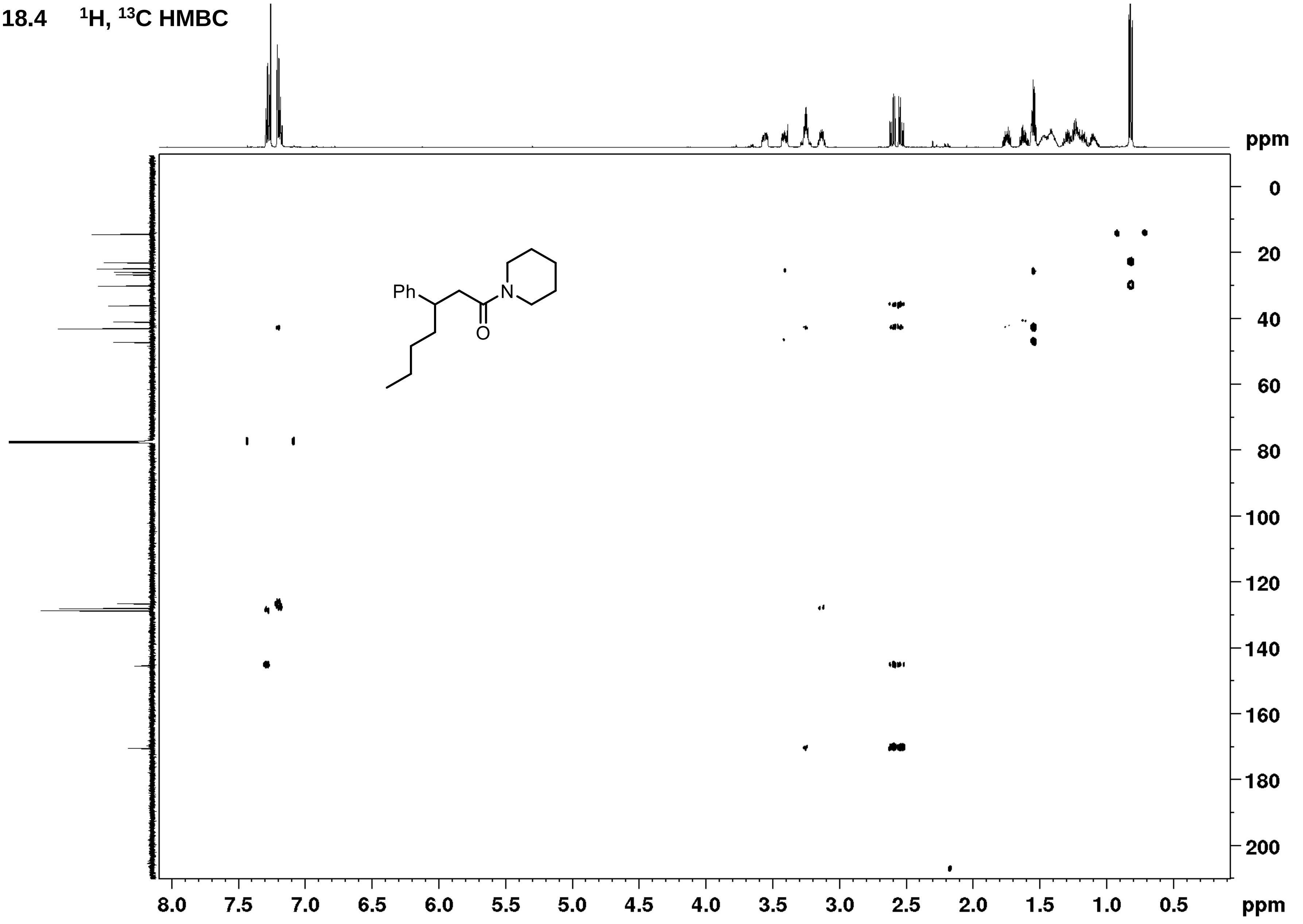

3.18.5 ¹H, ¹³C HSQC

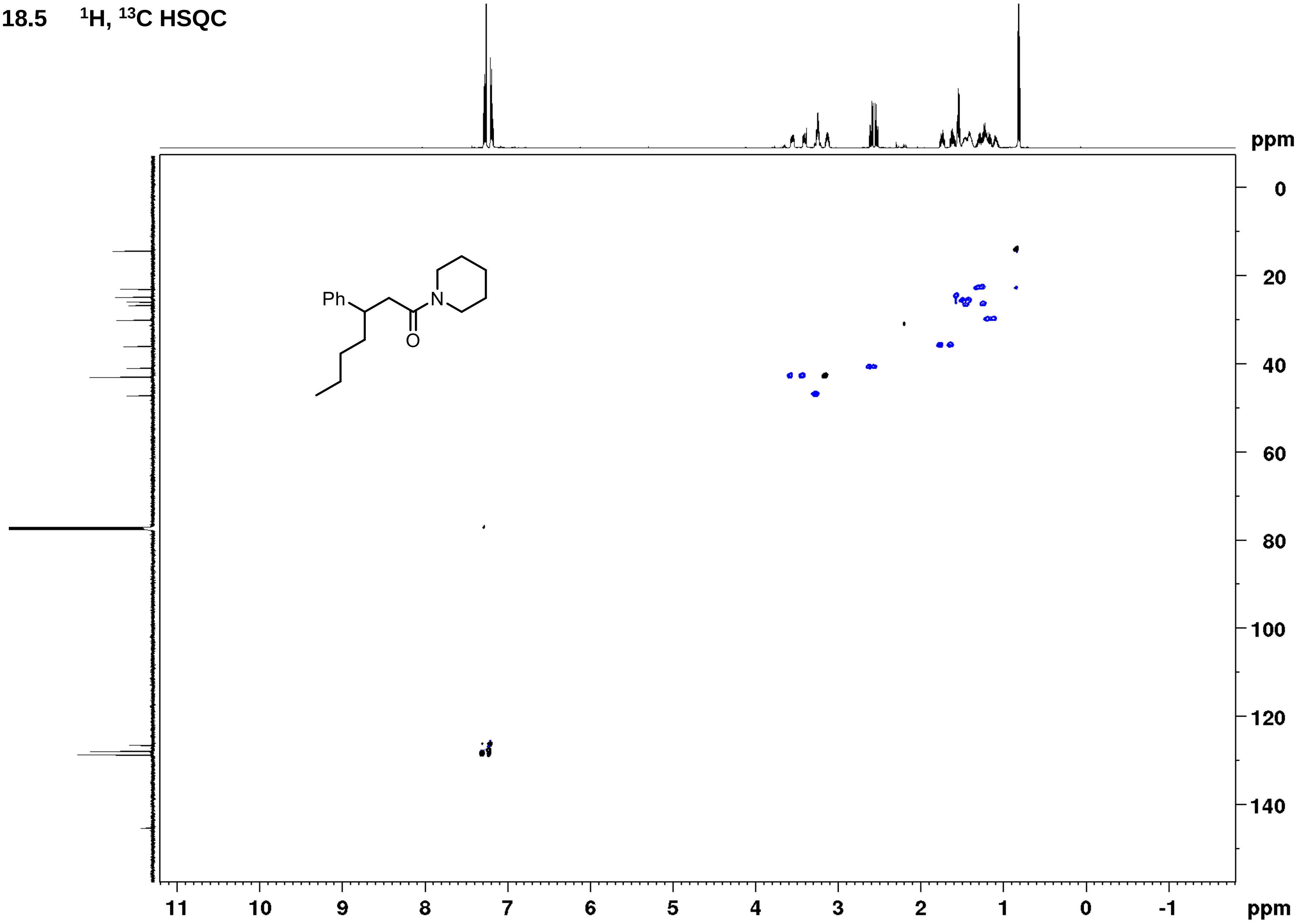

3.18.6 HRMS

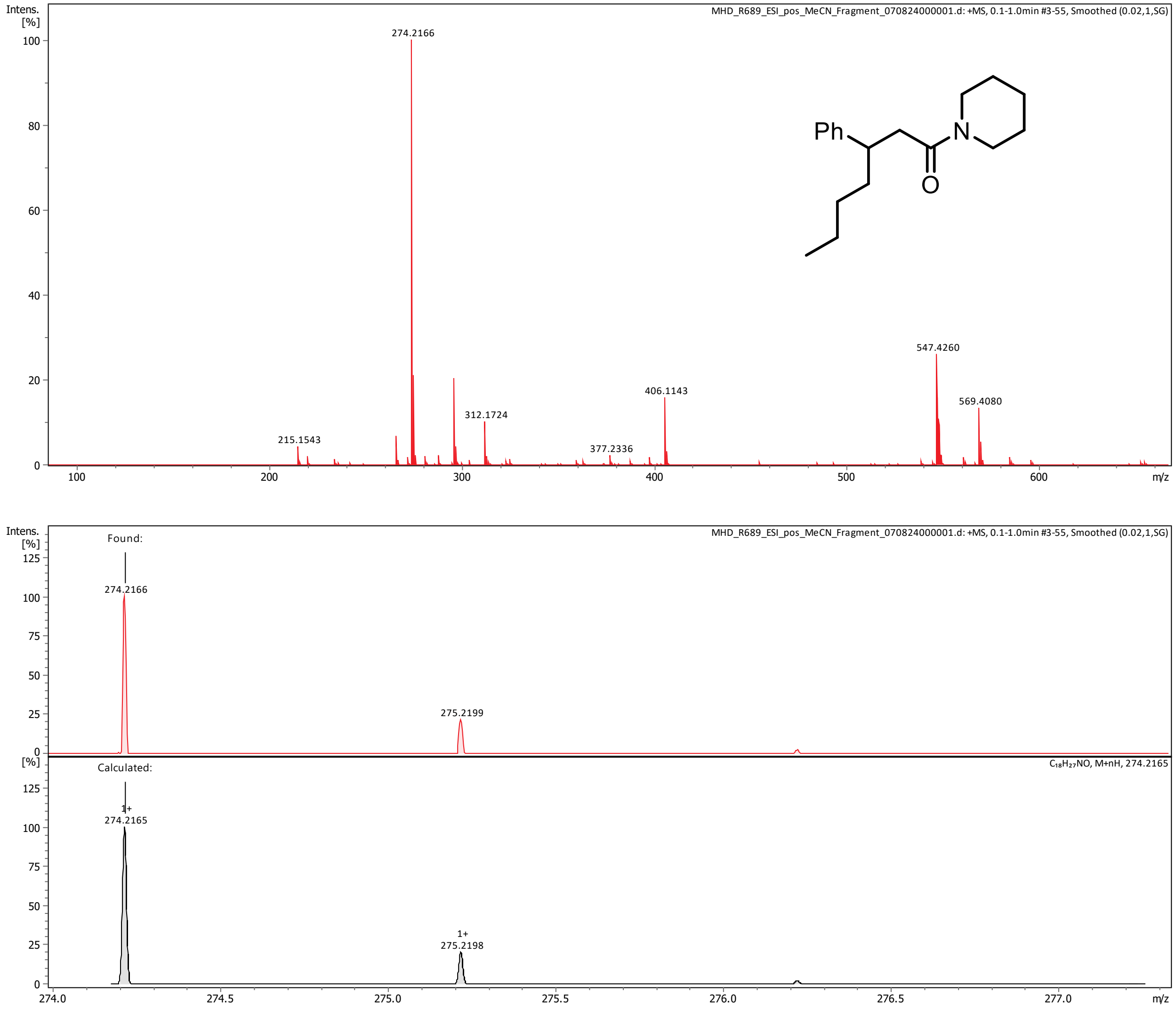

MHD_R689_ESI_pos_MeCN_Fragment_070824000001.d: +MS, 0.1-1.0min #3-55, Smoothed (0.02,1,SG)
Intens. [%]
274.2166
547.4260
569.4080
406.1143
312.1724
377.2336
215.1543
Ph
N
O
m/z
Found:
274.2166
275.2199
Calculated:
C18H27NO, M+nH, 274.2165
1+ 274.2165
1+ 275.2198

3.18.7 IR

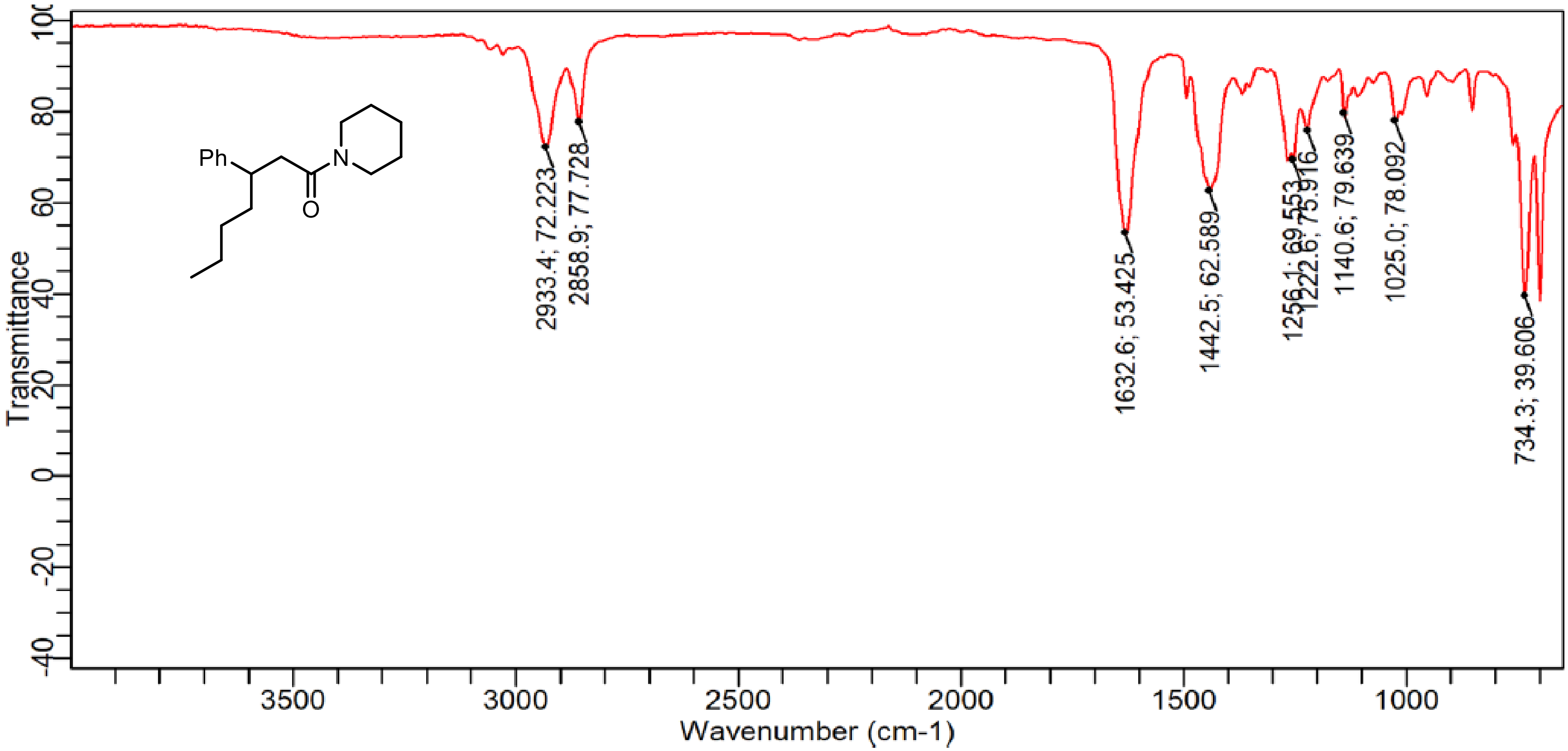

Ph
N
O
2933.4; 72.223
2858.9; 77.728
1632.6; 53.425
1442.5; 62.589
1256.1; 69.553
1222.6; 75.916
1140.6; 79.639
1025.0; 78.092
734.3; 39.606
Transmittance
Wavenumber (cm-1)

3.19 *N*,*N*-Diethyl-3-phenylbutanamide (12s) (600 MHz, $CDCl_3$)

3.19.1 ^{1}H NMR

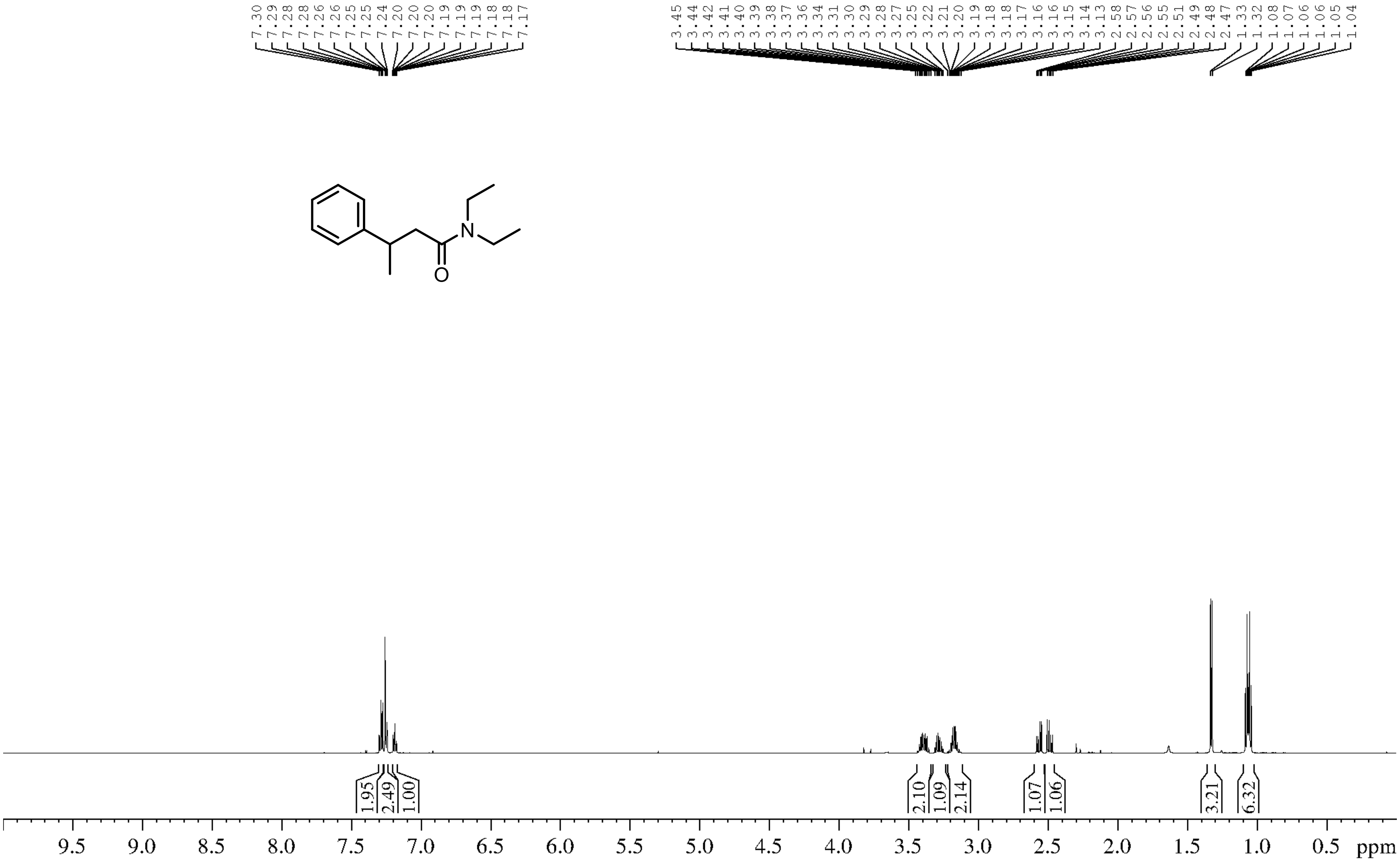

3.19.2 ^{13}C NMR

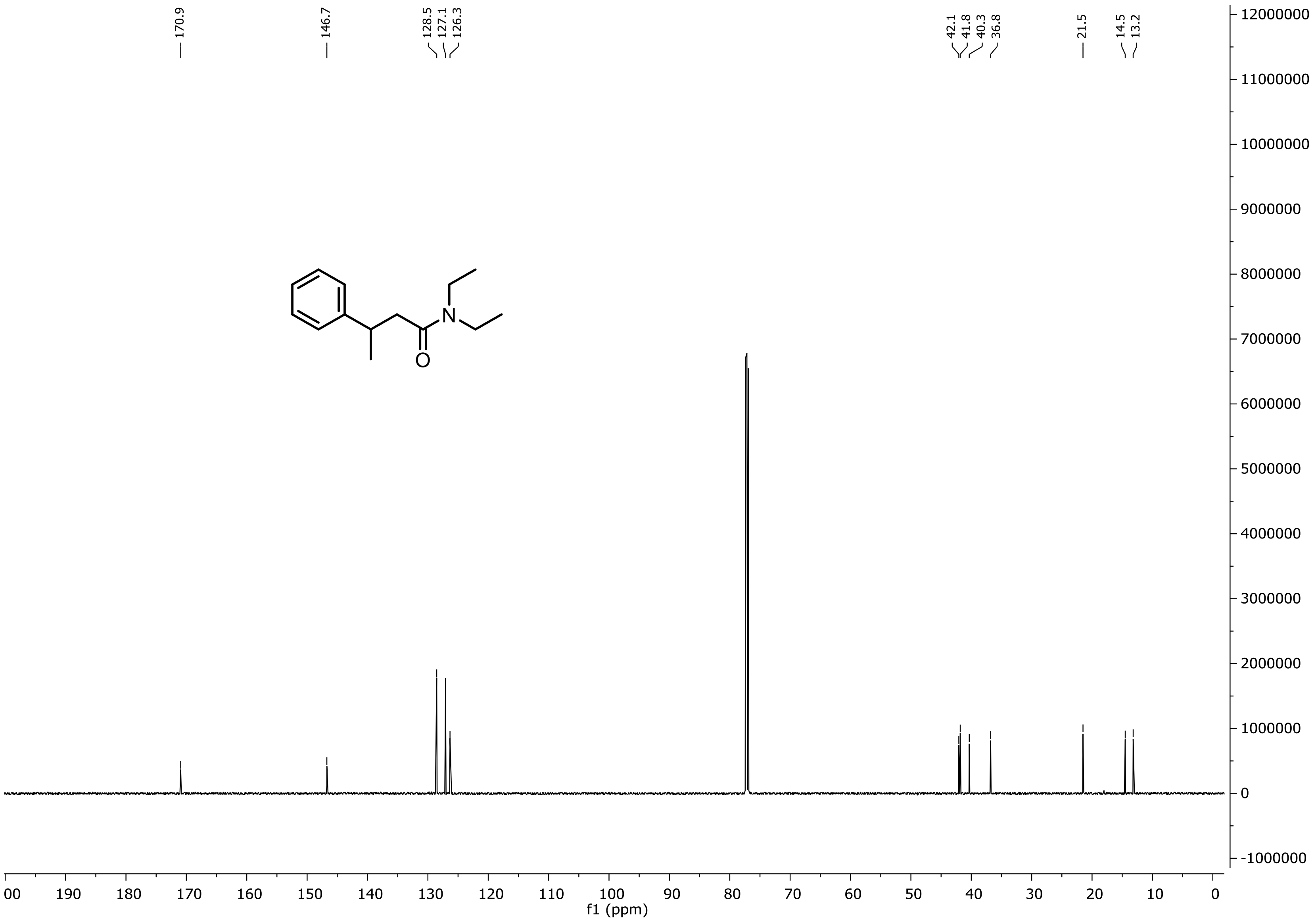

3.19.3 ^{1}H, ^{1}H COSY

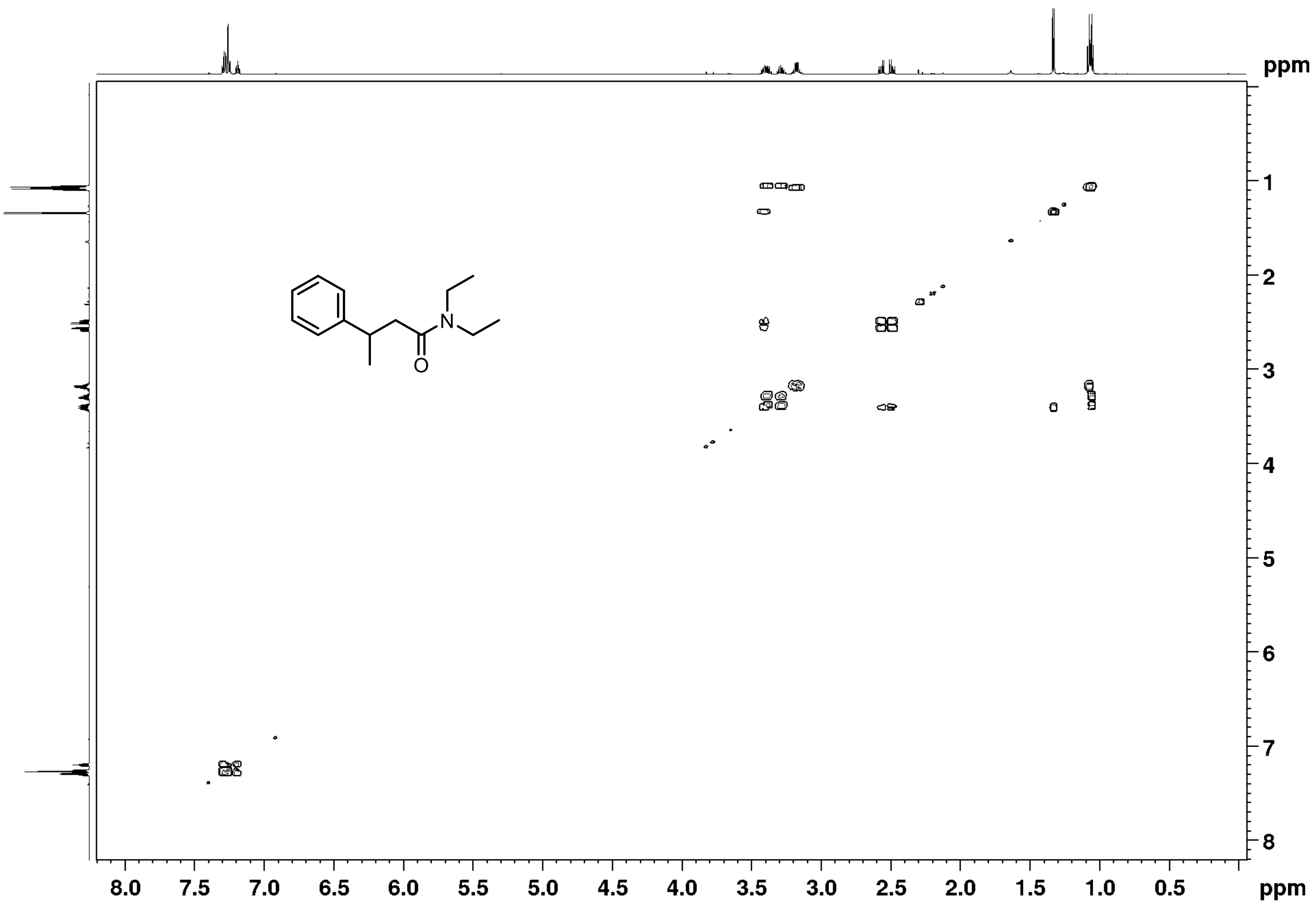

3.19.4 ^{1}H, ^{13}C HMBC

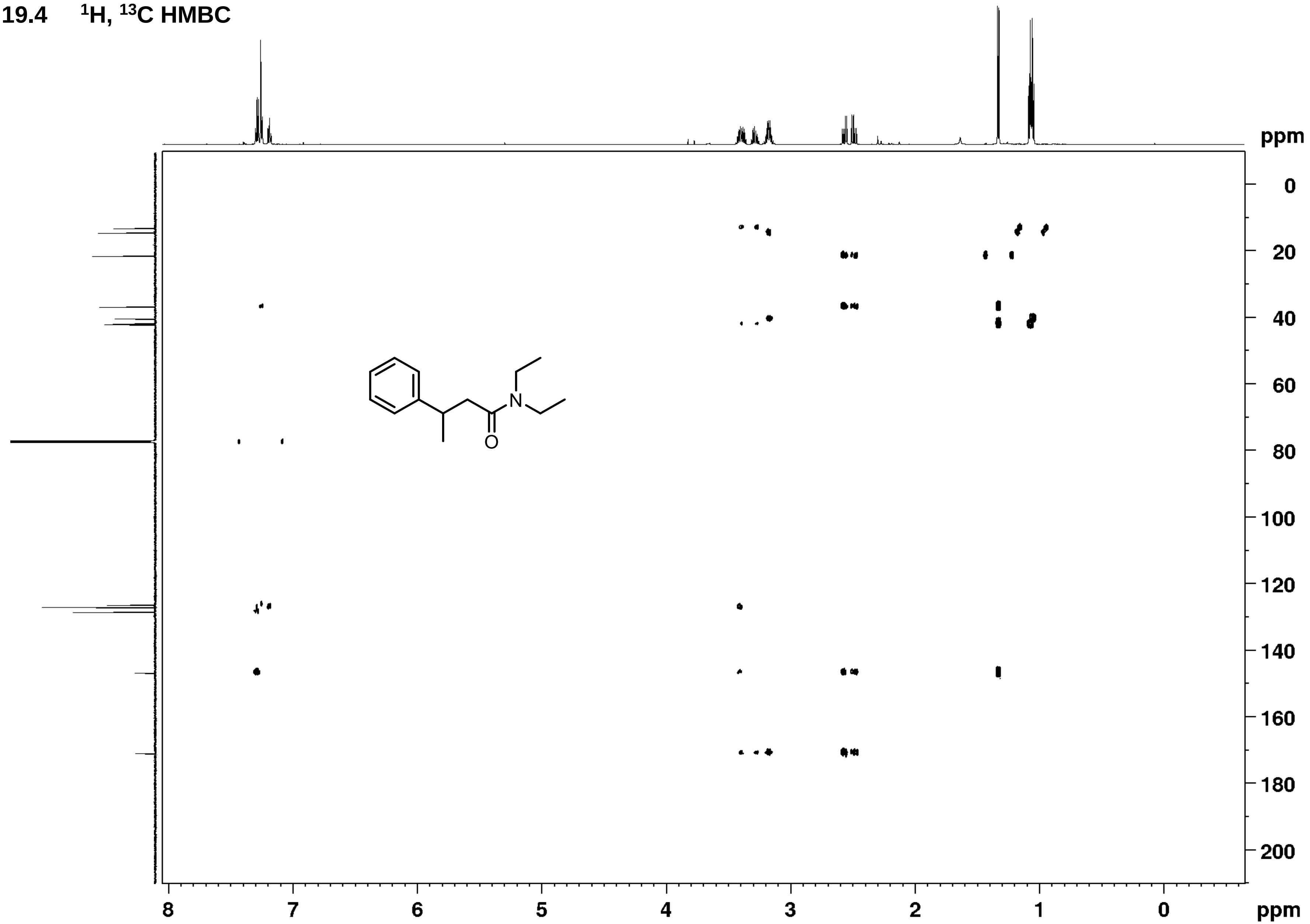

3.19.5 ^{1}H, ^{13}C HSQC

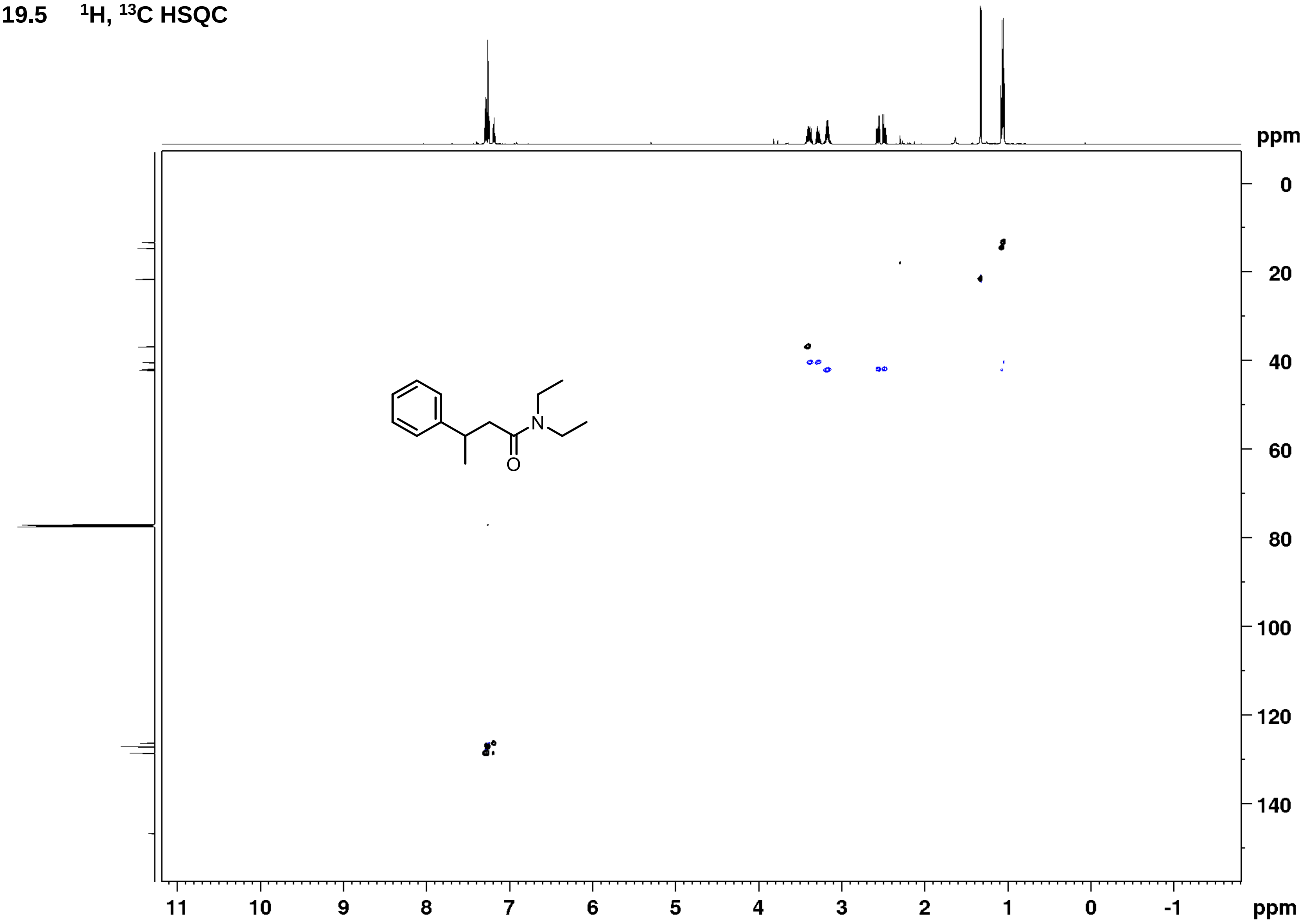

3.19.6 HRMS

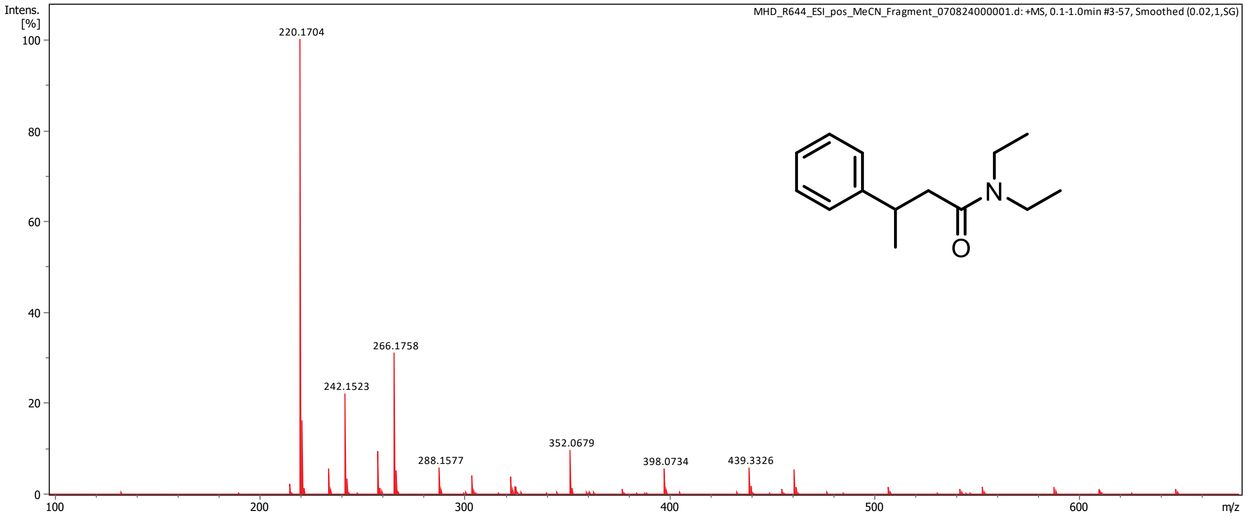

MHD_R644_ESI_pos_MeCN_Fragment_070824000001.d: +MS, 0.1-1.0min #3-57, Smoothed (0.02,1,SG)
Intens. [%]
220.1704
266.1758
242.1523
352.0679
288.1577
398.0734
439.3326
m/z
N
O

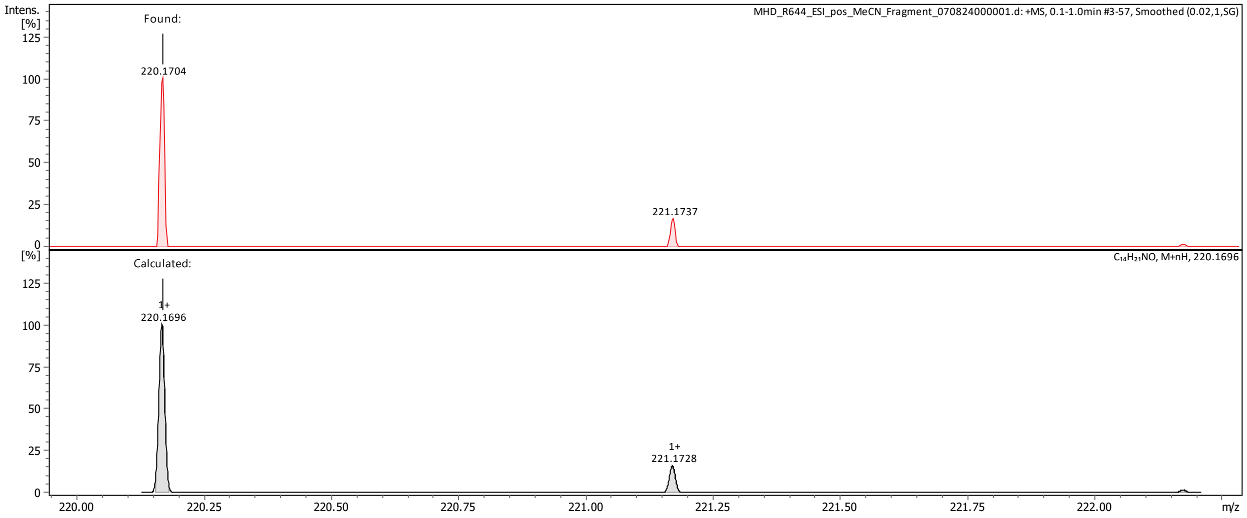

MHD_R644_ESI_pos_MeCN_Fragment_070824000001.d: +MS, 0.1-1.0min #3-57, Smoothed (0.02,1,SG)
Found:
220.1704
221.1737
Calculated:
C14H21NO, M+nH, 220.1696
1+
220.1696
1+
221.1728
m/z

3.19.7 IR

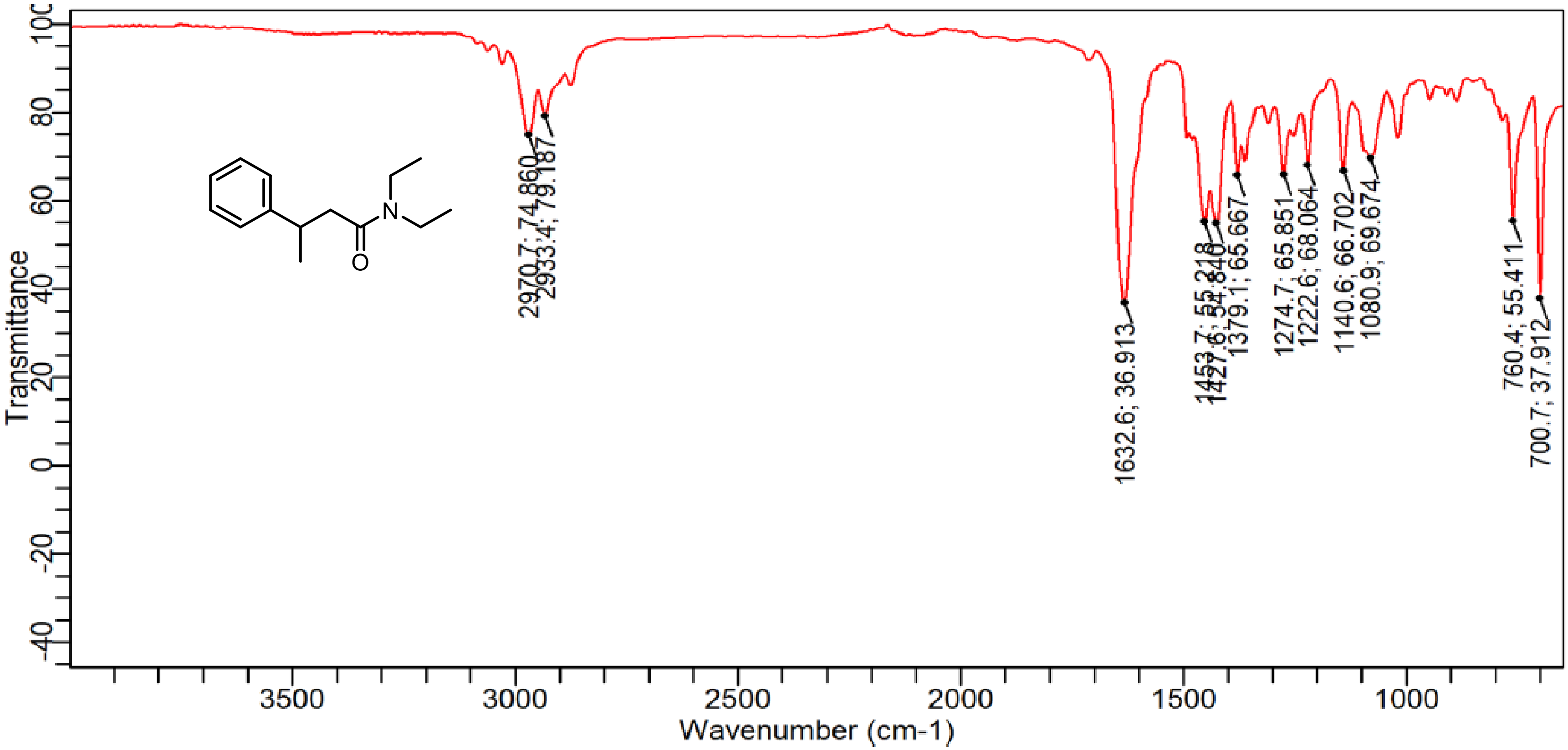

Transmittance
Wavenumber (cm-1)
2970.7; 74.860
2933.4; 79.187
1632.6; 36.913
1453.7; 55.218
1427.6; 54.840
1379.1; 65.667
1274.7; 65.851
1222.6; 68.064
1140.6; 66.702
1080.9; 69.674
760.4; 55.411
700.7; 37.912
N
O

3.20 3-Phenyl-1-(3-(trifluoromethyl)-5,6-dihydro-[1,2,4]triazolo[4,3-*a*]pyrazin-7(8*H*)-yl)butan-1-one (12t & 12t_rotamer) (600 MHz, $CDCl_3$)

3.20.1 ^{1}H NMR

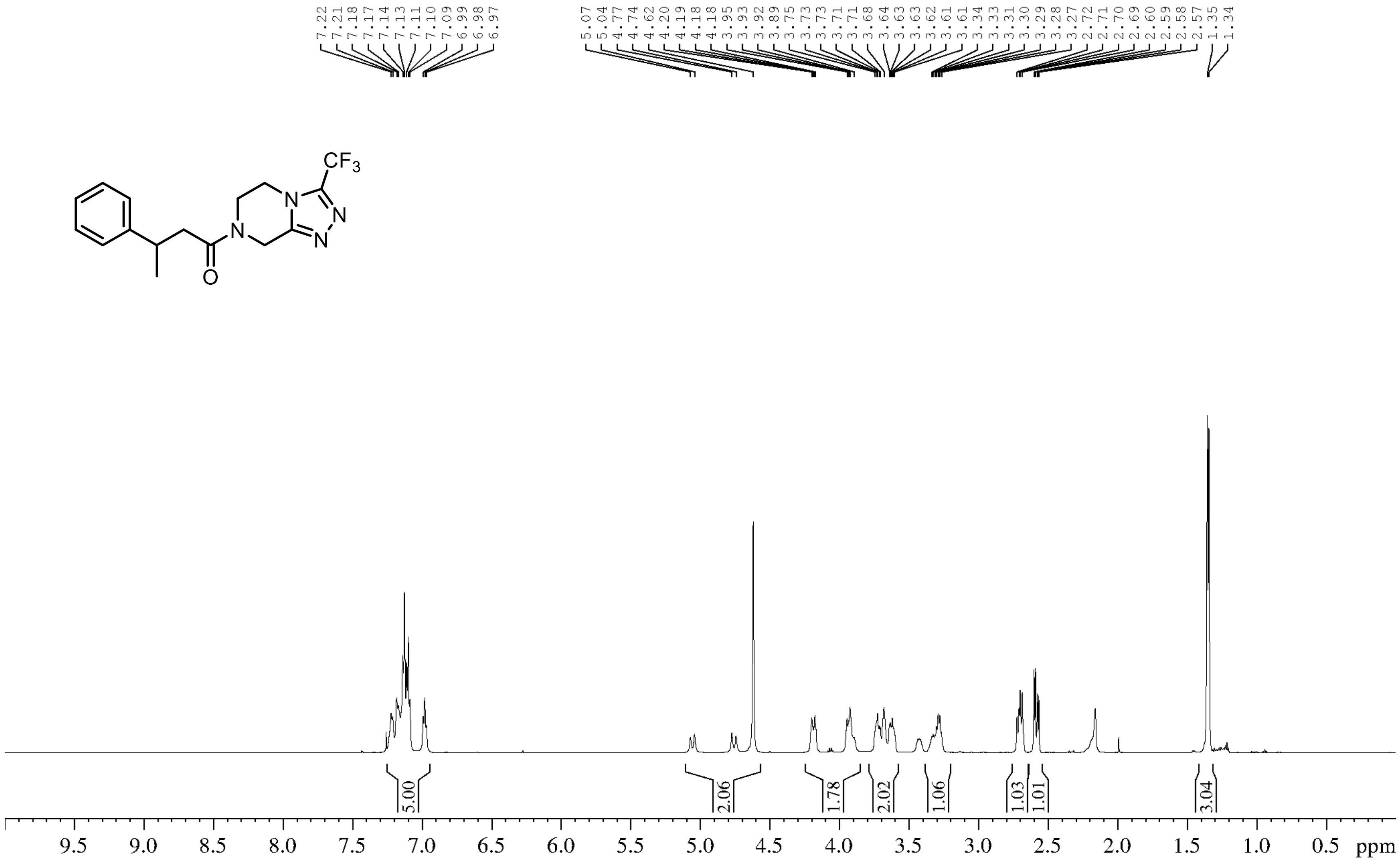

3.20.2 ^{13}C NMR

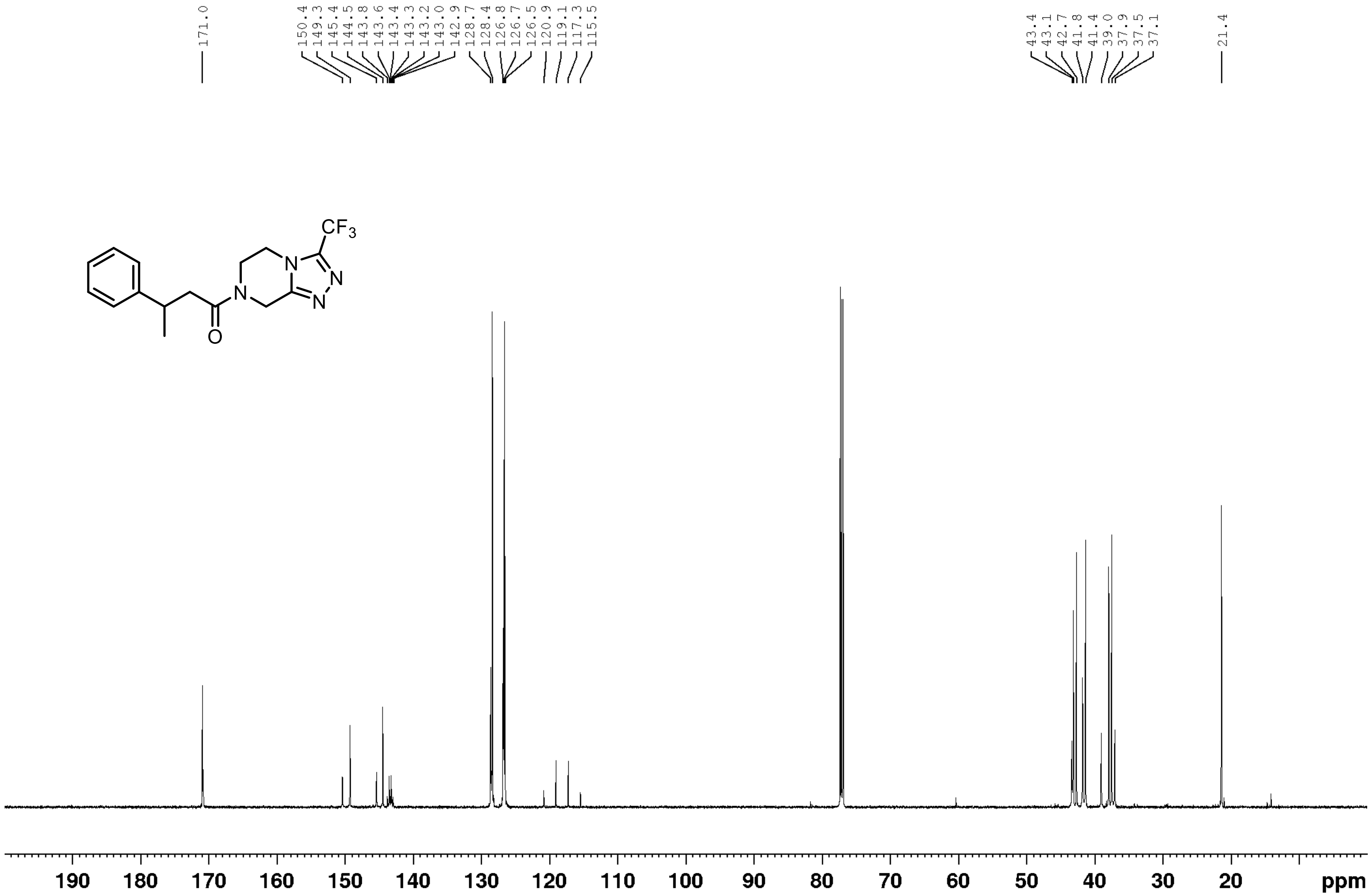

171.0
150.4
149.3
145.4
144.5
143.8
143.6
143.4
143.3
143.2
143.0
142.9
128.7
128.4
126.8
126.7
126.5
120.9
119.1
117.3
115.5
43.4
43.1
42.7
41.8
41.4
39.0
37.9
37.5
37.1
21.4
CF3
N
O
190
180
170
160
150
140
130
120
110
100
90
80
70
60
50
40
30
20
ppm

3.20.3 19F

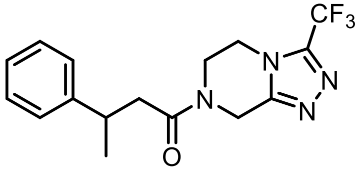

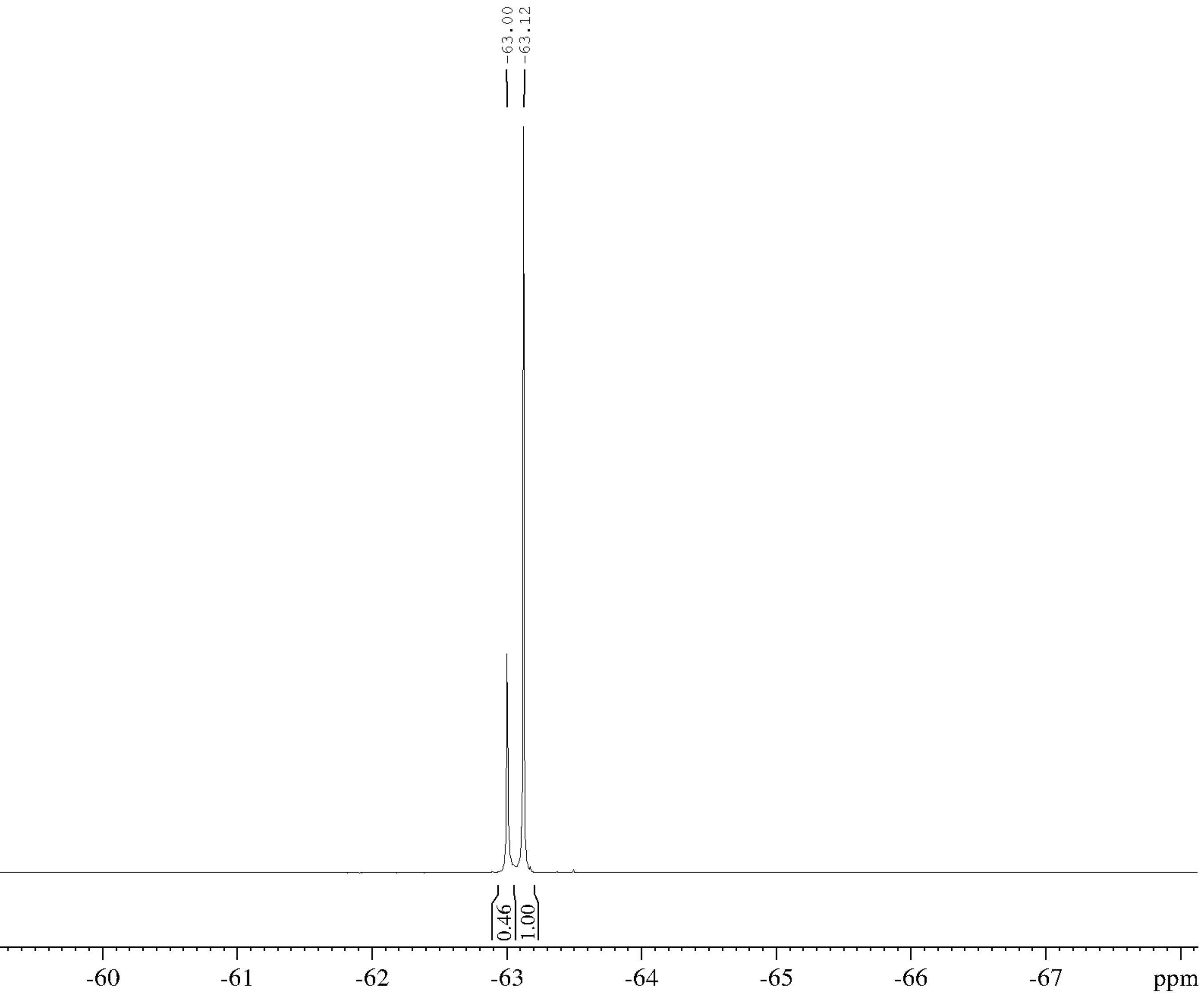

3.20.4 ^{1}H, ^{1}H COSY

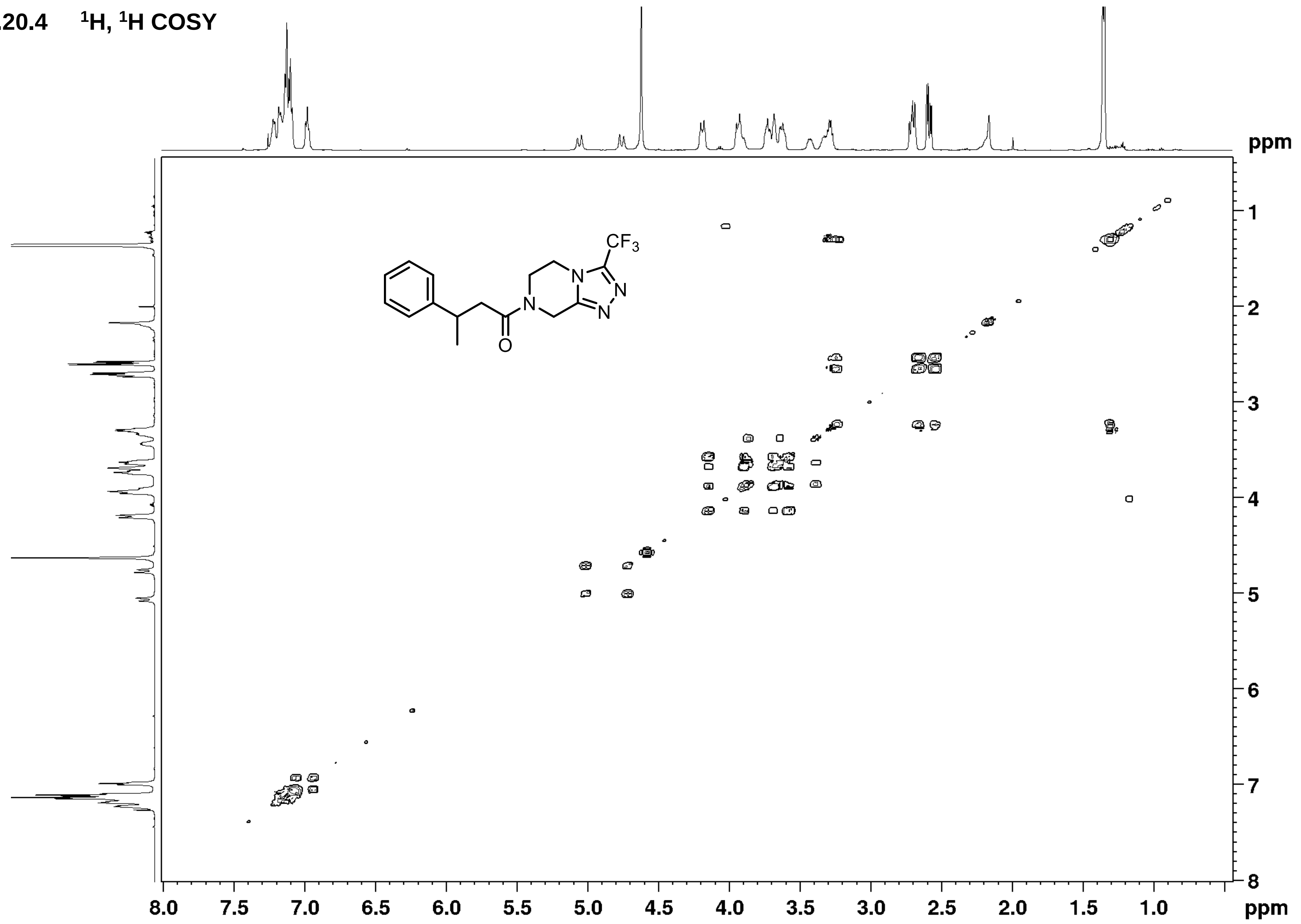

3.20.5 ^{1}H, ^{13}C HMBC

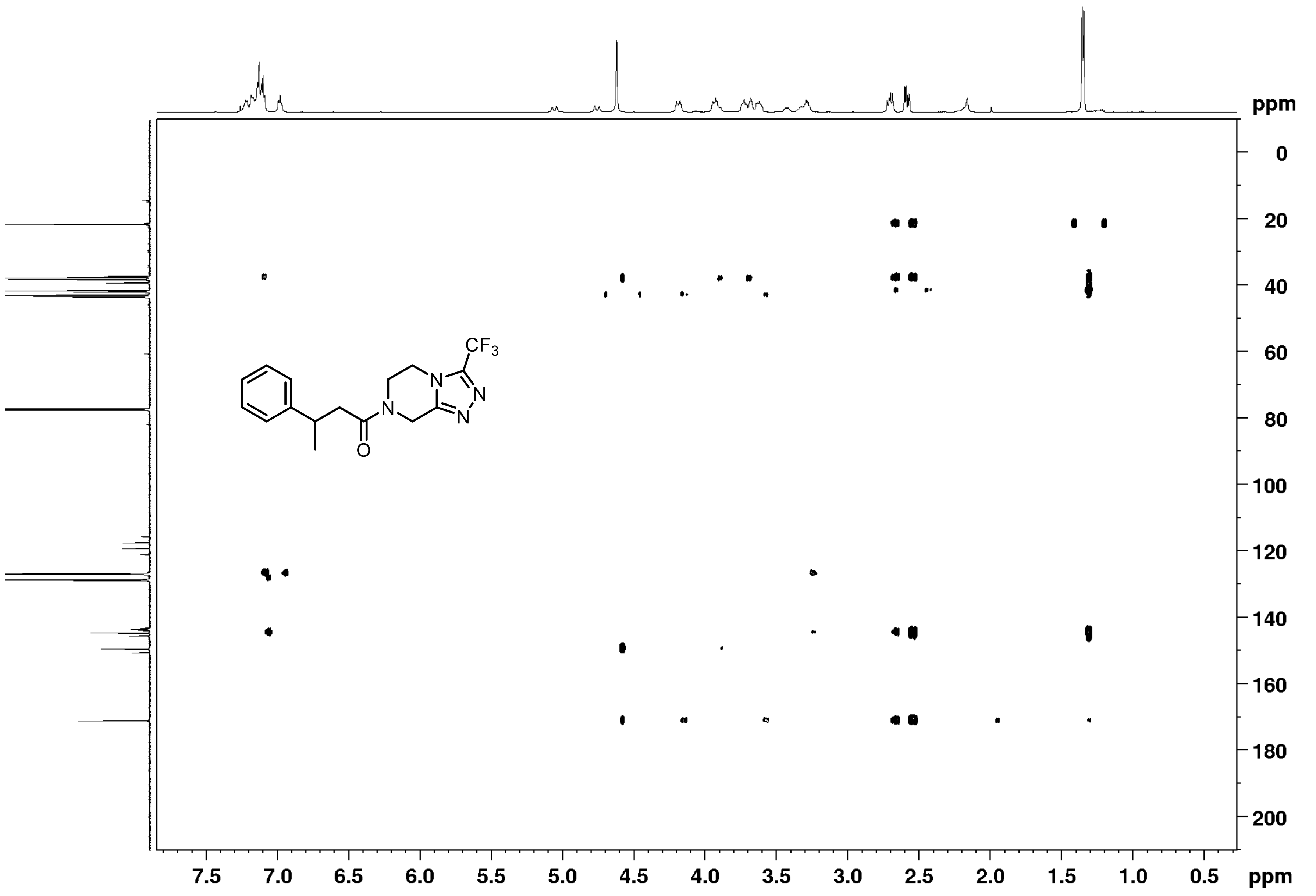

3.20.6 ^{1}H, ^{13}C HSQC

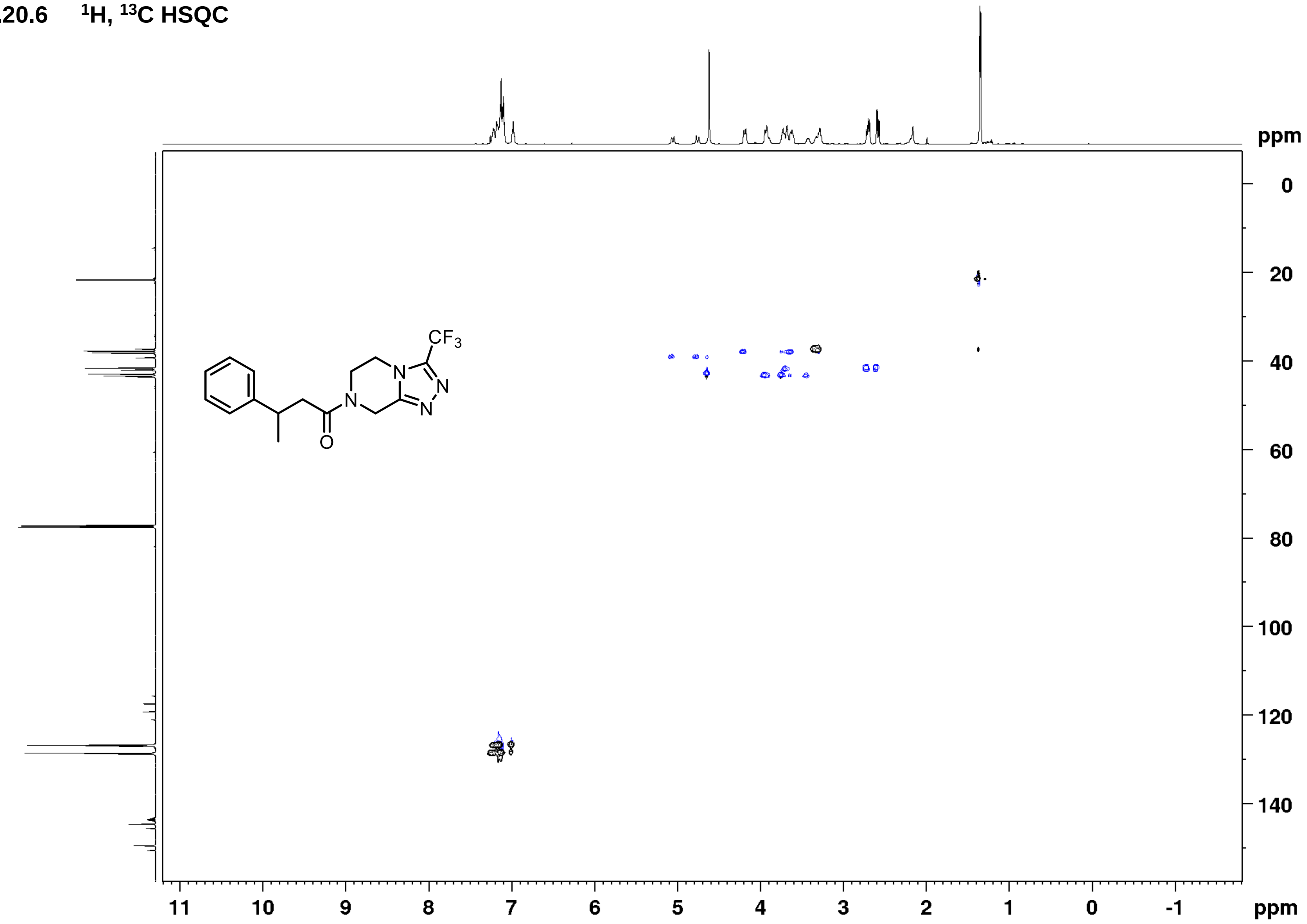

3.20.7 HRMS

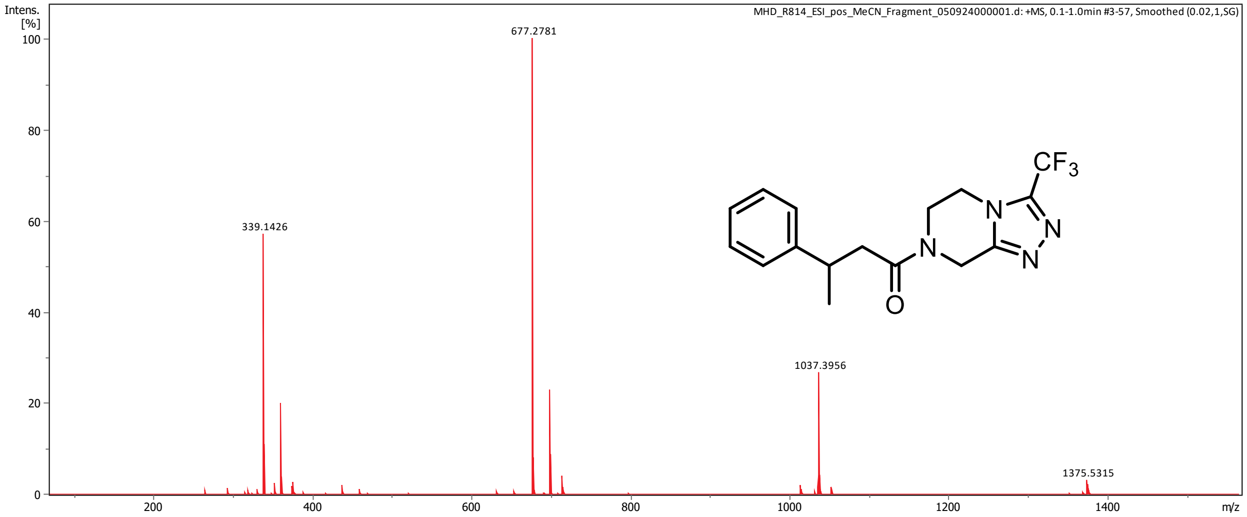
MHD_R814_ESI_pos_MeCN_Fragment_050924000001.d: +MS, 0.1-1.0min #3-57, Smoothed (0.02,1,SG)
Intens. [%]
677.2781
339.1426
1037.3956
1375.5315
m/z
CF3
N
O

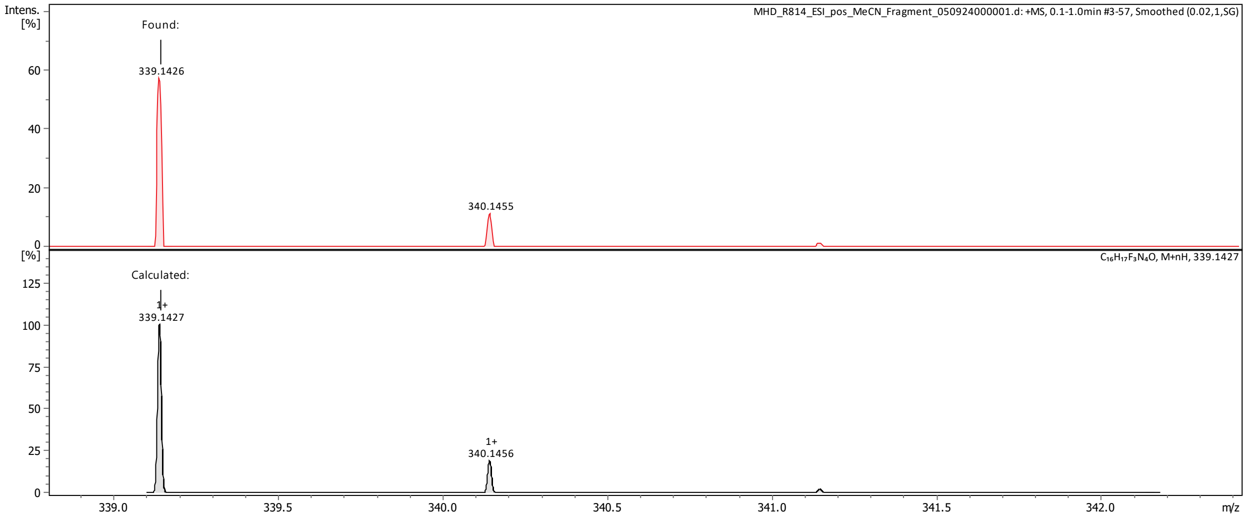
MHD_R814_ESI_pos_MeCN_Fragment_050924000001.d: +MS, 0.1-1.0min #3-57, Smoothed (0.02,1,SG)
Intens. [%]
Found:
339.1426
340.1455
C16H17F3N4O, M+nH, 339.1427
Calculated:
1+
339.1427
1+
340.1456
m/z

3.20.8 IR

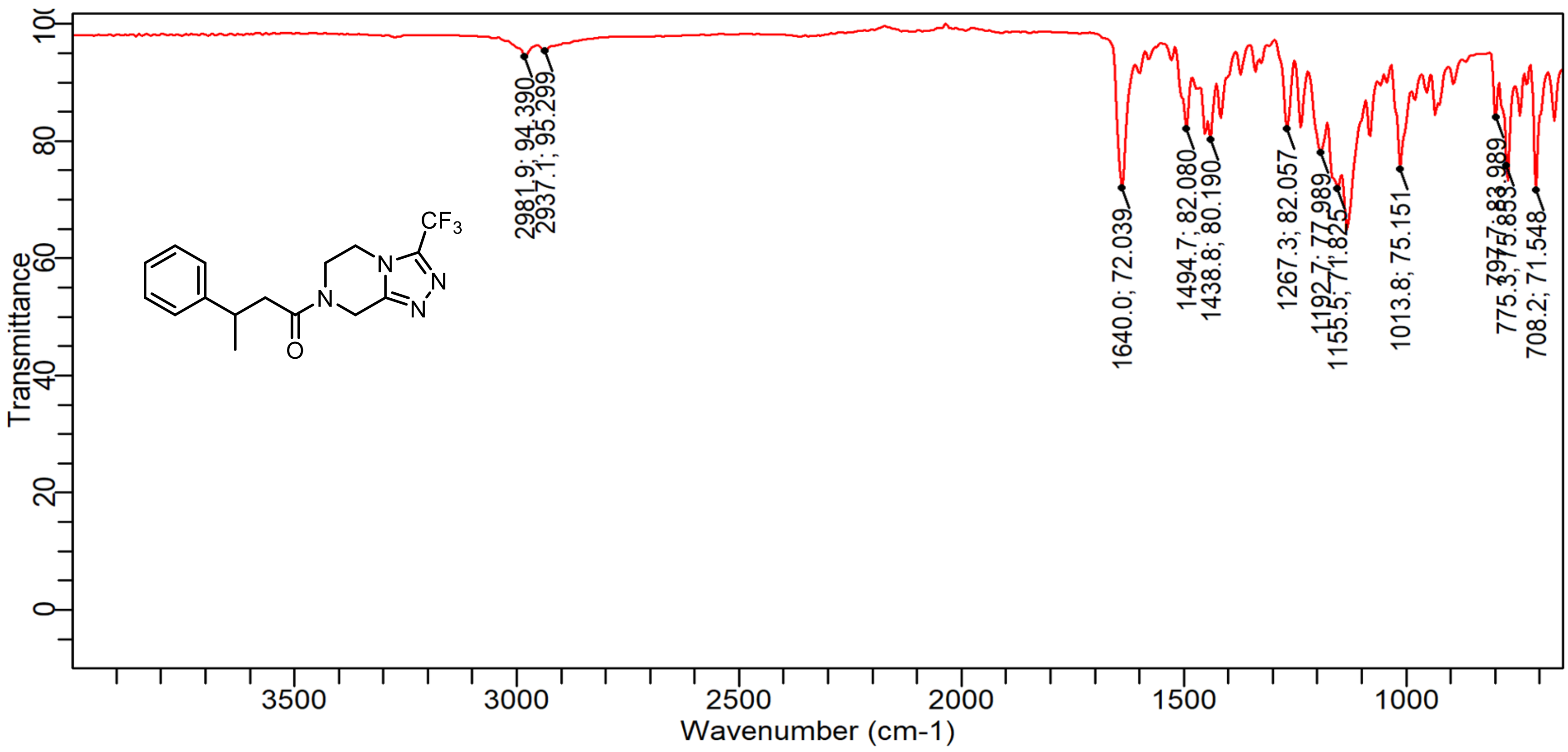

Transmittance
Wavenumber (cm-1)
2981.9; 94.390
2937.1; 95.299
1640.0; 72.039
1494.7; 82.080
1438.8; 80.190
1267.3; 82.057
1192.7; 77.989
1155.5; 71.825
1013.8; 75.151
797.7; 83.989
775.3; 75.853
708.2; 71.548
CF3
N
O

3.21 3 Ethyl 4-(3-phenylbutanoyl)-3,4-dihydro-2*H*-benzo[*b*][1,4]oxazine-2-carboxylate (12u & 12u_rotamer) (600 MHz, $CDCl_3$)

3.21.1 ^{1}H NMR

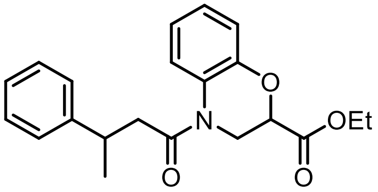

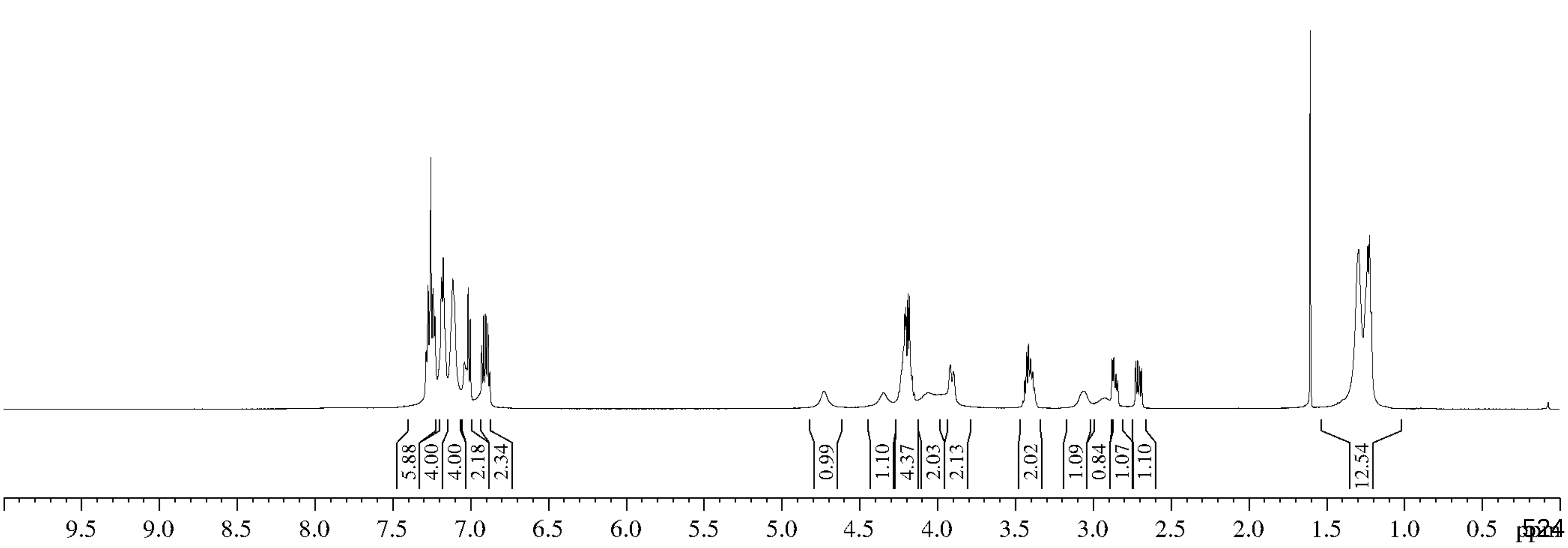

3.21.2 ^{13}C NMR

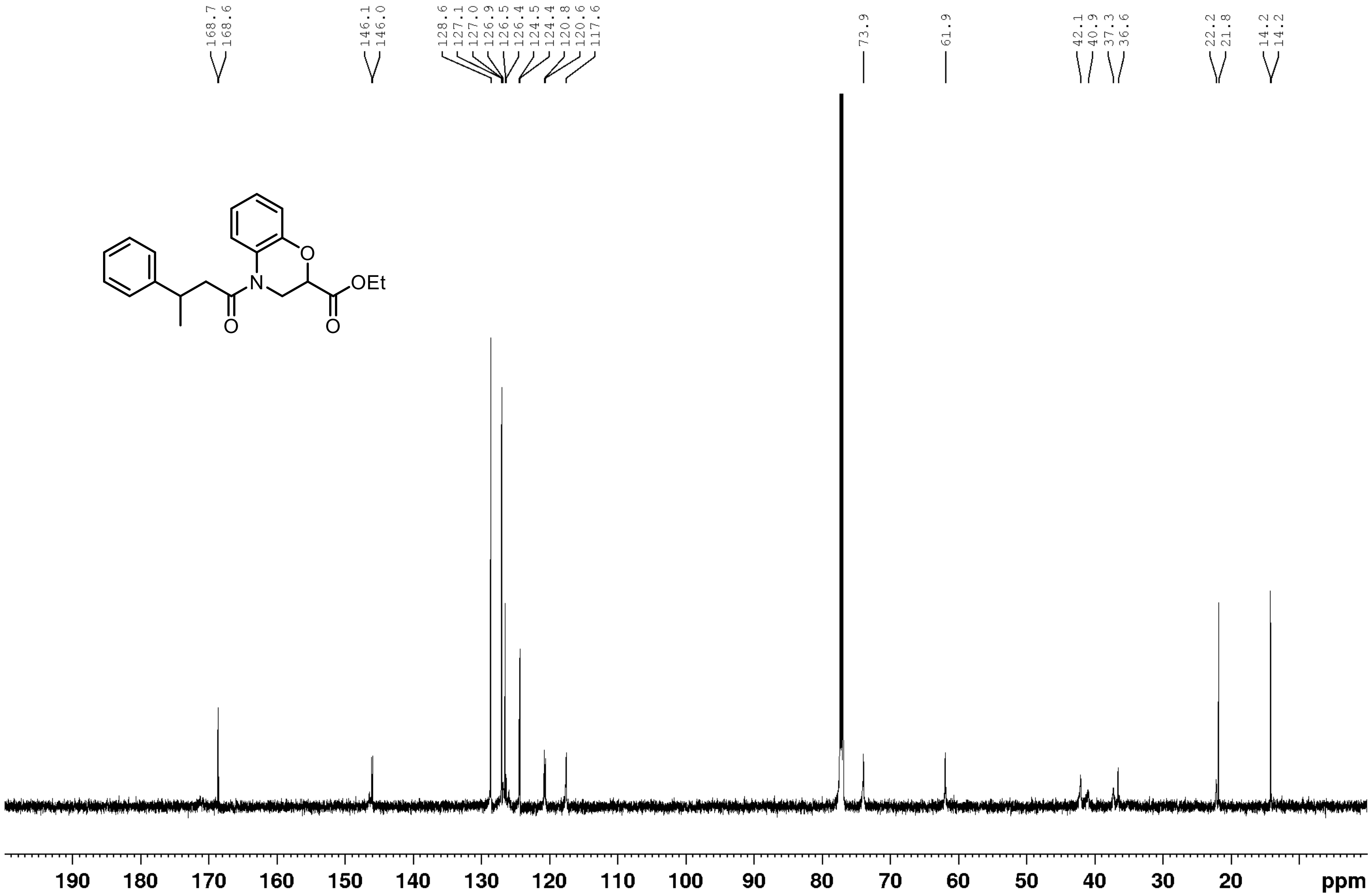
168.7
168.6
146.1
146.0
128.6
127.1
127.0
126.9
126.5
126.4
124.5
124.4
120.8
120.6
117.6
73.9
61.9
42.1
40.9
37.3
36.6
22.2
21.8
14.2
14.2
O
N
OEt
O
O
190
180
170
160
150
140
130
120
110
100
90
80
70
60
50
40
30
20
ppm

3.21.3 ^{1}H, ^{1}H COSY

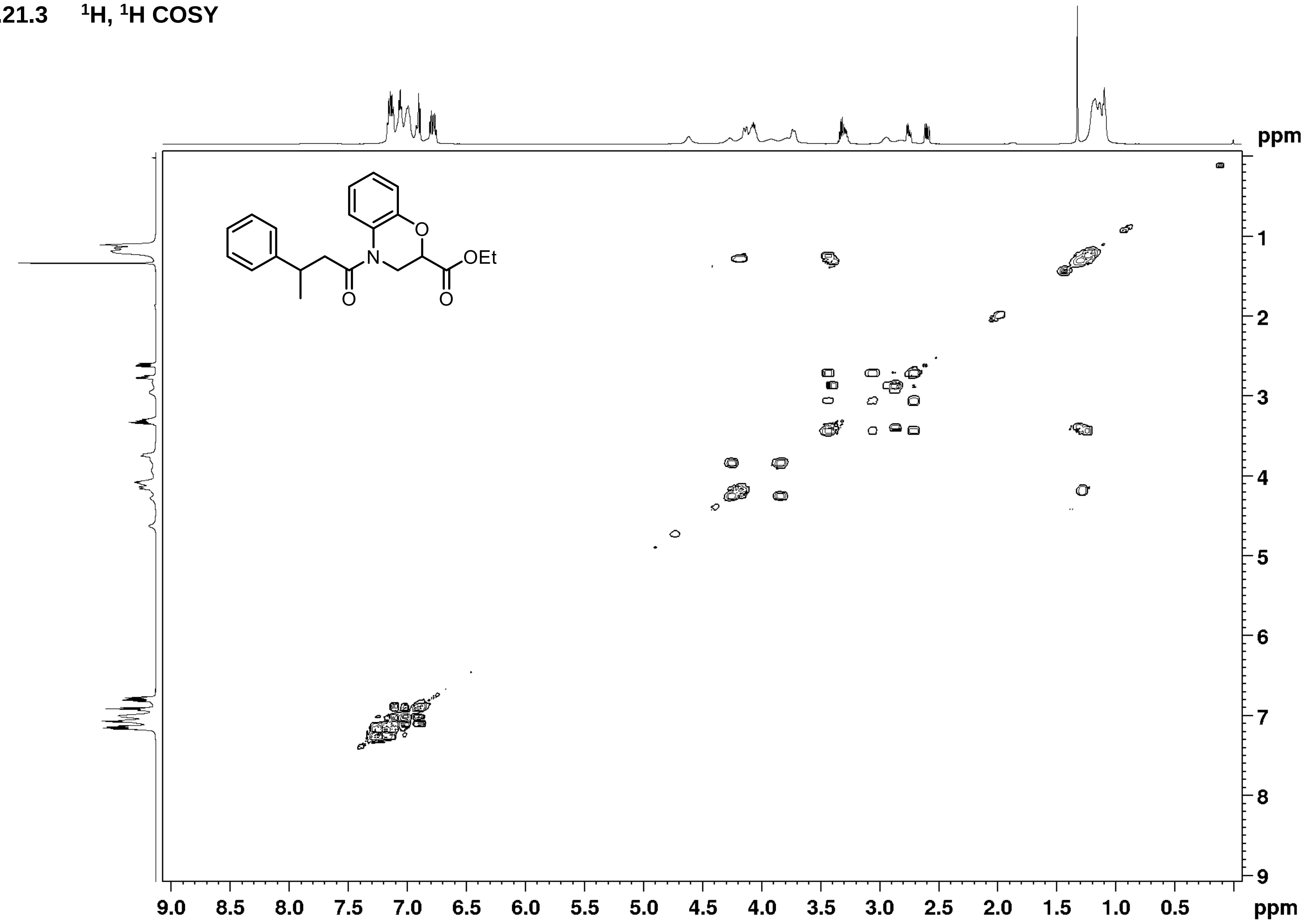

3.21.4 ^{1}H, ^{13}C HMBC

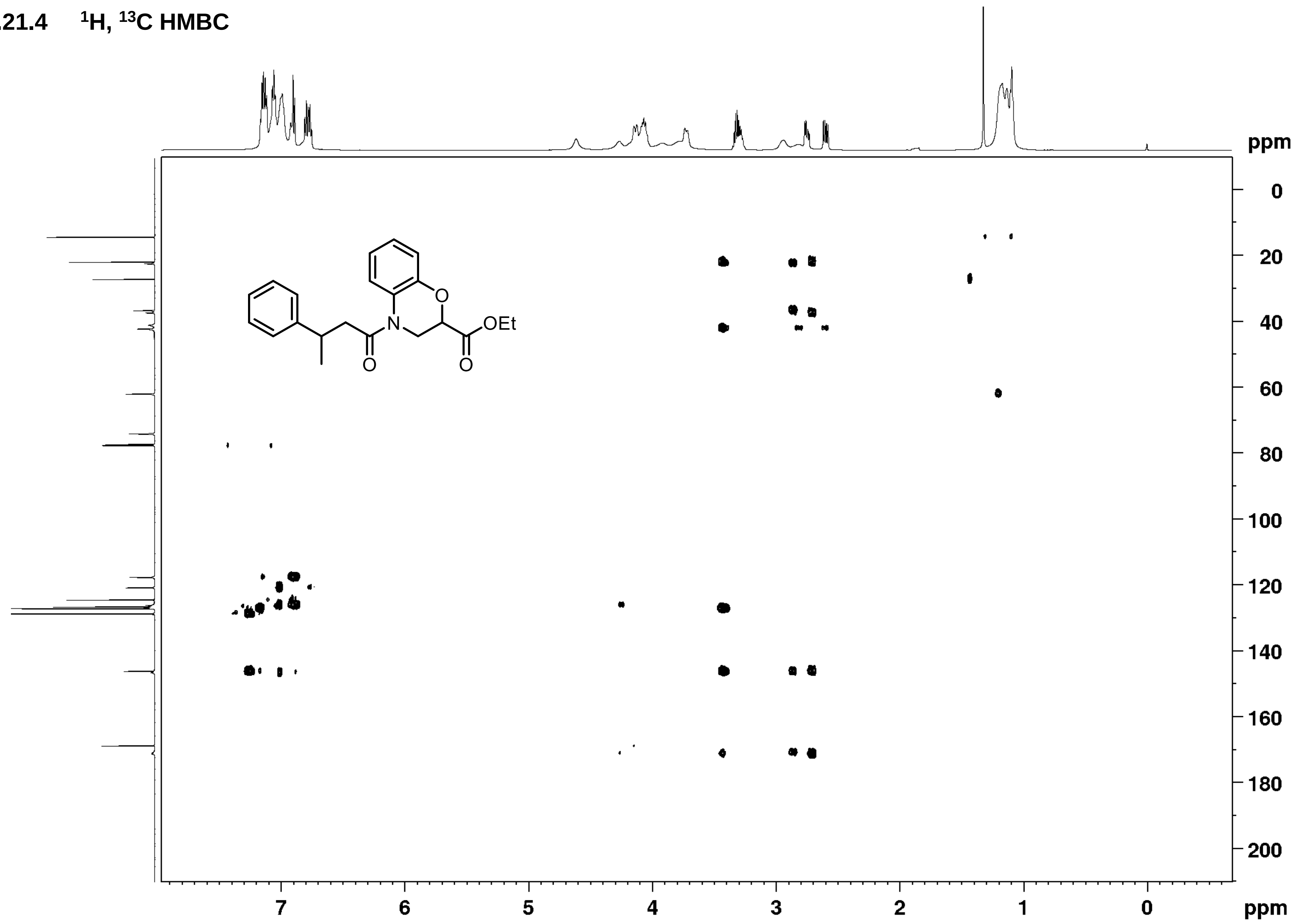

3.21.5 ^{1}H, ^{13}C HSQC

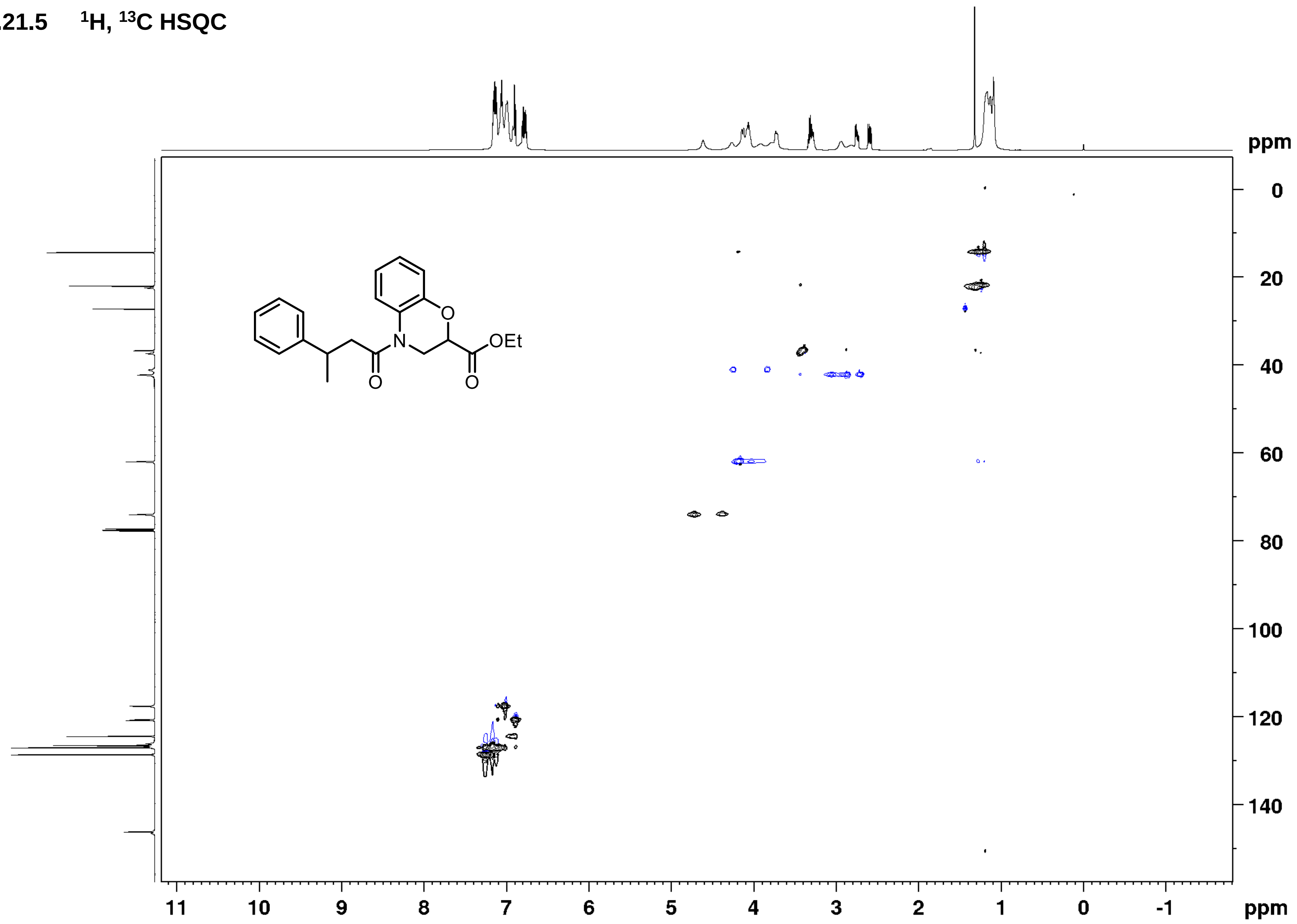

3.21.6 HRMS

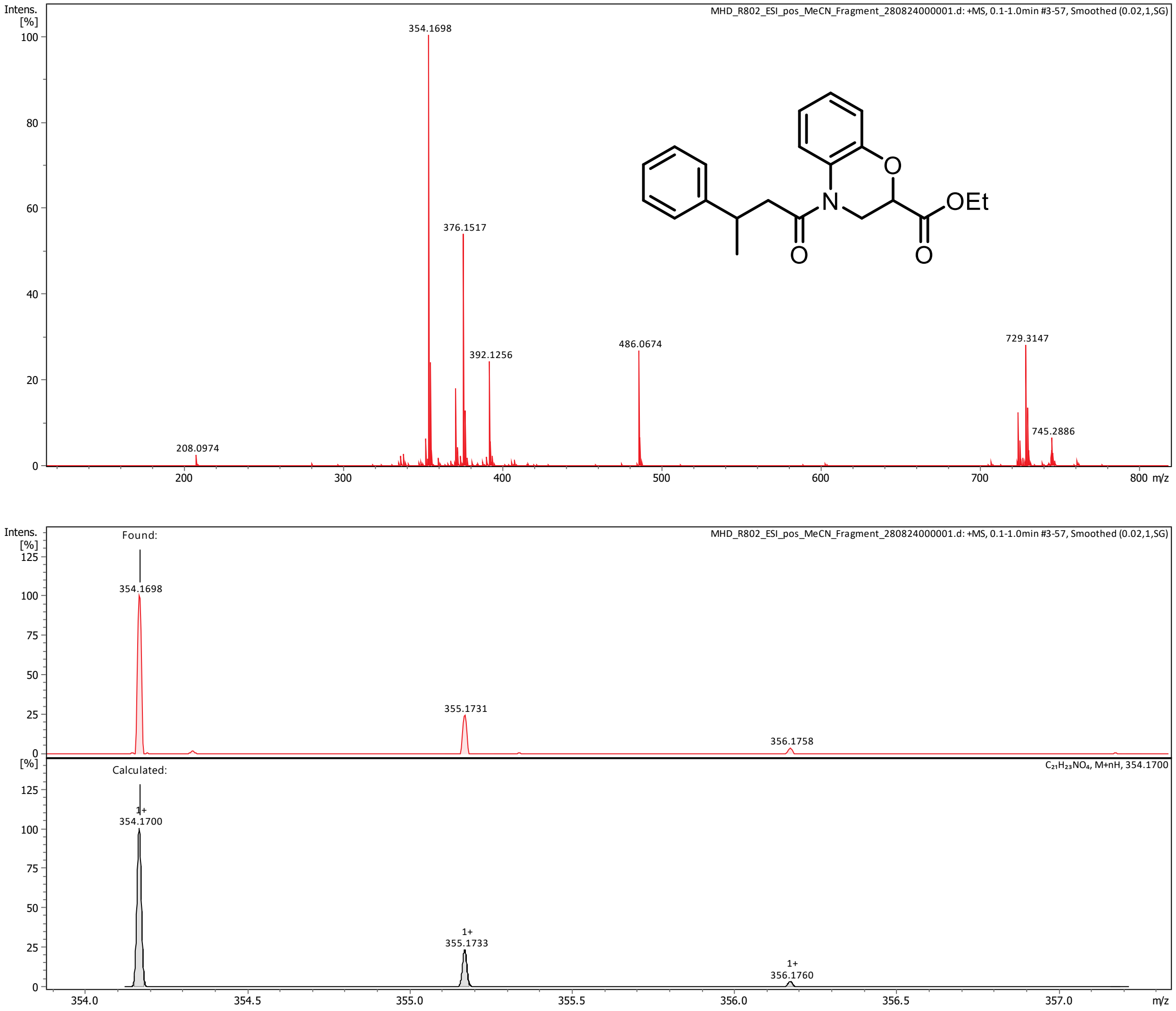

MHD_R802_ESI_pos_MeCN_Fragment_280824000001.d: +MS, 0.1-1.0min #3-57, Smoothed (0.02,1,SG)
Intens. [%]
354.1698
376.1517
392.1256
486.0674
729.3147
745.2886
208.0974
m/z
OEt
N
O
Found:
354.1698
355.1731
356.1758
Calculated:
$C_{21}H_{23}NO_4$, M+nH, 354.1700
1+ 354.1700
1+ 355.1733
1+ 356.1760

3.21.7 IR

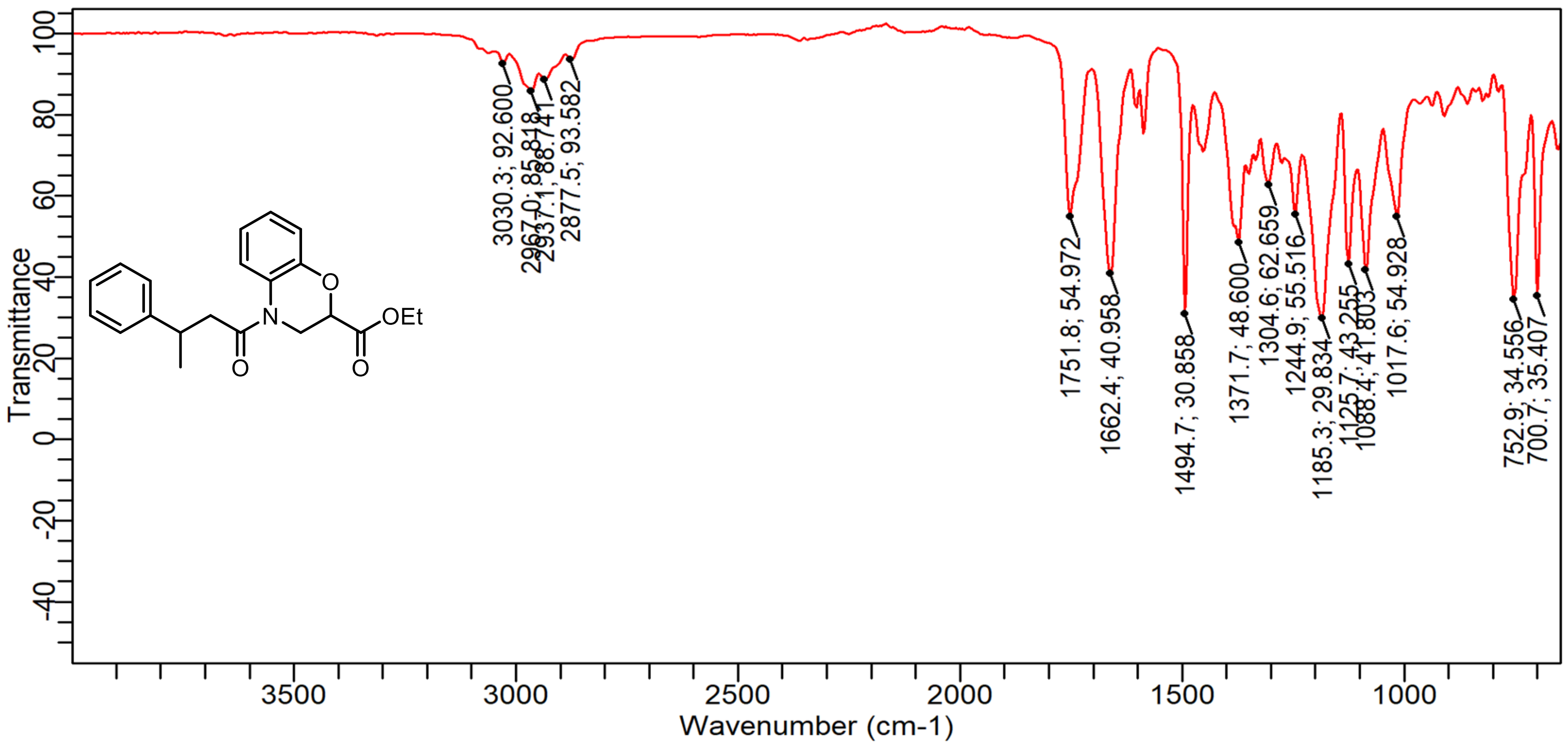

3030.3; 92.600
2967.0; 85.818
2937.1; 88.741
2877.5; 93.582
1751.8; 54.972
1662.4; 40.958
1494.7; 30.858
1371.7; 48.600
1304.6; 62.659
1244.9; 55.516
1185.3; 29.834
1125.7; 43.255
1088.4; 41.803
1017.6; 54.928
752.9; 34.556
700.7; 35.407
Transmittance
Wavenumber (cm-1)
OEt
O
N

3.22 *N*,*N*-Diethyl-1-(3-phenylbutanoyl)piperidine-3-carboxamide (12v & 12v_rotamer) (600 MHz, $CDCl_3$)

3.22.1 ^{1}H NMR

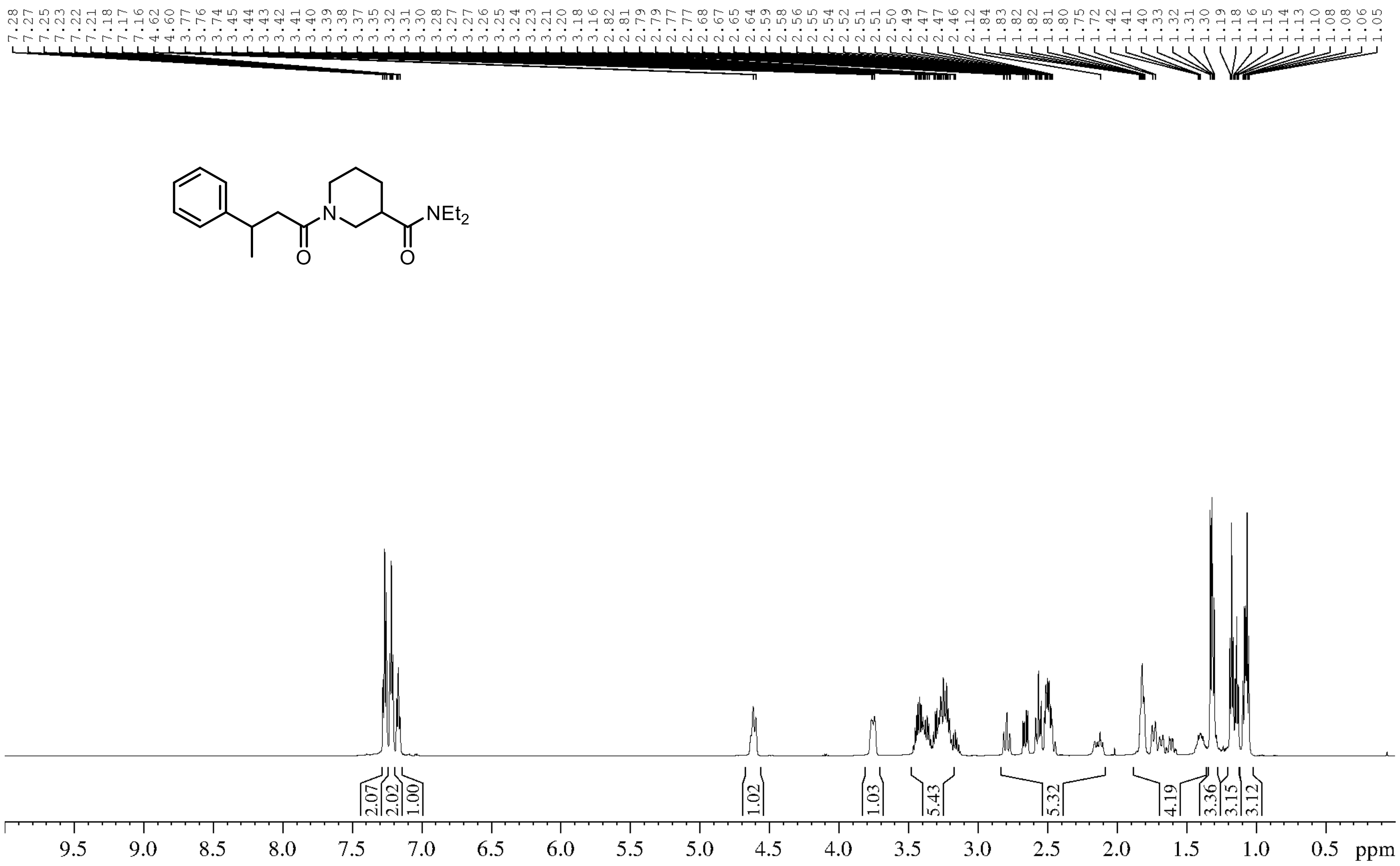

3.22.2 ^{13}C NMR

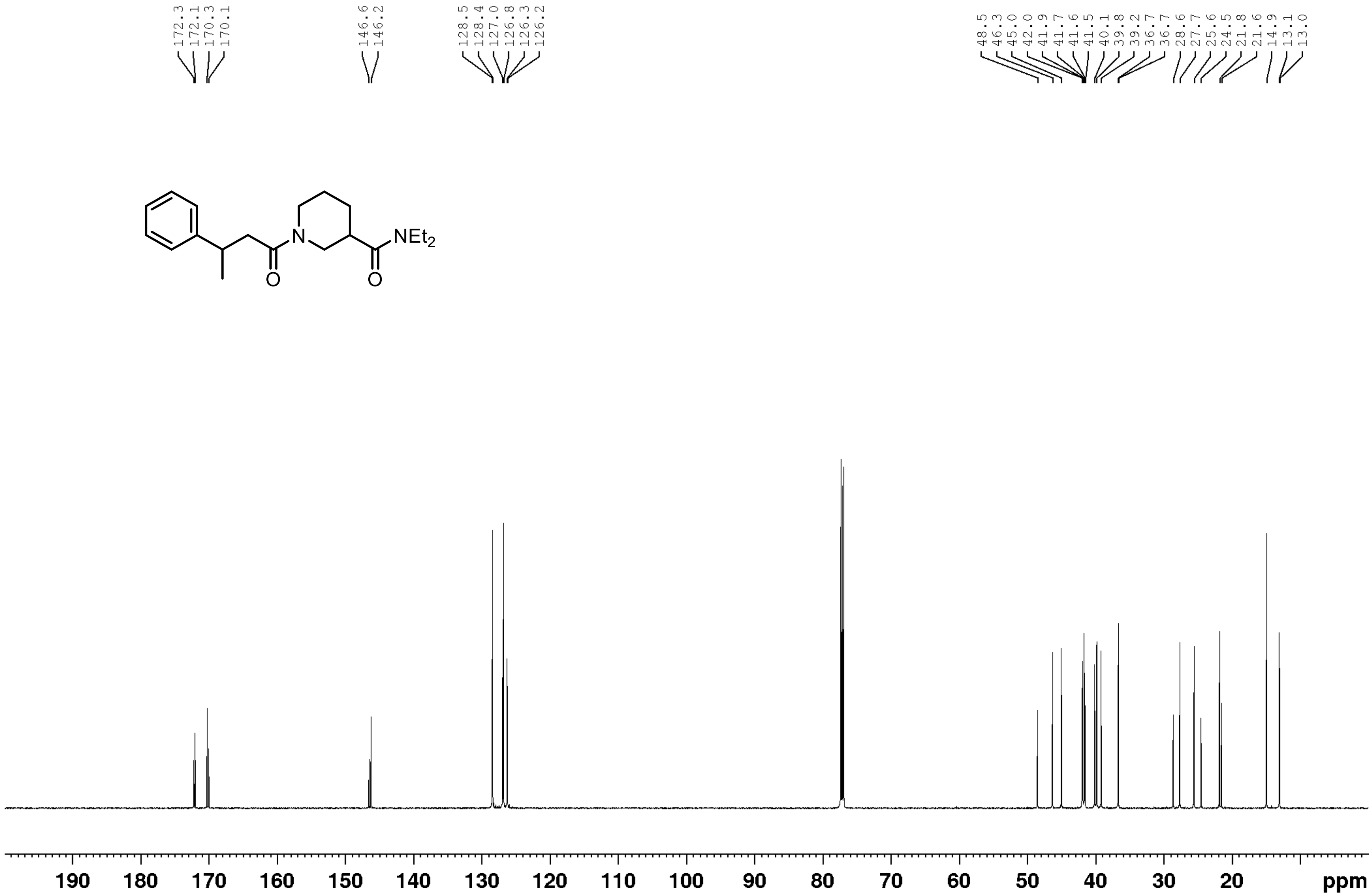
N
NEt2
O
O
172.3
172.1
170.3
170.1
146.6
146.2
128.5
128.4
127.0
126.8
126.3
126.2
48.5
46.3
45.0
42.0
41.9
41.7
41.6
41.5
40.1
39.8
39.2
36.7
36.7
28.6
27.7
25.6
24.5
21.8
21.6
14.9
13.1
13.0
190
180
170
160
150
140
130
120
110
100
90
80
70
60
50
40
30
20
ppm

3.22.3 ^{1}H, ^{1}H COSY

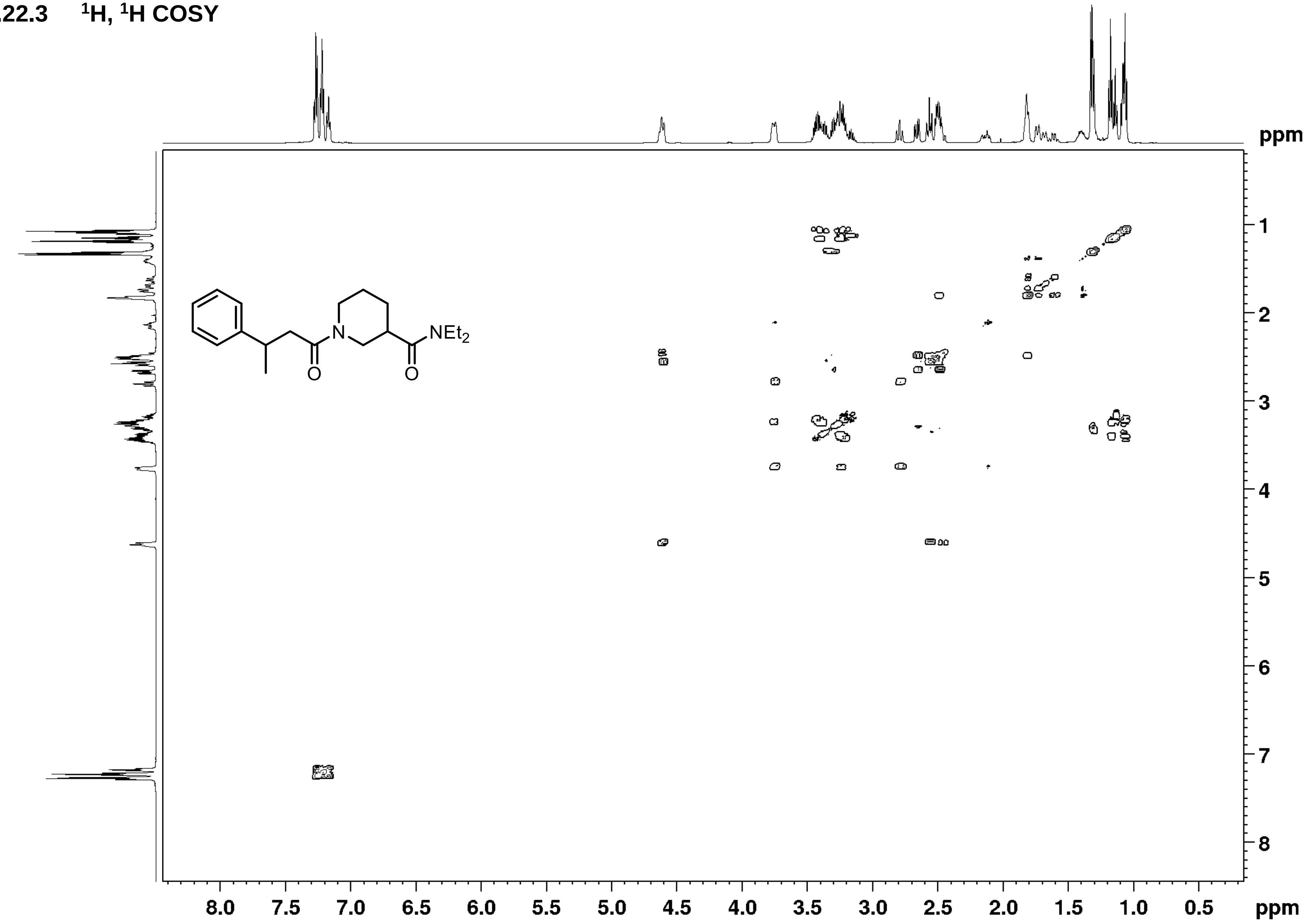

3.22.4 ^{1}H, ^{13}C HMBC

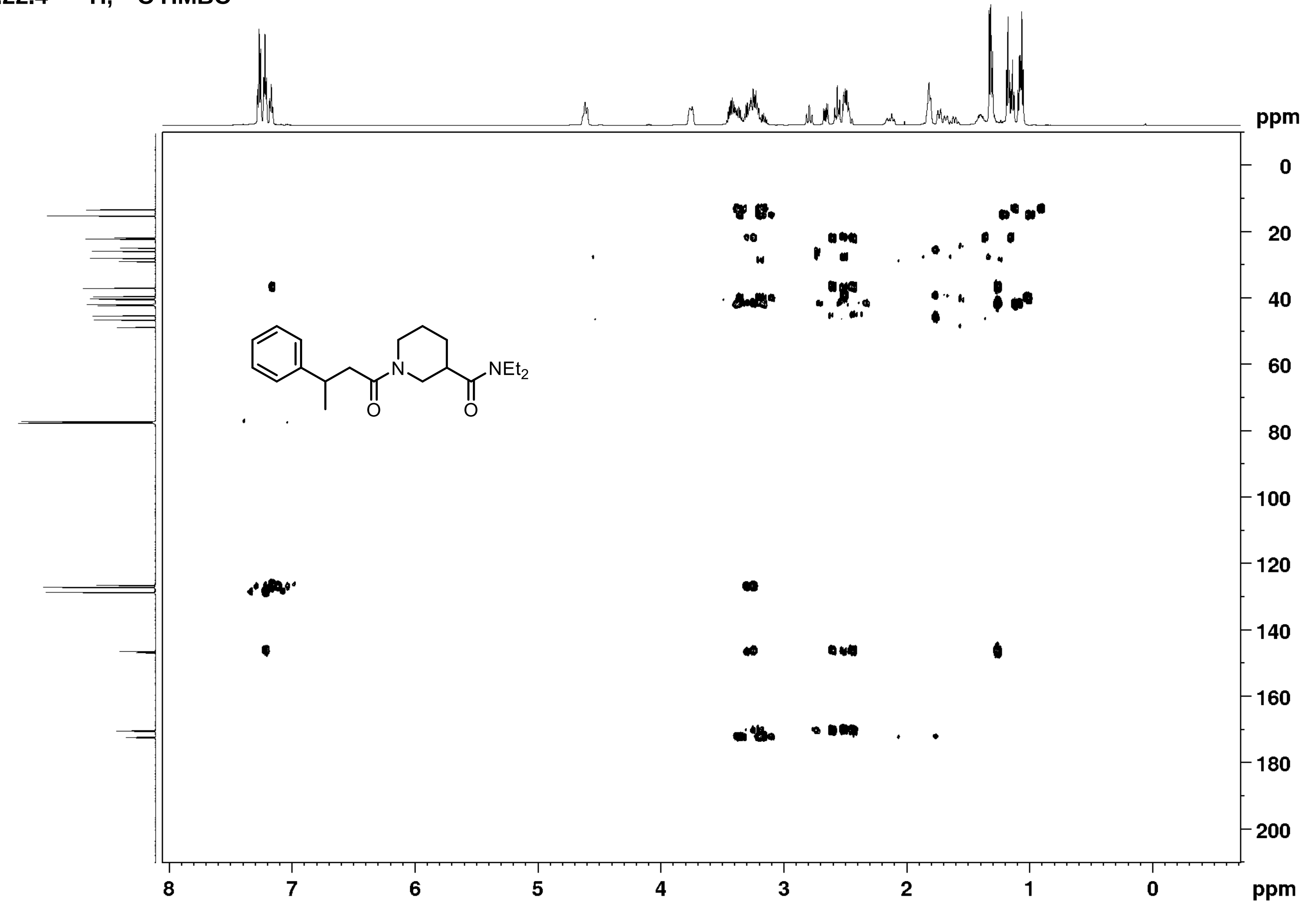

3.22.5 ^{1}H, ^{13}C HSQC

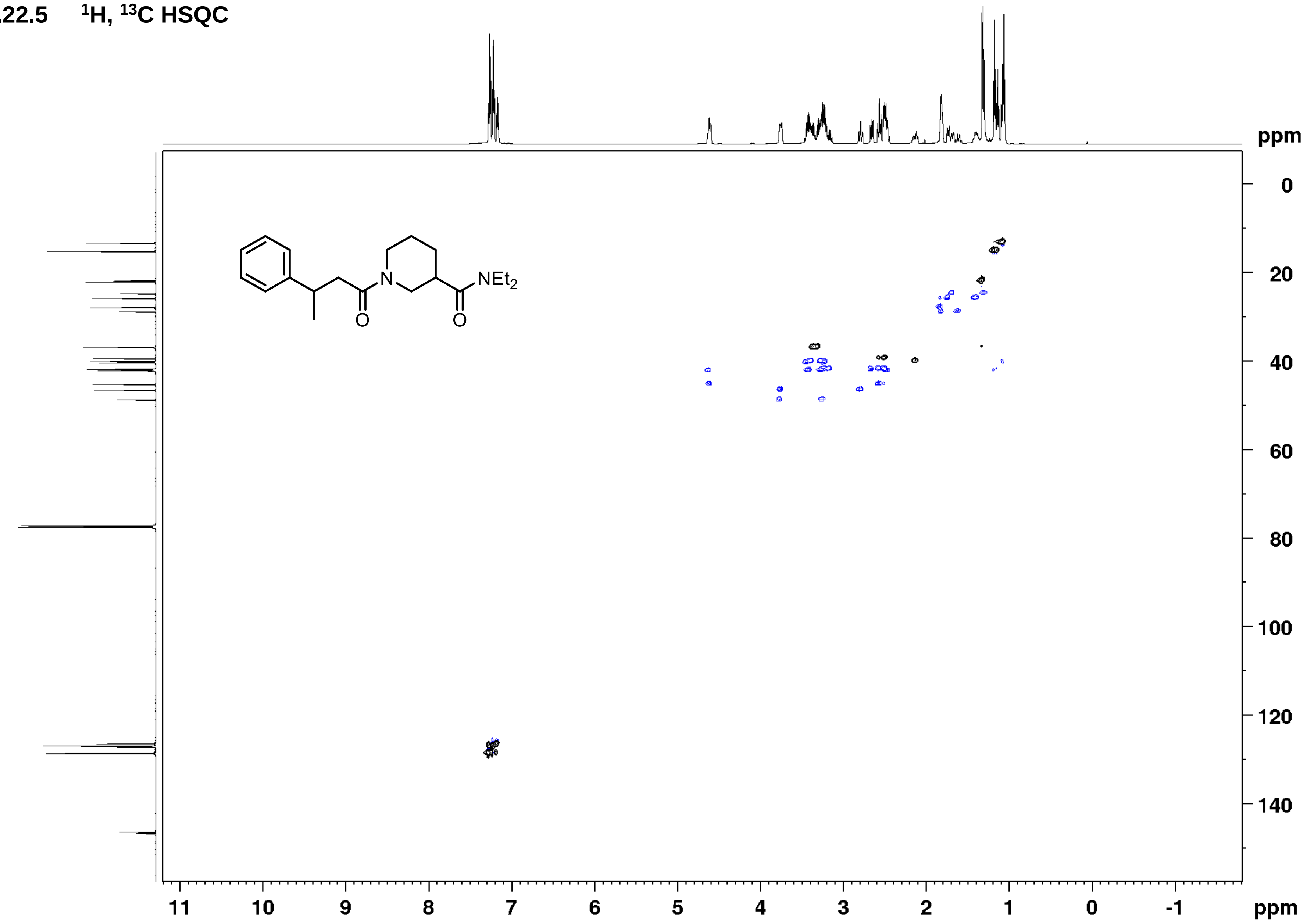

3.22.6 HRMS

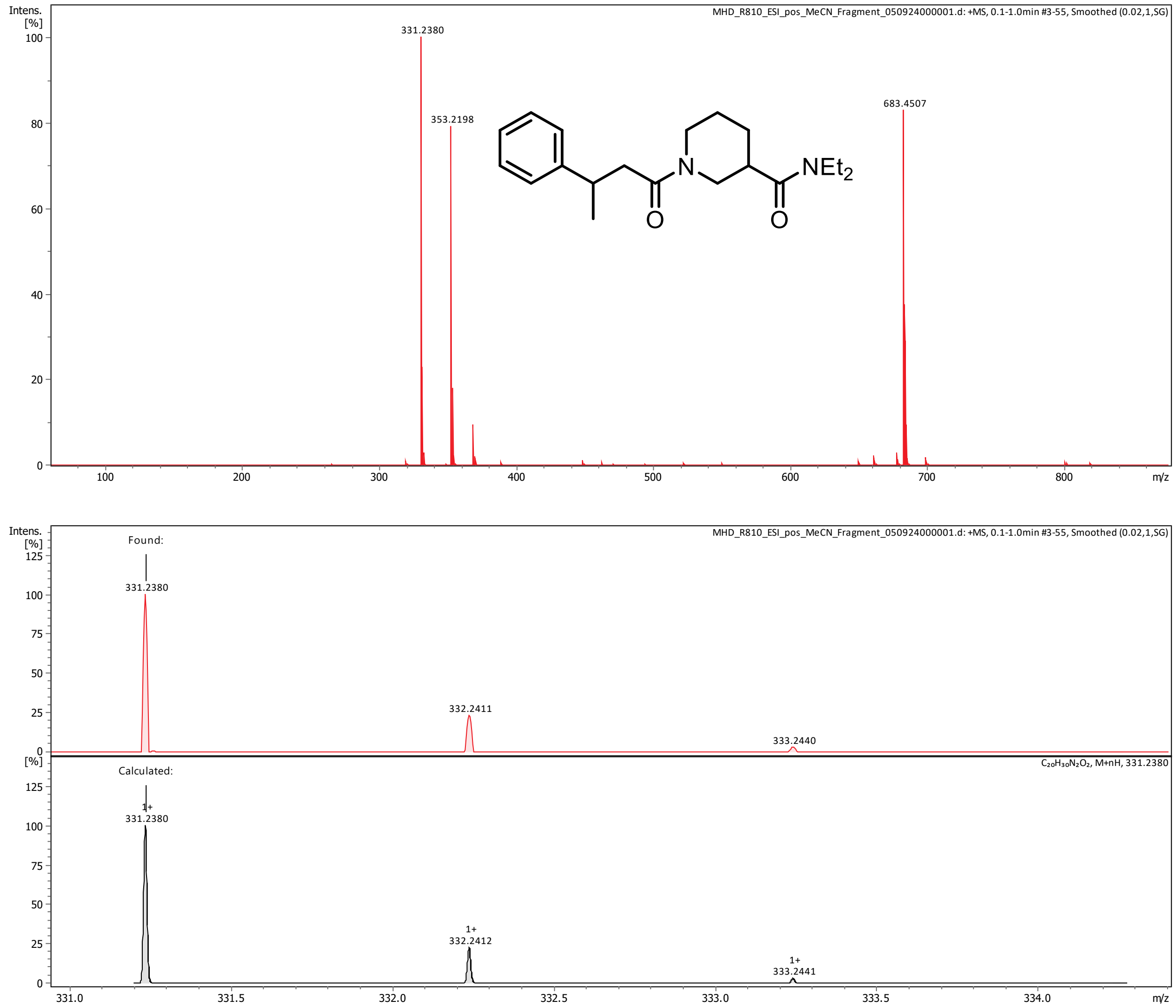

MHD_R810_ESI_pos_MeCN_Fragment_050924000001.d: +MS, 0.1-1.0min #3-55, Smoothed (0.02,1,SG)
Intens. [%]
331.2380
353.2198
683.4507
NEt2
N
O
O
m/z
Found:
331.2380
332.2411
333.2440
Calculated:
C20H30N2O2, M+nH, 331.2380
1+
331.2380
1+
332.2412
1+
333.2441

3.22.7 IR

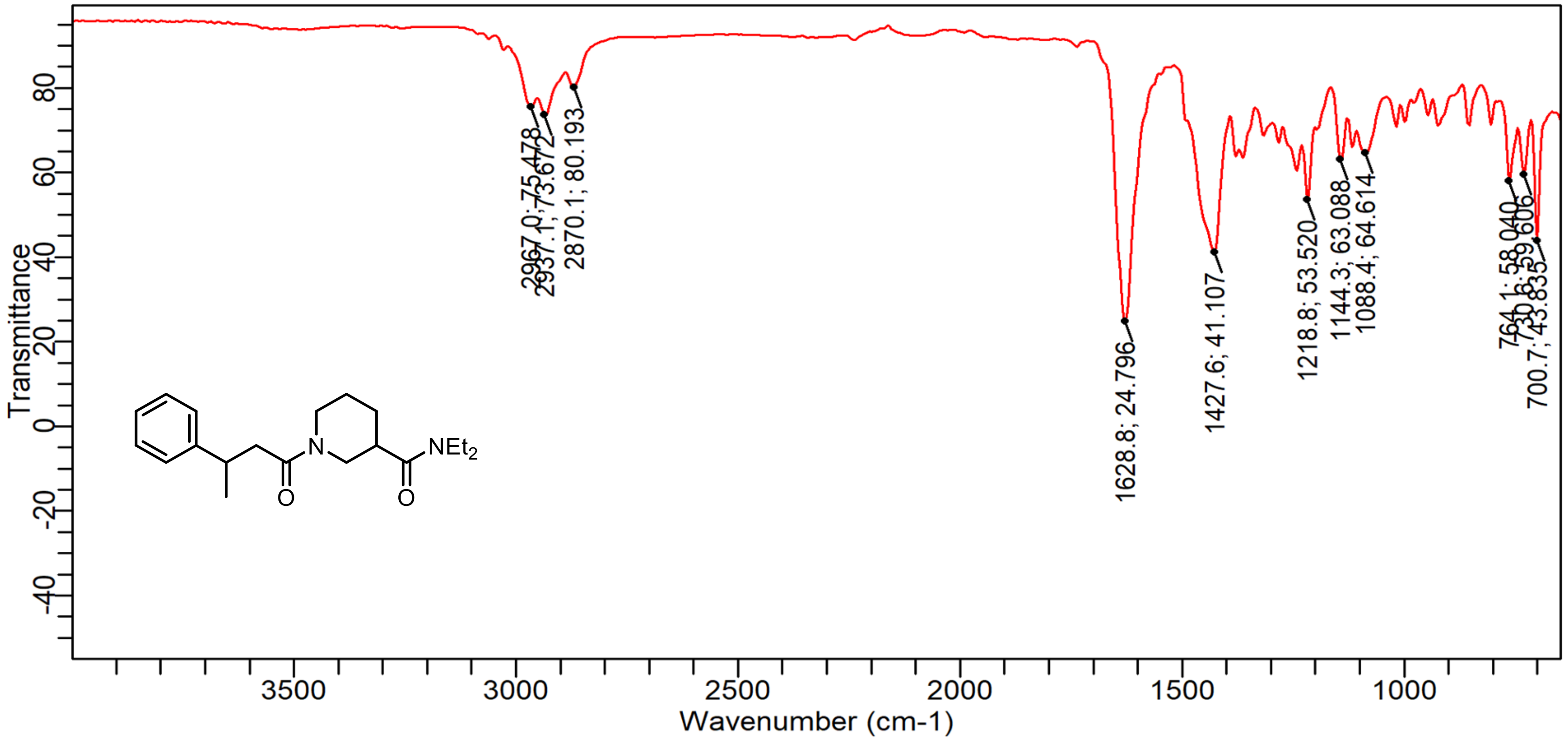

2967.0; 75.478
2937.1; 73.672
2870.1; 80.193
1628.8; 24.796
1427.6; 41.107
1218.8; 53.520
1144.3; 63.088
1088.4; 64.614
764.1; 58.040
730.8; 59.606
700.7; 43.835
Transmittance
Wavenumber (cm-1)
NEt2

3.23 Ethyl (3-phenylbutanoyl)-*L*-prolinate (12w) (600 MHz, $CDCl_3$)

3.23.1 ^1H NMR

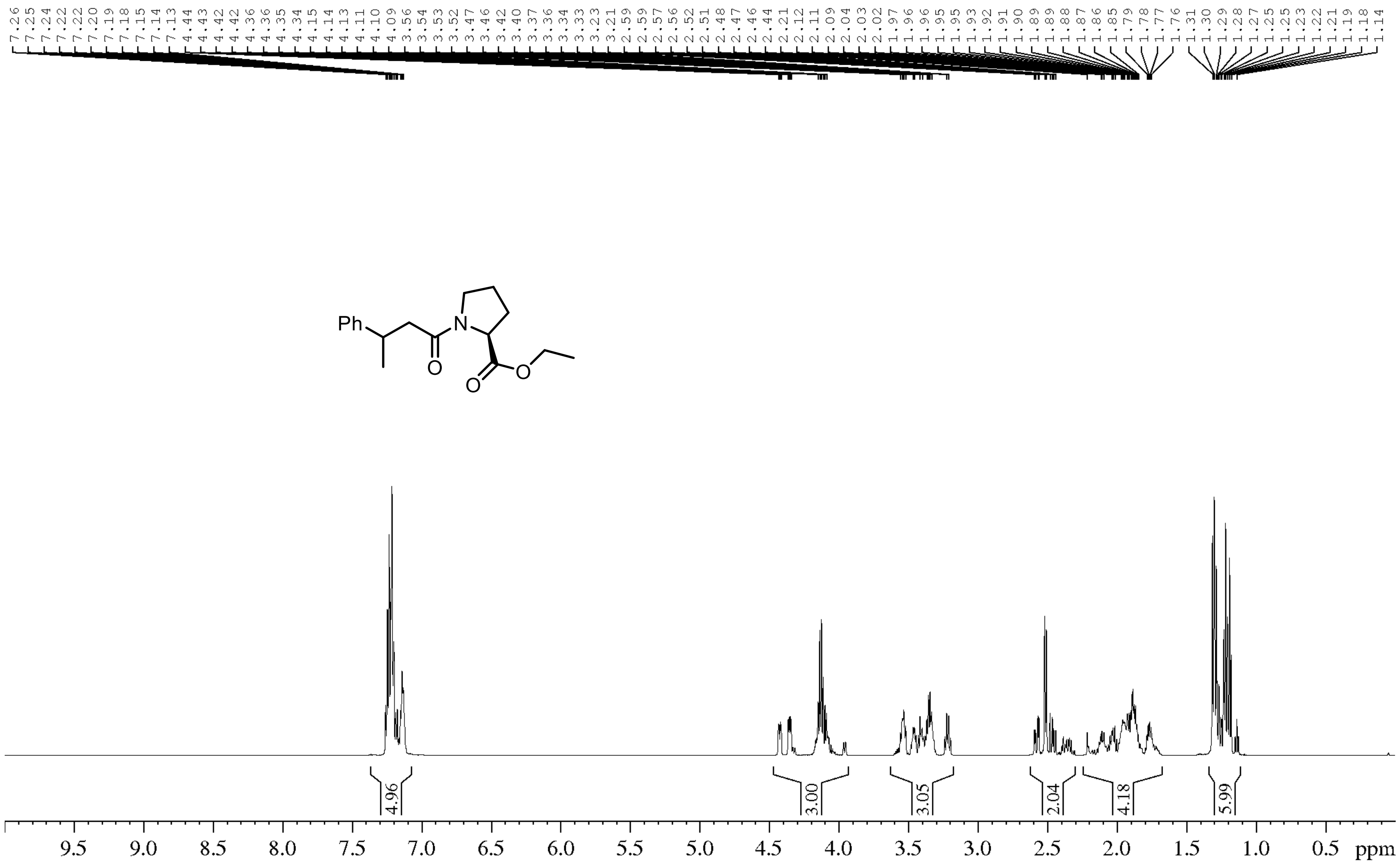

3.23.2 ^{13}C NMR

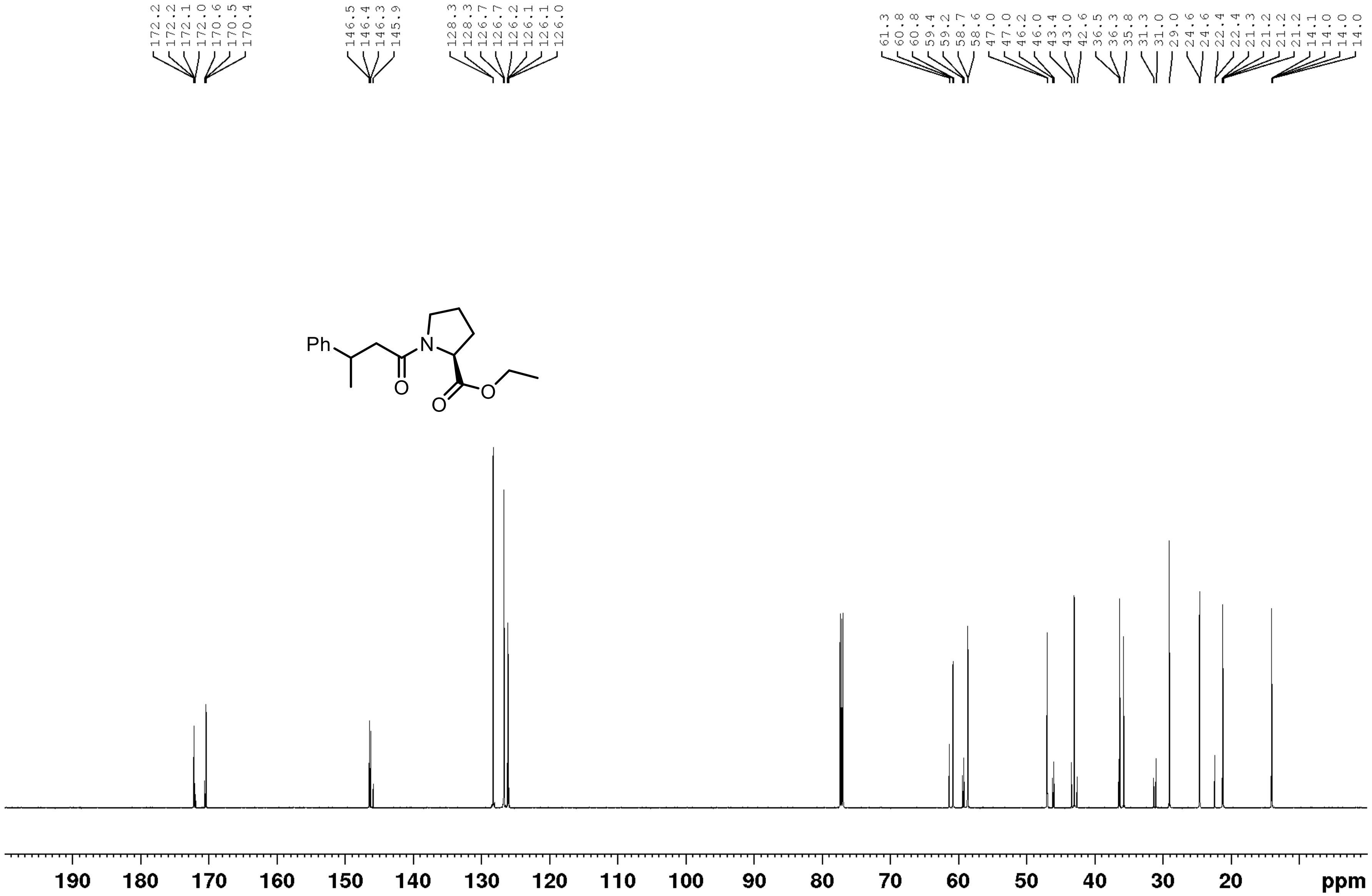
172.2
172.2
172.1
172.0
170.6
170.5
170.4
146.5
146.4
146.3
145.9
128.3
128.3
126.7
126.7
126.2
126.1
126.0
61.3
60.8
60.8
59.4
59.2
58.7
58.6
47.0
46.2
46.0
43.4
43.0
42.6
36.5
36.3
35.8
31.3
31.0
29.0
24.6
24.6
22.4
22.4
21.3
21.2
21.2
21.2
14.1
14.0
14.0
14.0
Ph
N
O
O
O
190
180
170
160
150
140
130
120
110
100
90
80
70
60
50
40
30
20
ppm

3.23.3 ^{1}H, ^{1}H COSY

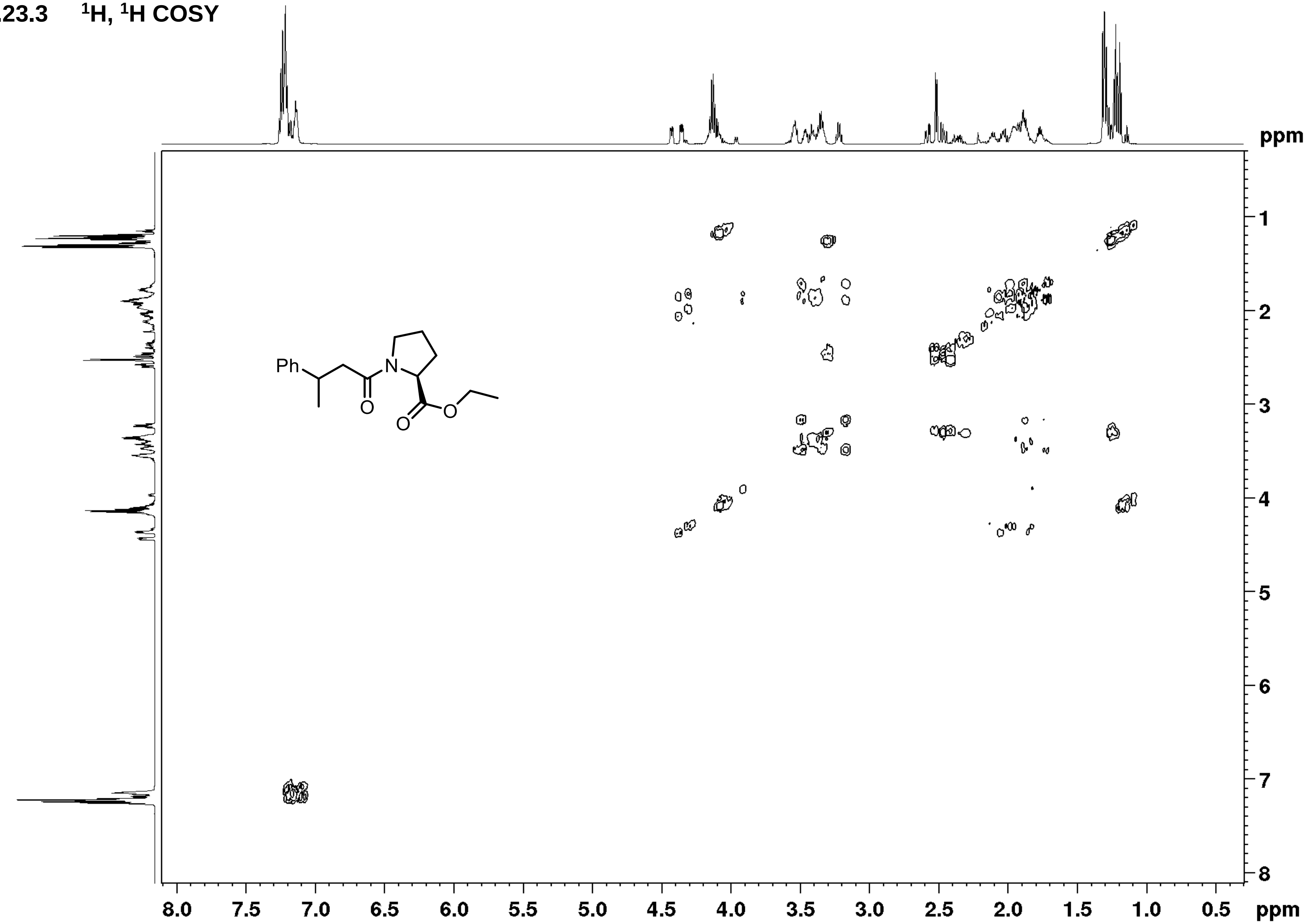

3.23.4 ^{1}H, ^{13}C HMBC

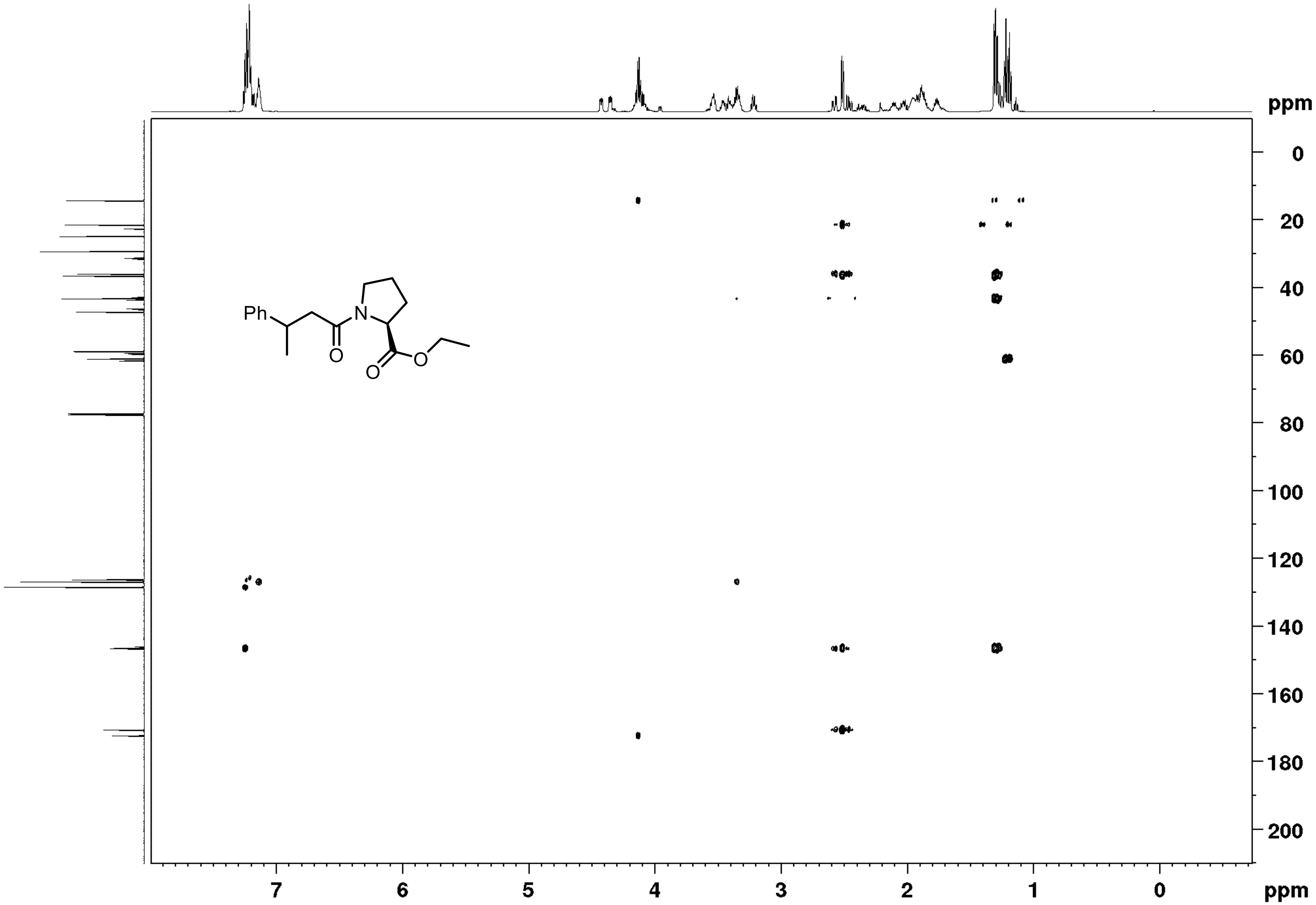

3.23.5 ^1H, ^{13}C HSQC

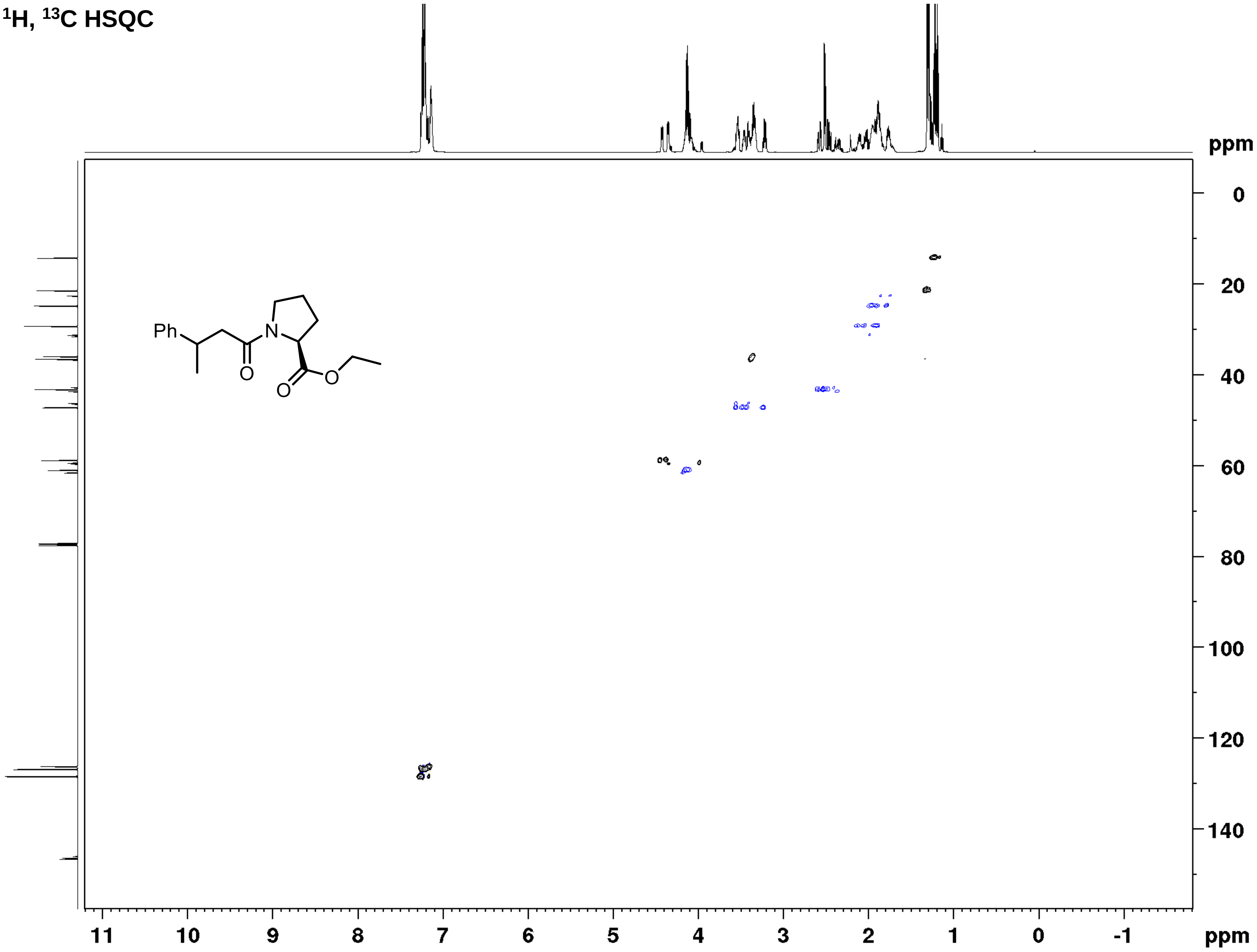

3.23.6 HRMS

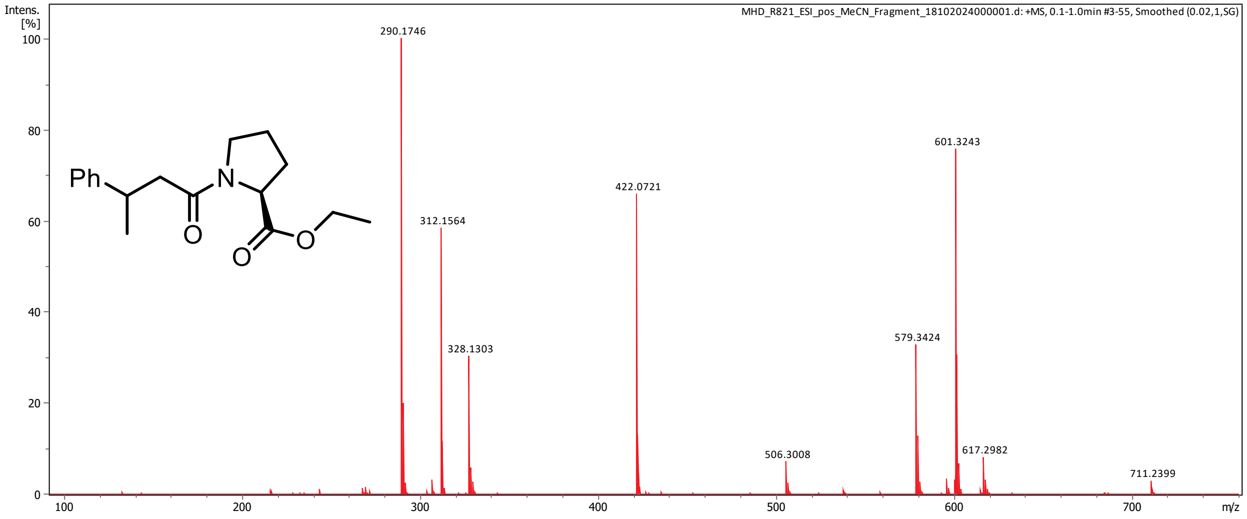
Intens. [%]
MHD_R821_ESI_pos_MeCN_Fragment_18102024000001.d: +MS, 0.1-1.0min #3-55, Smoothed (0.02,1,SG)
290.1746
312.1564
328.1303
422.0721
506.3008
579.3424
601.3243
617.2982
711.2399
Ph
N
O
O
O
m/z

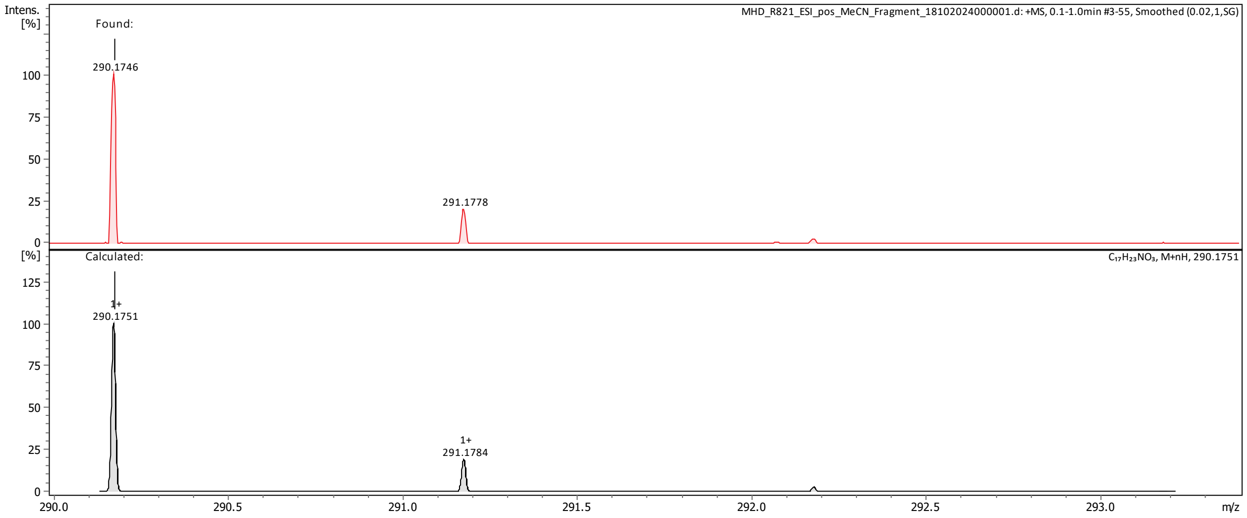
Intens. [%]
MHD_R821_ESI_pos_MeCN_Fragment_18102024000001.d: +MS, 0.1-1.0min #3-55, Smoothed (0.02,1,SG)
Found:
290.1746
291.1778
Calculated:
C17H23NO3, M+nH, 290.1751
1+
290.1751
1+
291.1784
m/z

3.23.7 IR

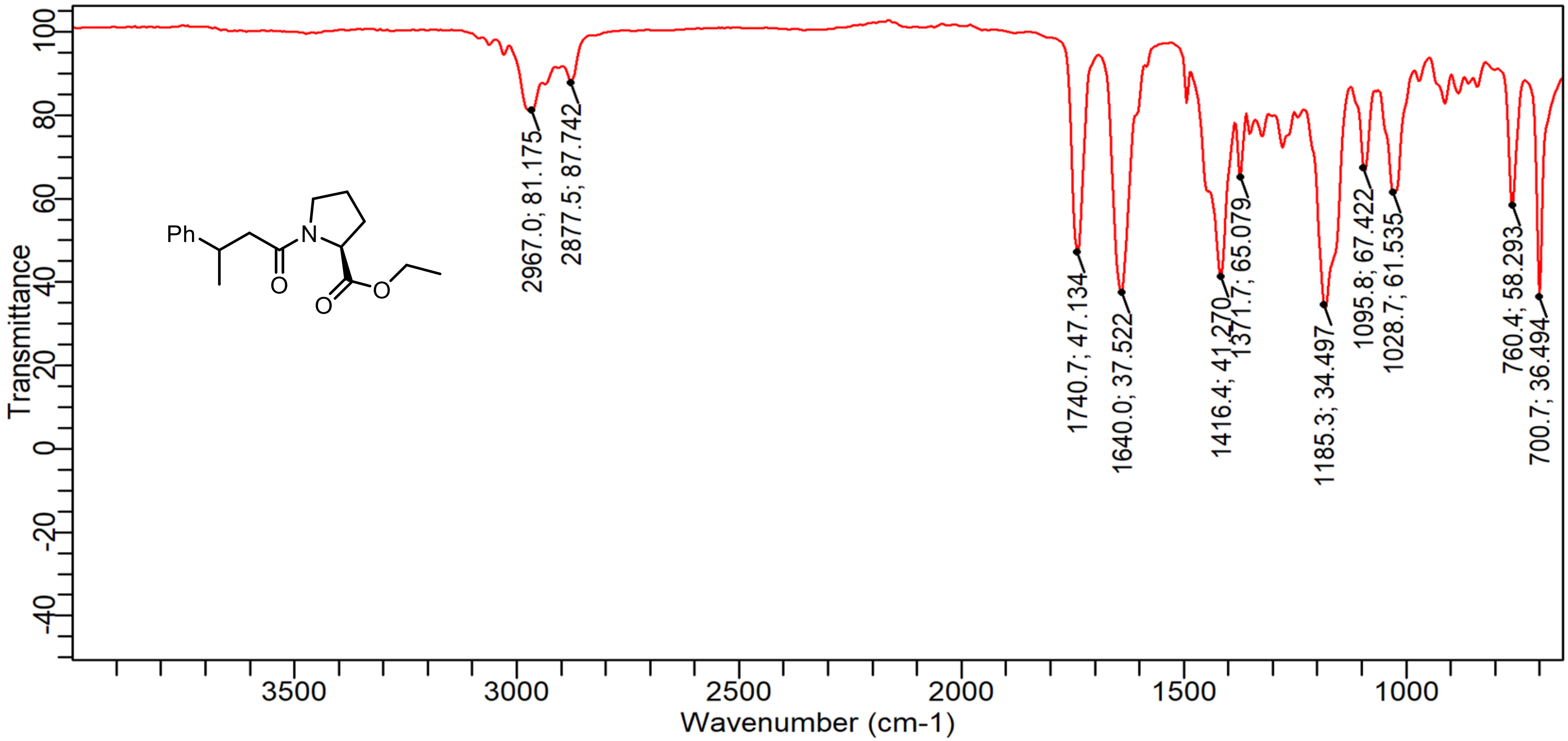

Transmittance
Wavenumber (cm-1)
2967.0; 81.175
2877.5; 87.742
1740.7; 47.134
1640.0; 37.522
1416.4; 41.270
1371.7; 65.079
1185.3; 34.497
1095.8; 67.422
1028.7; 61.535
760.4; 58.293
700.7; 36.494
Ph
N
O
O
O

3.24 Ethyl *N*-methyl-*N*-(3-phenylbutanoyl)-*L*-alaninate (12x) (600 MHz, $CDCl_3$)

3.24.1 ^{1}H NMR

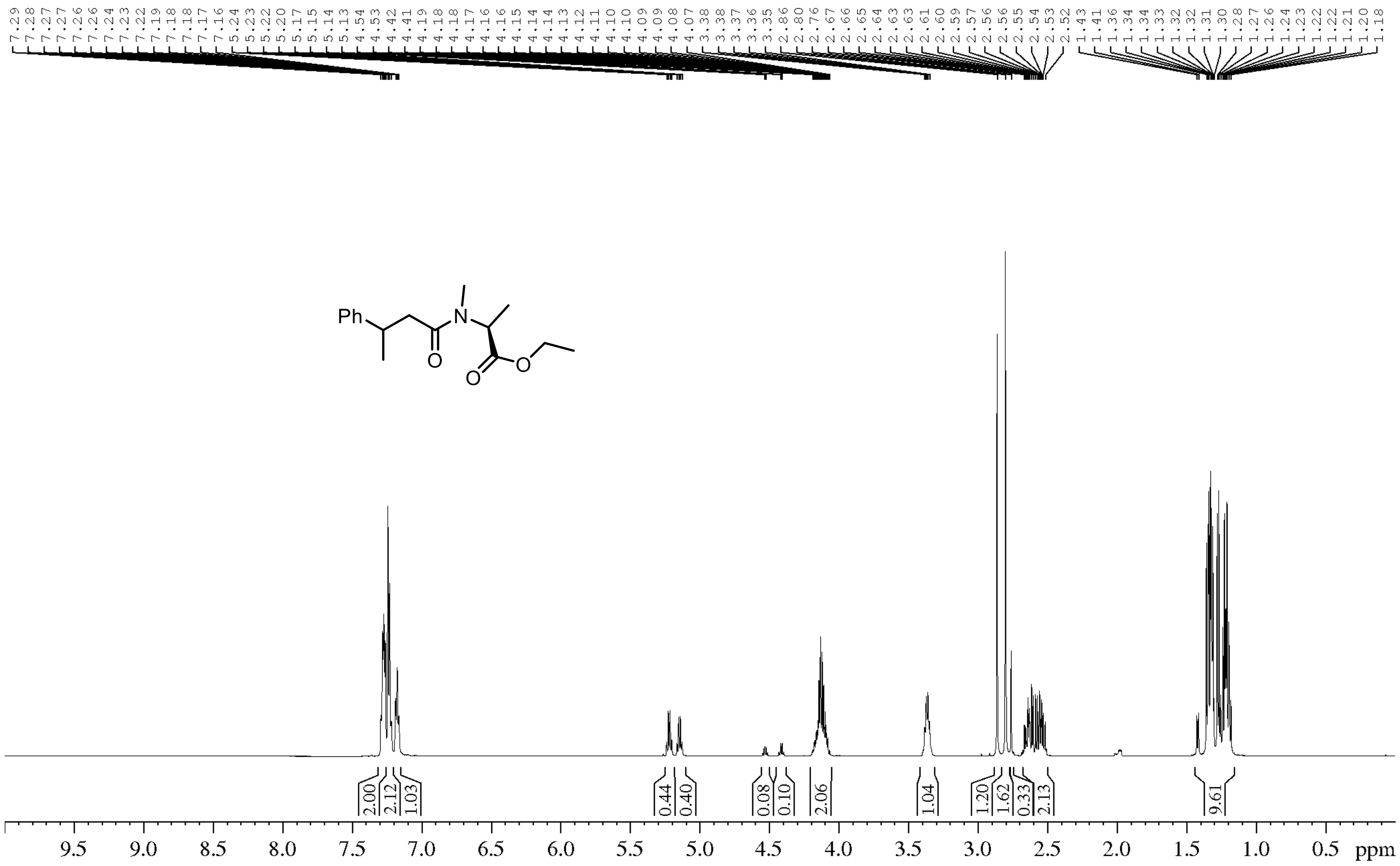

3.24.2 ^{13}C NMR

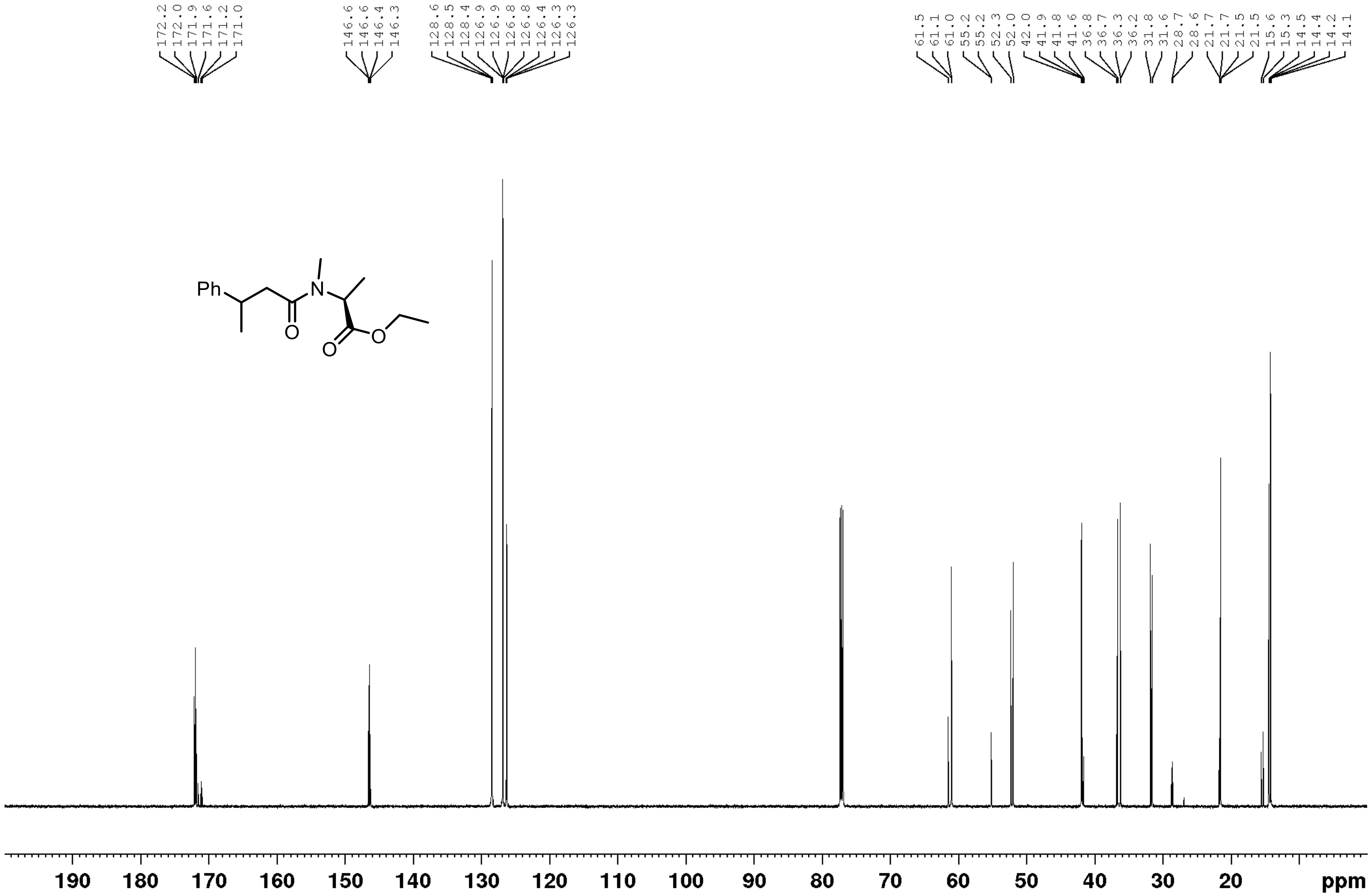

172.2
172.0
171.9
171.6
171.2
171.0
146.6
146.6
146.4
146.3
128.6
128.5
128.4
126.9
126.9
126.8
126.8
126.4
126.3
126.3
61.5
61.1
61.0
55.2
55.2
52.3
52.0
42.0
41.9
41.8
41.6
36.8
36.7
36.3
36.2
31.8
31.6
28.7
28.6
21.7
21.7
21.5
21.5
15.6
15.3
14.5
14.4
14.2
14.1
Ph
N
O
O
O
190
180
170
160
150
140
130
120
110
100
90
80
70
60
50
40
30
20
ppm

3.24.3 ^{1}H, ^{1}H COSY

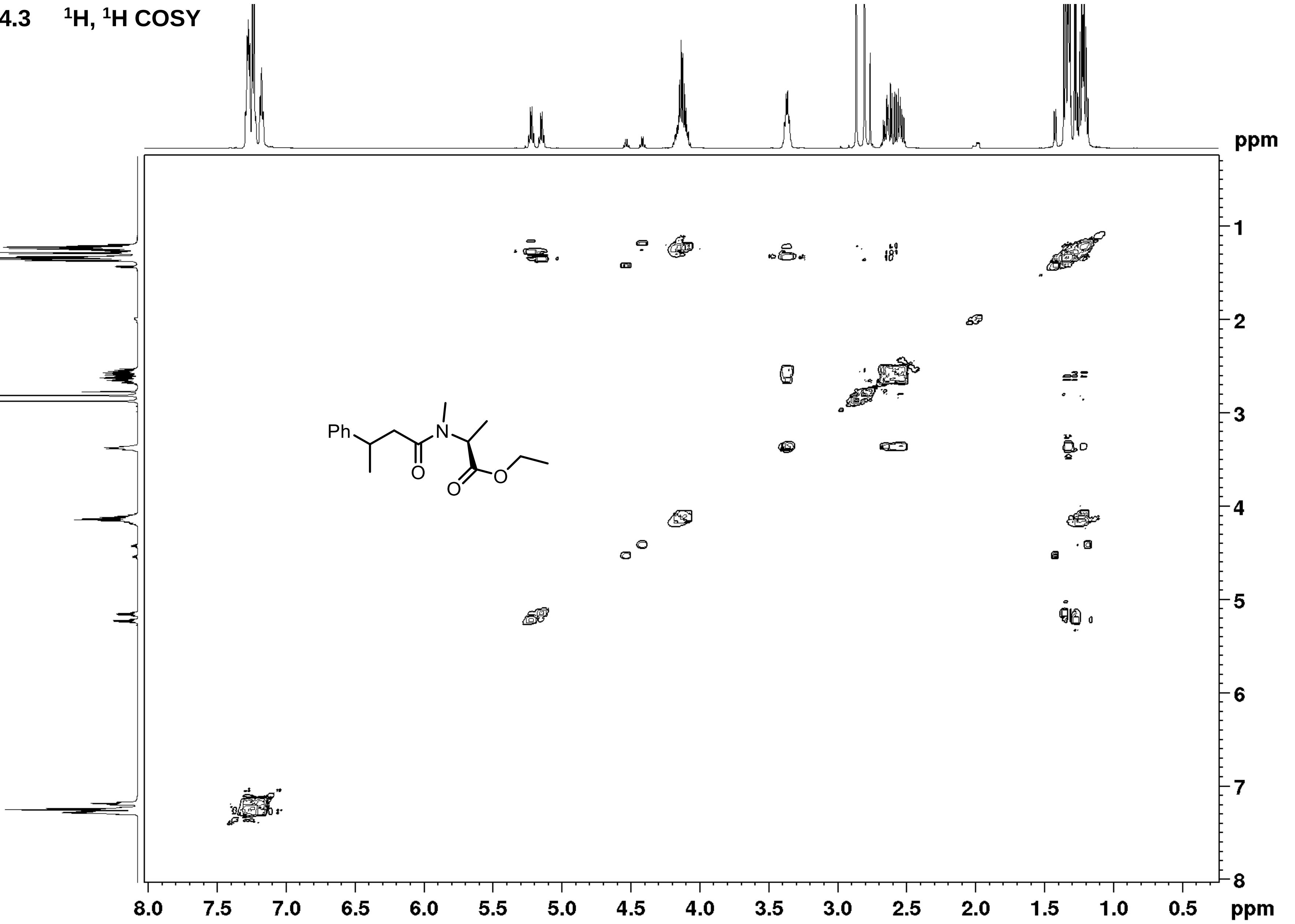

3.24.4 ^{1}H, ^{13}C HMBC

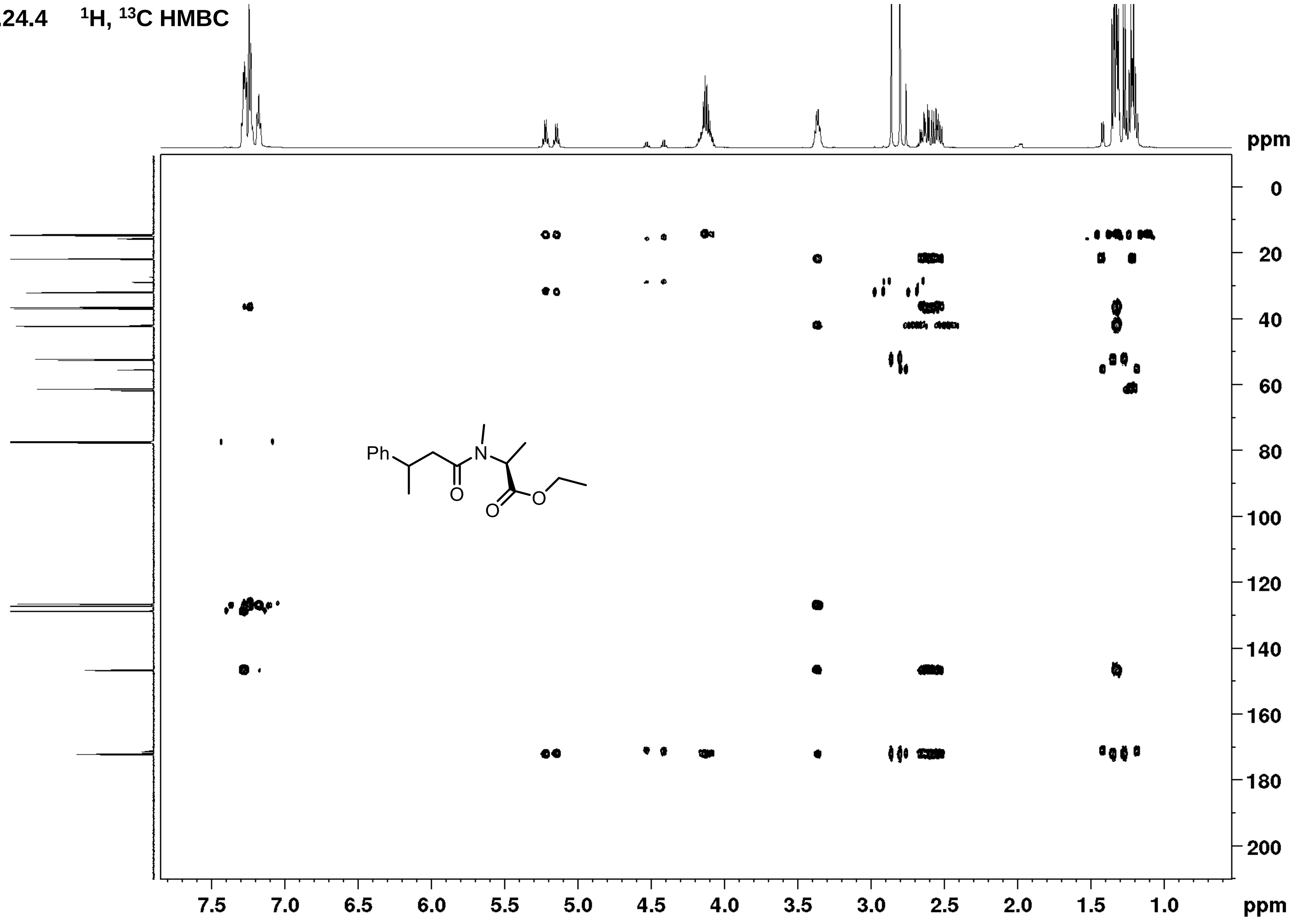

3.24.5 ^{1}H, ^{13}C HSQC

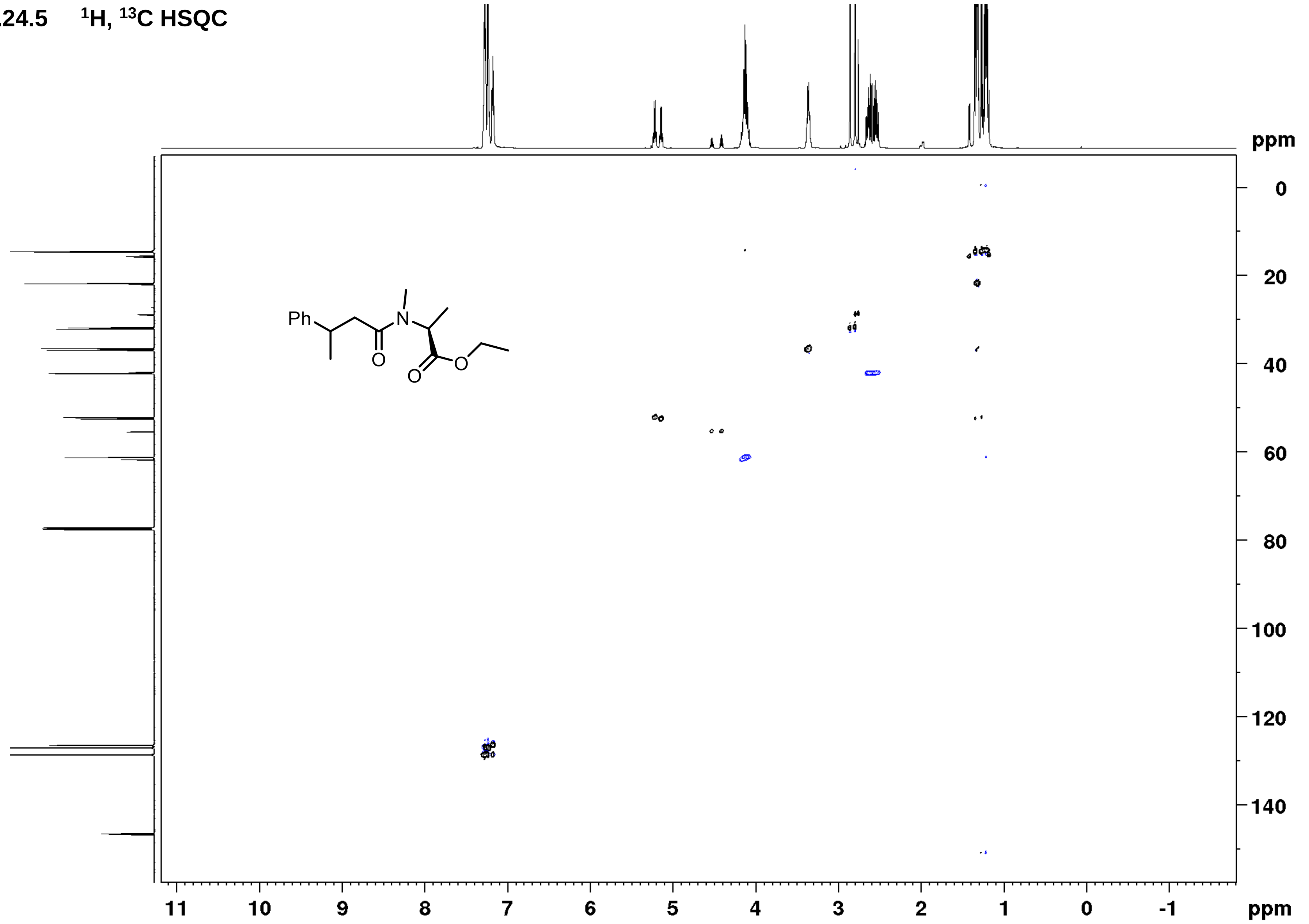

3.24.6 HRMS

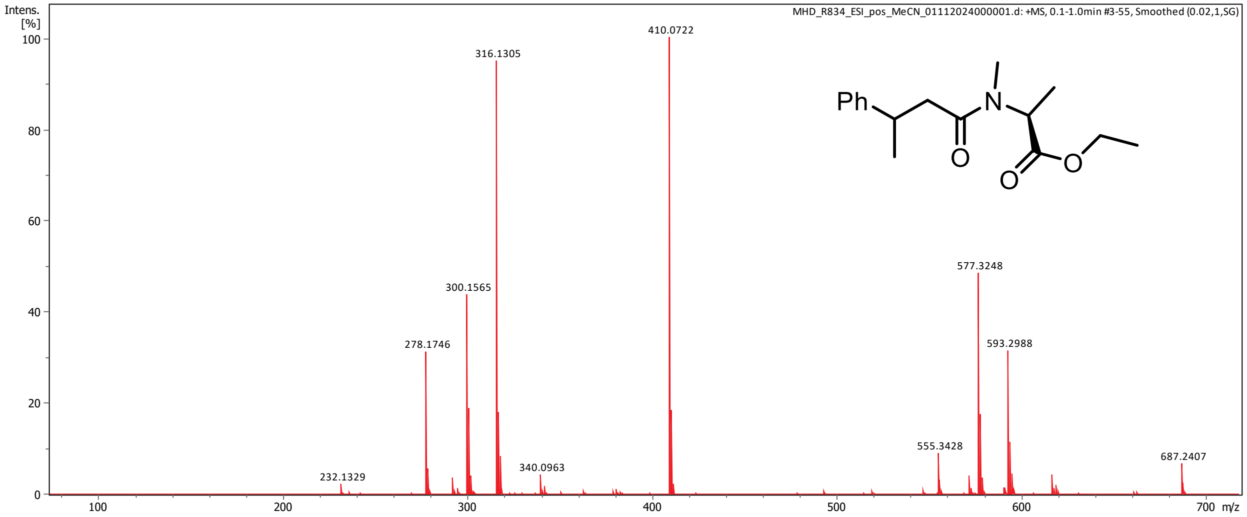
Intens.
[%]
MHD_R834_ESI_pos_MeCN_01112024000001.d: +MS, 0.1-1.0min #3-55, Smoothed (0.02,1,SG)
410.0722
316.1305
300.1565
278.1746
232.1329
340.0963
577.3248
593.2988
555.3428
687.2407
Ph
N
O
O
O
m/z

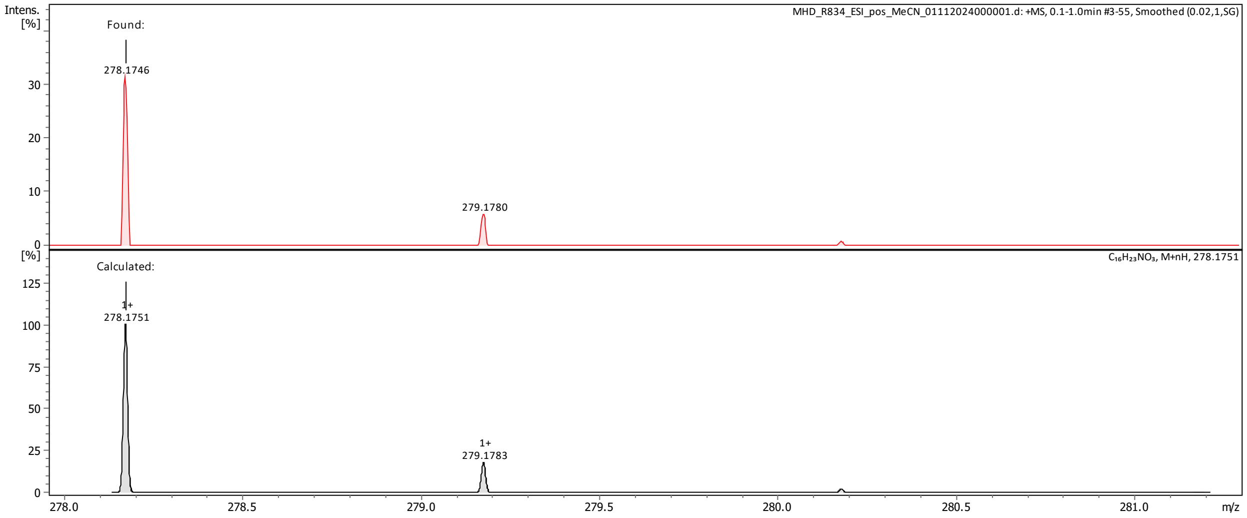
Intens.
[%]
Found:
278.1746
279.1780
MHD_R834_ESI_pos_MeCN_01112024000001.d: +MS, 0.1-1.0min #3-55, Smoothed (0.02,1,SG)
Calculated:
1+
278.1751
1+
279.1783
C16H23NO3, M+nH, 278.1751
m/z

3.24.7 IR

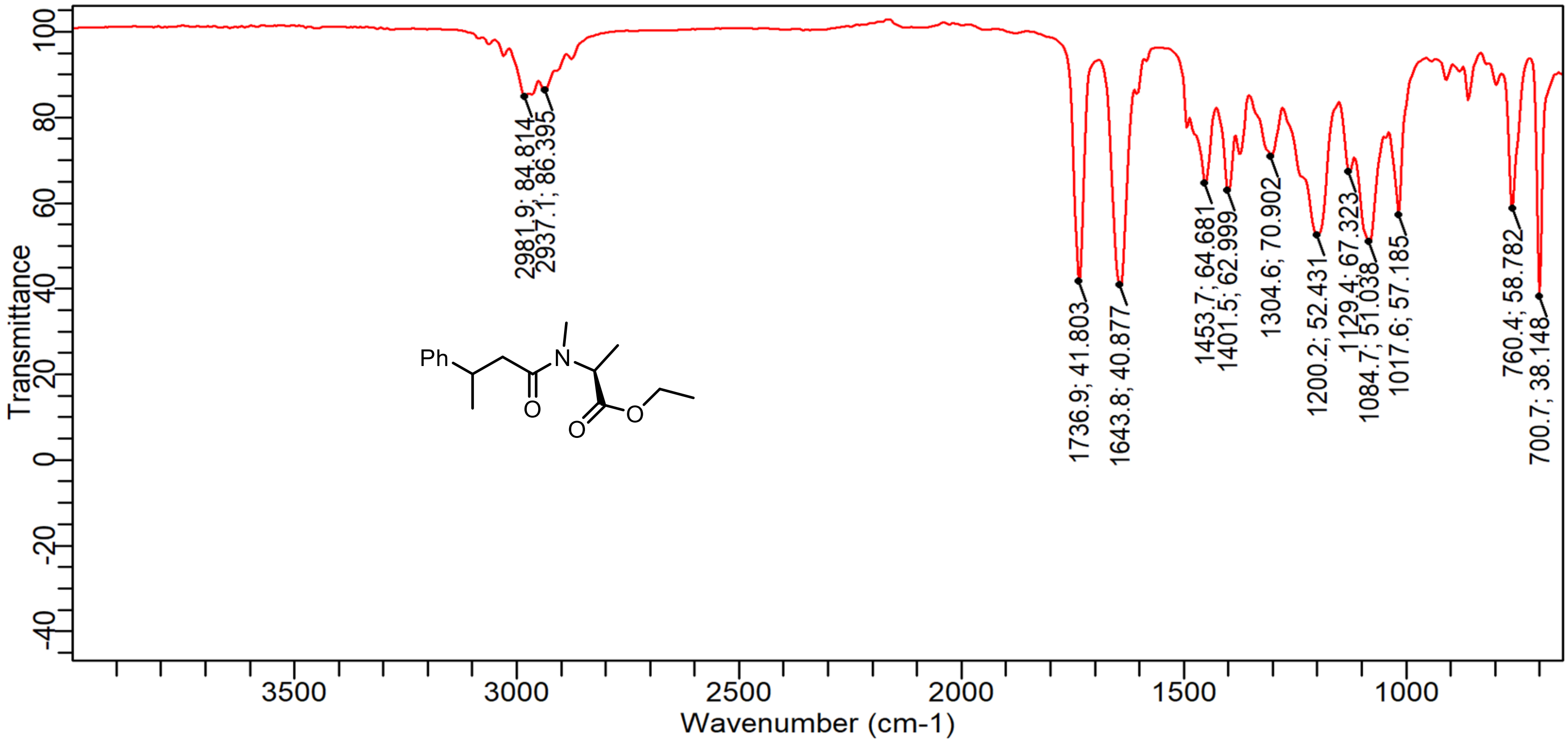

Transmittance
Wavenumber (cm-1)
2981.9; 84.814
2937.1; 86.395
1736.9; 41.803
1643.8; 40.877
1453.7; 64.681
1401.5; 62.999
1304.6; 70.902
1200.2; 52.431
1129.4; 67.323
1084.7; 51.038
1017.6; 57.185
760.4; 58.782
700.7; 38.148
Ph
N
O
O
O

3.25 1,3-Dimethyldihydropyrimidine-2,4(1*H*,3*H*)-dione (12y) (600 MHz, $CDCl_3$)

3.25.1 ^{1}H NMR

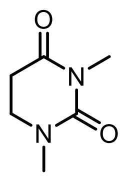

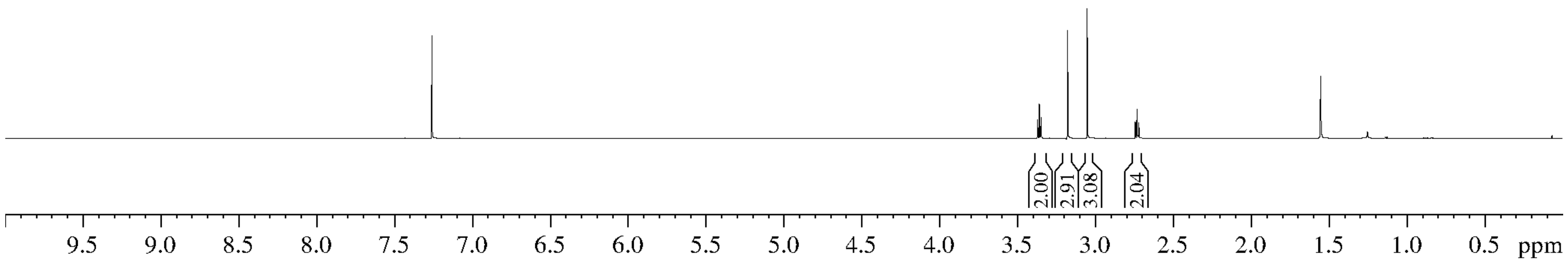

3.25.2 ^{13}C NMR

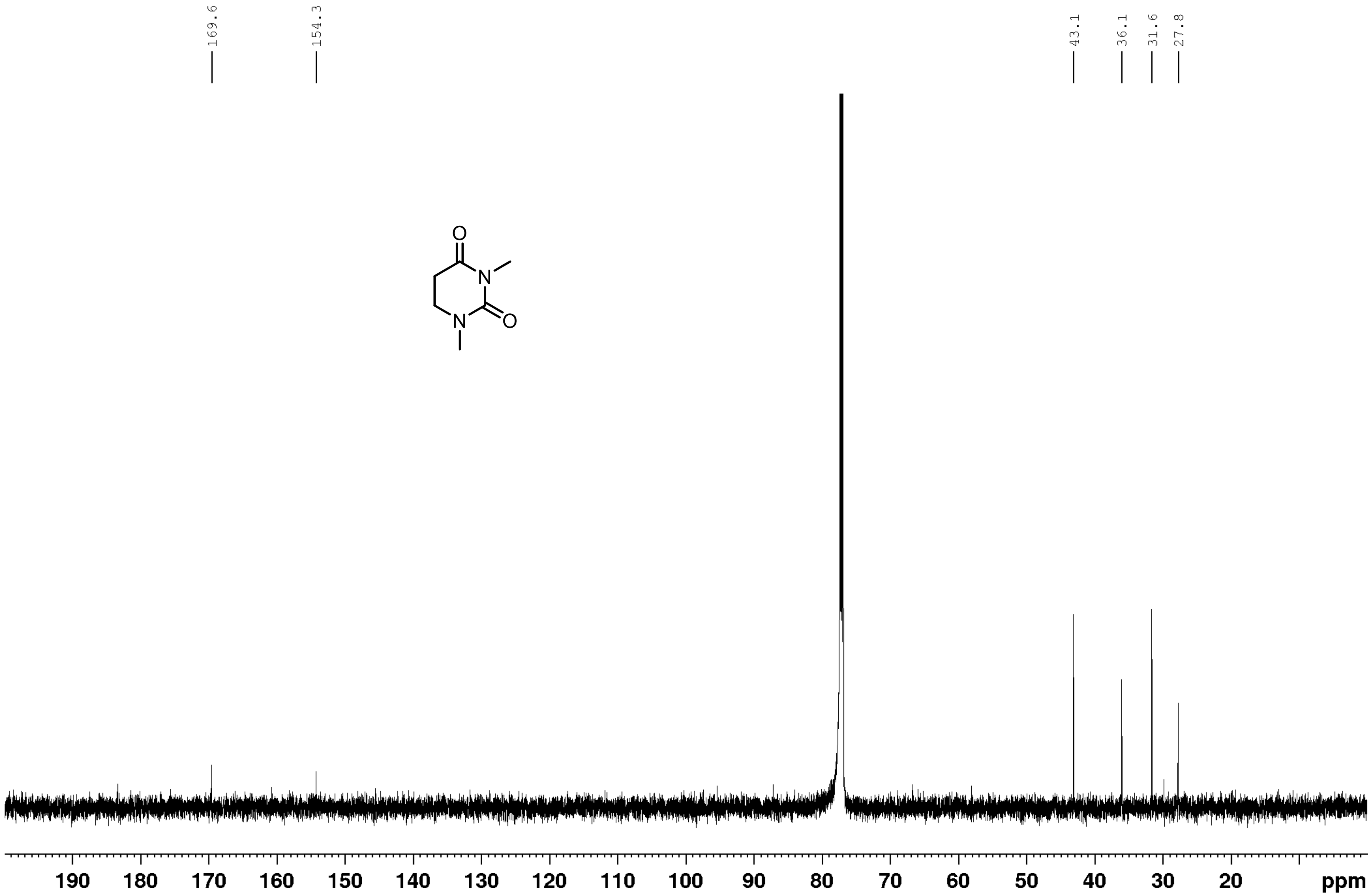
169.6
154.3
43.1
36.1
31.6
27.8
O
N
N
O
190
180
170
160
150
140
130
120
110
100
90
80
70
60
50
40
30
20
ppm

3.25.3 ^{1}H, ^{1}H COSY

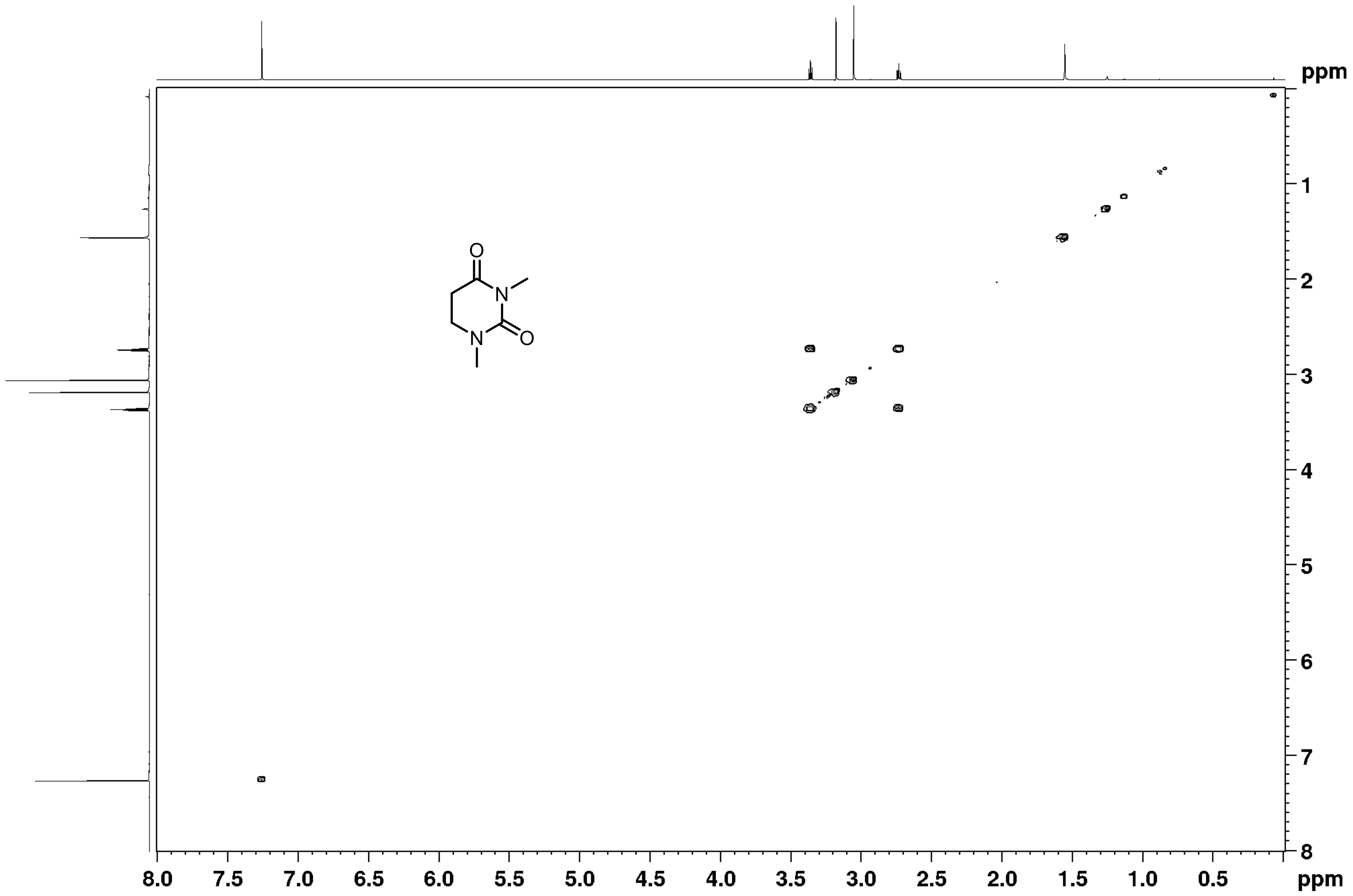

3.25.4 ¹H, ¹³C HMBC

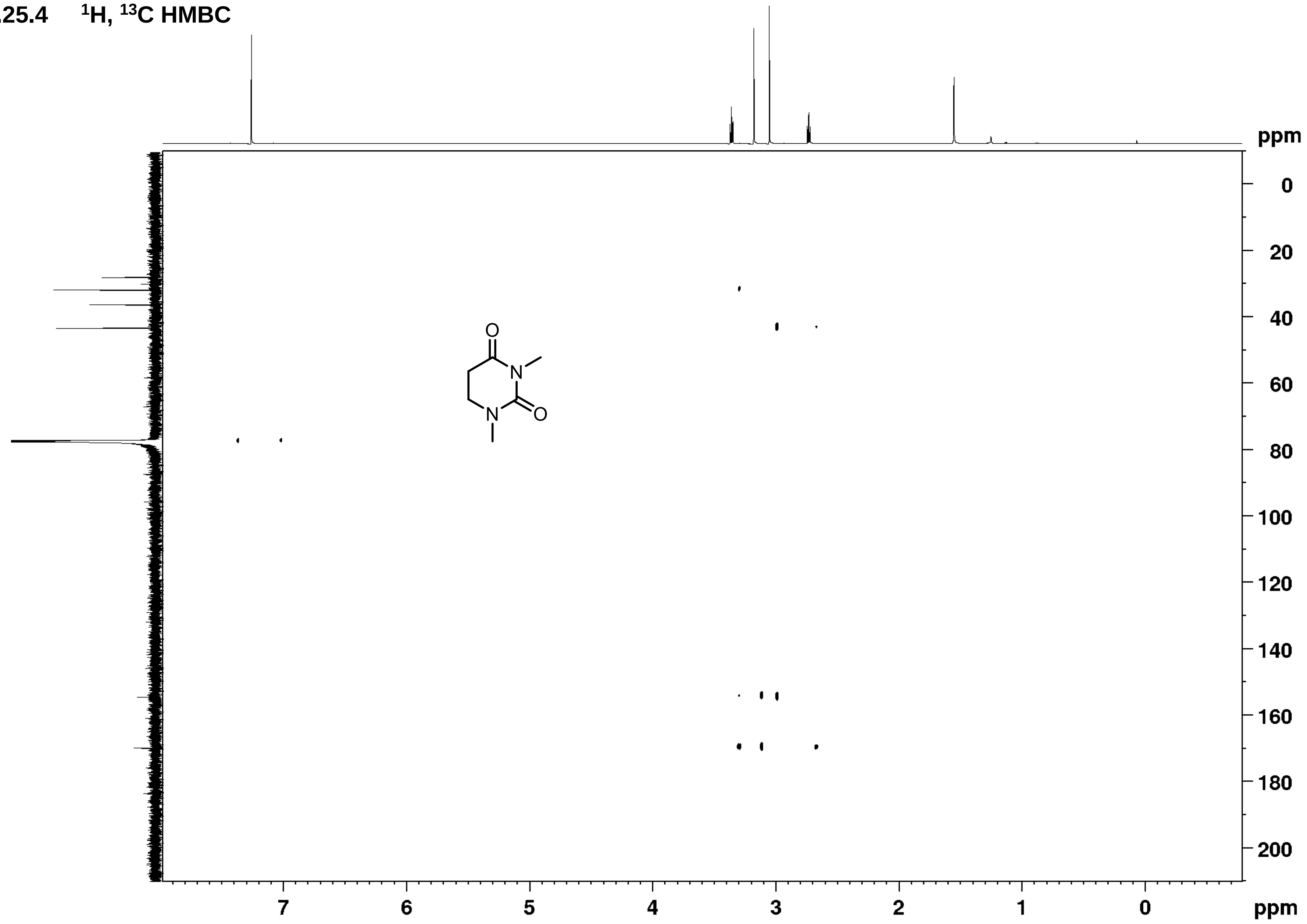

3.25.5 ^{1}H, ^{13}C HSQC

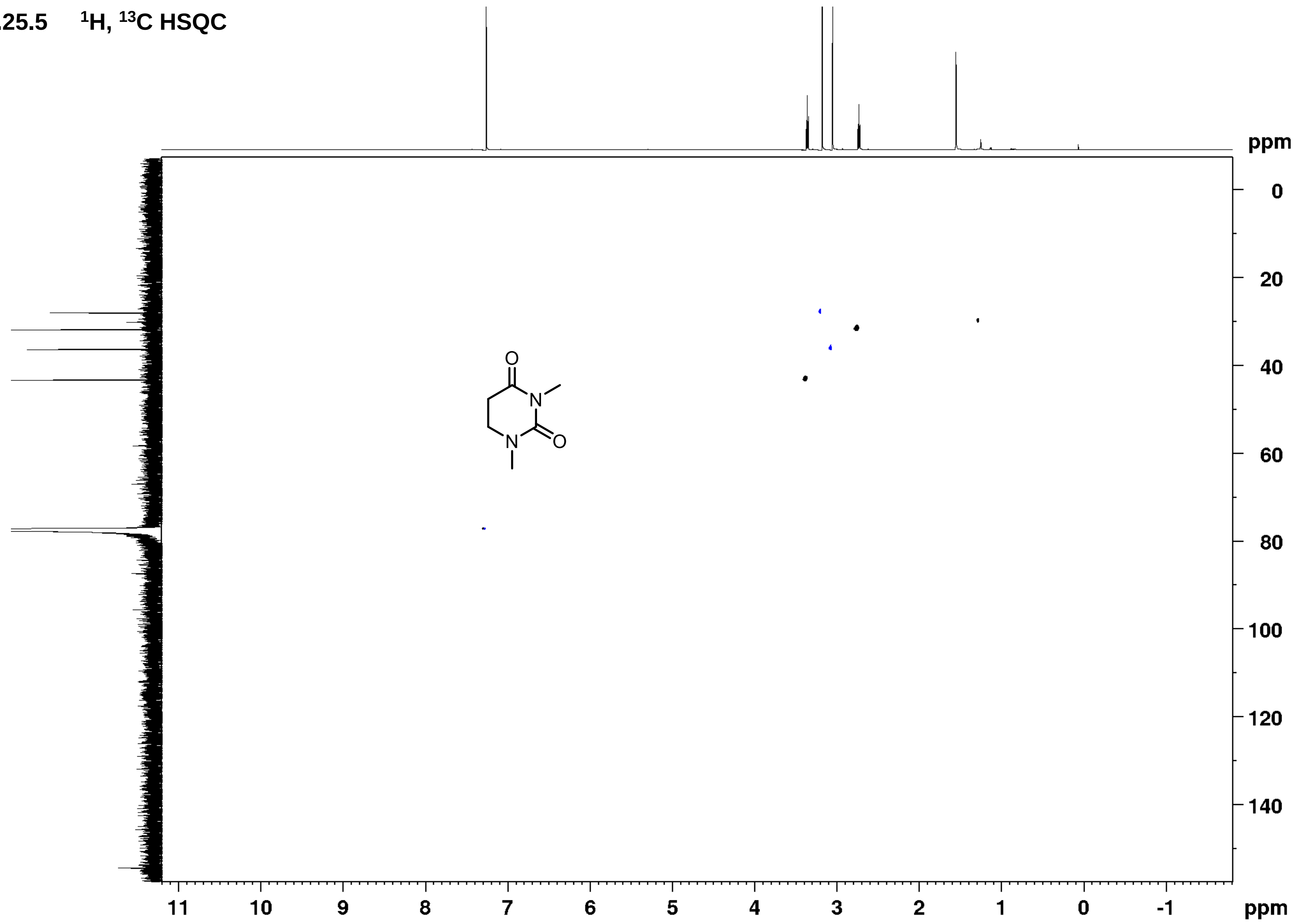

3.25.6 HRMS

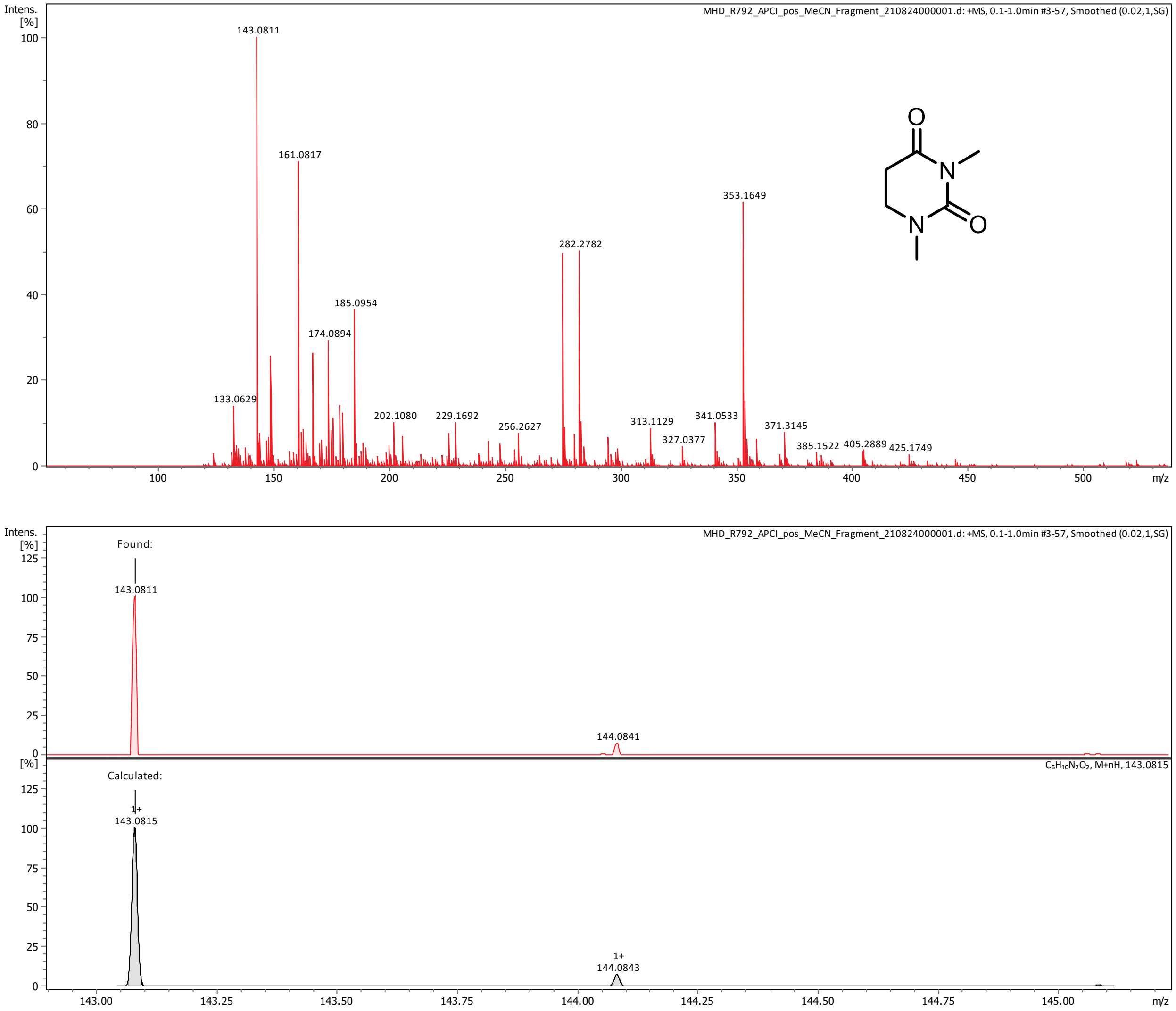

MHD_R792_APCI_pos_MeCN_Fragment_210824000001.d: +MS, 0.1-1.0min #3-57, Smoothed (0.02,1,SG)
Intens. [%]
143.0811
161.0817
185.0954
174.0894
133.0629
202.1080
229.1692
256.2627
282.2782
313.1129
327.0377
341.0533
353.1649
371.3145
385.1522
405.2889
425.1749
m/z
Found:
144.0841
Calculated:
1+
143.0815
144.0843
$C_6H_{10}N_2O_2$, M+nH, 143.0815

3.25.7 IR

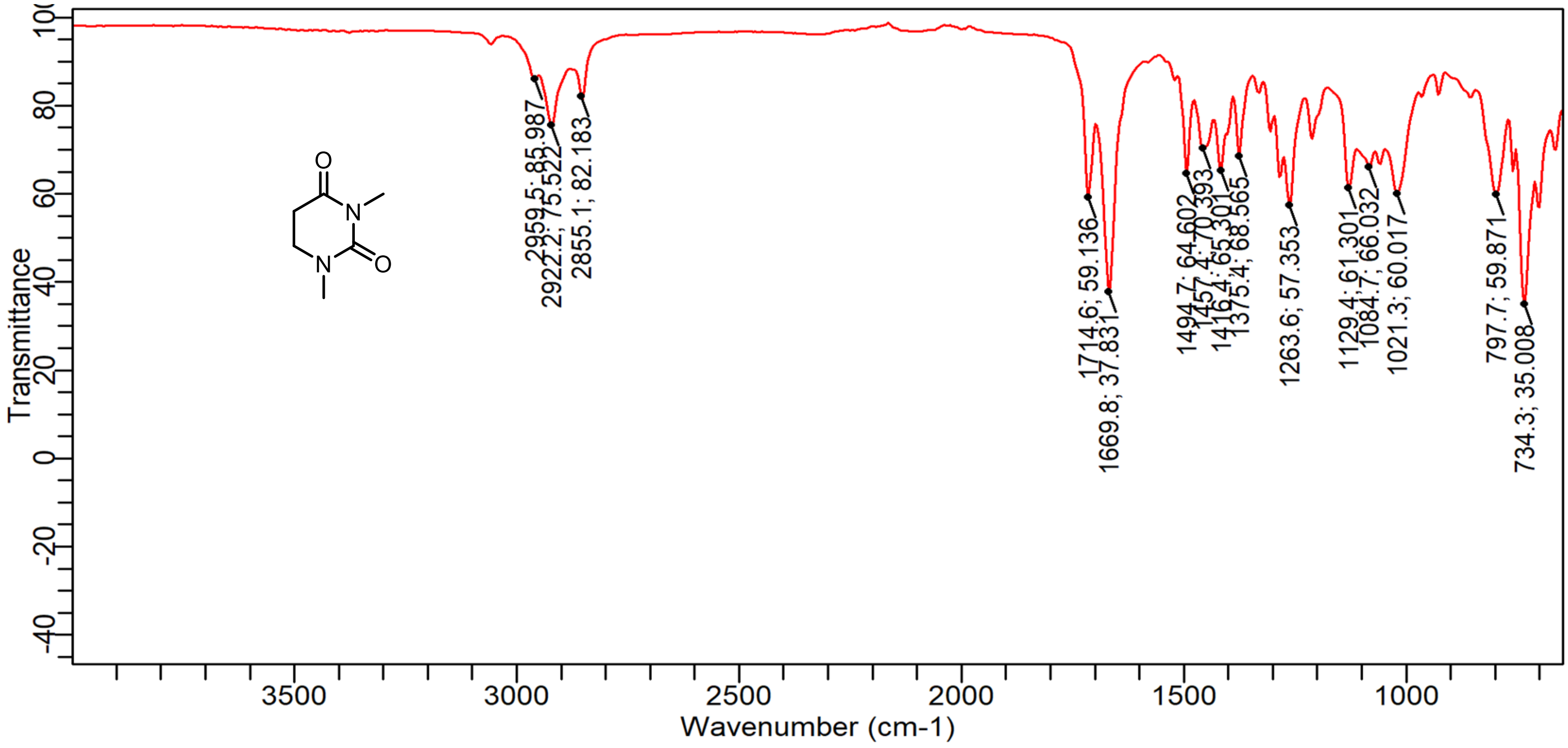

Transmittance
Wavenumber (cm-1)
2959.5; 85.987
2922.2; 75.522
2855.1; 82.183
1714.6; 59.136
1669.8; 37.831
1494.7; 64.602
1457.4; 70.393
1416.4; 65.301
1375.4; 68.565
1263.6; 57.353
1129.4; 61.301
1084.7; 66.032
1021.3; 60.017
797.7; 59.871
734.3; 35.008

3.26 Methyl-1-methyl-2-oxopiperidine-3-carboxylate (12z) (600 MHz, $CDCl_3$)

3.26.1 ^{1}H NMR

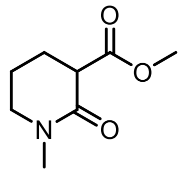

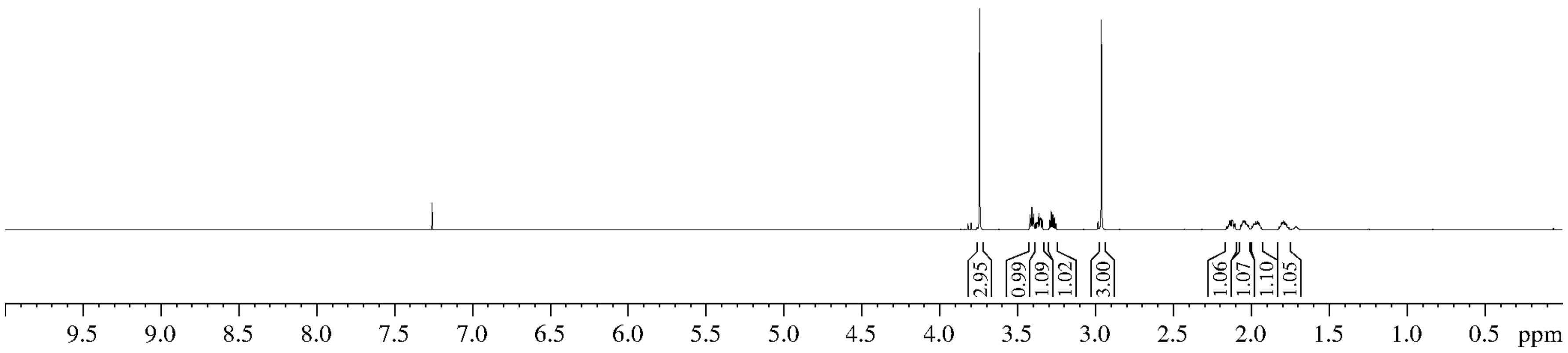

3.26.2 ^{13}C NMR

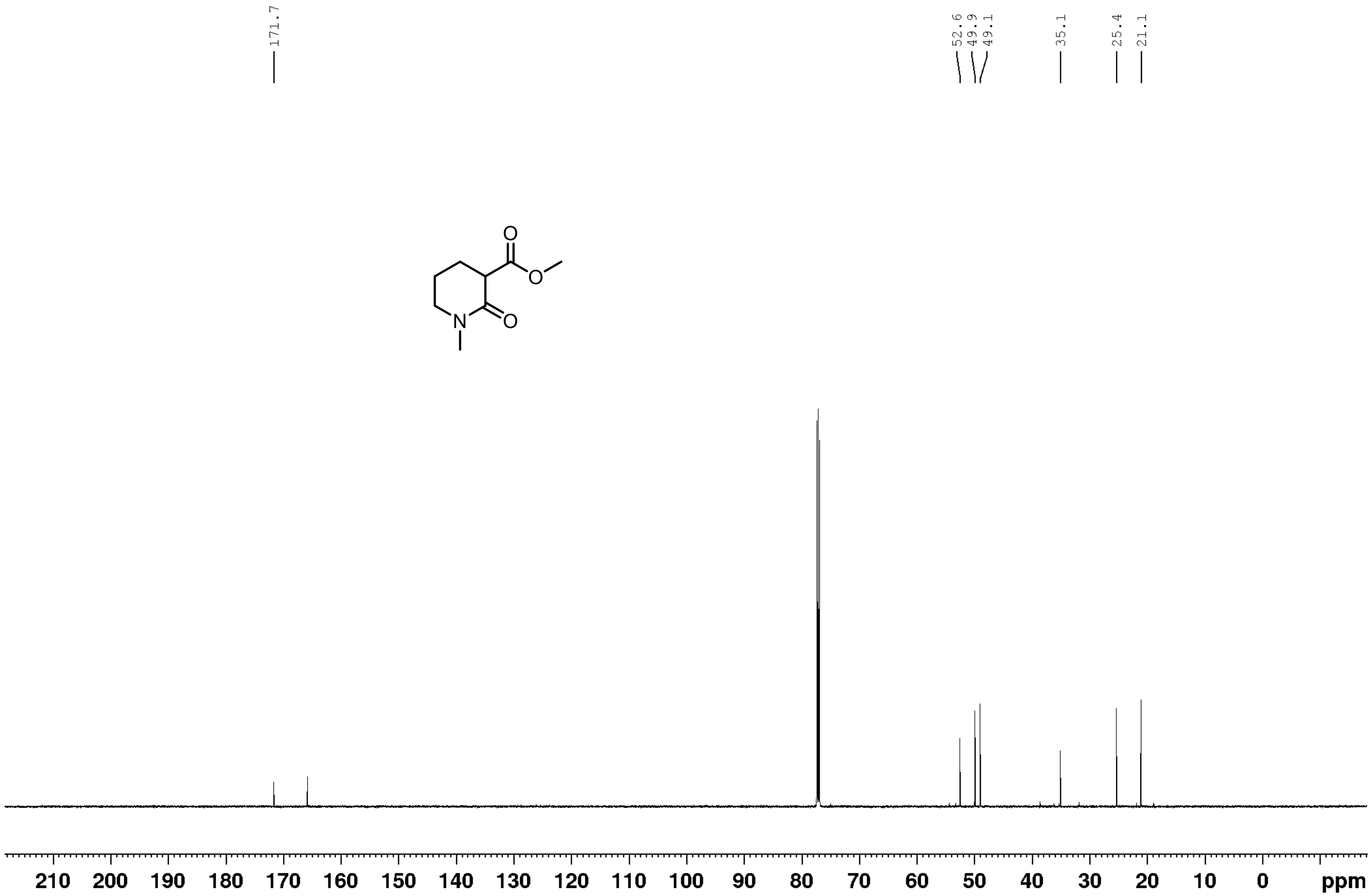
171.7
52.6
49.9
49.1
35.1
25.4
21.1
210 200 190 180 170 160 150 140 130 120 110 100 90 80 70 60 50 40 30 20 10 0 ppm

3.26.3 ^{1}H, ^{1}H COSY

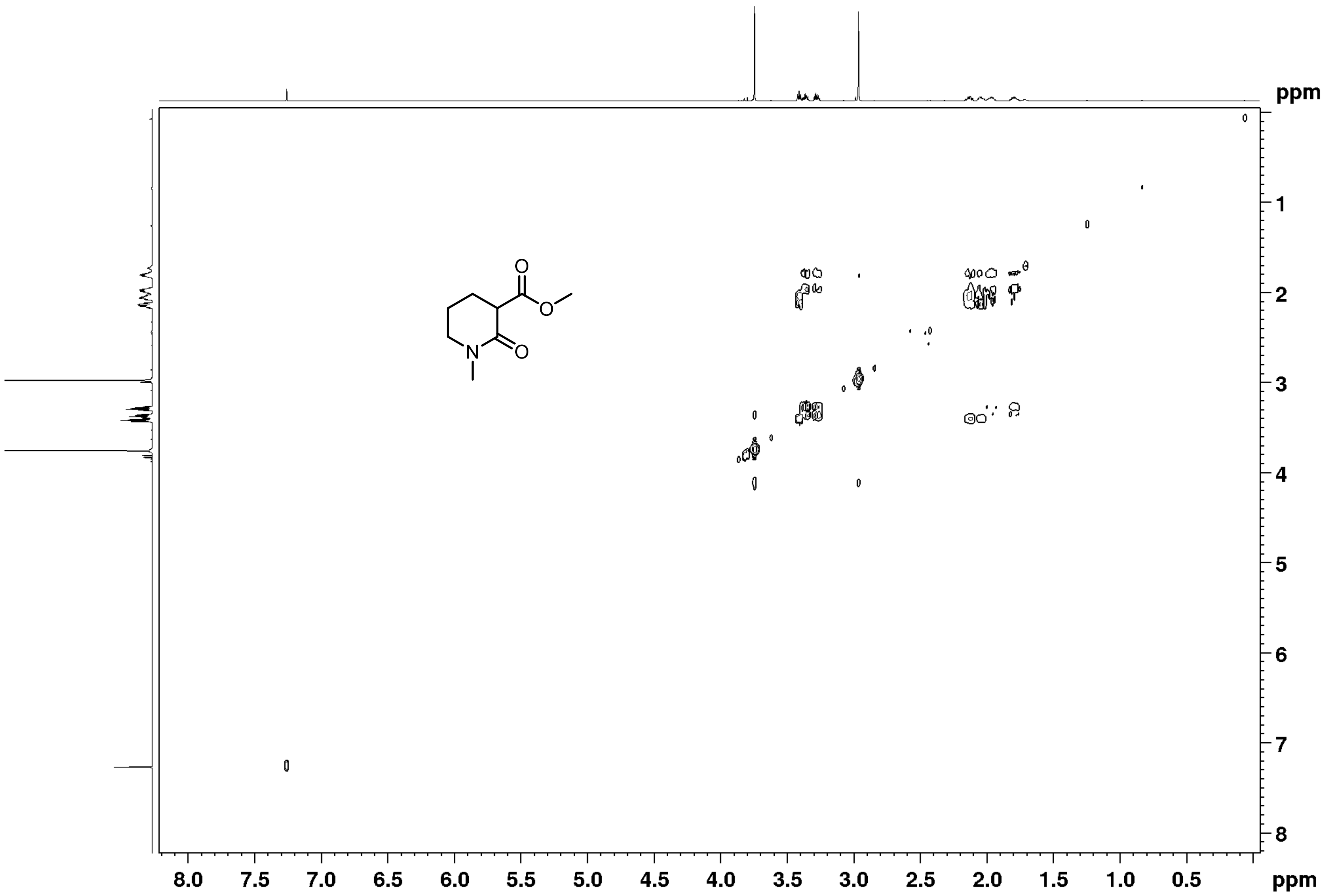

3.26.4 ¹H, ¹³C HMBC

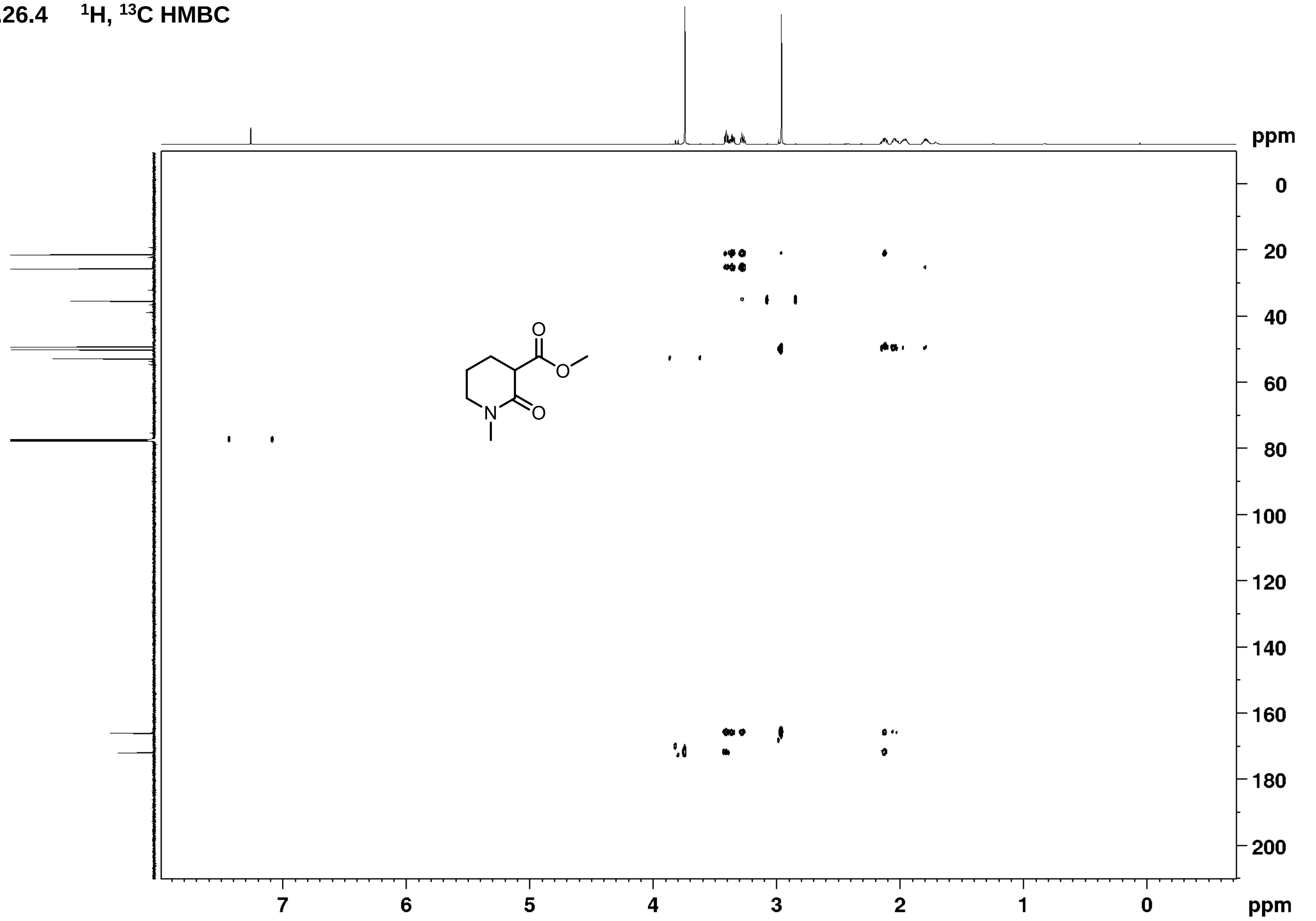

3.26.5 ^{1}H, ^{13}C HSQC

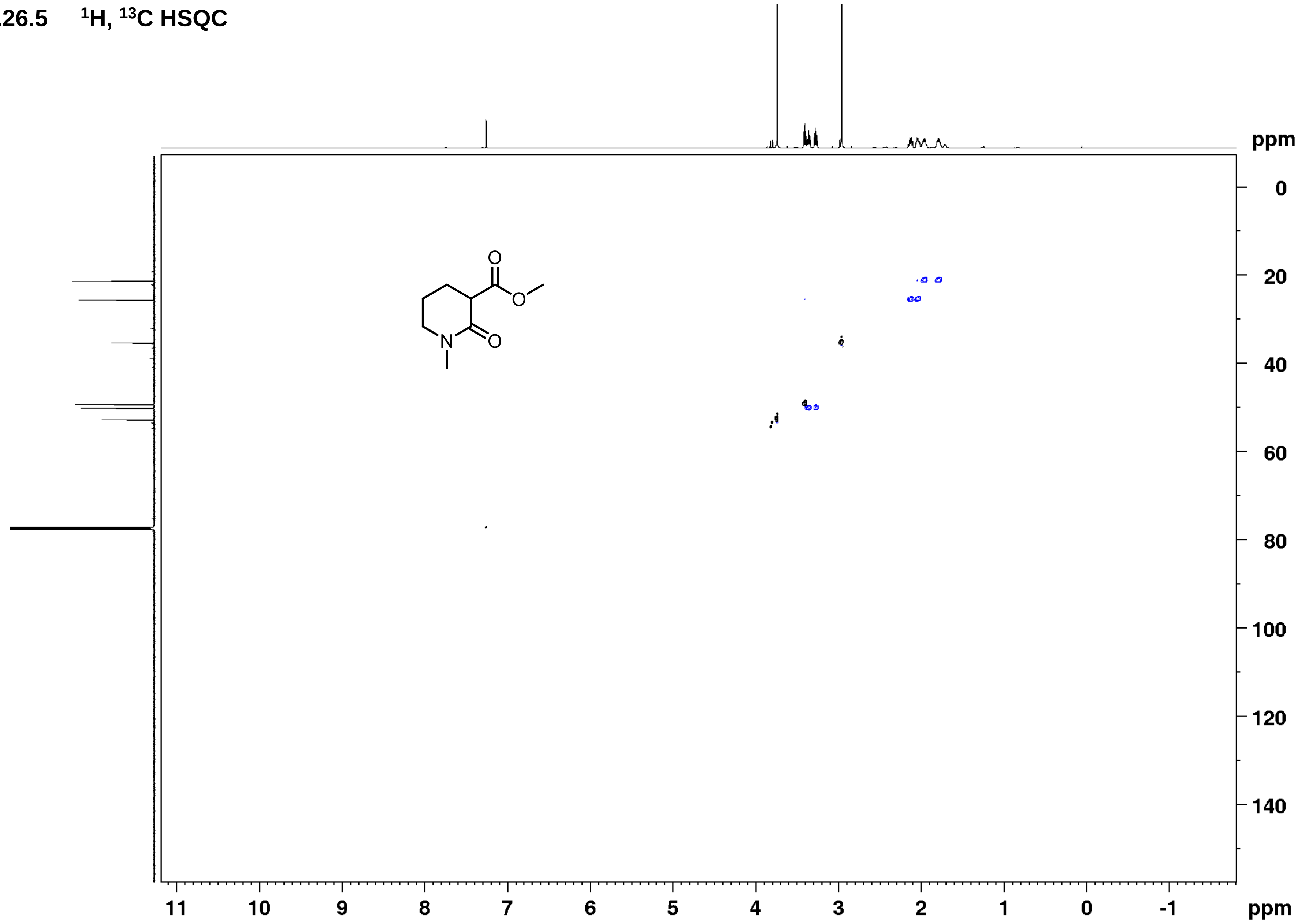

3.26.6 HRMS

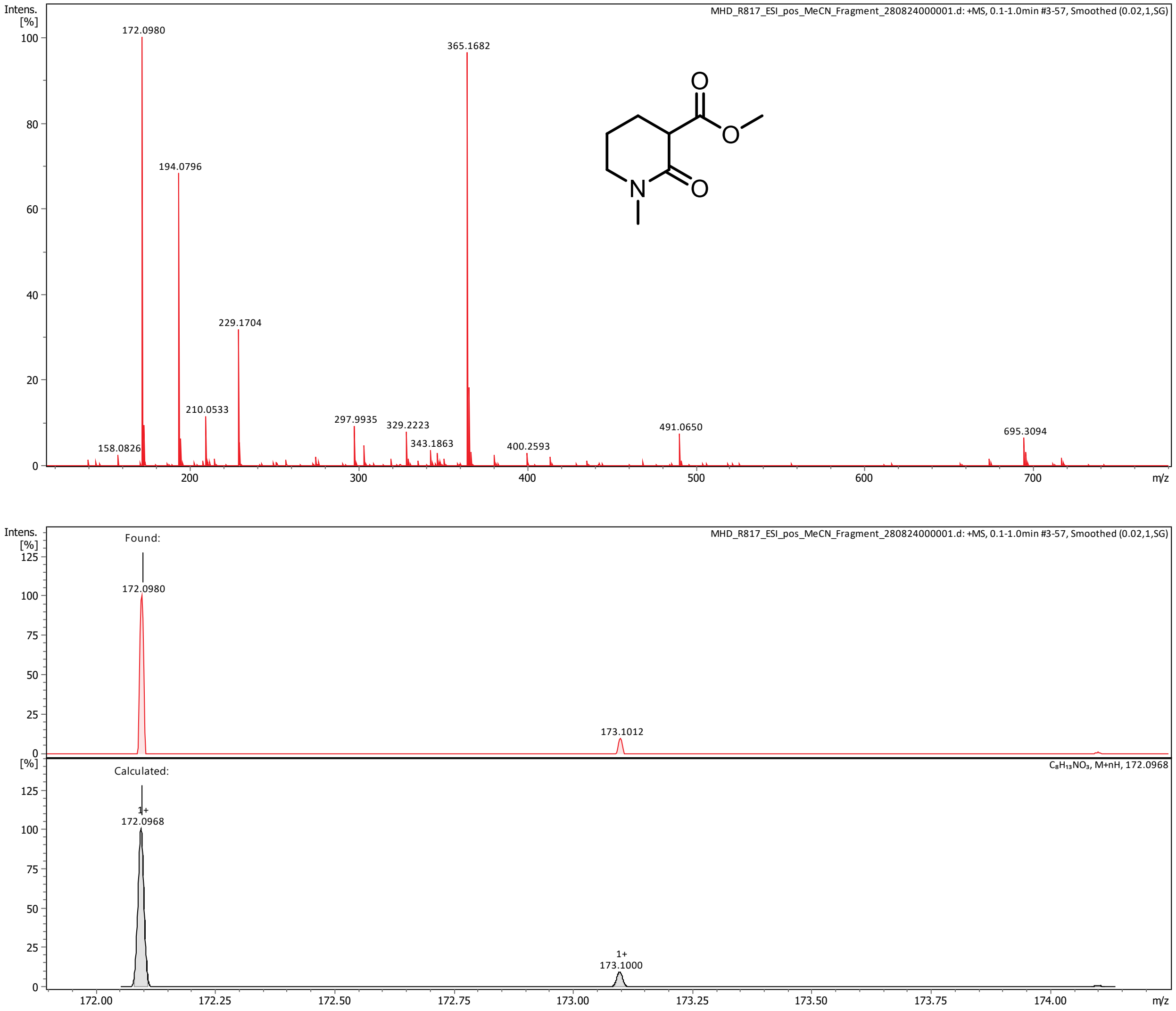

MHD_R817_ESI_pos_MeCN_Fragment_280824000001.d: +MS, 0.1-1.0min #3-57, Smoothed (0.02,1,SG)
Intens. [%]
172.0980
365.1682
194.0796
229.1704
210.0533
297.9935
329.2223
343.1863
158.0826
400.2593
491.0650
695.3094
m/z
Found:
172.0980
173.1012
Calculated:
C8H13NO3, M+nH, 172.0968
1+
172.0968
1+
173.1000

3.26.7 IR

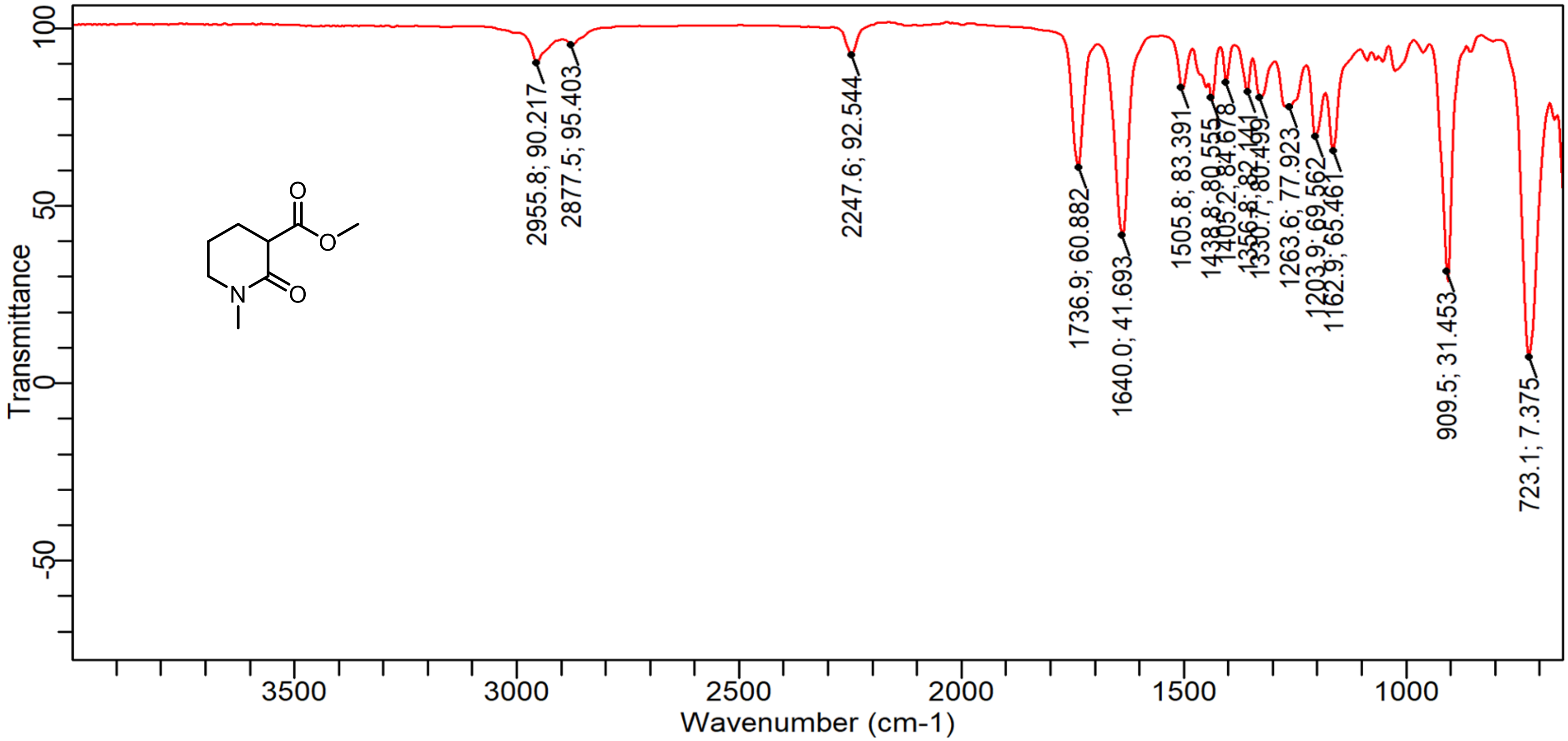

Transmittance
Wavenumber (cm-1)
2955.8; 90.217
2877.5; 95.403
2247.6; 92.544
1736.9; 60.882
1640.0; 41.693
1505.8; 83.391
1438.8; 80.555
1405.2; 84.678
1356.8; 82.141
1330.7; 80.499
1263.6; 77.923
1203.9; 69.562
1162.9; 65.461
909.5; 31.453
723.1; 7.375

3.27 3-phenylpropanamide (12aa) (600 MHz, $CDCl_3$)

3.27.1 ^{1}H NMR

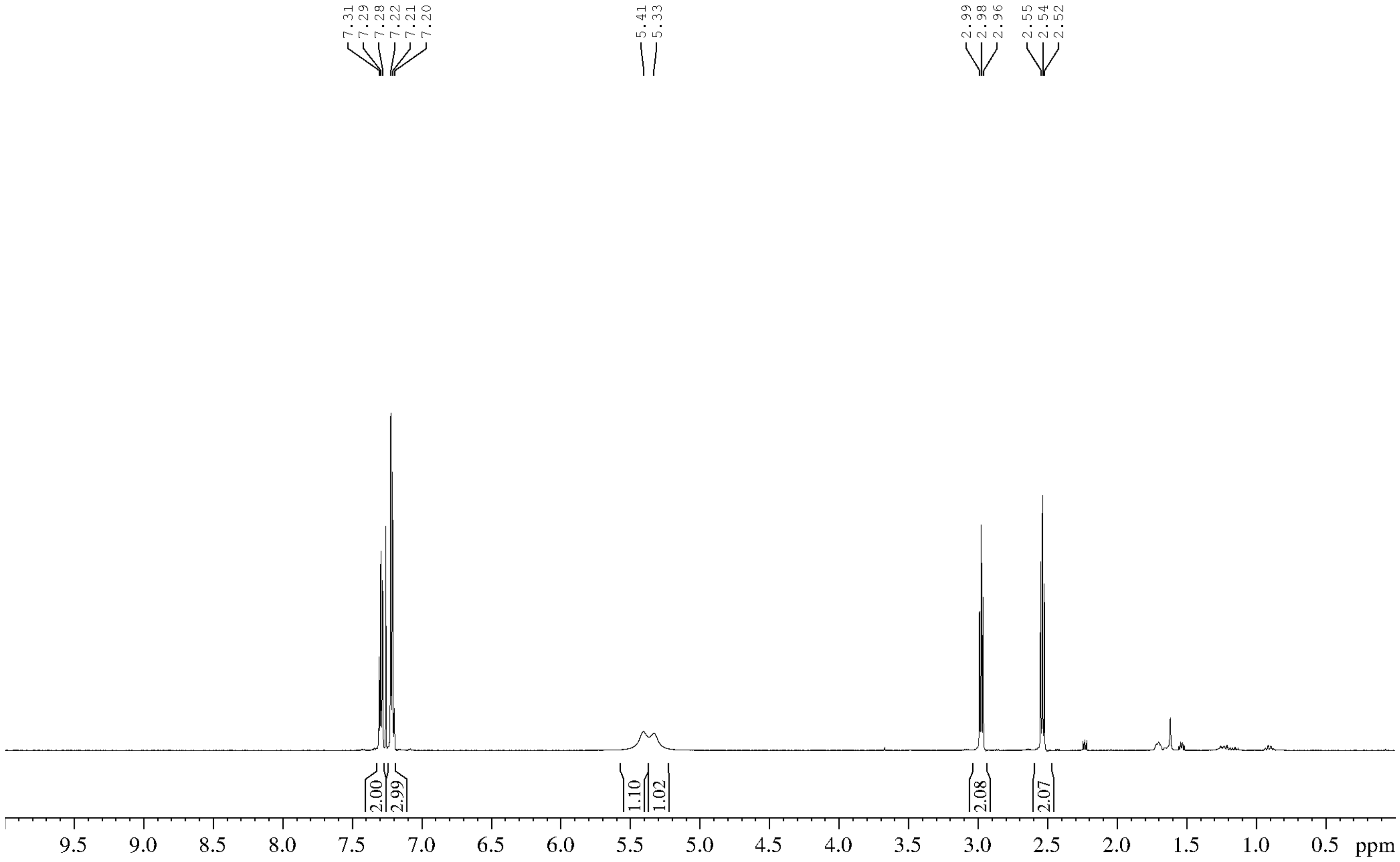

3.27.2 ^{13}C NMR

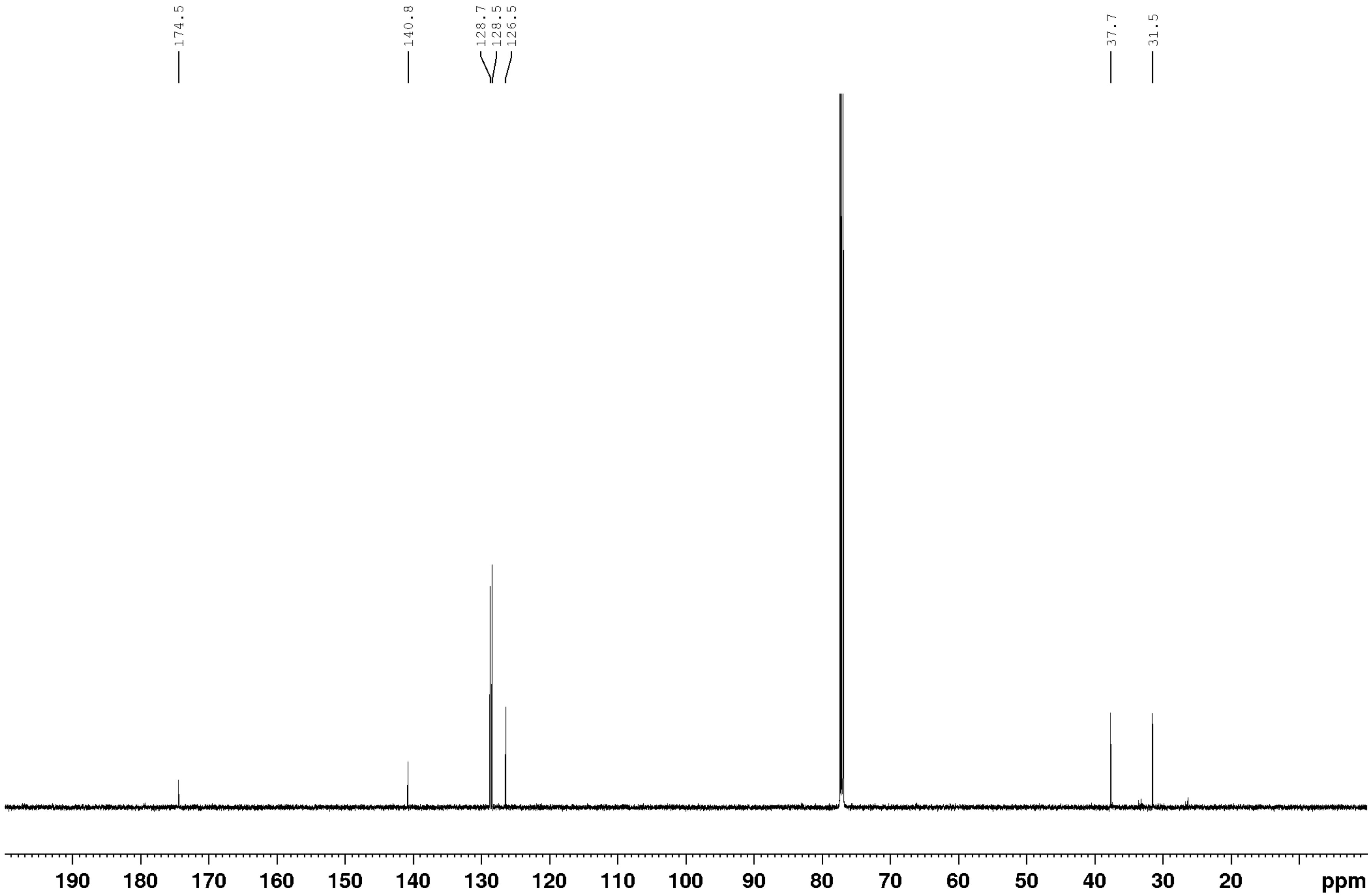
174.5
140.8
128.7
128.5
126.5
37.7
31.5
190
180
170
160
150
140
130
120
110
100
90
80
70
60
50
40
30
20
ppm

3.27.3 ^{1}H ^{1}H COSY

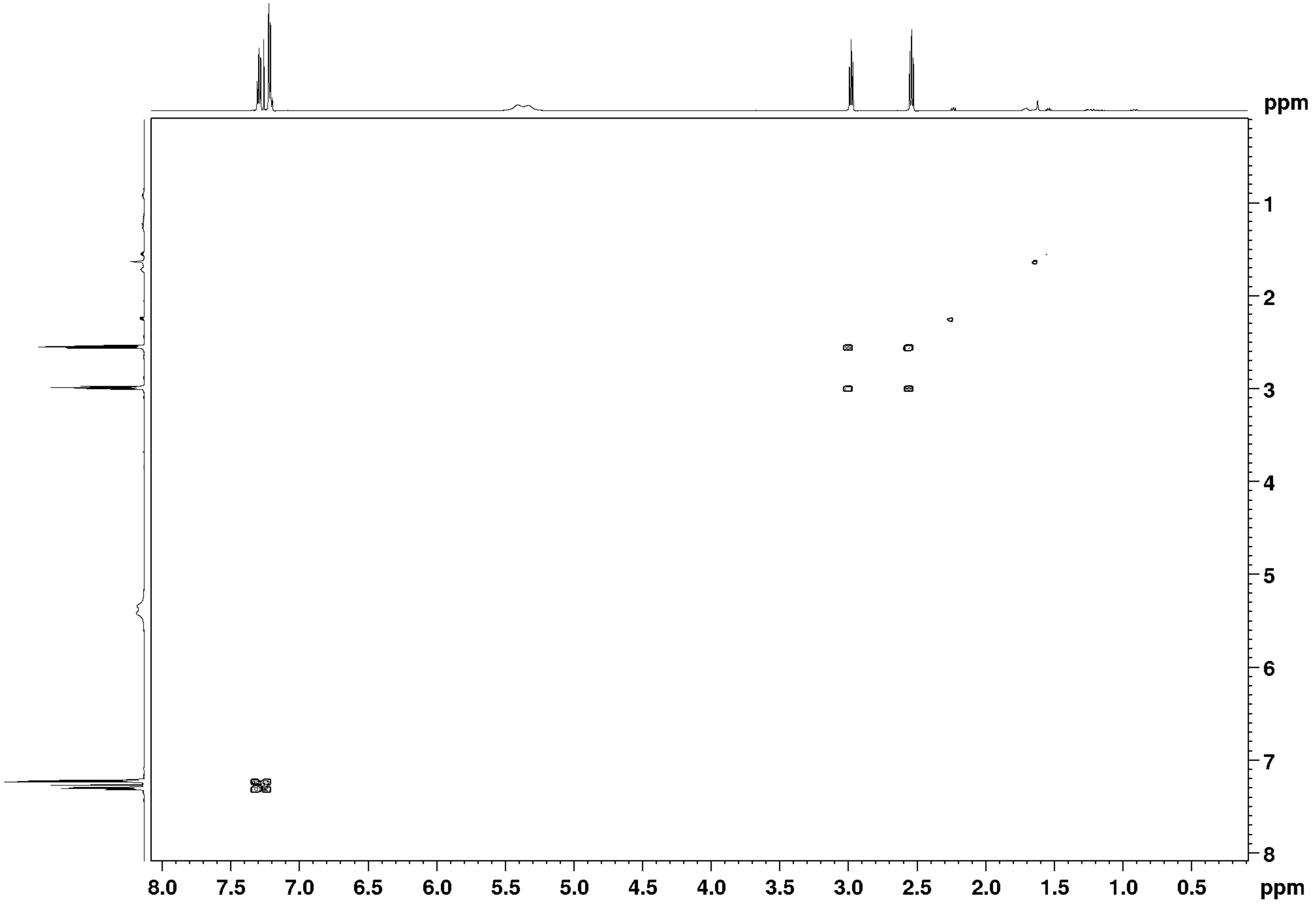

3.27.4 ^{1}H ^{13}C HMBC

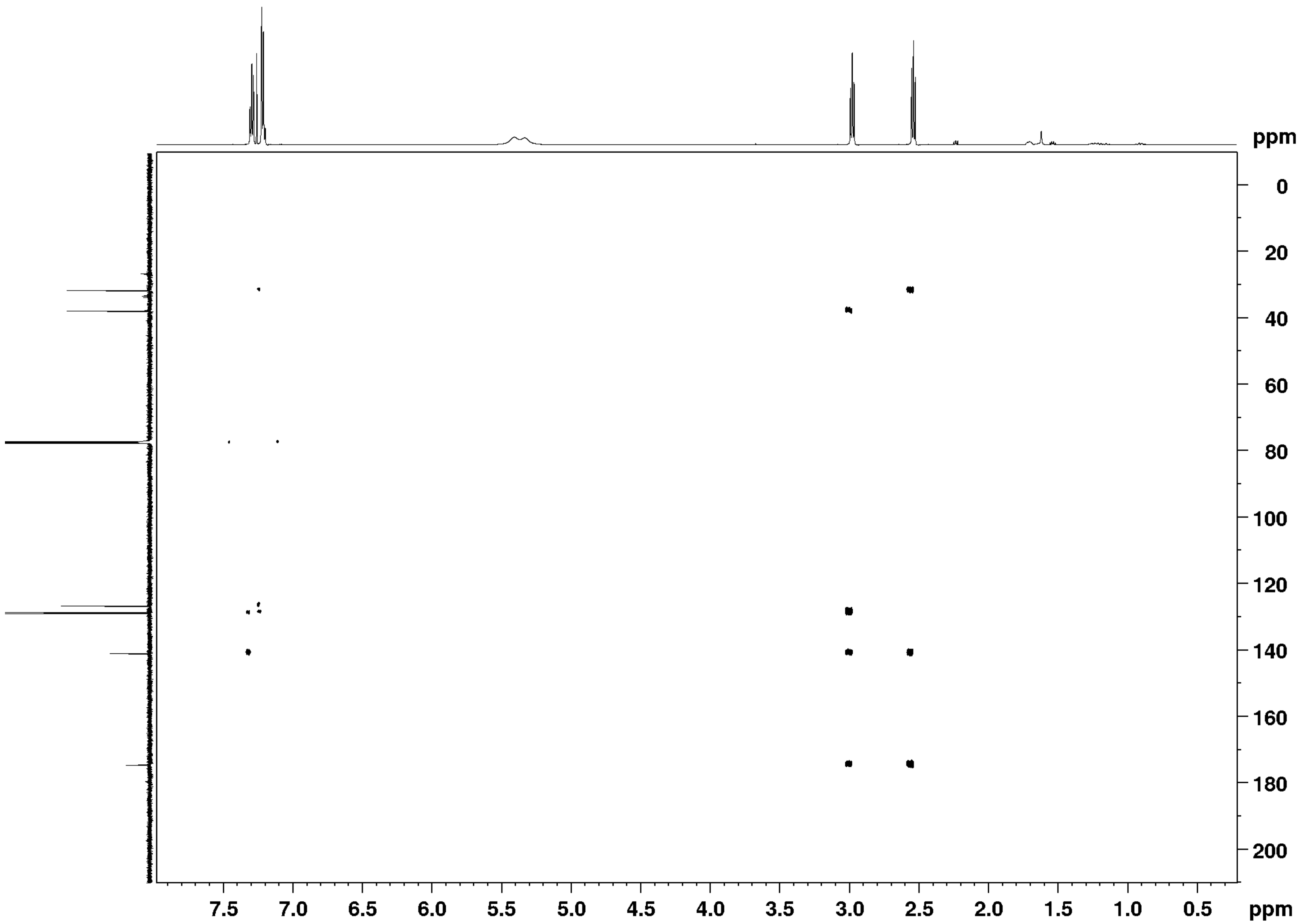

3.27.5 ^{1}H ^{13}C HSQC

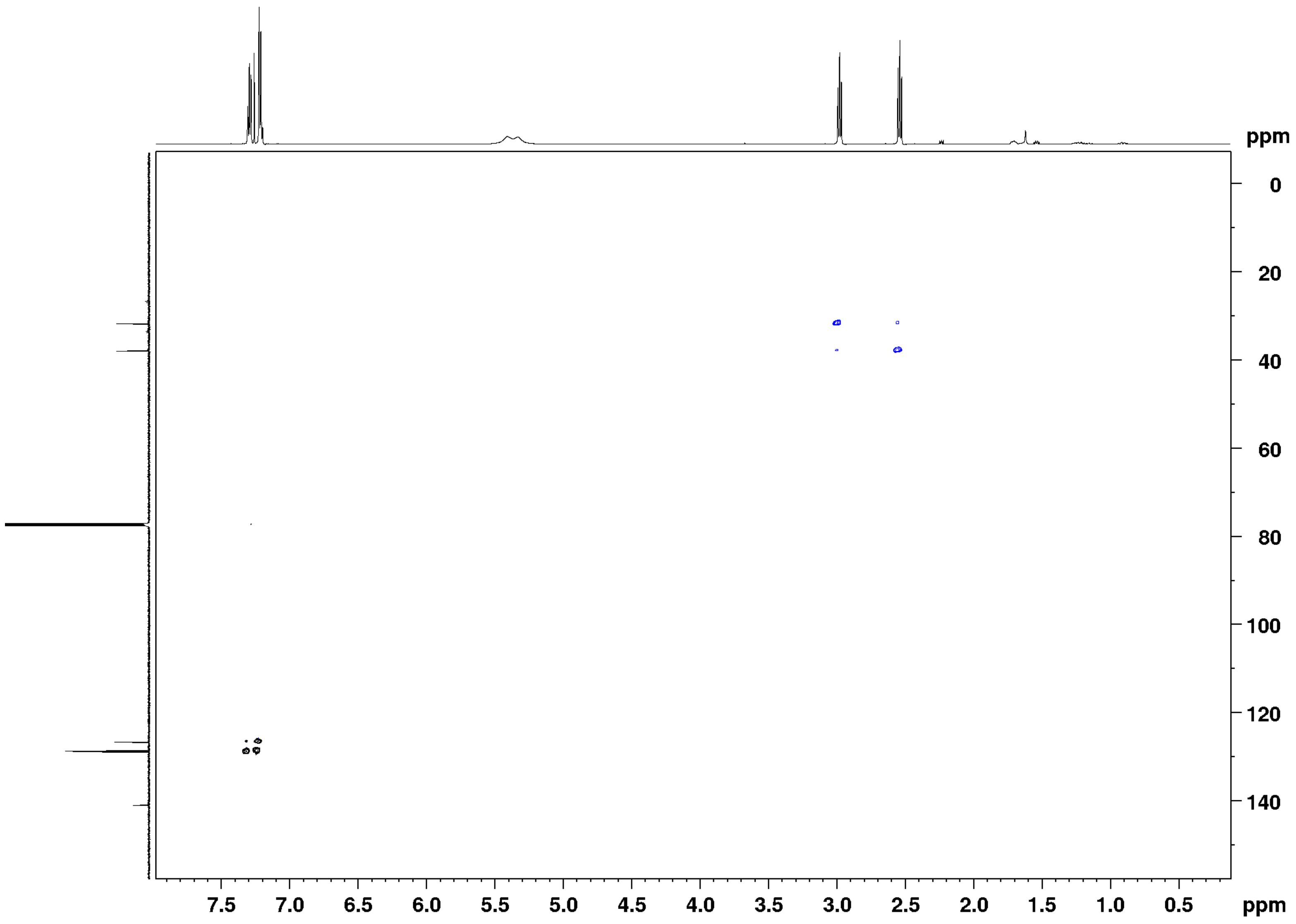

3.27.6 GC-MS

3.28 *N*-methyl-3-phenylpropanamide (12ab) (600 MHz, $CDCl_3$)

3.28.1 ^1H NMR

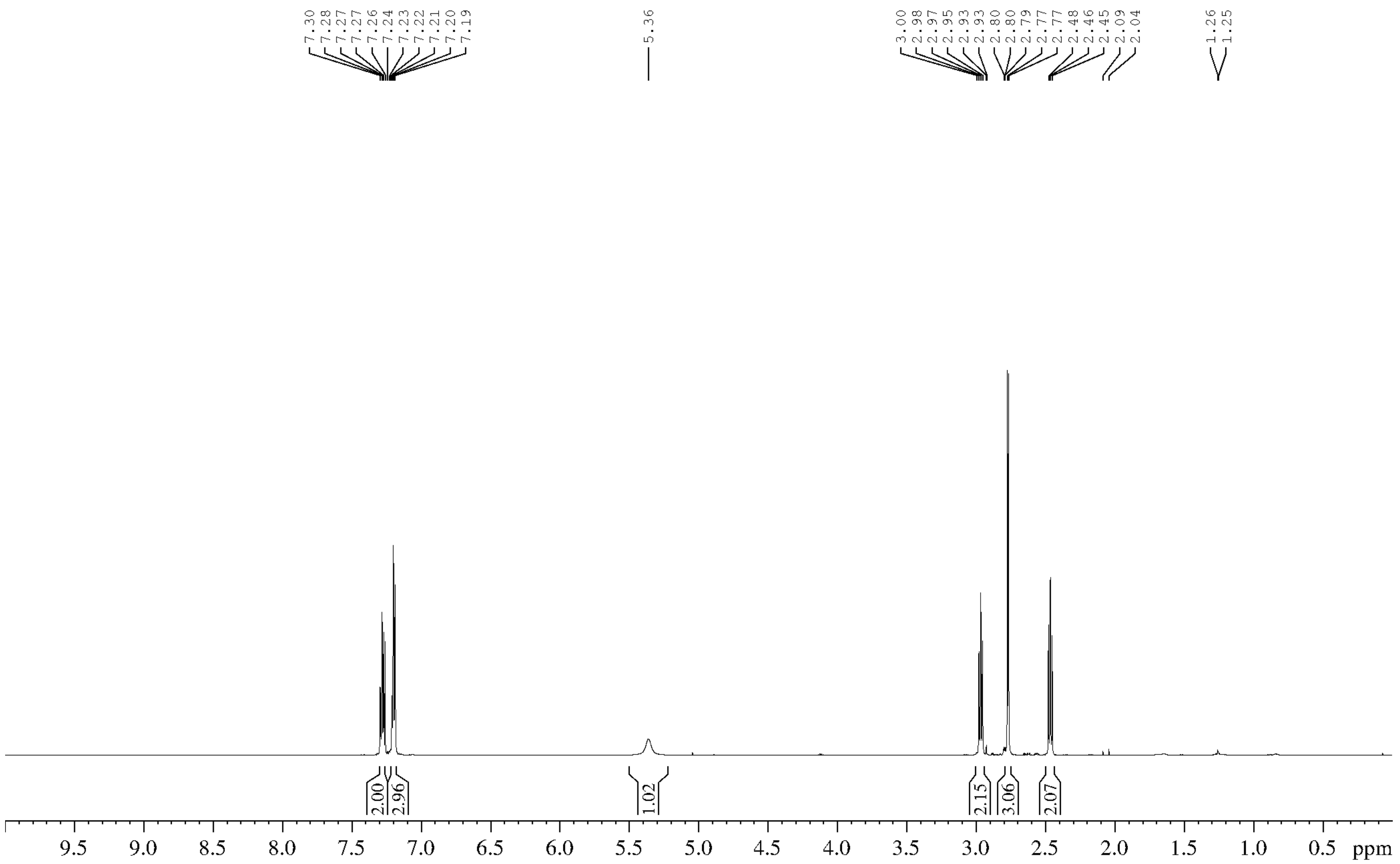

3.28.2 ^{13}C NMR

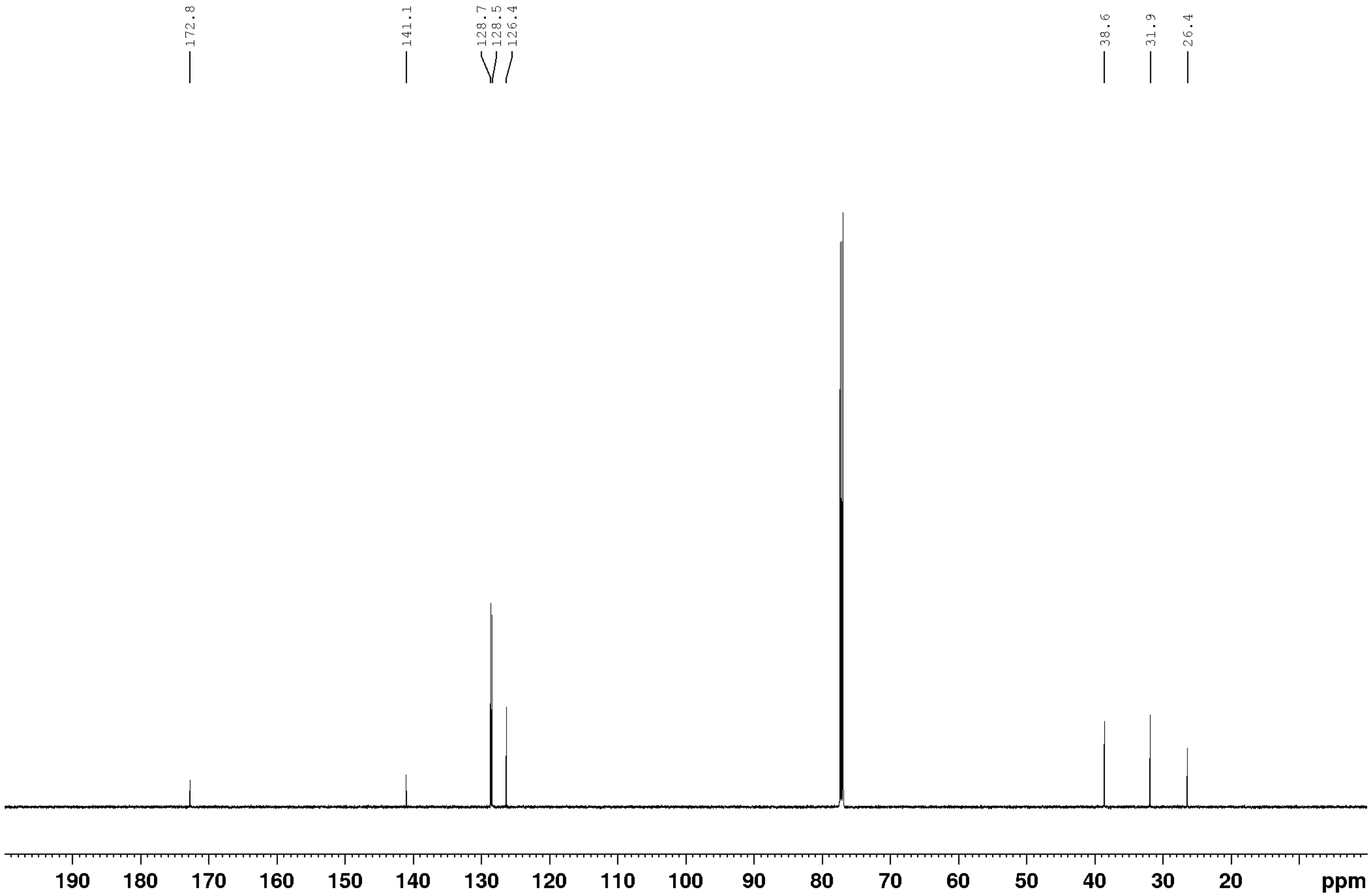
172.8
141.1
128.7
128.5
126.4
38.6
31.9
26.4
190 180 170 160 150 140 130 120 110 100 90 80 70 60 50 40 30 20
ppm

3.28.3 ^{1}H ^{1}H COSY

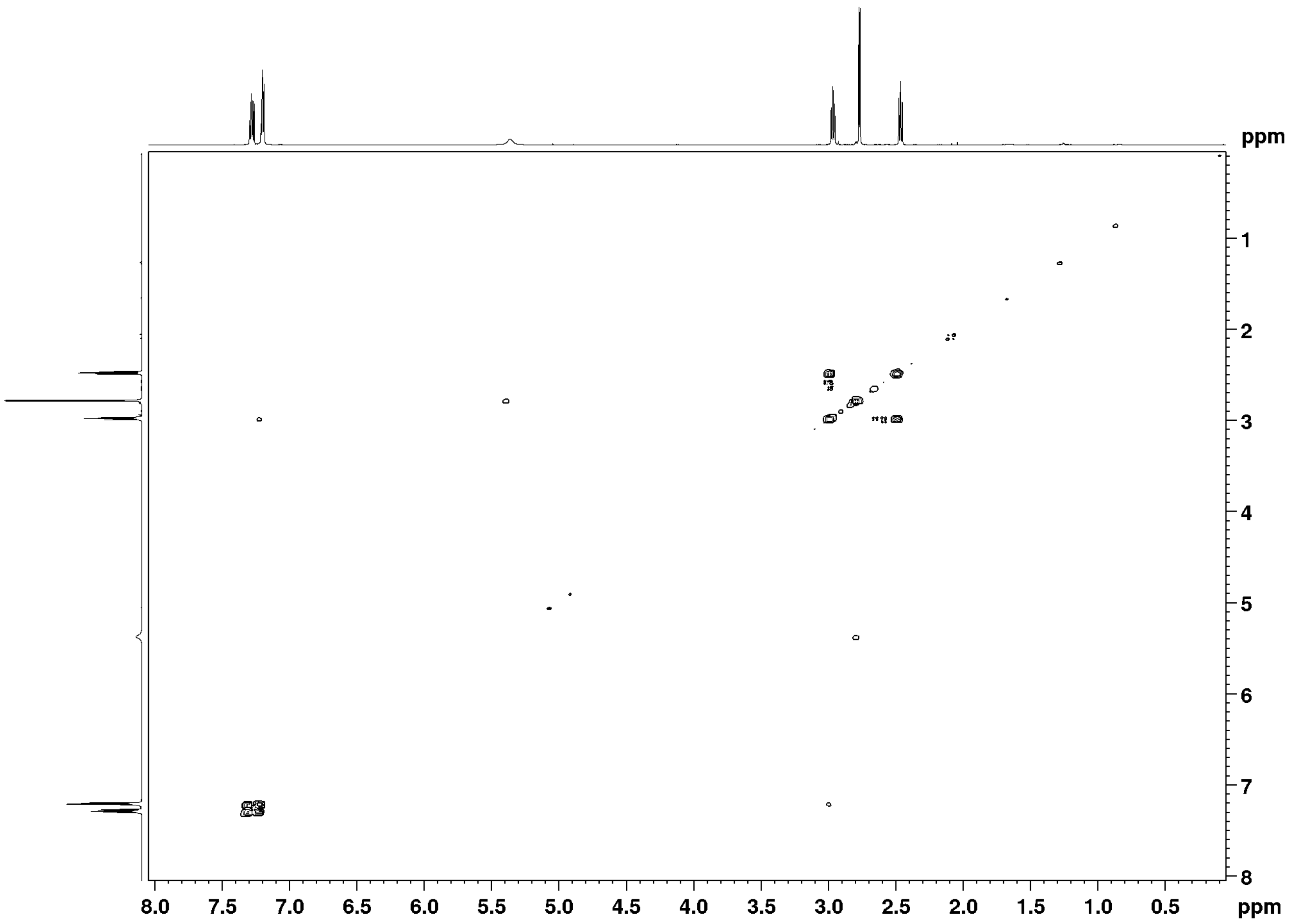

3.28.4 ^{1}H ^{13}C HMBC

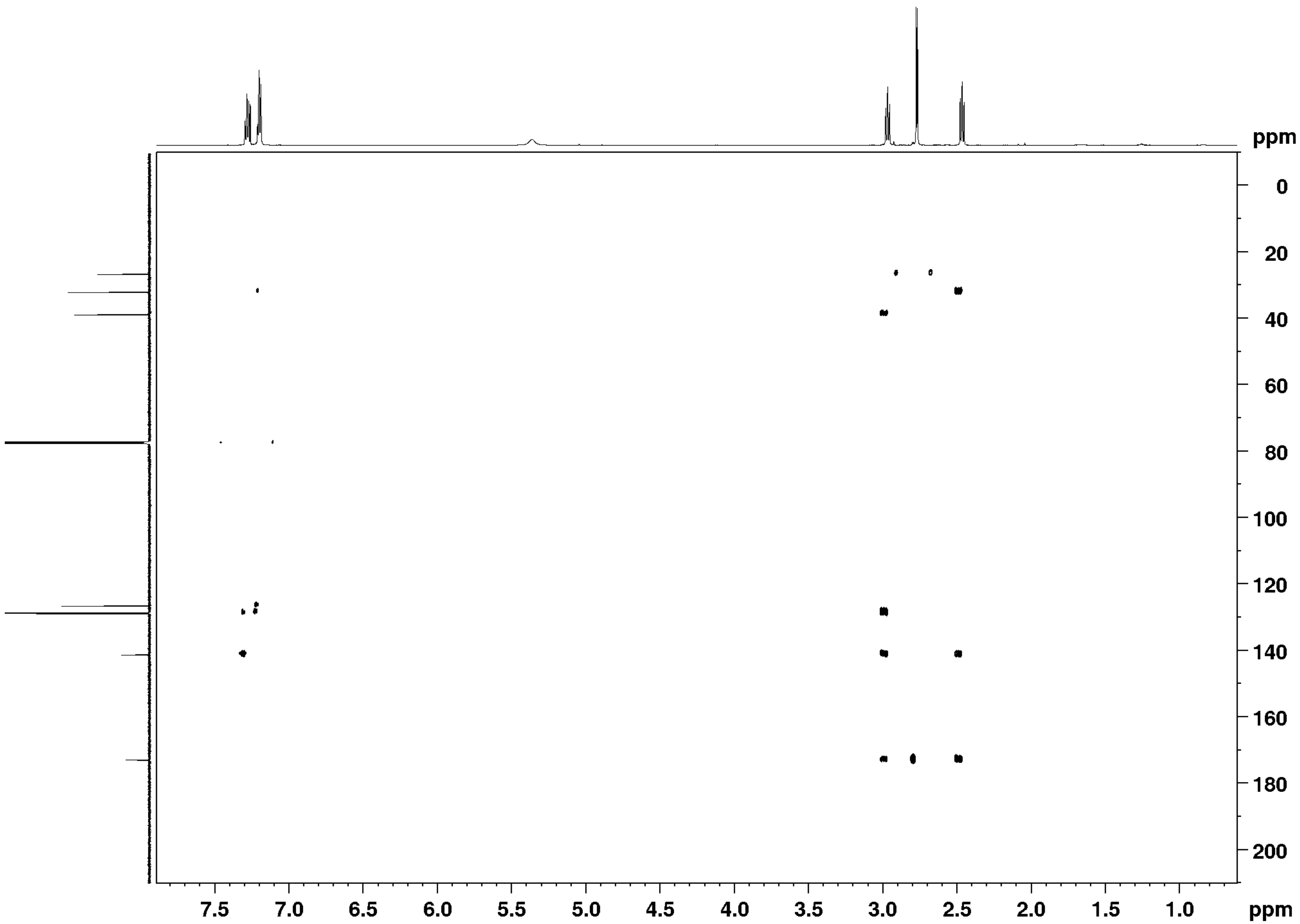

3.28.5 ^{1}H ^{13}C HSQC

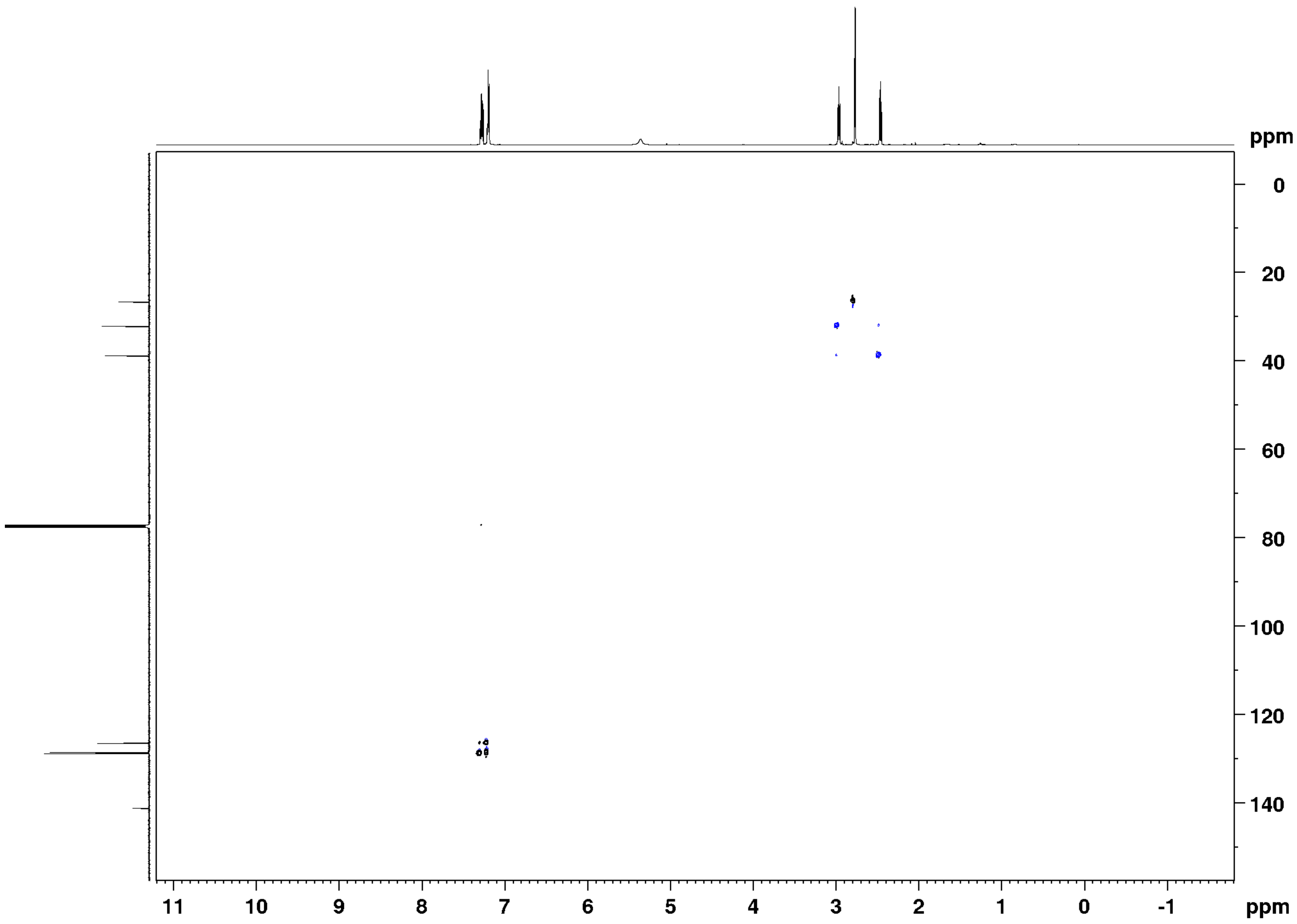

3.28.6 HRMS

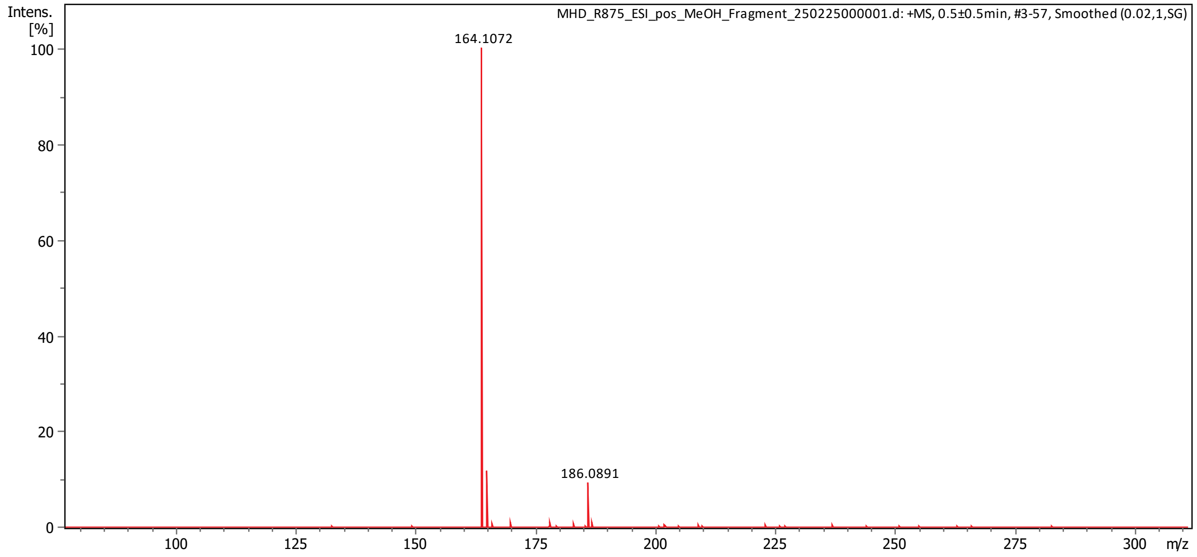

Intens. [%]
MHD_R875_ESI_pos_MeOH_Fragment_250225000001.d: +MS, 0.5±0.5min, #3-57, Smoothed (0.02,1,SG)
164.1072
186.0891
m/z

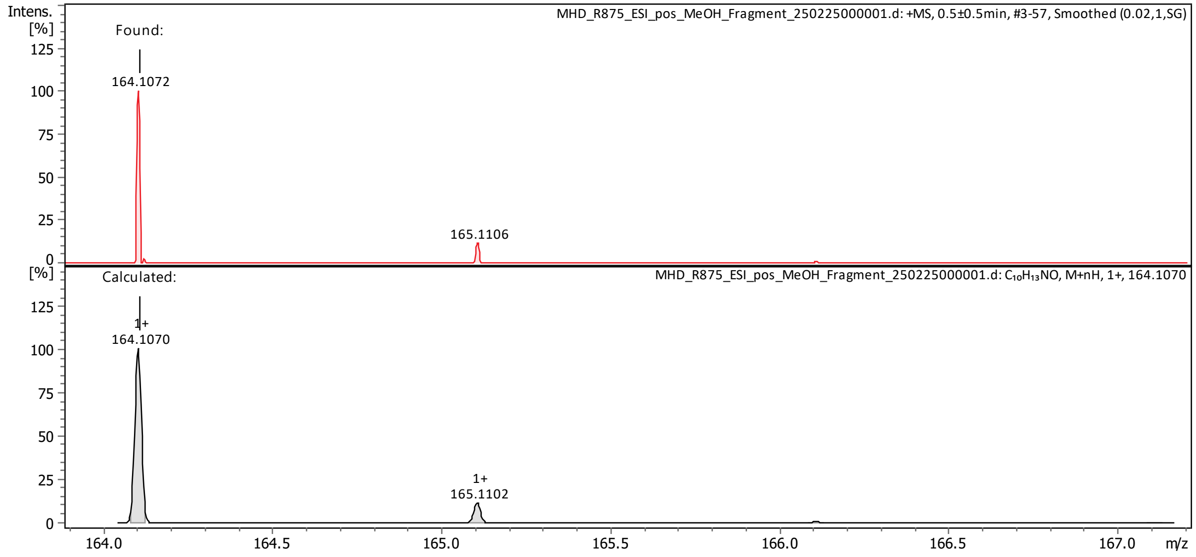

Intens. [%]
MHD_R875_ESI_pos_MeOH_Fragment_250225000001.d: +MS, 0.5±0.5min, #3-57, Smoothed (0.02,1,SG)
Found:
164.1072
165.1106
Calculated:
MHD_R875_ESI_pos_MeOH_Fragment_250225000001.d: C₁₀H₁₃NO, M+nH, 1+, 164.1070
1+
164.1070
1+
165.1102
m/z

3.29 *N*-(3,4-Dimethoxyphenethyl)-*N*-methyl-3-phenylpropanamide (12ba & 12ba_rotamer) (600 MHz, $CDCl_3$)

3.29.1 ^{1}H NMR

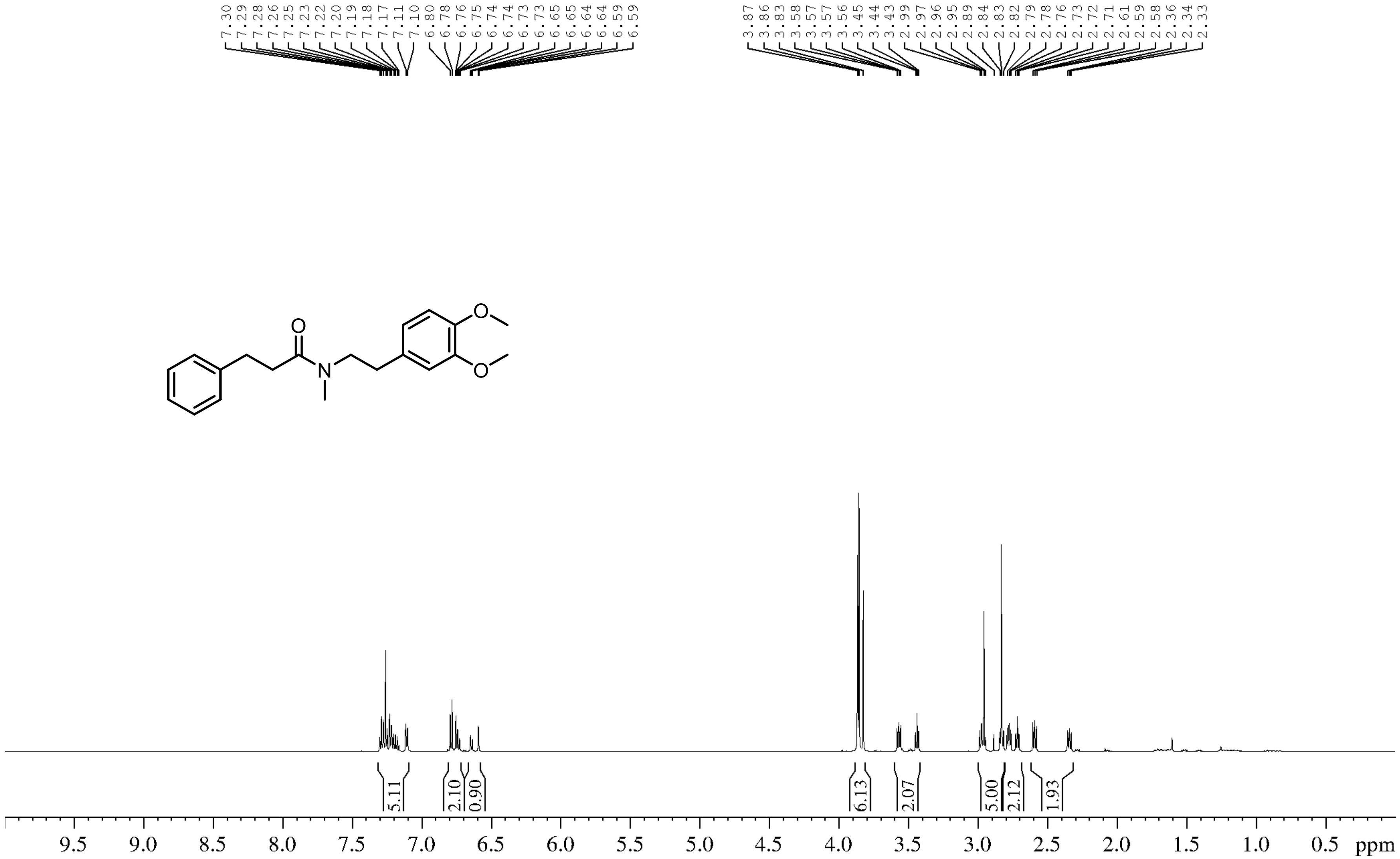

3.29.2 ^{13}C NMR

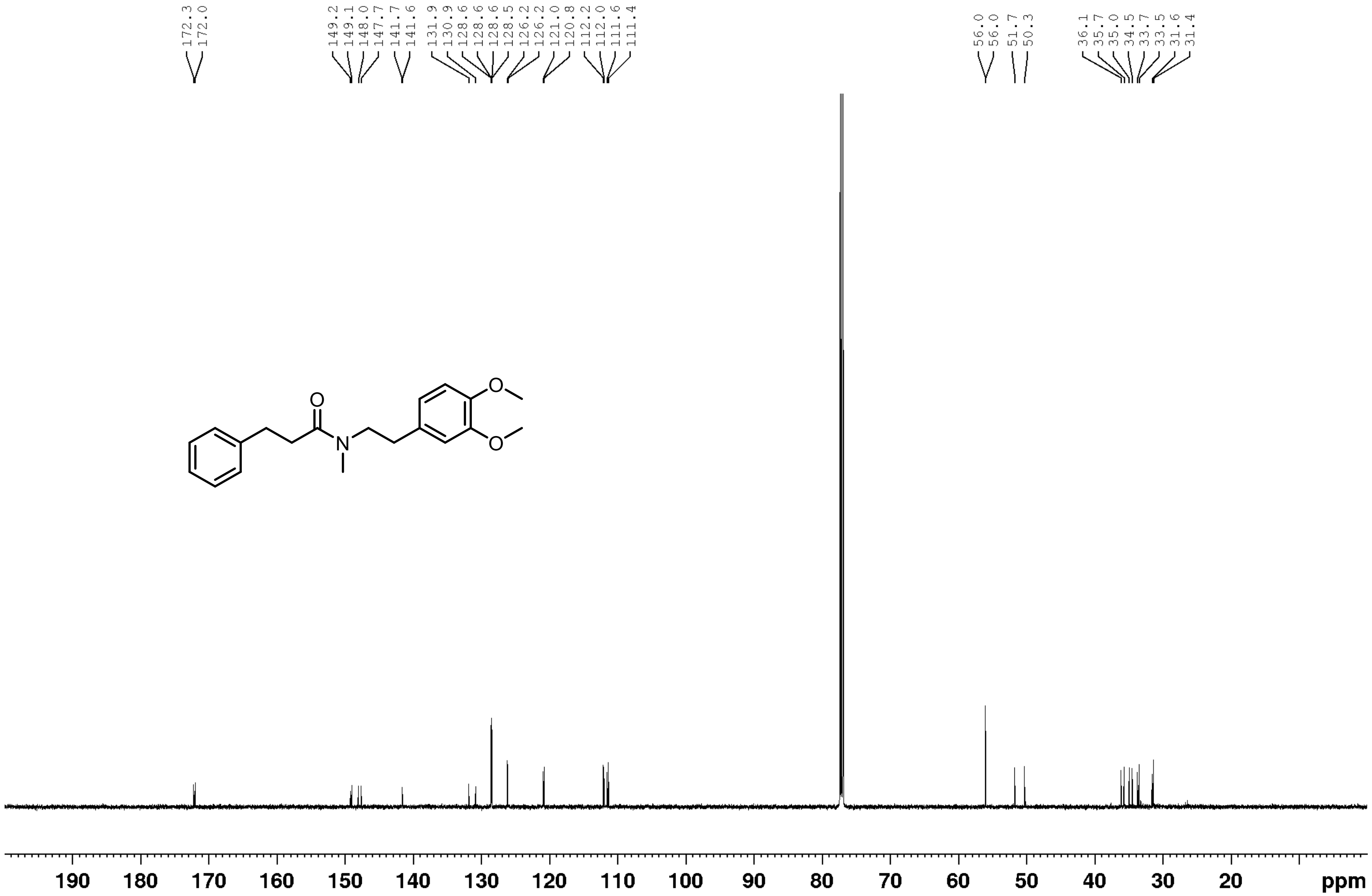

172.3
172.0
149.2
149.1
148.0
147.7
141.7
141.6
131.9
130.9
128.6
128.6
128.6
128.5
126.2
126.2
121.0
120.8
112.2
112.0
111.6
111.4
56.0
56.0
51.7
50.3
36.1
35.7
35.0
34.5
33.7
33.5
31.6
31.4
O
N
O
O
190 180 170 160 150 140 130 120 110 100 90 80 70 60 50 40 30 20 ppm

3.29.3 ^{1}H, ^{1}H COSY

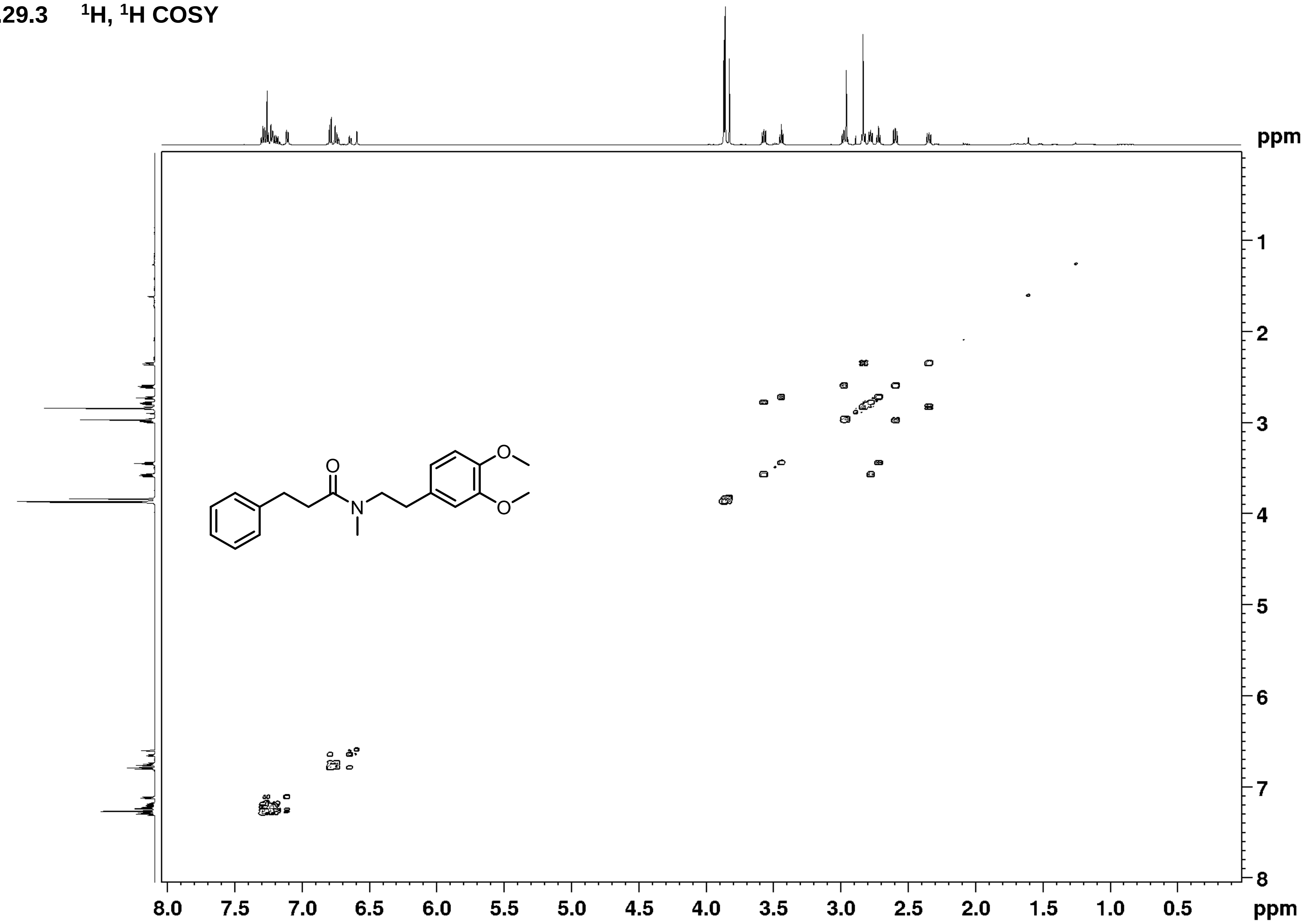

3.29.4 ^{1}H, ^{13}C HMBC

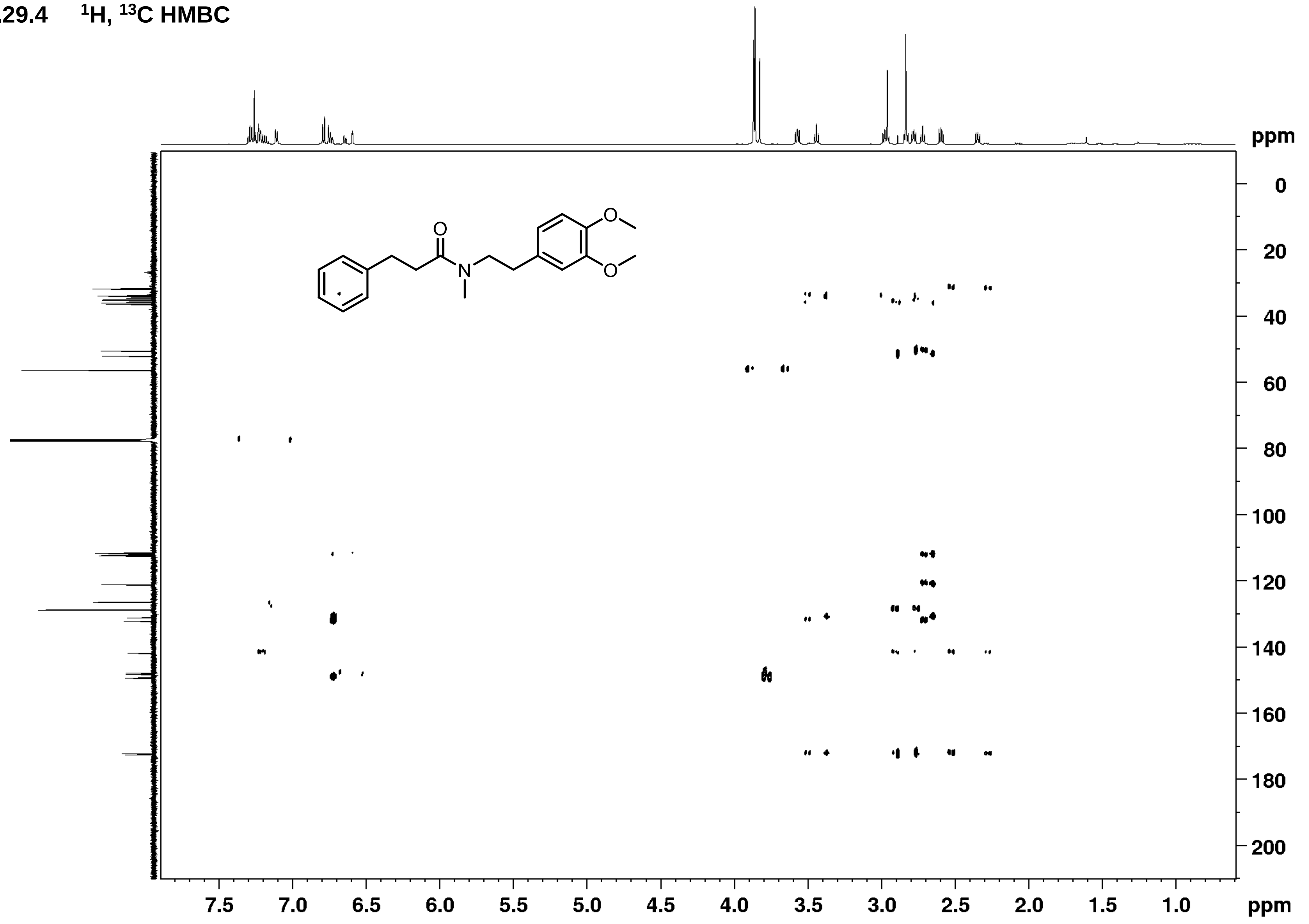

3.29.5 ^{1}H, ^{13}C HSQC

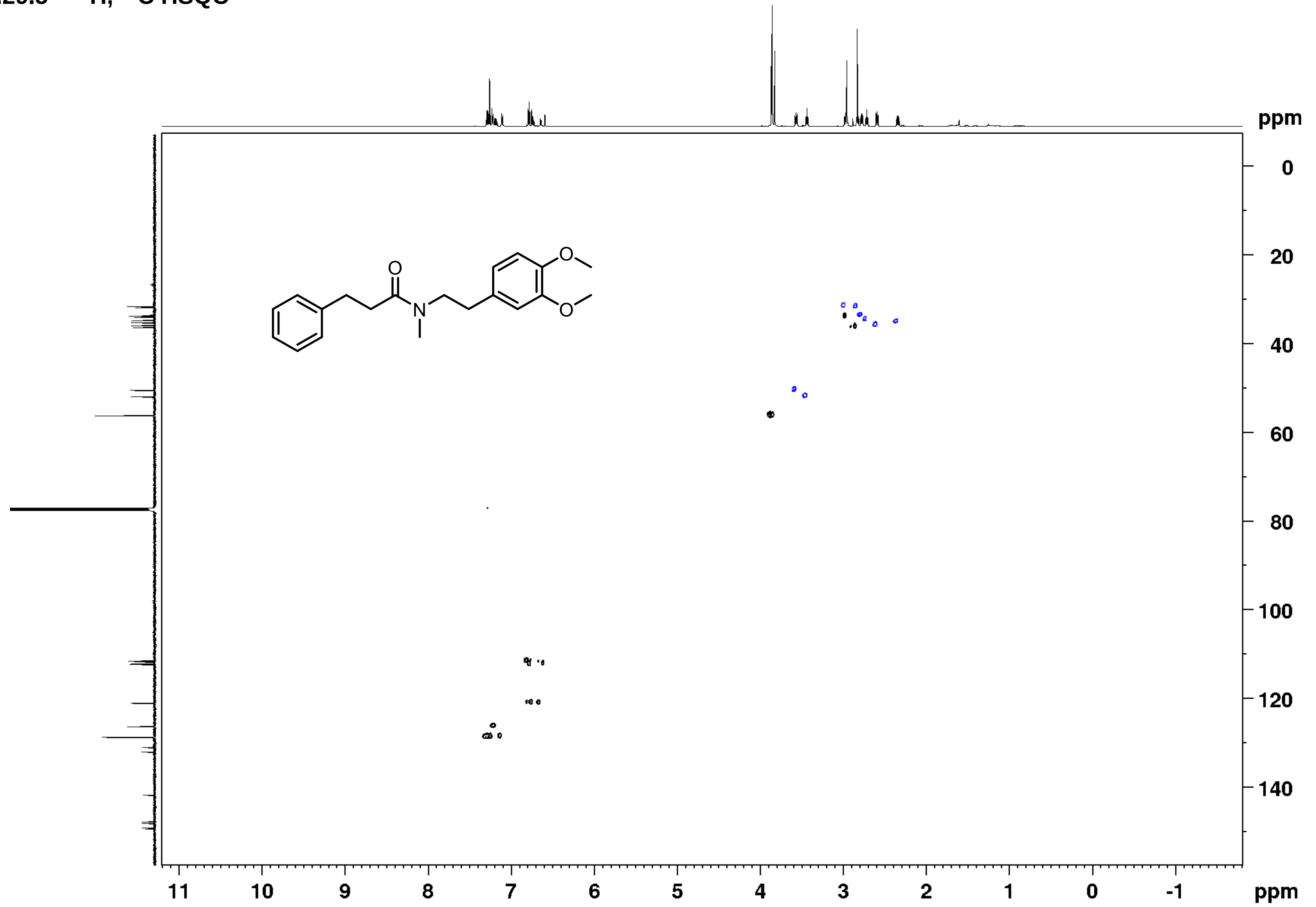

3.29.6 HRMS

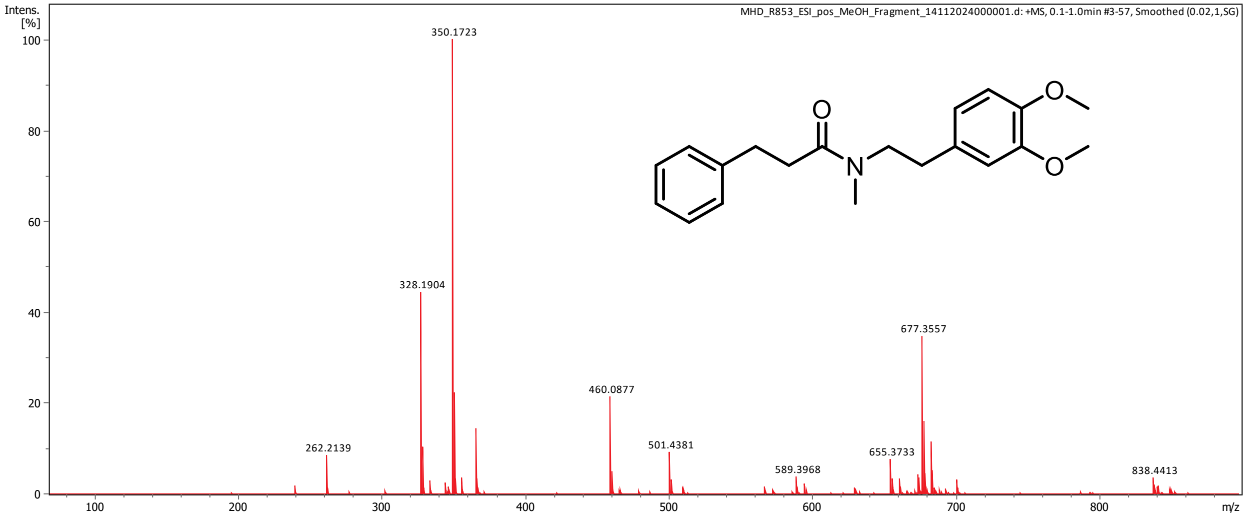
MHD_R853_ESI_pos_MeOH_Fragment_14112024000001.d: +MS, 0.1-1.0min #3-57, Smoothed (0.02,1,SG)
Intens. [%]
350.1723
328.1904
262.2139
460.0877
501.4381
589.3968
655.3733
677.3557
838.4413
m/z
O
N
O
O

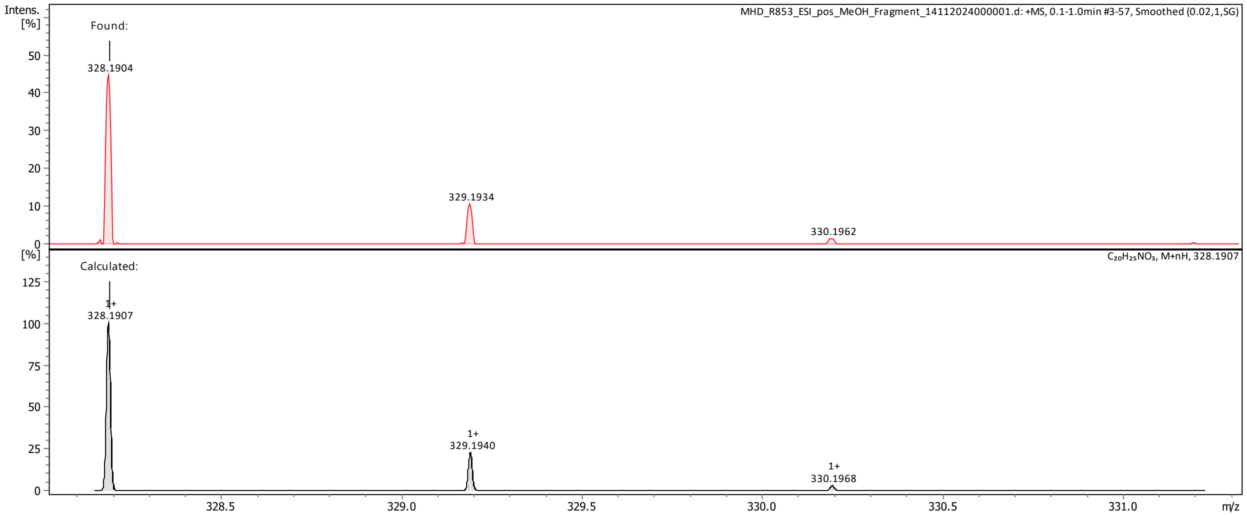
MHD_R853_ESI_pos_MeOH_Fragment_14112024000001.d: +MS, 0.1-1.0min #3-57, Smoothed (0.02,1,SG)
Intens. [%]
Found:
328.1904
329.1934
330.1962
C20H25NO3, M+nH, 328.1907
Calculated:
1+ 328.1907
1+ 329.1940
1+ 330.1968
m/z

3.29.7 IR

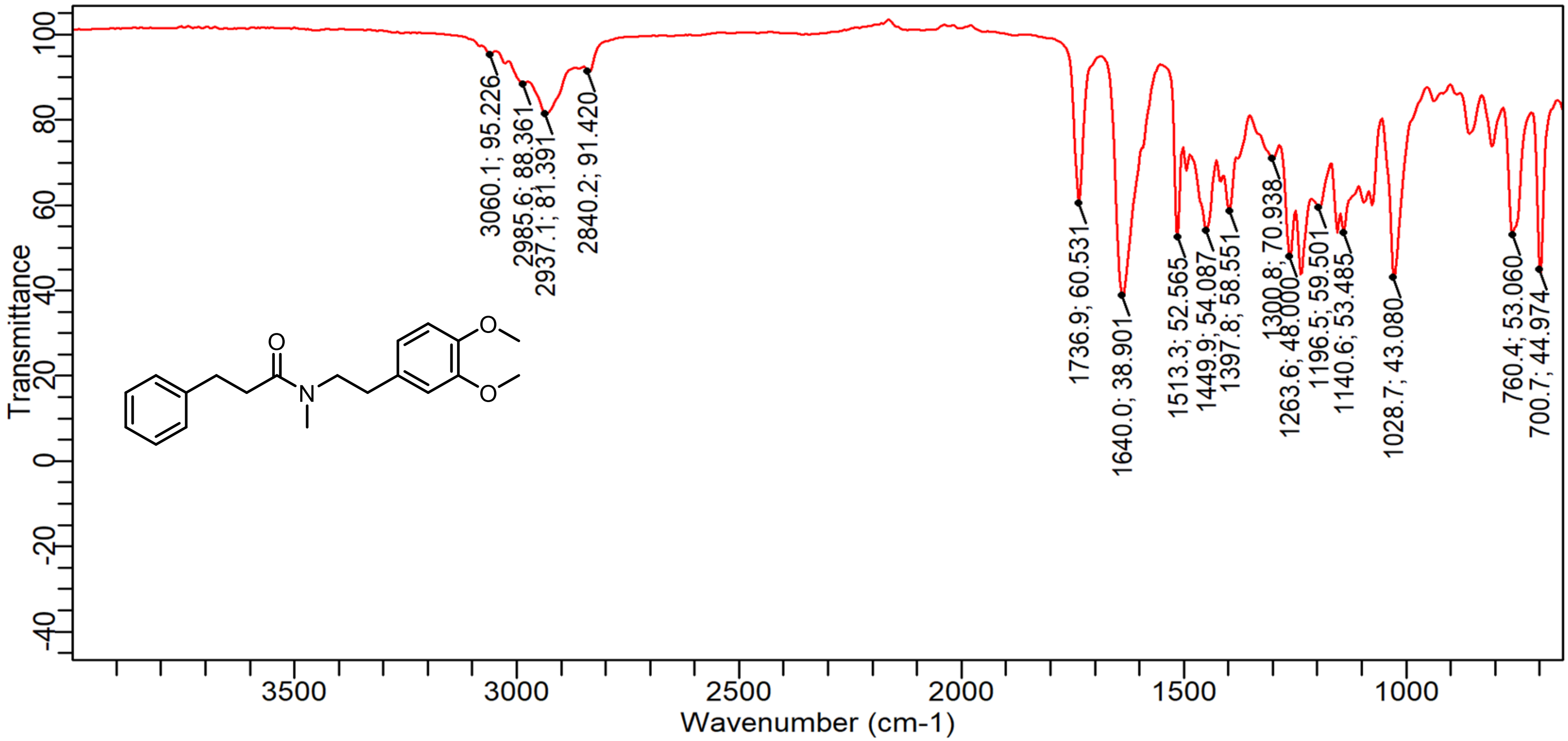
Transmittance
Wavenumber (cm-1)
3060.1; 95.226
2985.6; 88.361
2937.1; 81.391
2840.2; 91.420
1736.9; 60.531
1640.0; 38.901
1513.3; 52.565
1449.9; 54.087
1397.8; 58.551
1300.8; 70.938
1263.6; 48.000
1196.5; 59.501
1140.6; 53.485
1028.7; 43.080
760.4; 53.060
700.7; 44.974
O
N

3.30 (1*R*,5*S*)-3-(3-Phenylpropanoyl)-1,2,3,4,5,6-hexahydro-8*H*-1,5-methanopyrido[1,2-*a*][1,5]diazocin-8-one (12bb & 12bb_rotamer) (600 MHz, $CDCl_3$)

3.30.1 ^{1}H NMR

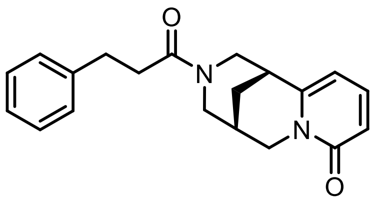

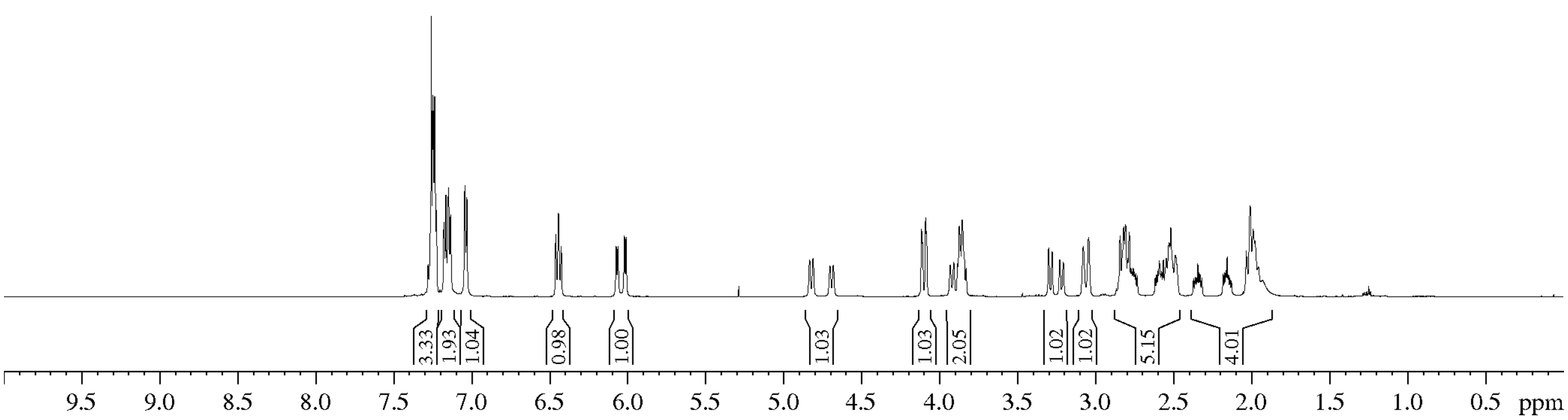

3.30.2 ^{13}C NMR

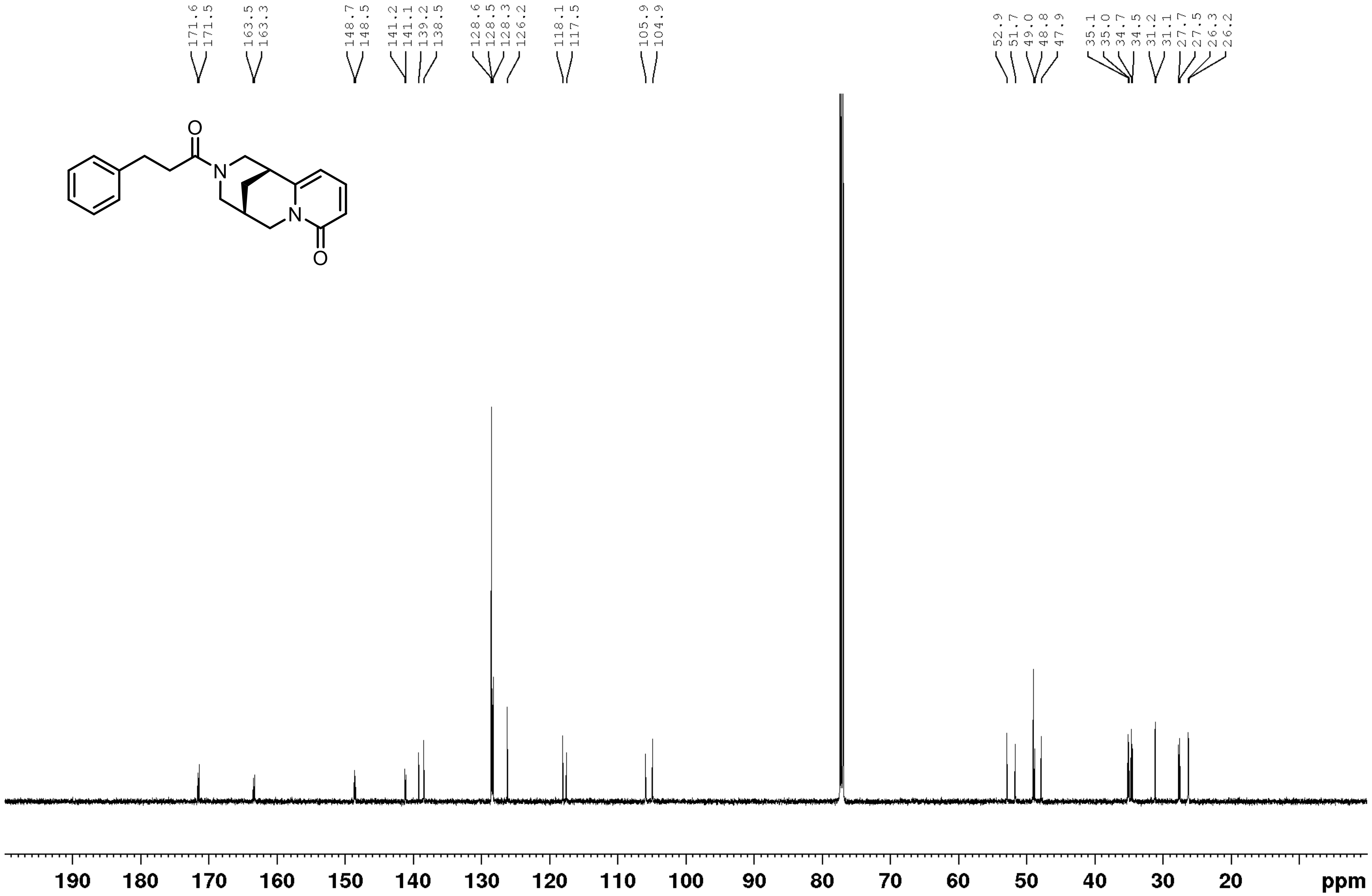

171.6
171.5
163.5
163.3
148.7
148.5
141.2
141.1
139.2
138.5
128.6
128.5
128.3
126.2
118.1
117.5
105.9
104.9
52.9
51.7
49.0
48.8
47.9
35.1
35.0
34.7
34.5
31.2
31.1
27.7
27.5
26.3
26.2
O
N
N
O
190
180
170
160
150
140
130
120
110
100
90
80
70
60
50
40
30
20
ppm

3.30.3 ^{1}H, ^{1}H COSY

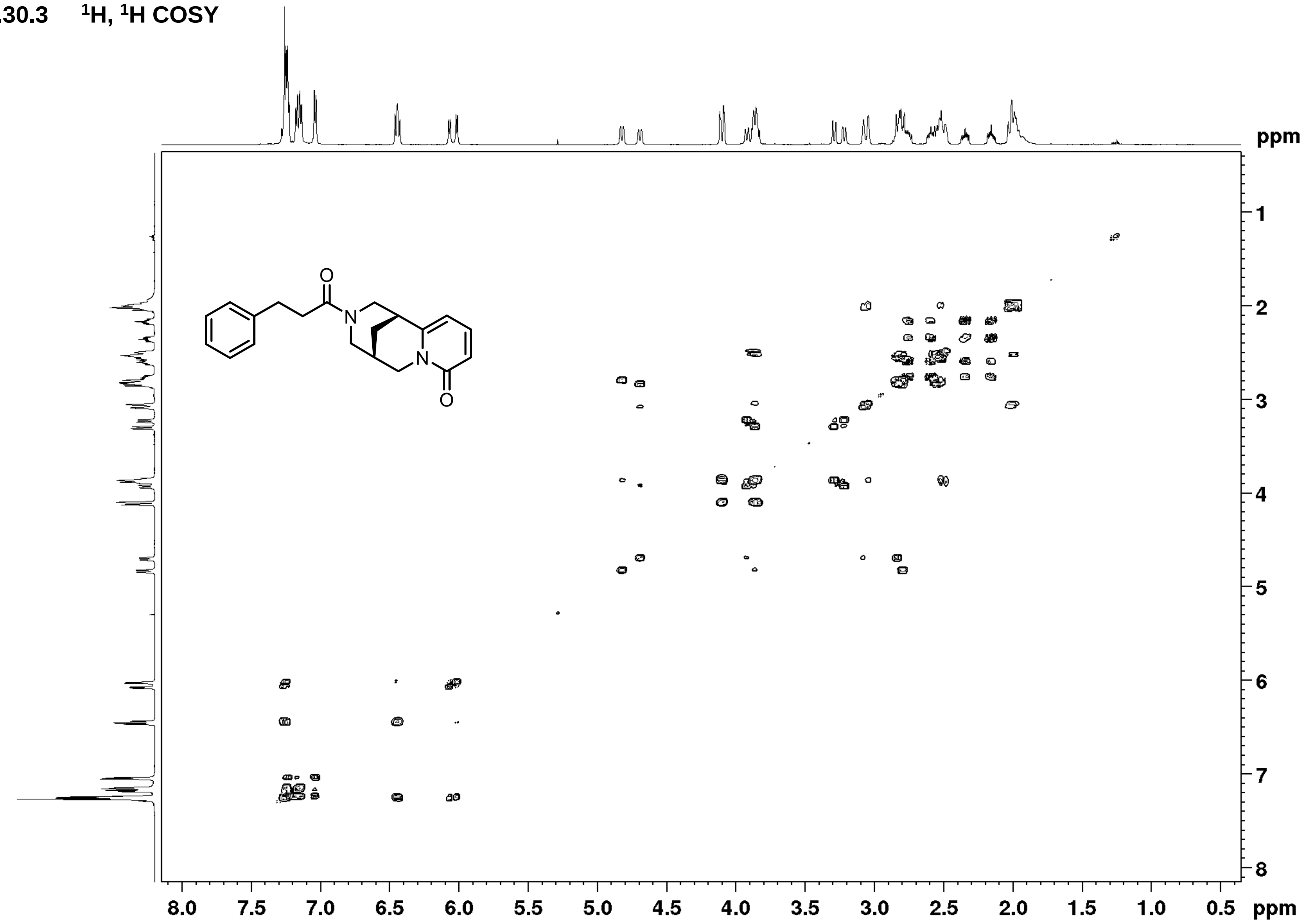

3.30.4 ^{1}H, ^{13}C HMBC

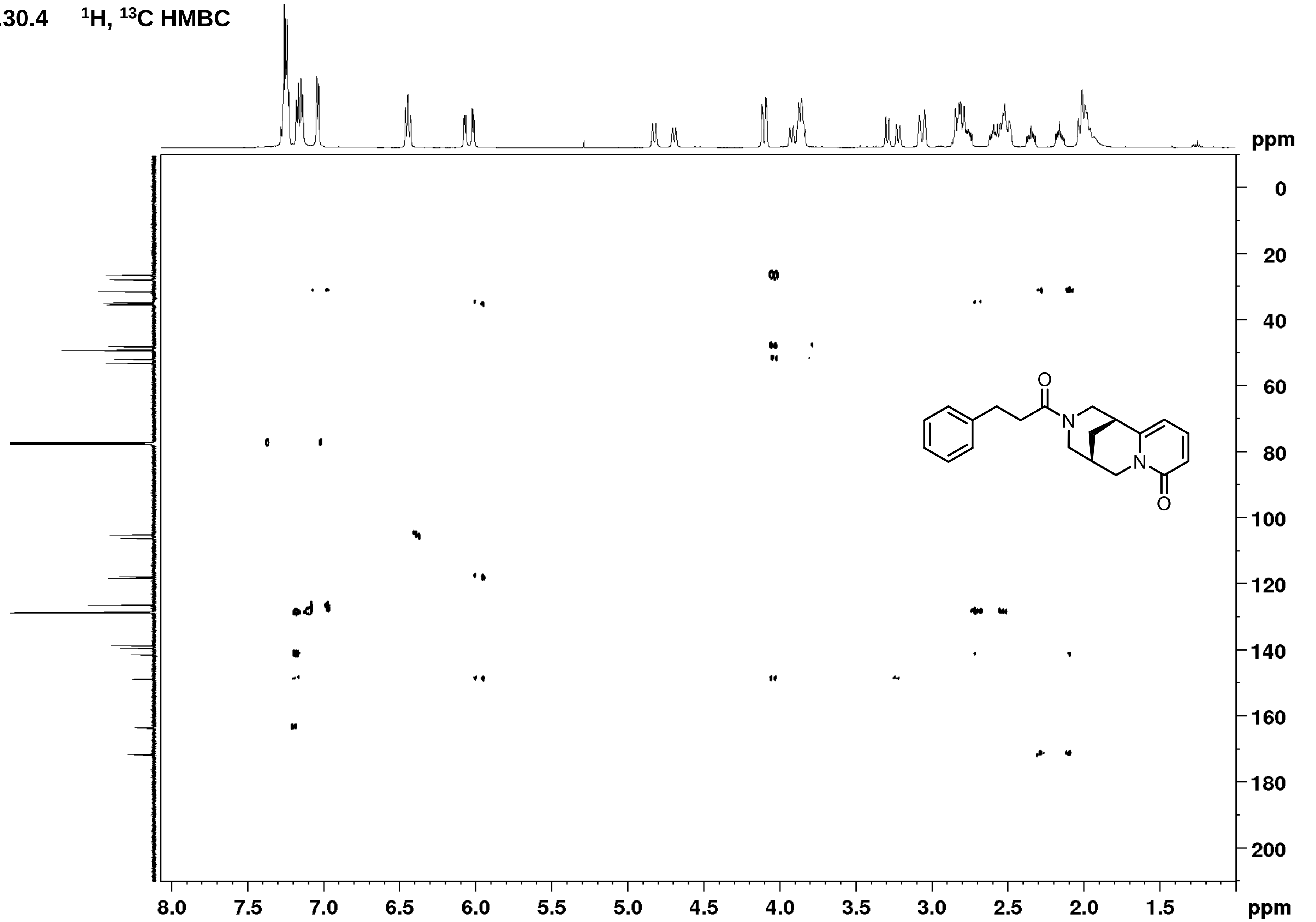

3.30.5 ^{1}H, ^{13}C HSQC

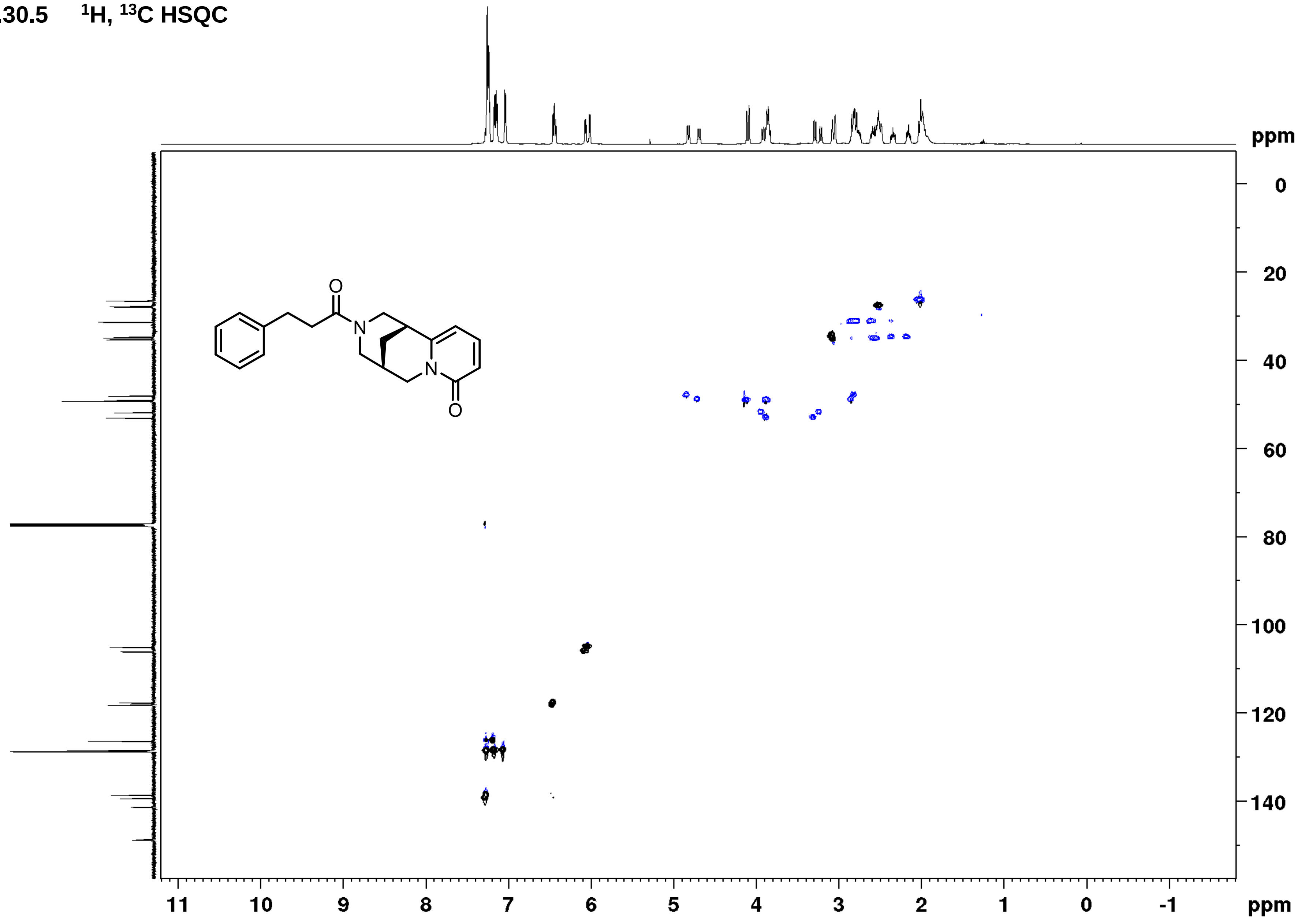

3.30.6 HRMS

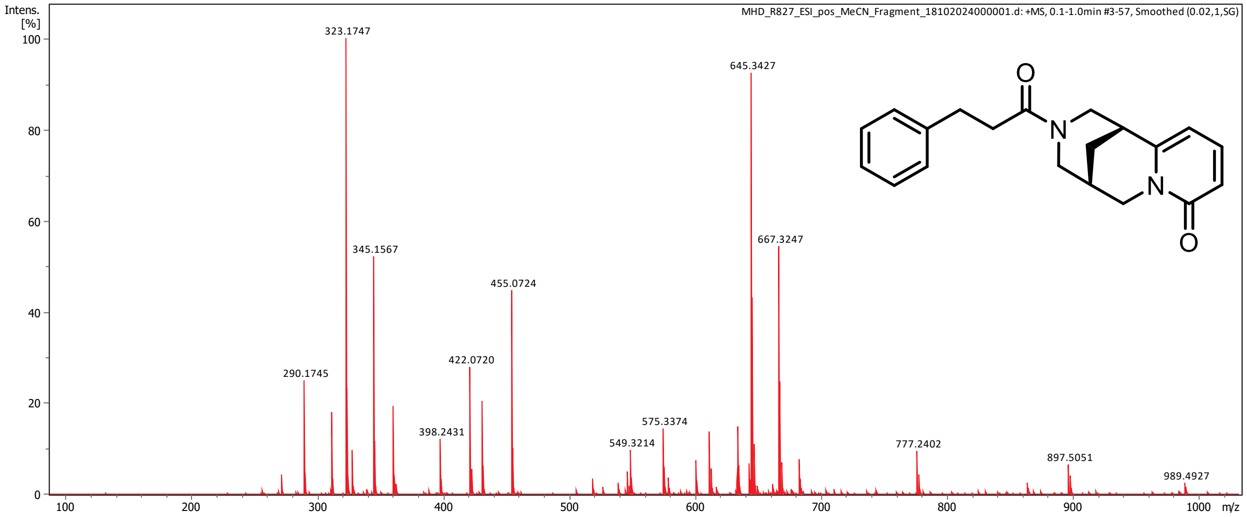

MHD_R827_ESI_pos_MeCN_Fragment_18102024000001.d: +MS, 0.1-1.0min #3-57, Smoothed (0.02,1,SG)
Intens. [%]
323.1747
645.3427
667.3247
345.1567
455.0724
422.0720
290.1745
398.2431
575.3374
549.3214
777.2402
897.5051
989.4927
m/z

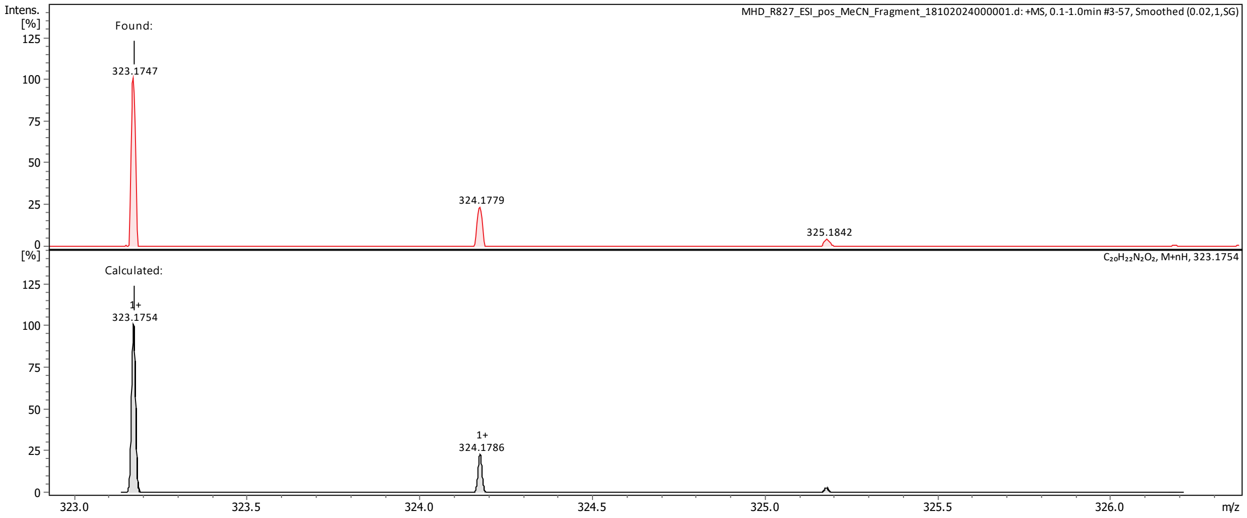

MHD_R827_ESI_pos_MeCN_Fragment_18102024000001.d: +MS, 0.1-1.0min #3-57, Smoothed (0.02,1,SG)
Intens. [%]
Found:
323.1747
324.1779
325.1842
Calculated:
C20H22N2O2, M+nH, 323.1754
1+
323.1754
1+
324.1786
m/z

3.30.7 IR

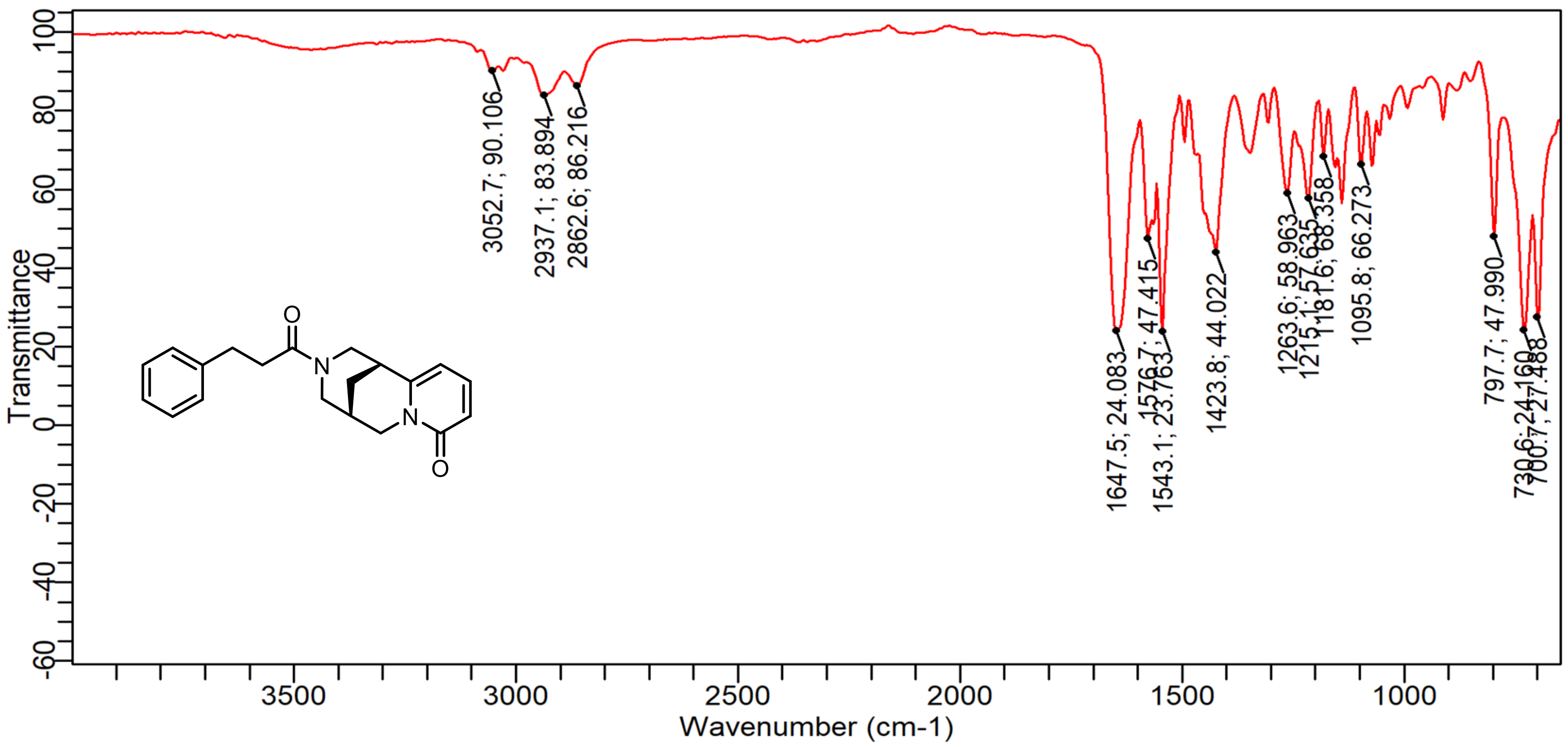

Transmittance
Wavenumber (cm-1)
3052.7; 90.106
2937.1; 83.894
2862.6; 86.216
1647.5; 24.083
1576.7; 47.415
1543.1; 23.763
1423.8; 44.022
1263.6; 58.963
1215.1; 57.635
1181.6; 68.358
1095.8; 66.273
797.7; 47.990
730.6; 24.160
700.7; 27.488

3.31 3-(4-(*tert*-Butyl)phenyl)-*N*-(2,3-dihydrobenzo[*b*][1,4]dioxin-6-yl)-*N*-methylpropanamide (12bc) (600 MHz, $CDCl_3$)

3.31.1 ^{1}H NMR

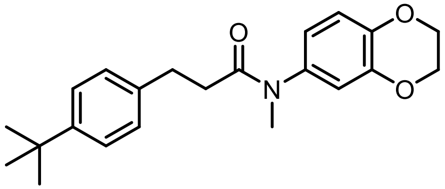

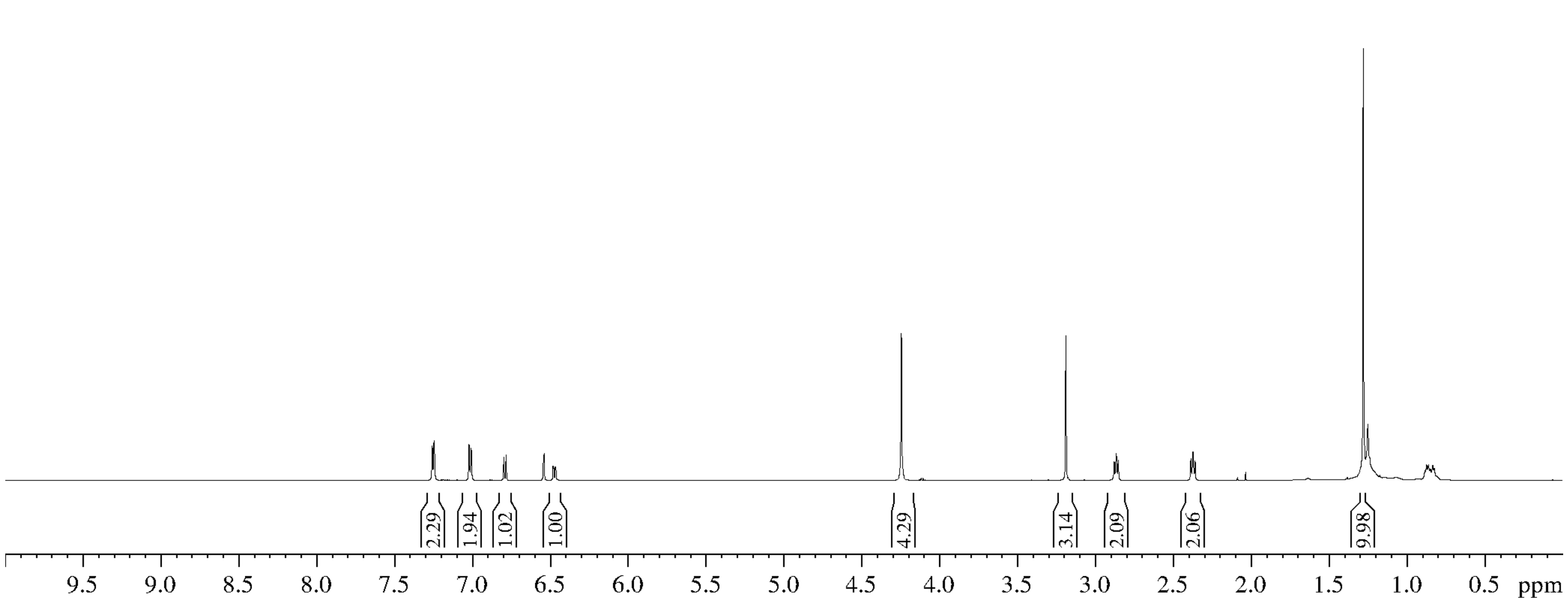

3.31.2 ^{13}C NMR

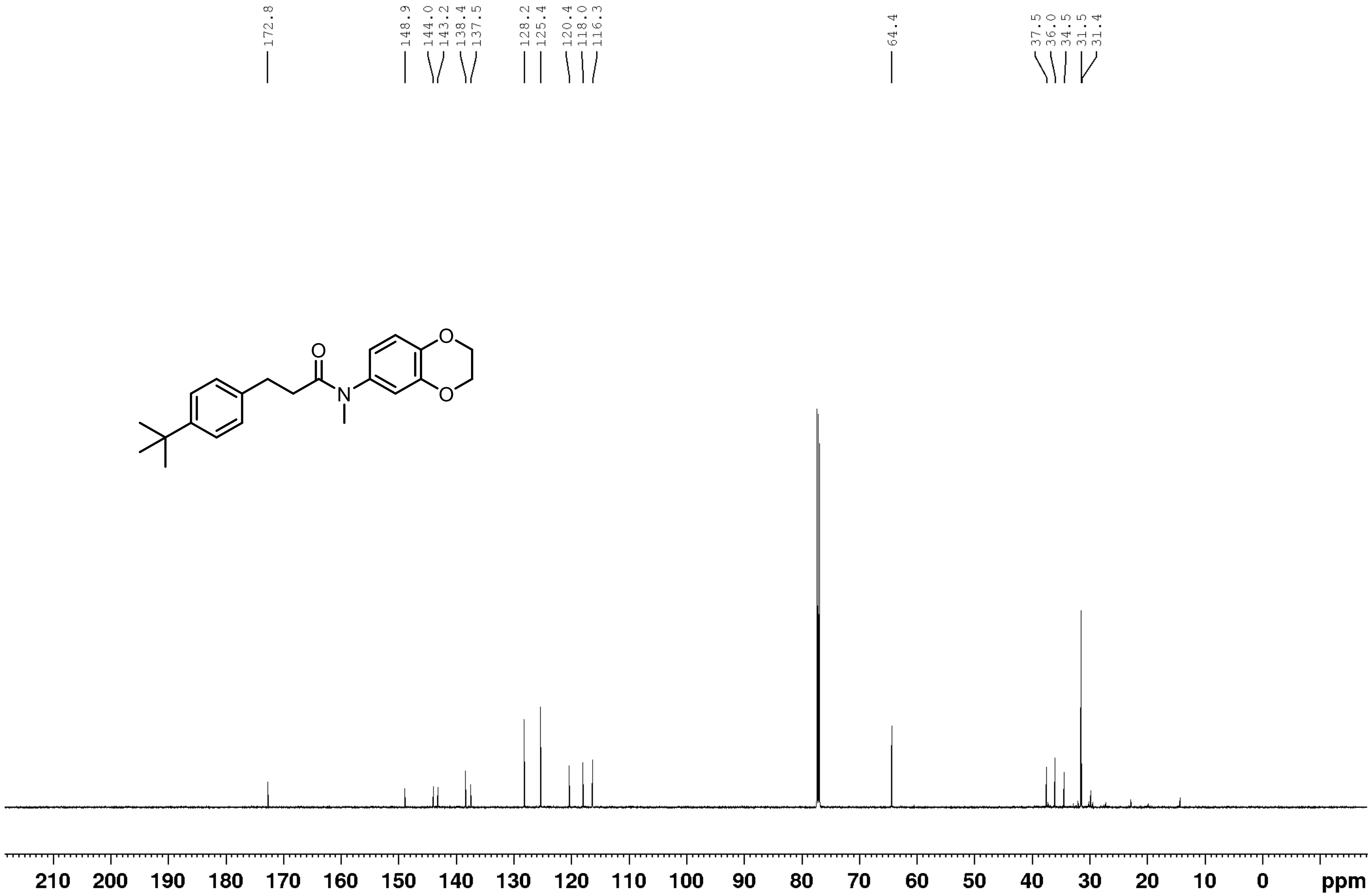

172.8
148.9
144.0
143.2
138.4
137.5
128.2
125.4
120.4
118.0
116.3
64.4
37.5
36.0
34.5
31.5
31.4
O
N
O
O
210 200 190 180 170 160 150 140 130 120 110 100 90 80 70 60 50 40 30 20 10 0 ppm

3.31.3 ^{1}H, ^{1}H COSY

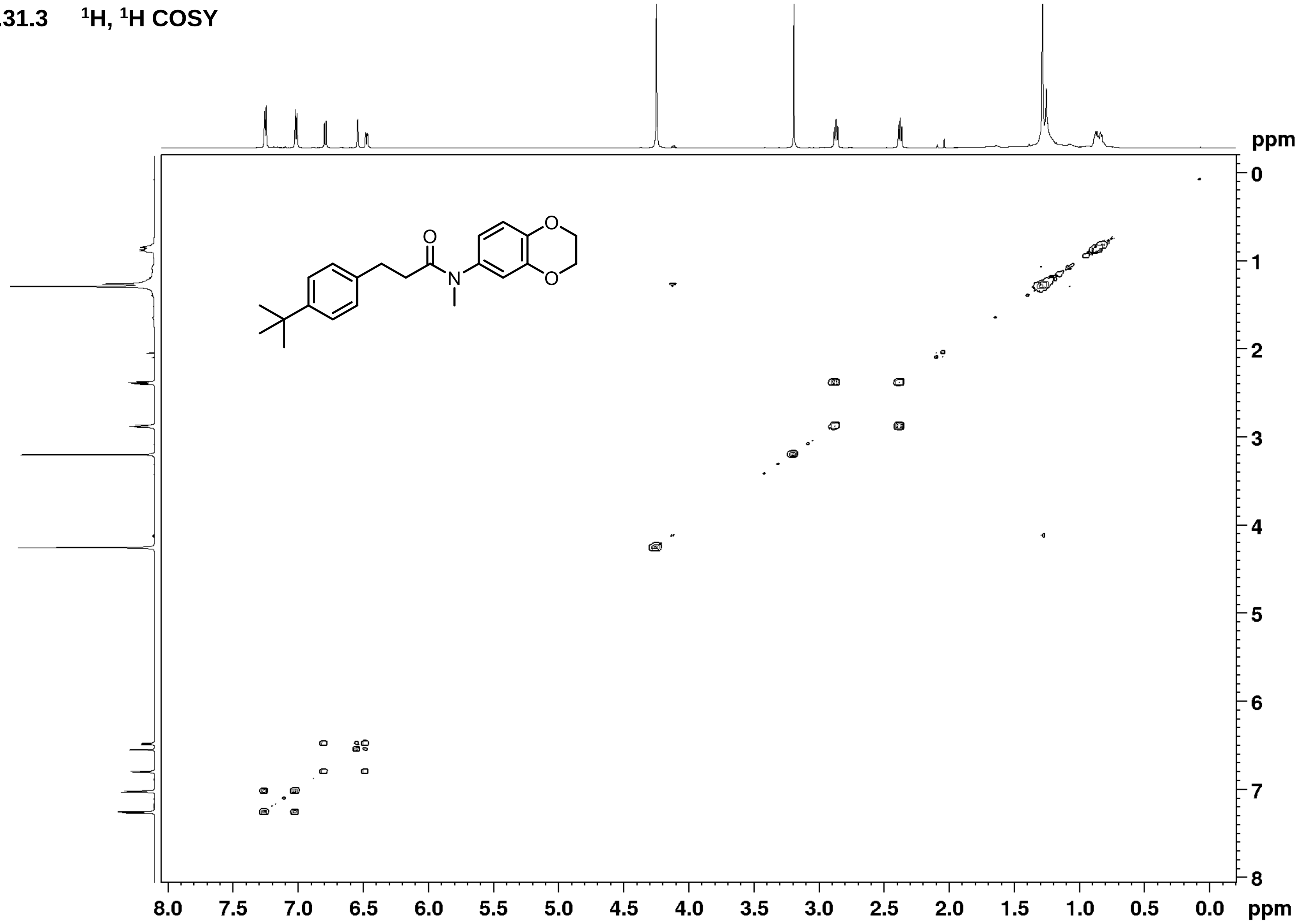

3.31.4 ^{1}H, ^{13}C HMBC

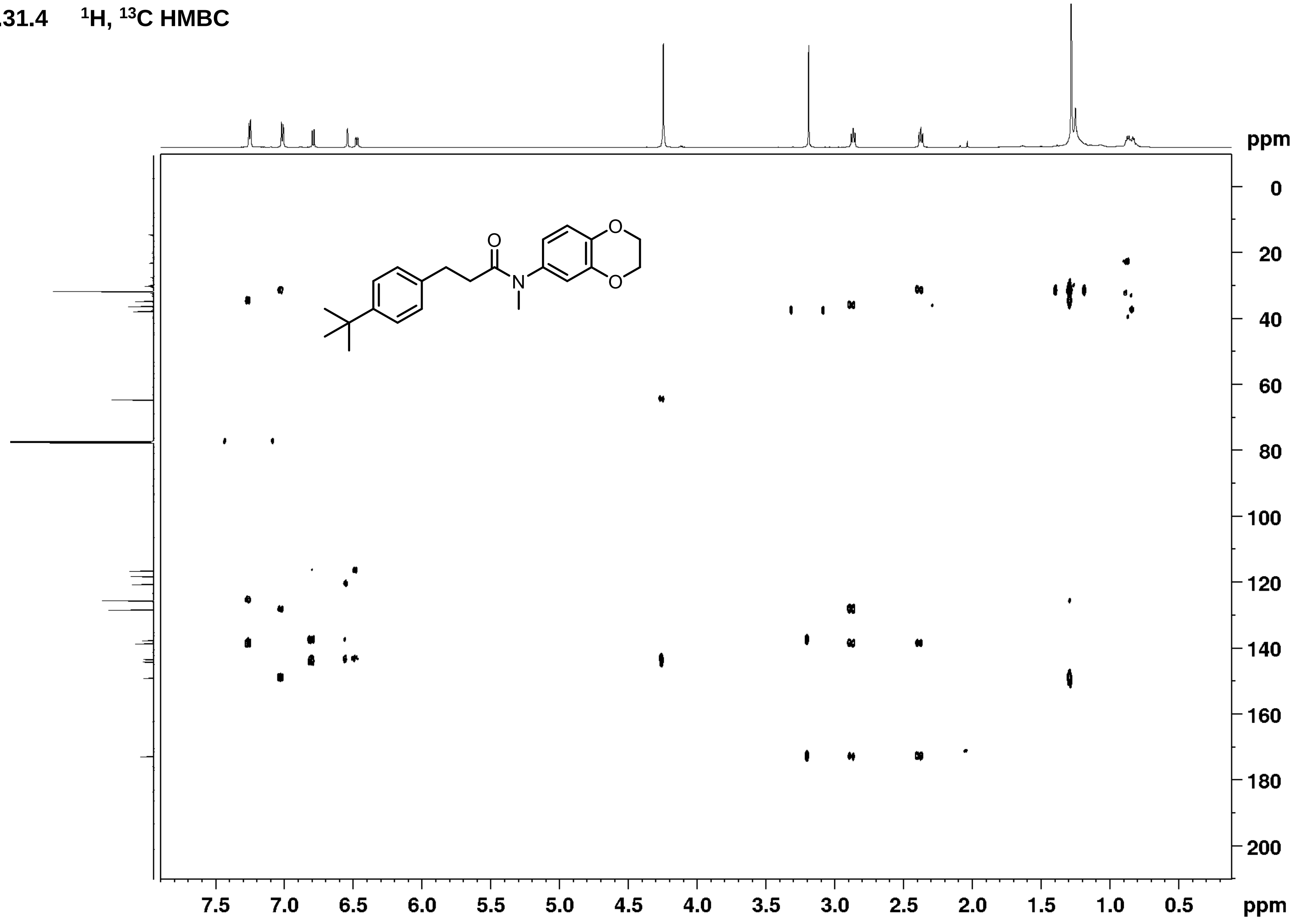

3.31.5 ^{1}H, ^{13}C HSQC

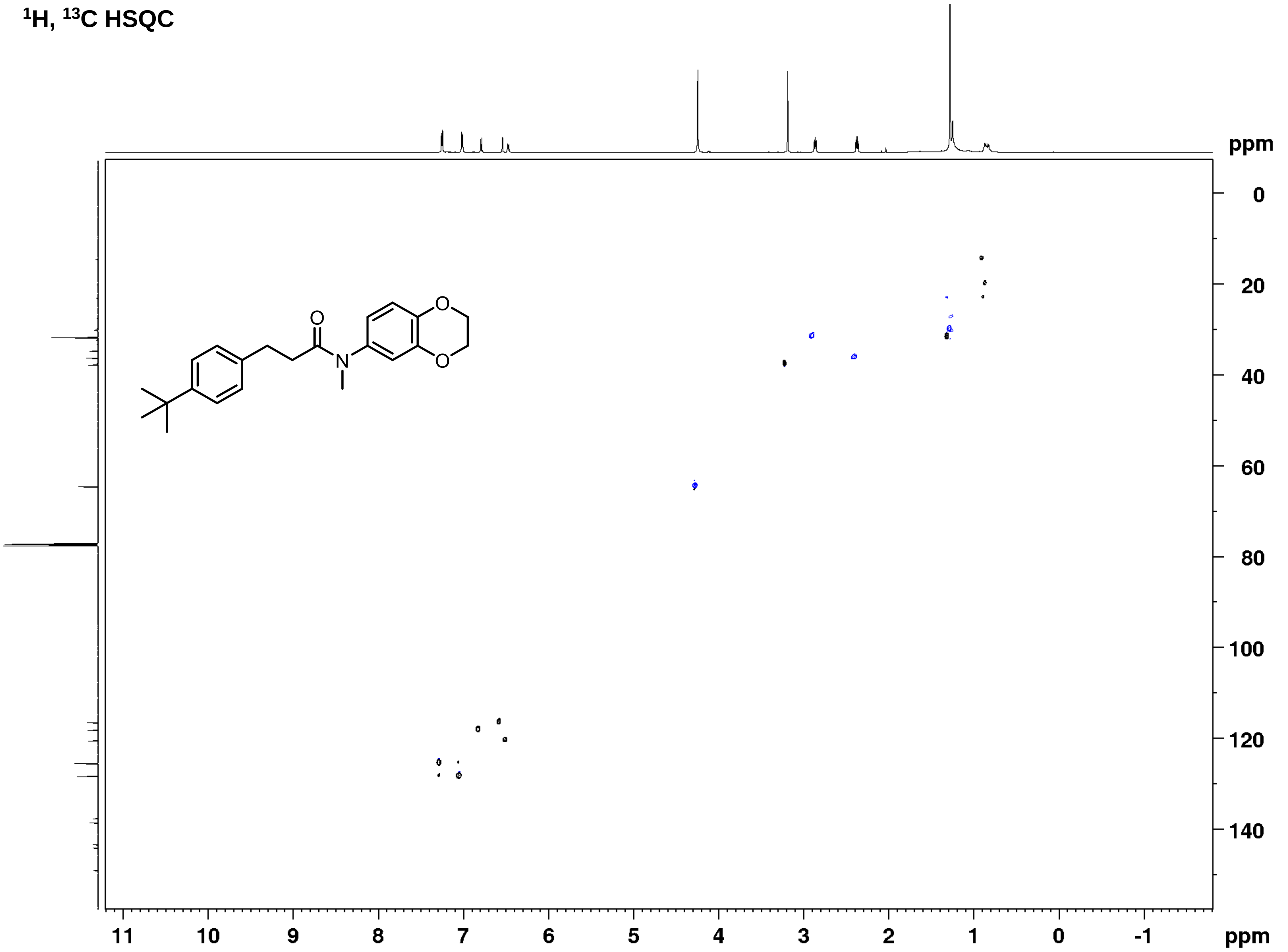

3.31.6 HRMS

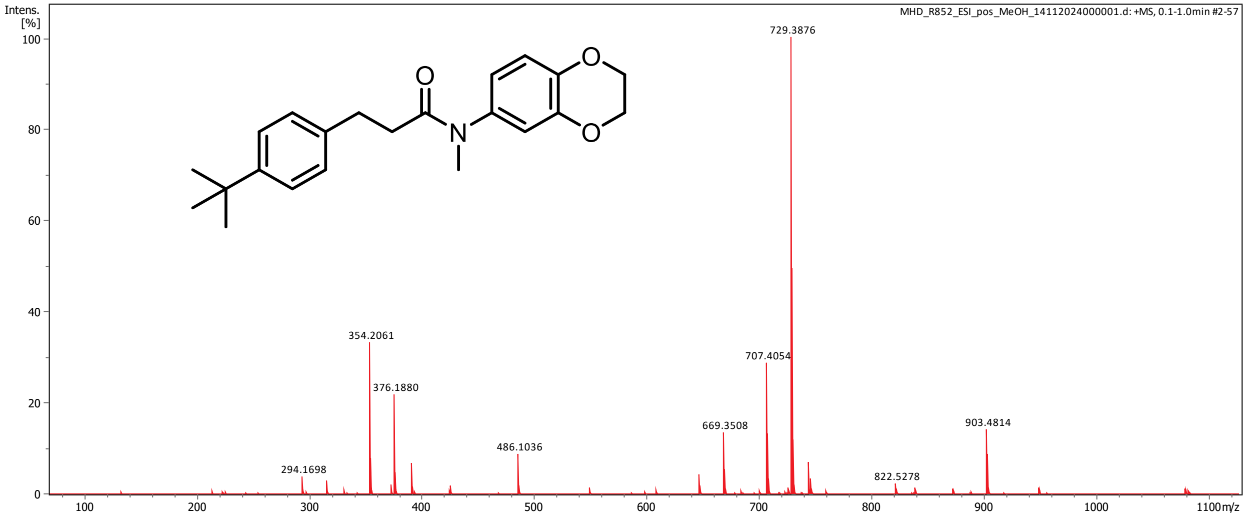

MHD_R852_ESI_pos_MeOH_14112024000001.d: +MS, 0.1-1.0min #2-57
Intens. [%]
729.3876
354.2061
376.1880
707.4054
669.3508
903.4814
486.1036
294.1698
822.5278
m/z

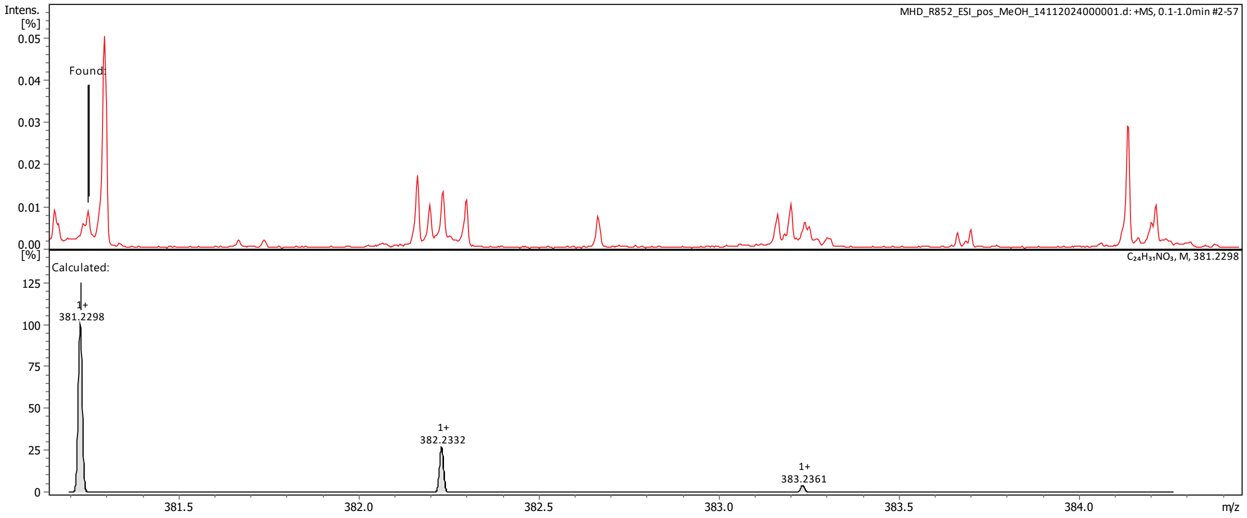

MHD_R852_ESI_pos_MeOH_14112024000001.d: +MS, 0.1-1.0min #2-57
Intens. [%]
Found:
Calculated:
$C_{24}H_{31}NO_3$, M, 381.2298
1+ 381.2298
1+ 382.2332
1+ 383.2361
m/z

3.31.7 IR

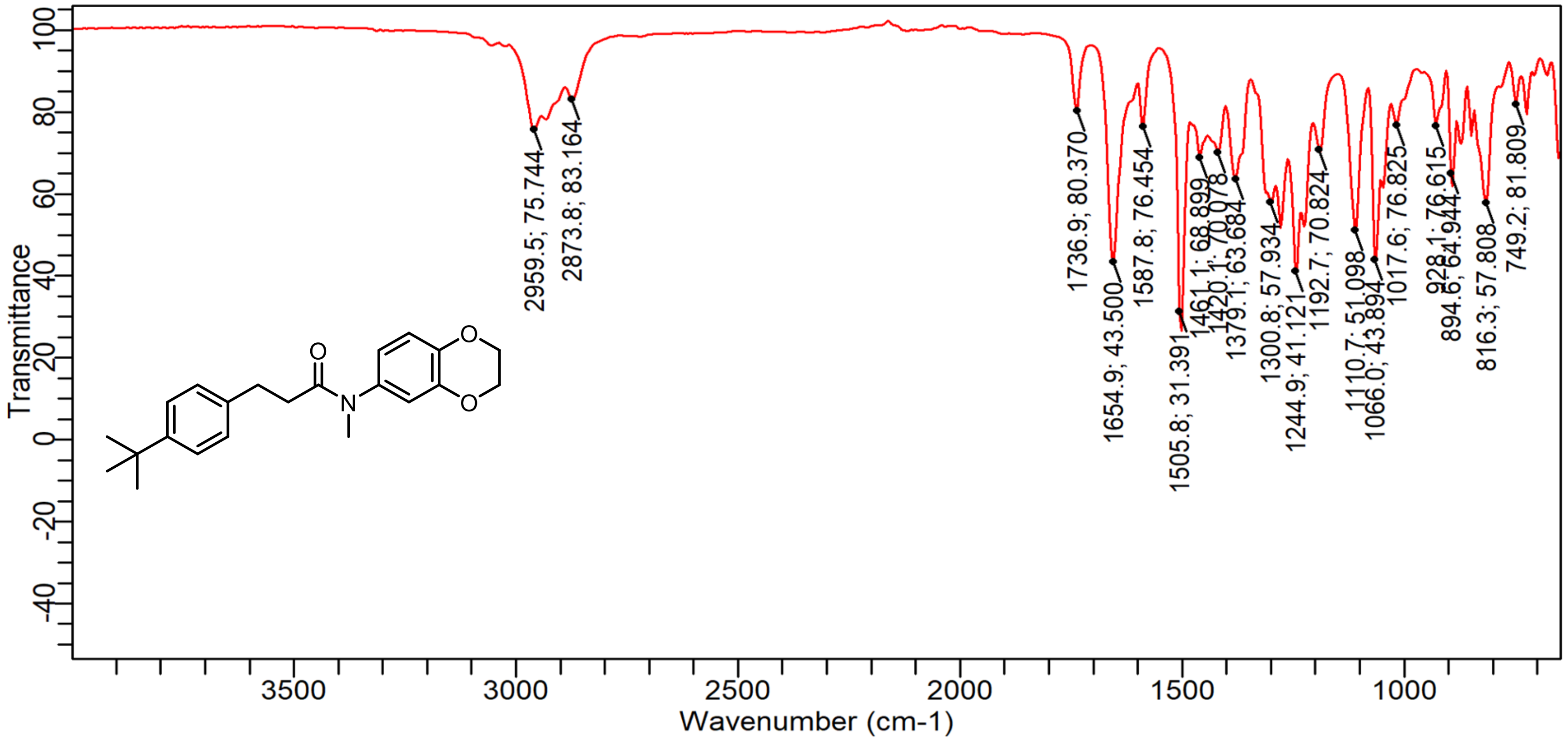

Transmittance
Wavenumber (cm-1)
2959.5; 75.744
2873.8; 83.164
1736.9; 80.370
1654.9; 43.500
1587.8; 76.454
1505.8; 31.391
1461.1; 68.899
1420.1; 70.078
1379.1; 63.684
1300.8; 57.934
1244.9; 41.121
1192.7; 70.824
1110.7; 51.098
1066.0; 43.894
1017.6; 76.825
928.1; 76.615
894.6; 64.944
816.3; 57.808
749.2; 81.809

3.32 1-(Piperidin-1-yl)-3-(3,4,5-trimethoxyphenyl)propan-1-one (12bd) (600 MHz, $CDCl_3$)

3.32.1 ^{1}H NMR

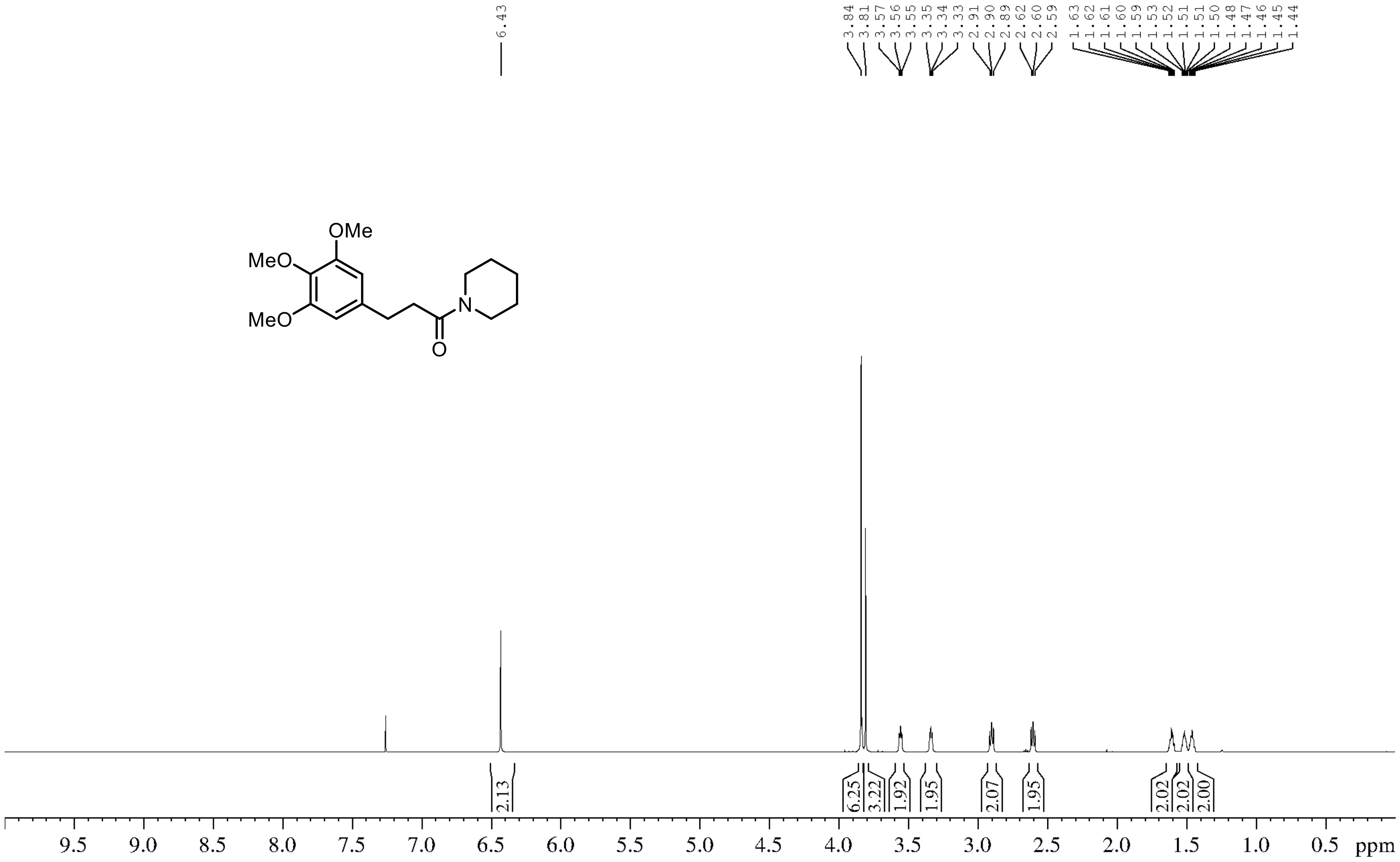

3.32.2 ^{13}C NMR

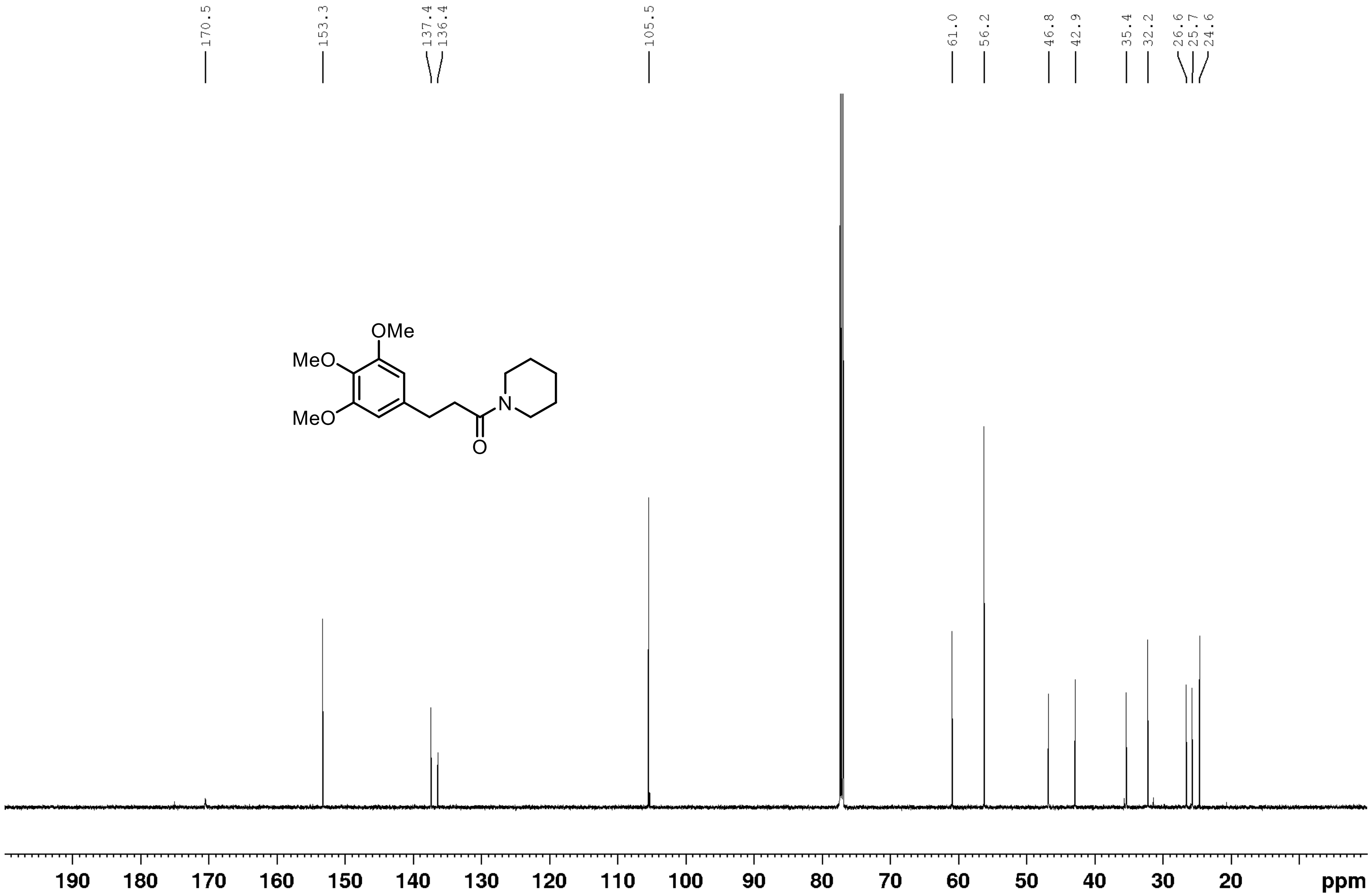
170.5
153.3
137.4
136.4
105.5
61.0
56.2
46.8
42.9
35.4
32.2
26.6
25.7
24.6
OMe
MeO
MeO
N
O
190 180 170 160 150 140 130 120 110 100 90 80 70 60 50 40 30 20 ppm

3.32.3 ^{1}H, ^{1}H COSY

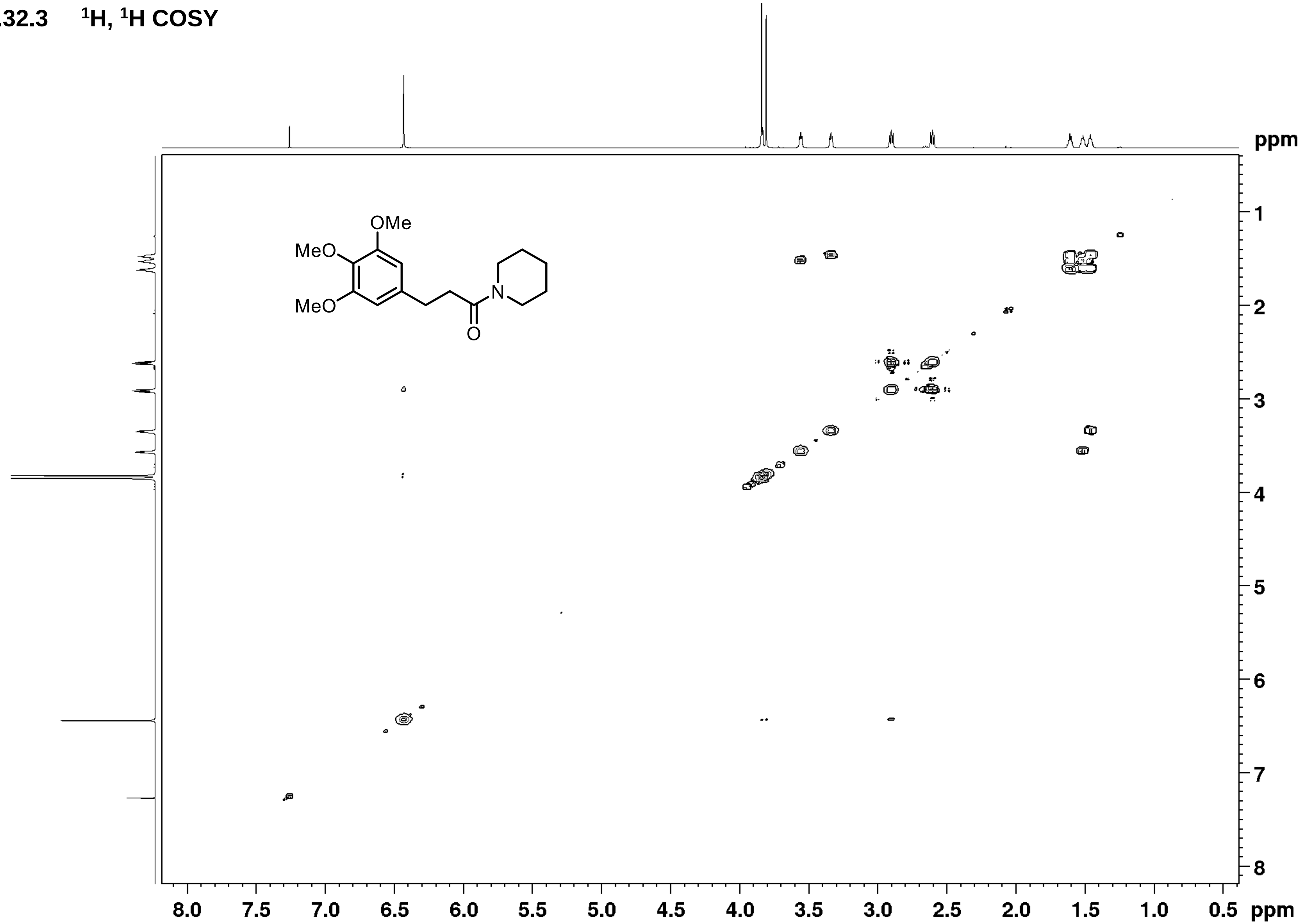

3.32.4 ^{1}H, ^{13}C HMBC

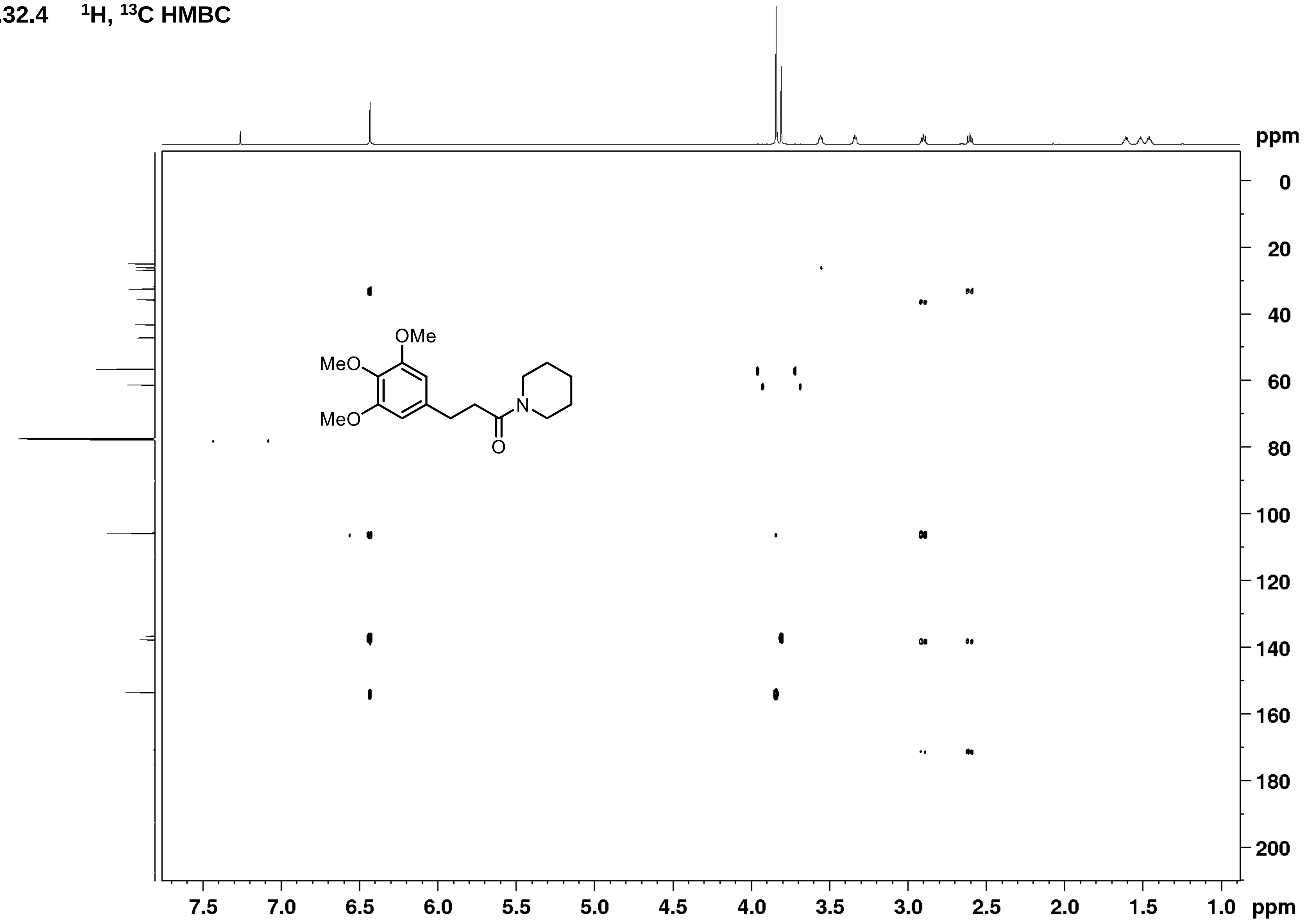

3.32.5 ¹H, ¹³C HSQC

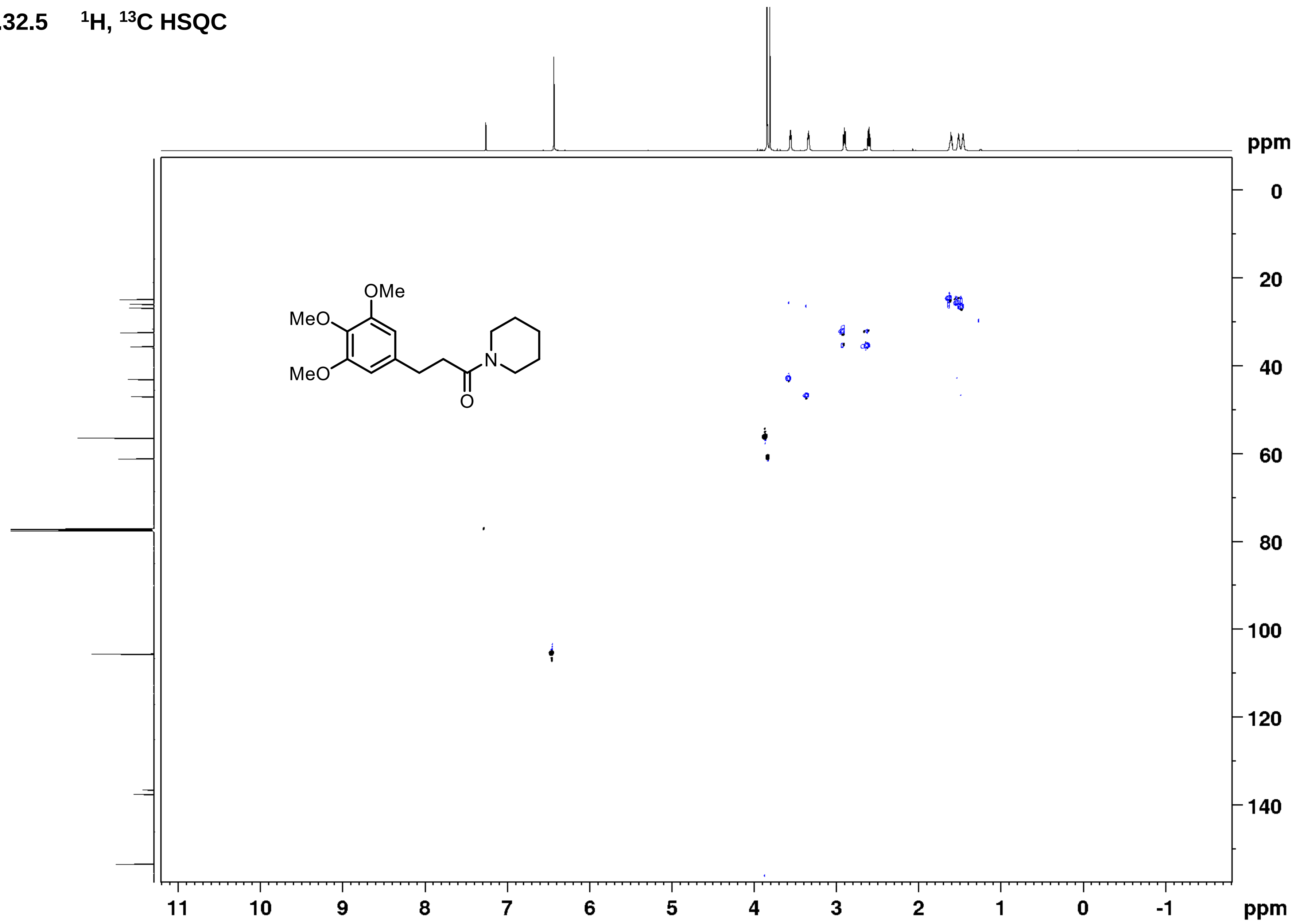

3.32.6 HRMS

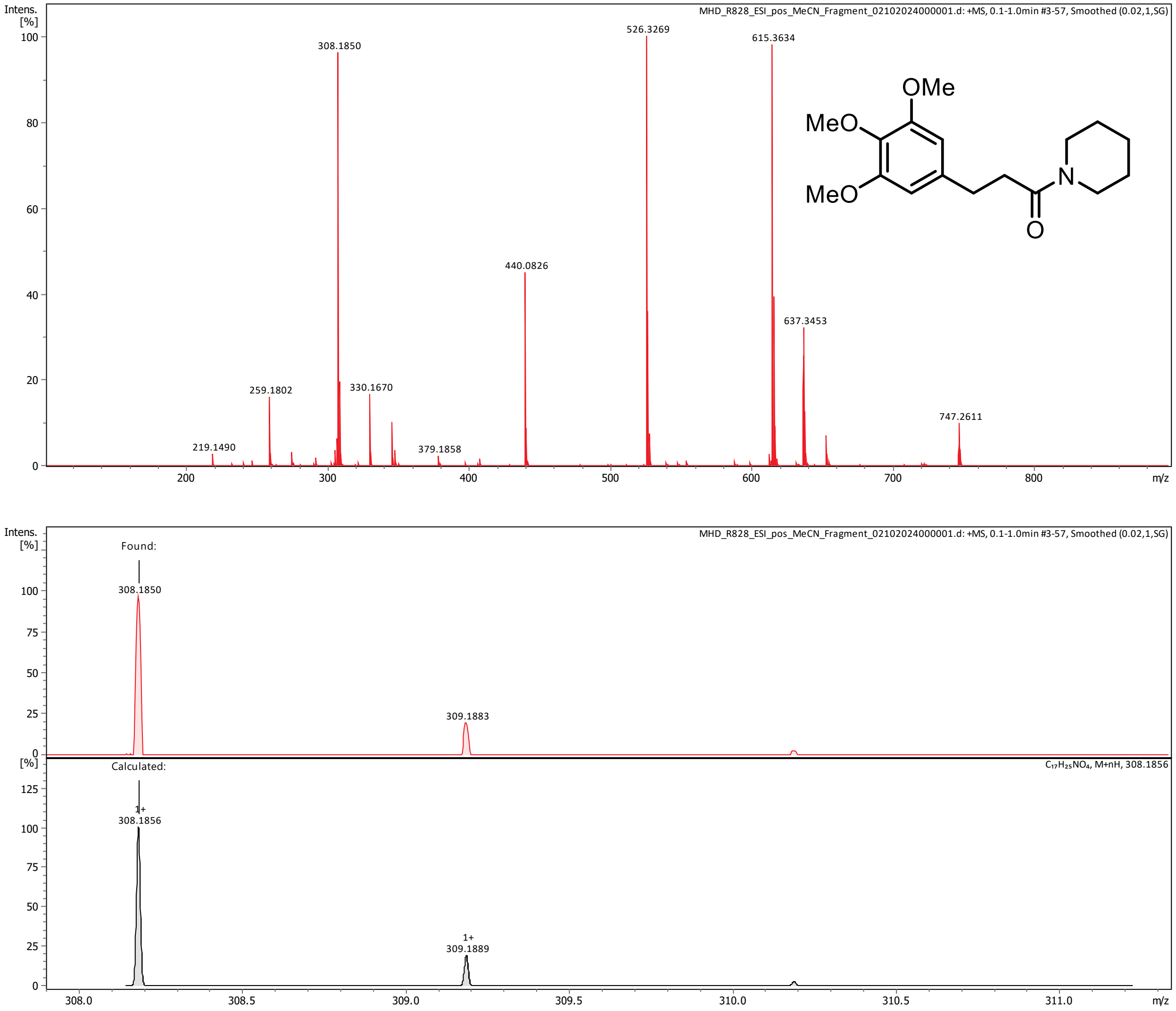
MHD_R828_ESI_pos_MeCN_Fragment_02102024000001.d: +MS, 0.1-1.0min #3-57, Smoothed (0.02,1,SG)
Intens. [%]
308.1850
526.3269
615.3634
440.0826
637.3453
259.1802
330.1670
219.1490
379.1858
747.2611
m/z
OMe
MeO
MeO
N
O
Found:
308.1850
309.1883
Calculated:
C₁₇H₂₅NO₄, M+nH, 308.1856
1+
308.1856
1+
309.1889

3.32.7 IR

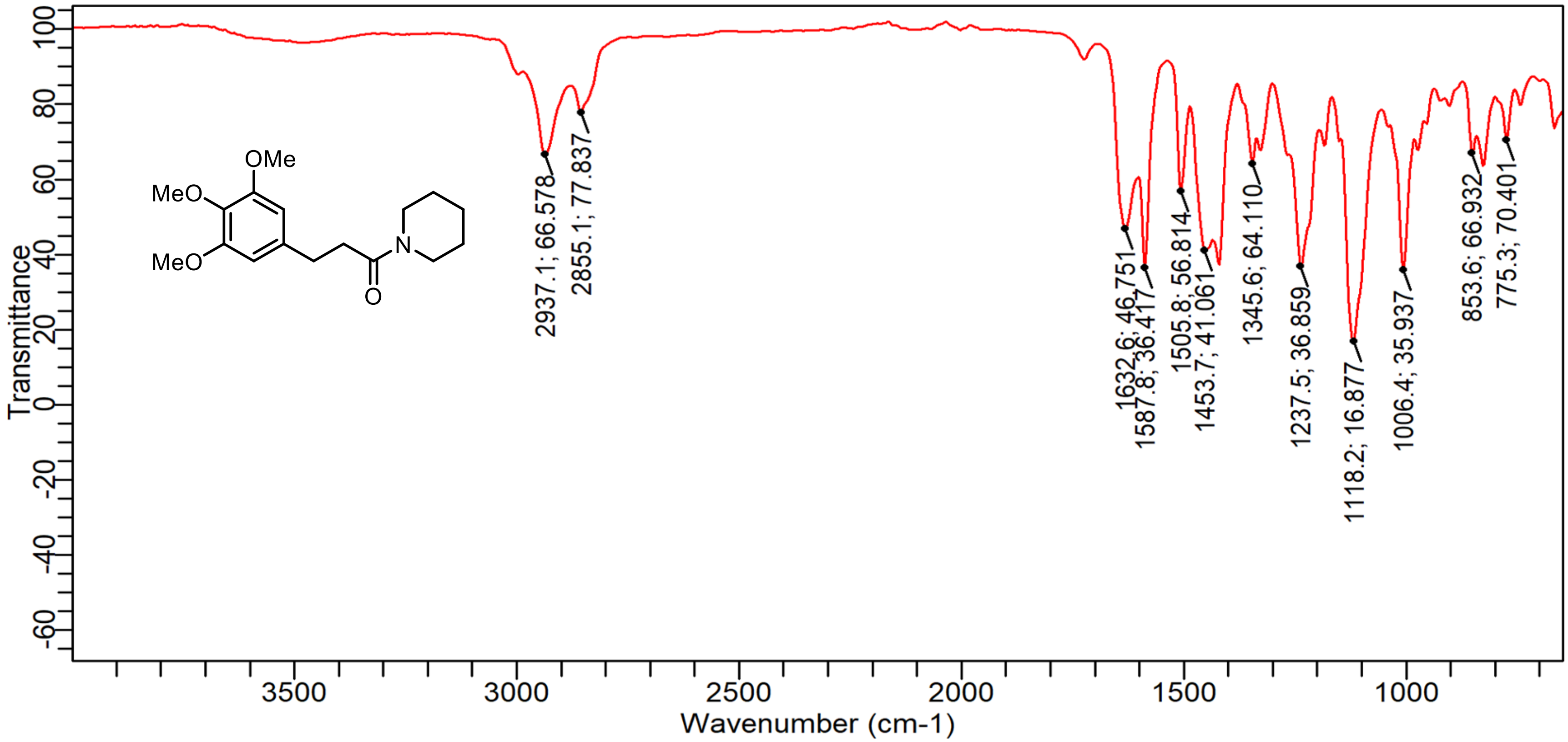

OMe
MeO
MeO
N
O
Transmittance
100
80
60
40
20
0
-20
-40
-60
2937.1; 66.578
2855.1; 77.837
1632.6; 46.751
1587.8; 36.417
1505.8; 56.814
1453.7; 41.061
1345.6; 64.110
1237.5; 36.859
1118.2; 16.877
1006.4; 35.937
853.6; 66.932
775.3; 70.401
3500
3000
2500
2000
1500
1000
Wavenumber (cm-1)

3.33 3-(Benzo[*d*][1,3]dioxol-5-yl)-1-(piperidin-1-yl)propan-1-one (12be) (600 MHz, $CDCl_3$)

3.33.1 ^1H NMR

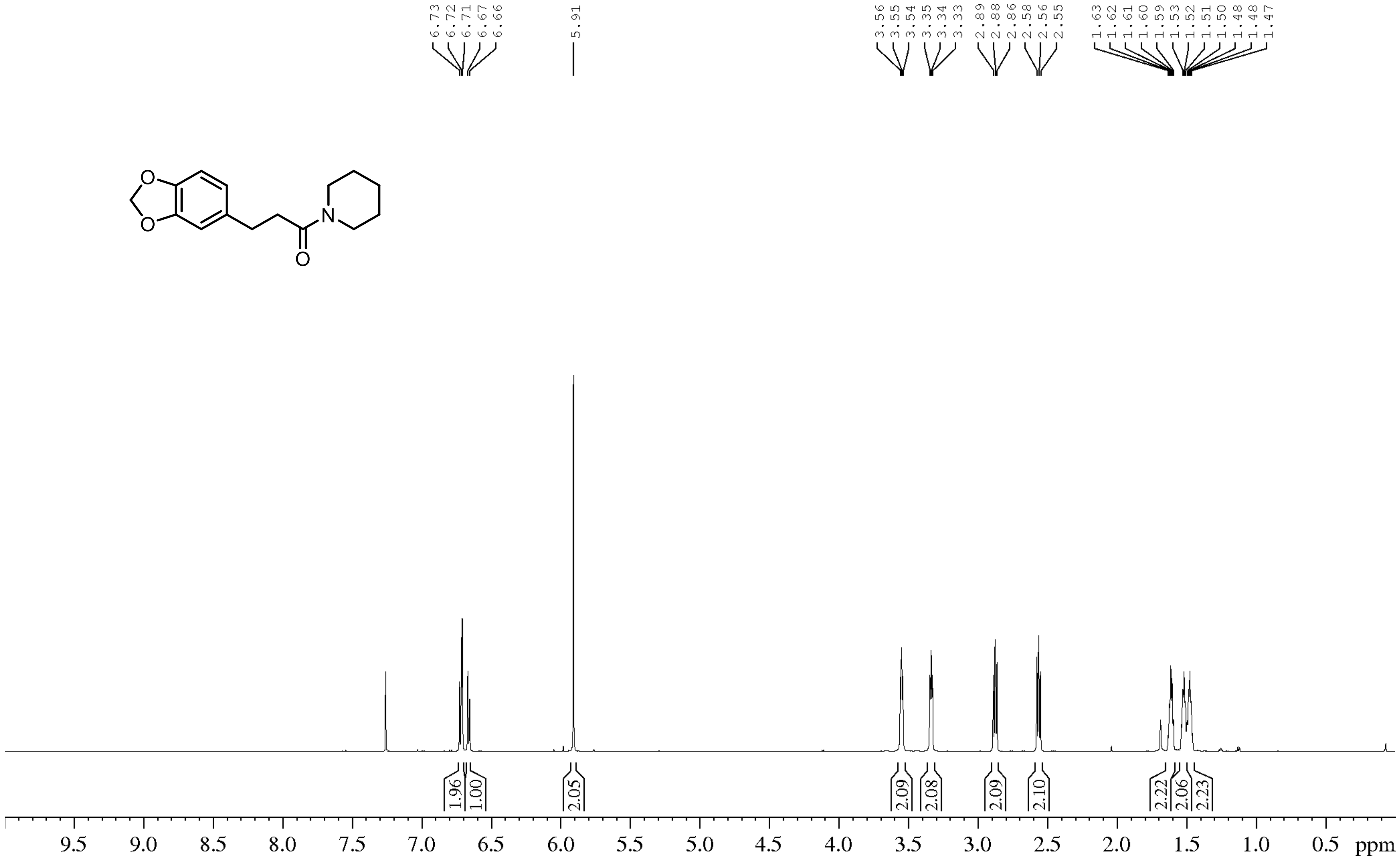

3.33.2 ^{13}C NMR

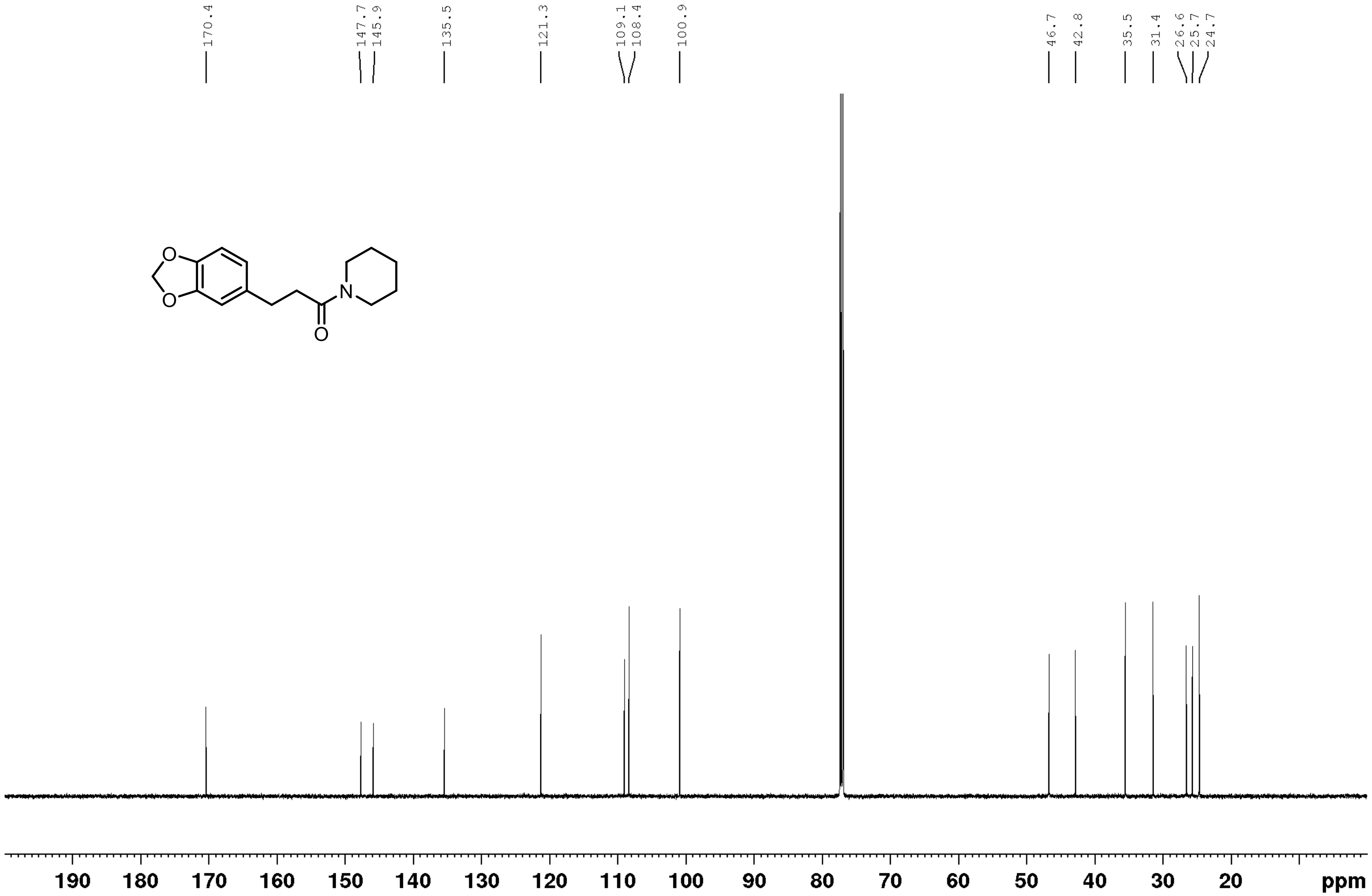

170.4
147.7
145.9
135.5
121.3
109.1
108.4
100.9
46.7
42.8
35.5
31.4
26.6
25.7
24.7
O
O
N
O
190 180 170 160 150 140 130 120 110 100 90 80 70 60 50 40 30 20 ppm

3.33.3 ^{1}H, ^{1}H COSY

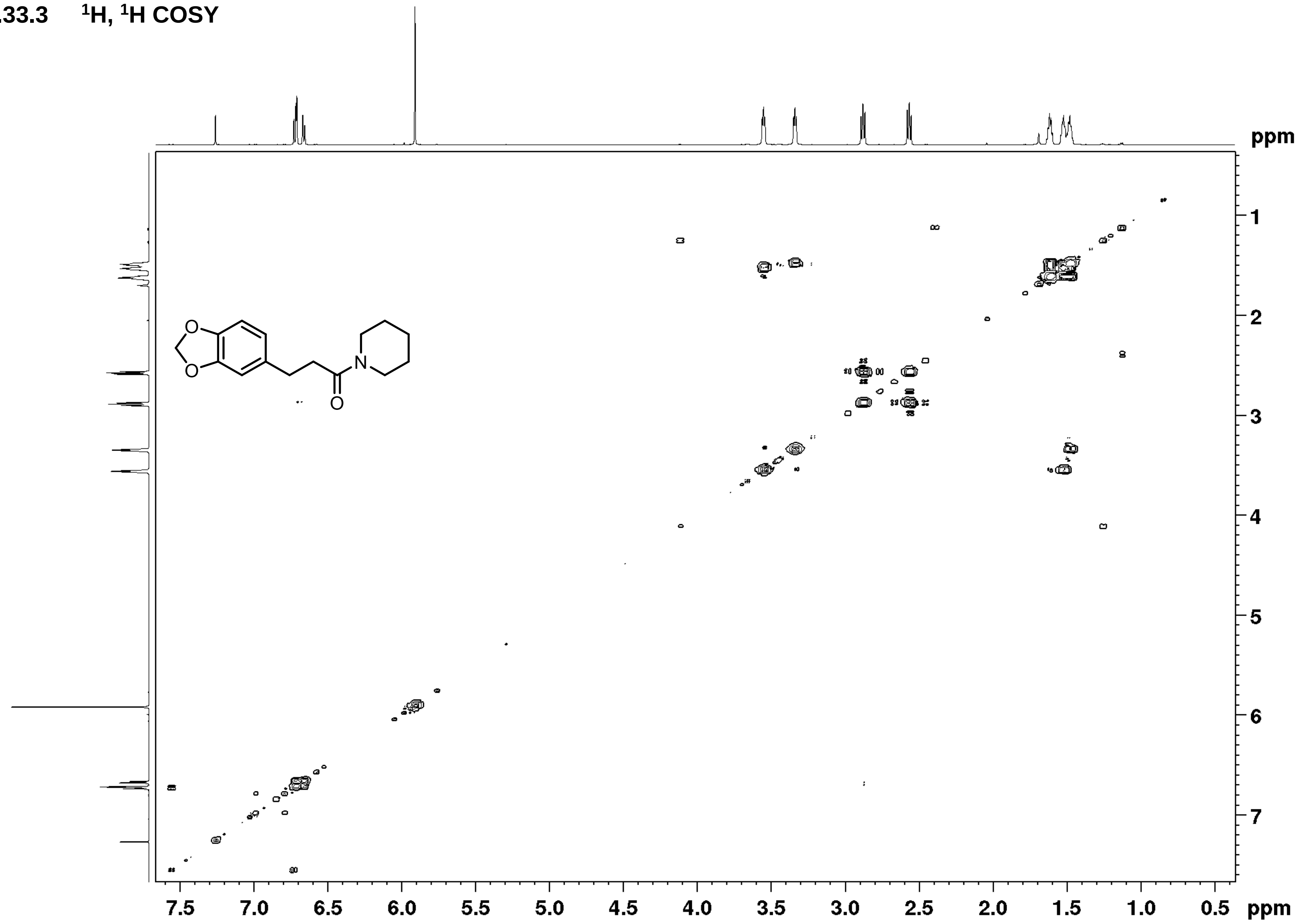

3.33.4 ^{1}H, ^{13}C HMBC

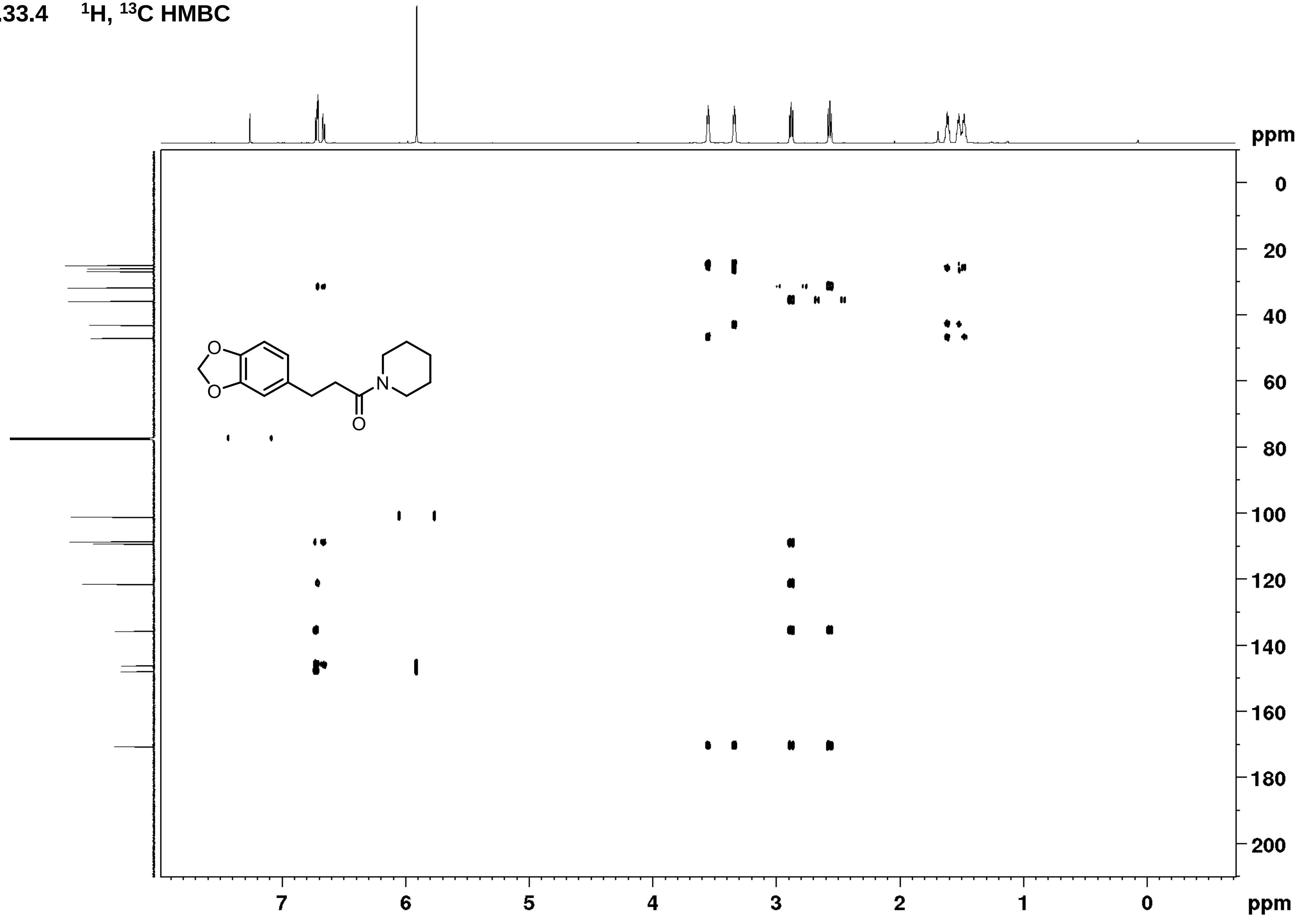

3.33.5 ^{1}H, ^{13}C HSQC

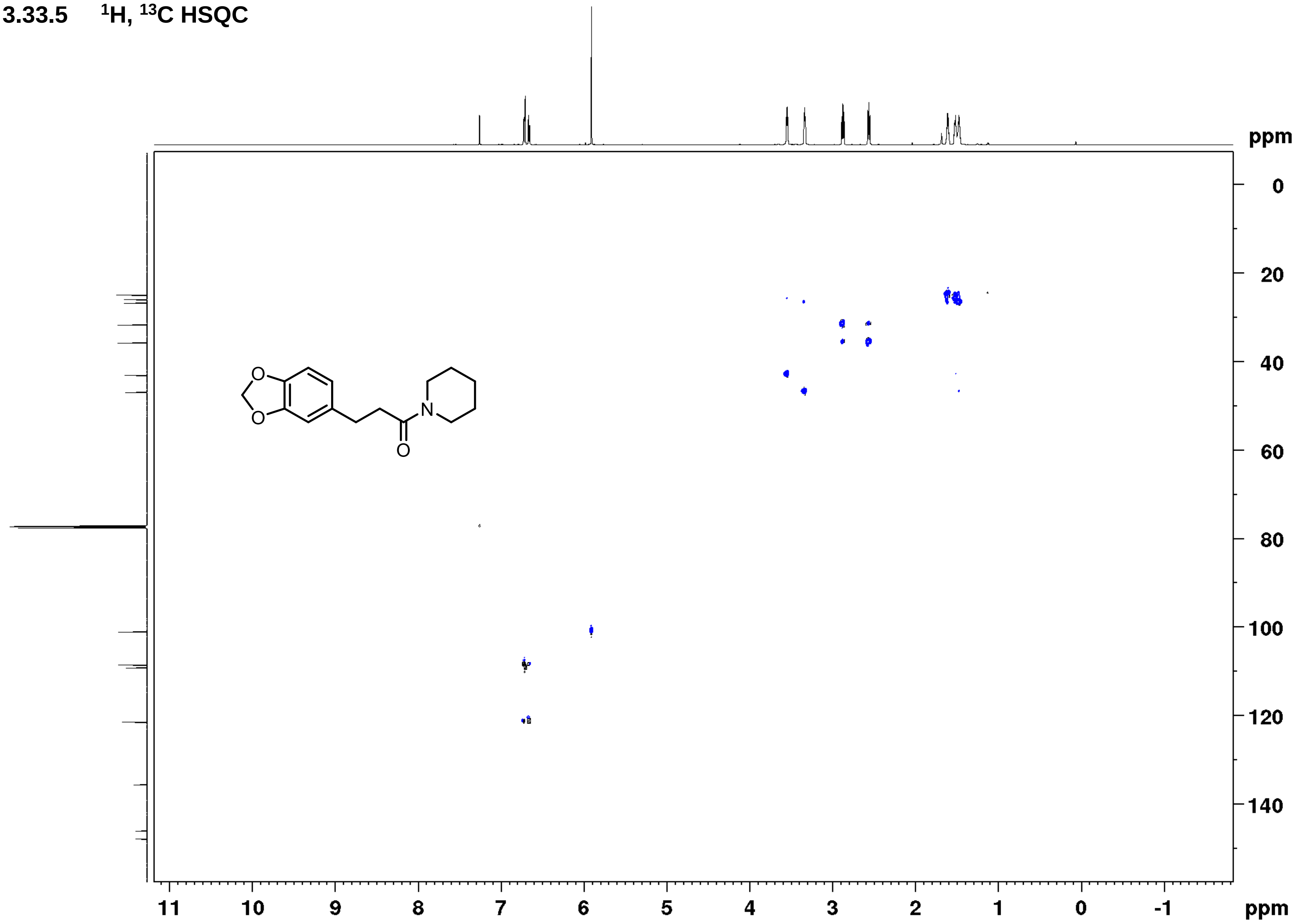

3.33.6 HRMS

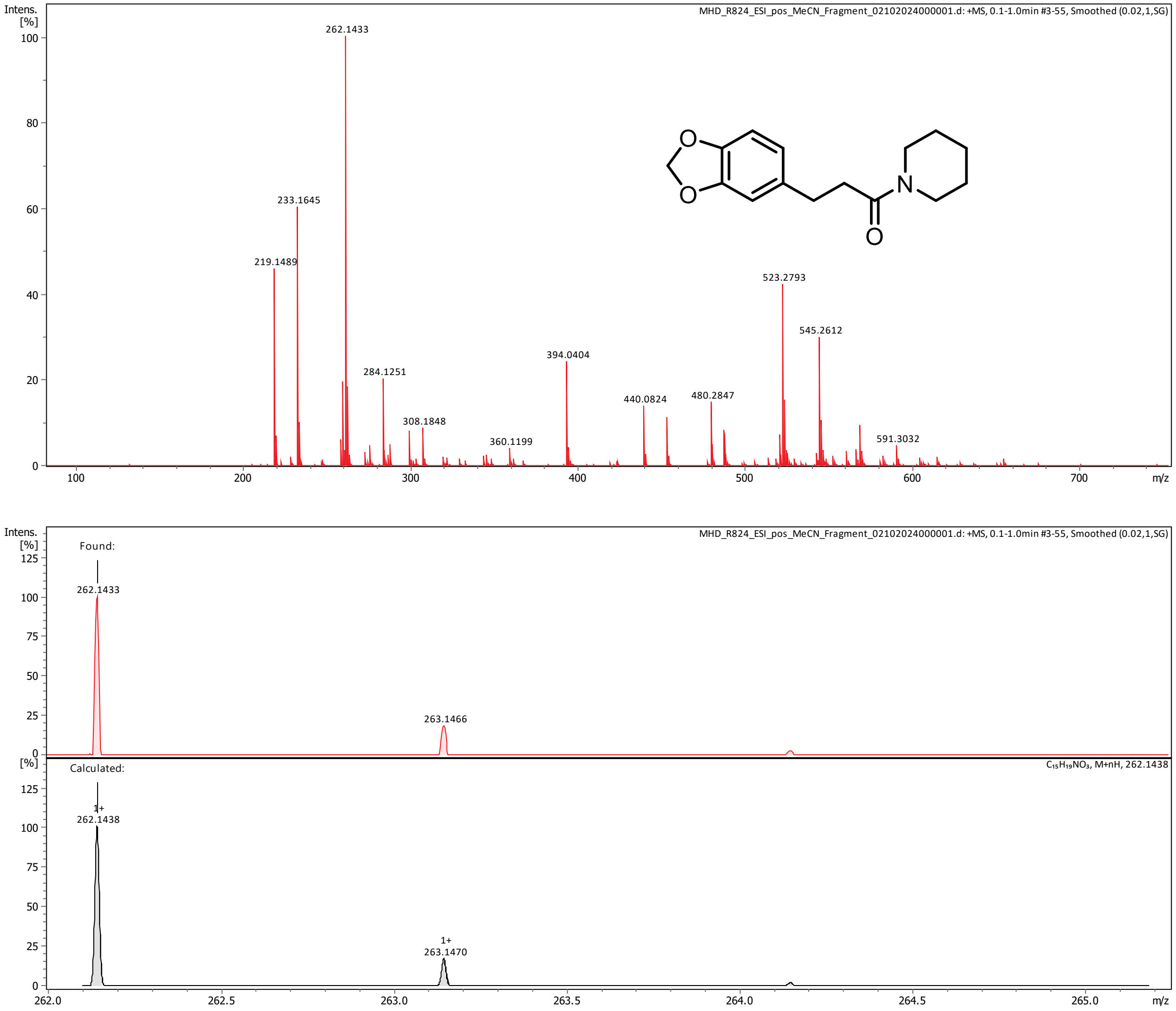

MHD_R824_ESI_pos_MeCN_Fragment_02102024000001.d: +MS, 0.1-1.0min #3-55, Smoothed (0.02,1,SG)
Intens. [%]
262.1433
233.1645
219.1489
523.2793
545.2612
394.0404
284.1251
440.0824
480.2847
308.1848
360.1199
591.3032
m/z
Found:
262.1433
263.1466
Calculated:
C15H19NO3, M+nH, 262.1438
1+ 262.1438
1+ 263.1470

3.33.7 IR

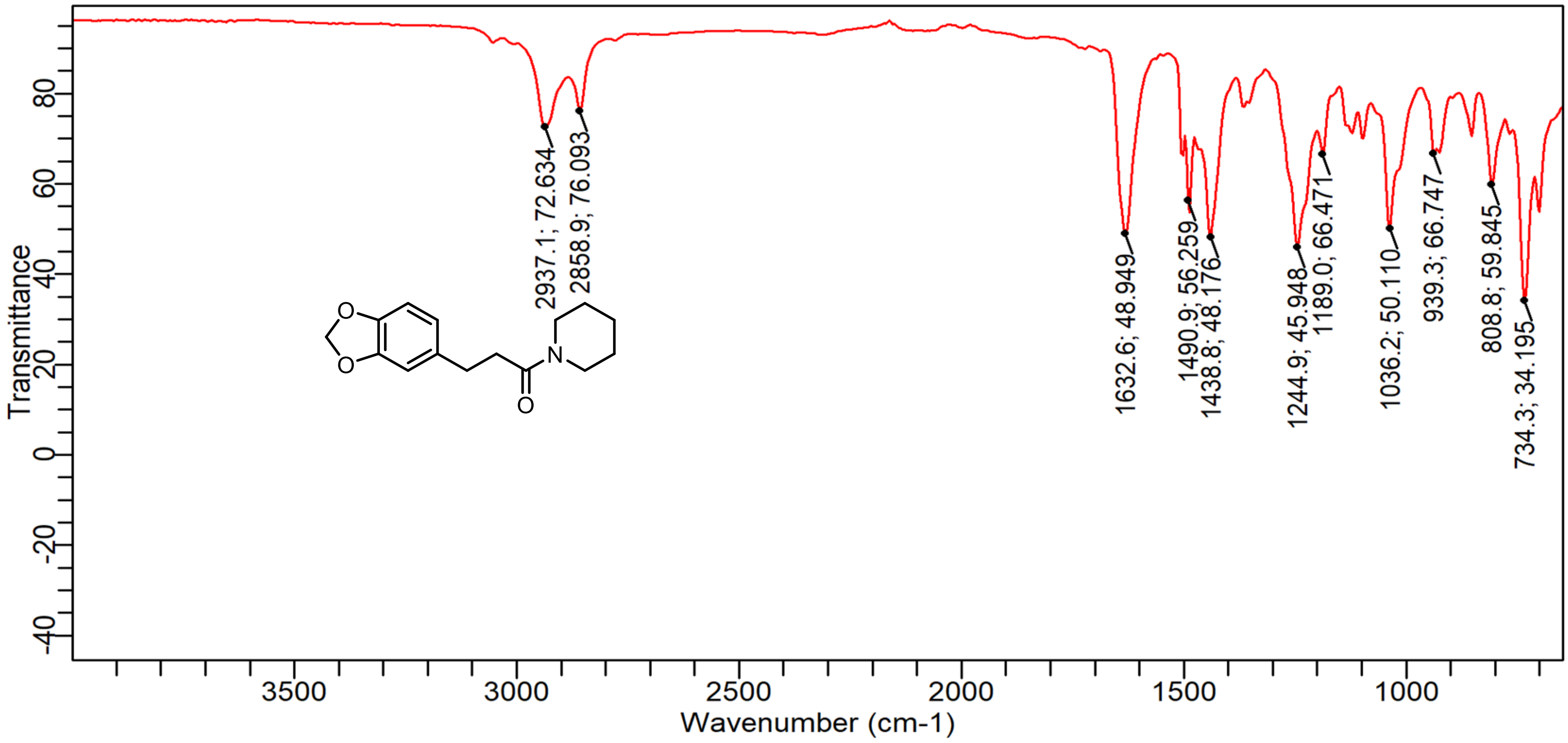

Transmittance
Wavenumber (cm-1)
2937.1; 72.634
2858.9; 76.093
1632.6; 48.949
1490.9; 56.259
1438.8; 48.176
1244.9; 45.948
1189.0; 66.471
1036.2; 50.110
939.3; 66.747
808.8; 59.845
734.3; 34.195

3.34 3-(4-Methoxyphenyl)-1-(pyrrolidin-1-yl)propan-1-one (12bf) (600 MHz, $CDCl_3$)

3.34.1 ^{1}H NMR

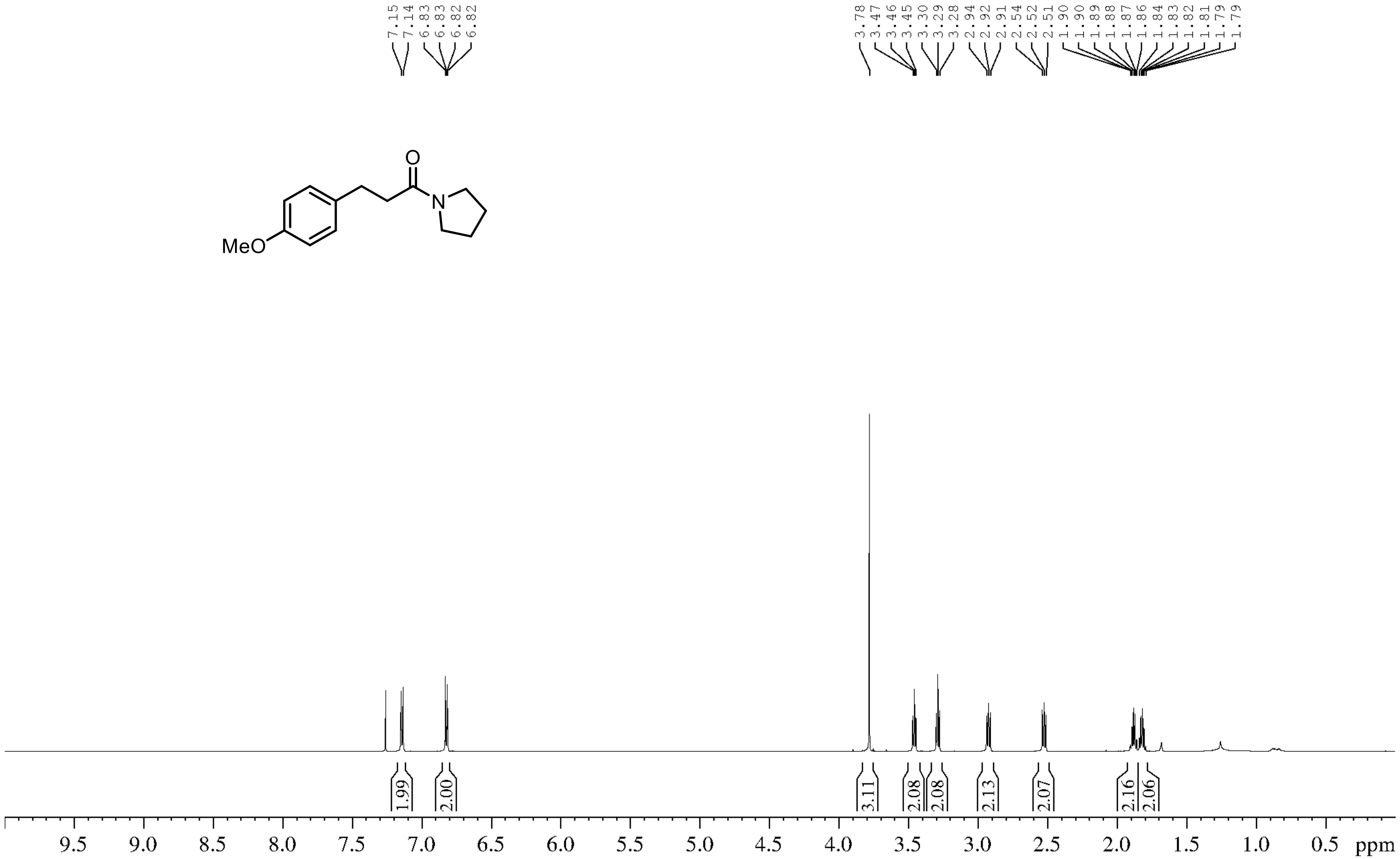

3.34.2 ^{13}C NMR

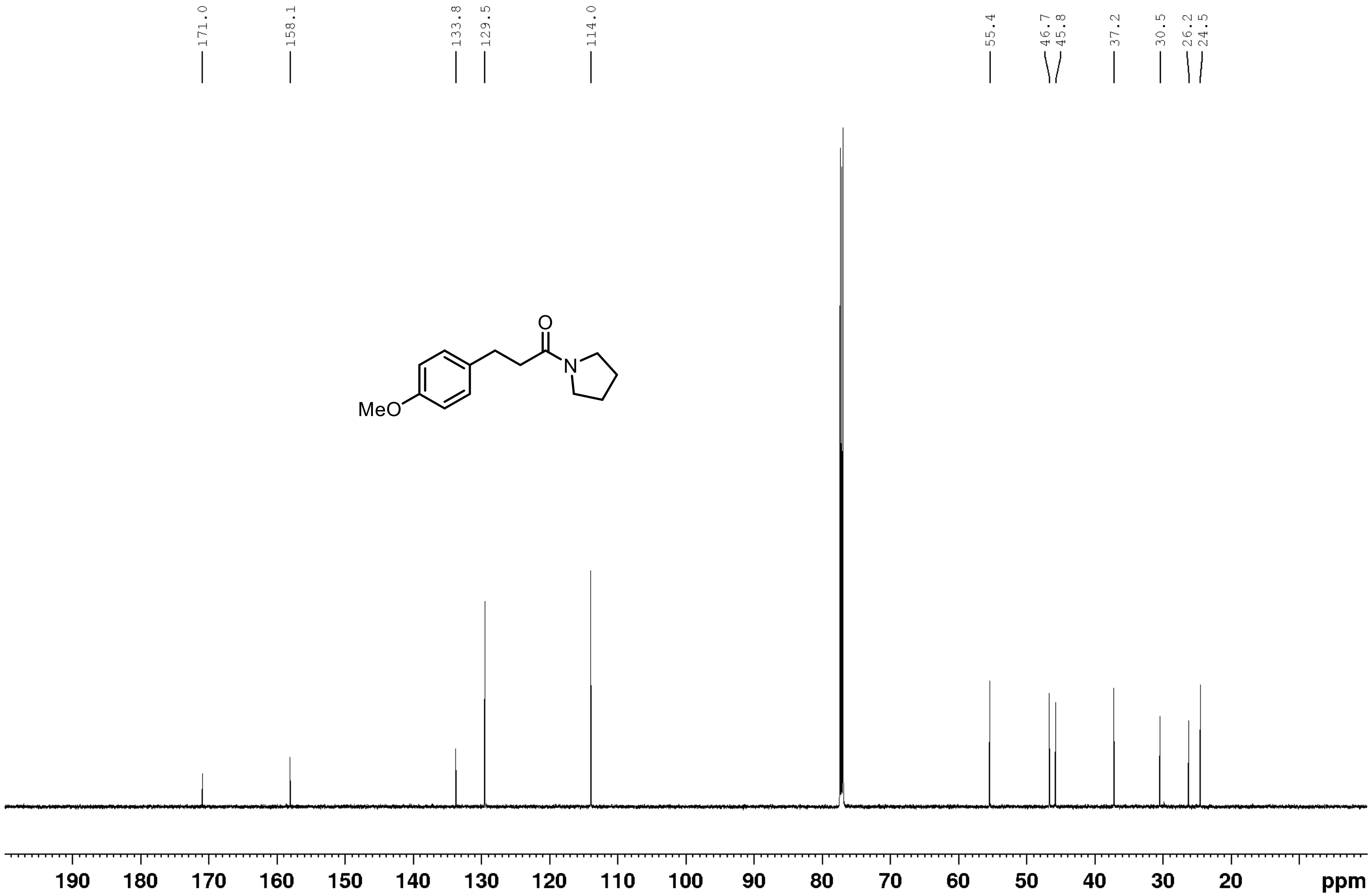

171.0
158.1
133.8
129.5
114.0
55.4
46.7
45.8
37.2
30.5
26.2
24.5
O
N
MeO
190
180
170
160
150
140
130
120
110
100
90
80
70
60
50
40
30
20
ppm

3.34.3 ¹H, ¹H COSY

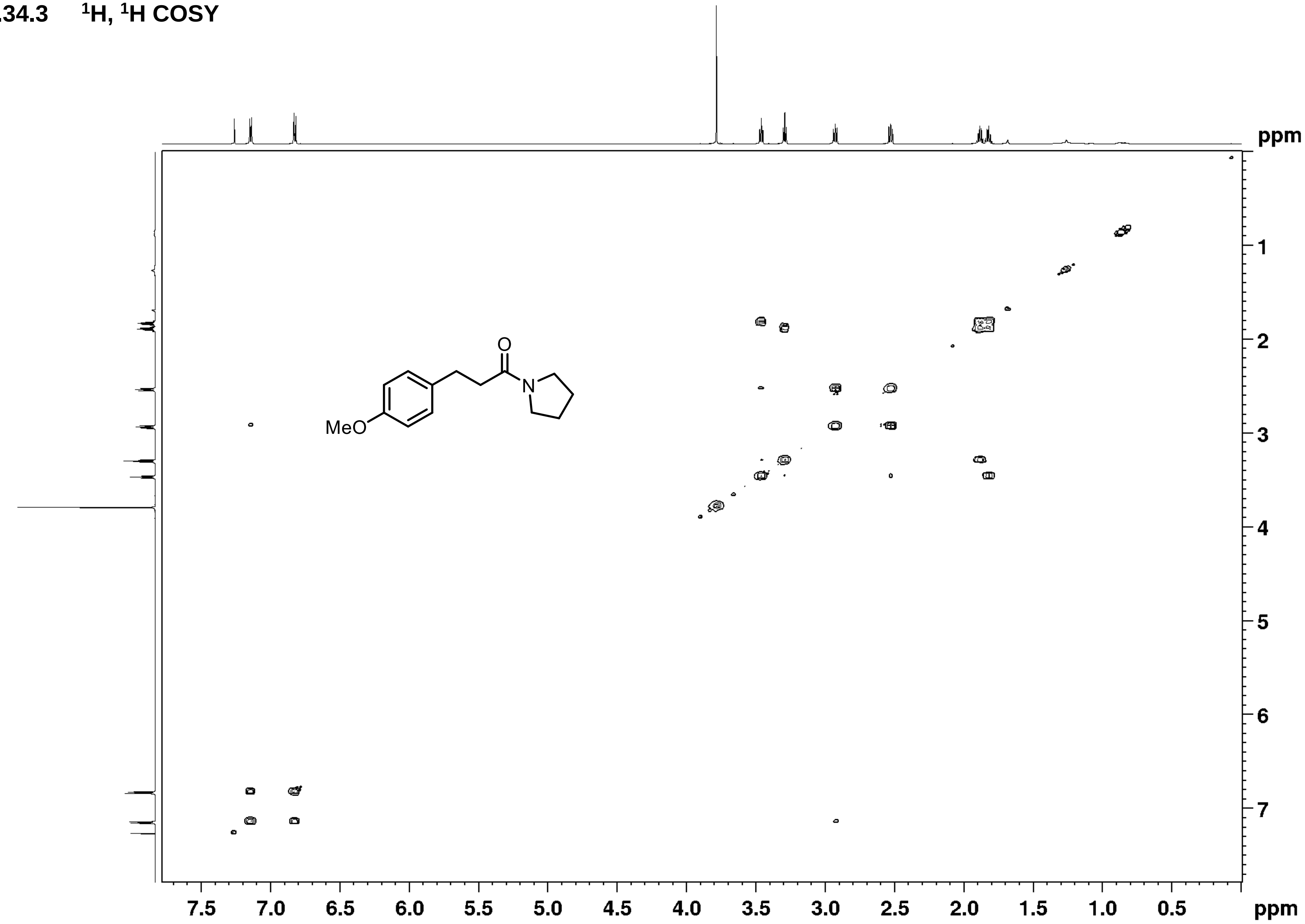

3.34.4 ^{1}H, ^{13}C HMBC

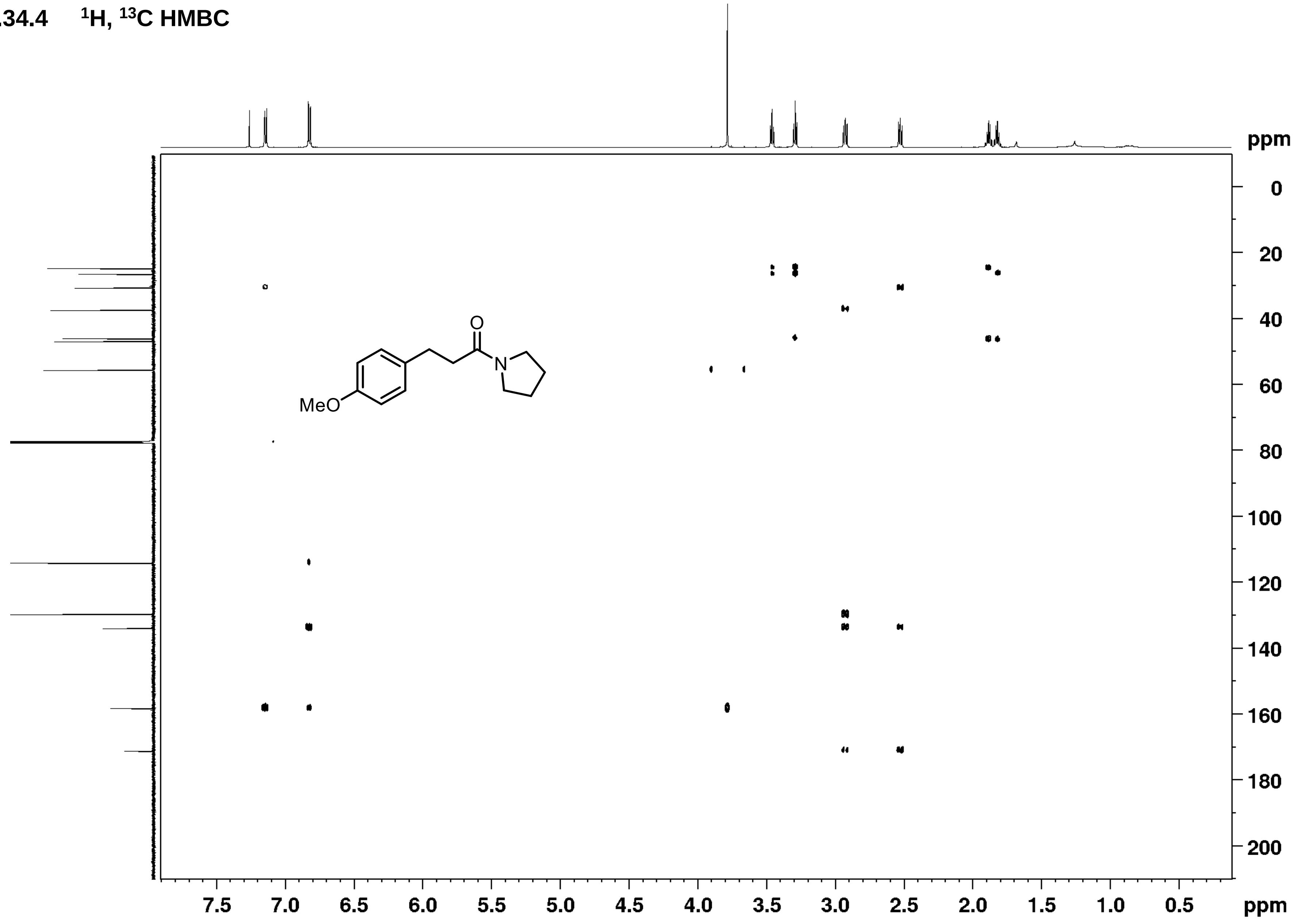

3.34.5 ^{1}H, ^{13}C HSQC

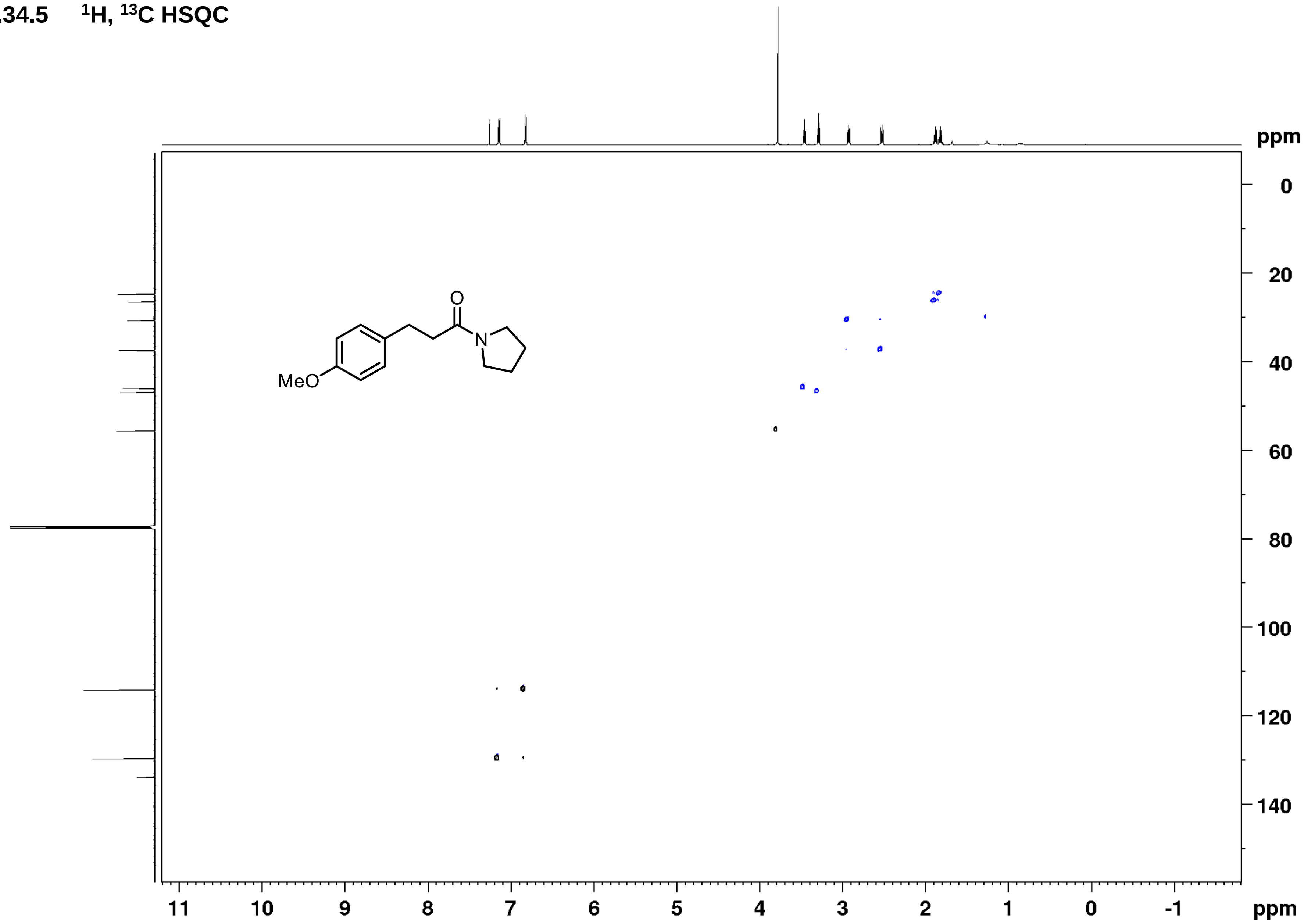

3.34.6 HRMS

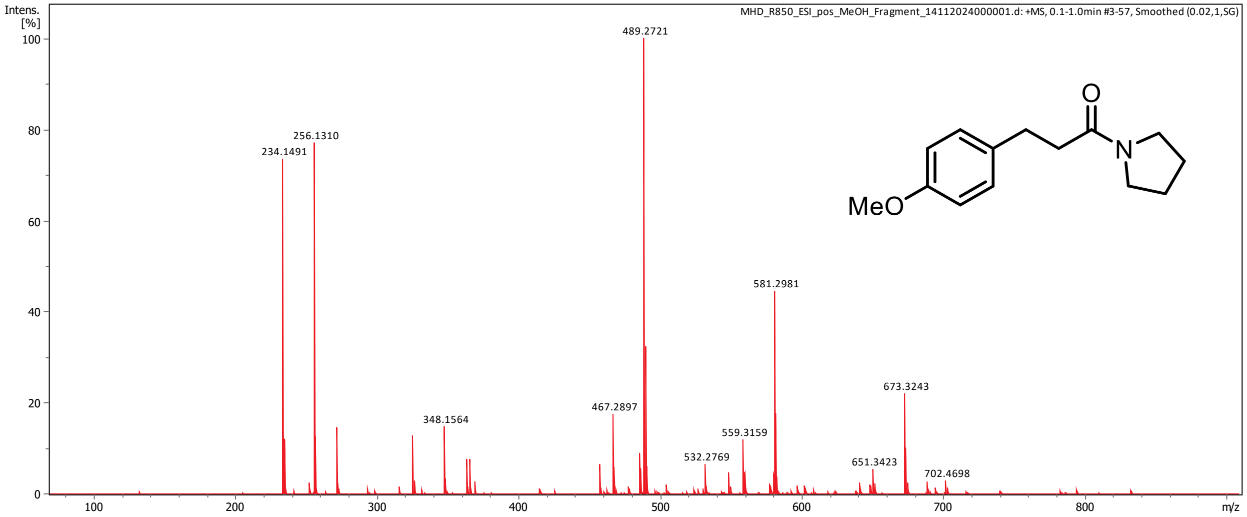

Intens. [%]
MHD_R850_ESI_pos_MeOH_Fragment_14112024000001.d: +MS, 0.1-1.0min #3-57, Smoothed (0.02,1,SG)
489.2721
256.1310
234.1491
581.2981
673.3243
348.1564
467.2897
559.3159
532.2769
651.3423
702.4698
MeO
O
N
m/z

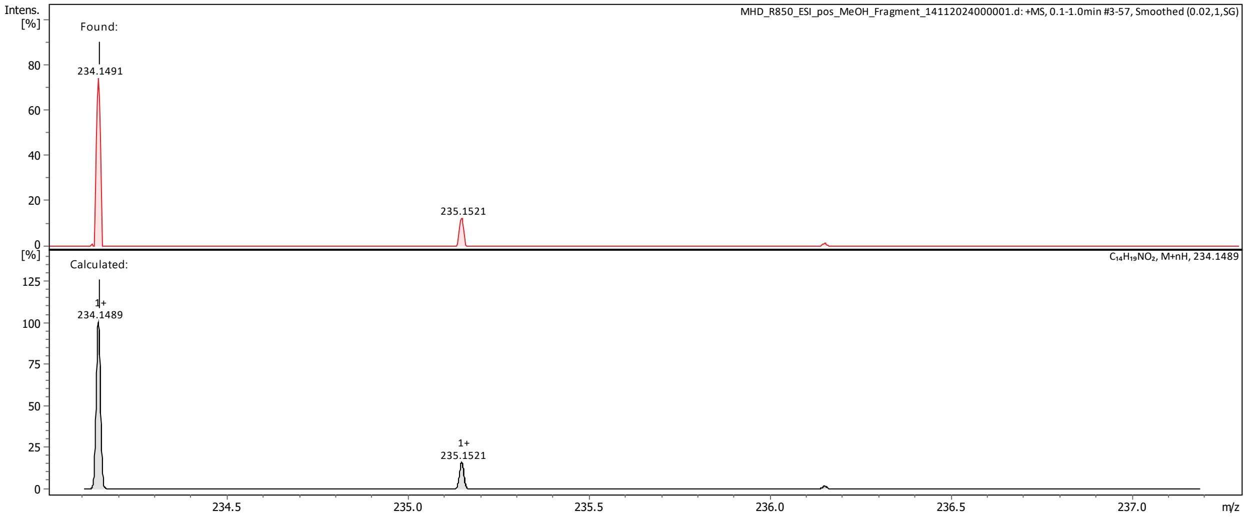

Intens. [%]
MHD_R850_ESI_pos_MeOH_Fragment_14112024000001.d: +MS, 0.1-1.0min #3-57, Smoothed (0.02,1,SG)
Found:
234.1491
235.1521
Calculated:
C14H19NO2, M+nH, 234.1489
1+
234.1489
1+
235.1521
m/z

3.34.7 IR

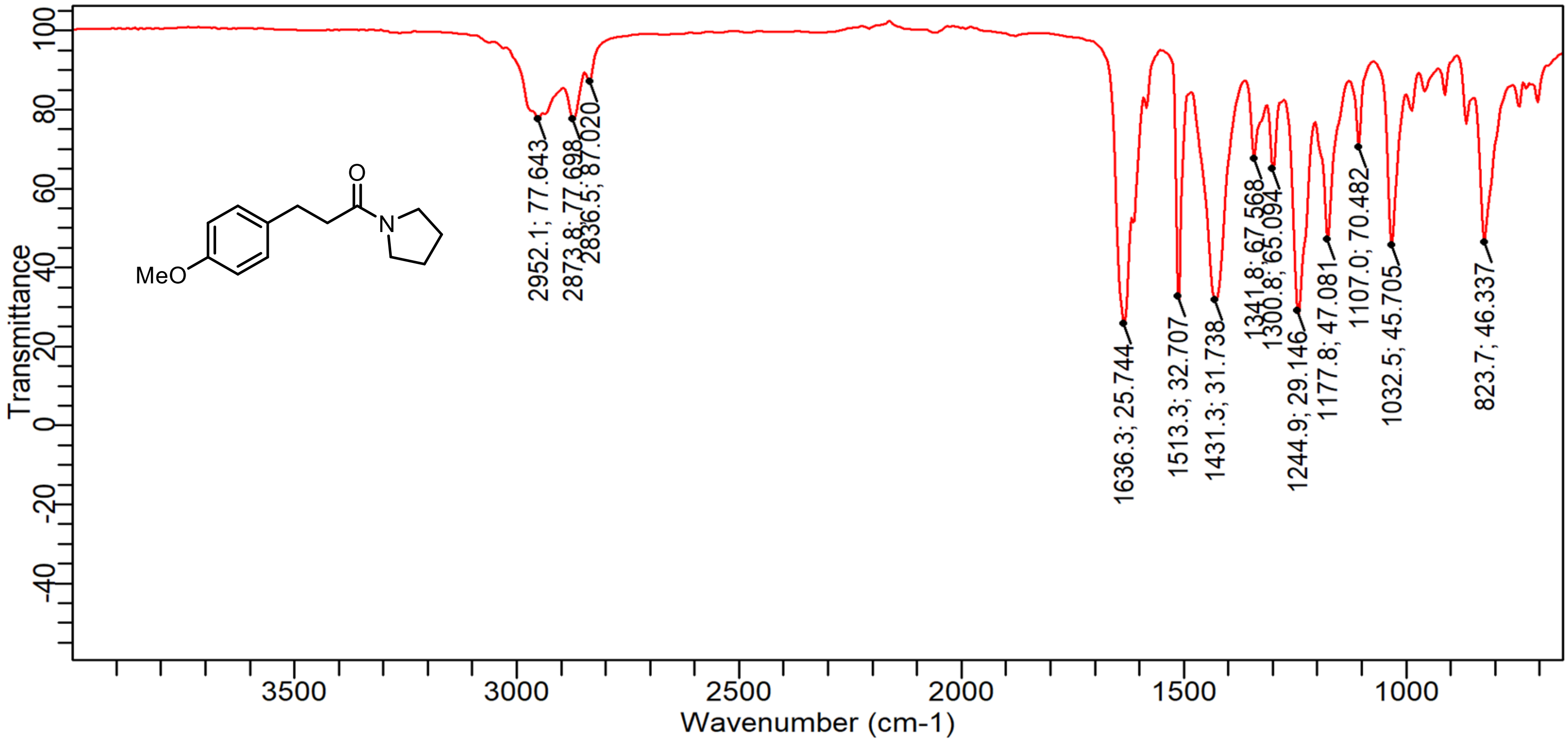

Transmittance
Wavenumber (cm-1)
MeO
O
N
2952.1; 77.643
2873.8; 77.698
2836.5; 87.020
1636.3; 25.744
1513.3; 32.707
1431.3; 31.738
1341.8; 67.568
1300.8; 65.094
1244.9; 29.146
1177.8; 47.081
1107.0; 70.482
1032.5; 45.705
823.7; 46.337

3.35 1-(Pyrrolidin-1-yl)-3-(3,4,5-trimethoxyphenyl)propan-1-one (12bg) (600 MHz, $CDCl_3$)

3.35.1 ^{1}H NMR

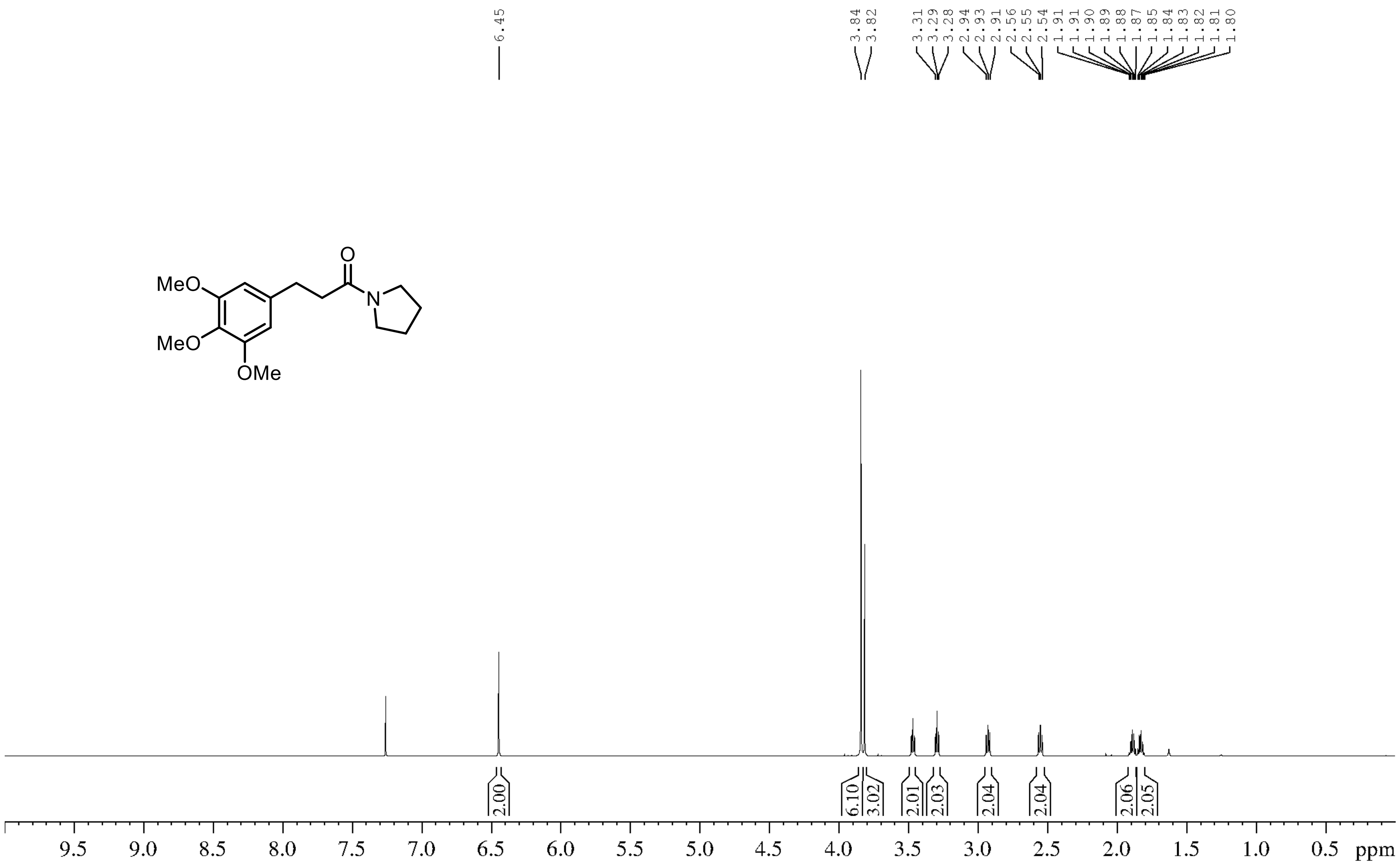

3.35.2 ^{13}C NMR

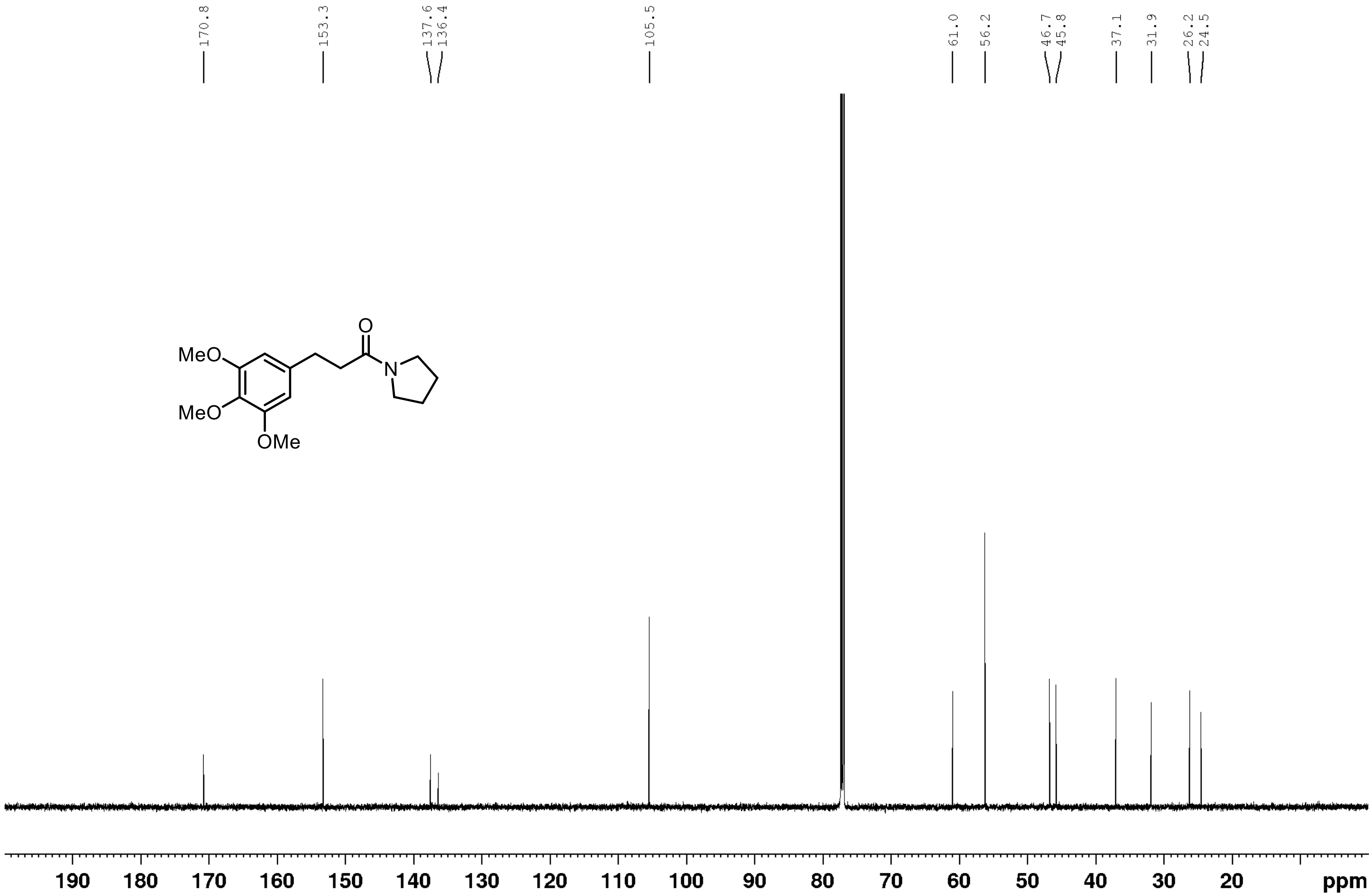
170.8
153.3
137.6
136.4
105.5
61.0
56.2
46.7
45.8
37.1
31.9
26.2
24.5
MeO
MeO
OMe
O
N
190 180 170 160 150 140 130 120 110 100 90 80 70 60 50 40 30 20 ppm

3.35.3 ¹H, ¹H COSY

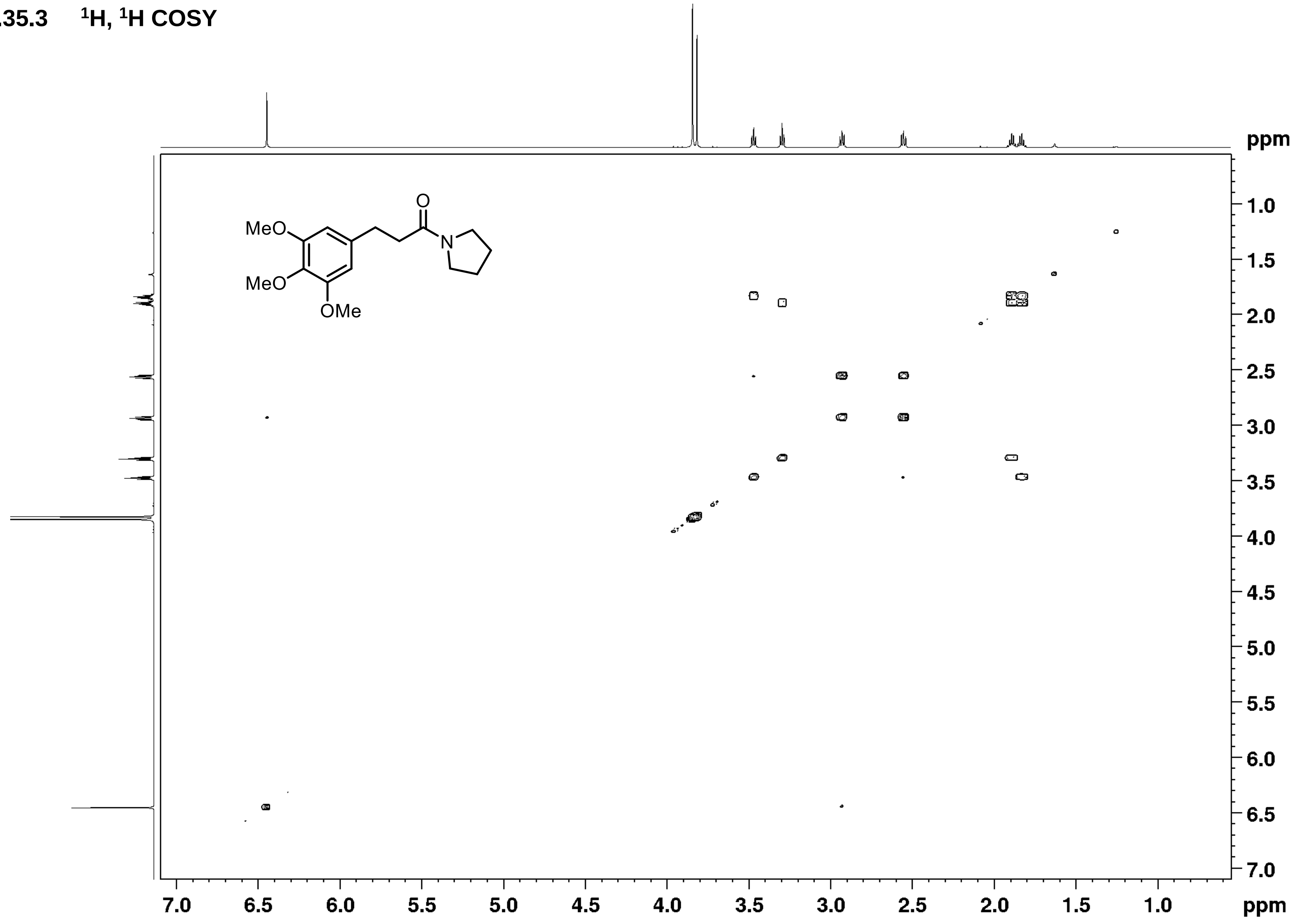

3.35.4 ^1H, ^{13}C HMBC

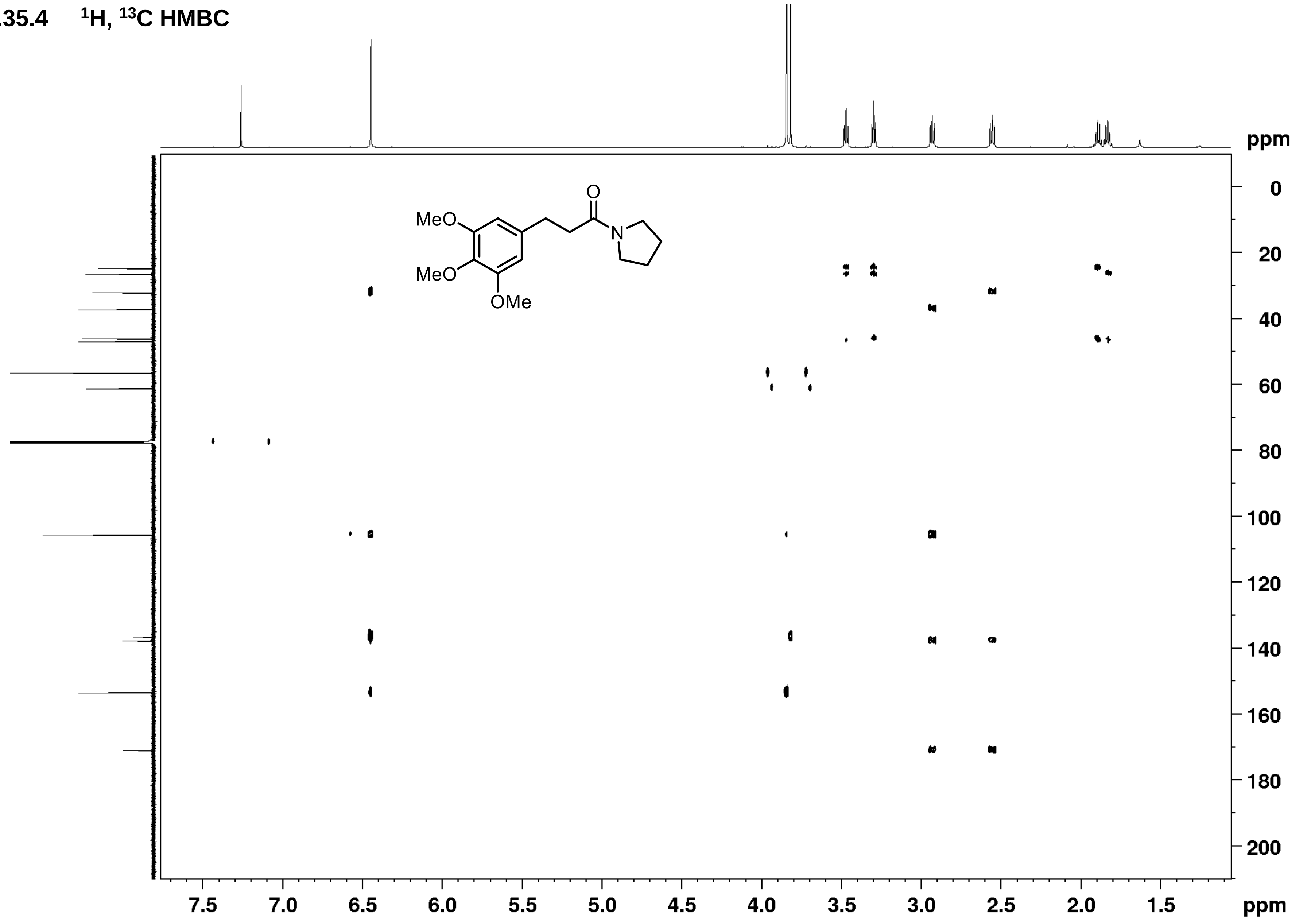

3.35.5 ^{1}H, ^{13}C HSQC

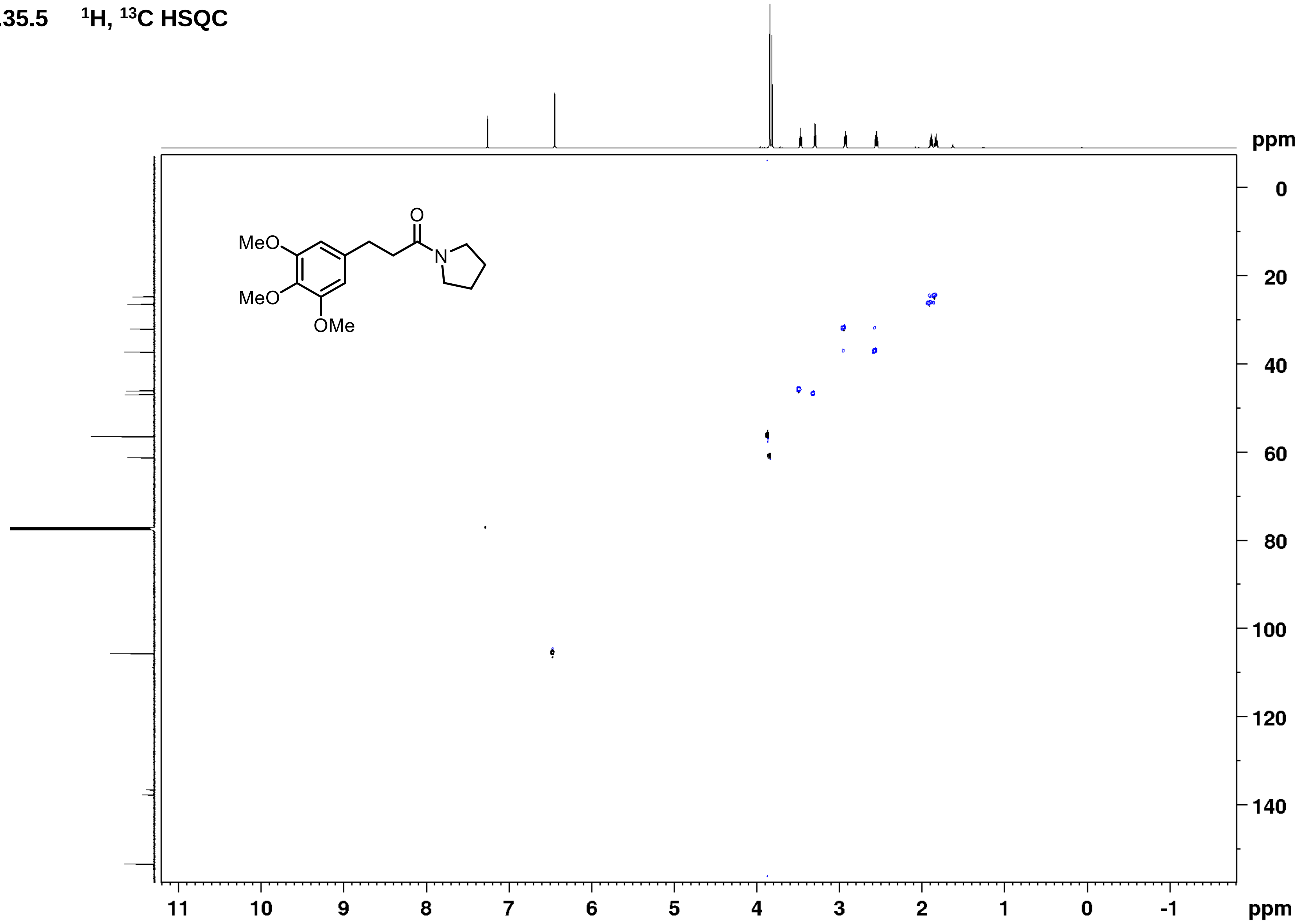

3.35.6 HRMS

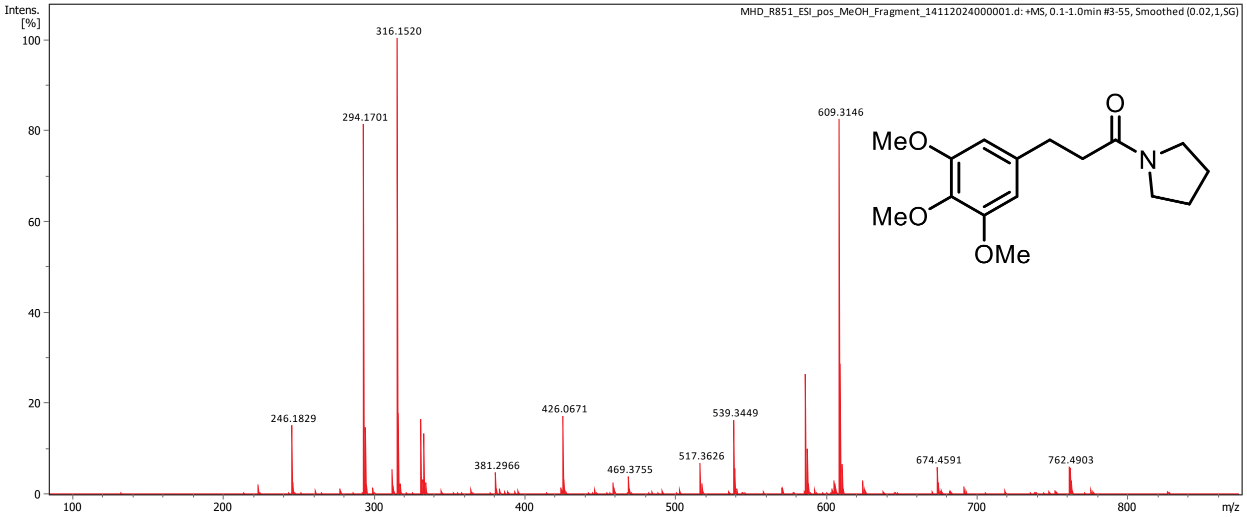
MHD_R851_ESI_pos_MeOH_Fragment_14112024000001.d: +MS, 0.1-1.0min #3-55, Smoothed (0.02,1,SG)
Intens. [%]
316.1520
294.1701
609.3146
246.1829
426.0671
539.3449
381.2966
469.3755
517.3626
674.4591
762.4903
MeO
MeO
OMe
O
N
m/z

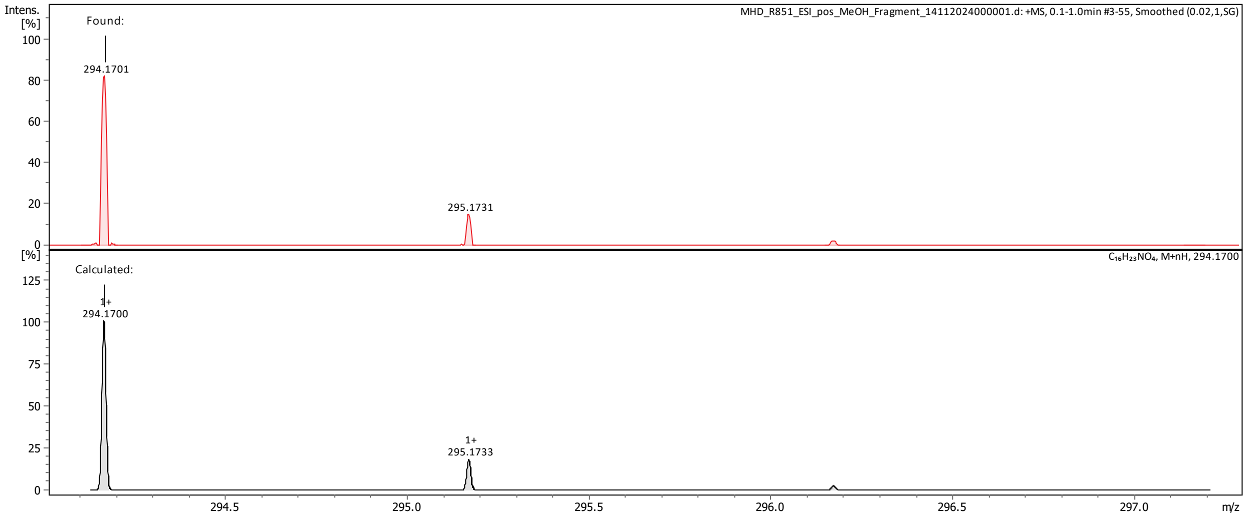
MHD_R851_ESI_pos_MeOH_Fragment_14112024000001.d: +MS, 0.1-1.0min #3-55, Smoothed (0.02,1,SG)
Found:
294.1701
295.1731
Calculated:
1+
294.1700
1+
295.1733
C16H23NO4, M+nH, 294.1700
m/z

3.35.7 IR

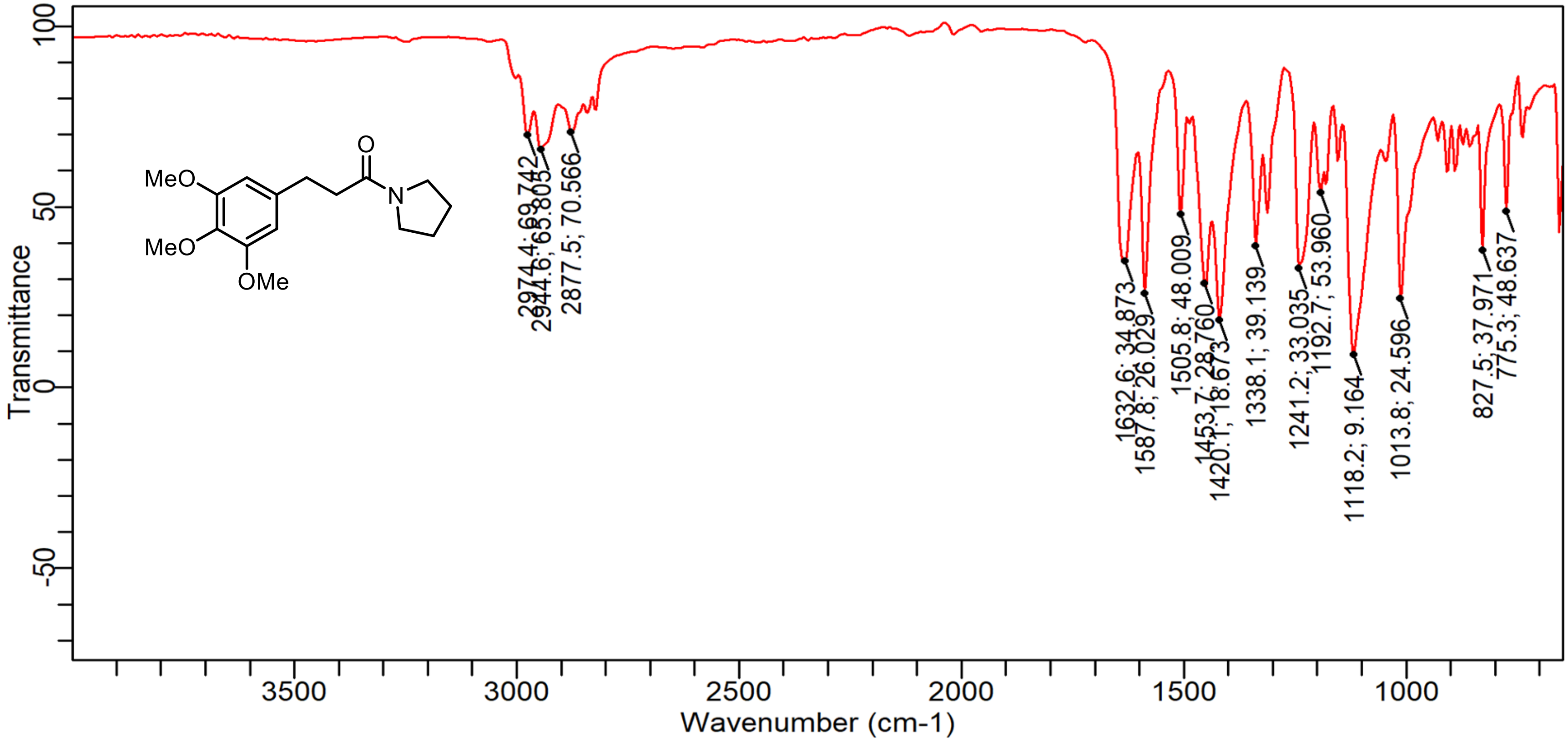

MeO
MeO
OMe
O
N
Transmittance
Wavenumber (cm-1)
2974.4; 69.742
2944.6; 65.805
2877.5; 70.566
1632.6; 34.873
1587.8; 26.029
1505.8; 48.009
1453.7; 28.760
1420.1; 18.673
1338.1; 39.139
1241.2; 33.035
1192.7; 53.960
1118.2; 9.164
1013.8; 24.596
827.5; 37.971
775.3; 48.637

3.36 1-(3-(3,4,5-Trimethoxyphenyl)propanoyl)piperidin-2-one (12bh) (600 MHz, $CDCl_3$)

3.36.1 ^{1}H NMR

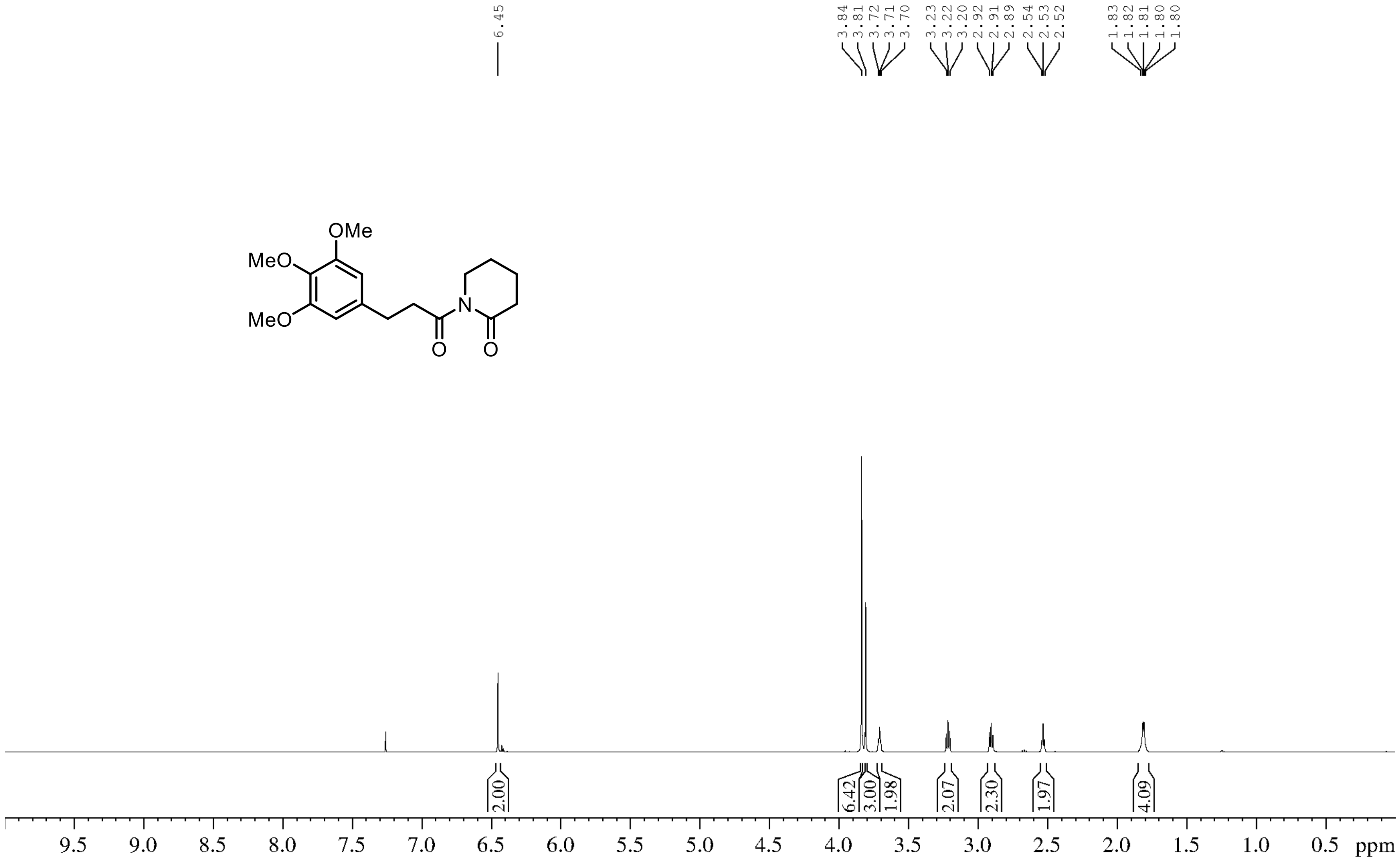

3.36.2 ¹³C NMR

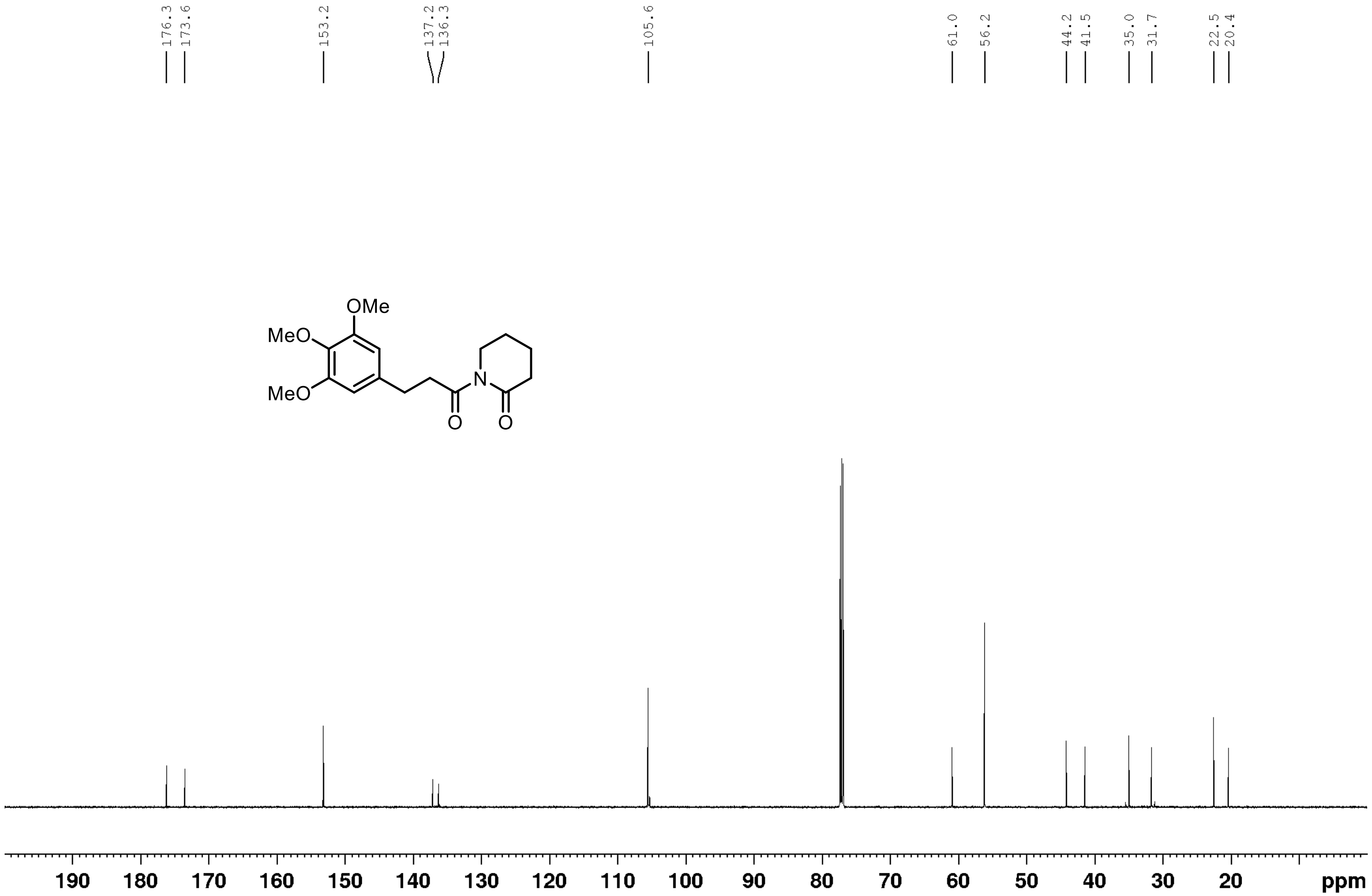
176.3
173.6
153.2
137.2
136.3
105.6
61.0
56.2
44.2
41.5
35.0
31.7
22.5
20.4
OMe
MeO
MeO
N
O
O
190
180
170
160
150
140
130
120
110
100
90
80
70
60
50
40
30
20
ppm

3.36.3 ^{1}H, ^{1}H COSY

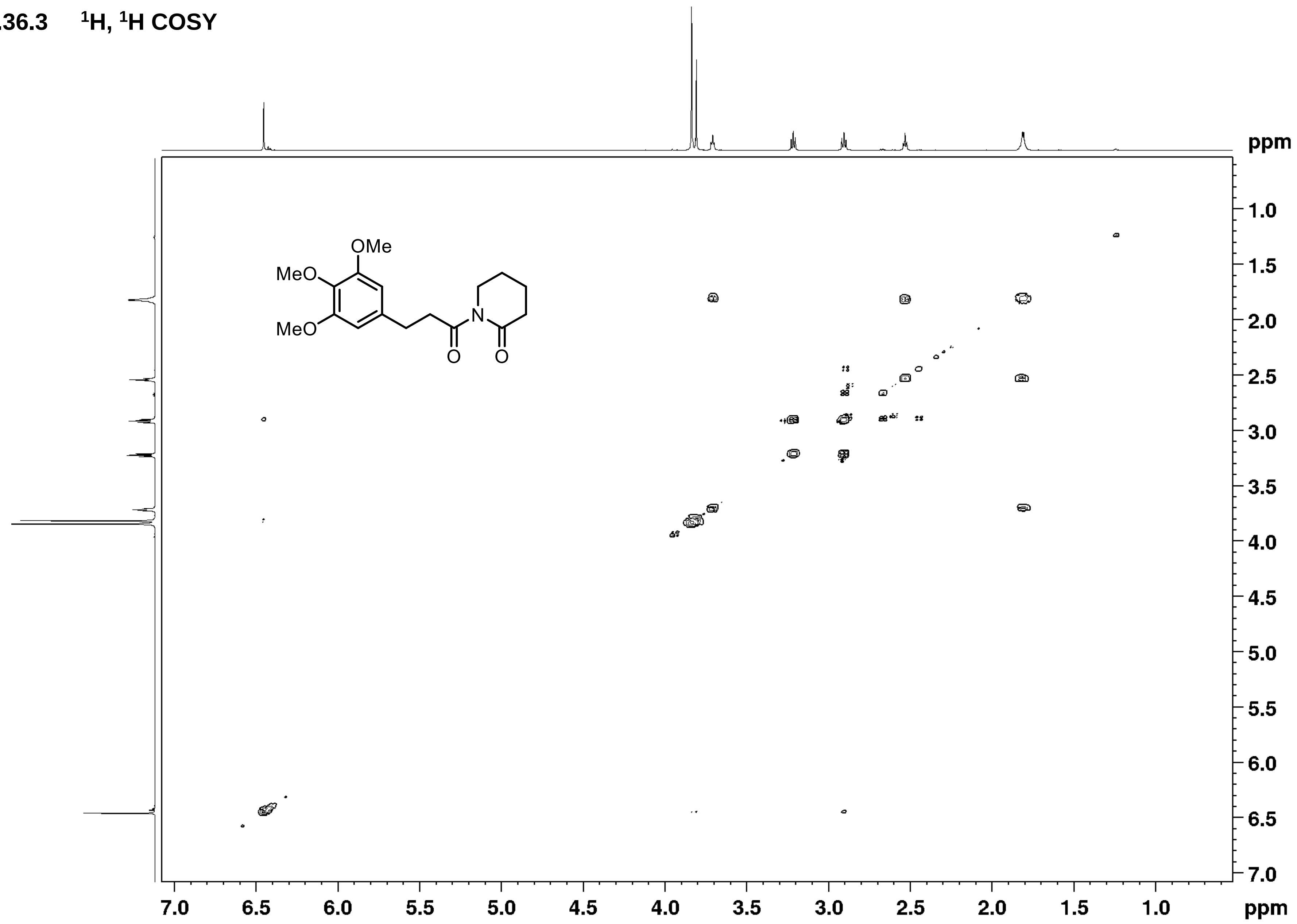

3.36.4 ^{1}H, ^{13}C HMBC

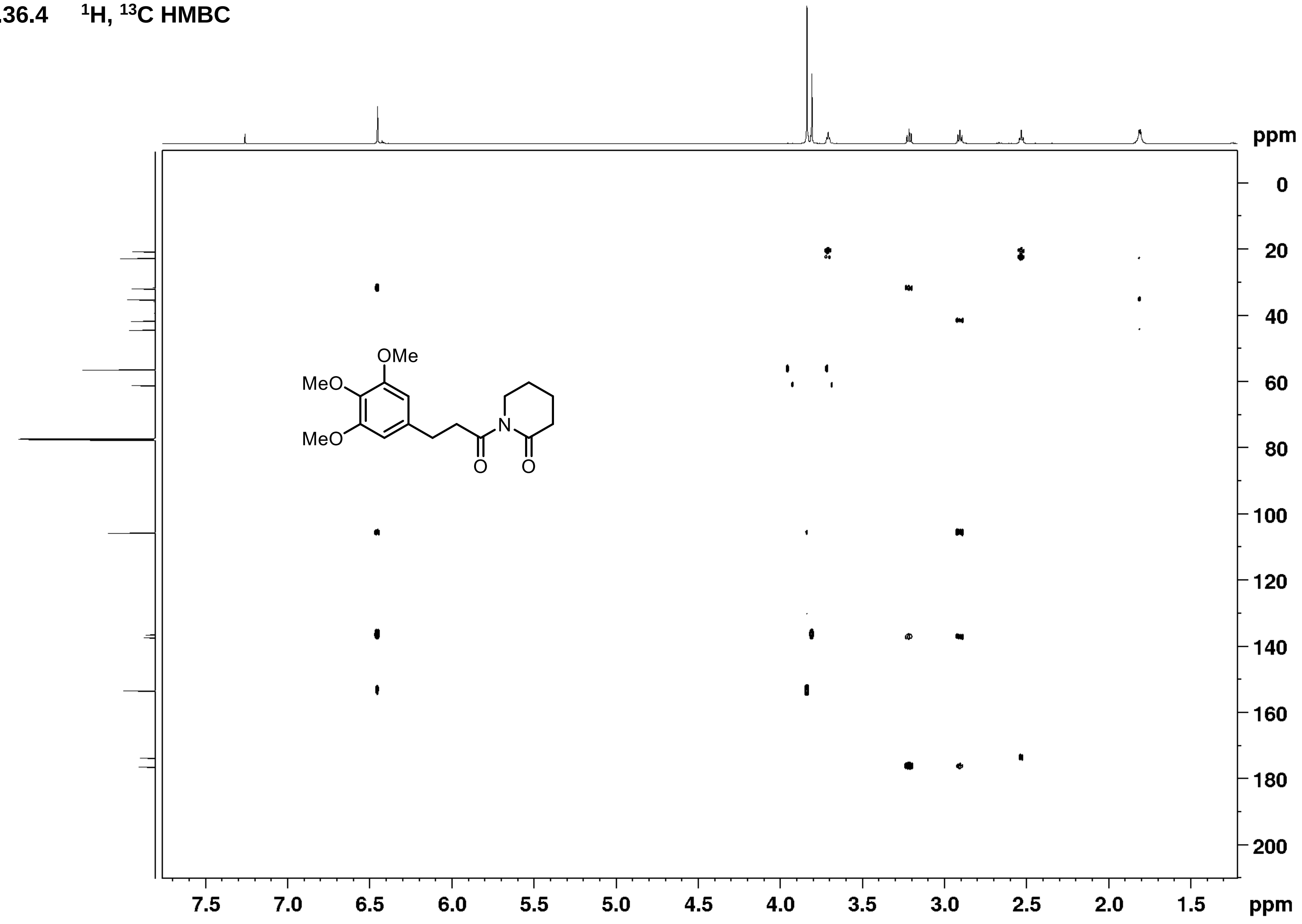

3.36.5 ^{1}H, ^{13}C HSQC

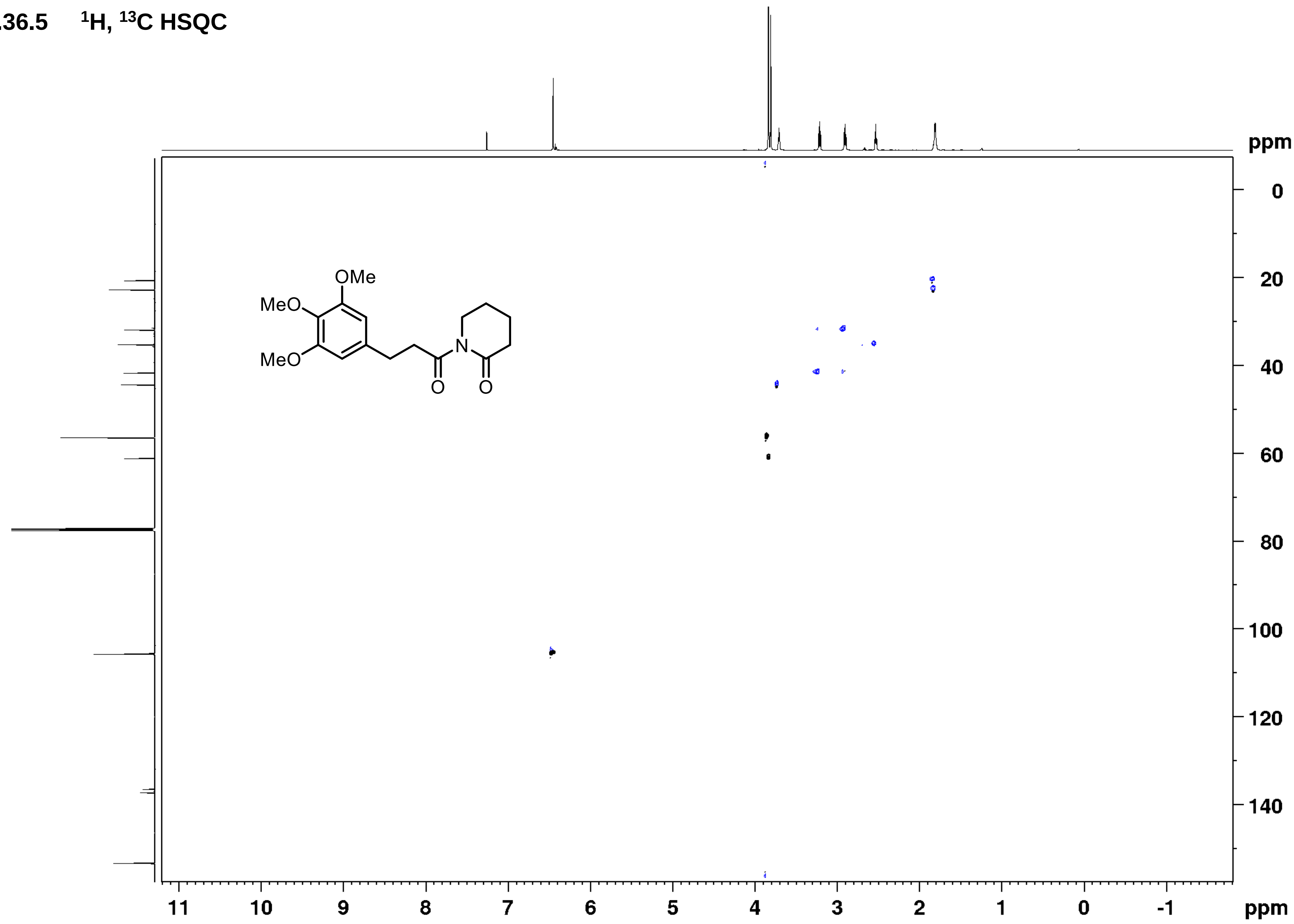

3.36.6 HRMS

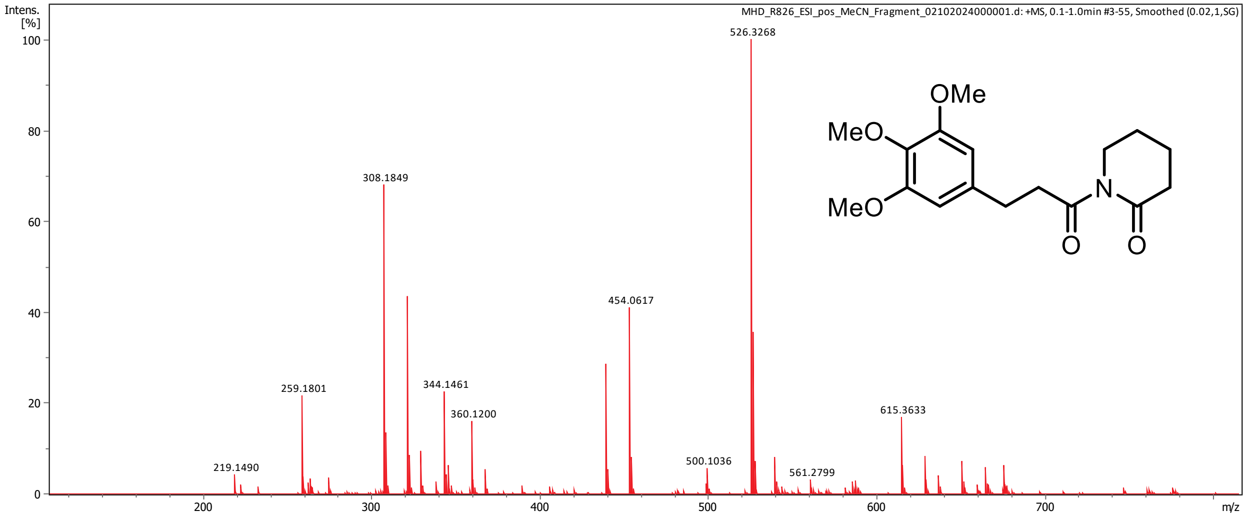

Intens. [%]
MHD_R826_ESI_pos_MeCN_Fragment_02102024000001.d: +MS, 0.1-1.0min #3-55, Smoothed (0.02,1,SG)
526.3268
308.1849
454.0617
259.1801
344.1461
360.1200
615.3633
219.1490
500.1036
561.2799
OMe
MeO
MeO
N
O
O
m/z

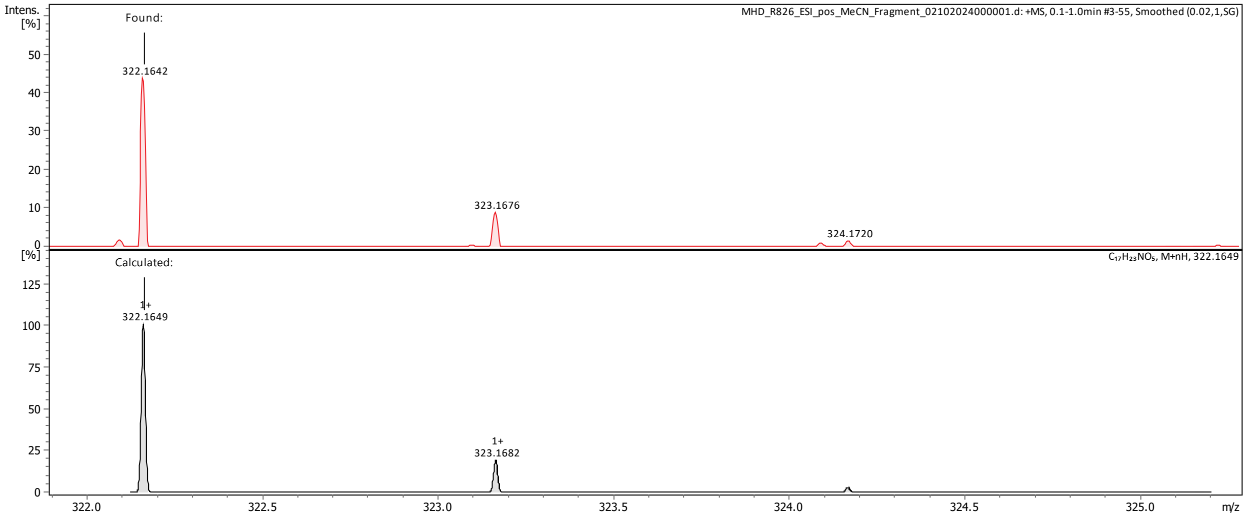

Intens. [%]
Found:
322.1642
323.1676
324.1720
MHD_R826_ESI_pos_MeCN_Fragment_02102024000001.d: +MS, 0.1-1.0min #3-55, Smoothed (0.02,1,SG)
Calculated:
1+
322.1649
1+
323.1682
C17H23NO5, M+nH, 322.1649
m/z

3.36.7 IR

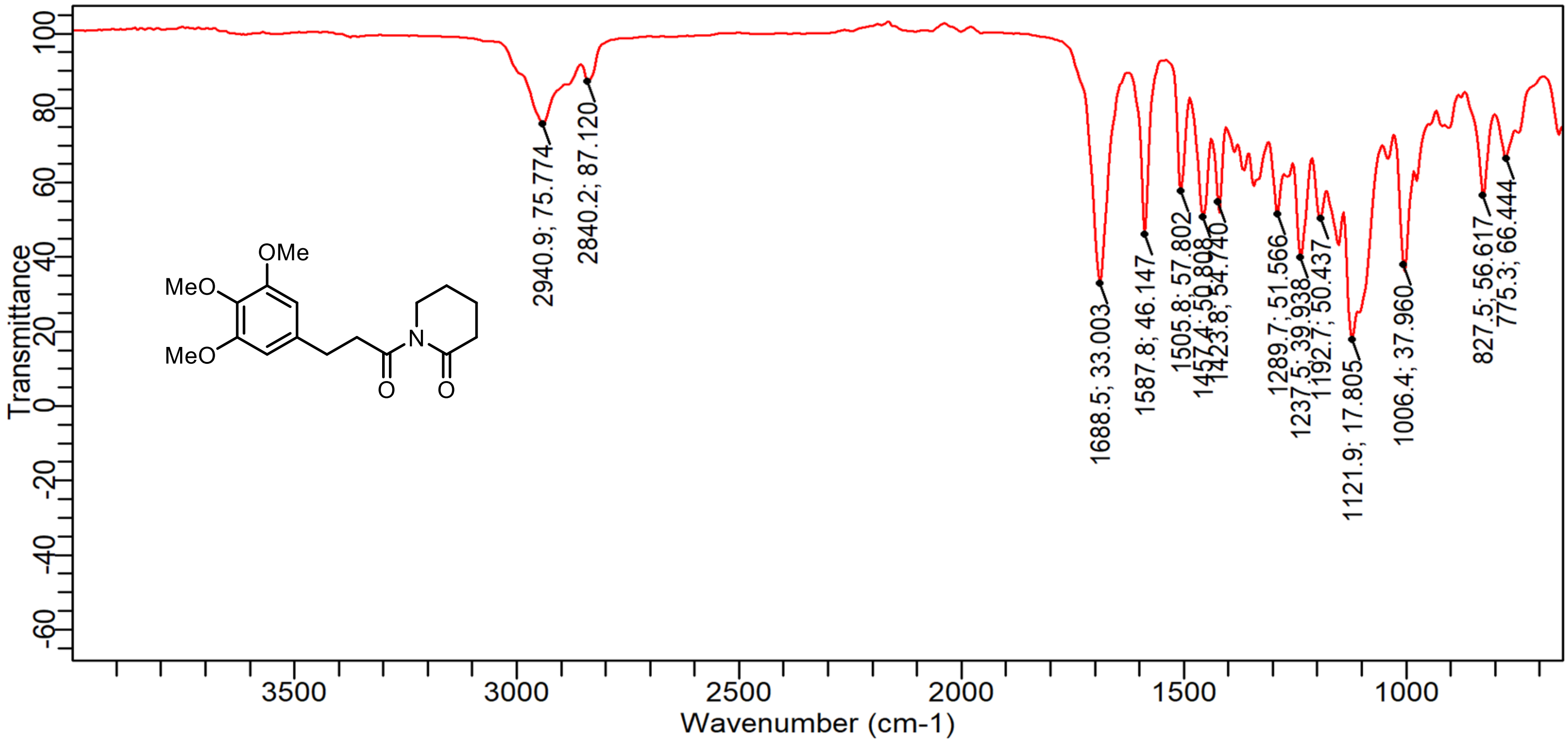

Transmittance
Wavenumber (cm-1)
2940.9; 75.774
2840.2; 87.120
1688.5; 33.003
1587.8; 46.147
1505.8; 57.802
1457.4; 50.808
1423.8; 54.740
1289.7; 51.566
1237.5; 39.938
1192.7; 50.437
1121.9; 17.805
1006.4; 37.960
827.5; 56.617
775.3; 66.444
OMe
MeO
MeO
N
O
O

3.37 5-(Benzo[*d*][1,3]dioxol-5-yl)-1-(piperidin-1-yl)pentan-1-one (12bi) (600 MHz, $CDCl_3$)

3.37.1 ^{1}H NMR

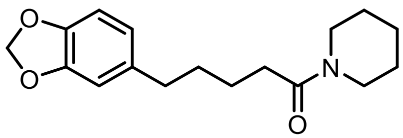

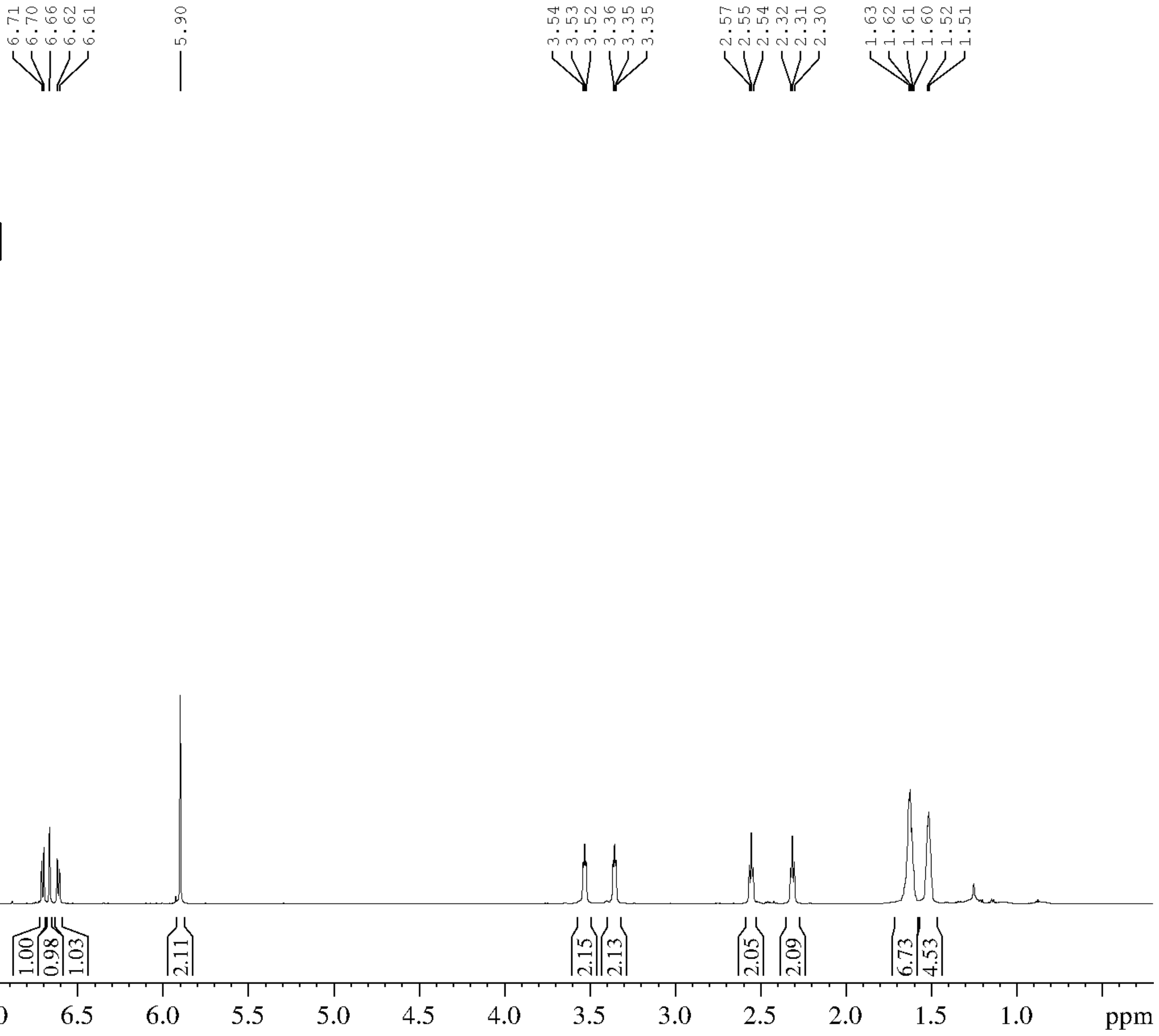

3.37.2 ^{13}C NMR

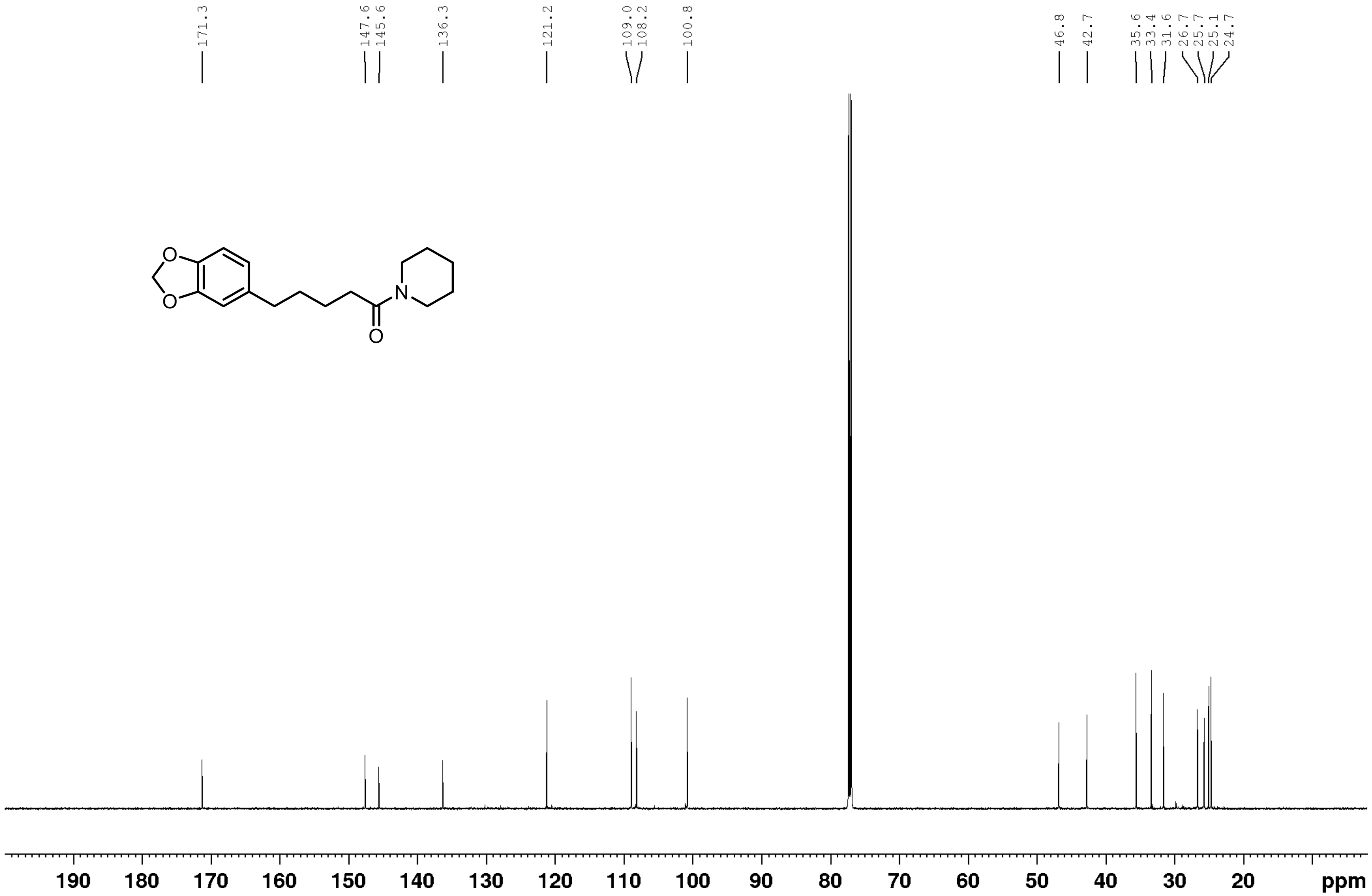

171.3
147.6
145.6
136.3
121.2
109.0
108.2
100.8
46.8
42.7
35.6
33.4
31.6
26.7
25.7
25.1
24.7
190 180 170 160 150 140 130 120 110 100 90 80 70 60 50 40 30 20 ppm

3.37.3 ^{1}H, ^{1}H COSY

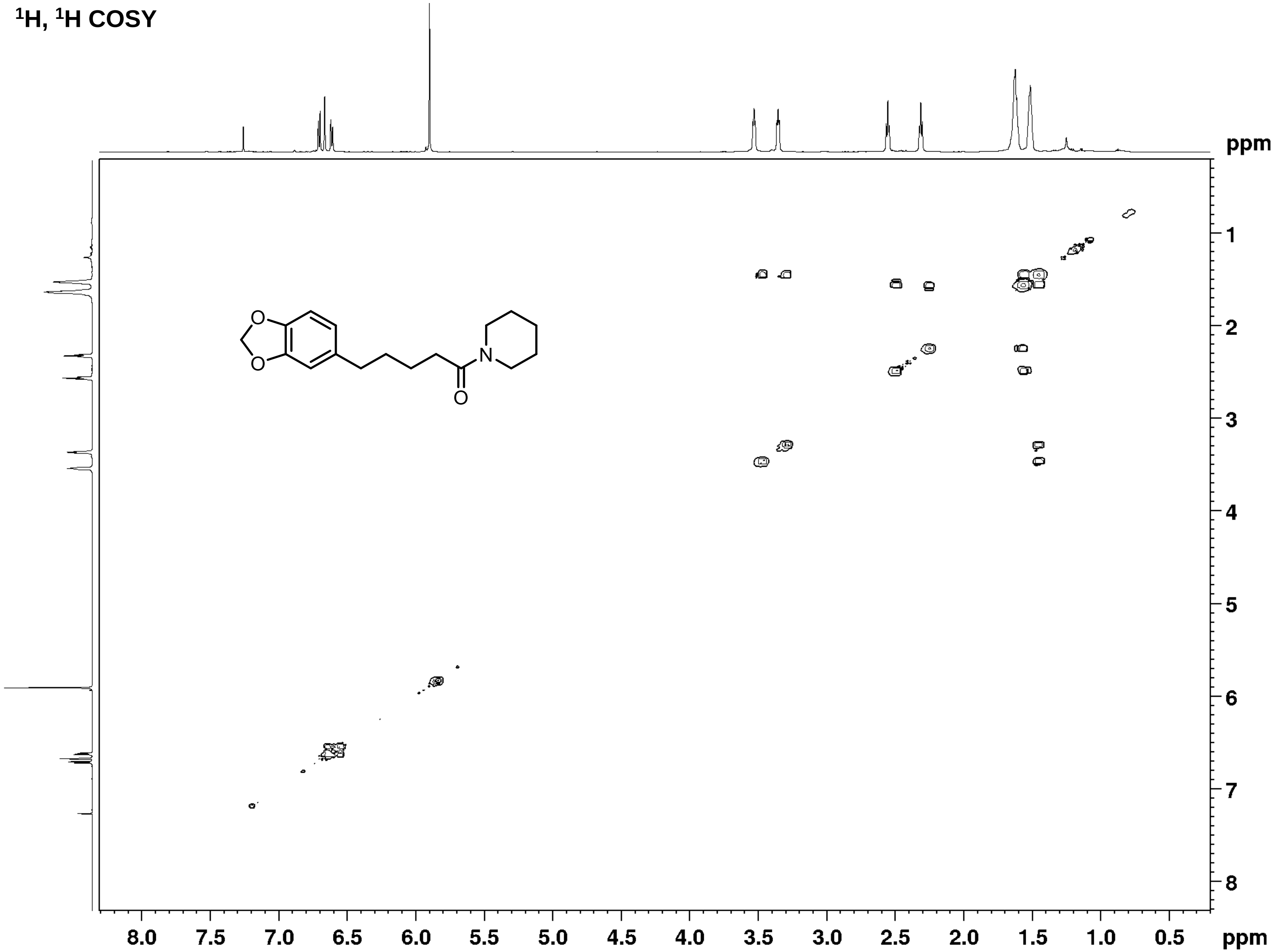

3.37.4 ^{1}H, ^{13}C HMBC

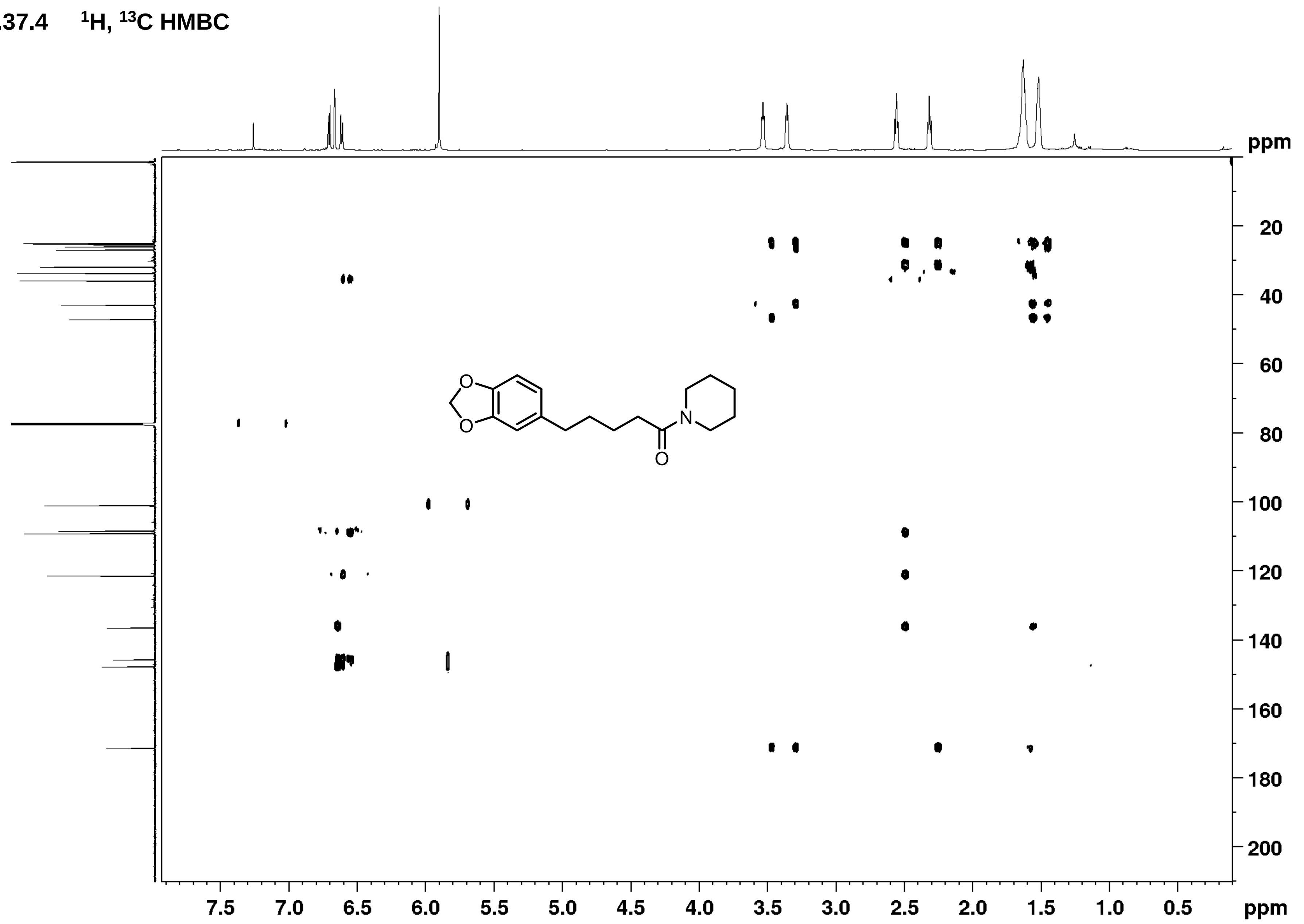

3.37.5 ^{1}H, ^{13}C HSQC

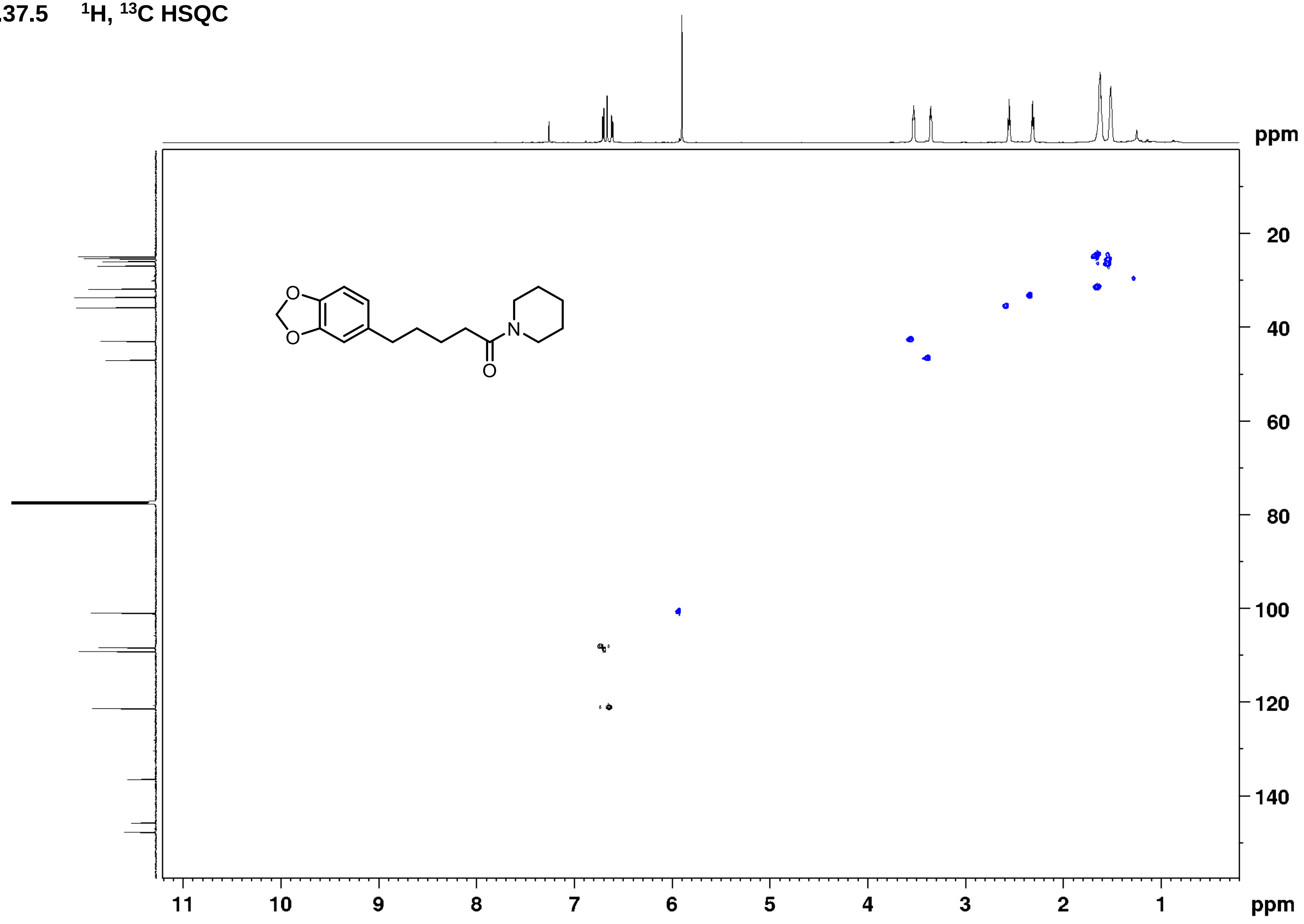

3.37.6 HRMS

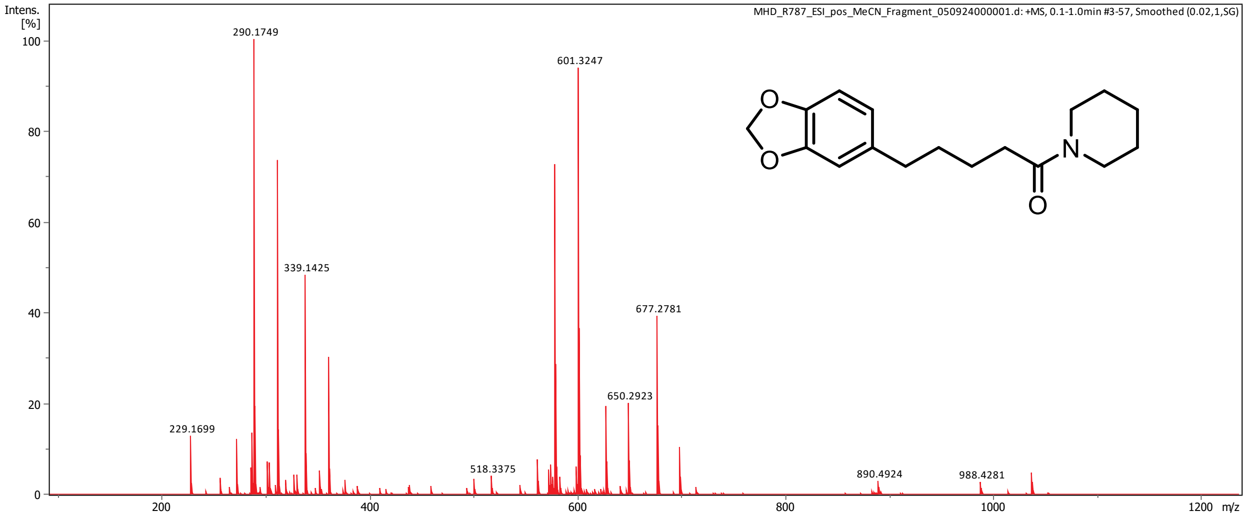
MHD_R787_ESI_pos_MeCN_Fragment_050924000001.d: +MS, 0.1-1.0min #3-57, Smoothed (0.02,1,SG)
Intens. [%]
290.1749
601.3247
339.1425
677.2781
650.2923
229.1699
518.3375
890.4924
988.4281
m/z
O
O
N
O

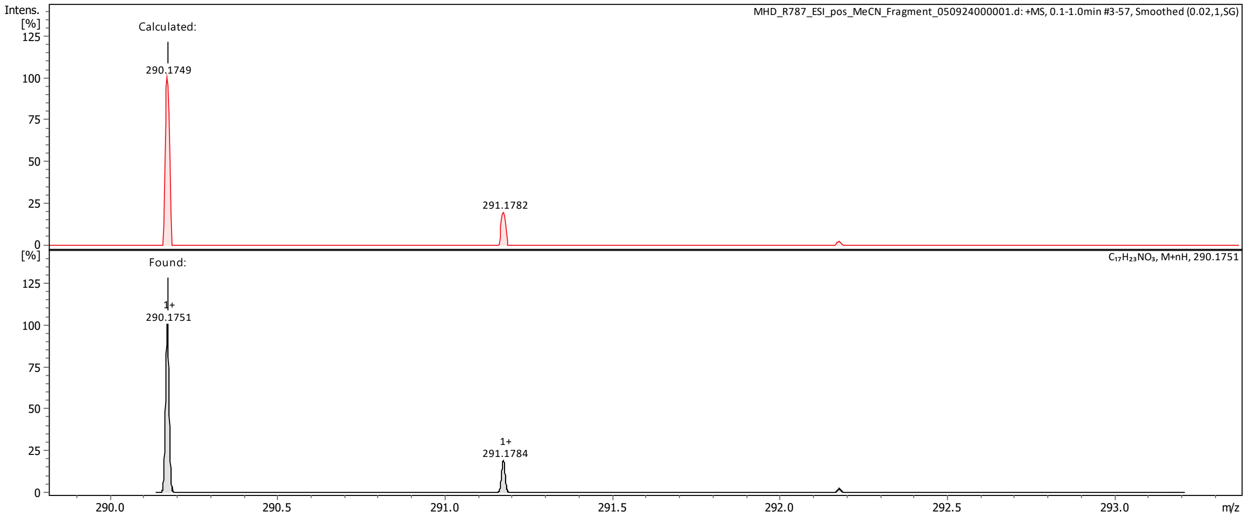
MHD_R787_ESI_pos_MeCN_Fragment_050924000001.d: +MS, 0.1-1.0min #3-57, Smoothed (0.02,1,SG)
Intens. [%]
Calculated:
290.1749
291.1782
$C_{17}H_{23}NO_3$, M+nH, 290.1751
Found:
1+
290.1751
1+
291.1784
m/z

3.37.7 IR

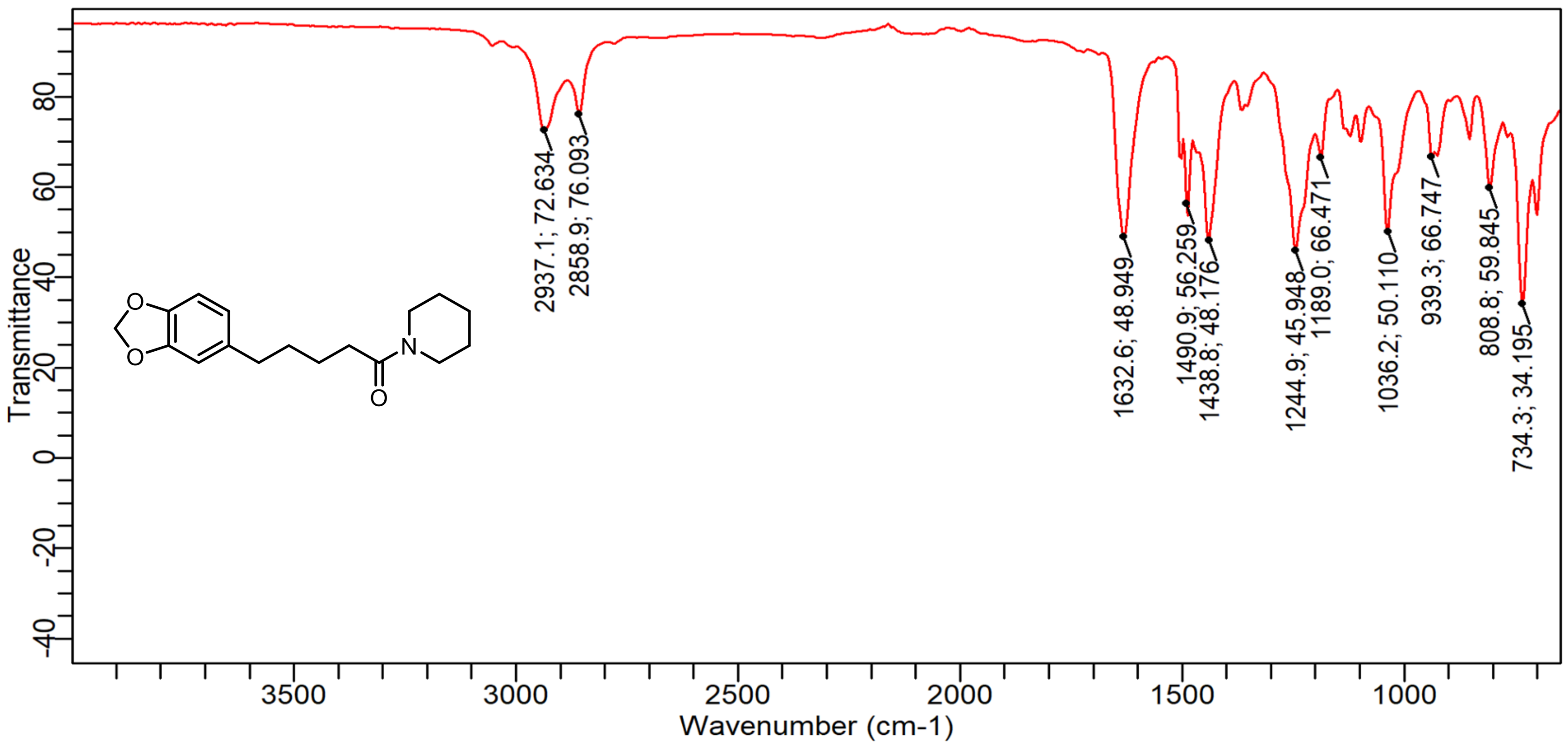

Transmittance
Wavenumber (cm-1)
2937.1; 72.634
2858.9; 76.093
1632.6; 48.949
1490.9; 56.259
1438.8; 48.176
1244.9; 45.948
1189.0; 66.471
1036.2; 50.110
939.3; 66.747
808.8; 59.845
734.3; 34.195
O
O
N
O

3.38 *N*-Isobutyl-*N*-methyldecanamide (12bj-1) (600 MHz, $CDCl_3$)

3.38.1 ^{1}H NMR

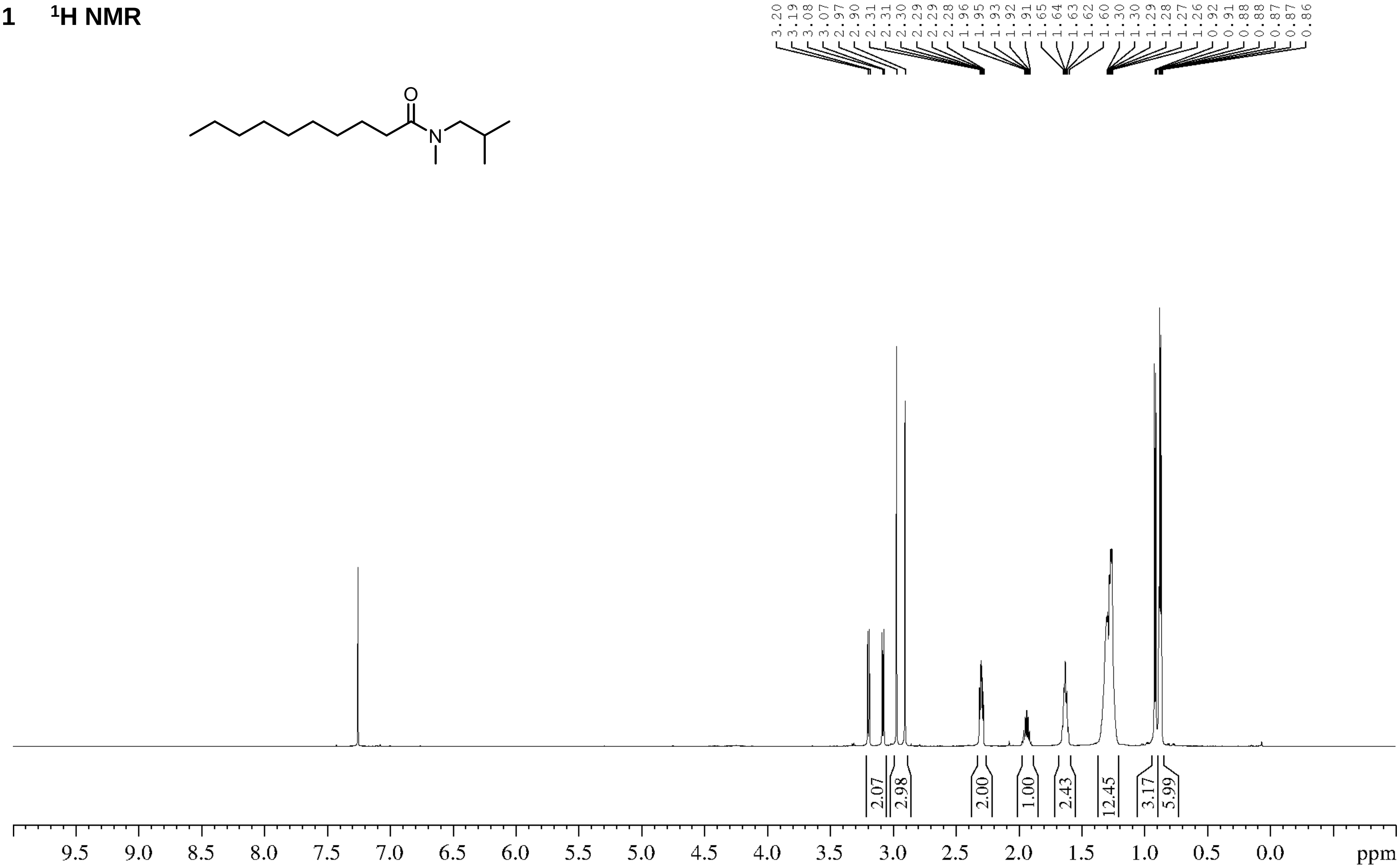

3.38.2 ^{13}C NMR

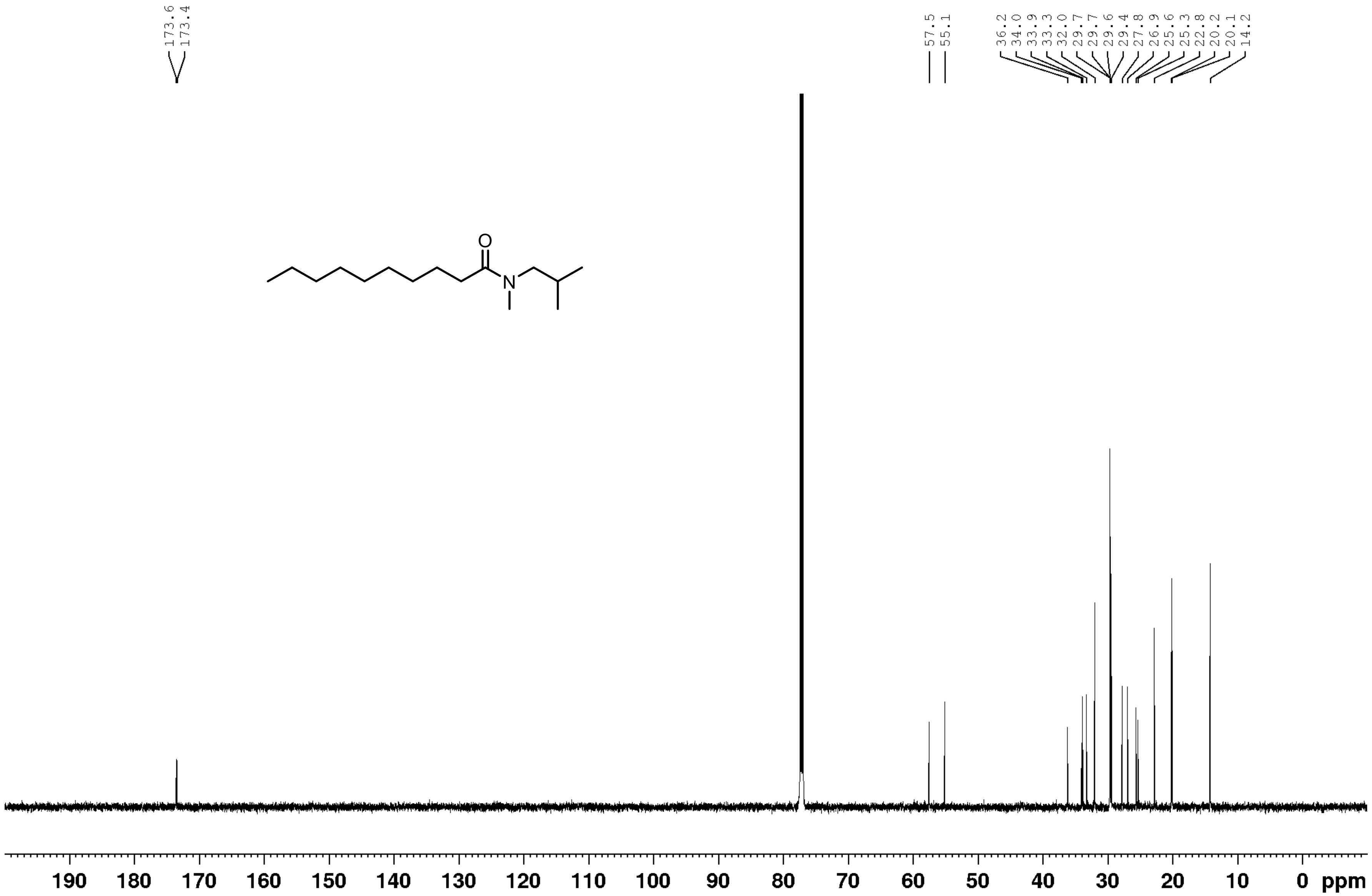

173.6
173.4
57.5
55.1
36.2
34.0
33.9
33.3
32.0
29.7
29.7
29.6
29.4
27.8
26.9
25.6
25.3
22.8
20.2
20.1
14.2
O
N
190 180 170 160 150 140 130 120 110 100 90 80 70 60 50 40 30 20 10 0 ppm

3.38.3 ^{1}H, ^{1}H COSY

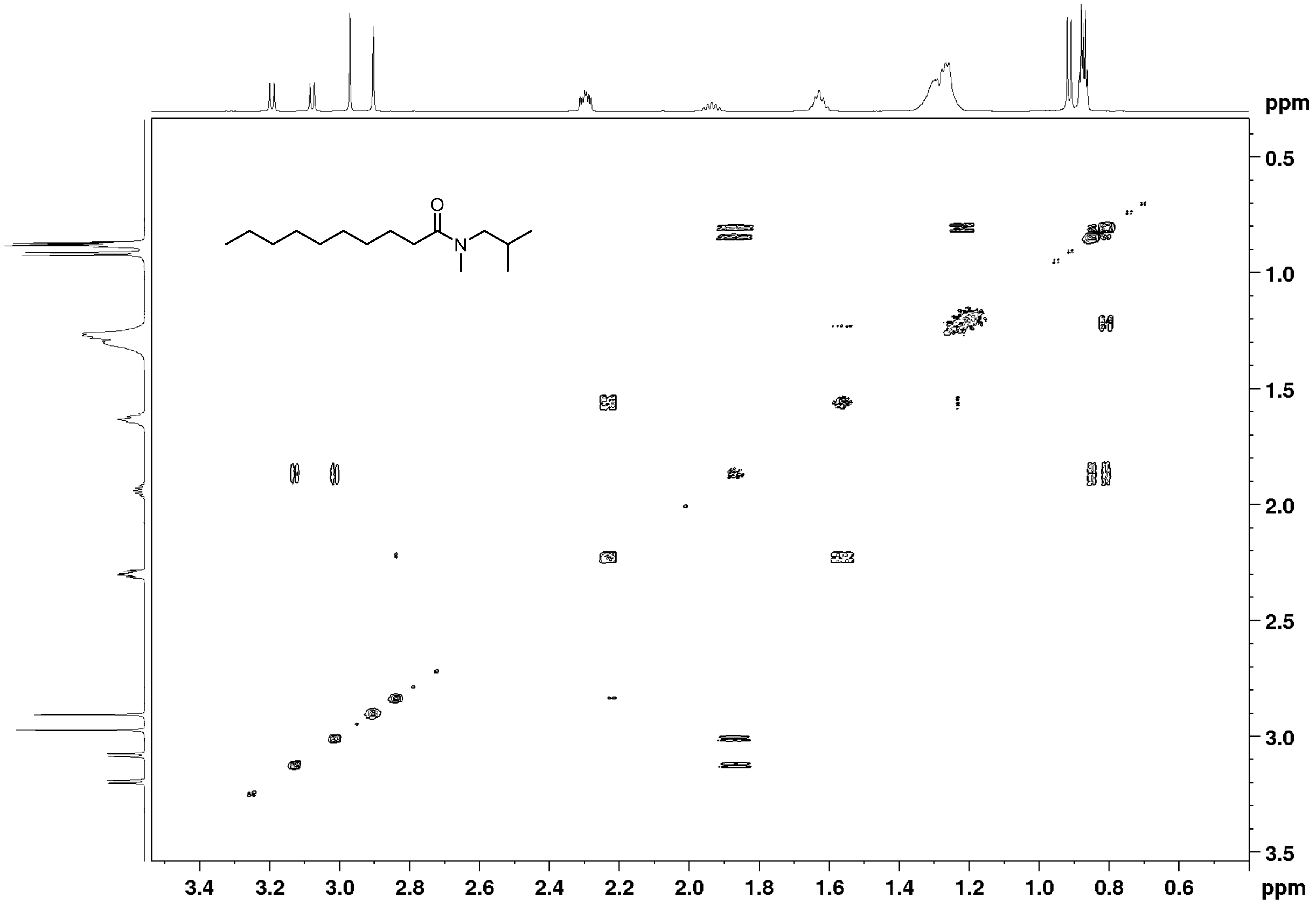

3.38.4 ^{1}H, ^{13}C HMBC

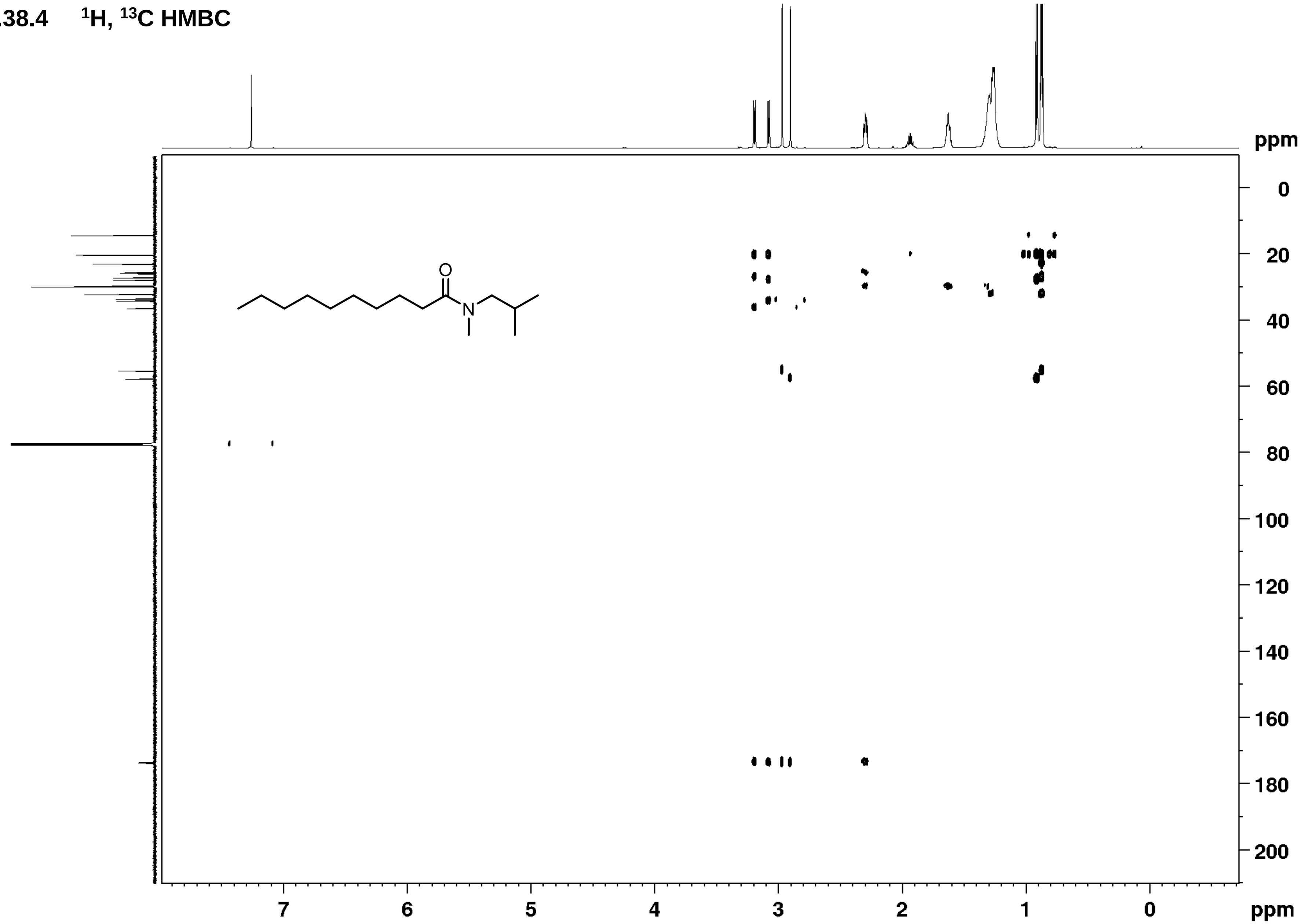

3.38.5 ¹H, ¹³C HSQC

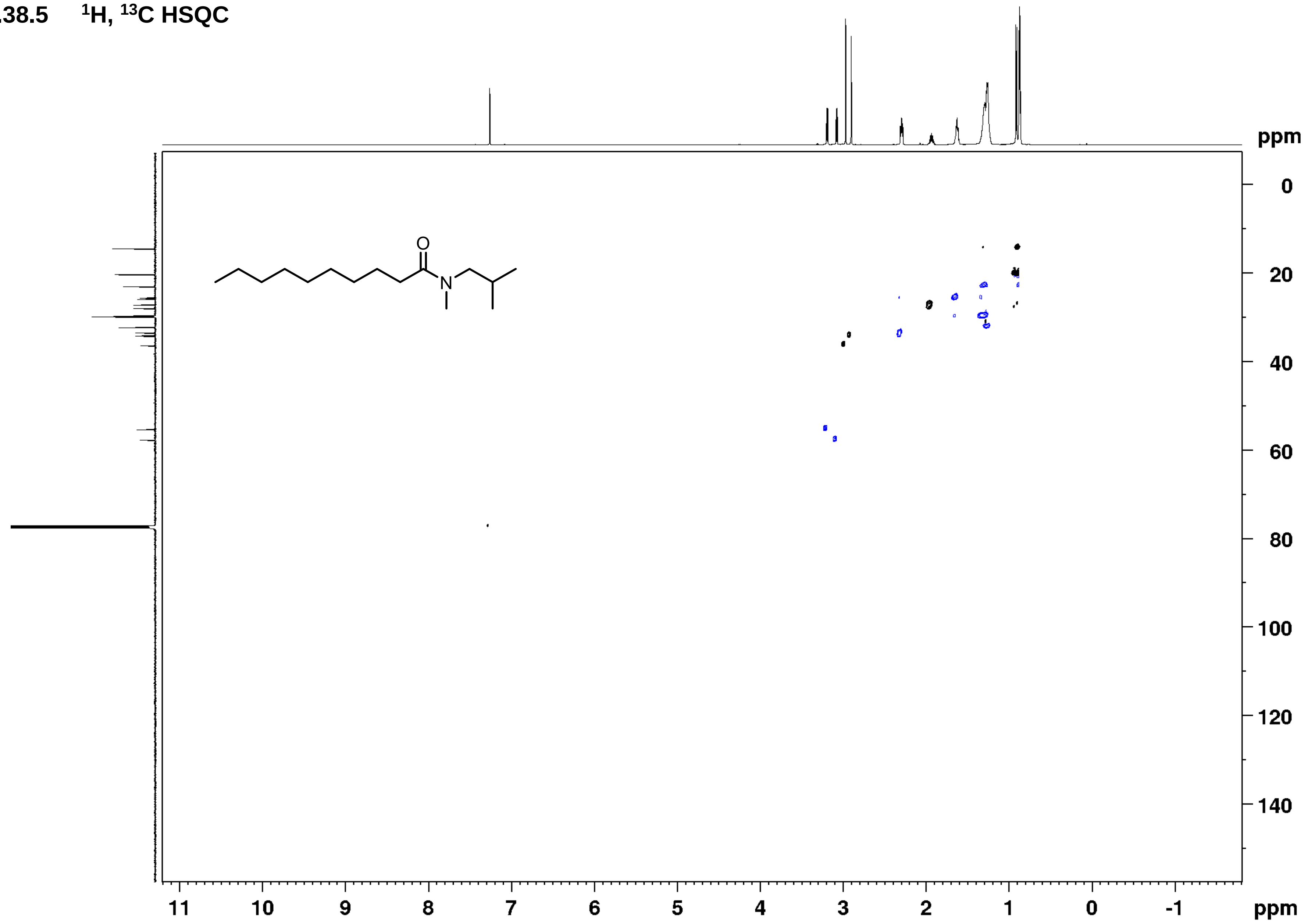

3.38.6 HRMS

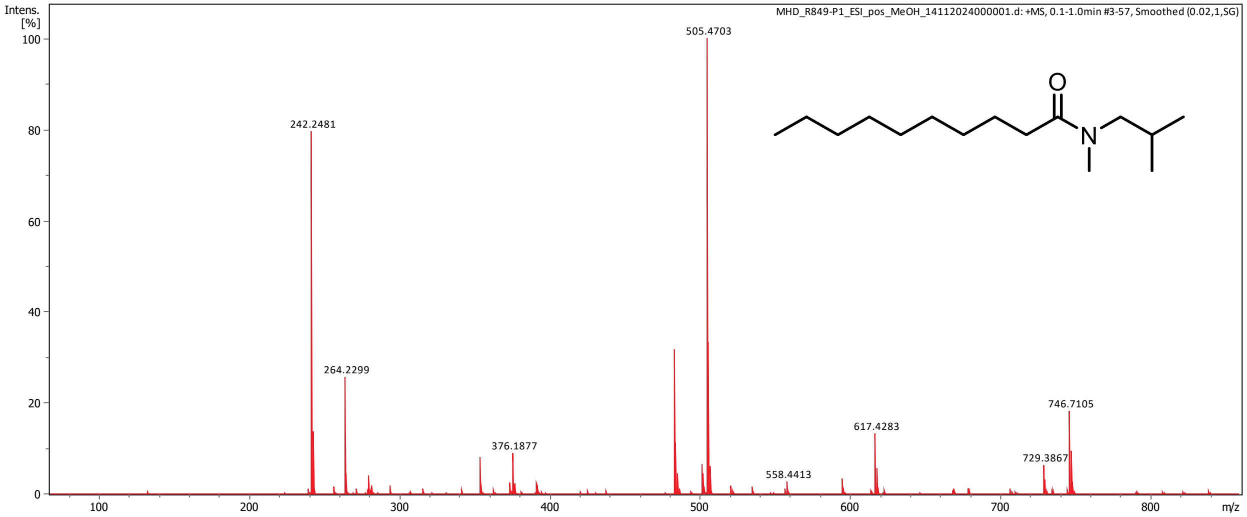

MHD_R849-P1_ESI_pos_MeOH_14112024000001.d: +MS, 0.1-1.0min #3-57, Smoothed (0.02,1,SG)
Intens. [%]
505.4703
242.2481
264.2299
376.1877
558.4413
617.4283
729.3867
746.7105
O
N
m/z

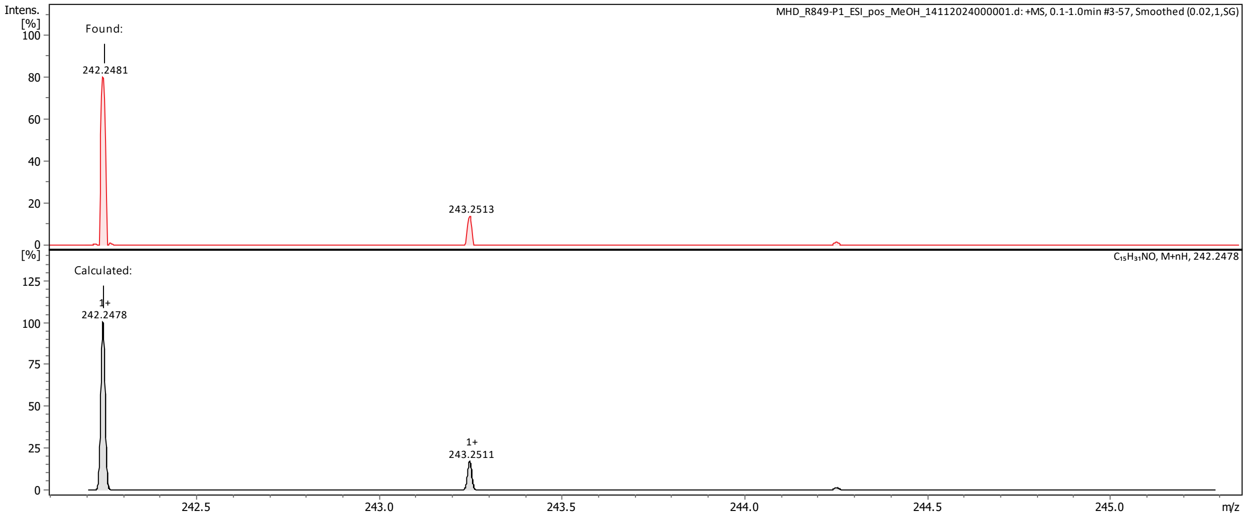

MHD_R849-P1_ESI_pos_MeOH_14112024000001.d: +MS, 0.1-1.0min #3-57, Smoothed (0.02,1,SG)
Intens. [%]
Found:
242.2481
243.2513
$C_{15}H_{31}NO$, M+nH, 242.2478
Calculated:
1+
242.2478
1+
243.2511
m/z

3.38.7 IR

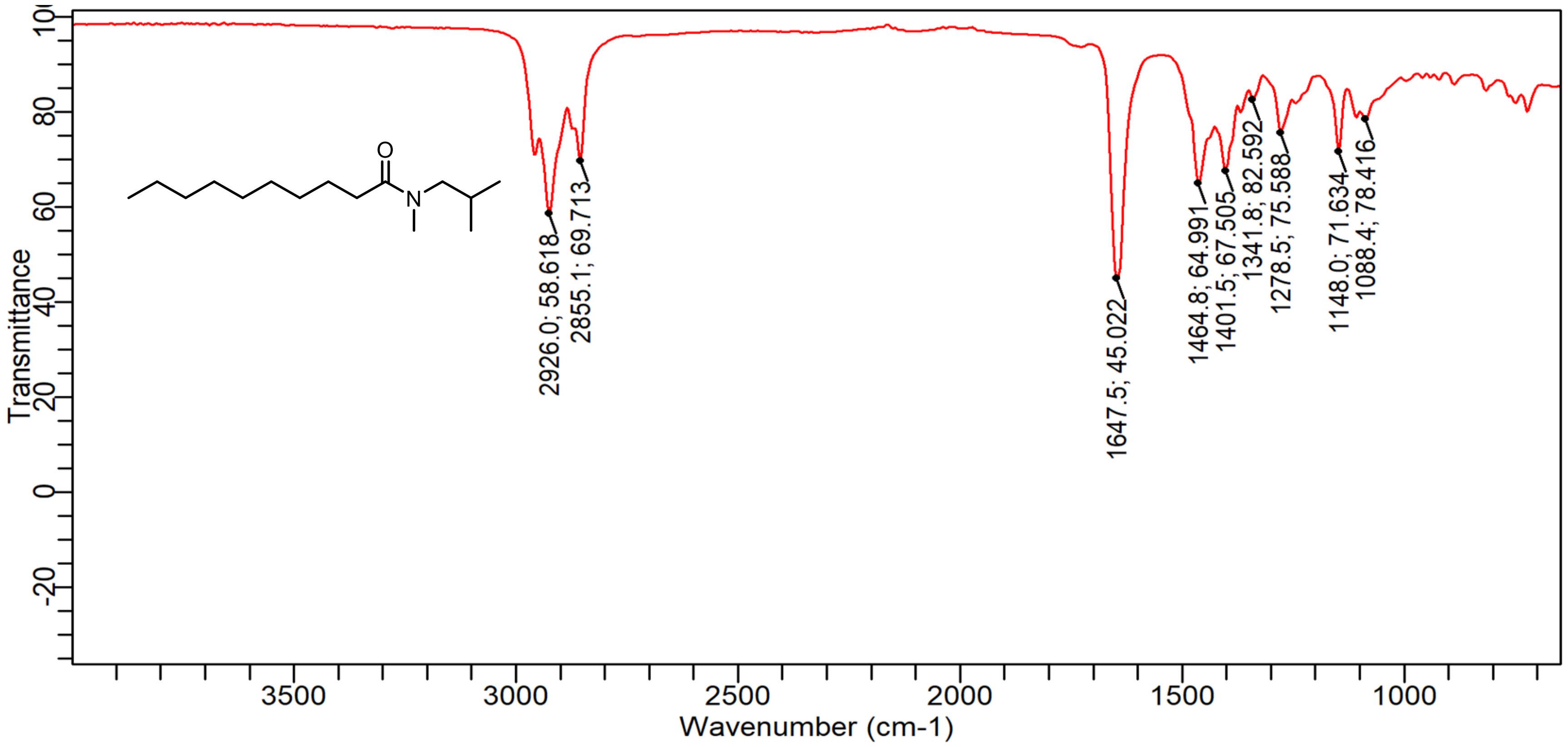

Transmittance
Wavenumber (cm-1)
2926.0; 58.618
2855.1; 69.713
1647.5; 45.022
1464.8; 64.991
1401.5; 67.505
1341.8; 82.592
1278.5; 75.588
1148.0; 71.634
1088.4; 78.416
O
N

3.39 (*E*)-1-(pyrrolidin-1-yl)dec-4-en-1-one (12bj-2) (600 MHz, $CDCl_3$)

3.39.1 ^{1}H NMR

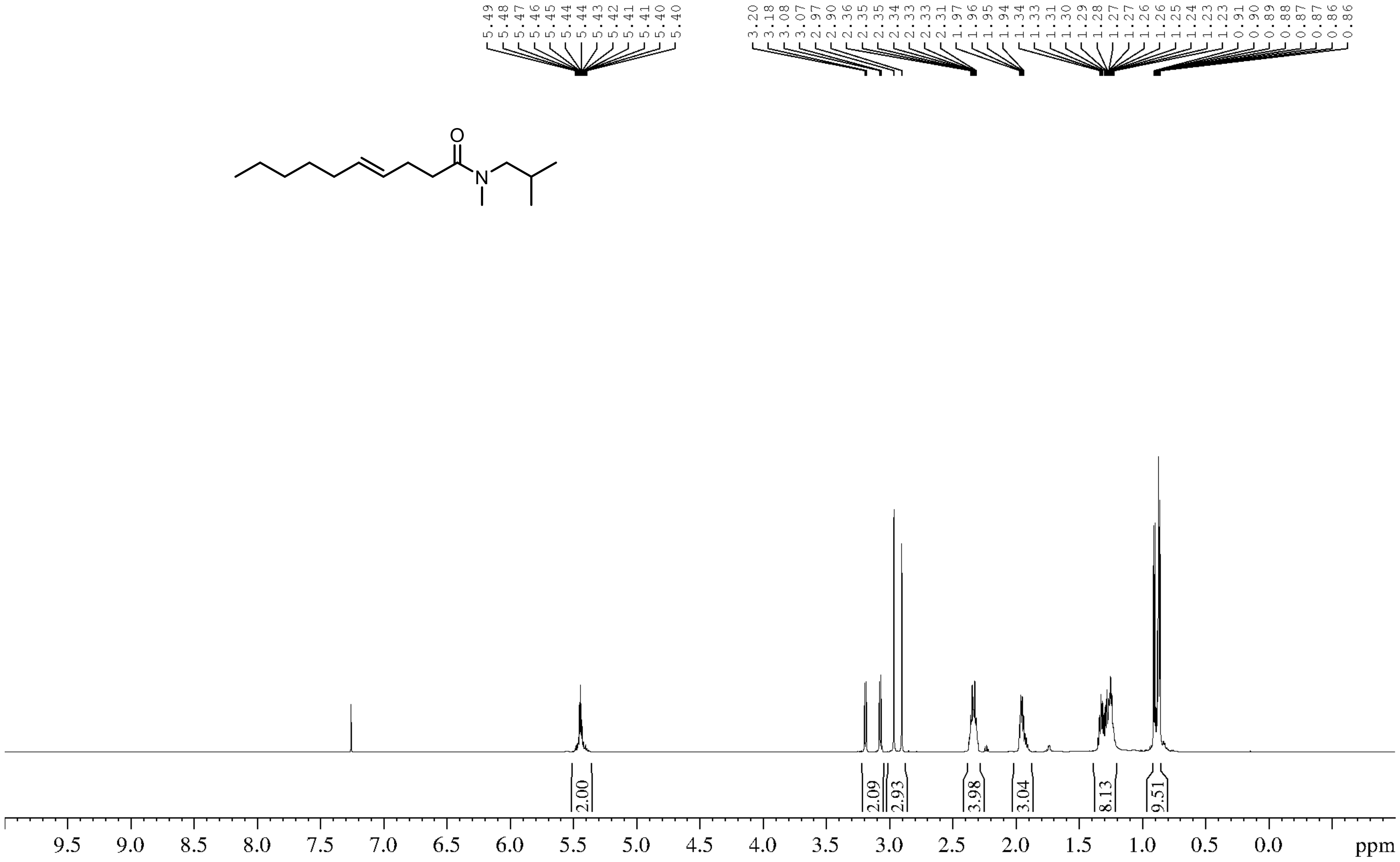

3.39.2 ^{13}C NMR

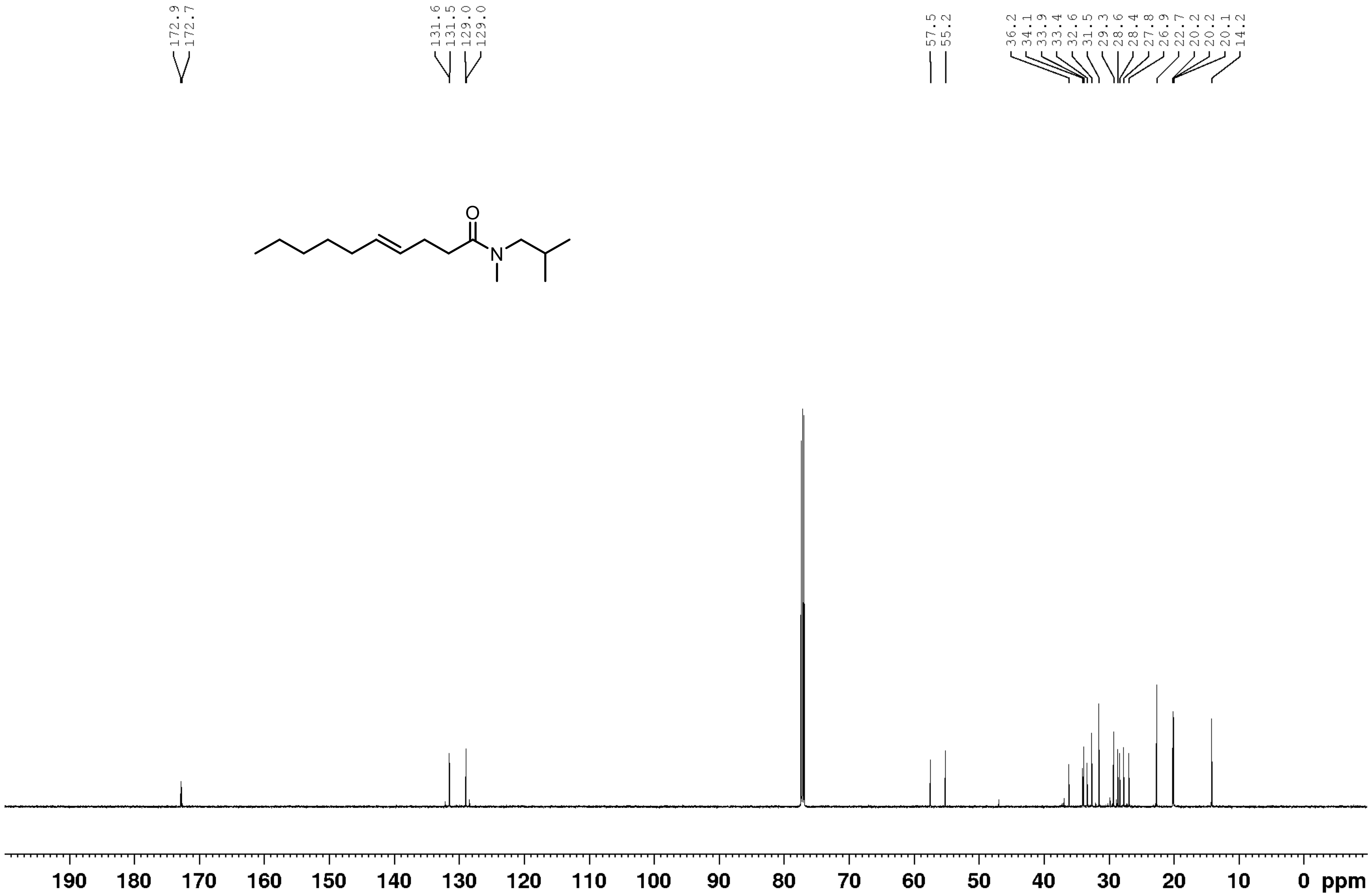
172.9
172.7
131.6
131.5
129.0
129.0
57.5
55.2
36.2
34.1
33.9
33.4
32.6
31.5
29.3
28.6
28.4
27.8
26.9
22.7
20.2
20.2
20.1
14.2
190 180 170 160 150 140 130 120 110 100 90 80 70 60 50 40 30 20 10 0 ppm

3.39.3 ^{1}H, ^{1}H COSY

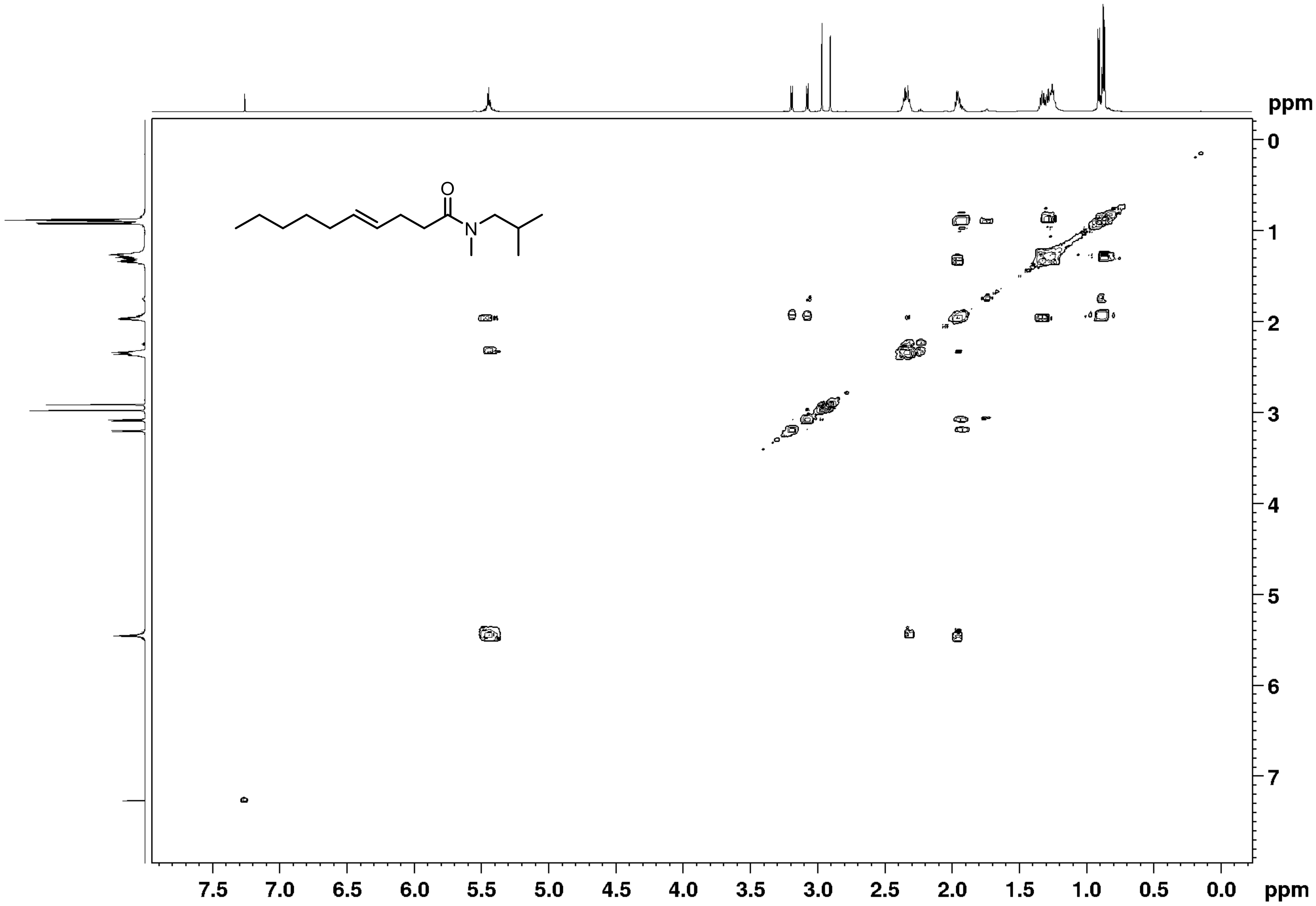

3.39.4 ^{1}H, ^{13}C HMBC

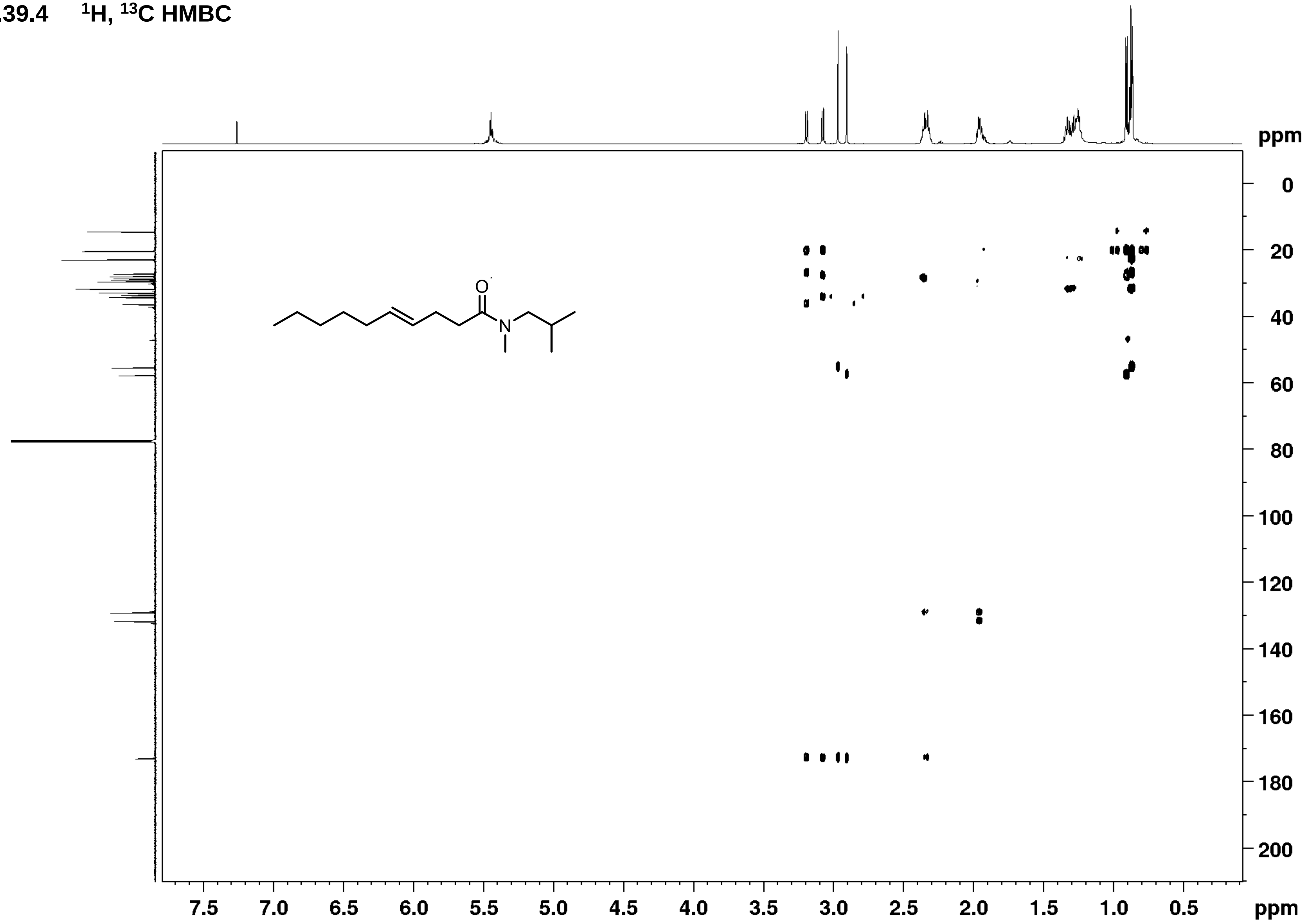

3.39.5 ^{1}H, ^{13}C HSQC

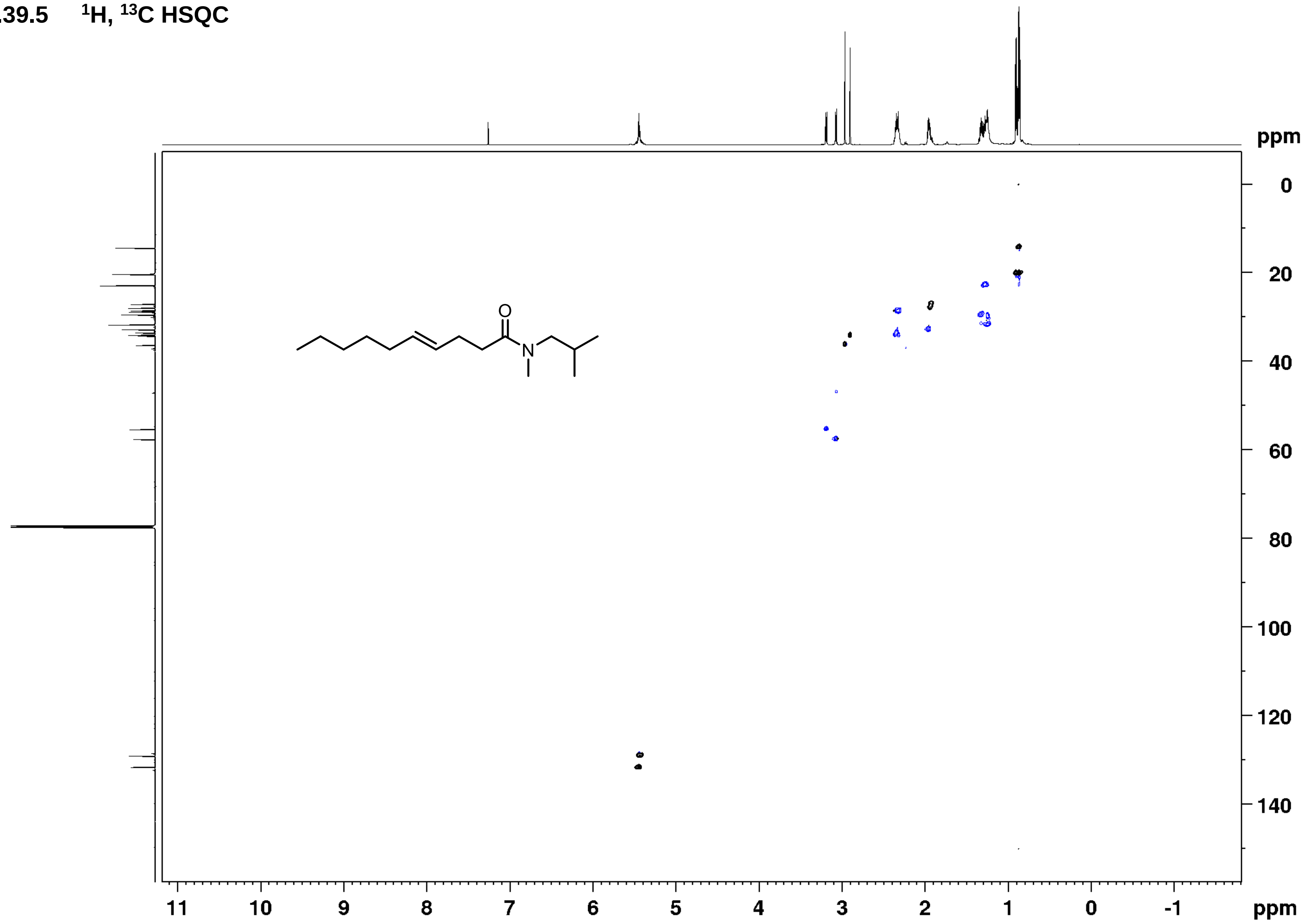

3.39.6 HRMS

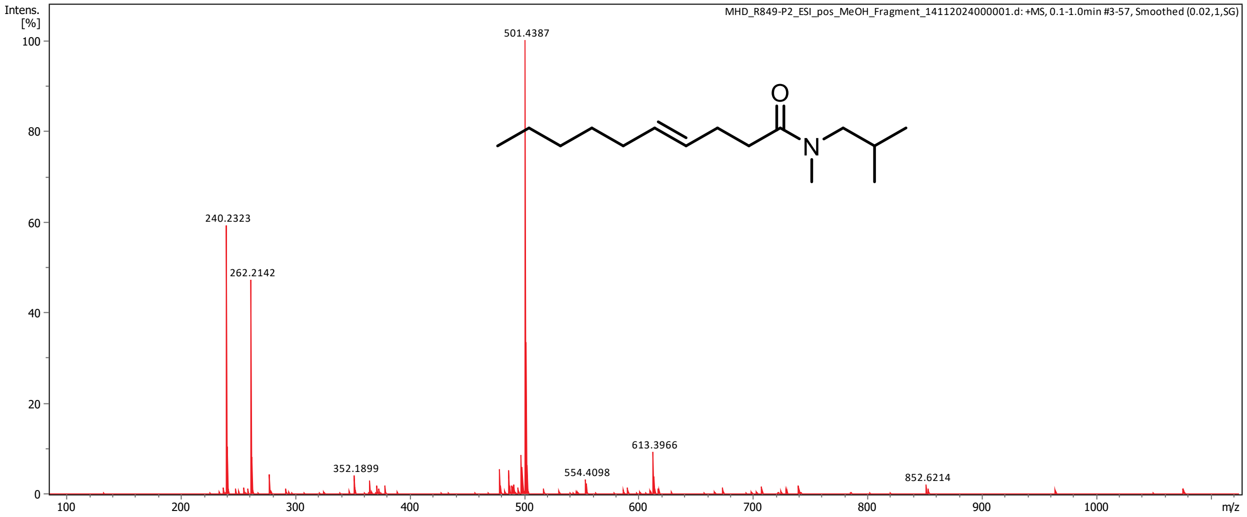

MHD_R849-P2_ESI_pos_MeOH_Fragment_14112024000001.d: +MS, 0.1-1.0min #3-57, Smoothed (0.02,1,SG)
Intens. [%]
501.4387
240.2323
262.2142
352.1899
554.4098
613.3966
852.6214
O
N
m/z

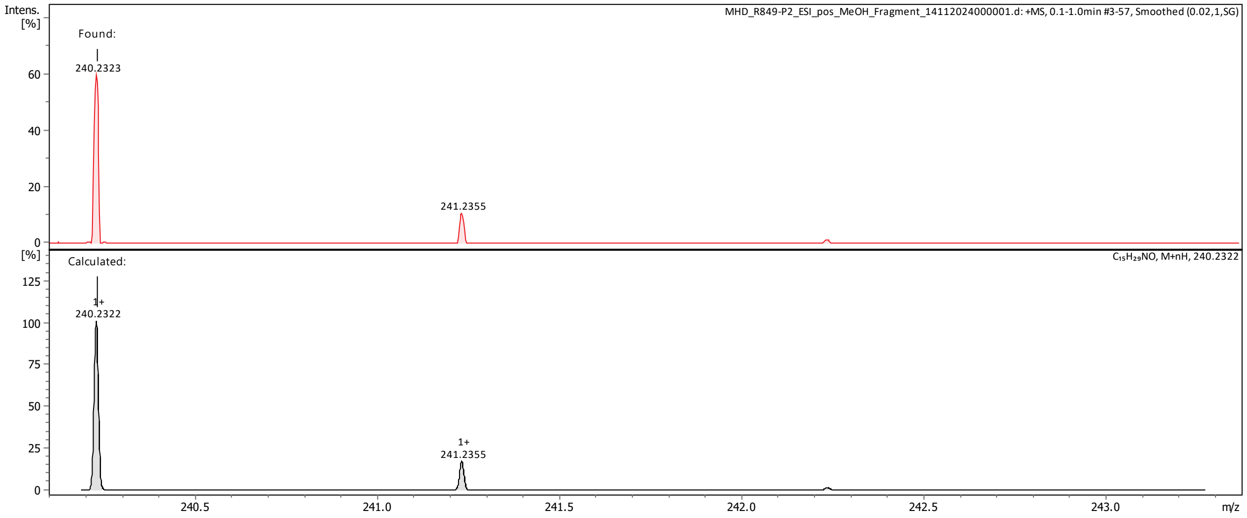

MHD_R849-P2_ESI_pos_MeOH_Fragment_14112024000001.d: +MS, 0.1-1.0min #3-57, Smoothed (0.02,1,SG)
Intens. [%]
Found:
240.2323
241.2355
Calculated:
C15H29NO, M+nH, 240.2322
1+ 240.2322
1+ 241.2355
m/z

3.39.7 IR

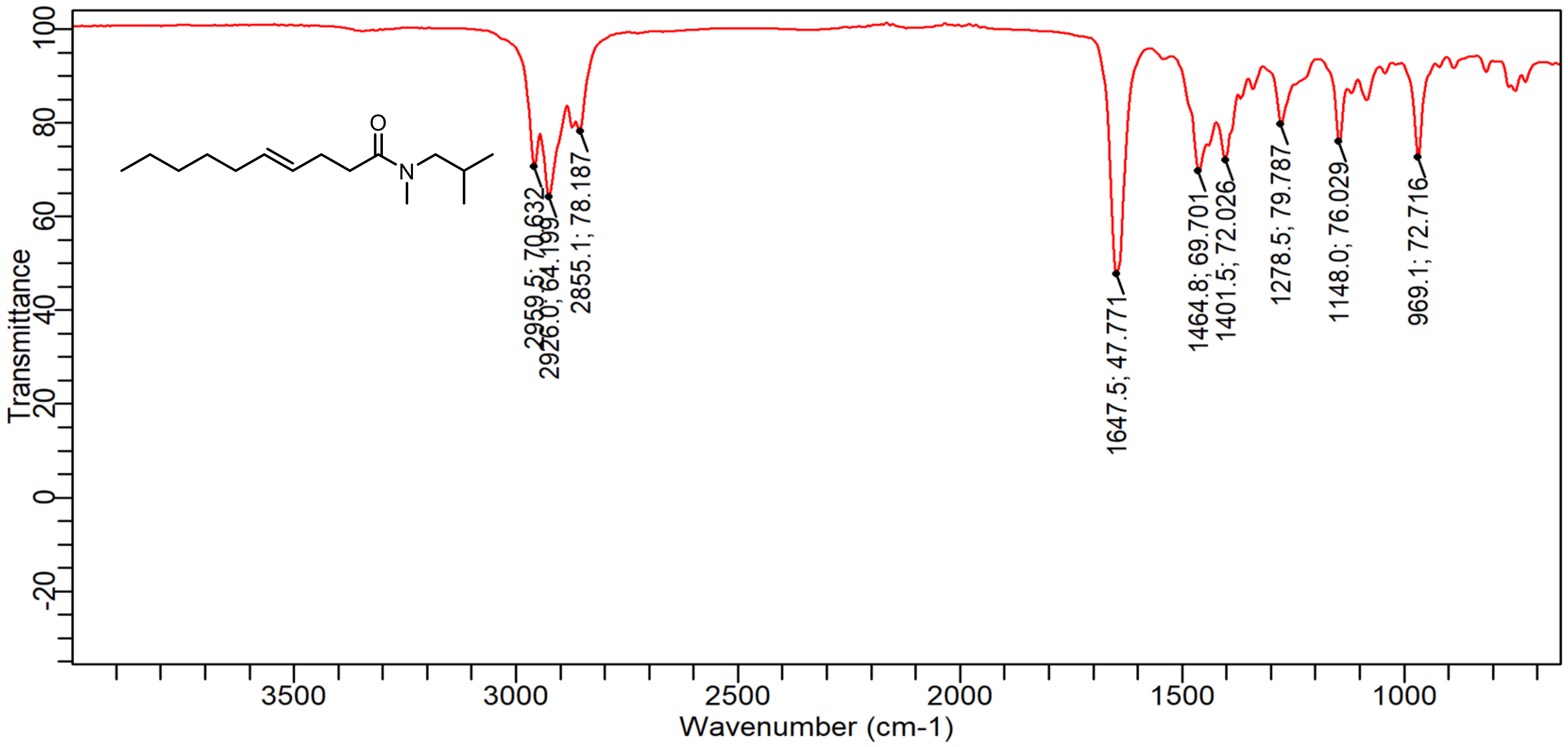

O
N
Transmittance
100
80
60
40
20
0
-20
2959.5; 70.632
2926.0; 64.199
2855.1; 78.187
1647.5; 47.771
1464.8; 69.701
1401.5; 72.026
1278.5; 79.787
1148.0; 76.029
969.1; 72.716
3500
3000
2500
2000
1500
1000
Wavenumber (cm-1)

3.40 1-(Pyrrolidin-1-yl)decan-1-one (12bk-1) (600 MHz, $CDCl_3$)

3.40.1 ^{1}H NMR

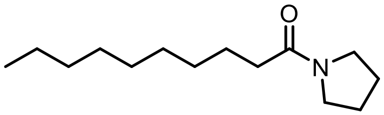

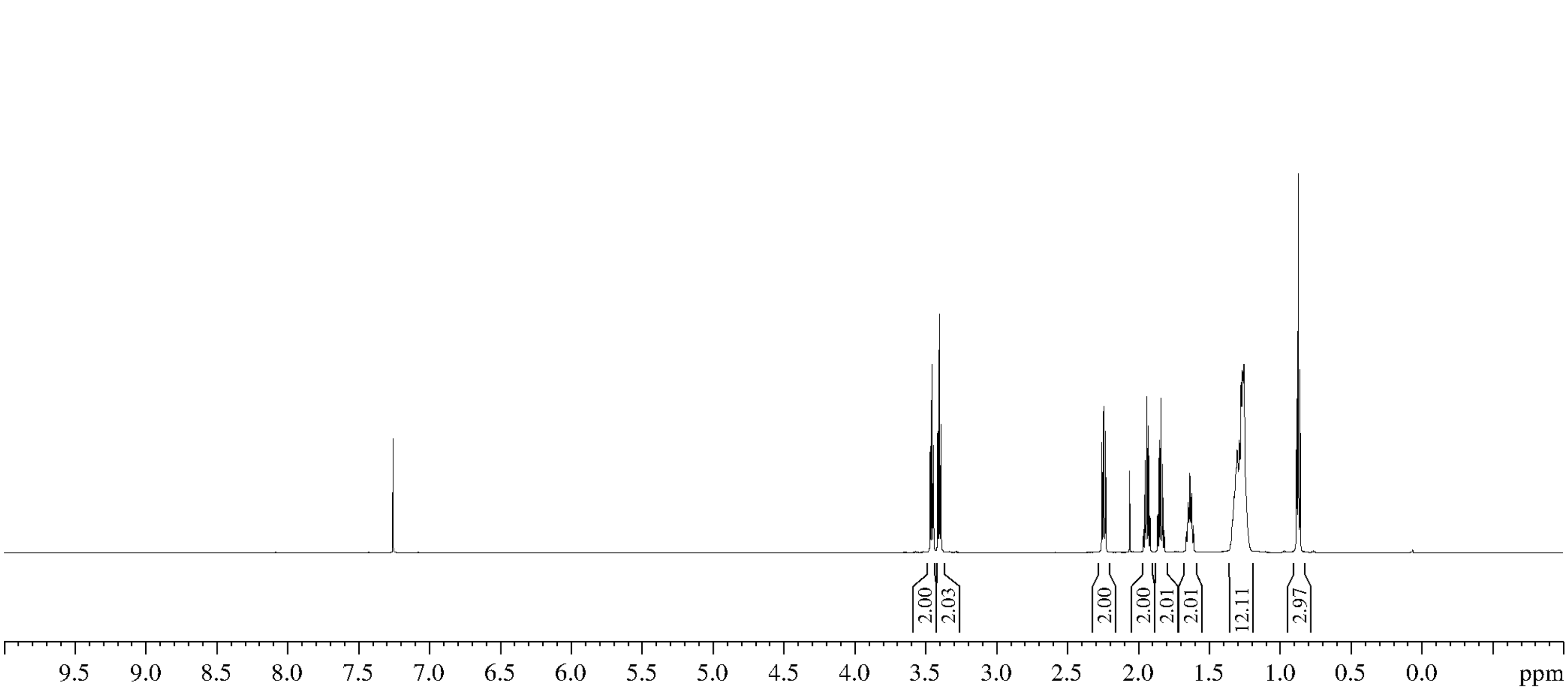

3.40.2 ^{13}C NMR

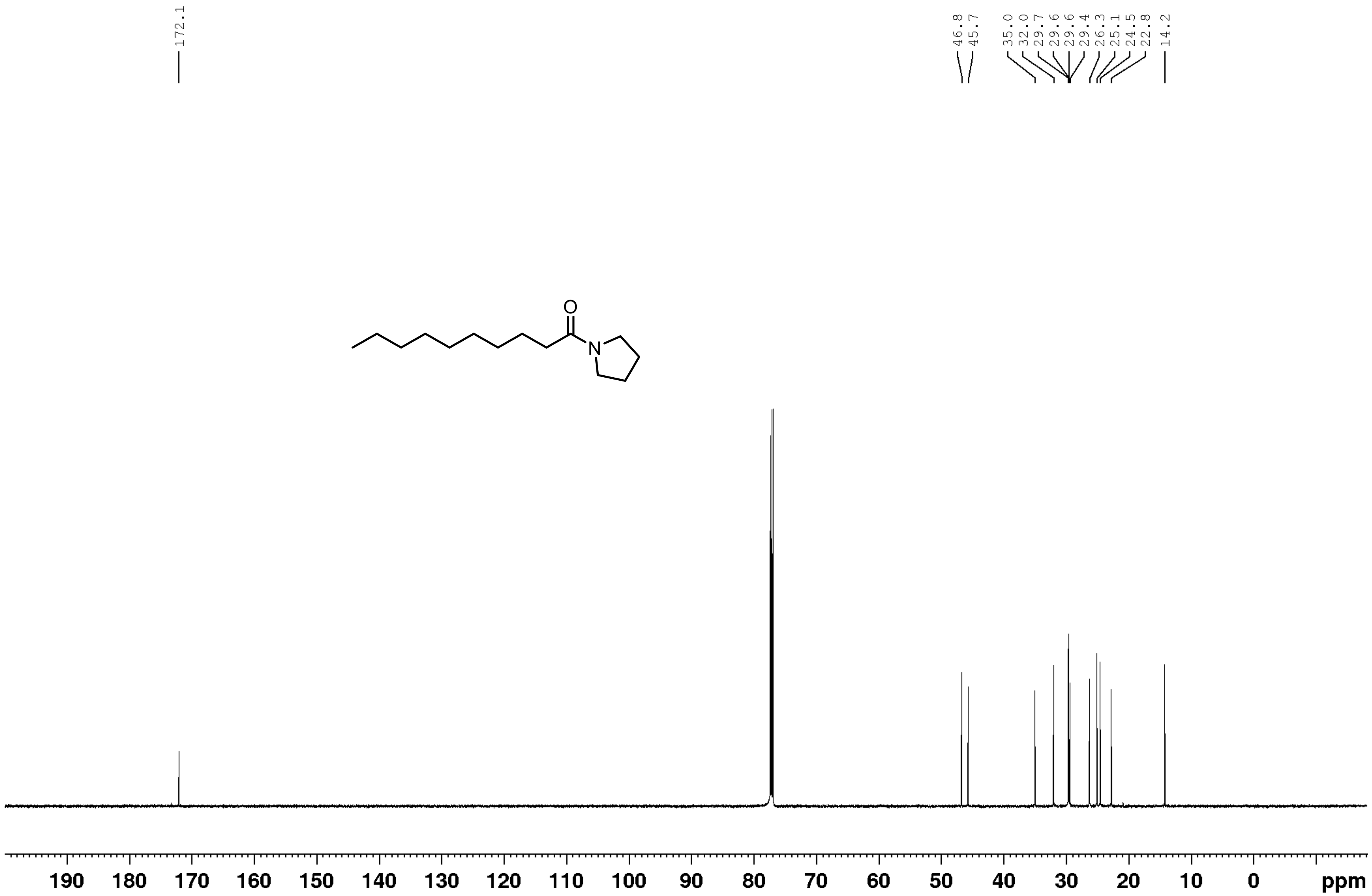

172.1
46.8
45.7
35.0
32.0
29.7
29.6
29.6
29.4
26.3
25.1
24.5
22.8
14.2
O
N
190
180
170
160
150
140
130
120
110
100
90
80
70
60
50
40
30
20
10
0
ppm

3.40.3 ^{1}H, ^{1}H COSY

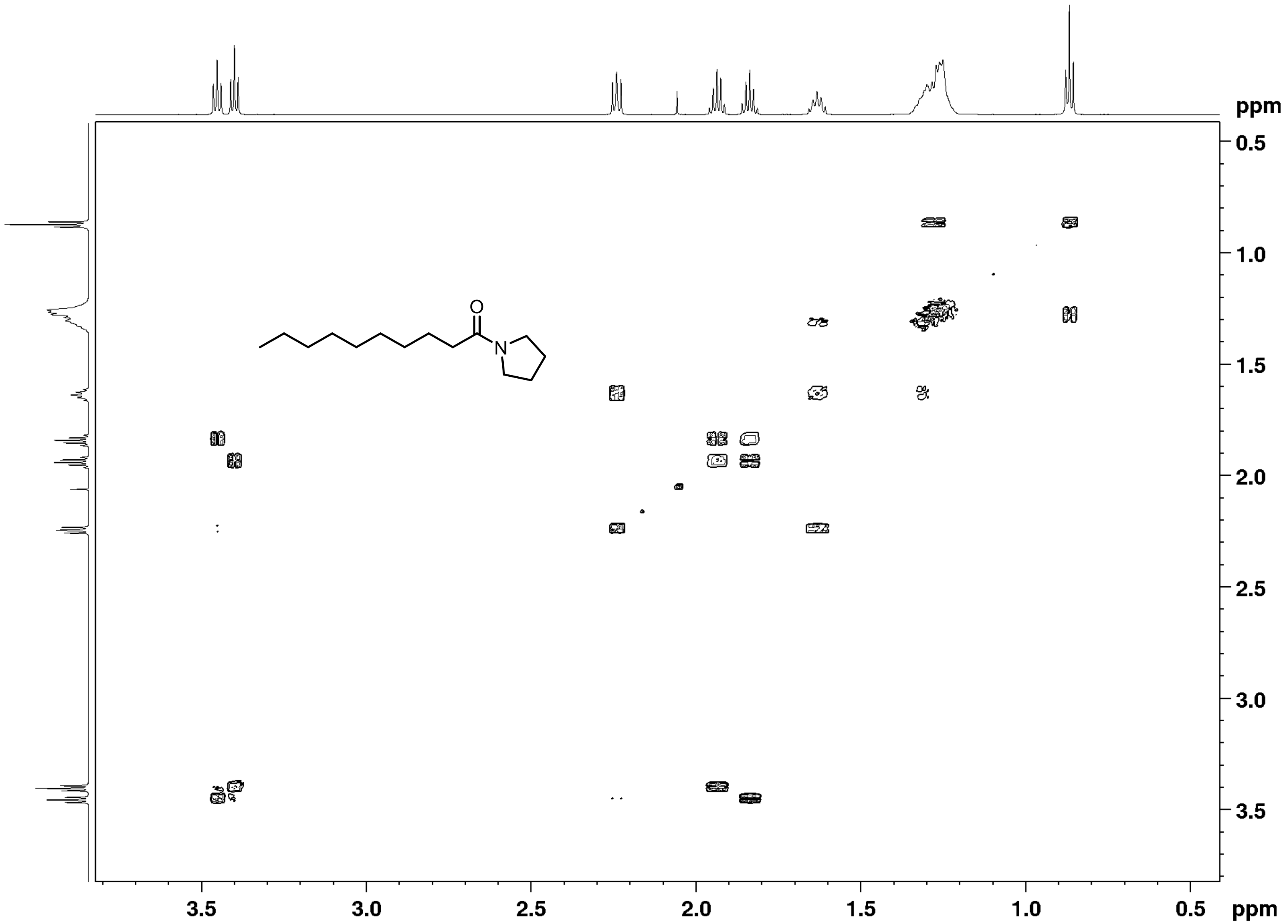

3.40.4 ^{1}H, ^{13}C HMBC

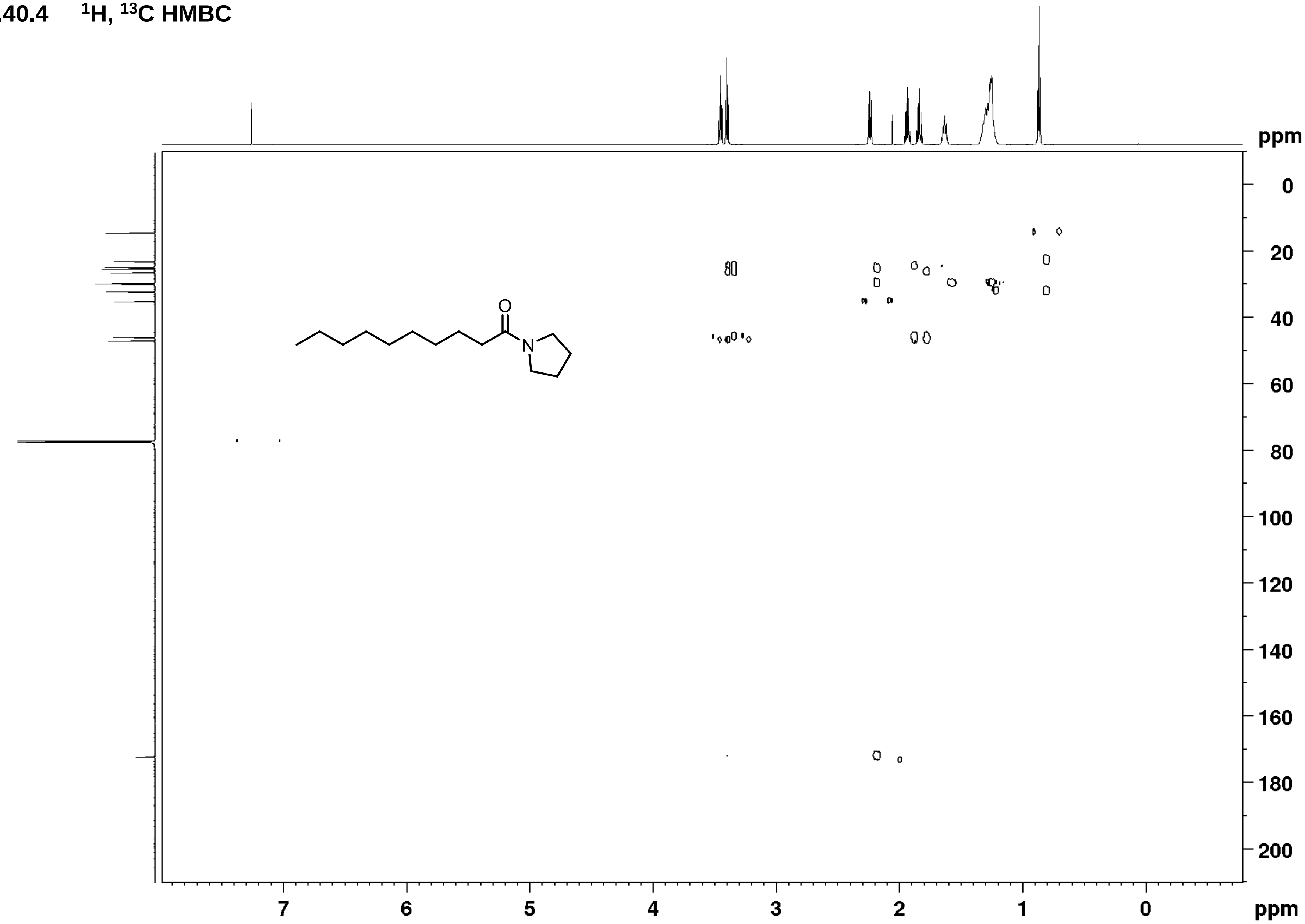

3.40.5 ^{1}H, ^{13}C HSQC

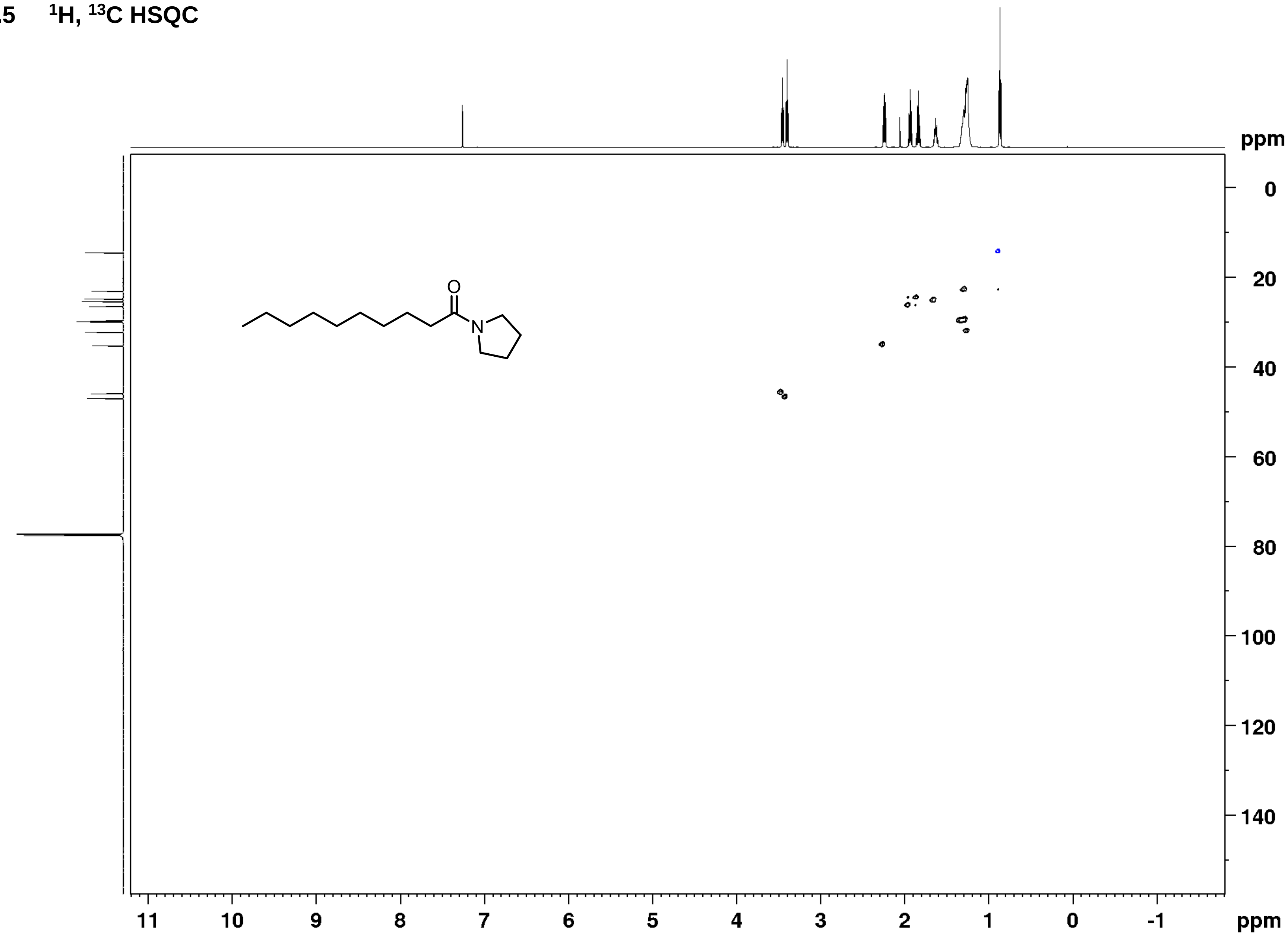

3.40.6 HRMS

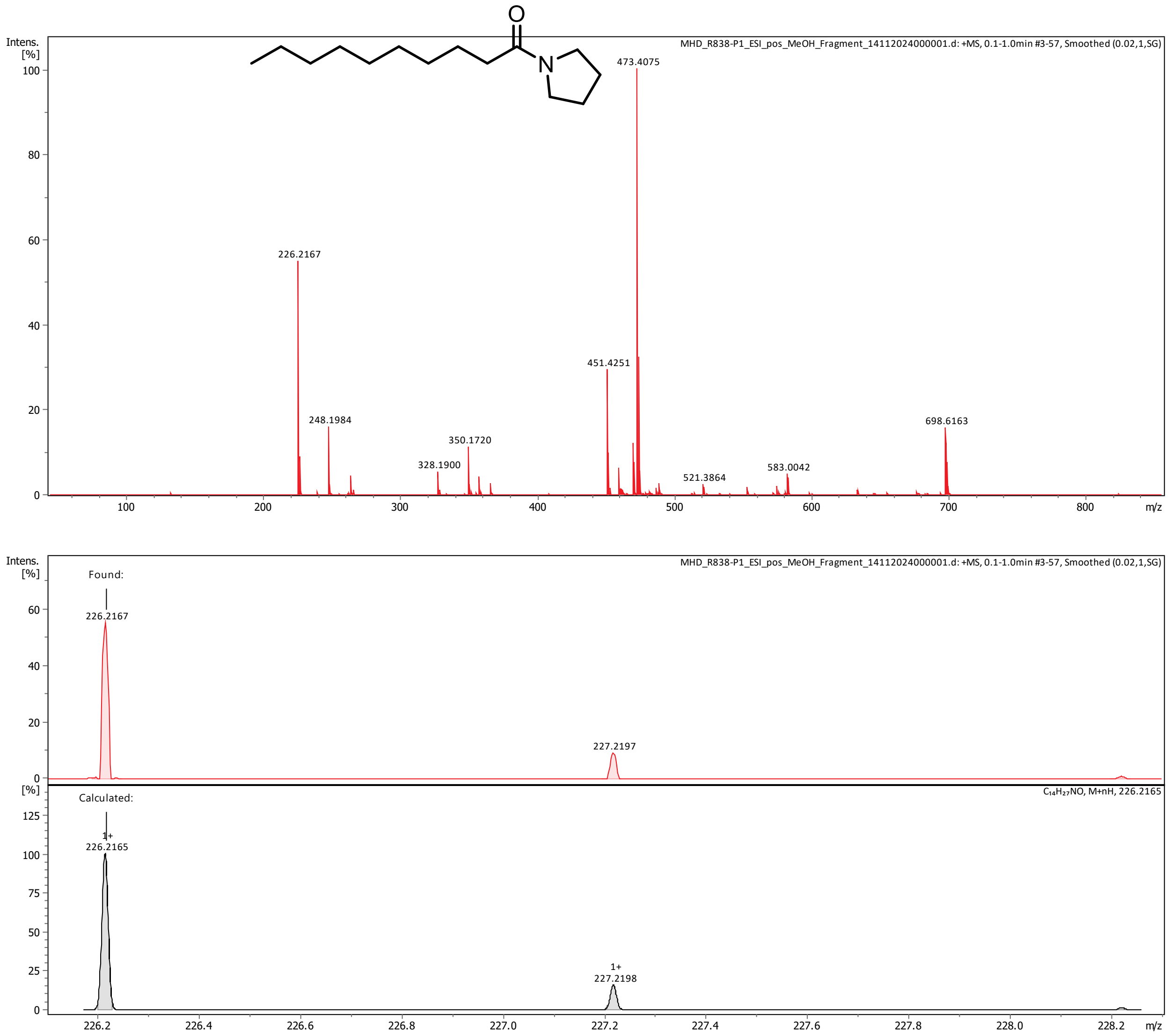

Intens. [%]
MHD_R838-P1_ESI_pos_MeOH_Fragment_14112024000001.d: +MS, 0.1-1.0min #3-57, Smoothed (0.02,1,SG)
473.4075
226.2167
451.4251
248.1984
350.1720
328.1900
698.6163
521.3864
583.0042
m/z
Found:
226.2167
227.2197
Calculated:
$C_{14}H_{27}NO$, M+nH, 226.2165
1+ 226.2165
1+ 227.2198

3.40.7 IR

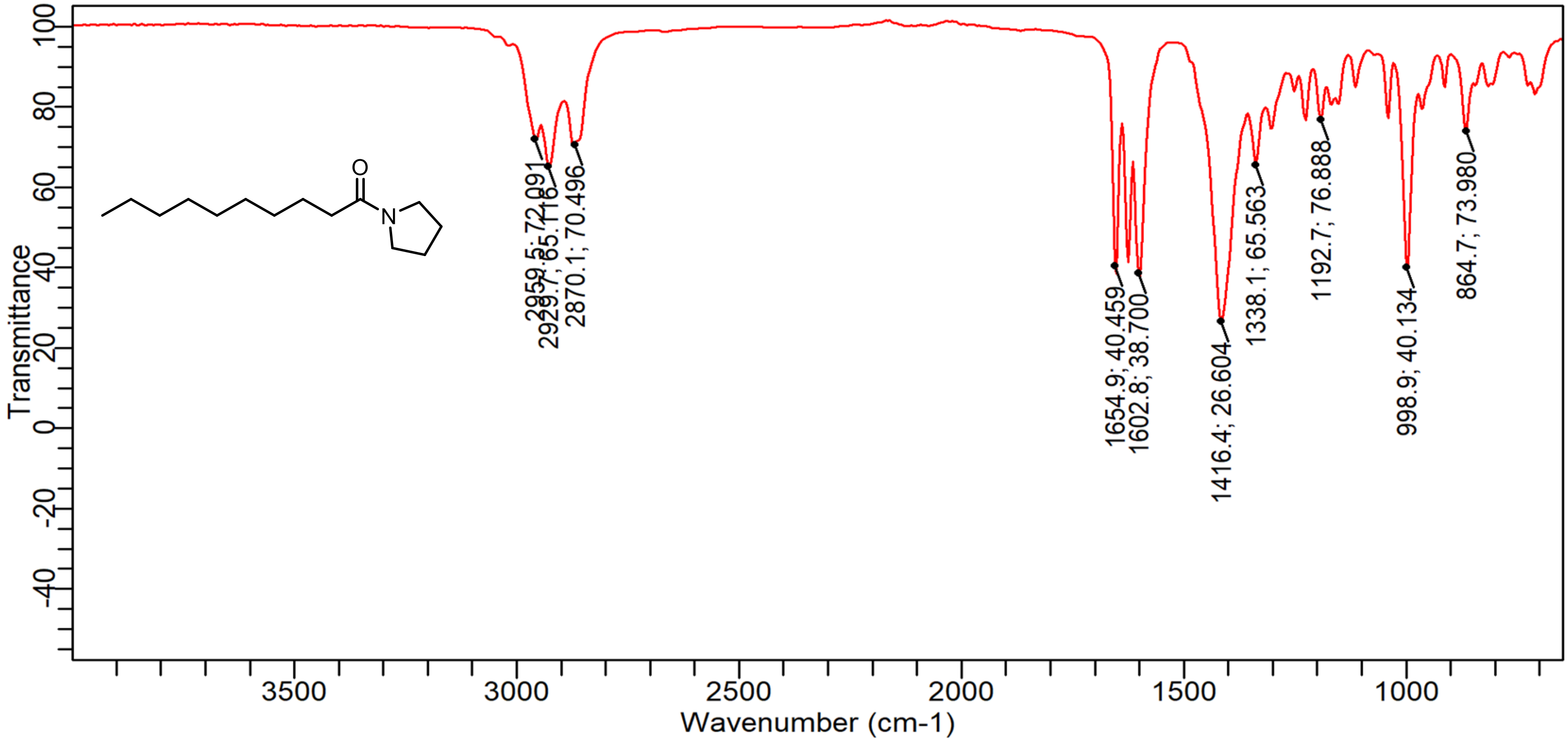
Transmittance
Wavenumber (cm-1)
2959.5; 72.091
2929.7; 65.116
2870.1; 70.496
1654.9; 40.459
1602.8; 38.700
1416.4; 26.604
1338.1; 65.563
1192.7; 76.888
998.9; 40.134
864.7; 73.980
O
N

3.41 (*E*)-1-(Pyrrolidin-1-yl)dec-4-en-1-one (12bk-2) (600 MHz, $CDCl_3$)

3.41.1 ^{1}H NMR

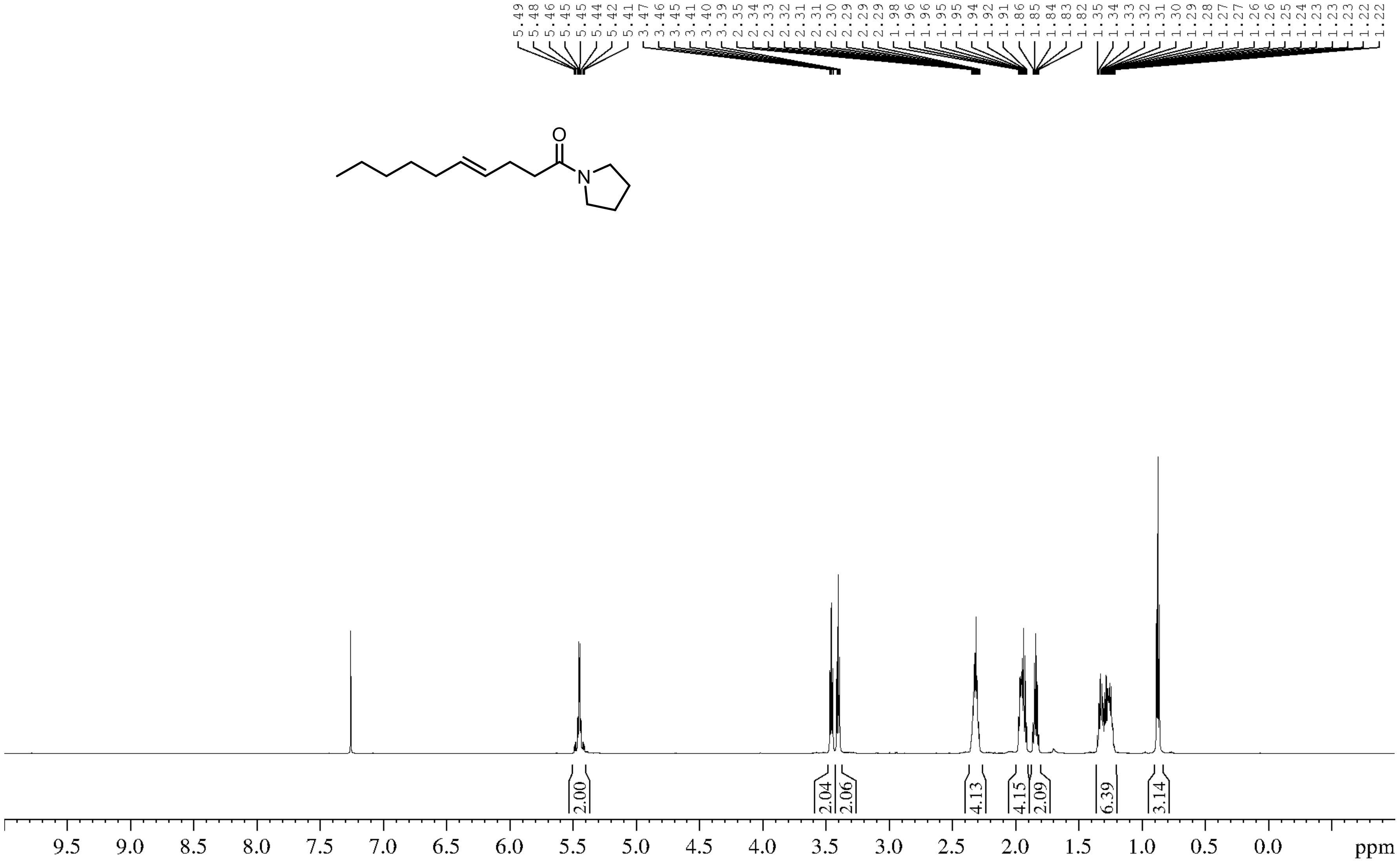

3.41.2 ^{13}C NMR

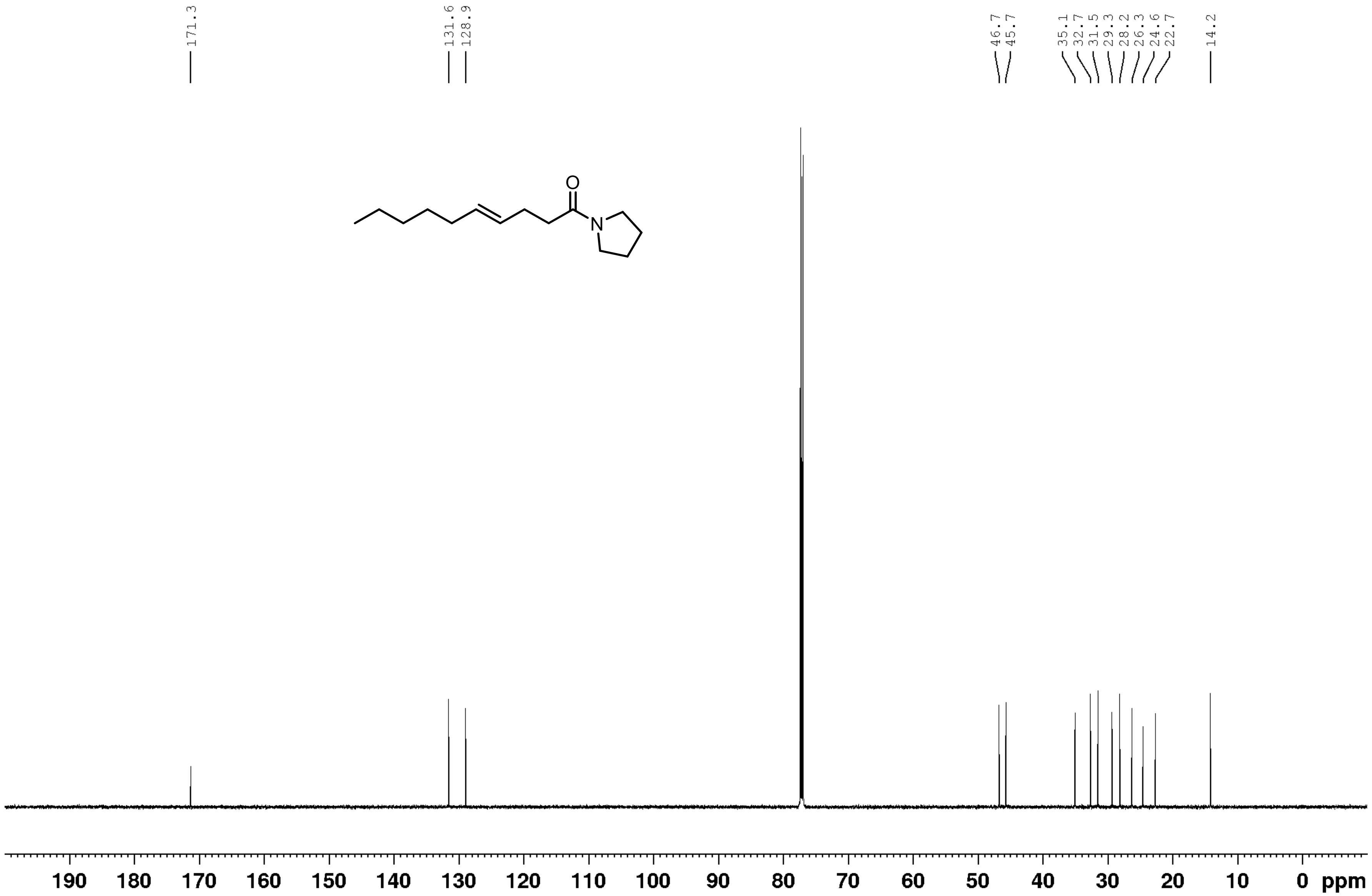
171.3
131.6
128.9
46.7
45.7
35.1
32.7
31.5
29.3
28.2
26.3
24.6
22.7
14.2
O
N
190 180 170 160 150 140 130 120 110 100 90 80 70 60 50 40 30 20 10 0 ppm

3.41.3 ^{1}H, ^{1}H COSY

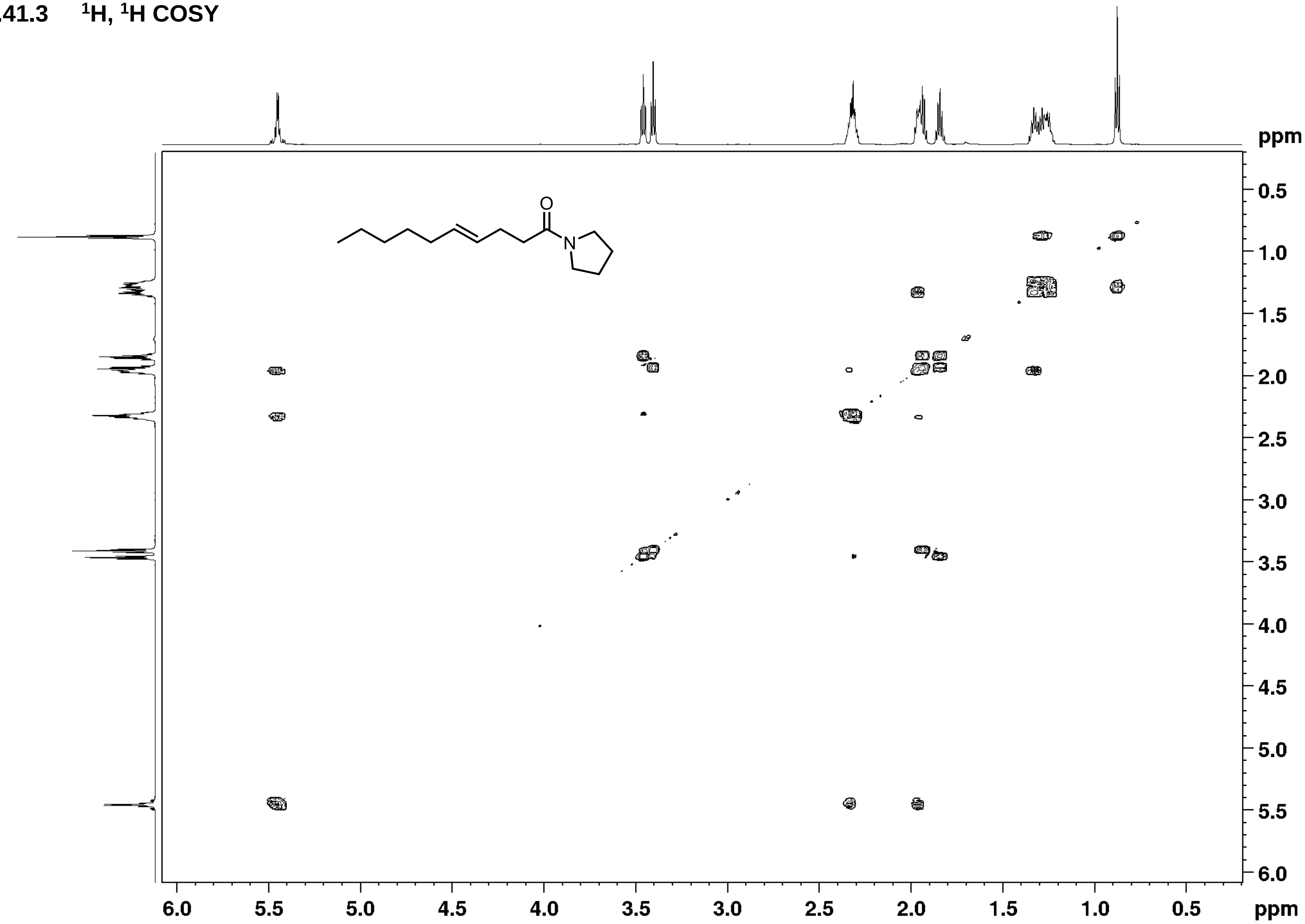

3.41.4 ¹H, ¹³C HMBC

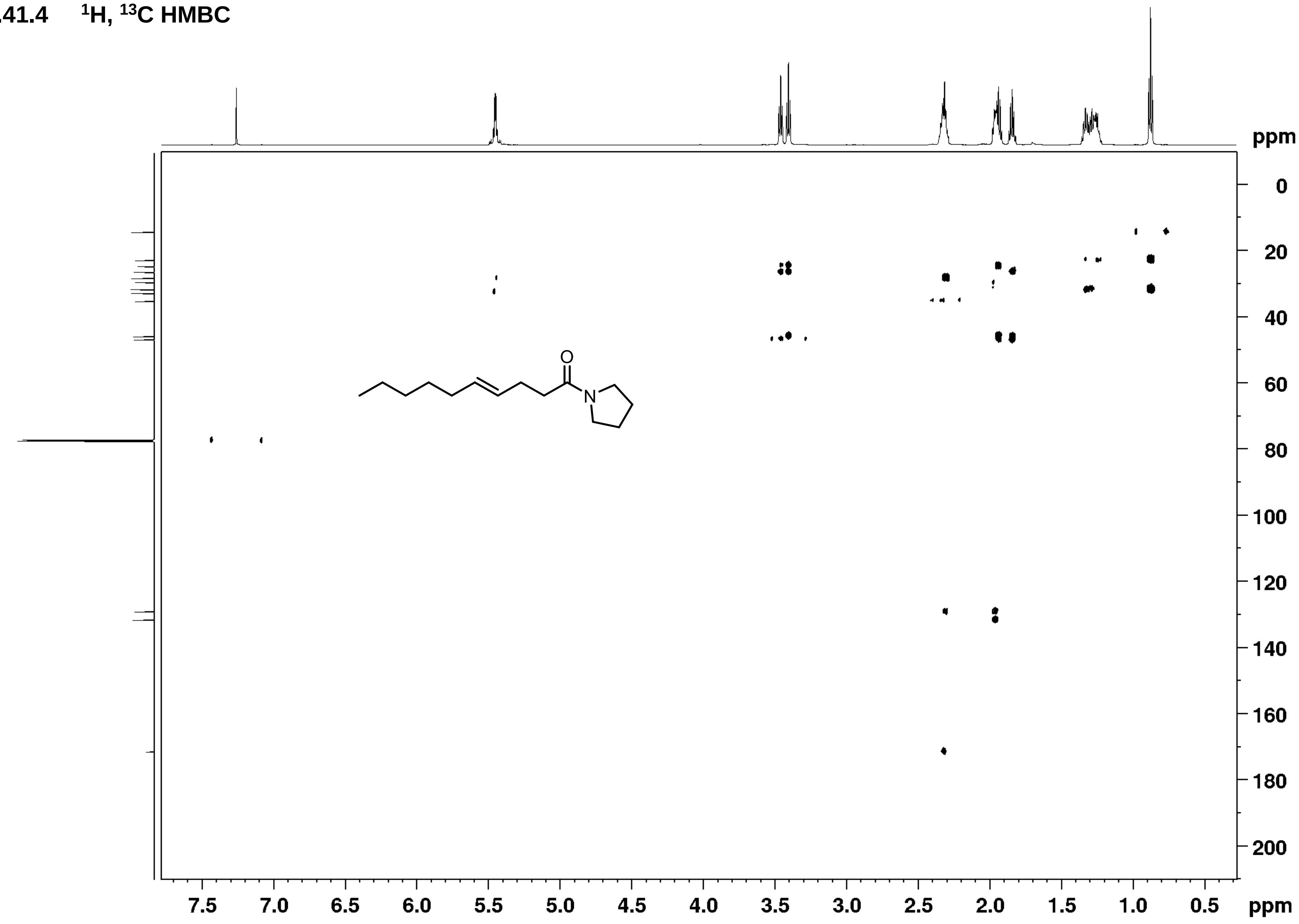

3.41.5 ^{1}H, ^{13}C HSQC

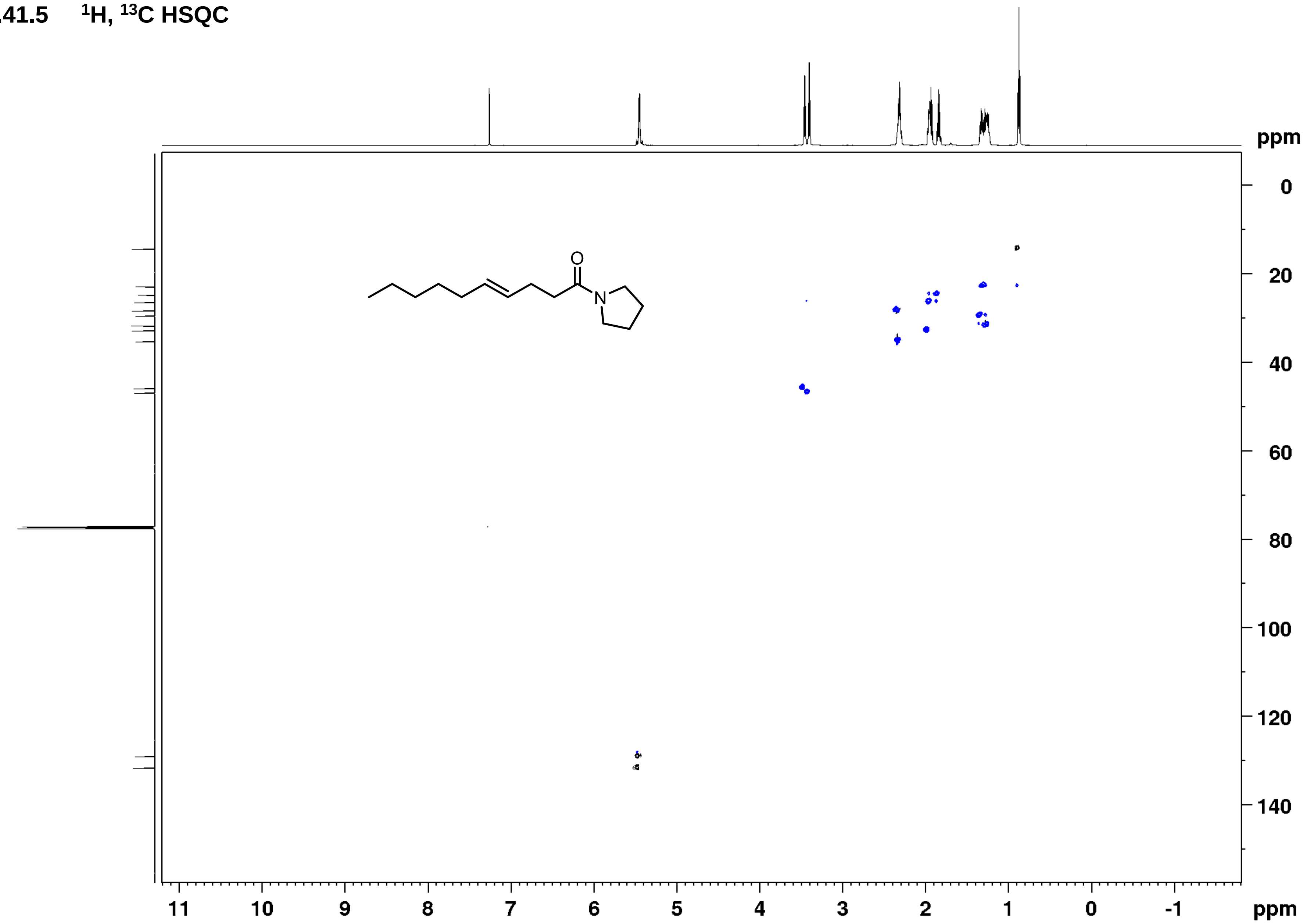

3.41.6 HRMS

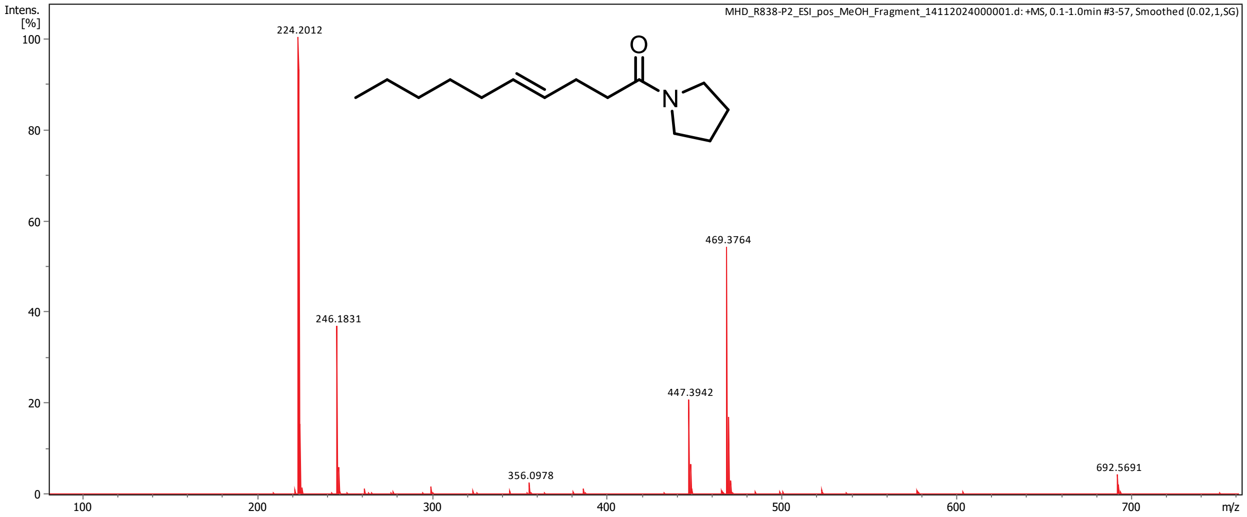

Intens. [%]
MHD_R838-P2_ESI_pos_MeOH_Fragment_14112024000001.d: +MS, 0.1-1.0min #3-57, Smoothed (0.02,1,SG)
224.2012
246.1831
356.0978
447.3942
469.3764
692.5691
O
N
m/z

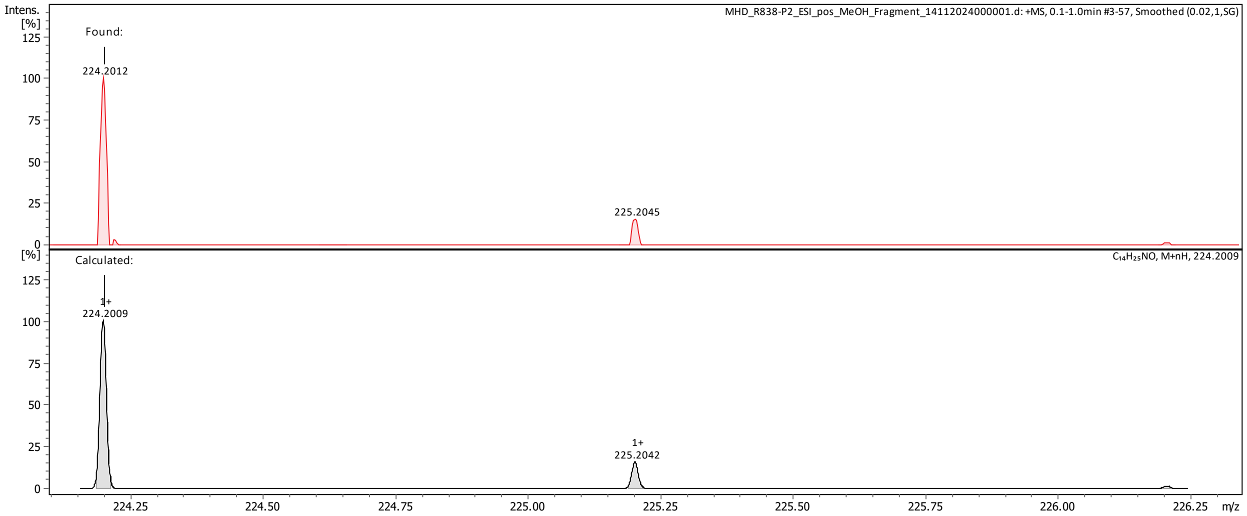

Intens. [%]
MHD_R838-P2_ESI_pos_MeOH_Fragment_14112024000001.d: +MS, 0.1-1.0min #3-57, Smoothed (0.02,1,SG)
Found:
224.2012
225.2045
Calculated:
C₁₄H₂₅NO, M+nH, 224.2009
1+
224.2009
1+
225.2042
m/z

3.41.7 IR

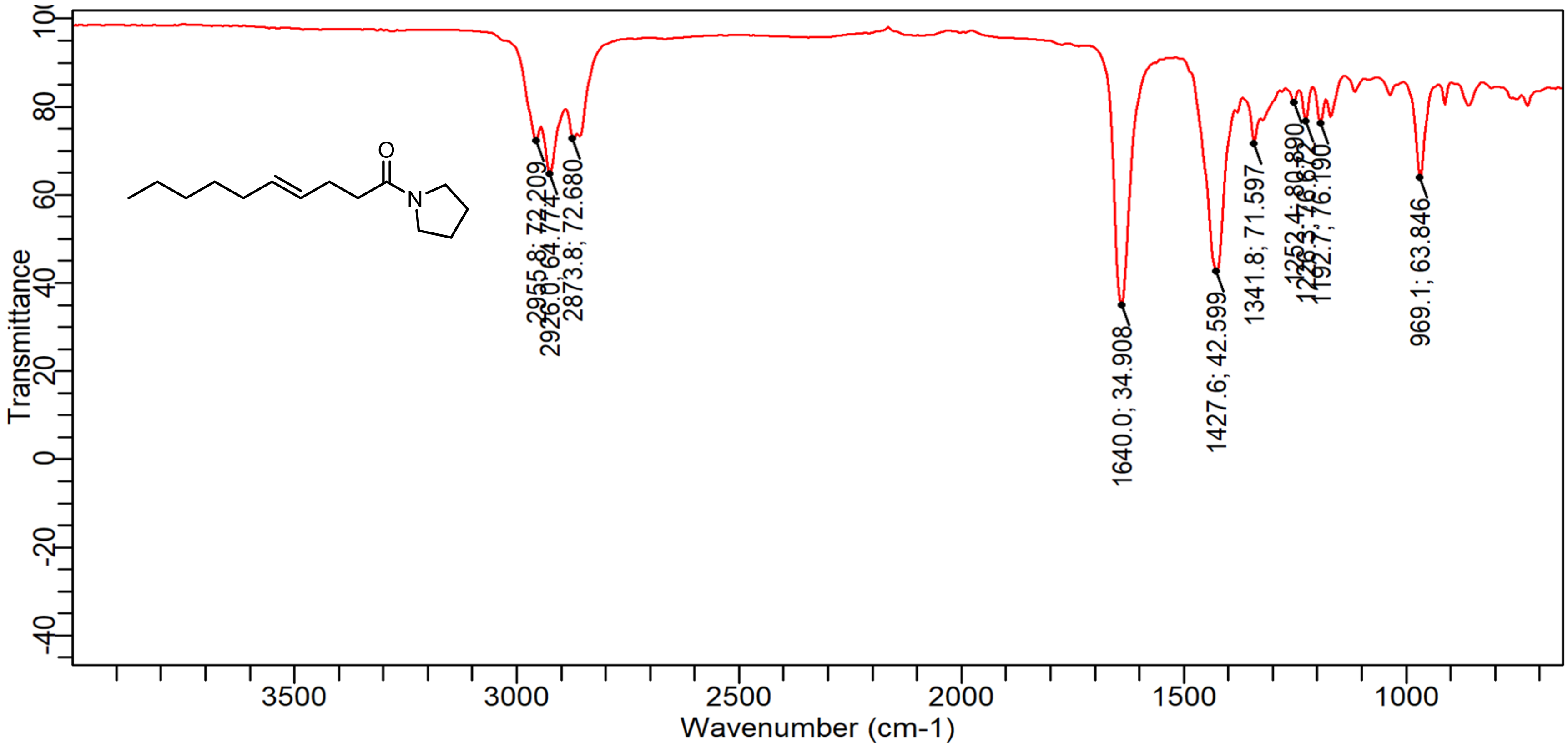

Transmittance
Wavenumber (cm-1)
2955.8; 72.209
2926.0; 64.774
2873.8; 72.680
1640.0; 34.908
1427.6; 42.599
1341.8; 71.597
1252.4; 80.890
969.1; 63.846

3.42 1-(Piperidin-1-yl)-3-(3,4,5-trimethoxyphenyl)propan-1-one-2,3-*d*$_2$ (12bd-*d*$_2$) (600 MHz, $CDCl_3$)

3.42.1 ^{1}H NMR

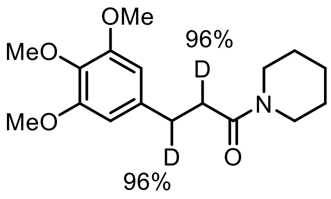

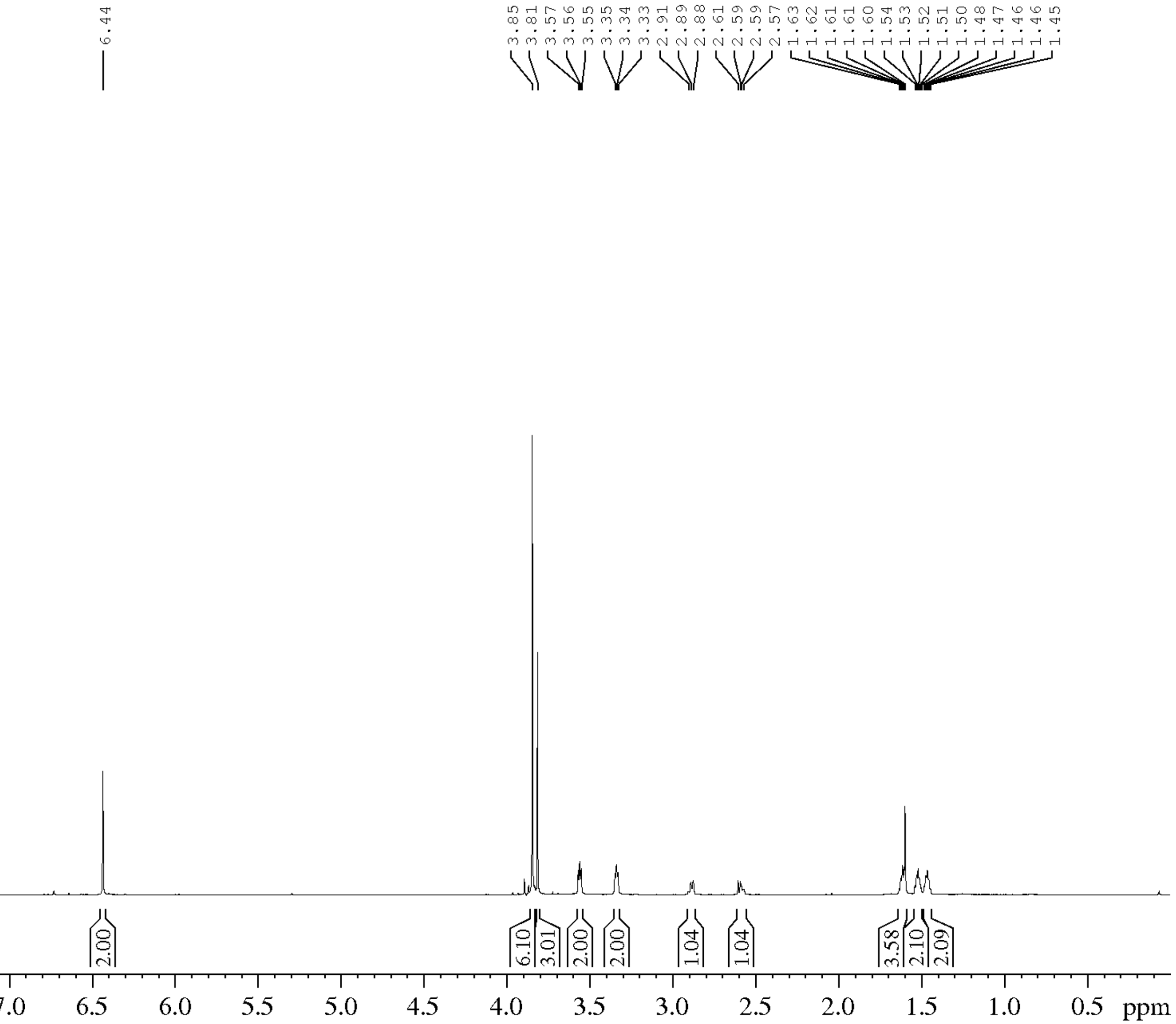

3.42.2 ^{2}H NMR

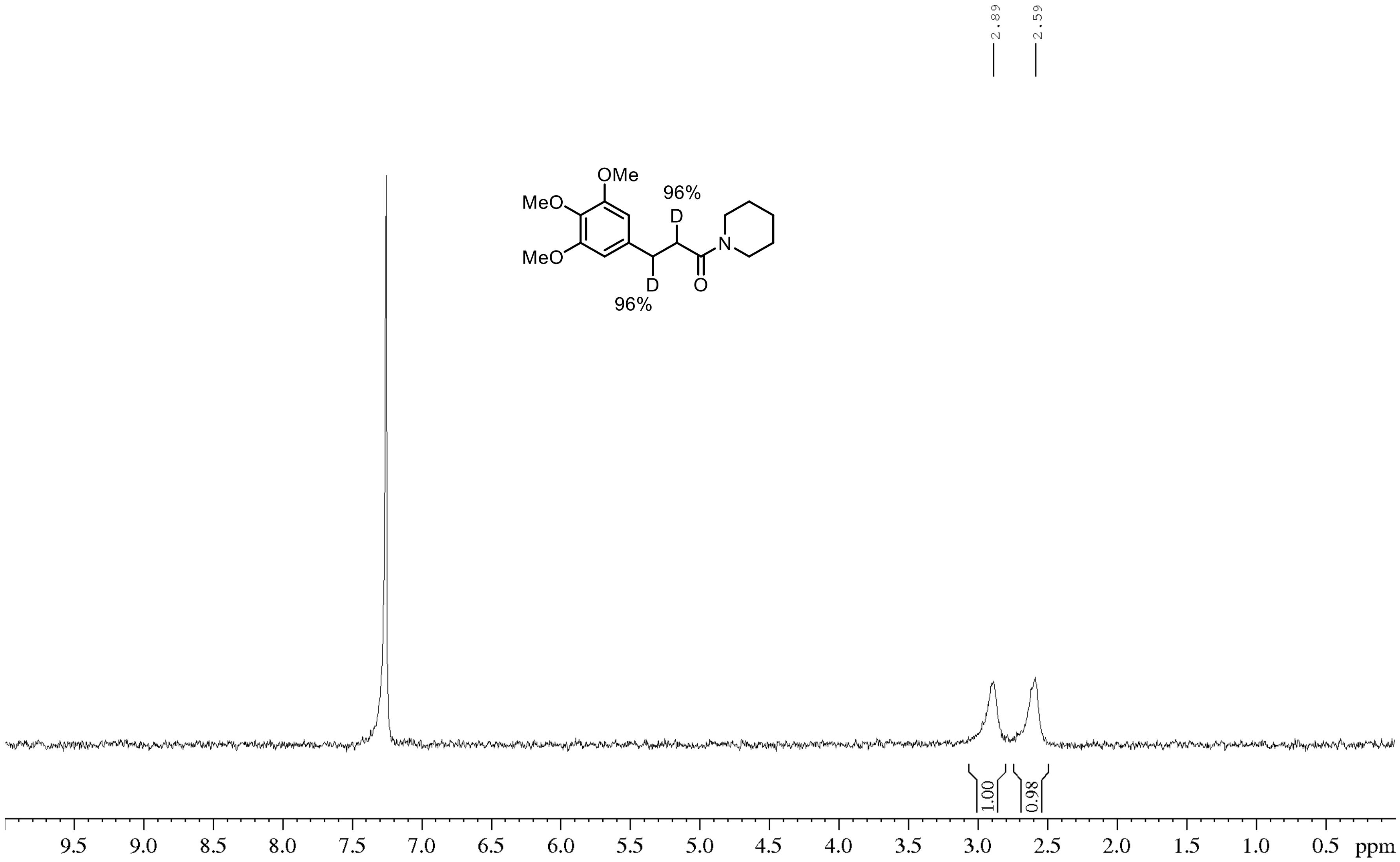

2.89
2.59
OMe
MeO
MeO
96%
D
N
D
O
96%
1.00
0.98
9.5
9.0
8.5
8.0
7.5
7.0
6.5
6.0
5.5
5.0
4.5
4.0
3.5
3.0
2.5
2.0
1.5
1.0
0.5
ppm

3.42.3 ^{13}C NMR

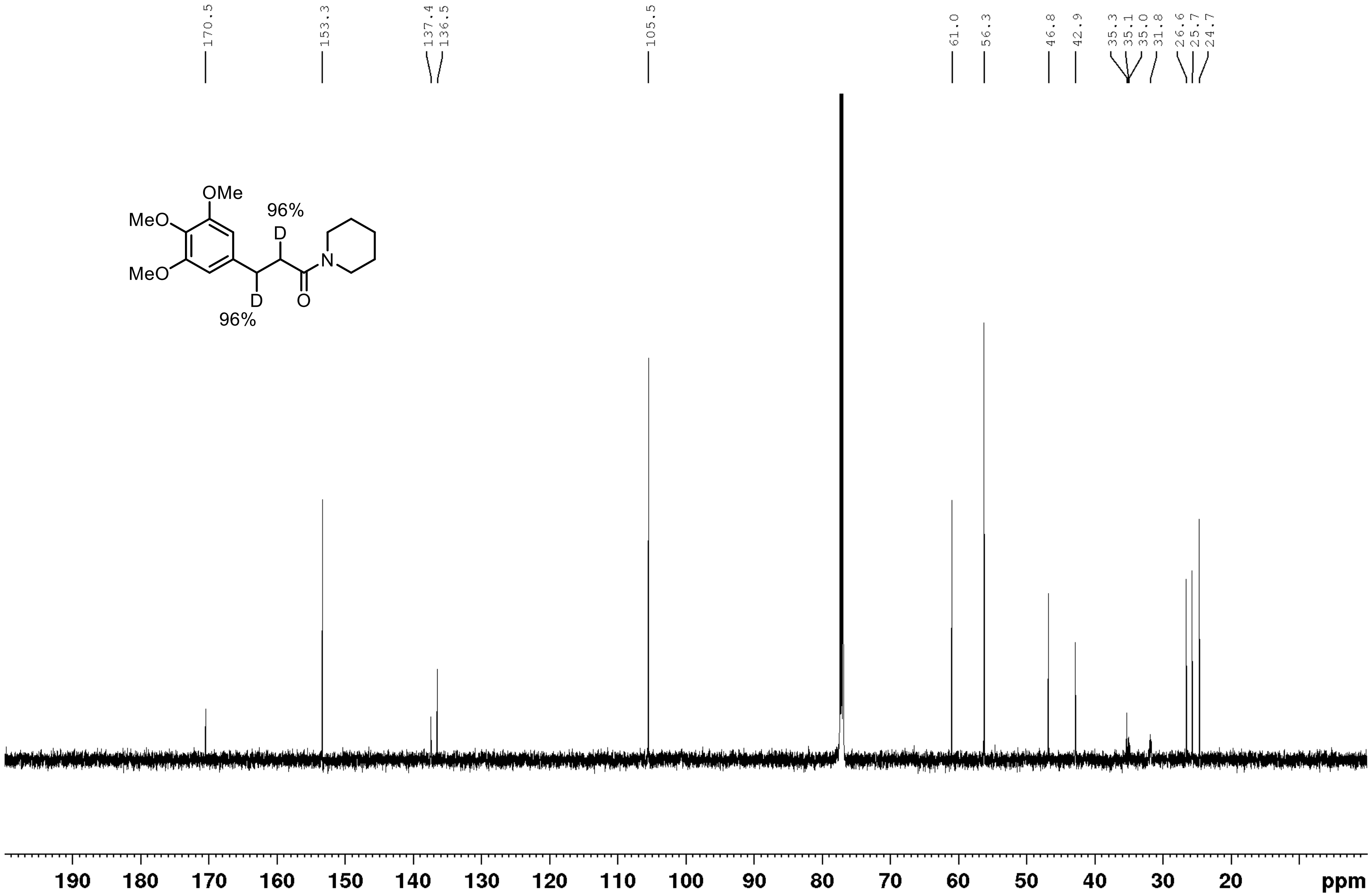

170.5
153.3
137.4
136.5
105.5
61.0
56.3
46.8
42.9
35.3
35.1
35.0
31.8
26.6
25.7
24.7
OMe
MeO
MeO
96%
D
N
D
O
96%
190 180 170 160 150 140 130 120 110 100 90 80 70 60 50 40 30 20
ppm

3.42.4 ¹H, ¹H COSY

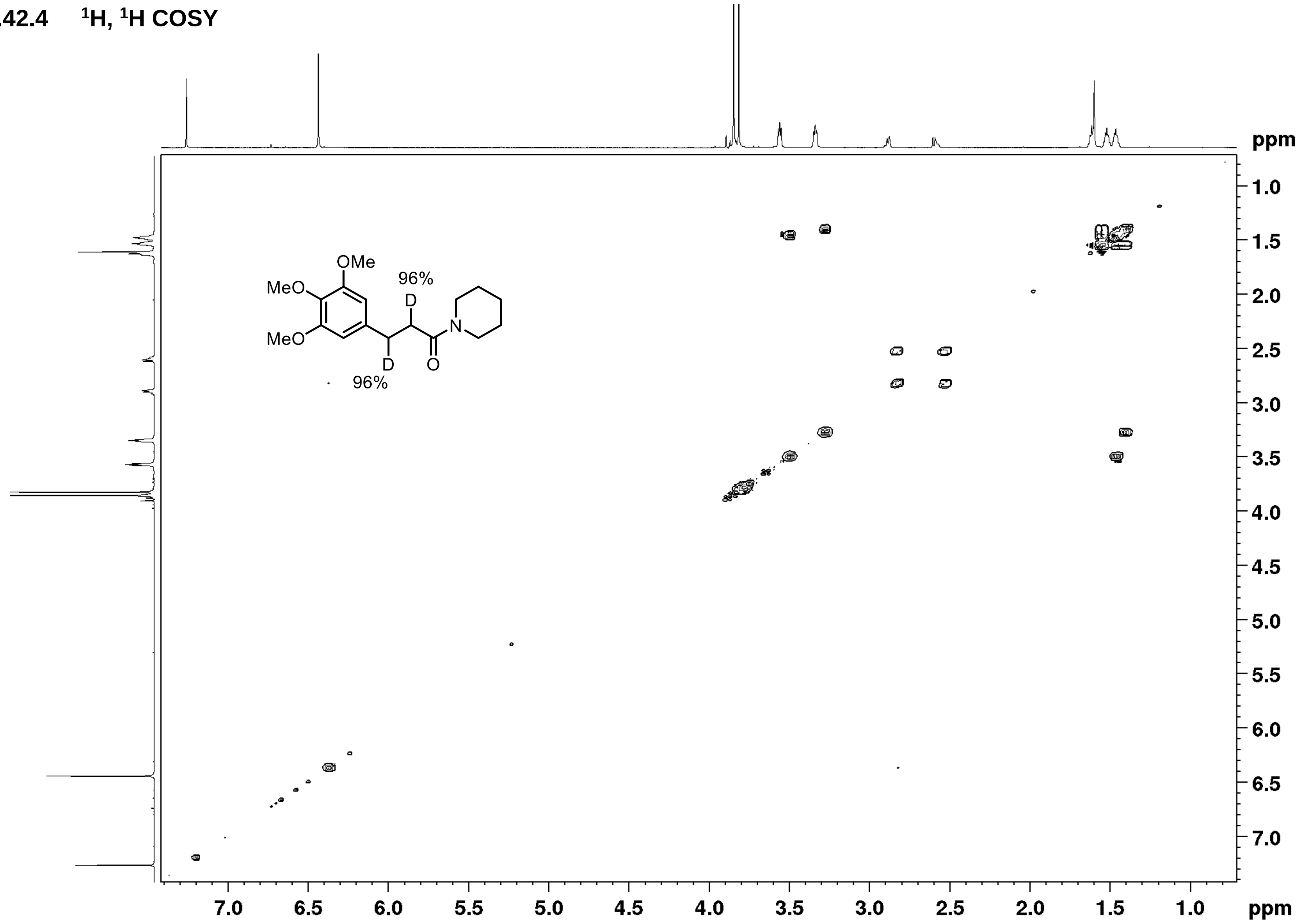

3.42.5 ^{1}H, ^{13}C HMBC

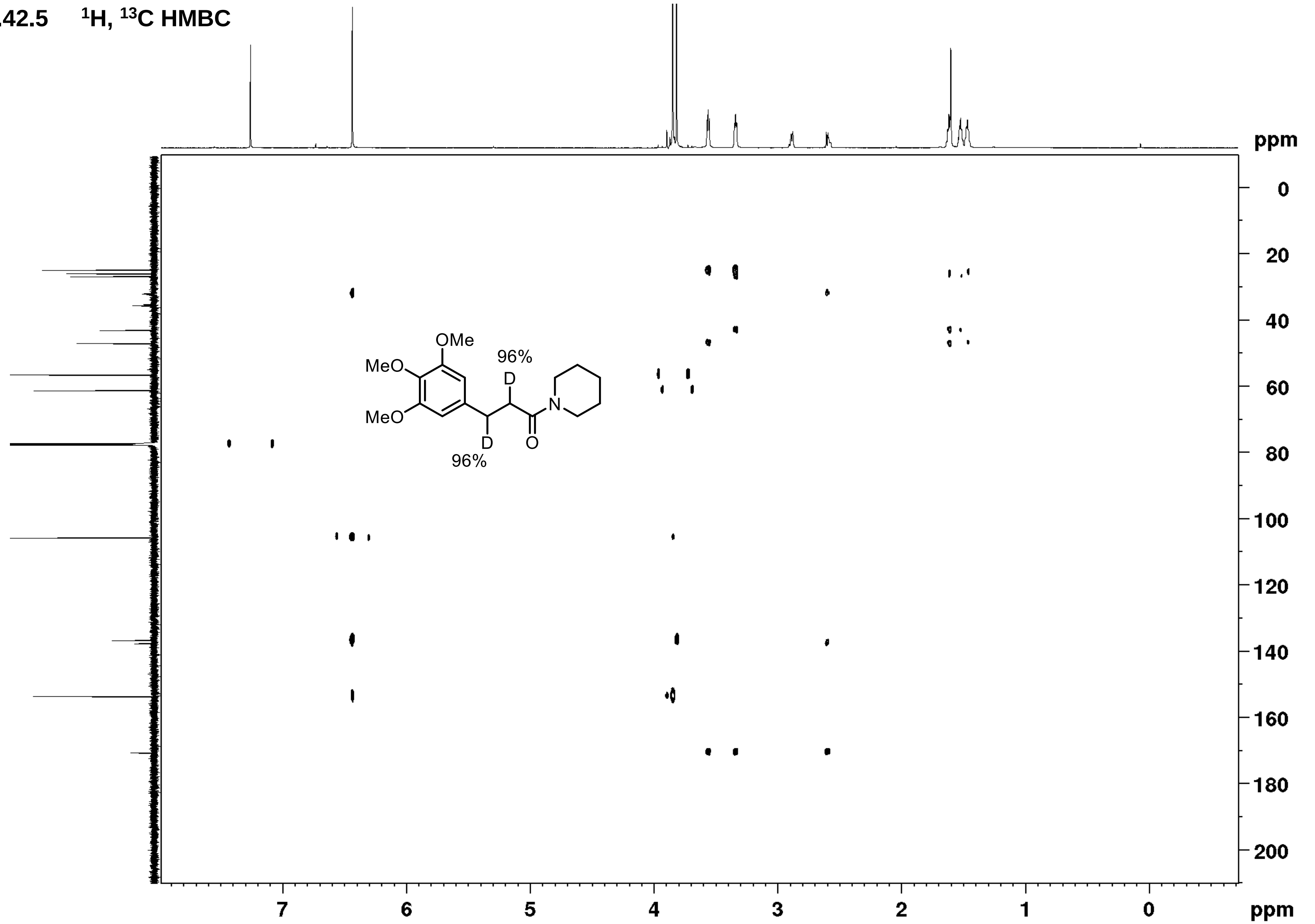

3.42.6 ^{1}H, ^{13}C HSQC

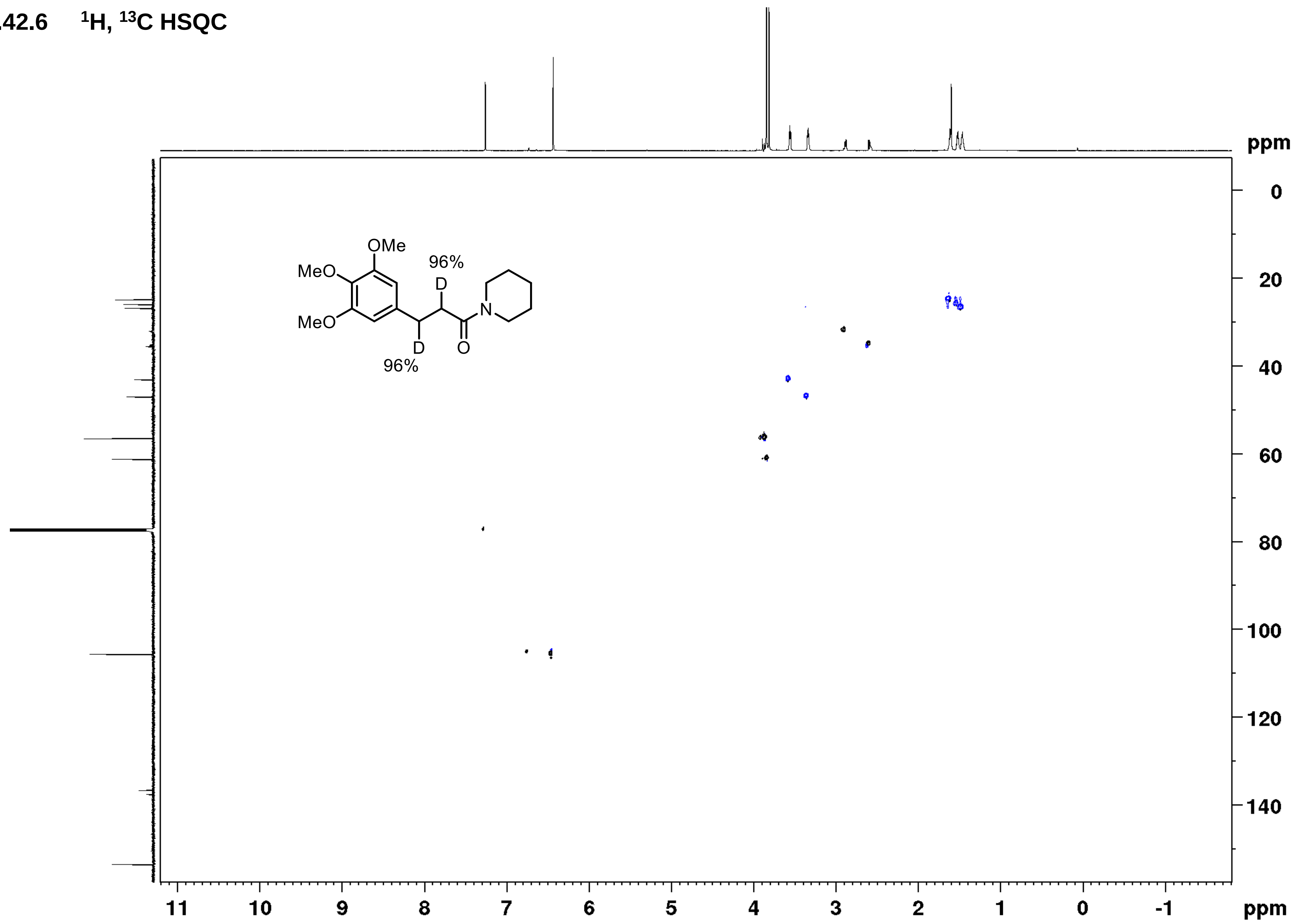

3.42.7 HRMS

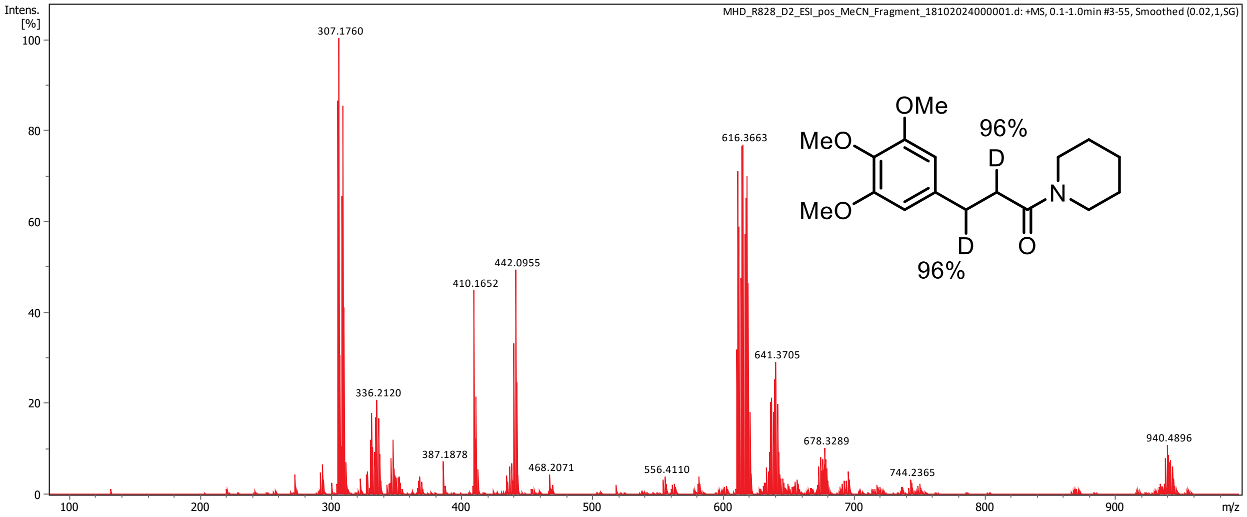

MHD_R828_D2_ESI_pos_MeCN_Fragment_18102024000001.d: +MS, 0.1-1.0min #3-55, Smoothed (0.02,1,SG)
307.1760
336.2120
387.1878
410.1652
442.0955
468.2071
556.4110
616.3663
641.3705
678.3289
744.2365
940.4896
OMe
MeO
MeO
96%
D
D
N
O
96%
Intens. [%]
m/z

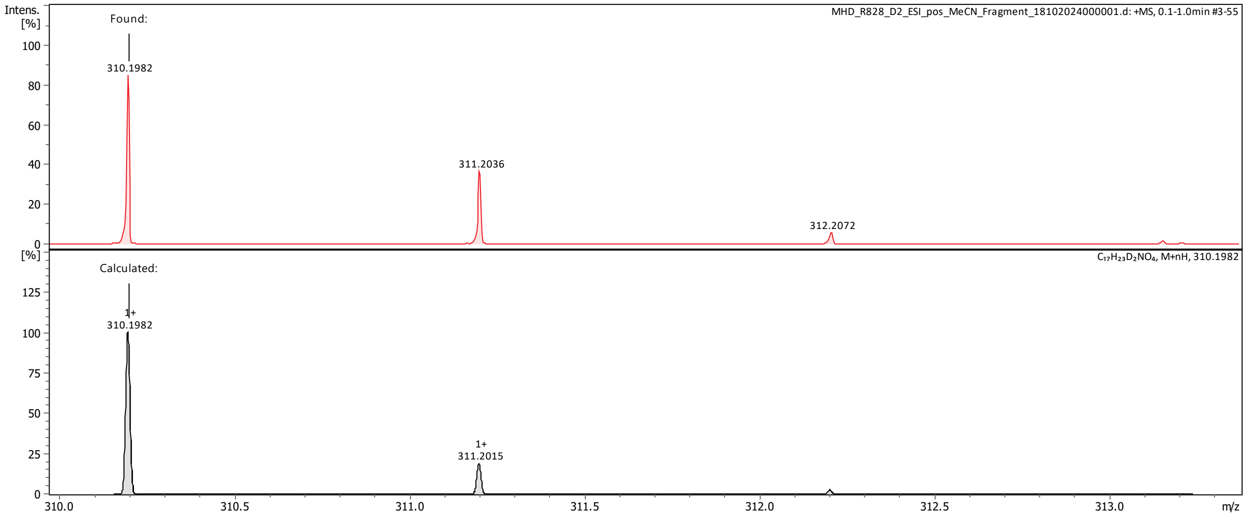

MHD_R828_D2_ESI_pos_MeCN_Fragment_18102024000001.d: +MS, 0.1-1.0min #3-55
Found:
310.1982
311.2036
312.2072
Calculated:
C17H23D2NO4, M+nH, 310.1982
1+
310.1982
1+
311.2015
Intens. [%]
m/z

3.42.8 IR

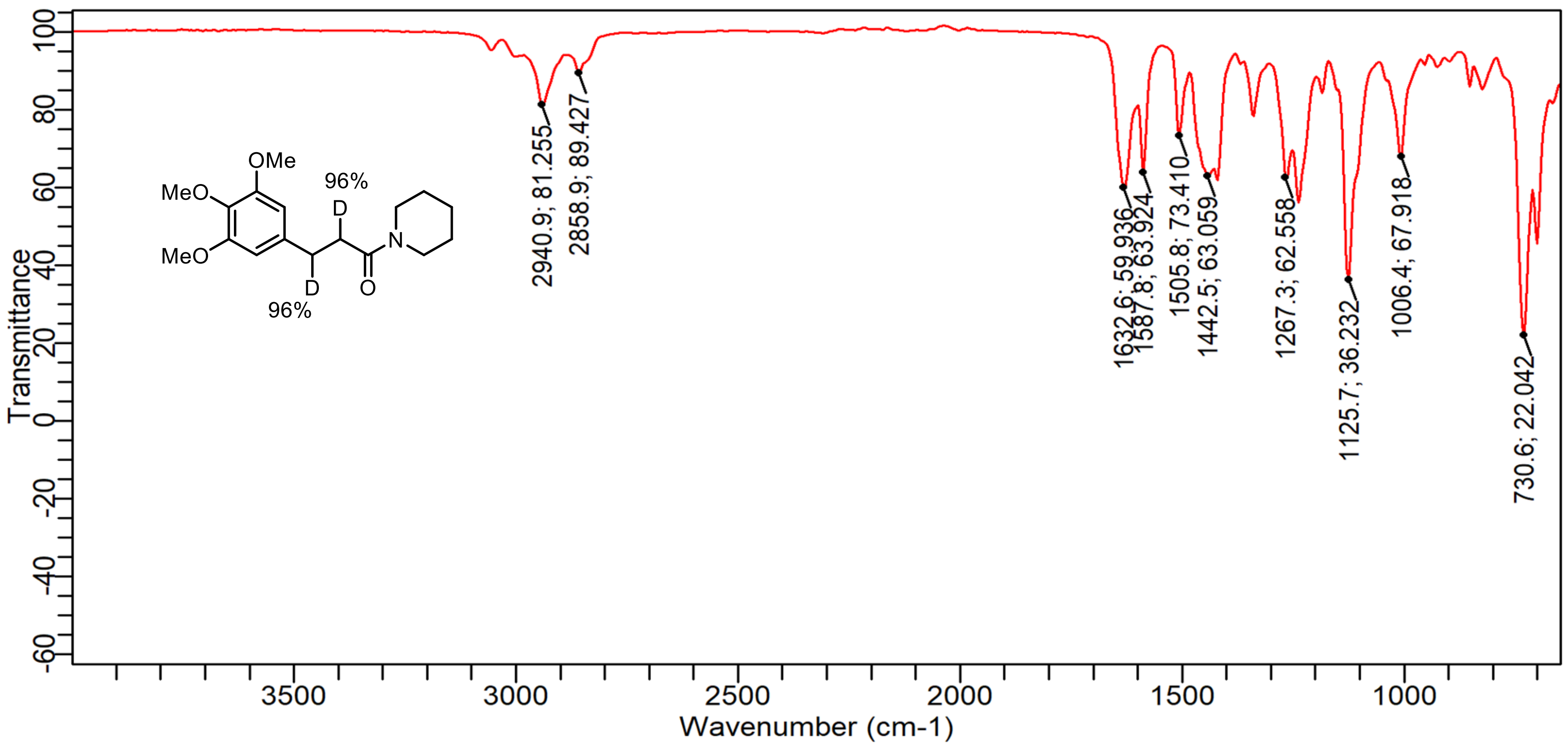

OMe
MeO
MeO
96%
D
D
96%
O
N
2940.9; 81.255
2858.9; 89.427
1632.6; 59.936
1587.8; 63.924
1505.8; 73.410
1442.5; 63.059
1267.3; 62.558
1125.7; 36.232
1006.4; 67.918
730.6; 22.042
Transmittance
Wavenumber (cm-1)

3.43 3-(Benzo[*d*][1,3]dioxol-5-yl)-1-(piperidin-1-yl)propan-1-one-2,3-d_2 (12be-d_4) (600 MHz, $CDCl_3$)

3.43.1 ^{1}H NMR

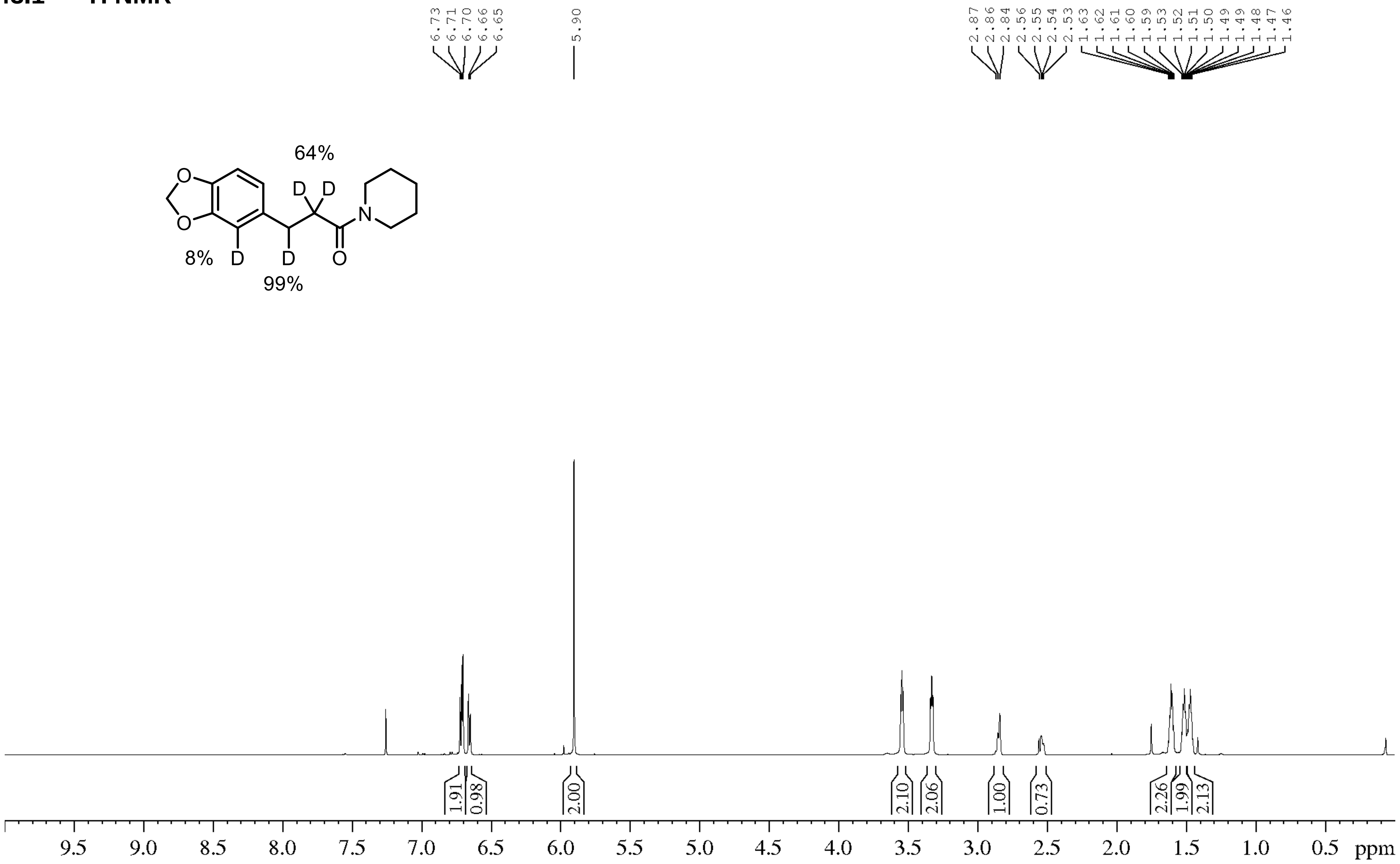

3.43.2 ^{2}H NMR

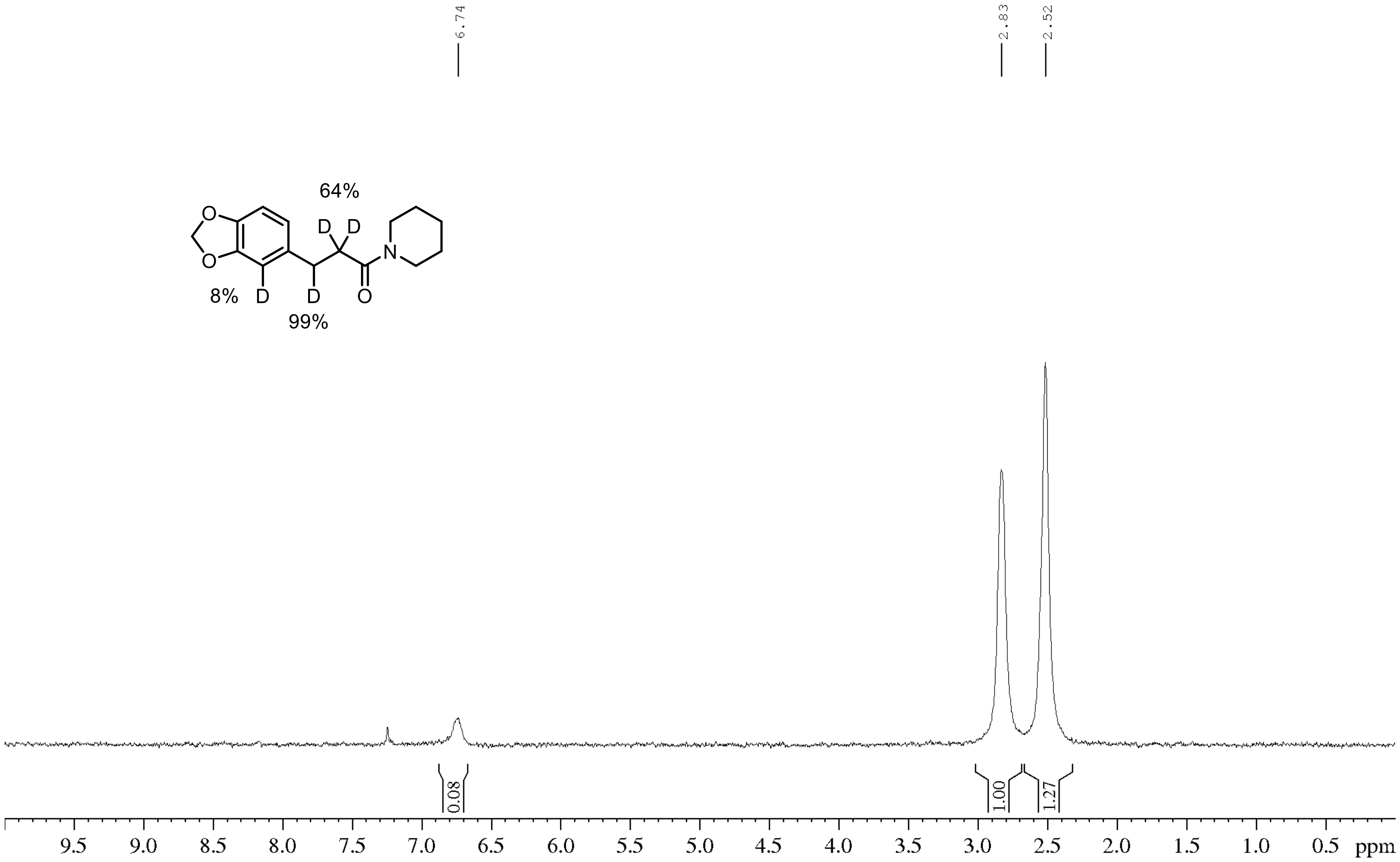

6.74
2.83
2.52
64%
8%
99%
D
O
N
0.08
1.00
1.27
9.5
9.0
8.5
8.0
7.5
7.0
6.5
6.0
5.5
5.0
4.5
4.0
3.5
3.0
2.5
2.0
1.5
1.0
0.5
ppm

3.43.3 ^{13}C NMR

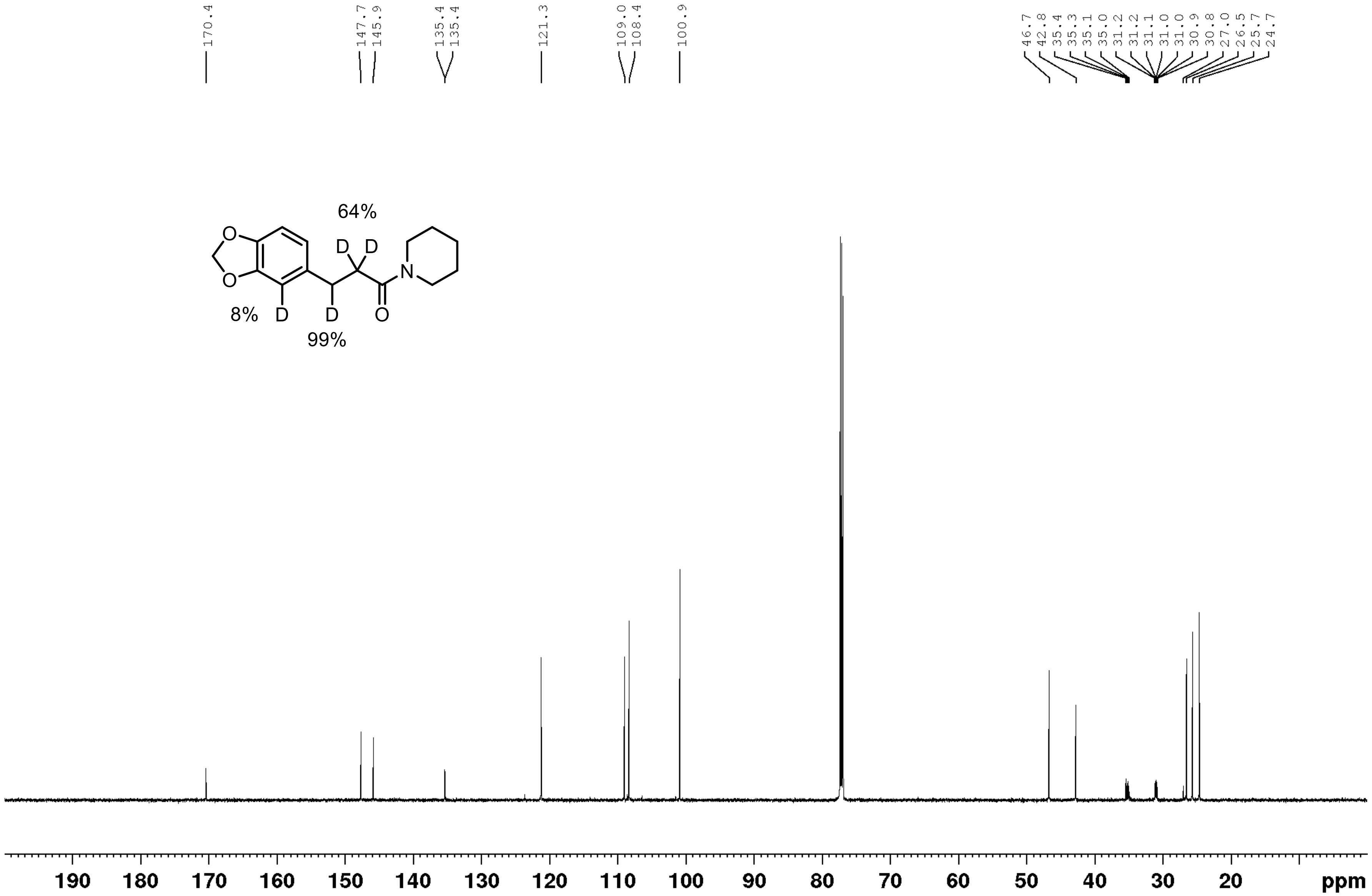
170.4
147.7
145.9
135.4
135.4
121.3
109.0
108.4
100.9
46.7
42.8
35.4
35.3
35.1
35.0
31.2
31.1
31.0
31.0
30.9
30.8
27.0
26.5
25.7
24.7
64%
8%
99%
190
180
170
160
150
140
130
120
110
100
90
80
70
60
50
40
30
20
ppm

3.43.4 ^{1}H, ^{1}H COSY

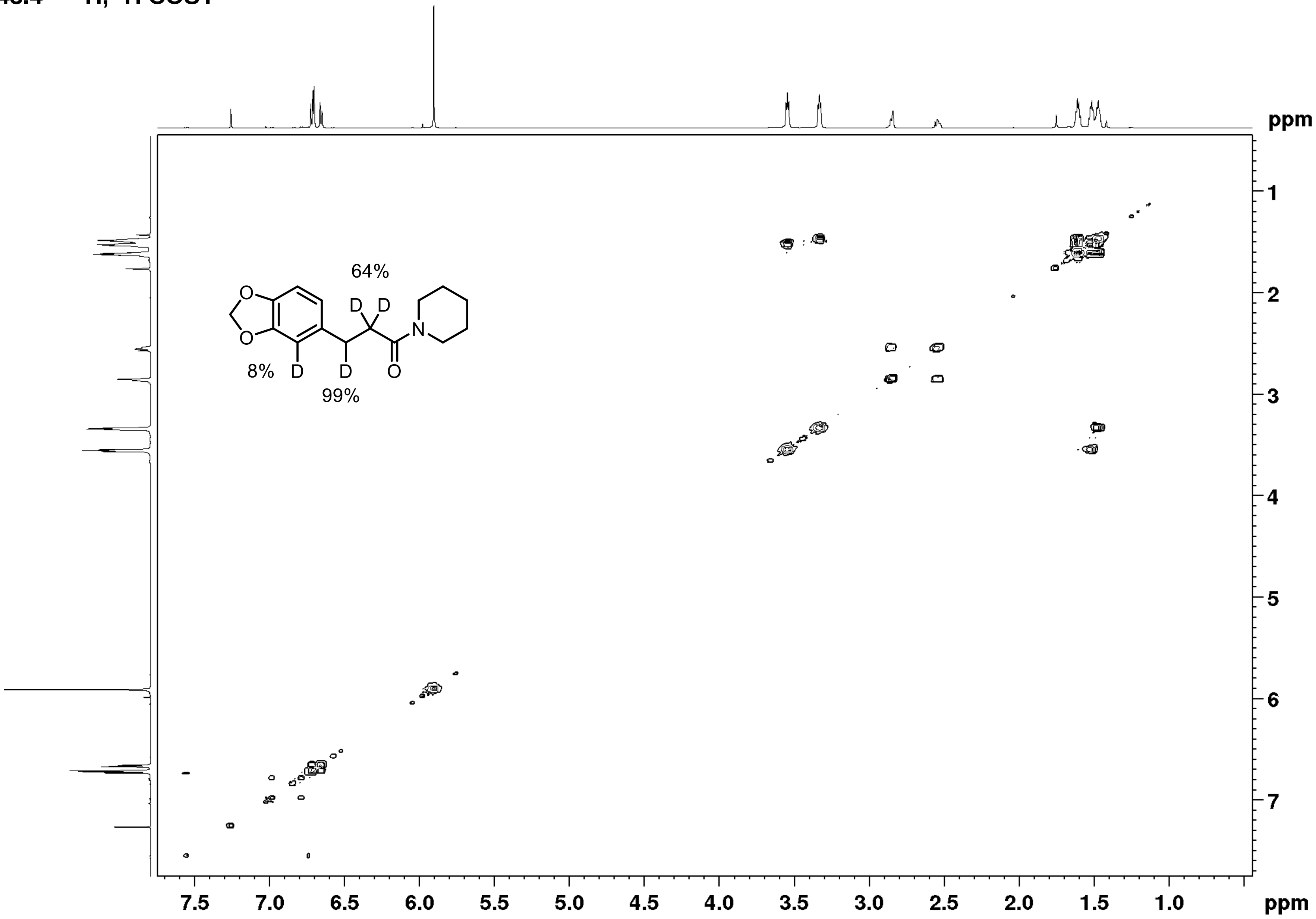

3.43.5 ^{1}H, ^{13}C HMBC

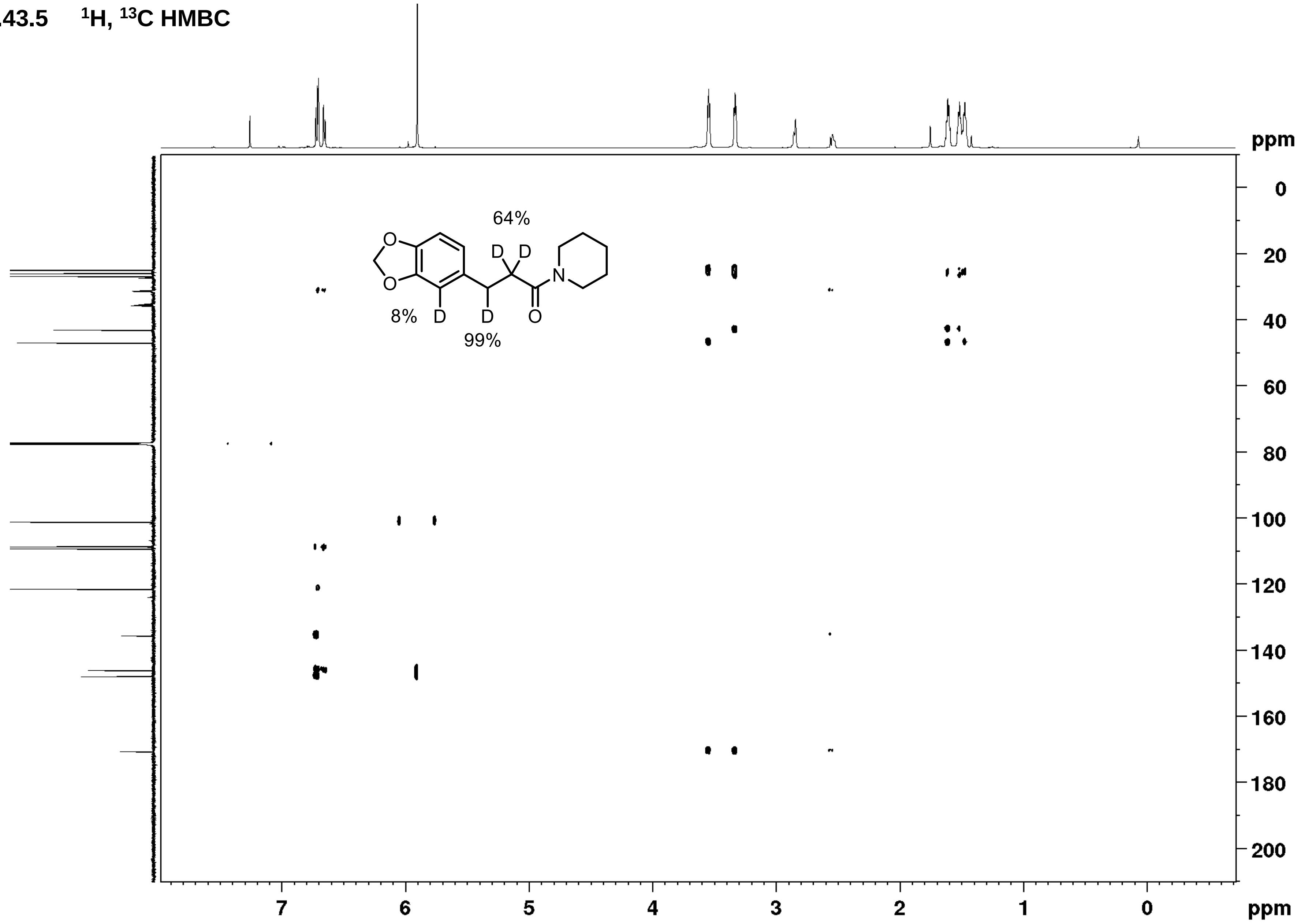

3.43.6 ^{1}H, ^{13}C HSQC

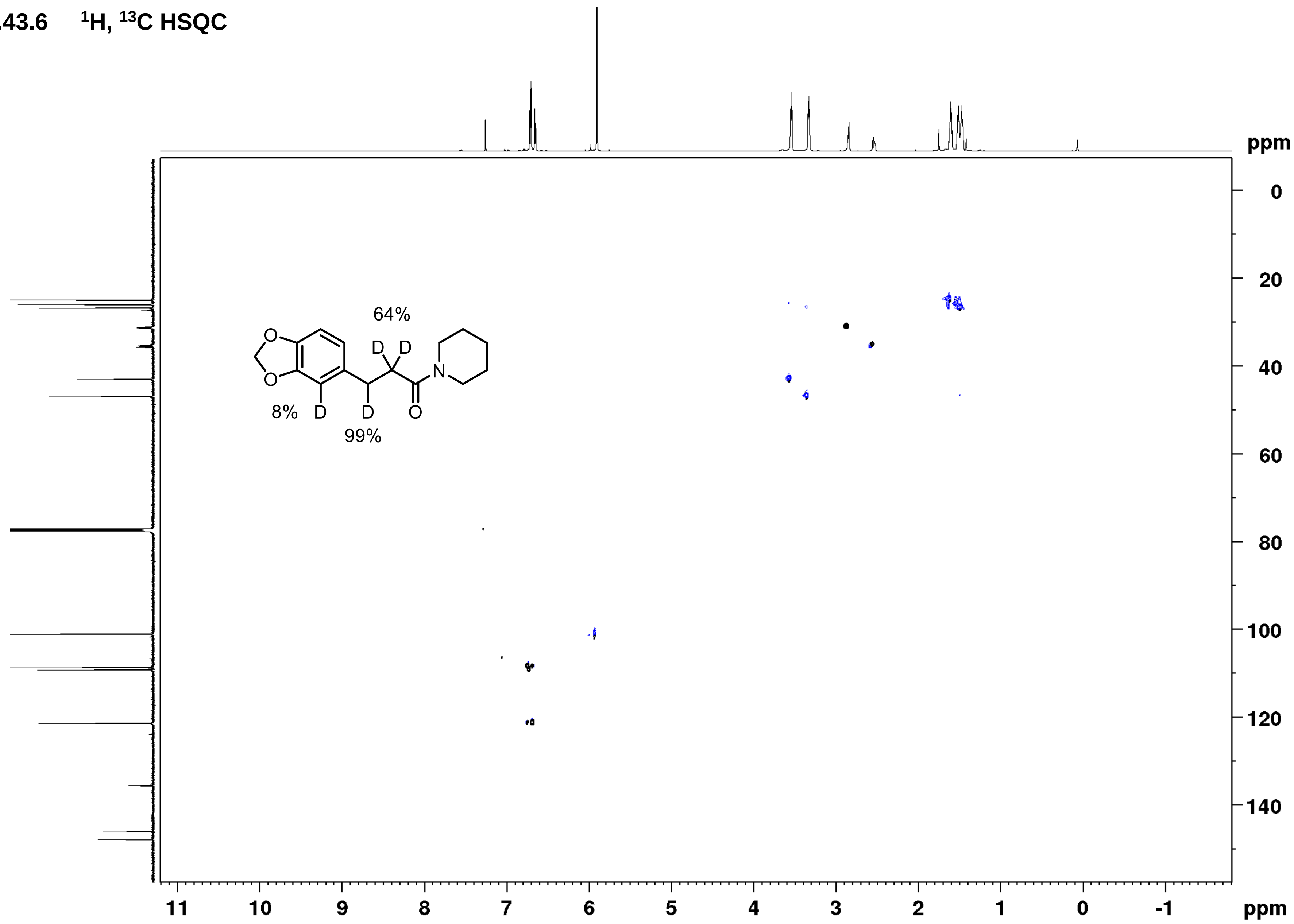

3.43.7 HRMS

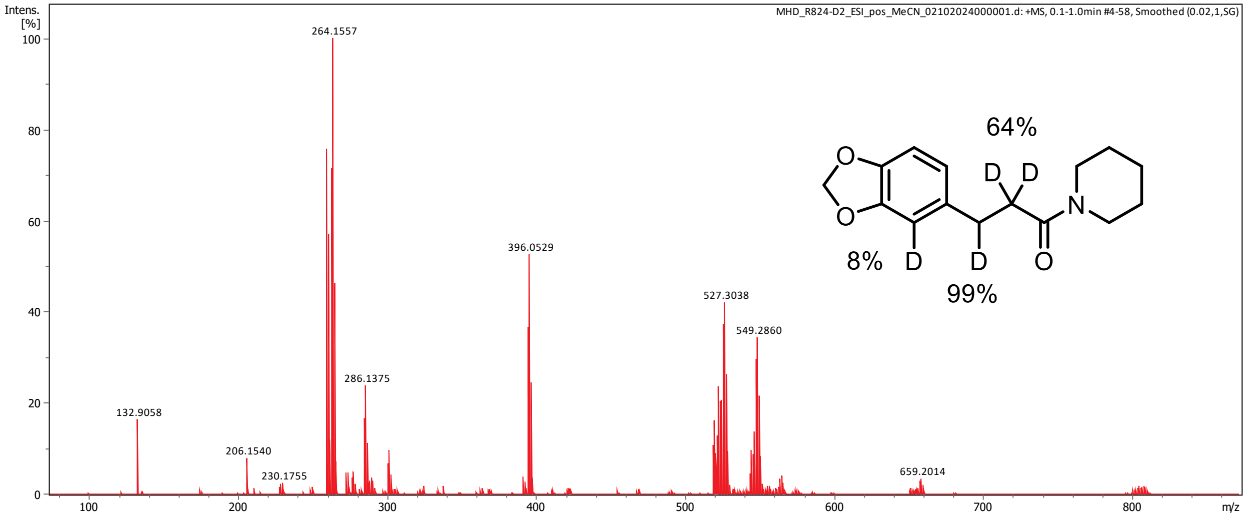

Intens. [%]
MHD_R824-D2_ESI_pos_MeCN_02102024000001.d: +MS, 0.1-1.0min #4-58, Smoothed (0.02,1,SG)
264.1557
396.0529
527.3038
549.2860
286.1375
132.9058
206.1540
230.1755
659.2014
64%
8%
99%
m/z

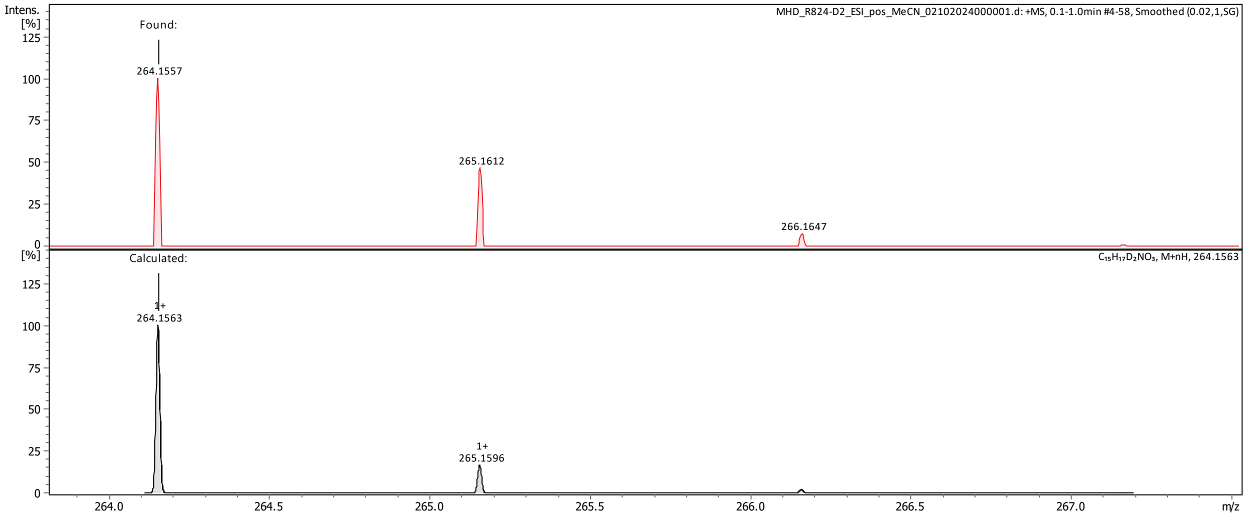

Intens. [%]
MHD_R824-D2_ESI_pos_MeCN_02102024000001.d: +MS, 0.1-1.0min #4-58, Smoothed (0.02,1,SG)
Found:
264.1557
265.1612
266.1647
Calculated:
C15H17D2NO3, M+nH, 264.1563
1+
264.1563
1+
265.1596
m/z

3.43.8 IR

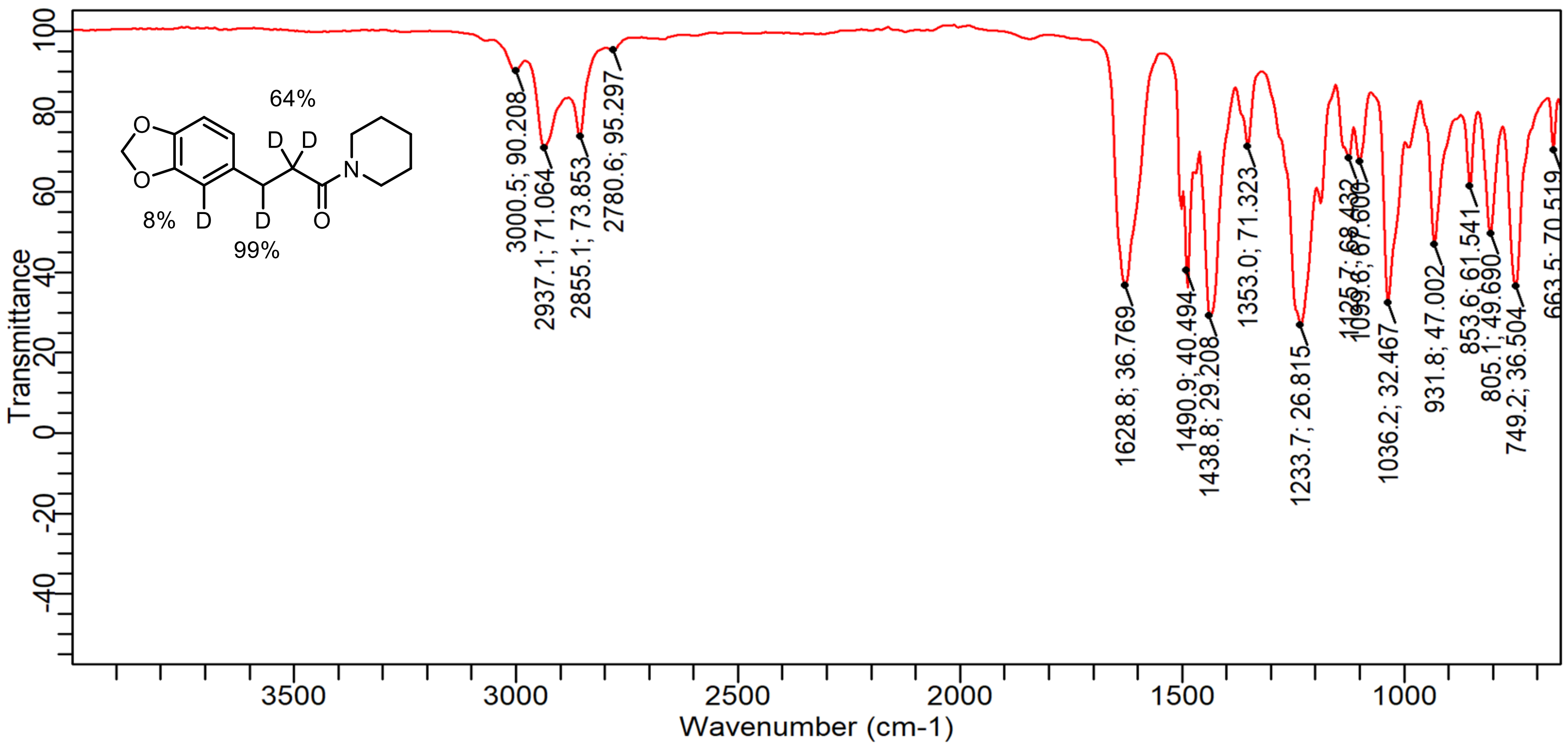

64%
8%
99%
D
D
D
D
O
O
O
N
3000.5; 90.208
2937.1; 71.064
2855.1; 73.853
2780.6; 95.297
1628.8; 36.769
1490.9; 40.494
1438.8; 29.208
1353.0; 71.323
1233.7; 26.815
1036.2; 32.467
931.8; 47.002
853.6; 61.541
805.1; 49.690
749.2; 36.504
Transmittance
Wavenumber (cm-1)

3.44 5-(Benzo[*d*][1,3]dioxol-5-yl)-1-(piperidin-1-yl)pentan-1-one-2,3,4,5-d_4 (12bi_d_4) (600 MHz, $CDCl_3$)

3.44.1 ^{1}H NMR

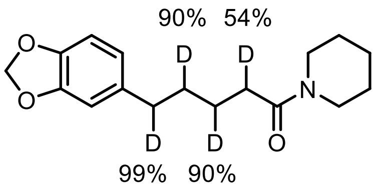

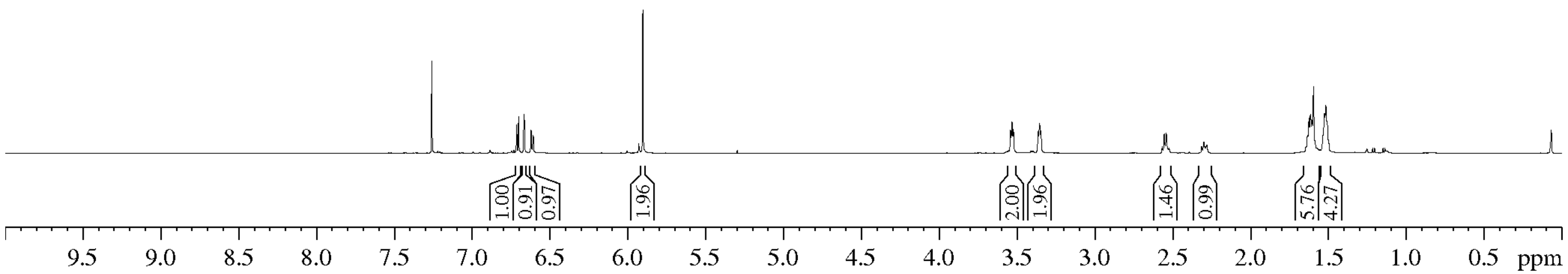

3.44.2 ^{2}H NMR

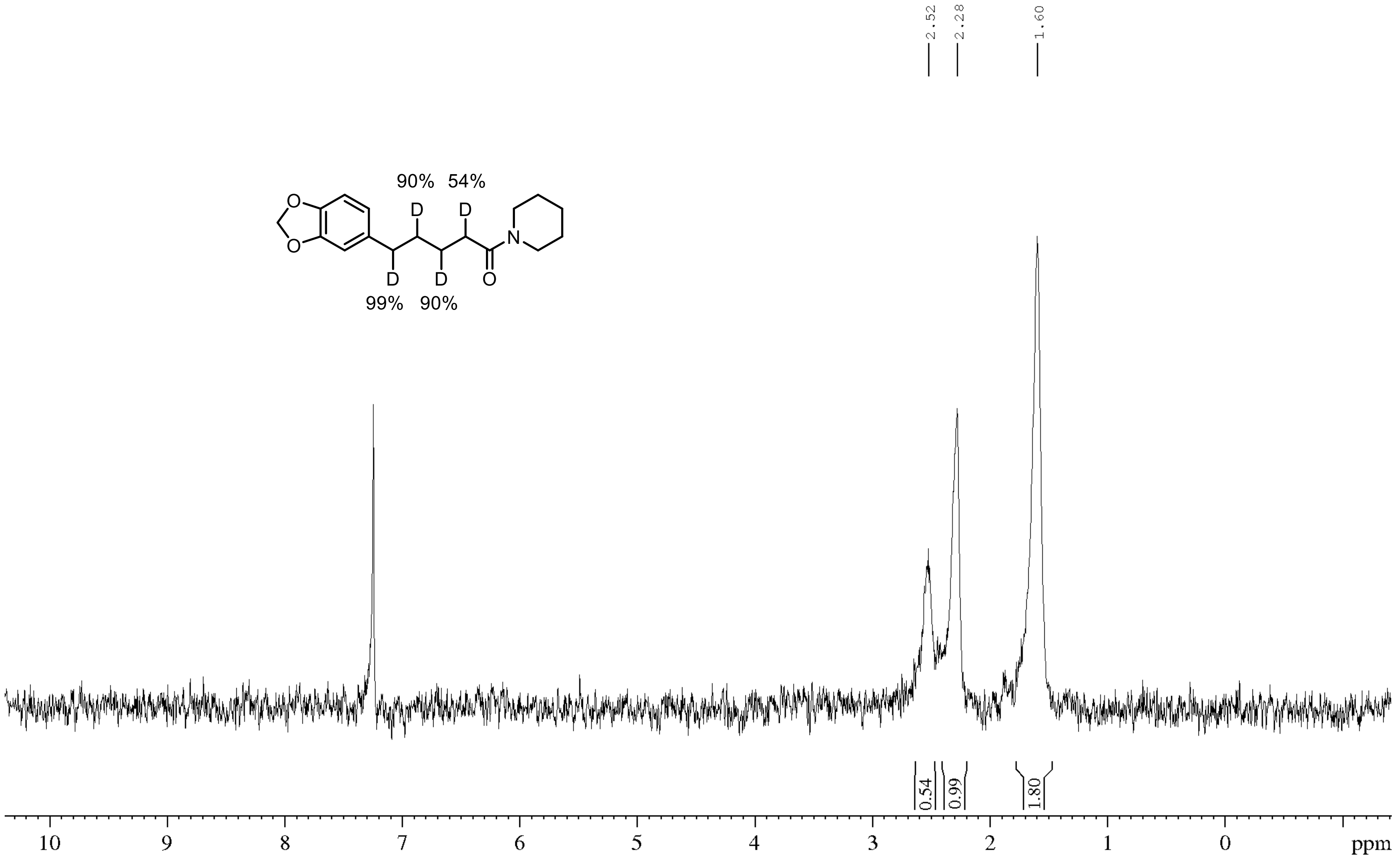

3.44.3 ^{13}C NMR

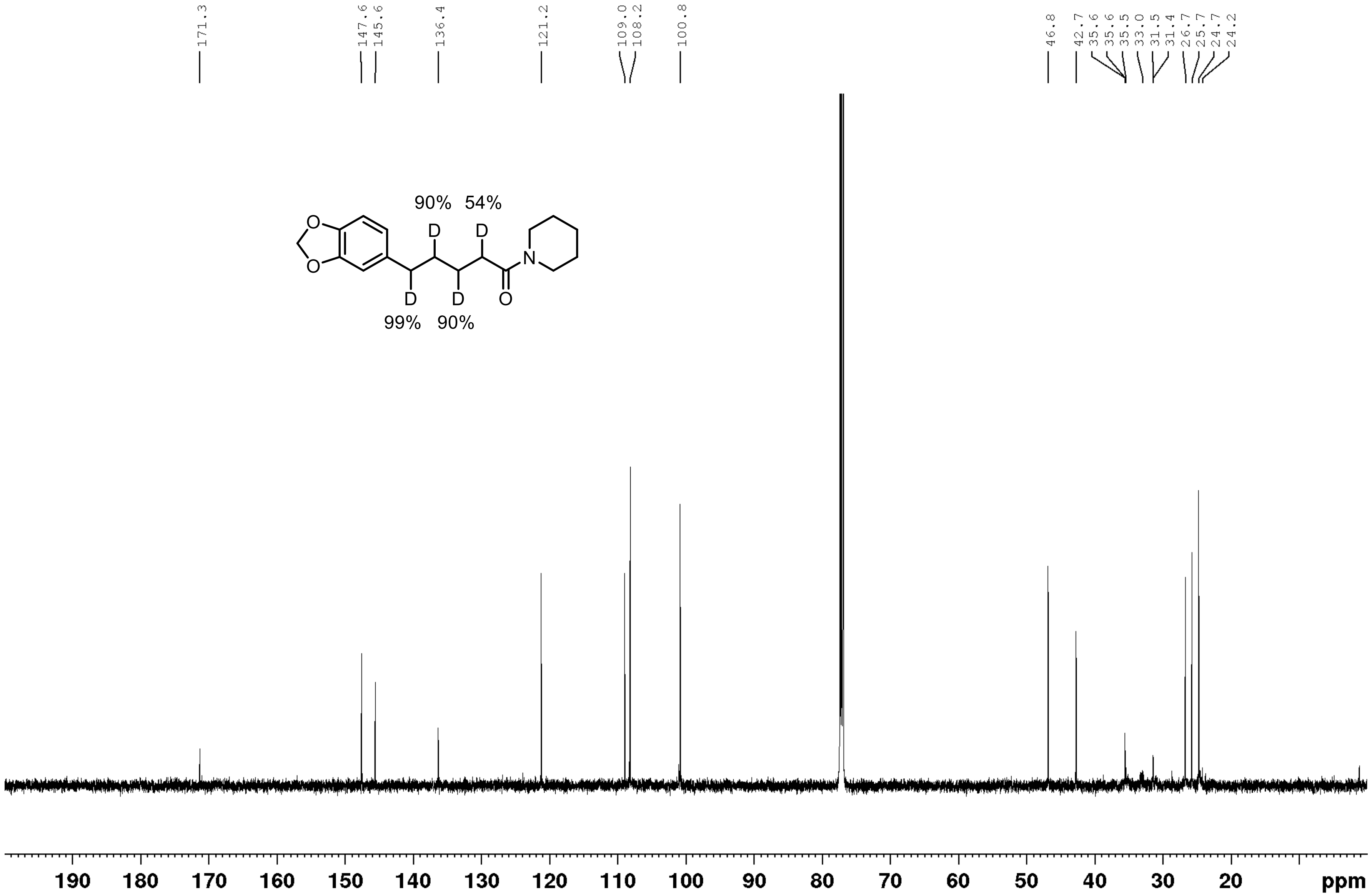

171.3
147.6
145.6
136.4
121.2
109.0
108.2
100.8
46.8
42.7
35.6
35.6
35.5
33.0
31.5
31.4
26.7
25.7
24.7
24.2
90% 54%
D
D
N
O
O
D
D
O
99% 90%
190 180 170 160 150 140 130 120 110 100 90 80 70 60 50 40 30 20 ppm

3.44.4 ^{1}H, ^{1}H COSY

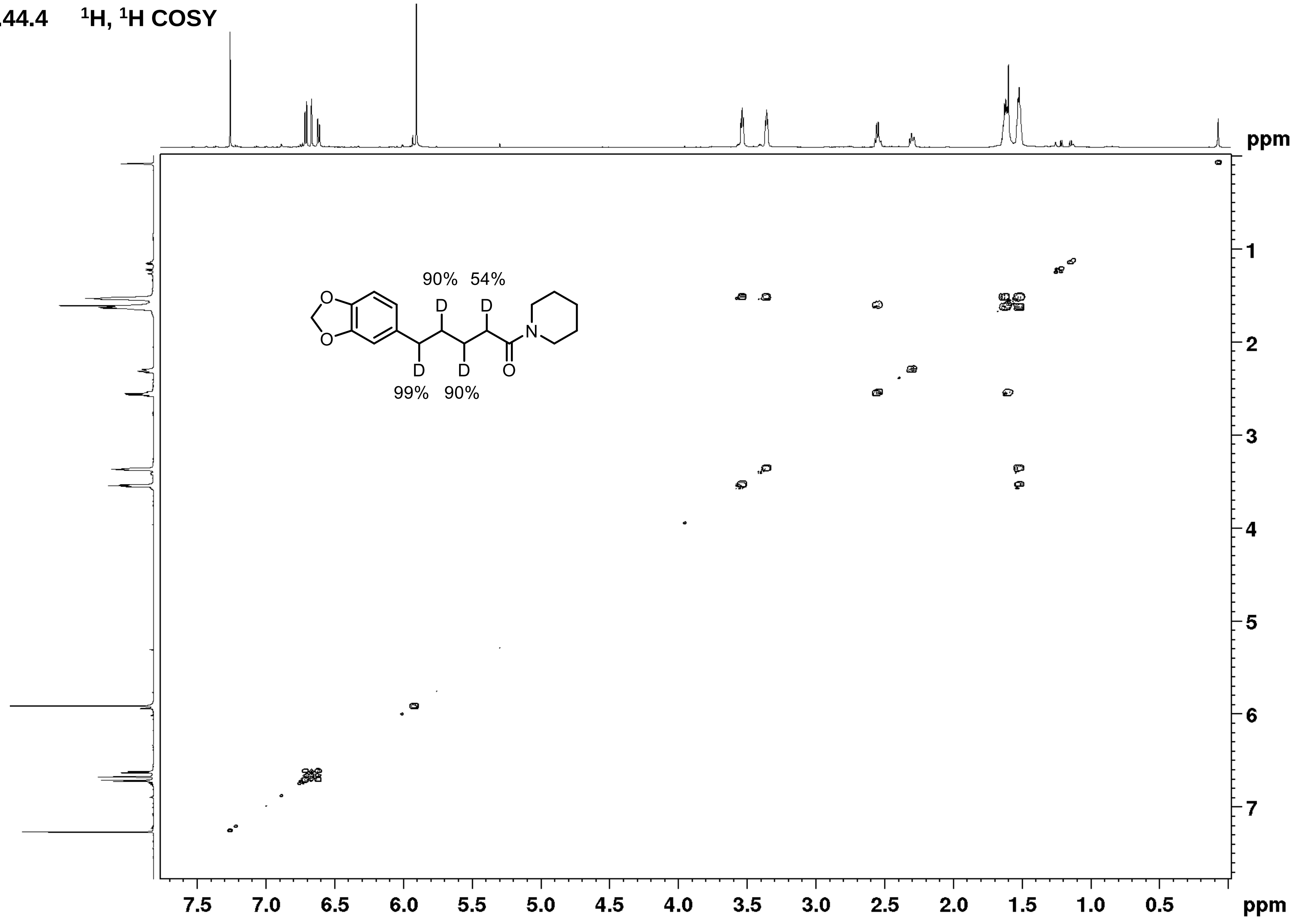

3.44.5 ^{1}H, ^{13}C HMBC

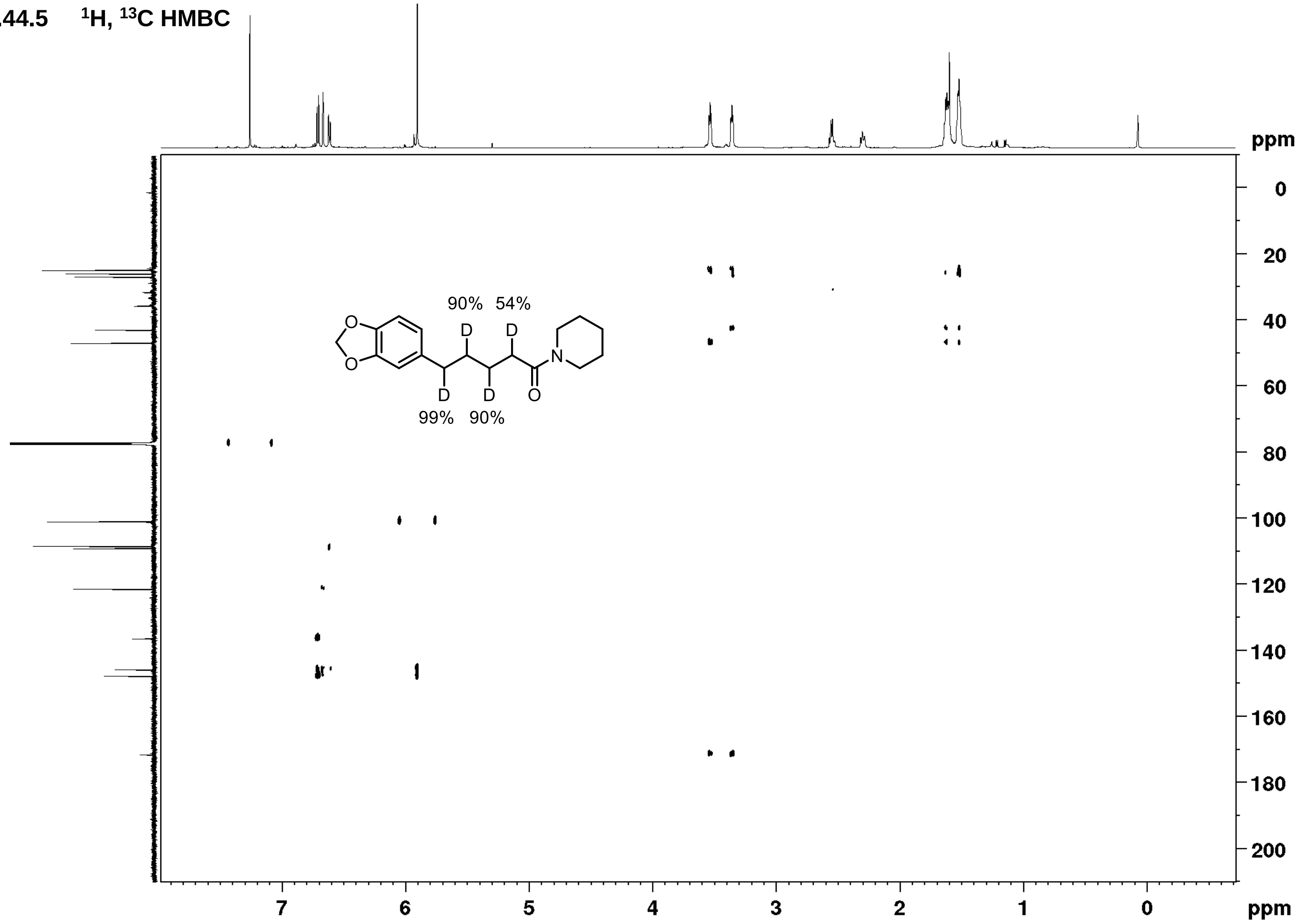

3.44.6 ^{1}H, ^{13}C HSQC

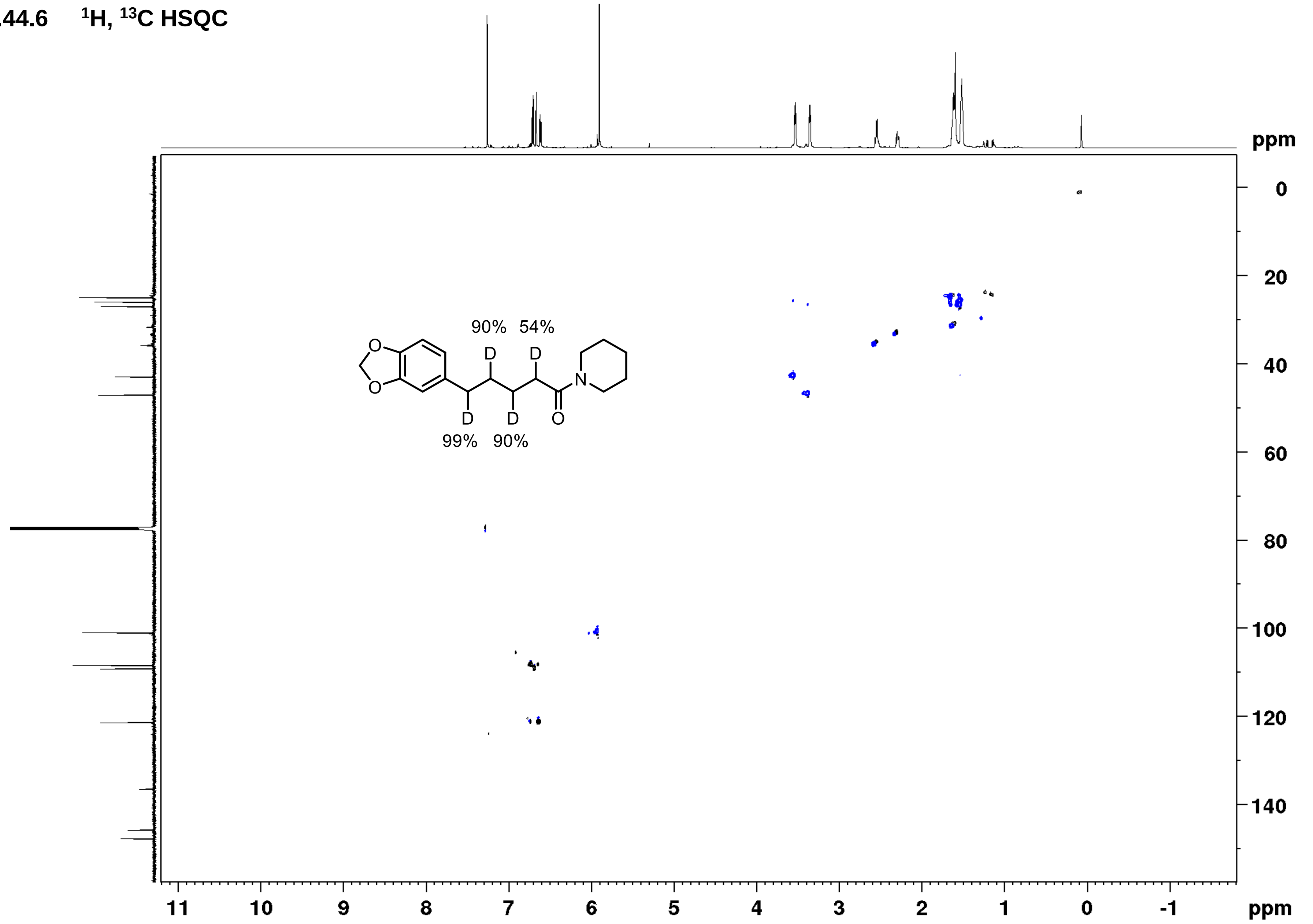

3.44.7 HRMS

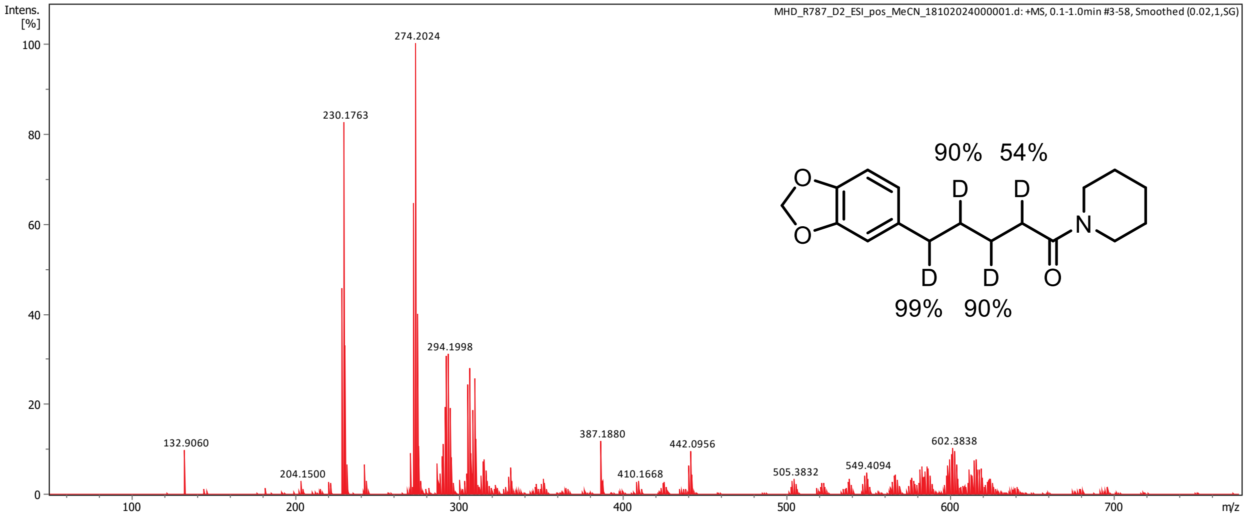
MHD_R787_D2_ESI_pos_MeCN_18102024000001.d: +MS, 0.1-1.0min #3-58, Smoothed (0.02,1,SG)
Intens. [%]
274.2024
230.1763
294.1998
132.9060
204.1500
387.1880
410.1668
442.0956
505.3832
549.4094
602.3838
m/z
90% 54%
99% 90%
D
O
N

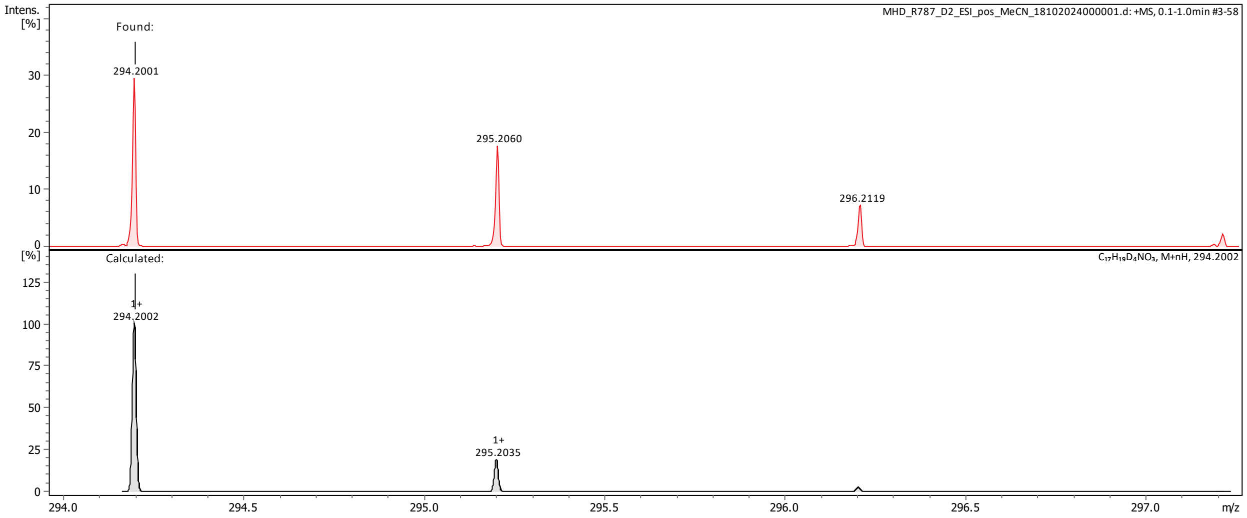
MHD_R787_D2_ESI_pos_MeCN_18102024000001.d: +MS, 0.1-1.0min #3-58
Found:
294.2001
295.2060
296.2119
Calculated:
$C_{17}H_{19}D_4NO_3$, M+nH, 294.2002
1+
294.2002
1+
295.2035
m/z

3.44.8 IR

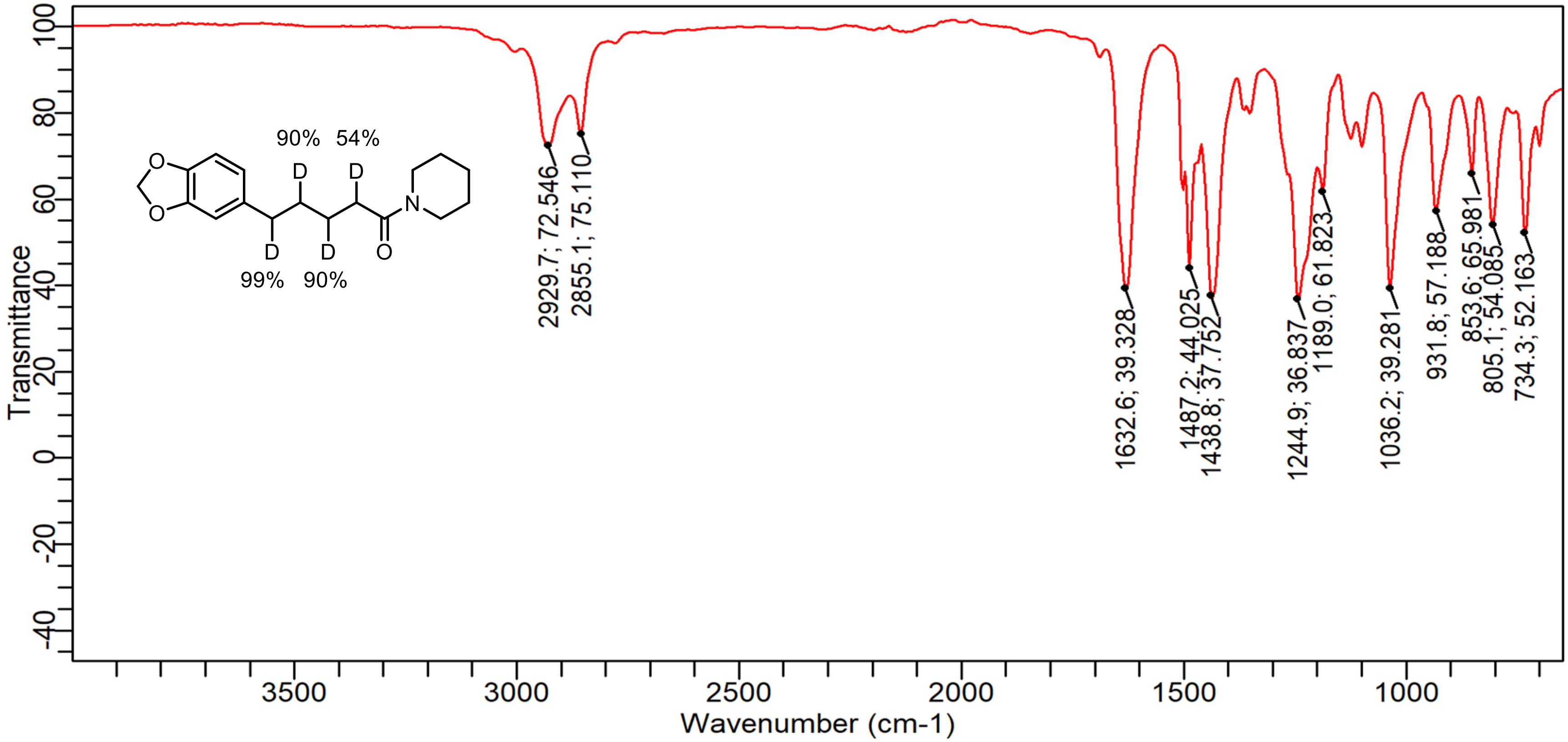

90%
54%
D
D
O
O
N
D
D
O
99%
90%
2929.7; 72.546
2855.1; 75.110
1632.6; 39.328
1487.2; 44.025
1438.8; 37.752
1244.9; 36.837
1189.0; 61.823
1036.2; 39.281
931.8; 57.188
853.6; 65.981
805.1; 54.085
734.3; 52.163
Transmittance
Wavenumber (cm-1)

3.45 1(1*R*,5*S*)-3-(3-Phenylpropanoyl-2,3-d_2)-1,2,3,4,5,6-hexahydro-8*H*-1,5-methanopyrido[1,2-*a*][1,5]diazocin-8-one (12bb-d_7) (600 MHz, $CDCl_3$)

3.45.1 ^{1}H NMR

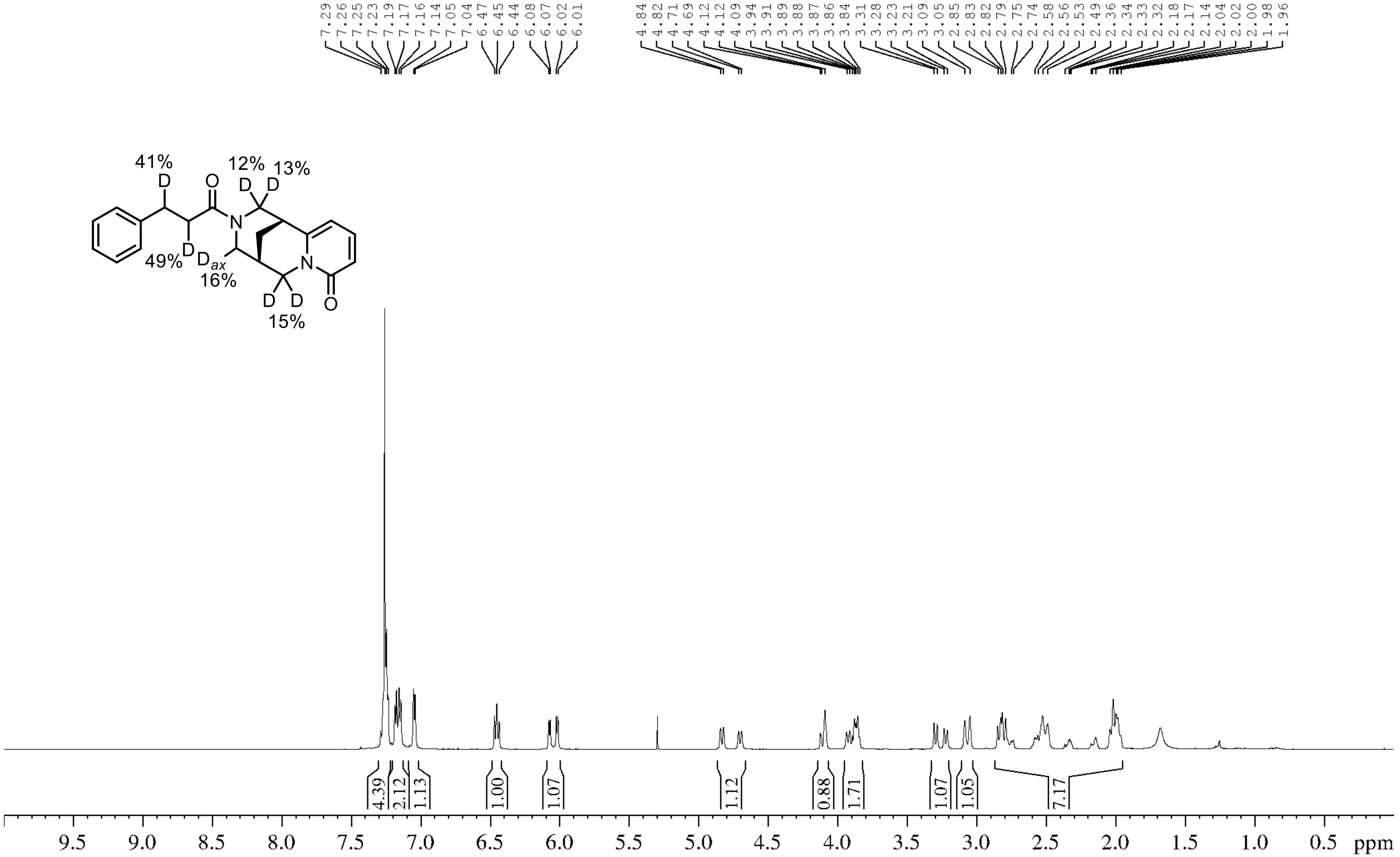

3.45.2 ^{2}H NMR

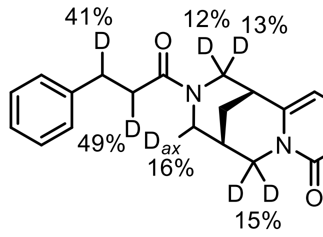
41%
D
O
12%
13%
D
D
N
N
O
49%
D
D$_{ax}$
16%
D
D
15%

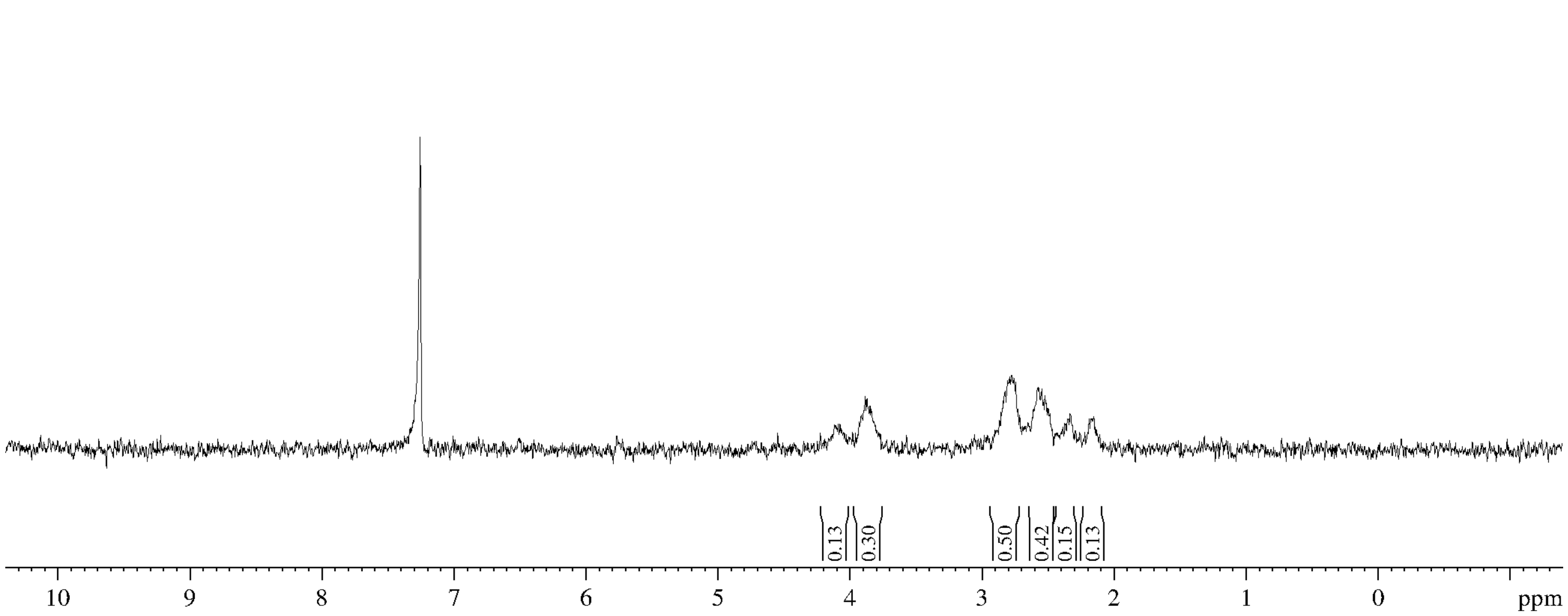
4.09
3.88
2.77
2.57
2.33
2.15
0.13
0.30
0.50
0.42
0.15
0.13
10
9
8
7
6
5
4
3
2
1
0
ppm

3.45.3 ^{13}C NMR

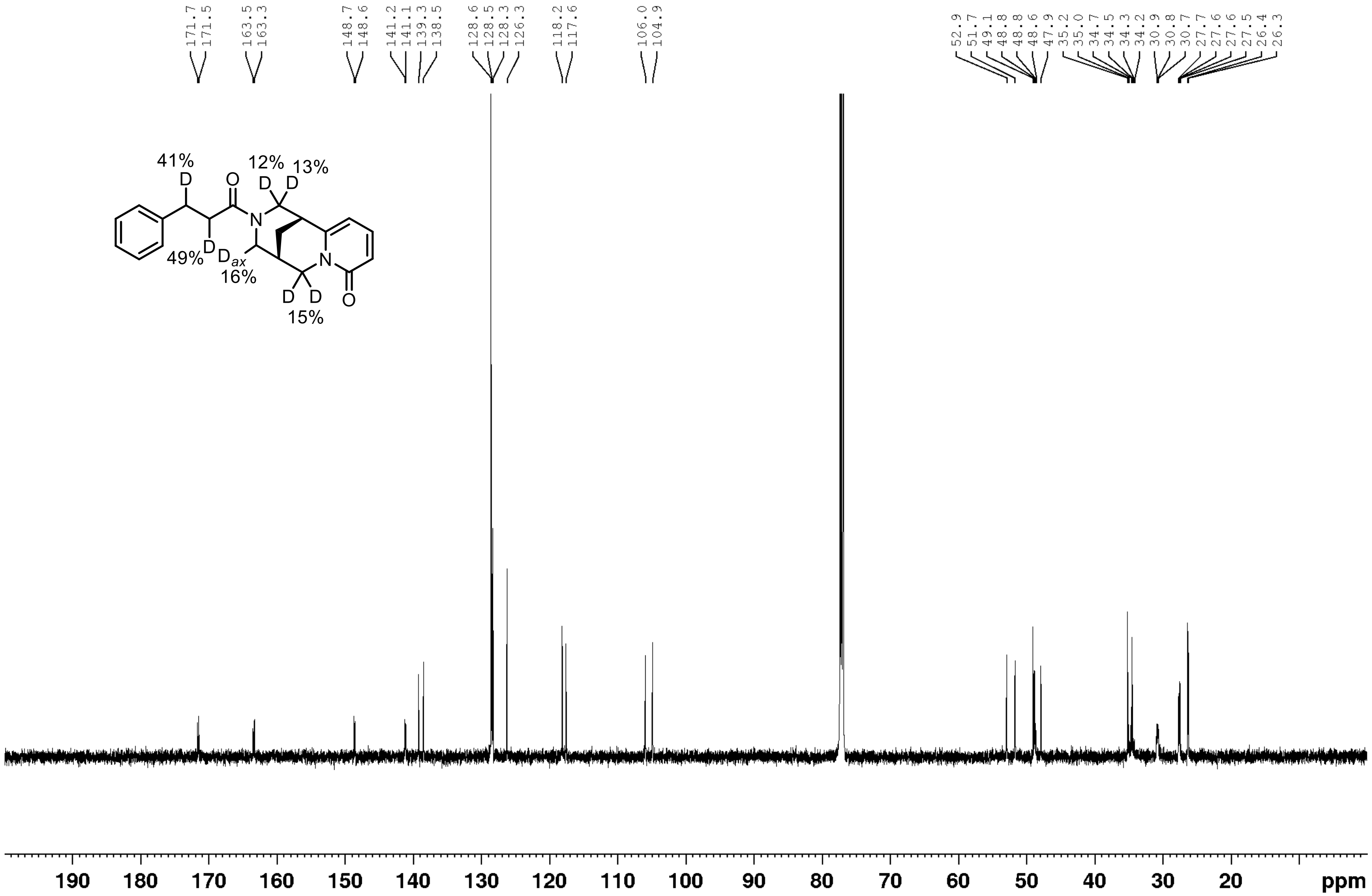
171.7
171.5
163.5
163.3
148.7
148.6
141.2
141.1
139.3
138.5
128.6
128.5
128.3
126.3
118.2
117.6
106.0
104.9
52.9
51.7
49.1
48.8
48.8
48.6
47.9
35.2
35.0
34.7
34.5
34.3
34.2
30.9
30.8
30.7
27.7
27.6
27.6
27.5
26.4
26.3
41%
49%
12%
13%
16%
15%
190
180
170
160
150
140
130
120
110
100
90
80
70
60
50
40
30
20
ppm

3.45.4 ^{1}H, ^{1}H COSY

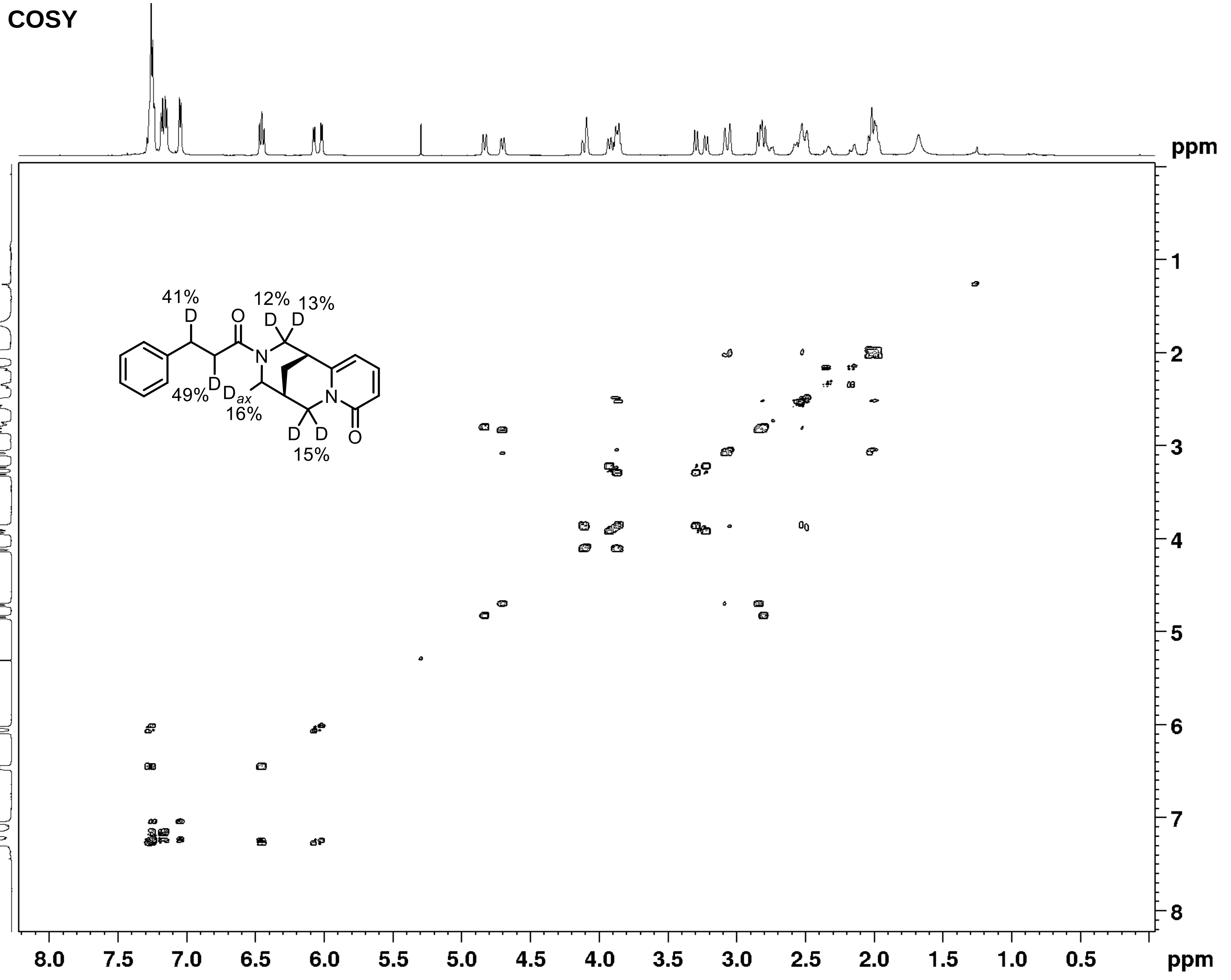

3.45.5 ^{1}H, ^{13}C HMBC

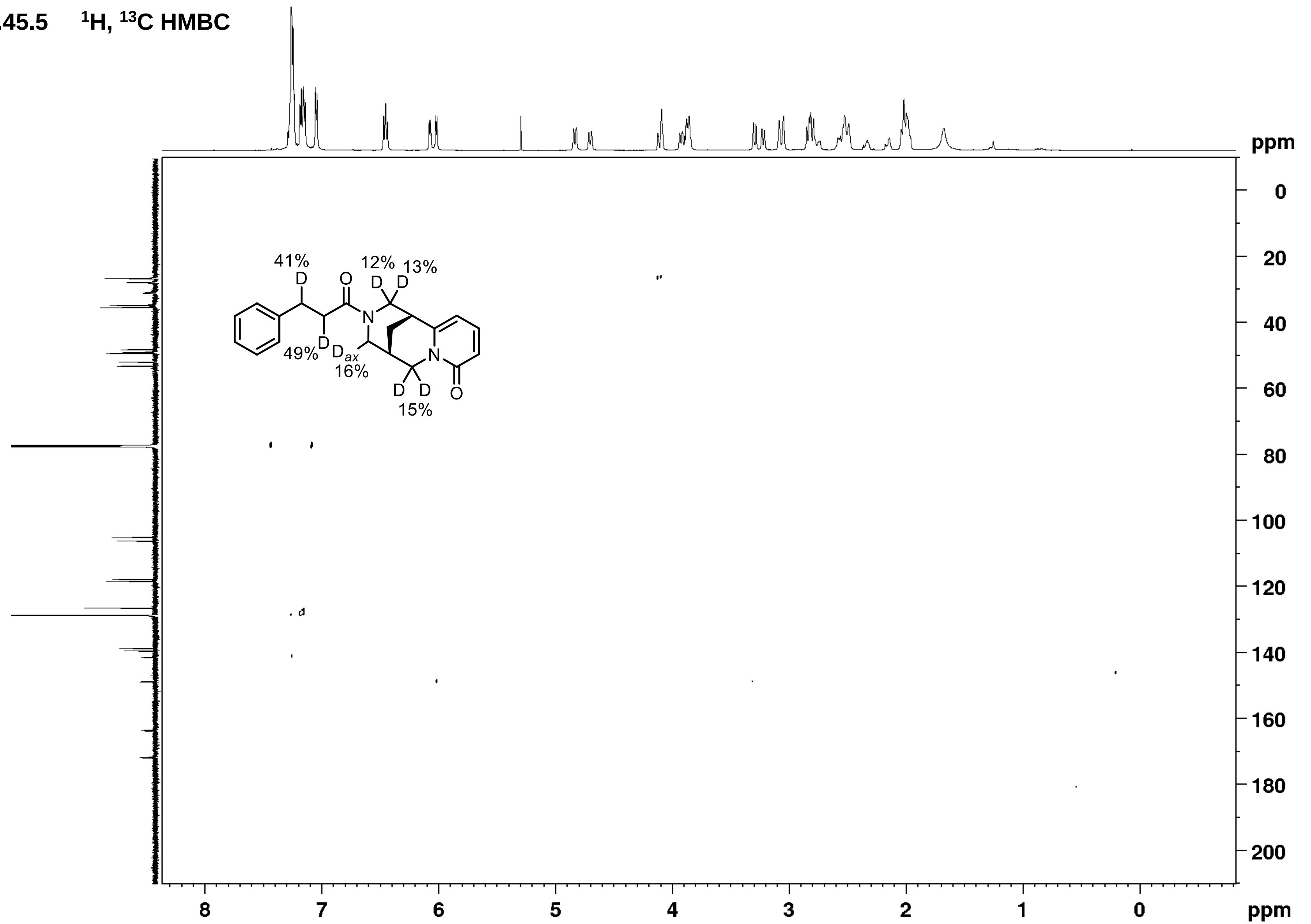

3.45.6 ^{1}H, ^{13}C HSQC

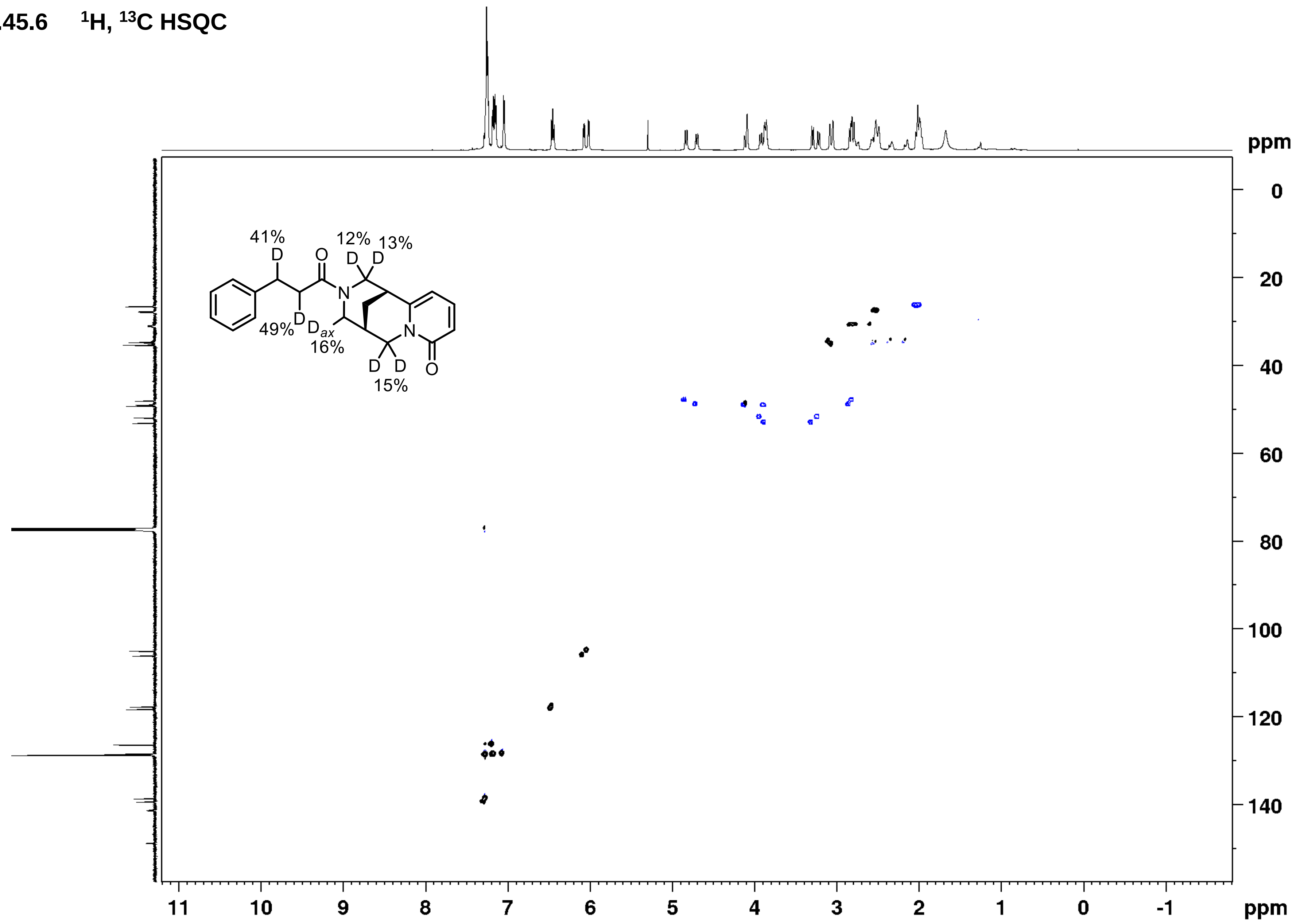

3.45.7 HRMS

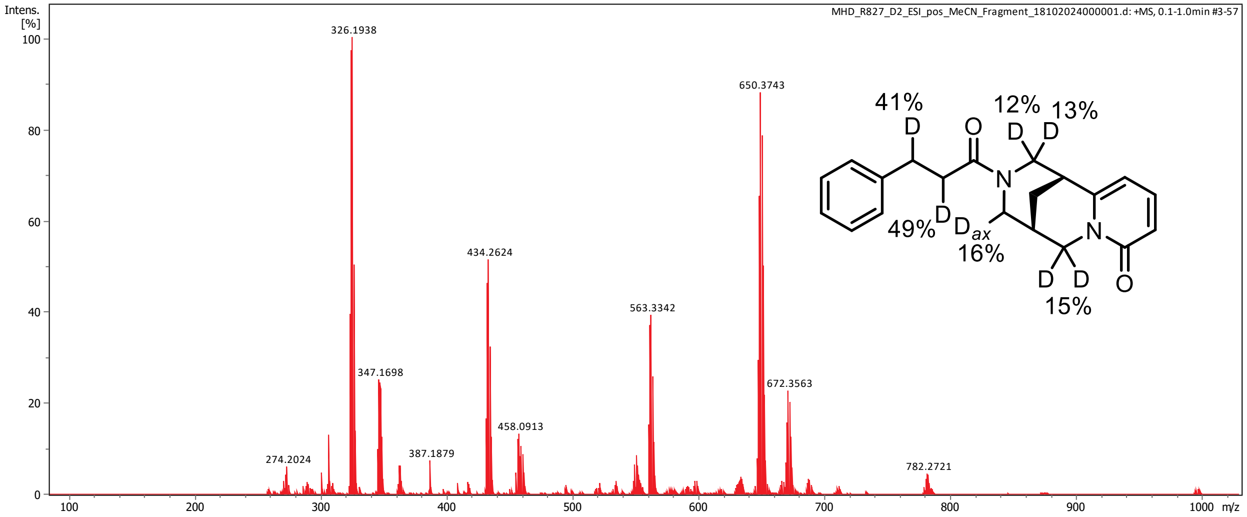

MHD_R827_D2_ESI_pos_MeCN_Fragment_18102024000001.d: +MS, 0.1-1.0min #3-57
Intens. [%]
326.1938
347.1698
274.2024
387.1879
434.2624
458.0913
563.3342
650.3743
672.3563
782.2721
m/z
41%
49%
12%
13%
16%
15%
D
D_ax
N
O

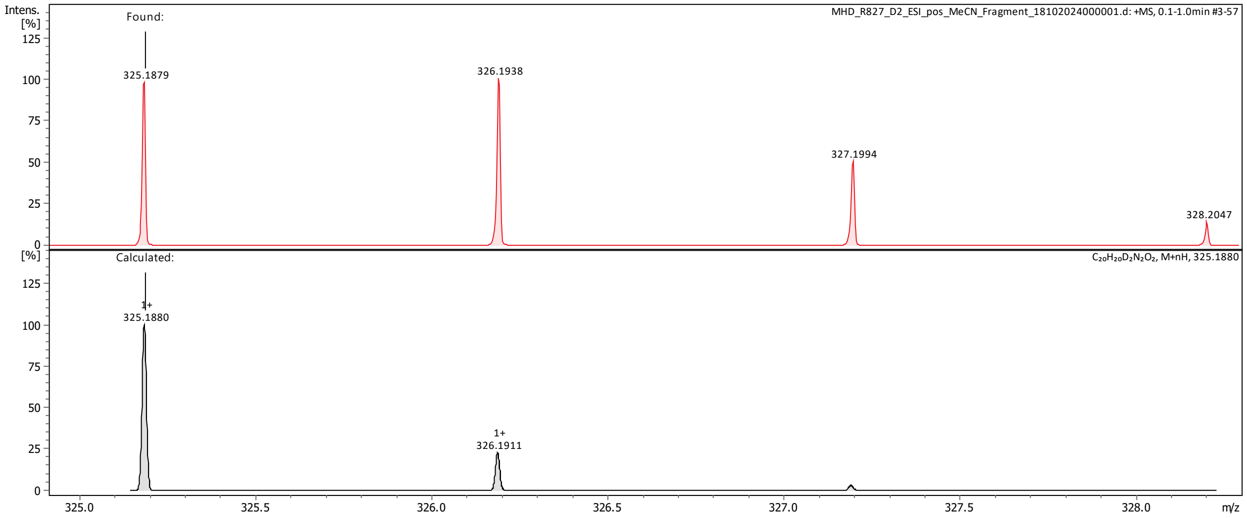

MHD_R827_D2_ESI_pos_MeCN_Fragment_18102024000001.d: +MS, 0.1-1.0min #3-57
Found:
325.1879
326.1938
327.1994
328.2047
Calculated:
C20H20D2N2O2, M+nH, 325.1880
1+
325.1880
1+
326.1911
Intens. [%]
m/z

3.45.8 IR

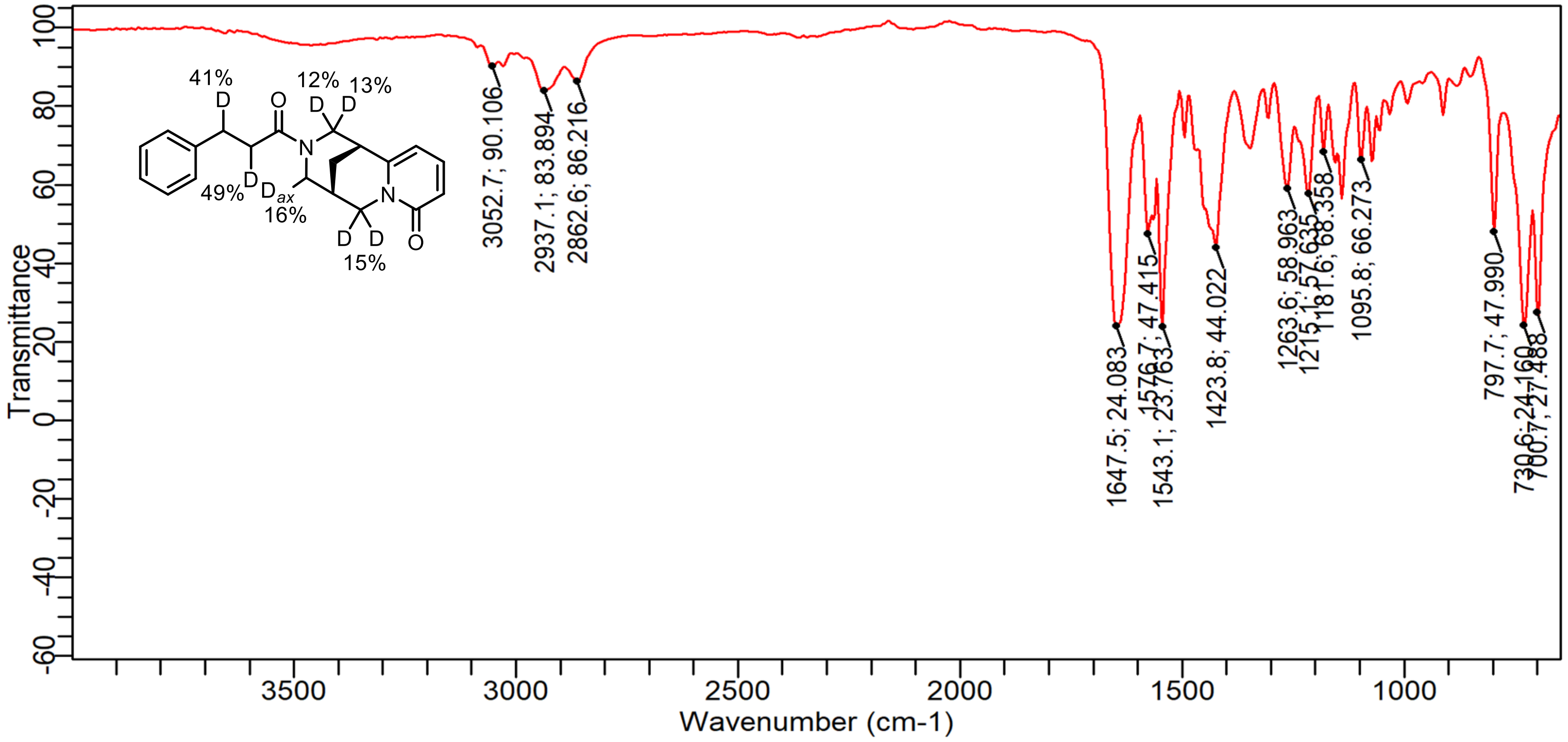

Transmittance
Wavenumber (cm-1)
3052.7; 90.106
2937.1; 83.894
2862.6; 86.216
1647.5; 24.083
1576.7; 47.415
1543.1; 23.763
1423.8; 44.022
1263.6; 58.963
1215.1; 57.635
1181.6; 68.358
1095.8; 66.273
797.7; 47.990
41%
12%
13%
49%
16%
15%

Spectral data of starting materials for alkyne semihydrogenation and conjugate reduction of unsaturated amides

4 Spectral data of alkyne semihydrogenation starting materials

4.1 1-(Benzyloxy)-4-(phenylethynyl)benzene (1b) (600 MHz, $CDCl_3$)

4.1.1 ^{1}H NMR

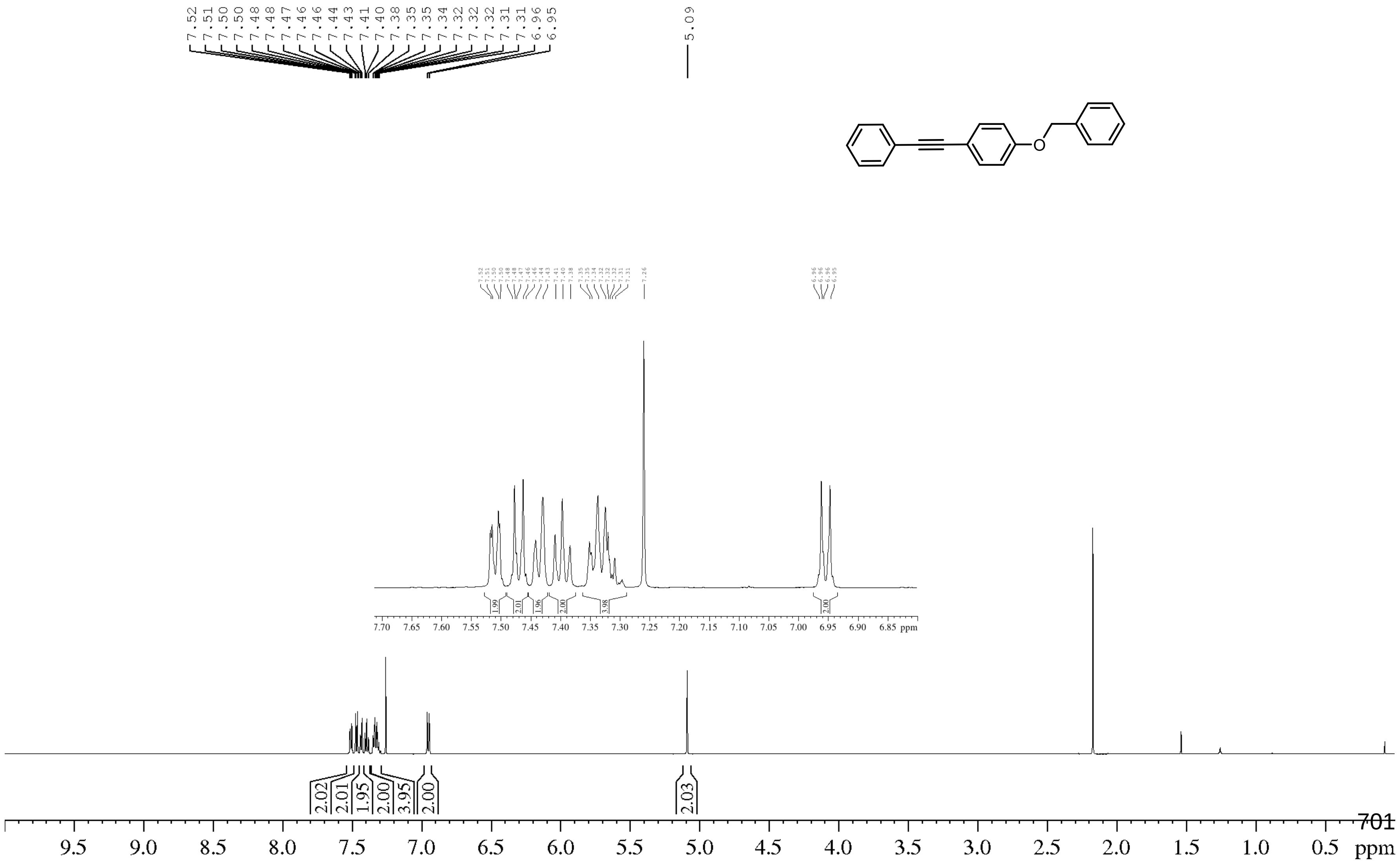

4.1.2 ^{13}C NMR

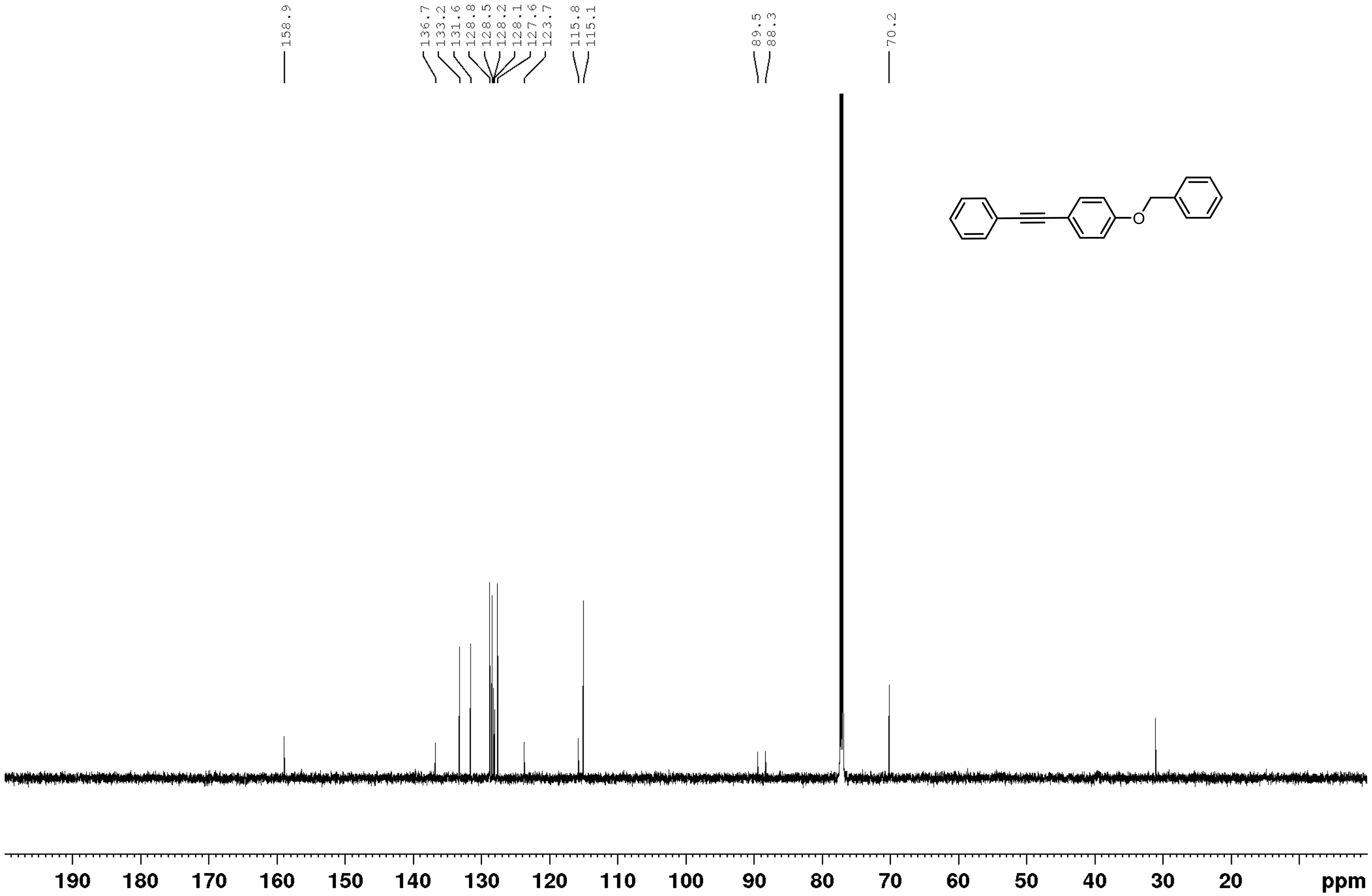
158.9
136.7
133.2
131.6
128.8
128.5
128.2
128.1
127.6
123.7
115.8
115.1
89.5
88.3
70.2
O
190
180
170
160
150
140
130
120
110
100
90
80
70
60
50
40
30
20
ppm

4.1.3 ^1H, ^1H COSY

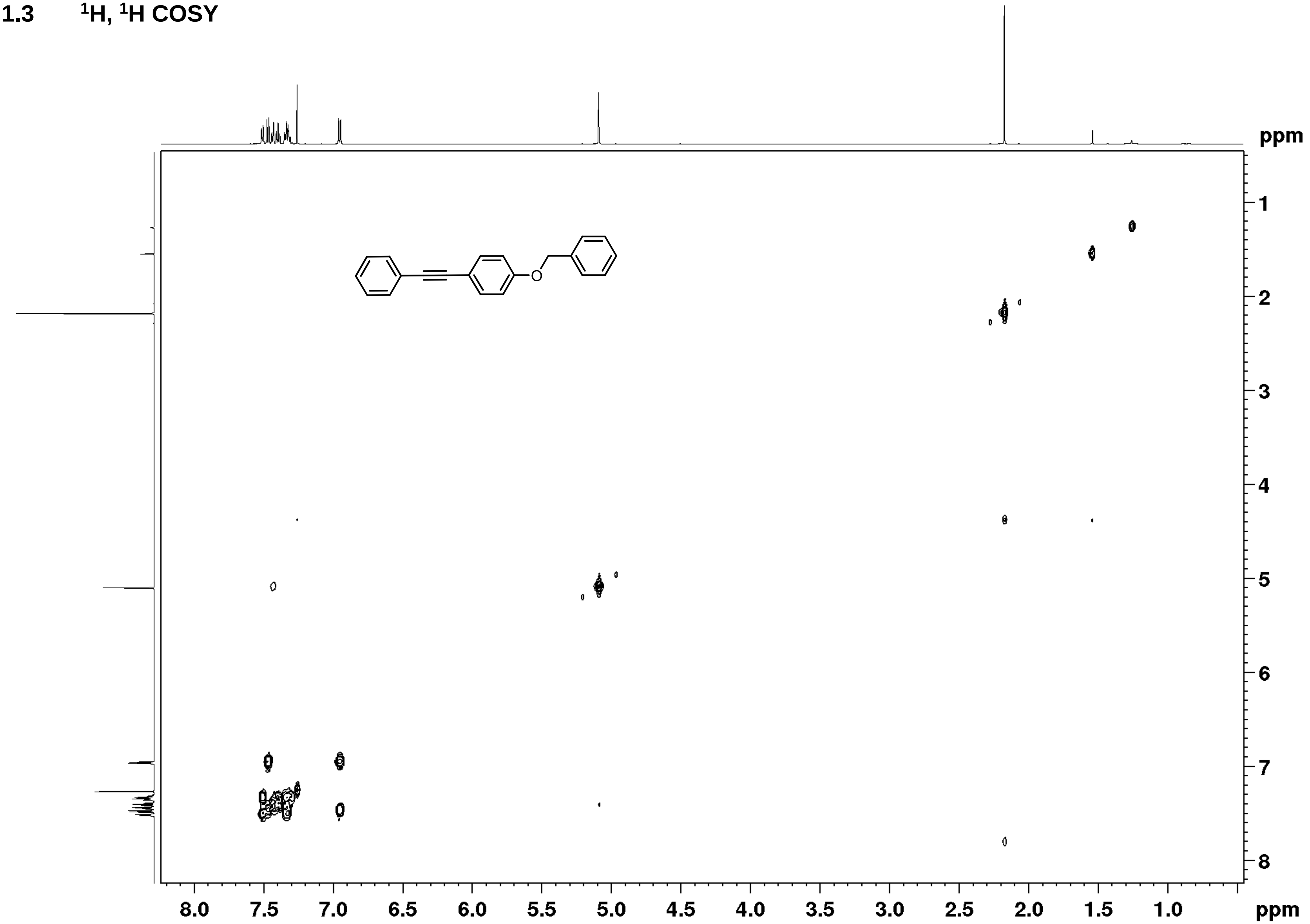

4.1.4 ^{1}H, ^{13}C HMBC

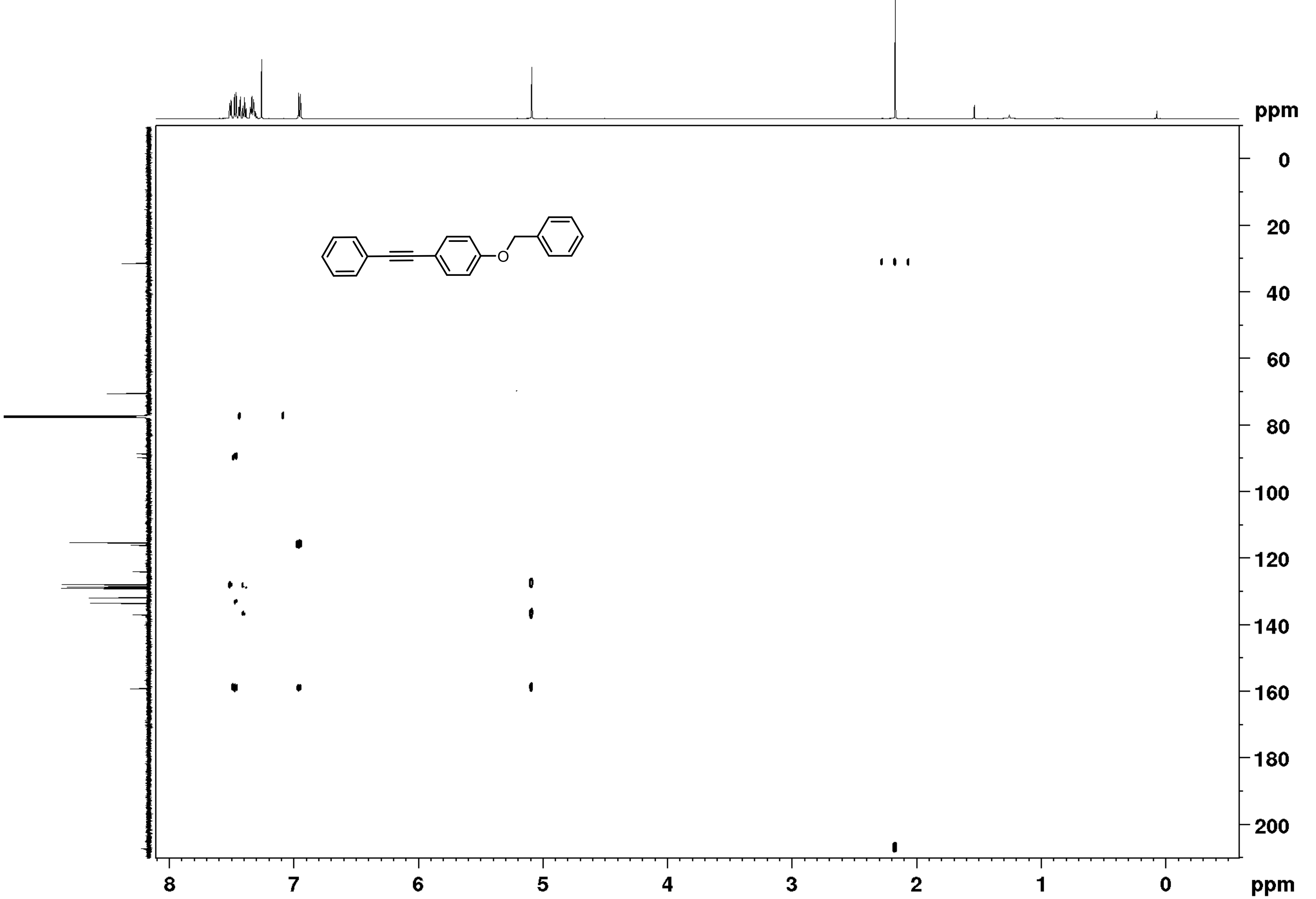

4.1.5 ^{1}H, ^{13}C HSQC

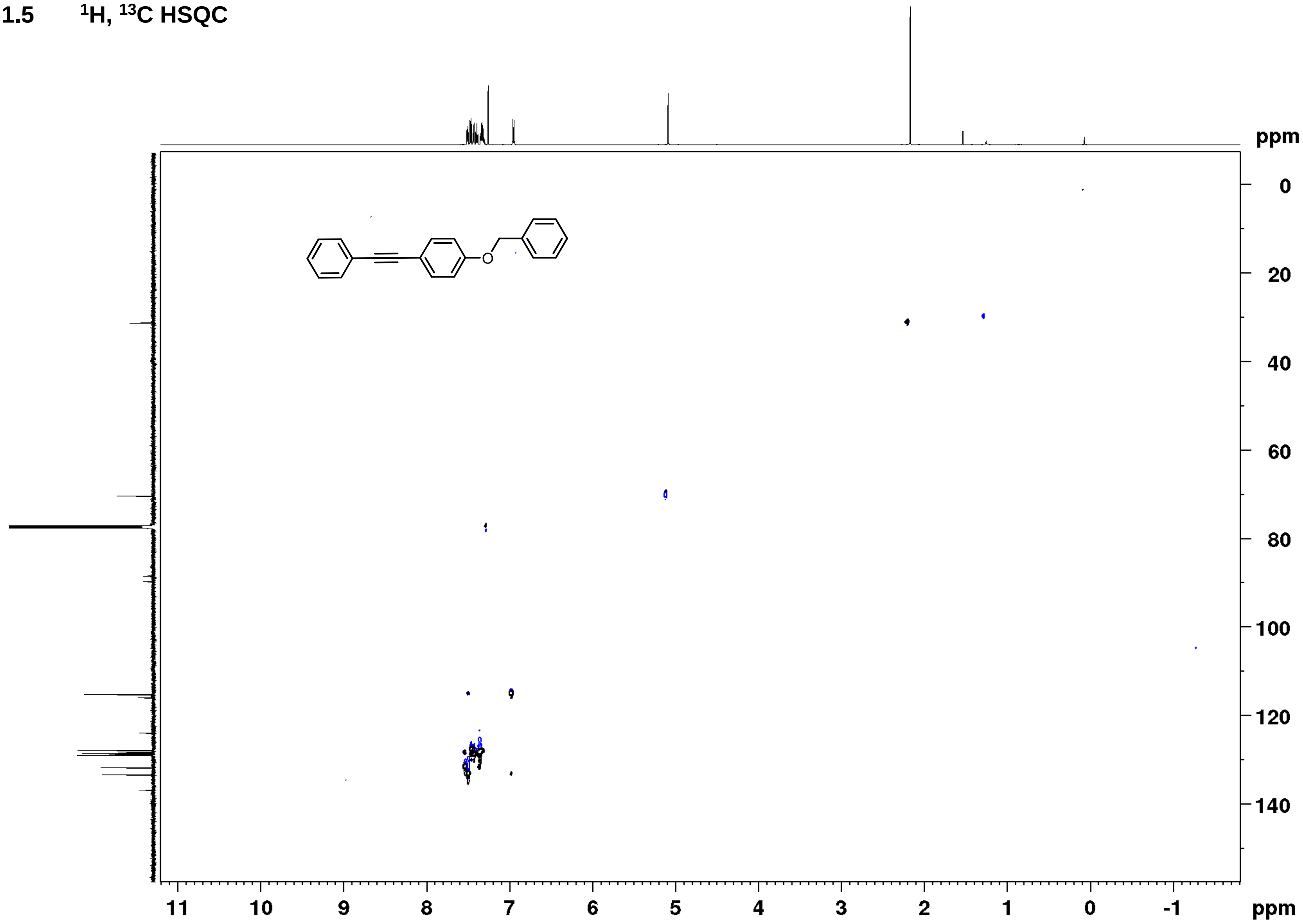

4.1.6 HRMS

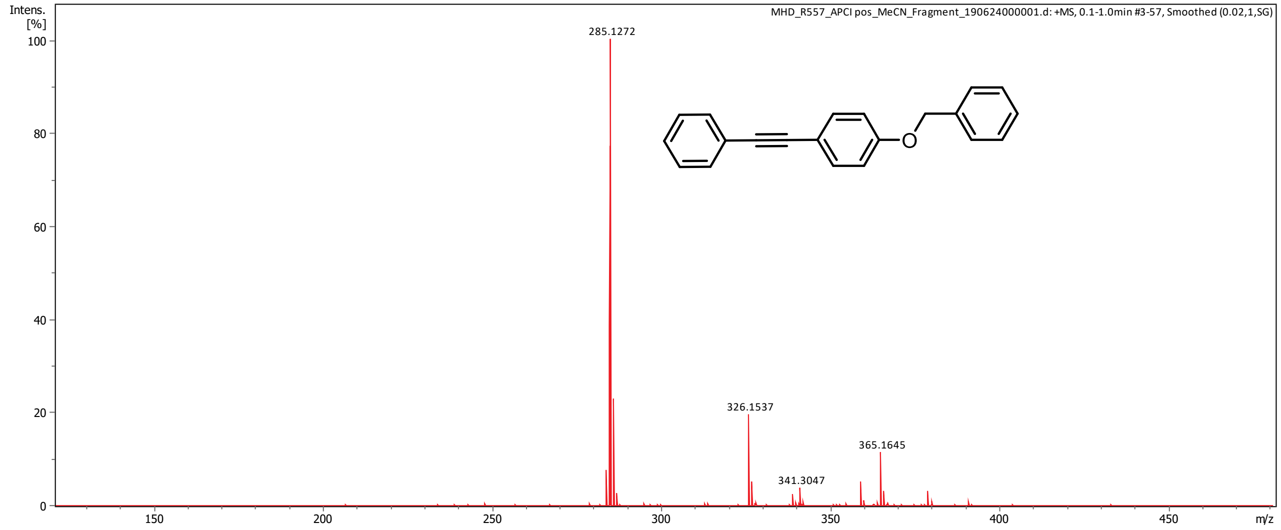

MHD_R557_APCI pos_MeCN_Fragment_190624000001.d: +MS, 0.1-1.0min #3-57, Smoothed (0.02,1,SG)
Intens. [%]
285.1272
326.1537
365.1645
341.3047
O
m/z

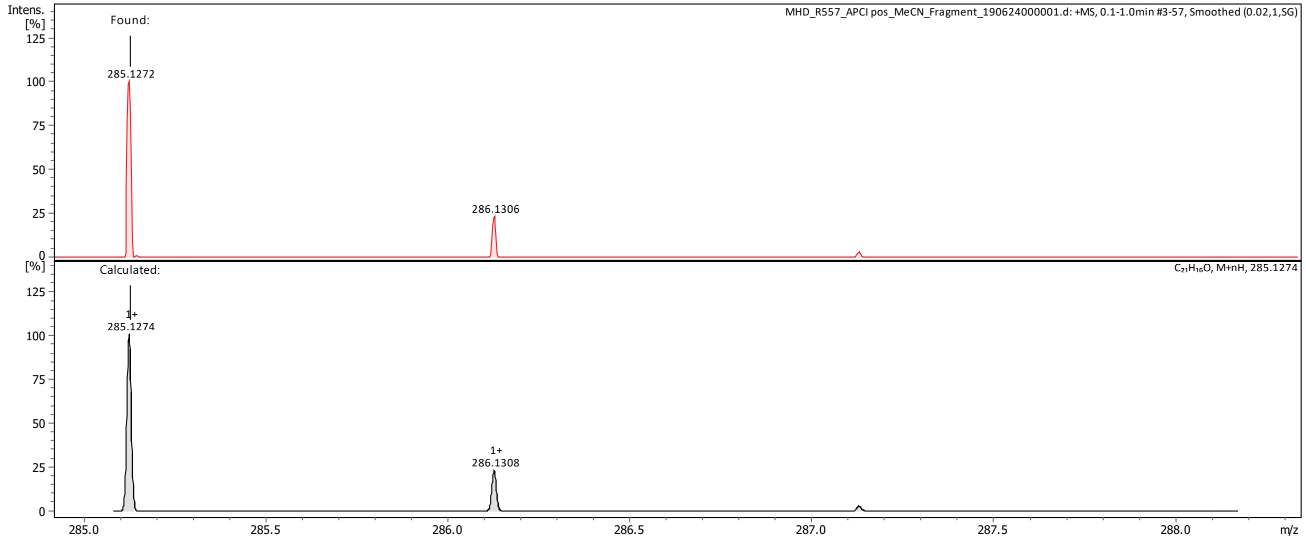

MHD_R557_APCI pos_MeCN_Fragment_190624000001.d: +MS, 0.1-1.0min #3-57, Smoothed (0.02,1,SG)
Intens. [%]
Found:
285.1272
286.1306
Calculated:
$C_{21}H_{16}O$, M+nH, 285.1274
1+
285.1274
1+
286.1308
m/z

4.2 1-Methoxy-4-((4-(trifluoromethyl)phenyl)ethynyl)benzene (1c) (600 MHz, $CDCl_3$)

4.2.1 ^{1}H NMR

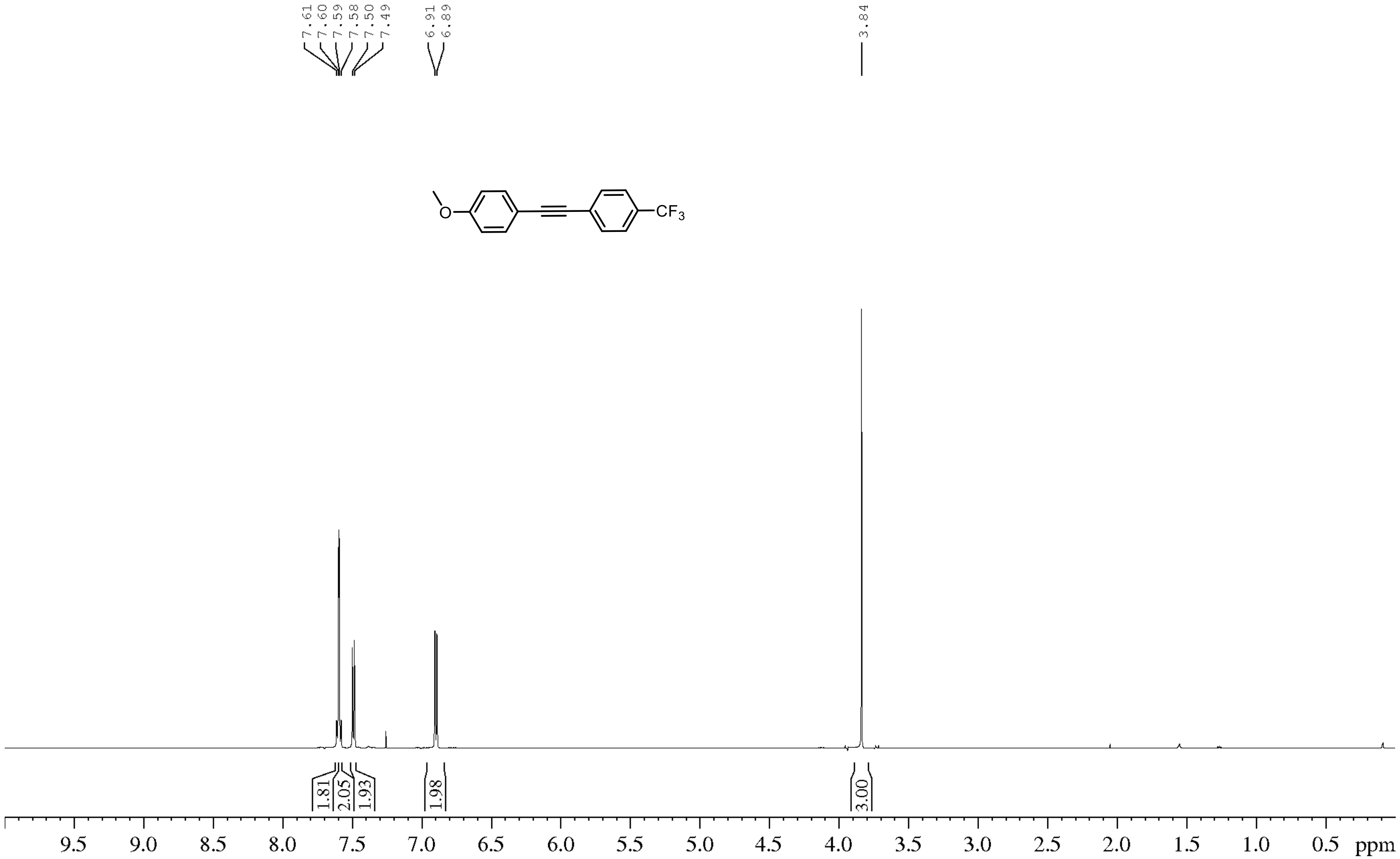

4.2.2 ^{13}C NMR

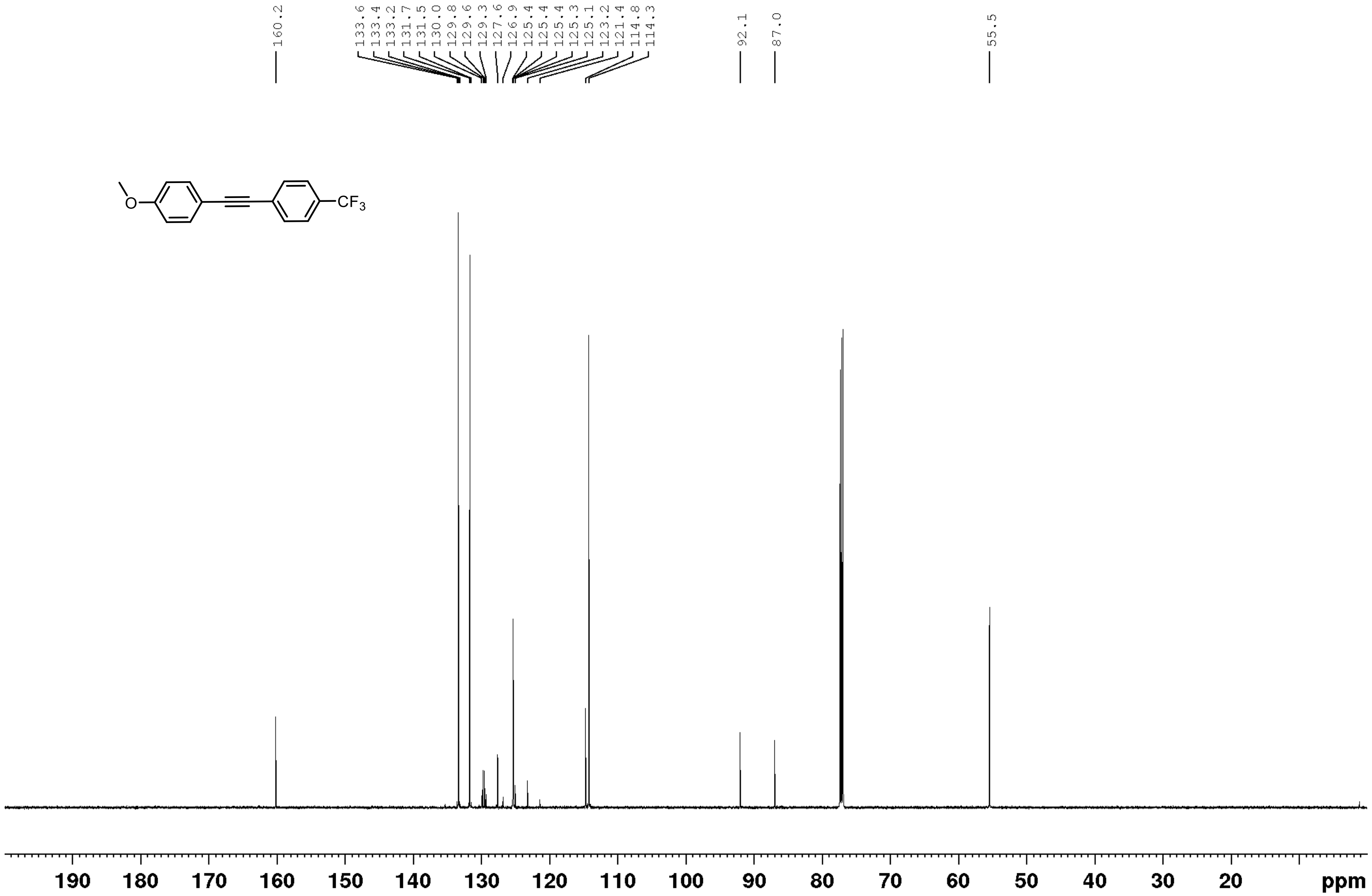

160.2
133.6
133.4
133.2
131.7
131.5
130.0
129.8
129.6
129.3
127.6
126.9
125.4
125.4
125.4
125.3
125.1
123.2
121.4
114.8
114.3
92.1
87.0
55.5
O
CF3
190
180
170
160
150
140
130
120
110
100
90
80
70
60
50
40
30
20
ppm

4.2.3 ^{1}H, ^{1}H COSY

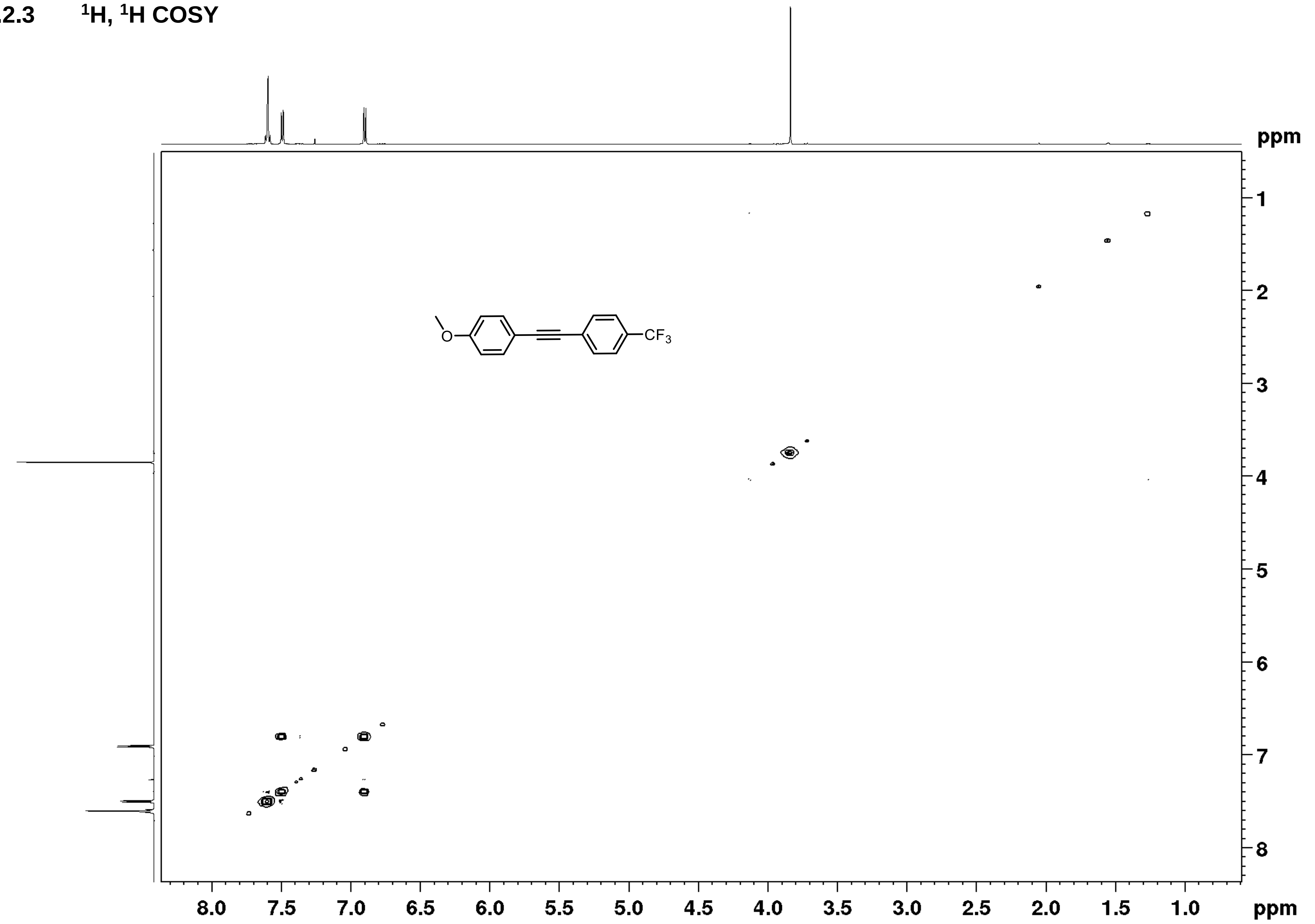

4.2.4 ^{1}H, ^{13}C HMBC

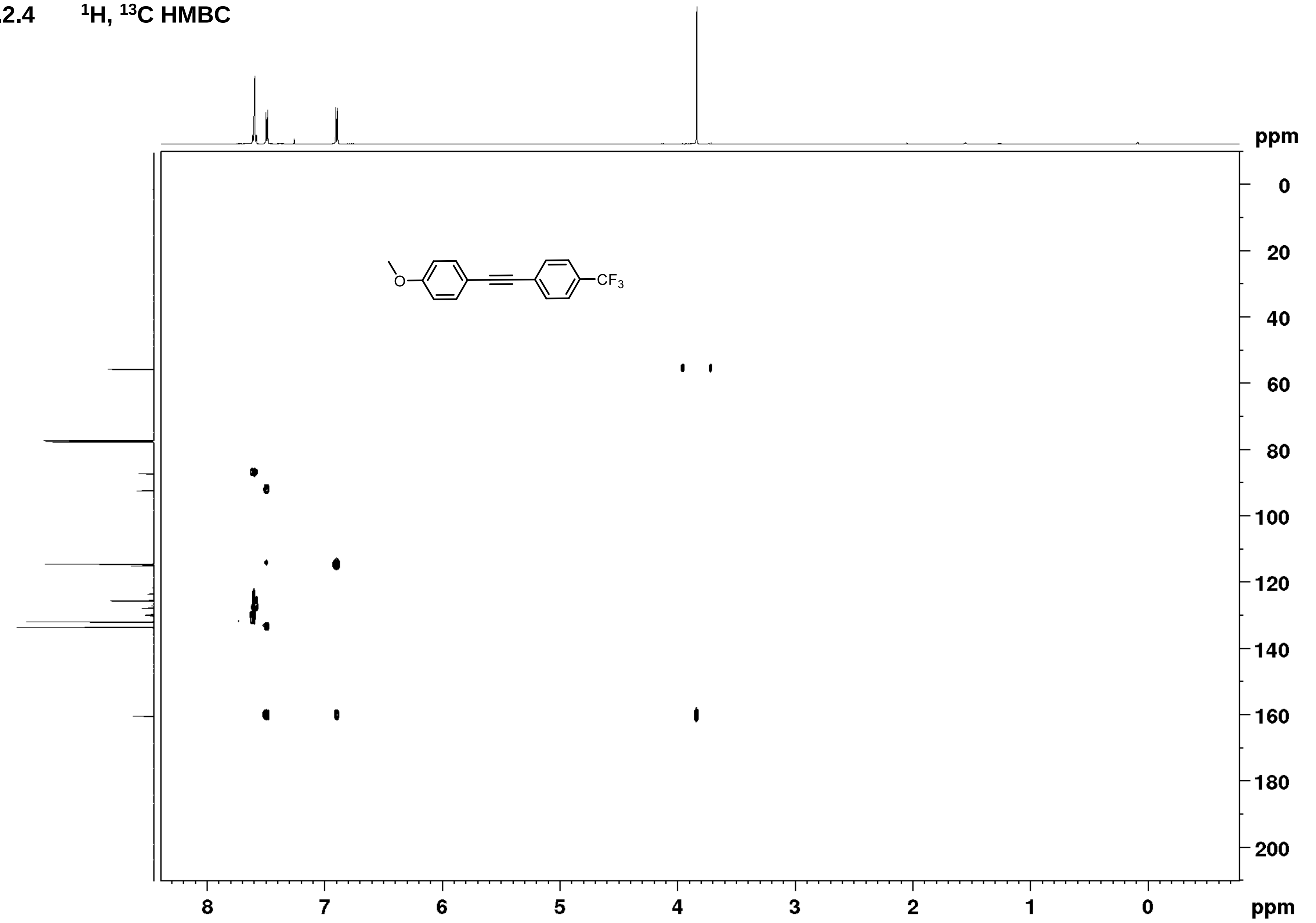

4.2.5 ¹H, ¹³C HSQC

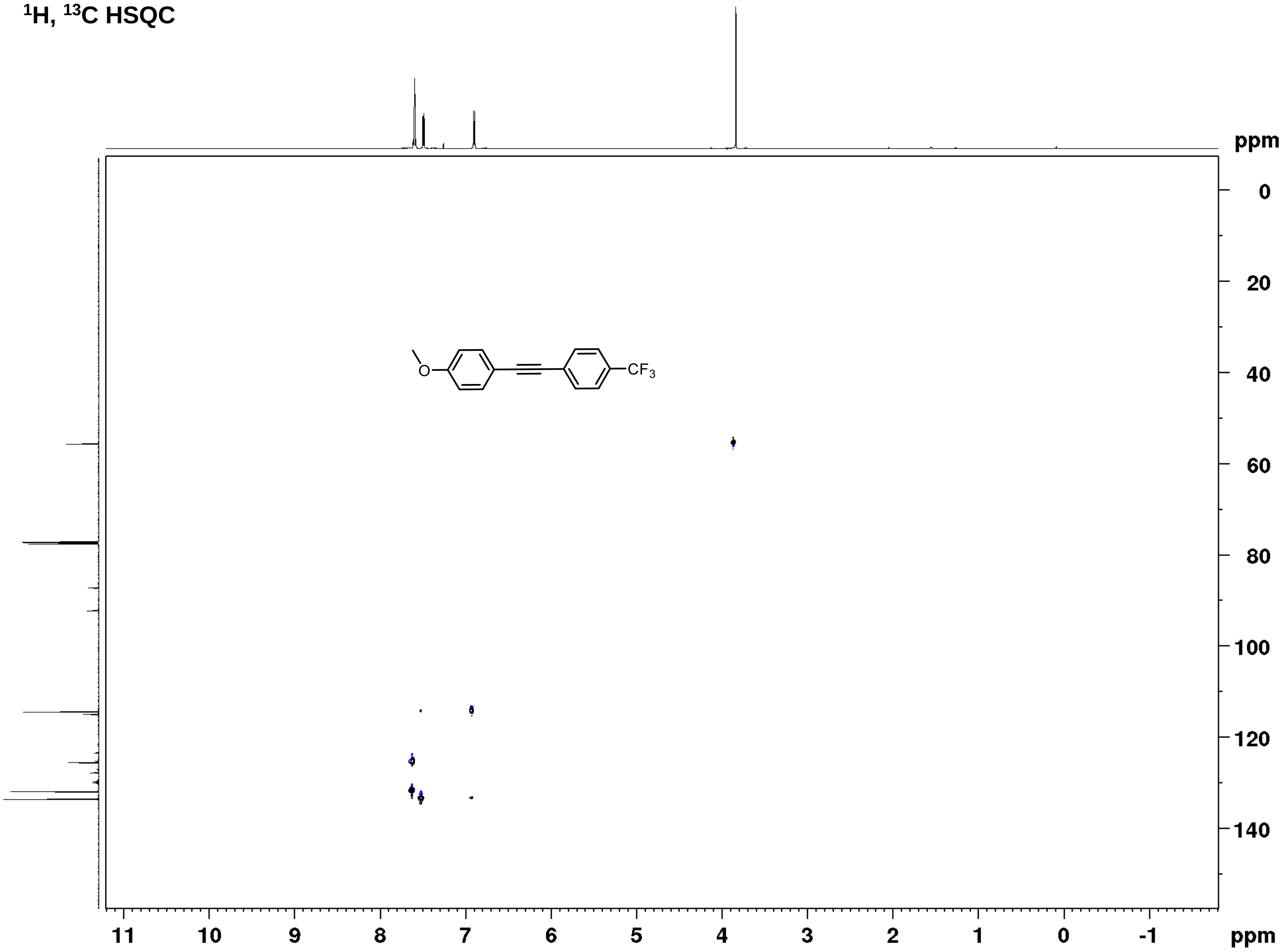

4.2.6 ^{19}F

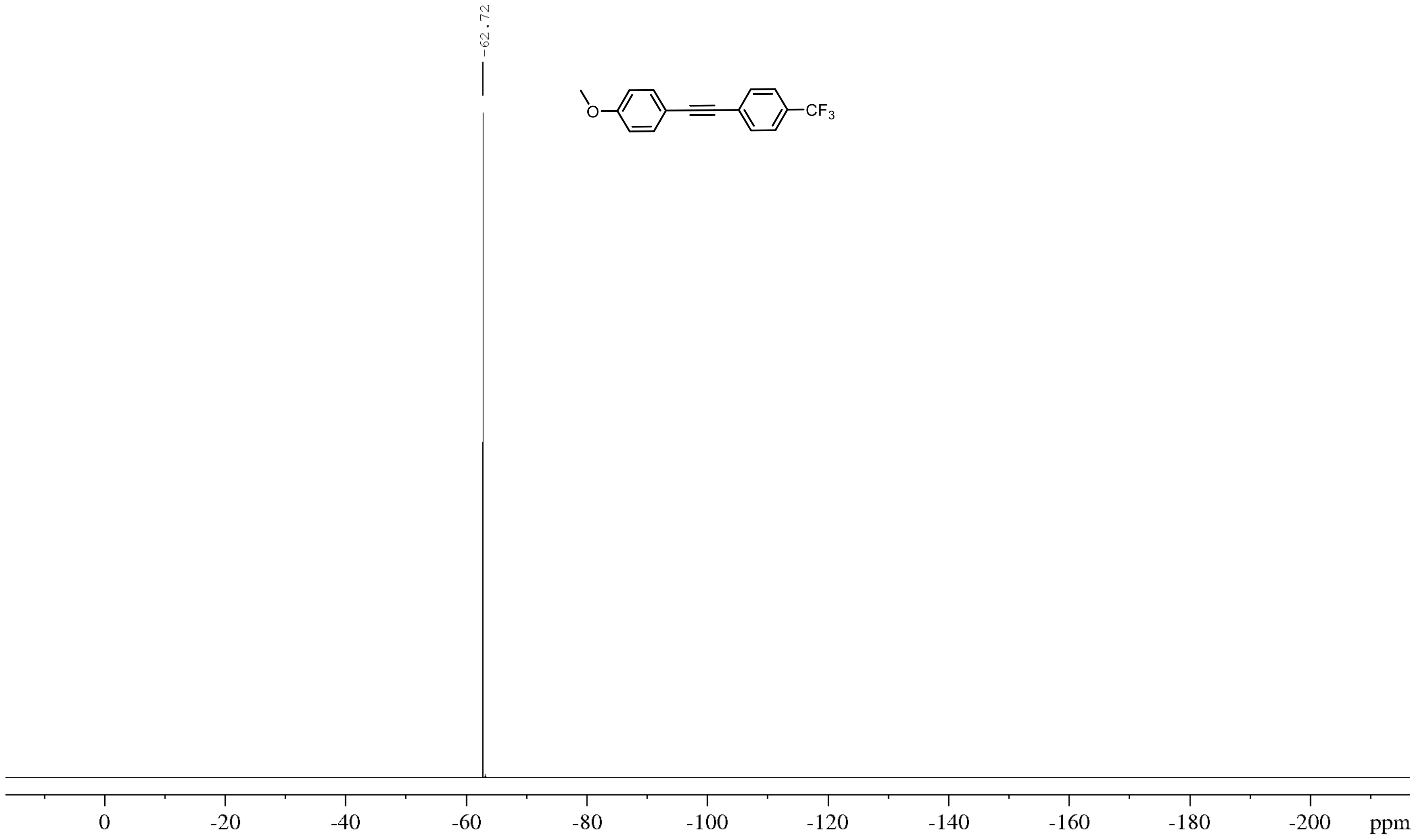

4.2.7 HRMS

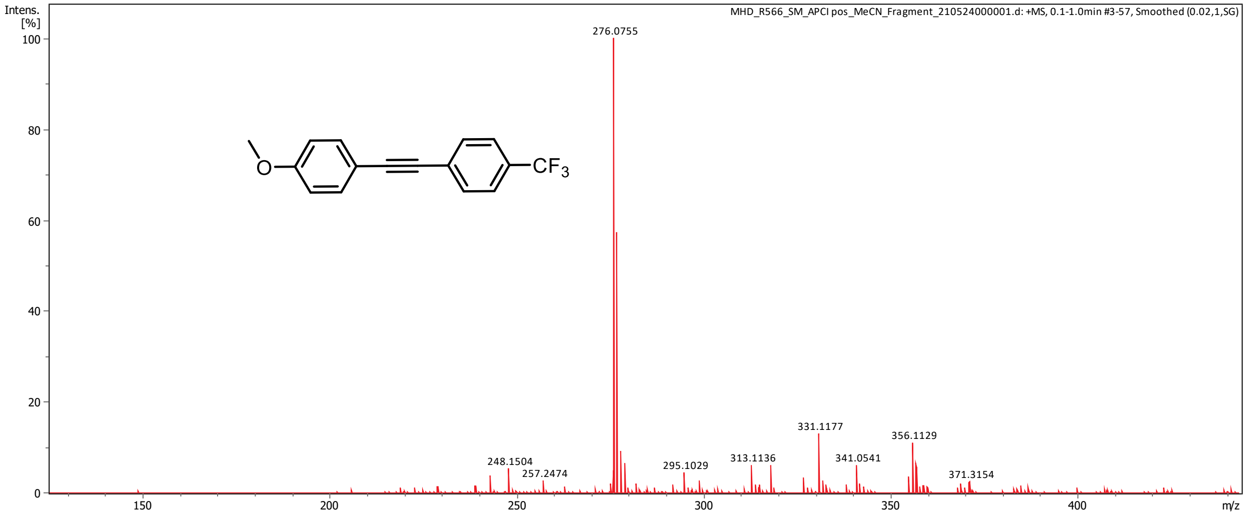

Intens. [%]
MHD_R566_SM_APCI pos_MeCN_Fragment_210524000001.d: +MS, 0.1-1.0min #3-57, Smoothed (0.02,1,SG)
276.0755
248.1504
257.2474
295.1029
313.1136
331.1177
341.0541
356.1129
371.3154
CF3
O
m/z

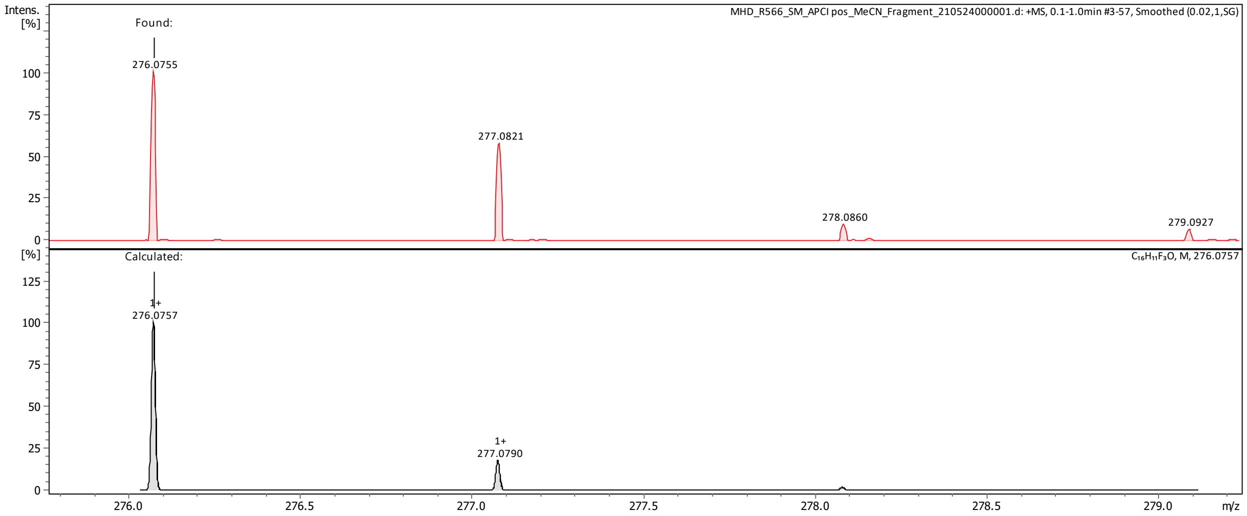

Intens. [%]
MHD_R566_SM_APCI pos_MeCN_Fragment_210524000001.d: +MS, 0.1-1.0min #3-57, Smoothed (0.02,1,SG)
Found:
276.0755
277.0821
278.0860
279.0927
Calculated:
C16H11F3O, M, 276.0757
1+
276.0757
1+
277.0790
m/z

4.3 4-((4-Methoxyphenyl)ethynyl)benzonitrile (1d) (600 MHz, $CDCl_3$)

4.3.1 ¹H NMR

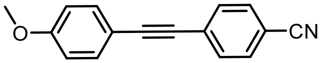

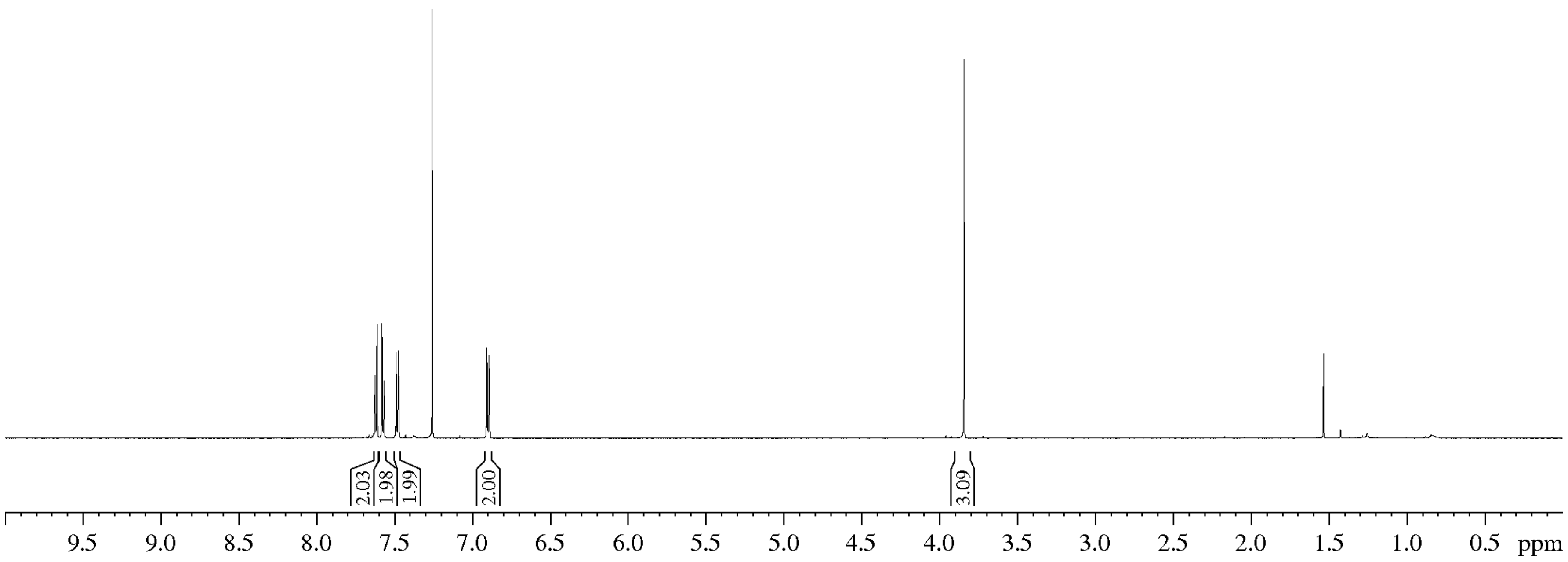

4.3.2 ^{13}C NMR

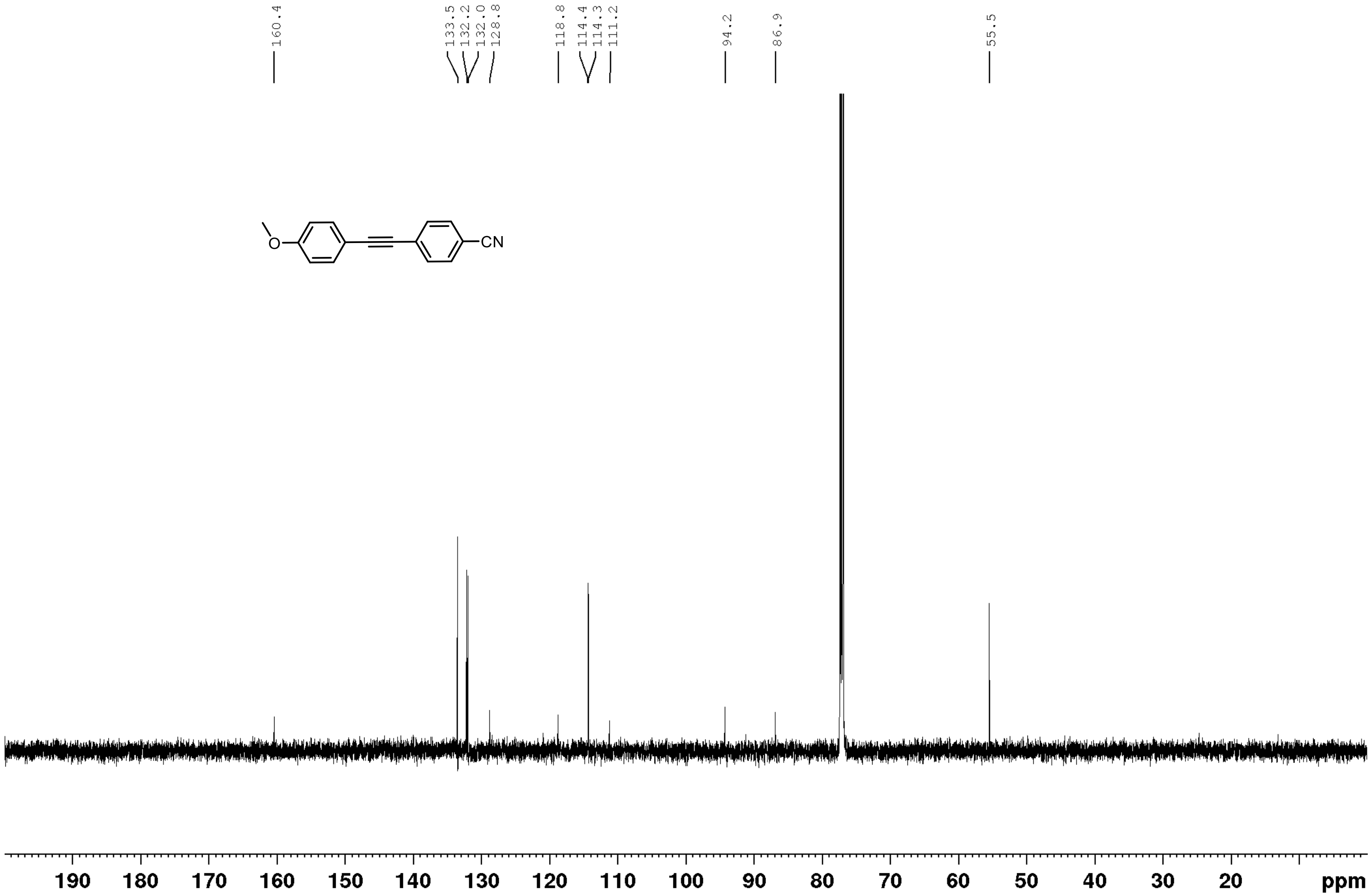

160.4
133.5
132.2
132.0
128.8
118.8
114.4
114.3
111.2
94.2
86.9
55.5
O
CN
190
180
170
160
150
140
130
120
110
100
90
80
70
60
50
40
30
20
ppm

4.3.3 ¹H, ¹H COSY

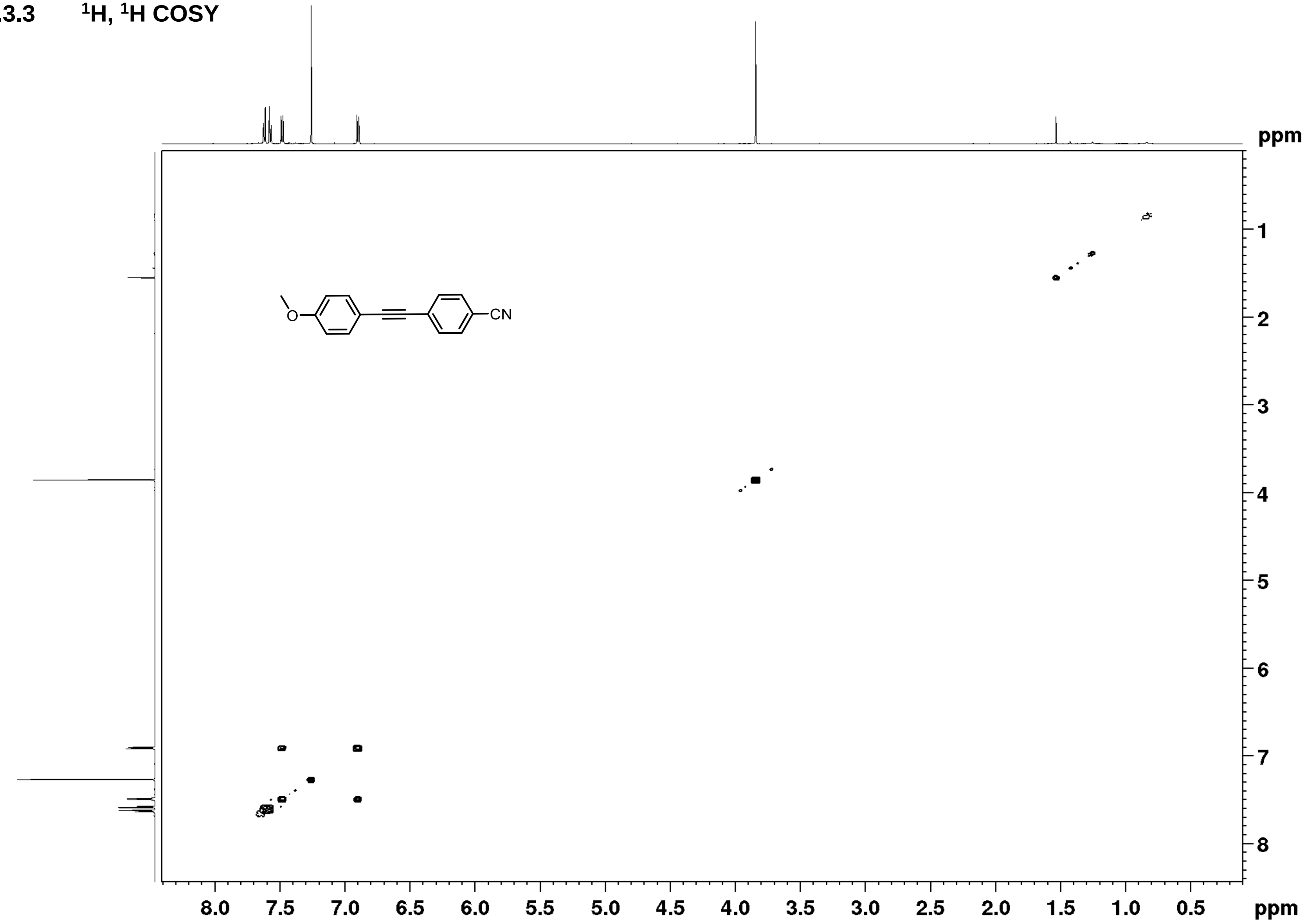

4.3.4 ^{1}H, ^{13}C HMBC

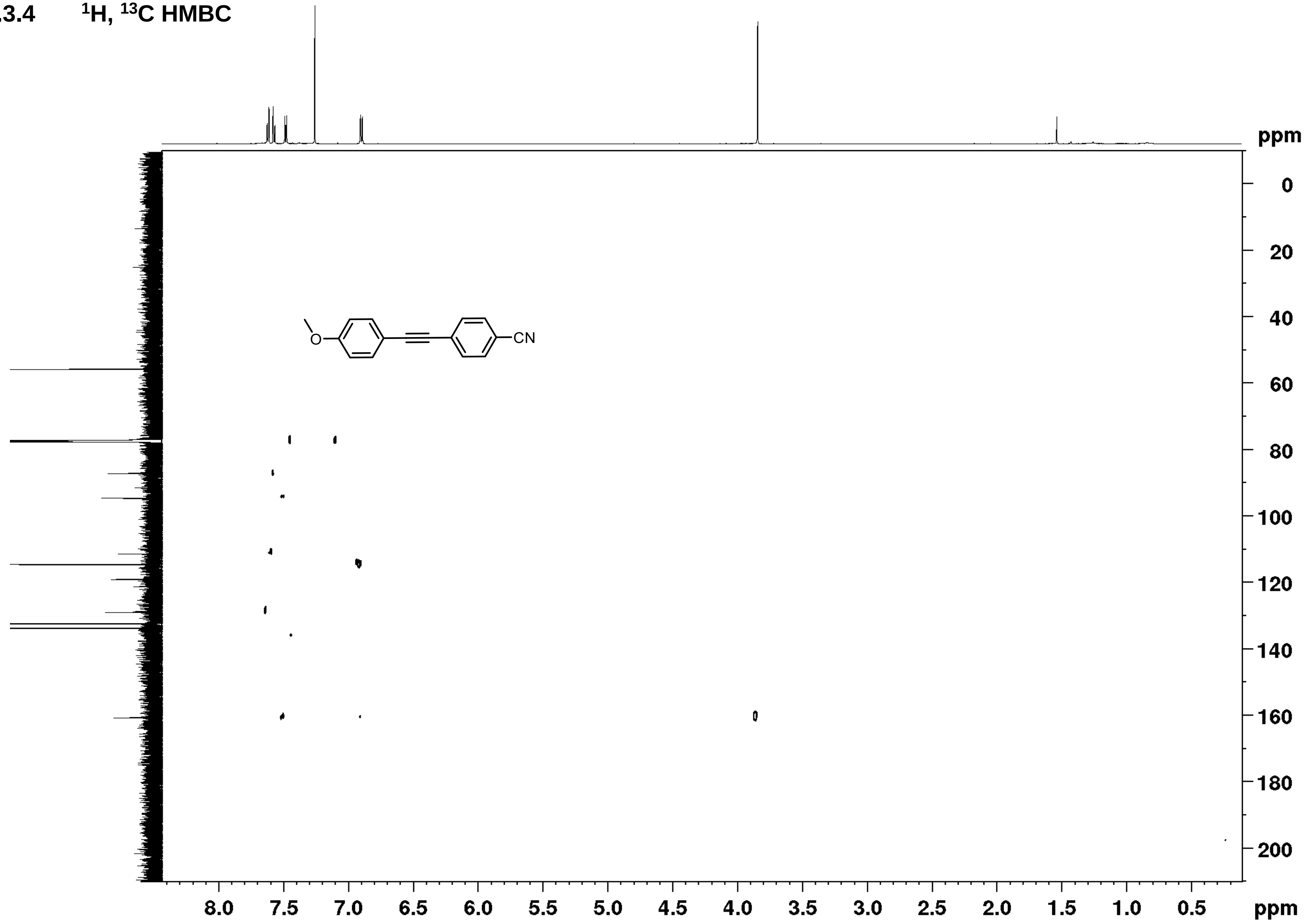

4.3.5 ^{1}H, ^{13}C HSQC

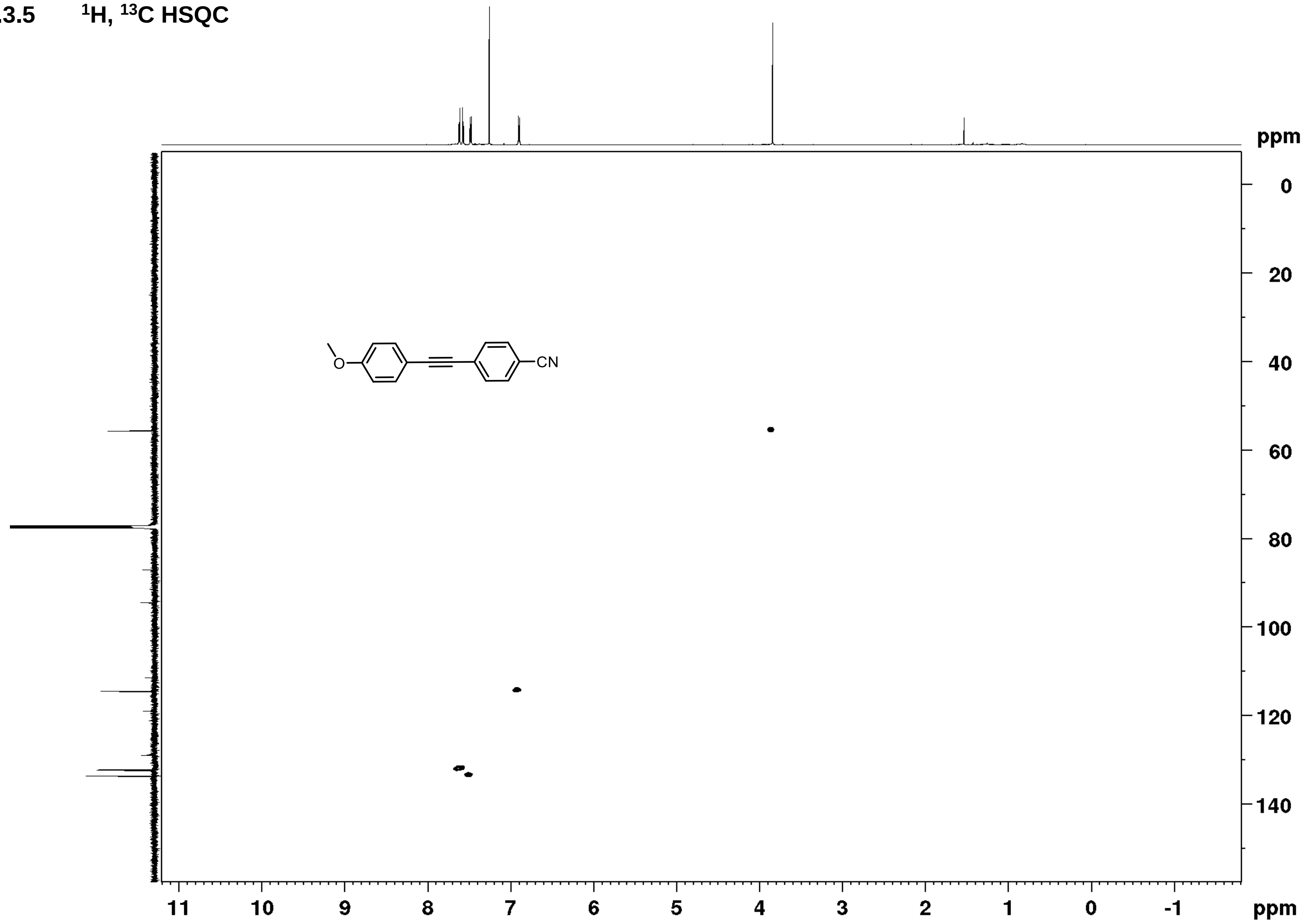

4.3.6 HRMS

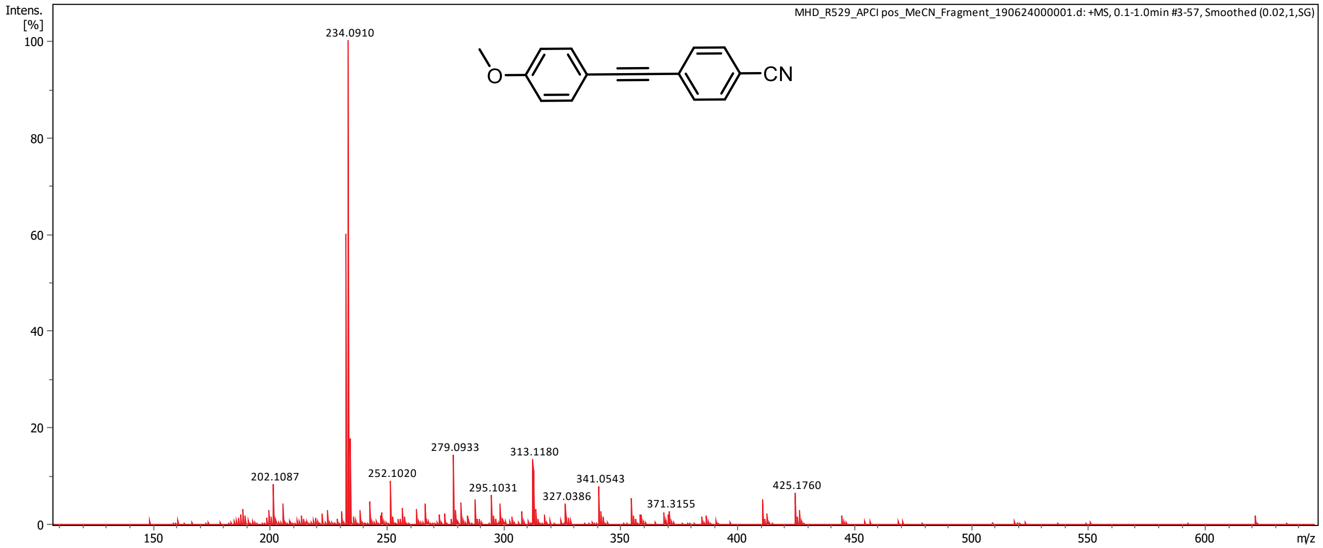

MHD_R529_APCI pos_MeCN_Fragment_190624000001.d: +MS, 0.1-1.0min #3-57, Smoothed (0.02,1,SG)
Intens. [%]
234.0910
202.1087
252.1020
279.0933
295.1031
313.1180
327.0386
341.0543
371.3155
425.1760
CN
O
m/z

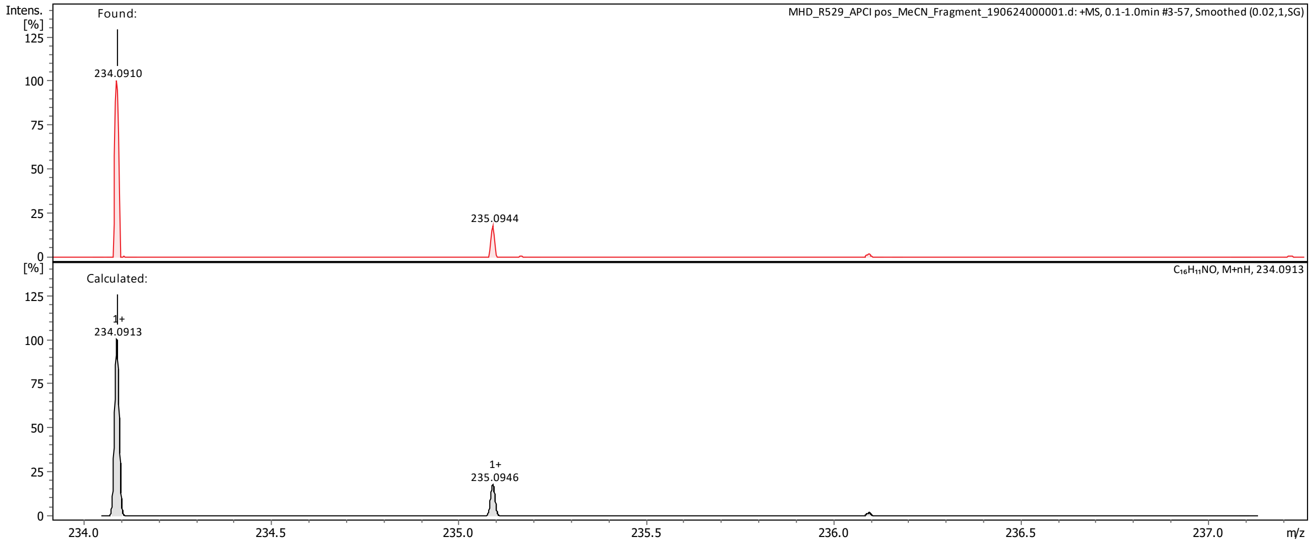

Found:
MHD_R529_APCI pos_MeCN_Fragment_190624000001.d: +MS, 0.1-1.0min #3-57, Smoothed (0.02,1,SG)
234.0910
235.0944
Calculated:
C16H11NO, M+nH, 234.0913
1+
234.0913
1+
235.0946
Intens. [%]
m/z

4.4 1-Fluoro-4-(phenylethynyl)benzene (1e) (600 MHz, $CDCl_3$)

4.4.1 ^{1}H NMR

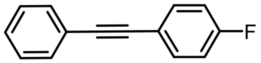

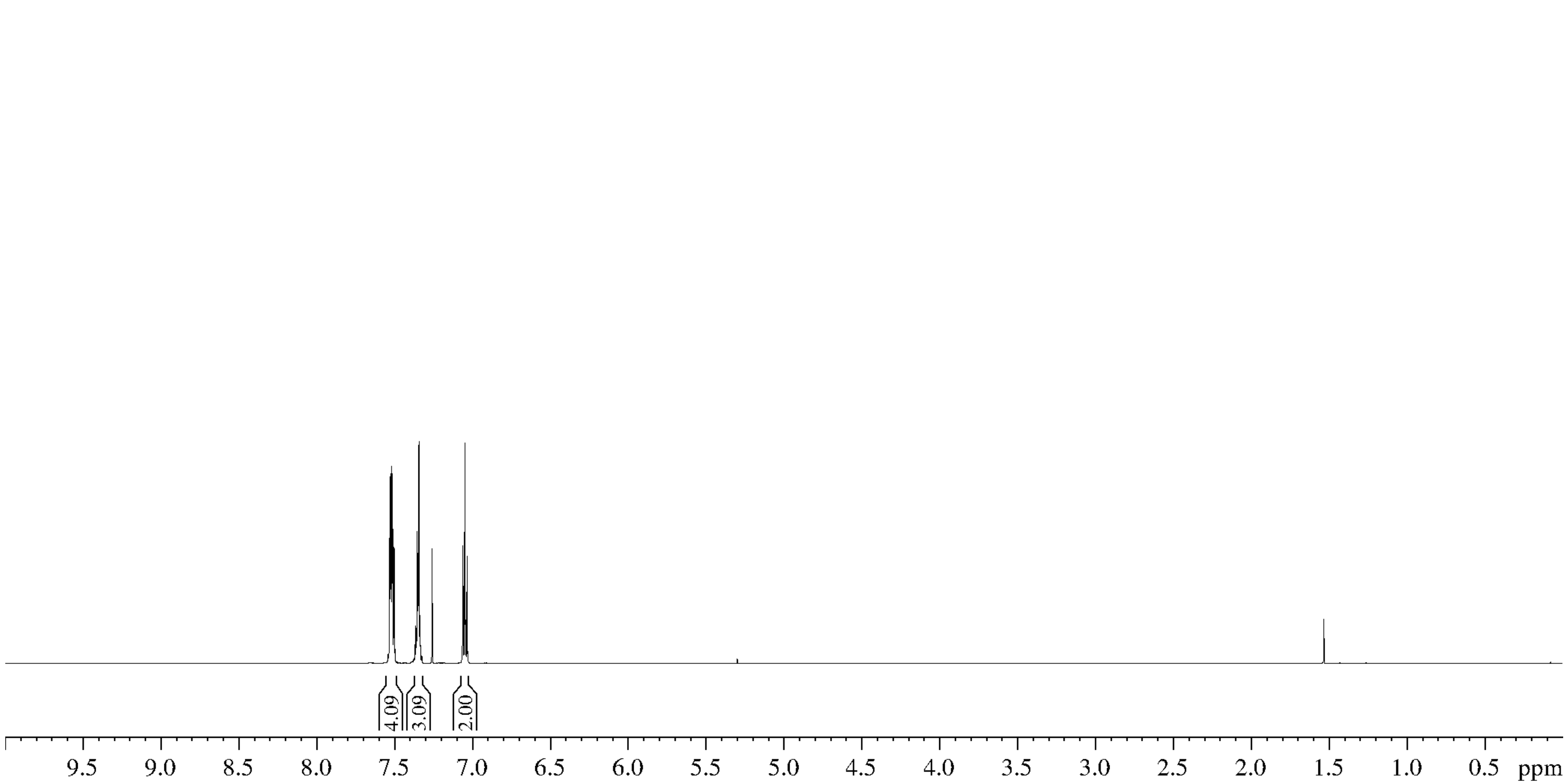

4.4.2 ^{13}C NMR

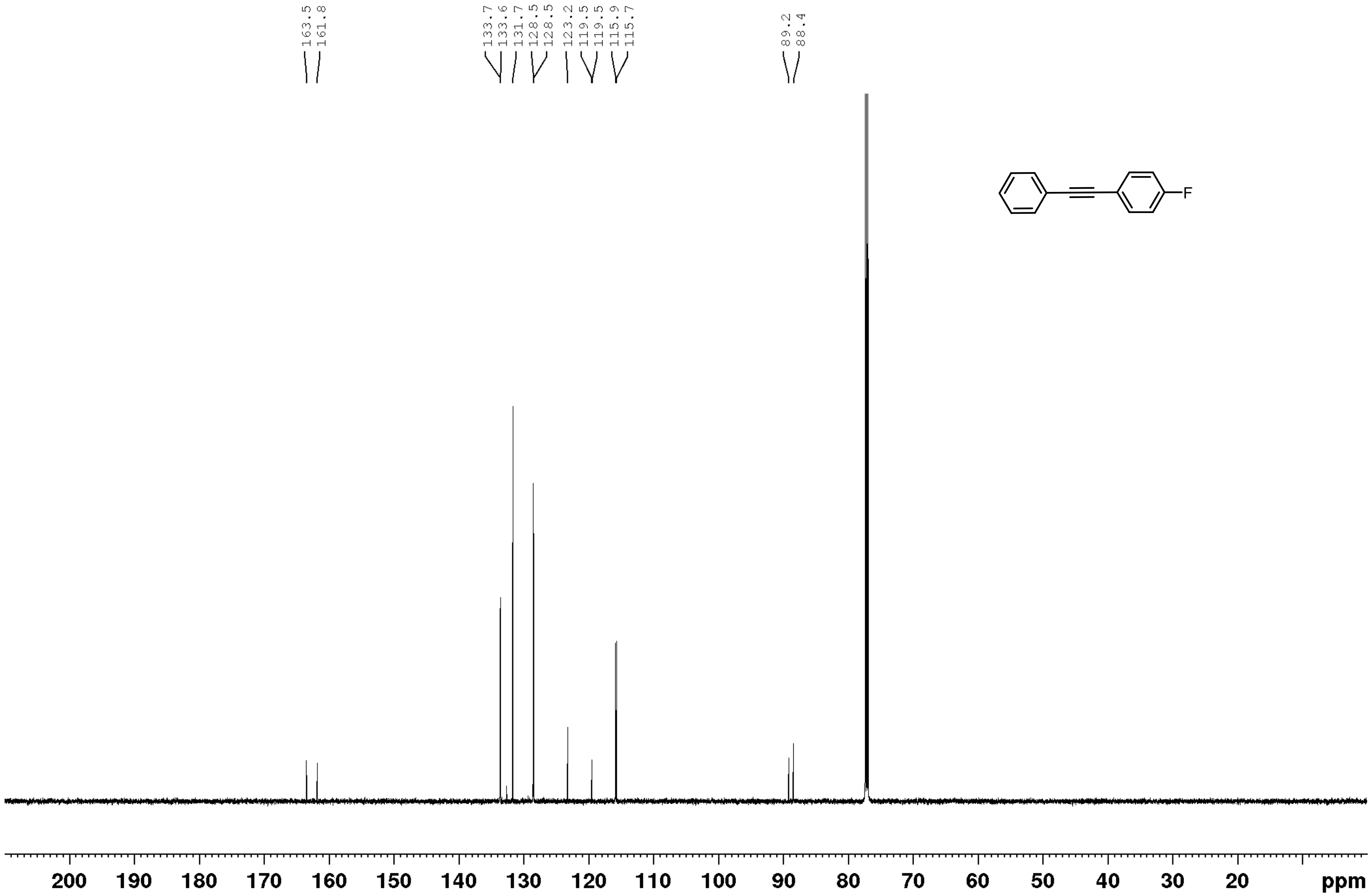
163.5
161.8
133.7
133.6
131.7
128.5
128.5
123.2
119.5
119.5
115.9
115.7
89.2
88.4
F
200 190 180 170 160 150 140 130 120 110 100 90 80 70 60 50 40 30 20 ppm

4.4.3 ^{1}H, ^{1}H COSY

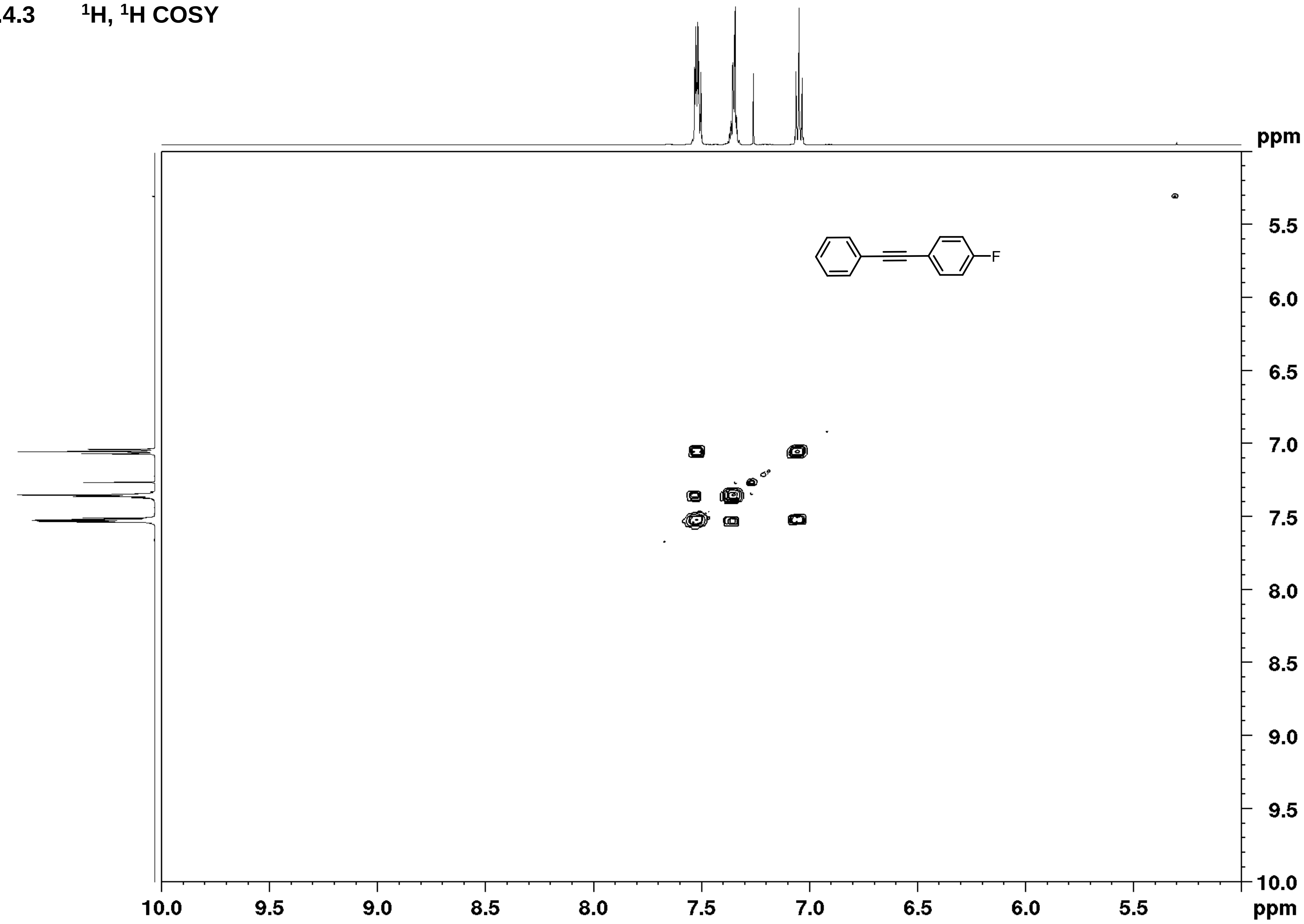

4.4.4 ^{1}H, ^{13}C HMBC

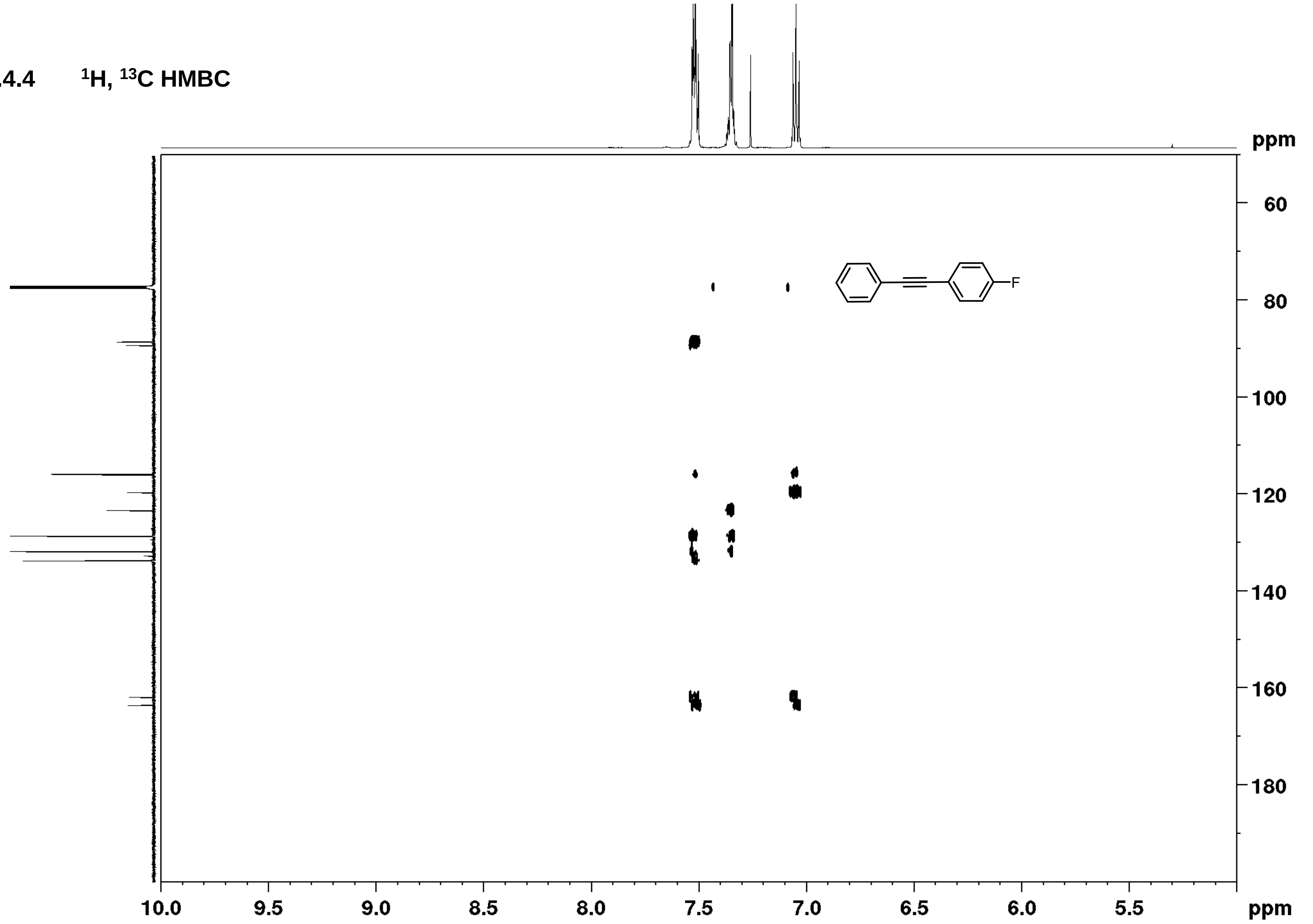

4.4.5 ^{1}H, ^{13}C HSQC

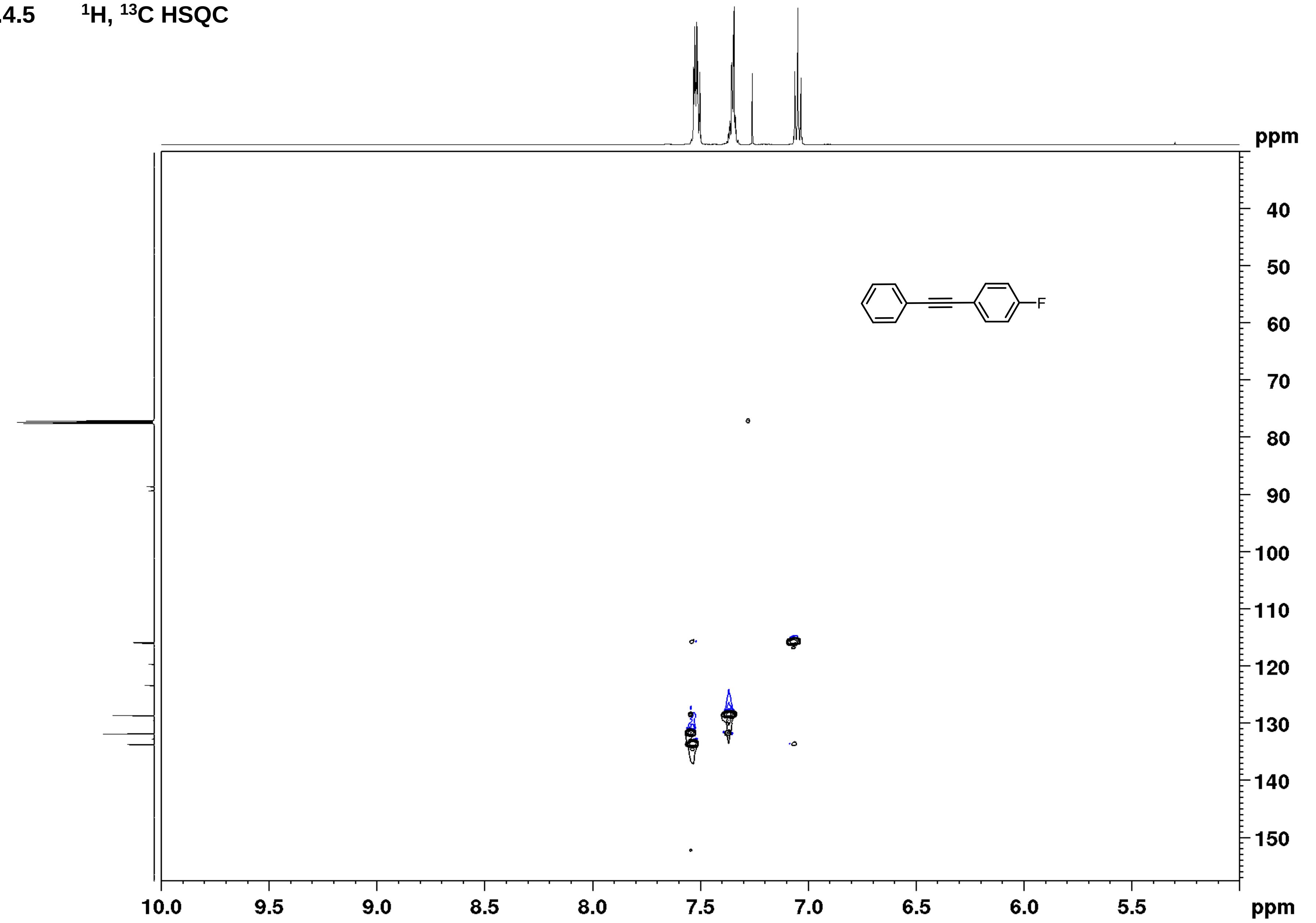

4.4.6 ^{19}F NMR

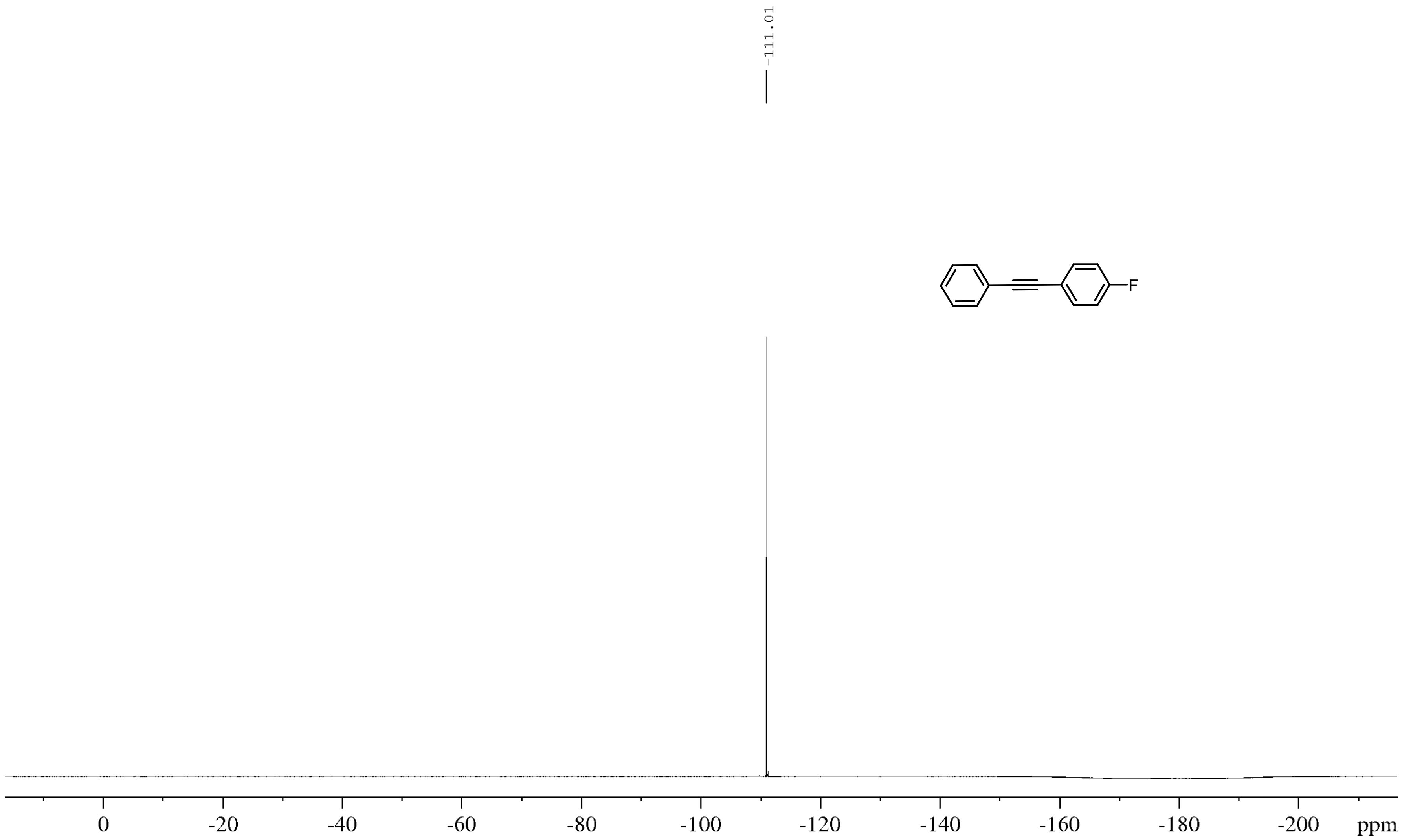

4.4.7 HRMS

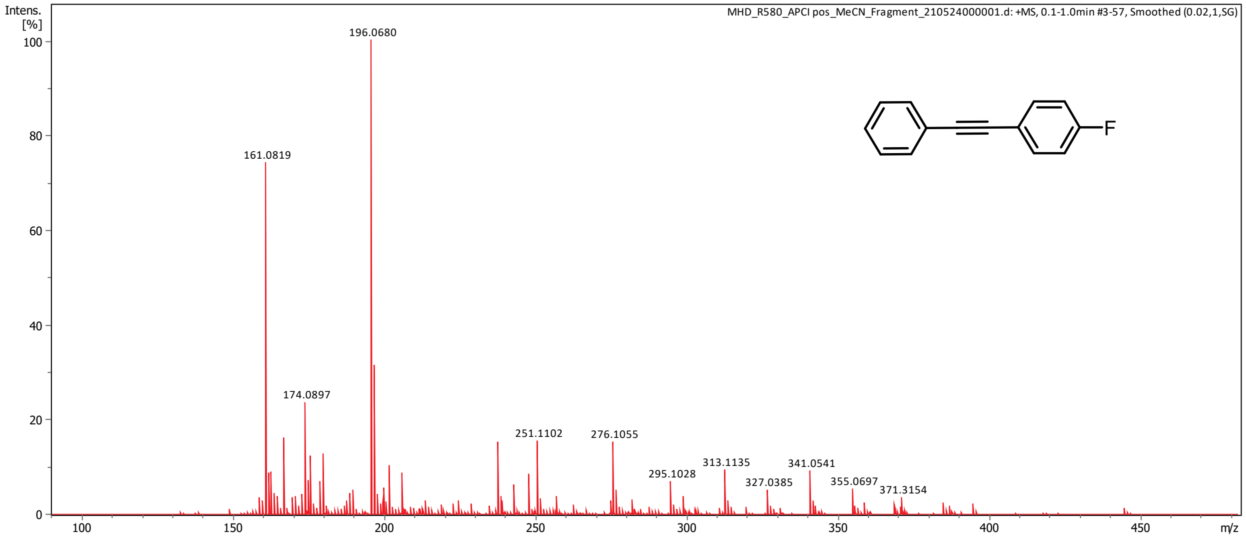
Intens. [%]
MHD_R580_APCI pos_MeCN_Fragment_210524000001.d: +MS, 0.1-1.0min #3-57, Smoothed (0.02,1,SG)
196.0680
161.0819
174.0897
251.1102
276.1055
295.1028
313.1135
327.0385
341.0541
355.0697
371.3154
F
m/z

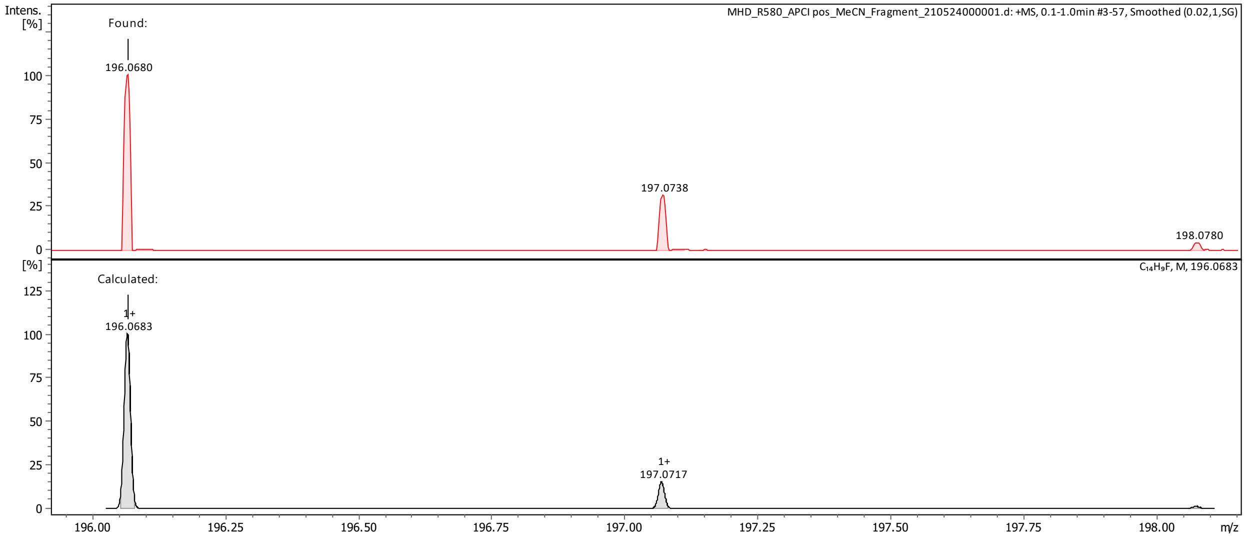
Intens. [%]
MHD_R580_APCI pos_MeCN_Fragment_210524000001.d: +MS, 0.1-1.0min #3-57, Smoothed (0.02,1,SG)
Found:
196.0680
197.0738
198.0780
Calculated:
C14H9F, M, 196.0683
1+
196.0683
1+
197.0717
m/z

4.5 1-Bromo-4-(phenylethynyl)benzene (1g) (600 MHz, $CDCl_3$)

4.5.1 ^{1}H NMR

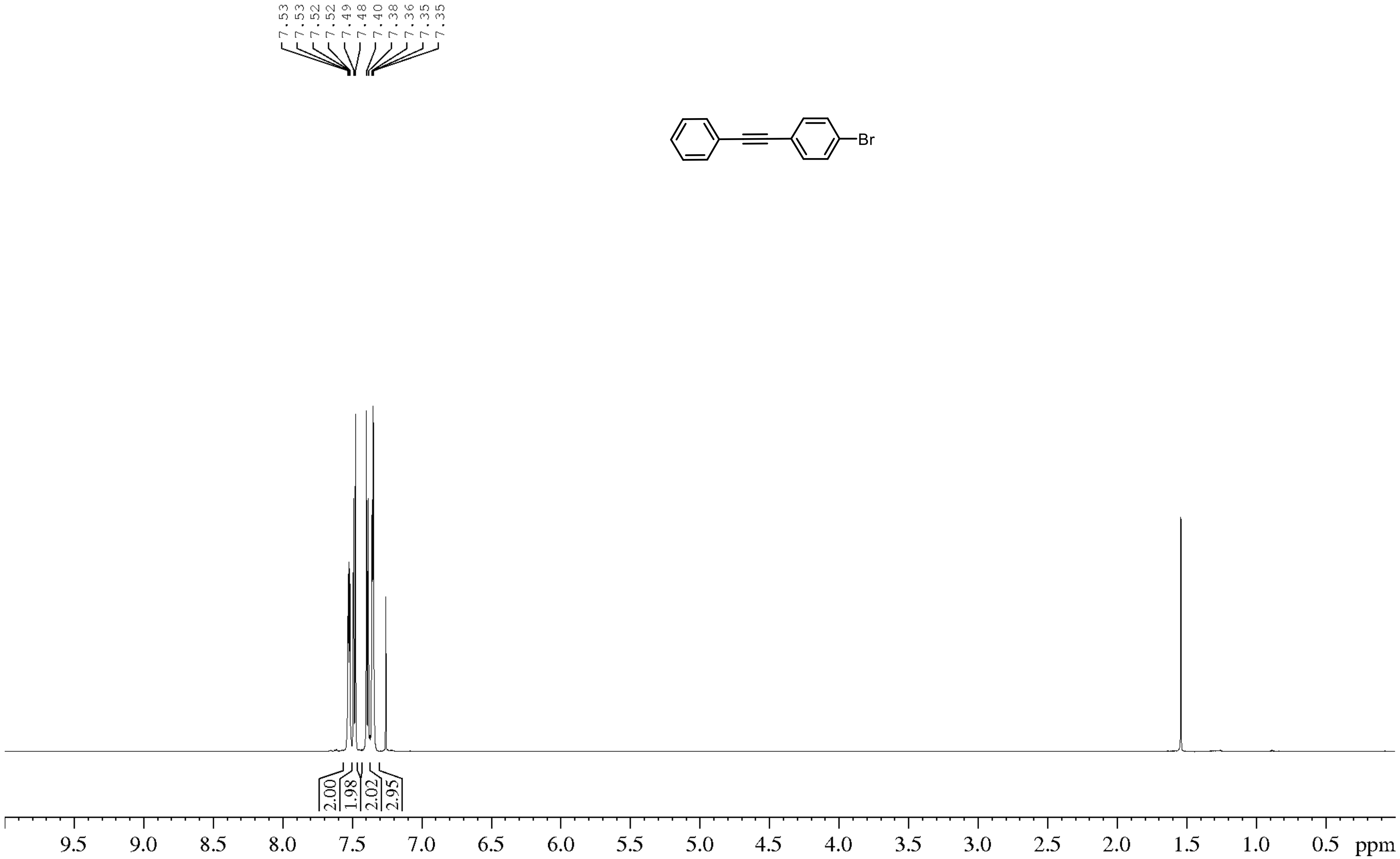

4.5.2 ^{13}C NMR

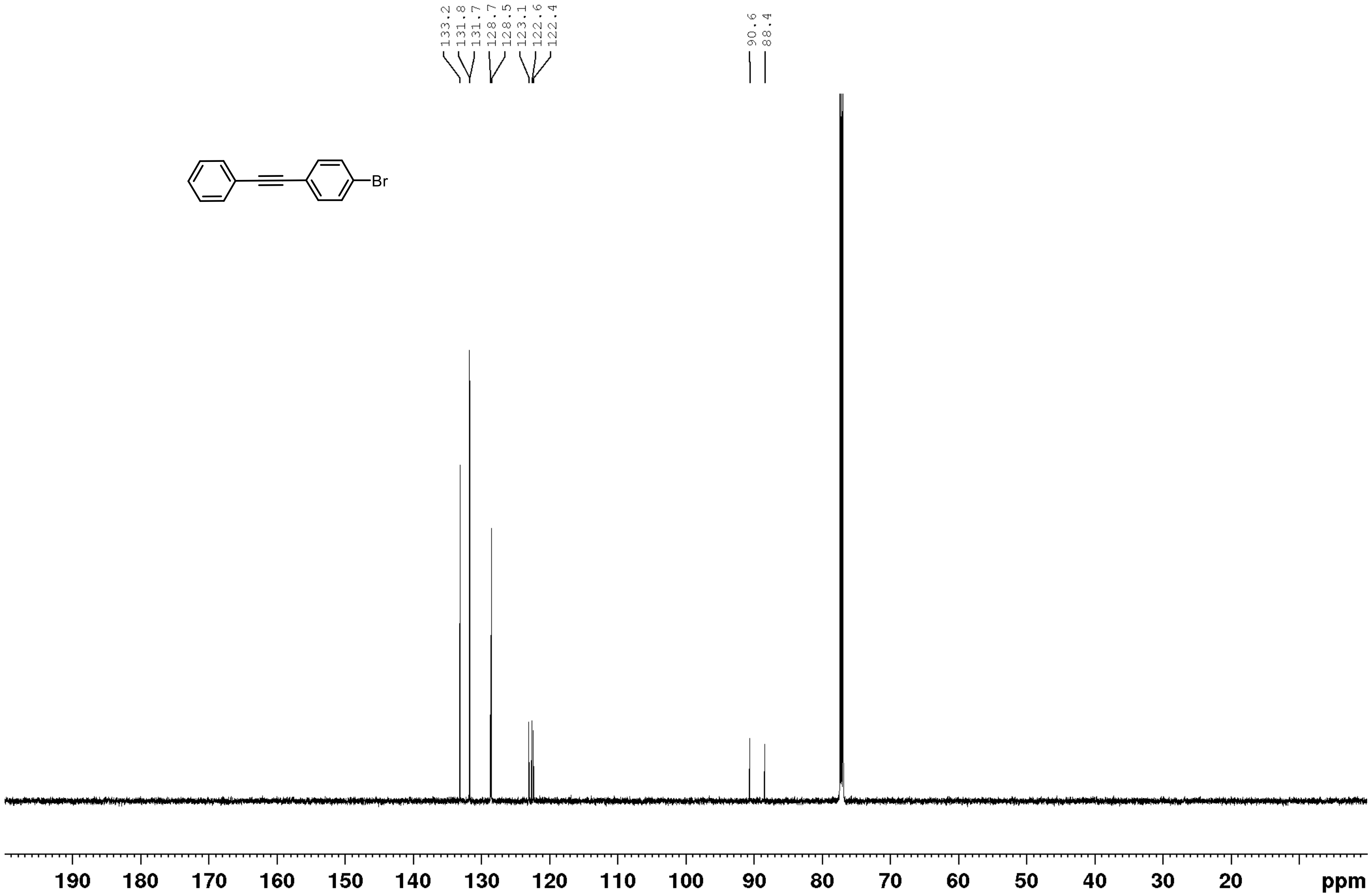
133.2
131.8
131.7
128.7
128.5
123.1
122.6
122.4
90.6
88.4
Br
190
180
170
160
150
140
130
120
110
100
90
80
70
60
50
40
30
20
ppm

4.5.3 ^{1}H, ^{1}H COSY

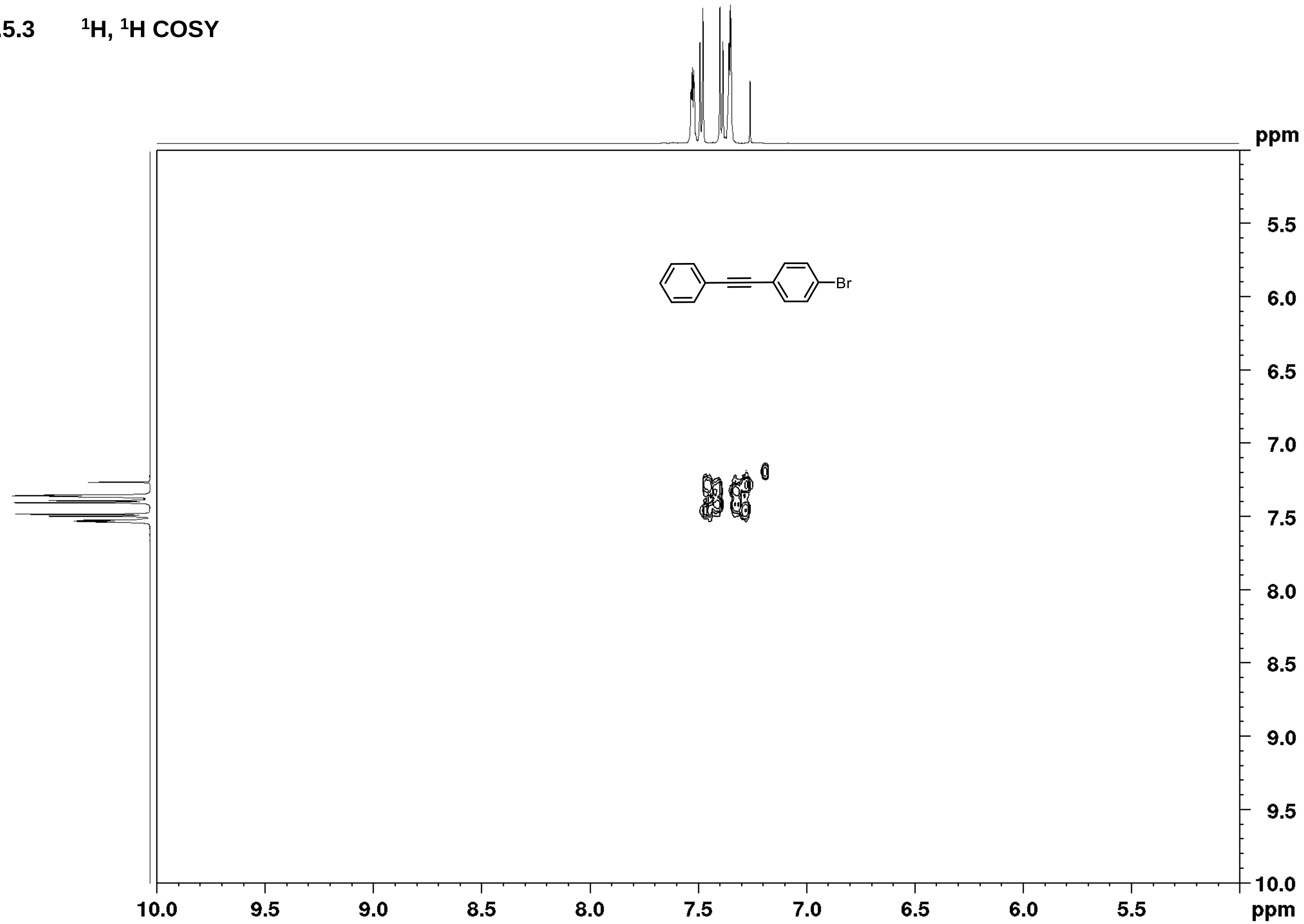

4.5.4 ¹H, ¹³C HMBC

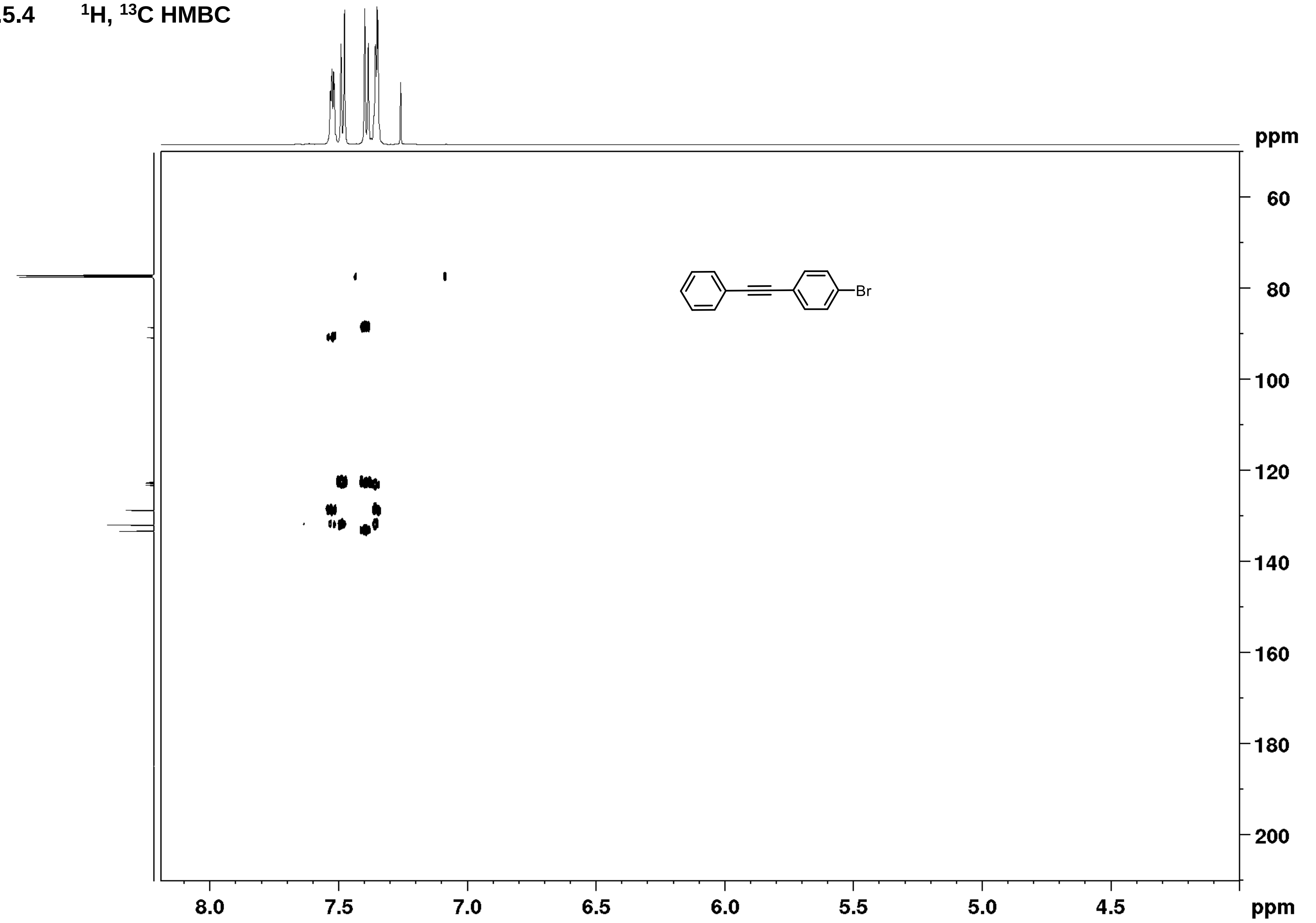

4.5.5 ^{1}H, ^{13}C HSQC

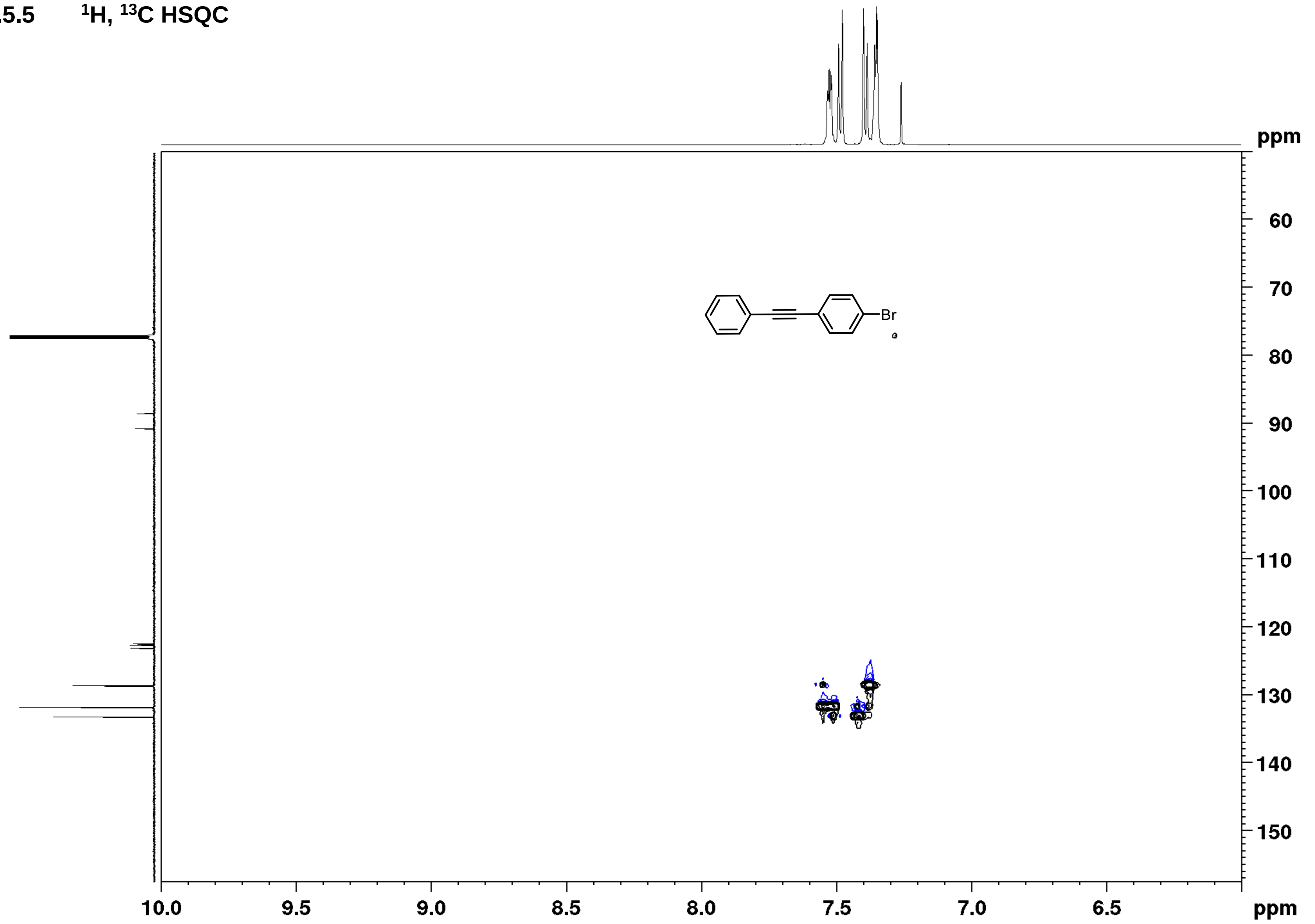

4.5.6 HRMS

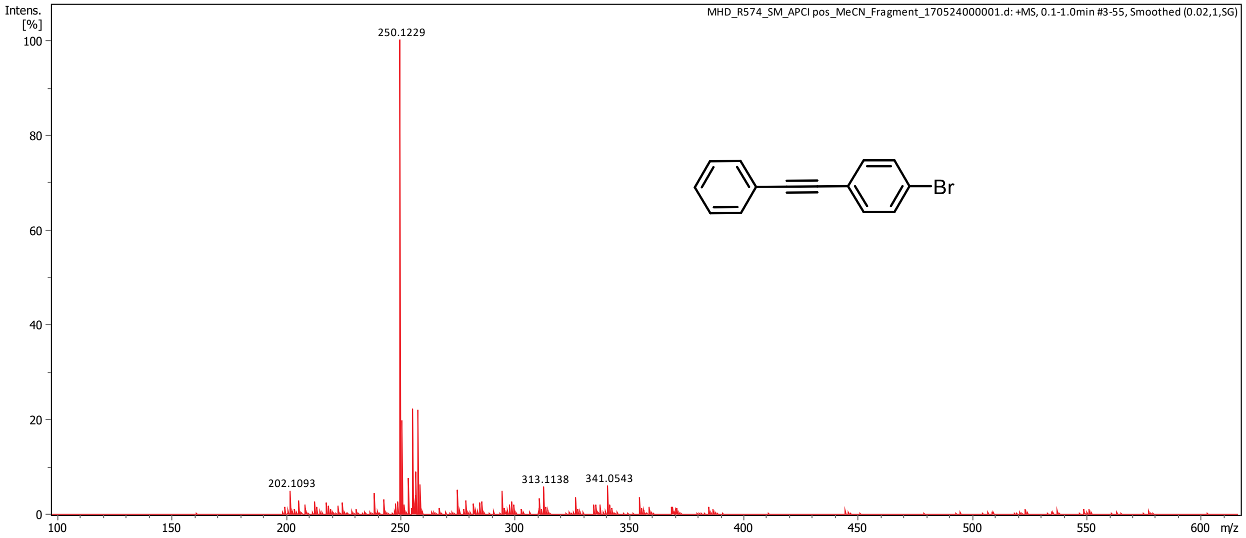
Intens.
[%]
MHD_R574_SM_APCI pos_MeCN_Fragment_170524000001.d: +MS, 0.1-1.0min #3-55, Smoothed (0.02,1,SG)
250.1229
202.1093
313.1138
341.0543
Br
m/z

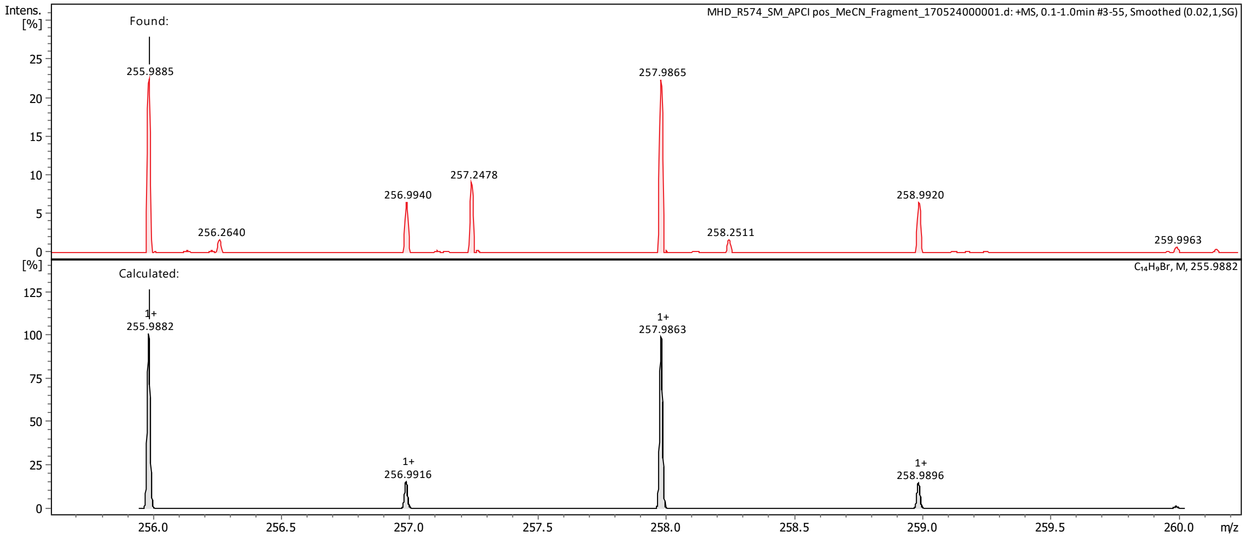
Intens.
[%]
MHD_R574_SM_APCI pos_MeCN_Fragment_170524000001.d: +MS, 0.1-1.0min #3-55, Smoothed (0.02,1,SG)
Found:
255.9885
257.9865
257.2478
256.9940
258.9920
256.2640
258.2511
259.9963
Calculated:
$C_{14}H_9Br$, M, 255.9882
1+
255.9882
1+
257.9863
1+
256.9916
1+
258.9896
m/z

4.6 1-Iodo-4-(phenylethynyl)benzene (1h) (600 MHz, $CDCl_3$)

4.6.1 ^{1}H NMR

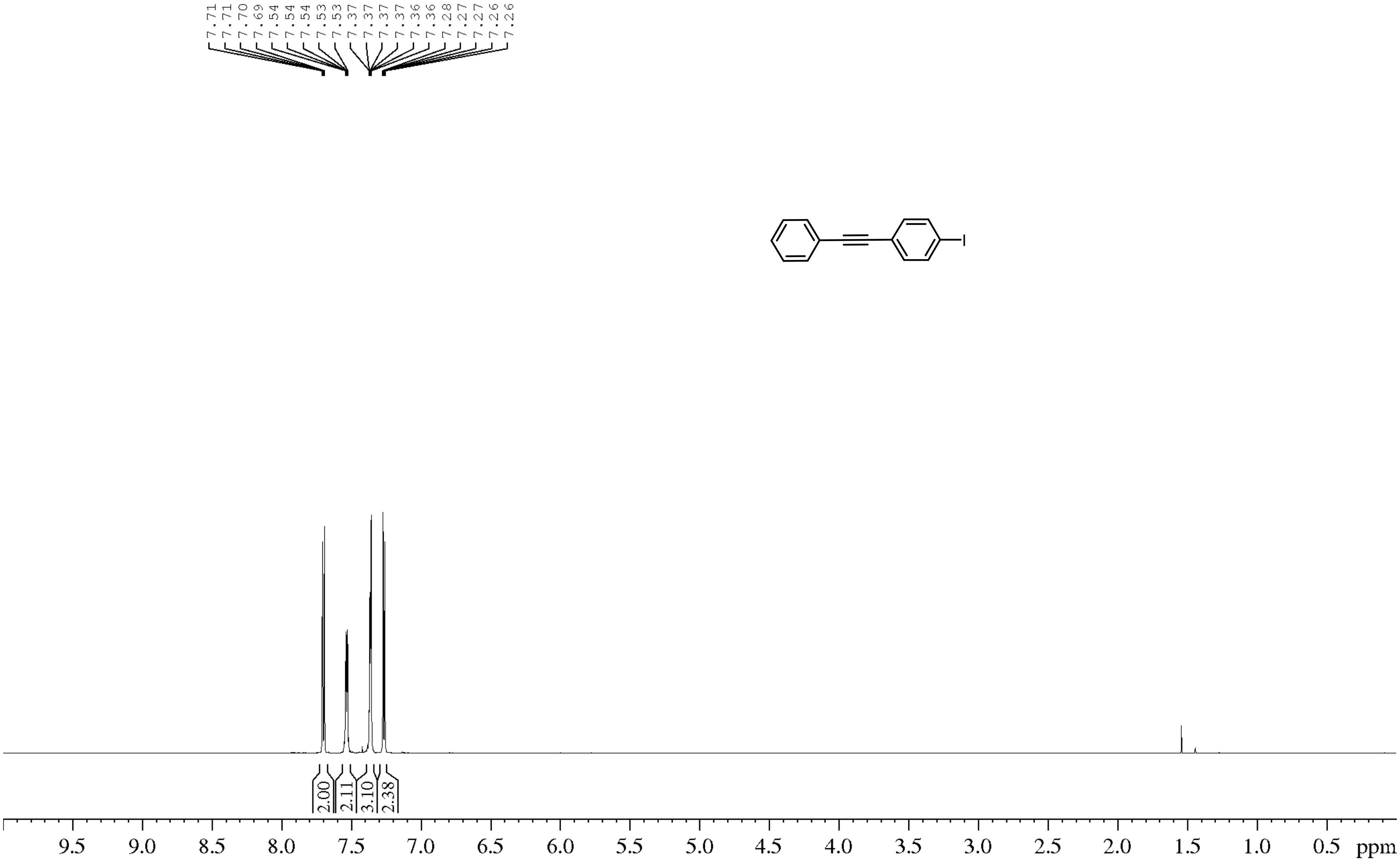

4.6.2 ^{13}C NMR

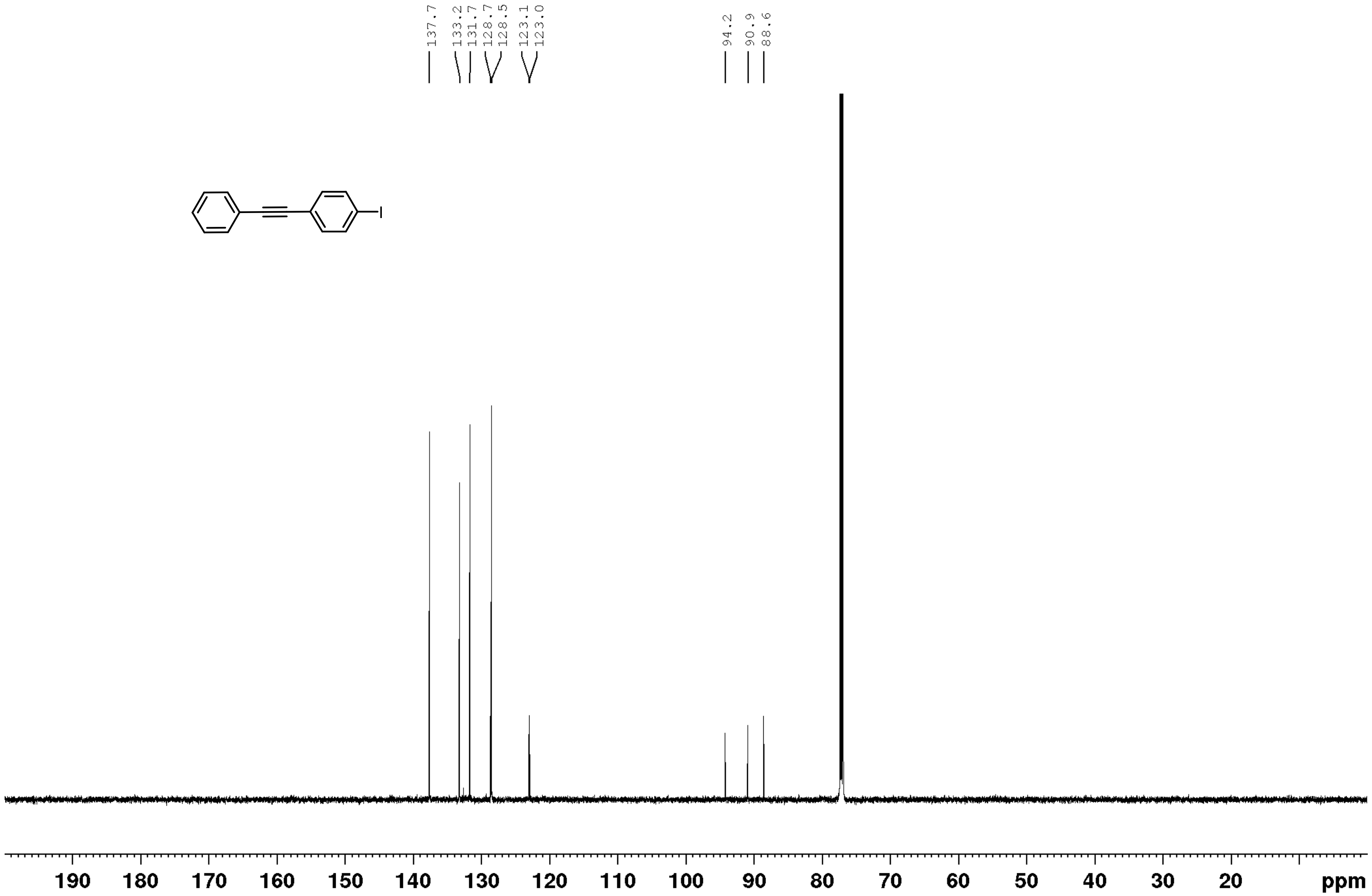
137.7
133.2
131.7
128.7
128.5
123.1
123.0
94.2
90.9
88.6
I
190
180
170
160
150
140
130
120
110
100
90
80
70
60
50
40
30
20
ppm

4.6.3 ^{1}H, ^{1}H COSY

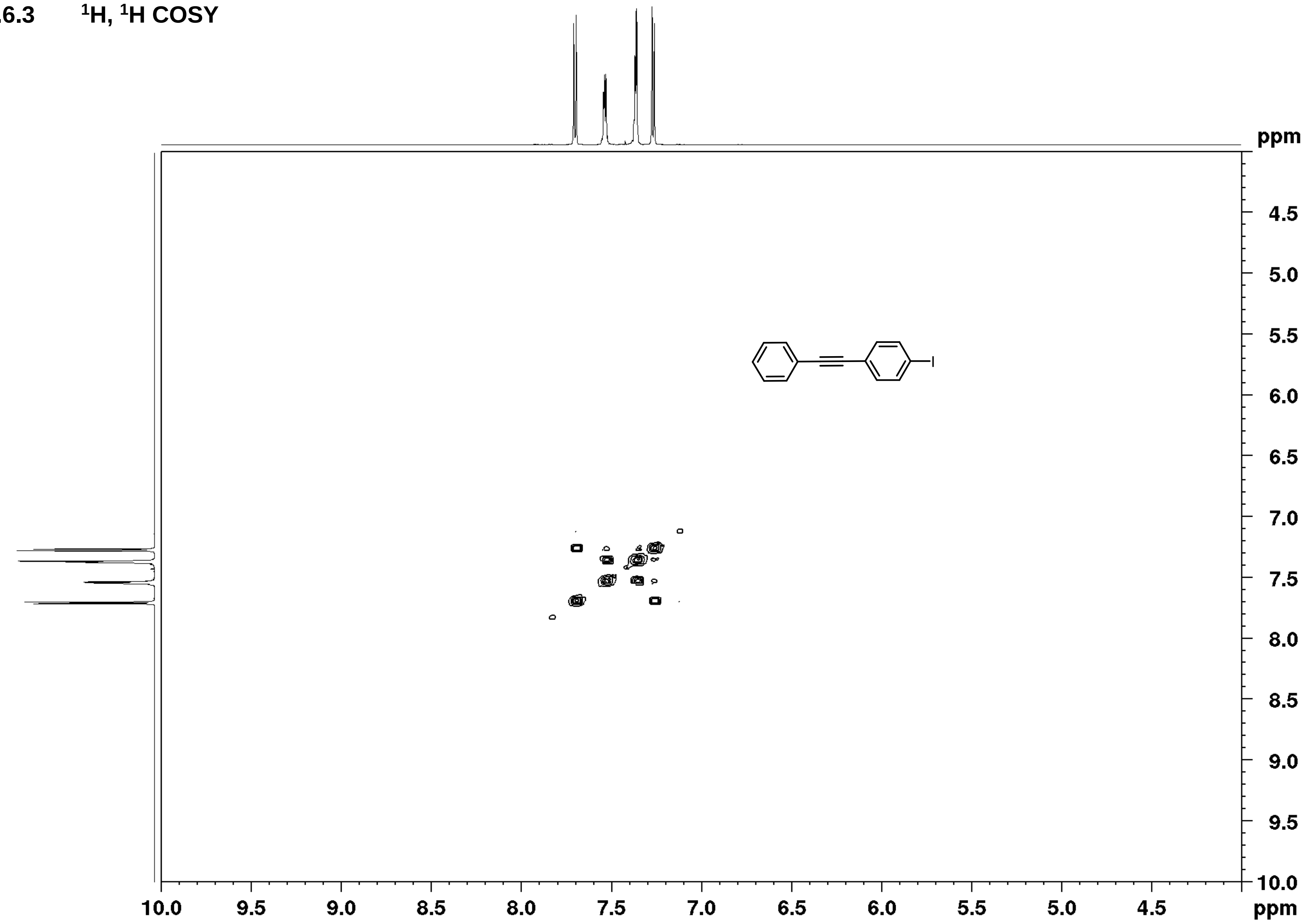

4.6.4 ^{1}H, ^{13}C HMBC

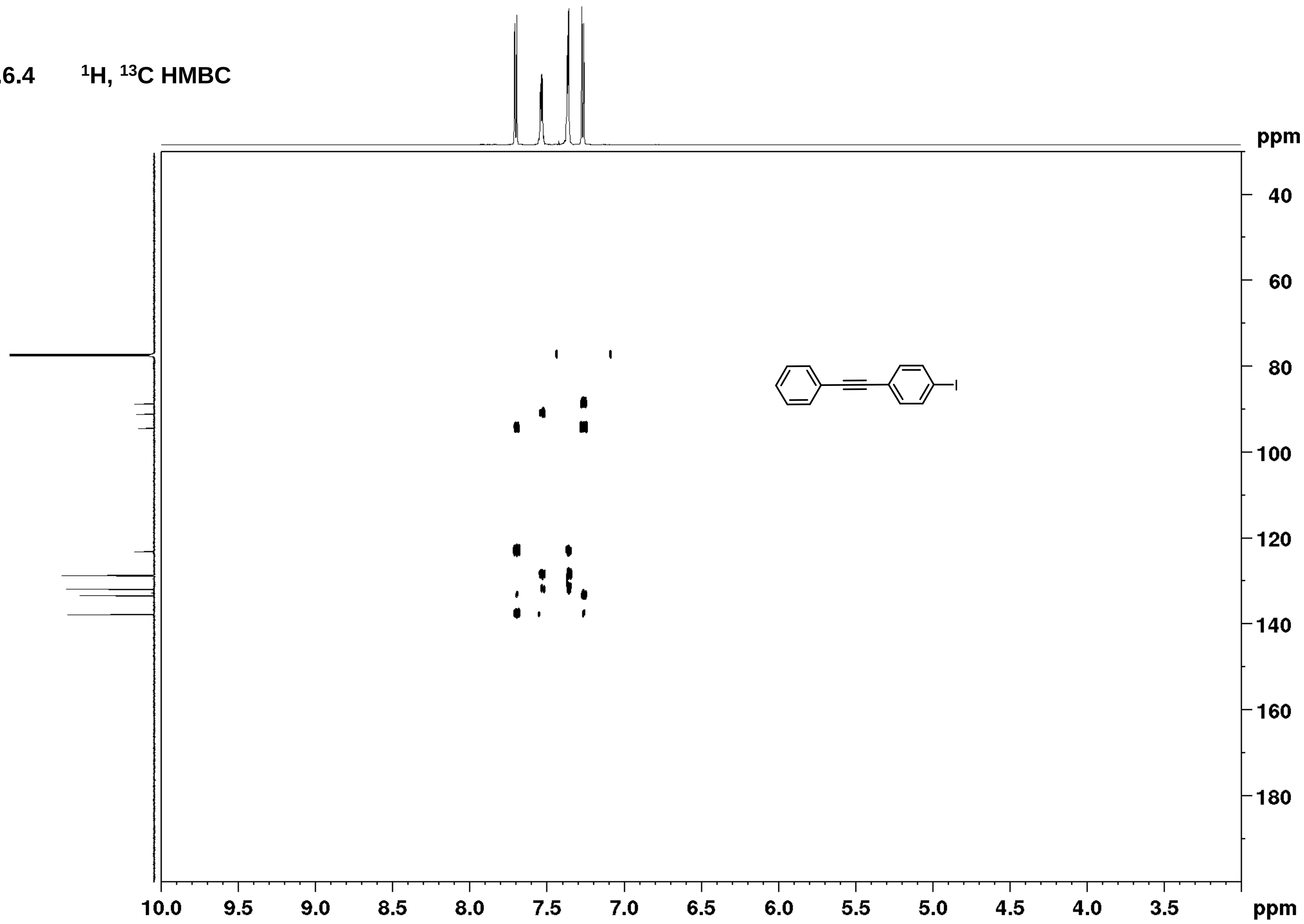

4.6.5 ^{1}H, ^{13}C HSQC

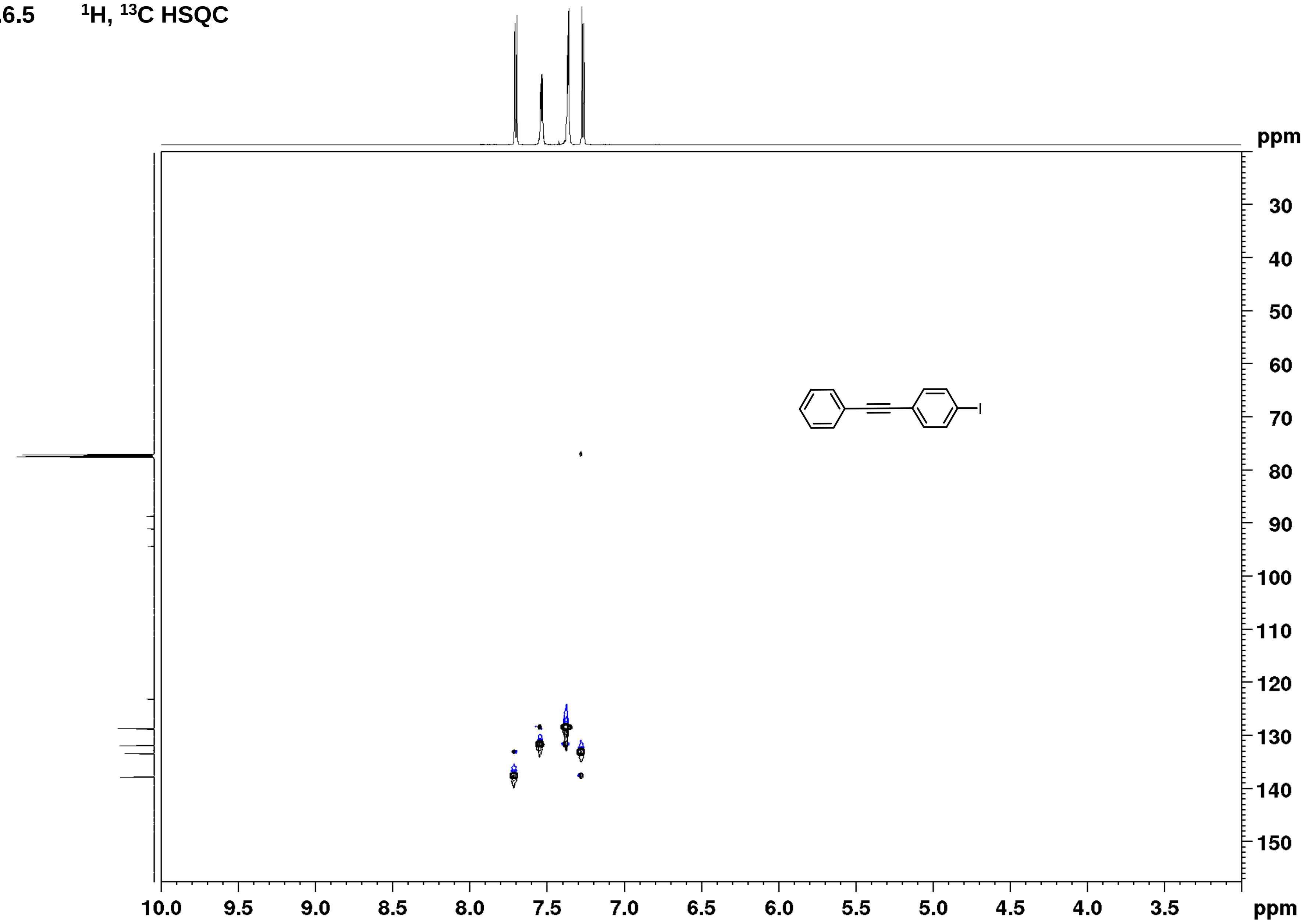

4.6.6 HRMS

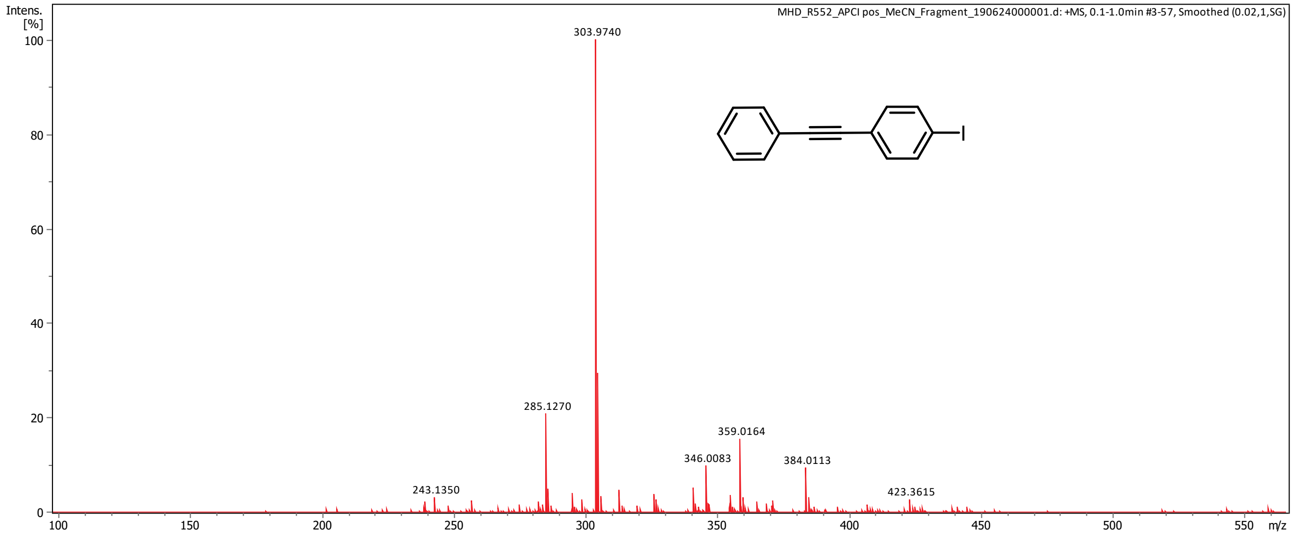
Intens. [%]
MHD_R552_APCI pos_MeCN_Fragment_190624000001.d: +MS, 0.1-1.0min #3-57, Smoothed (0.02,1,SG)
303.9740
285.1270
359.0164
346.0083
384.0113
243.1350
423.3615
m/z
I

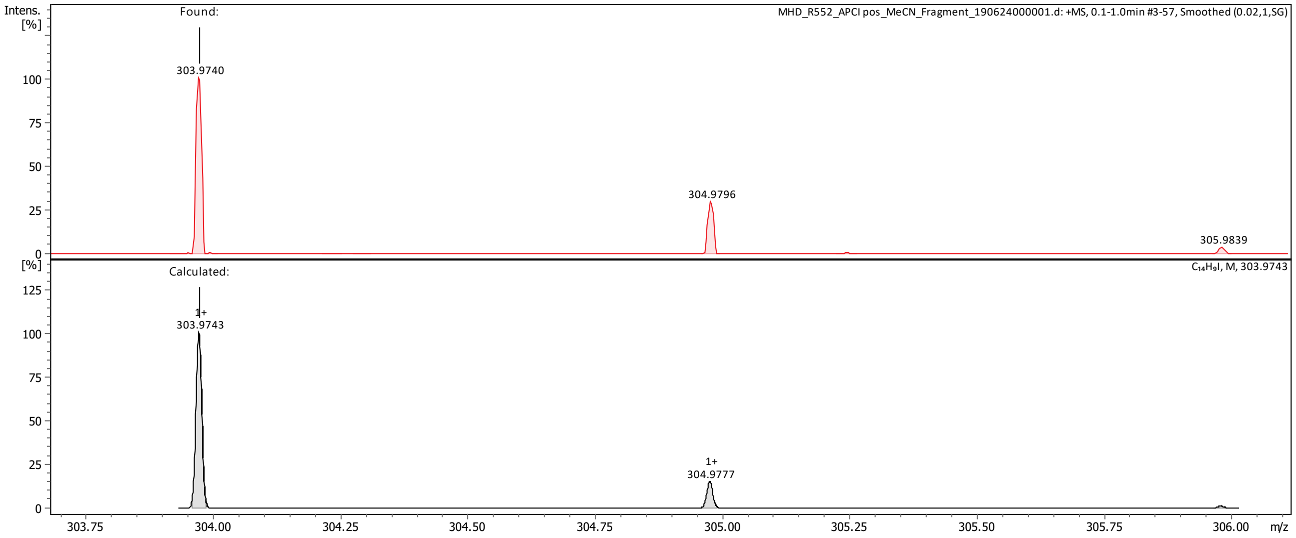
Intens. [%]
Found:
MHD_R552_APCI pos_MeCN_Fragment_190624000001.d: +MS, 0.1-1.0min #3-57, Smoothed (0.02,1,SG)
303.9740
304.9796
305.9839
Calculated:
C14H9I, M, 303.9743
1+
303.9743
1+
304.9777
m/z

4.7 3-(Phenylethynyl)benzaldehyde (1i) (600 MHz, $CDCl_3$)

4.7.1 ^{1}H NMR

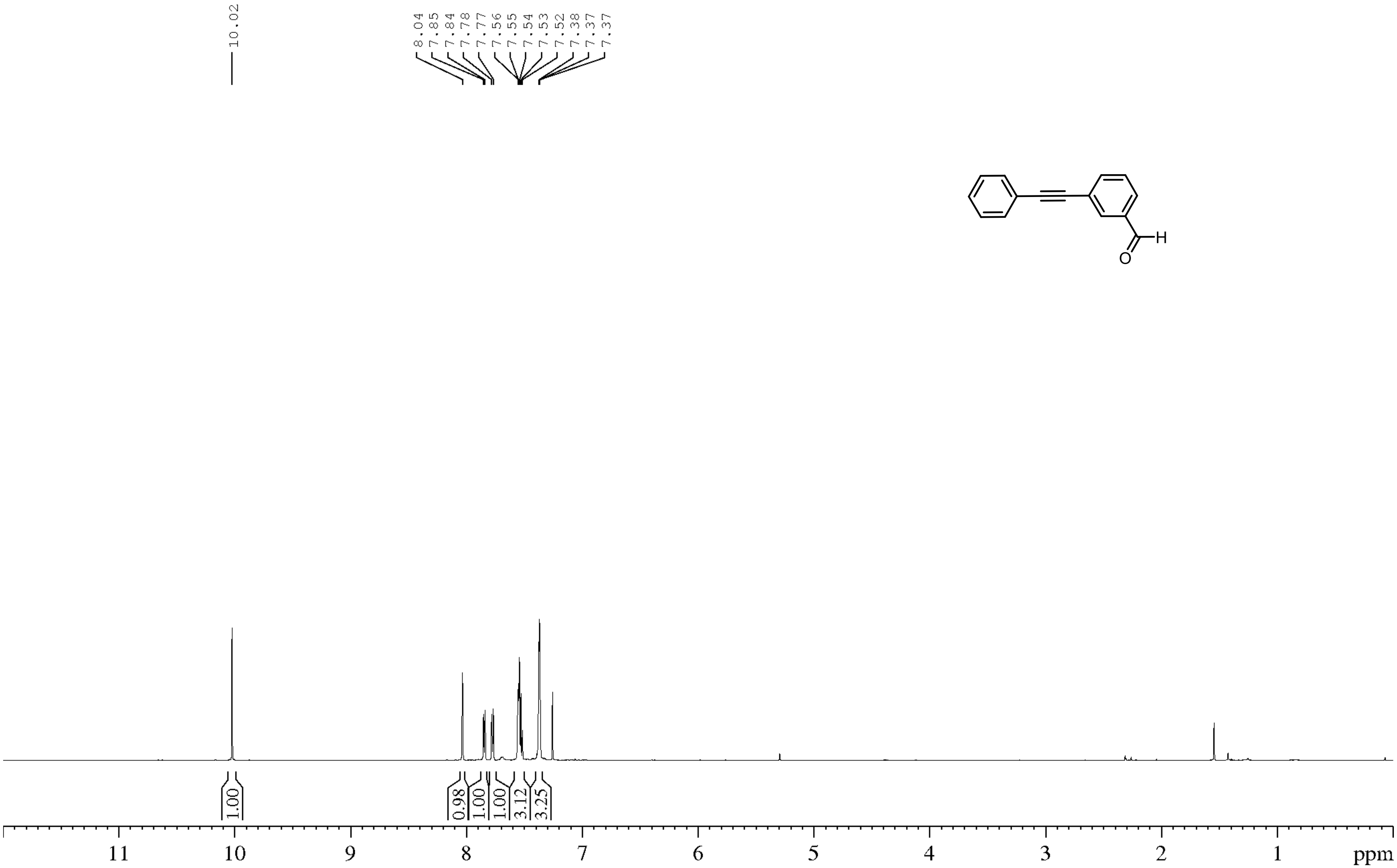

4.7.2 ^{13}C NMR

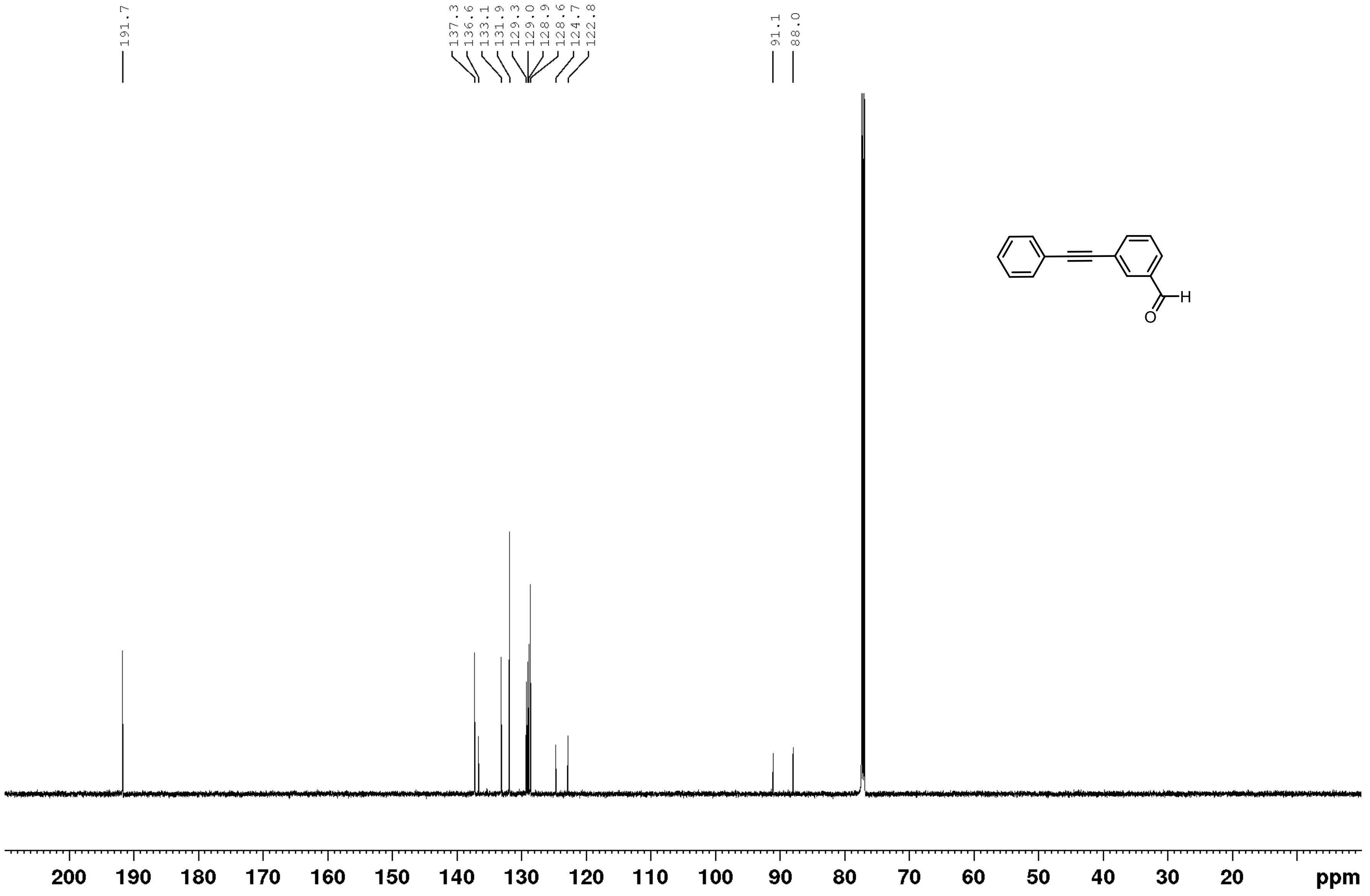

191.7
137.3
136.6
133.1
131.9
129.3
129.0
128.9
128.6
124.7
122.8
91.1
88.0
H
O
200 190 180 170 160 150 140 130 120 110 100 90 80 70 60 50 40 30 20 ppm

4.7.3 ^{1}H, ^{1}H COSY

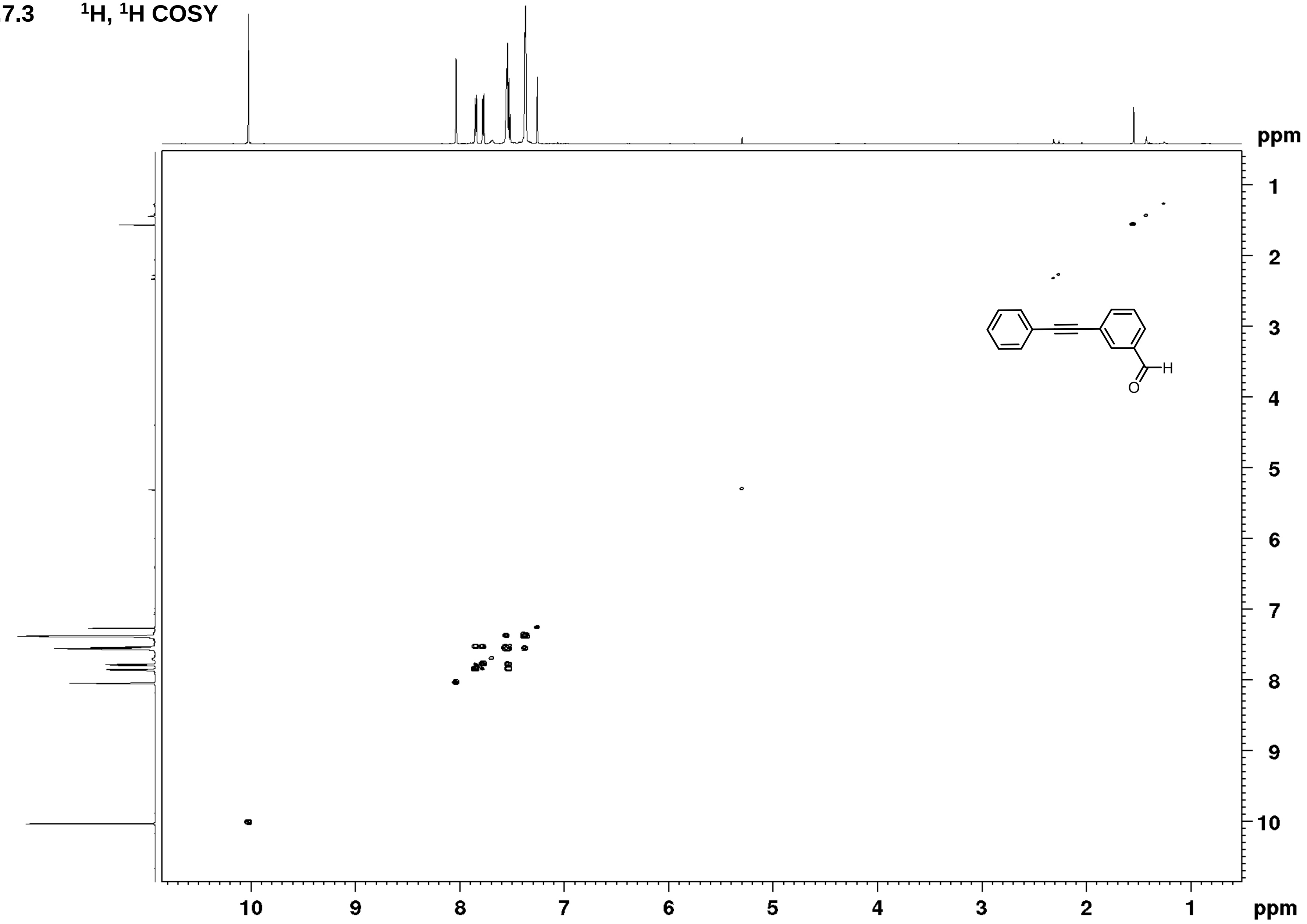

4.7.4 ^{1}H, ^{13}C HMBC

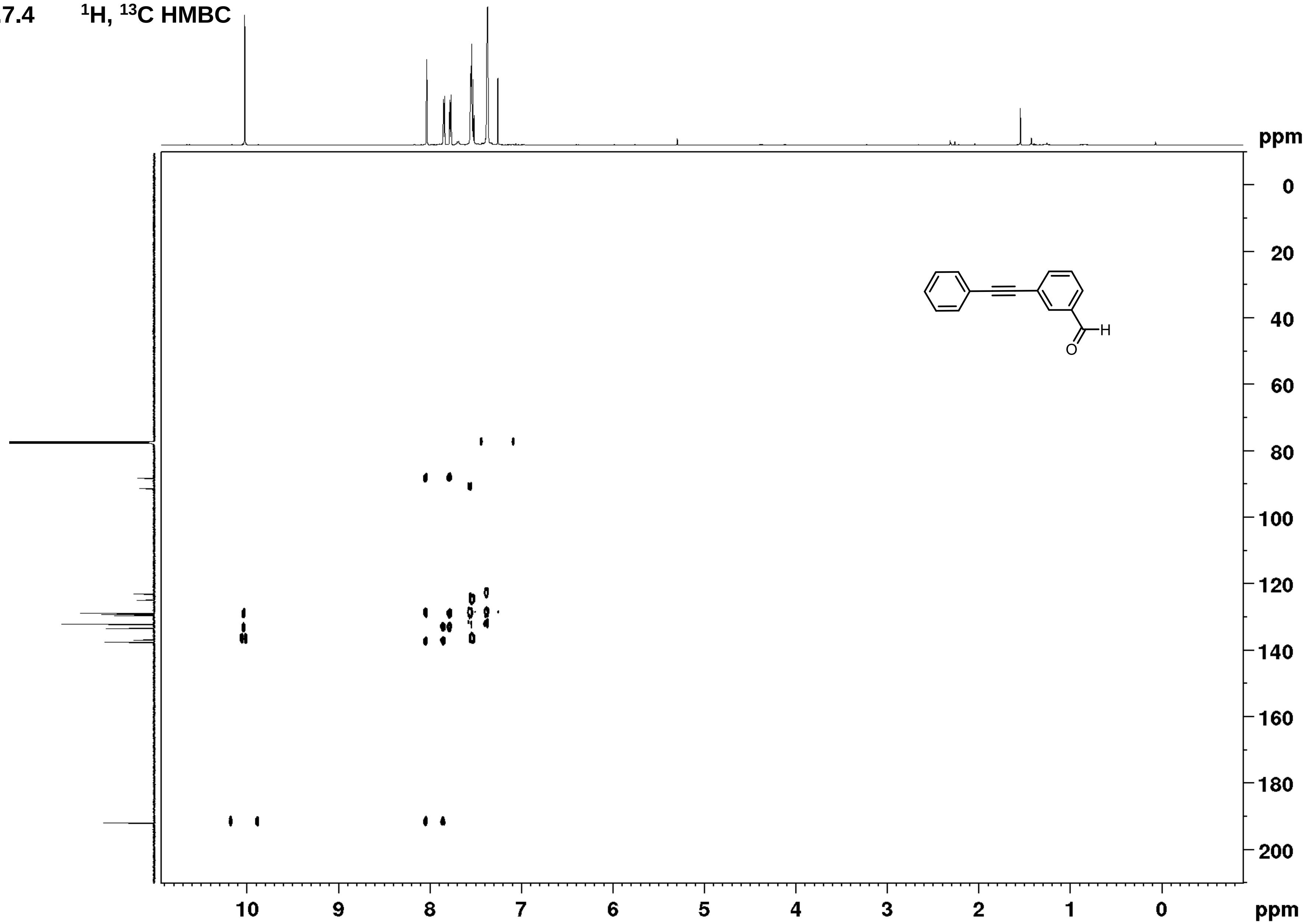

4.7.5 ^{1}H, ^{13}C HSQC

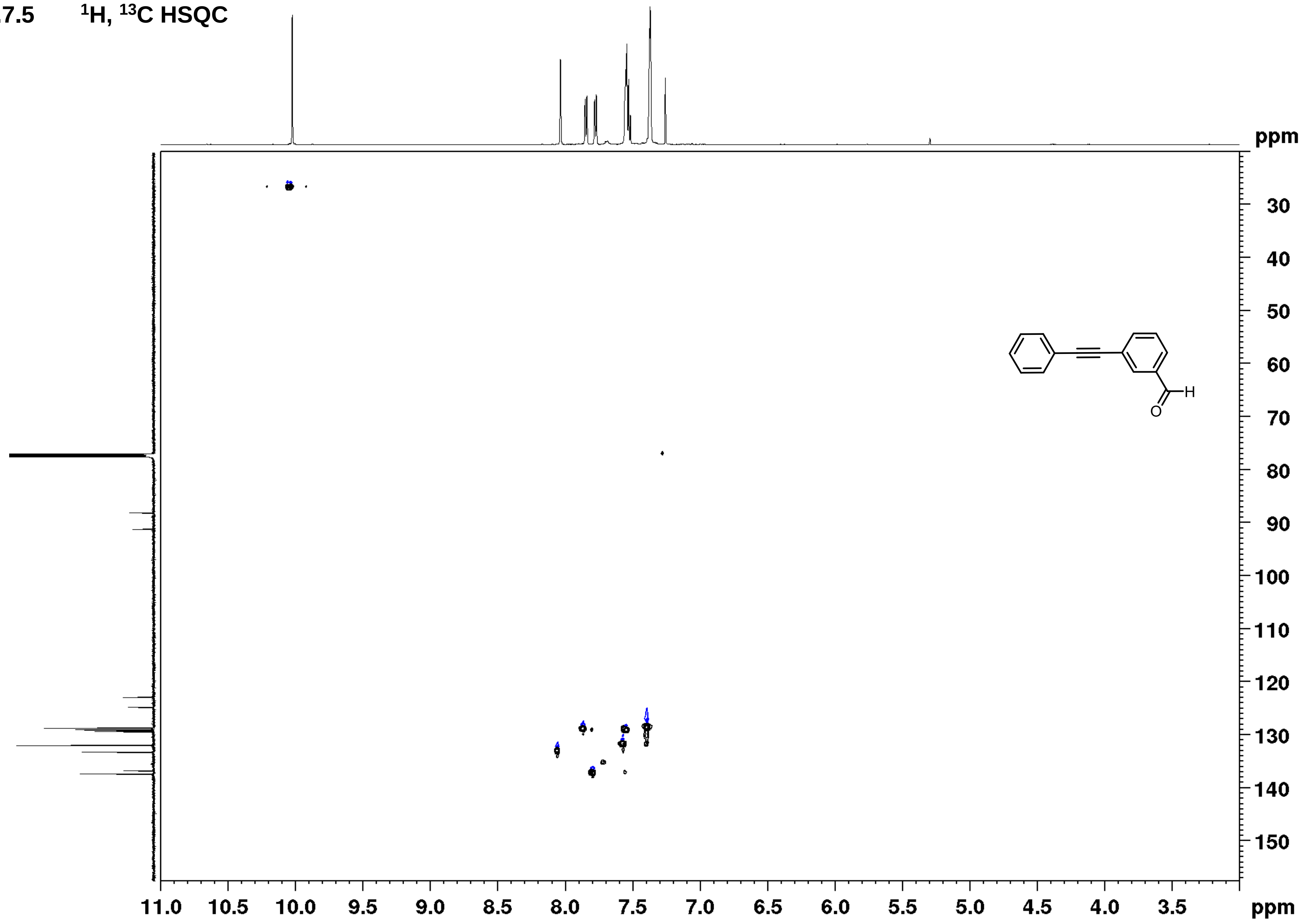

4.7.6 HRMS

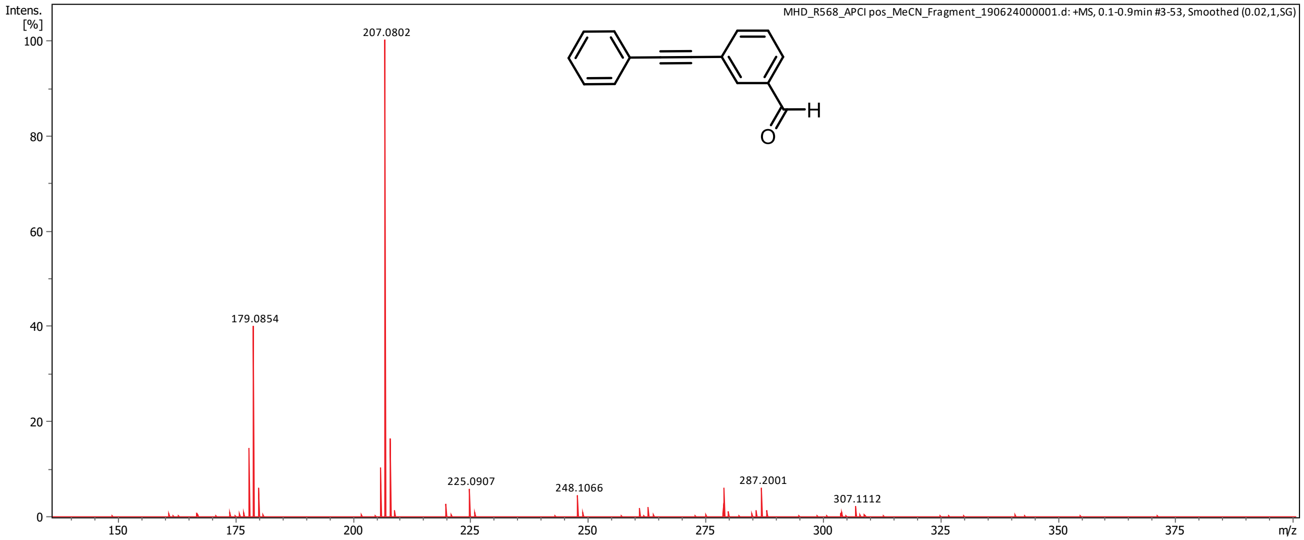
MHD_R568_APCI pos_MeCN_Fragment_190624000001.d: +MS, 0.1-0.9min #3-53, Smoothed (0.02,1,SG)
Intens. [%]
207.0802
179.0854
225.0907
248.1066
287.2001
307.1112
H
O
m/z

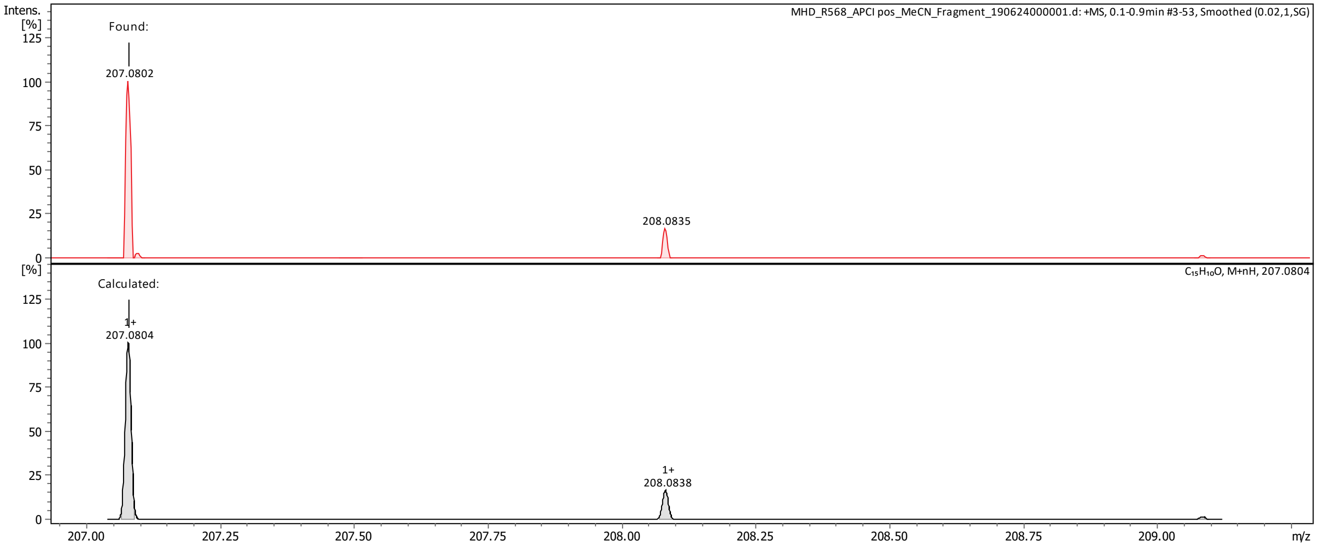
MHD_R568_APCI pos_MeCN_Fragment_190624000001.d: +MS, 0.1-0.9min #3-53, Smoothed (0.02,1,SG)
Intens. [%]
Found:
207.0802
208.0835
$C_{15}H_{10}O$, M+nH, 207.0804
Calculated:
1+
207.0804
1+
208.0838
m/z

4.8 1-(3-(Phenylethynyl)phenyl)ethan-1-one (1j) (600 MHz, $CDCl_3$)

4.8.1 ^1H NMR

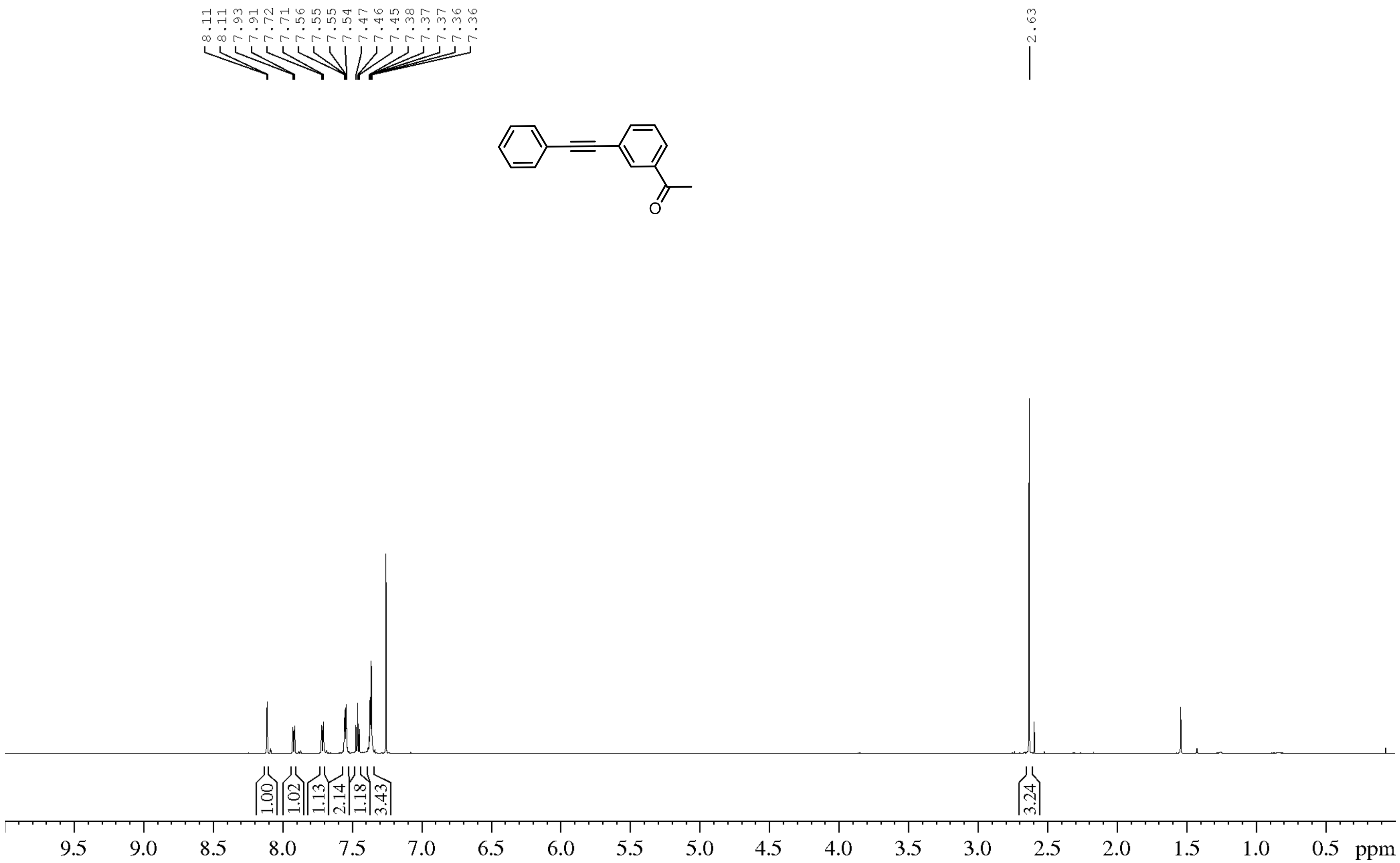

4.8.2 ^{13}C NMR

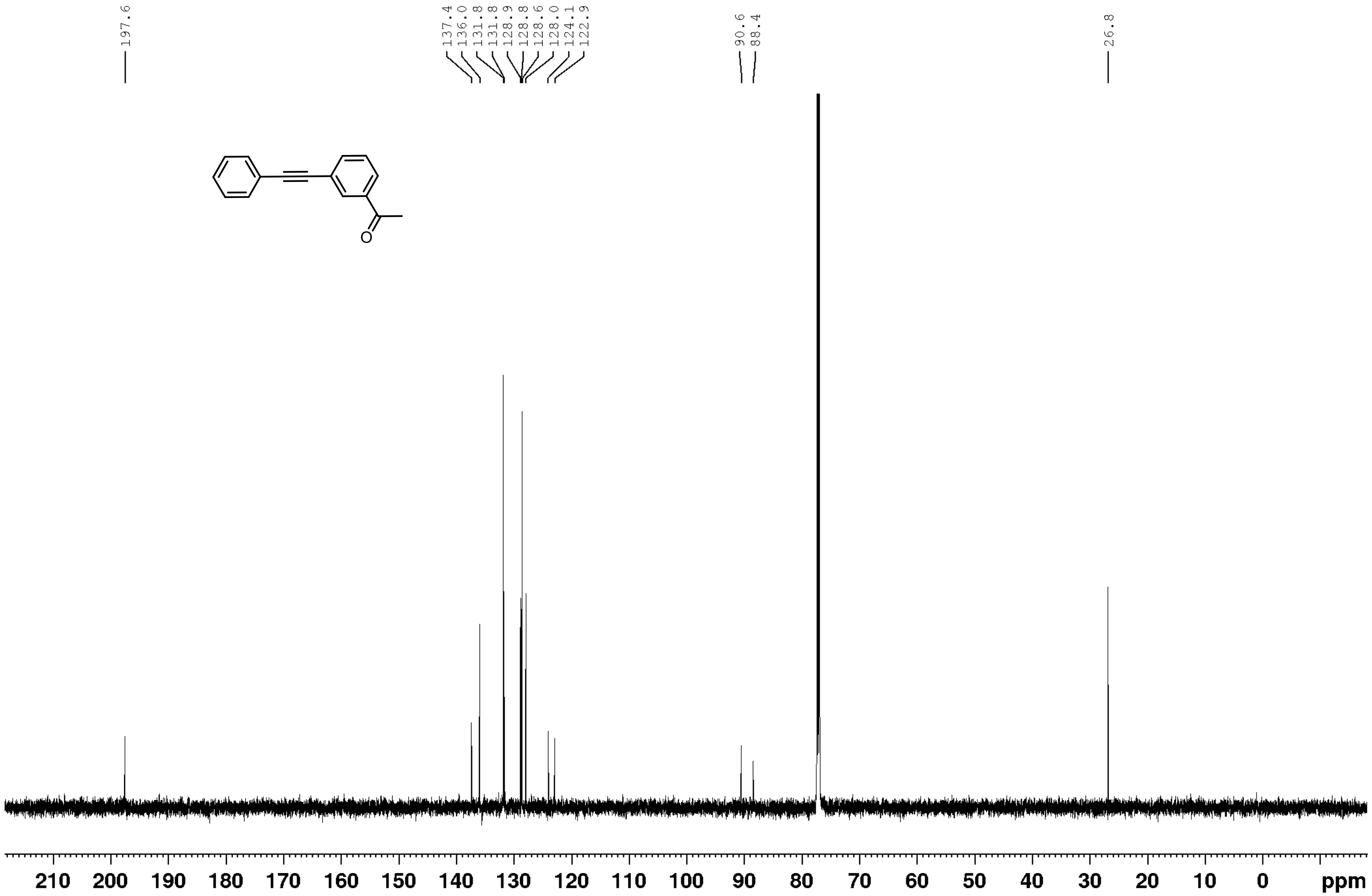

197.6
137.4
136.0
131.8
131.8
128.9
128.8
128.6
128.0
124.1
122.9
90.6
88.4
26.8
O
210 200 190 180 170 160 150 140 130 120 110 100 90 80 70 60 50 40 30 20 10 0 ppm

4.8.3 ^{1}H, ^{1}H COSY

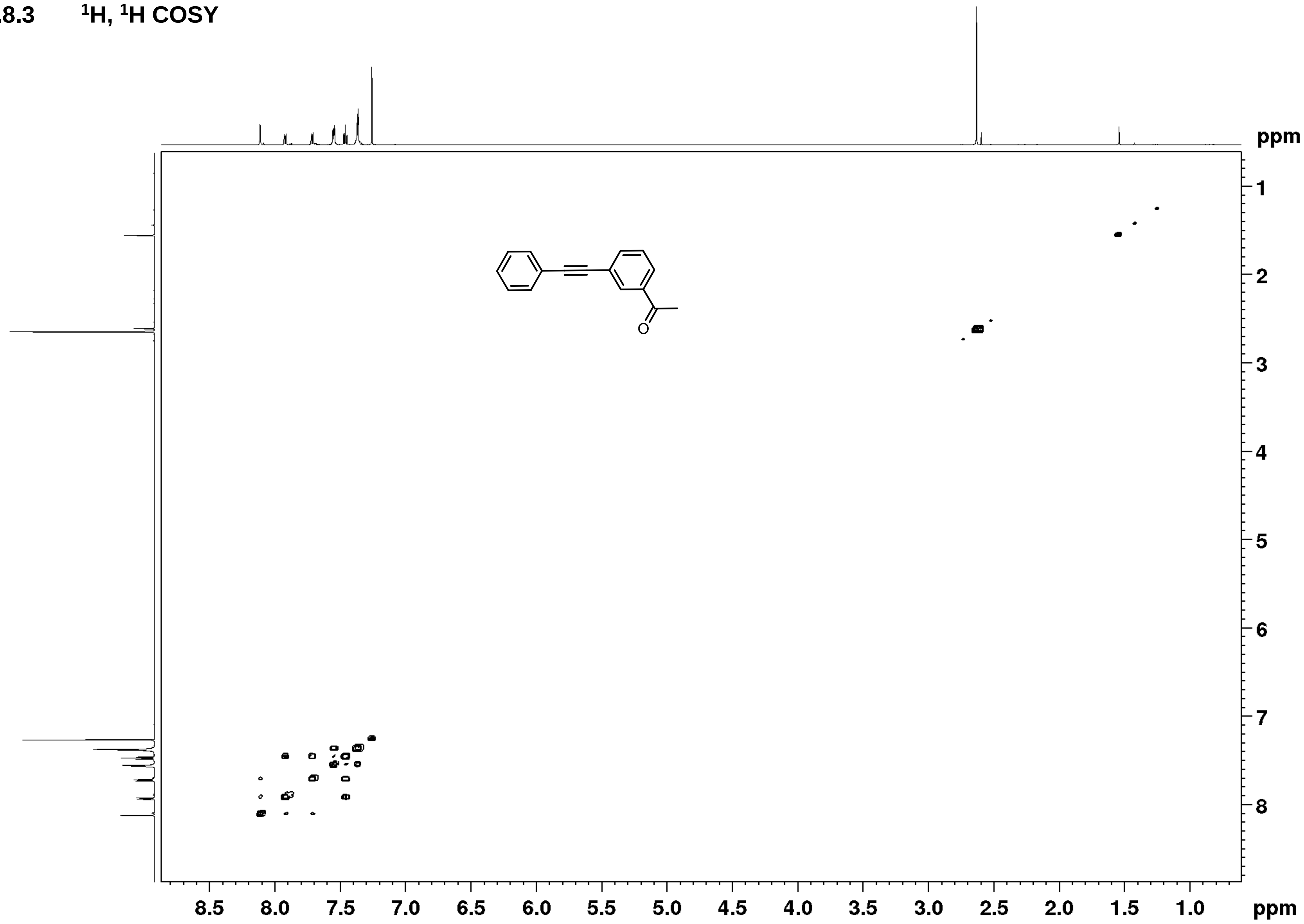

4.8.4 ^{1}H, ^{13}C HMBC

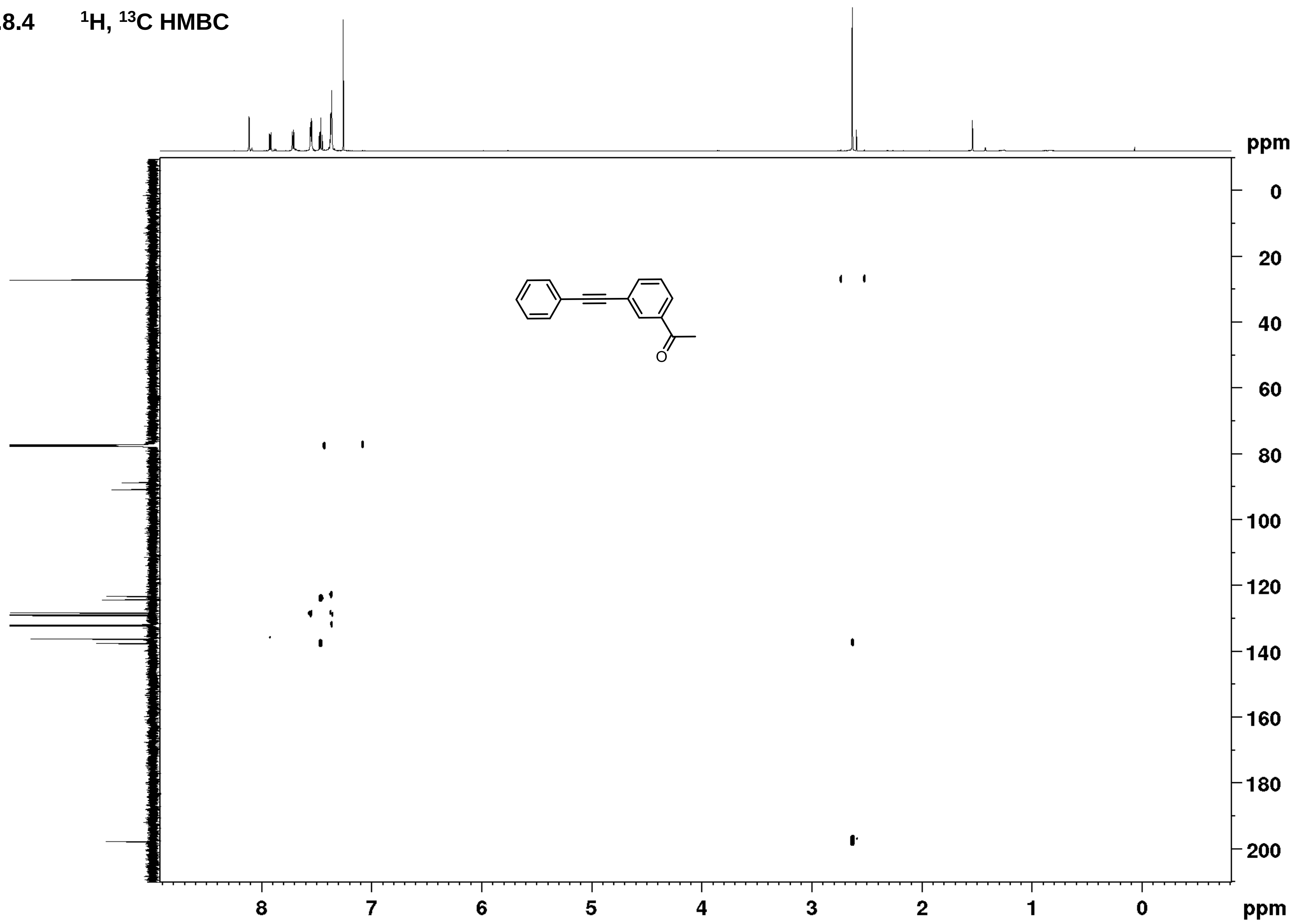

4.8.5 ^{1}H, ^{13}C HSQC

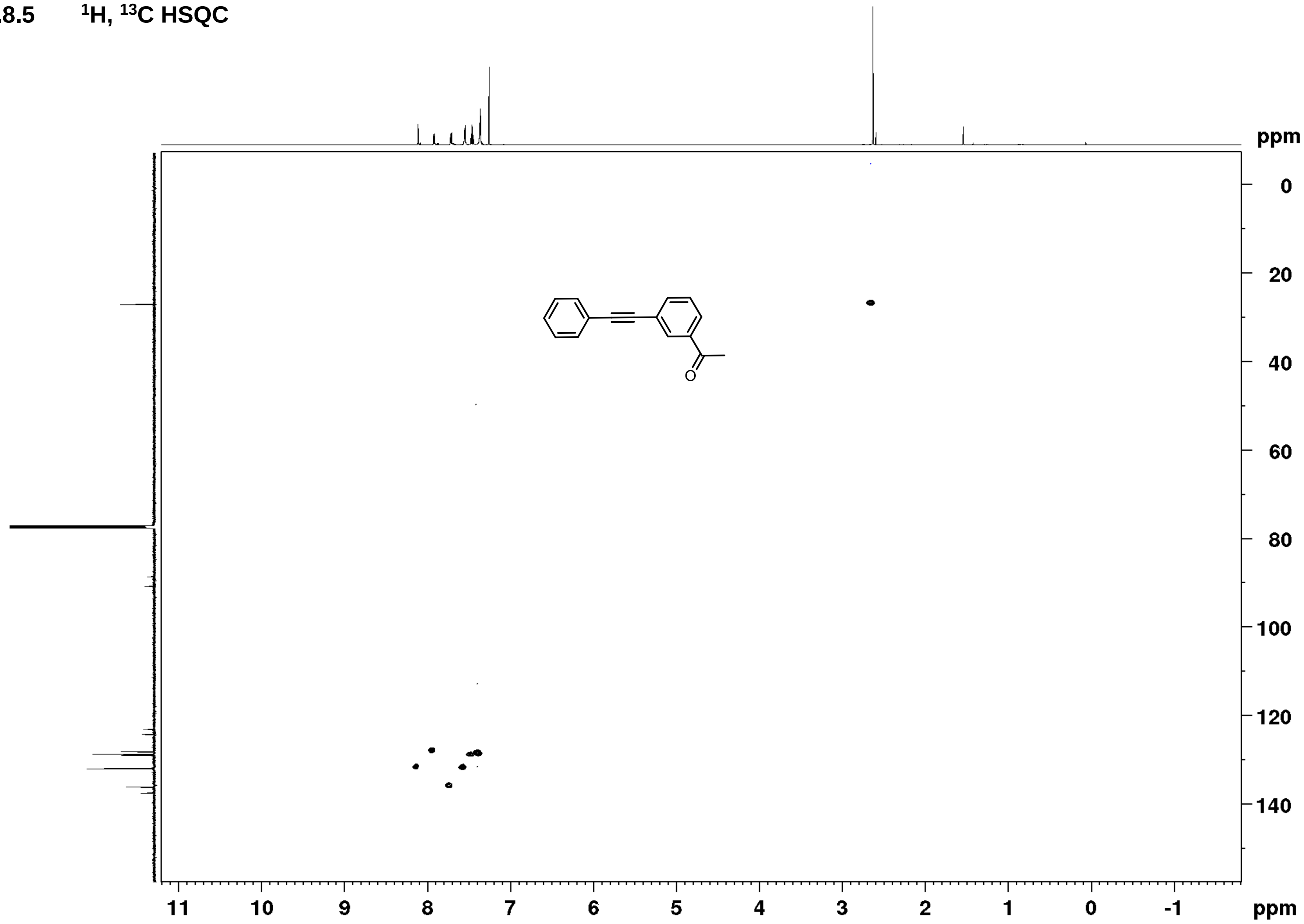

4.8.6 HRMS

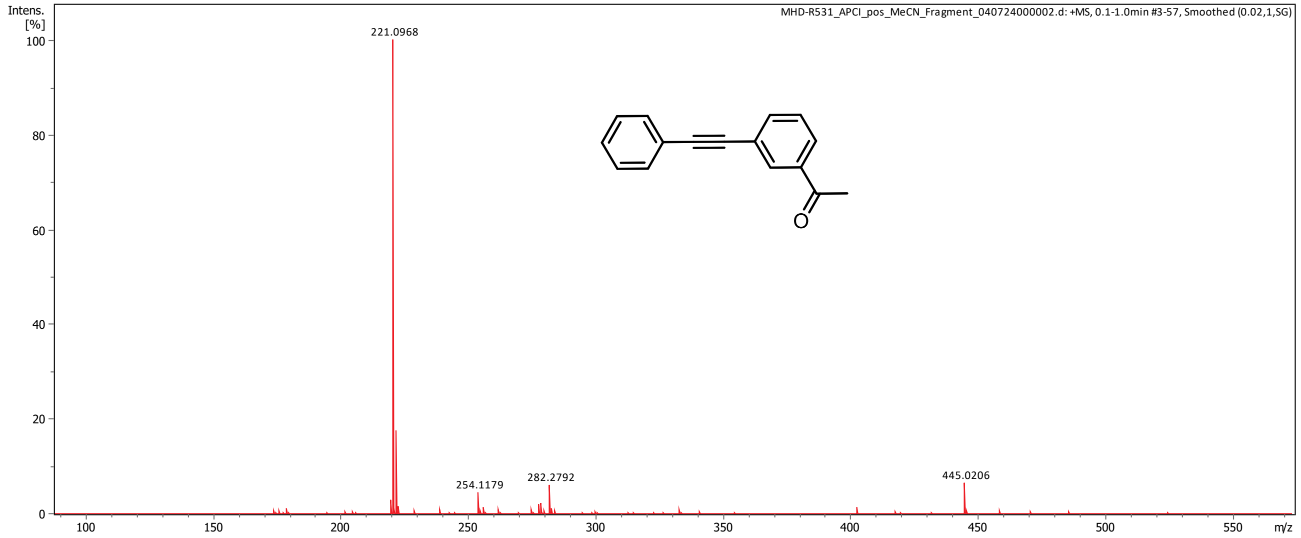

Intens. [%]
MHD-R531_APCI_pos_MeCN_Fragment_040724000002.d: +MS, 0.1-1.0min #3-57, Smoothed (0.02,1,SG)
221.0968
254.1179
282.2792
445.0206
m/z

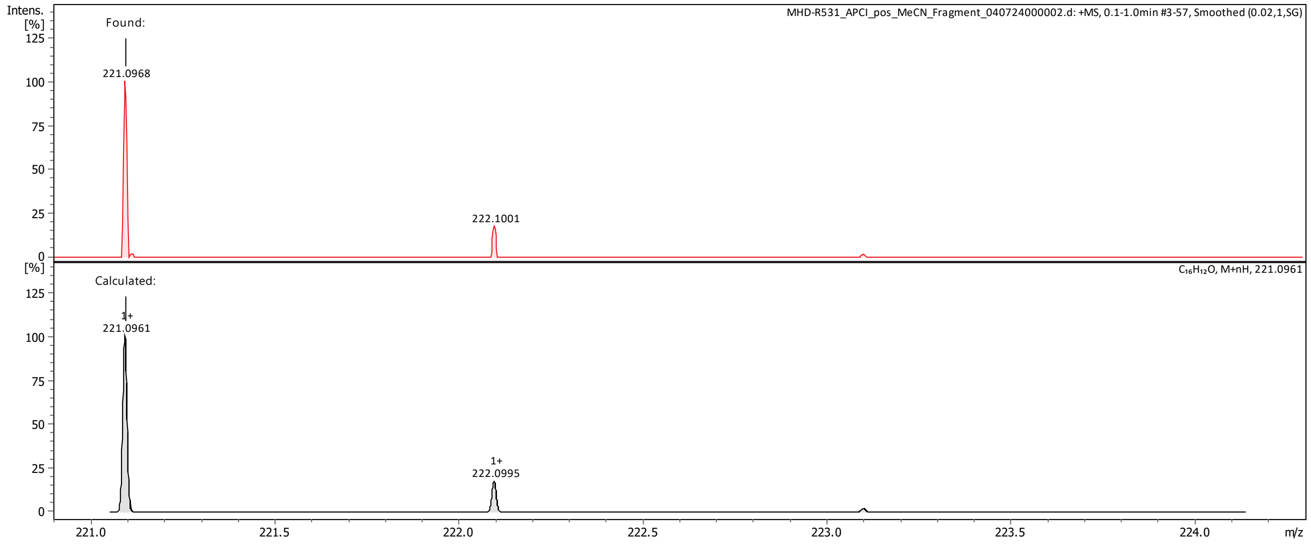

Intens. [%]
MHD-R531_APCI_pos_MeCN_Fragment_040724000002.d: +MS, 0.1-1.0min #3-57, Smoothed (0.02,1,SG)
Found:
221.0968
222.1001
Calculated:
$C_{16}H_{12}O$, M+nH, 221.0961
1+
221.0961
1+
222.0995
m/z

4.9 1-(3-(Phenylethynyl)phenyl)ethan-1-one *O*-methyl oxime (1k) (600 MHz, $CDCl_3$)

4.9.1 ^{1}H NMR

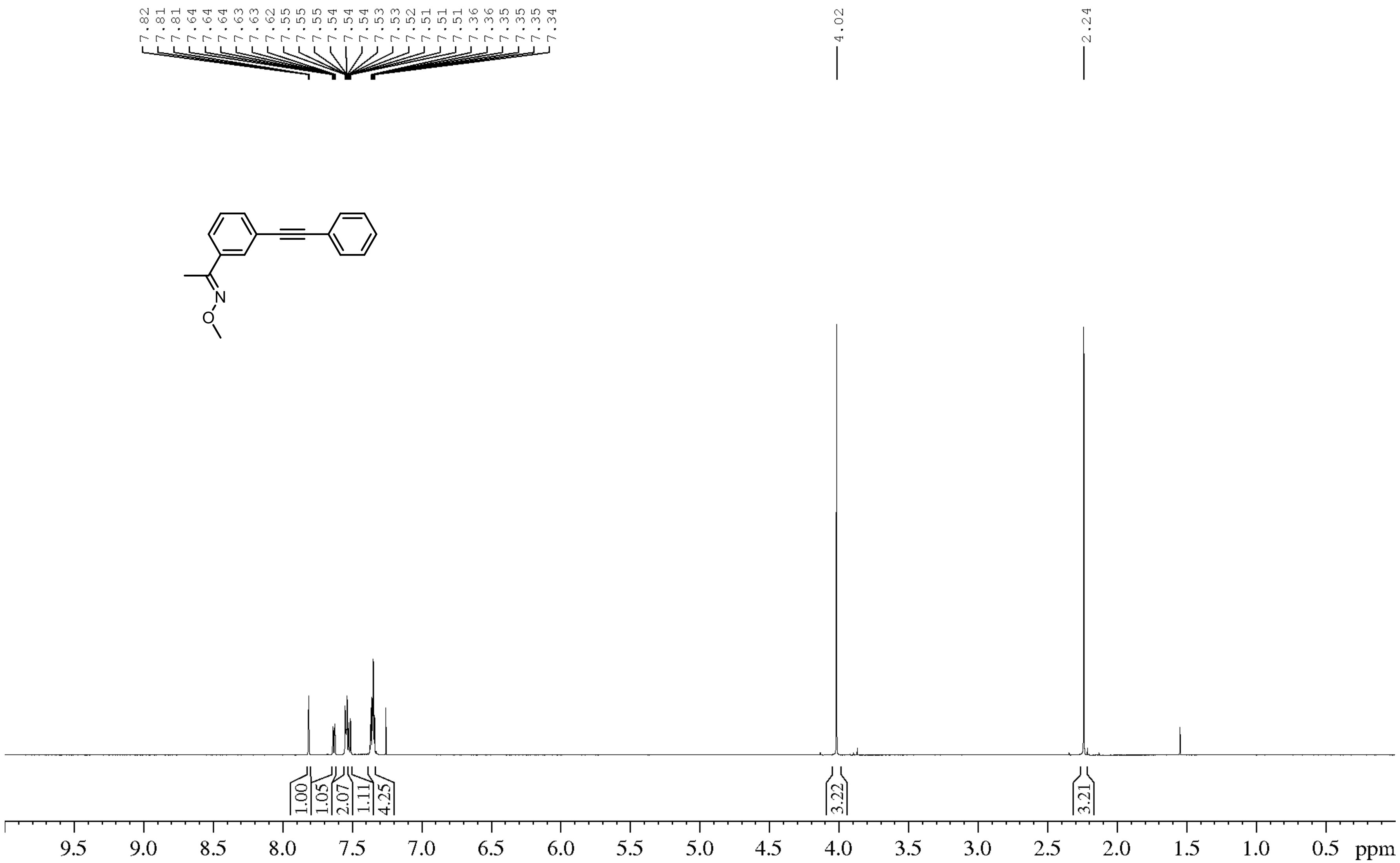

4.9.2 ^{13}C NMR

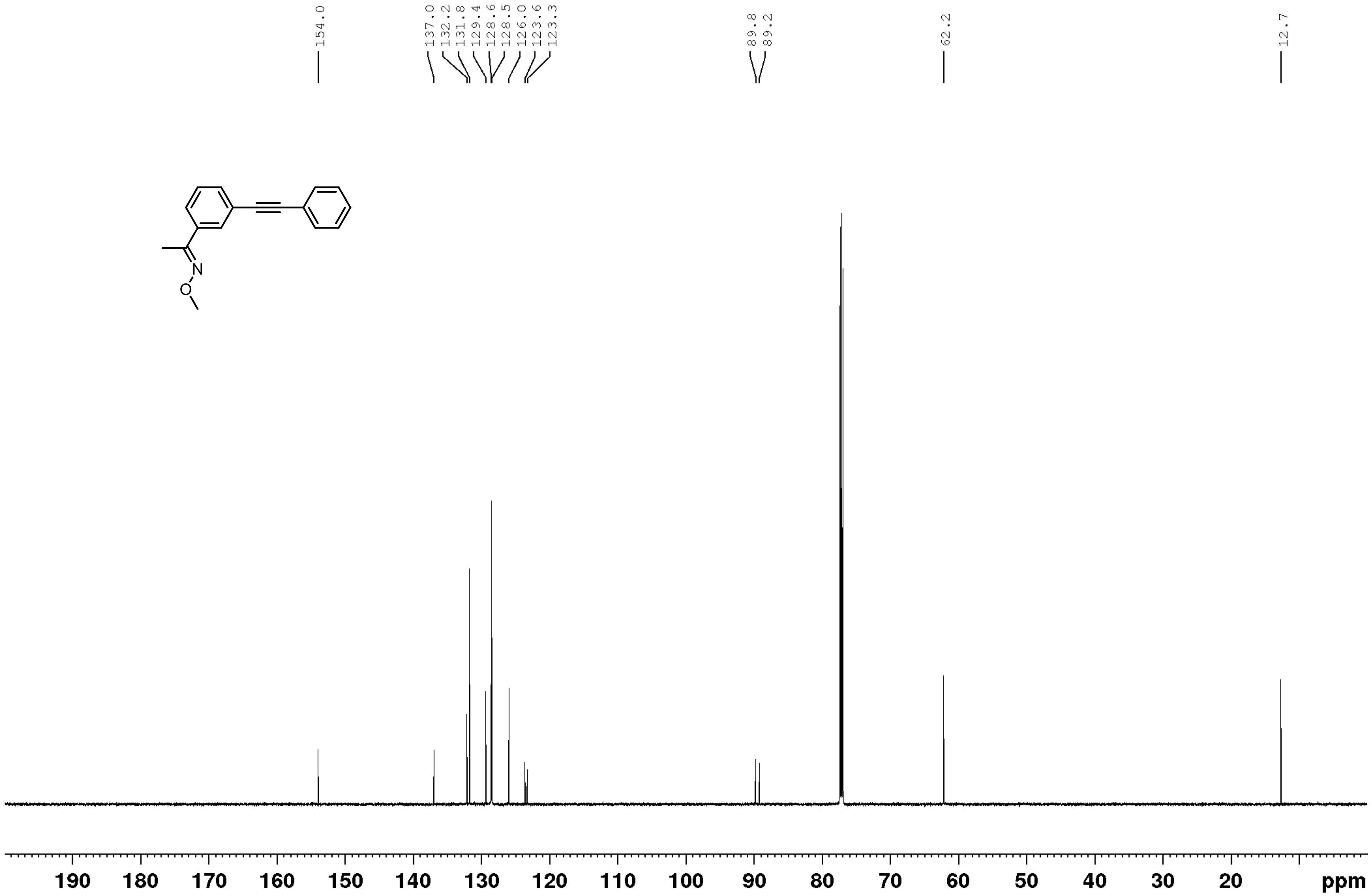

154.0
137.0
132.2
131.8
129.4
128.6
128.5
126.0
123.6
123.3
89.8
89.2
62.2
12.7
N
O
190
180
170
160
150
140
130
120
110
100
90
80
70
60
50
40
30
20
ppm

4.9.3 ^{1}H, ^{1}H COSY

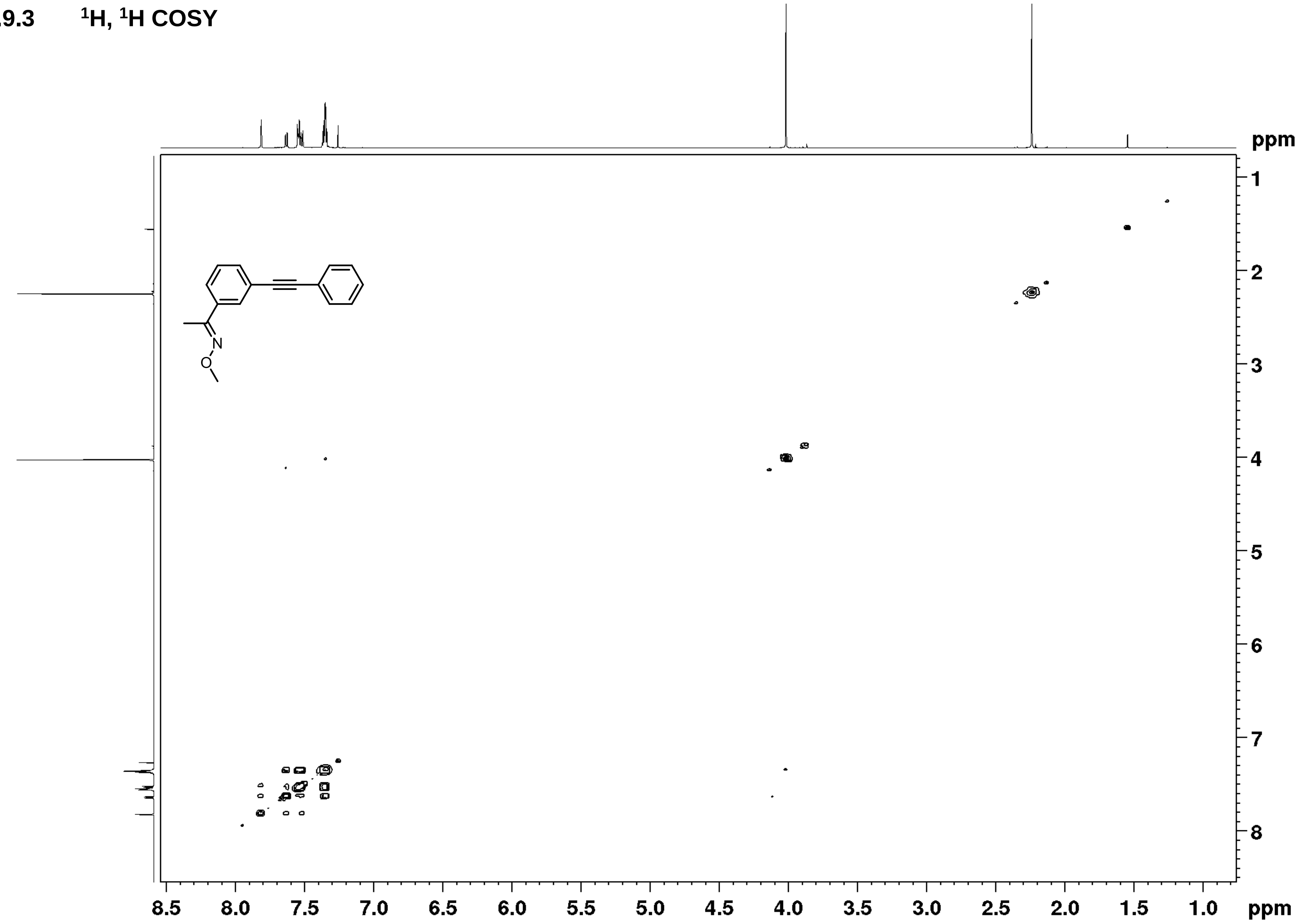

4.9.4 ^{1}H, ^{13}C HMBC

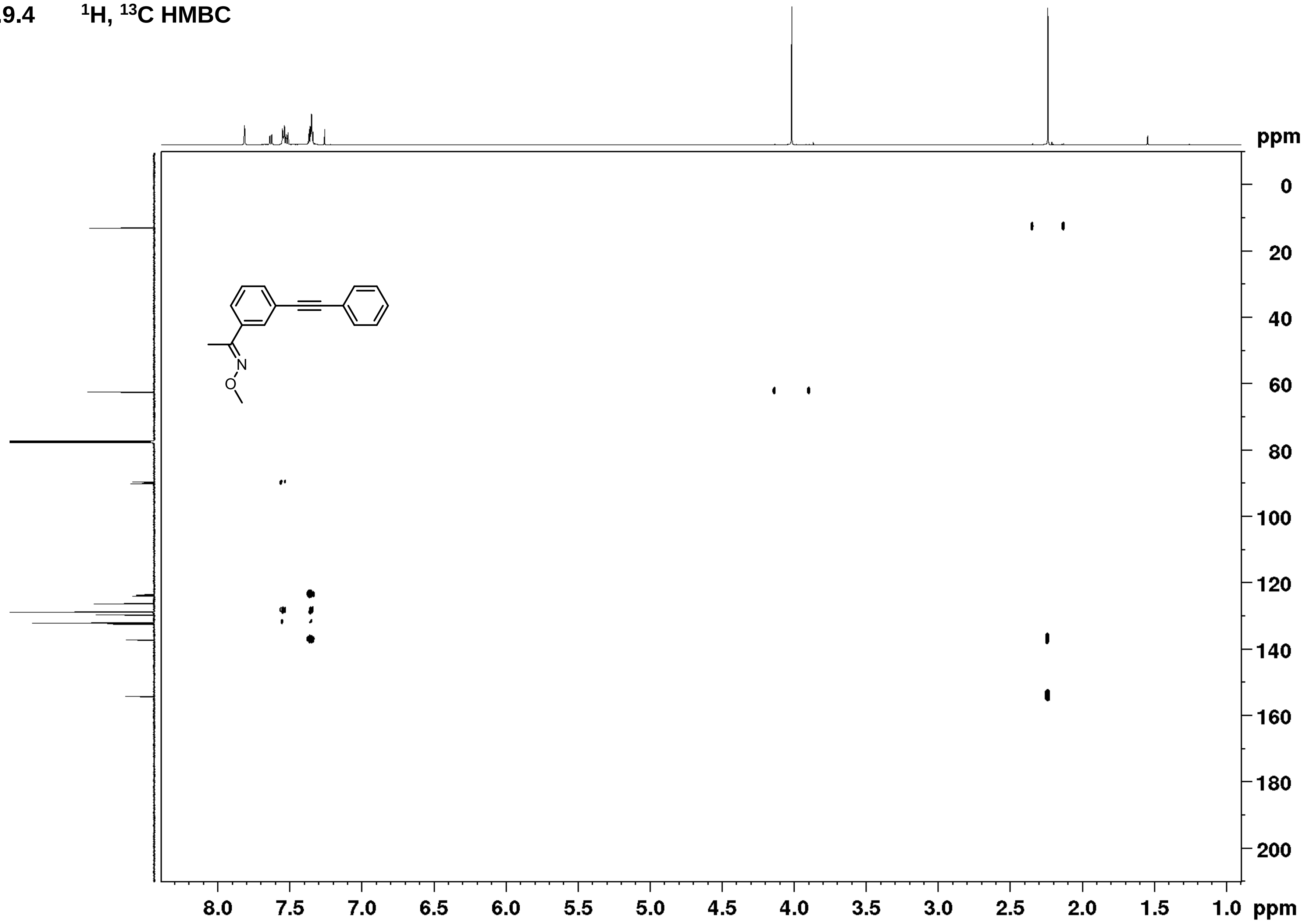

4.9.5 ^{1}H, ^{13}C HSQC

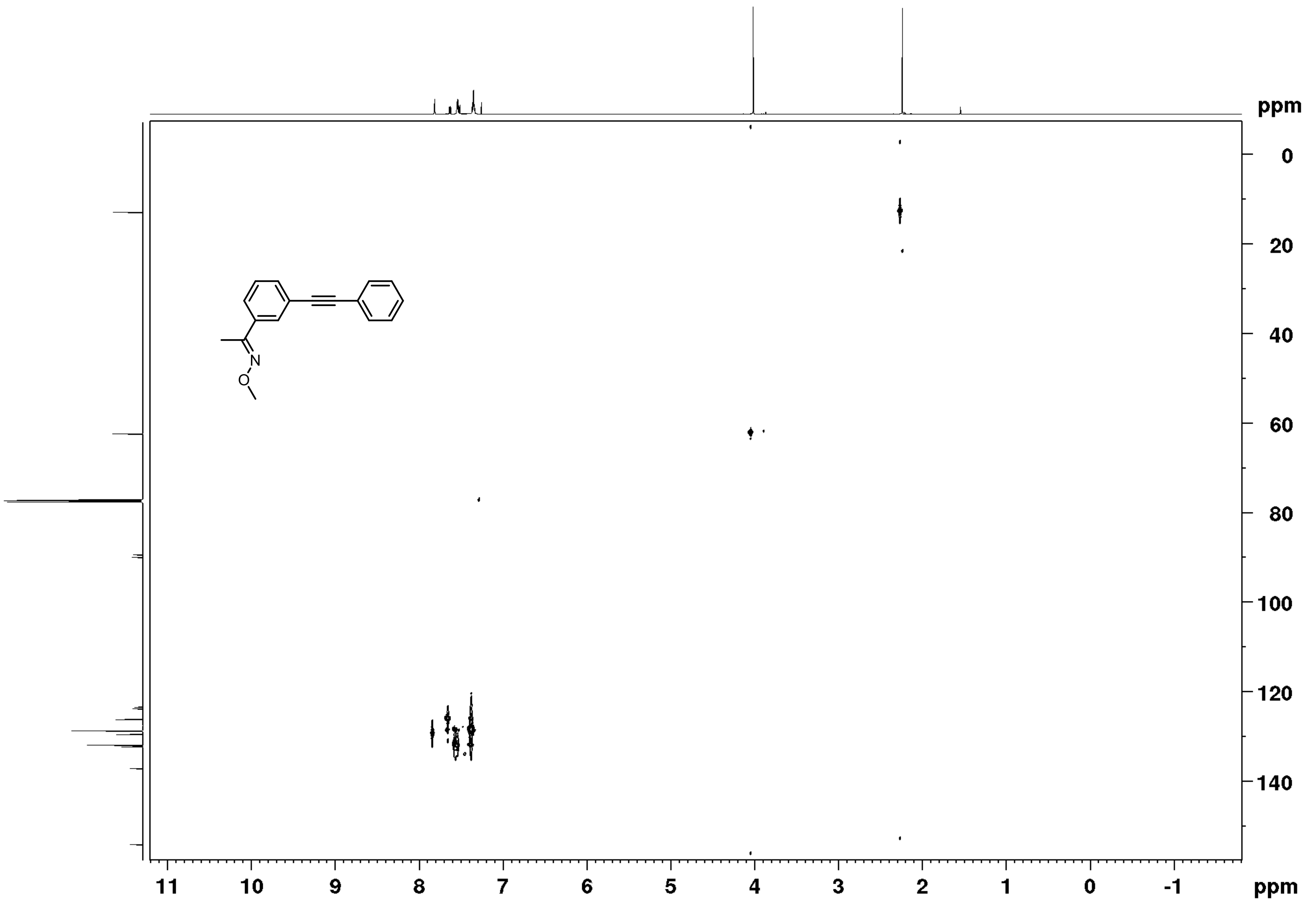

4.9.6 HRMS

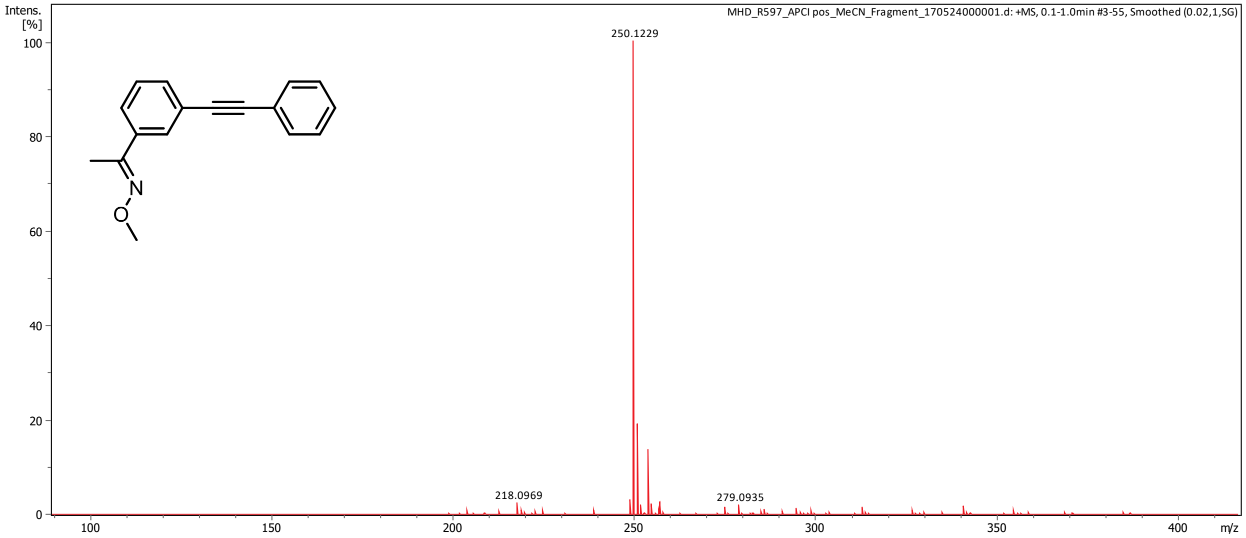

Intens. [%]
MHD_R597_APCI pos_MeCN_Fragment_170524000001.d: +MS, 0.1-1.0min #3-55, Smoothed (0.02,1,SG)
250.1229
218.0969
279.0935
m/z

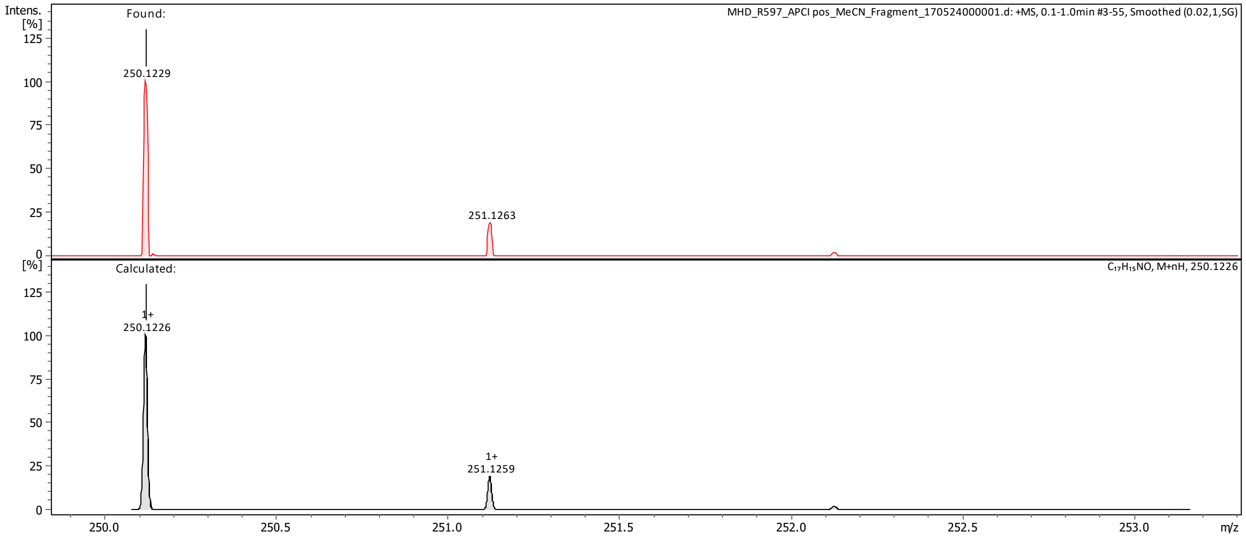

Intens. [%]
Found:
250.1229
251.1263
MHD_R597_APCI pos_MeCN_Fragment_170524000001.d: +MS, 0.1-1.0min #3-55, Smoothed (0.02,1,SG)
Calculated:
C17H15NO, M+nH, 250.1226
1+ 250.1226
1+ 251.1259
m/z

4.9.7 IR

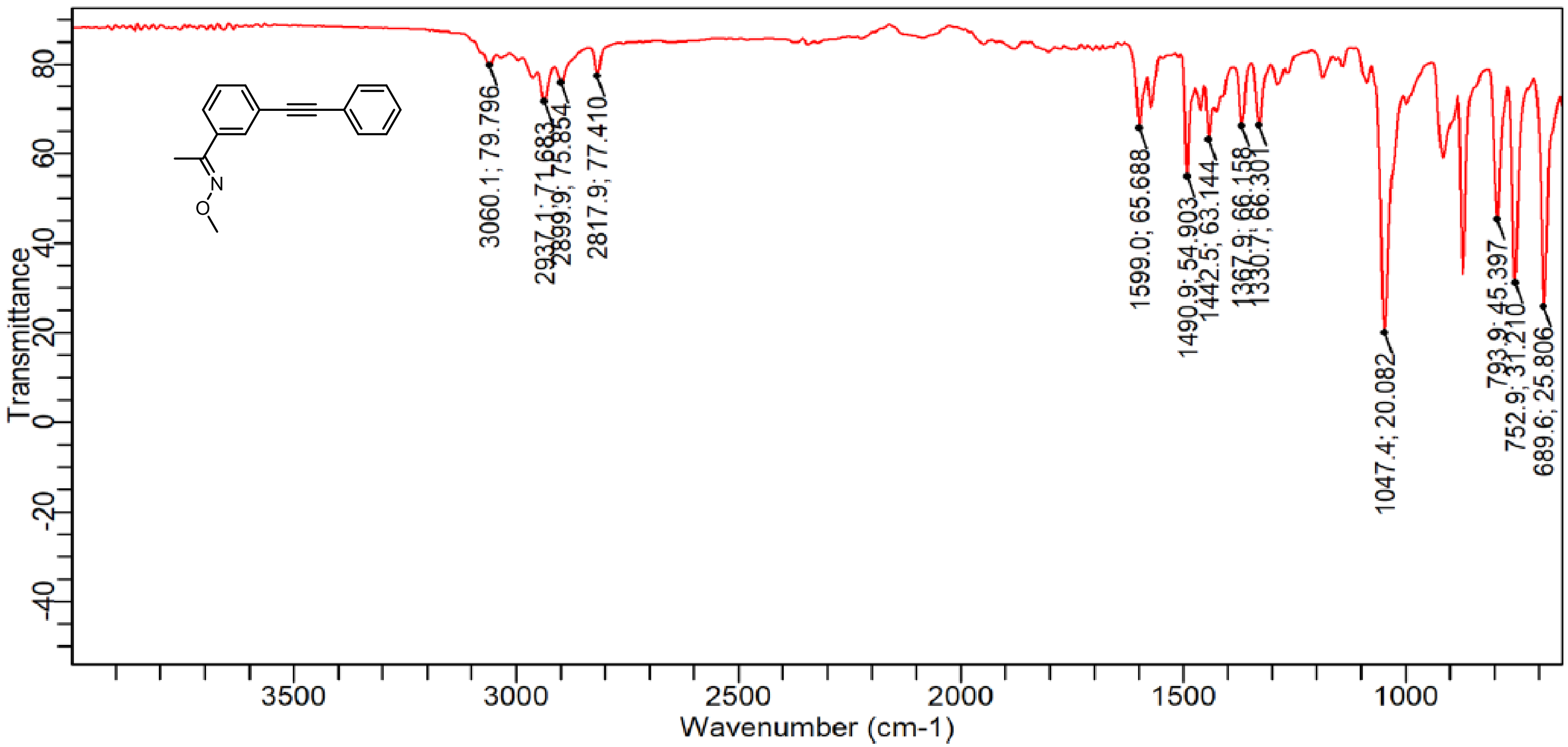

Transmittance
Wavenumber (cm-1)
3060.1; 79.796
2937.1; 71.683
2899.9; 75.854
2817.9; 77.410
1599.0; 65.688
1490.9; 54.903
1442.5; 63.144
1367.9; 66.158
1330.7; 66.301
1047.4; 20.082
793.9; 45.397
752.9; 31.210
689.6; 25.806
N
O

4.10 *N*-Methyl-1-(3-(phenylethynyl)phenyl)ethan-1-imine (1l) (600 MHz, $CDCl_3$)

4.10.1 ^{1}H NMR

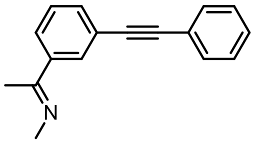

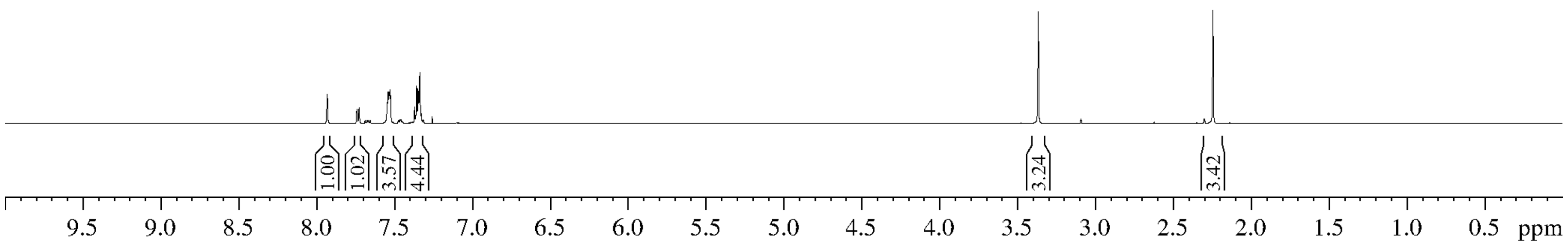

4.10.2 ¹³C NMR

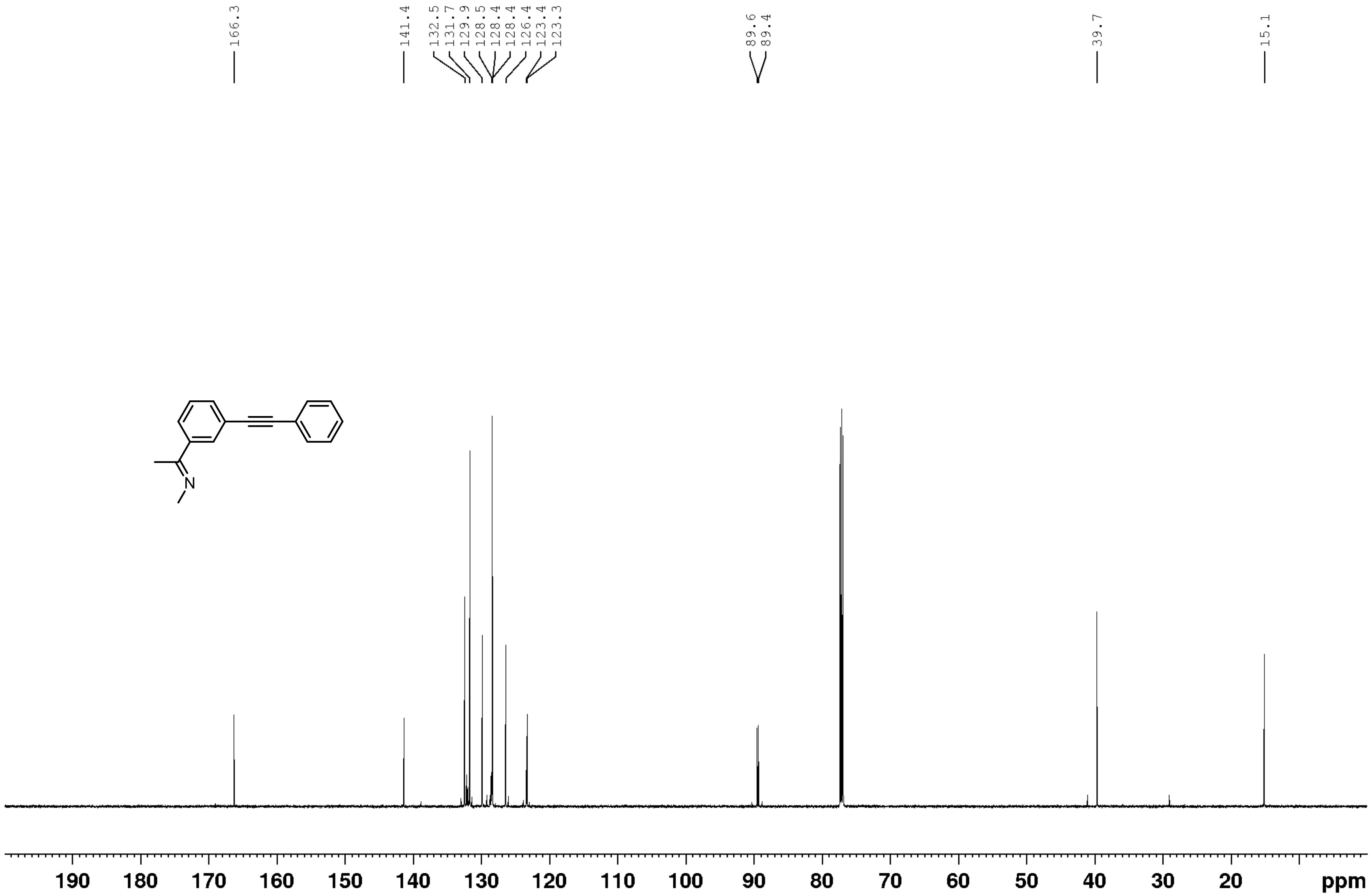
166.3
141.4
132.5
131.7
129.9
128.5
128.4
128.4
126.4
123.4
123.3
89.6
89.4
39.7
15.1
N
190 180 170 160 150 140 130 120 110 100 90 80 70 60 50 40 30 20
ppm

4.10.3 ^{1}H, ^{1}H COSY

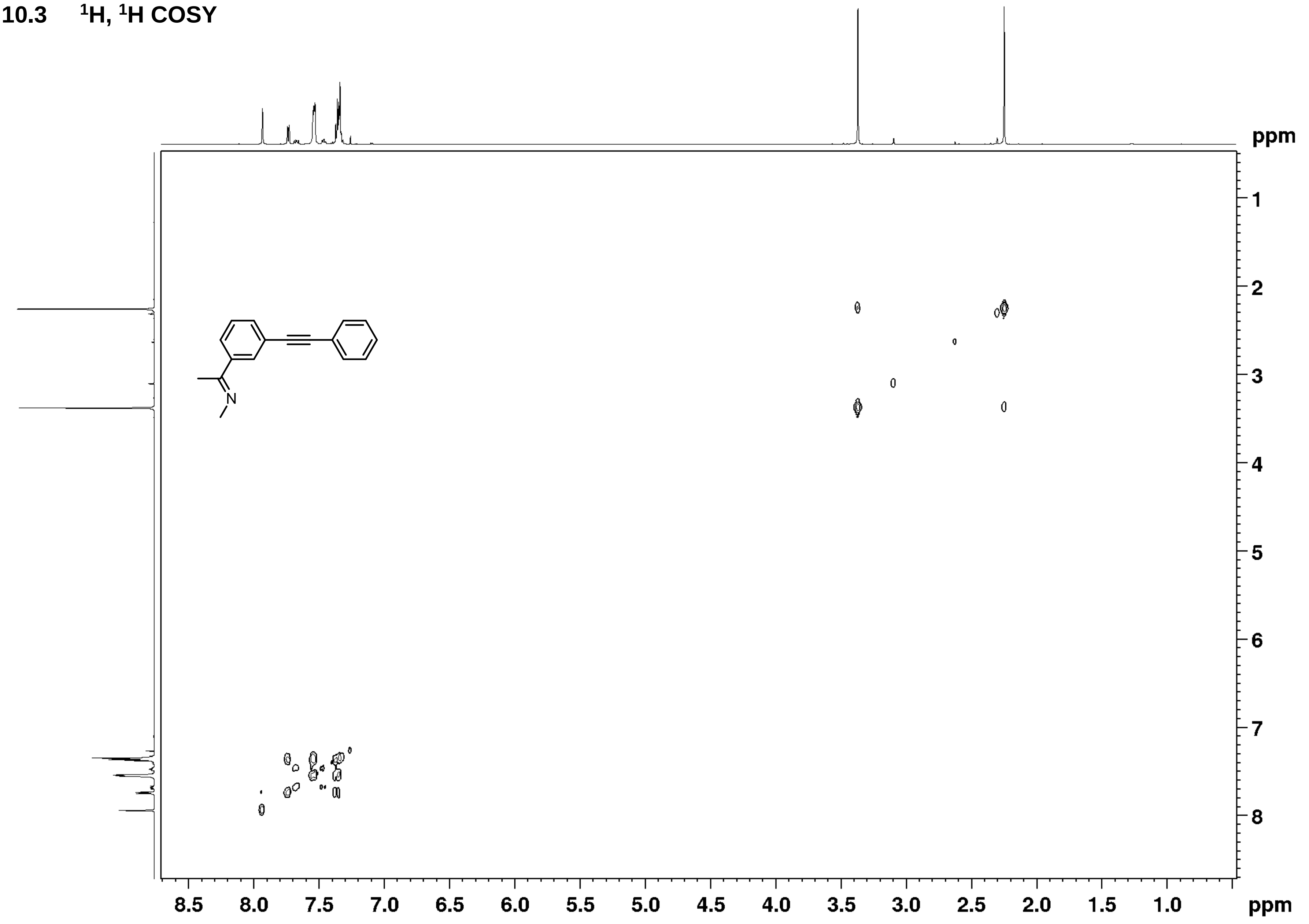

4.10.4 ^{1}H, ^{13}C HMBC

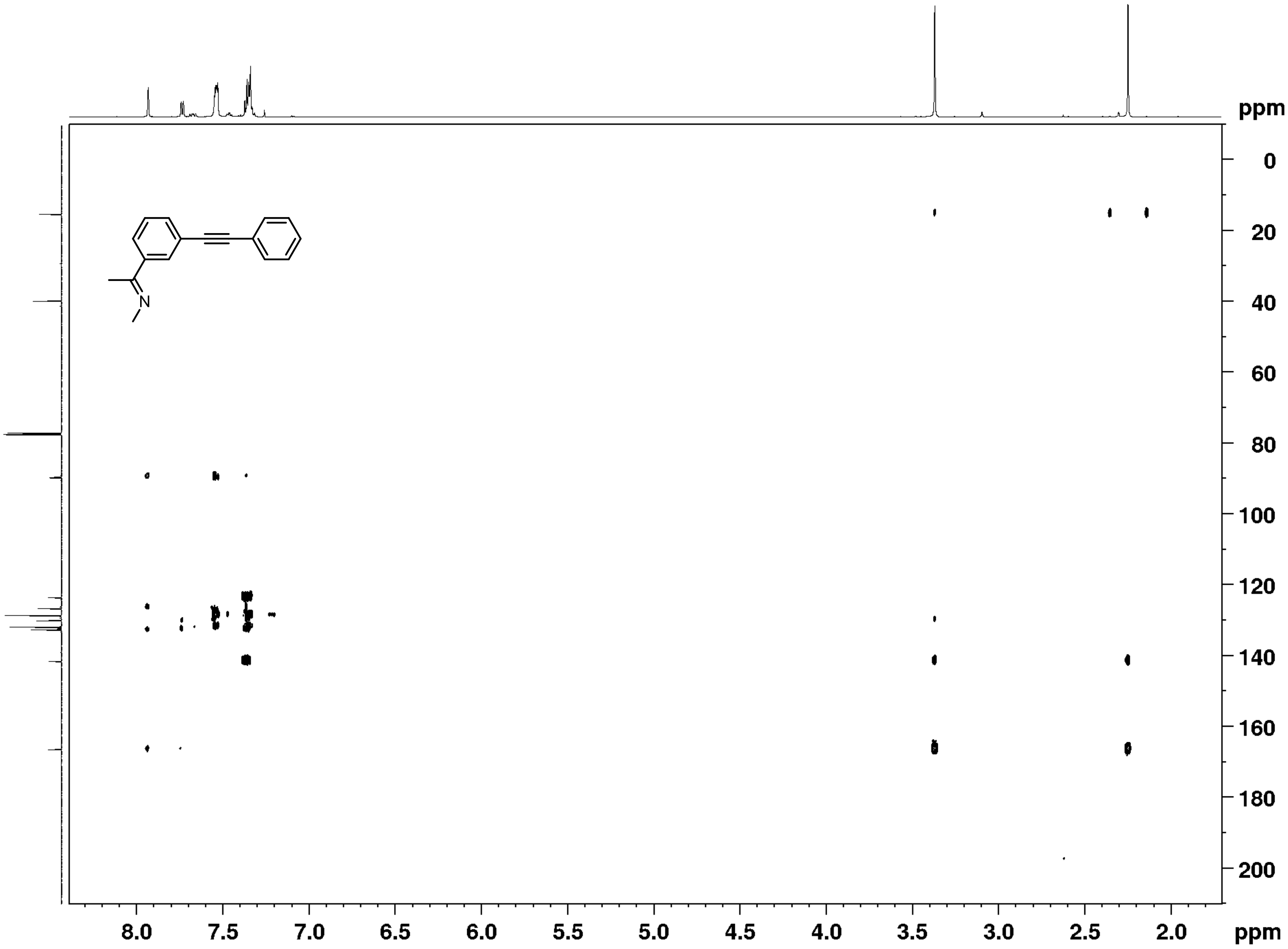

4.10.5 ^{1}H, ^{13}C HSQC

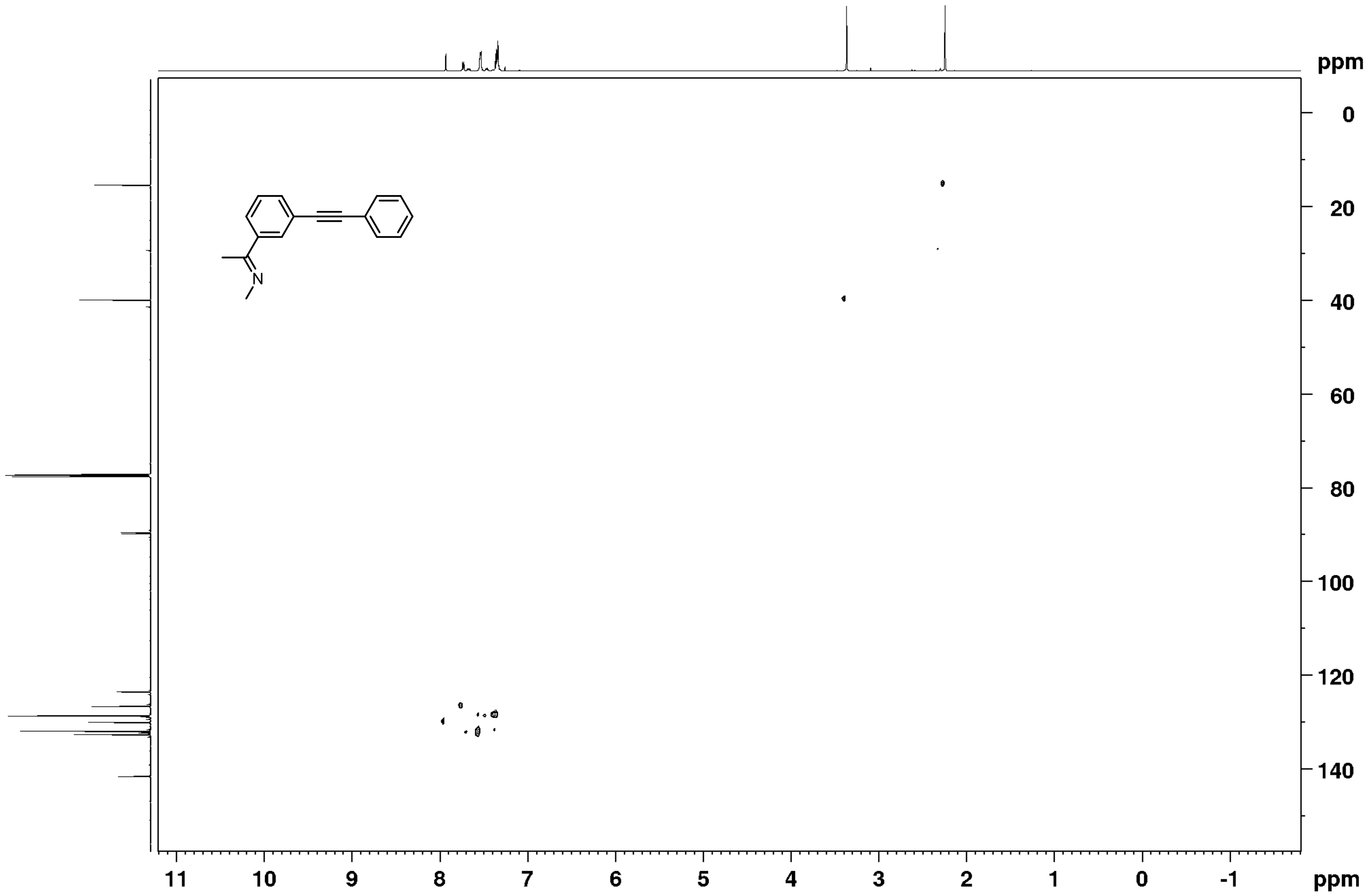

4.10.6 HRMS

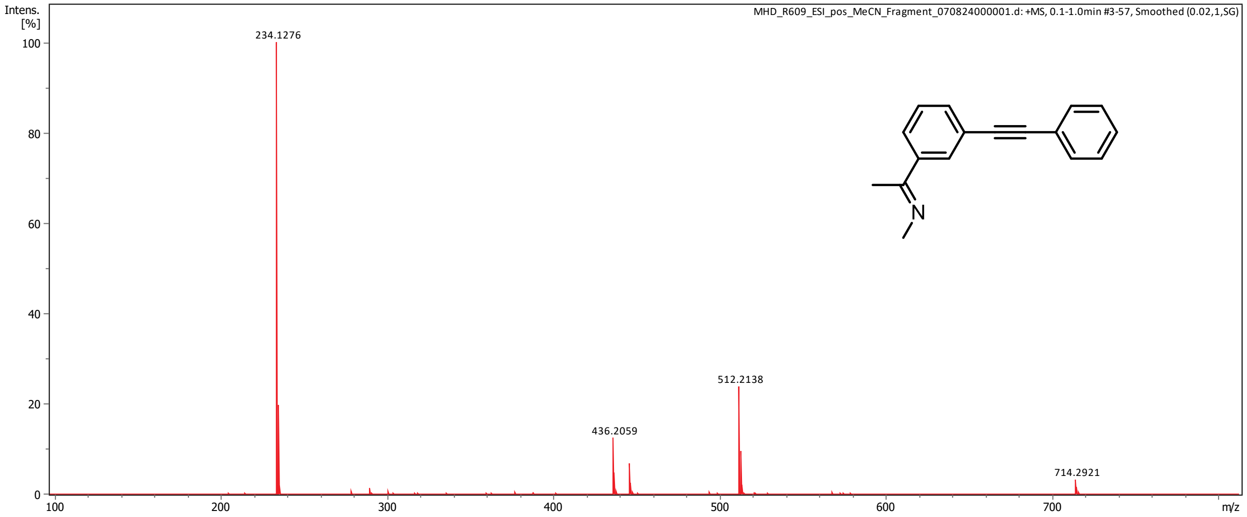

Intens. [%]
MHD_R609_ESI_pos_MeCN_Fragment_070824000001.d: +MS, 0.1-1.0min #3-57, Smoothed (0.02,1,SG)
234.1276
512.2138
436.2059
714.2921
N
m/z

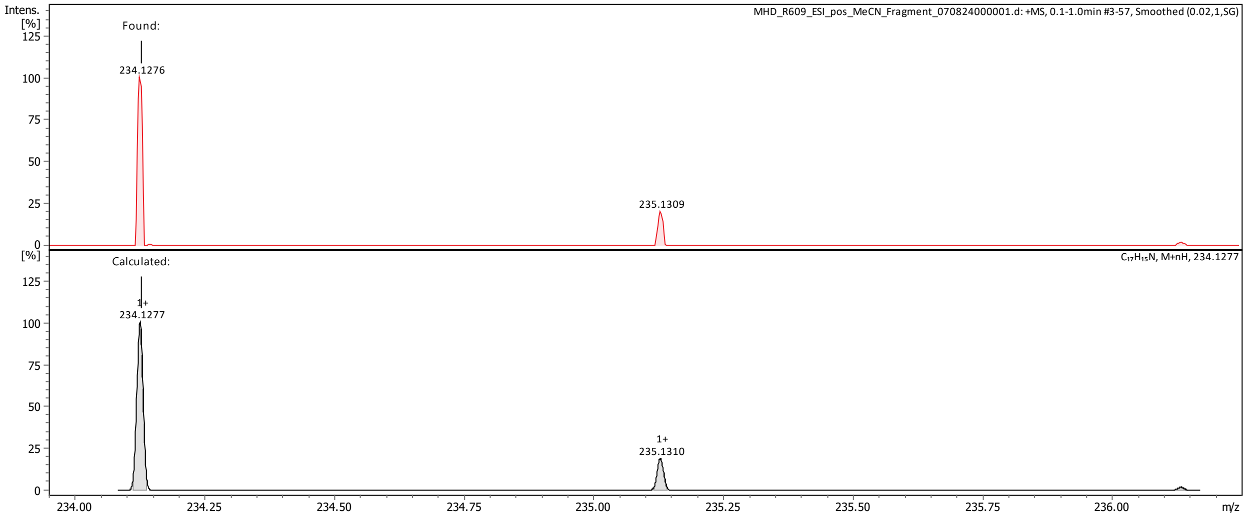

Intens. [%]
MHD_R609_ESI_pos_MeCN_Fragment_070824000001.d: +MS, 0.1-1.0min #3-57, Smoothed (0.02,1,SG)
Found:
234.1276
235.1309
Calculated:
C17H15N, M+nH, 234.1277
1+
234.1277
1+
235.1310
m/z

4.10.7 IR

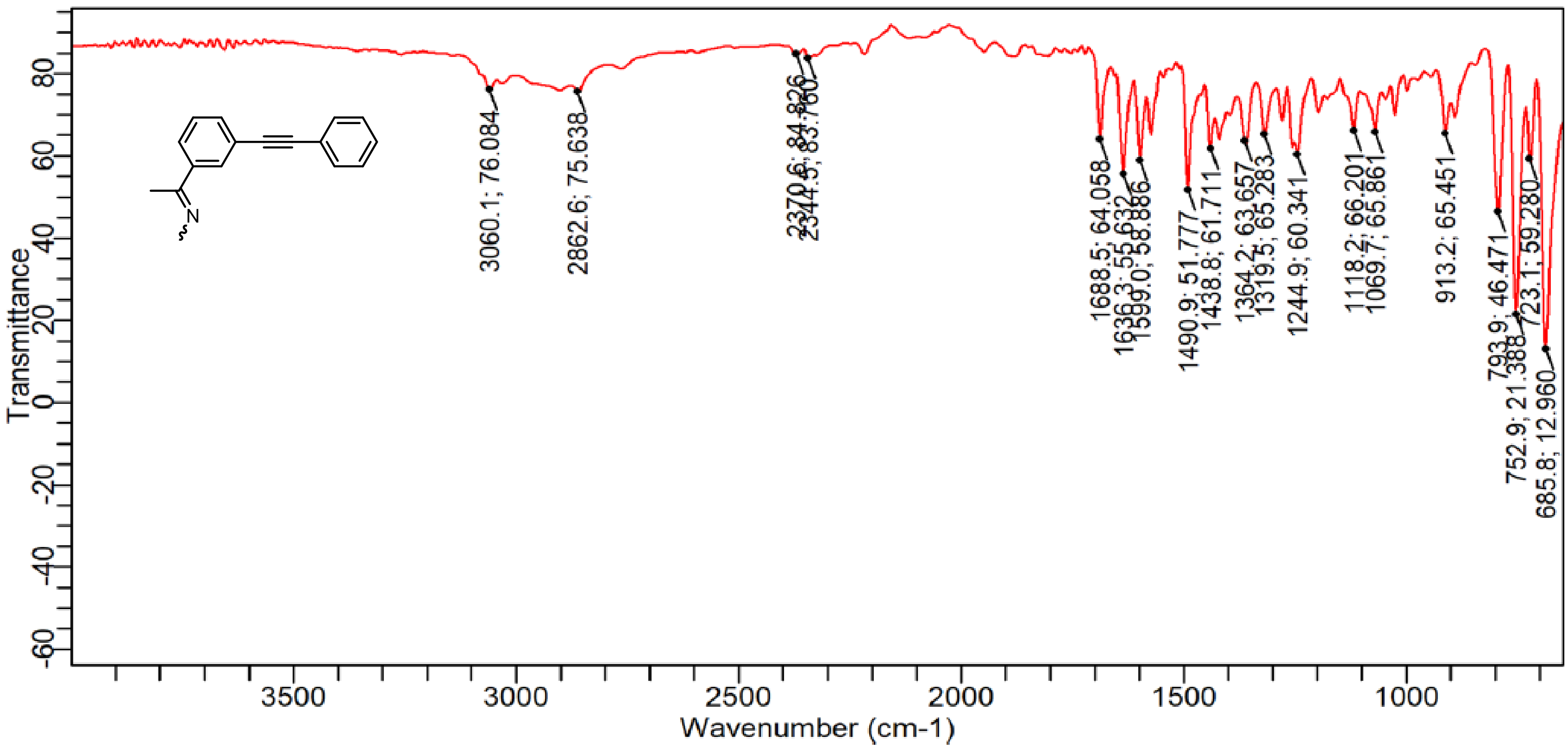

Transmittance
Wavenumber (cm-1)
3060.1; 76.084
2862.6; 75.638
2370.6; 84.826
2344.5; 83.760
1688.5; 64.058
1636.3; 55.632
1599.0; 58.886
1490.9; 51.777
1438.8; 61.711
1364.2; 63.657
1319.5; 65.283
1244.9; 60.341
1118.2; 66.201
1069.7; 65.861
913.2; 65.451
793.9; 46.471
752.9; 21.388
723.1; 59.280
685.8; 12.960
N

4.11 Ethyl 6-phenylhex-5-ynoate (1m) (600 MHz, $CDCl_3$)

4.11.1 ^1H NMR

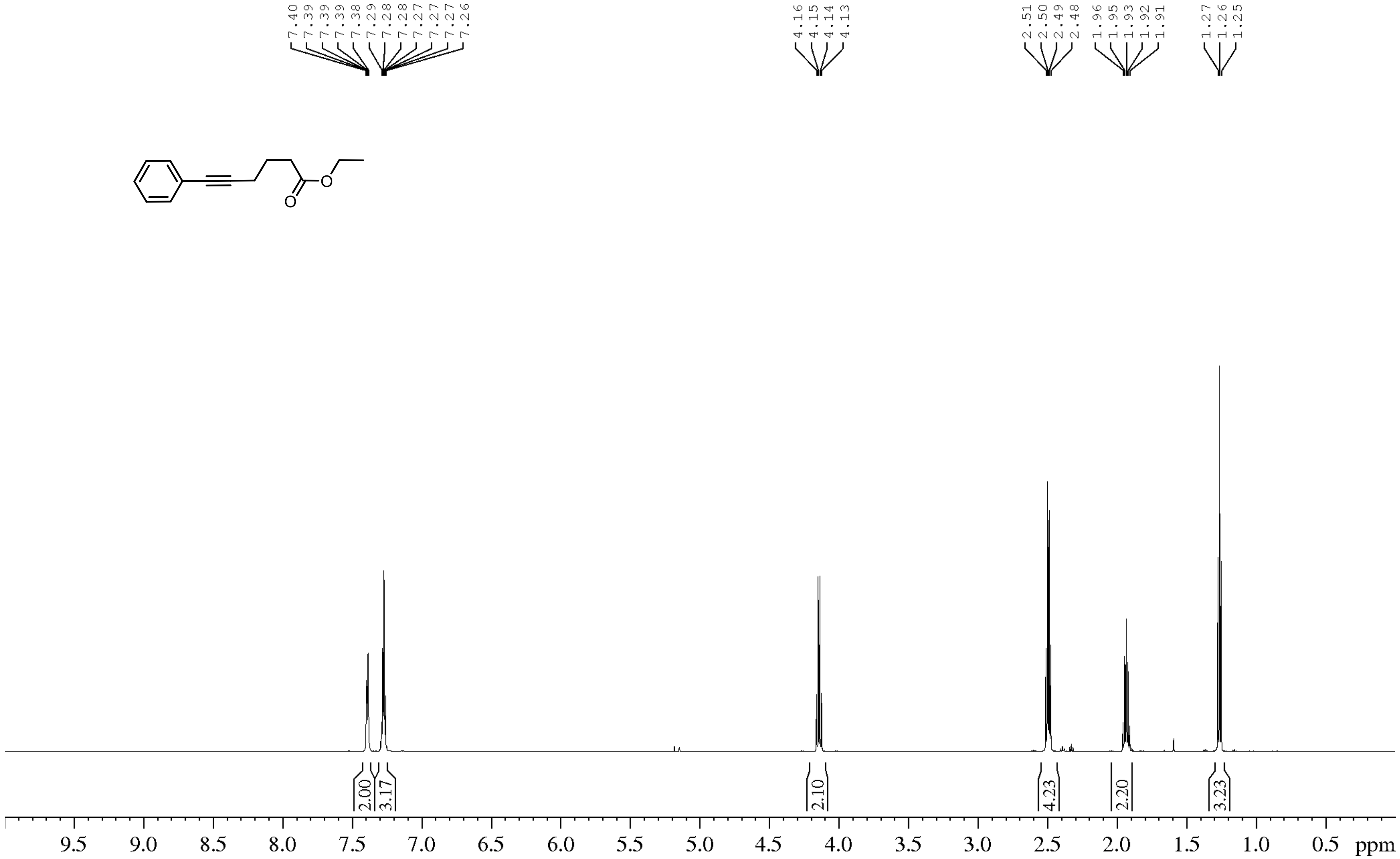

4.11.2 ^{13}C NMR

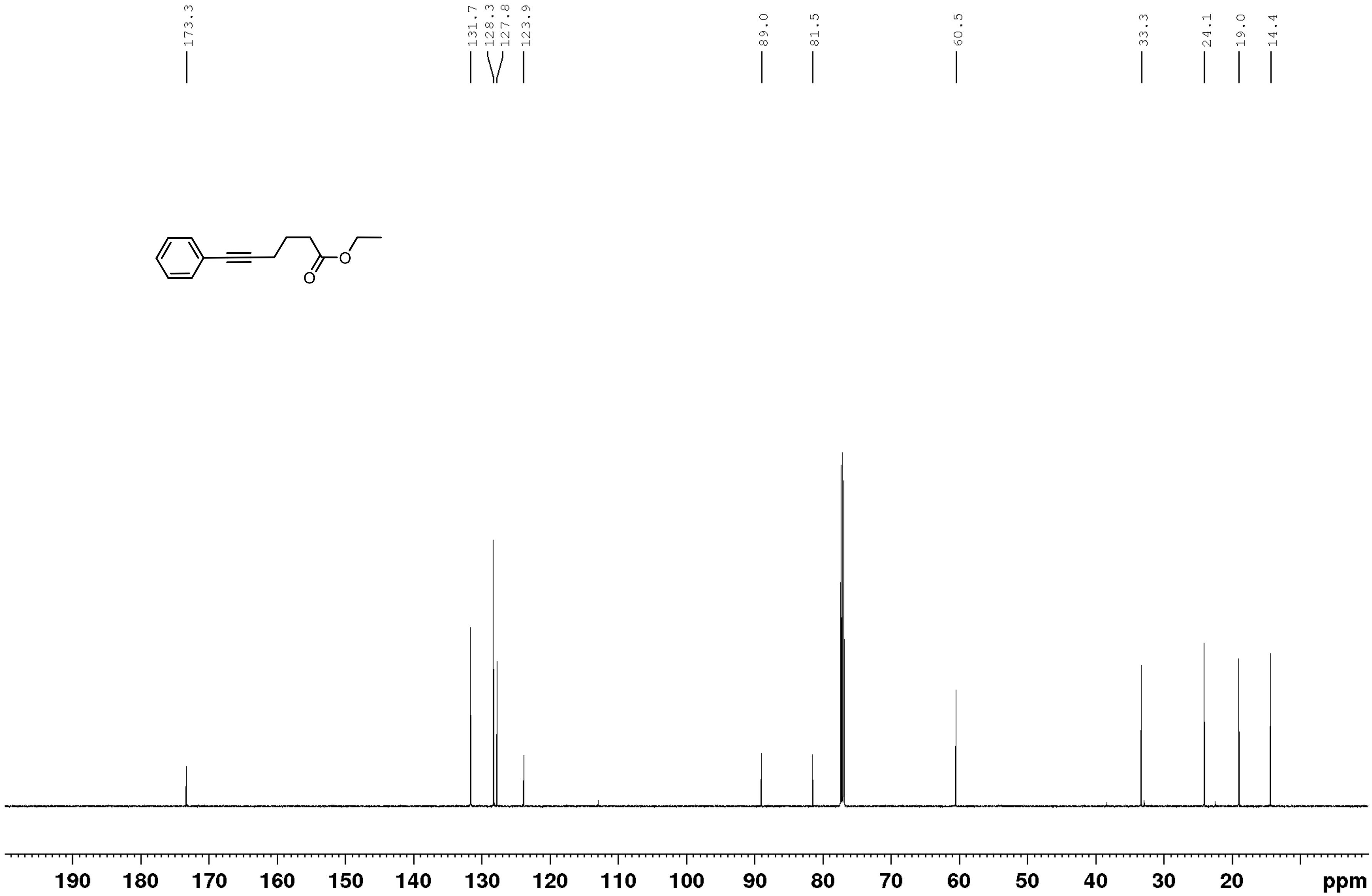

173.3
131.7
128.3
127.8
123.9
89.0
81.5
60.5
33.3
24.1
19.0
14.4
O
O
190
180
170
160
150
140
130
120
110
100
90
80
70
60
50
40
30
20
ppm

4.11.3 ^{1}H, ^{1}H COSY

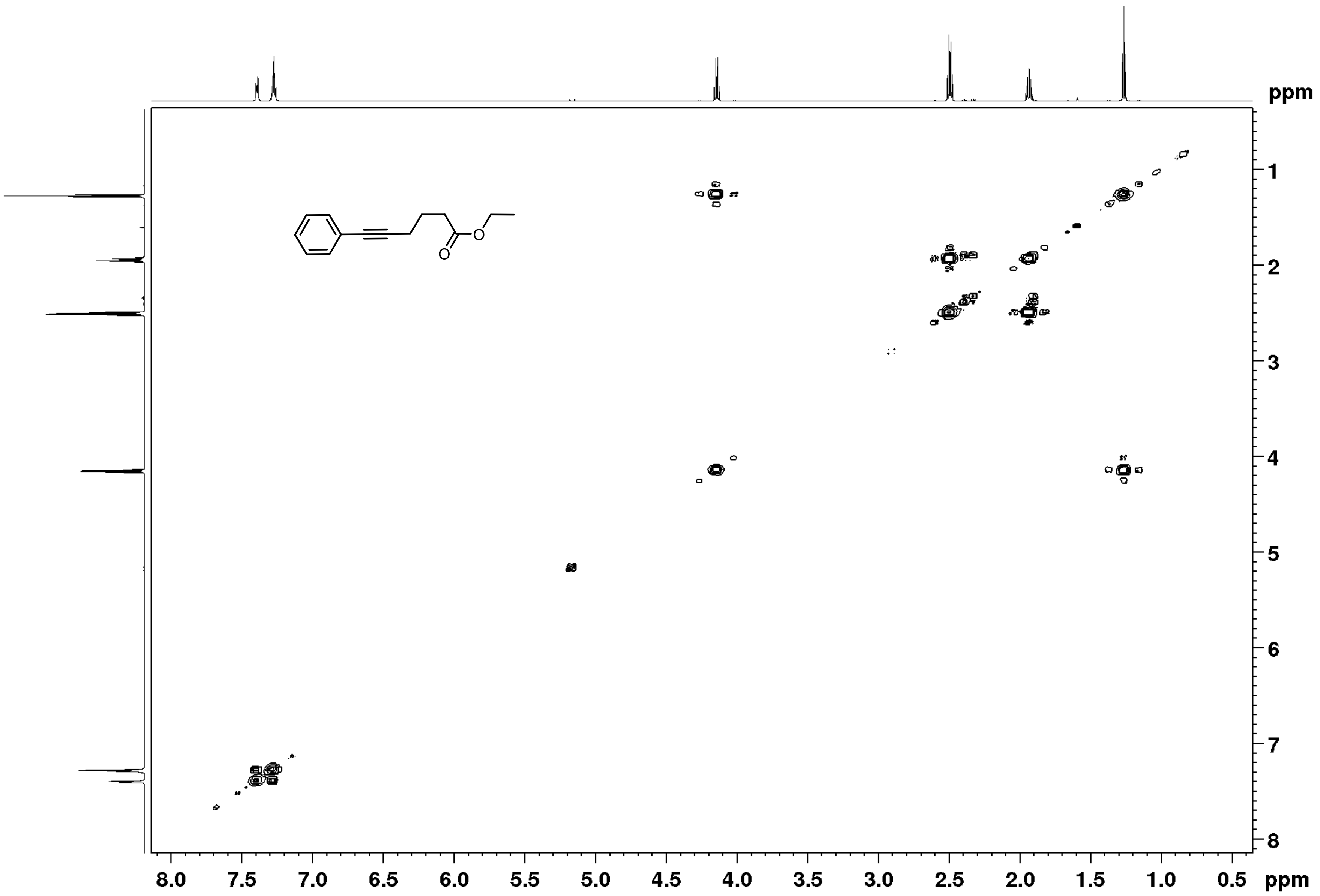

4.11.4 ^{1}H, ^{13}C HMBC

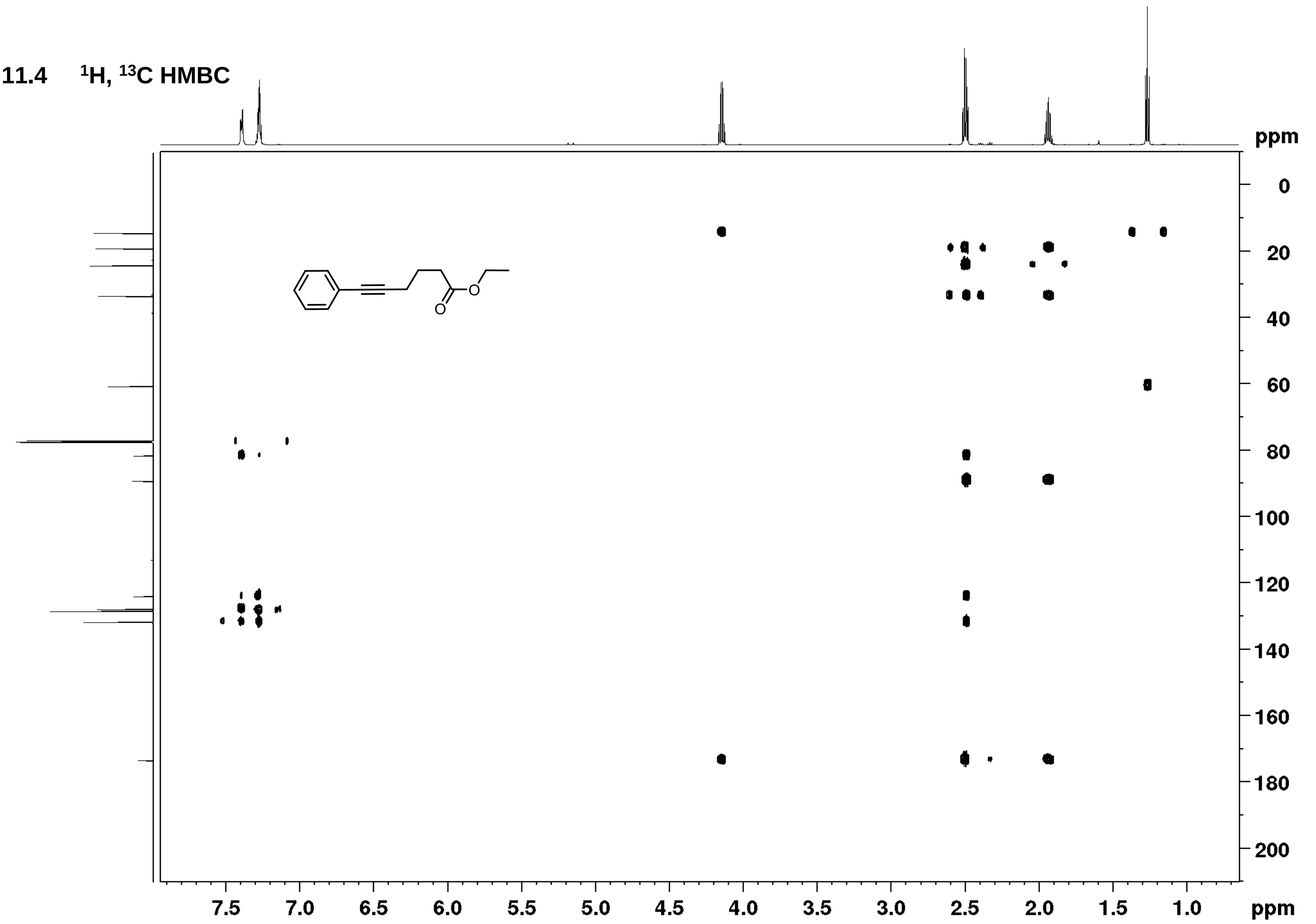

4.11.5 ^{1}H, ^{13}C HSQC

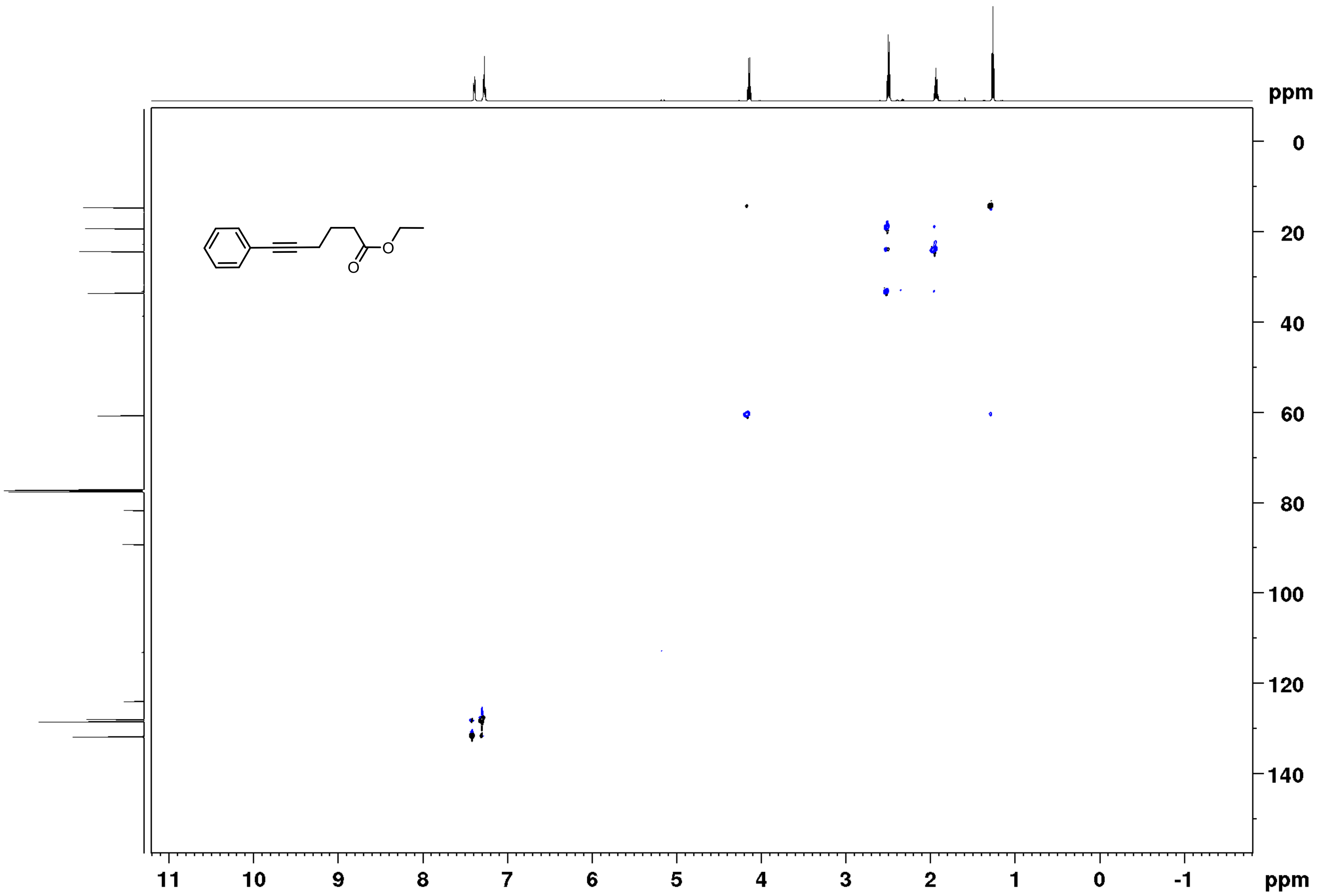

4.11.6 HRMS

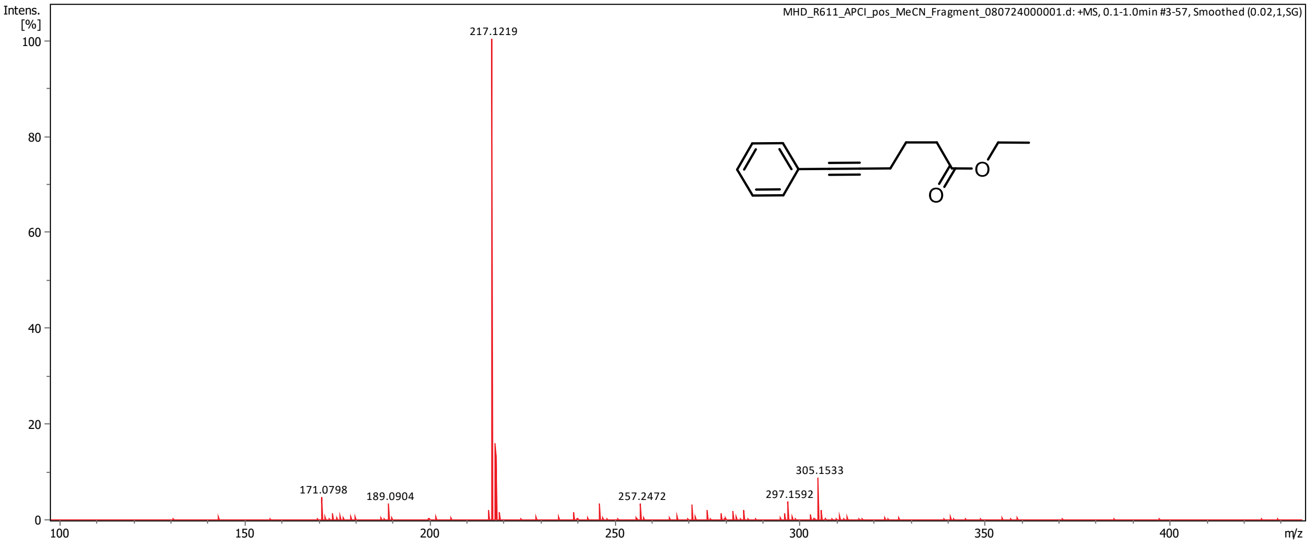

Intens.
[%]
MHD_R611_APCI_pos_MeCN_Fragment_080724000001.d: +MS, 0.1-1.0min #3-57, Smoothed (0.02,1,SG)
217.1219
171.0798
189.0904
257.2472
297.1592
305.1533
O
O
m/z

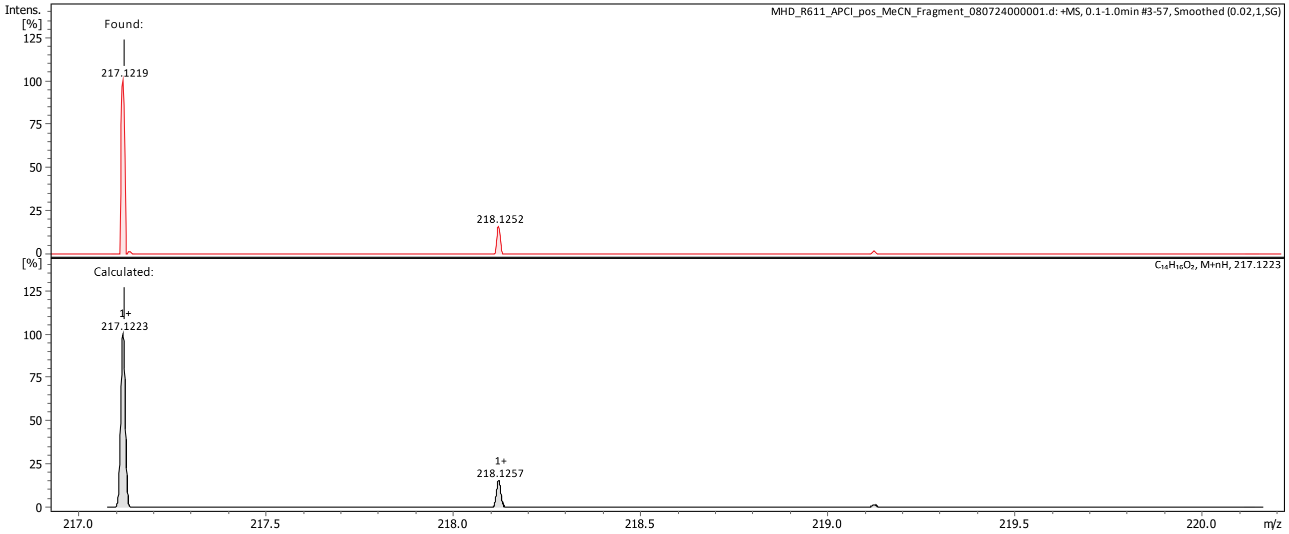

Intens.
[%]
Found:
217.1219
218.1252
MHD_R611_APCI_pos_MeCN_Fragment_080724000001.d: +MS, 0.1-1.0min #3-57, Smoothed (0.02,1,SG)
Calculated:
1+
217.1223
1+
218.1257
$C_{14}H_{16}O_2$, M+nH, 217.1223
m/z

4.12 1-(Phenylethynyl)-4-(prop-1-yn-1-yl)benzene (1o) (600 MHz, $CDCl_3$)

4.12.1 ^{1}H NMR

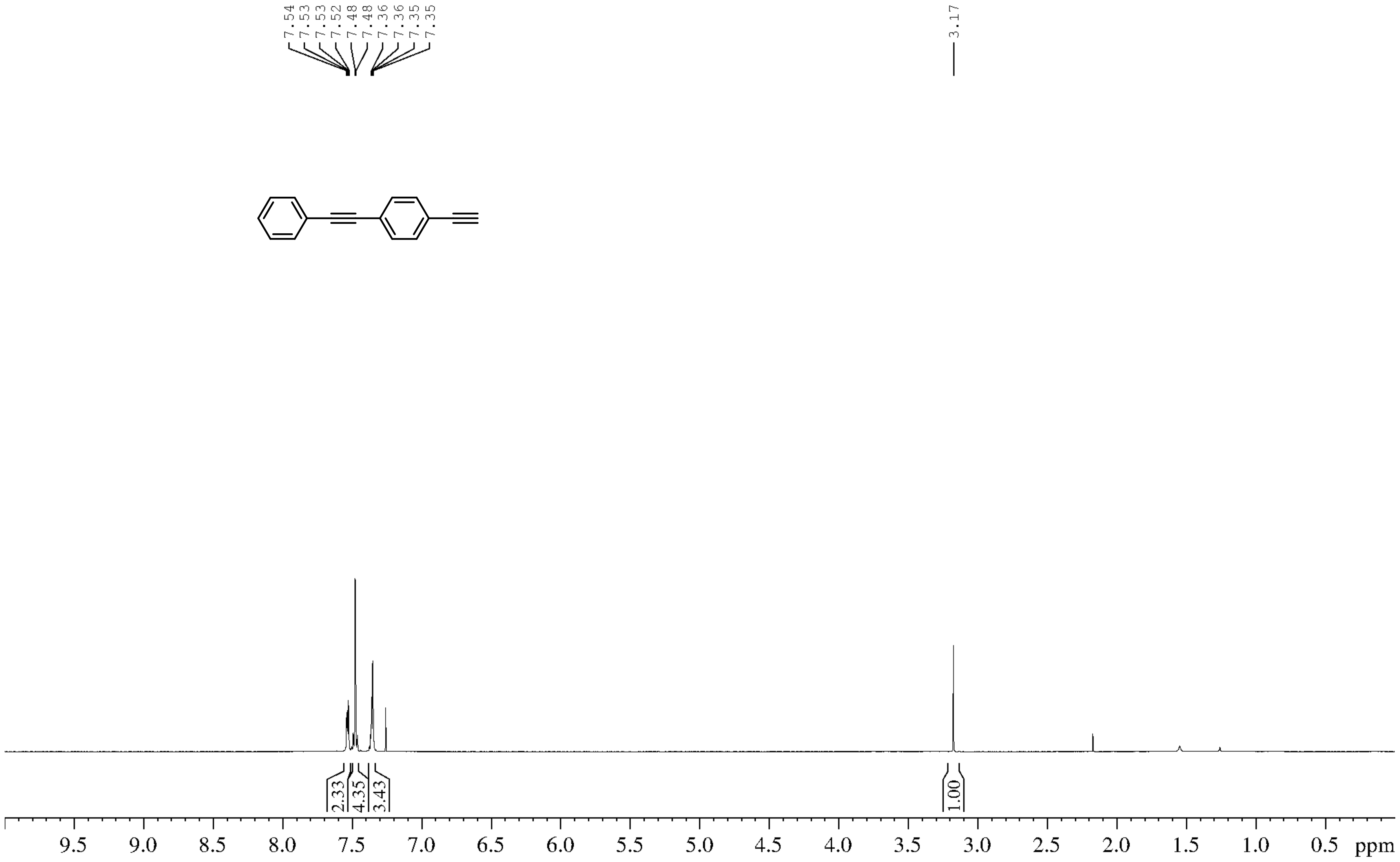

4.12.2 ^{13}C NMR

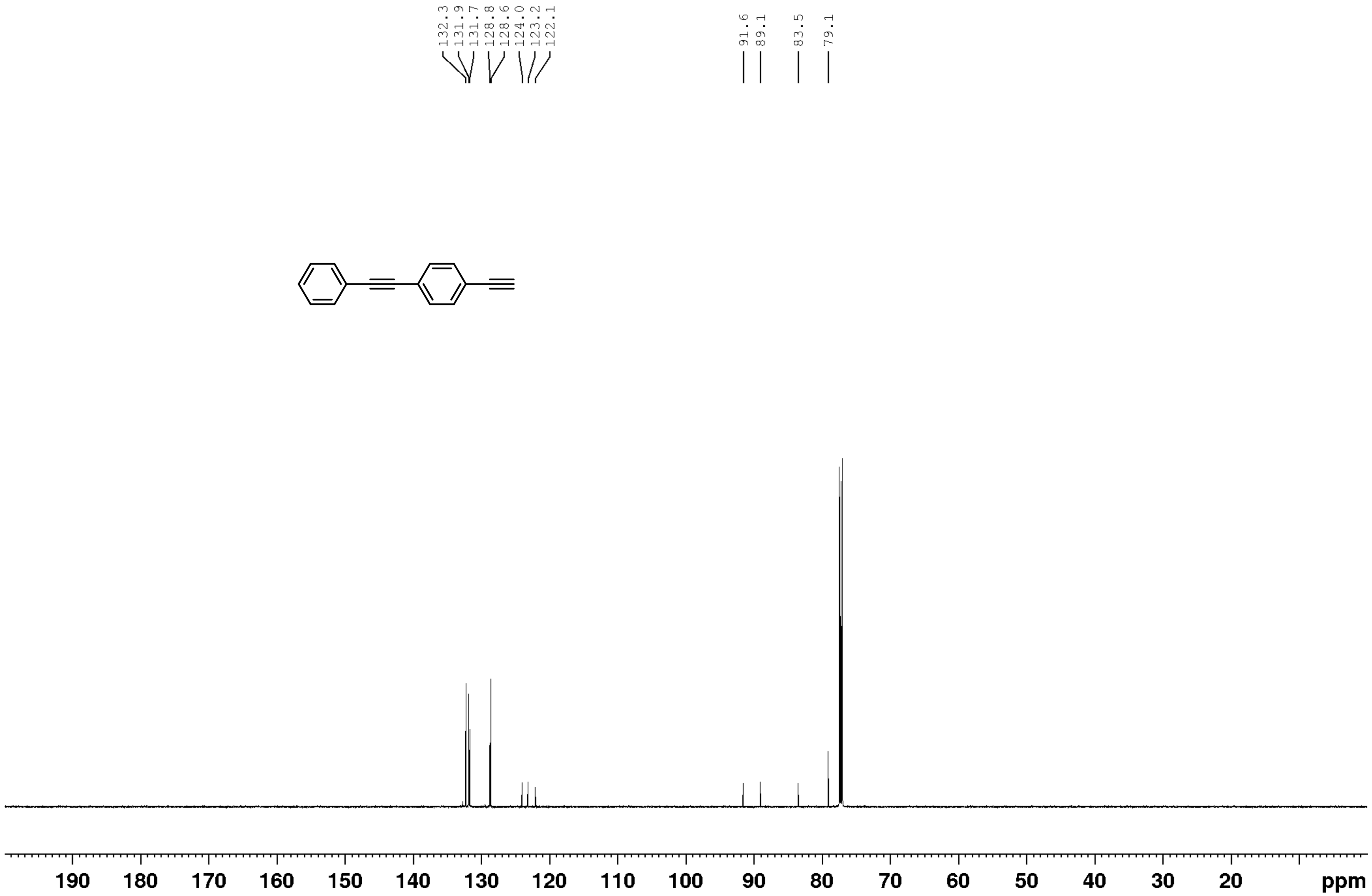

132.3
131.9
131.7
128.8
128.6
124.0
123.2
122.1
91.6
89.1
83.5
79.1
190
180
170
160
150
140
130
120
110
100
90
80
70
60
50
40
30
20
ppm

4.12.3 ^{1}H, ^{1}H COSY

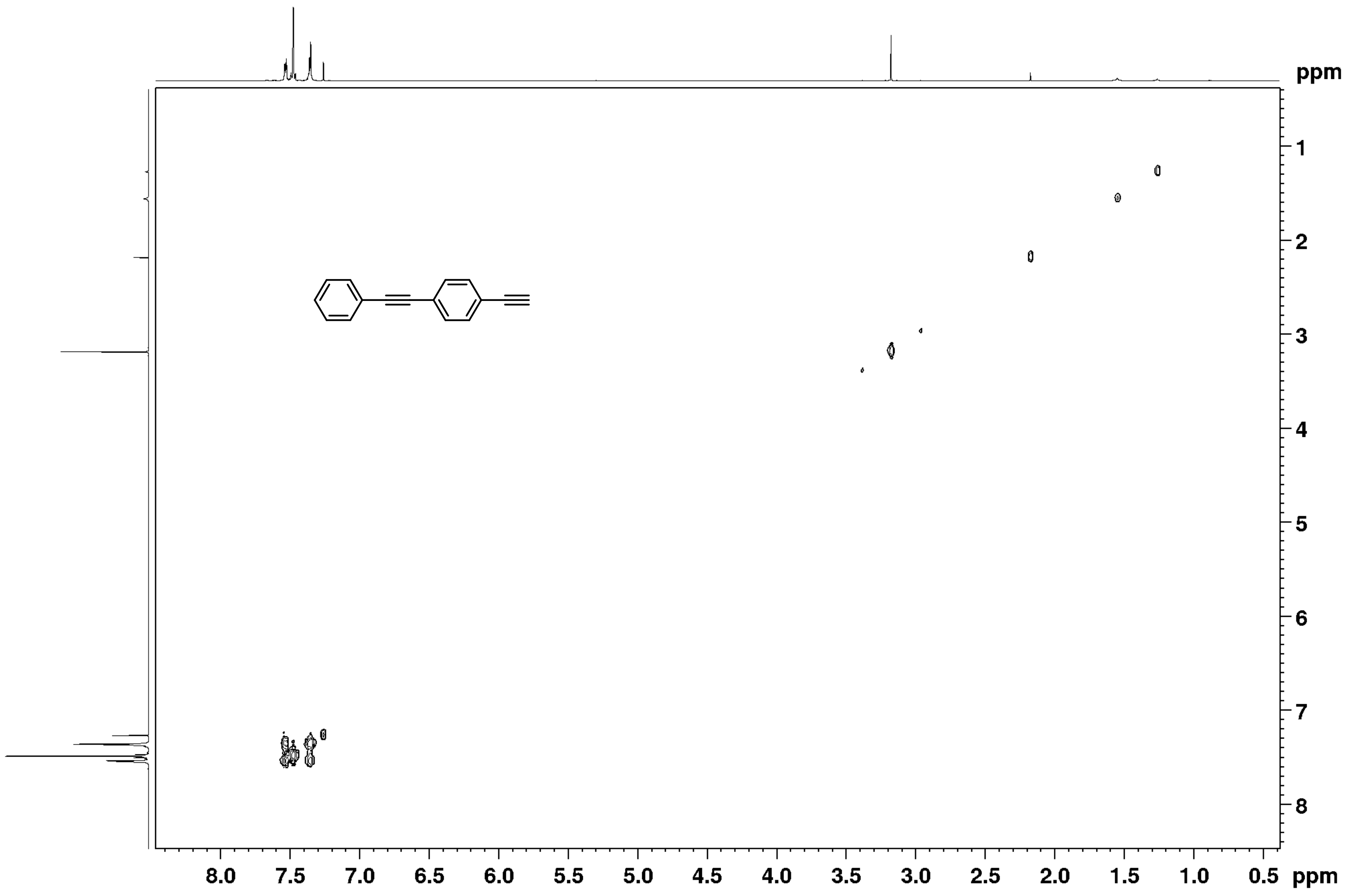

4.12.4 ^{1}H, ^{13}C HMBC

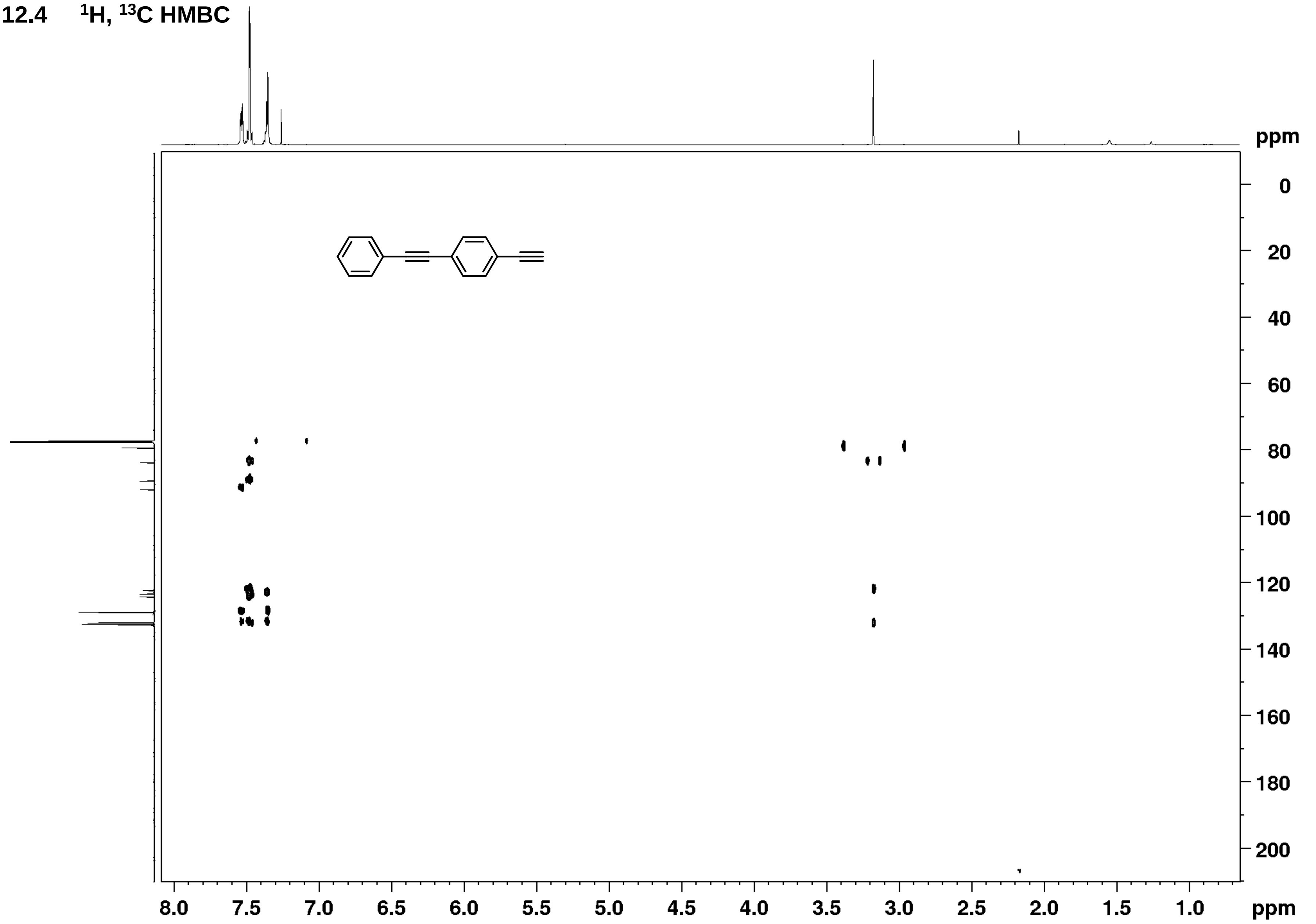

4.12.5 ^{1}H, ^{13}C HSQC

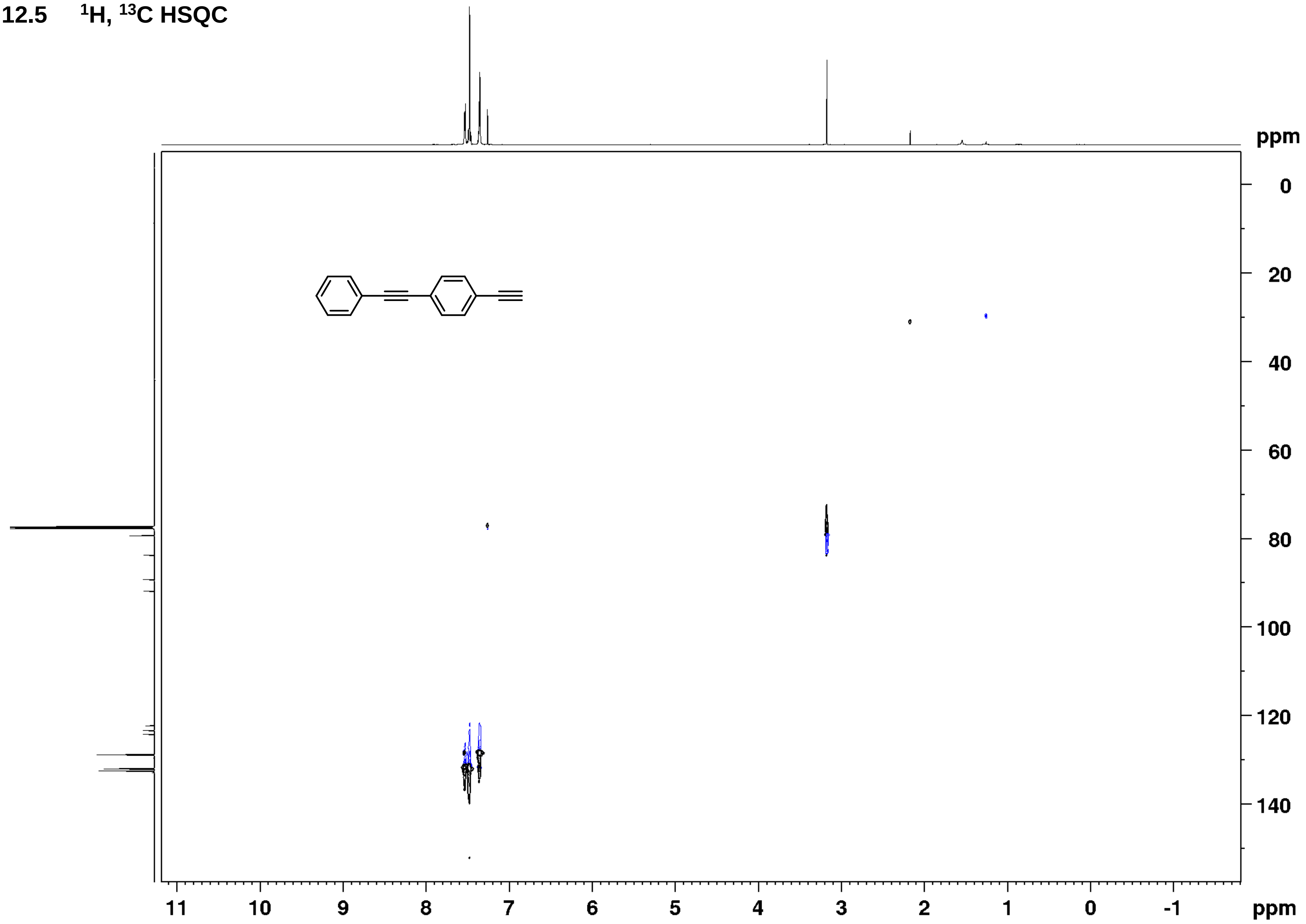

4.12.6 HRMS

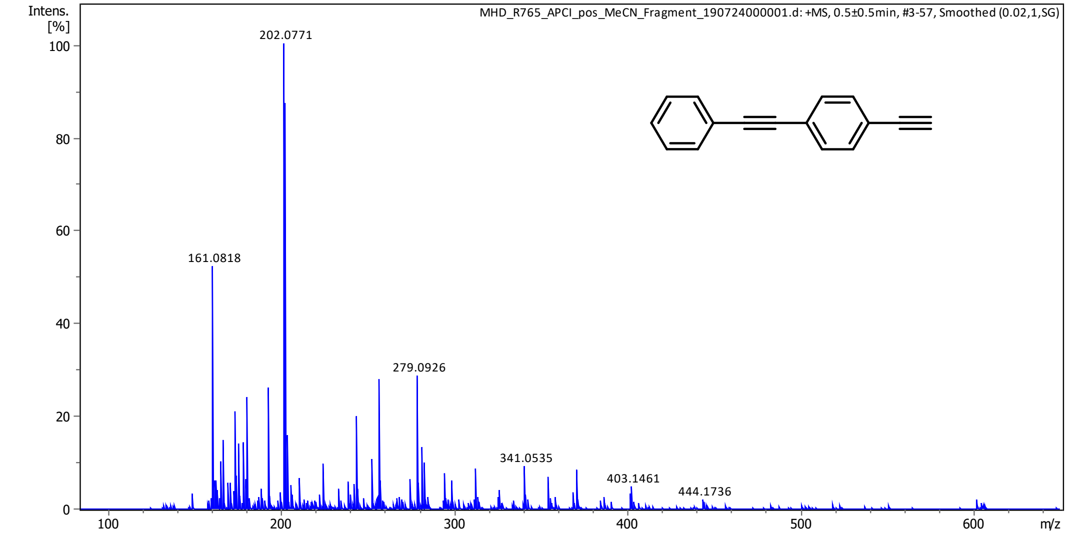

MHD_R765_APCI_pos_MeCN_Fragment_190724000001.d: +MS, 0.5±0.5min, #3-57, Smoothed (0.02,1,SG)
Intens. [%]
202.0771
161.0818
279.0926
341.0535
403.1461
444.1736
m/z

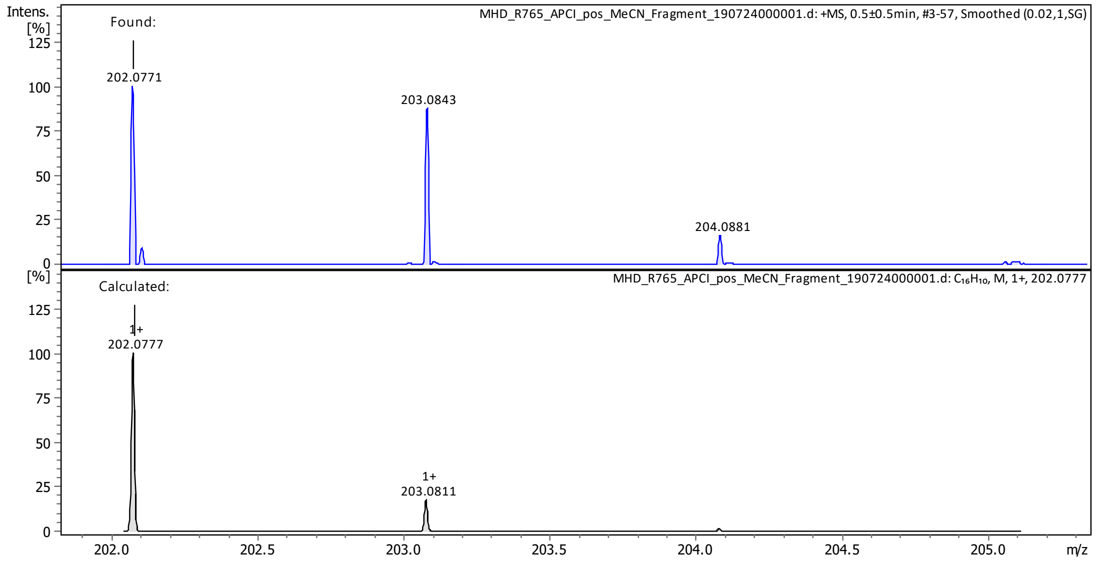

MHD_R765_APCI_pos_MeCN_Fragment_190724000001.d: +MS, 0.5±0.5min, #3-57, Smoothed (0.02,1,SG)
Intens. [%]
Found:
202.0771
203.0843
204.0881
MHD_R765_APCI_pos_MeCN_Fragment_190724000001.d: $C_{16}H_{10}$, M, 1+, 202.0777
Calculated:
1+
202.0777
1+
203.0811
m/z

4.13 1-(Cyclopropylethynyl)naphthalene (1q) (600 MHz, $CDCl_3$)

4.13.1 ^{1}H NMR

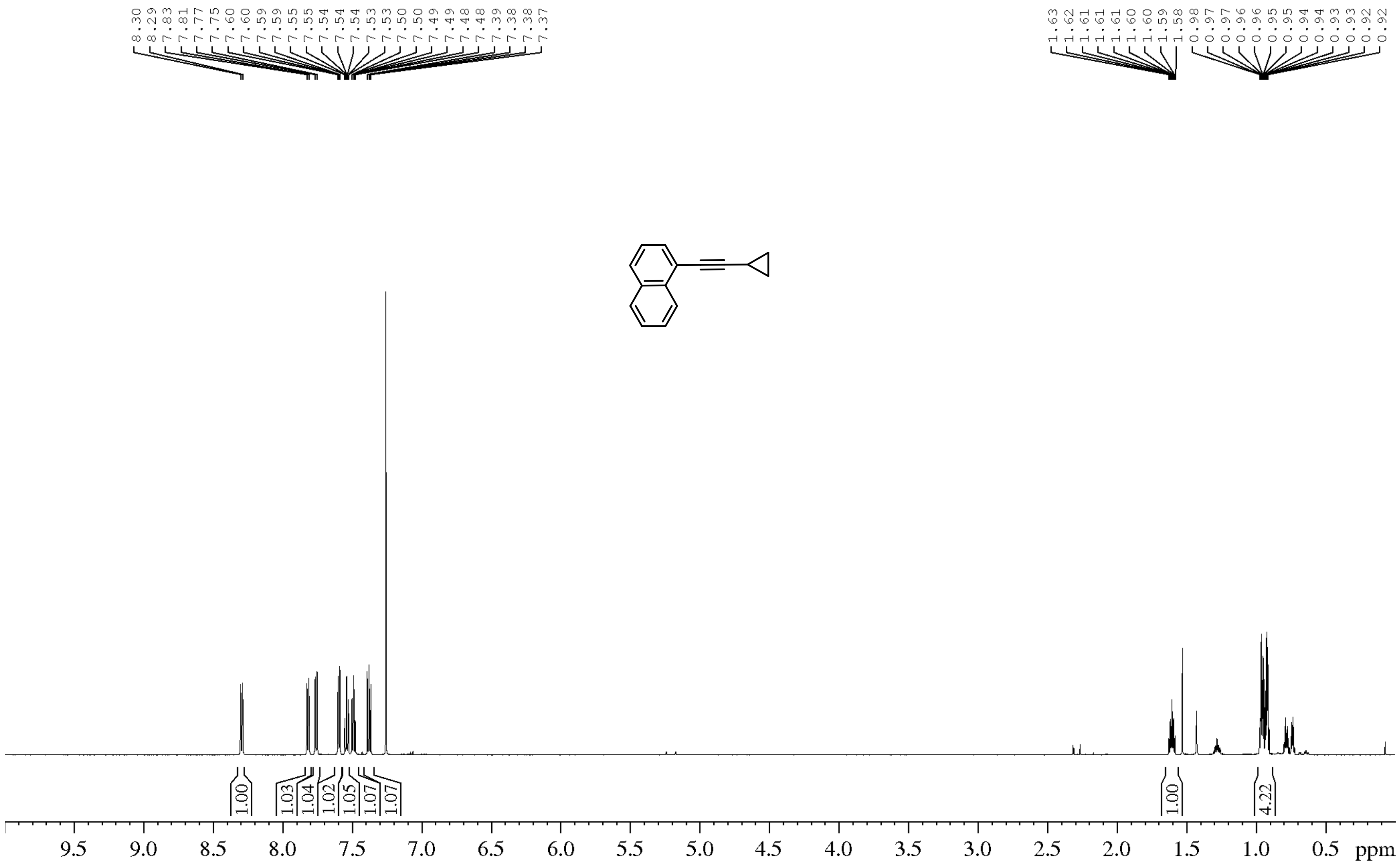

4.13.2 ^{13}C NMR

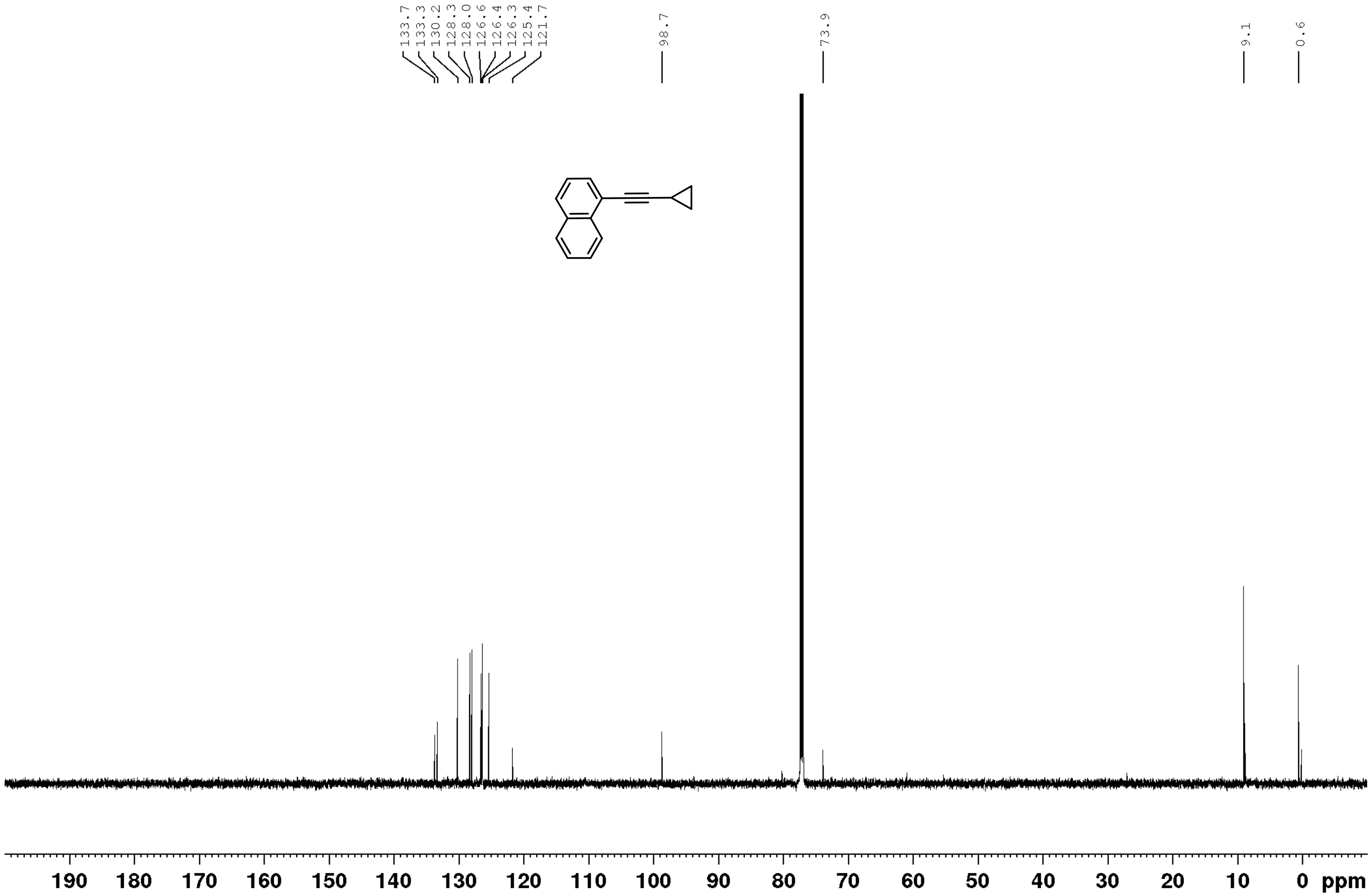
133.7
133.3
130.2
128.3
128.0
126.6
126.4
126.3
125.4
121.7
98.7
73.9
9.1
0.6
190 180 170 160 150 140 130 120 110 100 90 80 70 60 50 40 30 20 10 0 ppm

4.13.3 ^{1}H, ^{1}H COSY

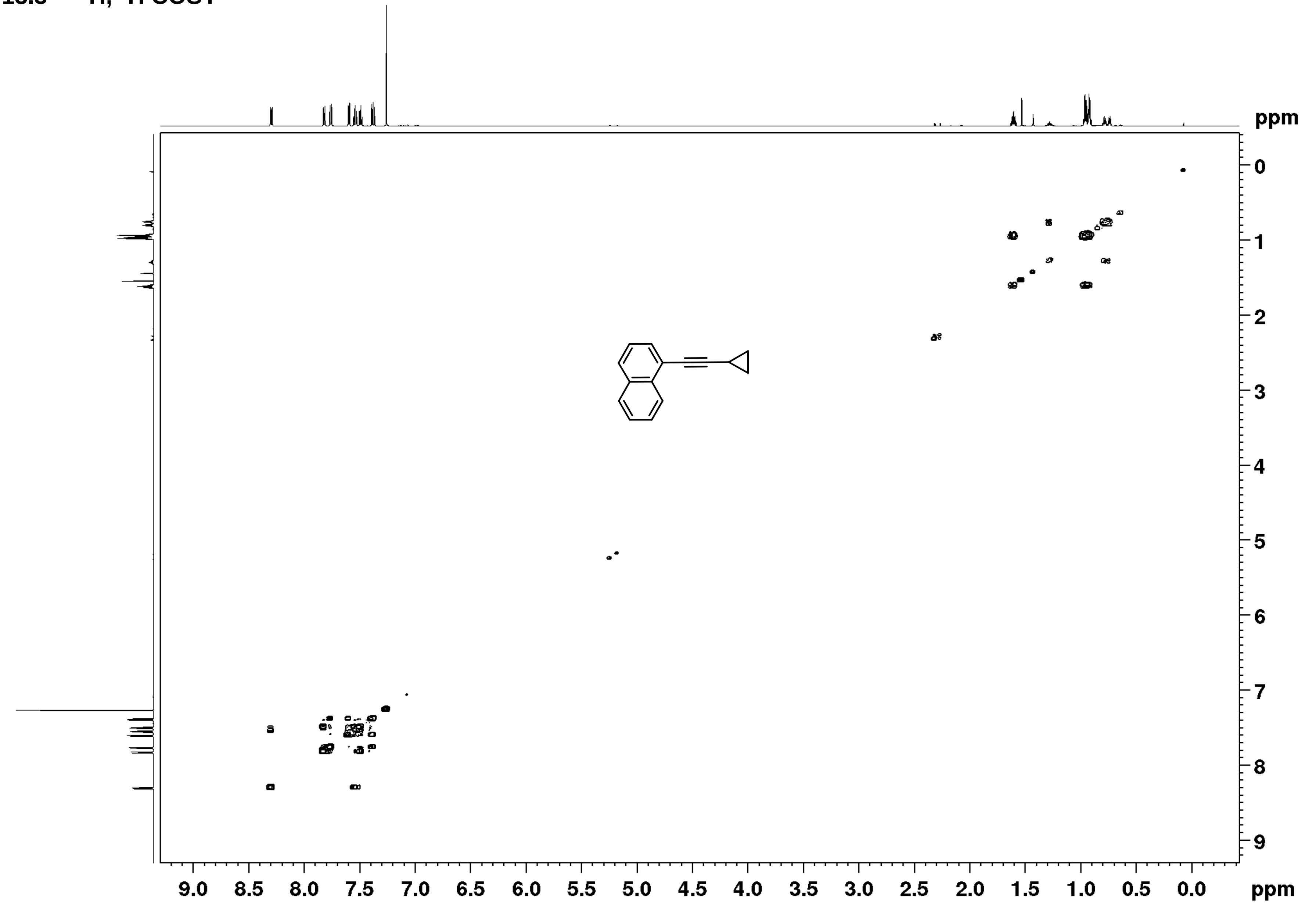

4.13.4 ^{1}H, ^{13}C HMBC

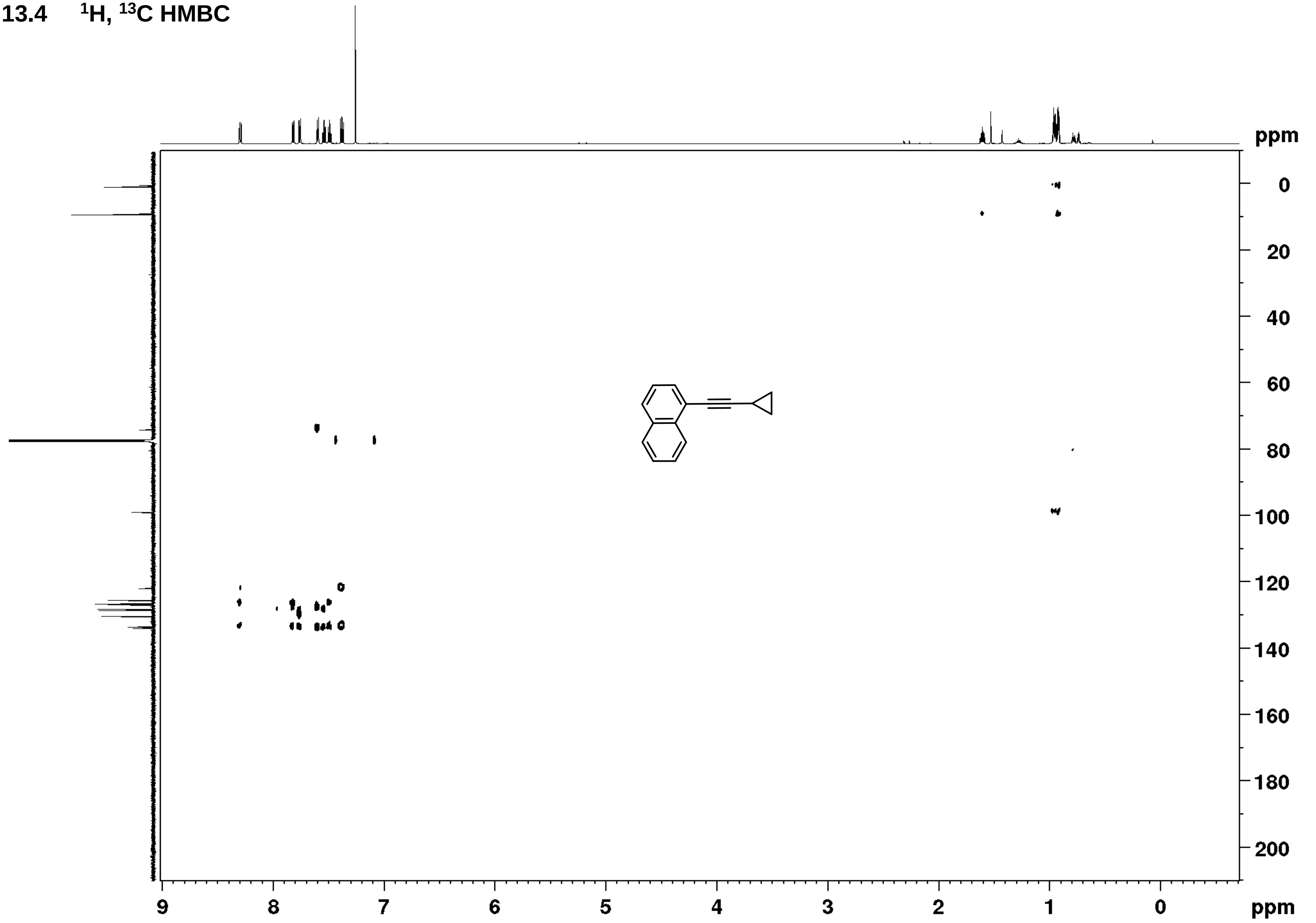

4.13.5 ^{1}H, ^{13}C HSQC

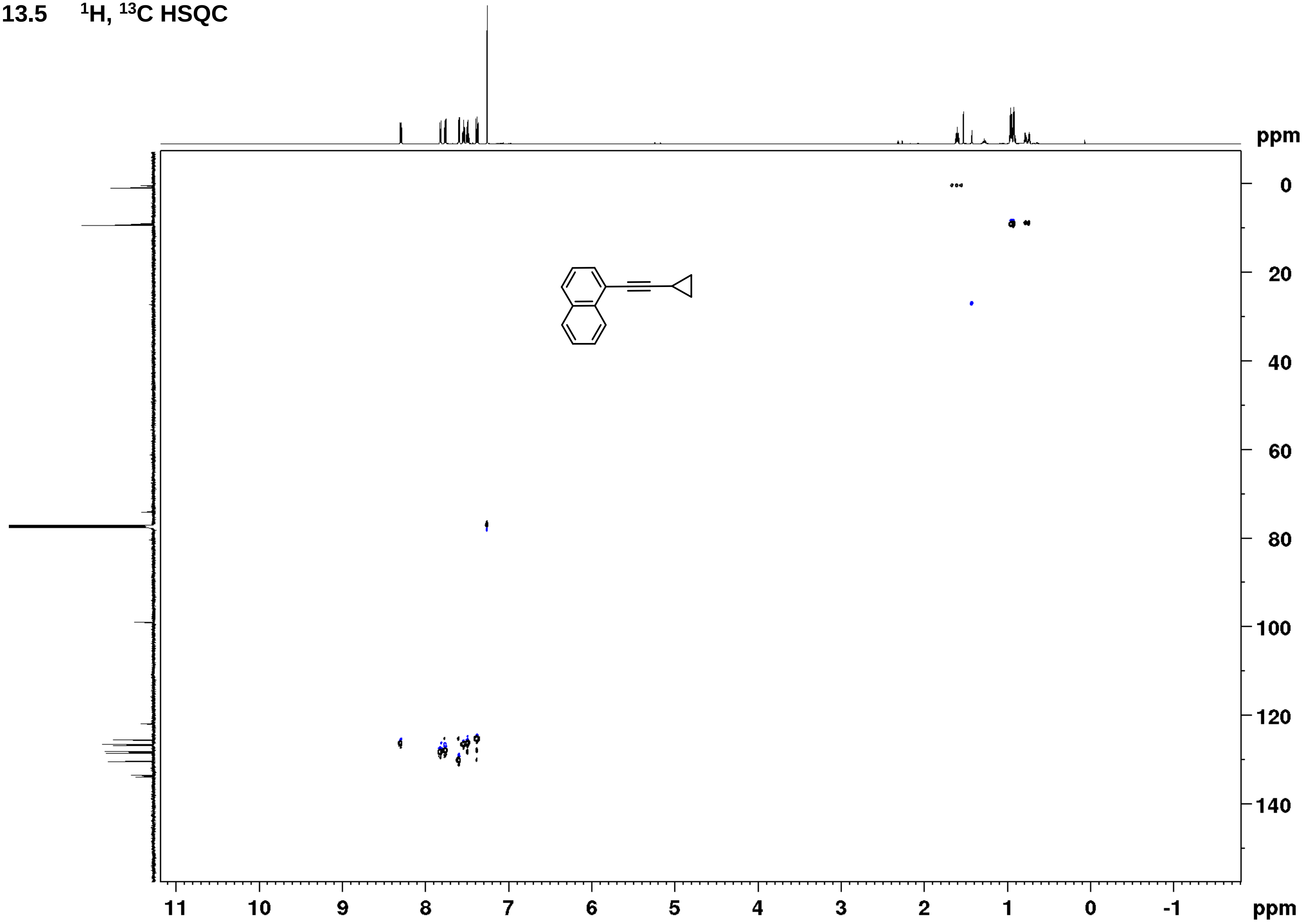

4.13.6 HRMS

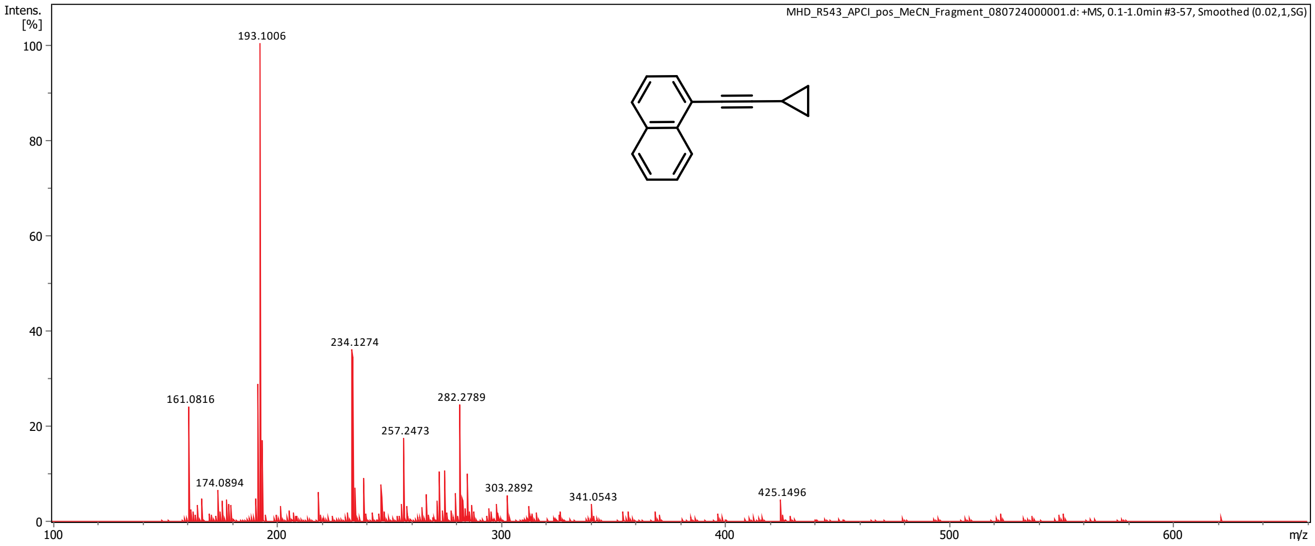

Intens. [%]
MHD_R543_APCI_pos_MeCN_Fragment_080724000001.d: +MS, 0.1-1.0min #3-57, Smoothed (0.02,1,SG)
193.1006
234.1274
161.0816
282.2789
257.2473
174.0894
303.2892
341.0543
425.1496
m/z

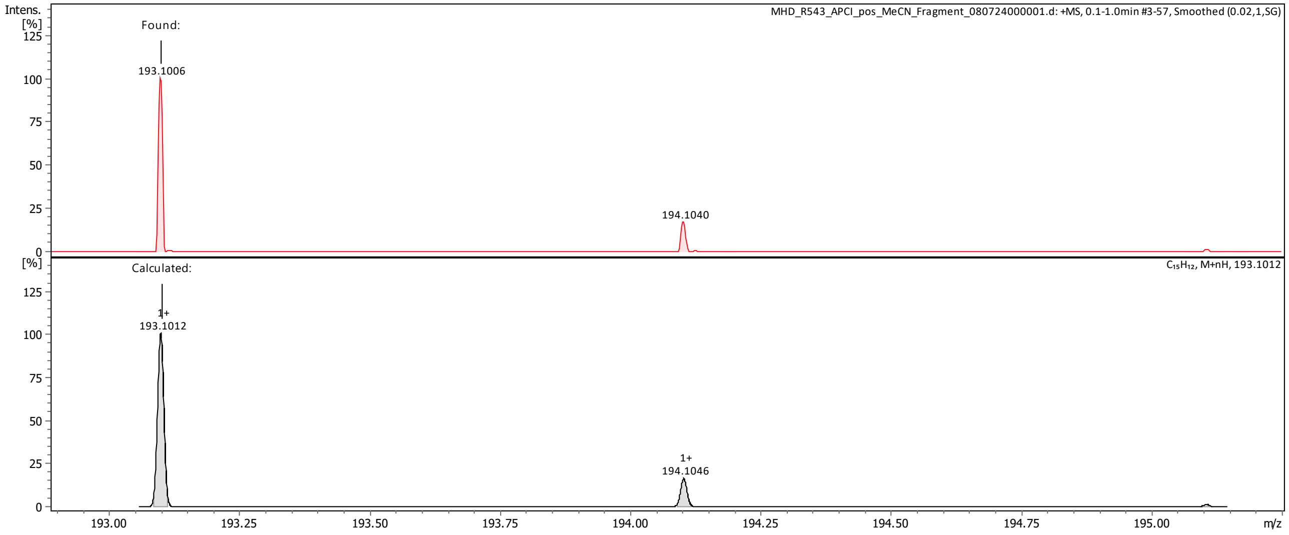

Intens. [%]
MHD_R543_APCI_pos_MeCN_Fragment_080724000001.d: +MS, 0.1-1.0min #3-57, Smoothed (0.02,1,SG)
Found:
193.1006
194.1040
Calculated:
$C_{15}H_{12}$, M+nH, 193.1012
1+
193.1012
1+
194.1046
m/z

4.14 1-(Phenylethynyl)adamantine (1s) (600 MHz, $CDCl_3$)

4.14.1 ^1H NMR

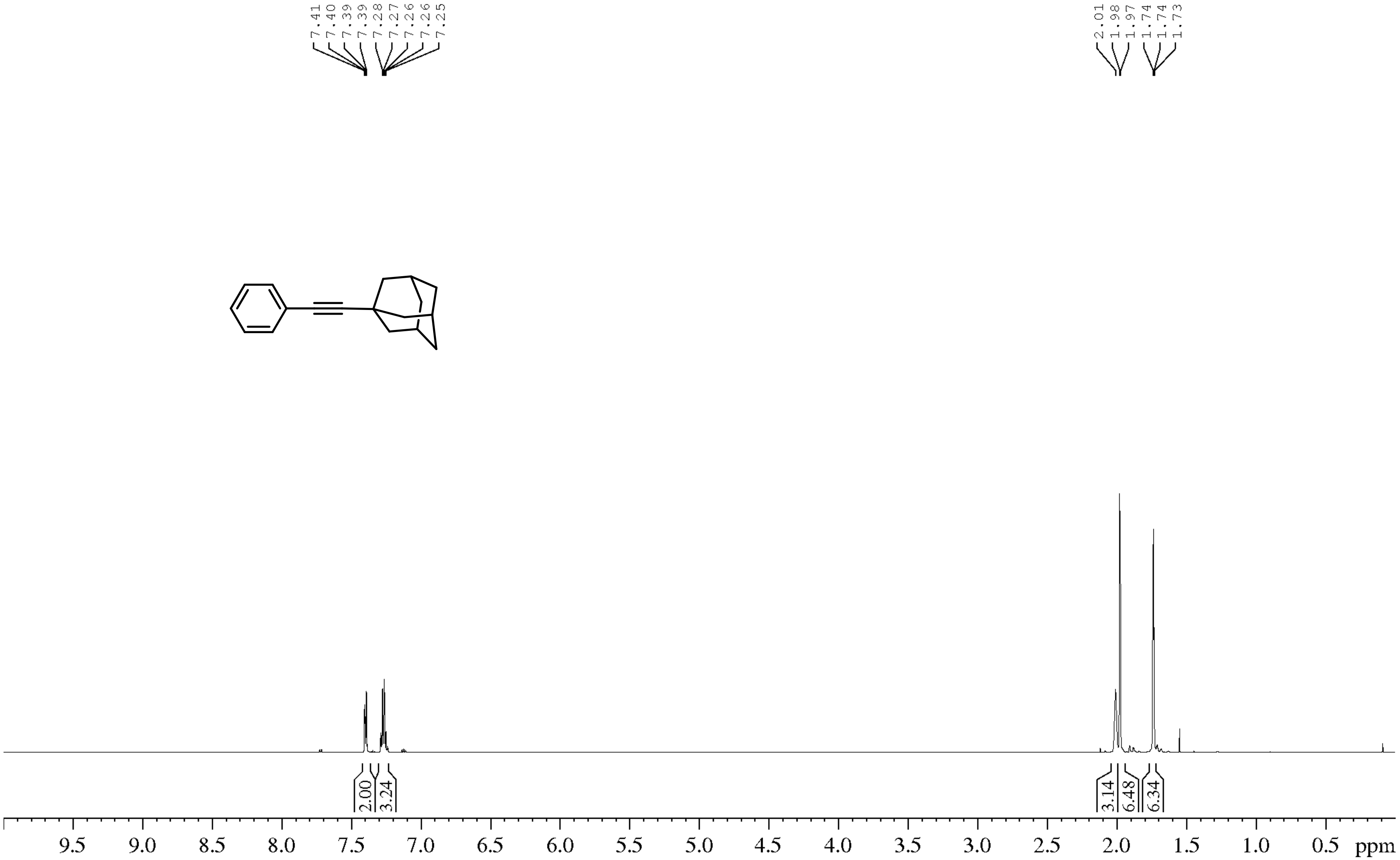

4.14.2 ^{13}C NMR

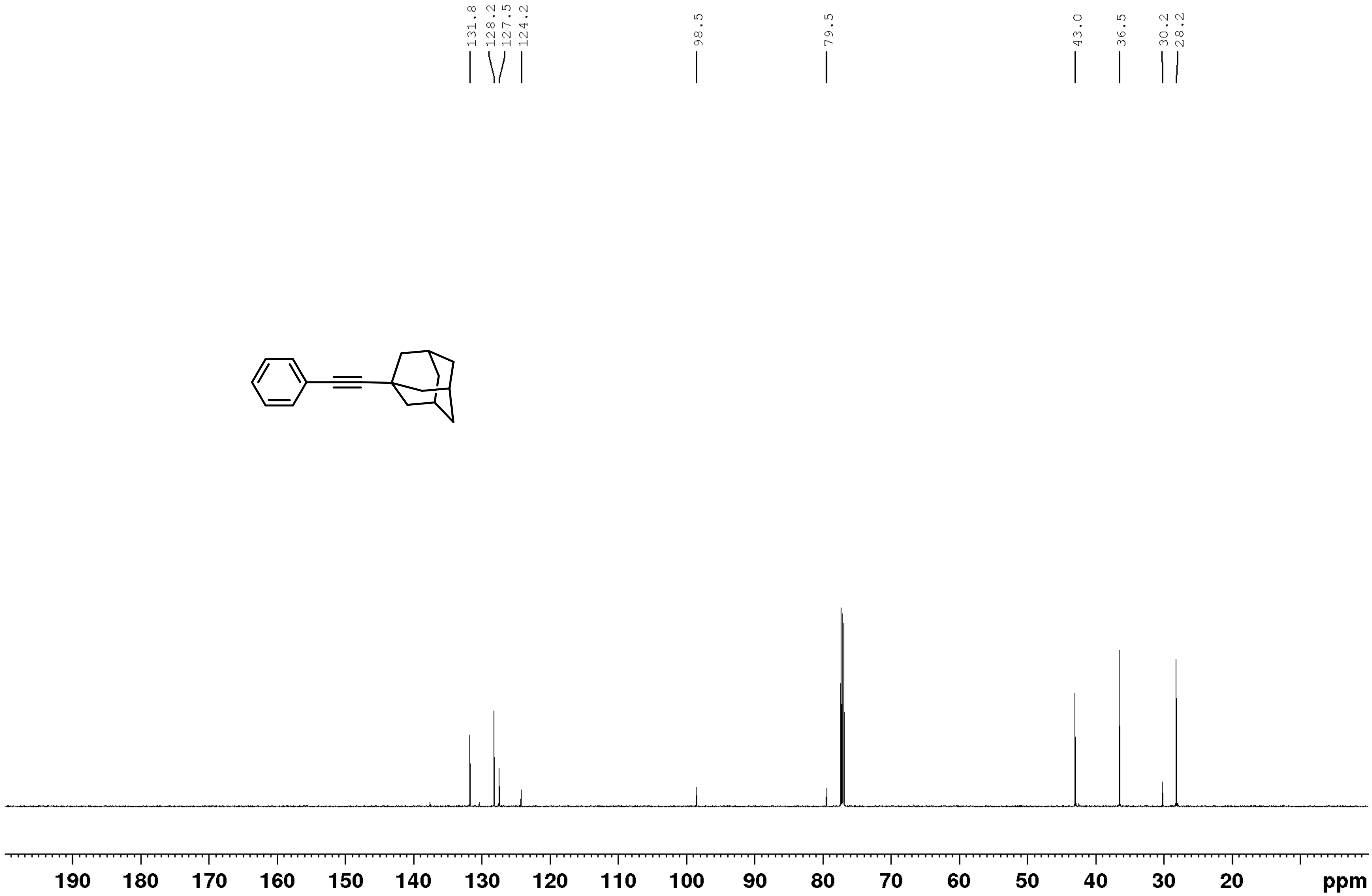
131.8
128.2
127.5
124.2
98.5
79.5
43.0
36.5
30.2
28.2
190
180
170
160
150
140
130
120
110
100
90
80
70
60
50
40
30
20
ppm

4.14.3 ^{1}H, ^{1}H COSY

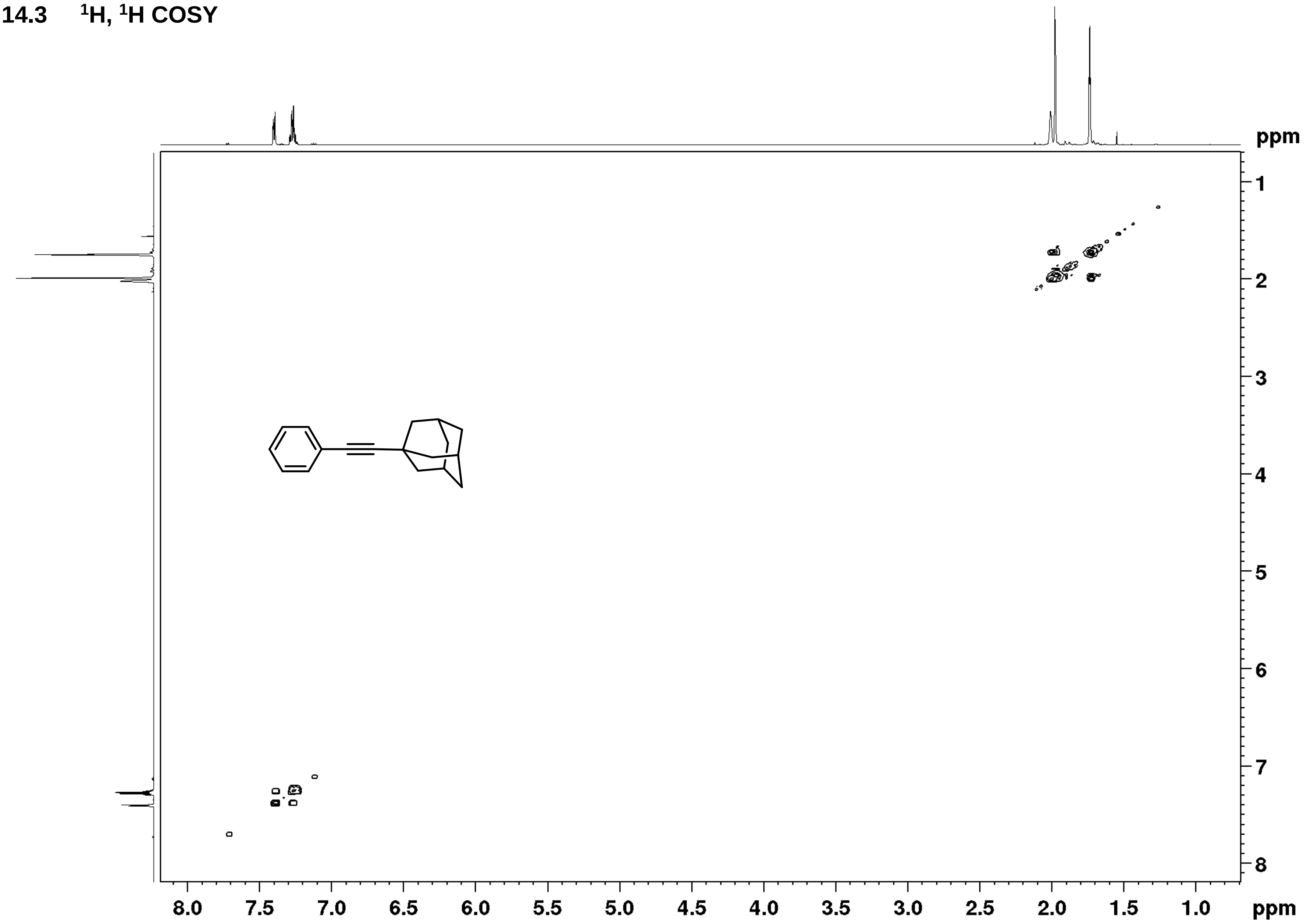

4.14.4 ^{1}H, ^{13}C HMBC

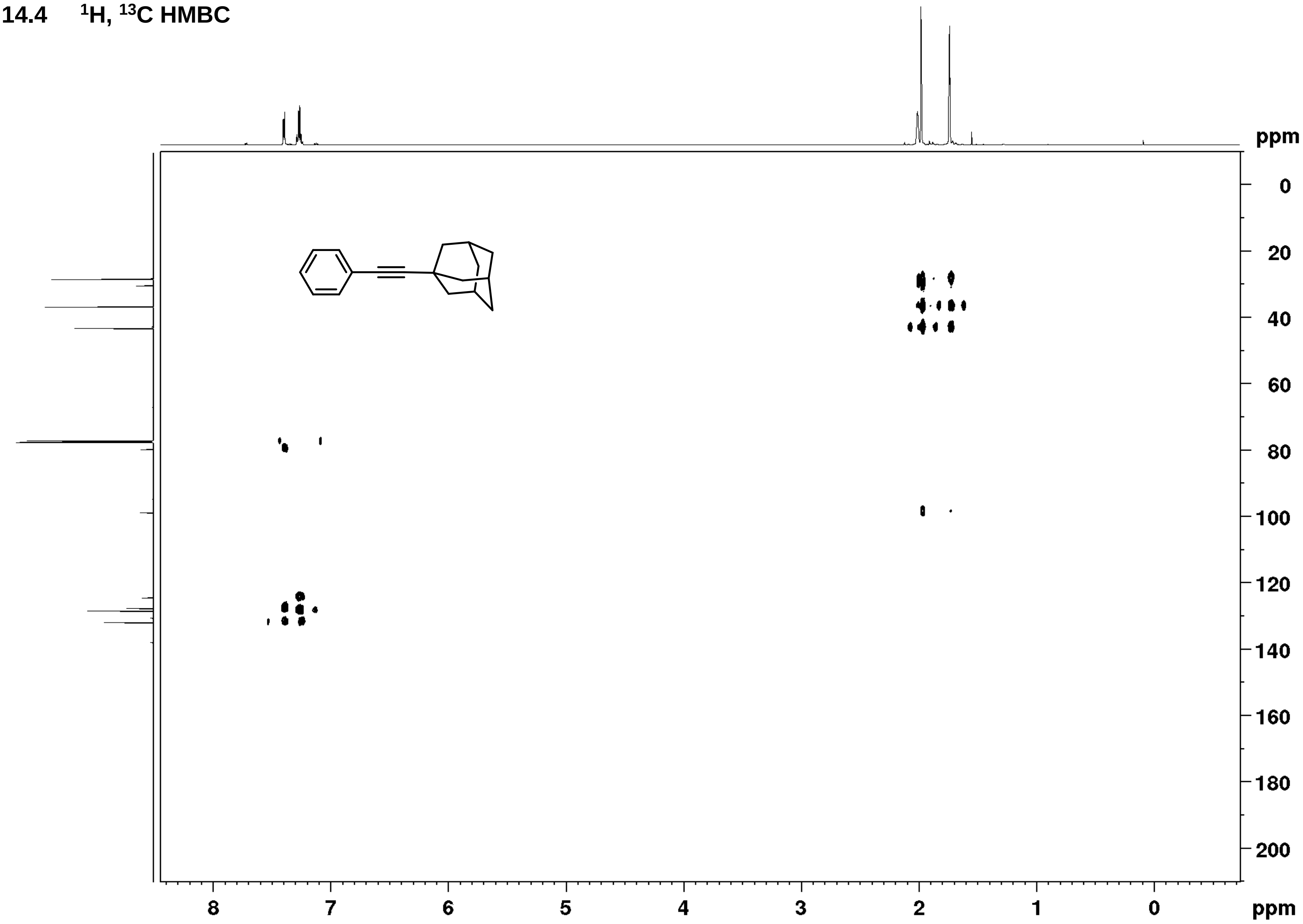

4.14.5 ^{1}H, ^{13}C HSQC

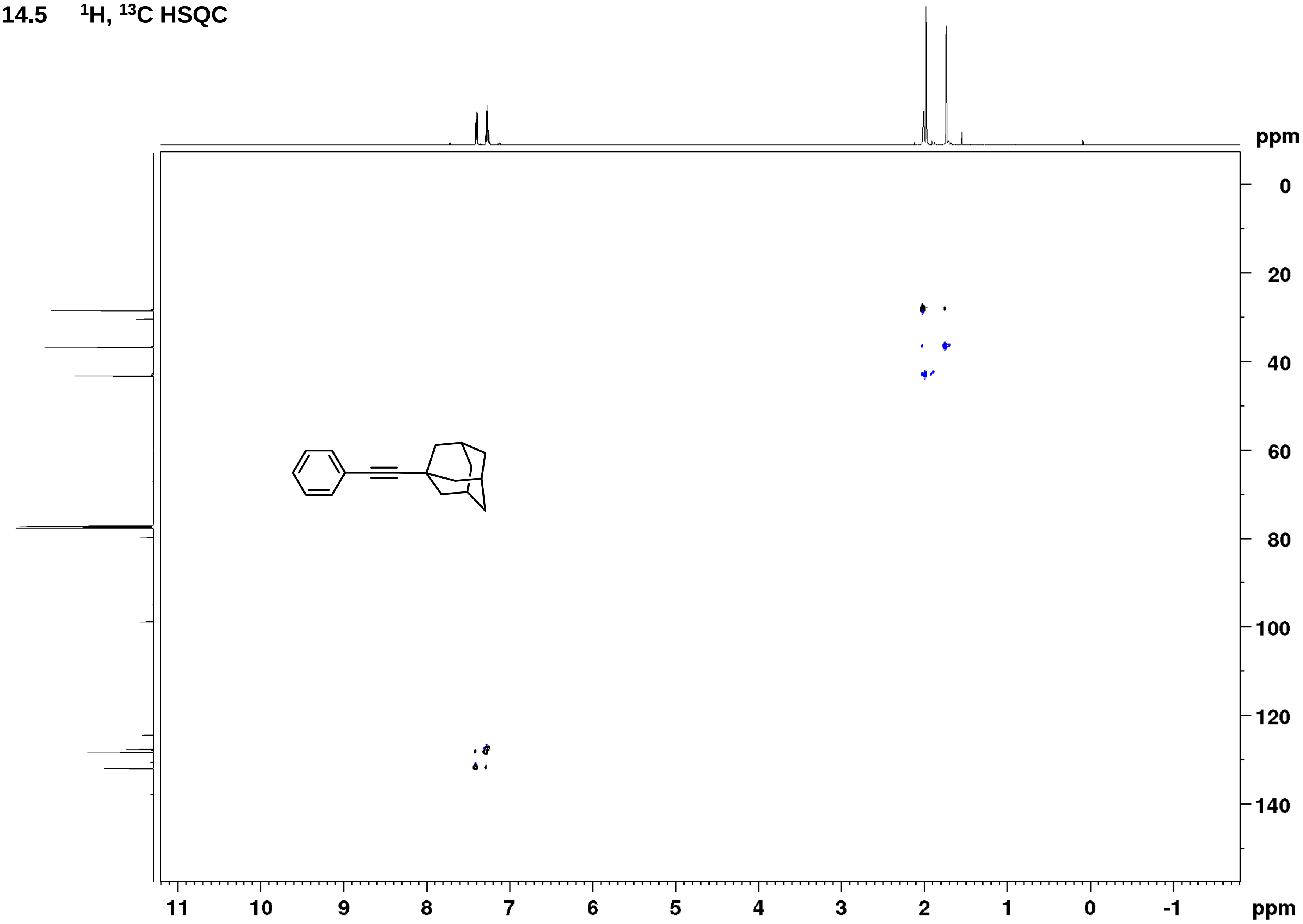

4.14.6 HRMS

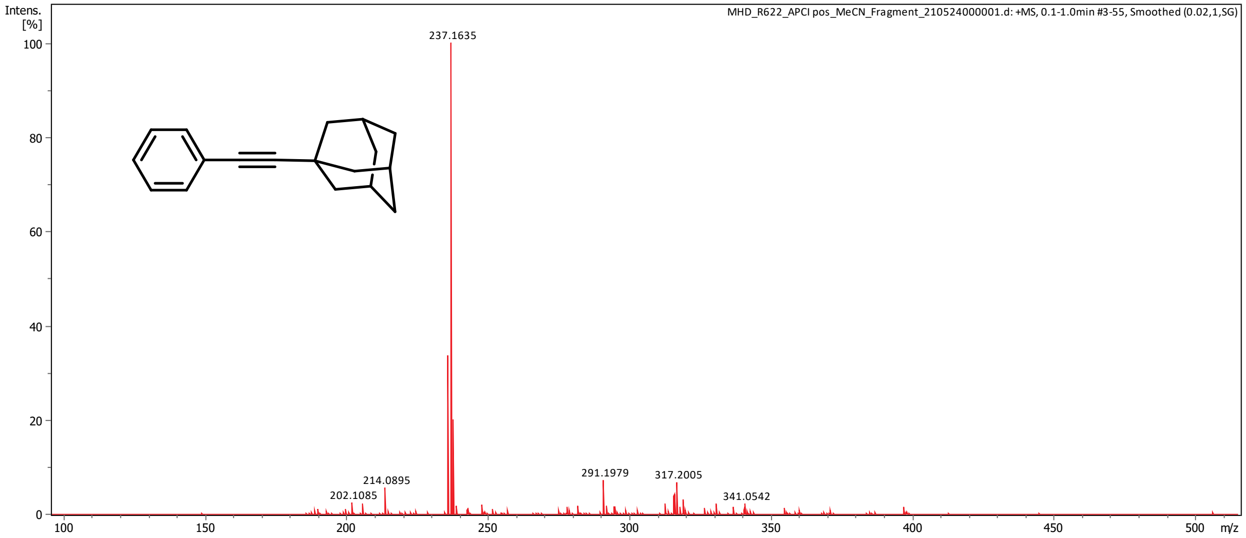
MHD_R622_APCI pos_MeCN_Fragment_210524000001.d: +MS, 0.1-1.0min #3-55, Smoothed (0.02,1,SG)
Intens.
[%]
237.1635
214.0895
202.1085
291.1979
317.2005
341.0542
m/z

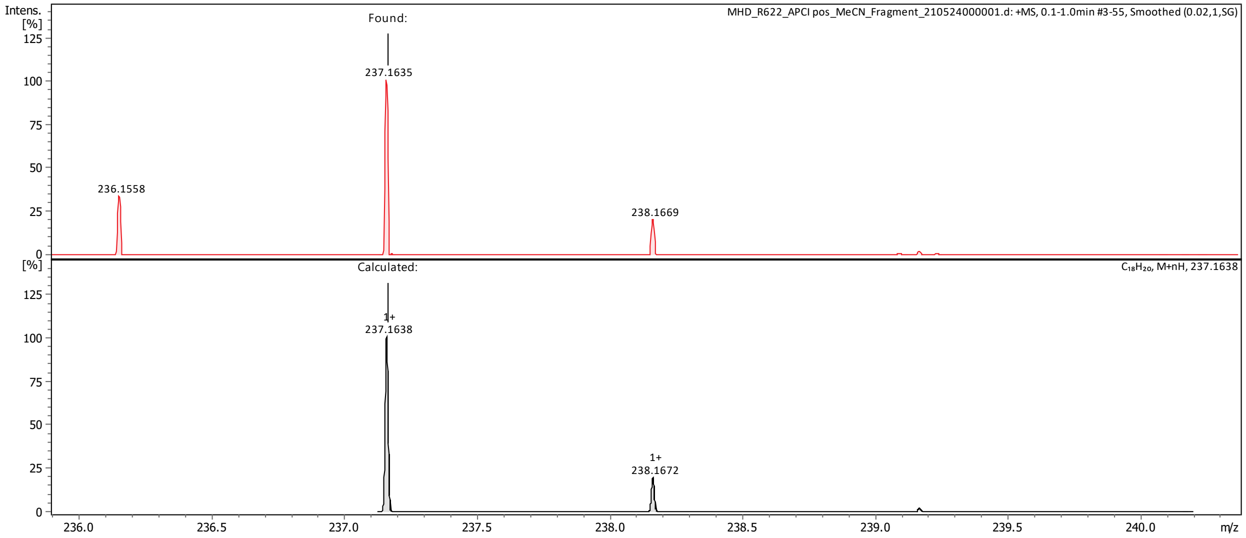
MHD_R622_APCI pos_MeCN_Fragment_210524000001.d: +MS, 0.1-1.0min #3-55, Smoothed (0.02,1,SG)
Intens.
[%]
Found:
237.1635
236.1558
238.1669
Calculated:
C18H20, M+nH, 237.1638
1+
237.1638
1+
238.1672
m/z

4.15 6-Phenylhex-5-yn-1-ol (1ab) (600 MHz, $CDCl_3$)

4.15.1 ^1H NMR

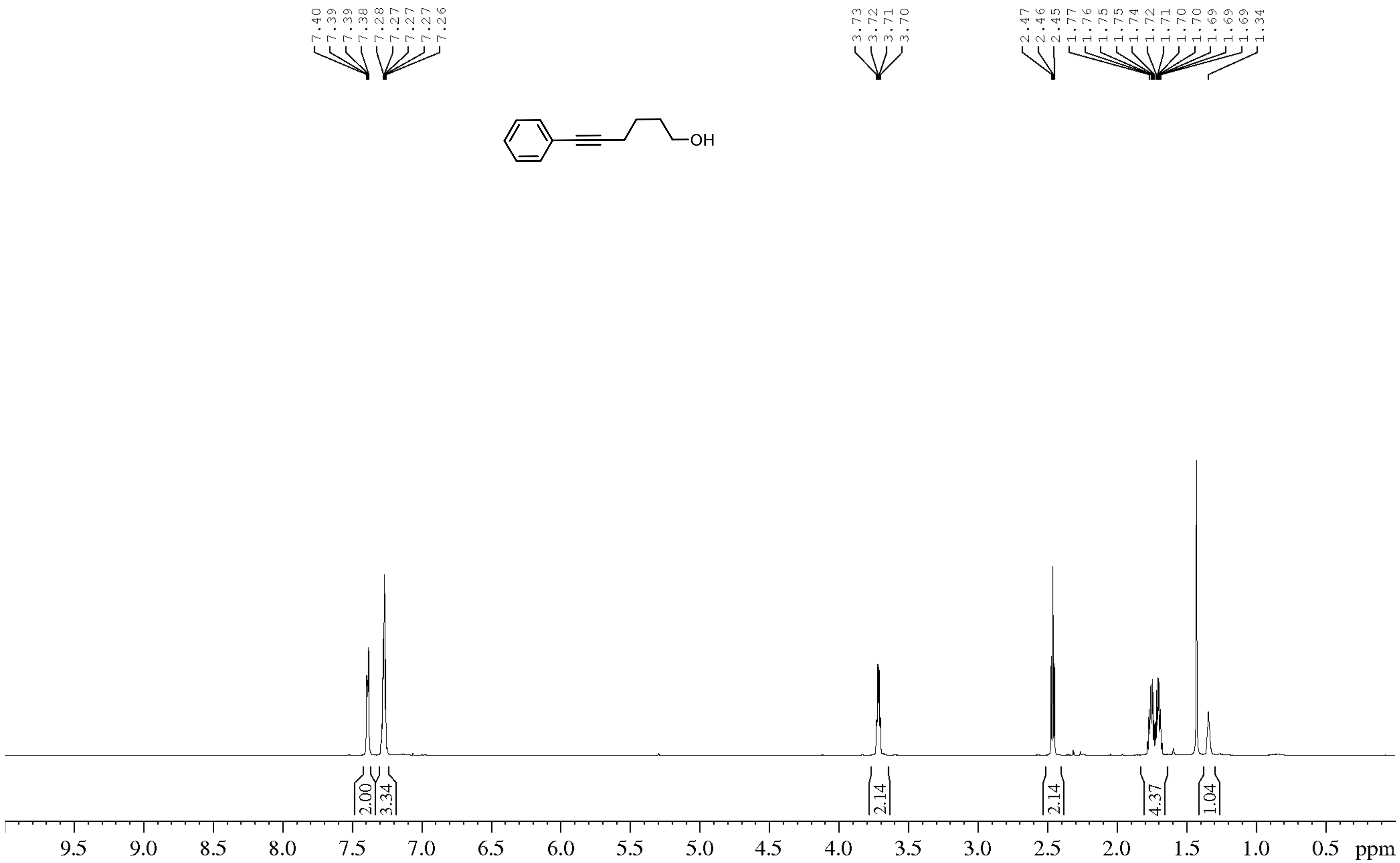

4.15.2 ^{13}C NMR

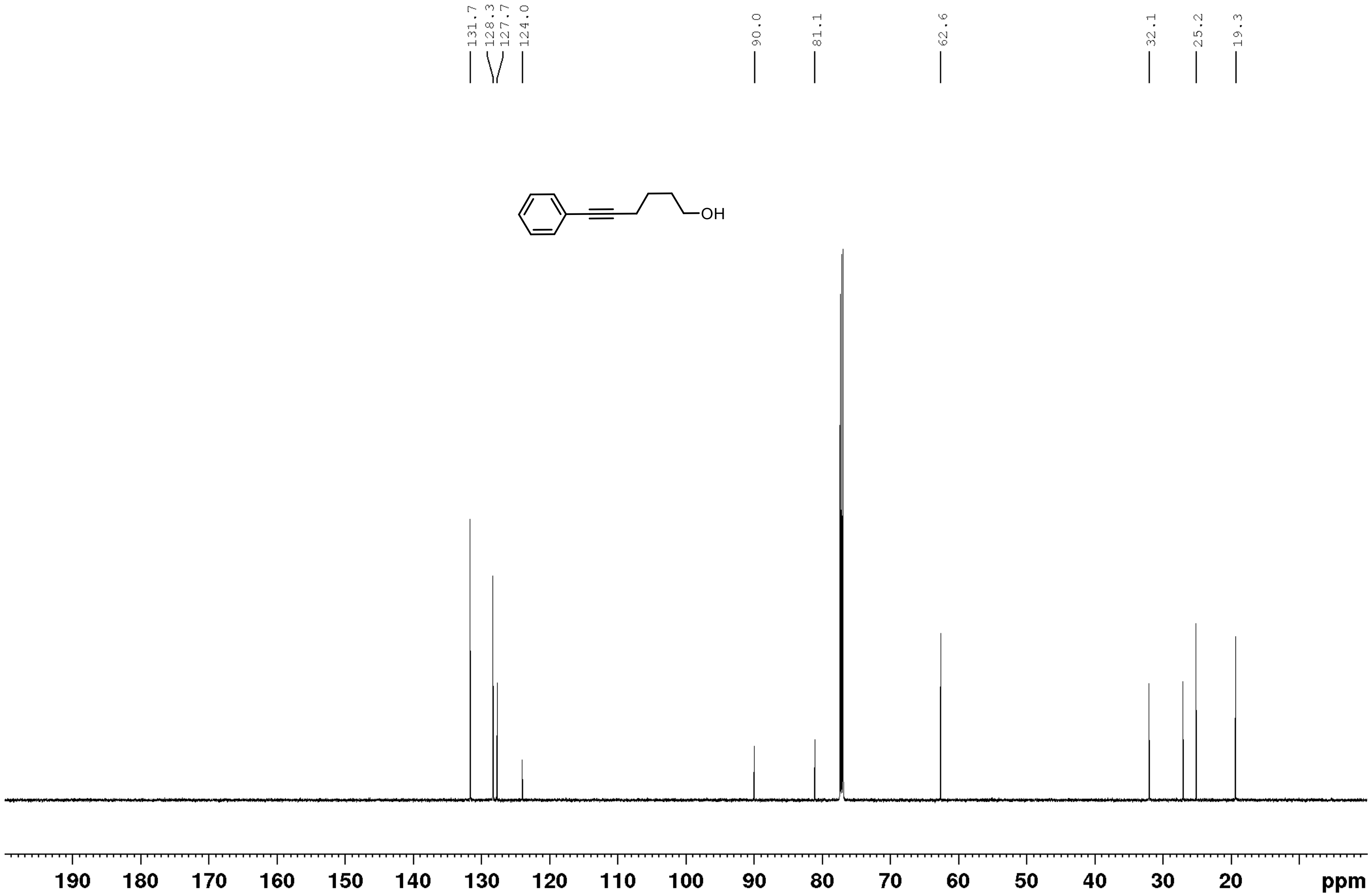
131.7
128.3
127.7
124.0
90.0
81.1
62.6
32.1
25.2
19.3
OH
190
180
170
160
150
140
130
120
110
100
90
80
70
60
50
40
30
20
ppm

4.15.3 ^{1}H, ^{1}H COSY

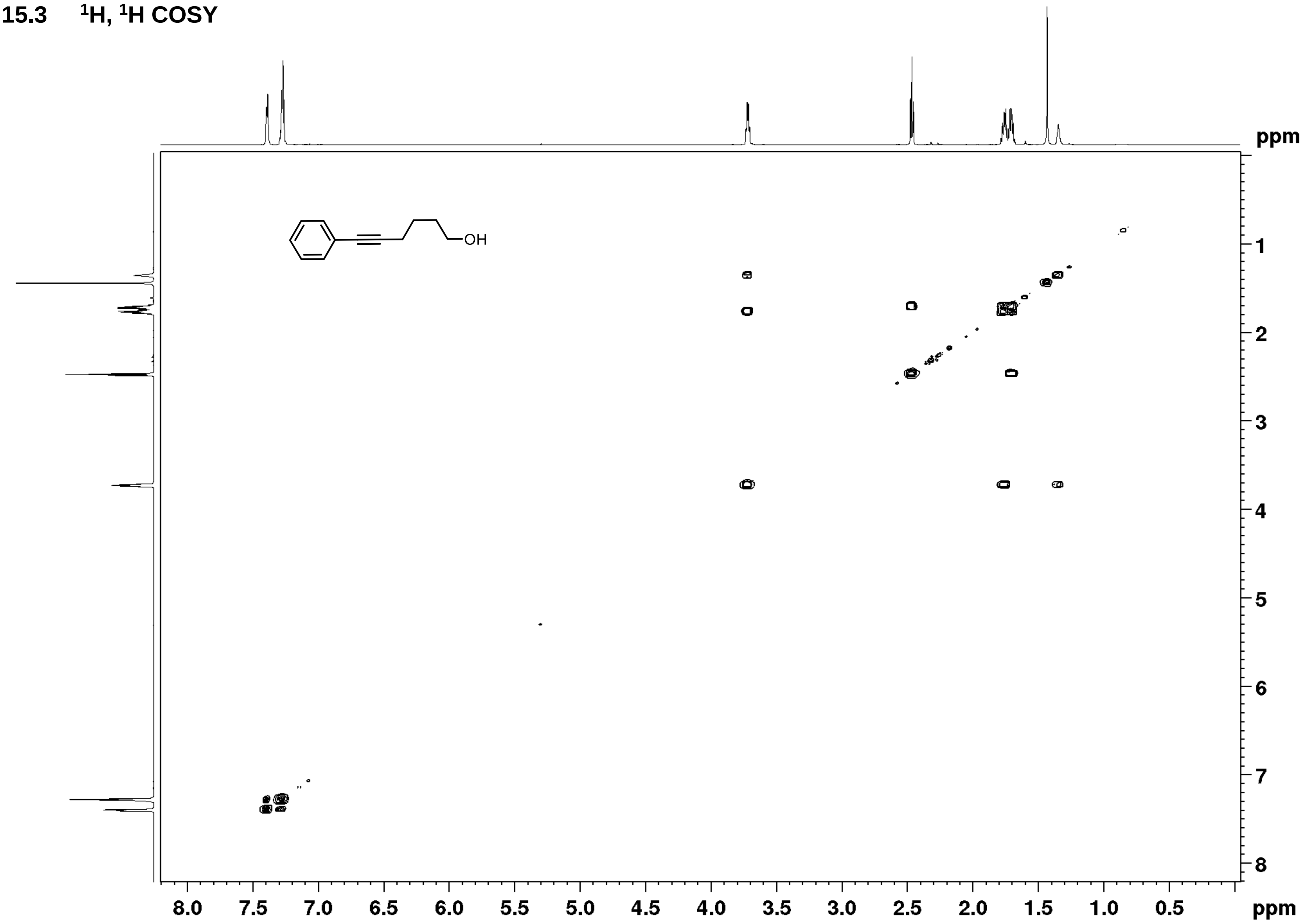

4.15.4 ¹H, ¹³C HMBC

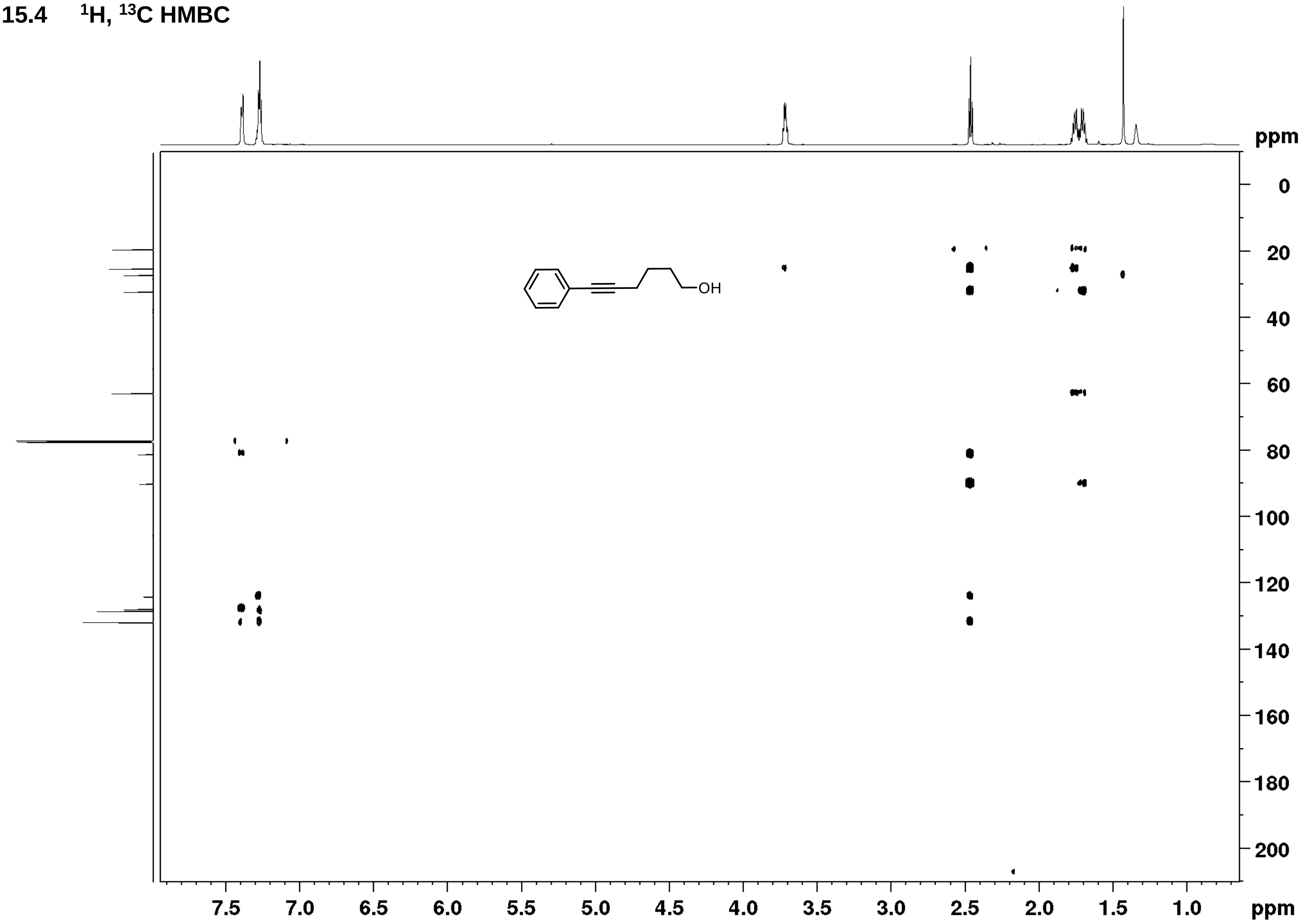

4.15.5 ^{1}H, ^{13}C HSQC

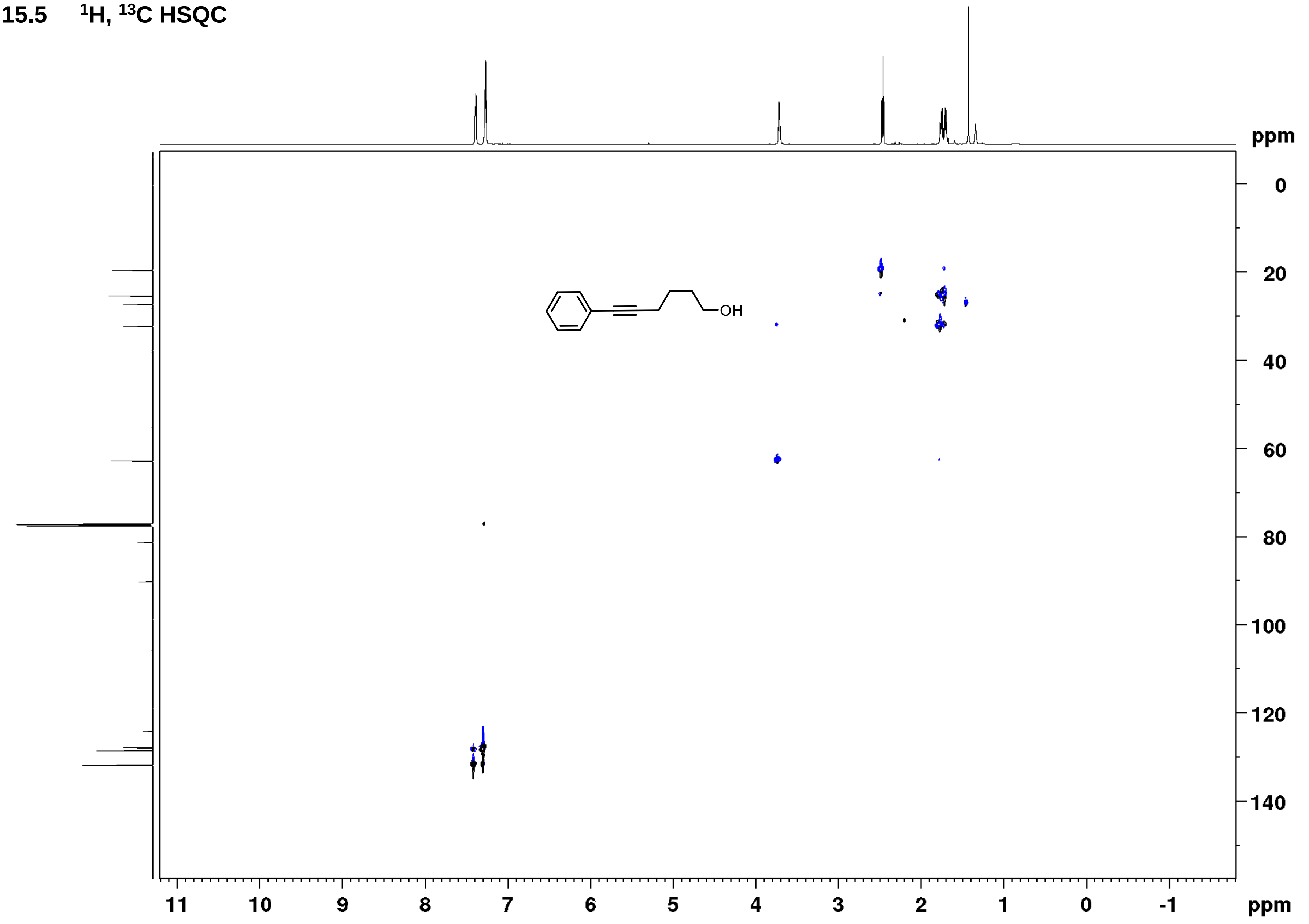

4.15.6 HRMS

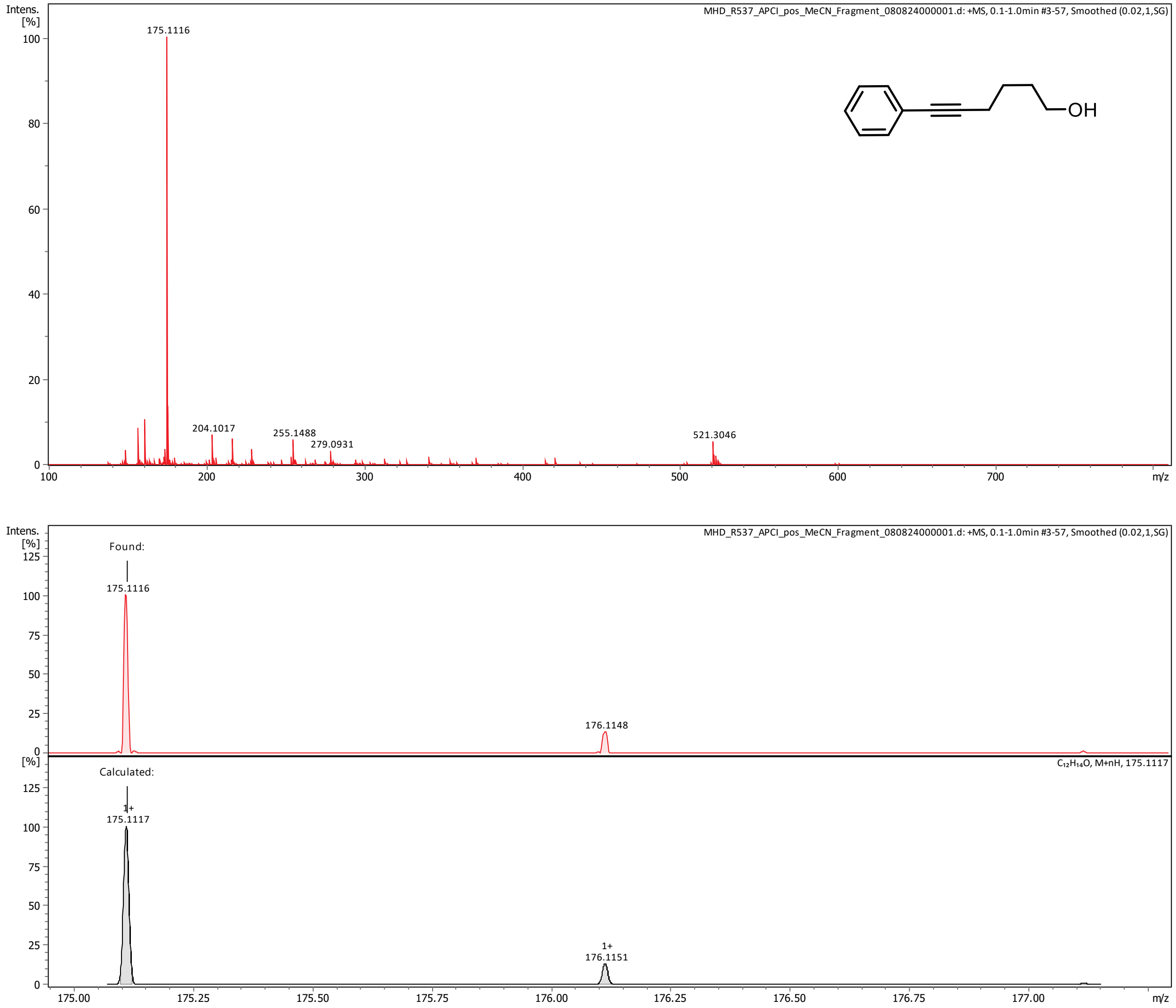
MHD_R537_APCI_pos_MeCN_Fragment_080824000001.d: +MS, 0.1-1.0min #3-57, Smoothed (0.02,1,SG)
Intens. [%]
175.1116
204.1017
255.1488
279.0931
521.3046
OH
m/z
Found:
175.1116
176.1148
Calculated:
1+
175.1117
176.1151
C12H14O, M+nH, 175.1117

4.16 4,4-Difluoro-1-(6-phenylhex-5-yn-1-yl)piperidine (1t) (600 MHz, $CDCl_3$)

4.16.1 ^{1}H NMR

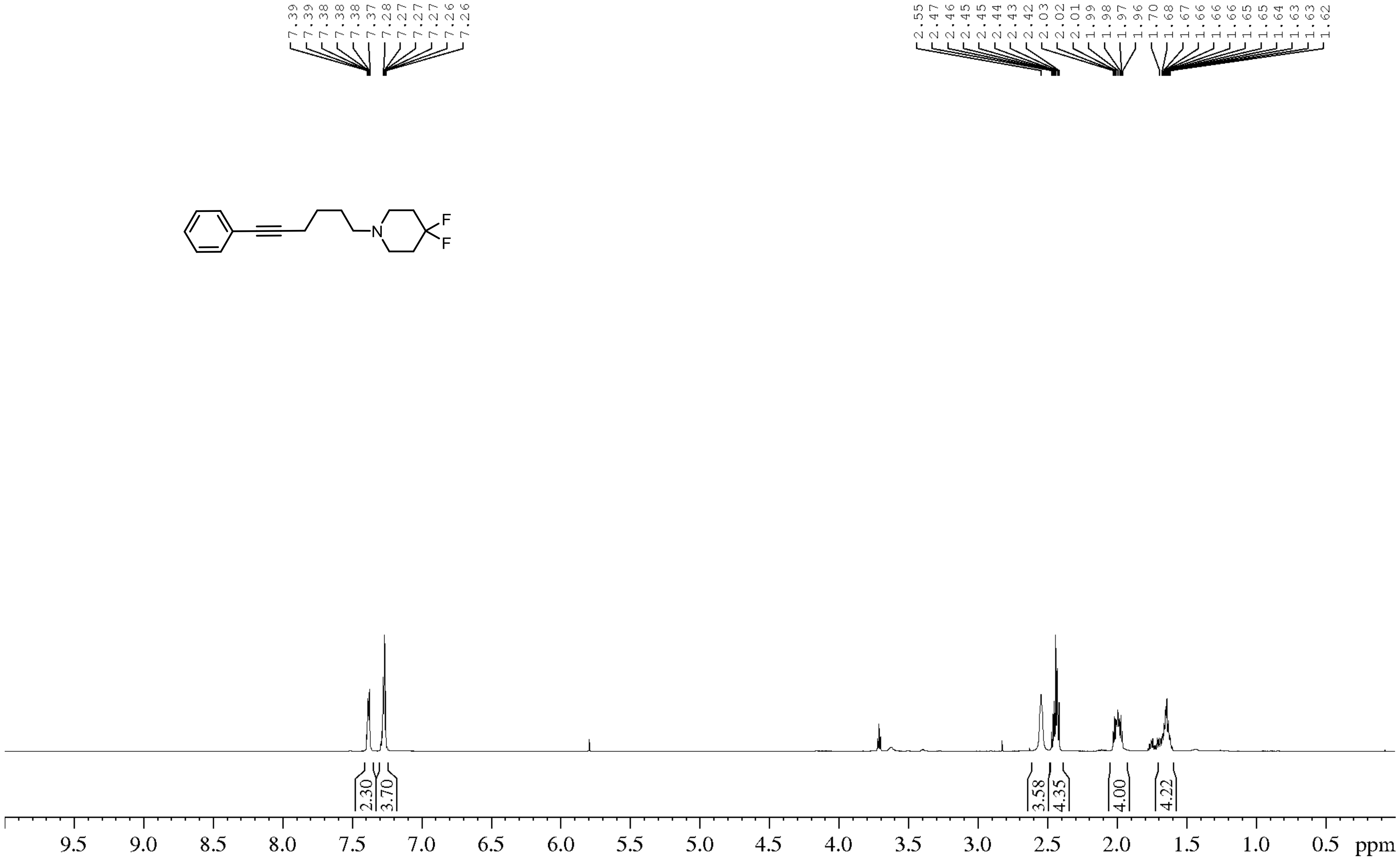

4.16.2 ^{13}C NMR

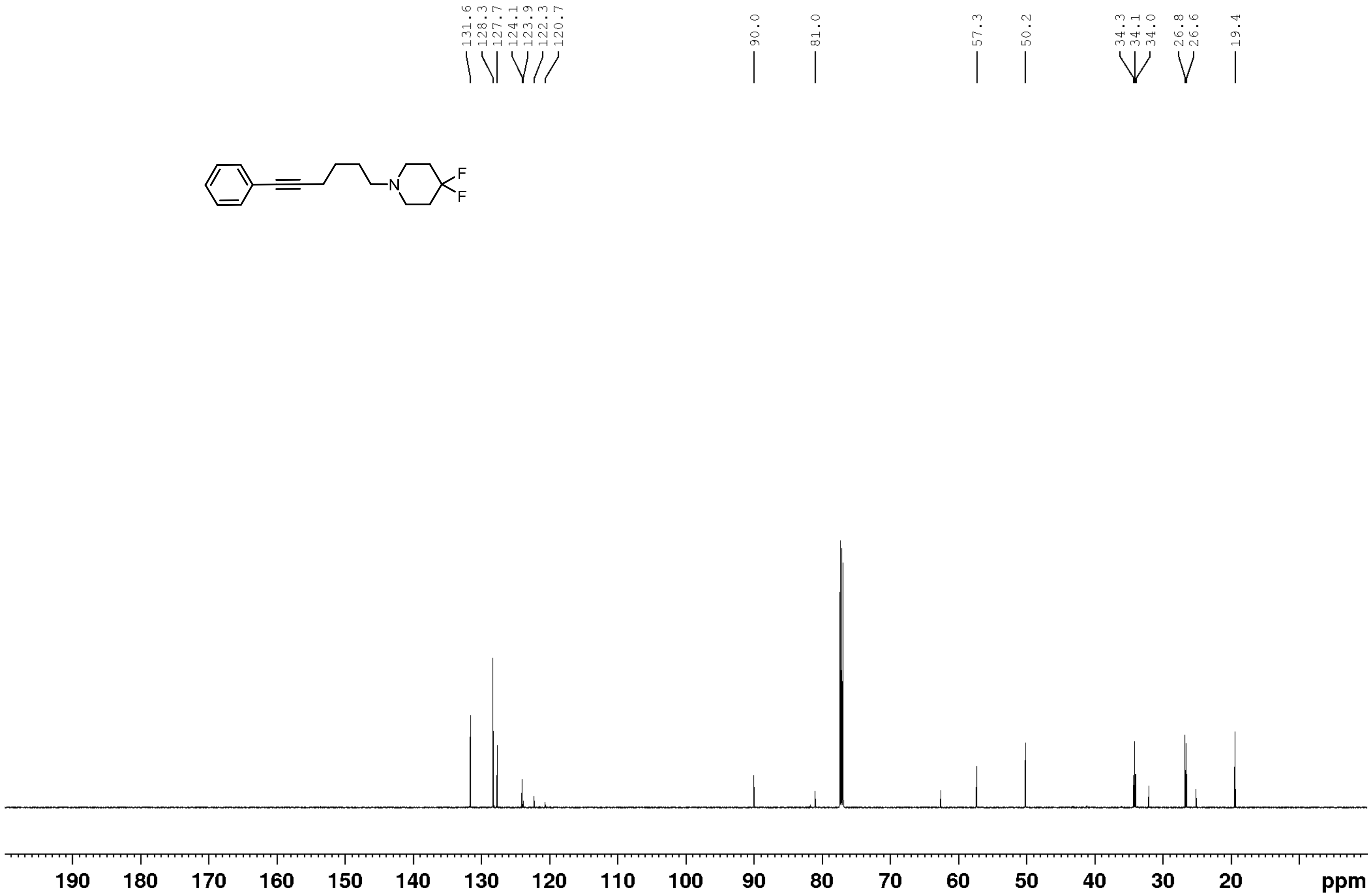
131.6
128.3
127.7
124.1
123.9
122.3
120.7
90.0
81.0
57.3
50.2
34.3
34.1
34.0
26.8
26.6
19.4
190
180
170
160
150
140
130
120
110
100
90
80
70
60
50
40
30
20
ppm

4.16.3 ^{1}H, ^{1}H COSY

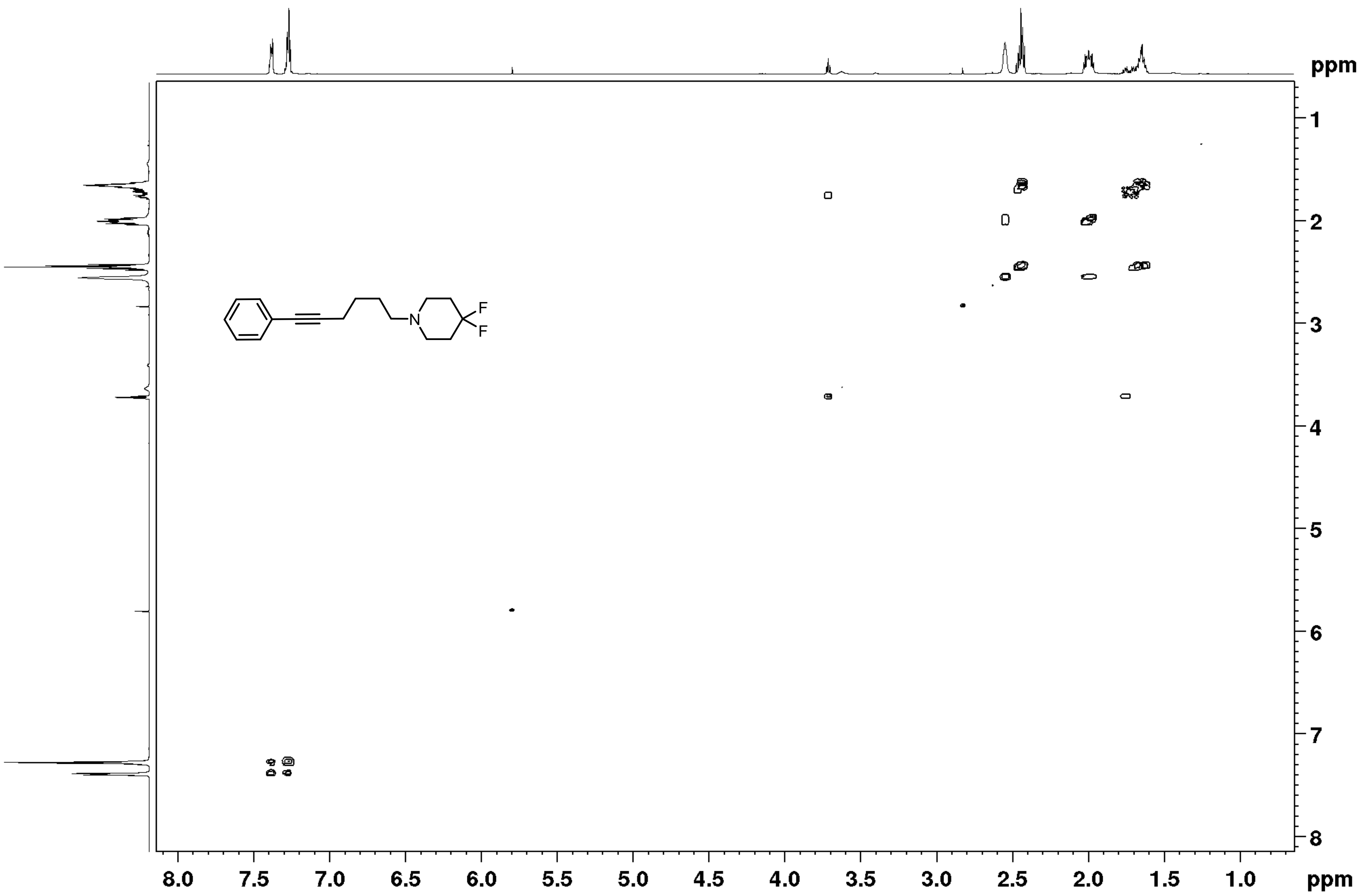

4.16.4 ^{1}H, ^{13}C HMBC

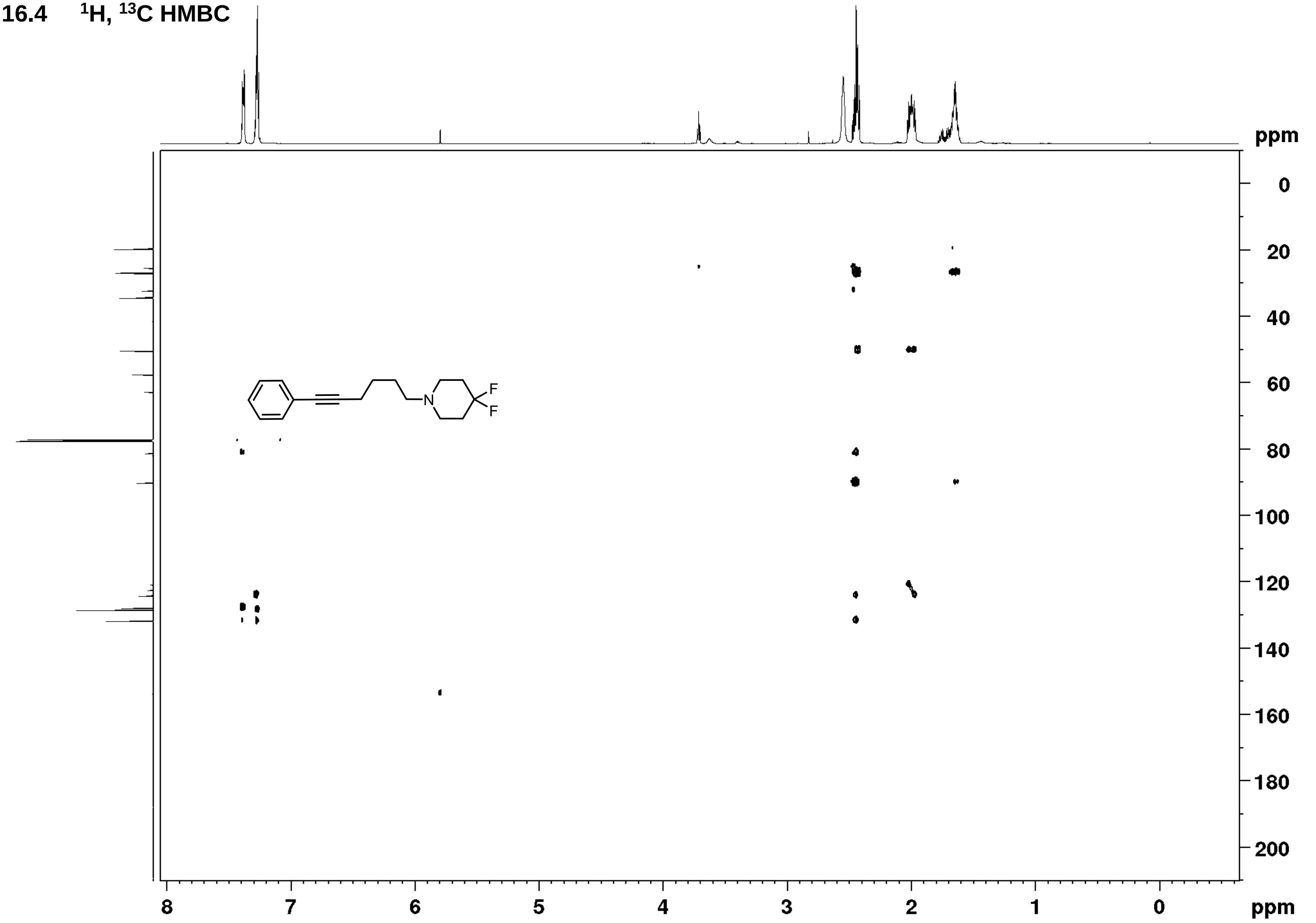

4.16.5 ^{1}H, ^{13}C HSQC

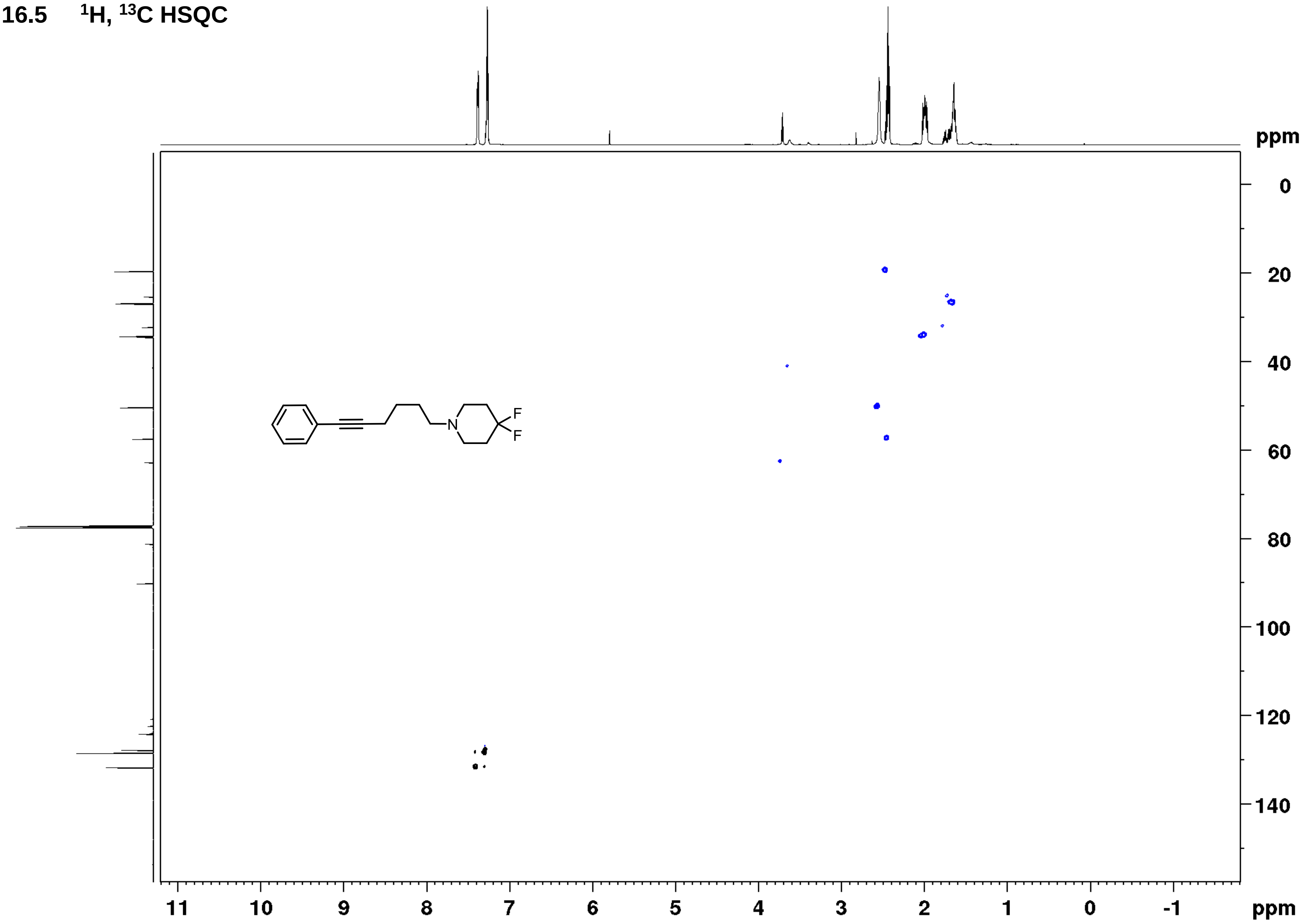

4.16.6 ^{19}F

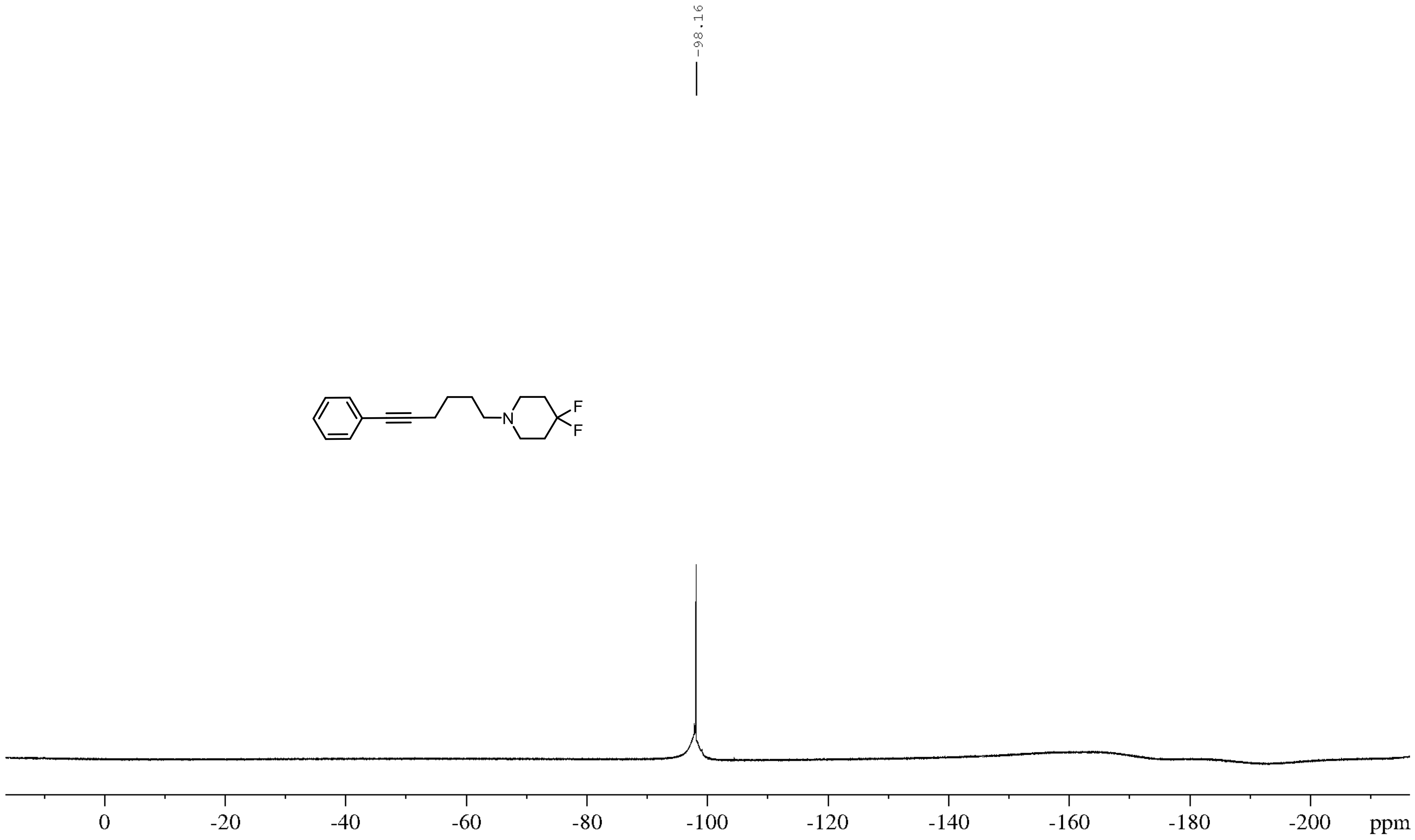

4.16.7 HRMS

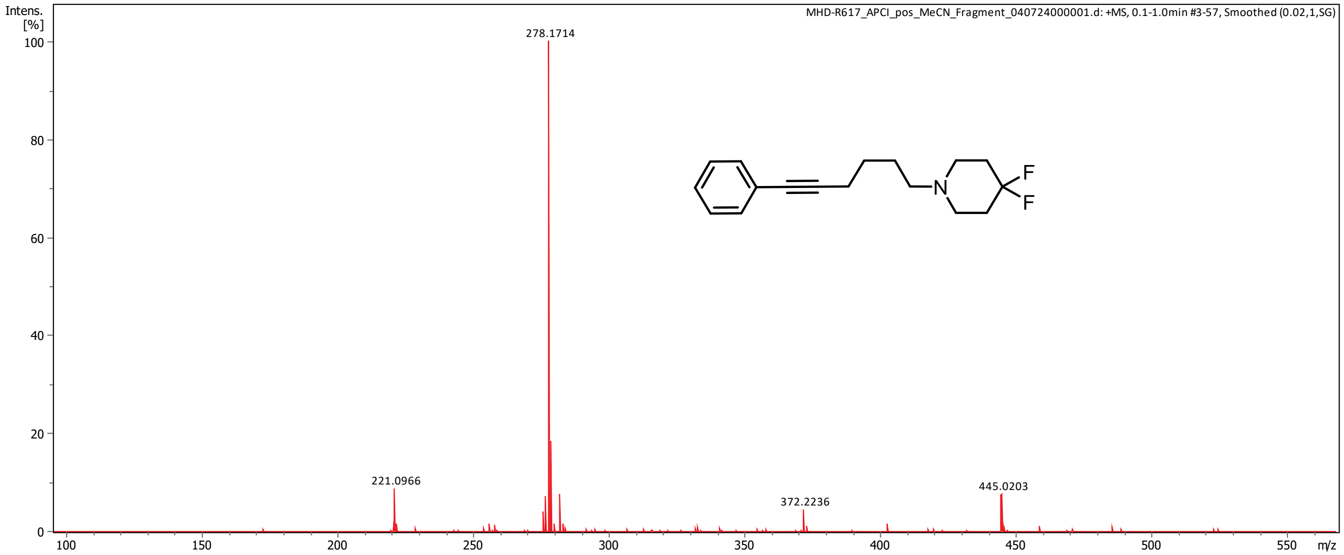

Intens. [%]
MHD-R617_APCI_pos_MeCN_Fragment_040724000001.d: +MS, 0.1-1.0min #3-57, Smoothed (0.02,1,SG)
278.1714
221.0966
372.2236
445.0203
m/z

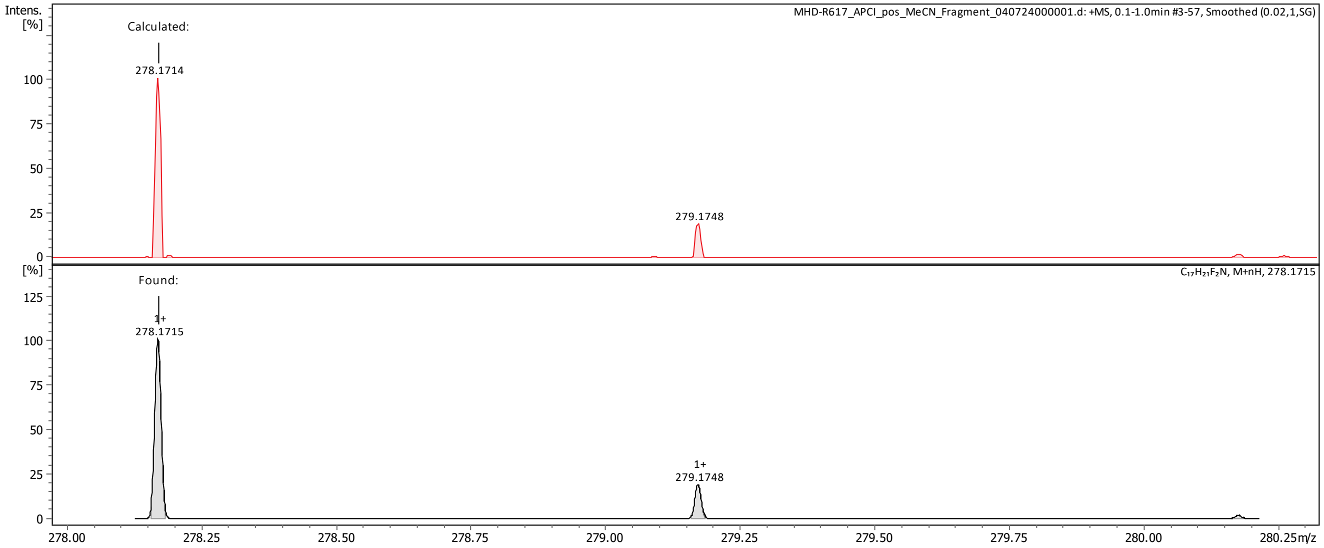

Intens. [%]
MHD-R617_APCI_pos_MeCN_Fragment_040724000001.d: +MS, 0.1-1.0min #3-57, Smoothed (0.02,1,SG)
Calculated:
278.1714
279.1748
C17H21F2N, M+nH, 278.1715
Found:
1+
278.1715
1+
279.1748
m/z

4.16.8 IR

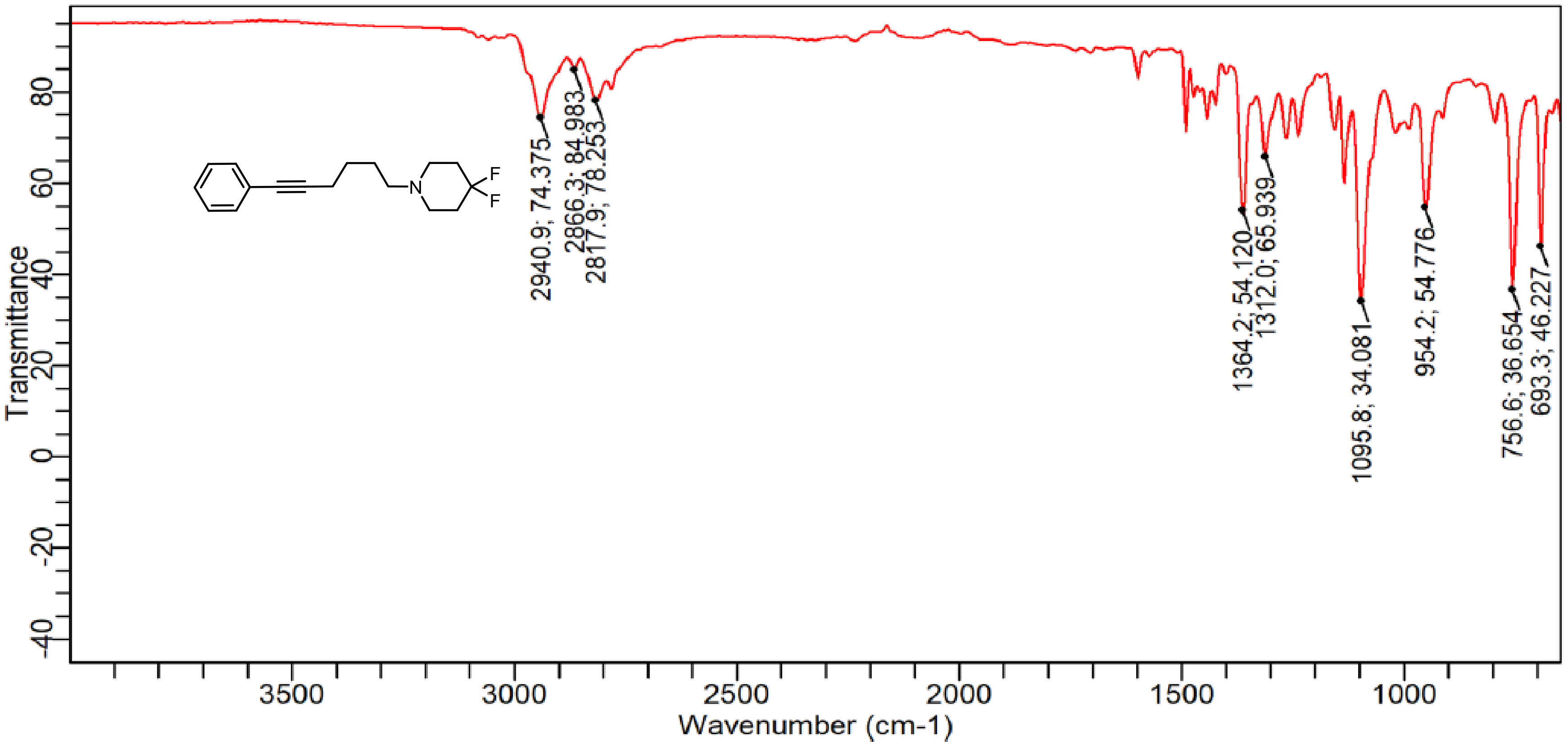

2940.9; 74.375
2866.3; 84.983
2817.9; 78.253
1364.2; 54.120
1312.0; 65.939
1095.8; 34.081
954.2; 54.776
756.6; 36.654
693.3; 46.227
N
F
F
Transmittance
Wavenumber (cm-1)

4.17 (6-chlorohex-1-yn-1-yl)benzene (1u) (600 MHz, $CDCl_3$)

4.17.1 ^{1}H NMR

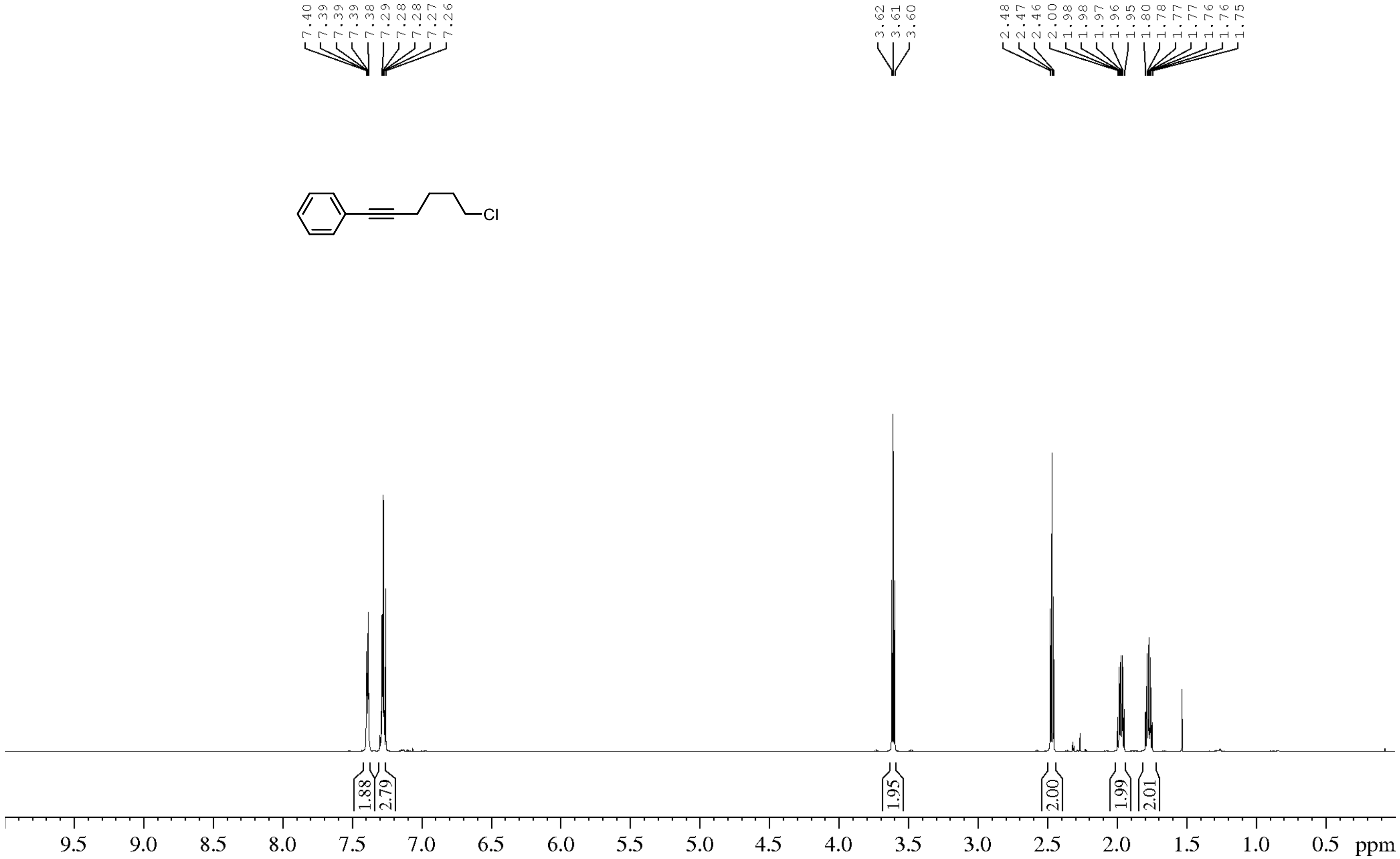

4.17.2 ^{13}C NMR

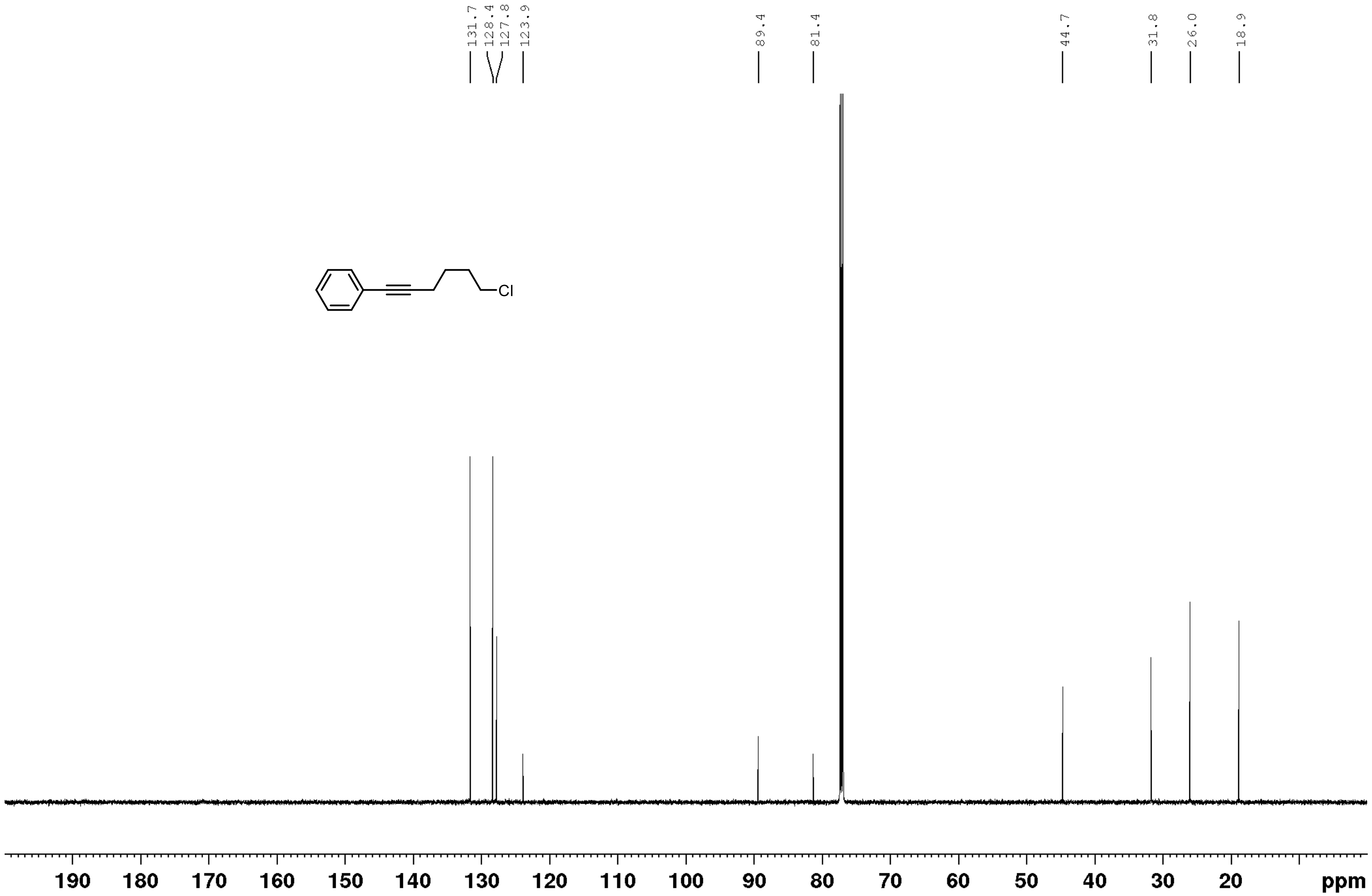

131.7
128.4
127.8
123.9
89.4
81.4
44.7
31.8
26.0
18.9
Cl
190
180
170
160
150
140
130
120
110
100
90
80
70
60
50
40
30
20
ppm

4.17.3 ^{1}H, ^{1}H COSY

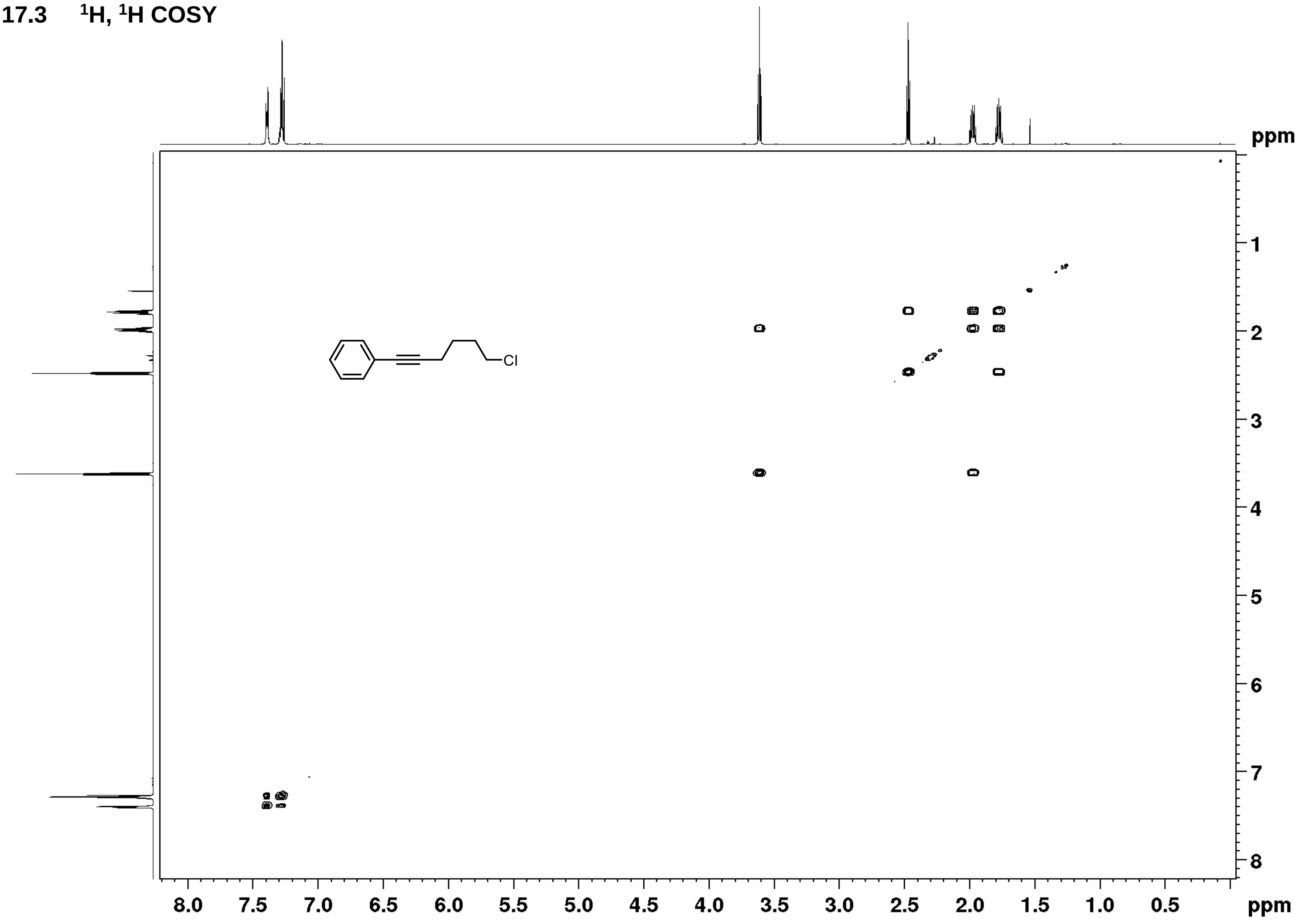

4.17.4 ^{1}H, ^{13}C HMBC

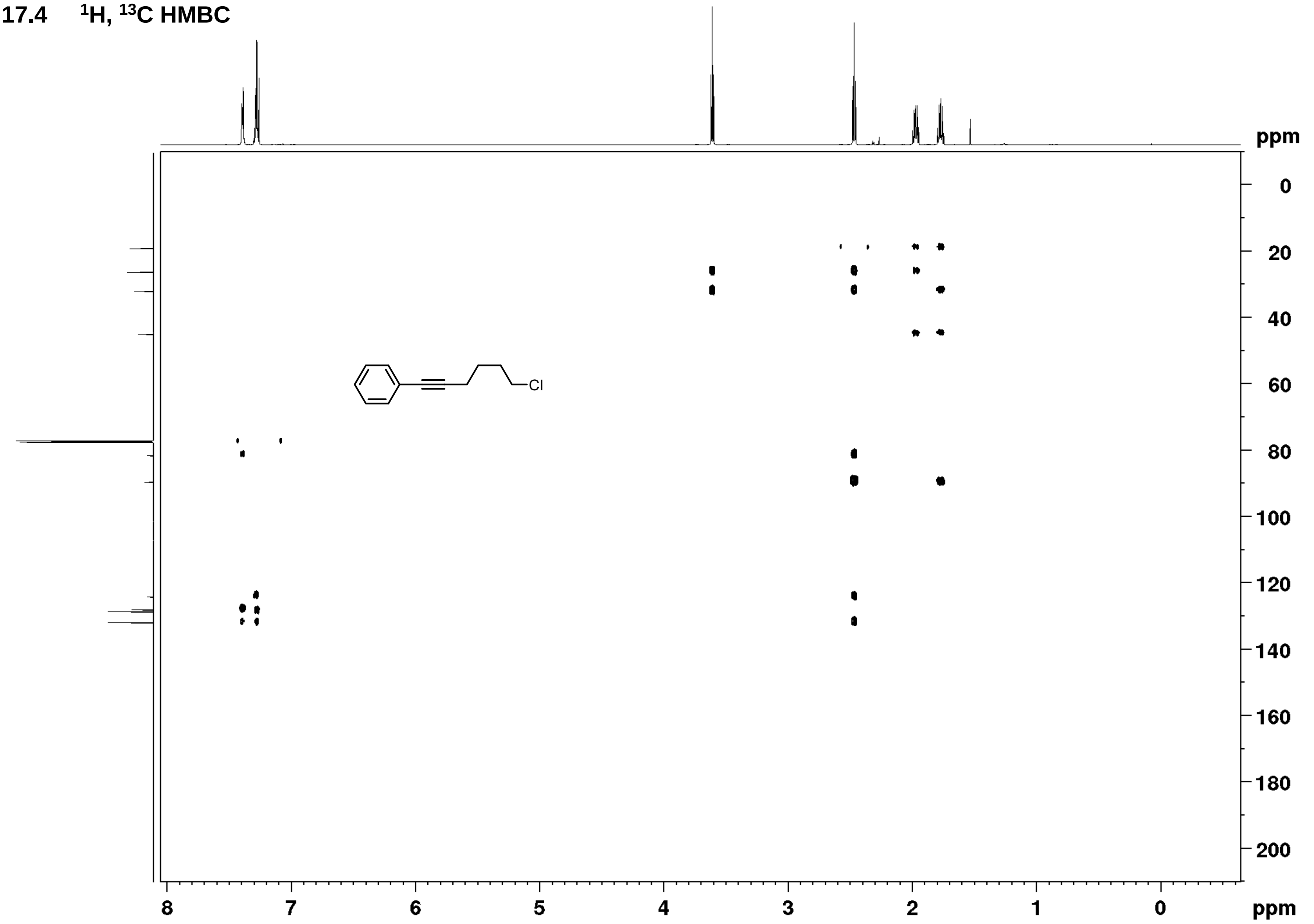

4.17.5 ^{1}H, ^{13}C HSQC

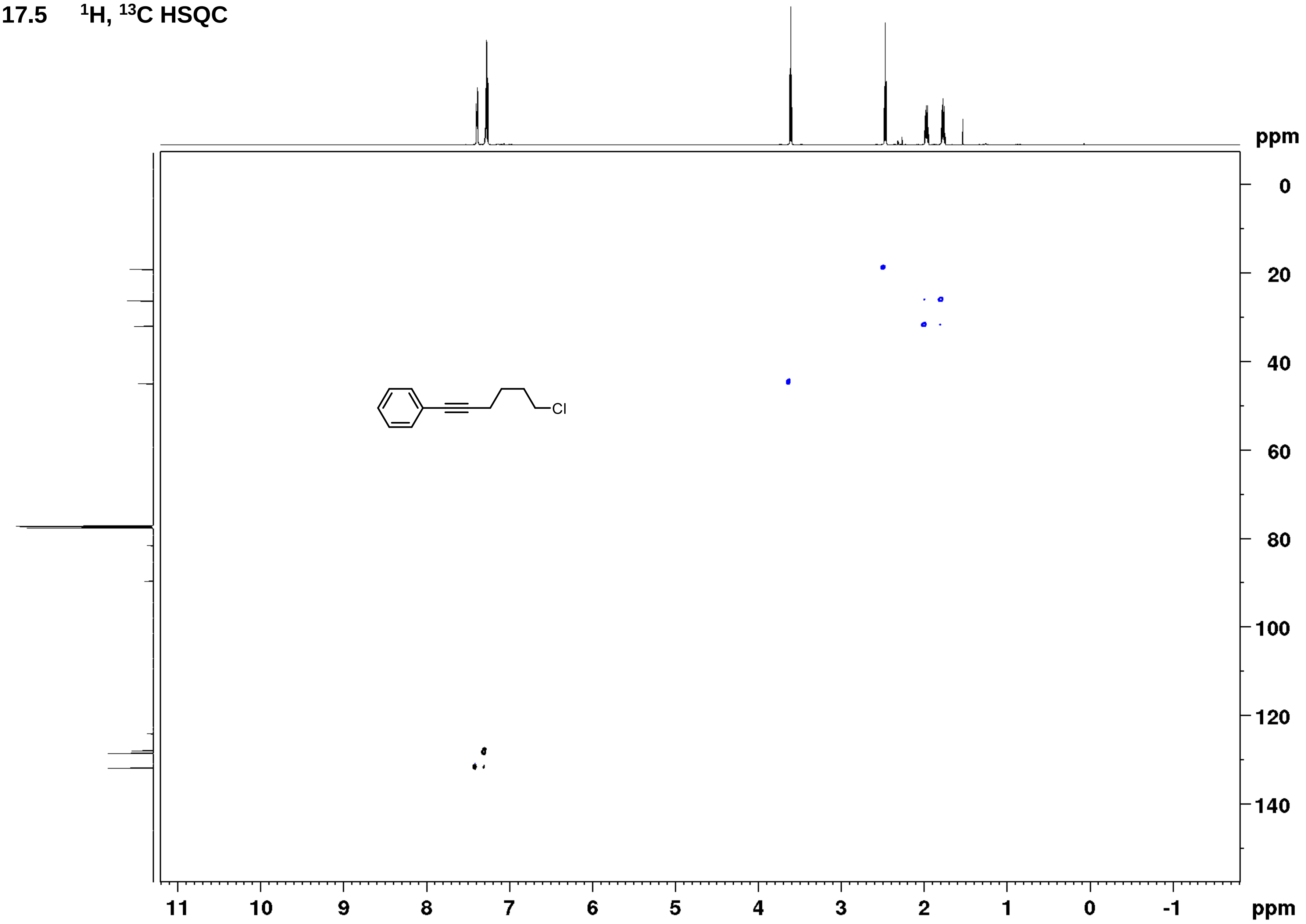

4.17.6 HRMS

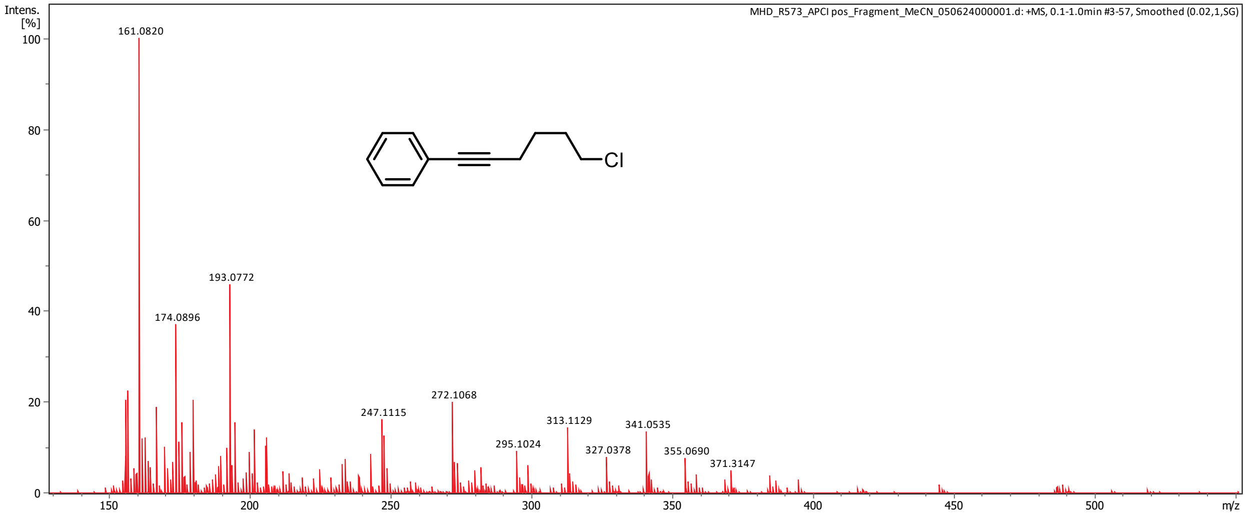

MHD_R573_APCI pos_Fragment_MeCN_050624000001.d: +MS, 0.1-1.0min #3-57, Smoothed (0.02,1,SG)
Intens. [%]
161.0820
193.0772
174.0896
272.1068
247.1115
313.1129
341.0535
295.1024
327.0378
355.0690
371.3147
Cl
m/z

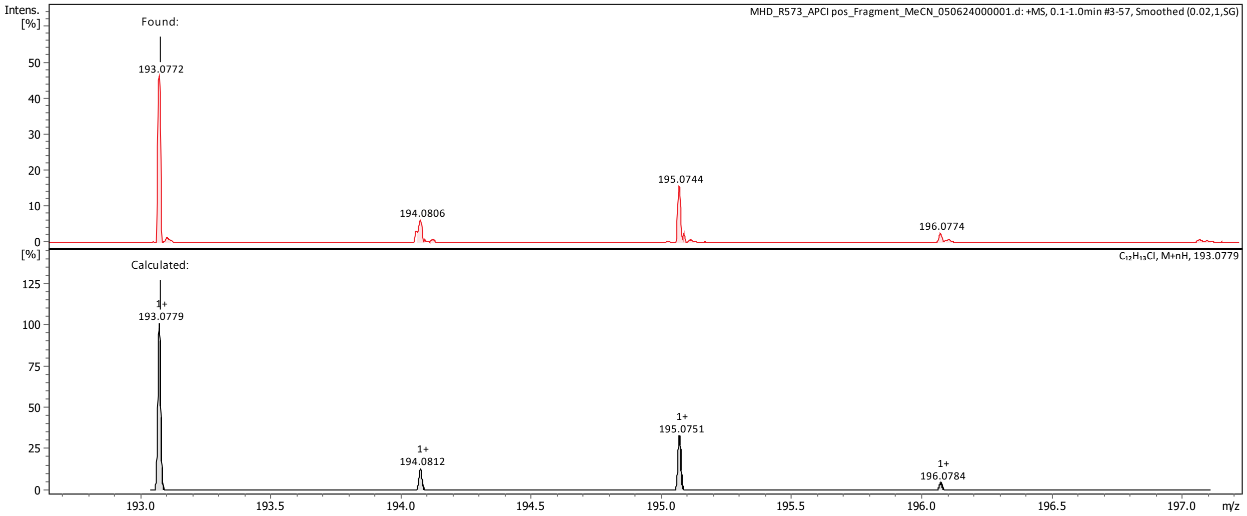

MHD_R573_APCI pos_Fragment_MeCN_050624000001.d: +MS, 0.1-1.0min #3-57, Smoothed (0.02,1,SG)
Found:
193.0772
194.0806
195.0744
196.0774
$C_{12}H_{13}Cl$, M+nH, 193.0779
Calculated:
1+ 193.0779
1+ 194.0812
1+ 195.0751
1+ 196.0784
m/z

4.18 2-((4-Methoxyphenyl)ethynyl)pyridine (1w) (600 MHz, $CDCl_3$)

4.18.1 ^{1}H NMR

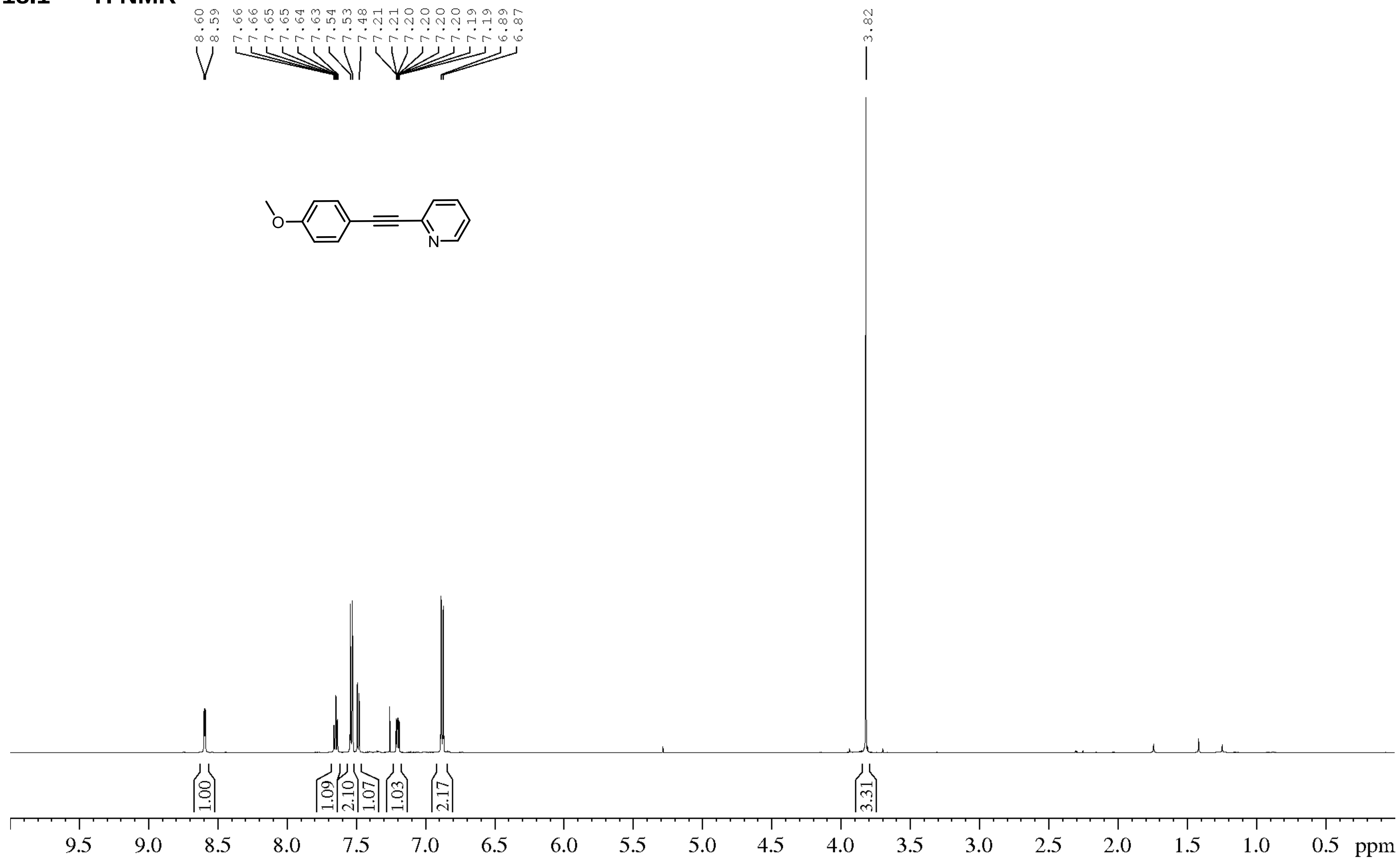

4.18.2 ^{13}C NMR

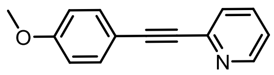

O
N

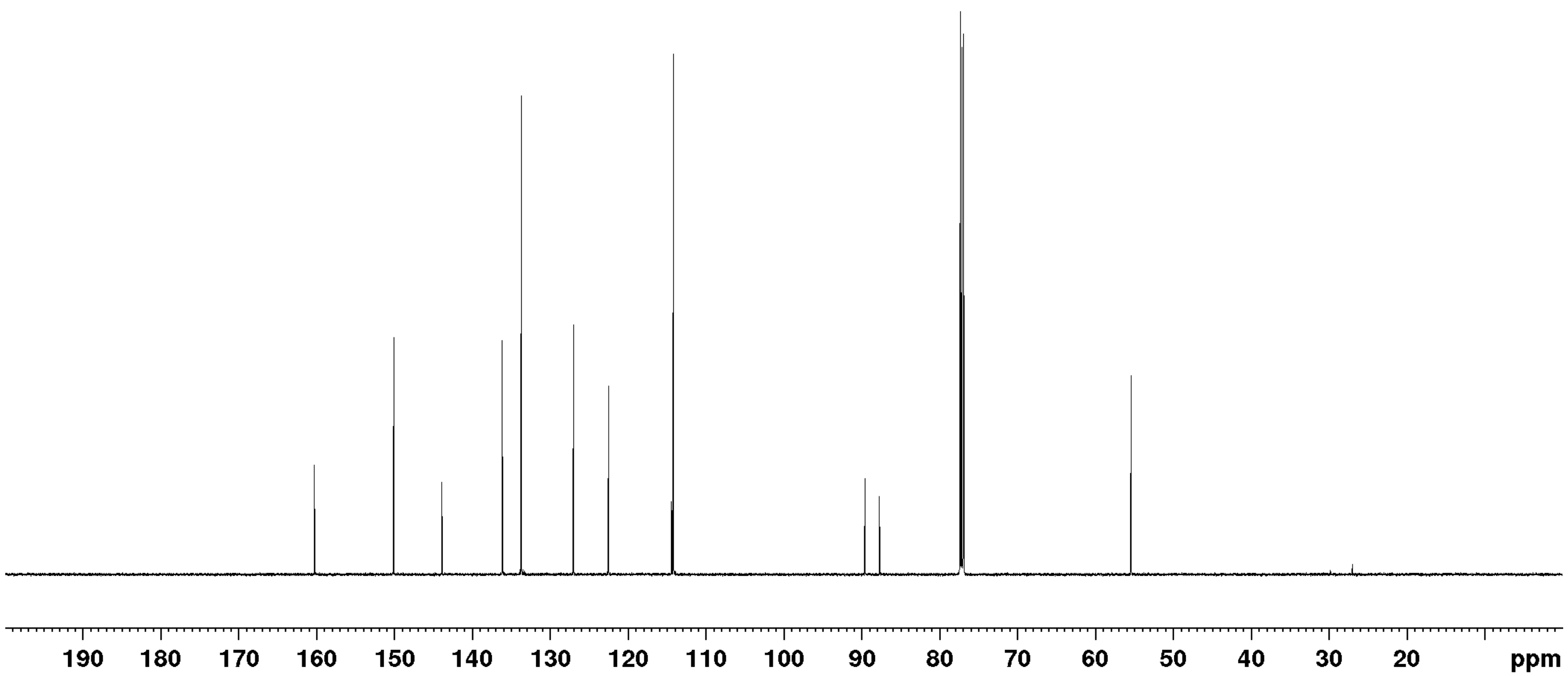

160.3
150.1
143.9
136.2
133.7
127.0
122.5
114.4
114.2
89.6
87.7
55.4
190 180 170 160 150 140 130 120 110 100 90 80 70 60 50 40 30 20 ppm

4.18.3 ^{1}H, ^{1}H COSY

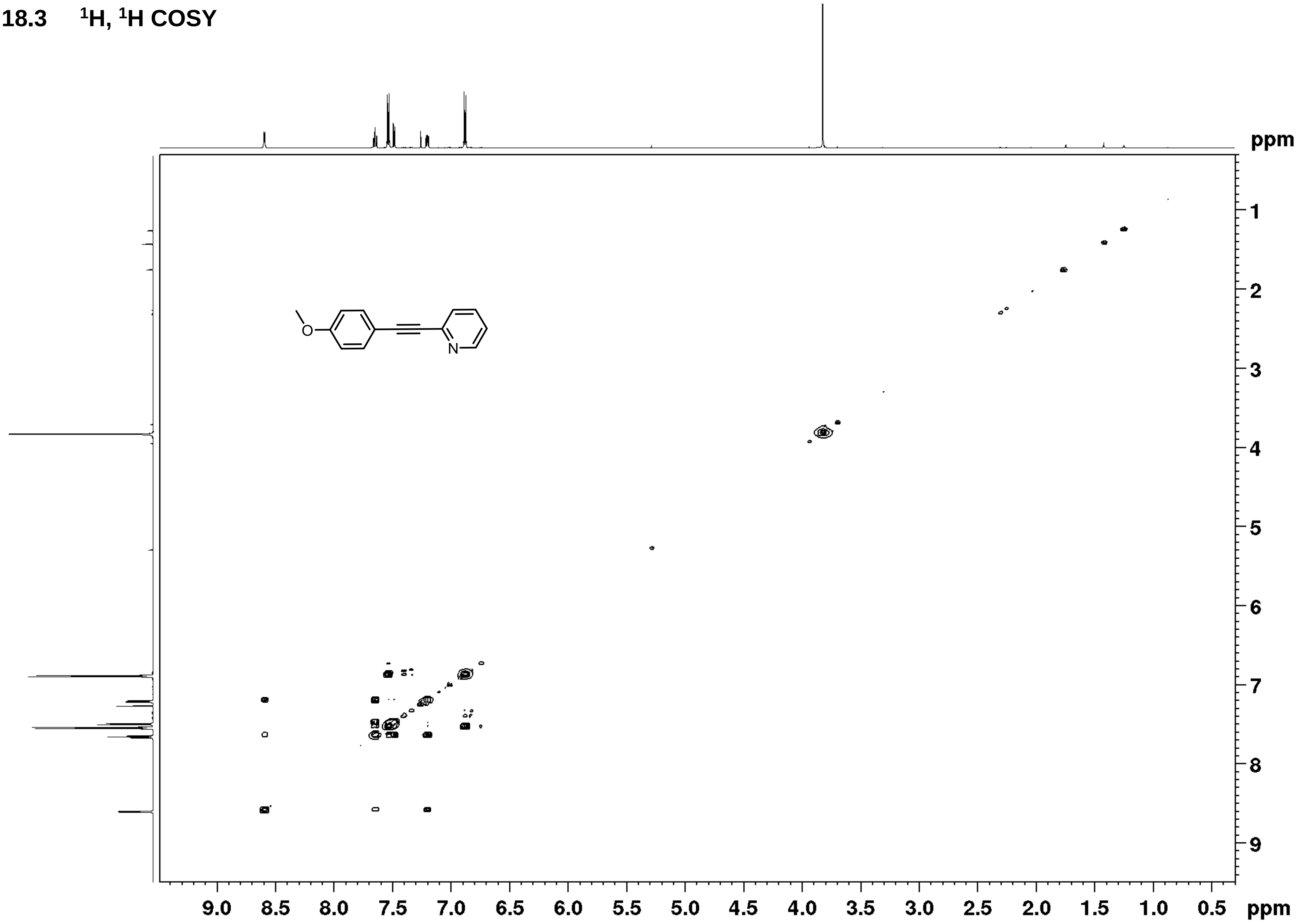

4.18.4 ^{1}H, ^{13}C HMBC

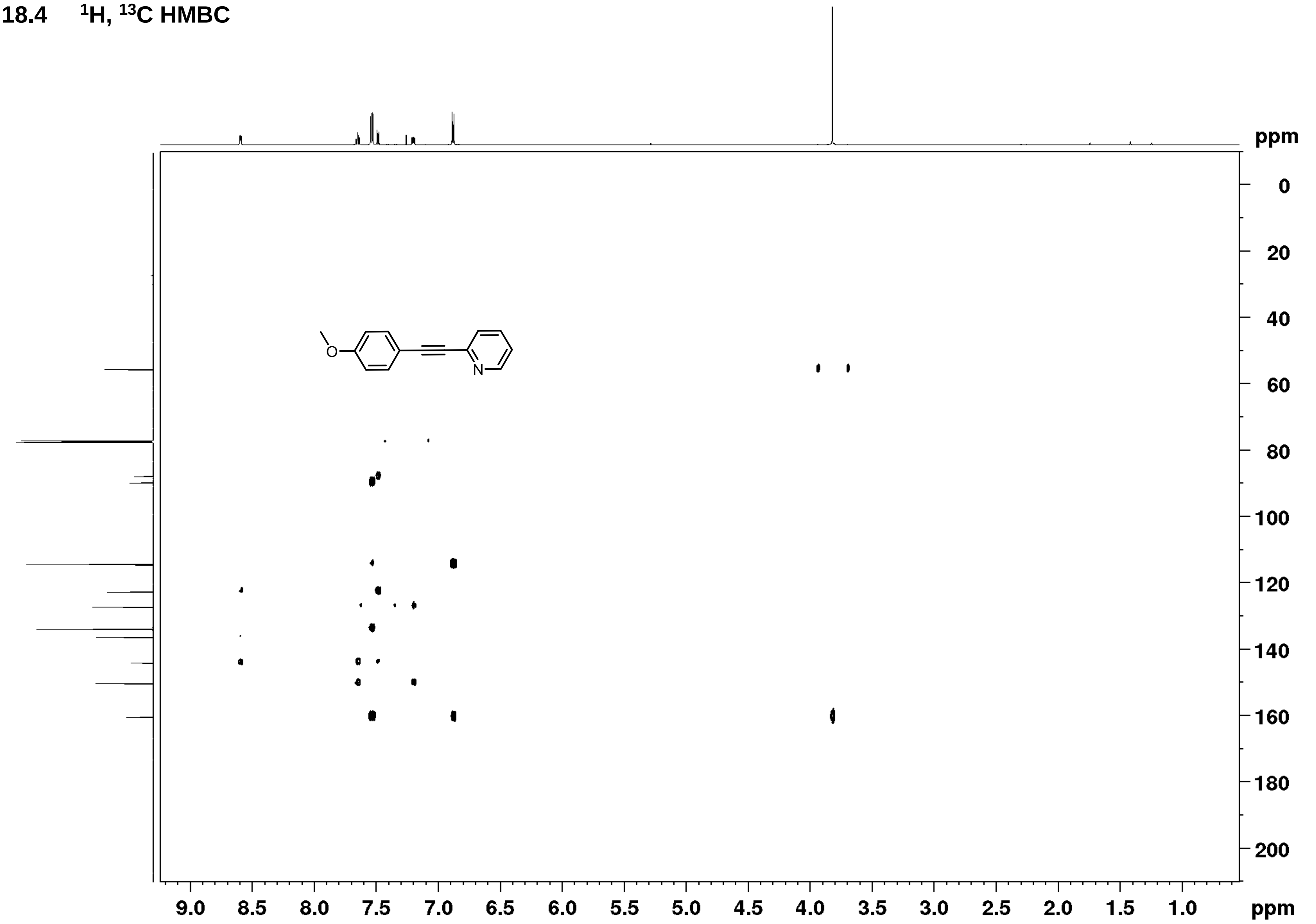

4.18.5 ^{1}H, ^{13}C HSQC

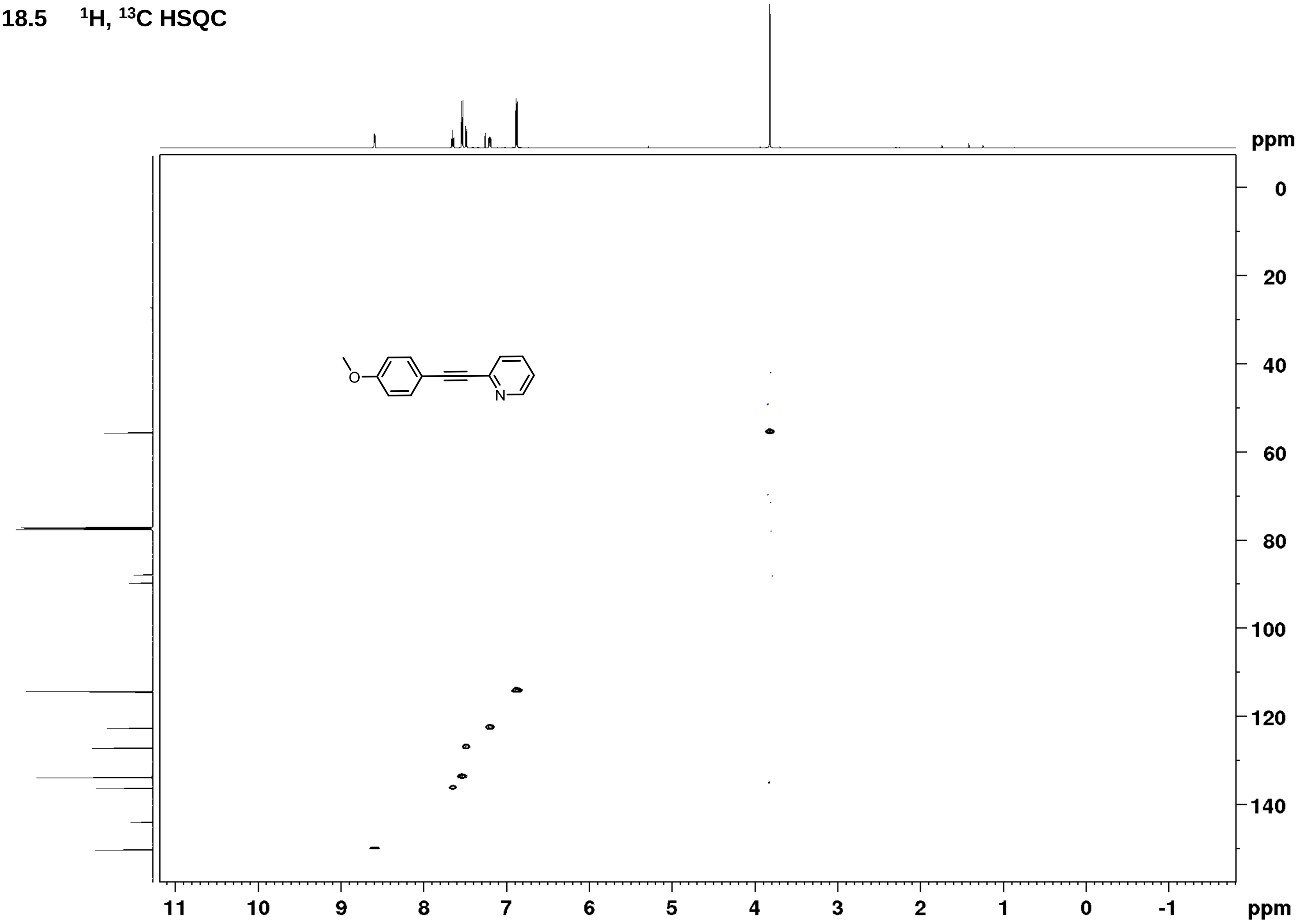

4.18.6 HRMS

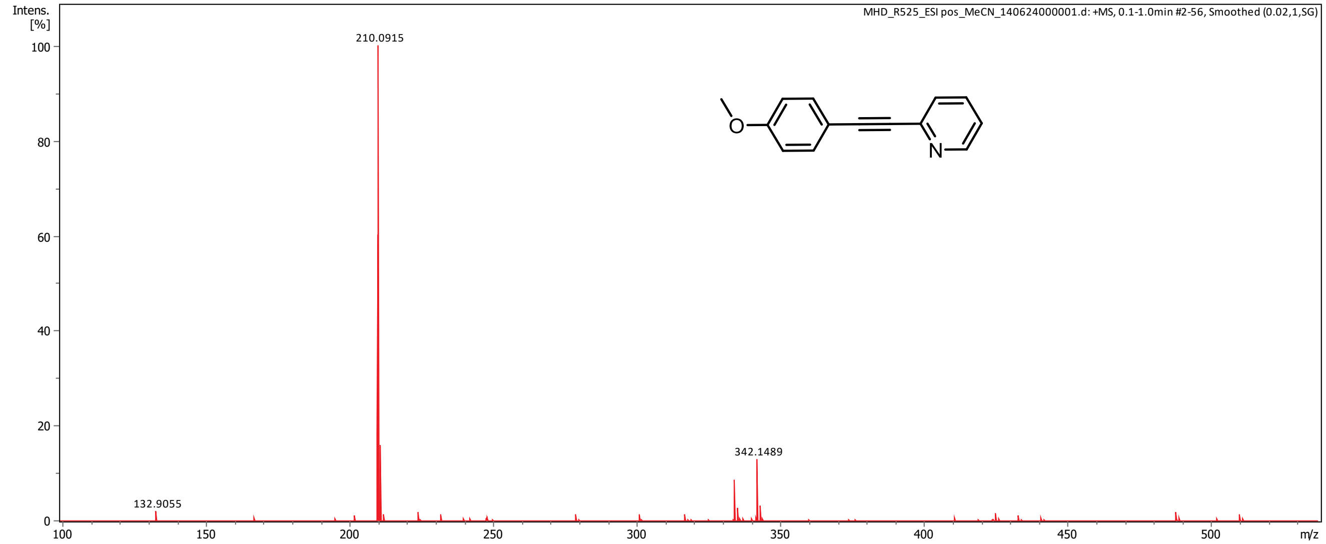

Intens. [%]
MHD_R525_ESI pos_MeCN_140624000001.d: +MS, 0.1-1.0min #2-56, Smoothed (0.02,1,SG)
210.0915
342.1489
132.9055
m/z

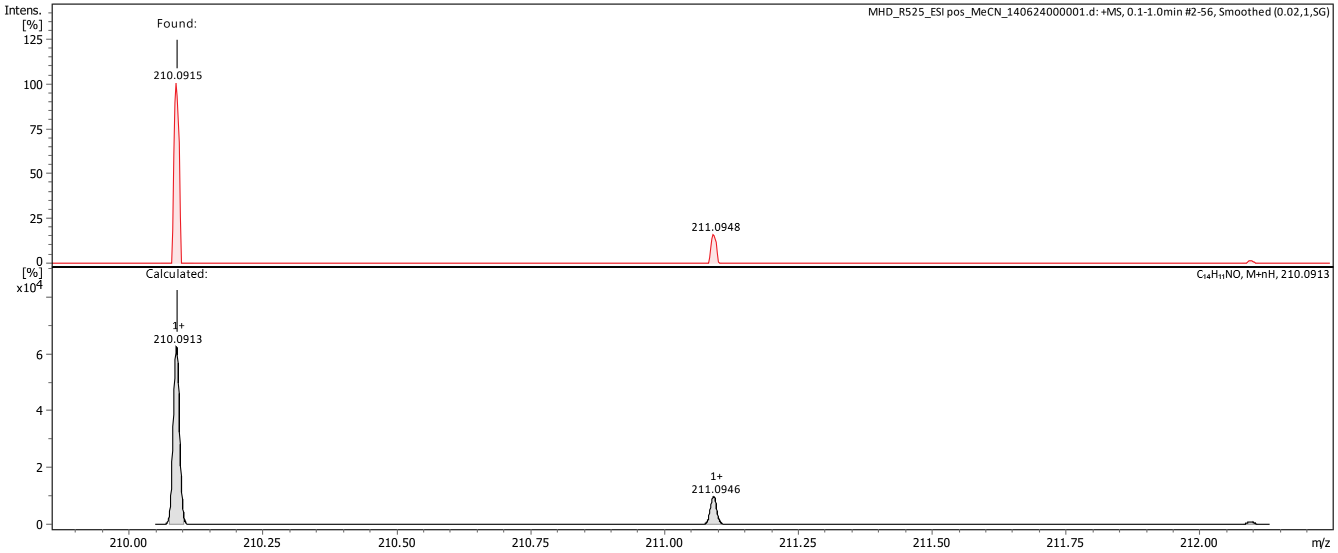

Intens. [%]
MHD_R525_ESI pos_MeCN_140624000001.d: +MS, 0.1-1.0min #2-56, Smoothed (0.02,1,SG)
Found:
210.0915
211.0948
[%] x10^4
Calculated:
C14H11NO, M+nH, 210.0913
1+ 210.0913
1+ 211.0946
m/z

4.19 3-((4-Methoxyphenyl)ethynyl)pyridine (1x) (600 MHz, $CDCl_3$)

4.19.1 ^{1}H NMR

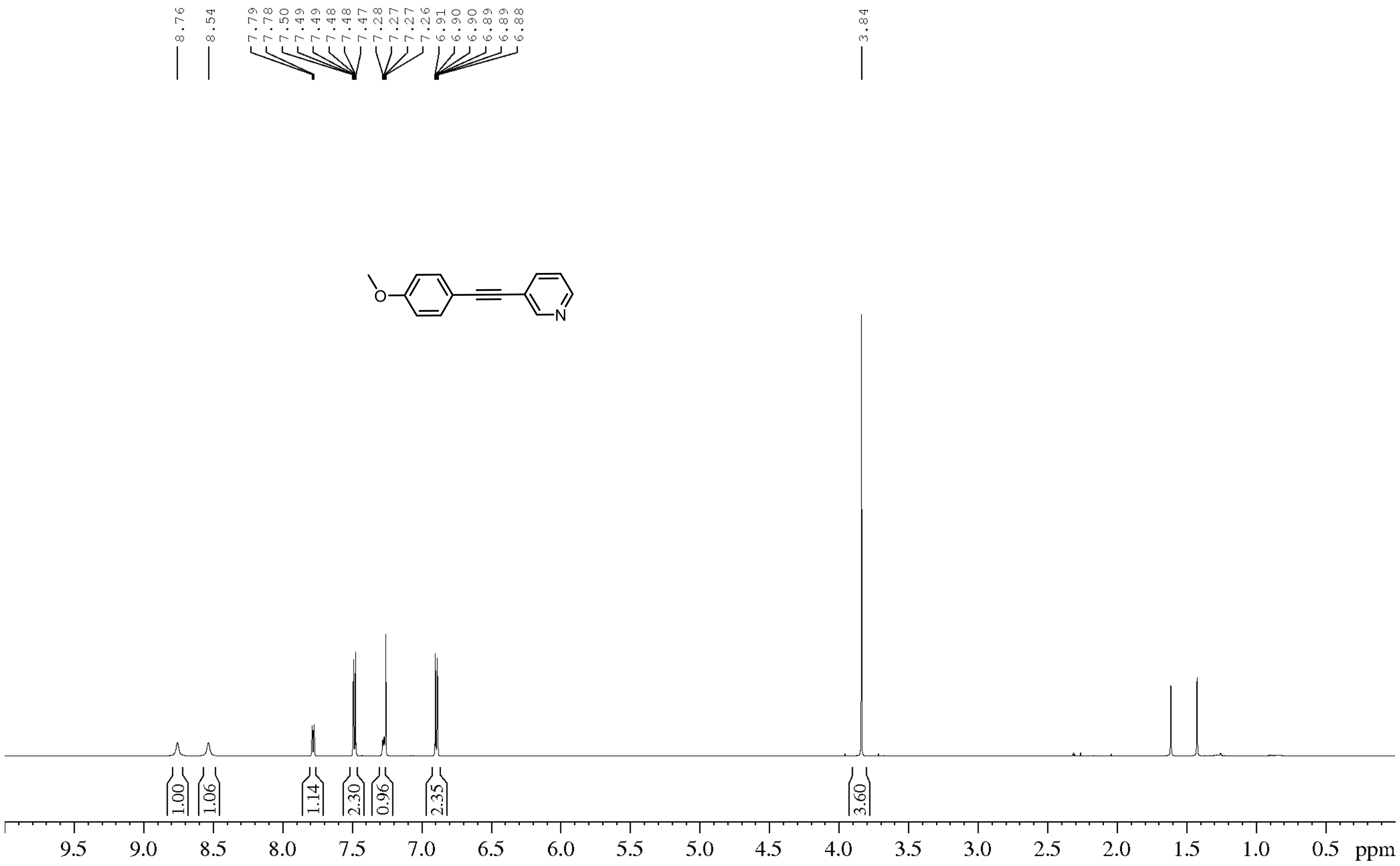

4.19.2 ^{13}C NMR

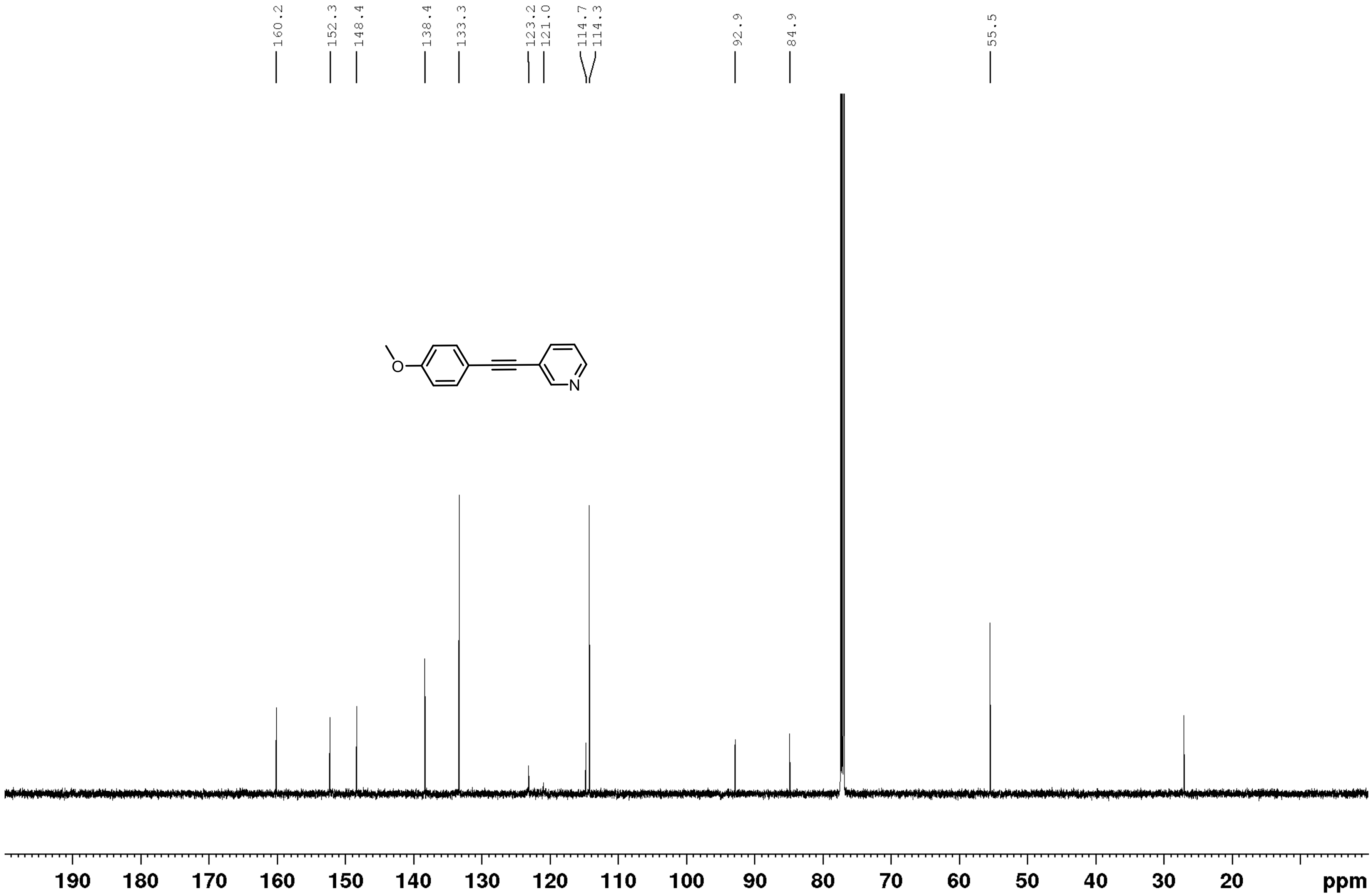

160.2
152.3
148.4
138.4
133.3
123.2
121.0
114.7
114.3
92.9
84.9
55.5
O
N
190 180 170 160 150 140 130 120 110 100 90 80 70 60 50 40 30 20 ppm

4.19.3 $^{1}H, ^{1}H$ COSY

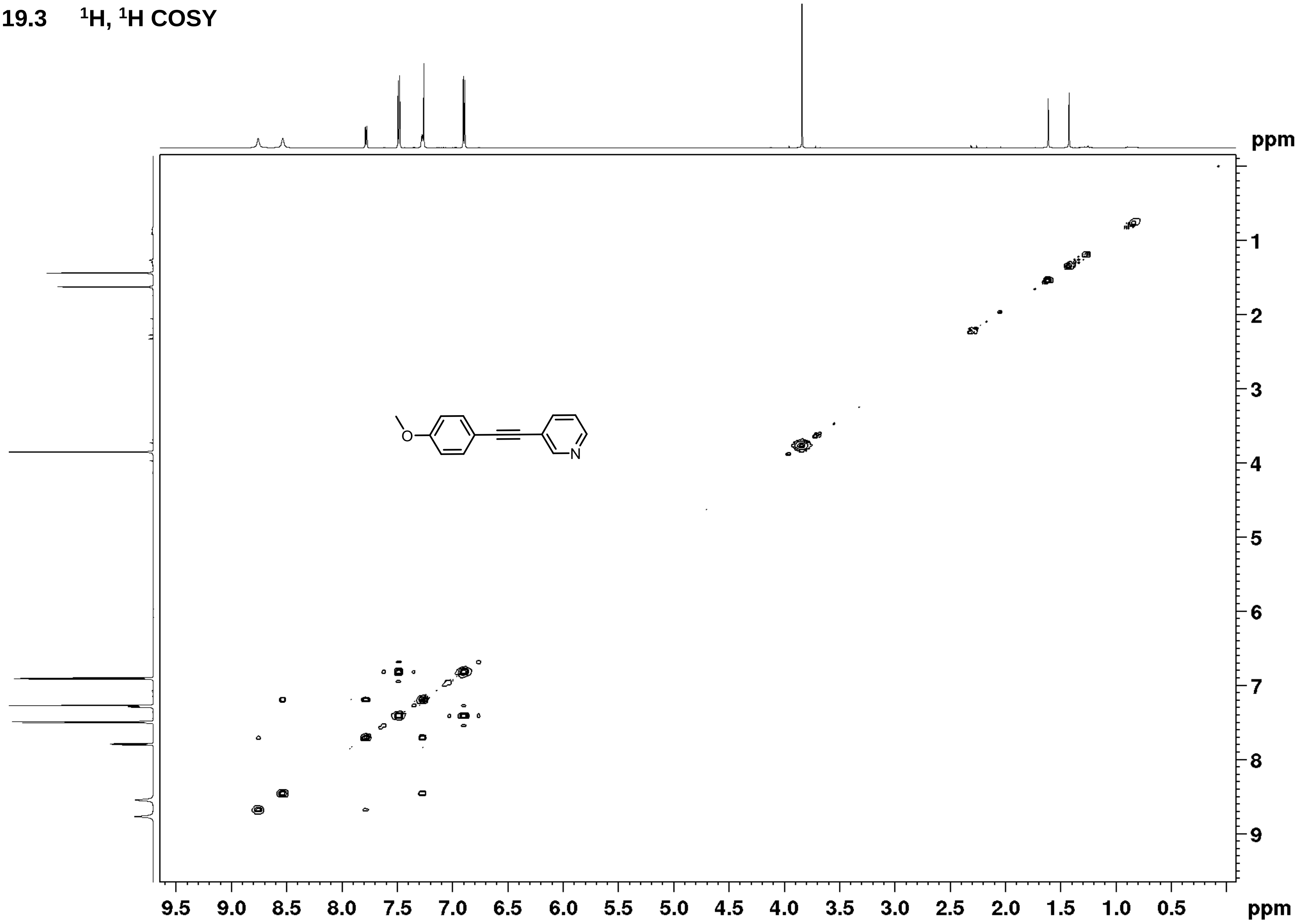

4.19.4 ^1H, ^{13}C HMBC

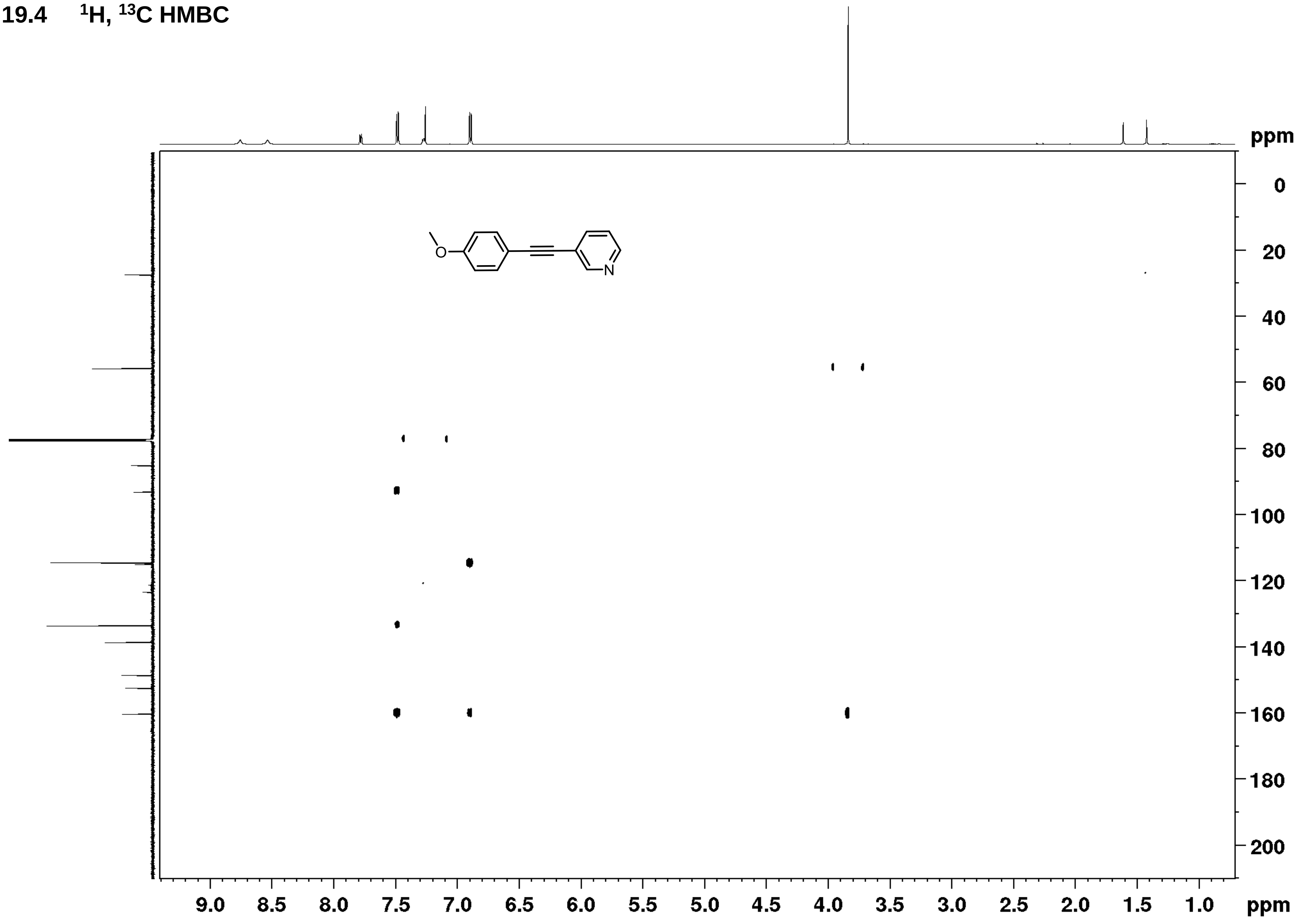

4.19.5 ^{1}H, ^{13}C HSQC

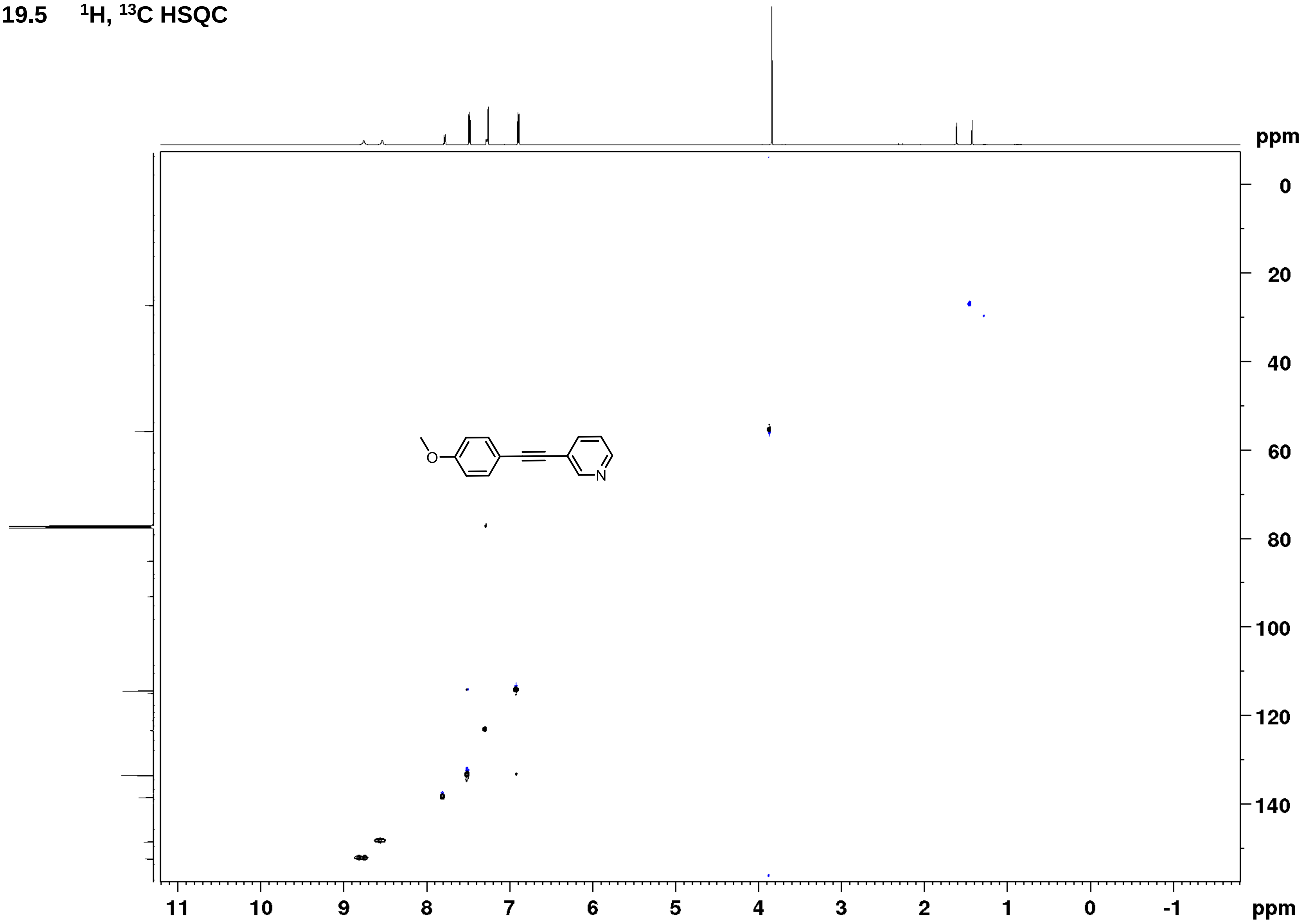

4.19.6 HRMS

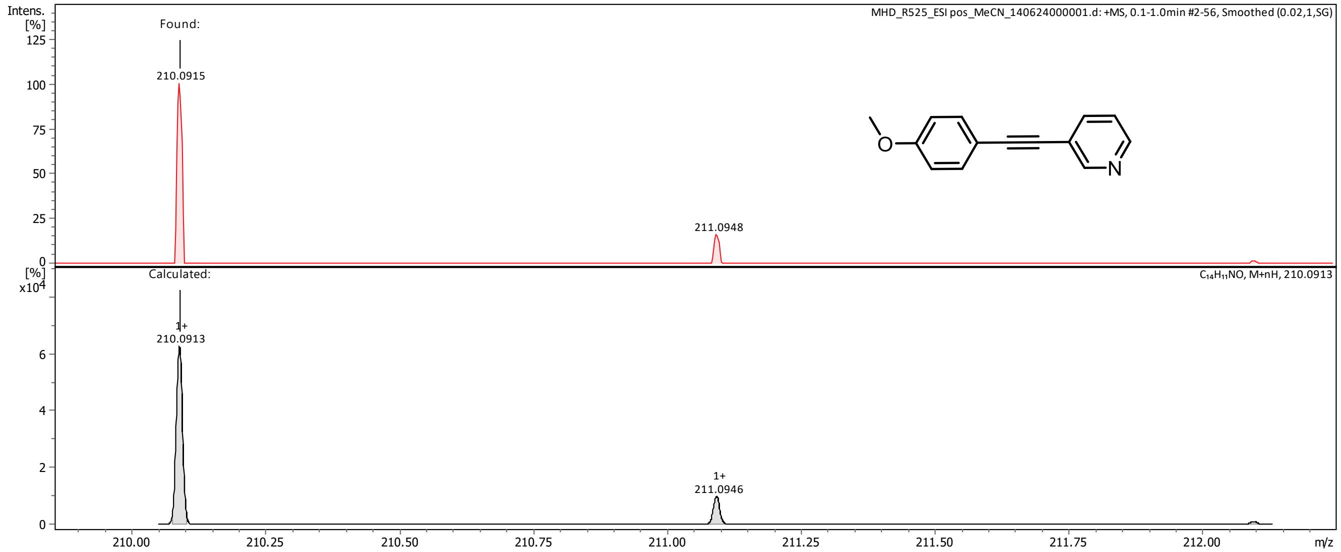

Intens. [%]
MHD_R525_ESI pos_MeCN_140624000001.d: +MS, 0.1-1.0min #2-56, Smoothed (0.02,1,SG)
Found:
210.0915
211.0948
O
N
Calculated:
$C_{14}H_{11}NO$, M+nH, 210.0913
1+
210.0913
1+
211.0946
[%]
x10^4
210.00
210.25
210.50
210.75
211.00
211.25
211.50
211.75
212.00
m/z

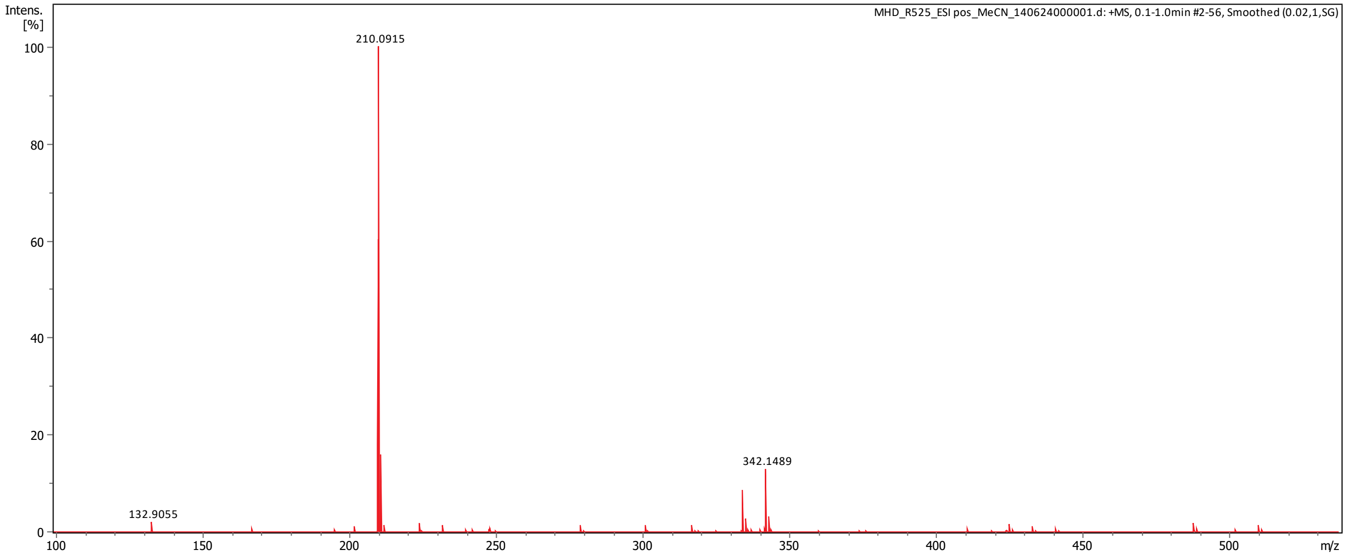

Intens. [%]
MHD_R525_ESI pos_MeCN_140624000001.d: +MS, 0.1-1.0min #2-56, Smoothed (0.02,1,SG)
210.0915
342.1489
132.9055
m/z

4.20 2-((4-Methoxyphenyl)ethynyl)-1-methyl-1*H*-imidazole (1y) (600 MHz, $CDCl_3$)

4.20.1 ^{1}H NMR

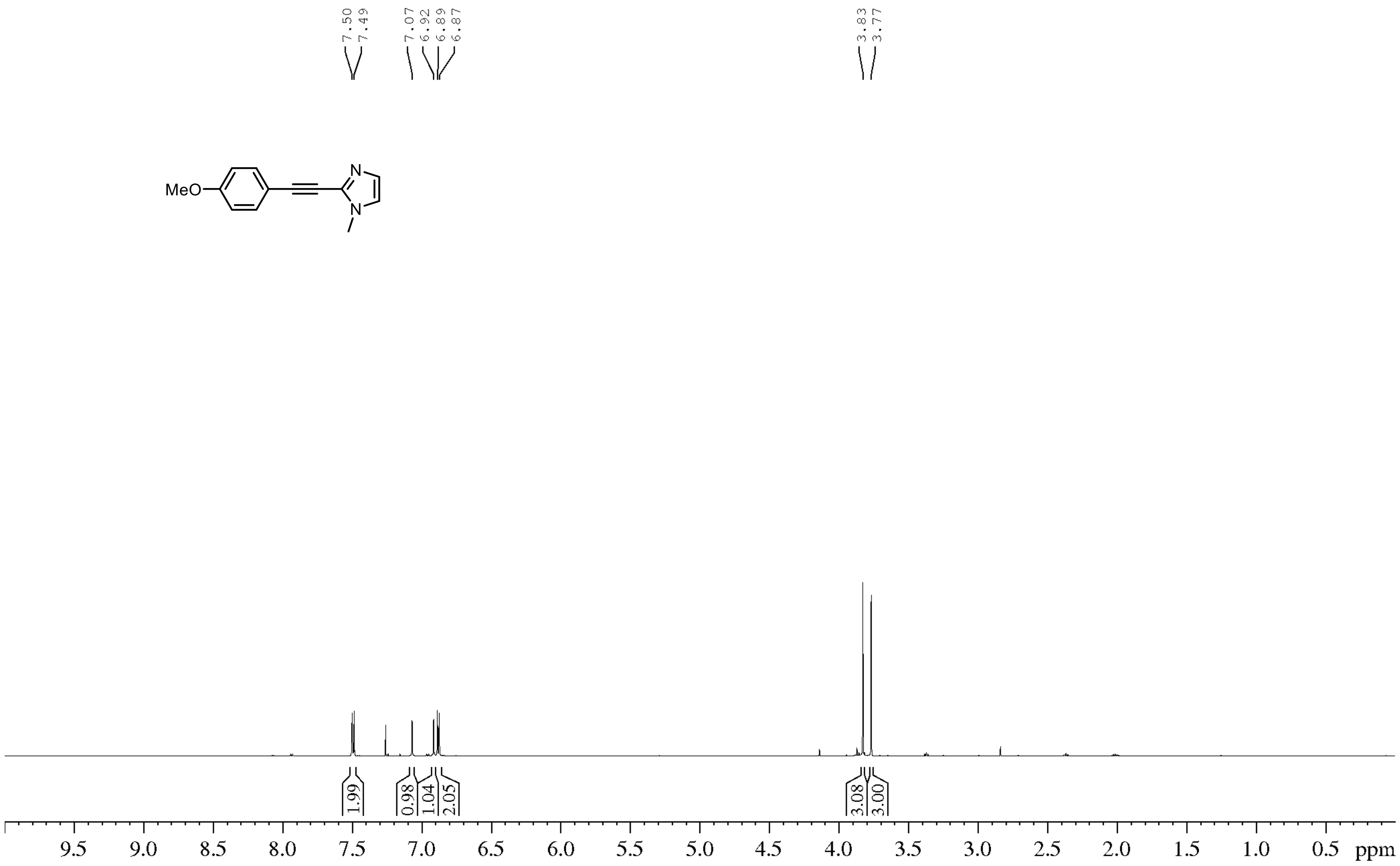

4.20.2 ^{13}C NMR

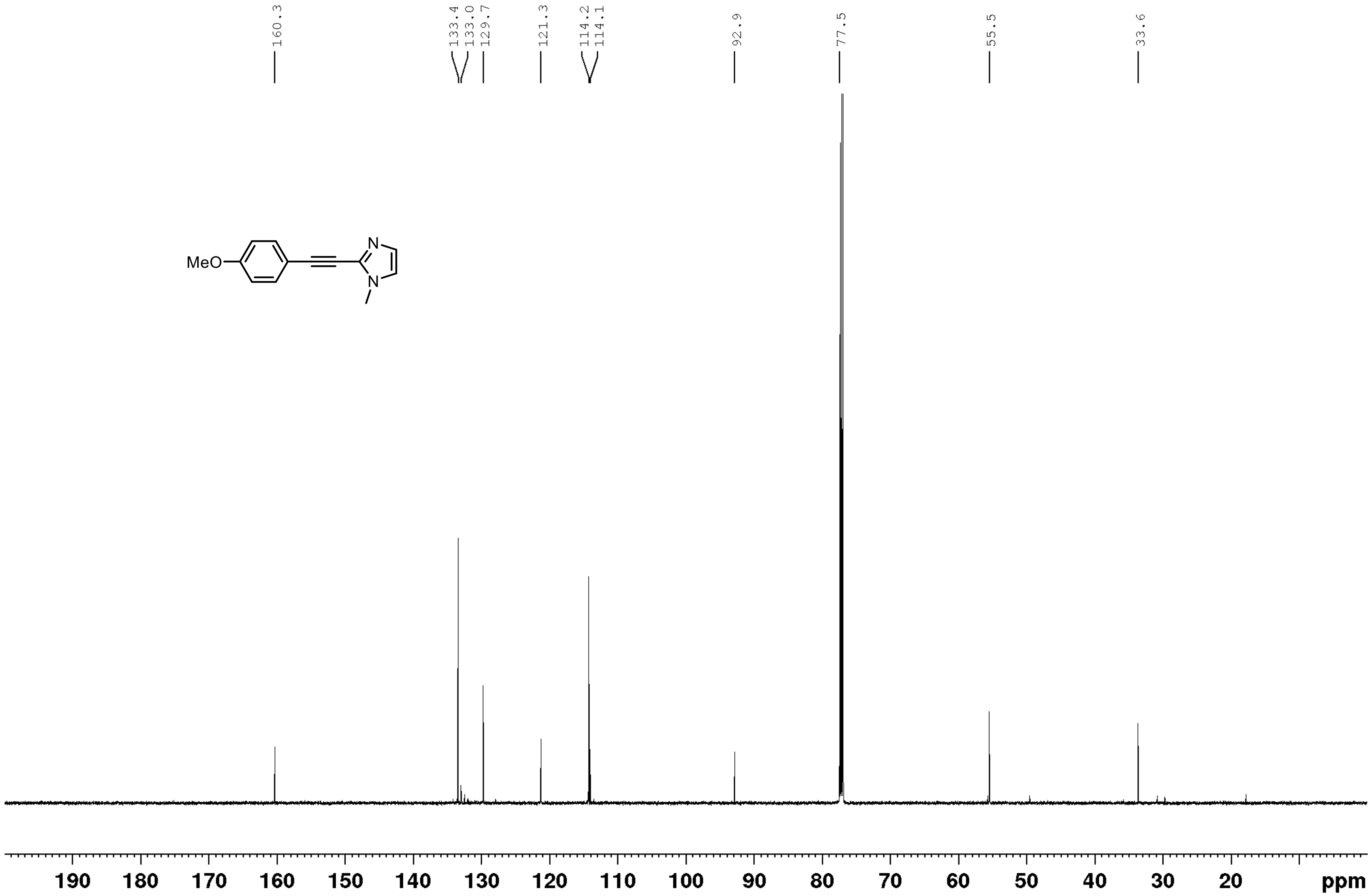

160.3
133.4
133.0
129.7
121.3
114.2
114.1
92.9
77.5
55.5
33.6
MeO
N
N
190 180 170 160 150 140 130 120 110 100 90 80 70 60 50 40 30 20 ppm

4.20.3 ^{1}H, ^{1}H COSY

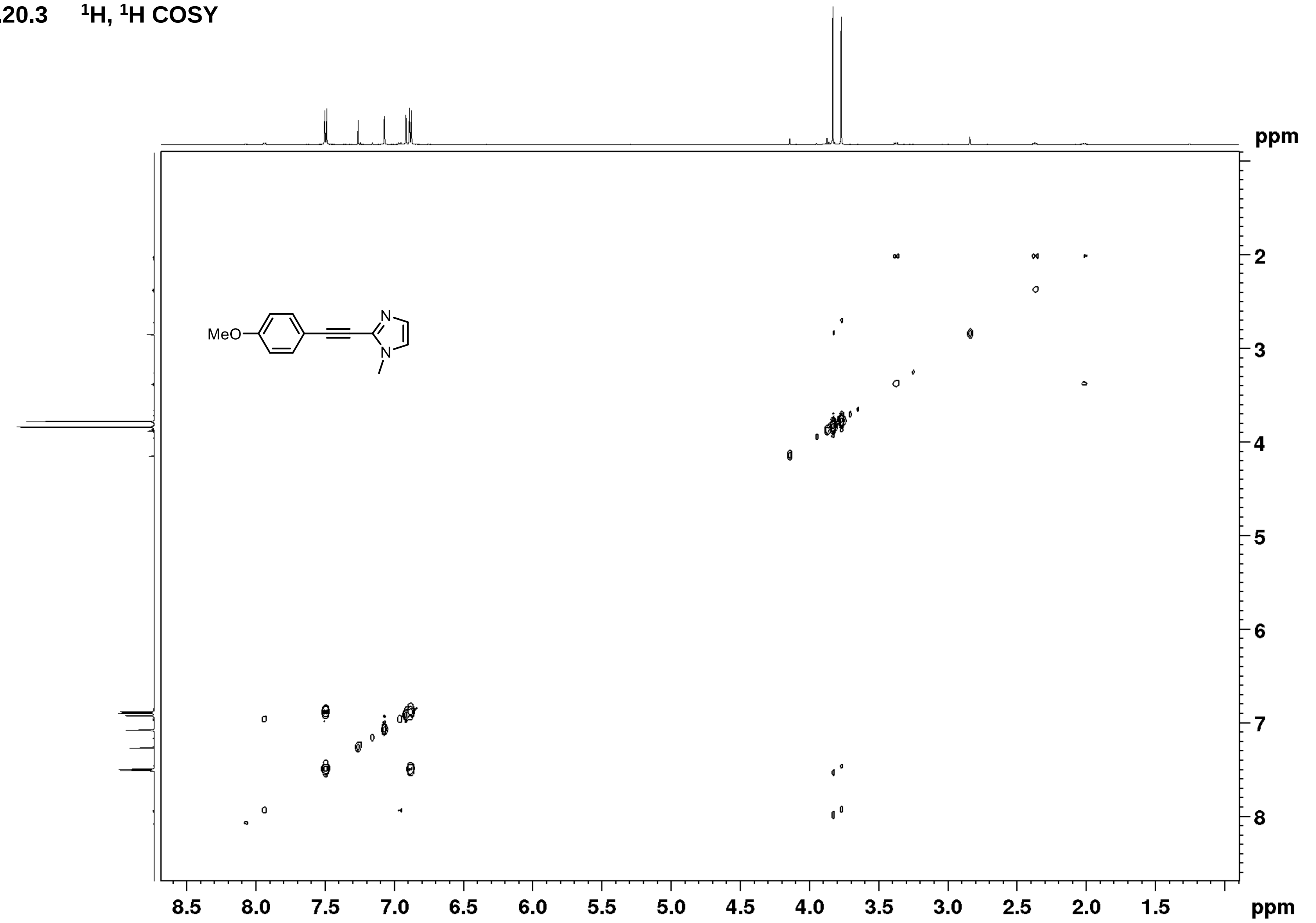

4.20.4 ^{1}H, ^{13}C HMBC

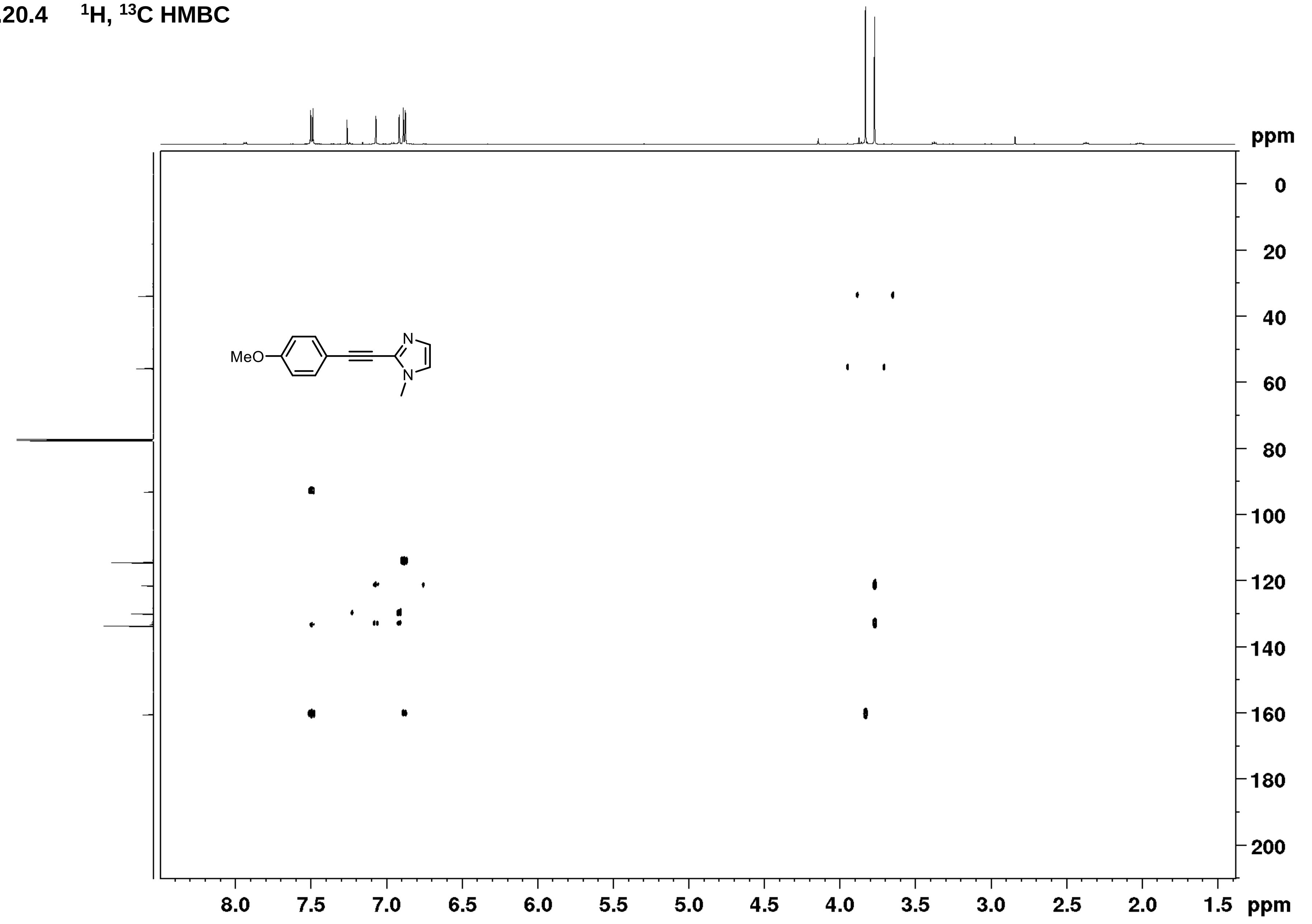

4.20.5 ^{1}H, ^{13}C HSQC

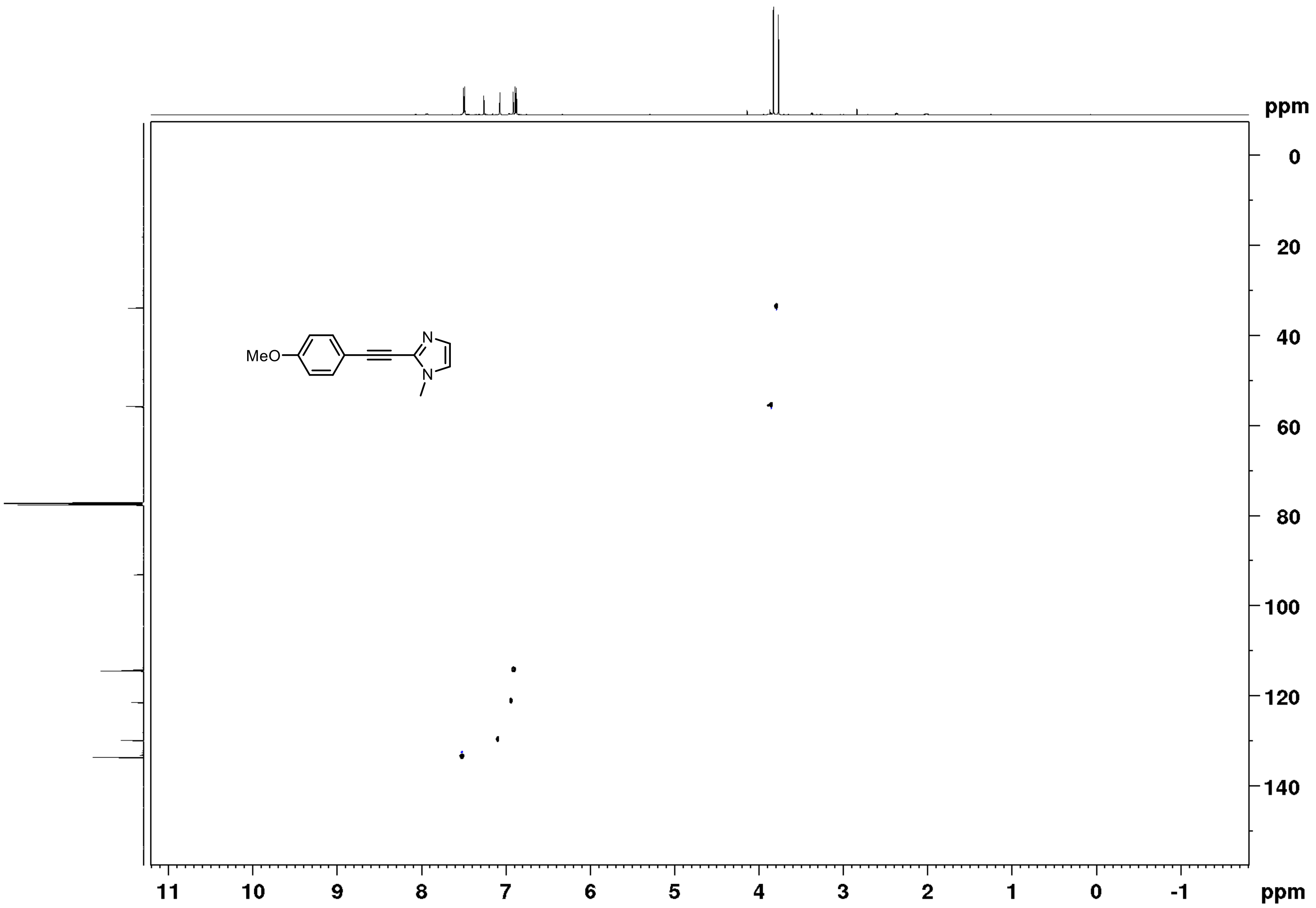

4.20.6 HRMS

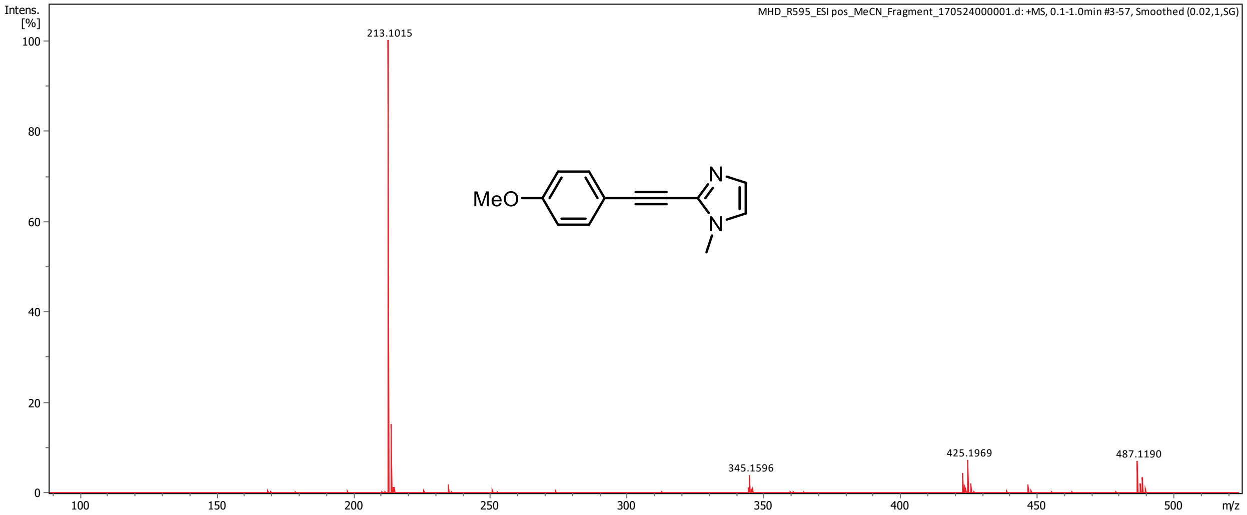
MHD_R595_ESI pos_MeCN_Fragment_170524000001.d: +MS, 0.1-1.0min #3-57, Smoothed (0.02,1,SG)
Intens. [%]
213.1015
345.1596
425.1969
487.1190
MeO
N
N
m/z

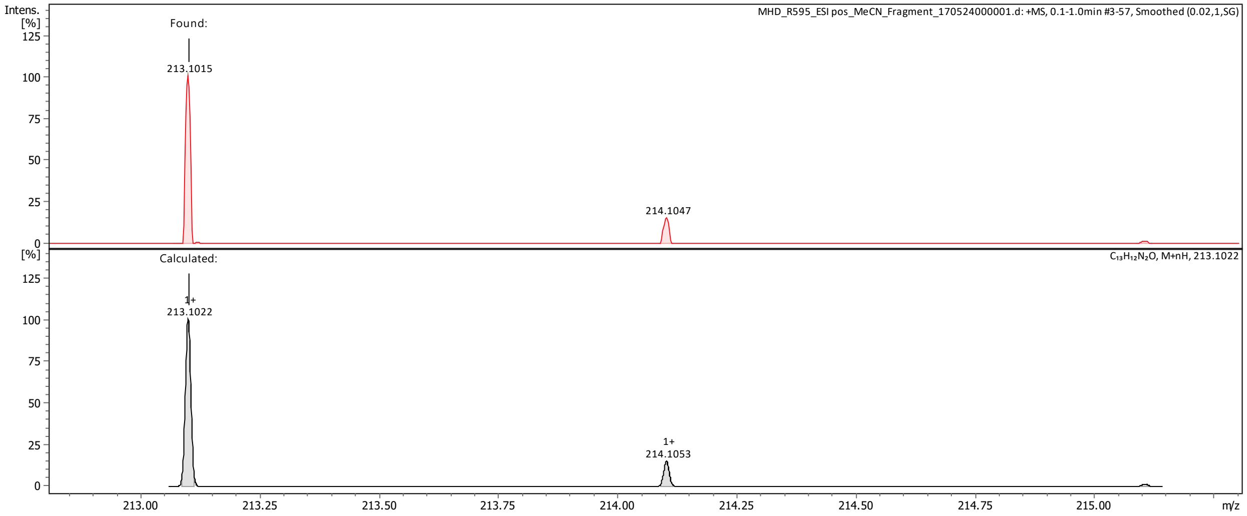
MHD_R595_ESI pos_MeCN_Fragment_170524000001.d: +MS, 0.1-1.0min #3-57, Smoothed (0.02,1,SG)
Intens. [%]
Found:
213.1015
214.1047
Calculated:
$C_{13}H_{12}N_2O$, M+nH, 213.1022
1+
213.1022
1+
214.1053
m/z

4.21 3-(Phenylethynyl)thiophene (1z) (600 MHz, $CDCl_3$)

4.21.1 ^{1}H NMR

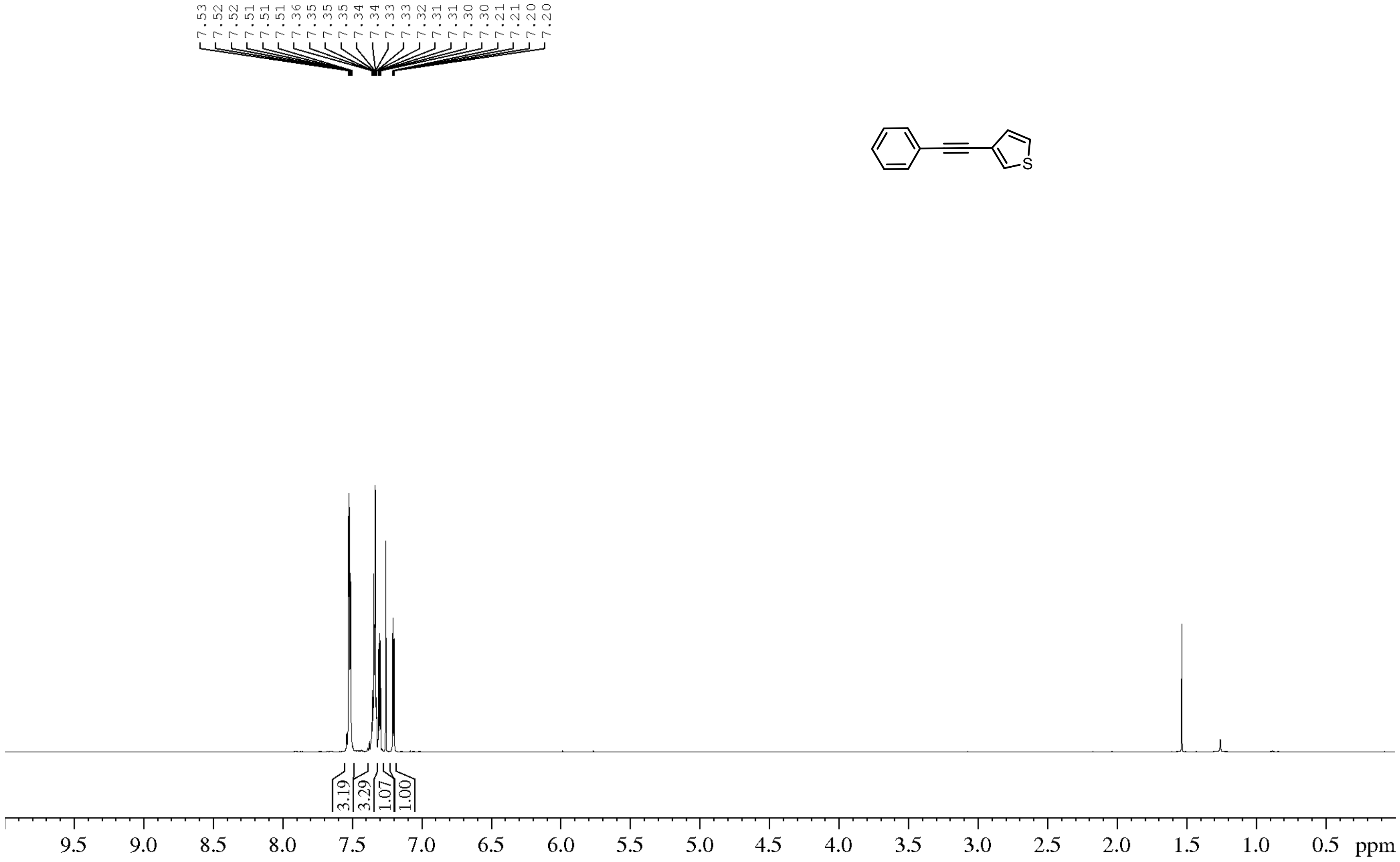

4.21.2 ^{13}C NMR

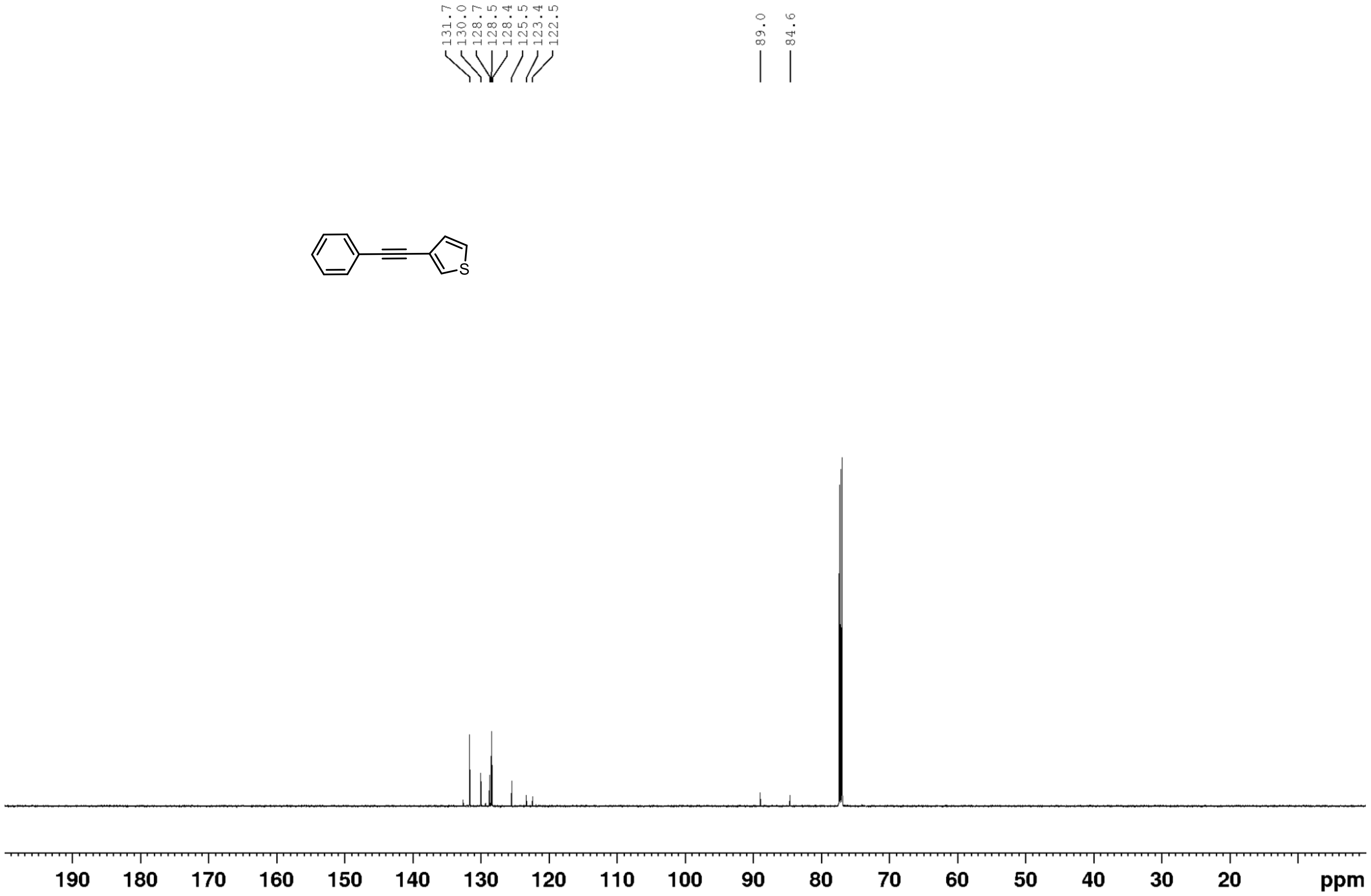
131.7
130.0
128.7
128.5
128.4
125.5
123.4
122.5
89.0
84.6
S
190
180
170
160
150
140
130
120
110
100
90
80
70
60
50
40
30
20
ppm

4.21.3 ¹H, ¹H COSY

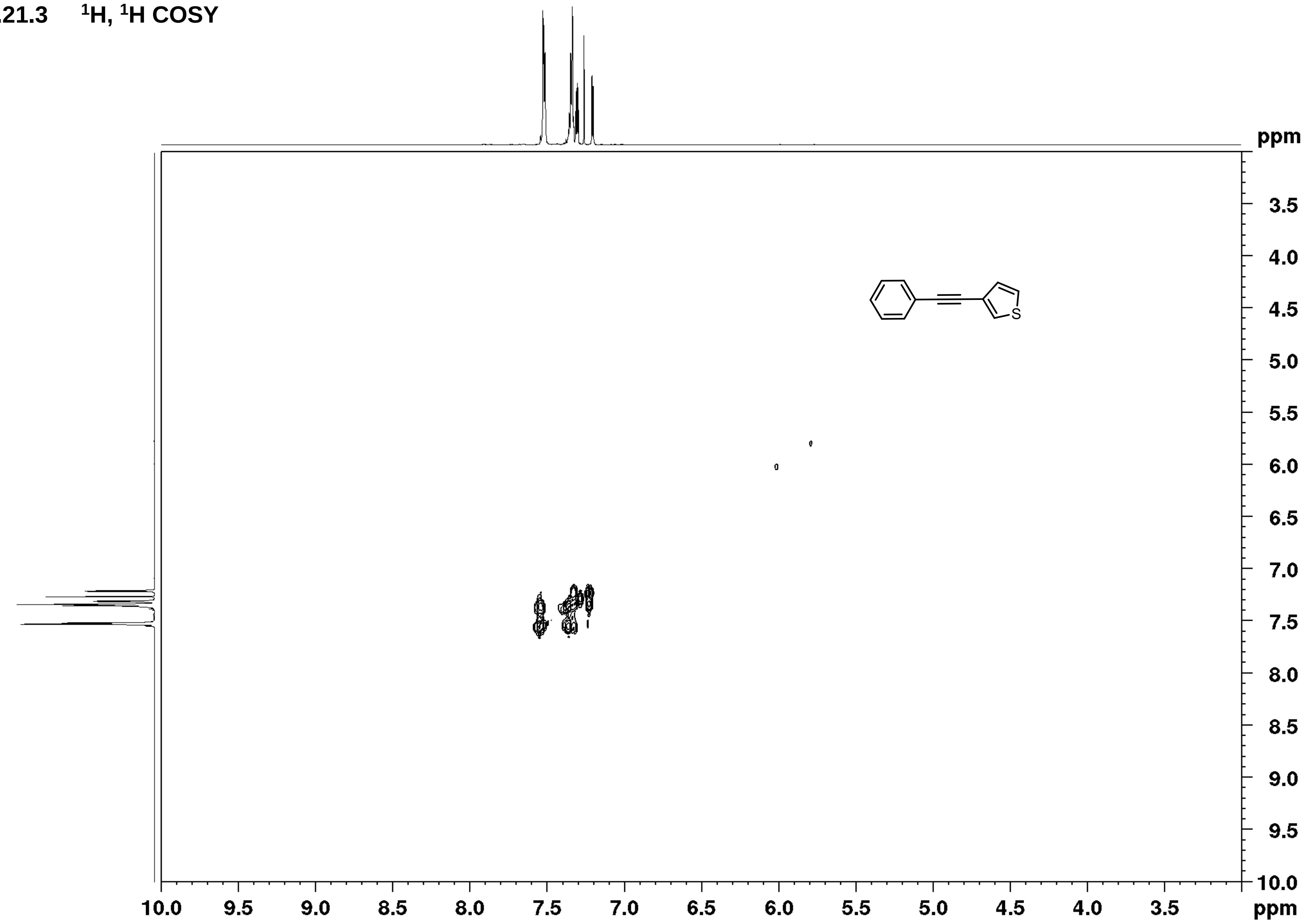

4.21.4 ^{1}H, ^{13}C HMBC

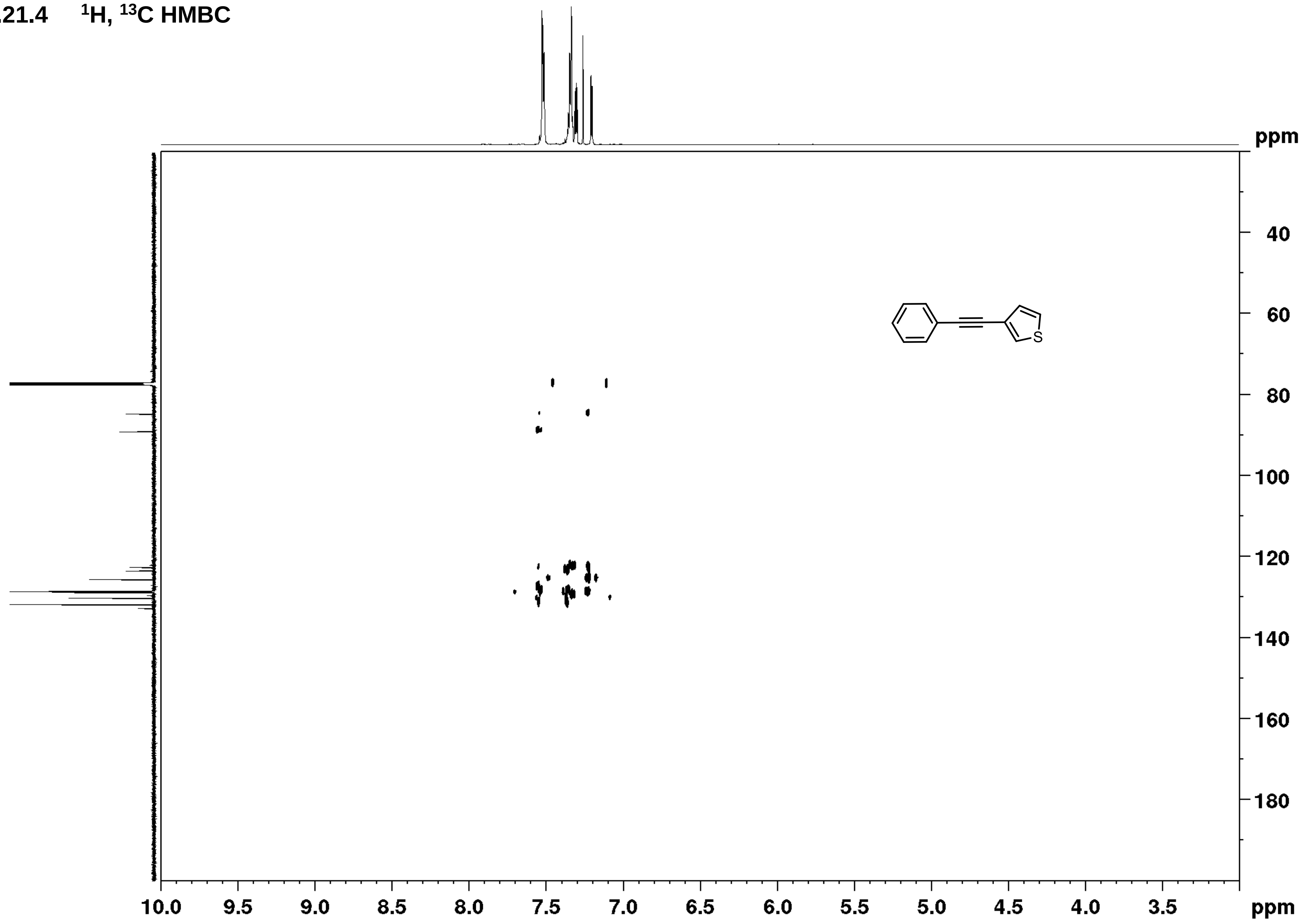

4.21.5 ¹H, ¹³C HSQC

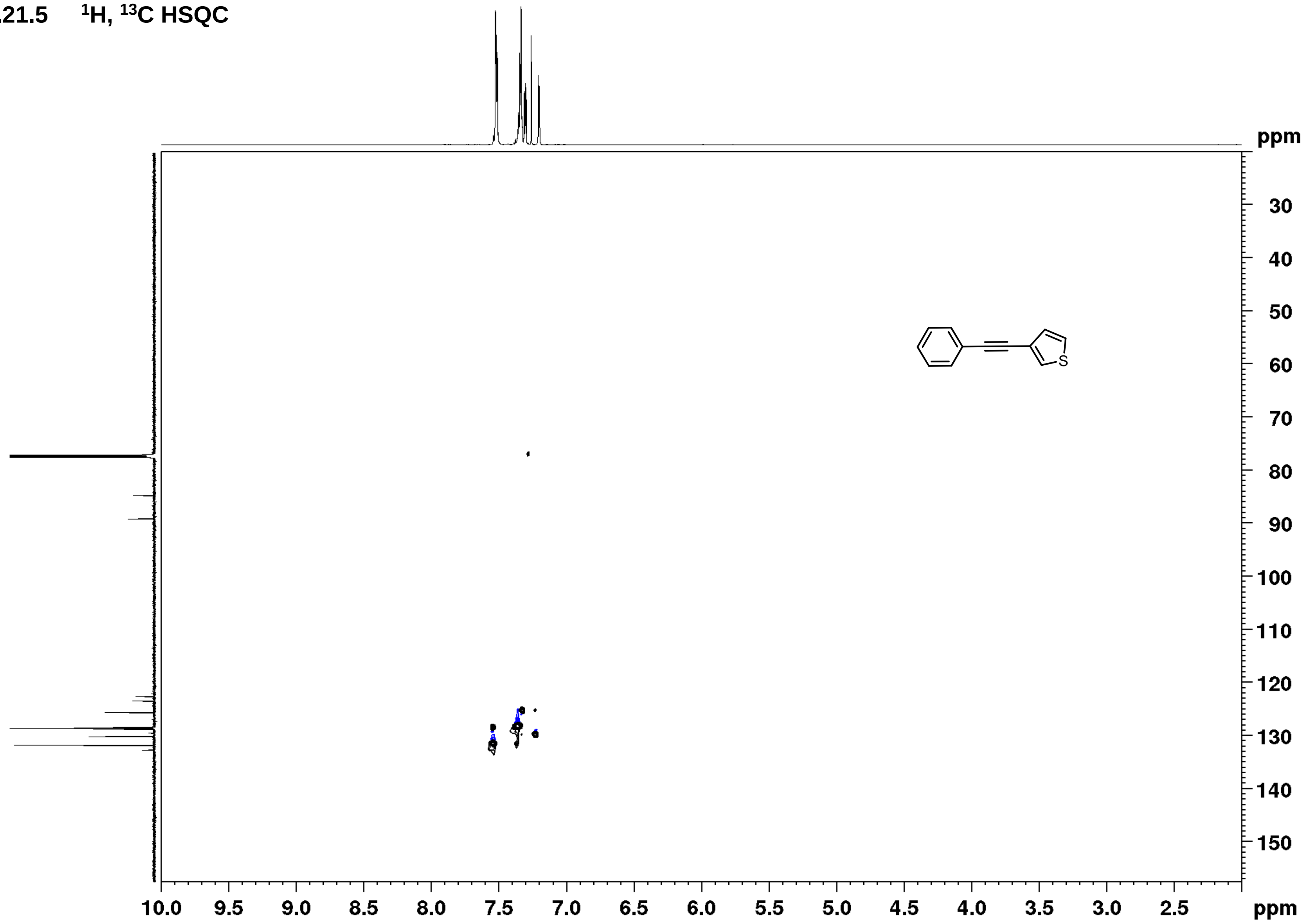

4.21.6 HRMS

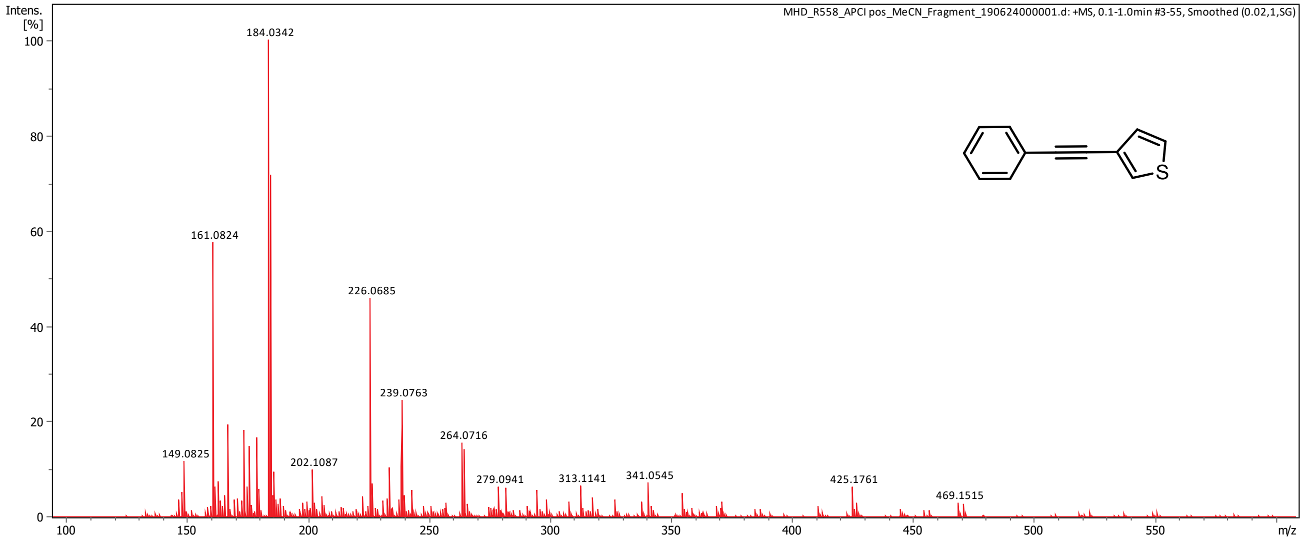

Intens. [%]
MHD_R558_APCI pos_MeCN_Fragment_190624000001.d: +MS, 0.1-1.0min #3-55, Smoothed (0.02,1,SG)
184.0342
161.0824
226.0685
239.0763
149.0825
202.1087
264.0716
279.0941
313.1141
341.0545
425.1761
469.1515
S
m/z

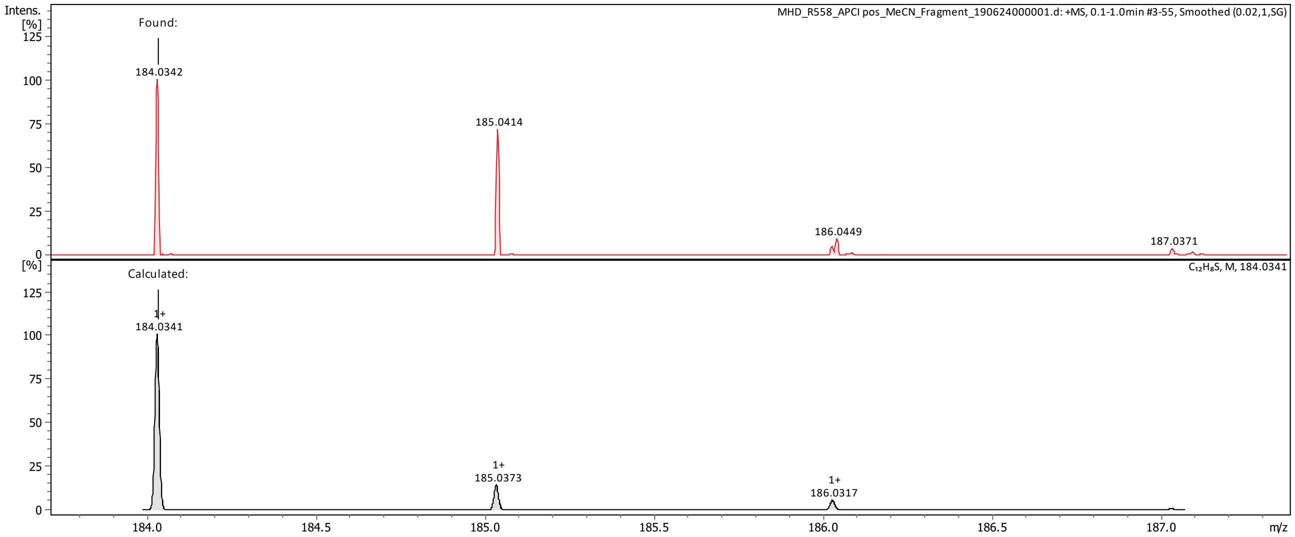

Intens. [%]
MHD_R558_APCI pos_MeCN_Fragment_190624000001.d: +MS, 0.1-1.0min #3-55, Smoothed (0.02,1,SG)
Found:
184.0342
185.0414
186.0449
187.0371
C₁₂H₈S, M, 184.0341
Calculated:
1+ 184.0341
1+ 185.0373
1+ 186.0317
m/z

4.22 1-Benzyl-5-(phenylethynyl)-1*H*-indole (1aa) (600 MHz, $CDCl_3$)

4.22.1 ^{1}H NMR

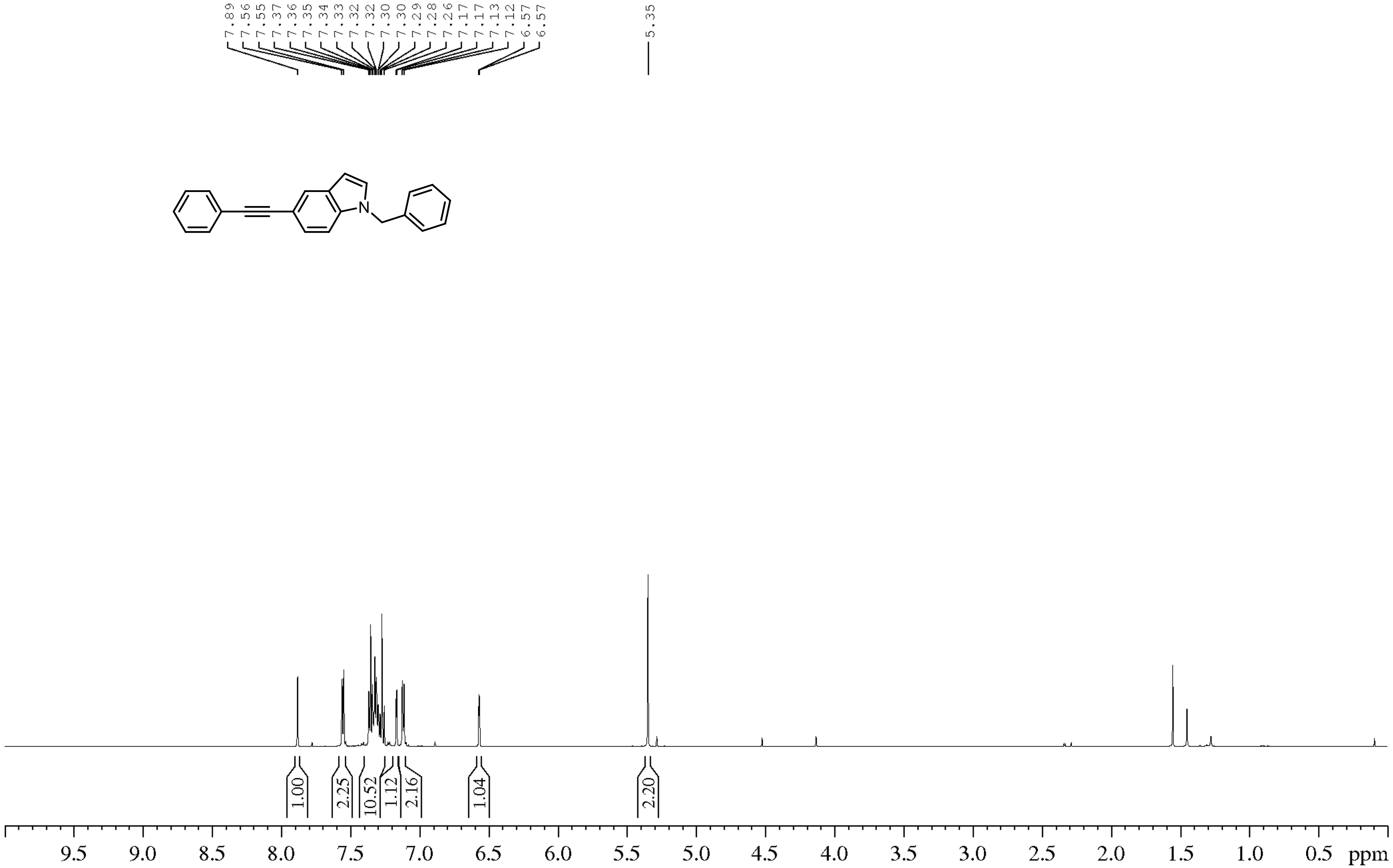

4.22.2 ^{13}C NMR

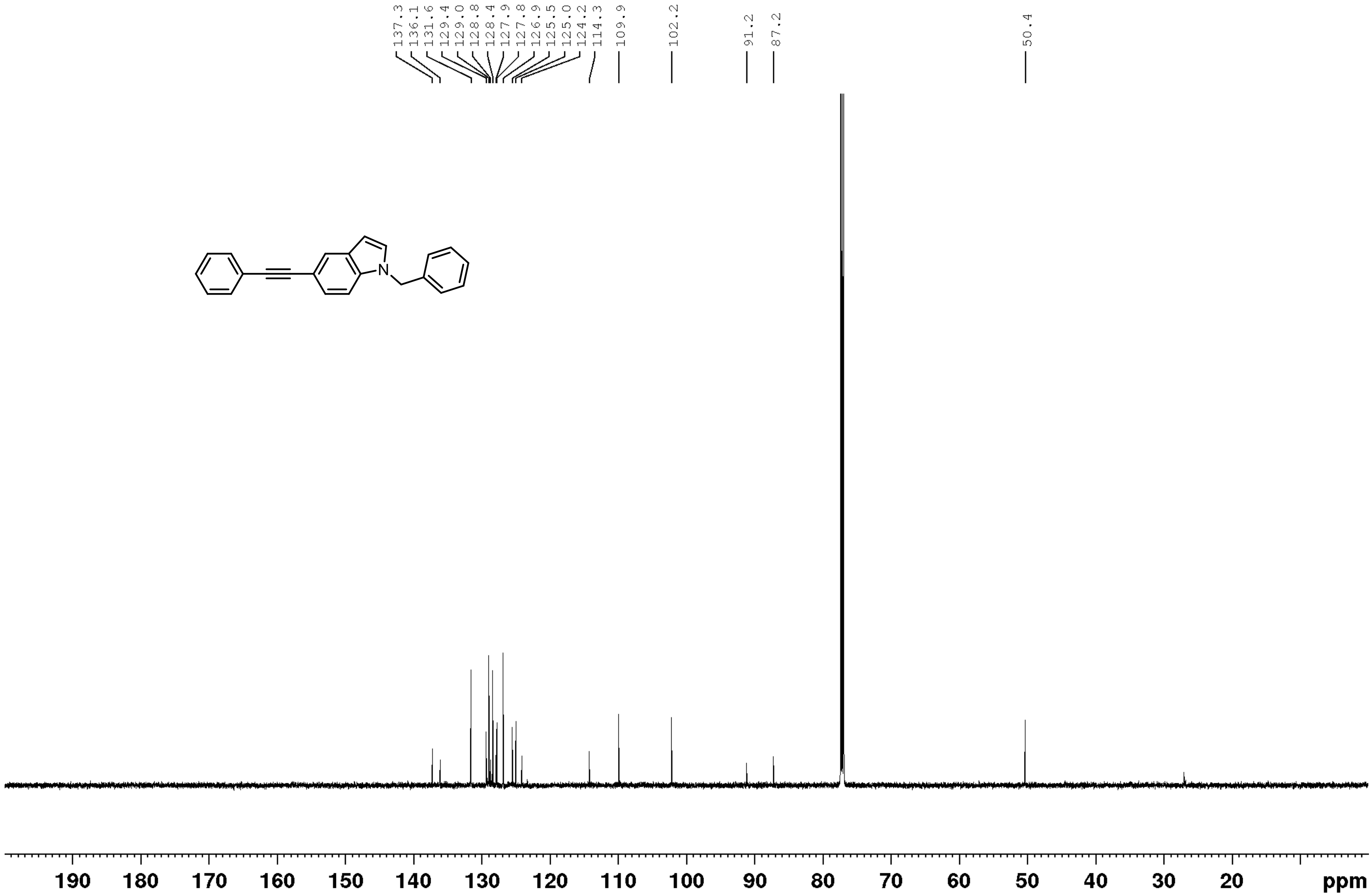
137.3
136.1
131.6
129.4
129.0
128.8
128.4
127.9
127.8
126.9
125.5
125.0
124.2
114.3
109.9
102.2
91.2
87.2
50.4
N
190
180
170
160
150
140
130
120
110
100
90
80
70
60
50
40
30
20
ppm

4.22.3 ^{1}H, ^{1}H COSY

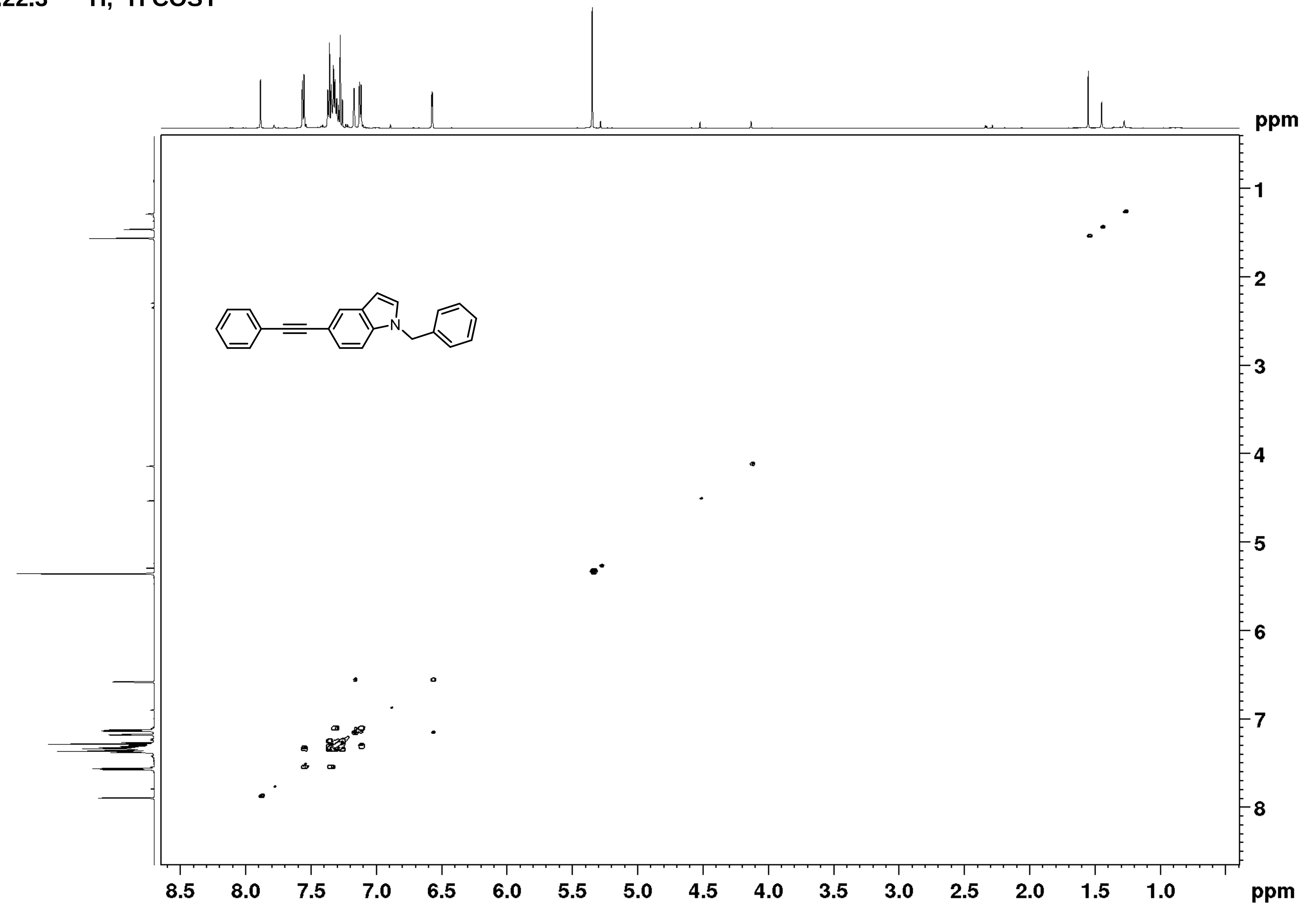

4.22.4 ^{1}H, ^{13}C HMBC

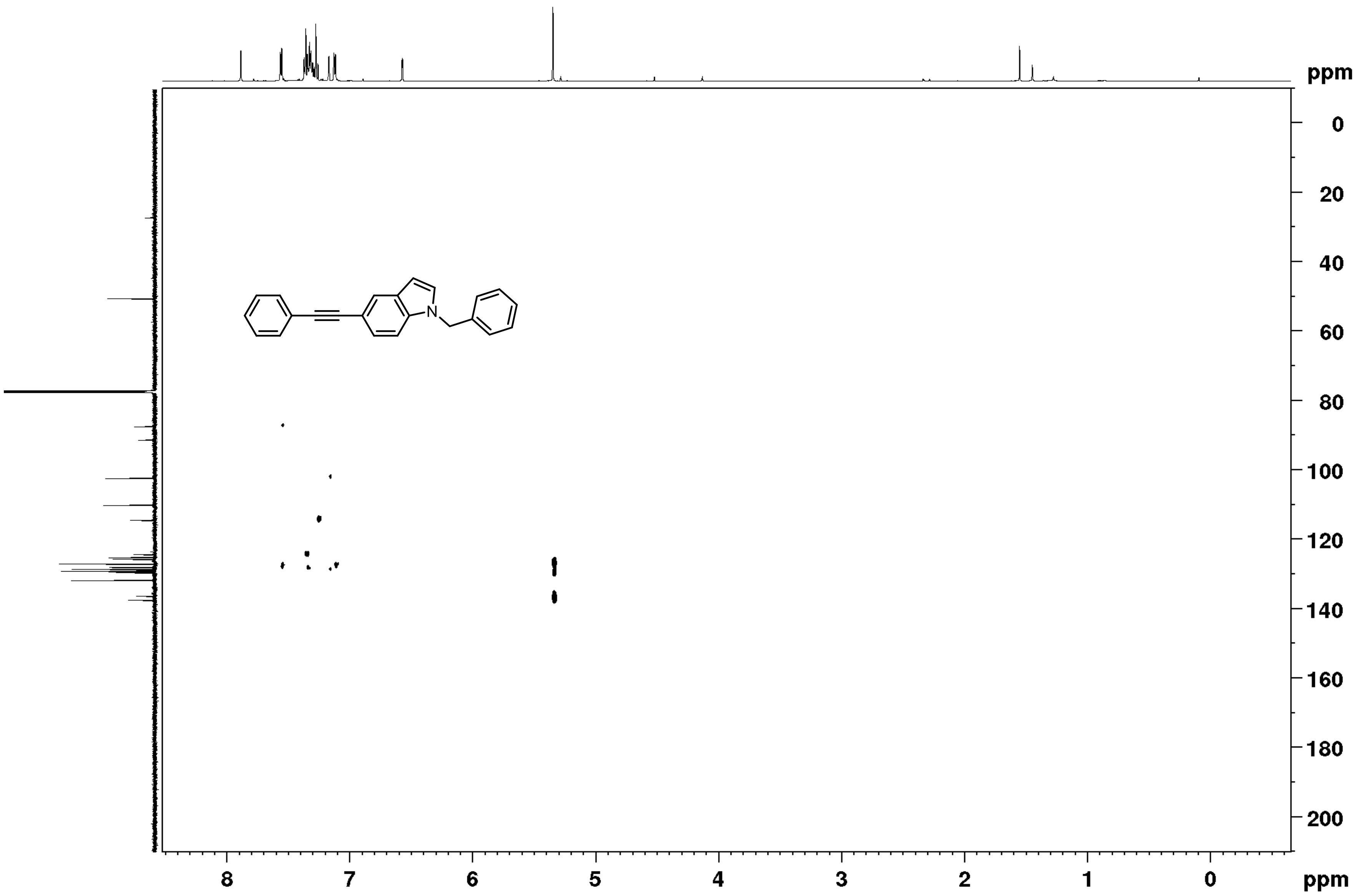

4.22.5 ^{1}H, ^{13}C HSQC

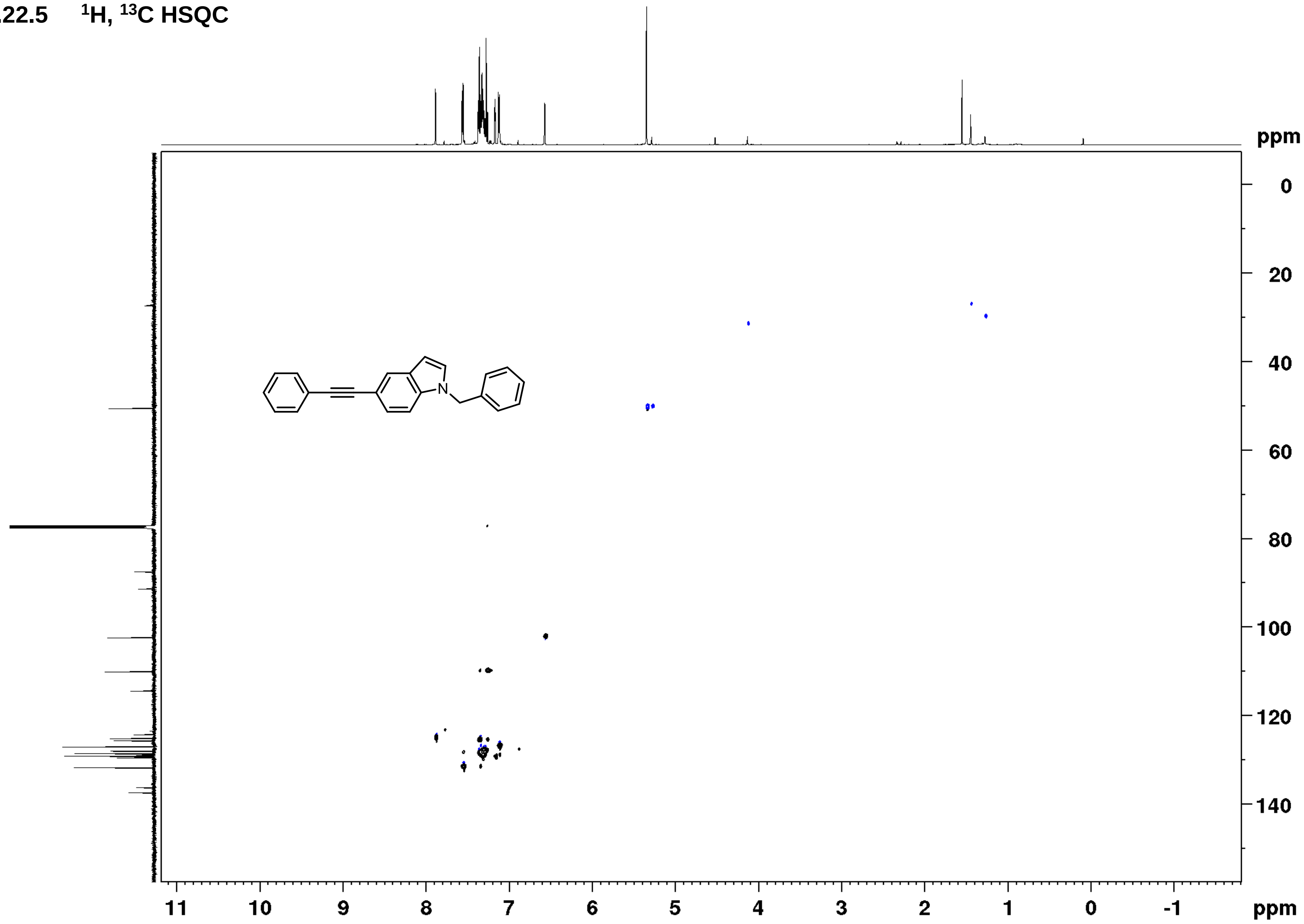

4.22.6 HRMS

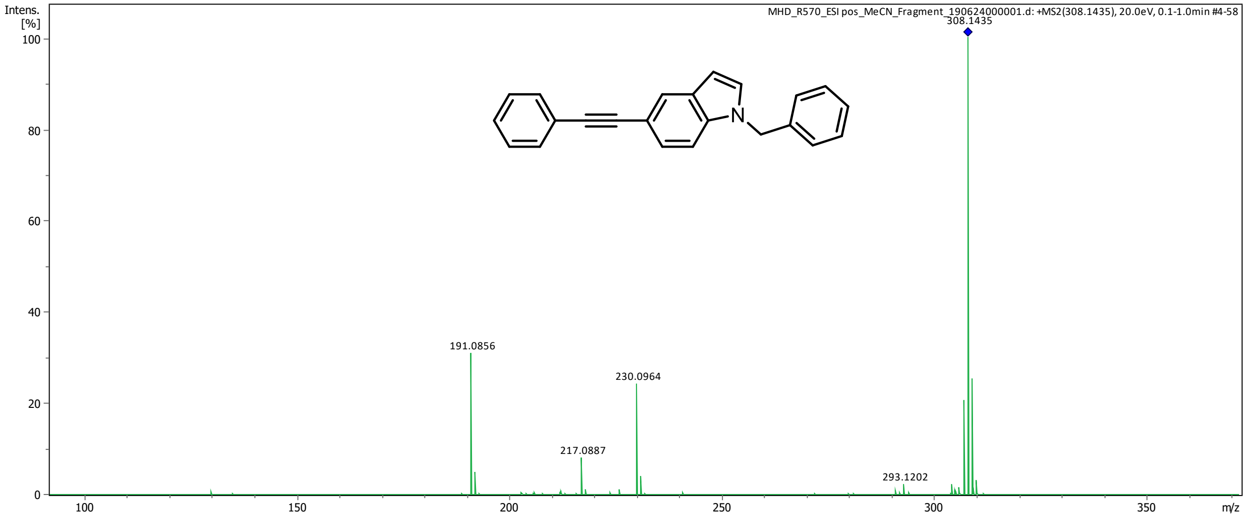
MHD_R570_ESI pos_MeCN_Fragment_190624000001.d: +MS2(308.1435), 20.0eV, 0.1-1.0min #4-58
Intens. [%]
308.1435
191.0856
230.0964
217.0887
293.1202
N
m/z

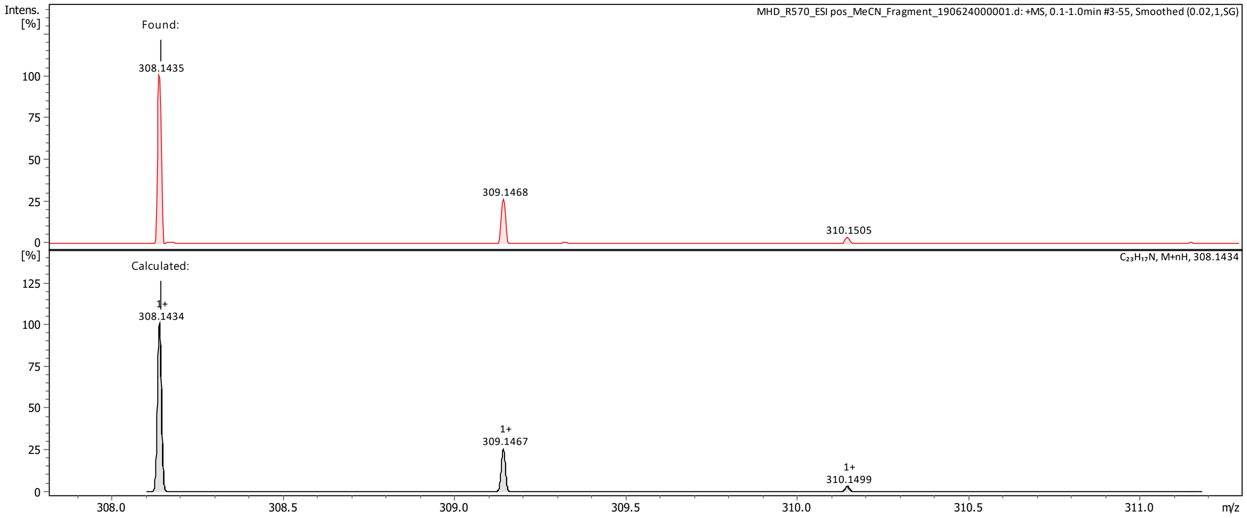
MHD_R570_ESI pos_MeCN_Fragment_190624000001.d: +MS, 0.1-1.0min #3-55, Smoothed (0.02,1,SG)
Intens. [%]
Found:
308.1435
309.1468
310.1505
Calculated:
C₂₃H₁₇N, M+nH, 308.1434
1+
308.1434
1+
309.1467
1+
310.1499
m/z

5 Spectral data of conjugated amide staring materials

5.1 (*E*)-3-Phenyl-1-(piperidin-1-yl)prop-2-en-1-one (11b) (600 MHz, $CDCl_3$)

5.1.1 ^{1}H NMR

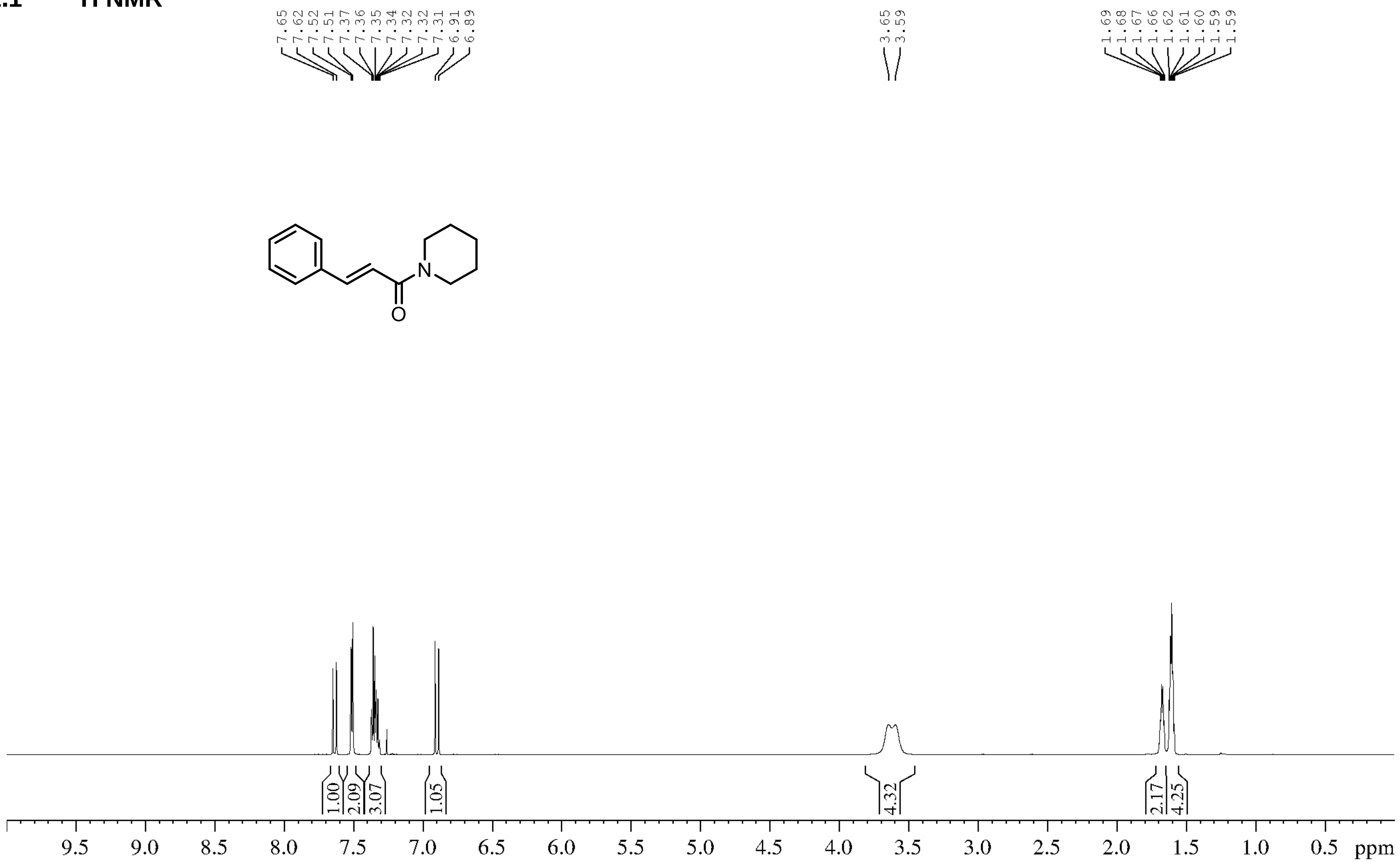

5.1.2 ^{13}C NMR

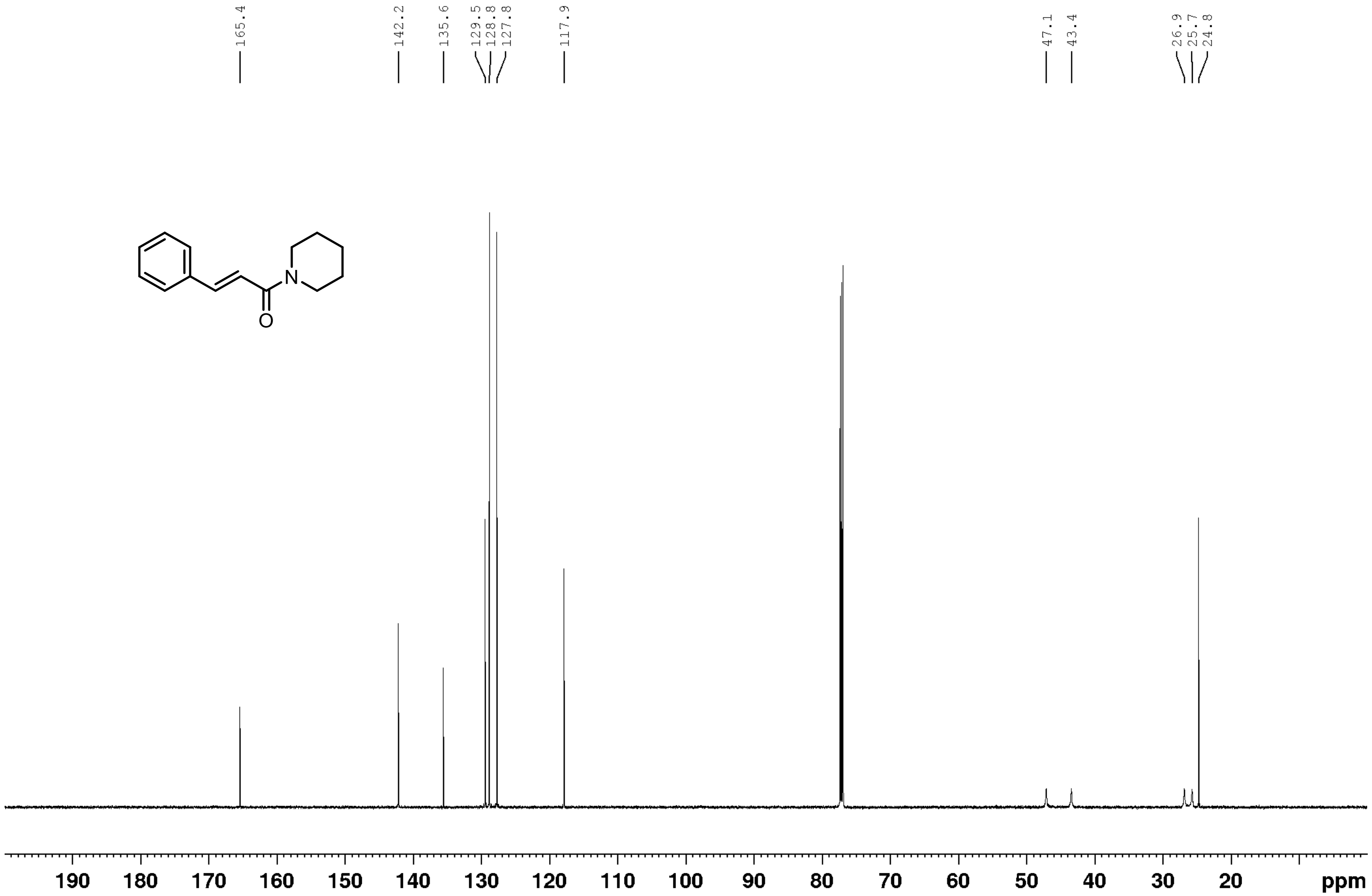

165.4
142.2
135.6
129.5
128.8
127.8
117.9
47.1
43.4
26.9
25.7
24.8
N
O
190
180
170
160
150
140
130
120
110
100
90
80
70
60
50
40
30
20
ppm

5.1.3 $^{1}H, ^{1}H$ COSY

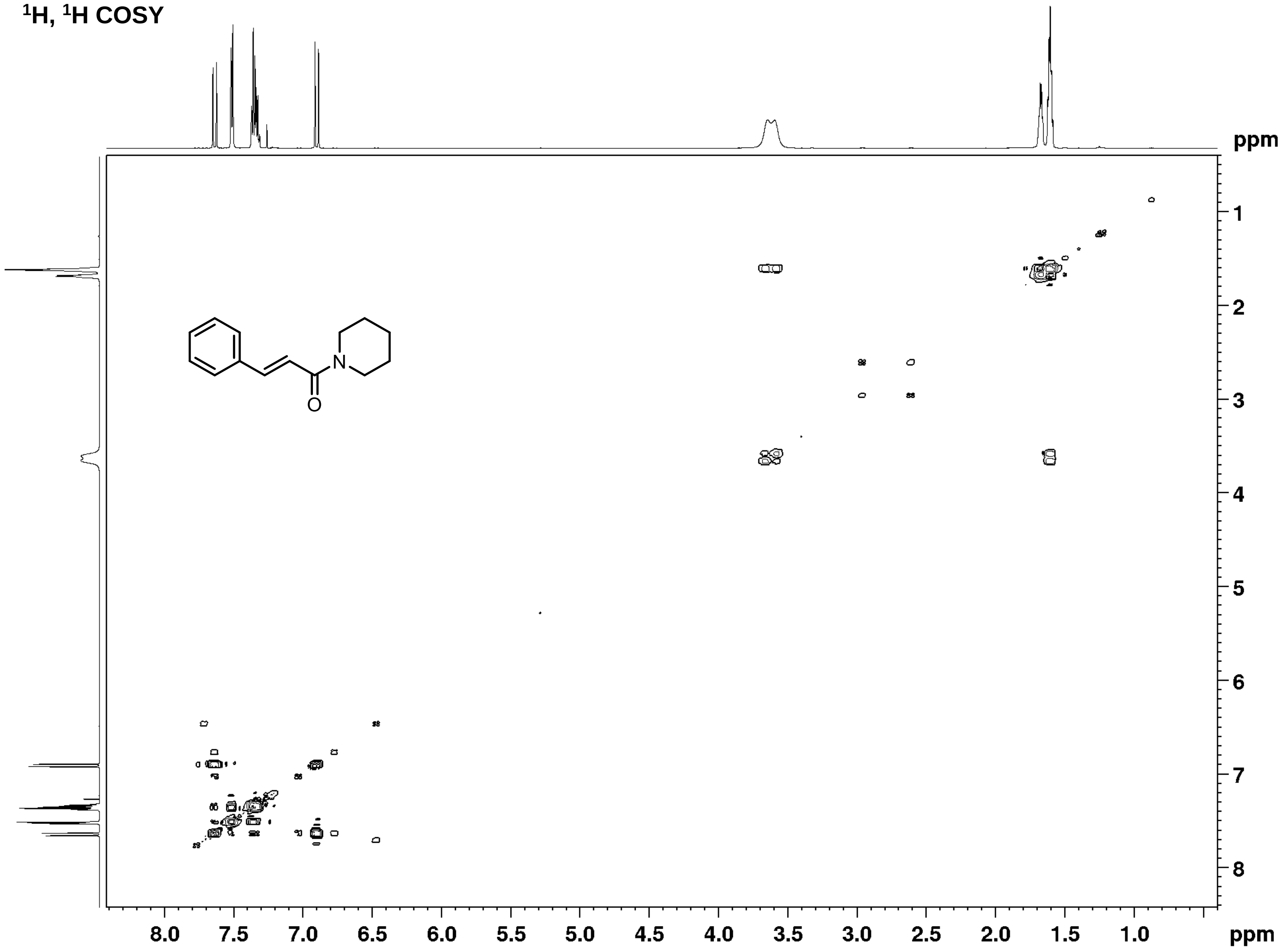

5.1.4 ^{1}H, ^{13}C HMBC

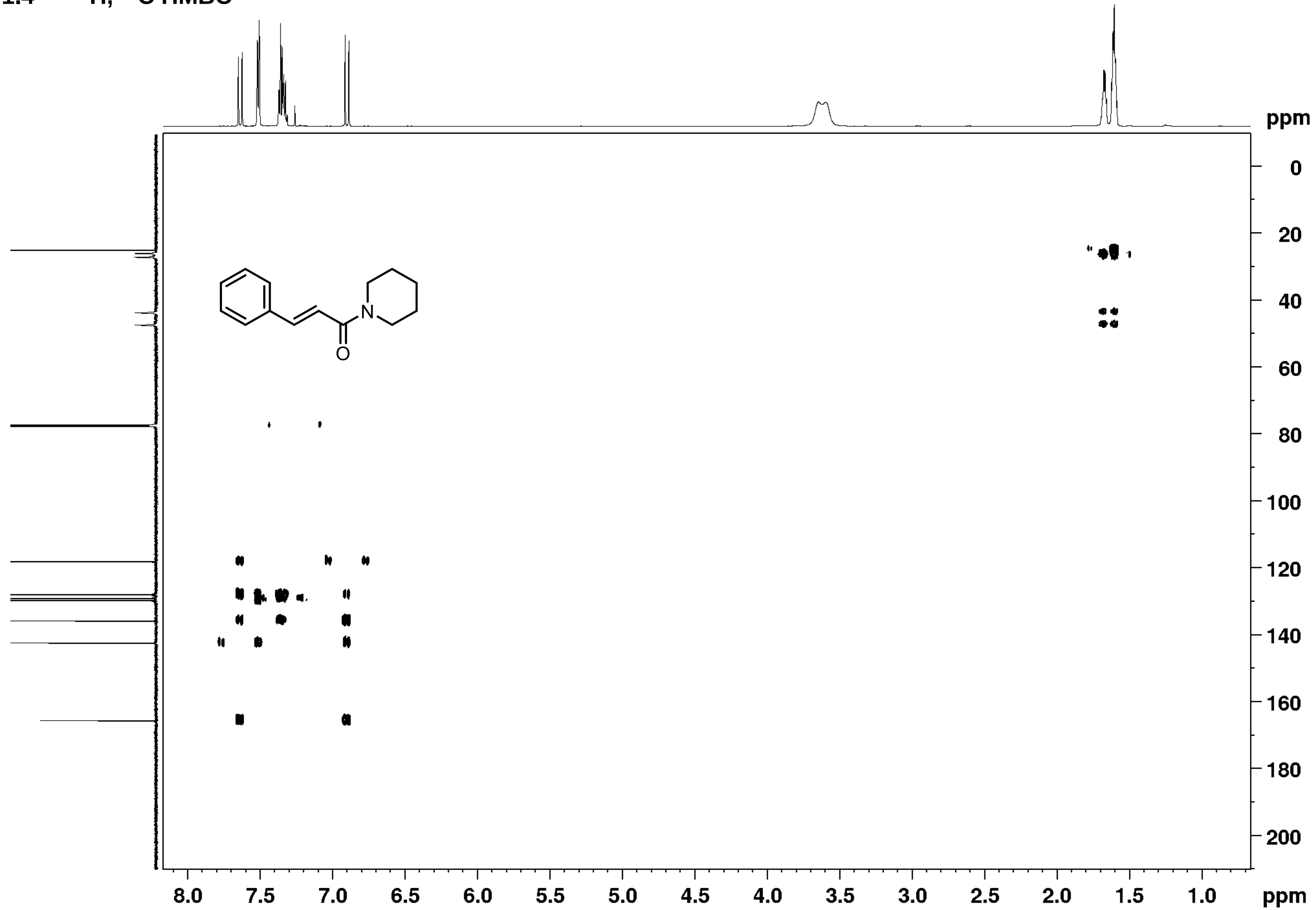

5.1.5 ^1H, ^{13}C HSQC

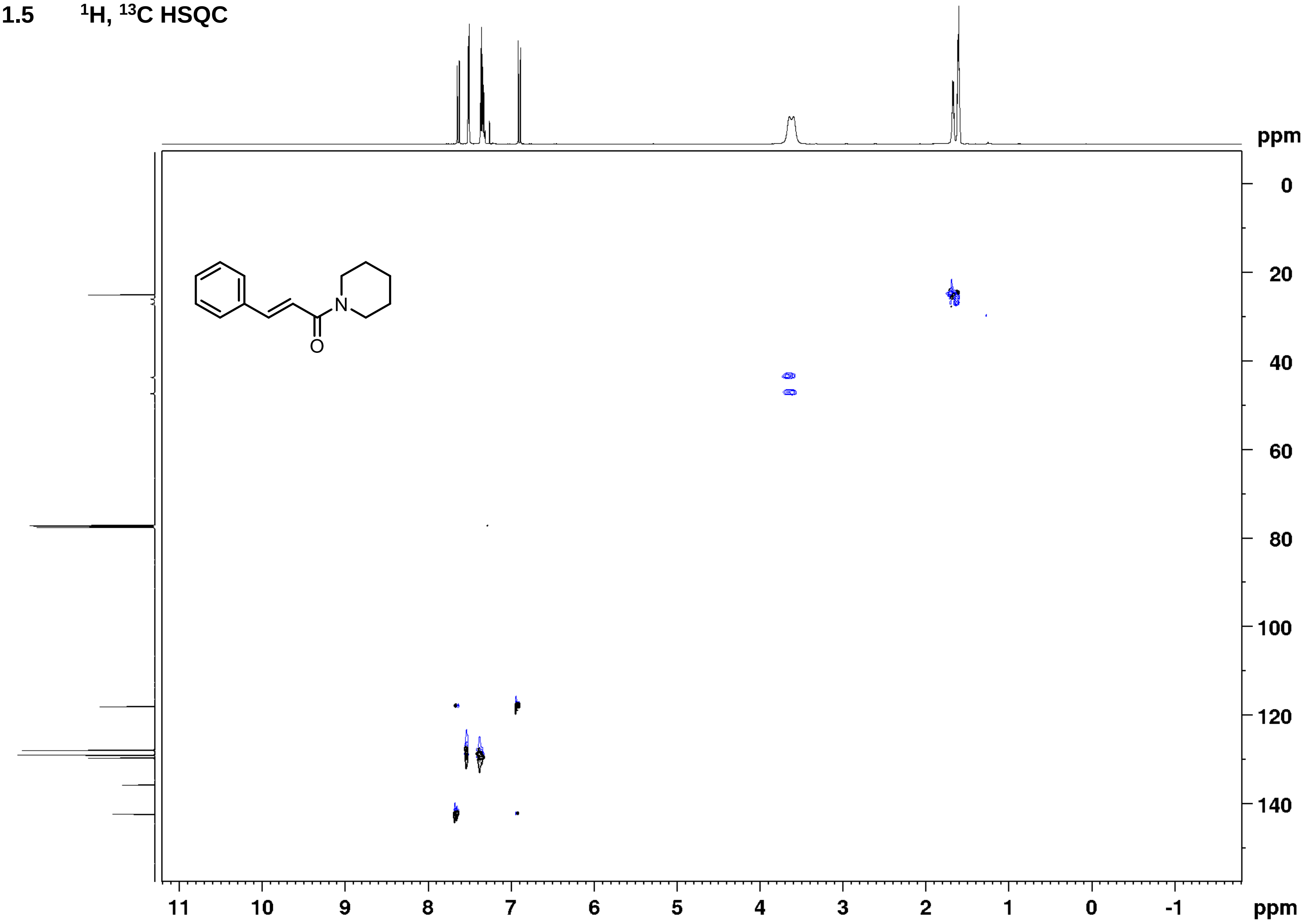

5.1.6 HRMS

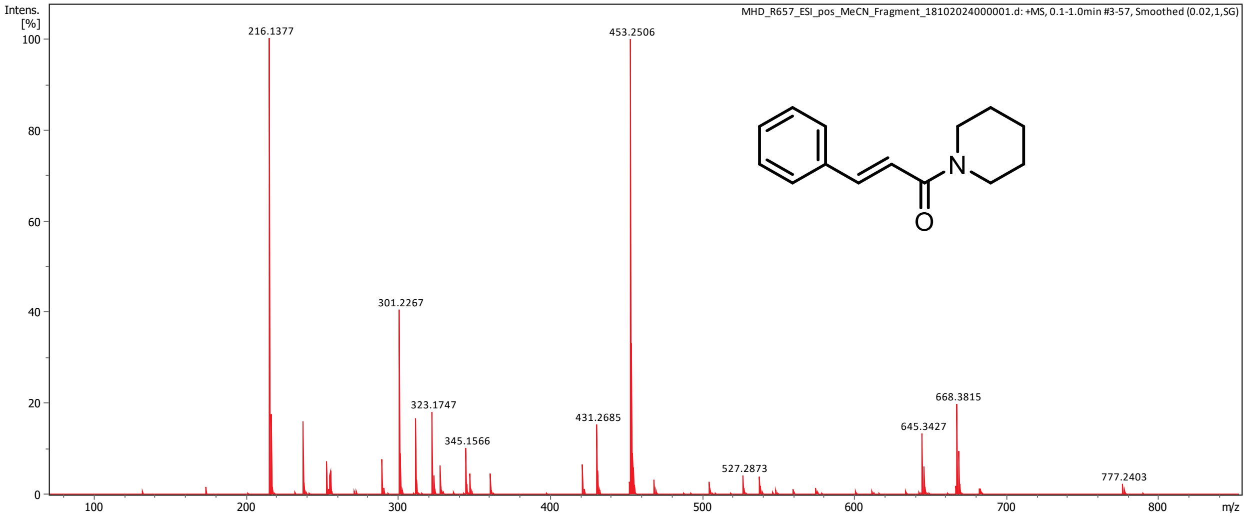
MHD_R657_ESI_pos_MeCN_Fragment_18102024000001.d: +MS, 0.1-1.0min #3-57, Smoothed (0.02,1,SG)
Intens. [%]
216.1377
453.2506
301.2267
323.1747
345.1566
431.2685
527.2873
645.3427
668.3815
777.2403
N
O
m/z

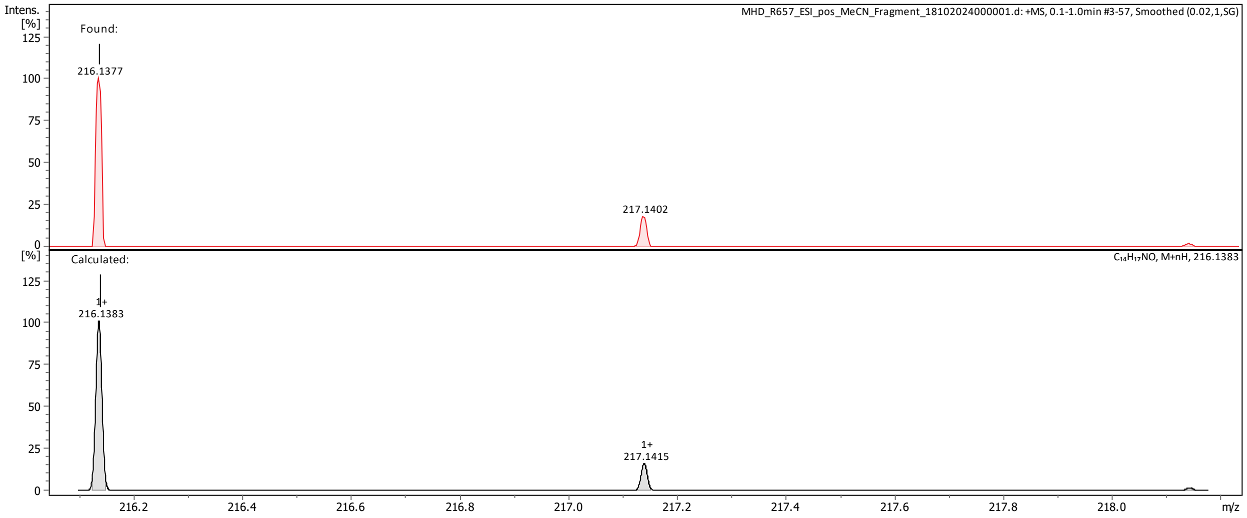
MHD_R657_ESI_pos_MeCN_Fragment_18102024000001.d: +MS, 0.1-1.0min #3-57, Smoothed (0.02,1,SG)
Intens. [%]
Found:
216.1377
217.1402
Calculated:
C14H17NO, M+nH, 216.1383
1+ 216.1383
1+ 217.1415
m/z

5.1.7 IR

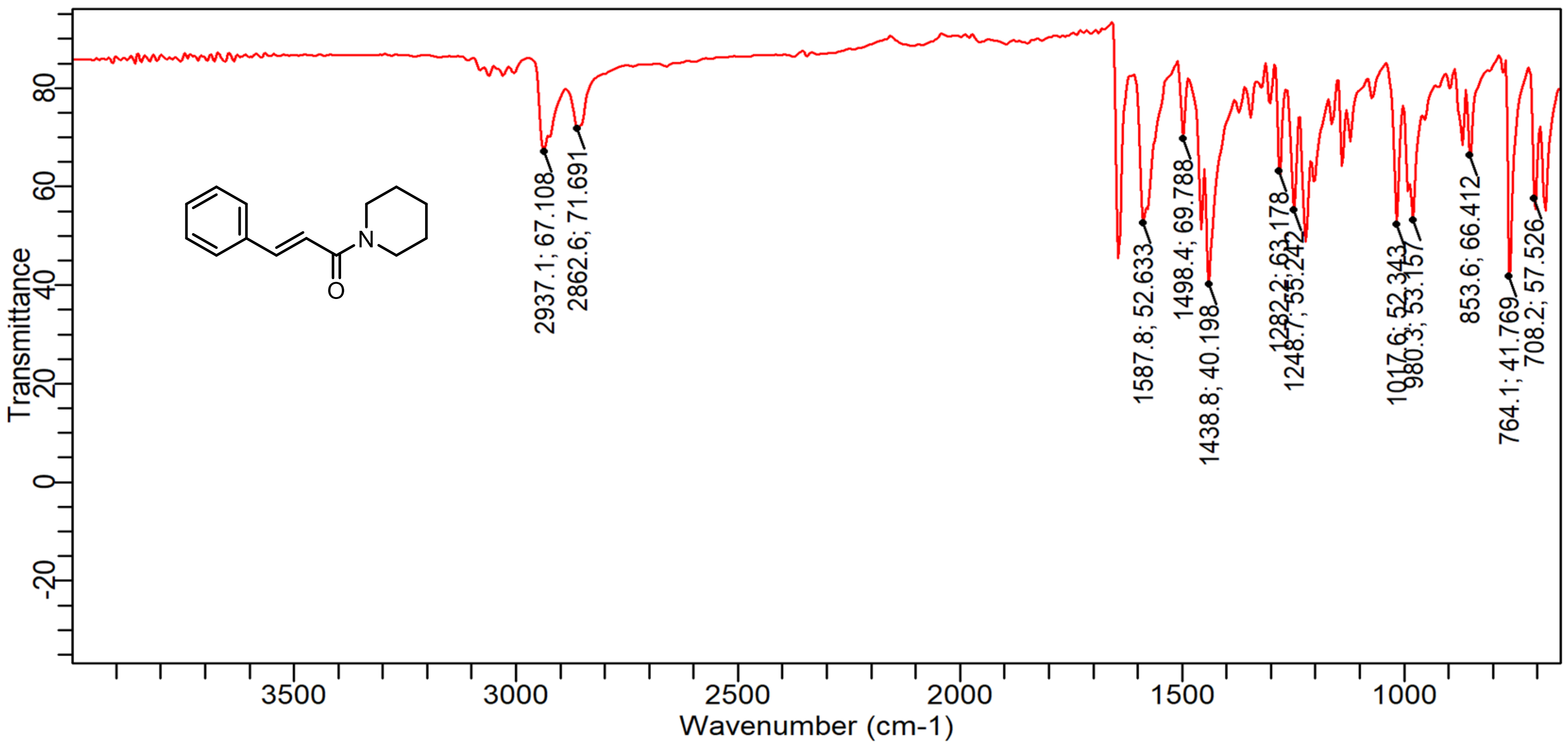
Transmittance
Wavenumber (cm-1)
2937.1; 67.108
2862.6; 71.691
1587.8; 52.633
1498.4; 69.788
1438.8; 40.198
1282.2; 63.178
1248.7; 55.242
1017.6; 52.343
980.3; 53.157
853.6; 66.412
764.1; 41.769
708.2; 57.526
N
O

5.2 (*E*)-1-(4,4-Difluoropiperidin-1-yl)-3-phenylprop-2-en-1-one (11c) (600 MHz, $CDCl_3$)

5.2.1 ^{1}H NMR

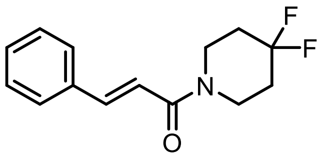

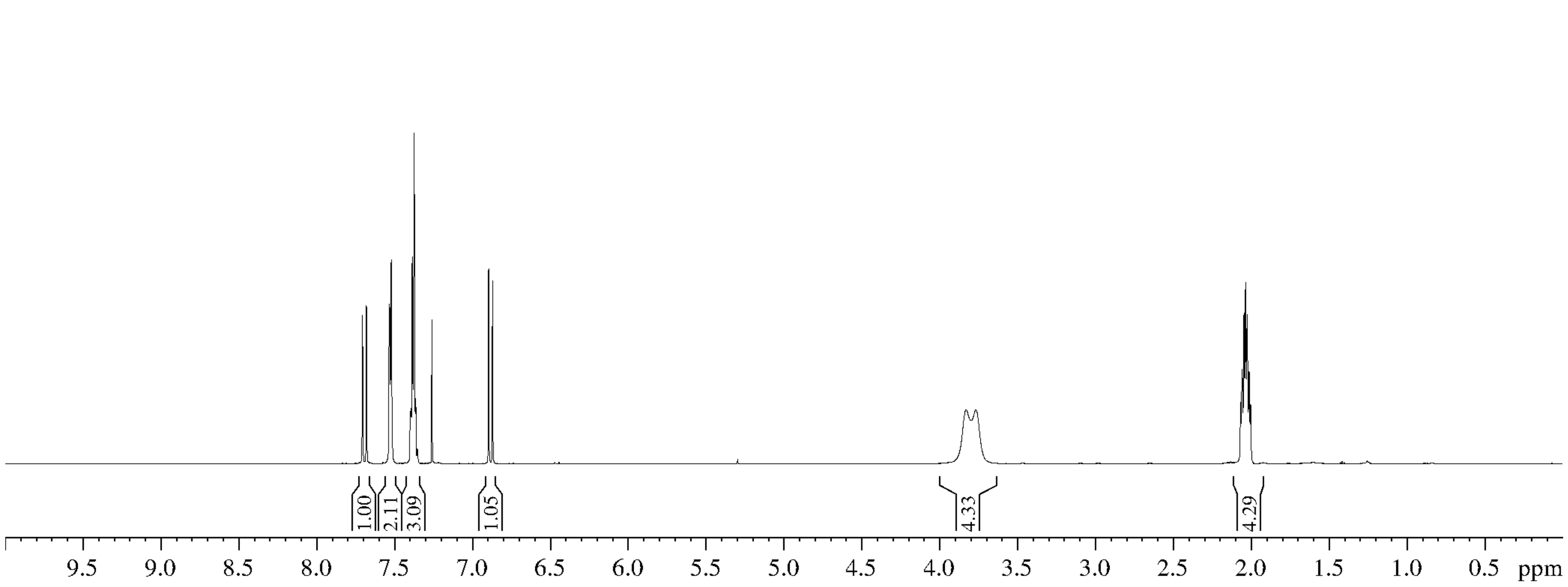

5.2.2 ^{13}C NMR

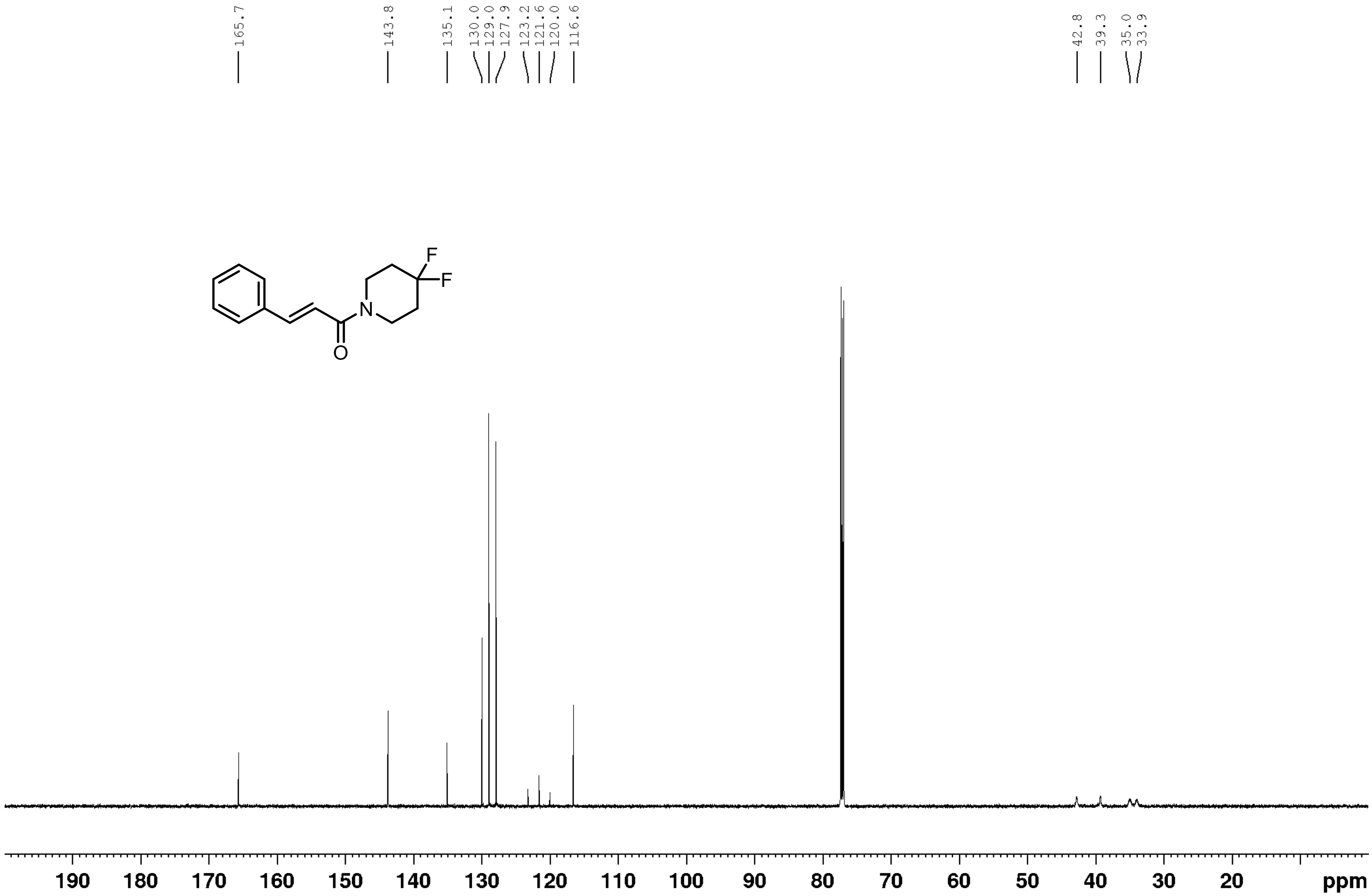

165.7
143.8
135.1
130.0
129.0
127.9
123.2
121.6
120.0
116.6
42.8
39.3
35.0
33.9
F
F
N
O
190
180
170
160
150
140
130
120
110
100
90
80
70
60
50
40
30
20
ppm

5.2.3 ^{19}F NMR

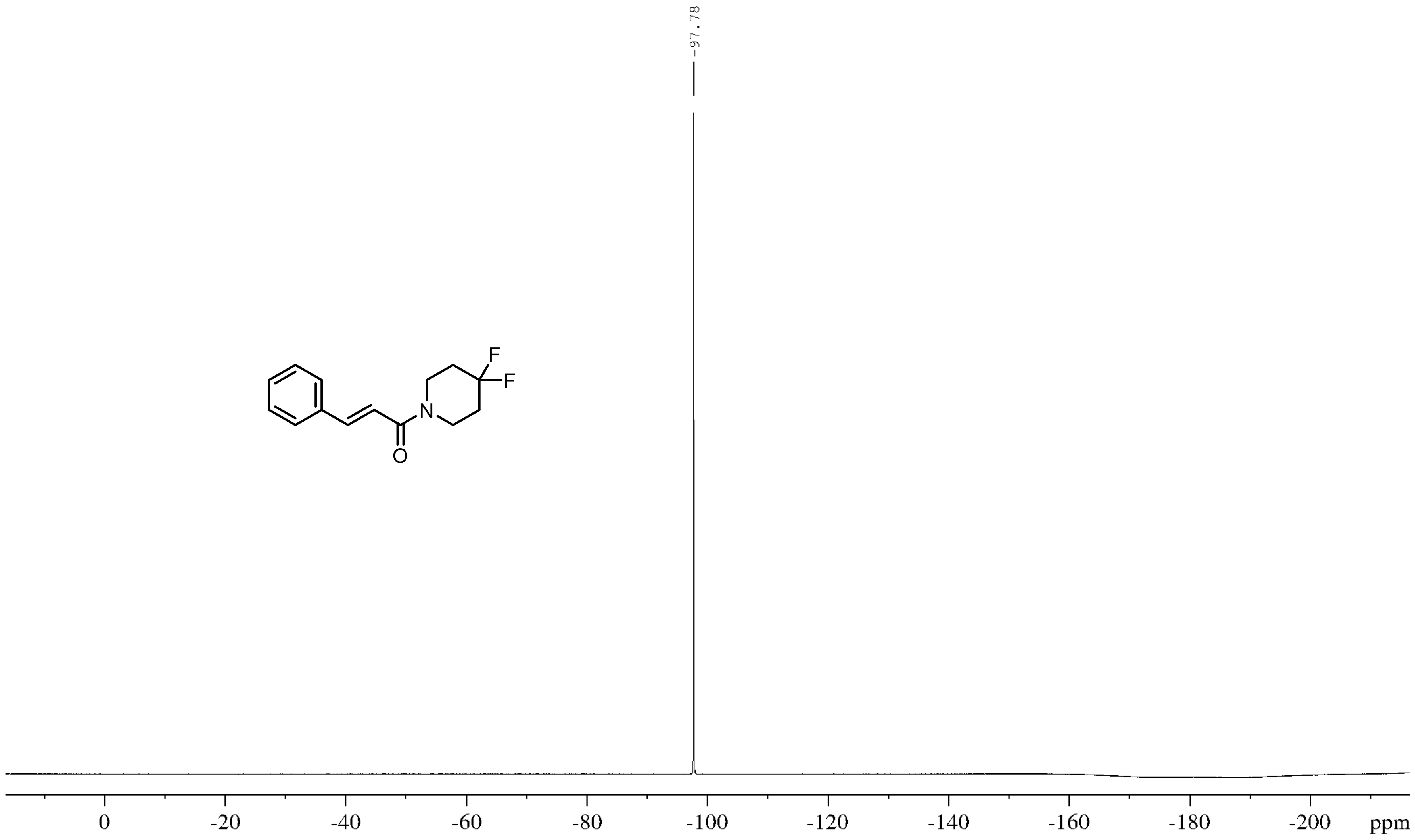

5.2.4 ^{1}H, ^{1}H COSY

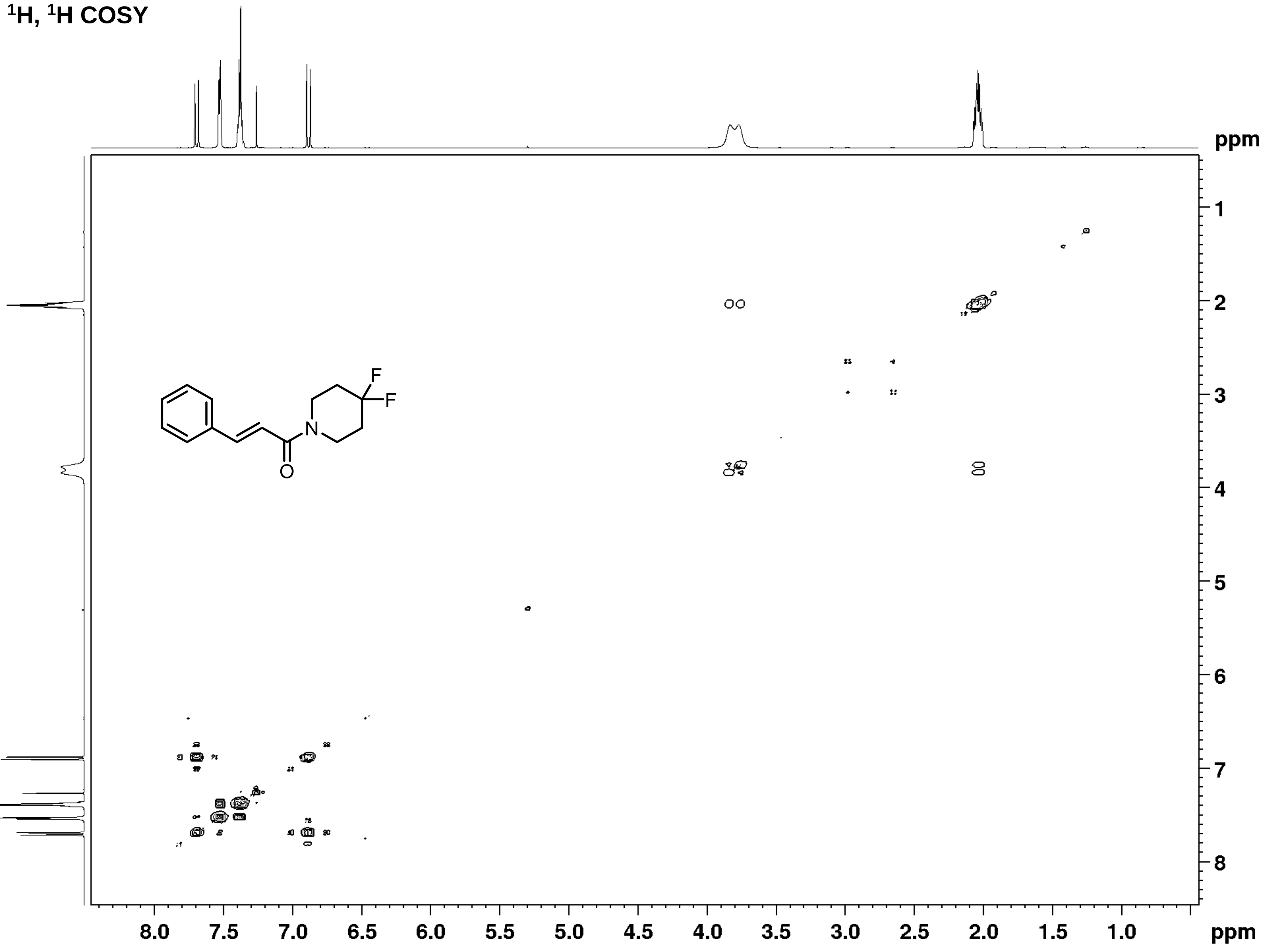

5.2.5 ^{1}H, ^{13}C HMBC

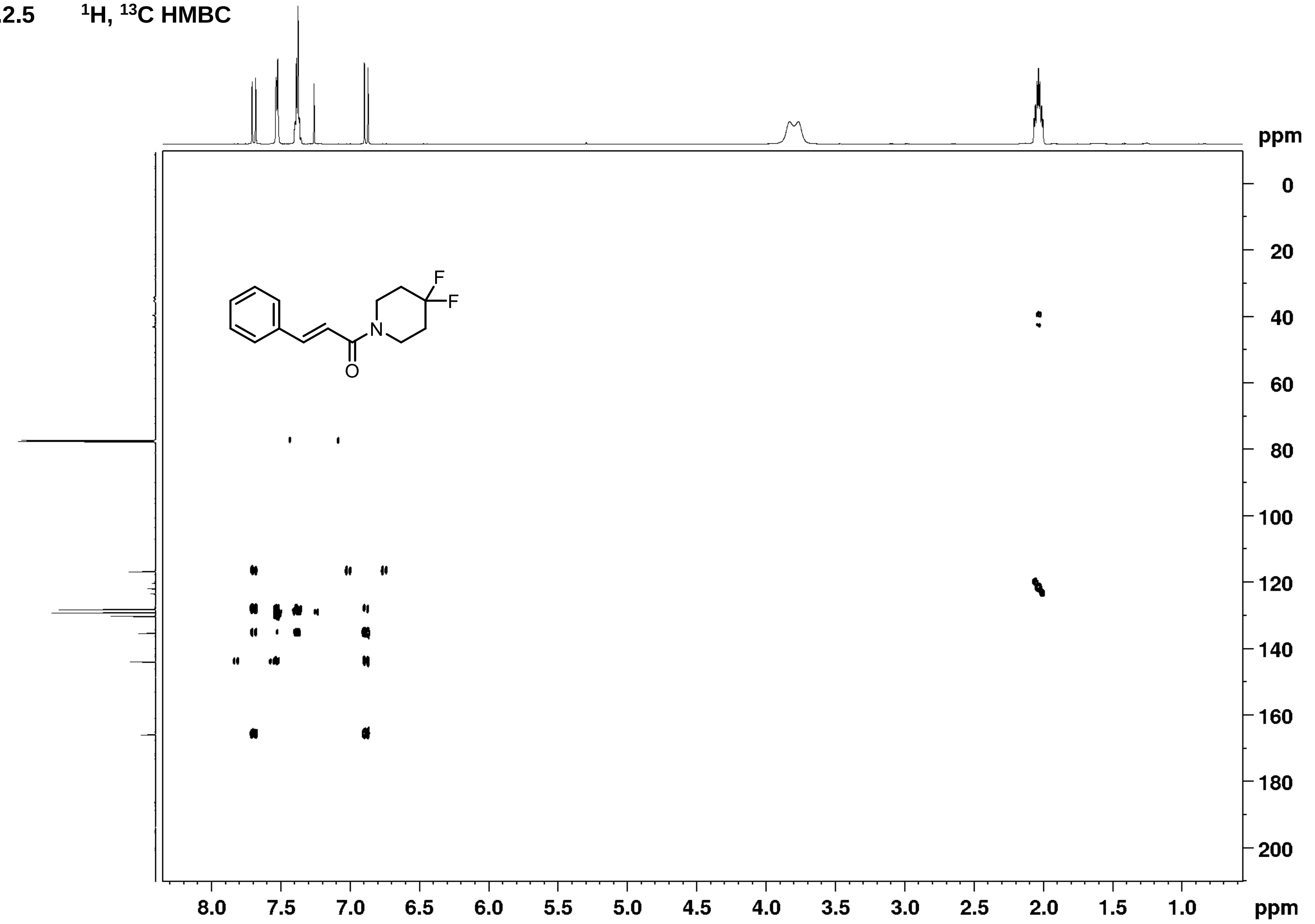

5.2.6 $^{1}H, ^{13}C$ HSQC

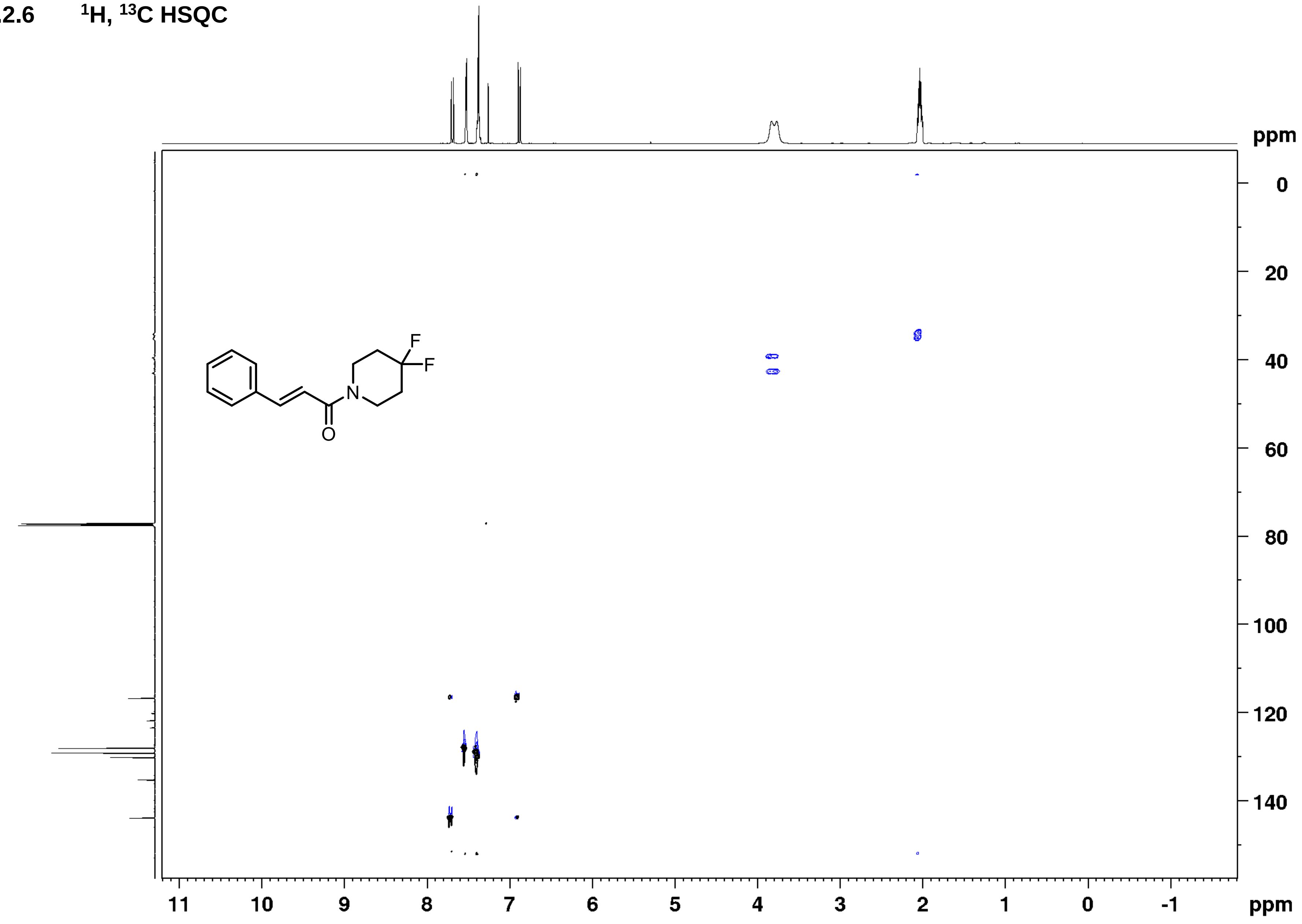

5.2.7 HRMS

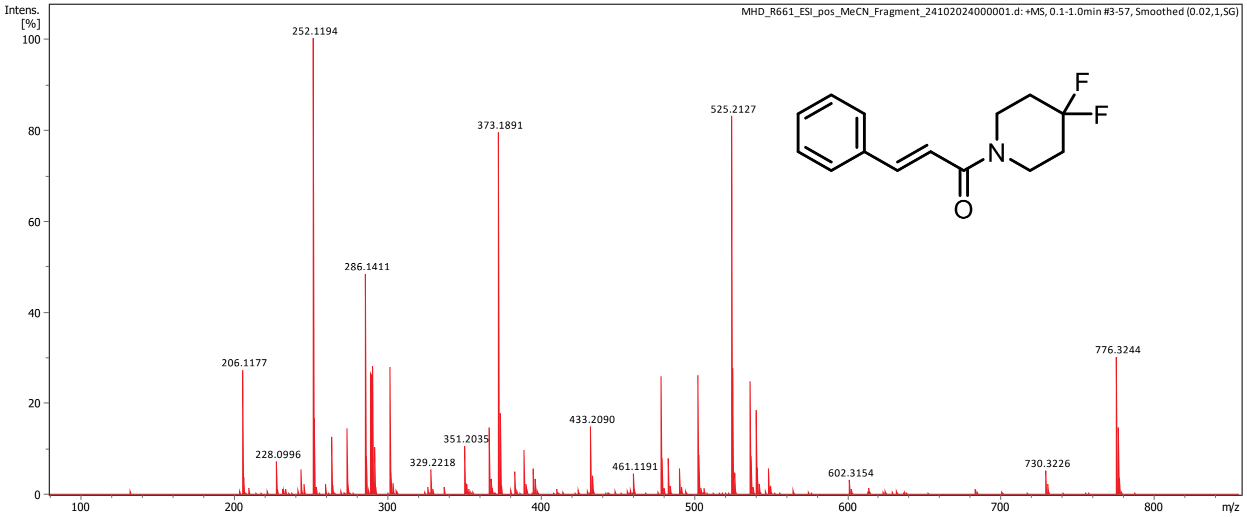

Intens. [%]
MHD_R661_ESI_pos_MeCN_Fragment_24102024000001.d: +MS, 0.1-1.0min #3-57, Smoothed (0.02,1,SG)
252.1194
373.1891
525.2127
286.1411
206.1177
776.3244
433.2090
351.2035
228.0996
329.2218
461.1191
602.3154
730.3226
m/z
F
F
N
O

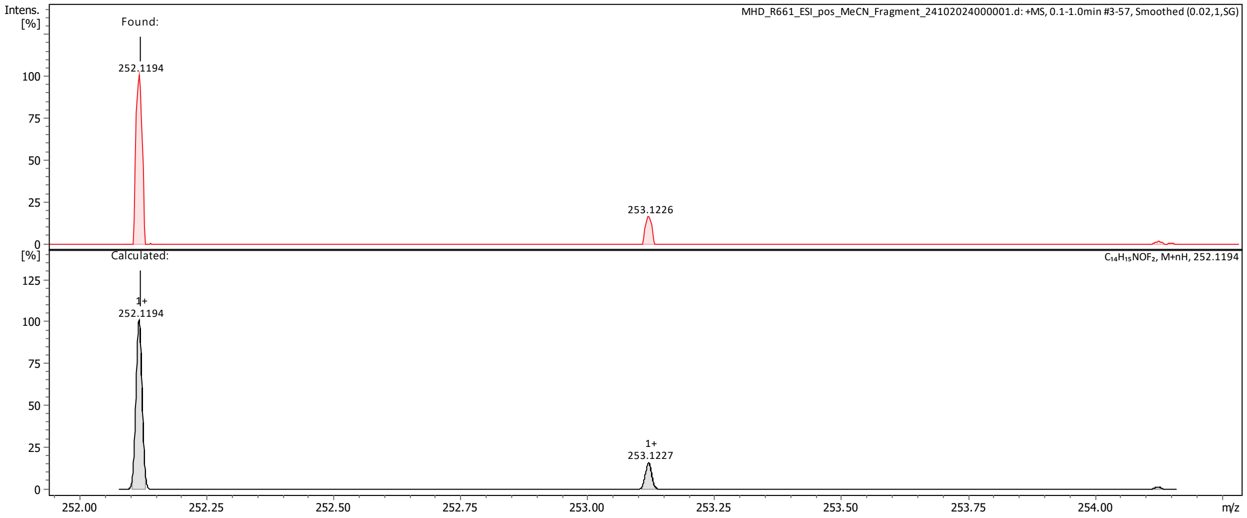

Intens. [%]
Found:
252.1194
253.1226
MHD_R661_ESI_pos_MeCN_Fragment_24102024000001.d: +MS, 0.1-1.0min #3-57, Smoothed (0.02,1,SG)
Calculated:
1+
252.1194
1+
253.1227
C14H15NOF2, M+nH, 252.1194
m/z

5.2.8 IR

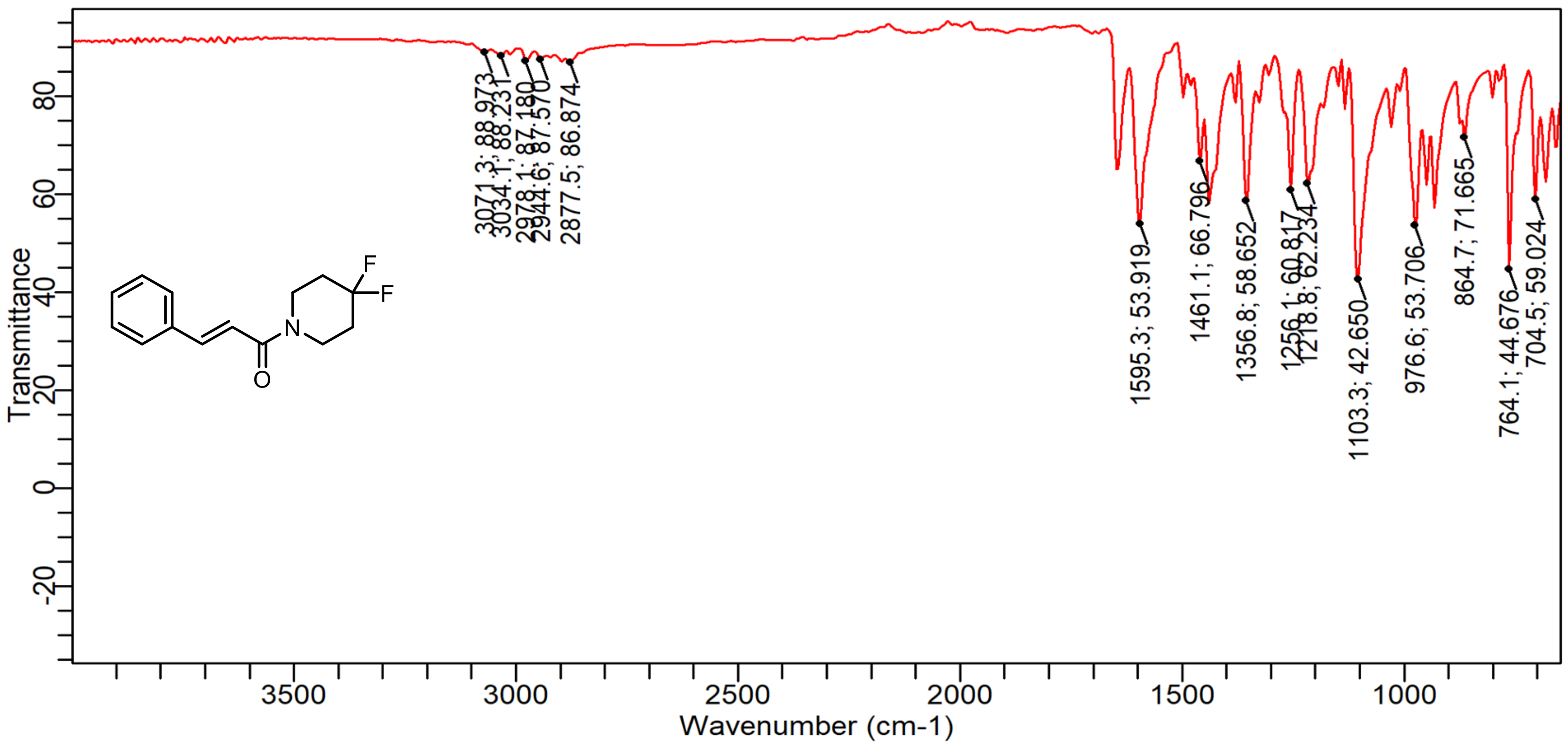

Transmittance
Wavenumber (cm-1)
3071.3; 88.973
3034.1; 88.231
2978.1; 87.180
2944.6; 87.570
2877.5; 86.874
1595.3; 53.919
1461.1; 66.796
1356.8; 58.652
1256.1; 60.817
1218.8; 62.234
1103.3; 42.650
976.6; 53.706
864.7; 71.665
764.1; 44.676
704.5; 59.024

5.3 (*E*)-1-Morpholino-3-phenylprop-2-en-1-one (11d) (600 MHz, $CDCl_3$)

5.3.1 ^{1}H NMR

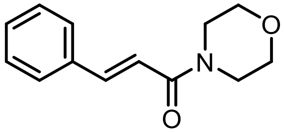

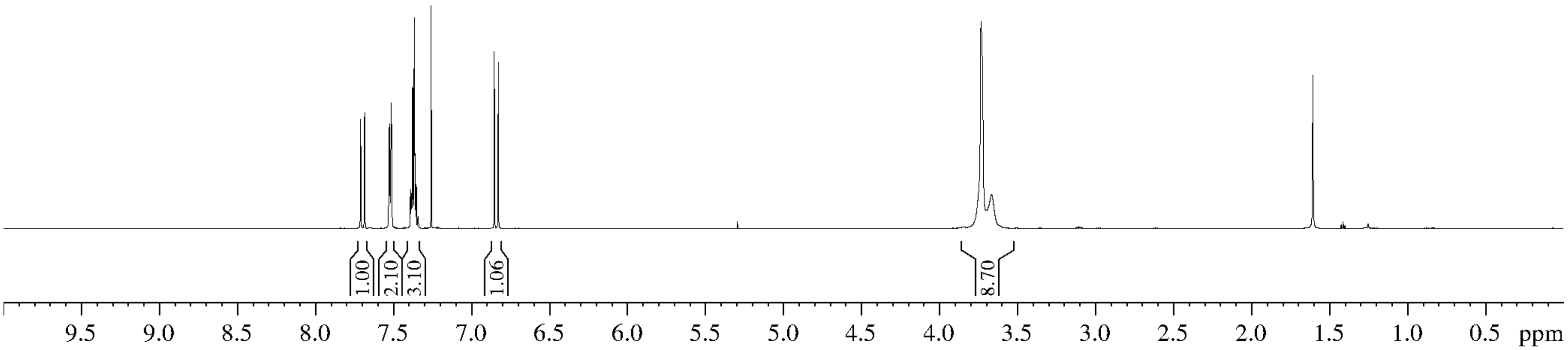

5.3.2 ^{13}C NMR

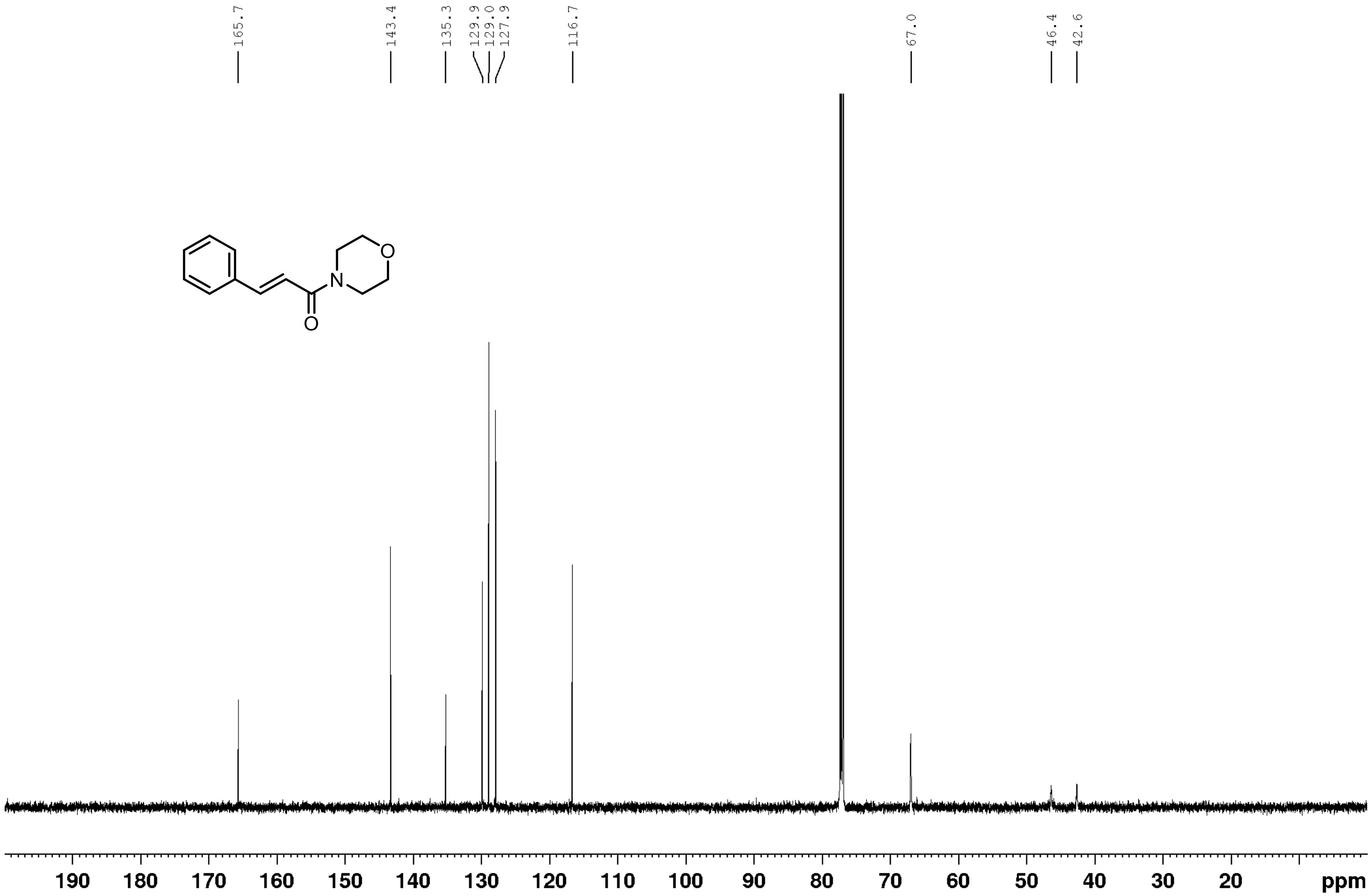

165.7
143.4
135.3
129.9
129.0
127.9
116.7
67.0
46.4
42.6
O
N
O
190
180
170
160
150
140
130
120
110
100
90
80
70
60
50
40
30
20
ppm

5.3.3 ^{1}H, ^{1}H COSY

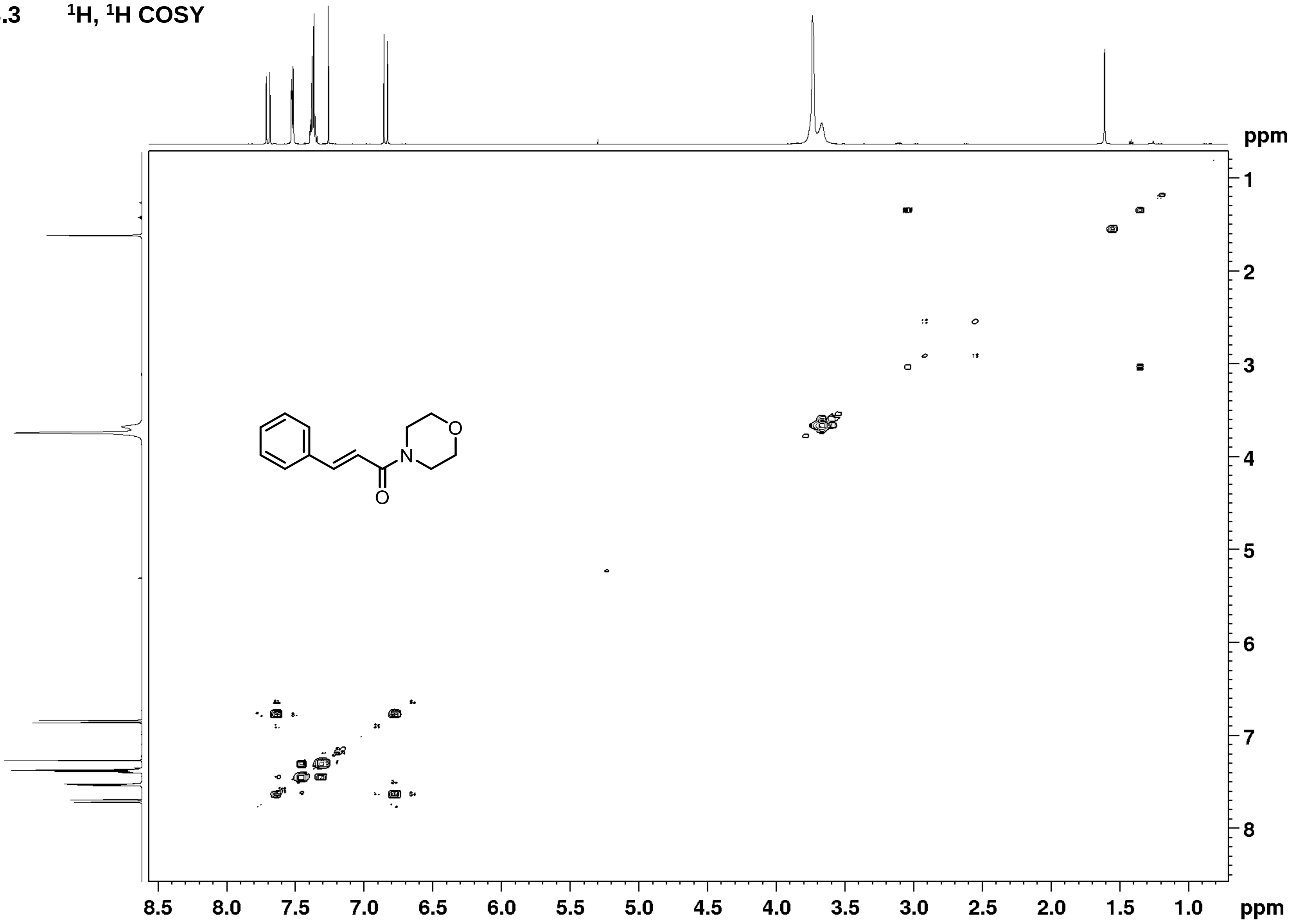

5.3.4 ^1H, ^{13}C HMBC

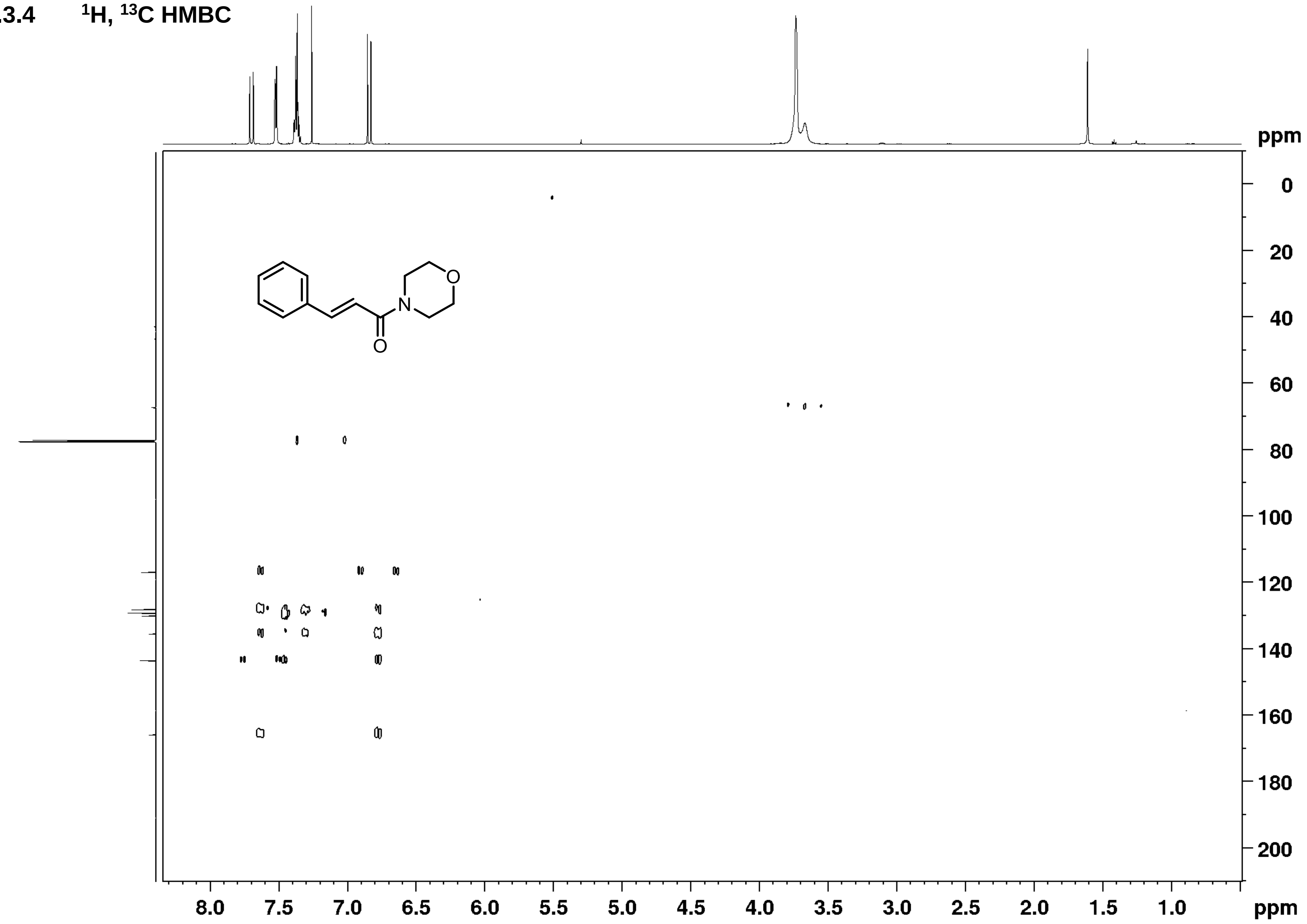

5.3.5 ^{1}H, ^{13}C HSQC

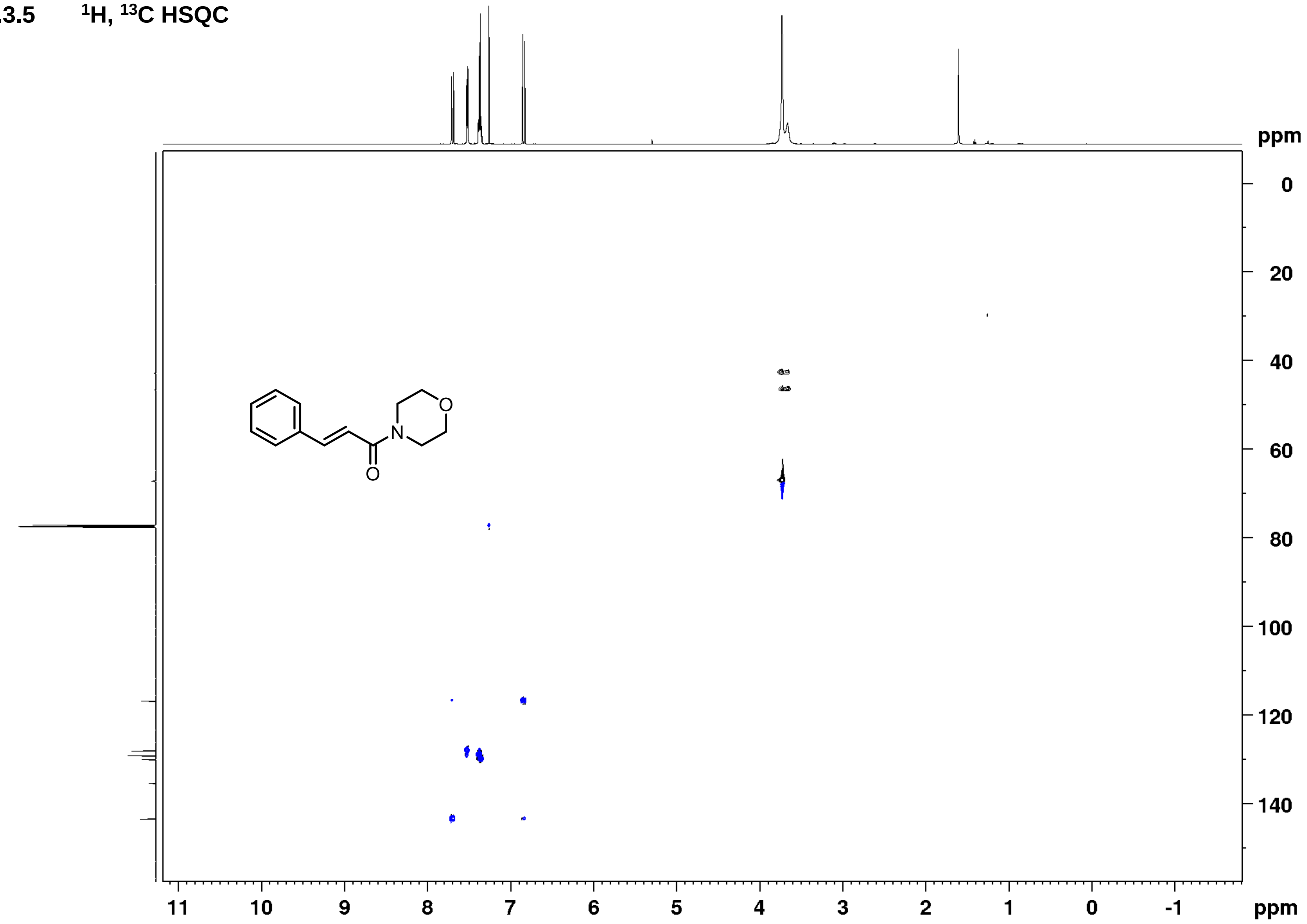

5.3.6 HRMS

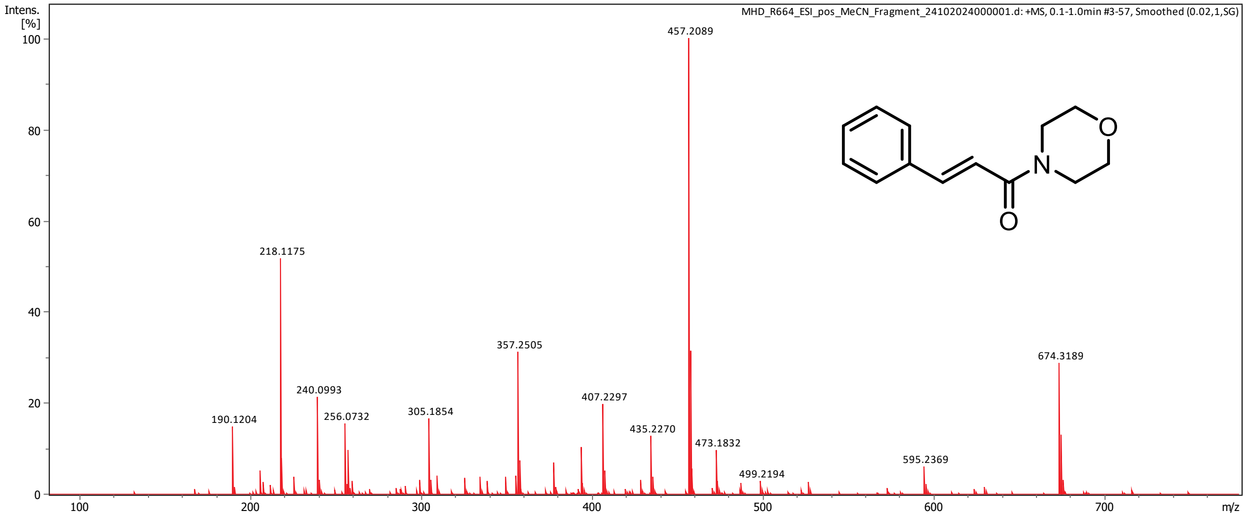
Intens. [%]
MHD_R664_ESI_pos_MeCN_Fragment_24102024000001.d: +MS, 0.1-1.0min #3-57, Smoothed (0.02,1,SG)
457.2089
218.1175
357.2505
674.3189
407.2297
240.0993
190.1204
256.0732
305.1854
435.2270
473.1832
595.2369
499.2194
N
O
O
m/z

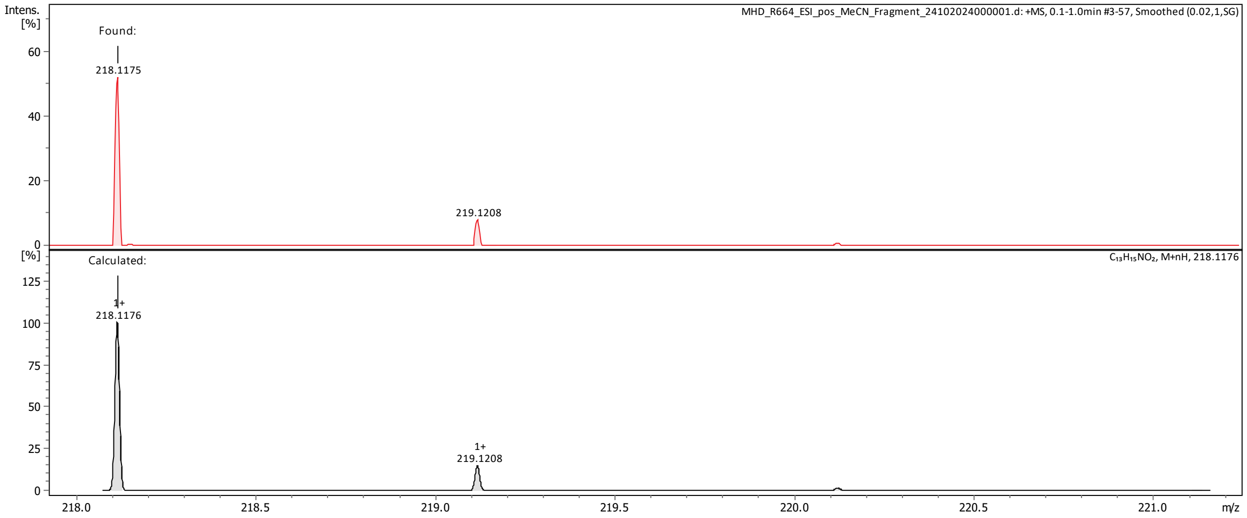
Intens. [%]
MHD_R664_ESI_pos_MeCN_Fragment_24102024000001.d: +MS, 0.1-1.0min #3-57, Smoothed (0.02,1,SG)
Found:
218.1175
219.1208
Calculated:
C₁₃H₁₅NO₂, M+nH, 218.1176
1+
218.1176
1+
219.1208
m/z

5.3.7 IR

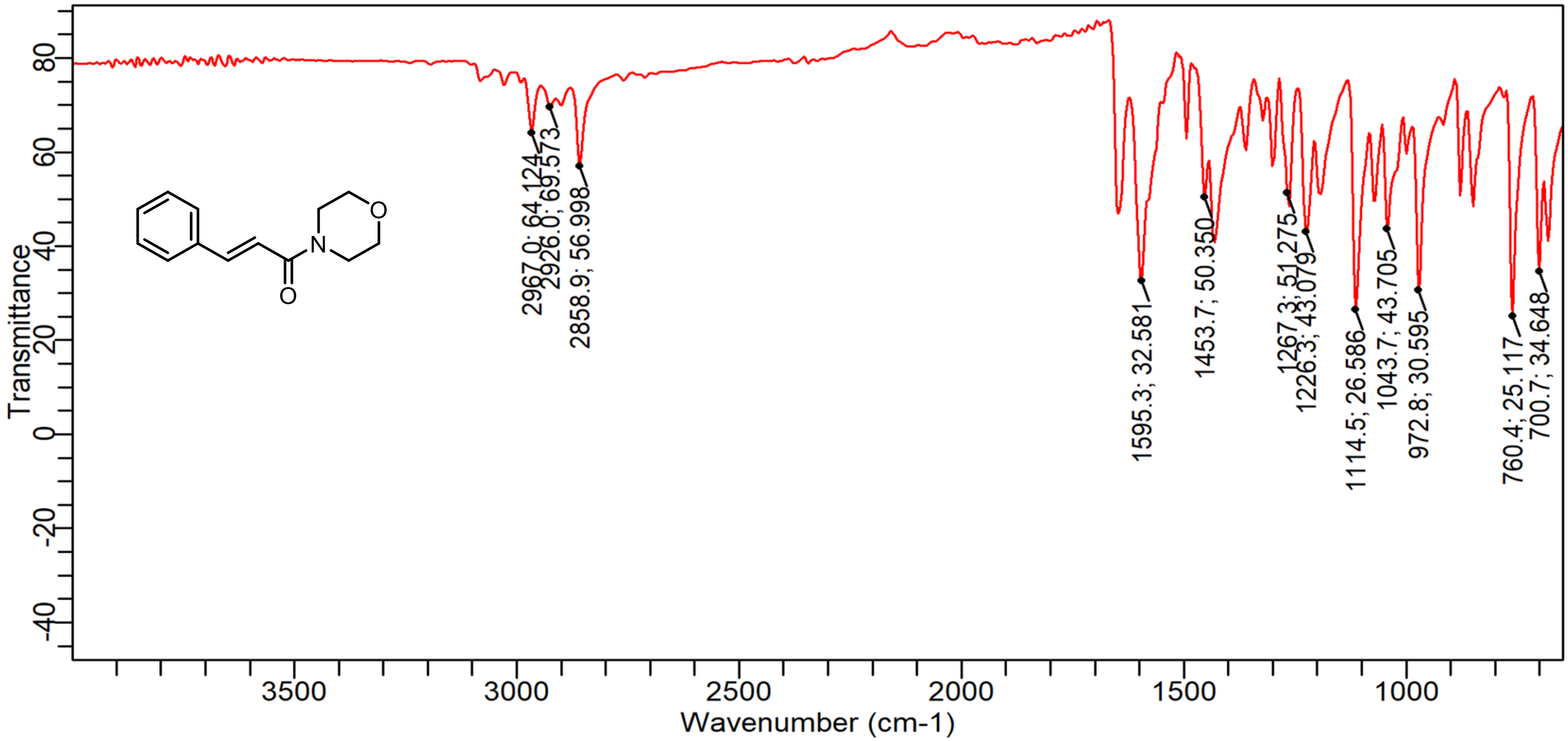

Transmittance
Wavenumber (cm-1)
2967.0; 64.124
2926.0; 69.573
2858.9; 56.998
1595.3; 32.581
1453.7; 50.350
1267.3; 51.275
1226.3; 43.079
1114.5; 26.586
1043.7; 43.705
972.8; 30.595
760.4; 25.117
700.7; 34.648
N
O
O

5.4 (*E*)-3-Phenyl-1-thiomorpholinoprop-2-en-1-one (11e) (600 MHz, $CDCl_3$)

5.4.1 ^{1}H NMR

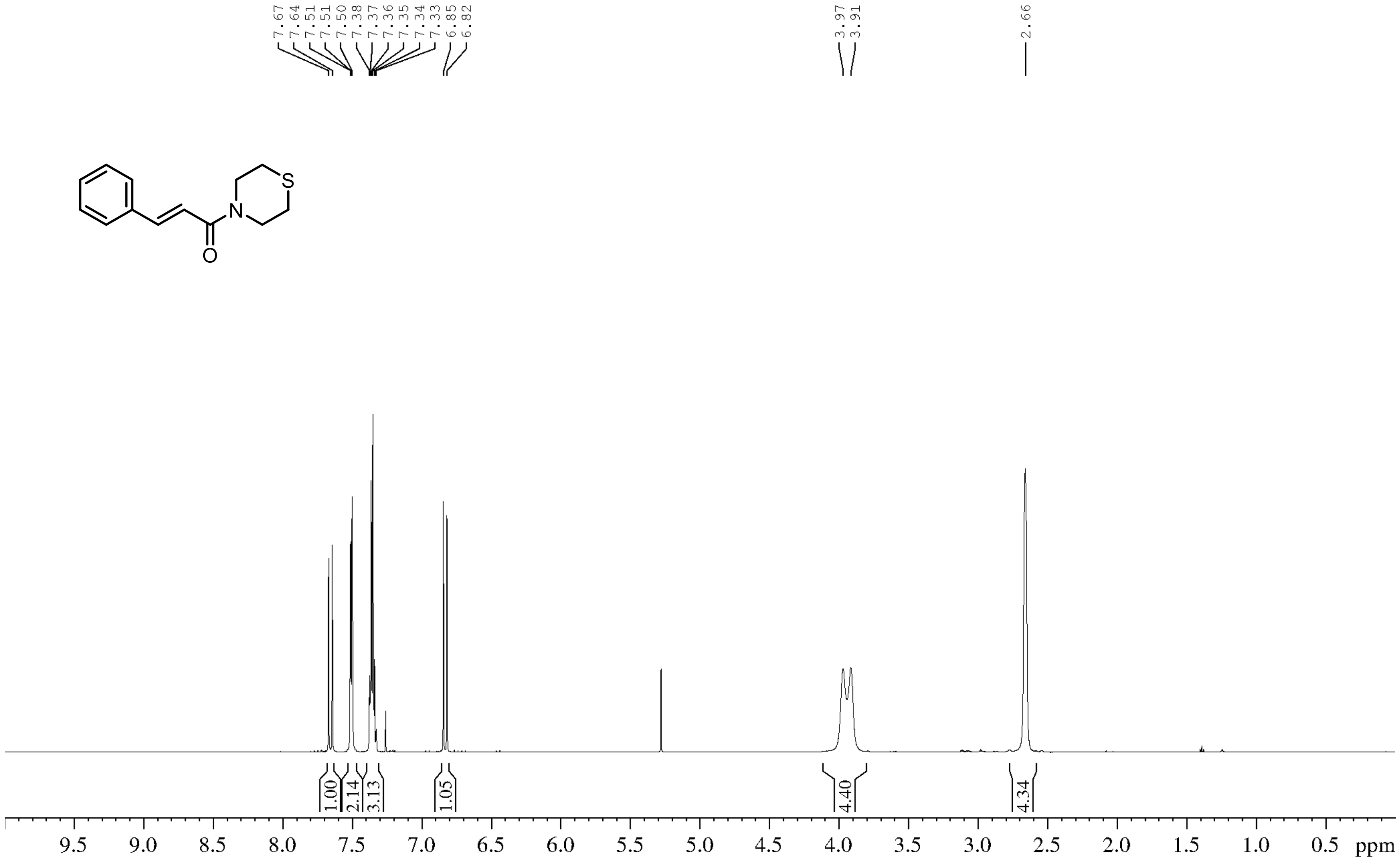

5.4.2 ^{13}C NMR

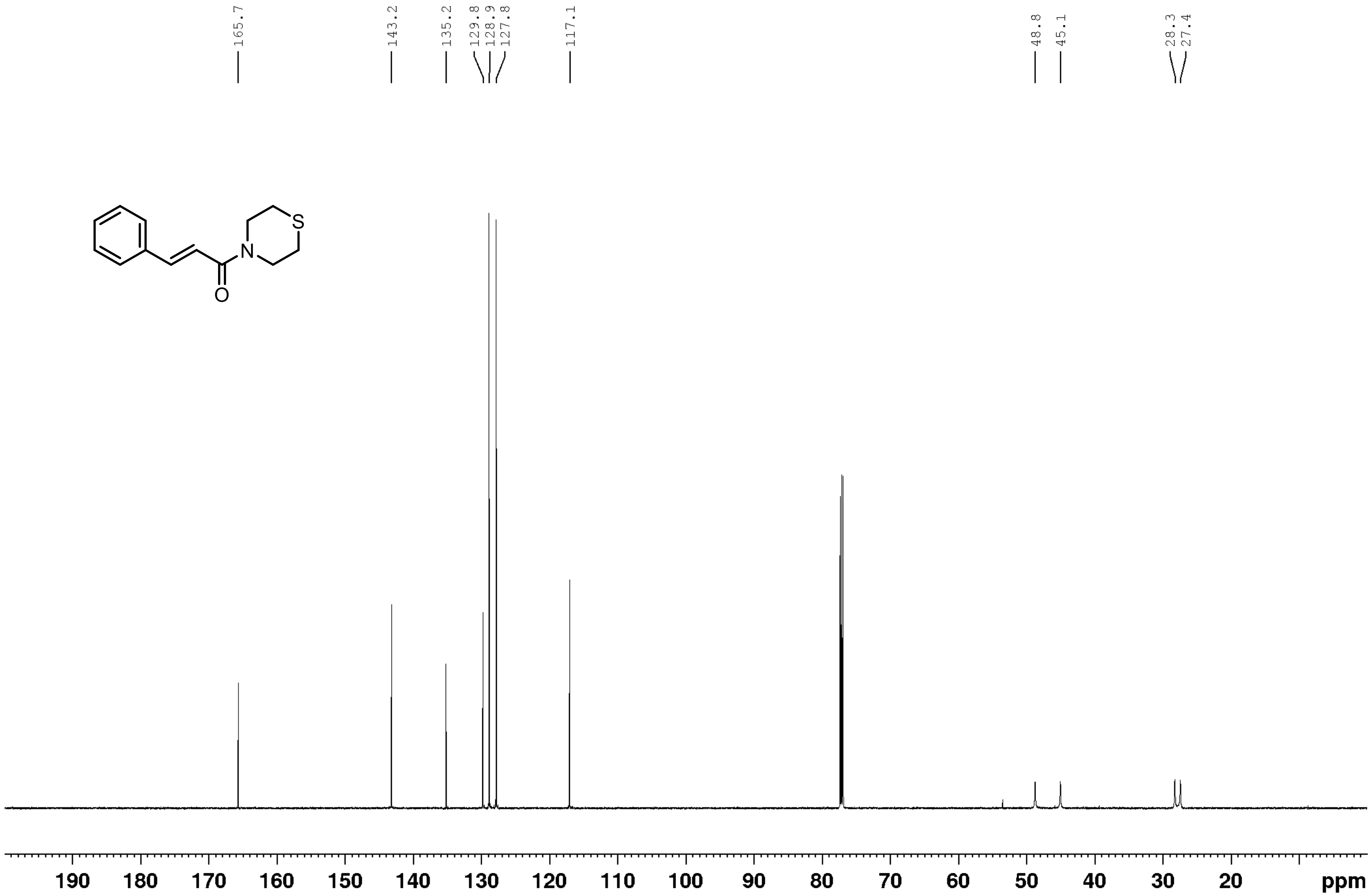
165.7
143.2
135.2
129.8
128.9
127.8
117.1
48.8
45.1
28.3
27.4
S
N
O
190
180
170
160
150
140
130
120
110
100
90
80
70
60
50
40
30
20
ppm

5.4.3 ^{1}H, ^{1}H COSY

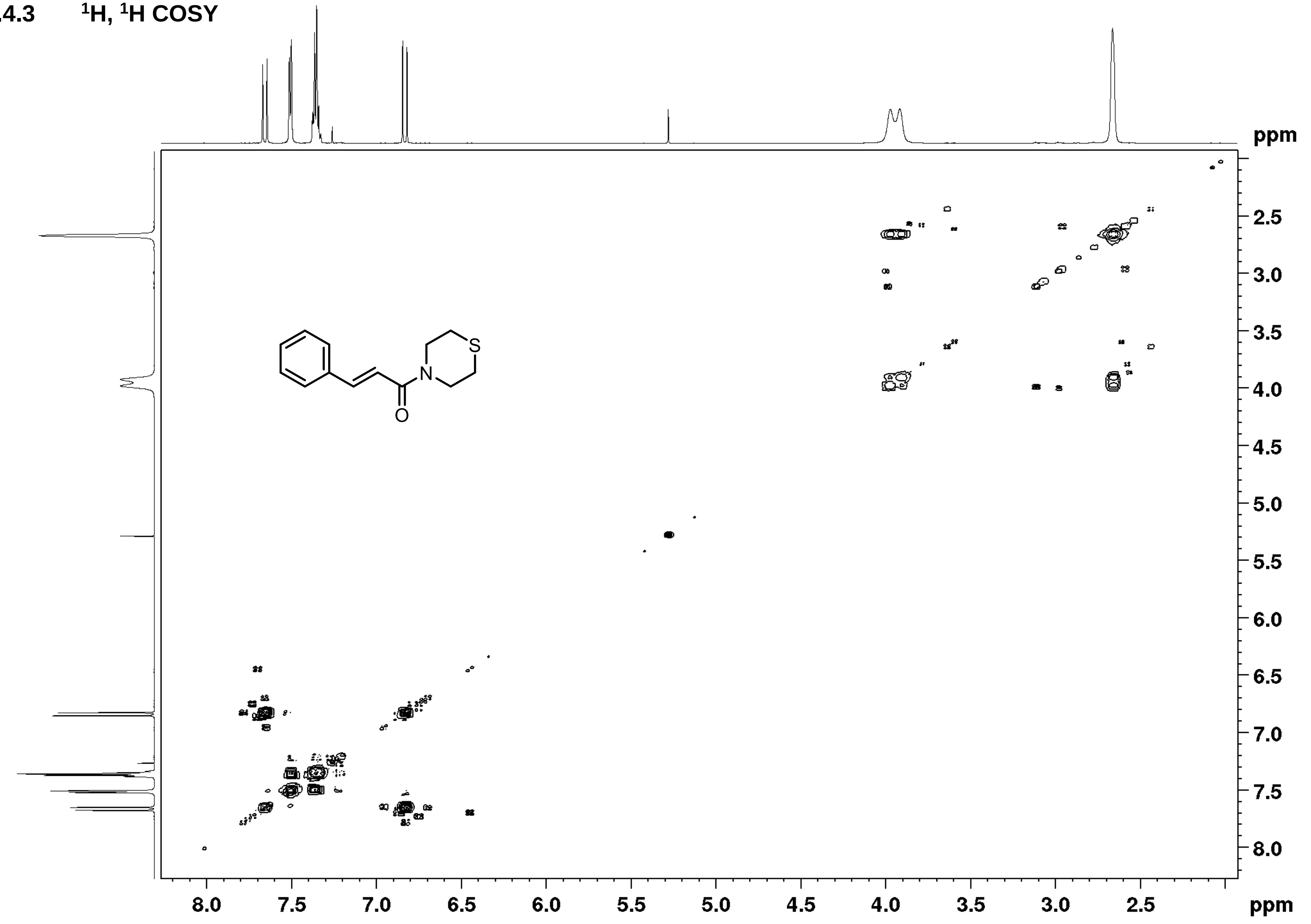

5.4.4 ^{1}H, ^{13}C HMBC

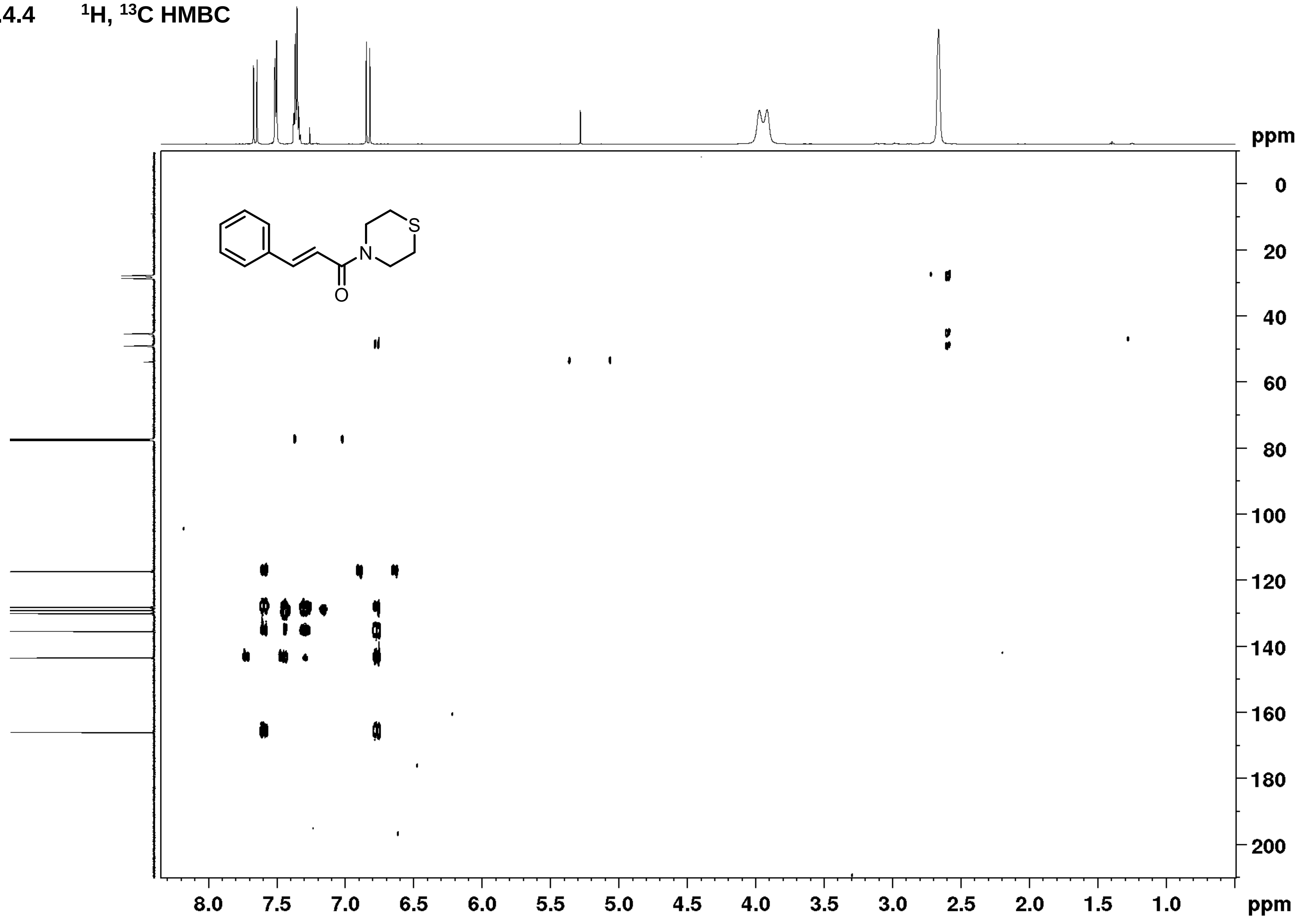

5.4.5 ^{1}H, ^{13}C HSQC

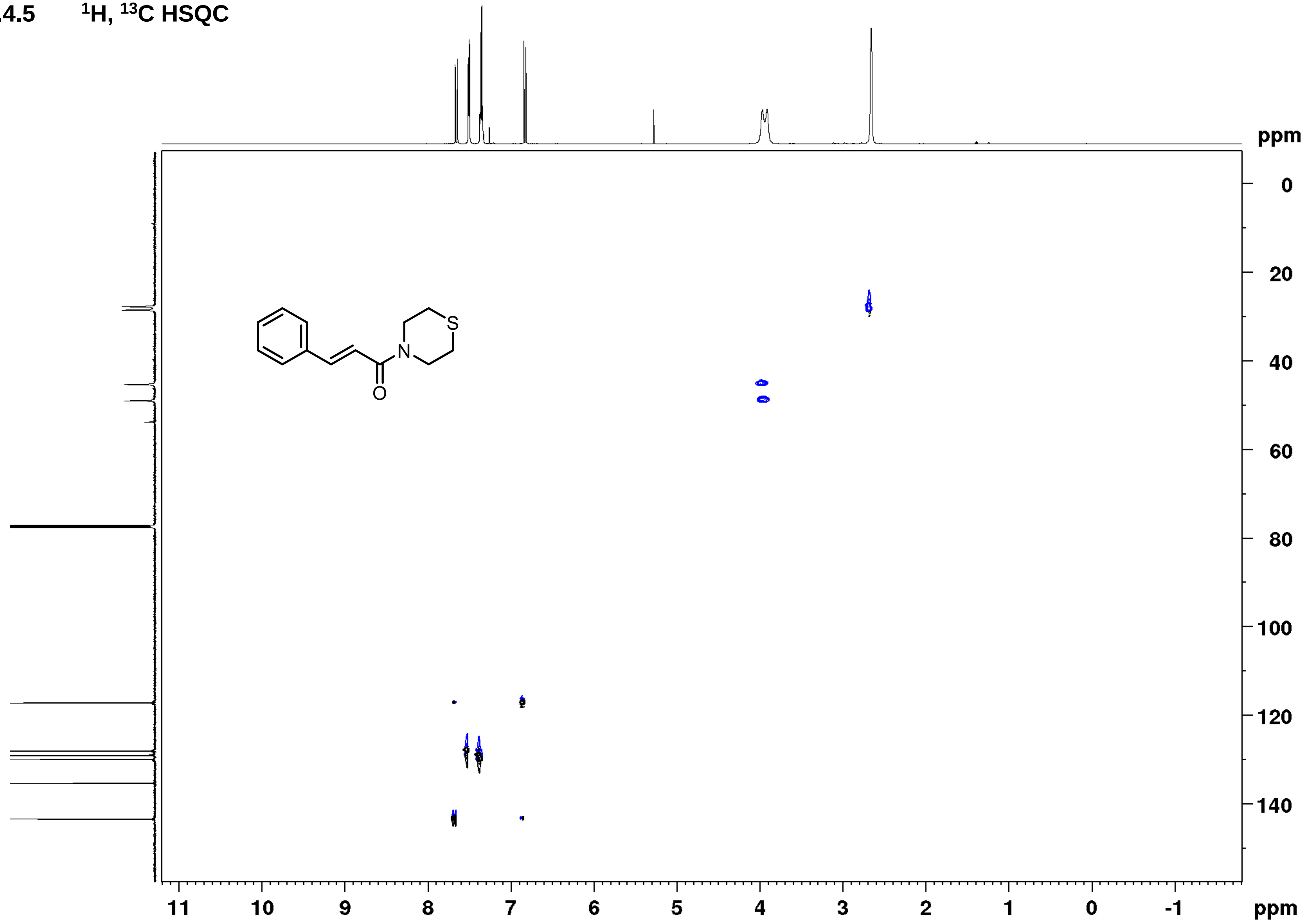

5.4.6 HRMS

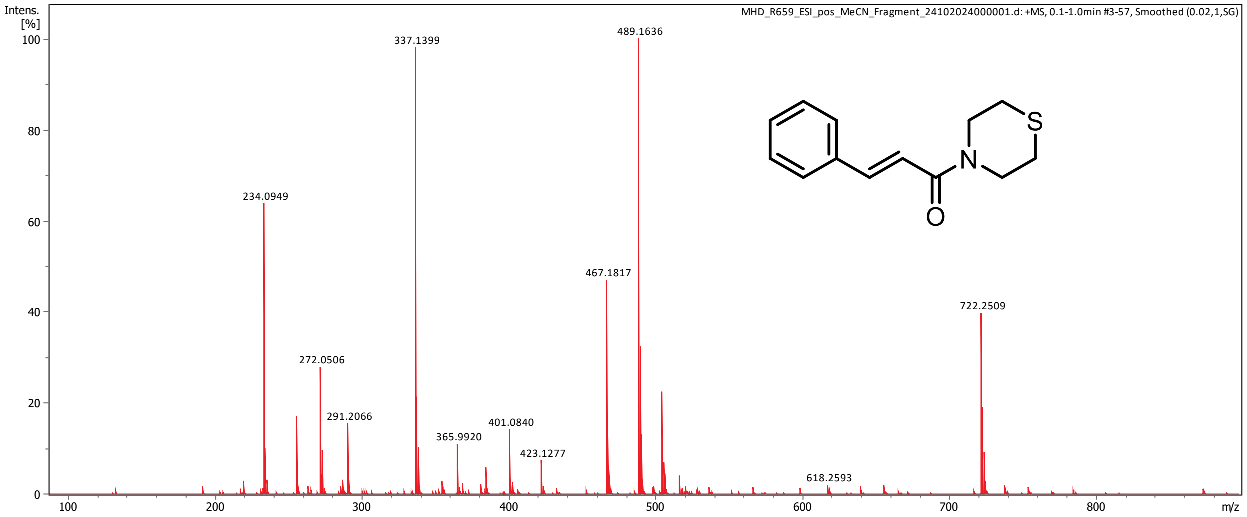

Intens. [%]
MHD_R659_ESI_pos_MeCN_Fragment_24102024000001.d: +MS, 0.1-1.0min #3-57, Smoothed (0.02,1,SG)
337.1399
489.1636
234.0949
467.1817
722.2509
272.0506
291.2066
365.9920
401.0840
423.1277
618.2593
m/z

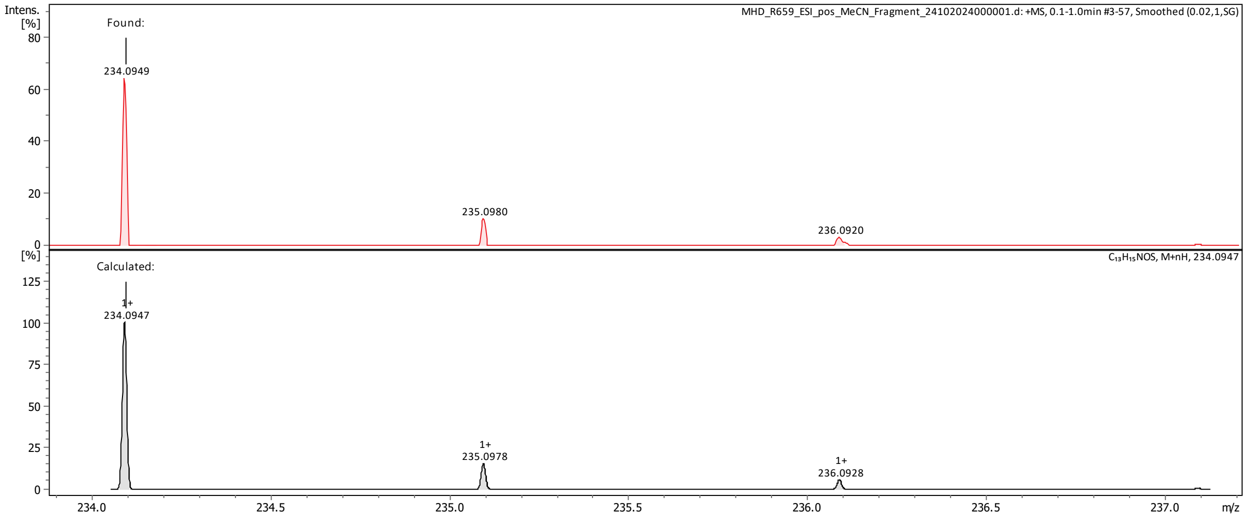

Intens. [%]
MHD_R659_ESI_pos_MeCN_Fragment_24102024000001.d: +MS, 0.1-1.0min #3-57, Smoothed (0.02,1,SG)
Found:
234.0949
235.0980
236.0920
Calculated:
C13H15NOS, M+nH, 234.0947
1+ 234.0947
1+ 235.0978
1+ 236.0928
m/z

5.4.7 IR

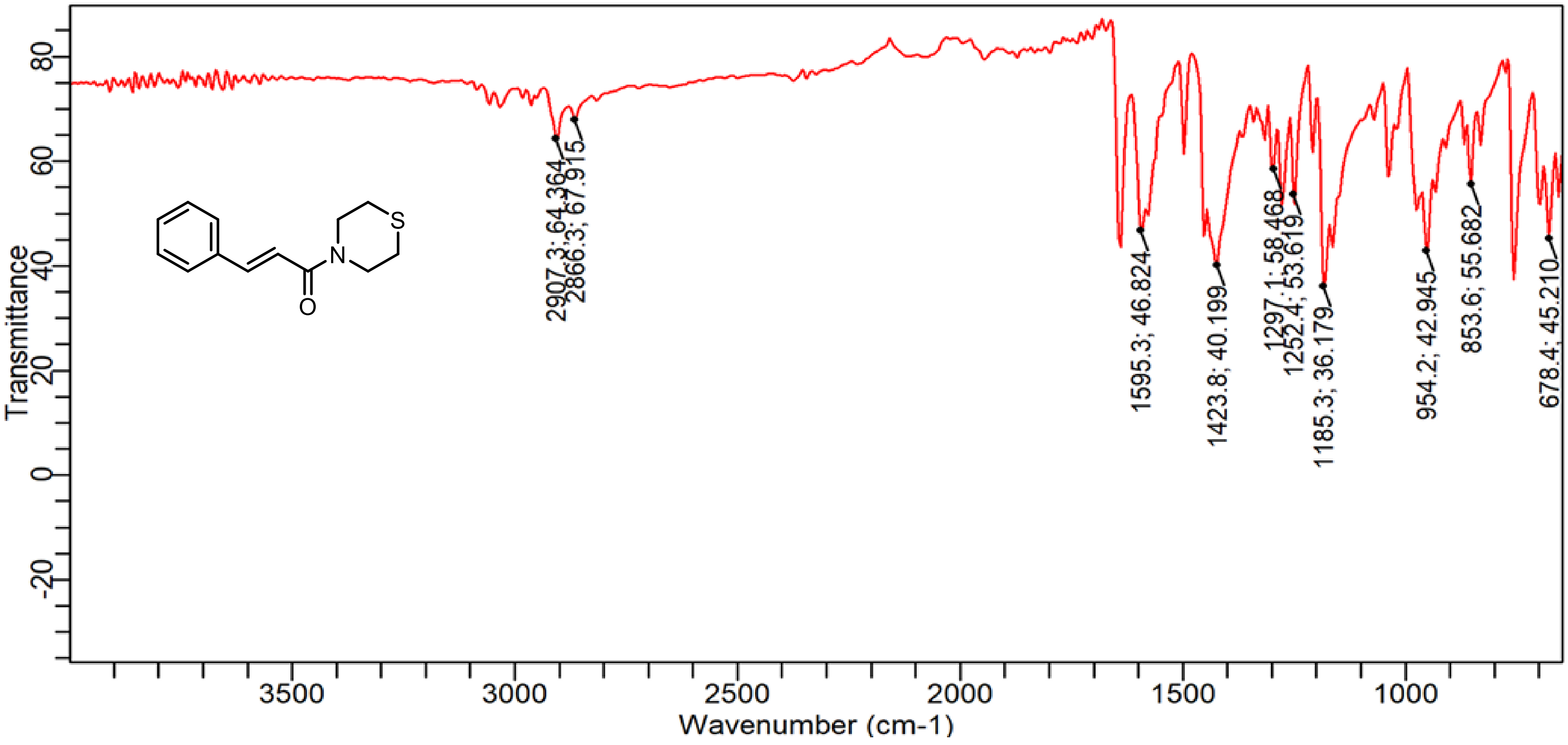

2907.3; 64.364
2866.3; 67.915
1595.3; 46.824
1423.8; 40.199
1297.1; 58.468
1252.4; 53.619
1185.3; 36.179
954.2; 42.945
853.6; 55.682
678.4; 45.210
Transmittance
Wavenumber (cm-1)

5.5 *N*,*N*-Bis(2-methoxyethyl)cinnamamide (11f) (600 MHz, $CDCl_3$)

5.5.1 ^{1}H NMR

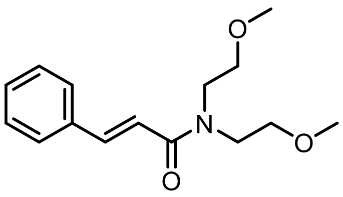

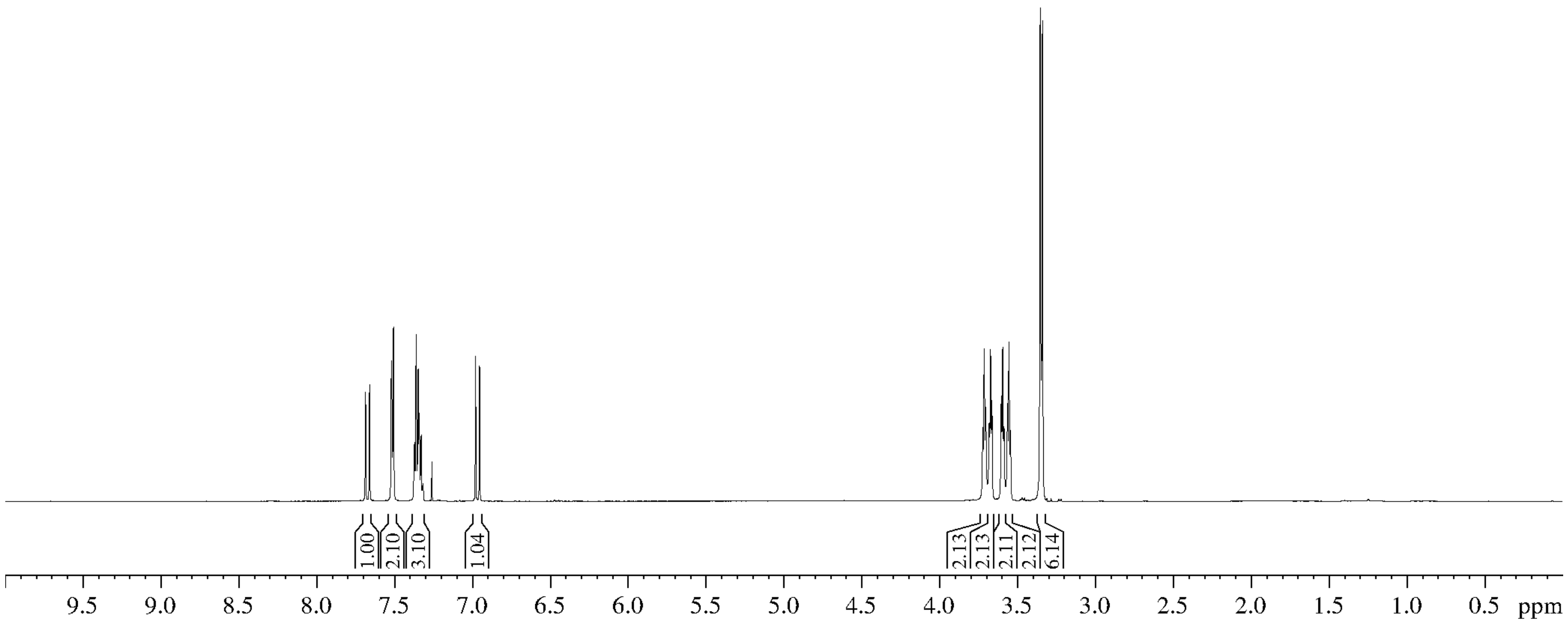

5.5.2 ^{13}C NMR

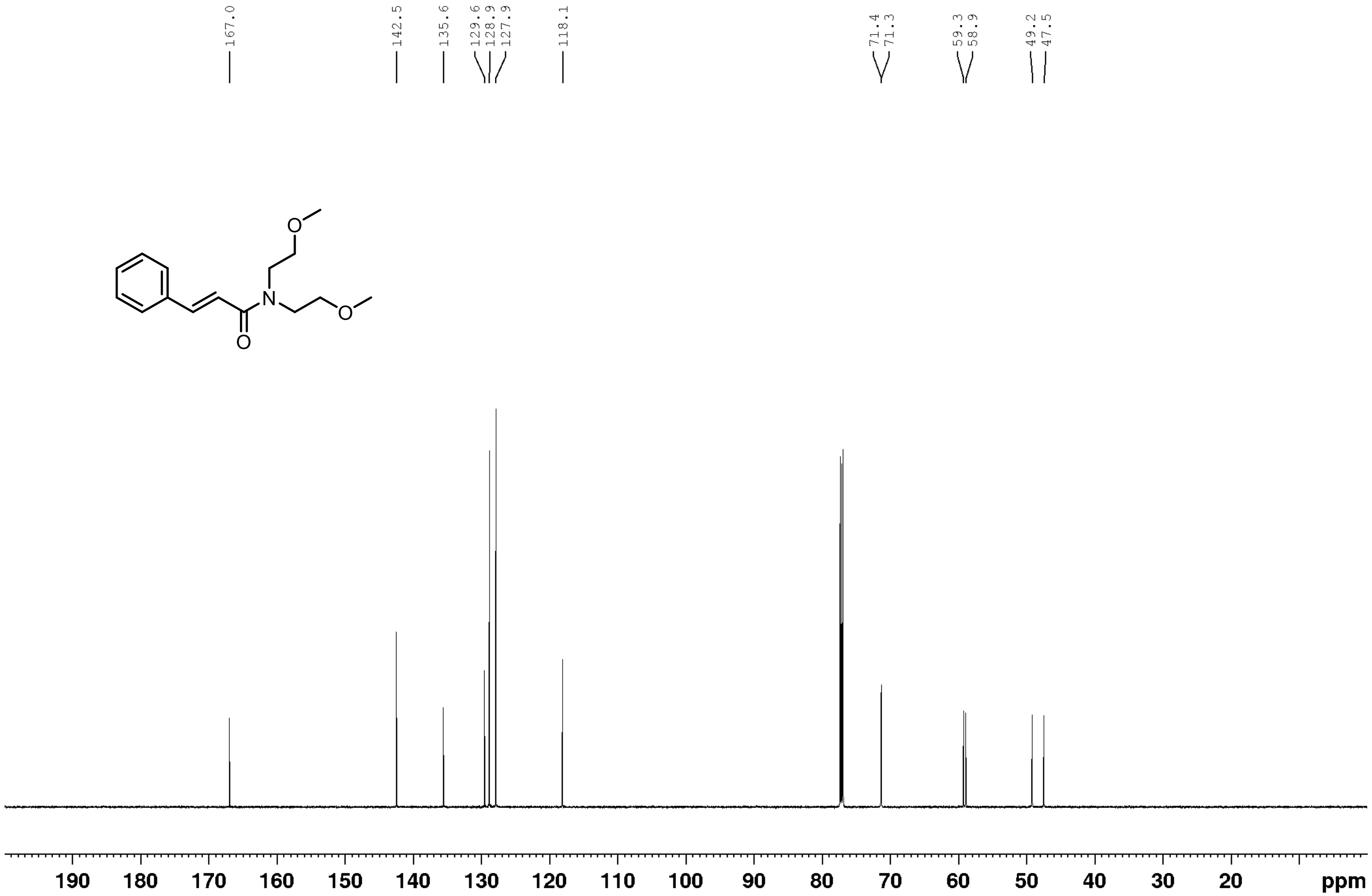
167.0
142.5
135.6
129.6
128.9
127.9
118.1
71.4
71.3
59.3
58.9
49.2
47.5
O
N
O
O
190
180
170
160
150
140
130
120
110
100
90
80
70
60
50
40
30
20
ppm

5.5.3 ¹H, ¹H COSY

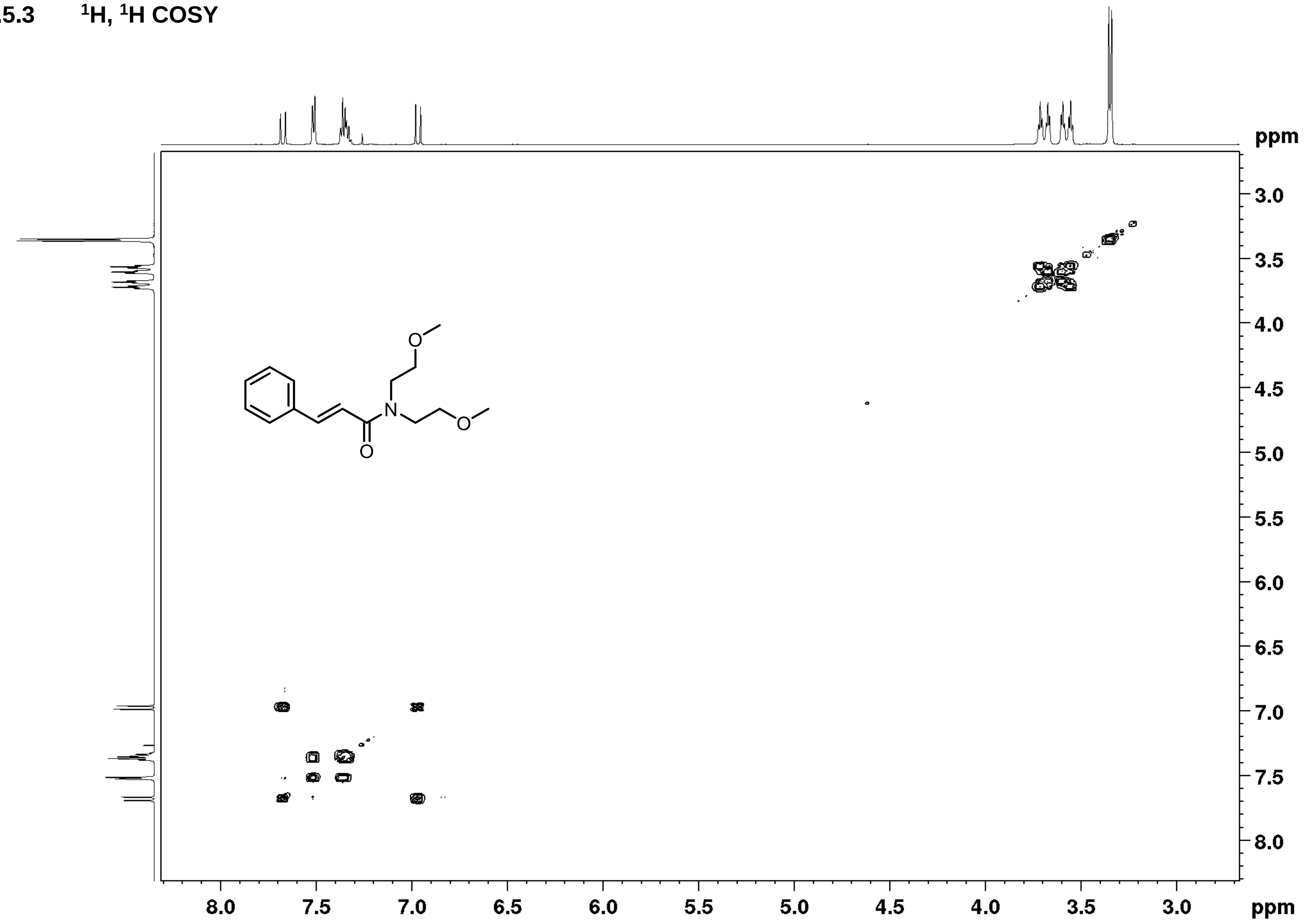

5.5.4 ^{1}H, ^{13}C HMBC

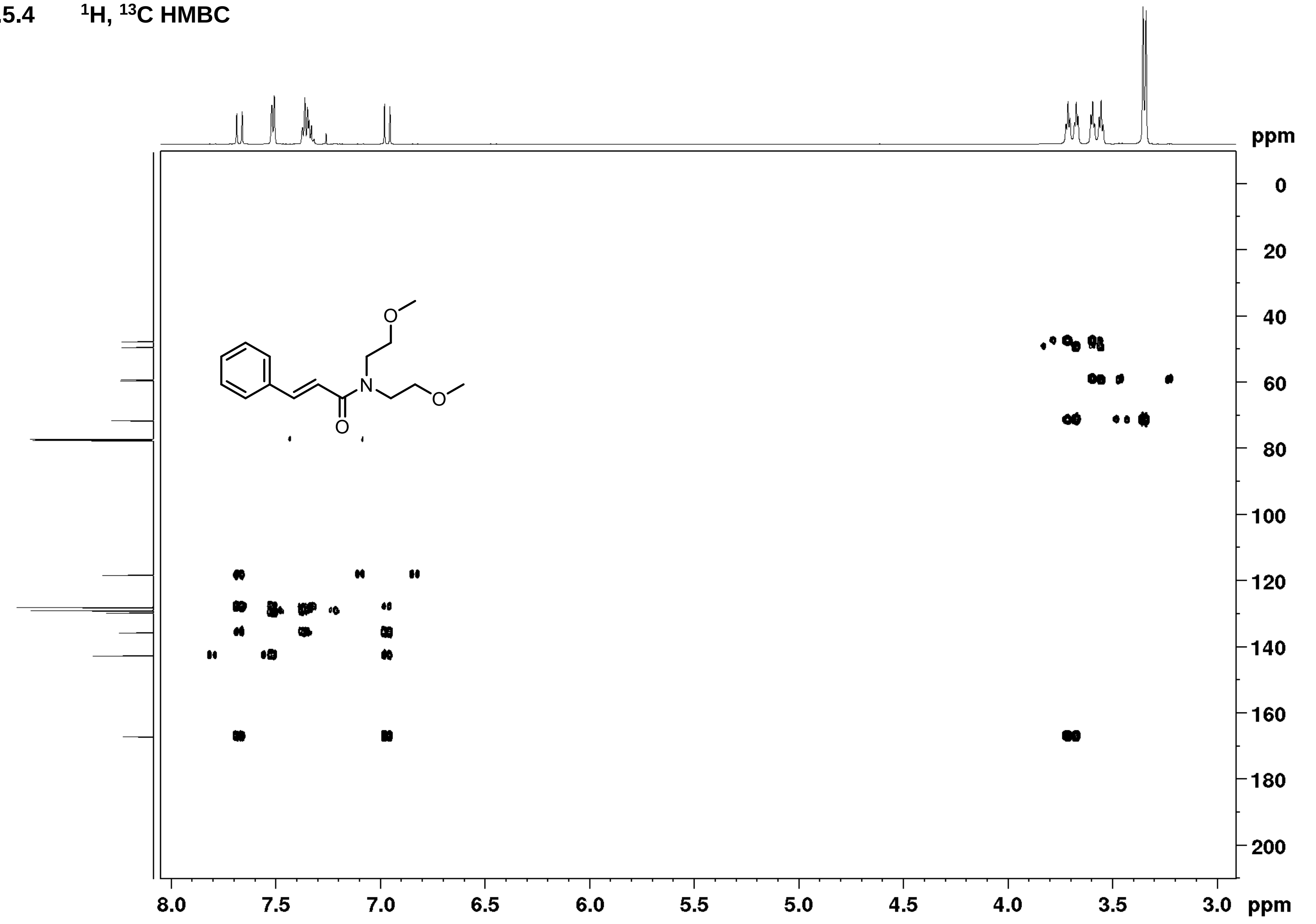

5.5.5 ^{1}H, ^{13}C HSQC

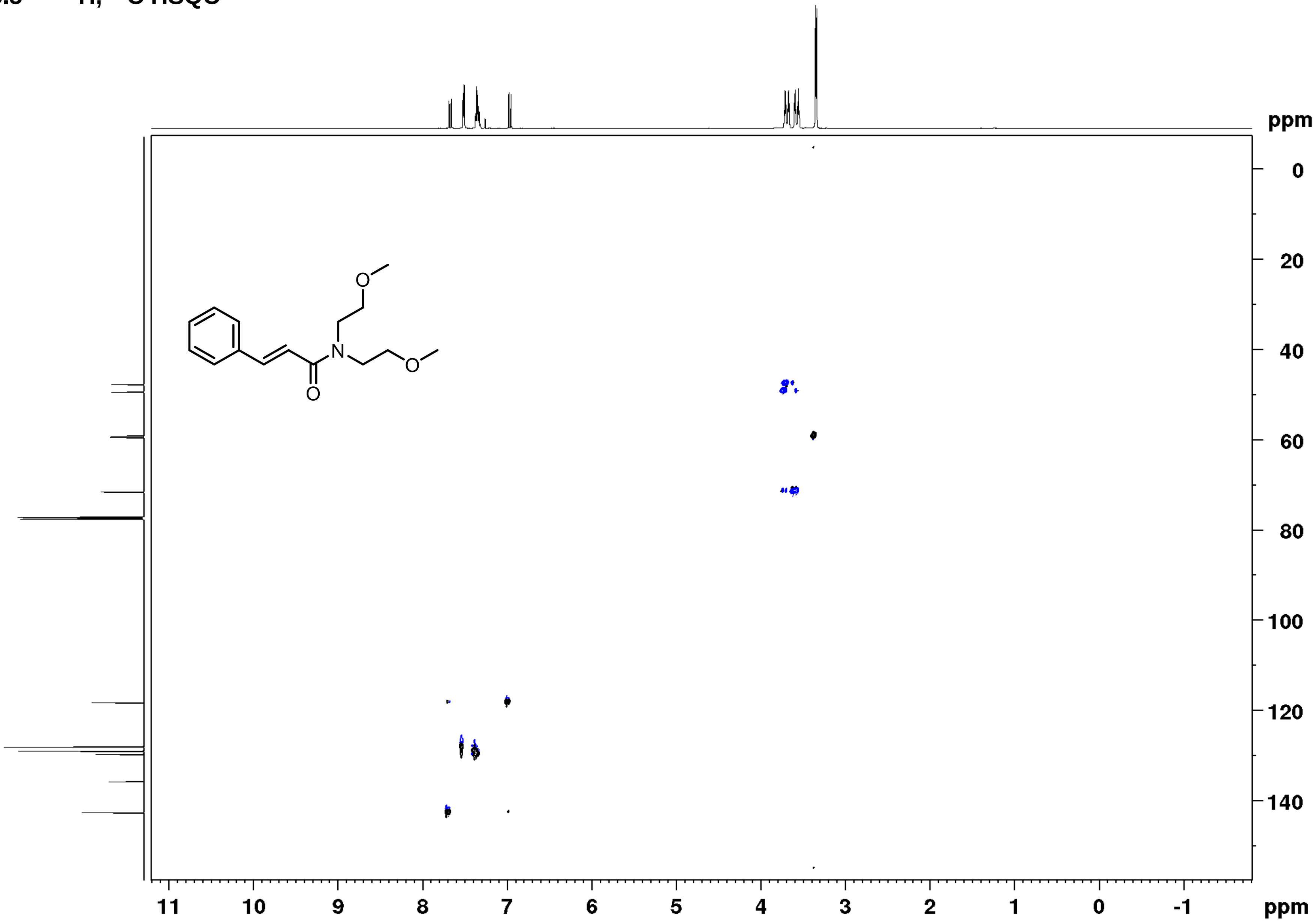

5.5.6 HRMS

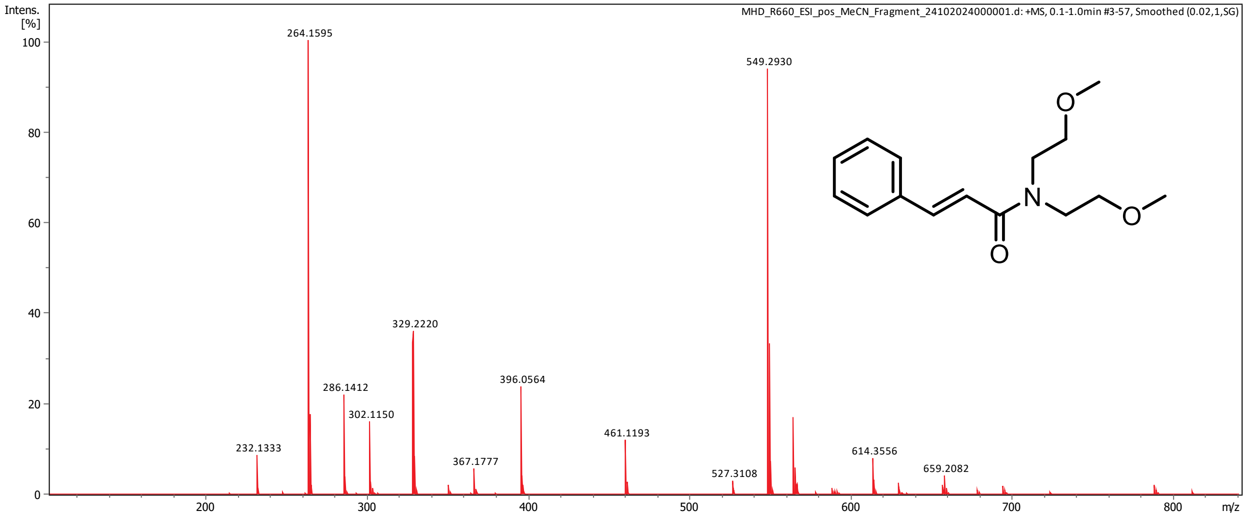
MHD_R660_ESI_pos_MeCN_Fragment_24102024000001.d: +MS, 0.1-1.0min #3-57, Smoothed (0.02,1,SG)
Intens. [%]
264.1595
549.2930
329.2220
286.1412
302.1150
396.0564
232.1333
367.1777
461.1193
527.3108
614.3556
659.2082
m/z

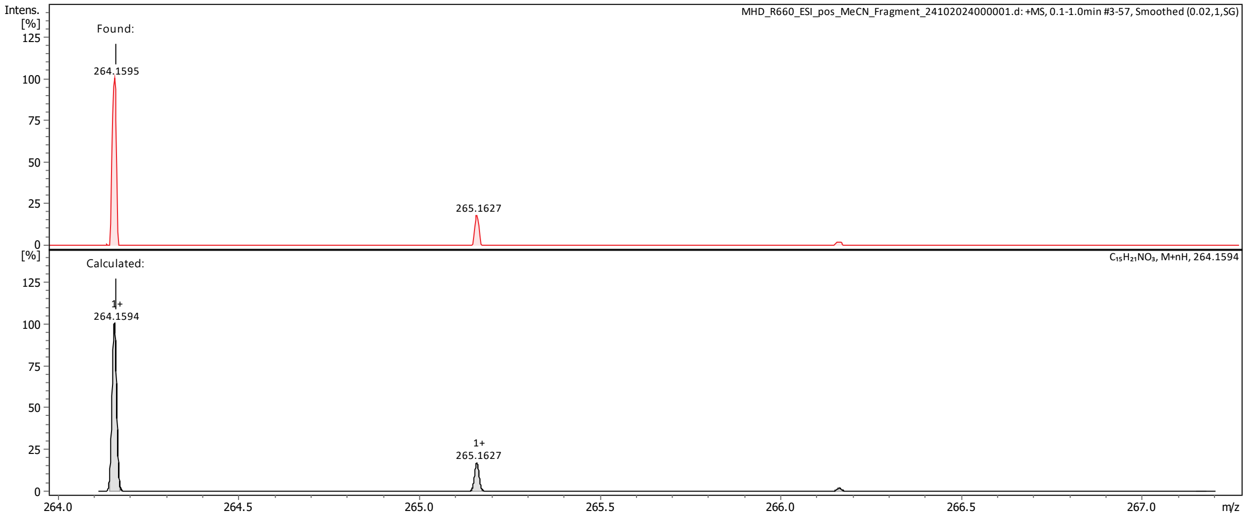
MHD_R660_ESI_pos_MeCN_Fragment_24102024000001.d: +MS, 0.1-1.0min #3-57, Smoothed (0.02,1,SG)
Intens. [%]
Found:
264.1595
265.1627
Calculated:
$C_{15}H_{21}NO_3$, M+nH, 264.1594
1+
264.1594
1+
265.1627
m/z

5.5.7 IR

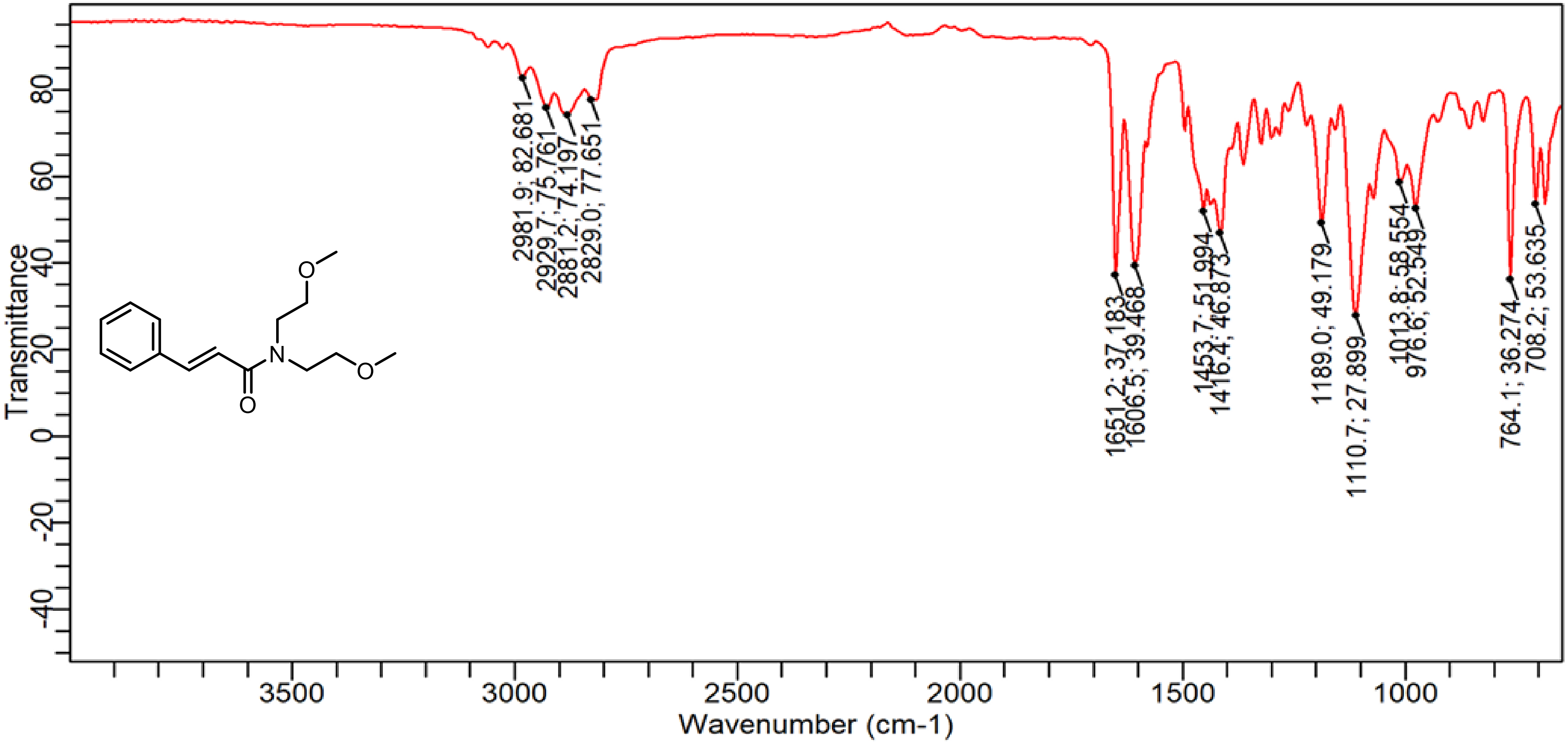

2981.9; 82.681
2929.7; 75.761
2881.2; 74.197
2829.0; 77.651
1651.2; 37.183
1606.5; 39.468
1453.7; 51.994
1416.4; 46.873
1189.0; 49.179
1110.7; 27.899
1013.8; 58.554
976.6; 52.549
764.1; 36.274
708.2; 53.635
Transmittance
Wavenumber (cm-1)
3500
3000
2500
2000
1500
1000
80
60
40
20
0
-20
-40
N
O
O
O

5.6 (*E*)-1-(4,7-dihydrothieno[2,3-c]pyridin-6(5*H*)-yl)-3-phenylprop-2-en-1-one (11g & 11g_rotamer) (600 MHz, $CDCl_3$)

5.6.1 ^{1}H NMR

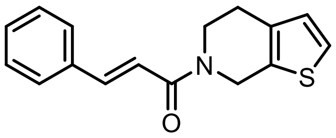

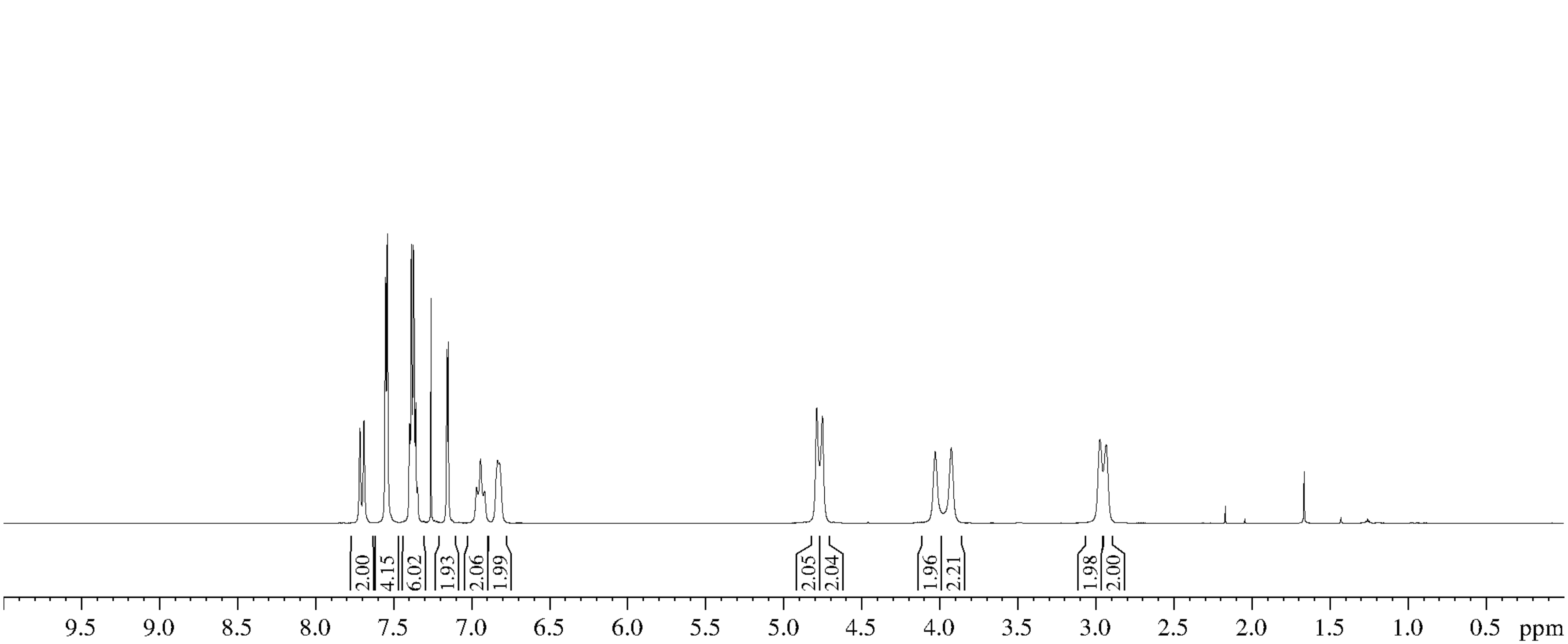

5.6.2 ^{13}C NMR

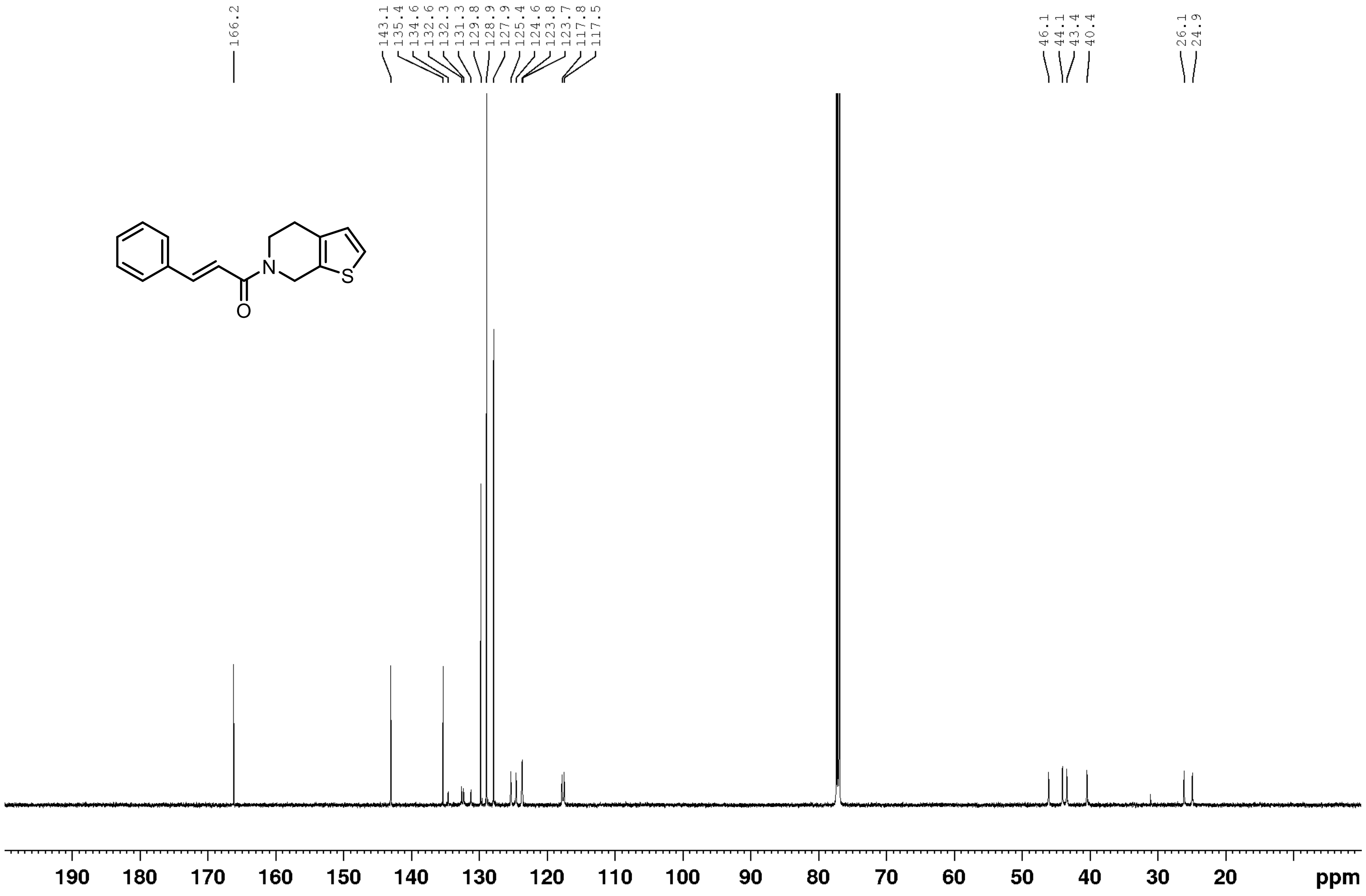

166.2
143.1
135.4
134.6
132.6
132.3
131.3
129.8
128.9
127.9
125.4
124.6
123.8
123.7
117.8
117.5
46.1
44.1
43.4
40.4
26.1
24.9
N
S
O
190 180 170 160 150 140 130 120 110 100 90 80 70 60 50 40 30 20
ppm

5.6.3 ^{1}H, ^{1}H COSY

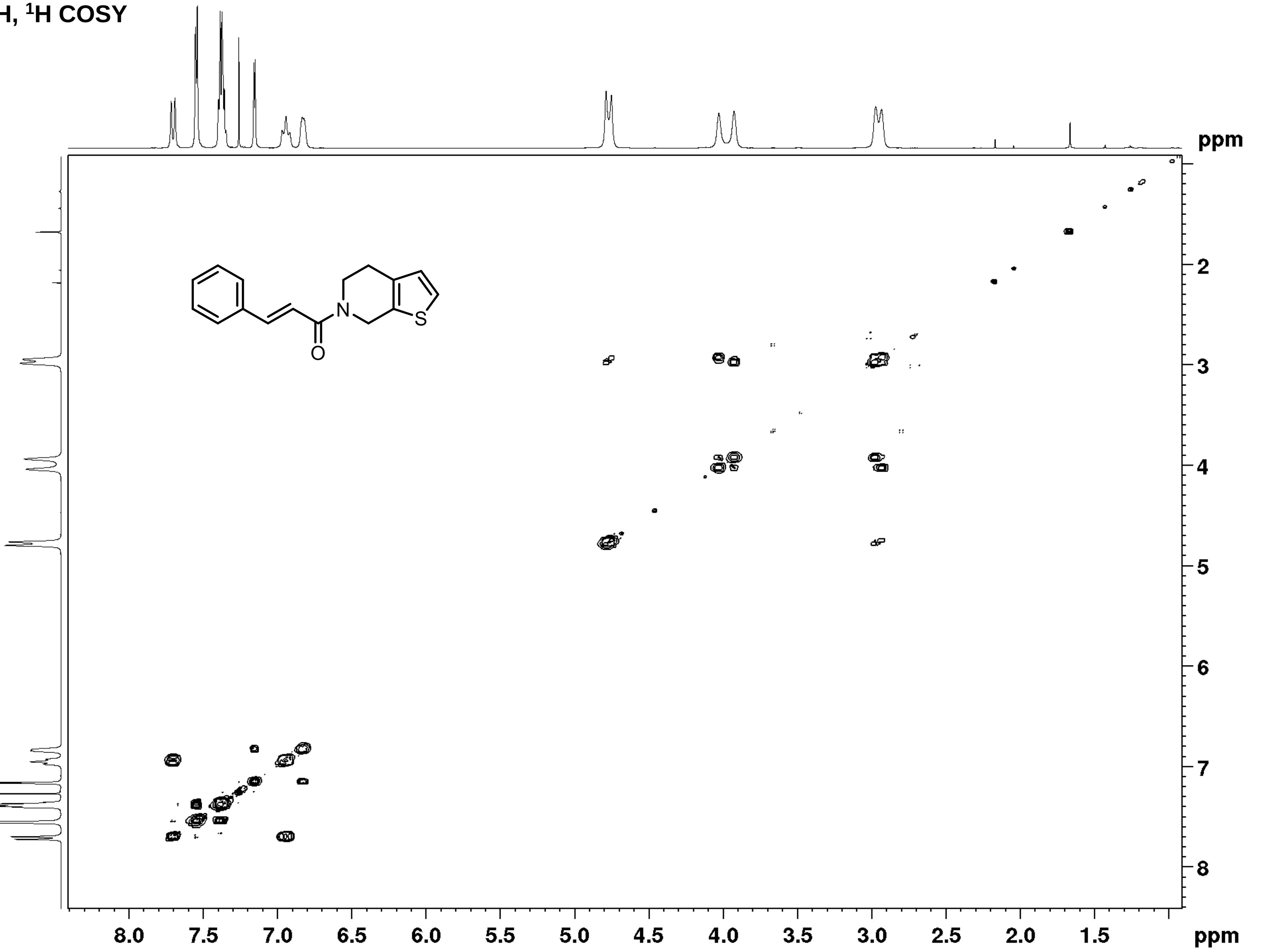

5.6.4 ^{1}H, ^{13}C HMBC

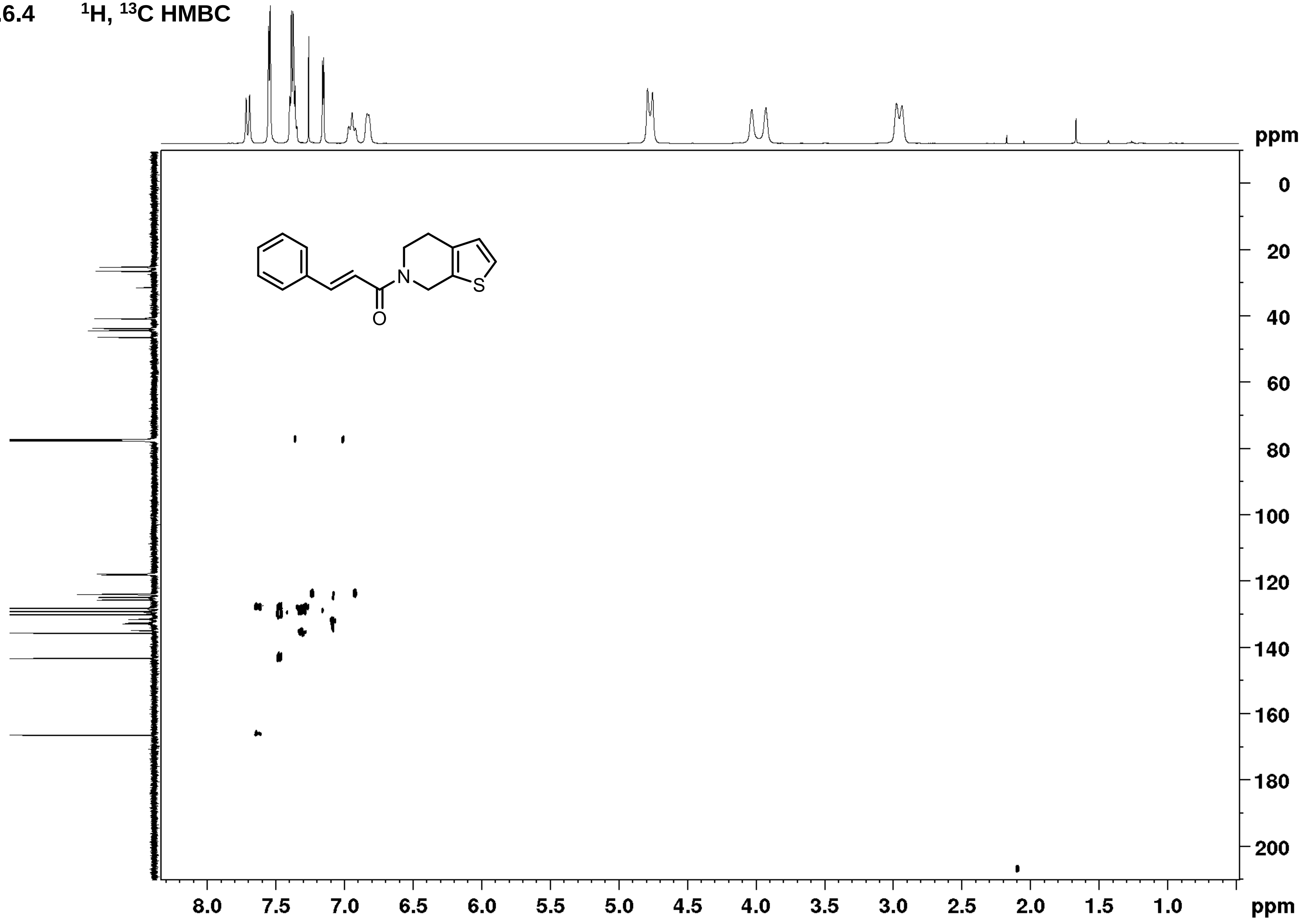

5.6.5 ^{1}H, ^{13}C HSQC

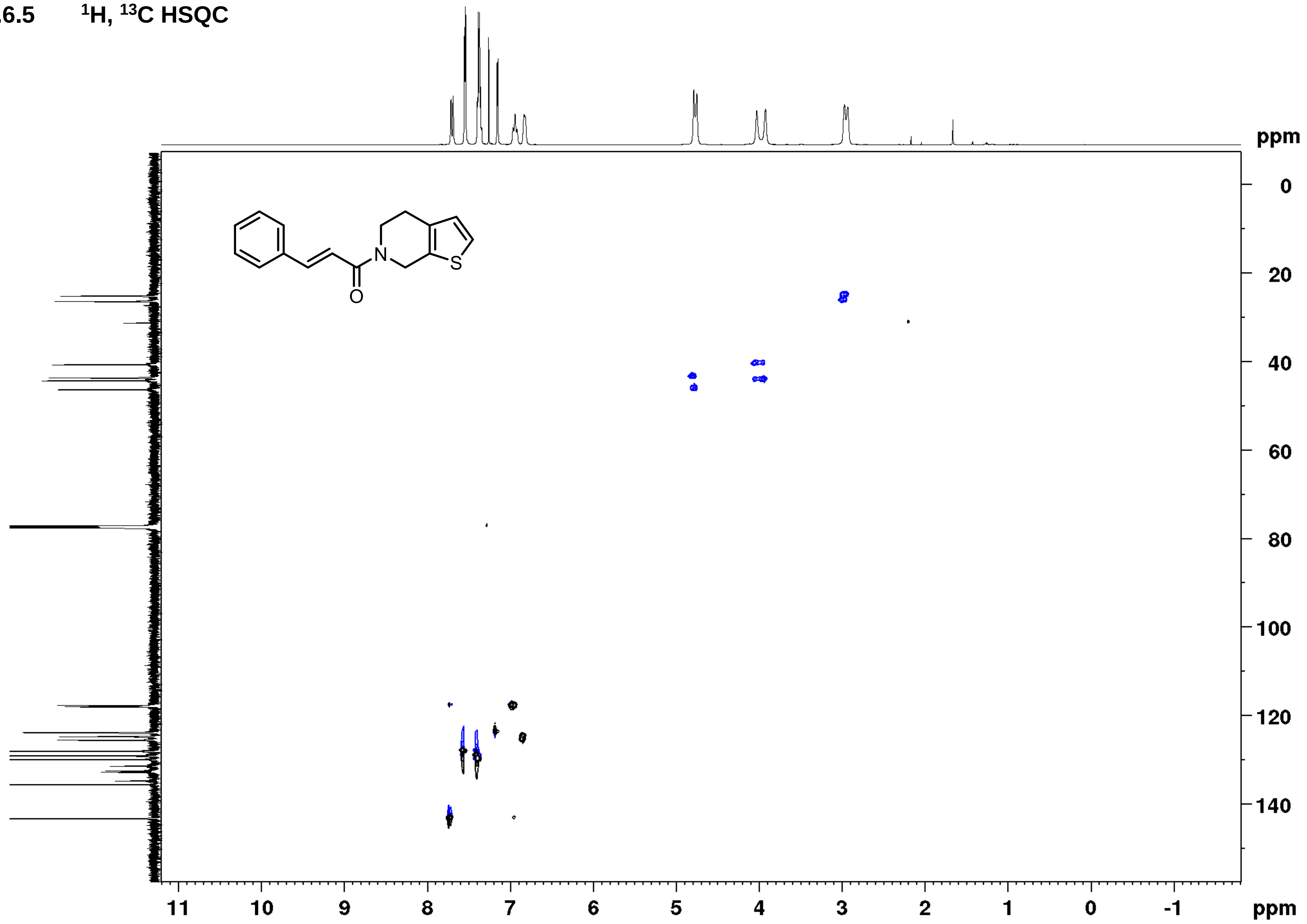

5.6.6 HRMS

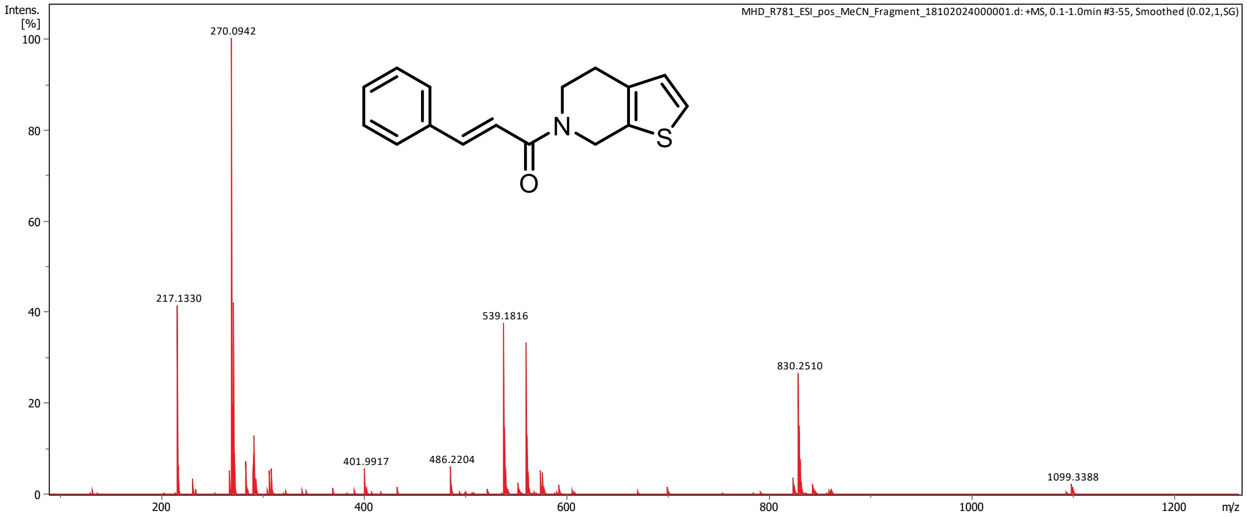
MHD_R781_ESI_pos_MeCN_Fragment_18102024000001.d: +MS, 0.1-1.0min #3-55, Smoothed (0.02,1,SG)
Intens. [%]
270.0942
217.1330
539.1816
830.2510
401.9917
486.2204
1099.3388
m/z

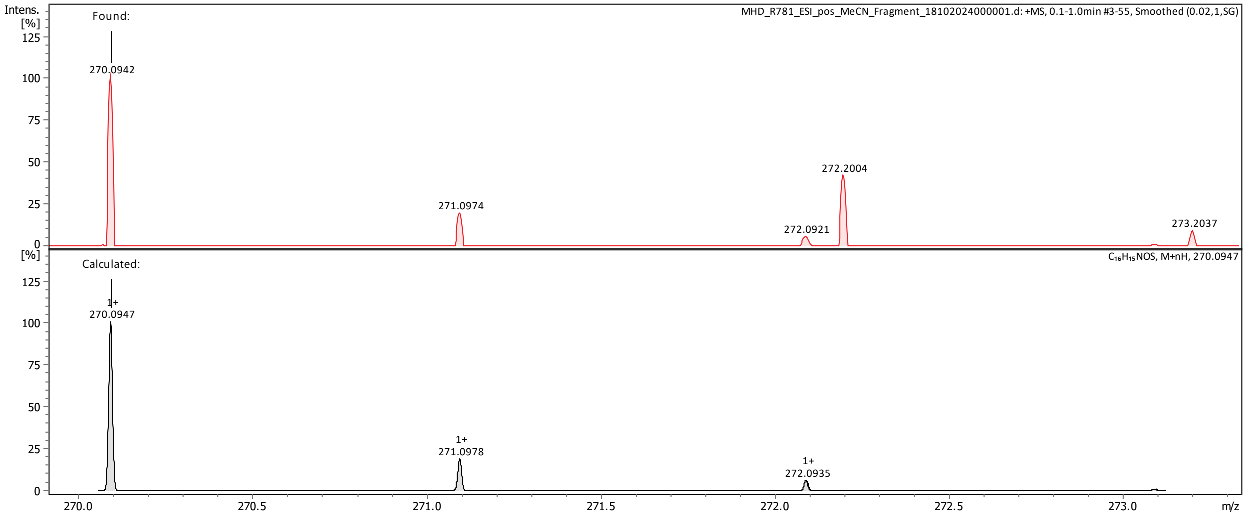
MHD_R781_ESI_pos_MeCN_Fragment_18102024000001.d: +MS, 0.1-1.0min #3-55, Smoothed (0.02,1,SG)
Found:
270.0942
271.0974
272.0921
272.2004
273.2037
Calculated:
C16H15NOS, M+nH, 270.0947
1+ 270.0947
1+ 271.0978
1+ 272.0935
m/z

5.6.7 IR

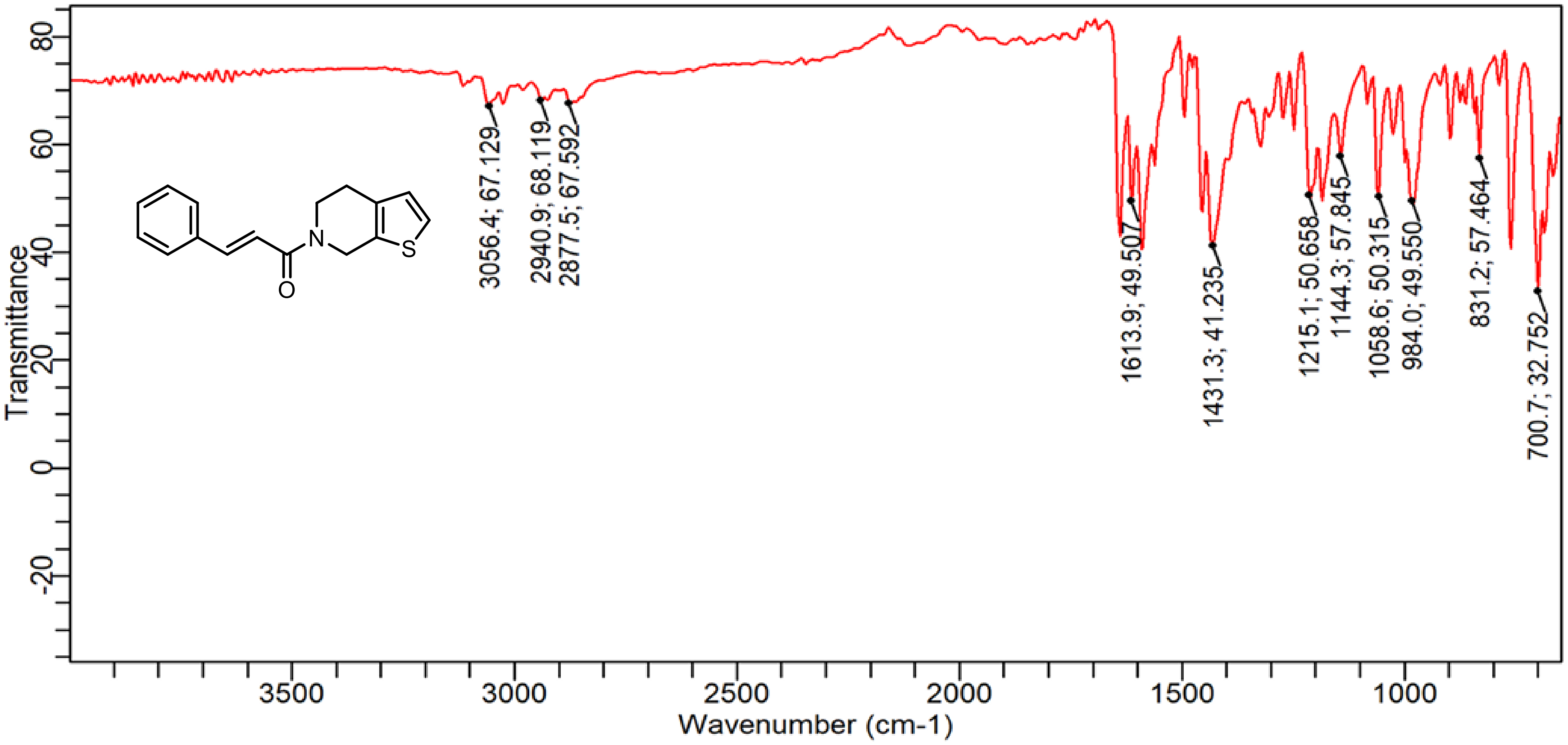

Transmittance
Wavenumber (cm-1)
3056.4; 67.129
2940.9; 68.119
2877.5; 67.592
1613.9; 49.507
1431.3; 41.235
1215.1; 50.658
1144.3; 57.845
1058.6; 50.315
984.0; 49.550
831.2; 57.464
700.7; 32.752
N
O
S

5.7 (*E*)-1-(4-(2,4-difluorobenzoyl)piperidin-1-yl)-3-phenylprop-2-en-1-one (11h) (600 MHz, $CDCl_3$)

5.7.1 ^{1}H NMR

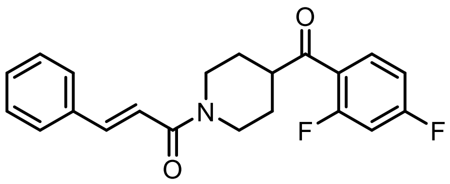

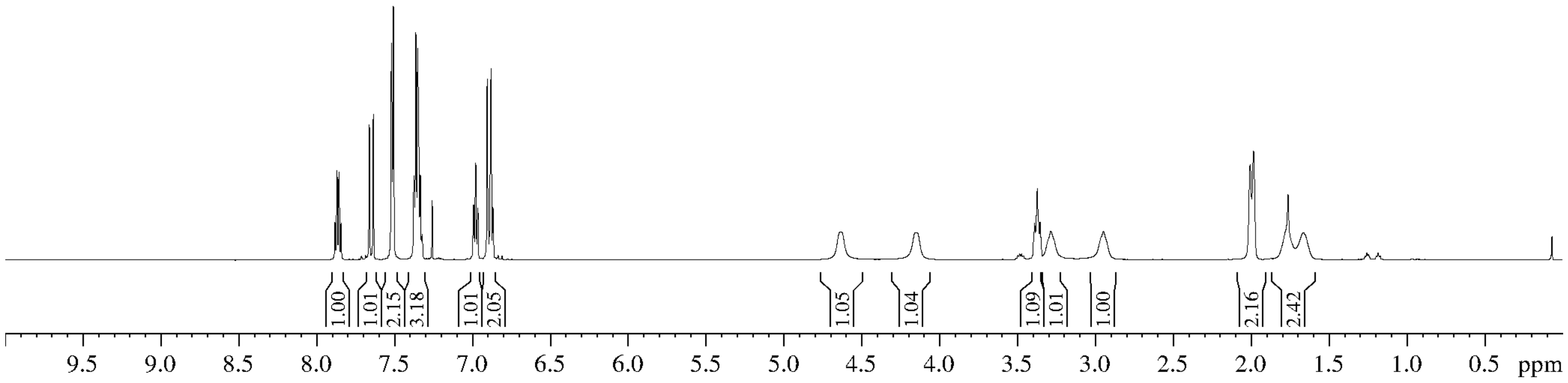

5.7.2 ^{13}C NMR

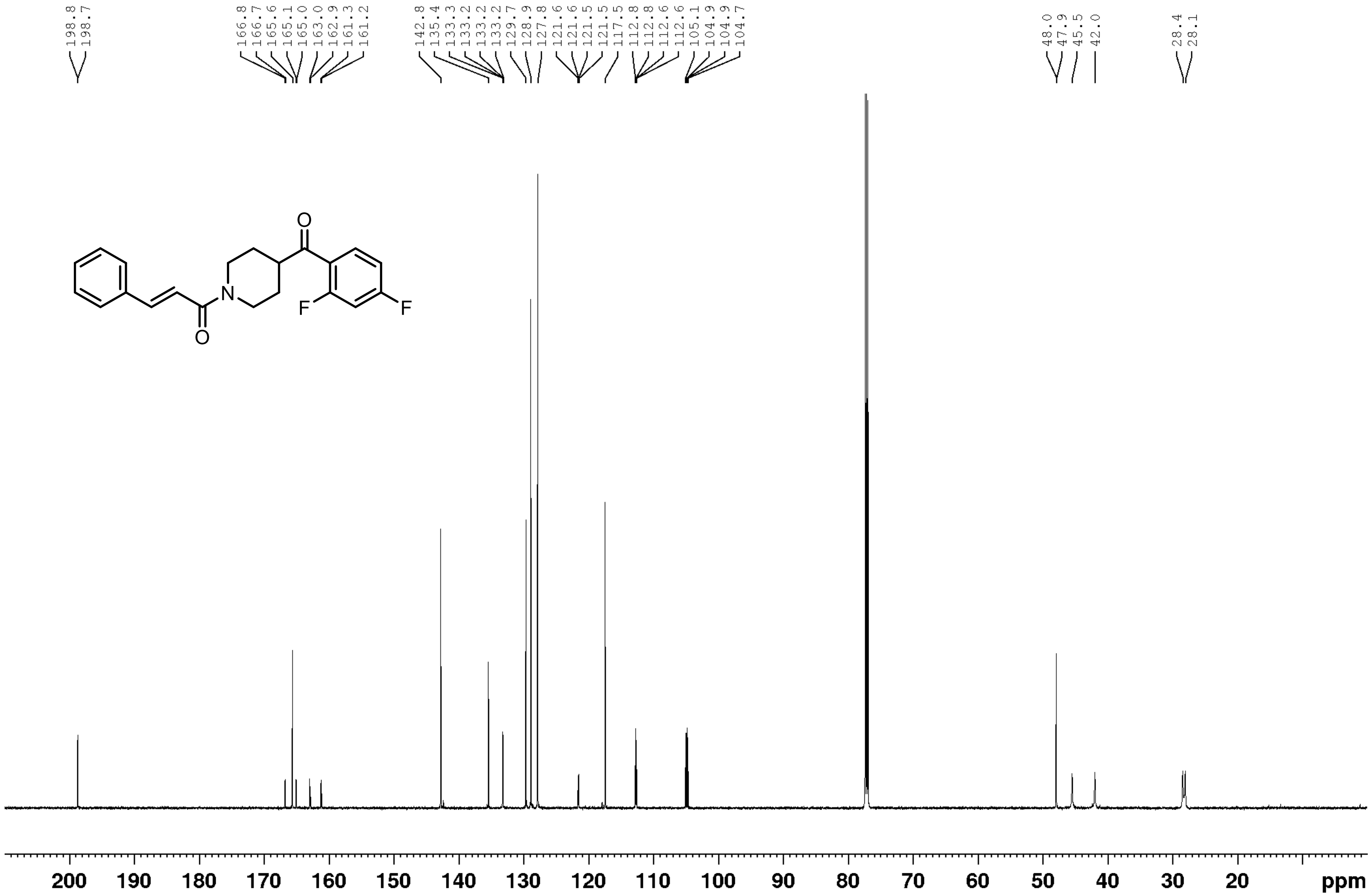
198.8
198.7
166.8
166.7
165.6
165.1
165.0
163.0
162.9
161.3
161.2
142.8
135.4
133.3
133.2
133.2
133.2
129.7
128.9
127.8
121.6
121.6
121.5
121.5
117.5
112.8
112.8
112.6
112.6
105.1
104.9
104.9
104.7
48.0
47.9
45.5
42.0
28.4
28.1
O
N
O
F
F
200
190
180
170
160
150
140
130
120
110
100
90
80
70
60
50
40
30
20
ppm

5.7.3 ^{19}F NMR

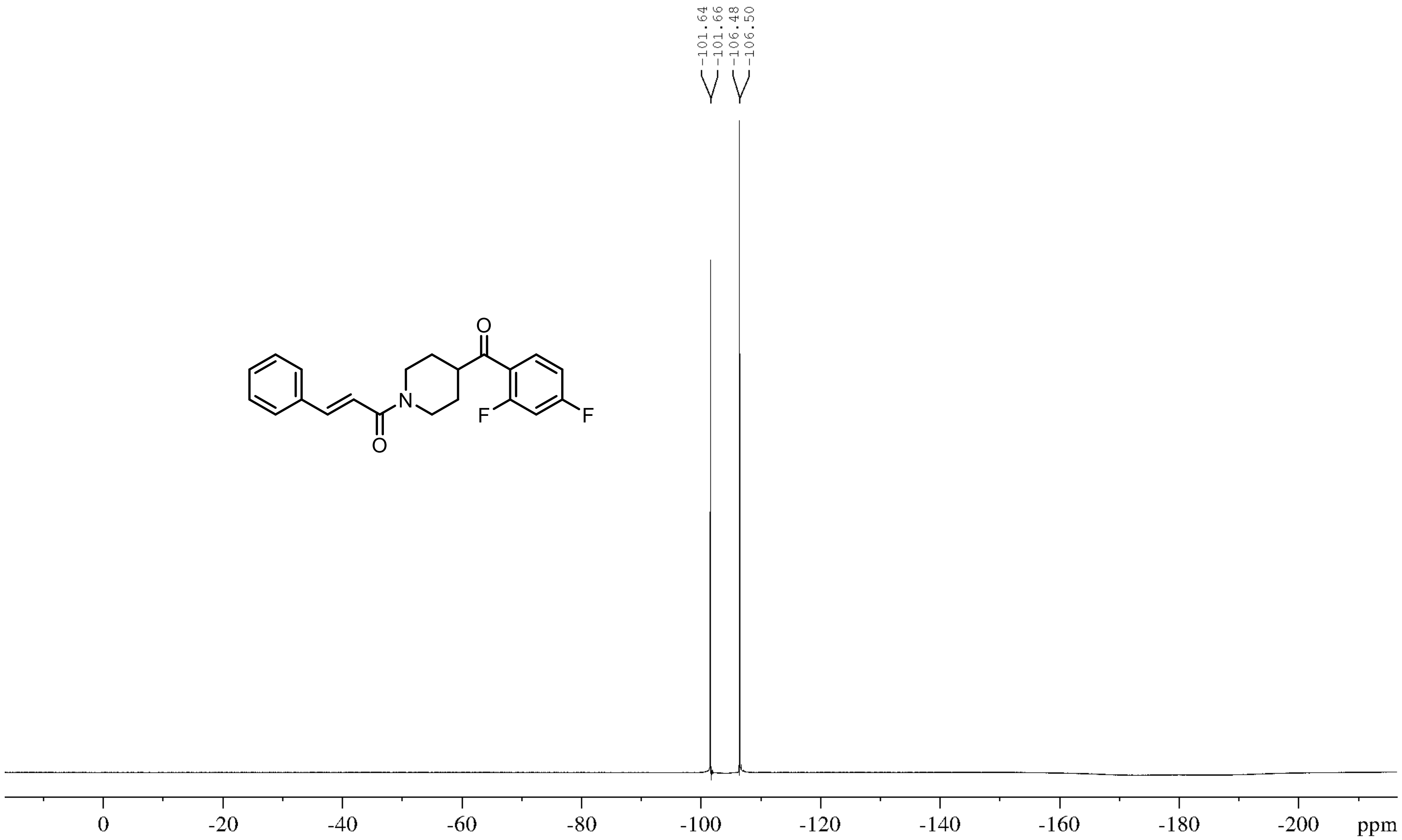

-101.64
-101.66
-106.48
-106.50
0
-20
-40
-60
-80
-100
-120
-140
-160
-180
-200
ppm

5.7.4 ^{1}H, ^{1}H COSY

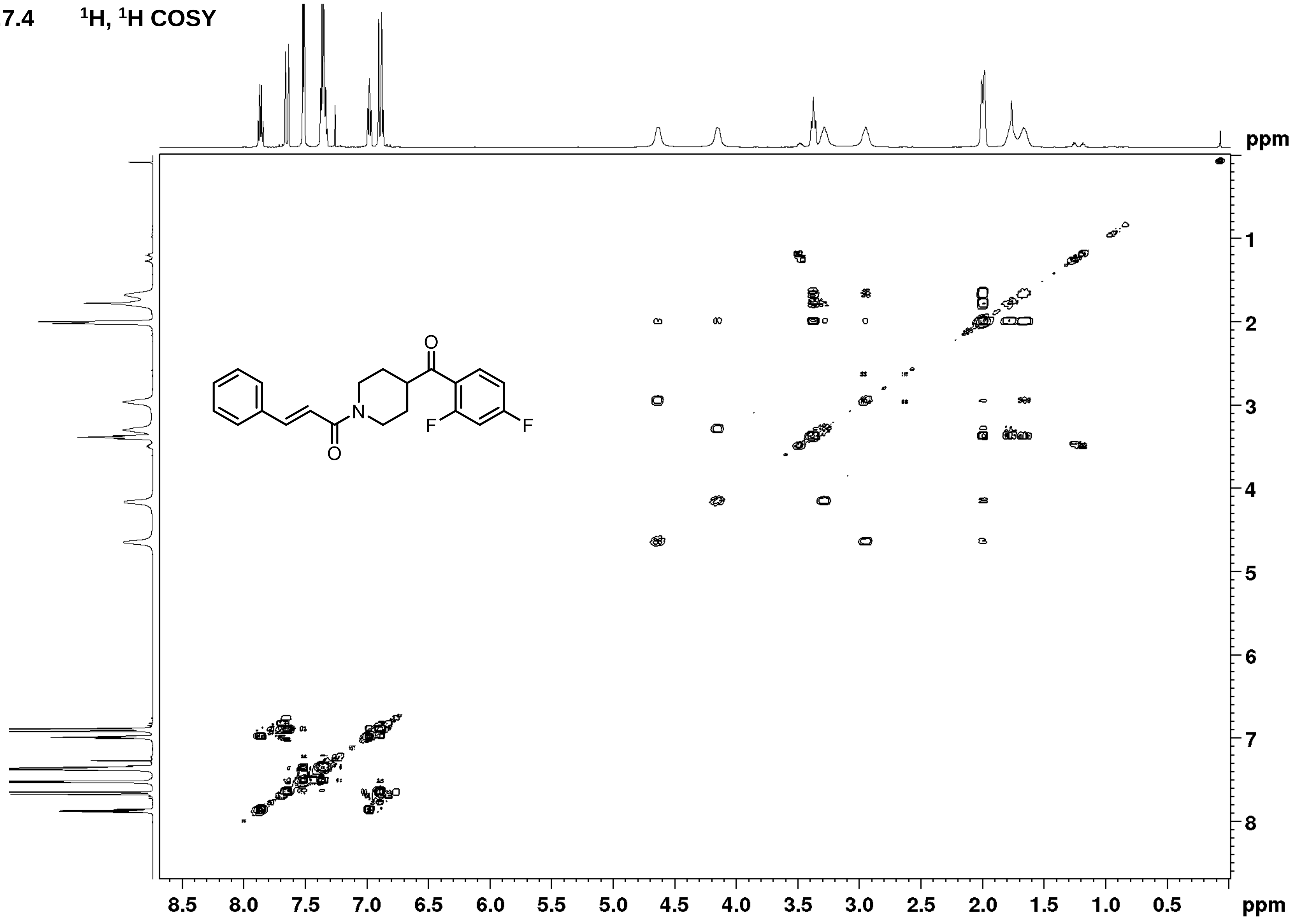

5.7.5 ^{1}H, ^{13}C HMBC

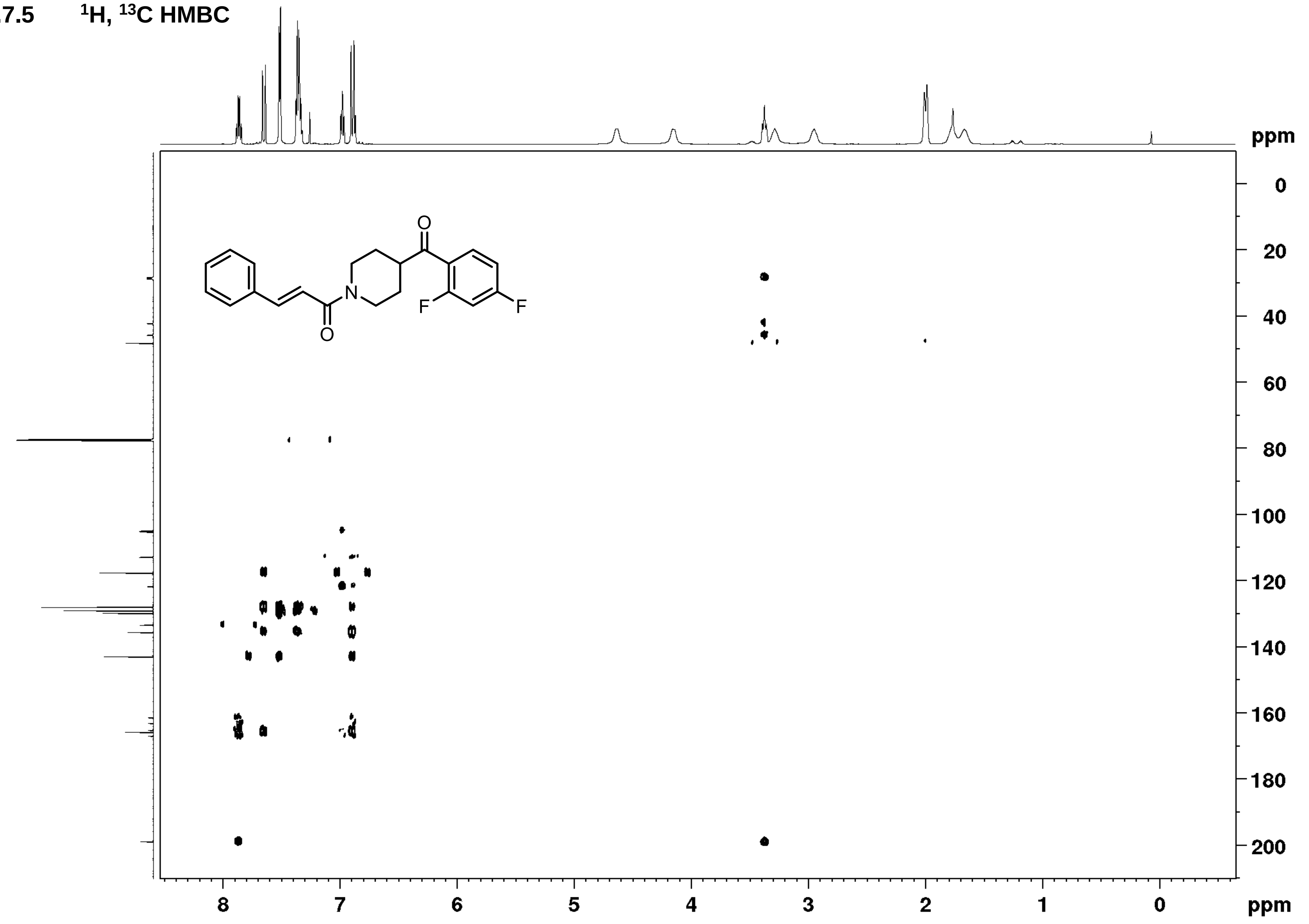

5.7.6 ^{1}H, ^{13}C HSQC

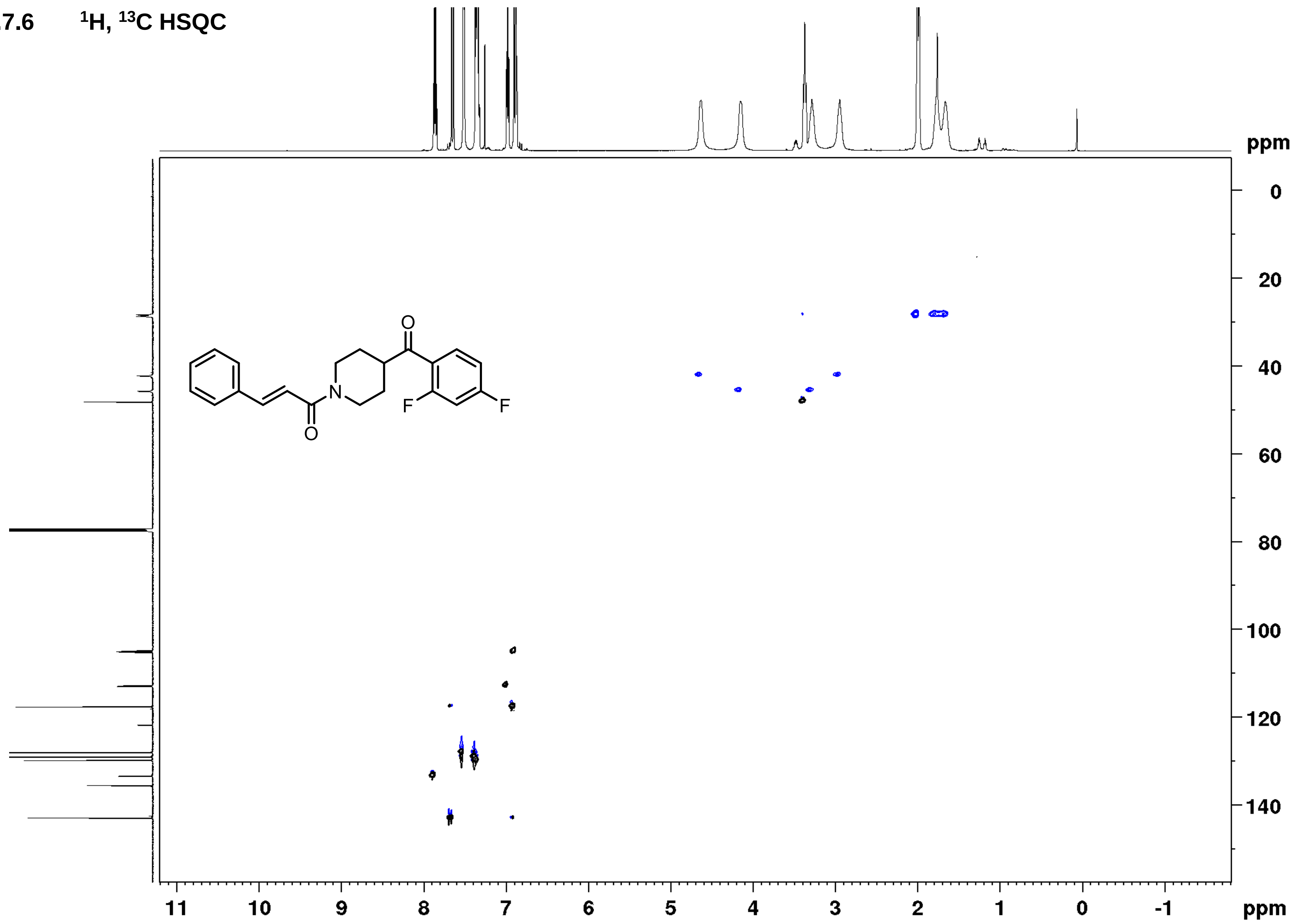

5.7.7 HRMS

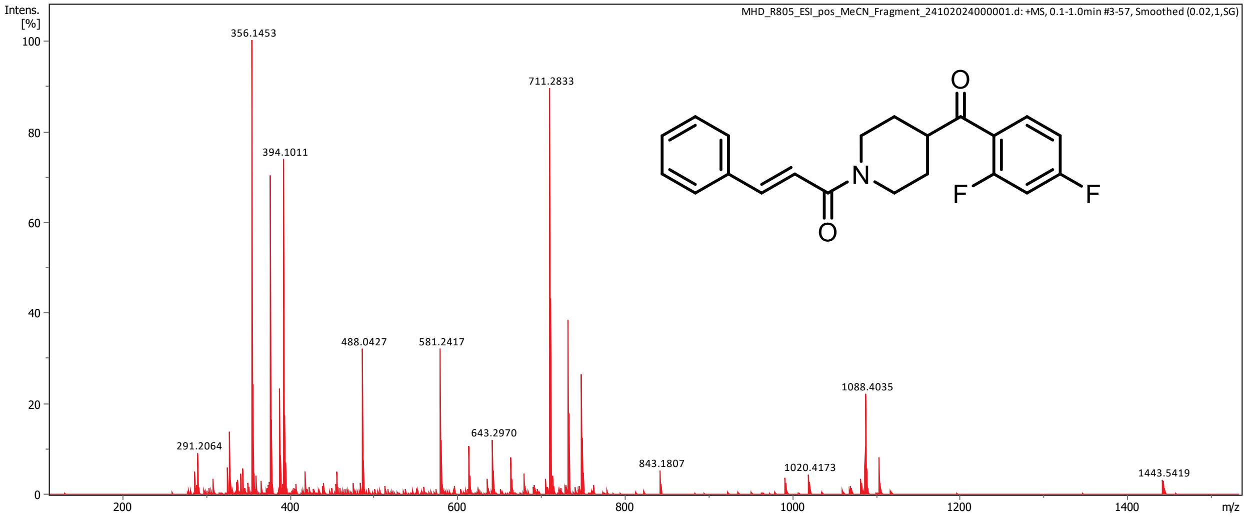

Intens. [%]
MHD_R805_ESI_pos_MeCN_Fragment_24102024000001.d: +MS, 0.1-1.0min #3-57, Smoothed (0.02,1,SG)
356.1453
711.2833
394.1011
488.0427
581.2417
643.2970
291.2064
843.1807
1020.4173
1088.4035
1443.5419
O
N
O
F
F
m/z

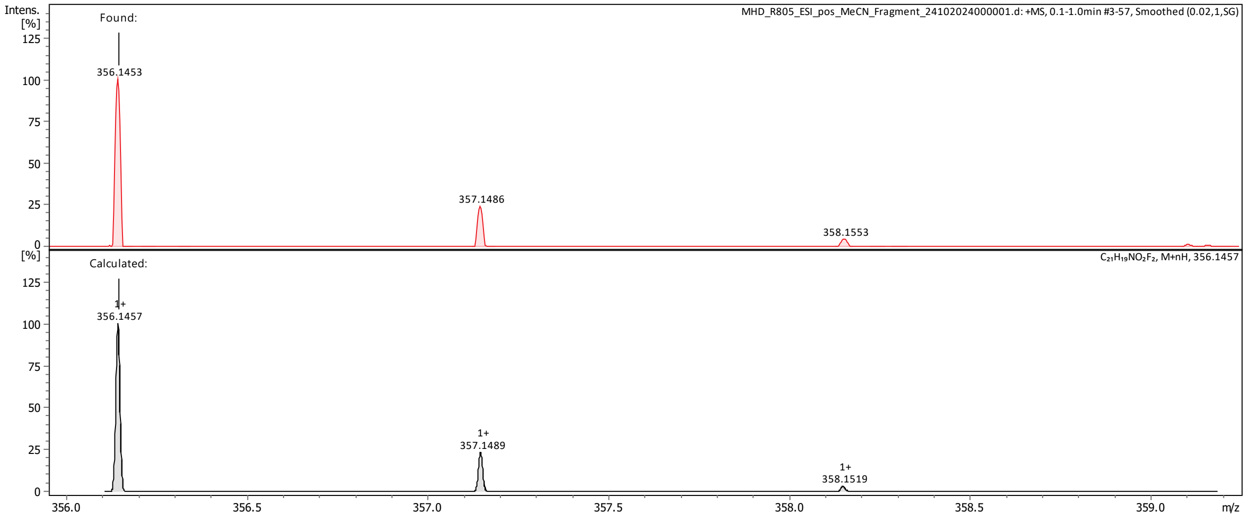

Intens. [%]
Found:
MHD_R805_ESI_pos_MeCN_Fragment_24102024000001.d: +MS, 0.1-1.0min #3-57, Smoothed (0.02,1,SG)
356.1453
357.1486
358.1553
Calculated:
$C_{21}H_{19}NO_2F_2$, M+nH, 356.1457
1+
356.1457
1+
357.1489
1+
358.1519
m/z

5.7.8 IR

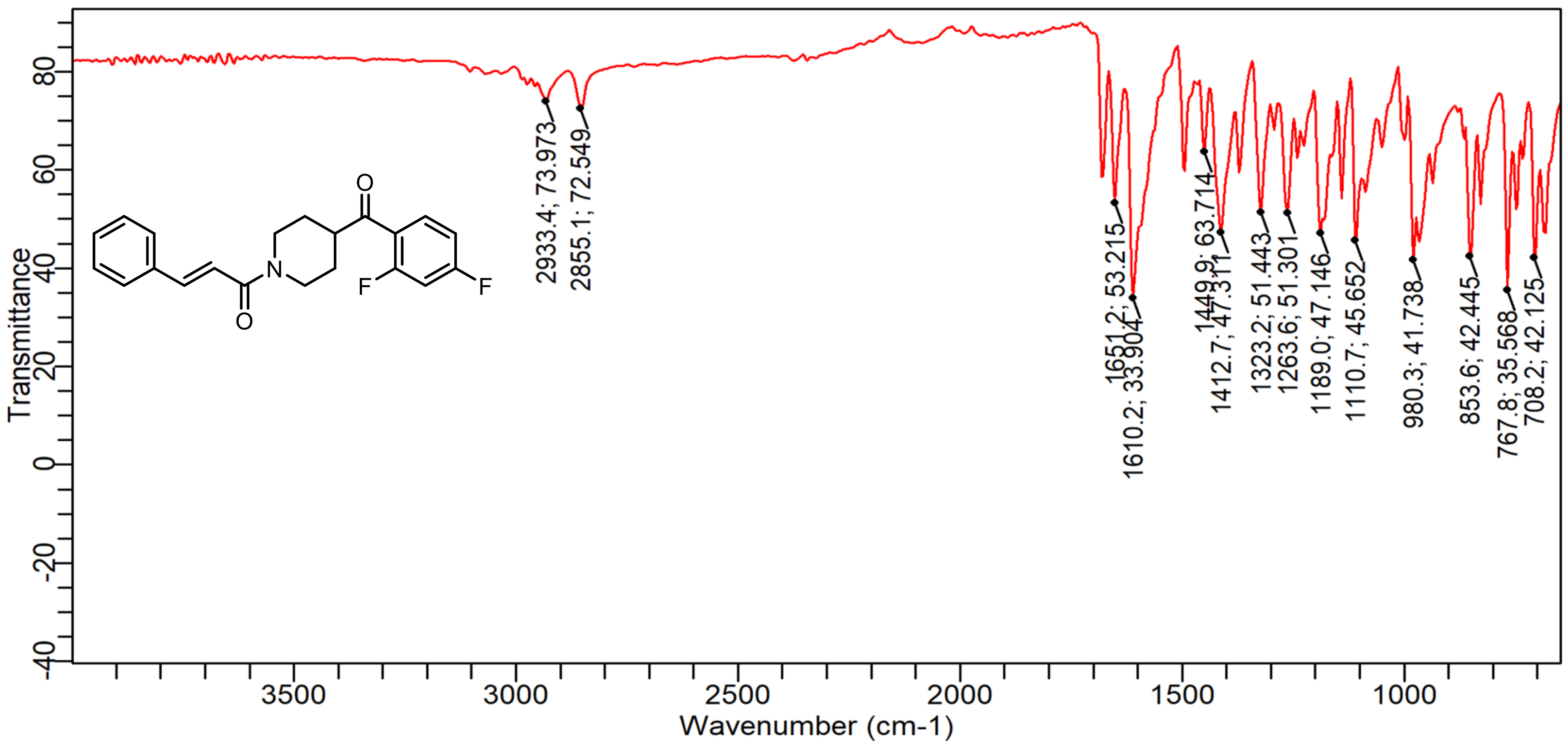

Transmittance
Wavenumber (cm-1)
2933.4; 73.973
2855.1; 72.549
1651.2; 53.215
1610.2; 33.904
1449.9; 63.714
1412.7; 47.311
1323.2; 51.443
1263.6; 51.301
1189.0; 47.146
1110.7; 45.652
980.3; 41.738
853.6; 42.445
767.8; 35.568
708.2; 42.125

5.8 (*E*)-1-(4-(-(2,4-difluorophenyl)(methoxyimino)methyl)piperidin-1-yl)-3-phenylprop-2-en-1-one (11i) (600 MHz, $CDCl_3$)

5.8.1 ^{1}H NMR

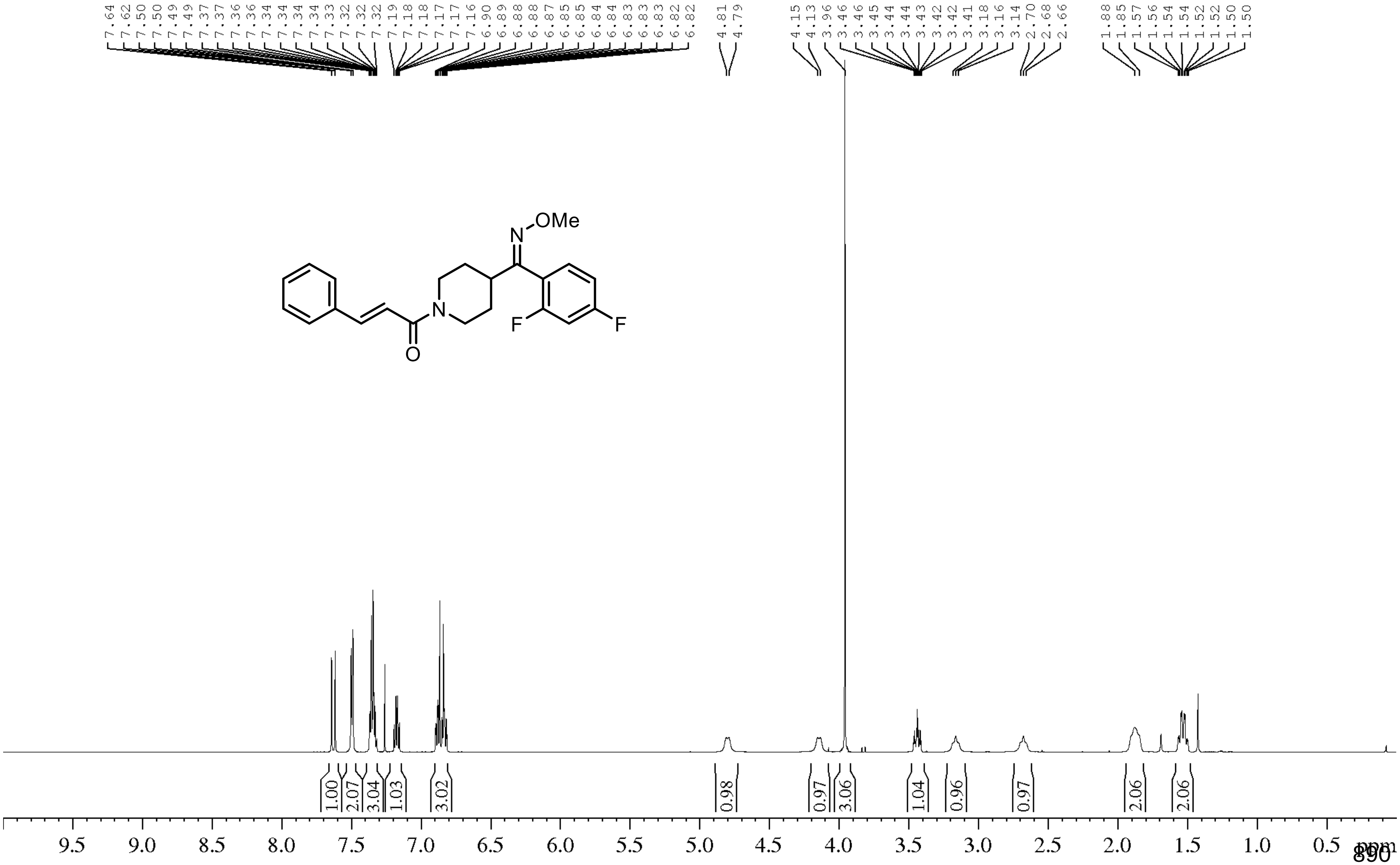

5.8.2 ^{13}C NMR

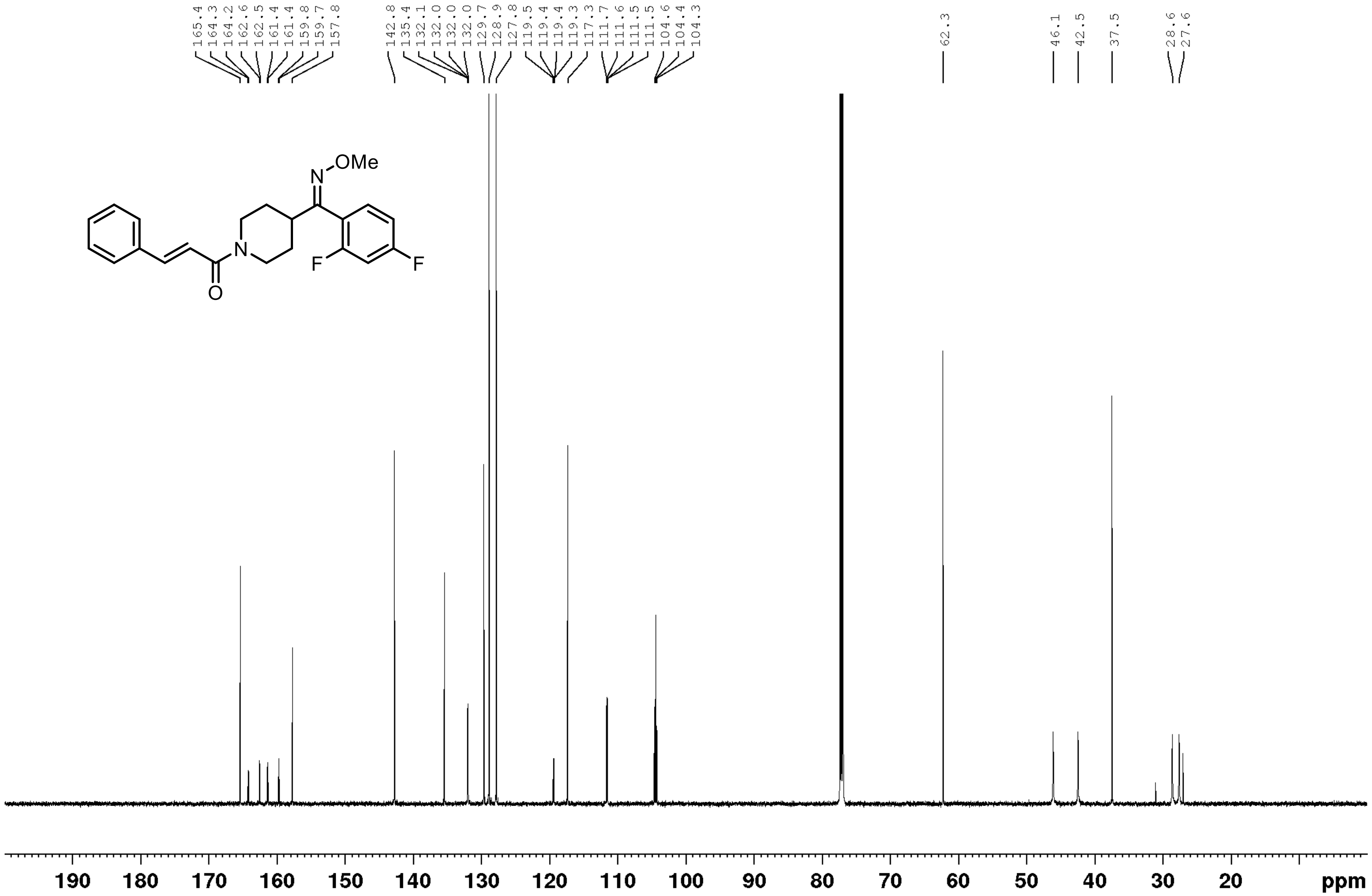
OMe
N
N
O
F
F
165.4
164.3
164.2
162.6
162.5
161.4
161.4
159.8
159.7
157.8
142.8
135.4
132.1
132.0
132.0
132.0
129.7
128.9
127.8
119.5
119.4
119.4
119.3
117.3
111.7
111.6
111.5
111.5
104.6
104.4
104.3
62.3
46.1
42.5
37.5
28.6
27.6
190
180
170
160
150
140
130
120
110
100
90
80
70
60
50
40
30
20
ppm

5.8.3 ¹⁹F NMR

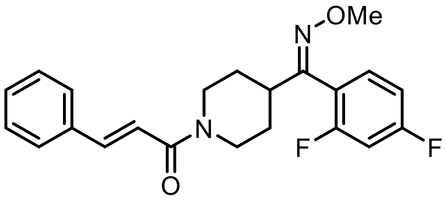

OMe
N
N
O
F
F

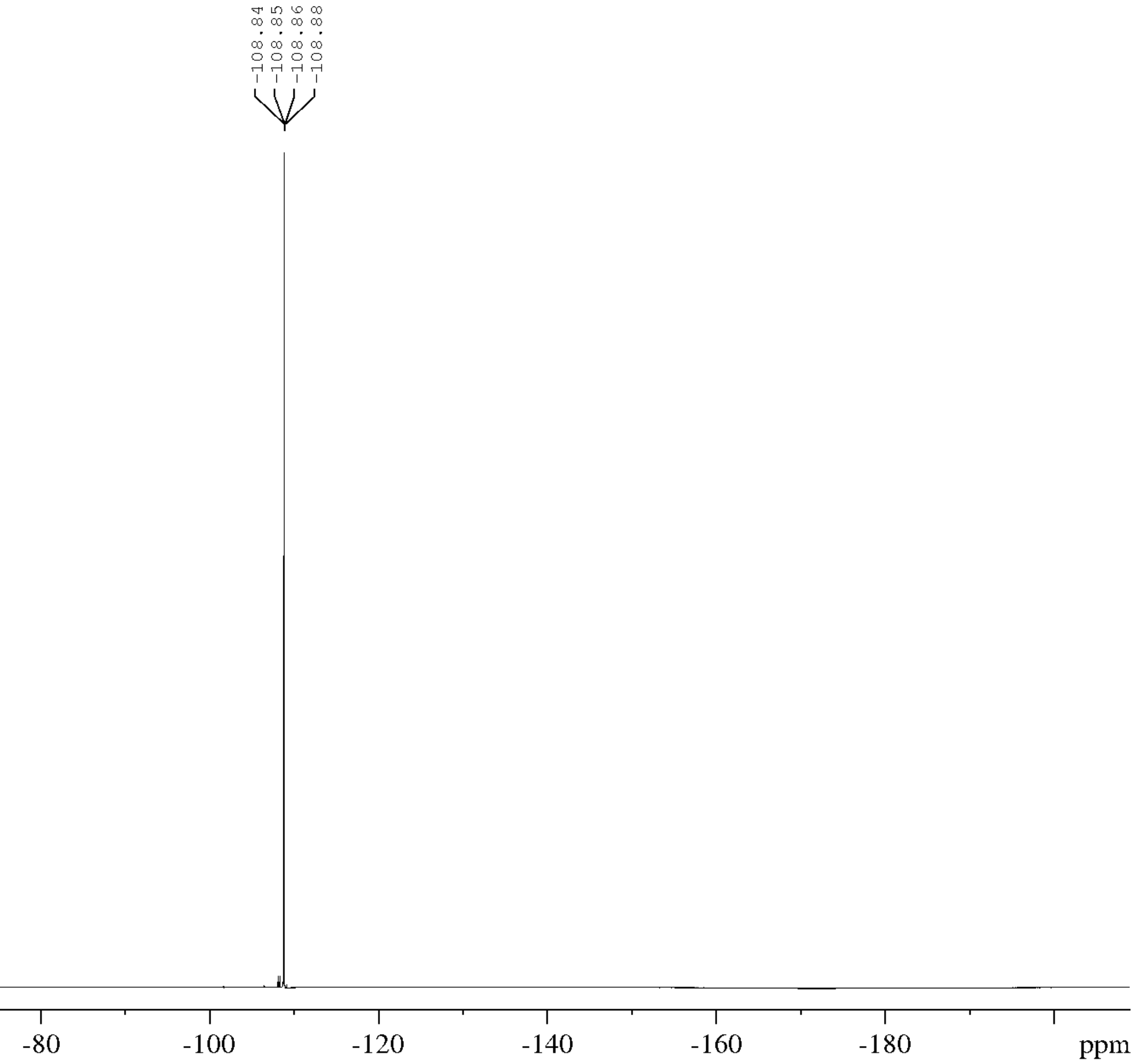

-108.84
-108.85
-108.86
-108.88
-20
-40
-60
-80
-100
-120
-140
-160
-180
ppm

5.8.4 ^{1}H, ^{1}H COSY

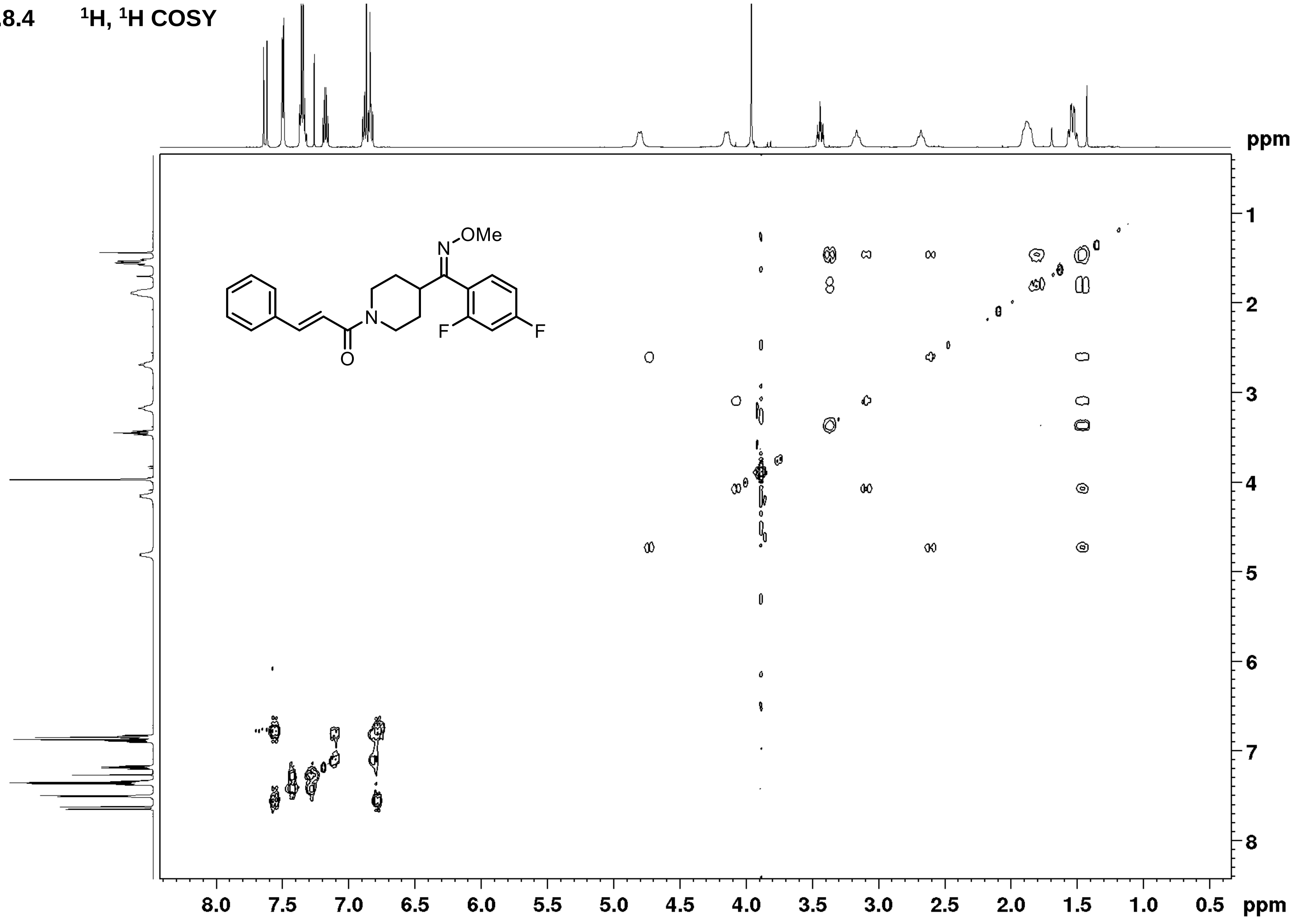

5.8.5 ^{1}H, ^{13}C HMBC

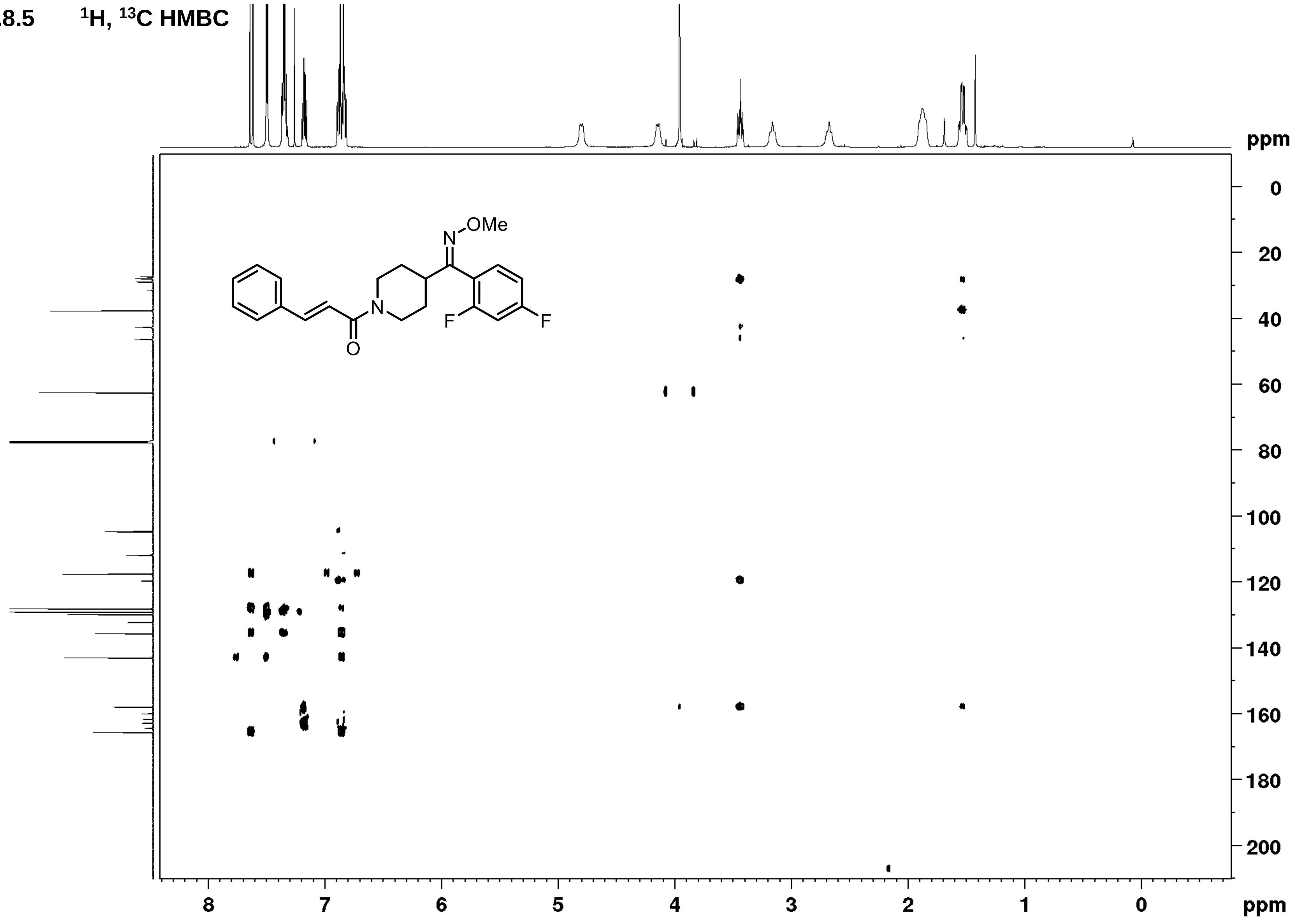

5.8.6 ^{1}H, ^{13}C HSQC

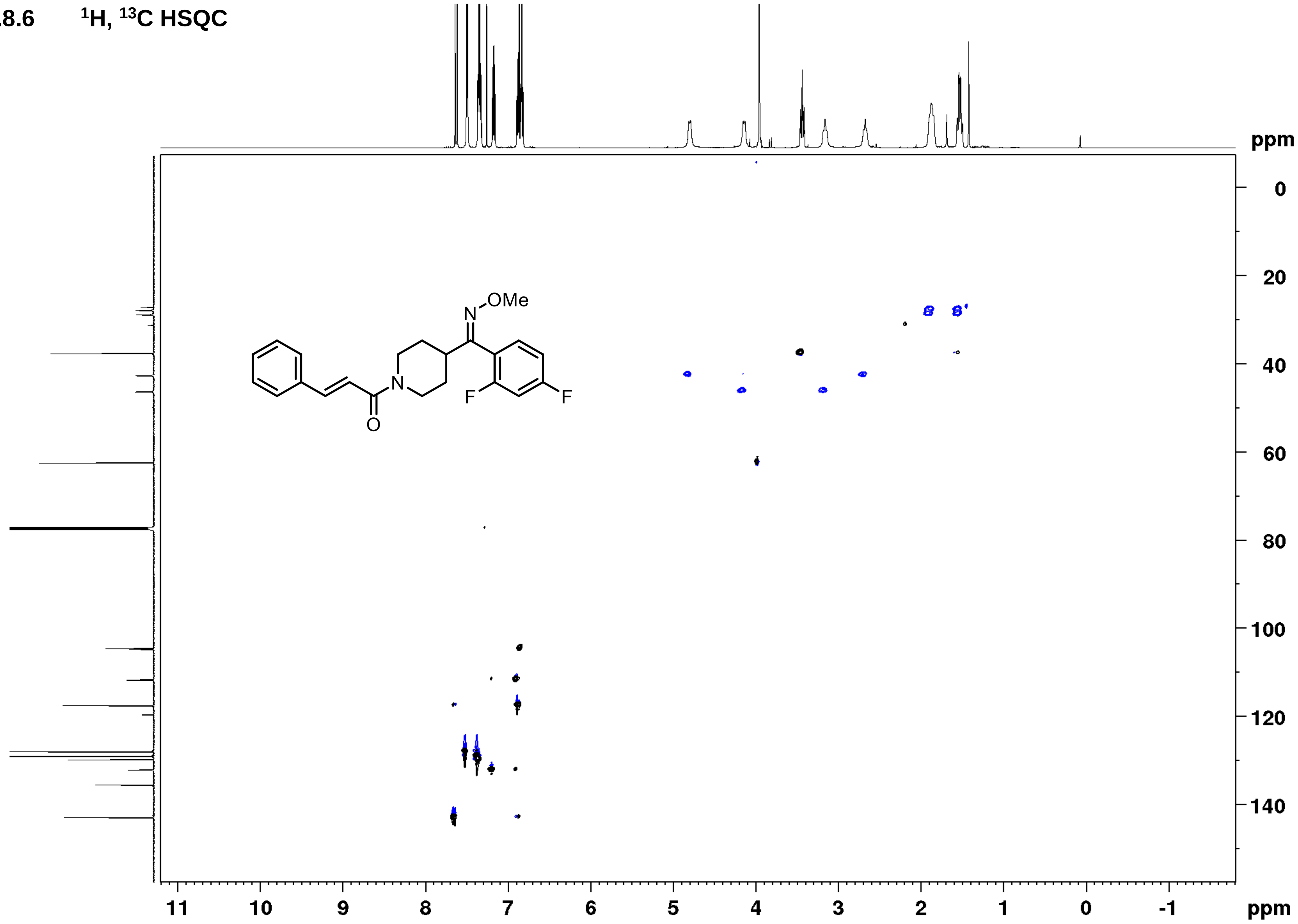

5.8.7 HRMS

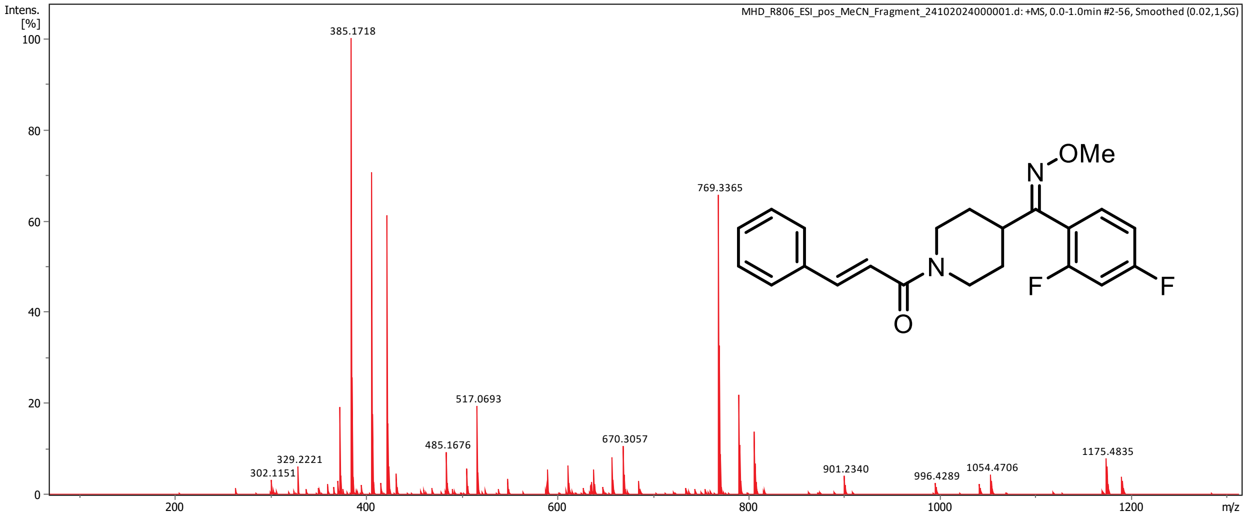

Intens. [%]
MHD_R806_ESI_pos_MeCN_Fragment_24102024000001.d: +MS, 0.0-1.0min #2-56, Smoothed (0.02,1,SG)
385.1718
769.3365
517.0693
485.1676
670.3057
329.2221
302.1151
901.2340
996.4289
1054.4706
1175.4835
m/z
N
OMe
N
O
F
F

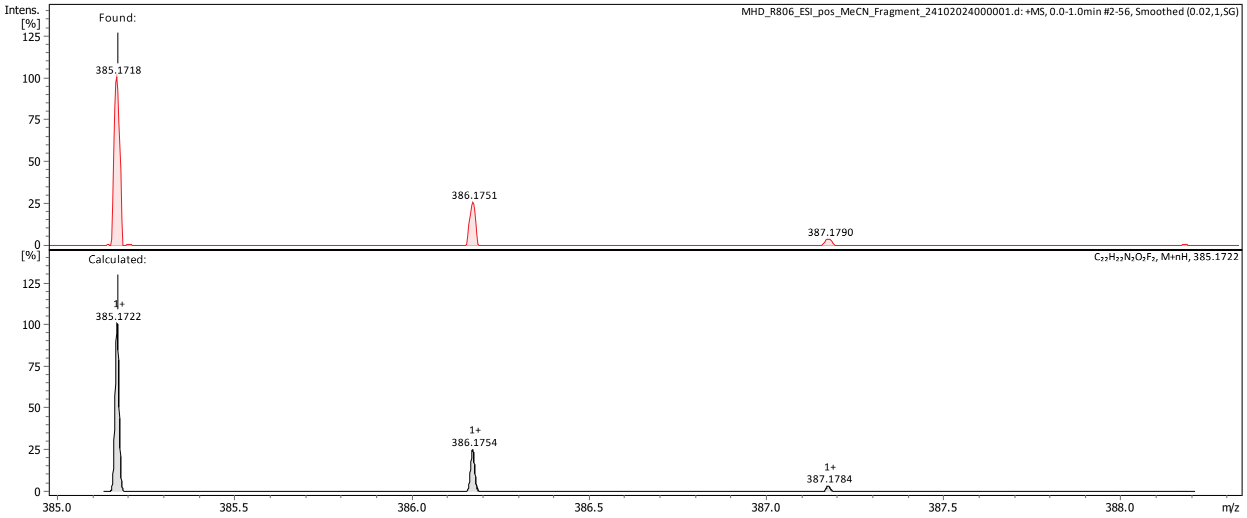

Intens. [%]
Found:
MHD_R806_ESI_pos_MeCN_Fragment_24102024000001.d: +MS, 0.0-1.0min #2-56, Smoothed (0.02,1,SG)
385.1718
386.1751
387.1790
Calculated:
$C_{22}H_{22}N_2O_2F_2$, M+nH, 385.1722
1+
385.1722
1+
386.1754
1+
387.1784
m/z

5.8.8 IR

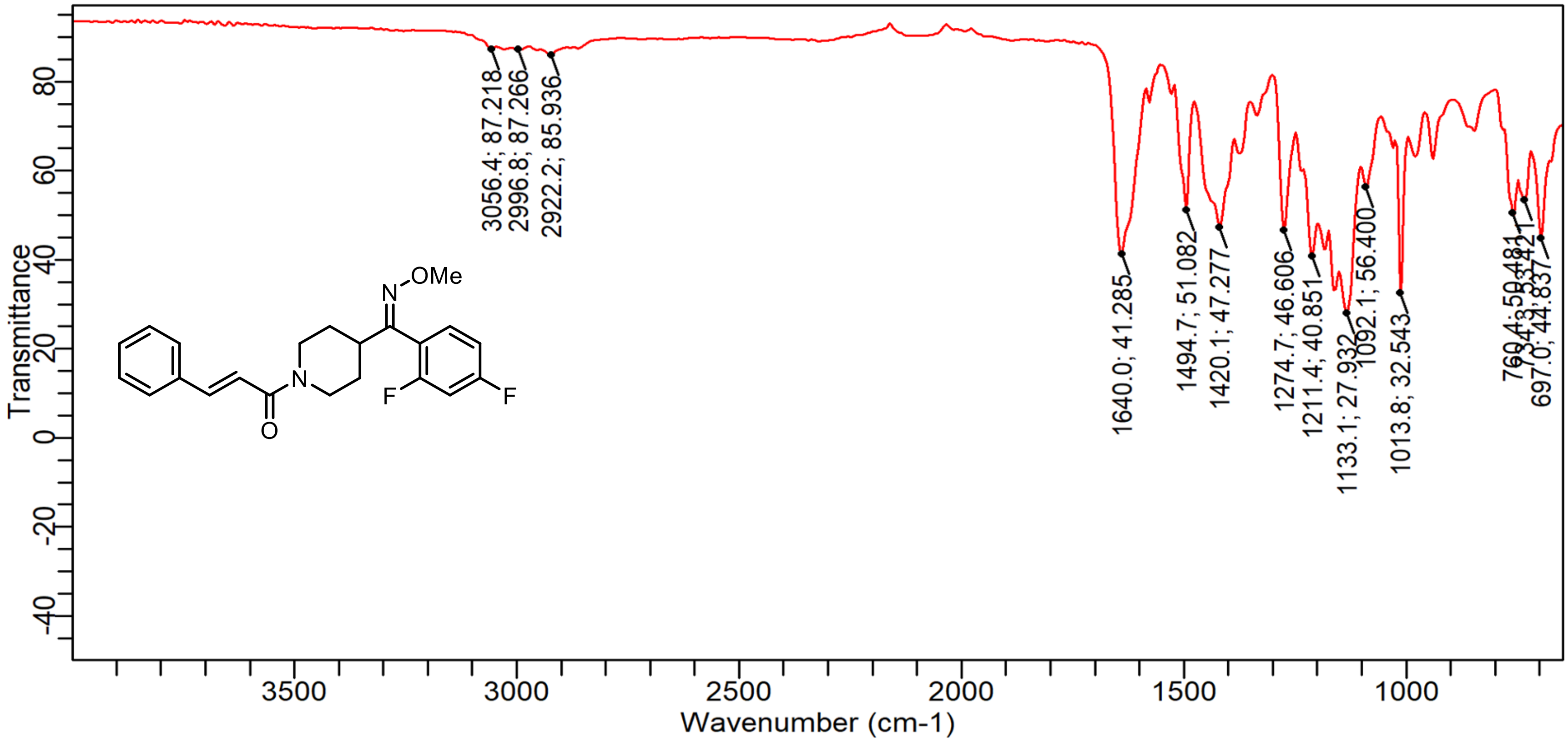

Transmittance
Wavenumber (cm-1)
3056.4; 87.218
2996.8; 87.266
2922.2; 85.936
1640.0; 41.285
1494.7; 51.082
1420.1; 47.277
1274.7; 46.606
1211.4; 40.851
1133.1; 27.932
1092.1; 56.400
1013.8; 32.543
697.0; 44.837
N
OMe
N
O
F
F

5.9 4-Cinnamoylpiperazine-1-carbaldehyde (11j) (600 MHz, $CDCl_3$)

5.9.1 ^1H NMR

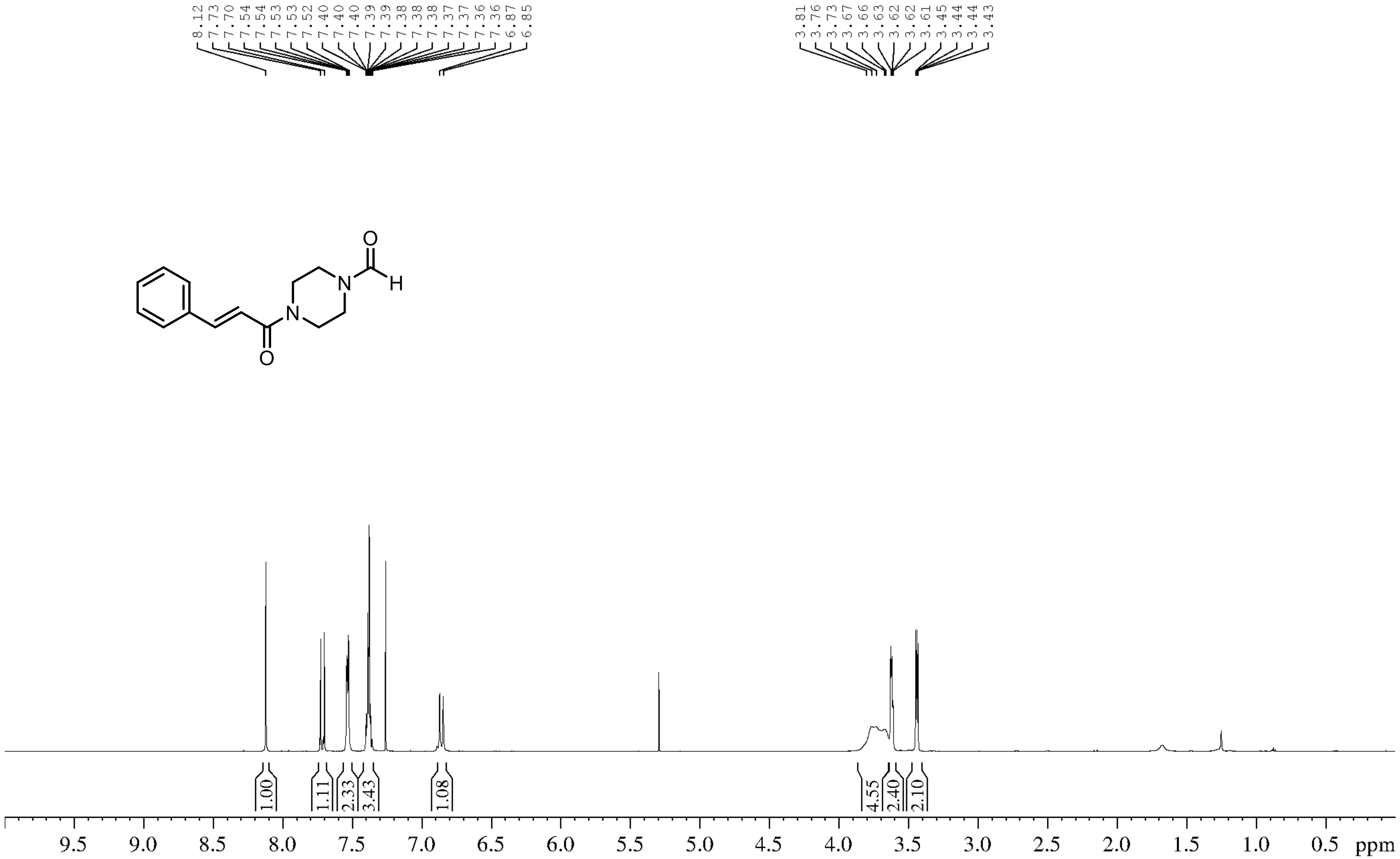

5.9.2 ^{13}C NMR

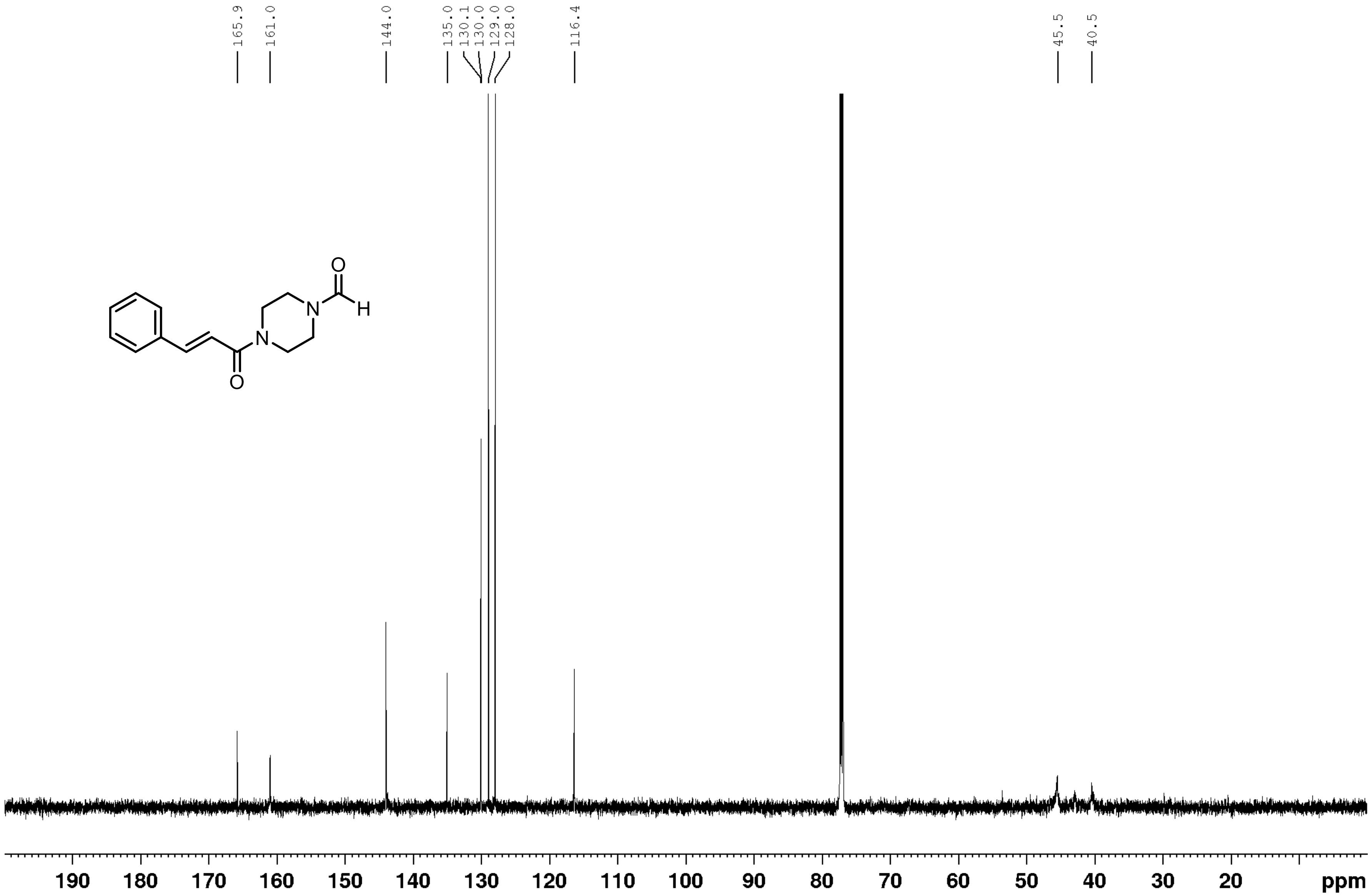

165.9
161.0
144.0
135.0
130.1
130.0
129.0
128.0
116.4
45.5
40.5
O
N
H
N
O
190
180
170
160
150
140
130
120
110
100
90
80
70
60
50
40
30
20
ppm

5.9.3 ¹H, ¹H COSY

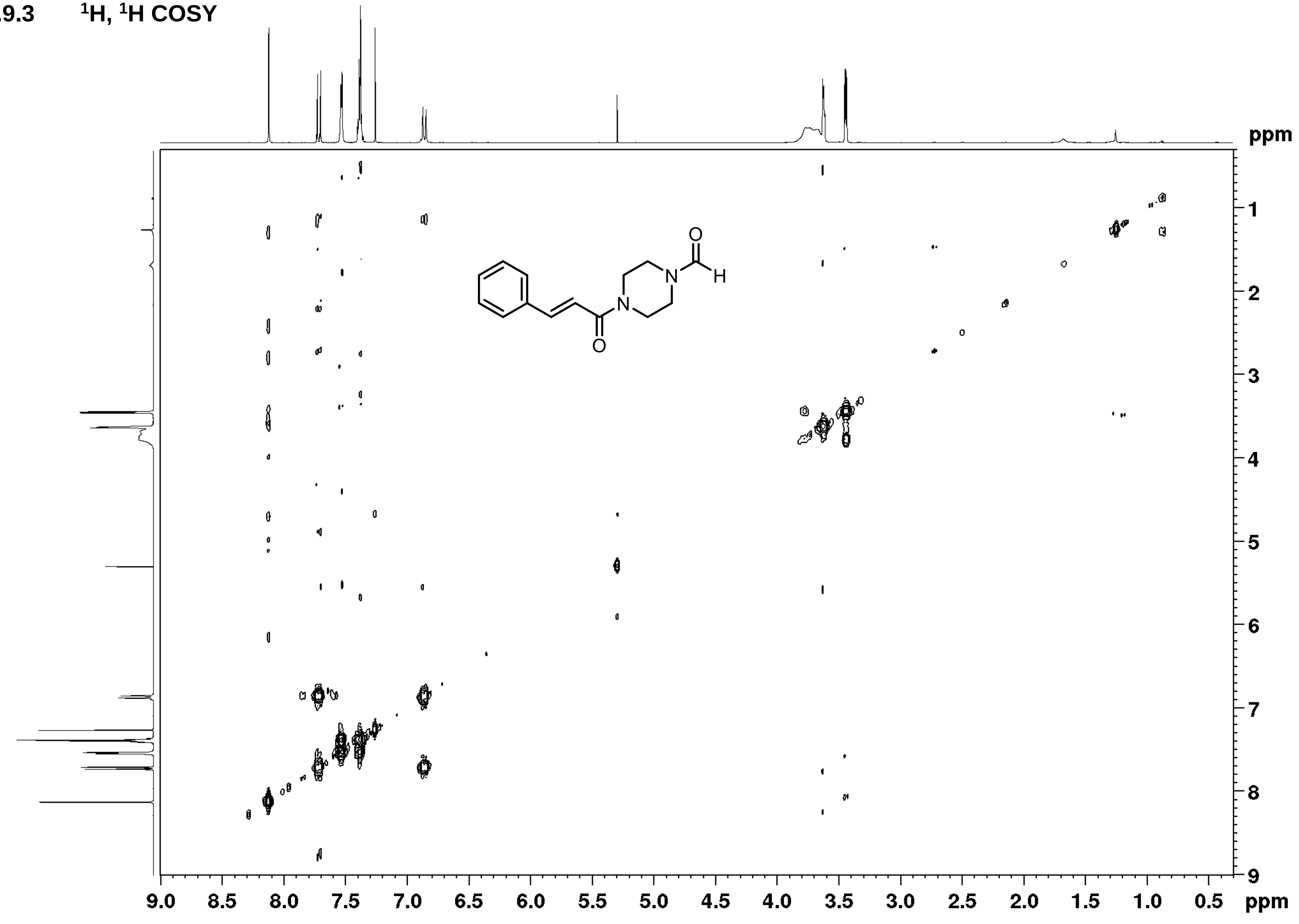

5.9.4 ^{1}H, ^{13}C HMBC

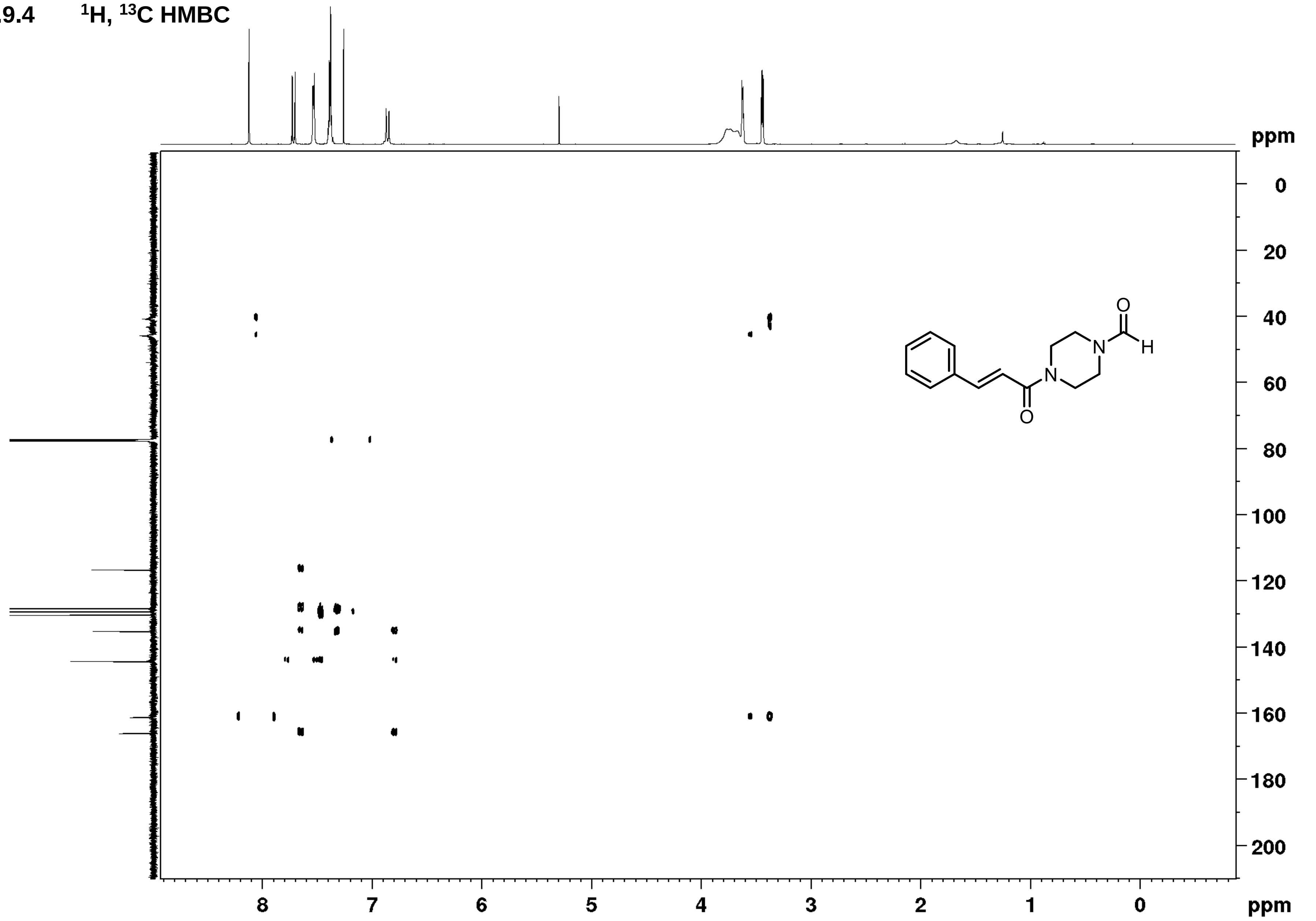

5.9.5 ^{1}H, ^{13}C HSQC

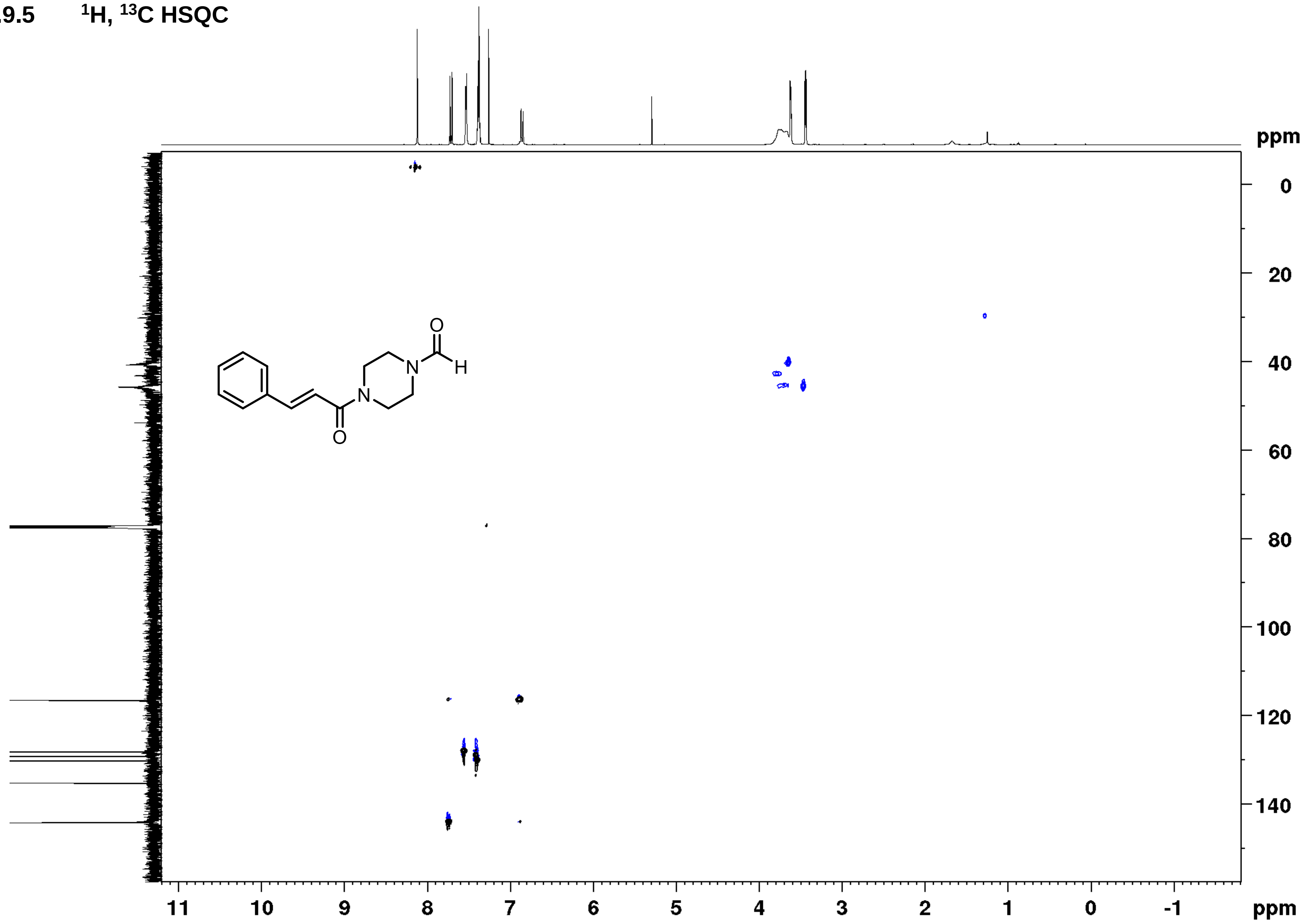

5.9.6 HRMS

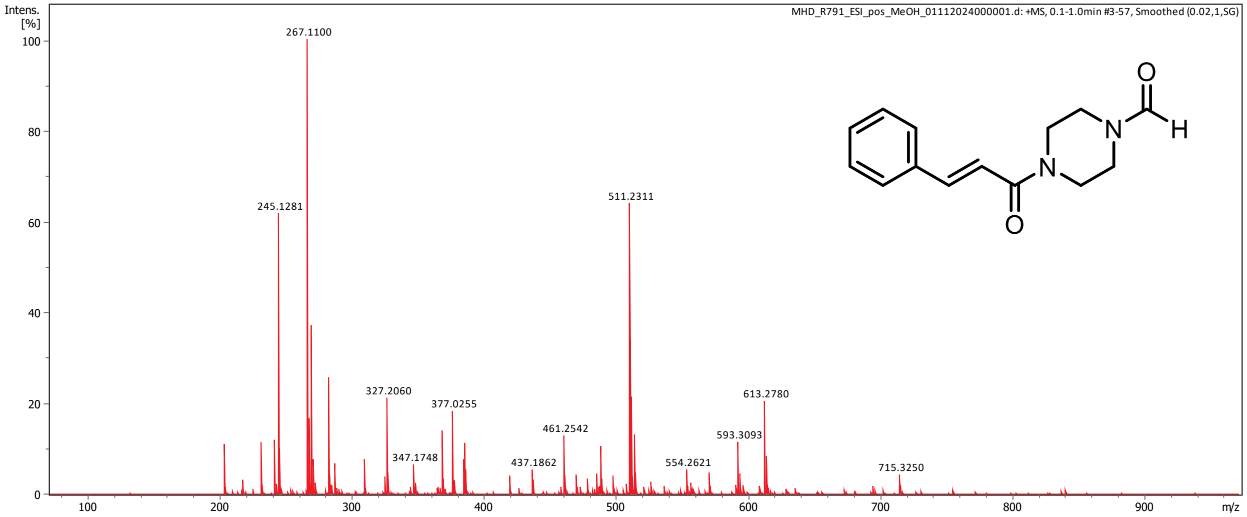

MHD_R791_ESI_pos_MeOH_01112024000001.d: +MS, 0.1-1.0min #3-57, Smoothed (0.02,1,SG)
Intens. [%]
267.1100
245.1281
511.2311
327.2060
377.0255
347.1748
461.2542
437.1862
613.2780
593.3093
554.2621
715.3250
m/z

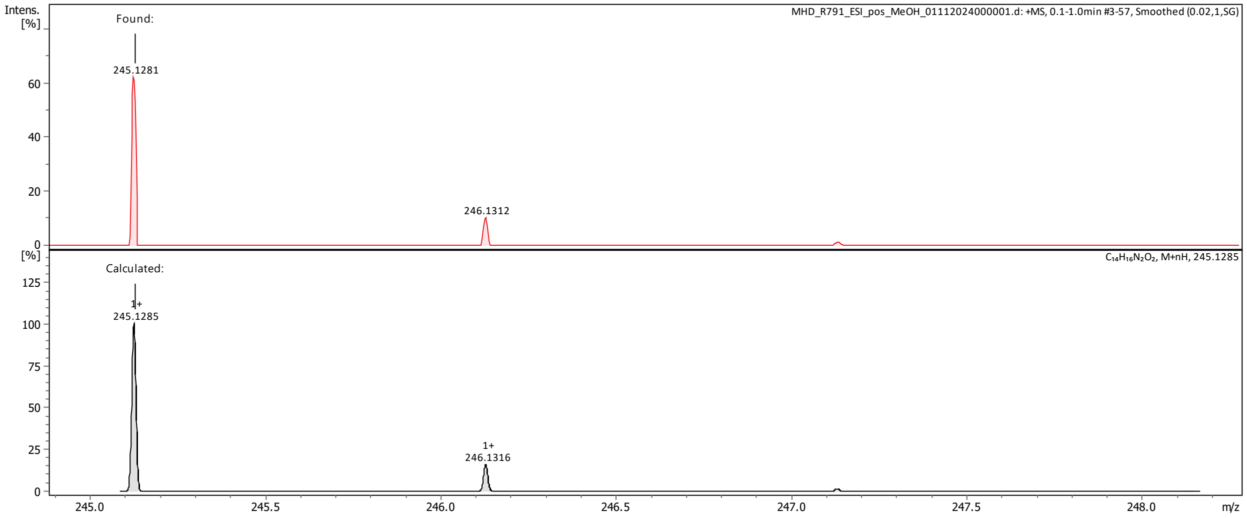

MHD_R791_ESI_pos_MeOH_01112024000001.d: +MS, 0.1-1.0min #3-57, Smoothed (0.02,1,SG)
Intens. [%]
Found:
245.1281
246.1312
C14H16N2O2, M+nH, 245.1285
Calculated:
1+
245.1285
1+
246.1316
m/z

5.9.7 IR

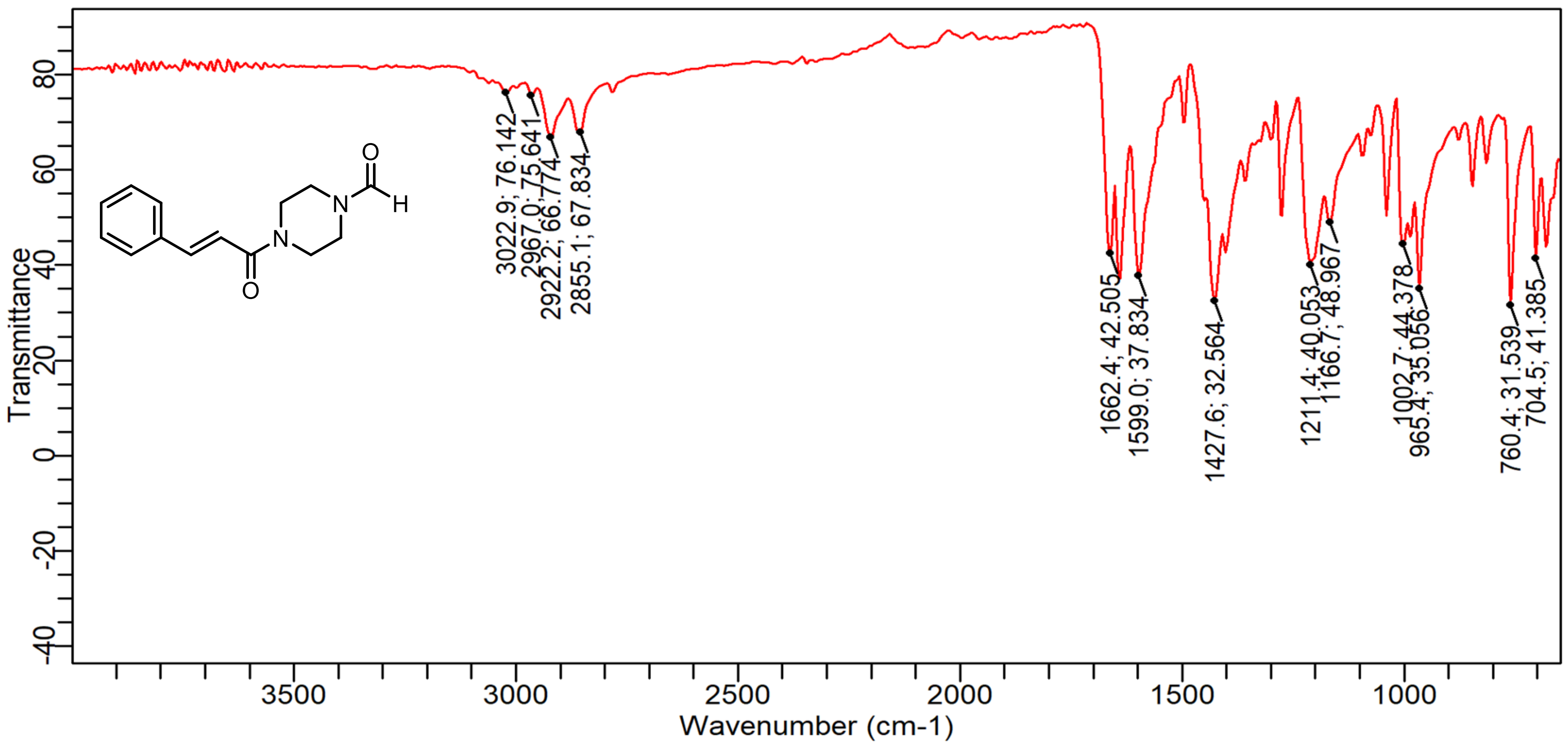

3022.9; 76.142
2967.0; 75.641
2922.2; 66.774
2855.1; 67.834
1662.4; 42.505
1599.0; 37.834
1427.6; 32.564
1211.4; 40.053
1166.7; 48.967
1002.7; 44.378
965.4; 35.056
760.4; 31.539
704.5; 41.385
Transmittance
Wavenumber (cm-1)
O
N
N
H
O

5.10 (*E*)-1-(4-(Oxetan-3-yl)piperazin-1-yl)-3-phenylprop-2-en-1-one (11k) (600 MHz, $CDCl_3$)

5.10.1 ^{1}H NMR

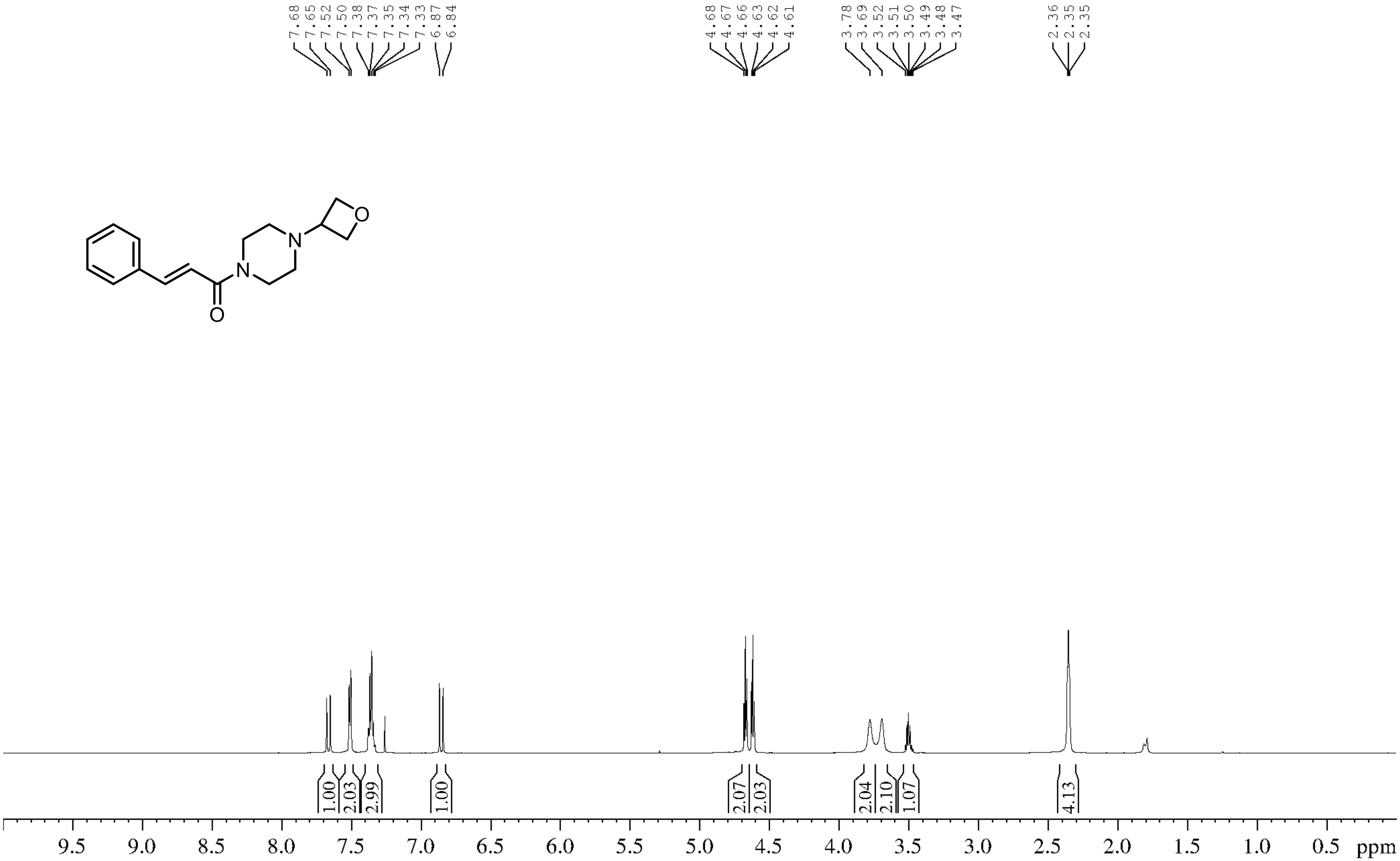

5.10.2 ^{13}C NMR

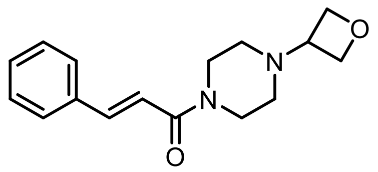

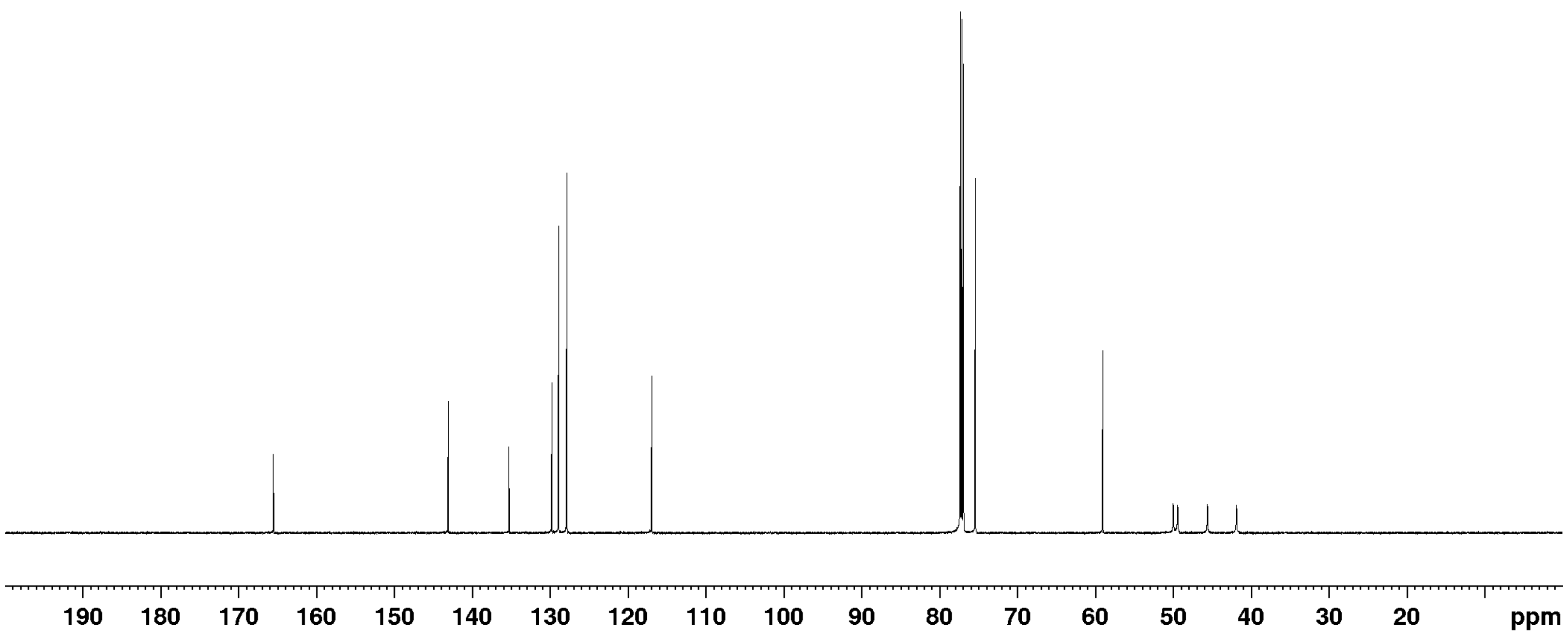
165.6
143.1
135.3
129.8
128.9
127.9
117.0
75.4
59.1
50.0
49.4
45.6
41.9
190
180
170
160
150
140
130
120
110
100
90
80
70
60
50
40
30
20
ppm

5.10.3 ^{1}H, ^{1}H COSY

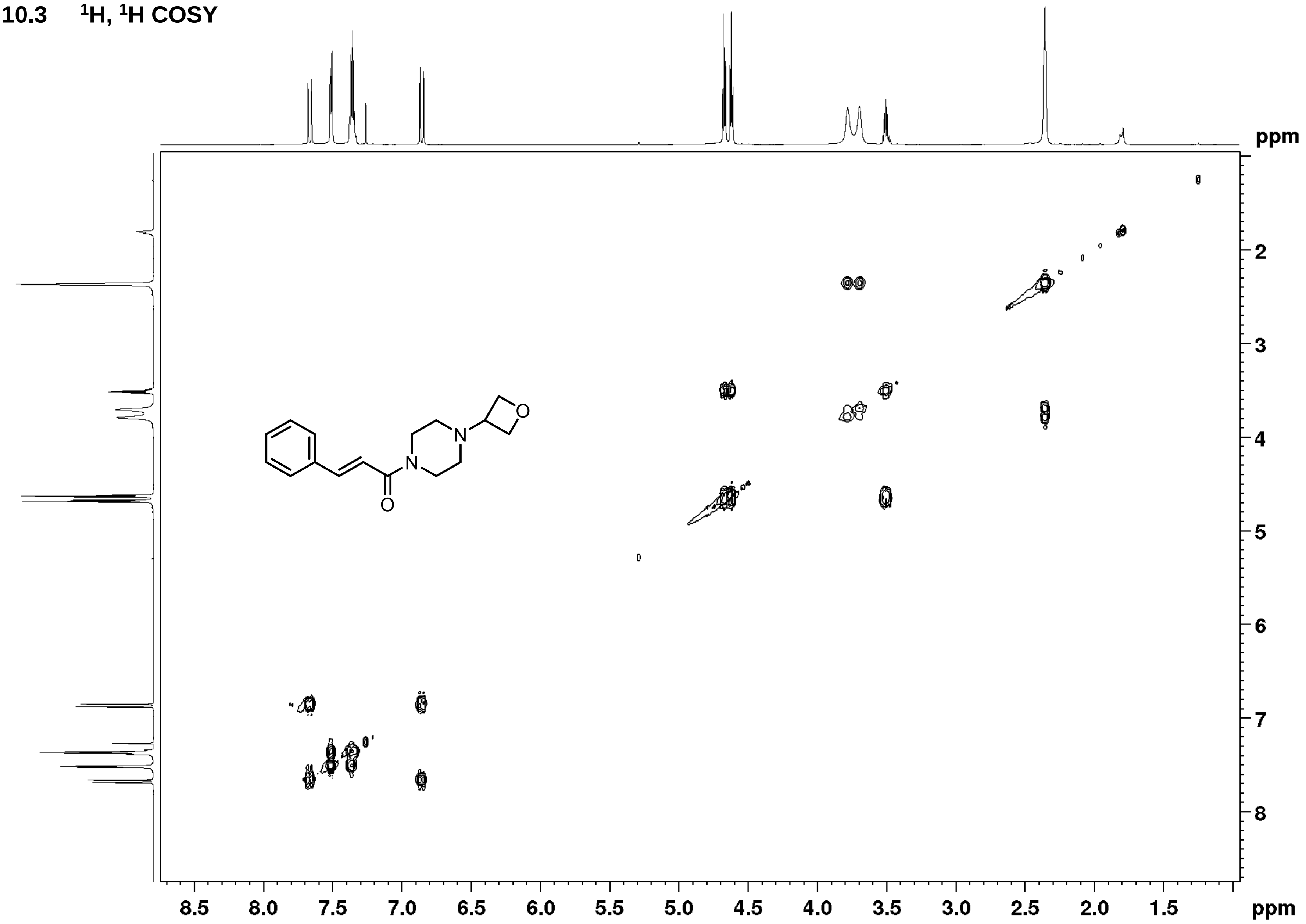

5.10.4 ^{1}H, ^{13}C HMBC

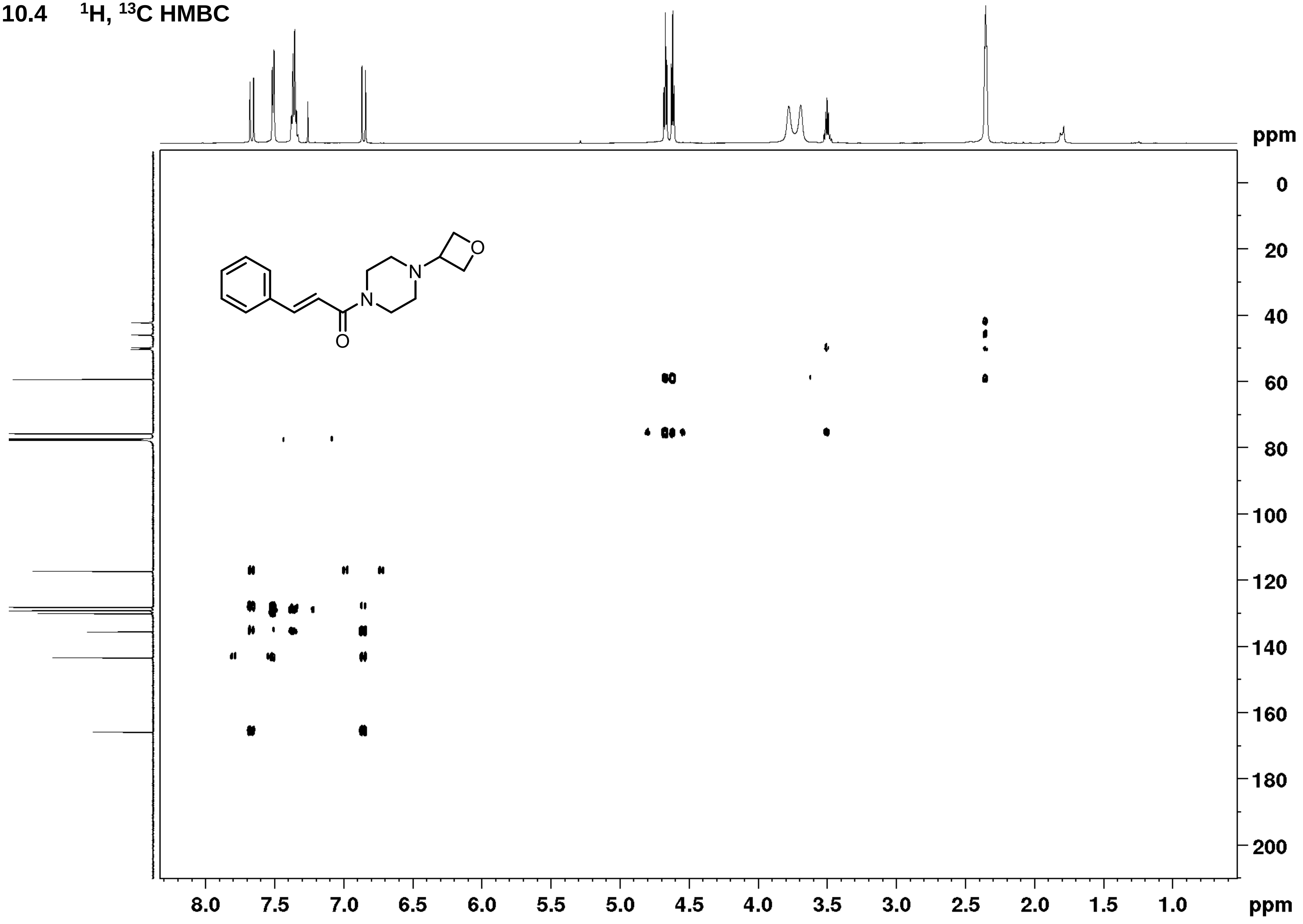

5.10.5 ^{1}H, ^{13}C HSQC

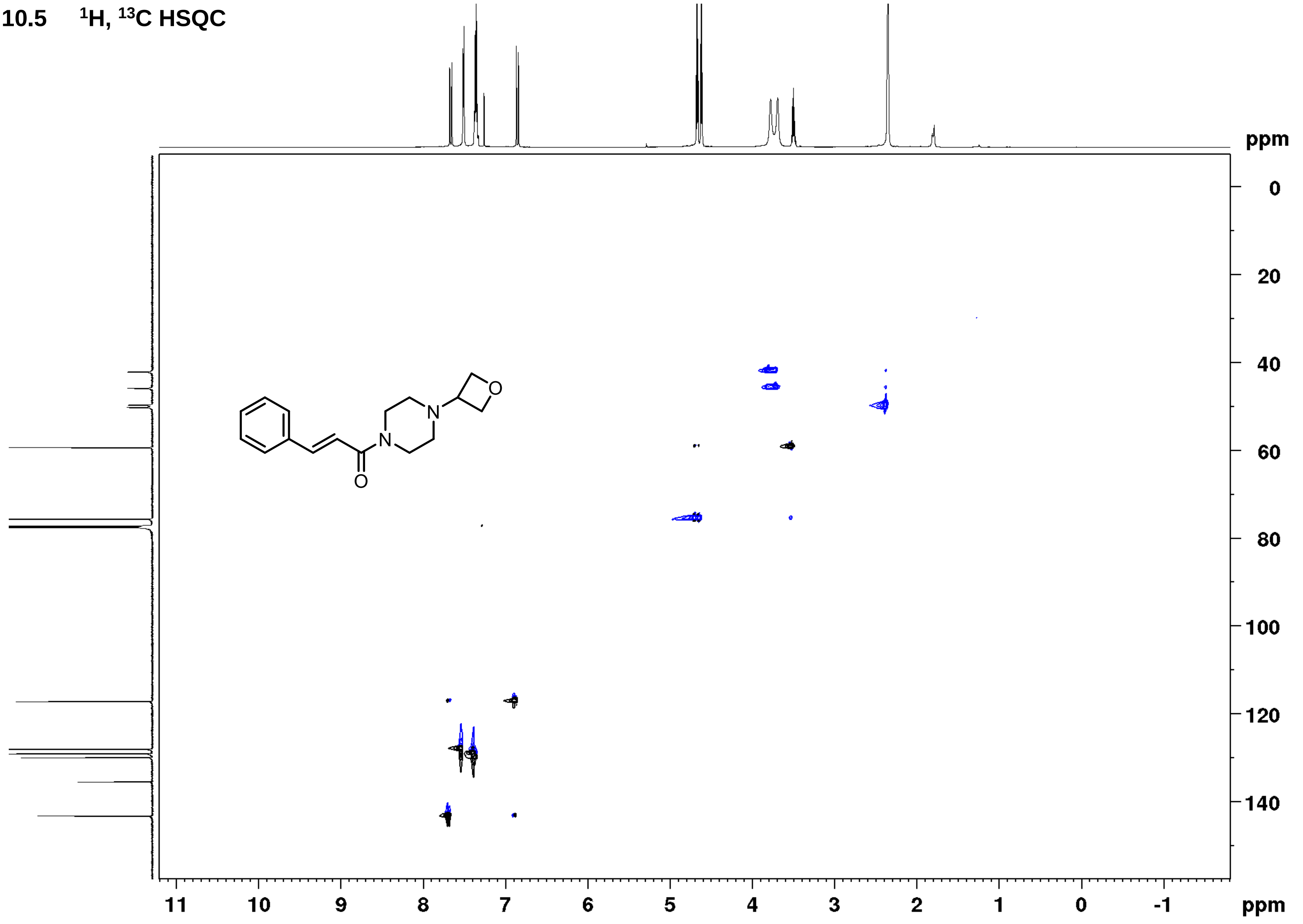

5.10.6 HRMS

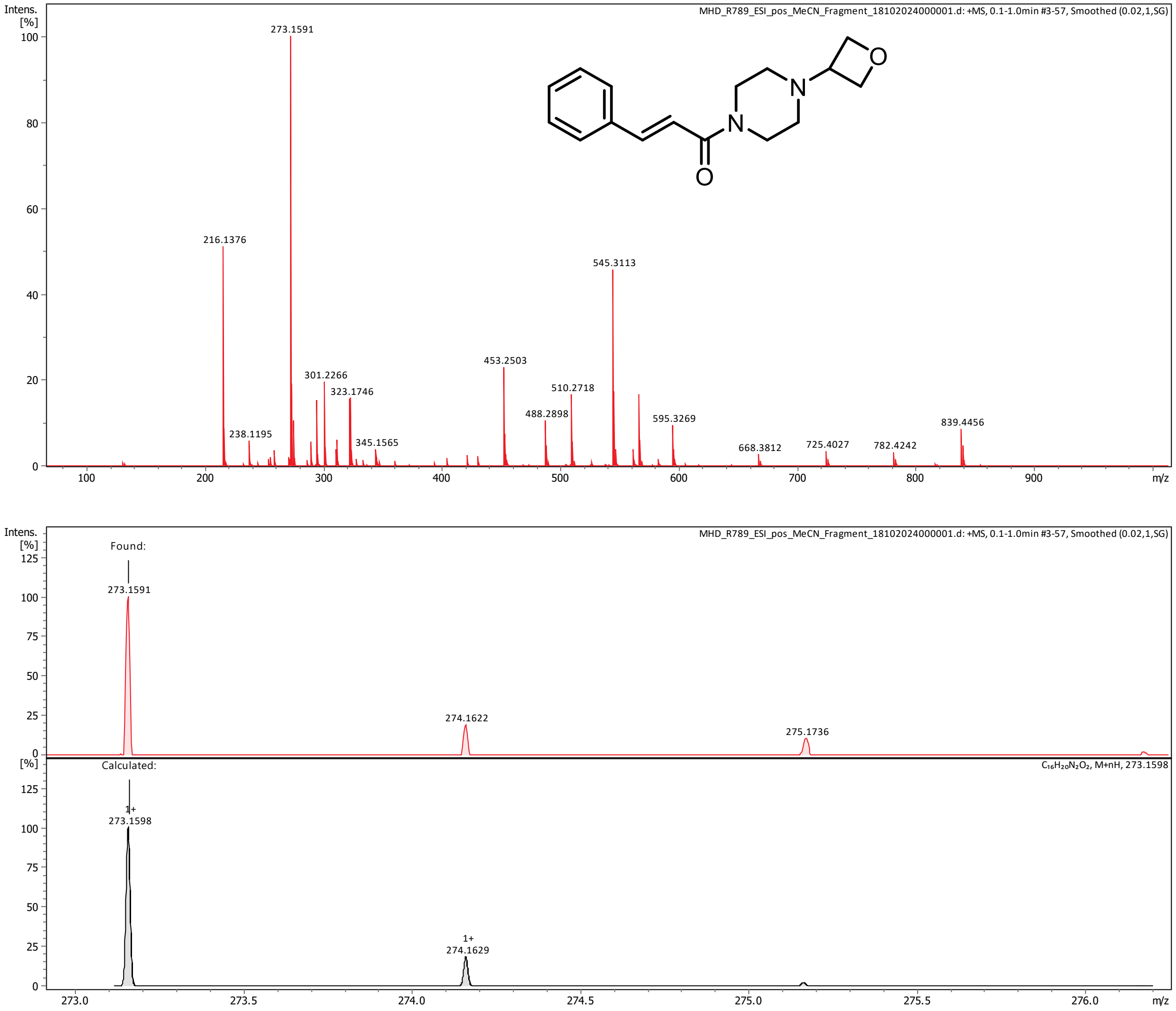

Intens. [%]
MHD_R789_ESI_pos_MeCN_Fragment_18102024000001.d: +MS, 0.1-1.0min #3-57, Smoothed (0.02,1,SG)
273.1591
216.1376
545.3113
453.2503
301.2266
323.1746
510.2718
488.2898
595.3269
238.1195
345.1565
839.4456
668.3812
725.4027
782.4242
m/z
Found:
273.1591
274.1622
275.1736
Calculated:
C16H20N2O2, M+nH, 273.1598
1+
273.1598
1+
274.1629

5.10.7 IR

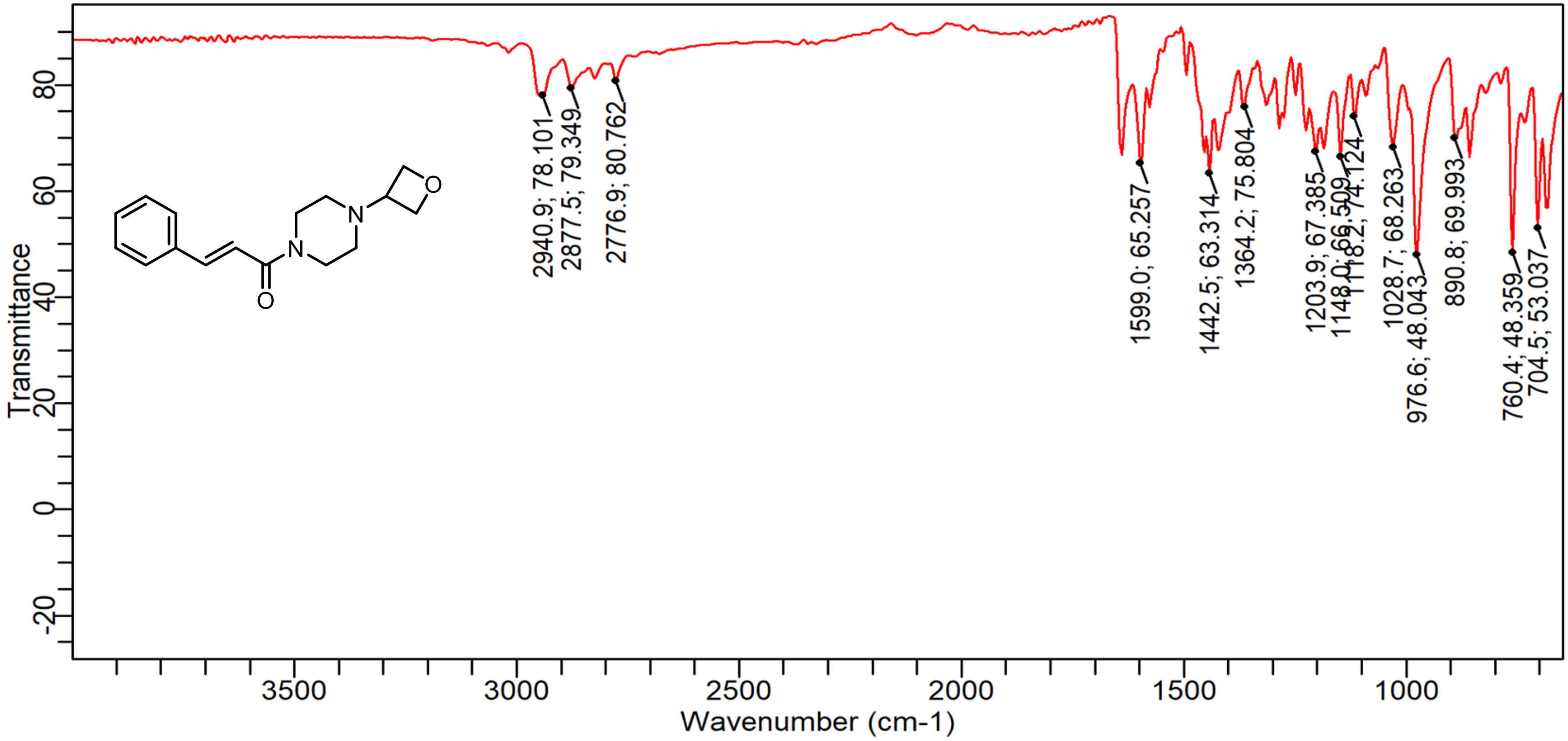

Transmittance
Wavenumber (cm-1)
2940.9; 78.101
2877.5; 79.349
2776.9; 80.762
1599.0; 65.257
1442.5; 63.314
1364.2; 75.804
1203.9; 67.385
1028.7; 68.263
976.6; 48.043
890.8; 69.993
760.4; 48.359
704.5; 53.037

5.11 (*E*)-1-(4-(6-fluorobenzo[*d*]isoxazol-3-yl)piperidin-1-yl)-3-phenylprop-2-en-1-one (11l) (600 MHz, $CDCl_3$)

5.11.1 ^1H NMR

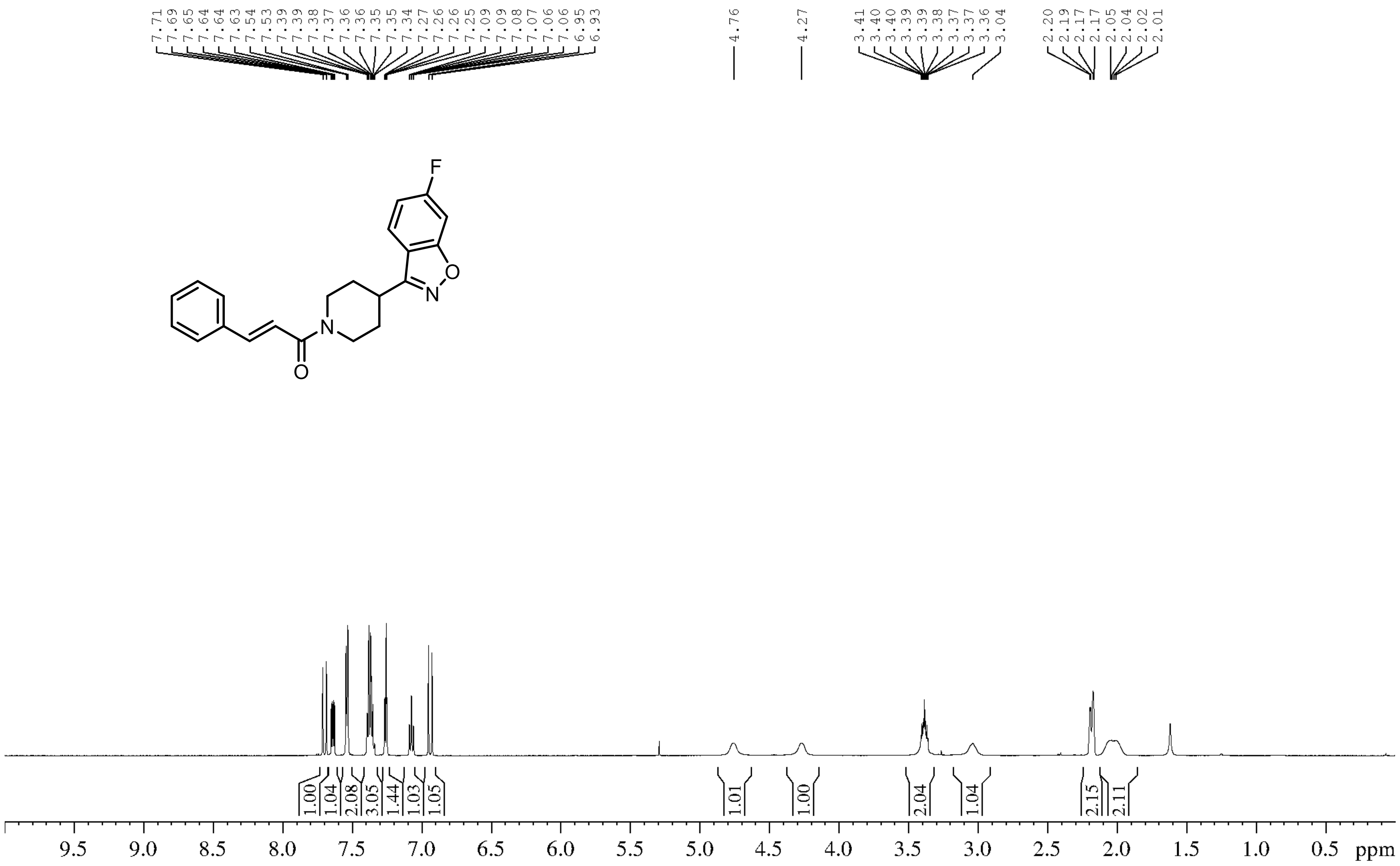

5.11.2 ^{13}C NMR

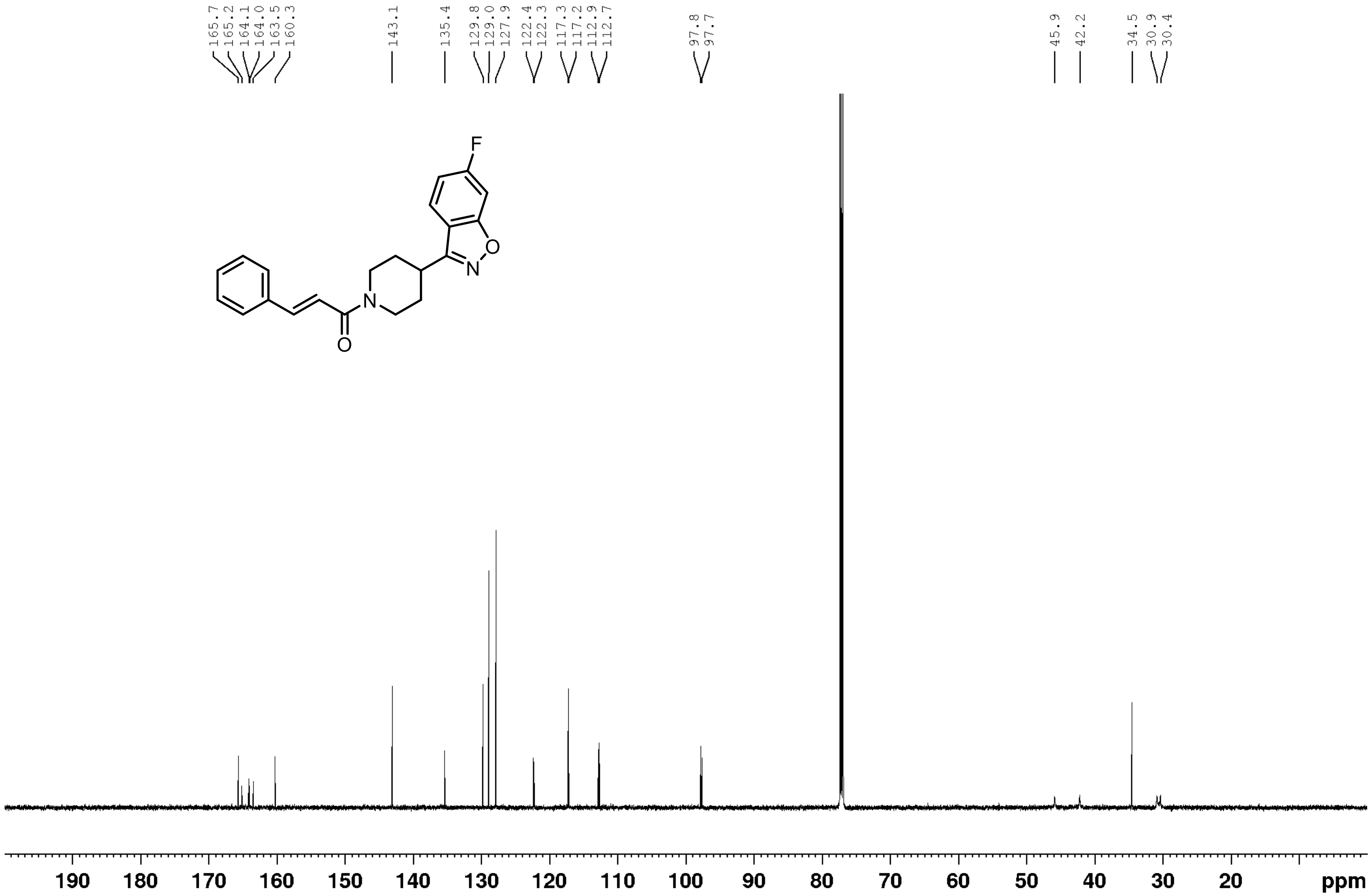
165.7
165.2
164.1
164.0
163.5
160.3
143.1
135.4
129.8
129.0
127.9
122.4
122.3
117.3
117.2
112.9
112.7
97.8
97.7
45.9
42.2
34.5
30.9
30.4
F
O
N
N
O
190
180
170
160
150
140
130
120
110
100
90
80
70
60
50
40
30
20
ppm

5.11.3 ^{19}F NMR

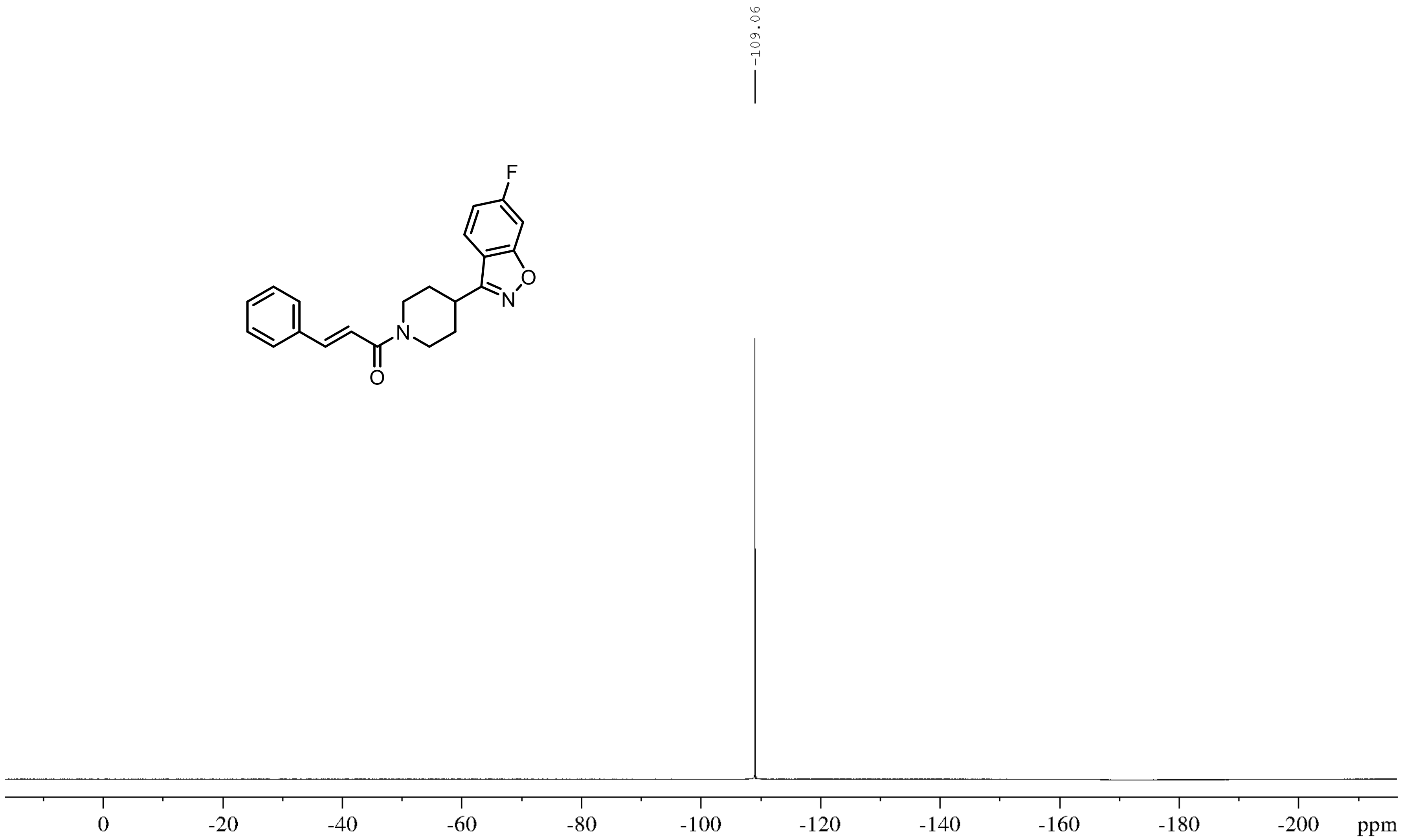

5.11.4 ^{1}H, ^{1}H COSY

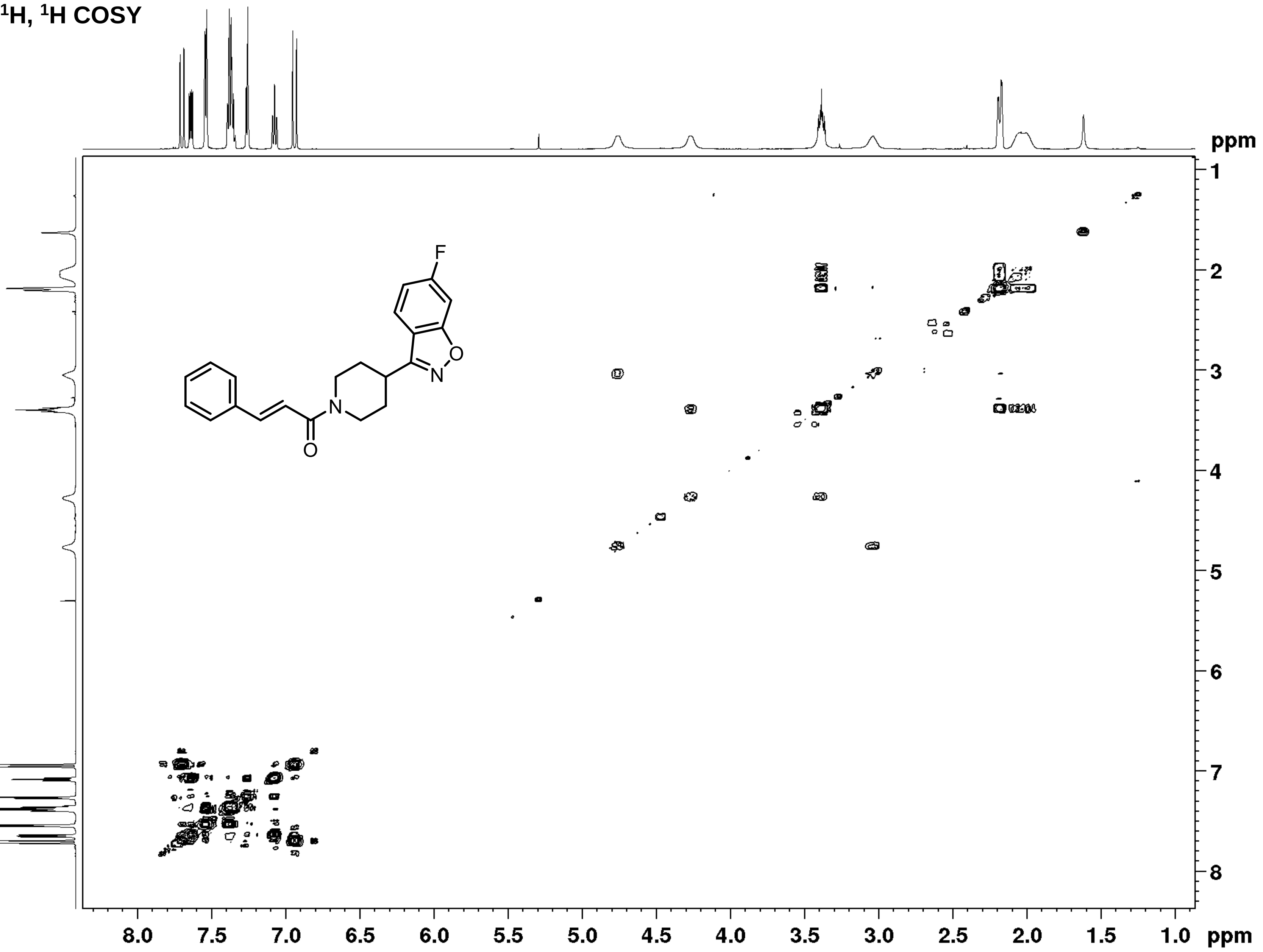

5.11.5 ^{1}H, ^{13}C HMBC

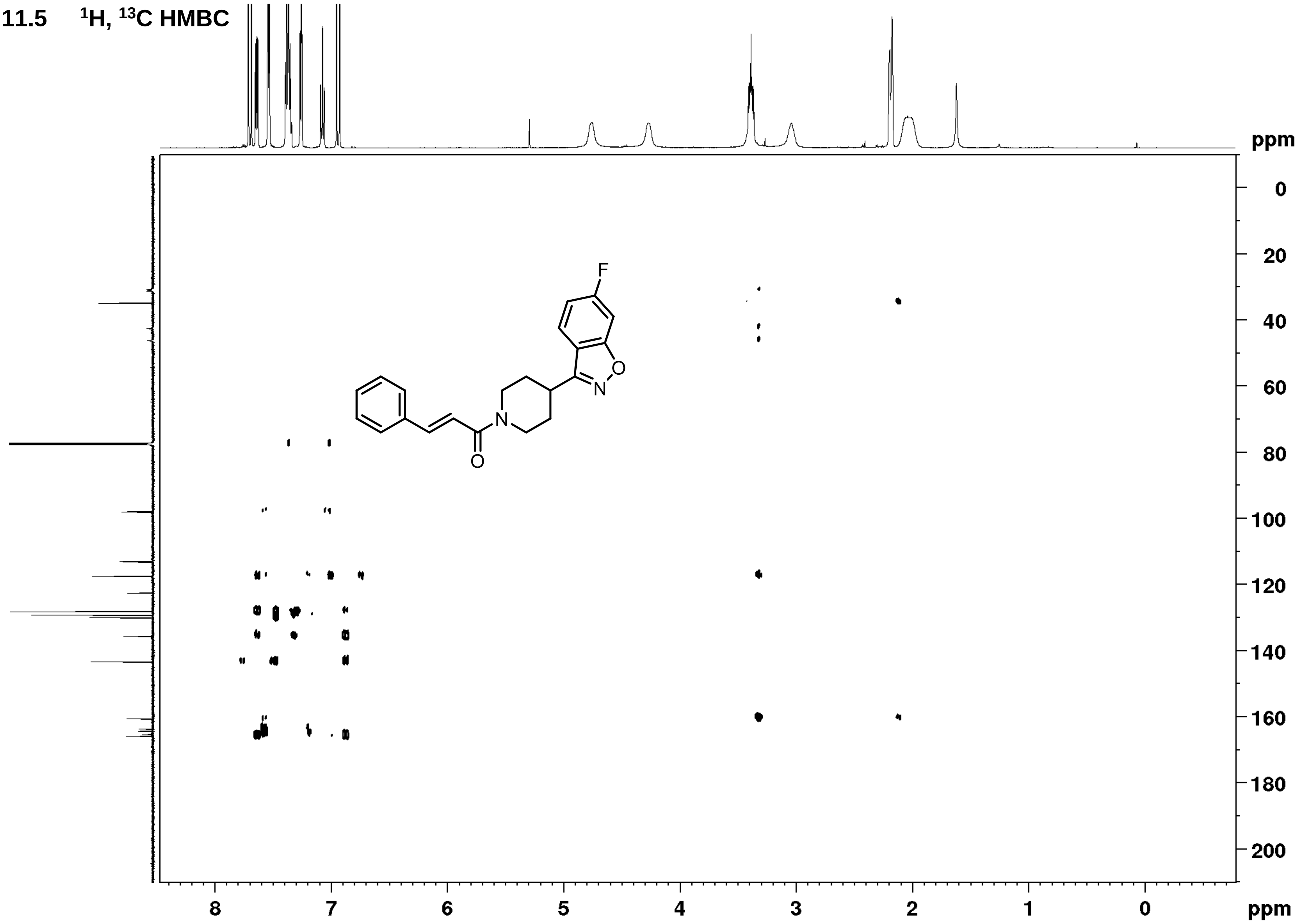

5.11.6 ^1H, ^{13}C HSQC

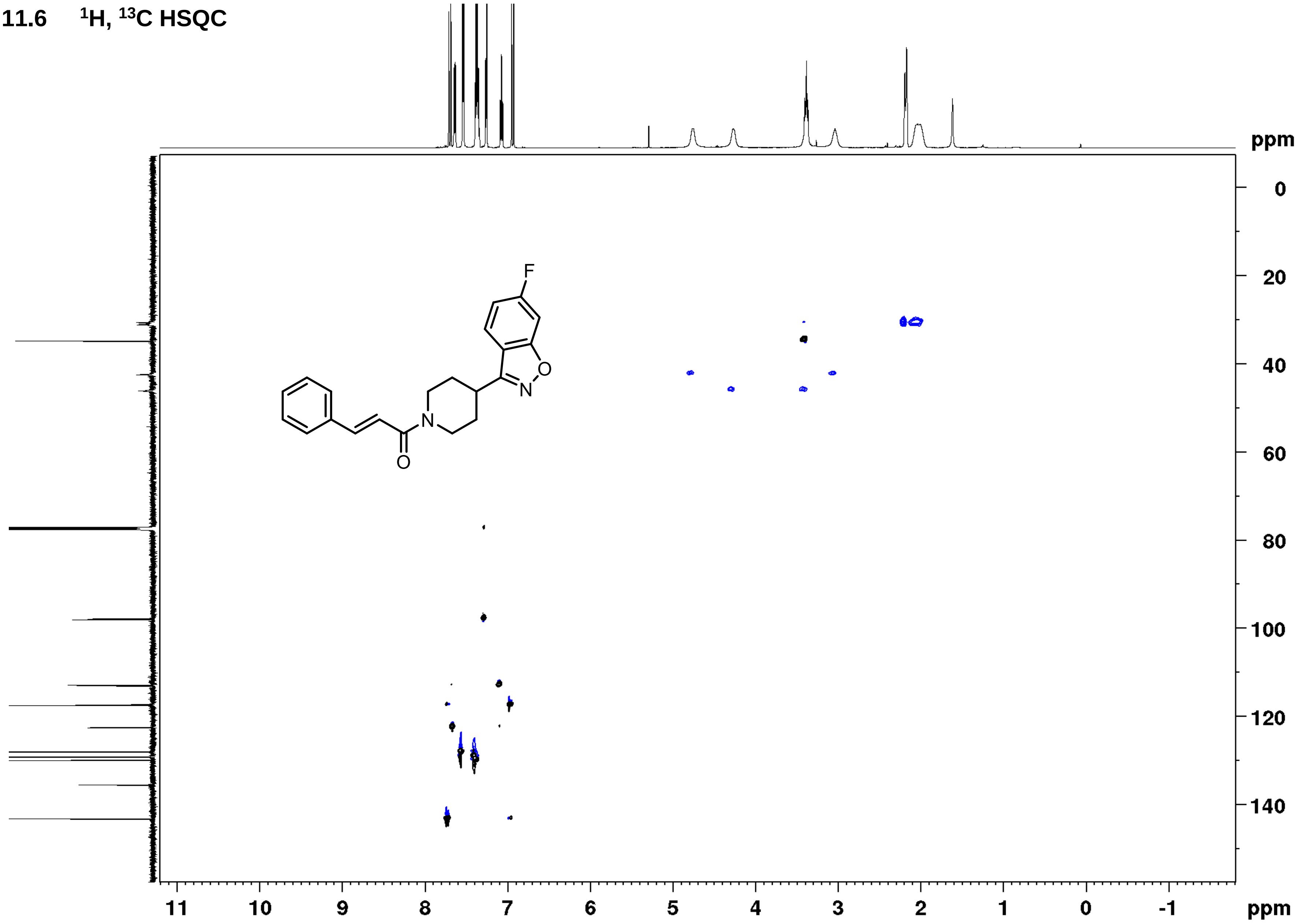

5.11.7 HRMS

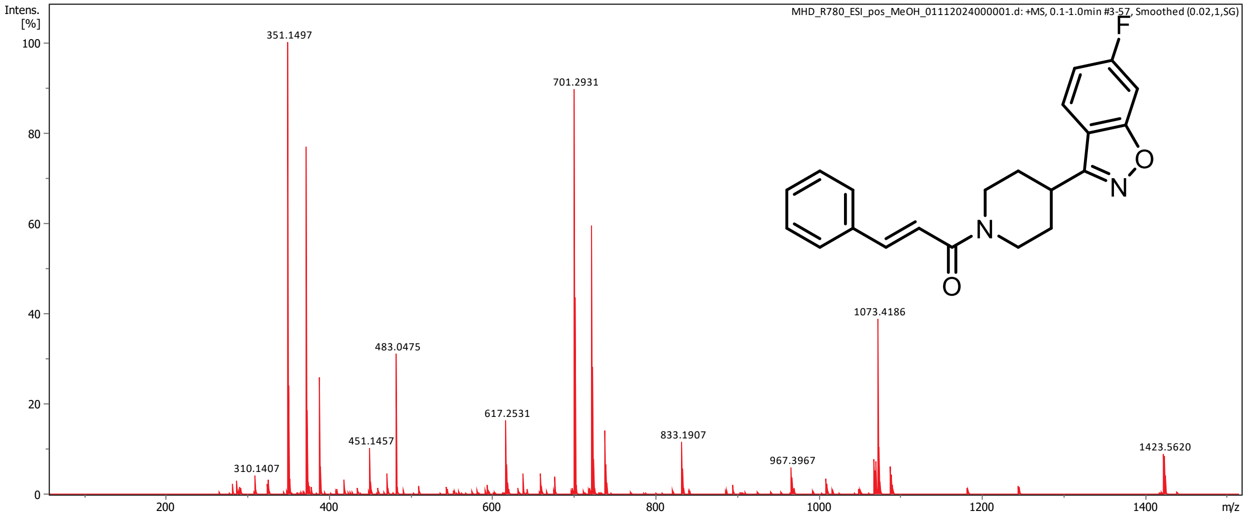

Intens. [%]
MHD_R780_ESI_pos_MeOH_01112024000001.d: +MS, 0.1-1.0min #3-57, Smoothed (0.02,1,SG)
351.1497
701.2931
483.0475
617.2531
451.1457
310.1407
833.1907
967.3967
1073.4186
1423.5620
m/z

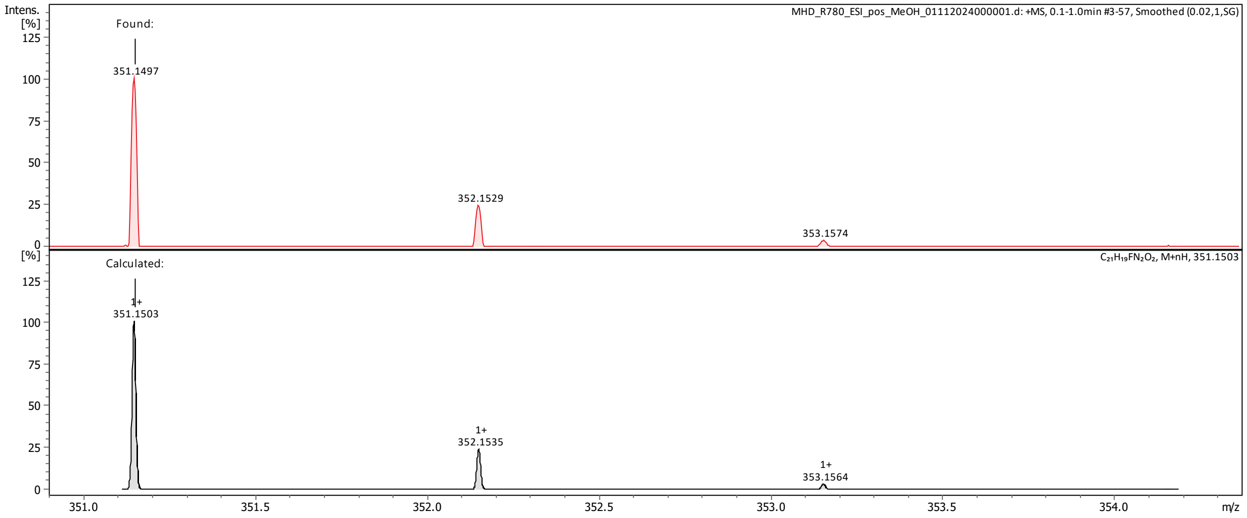

Intens. [%]
MHD_R780_ESI_pos_MeOH_01112024000001.d: +MS, 0.1-1.0min #3-57, Smoothed (0.02,1,SG)
Found:
351.1497
352.1529
353.1574
Calculated:
C21H19FN2O2, M+nH, 351.1503
1+ 351.1503
1+ 352.1535
1+ 353.1564
m/z

5.11.8 IR

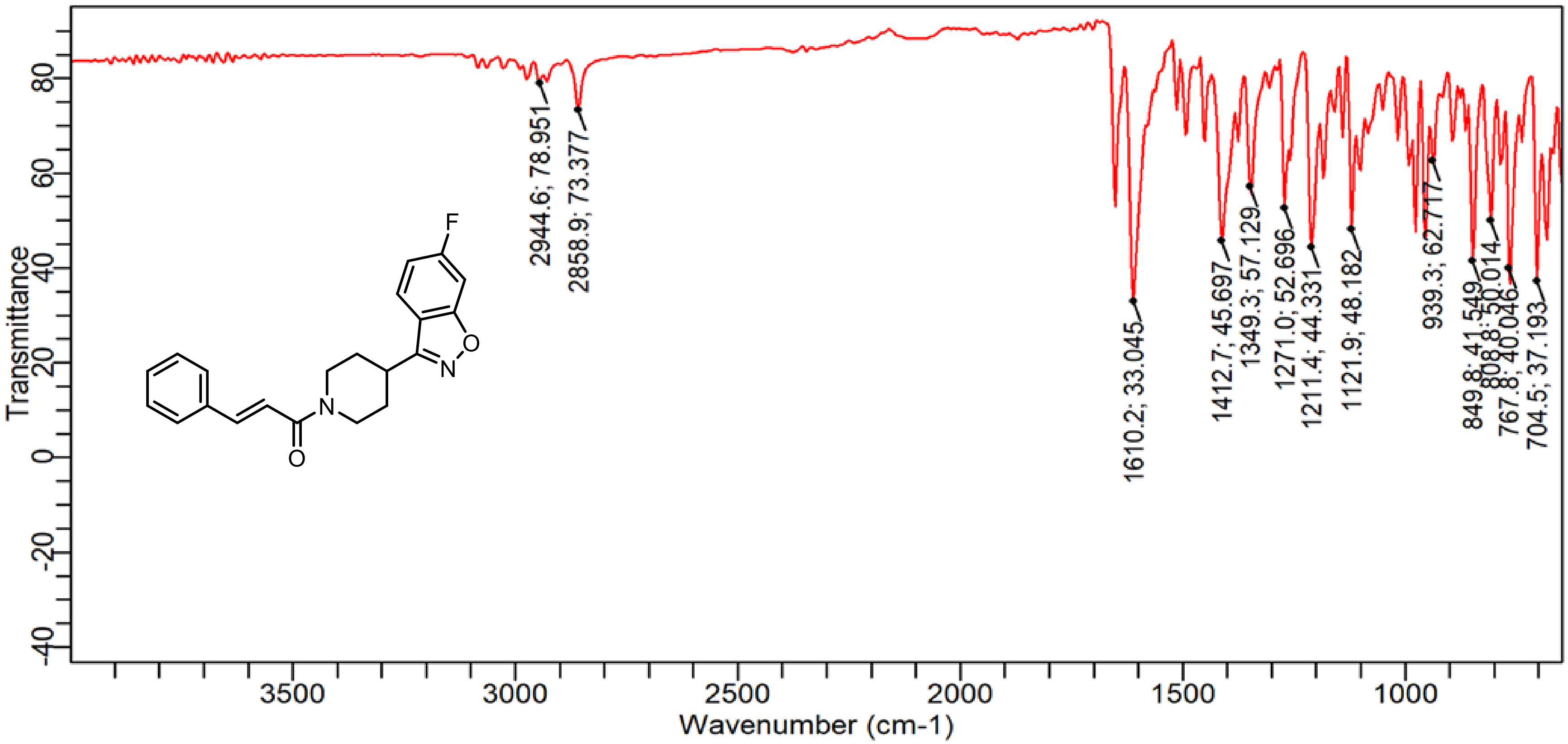

Transmittance
Wavenumber (cm-1)
2944.6; 78.951
2858.9; 73.377
1610.2; 33.045
1412.7; 45.697
1349.3; 57.129
1271.0; 52.696
1211.4; 44.331
1121.9; 48.182
939.3; 62.717
849.8; 41.549
767.8; 40.046
704.5; 37.193
F
O
N

5.12 (*E*)-1-(piperidin-1-yl)-3-(4-(piperidine-1-carbonyl)phenyl)prop-2-en-1-one (11m) (600 MHz, $CDCl_3$)

5.12.1 ^{1}H NMR

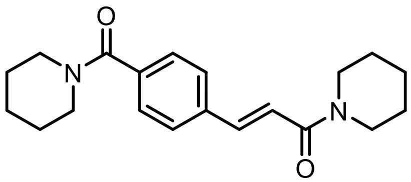

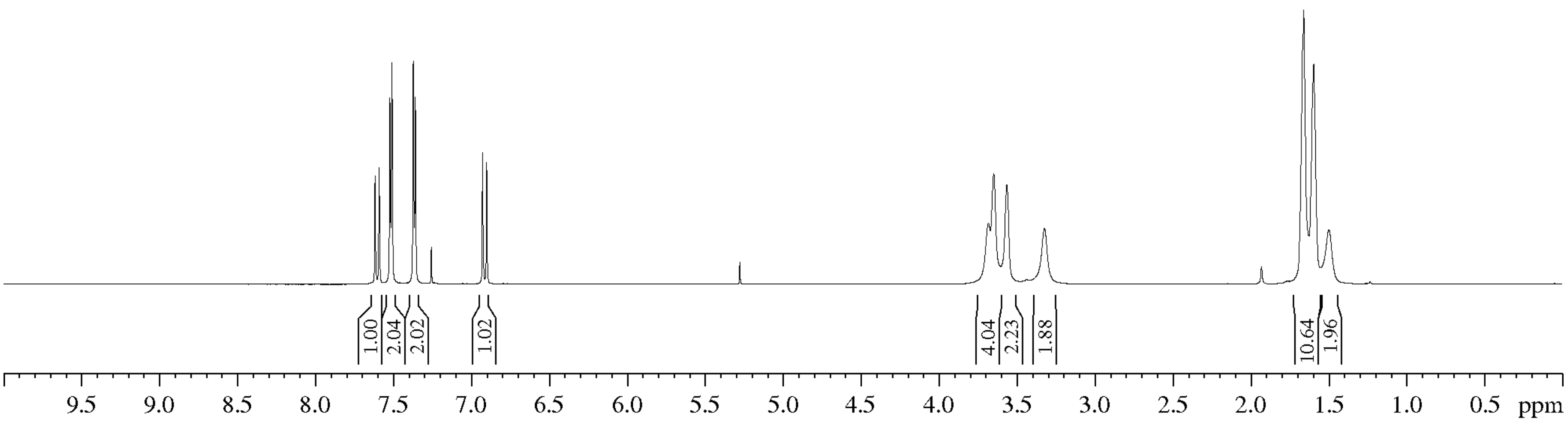

5.12.2 ^{13}C NMR

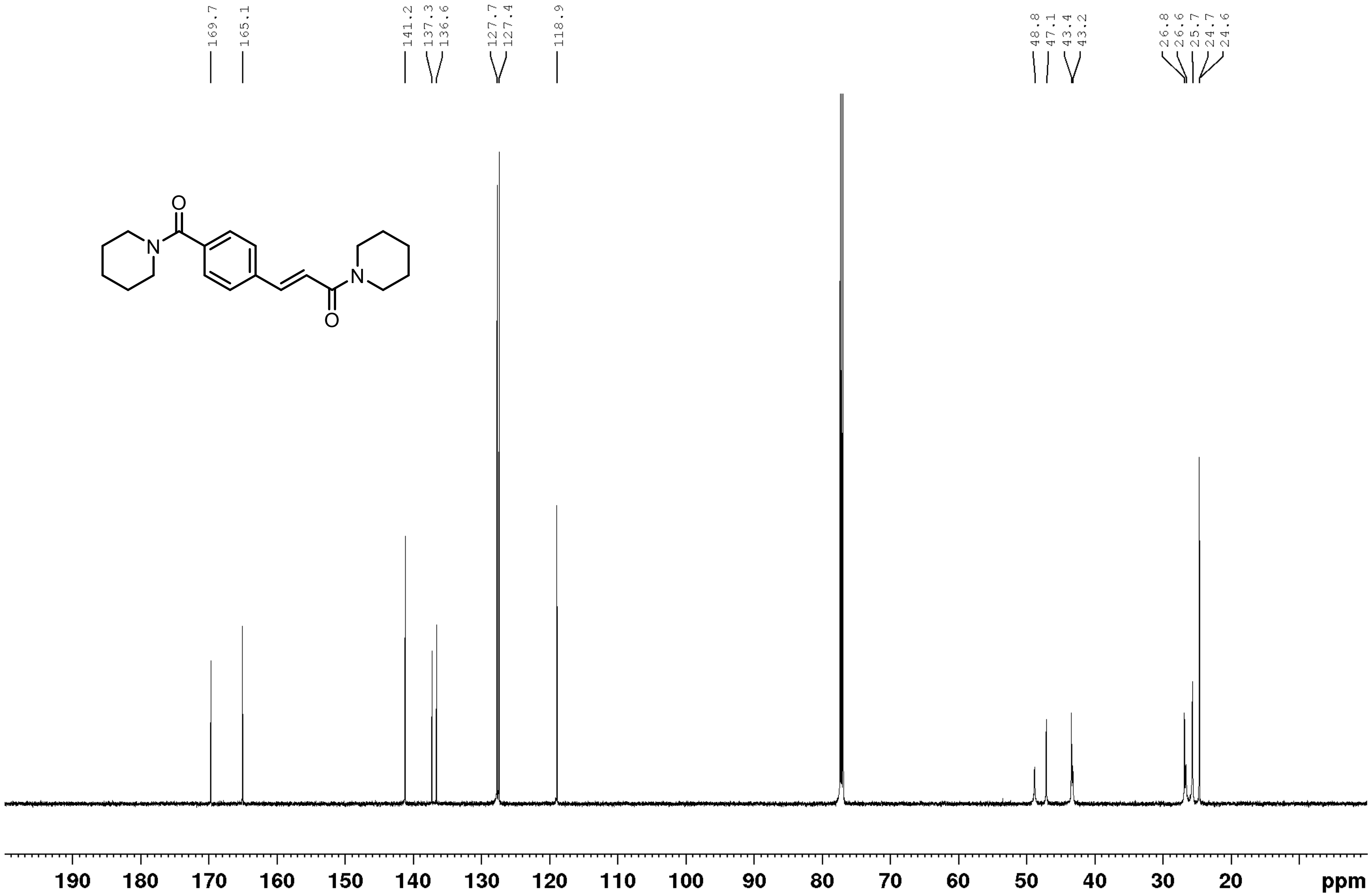
169.7
165.1
141.2
137.3
136.6
127.7
127.4
118.9
48.8
47.1
43.4
43.2
26.8
26.6
25.7
24.7
24.6
O
N
N
O
190 180 170 160 150 140 130 120 110 100 90 80 70 60 50 40 30 20
ppm

5.12.3 ¹H, ¹H COSY

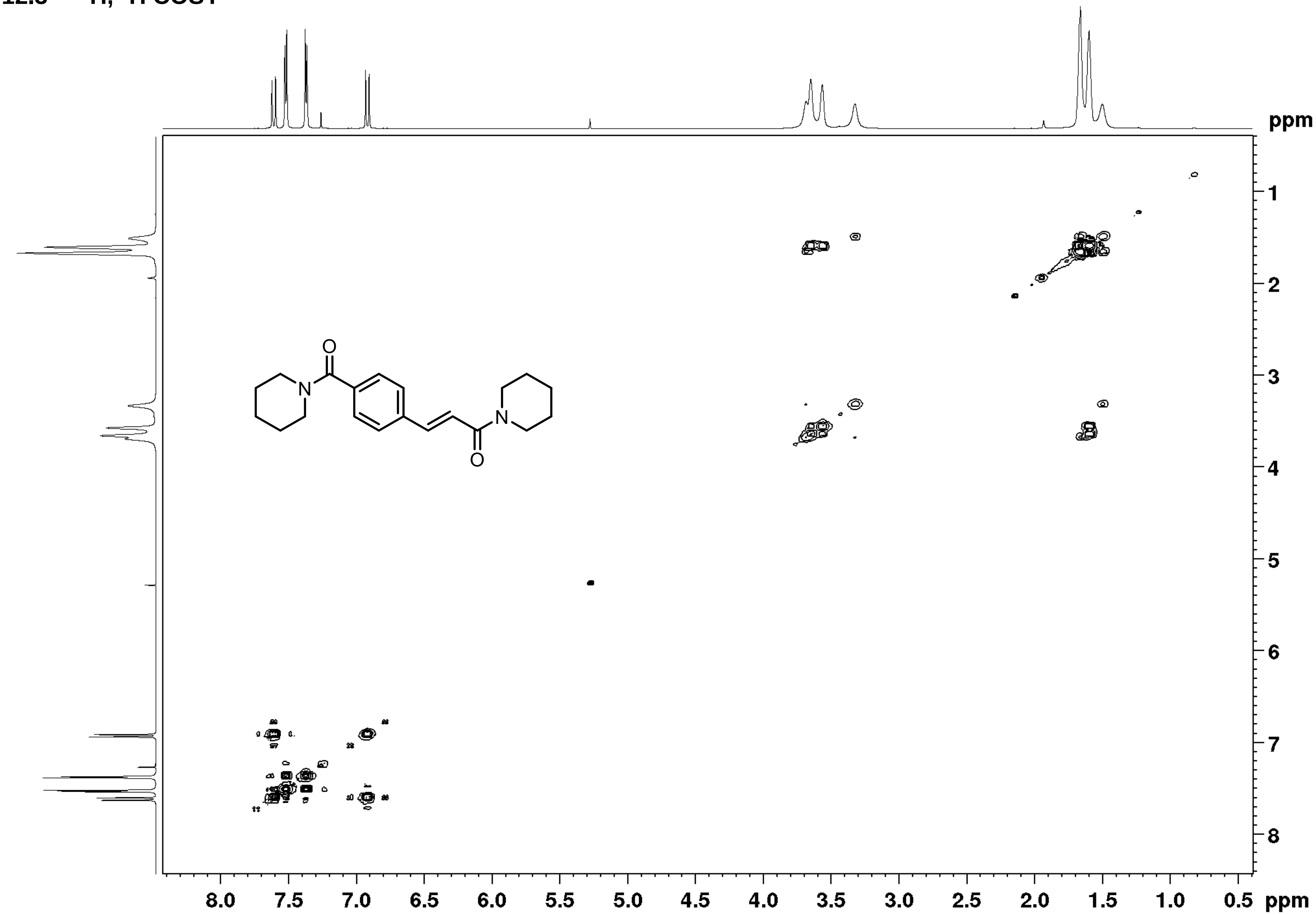

5.12.4 ^{1}H, ^{13}C HMBC

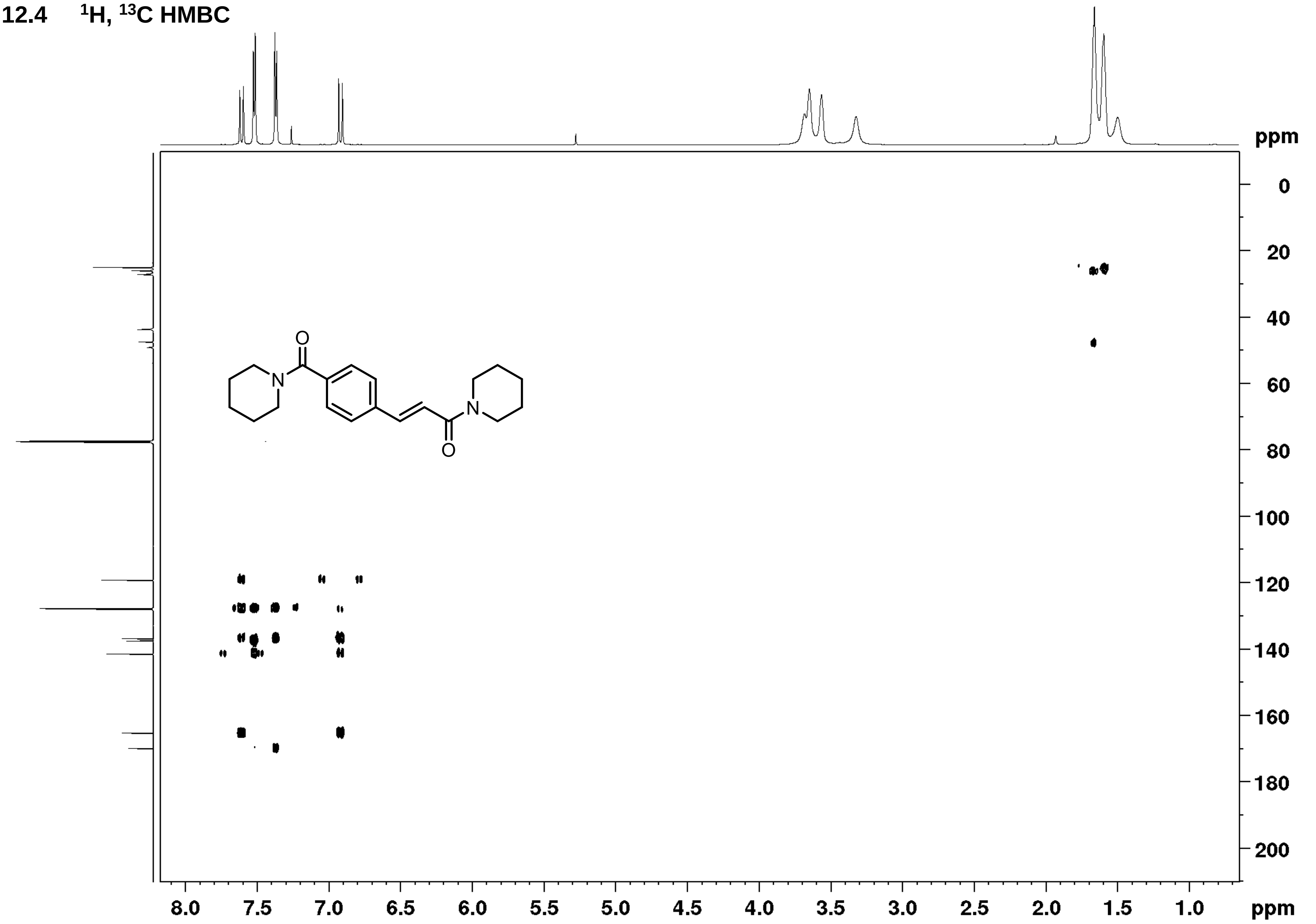

5.12.5 ^{1}H, ^{13}C HSQC

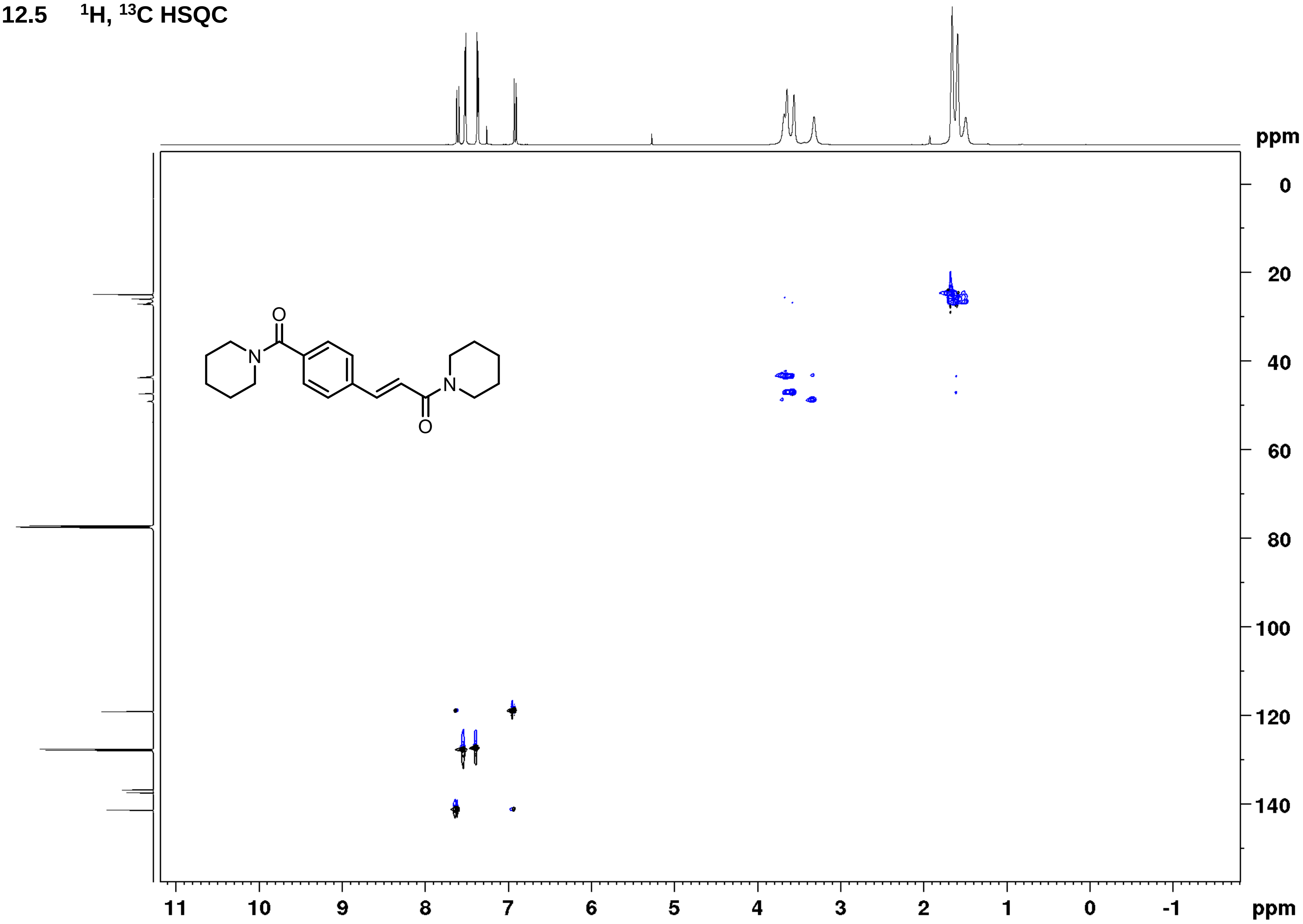

5.12.6 HRMS

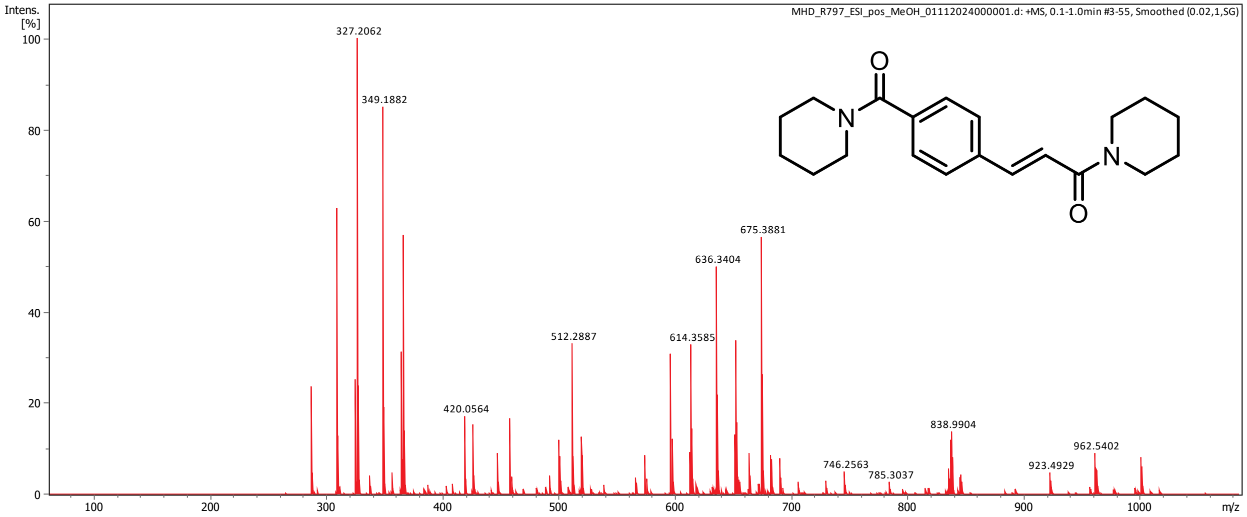

Intens. [%]
MHD_R797_ESI_pos_MeOH_01112024000001.d: +MS, 0.1-1.0min #3-55, Smoothed (0.02,1,SG)
327.2062
349.1882
675.3881
636.3404
512.2887
614.3585
420.0564
838.9904
962.5402
746.2563
923.4929
785.3037
m/z

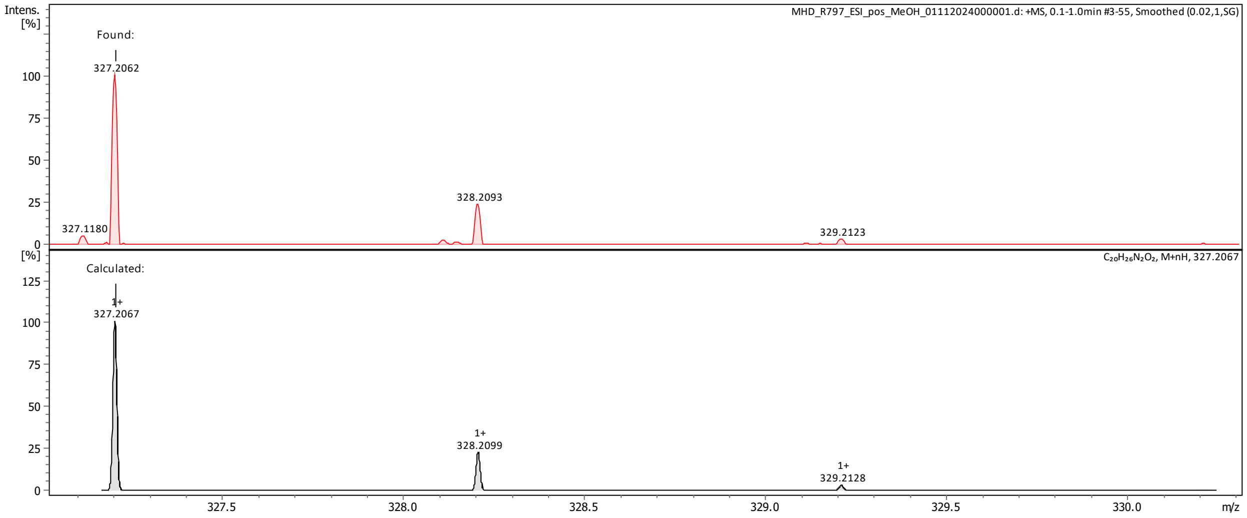

Intens. [%]
MHD_R797_ESI_pos_MeOH_01112024000001.d: +MS, 0.1-1.0min #3-55, Smoothed (0.02,1,SG)
Found:
327.2062
328.2093
327.1180
329.2123
Calculated:
$C_{20}H_{26}N_2O_2$, M+nH, 327.2067
1+
327.2067
1+
328.2099
1+
329.2128
m/z

5.12.7 IR

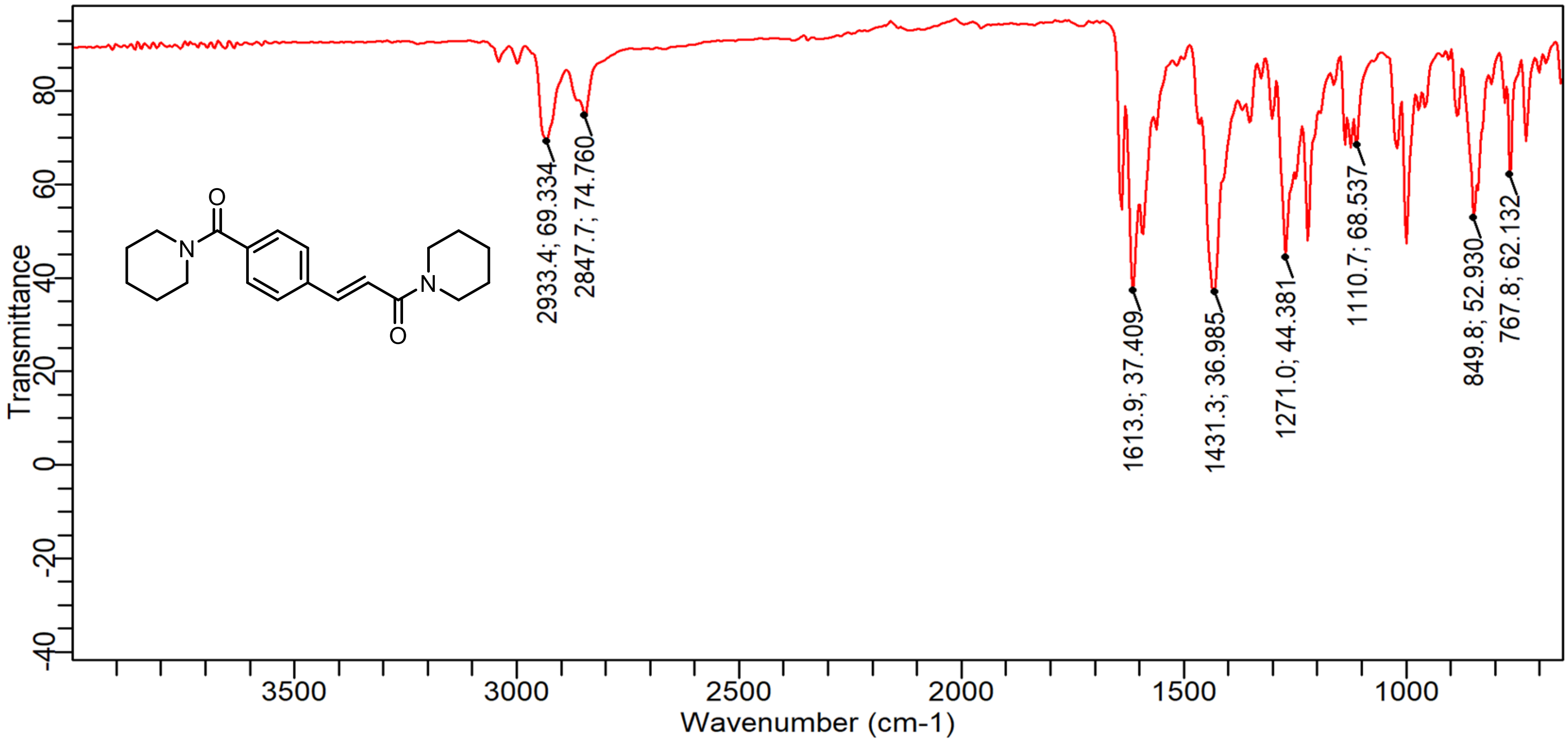

Transmittance
Wavenumber (cm-1)
2933.4; 69.334
2847.7; 74.760
1613.9; 37.409
1431.3; 36.985
1271.0; 44.381
1110.7; 68.537
849.8; 52.930
767.8; 62.132

5.13 (*E*)-1-(Piperidin-1-yl)-3-(4-(trifluoromethyl)phenyl)prop-2-en-1-one (11n) (600 MHz, $CDCl_3$)

5.13.1 ^{1}H NMR

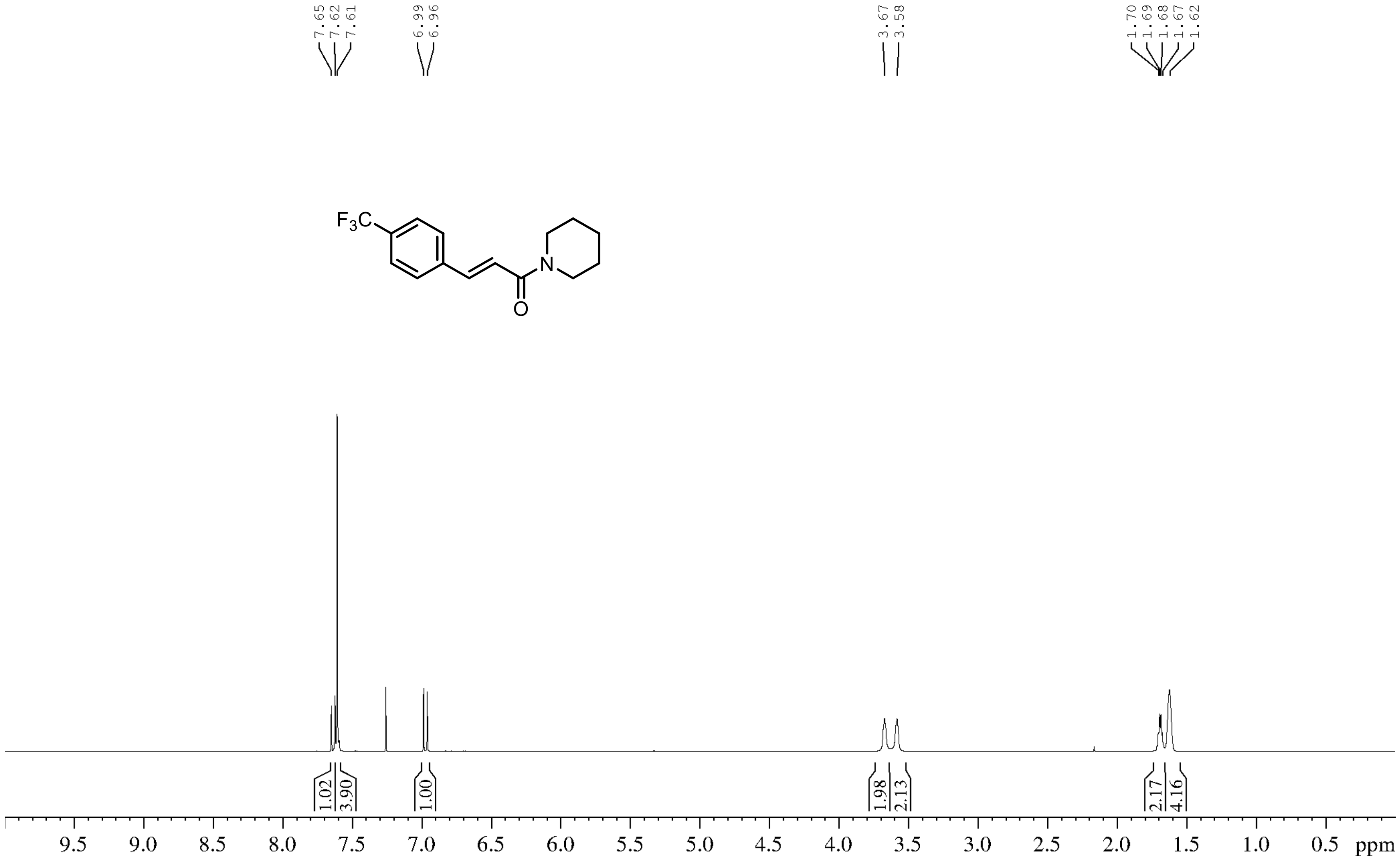

5.13.2 ^{13}C NMR

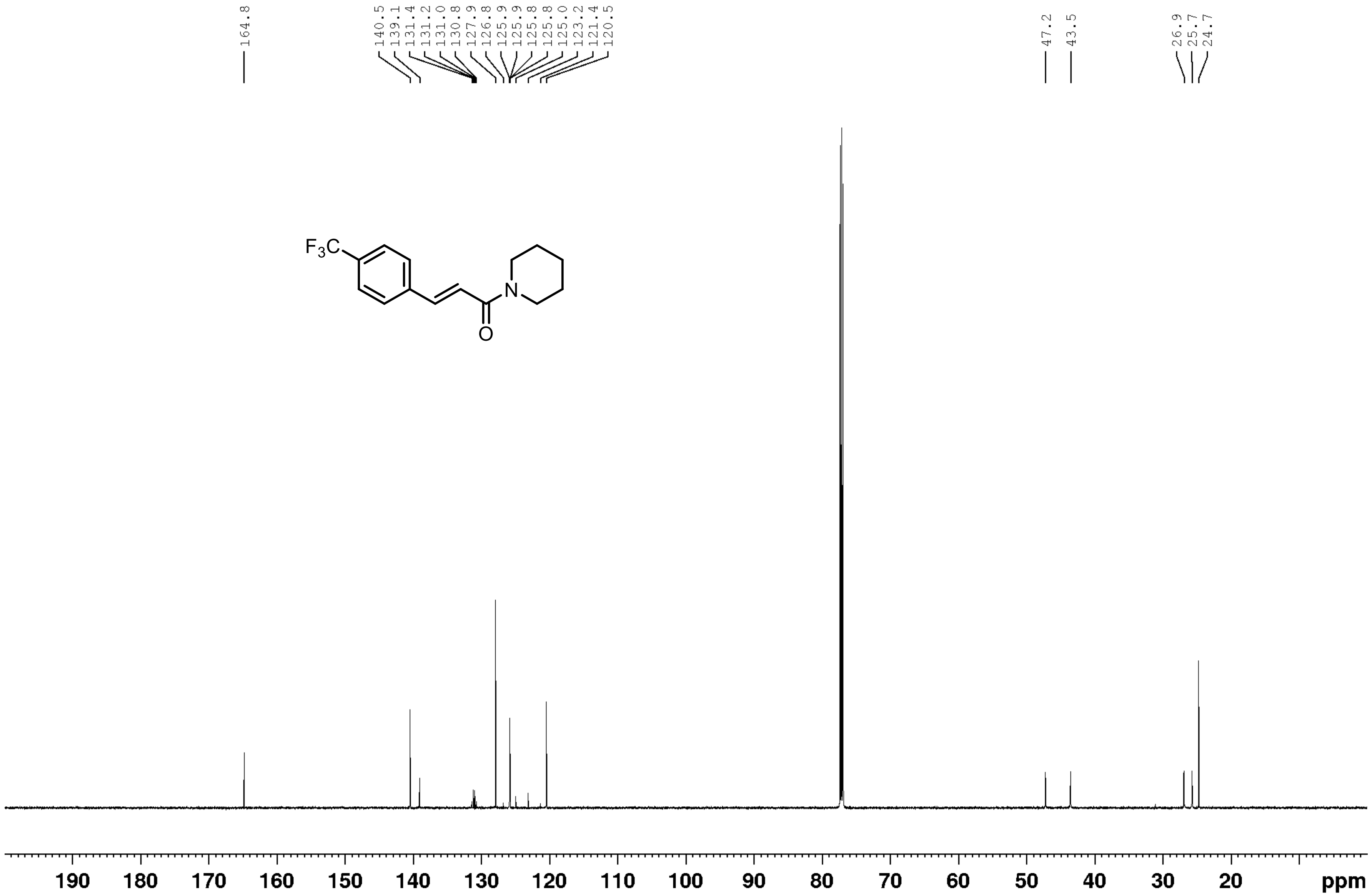
164.8
140.5
139.1
131.4
131.2
131.0
130.8
127.9
126.8
125.9
125.9
125.8
125.8
125.0
123.2
121.4
120.5
47.2
43.5
26.9
25.7
24.7
F3C
N
O
190
180
170
160
150
140
130
120
110
100
90
80
70
60
50
40
30
20
ppm

5.13.3 ^{19}F NMR

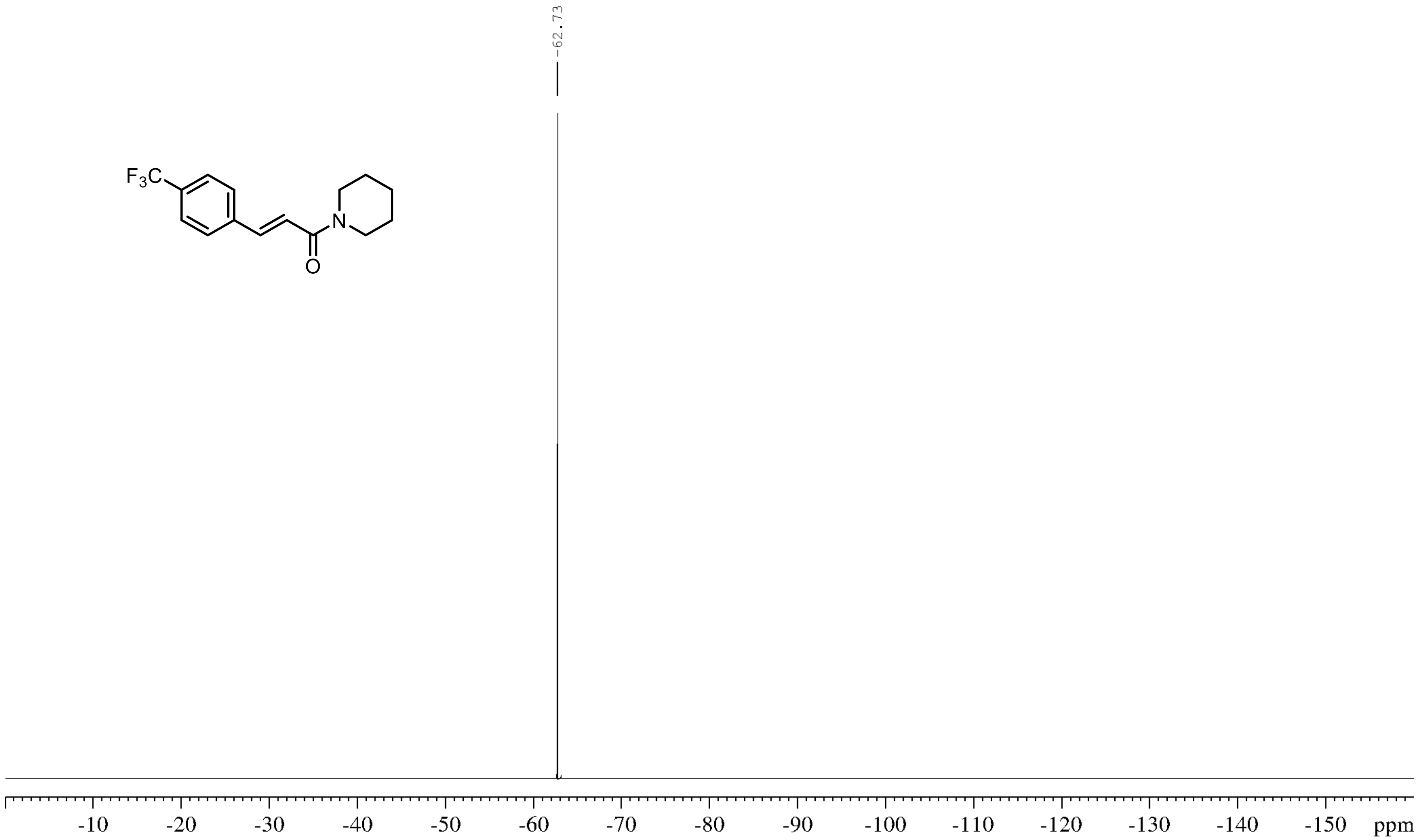

5.13.4 ^{1}H, ^{1}H COSY

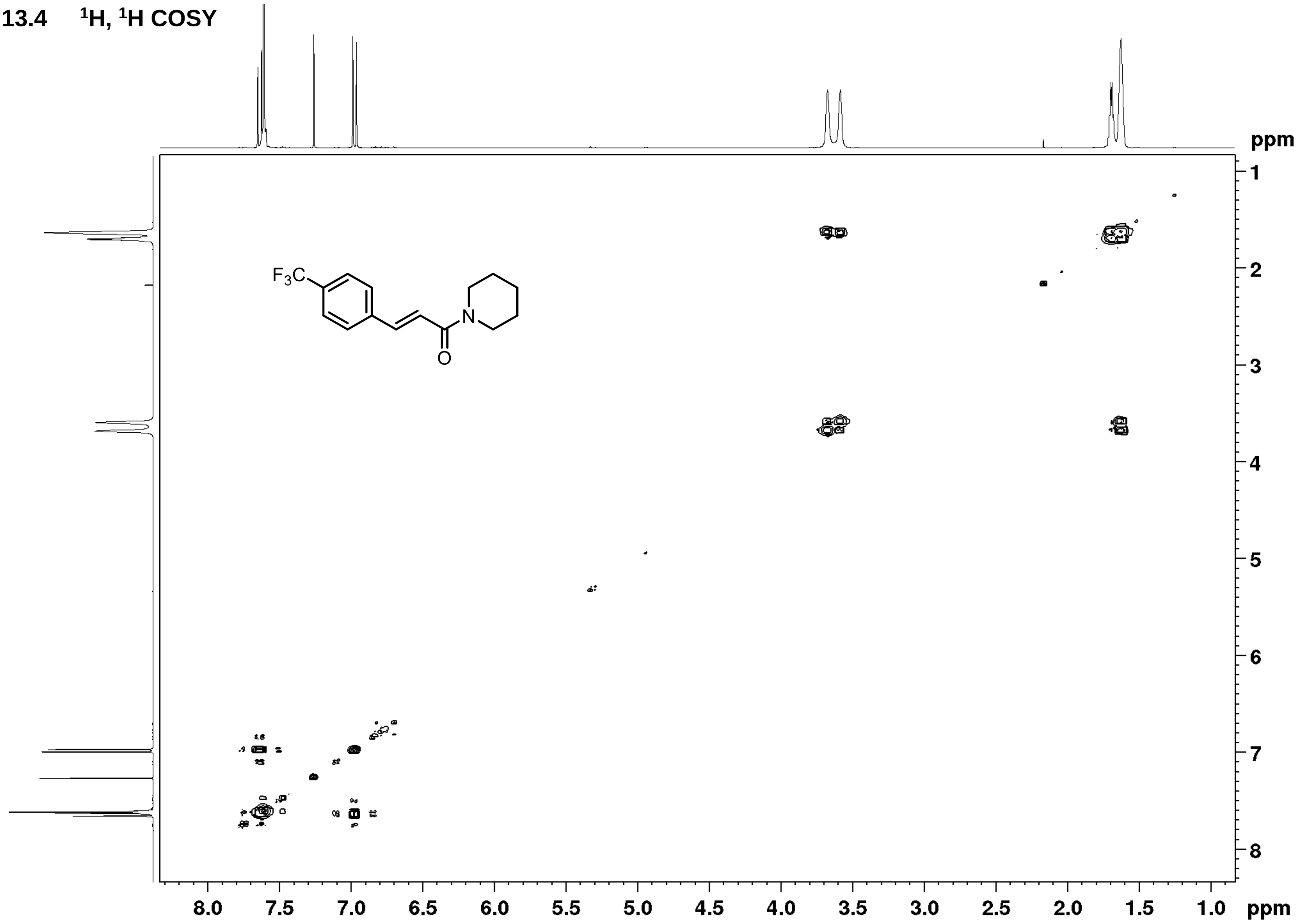

5.13.5 $^{1}H, ^{13}C$ HMBC

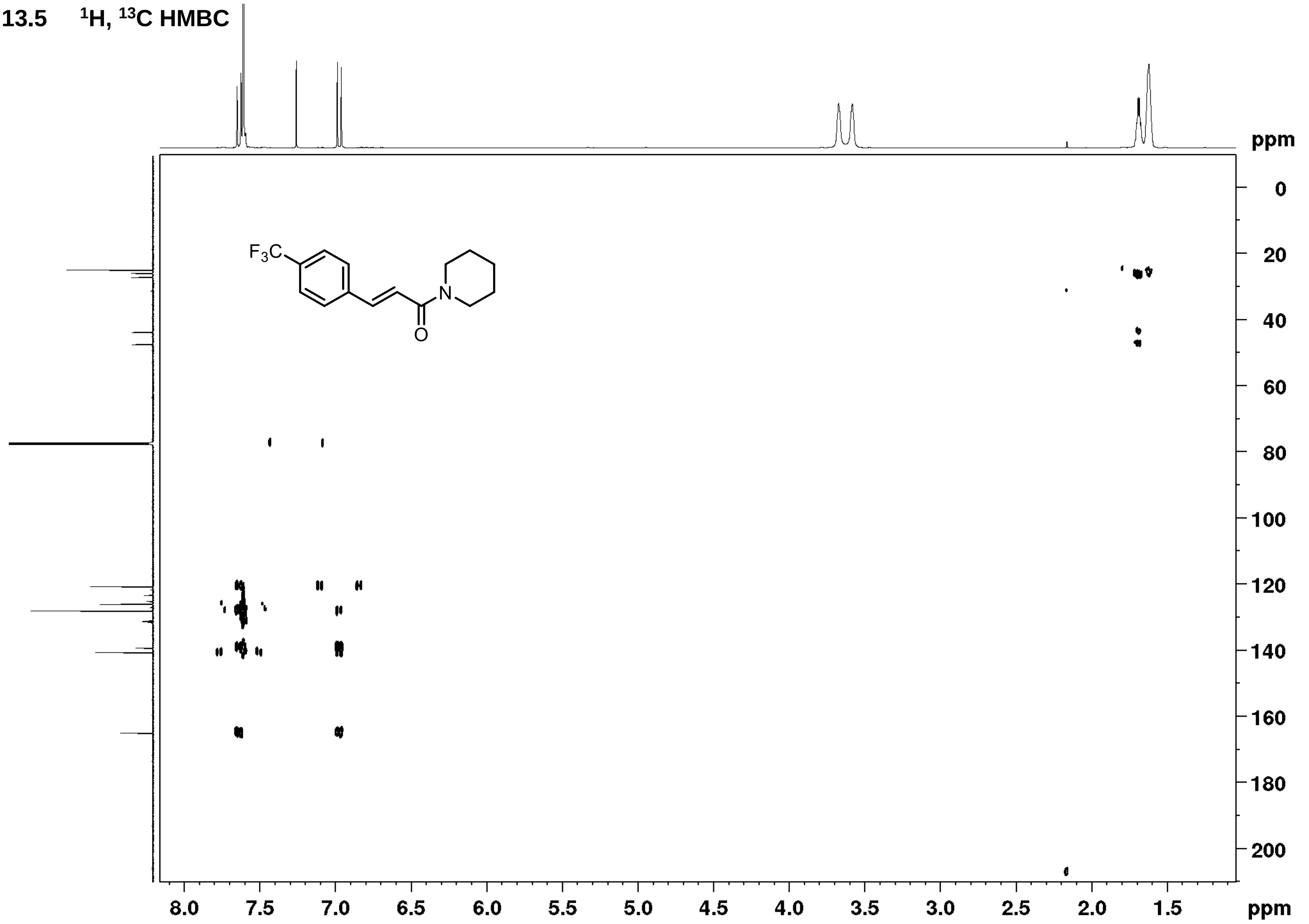

5.13.6 ^{1}H, ^{13}C HSQC

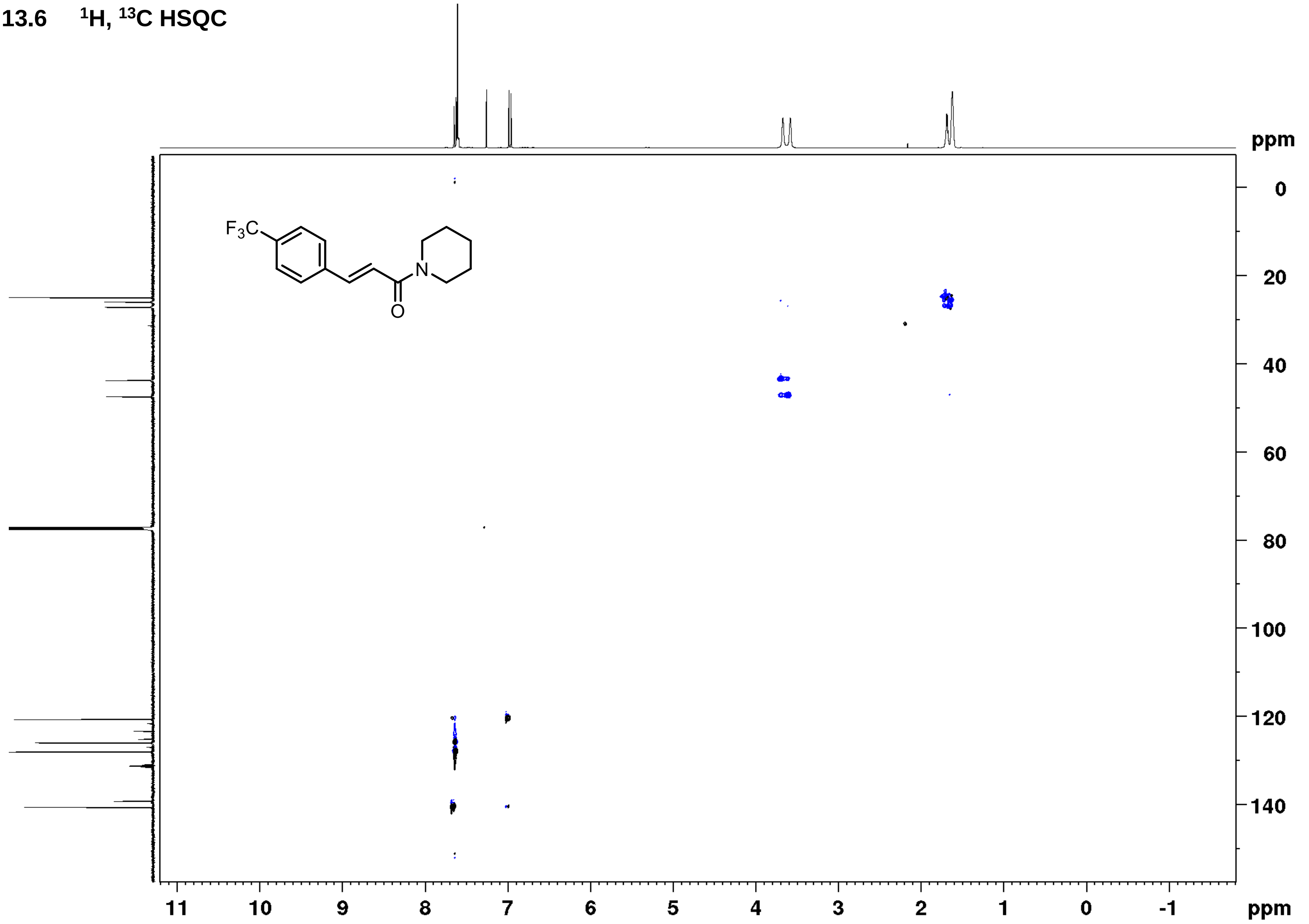

5.13.7 HRMS

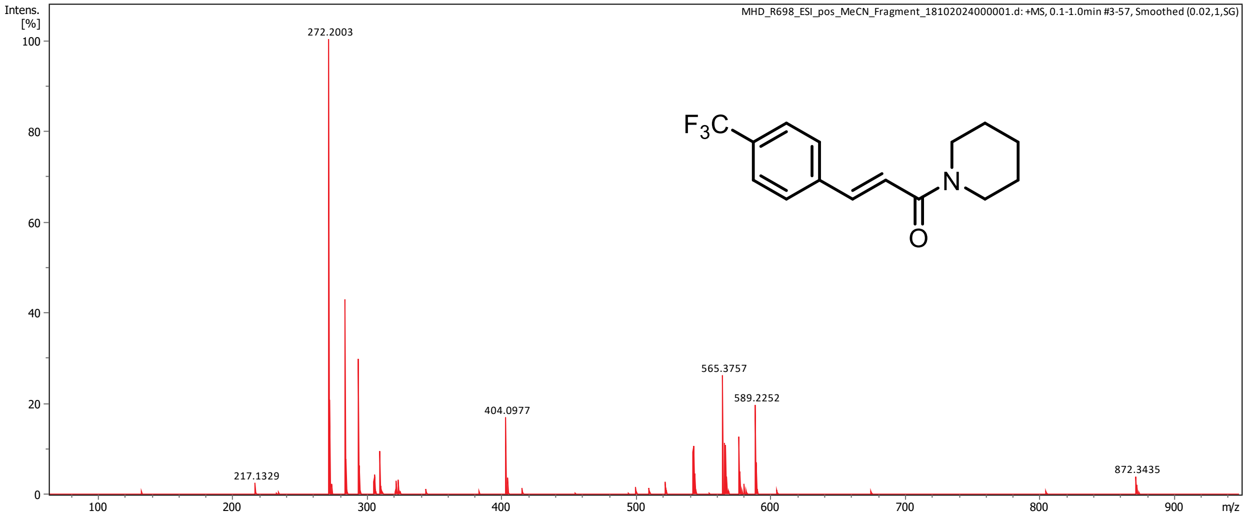

Intens. [%]
MHD_R698_ESI_pos_MeCN_Fragment_18102024000001.d: +MS, 0.1-1.0min #3-57, Smoothed (0.02,1,SG)
272.2003
217.1329
404.0977
565.3757
589.2252
872.3435
F3C
N
O
m/z

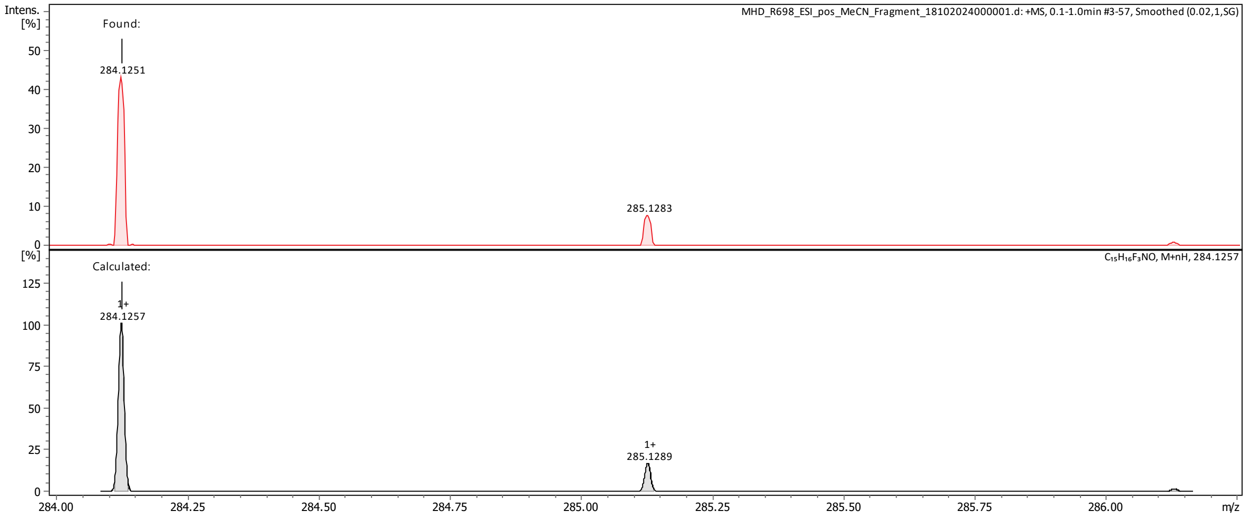

Intens. [%]
MHD_R698_ESI_pos_MeCN_Fragment_18102024000001.d: +MS, 0.1-1.0min #3-57, Smoothed (0.02,1,SG)
Found:
284.1251
285.1283
Calculated:
C15H16F3NO, M+nH, 284.1257
1+
284.1257
1+
285.1289
m/z

5.13.8 IR

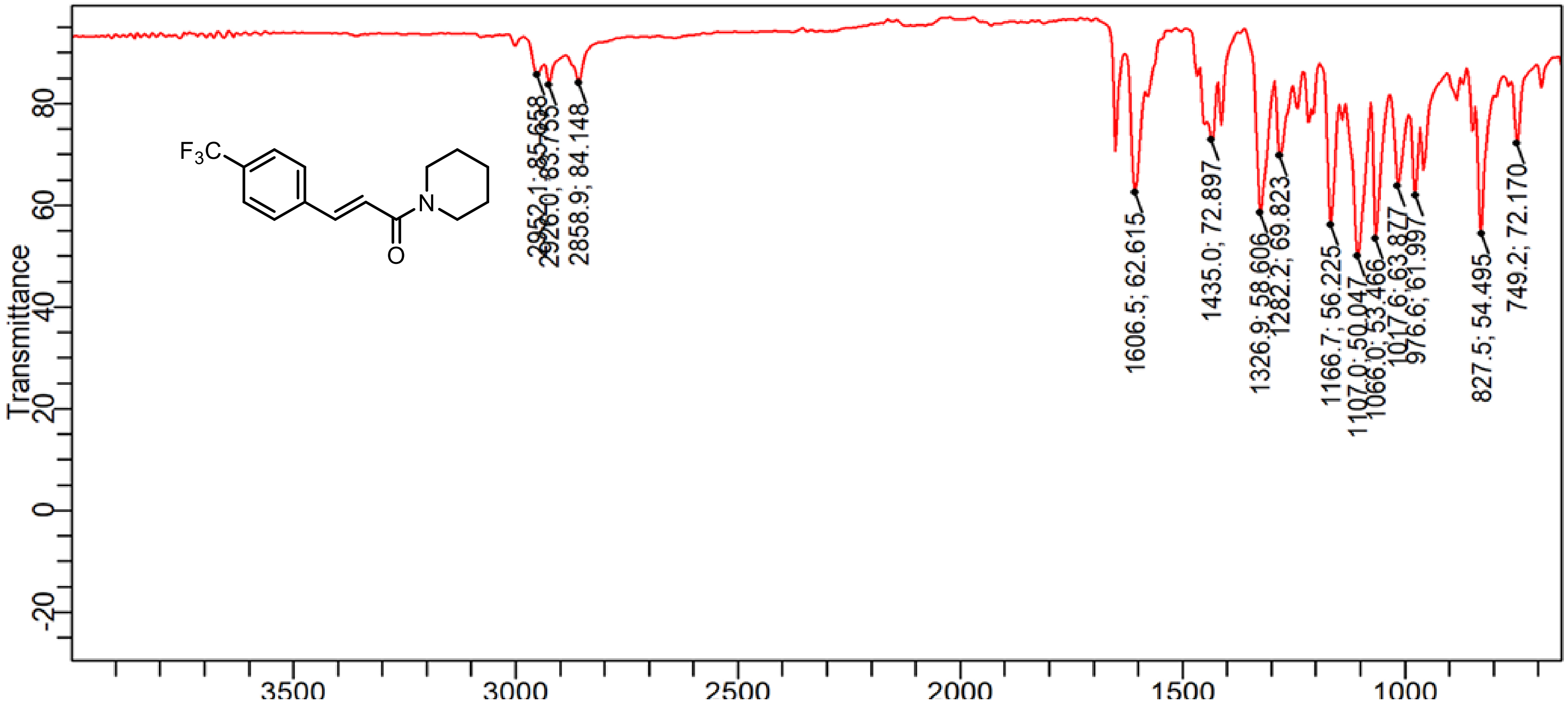

Transmittance
80
60
40
20
0
-20
3500
3000
2500
2000
1500
1000
F3C
N
O
2952.1; 85.658
2926.0; 83.755
2858.9; 84.148
1606.5; 62.615
1435.0; 72.897
1326.9; 58.606
1282.2; 69.823
1166.7; 56.225
1107.0; 50.047
1066.0; 53.466
1017.6; 63.877
976.6; 61.997
827.5; 54.495
749.2; 72.170

5.14 (*E*)-1-(Piperidin-1-yl)-3-(pyridin-3-yl)prop-2-en-1-one (11o) (600 MHz, $CDCl_3$)

5.14.1 ^1H NMR

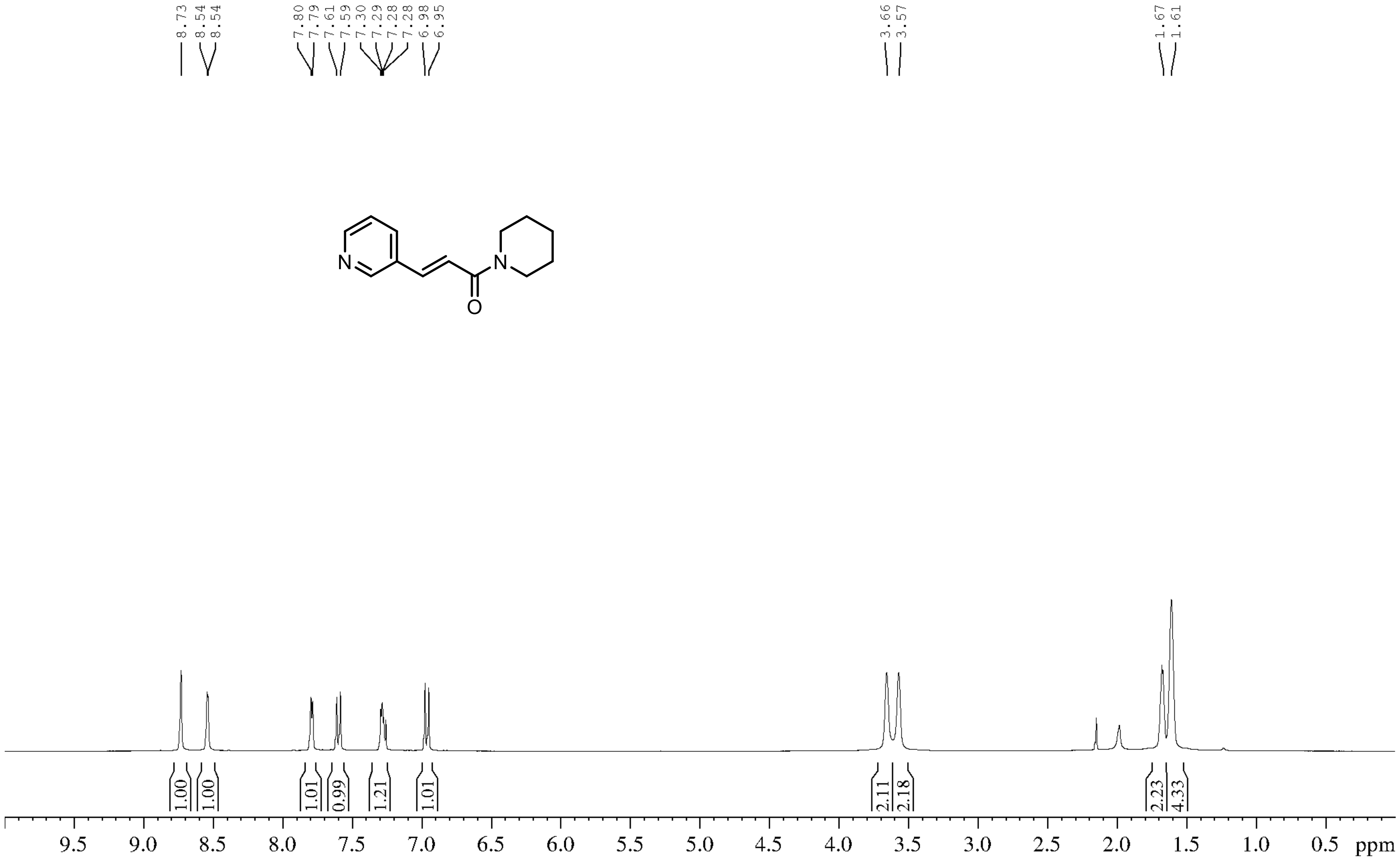

5.14.2 ^13^C NMR

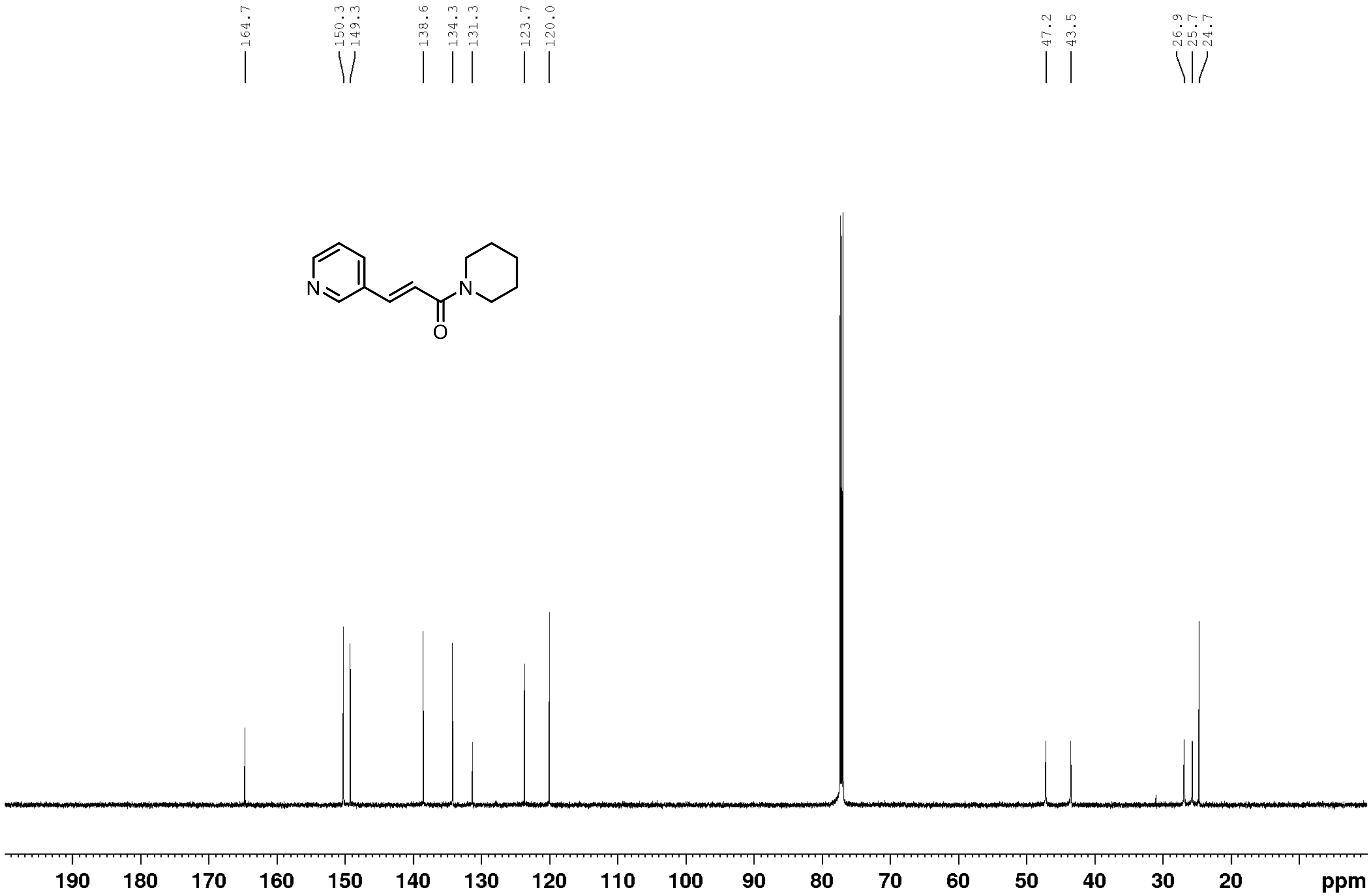

164.7
150.3
149.3
138.6
134.3
131.3
123.7
120.0
47.2
43.5
26.9
25.7
24.7
N
N
O
190
180
170
160
150
140
130
120
110
100
90
80
70
60
50
40
30
20
ppm

5.14.3 ^{1}H, ^{1}H COSY

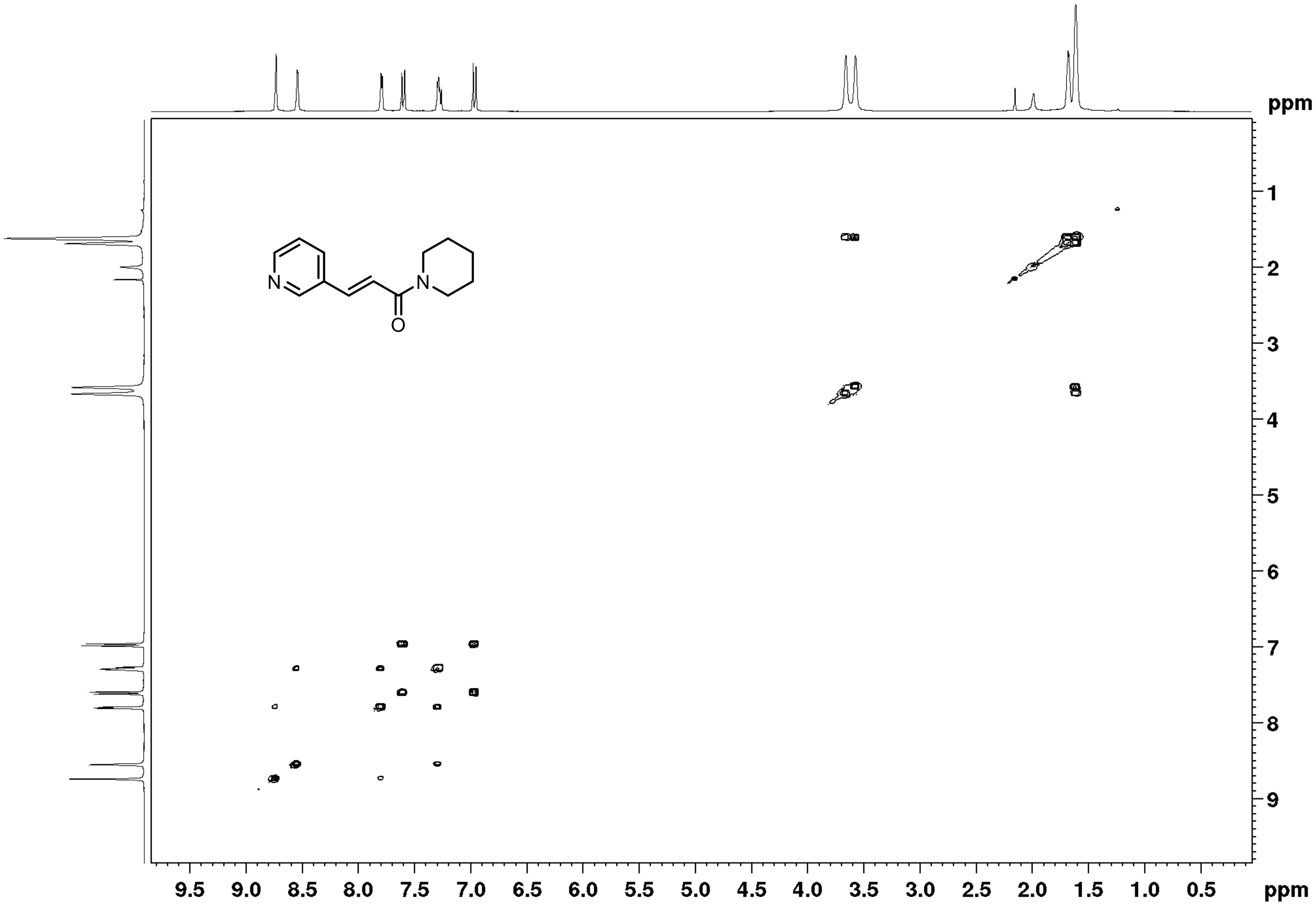

5.14.4 ^{1}H, ^{13}C HMBC

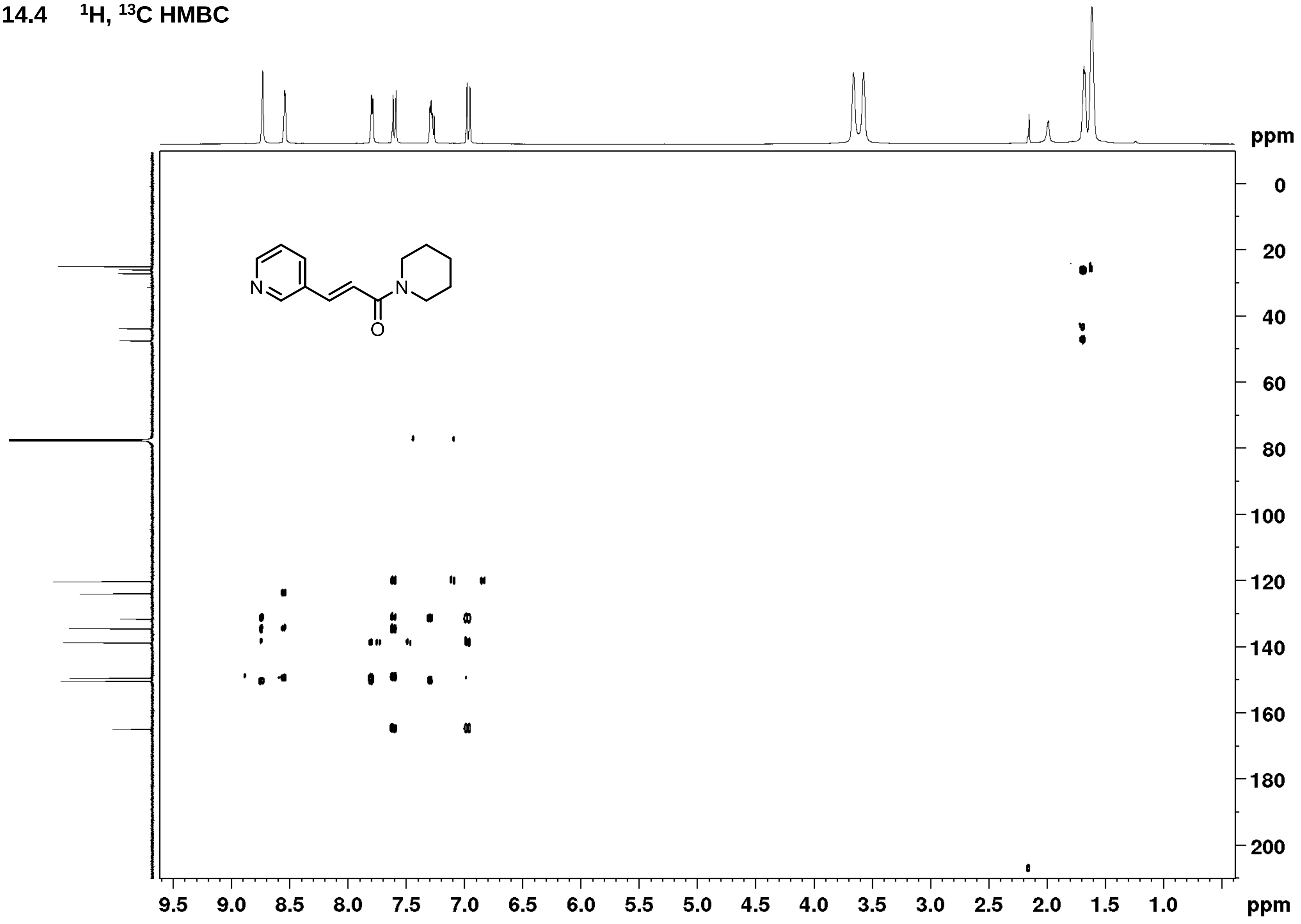

5.14.5 ^{1}H, ^{13}C HSQC

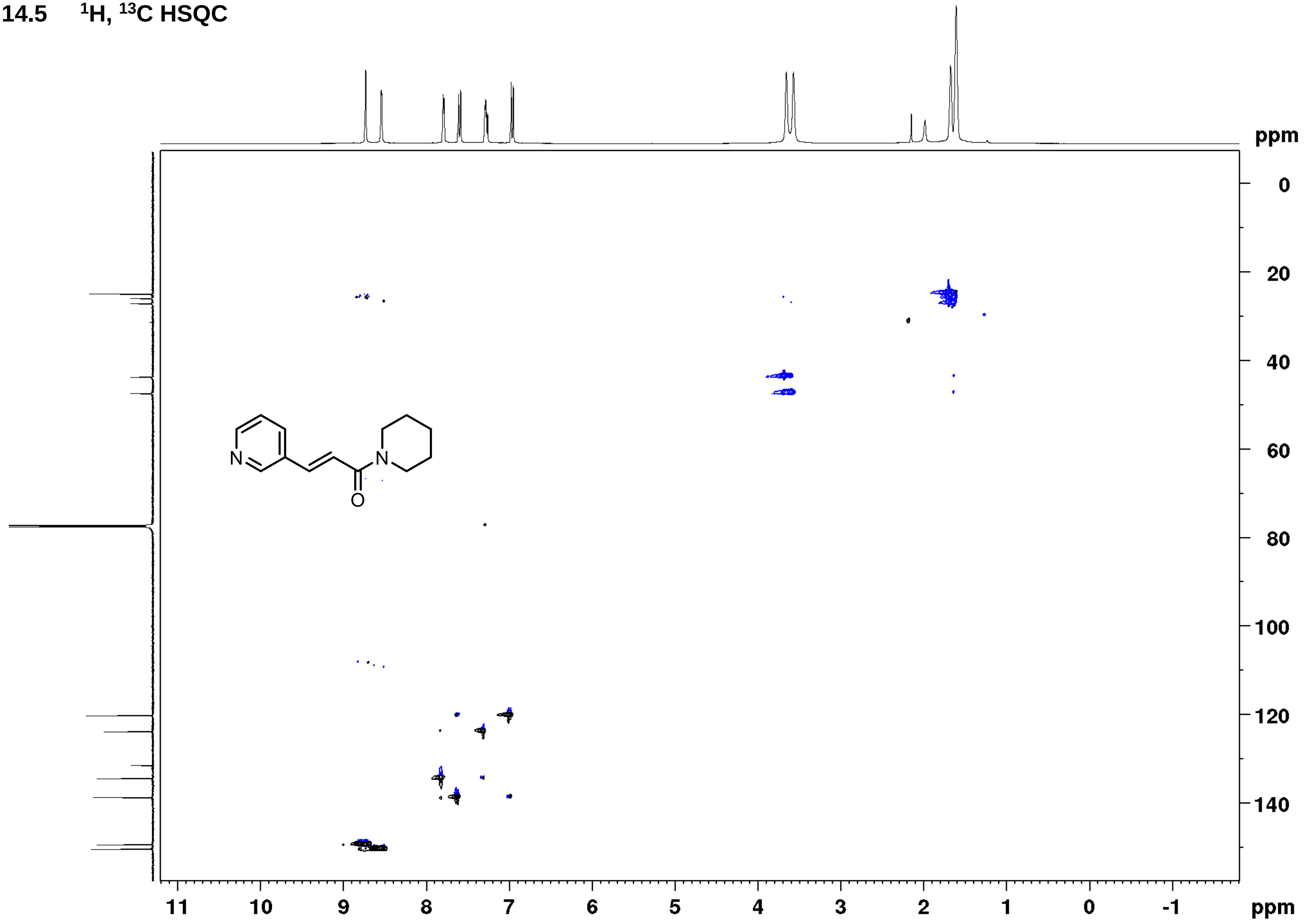

5.14.6 HRMS

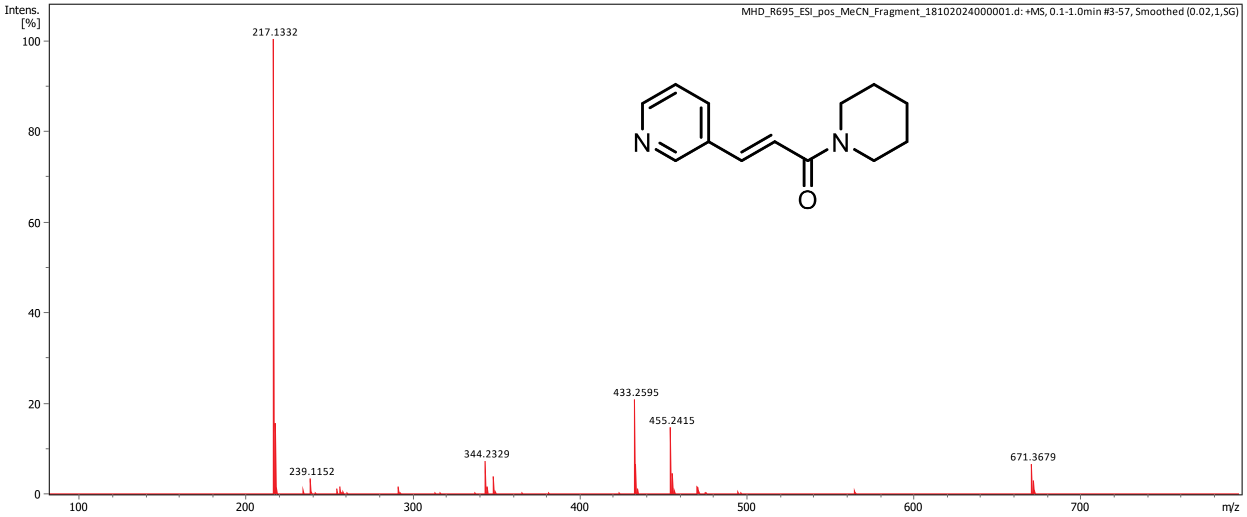

Intens.
[%]
MHD_R695_ESI_pos_MeCN_Fragment_18102024000001.d: +MS, 0.1-1.0min #3-57, Smoothed (0.02,1,SG)
217.1332
239.1152
344.2329
433.2595
455.2415
671.3679
m/z

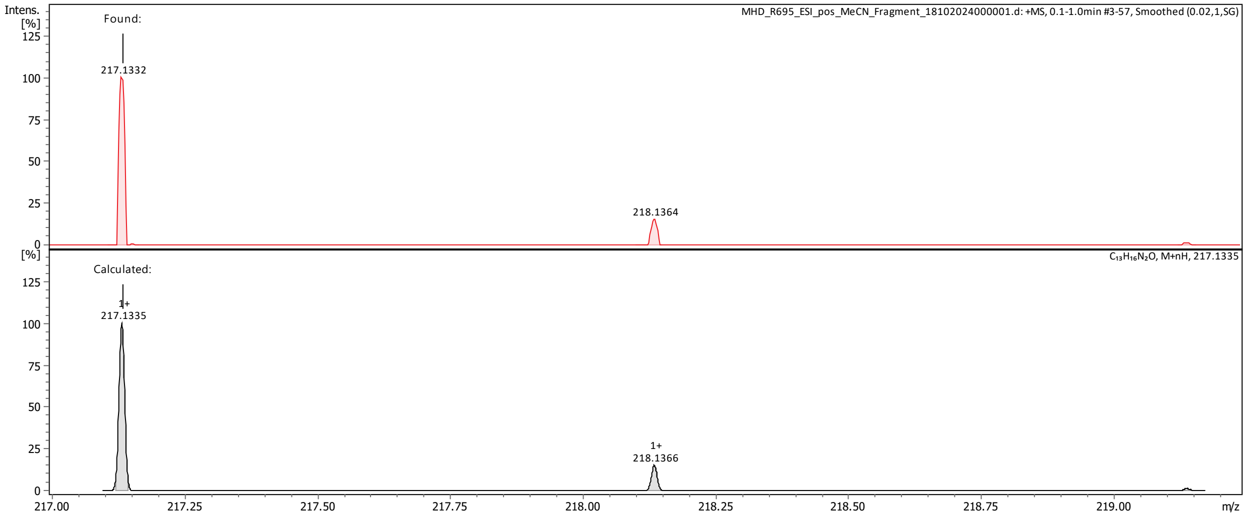

Intens.
[%]
Found:
217.1332
218.1364
MHD_R695_ESI_pos_MeCN_Fragment_18102024000001.d: +MS, 0.1-1.0min #3-57, Smoothed (0.02,1,SG)
Calculated:
1+
217.1335
1+
218.1366
C₁₃H₁₆N₂O, M+nH, 217.1335
m/z

5.14.7 IR

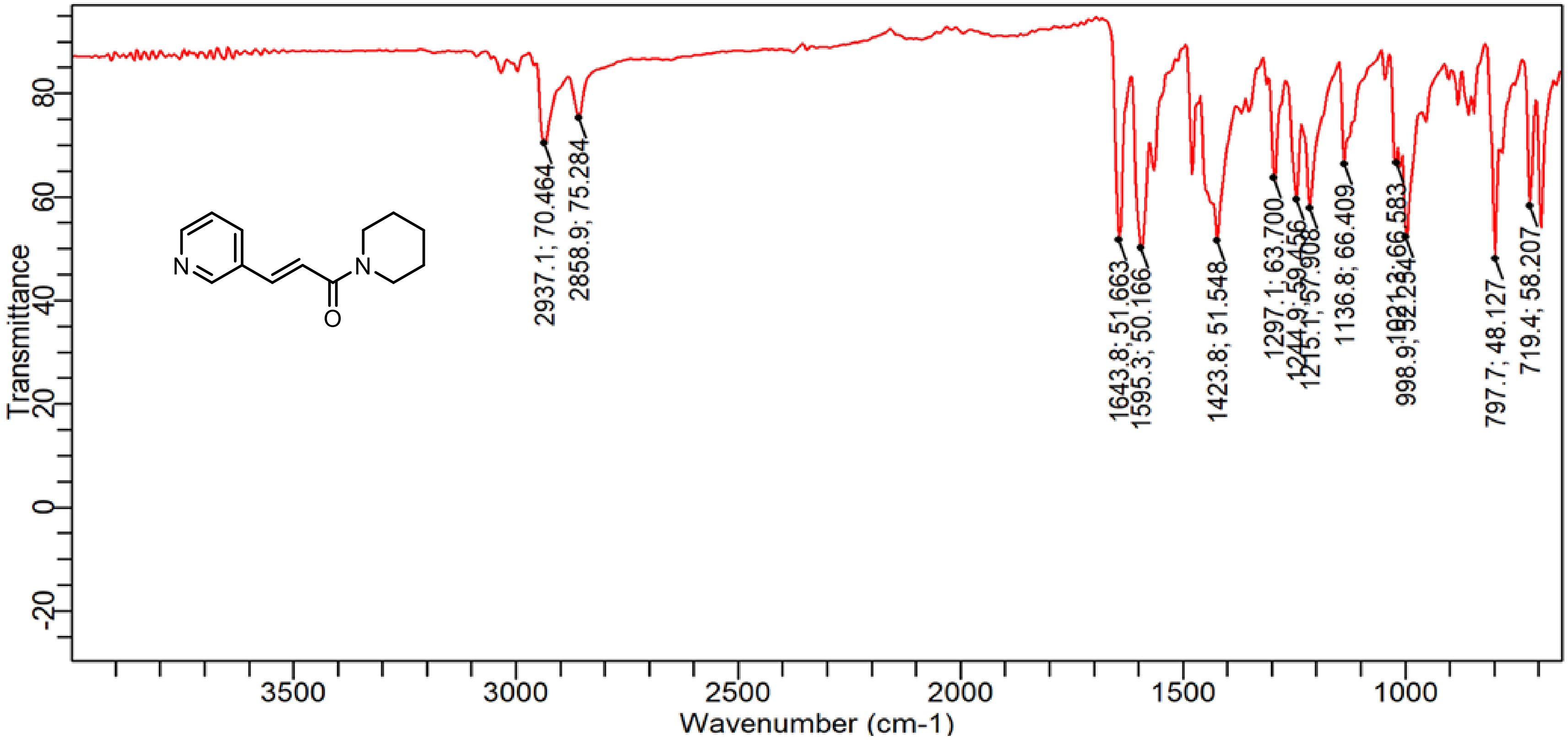

Transmittance
80
60
40
20
0
-20
3500
3000
2500
2000
1500
1000
Wavenumber (cm-1)
N
N
O
2937.1; 70.464
2858.9; 75.284
1643.8; 51.663
1595.3; 50.166
1423.8; 51.548
1297.1; 63.700
1244.9; 59.456
1215.1; 57.908
1136.8; 66.409
1021.3; 66.583
998.9; 52.254
797.7; 48.127
719.4; 58.207

5.15 (*E*)-3-(Furan-2-yl)-1-(piperidin-1-yl)prop-2-en-1-one (11p) (600 MHz, $CDCl_3$)

5.15.1 ^{1}H NMR

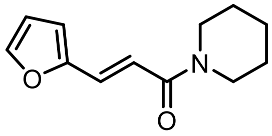

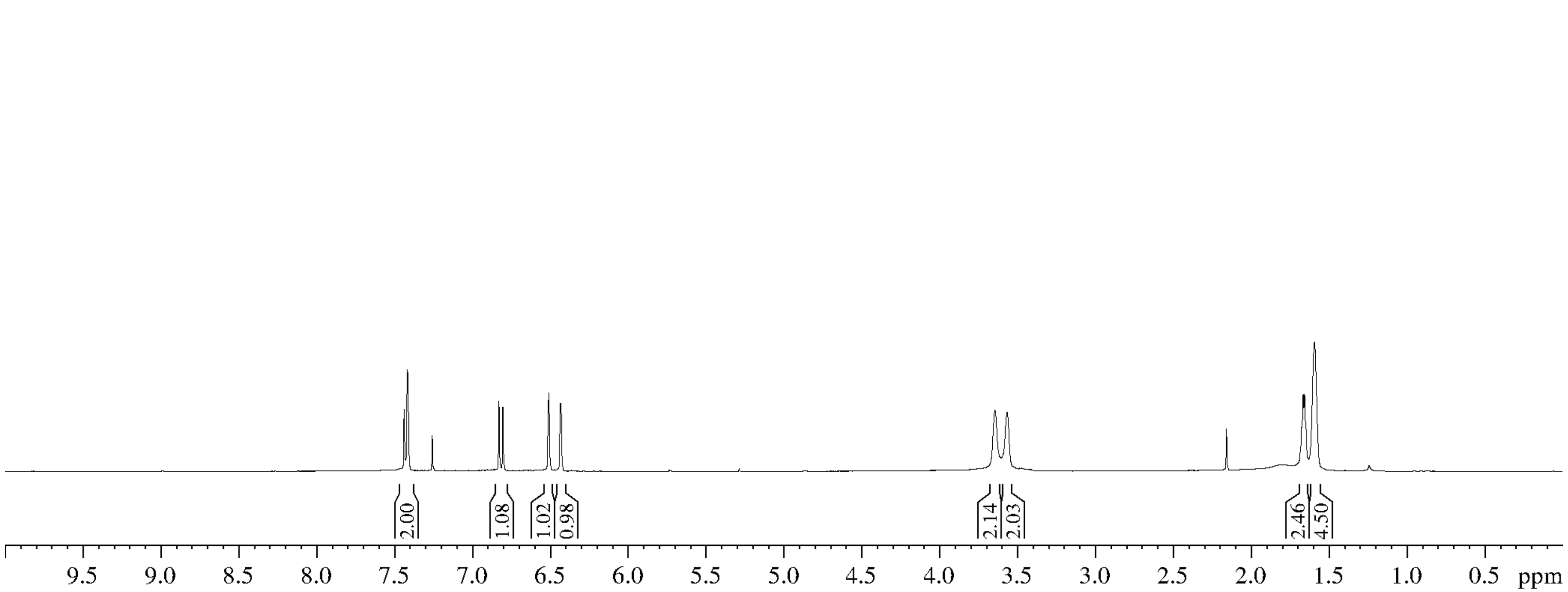

5.15.2 ^{13}C NMR

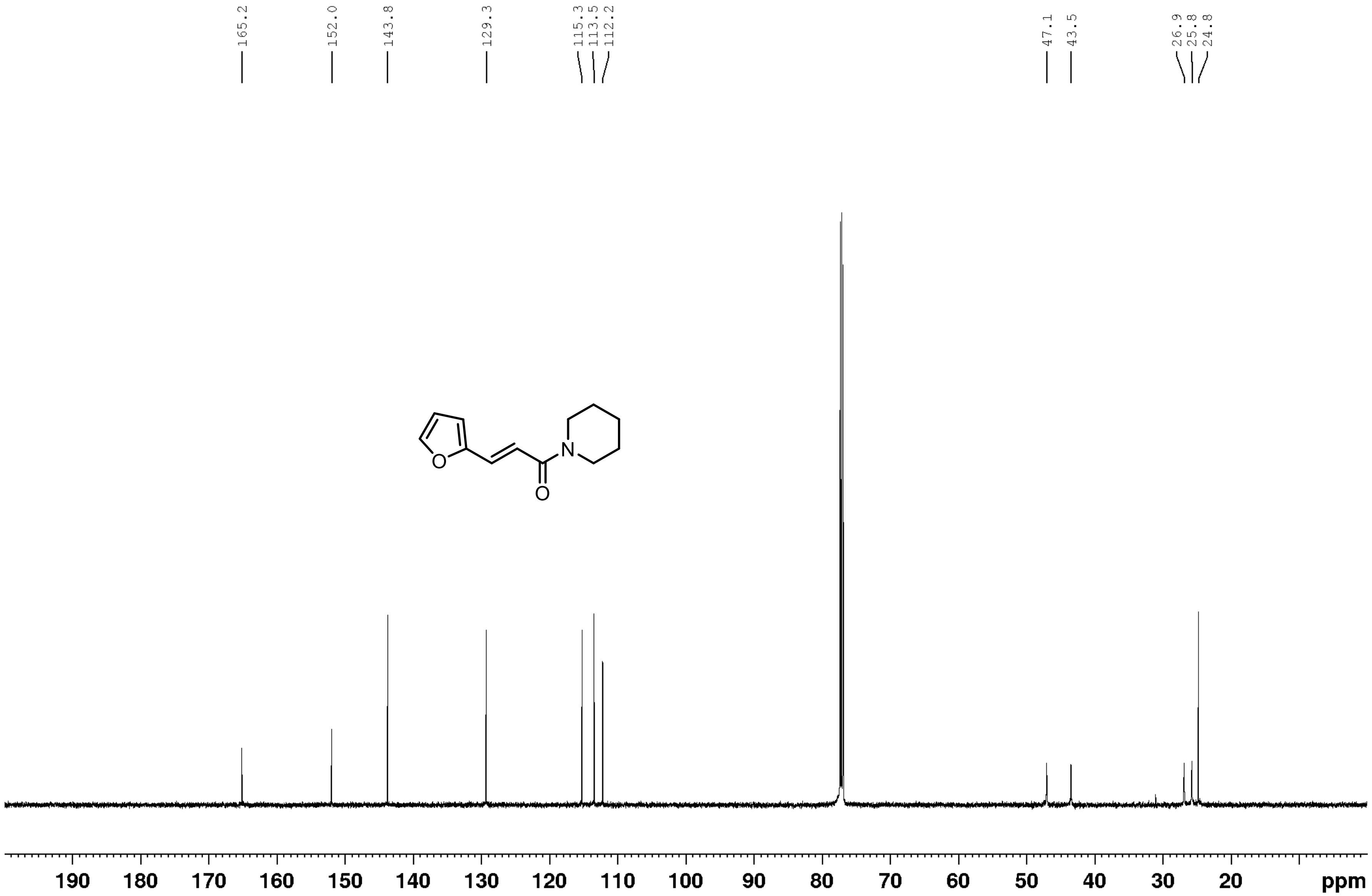
165.2
152.0
143.8
129.3
115.3
113.5
112.2
47.1
43.5
26.9
25.8
24.8
190 180 170 160 150 140 130 120 110 100 90 80 70 60 50 40 30 20
ppm
O
N
O

5.15.3 ^{1}H, ^{1}H COSY

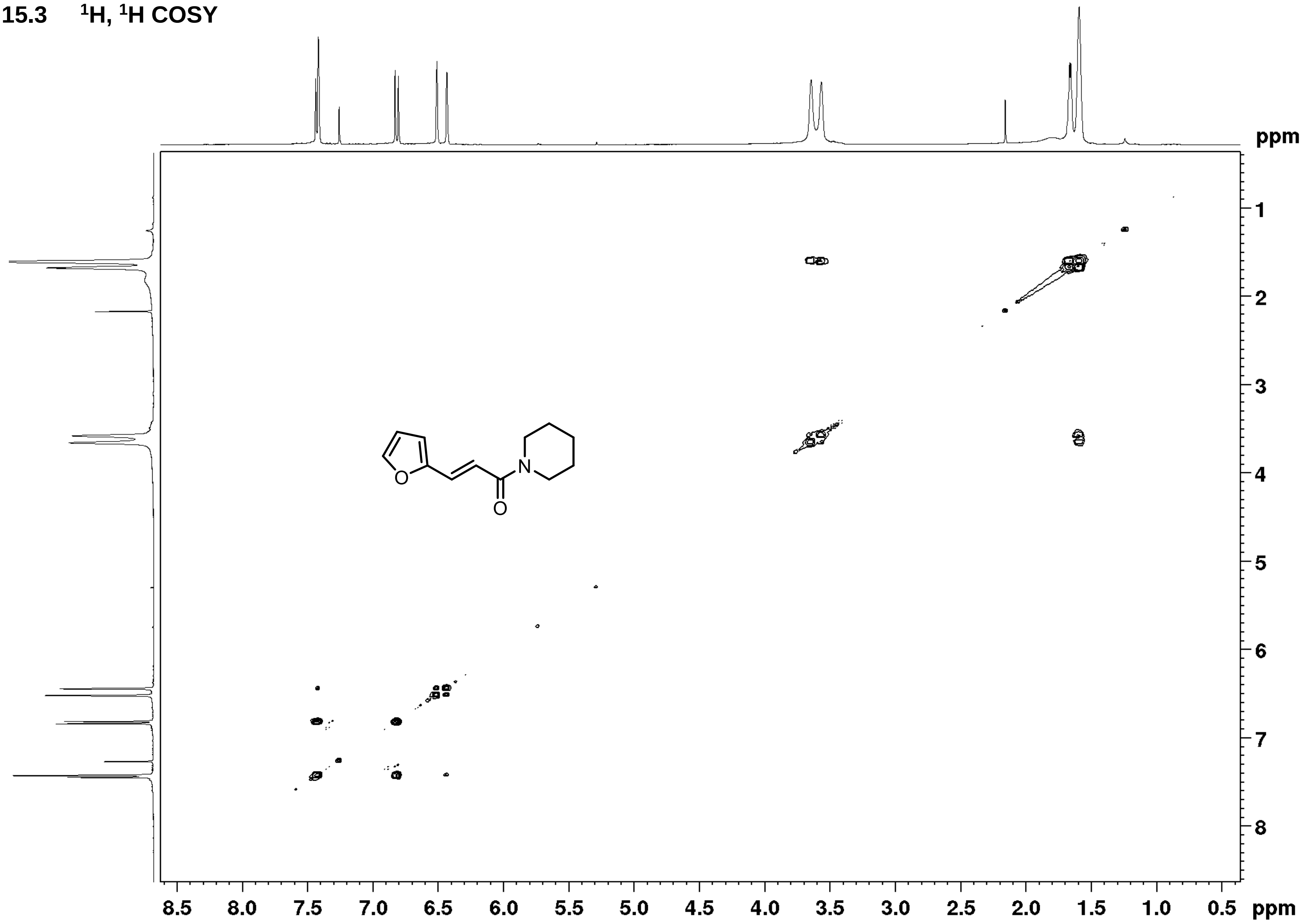

5.15.4 ^{1}H, ^{13}C HMBC

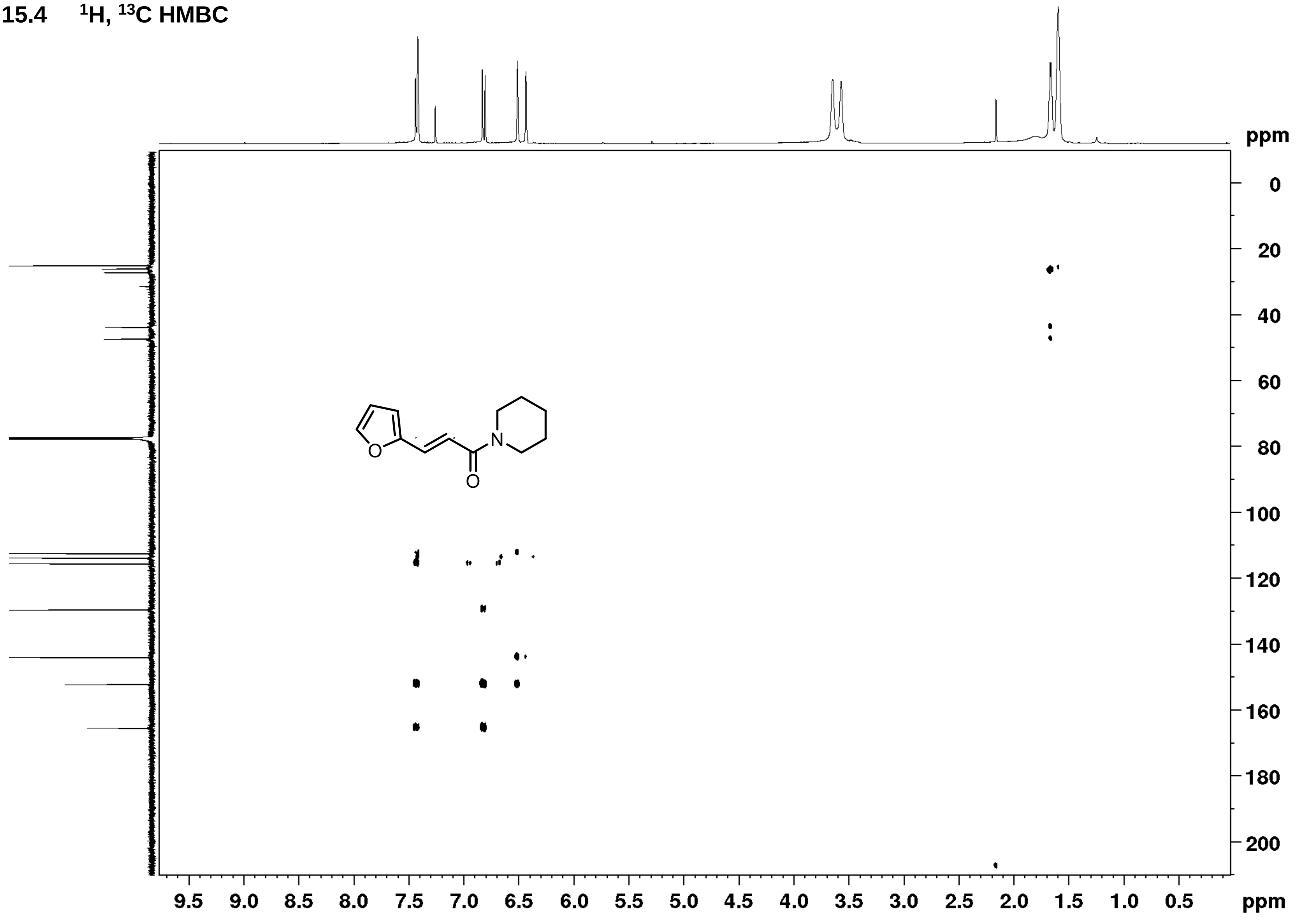

5.15.5 ^{1}H, ^{13}C HSQC

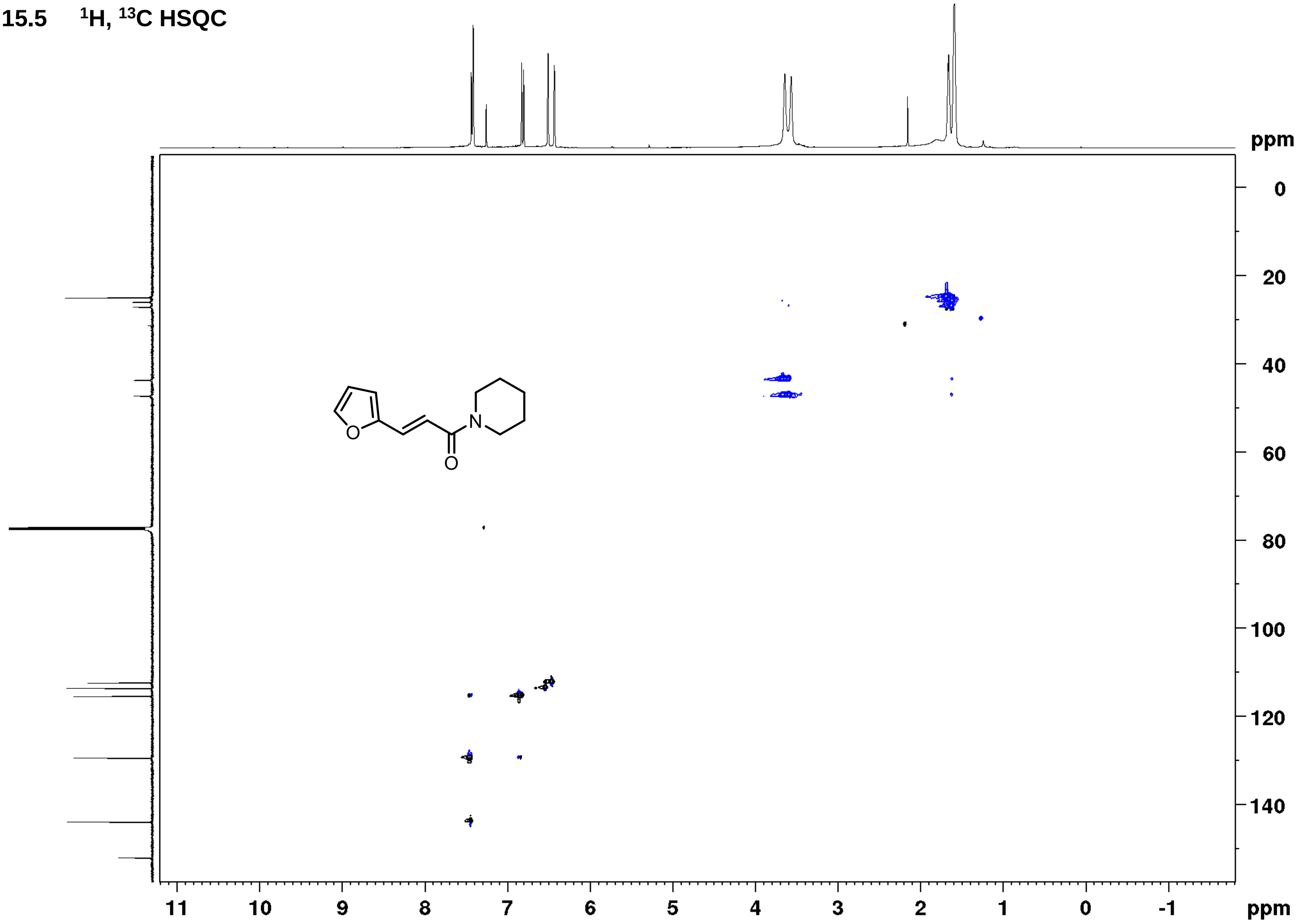

5.15.6 HRMS

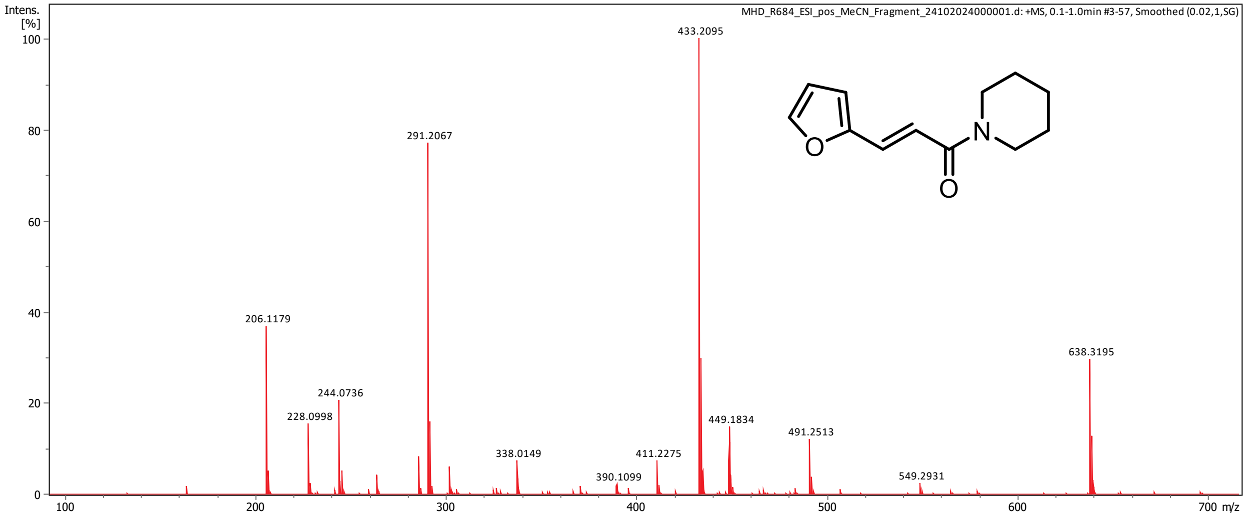
MHD_R684_ESI_pos_MeCN_Fragment_24102024000001.d: +MS, 0.1-1.0min #3-57, Smoothed (0.02,1,SG)
Intens. [%]
433.2095
291.2067
206.1179
244.0736
228.0998
338.0149
390.1099
411.2275
449.1834
491.2513
549.2931
638.3195
m/z

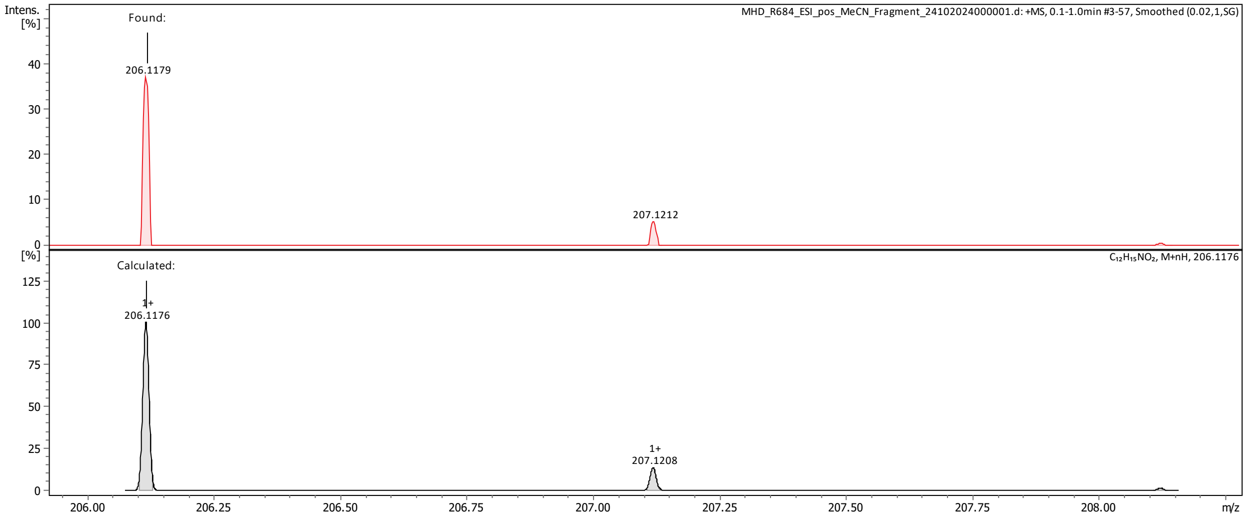
MHD_R684_ESI_pos_MeCN_Fragment_24102024000001.d: +MS, 0.1-1.0min #3-57, Smoothed (0.02,1,SG)
Intens. [%]
Found:
206.1179
207.1212
$C_{12}H_{15}NO_2$, M+nH, 206.1176
Calculated:
1+
206.1176
1+
207.1208
m/z

5.15.7 IR

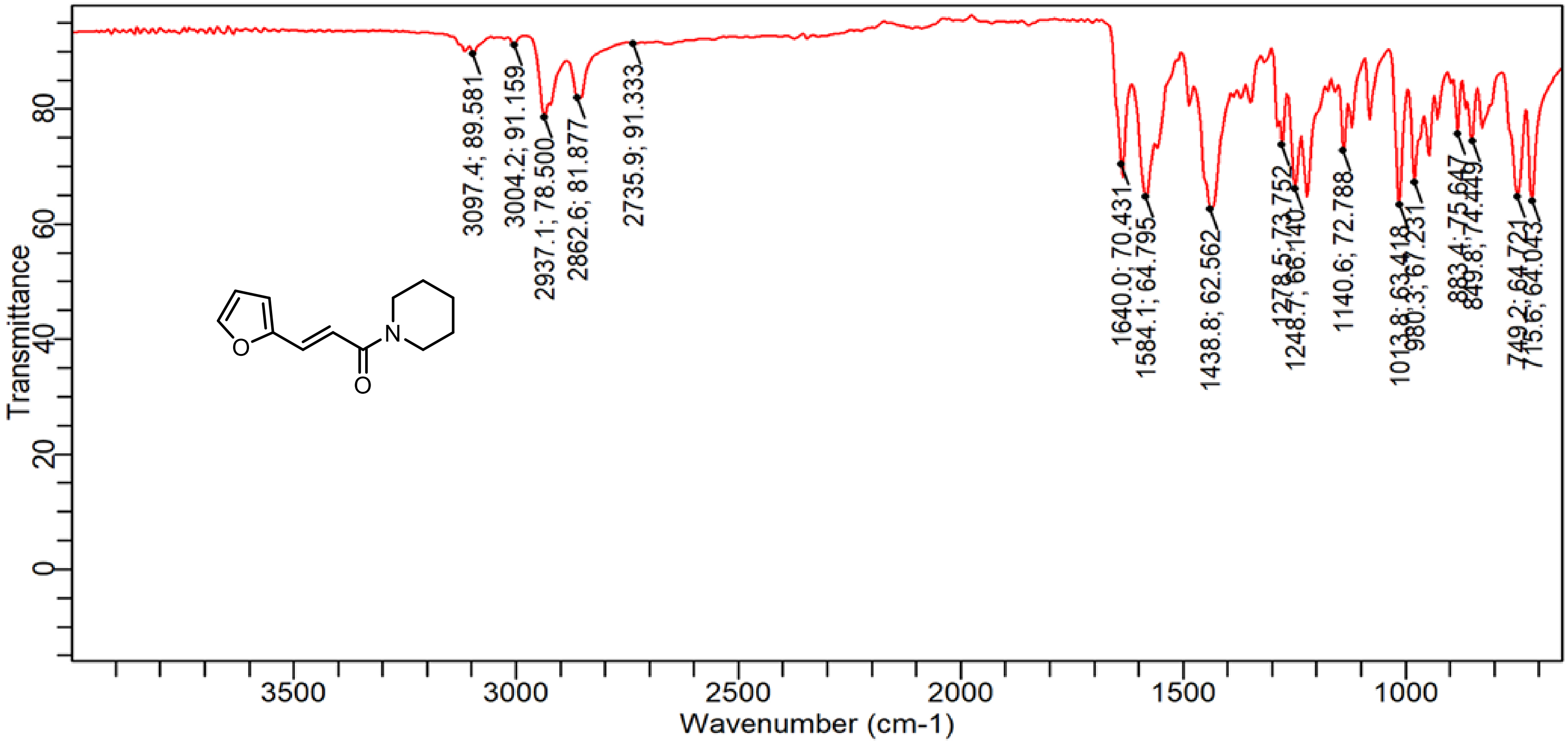

3097.4; 89.581
3004.2; 91.159
2937.1; 78.500
2862.6; 81.877
2735.9; 91.333
1640.0; 70.431
1584.1; 64.795
1438.8; 62.562
1278.5; 73.752
1248.7; 66.140
1140.6; 72.788
1013.8; 63.418
980.3; 67.231
883.4; 75.647
849.8; 74.449
749.2; 64.721
715.6; 64.043
Transmittance
Wavenumber (cm-1)
O
N
O

5.16 3,3-Diphenyl-1-(piperidin-1-yl)prop-2-en-1-one (11q) (600 MHz, $CDCl_3$)

5.16.1 ^{1}H NMR

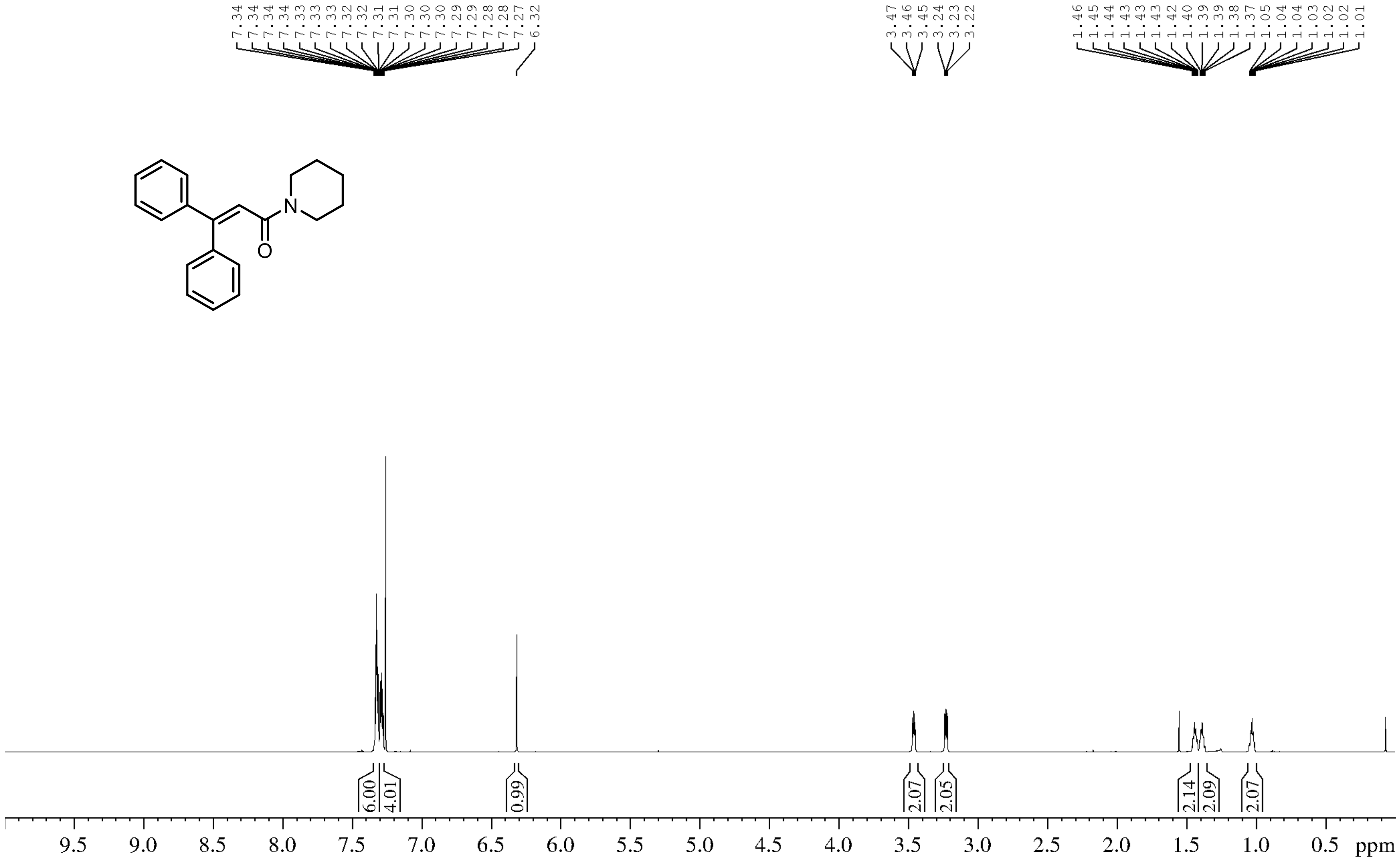

5.16.2 ^{13}C NMR

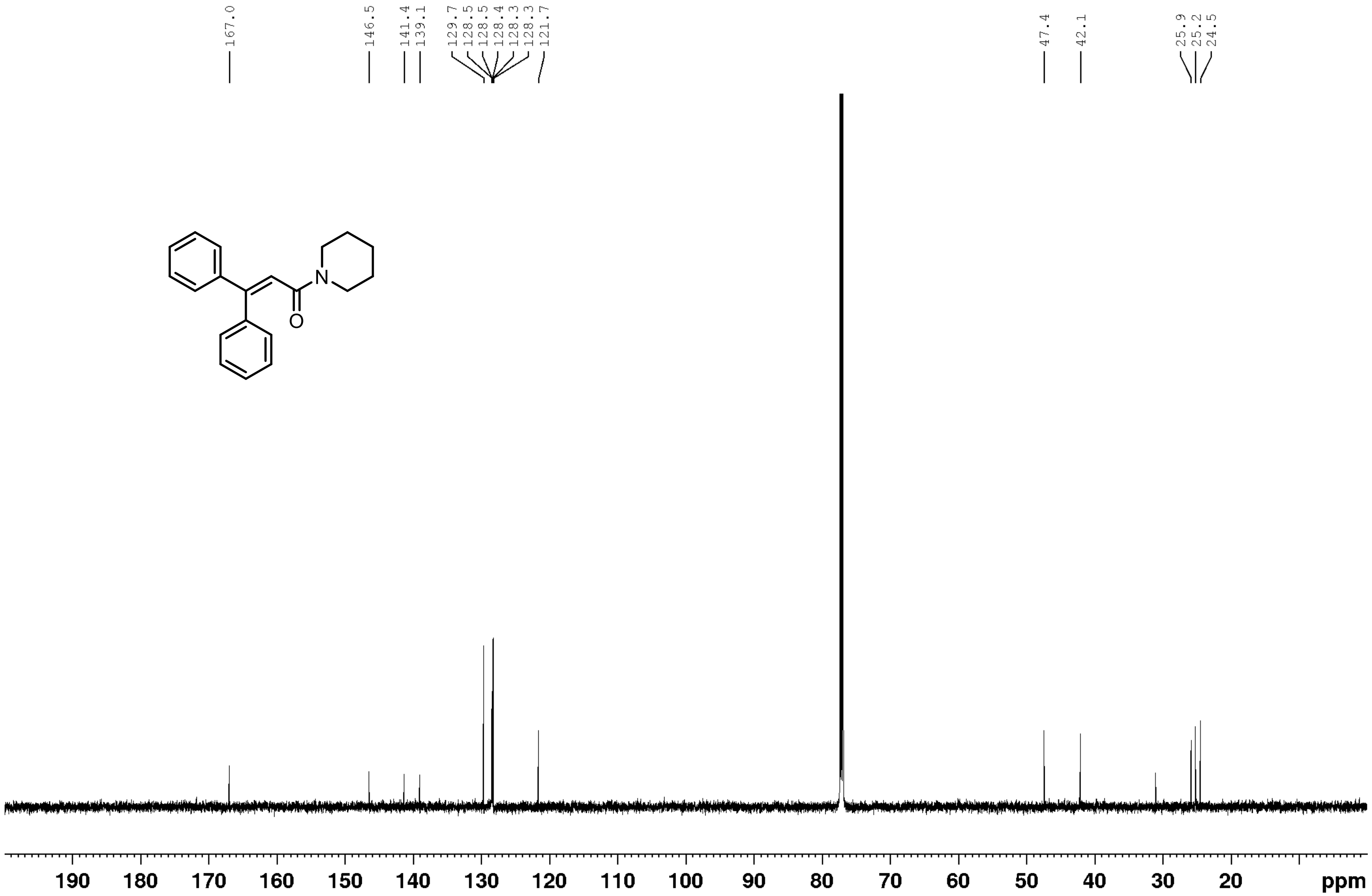

167.0
146.5
141.4
139.1
129.7
128.5
128.5
128.4
128.3
128.3
121.7
47.4
42.1
25.9
25.2
24.5
N
O
190 180 170 160 150 140 130 120 110 100 90 80 70 60 50 40 30 20 ppm

5.16.3 $^{1}H, ^{1}H$ COSY

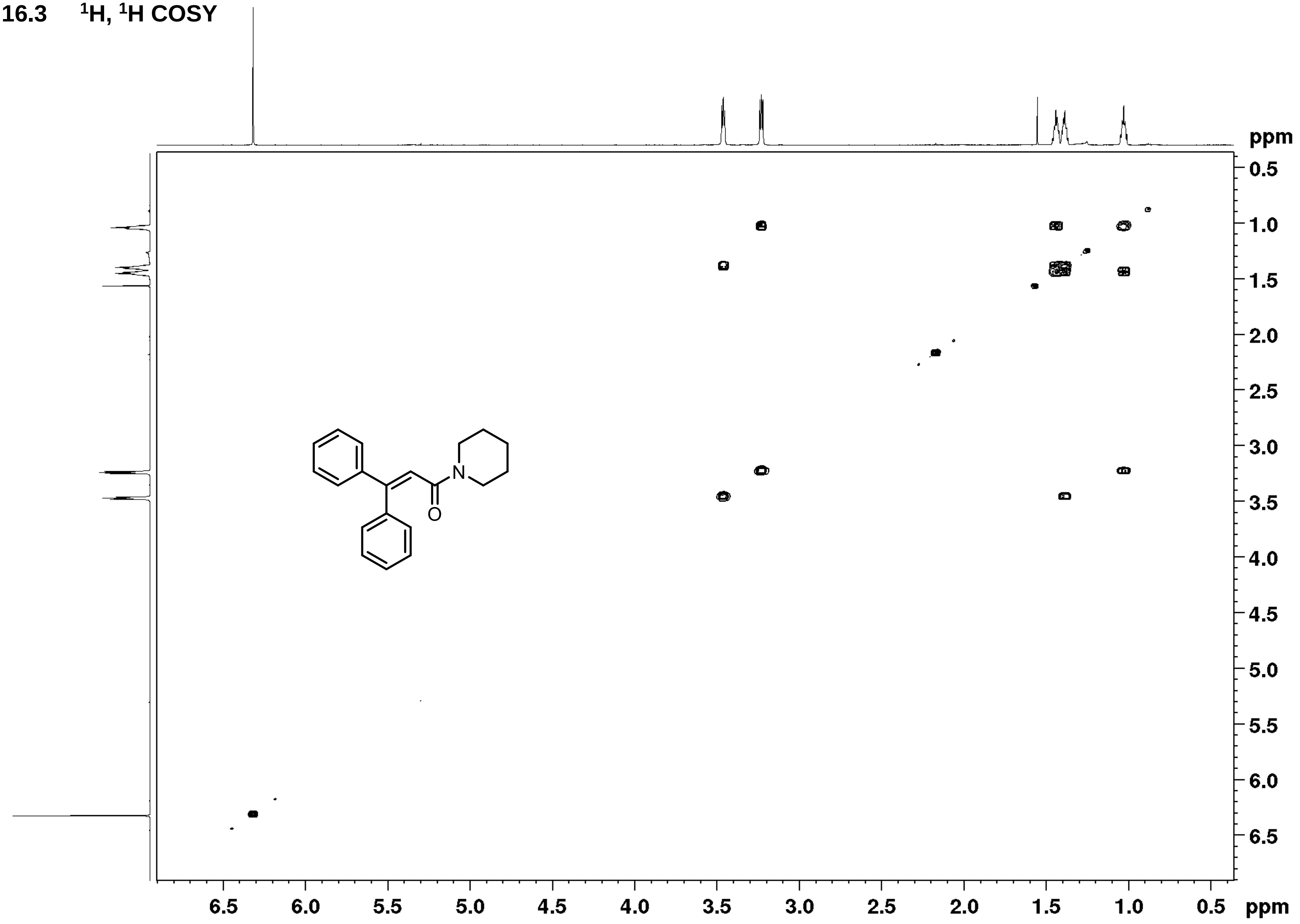

5.16.4 ^{1}H, ^{13}C HMBC

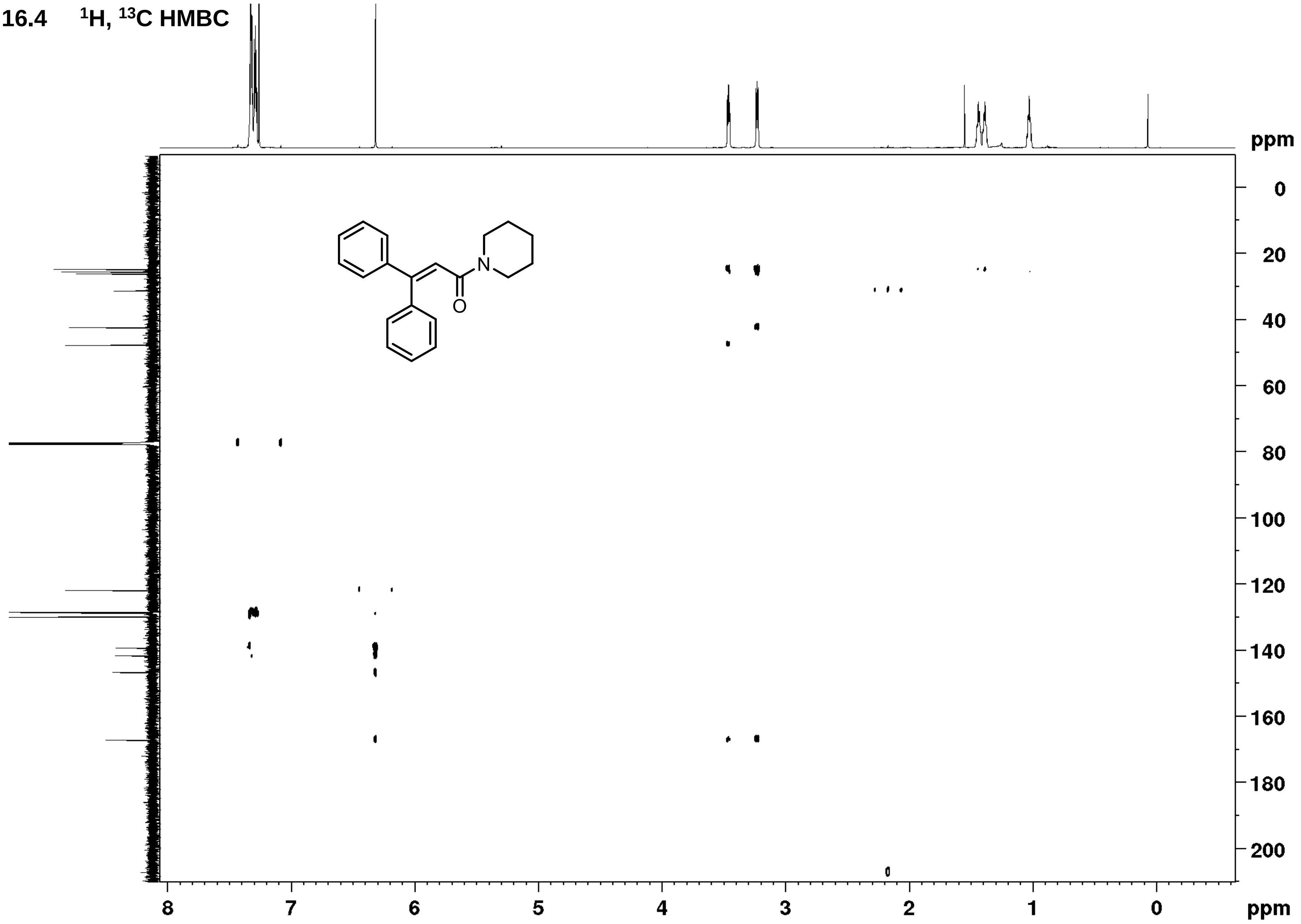

5.16.5 ^{1}H, ^{13}C HSQC

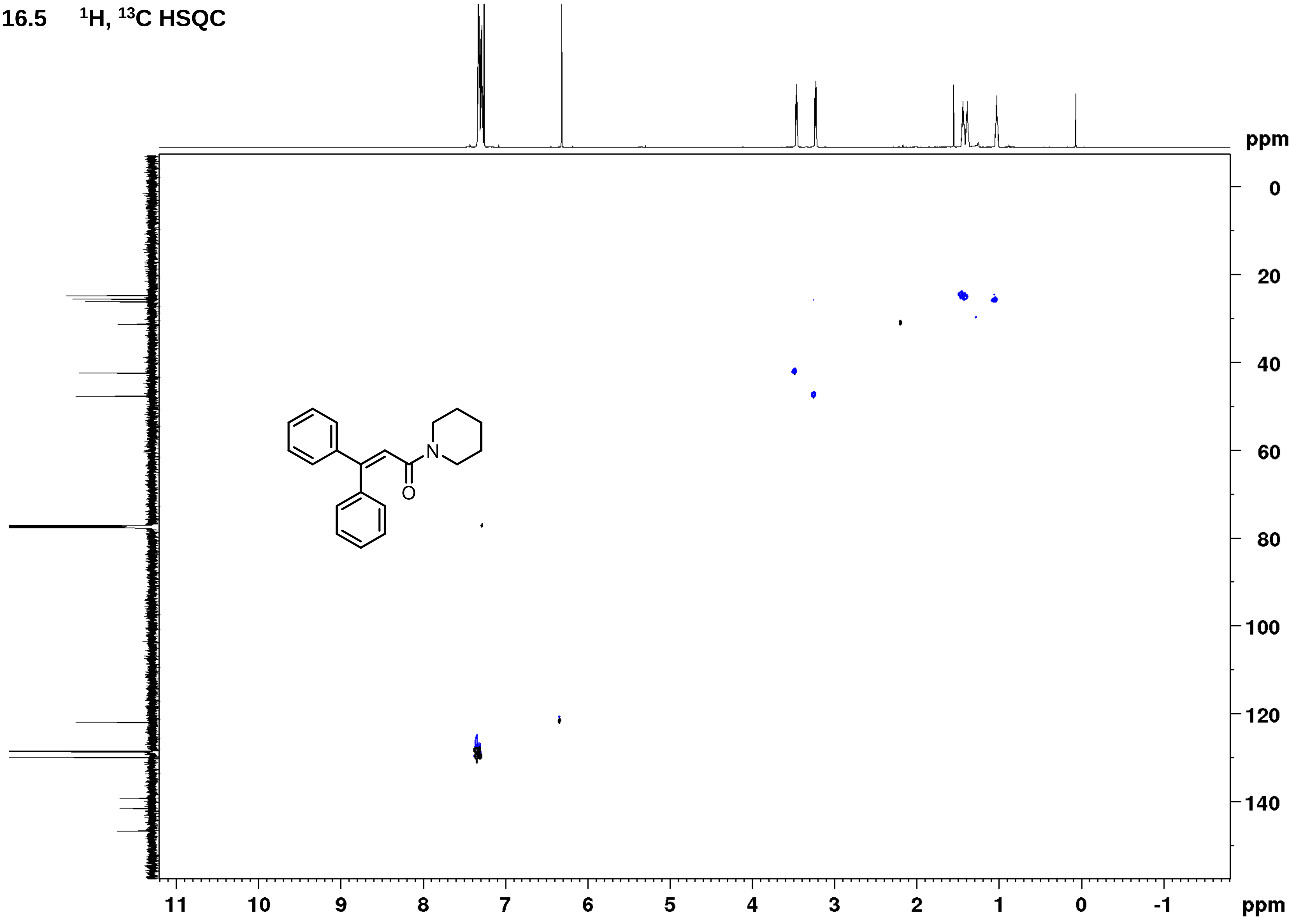

5.16.6 HRMS

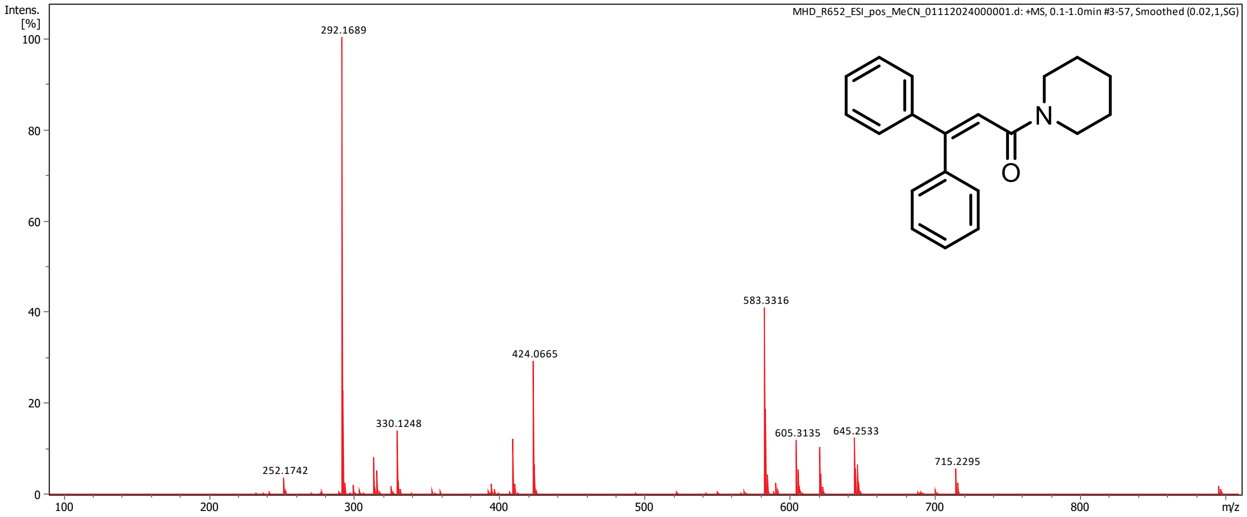

MHD_R652_ESI_pos_MeCN_01112024000001.d: +MS, 0.1-1.0min #3-57, Smoothed (0.02,1,SG)
Intens. [%]
292.1689
583.3316
424.0665
330.1248
605.3135
645.2533
715.2295
252.1742
N
O
m/z

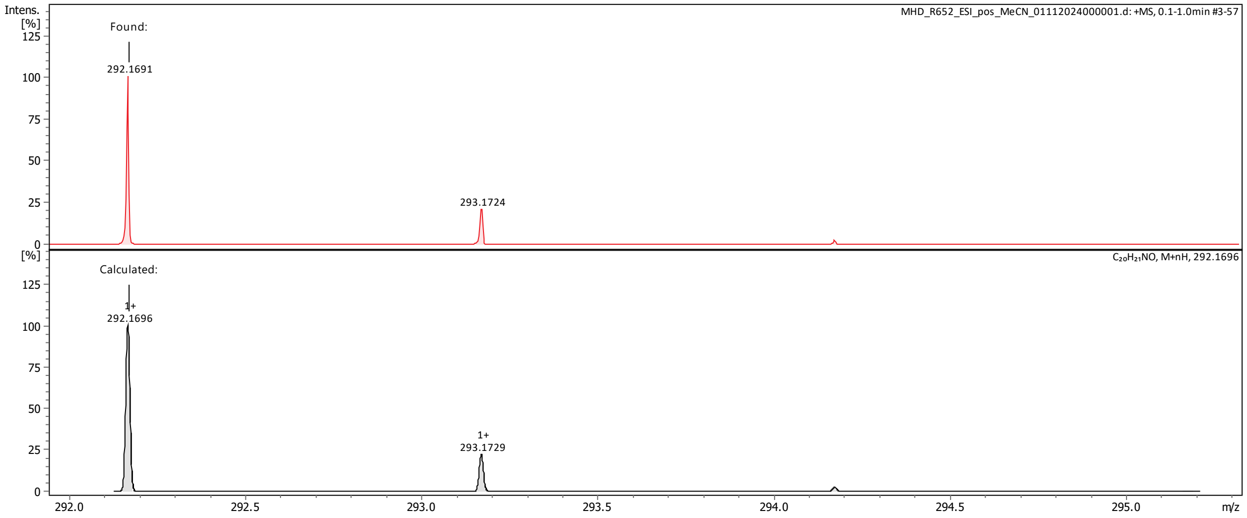

MHD_R652_ESI_pos_MeCN_01112024000001.d: +MS, 0.1-1.0min #3-57
Intens. [%]
Found:
292.1691
293.1724
$C_{20}H_{21}NO$, M+nH, 292.1696
Calculated:
1+
292.1696
1+
293.1729
m/z

5.16.7 IR

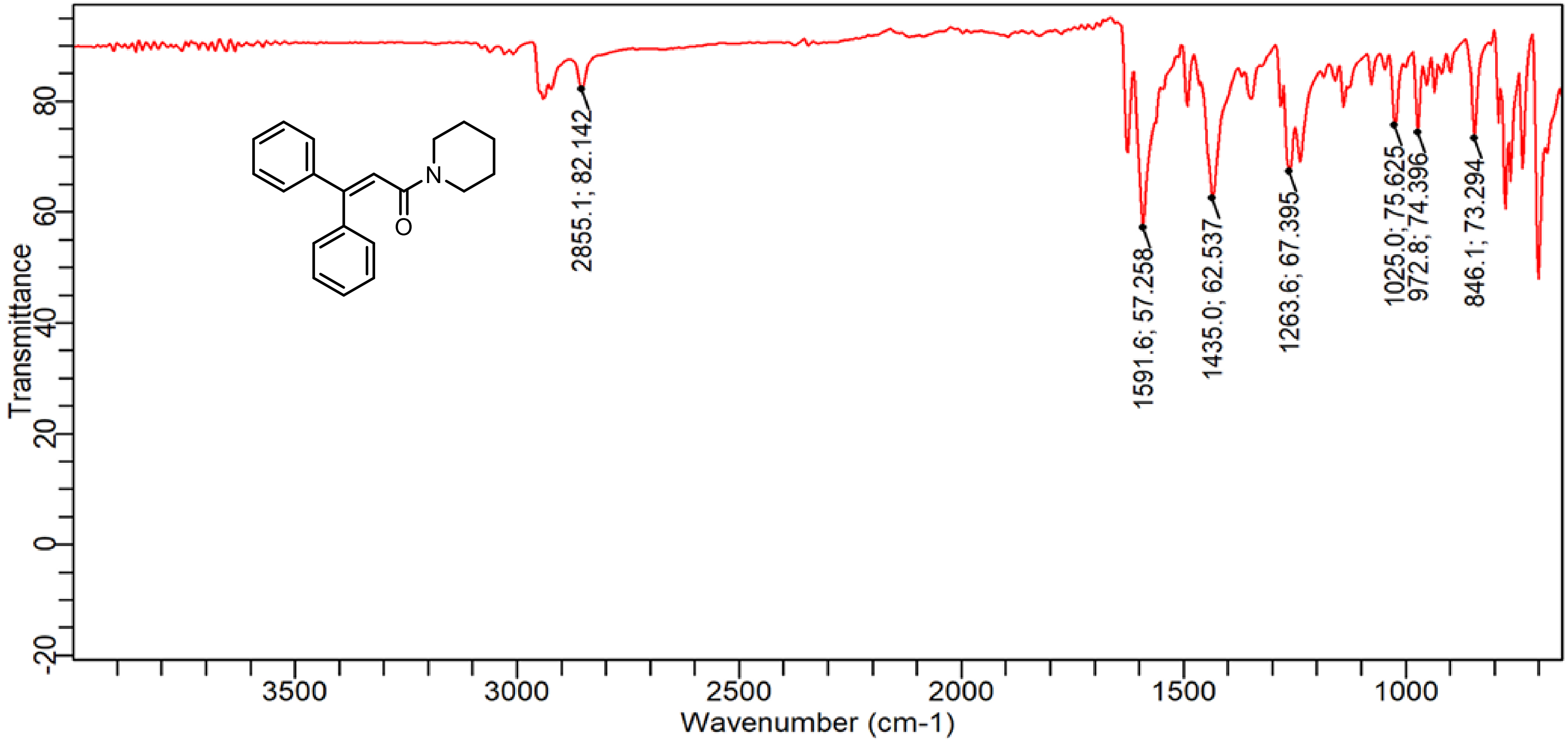

Transmittance
Wavenumber (cm-1)
2855.1; 82.142
1591.6; 57.258
1435.0; 62.537
1263.6; 67.395
1025.0; 75.625
972.8; 74.396
846.1; 73.294
N
O

5.17 (*E*)-3-phenyl-1-(piperidin-1-yl)hept-2-en-1-one (11r) (600 MHz, $CDCl_3$)

5.17.1 ^{1}H NMR

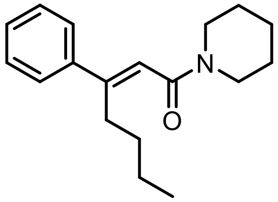

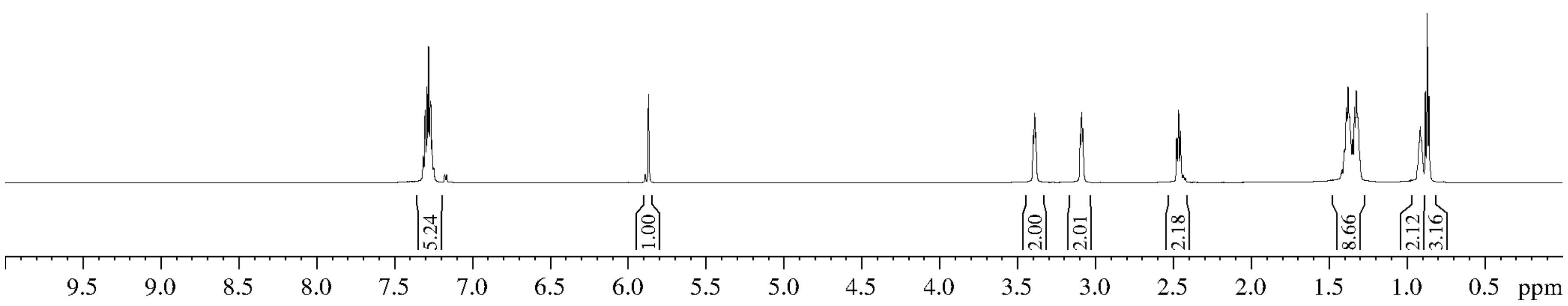

5.17.2 ^{13}C NMR

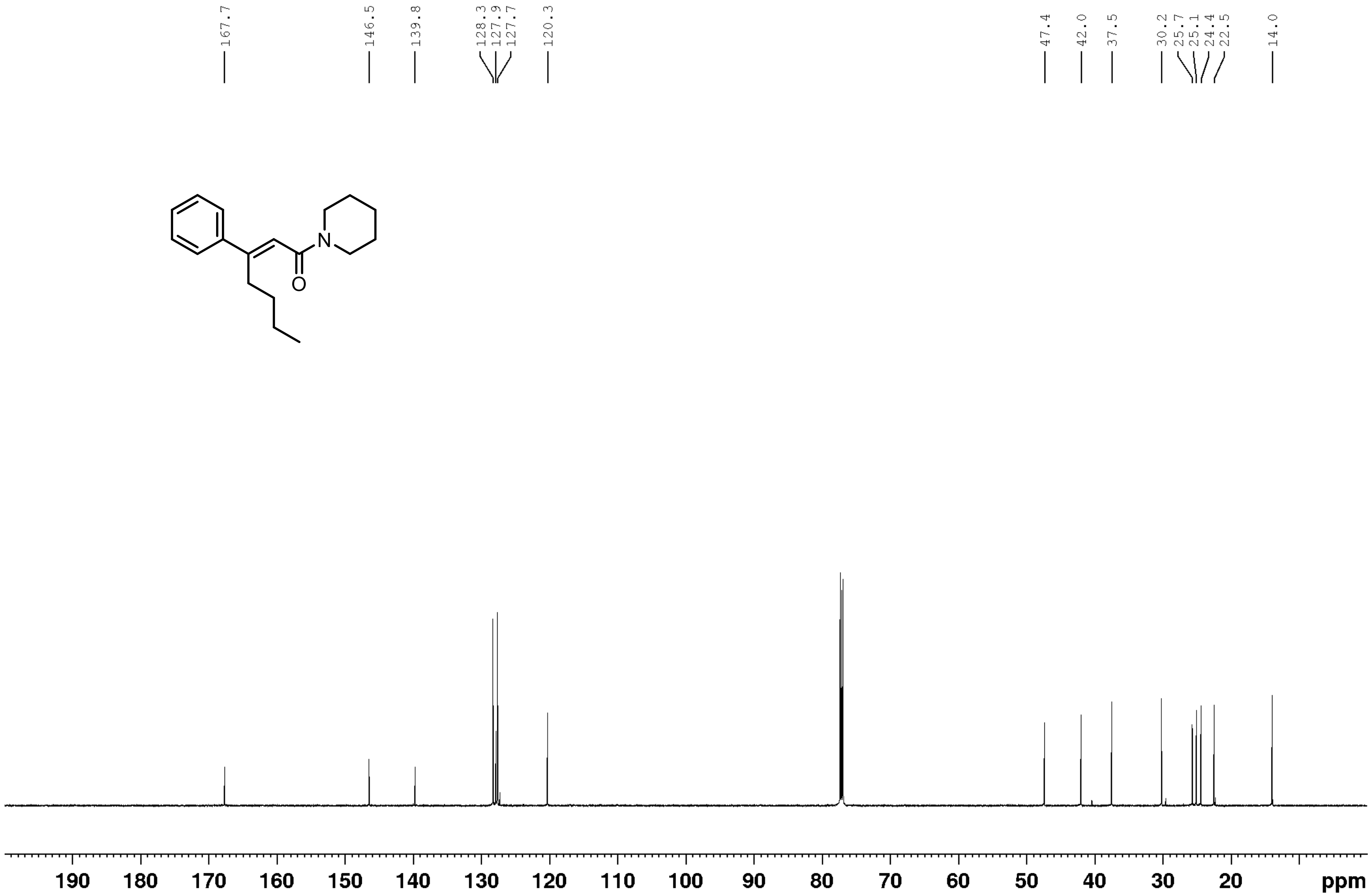
167.7
146.5
139.8
128.3
127.9
127.7
120.3
47.4
42.0
37.5
30.2
25.7
25.1
24.4
22.5
14.0
N
O
190 180 170 160 150 140 130 120 110 100 90 80 70 60 50 40 30 20
ppm

5.17.3 ^{1}H, ^{1}H COSY

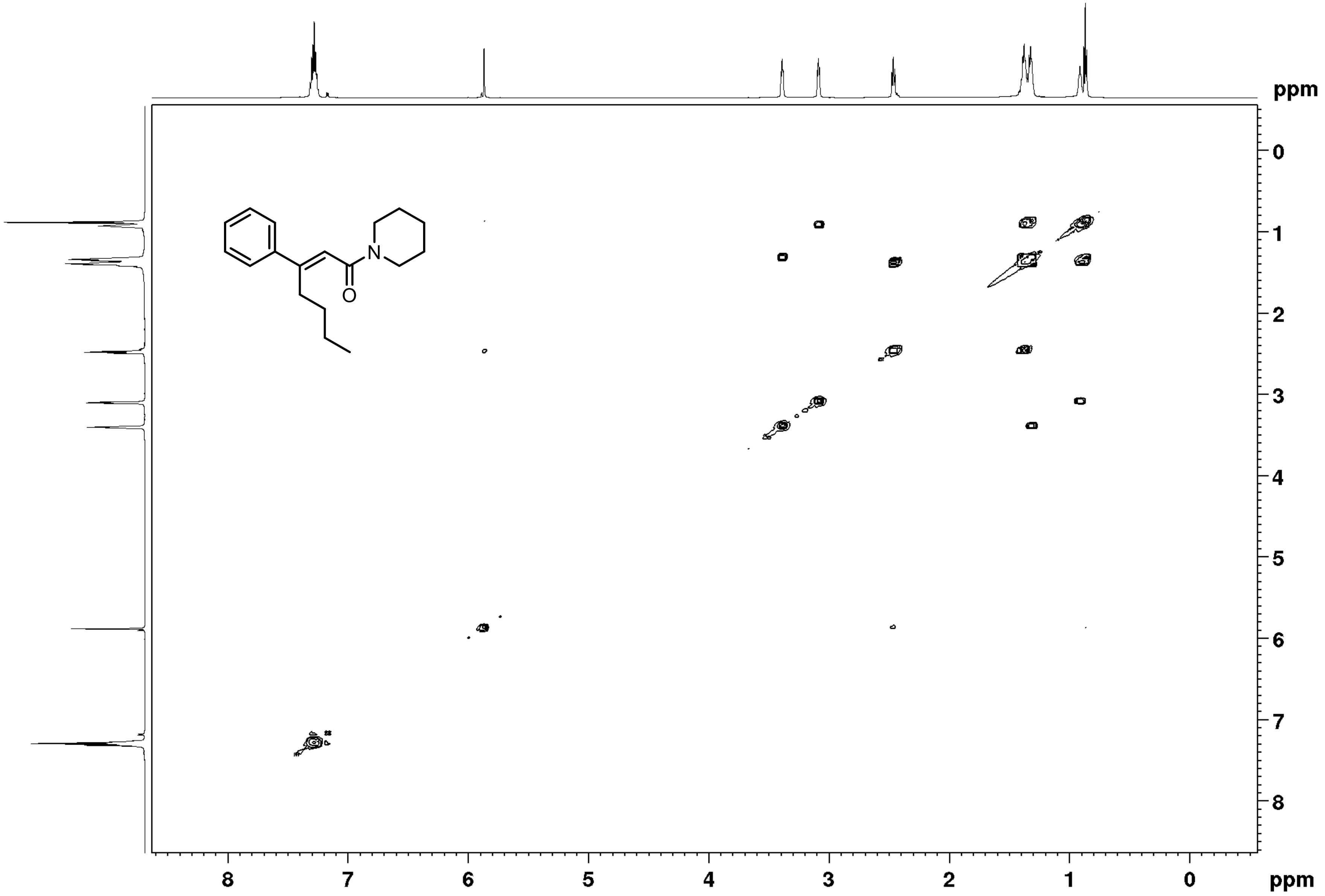

5.17.4 ¹H, ¹³C HMBC

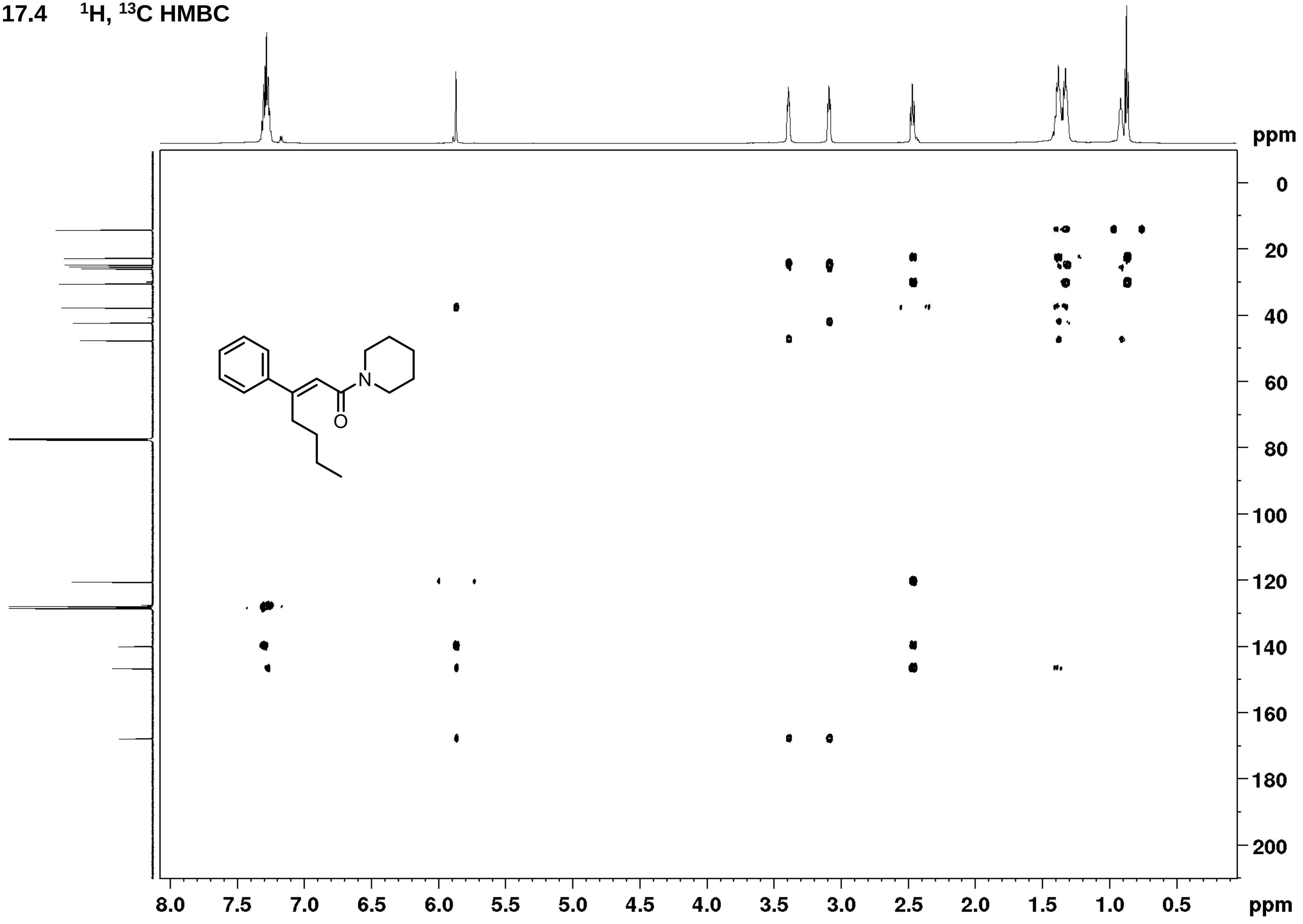

5.17.5 ^{1}H, ^{13}C HSQC

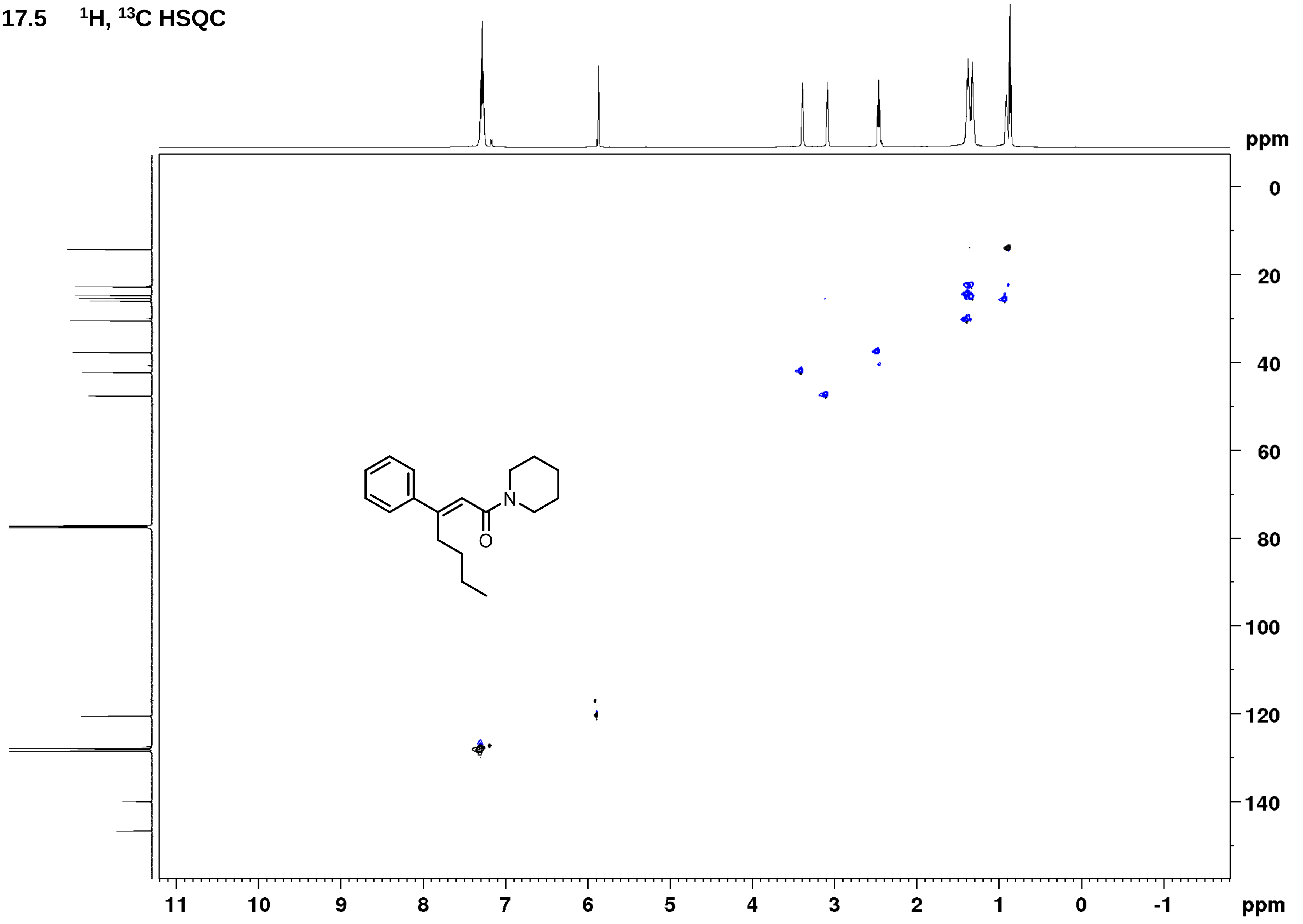

5.17.6 HRMS

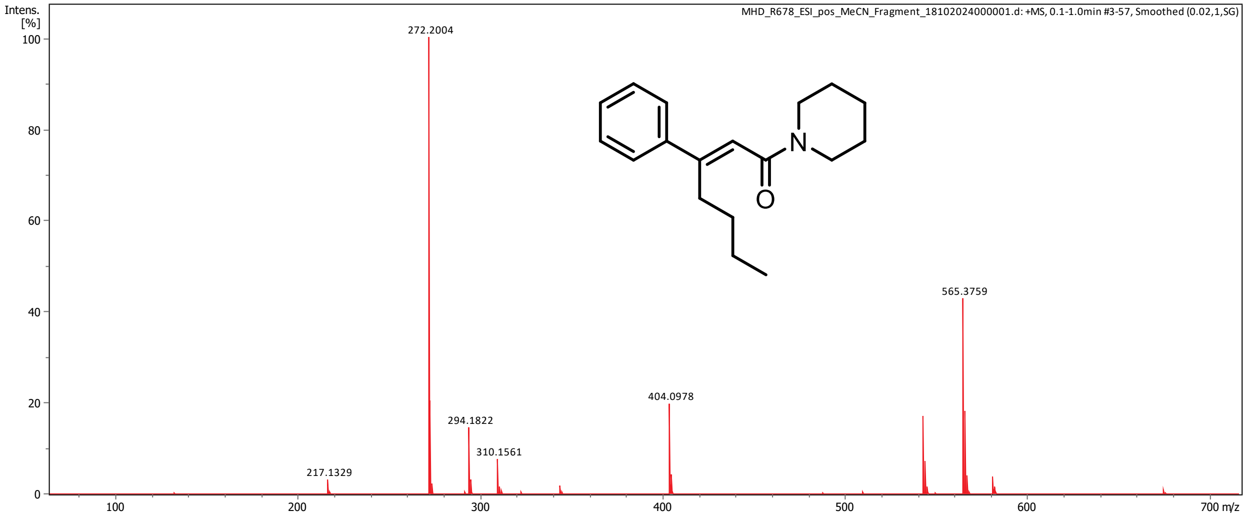
MHD_R678_ESI_pos_MeCN_Fragment_18102024000001.d: +MS, 0.1-1.0min #3-57, Smoothed (0.02,1,SG)
Intens. [%]
272.2004
565.3759
404.0978
294.1822
310.1561
217.1329
N
O
m/z

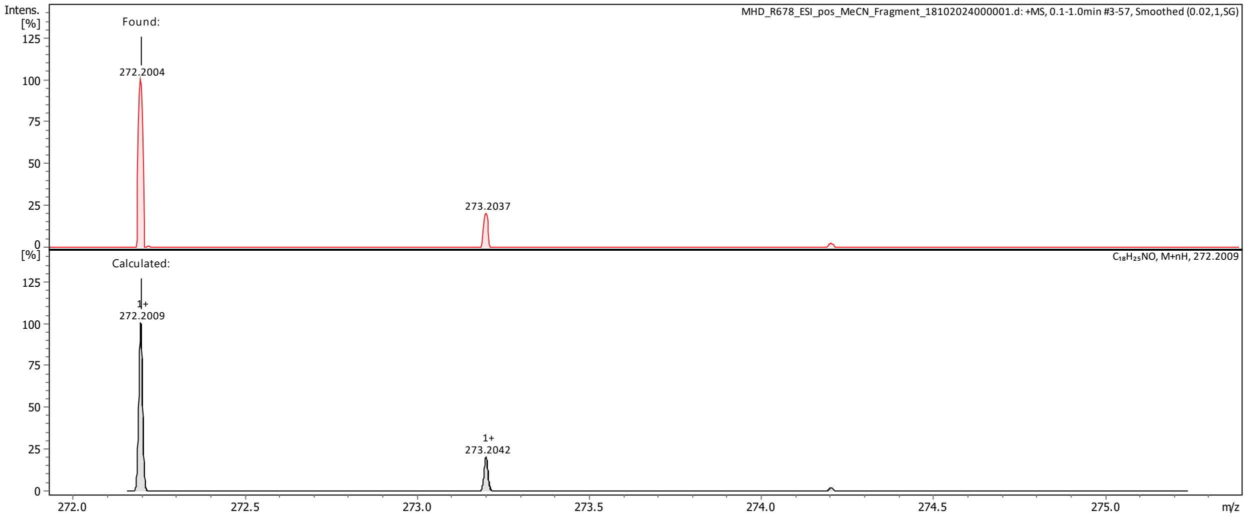
MHD_R678_ESI_pos_MeCN_Fragment_18102024000001.d: +MS, 0.1-1.0min #3-57, Smoothed (0.02,1,SG)
Intens. [%]
Found:
272.2004
273.2037
$C_{18}H_{25}NO$, M+nH, 272.2009
Calculated:
1+ 272.2009
1+ 273.2042
m/z

5.17.7 IR

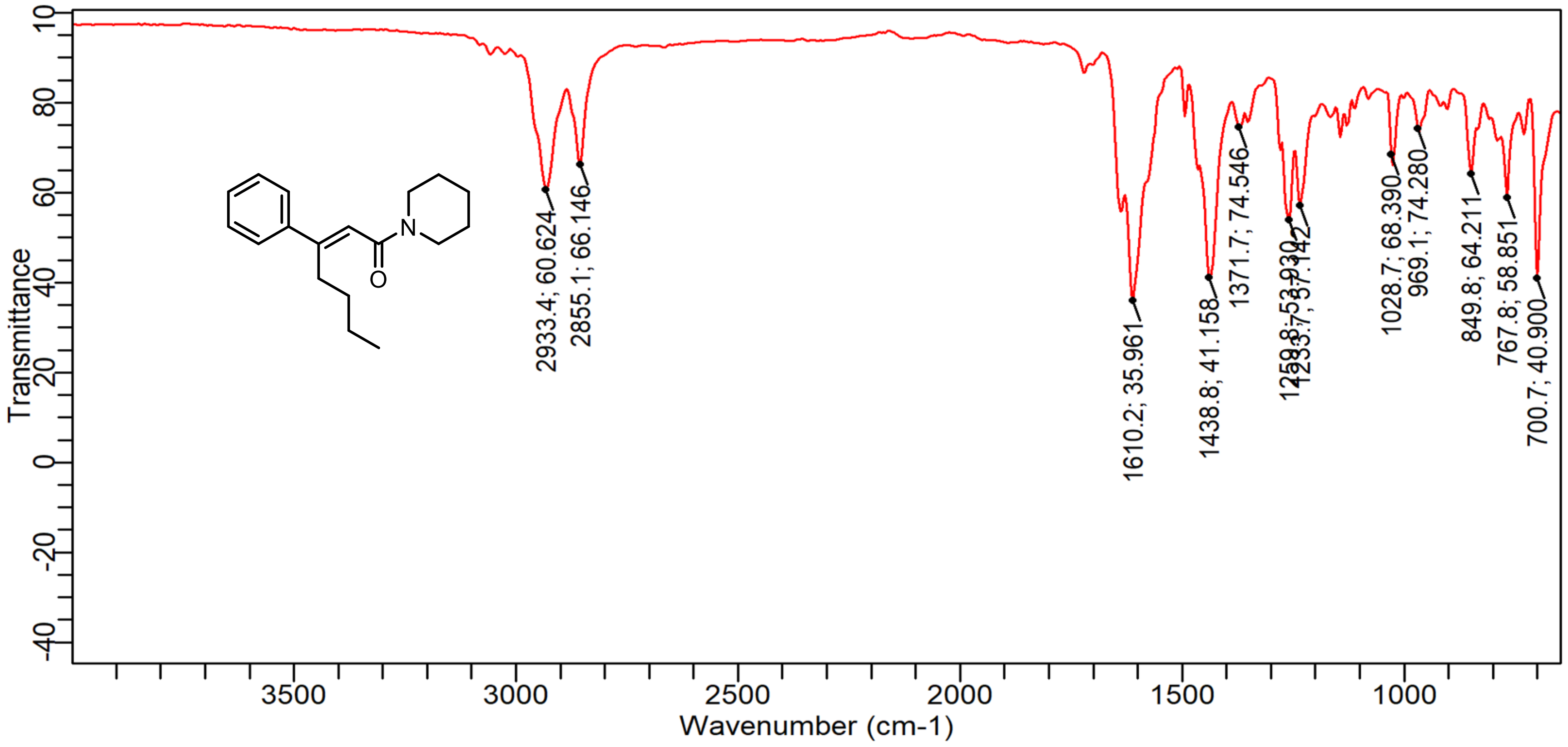

Transmittance
Wavenumber (cm-1)
2933.4; 60.624
2855.1; 66.146
1610.2; 35.961
1438.8; 41.158
1371.7; 74.546
1259.8; 53.930
1233.7; 57.142
1028.7; 68.390
969.1; 74.280
849.8; 64.211
767.8; 58.851
700.7; 40.900
N
O

5.18 (*E*)-*N*,*N*-diethyl-3-phenylbut-2-enamide (11s) (600 MHz, $CDCl_3$)

5.18.1 ^{1}H NMR

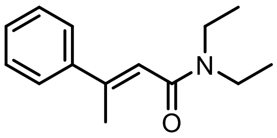

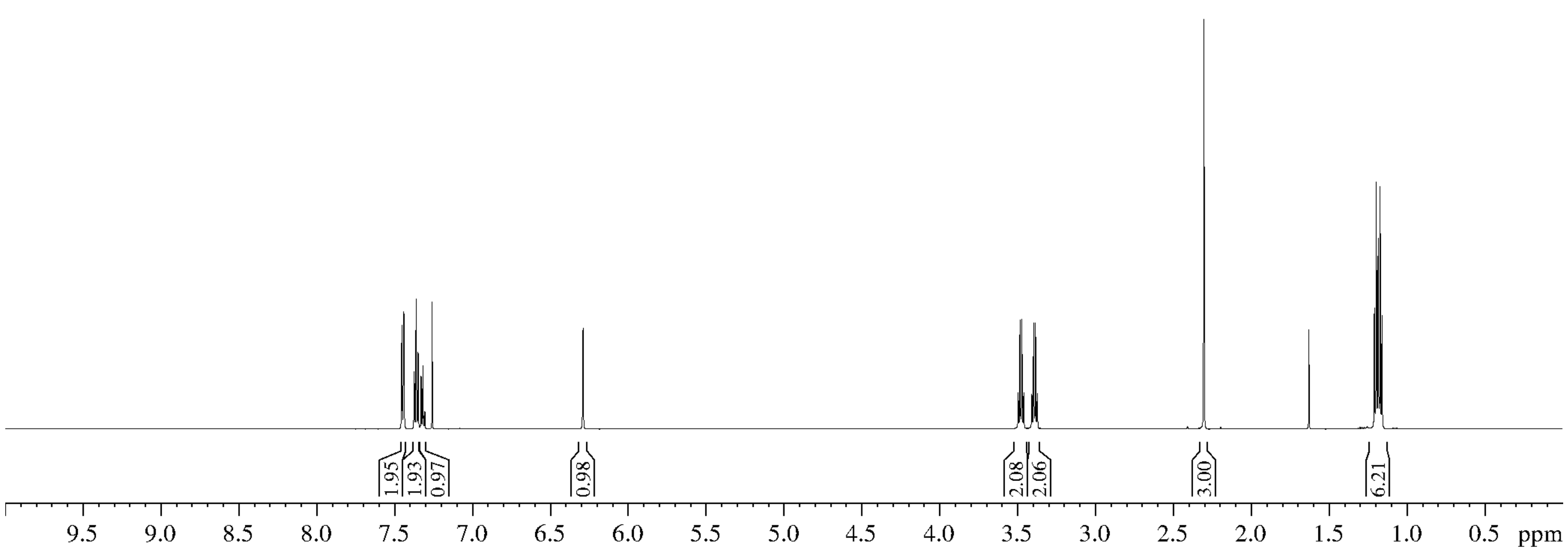

5.18.2 ^{13}C NMR

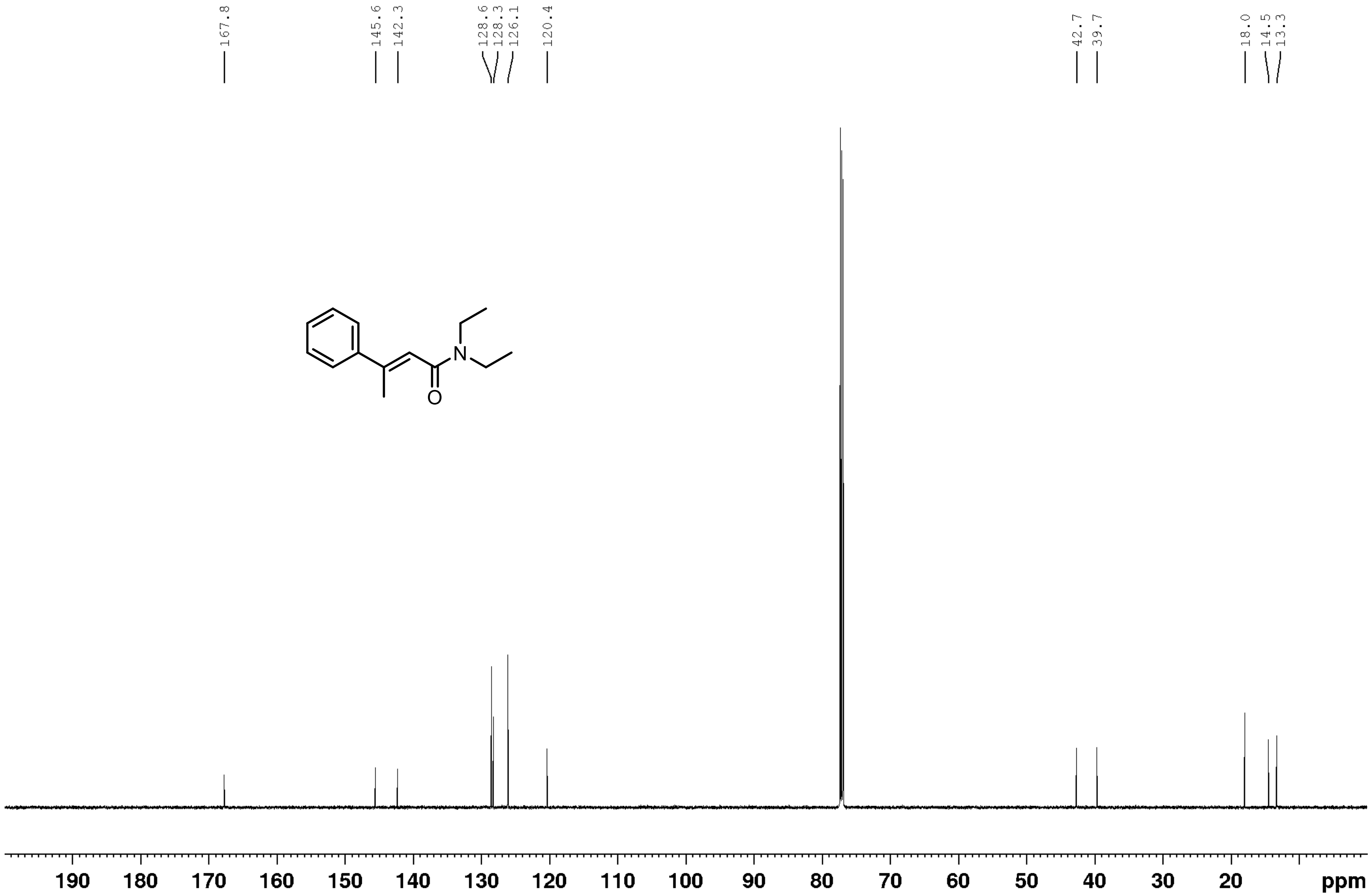
167.8
145.6
142.3
128.6
128.3
126.1
120.4
42.7
39.7
18.0
14.5
13.3
N
O
190
180
170
160
150
140
130
120
110
100
90
80
70
60
50
40
30
20
ppm

5.18.3 ^1H, ^1H COSY

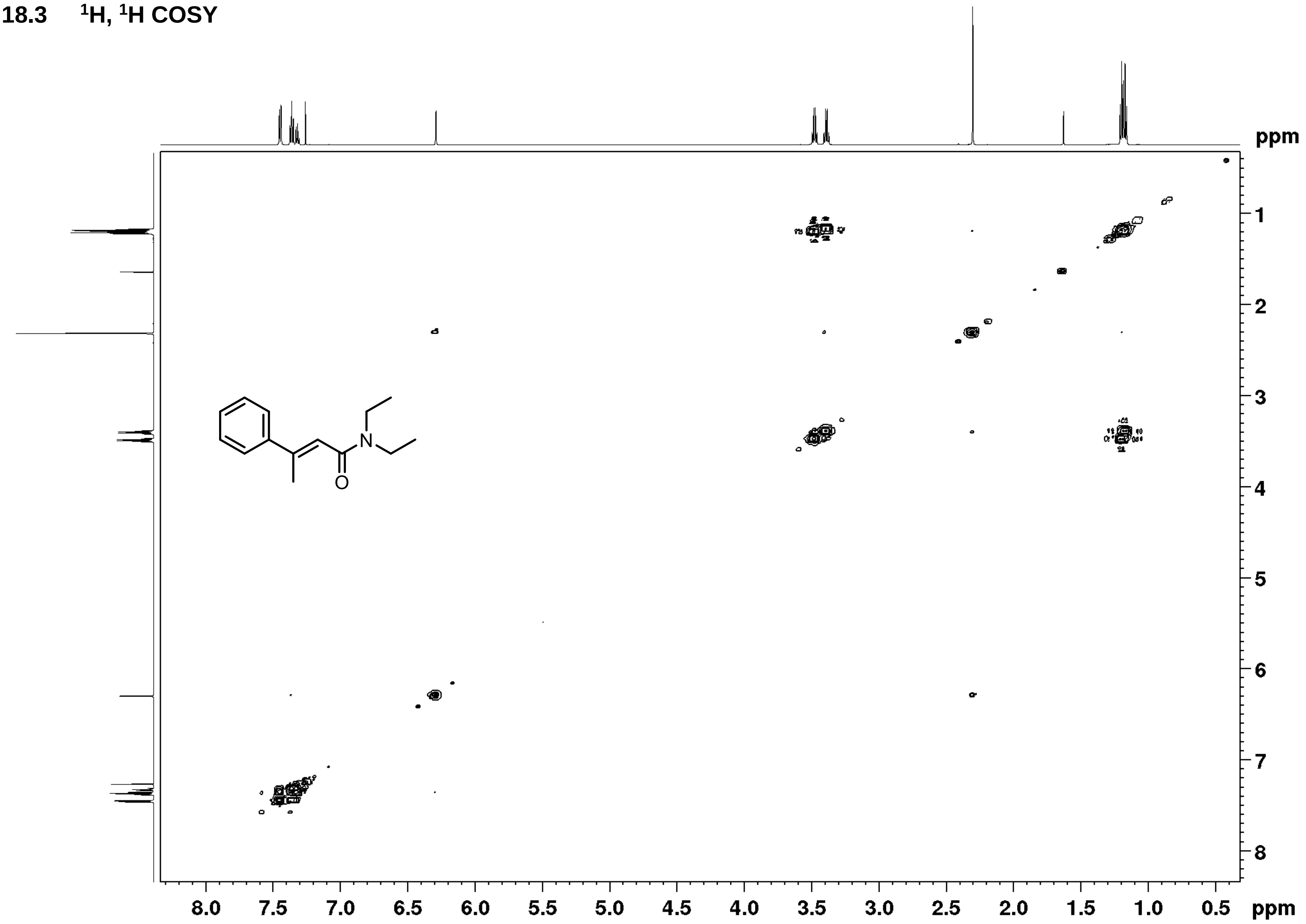

5.18.4 ^{1}H, ^{13}C HMBC

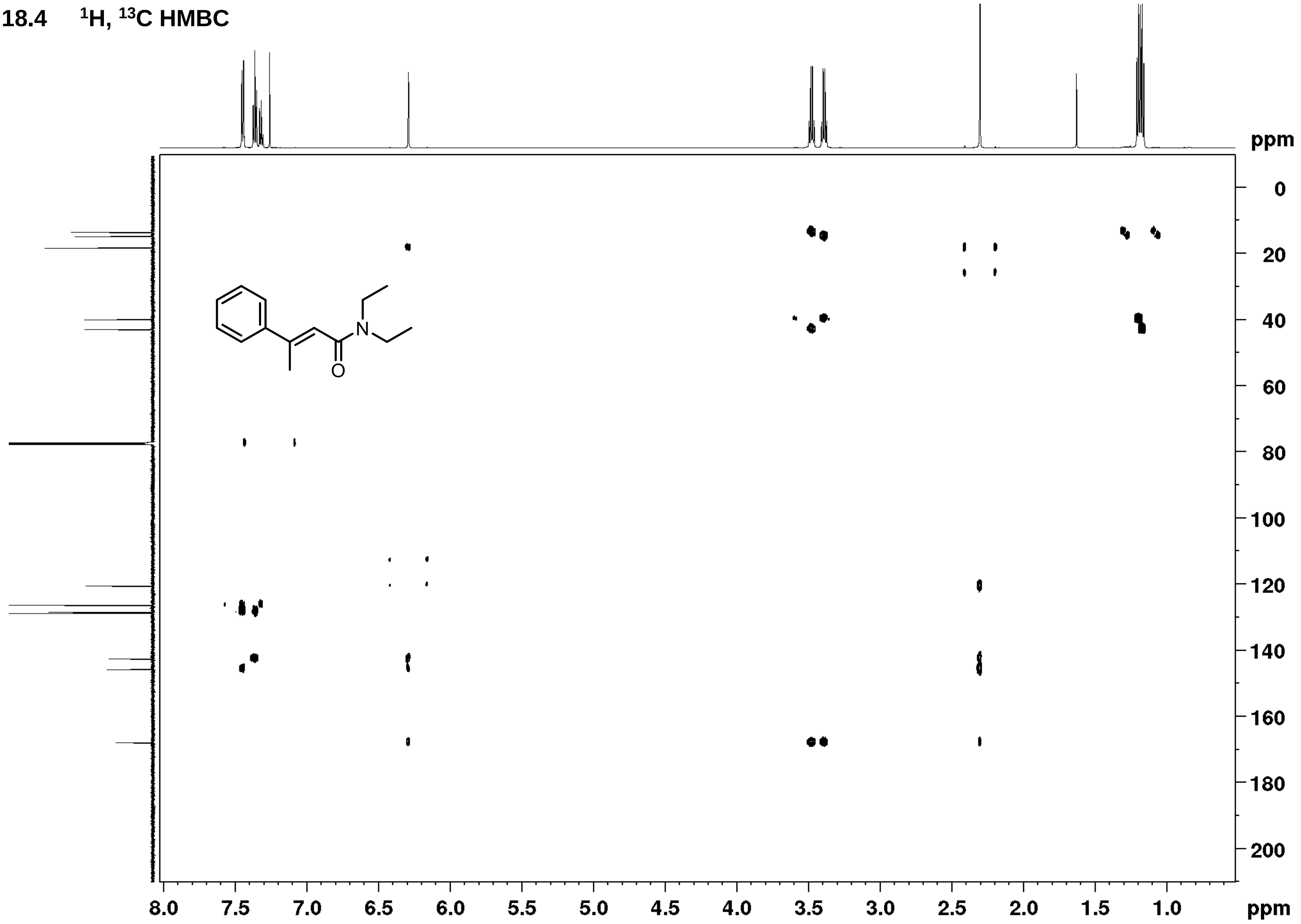

5.18.5 ¹H, ¹³C HSQC

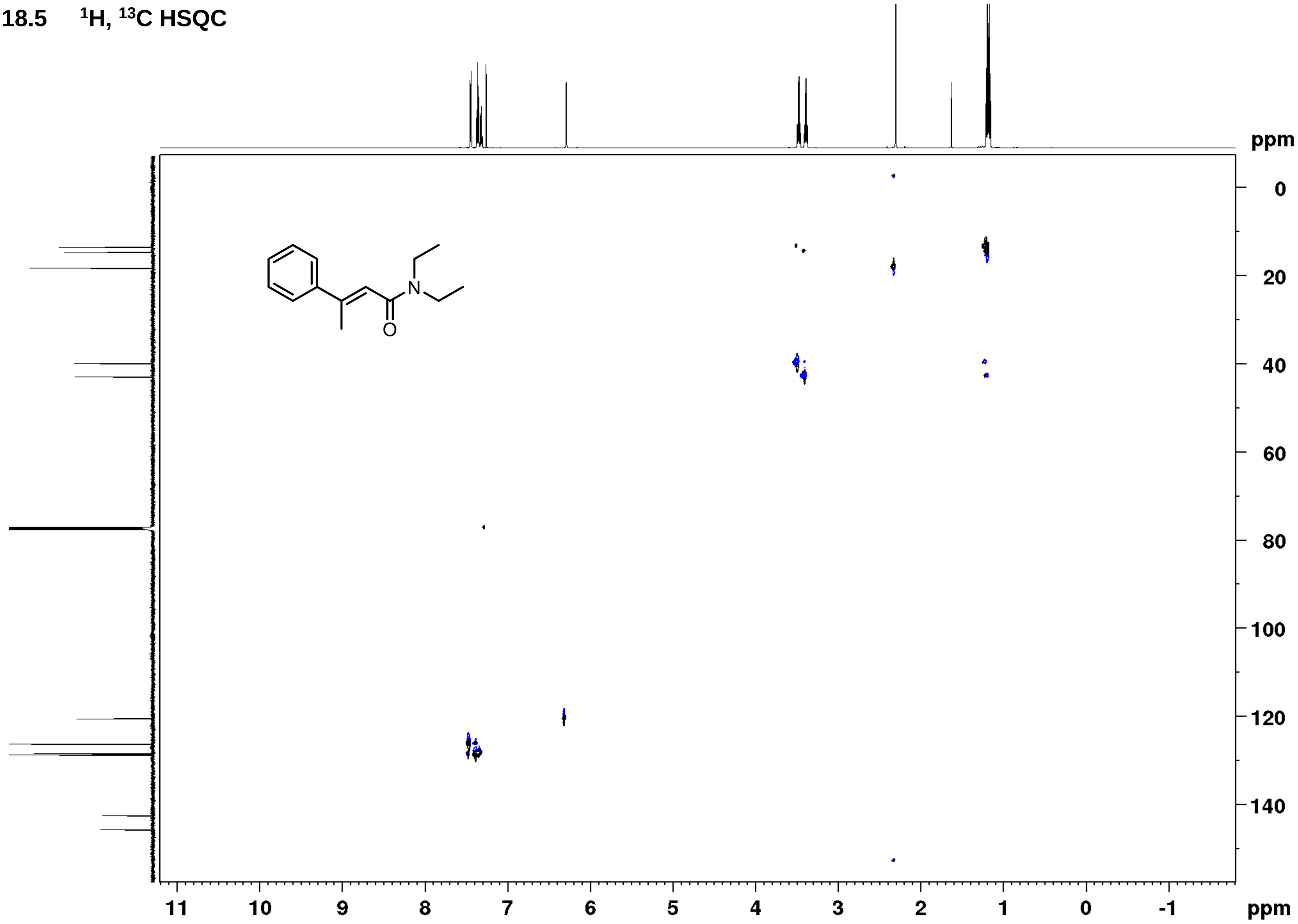

5.18.6 HRMS

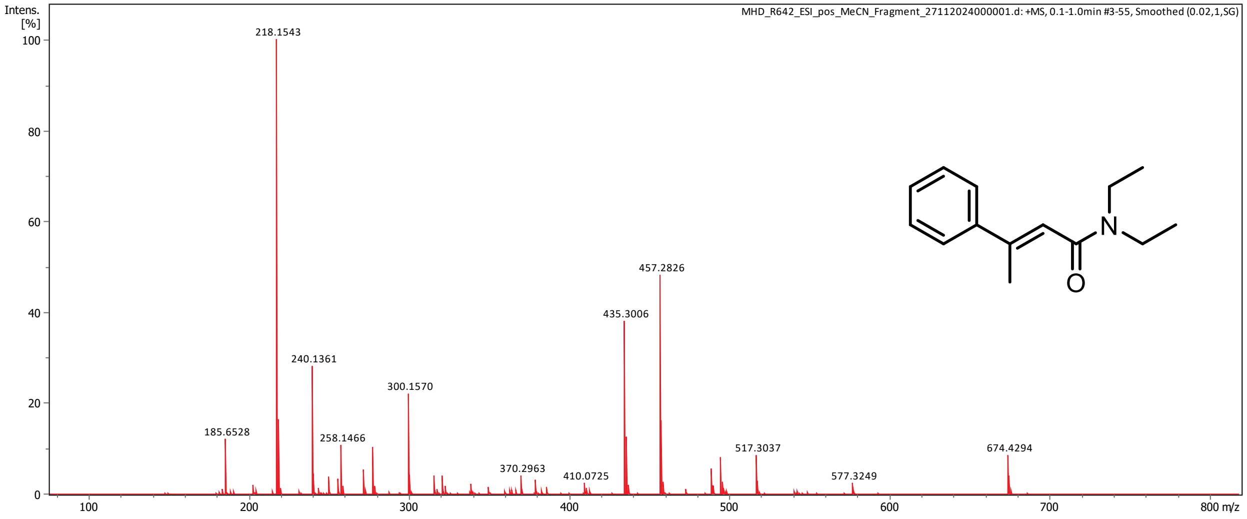

Intens. [%]
MHD_R642_ESI_pos_MeCN_Fragment_27112024000001.d: +MS, 0.1-1.0min #3-55, Smoothed (0.02,1,SG)
218.1543
185.6528
240.1361
258.1466
300.1570
370.2963
410.0725
435.3006
457.2826
517.3037
577.3249
674.4294
m/z
N
O

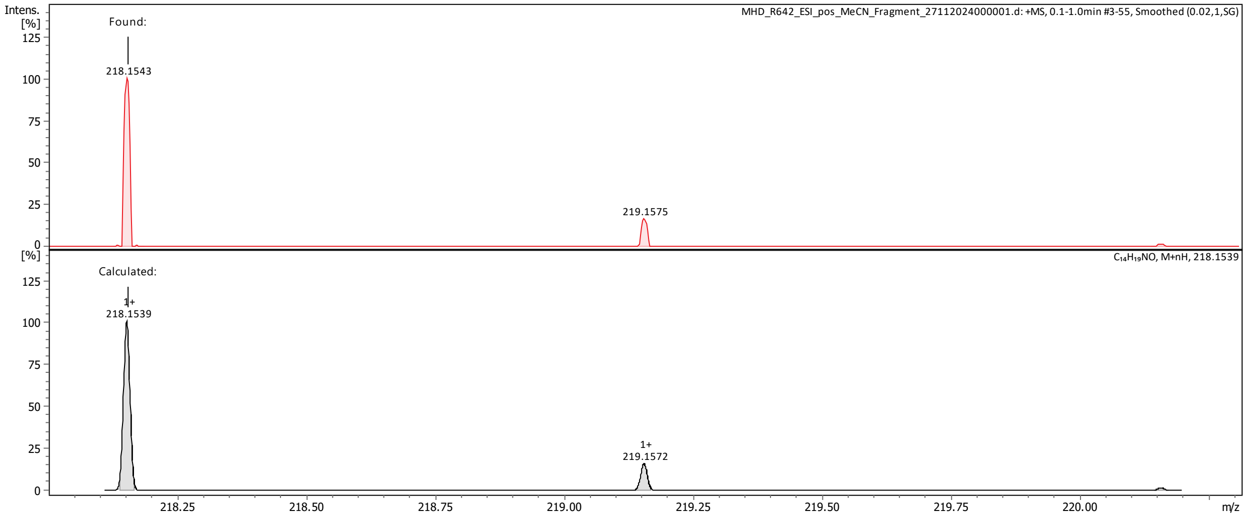

Intens. [%]
MHD_R642_ESI_pos_MeCN_Fragment_27112024000001.d: +MS, 0.1-1.0min #3-55, Smoothed (0.02,1,SG)
Found:
218.1543
219.1575
C14H19NO, M+nH, 218.1539
Calculated:
1+
218.1539
1+
219.1572
m/z

5.18.7 IR

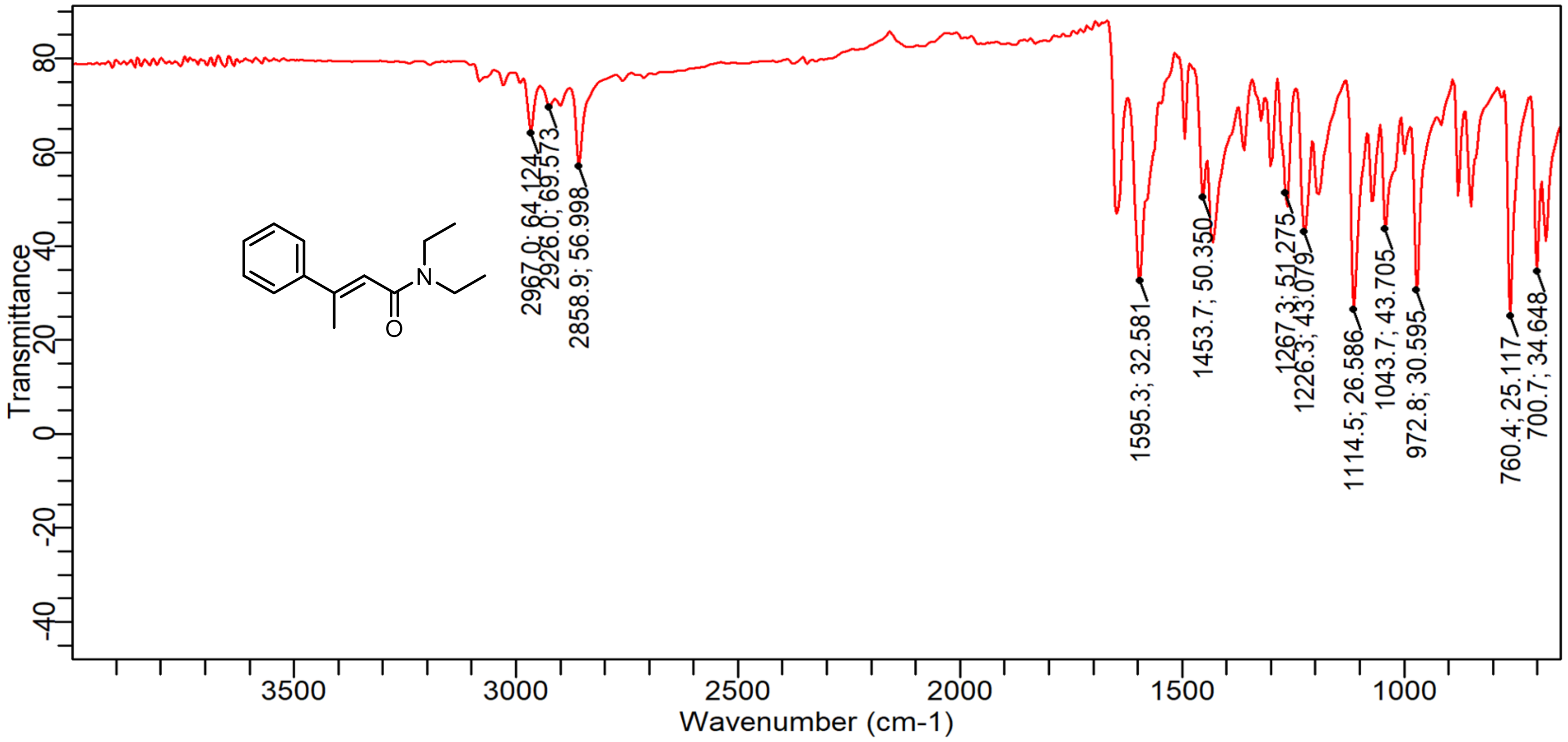

Transmittance
Wavenumber (cm-1)
2967.0; 64.124
2926.0; 69.573
2858.9; 56.998
1595.3; 32.581
1453.7; 50.350
1267.3; 51.275
1226.3; 43.079
1114.5; 26.586
1043.7; 43.705
972.8; 30.595
760.4; 25.117
700.7; 34.648
O
N

5.19 (*E*)-3-Phenyl-1-(3-(trifluoromethyl)-5,6-dihydro-[1,2,4]triazolo[4,3-*a*]pyrazin-7(8*H*)-yl)but-2-en-1-one (11t) (600 MHz, $CDCl_3$)

5.19.1 ^{1}H NMR

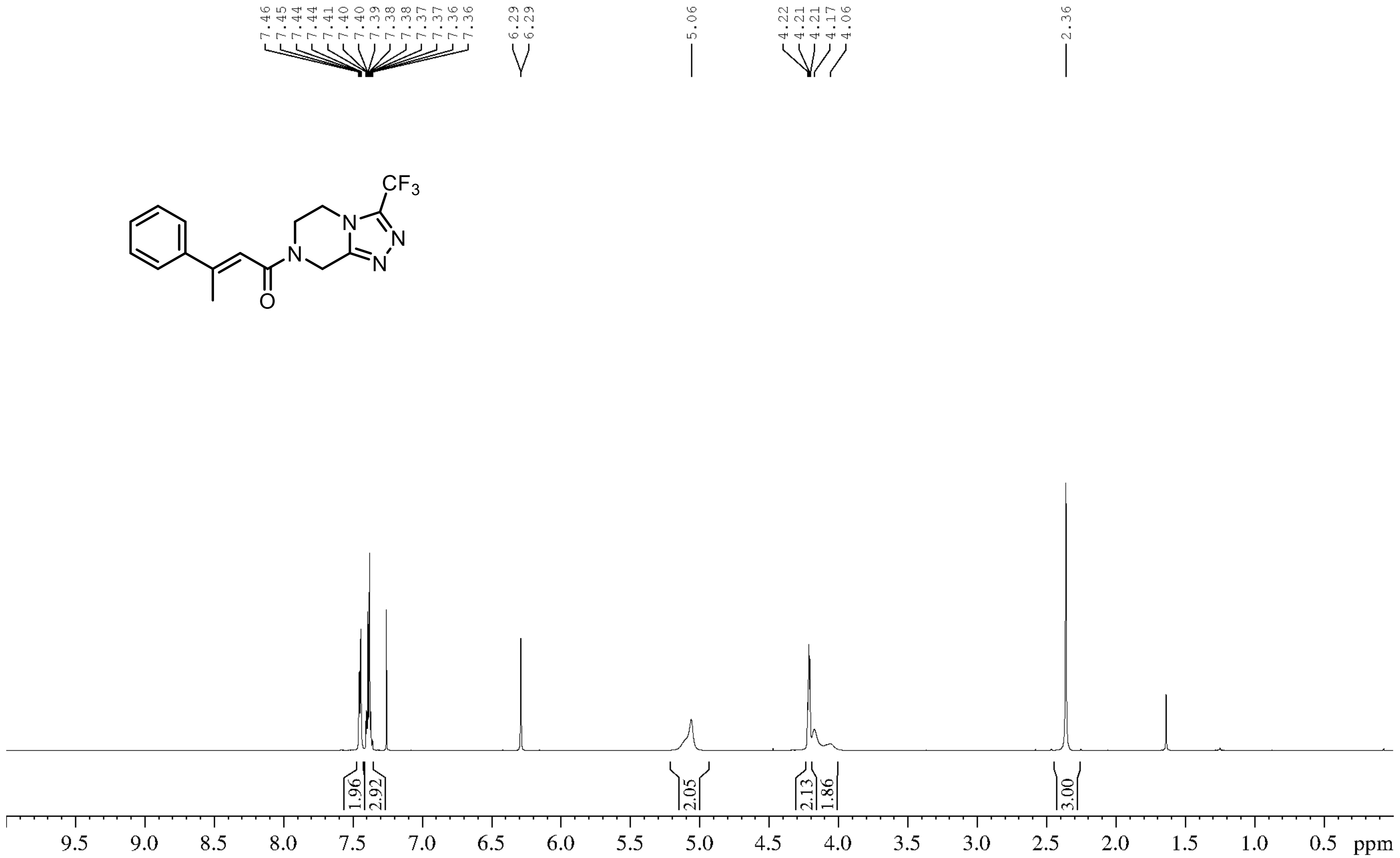

5.19.2 ^{13}C NMR

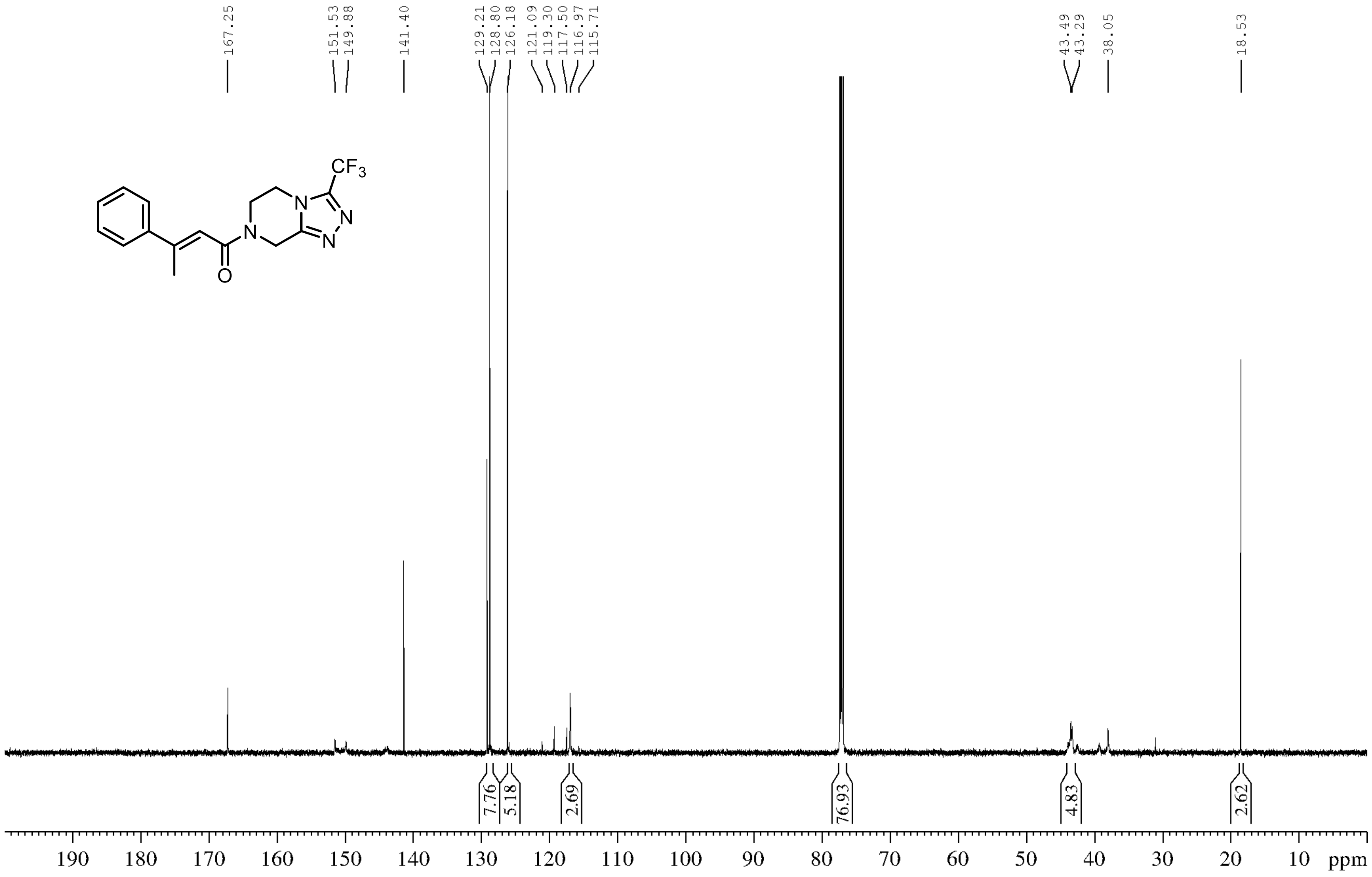

167.25
151.53
149.88
141.40
129.21
128.80
126.18
121.09
119.30
117.50
116.97
115.71
43.49
43.29
38.05
18.53
CF3
N
N
N
N
O
7.76
5.18
2.69
76.93
4.83
2.62
190
180
170
160
150
140
130
120
110
100
90
80
70
60
50
40
30
20
10
ppm

5.19.3 ^{19}F NMR

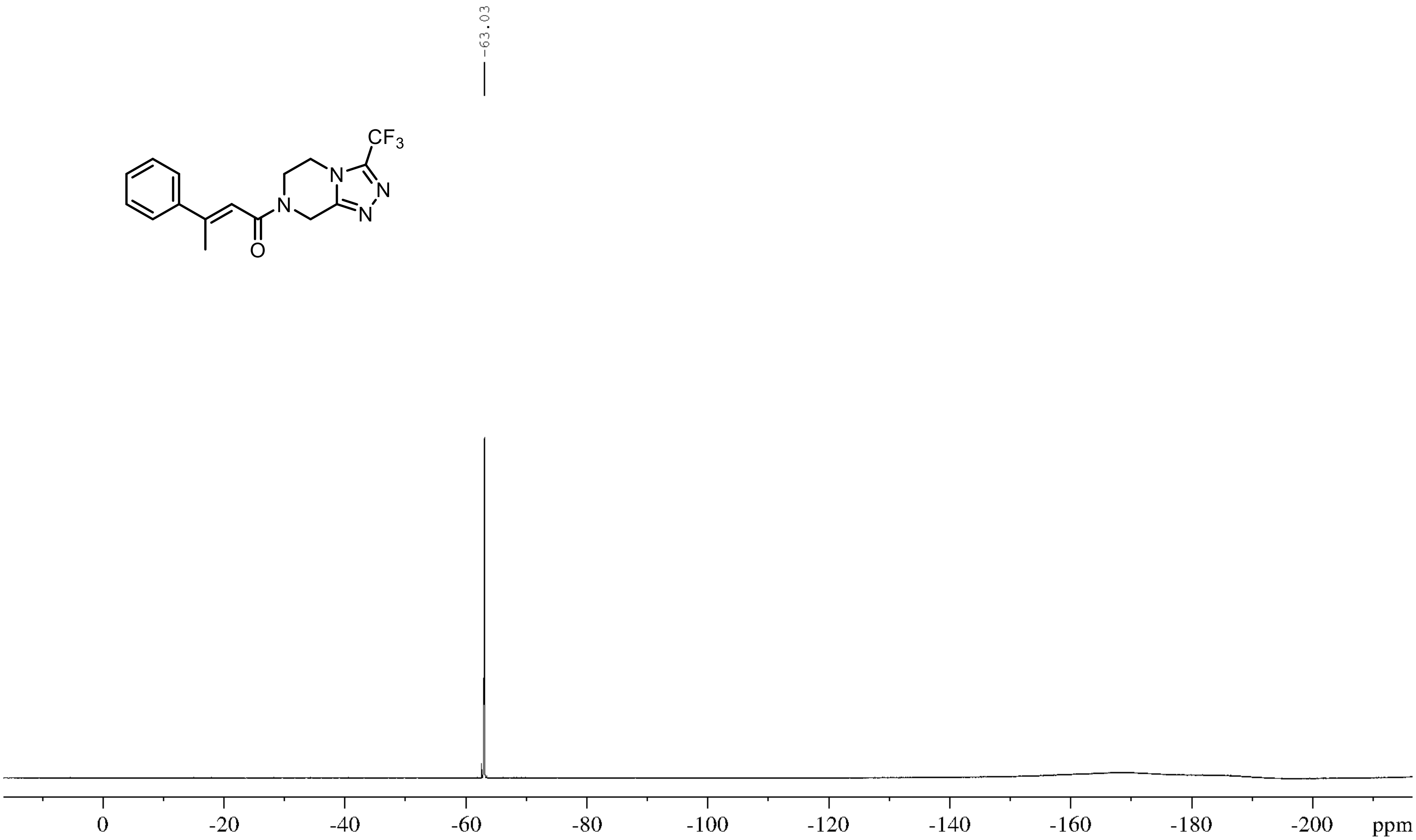

5.19.4 ^{1}H, ^{1}H COSY

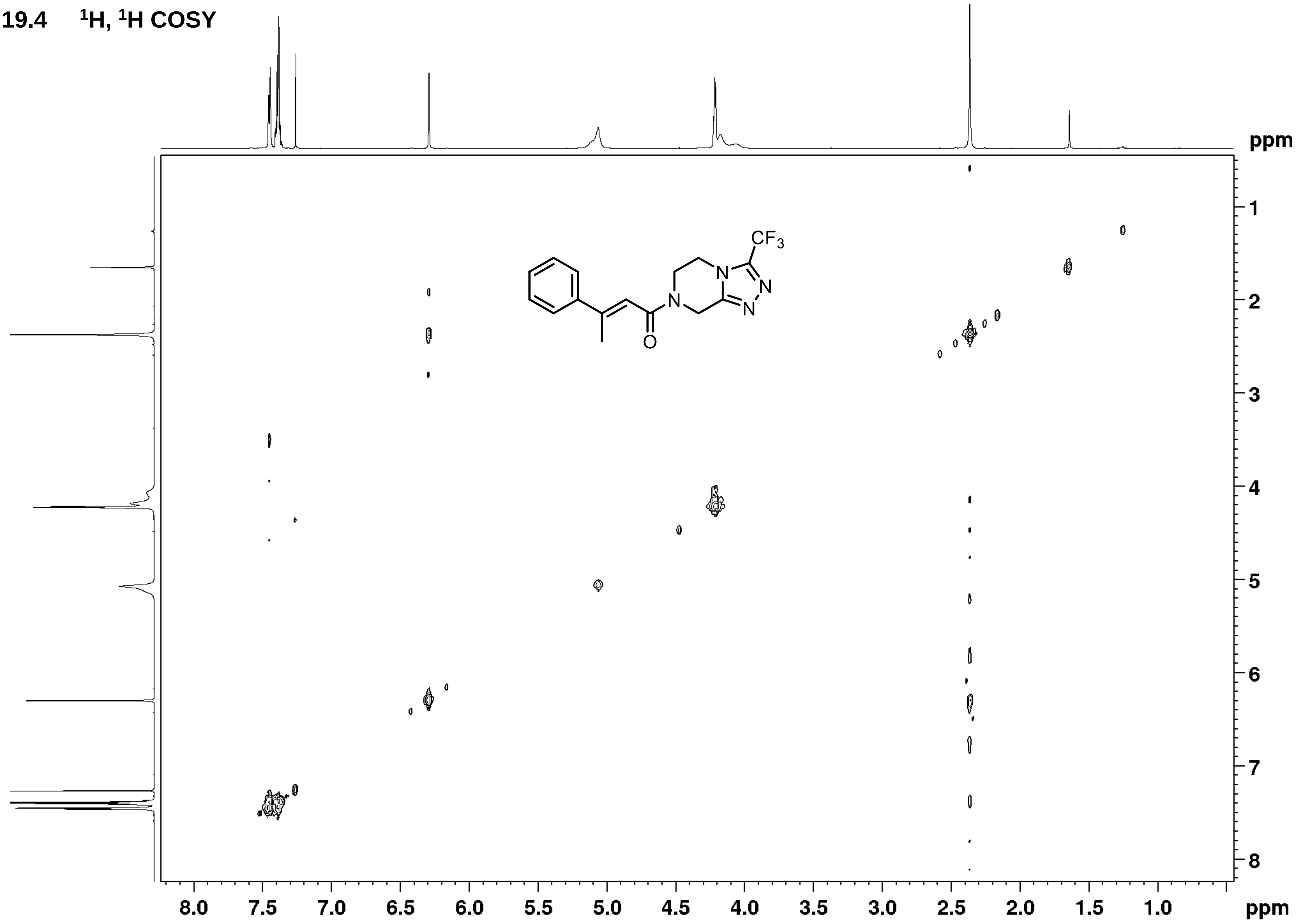

5.19.5 ^{1}H, ^{13}C HMBC

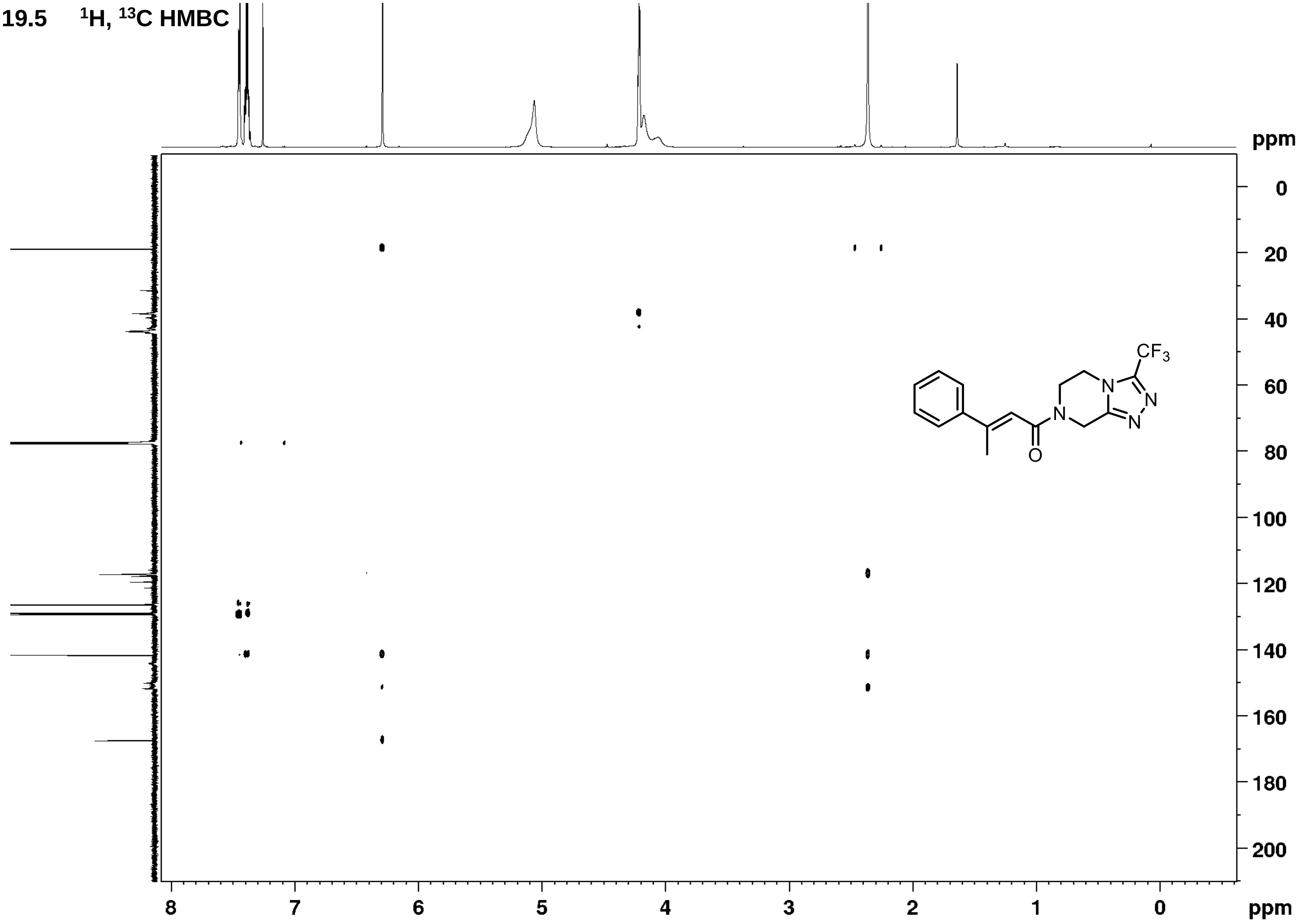

5.19.6 ^{1}H, ^{13}C HSQC

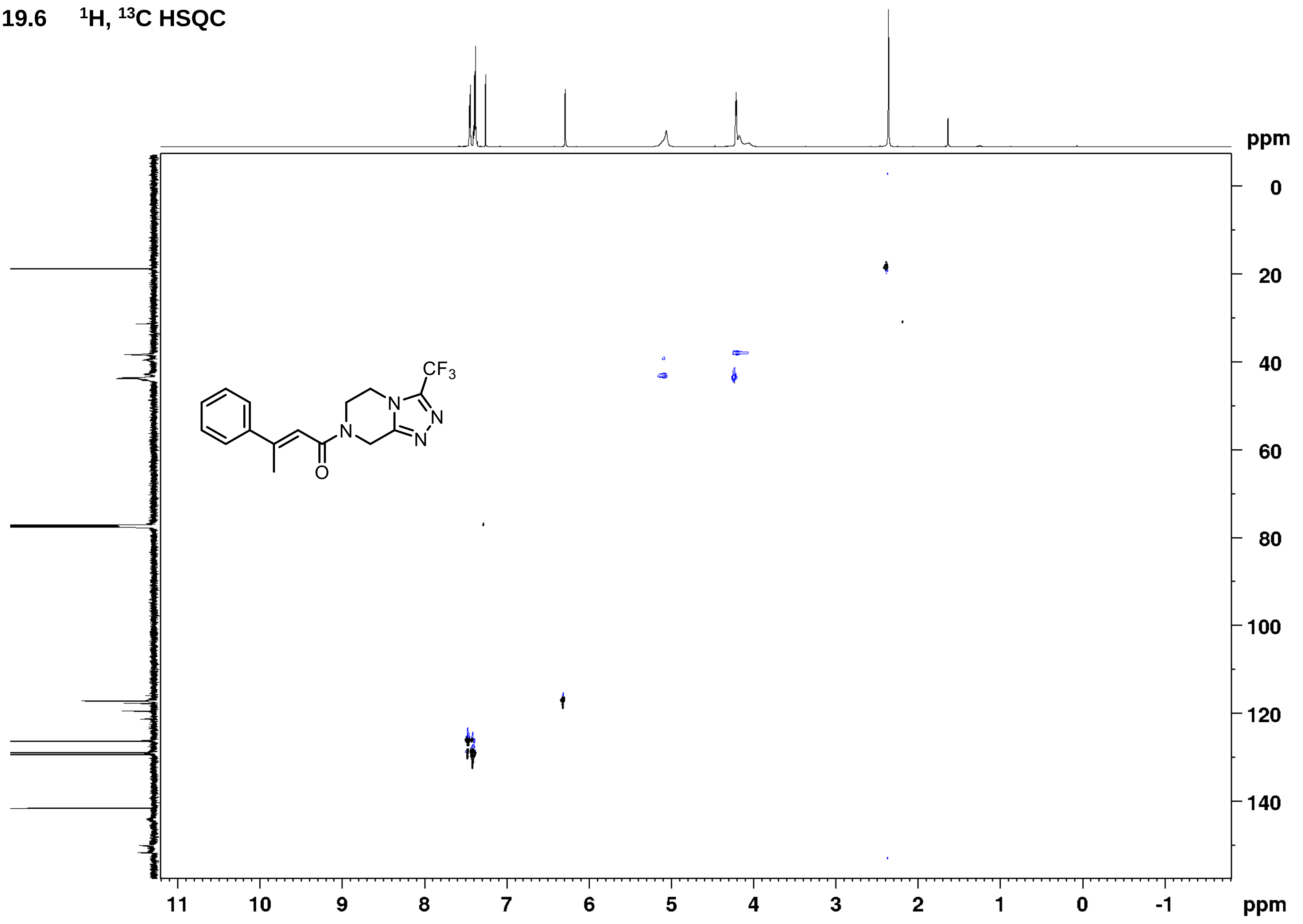

5.19.7 HRMS

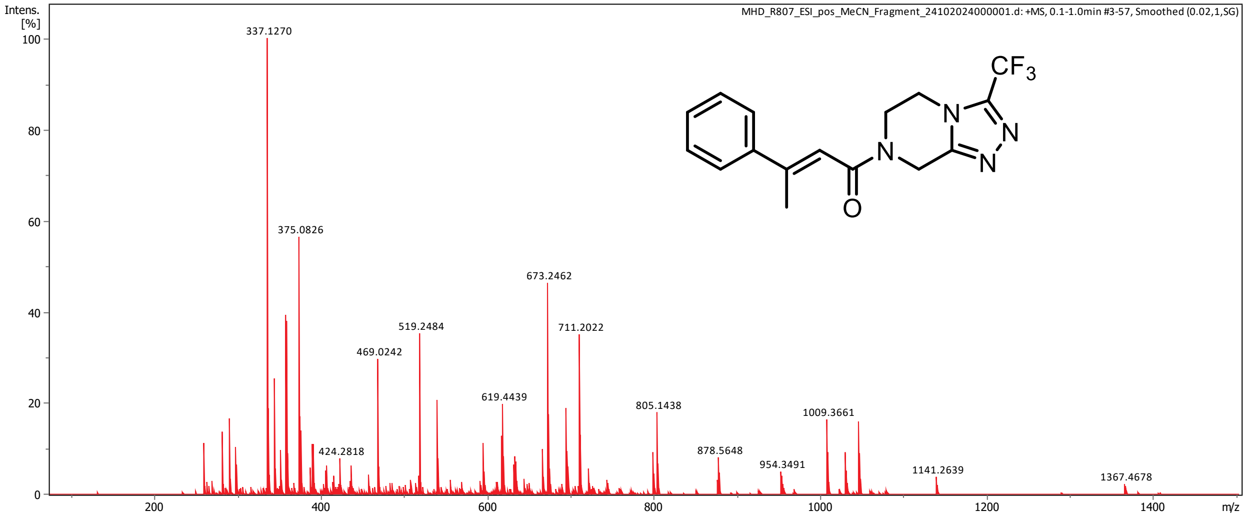
MHD_R807_ESI_pos_MeCN_Fragment_24102024000001.d: +MS, 0.1-1.0min #3-57, Smoothed (0.02,1,SG)
Intens. [%]
337.1270
375.0826
673.2462
519.2484
711.2022
469.0242
619.4439
805.1438
1009.3661
424.2818
878.5648
954.3491
1141.2639
1367.4678
m/z
CF3
N
O

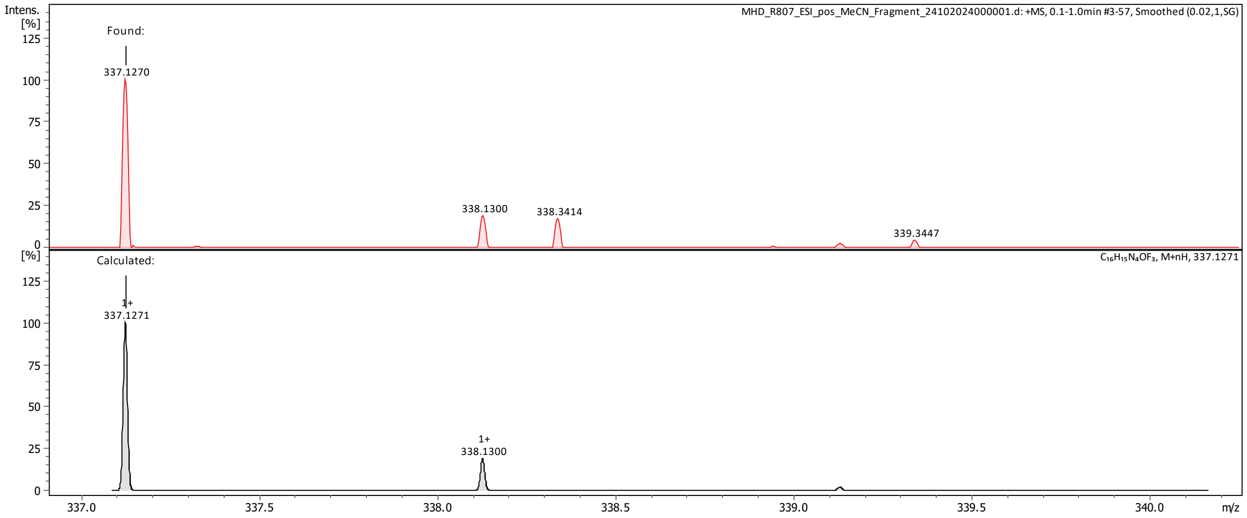
MHD_R807_ESI_pos_MeCN_Fragment_24102024000001.d: +MS, 0.1-1.0min #3-57, Smoothed (0.02,1,SG)
Intens. [%]
Found:
337.1270
338.1300
338.3414
339.3447
Calculated:
C16H15N4OF3, M+nH, 337.1271
1+
337.1271
1+
338.1300
m/z

5.19.8 IR

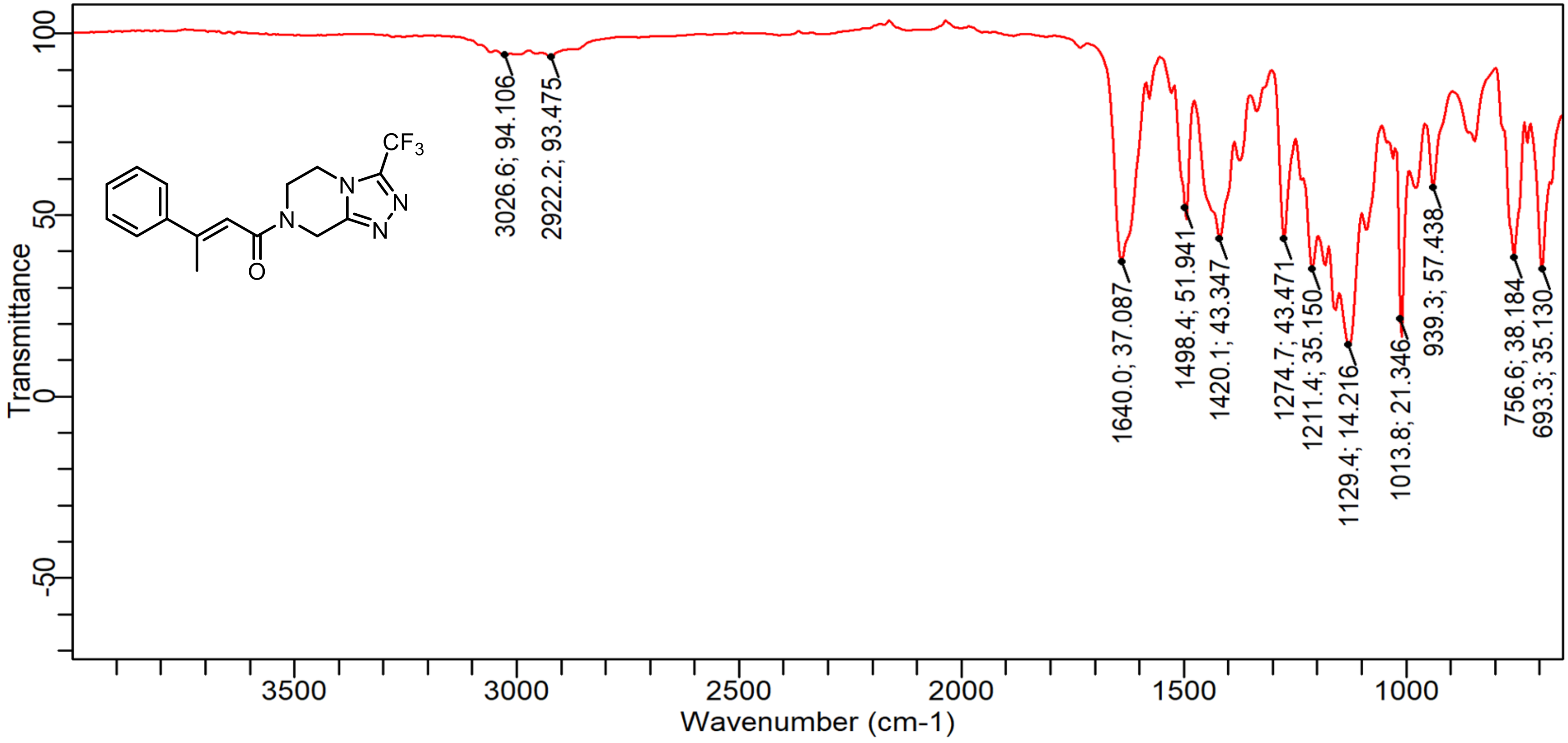

Transmittance
Wavenumber (cm-1)
100
50
0
-50
3500
3000
2500
2000
1500
1000
CF3
N
N
N
N
O
3026.6; 94.106
2922.2; 93.475
1640.0; 37.087
1498.4; 51.941
1420.1; 43.347
1274.7; 43.471
1211.4; 35.150
1129.4; 14.216
1013.8; 21.346
939.3; 57.438
756.6; 38.184
693.3; 35.130

5.20 Ethyl (*E*)-4-(3-phenylbut-2-enoyl)-3,4-dihydro-2*H*-benzo[*b*][1,4]oxazine-2-carboxylate (11u) (600 MHz, $CDCl_3$)

5.20.1 ^{1}H NMR

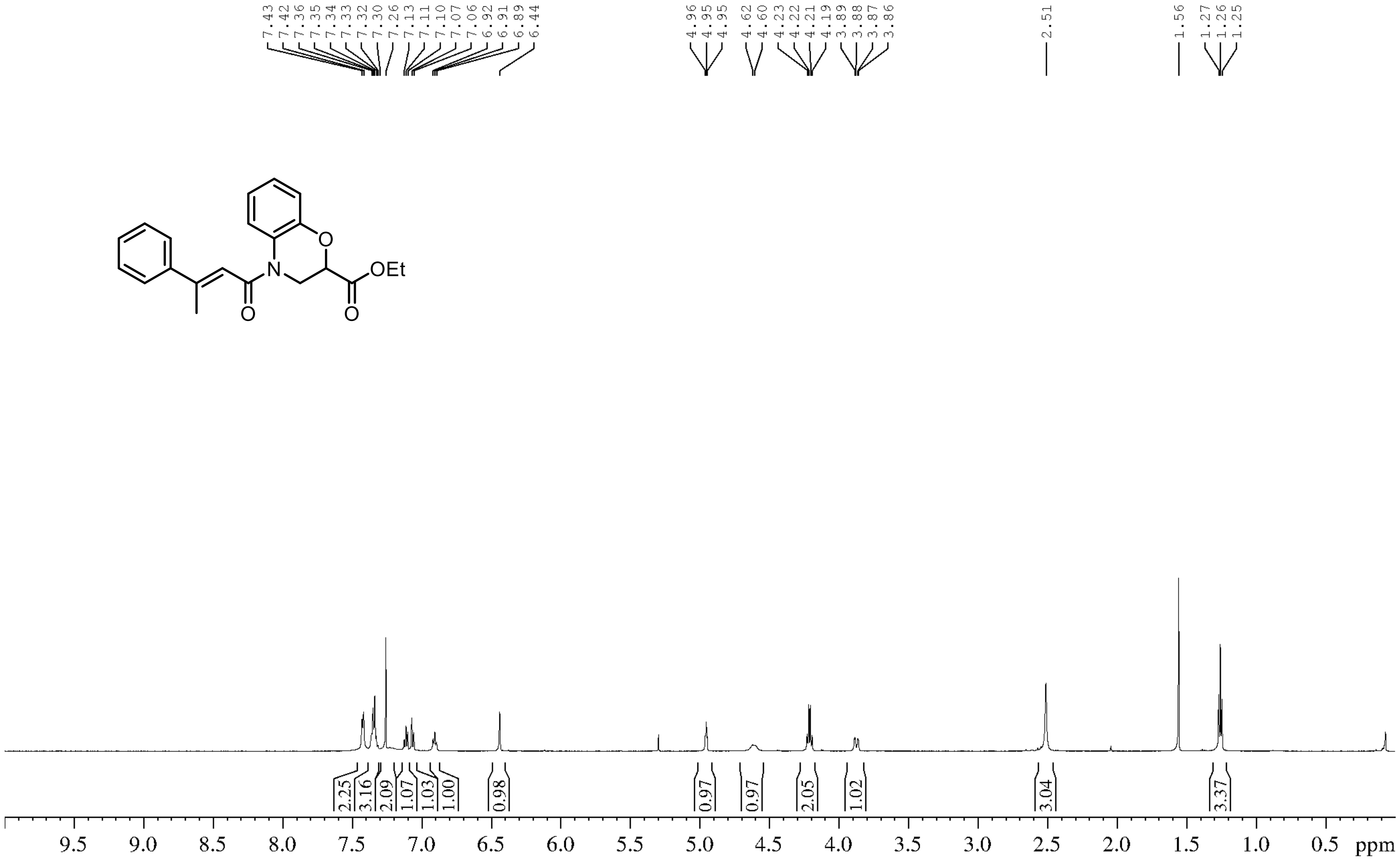

5.20.2 ^{13}C NMR

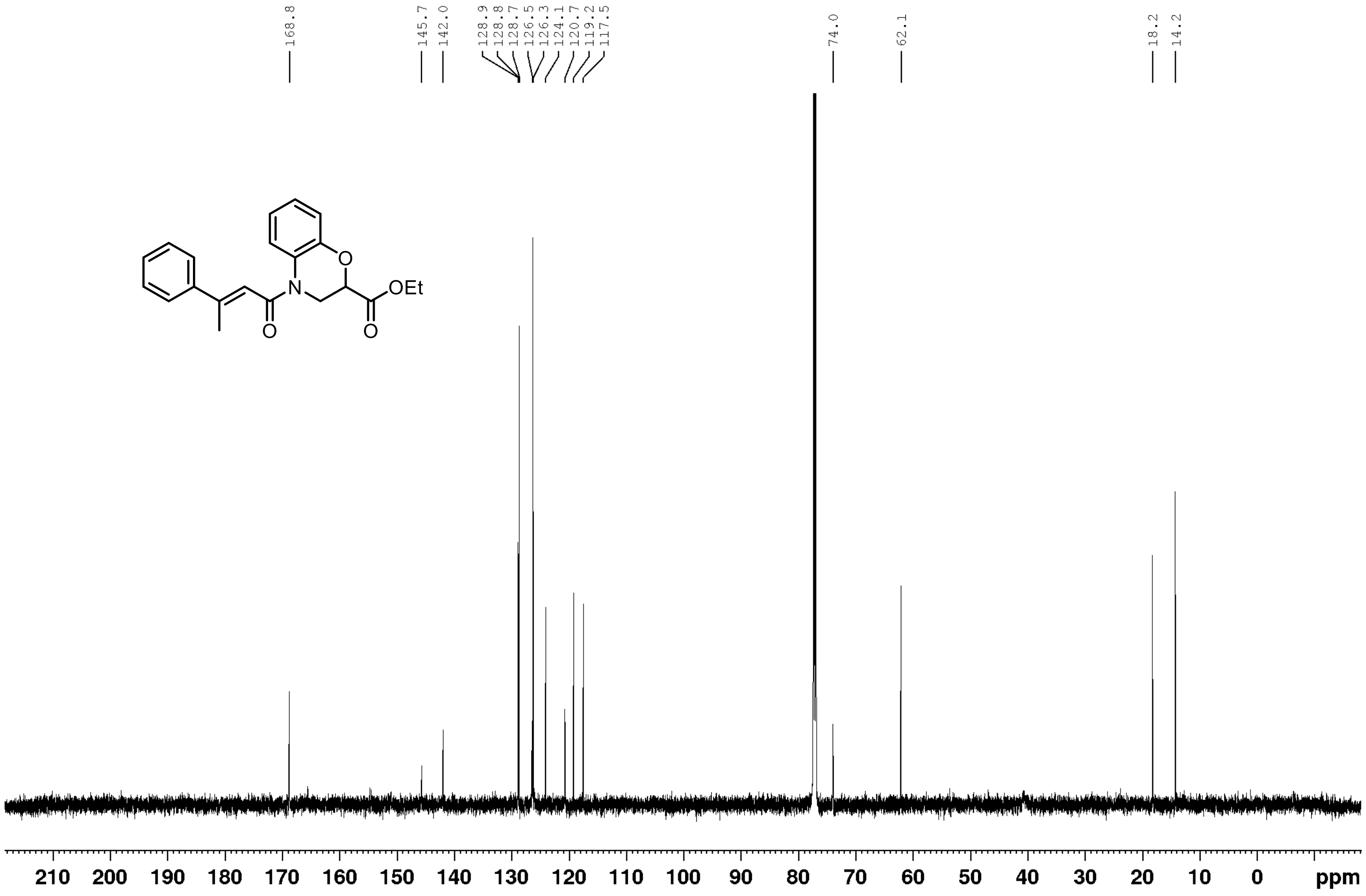
168.8
145.7
142.0
128.9
128.8
128.7
126.5
126.3
124.1
120.7
119.2
117.5
74.0
62.1
18.2
14.2
OEt
O
N
210 200 190 180 170 160 150 140 130 120 110 100 90 80 70 60 50 40 30 20 10 0 ppm

5.20.3 ^{1}H, ^{1}H COSY

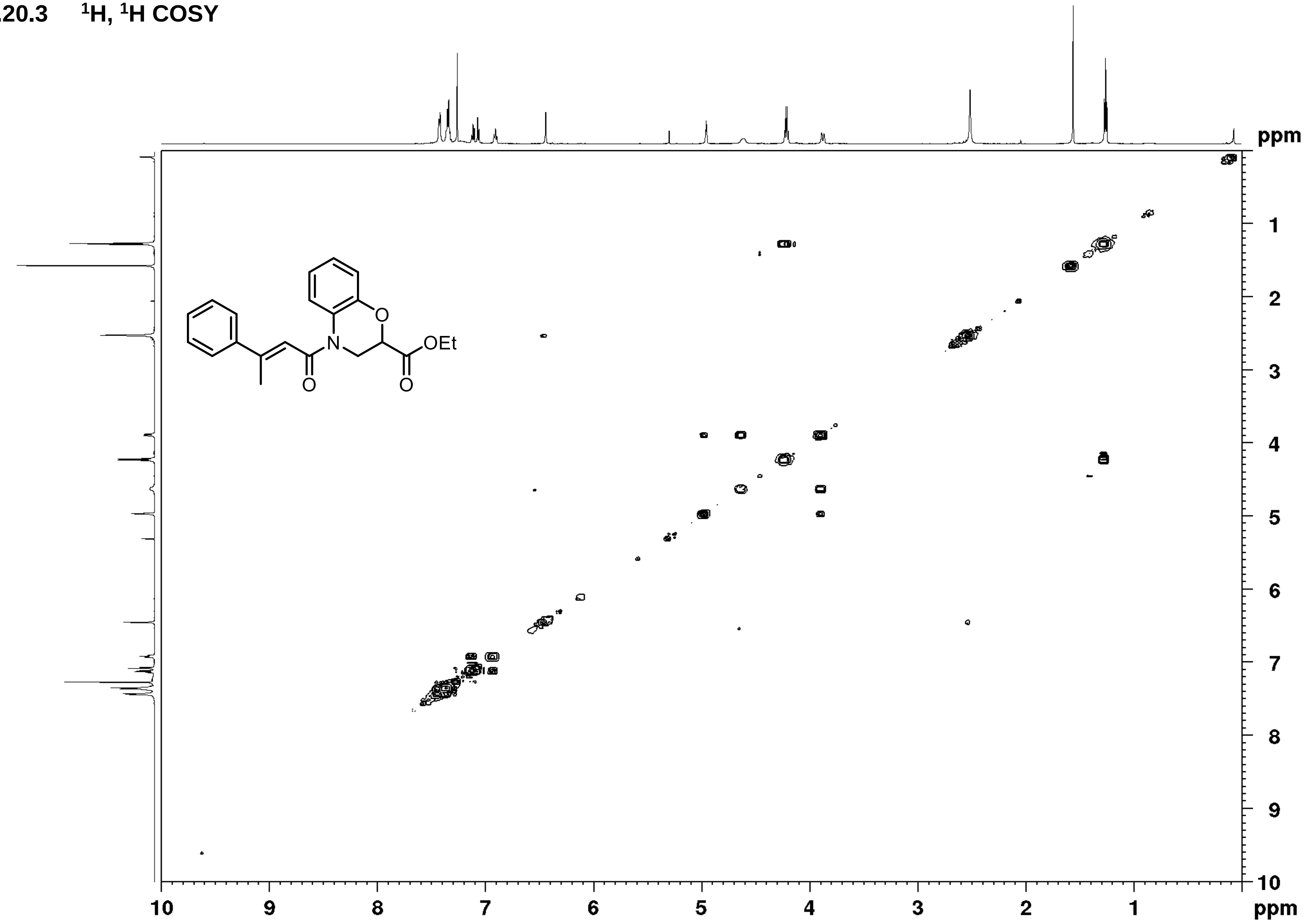

5.20.4 ^{1}H, ^{13}C HMBC

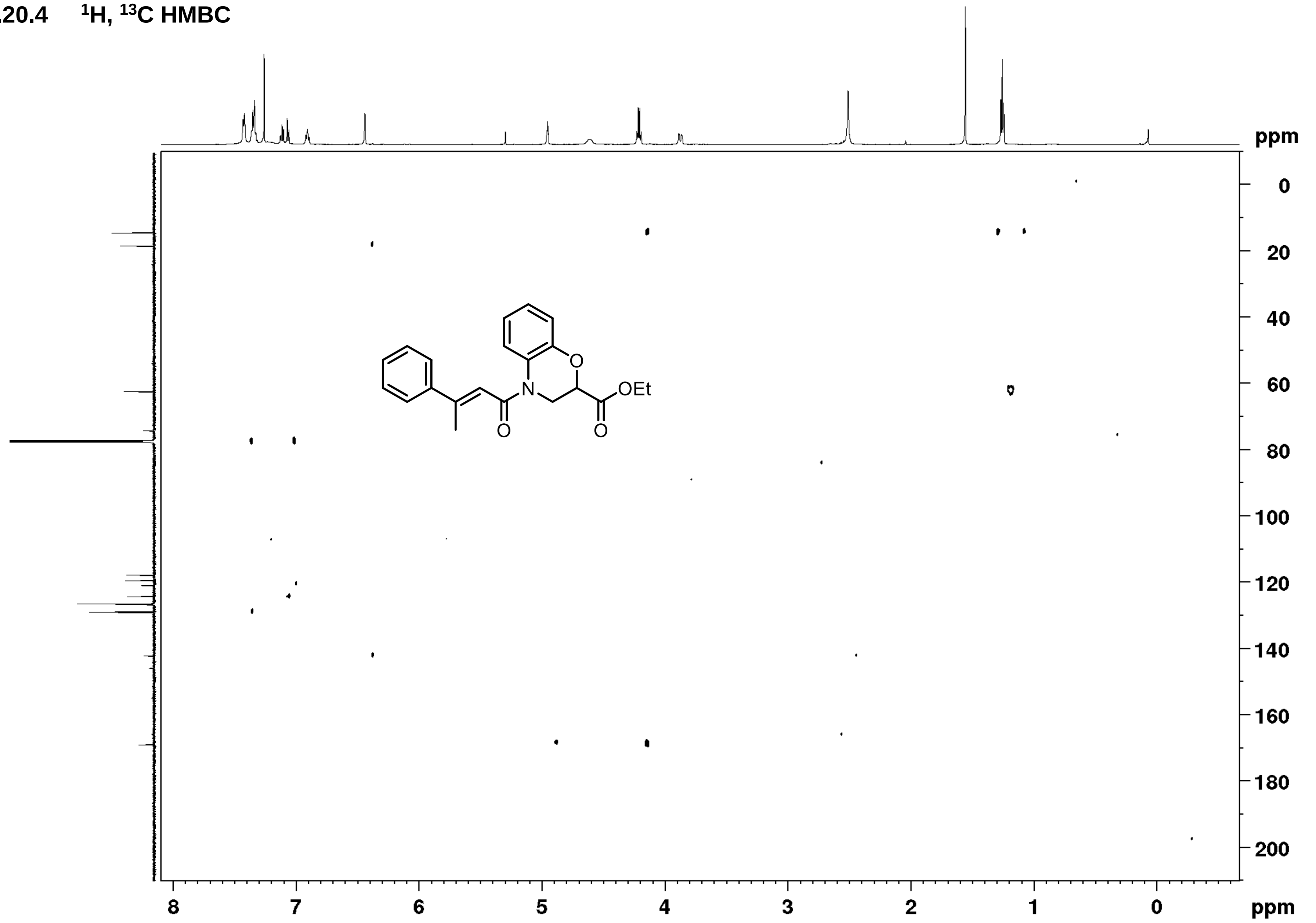

5.20.5 ^{1}H, ^{13}C HSQC

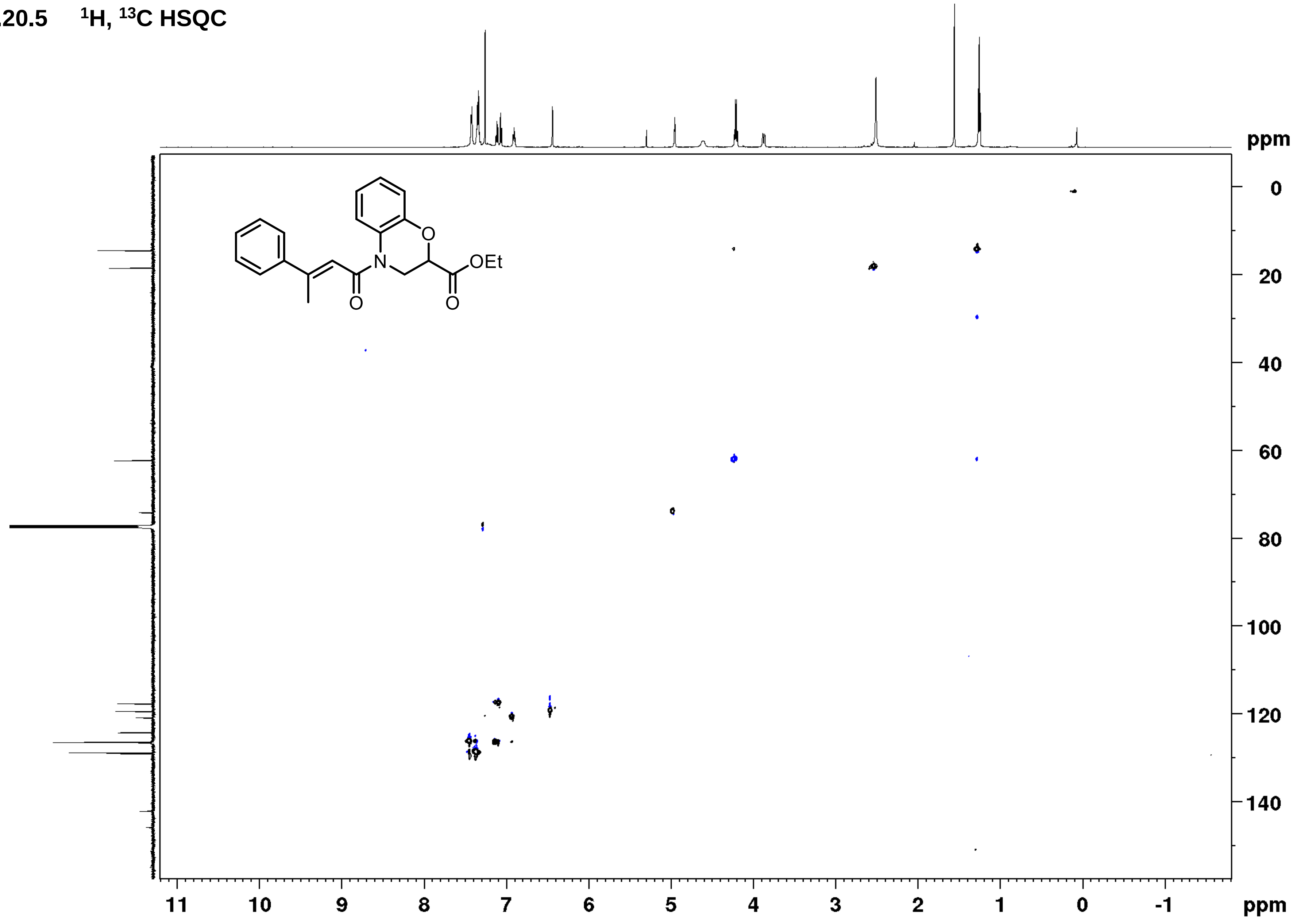

5.20.6 HRMS

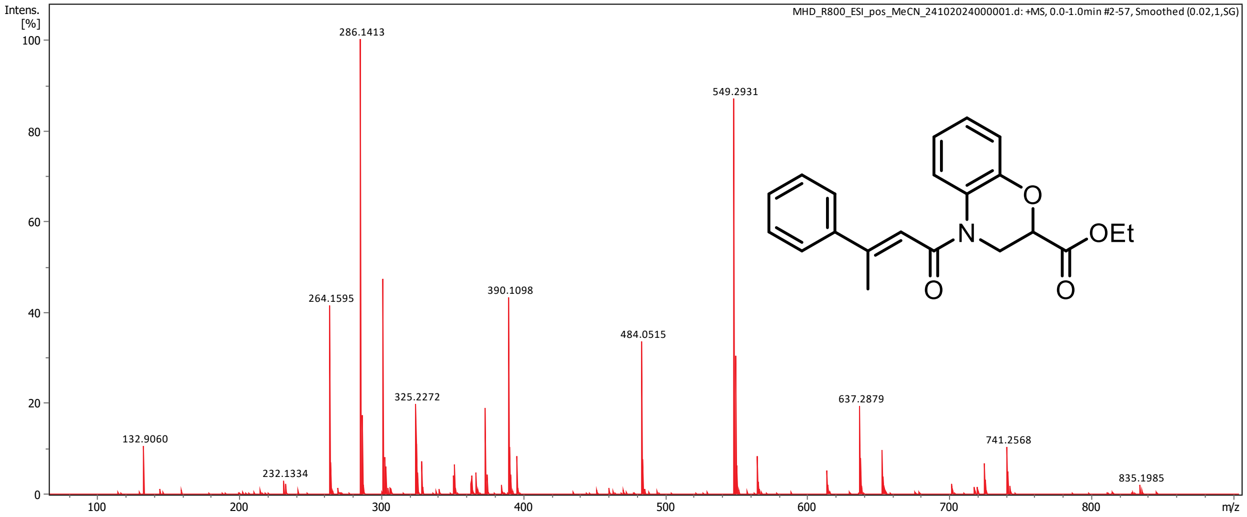

Intens. [%]
MHD_R800_ESI_pos_MeCN_24102024000001.d: +MS, 0.0-1.0min #2-57, Smoothed (0.02,1,SG)
286.1413
549.2931
264.1595
390.1098
484.0515
325.2272
637.2879
132.9060
741.2568
232.1334
835.1985
O
N
OEt
O
O
m/z

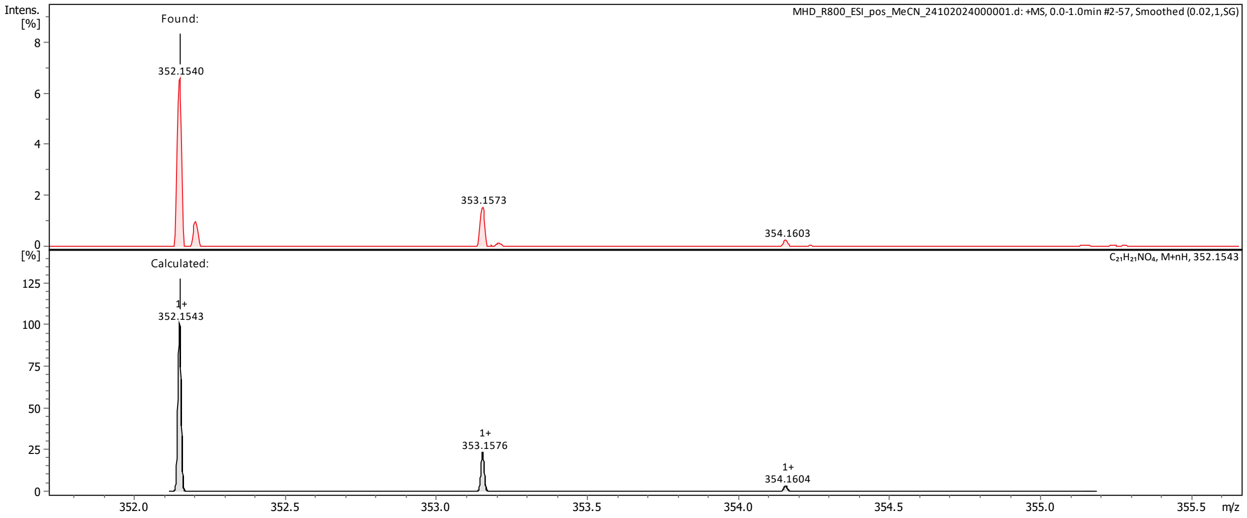

Intens. [%]
MHD_R800_ESI_pos_MeCN_24102024000001.d: +MS, 0.0-1.0min #2-57, Smoothed (0.02,1,SG)
Found:
352.1540
353.1573
354.1603
Calculated:
C21H21NO4, M+nH, 352.1543
1+ 352.1543
1+ 353.1576
1+ 354.1604
m/z

5.20.7 IR

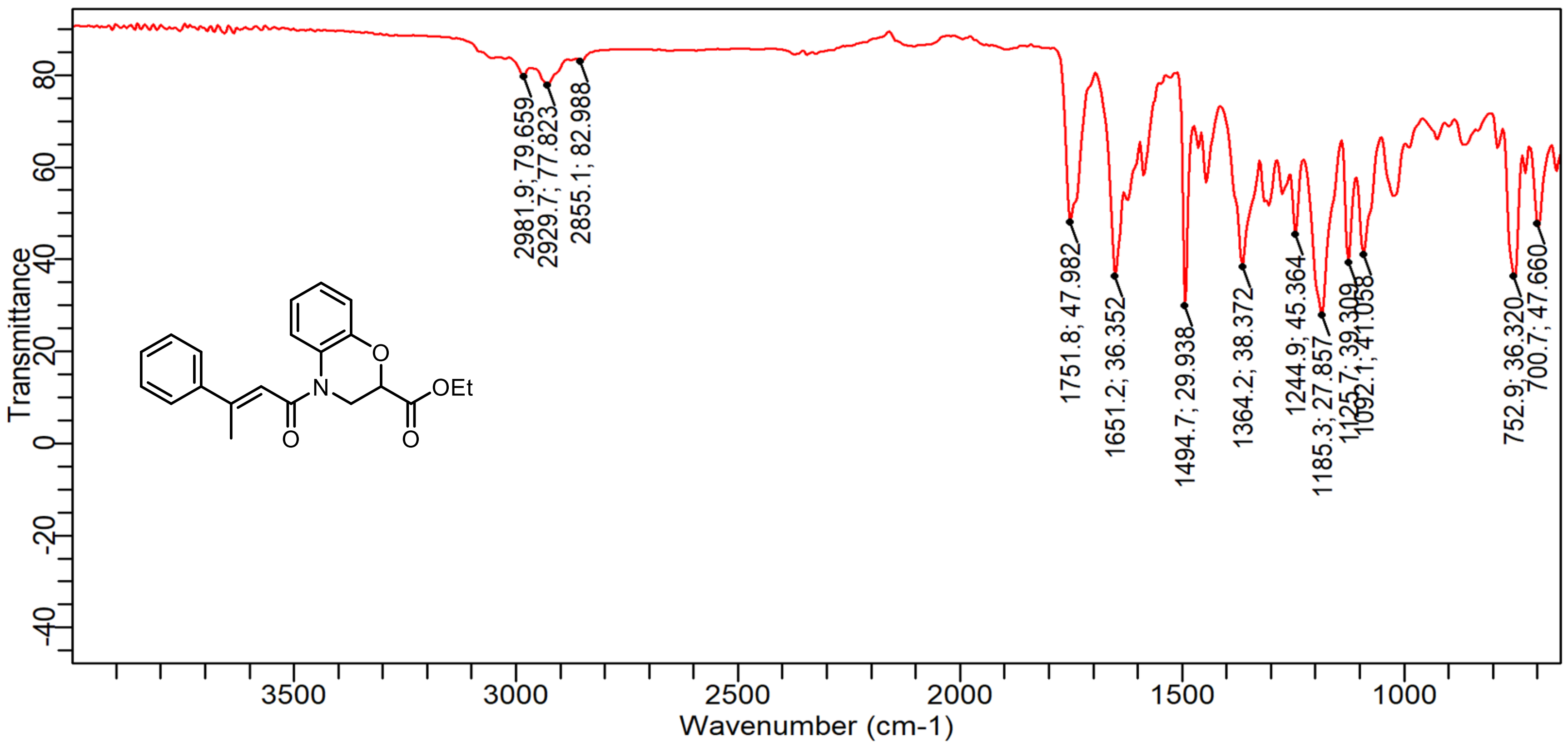

Transmittance
Wavenumber (cm-1)
2981.9; 79.659
2929.7; 77.823
2855.1; 82.988
1751.8; 47.982
1651.2; 36.352
1494.7; 29.938
1364.2; 38.372
1244.9; 45.364
1185.3; 27.857
1125.7; 39.309
1092.1; 41.058
752.9; 36.320
700.7; 47.660
OEt
N
O

5.21 (*E*)-*N*,*N*-Diethyl-1-(3-phenylbut-2-enoyl)piperidine-3-carboxamide (11v) (600 MHz, $CDCl_3$)

5.21.1 ^{1}H NMR

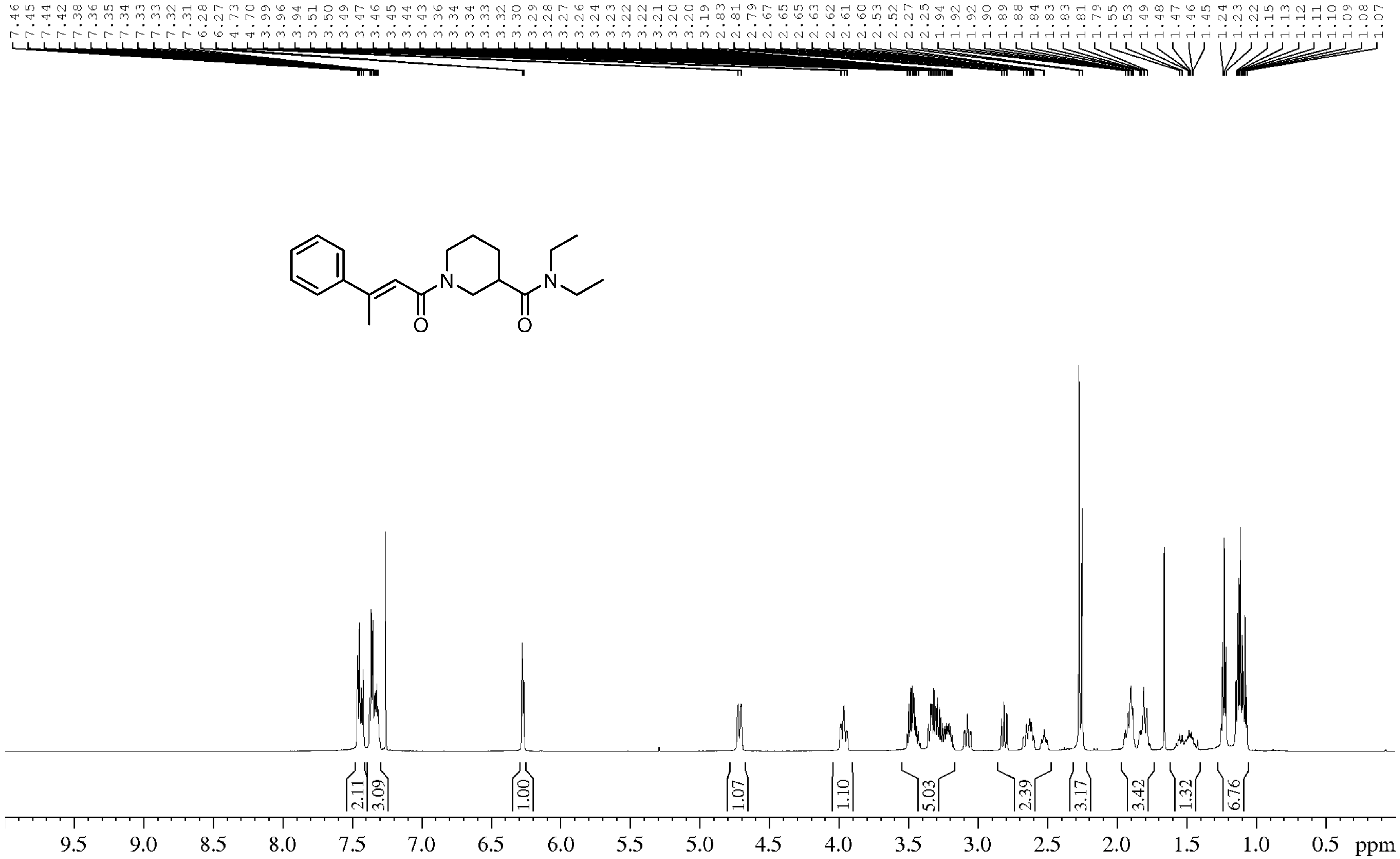

5.21.2 ^{13}C NMR

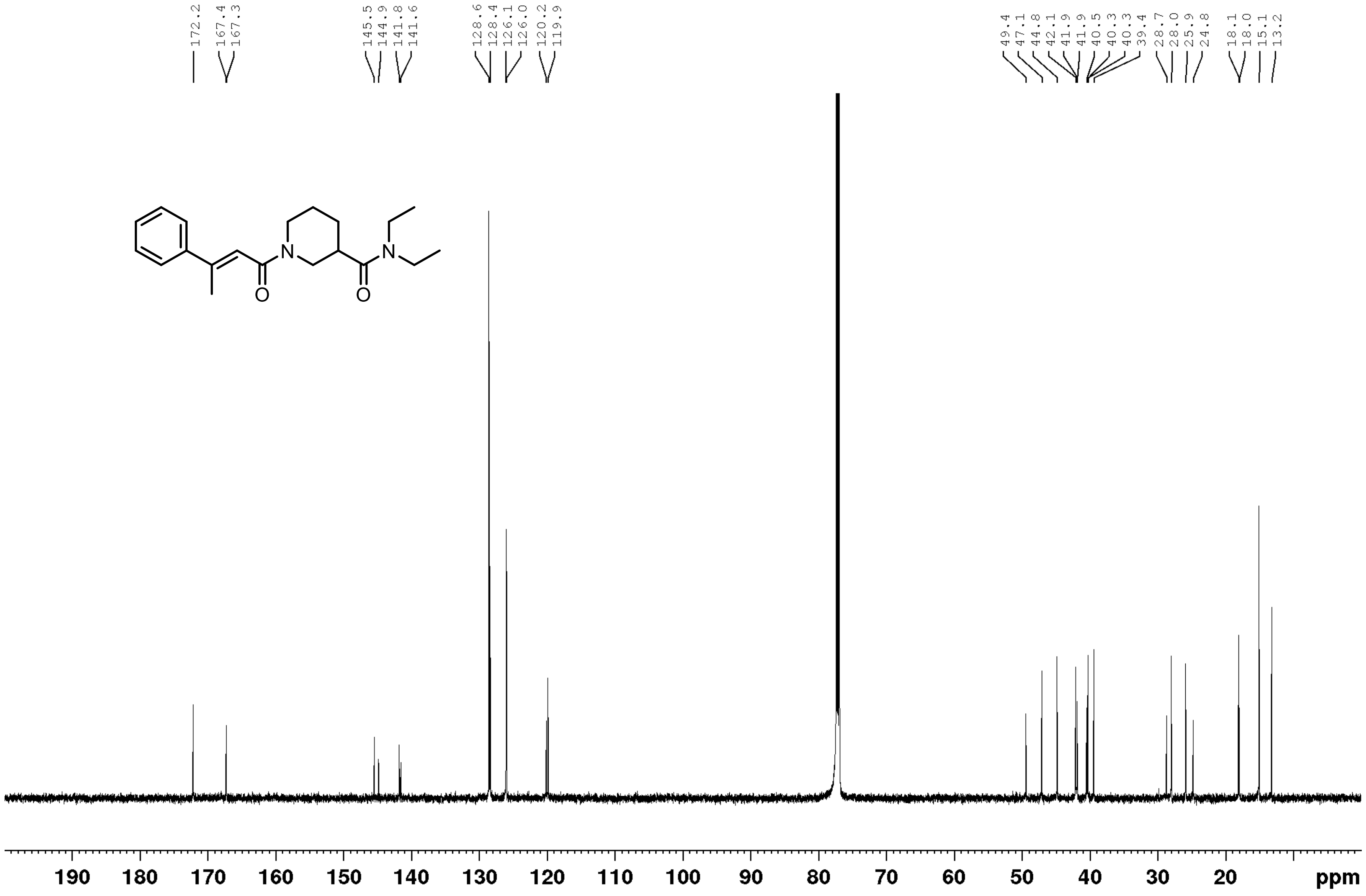
172.2
167.4
167.3
145.5
144.9
141.8
141.6
128.6
128.4
126.1
126.0
120.2
119.9
49.4
47.1
44.8
42.1
41.9
40.5
40.3
40.3
39.4
28.7
28.0
25.9
24.8
18.1
18.0
15.1
13.2
190 180 170 160 150 140 130 120 110 100 90 80 70 60 50 40 30 20 ppm

5.21.3 $^{1}H, ^{1}H$ COSY

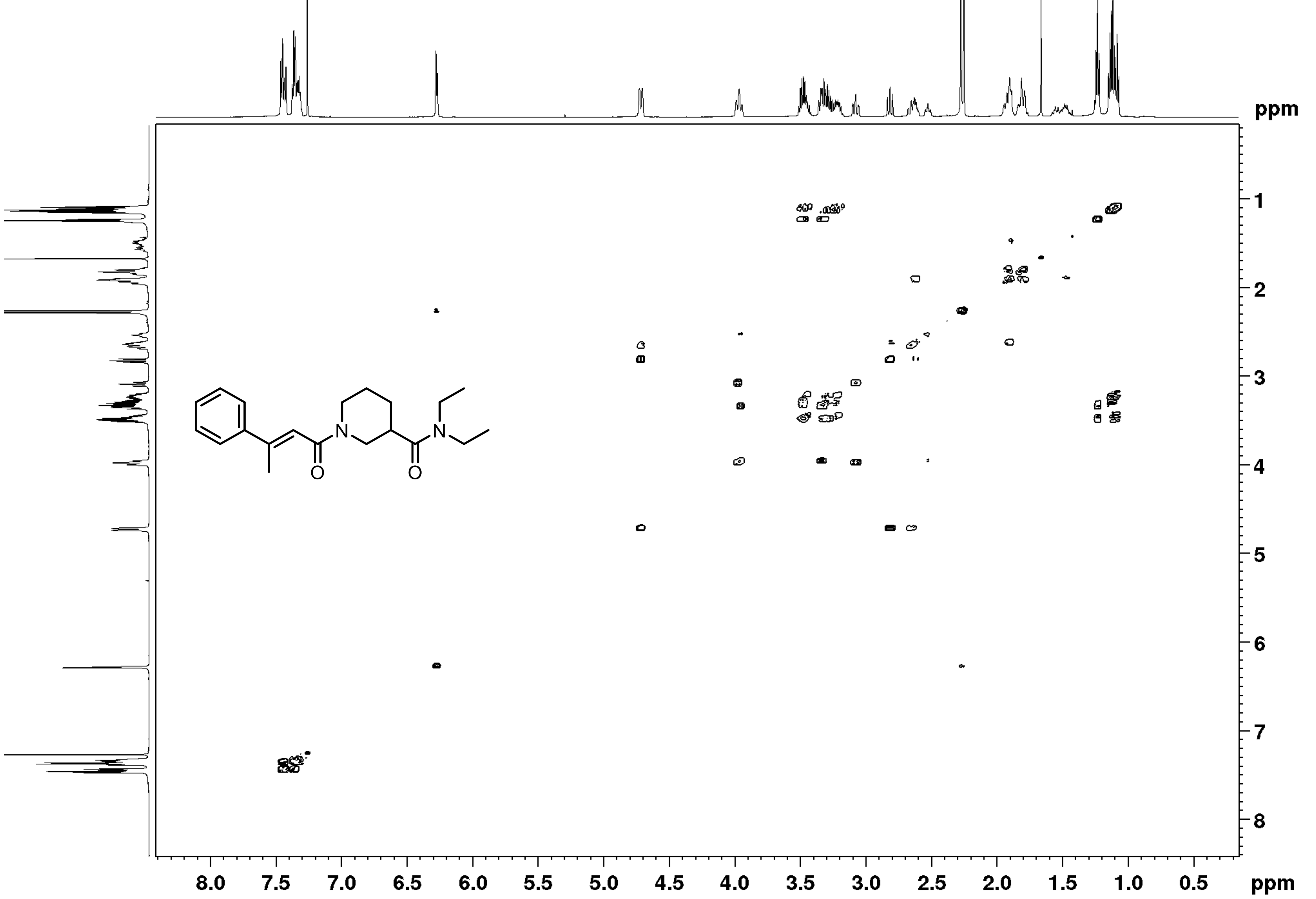

5.21.4 ^{1}H, ^{13}C HMBC

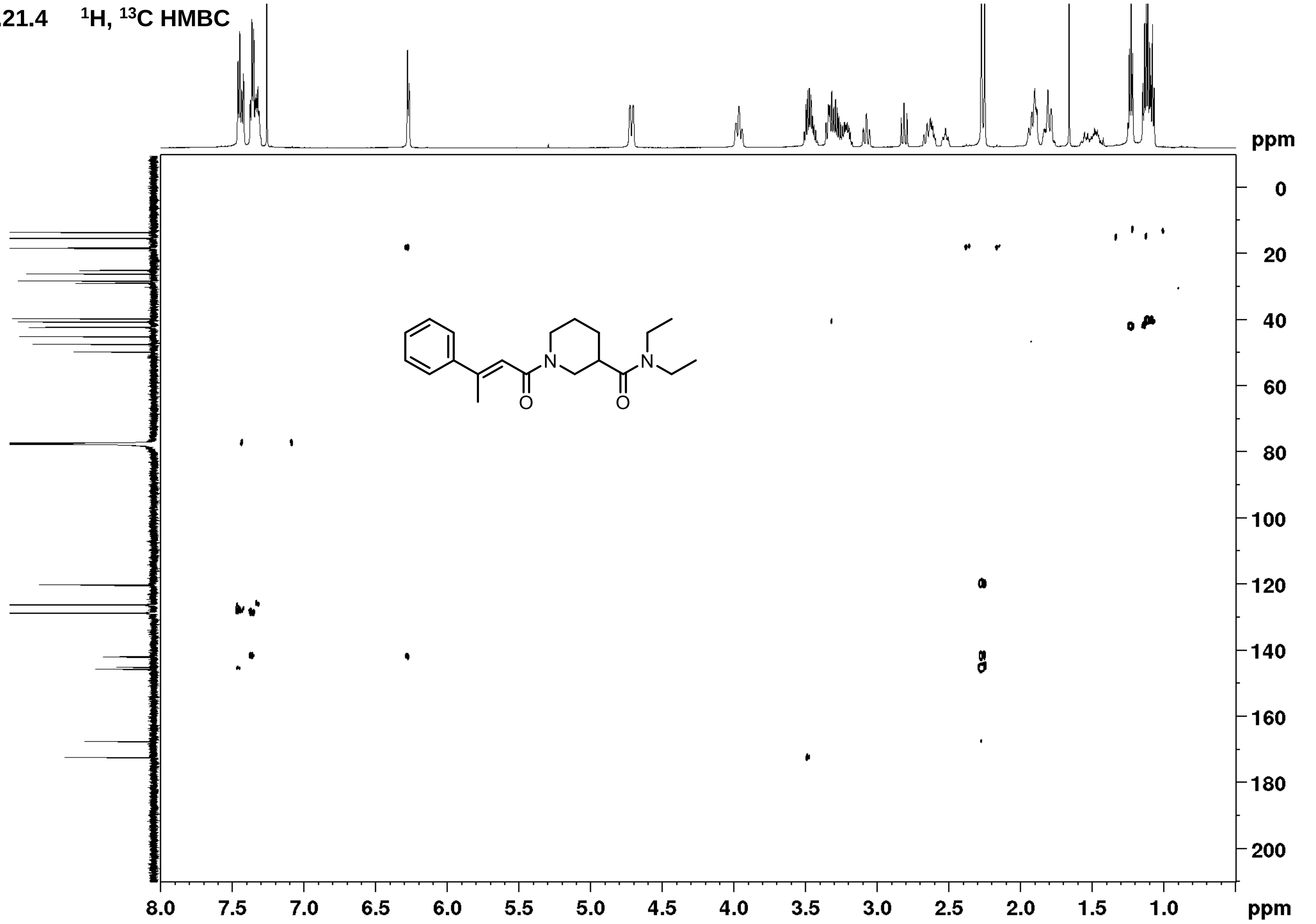

5.21.5 ^{1}H, ^{13}C HSQC

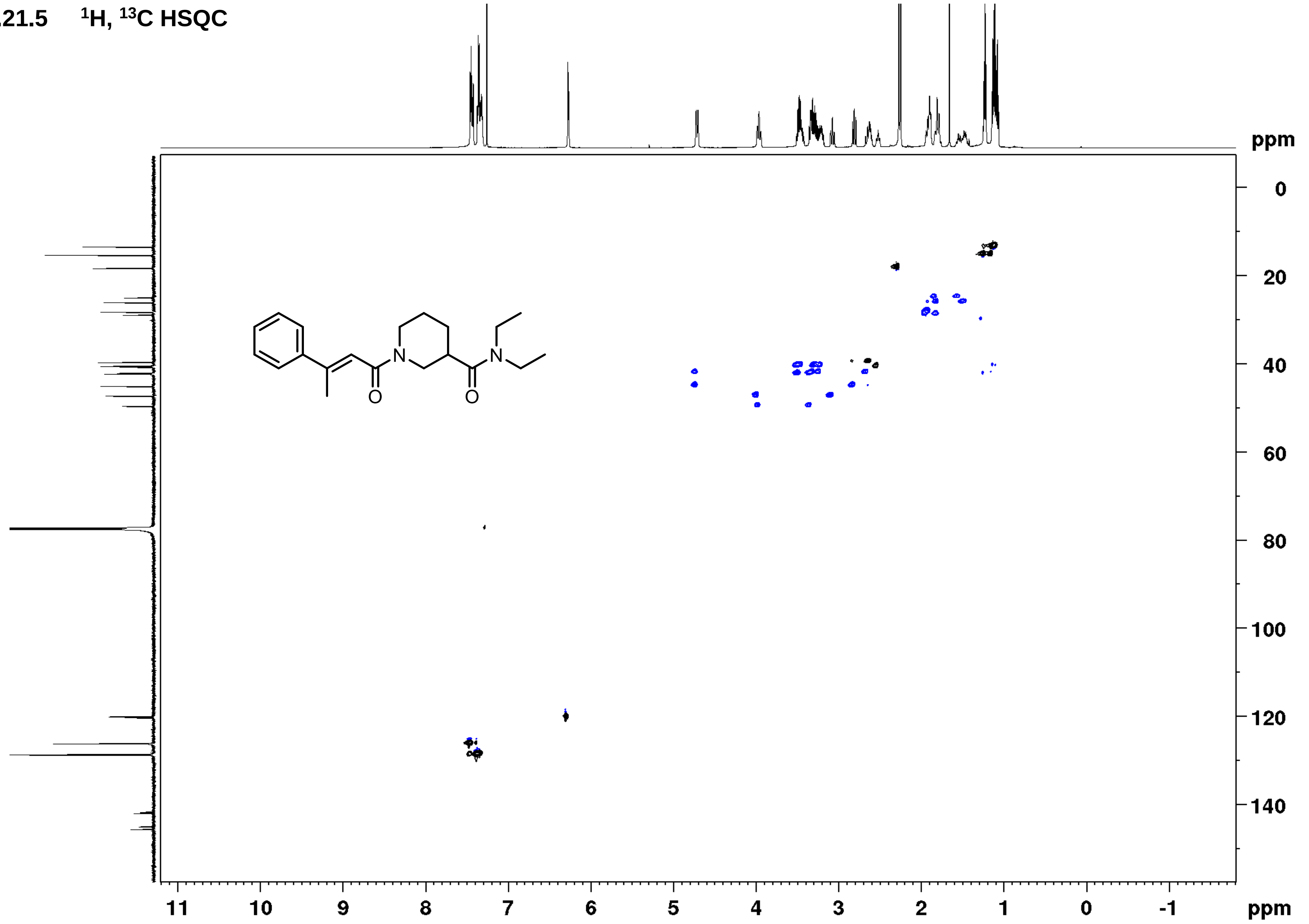

5.21.6 HRMS

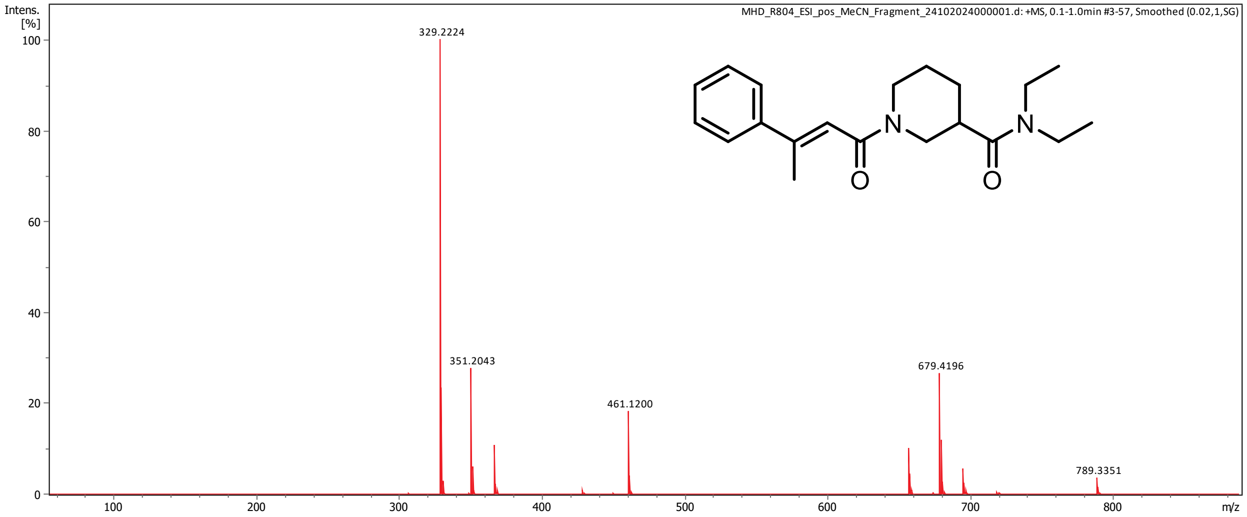
MHD_R804_ESI_pos_MeCN_Fragment_24102024000001.d: +MS, 0.1-1.0min #3-57, Smoothed (0.02,1,SG)
Intens. [%]
329.2224
351.2043
461.1200
679.4196
789.3351
m/z

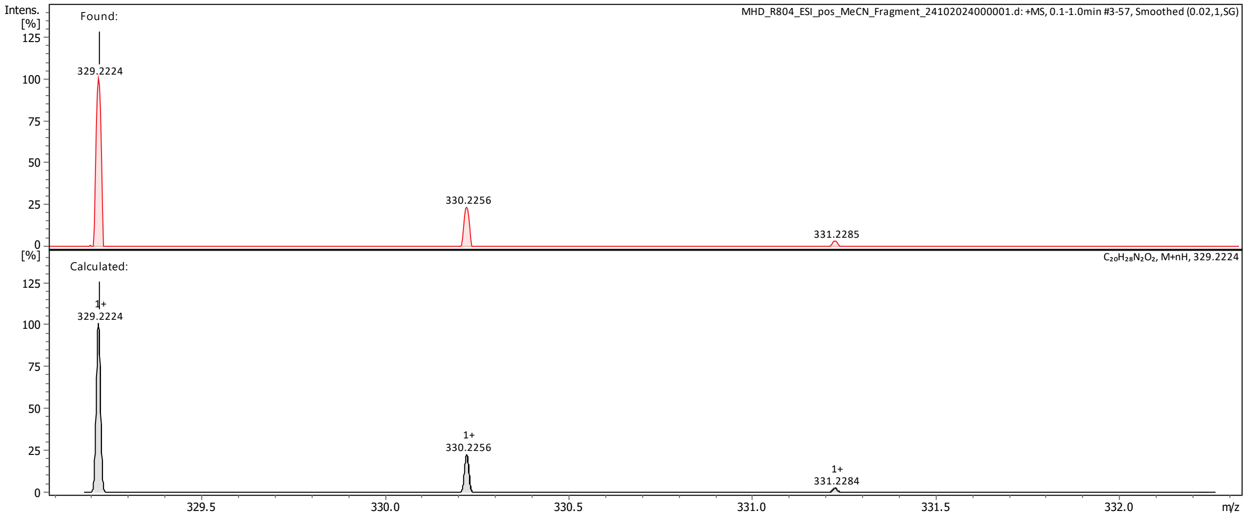
MHD_R804_ESI_pos_MeCN_Fragment_24102024000001.d: +MS, 0.1-1.0min #3-57, Smoothed (0.02,1,SG)
Intens. [%]
Found:
329.2224
330.2256
331.2285
Calculated:
$C_{20}H_{28}N_2O_2$, M+nH, 329.2224
1+ 329.2224
1+ 330.2256
1+ 331.2284
m/z

5.21.7 IR

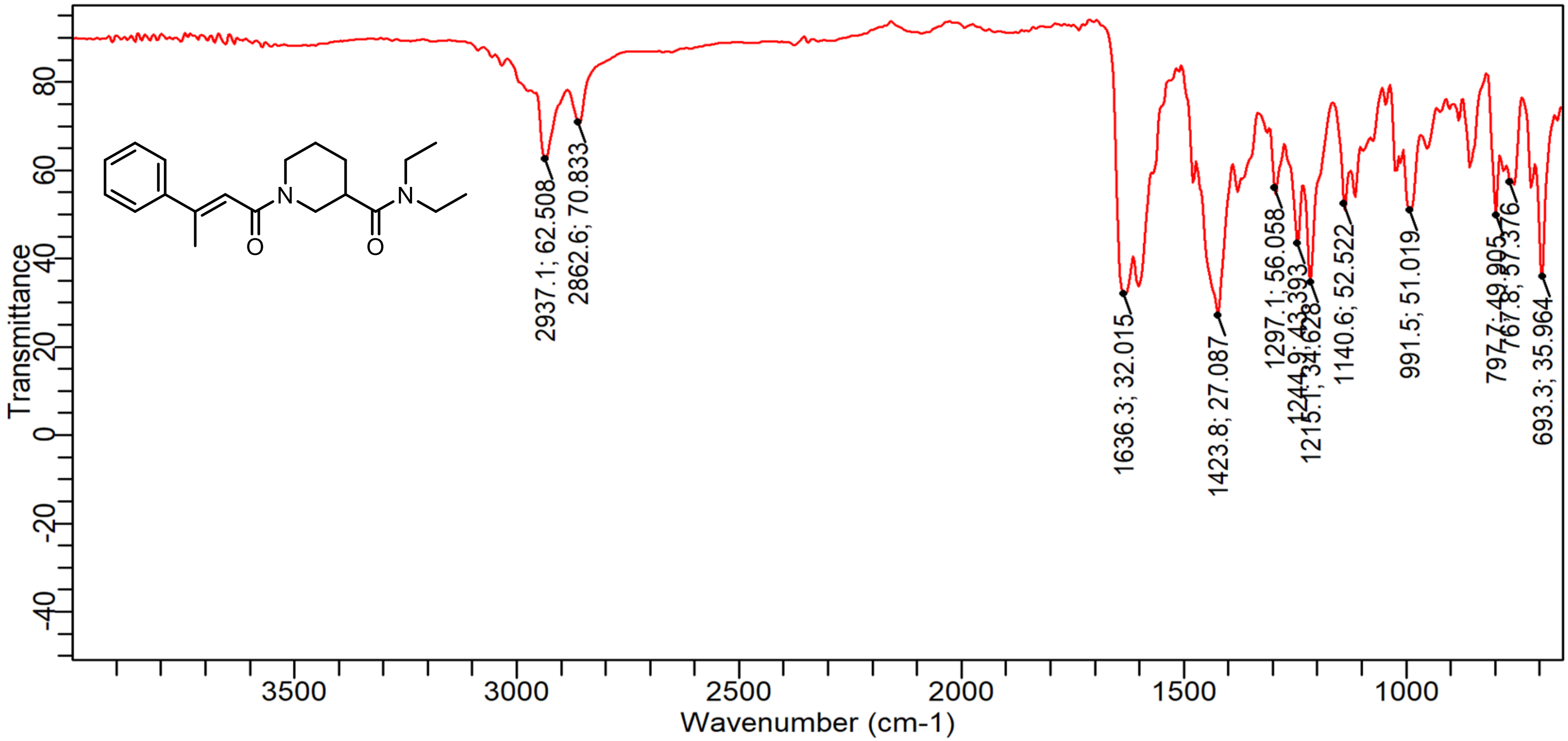
Transmittance
80
60
40
20
0
-20
-40
3500
3000
2500
2000
1500
1000
Wavenumber (cm-1)
2937.1; 62.508
2862.6; 70.833
1636.3; 32.015
1423.8; 27.087
1297.1; 56.058
1244.9; 43.393
1215.1; 34.628
1140.6; 52.522
991.5; 51.019
797.7; 49.905
767.8; 57.376
693.3; 35.964
N
O
N
O

5.22 Ethyl (*E*)-(3-phenylbut-2-enoyl)-L-prolinate (11w & 11w_rotamer) (600 MHz, $CDCl_3$)

5.22.1 ^{1}H NMR

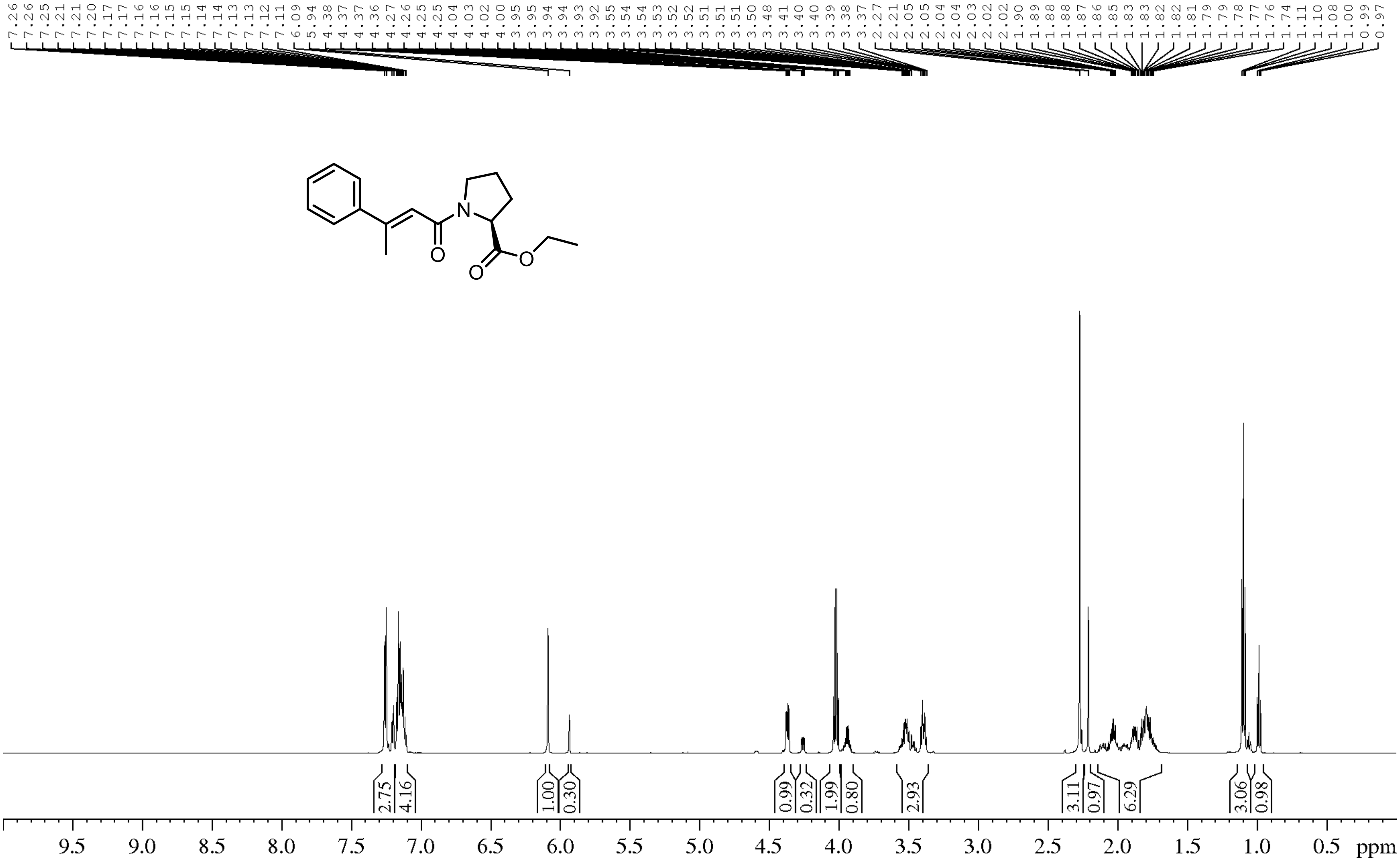

5.22.2 ^{13}C NMR

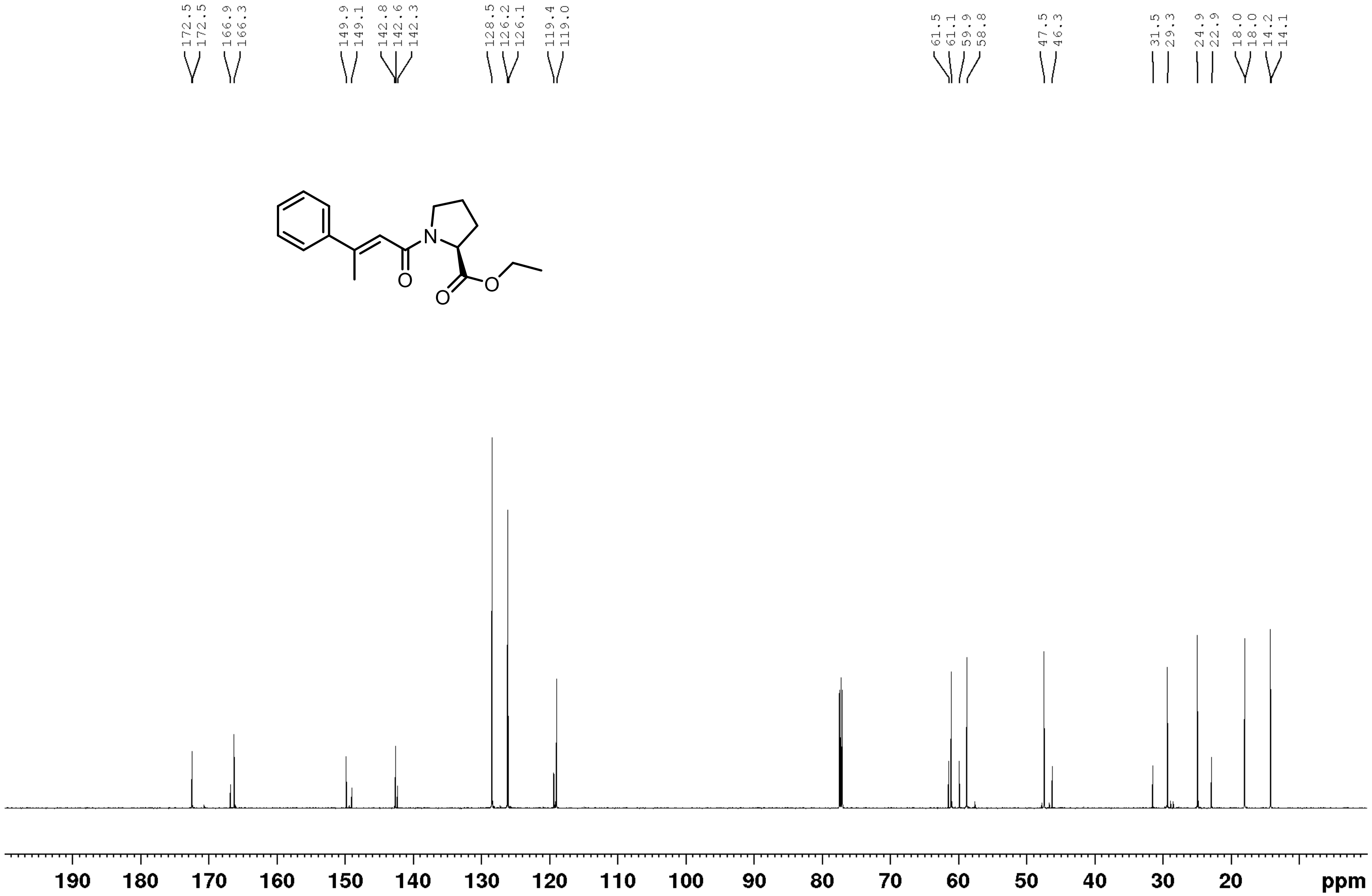

172.5
172.5
166.9
166.3
149.9
149.1
142.8
142.6
142.3
128.5
126.2
126.1
119.4
119.0
61.5
61.1
59.9
58.8
47.5
46.3
31.5
29.3
24.9
22.9
18.0
18.0
14.2
14.1
190 180 170 160 150 140 130 120 110 100 90 80 70 60 50 40 30 20 ppm

5.22.3 $^{1}H, ^{1}H$ COSY

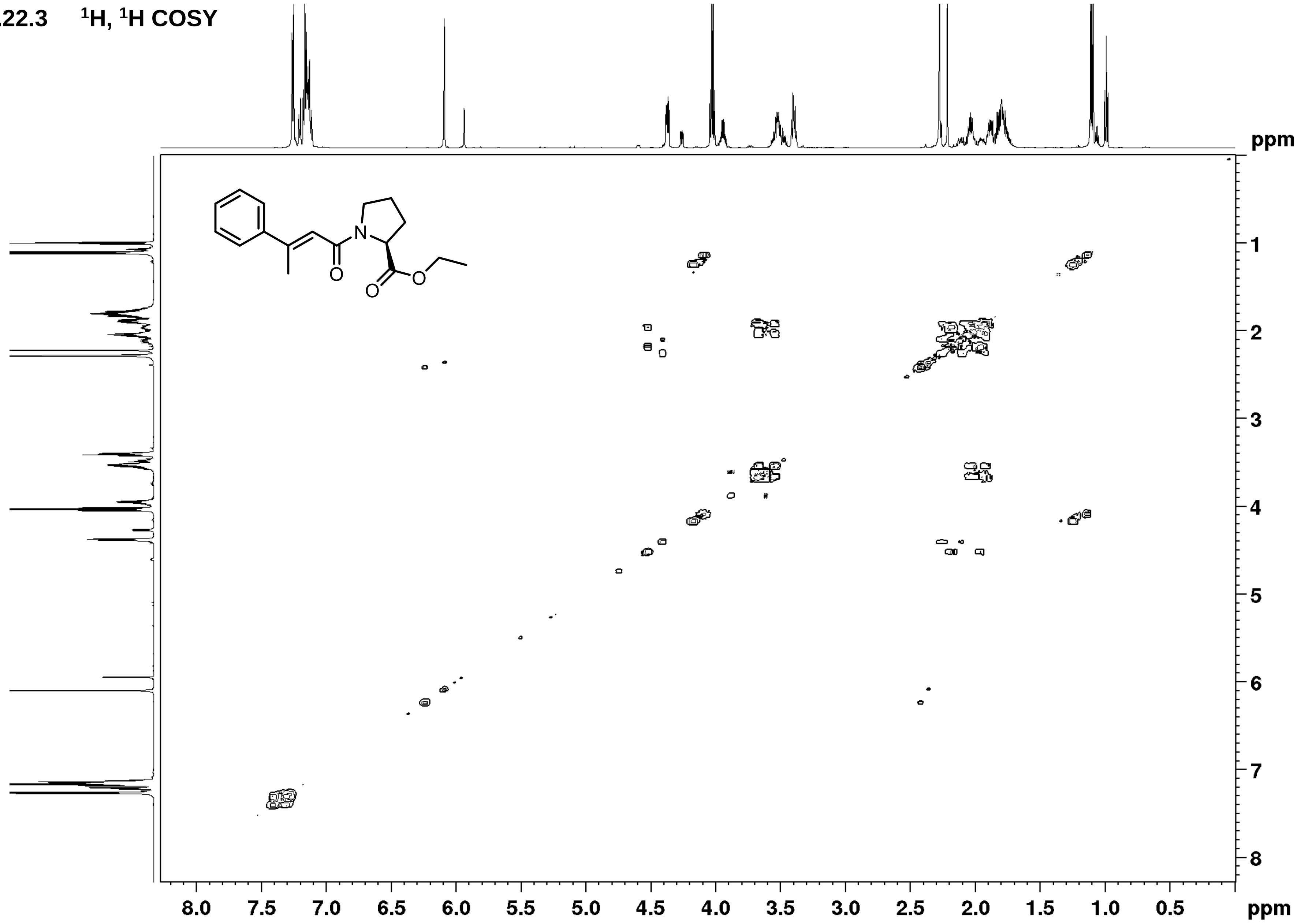

5.22.4 ^{1}H, ^{13}C HMBC

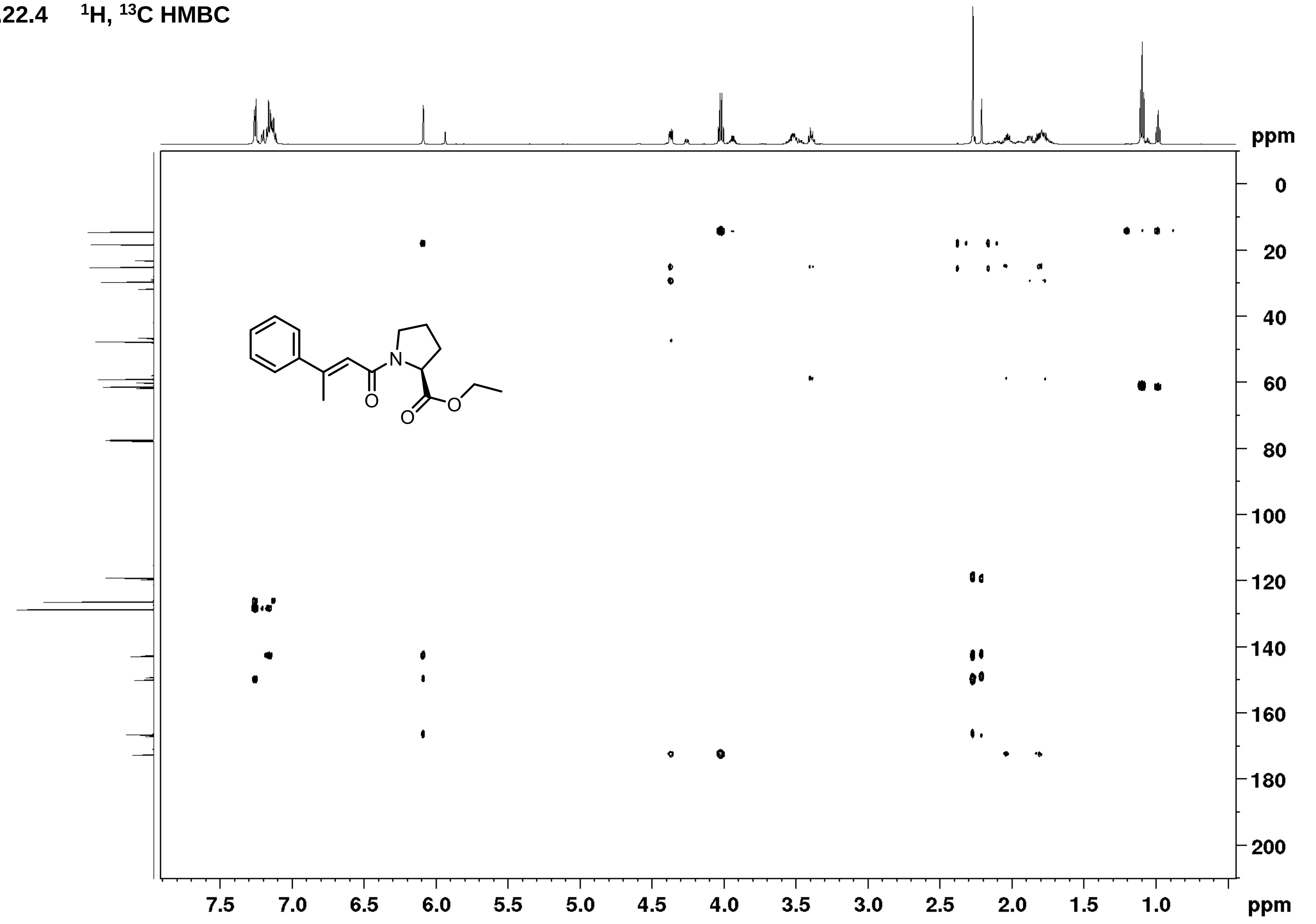

5.22.5 ^{1}H, ^{13}C HSQC

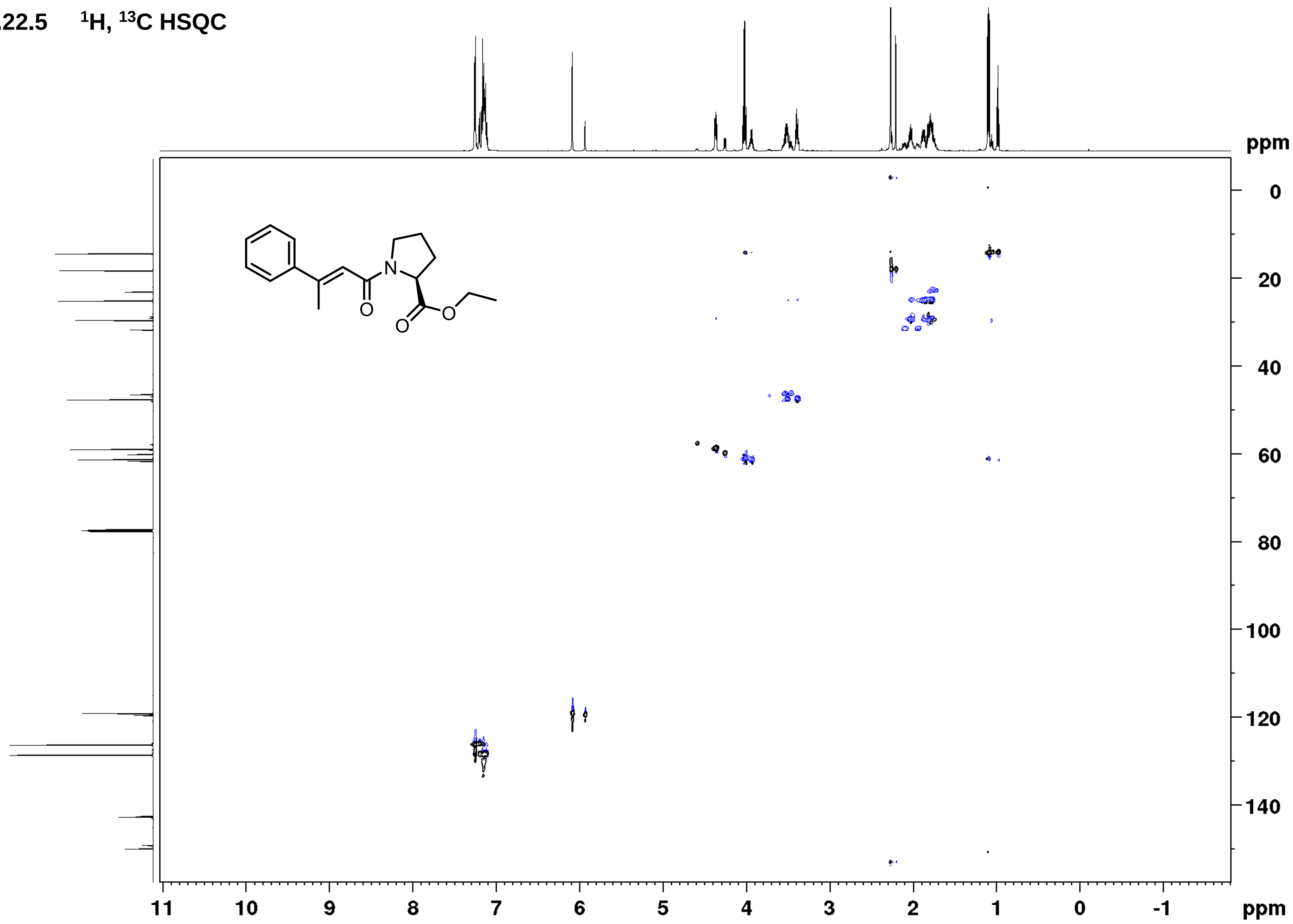

5.22.6 HRMS

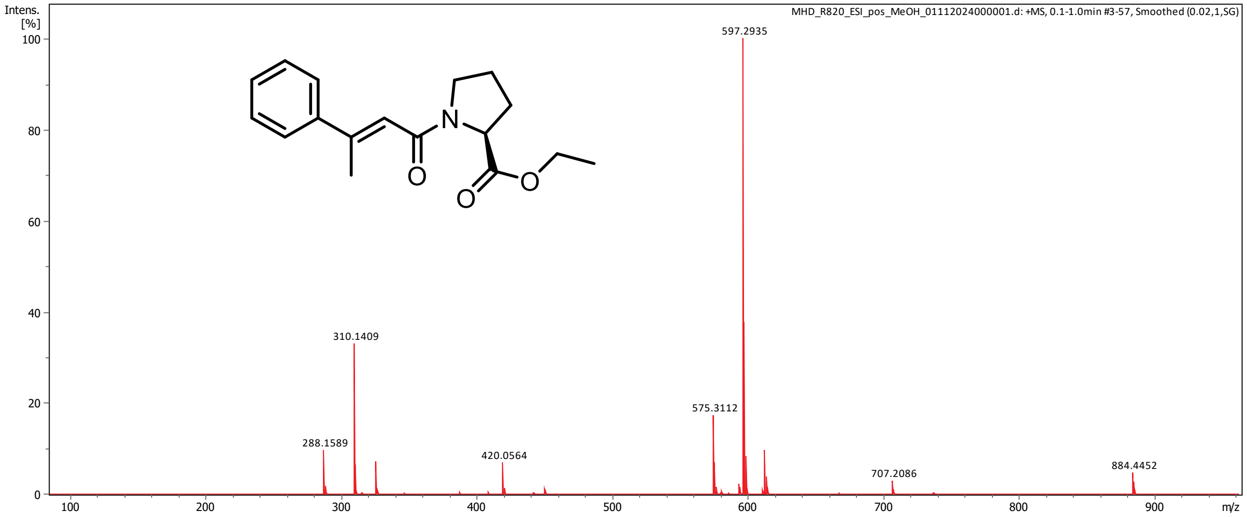

MHD_R820_ESI_pos_MeOH_01112024000001.d: +MS, 0.1-1.0min #3-57, Smoothed (0.02,1,SG)
Intens. [%]
597.2935
310.1409
575.3112
288.1589
420.0564
707.2086
884.4452
m/z

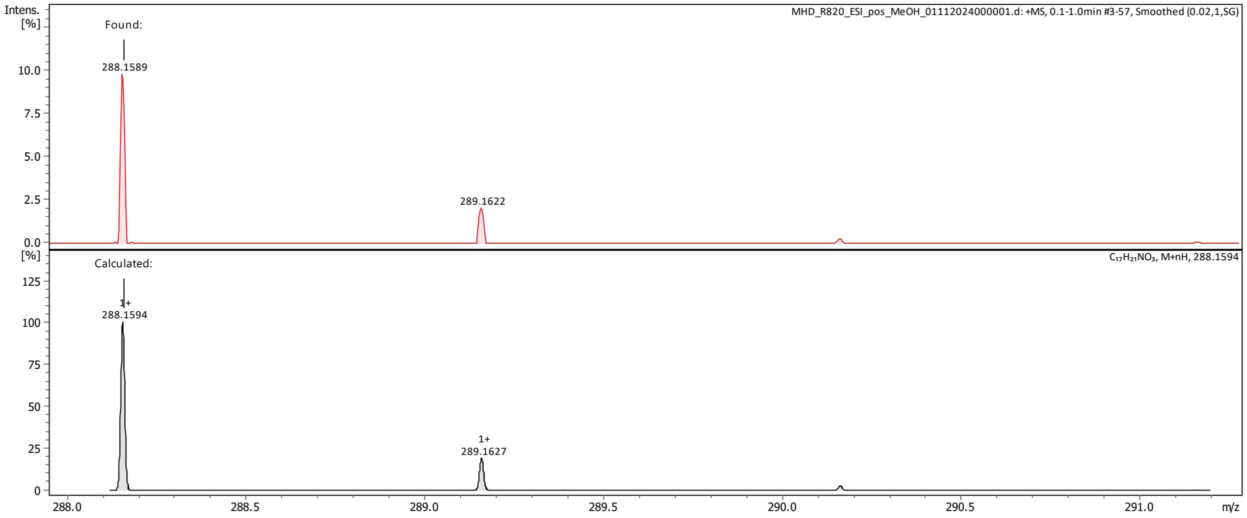

MHD_R820_ESI_pos_MeOH_01112024000001.d: +MS, 0.1-1.0min #3-57, Smoothed (0.02,1,SG)
Intens. [%]
Found:
288.1589
289.1622
Calculated:
C17H21NO3, M+nH, 288.1594
1+ 288.1594
1+ 289.1627
m/z

5.22.7 IR

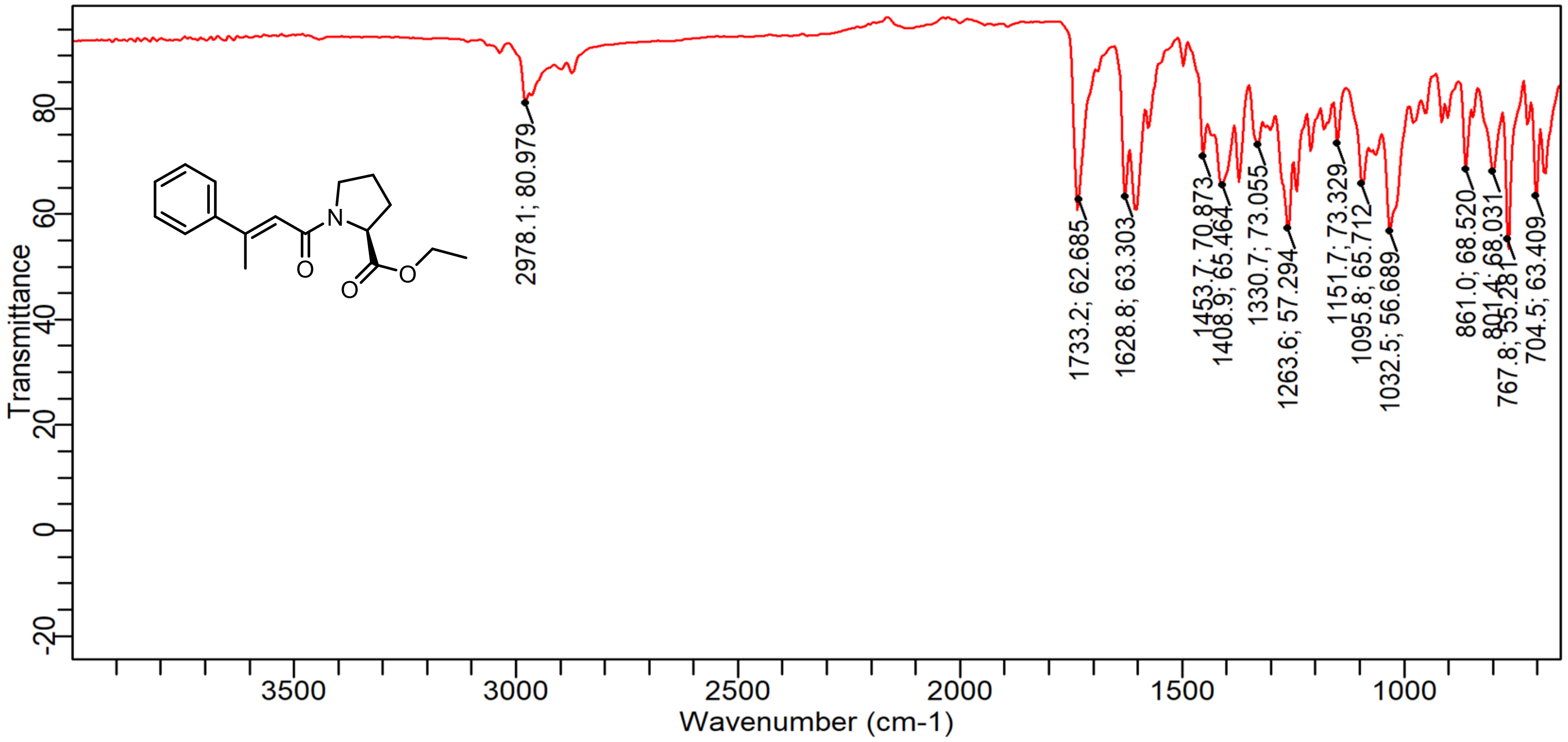

Transmittance
Wavenumber (cm-1)
2978.1; 80.979
1733.2; 62.685
1628.8; 63.303
1453.7; 70.873
1408.9; 65.464
1330.7; 73.055
1263.6; 57.294
1151.7; 73.329
1095.8; 65.712
1032.5; 56.689
861.0; 68.520
801.4; 68.031
767.8; 55.281
704.5; 63.409

5.23 Ethyl (*E*)-*N*-methyl-*N*-(3-phenylbut-2-enoyl)-*L*-alaninate (11x & 11x_rotamer) (600 MHz, $CDCl_3$)

5.23.1 ^{1}H NMR

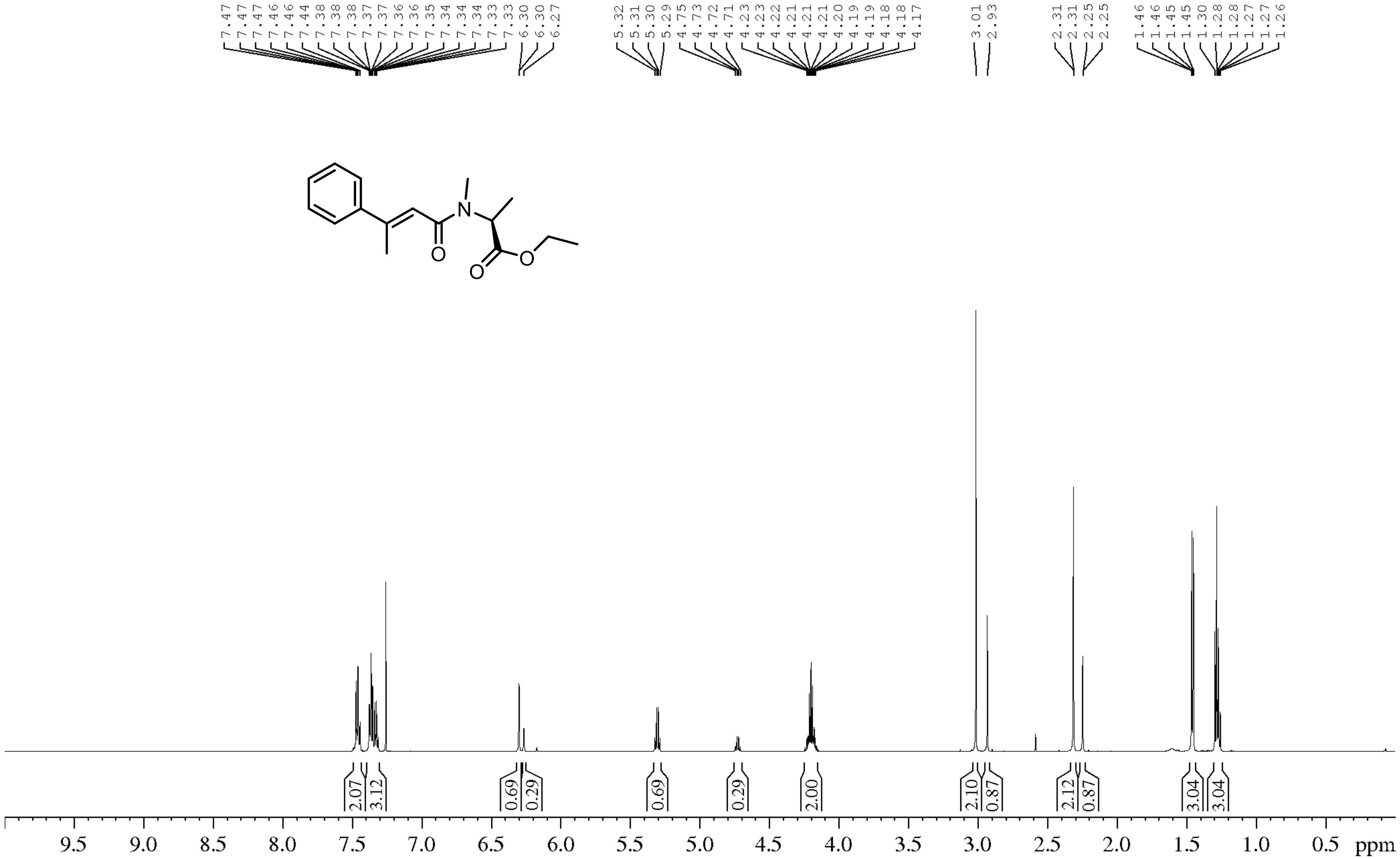

5.23.2 ^{13}C NMR

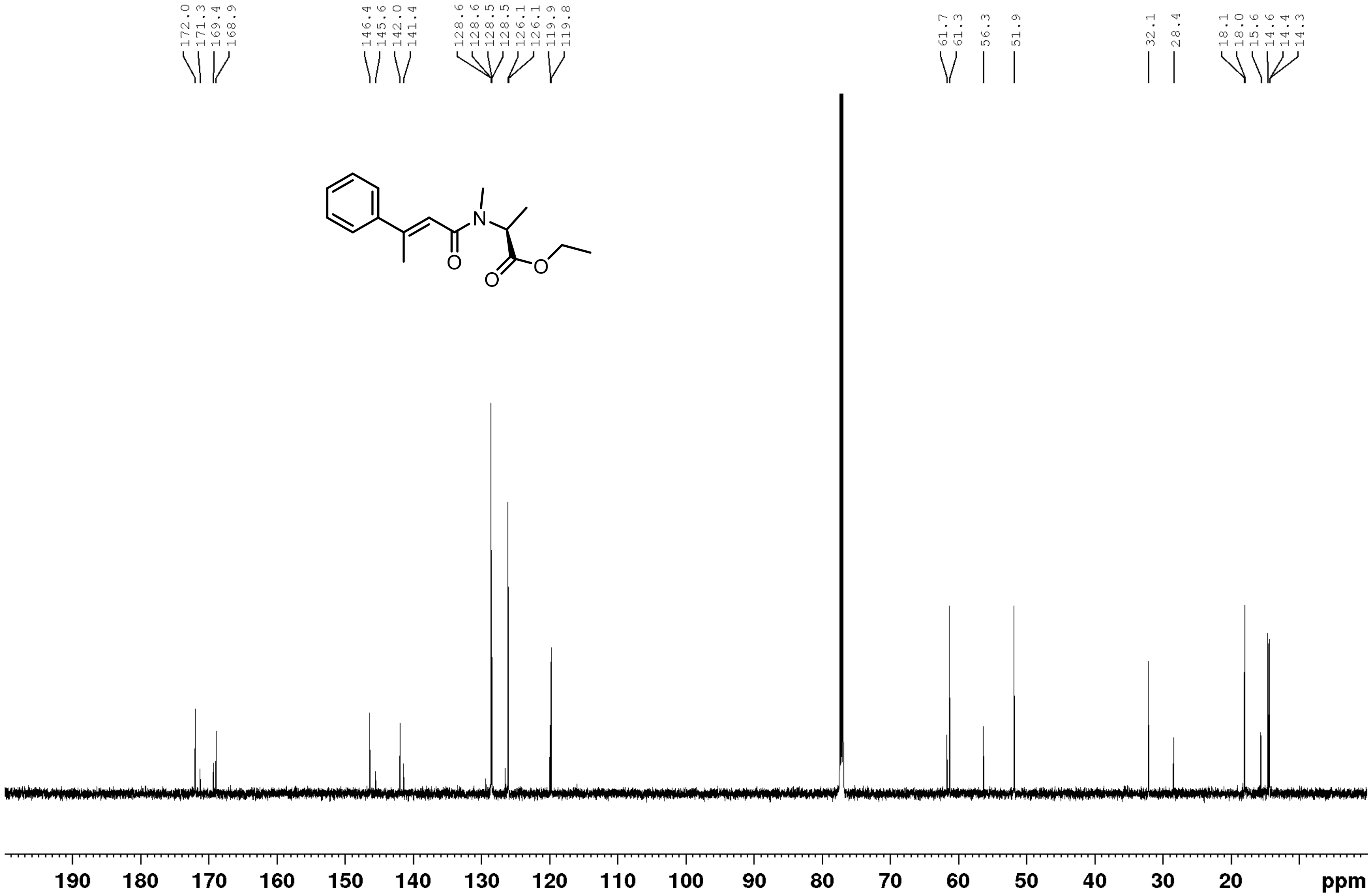

172.0
171.3
169.4
168.9
146.4
145.6
142.0
141.4
128.6
128.6
128.5
128.5
126.1
126.1
119.9
119.8
61.7
61.3
56.3
51.9
32.1
28.4
18.1
18.0
15.6
14.6
14.4
14.3
N
O
O
O
190
180
170
160
150
140
130
120
110
100
90
80
70
60
50
40
30
20
ppm

5.23.3 ^{1}H, ^{1}H COSY

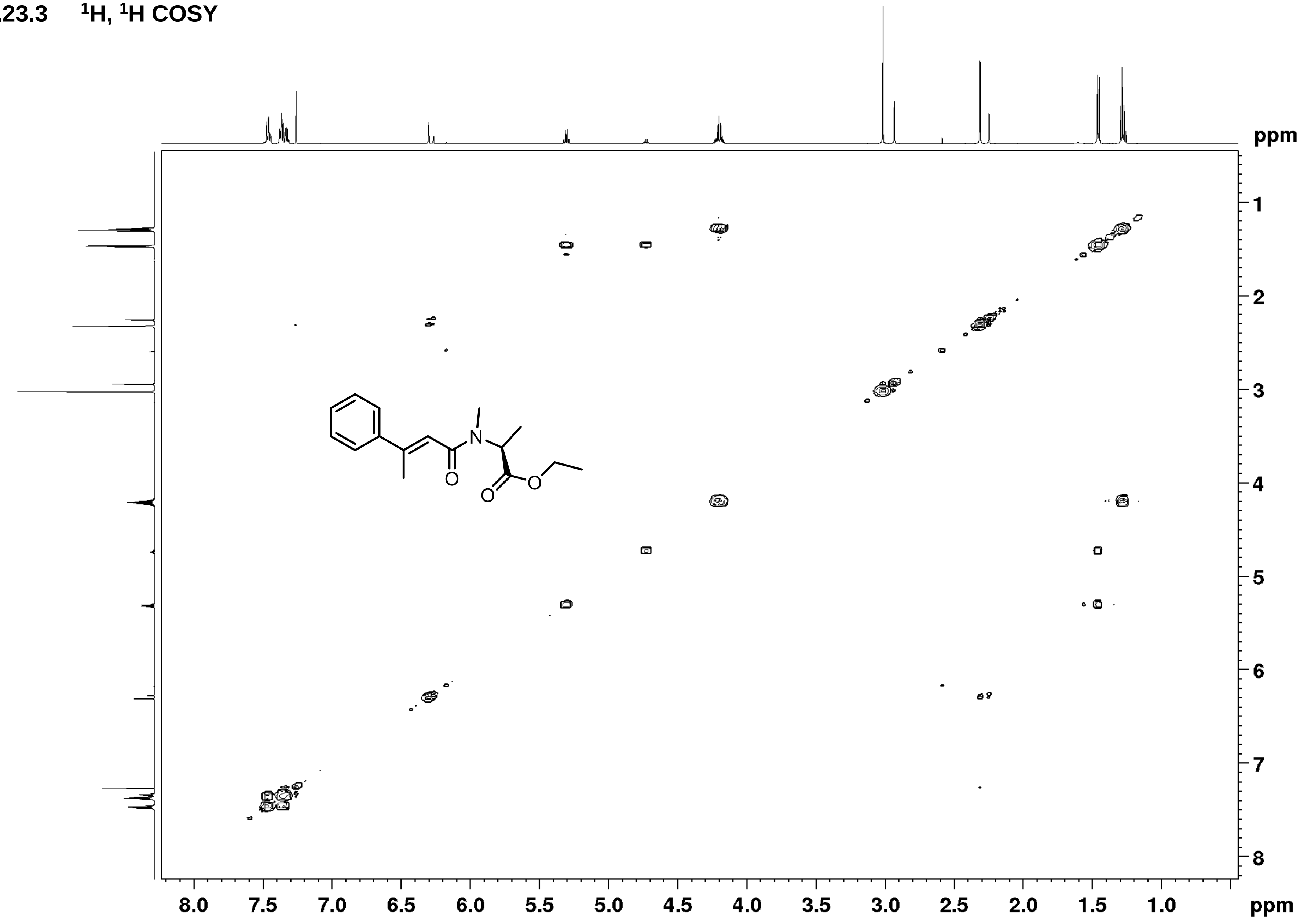

5.23.4 ^{1}H, ^{13}C HMBC

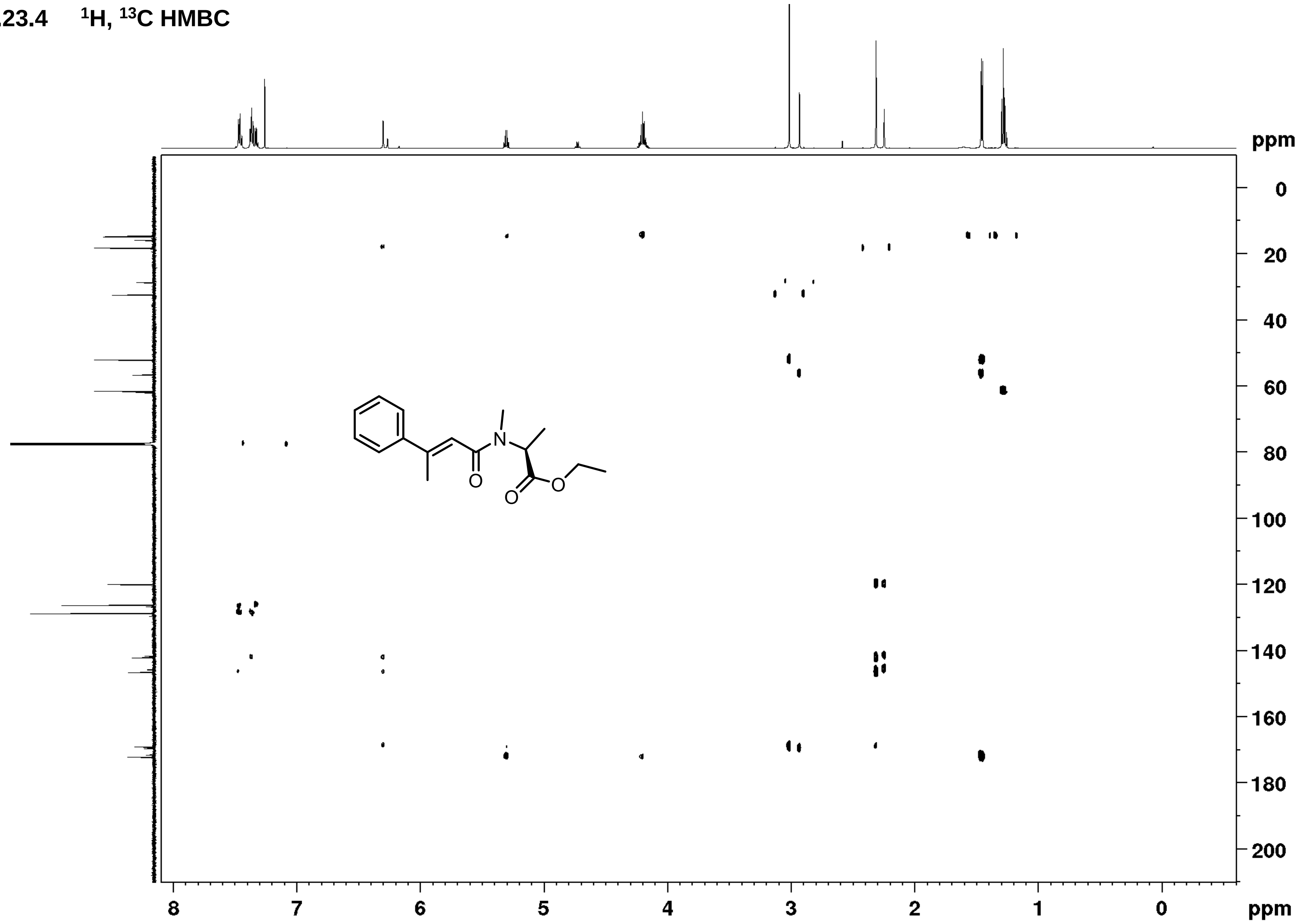

5.23.5 ^{1}H, ^{13}C HSQC

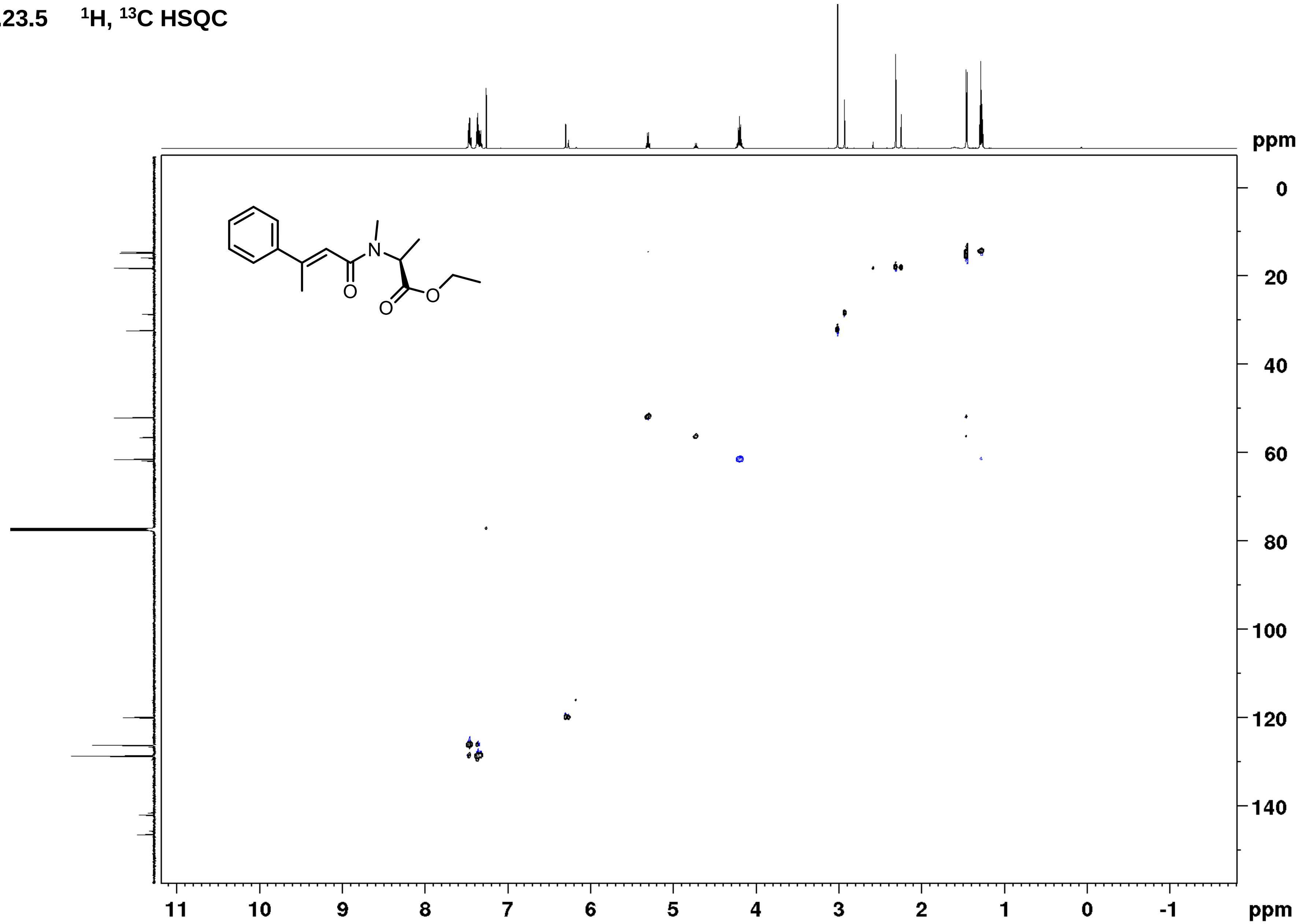

5.23.6 HRMS

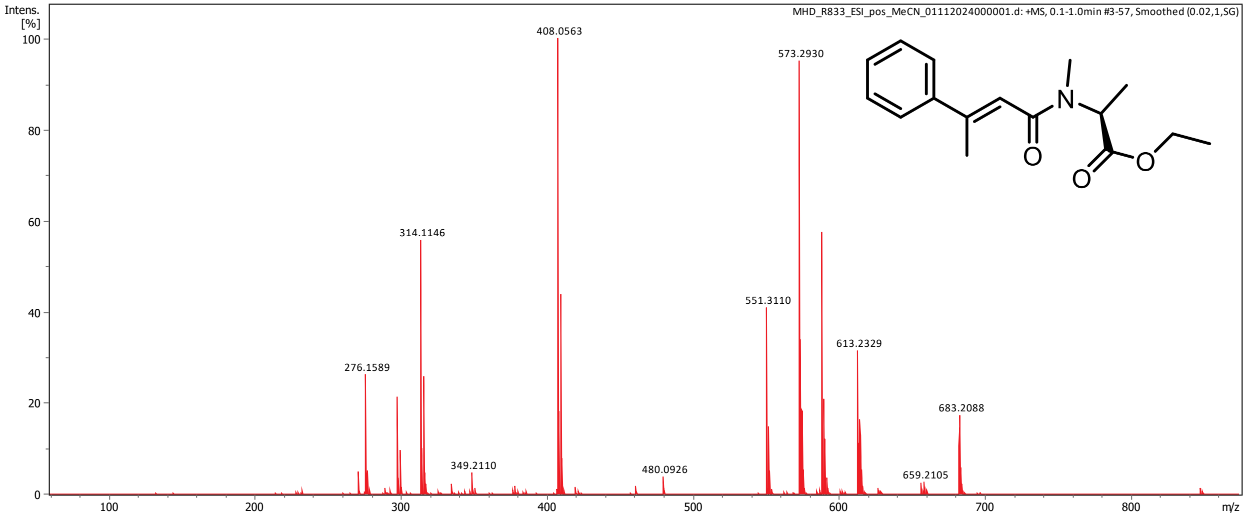
MHD_R833_ESI_pos_MeCN_01112024000001.d: +MS, 0.1-1.0min #3-57, Smoothed (0.02,1,SG)
Intens. [%]
408.0563
573.2930
314.1146
551.3110
613.2329
276.1589
683.2088
349.2110
480.0926
659.2105
m/z
N
O
O
O

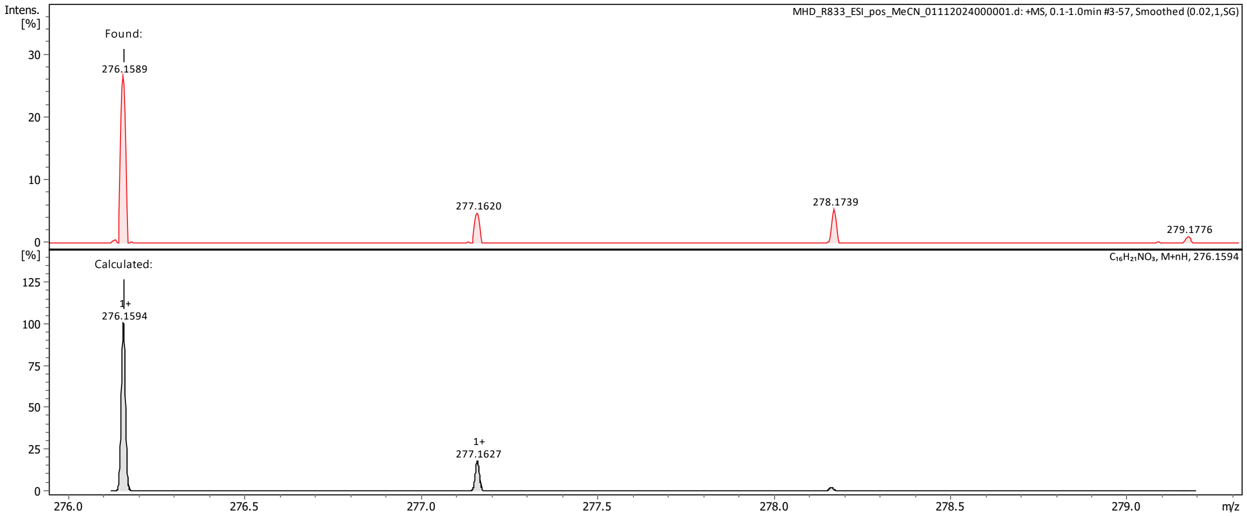
MHD_R833_ESI_pos_MeCN_01112024000001.d: +MS, 0.1-1.0min #3-57, Smoothed (0.02,1,SG)
Found:
276.1589
277.1620
278.1739
279.1776
Calculated:
$C_{16}H_{21}NO_3$, M+nH, 276.1594
1+
276.1594
1+
277.1627
m/z

5.23.7 IR

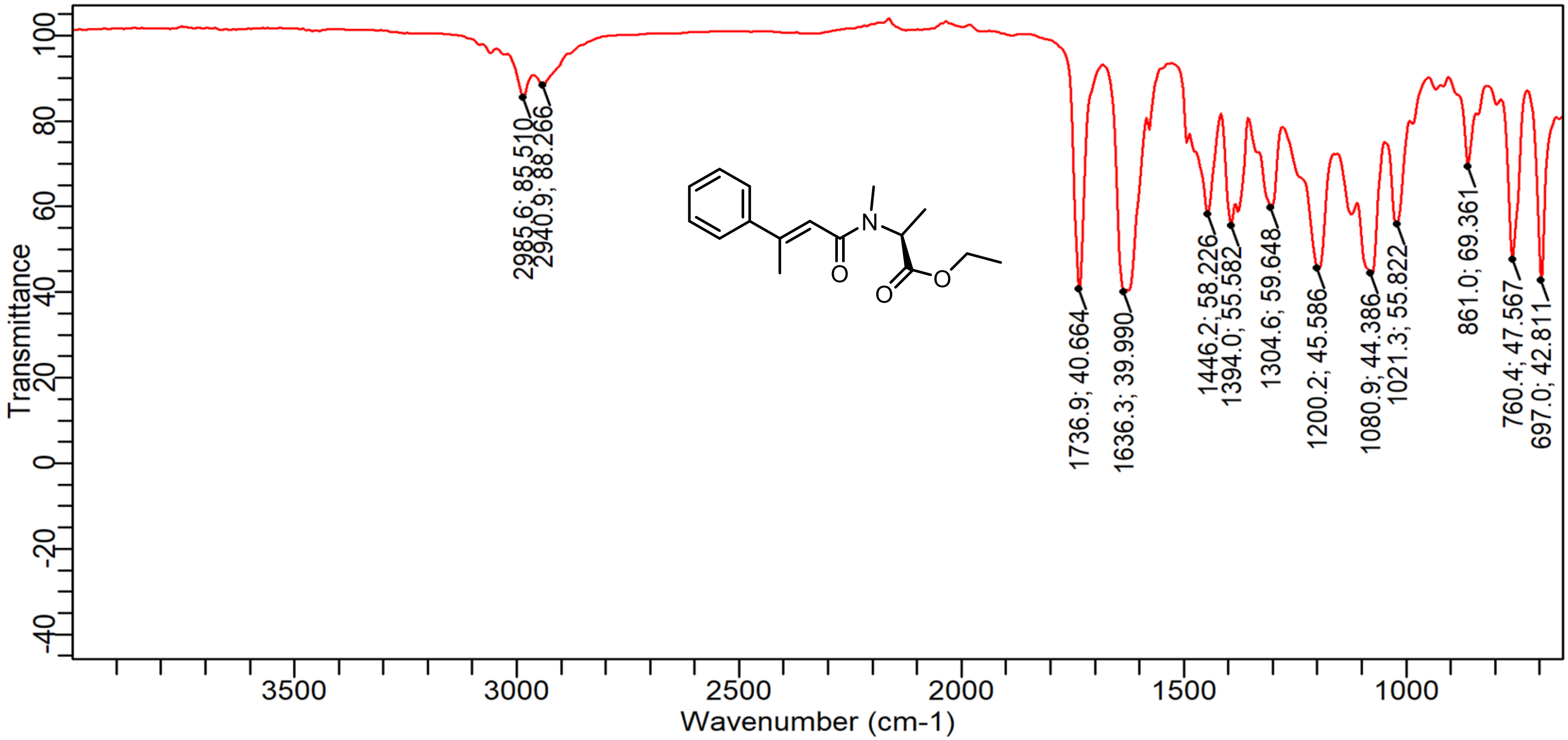

Transmittance
Wavenumber (cm-1)
2985.6; 85.510
2940.9; 88.266
1736.9; 40.664
1636.3; 39.990
1446.2; 58.226
1394.0; 55.582
1304.6; 59.648
1200.2; 45.586
1080.9; 44.386
1021.3; 55.822
861.0; 69.361
760.4; 47.567
697.0; 42.811

5.24 *N*-(3,4-Dimethoxyphenethyl)-*N*-methylcinnamamide (11ba & 11ba_rotamer) (600 MHz, $CDCl_3$)

5.24.1 ^{1}H NMR

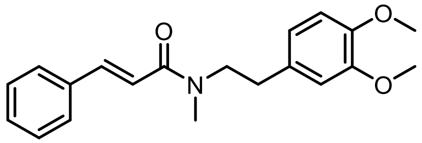

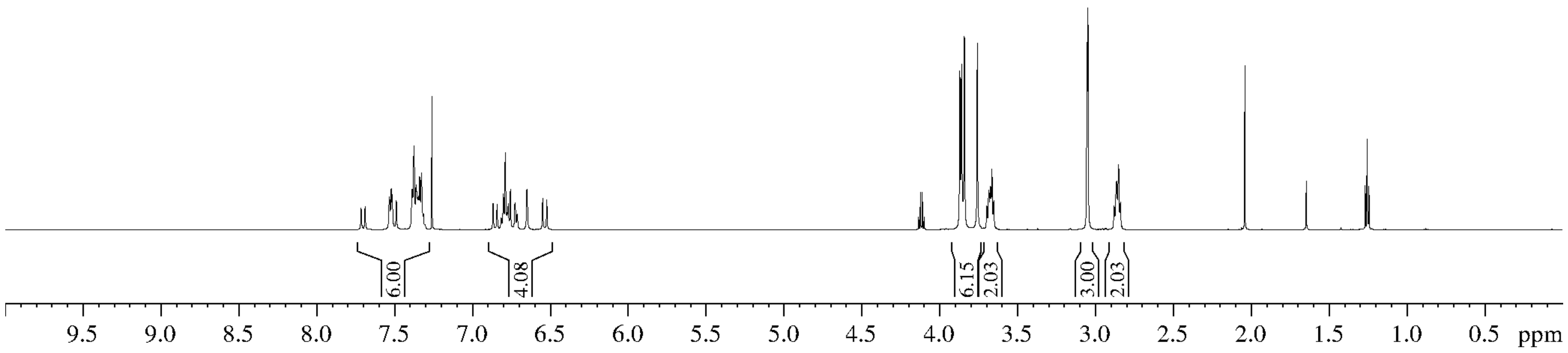

5.24.2 ^{13}C NMR

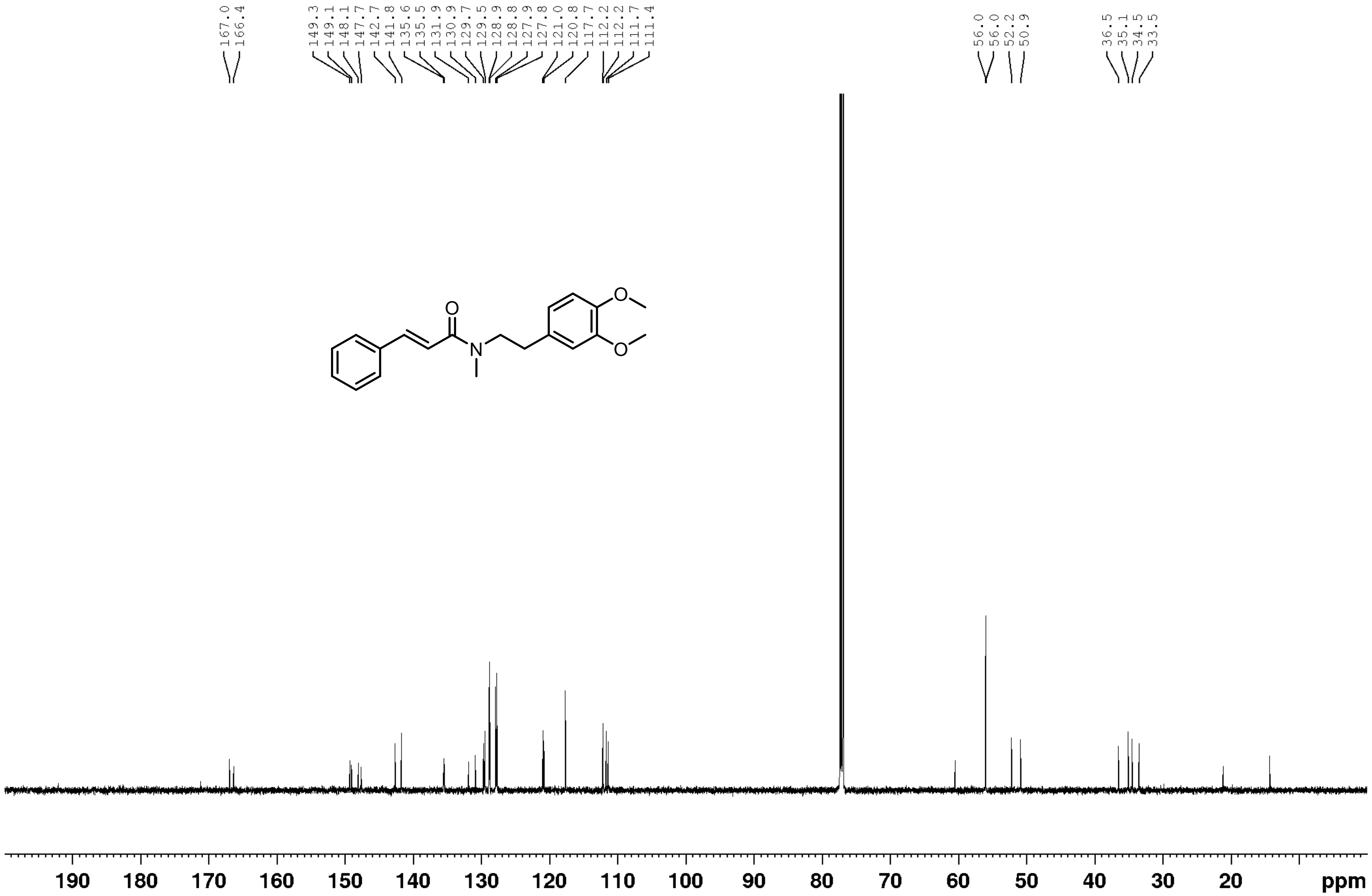
167.0
166.4
149.3
149.1
148.1
147.7
142.7
141.8
135.6
135.5
131.9
130.9
129.7
129.5
128.9
128.8
127.9
127.8
121.0
120.8
117.7
112.2
112.2
111.7
111.4
56.0
56.0
52.2
50.9
36.5
35.1
34.5
33.5
O
N
O
O
190 180 170 160 150 140 130 120 110 100 90 80 70 60 50 40 30 20 ppm

5.24.3 ^{1}H, ^{1}H COSY

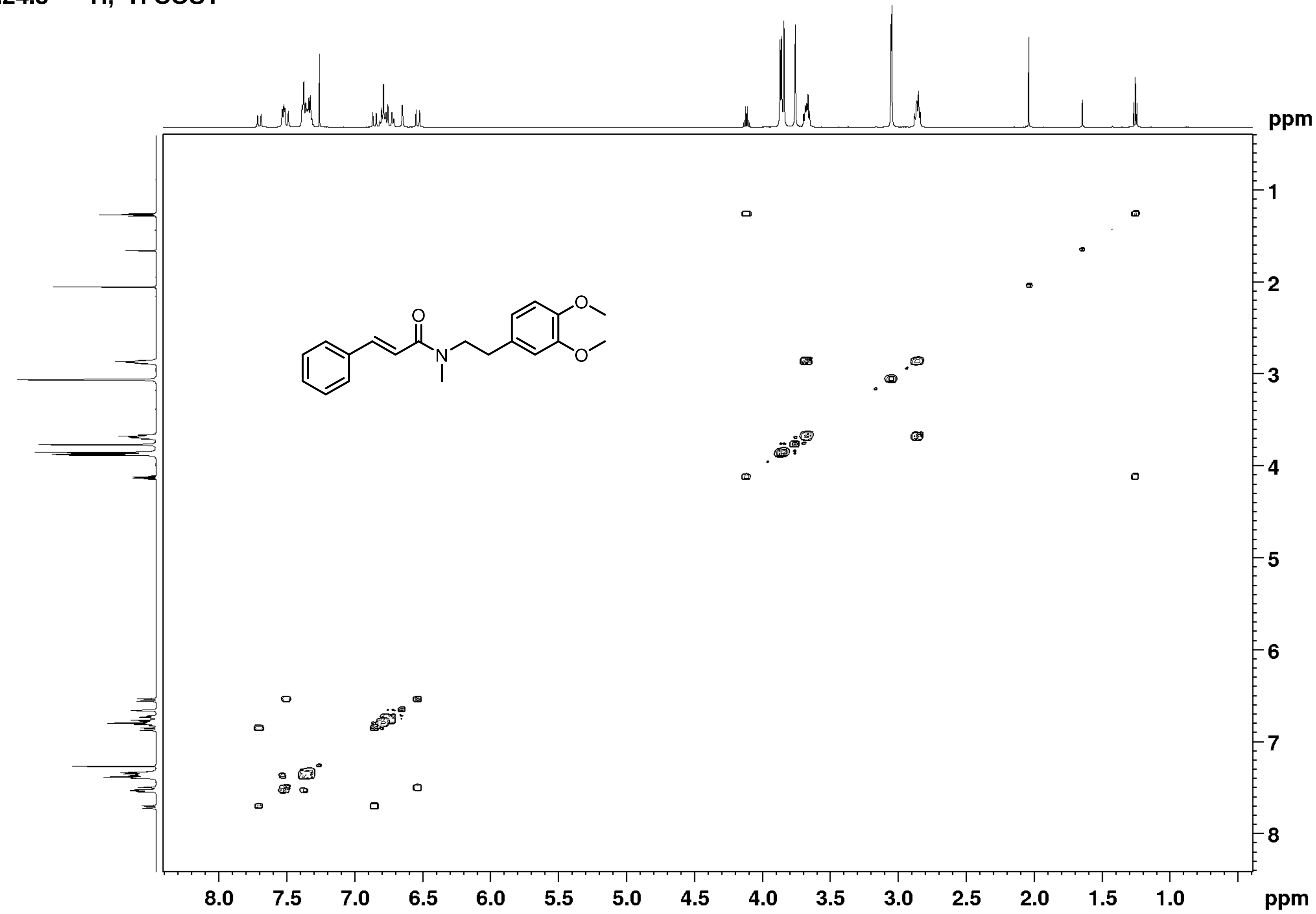

5.24.4 ^{1}H, ^{13}C HMBC

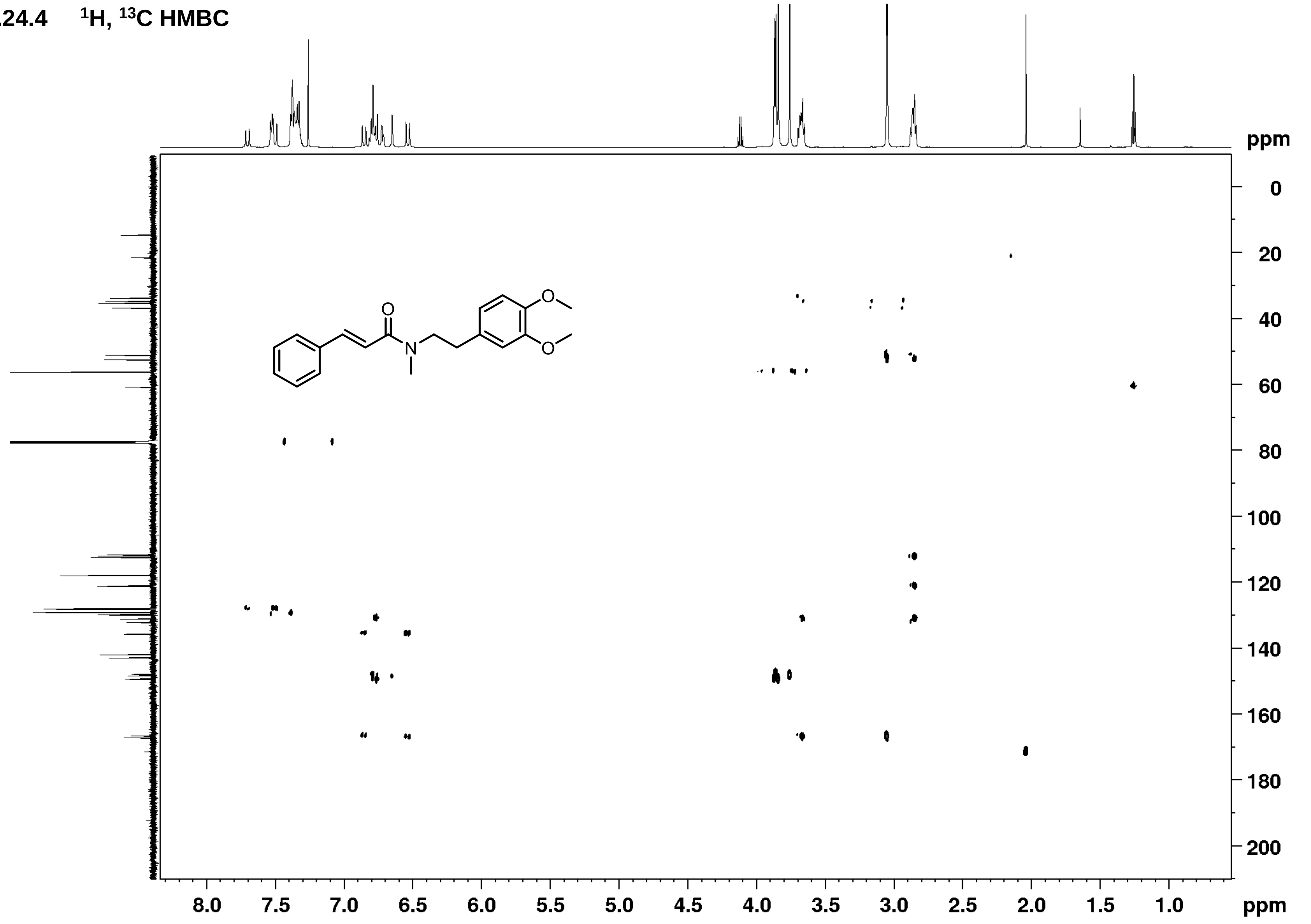

5.24.5 ^{1}H, ^{13}C HSQC

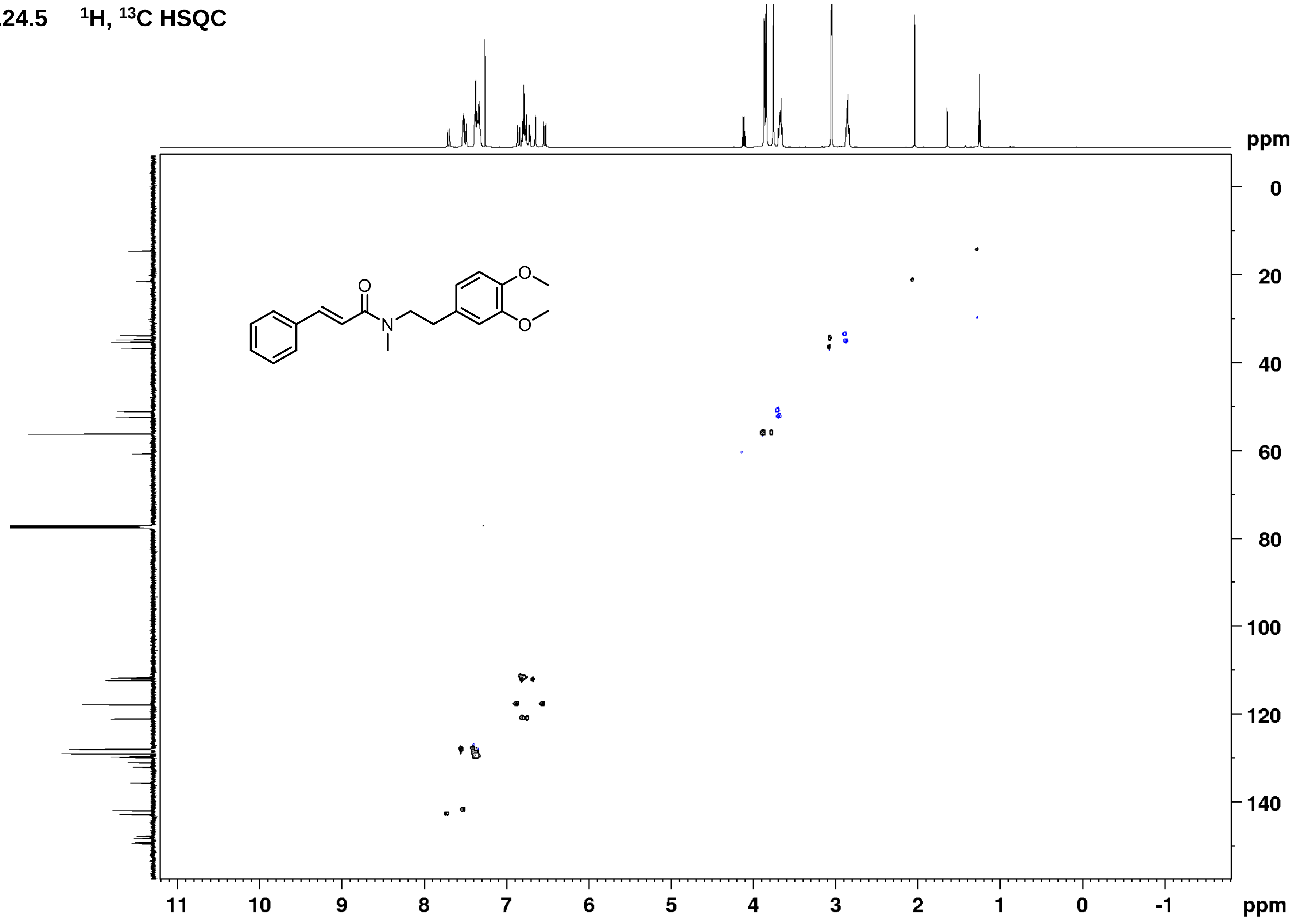

5.24.6 HRMS

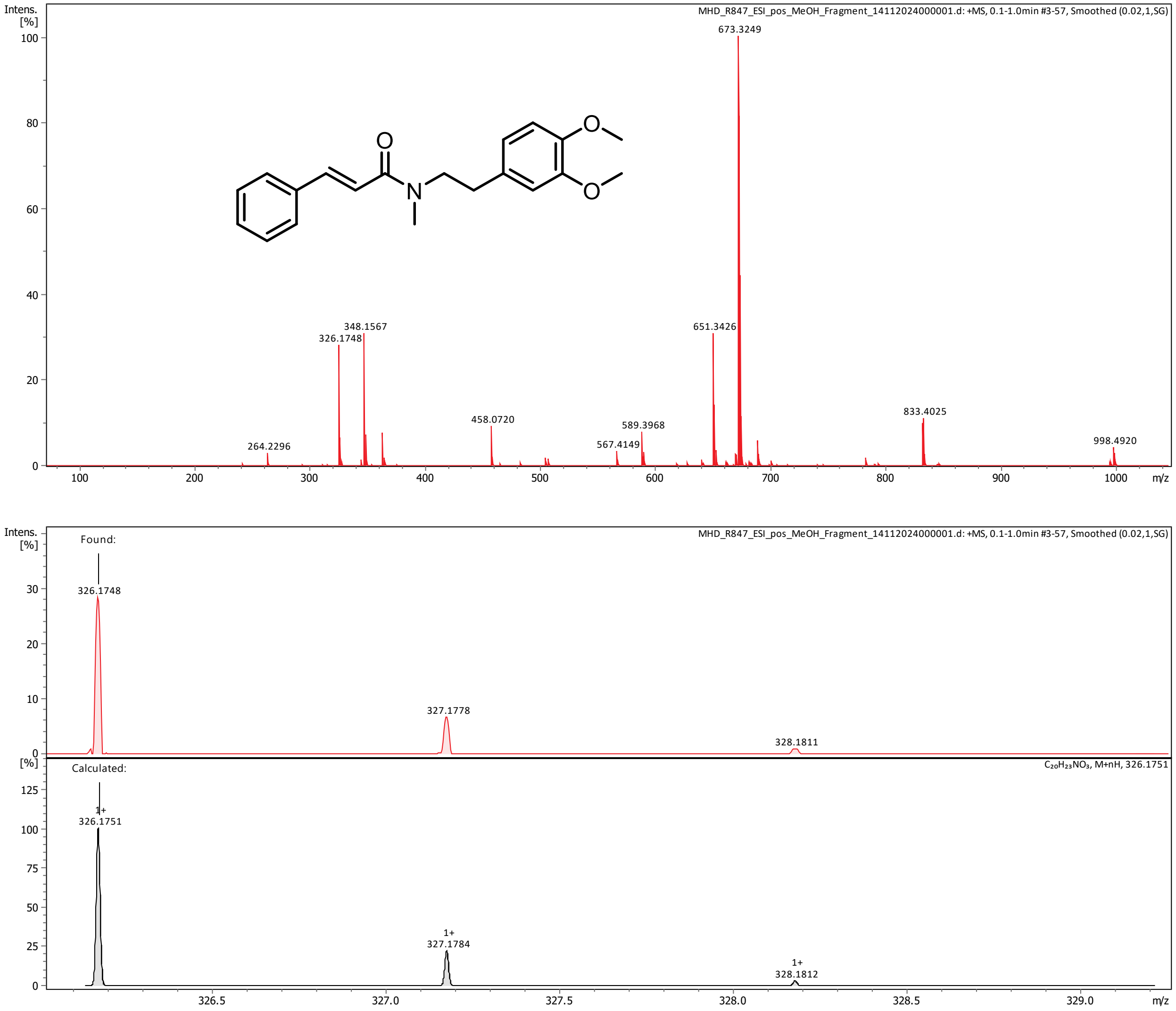
MHD_R847_ESI_pos_MeOH_Fragment_14112024000001.d: +MS, 0.1-1.0min #3-57, Smoothed (0.02,1,SG)
Intens. [%]
673.3249
651.3426
348.1567
326.1748
458.0720
589.3968
567.4149
264.2296
833.4025
998.4920
m/z
MHD_R847_ESI_pos_MeOH_Fragment_14112024000001.d: +MS, 0.1-1.0min #3-57, Smoothed (0.02,1,SG)
Found:
326.1748
327.1778
328.1811
Calculated:
C20H23NO3, M+nH, 326.1751
1+
326.1751
1+
327.1784
1+
328.1812

5.24.7 IR

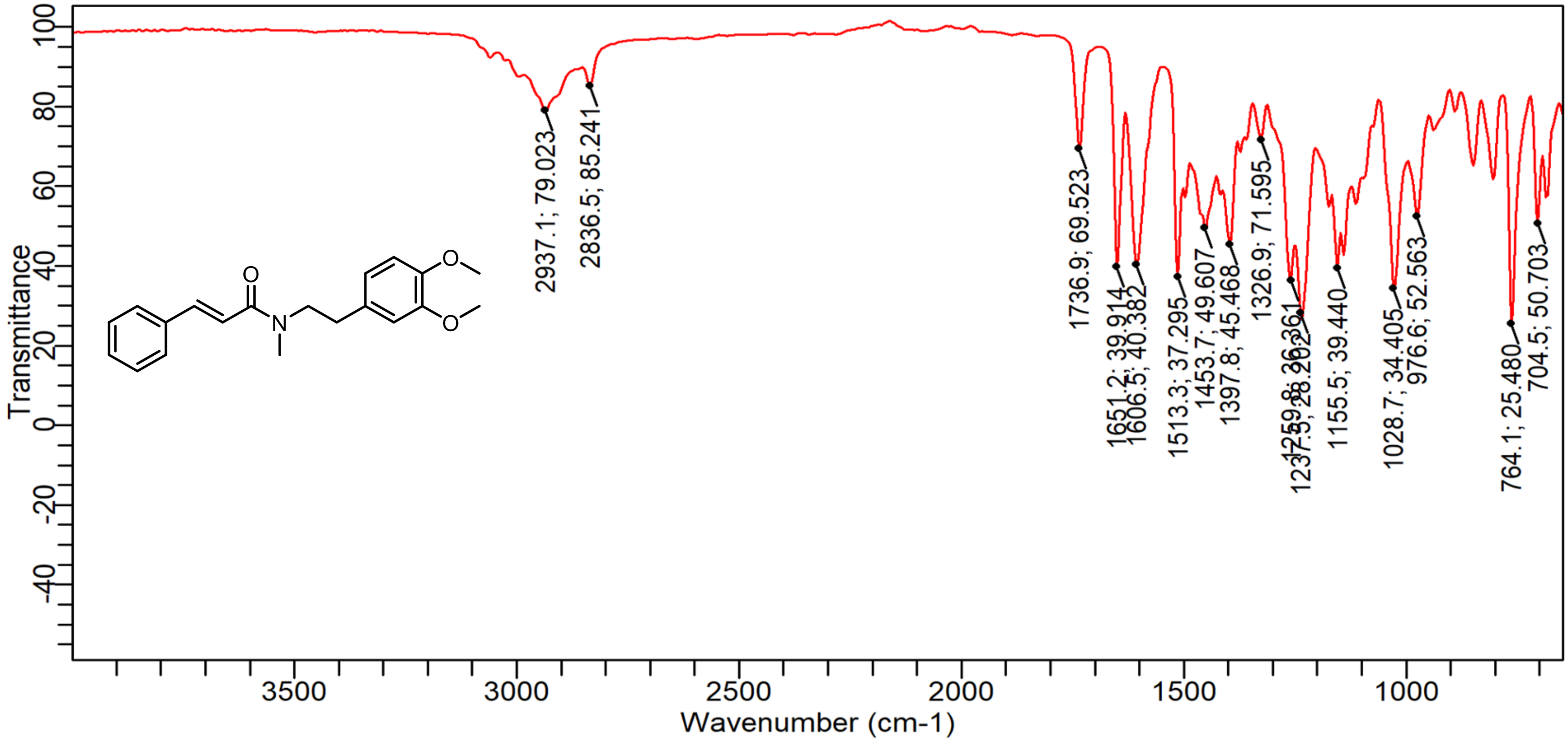

Transmittance
Wavenumber (cm-1)
100
80
60
40
20
0
-20
-40
3500
3000
2500
2000
1500
1000
2937.1; 79.023
2836.5; 85.241
1736.9; 69.523
1651.2; 39.914
1606.5; 40.382
1513.3; 37.295
1453.7; 49.607
1397.8; 45.468
1326.9; 71.595
1259.8; 36.361
1237.5; 28.202
1155.5; 39.440
1028.7; 34.405
976.6; 52.563
764.1; 25.480
704.5; 50.703
O
N
O
O

5.25 (*E*)-3-(4-(*tert*-Butyl)phenyl)-*N*-(2,3-dihydrobenzo[*b*][1,4]dioxin-6-yl)-*N*-methylacrylamide (11bc) (600 MHz, $CDCl_3$)

5.25.1 ^{1}H NMR

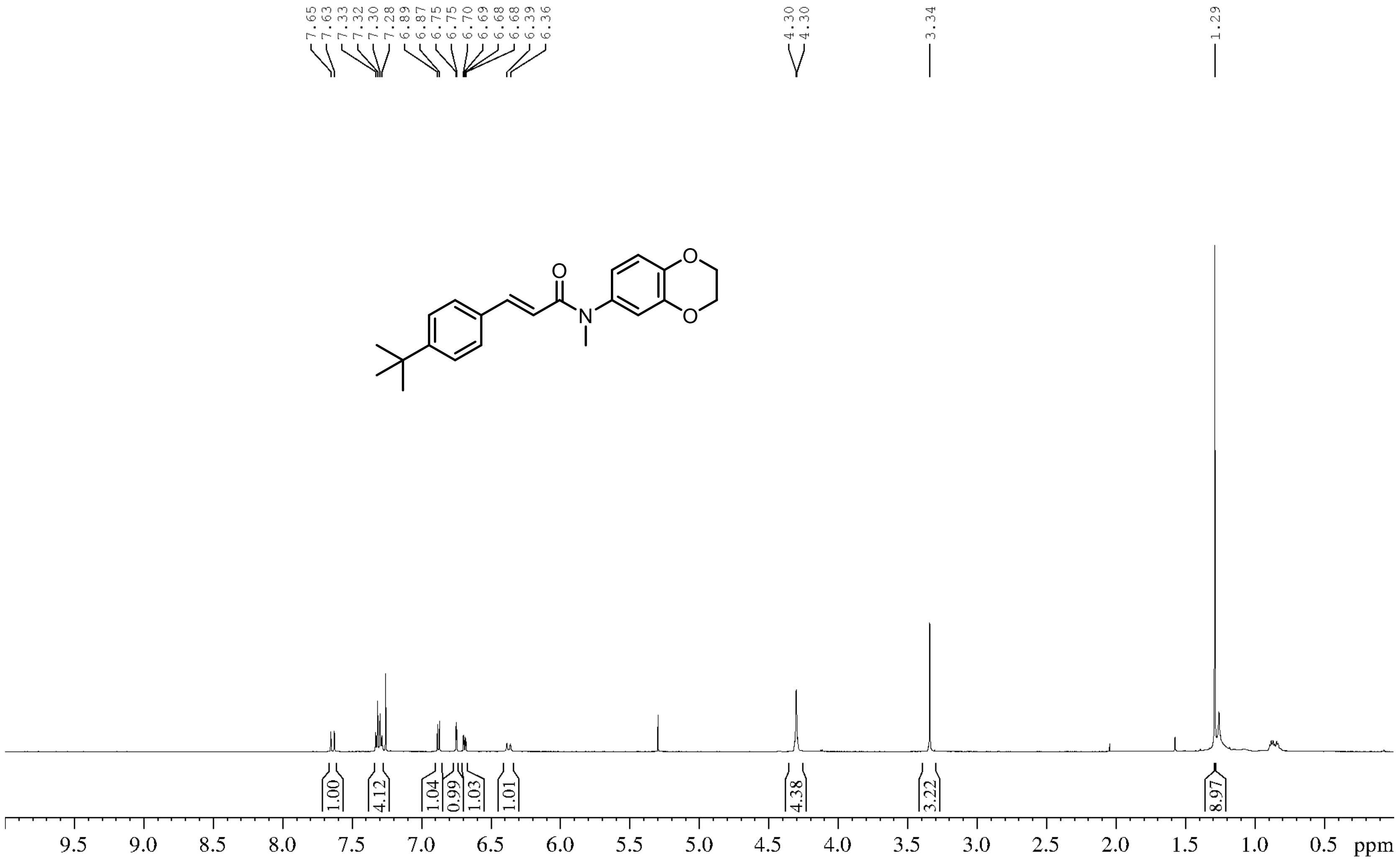

5.25.2 ^{13}C NMR

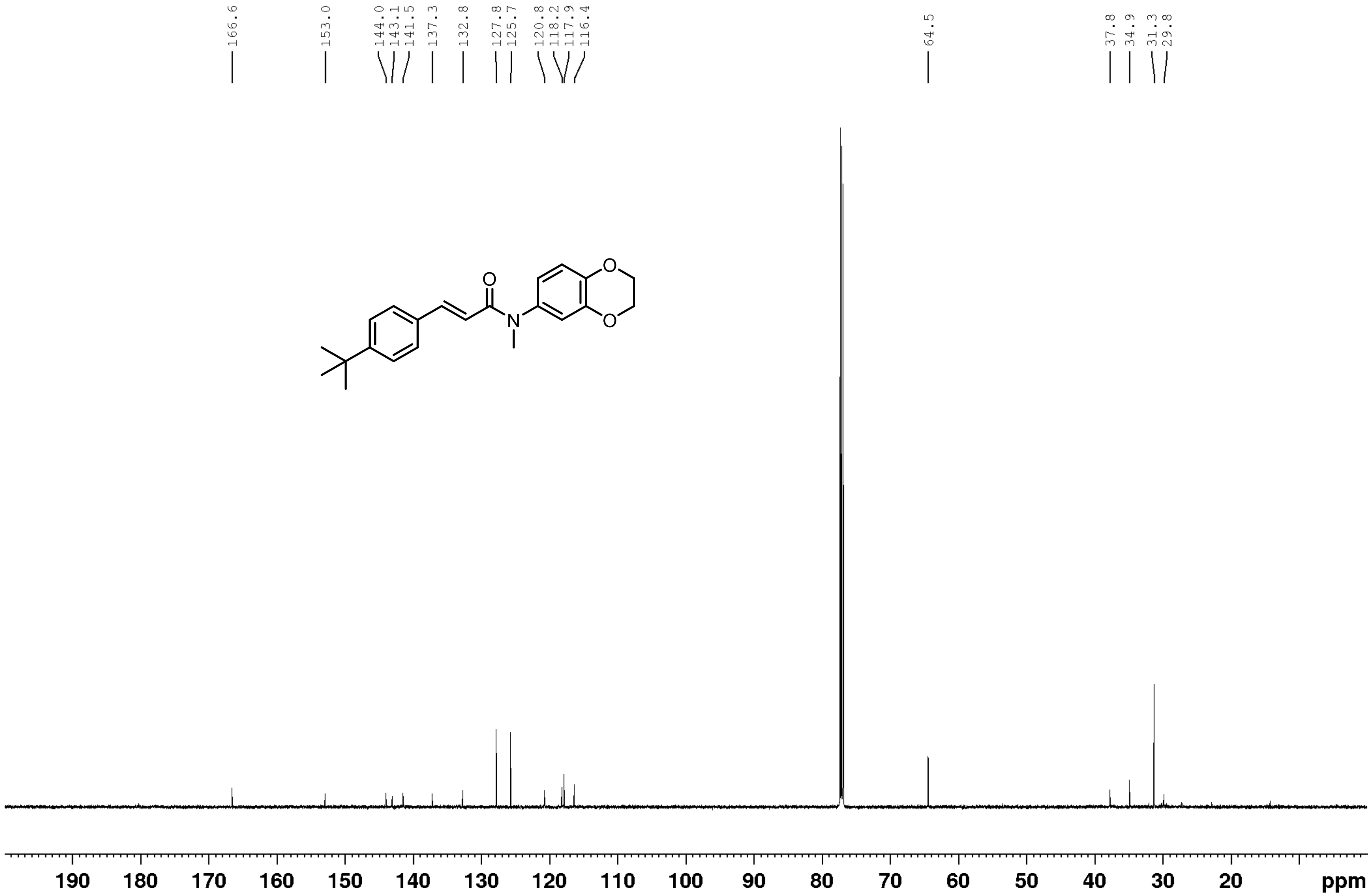
166.6
153.0
144.0
143.1
141.5
137.3
132.8
127.8
125.7
120.8
118.2
117.9
116.4
64.5
37.8
34.9
31.3
29.8
O
N
O
O
190
180
170
160
150
140
130
120
110
100
90
80
70
60
50
40
30
20
ppm

5.25.3 ^{1}H, ^{1}H COSY

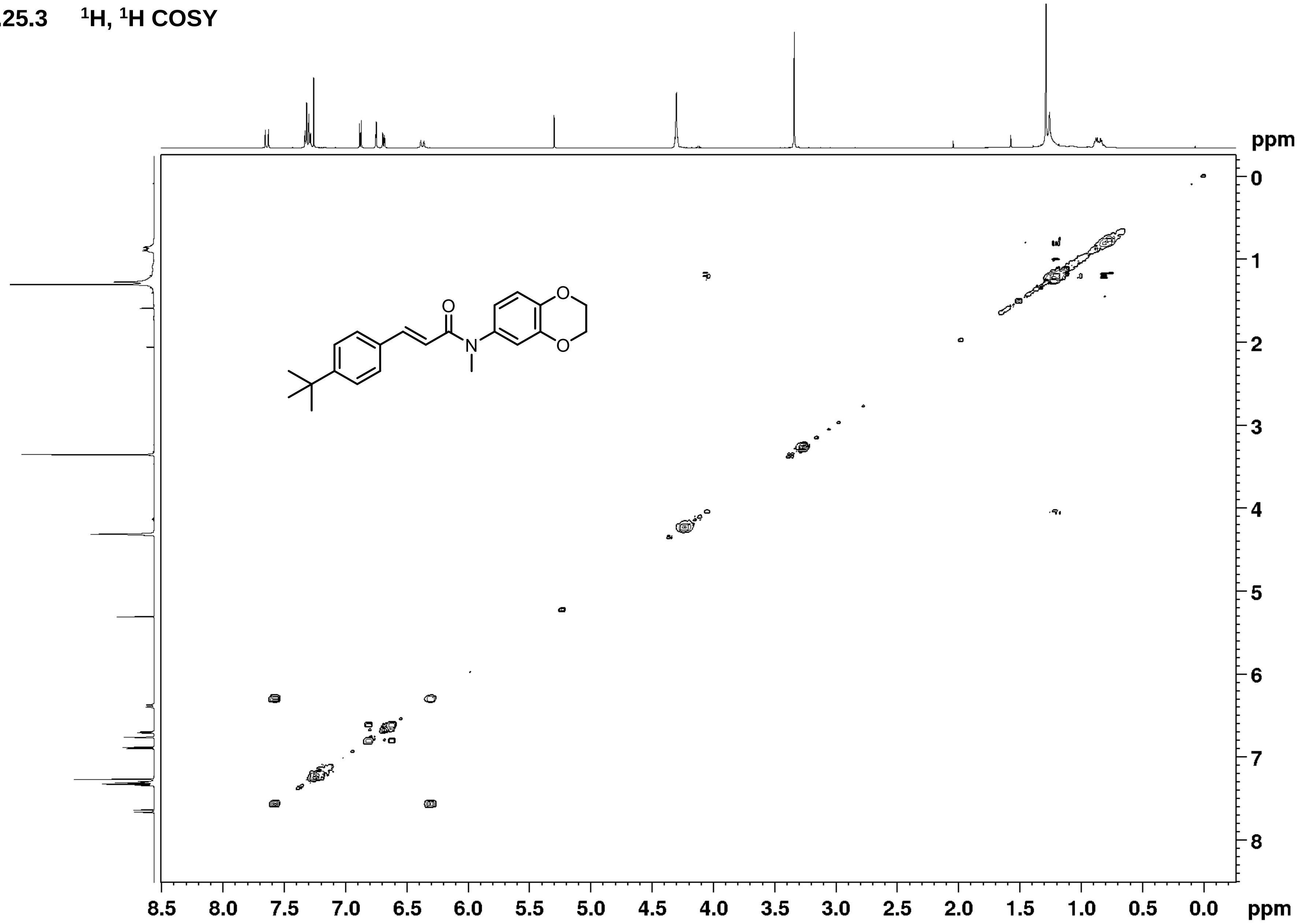

5.25.4 ^{1}H, ^{13}C HMBC

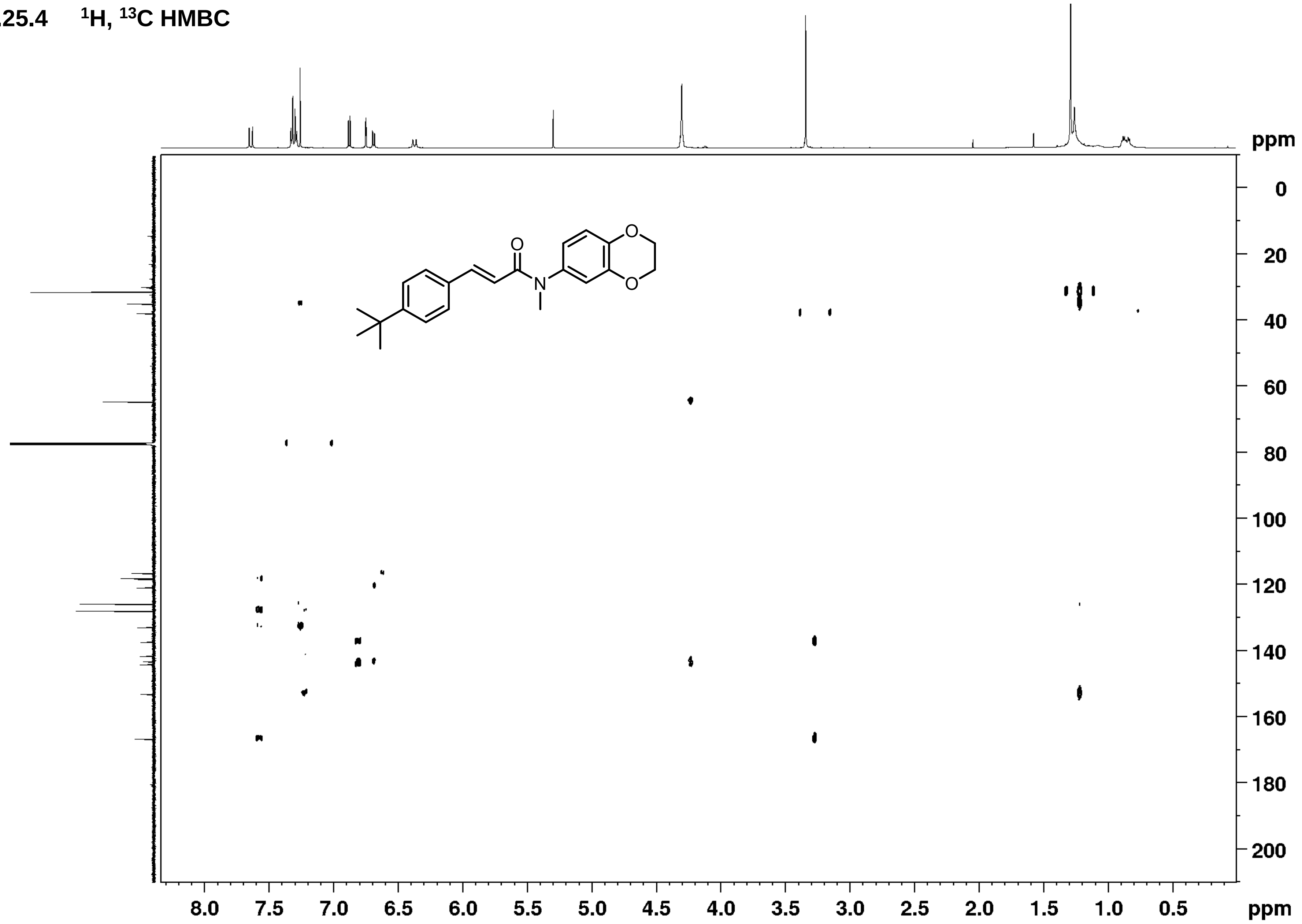

5.25.5 ^{1}H, ^{13}C HSQC

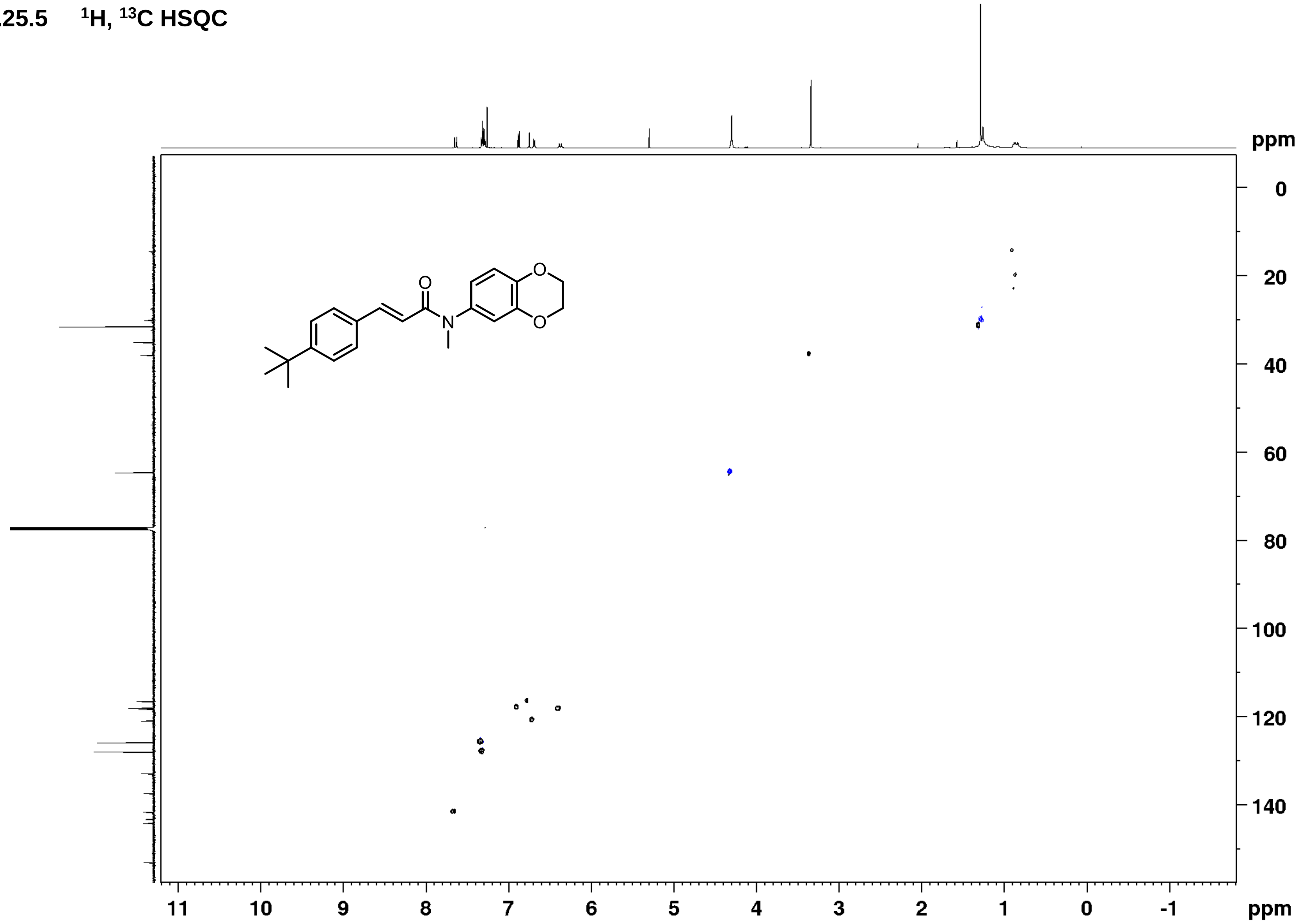

5.25.6 HRMS

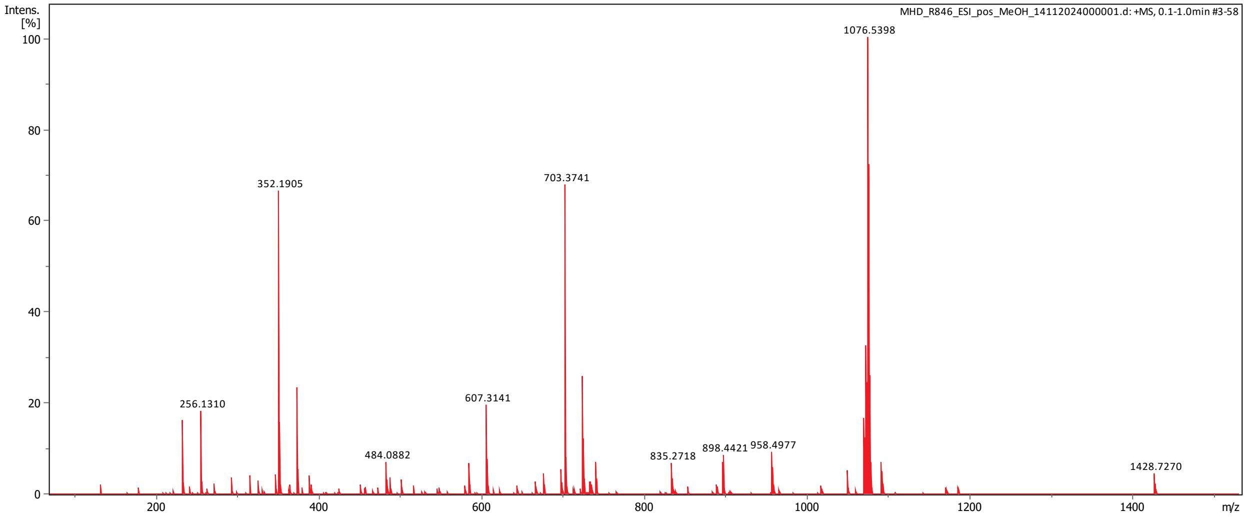

Intens. [%]
MHD_R846_ESI_pos_MeOH_14112024000001.d: +MS, 0.1-1.0min #3-58
1076.5398
352.1905
703.3741
256.1310
607.3141
484.0882
835.2718
898.4421
958.4977
1428.7270
m/z

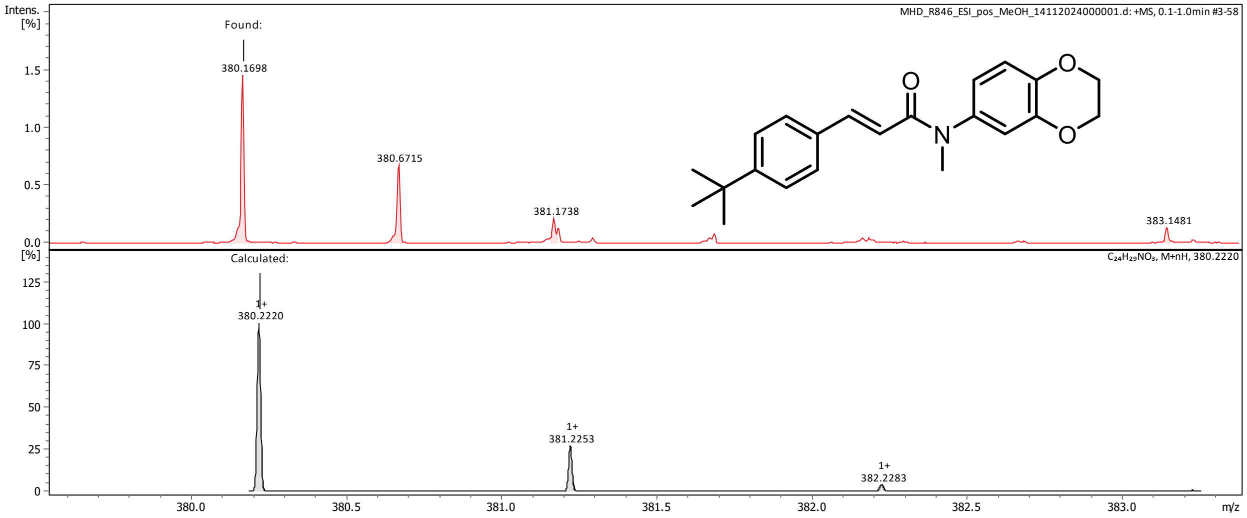

Intens. [%]
MHD_R846_ESI_pos_MeOH_14112024000001.d: +MS, 0.1-1.0min #3-58
Found:
380.1698
380.6715
381.1738
383.1481
Calculated:
C24H29NO3, M+nH, 380.2220
1+
380.2220
1+
381.2253
1+
382.2283
m/z

5.25.7 IR

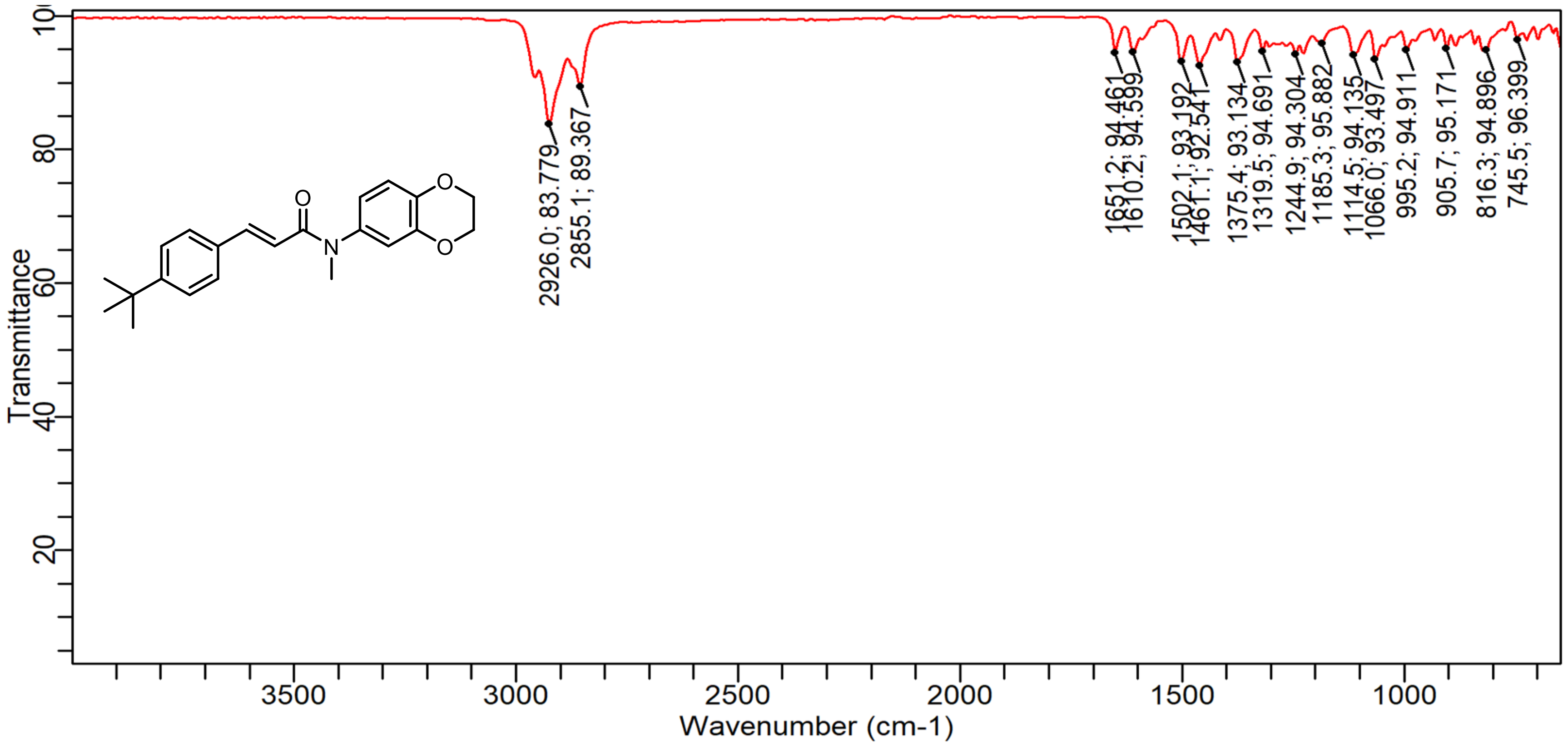
Transmittance
Wavenumber (cm-1)
100
80
60
40
20
3500
3000
2500
2000
1500
1000
O
N
O
O
2926.0; 83.779
2855.1; 89.367
1651.2; 94.461
1610.2; 94.599
1502.1; 93.192
1461.1; 92.541
1375.4; 93.134
1319.5; 94.691
1244.9; 94.304
1185.3; 95.882
1114.5; 94.135
1066.0; 93.497
995.2; 94.911
905.7; 95.171
816.3; 94.896
745.5; 96.399

5.26 (*E*)-3-(Benzo[*d*][1,3]dioxol-5-yl)-1-(piperidin-1-yl)prop-2-en-1-one (11be) (600 MHz, $CDCl_3$)

5.26.1 ^{1}H NMR

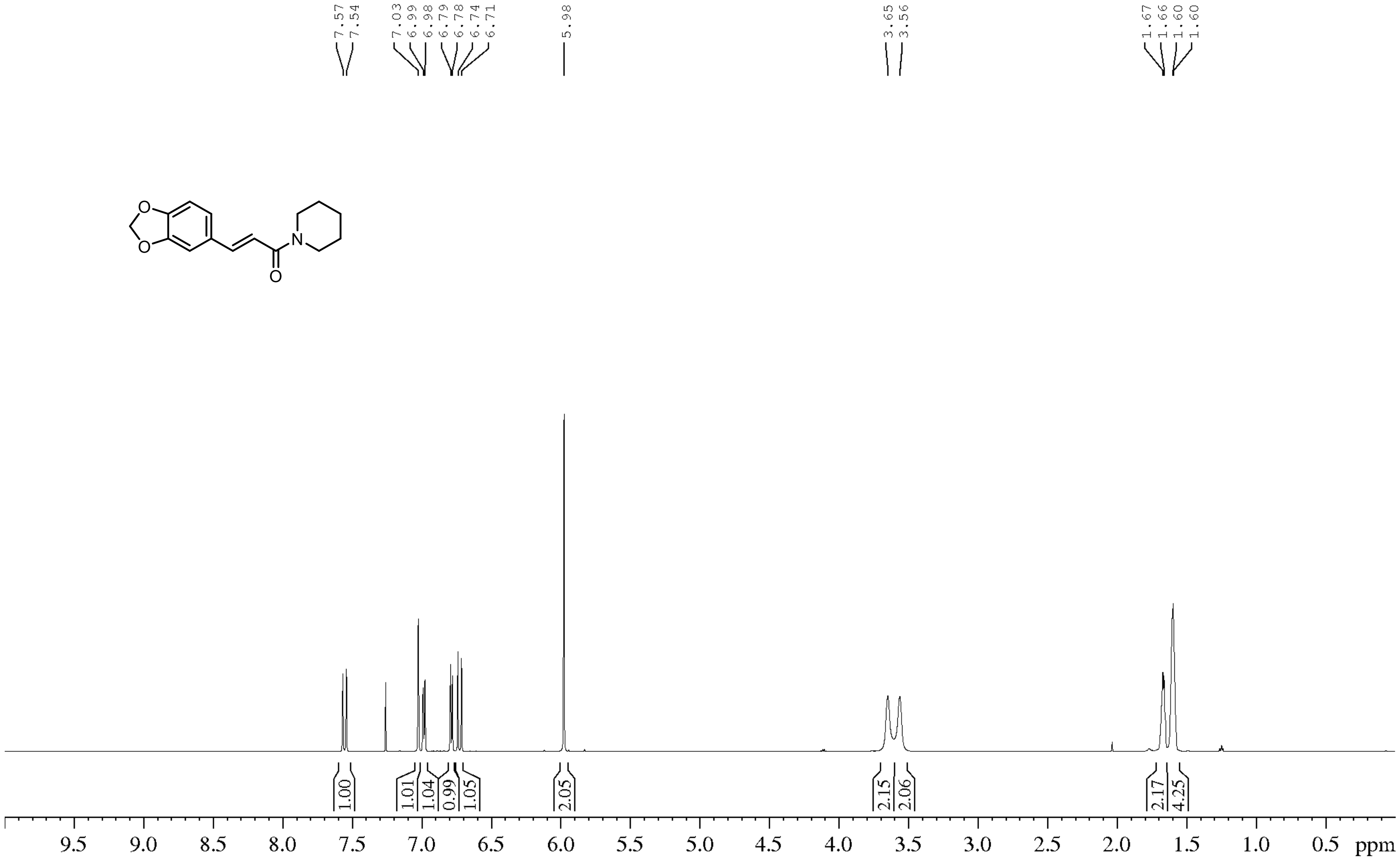

5.26.2 ^{13}C NMR

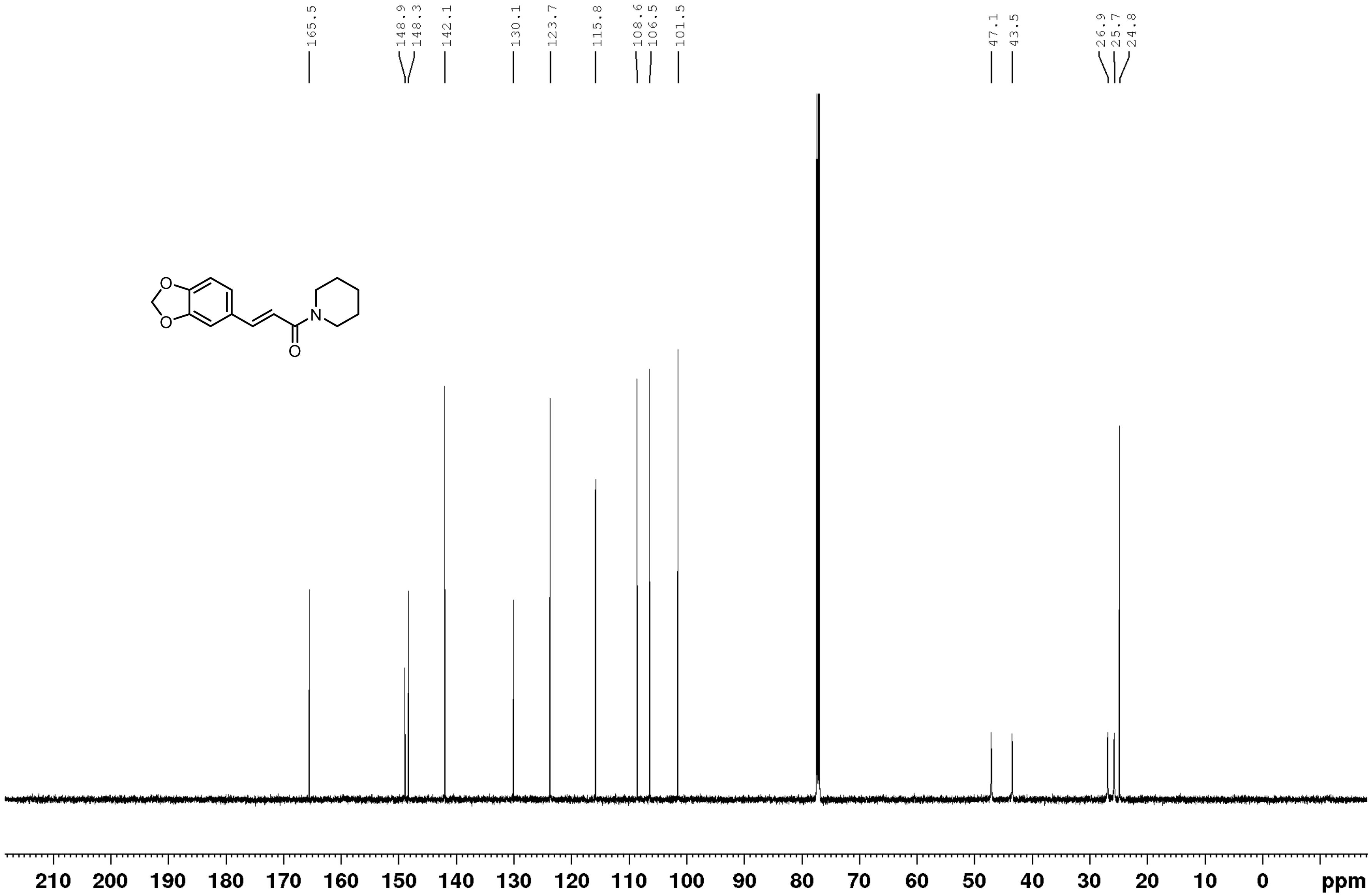
165.5
148.9
148.3
142.1
130.1
123.7
115.8
108.6
106.5
101.5
47.1
43.5
26.9
25.7
24.8
210 200 190 180 170 160 150 140 130 120 110 100 90 80 70 60 50 40 30 20 10 0 ppm

5.26.3 ^{1}H, ^{1}H COSY

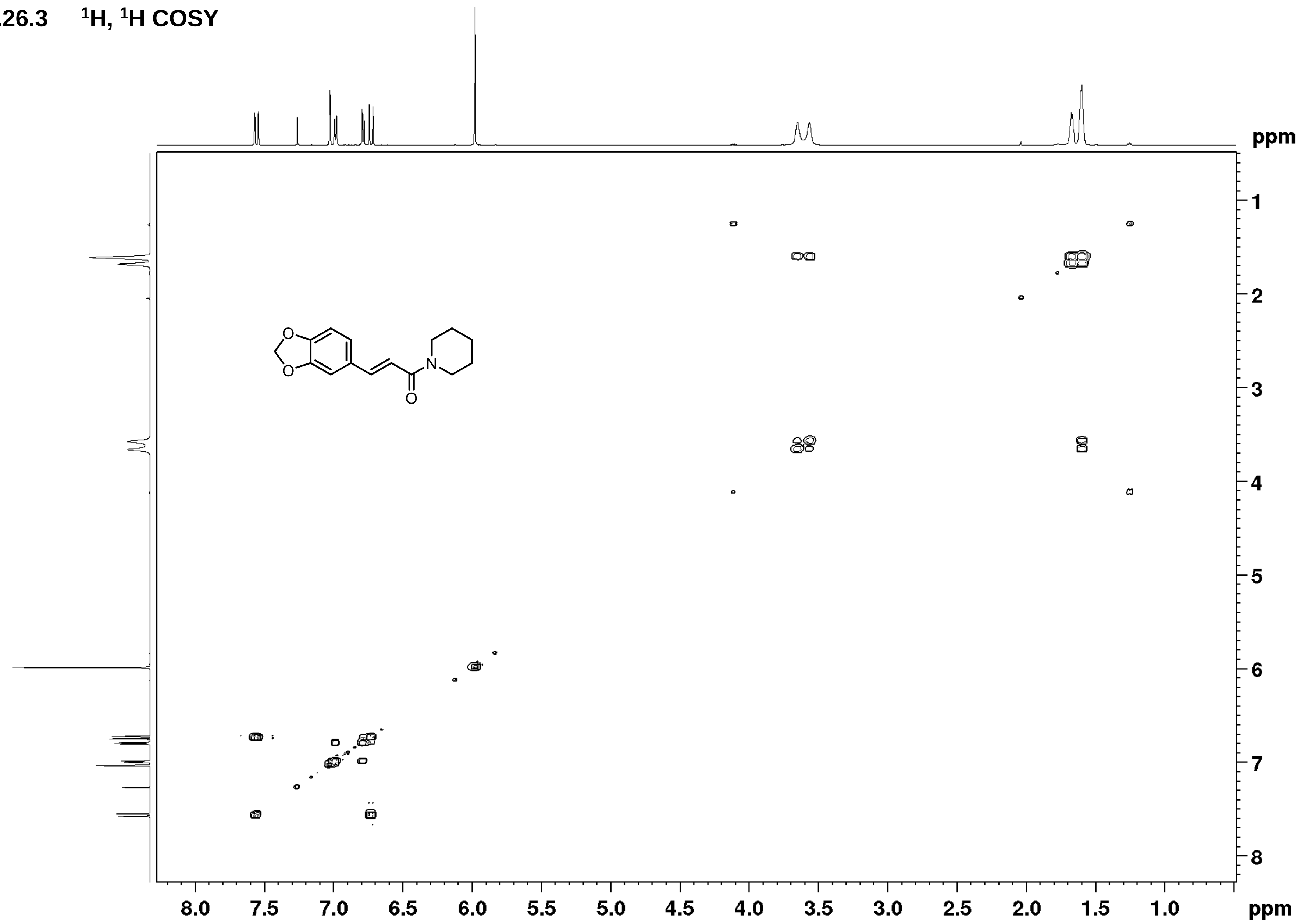

5.26.4 ^{1}H, ^{13}C HMBC

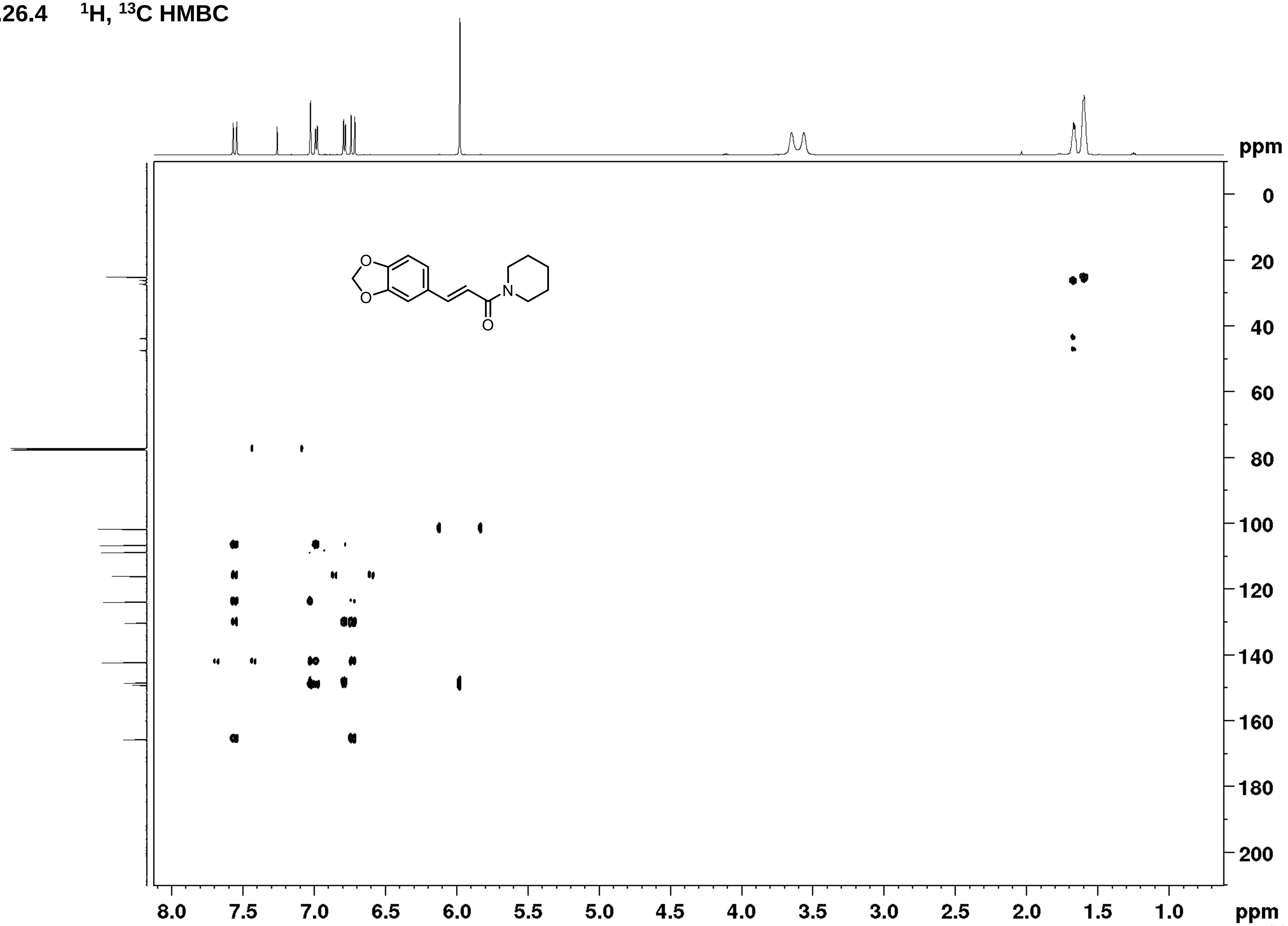

5.26.5 ^{1}H, ^{13}C HSQC

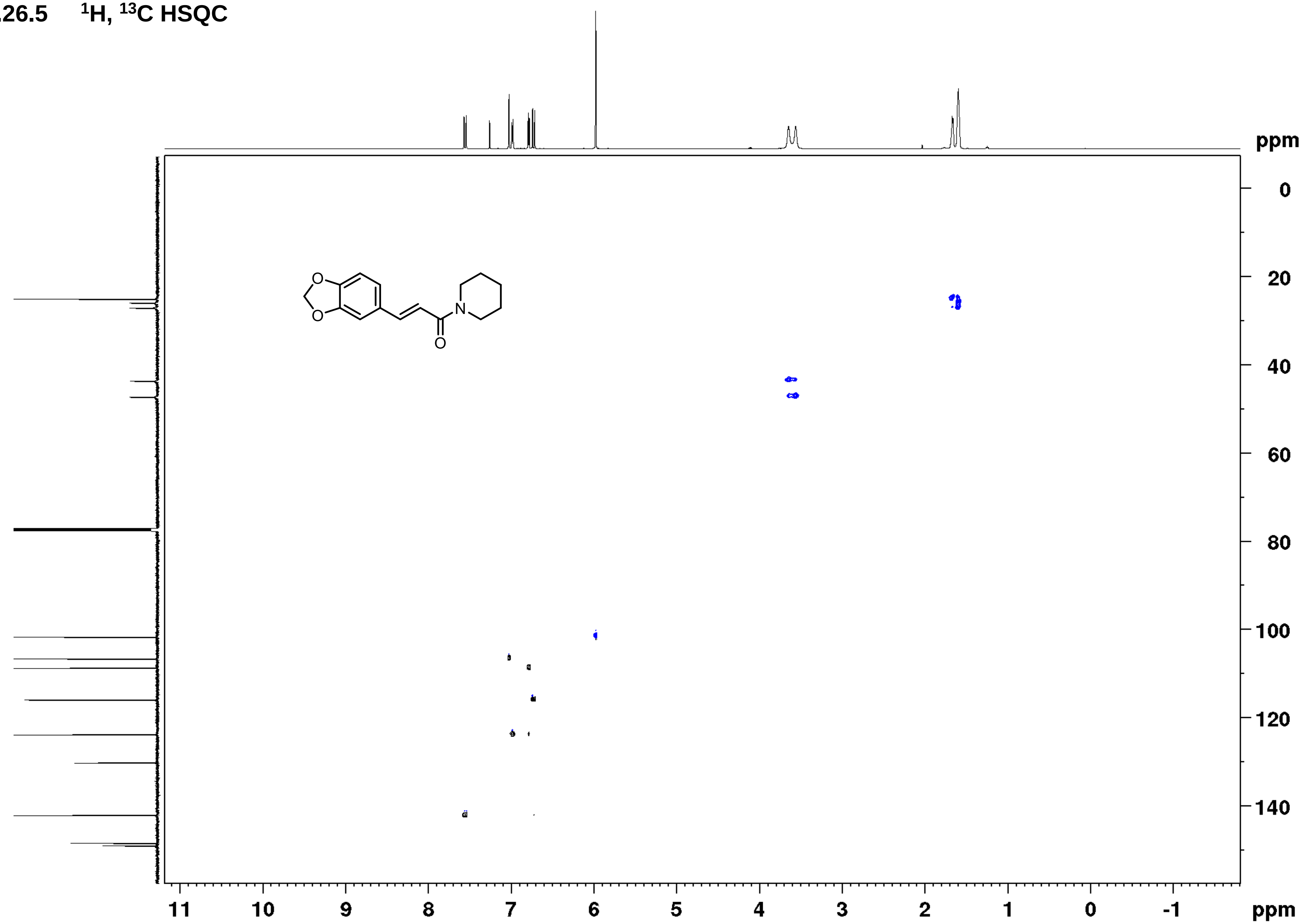

5.26.6 HRMS

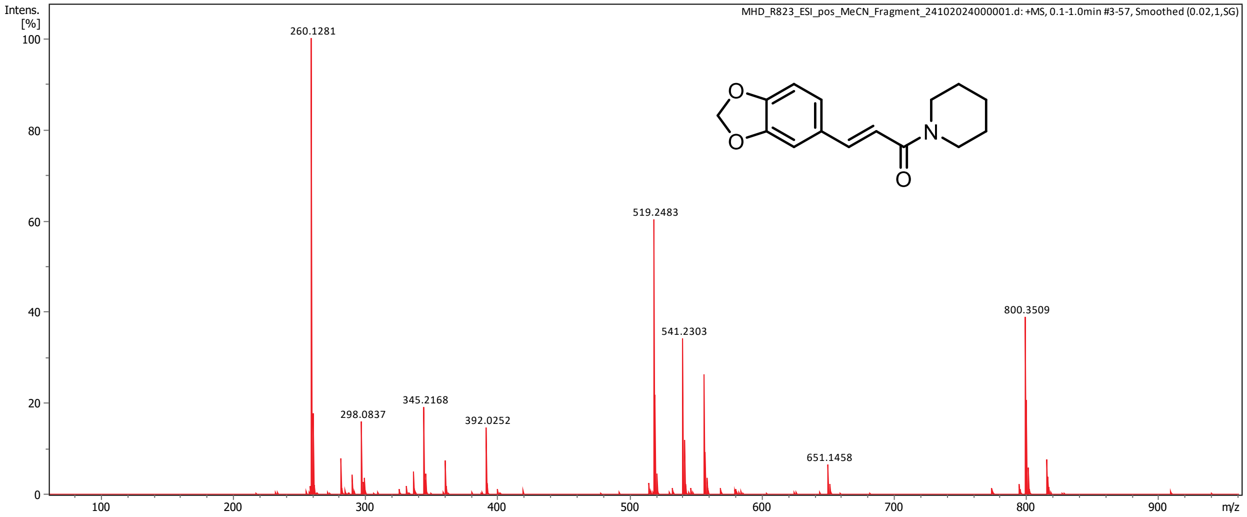

MHD_R823_ESI_pos_MeCN_Fragment_24102024000001.d: +MS, 0.1-1.0min #3-57, Smoothed (0.02,1,SG)
Intens. [%]
260.1281
298.0837
345.2168
392.0252
519.2483
541.2303
651.1458
800.3509
m/z

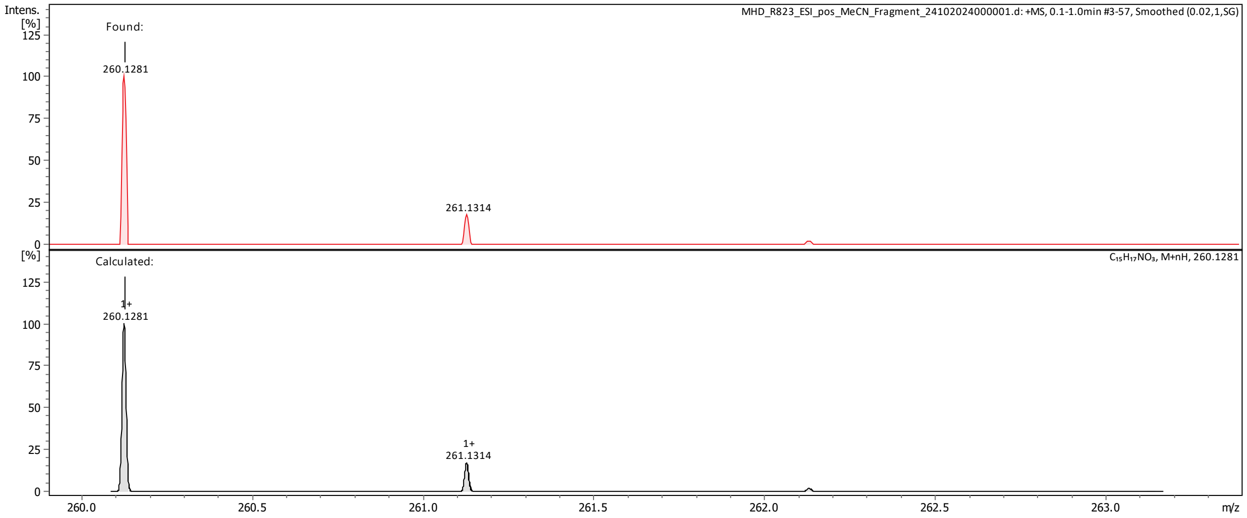

MHD_R823_ESI_pos_MeCN_Fragment_24102024000001.d: +MS, 0.1-1.0min #3-57, Smoothed (0.02,1,SG)
Intens. [%]
Found:
260.1281
261.1314
Calculated:
C15H17NO3, M+nH, 260.1281
1+ 260.1281
1+ 261.1314
m/z

5.27 (2*E*,4*E*)-*N*-Isobutyl-*N*-methyldeca-2,4-dienamide (11bj) (600 MHz, $CDCl_3$)

5.27.1 ^{1}H NMR

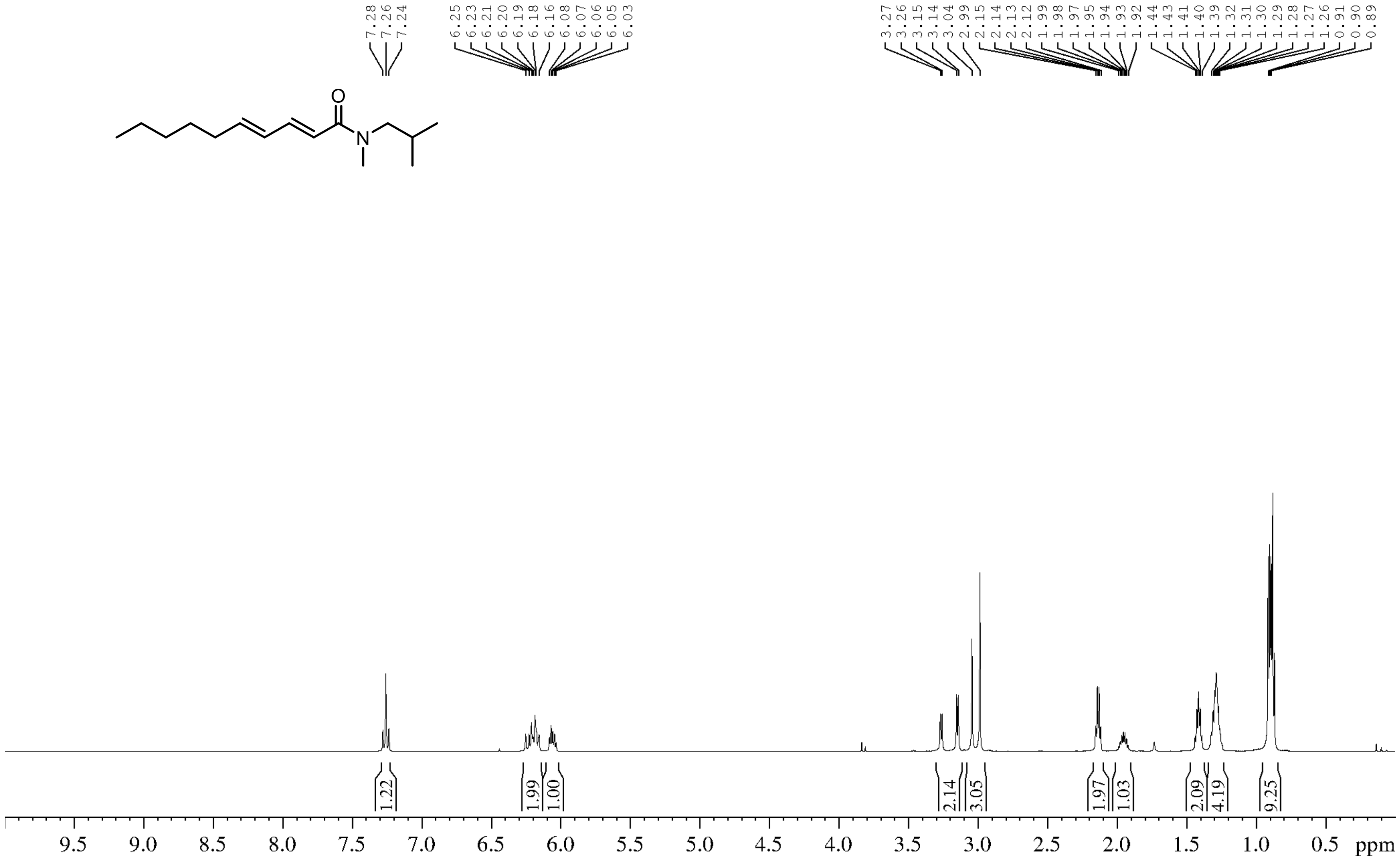

5.27.2 ^{13}C NMR

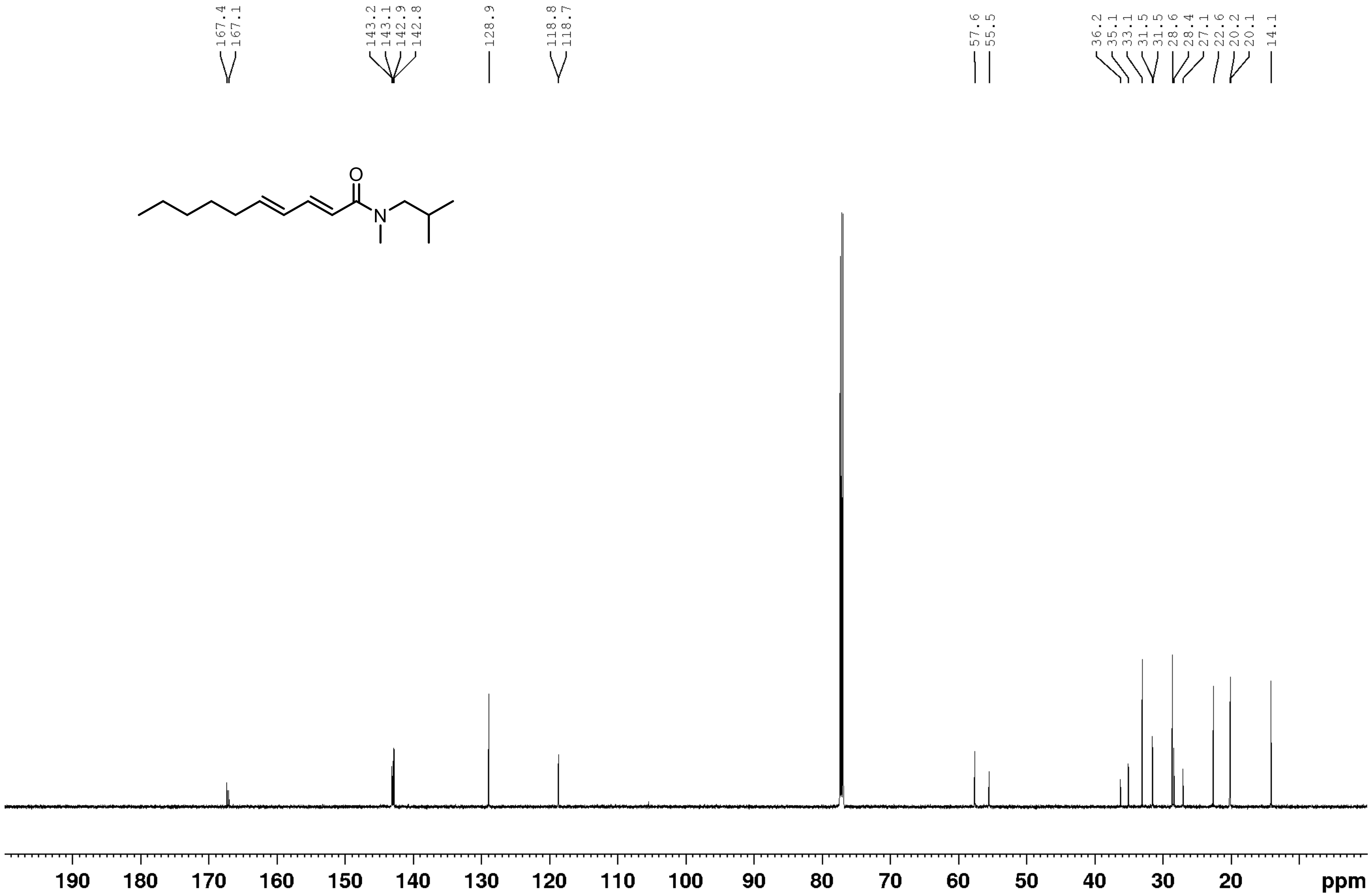
167.4
167.1
143.2
143.1
142.9
142.8
128.9
118.8
118.7
57.6
55.5
36.2
35.1
33.1
31.5
31.5
28.6
28.4
27.1
22.6
20.2
20.1
14.1
O
N
190 180 170 160 150 140 130 120 110 100 90 80 70 60 50 40 30 20 ppm

5.27.3 ^{1}H, ^{1}H COSY

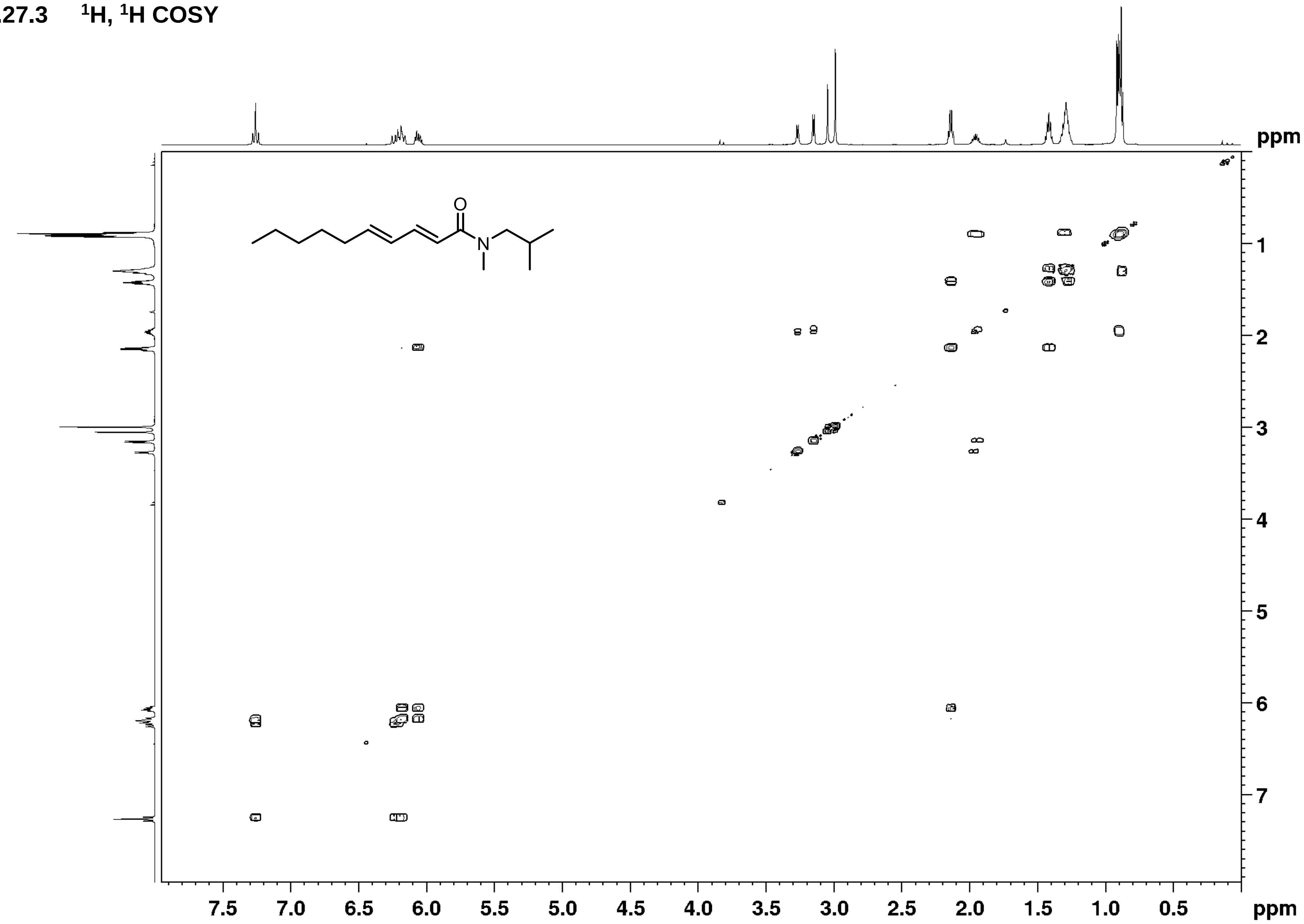

5.27.4 ^{1}H, ^{13}C HMBC

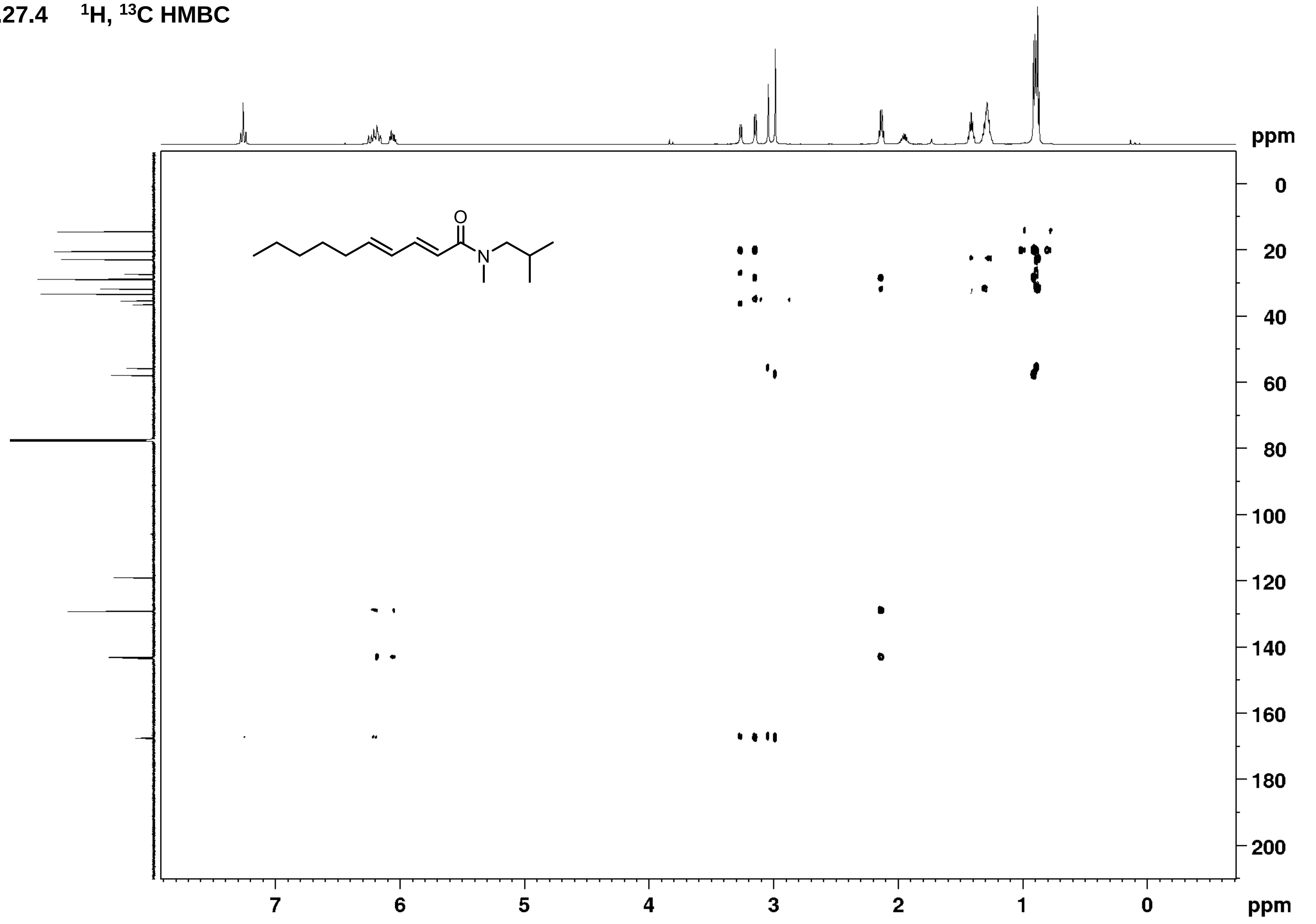

5.27.5 ^{1}H, ^{13}C HSQC

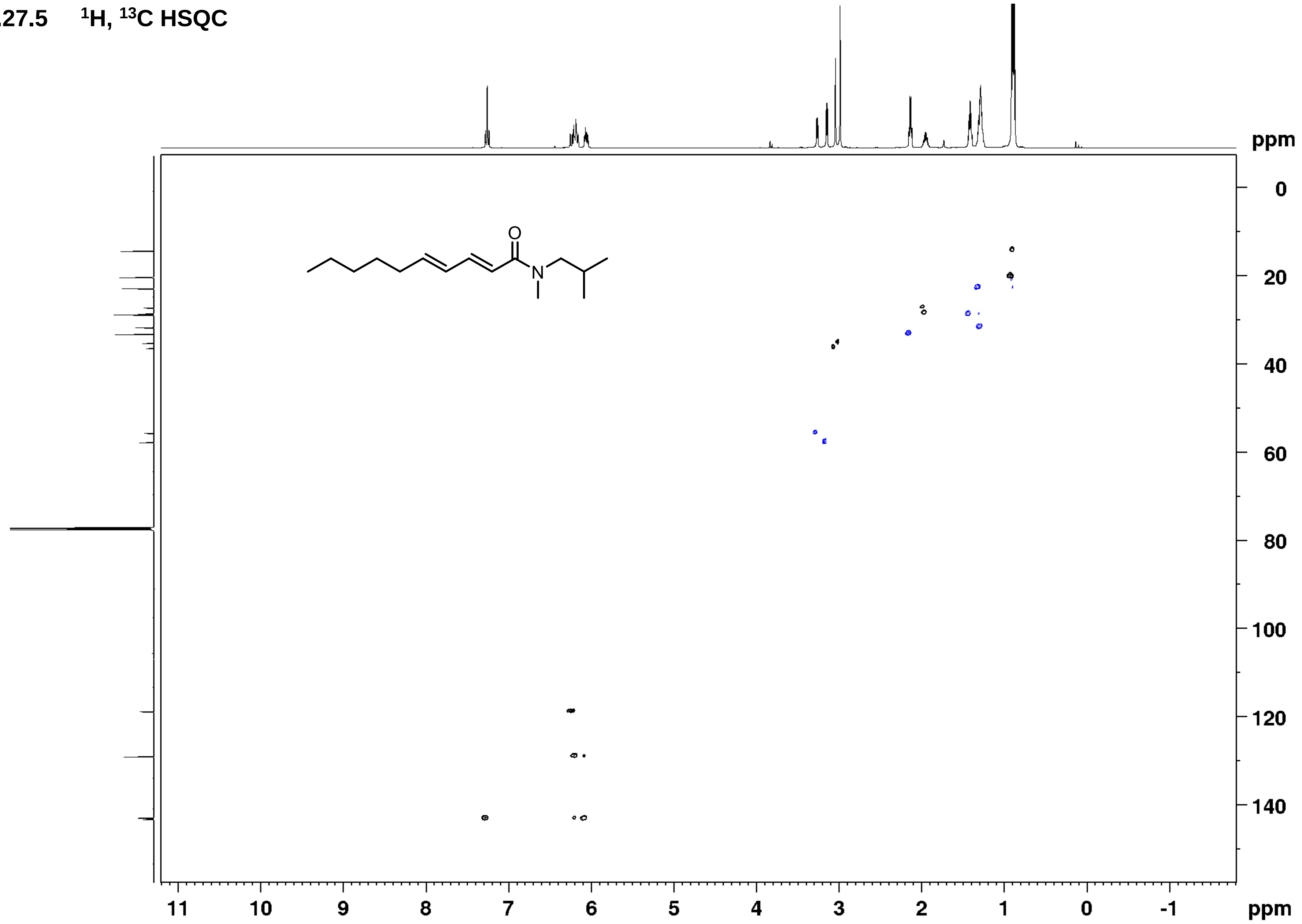

5.27.6 HRMS

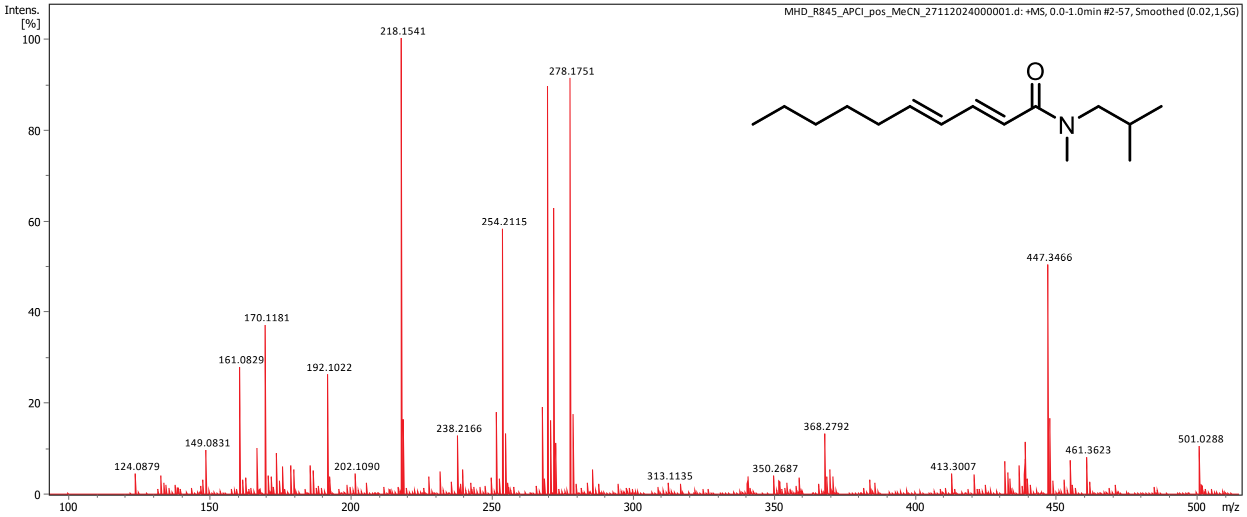

Intens. [%]
MHD_R845_APCI_pos_MeCN_27112024000001.d: +MS, 0.0-1.0min #2-57, Smoothed (0.02,1,SG)
218.1541
278.1751
254.2115
447.3466
170.1181
161.0829
192.1022
238.2166
368.2792
149.0831
124.0879
202.1090
313.1135
350.2687
413.3007
461.3623
501.0288
O
N
m/z

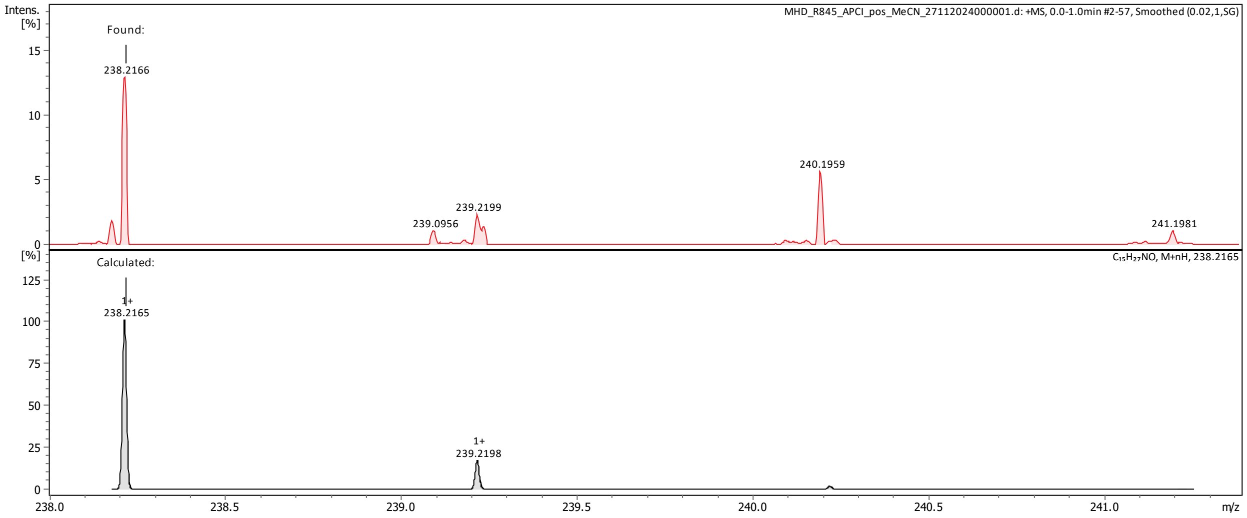

Intens. [%]
MHD_R845_APCI_pos_MeCN_27112024000001.d: +MS, 0.0-1.0min #2-57, Smoothed (0.02,1,SG)
Found:
238.2166
239.0956
239.2199
240.1959
241.1981
Calculated:
$C_{15}H_{27}NO$, M+nH, 238.2165
1+
238.2165
1+
239.2198
m/z

5.27.7 IR

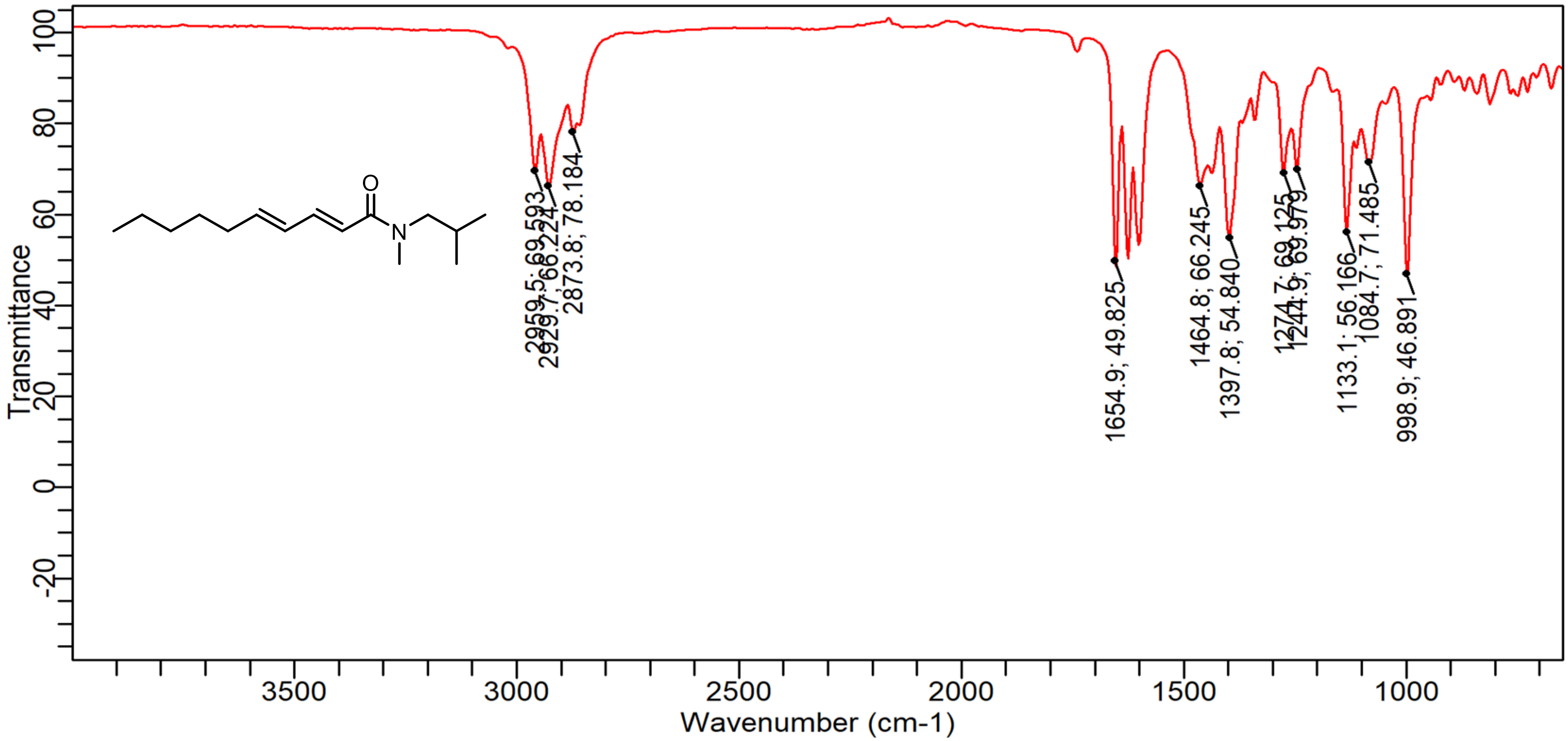

Transmittance
Wavenumber (cm-1)
2959.5; 69.593
2929.7; 66.224
2873.8; 78.184
1654.9; 49.825
1464.8; 66.245
1397.8; 54.840
1274.7; 69.125
1244.9; 69.979
1133.1; 56.166
1084.7; 71.485
998.9; 46.891
O
N

5.28 (2*E*,4*E*)-1-(Pyrrolidin-1-yl)deca-2,4-dien-1-one (11bk) (600 MHz, $CDCl_3$)

5.28.1 ^{1}H NMR

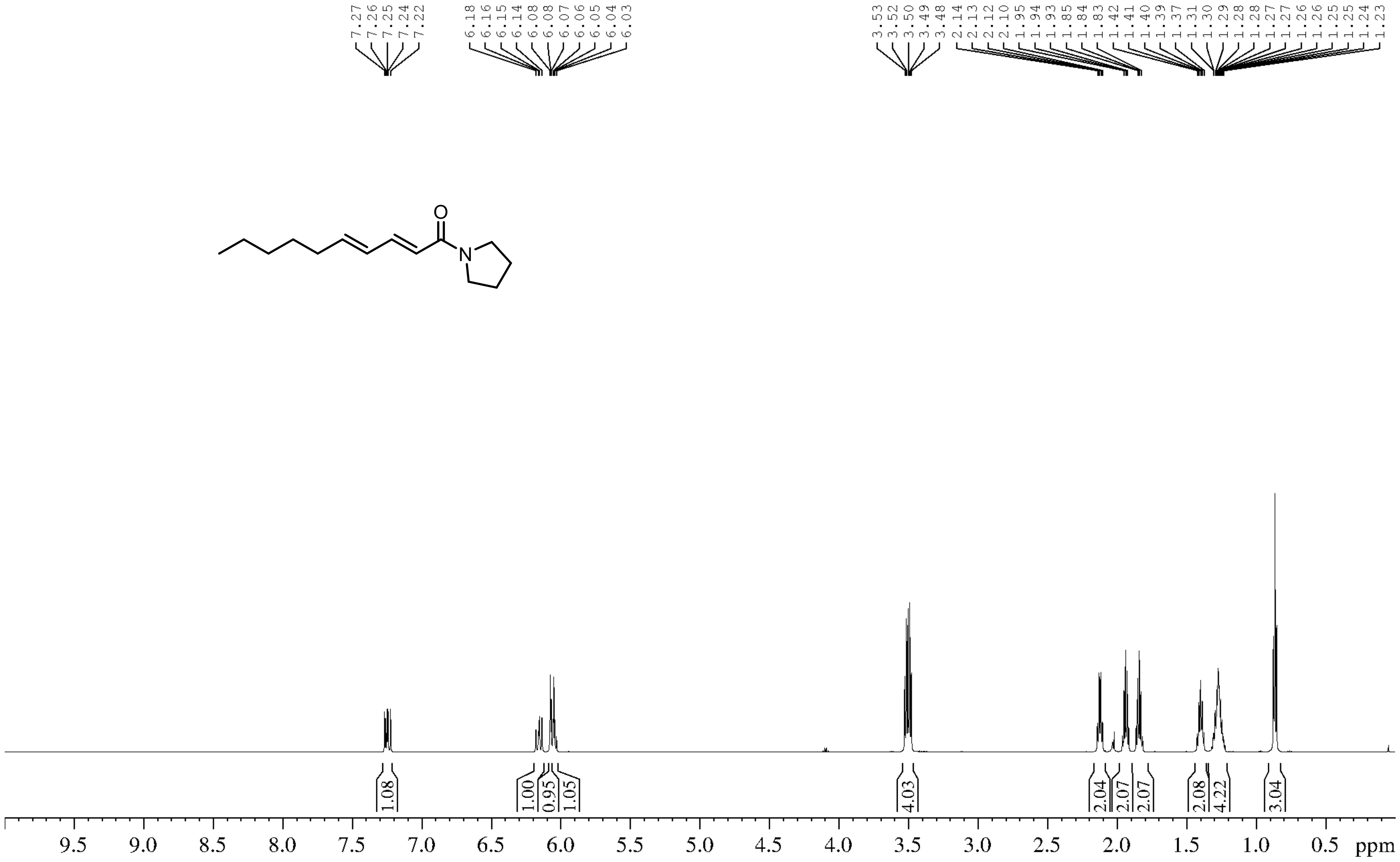

5.28.2 ^{13}C NMR

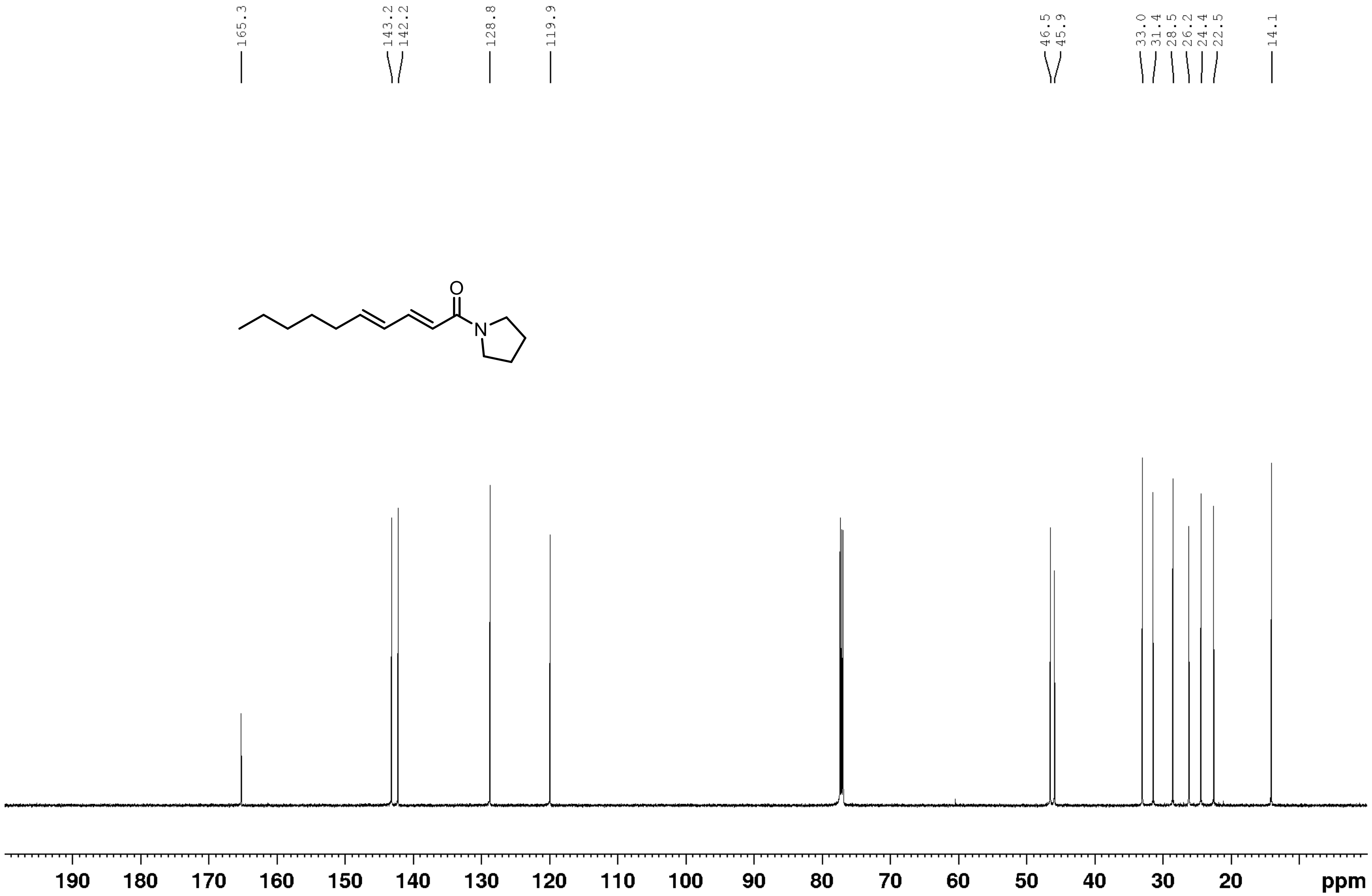
165.3
143.2
142.2
128.8
119.9
46.5
45.9
33.0
31.4
28.5
26.2
24.4
22.5
14.1
O
N
190
180
170
160
150
140
130
120
110
100
90
80
70
60
50
40
30
20
ppm

5.28.3 $^{1}H, ^{1}H$ COSY

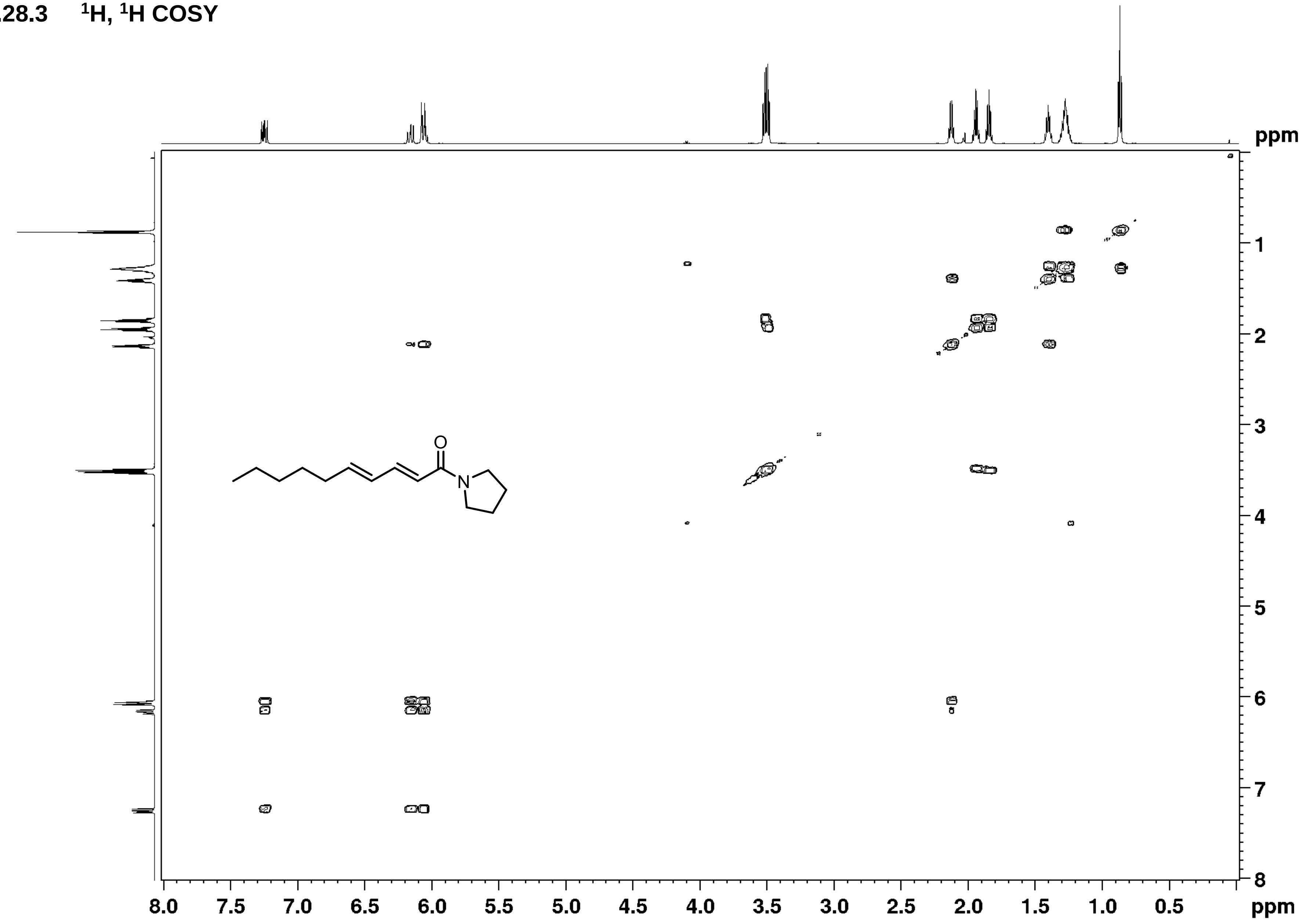

5.28.4 ^{1}H, ^{13}C HMBC

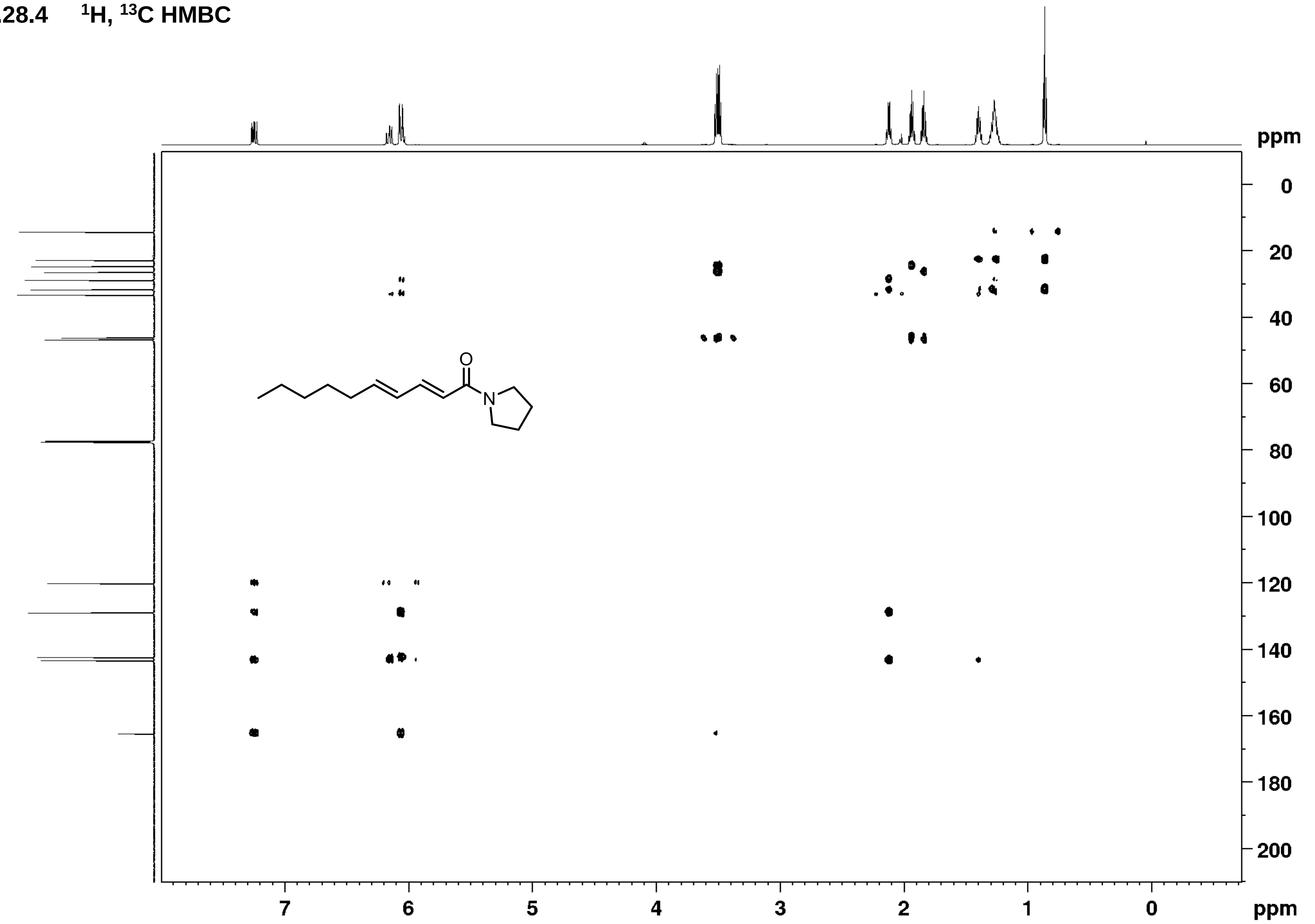

5.28.5 ^{1}H, ^{13}C HSQC

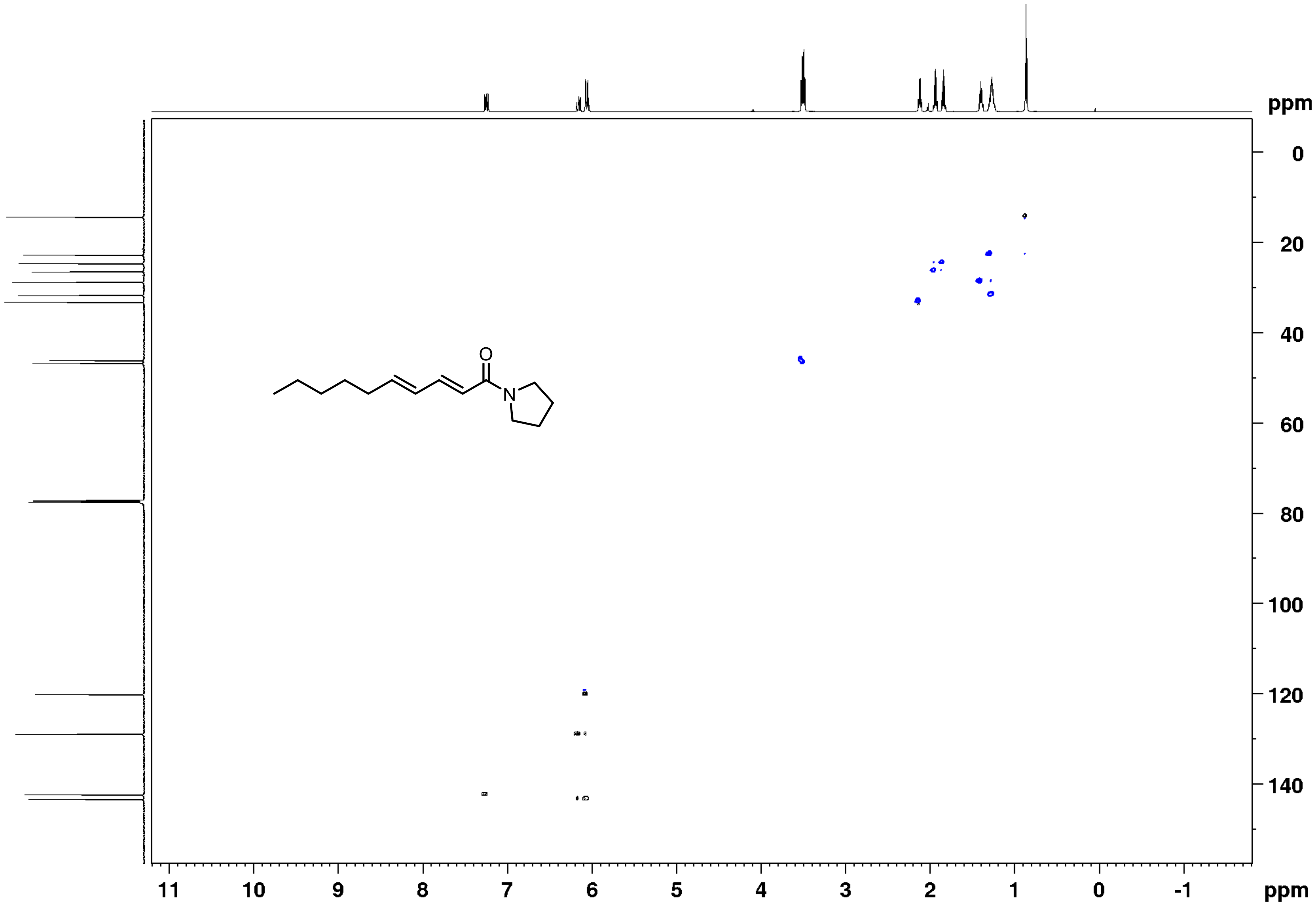

5.28.6 HRMS

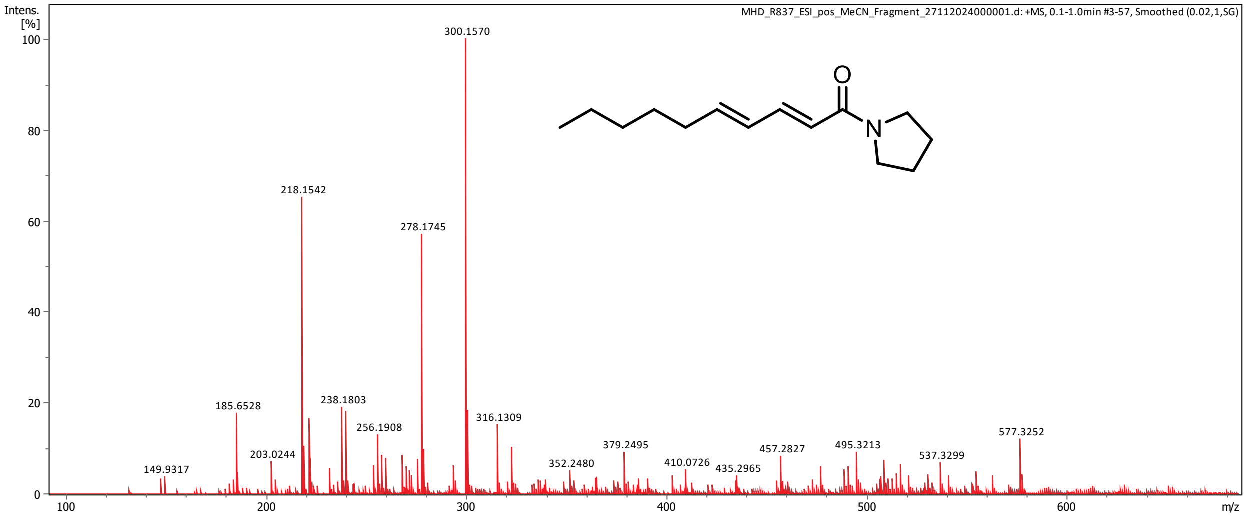

MHD_R837_ESI_pos_MeCN_Fragment_27112024000001.d: +MS, 0.1-1.0min #3-57, Smoothed (0.02,1,SG)
Intens. [%]
300.1570
218.1542
278.1745
185.6528
238.1803
256.1908
316.1309
203.0244
149.9317
352.2480
379.2495
410.0726
435.2965
457.2827
495.3213
537.3299
577.3252
m/z
O
N

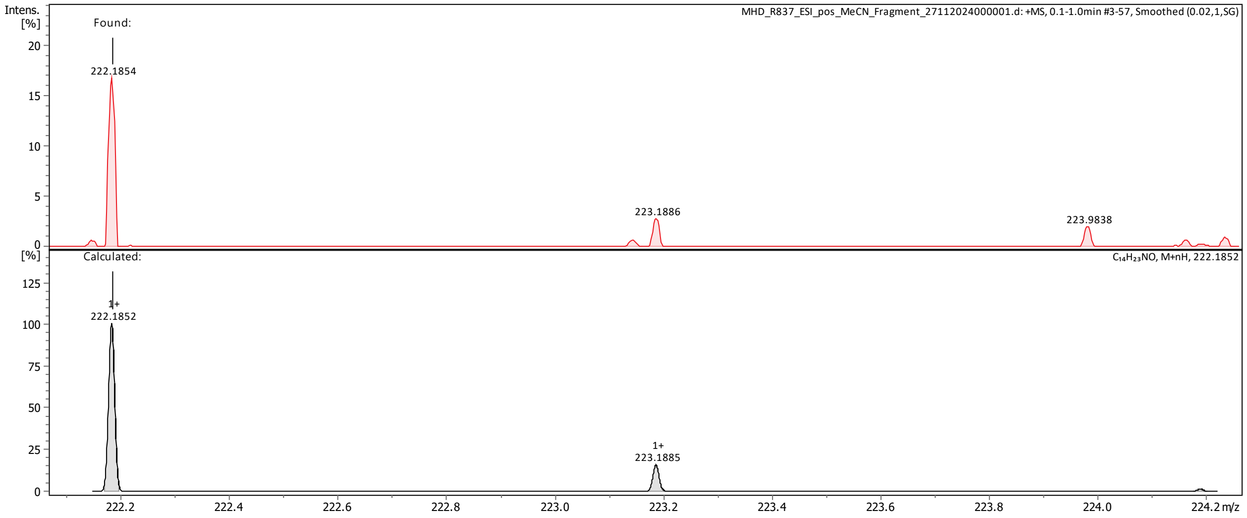

MHD_R837_ESI_pos_MeCN_Fragment_27112024000001.d: +MS, 0.1-1.0min #3-57, Smoothed (0.02,1,SG)
Intens. [%]
Found:
222.1854
223.1886
223.9838
Calculated:
C14H23NO, M+nH, 222.1852
1+
222.1852
1+
223.1885
m/z

5.28.7 IR

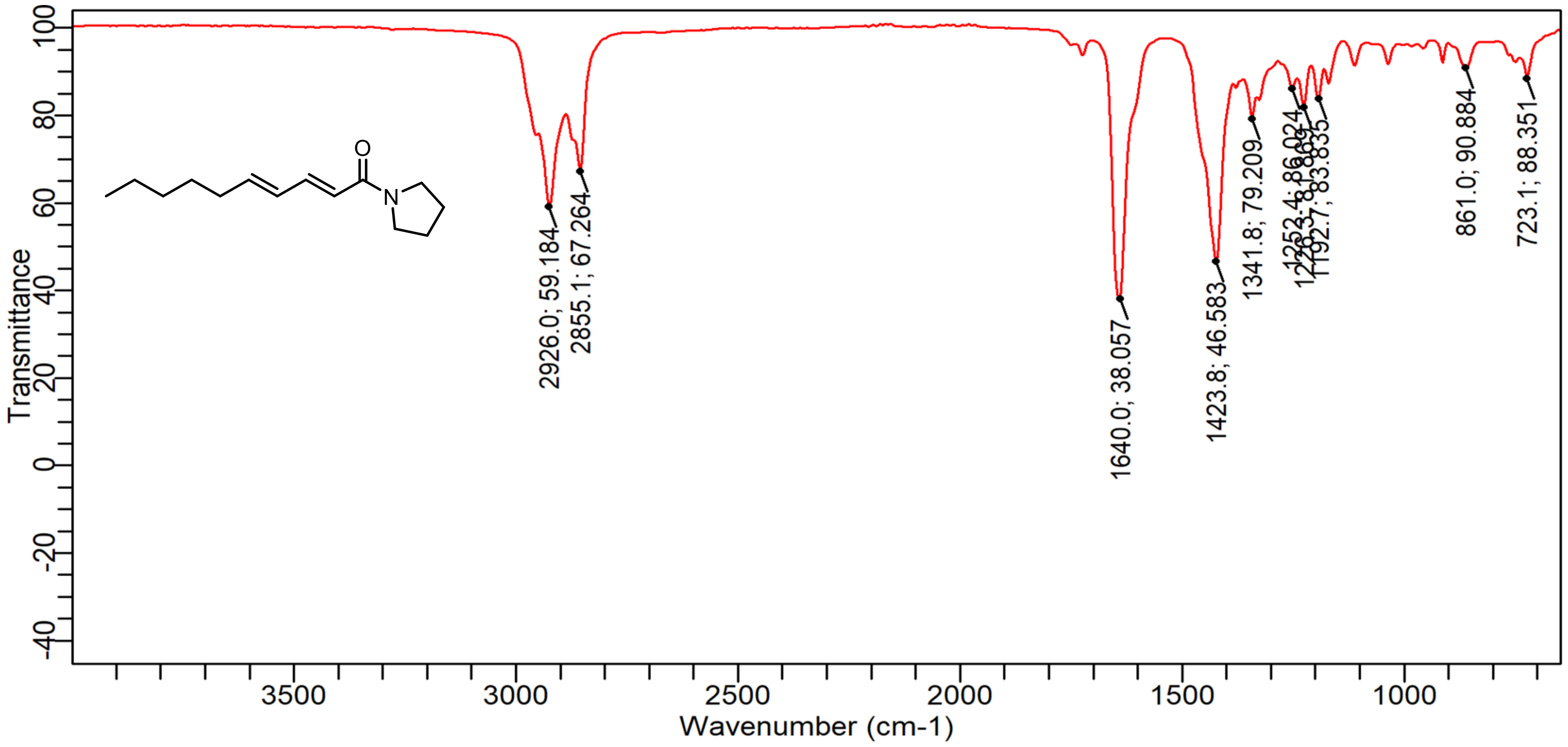

Transmittance
Wavenumber (cm-1)
100
80
60
40
20
0
-20
-40
3500
3000
2500
2000
1500
1000
O
N
2926.0; 59.184
2855.1; 67.264
1640.0; 38.057
1423.8; 46.583
1341.8; 79.209
1252.4; 86.024
1192.7; 83.835
861.0; 90.884
723.1; 88.351
